# Large Language Models Evaluation of Medical Licensing Examination Using GPT-4.0, ERNIE Bot 4.0, and GPT-4o

**DOI:** 10.3390/bioengineering13010113

**Published:** 2026-01-17

**Authors:** Luoyu Lian, Xin Luo, Kavimbi Chipusu, Muhammad Awais Ashraf, Kelvin K. L. Wong, Wenjun Zhang

**Affiliations:** 1 Department of Thoracic Surgery, Quanzhou First Hospital Affiliated to Fujian Medical University, Quanzhou 362000, China; 9202271515@fjmu.edu.cn; 2 Department of Cardiac and Thoracic Surgery, Longyan First Affiliated Hospital of Fujian Medical University, Longyan 364000, China; luoxin192@126.com; 3 Department of Mechanical Engineering, Division of Biomedical Engineering, University of Saskatchewan, Saskatoon, SK S7N 5A9, Canada; kavimbi.chipusu@usask.ca (K.C.); awais.ashraf@usask.ca (M.A.A.)

**Keywords:** large language models, medical licensing examination, GPT-4.0, ERNIE Bot 4.0, GPT-4o, multilingual AI

## Abstract

This study systematically evaluated the performance of three advanced large language models (LLMs)—GPT-4.0, ERNIE Bot 4.0, and GPT-4o—in the 2023 Chinese Medical Licensing Examination. Employing a dataset of 600 standardized questions, we analyzed the accuracy of each model in answering questions from three comprehensive sections: Basic Medical Comprehensive, Clinical Medical Comprehensive, and Humanities and Preventive Medicine Comprehensive. Our results demonstrate that both ERNIE Bot 4.0 and GPT-4o significantly outperformed GPT-4.0, achieving accuracies above the national pass mark. The study further examined the strengths and limitations of each model, providing insights into their applicability in medical education and potential areas for future improvement. These findings underscore the promise and challenges of deploying LLMs in multilingual medical education, suggesting a pathway towards integrating AI into medical training and assessment practices.

## 1. Introduction

In recent years, artificial intelligence (AI) and large language models (LLMs) have demonstrated significant potential in the medical field. Leveraging natural language processing (NLP) technologies, LLMs are capable of generating human-like text, which assists in medical decision-making and enhances the accuracy and efficiency of diagnoses [1,2]. For instance, GPT-3 and GPT-4, the latest generations of LLMs, excel in handling complex medical issues, providing decision support for clinicians and playing a vital role in medical education [2]. The application of AI technology in medical diagnostics, patient management, and medical education is continually expanding [3,4]. Studies have shown that AI models can significantly enhance the efficiency of medical work and reduce human errors [5,6,7].

Despite the need for further exploration of the performance and applicability of large language models (LLMs) in medical exams [2], numerous studies have assessed their effectiveness. In the United States Medical Licensing Examination (USMLE), research has shown that GPT-4 significantly outperforms GPT-3.5 in various medical exams [8]. For instance, Gilson et al. (2023) examined ChatGPT’s performance in the USMLE, finding its accuracy to exceed 60%, reaching the level of a third-year medical student [9]. In the National Eligibility cum Entrance Test (NEET) in India, Farhat et al. (2024) compared the performances of GPT-3.5 and GPT-4, showing that GPT-4’s accuracy significantly surpassed that of GPT-3.5, achieving over 70% [10].

GPT-4’s performance has also been extensively studied across different linguistic environments [11]. Takagi et al. (2023) evaluated GPT-4 in the Japanese National Medical Licensing Examination, finding that GPT-4’s accuracy was significantly higher than that of GPT-3.5, especially in complex questions and areas requiring specialized knowledge, demonstrating its reliability in non-English environments [12]. In South Korea, Jang et al. researched ChatGPT’s performance in the Korean National Medical Licensing Examination, revealing that its accuracy in answering parasitology questions was close to that of medical students [13].

In Germany, GPT-4 also exhibited outstanding performance. Meyer et al. (2024) studied GPT-4 in the written German Medical Licensing Examination, finding its accuracy reached 85%, significantly outperforming GPT-3.5’s 58% [14]. Overall, existing research demonstrates the significant potential of LLMs in medical exams, especially in handling standardized test questions. However, these models still have limitations in processing across different language environments and multimodal data. Chat-GPT’s performance on non-English questions is not as good as its performance on English questions [15,16], suggesting the need for further research to optimize LLMs’ applications in multilingual environments.

In the Chinese language environment, AI technology faces unique challenges and opportunities. Ernie Bot 4.0, developed by Baidu, is a Chinese large language model [17]. Although there is currently no specific study examining Ernie Bot 4.0’s performance in Chinese medical exams, its potential in processing Chinese medical texts is particularly noteworthy [18]. This potential provides direction for future research, especially in the application of Chinese medical education and examinations.

Additionally, the newly released GPT-4o in May 2024, as the latest large language model, has demonstrated its formidable capabilities in multilingual environments [19,20]. GPT-4o excels not only in handling medical questions in English and other languages but also shows significant improvements in Chinese contexts. Particularly, GPT-4o exhibits outstanding abilities in processing multimodal data, including text, images, and audio, enabling it to provide more comprehensive and accurate diagnostic support [21].

Recent studies such as CMB (Wang et al., 2023) and CMExam (Liu et al., 2024) have provided comprehensive benchmarks for evaluating large language models (LLMs) on Chinese medical exams [22,23]. CMB focuses on the integration of Traditional Chinese Medicine (TCM) with Western medicine, making it an invaluable resource for evaluating LLMs in a localized Chinese medical context [22]. CMExam, derived from the Chinese National Medical Licensing Examination, offers over 68,000 multiple-choice questions and a range of additional annotations for evaluating model reasoning and accuracy [23]. Our study extends these efforts by comparing the performance of GPT-4.0, ERNIE Bot 4.0, and GPT-4o on the 2023 Chinese Medical Licensing Examination, providing deeper insights into their strengths and limitations. In addition to GPT-4.0, ERNIE Bot 4.0, and GPT-4o, several other large language models have been developed for medical applications. MedPaLM, developed by Google, is specifically designed for medical reasoning and has demonstrated strong performance on medical benchmark datasets [24]. Qwen, an advanced multilingual model, also exhibits promising capabilities in non-English medical contexts [25]. While these models were not included in our direct evaluation, their performance in medical education and assessment remains an important topic for future research.

The central issue of this study was to evaluate the performance of GPT-4.0, ERNIE Bot 4.0, and GPT-4o in the 2023 Chinese Medical Licensing Examination. By systematically comparing these three large language models in the Chinese medical exam context, we aim to reveal their potential and limitations in medical exam applications, helping us understand their suitability and reliability in the Chinese medical education environment. By comparing the performance of different models, we can identify their respective strengths and limitations, providing data support for future model improvements and optimizations. Additionally, the newly released GPT-4o in May 2024, as the latest large language model, has demonstrated its significant capabilities in multilingual environments. GPT-4o excels not only in handling medical questions in English and other languages but also shows significant improvements in Chinese contexts. However, this study focuses solely on the text-based capabilities of these models, and their performance in image or audio-based tasks remains beyond the scope of our evaluation. This study distinguishes itself from previous evaluations through three key contributions: (1) Data Freshness: We utilized the 2023 exam dataset to mitigate ‘data leakage’ issues common in studies using older public datasets. (2) Geopolitical Benchmark: We provide a comparative analysis of ‘Cultural Fit,’ assessing how a top-tier domestic model (ERNIE Bot 4.0) competes with a US-based model (GPT-4o) specifically in localized Chinese medical knowledge. (3) Diagnostic Roadmap: We offer a section-wise diagnostic breakdown to identify specific medical sub-disciplines that require localized model fine-tuning.

## 2. Methods and Results

### 2.1. Data Sources

This study utilized all 600 original questions from the 2023 Chinese Medical Licensing Examination. Detailed content and reference answers can be found in the Supplementary Materials (English-Version All_Questions_Reference_Answers_and_Model_Responses). To minimize the risk of data contamination (memorization), we verified that the 2023 exam questions were not officially released by the National Medical Examination Center and were obtained from proprietary internal archives not publicly indexed on the open web. A random spot-check of 20 question stems showed that none of the models could verbatim autocomplete the questions, suggesting that they were generating answers based on reasoning rather than retrieval. These questions strictly adhered to the comprehensive medical examination syllabus, encompassing three main sections: Basic Medical Comprehensive, Clinical Medical Comprehensive, and Humanities and Preventive Medicine Comprehensive. The specific contents are as follows:

Basic Medical Comprehensive: This section includes foundational medical knowledge such as anatomy, biochemistry, physiology, medical microbiology, medical immunology, pathology, pathophysiology, and pharmacology.

Humanities and Preventive Medicine Comprehensive: This section covers the basic theories and behavioral norms of medical humanities disciplines such as medical psychology, medical ethics, and health law, as well as preventive medicine content like medical statistical methods, principles and methods of epidemiology, clinical preventive services, community public health, and health service systems and management.

Clinical Medical Comprehensive: This section primarily assesses comprehensive knowledge categorized by system diseases, involving the respiratory system, cardiovascular system, digestive system, urinary system (including male reproductive system), female reproductive system, hematological system, metabolic and endocrine systems, psychiatric and neurological systems, musculoskeletal system, rheumatic and immune diseases, pediatric diseases, infectious diseases, and sexually transmitted diseases.

All questions were standardized multiple-choice questions, including the following types:

Type A1 (Single Best Answer): Each question consists of a stem and five options, with only one best choice. Type A2 (Case Summary Best Choice): The stem is a brief case description, with five options among which only one is the best choice. Type B1 (Standard Matching): Five options are provided, with at least two questions, each requiring the selection of the most relevant answer. Type A3 (Cluster Case Best Choice): Centered on a patient scenario, presenting 2–3 related questions, each independent of the others. Type A4 (Sequential Case Best Choice): Centered on a single patient or family clinical scenario, gradually increasing information and posing 3–6 related questions.

### 2.2. Study Design

The process of this study is as follows:

All 600 questions from the 2023 Chinese Medical Licensing Examination were manually inputted into each model by the researchers. To ensure consistency, each question was presented with the following prompt: “You are currently participating in the Chinese Medical Licensing Examination. Here is an exam question. Please read carefully and choose the most correct answer based on your knowledge. Your answer should be based on existing medical knowledge and best practice guidelines”. All models responded within a Chinese-language environment to ensure consistency with the actual exam conditions. Each question was initiated in a new dialogue box to prevent interference between questions. Access to and use of each model were obtained in May 2024 through the purchase of the respective membership services. The responses from each model were recorded in an Excel file and organized into structured data for analysis.

**Figure 1:**
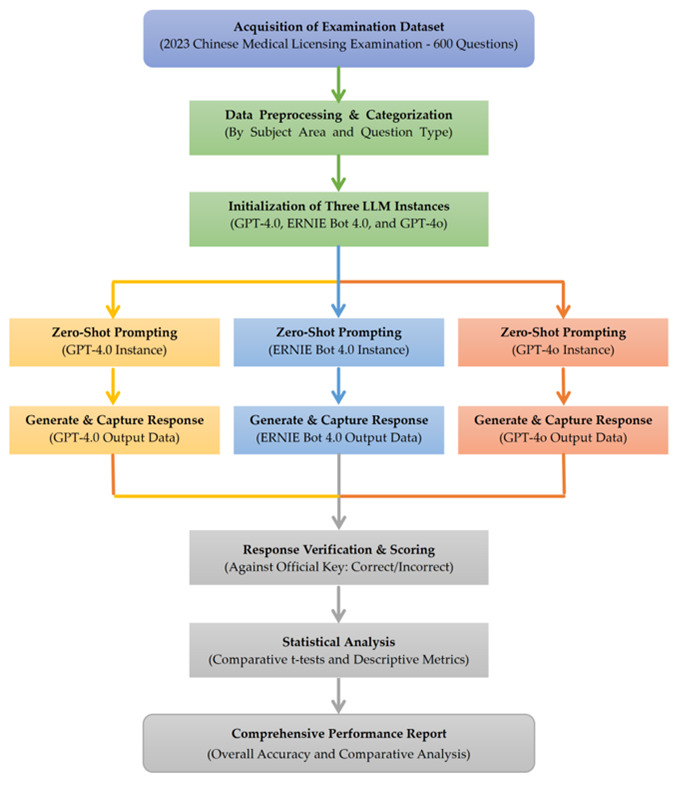
Study workflow for model performance evaluation on the 2023 Chinese Medical Licensing Examination.

In this study, we adopted a zero-shot prompting approach for all models, meaning that each question was presented to the model without any prior examples or contextual cues. We selected zero-shot prompting to simulate a real-world testing scenario where the model must rely solely on its pre-trained knowledge without external guidance. However, various alternative prompting techniques have been shown to enhance LLM performance, including: Few-shot prompting: Providing a few example questions with correct answers to guide the model’s response. Chain-of-Thought (CoT) prompting: Encouraging step-by-step reasoning by explicitly prompting the model to break down complex problems before reaching an answer [26]. Retrieval-Augmented Generation (RAG): Enhancing responses by incorporating relevant external information retrieved from a database [27]. While these techniques have demonstrated improved accuracy in certain tasks, we opted for zero-shot prompting to maintain consistency across models and align with a standardized examination format. This approach aligns with established protocols in recent authoritative medical LLM benchmarks (e.g., Nori et al., 2023 [28]; Kung et al., 2023 [29]), ensuring that the results reflect the models’ intrinsic knowledge retrieval capabilities while minimizing the variability introduced by prompt engineering bias. Future work could explore the impact of these techniques on medical question-answering performance. The overall study workflow is summarized in Figure 1.

### 2.3. Data Processing

The responses from each model were compared with the official correct answers. Based on the comparison results, responses were marked as correct (1) or incorrect (0). The results were stored in an Excel file.

### 2.4. Statistical Analysis

**Figure 2:**
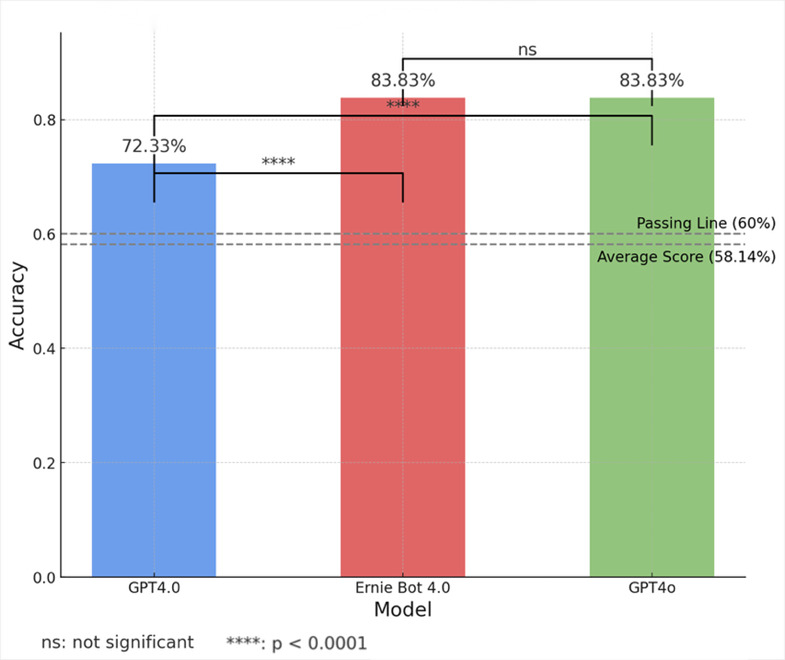
Accuracy comparison of GPT-4.0, Ernie Bot 4.0, and GPT-4o on the 2023 Chinese Medical Licensing Examination.

**Figure 3:**
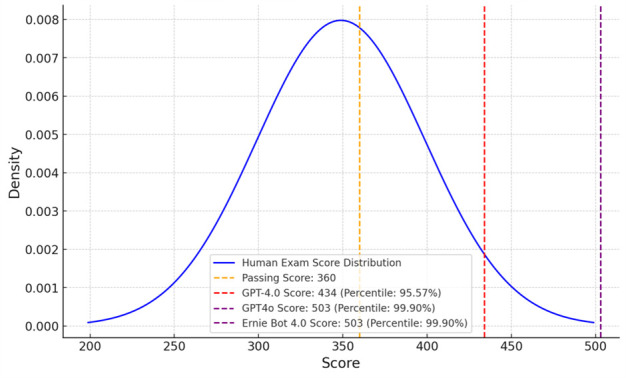
Normal Distribution of 2023 China Medical Licensing Exam Scores.

**Figure 4:**
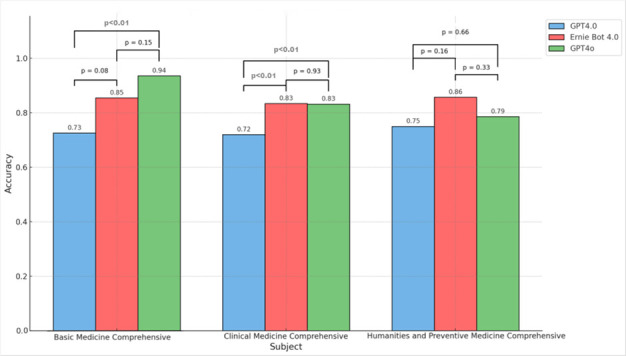
Model Accuracy Comparison by Subject with Statistical Significance.

**Figure 5:**
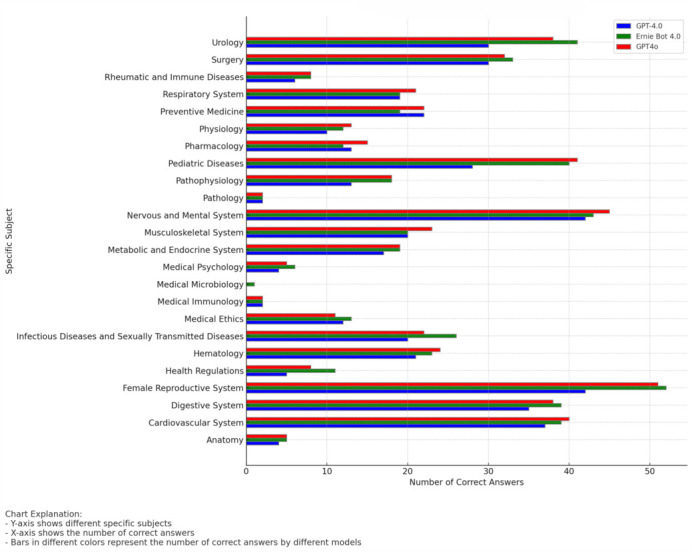
Comparison of Model Accuracy Across Medical Subjects.

**Figure 6:**
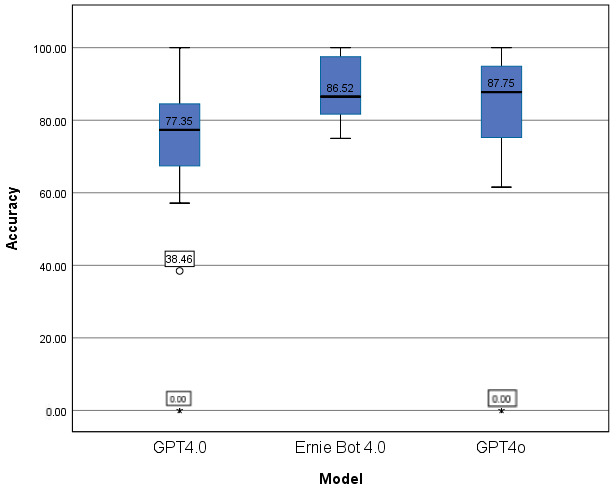
Box plot showing the distribution of accuracy for GPT-4.0, Ernie Bot 4.0, and GPT-4o across specific subjects. Data points represent the accuracy rates of the 18 specific medical sub-disciplines (e.g., Anatomy, Pathology, etc.) listed in Table 2.

Descriptive statistical analyses were conducted using Microsoft Excel 2016 (Microsoft Corp., Redmond, WA, USA), calculating the number of correct answers and the overall accuracy rate for each model. Subsequently, independent sample *t*-tests were performed using IBM SPSS Statistics 26.0 (IBM Corp., Armonk, NY, USA) to analyze the statistical differences between the models, with a *p*-value of less than 0.05 considered statistically significant. For the graphical section, Figure 2, Figure 3, Figure 4 and Figure 5 were generated by GPT-4o and validated through IBM SPSS Statistics 26.0 (IBM Corp., Armonk, NY, USA), while Figure 6 was produced using IBM SPSS Statistics 26.0 (IBM Corp., Armonk, NY, USA), ensuring the accuracy of all figures. Since the National Medical Examination Center does not publicly release the granular raw score distribution of all candidates due to privacy regulations, the distribution presented in Figure 3 is a statistical estimation reconstructed based on the officially disclosed mean score and pass rate.

## 3. Results

### 3.1. Main Results

All three models (GPT-4.0, Ernie Bot 4.0, and GPT-4o) passed the exam, with accuracies surpassing the national average score (58.14%) and the pass mark (60%). The specific performances were as follows: GPT-4.0 correctly answered 434 out of 600 questions, achieving an accuracy of 72.33%, while both Ernie Bot 4.0 and GPT-4o answered 503 questions correctly, with an accuracy of 83.83% each. Statistical analysis revealed that the performance of Ernie Bot 4.0 and GPT-4o was significantly better than that of GPT-4.0 (*p* < 0.0001), with no significant difference between Ernie Bot 4.0 and GPT-4o. To further validate the performance differences, McNemar’s tests on paired response data confirmed that the accuracy advantages of ERNIE Bot 4.0 and GPT-4o over GPT-4.0 were statistically significant (*p* < 0.05). The key statistical features are shown by Table 1 and Figure 2.

Scoring standards for the Chinese Medical Licensing Examination stipulate that each correct answer earns one point, with no points awarded for incorrect responses. According to the official score report, the average score for human candidates is 348.84, with a passing score threshold set at 360 points. Scoring 360 points ranks a candidate in the 57th percentile among human examinees. Based on the assumption of a normal distribution for human candidates’ scores, with a mean of 348.84 and a passing score of 360, the percentile rank of the AI models’ scores relative to the human candidates is estimated. GPT-4.0, with a score of 434, was estimated to surpass approximately 91.08% of human candidates, while both Ernie Bot 4.0 and GPT-4o, with a score of 503, were estimated to exceed 99.26% of human candidates. This percentile ranking was calculated based on the properties of the normal distribution, as illustrated in Figure 3.

### 3.2. Performance by Major Subject

The accuracy for each model in Basic Medical Comprehensive, Clinical Medical Comprehensive, and Humanities and Preventive Medicine Comprehensive are calculated. Independent sample t-tests were used to analyze the differences in accuracy between the models and their statistical significance. The results, can be presented by in Figure 4:

Basic Medical Comprehensive: GPT-4.0 achieved an accuracy of 72.58%, Ernie Bot 4.0 achieved 85.48%, and GPT-4o achieved 93.55%. GPT-4o was significantly superior to GPT-4.0 (*p* < 0.05); there was no significant difference between GPT-4.0 and Ernie Bot 4.0.

Clinical Medical Comprehensive: GPT-4.0 had an accuracy of 71.99%, Ernie Bot 4.0 had 83.40%, and GPT-4o had 83.20%. GPT-4.0 was significantly lower than both Ernie Bot 4.0 and GPT-4o (*p* < 0.05); there was no significant difference between Ernie Bot 4.0 and GPT-4o.

Humanities and Preventive Medicine Comprehensive: GPT-4.0 achieved an accuracy of 75.00%, Ernie Bot 4.0 achieved 85.71%, and GPT-4o achieved 78.57%. There were no significant differences between the three models.

These results suggest that Ernie Bot 4.0 and GPT-4o generally outperformed GPT-4.0, especially in Basic Medical and Clinical Medical subjects. Ernie Bot 4.0 showed a balanced performance across subjects. GPT-4o excelled particularly in Basic Medical Comprehensive. In Humanities and Preventive Medicine Comprehensive, which primarily involves law and computation, no significant differences were observed between the models.

### 3.3. Performance by Specific Subject

The accuracy of the three models—GPT-4.0, Ernie Bot 4.0, and GPT-4o—was further analyzed across various specific subjects within the major categories. The detailed performance for each specific subject is summarized in Table 2 and Figure 5.

Overall, Ernie Bot 4.0 and GPT-4o outperformed GPT-4.0, especially showing higher accuracy across multiple subjects. For instance, in critical subjects such as the female reproductive system, digestive system, and urinary system, the accuracy rates of Ernie Bot 4.0 and GPT-4o were significantly higher than those of GPT-4.0 (*p* < 0.05).

In terms of performance by specific subjects:

Anatomy: All models performed exceptionally well in anatomy, with Ernie Bot 4.0 and GPT-4o achieving a 100% accuracy, while GPT-4.0 also performed well with an 80% accuracy rate.

Cardiovascular System: GPT-4o had the highest accuracy rate at 92%, followed by Ernie Bot 4.0 at 83%, and GPT-4.0 at 75%.

Female Reproductive System: Ernie Bot 4.0 and GPT-4o substantially outperformed GPT-4.0, with accuracy rates of 86% and 82%, respectively, while GPT-4.0’s accuracy was 69%.

Health Regulations: Ernie Bot 4.0 had the best performance with an accuracy rate of 85%, GPT-4o scored 62%, and GPT-4.0 had the lowest rate at 38%.

Pediatric Diseases: The accuracy rates for GPT-4o and Ernie Bot 4.0 were similar, at 86% and 86%, respectively, while GPT-4.0’s rate was 57%.

The box plot (Figure 6) provides a detailed visualization of the accuracy distribution for each model (GPT-4.0, Ernie Bot 4.0, and GPT-4o) across all specific subjects.

From Table 3, it is evident that Ernie Bot 4.0 and GPT-4o demonstrated greater consistency and stability in accuracy compared to GPT-4.0. Among them, Ernie Bot 4.0 showed a more balanced performance across different subjects, while GPT-4o exhibited higher accuracy rates, indicating superior overall performance.

A detailed analysis of the performance of these three natural language processing models across various medical disciplines revealed that Ernie Bot 4.0 and GPT-4o generally outperformed GPT-4.0 in most subjects. These results suggest that, in the context of the Medical Licensing Examination, Ernie Bot 4.0 and GPT-4o may offer higher practical value and reliability.

### 3.4. Performance by Question Type

We compared the performance of GPT-4.0, Ernie Bot 4.0, and GPT-4o across different question types and calculated their accuracy rates along with the statistical differences between them (*p* values). The specific results are shown in Table 3.

Type A1: Ernie Bot 4.0 demonstrated the highest performance with an accuracy of 86% (179/207), showing statistically significant differences from GPT-4.0 at 73% (152/207) and GPT-4o at 85% (175/207), with *p*-values of 0.00 and 0.01, respectively. However, there was no significant difference between Ernie Bot 4.0 and GPT-4o (*p* = 0.68).

Type A2: GPT-4o (80%, 189/235) and Ernie Bot 4.0 (80%, 188/235) performed similarly, while GPT-4.0’s accuracy was 74% (174/235), with no statistically significant differences among them (*p* > 0.05).

Type A3/A4: GPT-4o showed the best performance with an accuracy of 86.87% (86/99), significantly different from GPT-4.0’s 71% (70/99) with a *p*-value of 0.01, but not significantly different from Ernie Bot 4.0 at 82% (81/99) with a *p*-value of 0.43.

Type B1: Ernie Bot 4.0 exhibited optimal performance at 93% (55/59), significantly better than GPT-4.0 at 64% (38/59) and GPT-4o at 90% (53/59), with *p*-values of 0.00.

Ernie Bot 4.0 excelled across most question types, particularly in Types A1 and B1. GPT-4o showed the best performance in Type A3/A4. GPT-4.0 had relatively poorer performance across all types. These results indicate that Ernie Bot 4.0 and GPT-4o significantly outperformed GPT-4.0 in different question types, highlighting the superior performance of Ernie Bot 4.0 and GPT-4o in the context of the medical licensing examination.

### 3.5. Efficiency Analysis

In addition to accuracy, we assessed the practical feasibility of these models for real-time medical education. While server-side metrics such as peak memory usage could not be measured due to the closed-source API nature of the tested models, we recorded the inference latency.

On average, GPT-4o and ERNIE Bot 4.0 generated responses within 3 to 6 s per question. This low latency indicates that these models are sufficiently responsive for deployment in interactive educational platforms, allowing for immediate feedback without significant user waiting time.

## 4. Discussion

This study systematically assessed the performance of GPT-4.0, ERNIE Bot 4.0, and GPT-4o in the 2023 Chinese Medical Licensing Examination, revealing that ERNIE Bot 4.0 and GPT-4o significantly outperformed GPT-4.0. Both ERNIE Bot 4.0 and GPT-4o correctly answered 503 out of 600 questions, achieving an accuracy rate of 84%, while GPT-4.0 only correctly answered 434 questions, with an accuracy rate of 72%.

Specifically, in the three major subjects (Basic Medical Comprehensive, Clinical Medical Comprehensive, and Humanities and Preventive Medicine Comprehensive), the performance of ERNIE Bot 4.0 and GPT-4o was superior to that of GPT-4.0 [30]. The balanced performance of Ernie Bot 4.0 across different subjects suggests a high degree of stability in processing native Chinese medical data. Meanwhile, the high scores of GPT-4o in basic medical sciences may indicate its robust capability in handling broad scientific knowledge. A more detailed analysis of specific subjects showed that ERNIE Bot 4.0 and GPT-4o excelled particularly in anatomy, cardiovascular system, digestive system, female reproductive system, health regulations, hematology, infectious diseases, and sexually transmitted diseases. Additionally, when addressing different types of questions (such as A1, A2, A3/A4, and B1), ERNIE Bot 4.0 and GPT-4o also demonstrated higher accuracy rates. In particular, when analyzing healthcare regulations-related questions, ERNIE Bot 4.0 showed a notable advantage over GPT-4 and GPT-4o, especially in areas where national regulations and specific legal frameworks differ. For instance, in questions regarding the prescription validity period, ERNIE Bot 4.0 accurately identified the 3-day validity period for regular prescriptions in China. Both GPT-4 and GPT-4o, however, provided incorrect answers, potentially due to their exposure to Western regulatory standards, where prescription validity periods tend to be longer. Beyond regulatory differences, distinct failure patterns were observed in other domains. In questions requiring multi-step logic, such as pediatric fluid therapy calculations, GPT-4.0 often retrieved the correct formula but failed the arithmetic execution, resulting in incorrect dosage recommendations. Furthermore, in the Traditional Chinese Medicine (TCM) module, GPT-4o occasionally generated ‘hallucinations’ by inventing plausible-sounding but non-existent herbal interactions, likely due to a lack of specialized training data in this localized domain. Similarly, in questions related to medical institution license verification periods, ERNIE Bot 4.0 demonstrated a better understanding of the Chinese system, where verification intervals are determined by hospital bed count—an approach differing from Western regulations. This highlights the critical role of considering regional legal and regulatory contexts when evaluating the performance of AI models, particularly in specialized domains such as medical law and healthcare regulations.

These results indicate that in the Chinese Medical Licensing Examination, ERNIE Bot 4.0 and GPT-4o perform significantly better than GPT-4.0 across various subjects and question types. ERNIE Bot 4.0 shows a more balanced performance across subjects, while GPT-4o exhibits higher accuracy, demonstrating superior overall performance. These findings highlight the practical value and reliability of ERNIE Bot 4.0 and GPT-4o in this context [31], suggesting that they could be more suitable tools for educational and preparatory purposes in the field of medical licensure in China.

Our research findings are consistent with existing studies, demonstrating the superior performance of advanced language models in medical examinations [32]. For instance, in an English-language examination environment, Gilson et al. (2023) discovered that ChatGPT-4.0’s accuracy in the United States Medical Licensing Examination (USMLE) exceeded 60%, equivalent to the level of a third-year medical student and surpassing GPT-3.5 [9]. Farhat et al. (2024) compared the performances of GPT-3.5, GPT-4, and Google Bard in the National Eligibility cum Entrance Test (NEET) in India, showing that GPT-4 achieved the highest overall score [10]. In non-English examination settings, ChatGPT-4 significantly outperformed GPT-3.5 and Google Bard in the Japanese National Dentist Examination (JNDE) [33]. GPT-4 scored an average of 85% in the German Medical Licensing Exam, ranking in the 92.8th, 99.5th, and 92.6th percentiles in the exams of October 2021, April 2022, and October 2022, respectively [14]. In the Iranian Medical Licensing Exam, GPT-4 also performed better than a random test group, showcasing its advantages in diagnostic accuracy and decision-making capabilities [34]. Our study found that GPT-4.0, Ernie Bot 4.0, and GPT-4o all passed the 2023 Chinese Medical Licensing Examination, achieving scores that, based on assumed statistical distributions, were higher than the estimated average performance of human candidates. However, it is important to note that the actual human performance distribution for this specific exam was not available for direct comparison. Thus, while these models demonstrated strong capabilities, their relative ranking against human candidates should be interpreted with caution.

This study found that Ernie Bot 4.0 and GPT-4o both outperformed GPT-4.0 in the 2023 Chinese Medical Licensing Examination. While Ernie Bot 4.0 is optimized for Chinese-language tasks, GPT-4.0 is also a multilingual model trained on diverse linguistic data, including Chinese medical literature. Therefore, the observed differences in performance may not solely be attributed to language-specific training but could also result from architectural optimizations, fine-tuning strategies, or domain-specific enhancements implemented in Ernie Bot 4.0 and GPT-4o [35]. Furthermore, as GPT-4o achieved results comparable to Ernie Bot 4.0, it suggests that advancements in multilingual and multimodal learning have contributed to its improved performance in this context. Additionally, while ChatGPT shows a decline in non-English language capabilities compared to English [15], in this study, GPT-4o scored comparably to Ernie Bot 4.0 across all aspects, particularly excelling in Basic Medical knowledge—a universally applicable medical field—with an accuracy of 93.55%, significantly higher than GPT-4.0. This indicates GPT-4o’s superior performance in universally applicable medical knowledge. In subjects related to Humanities and Preventive Medicine, neither Ernie Bot 4.0 nor GPT-4o showed a significant advantage, though Ernie Bot 4.0 had higher accuracy rates. This might be due to the differences in the content of Chinese versus English data concerning law, ethics, public health, and other issues.

Our study primarily focused on evaluating the performance of general-purpose LLMs (GPT-4.0, ERNIE Bot 4.0, and GPT-4o) on the 2023 Chinese Medical Licensing Examination. However, it is important to acknowledge the existence of domain-specific models such as MedPaLM [36], which is optimized for medical reasoning and has achieved strong results on global medical benchmarks [24]. Additionally, multilingual models such as Qwen may offer advantages in non-English medical assessments [25]. Future studies should consider direct comparisons between general-purpose and medical-specialized models to assess their respective strengths and limitations. While general-purpose models provide broader applicability across multiple domains, domain-specific models like MedPaLM might exhibit superior performance in highly specialized medical scenarios.

Our study utilized a zero-shot prompting strategy to ensure that each model’s response was based entirely on its internal knowledge without external context or guiding examples. However, prior research has indicated that alternative prompting methods [26,37,38], such as few-shot and chain-of-thought prompting, can significantly enhance LLM performance in complex reasoning tasks. Few-shot prompting, by providing the model with a small set of training examples, has been shown to improve contextual understanding and accuracy, particularly in domain-specific applications. Chain-of-Thought prompting encourages explicit step-by-step reasoning, which could be beneficial for complex clinical decision-making and case-based questions. Furthermore, Retrieval-Augmented Generation (RAG) enables models to retrieve real-time information from external databases, mitigating the risk of outdated or incorrect knowledge [27]. While zero-shot prompting allowed for a direct and unbiased comparison between models, future studies should systematically evaluate the effects of these prompting strategies on performance in medical licensing examinations. Investigating how these methods influence reasoning accuracy and domain-specific comprehension could provide deeper insights into optimizing LLMs for medical applications.

While the models performed well across all subject categories, it is essential to acknowledge the potential risks of data leakage. Many questions in the Chinese Medical Licensing Examination are available online, and some models, especially those trained on publicly available datasets, may have been exposed to this information. Future studies should incorporate more stringent safeguards, such as blind testing with newly created or unpublished questions, to prevent the influence of data leakage and ensure a more accurate evaluation of the models’ true capabilities. This study systematically assessed the performance of GPT-4.0, ERNIE Bot 4.0, and GPT-4o in the 2023 Chinese Medical Licensing Examination, but it is crucial to evaluate how these models might handle real-world clinical scenarios. In particular, their ability to process and generate appropriate responses for complex, multi-step medical cases, involving differential diagnosis and patient management, remains untested. Given the importance of clinical reasoning in medical practice, a deeper exploration of how AI models contribute to medical decision-making is needed.

In conclusion, compared with previous research, this study demonstrates that Ernie Bot 4.0 and GPT-4o perform better than GPT-4.0 in Chinese medical examinations. Future research could further explore how to optimize LLMs in multilingual environments, particularly through enhancing multilingual training data and improving model structures, to boost their application in various language settings. This could help facilitate the widespread application of LLMs in global medical education and clinical decision-making support.

Evaluating large language models (LLMs) in the context of the Chinese Medical Licensing Examination helps to understand their applicability and reliability in the Chinese medical education environment. Wang et al. (2023) noted that although ChatGPT 3.5 did not perform as well as medical students in Chinese medical exams, its explanatory and learning support capabilities still hold value [39]. Our research confirms the advantages of Ernie Bot 4.0 and GPT-4o in handling Chinese medical questions, providing a basis for their application in Chinese medical education. Li et al. (2024) discovered that ChatGPT performed exceptionally in the entrance examination for a Master’s degree in clinical medicine, especially in the medical humanities subjects [40]. This study further assesses the performance of Ernie Bot 4.0, GPT-4.0, and GPT-4o across different subjects and question types, revealing their unique strengths and areas for improvement in Chinese medical exams.

LLMs can serve as auxiliary tools to enhance the learning efficiency and diagnostic accuracy of medical students and clinicians [41], particularly in regions with limited resources where AI technology can offer additional educational support and compensate for the lack of teaching and medical resources. Due to Internet restrictions in mainland China, GPT-4.0 and GPT-4o are not usable, making Ernie Bot 4.0 (Wenxin Yiyi) a viable alternative. Its ability to process Chinese data gives it significant applicability in localized medical education and clinical practice.

This study still has several limitations: 1. Variability of Accuracy Over Time: The accuracy of large language models may vary over time [42]. This study only collected data from the three models in May 2024 and did not evaluate the performance of the models over different periods. This limitation restricts a continuous assessment of model consistency. 2. Multimodal Capabilities Limitation: Although all three models are multimodal, capable of processing various types of content including images, this examination did not involve image-related questions. Therefore, we could not assess the models’ capabilities in image processing, which may be critical for complete diagnostic assessments in some medical fields. 3. Lack of Comparison with Human Candidates’ Performance: Since the official distribution of scores for the 2023 Chinese Medical Licensing Examination was not published, we could not compare the models’ performance with human candidates who took the exam at the same time. This limitation restricts a comprehensive evaluation of how models perform relative to human levels. 4. Potential Data Leakage Issues: Some answers to the medical licensing examination may be available online, and these answers might have been included in the dataset used during the training of the large language models, potentially skewing the assessment of the models’ true capabilities. This could result in the models performing better than they would in real clinical scenarios. These limitations suggest that caution is needed when generalizing our research findings to all medical settings. Regarding computational efficiency, since this study evaluated closed-source commercial models via public APIs, server-side metrics were opaque. Future research utilizing open-weights models is necessary to benchmark hardware-level efficiency [43]. Furthermore, the robustness of medical AI remains a critical concern. Future work must address system reliability against perturbations. Recent studies have proposed methods such as classification score analysis for detecting adversarial attacks [44] and highlighted the importance of algorithmic efficiency [43]. Developing robust defenses in both medical text and imaging models [45,46] is essential for patient safety. Finally, this study utilized a zero-shot prompting strategy to establish a standardized baseline. We acknowledge that advanced prompting techniques, such as Chain-of-Thought (CoT), could further enhance medical reasoning capabilities, and future studies should explore these methods to unlock the full potential of LLMs.

Future research should focus on improving the robustness of the models in multilingual environments [47] while also incorporating enhanced data leakage detection methods. These methods could include randomizing exam questions, cross-validating responses with multiple independent evaluators, and using newly released content to ensure that the models’ performance is based on actual clinical knowledge, not pre-exposed data. As a Chinese model, Ernie Bot 4.0 requires additional research support for its application in Chinese materials. For GPT-4o, as a new model, its performance and stability in practical applications still need further validation.

## 5. Conclusions

In summary, this study systematically evaluated the performance of GPT-4.0, ERNIE Bot 4.0, and GPT-4o in the 2023 Chinese Medical Licensing Examination. The findings indicate that ERNIE Bot 4.0 and GPT-4o both outperformed GPT-4.0 overall and in specific subjects. ERNIE Bot 4.0 displayed a more balanced performance across various subjects, while GPT-4o exhibited higher accuracy rates, demonstrating superior overall performance. Future research should further verify the stability and reliability of these models in practical applications to provide stronger data support for advancing AI’s application in medical education.

## Figures and Tables

**Table 1:** Overall performance of each model.

Model	Correct Answers Count	Accuracy (%)
GPT-4.0	434	72.33
Ernie Bot 4.0	503	83.83
GPT-4o	503	83.83

**Table 2:** Accuracy of GPT-4.0, Ernie Bot 4.0, and GPT-4o across specific subjects.

Specific Subject	GPT-4.0 (Correct/Total, %)	Ernie Bot 4.0 (Correct/Total, %)	GPT-4o (Correct/Total, %)
Anatomy (20)	16/20 (80%)	20/20 (100%)	20/20 (100%)
Cardiovascular System (24)	18/24 (75%)	20/24 (83%)	22/24 (92%)
Digestive System (26)	18/26 (69%)	22/26 (85%)	19/26 (73%)
Female Reproductive System (22)	15/22 (68%)	19/22 (86%)	18/22 (82%)
Health Regulations (13)	5/13 (38%)	11/13 (85%)	8/13 (62%)
Hematology (27)	21/27 (78%)	23/27 (85%)	24/27 (89%)
Infectious Diseases and Sexually Transmitted Diseases (15)	10/15 (67%)	13/15 (87%)	11/15 (73%)
Medical Ethics (14)	12/14 (86%)	13/14 (93%)	11/14 (79%)
Medical Immunology (6)	6/6 (100%)	6/6 (100%)	6/6 (100%)
Medical Microbiology (2)	0/2 (0%)	2/2 (100%)	0/2 (0%)
Medical Psychology (3)	2/3 (67%)	3/3 (100%)	2/3 (67%)
Metabolic and Endocrine System (20)	17/20 (85%)	19/20 (95%)	19/20 (95%)
Musculoskeletal System (25)	20/25 (80%)	20/25 (80%)	23/25 (92%)
Nervous and Mental System (25)	21/25 (84%)	23/25 (92%)	20/25 (80%)
Pathology (6)	6/6 (100%)	6/6 (100%)	6/6 (100%)
Pathophysiology (19)	13/19 (68%)	18/19 (95%)	18/19 (95%)
Pediatric Diseases (21)	12/21 (57%)	18/21 (86%)	18/21 (86%)
Pharmacology (16)	13/16 (81%)	12/16 (75%)	15/16 (94%)
Physiology (8)	8/8 (100%)	7/8 (88%)	8/8 (100%)
Preventive Medicine (25)	22/25 (88%)	19/25 (76%)	22/25 (88%)
Respiratory System (24)	19/24 (79%)	19/24 (79%)	21/24 (88%)
Rheumatic and Immune Diseases (13)	10/13 (77%)	13/13 (100%)	13/13 (100%)
Surgery (35)	25/35 (71%)	28/35 (80%)	27/35 (77%)
Urology (18)	11/18 (61%)	14/18 (78%)	13/18 (72%)

**Table 3:** Accuracy Rates and Statistical Differences Analysis by Question Type.

Question Type	GPT-4.0 (Correct/Total, %)	Ernie Bot 4.0 (Correct/Total, %)	GPT-4o (Correct/Total, %)	GPT-4.0 vs. Ernie Bot 4.0 *p*-Value	GPT-4.0 vs. GPT-4o *p*-Value	Ernie Bot 4.0 vs. GPT-4o *p*-Value
A1	152/207 (73%)	179/207 (86%)	175/207 (85%)	<0.01	0.01	0.68
A2	174/235 (74%)	188/235 (80%)	189/235 (80%)	0.15	0.12	1.00
A3/A4	70/99 (71%)	81/99 (82%)	86/99 (87%)	0.09	0.01	0.43
B1	38/59 (64%)	55/59 (93%)	53/59 (90%)	<0.01	<0.01	0.74

## Data Availability

Data are provided within the manuscript or supplementary information files.

## Supplementary Materials

The following supporting information is available online: https://www.mdpi.com/article/10.3390/bioengineering13010113/s1. The complete set of examination questions and the detailed, raw response data generated by the three Large Language Models (GPT-4.0, ERNIE Bot 4.0, and GPT-4o) used in this study.

## Abbreviations

**AI**	Artificial Intelligence
**LLM**	Large Language Model
**NLP**	Natural Language Processing
**GPT**	Generative Pre-trained Transformer
**USMLE**	United States Medical Licensing Examination
**NEET**	National Eligibility cum Entrance Test
