# Supplementary material for: Large Language Models Evaluation of Medical Licensing Examination Using GPT-4.0, ERNIE Bot 4.0, and GPT-4o
Source: Bioengineering (Basel). 2026 Jan 17;13(1):113. doi: 10.3390/bioengineering13010113 (PMC12837214; doi:10.3390/bioengineering13010113)
Supplement: Supplementary file 1 [file bioengineering-13-00113-s001.zip › All_Questions_Reference_Answers_and_Model_Responses.pdf]

所有题目及各模型答案（白底黑字为正确答案，下方彩色字体为 gpt4.0 答案，再下方黄底字体为文心一言 4.0 答案，再下方彩色字体为 gpt4o 答案，所有彩色字体，绿色为正确答案，红色为错误答案）

2023 第一单元

一、A1 型选择题 （每一道考题下面有 A 、B 、C 、D 、E 五个备选答 案。请从中选择一个最佳答案。）

1、 (A1 型题)

医疗机构临时抽血时，下列哪一项不是医疗机构临时抽血的规定要求

A.甲肝表面抗体

B.乙肝表面抗原

C.丙肝表面抗体

D.HIV 抗体

E.梅毒抗体

参考答案： A 您的答案：

图文解析： 【解析】医疗机构临时采血时应当具备开展交叉配血及乙型肝炎病毒表面抗原、丙型肝炎
病毒抗体、艾滋病病毒抗体和梅毒螺旋体抗体的检测能力。故本题选 A。
【避错】为保证应急用血，医疗机构可以临时采集血液，但必须同时符合以下条件：
（1）危及患者生命，急需输血；（2）所在地血站无法及时提供血液，且无法及时从其他医疗机构调剂血液，而其他医疗措施不能替代输血治疗；（3）具备开展交叉配血及乙型肝炎病毒表面抗原、丙型肝炎病毒抗体、艾滋病病毒抗体和梅毒螺旋体抗体的检测能力；（4）遵守采供血相关操作规程和技术标准。医疗机构应当在临时采集血液后 10 日内将情况报告县级以上人民政府卫生行政部门。

在医疗机构进行临时抽血时，通常要求进行一系列传染病的血液检测，以确保安全和防止疾病传播。选项 A、B、C、D 和 E 分别提到了甲型肝炎表面抗体、乙型肝炎表面抗原、丙型肝炎表面抗体、HIV 抗体和梅毒抗体。这些检测通常是为了识别可能的传染性疾病，以防止其在医疗过程中传播。

然而，题目询问的是“不是医疗机构临时抽血的规定要求”，这意味着我们需要找到一个在临时抽血时通常不必要进行的检测。在上述选项中，甲型肝炎表面抗体（选项 A）通常不是临时抽血必须检测的项目，因为甲型肝炎更多是通过口粪途径传播，而非血液传播。因此，与乙型肝炎、丙型肝炎、HIV 和梅毒相比，甲型肝炎在血液传播方面的风险较低。

基于这个逻辑，最正确的答案应该是 A.甲肝表面抗体。

解析：
在医疗机构临时采集血液时，需对采集的血液进行必要的传染病学检查，以确保血液安全，减少疾病传播风险。选项中涉及的检验项目与疾病对应关系如下：
选项 A 甲肝表面抗体可判断患者是否感染甲型肝炎病毒，或既往是否接种过甲肝疫苗，是医疗机构临时采集血液时的必要检测项目。
选项 B 乙肝表面抗原是乙肝病毒的外壳蛋白，本身不具有传染性，但它的出现常伴随乙肝病毒的存在，是感染乙肝病毒的标志，也是医疗机构临时采集血液时的必要检测项目。

丙型肝炎病毒无表面抗体，因此无法检测丙肝表面抗体，通常是通过检测丙肝抗体来判断是否有丙肝病毒感染。故选项 C 丙肝表面抗体不是医疗机构临时抽血的检测项目，为本题正确答案。

选项 D：HIV 抗体是检测艾滋病病毒感染的重要指标，是医疗机构临时采集血液时的必要检测项目。

选项 E 梅毒抗体检测可帮助判断是否有梅毒螺旋体感染，是医疗机构临时采集血液时的必要检测项目。

综上所述，正确答案是：C.丙肝表面抗体。

医疗机构临时抽血时，下列哪一项不是医疗机构临时抽血的规定要求：

A. 甲肝表面抗体
B. 乙肝表面抗原
C. 丙肝表面抗体
D. HIV 抗体
E. 梅毒抗体

根据现有的医学知识和最佳实践指南，医疗机构在进行临时抽血时通常会检测乙肝表面抗原（HBsAg）、丙肝抗体（HCV Ab）、HIV 抗体和梅毒抗体，因为这些都是传染性较高且需要及时筛查的病原体。甲肝表面抗体（HAV Ab）通常不在临时抽血的常规筛查项目中，因为甲肝主要通过消化道传播，而不是血液传播。

因此，答案是：

A. 甲肝表面抗体

2、 (A1 型题)

有权保存医师处方签名或有关签章的机构是

A.医师执业地的卫生行政部门
B.医师注册的执业机构
C.医师注册地的监督管理部门
D.省级卫生管理部门
E.医师所在科室

参考答案： B 您的答案：

图文解析： 【解析】医师须在注册的医疗、预防、保健机构签名留样及专用签章备案后方可开具处方。故本题选 B。

【避错】《处方管理办法》第五条关于处方权的规定：经注册的执业助理医师开具的处方须经所在执业地点执业医师签字或加盖专用签章后方有效。经注册的执业助理医师在乡、民族乡、镇的医疗、预防、保健机构执业，在注册的执业地点取得相应的处方权。试用期的医师开具处方，须经所在医疗、预防、保健机构有处方权的执业医师审核、并签名或加盖专用签章后方有效。医师须在注册的医疗、预防、保健机构签名留样及专用签章备案后方可开具处方。医师被责令暂停执业、被责令离岗培训期间或被注销、吊销执业证书后，其处方权即被取消。

在中国，医师处方签名或有关签章的保存通常是由医师注册的执业机构负责的。因此，最合适的选择应该是 B.医师注册的执业机构。这些机构负责管理医师的执业活动，包括处方签名和相关文件的保存，以确保医疗服务的质量和法律合规性。因此，我的答案是 B.医师注册的执业机构。

首先，我们要明确这个问题是关于哪个机构有权保存医师处方签名或有关签章。接下来，我们可以逐一分析各个选项：

A. 医师执业地的卫生行政部门：卫生行政部门主要负责公共卫生和医疗服务的监督和管理，虽然他们有一定的监管权力，但他们通常不负责具体医疗机构的内部管理事务，如保存医师的处方签名等。

B. 医师注册的执业机构：医师注册的执业机构是医师日常工作的场所，也是他们开具处方的地方。根据相关规定，医师处方签名或有关签章应由其注册的执业机构妥善保存，以备查证。

C. 医师注册地的监督管理部门：这个部门主要负责医师的注册和管理，虽然他们保存有医师的注册信息，但并不涉及处方签名的保存。

D. 省级卫生管理部门：这是一个更高级别的管理部门，主要负责全省的卫生政策和管理，不直接参与医疗机构的内部管理。

E. 医师所在科室：科室是医院内部的一个单位，虽然医师在科室工作，但处方签名或签章的保存通常是由医院或执业机构统一进行的，而不是由单个科室负责。

综上所述，根据医疗机构的内部管理规定和相关法规，医师处方签名或有关签章应由其注册的执业机构负责保存。这样可以确保处方的真实性和可追溯性，有助于保障患者的用药安全和医疗质量。

因此，正确答案是 B.医师注册的执业机构。

根据现有的医学知识和最佳实践指南，有权保存医师处方签名或有关签章的机构是：

B. 医师注册的执业机构

3、 (A1 型题)

处方在特殊情况下可以延长有效期，但有效期最长不得超过

A.2 天

B.3 天

C.5 天

D.7 天

E.10 天

参考答案： B 您的答案：

图文解析： 【解析】处方为开具当日有效。特殊情况下需延长有效期的，由开具处方的医师注明有效

期限，但有效期最长不得超过 3 天。
【避错】记忆性题目，出自《处方管理办法》。
在中国，处方的有效期通常由相关的医疗法规和政策决定。处方的有效期可能因地区和具体情况而有所不同，但一般来说，普通处方的有效期通常不超过 7 天。对于一些特殊情况，如慢性病患者的长期用药处方，可能会有更长的有效期，但这通常需要特别的规定或批准。

在没有具体国家或地区的详细规定的情况下，选择“D.7 天”作为处方最长有效期的选项是比较合理的，因为这是常见的处方有效期上限。然而，实际操作中应依据当地的医疗规定和政策。

因此，最合适的答案是 D.7 天。不过，建议查阅最新的医疗法规和指南，以获取最准确的信息。

解析：
处方是医疗和药剂配制的重要书面文件，当医师诊治病人的时候通常会开具处方，然后由药师审核、调配、核对，最后发于患者的药剂调配的凭证，处方药的有效期一般为 3 天，但处方在特殊情况下需要延长有效期的，由开具处方的医师注明有效期限，但有效期最长不得超过 3 天。

综上所述，正确答案是：B.3 天。

根据《处方管理办法》规定，在特殊情况下，处方有效期最长不得超过 7 天。因此，正确答案是：

D. 7 天
4、 (A1 型题)
医生开具麻醉药品、第一类精神药品处方必须符合的法定的资格是
A.具备开立麻醉药品、第一类精神药品处方资格
B.具备开立麻醉药品、第一类精神药品临床使用资格
C.参加麻醉药品、第一类精神药品的省级培训班
D.具备开立麻醉药品、第一类精神药品的临床经验
E.取得麻醉药品、第一类精神药品的印鉴卡
参考答案： A 您的答案：
图文解析： 【解析】医疗机构应当按照国务院卫生主管部门的规定，对本单位执业医师进行有关麻醉药品和精神药品使用知识的培训、考核，经考核合格的，授予麻醉药品和第一类精神药品处方资格。故本题选 A。
【避错】医疗机构需要使用麻醉药品和第一类精神药品的，应当经所在地设区的市级人民政府卫生主管部门批准，取得麻醉药品、第一类精神药品购用印鉴卡。
在中国，医生开具麻醉药品、第一类精神药品处方必须具备相应的法定资格。这通常要求医生不仅有相关的临床经验，而且还需要通过特定的培训并取得官方批准的资格或认证。选项中提到了“具备开立麻醉药品、第一类精神药品处方资格”、“临床使用资格”、“参加培训班”、“临床经验”以及“取得印鉴卡”。

在这些选项中，A 选项“具备开立麻醉药品、第一类精神药品处方资格”最直接地指向了法

定要求，因为这表明医生已经获得了官方认证，可以合法开具这些药品的处方。虽然其他选项如培训、临床经验和印鉴卡都可能是获取这种资格的一部分或与之相关，但最根本的是医生必须具备正式的处方资格。

因此，正确答案应该是 A.具备开立麻醉药品、第一类精神药品处方资格。

解析：
麻醉药品和精神药品是国家实行特殊管理的药品，医疗机构需要使用麻醉药品和第一类精神药品的，应当经所在地设区的市级药品监督管理部门批准，取得麻醉药品、第一类精神药品购用印鉴卡。医疗机构应当按照国务院卫生主管部门制定的麻醉药品和精神药品临床应用指导原则，开具麻醉药品、第一类精神药品处方。而具有麻醉药品和第一类精神药品处方权的执业医师，需参加麻醉药品和精神药品使用知识和规范化管理的培训并经考核合格后，取得麻醉药品和第一类精神药品的处方权。
选项 A 具备开立麻醉药品、第一类精神药品处方资格；选项 B 具备开立麻醉药品、第一类精神药品临床使用资格；选项 D 具备开立麻醉药品、第一类精神药品的临床经验；以上三个选项表述均不够准确，具有处方权资格或临床经验并不代表有开具麻醉药品和精神药品的处方权，且临床使用资格不是法律规定的必要条件，可一并排除。
选项 C 参加麻醉药品、第一类精神药品的省级培训班，表述不准确，参加培训班是取得处方权的一个环节，但并非唯一条件，还需要经考核合格，故不选。

综上所述，正确答案是：E.取得麻醉药品、第一类精神药品的印鉴卡。

在中国执业医师考试中，关于医生开具麻醉药品和第一类精神药品处方所需的法定资格，最符合规定的答案是：

A. 具备开立麻醉药品、第一类精神药品处方资格

依据我国相关法律法规，医生需要具备开立麻醉药品和第一类精神药品处方的专门资格，才能合法开具这些药品的处方。这个资格通常通过相关部门的认证和培训获得。
5、 (A1 型题)
以下为劣药的情形是
A.以非药品冒充药品的
B.变质的
C.药品成分与国家规定的成分不符的
D.标明的功能主治范围超出药品的治疗范围
E.超出有效期
参考答案： E 您的答案：
图文解析： 【解析】属于劣药的情况有：（1）药品成份的含量不符合国家药品标准；（2）被污染的
药品；（3）未标明或者更改有效期的药品；（4）未注明或者更改产品批号的药品；（5）超过有效期的药品；（6）擅自添加防腐剂、辅料的药品；（7）其他不符合药品标准的药品。
【避错】属于假药的情况有：药品所含成份与国家药品标准规定的成份不符；以非药品冒充药品或者以他种药品冒充此种药品；变质的药品；药品所标明的适应证或者功能主治超

出规定范围。

在中国执业医师考试中，劣药的定义是指那些在质量上不符合国家标准的药品。根据这个定义，以下情形可以被认为是劣药：

A. 以非药品冒充药品的：这种情形涉及到假冒，但并不直接关系到药品本身的质量，因此虽然这是一个严重的违规行为，但不一定符合劣药的定义。

B. 变质的：变质的药品肯定是质量不符合标准的，因此可以被认为是劣药。

C. 药品成分与国家规定的成分不符的：这种情况直接违反了药品质量标准，因此这种药品可以被认为是劣药。

D. 标明的功能主治范围超出药品的治疗范围：这涉及到药品标签和广告的问题，虽然这是不合规的，但不一定意味着药品本身的质量问题。

E. 超出有效期：超出有效期的药品可能在化学成分和疗效上发生变化，不再符合原有的质量标准，因此可以被认为是劣药。

考虑到劣药的定义主要与药品质量相关，选项 B（变质的）和 C（药品成分与国家规定的成分不符的）以及 E（超出有效期）都符合劣药的条件。但是，如果要选择一个“最正确”的答案，我们通常会寻找那个最直接反映药品质量问题的选项。在这种情况下，选项 C（药品成分与国家规定的成分不符的）可能是最直接指出药品为劣质的情况，因为它直接违反了药品的成分规定。

因此，最合适的答案应该是 C. 药品成分与国家规定的成分不符的。

这是一道关于药品分类的问题，我们需要从给定的选项中找出属于劣药的情形。

理解劣药的定义是解决这个问题的关键。劣药指的是药品成分含量不符合国家药品标准规定的药品。这通常包括药品成分的含量不符合国家药品标准，或被污染，或未标明或更改有效期、生产批号等关键信息，以及其他不符合药品标准的情形。

分析选项：

A 选项（以非药品冒充药品的）：这实际上是假药的一种情形，不是劣药。
B 选项（变质的）：变质药品属于假药，因为它们的性质已经发生了变化，可能对人体有害。
C 选项（药品成分与国家规定的成分不符的）：这同样被视为假药，因为它与规定的标准不符。
D 选项（标明的功能主治范围超出药品的治疗范围）：这种情况可能涉及虚假宣传，但不直接指向劣药。
E 选项（超出有效期）：这是劣药的一种情形。超过有效期的药品可能无法保证其安全性和有效性，因此被视为劣药。
综上所述，超出有效期的药品，其安全性和有效性可能受到影响，符合劣药的定义。

因此，答案是 E.超出有效期。

题目问的是劣药的情形，下列选项中哪一个是正确的。

分析选项：
- A. 以非药品冒充药品的：这是假药的情形，不是劣药。
- B. 变质的：药品变质属于劣药的范畴。
- C. 药品成分与国家规定的成分不符的：这是假药的情形，不是劣药。
- D. 标明的功能主治范围超出药品的治疗范围：这属于广告虚假宣传的范畴，不是劣药。
- E. 超出有效期：药品超出有效期属于劣药的范畴。

所以，正确答案是：
B. 变质的
6、 (A1 型题)
疫情防控是由哪个部门发布的
A.省级三甲医院
B.卫生管理部门
C.省级疫情防控中心
D.方舱医院
E.国务院
参考答案： E 您的答案：
图文解析： 【解析】疫情防控由国务院卫生行政部门发布，故本题选 E。
【避错】省级三甲医院、卫生管理部门、省级疫情防控中心、方舱医院均为配合疫情防控实施的单位。
疫情防控通常是由具有权威性和管理责任的官方部门发布的。在中国，这类信息通常由国家或地方的卫生健康委员会或卫生管理部门负责发布。因此，最合适的答案是：

B. 卫生管理部门

这是一道关于疫情防控信息发布部门的选择题。我们来一一分析各个选项：

A. 省级三甲医院：三甲医院主要负责医疗服务，包括疾病的诊断和治疗。它们并不直接负责发布疫情防控信息，因此可以排除。

B. 卫生管理部门：卫生管理部门通常负责公共卫生政策的制定和执行，但它们通常不是疫情防控信息的直接发布者，尤其是在国家层面。

C. 省级疫情防控中心：虽然省级疫情防控中心与疫情防控密切相关，但它们通常执行由上级部门制定的政策，并非信息的原发布者。

D. 方舱医院：方舱医院主要用于隔离和治疗病患，不负责发布疫情防控信息。

E. 国务院：在中国，国务院是最高行政机关，负责国家层面的政策制定和发布。疫情防控作为国家级的紧急公共卫生事件，其相关信息的发布通常由国务院进行。

综上所述，疫情防控信息的发布是由国务院负责的，因此正确答案是 E.国务院。

疫情防控的相关政策和指导是由卫生管理部门发布的。在中国，具体执行和管理疫情防控工作的主要机构是各级卫生健康委员会，这些机构负责制定和发布防控措施、指南和政策。因此，最正确的答案是：

B. 卫生管理部门
7、 (A1 型题)
确定对某传染病接触者留验、检疫或医学观察期限，主要依据该传染病的
A.潜伏期
B.传染期
C.病原携带期
D.恢复期
E.临床诊断期
参考答案： A 您的答案：
图文解析： 【解析】潜伏期（A 对）为从病原体侵入人体起，至开始出现临床症状为止的时期。传染
病病种的潜伏期不尽相同，检疫工作可通过结合潜伏期确定留观人群、时间等具体情况，以免漏检。
【避错】每一个传染病的潜伏期都有一个范围（最短、最长），并呈常态分布，是检疫工作观察、留验接触者的重要依据。
在确定某传染病接触者的留验、检疫或医学观察期限时，主要依据的是该传染病的潜伏期。潜伏期是指从个体受到感染到开始出现症状之间的时间段。这个期间对于确定可能的感染者进行隔离和观察至关重要，以防止病毒的进一步传播。因此，正确答案是 A.潜伏期。

解析：
传染病接触者是指与传染源有过接触并有可能受感染者。留验、检疫或医学观察的期限需要根据接触者可能的感染时间来确定。而潜伏期是从病原体侵入人体起，至开始出现临床症状为止的时期，因此可以依据潜伏期来确定传染病接触者的留验、检疫或医学观察期限。
选项 B 传染期是指感染者或病人有能力把病毒传染给其他人的那段时间，与传染病接触者的观察期限无关，故不选。
选项 C 病原携带期是指在临床症状消失之后，病人体内仍保留病原体，并不再出现临床症状，病原携带者不具备传染性，故不考虑。
选项 D 恢复期是指当病原体的致病力减弱或消失，而机体的免疫反应逐渐加强，病理学变化逐渐减轻，病人症状逐渐好转，此时传染性逐渐消失，与本题无关。
选项 E 临床诊断期是临床医生根据临床经验结合患者的病史、临床症状、体征、医学影像以及血生化、免疫学检查或细胞、组织病理学检查等辅助检查资料，对病人提出的疾病诊断，非传染病学概念，可排除。

综上所述，正确答案是：A.潜伏期。

确定对某传染病接触者留验、检疫或医学观察期限，主要依据该传染病的潜伏期。潜伏期是指从病原体侵入人体到出现临床症状的时间。因为潜伏期内虽然没有症状，但可能已经具有

传染性，所以确定潜伏期是防止传染病扩散的重要依据。

正确答案是：A. 潜伏期

8、 (A1 型题)

下列属于乙类传染病的是

A.鼠疫

B.霍乱

C.风疹

D.艾滋病

E.流行性腮腺炎

参考答案： D 您的答案：

图文解析： 【解析】乙类包括:传染性非典型肺炎(严重急性呼吸综合征)、艾滋病(D 对)、病毒性

肝炎、脊髓灰质炎、人感染高致病性禽流感、麻疹、肾综合征出血热狂犬病、流行性乙型脑炎、登革热、炭疽、细菌性和阿米巴痢疾、肺结核、伤寒和副伤寒、流行性脑脊髓膜炎、百日咳、白喉、新生儿破伤风、猩红热、布鲁菌病、淋病、梅毒、钩端螺旋体病、血吸虫病、疟疾,2013 年 11 月增加了人感染 H7N9 禽流感。

【避错】甲类传染病包括鼠疫和霍乱。丙类传染病包括流行性腮腺炎和风疹等。

根据中国的传染病分类,乙类传染病包括一些较为严重但相比甲类传染病传播性或危害性稍低的疾病。选项中：

A. 鼠疫属于甲类传染病。

B. 霍乱也属于甲类传染病。

C. 风疹属于乙类传染病。

D. 艾滋病属于乙类传染病。

E. 流行性腮腺炎则通常不归类为甲乙类传染病。

因此，最正确的答案是 D. 艾滋病。

首先，我们要了解中国的传染病分类系统。在中国，根据传染病的传播方式、传播速度、流行强度以及对人体健康、对社会和经济的危害程度，将 39 种法定传染病依据其传播方式、危害程度等，分为甲类传染病、乙类传染病和丙类传染病。

接下来，我们分析各个选项：

A. 鼠疫 - 鼠疫是甲类传染病，具有极高的致死率和传染性，因此不符合题目中乙类传染病的要求。

B. 霍乱 - 霍乱同样被分类为甲类传染病，其传播速度和危害性都非常高，所以也不是乙类传染病。

C. 风疹 - 风疹并不在甲类或乙类传染病的列表中，而是属于丙类传染病，因此可以排除。

D. 艾滋病 - 艾滋病被明确列为乙类传染病。它主要通过性传播、血液传播和母婴传播，对人体健康有严重危害，但传播速度和方式不同于甲类传染病。

E. 流行性腮腺炎 - 流行性腮腺炎也是丙类传染病，主要通过飞沫传播，其传播速度和危害性相对较低。

综上所述，根据中国的传染病分类，艾滋病是唯一符合乙类传染病定义的疾病。

因此，正确答案是 D.艾滋病。

根据中华人民共和国《传染病防治法》，乙类传染病包括艾滋病、肺结核、梅毒、乙型病毒性肝炎等。以下是各类传染病的分类：

- 甲类传染病：鼠疫、霍乱
- 乙类传染病：艾滋病、流行性腮腺炎、风疹等
- 丙类传染病：流行性感冒、手足口病等

题目中要求选择乙类传染病。选项中只有艾滋病（D）属于乙类传染病。

因此，最正确的答案是：

D. 艾滋病

9、 (A1 型题)

用药过程中药品导致的死亡，上报时限是

A.15 小时

B.5 小时

C.3 小时

D.立即

E.2 小时

参考答案： D 您的答案：

图文解析： 【解析】用药过程中药品导致的死亡，应立即上报，故本题选 D。

【避错】死亡立即上报，新的、严重不良反应 15 天内上报， 一般不良反应 30 天内上报。在用药过程中，如果药品导致了死亡，这种严重的不良事件应该被立即上报。因此，正确的答案是 D.立即。这种上报的及时性对于防止进一步的不良事件和采取必要的措施至关重要。

这是一道关于药品不良事件上报时限的问题。我们需要确定在用药过程中，如果药品导致患者死亡，应该在多长时间内上报这一严重不良事件。

理解问题的关键在于认识到药品不良事件，特别是导致死亡的事件，对于药品监管和患者安全至关重要。因此，及时上报这类事件对于预防类似事件的再次发生以及保护更多患者的安全具有重要意义。

现在，我们来分析各个选项：

A. 15 小时：这个时间相对较长，可能无法及时提醒药品监管机构采取必要的行动。

B. 5 小时：虽然比 15 小时短，但仍然可能不是最迅速的反应时间，特别是在处理可能导致死亡的严重药品不良事件时。

C. 3 小时：这个时间相对较短，但仍然不是最立即的反应。

D. 立即：这个选项强调了不良事件上报的紧迫性，特别是在处理可能致命的药品不良事件时，需要立即采取行动以保护患者安全。

E. 2 小时：虽然比 3 小时更短，但仍然不是“立即”上报，可能不适用于处理最紧急的情况。

综上所述，当涉及到药品导致的死亡等严重不良事件时，最重要的是立即上报，以便药品监管机构能够尽快采取行动，防止类似事件的再次发生。

因此，正确答案是 D.立即。

用药过程中药品导致的死亡属于严重不良反应，必须立即上报以确保及时处理和避免进一步的危害。因此，最正确的答案是：

D. 立即
10、 (A1 型题)
医疗机构执业许可证校验时间为三年，则该医院是
A.诊所
B.三级医院
C.村卫生室
D.门诊部
E.社区服务中心
参考答案： B 您的答案：
图文解析： 【解析】《医疗机构管理条例》规定，床位在 100 张以上的医疗机构，其《医疗机构执业
许可证》每 3 年校验 1 次。床位在 100 张以上的有二级医院（100~500）和三级医院(>500)，故本题选 B。
【避错】床位不满 100 张的医疗机构，其《医疗机构执业许可证》每年校验 1 次。
在中国，医疗机构的执业许可证校验时间根据医疗机构的类型和级别有所不同。一般来说，较大的医院，如三级医院，执业许可证的有效期较长，可能是五年。而较小的医疗单位，如诊所、村卫生室、门诊部和社区服务中心，可能需要更频繁的校验，比如每三年一次。

根据这些信息，最符合三年一次校验时间的医疗机构类型可能是诊所、村卫生室、门诊部或社区服务中心。但在中国，三级医院通常有更长的校验周期，因此 B 选项（三级医院）应该不是正确答案。

由于题目要求选择最佳答案，我们需要进一步确定哪种类型的医疗机构的校验周期明确为三年。通常，相对较小的医疗机构如诊所（A 选项）或社区服务中心（E 选项）可能有更短的许可证有效期，因此我倾向于选择其中之一。

因此，最可能的答案是 A.诊所或 E.社区服务中心。然而，具体答案可能取决于当地的法规和政策。如果没有具体的法律文本或政策作为参考，确定确切的答案可能有些困难。如果必须选择一个，我可能会选择 A.诊所，因为它通常是规模较小的医疗机构，可能需要较频繁的校验。

解析：
医疗机构执业许可证校验，是指卫生行政部门依法对医疗机构的基本条件和执业状况进行检查、评估、审核，并依法作出相应结论的过程。根据《医疗机构管理条例实施细则》规定，医疗机构应当于校验期满前三个月向登记机关申请办理校验手续，校验期按照医疗机构评审办法和有关规定进行校验。其中，床位在 100 张以上的综合医院、中医医院、中西医结合医院、民族医医院以及专科医院、疗养院、康复医院、妇幼保健院、急救中心、临床检验中心和专科疾病防治机构的校验期为 3 年。三级医院床位数一般都在 100 张以上，因此其医疗机构执业许可证校验时间为三年。
选项 A 诊所，选项 C 村卫生室，选项 D 门诊部，选项 E 社区服务中心，均属于床位不满 100 张的医疗机构，校验期应为 1 年，可一并排除。

综上所述，正确答案是：B.三级医院。

根据中国的《医疗机构管理条例》和相关规定，医疗机构执业许可证的校验时间因不同类型的医疗机构而有所不同。三级医院的执业许可证通常为三年校验一次。因此，该医院是：

B. 三级医院
11、 (A1 型题)
某医院进行了医院管理制度、接诊转诊过程、心理咨询、健康体检等新型管理，保证了对医务人员的关心、关注、公平。上述措施
A.有利于提高医学科学的发展水平
B.有利于构建和谐的医患沟通
C.有利于患者诊疗和公平治疗
D.有利于医学保健科室的利用
E.有利于发挥医院的整体效应
参考答案： E 您的答案：
图文解析： 【解析】某医院进行了一系列新型管理，保证了对医务人员的关心、关注、公平，这有利
于发挥医院的整体效应。
【避错】选项 ABCD 均未体现。
这个问题考察的是医院管理制度改革对医院运作和医疗服务的影响。选项包含了不同方面的好处，但需要选出最符合题目描述的答案。根据题目描述，医院进行了包括管理制度、接诊转诊过程、心理咨询、健康体检等方面的改革，重点在于保障了对医务人员的关心、关注、公平，进而推断这些改革的直接效果。

A. 有利于提高医学科学的发展水平：虽然好的管理可以间接促进科学发展，但题目并未直接涉及科学研究活动。
B. 有利于构建和谐的医患沟通：此选项符合题目对于改善医院管理、提高医务人员工作满意度的描述，可能直接影响医患沟通。

C. 有利于患者诊疗和公平治疗：这个选项也符合题意，特别是在强调了公平性的背景下。
D. 有利于医学保健科室的利用：这个选项太具体，而且不直接关联到题目描述的管理措施。
E. 有利于发挥医院的整体效应：这是一个较为宽泛的选项，虽然改进管理制度确实可以提高医院整体效能，但不够具体。
综上，最佳答案应当是那个最直接反映医院管理改革结果的选项。我倾向于选择 B. 有利于构建和谐的医患沟通，因为它直接关联到改善医院管理和提升医务人员满意度，进而促进和谐的医患关系。然而，C 选项也有一定的相关性，因为改革确保了公平，可能直接影响到患者诊疗和治疗。在这种情况下，B 选项更具体地对应了题目中描述的改革措施和预期效果。

解析：
该医院进行了管理制度、接诊转诊、心理咨询、健康体检等多方面的改革，这些措施不仅涉及医疗服务流程的优化，还关注医务人员的心理和工作状态，显然是一个全面的医院管理改革。医院通过优化接诊转诊过程，可以提升患者就医体验，确保患者得到及时有效的诊疗。同时，对医务人员的关心、关注、公平，可以提升医务人员的工作满意度和效率，从而发挥医院的整体效应。
选项 A 有利于提高医学科学的发展水平通常与医学研究、技术创新和教育培训等相关，与该医院的管理改革关系不大，故排除。
虽然优化接诊转诊过程和提供心理咨询可能有助于改善医患沟通，但题干中并未明确提及医患沟通方面的具体措施，因此选项 B 有利于构建和谐的医患沟通不是最佳选项。
题干中的措施更多关注于医院管理和医务人员关怀，并未直接针对患者的诊疗和公平治疗进行具体改革，因此选项 C 有利于患者诊疗和公平治疗，可排除。
虽然健康体检可能涉及医学保健科室，但题干中的改革措施并未特别强调医学保健科室的利用，因此选项 D 有利于医学保健科室的利用，与题意不符。

综上所述，正确答案是：E.有利于发挥医院的整体效应。

这道题目考察的是医院管理措施的效应。题目中提到的管理制度和措施，如医院管理制度、接诊转诊过程、心理咨询、健康体检等，旨在关心和关注医务人员，同时保证公平。

在这种背景下，这些措施最直接和明显的效应是促进医患关系的和谐，因为医务人员在得到关心和公平对待后，更能以积极的态度对待患者，从而提高医患沟通的质量。

因此，最佳答案是：

B. 有利于构建和谐的医患沟通
12、 (A1 型题)
某医师定期考核不合格，经过培训后再次考核仍不合格，当地卫生行政部门依据《执业医师法》对其进行处理，该处理是
A.注销注册，收回医师执业证书
B.暂停执业活动
C.吊销执业医师资格证
D.再次培训后考核
E.终身禁业
参考答案： A 您的答案：

图文解析：　　　【解析】《执业医师法》规定，对考核不合格的医师，县级以上人民政府卫生行政部门可
以责令其暂停执业活动 3 个月至 6 个月，并接受培训和继续教育，经考核仍不合格的，则注销注册，收回医师执业证书。
【避错】《执业医师法》规定，县级以上人民政府卫生行政部门负责指导、检查和监督医师考核工作。受县级以上人民政府卫生行政部门委托的机构或组织，应当按照医师执业标准，对医师的业务水平、工作成绩和职业道德状况进行定期考核。
在处理执业医师考核不合格的情况时，通常会根据具体的法规和情况来确定适当的措施。考虑到这种情况表明医师连续两次考核不合格，并且考虑到《执业医师法》的规定，最严厉的措施如吊销执业医师资格证或终身禁业可能适用于更严重的违规行为。通常，在经过再次培训后仍然不合格的情况下，可能会选择一种较为严格但有一定限度的措施，如暂停执业活动。因此，根据这些考虑，最合适的答案可能是：

B. 暂停执业活动

这样既体现了对不合格医师的处罚，又留有改正的余地，便于在将来某个时点经过进一步的评估和可能的培训后重新考核。

解析：
本题考察的是执业医师考核不合格的处理措施。

综上所述，正确答案是：A.注销注册，收回医师执业证书。

根据《执业医师法》规定，对考核不合格的医师，县级以上人民政府卫生行政部门可以责令其暂停执业活动三至六个月，并接受培训和继续教育。经考核仍不合格的，将注销注册，收回医师执业证书。本题中，某医师定期考核不合格，且经过培训后再次考核仍不合格，应注销其注册，收回医师执业证书。因此选项 A 是正确答案。

选项 B 暂停执业活动是对考核不合格的医师的初步处理措施，但题干描述该医师经过培训后再次考核仍不合格，故不再适用暂停执业的处罚，可排除。

《执业医师法》并未赋予卫生行政部门吊销执业医师资格证的权力，因此选项 C 吊销执业医师资格证，不符合《执业医师法》的规定，故排除。

该医师已经过培训并再次考核不合格，因此选项 D 再次培训后考核，不符合题干描述，可排除。

《执业医师法》中并未规定终身禁业的处罚，因此选项 E 终身禁业，不符合法律规定，与本题不符。

综上所述，正确答案是：A.注销注册，收回医师执业证书。

根据《中华人民共和国执业医师法》第三十六条规定，医师在定期考核中不合格的，应当进行培训，经培训后再次考核仍不合格的，由卫生行政部门取消其注册或者降低其执业类别。

根据这一规定，对该医师的处理应当是注销注册，收回医师执业证书。

因此，最正确的答案是：

A. 注销注册，收回医师执业证书
13、　(A1 型题)
在动物实验中，为了获取活体小鼠血液，实验室人员直接剪掉实验小白鼠的头部，按压残余肢体，使其剩余血液从颈部大动脉流出。该实验室人员违背的原则为
A.给予实验室动物良好的生存环境
B.给予实验动物充足的生活保障
C.最大程度减轻受实验动物的痛苦
D.尽量使用实验材料替代活体动物实验
E.减少实验动物不必要数量
参考答案：　C　您的答案：
图文解析：　【解析】本题干中，该实验室人员直接剪掉动物头部，并按压肢体来获得血液，此行为没
有最大程度减轻受实验动物的痛苦。故本题选 C。
【避错】动物实验应遵循的伦理原则包括：替代、减少、优化和责任。替代是指使用没有知觉的实验材料代替活体动物，或使用低等动物替代高等动物进行试验。减少就是在动物实验时尽量减少动物的使用量，使用较少量的动物获取同样多的试验数据或使用一定数量的动物能获得更多的试验数据。优化是指在必须使用动物进行实验时，尽量减少非人道程序对动物的影响范围和程度。动物福利是指人类应该避免对动物造成不必要的伤害，反对并防止虐待动物，让动物在康乐的状态下生存。
该实验室人员违反的原则是 C.最大程度减轻受实验动物的痛苦。动物福利原则要求在实验中应尽可能减少动物的痛苦和压力，且应采取人道的方法获取动物血液，例如使用针头抽血等方式，以减轻动物的痛苦。直接剪掉动物的头部是极不人道的，严重违反了减轻实验动物痛苦的原则。

这是一道医学伦理学的选择题，旨在考察实验室动物伦理原则的理解。题目描述了一个实验室人员为了获取活体小鼠血液而采取的直接剪掉实验小白鼠头部的行为，我们需要分析这种行为违背了哪一项实验室动物使用的伦理原则。

首先，我们分析题目并罗列出重要信息：

实验室人员为了获取活体小鼠血液。
直接剪掉实验小白鼠的头部。
按压残余肢体，使剩余血液流出。
接着进行解释和推理：

实验室动物伦理原则主要包括：提供良好的生存环境、充足的生活保障、最大程度减轻动物的痛苦、尽量使用实验材料替代活体动物实验、减少实验动物的不必要数量。
在本题中，实验室人员通过直接剪掉小白鼠的头部来获取血液，这种方式显然给动物带来了巨大的痛苦，与“最大程度减轻受实验动物的痛苦”的原则相违背。
现在我们对比选项：

A. 给予实验室动物良好的生存环境：题目并未提及实验动物的生活环境，因此这个选项与问题描述不符。
B. 给予实验动物充足的生活保障：同样，题目中并未提及生活保障的问题，所以此选项也不适用。
C. 最大程度减轻受实验动物的痛苦：实验室人员通过极端手段获取血液，显然没有遵循减轻动物痛苦的原则，因此这个选项是符合题意的。
D. 尽量使用实验材料替代活体动物实验：题目并未提及是否使用了替代材料，因此这个选项与问题描述不符。
E. 减少实验动物不必要数量：题目中并未提及实验动物的数量问题，所以此选项也不适用。

综上所述，实验室人员通过直接剪掉小白鼠的头部来获取血液，违背了“最大程度减轻受实验动物的痛苦”的原则。

因此，正确答案是：C.最大程度减轻受实验动物的痛苦。

该实验室人员的行为违背的原则是：

C. 最大程度减轻受实验动物的痛苦

解释：在动物实验中，伦理和人道主义的原则要求研究人员应尽可能减少实验动物的痛苦和不适。剪掉小鼠头部并按压其肢体获取血液是一种造成极大痛苦的方法，这与减轻实验动物痛苦的原则相违背。因此，正确答案是 C。
14、 (A1 型题)
以下关于脑死亡标准说法正确的是
A.有利于提供评判患者死亡的科学依据
B.目的是节约卫生资源
C.实施器官捐献的必然要求
D.心肺功能必须完全丧失
E.判断患者死亡的唯一标准
参考答案： A 您的答案：
图文解析： 【解析】实施脑死亡标准的直接伦理目的是：①使死亡标准更趋于科学化，指导医生正确
地实施复苏与抢救，确定准确的死亡时间，减少法律纠纷；②在伦理学上维护死者的尊严（故本题选 A，BC 错误）。脑死亡是脑细胞广泛、永久地丧失了全部功能，范围涉及大脑、小脑、桥脑和延髓。即发生全脑死亡后，虽心跳尚存，但脑复苏已不可能，个体死亡已经发生且不可避免（D 错）。脑死亡不是判断患者死亡的唯一标准（E 错）。
【避错】脑死亡诊断标准的伦理价值和意义主要为五方面：科学准确判断死亡，维护逝者身后尊严，节约社会资源，减轻家庭负担，利于实施器官移植。
关于脑死亡标准的正确说法是 A："有利于提供评判患者死亡的科学依据"。脑死亡是指大脑功能完全且不可逆转地丧失，包括大脑皮层和脑干的功能。这一概念有助于医学和法律界提供评判个体死亡的明确和科学的依据。虽然脑死亡常与器官捐献相关联（选项 C），但器官捐献不是脑死亡标准制定的必然要求。心肺功能的丧失（选项 D）是心肺死亡的标准，而不是脑死亡。因此，选项 A 是最准确的答案。

这是一道关于脑死亡标准的选择题。我们需要先理解脑死亡的定义和含义，然后分析每个选项，找出符合脑死亡标准的说法。

理解背景信息：脑死亡指的是包括脑干在内的全脑功能完全、不可逆转的丧失，即死亡。

理解问题核心：分析每个选项，找出符合脑死亡标准的说法。

分析选项：

A 选项（有利于提供评判患者死亡的科学依据）：脑死亡标准提供了一个科学的、客观的死亡判定依据，这与选项 A 的描述相符。
B 选项（目的是节约卫生资源）：脑死亡标准并非为了节约卫生资源而设立，而是为了更科学地定义死亡，因此 B 选项不正确。
C 选项（实施器官捐献的必然要求）：虽然脑死亡标准与器官捐献有关联，但它并不是器官捐献的必然要求，因此 C 选项不正确。
D 选项（心肺功能必须完全丧失）：脑死亡标准并不要求心肺功能完全丧失，而是指全脑功能的完全、不可逆转的丧失，因此 D 选项不正确。
E 选项（判断患者死亡的唯一标准）：虽然脑死亡是一种科学的死亡判定标准，但它并不是唯一的标准，因此 E 选项不正确。
综上所述，答案是 A.有利于提供评判患者死亡的科学依据。

14. 以下关于脑死亡标准说法正确的是：

A. 有利于提供评判患者死亡的科学依据

解析：

脑死亡是指大脑及脑干的功能完全和不可逆的丧失，是判断一个人死亡的一种标准。其目的是为医疗决策提供一个科学的依据。脑死亡标准在许多国家被广泛接受和使用，其科学性和可靠性已被大量研究证实。

- **A. 有利于提供评判患者死亡的科学依据**：这是正确的，因为脑死亡标准提供了评判患者死亡的科学依据。
- **B. 目的是节约卫生资源**：虽然脑死亡判定可以影响卫生资源的使用，但这不是其主要目的。
- **C. 实施器官捐献的必然要求**：脑死亡判定通常与器官捐献有关，但并不是实施器官捐献的唯一或必然要求。
- **D. 心肺功能必须完全丧失**：脑死亡判定并不要求心肺功能完全丧失。
- **E. 判断患者死亡的唯一标准**：脑死亡是判断死亡的一种标准，但不是唯一标准，心脏死亡也是一种常见的标准。

因此，正确答案是 A。
15、 (A1 型题)
在临床问诊中，医师应避免无精打采，漫无边际的反复提问或过度情绪化的语言等现象，其

体现的诊疗伦理要求是
A.尊重患者，公正无私
B.全神贯注，语言得当
C.全面系统，认真细致
D.举止端庄，态度热情
E.耐心倾听，正确引导
参考答案：　　B　您的答案：
图文解析：　　【解析】在询问病史过程中，医生应遵循以下伦理要求：举止端庄，态度热情；全神贯
注，语言得当；耐心倾听，正确引导。本案例中，医生避免无精打采、反复提问等所体现的伦理要求是全神贯注，语言得当，故本题选 B。
【避错】体格检查的伦理要求：全面系统，认真细致；关心体贴，减少痛苦；尊重患者，心正无私。
在临床问诊中，医师应避免的现象如无精打采，漫无边际的反复提问或过度情绪化的语言，体现了医师应全神贯注于病人，并且使用得体的语言进行交流。因此，最适合的答案应该是：

B.全神贯注，语言得当

这个选项强调了医生在问诊过程中应该保持专注，并且使用适当的语言，这有助于建立与患者的良好沟通，从而提高诊疗的质量和效率。

解析：
在临床问诊中，医师应避免无精打采，漫无边际的反复提问或过度情绪化的语言等现象，要求医师全神贯注，语言得当，这是最基本的医德情感内容中低层次的责任感，是责任感内容中低层次的医德情感内容。
选项 A 尊重患者，公正无私是要求医务人员为患者诊治、护理时，态度要严肃、认真，一丝不苟，尊重患者的人格尊严及其自主性，同时公正地、无私地对待每一位患者，与题干不符。
选项 C 全面系统，认真细致是指医生问诊时要全面系统地了解患者的病史，不遗漏任何与疾病相关的信息，同时对患者的主诉要仔细分析，与题干不符。
选项 D 举止端庄，态度热情是指医务人员的仪容、服饰、姿态等均应体现专业人员的良好形象，同时医务人员要热情为患者诊治，但题干描述中并未体现，故不选。
选项 E 耐心倾听，正确引导是要求医务人员为患者诊治时，对患者的病史要耐心倾听，对患者的心理问题进行正确的引导，与题干描述不符。

综上所述，正确答案是：B.全神贯注，语言得当。

在临床问诊中，医师应避免无精打采，漫无边际的反复提问或过度情绪化的语言等现象，其体现的诊疗伦理要求是 **B. 全神贯注，语言得当**。

解析：
- **A. 尊重患者，公正无私**：虽然尊重患者和公正无私是医师应遵循的伦理原则，但它们主要强调的是医师在对待患者时的公平和尊重，而不是专注和语言的准确性。
- **B. 全神贯注，语言得当**：这一选项强调了医师在问诊时需要保持专注，并使用得当的

语言，避免无精打采和过度情绪化的语言，符合题干的要求。
- **C. 全面系统，认真细致**：这一选项强调的是医师在问诊过程中要全面、系统、认真和细致，但与题干所描述的现象不完全匹配。
- **D. 举止端庄，态度热情**：这一选项强调的是医师的举止和态度，但与题干中避免无精打采和过度情绪化的语言现象的对应不够直接。
- **E. 耐心倾听，正确引导**：这一选项强调的是医师应耐心倾听和正确引导患者，但与题干中的“避免无精打采、漫无边际的反复提问或过度情绪化的语言”等现象不完全对应。

因此，最符合题干要求的答案是 **B. 全神贯注，语言得当**。
16、 (A1 型题)
某医院心血管内科主任被其博士生举报“每安装一个支架回扣 1 万元”，经调查情况属实，该主任违背了什么原则
A.公平原则
B.廉洁自律
C.保密原则
D.最优化原则
E.知情同意原则
参考答案： B 您的答案：
图文解析： 【解析】医学伦理学要求医师应具备基本的职业道德，是否有利于病人疾病的缓解和康复；是否有利于人类生存和环境的保护和改善；是否有利于优生优育和人群的健康长寿；是否有利于医学科学的发展和社会进步。其中是否有利于患者疾病的缓解和康复为医学道德评价的首要、至上标准。该主任收取病人回扣，违反了医学道德中的廉洁自律原则，故本题选 B。
【避错】知情同意是指患者或者家属有权知晓患者的病情，并对医务人员采取的防治措施决定取舍的自主权。保密原则指医务人员在防病治病中应当保守医疗秘密，不得随意泄露病人的疾病情况等个人隐私，以防对病人造成不必要的伤害。临床诊疗道德的最优化原则是指认真、仔细地选择使病人受益与代价比例适当的优化治疗措施的临床伦理准则，其内容主要包括疗效最好、安全无害、痛苦最小和耗费最少。
这个问题涉及的是医疗伦理问题。从所给的选项来看，这位心血管内科主任接受回扣的行为违背了医疗伦理中的“廉洁自律”原则。廉洁自律原则要求医务人员在职业活动中应保持诚信，避免因个人利益而影响医疗决策和患者护理。因此，最合适的答案是：

B 廉洁自律

这是一道医学伦理的问题。我们需要先理解题目背景信息和问题核心，再仔细分析每个选项，并将其与题中给出的信息进行对比。

理解问题背景信息：题目描述了一个场景，医院心血管内科主任被举报因为安装支架而收受回扣。

理解问题的核心：我们需要基于这个关系，判断心血管内科主任违背了哪一个伦理原则。

分析选项：

A 选项（公平原则）：公平原则强调对所有患者应公平对待，不偏不倚。这个原则并未直接涉及到医生收受回扣的问题，因此可以排除。

B 选项（廉洁自律）：廉洁自律是指医务人员在医疗服务中应坚守廉洁自律的原则，不收受任何形式的回扣或好处。在这个案例中，心血管内科主任因为安装支架而收受回扣，明显违背了廉洁自律的原则。

C 选项（保密原则）：保密原则主要涉及对患者信息的保护，与本案例无直接关联，故可排除。

D 选项（最优化原则）：最优化原则是指在医疗行为中选择对患者最有利的治疗方案。这个原则并未涉及到医生收受回扣的问题，因此也可以排除。

E 选项（知情同意原则）：知情同意原则强调在医疗行为中应获得患者的充分知情和同意。这个原则与本题中的情况无直接关联，故不考虑。

综上所述，医院心血管内科主任被举报“每安装一个支架回扣 1 万元”，这明显违背了廉洁自律的原则。因此，正确答案是 B.廉洁自律。

根据题目描述，该心血管内科主任每安装一个支架获得 1 万元的回扣，这种行为违背了医生职业道德和医德规范。具体来看，这是违反了“廉洁自律”的原则。

B. 廉洁自律
17、 (A1 型题)
以行动产生的实际效果为道德评判基础的是
A.道义论
B.结果论
C.义务论
D.后果论
E.德行论
参考答案： D 您的答案：
图文解析： 【解析】以行动产生的实际效果为道德评判基础的是后果论，又被称为目的论或效果论，
是以道德行为后果作为确定道德规范的最终依据的伦理学理论，故本题选 D。
【避错】记忆性知识点。
这个问题考察的是伦理学中不同的道德理论。根据题目描述，需要找到一个将行动产生的实际效果作为道德评判基础的理论。选项中的"B.结果论"和"D.后果论"都与行动的结果相关，但在伦理学中通常使用的术语是"后果论"（consequentialism），它强调行为的结果是评判行为道德性的最主要标准。因此，最正确的答案是 D.后果论。
首先，我们要明确这个问题考察的是医学伦理学中的道德评判基础。
现在，我们逐一分析选项：
A. 道义论（道义主义）：道义论主张人的行为应遵循道德原则和道德义务，不论结果如何。它侧重于行为的正当性，而不是结果的好坏。
B. 结果论：结果论主张道德评价应以行为的结果为标准，即行为的好坏取决于其产生的结

果。然而，在伦理学理论中，更常见的术语是“效果论”，而不是“结果论”。效果论认为，道德的价值取决于行为的结果或效果，与本题中的“以行动产生的实际效果为道德评判基础”相符，但选项中没有直接使用“效果论”这一术语。
C. 义务论（去行动主义）：与道义论相似，义务论强调道德义务和原则的重要性，而不只是看重结果。
D. 后果论：这是一个与效果论相似的概念，也是以行为的后果作为道德评判的标准。在伦理学中，后果论和效果论常常被交替使用，均指以结果为导向的道德评价。
E. 德行论：德行论关注的是人的品德和道德品质，而不是单一行为的后果。
综上所述，虽然“效果论”和“后果论”在伦理学中都指以行为的结果或后果作为道德评价的标准，但本题中更准确的术语应是“效果论”。由于选项中未直接给出“效果论”，我们可以选择与其意义最为接近的“后果论”。
因此，正确答案是 D.后果论。这一选项最符合题目中“以行动产生的实际效果为道德评判基础”的描述。

以行动产生的实际效果为道德评判基础的理论是**结果论**，也称为后果论。这种伦理学理论认为行为的道德价值取决于其产生的结果或后果。换句话说，一个行为的好坏要根据它带来的后果是否有利来判断。

所以，正确答案是：

B. 结果论
18、 (A1 型题)
下列需要强制就医的情况是
A.糖尿病
B.婴幼儿发热
C.老年痴呆
D.冠心病
E.有暴力倾向的人
参考答案： E 您的答案：
图文解析： 【解析】强制就医一般包括性病、吸毒、有暴力倾向的精神障碍、严重传染性疾病等，故
本题选 E。
【避错】强制医疗，是指非自愿性强制治疗，是指国家为避免公共健康危机，通过强制对患者疾病的治疗，达到治愈疾病、防止疾病传播、维护公众健康利益，具有强制性、非自愿性、公益性的特点。
在选择需要强制就医的情况时，通常考虑的是那些可能对患者自身或他人构成直接危险的情况。在这些选项中，最符合这一标准的是：

E. 有暴力倾向的人

此类个体可能对自己或他人构成伤害，因此在某些情况下，法律可能要求这类人士进行强制医疗评估或治疗，以防止潜在的伤害发生。因此，选项 E 是最合适的答案。其他选项如糖尿病、婴幼儿发热、老年痴呆和冠心病虽然需要医疗关注，但通常不需要强制就医。

解析：
强制医疗，是指非自愿性强制治疗，是指国家为避免公共健康危机，通过强制对患者疾病的治疗，达到治愈疾病、防止疾病传播、维护社会公众健康利益，同时强制患者进行疾病治疗的一种制度。需要强制就医的人一般是精神疾病患者，他们不能辨认或不能控制自己的行为，有可能发生伤害自身、危害他人安全的行为及扰乱医疗秩序、妨害医务人员工作秩序等行为的人。在给出的选项中，有暴力倾向的人可能会出现伤害自身或危害他人的行为，因此需要强制就医。
选项 A 糖尿病，选项 C 老年痴呆，选项 D 冠心病，这三种疾病的患者大多不会伤害自己或危害他人，因此不属于需要强制就医的范畴，故一并排除。
选项 B 婴幼儿发热的患儿一般不具有伤害自己的能力，且婴幼儿不具有自理能力，往往需要家长全程陪同，因此不需强制就医，故不选。

综上所述，正确答案是：E.有暴力倾向的人。

这道题目考查的是在某些情况下需要强制就医的情况。以下是每个选项的分析：

A. **糖尿病**：尽管糖尿病是一种需要长期管理和治疗的疾病，但通常不需要强制就医，除非出现急性并发症（如糖尿病酮症酸中毒或高渗性昏迷）。

B. **婴幼儿发热**：婴幼儿发热是一种常见现象，虽然需要密切观察和适当处理，但通常不需要强制就医。

C. **老年痴呆**：老年痴呆是一种慢性进行性疾病，患者需要长期护理和管理，但一般情况下不需要强制就医。

D. **冠心病**：冠心病患者需要长期的药物治疗和生活方式管理，急性冠状动脉综合征（如心肌梗死）需要紧急医疗干预，但通常不涉及强制就医。

E. **有暴力倾向的人**：具有暴力倾向的人可能对自己或他人构成威胁，需要立即强制就医进行评估和管理，以确保安全。

综上所述，**E. 有暴力倾向的人**是唯一一个需要强制就医的情况。

因此，最佳答案是 **E**。
19、 (A1 型题)
昏迷、休克患者使用的医疗模式为
A.主动-合作型
B.主动-参与型
C.共同参与型
D.主动-被动型
E.指导-合作型
参考答案： D 您的答案：
图文解析： 【解析】主动-被动模式：这种模式适用于昏迷、休克、精神病患者发作期、严重智力低

下者以及婴幼儿等一些难以表达主观意志的患者(D 对)。
【避错】指导合作模式：这种模式适用于大多数患者；共同参与模式：这种模式适用于具有一定医学知识背景或长期的慢性病患者。
对于昏迷、休克患者的医疗模式，选择应该是 D.主动-被动型。这是因为昏迷或休克的患者通常无法参与到他们的治疗过程中，因此需要医护人员主动进行治疗和护理，而患者处于被动接受治疗的状态。所以，最合适的医疗模式应该是主动-被动型。
解析：
医患关系分为三种类型：主动-被动型、指导-合作型、共同参与型。其中主动-被动型指医师主动命令，病人被动服从，适合难以表述自己主观意见的病人，如麻醉、严重外伤昏迷的病人等，因此昏迷、休克患者使用的医疗模式为主动-被动型。
选项 A 主动-合作型，选项 E 指导-合作型，均属于干扰项，医患关系中不存在这两种模式，可一并排除。
选项 B 主动-参与型，选项 C 共同参与型，均属于患者主动参与到自身治疗中，与医生共同商量治疗方案等，昏迷休克患者无法表达自己的主观意见，故这两种模式不适合此类患者，因此可一并排除。

综上所述，正确答案是：D.主动-被动型。

昏迷、休克患者使用的医疗模式为：

D. 主动-被动型

解释：在昏迷或休克状态下，患者无法主动参与或配合医疗决策和治疗，因此医生需要主动采取措施，而患者处于被动接受治疗的状态。这种医疗模式强调医生的主动性和决策权，适用于患者无法表达自己意愿或无法配合治疗的紧急情况下。
20、 (A1 型题)
某来访者因经常与人发生冲突到心理门诊就诊，经心理医生了解，发现其与人交往过程中，往往只考虑自己的快乐，不顾及“现实标准”，根据精神分析理论，该来访者 占据优势地位的是
A.超我
B.本我
C.主体自我
D.自我
E.客体自我
参考答案： B 您的答案：
图文解析： 【解析】位于无意识中的本能、冲动与欲望构成本我，是人格的生物面，遵循“快乐原
则，故 B 选项正确。
【避错】自我介于本我与外部世界之间，是人格的心理面，自我的作用是一方面能使个体意识到其认识能力；另一方面使个体为了适应现实而对本我加以约束和压抑，遵循的是“现实原则”。超我是人格的社会面，是“道德化的自我”，由“良心”和“自我理想”组成，超我的力量是指导自我、限制本我，遵循“理想原则”。
在精神分析理论中，人格结构被划分为三个部分：本我（id）、自我（ego）和超我（superego）。本我是与生俱来的部分，代表了本能冲动和欲望，追求即时满足，不考虑现实或社会规范。

自我是理性的部分，努力满足本我的需求，同时考虑现实和可行性。超我代表了道德标准和理想，力图抑制本我的冲动，符合社会和道德规范。

根据题干描述，这位来访者在人际交往中只考虑自己的快乐，不顾现实标准，这符合本我（id）的特点，因为本我追求即时的满足，不考虑后果或他人的需求。

因此，正确答案是 B.本我。

解析：

来访者在与人交往过程中，往往只考虑自己的快乐，不顾及“现实标准”，这是典型的“本我”至上的表现。在精神分析的理论中，人的心理包含着两个主要的部分，即本我和自我。后来又加入了超我的概念，即约束、压抑本我的一个正向力量。其中本我代表着各种原始本能和欲望，它按照“快乐原则”行事，毫无理性，一味追求满足。来访者与人交往过程中，只考虑自己的快乐，正是“本我”占据优势地位的表现。

选项 A 超我代表道德和良心，处于“道德原则”的支配下，抑制本我的冲动，对本我进行约束，迫使其延迟满足或消除冲动，故不选。

选项 C 主体自我是指个体对自己存在状态的认知，是个体对其社会角色进行自我评价的结果，与题干描述不符，故排除。

选项 D 自我代表理性和常识，处于“现实原则”的支配下，根据现实情况允许本我冲动有限的表现，与来访者表现不符，故不考虑。

选项 E 客体自我是指作为社会客体的自我，是社会关系的反映，被他人所认识和评价，与题干描述不符，故不选。

综上所述，正确答案是：B.本我。

根据精神分析理论，弗洛伊德提出了人格的三个组成部分：本我（Id）、自我（Ego）和超我（Superego）。本我代表原始的、冲动的欲望和本能，寻求立即满足，而不考虑现实和道德。自我是负责现实感知和行为调节的部分，能够调和本我的欲望和超我的道德标准。超我代表内化的社会规范和道德标准，追求完美和理想。

题目中的来访者往往只考虑自己的快乐，不顾及“现实标准”，这说明其行为主要受本能和欲望驱动，缺乏现实和道德约束。根据这个描述，来访者占据优势地位的是本我。

因此，正确答案是：

B. 本我

21、 (A1 型题)

某高校心理健康中心为对抑郁高危学生的抑郁程度定量评估，最宜选用的方式是

A.主题统觉测验

B.特性应对方式问卷

C.适应行为评估量表

D.SAS 自评量表

E.SDS 自评量表

参考答案： E 您的答案：

图文解析： 【解析】抑郁自评量表（SDS）能相当直观地反映病人抑郁的主观感受及严

重程度，故本
题选 E。
【避错】TAT（主题统觉测验）（A 错）用于人格测量。特性应对方式问卷（B 错）用于反映被试者面对困难挫折时的积极与消极的态度和行为特征。适应行为评估量表（C 错） 一般是指《儿童适应行为评定量表》，多是用于对儿童社会适应行为的评量测试。焦虑自评量表（SAS）测量有无焦虑症状及其严重程度（D 错）。
最宜选用的方式是 E. SDS 自评量表。

SDS（抑郁自评量表）是用来定量评估抑郁程度的一种常用工具。这个量表专门设计用于评估抑郁症的临床症状，包括情绪低落、兴趣减少、能量下降等。它是一种自评量表，意味着个体可以根据自己的感受和经历来回答问题，这样可以更直接地反映其心理状态。因此，在心理健康中心对抑郁高危学生进行抑郁程度的定量评估时，SDS 自评量表是非常合适的选择。
解析：
对抑郁高危学生的抑郁程度定量评估，需选择评估抑郁程度的量表。选项中涉及的评估量表各有侧重，只有 SDS 自评量表即抑郁自评量表，能直观反映病人抑郁的主观感受及其在治疗中的变化，适用于具有抑郁症状的成年人，特别适用于发现抑郁症病人，也可用于心理咨询门诊及精神科门诊，用以判断有无抑郁症状，并评定抑郁程度的轻重，特别适用于对抑郁高危学生的抑郁程度定量评估。
选项 A 主题统觉测验简称 TAT，属于投射法人格测验，主要用来确定人们的需要和成就动机，不能定量评估抑郁程度，故不选。
选项 B 特性应对方式问卷简称 CSQ，是一种自评问卷，旨在测查个体具有哪些特质应对方式，以解释个体面对应激时为何有不同的反应方式，与抑郁程度定量评估无关，故排除。
选项 C 适应行为评估量表简称 ABAS，主要用于评估儿童和青少年的适应性行为，不用于抑郁程度定量评估，故不选。
选项 DSAS 自评量表即焦虑自评量表，主要用于评定病人焦虑的主观感受及其在治疗中的变化，不能用于抑郁程度定量评估，故排除。

综上所述，正确答案是：E.SDS 自评量表。

针对该题目，要为抑郁高危学生的抑郁程度进行定量评估，选择适当的量表非常重要。以下是各选项的简介：

A. **主题统觉测验 (Thematic Apperception Test, TAT)**：一种投射测验，通过图片让受试者讲述故事，以揭示潜在的情感和动机。它不专门用于定量评估抑郁。

B. **特性应对方式问卷 (Trait Coping Style Questionnaire)**：用于评估个体在面对压力时的应对方式，非特定针对抑郁评估。

C. **适应行为评估量表 (Adaptive Behavior Scale)**：评估个体的适应行为，不专门用于抑郁评估。

D. **SAS 自评量表 (Self-Rating Anxiety Scale, SAS)**：由 Zung 开发的用于评估焦虑的自评量表，不适用于专门评估抑郁。

E. **SDS 自评量表 (Self-Rating Depression Scale, SDS)**：也是由 Zung 开发的，用于评估抑郁的自评量表。该量表可以定量评估抑郁程度，是针对抑郁评估的合适工具。

基于这些信息，最适合用于对抑郁高危学生进行抑郁程度定量评估的量表是 **E. SDS 自评量表**。

因此，正确答案是：**E. SDS 自评量表**。
22、 (A1 型题)
黄曲霉素急性中毒受损最严重的器官是
A.肝
B.肾
C.脾脏
D.卵巢
E.大脑
参考答案： A 您的答案：
图文解析： 【解析】黄曲霉菌广泛存在于霉变食品中。霉变的花生、玉米及谷类含量最多。黄曲霉毒
素有多种，其中黄曲霉毒素 B1 致癌性最强。黄曲霉毒素 B1 是异环芳烃，在肝脏代谢为环氧
化物，可使肿瘤抑制基因 p53 发生点突变而失去活性。这种毒素可诱发肝细胞癌。故本题选 A。
【避错】乙型肝炎病毒（HBV）感染导致肝细胞慢性损伤和再生，可能给黄曲霉毒素 B1 的致突变作用提供了条件。HBV 感染与黄曲霉毒素 B1 的协同作用可能是我国肝癌高发地区的重要致肝癌因素。
黄曲霉素，也称为黄曲霉毒素，是由黄曲霉菌产生的一种有毒代谢物。这种毒素主要影响肝脏，因此在黄曲霉素急性中毒的情况下，受损最严重的器官是肝脏。因此，正确答案是 A. 肝。
首先，我们需要了解黄曲霉素对人体的影响。黄曲霉素是一种强烈的肝毒素，主要损害的是肝脏。当人体摄入被黄曲霉素污染的食物后，这种毒素主要被肝吸收，通过抑制肝细胞内混合功能氧化酶系的活性和损伤其他有关物质引起肝损伤。当大量摄入黄曲霉素时，可发生急性中毒，出现急性肝炎、出血性坏死、肝细胞脂肪变性和胆管增生。脾脏、肾、大脑和卵巢虽然也是黄曲霉素作用的靶器官，但其主要损害还是集中在肝脏。

现在，我们逐一分析各个选项：

A. 肝：如前所述，黄曲霉素主要损害的是肝脏，可引起急性肝炎、出血性坏死等严重肝损伤。因此，这是一个合理的答案选项。

B. 肾：虽然黄曲霉素也可能对肾脏产生一定影响，但其主要损害器官是肝脏，而非肾脏。

C. 脾脏：脾脏虽然也会受到一定影响，但并不是黄曲霉素主要损害的器官。

D. 卵巢：卵巢也可能受到黄曲霉素的影响，但相对于肝脏来说，其影响较小。

E. 大脑：大脑不是黄曲霉素主要损害的器官。

综上所述，黄曲霉素急性中毒受损最严重的器官是肝脏。因此，正确答案是：

A. 肝

黄曲霉素是一种由黄曲霉菌产生的毒素，其主要毒性作用是损害肝脏。急性中毒时，黄曲霉素会引起肝细胞坏死，导致急性肝炎、黄疸，甚至肝衰竭。因此，黄曲霉素急性中毒受损最严重的器官是肝脏。

所以，正确答案是：

A. 肝

23、 (A1 型题)

不能作为诊断职业病需要综合分析的因素

A.职业的危险因素

B.辅助检查结果

C.职业病危险因素接触史

D.既往职业病史

E.地区的职业病发病率

参考答案： E 您的答案：

图文解析： 【解析】《中华人民共和国职业病防治法》种职业病诊断要求应当综合分析下列因素：

（1）病人的职业史；（2）职业病危害接触史和工作场所职业病危害因素情况；（3）临床表现以及辅助检查结果等。而地区的职业病发病率不作为综合分析的因素，故本题选E。

【避错】记忆性题目。

在诊断职业病时，需要综合分析多种因素，包括职业的危险因素、辅助检查结果、职业病危险因素接触史、既往职业病史等。这些都是重要的信息点，有助于医生判断一个人是否患有职业病。然而，地区的职业病发病率虽然可以提供一定的背景信息，但它不是诊断个体是否患病的决定性因素。因此，最不可能作为诊断个别病例职业病的需要综合分析的因素是：

E 地区的职业病发病率

首先，我们要明确题目要求的是找出那个不能作为诊断职业病需要综合分析的因素。接下来，我们逐一分析每个选项。

A. 职业的危险因素：这是诊断职业病时必须考虑的重要因素。了解患者所处职业中可能存在的有害因素，对于确定是否患有职业病至关重要。

B. 辅助检查结果：辅助检查，如血液检测、影像学检查等，可以提供关于患者健康状况的客观证据，也是诊断职业病时不可或缺的一部分。

C. 职业病危险因素接触史：了解患者是否有长期接触可能导致职业病的危险因素，是诊断

过程中的关键环节。

D. 既往职业病史：患者的既往职业病史，特别是与当前职业病相关的病史，对于诊断和治疗都非常重要。

E. 地区的职业病发病率：虽然地区职业病发病率可以提供一定区域内职业病发生的普遍情况，但它并不能直接作为诊断个体是否患有职业病的依据。每个个体的具体情况还需根据其职业危险因素接触史、临床表现和辅助检查结果来综合分析。

综上所述，虽然地区职业病发病率对于了解职业病在该地区的流行情况有帮助，但它并不能直接用于诊断个体是否患有职业病。因此，不能作为诊断职业病需要综合分析的因素是 E. 地区的职业病发病率。

因此，正确答案是 E.地区的职业病发病率。

题目：不能作为诊断职业病需要综合分析的因素

A. 职业的危险因素
B. 辅助检查结果
C. 职业病危险因素接触史
D. 既往职业病史
E. 地区的职业病发病率

答案：E. 地区的职业病发病率

解析：诊断职业病时，通常需要综合分析多方面的信息，包括职业的危险因素、辅助检查结果、职业病危险因素接触史以及既往的职业病史等。然而，地区的职业病发病率虽然可以作为参考，但它并不能直接作为诊断个体职业病的因素。因此，选项 E 是正确答案。

24、 (A1 型题)

氯氟羟对环境的危害是

A.对大自然植被造成破坏

B.对臭氧层的破坏

C.酸雨

D.破坏生态平衡

E.产生温室效应

参考答案： B 您的答案：

图文解析： 【解析】氯氟烃破坏臭氧层最严重。其特点是穿透力很强，能很快穿过大气对流层，进入

平流层，从而破坏臭氧层。

【避错】二氧化碳是典型的温室气体，它会强烈吸收地面辐射出的红外线。红外辐射主要表现为热效应，因此，这一过程会使大气增温。氯氟烃类物质，在过去常用作制冷剂。由于它制作工艺简单，价格低廉，在曾经风靡一时的制冷剂“氟利昂”中，氯氟烃是最有效的成分。当时的冰箱、冰柜、空调中，大量添加氯氟烃类氟利昂。

在这道题目中，我们要识别氯氟烃（Chlorofluorocarbons，简称 CFCs，即题目中提到的“氯

氟羟”可能是指的这种物质）对环境的主要危害。氯氟烃最著名的环境影响是对臭氧层的破坏。它们在大气中上升到平流层后，由于太阳辐射的作用释放出氯原子，破坏臭氧分子，导致臭氧层变薄，这个过程被称为臭氧层耗竭。

因此，最正确的答案是：

B. 对臭氧层的破坏
首先，我们需要理解氯氟羟对环境的危害。氯氟烃是一类人工合成的有机化合物，常被用作制冷剂、发泡剂等。由于其化学稳定性好，在对流层中不会分解，可以上升到平流层，并在强烈紫外线的作用下被分解，产生氯原子，氯原子可作为臭氧分解的催化剂，破坏臭氧层。

接下来，我们来分析各个选项：

A. 对大自然植被造成破坏：这个选项并不特指氯氟羟的影响，多种环境污染和人为因素都可能导致大自然植被的破坏。

B. 对臭氧层的破坏：如前所述，氯氟羟能够上升到平流层并在紫外线的作用下分解产生氯原子，这些氯原子会催化臭氧的分解，从而破坏臭氧层。这是氯氟羟特有的环境影响。

C. 酸雨：酸雨主要由二氧化硫和氮氧化物等污染物导致，与氯氟羟无直接关系。

D. 破坏生态平衡：虽然环境污染和气候变化最终可能影响生态平衡，但这个选项过于宽泛，不特指氯氟羟的影响。

E. 产生温室效应：温室气体主要包括二氧化碳、甲烷等，氯氟羟不是主要的温室气体。

综上所述，氯氟羟对环境的特定危害是对臭氧层的破坏。因此，最符合题意的答案是：

B.对臭氧层的破坏。

题目 24：氯氟羟对环境的危害是

氯氟羟（chlorofluorohydrocarbons，CFCs）是一类含有氯、氟和碳的化合物，通常用于制冷剂、发泡剂和清洁剂。其主要的环境危害是对臭氧层的破坏。

答案：B.对臭氧层的破坏

氯氟烃进入大气层后，在高空中的紫外线照射下分解出氯原子，氯原子再与臭氧发生反应，导致臭氧层的破坏。这种破坏会导致更多的紫外线到达地球表面，从而引发一系列环境和健康问题。因此，氯氟烃对臭氧层的破坏是其最主要的环境危害。
25、 (A1 型题)
吸烟对人体成瘾最大的成分是
A.焦油
B.CO

C.尼古丁
D.CO2
E.NO2
参考答案：　　C　您的答案：
图文解析：　　【解析】尼古丁（C 对）是烟草成瘾的主要物质。由于大脑由于长期处于被尼古丁激活的
状态，对于尼古丁的敏感性下降，导致大部分人吸烟量逐步增加，日久形成对尼古丁的依赖即成瘾性。
【避错】焦油、烟草烟雾的主要成分 CO 与肺癌的发生密切相关。
吸烟对人体成瘾最大的成分是 C.尼古丁。尼古丁是烟草中的主要成瘾物质，能够迅速进入大脑并刺激多巴胺的释放，产生愉悦感，从而导致依赖和成瘾。焦油、一氧化碳（CO）、二氧化碳（CO2）和二氧化氮（NO2）虽然也存在于烟草烟雾中，并对人体有害，但它们并不是导致成瘾的主要因素。
这是一道涉及烟草成分及其成瘾性的选择题。我们需要确定吸烟对人体成瘾性最大的成分。

我们来分析各个选项：

A. 焦油：焦油是烟草燃烧的产物，主要对人体造成健康危害，但并不具有成瘾性。

B. CO（一氧化碳）：CO 是烟草燃烧时产生的一种有害气体，它与血红蛋白结合能力强，可能导致机体缺氧，但并不具有成瘾性。

C. 尼古丁：尼古丁是烟草中的主要成分之一，具有强烈的成瘾性。它能与中枢神经系统的尼古丁受体结合，产生兴奋、镇静、愉悦等效果，是导致吸烟成瘾的关键因素。

D. CO2（二氧化碳）：CO2 是呼吸作用的正常产物，对人体没有成瘾性。

E. NO2（二氧化氮）：NO2 是一种有害气体，对人体有害，但并不具有成瘾性。

综上所述，尼古丁是导致吸烟成瘾的主要成分。因此，正确答案是 C.尼古丁。

吸烟对人体成瘾最大的成分是：

C. 尼古丁
26、　(A1 型题)
为提升某山区产妇住院分娩率，某县建立产妇住院分娩绿色通道，该措施属于
A.调整卫生服务方向
B.加强社区行动
C.创造支持环境
D.发展个人健康技能
E.制定促进健康的公共政策
参考答案：　　E　您的答案：
图文解析：　　【解析】健康促进的活动领域包括：制定能促进健康的公共政策，创造支持的环境，加强社区的行动，发展个人技能，调整卫生服务方向。明确要求相关部门建立和实

行健康促进政策，其目的就是要使人们更容易作出更有利健康的抉择，本案例中医院建立产妇住院分娩绿色通道属于此项，故本题选 E。

【避错】创造支持环境指创造安全、舒适、满意、愉快的工作和生活环境；加强社区行动是发动社区力量，利用社区资源，增进自我帮助和社会支持；发展个人技能是通过提供健康信息和教育，帮助人们提高健康决策的能力；调整卫生服务方向指卫生部门不仅提供临床治疗服务，还应提供预防和健康促进服务。

这个问题是关于公共卫生干预措施的分类。产妇住院分娩绿色通道的建立旨在提高某山区产妇的住院分娩率，这样的措施通常涉及改善访问条件、提供便利和支持环境，使产妇更容易接受专业的分娩服务。因此，最合适的选项应该是“C.创造支持环境”。这一选项指的是通过提供有利的条件和资源，如便捷的交通、经济支持和医疗资源，来促进健康行为和服务的使用。

解析：

某县建立产妇住院分娩绿色通道，旨在通过政策手段，提高产妇住院分娩的可及性，降低分娩风险，提高母婴健康水平。这一措施体现了政府对公共健康的重视和投入，属于制定促进健康的公共政策。

选项 A 调整卫生服务方向主要是指将卫生服务的重点从治疗转向预防，从城市转向农村，从上层转向基层，与题意不符，故不选。

选项 B 加强社区行动主要是指社区组织通过自我管理和参与式研究等方式，提高社区居民的健康意识和健康行为，与产妇住院分娩绿色通道的建立不直接相关，故排除。

选项 C 创造支持环境是指从社会、经济、文化等方面创造一个有利于健康的环境，虽然与产妇住院分娩绿色通道有一定的联系，但不是最直接相关的选项，故不选。

选项 D 发展个人健康技能是指通过健康教育、健康促进等方式，提高个人的健康意识和技能，虽然重要，但与产妇住院分娩绿色通道的建立不直接相关，故排除。

综上所述，正确答案是：E.制定促进健康的公共政策。

26. 为提升某山区产妇住院分娩率，某县建立产妇住院分娩绿色通道，该措施属于

A. 调整卫生服务方向
B. 加强社区行动
C. 创造支持环境
D. 发展个人健康技能
E. 制定促进健康的公共政策

答案：C. 创造支持环境

解析：建立产妇住院分娩绿色通道是通过提供便捷的医疗服务来支持产妇住院分娩，从而提升住院分娩率。这种措施属于提供便利条件和资源，营造有利于健康行为的环境，因此选项 C“创造支持环境”是最符合的答案。

27、 (A1 型题)

国家选择一定范围地区的艾滋病流行情况，通过针对该地区总人群进行统一的方式和方法的调查方法，该统计方法属于

A.被动监测
B.死因监测

C.症状监测
D.哨点监测
E.行为学监测
参考答案：　　D　您的答案：
图文解析：　　【解析】哨点监测：是指为了更清楚地掌握特定的公共卫生问题，依据疾病、健康状况或
危险因素分布特点，选择若干有代表性的地区和（或）人群作为监测点，按照统一规定要求收集资料（D 对）。
【避错】在检测过程中，下级单位按照常规上报监测资料，而上级检测单位被动接受，称为被动监测（A 错）；上级监测单位专门组织调查或者要求下级检测单位严格按照规定收集资料，称为主动监测。死因监测（B 错）可了解人群各类疾病(包括伤害）的死亡率和死因分布，掌握人群健康水平，确定不同时期主要死因及疾病防治重点。症状监测（C 错）又称为综合征监测或症候群监测，它特别适用于一些新发疾病、病因不明疾病,临床上尚无明确诊断方法的疾病。行为学监测（E 错）适用于某病的患病群体行为监测，例如对艾滋病患者的静脉注射行为、性行为等的监测。
该题目考查的是流行病学监测的不同类型。选项中的“被动监测”、“死因监测”、“症状监测”、“哨点监测”和“行为学监测”都是监测公共健康问题，特别是传染病的不同方法。

A. 被动监测是指依赖医疗机构在日常工作中报告病例的监测方式。
B. 死因监测专注于收集死亡原因的数据。
C. 症状监测通常关注特定症状的出现情况，以便及时发现疾病爆发。
D. 哨点监测是指在特定地点或机构进行的有目的、系统的疾病监测，用以代表整个人群或特定群体的健康状况。
E. 行为学监测关注的是人们的行为方式及其对健康的影响。
题目描述的是国家在一定范围内对艾滋病流行情况进行的统一方式和方法的调查，这种方法涉及到针对特定人群的有目的和系统的监测，最符合“哨点监测”的定义。因此，最佳答案是：

D. 哨点监测
解析：
公共卫生监测包括传染病监测和非传染病监测，其中传染病监测是疾病监测的主体。传染病监测是长期地、连续地收集、核对、分析疾病的动态分布和影响因素的资料，并将信息及时上报和反馈，以便及时采取干预措施。其类型包括医院为基础的监测、实验室监测、哨点监测和人群调查等。题干中国家选择一定范围地区的艾滋病流行情况，通过针对该地区总人群进行统一的方式和方法的调查方法，这种针对特定疾病，对特定人群进行的一种系统、连续的监测，该统计方法属于哨点监测。
选项 A 被动监测是指下级单位按照常规上报资料，上级单位被动接受，不属于针对特定疾病和人群的特定监测，故不选。
选项 B 死因监测指对一个地区或人群的死亡原因进行监测，题干描述的是针对艾滋病这一特定疾病的监测，与题意不符。
选项 C 症状监测是监测体系的一部分，指针对特定症状进行监测，但题干中明确监测的是艾滋病流行情况，而不仅仅是艾滋病症状，故排除。
选项 E 行为学监测通常用于研究特定人群的行为模式，而非特定疾病的流行情况，不符合题意。

综上所述，正确答案是：D.哨点监测。

国家选择一定范围地区的艾滋病流行情况，通过针对该地区总人群进行统一的方式和方法的调查方法，该统计方法属于：

D. 哨点监测

哨点监测是指在特定的地点和人群中，系统地、持续地收集、分析和解释某些疾病或健康相关事件的数据，以便及时发现和控制疾病的传播。这种方法通常用于传染病的监测，包括艾滋病在内。哨点监测可以通过在某些高风险地区或特定人群中进行定期调查来了解疾病的流行情况。

28、 (A1 型题)

现况调查哪个数据最适合收集

A.发病率

B.患病率

C.罹患率

D.死亡率

E.续发率

参考答案： B 您的答案：

图文解析： 【解析】现况调查也称横断面调查、患病率调查等。某特定时间内，出现患某病者(包括

新旧病例)占总人口的比例。多用于普查。故选择 B。

【避错】发病率=(新发病例数/同时期暴露人口数)×k(k= 100 %， 1000 ‰，或 100000/10 万)，故 A 错。罹患率用于小范围、短时间内疾病频率的测量，常用于疾病暴发或流行时的调查，故 C 错。死亡率是单一病种因该病死亡的频率，故 D 错。续发率多指一个家庭内、病房、集体宿舍、托儿所、幼儿园班组中第一个病例发生后，在该病最短与最长潜伏期之间出现的病例称续发病例，故 E 错。

这道题目问的是在进行现况调查时，哪个数据最适合收集。现况调查通常指的是在某一特定时间点对一定人群进行的健康状况的横断面研究。因此，关键在于确定哪种率最能反映研究人群在特定时间点的健康状况。

A. 发病率：指在一定时间内新发病例的数量，通常用来反映疾病的发生情况。

B. 患病率：指在特定时间点或期间内，某病在一定人群中的存在情况，反映了新旧病例的总和。

C. 罹患率：这不是一个常用的流行病学术语，可能是患病率的同义词。

D. 死亡率：指在一定时间内，因特定原因死亡的人数与总人口的比例。

E. 续发率：通常指已有某疾病的人群中发展出新的、次要的病状或疾病的比例。

对于现况调查，我们通常关注的是在特定时间点人群中疾病的存在情况，因此患病率（B）是最适合收集的数据，因为它能够提供某个时间点人群中疾病的普遍性信息。所以最正确的答案是 B. 患病率。

首先，我们需要明确这是一道关于流行病学调查方法选择的问题。题目问的是现况调查最适合收集哪种数据，这需要我们了解现况调查的特点和各种率的概念。

现况调查，又称为横断面研究，是在特定时间点对特定人群的健康状况、有关因素或疾病进行的调查。它的目的是收集某一时间断面上的人群健康状况及有关因素的资料，并对资料的分布特征加以描述。

接下来，我们来分析各个选项：

A. 发病率：表示在一定期间内，一定人群中某病新发生的病例出现的频率。这是一个时期指标，通常用于疾病监测和描述疾病的分布状态，不适用于现况调查。

B. 患病率：表示某一时点某一人群中患病人数与该时点人口数的比值。这是一个时点指标，非常适合用于现况调查，可以反映人群中现患病人数的指标。

C. 罹患率：与发病率相似，也是反映新发病例的指标，但它适用于小范围、短时间内疾病频率的测量，不适合现况调查。

D. 死亡率：表示某时期因某病死亡人数与同期患某病的病人数的比值，它主要用于衡量疾病的严重程度和医院的医疗、诊断水平，不适用于现况调查。

E. 续发率：指某传染病易感接触者中，在最短潜伏期与最长潜伏期之间发病的人数占所有易感接触者总数的百分率，更多用于疾病的传播和感染控制研究，并不适合现况调查。

综上所述，患病率是最能反映现况调查目的和特点的指标，因为它代表了在某一特定时间点，患某种疾病的人数占总人数的比例，非常适合用于描述某一时间点上人群的健康状况。

因此，正确答案是 B.患病率。

现况调查（cross-sectional study）是一种在某一特定时间点上对某个特定人群进行的调查，主要目的是了解该人群某些特定健康指标的分布情况。因此，现况调查最适合收集**患病率**的数据。患病率指的是在某一特定时间点上，特定人群中某种疾病或健康状态的现有病例数占该人群总人数的比例。

正确答案是：**B. 患病率**。
29、 (A1 型题)
以下是二级预防的是
A.缓解患者心绞痛患者疼痛
B.通过血糖筛查糖尿病患者
C.监测环境中的有害气体
D.开展健康教育
E.吸烟者开始戒烟
参考答案： B 您的答案：
图文解析： 【解析】第二级预防包括早期发现、早期诊断、早期治疗，其中早期发现疾病可通过普
查、筛查（B 对）、定期健康检查、高危人群重点项目检查及设立专科门诊等。
【避错】一级预防是针对健康个体的措施；三级预防是针对已患病者采取及时的、有效的

治疗措施，终止疾病的发展，防止病情恶化。
二级预防的目的是在疾病初期进行诊断和治疗，以阻止其进展和减轻其影响。它旨在早期发现病症并进行干预，以减少疾病的严重程度和避免潜在的健康问题。

选项 A（缓解患者心绞痛疼痛）和 E（吸烟者开始戒烟）更接近一级预防或治疗措施，不属于二级预防。选项 C（监测环境中的有害气体）和 D（开展健康教育）更像是一级预防，因为它们旨在防止疾病发生。

选项 B（通过血糖筛查糖尿病患者）属于二级预防，因为这是一种筛查措施，旨在早期发现疾病，即使在症状出现之前也能诊断糖尿病，从而可以及早开始治疗和管理，阻止病情恶化。

因此，最正确的答案是 B.通过血糖筛查糖尿病患者。
这是一道选择题，需要我们分析“二级预防”的定义，并从提供的选项中找出符合“二级预防”定义的答案。

理解二级预防：二级预防是指在疾病的早期阶段进行干预，以阻止或减缓疾病的发展，减少并发症和后遗症的发生。这通常包括早期诊断和适当的治疗。

分析选项：

A 选项（缓解患者心绞痛患者疼痛）：这更像是对已经确诊疾病的治疗措施，属于三级预防的范畴，即针对已患病的个体采取措施以防止疾病恶化和并发症。
B 选项（通过血糖筛查糖尿病患者）：这符合二级预防的定义。通过筛查，可以在糖尿病的早期阶段进行诊断和治疗，防止病情恶化和并发症的发生。
C 选项（监测环境中的有害气体）：这属于一级预防，即针对整个群体的预防措施，目的是防止疾病的发生。
D 选项（开展健康教育）：健康教育主要是为了提高公众的健康意识和知识水平，属于一级预防的范畴。
E 选项（吸烟者开始戒烟）：这同样属于一级预防，通过改变生活方式来预防疾病的发生。
综上所述，只有 B 选项（通过血糖筛查糖尿病患者）符合二级预防的定义，即在疾病的早期阶段进行诊断和干预，以防止病情恶化和并发症的发生。

因此，答案是 B.通过血糖筛查糖尿病患者。

二级预防是指在疾病早期，通过筛查、早期诊断和早期治疗等措施，预防疾病的进一步发展或并发症的发生。

在上述选项中：

A. 缓解患者心绞痛患者疼痛 - 这是治疗措施，不是预防措施。
B. 通过血糖筛查糖尿病患者 - 这是早期筛查和诊断糖尿病，属于二级预防。
C. 监测环境中的有害气体 - 这是一级预防的内容，目的是减少有害因素的暴露。
D. 开展健康教育 - 这是一级预防的内容，目的是提高人群健康水平，预防疾病发生。
E. 吸烟者开始戒烟 - 这是一级预防的内容，目的是预防吸烟相关疾病的发生。

因此，最正确的答案是：

B. 通过血糖筛查糖尿病患者
30、 (A1 型题)
某患者长期使用免疫抑制剂，在医院最可能患的感染是
A.带菌感染
B.外源性感染
C.交叉感染
D.输血感染
E.疫区感染
参考答案： C 您的答案：
图文解析： 【解析】医院感染的发生既可以由皮肤、鼻腔、消化道等部位的内源性常驻菌引起，也可以由医疗环境、医疗设备和器械、访客、医务人员带给患者的外源性病原体引起，亦称交叉感染（C 对)。【避错】带菌感染（A 错）指正常健康人中潜伏着病原菌， 一旦宿主免疫功能降低，病原菌得以繁殖而致病。外源性感染（B 错）指来自宿主体外的病原菌所引起的感染。疫区感染（E 错）指病原体存在于传染源的体内或体表，通过不同的传播途径传染给其他人。
患者长期使用免疫抑制剂会增加感染的风险，尤其是在医院环境中。免疫系统被抑制后，患者对各种病原体的抵抗力减弱。在这种情况下，最常见的是医院获得性感染，也称为院内感染。

选项中：

A. 带菌感染通常指的是个体内部已存在的微生物引起的感染。
B. 外源性感染指的是来源于外部环境的感染。
C. 交叉感染指的是从一个患者或其环境到另一个患者的传播。
D. 输血感染是通过输血传播的感染。
E. 疫区感染指的是在特定疫区内或与之相关的感染。
考虑到这些患者在医院环境中更易受到感染，尤其是免疫系统受抑制时，交叉感染（C. 交叉感染）风险较高，因为医院中可以通过各种途径，如手卫生不当、医疗设备或环境表面，进行微生物的传播。因此，最可能的答案是 C. 交叉感染。
解析：
患者长期使用免疫抑制剂，免疫功能低下，容易感染。外源性感染指病原体来自患者体外，通过不同途径由外界侵入机体而引发的感染。对于免疫功能低下的患者，医院环境中存在大量的微生物，很容易侵入机体而引起外源性感染。
选项 A 带菌感染是指处于带菌状态的细菌引起的感染，带菌者一般无症状，但可不定期排菌，成为传染源，但不是免疫抑制剂使用者最易发生的感染，故不选。
选项 C 交叉感染是指医疗单位内病人之间发生的相互感染，与免疫抑制剂使用者关系不大，故不考虑。
选项 D 输血感染是指通过输血或血液制品而引起的感染，患者无输血史，故排除。
选项 E 疫区感染指的是患者在某种传染病的疫区被感染，题干中并未提及患者在疫区，故不选。

综上所述，正确答案是：B.外源性感染。

对于长期使用免疫抑制剂的患者，最可能患的感染类型是 **外源性感染**（选项 B）。免疫抑制剂会削弱患者的免疫系统，使其对来自外部环境的病原体更加易感。因此，这类患者在医院环境中最容易受到外界病原体的感染。选项解释如下：

A. **带菌感染**：通常是指患者本身携带的微生物在免疫力下降时引起感染，但不如外源性感染常见。

B. **外源性感染**：是指病原体来自外部环境，通过空气、接触等途径进入人体，这在免疫抑制患者中非常常见，因为他们的免疫系统无法有效抵御外界病原体。

C. **交叉感染**：是指通过医护人员、器械或其他患者之间传播的感染，虽然在医院环境中也常见，但其主要因素还是来自外源的病原体。

D. **输血感染**：是通过输血传播的感染，一般在输血筛查严格的情况下发生率较低。

E. **疫区感染**：是指在流行病疫区感染的情况，这与患者所在的环境有直接关系，不如外源性感染常见。

因此，答案是 **B. 外源性感染**。
31、 (A1 型题)
某患者，考虑维生素 B12 缺乏性贫血，若对其进行治疗，以下哪项是正确的
A.如果叶酸吸收障碍则静脉滴注叶酸
B.如果维生素 B12 吸收不良则肌注维生素 B12 的时间延长
C.叶酸不能和抗氧化剂维生素 C 同时口服
D.因为维生素 B12 会影响叶酸的吸收所以不能一起口服
E.因为叶酸会促进维生素 B12 的合成所以要先大量口服叶酸
参考答案： B 您的答案：
图文解析： 【解析】身体贮存维生素 B12 的能力有限，如果维生素 B12 吸收不良则肌注维生素 B12 的时间相应延长，以改善体内维生素 B12 储存情况,故选择 B。
【避错】叶酸吸收障碍可以肌注叶酸（但不可作为维持治疗，除非是患肠道吸收不良的病人），不可滴注，故 A 错。叶酸和维生素 C 同时服用可以促进吸收，故可以同时口服，故 C 错。叶酸可以和维生素 B12 同时口服，对吸收无影响,故 D 错。叶酸是一种水溶性 B 族维生素，经二氢叶酸还原酶及维生素 B12 的作用，形成四氢叶酸，后者与多种一碳单位（包括 CH_3、CH_2、CHO 等）结合成四氢叶酸类辅酶，传递一碳单位，参与体内很多重要反应及核酸和氨基酸的合成，而不是促进维生素 B12 合成，故 E 错。
对于维生素 B12 缺乏性贫血的治疗，正确的做法是如果维生素 B12 的吸收不良，则应该通过肌肉注射的方式给予维生素 B12，以确保患者可以直接吸收和利用这种维生素。因此，选项 B（如果维生素 B12 吸收不良则肌注维生素 B12 的时间延长）是正确的。这是因为维生素 B12 的吸收不良常常需要通过直接给予维生素 B12 的方式来补充，而非依赖于口服途径，这样可以绕过胃肠道的吸收障碍。

其他选项如 A、C、D 和 E 都不是维生素 B12 缺乏性贫血治疗的标准做法或者是基于错误的

理解。特别是 E 选项提到的叶酸促进维生素 B12 合成是不准确的；实际上，过量的叶酸可以掩盖维生素 B12 缺乏症的血液学表现，但不会促进维生素 B12 的合成。因此，最正确的答案是 B。
解析：
维生素 B12 缺乏性贫血又称为恶性贫血，是由于内因子缺乏导致维生素 B12 吸收不良而引起的贫血。其治疗原则是补充维生素 B12，若存在维生素 B12 吸收不良，则需延长维生素 B12 的疗程，即如果维生素 B12 吸收不良则肌注维生素 B12 的时间延长。
叶酸吸收障碍者，可口服叶酸，故选项 A 如果叶酸吸收障碍则静脉滴注叶酸，可排除。
叶酸和维生素 C 可以一起服用，二者之间并不会发生相互作用，并不会对人体健康造成影响，故选项 C 叶酸不能和抗氧化剂维生素 C 同时口服，可排除。
叶酸和维生素 B12 之间并无相互影响，两者可以一起服用，故选项 D“因为维生素 B12 会影响叶酸的吸收所以不能一起口服”，选项 E“因为叶酸会促进维生素 B12 的合成所以要先大量口服叶酸”，可一并排除。

综上所述，正确答案是：B.如果维生素 B12 吸收不良则肌注维生素 B12 的时间延长。

维生素B12缺乏性贫血的治疗主要是补充维生素B12。根据现有的医学知识和最佳实践指南，以下是对各个选项的分析：

A. **如果叶酸吸收障碍则静脉滴注叶酸** - 叶酸吸收障碍的治疗确实可以通过静脉滴注叶酸，但这与维生素 B12 缺乏性贫血的直接治疗没有直接关系。

B. **如果维生素 B12 吸收不良则肌注维生素 B12 的时间延长** - 维生素 B12 吸收不良（如恶性贫血患者）通常需要长期肌注维生素 B12，最初每天注射，然后逐渐减少频率。这个选项是正确的。

C. **叶酸不能和抗氧化剂维生素 C 同时口服** - 叶酸和维生素 C 可以同时口服，没有证据表明两者不能一起服用。

D. **因为维生素 B12 会影响叶酸的吸收所以不能一起口服** - 维生素 B12 和叶酸可以一起服用，它们在体内协同作用于造血过程，没有证据表明维生素 B12 会影响叶酸的吸收。

E. **因为叶酸会促进维生素B12的合成所以要先大量口服叶酸** - 叶酸不会促进维生素B12的合成，维生素 B12 是需要通过饮食或补充来获取的，两者的补充方式和途径不同。

所以，正确答案是：

B. 如果维生素 B12 吸收不良则肌注维生素 B12 的时间延长
32、 (A1 型题)
股骨头的营养动脉不包括
A.旋髂深动脉
B.股骨干滋养动脉升支
C.旋股外侧动脉分支
D.小凹动脉

E.旋股内侧动脉分支
参考答案：　　A　您的答案：
图文解析：　　　【解析】股骨头血液供应来源有小凹动脉、股骨干滋养动脉升支和旋股内、外侧动脉。
（A 错，为本题正确答案）
【避错】旋股内侧动脉又可分为骺外侧动脉、干骺端上侧动脉、干骺端下侧动脉，其中骺外侧动脉供应股骨头 2/3～4/5 区域的血液循环，是股骨头最主要的供血来源。
股骨头的主要血供来源于旋股内侧动脉、旋股外侧动脉的分支以及股骨干滋养动脉的升支。旋髂深动脉主要供血于髋关节周围的软组织，而不直接供血于股骨头。因此，在这些选项中，不包括股骨头营养动脉的是：

A. 旋髂深动脉

所以正确答案是 A。
解析：
股骨头即股骨上端，呈球形，朝向内上，与髋臼构成髋关节。股骨头的血液供应来源有小凹动脉、股骨干滋养动脉升支和旋股内、外侧动脉。其中髂腰动脉发出的旋髂深动脉主要营养髂骨，并不直接为股骨头供血。
选项 B 股骨干滋养动脉升支，选项 C 旋股外侧动脉分支，选项 D 小凹动脉，选项 E 旋股内侧动脉分支，均是股骨头的营养动脉，可一并排除。

综上所述，正确答案是：A.旋髂深动脉。

32. 股骨头的营养动脉不包括

A. 旋髂深动脉

B. 股骨干滋养动脉升支

C. 旋股外侧动脉分支

D. 小凹动脉

E. 旋股内侧动脉分支

正确答案是 A. 旋髂深动脉。

解释：股骨头的主要营养动脉包括旋股内侧动脉分支、小凹动脉、股骨干滋养动脉升支和旋股外侧动脉分支。旋髂深动脉并不参与股骨头的血供，因此不包括在内。
33、　(A1 型题)
某天医生查房时发现一位患者脸色苍白、四肢湿冷、出冷汗、脉搏加快、烦躁不安，呼吸困难，立刻判断该患者为休克。医生这一心理活动过程属于
A.感觉
B.知觉

C.想象
D.思维
E.人格
参考答案：　　D　您的答案：
图文解析：　　【解析】思维是人脑间接地概括地对客观事物的反映。人的思维是借助概念、表象和动作，在感性认识的基础上认识事物的一般的和本质的特征和规律性联系的心理过程。医生通过观察患者的客观表现，认识到患者处于休克状态，这一过程属于思维，故本题选 D。
【避错】心理现象分为心理过程和个性特征。心理过程包括认知过程、情感过程与意志过程。认知过程包括感觉、知觉、记忆、思维、想象等。
该题目是关于心理活动过程的判断。根据题目描述，医生在查房时通过观察患者的外在症状（脸色苍白、四肢湿冷、出冷汗、脉搏加快、烦躁不安、呼吸困难）迅速判断患者为休克。这个过程是通过对患者症状的接收和解释来完成的，侧重于对信息的整合和理解，从而作出判断。

在这些选项中，感觉（A）主要是指通过感官接收外界信息的过程；知觉（B）是指对感觉到的信息进行加工和解释的过程，形成对事物的认识；想象（C）是指在大脑中构建事物形象的过程，通常与现实观察无直接联系；思维（D）是指对信息进行加工、分析、综合、判断或解决问题的心理过程；人格（E）是指个体心理特征的总和，与本题的心理活动过程判断不直接相关。

因此，最符合题目描述的是“知觉”（B），因为医生需要对观察到的症状进行解释和加工，以判断患者处于休克状态。所以正确答案应该是 B.知觉。
首先，我们来仔细分析这个问题。题目描述了一个医生查房时，通过观察患者的具体症状（脸色苍白、四肢湿冷等），迅速做出了患者处于休克状态的判断。这个过程涉及到医生对患者症状的感知和理解，并从中得出结论。

现在，我们逐一分析各个选项：

A. 感觉：感觉是对客观事物个别特性的反映，如看到颜色、听到声音等。在这个案例中，医生确实在感觉患者的症状，但单纯的感觉并不足以形成休克的判断。

B. 知觉：知觉是对感觉信息的整合和解释，比感觉更高一级。虽然医生在整合患者的各种症状，但知觉本身并不包含对信息的深入分析和判断。

C. 想象：想象是对头脑中已有的表象进行加工、改造，形成新形象的过程。这个选项与医生的判断过程关系不大。

D. 思维：思维是人脑对客观事物本质和事物之间内在联系的认识。在这个案例中，医生通过观察患者的症状，运用自己的医学知识，迅速判断患者处于休克状态。这个过程涉及到了深入的分析和推理，正是思维能力的体现。

E. 人格：人格是一个人的心理品质的统称，与这个具体的判断过程关系不大。

综上所述，医生通过观察患者的多种症状，结合自己的医学知识，迅速做出患者休克的判断。这个过程主要体现了医生的思维能力，即选项 D。在这个过程中，医生不仅感知了患者的症状，还对这些症状进行了深入的分析和判断，这是单纯的感觉或知觉所无法做到的。

因此，正确答案是 D.思维。

根据题目中描述的医生的心理活动过程，这种活动涉及对患者症状的综合判断和分析，并做出诊断，这属于思维过程。因此，最正确的答案是：

D. 思维

34、 (A1 型题)

确定尿道损伤的部位、深度及严重程度的检查是

A.导尿管

B.超声检查

C.尿道 CT

D.逆行尿道造影

E.输尿管镜

参考答案： D 您的答案：

图文解析： 【解析】逆行尿道造影可显示尿道外伤部位和程度（第 9 版《外科学》）（D 对）。

【避错】虽然尿道外伤可以进行诊断性导尿，但只能了解尿道的完整性和连续性（A 错）。超声在泌尿外科主要应用于确定肾肿块的性质、结石和肾积水，测定残余尿、测量前列腺体积，检查阴囊、睾丸、附睾的情况等（B 错）。CT 在泌尿外科主要用于鉴别肾囊肿和肾实质性病变，确定肾损伤范围和程度和肿瘤的诊断和分期等（C 错）。输尿管镜无法判断损伤深度（E 错）。

确定尿道损伤的部位、深度及严重程度最准确的方法是进行逆行尿道造影（Retrograde Urethrography, RUG）。这种检查能够清晰地显示尿道的轮廓，帮助确定损伤的具体位置和程度。因此，最佳选项是 D.逆行尿道造影。

解析：

尿道损伤是泌尿系统最常见的损伤，多见于男性。在解剖上男性尿道以尿生殖膈为界，分为前、后两段。前尿道包括球部和阴茎部，后尿道包括前列腺部和膜部。前尿道损伤多发生于球部，这段尿道的管径较大，为比较固定的尿道部分，而膜部尿道穿过尿生殖膈，位置比较固定，外伤性尿道断裂易在此处发生。确定尿道损伤的部位、深度及严重程度的检查是逆行尿道造影，可以清晰显示出尿道损伤的部位、程度以及尿外渗的范围。

选项 A 导尿管，主要用于术前术后导尿或用于尿道损伤后探查尿道是否连续、完整，但不能判断尿道损伤的部位、深度及严重程度，故不选。

选项 B 超声检查可以发现尿外渗、膀胱周围血肿，但难以发现后尿道损伤，因此不能作为确定尿道损伤的部位、深度及严重程度的检查方法，故排除。

选项 C 尿道 CT 可以显示骨盆骨折，但不能清晰显示出尿道损伤的部位，且价格昂贵，故不选。

选项 E 输尿管镜主要用于肾盂、输尿管疾病的诊断与治疗，对尿道损伤的诊断价值不大，故排除。

综上所述，正确答案是：D.逆行尿道造影。

34. 确定尿道损伤的部位、深度及严重程度的检查是

最佳答案是：

D. 逆行尿道造影

逆行尿道造影是评估尿道损伤的标准检查方法，可以详细显示尿道的部位、深度和严重程度。这种检查方法通过逆行注入造影剂并拍摄 X 线片，能够清晰地显示尿道的形态和损伤情况。

35、 (A1 型题)

碱性尿液中形成的结石是

A.尿酸结石

B.草酸结石

C.胱氨酸结石

D.磷酸盐结石

E.黄嘌呤结石

参考答案： D 您的答案：

图文解析： 【解析】碱性尿有利于磷酸盐形成；酸性尿内易形成草酸结石、尿酸结石、胱氨酸结石、黄嘌呤结石（D 对）。

在碱性尿液中最常形成的结石是磷酸盐结石。因此，正确答案是 D.磷酸盐结石。磷酸盐结石主要包括磷酸钙和磷酸镁铵结石，它们在碱性环境中更容易形成。其他类型的结石，如尿酸结石和胱氨酸结石，更常在酸性尿液中形成。

解析：

碱性尿液中容易形成的结石类型是磷酸盐结石，因其溶解度在碱性尿液中较低，故易析出形成结石。

选项 A 尿酸结石主要在酸性尿液中形成，故不选。

选项 B 草酸结石，选项 C 胱氨酸结石，选项 E 黄嘌呤结石，这三种结石与尿液酸碱度的关系不大，可在酸性或碱性尿液中形成，但碱性尿液中并不比其他酸碱度环境下更易形成，故一并排除。

综上所述，正确答案是：D.磷酸盐结石。

碱性尿液中形成的结石通常是磷酸盐结石。因此，最佳答案是：

D. 磷酸盐结石

36、 (A1 型题)

慢性肾衰竭的情况下，造成的钙磷代谢异常情况是

A.血磷正常，血钙升高

B.血磷降低，血钙升高

C.血磷降低，血钙降低

D.血磷升高，血钙降低

E.血磷升高，血钙升高

参考答案： D 您的答案：

图文解析： 【解析】在慢性肾衰竭早期，血钙、血磷仍能维持在正常范围内。随着病情进展，肾脏排磷减少，出现高磷血症、低钙血症（D 对）。低钙血症主要与钙摄入不足、活性

维生素 D 缺乏、高磷血症、代谢性酸中毒等有关。（第 9 版《内科学》）
在慢性肾衰竭的情况下，通常会出现血磷升高和血钙降低的情况。这是因为肾脏功能减退导致磷排泄减少，从而引起血磷升高；同时，肾脏对钙的调节受损，导致血钙降低。此外，肾脏对维生素 D 的激活减少，也影响了钙的吸收和代谢，进一步降低血钙水平。因此，最佳答案是 D. 血磷升高，血钙降低。
解析：
慢性肾衰竭时，由于肾单位损坏，肾脏排泄固定酸的能力减弱或丧失，导致酸性代谢产物排泄障碍，引发酸性中毒，并可导致肾小管性酸中毒，机体为维持体液酸碱平衡，会动员骨钙以中和酸性代谢产物，导致血钙降低。同时，由于肾小管的分泌 H^+障碍，H^+与 HPO_4^{2-}结合成 $H_2PO_4^-$减少，导致 HPO_4^{2-}重吸收增加，血磷升高。故慢性肾衰竭可导致的钙磷代谢异常情况通常为血磷升高，血钙降低。
选项 A“血磷正常，血钙升高”，选项 B“血磷降低，血钙升高”，选项 E“血磷升高，血钙升高”，三者中均包含血钙升高，而慢性肾衰竭时，为维持体液酸碱平衡，会动员骨钙以中和酸性代谢产物，导致血钙降低，故可一并排除。
选项 C 血磷降低，血钙降低可见于甲状旁腺功能减退的患者，不符合慢性肾衰竭的病理生理，故不选。
综上所述，正确答案是：D.血磷升高，血钙降低。

慢性肾衰竭（CKD）时，由于肾脏功能下降，磷的排泄减少，导致血磷水平升高。同时，肾脏合成活性维生素 D（1,25-二羟基维生素 D）的能力下降，导致钙的吸收减少，血钙水平降低。这种情况会刺激甲状旁腺分泌更多的甲状旁腺激素（PTH），试图通过骨吸收来提高血钙水平，但通常还是会导致高磷低钙的状态。

因此，正确答案是：

D. 血磷升高，血钙降低
37、 (A1 型题)
消化道出血导致的肝性脑病的神经机制
A.苯乙胺代谢增强
B.谷氨酰胺代谢增强
C.乙酰胆碱合成增多
D.锰透过血脑屏障进脑
E.γ-氨基丁酸分解增多
参考答案： B 您的答案：
图文解析： 【解析】肝性脑病时中枢神经系统的机能障碍主要是代谢性的或功能性的，是多种发病因素综合作用的结果，目前主流学说主要认为与血氨增多有关。正常时血氨含量很少，脑组织内少量的氨与α-酮戊二酸在谷氨酸脱氢酶的作用下形成谷氨酸。谷氨酸在谷氨酰合成酶及 ATP、Mg2+的参与下，再与氨结合形成谷氨酰胺。谷氨酰胺是运送氨的一种形式，在肝及肾内经谷氨酰胺酶的作用，再分解成谷氨酸和氨。氨在肝内经鸟氨酸循环形成尿素，在肾内形成的 NH4+，由肾排出。血氨增多时，血氨通过血脑屏障进入脑组织的氨增多，进而损伤中枢神经系统机能。故本题选 B。
消化道出血导致的肝性脑病的一个重要神经机制是由于血氨水平的升高。氨是肝性脑病发病的关键因素，因为肝脏受损无法有效地将氨转化为尿素，导致氨在血液中积累。然而，在给定的选项中，没有直接提到氨，因此我们需要考虑与氨代谢最相关的选项。

A. 苯乙胺代谢增强
B. 谷氨酰胺代谢增强
C. 乙酰胆碱合成增多
D. 锰透过血脑屏障进脑
E. γ-氨基丁酸（GABA）分解增多
在这些选项中，选项 B "谷氨酰胺代谢增强" 与氨的代谢最相关。在肝性脑病中，由于氨的积累，大脑中的谷氨酸会与氨结合形成谷氨酰胺，导致谷氨酰胺的代谢增强。这一过程有助于减少氨的毒性，但同时会导致大脑代谢失衡，进而促进肝性脑病的发展。
因此，最合适的答案是 B. 谷氨酰胺代谢增强。
解析：
消化道出血导致的肝性脑病多由于消化道出血后，大量血液蛋白质的消化产物在肠道被细菌分解，产生大量的氨，吸收入血后引起氨中毒，导致肝性脑病。其中消化道出血导致的肝性脑病的神经机制是消化道出血后，肠道细菌分解血液，产生大量的氨，氨入血后可通过血脑屏障进入脑组织，引起脑水肿，氨在脑中与α-酮戊二酸结合，形成谷氨酸，后者再与氨结合生成谷氨酰胺，并消耗大量的 ATP 和其他辅酶，引起脑细胞功能紊乱，导致肝性脑病。
选项 A 苯乙胺代谢增强与肝性脑病的发生无关，故排除。
选项 C 乙酰胆碱合成增多与重症肌无力发病机制有关，与肝性脑病无直接关系，故不选。
锰在正常情况下不会通过血脑屏障，只有在肝功能受损时才可通过血脑屏障进入脑组织，但这不是消化道出血导致的肝性脑病的神经机制，故选项 D 锰透过血脑屏障进脑，不符合题意。
选项 E：γ-氨基丁酸分解增多与肝性脑病无关，事实上，肝性脑病时抑制性神经递质γ-氨基丁酸合成增多，故不选。
综上所述，正确答案是：B.谷氨酰胺代谢增强。

消化道出血导致的肝性脑病的神经机制主要与氨代谢紊乱和毒性物质的积累有关。肝性脑病（HE）是由于肝功能衰竭或门静脉高压引起的肝脏解毒功能下降，使有毒物质（如氨）在血液中积累并进入大脑，导致神经功能障碍。

在消化道出血时，大量的血红蛋白分解产生氨，肝脏无法有效解毒，这些氨通过血脑屏障进入大脑，进一步影响神经系统。其中谷氨酰胺的代谢增强是一个关键机制，因为谷氨酰胺是氨和谷氨酸结合的产物，过多的氨会导致大脑中的谷氨酰胺水平增加，从而导致神经毒性。

因此，最合适的答案是：

B. 谷氨酰胺代谢增强
38、 (A1 型题)
不属于危险三角区的静脉血供是
A.内眦静脉
B.眼上静脉
C.眼下静脉
D.颞浅静脉
E.面深静脉
参考答案： D 您的答案：

图文解析：　　【解析】危险三角为两侧口角至鼻根连线所形成的三角形区。面静脉经内眦静脉、眼静脉（眼的静脉有：视网膜中央静脉、眼上静脉、眼下静脉和眼中静脉）与海绵窦交通，也可经面深静脉、翼丛等与海绵窦交通。故 ABCE 对，D 错。
在医学领域，危险三角区通常指的是面部从鼻梁延伸至嘴角两侧的三角形区域。该区域内的静脉与颅内静脉窦有直接或间接的静脉连接，因此感染有可能通过这些静脉通道传播到颅内，造成严重并发症。
A. 内眦静脉属于危险三角区的静脉血供。
B. 眼上静脉也属于危险三角区的静脉血供。
C. 眼下静脉同样属于危险三角区的静脉血供。
D. 颞浅静脉不直接属于危险三角区的静脉血供，它位于颞部。
E. 面深静脉属于危险三角区的静脉血供。
因此，不属于危险三角区的静脉血供是 D.颞浅静脉。所以，正确答案是 D. 颞浅静脉。
解析：
危险三角区通常指的是两侧口角至鼻根连线所形成的三角形区域，由于面部的静脉没有静脉瓣，这个区域的感染有可能经过内眦静脉、眼静脉等进入颅内的海绵状静脉窦，引起化脓性海绵状静脉窦炎，出现眼部及其周围组织的红肿和硬结，伴有疼痛和压痛，并有寒战、发热、头痛等，病情严重者甚至会发生败血症、毒血症，危及生命。因此，切忌对于面部“危险三角区”的脓点，切勿挤压，搔抓挤压或者挑刺，以免炎症扩散，引起更严重的并发症。面部“危险三角区”的静脉血供包括面前静脉、面深静脉、内眦静脉、眼上静脉和眼下静脉等，颞浅静脉属于颈外静脉的分支，不属于危险三角区的静脉血供。
选项 A 内眦静脉，选项 B 眼上静脉，选项 C 眼下静脉，选项 E 面深静脉，均属于危险三角区的静脉血供，因此可一并排除。
综上所述，正确答案是：D.颞浅静脉。

在人体解剖学中，面部的危险三角区是指由内眦到口角之间的区域，这一区域的静脉血供与颅内静脉系统相连，如果感染扩散，可能会导致严重的颅内感染。危险三角区内的静脉包括内眦静脉、眼上静脉和面深静脉。颞浅静脉则不在危险三角区的静脉血供范围内。

因此，正确答案是：

D. 颞浅静脉
39、　(A1 型题)
心直视手术钳夹主动脉和肺动脉血流时，涉及的解剖结构有
A.心包斜窦
B.心包前下窦
C.腔静脉窦
D.冠状窦
E.心包横窦
参考答案：　E　您的答案：
图文解析：　【解析】心包横窦是心包腔在升主动脉、肺动脉干与上腔静脉、左心房前壁之间的间隙，当心直视手术要阻断主动脉、肺动脉血流时，可通过此窦从前后钳夹大动脉。故本题选 E。
心直视手术中钳夹主动脉和肺动脉血流通常涉及的解剖结构是心包横窦。因为心包横窦位于主动脉和肺动脉的后方，是一个重要的解剖标志，手术中常常需要识别这个区域以避免损伤

邻近的结构。因此，最合适的选项是 E.心包横窦。
解析：
在心直视手术中，为了暂时阻断主动脉和肺动脉的血流，需要穿过心包横窦来钳夹主动脉和肺动脉。心包横窦是心包腔在主动脉、肺动脉升部和上腔静脉、左心房前壁之间的间隙，是心脏手术时阻断主动脉和肺动脉血流的必经之路。
选项 A 心包斜窦位于左心房后壁、左右肺静脉、下腔静脉与心包后壁之间的心包腔。手术需要阻断下腔静脉回流时才经过此间隙，与题意不符。
选项 B 心包前下窦位于心包前壁的前下部、胸骨柄的后方和膈神经前方的心包腔。心包穿刺时可经此部位刺入，与钳夹主动脉和肺动脉血流无关，故排除。
选项 C 腔静脉窦位于上、下腔静脉与右心房的上腔静脉口和下腔静脉口之间，是心包腔中最低的位置，当人体直立时，心包腔内如果有液体将首先积聚于此，与题干描述不符。
选项 D 冠状窦开口于右心房，汇集来自于心脏的静脉血液，注入右心房，手术时不会涉及此结构，故不选。
综上所述，正确答案是：E.心包横窦。

在心脏直视手术中，当需要钳夹主动脉和肺动脉血流时，所涉及的解剖结构是心包横窦（transverse pericardial sinus）。心包横窦位于升主动脉和肺动脉之间，是手术过程中便于钳夹这些大血管的一个重要解剖标志。因此，正确答案是：

E. 心包横窦

40、 (A1 型题)
维持子宫前倾的韧带是
A.子宫圆韧带
B.子宫阔韧带
C.子宫主韧带
D.卵巢固有韧带
E.卵巢悬韧带
参考答案： A 您的答案：
图文解析： 【解析】子宫韧带共包括 4 条韧带，分别是：子宫主韧带、子宫阔韧带、子宫圆韧带、和骶子宫韧带。子宫阔韧带可防止子宫侧方移位，维持子宫正中位。子宫圆韧带主要维持保持子宫前倾位。子宫主韧带固定子宫颈的位置，防止子宫不致脱垂。子宫骶骨韧带间接维持子宫前倾位。故本题选 A。
维持子宫前倾的韧带是子宫圆韧带。所以正确答案是 A. 子宫圆韧带。子宫圆韧带是一对结构，起源于子宫两侧，并通过骨盆延伸至大腿内侧，有助于维持子宫的位置和前倾状态。
解析：
子宫韧带共有 4 对，包括圆韧带、阔韧带、主韧带和宫骶韧带，其中子宫圆韧带起自宫角的前面、输卵管近端的稍下方，在腹股沟管浅环附近斜向前下，经腹股沟管止于大阴唇前端。圆韧带在维持子宫前倾位方面起重要作用。
选项 B 子宫阔韧带是覆盖子宫前后壁的腹膜自子宫侧缘向两侧延伸达盆壁而形成的双层腹膜皱襞，其作用是固定宫颈，保持子宫位于盆腔中央，故不选。
选项 C 子宫主韧带是阔韧带下部增厚形成的横行纤维束，又称子宫颈横韧带，是固定子宫颈位置、防止子宫下垂的主要结构，与维持子宫前倾无关，故排除。
选项 D 卵巢固有韧带是阔韧带在卵巢内侧与宫角之间增厚形成的，其作用是连接卵巢和子宫，对子宫的位置无影响，故不考虑。

选项 E 卵巢悬韧带是阔韧带在卵巢的动静脉附近形成的结构，其内有卵巢动静脉穿过，是手术时寻找卵巢动静脉的标志，对子宫的位置亦无影响，可排除。
综上所述，正确答案是：A.子宫圆韧带。

维持子宫前倾的韧带是：

A. 子宫圆韧带

子宫圆韧带的作用是通过固定子宫的前倾位置，使其保持在正常的解剖位置上。因此，在这道题中，最正确的答案是 A. 子宫圆韧带。
41、 (A1 型题)
胰岛素降糖的主要机制是
A.促进糖异生
B.促进肌肉、脂肪、蛋白质对葡萄糖的吸收
C.抑制大脑对近端肾小管对葡萄糖的吸收
D.抑制肝糖原、肌糖原的合成抑制葡萄糖的分解
参考答案： B 您的答案：
图文解析： 【解析】胰岛素降低血糖的机制主要是胰岛素对糖代谢的作用，促进外周组织细胞对葡萄糖的摄取和利用（B 对），促进肌糖原、肝糖原的合成（D 错），抑制糖异生（A 错），从而使血糖降低。
胰岛素是一种调节血糖水平的重要激素，它通过多种机制降低血糖浓度。根据提供的选项，胰岛素的主要作用机制是促进肌肉、脂肪和蛋白质对葡萄糖的吸收。因此，最正确的答案是：
B. 促进肌肉、脂肪、蛋白质对葡萄糖的吸收
这反映了胰岛素如何帮助细胞吸收血液中的葡萄糖并将其用作能量，从而降低血糖水平。
解析：
胰岛素是由胰岛 B 细胞分泌的，具有降低血糖作用的蛋白质类激素。胰岛素降糖的主要机制是促进肌肉、脂肪、蛋白质对葡萄糖的吸收，从而降低血糖水平。
选项 A 促进糖异生，选项 D 抑制肝糖原、肌糖原的合成抑制葡萄糖的分解，均是胰高血糖素的作用机制，与胰岛素的作用相反，故可一并排除。
胰岛素通过促进肌肉和脂肪组织对葡萄糖的摄取和利用来降低血糖，而不是通过抑制大脑对近端肾小管对葡萄糖的吸收。故选项 C 抑制大脑对近端肾小管对葡萄糖的吸收，与题意不符。
综上所述，正确答案是：B.促进肌肉、脂肪、蛋白质对葡萄糖的吸收。

胰岛素降糖的主要机制是促进肌肉、脂肪、蛋白质对葡萄糖的吸收。胰岛素是由胰腺的β细胞分泌的一种激素，主要作用是降低血糖水平。它通过促进细胞对葡萄糖的吸收和利用，抑制肝脏葡萄糖的生成以及促进肝糖原和肌糖原的合成来实现这一作用。因此，最正确的答案是：

B. 促进肌肉、脂肪、蛋白质对葡萄糖的吸收
42、 (A1 型题)
小剂量可以增加肾动脉血流量的是
A.肾上腺素
B.去甲肾上腺素
C.异丙肾上腺素

D.多巴胺
E.氨茶碱
参考答案： D 您的答案：
图文解析： 【解析】小剂量多巴胺能够增加肾动脉血流量，主要是因为抑制近曲小管 Na＋-K＋-ATP 酶的活性，减少钠的重吸收有关，D 对。肾上腺素主要用于心肺复苏和过敏性休克的抢救（A 错）。去甲肾上腺素有很强的α-肾上腺素能兴奋作用，是一种非常有效的升压药物，对于正常或低血容量性休克的机体，去甲肾上腺素会减少肾血流量和尿量（B 错）。异丙肾上腺素激动血管平滑肌的β2 受体（骨骼肌、肝脏、冠脉血管为主），使血管舒张（C 错）。氨茶碱在临床上主要的作用是解除支气管痉挛（E 错）。
在选择能增加肾动脉血流量的药物时，需要考虑药物的生理作用。肾上腺素、去甲肾上腺素、异丙肾上腺素主要作用是对心脏和血管的α和β受体，而多巴胺和氨茶碱有不同的作用机制。
肾上腺素（A）主要作用于α和β受体，会导致血管收缩，从而影响肾动脉血流。
去甲肾上腺素（B）主要对α受体有较强的作用，也会引起血管收缩，不利于增加肾动脉血流量。
异丙肾上腺素（C）是一个强心剂，主要作用于心脏的β1 受体，对肾动脉血流的直接影响较小。
多巴胺（D）在小剂量时主要作用于多巴胺受体，能够增加肾脏血流量，是用于治疗急性肾功能不全的药物之一。
氨茶碱（E）主要用于治疗哮喘，通过松弛平滑肌增加气道开放，对肾动脉血流的直接影响不大。
因此，最佳答案是 D. 多巴胺，因为它在小剂量时能增加肾脏血流量。
解析：
本题主要考察血管活性药物对肾动脉血流量的影响。小剂量多巴胺可以激动肾脏的多巴胺受体（D_1受体），使肾血管扩张，肾血流量增加，肾小球的滤过率也增加，尿量增加，同时有排钠利尿的作用。故多巴胺小剂量使用时可增加肾动脉血流量。
选项 A 肾上腺素可与α和β两类肾上腺素能受体结合，产生强烈快速而短暂的兴奋α和β型效应，对心脏β_1受体的兴奋，可使心肌收缩力增强，心率加快，心肌耗氧量增加，增加肾动脉血流量作用不明显，故排除。
选项 B 去甲肾上腺素可与心肌β_1肾上腺素能受体结合，从而增强心肌收缩力而使心率加速，也可与血管平滑肌α受体结合，使血管收缩，与题意不符。
选项 C 异丙肾上腺素是一种β受体激动剂，主要用于支气管哮喘及心脏房室传导阻滞，对肾动脉血流量无明显影响，故不选。
选项 E 氨茶碱是磷酸二酯酶抑制剂，使细胞内 cAMP 水平升高而舒张支气管，对肾动脉血流量影响较小，可排除。
综上所述，正确答案是：D.多巴胺。

小剂量可以增加肾动脉血流量的是：

D. 多巴胺

解析：
多巴胺是一种多巴胺受体激动剂，可以引起肾血管的扩张。小剂量的多巴胺主要作用于多巴胺受体（D1 受体），能够扩张肾动脉，增加肾血流量。因此，多巴胺在小剂量时可以用于治

疗急性肾衰竭或其他需要增加肾血流量的情况。其他选项如肾上腺素、去甲肾上腺素和异丙肾上腺素主要作用于 α 和 β 肾上腺素受体，通常会引起血管收缩和心脏刺激作用，不适用于增加肾血流量。而氨茶碱主要是通过抑制磷酸二酯酶，提高细胞内 cAMP 的水平，主要用于治疗哮喘和慢性阻塞性肺病。

43、 (A1 型题)

HIV 感染的主要细胞是

A.树突状细胞

B.T 细胞

C.CD4^{+}T 细胞

D.B 淋巴细胞

E.CD8^{+}T 细胞

参考答案： C 您的答案：

图文解析： 【解析】CD4+T 淋巴细胞是 HIV 感染最主要的靶细胞，HIV 感染人体后，出现 CD4+T 淋巴细胞进行性减少，CD4+/CD8+T 淋巴细胞比值倒置，细胞免疫功能受损，C 对。

HIV（人类免疫缺陷病毒）主要感染的细胞是 CD4^{+}T 细胞。因此，最正确的答案是 C. CD4^{+}T 细胞。

解析：

HIV 即人类免疫缺陷病毒，是造成人类免疫系统缺陷的一种逆转录病毒。HIV 主要侵犯人体的免疫系统，包括 CD4^{+}T 细胞、巨噬细胞和树突状细胞。但 HIV 感染人体后，会选择性地吸附于靶细胞的 CD4 受体上，在辅助受体的帮助下进入宿主细胞，主要感染 CD4^{+}T 细胞，并在其中复制、扩增，最终导致 CD4^{+}T 细胞破裂、死亡。

选项 A 树突状细胞虽也是 HIV 侵犯的靶细胞，但不是其主要感染对象，故不选。

选项 B：T 细胞，选项 D：B 淋巴细胞，选项 E：CD8^{+}T 细胞，均不是 HIV 的主要感染对象，故可一并排除。

综上所述，正确答案是：C.CD4^{+}T 细胞。

HIV（人类免疫缺陷病毒）感染的主要靶细胞是 CD4^{+}T 细胞。这些细胞是免疫系统中非常重要的一部分，HIV 病毒通过结合 CD4 受体进入这些细胞并进行复制，从而导致免疫系统的破坏。因此，最正确的答案是：

C. CD4^{+}T 细胞

44、 (A1 型题)

HIV 主要感染的细胞不包括

A.库普弗细胞

B.T 细胞

C.单核巨噬细胞

D.B 淋巴细胞

E.神经胶质细胞

参考答案： A 您的答案：

图文解析： 【解析】HIV 侵入人体后,在直接细胞毒性作用和免疫病理作用下,以 CD4+淋巴细胞为主的多种细胞(CD8+T 细胞、单核-巨噬细胞、B 淋巴细胞、NK 细胞、小神经胶质细胞等)功能受损和大量破坏,导致机体细胞免疫缺陷,促使机体并发各种严重的机会性感染和肿瘤的发生。故本题选 A，不包括库普弗细胞。

HIV（人类免疫缺陷病毒）主要感染免疫系统中的细胞，尤其是具有 CD4 受体的细胞。考虑

以下细胞类型：
A. 库普弗细胞 - 这些是肝脏中的巨噬细胞，虽然它们可以被 HIV 感染，但并不是 HIV 的主要目标细胞。
B. T 细胞 - HIV 主要感染 CD4+ T 细胞，这是 HIV 生命周期中的关键环节。
C. 单核巨噬细胞 - HIV 也可以感染单核巨噬细胞，这是病毒传播和持续存在的重要因素。
D. B 淋巴细胞 - 虽然 B 细胞不是 HIV 的主要目标，但它们可以受到影响，尤其是在 HIV 感染晚期。
E. 神经胶质细胞 - HIV 可以感染中枢神经系统的细胞，包括微胶质细胞，但神经胶质细胞（如星形细胞和少突胶质细胞）并不是 HIV 的主要目标细胞。
因此，最不可能被 HIV 主要感染的细胞是 E. 神经胶质细胞。
首先，我们需要了解 HIV 即人类免疫缺陷病毒主要感染哪些细胞。HIV 主要通过与细胞表面的 CD4 分子结合并进入细胞内部，然后利用细胞的生物合成系统复制自身的 RNA 和蛋白质，并组装成新的病毒颗粒。接下来，我们逐一分析每个选项：
A. 库普弗细胞：库普弗细胞是肝脏中的巨噬细胞，主要功能是清除血液中的有害物质。HIV 并不主要感染这些细胞，虽然巨噬细胞可以被 HIV 感染，但库普弗细胞作为特定类型的巨噬细胞，并非 HIV 的主要感染目标。
B. T 细胞：HIV 主要通过感染 CD4+ T 细胞来复制和传播。这些细胞在免疫系统中起着关键作用，是 HIV 的主要攻击目标。
C. 单核巨噬细胞：这些细胞也属于免疫系统的一部分，并且可以被 HIV 感染。虽然它们不是 HIV 的主要目标，但仍然可以被病毒利用进行复制。
D. B 淋巴细胞：虽然 B 淋巴细胞不是 HIV 的主要感染目标，但它们在某些情况下也可以被 HIV 感染。
E. 神经胶质细胞：HIV 在晚期可以侵入神经系统，感染神经胶质细胞和神经元，导致艾滋病痴呆综合征等神经系统疾病。
综上所述，HIV 并不主要感染库普弗细胞，尽管这些细胞在理论上可以被感染。因此，最符合题意的答案是 A.库普弗细胞。

HIV 主要感染的细胞不包括以下哪种细胞？我们知道，HIV（人类免疫缺陷病毒）主要攻击和破坏人体的免疫系统，尤其是攻击以下几种细胞：T 细胞（特别是 CD4+ T 细胞）、单核巨噬细胞和神经胶质细胞。库普弗细胞是肝脏中的巨噬细胞，也可能受到 HIV 的感染。然而，B 淋巴细胞并不是 HIV 的主要目标细胞。

因此，最正确的答案是：

D. B 淋巴细胞
45、 (A1 型题)
艾滋病晚期淋巴结病理变化有
A.淋巴滤泡增生
B.副皮质区增生
C.窦组织细胞增生
D.淋巴细胞消失殆尽
E.副皮质区变窄
参考答案： D 您的答案：
图文解析： 【解析】艾滋病患者淋巴组织的变化：（1）早期，淋巴结肿大；镜下淋巴滤

泡明显增生（生发中心活跃，可见“满天星”现象），髓质内较多浆细胞；电镜下可见 HIV 颗粒位于生发中心内，主要集中于滤泡树突状细胞，也可出现于巨噬细胞及 CD4+T 细胞内；（2）随着病变的发展，滤泡外层淋巴细胞减少或消失，小血管增生，生发中心被零落分割；副皮质区 CD4+T 细胞进行性减少，代之以浆细胞浸润；（3）晚期，淋巴结一片荒芜，淋巴细胞几乎消失殆尽，仅残留少许巨噬细胞和浆细胞。故本题选 D。
艾滋病晚期的淋巴结病理变化特征是淋巴细胞的消失。艾滋病是由人类免疫缺陷病毒（HIV）引起的，该病毒破坏人体的免疫系统，特别是破坏 CD4+ T 细胞。随着疾病的进展，淋巴细胞数量会显著减少，特别是在淋巴结中。
因此，最正确的答案是：
D. 淋巴细胞消失殆尽
解析：
艾滋病晚期淋巴结病理变化主要特点为：淋巴结呈现荒芜状态，淋巴细胞消失殆尽。这是因为艾滋病病毒侵犯 $CD4^+T$ 淋巴细胞，导致细胞免疫缺陷，从而继发机会性感染和恶性肿瘤，使淋巴结结构破坏，淋巴细胞大量减少。
选项 A 淋巴滤泡增生是滤泡性淋巴瘤的病理特点，与艾滋病无关，故不选。
选项 B 副皮质区增生，选项 E 副皮质区变窄，均可见于艾滋病早期，但在艾滋病晚期不会出现，可一并排除。
选项 C 窦组织细胞增生是 Rosai-Dorfman 病（RDD）的病理特点，而非艾滋病，故不考虑。
综上所述，正确答案是：D.淋巴细胞消失殆尽。

艾滋病（AIDS）是由人类免疫缺陷病毒（HIV）引起的慢性感染性疾病，其晚期常表现为免疫系统的严重破坏，导致多种机会性感染和肿瘤的发生。艾滋病晚期的淋巴结病理变化具有特征性的表现。

在艾滋病的早期和中期，淋巴结通常会出现淋巴滤泡增生和副皮质区增生。然而，在艾滋病的晚期，由于免疫系统的进一步衰竭，淋巴结的结构会发生显著改变。具体表现为淋巴结中的淋巴细胞显著减少甚至消失，导致淋巴组织的萎缩和消退。

因此，针对艾滋病晚期淋巴结病理变化，最正确的答案是：

D. 淋巴细胞消失殆尽
46、 (A1 型题)
乳腺纤维腺瘤的病理特征是
A.由增生的纤维间质和腺体组成，增生的腺体被纤维挤压呈裂隙状
B.癌细胞单行串珠状浸润于纤维质之间
C.痛性孤立肿块
D.乳腺纤维腺瘤好发于乳房内上象限，呈圆形或卵圆形
E.女性乳腺最常见的恶性肿瘤
参考答案： A 您的答案：
图文解析： 【解析】乳腺纤维腺瘤是女性最常见的乳腺良性肿瘤（E 错），由腺上皮和纤维组织两种成分混合组成，各年龄段均可发病，更常见于 15~35 岁女性。主要为乳房无痛性肿块（C 错），好发于乳房外上象限，临床触诊多为椭圆形、橡皮样肿块，边界清楚（D 错），活动度好。大体表现为质实、界限清楚的卵圆形结节，切面灰褐色、膨出、分叶状，常有肉眼可见的裂隙样腔隙（A 对）。

乳腺纤维腺瘤是一种常见的良性乳房肿瘤，其病理特征主要包括增生的纤维组织和腺体组织。这些增生的腺体被纤维组织挤压，通常呈现出裂隙状的结构。因此，针对这个问题，最正确的答案是：

A. 由增生的纤维间质和腺体组成，增生的腺体被纤维挤压呈裂隙状

解析：

乳腺纤维腺瘤是乳腺最常见的良性肿瘤，由上皮和间叶组织构成的良性肿瘤，其病理特征是由增生的纤维间质和腺体组成，增生的腺体被纤维挤压呈裂隙状。

选项 B 癌细胞单行串珠状浸润于纤维质之间为浸润性小叶癌的病理特点，与乳腺纤维腺瘤无关，故不选。

乳腺纤维腺瘤多数为无痛性肿块，仅 14%有轻度疼痛，因此选项 C 痛性孤立肿块不是乳腺纤维腺瘤的病理特征，故排除。

乳腺纤维腺瘤好发于乳房外上象限，故选项 D 乳腺纤维腺瘤好发于乳房内上象限，呈圆形或卵圆形，可排除。

选项 E 女性乳腺最常见的恶性肿瘤是浸润性导管癌，不是乳腺纤维腺瘤，故不选。

综上所述，正确答案是：A.由增生的纤维间质和腺体组成，增生的腺体被纤维挤压呈裂隙状。

46. 乳腺纤维腺瘤的病理特征是

A. 由增生的纤维间质和腺体组成，增生的腺体被纤维挤压呈裂隙状

答案: A

解释: 乳腺纤维腺瘤是由增生的纤维间质和腺体组成的良性肿瘤，其病理特征是腺体增生并被纤维组织挤压呈裂隙状排列。这种结构区别于其他病变，如乳腺癌的浸润性特点和痛性肿块。

47、 (A1 型题)

胰尾癌最常见的转移途径是

A.经淋巴转移至腹膜后淋巴结

B.经血行转移至腰椎

C.经门静脉转移至肝脏

D.经直接蔓延转移至腹膜

E.经血行转移至肝脏

参考答案： E 您的答案：

图文解析： 【解析】胰尾癌最常见转移部位为肝脏，胰腺血流通过门静脉回到肝脏。故本题选 E。

胰尾癌最常见的转移途径是经血行转移至肝脏。因此，最佳答案是 E.经血行转移至肝脏。

胰腺癌（包括胰尾部的癌症）通常通过血液循环传播，其中肝脏是最常见的远处转移部位。

解析：

胰腺癌的恶性程度高，容易发生转移，主要的转移途径包括淋巴转移、血行转移、直接浸润、沿神经周围转移等。胰腺癌淋巴转移发生较早，其转移途径通常是经过淋巴管，先转移至腹膜后，再转移至锁骨上淋巴结。胰尾癌最常见的转移途径是经淋巴转移至腹膜后淋巴结。

胰尾癌的淋巴转移是其主要转移途径，而血行转移相对较少见，因此选项 B“经血行转移至腰椎”，选项 E“经血行转移至肝脏”，可一并排除。

选项 C 经门静脉转移至肝脏常见于肝细胞癌，不是胰尾癌的转移途径，故不选。

胰腺癌晚期可直接侵犯邻近的胆总管下端、门静脉、下腔静脉及肠系膜上动静脉等，但胰尾癌晚期不常直接蔓延至腹膜，故选项 D 经直接蔓延转移至腹膜，不符合胰尾癌转移特点，故排除。
综上所述，正确答案是：A.经淋巴转移至腹膜后淋巴结。

胰尾癌（pancreatic tail cancer）最常见的转移途径是通过血行转移至肝脏（hepatic metastasis via hematogenous spread）。因此，最佳答案是：

C. 经门静脉转移至肝脏
48、 (A1 型题)
小叶性肺炎的主要渗出物是
A.纤维素
B.浆液
C.中性粒细胞
D.淋巴细胞
E.嗜酸性粒细胞
参考答案： C 您的答案：
图文解析： 【解析】小叶性肺炎以细支气管为中心的肺组织化脓性炎症为主，以肺组织中性粒细胞侵
入为主，C 对。
小叶性肺炎（也称为支气管肺炎）通常以支气管和肺泡中的渗出物为特征，这些渗出物主要由中性粒细胞组成。因此，对于问题 "小叶性肺炎的主要渗出物是什么?"，最合适的答案是：
C. 中性粒细胞
解析：
小叶性肺炎主要由化脓性细菌引起，以肺小叶为病变单位的灶状急性化脓性炎症。病变起始于支气管，并向其周围所属肺泡蔓延。小叶性肺炎多见于小儿和年老体弱者。临床上以发热、咳嗽、咳痰为主要症状，听诊肺部有湿性啰音，X 线胸片可见大小不等的灶状阴影。末梢血白细胞总数和中性粒细胞数增高。小叶性肺炎的病变特征是肺组织内散布一些以细支气管为中心的化脓性炎症病灶。严重者，病灶互相融合甚或累及全肺，形成肺炎链球菌性肺炎。镜下，病灶中主要渗出物是中性粒细胞。
选项 A 纤维素主要见于大叶性肺炎，与小叶性肺炎不符，故排除。
选项 B 浆液是浆液性炎的主要渗出物，小叶性肺炎为化脓性炎，故不选。
选项 D 淋巴细胞多见于慢性炎症和病毒性感染，非小叶性肺炎的主要渗出物，可排除。
选项 E 嗜酸性粒细胞多见于寄生虫感染及 IgE 介导的炎症反应，非小叶性肺炎的主要渗出物，与题意不符。
综上所述，正确答案是：C.中性粒细胞。

小叶性肺炎（又称支气管肺炎）主要是由细菌感染引起的，其特征是肺泡和支气管周围组织的局灶性炎症。其主要渗出物通常是中性粒细胞，这是由于中性粒细胞是身体在应对细菌感染时最主要的防御细胞之一。

因此，答案是：

C. 中性粒细胞

49、 (A1 型题)
能够向人 CD8+T 细胞提呈抗原肽的分子是
A.B7
B.CD1
C.HLA-2
D.HLA-1
E.CD4
参考答案： D 您的答案：
图文解析： 【解析】HLA 又称为 MHC，主要组织相容性复合物，是存在于抗原呈递细胞表面负责抗原呈递的蛋白分子。HLA-1 与抗原结合后可以被呈递至细胞表面并将其传递给 CD8+的 T 细胞，进而激活 CD8+细胞，故 D 对。
在人体中，CD8+ T 细胞识别和响应的抗原是通过主要组织相容性复合体（MHC）类 I 分子呈递的。HLA-1（人类白细胞抗原-1）是 MHC 类 I 分子的一种，在这种背景下，它负责将抗原肽呈递给 CD8+ T 细胞。因此，最佳答案是：

D.HLA-1
解析：
抗原肽是指由 MHC 分子提呈的抗原短肽，能够被 T 细胞受体所识别。MHC-Ⅰ类分子可与内源性抗原肽结合，提呈给 $CD8^+$细胞毒性 T 细胞，MHC-Ⅱ类分子可与外源性抗原肽结合，提呈给 $CD4^+$辅助性 T 细胞。其中，MHC-Ⅰ类分子在人类又被称为 HLA-Ⅰ类分子，包括 HLA-A、HLA-B 和 HLA-C。因此，能够向人 CD8+T 细胞提呈抗原肽的分子是 HLA-1。
选项 A：B7 为协同刺激分子，在自身免疫监控、体液免疫应答及移植反应中发挥重要作用，但不参与抗原肽的提呈，故不选。
选项 B：CD1 是跨膜糖蛋白，与 MHC-Ⅰ类分子结构相似，可提呈脂类抗原给 T 细胞，但不向 CD8+T 细胞提呈抗原肽，故排除。
选项 C：HLA-2 并不是 MHC 分子的分型，为干扰项，可优先排除。
选项 E：CD4 是 T 细胞表面的标志分子，可表达于部分 T 细胞、胸腺细胞和某些巨噬细胞表面，不参与抗原肽的提呈，与题意不符。
综上所述，正确答案是：D.HLA-1。

正确答案是 D. HLA-1。

解释：CD8+ T 细胞通过与主要组织相容性复合体 I 类分子 (MHC I，也称为 HLA-I) 结合来识别抗原肽。HLA-1 分子是 MHC I 类分子，能够向 CD8+ T 细胞提呈抗原肽，而 HLA-2（MHC II 类）则是向 CD4+ T 细胞提呈抗原肽的分子。因此，能够向人 CD8+ T 细胞提呈抗原肽的分子是 HLA-1。
50、 (A1 型题)
组胺的合成部位
A.肥大细胞
B.内皮细胞
C.红细胞
D.白细胞
E.巨噬细胞
参考答案： A 您的答案：

图文解析：　　【解析】组胺主要由肥大细胞合成，A 对。血管内皮的主要功能是屏障作用，同时血管内皮具有重要的内分泌功能，可合成和释放多种内皮衍生的血管活性因子（B 错）。巨噬细主要作用是胞吞噬碎片、损伤细胞、死亡细胞和凋亡细胞，抑制巨噬细胞产生促炎细胞因子，E 错。

【避错】

组胺是一种生物胺，主要在体内由肥大细胞和嗜碱细胞合成和储存。因此，针对题目中的"组胺的合成部位"，最正确的答案是：

A. 肥大细胞

解析：

组胺广泛的存在于人体组织中，具有强大的促炎作用，在外伤、炎症等刺激下，肥大细胞内的组胺酸脱羧酶会被激活，催化组胺酸脱羧，产生组胺。所以组胺的合成部位是肥大细胞。选项 B 内皮细胞，选项 C 红细胞，选项 D 白细胞，选项 E 巨噬细胞，均不产生组胺，可一并排除。

综上所述，正确答案是：A.肥大细胞。

组胺（Histamine）是一种重要的生物活性胺，在过敏反应和免疫反应中发挥重要作用。它主要由肥大细胞和嗜碱性粒细胞合成和储存。以下是对各个选项的分析：

A. **肥大细胞**：肥大细胞是合成和释放组胺的主要细胞类型，尤其是在过敏反应和炎症过程中。

B. 内皮细胞：内皮细胞主要功能是构成血管内壁，不是组胺的主要合成部位。

C. 红细胞：红细胞的主要功能是运输氧气和二氧化碳，不合成组胺。

D. 白细胞：白细胞包括多种类型细胞，如中性粒细胞、淋巴细胞等，虽然在免疫反应中起作用，但不是组胺的主要合成部位。

E. 巨噬细胞：巨噬细胞主要功能是吞噬和清除体内的病原体和细胞残骸，也不是组胺的主要合成部位。

综上所述，组胺的主要合成部位是肥大细胞。因此，最正确的答案是：

A. 肥大细胞

51、　(A1 型题)

属于中枢免疫器官的是

A.骨髓

B.阑尾

C.淋巴结

D.脾脏

E.扁桃体

参考答案：　　A　您的答案：

图文解析：　　【解析】中枢免疫器官：骨髓和胸腺。外周免疫器官：脾脏、淋巴结、扁桃体、小肠集合

淋巴结、阑尾等。故本题 A 对。
【避错】
中枢免疫器官是指那些负责生成和/或成熟免疫细胞的器官。根据这个定义，骨髓属于中枢免疫器官，因为它是所有类型的血细胞，包括免疫细胞（如 B 细胞和 T 细胞）的生成地。因此，正确答案是：
A. 骨髓
这是一道关于免疫系统组成的选择题，需要我们准确区分中枢免疫器官和外周免疫器官。
首先，我们梳理一下题目中的关键信息：题目要求识别出属于中枢免疫器官的选项。
接下来，我们分析每个选项：
A. 骨髓：骨髓是各类血细胞和免疫细胞发生及成熟的场所，是机体重要的中枢免疫器官。它可以产生各种免疫细胞，并将其输送到外周免疫器官，因此符合题目要求。
B. 阑尾：阑尾是淋巴组织丰富的管状器官，但它主要作为外周免疫器官，与中枢免疫器官不同，因此排除。
C. 淋巴结：淋巴结是重要的外周免疫器官，过滤淋巴液并参与免疫反应，但不属于中枢免疫器官，故不选。
D. 脾脏：脾脏同样是外周免疫器官，主要功能是过滤血液并移除病原体，不符合中枢免疫器官的定义，故排除。
E. 扁桃体：扁桃体也属于外周免疫器官，是咽喉部位的重要淋巴组织，但不属于中枢免疫器官，故不选。
综上所述，骨髓是中枢免疫器官，负责产生和输出免疫细胞。因此，正确答案是 A.骨髓。

属于中枢免疫器官的是：

A. 骨髓

解释：
中枢免疫器官是指产生和发育免疫细胞的器官，主要包括骨髓和胸腺。骨髓是血液和免疫细胞生成的主要场所，因此属于中枢免疫器官。阑尾、淋巴结、脾脏和扁桃体都属于外周免疫器官，主要功能是执行免疫反应。
52、 (A1 型题)
属于人和动物共患的最常见病毒的形态是
A.球形或类球形
B.砖形
C.线条状
D.蝌蚪形
E.弹状
参考答案： A 您的答案：
图文解析： 【解析】病毒的基本形态为球状（腺病毒）、杆状（烟草花叶病毒)、蝌蚪状。也有子弹状、丝状、砖状等形态。最常见的由病毒引起的人畜共患病是流行性乙型脑炎，乙脑病毒属于球状病毒，A 对。
最常见的人和动物共患的病毒通常是球形或类球形的，因此正确答案是 A.球形或类球形。这种形态的病毒包括许多呼吸道病毒和一些肠道病毒，这些病毒可以在人和动物之间传播。
解析：
病毒颗粒的形态多种多样，常见形态有球形、杆状、蝌蚪状、砖状、丝状等。其中，球形或

类球形是病毒体最常见的形态，人和动物共患的最常见病毒如流感病毒、狂犬病毒等均为球形或类球形。
选项 B 砖形病毒，如痘病毒，主要感染动物，感染人类的病例较少，故不选。
选项 C 线条状病毒，如烟草花叶病毒，仅感染植物，不感染动物和人类，故排除。
选项 D 蝌蚪形病毒，如噬菌体，主要感染细菌，与人类和动物的病毒感染关系不大，故不选。
选项 E 弹状病毒，如狂犬病毒、麻疹病毒，为球形或类球形，而不是弹状，故排除。
综上所述，正确答案是：A.球形或类球形。

人和动物共患的最常见病毒的形态是：

A. 球形或类球形

这种形态的病毒包括许多常见的动物传染给人的病毒，如流感病毒、冠状病毒等。因此，答案是：

A. 球形或类球形
53、 (A1 型题)
不属于鸟型分枝杆菌的特点
A.细胞壁有磷壁酸
B.机会性致病菌
C.药物治疗效果差
D.生长速度较快
E.抗酸染色阳性
参考答案： A 您的答案：
图文解析： 【解析】鸟型分枝杆菌是最常见的机会性病原菌，与非结核性分枝杆菌因两种病原菌表型极相似，生长速度快，且药物治疗效果差，抗酸染色阳性，因此早期很难与结核病区分，BCDE 均为鸟型分枝杆菌的特点。该菌细菌壁既没有革兰阳性菌的磷壁酸，也没有革兰阴性菌的脂多糖。本题选 A。
鸟型分枝杆菌（Mycobacterium avium complex, MAC）的特点主要包括：
A. 细胞壁有磷壁酸：这是分枝杆菌属的一个共有特征，细胞壁中含有磷壁酸，有助于细菌抵抗抗生素等药物的侵袭。
B. 机会性致病菌：MAC 是一种机会性致病菌，通常在免疫力低下的个体中引发疾病。
C. 药物治疗效果差：由于细胞壁结构的特殊性，使得对 MAC 的药物治疗效果不佳，治疗过程较为复杂和长期。
D. 生长速度较快：这个选项是不正确的。实际上，分枝杆菌的生长速度通常较慢，尤其是 MAC，它们的生长和繁殖速度慢于许多其他细菌。
E. 抗酸染色阳性：分枝杆菌，包括 MAC，是抗酸染色阳性的，这意味着它们能够在抗酸染色过程中保持染料，这是诊断这类细菌感染的一个重要特征。
因此，最不属于鸟型分枝杆菌特点的选项是 **D. 生长速度较快**。
解析：
鸟型分枝杆菌属于非结核分枝杆菌的一种，非结核分枝杆菌的细胞壁中既无磷壁酸，也无大量脂质，因此与结核分枝杆菌的毒力和致病性有所不同。
选项 B 机会性致病菌是指有些微生物在通常情况下不致病，只在特定条件下致病的微生物，

鸟型分枝杆菌属于机会性致病菌，可排除。
鸟型分枝杆菌感染后，目前尚无特异高效的抗菌药物，因此治疗效果较差，故选项 C 药物治疗效果差属于鸟型分枝杆菌的特点，与题意不符。
鸟型分枝杆菌的生长速度比其他非结核分枝杆菌快，因此选项 D 生长速度较快是鸟型分枝杆菌的特点，可排除。
选项 E 抗酸染色阳性是分枝杆菌的特点，故不考虑。
综上所述，正确答案是：A.细胞壁有磷壁酸。

题目：不属于鸟型分枝杆菌的特点

A. 细胞壁有磷壁酸
B. 机会性致病菌
C. 药物治疗效果差
D. 生长速度较快
E. 抗酸染色阳性

鸟型分枝杆菌（Mycobacterium avium complex，MAC）是一种机会性致病菌，常见于免疫功能低下的患者，如艾滋病患者。它们的细胞壁含有大量脂质，抗酸染色阳性，且药物治疗效果一般较差。然而，它们的生长速度较慢，这与其他一些快速生长的分枝杆菌不同。因此，选项 D“生长速度较快”是不正确的。

正确答案是：D. 生长速度较快
【避错】
54、 (A1 型题)
卵巢性索间质细胞最常分泌的激素是
A.孕激素
B.雌激素
C.HCG
D.LH
E.FSH
参考答案： B 您的答案：
图文解析： 【解析】卵巢性索间质细胞最常分泌雌激素。孕激素是由卵巢黄体细胞分泌的一种类固醇激素。HCG 是由胎盘的滋养层细胞分泌的一种糖蛋白。黄体生成素（LH）是由垂体产生的一种激素。促卵泡激素（FSH）是由人脑垂体前叶嗜碱性细胞合成与分泌的一种糖蛋白类促性腺激素。故本题选 B。
卵巢性索间质细胞最常分泌的激素是雌激素。因此，正确答案是 B.雌激素。卵巢由多种细胞类型组成，包括性索间质细胞，这些细胞主要负责合成和分泌雌激素，尤其是雌二醇。雌激素在女性的生殖系统和其他多个生理过程中发挥重要作用。
解析：
卵巢性索间质细胞是卵巢肿瘤的一种，起源于原始性腺中的性索和间质组织，分别在男性和女性衍化成各自不同类型细胞，并形成各自不同的肿瘤。在女性中，卵巢性索间质细胞最常分泌的激素是雌激素。
选项 A 孕激素主要由黄体细胞分泌，而不是卵巢性索间质细胞，故不选。
选项 C：HCG，选项 D：LH，选项 E：FSH，均是由腺垂体分泌的，而不是卵巢性索间质细胞

分泌，故可一并排除。
综上所述，正确答案是：B.雌激素。

卵巢性索间质细胞主要包括卵泡膜细胞和颗粒细胞，它们在卵巢中发挥重要的内分泌功能。卵巢性索间质细胞最常分泌的激素是雌激素。

因此，最正确的答案是：

B. 雌激素
55、 (A1 型题)
FSH 作用于睾丸的细胞是
A.精原细胞
B.支持细胞
C.间质细胞
D.生精细胞
E.精母细胞
参考答案： B 您的答案：
图文解析： 【解析】FSH 作用于支持细胞，与其特异性受体结合后，使支持细胞产生雄激素结合蛋白，后者可与睾酮结合，将其转运至生精小管内以维持管腔内的睾酮浓度，促进各级生精上皮细胞的生长发育。故本题 B 对。
FSH（促卵泡激素）在男性体内主要作用于睾丸的支持细胞（也称为 Sertoli 细胞），它们负责维持精子生成的适宜环境。因此，最正确的答案是：
B. 支持细胞
解析：
卵泡刺激素（FSH）是一种由脑垂体分泌的促性腺激素，主要作用于睾丸的支持细胞，即 Sertoli 细胞，刺激其合成和分泌雄激素结合蛋白，促进精子的发生和成熟。因此 FSH 作用于睾丸的细胞是支持细胞。
选项 A 精原细胞是精子的原始细胞，是形成精子的干细胞，其发育受促性腺激素释放激素（GnRH）的影响，FSH 并不直接作用于此类细胞，故排除。
选项 C 间质细胞，又称 Leydig 细胞，能合成和分泌雄激素，并受黄体生成素（LH）的影响，而非 FSH，故不选。
选项 D 生精细胞包括精原细胞、初级精母细胞、次级精母细胞、精子细胞和精子，是精子发生的不同阶段，其发育成熟受多种激素的共同作用，FSH 并不直接作用于此类细胞，故不考虑。
选项 E 精母细胞是生精细胞发育的一个阶段，同样受多种激素的共同作用，FSH 并不直接作用于此类细胞，可排除。
综上所述，正确答案是：B.支持细胞。

FSH（促卵泡激素）在男性生殖系统中的主要作用是刺激睾丸中的支持细胞（Sertoli 细胞），促进精子发生和睾丸功能。因此，FSH 作用于睾丸的细胞是支持细胞。

正确答案是：B. 支持细胞
56、 (A1 型题)
小肠特有运动形式是

A.紧张性收缩
B.分节运动
C.集团运动
D.节段运动
E.蠕动
参考答案： B 您的答案：
图文解析： 【解析】小肠的运动方式主要有紧张性收缩、分节运动和蠕动。其中分节运动是小肠所特有的运动形式（B 对）。分节运动是一种以肠壁环行肌为主的节律性收缩和舒张运动，可将食物反复分成许多节段，其意义是：（1）使食糜与消化液充分混合，便于化学性消化；（2）使食糜与肠壁密切接触，为吸收创造良好条件；（3）挤压肠壁，有利于血液和淋巴液的回流。
小肠特有的运动形式是 B. 分节运动。分节运动是小肠的一种运动方式，其目的是通过肌肉的收缩和放松将食物混合和推进，以促进消化吸收。这种运动能够将肠内容物分段并进行搅动，有助于食物与消化液的充分接触。因此，正确答案是 B. 分节运动。
这是一道考察小肠运动形式的医学问题。首先，我们需要了解小肠的运动特点，并从提供的选项中选出小肠的特有运动形式。
首先，我们分析各选项代表的运动形式：
A. 紧张性收缩：这是一种普遍存在于消化道平滑肌的基础性收缩，不是小肠特有的。
B. 分节运动：这是小肠的特有运动形式，它以环行肌为主的节律性收缩和舒张运动，使小肠内容物不断分节推动，有利于食糜与消化液充分混合，并促进肠壁血液淋巴回流。
C. 集团运动：这是大肠的一种推进性运动，也不是小肠特有的。
D. 节段运动：这种运动形式并不特指小肠，且不是其特有运动。
E. 蠕动：这是消化道共有的运动形式，通过蠕动使内容物向肛门方向推进，不是小肠特有的。
综上所述，小肠的特有运动形式是分节运动，它有助于食糜与消化液的充分混合，同时促进小肠壁的血液和淋巴回流。
因此，答案是 B. 分节运动。

B
57、 (A1 型题)
不具有内分泌功能的细胞是
A.胰腺导管细胞
B.肾上腺髓质细胞
C.甲状腺细胞
D.甲状旁腺细胞
E.胃黏膜 G 细胞
参考答案： A 您的答案：
图文解析： 【解析】肾上腺髓质细胞主要分泌肾上腺素和去甲肾上腺素，甲状腺内的滤泡细胞产生两种主要的甲状腺激素，四碘甲状腺原氨酸（甲状腺素,T4）和三碘甲状腺原氨酸（T3）。甲状旁腺主要的作用是合成和分泌甲状旁腺激素。胃黏膜 G 细胞主要分泌胃泌素。综上，
本题选 A。
不具有内分泌功能的细胞是 A.胰腺导管细胞。

胰腺由两部分组成：外分泌部分和内分泌部分。胰腺导管细胞属于外分泌部分，它们的功能是运输消化酶到小肠。这些细胞本身不具有内分泌功能，也就是说，它们不会将激素分泌到血液中。相反，内分泌部分的细胞（如胰岛细胞）负责生产和分泌激素，如胰岛素和胰高血糖素。

其他选项中的细胞都具有内分泌功能：

- B. 肾上腺髓质细胞分泌肾上腺素和去甲肾上腺素。
- C. 甲状腺细胞分泌甲状腺激素。
- D. 甲状旁腺细胞分泌甲状旁腺激素。
- E. 胃黏膜 G 细胞分泌胃泌素。

首先，我们需要明确什么是内分泌功能。内分泌是指体内的一些细胞或组织能够分泌激素，通过血液传递来调节身体的各种生理活动。接下来，我们分析各个选项：

A. 胰腺导管细胞：胰腺导管细胞主要是外分泌细胞，它们负责将胰液输送到小肠以帮助消化。这些细胞并不具有内分泌功能。

B. 肾上腺髓质细胞：这些细胞能够分泌肾上腺素和去甲肾上腺素，这两种激素在调节心跳、血压和应激反应中起着关键作用。因此，这些细胞具有内分泌功能。

C. 甲状腺细胞：甲状腺细胞能够分泌甲状腺激素，如 T3 和 T4，这些激素对身体的代谢率、蛋白质合成和其他多种生理功能有重要影响。所以，这些细胞也具有内分泌功能。

D. 甲状旁腺细胞：甲状旁腺细胞能够分泌甲状旁腺激素，这种激素在调节血钙和血磷水平中起着重要作用。这显示了这些细胞的内分泌功能。

E. 胃黏膜 G 细胞：胃黏膜中的 G 细胞能够分泌胃泌素，这是一种刺激胃酸分泌的激素。因此，这些细胞也具有内分泌功能。

综上所述，不具有内分泌功能的细胞是胰腺导管细胞，因为它们主要是外分泌细胞，负责胰液的输送，而不是激素的分泌。所以，正确答案是 A.胰腺导管细胞。

不具有内分泌功能的细胞是：

A. 胰腺导管细胞

解释：胰腺导管细胞主要负责分泌消化酶进入小肠，而不是分泌激素，因此它们不具有内分泌功能。其余选项中的细胞都具有内分泌功能：肾上腺髓质细胞分泌肾上腺素和去甲肾上腺素，甲状腺细胞分泌甲状腺激素，甲状旁腺细胞分泌甲状旁腺激素，胃黏膜 G 细胞分泌胃泌素。

58、 (A1 型题)

解剖性分流最可能见于

A.阻塞性肺气肿

B.慢性支气管炎

C.支气管哮喘

D.支气管扩张

E.肺栓塞

参考答案： D 您的答案：

图文解析： 【解析】肺泡换气功能障碍包括弥散障碍、肺泡通气与血流比例失调、解剖分流。生理情况下，肺内有一部分静脉血经支气管静脉和极少的肺内动-静脉交通支直接流入肺动脉，称为解剖分流。病理情况(如支气管扩张等)下，支气管血管扩张和肺内动-静脉短路开放，使解剖分流增加，静脉血掺杂异常增多，而导致呼吸衰竭。解剖分流的血液完全未

经气体交换过程，故称为真性分流。在肺实变和肺不张时，病变肺泡完全失去通气功能，但仍有血流，流经的血液完全未进行气体交换而进入动脉，属于功能性分流。综上，本题选D。

解剖性分流是指血液从心脏的右侧直接流向左侧而绕过肺部气体交换的过程。这种情况通常发生在存在某些心脏或肺部结构异常的情况下。考虑到选项中的疾病，肺栓塞（E）最有可能导致解剖性分流。肺栓塞可以导致血流绕过阻塞的区域，造成未经过肺部气体交换的血液直接进入全身循环。

因此，正确答案是 E.肺栓塞。

解析：

解剖分流指肺动脉内的静脉血未经氧合直接流入肺静脉，导致血氧分压降低，是通气/血流比例失调的特例。常见于肺动-静脉瘘，即肺动脉内的静脉血未经氧合直接流入肺静脉，导致血氧分压降低，是一种先天性心脏病。肺栓塞时，栓子阻塞肺动脉及其分支引起血流阻力增加，形成肺动脉高压，同时造成右心负荷增大，甚至右心衰竭，部分血液未经氧合直接流入肺静脉，形成解剖性分流，造成低氧血症。

选项 A 阻塞性肺气肿，选项 B 慢性支气管炎，选项 C 支气管哮喘，均存在持续性气流受限，随着病情的发展，肺组织弹性日益减退，肺泡持续扩大，残气量增多，引起肺通气功能障碍，此时部分肺泡内的血流无气体交换，形成了功能性分流，不属于解剖性分流，故一并排除。

选项 D 支气管扩张是由于支气管及其周围肺组织慢性化脓性炎症和纤维化，使支气管壁的肌肉和弹性组织破坏，导致支气管变形及持久扩张。典型的症状有慢性咳嗽、咳大量脓痰和反复咯血，不出现解剖性分流，故不选。

综上所述，正确答案是：E.肺栓塞。

解剖性分流（anatomic shunt）是指肺部血液流动中未经过肺泡进行气体交换的那部分血流。这种情况通常见于某些特定的肺部病理状态。让我们逐一分析选项：

A. **阻塞性肺气肿**：阻塞性肺气肿主要是由于肺泡壁的破坏和气道阻塞引起的，这会导致气体交换效率降低，但不一定产生解剖性分流。

B. **慢性支气管炎**：慢性支气管炎主要表现为气道的炎症和分泌物增加，也不太可能引起解剖性分流。

C. **支气管哮喘**：支气管哮喘是由于气道的可逆性阻塞引起的喘息和呼吸困难，同样不涉及解剖性分流。

D. **支气管扩张**：支气管扩张是由于气道的永久性扩张和炎症引起的，虽然会导致肺部感染和分泌物滞留，但不一定会形成解剖性分流。

E. **肺栓塞**：肺栓塞是指肺动脉或其分支被栓子阻塞，导致部分肺组织失去血供。由于血流被阻断，受阻区域的血液不会参与气体交换，这可以导致解剖性分流。

因此，解剖性分流最可能见于 **E. 肺栓塞**。

59、 (A1 型题)

心脏电活动中传导速度最快的是

A.窦房结细胞

B.心房肌细胞

C.心室肌细胞

D.房室结

E.浦肯野纤维

参考答案： E 您的答案：

图文解析： 【解析】浦肯野细胞兴奋时产生的动作电位是快反应动作电位,0 期去极化由

Na+内流引起，由于浦肯野细胞细胞膜上 INa 通道密度高，所以 0 期去极化速率快，所以该细胞传导速率最快。故本题选 E。
心脏电活动中传导速度最快的是**E.浦肯野纤维**。
浦肯野纤维（Purkinje fibers）是心脏内部的一部分，它们负责将电脉冲高速传导至心室肌细胞，使心室快速且协调地收缩。浦肯野纤维的传导速度非常快，这有助于心室能够有效地泵血至全身。因此，在给出的选项中，浦肯野纤维是传导速度最快的。
首先，我们需要理解心脏电活动中不同部分的传导速度。心脏电活动的传导速度主要取决于心肌细胞的类型和特性。
现在我们逐项分析：
A. 窦房结细胞：这是心脏电活动的起搏点，但其传导速度并不是最快的。窦房结细胞主要负责产生心脏的电信号，而不是快速传导。
B. 心房肌细胞：虽然心房肌细胞能够传导电信号，但其传导速度也不是最快的。心房肌细胞主要负责心房的收缩。
C. 心室肌细胞：心室肌细胞负责心室的收缩，虽然其也具有一定的电信号传导能力，但并非最快。
D. 房室结：房室结在心脏电信号传导中起到关键作用，但其传导速度同样不是最快的。
E. 浦肯野纤维：浦肯野纤维是心脏传导系统中传导速度最快的部分。这些特殊的心肌细胞能够迅速将电信号从心房传到心室，确保心脏的同步收缩。
综上所述，心脏电活动中传导速度最快的是浦肯野纤维。
因此，正确答案是 E.浦肯野纤维。

心脏电活动中传导速度最快的是

E. 浦肯野纤维

浦肯野纤维是心脏传导系统的一部分，它们位于心室内，负责快速传导电信号，以确保心室肌肉能够协调一致地收缩。浦肯野纤维的传导速度显著高于其他心脏结构，因此是心脏电活动中传导速度最快的部分。
60、 (A1 型题)
在活体的心脏或者血管内，血液发生凝固或血液中某些有形成分相互聚集形成的固体质块是
A.栓子
B.血栓
C.栓塞
D.淤血
E.凝血
参考答案： B 您的答案：
图文解析： 【解析】在活体的心脏和血管内血液发生凝固或血液中某些有形成分凝集形成固体质块的过程，称为血栓形成，所形成的固体质块称为血栓。故本题选 B。
在活体的心脏或者血管内，血液发生凝固或血液中某些有形成分相互聚集形成的固体质块被称为血栓。因此，正确的答案是 B.血栓。
- **A. 栓子**：通常指脱落并通过血流迁移的物质，可以是气体、液体或固体，它们可以在体内的其他位置导致栓塞。
- **B. 血栓**：血液在血管内部发生局部凝固形成的固体质块，它通常附着在血管壁上。
- **C. 栓塞**：是由栓子引起的血管阻塞现象，栓子可能是血栓，但也可能是其他物质。

- **D. 淤血**：指血液流动减慢或停滞，导致血液积聚在某部位的现象。
- **E. 凝血**：是指血液由液态转变为凝固状态的过程，是止血的一部分。
解析：
血液在心脏或血管内发生凝固，或血液中的有形成分互相粘集，形成固体质块的过程称为血栓形成，所形成的固体质块称为血栓。
选项 A 栓子是指在循环血液中阻塞血管的异常物质，可以是固体、液体或气体，与题干描述不符，故不选。
选项 C 栓塞是指不溶于血液的异常物质出现于循环血液中，并随血液流动，进而阻塞血管管腔的现象，与题干描述差异较大。
选项 D 淤血是指因静脉血液回流受阻，血液淤积于小静脉和毛细血管内，与题干描述差异明显。
选项 E 凝血是生理性止血的重要环节，是血液由流动的液体状态变成不能流动的凝胶状态的过程，是生理性止血的一项，而题干描述的是病理性过程，故排除。
综上所述，正确答案是：B.血栓。

在活体的心脏或者血管内，血液发生凝固或血液中某些有形成分相互聚集形成的固体质块称为血栓。

因此，最正确的答案是：

B. 血栓
61、 (A1 型题)
属于脂溶性维生素的是
A.维生素 PP
B.维生素 B
C.维生素 C
D.维生素 A
E.维生素 B12
参考答案： D 您的答案：
图文解析： 【解析】脂溶性维生素是指不溶于水而溶于脂肪及非极性有机溶剂(如苯、乙醚及氯仿等)的一类维生素，包括维生素 A、维生素 D、维生素 E、维生素 K 等。故本题选 A。水溶性维生素溶于水，进入体内后，会被小肠吸收，运到身体所需的部位，剩下部分会随着排泄物排出，主要是 B 族维生素和维生素 C。
脂溶性维生素包括维生素 A、D、E 和 K。根据这些信息，属于脂溶性维生素的是 D 选项，维生素 A。所以正确答案是：
D.维生素 A
这是一道关于维生素分类的问题。我们需要识别哪种维生素是脂溶性的。维生素按其溶解性可分为两大类：脂溶性维生素和水溶性维生素。

接下来，我们逐一分析每个选项：
A. 维生素 PP（烟酸、烟酰胺）：这是一种水溶性维生素，也称为维生素 B3，与脂溶性不符。
B. 维生素 B：维生素 B 族中的大部分成员，如维生素 B1、B2、B6 等，都是水溶性的。
C. 维生素 C：维生素 C 是一种典型的水溶性维生素，具有抗氧化作用。
D. 维生素 A：维生素 A 是一种脂溶性维生素，它在体内起着重要的视觉功能和细胞生长调

节作用。
E. 维生素 B12：尽管维生素 B12 在结构上含有脂溶性部分（即含有烷基链），但它通常被分类为水溶性维生素，因为它在水溶液中具有较高的溶解度。
综上所述，属于脂溶性维生素的是维生素 A。因此，正确答案是 D.维生素 A。

属于脂溶性维生素的是：

D. 维生素 A
62、 (A1 型题)
在基因工程中，质粒的作用常是
A.供体
B.目的基因的载体
C.作为目的基因
D.受体
E.工具酶
参考答案： B 您的答案：
图文解析： 【解析】质粒是细菌中常见的小型环状 DNA，区分于拟核部分的大型环状 DNA，其中有基因可以表达。质粒在基因工程中常用作载体携带目的基因，B 对。
在基因工程中，质粒最常见的作用是作为目的基因的载体，因为它们可以携带外来的 DNA 进入宿主细胞。因此，最正确的答案是 B.目的基因的载体。
首先，我们来理解题目中的关键概念。本题询问的是在基因工程中质粒的作用。质粒是细菌中的小型环状 DNA 分子，它们在基因工程中常被用作载体，因为它们可以容易地接受并携带外源 DNA。
接下来，我们逐一分析选项：
A. 供体：在基因工程中，供体通常指的是提供目的基因的原始生物或 DNA 片段，而质粒不是供体，它是一个用于携带这些基因的载体。因此，A 选项不正确。
B. 目的基因的载体：质粒在基因工程中常被用作携带目的基因的载体。它们可以被改造以包含特定的目的基因，并随后被导入到受体细胞中。这是质粒在基因工程中的主要作用。因此，B 选项是正确的。
C. 作为目的基因：质粒本身并不作为目的基因。它们是用来携带和转移目的基因的工具，而不是目的基因本身。因此，C 选项不正确。
D. 受体：在基因工程中，受体通常是指接受外源 DNA 的细胞或生物体，而不是质粒。质粒是携带基因的载体，而不是接受基因的受体。因此，D 选项不正确。
E. 工具酶：工具酶通常指的是在基因工程中用于切割、连接或修饰 DNA 的酶，如限制性内切酶、DNA 连接酶等。质粒不是酶，而是 DNA 的载体。因此，E 选项不正确。
综上所述，质粒在基因工程中的主要作用是作为目的基因的载体，用于携带和转移基因到受体细胞中。因此，正确答案是 B.目的基因的载体。

在基因工程中，质粒的作用常是

B. 目的基因的载体

质粒是一种常用于基因工程的环状 DNA 分子，可以携带外源基因并将其引入宿主细胞。质粒作为载体，可以帮助目的基因在宿主细胞中复制和表达，因此选项 B 是正确答案。

63、　(A1 型题)
痛风相关的代谢途径是
A.嘌呤核苷酸代谢
B.磷酸戊糖代谢
C.氨基酸代谢
D.嘧啶核苷酸代谢
E.甘油三酯代谢
参考答案：　A　您的答案：
图文解析：　【解析】痛风是一种由于嘌呤生物合成代谢增加，尿酸产生过多或因尿酸排泄不良而致血中尿酸升高，尿酸盐结晶沉积在关节滑膜、滑囊、软骨及其他组织中引起的反复发作性炎性疾病。因此本题选 A。磷酸戊糖途径是葡萄糖氧化分解的一种方式。蛋白质分解代谢首先在酶的催化下水解为氨基酸，而后各氨基酸进行分解代谢，或转变为其他物质、或参与新的蛋白质的合成。因此氨基酸代谢是蛋白质分解代谢的中心内容。嘧啶核苷酸的分解代谢是先去除磷酸和核糖生成嘧啶碱，嘧啶碱在肝内降解，降解产物易溶于水。
痛风是由体内尿酸水平升高引起的一种疾病，其根本原因在于嘌呤的代谢异常。因此，与痛风相关的代谢途径是嘌呤核苷酸代谢。所以正确答案是：
A. 嘌呤核苷酸代谢
解析：
痛风是由单钠尿酸盐沉积所致的晶体相关性关节病，与嘌呤核苷酸代谢紊乱和（或）尿酸排泄减少所致的高尿酸血症直接相关，特指急性特征性关节炎和慢性痛风石疾病，主要包括急性发作性关节炎、痛风石形成、痛风石性慢性关节炎、尿酸盐肾病和尿酸性尿路结石，重者可出现关节残疾和肾功能不全。痛风常伴腹型肥胖、高脂血症、高血压、2 型糖尿病及心血管病等表现。
选项 B 磷酸戊糖代谢，选项 C 氨基酸代谢，选项 D 嘧啶核苷酸代谢，选项 E 甘油三酯代谢，均与痛风的发生无明显关联，可一并排除。
综上所述，正确答案是：A.嘌呤核苷酸代谢。

痛风是一种由尿酸盐结晶沉积在关节和组织中引起的疾病。尿酸是嘌呤代谢的终产物，因此痛风的相关代谢途径是嘌呤核苷酸代谢。

正确答案是：

A. 嘌呤核苷酸代谢
64、　(A1 型题)
下列属于人体必需氨基酸的是
A.甘氨酸
B.苯丙氨酸
C.精氨酸
D.丝氨酸
E.脯氨酸
参考答案：　B　您的答案：
图文解析：　【解析】人体必需氨基酸有以下 9 种：赖氨酸、色氨酸、苯丙氨酸、甲硫氨酸、苏氨酸、异亮氨酸、亮氨酸、缬氨酸、组氨酸。故本题选 B。
在人体必需氨基酸中，体内不能合成，必须通过饮食摄取。根据这个定义，选项中的必需氨

基酸是：
B.苯丙氨酸
因此，正确答案是 B.苯丙氨酸。其他氨基酸如甘氨酸、精氨酸、丝氨酸和脯氨酸，人体可以自行合成，因此它们不被归类为必需氨基酸。
这是一道生物化学问题，我们需要从给定的选项中识别出哪一种是人体必需氨基酸。
首先，我们需要明确什么是必需氨基酸。必需氨基酸是指人体不能自身合成或合成速度不能满足人体需要，必须从食物中摄取的氨基酸。
现在我们来分析每个选项：
A. 甘氨酸：甘氨酸是一种非必需氨基酸，人体可以自行合成，因此不符合题目要求。
B. 苯丙氨酸：苯丙氨酸是人体不能合成的，必须从食物中获取的必需氨基酸之一。这符合题目要求。
C. 精氨酸：虽然精氨酸在某些情况下被认为是半必需氨基酸，但在成人中，它通常被认为是非必需的，因为人体能够合成足够的量。
D. 丝氨酸：丝氨酸是一种非必需氨基酸，人体可以自行合成。
E. 脯氨酸：脯氨酸也是一种非必需氨基酸，人体可以自行合成。
综上所述，只有选项 B.苯丙氨酸是人体不能自身合成，必须从食物中获取的必需氨基酸。
因此，正确答案是 B.苯丙氨酸。
下列属于人体必需氨基酸的是：

B. 苯丙氨酸
65、 (A1 型题)
酮症酸中毒时血液中浓度增加的分子是
A.甘油三酯
B.乳酸
C.丙酮酸
D.尿酸
E.乙酰乙酸
参考答案： E 您的答案：
图文解析： 【解析】糖尿病患者在各种诱因下，由于严重的胰岛素缺乏，与胰岛素作用相反的激素如胰高血糖素、儿茶酚胺、生长激素、肾上腺皮质激素对代谢的影响变得更加显著，使脂肪分解加速。脂肪酸在肝脏内经β-氧化产生大量酮体(包括乙酰乙酸、β-羟丁酸和丙酮)，当酮体生成大于组织利用和肾脏排泄时，可使血酮体和尿酮体升高，称为酮症。乙酰乙酸、β-羟丁酸消耗体内大量碱储备，容易导致代谢性酸中毒，故称酮症酸中毒。综上，本题选 E。
酮症酸中毒时血液中浓度增加的分子是 E.乙酰乙酸。酮症酸中毒是一种由于体内糖原耗尽，脂肪分解加快而导致的酮体（主要是乙酰乙酸和β-羟基丁酸）在血液中积聚的病理状态。因此，乙酰乙酸的浓度在这种情况下会增加。
解析：
酮体主要包括乙酰乙酸、β-羟丁酸和丙酮。酮症酸中毒时机体内胰岛素绝对或相对不足，肝糖原异生增加，糖酵解增强，导致脂肪酸β-氧化增强，从而生成大量乙酰 CoA，乙酰 CoA 堆积促进了酮体的生成，导致酮体血症和酮尿症，同时由于肝外组织利用酮体的能力降低，导致血中乙酰乙酸浓度升高，病情进一步恶化，便发生酮症酸中毒。
选项 A 甘油三酯在酮症酸中毒时一般无明显变化，故不选。
选项 B 乳酸在机体缺氧或剧烈运动时，由于无氧糖酵解增强，会导致乳酸浓度升高，与酮症

酸中毒无关，故排除。
选项 C 丙酮酸为糖酵解的中间产物，在正常情况下丙酮酸可转化为乙酰 CoA 进入三羧酸循环，在酮症酸中毒时无明显变化，故不考虑。
选项 D 尿酸为嘌呤代谢的终产物，体内尿酸生成过多和（或）排泄过少时，可导致高尿酸血症，与酮症酸中毒无关，故不选。
综上所述，正确答案是：E.乙酰乙酸。

在酮症酸中毒（Diabetic Ketoacidosis, DKA）时，身体无法使用葡萄糖作为主要的能量来源，转而通过脂肪分解产生能量。脂肪分解产生的脂肪酸在肝脏中被转化为酮体，其中包括乙酰乙酸、β-羟丁酸和丙酮。这些酮体的生成和积累会导致血液中的酮体浓度升高，从而引起酮症酸中毒。

因此，在酮症酸中毒时，血液中浓度增加的分子是酮体，其中包括乙酰乙酸。

答案是：E.乙酰乙酸
66、 (A1 型题)
呼吸链抑制剂鱼藤酮可以抑制电子传递，其抑制对象是
A.复合体Ⅰ
B.复合体Ⅱ
C.复合体Ⅲ
D.复合体Ⅳ
E.复合体Ⅴ
参考答案： A 您的答案：
图文解析： 【解析】鱼藤酮是一种线粒体电子传递链复合物 I 抑制剂，通过促进线粒体活性氧的产生进而诱导细胞的凋亡。故 A 对。
鱼藤酮是一种已知的呼吸链抑制剂，它通过抑制线粒体内电子传递链中的复合体Ⅰ（NADH 脱氢酶）来发挥作用。因此，它阻断了电子从 NADH 传递到辅酶 Q 的过程，从而抑制线粒体的氧化磷酸化和 ATP 的产生。
所以，正确答案是 **A. 复合体Ⅰ**。
解析：
呼吸链，也被称为氧化呼吸链或电子传递链，是位于线粒体内膜上的一组递氢反应和递电子反应按一定顺序排列的组合，能够将代谢物脱下的成对氢原子交给氧，从而生成水并同时产生 ATP。这个过程中涉及的关键酶或分子被称为复合体，其中复合体Ⅰ即 NADH-Q 还原酶，其功能是将电子从 NADH 传递给泛醌，同时将质子从线粒体基质泵入膜间隙。鱼藤酮是一种呼吸链抑制剂，其抑制作用的主要对象是复合体Ⅰ。
综上所述，正确答案是：A.复合体Ⅰ。
选项 B 复合体Ⅱ即琥珀酸-Q 还原酶，其功能是将电子从琥珀酸传递给泛醌，同时将质子从线粒体基质泵入膜间隙。其特异性抑制剂为萎锈灵、粉锈宁、丙二酸等物质，鱼藤酮并不能抑制其功能，故不选。
选项 C 复合体Ⅲ即 Q-细胞色素 c 还原酶，是线粒体内膜上的一个大型蛋白质复合物，可将电子从还原型泛醌传递给细胞色素 c。其特异性抑制剂为抗霉素 A 等物质，鱼藤酮对其并无抑制作用，故排除。
选项 D 复合体Ⅳ即细胞色素 c 氧化酶，是线粒体内膜呼吸链电子传递的终末复合物，是氧化呼吸链中电子传递的最终环节，可将电子从细胞色素 c 传递给氧分子。其特异性抑制剂为氰

化物、硫化氢、叠氮化钠等物质，鱼藤酮并不是其抑制剂，故不考虑。
选项 E 复合体Ⅴ即 ATP 合酶，是线粒体内膜上的一个酶复合物，能够利用质子电化学梯度催化 ADP 磷酸化生成 ATP，其抑制剂为寡霉素等物质，鱼藤酮对其并无抑制作用，可排除。

呼吸链抑制剂鱼藤酮（rotenone）是一种常用的呼吸链抑制剂，它能够抑制电子传递链中的复合体Ⅰ（NADH 脱氢酶）。通过抑制复合体Ⅰ，鱼藤酮阻止 NADH 生成的电子进入呼吸链，从而抑制 ATP 的生成。

因此，正确答案是：
A. 复合体Ⅰ
67、 (A1 型题)
储存并传递遗传信息的核酸分子是
A.DNA
B.mRNA
C.tRNA
D.rRNA
E.hnRNA
参考答案： A 您的答案：
图文解析： 【解析】DNA 是储存、复制和传递遗传信息的主要物质基础，故本题选 A。RNA 在蛋白质合成过程中也起着重要作用，其中转移核糖核酸也即 tRNA，起着携带和转移活化氨基酸的作用；信使核糖核酸也即 mRNA，是合成蛋白质的模板；核糖体的核糖核酸也即 rRNA，是细胞合成蛋白质的主要场所。核不均一 RNA 即 hnRNA，是 mRNA 的前体，经过剪切修饰之后成为 mRNA。
储存并传递遗传信息的核酸分子是 DNA。因此，最正确的答案是 A. DNA。
DNA（脱氧核糖核酸）负责储存生物体内的遗传信息，并在细胞分裂时将这些信息传递给后代。其他选项如 mRNA（信使核糖核酸）、tRNA（转运核糖核酸）、rRNA（核糖体核酸）和 hnRNA（异核核糖核酸）虽然都与遗传信息的处理和翻译过程有关，但不是主要的遗传信息存储介质。
解析：
核酸包括 DNA 和 RNA，DNA 即脱氧核糖核酸，RNA 即核糖核酸。DNA 是遗传信息的载体，是主要的遗传物质，由脱氧核苷酸组成的大分子聚合物，DNA 分子结构中，两条脱氧核苷酸单链围绕着一个共同的中心轴盘绕，构成双螺旋结构。具有储存和传递遗传信息的功能，因此储存并传递遗传信息的核酸分子是 DNA。
选项 B：mRNA 即信使 RNA，是指导蛋白质生物合成的直接模板，并不储存遗传信息，故不选。
选项 C：tRNA 即转运 RNA，主要作用是携带氨基酸进入核糖体，在 mRNA 指导下合成蛋白质，并不传递遗传信息，故排除。
选项 D：rRNA 即核糖体 RNA，主要功能是与核糖体蛋白组成核糖体，作为蛋白质的合成场所，与题干不符。
选项 E：hnRNA 即不均一核 RNA，为 mRNA 的前体，真核生物中转录形成的直接产物，需经过不同的加工修饰才能成为 mRNA，本身并不储存遗传信息，可排除。
综上所述，正确答案是：A.DNA。

储存并传递遗传信息的核酸分子是

A. DNA
68、 (A1 型题)
不属于蛋白质变性的特征的是
A.黏性增加
B.溶解度降低
C.结晶能力增强
D.更容易被酶水解
E.生物活性降低
参考答案： C 您的答案：
图文解析： 【解析】蛋白质的变性是指在某些物理和化学因素作用下，蛋白质特定的空间构象被破坏，从而导致其理化性质和生物学性质的改变，如溶解度降低、黏度增加、结晶能力消失、生物学活性丧失、易被蛋白酶水解。蛋白质的变性主要发生二硫键和非共价键的破坏，分子量不发生改变。
在这个问题中，我们需要确定哪一项不是蛋白质变性的特征。蛋白质变性通常意味着蛋白质的三维结构发生改变，但其一级结构即氨基酸序列保持不变。这种结构的改变通常导致蛋白质的某些物理和化学性质的改变，例如溶解度降低、生物活性降低，以及对热或其他因素的敏感性增加。根据这些信息，我们可以分析各个选项：
A. 黏性增加 - 变性后的蛋白质可能会因结构的变化而导致黏性增加。
B. 溶解度降低 - 这是蛋白质变性常见的结果，因为其三维结构的改变可能会影响其在水中的溶解性。
C. 结晶能力增强 - 通常，变性蛋白质的结晶能力会降低，因为其规则的结构被破坏。
D. 更容易被酶水解 - 变性蛋白质可能更容易或更难被酶水解，这取决于其结构变化如何影响酶的作用位点。
E. 生物活性降低 - 这通常是蛋白质变性的直接后果，因为其活性往往依赖于其特定的三维结构。
因此，不属于蛋白质变性特征的是 **C. 结晶能力增强**，因为变性通常会损害蛋白质的结晶能力，而不是增强它。
解析：
蛋白质变性指的是在某些物理和化学因素作用下，蛋白质的空间结构被破坏，从而导致其理化性质改变和生物活性的丧失。蛋白质变性后，其理化性质及生物学性质发生改变，如溶解度降低，黏度增加，结晶能力消失，生物学活性消失，易被蛋白酶水解等。故结晶能力不会增强，反而会消失。
选项 A 黏性增加是由于蛋白质变性后，蛋白质分子内部的水化层被破坏，多肽链伸展松散，更易形成网状结构，从而使溶液黏度增加，符合蛋白质变性的特征。
选项 B 溶解度降低是由于蛋白质变性后，原本埋藏在分子内部的疏水基团暴露，使蛋白质溶解性降低，符合蛋白质变性的特征。
选项 D 更容易被酶水解是由于蛋白质变性后，其特定的空间结构被破坏，肽键更易暴露，从而更容易被酶水解，符合蛋白质变性的特征。
选项 E 生物活性降低是由于蛋白质变性后，其特定的空间结构被破坏，生物学活性消失，符合蛋白质变性的特征。
综上所述，正确答案是：C.结晶能力增强。

68、 不属于蛋白质变性的特征的是

A. 黏性增加
B. 溶解度降低
C. 结晶能力增强
D. 更容易被酶水解
E. 生物活性降低
蛋白质变性是指蛋白质分子的结构发生变化，从而丧失其天然构象和功能。变性通常会导致以下特征：
- 溶解度降低（B）：变性后的蛋白质通常会聚集，导致溶解度下降。
- 更容易被酶水解（D）：变性会使蛋白质结构松散，更容易被蛋白酶分解。
- 生物活性降低（E）：由于结构变化，蛋白质通常失去其生物功能。
- 黏性增加（A）：变性后的蛋白质分子可能形成更大的聚集体，导致溶液黏性增加。
然而，结晶能力增强（C）不是蛋白质变性的典型特征。变性通常会导致蛋白质失去其有序结构，反而不容易结晶。因此，答案是：C. 结晶能力增强
69、 (A1 型题)
湿性坏疽常发生在
A.脑、脾、肝
B.脑、肠、子宫
C.肺、肠、子宫
D.肺、肠、肝
E.肺、肠、子宫
参考答案： E 您的答案：
图文解析： 【解析】湿性坏疽是坏疽的一种，多发生于与外界相通的内脏（肠、子宫、肺等），也可见于四肢（伴有淤血水肿时）。常见的湿性坏疽有坏疽性阑尾炎、肠坏疽、肺坏疽及产后坏疽性子宫内膜炎等。故本题选 E。
湿性坏疽（wet gangrene）是由于细菌感染导致的坏死组织中有液体积聚。这种类型的坏疽通常发生在体内的器官或肢体的末端，尤其是在血流受阻的情况下。在给定的选项中，我们应该考虑那些容易受到血流不良影响并且容易发生细菌感染的器官。
选项 A（脑、脾、肝）、B（脑、肠、子宫）和 D（肺、肠、肝）中包含了一些容易受到感染的器官，但湿性坏疽更倾向于发生在有明显感染和血流不良的部位，如肠。因此，我们可以排除包含脑和脾的选项，因为这些器官不是湿性坏疽最常见的部位。
选项 C（肺、肠、子宫）和 E（肺、肠、子宫）看起来很相似，但实际上选项 E 是重复的，可能是输入错误。考虑到湿性坏疽常见于肢体的末端以及内脏器官，特别是那些容易出现血流障碍和细菌感染的部位，肠是其中一个常见部位。然而，在这些选项中没有直接指向四肢的选择，我们只能从给出的器官中进行选择。
因此，最合适的选择似乎是 D（肺、肠、肝），因为这些部位相对于脑和脾，更可能与湿性坏疽的特点相符，尤其是考虑到肠的血流问题和感染倾向。然而，这种类型的坏疽通常与四肢末端更为相关，但基于题目给出的选项，D 似乎是最接近的答案。
题目有问题！
解析：
湿性坏疽是坏疽的一种，指组织坏死后因含水分较多，腐败菌感染严重，局部明显肿胀，呈暗绿色或污黑色。由于腐败菌分解蛋白质，产生吲哚、粪臭素等，造成恶臭。湿性坏疽常发生在肺、肠、子宫、阑尾及胆囊等器官，也可发生于动脉阻塞但静脉回流尚畅通的四肢。
选项 A“脑、脾、肝”，选项 B“脑、肠、子宫”，选项 D“肺、肠、肝”，中均有脑，而脑组织坏死后由于缺血缺氧，一般属于凝固性坏死，而非湿性坏疽，故一并排除。

选项 D 肺、肠、肝中肝脏属于实质器官，该器官坏死后多为凝固性坏死，而非湿性坏疽，故不选。
综上所述，正确答案是：E.肺、肠、子宫。

湿性坏疽（Wet gangrene）通常是由细菌感染引起的，它最常见于含水量较高的器官和组织，特别是那些容易受到感染的部位。湿性坏疽常见的部位包括肠、肺、和子宫。

因此，根据现有的医学知识和最佳实践指南，湿性坏疽常发生在肺、肠和子宫。

正确答案是：C. 肺、肠、子宫
70、 (A2 型题)
男，47 岁。既往慢性乙型肝炎病史 10 余年，1 月前出现右上腹隐痛不适。查体：右腹部膨隆，可扪及质地坚硬，表面凹凸不平的肿块，移动性浊音阳性，腹腔积液血性。 是
A.肝包虫病
B.原发性肝癌
C.肝囊肿
D.肝脓肿
E.肝血管瘤
参考答案： B 您的答案：
图文解析： 【解析】肝炎、肝硬化、肝癌三部曲。血性腹水、肿块表面不平提示为恶性肿瘤。
这个病例描述的是一名 47 岁男性，有超过十年的慢性乙型肝炎病史，最近一月出现右上腹隐痛，并发现右腹部有质地坚硬、表面凹凸不平的肿块，伴有移动性浊音阳性和腹腔积液血性。根据这些症状，我们可以逐一排查备选答案：
A. 肝包虫病：虽然可以引起肝脏肿块，但一般伴随有特定的流行病学史，且肿块通常不会导致血性腹水。
B. 原发性肝癌（肝细胞癌）：在慢性乙型肝炎患者中较为常见，可以引起坚硬的肿块，表面凹凸不平，并且可能伴有血性腹水，符合病例描述。
C. 肝囊肿：通常为良性病变，肿块表面平滑，不太可能导致血性腹水。
D. 肝脓肿：虽然会造成腹痛和肿块，但肿块通常较软，且症状会有明显的急性炎症表现，如发热、寒战等。
E. 肝血管瘤：通常是无症状的良性肿瘤，很少引起血性腹水或腹痛。
因此，根据病例描述和相关症状，最合适的答案是 B.原发性肝癌。
这是一道涉及疾病诊断的问题，需要根据患者的病史、症状和体征来确定最可能的疾病。
首先，我们梳理一下题目中给出的关键信息：
患者为中年男性，有 10 余年的慢性乙型肝炎病史。
1 月前出现右上腹隐痛不适。
查体发现右腹部膨隆，可扪及质地坚硬，表面凹凸不平的肿块。
腹腔积液为血性，且移动性浊音阳性，提示有大量腹水。
接下来，我们分析各选项对应的疾病及其特点：
A. 肝包虫病：这是一种寄生虫病，主要表现为腹部肿块，但通常不会伴有腹水，且病史与患者描述不符。
B. 原发性肝癌：与乙型肝炎病史密切相关，常表现为肝区疼痛、肝大、质地变硬，并可能出现腹水。患者的病史、症状和体征与此相符。

C. 肝囊肿：通常无症状，除非囊肿非常大，否则不太可能引起腹水和疼痛。
D. 肝脓肿：主要表现为寒战、高热、肝区疼痛和肝大，但不会出现血性腹水和质地坚硬的肿块。
E. 肝血管瘤：这是一种良性肿瘤，通常无症状，除非血管瘤非常大或破裂，否则不太可能引起疼痛和腹水。
综上所述，结合患者的乙型肝炎病史、右上腹隐痛、腹部膨隆、质硬凹凸不平的肿块以及血性腹水等症状，最可能的诊断是原发性肝癌。
此，正确答案是 B.原发性肝癌。

根据题目描述，患者 47 岁，有慢性乙型肝炎病史 10 余年，1 月前出现右上腹隐痛不适。体检发现右腹部膨隆，可触及质地坚硬、表面凹凸不平的肿块，移动性浊音阳性，并有血性腹腔积液。这些症状和体征高度提示原发性肝癌。
慢性乙型肝炎患者是原发性肝癌的高危人群，而题目中描述的肿块特征和血性腹腔积液也是原发性肝癌的常见表现。
因此，最可能的诊断是：B. 原发性肝癌
71、 (A2 型题)
某男，52 岁，2 型糖尿病，BMI 值为 32kg/m2，腰围 90cm，下列适合该男子的运动方式是
A.举重
B.游泳
C.跳绳
D.拔河
E.长跑
参考答案： B 您的答案：
图文解析： 【解析】中老年男性患者，BMI 值为 32kg/m2，肥胖，最适合对关节压力较小的中低强度运动，例如游泳（B 对）。
【避错】举重、拔河等对抗性运动较为剧烈，不适合此患者。而跳绳、长跑对下肢大关节造成的压力较大，因此也不适合。
考虑到这位 52 岁的 2 型糖尿病患者的 BMI 值为 32kg/m2，表明他属于肥胖，因此适宜的运动方式应该是低至中等强度、低冲击的，能够帮助其改善血糖控制和减少体重。根据这些信息，最合适的选项是：
B. 游泳
游泳是一种全身运动，对关节的冲击小，适合肥胖和 2 型糖尿病患者，可以帮助改善心肺功能、控制血糖和减轻体重。与其他选项相比，游泳为低冲击性运动，适合于 BMI 较高的患者，且能有效控制血糖水平，减少体重，因此是最佳选择。
解析：
中年男性患者，诊断为 2 型糖尿病，体重指数（BMI）提示肥胖，腰围较大。糖尿病患者进行规律的运动锻炼可降低血糖、血脂，增加能量消耗，减轻胰岛素抵抗，降低糖尿病心血管病变和微血管并发症的风险，提高整体健康水平。在选择运动方式时，应选择中、低强度的有氧运动，如游泳，对不能负重或由于并发症所限不能行走的患者尤为适合。
选项 A 举重，选项 C 跳绳，选项 D 拔河，选项 E 长跑，均属于高强度运动，运动强度较大，不适宜该患者。且拔河运动还需要团队协作，不适合患者单独进行。
综上所述，正确答案是：B.游泳。

对于一名 52 岁、患有 2 型糖尿病且 BMI 值为 32 kg/m^2 的男性来说，适合的运动方式应该是

安全、可持续且能够帮助控制血糖水平和体重。以下是每种运动方式的分析：

- **举重**：虽然举重可以增加肌肉质量和力量，但对于糖尿病患者，尤其是刚开始锻炼的人来说，可能存在过大的风险，尤其是心血管负担。
- **游泳**：游泳是一种低冲击、高效的全身运动，有助于心肺健康，减少体重，并且对关节压力较小。对于糖尿病患者来说，是一种非常适合的运动方式。
- **跳绳**：跳绳是一种高强度的有氧运动，虽然有助于心肺功能，但对关节的冲击较大，且不易长期坚持，可能不太适合 52 岁并且体重较大的糖尿病患者。
- **拔河**：拔河是一种高强度的短时运动，参与者需要爆发力和较大的体力，对糖尿病患者并不推荐。
- **长跑**：长跑可以有效提高心肺功能和代谢率，但对于体重较大的糖尿病患者来说，长时间的跑步可能会对膝关节和其他关节造成较大压力，容易引发损伤。

综上所述，适合该男子的运动方式是 **B. 游泳**。
72、 (A2 型题)
女，32 岁，怕冷，嗜睡两个月余。查体：脉搏 56 次/分，眼睑水肿，甲状腺弥漫性肿大，质地韧。最可能的甲状腺功能表现是
A.TT3 正常，TT4 正常，TSH 减少
B.TT3 下降，TT4 下降，TSH 增加
C.TT3 增加，TT4 增加，TSH 减少
D.TT3 增加，TT4 增加，TSH 增加
E.TT3 正常，TT4 正常，TSH 增加
参考答案： B 您的答案：
图文解析： 【解析】中年女性患者，怕冷、嗜睡，眼睑水肿，甲状腺弥漫性肿大，均属于甲状腺功能减退的典型表现，诊断为甲状腺功能减退。甲状腺功能减退症血清 TSH 增高，TT4 和 FT4 均降低。血清 TT3、FT3 早期正常，晚期减低。故本题选 B。
【避错】原发性甲减由于甲状腺腺体本身病变，引起甲状腺分泌甲状腺激素减少，对于下丘脑、腺垂体的负反馈抑制减弱，TSH 分泌增加。TSH 变化比甲状腺激素（TT4、FT4）更为灵敏。
这位 32 岁的女性患者出现怕冷、嗜睡、脉搏减慢（56 次/分）、眼睑水肿和甲状腺弥漫性肿大的症状，这些都是低甲状腺功能（甲状腺功能减退症）的典型表现。低甲状腺功能时，甲状腺激素（TT3 和 TT4）的水平会降低，而促甲状腺激素（TSH）的水平会增加，因为下丘脑-垂体-甲状腺轴试图通过增加 TSH 来刺激甲状腺产生更多的甲状腺激素。
根据这些信息，最可能的甲状腺功能表现是：
- TT3 下降
- TT4 下降
- TSH 增加
因此，最正确的答案是 B.TT3 下降，TT4 下降，TSH 增加。
解析：
患者青年女性，怕冷，嗜睡两个月余，查体见心动过缓，眼睑水肿，甲状腺弥漫性肿大，质地韧，考虑诊断为甲状腺功能减退。甲状腺功能减退时，甲状腺激素分泌不足，下丘脑和垂体会增加 TSH 的分泌来促进甲状腺激素的分泌，因此血清 TSH 水平升高。同时，甲状腺激素包括 TT_3和 TT_4生成减少，故甲状腺功能减退时，患者最可能的甲状腺功能表现为 TT3 下降，TT4 下降，TSH 增加。

甲状腺功能减退时，血清 TT_3和 TT_4水平下降，TSH 水平升高。故选项 A“TT3 正常，TT4 正常，TSH 减少”，选项 C“TT3 增加，TT4 增加，TSH 减少”，选项 D“TT3 增加，TT4 增加，TSH 增加”，选项 E“TT3 正常，TT4 正常，TSH 增加”，均与甲状腺功能减退时甲状腺功能不符，可一并排除。
综上所述，正确答案是：B.TT3 下降，TT4 下降，TSH 增加。
这位 32 岁的女性患者表现出怕冷、嗜睡等症状，并且查体发现脉搏缓慢（56 次/分），眼睑水肿和甲状腺弥漫性肿大。这些症状和体征提示她可能患有甲状腺功能减退（甲减）。

甲状腺功能减退时，甲状腺激素分泌减少，导致机体代谢减慢，表现为怕冷、嗜睡、脉搏缓慢等症状。由于甲状腺激素（TT3 和 TT4）水平下降，负反馈作用减弱，促甲状腺激素（TSH）分泌增加以刺激甲状腺产生更多的激素。

因此，最可能的甲状腺功能表现是：
B. TT3 下降，TT4 下降，TSH 增加答案：B
73、　(A2 型题)
女性，30 岁，孕期 34 周，突发持续腹部疼痛伴阴部流血 2 小时，查体：体温 36.2℃,P120 次/分，R：26 次/分，血压：80/60mmhg，神志清，四肢湿冷，眼球及四肢多发瘀　查：Hb70g/L，RBC：2.7×1012/L，Plt：85×109/L，Fg：1.48g/L，D-二聚体：10.8mg/L，外周血有裂细胞，B 超显示盆腔和子宫内膜之间有界限不清楚的低密度回声影，　的影响不正确的是
A.微血栓导致回心血量减少
B.激肽基酶增加导致外周阻力减少
C.补体激活导致外周阻力减少
D.裂细胞增多导致冠状动脉收缩
E.前负荷减少导致心排出血量减少
参考答案：　D　您的答案：
图文解析：　【解析】患者孕 34 周，突发突发持续腹部疼痛伴阴部流血 2 小时，伴循环障碍低血容量休克，诊断考虑弥漫性血管内溶血（DIC）。裂口红细胞红细胞中央有裂缝，中心苍白区呈翩平状，颇似张开的口形或鱼口，可见于 DIC，但并不导致冠状动脉收缩，冠状动脉收缩主要是微血栓形成导致，本题问的是对血压影响不正确的选项，故选择 D。
【避错】DIC 常见微血栓形成，导致回心血量减少（选项 A），进而前负荷减少导致心排血量减少（选项 E）。DIC 可以直接或间接激活激肽系统和补体系统，以致激肽和补体生成增多，激肽能使微动脉和毛细血管前括约肌舒张，毛细血管通透性增高，外周阻力减少（选项 B）。补体使肥大细胞脱颗粒，通过释放组胺，发挥与激肽类似的作用，使外周阻力减少（选项 C）。
这道题目是关于一位孕妇在孕期出现的急症情况，考查的是对相关临床表现及其背后生理机制的理解。基于题干信息，这位孕妇出现了严重的症状，包括持续腹痛、阴部流血、显著的贫血（Hb 70g/L）、血小板减少、凝血功能障碍（低纤维蛋白原和高 D-二聚体）、以及裂细胞的存在，这些迹象暗示可能的重度 DIC（弥散性血管内凝血）和血栓性微血管病变。
选项的正确性如下：
A. 微血栓导致回心血量减少：可能正确，因为 DIC 和微血管血栓可以减少有效循环血量。
B. 激肽基酶增加导致外周阻力减少：可能正确，因为激肽系统的激活可以导致血管舒张，从而减少外周阻力。
C. 补体激活导致外周阻力减少：可能正确，补体激活在炎症反应中可以导致血管舒张。
D. 裂细胞增多导致冠状动脉收缩：这个选项似乎不太合理，因为裂细胞的存在主要指示了

红细胞的机械性破坏和微血管病变，并不直接导致冠状动脉收缩。
E. 前负荷减少导致心排出血量减少：可能正确，因为贫血和血容量减少可以减少心脏的前负荷，从而减少心排血量。
因此，选项 D“裂细胞增多导致冠状动脉收缩”是不正确的描述，这与裂细胞的出现和相关的病理生理机制不符。所以最正确的答案应该是 D。
解析：
患者青年女性，孕期突发持续腹痛伴阴道流血，查体见休克血压，四肢湿冷，眼球及四肢多发瘀斑，提示可能出现 DIC，实验室检查见血红蛋白偏低，提示贫血，血小板偏低，纤维蛋白原偏低，D-二聚体偏高，均符合 DIC 的实验室诊断，且外周血有裂细胞，B 超显示有羊膜后血肿，因此可诊断为胎盘早剥并发 DIC。
选项 A 微血栓导致回心血量减少是由于 DIC 形成大量微血栓，导致全身微循环障碍，组织缺血缺氧，从而回心血量减少，符合 DIC 对机体的影响。
选项 B 激肽基酶增加导致外周阻力减少是由于在 DIC 过程中，激肽释放酶原被激活，转变为激肽释放酶，后者催化缓激肽原转变为缓激肽和赖氨酰舒缓激肽，可使细动脉扩张、血管通透性增加、支气管平滑肌收缩，并可引起疼痛，故可使外周阻力减少。
选项 C 补体激活导致外周阻力减少是由于 DIC 过程中，补体系统被激活，产生过敏毒素 C3a 和 C5a，可使肥大细胞或嗜碱性粒细胞释放组胺，引起血管扩张，增加血管通透性，使外周阻力减少。
选项 D 裂细胞增多导致冠状动脉收缩说法错误，因为冠状动脉收缩主要与交感-肾上腺髓质系统兴奋有关，而与裂细胞无直接关系，且裂细胞增多主要是由于 DIC 导致红细胞受损。
选项 E 前负荷减少导致心排出血量减少是由于 DIC 过程中，微血栓形成，导致回心血量减少，即心脏的前负荷减少，从而使心排出量减少。
综上所述，正确答案是：D.裂细胞增多导致冠状动脉收缩。

这道题目描述了一位孕 34 周的女性，突发持续腹部疼痛伴阴部流血，检查发现其生命体征不稳定，血压低，血红蛋白和血小板降低，D-二聚体显著升高，外周血有裂细胞。根据这些信息，可以推测患者可能发生了胎盘早剥并伴有弥散性血管内凝血（DIC）。我们需要选择不正确的影响因素。

让我们逐项分析选项：

A. **微血栓导致回心血量减少** - 这是正确的。DIC 会导致微血栓形成，进而减少回心血量。
B. **激肽基酶增加导致外周阻力减少** - 这是正确的。激肽基酶增加会导致血管扩张，外周阻力减少。
C. **补体激活导致外周阻力减少** - 这是正确的。补体系统的激活会导致血管扩张和外周阻力减少。
D. **裂细胞增多导致冠状动脉收缩** - 这是不正确的。裂细胞的增多主要是由于红细胞被破坏，并不直接导致冠状动脉收缩。
E. **前负荷减少导致心排出血量减少** - 这是正确的。由于出血和微血栓形成，前负荷减少会导致心排出量减少。

综合上述分析，选项 D“裂细胞增多导致冠状动脉收缩”是不正确的，因此正确答案是：

D. 裂细胞增多导致冠状动脉收缩

74、 (A2 型题)
男，45 岁，因车祸导致脊髓胸段受损。2 个月后，患者最有可能出现的排尿障碍是
A.尿频
B.多尿
C.尿失禁
D.膀胱颈痉挛
E.无张力膀胱
参考答案： C 您的答案：
图文解析： 【解析】脊髓损伤 2~4 周后，反射活动逐渐恢复，转变为中枢性瘫痪，出现肌张力增高、反射亢进、病理征阳性和反射性排尿等（C 对）。
【避错】脊髓损伤急性期往往出现脊髓休克症状，包括损害平面以下弛缓性瘫痪，肌张力减低,腱反射减弱，病理反射阴性及尿潴留。
在脊髓损伤后，尤其是胸段损伤，患者可能会出现不同的排尿功能障碍，这取决于损伤的程度和位置。脊髓损伤常导致下尿路功能失调，尤其是在急性和亚急性阶段。初始阶段（急性期）通常表现为脊髓休克，此时可能出现无张力膀胱，表现为无法自主排尿。但随着时间的推移，尤其是 2 个月后，情况可能会发生变化，患者可能出现不同的症状。
在这种情况下，最有可能出现的排尿障碍是**无张力膀胱（E）**。无张力膀胱是由于神经控制缺失导致膀胱肌肉无法正常收缩，造成排尿困难或无法自主排尿。然而，应注意，随着时间的推移和损伤的部位，患者的症状可能向其他类型的排尿障碍转变，例如神经源性膀胱，可能出现尿频、尿失禁或膀胱颈痉挛。

因此，根据题目描述，2 个月后患者最有可能出现的排尿障碍是**无张力膀胱（E）**。
解析：
患者中年男性，因车祸导致脊髓胸段受损。脊髓受损部位不同，患者可出现不同类型的膀胱功能障碍。脊髓排尿中枢位于腰骶段，当脊髓损伤发生在胸段或以上时，排尿过程将不受脊髓中低级排尿中枢控制，排尿过程将受脑干和大脑皮质上组织控制，形成无张力膀胱，即膀胱的传入神经受损，膀胱充盈的传入信号不能被感知，脊髓中的排尿反射中枢活动发生障碍，膀胱逼尿肌收缩无力，导致大量残余尿、尿潴留。
选项 A 尿频是膀胱刺激征的主要表现，多见于尿路感染和肾结核，故排除。
选项 B 多尿多见于糖尿病、尿崩症等，与脊髓损伤无关，故不选。
选项 C 尿失禁分为真性尿失禁、假性尿失禁和压力性尿失禁，多与膀胱括约肌受损、神经功能失调和产伤有关，而该患者并未损伤腰骶段，所以不会出现尿失禁，故不考虑。
选项 D 膀胱颈痉挛多见于急性细菌性膀胱炎，故不选。
综上所述，正确答案是：E.无张力膀胱。

对于这道题目，我们需要考虑脊髓损伤对排尿功能的影响。脊髓胸段损伤可能会导致神经控制中断，进而影响膀胱的功能。

在脊髓损伤后，排尿障碍通常取决于损伤的部位和程度。胸段脊髓损伤常常会导致膀胱功能障碍，具体表现为尿失禁或无张力膀胱。两个月后，患者最有可能出现的是自主神经功能失调，导致膀胱不能正常收缩或排尿。

综上所述，最有可能出现的排尿障碍是：

E.无张力膀胱
75、 (A2 型题)
男 55 岁，甲状腺癌颈部淋巴结清扫术后，出现左肩下垂，左上肢上举受限，术后可能损伤的神经是
A.膈神经
B.迷走神经
C.副神经
D.枕小神经
E.耳大神经
参考答案： C 您的答案：
图文解析： 【解析】当一侧副神经发生损伤时，会导致同侧斜方肌与胸锁乳突肌功能障碍，造成肌肉瘫痪，同时伴有肌肉萎缩。此时表现为向对侧转动颈部时无力，以及伴有同侧肩部下垂，无法耸肩，长此以往还容易出现相应肌肉发生萎缩。肌肉萎缩后容易出现肩胛骨移位，使得臂丛神经受到牵拉，导致患者的患侧上肢无法上举，肢体外展功能受限，后期还会出现挛性挛缩以及畸形。故本题选 C。
【避错】膈神经损伤会出现膈肌麻痹、自主呼吸受限、膈肌痉挛。迷走神经损伤时可出现声音嘶哑、呛咳等。枕小神经受损时枕部皮肤感觉功能障碍。耳大神经损伤可出现耳郭皮肤麻木。
这位患者在甲状腺癌颈部淋巴结清扫术后出现左肩下垂和左上肢上举受限，这些症状表明可能损伤的是副神经。副神经（Accessory nerve，又称 XI 颅神经）主要支配斜方肌和胸锁乳突肌，其功能与肩部的运动紧密相关。因此，副神经损伤后常见的表现包括肩部无力、下垂和上肢抬高受限。
选项 A 的膈神经（Phrenic nerve）主要控制膈肌，与呼吸相关，而非直接影响肩部或上肢的运动。
选项 B 的迷走神经（Vagus nerve）影响的是内脏功能，包括心脏、肺部和消化系统，不直接控制肩部或上肢的运动。
选项 D 的枕小神经（Lesser occipital nerve）主要与头皮的感觉有关。
选项 E 的耳大神经（Great auricular nerve）影响的是耳部和面部的感觉。
因此，最符合这个症状的是 C.副神经。
这是一道医学知识的问题。我们需要先理解题目背景信息和问题核心，再仔细分析每个选项，并将其与题中给出的信息进行对比。
理解题目背景信息：首先，题目描述了一位 55 岁的男性患者，甲状腺癌颈部淋巴结清扫术后，出现了左肩下垂，左上肢上举受限的症状。我们需要根据这些症状判断手术可能损伤的神经。
理解问题核心：我们需要基于这些症状，从选项中找出术后可能损伤的神经。
分析选项：
A 选项（膈神经）：膈神经主要负责膈肌的运动，损伤后会导致膈肌功能异常，出现呼吸困难等症状，与题目描述的症状不符。
B 选项（迷走神经）：迷走神经主要负责心、肺、胃肠道等多个器官的运动和感觉，损伤后可能导致相应器官功能障碍，但不会出现左上肢运动受限的症状。
C 选项（副神经）：副神经主要支配胸锁乳突肌和斜方肌，使胸锁乳突肌和斜方肌收缩，损伤后会导致胸锁乳突肌和斜方肌瘫痪，出现患侧胸锁乳突肌和斜方肌萎缩，患者向患侧转头无力，同侧胸锁乳突肌和斜方肌萎缩，同侧胸锁乳突肌和斜方肌麻痹，因此会导致左肩下垂，左上肢上举受限，这与题目描述的症状相符。

D 选项（枕小神经）：枕小神经主要负责枕部的感觉，损伤后会出现枕部感觉异常，与题目描述的症状不符。
E 选项（耳大神经）：耳大神经主要负责耳廓后部的感觉，损伤后会出现相应区域的感觉异常，与题目描述的症状不符。
综上所述，术后可能损伤的神经是 C.副神经。

对于题目所描述的情况，即甲状腺癌颈部淋巴结清扫术后出现左肩下垂和左上肢上举受限，最可能损伤的神经是副神经（Accessory nerve）。副神经支配斜方肌，斜方肌的功能是抬肩和上举上肢，因此副神经受损会导致患者肩膀下垂和上肢上举受限。

所以正确答案是：C. 副神经
76、 (A2 型题)
女，50 岁，腹痛、腹胀停止排气排便，腹股沟韧带下可触及一半圆形肿块，触之疼痛，该肿块内侧组织结构为
A.髂耻弓
B.股静脉
C.子宫圆韧带
D.腹股沟韧带
E.腔隙韧带
参考答案： E 您的答案：
图文解析： 【解析】患者女性，50 岁，有梗阻症状（腹痛、腹胀停止排气排便），腹股沟韧带下可触及一半圆形肿块，触之疼痛，诊断考虑股疝。股疝通过股环、股管、卵圆窝向大腿根部突出的腹外疝。股管由四壁两口构成:内侧壁是腔隙韧带(也称陷窝韧带)；外侧壁是股鞘内股静脉旁的纤维隔(也有说股静脉)；前壁是腹股沟韧带；后壁是耻骨梳韧带(Cooper 韧带)；两口为股环口和股内侧的隐静脉裂孔。故肿块内侧的组织结构考虑为腔隙韧带，故选择 E。

【避错】髂耻弓是分隔腹股沟韧带与髋骨之间间隙的结构， 一端连于腹股沟韧带，另一端附着于髋骨的髂耻隆起，与股疝无关，故 A 错。股静脉是股疝的外侧壁，故 B 错。子宫圆韧
带是维持子宫前倾的作用，故 C 错。腹股沟韧带是股疝的前壁，故 D 错。
这个病例描述的是一位 50 岁女性患者出现腹痛、腹胀、停止排气排便，并在腹股沟韧带下可触及一个半圆形的疼痛肿块。这些症状和体征提示可能是腹股沟疝。在女性中，腹股沟疝可能包含子宫圆韧带。

选项中的各个结构位于不同的解剖位置，其中子宫圆韧带是通向女性腹股沟管的结构之一，尤其是在腹股沟疝的情况下，子宫圆韧带可能会突出并形成肿块。

因此，最合适的答案是：
C. 子宫圆韧带
解析：
患者中年女性，因腹痛、腹胀停止排气排便就诊，腹股沟韧带下可触及一半圆形肿块，触之疼痛，考虑为股疝。股疝的疝囊通过股环、经股管向卵圆窝突出的疝，在腹股沟韧带下方卵圆窝处表现为一半球形的突起，平卧回纳内容物后，疝块有时并不完全消失。由于疝囊外有

很多韧带，所以其内侧组织结构为腔隙韧带，又名陷窝韧带，是腹股沟韧带内侧端的纤维性增厚部分，是股环的内缘。
选项 A 髂耻弓是判断股疝和斜疝的一个标志，为腹股沟韧带内上方由腹内斜肌和腹横肌的弓状下缘与腹股沟韧带围成，位于股疝的外侧，故不选。
选项 B 股静脉位于股疝的外侧，因此排除。
选项 C 子宫圆韧带起自宫角的前面、输卵管近端的稍下方，在阔韧带前叶的覆盖下向前外侧走行，到达两侧骨盆侧壁后，经腹股沟管止于大阴唇的前端，与股疝位置相邻，但不是其内侧结构，故排除。
选项 D 腹股沟韧带是腹外斜肌腱膜在耻骨嵴上外缘增厚卷曲而形成的韧带样结构，位于股疝的外侧，所以不选。
综上所述，正确答案是：E.腔隙韧带。

根据题干描述，该患者是 50 岁的女性，出现腹痛、腹胀、停止排气排便，并且在腹股沟韧带下方可触及半圆形肿块且触之疼痛。这个描述符合腹股沟疝的特征。

题目问的是肿块内侧的组织结构是什么。解剖学上，腹股沟疝的解剖结构非常明确，肿块内侧通常是股静脉。因此，最有可能的答案是：B. 股静脉

77、 (A2 型题)
女，32 岁。双下肢水肿，关节痛，口腔溃疡。血常规：Hb100g/L,尿蛋白（+++），抗 dsDNA（+），ANCA1:1000，与该患者最相关的免疫物是
A.IgA
B.IgE
C.免疫复合物
D.免疫分子
E.IgD
参考答案： C 您的答案：
图文解析： 【解析】患者双下肢水肿，关节痛，口腔溃疡，实验室检查：dsDNA（+）（是诊断 SLE 的 特异性抗体），ANCA（+），尿蛋白（+++）（提示系统性红斑狼疮），故根据患者的临床 表现和实验室检查，诊断为系统性红斑狼疮疾病。抗 dsDNA 抗体（C 正确）：是诊断 SLE 的 特异性抗体，为 SLE 的标记抗体；多出现在 SLE 的活动期，抗 dsDNA 抗体的滴度与疾病活动 性密切相关，稳定期的病人如抗 dsDNA 滴度增高，提示复发风险较高，需要更加严密的监 测。
【避错】类风湿关节炎常有 RF（+）、抗 CCP 抗体（+）；干燥综合征常有 ANA（+）、抗 SSA （+）、抗 SSB（+）。
这个病例的症状，包括双下肢水肿、关节痛和口腔溃疡，以及实验室检查结果（Hb 100g/L，尿蛋白+++，抗 dsDNA 阳性，ANCA 1:1000）指向系统性红斑狼疮（Systemic Lupus Erythematosus, SLE）或相关的自身免疫性疾病。
在 SLE 中，最典型的免疫学特征是针对不同自身抗原的自身抗体的产生，尤其是抗双链 DNA（dsDNA）抗体，这在实验室检查中已经证实为阳性。SLE 的一个重要特征是免疫复合物的形成和沉积，这些免疫复合物可以在全身多个器官和组织中引发炎症和损伤。
因此，与该患者最相关的免疫物是 C.免疫复合物。
解析：
青年女性患者，出现双下肢水肿、关节痛、口腔溃疡，血红蛋白降低，大量蛋白尿，抗 dsDNA

阳性，ANCA 滴度显著增高，考虑诊断为系统性红斑狼疮。该病是一种多发于青年女性的累及多脏器的自身免疫性炎症性结缔组织病，早期、轻型和不典型的病例日渐增多。其发病机制是免疫复合物形成与沉积，从而引起一系列的免疫反应，导致组织损伤。所以，与该患者最相关的免疫物是免疫复合物。
选项 A：IgA 肾病是最为常见的一种原发性肾小球疾病，是指肾小球系膜区以 IgA 或 IgA 沉积为主，伴或不伴有其他免疫球蛋白在肾小球系膜区沉积的原发性肾小球病，与该患者诊断不符，故排除。
选项 B：IgE 是指人体的一种抗体，存在于血液中，是一种亲细胞性抗体，与变态反应，寄生虫病与皮肤过敏有关，与本例不符，因此不选。
选项 D：免疫分子是参与免疫应答和免疫调节的多种分子，包括细胞因子、抗体、补体、MHC 分子等，是免疫系统中发挥重要作用的活性物质，其概念过于宽泛，不是最佳选项，故不选。
选项 E：IgD 抗体是人类 B 淋巴细胞在抗原刺激下转化为浆细胞，产生能与相应抗原发生特异性结合的抗体，IgD 仅占血清免疫球蛋白的 0.6%。IgD 由 B 淋巴细胞在抗原刺激下转化为浆细胞时产生，可能与启动 B 细胞产生抗体有关，还可能与某些超敏反应有关，但与该患者关系不大，因此排除。
综上所述，正确答案是：C.免疫复合物。

题目描述了一位 32 岁女性患者，症状包括双下肢水肿、关节痛和口腔溃疡。实验室检查显示贫血（Hb 100g/L）、尿蛋白（+++），抗 dsDNA 抗体阳性（+），ANCA 滴度 1:1000。这些体征和实验室结果提示可能存在系统性红斑狼疮（SLE）。

系统性红斑狼疮是一种自身免疫性疾病，常表现为关节痛、皮疹、口腔溃疡和肾炎（尿蛋白）。抗 dsDNA 抗体阳性是 SLE 的重要诊断标志之一，ANCA 通常与其他自身免疫性疾病如抗中性粒细胞胞浆抗体相关性血管炎相关，但也可在 SLE 中见到。SLE 的病理机制通常涉及免疫复合物的形成和沉积，这些免疫复合物会在肾小球等部位引起炎症和损伤，导致相应的临床症状。

因此，与该患者最相关的免疫物是：
C. 免疫复合物

免疫复合物的沉积是 SLE 病理机制中的重要环节，可以解释该患者的临床表现和实验室检查结果。
78、 (A2 型题)
夏天建筑工人于工地干活时突然头晕、头痛，大量出汗后无汗，皮肤干热，体温超过 40℃，血压 88/60mmHg，伴意识障碍，请该患者可能诊断为
A.休克
B.热痉挛
C.热射病
D.热衰竭
E.中暑
参考答案： C 您的答案：
图文解析： 【解析】夏季高温室外劳动时，体温超过 40℃,血压 88/60mmHg，伴意识障碍，诊断为热 射病，故本题选 C。

【避错】热痉挛无明显体温升高，无神志障碍；热衰竭多见于老年人、儿童和慢性病患 者，体温升高多不超过 40℃,无神志障碍。
这道题描述的是一个建筑工人在夏天高温环境下工作，出现了头晕、头痛、大量出汗后无汗、皮肤干热、体温超过 40° C、血压下降和意识障碍的症状。这些症状表明患者可能遭受了严重的热相关疾病。
选项分析如下：
- A. 休克：虽然血压下降可能与休克相关，但休克的原因很多，且此处描述的情境和症状更加符合热相关的疾病。
- B. 热痉挛：通常由于大量出汗和电解质流失引起肌肉痉挛，但不会导致高热或意识障碍。
- C. 热射病：是高温环境下最严重的热相关疾病，特点包括体温急剧升高（通常超过 40° C）、中枢神经系统功能障碍（如意识障碍）和皮肤干热无汗。这与题目描述的症状非常吻合。
- D. 热衰竭：是由于水分和盐分不足引起的，表现为大量出汗、虚弱、疲乏、可能有轻微的体温升高，但通常不会像热射病那样体温急剧升高。
- E. 中暑：这是一个通用术语，涵盖由于高温导致的各种健康问题，但在医学分类中，更具体的条件（如热衰竭或热射病）是被单独诊断的。
综上所述，最符合这种情况的诊断是**C. 热射病**。热射病是一种紧急医疗状况，需要立即治疗以降低高体温并处理潜在的生命威胁。
解析：
患者夏天于工地干活时突然头晕、头痛，大量出汗后无汗，皮肤干热，体温超过 40℃，血压下降，伴意识障碍，考虑诊断为热射病。热射病是最严重的中暑类型，主要表现为高热、无汗、意识障碍，多器官功能损伤。
选项 A 休克是机体遭受强烈的致病因素侵袭后，由于有效循环血量锐减，组织血流灌注广泛、持续、显著减少，致全身微循环功能不良，生命重要器官严重障碍的综合症候群，但一般不会有高热，无汗，皮肤干热等表现，故排除。
选项 B 热痉挛是一种高温中暑现象，在干热环境条件下劳动，出汗过度，随汗液排出很多 NaCl，发生肢体和腹壁肌肉的痉挛现象，患者体温并不升高，不符合题意。
选项 D 热衰竭多由于大量出汗导致失水失钠过多引起血容量不足所致，表现为头昏，头痛，恶心，呕吐，心慌及血压下降等，但一般不会有高热，无汗，皮肤干热等表现，可排除。
选项 E 中暑是指在温度或湿度较高、不透风的环境下，因体温调节中枢功能障碍或汗腺功能衰竭，以及水、电解质丢失过多，从而发生的以心血管和中枢神经系统功能障碍为主要表现的急性综合病征，症状较轻，不符合题意。
综上所述，正确答案是：C.热射病。

根据题目描述，该患者在夏天建筑工地干活时突然出现头晕、头痛、大量出汗后无汗，皮肤干热，体温超过 40℃，血压 88/60 mmHg，并伴有意识障碍。这些症状高度提示热射病。

热射病（Heat Stroke）是一种严重的热相关疾病，通常发生在高温环境下，体温调节失常。其典型表现包括高热（体温通常超过 40℃）、中枢神经系统功能异常（如意识障碍、癫痫等）以及皮肤干热无汗（在经典型热射病中，运动型热射病可能仍有出汗）。如果不及时处理，热射病可能导致多器官功能衰竭和死亡。

其他选项分析：
- **A. 休克**：患者的血压较低，但休克的诊断需要结合更多的临床表现和原因，题干中更突出的是高热和意识障碍。

- **B. 热痉挛**：主要表现为肌肉痉挛，通常体温不会达到 40℃，也不会有明显的意识障碍。
- **D. 热衰竭**：也称为热衰竭综合征，主要表现为大量出汗、虚弱、乏力、头晕等，但不会有明显的高热和意识障碍。
- **E. 中暑**：是一个广义的术语，可以包括热痉挛、热衰竭和热射病，但题干中的具体症状更符合热射病的特征。

因此，最合适的答案是：

C. 热射病

79、 (A2 型题)

某化工厂工人，在一次事故中出现恶心、呕吐、震颤等急诊住院，查体：皮肤黏膜呈樱桃红色，呼出气中有苦杏仁味，疑为急性职业中毒，最可能是

A.一氧化碳

B.硫化氢

C.砷化氢

D.苯胺

E.氰化物

参考答案： E 您的答案：

图文解析： 【解析】氰化物中毒时，呼吸气味有苦杏仁味（E 对）。

【避错】硫化氢中毒时表现为上呼吸道黏膜的刺激症状，心悸、肺水肿、昏迷等；砷化氢 中毒时出现血红蛋白尿、贫血，重者急性肾衰竭；苯的急性毒作用主要表现为对中枢神经 系统的麻醉作用。

该题目描述的症状，包括恶心、呕吐、震颤、皮肤黏膜樱桃红色，以及呼出气中有苦杏仁味，最符合氰化物中毒的临床表现。氰化物中毒是一种紧急情况，需要立即治疗，因为它会迅速导致细胞呼吸的阻断和组织缺氧。

答案选 E.氰化物。

解析：

患者化工厂工人，因职业暴露后出现恶心、呕吐、震颤，查体显示皮肤黏膜呈樱桃红色，呼出气中有苦杏仁味，根据患者的职业、临床表现、体征，疑诊为氰化物中毒。氰化物中毒主要由呼吸道吸入、皮肤吸收或误食等，导致组织缺氧而窒息，其临床表现为前期乏力、头昏、头痛、恶心，之后出现强直性和阵发性抽搐，直至角弓反张，血压下降，尿、便失禁，常伴发脑水肿和呼吸衰竭，其特有的表现为黏膜和皮肤呈鲜红色，呼出气中有苦杏仁味。

选项 A 一氧化碳中毒常表现为口唇呈樱桃红色，但呼出气无特殊气味，与题干描述不符，故排除。

选项 B 硫化氢中毒常有畏光、流泪、眼刺痛、异物感等症状，且呼出气中有臭鸡蛋味，与题干描述不符，故不选。

选项 C 砷化氢中毒主要表现为不同程度的急性溶血和肾脏损害，呼出气中无特殊气味，与题干描述不符，可排除。

选项 D 苯胺中毒主要损害血液系统，引起溶血性贫血和肝、肾损害，皮肤可呈淡青紫色，但不会呼出苦杏仁味，故不选。

综上所述，正确答案是：E.氰化物。

根据题目描述，该化工厂工人在事故中出现恶心、呕吐、震颤等症状，查体发现皮肤黏膜呈

樱桃红色，呼出气中有苦杏仁味，这些特征提示急性中毒的可能性很大。

结合这些症状和体征，最可能的原因是氰化物中毒。氰化物中毒的典型特征包括：

- **樱桃红色皮肤黏膜**：氰化物通过抑制细胞色素氧化酶，导致组织缺氧，尽管血液中含氧量高，但组织无法利用氧气，从而出现樱桃红色皮肤黏膜。
- **呼出气中有苦杏仁味**：这是氰化物中毒的典型气味特征。

其他选项的特征：

- 一氧化碳（A）：通常引起头痛、眩晕、乏力、意识障碍，皮肤可呈樱桃红色，但没有苦杏仁味。
- 硫化氢（B）：有臭鸡蛋味，主要表现为呼吸道刺激症状。
- 砷化氢（C）：通常表现为溶血性贫血、黄疸、血红蛋白尿。
- 苯胺（D）：可引起高铁血红蛋白血症，导致皮肤和粘膜呈蓝色或灰色。

综上所述，最符合描述的答案是：

E. 氰化物

80、 (A2 型题)

男，18 岁，因“感冒”后自行服用抗菌药物半小时后突然出汗、面色苍白。查体：血压 40/20mmHg，神志不清，面色苍白，脉搏细速，四肢冰冷，发绀，该患者首要治疗

A.多巴胺静滴

B.肾上腺素肌肉注射

C.地塞米松静滴

D.异丙肾上腺素肌内注射

E.吸氧、心电监护

参考答案： B 您的答案：

图文解析： 【解析】青少年男性患者，自行服用抗菌药物后出现血压 40/20mmHg，神志不清，面色苍白，脉搏细速，四肢冰冷，发绀等一系列休克表现，考虑为过敏性休克，此时应紧急行肾上腺素肌内注射，故本题选 B。

【避错】过敏性休克表现为小血管扩张和毛细血管通透性增加，出现循环血量降低，血压下降，同时支气管平滑肌痉挛，出现呼吸困难，使用肾上腺素治疗过敏性休克，其激动 α1 受体，收缩小动脉和毛细血管前括约肌，而降低后者的通透性，激动 β1 受体改善心功能出现心肌收缩力增强、心率增快，减少过敏介质释放，扩张冠状动脉，因此可迅速缓解过敏性休克的临床症状，是治疗过敏性休克的首选药物。

这位 18 岁的男性患者在自行服用抗菌药物后出现突然出汗、面色苍白、血压下降（40/20 mmHg）、神志不清、脉搏细速、四肢冰冷和发绀，这些症状暗示他可能发生了严重的过敏反应或休克，特别是药物引起的过敏性休克。

首要治疗应该是立即稳定患者的血流动力学状态和改善氧合。考虑到他的症状严重性，首选的治疗应是肾上腺素，因为它能快速提高血压，缓解过敏反应。肾上腺素通常通过肌肉注射给药以确保迅速吸收和起效，特别是在急救情况下。

因此，最合适的选项是：B.肾上腺素肌肉注射。

解析：

青少年男性患者，自服抗菌素后出现低血压、神志不清，面色苍白，脉搏细速，四肢冰冷，发绀等休克的症状，考虑过敏性休克。肾上腺素能激动 α 和 β 受体，从而增强心肌收缩力，增加心排出量，加快心率，收缩血管，松弛支气管平滑肌，是救治过敏性休克的首选药。故此时患者应首选肾上腺素肌肉注射。
选项 A 多巴胺静滴主要用于心肌梗死、创伤、内毒素败血症等情况引起的休克综合征，但不作为过敏性休克的首选，故排除。
选项 C 地塞米松静滴是常用的抗过敏药物，但其起效较慢，作用较弱，一般不作为过敏性休克的首选，故不选。
选项 D 异丙肾上腺素肌内注射为 β 受体激动剂，能增强心肌收缩力，加快心率，扩张周围血管及支气管，但对 α 受体无作用，因此升压效果不明显，故不考虑。
选项 E 吸氧、心电监护是休克患者的一般辅助治疗措施，并非首要治疗措施，故不符合题意。
综上所述，正确答案是：B.肾上腺素肌肉注射。
该患者在服用抗菌药物后出现急性过敏反应，表现为休克的症状。休克是一种危及生命的紧急情况，必须迅速处理。根据现有医学指南和最佳实践，过敏性休克（过敏性反应引起的休克）的首选治疗是肾上腺素注射。

在这种情况下，最正确的答案是：

B. 肾上腺素肌肉注射

肾上腺素可以快速收缩血管，增加心输出量，逆转休克状态，并减少过敏反应的严重性。虽然多巴胺和异丙肾上腺素也有一定的升压作用，但它们并不是过敏性休克的首选药物。地塞米松是一种糖皮质激素，可以辅助治疗过敏反应，但不是急救的首选药物。吸氧和心电监护是支持治疗，但无法迅速逆转休克。
81、 (A2 型题)
患者入院后，出现特殊情况，需紧急处理，无法取得患者同意又无相联系人或家属在场，管床医师需行处理，需经得谁批准后开展
A.病房负责人
B.科室负责人
C.上级医师
D.医院监督管理部门
E.医院负责人
参考答案： E 您的答案：
图文解析： 【解析】在施行手术、特殊检查或者特殊治疗时，应当向患者做必要的解释，征得患者同意，并取得其家属或者关系人同意及签字；无法取得患者意见时，应当取得其家属或者关系人同意及签字；无法取得患者意见又无家属或者关系人在场，或者遇到其他特殊情况时，经治医师应当提出医疗处置方案，在取得医疗机构负责人（E 对）或者被授权负责人的批准后实施。
【避错】记忆性题目。
在紧急情况下，如果患者无法给予同意并且没有联系人或家属在场，处理这种情况通常需要遵循医院的紧急医疗干预政策。虽然这可能因国家和医院政策而异，但通常在紧急情况下，需要上级医师或医院负责人的批准来进行治疗，特别是当涉及到可能有生命危险的紧急情况时。
在中国，紧急医疗服务法律通常规定，当患者生命处于危险并且无法及时获取同意时，医生

有责任采取必要的治疗措施。在没有明确规定的情况下，上级医师通常具有做出这类紧急决策的权力。
考虑到这些因素，最佳答案可能是：
C. 上级医师
这是因为在紧急情况下，上级医师通常负责做出迅速的医疗决定，尤其是在没有其他授权人员可联系的情况下。
解析：
《医疗机构管理条例》规定，在紧急情况下为抢救垂危患者生命而采取紧急医学措施造成不良后果的不属于医疗事故，医疗机构及其医务人员在医疗活动中，必须严格遵守医疗卫生管理法律、行政法规、部门规章和诊疗护理规范、常规，恪守医疗服务职业道德。医师在执业活动中，应当遵守法律、法规，遵守技术操作规范。对于患者的紧急情况，需紧急处理，无法取得患者同意又无相关联系人或家属在场时，管床医师需经医院负责人或者被授权负责人员的批准后实施相应的医疗救治措施。
综上所述，正确答案是：E.医院负责人。

在患者入院后出现特殊情况，需要紧急处理且无法取得患者同意，又无相关联系人或家属在场时，根据中国的医疗法规和最佳实践指南，通常需要由上级医师批准后开展紧急处理。这是因为上级医师通常具备更多的经验和权威，能够迅速做出准确的决策以保障患者的安全和健康。因此，答案是：

C. 上级医师
82、 (A2 型题)
男，65 岁。腹泻一周，心悸 2 天。既往有高血压、房颤病史。口服华法林，培哚普利，硝酸酯类，地高辛治疗。急诊心电图:频繁室性期前收缩，短阵室性心动过速。如果 病情转变的原因，应选择哪项检查
A.地高辛浓度测定
B.凝血时间测定
C.D-二聚体测定
D.心肌坏死标志物测定
E.脑钠肽
参考答案： A 您的答案：
图文解析： 【解析】老年男性患者，口服华法林、培哚普利、硝酸酯类、地高辛治疗。腹泻 1 周，心悸两天，急诊心电图:频繁室性期前收缩（洋地黄中毒，其最重要的表现为各类心律失常，以室性期前收缩常见），短阵室性心动过速，根据患者检查及临床表现考虑洋地黄中毒，需行地高辛浓度测定，故本题选 A。
【避错】洋地黄中毒的胃肠道表现如恶心、呕吐，以及神经系统症状如视物模糊、黄视、绿视，定向力障碍、意识障碍等则较少见。
在这个病例中，患者有高血压和房颤病史，同时使用华法林（一种抗凝血药物）、培哚普利（降血压药）、硝酸酯类（用于治疗心绞痛）和地高辛（用于心脏功能问题）。急诊心电图显示频繁室性期前收缩和短阵室性心动过速，这可能表明地高辛毒性，特别是考虑到患者有腹泻史，腹泻可能导致电解质失衡，增加地高辛毒性的风险。
因此，正确答案应该是 **A. 地高辛浓度测定**。这项检查可以确定是否存在地高辛毒性，这是考虑到患者的病史和临床表现后最有可能的原因。其他选项虽然在某些情况下可能相关，但针对这个特定病例和患者的症状，测定地高辛浓度是最直接和相关的检查。

解析：
老年男性患者，腹泻一周，心悸 2 天，心电图显示频繁室性期前收缩，短阵室性心动过速。由于患者长期口服地高辛，腹泻一周，疑为地高辛中毒。地高辛安全范围窄，有效量和中毒量很接近，使用时应进行血药浓度监测，所以应做地高辛浓度测定。
选项 B 凝血时间测定主要用于监测华法林抗凝效果，与题意不符。
选项 C：D-二聚体测定可以作为纤溶亢进和血栓形成的辅助诊断，不能反映地高辛中毒，故不选。
选项 D：心肌坏死标志物测定用于诊断心肌梗死，该患者并无心肌梗死，故排除。
选项 E：脑钠肽主要用于诊断心衰，该患者未出现心衰，故不考虑。
综上所述，正确答案是：A.地高辛浓度测定。

根据题目描述，该患者 65 岁，有高血压和房颤病史，并且正在服用华法林、培哚普利、硝酸酯类和地高辛。出现了腹泻一周和心悸 2 天的症状，急诊心电图显示频繁室性期前收缩和短阵室性心动过速。

在这种情况下，需要考虑患者的药物是否导致了当前症状的恶化。特别是，地高辛是一种用于治疗心力衰竭和房颤的药物，但其治疗窗口较窄，容易出现中毒情况。地高辛中毒的表现包括胃肠道症状（如腹泻）和心脏症状（如室性心律失常）。

因此，最合适的检查是 **A.地高辛浓度测定**。

这将有助于判断患者的地高辛水平是否过高，从而引起当前的症状变化。其他选项如凝血时间测定、D-二聚体测定和心肌坏死标志物测定虽然在某些临床情况中有用，但在此具体案例中，地高辛浓度测定是最直接和相关的检查。

答案：**A.地高辛浓度测定**
83、 (A2 型题)
男，76 岁。尿频、尿急、夜尿增多，排尿困难两个月，病变累及结构为
A.前列腺囊
B.前列腺外周区
C.前列腺纤维肌性基质
D.前列腺中央区
E.前列腺移行区
参考答案： E 您的答案：
图文解析： 【解析】老年男性患者，尿频、尿急、夜尿增多，排尿困难两个月，诊断为良性前列腺增生。前列腺增生主要发生于尿道周围移行带（E 对）。
【避错】前列腺癌主要为腺癌，多发于外周带。
76 岁男性患者出现尿频、尿急、夜尿增多和排尿困难的症状，最可能受累的前列腺结构是前列腺移行区。这些症状通常与良性前列腺增生（BPH）相关，而良性前列腺增生主要发生在前列腺的移行区。因此，最佳答案是：
C. 前列腺移行区
首先，我们要了解该患者的主要症状：老年男性，有尿频、尿急、夜尿增多，以及排尿困难的症状，这些症状均指向前列腺增生症。
现在，我们逐一分析选项：

A. 前列腺囊：这不是前列腺增生症主要涉及的区域，故排除。
B. 前列腺外周区：虽然这也是前列腺的一部分，但它不是前列腺增生症主要累及的区域，故不选。
C. 前列腺纤维肌性基质：这个部分虽然与前列腺功能和结构有关，但不是前列腺增生症的主要病变区域，故排除。
D. 前列腺中央区：同样，这个区域不是前列腺增生症的主要病变部位，故不选。
E. 前列腺移行区：这是前列腺增生症最常累及的区域。增大的前列腺组织主要来源于前列腺移行区的腺体组织和基质组织的增生。这种增生会对尿道产生压迫，从而引发尿频、尿急、夜尿增多以及排尿困难等症状。
综上所述，该患者病变累及的结构最可能是前列腺移行区。
因此，正确答案是 E.前列腺移行区。

这道题目描述了一名 76 岁的男性，症状包括尿频、尿急、夜尿增多和排尿困难。这些症状提示可能存在前列腺相关的病变。根据临床表现，最可能累及的前列腺区域是：
A. 前列腺囊
B. 前列腺外周区
C. 前列腺纤维肌性基质
D. 前列腺中央区
E. 前列腺移行区
结合患者的症状和前列腺病变的常见区域，前列腺移行区是前列腺增生（BPH）的常见部位，而 BPH 通常会引起上述尿路症状。因此，最有可能的答案是：E. 前列腺移行区

84、 (A2 型题)
足月儿，出生后 12h，出现巩膜黄染 6h，神志欠佳。其母亲有流产史，B 型血，Rh 阴性，其父亲 A 型血，Rh 阳性，查血：Hb142g/L，造成该患儿疾病相关的抗体是
A.IgM
B.IgE
C.IgG
D.IgD
E.IgA
参考答案： C 您的答案：
图文解析： 【解析】足月儿，出生后 12h，出现巩膜黄染 6h，其母有流产史 B 型血，Rh 阴性，其父亲 A 型血，Rh 阳性，考虑为新生儿溶血，血型为 Bh 的母亲由于输血、流产或分娩等原因接受 Rh+红细胞刺激后，可产生抗 Rh 的 1gG 类抗体。故本题选 C。
【避错】ABO 血型系统的天然抗体为 IgM，血型不符的输血，可导致严重溶血反应
这个考题涉及到新生儿溶血性疾病，特别是与 Rh 血型不合引起的。在这种情况下，母亲是 Rh 阴性，而父亲是 Rh 阳性，可能导致胎儿为 Rh 阳性。如果母亲产生针对 Rh 阳性红细胞的抗体，这些抗体可以穿过胎盘，攻击胎儿的红细胞，引起溶血。
对于新生儿，出生后不久出现黄疸（如题中的巩膜黄染）和神志不佳，可能是由于溶血性疾病导致的高胆红素血症。母亲的 Rh 阴性与父亲的 Rh 阳性血型不匹配增加了这种可能性。
能够穿过胎盘并在新生儿中引起溶血的抗体类型是 IgG。因此，最可能导致这个新生儿疾病的相关抗体是 IgG。
所以，正确答案是 C.IgG。
解析：

足月患儿，出生后 12 小时出现巩膜黄染，应考虑为新生儿溶血性疾病。其母为 B 型血，Rh 阴性，有流产史，其父为 A 型血，Rh 阳性，根据双亲的血型可推断患儿的血型可能为 A 型，Rh 阳性，因此可造成母体内产生免疫性 Rh 抗体，再次怀孕时经胎盘进入胎儿体内可引起溶血。由于免疫性 Rh 抗体属于 IgG 型抗体，分子量较小，因此可以通过胎盘进入胎儿体内引起溶血。
选项 A：IgM 分子量较大，不能透过胎盘，不会对胎儿造成溶血性疾病，故排除。
选项 B：IgE 与机体的抗寄生虫免疫与皮肤过敏有关，不参与新生儿溶血性疾病的发生，因此排除。
选项 D：IgD 的功能尚不清楚，可能与启动 B 细胞产生抗体有关，还可能与某些超敏反应有关，与题意不符。
选项 E：IgA 主要存在于乳汁、唾液、泪液，以及呼吸道、消化道和泌尿生殖道等黏膜表面，参与机体的黏膜免疫，与本题不符。
综上所述，正确答案是：C.IgG。

这道题描述了一名足月儿出生后 12 小时出现黄疸，且母亲有流产史，血型为 B 型 Rh 阴性，而父亲为 A 型 Rh 阳性。结合这些信息，这名新生儿很可能患有新生儿溶血病（新生儿黄疸的一种），这是由于母婴血型不合引起的免疫反应，尤其是 Rh 不合。

在 Rh 血型不合的情况下，母亲的 Rh 阴性血液可能会对胎儿的 Rh 阳性血液产生免疫反应，生成针对胎儿红细胞的抗体，这些抗体通过胎盘进入胎儿体内，导致胎儿红细胞被破坏，从而引发溶血和黄疸。这类抗体是 IgG 型，因为 IgG 是唯一一种可以通过胎盘屏障的抗体。

因此，正确答案是：

C. IgG
85、 (A2 型题)
某研究员获得了我市北方某城市 1998 年到 2017 年的 2 型糖尿病患病率的数据，想要描述该市 20 年来 2 型糖尿病患病率与时间的变化趋势，应该用哪种统计图
A.线图
B.圆图
C.直条图
D.直方图
E.散点图
参考答案： A 您的答案：
图文解析： 【解析】线图（A 对）是用线段的升降来表示指标（变量）的连续变化情况，适用于描述一个变量随另一个变量变化趋势。
【避错】圆图（B 错）中把圆的总面积作为 100%，表示事物的全部，而圆内各扇形面积用来表示全体中各部分所占的比例。直条图（C 错）又称条图，用等宽直条的长短来表示相互独立的统计指标数值大小和它们之间的对比关系。直方图（D 错）用直条矩形面积代表各组频数，各矩形面积总和代表频数的总和。它主要用于表示连续变量频数分布情况。散点图（E 错）用点的密集程度和变化趋势表示两指标之间的直线或曲线关系。
为了描述该市 20 年来 2 型糖尿病患病率与时间的变化趋势，最适合使用线图（A 选项）。线图能够清晰地展示数据随时间的变化趋势，是表达连续数据变化，如患病率随时间变化的理想选择。圆图、直条图、直方图和散点图虽然能展示数据分布或比例，但不如线图直观有效

地显示长时间跨度内的趋势变化。因此，最佳答案是 A.线图。
这是一道关于数据可视化的选择题。要根据数据的特性和展示需求，选择最合适的统计图。
我们先来分析题目中的关键信息：
首先，理解题目背景和要求：研究员获得了某市 20 年的 2 型糖尿病患病率数据，目的是要展示患病率随时间的变化趋势。
接下来，我们分析每个选项的适用性：
A.线图：线图是用线段的升降来表示变量的连续变化情况，适用于描述一个变量随另一个变量变化趋势，通常用于描述统计指标随时间的变化趋势。
B.圆图：圆图主要用于表示事物的各部分所占的比例，不适用于展示时间序列数据。
C.直条图：直条图主要用于表示相互独立的统计指标数值大小和它们之间的对比关系，不适合展示连续变化趋势。
D.直方图：直方图主要用于表示连续变量频数分布情况，不适用于本案例。
E.散点图：散点图主要用于表示两指标间的关系，虽然可以展示数据点，但不如线图直观展示变化趋势。
结合题目要求，要描述的是 2 型糖尿病患病率与时间的变化趋势，线图最适合用于展示这种连续性的变化趋势。
综上所述，正确答案是：A.线图。

描述某城市 20 年来 2 型糖尿病患病率与时间的变化趋势，最适合的统计图是线图。线图可以清晰地显示出随时间变化的趋势，帮助研究员观察患病率的增加或减少情况。

正确答案：A. 线图
86、 (A2 型题)
若要研究 2~7 岁儿童年龄 X 与平均体重 Y（kg）的关系可表示为 Y＝7+2X，则以下正确的是
A.0 岁儿童的平均体重为 7kg
B.11 岁儿童平均体重为 29kg
C.5 岁儿童平均体重为 17kg
D.6 岁比 5 岁平均体重多 7kg
E.1 岁到 2 岁增长 2kg
参考答案： C 您的答案：
图文解析： 【解析】2~7 岁（A、B、E 错，0 岁、1 岁及 11 岁儿童不适用于此公式）儿童年龄 X 与平均体重 Y（kg）的关系可表示为 Y＝7+2X，根据公式可得 5 岁儿童平均体重为 17kg，6 岁儿童平均体重为 19kg（D 错），故本题选 C。
【避错】注意公式中 X 的取值范围。
根据给定的线性方程 Y = 7 + 2X，其中 X 代表年龄，Y 代表平均体重（kg），我们可以计算不同年龄儿童的平均体重。通过替换 X 的值，我们能找出哪个选项是正确的。
- 对于 A 选项，当 X=0 时，Y=7+2*0=7，因此 0 岁儿童的平均体重为 7kg，这看起来是正确的。
- 对于 B 选项，当 X=11 时，Y=7+2*11=29，所以 11 岁儿童的平均体重是 29kg，这也是正确的。
- 对于 C 选项，当 X=5 时，Y=7+2*5=17，所以 5 岁儿童的平均体重是 17kg，这也是正确的。
- 对于 D 选项，比较 6 岁与 5 岁儿童的体重差异，6 岁时 Y=7+2*6=19，5 岁时 Y=17，6 岁比 5 岁多 2kg，不是 7kg，因此这个选项是错误的。
- 对于 E 选项，从 1 岁到 2 岁，增长的体重为 2*(2-1)=2kg，因此这个选项也是正确的。

在这些选项中，A、B、C 和 E 都是正确的，但题目要求选择一个最佳答案。由于这是关于线性关系的数学计算，而所有这些答案都可以直接从给定的方程中算出，没有明确的“最佳”答案，因此我们需要找到一个在实际情况中最合理的答案。
考虑到实际情况，0 岁儿童的平均体重为 7kg（选项 A）似乎是最合理的，因为它基于方程设定的基线。因此，虽然多个选项在数学上是正确的，但 A 选项可能是最符合题目要求的答案。
这是一道关于儿童年龄与平均体重关系的推理题。

首先，我们要理解题目中给出的公式：Y = 7 + 2X，其中 Y 代表平均体重，X 代表儿童的年龄。这个公式表明，儿童的平均体重与他们的年龄之间存在线性关系，具体来说，每增加一岁，平均体重增加 2kg，并且基础体重（即 0 岁时的体重）为 7kg。
现在，我们逐一分析各个选项：
A. 0 岁儿童的平均体重为 7kg：根据公式 Y = 7 + 2X，当 X=0 时，Y=7。这意味着 0 岁儿童的平均体重是 7kg。此选项正确。
B. 11 岁儿童平均体重为 29kg：将 X=11 代入公式，得到 Y = 7 + 2*11 = 29。但题目明确指出研究范围是 2~7 岁的儿童，所以 11 岁不在研究范围内，此选项虽然数学上正确，但与题意不符，因此错误。
C. 5 岁儿童平均体重为 17kg：将 X=5 代入公式，得到 Y = 7 + 2*5 = 17。这符合题意，但与正确答案 A 相比，不是本题的直接答案。
D. 6 岁比 5 岁平均体重多 7kg：根据公式，6 岁和 5 岁儿童的体重差应为 2kg（因为每增加一岁体重增加 2kg），而不是 7kg。此选项错误。
E. 1 岁到 2 岁增长 2kg：这个选项是正确的，因为根据公式，每增加一岁体重增加 2kg。但与正确答案 A 相比，不是本题的直接答案。
综上所述，正确答案是 A：0 岁儿童的平均体重为 7kg。

根据题干，给出了 2~7 岁儿童的年龄 X 与平均体重 Y（kg）之间的关系，可以用公式 Y＝7+2X 表示。

让我们逐个选项进行分析：

A. 0 岁儿童的平均体重为 7kg
题干要求研究的是 2~7 岁儿童的体重关系，0 岁儿童不在研究范围内。这个选项的假设不在题干范围内，因此不符合题意。

B. 11 岁儿童平均体重为 29kg
题干只研究 2~7 岁的儿童，11 岁超出了研究范围，无法用给定公式计算其体重，因此这个选项不正确。
C. 5 岁儿童平均体重为 17kg
代入公式 Y＝7+2X，X＝5，即 Y＝7+2×5＝7+10＝17kg。这是正确的计算结果。
D. 6 岁比 5 岁平均体重多 7kg
计算 6 岁儿童的体重：Y＝7+2×6＝7+12＝19kg。
计算 5 岁儿童的体重：Y＝7+2×5＝7+10＝17kg。
6 岁比 5 岁多的体重：19kg - 17kg = 2kg。因此这个选项错误。
E. 1 岁到 2 岁增长 2kg

题干给出的研究范围是 2~7 岁，不涉及 1 岁的情况。这个选项超出了题干的范围，因此不符合题意。

综上所述，正确答案是：

C. 5 岁儿童平均体重为 17kg
87、 (A2 型题)
要分析甲乙两地钩虫病患病率，已知男性患病率高于女性，甲地男性比女性多，乙地女性比男性多，今欲比较甲乙两地钩虫的患病率，下列说法正确的是
A.先对性别进行标准化后再计算
B.无法比较
C.分性别进行比较
D.卡方检验
E.Z 检验
参考答案： A 您的答案：
图文解析： 【解析】在比较两不同人群的患病率、发病率、死亡率等资料时，为消除其内部构成（如年龄、性别、工龄、病程长短、病情轻重等）对率的影响，可以使用标准化率（A 对）。
【避错】χ2 检验主要目的是推断两个或多个总体率或构成比之间有无差别，Z 检验、χ2 检验均只是一些检验方法，无法消除其内部构成产生的误差。
正确答案是 A。要比较甲乙两地的钩虫病患病率时，需要考虑性别构成的差异，因为已知男性患病率高于女性。甲地男性比女性多，乙地女性比男性多，这种性别比例的差异会影响患病率的直接比较。因此，应该先对性别进行标准化，以消除性别结构差异对患病率比较的影响，再计算和比较两地的标准化患病率。这样可以更公平、准确地比较两地的钩虫病患病率。
解析：
本题需对甲乙两地钩虫患病率进行比较，而甲乙两地男女比例不一致，故应先调整两地性别比例，即先对性别进行标准化后再计算患病率，以消除性别构成不同的影响。
若直接对甲乙两地钩虫患病率进行比较，会由于甲乙两地男女比例不一致而导致结果不准确，故选项 B 无法比较不选。
选项 C 分性别进行比较只能得出不同性别患病率的差异，而不能得出甲乙两地的患病率差异，故不选。
选项 D 卡方检验是用于推断两个或多个总体率或构成比之间有无差别，与本题无关。
选项 E 的 Z 检验适用于大样本时两组均数之间比较，不适用于患病率的比较，故不考虑。
综上所述，正确答案是：A.先对性别进行标准化后再计算。

要比较甲乙两地钩虫病患病率，已知甲地男性比女性多，乙地女性比男性多，并且男性的患病率高于女性，这样会使得直接比较两地的患病率结果失真。为了得到更准确的比较结果，应该先对性别进行标准化后再计算。因此，最正确的答案是：

A. 先对性别进行标准化后再计算
88、 (A2 型题)
某地区对 500 名沙门氏菌感染患者的感染情况进行调查，最能反映该地区沙门氏菌时长平均情况的是

A.平均数
B.几何平均数
C.中位数
D.P75
E.标准差
参考答案：　　C　您的答案：
图文解析：　　【解析】中位数是一组数据按大小顺序排列后的中间数，通常用于表示一组数据的“中位数”水平。由于沙门氏菌感染情况为等级资料，且中位数可反映一组数据的集中趋势，故最能反映该地区沙门氏菌感染情况平均情况的是中位数（C 对）。
【避错】平均数是一组数的总和除以这组数的数量，通常用于表示一组数据的“平均”水平（A 错）。几何平均数是一组数的乘积的 n 次方根，通常用于表示一组数据的“平均变化率”（B 错）。P75 表示第 75 百分位数，即数据按大小顺序排列后的第 75 百分位的数值，通常用于表示一组数据的“上边缘”水平（D 错）。标准差表示一组数据的离散程度，通常用于表示一组数据的“波动”情况（E 错）。
在这种情况下，为了最能反映该地区沙门氏菌感染时长的平均情况，最合适的统计量是中位数（选项 C）。中位数是将数据集中间的值，并且当数据分布不对称或包含异常值时，中位数比平均数更能准确地反映数据的中心趋势。沙门氏菌感染的持续时间可能因个体差异而极端变化，因此中位数在这种情况下是更稳健的度量标准，它不会被极端值影响。所以，正确答案是 C.中位数。
解析：
本题中，我们要找出一个统计量，能最准确地反映沙门氏菌在患者体内的平均感染时长。考虑到感染时长的数据可能呈现偏态分布，特别是当存在极端值时，算术平均数可能无法准确反映大多数患者的情况。而中位数是将一组数据从小到大排列后，位于中间位置的数。在偏态分布中，中位数能更好地代表大多数数据的情况，因为它不受极端值的影响。因此，对于沙门氏菌感染时长的偏态分布数据，中位数是最能反映大多数患者感染时长的统计量。
综上所述，正确答案是：C.中位数。
选项 A 平均数即算术平均数，适用于数据呈现正态分布或近似正态分布的情况，且容易受到极端值的影响，而本题中沙门氏菌感染时长可能呈现偏态分布，故不选。
选项 B 几何平均数通常用于反映一组经对数转换后呈正态分布的数据的平均水平，或是计算平均增长率，故不考虑。
选项 DP75 是一个百分位数，表示有 75%的数据小于或等于这个值，但它并不能全面反映整体数据的平均水平，故排除。
选项 E 标准差用于测量数据的离散程度，而不是平均水平，故不选。

在统计学中，用于描述一组数据的集中趋势的指标有很多种。对于沙门氏菌感染患者的感染情况，如果要反映时长的平均情况，选择适当的集中趋势指标是非常重要的。

1. **平均数（A）**：即算术平均数，是所有数据值的总和除以数据的个数。它受极端值影响较大，如果数据分布有偏或存在异常值，平均数可能会被拉得过高或过低。

2. **几何平均数（B）**：几何平均数适用于乘积关系的数据，例如生长率等。它在沙门氏菌感染时长中不常使用，因为时间数据通常不是以乘法关系呈现的。

3. **中位数（C）**：中位数是将数据按大小顺序排列后，处于中间位置的值。如果数据有

偏或存在极端值，中位数不会像平均数那样受到极端值的影响。因此，中位数更能代表一组偏态数据的中心趋势。

4. **P75（D）**：第 75 百分位数，表示 75%的数据值小于或等于该值。它主要用来描述数据的分布，而不是集中趋势。

综上所述，对于反映某地区沙门氏菌感染患者的感染时长平均情况，最能代表数据集中趋势且不受极端值影响较大的是**中位数（C）**。

因此，最佳答案是：
C. 中位数
89、 (A2 型题)
某尿路感染药物疗效检测共收集了 87 例患者，其中 43 例治愈，17 例显效，6 例改善，21 例无效。该资料属于
A.连续型变量资料
B.有序多分类变量资料
C.无序多分类变量资料
D.定量资料
E.定性资料
参考答案： B 您的答案：
图文解析： 【解析】等级资料，又称为有序多分类资料（B 对）是变量的观测结果是定性的，但各类别（属性）之间有程度或顺序上的差别，如药物的治疗效果按照“显效，有效，好转，无效”进行分类等。
【避错】定性资料说明的是研究对象的品质特征，表现为互不相容的类别。定量资料是变量的观测结果是数值型的，用来说明研究对象的数量特征，其特点是能够用数值大小衡量观察单位不同特征水平的高低， 一般有计量单位。
在这个尿路感染药物疗效检测的案例中，我们看到患者的疗效被分为“治愈”、“显效”、“改善”和“无效”四种类别。这些类别是有顺序的，从“治愈”到“无效”表示疗效的逐渐降低。因此，这种数据类型是根据疗效的不同级别进行分类的，并且这些分类是有序的。
选项解析如下：
A. 连续型变量资料指的是可以无限细分的数值数据，例如身高、体重等，这里不适用。
B. 有序多分类变量资料是指数据分为几个有序的类别，适用于此案例。
C. 无序多分类变量资料是指数据分为几个没有内在顺序的类别，例如血型、职业等，这里不适用。
D. 定量资料是指可以量化的数值数据，通常是连续的或可数的，这里不适用。
E. 定性资料是指不能以数值形式表示的分类数据，虽然这个案例的数据是分类的，但更具体地属于有序分类。
因此，最合适的答案是 **B. 有序多分类变量资料**。
这是一道关于数据类型识别的问题。首先，我们需要了解题目中给出的数据及其分类，然后结合各选项的定义，来判断哪种类型最符合题目描述的数据。

理解背景信息：题目描述了一个关于尿路感染药物治疗效果的研究，涉及 87 例患者，其治疗效果被分类为治愈、显效、改善和无效四种。
理解问题核心：我们需要根据这些数据的特点，从给定的选项中选择最符合的类型。

现在，我们逐一分析每个选项：
A. 连续型变量资料：这指的是可以取任一实数值的变量，如身高、体重等。本题中的数据是分类的，不是连续的数值，因此 A 选项不符合。
B. 有序多分类变量资料：这指的是变量有多个分类，并且这些分类之间有一定的顺序关系。本题中的治疗效果（治愈、显效、改善、无效）确实存在一种从好到差的顺序关系，因此 B 选项看似合理。
C. 无序多分类变量资料：指的是分类之间没有明确的顺序关系，如血型（A、B、O、AB）。本题中的分类是有序的，所以 C 选项不正确。
D. 定量资料：这指的是可以量化的数据，如身高、体重等连续型变量。虽然治疗效果可以量化（如治愈=4，显效=3，改善=2，无效=1），但原始数据本身不是以数量形式存在的，因此 D 选项也不符合。
E. 定性资料：指的是描述性质的资料，不能进行数学运算。虽然治疗效果可以量化转换，但其本质仍是描述性质的，且题目未给出具体的量化数值，因此可以看作是定性资料。
综合考虑，虽然治疗效果看似有序（治愈、显效、改善、无效），但在这个问题中，我们更关注的是治疗效果的“质”的差异，而不是“量”的差异。即，我们关注的是患者是否治愈，是否有效，而不是治疗效果的“量”有多少。因此，该资料更符合定性资料的特征。
因此，最正确的答案是 E.定性资料。

根据题目描述，该尿路感染药物疗效检测的结果包括“治愈”、“显效”、“改善”和“无效”四种情况。此类资料的特征是有序的分类变量，即可以对这些类别进行排序，但不能进行定量测量。因此，最正确的答案是：

B. 有序多分类变量资料
90、 (A2 型题)
一烧伤患者，来门诊换药，质疑医生专业能力，说为什么不将两种药一起用，医生耐心解释道其中一种药会使另一种药的药效减弱，不能一起用。该过程体现了医师用药 则是
A.严守法规
B.节约费用，公正分配
C.合理配伍，适时调整
D.合理用药，细致观察
E.对症下药，因人施治
参考答案： C 您的答案：
图文解析： 【解析】医生向烧伤患者解释为什么两种药物不能一起用，该过程体现了医师用药过程中的合理配伍，适时调整原则，故本题选 C。
【避错】癫痫患者用药原则应为：按类型用药、单药用药、尽早用药、小量起始、长期治疗。
此题考查的是医生在用药过程中的原则和考虑。医生解释不能将两种药物一起使用是因为其中一种会降低另一种的药效，这反映了医生在用药时的合理配伍和适时调整的原则。因此，正确答案应该是：
C.合理配伍，适时调整
这个选项强调了医生根据药物间相互作用的知识，合理地选择和调整药物，以确保治疗的有效性和安全性。
这是一道医学伦理和用药原则的选择题。我们需要先理解题目背景信息和问题核心，再仔细

分析每个选项，并将其与题中给出的信息进行对比。
理解背景信息：医生向患者解释，两种药物不能同时使用，因为其中一种药物会使另一种药物的药效减弱。
理解问题的核心：我们需要确定哪个选项最能体现这一用药原则。
分析选项：
A. 严守法规：虽然医师在执业过程中应遵守相关法规，但本题中医生的解释并未直接体现这一点。
B. 节约费用，公正分配：这一选项涉及到医疗资源的分配和经济考虑，与题目中的情况不直接相关。
C. 合理配伍，适时调整：这个选项直接对应了题目中的情况，医生解释了为什么不能同时使用两种药物，即药物之间存在相互作用，需要合理配伍。
D. 合理用药，细致观察：虽然合理用药是医师用药的一个重要原则，但本题中的重点在于药物之间的相互作用和配伍问题，而非单纯的合理用药和观察。
E. 对症下药，因人施治：这个选项强调的是针对个体差异进行治疗，与题目中的药物配伍问题不直接相关。
综上所述，医生的解释直接体现了在用药过程中需要注意药物的合理配伍，避免药物之间的不良相互作用，因此选项 C“合理配伍，适时调整”是最符合题目描述的选择。
因此，答案是 C.合理配伍，适时调整。

这道题目描述了一位医生向患者解释为什么不能将两种药物一起使用的情况，因为其中一种药物会减弱另一种药物的药效。这体现了医生在用药过程中遵循合理配伍、适时调整的原则。合理配伍是指药物之间的相互作用，确保药物组合的安全性和有效性，避免不良反应或药效减弱。适时调整则指根据患者的具体情况对治疗方案进行调整，以达到最佳治疗效果。
因此，最正确的答案是：C. 合理配伍，适时调整
91、 (A2 型题)
一老人因为牙口不好，经常以大米粥，稀饭等为主食，也经常食用肉类、蛋类，但经常出现便秘，考虑老人便秘的原因为
A.蔬菜进食过少
B.以谷类食物为主
C.肉类进食过少
D.豆制品进食过多
E.以面食为主食
参考答案： A 您的答案：
图文解析： 【解析】该患者饮食简单，缺粗纤维，使粪便体积缩小，黏滞度增加，在肠内运动减慢，水分过度吸收而致便秘。故本题选 A。
【避错】便秘是指排便次数减少，同时排便困难、粪便干结。正常人每日排便 1～2 次或 1 ~2 日排便 1 次，便秘患者每周排便少于 3 次，并且排便费力，粪质硬结、量少。
便秘的原因可能有多种，但根据题干信息，这位老人主要以大米粥、稀饭为主食，并且也经常食用肉类和蛋类。没有提到蔬菜的摄入情况，可以推测蔬菜的摄入可能不足。蔬菜富含纤维，对于促进肠蠕动和防止便秘非常重要。因此，最可能的原因是蔬菜进食过少。
选项 A“蔬菜进食过少”最符合这位老人便秘的可能原因，所以正确答案应该是 A。
解析：
老年患者的饮食习惯以大米粥，稀饭等为主食，也经常食用肉类、蛋类，但经常出现便秘。分析该患者的饮食结构，主食以谷类为主，蛋白质来源以肉类蛋类为主，缺乏富含膳食纤维

的蔬菜，而膳食纤维具有亲水性，能吸收水分，使食物残渣膨胀并形成润滑凝胶，容易推进粪便，且膳食纤维还能刺激肠壁，加快肠道蠕动，因此膳食纤维摄入过少是导致患者便秘的主要原因。
选项 B 以谷类食物为主，选项 E 以面食为主食，均能提供机体所需的碳水化合物等，且对肠道具有一定的刺激作用，能促进排便，不是便秘的原因，故可一并排除。
选项 C 肉类进食过少一般会导致蛋白质摄入不足，不会导致便秘，故不选。
豆制品中多含有低聚糖等物质，该类物质与水结合后体积可膨胀，能对肠道造成刺激，加快肠道蠕动，从而可缓解便秘，选项 D 豆制品进食过多不仅不会导致便秘，反而有助于排便，故排除。
综上所述，正确答案是：A.蔬菜进食过少。

这道题目中，一位老人经常以大米粥和稀饭为主食，同时也食用肉类和蛋类，但经常出现便秘。为了找出便秘的原因，我们需要考虑他的饮食习惯。

根据题目描述，老人的饮食中蔬菜摄入较少，而蔬菜是膳食纤维的重要来源，纤维有助于促进肠蠕动，防止便秘。因此，便秘的主要原因可能是因为蔬菜摄入不足。

所以，最可能的答案是：

A. 蔬菜进食过少
92、 (A2 型题)
某医生对某地区 1160 名群众进行糖尿病病人筛查，共发现 140 名糖尿病患者，对这些人进行空腹血糖检测，以空腹血糖≥6.1 为阳性，结果如下，问阳性检测率是多少

A.27/85
B.87/89
C.31/112
D.124/140
E.58/85
参考答案： D 您的答案：
图文解析： 【解析】对 140 名糖尿病患者进行空腹血糖检测，以空腹血糖≥6.1mmol/L 为阳性，检查结果为阳性：124，阴性：16，则该标准的阳性检测率为 124/140，故本题选 D。【避错】灵敏度又称敏感度或真阳性率,指金标准确诊的病例中待评价试验也判断为阳性者所占的百分比。它可反映待评价试验能将实际患病的病例正确地判断为患某病的能力。特异度又称真阴性率，指金标准确诊的非病例中待评价试验也判断为阴性者所占的百分比。它可反映待评价试验能将实际未患某病的研究对象正确地判断为未患某病的能力。
在这个问题中，我们需要确定阳性检测率，即被检测出空腹血糖≥6.1 的人数除以总共进行空腹血糖检测的糖尿病患者人数。
给定信息中并没有提到具体多少人的空腹血糖≥6.1，只知道总共发现了 140 名糖尿病患者并对这些人进行了空腹血糖检测。通常，阳性检测率是指所有测试对象中测试结果为阳性的比例，所以我们应该计算的是阳性结果的人数除以总测试人数（140 名糖尿病患者）。
选项中的数字应该代表了不同的阳性检测结果和测试总数的组合。这里最合理的推测是阳性人数应接近 140 名，因为这是总共检测的糖尿病患者人数。
因此，最接近 140 名阳性的选项是 D. 124/140。这表示有 124 名糖尿病患者的空腹血糖测量

结果是阳性的，总共检测了 140 名糖尿病患者。
所以，阳性检测率应该是 124/140，选择 D 作为答案。
这是一道关于糖尿病筛查的问题，需要我们计算空腹血糖检测的阳性率。
理解题目背景信息：这个问题涉及到对 1160 名群众进行糖尿病筛查，共发现 140 名糖尿病患者。接下来，对这些糖尿病患者进行了空腹血糖检测，以空腹血糖≥6.1 为阳性。
理解问题的核心：我们需要基于这些数据来计算阳性检测率。
分析选项：
A. 27/85：这个比例与题目中提供的数据不匹配。
B. 87/89：同样，这个比例也不符合题目中的数据。
C. 31/122：这个比例也不符合题目中的数据。
D. 124/140：根据题目描述，空腹血糖≥6.1 被视为阳性，而题目中明确指出空腹血糖≥6.1 的人数为 124 人。总检测人数为 140 人，因此阳性检测率应该是 124/140。
E. 58/85：这个比例与题目中的数据不匹配。
综上所述，空腹血糖检测的阳性率应该是检测呈阳性的人数除以总检测人数，即 124/140。因此，正确答案是 D.124/140。

要计算阳性检测率，我们需要了解定义：阳性检测率是指筛查结果中阳性结果的比率。在这道题中，我们关注的是空腹血糖检测的阳性结果。

从题目中我们得知：对 1160 名群众进行筛查，发现 140 名糖尿病患者。对这些糖尿病患者进行空腹血糖检测，并以空腹血糖≥6.1 为阳性。

因此，我们需要找到以下比例：

$$\text{阳性检测率} = \frac{\text{阳性结果数量}}{\text{检测总人数}}$$

题目提供的选项中只有 D 选项的分母是 140（检测总人数），所以我们只需看阳性结果数量是不是 124。

答案是：

$$D. 124/140$$

所以阳性检测率是：

$$\frac{124}{140} = 0.8857$$

约等于 88.57%。

正确答案是 D. 124/140。
93、 (A2 型题)
某医院选取 117 名孕妇，研究孕妇注射催产素后对新生儿黄疸有无影响。其中 60 名孕妇给予药物治疗，57 名孕妇不给与药物治疗，观察新生儿是否出现新生儿黄疸，从而 与新生儿黄疸的联系。此种研究方法为

A.病例对照研究
B.队列研究
C.现况研究
D.实验研究
E.横向研究
参考答案：　　D　您的答案：
图文解析：　　【解析】临床试验（D 对）是将来自同一总体的研究对象随机分为实验组和对照组，实验组给予实验因素，对照组不给予该因素，然后前瞻性地随访各组的结局并比较其差别的程度从而判断实验因素的效果。
【避错】病例对照研究（A 错）是选择患有和未患有某特定疾病的人群分别作为病例组和对照组，调查各组人群过去暴露于某种或某些可疑危险因素的比例或水平，通过比较各组之间暴露比例或水平的差异，判断暴露因素是否与研究的疾病有关联及其关联程度大小的一种观察性研究方法。队列研究（B 错）是将一个范围明确的人群按是否暴露于某可疑因素或暴露程度分为不同的亚组，追踪各组的结局并比较其差异，从而判定暴露因素与结局之间有无关联及关联程度大小的一种观察性研究方法。现况研究和横断面研究（CE 错）是描述性研究中应用最为广泛的一种方法。它是应用普查或抽样调查的方法收集特定时间、特定人群中疾病、健康状况及有关因素的资料，并对资料的分布特征加以描述。
此题考查的是不同类型研究设计的识别。病例对照研究（A）通常是回顾性的，比较有某种疾病（或结果）的人和没有该疾病的人之间过去的暴露情况。队列研究（B）是前瞻性或回顾性的，追踪两组（暴露组和非暴露组）人群，看他们是否发展出某种疾病或结果。现况研究（C）是指在某一时间点上对特定人群的健康状况或某些变量进行的横断面研究。实验研究（D）涉及到研究者对研究对象进行某种干预措施的研究设计，如随机对照试验。横向研究（E）是另一种说法的现况研究，也是在某一时间点上进行的。
根据题干信息，研究者选取孕妇，一组接受催产素治疗，另一组不接受，以比较新生儿黄疸的发生率。这里有明确的干预（注射催产素），并且是前瞻性地观察两组孕妇的新生儿结果。这符合实验研究的特征。
因此，正确答案是 **D.实验研究**。
首先，我们简要梳理题干中的关键信息：这是一个关于孕妇注射催产素后对新生儿黄疸影响的研究。研究中，117 名孕妇被分为两组，一组给予药物治疗（催产素），另一组不给药物治疗。目的是观察新生儿是否出现黄疸，并探究与催产素的联系。
接下来，我们分析各选项：
A. 病例对照研究：这种方法通常用于判断暴露因素是否与研究的疾病有关联及其关联程度大小。它通常是比较患有和未患有某病的两组人群过去暴露于某种可能危险因素的比例，以此来分析和推断该因素与疾病的关联。题干描述的研究并不是基于已有的病例进行回溯性研究，所以 A 选项不正确。
B. 队列研究：队列研究是将一个范围明确的人群按是否暴露于某可疑因素或暴露程度分为不同的亚组，追踪各组的结局并比较其差异，从而判定暴露因素与结局之间有无因果关联及关联程度大小的一种观察性研究方法。题干描述的研究正是按照是否给予药物治疗（催产素）将孕妇分组，并追踪观察新生儿的黄疸情况，这与队列研究的定义相符。
C. 现况研究：这种方法是普查或抽样调查的方法收集一定的数据，并对数据分布特征进行描述。它通常用于了解某一时间横断面上的疾病或健康状况，而不是进行因果关系的推断。题干描述的研究明显涉及到因果关系的探究，所以 C 选项不正确。
D. 实验研究：虽然题干中有实验组和对照组的设置，但实验研究通常涉及到对实验条件的严格控制，以及对实验对象的随机分配。题干没有明确提及这些实验设计的关键要素，所以

不能完全确定这是实验研究。
E. 横向研究：这通常是指在同一时间内对不同年龄组的被试进行心理实验研究或调查的研究方法，与题干描述的研究不符。
综上所述，根据题干描述的研究设计和目的，最符合的是队列研究。这种方法能够前瞻性地观察不同组别（暴露组和非暴露组）在一段时间后的健康状况，并据此判断暴露因素与疾病之间的因果关系。
因此，正确答案是 B.队列研究。

根据题目描述，这种研究方法属于**实验研究**。

解释如下：

- **实验研究**：研究者干预某些变量并观察其结果。在本题中，研究者通过干预（给予催产素）来观察其对新生儿黄疸的影响。

- **病例对照研究**：将患有某病的个体（病例组）与不患该病的个体（对照组）进行比较，以寻找可能的致病因素。该研究通常是回顾性的。

- **队列研究**：将一组没有某病的个体（队列）按是否暴露于某致病因素分成两组，随后前瞻性地观察两组的发病情况。

- **现况研究**：在某一特定时间点上对某一人群进行观察，以了解某病的现状或分布情况。

- **横向研究**：在某一特定时间点上对某一人群进行观察，通常用于描述疾病或健康相关因素在某一时点的分布情况，类似于现况研究。

题目中的描述是对 117 名孕妇进行分组，分别给予或不给予催产素，并观察新生儿黄疸的发生情况，这是典型的实验研究设计。因此，正确答案是：

D. 实验研究
94、 (A2 型题)
某研究者采取随机单盲试验，对比甲药和乙药对轻中度原发性高血压患者的降压疗效，在此实验中，不知道试验分组情况的最可能
A.实验设计人员
B.统计分析人员
C.接收治疗的患者
D.实施治疗的医生
E.测量血压的护士
参考答案： C 您的答案：
图文解析： 【解析】单盲：研究对象（C 对）不知道自己被分在哪组和接受干预措施的具体内容称为单盲。
【避错】如果是研究对象和观察者均不知道分组情况和接受治疗措施的具体内容则为双盲设计，临床试验中的观察者可以是护士或者医生。研究对象、观察者和资料分析者均不知患者的分组情况和接受治疗措施的具体内容称为三盲。

在随机单盲试验中，通常保持一组人员对试验分组情况不了解，以保证实验的客观性和公正性。单盲实验意味着实验中的某一方（通常是参与者本人）不知道自己是否接受了实验治疗或对照治疗。因此，在此实验中，最可能不知道试验分组情况的是接收治疗的患者。
选项 C（接收治疗的患者）是最正确的答案。
解析：
随机单盲试验是指研究对象不知道自己被分配在哪一组，研究者知道分组情况，单盲主要是为了避免研究对象的主观因素所可能带来的种种影响。根据题意，对比甲药和乙药对轻中度原发性高血压患者的降压疗效，在此试验中，采取的是随机单盲的研究方法，最可能不知道分组情况的是接受治疗的患者。
随机单盲试验中，研究者是知道分组情况的，因此选项 A 实验设计者，选项 B 统计分析人员，选项 D 实施治疗的医生，均会知道分组情况，可一并排除。
选项 E 测量血压的护士属于研究人员，在单盲试验中也会知道分组情况，故不选。
综上所述，正确答案是：C.接收治疗的患者。

在随机单盲试验中，不知道试验分组情况的通常是患者。这种设计是为了防止患者的预期或偏见影响试验结果。患者不知道自己接受的是甲药还是乙药，从而避免了心理因素对结果的影响。

因此，最可能不知道试验分组情况的是：

C. 接收治疗的患者
95、 (A2 型题)
病例对照研究资料进行分层分析的目的是
A.控制选择偏移
B.控制信息偏移
C.控制混杂偏倚
D.控制回忆偏倚
E.控制无应答偏倚
参考答案： C 您的答案：
图文解析： 【解析】分层分析是把研究人群根据某特征或因素分为不同层,然后分别分析各层中暴露与疾病的关联。用以分层的因素是可能的混杂因素,通过分层分析,可以判断该因素是否起混杂作用,如果起混杂作用,通过分层分析可以加以控制,故分层分析的目的是控制混杂偏倚（C 对）。
【避错】常见的选择偏倚：(1)入院率偏倚，(2)检出症候偏倚，（3）现患病例-新发病例偏倚，（4）无应答偏倚，（5）易感性偏倚；常见的信息偏倚：（1）回忆偏倚，（2）报告偏倚，（3）暴露怀疑偏倚，（4）诊断怀疑偏倚，（5）测量偏倚，（6）发表偏倚。
病例对照研究中进行分层分析的主要目的是控制混杂偏倚。因此，正确答案是 C.控制混杂偏倚。混杂偏倚发生在观察性研究中，当一个或多个额外变量与研究的暴露和结果同时相关时，这可能会影响结果，造成错误的关联。分层分析是一种统计方法，可以在分析中考虑这些混杂因素，从而减少它们对研究结果的影响。
首先，我们来解析这道题目并指出关键信息。本题询问的是病例对照研究中进行分层分析的目的。
接下来，对每个选项进行逐一分析：
A. 控制选择偏移：选择偏移主要是由于研究对象的选取方法或标准导致的偏差。病例对照

研究中的分层分析并不是为了控制选择偏移。
B. 控制信息偏移：信息偏移主要是由于测量或信息收集过程中的误差导致的。分层分析主要是用于控制混杂因素，而不是信息偏移。
C. 控制混杂偏倚：混杂偏倚是由于其他因素的干扰，使得研究结果偏离真实情况。分层分析正是通过将研究对象按照潜在的混杂因素进行分层，以控制这些混杂因素对结果的影响。因此，这个选项是正确的。
D. 控制回忆偏倚：回忆偏倚主要是由于研究对象在回忆信息时产生的误差。虽然病例对照研究可能会受到回忆偏倚的影响，但分层分析并不是为了控制这种偏倚。
E. 控制无应答偏倚：无应答偏倚是由于部分研究对象没有回应调查或研究而导致的偏差。分层分析与此无关。
综上所述，病例对照研究中进行分层分析的主要目的是控制混杂偏倚，即选项 C。这种方法通过将研究对象按照潜在的混杂因素（如年龄、性别等）进行分层，从而更准确地评估暴露因素与疾病之间的关系。
因此，正确答案是 C.控制混杂偏倚。

病例对照研究是一种观察性研究设计，常用于探索疾病与潜在风险因素之间的关联。分层分析是一种统计方法，用于控制混杂变量对研究结果的影响。

混杂偏倚（混杂因素）是指在研究中存在一个或多个与暴露和结果均有关联的因素，这些因素可能会混淆暴露与结果之间的真实关系。通过分层分析，可以在各个层次上分别分析暴露与结果的关系，从而控制这些混杂因素的影响。

因此，病例对照研究资料进行分层分析的目的是：

C. 控制混杂偏倚
96、 (A2 型题)
某学者欲采用病例对照研究的方法，探讨冠心病的影响因素，选择病例时最不适宜的是
A.尽量选择新发的病例
B.尽量选择国内外通用的诊断标准确诊的病例
C.尽量选择纳入不同医院的病例
D.尽量选择社区来源的病例
E.尽量从大医院选择病例
参考答案： E 您的答案：
图文解析： 【解析】由于利用医院病人作为病例和对照，病人对医院，医院对病人都有选择性，病例不是全体病人的随机样本，因此选择开展以医院为基础的病例对照研究，易产生入院率偏倚。大医院来源的病人症状多较重，因此最不适宜选用，故本题选 E。
【避错】本题 C 选项为易混淆选项，冠心病为慢性长期疾病，社区病例有轻有重，但没有危急重症，因此可作为研究对象。
在病例对照研究中，研究者通常旨在比较有某种疾病（病例）和没有这种疾病（对照）的人群，以识别疾病的风险因素。选择病例时，应考虑多个因素以确保研究的有效性和代表性。
选项 A 指的是尽量选择新发的病例，这通常是合适的，因为它可以减少回忆偏差和选择偏差，更准确地反映当前的病情和风险因素。
选项 B 提到的是尽量选择国内外通用的诊断标准确诊的病例，这也是适当的，因为使用标准化的诊断标准有助于确保病例的准确性和研究结果的可比性。

选项 C 的意思是尽量选择纳入不同医院的病例，这可以增加研究样本的多样性和代表性，从而提高研究结果的外推性。
选项 D 是尽量选择社区来源的病例，这有助于确保研究样本不仅限于特定医疗机构，从而增加了样本的代表性。
选项 E 是尽量从大医院选择病例，这可能导致选择偏差，因为大医院的病例可能并不代表一般人群，尤其是如果研究关注的是整个社区或人群的状况。
因此，最不适宜的选择是 E，尽量从大医院选择病例，因为这可能会引入选择偏差，限制了研究的代表性和普遍性。
解析：
病例对照研究亦称回顾性研究，是比较患某病者与未患某病的对照者暴露于某可能危险因素的百分比差异，分析这些因素是否与该病存在联系。是分析流行病学方法中最基本的、最重要的研究类型之一。在选择病例时，若尽量从大医院选择病例，可能会引入选择偏倚，这是因为大医院的病例可能病情更重、更复杂，且可能包含了来自小医院转诊的病例，因此不具有代表性，不能反映冠心病患者的整体情况。
选项 A 尽量选择新发病例可以减少回忆偏倚，保证结果的真实性，是病例选择时的理想选择。
选项 B 尽量选择国内外通用的诊断标准确诊的病例可以确保病例的诊断准确性，使研究结果更可靠。
选项 C 尽量选择纳入不同医院的病例可以扩大样本来源，使研究结果更具有代表性。
选项 D 尽量选择社区来源的病例可以反映冠心病在基层患者中的真实情况，增加研究的外部有效性。
综上所述，正确答案是：E.尽量从大医院选择病例。

病例对照研究是一种回顾性研究，通过比较病例组（有疾病的个体）和对照组（无疾病的个体）的特征来确定疾病的风险因素。在选择病例时，以下是一些考虑因素：

- **新发病例（A 选项）**：选择新发病例有助于减少选择偏倚，因为新发病例更能代表当前的疾病特征和风险因素。
- **诊断标准确诊的病例（B 选项）**：使用国内外通用的诊断标准确诊的病例确保了病例的同质性和诊断准确性，有助于提高研究的内部效度。
- **纳入不同医院的病例（C 选项）**：选择来自不同医院的病例可以增加样本的代表性，减少由于单一医院特征导致的偏倚。
- **社区来源的病例（D 选项）**：社区来源的病例通常能更好地代表普通人群，从而提高研究结果的外部效度。
- **大医院选择的病例（E 选项）**：尽量从大医院选择病例可能导致选择偏倚，因为大医院的患者往往病情较重，不能很好地代表一般人群的患病情况。

综上所述，在病例对照研究中，选择病例时最不适宜的是 **E. 尽量从大医院选择病例**。

所以，答案是：
E.尽量从大医院选择病例
97、 (A2 型题)
某市 2006 年对中小学进行视力健康调查，发现中小学生视力健康不好。对全市中小学进行分组，分为实验组和对照组，实验组实施眼保健宣讲和用眼不良行为制止，为期 后分析结

果。该研究为
A.实验研究
B.社区干预实验
C.队列研究
D.病例对照研究
E.个体实验
参考答案：　　A　您的答案：
图文解析：　　【解析】实验研究（A 对）是将来自同一总体的研究对象随机分为实验组和对照组，实验组给予实验因素，对照组不给予该因素，然后前瞻性地随访各组的结局并比较其差别的程度从而判断实验因素的效果。
【避错】病例对照研究是选择患有和未患有某特定疾病的人群分别作为病例组和对照组，调查各组人群过去暴露于某种或某些可疑危险因素的比例或水平，通过比较各组之间暴露比例或水平的差异，判断暴露因素是否与研究的疾病有关联及其关联程度大小的一种观察性研究方法。队列研究是将一个范围明确的人群按是否暴露于某可疑因素或暴露程度分为不同的亚组，追踪各组的结局并比较其差异，从而判定暴露因素与结局之间有无关联及关联程度大小的一种观察性研究方法。
这道题目描述了一个对中小学生进行的视力健康调查，然后将学生分为实验组和对照组，实验组采取了眼保健宣讲和用眼不良行为制止的干预措施。这里的关键点是有一个明确的干预措施（眼保健宣讲和用眼不良行为的制止）和一个对照组，这是干预研究的特征。
A 选项“实验研究”通常指的是在严格控制条件下进行的研究，可以是实验室环境下的也可以是社区环境下的，但这个选项过于宽泛。

B 选项“社区干预实验”指的是在社区层面上实施的干预措施来观察其对某些健康结果的影响，这符合题目中描述的情境。
C 选项“队列研究”通常是观察性研究，跟踪两组或以上的人群来比较他们的暴露因素和健康结果，没有主动的干预措施。
D 选项“病例对照研究”通常是回顾性研究，比较有疾病的个体（病例）和没有疾病的个体（对照）的暴露情况。
E 选项“个体实验”没有在这个上下文中提供足够的信息，而且不符合整个群体干预的描述。
综上所述，最合适的答案是 B.社区干预实验，因为这项研究在社区（学校）环境中实施了明确的干预措施，并设立了对照组进行比较。
首先，我们来看题目描述的实验设计。在这个研究中，研究者首先发现了中小学生视力健康不佳的问题，然后对全市的中小学生进行了分组，实验组和对照组。实验组接受了眼保健宣讲和用眼不良行为的制止。这个设计的核心是在一个特定的社区（中小学）中，对一部分人群（实验组）实施了某种干预（眼保健宣讲和用眼不良行为制止），然后观察这种干预的效果。
接下来，我们逐一分析选项：
A. 实验研究：虽然该研究涉及实验分组，但“实验研究”这一术语较为宽泛，不足以准确描述该研究的特性。
B. 社区干预实验：这个选项与题目描述高度吻合。研究在一个特定的社区（中小学）进行，对实验组进行了特定的干预（眼保健宣讲和用眼不良行为制止），然后评估这种干预的效果。这是一个典型的社区干预实验设计。
C. 队列研究：队列研究通常涉及对特定人群进行长期跟踪，以观察某种暴露因素（如吸烟、饮食习惯等）与健康结果（如疾病发病率）之间的关系。本题中的研究设计与此不符。

D. 病例对照研究：这种研究设计通常用于评估某种暴露因素是否与某种疾病有关联。它涉及选择已患病（病例组）和未患病（对照组）的个体，然后回顾性地比较他们的暴露历史。本题中的设计并非此类研究。
E. 个体实验：这个选项强调的是在个体水平上的实验，而本题中的研究是在社区（学校）层面上进行的。
综上所述，该研究最符合“社区干预实验”的定义。在这类实验中，研究者会在一个特定的社区内对一部分人群实施特定的干预措施，然后评估这种干预对人群健康的影响。
因此，正确答案是 B.社区干预实验。

根据题目描述，该研究涉及对中小学学生进行分组并实施干预（眼保健宣讲和用眼不良行为制止），然后分析结果。这种研究设计的特点符合实验研究的定义，因为它包括干预措施和对比组。以下是对选项的分析：

A. 实验研究：确实是一种实验研究，因为它包括干预措施和实验对照组。
B. 社区干预实验：虽然这个研究涉及社区，但它的具体干预对象是学生，因此更适合称为实验研究。
C. 队列研究：队列研究通常涉及长期随访一组没有特定干预的个体，这里不符合。
D. 病例对照研究：这种研究设计用于比较有病和无病个体的过去暴露情况，不符合此处描述。
E. 个体实验：个体实验是对单个个体的干预研究，而此处研究对象是群体。

因此，最合适的答案是：

A. 实验研究
98、 (A2 型题)
美国在“行为研究”中选取 300 名健康老年男性雇员，跟踪随访 10 年得出 A 型人格是心血管疾病原因的结论，此研究方法是
A.前瞻性研究
B.回顾性研究
C.试验性研究
D.队列研究
E.观察性研究
参考答案： A 您的答案：
图文解析： 【解析】前瞻性的研究是以现在为起点追踪到将来的研究方法，可弥补回顾性研究的缺陷。本题中对于 300 名健康老年男性雇员，跟踪随访 10 年的研究属于前瞻性研究，故本题选 A。
【避错】回顾性研究是以现在为结果，回溯到过去的研究。实验研究是将来自同一总体的研究对象随机分为实验组和对照组，实验组给予实验因素，对照组不给予该因素，然后前瞻性地随访各组的结局并比较其差别的程度从而判断实验因素的效果。队列研究是将一个范围明确的人群按是否暴露于某可疑因素或暴露程度分为不同的亚组，追踪各组的结局并比较其差异，从而判定暴露因素与结局之间有无关联及关联程度大小的一种观察性研究方法。观察性研究是应用普查或抽样调查的方法收集特定时间、特定人群中疾病、健康状况及有关因素的资料，并对资料的分布特征加以描述。
本题描述了一项研究，其中 300 名健康老年男性雇员被跟踪随访 10 年，以确定 A 型人格与

心血管疾病之间的关联。研究方法涉及追踪一个特定群体随时间的变化，没有提及任何干预措施。这种类型的研究设计通常被分类为队列研究。
队列研究是一种观察性研究方法，用于研究暴露与结果之间的关联。它涉及选择一群未患病但暴露于某种因素（本例中为 A 型人格）的人群，并随时间跟踪他们，以观察和记录结果（如心血管疾病）的发生。
因此，最合适的答案是：D. 队列研究
解析：
本题中，美国在“行为研究”中选取 300 名健康老年男性雇员，跟踪随访 10 年得出 A 型人格是心血管疾病原因的结论，该研究根据是否暴露 A 型人格特征进行分组，经过长达 10 年的前瞻性观察，比较两组之间心血管疾病的发生率，从而得出 A 型人格是心血管疾病原因的结论，这种研究方法称为前瞻性队列研究。前瞻性队列研究是指将明确无某疾病的人群按是否暴露于某可疑因素或暴露程度分组，追踪观察其各自的结局，比较其差异，从而判定暴露因素与结局之间有无因果关联及关联程度的一种观察性研究方法。
选项 B 回顾性研究是以现在为结果，回溯到过去的研究，与题干描述的研究方法不符，故不选。
选项 C 试验性研究是研究者根据研究目的，合理地控制或创设一定条件，人为地变革研究对象，从而验证假设，探讨教育现象因果关系的一种研究方法，与题干描述不符，故排除。
选项 D 队列研究是将某一特定人群按是否暴露于某可疑因素或暴露程度分为不同的亚组，追踪观察两组或多组成员结局（如疾病）发生的情况，比较各组之间结局发生率的差异，从而判定这些因素与该结局之间有无因果关联及关联程度的一种观察性研究方法，包括前瞻性队列研究和历史性队列研究。前瞻性队列研究是队列研究的一种，选项表述不够确切，故不选。
选项 E 观察性研究是研究者在没有任何干预措施的情况下，客观地观察和记录研究对象的行为或现状信息，以了解人群中疾病、健康状况及有关因素的分布特征，不给予研究对象任何干预措施，与题干描述不符，故不选。
综上所述，正确答案是：A.前瞻性研究。

这道题的描述涉及对一组健康老年男性雇员进行长期随访，以得出 A 型人格与心血管疾病之间关系的研究结论。根据题目内容，这是一种长期跟踪观察研究。

题干中提到的关键点有：
1. 研究对象是 300 名健康老年男性雇员。
2. 研究方法是跟踪随访 10 年。
3. 研究结论是 A 型人格是心血管疾病的原因。

结合这些信息，我们可以分析各个选项：
- **前瞻性研究**：这种研究方法是从现在开始收集数据，未来进行随访观察。
- **回顾性研究**：这种研究方法是回顾过去的数据或病例，来进行分析。
- **试验性研究**：这种研究方法是通过设定试验和对照组，进行干预试验。
- **队列研究**：这是一种前瞻性研究类型，涉及对一组特定人群（队列）进行长期随访。
- **观察性研究**：这是一种不干预，仅观察的研究方法，可以包括前瞻性和回顾性研究。

结合题干，选项 A （前瞻性研究）和 D （队列研究）看起来都符合描述，但更为准确的答案是：

D. **队列研究**

因为题干描述的是对一组特定人群进行长期随访，以观察其健康变化并得出结论，这正是队列研究的定义。

99、 (A2 型题)

男，40 岁，1 周前去外地出差吃街边大排档后，出现发热、巩膜轻度黄染，AST,ALT 升高，HAVIgM(+),HBsAg(+),对于该患者所患病原体说法正确的是

A.容易隐性感染

B.可以母婴传播

C.可以引起二次病毒血症

D.是 DNA 病毒

E.无包膜病毒

参考答案： A 您的答案：

图文解析： 【解析】抗 HAVIgM 阳性是早期诊断甲型肝炎的特异性指标。血 HBsAg（+）提示无症状携带者或慢性乙肝患者，综合患者的不洁食物食用史和临床表现诊断为甲型病毒性肝炎。甲型肝炎病毒为小 RNA 病毒科嗜肝病毒属。人类感染 HAV 后，大多表现为亚临床或隐性感染，仅少数人表现为急性甲型肝炎。 一般可完全恢复，不转为慢性肝炎，亦无慢性携带者。

【避错】抗-HAV-IgG 阳性是既往感染 HAV 或免疫接种后的标志，可长期存在，不能作为甲肝的诊断依据。

该患者的症状包括发热、巩膜轻度黄染，以及 AST 和 ALT 升高，HAV IgM（肝炎 A 病毒免疫球蛋白 M）阳性，HBsAg（乙型肝炎表面抗原）也阳性。HAV IgM 阳性意味着患者近期有肝炎 A 病毒感染，而 HBsAg 阳性则表明存在乙型肝炎病毒感染。

- A. 容易隐性感染：更常见于乙型肝炎病毒（HBV）。
- B. 可以母婴传播：这是乙型肝炎病毒的特征，可以通过母婴传播。
- C. 可以引起二次病毒血症：这在肝炎 A 病毒中不是常见特征。
- D. 是 DNA 病毒：乙型肝炎病毒是 DNA 病毒，而肝炎 A 病毒是 RNA 病毒。
- E. 无包膜病毒：肝炎 A 病毒是无包膜的 RNA 病毒。

考虑到患者同时有肝炎 A 和乙型肝炎的标志，这道题稍有复杂性。但根据提供的信息和选项，我们更倾向于乙型肝炎病毒的特征，因为它是题目中的关键病原体（特别是 HBsAg 阳性）。因此，最适合的答案选项是 **B. 可以母婴传播**，因为这是乙型肝炎病毒的一个明确特征。

解析：

患者中年男性，因吃街边大排档后，出现发热、巩膜轻度黄染，AST，ALT 升高，HAVIgM 阳性，HBsAg 阳性，考虑诊断为甲型肝炎合并乙型肝炎。乙型肝炎是由乙型肝炎病毒引起的，乙型肝炎病毒是 DNA 病毒，感染人体后可引起急性或慢性乙型肝炎，慢性肝炎易发展为肝硬化、肝癌。

选项 A 容易隐性感染的是甲型肝炎病毒，甲型肝炎病毒是 RNA 病毒，以粪-口途径传播为主，人群普遍易感，感染后可获得持久免疫，隐性感染极为常见，不符合题意。

选项 B 可以母婴传播的病原体是艾滋病病毒，乙型肝炎病毒虽可母婴传播，但相比艾滋病母婴传播的可能性较低，故排除。

选项 C 可以引起二次病毒血症的是登革病毒，乙型肝炎病毒无此特性，故不选。

选项 E 无包膜病毒是脊髓灰质炎病毒，而乙型肝炎病毒是有包膜的病毒，因此可以优先排除。

综上所述，正确答案是：D.是 DNA 病毒。

根据题干信息，这名 40 岁男性患者在外地吃大排档后出现发热和巩膜轻度黄染，AST 和 ALT 升高，HAV IgM(+) 和 HBsAg(+)。这些提示患者同时感染了甲型肝炎病毒（HAV）和乙型肝炎病毒（HBV）。

我们来分析各个选项：

A. 容易隐性感染：甲型肝炎病毒（HAV）通常是显性感染，即患者会出现明显的症状，隐性感染不常见。

B. 可以母婴传播：乙型肝炎病毒（HBV）可以通过母婴传播。

C. 可以引起二次病毒血症：二次病毒血症一般与慢性病毒感染相关，甲型肝炎病毒（HAV）通常是急性自限性疾病，不引起二次病毒血症。

D. 是 DNA 病毒：乙型肝炎病毒（HBV）是 DNA 病毒，而甲型肝炎病毒（HAV）是 RNA 病毒。

E. 无包膜病毒：甲型肝炎病毒（HAV）是无包膜的病毒，而乙型肝炎病毒（HBV）是有包膜的病毒。

结合题意患者的 HAV IgM(+) 和 HBsAg(+)，我们需要选择一个适用于这两种病毒的特点。

正确答案是：
B. 可以母婴传播

因为乙型肝炎病毒（HBV）可以通过母婴传播。
100、 (A2 型题)
女，28 岁，外阴瘙痒 1 周，无发热。妇科检查：外阴可见豆腐渣样分泌物。引起该病的病原体特点不包括
A.可在沙保弱培养基中生长
B.为单细胞微生物
C.可引起深层组织器官损伤
D.为机会致病菌
E.为革兰氏染色阴性菌
参考答案： E 您的答案：
图文解析： 【解析】青年女性，外阴瘙痒 1 周，无发热。妇科检查可见豆腐渣样分泌物，患者最可能的诊断为外阴阴道假丝酵母菌病，假丝酵母菌为革兰阳性细菌，故本题选 E。【避错】白假丝酵母菌在普通琼脂、血琼脂和沙保培养基上均生长良好（A 对），为单细胞微生物（B 对），可感染皮肤、黏膜、内脏等（C 对，）外阴阴道假丝酵母菌病主要为内源性传染，假丝酵母菌作为机会致病菌（D 对），除阴道外，也可寄生于人的口腔、肠道，这了个部位的假丝酵母菌可互相传染，也可通过性交直接传染。
这道题目描述的是一位 28 岁女性患者，主诉外阴瘙痒 1 周，妇科检查发现外阴有豆腐渣样分泌物。根据这些症状，最可能的病原体是白色念珠菌（Candida albicans），这是一种常见

的念珠菌感染病原体。
对于选项中的病原体特点，我们可以进行分析：
A. 白色念珠菌可以在沙保弱培养基中生长，因此 A 选项是正确的特征。
B. 白色念珠菌是一种单细胞真菌，所以 B 选项也是正确的。
C. 白色念珠菌通常引起皮肤和粘膜的感染，较少引起深层组织或器官的损伤，但在免疫力低下的情况下可以引起深层组织损伤。因此，C 选项在某种程度上是正确的。
D. 白色念珠菌是一种机会致病菌，通常在正常菌群失衡或免疫力下降时引起感染，因此 D 选项正确。
E. 白色念珠菌是革兰氏染色阳性菌，因此 E 选项描述不符合白色念珠菌的特点。
根据上述分析，正确答案应该是 **E.为革兰氏染色阴性菌**，因为引起该病的病原体白色念珠菌实际上是革兰氏染色阳性的。
解析：
青年女性患者，外阴瘙痒，外阴可见豆腐渣样分泌物，最可能的诊断是外阴阴道假丝酵母菌病，病原体为白假丝酵母菌，为真菌的一种。而真菌的细胞壁含有几丁质和β-葡聚糖，革兰氏染色时易着色，因此白假丝酵母菌为革兰氏染色阳性菌。
白假丝酵母菌为真菌，可在沙保弱培养基中生长，故选项 A 可在沙保弱培养基中生长属于该菌的特点。
白假丝酵母菌为真菌，真菌是真核生物，具有真正的细胞核和细胞器，属于单细胞微生物，故选项 B 为单细胞微生物是其特点。
白假丝酵母菌除了可以侵犯皮肤和黏膜外，还能引起内脏的急、慢性感染，故选项 C 可引起深层组织器官损伤合其特点。
白假丝酵母菌为机会致病菌，主要为内源性传染，除作为条件致病菌寄生阴道外，也可寄生于口腔、肠道，一旦条件适宜，可引起感染，故选项 D 为机会致病菌是其特点。
综上所述，正确答案是：E.为革兰氏染色阴性菌。

根据题干提供的病例信息，患者为 28 岁女性，外阴瘙痒 1 周，无发热，妇科检查发现外阴有豆腐渣样分泌物。根据这些症状和体征，最可能的诊断是**外阴阴道假丝酵母菌感染**（即阴道念珠菌病）。

念珠菌是一种常见的酵母菌感染，尤其在女性中多见，典型症状包括外阴瘙痒和豆腐渣样白带。

现在我们来分析每一个备选答案：

A. **可在沙保弱培养基中生长**：正确。念珠菌可以在沙保弱培养基中生长，这是一种常用于培养真菌的培养基。

B. **为单细胞微生物**：正确。念珠菌是单细胞微生物，属于真菌的一种。

C. **可引起深层组织器官损伤**：错误。虽然念珠菌在免疫功能低下的患者中可能引起系统性侵袭性感染，但在免疫功能正常的患者中通常只引起表层感染，如皮肤和粘膜感染。深层组织和器官损伤并不是其典型特征。

D. **为机会致病菌**：正确。念珠菌通常作为机会致病菌存在，平时寄生在人体粘膜和皮

肤上，当人体免疫力下降或局部环境改变时才引起感染。

E. **为革兰氏染色阴性菌**：错误。念珠菌是革兰氏染色阳性菌。

因此，**最正确的答案是 C. 可引起深层组织器官损伤**。
101、 (A2 型题)
某男发现其妻子出轨，第二天双下肢不能行走，并出现癫痫症状，双下肢无力，检查神经系统阴性，用明尼苏达人格测验，该患者属于
A.疑病
B.妄想症
C.抑郁症
D.癔症
E.精神分裂症
参考答案： D 您的答案：
图文解析： 【解析】癔症是指一种以分离症状（部分或完全丧失对自我身份识别和对过去的记忆）和转换症状（在遭遇无法解决的问题和冲突时产生的不快心情，以转化成躯体症状的方式出现）为主的精神障碍，其中癔症转换症状包括癔症性失明、癔症性瘫痪、癔症性失声、癔症性抽搐。故本题选 D。
【避错】癔症又称歇斯底里、分离障碍。癔症性癫痫，首选的心理治疗方法是催眠暗示疗法。疑病症（A 错）患者毫无根据地坚信自己患了某种严重的躯体疾病或不治之症。妄想症（B 错）指抱有一个或多个非怪诞性的妄想，同时不存在任何其他精神病症状抑郁发作（C 错）概括为情绪低落、思维迟缓、意志活动减退。精神分裂症（E 错） 一般有幻觉、妄想、激越行为、社交不良、兴趣缺失、意志力减退等症状。
这个题目描述了一个患者在经历情感创伤后出现的身体症状，如双下肢无力且不能行走，并有癫痫症状，但神经系统检查未发现异常。这种情况下，症状很可能是心理原因引起的。根据题目描述和选项，最合适的答案是 D.癔症。
癔症，也称为转换障碍，是一种心理疾病，在这种情况下，患者表现出与神经系统有关的症状，如麻痹、行走困难或癫痫发作，但这些症状不能由医学检查所解释。这类症状通常与心理因素有关，如在经历极端压力或情绪创伤后发生。因此，根据题目描述的情况和症状，选择 D.癔症是最合适的。
解析：
中年男性患者，在发现妻子出轨后，突然双下肢不能行走，并出现类似癫痫的症状，双下肢无力，神经系统查体未见异常，明尼苏达人格测验可辅助诊断，根据患者症状、查体，该患者最可能的诊断是癔症。癔症是由精神因素，如生活事件、内心冲突、暗示或自我暗示，作用于易病个体引起的精神障碍，主要表现有分离症状和转换症状两种。分离，是指对过去经历与当今环境和自我身份的认知完全或部分不相符合。转换，是指精神刺激引起的情绪反应，接着出现躯体症状，一旦躯体症状出现，情绪反应便褪色或消失。该患者受精神刺激后出现双下肢无力和癫痫样症状，为癔症的转换症状。
选项 A 疑病是一种对躯体情况的过分或不相称的关注，主要表现为患者认为自己患了某种或多种严重的进行性的躯体障碍。该患者并没有认为自己患病，因此不选。
选项 B 妄想症又称偏执性精神障碍，是一种精神疾病，通常涉及对现实情况的扭曲感知，坚信自己受到迫害、欺骗或伤害，而题干中患者并未表现出此类症状，故不选。
选项 C 抑郁症以显著而持久的心境低落为主要特征，临床表现从闷闷不乐到悲痛欲绝，可有消极自杀企图或行为，甚至发生木僵，与该患者症状不符，故排除。

选项 E 精神分裂症是一组病因未明的慢性疾病，多在青壮年缓慢或亚急性起病，临床上往往表现为症状各异的综合征，涉及感知觉、思维、情感和行为等多方面的障碍以及精神活动的不协调。该患者并未见上述症状，故不考虑。
综上所述， 正确答案是：D.癔症。

根据题目描述，该患者在发现其妻子出轨后，第二天出现双下肢不能行走、癫痫症状和双下肢无力，但神经系统检查无异常。这种情况提示可能是心理因素引起的症状。

明尼苏达人格测验（MMPI）是一种心理测验，用于评估个体的心理健康状态和人格特质。题目中描述的症状突然出现且与心理创伤事件（妻子出轨）有关，且神经系统检查无异常，考虑可能是心理障碍引起的症状。

根据这些信息，最佳答案是：

D. 癔症（又称转换障碍）

癔症是一种心理障碍，常常表现为突然出现的、无法通过医学检查解释的神经系统症状，如瘫痪、癫痫发作等，通常与心理压力或创伤事件有关。
102、 (A2 型题)
男，52 岁，反复上腹部不适多年，经胃镜检查拟诊为胃溃疡，经抗溃疡药物治疗后好转不明显。后经医师询问，自诉压力大或心情紧张时症状加重。给予的治疗是
A.电力休克治疗
B.心理疗法
C.予抗精神分裂症药物治疗
D.予镇静药物治疗
E.加大抗溃疡药物
参考答案： B 您的答案：
图文解析： 【解析】胃溃疡属于心身疾病的范畴，对心身疾病实施心理干预应围绕消除心理社会刺激因素、消除心理学病因和消除生物学症状为主要目标。主要原则是心、身同治，但对于具体病例应有所侧重，主要包括：对于急性发病而又躯体症状严重的病人,应以躯体对症治疗为主,辅以心理治疗；对于以心理症状为主、辅以躯体症状的疾病，或虽然以躯体症状为主但已呈慢性化的心身疾病,则可在实施常规躯体治疗的同时，重点安排好心理治疗。故本题选 B。
【避错】心身疾病指心理社会因素在疾病的发生、发展过程中起重要作用的躯体器质性疾病和躯体功能性障碍。（1）心血管系统的心身疾病包括冠状动脉粥样硬化性心脏病、阵发性心动过速、心律不齐、原发性高血压、原发性低血压、雷诺病等；（2）呼吸系统疾病的心身疾病包括支气管哮喘、过度换气综合征、神经性咳嗽等；（3）消化系统的心身疾病包括胃、十二指肠溃疡、神经性厌食、神经性呕吐、溃疡性结肠炎、肠道易激综合征等；（4）皮肤系统的心身疾病包括神经性皮炎、斑秃、银屑病、多汗症、慢性荨麻疹、湿疹等。
对于这位 52 岁男性患者，他长期反复出现上腹部不适，胃镜检查提示胃溃疡，但对抗溃疡药物治疗反应不明显。患者还报告在压力大或心情紧张时症状加重，这暗示可能存在心理或精神压力因素的影响。
在选项中，**B. 心理疗法**可能是最合适的选择。这是因为患者的症状与心理压力有关，

心理疗法可以帮助患者学会如何管理压力和情绪，从而可能减轻症状。虽然增加抗溃疡药物剂量（选项 E）也是一个可能的选择，但鉴于患者对初步治疗的反应不佳，考虑到心理因素的治疗可能更加合理。
电力休克治疗、抗精神分裂症药物治疗和镇静药物治疗通常用于更严重的精神病学疾病，对于本病例描述的情况来说，这些治疗措施似乎不是首选。因此，最适合的选择是 B. 心理疗法。
首先，我们分析题干。
中年男性患者，长期上腹部不适，诊断为胃溃疡。关键信息是，患者的症状加重与压力大或心情紧张有关，这表明心理因素可能在疾病过程中起到了重要作用。
接下来，我们逐一分析选项：
A. 电力休克治疗：这是一种通过电流刺激大脑以引起暂时性意识和记忆丧失的疗法，通常用于治疗某些精神疾病，如抑郁症、精神分裂症和双相情感障碍。在此案例中，患者的主要问题是胃溃疡，且心理因素是加重因素，而非主要病因，因此电力休克治疗并不适用。
B. 心理疗法：鉴于患者的症状加重与心理压力有关，心理疗法可能是一个有效的治疗方法。心理疗法可以帮助患者学会应对压力的方法，减轻焦虑和紧张，从而可能有助于减轻胃溃疡的症状。
C. 予抗精神分裂症药物治疗：患者并未被诊断为精神分裂症，因此使用抗精神分裂症药物是不合理的。
D. 予镇静药物治疗：虽然镇静药物可能有助于缓解患者的紧张和焦虑，但它们通常不是长期解决方案，且可能有副作用。此外，它们并不直接针对胃溃疡的治疗。
E. 加大抗溃疡药物：虽然抗溃疡药物是治疗胃溃疡的主要手段，但在此案例中，患者的症状加重主要与心理压力有关，因此单纯加大药物剂量可能不是最有效的解决方案。
综上所述，考虑到患者的症状加重与心理压力密切相关，心理疗法可能是最合适的治疗方法。心理疗法可以帮助患者学会更有效的应对压力的方法，从而可能减轻胃溃疡的症状。
因此，正确答案是 B.心理疗法。

根据题目描述，该 52 岁男性反复上腹部不适多年，经胃镜检查拟诊为胃溃疡，并且抗溃疡药物治疗效果不明显。同时患者自述压力大或心情紧张时症状加重，说明他的胃溃疡可能与心理因素有关。因此，最适合的治疗方案应考虑心理因素的影响。

A. 电力休克治疗主要用于严重的精神障碍或难治性抑郁症，不适用于此情况。
B. 心理疗法可以帮助患者应对压力和情绪紧张，从而可能缓解与此相关的胃溃疡症状。
C. 抗精神分裂症药物治疗不适用于此患者的症状。
D. 镇静药物治疗可以暂时缓解焦虑，但不是解决心理因素引起的根本问题的最佳选择。
E. 加大抗溃疡药物的剂量在此情况下未必能解决问题，且有可能产生副作用。

基于以上分析，最适合的治疗方案是 **B. 心理疗法**。
103、 (A2 型题)
一名医生接待了一位患者，看了其 CT 结果及检验学结果，并结合患者症状，给出患者患有肺癌的结论。该思维过程属于
A.聚合思维
B.逻辑思维
C.发散思维
D.创新思维

E.抽象思维
参考答案： A 您的答案：
图文解析： 【解析】聚合思维是指从已知信息中产生逻辑结论，从现成资料中寻求正确答案的一种有方向、有条理的思维方式。本题中医生根据检查及检验结果，结合症状给出肺癌的诊断，属于聚合思维（A 对）。
【避错】发散思维（C 错），又称辐射思维、放射思维、扩散思维或求异思维，是指大脑在思维时呈现的一种扩散状态的思维模式。抽象思维（BE 错），又称词的思维或者逻辑思维，是指用词进行判断、推理并得出结论的过程，抽象思维以词为中介来反映现实。创新思维（D 错）是重新组织已有的知识经验，产生新颖的、前所未有的、有创造性想象参加的思维。
此题目考察的是医生如何通过不同信息（CT 结果、检验学结果和患者症状）来诊断疾病。在这种情况下，医生必须从各种检查结果中提取相关信息，然后应用其医学知识和经验来综合这些信息，从而得出诊断结论。
选项分析如下：
- A. 聚合思维：指的是将不同信息、数据或事实汇聚在一起，形成一个整体的过程。在医学诊断中，聚合思维可能涉及将不同的检查结果和临床表现综合起来考虑。
- B. 逻辑思维：指用逻辑方法进行思考，推理出合理的结论。在医疗领域，逻辑思维用于根据病人的症状和检查结果来推断可能的疾病。
- C. 发散思维：是一种思考方式，旨在通过探索许多可能的解决方案来生成创造性思想。在此背景下，它可能不是最适合的，因为医生需要根据具体的证据得出明确的结论。
- D. 创新思维：涉及创造新的想法或概念。在诊断过程中，虽然创新很重要，但更多的是在治疗方法或解决问题时应用。
- E. 抽象思维：是理解复杂概念并在没有具体信息的情况下进行思考的能力。它在诊断过程中不是最直接相关的思维方式。
综合考虑，最适合描述医生通过分析 CT 结果、检验结果和患者症状来诊断疾病的思维过程是聚合思维（A 选项）。因此，本题的最佳答案应该是 A. 聚合思维。
首先，我们分析题目并罗列出重要信息。本题描述的是医生在综合了患者的 CT 结果、检验学结果以及患者的症状后，给出了患者患有肺癌的结论。
接下来，我们需要对每个选项进行分析，以判断哪种思维过程与题目描述最为吻合：
A. 聚合思维：这种思维方式是指从已知的信息出发，通过逻辑推理和综合分析，得出一个集中的结论。这与题目中医生综合多种检查结果和患者症状后得出结论的过程相符。
B. 逻辑思维：虽然医生的推理过程中包含了逻辑成分，但逻辑思维更侧重于推理的严密性和形式化，而不仅仅是综合信息得出结论。
C. 发散思维：这种思维方式强调从一个中心点出发，产生多种不同的想法或解决方案。这与题目描述的医生综合信息后得出单一结论的过程不符。
D. 创新思维：这种思维方式侧重于产生新颖、独特的想法或解决方案。题目中并未体现创新或新颖的元素。
E. 抽象思维：这种思维方式强调从具体事物中抽取出其共同的本质特征。虽然医生的诊断过程包含了一定的抽象思维，但题目描述的重点在于综合信息得出结论，而非抽象出共同特征。
综上所述，医生的思维过程最符合聚合思维的定义，即从多种信息和检查结果出发，综合分析后得出一个集中的结论。
因此，正确答案是 A.聚合思维。

在该案例中，医生通过查看患者的 CT 结果和检验学结果，并结合患者的症状，得出患者患有肺癌的结论。这个过程涉及综合分析各种信息，并进行推理以得出结论。这种思维过程最符合**逻辑思维**。

逻辑思维指的是基于已知事实和证据，通过推理和分析来得出结论的过程。在医学诊断中，医生经常使用逻辑思维来综合各种检查和检验结果，结合患者的症状，最终做出诊断。

因此，正确答案是：

B. 逻辑思维
104、 (A2 型题)
工作人员调查某村儿童健康情况，发现该村人工喂养的婴儿近期出现腹泻、呕吐伴发绀，检查发现儿童血液中高铁血红蛋白增高，考虑原因是
A.亚硝酸盐中毒
B.甲基汞中毒
C.饮用水含氯高
D.燃烧劣质煤炭
E.奶瓶材质含双酚
参考答案： A 您的答案：
图文解析： 【解析】人工喂养（怀疑引用不洁水）的婴儿近期出现腹泻、呕吐 伴发绀（亚硝酸盐中毒者最先发生的症状），检查发现儿童血液中高铁血红蛋白增高（提示亚硝酸盐中毒），考虑诊断为亚硝酸盐中毒，故本题选 A。
【避错】亚硝酸盐多存在于腌制的咸菜、肉类、不洁井水和变质腐败蔬菜等。
在这个案例中，儿童出现的腹泻、呕吐和发绀症状，结合血液中高铁血红蛋白（methemoglobin）增高，最有可能的原因是亚硝酸盐中毒。亚硝酸盐中毒可以导致血液中铁离子从+2 价氧化成+3 价，形成高铁血红蛋白，减少血液携氧能力，从而引发腹泻、呕吐和发绀等症状。这种中毒通常与摄入了含有较高量亚硝酸盐的水或食物有关。
因此，最佳答案是 A.亚硝酸盐中毒。
解析：
该村人工喂养的儿童近期集体出现腹泻、呕吐伴发绀，同时检查发现儿童血液中高铁血红蛋白增高，是亚硝酸盐中毒的典型表现。亚硝酸盐中毒是由于误食亚硝酸盐或饮用亚硝酸盐含量高的井水或腌制不充分的蔬菜而引起的中毒，也可因胃肠功能紊乱时，胃肠道内硝酸盐还原菌大量繁殖，食入富含硝酸盐的蔬菜后，硝酸盐在肠道内被还原菌转化为亚硝酸盐，从而引起中毒。亚硝酸盐能将血液中正常的携氧的低铁血红蛋白氧化成高铁血红蛋白，因而失去携氧能力而引起组织缺氧，出现发绀的症状。
选项 B 甲基汞中毒常见于长期接触汞作业的人员，临床上精神-神经异常、齿龈炎、震颤为主要症状，与题干描述不符，故不选。
选项 C 饮用水含氯高可造成腹泻等症状，但无法解释高铁血红蛋白增高的原因，故排除。
选项 D 燃烧劣质煤炭可能导致一氧化碳中毒，但不会导致血液中高铁血红蛋白增高，不符合题意。
选项 E 奶瓶材质含双酚可能会影响婴儿的健康，但与高铁血红蛋白增高没有直接关系，故不选。
综上所述，正确答案是：A.亚硝酸盐中毒。

根据题目提供的信息，人工喂养的婴儿出现腹泻、呕吐伴发绀，并且检查发现儿童血液中高铁血红蛋白增高，考虑原因是亚硝酸盐中毒。

亚硝酸盐中毒会导致高铁血红蛋白血症（Methemoglobinemia），使血液中的高铁血红蛋白增高，进而导致组织缺氧，表现为发绀（皮肤和黏膜发青）。人工喂养婴儿有时可能通过受污染的水源或食物摄入亚硝酸盐。

因此，最可能的答案是：

A. 亚硝酸盐中毒

105、 (A2 型题)

王某和其朋友外出聚餐，点了扬州肴肉、金枪鱼、泡菜、四季豆和蕈类食物，用餐十分钟左右王某和其朋友均出现头痛，胸闷，心率加快，全身不适，头部颈部胸部出现 灼热和刺痛，其中一位朋友哮喘发作，出现呼吸困难，分析其发生主要原因是

A.毒蕈碱中毒

B.组胺中毒

C.氰苷类食物中毒

D.四季豆中毒

E.亚硝酸盐中毒

参考答案： B 您的答案：

图文解析： 【解析】服用青皮红肉鱼后出现头痛，胸闷，心率加快，全身不适，头部颈部胸部出现潮红，伴有灼热和刺痛，考虑诊断为组胺中毒，故本题选 B。

【避错】当摄入海产鱼中的青皮红肉鱼类（富含组氨酸），如鲣鱼、鲐鱼、秋刀鱼、沙丁鱼、鲭鱼、竹荚鱼、金枪鱼等鱼类可引起组胺中毒。

这道题描述了一群人在进食扬州肴肉、金枪鱼、泡菜、四季豆和蕈类食物后出现的一系列症状，如头痛、胸闷、心率加快、全身不适、灼热和刺痛感，以及其中一位朋友的哮喘发作和呼吸困难。根据这些症状，我们需要考虑哪种食物引起的中毒可能导致了这些反应。

A 选项是毒蕈碱中毒，通常与食用野生蕈类有关，但蕈类中毒的症状更多关联于胃肠道症状和神经系统症状，如恶心、呕吐、腹痛、视觉扭曲和幻觉。

B 选项是组胺中毒，这种中毒通常与食用不新鲜的海鲜（如金枪鱼）有关，可导致头痛、胸闷、心率加快、全身不适等症状，与本案例描述的症状较为匹配。

C 选项是氰苷类食物中毒，虽然某些食物中含有氰苷，但其中毒症状主要包括头晕、头痛、呼吸困难、血压下降等，与本案例不完全吻合。

D 选项是四季豆中毒，四季豆如果未煮熟可能含有植物血凝素，会引起腹痛、呕吐等症状，但不太可能导致这种广泛的神经和呼吸系统症状。

E 选项是亚硝酸盐中毒，虽然亚硝酸盐中毒可以引起一些严重的症状，但它更多与摄入处理不当的加工食品有关，并且其典型症状是引起血液中的氧传输能力降低。

综上所述，最符合这些症状的是 B 选项，组胺中毒，尤其是与食用了可能未充分新鲜的金枪鱼有关。

解析：

王某和其朋友外出聚餐，点了多种食物，用餐后十分钟左右均出现头痛，胸闷，心率加快，全身不适，头部颈部胸部出现灼热和刺痛，其中一位朋友哮喘发作，出现呼吸困难。考虑可能为组胺中毒。组胺中毒的特点是发病快、症状轻、恢复快，少有死亡发生。主要表现为皮

肤潮红，结膜充血，似醉酒样，头晕，头痛，心跳加快，胸闷和呼吸急促，部分病人出现结膜充血，眼睑水肿，咽部灼痛，吞咽困难，甚至出现荨麻疹，呕吐，腹泻等，重者可出现支气管痉挛，瞳孔散大，呼吸困难，血压下降。
选项 A 毒蕈碱中毒主要表现为副交感神经末梢过度兴奋，出现类似毒蕈碱样症状，表现为平滑肌痉挛和腺体分泌增加，如恶心、呕吐、腹痛、多汗、瞳孔缩小、心率减慢、流涎、大小便失禁等，严重者可出现肺水肿、呼吸困难、昏迷、抽搐等，与题意不符。
选项 C 氰苷类食物中毒是指含氰甙植物中毒，如木薯中毒等，食后可在胃酸作用下释放出氢氰酸，导致中毒，主要症状为神经及消化道损伤，出现恶心、呕吐、腹痛、腹泻，有的出现眩晕、头疼、口中苦杏仁味，瞳孔散大，严重者昏迷，最后因呼吸麻痹和心脏停止而死亡，与题干描述不符。
选项 D 四季豆中毒是因食用四季豆引起的中毒，四季豆中含有毒蛋白和皂素，如果没有煮熟煮透，人吃后 1～5 小时就会引起中毒，轻者头痛头昏，重者恶心、呕吐、腹痛，与题干不符。
选项 E 亚硝酸盐中毒常表现为组织缺氧而致的发绀，有条件时可做血液中高铁血红蛋白的定量检验和剩余食物中亚硝酸盐的定量检验，与王某和其朋友的表现不符，故排除。
综上所述，正确答案是：B.组胺中毒。

根据王某和其朋友的症状，包括头痛、胸闷、心率加快、全身不适以及头部颈部胸部的灼热和刺痛，并且其中一位朋友出现哮喘发作和呼吸困难，这些症状非常典型地符合组胺中毒的表现。组胺中毒常见于食用含有高组胺的食物如金枪鱼、泡菜等。尤其是在食物处理不当或保存不当的情况下，组胺含量可能会显著增加。

因此，最有可能的答案是：B. 组胺中毒
106、 (A2 型题)
2017 年某市卫生局开创评选“先进科室”，共评选出 30 个“优秀先进科室”，在这个良好环境下各医院科室均争做“先进科室”，在这个案例中体现的心理促进因素是
A.舆论扬抑，以境育人
B.案例分析，以理服人
C.技术过关，实力至上
D.以典为先，以形感人
E.知识教育，以德育人
参考答案： D 您的答案：
图文解析： 【解析】开创评选先进科室，为树立典型，以典为先，以形感人，使各医院科室在良好的氛围中均争做“先进科室”，故本题选 D。
【避错】常识性题目。
在这个案例中，由于各医院科室都在努力成为“先进科室”，这反映了一种心理促进因素，即通过创设优秀的典范或榜样来鼓励和激励他人。这种方式通过展示可望达到的标准或成就来激发其他人或团体的积极性。因此，最合适的答案是：
D.以典为先，以形感人
这个选项意味着通过树立典型的榜样来感染和激励人，使他们受到积极影响，从而努力成为类似的“先进科室”。
这是一道涉及医学心理学和健康教育学的选择题，旨在分析一个实际案例中的心理促进因素。
首先，我们要理解题目背景：某市卫生局评选“先进科室”，并成功评选出 30 个“优秀先进

科室”。这个活动激发了各医院科室争做“先进科室”的积极性。
现在，我们逐一分析选项：
A. 舆论扬抑，以境育人：虽然案例中提到了评选活动创造了一个良好的环境，但并未明确体现舆论的扬抑作用，因此这个选项可能不是最直接相关的。
B. 案例分析，以理服人：案例中并未提及通过案例分析来激励或说服他人，因此这个选项不符合题意。
C. 技术过关，实力至上：虽然技术实力对于评选“先进科室”可能很重要，但案例中并未特别强调技术因素，因此这个选项不是最直接相关的。
D. 以典为先，以形感人：这个选项与案例高度相关。评选出的 30 个“优秀先进科室”实际上成为了榜样（典型），它们的表现激发了其他科室的效仿欲望。这正是“以典为先”的体现，通过树立典型来影响和激励他人。
E. 知识教育，以德育人：案例中并未明确提及通过知识教育来培育品德，因此这个选项与题意不符。
综上所述，答案是 D.以典为先，以形感人。这个选项最直接地体现了案例中各医院科室争做“先进科室”的心理促进因素，即通过树立优秀典型来影响和激励其他科室。

根据题目中的描述，某市卫生局评选“先进科室”，营造了一个良好的环境，鼓励各医院科室争做“先进科室”。这种做法利用了榜样的力量和典型的示范效应来激励其他科室提升自己，争取成为“先进科室”。

因此，在这个案例中体现的心理促进因素是：

D.以典为先，以形感人

这种方式通过树立先进典型，以实际行动和成就来激励和感染他人，促使大家争做先进，营造良好的竞争和发展环境。

107、 (A2 型题)
男，11 岁，因发生车祸，临床诊断脑死亡。患者父母极其伤心，但接受器官捐献的观点，要求与受体者见面，并与之认为亲戚。器官捐献医师坚持拒绝其见面，患者父母 献。本案例中，体现的医学伦理是
A.供者的尊严与保护
B.患者健康利益至上
C.无偿与非商业化
D.知情同意
E.保密
参考答案： E 您的答案：
图文解析： 【解析】器官捐献医师坚持拒绝供者与受者的见面体现了人体器官移植的保密原则（E 对）。
【避错】我国人体器官移植的伦理原则：（1）患者利益至上原则，（2）自愿、无偿、禁止买卖原则，（2）知情同意原则，（4）尊重和保护供体原则，（5）保密原则，（6）公平、公正、公开原则。
本案例中体现的医学伦理原则是 A.供者的尊严与保护。这里的情境是围绕着一个脑死亡患者的器官捐献问题，强调了对患者（即供者）尊严的保护。虽然患者的父母希望见到器官受体者并与其建立联系，医师拒绝这一请求是为了保护供者的隐私和尊严，防止可能的社会和

心理问题。此外，这也涉及到了保护未成年供者的特殊性，确保其尊严不受侵犯，即使在其生命结束后。因此，选项 A 是最符合此情境的答案。

这是一道医学伦理问题。我们首先需要理解并分析题目中的关键信息，再结合这些信息，对给出的选项进行逻辑推理。

关键信息分析：

11 岁男孩因车祸脑死亡。

患者父母虽然伤心，但接受器官捐献的观点。

患者父母要求与受体者见面，并希望与受体者建立亲戚关系。

器官捐献医师拒绝了他们与受体者见面的要求。

选项分析：

A. 供者的尊严与保护：虽然供者的尊严和保护是重要的伦理原则，但在这个案例中，问题的焦点更多在于保护受体者和捐献过程的匿名性，而非直接涉及供者的尊严。因此，这个选项可能不是最直接相关的。

B. 患者健康利益至上：这一原则主要关注的是患者的健康利益。在这个案例中，虽然器官捐献是为了拯救其他患者的生命，但问题的核心在于捐献过程中的伦理问题，而非直接关注患者的健康利益。因此，这个选项可能不是最相关的。

C. 无偿与非商业化：这个原则强调器官捐献应该是无偿和非商业化的。然而，本案例的焦点是保护捐献过程的匿名性和保护捐献双方的身份，与无偿和非商业化无直接关系。

D. 知情同意：虽然知情同意是医学伦理中的重要原则，但在这个案例中，问题的关键并不在于是否获得了知情同意，而是关于捐献过程中的隐私和匿名性问题。

E. 保密：在这个案例中，器官捐献医师拒绝患者父母与受体者见面的要求，很可能是为了维护双方的隐私和匿名性。这是为了保护供体和受体双方免受不必要的压力和干扰，确保捐献过程的公正性和客观性。因此，这个选项与案例中的情境高度相关。

综上所述，最符合本案例医学伦理原则的是选项 E：保密。这一原则体现了对供体和受体双方隐私的保护，以及确保器官捐献过程的公正性和客观性。

本题描述的是一个涉及脑死亡患者器官捐献的案例，涉及多个医学伦理原则。题目要求选择一个最能体现该案例中的伦理原则。

题目中，患者父母要求与受体者见面，并与之认为亲戚，而器官捐献医师拒绝了这个请求。拒绝的理由可能涉及到保护供者的隐私和受体者的隐私，这与医学伦理中的保密原则（E. 保密）密切相关。保密原则强调在医患关系中保护患者的隐私和信息安全。

综上所述，本案例中最能体现的医学伦理原则是：

E. 保密

108、　(A2 型题)

小儿出生以来母乳喂养，未添加辅食，近来出现食欲下降，面色苍白。查体：P125 次/分，肝肋下 3cm，脾肋下 1cm，血红蛋白 87g/L。外周血涂片：血象中央淡染区扩大。 不等，以小为主。婴儿体内最可能缺乏的氨基酸类型是

A.色氨酸

B.甘氨酸

C.苏氨酸

D.亮氨酸

E.异亮氨酸
参考答案：　　A　您的答案：
图文解析：　　【解析】小儿患者有贫血症状。色氨酸是一种必需氨基酸，它是合成血清素的前体，血清素在调节情绪、睡眠和食欲方面具有重要作用。由于母乳喂养的婴儿通常无法从母亲的饮食中获取足够的色氨酸，因此容易出现生长发育迟缓、食欲不振等症状。此外，婴儿体内的异亮氨酸（E 选项）和甘氨酸（B 选项）水平也可能受到影响，但相对于色氨酸来说，它们的影响较小。
【避错】
这道题考察的是婴儿营养不良相关的氨基酸缺乏问题。婴儿出现食欲下降、面色苍白，查体发现贫血（血红蛋白 87g/L）和肝脾轻度增大，外周血涂片显示中央淡染区扩大，这些都是贫血的表现。贫血可能与多种营养素缺乏有关，包括铁、维生素 B12、叶酸和某些氨基酸。考虑到婴儿是纯母乳喂养且未添加辅食，最可能出现的是必需氨基酸的缺乏，因为母乳虽然是婴儿的理想食物，但如果不添加辅食，可能无法满足生长迅速的婴儿对某些营养素的需求。在给出的选项中，亮氨酸（Leucine）和异亮氨酸（Isoleucine）是支链氨基酸，对儿童生长发育非常重要。
亮氨酸是一种必需氨基酸，人体无法自行合成，必须通过食物获取。亮氨酸对于蛋白质合成、肌肉修复和血糖调节具有重要作用。婴儿和儿童在快速生长发育阶段，对亮氨酸的需求尤为重要。
根据这些信息，最可能缺乏的氨基酸类型应该是与生长发育密切相关的一种。在这些选项中，D.亮氨酸（Leucine）是较合理的选择，因为它是一种重要的必需氨基酸，特别是在儿童的生长发育中发挥着关键作用。因此，选择 D.亮氨酸作为答案是合理的。
解析：
患儿母乳喂养，未添加辅食，近来出现食欲下降，面色苍白。查体见肝脾肿大，心率增快，血红蛋白明显降低，贫血。外周血涂片显示血象中央淡染区扩大，细胞大小不等，以小为主，考虑诊断为营养性缺铁性贫血。缺铁性贫血时，血红蛋白合成减少，而血红蛋白的合成原料包括铁和珠蛋白，其中珠蛋白的合成原料为氨基酸，尤其是甘氨酸，为珠蛋白合成的必需氨基酸。因此，该患儿体内最可能缺乏甘氨酸。
选项 A 色氨酸，选项 C 苏氨酸，选项 D 亮氨酸，选项 E 异亮氨酸，均为人体必需氨基酸，但在珠蛋白的合成过程中并不是必需的，故可一并排除。
综上所述，正确答案是：B.甘氨酸。

这道题目描述的是一个母乳喂养的小儿，未添加辅食，近期出现食欲下降和面色苍白，体检发现心率快、肝脾肿大，血红蛋白降低，外周血涂片显示中央淡染区扩大，提示贫血。结合以上症状和体征，婴儿最可能缺乏的氨基酸类型是什么？

考虑到母乳喂养的小儿没有添加辅食，很可能会缺乏某些必要的营养素，包括铁、维生素 D、维生素 B12 等。题干中提到的血红蛋白低和外周血涂片的中央淡染区扩大是缺铁性贫血的典型表现。缺铁性贫血的主要原因是缺乏铁，而不是某种特定的氨基酸。

不过，如果题目要求我们选择一种氨基酸的话，可以考虑到氨基酸在体内的代谢和营养特点。对于婴儿来说，必须氨基酸是必不可少的，因为身体不能合成这些氨基酸，必须通过饮食摄取。色氨酸（A）、甘氨酸（B）、苏氨酸（C）、亮氨酸（D）和异亮氨酸（E）都是人体所需的氨基酸，但考虑到母乳中的氨基酸组成情况，婴儿缺乏色氨酸的可能性较低，因为母乳中含有足够的色氨酸。

基于这一推理，母乳喂养的婴儿在没有添加辅食的情况下，最可能缺乏的氨基酸类型是苏氨酸（C）。因为苏氨酸是必需氨基酸，且在快速生长期的婴儿体内需求量较高。

因此，最正确的答案是：
C. 苏氨酸
109、 (A2 型题)
男，67 岁，1 小时前被银环蛇咬伤，随后出现四肢无力，呼吸困难，眼睑下垂，查体见皮肤局部有 2 个齿痕，针尖大小，与之相关的神经-肌肉接头处的乙酰胆碱变化为
A.在间隙弥漫受阻
B.与受体结合受阻
C.合成受阻
D.释放受阻
E.降解受阻
参考答案： B 您的答案：
图文解析： 【解析】银环蛇具有α-、β-bungarotoxin 两种神经毒素， 一类为突触后神经毒素或α-神经毒素，这类毒素竞争性的与神经肌肉接头处的乙酰胆碱受体结合，阻断神经递质的传导（故选 B）；另一类为突触前神经毒素或β-神经毒素，其直接作用于运动神经突触前膜，阻断乙酰胆碱的释放，使骨骼肌失去收缩功能而麻痹。
【避错】
银环蛇咬伤可以引起神经-肌肉接头处的乙酰胆碱变化，这通常涉及神经传递物质的释放受阻。银环蛇毒素主要影响神经-肌肉接头，阻断神经冲动传递到肌肉的能力，导致肌肉无力、呼吸困难和眼睑下垂等症状。这种毒素阻碍了乙酰胆碱的正常释放到神经-肌肉接头的突触间隙中，从而影响了肌肉的正常收缩。
因此，对于这个问题，最合适的答案是：D. 释放受阻
解析：
老年男性患者，银环蛇咬伤后出现四肢无力，呼吸困难，眼睑下垂，查体见皮肤局部有 2 个齿痕，针尖大小，可诊断为银环蛇咬伤。银环蛇毒素主要损害神经系统，造成神经肌肉麻痹和呼吸麻痹，为神经毒素。银环蛇咬伤后，其神经毒素作用于神经-肌肉接头处的乙酰胆碱受体，导致乙酰胆碱与受体结合受阻，从而阻断神经-肌肉接头的神经传导，造成骨骼肌弛缓性麻痹。
神经-肌肉接头处的乙酰胆碱在间隙中弥散并不受银环蛇毒素的影响，故选项 A 在间隙弥漫受阻可排除。
银环蛇毒素并不影响乙酰胆碱的合成，故选项 C 合成受阻与题意不符。
银环蛇毒素对乙酰胆碱的释放无影响，故选项 D 释放受阻不考虑。
银环蛇毒素影响的是乙酰胆碱受体，不影响乙酰胆碱的降解，故选项 E 降解受阻可排除。
综上所述，正确答案是：B.与受体结合受阻。

根据题目描述，这名 67 岁的男性被银环蛇咬伤，随后出现了四肢无力、呼吸困难、眼睑下垂等症状。银环蛇毒主要是神经毒素，影响神经-肌肉接头的乙酰胆碱功能。

银环蛇毒素通过阻断乙酰胆碱与受体的结合，导致神经信号无法传递，产生类似重症肌无力的症状。因此，与之相关的神经-肌肉接头处的乙酰胆碱变化应为：

B.与受体结合受阻
110、　(A2 型题)
患者男，50 岁，因高脂血症进行低脂饮食，近日自觉视觉感受力下降，应补充以下哪种食物
A.面包
B.绿豆
C.胡萝卜
D.冬瓜
E.苹果
参考答案：　C　您的答案：
图文解析：　【解析】低脂饮食时出现视觉感受力下降，多考虑为维生素 A 的缺乏，因此应补充含维生素 A 多的食物，例如胡萝卜和动物肝脏，故本题选 C。
【避错】维生素 A 缺乏时引起夜盲症。癞皮病是由维生素 PP 缺乏引起的；脚气病是由维生素 B1 缺乏引起的；维生素 C 缺乏病是由于维生素 C 缺乏引起的；佝偻病是由维生素 D 缺乏引起的。
这个题目涉及到视觉感受力下降的问题，可能与维生素 A 的缺乏有关，因为维生素 A 对视觉特别是在光线较暗的环境中看东西非常重要。维生素 A 缺乏可以导致夜盲症和视觉感受力减弱。
在所给的食物选项中，胡萝卜是著名的维生素 A 来源，因为它们富含β-胡萝卜素，人体可以将其转化为维生素 A。因此，对于一个因低脂饮食而可能导致维生素 A 摄入不足，从而感觉视觉感受力下降的患者来说，补充胡萝卜是有益的。
所以，最正确的答案是 **C. 胡萝卜**。
解析：
患者中年男性，因高脂血症进行低脂饮食，近日自觉视觉感受力下降，考虑可能为维生素 A 缺乏导致的夜盲症。维生素 A 缺乏病又称蟾皮病，系因体内维生素 A 缺乏而引起的以眼和皮肤病变为主的全身性疾病，多见于 1～4 岁小儿，最早的症状是暗适应差，眼结膜及角膜干燥，以后发展为角膜软化且有皮肤干燥和毛囊角化，故又称夜盲症、干眼病、角膜软化症。高脂血症患者低脂饮食时，若过度限制脂肪类食物的摄入，会影响脂溶性维生素 A 的吸收，从而出现维生素 A 缺乏的表现。故饮食中可适当增加富含维生素 A 的食物，如胡萝卜，它含有丰富的β-胡萝卜素，摄入人体消化器官后，可以转化成维生素 A，是目前最安全补充维生素 A 的产品　（单纯补充化学合成维生素 A，过量时会使人中毒）。
选项 A 面包，选项 B 绿豆，选项 D 冬瓜，选项 E 苹果，以上食物维生素 A 含量均较低，对该患者视觉感受力下降的改善作用有限，故可一并排除。
综上所述，正确答案是：C.胡萝卜。

根据题目中的信息，患者因高脂血症进行低脂饮食，近日自觉视觉感受力下降。这种情况下，考虑到可能的营养缺乏，特别是与视觉相关的营养素。

胡萝卜富含维生素 A（以β-胡萝卜素的形式），而维生素 A 对于维持正常的视觉功能是非常重要的。维生素 A 的缺乏可能导致夜盲症和其他视力问题。因此，建议补充富含维生素 A 的食物。

正确答案是：

C. 胡萝卜

111、 (A2 型题)

女，70 岁，呕吐两天， 一天 5～6 次，腹泻一天， 一天 10 余次，尿量减少 1 天，查体：36.5℃,心率 110 次/分，脉搏 26 次/分，血压 85/60mmHg。神情淡漠，口腔黏膜苍白，300ml/24h。心率 110 次/分，心律齐，造成血压改变的原因是

A.血管运动系统兴奋

B.有效循环血量减少

C.儿茶酚胺分泌增加

D.微静脉扩张

E.回心血量减少

参考答案： B 您的答案：

图文解析： 【解析】老年女性，频繁呕吐、腹泻（大量的体液丢失），尿量减少 1 天，心率 110 次/分，脉搏 26 次/分，血压 85/60mmHg，神情淡漠，提示存在休克。故该患者少尿的原因是由于大量体液丢失，而引起的有效循环血量减少，故本题选 B。

【避错】休克是机体有效血容量减少、组织灌注不足、细胞代谢紊乱和功能受损的病理过程，其根本变化是组织灌注不足。

这位 70 岁女性患者表现出呕吐、腹泻、尿量减少、心率加快（110 次/分），血压降低（85/60mmHg），以及口腔黏膜苍白等症状。这些临床表现提示该患者可能存在体液丢失，导致血容量减少。血压下降可能是由于有效循环血量减少。因此，在这种情况下，最可能的原因是有效循环血量减少。所以，选择 B.有效循环血量减少 作为最佳答案。

首先，我们分析题干：

老年女性患者，主要症状为呕吐、腹泻，且尿量减少，查体显示体温正常，但心率偏快，血压偏低，处于休克血压范围，且有神情淡漠和口腔黏膜苍白的症状。这些信息提示患者可能存在因呕吐和腹泻导致的体液丢失，进而引发的低血容量性休克。

接下来，我们逐一分析选项：

A. 血管运动系统兴奋：虽然血管运动系统的状态可能影响血压，但在此情境下，由于体液的大量丢失，血管运动系统的兴奋不足以维持正常血压，故不选。

B. 有效循环血量减少：考虑到患者的呕吐、腹泻和尿量减少，这很可能导致体液的大量丢失，从而减少有效循环血量，进而影响血压。这是一个合理的解释。

C. 儿茶酚胺分泌增加：儿茶酚胺的分泌增加通常与应激反应有关，但它不是导致血压下降的直接原因，特别是在体液大量丢失的情况下，故排除。

D. 微静脉扩张：虽然血管的扩张和收缩会影响血压，但在此情境下，它不是导致血压下降的主要原因，与题干描述不符。

E. 回心血量减少：尽管体液丢失可能导致回心血量减少，但它更多地是有效循环血量减少的一个结果，而不是导致血压改变的直接原因，可排除。

综上所述，最合理的解释是患者因呕吐和腹泻导致体液大量丢失，进而引发有效循环血量的显著减少，这是导致血压下降的主要原因。

因此，正确答案是 B.有效循环血量减少。

这道题描述了一位 70 岁女性患者，出现呕吐和腹泻，并且伴有尿量减少、低血压、心率增快等症状。这些症状表明患者可能处于休克状态，特别是由于体液丢失导致的低血容量性休克。

以下是对选项的分析：

A. 血管运动系统兴奋：这种情况一般会导致血压升高，而不是降低。

B. 有效循环血量减少：呕吐和腹泻会导致体液丢失，从而减少有效循环血量，导致低血压。

C. 儿茶酚胺分泌增加：儿茶酚胺的增加会引起心率加快和血管收缩，通常会导致血压升高。

D. 微静脉扩张：微静脉扩张会引起血液在外周聚集，可能会导致血压下降，但不是这种急性失液引起的主要原因。

E. 回心血量减少：虽然回心血量减少可以导致血压下降，但这里的主要原因是由于呕吐和腹泻引起的体液丢失，直接导致有效循环血量减少。

综合分析，最可能的答案是：

B. 有效循环血量减少
112、 (A2 型题)
某医生在给患者接种疫苗的时候并未告诉患者相关注意事项，被当地县级卫生部门知道后，责令其整改，其未整改且继续违反接种相关规定，当地卫生管理部门停止其执 照相关规定，其停止其执业的时间是
A.1 年以上 15 个月以下
B.1 年以上 18 个月以下
C.6 个月以下
D.3 个月以下
E.6 个月以上 12 个月以下
参考答案： E 您的答案：
图文解析： 【解析】《执业医师法》规定，医师在执业活动中违反卫生行政规章制度造成严重后果的，由县级以上人民政府卫生行政部门给予警告或者责令暂停 6 个月以上 1 年以下（E 对）执业活动。
【避错】医师在执业活动中，违反本法规定，有下列行为之一的，由县级以上人民政府卫生行政部门给予警告或者责令暂停 6 个月以上 1 年以下执业活动；情节严重的，吊销其执业证书；构成犯罪的，依法追究刑事责任：①违反卫生行政规章制度或技术操作规范，造成严重后果的；②由于不负责任延误急危患者的抢救和诊治，造成严重后果的；③造成医疗责任事故的；④未经亲自诊查、调查，签署诊断、治疗、流行病学等证明文件或有关出生、死亡等证明文件的；⑤隐匿、伪造或者擅自销毁医学文书及有关资料的；⑥使用未经批准使用的药品、消毒药剂和医疗器械的；⑦不按照规定使用麻醉药品、医疗用毒性药品、精神药品和放射性药品的；⑧未经患者或家属同意，对患者进行实验性临床医疗的；⑨泄露患者隐私，造成严重后果的；⑩利用职务之便，索取、非法收受患者财物或牟取其他不正当利益的；（11）发生自然灾害、传染病流行、突发重大伤亡事故以及其他严重威胁人民生命健康的紧急情况时，不服从卫生行政部门调遣的；（12）发生医疗事故或发现传染病疫情及患者涉嫌伤害事件或非正常死亡，不按照规定报告的。
在处理违反疫苗接种规定的医生执业许可问题时，通常考虑的是该医生的违规行为的严重性和持续时间。由于题目描述中提到该医生未经整改继续违反接种相关规定，这表明违规行为是重复且未得到纠正的。在中国的相关法律和规章制度中，对于此类重复违规行为，执业许

可的暂停时间通常会较长。
虽然具体的暂停时间可能会根据实际情况和地方政策有所不同，但考虑到违规的严重性和重复性，较长的暂停期是合理的。因此，选项 A 和 B 看起来较为合适。然而，没有具体的法律条文作为参考，我倾向于选择一个中等长度的暂停期来反映严重的违规行为但也留有一定的调整空间。
基于上述分析，我会选择 **E. 6 个月以上 12 个月以下** 作为最合适的答案。这个选项反映了对医生违规行为的严肃处理，同时考虑到了可能的法律和规章制度中的灵活性。然而，具体答案还需要根据当地具体的法律和规定来确定。
解析：
《疫苗管理法》规定，医疗卫生人员在实施接种前，应当按照预防接种工作规范的要求，严格核对受种者信息、检查疫苗、注射器和针头，并告知受种者或其监护人所接种疫苗的品种、作用、禁忌、不良反应及注意事项等。若未按照规定告知受种者或其监护人，由县级以上人民政府卫生健康主管部门责令改正，给予警告；拒不改正的，对责任单位处五千元以上五万元以下的罚款，对负有直接责任的医疗卫生人员，处三千元以下的罚款或者责令暂停 6 个月以上 1 年以下执业活动。
综上所述，正确答案是：E.6 个月以上 12 个月以下。

根据《中华人民共和国执业医师法》第三十七条的规定，医生如果违反卫生行政规章制度，情节严重的，可以由卫生行政部门责令其暂停执业活动，时间为六个月以上一年以下。因此，正确答案是：

E. 6 个月以上 12 个月以下
113、　(A2 型题)
婴儿，7 个月大，按计划接种疫苗，下列属于还没接种的疫苗是
A.麻腮风疫苗
B.脊髓灰质炎疫苗
C.乙肝疫苗
D.百白破疫苗
E.卡介苗
参考答案：　A　您的答案：
图文解析：　【解析】麻腮风疫苗初种为 18~24 月龄（A 对）。
【避错】乙肝疫苗接种的时间为出生、1 月龄和 6 月龄。卡介苗初种为出生时。麻疹疫苗初种为八月龄。百白破疫苗的时间为出生 3、4、5 月龄进行注射。
对于 7 个月大的婴儿，根据常规的疫苗接种计划：

- A. 麻腮风疫苗（麻疹、腮腺炎和风疹疫苗），通常在婴儿 12 个月大时接种。
- B. 脊髓灰质炎疫苗，通常在出生后的前几个月内开始接种，并在婴儿期间完成多次加强。
- C. 乙肝疫苗，通常在出生时、一个月和六个月大时接种。
- D. 百白破疫苗（百日咳、白喉、破伤风疫苗），通常在出生后的 2、4、6 个月内接种。
- E. 卡介苗（针对结核病），在中国，通常在婴儿出生后不久接种。

根据上述信息，7 个月大的婴儿应该已经接种了脊髓灰质炎疫苗、乙肝疫苗、百白破疫苗和卡介苗。因此，还没有接种的疫苗是**麻腮风疫苗**。
选项**A. 麻腮风疫苗**是正确答案。

这是一道关于婴儿疫苗接种计划的问题。首先，我们要了解在中国，婴儿疫苗接种有一个明确的计划表，根据年龄来规定何时接种哪种疫苗。接下来，我们将根据这个背景信息逐一分析选项，以确定哪个疫苗是 7 个月大的婴儿还未接种的。

A. 麻腮风疫苗：根据中国疫苗接种计划，麻腮风疫苗（MMR，包括麻疹、腮腺炎和风疹疫苗）通常在婴儿 18 个月时接种。因此，对于一个 7 个月大的婴儿来说，这种疫苗还未到接种时间。

B. 脊髓灰质炎疫苗：这个疫苗通常在婴儿 2 个月、3 个月和 4 个月大时接种，所以对于一个 7 个月大的婴儿来说，应该已经接种完毕。

C. 乙肝疫苗：乙肝疫苗在出生时、1 个月和 6 个月时接种，所以 7 个月大的婴儿应该已经完成接种。

D. 百白破疫苗：这种疫苗在婴儿 3 个月、4 个月和 5 个月大时接种，因此 7 个月大的婴儿也应该已经接种完成。

E. 卡介苗：卡介苗通常在出生时接种，用于预防结核病，所以 7 个月大的婴儿应该已经接种。

综上所述，对于一个 7 个月大的婴儿来说，麻腮风疫苗是还未接种的疫苗，因为它通常在婴儿 18 个月时接种。

因此，正确答案是 A.麻腮风疫苗。

婴儿疫苗接种时间表根据中国的《国家免疫规划疫苗儿童免疫程序及说明》来安排。根据这个程序，7 个月大的婴儿通常已经接种了以下几种疫苗：

1. **乙肝疫苗**（出生时、1 个月、6 个月接种）
2. **卡介苗**（出生时接种）
3. **脊髓灰质炎疫苗**（2、3、4 个月接种）
4. **百白破疫苗**（3、4、5 个月接种）

但 7 个月大的婴儿通常还没有接种以下疫苗：

5. **麻腮风疫苗**（麻疹、流行性腮腺炎、风疹联合疫苗，通常在 8 个月大时开始接种）

因此，正确答案是：

A. 麻腮风疫苗

114、 (A2 型题)

患者，女，16 岁。近期情绪低落，并偷偷藏有一大瓶安眠药。父母发现后，将其送往某精神病院就诊。接诊医生将其诊断为抑郁症，建议住院治疗，但其父母不同意。此 采取的是

A.自愿住院

B.强迫住院

C.检查评估

D.医学鉴定

E.再次诊断

参考答案： C 您的答案：

图文解析： 【解析】患者发生危害自身行为，需经监护人同意方可实施住院治疗，本题目中患者已经有伤害自己的计划，但并未实施，且监护人拒绝住院治疗，因此需进一步检查

评估后再做决定（C 对）。
【避错】强制医疗，是指非自愿性强制治疗，是指国家为避免公共健康危机，通过强制对患者疾病的治疗，达到治愈疾病、防止疾病传播、维护公众健康利益，具有强制性、非自愿性、公益性的特点。 一般包括性病、吸毒、有暴力倾向的精神障碍、严重传染性疾病等。
针对这个情况，选项 C“检查评估”可能是最合适的选择。虽然接诊医生建议住院治疗，但如果病人或其家属不同意，通常需要进行进一步的评估来确定患者的精神状态和治疗需求。这种评估可以帮助决定是否需要采取其他措施，如强迫住院或其他干预措施。因此，选择 C 是在这种情况下进行的适当临床操作。
解析：
青年女性患者，诊断为抑郁症，医生建议住院治疗，父母未同意，表明患者此时并未办理住院手续，故患者此时并未住院，而处于门诊检查评估状态。
选项 A 自愿住院指患者自知力完整，主动要求住院的情况，而题干中明确说明患者父母并不同意住院治疗，故排除。
选项 B 强迫住院指患者自知力不完整，在监护人同意的情况下由医院对患者实施强制性住院治疗，但题干中父母并不同意其住院治疗，故不选。
因患者此时并未办理住院手续，处于门诊阶段，故选项 D 医学鉴定，选项 E 再次诊断，可一并排除。
综上所述，正确答案是：C.检查评估。

根据题干提供的信息，这位 16 岁的女性患者近期情绪低落，并且藏有大量安眠药，显示出自杀风险。医生诊断她患有抑郁症并建议住院治疗。然而，父母不同意住院。在这种情况下，如果患者有明显的自杀倾向或对自己或他人构成危险，医疗机构有权采取强制措施以保障患者的生命安全。

因此，最佳答案是：

B. 强迫住院
115、 (A2 型题)
女，30 岁。每次喝牛奶后出现腹痛、腹胀、腹泻症状，怀疑是某种酶的缺乏，经检查发现是某种酶活性降低，为减轻症状，可以建议选择哪种牛奶
A.复合奶
B.脱脂奶粉
C.酸奶
D.调制奶粉
E.淡炼乳
参考答案： C 您的答案：
图文解析： 【解析】每次喝牛奶后出现腹痛、腹胀、腹泻症状，考虑乳糖酶缺乏，导致牛奶中的乳糖不能被分解。可以适量饮用酸奶，因为酸奶中的乳酸菌本身会产生大量的乳糖酶，可以帮助身体更好的消化乳糖，故本题选 C。
【避错】酸奶为鲜牛奶加乳酸杆菌发酵制成，其凝块细、酸度高，有利于消化吸收。甜炼乳是将鲜乳经真空浓缩或其他方法除去大部分的水分，浓缩至原体积 25%～40%左右的乳制品，再加入 40%的蔗糖装罐制成的，糖类含量过高。
这位 30 岁女性出现喝牛奶后的腹痛、腹胀、腹泻症状，这些症状通常与乳糖不耐受有关，

这是由于乳糖酶的活性降低或缺乏导致。乳糖酶是一种酶，负责将乳糖分解成葡萄糖和半乳糖。如果体内缺乏乳糖酶，未被消化的乳糖进入大肠后会被细菌发酵，产生气体和其他物质，引起腹痛、腹胀和腹泻。
为减轻症状，可以选择酸奶，因为酸奶在发酵过程中由乳酸菌产生的乳酸会帮助分解乳糖，从而减少乳糖进入大肠的量，减轻乳糖不耐受症状。因此，最佳选项是：C. 酸奶
首先，我们需要明确问题的关键点：这位 30 岁的女性在饮用牛奶后出现腹痛、腹胀和腹泻，这通常是由于乳糖不耐受所导致的。乳糖不耐受是因为体内缺乏乳糖酶，无法分解牛奶中的乳糖，从而导致上述症状。
接下来，我们来分析各个选项：
A. 复合奶：通常含有多种营养成分，但并不能解决乳糖不耐受的问题，因为乳糖仍然存在。
B. 脱脂奶粉：虽然去除了牛奶中的脂肪，但乳糖含量并未减少，因此不适用于乳糖不耐受患者。
C. 酸奶：酸奶在制作过程中，乳酸菌会将牛奶中的乳糖大部分分解为乳酸，从而显著降低乳糖含量。因此，酸奶是乳糖不耐受患者的良好选择，可以减轻或避免乳糖不耐受的症状。
D. 调制奶粉：这种奶粉可能经过了一些加工，但如果不特别说明是低乳糖或无乳糖，那么对于乳糖不耐受患者来说，并不是一个好的选择。
E. 淡炼乳：淡炼乳也是牛奶制品，乳糖含量并未显著减少，因此不适用于乳糖不耐受患者。
综上所述，对于乳糖不耐受的患者，酸奶是最佳选择，因为它显著降低了乳糖含量，能够减轻乳糖不耐受引起的症状。
因此，正确答案是 C.酸奶。

该患者每次喝牛奶后出现腹痛、腹胀、腹泻症状，怀疑是由于乳糖不耐受引起的。乳糖不耐受是由于体内乳糖酶活性不足，不能有效分解乳糖所致。针对这种情况，可以选择乳糖含量较低或经过乳糖处理的奶制品以减轻症状。

以下是每种牛奶的分析：
A. 复合奶：含有多种成分，但不一定是低乳糖的。
B. 脱脂奶粉：脱脂奶粉主要去除了脂肪，但乳糖含量仍然较高。
C. 酸奶：酸奶中的乳糖在发酵过程中被部分分解成乳酸，乳糖含量较低，因此适合乳糖不耐受者。
D. 调制奶粉：成分复杂，但不一定是低乳糖的。
E. 淡炼乳：是一种浓缩牛奶，乳糖含量依然较高。

因此，最适合乳糖不耐受患者选择的是酸奶，因为酸奶中乳糖已经部分分解，可以减轻症状。

正确答案是：C. 酸奶
116、　(A2 型题)
女，62 岁，胸闷气短 1 个月，加重伴少尿 3 天。既往高血压、糖尿病肾病病史。血清钠 138mmol/L，钾 7.8mmol/L，心电图 T 波高尖。导致心电图变化的机制是心室肌细胞的
A.0 期 Ca^{2+}离子内流减慢
B.2 期 Ca^{2+}离子内流加速
C.3 期 K^{+}离子外流加速
D.4 期 K^{+}离子外流减慢
E.0 期 Na^{+}离子内流加速

参考答案： C 您的答案：
图文解析： 【解析】中老年女性患者，既往高血压、糖尿病肾病病史，血钾 7.8mmol/L（大于 5.5mmol/L），心电图 T 波高尖，考虑诊断为高钾血症。在血钾浓度增高的情况下，细胞内外钾离子浓度差减小，钾离子平衡，阈电位降低，造成细胞膜对钾离子通透性增加，导致动作电位时程的缩短。同时是心肌细胞 3 期复极化速度加快，引起心电图表现中的 T 波高尖及 QT 间期缩短的表现。故本题选 C。
【避错】高钾血症时肌肉轻度震颤，手足感觉异常，肢体软弱无力，腱反射减退或消失，甚至出现延缓性麻痹。高钾血症可以引起窦性心动过缓、房室传导阻滞或快速性心律失常，最危险的是心室颤动或心搏骤停。高钾血症常有心电图异常变化，早期改变为 T 波高而尖,Q-T 间期缩短，QRS 波增宽伴幅度下降,P 波波幅下降并逐渐消失。
这个问题涉及到心电图变化的机制，特别是在一个患有高血压、糖尿病肾病且出现高钾血症（钾 7.8mmol/L）的患者中。心电图显示 T 波高尖，这通常与高钾血症相关。在心脏肌细胞的动作电位中，各个相位的离子流动特性对心电图的波形有直接影响。特别是，钾离子的流动在动作电位的第 3 期（复极阶段）至关重要。
A、B、E 选项讨论的是钙离子和钠离子的流动，这些在心室肌细胞动作电位的第 0 期和第 2 期更为重要，而这些阶段不是高钾血症影响 T 波形态的关键因素。
C 选项提到了第 3 期 K^+离子外流加速，这是复极的一个重要部分，但在高钾血症情况下，外流并不会加速；相反，它会因为细胞外钾浓度增高而减慢。
D 选项指出的第 4 期 K^+离子外流减慢与高钾血症引起的心电图 T 波高尖现象相符。因为在高钾血症中，细胞外的钾浓度增高，导致心室肌细胞的钾离子外流减慢，这会导致动作电位持续时间延长，最终表现为心电图上的 T 波高尖。
因此，正确答案是 D：4 期 K^+离子外流减慢。
解析：
老年女性患者，既往有糖尿病肾病病史，血钾显著升高，心电图示 T 波高尖，为典型的高钾血症心电图表现。高钾血症时，细胞外 K^+浓度升高，K^+外流减少，使心室肌细胞 4 期自动去极化速度减慢，即 K^+外流减慢，从而影响心肌细胞的自律性，导致心电图发生变化。
选项 A：0 期 Ca^{2+}离子内流减慢主要见于低钙血症，与高钾血症无关，故不选。
选项 B：2 期 Ca^{2+}离子内流加速，选项 C：3 期 K^+离子外流加速，均会缩短心室肌细胞的动作电位时程，与高钾血症时心电图的表现不符，故一并排除。
选项 E：0 期 Na^+离子内流加速是心肌细胞受刺激兴奋的表现，与高钾血症无直接关系，故不考虑。
综上所述，正确答案是：D.4 期 K^+离子外流减慢。

根据题干中的信息，62 岁女性患者有胸闷气短 1 个月，近 3 天加重伴少尿，既往有高血压和糖尿病肾病病史。实验室检查显示血清钾 7.8mmol/L，心电图显示 T 波高尖。这提示患者存在高钾血症，而高钾血症常引起心电图上的特征性变化。

高钾血症对心电图的影响主要体现在以下几个方面：
1. **T 波高尖**：这是高钾血症的早期特征性表现，通常在血清钾超过 5.5 mmol/L 时出现。
2. P 波消失或振幅降低。
3. QRS 波群增宽。
4. 心率减慢，最终可能导致心脏停搏。

高钾血症导致的 T 波高尖主要是由于心室肌细胞的复极化过程（即心电图中的 3 期）受到了

影响。具体机制如下：

高钾血症使得细胞外钾离子浓度增高，导致复极化过程中 K^+离子外流增加。由于复极化（3期）是由 K^+离子外流主导的，因此血清钾升高会加速这一过程，导致 T 波高尖。

因此，题干中患者的心电图变化是由于心室肌细胞的**3 期 K^+离子外流加速**所致。正确答案是：

C. 3 期 K^+离子外流加速

117、 (A2 型题)

女，26 岁。多饮，多尿，易饥，体重下降 6 个月，检查：空腹血糖 14.2mmol/L，HbAlc8.6%，空腹血清胰岛素 7U/L（参考值 10～20mU/L），尿糖（+++）。该患者体内可能 化是

A.三羧酸循环增加

B.糖原合成增加

C.组织用糖减少

D.葡萄糖分解增多

E.糖异生减少

参考答案： C 您的答案：

图文解析： 【解析】青年女性患者，多饮，多尿，易饥，体重下降 6 个月（糖尿病典型临床表现），结合检查提示血糖增高（空腹血糖 14.2mmol/L）、胰岛素不足（HbAlc8.6%，空腹血清胰岛素 7U/L），诊断考虑为 1 型糖尿病。1 型糖尿病主要因为胰腺不能分泌足够的胰岛素，细胞质中的葡萄糖无法进入线粒体进行氧化分解，葡萄糖分解减少（D 错），导致三羧酸循环的底物减少，从而影响了三羧酸循环的进行（A 错）。同时导致糖原合成减少、分解增加（B 错）。肌肉和脂肪组织用糖减少（C 对）。糖异生增多（E 错）。故此题选 C。

这位 26 岁女性患者出现了多饮、多尿、易饥和体重下降的症状，伴有高血糖（空腹血糖 14.2mmol/L）和高糖化血红蛋白（HbAlc8.6%），同时空腹血清胰岛素水平低（7U/L，参考值 10～20mU/L），并有尿糖（+++）。这些临床表现和实验室检查结果符合糖尿病的诊断。

在糖尿病状态下，由于胰岛素作用不足或细胞对胰岛素的反应减弱，葡萄糖无法有效进入细胞利用，导致细胞内葡萄糖利用减少，而血糖水平升高。此外，由于细胞感受到能量不足，会通过增加糖异生（从非碳水化合物前体合成葡萄糖）和脂肪酸氧化来补充能量，而不是通过增加糖原合成。因此，这种情况下不会出现糖原合成增加。同时，由于糖尿病患者的葡萄糖利用减少，体内的葡萄糖分解过程实际上是增多的，以试图补充能量。

基于以上分析，最合适的选项是 D.葡萄糖分解增多。这反映了糖尿病中胰岛素不足导致的细胞葡萄糖利用减少，同时机体尝试通过增加葡萄糖分解来满足能量需求。

首先，我们梳理一下题目中的关键信息：

（1）青年女性患者，主要症状为多饮、多尿、易饥、体重下降，持续 6 个月。

（2）空腹血糖 14.2mmol/L（正常值应小于 6.1mmol/L），HbA1c 8.6%（正常参考值为 4%～6%），这两个指标均显示患者血糖控制不佳，糖尿病的可能性极大。

（3）空腹血清胰岛素 7U/L，低于参考值 10～20mU/L，表明患者体内胰岛素水平低下。

（4）尿糖（+++），说明尿中葡萄糖含量高，与高血糖状态一致。

接下来，我们分析各选项：

A. 三羧酸循环增加：虽然三羧酸循环是葡萄糖有氧氧化的重要环节，但题干信息并未提及与此直接相关的任何生化指标变化，故无法判断三羧酸循环是否增加。

B. 糖原合成增加：在胰岛素缺乏的情况下，糖原合成通常会减少，因为胰岛素是促进糖原合成的主要激素。患者空腹血清胰岛素低于正常值，因此糖原合成增加的可能性不大。
C. 组织用糖减少：由于胰岛素水平低下，组织细胞对葡萄糖的摄取和利用会减少，这与糖尿病的典型病理生理过程相符。在胰岛素缺乏时，细胞对葡萄糖的摄取受阻，导致组织用糖减少。
D. 葡萄糖分解增多：题干中并未提供与葡萄糖分解直接相关的信息，且在高血糖状态下，通常葡萄糖的分解不会增多，反而可能因为胰岛素缺乏而减少。
E. 糖异生减少：糖异生是体内葡萄糖生成的重要途径，特别是在饥饿或长时间禁食状态下。在糖尿病的情况下，糖异生可能实际上会增加，因为体内需要尝试维持血糖水平。

综上所述，考虑到患者的高血糖状态和低胰岛素水平，最合理的解释是组织细胞对葡萄糖的摄取和利用减少，导致组织用糖减少。这是糖尿病状态下，胰岛素缺乏导致的直接后果。因此，正确答案是 C.组织用糖减少。

根据题目提供的信息，患者为 26 岁女性，出现多饮、多尿、易饥、体重下降的症状，并且检查结果显示空腹血糖 14.2mmol/L，HbA1c 8.6%，空腹血清胰岛素 7U/L（参考值 10～20mU/L），尿糖（+++）。这些结果提示该患者可能患有糖尿病，尤其是 1 型糖尿病或晚期的 2 型糖尿病，表现出明显的高血糖和胰岛素缺乏。

在糖尿病患者中，由于胰岛素不足或胰岛素作用受阻，导致葡萄糖不能被有效利用，以下情况可能发生：

- **三羧酸循环增加**：不符合，糖尿病患者葡萄糖不能被有效利用，三羧酸循环活动反而可能减少。
- **糖原合成增加**：不符合，糖尿病患者糖原合成减少，因为肝脏和肌肉细胞无法有效利用葡萄糖。
- **组织用糖减少**：符合，由于胰岛素不足或抵抗，组织对葡萄糖的利用显著减少。
- **葡萄糖分解增多**：不符合，尽管葡萄糖水平高，但由于胰岛素作用受限，细胞内的葡萄糖分解不会增加。
- **糖异生减少**：不符合，糖尿病患者肝脏糖异生反而增加，导致血糖进一步升高。

综上所述，最可能的答案是：

C. **组织用糖减少**
118、 (A2 型题)
男，15 岁，突发乏力，恶心、呕吐、厌油腻食物，学校 30 余人有同样症状，无黄疸，肝肋下 1cm，压痛。最可能的是
A.伤寒、副伤寒
B.甲型肝炎
C.霍乱
D.乙型肝炎
E.戊型肝炎
参考答案： B 您的答案：
图文解析： 【解析】学校群体性出现突发乏力，恶心、呕吐、厌油腻食物、无黄疸（甲

型肝炎病程中，传染性最强的阶段是黄疸前期），根据症状及体格检查最可能诊断为甲型肝炎（B 对）。
【避错】伤寒常见的临床表现有持续发热、相对缓脉、玫瑰疹、肝脾大。霍乱的典型临床表现是霍乱肠毒素导致肠道痉挛，进而诱发肠道痉挛，出现大量水样便和剧烈呕吐，其中腹泻特点为无发热，无里急后重感，多数不伴腹痛，排便后自觉轻松感。
这个案例描述的是一个 15 岁男孩和他的学校中 30 多人出现突发乏力、恶心、呕吐和厌油腻食物的症状，这些都是急性肝炎的典型症状。病例中未提及黄疸，但提到了肝肋下 1cm 处有压痛，这可能表明肝脏有所受累。
根据症状和学校中多人同时出现类似症状的情况，最可能的是病毒性肝炎，尤其是通过饮食传播的类型。甲型肝炎是通过口粪传播，通常与食物或水的卫生状况有关，可能会在群体中迅速传播，这符合多人同时出现症状的情况。
因此，最可能的答案是 **B.甲型肝炎**。甲型肝炎通常会引起急性肝炎症状，如乏力、恶心、呕吐和食欲减退，尤其是厌油腻食物，而不一定伴随黄疸。此外，它经常在密集居住条件下爆发，例如学校，这与题目描述的情况相符。
首先，我们提取题干关键信息：患者为 15 岁男性，主要症状为突发乏力、恶心、呕吐、厌油腻食物。此外，同一学校有 30 余人出现相同症状，这是一个重要的流行病学线索，可能指向食物中毒或某种传染性疾病。体检发现肝脏肋下 1cm，有压痛，但无黄疸。
接下来，我们分析各选项对应的疾病特点：
A. 伤寒、副伤寒：主要表现为持续发热、表情淡漠、相对缓脉、玫瑰疹、肝脾大和白细胞减少，与该患者的症状不符。
B. 甲型肝炎：主要通过粪-口途径传播，常因食用被污染的食物或水源而爆发流行。其潜伏期大约为 2～6 周，平均 4 周。临床表现分为急性黄疸型和无黄疸型，后者更为多见。急性期常表现为发热、乏力、恶心、呕吐、厌油腻食物等症状，与题干描述高度符合。
C. 霍乱：主要表现为剧烈腹泻、呕吐，以及由此引起的脱水、肌肉痉挛，严重者导致循环衰竭和急性肾衰竭，与该患者症状不符。
D. 乙型肝炎：主要通过血液、性接触、母婴传播，一般不会通过食物或水源传播，且潜伏期相对较长，平均为 3 个月，因此可以排除。
E. 戊型肝炎：虽然也是通过粪-口途径传播，但其潜伏期约为 2～9 周，平均 6 周，且临床表现与甲型肝炎相似，但相比甲型肝炎，戊型肝炎的爆发流行较为少见。
综合以上信息，考虑到患者学校内有 30 余人出现相同症状，以及患者的临床表现，最可能的诊断是甲型肝炎。因此，正确答案是 B.甲型肝炎。

根据题干中提供的信息，15 岁男孩和学校 30 余人都有突发乏力、恶心、呕吐、厌油腻食物等症状，且没有黄疸，肝肋下 1cm，压痛。这些症状和体征指向一种可能的传染病，结合患者和周围人群的集体发病情况，最可能的诊断是甲型肝炎。

甲型肝炎通常通过粪口途径传播，具有较强的传染性，常见于学校、幼儿园等集体场所。症状包括乏力、恶心、呕吐、食欲不振、厌油腻食物等。虽然部分患者可能出现黄疸，但并不是所有病例都有黄疸。

因此，最可能的答案是：

B. 甲型肝炎
119、 (A2 型题)

患者，男，38 岁。发热伴全身酸痛 7 天，查体：T39.6℃,P95 次/分，R31 次/分，腹股沟淋巴结肿大，腓肠肌压痛(+)，尿常规：尿蛋白(+++)，尿沉渣镜检：WBC（+）、RB 一步的检查是

A. 血液涂片

B. 粪便常规

C.血培养

D.粪培养

E.显微凝集试验

参考答案： E 您的答案：

图文解析： 【解析】男，38 岁（青壮年为主，男性高于女性），发热伴全身酸痛 7 天（全身感染中毒表现），腹股沟淋巴结肿大，腓肠肌压痛（钩体败血症期典型临床表现），结合该患者病史、临床表现、查体，该患者诊断考虑为钩端螺旋体病，该病由致病性钩端螺旋体（简称钩体）引起，显微镜凝集溶解试验（E 对）是用钩端螺旋体标准株或者当地流行菌株的活体做特异性抗原，分别与病人不同稀释度的血清混合，在 37℃孵育 2 小时后用暗视野显微镜观察，若待测血清中有相应抗体，则可见钩端螺旋体凝集成小蜘蛛状。

【避错】肥达试验是一种试管凝集反应，用已知的伤寒杆菌 O、H 抗原和甲、乙型副伤寒杆菌 H 抗原，与待测血清作试管或微孔板凝集实验，以测定血清中有无相应抗体存在，作为伤寒、副伤寒诊断的参考。外斐反应用于辅助诊断立克次体病，变形杆菌属菌株的菌体 O 抗原与斑疹伤寒立克次体和恙虫病的虫体有共同抗原，故可用这些菌株的 O 抗原代替立克次体抗原与患者血清进行交叉凝集反应，检测患者血清中相应抗体。

这名 38 岁的男性患者有发热、全身酸痛、腹股沟淋巴结肿大、腓肠肌压痛、尿蛋白阳性和少量白细胞及红细胞出现在尿沉渣中的症状。这些症状可能指向多种病因，包括感染（细菌、病毒、寄生虫等）、炎症性疾病或其他全身性疾病。

根据这些症状和体征，尤其是高热、腹股沟淋巴结肿大和尿蛋白阳性，一个重要的考虑是可能的感染性疾病。因此，我们应该寻找可以帮助诊断感染性疾病的检查。

A. 血液涂片：可以用来检查疟疾寄生虫或其他血液寄生虫。

B. 粪便常规：用于检测肠道感染，如细菌、病毒或寄生虫感染。

C. 血培养：可以检测血流感染，帮助确定感染的类型和引起感染的细菌或真菌。

D. 粪培养：用于确诊特定的肠道病原体。

E. 显微凝集试验：用于检测某些细菌感染，如钩端螺旋体病（例如鼠疫）。

考虑到患者的症状，如发热、腹股沟淋巴结肿大和尿蛋白阳性，显微凝集试验（E）可能是诊断钩端螺旋体病（例如回归热或鼠疫）的有效方法。然而，考虑到症状的全面性和严重性，进行血培养（C）以检测可能的血流感染似乎是更为直接和紧迫的需要，因为这可以指示系统性感染，需要迅速识别和治疗。

因此，我会选择 **C. 血培养** 作为下一步检查，以便查明感染的原因，并为治疗提供指导。

解析：

青壮年男性患者，发热、全身酸痛，查体高热，心率偏快，呼吸加快，腹股沟淋巴结肿大**，腓肠肌压痛**，实验室检查见尿蛋白强阳性，尿白细胞阳性，红细胞阳性，根据患者的病史、临床表现、查体及实验室检查，考虑诊断为钩端螺旋体病。钩端螺旋体病是由致病性钩端螺旋体引起的动物源性传染病，为确诊，可进行显微镜下凝集试验，其是检测血清中存在特异性抗体的一种血清学方法，是目前国内最常用的钩体血清学诊断方法。

选项 A 血液涂片主要用于检查寄生虫等，对钩端螺旋体感染的诊断意义不大，故排除。

选项 B 粪便常规，选项 D 粪培养，一般用于诊断消化道感染性疾病，而钩端螺旋体病主要

通过接触带菌的野生动物和家畜感染，故可一并排除。
选项 C 血培养主要用于诊断细菌感染性疾病，但钩端螺旋体体外培养的难度较大，国内很少开展，故不考虑。
综上所述，正确答案是：E.显微凝集试验。

根据患者的症状和体征，38 岁的男性患者发热伴全身酸痛 7 天，查体发现高热、快速心率、呼吸频率增加、腹股沟淋巴结肿大、腓肠肌压痛，尿常规显示尿蛋白增多和白细胞阳性。这些症状提示可能存在严重感染。

结合这些症状，特别是高热和腹股沟淋巴结肿大，考虑细菌感染的可能性较大。显微凝集试验（MAT）通常用于诊断钩端螺旋体病（钩体病），而血培养有助于检测血液中的细菌。

最佳答案应该是：

C. 血培养

血培养可以帮助检测是否存在血液中的细菌，明确感染源头。
120、 (A2 型题)
女，16 岁，随旅行团到边远地区旅游，晚饭后发生腹痛、腹泻， 一天腹泻 4 次，于当地卫生院治疗后好转，第二天烈日下阳光照射后皮肤出现红斑，瘙痒。可能是什么药
A.头孢他啶
B.庆大霉素
C.盐酸小檗碱
D.氧氟沙星
E.吡喹酮
参考答案： D 您的答案：
图文解析： 【解析】服用光敏感性药物后，第二天烈日下阳光照射后皮肤出现红斑、瘙痒。常见的光敏感性药物为喹诺酮类（D 对）、氢氯噻嗪、氯丙嗪、四环素等。
【避错】庆大霉素属于氨基糖苷类（耳毒肾毒肌肉毒、过敏）、吡喹酮是抗血吸虫。
这个问题描述的是一名 16 岁女性在旅行中经历了腹痛和腹泻，治疗后在阳光照射下皮肤出现红斑和瘙痒。这些症状可能与她接受的治疗有关。
根据描述的症状和情境，我们可以逐个排查这些药物：
- A. 头孢他啶：这是一种抗生素，主要用于治疗细菌感染。它通常不会引起阳光敏感。
- B. 庆大霉素：也是一种抗生素，主要用于治疗严重的细菌感染，同样也不太可能引起阳光敏感。
- C. 盐酸小檗碱：用于治疗各种疾病，包括疟疾和某些皮肤病，但通常不会导致阳光敏感。
- D. 氧氟沙星：这是一种广谱抗生素，属于氟喹诺酮类，它可能导致光敏感性反应，使得皮肤对阳光更加敏感，可能引起阳光下的红斑和瘙痒。
- E. 吡喹酮：用于治疗寄生虫感染，如疟疾，通常不与阳光敏感相关联。
综上所述，最可能的药物是 **D. 氧氟沙星**，因为它已知可能引起光敏感性反应。
解析：
青少年女性患者，旅游后出现腹痛腹泻，考虑诊断为旅行者腹泻，经治疗后好转，第二天日晒后皮肤出现红斑、瘙痒，考虑诊断为药物光敏反应。根据该患者的病史和临床表现，考虑腹泻时使用的药物可能为盐酸小檗碱，服用此药后如受阳光照射，可能出现光敏反应，导致

皮肤红斑、瘙痒。
选项 A 头孢他啶，选项 B 庆大霉素，选项 D 氧氟沙星，均属于抗生素，可用于旅行者腹泻的治疗，但一般不引起光敏反应，故一并排除。
选项 E 吡喹酮是治疗血吸虫病的首选用药，而该患者并无血吸虫感染征象，故不选。
综上所述，正确答案是：C.盐酸小檗碱。根据题目描述，该女性在旅行期间服药后出现了皮肤红斑和瘙痒的症状，这些症状在阳光照射后出现，提示可能是药物的光敏反应。许多抗菌药物都可能引起光敏反应，但氧氟沙星（D.氧氟沙星）是最常见的引起光敏反应的药物之一。

因此，最可能的药物是：

D. 氧氟沙星

121、 (A2 型题)
女，45 岁，月经量增多，延长 2 年， 心悸，头晕 2 个月， 查体： 子宫如孕 14 周大小，子宫多发肌瘤，最大者 8×7×6cm，血常规：Hb71g/L，WBC2.45×1012/L， PaO2 82.3mm，pH7.43。符合该患者血氧变化的是
A.动脉血氧饱和度下降
B.动脉血氧分压下降
C.动脉血氧含量下降
D.动静脉氧分压差增大
E.氧离曲线左移
参考答案： B 您的答案：
图文解析： 【解析】患者月经量增多、延长，心悸，头晕 2 个月（贫血常见临床表现），子宫如孕 14 周大小，子宫多发肌瘤，Hb71g/L，WBC2.45×1012/L，PaO282.3mmHg（血氧分压降低，B 对），综合患者病史及临床表现，考虑诊断为贫血、子宫肌瘤。
【避错】PaO2（血氧分压）指动脉血中物理溶解的氧分子所产生的张力，反映机体缺氧敏感指标，主要是判断机体是否缺氧及其程度。动脉血氧分压检测，主要用于缺氧或氧中毒性疾病的诊断，正常值 95~100mmHg。血氧饱和度（SaO2）是血液中被氧结合的氧合血红蛋白（HbO2）的容量占全部可结合的血红蛋白容量的百分比，即血液中血氧的浓度，其代表的是红细胞的携氧能力。
这位 45 岁的女性患者有多发性子宫肌瘤、月经量增多、心悸、头晕等症状，血常规检查显示低血红蛋白(Hb 71g/L)，这表明她可能有贫血。贫血会影响到血氧携带能力，因此我们需要考虑哪个选项最符合这种情况。
A. 动脉血氧饱和度下降 - 血氧饱和度是指血液中的血红蛋白与氧结合的程度。虽然贫血会减少血液中的血红蛋白总量，从而降低氧的总携带量，但并不一定会影响饱和度，除非也有氧合不良。
B. 动脉血氧分压下降 - 这指的是血液中氧气的分压，与肺的气体交换能力相关。这位患者的 PaO2 为 82.3mmHg，这在正常范围内，所以不是最佳选项。
C. 动脉血氧含量下降 - 这似乎是一个合理的选项，因为贫血减少了血红蛋白的总量，进而降低了血氧含量。
D. 动静脉氧分压差增大 - 这通常反映组织的氧需求和摄取，与本案例中的情况关系不大。
E. 氧离曲线左移 - 这表示血红蛋白对氧的亲和力增加，与贫血本身无直接关系。
综上所述，最符合这位患者症状和检查结果的是选项 C，动脉血氧含量下降。因此，答案应

该是 C。
首先，我们分析题干。
患者中年女性，长期月经量增多并延长，查体发现子宫异常增大，诊断为子宫多发肌瘤。血常规结果显示中度贫血，血氧分压（PaO2）为 82.3mmHg，处于正常范围内（正常值为 80-100mmHg）。
接下来，我们逐项分析选项：
A. 动脉血氧饱和度下降：血氧饱和度通常与血氧分压直接相关。由于患者的血氧分压处于正常范围，因此血氧饱和度也应是正常的，故 A 选项不正确。
B. 动脉血氧分压下降：题干已明确指出患者的血氧分压为 82.3mmHg，这在正常范围内，因此 B 选项不正确。
C. 动脉血氧含量下降：动脉血氧含量是指每 100ml 血液中血红蛋白（Hb）实际结合的氧量。由于患者血红蛋白显著降低（Hb 71g/L，正常值女性为 110-150g/L），尽管血氧分压正常，但血液中能结合的氧的总量会减少，因此动脉血氧含量会下降。故 C 选项正确。
D. 动静脉氧分压差增大：动静脉氧分压差通常反映组织对氧的利用情况。题干中未提及静脉血氧分压，且贫血主要影响的是动脉血氧含量，而非动静脉氧分压差，故 D 选项不正确。
E. 氧离曲线左移：氧离曲线描述的是血氧分压与血氧饱和度的关系。在贫血情况下，由于血红蛋白减少，血液携氧能力下降，但这并不直接导致氧离曲线左移。实际上，在贫血情况下，由于血红蛋白减少，可能导致在相同的血氧分压下，血氧饱和度相对更低，表现为氧离曲线右移。故 E 选项不正确。
综上所述，正确答案是：C.动脉血氧含量下降。

对于这道题目，我们需要仔细分析患者的情况和各个选项的含义。

患者，45 岁，女性，有月经量增多和延长的病史，并且最近 2 个月出现心悸和头晕症状。查体发现子宫如孕 14 周大小，子宫多发肌瘤，最大的肌瘤为 8×7×6cm。血常规显示 Hb 71g/L，提示严重贫血，WBC 2.45×10^9/L，提示可能有白细胞减少。动脉血气分析显示 PaO2 82.3mmHg，pH 7.43。

根据上述情况，患者有严重贫血（Hb 71g/L），可能引起心悸、头晕等症状。贫血会导致动脉血氧含量下降，因为血红蛋白是氧气的主要携带者。当血红蛋白水平降低时，即使动脉血氧分压正常，血氧含量也会降低。因此，最符合该患者血氧变化的选项是：

C. 动脉血氧含量下降
题目材料　　男，28 岁。婚前到某医疗保健机构进行婚检。经检查，医疗保健机构为其出具了医学婚前检查证明，其中列明了患者目前所患疾病，并将 其女友，引发男方强烈不满。
122、　(A3/A4 型题)　题
证明单上不应列出的疾病为
A.医学上不建议结婚的疾病
B.在传染期内的特定传染病
C.发病中的精神疾病
D.对生育有严重影响的遗传性疾病
E.自限性的传染病
参考答案：　E　您的答案：
图文解析：　【解析】婚前医学检查包括对下列疾病的检查：（1）严重遗传性疾病：　由

于遗传因素先天形成，患者全部或部分丧失自主生活能力，子代再现风险高，医学上认为不宜生育的疾病（A、D 错） 。(2）指定传染病：《中华人民共和国传染病防治法》 中规定的艾滋病、淋病、梅毒以及医学上认为影响结婚和生育的其他传染病。(3）有关精神病：精神分裂症、躁狂抑郁型精神病以及其他重型精神病（C 错） 。(4）其他与婚育有关的疾病，如重要脏器疾病和生殖系统疾病等。经婚前医学检查，对患指定传染病在传染期内或者有关精神病在发病期内的，医师应当提出

医学意见；准备结婚的男女双方应当暂缓结婚，B 错。综上，本题选 E。

【避错】

122、题目询问的是证明单上不应列出的疾病。这里的关键是考虑哪种疾病的信息在婚前检查中没有必要公开，因为它可能不会长期影响个体健康或婚姻生活。根据这个标准，E.自限性的传染病 是最合适的选择，因为自限性疾病通常会自我解决，不需要特别的治疗或可能不会对双方的长期健康造成影响。

正确答案是：A.医学上不建议结婚的疾病。

根据《婚姻法》规定，婚检应检查是否存在严重的遗传性疾病、指定的传染病以及有关精神病。自限性疾病通常不需要特殊治疗，可以自行恢复，因此婚检证明中无需特别列出。而医学上不建议结婚的疾病不是一个明确的医学分类，且可能包含了许多不影响结婚的疾病，因此不应列在婚检证明上。故 A 选项“医学上不建议结婚的疾病”是婚检证明上不应列出的。

122. 证明单上不应列出的疾病为

A. 医学上不建议结婚的疾病

B. 在传染期内的特定传染病

C. 发病中的精神疾病

D. 对生育有严重影响的遗传性疾病

E. 自限性的传染病

正确答案: E. 自限性的传染病

解析:

自限性的传染病是一类可以自我限制、会自然痊愈的疾病，一般不构成长期威胁或婚姻障碍，不应该列在婚检证明单上。

123、 (A3/A4 型题) 题

从医学心理学角度分析，该男士觉得未满足的需求是

A.生存需求

B.交往的需求

C.接纳的需求

D.尊重的需求

E.自我实现的需求

参考答案： D 您的答案：

图文解析： 【解析】医师在提出“不宜结婚 ”“不宜生育 ”和“暂缓结婚 ”等医学意见时，应充分尊重服务对象的意愿，耐心、细致地讲明科学道理，对可能产生的后果给予重点解释。D 对。

【避错】

123、题目考查的是医学心理学的知识。在这个情境中，男士对于医疗保健机构公开他的健康状况给他的女友感到强烈不满，这可能违背了他的 D.尊重的需求。尊重的需求包括自尊、

地位、认可和尊重他人的需求，这里的男士可能感觉他的地位和自尊受到了威胁。
正确答案是：D.尊重的需求
该男士在婚检过程中，其疾病情况被列明并告知了其女友，引发了他的强烈不满。从医学心理学角度分析，这种不满主要源于他觉得自己的隐私权和尊严受到了侵犯，即他的尊重需求没有得到满足。尊重需求包括对自己能力和成就的自豪，以及对独立、自由和尊严的追求。因此，D 选项“尊重的需求”是正确的。
123. 从医学心理学角度分析，该男士觉得未满足的需求是
A. 生存需求
B. 交往的需求
C. 接纳的需求
D. 尊重的需求
E. 自我实现的需求

正确答案: D. 尊重的需求

解析:
在该案例中，男士因为其个人健康信息被泄露而感到不满和愤怒，这主要是因为他的隐私和尊严没有得到尊重，这属于尊重需求的范畴。
124、 (A3/A4 型题) 题
本案例中，接诊医师违反的主要医师道德义务是
A.为患者进行健康教育
B.保护患者的隐私
C.遵循医学科学规律
D.医疗文书规范书写
E.遵守操作规范
参考答案： B 您的答案：
图文解析： 【解析】婚前医学检查表应妥善保存，对个人隐私保密。本题的题干主要是强调医生未经该患者同意，就将疾病情况通知给女友，据此本题选 B 比较合适。
【避错】
124、题目询问的是接诊医师违反的主要医师道德义务。在此案例中，医生未能保护患者的隐私信息，向未经同意的第三方透露了个人健康状况，所以违反的是 B.保护患者的隐私。这是医师职业道德中的重要一环，要求医师保护患者的隐私和保密信息。
正确答案是：B.保护患者的隐私
在案例中，接诊医师未经患者同意就将其疾病情况告知了其女友，这违反了患者的隐私权。保护患者隐私是医师的重要道德义务之一，医师应当尊重患者的隐私权和保密权，不得无故泄露患者的个人信息和健康状况。因此，接诊医师违反的主要医师道德义务是保护患者的隐私。其他选项如为患者进行健康教育、遵循医学科学规律、医疗文书规范书写、遵守操作规范等，虽然也是医师应遵守的道德规范，但在此案例中并未直接涉及到。
124. 本案例中，接诊医师违反的主要医师道德义务是
A. 为患者进行健康教育
B. 保护患者的隐私
C. 遵循医学科学规律
D. 医疗文书规范书写
E. 遵守操作规范

正确答案: B. 保护患者的隐私

解析:
根据《执业医师法》和相关医学伦理要求，医师应当保护患者的隐私权。在本案例中，接诊医师未能保护患者的隐私，导致患者的疾病信息被公开，从而引发不满，违反了保护患者隐私的医师道德义务。

题目材料　　某市随机抽取 206 名成年男性和 201 名成年女性，了解其 HBsAg 携带情况，其中男性阳性人数为 33 人，阳性率为 16.02%，女性阳性人 人，阳性率 10.94%，已知全省男性 HBsAg 阳性携带率为 17.3%，比较男女性别携带率，P＞0.05，按照α=0.05 标准。

125、　(A3/A4 型题)　题

若该市男性 HBsAg 携带率与全省男性 HBsAg 阳性携带率比较得 P＞0.05，则该市男性样本率与全省男性样本率的不同取决于

A.样本数太大

B.系统误差的影响

C.计算误差的影响

D.总体不同

E.抽样误差的影响

参考答案：　E　您的答案：

图文解析：　【解析】P＞0.05，即表示二者没有显著差异，该市和全省一样。大样本量给出的估计量更接近于总体参数，样本量越大，误差越小，A 错。系统误差是由于测量方法本身所造成的误差，根据题干和目前医疗水平，　乙肝的测定应该不至于有太大差错，因此排除 B 选项。没有计算误差的说法，相近的有统计误差，也称统计数据误差，是统计工作实际获取数据结果与相应客观真值之间的差距，在统计乙肝患者时不太容易出现明显的错误，C 错。总体不同，导致该市和全省统计结果一样，这个逻辑不对，D 错。该市的结果是通过随机抽取而来的，这个过程中很容易出现抽样误差，故本题选 E。

【避错】

正确答案是 E。在统计分析中，当 P 值大于 0.05 时，表明样本观察到的差异可能是由于抽样误差而不是总体间真实差异导致的。这里，男性 HBsAg 携带率与全省男性 HBsAg 阳性携带率比较得 P 值大于 0.05，表明两个率之间的差异可能仅仅是因为抽样误差。

E.抽样误差的影响

解析：

若该市男性 HBsAg 携带率与全省男性 HBsAg 阳性携带率比较得 P＞0.05，说明两者间没有统计学差异。而样本率和总体率之间的差异往往是由于抽样误差所导致的，即样本并不能完美地代表总体。这种误差是随机的，并随着样本量的增大而减小，但不可能完全消除。

125. 若该市男性 HBsAg 携带率与全省男性 HBsAg 阳性携带率比较得 P＞0.05，则该市男性样本率与全省男性样本率的不同取决于

A. 样本数太大

B. 系统误差的影响

C. 计算误差的影响

D. 总体不同

E. 抽样误差的影响

答案：E. 抽样误差的影响

解析：当 P＞0.05 时，说明没有足够的统计证据表明该市男性 HBsAg 携带率与全省男性 HBsAg 携带率之间存在显著差异。这种差异可能是由于抽样误差引起的，而不是系统误差、计算误差或总体的实际差异。

126、 (A3/A4 型题)

下列结论正确的是 题

A.男女性别携带率差异具有统计学意义

B.男性携带率＞女性

C.男性携带率＜女性

D.男性携带率＝女性

E.尚不能认为男女携带率不同

参考答案： E 您的答案：

图文解析： 【解析】根据题干， 比较男女性别差异的 P＞0.05，表明差异没有统计学意义，A 错。 由于没有男女之间并没有显著差异，因此也就没有男性携带率高于女性、男性携带率低于女性的说法，BCD 错。综上，本题选 E。

【避错】

正确答案是 E。由于 P 值大于 0.05，我们不能拒绝零假设，这意味着没有足够的证据表明男性和女性的 HBsAg 携带率存在统计学上的显著差异，因此不能认为男女携带率不同。

E.尚不能认为男女携带率不同

解析：

题中明确给出“比较男女性别携带率，P＞0.05”，说明男女 HBsAg 携带率之间的差异没有统计学意义，因此我们不能认为男女携带率不同。

126. 下列结论正确的是

A. 男女性别携带率差异具有统计学意义

B. 男性携带率＞女性

C. 男性携带率＜女性

D. 男性携带率＝女性

E. 尚不能认为男女携带率不同

答案：E. 尚不能认为男女携带率不同

解析：题目中提到比较男女性别携带率，P＞0.05，这表明在统计学上没有足够的证据证明男女携带率之间存在显著差异。因此，正确的结论是尚不能认为男女携带率不同。

127、 (A3/A4 型题) 题

用 Z 检验比较两率，下列说法正确的是

A.P 越大，越有理由说明两个总体率不同

B.P 越小，越说明两样本率差别越小

C.P 越小，越有理由说明两总体率不相同

D.P 越大，越有理由说明两个样本率不同

E.P 越小，越有理由说明两个样本率不同

参考答案： C 您的答案：

图文解析：　　【解析】Z 检验属于假设检验，在假设检验中，P 值代表在假设原假设 H0 正确时，出现当前证据或更强的证据的概率。P 值越小，说明样本提供的证据越强，对原假设越不利，越支持备择假设。在本题中，备择假设应该是男女两总体率不同，故本题选 C。注意，其他选项是比较样本率，　比较样本的率直接比大小就行了，假设检验的意义在于通过随机抽取的样本率来估算整个总体的率，因此 BDE 错。
【避错】
正确答案是 C。在使用 Z 检验比较两个比率时，较小的 P 值（通常小于 0.05）表示两个样本之间的差异在统计学上是显著的，因此更有理由认为两个总体率不相同。
C.P 越小，越有理由说明两总体率不相同
解析：
Z 检验是一种用于比较两个比例或率的统计方法。在此检验中，P 值越小，意味着观察到的数据在假设两总体率相同的情况下出现的概率越小，因此越有理由拒绝这一假设，即认为两总体率是不同的。所以选项 C“P 越小，越有理由说明两总体率不相同”是正确的。而 P 值越大，说明在假设两总体率相同的情况下，观察到的数据出现的概率越大，因此我们不能拒绝这一假设。所以，A、B、D 和 E 选项都是错误的。

127. 用 Z 检验比较两率，下列说法正确的是
A. P 越大，越有理由说明两个总体率不同
B. P 越小，越说明两样本率差别越小
C. P 越小，越有理由说明两总体率不相同
D. P 越大，越有理由说明两个样本率不同
E. P 越小，越有理由说明两个样本率不同

答案：C. P 越小，越有理由说明两总体率不相同

解析：在 Z 检验中，P 值越小，说明样本率之间的差异越显著，因此有更充分的理由认为两个总体率不相同。
题目材料　　男，22 岁，3 年来无明显诱因出现孤僻，不愿外出与人交往，白天窗帘紧闭，生活懒散，夏天可长达半个月不洗澡，头发很长也不理发，　见明显异常，精神检查：表情淡漠，话少，否认幻觉，思维贫乏，情感淡漠，自知力差。
128、　(A3/A4 型题)　题
该患者考虑诊断为
A.精神分裂症
B.广泛性焦虑
C.自闭症
D.抑郁症
E.社交回避性人格障碍
参考答案：　A　您的答案：
图文解析：　　【解析】本题难点在于精神分裂与抑郁症的鉴别诊断，主要鉴别点是精神分裂患者可出现精神活动与环境明显不协调，而抑郁症患者不会。本例患者白天窗帘紧闭、夏天可长达半个月不洗澡等表现属于精神活动与环境不协调，故考虑是精神分裂而非抑郁症。精神分裂症大多数患者在年富力强的青年时期起病，　以 25 岁左右为最多见。其中的单纯型精神分裂的临床表现为思维贫乏、情感淡漠，或意志减退等“　阴性症状　”为主，早期可表现为类似神经衰弱症状，如精神萎靡、注意力涣散、头昏、失眠等，然后逐渐出现孤僻、懒

散、兴致缺失、情感淡漠和行为古怪， 以至无法适应社会需要，但没有妄想、幻觉等明显的“ 阳性症状 ”。病程至少 2 年。本患者临床表现符合，A 对。广泛性焦虑是以持续的显著紧张不安，伴有自主神经功能兴奋和过分警觉为特征的一种慢性焦虑障碍，患者常具有特征性的外貌，如面肌扭曲、眉头紧锁、姿势紧张，并且坐立不安，甚至有颤抖，皮肤苍白，手心、脚心以及腋窝汗水淋漓。B 不符合。 自闭症起病于婴幼儿时期，C 错。回避型人格障碍的特征在于避免涉及拒绝，批评或屈辱风险的社交场合或互动，与本例患者不相符，E 错。

128 题：患者表现出的症状符合精神分裂症的特征，如社交退缩、情感淡漠、自知力差等。因此，最合适的诊断为：A. 精神分裂症

解析：

青年男性患者，近年来无明显诱因出现孤僻，不愿外出与人交往，生活懒散，精神检查发现表情淡漠，话少，否认幻觉，思维贫乏，情感淡漠，自知力差，根据其症状及体征，考虑诊断为精神分裂症。精神分裂症是一种病因未明的重性精神病，多在青壮年缓慢或亚急性起病，临床上往往表现为症状各异的综合征，涉及感知觉、思维、情感和行为等多方面的障碍以及精神活动的不协调。患者一般意识清楚，智能基本正常，但部分患者在疾病过程中会出现认知功能的损害。

选项 B 广泛性焦虑是以持续的显著紧张不安，伴有自主神经功能兴奋和过分警觉为特征的一种慢性焦虑障碍，与本题患者症状不符，故不选。

选项 C 自闭症多起病于婴幼儿期，主要特征为不同程度的言语发育障碍、人际交往障碍、兴趣狭窄和行为方式刻板，与本题患者年龄及症状不符，故排除。

选项 D 抑郁症以显著而持久的心境低落为主要特征，临床表现从闷闷不乐到悲痛欲绝，可有消极自杀企图或行为，甚至发生木僵，与本题无关。

选项 E 社交回避性人格障碍又叫逃避型人格，其最大特点是行为退缩、心理自卑，面对挑战多采取回避态度或无能应付，与本题不符。

综上所述，正确答案是：A.精神分裂症。

诊断分析：

精神分裂症 (A)：符合孤僻、不愿交往、情感淡漠、思维贫乏、自知力差等表现，最为符合患者的症状。

广泛性焦虑 (B)：一般表现为持续的紧张和担忧，不符合此案例中患者的主要表现。

自闭症 (C)：多发生于儿童期，主要表现为社会交往和沟通障碍，不完全符合此成年患者的表现。

抑郁症 (D)：虽然有生活懒散和情感淡漠的症状，但思维贫乏、自知力差等更倾向于精神分裂症。

社交回避性人格障碍 (E)：主要表现为对社交的极度恐惧和回避，但不一定伴随情感淡漠和思维贫乏等精神症状。

因此，最佳诊断为 A.精神分裂症。

129、 (A3/A4 型题) 题

治疗应选择的药物是

A.抗抑郁药物

B.心境稳定剂

C.精神兴奋剂

D.抗精神病药物

E.抗焦虑药物

参考答案：　　D　您的答案：
图文解析：　　【解析】针对精神分裂的药物治疗，应选择抗精神病药物，D 对。
129 题：治疗精神分裂症通常需要使用抗精神病药物，因为它们能够帮助控制幻觉、妄想以及思维和行为的混乱。所以，适合的药物是：D. 抗精神病药物
129、解析：
由于该患者考虑为精神分裂症，因此治疗应选择的药物是抗精神病药物。这类药物能有效地控制精神分裂症的精神病理学症状，明显改善患者的生活质量和预后。
选项 A 抗抑郁药物主要用于治疗抑郁症，故排除。
选项 B 心境稳定剂即抗躁狂药，主要用于治疗躁狂症，与本题不符。
选项 C 精神兴奋剂不是专业的医学术语，在精神医学中，通常用“兴奋剂”来描述某些能刺激中枢神经系统的药物，但这些药物通常不用于治疗精神分裂症，故不选。
选项 E 抗焦虑药物主要用于治疗广泛性焦虑、惊恐障碍等，不符合题意。
综上所述，正确答案是：D.抗精神病药物。

治疗选择：
抗抑郁药物 (A)：主要用于治疗抑郁症，不适合此患者。
心境稳定剂 (B)：用于双相情感障碍，不适合此患者。
精神兴奋剂 (C)：用于治疗注意缺陷多动障碍（ADHD）等，不适合此患者。
抗精神病药物 (D)：适用于精神分裂症患者，能够有效控制症状。
抗焦虑药物 (E)：用于焦虑症，不适合此患者。
因此，最佳药物选择为 D.抗精神病药物。
130、　(A3/A4 型题)
该患者服用精神药物后，症状明显好转，但出现静不能坐，手抖等症状，下一步的治疗应该是　题
A.加量使用该精神药物
B.该用其他不同机制作用的精神药物
C.减量使用该精神药物
D.停用该精神药物
E.加用苯海索治疗
参考答案：　　E　您的答案：
图文解析：　　【解析】患者静坐不能、手抖的症状属于锥体外系反应。 由抗精神病要引起的锥体外系反应， 比较常见的有 5 种不同形式，主要有：急性肌张力障碍、静坐不能、药源性的帕金森氏综合征、兔唇综合征、迟发性运动障碍。苯海索常与抗精神病药联用，用于改善甚至预防此类药物所致锥体外系反应，E 对。
130 题：描述的症状，如静不能坐和手抖，可能是药物副作用，特别是抗精神病药物引起的锥体外系副作用。苯海索（Benzhexol）通常用于治疗这种副作用。因此，下一步治疗应该是：E. 加用苯海索治疗
130、解析：
患者服用精神药物后，症状明显好转，但出现静不能坐，手抖等症状，这是药物引起的椎体外系反应，因此下一步的治疗应该是加用苯海索治疗。苯海索为中枢抗胆碱抗帕金森病药，临床用于帕金森病、帕金森综合征，也可用于药物引起的锥体外系疾患。
选项 A 加量使用该精神药物可能会加重患者的药物副作用，故不选。
选项 B 改用其他不同机制作用的精神药物并不能确切解决当前的锥体外系反应问题，且可能带来其他不确定的副作用，可排除。

选项 C 减量使用该精神药物可能会影响治疗效果，不符合题意。
选项 D 停用该精神药物可能会导致患者病情反复，与本题不符。
综上所述，正确答案是：E.加用苯海索治疗。
处理药物副作用：
患者服用精神药物后，症状好转，但出现静不能坐、手抖等副作用，可能是药物诱发的锥体外系反应（如静坐不能、震颤）。

加量使用该精神药物 (A)：不适合，可能加重副作用。
更换其他不同机制作用的精神药物 (B)：有一定合理性，但需要考虑疗效和副作用的平衡。
减量使用该精神药物 (C)：可以减轻副作用，可能是合理选择。
停用该精神药物 (D)：不推荐，可能导致症状复发。
加用苯海索治疗 (E)：苯海索是抗胆碱药物，可以缓解锥体外系反应。
因此，最佳处理方案为 E.加用苯海索治疗。
题目材料　　男，68 岁，急性心肌梗死入院治疗。3 周后，患者除了感觉活动后心前区稍不适之外其他症状均消失，实验室检查均阴性，经治医生简单 提前出院。1 个月后，患者心绞痛来该医院急诊科就诊，5 小时后室颤死亡。患者家属认为患者死亡与上次医生劝其出院有关，与医院发生纠纷，提出
131、　(A3/A4 型题)　题
医院具备尸体冻存条件，其尸检的法定最长时限是
A.患者死亡后 48 小时
B.患者死亡后 3 天
C.患者死亡后 7 天
D.患者近亲属签字后 48 小时
E.患者近亲属签字后 3 天
参考答案：　C　您的答案：
图文解析：　【解析】参照《医疗事故处理条例》第十八条，患者死亡，医患双方当事人不能确定死因或者对死因有异议的，应当在患者死亡后 48 小时内进行尸检。具备尸体冻存条件的，可以延长至 7 日，C 对。
关于尸体冻存的法定最长时限，根据我所了解的法律规定，选项中并不完全符合任何国家的标准法律规定，因为尸检的时间限制因国家或地区法律而异。不过，通常情况下，尸检应尽早进行以避免尸体的进一步变化，影响尸检结果。如果必须选择一个选项，我倾向于选择 B.患者死亡后 3 天，因为这个时间段相对合理，可以让家属做出决定并进行必要的法律和医学准备。
131、正确答案是：C.患者死亡后 7 天
根据《医疗事故处理条例》规定，患者死亡，医患双方当事人不能确定死因或对死因有异议的，应当在患者死亡后 48 小时内进行尸检；具备尸体冷冻条件的，可以延长至 7 日。因此，医院具备尸体冻存条件，其尸检的法定最长时限是患者死亡后 7 天，选项 C 正确。

131. 医院具备尸体冻存条件，其尸检的法定最长时限是
A. 患者死亡后 48 小时
B. 患者死亡后 3 天
C. 患者死亡后 7 天
D. 患者近亲属签字后 48 小时
E. 患者近亲属签字后 3 天

最佳答案: C. 患者死亡后 7 天

尸检的法定最长时限通常规定为患者死亡后 7 天内。这是为了确保尸检的及时性和准确性。

132、 (A3/A4 型题) 题

家属行为属于

A. H 型行为

B. D 型行为

C. X 型行为

D. B 型行为

E. A 型行为

参考答案： E 您的答案：

图文解析： 【解析】A 型行为主要体现为极端好胜、富有攻击性、缺乏耐心、有时间急迫忌和怀有敌意，通常对生活中的某些核心方面感到不满，极富竞争性。A 选项符合。B 型行为较少竞争性、较少敌意。D 型行为主要体现为悲观。

关于家属行为的类型，这些选项似乎指向特定的行为模式或类型，但这些类型并不是广泛认可或定义明确的医学或心理学术语。如果是根据常规理解，可能没有足够的信息来判断家属的行为属于哪种类型。因此，没有明确的答案，但如果必须选择，我会倾向于选择 C. X 型行为，这里假设 X 型行为指的是一种特殊或未明确定义的行为类型。

132、正确答案是：C. X 型行为

X 型行为模式是一种“交换型”应对社会压力的行为模式，指在社会竞争中，有的人会采取一种利用性、反抗性、调节性的行为或交换来处理冲突。患者家属因患者死亡与上次医生劝其出院有关而与医院发生纠纷，体现了患者家属采取反抗性行为来处理冲突，属于 X 型行为模式，因此选项 C 正确。

A 型行为模式表现为有较高的成就欲望，富于挑战和竞争精神，容易发生无端敌意、争强好胜、不耐烦、有时间紧迫感等，与题意不符，故选项 A 可排除。

B 型行为是与 A 型行为相反的一种人格特征，属较松散、与世无争，对任何事皆处之泰然，与本题不符，故选项 B 可排除。

D 型行为是孤僻型行为，表现为沉默寡言，孤僻内向，极端害羞，不善于社交，固执，情绪不稳，这类人容易患心血管疾病和恶性肿瘤，并可伴有变态心理，与本题无关，故选项 D 可排除。

临床上并无 H 型行为模式，故选项 E 可优先排除。

132. 家属行为属于

A. H 型行为

B. D 型行为

C. X 型行为

D. B 型行为

E. A 型行为

最佳答案: B. D 型行为

D 型行为通常指的是情绪容易激动，易受压力影响，并可能因此采取极端行为的行为类型。在这种情况下，患者家属因对医生的决策不满而与医院发生纠纷，属于 D 型行为。

133、 (A3/A4 型题)

患者家属对当时主治医师要求提前出院决定有异议，认为该行为造成患者死亡，下列说法哪项符合医学伦理 题

A.主治医生应该与患者家属充分沟通解释当时要求提前出院的原因和状况

B.患者家属无权质疑主治医生当时的决策

C.医院应对主治医生进行问责调查

D.患者可以在医院闹事

E.主治医生应该患者的死亡负责

参考答案： A 您的答案：

图文解析： 【解析】患者及家属有权质疑主治医生的决策，但在医院闹事属于违反相关法律部法规。医院的医护人员在对患者进行诊疗过程中存在过错的，医院对患者的死亡要承担一定的责任。 由于本例目前并未确定医生及医院是否存在过错。综上，BCDE 错。

对于第三题，符合医学伦理的是 A.主治医生应该与患者家属充分沟通解释当时要求提前出院的原因和状况。这体现了医学伦理中的透明度和沟通原则，确保患者及其家属理解治疗决策和出院决定，同时有助于避免误解和不信任。

133、正确答案是：A.主治医生应该与患者家属充分沟通解释当时要求提前出院的原因和状况

从医学伦理的角度来看，主治医生在面对患者家属的异议时，应该选择坦诚沟通，解释当时要求提前出院的医学理由和患者的具体状况，这样有助于消除误解，维护医患关系的和谐。因此，选项 A 符合医学伦理的要求。

选项 B“患者家属无权质疑主治医生当时的决策”违背了患者的知情同意权，患者及家属有权利对医生的医疗决策提出质疑，故排除。

选项 C“医院应对主治医生进行问责调查”过于武断，没有充分沟通了解情况就直接问责调查，不符合公正原则，故不选。

选项 D“患者可以在医院闹事”明显违反了社会秩序和医疗环境的要求，不仅不能解决问题，还可能造成更多的混乱和伤害，可排除。

选项 E“主治医生应该患者的死亡负责”的说法过于绝对，患者的死亡是否由医生的决策直接导致，需要经过专业的医疗事故鉴定才能确定，不能简单归责于医生，因此不符合题意。

133. 患者家属对当时主治医师要求提前出院决定有异议，认为该行为造成患者死亡，下列说法哪项符合医学伦理

A. 主治医生应该与患者家属充分沟通解释当时要求提前出院的原因和状况

B. 患者家属无权质疑主治医生当时的决策

C. 医院应对主治医生进行问责调查

D. 患者可以在医院闹事

E. 主治医生应该对患者的死亡负责

最佳答案: A. 主治医生应该与患者家属充分沟通解释当时要求提前出院的原因和状况

题目材料 腹部闭合性损伤患者伴休克，腹部穿刺抽出不凝血。

134、 (A3/A4 型题)

在尚未手术前应采用的最主要的治疗环节是

A.保持安静

B.止痛

C.防治休克

D.胃肠减压
E.应用抗生素
参考答案：　　C　您的答案：
图文解析：　　【解析】腹腔穿刺抽出不凝血提示腹腔内出血，有脏器破裂。 内脏损伤的伤者很容易并发休克，故防治休克是治疗的重要环节。腹腔进行性出血患者，在抗休克的同时，迅速剖腹止血。
134 题关注的是在尚未进行手术前，应该采用的最主要治疗环节。给出的选项包括保持安静、止痛、防治休克、胃肠减压和应用抗生素。在这种情况下，最紧急的事情是处理休克，因为休克是生命威胁的状态，需要立即处理以维持循环和组织灌注。因此，正确答案是 C.防治休克。
在尚未手术前应采用的最主要的治疗环节是：
对于这道题，考虑到患者已经出现了休克，并且腹部穿刺抽出不凝血，这通常意味着腹腔内出血。在这种情况下，最主要的治疗环节应当是迅速且有效地处理休克状态。
A.保持安静 - 虽然对于患者来说保持安静是重要的，但它不是治疗休克的主要环节。
B.止痛 - 止痛是重要的，但在处理休克时不是最主要的环节。
C.防治休克 - 这是最关键的。休克是一个紧急状况，需要迅速处理以确保患者的生命安全。
D.胃肠减压 - 虽然在某些腹部损伤的情况下可能是必要的，但不是治疗休克的主要方法。
E.应用抗生素 - 这主要用于预防感染，不是治疗休克的主要手段。
因此，答案是：C.防治休克

对于腹部闭合性损伤患者伴休克，腹部穿刺抽出不凝血的情况，以下是最佳答案的分析和选择：

134、 (A3/A4 型题)
在尚未手术前应采用的最主要的治疗环节是：
C. 防治休克

休克是一个危及生命的紧急情况，必须立即处理以维持患者的生命体征和器官灌注。保持安静（A）、止痛（B）、胃肠减压（D）和应用抗生素（E）虽然在某些情况下也很重要，但在面对休克时，首先要采取的主要措施是防治休克。
135、　(A3/A4 型题)　题
应采取的治疗原则是
A.输血补液，并用升压药物抗休克
B.抗休克待血压好转后手术
C.先抗休克后手术
D.抗休克同时手术
E.用大量止血药物不手术
参考答案：　　D　您的答案：
图文解析：　　【解析】腹腔穿刺抽出不凝血提示腹腔内出血，有脏器破裂。 内脏损伤的伤者很容易并发休克，故防治休克是治疗的重要环节。腹腔进行性出血患者，在抗休克的同时，迅速剖腹止血。
135 题关注的是应采取的治疗原则。给出的选项有输血补液并用升压药物抗休克、抗休克待血压好转后手术、先抗休克后手术、抗休克同时手术、用大量止血药物不手术。对于腹部闭

合性损伤伴随休克的患者，首要任务是稳定患者的血流动力学状态，但也不能延迟手术治疗，因为内脏可能受损。因此，应同时进行抗休克治疗（如输血补液、升压药物）和手术处理，以控制内出血并解决损伤源头。所以，正确答案是 D.抗休克同时手术。
应采取的治疗原则是：
在处理伴有休克的腹部闭合性损伤患者时，治疗原则需要综合考虑患者的整体状况。
A.输血补液，并用升压药物抗休克 - 这只是治疗休克的一部分，但不涉及对腹部损伤的处理。
B.抗休克待血压好转后手术 - 这可能延误对腹部损伤的治疗。
C.先抗休克后手术 - 同 B 选项，可能导致治疗延误。
D.抗休克同时手术 - 这是一个综合的治疗方法，既处理了休克，也处理了腹部损伤。
E.用大量止血药物不手术 - 仅依赖止血药物而不进行手术可能不足以处理腹部损伤。
综合考虑，最合适的治疗原则是同时进行抗休克治疗和手术。因此，答案是：D.抗休克同时手术
135、 (A3/A4 型题)
应采取的治疗原则是：
D. 抗休克同时手术

对于这种情况下的腹部闭合性损伤患者，因腹腔内出血需要立即手术控制出血源，同时需要抗休克治疗以维持血压和器官灌注。因此，最佳的治疗原则是抗休克同时手术（D)。
题目材料　　患者，女，28 岁，孕 34 周。10 天前开始感觉乏力，食欲差，近 5 天病情加重，伴呕吐，巩膜发黄，神志欠清而入院，血压 135/90mmHg，ALT35 素 176μmol/L，尿蛋白(一)。
136、　(A3/A4 型题)　题
首先选择的检查是
A.全血细胞计数
B.碱性磷酸酶
C.胆酸
D.肝炎病毒抗原抗体七项
E.血糖
参考答案：　D　您的答案：
图文解析：　【解析】妊娠的任何时期都有被肝炎病毒感染的可能，其中乙型肝炎病毒感染最常见。临床表现： 出现不能以妊娠反应或其他原因解释的消化系统症状，如食欲减退，肝区痛，乏力，腹胀；部分患者可有皮肤黏膜黄染，尿色深。血清胆红素多在 17μmol/L 以上。ALT、AST 多在正常范围内。
136 题首先选择的检查是，考虑到患者的症状和孕期，我们需要排除最紧急且可能对母婴有重大影响的情况。A 项全血细胞计数可以帮助评估是否有感染或血液系统的问题；B 项碱性磷酸酶升高可能指向肝脏或胆道疾病；C 项胆酸检测对妊娠相关的肝病诊断较为特异；D 项肝炎病毒抗原抗体七项可以排除肝炎；E 项血糖对于排查孕妇的糖尿病状态也是重要的。在这里，考虑到患者的黄疸和肝功能异常，C 项胆酸的检查对于诊断妊娠相关的肝病更为关键，因此选择 C 最为合适。
正确答案是：D.肝炎病毒抗原抗体七项。
青年女性患者，孕 34 周，出现消化道症状及黄疸，ALT 升高，提示肝功能受损，应首先排除病毒性肝炎，因此首选的检查是肝炎病毒抗原抗体七项，以明确是否为病毒性肝炎及肝炎类型。选项 A 全血细胞计数虽然为常规检查，但对肝炎无诊断意义，故排除。选项 B 碱性磷酸酶主要用于骨骼、肝胆系统疾病的诊断和鉴别诊断，尤其是黄疸的鉴别诊断，但不是诊

断肝炎的首选检查，因此不选。选项 C 胆酸主要用于妊娠期肝内胆汁淤积症的诊断，本题患者无瘙痒表现，故排除。选项 E 血糖为常规检查，但对肝炎无诊断价值，故不选。

136、首先选择的检查是
A. 全血细胞计数
B. 碱性磷酸酶
C. 胆酸
D. 肝炎病毒抗原抗体七项
E. 血糖
解析：
全血细胞计数：用于筛查贫血或感染，但不是首先考虑的检查。
碱性磷酸酶：用于评估肝胆疾病，但不能确诊特定疾病。
胆酸：妊娠期肝内胆汁淤积症（ICP）可引起胆酸升高，但不是首选检查。
肝炎病毒抗原抗体七项：用于筛查病毒性肝炎。
血糖：筛查糖尿病或妊娠期糖尿病。
由于患者表现为黄疸和肝功能异常，结合妊娠期的特异情况，首先考虑病毒性肝炎的可能性较大，因此选择 D. 肝炎病毒抗原抗体七项 作为首先选择的检查。

答案：D. 肝炎病毒抗原抗体七项
137、 (A3/A4 型题) 题
最佳诊断是
A.妊娠脂肪肝
B.妊娠肝内胆汁淤积症
C.妊娠高血压肝损害
D.药物性肝损害
E.妊娠合并重症肝炎
参考答案： E 您的答案：
图文解析： 妊娠的任何时期都有被肝炎病毒感染的可能，其中乙型肝炎病毒感染最常见。
临床表现： 出现不能以妊娠反应或其他原因解释的消化系统症状，如食欲减退，肝区痛，乏力，腹胀；部分患者可有皮肤黏膜黄染，尿色深。血清胆红素多在 17μmol/L 以上。ALT、AST 多在正常范围内。
137 题最佳诊断是，根据患者的临床表现，需要考虑与孕期相关的肝脏疾病，包括妊娠脂肪肝、妊娠肝内胆汁淤积症、妊娠高血压肝损害、药物性肝损害和妊娠合并重症肝炎。其中，妊娠脂肪肝通常表现为严重的肝功能损害、凝血功能障碍；妊娠肝内胆汁淤积症主要表现为皮肤瘙痒和黄疸；妊娠高血压肝损害常伴有高血压和蛋白尿；药物性肝损害需要有相关药物史；妊娠合并重症肝炎则表现为肝炎症状。考虑到本病例中的临床表现和实验室指标，尤其是意识障碍和显著的黄疸，最有可能的是 A.妊娠脂肪肝。
正确答案是：E.妊娠合并重症肝炎。
青年女性患者，孕 34 周，10 天前开始感觉乏力，食欲差，近 5 天病情加重，伴呕吐，巩膜发黄，提示出现黄疸，应高度怀疑病毒性肝炎，且患者神志欠清，ALT 及胆红素升高，根据临床表现和实验室检查，该患者最可能的诊断是妊娠合并重症肝炎。选项 A 妊娠脂肪肝常表现为持续性恶心呕吐、乏力、上腹痛或头痛，数天至 1 周出现黄疸且进行性加深，与本题患者症状不符，故不选。选项 B 妊娠肝内胆汁淤积症以皮肤瘙痒和胆酸高值为特征，本题患者无瘙痒表现，且未查见胆酸值，故排除。选项 C 妊娠高血压肝损害应有高血压、蛋白尿及

水肿等妊高症表现，本题患者血压正常，无蛋白尿及水肿表现，可排除。选项 D 药物性肝损害应有服药史，本题中未提及，故不选。

137、最佳诊断是
A. 妊娠脂肪肝
B. 妊娠肝内胆汁淤积症
C. 妊娠高血压肝损害
D. 药物性肝损害
E. 妊娠合并重症肝炎
解析：
妊娠脂肪肝：通常在妊娠晚期发生，表现为乏力、黄疸、肝功能异常，但 ALT 通常显著升高。
妊娠肝内胆汁淤积症（ICP）：主要表现为瘙痒和胆红素升高，但 ALT 较轻度升高。
妊娠高血压肝损害：常见于妊娠高血压疾病，ALT 通常升高显著。
药物性肝损害：需要明确药物史，不符合该患者情况。
妊娠合并重症肝炎：表现为严重乏力、黄疸、肝功能明显异常，ALT 通常升高，但此患者 ALT 仅为 35 U/L。
患者的症状和实验室结果（ALT 35 U/L，总胆红素 176 μmol/L）更符合 妊娠肝内胆汁淤积症（ICP） 的诊断。

答案：B. 妊娠肝内胆汁淤积症
题目材料 30 岁男性患者，病程 4 个月。头痛发病入院前出现左侧肢体无力和呕吐。入院查体：意识清，眼底视神经乳头水肿，左上下肢肌力Ⅳ级， 跃，病理征（＋）。
138、 (A3/A4 型题) 题
该患者的诊断是
A.脑梗死
B.脑出血
C.蛛网膜下隙出血
D.脑水肿
E.颅内高压
参考答案： E 您的答案：
图文解析： 【解析】颅内高压的典型表现：头痛，呕吐，视神经乳头水肿。应该尽快找出颅内高压的原因，从根本上解决颅内高压问题。CT 是诊断颅内病变首选检查。
38 题分析：
这位 30 岁男性患者的病史包含了头痛、左侧肢体无力、呕吐、意识清楚、眼底视神经乳头水肿、左上下肢肌力Ⅳ级和跃动性病理征，这些症状表明了可能的神经系统损害和颅内压增高。颅内高压的直接迹象是眼底视神经乳头水肿。因此，最符合这些临床表现的诊断是 E. 颅内高压。
题目 138 解析：
中年男性患者，病程较长，伴有头痛、呕吐，查体见眼底视神经乳头水肿，左上下肢肌力下降，病理征阳性，这些症状和体征均提示颅内高压。颅内高压的三大主症就是头痛、呕吐和视神经乳头水肿。
选项 A 脑梗死通常起病急骤，多数无明显头痛、呕吐及视乳头水肿等颅内压增高症状，主要表现为三偏症状（偏瘫、偏盲、偏身感觉障碍），与题干描述不符。
选项 B 脑出血通常有明显的高血压病史，起病急骤，多在情绪激动或活动中突然发病，与题

干描述不一致。
选项 C 蛛网膜下隙出血起病急骤，多因动脉瘤破裂、畸形脑血管破裂等原因所引起，常伴有明显的头痛、脑膜刺激征和眼部症状，与题干中患者表现不符。
选项 D 脑水肿通常指脑组织内水分增多、脑体积增大的一种病理现象，是多种疾病伴发的一种病理状态，并不特指某一疾病，且一般不会单独出现，因此不选。

问题 138：该患者的诊断是
A. 脑梗死
B. 脑出血
C. 蛛网膜下隙出血
D. 脑水肿
E. 颅内高压

分析：
患者有头痛、呕吐、左侧肢体无力等症状，入院查体发现眼底视神经乳头水肿，这提示颅内压增高。病程较长（4 个月），且有病理征阳性，这些特点提示可能有颅内占位性病变。综合考虑，最可能的诊断是颅内高压。

最佳答案：E. 颅内高压
139、 (A3/A4 型题) 题
应采用的检查是
A.X 线颅片
B.脑电图
C.脑血管造影
D.CT
E.ECT
参考答案： D 您的答案：
图文解析： 【解析】颅内高压的典型表现：头痛，呕吐，视神经乳头水肿。应该尽快找出颅内高压的原因，从根本上解决颅内高压问题。CT 是诊断颅内病变首选检查。
139 题分析：
对于颅内高压的病因诊断，需要进行影像学检查以查看大脑内部结构。CT 扫描是诊断颅内出血、肿瘤、脑水肿等导致颅内压升高的常用和快速方法。因此，最合适的检查方法是 D. CT。
题目 139 解析：
考虑到患者的诊断是颅内高压，为了明确其病因，需要进行进一步的检查。
选项 A：X 线颅片主要用于颅骨病变的诊断，对颅内高压的病因诊断帮助不大，故不选。
选项 B：脑电图主要用于癫痫等疾病的诊断，对于颅内高压的病因诊断价值有限，故排除。
选项 C：脑血管造影虽然可以显示脑血管的形态和结构，但并不是颅内高压的首选检查方法，故不选。
选项 D：CT 扫描可以清晰显示脑实质、脑室和脑池的形态和大小，是诊断颅内高压及其病因的首选影像学检查方法，故为正确答案。
选项 E：ECT（发射单光子计算机断层扫描）主要用于甲状腺、骨骼等部位肿瘤的检查，与颅内高压的诊断关系不大，故排除。
问题 139：应采用的检查是
A. X 线颅片

B. 脑电图
C. 脑血管造影
D. CT
E. ECT

分析：
为进一步明确诊断，CT 可以快速、有效地显示颅内占位性病变、脑水肿和脑内出血等情况。因此，CT 是首选的检查方法。

最佳答案：D. CT
140、　(B1 型题)
肉芽组织取代坏死组织、血栓以及渗出物的过程称为
A.化生
B.机化
C.分化
D.再生
E.增生
参考答案：　B　您的答案：
图文解析：
肉芽组织取代坏死组织、血栓以及渗出物的过程称为 D. 再生。
- 这个描述符合肉芽组织在愈合过程中的作用，通常出现在炎症后期，肉芽组织形成是修复过程的一部分，但更准确的说法是“替代”坏死组织的过程被称为修复或增生。因此，这里可能有一点歧义，需要根据实际情况考虑。如果考虑到肉芽组织的形成和作用，选择 E. 增生也是合理的。
140、正确答案是：B.机化
解析：
肉芽组织取代坏死组织、血栓以及渗出物的过程称为机化。机化是指组织和细胞损伤后，新生肉芽组织长入并取代坏死组织、血栓、脓液、异物等的过程。肉芽组织在机化过程中起着关键作用，它可以逐渐转化为瘢痕组织，从而使受损组织得到修复。
选项 A 化生是指一种分化成熟的细胞类型被另一种分化成熟的细胞类型所取代的过程，与题意不符。
选项 C 分化是指同一来源的细胞逐渐产生出形态结构、功能特征各不相同的细胞类群的过程，可排除。
选项 D 再生是指机体出现损伤时，以损伤周围的同种细胞对缺损进行修补恢复的过程，与题干描述不符。
选项 E 增生是指细胞有丝分裂活跃，导致组织或器官内细胞数目增多的现象，可排除。
140. (B1 型题)
肉芽组织取代坏死组织、血栓以及渗出物的过程称为：
B. 机化
141、　(B1 型题)
组织损伤后，由其邻近的健康细胞分裂增生完成修复的过程称为
A.化生
B.机化
C.分化

D.再生
E.增生
参考答案： D 您的答案：
图文解析： 【解析】再生是指组织和细胞损伤后，由周围同种细胞来完成修复的过程。
组织损伤后，由其邻近的健康细胞分裂增生完成修复的过程称为 D. 再生。
- 再生是指损伤后，组织的细胞通过分裂增生来恢复正常结构和功能的过程。
141、正确答案是：D.再生
解析：
组织损伤后，由其邻近的健康细胞分裂增生完成修复的过程称为再生。再生是机体在受到损伤后以损伤周围的同种细胞对缺损进行修补恢复的过程。在这个问题中，关键词是“健康细胞分裂增生”，这恰好是再生的定义。
选项 A 化生是指一种分化成熟的细胞类型被另一种分化成熟的细胞类型所取代的过程，可排除。
选项 B 机化是指组织和细胞损伤后，新生肉芽组织长入并取代坏死组织、血栓、脓液、异物等的过程，不符合题意。
选项 C 分化是指同一来源的细胞逐渐产生出形态结构、功能特征各不相同的细胞类群的过程，故不考虑。
选项 E 增生是指细胞有丝分裂活跃导致组织或器官内细胞数目增多的现象，故不选。
141. (B1 型题)
组织损伤后，由其邻近的健康细胞分裂增生完成修复的过程称为：
D. 再生
142、 (B1 型题)
同一胚层分化成熟组织转化为另一成熟组织的过程称为
A.化生
B.机化
C.分化
D.再生
E.增生
参考答案： A 您的答案：
图文解析：
同一胚层分化成熟组织转化为另一成熟组织的过程称为 B. 机化。
- 这个描述类似于转化的概念，通常用于描述一个成熟的组织类型变成另一种成熟的组织类型。但在医学术语中，“机化”一词通常用于描述血栓等组织在病理过程中转变为纤维组织的过程。因此，如果要准确匹配，可能需要考虑更多上下文。如果问题指的是正常的组织发生类型的改变，这可能是“分化”（C），但这种情况通常不会发生于成熟组织。
142、正确答案是：A.化生
解析：
同一胚层分化成熟组织转化为另一成熟组织的过程称为化生。化生是一种分化成熟的组织转变成另一种成熟组织可逆的病理过程，通常只出现在分裂增殖能力较活跃的细胞类型中。
选项 B 机化是指组织和细胞损伤后，新生肉芽组织长入并取代坏死组织、血栓、脓液、异物等的过程，与题意不符。
选项 C 分化是指同一来源的细胞逐渐产生出形态结构、功能特征各不相同的细胞类群的过程，可排除。
选项 D 再生是指机体出现损伤时，以损伤周围的同种细胞对缺损进行修补恢复的过程，不

符合题意。
选项 E 增生是指细胞有丝分裂活跃导致组织或器官内细胞数目增多的现象，故不考虑。
142. (B1 型题)
同一胚层分化成熟组织转化为另一成熟组织的过程称为：
A. 化生
143、 (B1 型题)
未成熟型畸胎瘤属于
A.交界性肿瘤
B.早期癌
C.良性肿瘤
D.恶性肿瘤
E.癌前病变
参考答案： D 您的答案：
图文解析：
143、未成熟型畸胎瘤属于
答案: D.恶性肿瘤
解释: 未成熟型畸胎瘤是一种恶性肿瘤，因为它们包含未成熟的、胚胎样的细胞，并有较高的恶化风险。

143、未成熟型畸胎瘤属于
D. 恶性肿瘤

解释：未成熟型畸胎瘤含有未分化的细胞成分，具有恶性潜能，通常需要进行进一步的治疗和监测。
144、 (B1 型题)
仅浸润黏膜层及黏膜下层的胃肠癌称
A.交界性肿瘤
B.早期癌
C.良性肿瘤
D.恶性肿瘤
E.癌前病变
参考答案： B 您的答案：
图文解析：
144、仅浸润黏膜层及黏膜下层的胃肠癌称
答案: B.早期癌
解释: 仅浸润黏膜层及黏膜下层的胃肠癌被称为早期癌，因为它还没有穿透肌层或扩散到远处组织。
144、仅浸润黏膜层及黏膜下层的胃肠癌称
B. 早期癌

解释：早期癌指癌细胞仅限于黏膜层及黏膜下层，未侵及更深层次的组织和器官。早期发现和治疗可提高治愈率。
145、 (B1 型题)

直、结肠家族型多发性腺瘤性息肉属于

A.交界性肿瘤

B.早期癌

C.良性肿瘤

D.恶性肿瘤

E.癌前病变

参考答案： E 您的答案：

图文解析：

145、直、结肠家族型多发性腺瘤性息肉属于

答案: E.癌前病变

解释: 家族性腺瘤性息肉病（FAP）是一种遗传性条件，涉及直、结肠中形成多发性腺瘤性息肉。这些息肉本身是良性的，但如果不治疗，有很高的风险恶变成结直肠癌。因此，它们被视为癌前病变。

首先，我们来理解一下每个选项的含义：

A. 交界性肿瘤：这是一种组织形态和生物学行为介于良性与恶性之间的肿瘤。

B. 早期癌：通常指的是癌症的早期阶段，一般病灶较小，未出现转移。

C. 良性肿瘤：指的是生长缓慢，不具备侵袭和转移能力的肿瘤。

D. 恶性肿瘤：指的是具有侵袭性和转移能力的肿瘤，对健康危害较大。

E. 癌前病变：指的是某些具有癌变潜在可能性的病变或疾病。

接下来，我们逐一分析每个问题：

未成熟型畸胎瘤通常具有较强的侵袭性和转移性，因此它属于恶性肿瘤。所以答案是 D.恶性肿瘤。

对于仅浸润黏膜层及黏膜下层的胃肠癌，这通常被认为是癌症的早期阶段，因此答案选 B.早期癌。

直、结肠家族型多发性腺瘤性息肉，虽然本身是良性的，但由于它具有较高的恶变潜能，通常被视为一种癌前病变。所以答案是 E.癌前病变。

综上所述，答案是：143. D.恶性肿瘤 144. B.早期癌 145. E.癌前病变

145、直、结肠家族型多发性腺瘤性息肉属于

E. 癌前病变

解释：家族性腺瘤性息肉病（FAP）是一种遗传性疾病，其特征是在结肠和直肠中形成大量腺瘤性息肉。若不治疗，这些息肉极有可能演变为结直肠癌，因而被认为是癌前病变。

146、 (B1 型题)

提示血容量不足

A.中心静脉压很低，尿量多

B.中心静脉压偏低，尿量少

C.中心静脉压偏低，尿量多

D.中心静脉压很高，尿量多

E.中心静脉压很高，尿量少

参考答案： B 您的答案：

图文解析：

146、提示血容量不足

答案：B. 中心静脉压偏低，尿量少

解释：血容量不足时，中心静脉压通常会下降，因为体内循环血量减少。同时，由于血容量

减少导致肾脏灌注不足，尿量会减少。
146、提示血容量不足。
答案：B.中心静脉压偏低，尿量少
解析：中心静脉压代表了右心房或者胸腔段腔静脉内压力的变化，可反映全身血容量与右心功能之间的关系，是临床观察血液动力学的主要指标之一。中心静脉压偏低通常表示血容量不足，而尿量少进一步印证了血容量不足，肾脏灌注不足。因此，中心静脉压偏低，尿量少提示血容量不足。

146、提示血容量不足
B.中心静脉压偏低，尿量少

解释：血容量不足时，中心静脉压（CVP）会偏低，因而导致肾脏灌注不足，尿量减少。
147、 (B1 型题)
说明液体量已补充足
A.中心静脉压很低，尿量多
B.中心静脉压偏低，尿量少
C.中心静脉压偏低，尿量多
D.中心静脉压很高，尿量多
E.中心静脉压很高，尿量少
参考答案： D 您的答案：
图文解析：
147、说明液体量已补充足
答案：E. 中心静脉压很高，尿量少
解释：这个选择略显复杂，但如果液体过度补充导致中心静脉压显著升高，可能会暂时出现尿量减少。这种情况可以发生在过度快速输液或心功能不全患者中。然而，正常情况下，液体补充足够后，尿量应该增加。因此，这个问题可能是在考察过度液体补充的情况。
147、说明液体量已补充足。
答案：D.中心静脉压很高，尿量多
解析：中心静脉压的大小取决于心脏射血能力和静脉回心血量之间的相互关系。中心静脉压很高，说明血容量充足，尿量多说明肾脏灌注充足，肾功能良好。因此，中心静脉压很高，尿量多可以说明液体量已补充足。

147、说明液体量已补充足
D.中心静脉压很高，尿量多

解释：液体量已补充足时，中心静脉压升高，并且肾脏灌注良好，尿量增加。
148、 (B1 型题)
可能有心功能不全存在
A.中心静脉压很低，尿量多
B.中心静脉压偏低，尿量少
C.中心静脉压偏低，尿量多
D.中心静脉压很高，尿量多
E.中心静脉压很高，尿量少
参考答案： E 您的答案：

图文解析：
148、可能有心功能不全存在
答案：E. 中心静脉压很高，尿量少
解释：心功能不全时，心脏泵血能力下降，导致静脉回流和中心静脉压增高。同时，肾脏灌注减少，可能导致尿量减少。这种情况反映了心功能不全和肾功能相互影响的结果。
148、可能有心功能不全存在。
答案：E.中心静脉压很高，尿量少
解析：中心静脉压的大小取决于心脏射血能力和静脉回心血量之间的相互关系。如果中心静脉压很高，但尿量少，这可能意味着尽管血容量充足，但心脏射血功能可能受限，导致肾脏灌注不足，尿量减少。这种情况可能提示心功能不全。因此，中心静脉压很高，尿量少可能表明存在心功能不全。
148、可能有心功能不全存在
E.中心静脉压很高，尿量少

解释：心功能不全时，心脏无法有效泵血，导致静脉回流受阻，中心静脉压升高，但由于肾脏灌注不足，尿量减少。
149、 (B1 型题)
胃术后低血糖综合征
A.呕吐物为食物，无胆汁
B.呕吐物为胆汁，无食物
C.呕吐物既有胆汁，又有食物
D.呕吐物呈酸臭味，有宿食
E.无呕吐
参考答案： E 您的答案：
图文解析：
对于胃术后低血糖综合征（149 题），常见的症状包括餐后早期饱胀感、腹痛、震颤、出汗、心慌等，而不是特别强调呕吐的特性。但如果要在给定的选项中选择，可以考虑与低血糖相关的症状，如饥饿感或消化不良的表现。因此，我倾向于选择 D（呕吐物呈酸臭味，有宿食），因为这可能与食物过快进入小肠并被迅速吸收，导致血糖水平下降有关。

149. 胃术后低血糖综合征
胃术后低血糖综合征常见于胃切除术后，由于进食后胃内容物快速进入小肠，导致胰岛素分泌过多，出现低血糖症状。此情况下的呕吐通常不是其主要症状，因此 E. 无呕吐是最可能的答案。
150、 (B1 型题)
胃术后输出袢梗阻
A.呕吐物为食物，无胆汁
B.呕吐物为胆汁，无食物
C.呕吐物既有胆汁，又有食物
D.呕吐物呈酸臭味，有宿食
E.无呕吐
参考答案： C 您的答案：
对于胃术后输出袢梗阻（150 题），呕吐是常见的症状，尤其是当食物不能正常通过胃小肠

接口时。输出袢梗阻通常会导致呕吐物中含有未消化的食物和胃酸。因此，我倾向于选择 A（呕吐物为食物，无胆汁），因为输出袢梗阻通常阻塞在胆汁排入肠道的位置之前，导致呕吐物中主要是食物而不是胆汁。

这是一道关于胃部手术后的不同并发症及其表现的考题。我们需要根据每个并发症的特点，选出最符合的呕吐物描述。

首先，我们分析胃术后低血糖综合征。这种情况下，胃的排空过快，导致葡萄糖被迅速吸收，胰岛素分泌过多，随后发生反应性低血糖。这通常不会导致呕吐，因此关键在于识别哪种症状与这种综合征最不相关。

对于胃术后输出袢梗阻，它通常发生在胃大部切除毕 II 式吻合术后。这种情况下，由于输出袢梗阻，使得胆汁与胰液等消化液积聚在梗阻近端，而食物残渣则无法通过。因此，当发生呕吐时，呕吐物中会同时含有胆汁和食物。

现在，我们来逐一分析选项：

胃术后低血糖综合征：

A. 呕吐物为食物，无胆汁 - 这更多地与输入袢梗阻有关。

B. 呕吐物为胆汁，无食物 - 这并不符合低血糖综合征的表现。

C. 呕吐物既有胆汁，又有食物 - 同样不符合。

D. 呕吐物呈酸臭味，有宿食 - 这通常与幽门梗阻相关。

E. 无呕吐 - 鉴于低血糖综合征与呕吐无直接关联，这是最符合的选项。

因此，对于 149 题，最合适的选择是 E. 无呕吐。

胃术后输出袢梗阻：

A. 呕吐物为食物，无胆汁 - 不符合输出袢梗阻的表现。

B. 呕吐物为胆汁，无食物 - 这更像是输入袢梗阻的表现。

C. 呕吐物既有胆汁，又有食物 - 这正是输出袢梗阻的典型表现，因为胆汁和食物都会在梗阻的近端积聚。

D. 呕吐物呈酸臭味，有宿食 - 与输出袢梗阻不符。

E. 无呕吐 - 显然不是这种情况，因为输出袢梗阻通常会导致呕吐。

因此，对于 150 题，最合适的选择是 C. 呕吐物既有胆汁，又有食物。

综上所述，正确答案是：149. E. 无呕吐；150. C. 呕吐物既有胆汁，又有食物。

150. 胃术后输出袢梗阻

胃术后输出袢梗阻是指胃术后输出袢的机械性梗阻。此时，呕吐物通常包含胆汁而无食物，因为食物无法通过梗阻部位到达小肠。最合适的答案是 B. 呕吐物为胆汁，无食物。

2023 第二单元

1、 (A1 型题)

不具有内分泌功能的细胞是

A.胰腺导管细胞

B.睾丸间质细胞

C.甲状旁腺主细胞

D.肾上腺髓质细胞

E.胃黏膜的 G 细胞

参考答案： A 您的答案：

图文解析： 【解析】胰腺导管细胞分泌胰液，属于胰腺的外分泌功能，不具有内分泌功

能（A 错，为本题的正确答案）。
【避错】肾上腺髓质激素分泌儿茶酚胺，甲状旁腺主细胞分泌甲状旁腺激素，胃黏膜的 G 细胞分泌胃泌素，睾丸间质细胞分泌雄激素。
在这个问题中，我们需要识别哪种细胞没有内分泌功能。内分泌细胞通常涉及到在血液中分泌激素的过程。让我们来看一下每个选项：
- A. 胰腺导管细胞：这些细胞参与胰腺液的输送，而不是直接参与内分泌激素的生产。
- B. 睾丸间质细胞：这些细胞分泌睾酮，属于内分泌细胞。
- C. 甲状旁腺主细胞：它们分泌甲状旁腺素，具有内分泌功能。
- D. 肾上腺髓质细胞：它们分泌肾上腺素和去甲肾上腺素，也具有内分泌功能。
- E. 胃黏膜的 G 细胞：它们分泌胃泌素，具有内分泌功能。
因此，最正确的答案是 **A. 胰腺导管细胞**，因为它们主要负责传输胰腺液到小肠，而不直接参与内分泌激素的生产。
首先，我们要明确内分泌细胞是指能合成并分泌生物活性物质，并通过血液或组织液将这些物质运送到其他器官或细胞的细胞。这些生物活性物质通常被称为激素。
现在，我们来看每一个选项：
A. 胰腺导管细胞：胰腺导管细胞主要功能是输送胰液，它们并不具备分泌激素的能力，因此不具有内分泌功能。
B. 睾丸间质细胞：这些细胞能分泌雄激素，这是一种内分泌功能。
C. 甲状旁腺主细胞：这些细胞能分泌甲状旁腺激素，这也属于内分泌功能。
D. 肾上腺髓质细胞：这些细胞可以分泌肾上腺素和去甲肾上腺素，显然具有内分泌功能。
E. 胃黏膜的 G 细胞：这些细胞分泌胃泌素，同样具有内分泌功能。
综上所述，只有胰腺导管细胞不具有内分泌功能。因此，答案是 A.胰腺导管细胞。

这道题目考察的是不同细胞的内分泌功能。以下是每个选项的分析：

A. **胰腺导管细胞**：主要功能是分泌碳酸氢盐（HCO_3^-）到胰管中，帮助中和胃酸，不具有内分泌功能。

B. **睾丸间质细胞**：又称 Leydig 细胞，分泌雄激素（如睾酮），具有内分泌功能。

C. **甲状旁腺主细胞**：分泌甲状旁腺激素（PTH），具有内分泌功能。

D. **肾上腺髓质细胞**：分泌肾上腺素和去甲肾上腺素，具有内分泌功能。

E. **胃黏膜的 G 细胞**：分泌胃泌素，具有内分泌功能。

综上所述，答案是：

A. **胰腺导管细胞**
2、　(A1 型题)
苯妥英钠的不良反应不包括
A.牙龈损害
B.共济失调
C.肾损害

D.过敏反应
E.贫血
参考答案： C 您的答案：
图文解析： 【解析】苯妥英钠常见不良反应为：共济失调、烦躁易怒、牙龈增生、贫血、过敏反应、肝脏毒性等；罕见不良反应：黄疸、维生素 D 和钙代谢紊乱，骨折、骨质异常等。本药物通过肝脏代谢，故一般不造成肾损害，故本题选 C。
【避错】苯妥英钠是一种抗癫痫药，也是抗惊厥药。用于治疗全身强直-阵挛性发作、复杂部分性发作(精神运行性发作、颞叶癫痫)、单纯部分性发作（局限性发作）和癫痫持续状态。
苯妥英钠是一种常用的抗白喉药物，其不良反应可能包括牙龈增生（牙龈损害）、共济失调、过敏反应和贫血。然而，肾损害不是苯妥英钠的常见不良反应。因此，最正确的答案是：C. 肾损害
首先，我们来详细了解一下苯妥英钠及其可能的不良反应。
苯妥英钠是一种抗癫痫和抗心律失常的药物，它可能引发多种不良反应。其中，牙龈增生是其常见的不良反应之一，这可能与药物对牙龈组织的直接刺激有关。此外，苯妥英钠还可能影响神经系统，引发如眩晕、震颤、复视、共济失调等不良反应。在少数情况下，苯妥英钠还可能导致过敏反应，如皮疹、发热和剥脱性皮炎等。
现在，我们根据这些信息来分析各个选项：
A. 牙龈损害：这是苯妥英钠的一个已知不良反应，因此这个选项是不正确的。
B. 共济失调：如上所述，苯妥英钠可能影响神经系统，导致共济失调等不良反应，所以这个选项也是不正确的。
C. 肾损害：虽然苯妥英钠主要经肝脏代谢，但其也可能对肾脏造成一定损害，特别是在长期使用或大剂量使用时。因此，这个选项同样是不正确的。
D. 过敏反应：如前所述，苯妥英钠可能引发过敏反应，所以这个选项也不正确。
E. 贫血：在现有的医学文献中，并没有明确报道苯妥英钠会导致贫血的不良反应。虽然长期使用苯妥英钠可能会影响造血系统，但这种影响通常表现为白细胞或血小板减少，而非贫血。
综上所述，苯妥英钠的不良反应不包括贫血。因此，正确答案是 E.贫血。

苯妥英钠是一种广泛使用的抗癫痫药物，其常见的不良反应包括牙龈损害（A）、共济失调（B）、过敏反应（D）和贫血（E）。然而，肾损害（C）并不是苯妥英钠的常见不良反应。

因此，正确答案是：

C. 肾损害
3、 (A1 型题)
储存铁的指标是
A.血清铁
B.血清铁蛋白
C.转铁蛋白饱和度
D.肌红蛋白铁
E.红细胞内铁
参考答案： B 您的答案：
图文解析： 【解析】在正常的生理条件下，血清铁蛋白测定（B 对）可以敏感的反映体内

贮存铁量。
【避错】血清铁测定、转铁蛋白饱和度测定，反映了血浆中的铁含量；红细胞内铁、肌红蛋白铁为体内的功能铁。
储存铁主要通过血清铁蛋白来反映，因为血清铁蛋白是体内储存铁的主要形式。因此，最正确的答案是：B.血清铁蛋白
首先，我们需要明确题目所问的是储存铁的指标。储存铁指的是体内存储的铁质，它并不直接参与血红蛋白的合成或其他生理活动，而是作为体内的铁储备存在。
接下来，我们逐一分析各个选项：
A. 血清铁：这主要反映的是血液中与转铁蛋白结合的铁量，更多体现的是血液中可用的铁，而非储存铁。
B. 血清铁蛋白：铁蛋白是体内储存铁的主要形式。血清铁蛋白的浓度可以直接反映体内储存铁的情况，是一个很好的储存铁指标。
C. 转铁蛋白饱和度：这是指血清中转铁蛋白的浓度与血清中转铁蛋白的最大结合能力的比值。它反映的是转铁蛋白的运载能力和血中铁离子的结合程度，并不直接代表储存铁。
D. 肌红蛋白铁：肌红蛋白主要存在于心肌和骨骼肌中，其铁含量虽然与某些疾病有关，但并不直接反映体内储存铁的状态。
E. 红细胞内铁：这主要反映的是红细胞中的铁含量，与血红蛋白的合成和功能有关，而不是储存铁。
综合上述分析，我们可以确定血清铁蛋白是反映体内储存铁状态的直接指标。
因此，正确答案是 B.血清铁蛋白。
储存铁的指标是：

B. 血清铁蛋白
4、 (A1 型题)
对于运动系统慢性损伤非甾体抗炎药使用，下列说法正确的是
A.为了减轻肝功能损害可联合使用吲哚美辛和阿司匹林
B.局灶浅表性病变可使用非甾体抗炎药口服液
C.为了减轻胃肠功能损害可使用 COX-1
D.非甾体抗炎药可多种合用，加强疗效
E.短期最低有效药物剂量
参考答案： E 您的答案：
图文解析： 【解析】非甾体抗炎药，是治疗运动系统慢性损伤的常用药物。使用时应注意以下几点：①短期用药（E 对）；②病灶局限且较表浅者使用非甾体抗炎药的外用剂型（B 错）；③为减少对胃肠道损害可用选择性环氧化酶 2（COX-2）抑制剂（C 错）；④对肾功能不全者可选用短半衰期、对肾血流量影响较小的药物；⑤为减少对肝功能的影响可选用结构简单、不含氮的药物，避免使用吲哚美辛和阿司匹林（A 错）；⑥非甾体抗炎药应单用（D 错），合用的抗炎镇痛效果不但不会增加反而会使药物副作用倍增。
【避错】运动系统慢性损伤的原因复杂，常见原因有：①全心全身疾病造成的局部组织病理性紧张、痉挛；②由于环境温度变化引起局部血管痉挛，循环供给下降，局部代谢产物积聚；③长期、反复、持续地重复同一个姿势，工作、学习和职业动作，超越了人体局部的代偿能力，造成组织损伤并得不到及时修复；④操作中技术不熟练、注意力不集中、姿势不正确，使局部产生异常应力；⑤身体生理结构或姿态性异常，应力分布不均；⑥急性损伤后未得到正确的康复转为慢性损伤。
对于运动系统慢性损伤，非甾体抗炎药（NSAIDs）是常用的治疗手段。选择最佳答案需要考

虑非甾体抗炎药的作用机制、副作用以及临床使用原则。
A. 吲哚美辛和阿司匹林都是 NSAIDs，两者联合使用可能增加胃肠道出血的风险，并不会减轻肝功能损害。
B. 局灶浅表性病变可能不需要口服 NSAIDs，可以使用外用制剂。
C. COX-1 抑制剂（如阿司匹林）可能会增加胃肠功能损害，而不是减轻。
D. NSAIDs 多种合用并不推荐，因为这会增加副作用的风险，尤其是对肾脏和胃肠道。
E. 使用最低有效剂量的 NSAIDs 并且短期使用是减少副作用和提高安全性的推荐做法。
因此，最佳答案是 E，即短期使用最低有效药物剂量来治疗慢性运动系统损伤。这种做法可以最小化副作用，特别是对胃肠道和肾脏的影响。
解析：
非甾体抗炎药是临床上常用的解热镇痛抗炎药，主要通过抑制前列腺素的合成发挥作用，具有解热、镇痛、抗炎、抗风湿、抗凝等作用。但在使用时，应短期使用最低有效药物剂量，以避免长期大量用药导致的副作用。
选项 A 为了减轻肝功能损害可联合使用吲哚美辛和阿司匹林这一说法是错误的，因为阿司匹林等非甾体抗炎药在肝脏代谢，同时使用两种或两种以上的非甾体抗炎药，会增加不良反应的发生率，也会增加肝脏的毒性，因此排除。
非甾体抗炎药有多种剂型，其中乳胶剂、膏剂、贴剂和外敷药水主要适用于局部用药，尤其对于节段的浅表性慢性疼痛，故选项 B 局灶浅表性病变可使用非甾体抗炎药口服液，可排除。
非甾体抗炎药通过抑制 COX，从而减少 PG 的合成而发挥作用，其中 COX-1 是正常组织细胞用来合成对身体有益的前列环素所必须的，因此长期大量使用 COX-1 抑制剂可能对消化道产生明显的损害作用，如诱发胃溃疡、胃出血、胃穿孔等，因此选项 C 为了减轻胃肠功能损害可使用 COX-1，不符合题意。
由于非甾体抗炎药之间的不良反应可以相加，同时使用两种或两种以上的非甾体抗炎药，会显著增加不良反应的发生率，故选项 D 非甾体抗炎药可多种合用，加强疗效，可排除。
综上所述，正确答案是：E.短期最低有效药物剂量。

对于运动系统慢性损伤非甾体抗炎药使用，下列说法正确的是：

E. 短期最低有效药物剂量

解释：非甾体抗炎药（NSAIDs）常用于缓解运动系统慢性损伤的疼痛和炎症。然而，这些药物的使用需要谨慎，以尽量减少副作用。通常的建议是使用最低有效剂量且尽可能短期，以减少对胃肠道、肝脏和肾脏等器官的潜在损害。联合使用多种 NSAIDs 可能增加不良反应的风险，因此不推荐。
5、 (A1 型题)
急性心肌梗死行 PCI 手术治疗的抗血小板药物用量用法
A.阿司匹林 300mg+氯吡格雷 450mg
B.阿司匹林 100mg+氯吡格雷 300mg
C.阿司匹林 100mg+氯吡格雷 600mg
D.阿司匹林 300mg+氯吡格雷 300mg
E.阿司匹林 300mg+氯吡格雷 600mg
参考答案： E 您的答案：
图文解析： 【解析】无禁忌证的 STEMI 患者均应立即嚼服肠溶阿司匹林 150～300mg 负荷剂量（BC

错），继以 75～100mg/d 长期维持。在替格瑞洛无法获得或有禁忌证时可选用氯吡格雷 600mg 负荷剂量（E 对），仅对年龄＞75 岁患者，采用氯吡格雷负荷量 300mg（D 错）。

【避错】与金属裸支架相比，冠心病患者优先选择植入药物洗脱支架，为控制缺血症状、降低心肌梗死和死亡的发生，需要接受阿司匹林及氯吡格雷抗血小板治疗的时间至少为 12 个月。

对于运动系统慢性损伤，非甾体抗炎药（NSAIDs）是常用的治疗手段。选择最佳答案需要考虑非甾体抗炎药的作用机制、副作用以及临床使用原则。

A. 吲哚美辛和阿司匹林都是 NSAIDs，两者联合使用可能增加胃肠道出血的风险，并不会减轻肝功能损害。

B. 局灶浅表性病变可能不需要口服 NSAIDs，可以使用外用制剂。

C. COX-1 抑制剂（如阿司匹林）可能会增加胃肠功能损害，而不是减轻。

D. NSAIDs 多种合用并不推荐，因为这会增加副作用的风险，尤其是对肾脏和胃肠道。

E. 使用最低有效剂量的 NSAIDs 并且短期使用是减少副作用和提高安全性的推荐做法。

因此，最佳答案是 E，即短期使用最低有效药物剂量来治疗慢性运动系统损伤。这种做法可以最小化副作用，特别是对胃肠道和肾脏的影响。

解析：

急性心肌梗死是指因冠状动脉出现急性阻塞，心脏肌肉因缺乏血液供应出现坏死，使得心脏功能受损的一种可能危及生命的急性病症。PCI 手术，即经皮冠状动脉介入治疗，是指经心导管技术疏通狭窄甚至闭塞的冠状动脉管腔，从而改善心肌的血流灌注的治疗方法。PCI 术后应给予双重抗血小板治疗，即阿司匹林联合氯吡格雷，具体用量用法是阿司匹林 300mg 嚼服+氯吡格雷 300mg 口服。

阿司匹林是一种抗血小板聚集药物，可抑制血小板在动脉粥样硬化斑块上的聚集，有利于内源性纤溶活性物质释放并抑制血栓形成，是预防心脑血管疾病发生的基石。氯吡格雷是一种 ADP 受体拮抗剂，可抑制血小板与 ADP 受体相结合，从而减少血小板聚集。氯吡格雷的常规用量是每日 75mg，但对于急性心梗行 PCI 的患者，应给予负荷量 300mg，故选项 A“阿司匹林 300mg+氯吡格雷 450mg”；选项 C“阿司匹林 100mg+氯吡格雷 600mg”；选项 E“阿司匹林 300mg+氯吡格雷 600mg”；可一并排除。

阿司匹林抗血小板聚集时的用量一般为每日 100mg，但对于急性心梗行 PCI 的患者，应给予负荷量 300mg，故选项 B“阿司匹林 100mg+氯吡格雷 300mg”可排除。

综上所述，正确答案是：D.阿司匹林 300mg+氯吡格雷 300mg。

在急性心肌梗死（AMI）患者进行经皮冠状动脉介入治疗（PCI）时，推荐的抗血小板药物使用方案是为了迅速抑制血小板的聚集，防止血栓形成。根据当前的医学指南和最佳实践，阿司匹林和氯吡格雷的用量和用法如下：

- **阿司匹林**：急性期一般使用 300mg 的负荷剂量。
- **氯吡格雷**：急性期推荐使用 600mg 的负荷剂量。

因此，最合适的选项是：

E. 阿司匹林 300mg+氯吡格雷 600mg

6、 (A1 型题)

高血压伴双侧肾动脉狭窄，禁用下列哪项药物

A.缬沙坦

B.美托洛尔

C.地尔硫䓬
D.洋地黄
E.呋塞米
参考答案：　　A　您的答案：
图文解析：　　【解析】合并双侧肾动脉狭窄的高血压患者降压不宜选用血管紧张素转换酶抑制剂、血管紧张素受体Ⅱ拮抗剂，例如卡托普利、氯沙坦、缬沙坦等，故本题选 A。
【避错】ACEI、ARB 类药物同样禁用于高血钾症、妊娠妇女。ACEI 类药物不良反应为刺激性干咳和血管性水肿，ARB 类直接与药物有关的不良反应较少，　一般不引起刺激性干咳。
高血压伴双侧肾动脉狭窄的患者，在治疗时需特别小心选择药物，以避免进一步损害肾功能。在这些选项中，呋塞米（E 选项）是一种利尿剂，通常用于治疗高血压，但它会增加肾脏负担，尤其是在肾动脉狭窄的情况下，可能导致肾功能进一步恶化。
然而，更为关键的是选择 C，地尔硫䓬（硫酸镁），在高血压伴双侧肾动脉狭窄的情况下通常是禁用的，因为它可能导致肾血流量减少，加剧肾脏缺血。但地尔硫䓬在临床上主要用于治疗严重的心脏病和某些情况下的镇静，而不常用于高血压治疗。
实际上，更符合题意的药物是血管紧张素转换酶抑制剂（ACEI）或血管紧张素受体拮抗剂（ARB），例如缬沙坦（A 选项），在双侧肾动脉狭窄的患者中通常是禁用的，因为它们会减少肾脏产生的血液量，可能导致急性肾功能衰竭。因此，在这个情境下，最合适的答案应该是 **A.缬沙坦**。
解析：
高血压伴双侧肾动脉狭窄是一种特殊类型的高血压，其治疗需考虑降压药物对肾脏血流量的影响。双侧肾动脉狭窄患者，肾血流量的灌注已降低，若使用血管紧张素Ⅱ受体阻滞剂，可使出球小动脉扩张，肾小球内囊压力下降，滤过压降低，肾小球滤过率下降，从而加重肾损害，甚至发生肾衰竭。因此，高血压伴双侧肾动脉狭窄患者禁用缬沙坦。缬沙坦属于血管紧张素Ⅱ受体阻滞剂。
选项 B 美托洛尔属于β受体阻滞剂，主要通过抑制中枢和周围 RAAS，抑制心肌收缩力和降低心输出量来达到降压效果，对肾脏功能无影响，可用于肾动脉狭窄患者，故排除。
选项 C 地尔硫䓬属于钙通道阻滞剂，主要通过阻滞细胞外钙离子经电压依赖 L 型钙通道进入血管平滑肌细胞内，减弱兴奋收缩偶联，降低阻力血管的收缩反应，从而达到降压效果，对肾脏功能无影响，可用于肾动脉狭窄患者，故不选。
选项 D 洋地黄属于强心药，主要用于心力衰竭患者，可增强心肌收缩力，对肾脏功能无影响，可用于肾动脉狭窄患者，与本题无关。
选项 E 呋塞米属于利尿剂，主要通过排钠，减少血容量，降低外周血管阻力达到降压效果，对肾脏功能无影响，可用于肾动脉狭窄患者，故不考虑。
综上所述，正确答案是：A.缬沙坦。
高血压伴双侧肾动脉狭窄的患者禁用的药物是缬沙坦。缬沙坦是一种血管紧张素Ⅱ受体拮抗剂（ARB），这种药物会通过抑制血管紧张素Ⅱ的作用来降低血压。由于血管紧张素Ⅱ在维持肾脏灌注压和肾小球滤过率方面起重要作用，使用 ARB 可能会导致双侧肾动脉狭窄患者的肾功能进一步恶化。

所以，正确答案是：

A. 缬沙坦
7、　(A1 型题)
急性白血病患者最不可能出现的临床表现是

A.匙状甲
B.脸色苍白
C.牙龈出血
D.脾脏肿大
E.间断性血尿
参考答案： A 您的答案：
图文解析： 【解析】匙状甲为缺铁性贫血的表现（A 错，为本题正确答案）。
【避错】AL 起病急缓不一。急者可以突然高热，类似“感冒”，也可以是严重的出血。缓慢者常为脸色苍白（B 对）、皮肤紫癜，月经过多或拔牙后出血难止（C 对）而就医时被发现。以出血为早期表现者近 40%，出血可发生在全身各部位（E 对），以皮肤瘀点、瘀斑、鼻出血、牙龈出血、月经过多为多见。白血病细胞增殖浸润表现为淋巴结和肝脾肿大（D 对）、关节骨骼疼痛等。
急性白血病是一种癌症，它迅速发展，主要影响骨髓和血液。它的症状可能因白血病类型及其他因素而异，但通常包括疲劳、发热、出血倾向（如牙龈出血）、以及因骨髓空间受到白血病细胞占据而导致的正常血细胞减少的相关症状。
- A. 匙状甲：通常与铁缺乏性贫血有关，并不常见于急性白血病。
- B. 脸色苍白：可能出现在急性白血病患者中，因为白血病会导致红细胞减少，引起贫血。
- C. 牙龈出血：在急性白血病中很常见，因为血小板减少导致出血倾向。
- D. 脾脏肿大：急性白血病中可能发生，尤其是某些亚型，如急性髓系白血病。
- E. 间断性血尿：虽然急性白血病可导致全身性出血倾向，但间断性血尿不是典型症状。
考虑到上述信息，最不可能出现的临床表现是 A. 匙状甲，因为它通常与其他病理状态相关，特别是与铁缺乏性贫血相关，而不是急性白血病。
解析：
急性白血病是一类造血干细胞异常的克隆性恶性疾病。克隆中的白血病细胞失去进一步分化成熟的能力而停滞在细胞发育的不同阶段，在骨髓和其他造血组织中大量增生累积，并浸润其他器官和组织，而正常造血受抑制。其症状包括贫血、出血、感染及白血病细胞浸润等。
选项 A 匙状甲是一种甲畸形，表现为甲变薄，中央凹陷而四周隆起，呈匙状，常见于缺铁性贫血、甲癣等疾病，与白血病无直接关系，为本题正确答案。
选项 B 脸色苍白为贫血表现，白血病细胞增殖会抑制骨髓生成正常血细胞，红细胞生成减少可引起贫血，出现面色苍白等症状，是白血病患者的常见表现。
选项 C 牙龈出血可能为血小板数量减少或凝血功能障碍所导致，白血病时骨髓中异常的白血病细胞增殖而影响了正常的血细胞生成，可出现血小板数量减少或功能障碍，从而引起出血，是白血病患者的常见表现。
选项 D 脾脏肿大是由于白血病细胞增殖导致的脾脏反应性增生或白血病细胞浸润，符合白血病的临床表现。
选项 E 间断性血尿可能为白血病细胞浸润泌尿系统所致，是白血病患者的常见症状。
综上所述，正确答案是：A.匙状甲。
急性白血病患者最不可能出现的临床表现是：

A. 匙状甲

急性白血病的常见临床表现包括贫血（导致脸色苍白）、出血（如牙龈出血）、脾脏肿大以及各种感染和出血症状（如间断性血尿）。然而，匙状甲（指甲呈现凹陷形状）通常与慢性贫血，特别是缺铁性贫血有关，而不是急性白血病的典型表现。因此，急性白血病患者最不可

能出现的临床表现是匙状甲。
8、　(A1 型题)
疟疾患者血常规的典型表现为
A.白细胞升高
B.粒细胞升高
C.网织红细胞升高
D.红细胞、血红蛋白减少
E.血小板减少
参考答案：　D　您的答案：
图文解析：　【解析】疟疾患者反复发作造成大量红细胞破坏，可使患者出现不同程度的贫血（D 对）和脾肿大。网织红细胞增多（C 错），血小板可稍降低（E 错），但二者均不是典型表现。白细胞总数初发时可稍增高，后正常或稍低（AB 错）。
【避错】疟疾的典型症状为突发寒战、高热和大量出汗。寒战常持续 20 分钟~1 小时。随后体温迅速上升,通常可达 40℃以上，伴头痛、全身酸痛、疲乏，但神志清楚。发热常持续 2~6 小时。随后开始大量出汗,体温骤降，持续时间为 30 分钟~1 小时。此时，患者自觉明显好转，但常感无力、口干。各种疟疾的两次发作之间都有一定的间歇期。病程早期的间歇期可不规则，但经数次发作后即逐渐变得规则。间日疟和卵形疟的间歇期约为 48 小时,三日疟约为 72 小时。恶性疟为 36~48 小时。
疟疾患者的血常规典型表现是红细胞和血红蛋白的减少。疟疾是由寄生虫感染造成的，主要影响红细胞，导致溶血性贫血。因此，最佳答案是：D.红细胞、血红蛋白减少
解析：
疟疾是经按蚊叮咬或输入带疟原虫者的血液而感染疟原虫所引起的虫媒传染病。寄生于人体的疟原虫共有四种，即间日疟原虫，三日疟原虫，恶性疟原虫和卵形疟原虫。不同的疟原虫分别引起间日疟、三日疟、恶性疟及卵形疟。疟疾主要表现为周期性规律发作，全身发冷、发热、多汗，长期多次发作后，可引起贫血和脾肿大。因此，疟疾患者血常规的典型表现为红细胞、血红蛋白减少。
选项 A 白细胞升高常见于急性细菌性感染、严重组织损伤、大出血、中毒和白血病等，与疟疾无直接关联，故不选。
选项 B 粒细胞升高常见于感染、物理或化学刺激、免疫性疾病以及血液系统疾病，并非疟疾的典型血常规表现，故不考虑。
选项 C 网织红细胞升高主要见于溶血性贫血或急性失血等，并非疟疾的典型血常规表现，故不符合。
选项 E 血小板减少常见于血小板的生成障碍、血小板破坏或消耗增多、血小板分布异常等，并非疟疾的典型血常规表现，故排除。
综上所述，正确答案是：D.红细胞、血红蛋白减少。
疟疾是一种由疟原虫感染引起的疾病，常见于热带和亚热带地区。其临床表现包括周期性发作的高热、寒战、头痛、乏力等症状。疟疾对血液系统有显著影响，典型的血常规变化可以为诊断提供线索。

疟疾患者的血常规检查通常会发现：

- 红细胞和血红蛋白减少：由于疟原虫在红细胞内寄生，引起红细胞破坏，导致贫血。
- 血小板减少：这是疟疾常见的血液学改变之一。

基于以上知识，疟疾患者血常规的典型表现为：

D. 红细胞、血红蛋白减少
9、 (A1 型题)
锁骨骨折不需要手术的是
A.锁骨中部 2/3 对位
B.锁骨远端伴喙突韧带损伤
C.不能耐受八字绷带包扎
D.开放性损伤后三小时
E.陈旧性骨折不愈合
参考答案： A 您的答案：
图文解析： 【解析】一般认为 80%～90%锁骨中段骨折（A 对）可采取非手术的方法进行治疗，即手法复位，横形“8”字绷带固定。
【避错】锁骨骨折在以下情况时，可考虑行切开复位内固定：①患者不能忍受“8”字绷带固定的痛苦（C 错）；②复位后再移位，影响外观；③合并神经、血管损伤；④开放性骨折（D 错）；⑤陈旧骨折不愈合（E 错）；⑥锁骨外端骨折，合并喙锁韧带断裂（B 错）
对于锁骨骨折的治疗，通常非手术治疗适用于未移位或轻微移位的锁骨中部骨折。因此，选项 A“锁骨中部 2/3 对位”（即中段锁骨骨折，且骨折断端对位良好）通常不需要手术治疗。这是因为中段锁骨骨折大多可以通过保守治疗，如使用八字带或锁骨带进行固定，来实现良好的愈合。
选项 B、C、D 和 E 中提到的情况通常需要更进一步的评估或治疗，可能包括手术。特别是当伴有喙突韧带损伤、无法耐受保守固定方法、开放性骨折或骨折不愈合时，通常需要考虑手术治疗。综上，最佳答案是 A.锁骨中部 2/3 对位。
首先，我们逐一分析各个选项：
A. 锁骨中部 2/3 对位：锁骨骨折中，大部分发生在锁骨的中外 1/3 交界处。如果锁骨中部 2/3 的骨折端对位良好，说明骨折的位置和稳定性都相对较好，这种情况下通常不需要手术治疗，可以通过保守治疗如八字绷带包扎来达到愈合。
B. 锁骨远端伴喙突韧带损伤：当锁骨远端骨折伴有喙突韧带损伤时，这通常意味着骨折较为严重，可能影响到肩锁关节的稳定性。这种情况下，手术可能是必要的，以确保关节的正常功能和稳定性。
C. 不能耐受八字绷带包扎：对于那些不能耐受八字绷带包扎的患者，保守治疗可能不是最佳选择。此时，手术可能是一个更好的解决方案，以确保骨折的正确对位和稳定。
D. 开放性损伤后三小时：开放性骨折由于存在感染的高风险，通常需要进行彻底的清创和固定。因此，手术治疗在这种情况下通常是必要的。
E. 陈旧性骨折不愈合：对于陈旧性骨折不愈合的情况，手术可能是促进骨折愈合的必要手段。
综上所述，只有当锁骨中部 2/3 的骨折端对位良好时，才不需要手术治疗。其他选项中描述的情况通常都需要手术干预。
因此，正确答案是：A.锁骨中部 2/3 对位。
锁骨骨折不需要手术的情况是：

A. 锁骨中部 2/3 对位

锁骨中部 2/3 的骨折如果对位良好，通常可以通过保守治疗如八字绷带包扎进行处理，而不

需要手术。其他选项则提示需要手术干预，例如 B（伴喙突韧带损伤），C（不能耐受包扎），D（开放性损伤），E（陈旧性骨折不愈合）。

10、　(A1 型题)

有助于确诊 PNH 的实验室检查是

A.骨髓细胞学检查

B.网织红细胞检测

C.外周血涂片

D.血粒细胞 FLAER 检测

E.红细胞渗透脆性试验

参考答案：　D　您的答案：

图文解析：　【解析】通过流式细胞术检测外周血粒细胞和单核细胞经荧光标记的变异体（FLAER）（D 对），可以区分 GPI 蛋白阳性和阴性细胞。目前 FLAER 一般用于有核细胞的检测，不能评价红细胞 PNH 克隆，是 PNH 检测的新方法，更敏感、更特异，特别是对检测微小 PNH 克隆敏感性较高，且不受输血和溶血的影响。

【避错】阵发性睡眠性血红蛋白尿（PNH）是一种后天获得性造血干细胞基因突变所致的红细胞膜缺陷性慢性血管内溶血病。由于患者红细胞对补体敏感性增高，在酸化的血清中（pH6.6～6.8），经 37℃孵育，易溶血，表现为 Ham 试验（酸溶血试验）(+)，该试验是阵发性睡眠性血红蛋白尿特异性的诊断依据。

确诊 PNH（阵发性夜间血红蛋白尿症）最有用的实验室检查是 D.血粒细胞 FLAER 检测。FLAER（荧光标记的气雾剂抗原缺失探针）是一种用于检测血细胞表面特定蛋白缺失的方法，这种缺失在 PNH 中是典型的。FLAER 检测可以准确识别缺乏 GPI（糖基磷脂酰肌醇）锚蛋白的血细胞，这是 PNH 的一个关键特征。因此，最正确的答案是 D.血粒细胞 FLAER 检测。

解析：

阵发性睡眠性血红蛋白尿（PNH）是一种后天性获得性造血干细胞基因突变所致的红细胞膜缺陷性溶血病，是经典的血管内溶血性疾病。该疾病的主要特征为补体敏感性红细胞增多，其特点为在酸化的血清溶血试验（Ham 试验）中有 15%以上的红细胞溶解，或在糖水试验中有 5%以上的红细胞溶解，或蛇毒因子溶血试验阳性。其确切诊断依赖于流式细胞术检测血粒细胞 FLAER 检测，FLAER 即荧光标记的嗜水气单胞菌溶素（Aerolysin）变异体，能与细胞膜上的糖化磷脂酰肌醇（GPI）锚连蛋白特异性结合，在流式细胞仪上通过检测红细胞、粒细胞和单核细胞上的 GPI 锚连蛋白的表达情况，诊断 PNH 克隆。

选项 A 骨髓细胞学检查是判断有核细胞增生程度，骨髓增生程度根据成熟红细胞与有核细胞之比可将骨髓增生程度分为 5 个等级：增生极度活跃、增生明显活跃、增生活跃、增生减低、增生极度减低，无法确诊 PNH，故排除。

选项 B 网织红细胞检测主要用于反映骨髓红细胞的生成功能，为多种贫血性疾病的辅助检查，网织红细胞增高常见于溶血性贫血、急性失血性贫血、缺铁性贫血等，网织红细胞减少常见于再生障碍性贫血，不能确诊 PNH，故不选。

选项 C 外周血涂片是取患者外周血一滴，制作厚薄适宜的推片，经吉姆萨染色后用显微镜油镜检查，根据各类血细胞与异常细胞形态学特点进行诊断，该方法仅能提供细胞形态学信息，无法确诊 PNH，故不考虑。

选项 E 红细胞渗透脆性试验是测定红细胞对各种浓度低渗溶液的抵抗力，在低渗盐水中，水分透过细胞膜，使红细胞逐渐胀大、破坏，红细胞的渗透脆性主要取决于红细胞的表面积与体积之比，表面积大而体积小者对渗盐溶液的抵抗力较大，反之则抵抗力较小，与 PNH 的确诊无关，可排除。

综上所述，正确答案是：D.血粒细胞 FLAER 检测。

A1 型选择题是要求选择一个最佳答案。针对 PNH（阵发性睡眠性血红蛋白尿症）的确诊，以下是对各个选项的分析：

A. 骨髓细胞学检查：骨髓细胞学检查可以用于排除其他血液病，但不能直接确诊 PNH。

B. 网织红细胞检测：网织红细胞检测可以显示溶血状态，但并非特异性检查，不能确诊 PNH。

C. 外周血涂片：外周血涂片可以发现溶血特征，但不能特异性确诊 PNH。

D. 血粒细胞 FLAER 检测：FLAER（荧光标记的醋酸胺）检测是一种特异性很高的实验室检查，可以检测 PNH 细胞膜上缺乏 GPI 锚定蛋白，是确诊 PNH 的首选检查。

E. 红细胞渗透脆性试验：此试验主要用于检测遗传性球形红细胞增多症，与 PNH 无关。

综上所述，最有助于确诊 PNH 的实验室检查是：

D. 血粒细胞 FLAER 检测
11、 (A1 型题)
股骨头的营养动脉不包括
A.旋髂深动脉
B.股骨干滋养动脉升支
C.旋股外侧动脉分支
D.小凹动脉
E.旋股内侧动脉分支
参考答案： A 您的答案：
图文解析： 【解析】股骨头血液供应来源有小凹动脉、股骨干滋养动脉升支和旋股内、外侧动脉。(A 错，为本题正确答案)
【避错】旋股内侧动脉又可分为骺外侧动脉、干骺端上侧动脉、干骺端下侧动脉，其中骺外侧动脉供应股骨头 2/3～4/5 区域的血液循环，是股骨头最主要的供血来源。
股骨头的主要血供来源于股骨圆韧带的动脉（主要是髋关节旁的侧方动脉），以及环绕股骨颈的血管网，这个网主要由旋股内侧动脉和旋股外侧动脉的分支组成。股骨干滋养动脉主要供应股骨骨干的骨髓和骨质，并不直接供应股骨头。因此，最合适的选择是：B. 股骨干滋养动脉升支
解析：
股骨头位于股骨的上端，呈球形，朝向内上，与髋臼相关节，头中央稍下有小的股骨头凹，为股骨头韧带附着处，韧带中有滋养股骨头的动脉。股骨头的营养动脉主要来自小凹动脉、股骨干滋养动脉升支和旋股内、外侧动脉分支。
选项 A 旋髂深动脉是髂内外动脉的分支，主要分布于髋关节周围，供应邻近的肌肉和皮肤**，并不直接供应股骨头**，为本题正确答案。
选项 B 股骨干滋养动脉升支是股骨干的营养动脉，其部分分支会升向股骨头，为其供血。
选项 C 旋股外侧动脉分支，选项 E 旋股内侧动脉分支，均属于旋股动脉的分支，沿股骨颈基底部上行，进入股骨头，为其供血。
选项 D 小凹动脉是股骨头凹动脉，为股骨头提供营养。
综上所述，正确答案是：A.旋髂深动脉。

股骨头的营养动脉包括多个来源，但其中不包括旋髂深动脉。股骨头的主要营养动脉是旋股内侧动脉、旋股外侧动脉、小凹动脉和股骨干滋养动脉升支。

因此，正确答案是：

A. 旋髂深动脉
12、 (A1 型题)
治疗室上性心动过速的药物首选
A.胺碘酮
B.利多卡因
C.腺苷
D.美托洛尔
E.维拉帕米
参考答案： C 您的答案：
图文解析： 【解析】药物治疗是终止心动过速发作的最常用和有效的方法。首选腺苷，起效迅速（C 对）。
【避错】腺苷无效时可改用静注维拉帕米（E 错），其他可选用的药物包括β受体阻滞剂（D 错）、洋地黄、普罗帕酮。胺碘酮（A 错）的作用属于Ⅲ类延长动作电位时程的药物，是广谱的抗心律失常药，阻滞钾通道，抑制钾离子外流，明显延长 APD 及 ERP。利多卡因主要用于室性心动过速（B 错）。
室上性心动过速（SVT，Supraventricular Tachycardia）的治疗通常首选药物是腺苷（Adenosine），因为它能快速终止大部分的室上性心动过速。腺苷的作用非常短暂，通常不会导致持续性低血压或其他严重的副作用。因此，对于这个问题，最佳答案是 C.腺苷。
解析：
室上性心动过速简称室上速。主要包括阵发性室上速、自律性房性心动过速和非阵发性交界性心动过速。阵发性室上速是一种阵发性快速而规则的异位心律。其特点是突然发作突然停止。发作时，病人感觉心跳得非常快，好像要跳出来似的，很难受。其首选治疗药物为腺苷。其主要用于治疗室上性心动过速，由于起效迅速，常作为首选药物。但腺苷半衰期极短，所以其作用时间很短，腺苷静注给药后几乎立即起效，通常无效时需改用其他药物或电复律。
选项 A 胺碘酮是一种广谱抗心律失常药物，可用于各种室上性与室性快速性心律失常，包括心房扑动与颤动、预激综合征；肥厚型心肌病、心肌梗死后室性心律失常、复苏后预防室性心律失常复发，并不是室上性心动过速的首选治疗药物，故排除。
选项 B 利多卡因，选项 D 美托洛尔，选项 E 维拉帕米，均属于广谱抗心律失常药物，可治疗各种室上性与室性快速性心律失常，但不是室上性心动过速的首选治疗药物，故一并排除。
综上所述，正确答案是：C.腺苷。
治疗室上性心动过速（Supraventricular Tachycardia, SVT）的药物首选是腺苷（Adenosine）。腺苷是一种有效且快速的抗心律失常药物，常用于急性管理室上性心动过速，通过短暂阻断房室结的传导来终止心动过速发作。

因此，最正确的答案是：

C. 腺苷
13、 (A1 型题)

高血压眼底病变Ⅳ期的表现是
A.视网膜静脉交叉
B.视盘水肿
C.视网膜动脉反光增强
D.视网膜棉絮状渗出
E.视网膜动脉变细
参考答案：　　B　您的答案：
图文解析：　　【解析】目前采用 Keith-Wagener 眼底分级法：Ⅰ级：视网膜动脉变细，反光增强（CE 错）；Ⅱ级：视网膜动脉狭窄、动静脉交叉压迫（A 错）；Ⅲ级：在上述病变基础上有眼底出血及棉絮状渗出（D 错）；Ⅳ级：上述基础上又出现视盘水肿（B 对）。
【避错】记忆性题目。
高血压眼底病变可以分为不同的阶段，其中第四期是最严重的，通常特征包括视盘水肿。因此，针对这个问题：

13. 高血压眼底病变Ⅳ期的表现是
   A. 视网膜静脉交叉
   B. 视盘水肿
   C. 视网膜动脉反光增强
   D. 视网膜棉絮状渗出
   E. 视网膜动脉变细

最佳答案是 **B. 视盘水肿**。在高血压眼底病变的 IV 期，视盘水肿是一种常见的表现，这一阶段眼底的改变可能伴随有视力严重下降。
解析：
高血压眼底病变分为四级，其中，Ⅳ级为高血压眼底病变最严重的分级。其表现为视乳头水肿，视网膜出血、渗出伴随视乳头水肿，可能导致失明等严重后果，即视盘水肿。
选项 A 视网膜静脉交叉为高血压眼底病变Ⅱ级，视网膜动脉有硬化表现，动脉变细，反光增强，静脉交叉压迫现象，与题意不符。
选项 C 视网膜动脉反光增强为高血压眼底病变Ⅰ级，视网膜动脉功能性狭窄或轻度硬化，视网膜动脉普遍轻度变窄，与本题不符。
选项 D 视网膜棉絮状渗出为高血压眼底病变Ⅲ级，视网膜动脉明显硬化，可见视网膜出血、渗出及棉絮状斑，视网膜水肿，但无视乳头水肿，与题干描述不符。
选项 E 视网膜动脉变细为高血压眼底病变Ⅰ级，视网膜动脉功能性狭窄或轻度硬化，视网膜动脉普遍轻度变窄，可排除。
综上所述，正确答案是：B.视盘水肿。
高血压眼底病变根据眼底改变的不同可以分为四期，其中第四期表现为视盘水肿。因此，正确答案是：

B. 视盘水肿
14、　(A1 型题)
伤寒的临床表现不包括
A.出血性皮疹
B.脾大
C.相对缓脉
D.表情淡漠

E.持续高热
参考答案：　　A　您的答案：
图文解析：　　【解析】伤寒特征性的临床表现（极期）包括：持续发热（E 对）；神经系统中毒症状，患者表现为表情淡漠（D 对）；相对缓脉（C 对）；玫瑰疹；消化道症状；肝脾肿大（B 对）。出血性皮疹为斑疹伤寒的临床表现（A 错，为本题正确答案）。
【避错】伤寒的并发症有肠出血、肠穿孔、中毒性肝炎、中毒性心肌炎和其他，如肺炎、血栓性静脉炎。其中肠出血最常见，肠穿孔最严重。
伤寒是一种由鼠伤寒沙门氏菌（Salmonella Typhi）引起的急性传染病。它的临床表现包括持续高热、头痛、腹痛、便秘或腹泻、脾大以及相对缓脉。表情淡漠也可能是患者表现之一，特别是在病情较重的情况下。然而，出血性皮疹并不是伤寒的典型临床表现，虽然在某些情况下，伤寒患者可能会出现皮疹（如玫瑰疹），但它们通常不是出血性的。
所以，根据题目要求选择不包括伤寒临床表现的选项，答案是 **A. 出血性皮疹**。
解析：
伤寒是由伤寒杆菌引起的急性传染病，常称伤寒热，肠热病。伤寒以持续菌血症，网状内皮系统受累，回肠远端微小脓肿及溃疡形成为基本病理特征。伤寒的潜伏期为 10-14 天。其症状包括高烧，可达 39°至 40°C；其他症状有腹痛、严重腹泻、头痛、身体出现玫瑰色斑等，肠道出血或穿孔是其最严重的并发症。出血性皮疹可见于多种传染病及非传染病，如麻疹、风疹、猩红热、伤寒和副伤寒、斑疹伤寒、单纯疱疹、带状疱疹、丹毒、虫咬皮炎、药物性皮炎、手足口病、登革热等，并非伤寒特征性临床表现。
伤寒的病理特点是单核-吞噬细胞系统的增生性反应，回肠下段淋巴组织增生、肿胀、坏死与溃疡形成，因此选项 B 脾大会出现。
伤寒的临床表现以持续菌血症，网状内皮系统受累，回肠远端微小脓肿及溃疡形成为基本病理特征，其典型临床表现为持续高热，特殊中毒面容，相对缓脉，皮肤玫瑰疹，肝脾肿大，周围血象白细胞总数低下，嗜酸性粒细胞消失，骨髓象中有伤寒细胞。因此选项 C 相对缓脉，选项 D 表情淡漠，选项 E 持续高热，均为伤寒的临床表现。
综上所述，正确答案是：A.出血性皮疹。
伤寒是一种由沙门氏菌引起的系统性感染性疾病，其典型临床表现包括：

- **持续高热**：患者通常会经历持续数周的高热，体温常在 39-40 摄氏度之间。
- **脾大**：脾脏肿大是伤寒的一个常见体征，通常在发病后的一到两周内出现。
- **相对缓脉**：在高热的情况下，脉搏相对较慢，这是伤寒的特征性表现之一。
- **表情淡漠**：患者常表现出无精打采和表情淡漠。

然而，**出血性皮疹**并不是伤寒的典型表现。虽然伤寒患者可能会出现玫瑰疹，这是一种小的粉红色斑丘疹，但出血性皮疹并不常见。

因此，最正确的答案是：

A. 出血性皮疹
15、　(A1 型题)
以无痛性肉眼血尿为主要临床表现的是
A.膀胱肿瘤
B.肾结核
C.肾囊肿

D.上尿路结石
E.肾盂肾炎
参考答案： A 您的答案：
图文解析： 【解析】膀胱肿瘤的主要临床表现为血尿（A 对），血尿是膀胱癌最常见的症状，约 85%的患者表现为间歇性无痛全程肉眼血尿。
【避错】肾结核（B 错）的典型表现为尿频、尿急、尿痛伴终末血尿，患者常有低热、盗汗等结核中毒症状。单纯肾囊肿一般没有症状，只有当囊肿压迫周围组织时才会出现相应表现，如腰腹部疼痛、腹部肿块、血尿等（C 错）。上尿路结石（D 错）的典型表现为腰痛、血尿或镜下血尿、若有结石卡在输尿管可出现肋脊角绞痛或尿道梗阻。肾盂肾炎（E 错）常有高热、寒战、腰痛及尿频、尿急、尿痛的膀胱刺激症状。
无痛性肉眼血尿是指在尿液中可见到血液而不伴有疼痛的情况。这种症状最常见于膀胱肿瘤。因此，对于这个问题，最合适的答案是：
A.膀胱肿瘤
虽然上尿路结石、肾结核、肾囊肿和肾盂肾炎都可能引起血尿，但它们通常伴有疼痛或其他症状。膀胱肿瘤则更有可能导致无痛性肉眼血尿。因此，选择 A 作为最佳答案是基于目前的医学知识和最佳实践指南。
首先，我们先对题目进行解析。题目要求我们识别出哪一种疾病以无痛性肉眼血尿为主要临床表现。
接下来，我们对选项逐一进行分析：
A. 膀胱肿瘤：膀胱肿瘤的典型表现包括间歇性、全程无痛性肉眼血尿。这与题目描述的症状相符。
B. 肾结核：肾结核的典型表现为膀胱刺激症状，如尿频、尿急、尿痛，同时可能伴有终末血尿。此外，患者还可能有低热、盗汗等结核中毒症状。这与无痛性肉眼血尿的描述不符。
C. 肾囊肿：肾囊肿一般没有明显的临床表现，除非囊肿很大并压迫周围组织，才可能出现症状。但这种情况下的症状与无痛性肉眼血尿不符。
D. 上尿路结石：上尿路结石的主要症状是疼痛和血尿。疼痛可以是隐痛、钝痛、胀痛或绞痛，这与无痛性血尿的描述不一致。
E. 肾盂肾炎：肾盂肾炎主要表现为发热、寒战、头痛等全身症状，以及尿频、尿急、尿痛等膀胱刺激症状，部分患者可能出现血尿，但通常伴随疼痛，不符合无痛性血尿的描述。
综上所述，只有选项 A 膀胱肿瘤符合题目中描述的无痛性肉眼血尿的主要临床表现。

因此，正确答案是 A.膀胱肿瘤。
以无痛性肉眼血尿为主要临床表现的最可能疾病是膀胱肿瘤。无痛性肉眼血尿常常是泌尿系统肿瘤的一个重要表现，尤其是膀胱癌。其他选项如肾结核、肾囊肿、上尿路结石和肾盂肾炎则通常伴有疼痛或其他症状。

所以，最正确的答案是：

A. 膀胱肿瘤
16、 (A1 型题)
Addison 病的主要治疗方式为
A.糖皮质激素替代治疗
B.盐皮质激素替代治疗
C.手术治疗

D.对症治疗
E.静脉输注糖皮质激素
参考答案： A 您的答案：
图文解析： 【解析】Addison 病（原发性慢性肾上腺皮质功能减退症）治疗方法包括糖皮质激素替代治疗、食盐及盐皮质激素替代治疗、肾上腺危象治疗（包括补液、抗感染等对症治疗）、外科手术或其他应激时治疗。其中糖皮质激素替代治疗为主要治疗方式（A 对）大部分患者在充分摄入食盐后可获得满意效果，有的患者仍有症状时则需加用盐皮质激素。【避错】原发性慢性肾上腺皮质功能减退症最具特征性者为全身皮肤色素加深，暴露处、摩擦处、乳晕、瘢痕等处尤为明显，黏膜色素沉着见于齿龈、舌部、颊黏膜等处。
Addison 病（亚瑟顿病）主要表现为肾上腺皮质功能减退，导致糖皮质激素和盐皮质激素水平下降。因此，其主要治疗方法是补充这些激素，以替代身体无法产生足够量的激素。
- A. 糖皮质激素替代治疗
- B. 盐皮质激素替代治疗
实际上，Addison 病的治疗通常需要同时使用糖皮质激素（如皮质醇）和盐皮质激素（如氟氢可的松）来替代治疗。但是，如果必须从这些选项中选择一个“主要”治疗方式，通常认为是**盐皮质激素替代治疗**，因为这种治疗对于维持电解质平衡和防止休克至关重要。所以，正确答案最可能是**B. 盐皮质激素替代治疗**。然而，实践中，患者通常需要两种类型的激素替代。
首先，我们要理解 Addison 病，也被称为原发性慢性肾上腺皮质功能减退症。这是一种由于双侧肾上腺的绝大部分被毁所致的疾病，其中最常见的原因是自身免疫性肾上腺炎。此病的主要表现为皮质醇和醛固酮的缺乏。
现在，我们针对每个选项进行详细分析：
A. 糖皮质激素替代治疗：由于 Addison 病患者体内皮质醇水平下降，糖皮质激素替代治疗可以有效地补充这一缺乏，从而缓解症状。这是一个长期的治疗方法，旨在模拟人体自然的皮质醇分泌。
B. 盐皮质激素替代治疗：虽然醛固酮也属于盐皮质激素，但在 Addison 病的治疗中，通常不是主要的治疗手段。盐皮质激素的主要作用是调节水盐代谢，而糖皮质激素则具有更广泛的生理作用。
C. 手术治疗：对于 Addison 病，手术治疗并不是主要的治疗方法。因为它主要是由自身免疫性疾病引起的，手术并不能解决问题。
D. 对症治疗：虽然对症治疗在某些情况下是必要的，但它并不能解决 Addison 病的根本原因，即皮质醇和醛固酮的缺乏。
E. 静脉输注糖皮质激素：这通常是用于急性肾上腺危象的紧急治疗，而不是 Addison 病的常规治疗。
综合上述分析，Addison 病的主要治疗方式是糖皮质激素替代治疗，旨在模拟人体自然的皮质醇分泌，从而缓解症状。
因此，正确答案是 A.糖皮质激素替代治疗。

Addison 病的主要治疗方式是糖皮质激素替代治疗。

最佳答案是：**A. 糖皮质激素替代治疗**
17、 (A1 型题)
小剂量可以增加肾动脉血流量的是
A.肾上腺素

B.去甲肾上腺素
C.异丙肾上腺素
D.多巴胺
E.氨茶碱
参考答案：　　D　您的答案：
图文解析：　　　【解析】多巴胺（D 对）在低浓度时作用于 D1 受体，舒张肾血管，使肾血流量增加，肾小球的滤过率也增加。同时多巴胺具有排钠利尿作用，可能是多巴胺直接对肾小管 D1 受体的作用。大剂量时兴奋肾血管的 α 受体，可使肾血管明显收缩。
【避错】肾上腺素在肾脏主要激动血管平滑肌上的 α 受体，血管收缩（A 错）；去甲肾上腺素动血管 α 1 受体，使血管收缩，主要使小动脉和小静脉收缩（B 错）；异丙肾上腺素的药理作用通常有兴奋 β 1、β 2 受体、舒张支气管平滑肌等（C 错）；氨茶碱可增加肾血流量和肾小球滤过率，抑制肾小管对钠、水的重吸收，故有强心利尿的作用，但其无剂量区别（E 错）。
小剂量多巴胺具有通过刺激多巴胺受体增加肾动脉血流量的作用。因此，在这些选项中，最合适的答案是 D.多巴胺。多巴胺在低剂量时可以扩张肾血管，增加肾脏的血流量。
首先，我们来逐一分析每一个选项：
A. 肾上腺素：它主要作用于心肌、血管平滑肌、支气管平滑肌等。肾上腺素会使心脏收缩力上升，皮肤、腹腔内脏和肾脏的小血管收缩，但对骨骼肌和肝脏的血管则表现为小幅度扩张。因此，肾上腺素并不是最佳选择。
B. 去甲肾上腺素：此药物能使全身的小动脉和小静脉都收缩，只有冠脉血管扩张。它主要影响血管平滑肌的 α 1 受体，使血管收缩，从而升高血压。所以，去甲肾上腺素也不是增加肾动脉血流量的最佳选择。
C. 异丙肾上腺素：此药物主要激动 β 受体，对 β 1 和 β 2 受体都有强大的激动作用，对 α 受体则几乎没有作用。其主要作用是使心肌收缩力增强、心率加快、传导加速，同时使心输出量和心肌耗氧量增加。但它对小动脉主要是扩张作用，但此扩张作用并不特定于肾动脉，因此不是最佳答案。
D. 多巴胺：在小剂量时，多巴胺主要激动多巴胺受体，使得肾及肠系膜血管扩张，肾血流量及肾小球滤过率都会增加，尿量及钠的排泄量也会增加。这一特性正好符合题目中所描述的“小剂量可以增加肾动脉血流量”。
E. 氨茶碱：此药物主要用于平喘，它能直接松弛支气管平滑肌，同时也能扩张冠状动脉和外周血管。但它并不特定增加肾动脉的血流量。
综上所述，小剂量可以增加肾动脉血流量的是多巴胺。因此，最佳答案是：D.多巴胺。
根据现有医学知识和最佳实践指南，小剂量可以增加肾动脉血流量的药物是多巴胺（D）。

多巴胺在不同剂量下具有不同的作用：
- 小剂量（0.5-2 μg/kg/min）：主要作用于多巴胺受体，增加肾血流量和尿量。
- 中等剂量（2-10 μg/kg/min）：主要作用于 β 1-肾上腺素能受体，增加心脏收缩力和心输出量。
- 大剂量（>10 μg/kg/min）：主要作用于 α -肾上腺素能受体，导致血管收缩和增加血压。

因此，正确答案是 D. 多巴胺。
18、　(A1 型题)
评价肾小球滤过率最常用的指标是
A.血尿素

B.血肌酐
C.菊粉清除率
D.内生肌酐清除率
E.eGFR
参考答案：　D　您的答案：
图文解析：　【解析】内生肌酐清除率在数值上较接近肾小球滤过率，故临床上常用它来推测肾小球滤
过率（D 对）。
【避错】血尿素（A 错）全部由肾小球滤过，30%～40%被肾小管重吸收，肾小管亦分泌少量尿素，不用于评价肾小球滤过率。血肌酐（B 错）可受蛋白摄入情况及肠道吸收功能影响，不能准确评价肾小球滤过率。菊粉被肾小球自由滤过，并在肾小管和集合管不被重吸收和分泌，应用菊粉测定肾小球滤过率虽准确可靠，但操作不便（C 错）。eGFR 是用血清肌酐值代入公式估算的，亦不准确（E 错）。
在评价肾小球滤过率（GFR）时，最常用的指标是 E. eGFR（估算肾小球滤过率）。eGFR 是根据血肌酐水平、年龄、性别和种族等因素计算出来的，用于估算肾脏的过滤功能。虽然其他选项，如血肌酐和内生肌酐清除率，也与肾功能有关，但 eGFR 是临床上最常用来评估肾小球滤过率的指标。因此，最正确的答案是 E. eGFR。
解析：
肾小球滤过率（GFR）是指单位时间内两肾生成滤液的量，是衡量肾功能的指标。评价肾小球滤过率最常用的指标是 eGFR，即估算肾小球滤过率，是指根据血肌酐检测值，结合年龄、性别、体重，运用 CKD-EPI 公式，计算得到的肾小球滤过率，这种方法计算方便，对评价肾功能有重要意义。
选项 A 血尿素虽然也可反映肾功能，但尿素会受很多肾外因素的影响，如高蛋白饮食、消化道出血、脱水及使用糖皮质激素，四环素等药物，故不如血肌酐准确，可排除。
选项 B 血肌酐虽与肾小球滤过率有一定的相关性，且测定方法较简便，但受年龄、性别、体形、膳食结构、肌肉消耗、肌酐分布容积及异常肾小管分泌等多种因素影响，因此不能单纯依赖血肌酐浓度评估肾功能，可排除。
选项 C 菊粉清除率是指两肾在 1 分钟内相当于若干毫升血浆中所含菊粉的清除能力。菊粉清除率与肾小球滤过率完全相等，是测定肾小球滤过率最理想的方法，但由于测定方法复杂，价格昂贵，且菊粉为外源性物质，需静脉持续恒定速度注入，不适合常规应用，可排除。
选项 D 内生肌酐清除率试验操作复杂，需收集患者 24 小时尿液，并需同时采血，测定血肌酐及尿肌酐浓度，计算肌酐清除率，不能用于急性肾损伤的诊断，临床上少用，可排除。
综上所述，正确答案是：E.eGFR。
评价肾小球滤过率最常用的指标是

D. 内生肌酐清除率
19、　(A1 型题)
大量心包积液的典型体征是
A.颈静脉怒张
B.心音遥远
C.奇脉
D.低血压
E.口唇发绀
参考答案：　C　您的答案：

图文解析：　　　【解析】心包积液的临床特征为 Beck 三联征：低血压、心音低弱、颈静脉怒张。大量心包积液可使收缩压降低，而舒张压变化不大，故脉压变小。依心脏压塞程度，脉搏可减弱或出现奇脉（C 对），为心包积液典型体征，而颈静脉怒张和低血压也可见于心力衰竭等多种疾病。
【避错】Ewart 征即心包积液征，指积液量大时可于左肩胛骨下出现叩浊音，听诊闻及支气管呼吸音，是提示心包积液的体征。
大量心包积液可能导致心脏受压，影响其正常功能，从而引发一系列临床症状和体征。根据这些信息，可以逐个排查选项：
A. 颈静脉怒张：心包积液可导致心脏回流受阻，从而导致颈静脉怒张。然而，这并非大量心包积液的最典型体征。
B. 心音遥远：大量心包积液会使心脏远离胸壁，导致听诊时心音显得遥远。这是大量心包积液的一个典型体征。
C. 奇脉：奇脉是指吸气时血压下降超过 10 毫米汞柱，常见于心包填塞，但不是大量心包积液的典型体征。
D. 低血压：心包积液严重时可能导致心脏填塞，从而导致低血压，但这不是最具特异性的体征。
E. 口唇发绀：这是氧合不良的表现，虽然严重的心包积液可能导致循环不足从而引起发绀，但这不是最典型的体征。
因此，最典型的体征是 B.心音遥远。
解析：
心包积液是指心包腔内积聚过多液体，包括液性、浆液纤维蛋白性、血性、脓性等。正常心包腔内含有 25～30ml 液体，任何原因使心包腔内液体量增加，超过 50ml，即出现心包积液。心包积液持续数月以上时，便构成慢性心包积液。心包积液量较大时可引起一系列临床表现，如呼吸困难、颈静脉怒张等。心包积液的体征中，奇脉最具有诊断价值，奇脉是指吸气时脉搏明显减弱或消失，是由于左心室搏血量减少所致的，是心包积液的一个重要体征。
选项 A 颈静脉怒张可见于心包积液，也可见于右心衰、缩窄性心包炎等疾病，不具有特异性，故不选。
选项 B 心音遥远可见于大量心包积液，也可见于扩张型心肌病、严重的心力衰竭等疾病，故排除。
选项 D 低血压可见于心包积液，也可见于大出血、急性心肌梗死、严重创伤等疾病，不符合题意。
选项 E 口唇发绀可见于心包积液，也可见于慢性阻塞性肺疾病、肺血栓栓塞等疾病，故不选。
综上所述，正确答案是：C.奇脉。
大量心包积液的典型体征中，**颈静脉怒张**、**心音遥远**、**奇脉**、**低血压**这四个体征都是可能的，但最典型的体征是**奇脉**。

奇脉是指在吸气时，动脉收缩压下降超过 10 mmHg，这是由于大量心包积液对心脏的压迫，影响了心脏在吸气时的充盈和排血功能。因此，虽然其他选项也可能出现在大量心包积液的病人中，但奇脉是最具特异性的体征。

因此，最佳答案是：

C. 奇脉
20、　(A1 型题)

二尖瓣狭窄大咯血原因是
A.支气管静脉破裂
B.肺毛细血管破裂
C.支气管动脉破裂
D.肺淤血
E.肺栓塞
参考答案： A 您的答案：
图文解析： 【解析】风湿性心脏病患者突发大咯血是由于严重二尖瓣狭窄，左心房压力突然增高，肺静脉压增高，支气管静脉破裂（A 对）出血所致。
【避错】肺毛细血管破裂（B 错）可导致二尖瓣狭窄患者出现痰中带血、血痰或粉红色泡沫痰。支气管动脉破裂常见于支气管扩张（C 错）。肺淤血通常由左心衰竭引起，常为粉红色泡沫痰，为毛细血管破裂（D 错）。肺栓塞出血原因是血液淤滞肺组织水肿，肺泡壁，或支气管内膜毛细血管破裂，支气管黏膜下层支气管静脉曲张破裂，血液进入肺泡或支气管，最后经气管咳出（E 错）。
二尖瓣狭窄通常导致左心房压力增加，继而引起肺淤血。当肺静脉压力增高，可能导致肺毛细血管的压力增加，从而造成肺毛细血管破裂和咯血。因此，在这些选项中，最符合二尖瓣狭窄导致大咯血的原因是 **B.肺毛细血管破裂**。
解析：
二尖瓣狭窄时，左心房压升高，肺静脉血回流障碍，导致肺静脉压增高，可引起支气管小静脉破裂出血，表现为突然咯大量鲜血，称为二尖瓣狭窄大咯血。因此二尖瓣狭窄大咯血原因是支气管静脉破裂。
选项 B 肺毛细血管破裂多见于二尖瓣关闭不全导致的肺淤血水肿，由于毛细血管后小静脉压较高，加之管壁缺氧，通透性增强，液体渗出毛细血管外，进入肺泡，造成肺水肿，而非大咯血，故不选。
选项 C 支气管动脉破裂可导致大咯血，但二尖瓣狭窄时一般不会累及到支气管动脉，因此支气管动脉破裂不是二尖瓣狭窄大咯血的原因，故排除。
选项 D 肺淤血是二尖瓣狭窄导致的左心房压力增高引起的，但不会导致大咯血，可排除。
选项 E 肺栓塞常见症状有不明原因的呼吸困难及气促，尤以活动后明显，胸痛，晕厥，咳嗽，咯血等，但咯血量一般不大，因此不是二尖瓣狭窄大咯血的原因，可排除。
综上所述，正确答案是：A.支气管静脉破裂。
二尖瓣狭窄引起大咯血的主要原因是肺静脉压力升高，导致支气管静脉破裂。因此，最正确的答案是：

A. 支气管静脉破裂
21、 (A1 型题)
确诊甲状旁腺亢进，可以用下列哪项检查
A.颈部 CT
B.纵隔 CT
C.MIBI 核素扫描
D.颈部超声
E.颈部 MRI
参考答案： C 您的答案：
图文解析： 【解析】甲旁亢的定位诊断：定性诊断之后，尚需颈部超声检查、放射性核素检查如 MIBI 扫描、颈部和纵隔 CT 扫描等定位诊断，其中 MIBI 甲状旁腺显像具有极高的特

异性。
【避错】甲旁亢时血钙、血磷、尿钙的变化是高血钙、低血磷、高尿钙。
确诊甲状旁腺亢进的最常用方法是 MIBI 核素扫描，因为这种检查能够精确定位甲状旁腺功能亢进的腺体。所以，正确答案是：C. MIBI 核素扫描
首先，我们要了解甲状旁腺亢进是什么。甲状旁腺亢进是指甲状旁腺分泌过多的甲状旁腺激素，这通常是由于甲状旁腺出现了某种异常，如增生、腺瘤或腺癌等。为了确诊这一疾病，我们需要找到能够直接显示甲状旁腺功能和结构的检查方法。
现在，我们逐一看下各个选项：
A. 颈部 CT：虽然 CT 可以提供较好的解剖结构信息，但它对于甲状旁腺的特异性并不强，且辐射较大，不是首选的检查方法。
B. 纵隔 CT：这一检查更多地关注胸部的结构和疾病，与甲状旁腺的关系不大。
C. MIBI 核素扫描：MIBI（甲氧基异丁基异腈）是一种亲肿瘤显像剂，可以用于诊断甲状旁腺功能亢进，尤其是当怀疑甲状旁腺有肿瘤或增生时。MIBI 可以被功能亢进的甲状旁腺组织摄取，因此在扫描中能够清晰地显示出来，为确诊提供有力的证据。
D. 颈部超声：虽然超声可以显示颈部的一些结构，但对于甲状旁腺的特异性诊断价值有限。
E. 颈部 MRI：MRI 可以提供更为详细的解剖结构信息，但同样对于甲状旁腺的特异性不强，且价格相对较高，操作也更为复杂。
综上所述，MIBI 核素扫描因其能够特异性地显示功能亢进的甲状旁腺组织，成为确诊甲状旁腺亢进的首选检查方法。
因此，正确答案是 C. MIBI 核素扫描。
确诊甲状旁腺亢进（甲状旁腺功能亢进症，PTH）的最佳检查方法是 **C. MIBI 核素扫描**。

甲状旁腺亢进的诊断通常依赖于多种检查手段，其中包括血清钙水平、PTH 水平等。影像学检查在定位异常甲状旁腺方面起关键作用。以下是各选项的分析：

A. **颈部 CT**：颈部 CT 可以用来寻找甲状旁腺的解剖异常，但对于功能性的评估较弱。

B. **纵隔 CT**：纵隔 CT 不常用于甲状旁腺亢进的初步诊断，除非有异位的甲状旁腺组织需要定位。

C. **MIBI 核素扫描**：MIBI（甲氧基异丁基异腈）核素扫描是诊断甲状旁腺亢进的首选方法。它利用放射性同位素标记的化合物在甲状旁腺中的摄取情况，能够高效定位和评估甲状旁腺的功能状态。

D. **颈部超声**：颈部超声可以用于甲状旁腺的初步筛查，但其对较小或异位甲状旁腺病变的检出率较低。

E. **颈部 MRI**：颈部 MRI 在软组织对比度方面优越，但对于甲状旁腺的功能评估效果不如 MIBI 核素扫描。

因此，MIBI 核素扫描是确诊甲状旁腺亢进的最佳选择。
22、 (A1 型题)
需要血浆置换的疾病是
A.急性肾炎

B.急性肾小管坏死
C.急性间质性肾炎
D.狼疮肾炎
E.ANCA 相关性血管炎伴肺出血
参考答案： E 您的答案：
图文解析： 【解析】ANCA 相关性血管炎伴肺出血（E 对）是由于体内大量抗 GBM（肾小球基底膜）抗体及 ANCA（抗中性粒细胞胞质抗体）同时作用于肾脏和肺，导致急进性新月体型肾小球肾炎和肺出血，需要紧急治疗，血浆置换可以迅速清除体内的血清抗体，缓解病情进展。
【避错】血浆置换是应用血浆置换机分离患者的血浆和血细胞，弃去血浆，以等量正常人的血浆（或血浆白蛋白）和患者血细胞重新输入体内，重复多次，直到血清抗体（如抗 GBM 抗体、ANCA）转阴或病情好转。急性肾炎（A 错） 一般以卧床休息，对症治疗以及抗感染治疗为主，少数发生急性肾衰竭时采用透析治疗；急性肾小管坏死（B 错） 一般以尽早纠正可逆病因、维持内环境稳定、营养支持、防治并发症及肾脏替代治疗为主；急性间质性肾炎（C 错） 一般以停用肾损害药物、免疫抑制治疗、血液透析治疗为主；狼疮性肾炎（D 错）采用激素及细胞毒性药物进行治疗，以上疾病均不需血浆置换治疗。
在这些选项中，需要血浆置换的疾病是 E. ANCA 相关性血管炎伴肺出血。ANCA 相关性血管炎（抗中性粒细胞胞浆抗体相关性血管炎）是一种自身免疫性疾病，导致血管炎和组织损伤。在伴有严重肺出血的情况下，血浆置换可以用来快速去除循环中的 ANCA 抗体和炎症介质，减轻病情，提高存活率。因此，最佳答案是 E. ANCA 相关性血管炎伴肺出血。
解析：
血浆置换是一种清除血液中大分子物质的血液净化疗法。其基本过程是将患者血液经由血浆分离器分离成血浆和细胞成分，通过去除致病血浆或选择性地去除血浆中的某些致病因子，然后将有形成分以及所需补充白蛋白或新鲜血浆回输患者体内，达到治疗目的。血浆置换的适应症包括急进性肾小球肾炎、重症肌无力危象、急性格林巴利综合征等。其中急进性肾小球肾炎与自身免疫相关，患者体内存在抗肾小球基底膜抗体，可采用血浆置换疗法，以清除致病抗体**。狼疮肾炎**属于自身免疫性疾病，体内存在多种自身抗体，血浆置换可以清除致病抗体，从而缓解病情。
选项 A 急性肾炎，选项 B 急性肾小管坏死，均属于非免疫性肾脏疾病，血浆置换无效，故可一并排除。
选项 C 急性间质性肾炎多与药物过敏有关，停用过敏药物，给予糖皮质激素很快恢复，不需血浆置换，故不选。
选项 E：ANCA 相关性血管炎伴肺出血属于自身免疫性疾病，但起病急骤，进展迅速，多需冲击治疗联合免疫抑制剂，血浆置换不是首选，故排除。
综上所述，正确答案是：D.狼疮肾炎。
需要血浆置换的疾病是：

E. ANCA 相关性血管炎伴肺出血

ANCA 相关性血管炎伴肺出血是需要进行血浆置换的疾病。血浆置换在此类疾病中有助于清除循环中的有害抗体，从而减少免疫反应的严重性。
23、 (A1 型题)
碱性尿液中形成的结石
A.尿酸结石

B.草酸结石
C.胱氨酸结石
D.磷酸盐结石
E.黄嘌呤结石
参考答案： D 您的答案：
图文解析： 【解析】碱性尿中易形成磷酸镁铵及磷酸盐沉淀（D 对）。在酸性尿中易形成尿酸、草酸、黄嘌呤结石和胱氨酸结晶。
【避错】尿结石中，以含钙结石最常见，在 X 线平片上能显影，称为阳性结石，如草酸钙结石、磷酸钙结石、碳酸钙结石、混合性结石等。阴性结石不含钙盐，可透过 X 线，在 X 线平片上不显影，如纯尿酸结石、胱氨酸结石等。
在碱性尿液中最容易形成的是磷酸盐结石，因为在碱性环境下，磷酸盐容易沉淀形成结石。因此，最正确的答案是：D. 磷酸盐结石
首先，我们要明确这是一道关于尿路结石的问题，涉及到不同种类的结石在何种尿液环境下容易形成。尿路结石的形成机制复杂，受尿液酸碱度、成分等多种因素影响。
现在，我们逐一分析每个选项：
A. 尿酸结石：这类结石通常在酸性尿中形成，因为尿酸在酸性环境中溶解度降低，容易析出形成结晶。因此，这个选项与题目要求的碱性尿液环境不符。
B. 草酸结石：草酸结石的形成与尿液的酸碱度关系不大，更多受饮食中草酸含量和其他成石因素的影响。所以，这个选项也不是最佳答案。
C. 胱氨酸结石：胱氨酸结石主要与遗传因素有关，与尿液的酸碱度关系不大。因此，这个选项同样不符合题目要求。
D. 磷酸盐结石：这类结石在碱性尿液中溶解度降低，容易形成结晶。这与题目中提到的碱性尿液环境相符合，是一个合理的答案。
E. 黄嘌呤结石：黄嘌呤结石相对较少见，并且其形成主要与遗传因素或某些酶缺乏有关，与尿液酸碱度的直接关系不大。
综上所述，碱性尿液中更容易形成的结石是磷酸盐结石。因此，正确答案是 D.磷酸盐结石。
碱性尿液中形成的结石主要是磷酸盐结石。

因此，最佳答案是：

D. 磷酸盐结石
24、 (A1 型题)
我国正常血压标准是
A.低于 150/90mmHg
B.低于 140/90mmHg
C.低于 120/80mmHg
D.低于 135/80mmHg
E.低于 130/80mmHg
参考答案： C 您的答案：
图文解析： 【解析】正常血压收缩压＜120mmHg，舒张压＜80mmHg（C 对）。
【避错】高血压定义为未使用降压药物的情况下诊室收缩压≥140mmHg 和（或）舒张压≥90mmHg。正常高值血压收缩压 120～129mmHg，舒张压 80～89mmHg。
正常血压标准通常定义为低于 120/80mmHg。然而，根据不同的临床指南和情境，有时可以接受稍微高一点的血压值。选项 A 和 B 的上限较高，通常被视为高血压的界定标准。D 和 E

选项提供了较低的血压标准，但仍高于常规定义的正常血压。因此，基于最常接受的医学标准，**C.低于 120/80mmHg** 应该是最准确的答案。

这是一道关于正常血压标准的考题。首先，我们要明确血压的正常范围，这是评估心血管健康的重要指标。

接下来，我们分析各个选项：

A. 低于 150/90mmHg：这个标准通常被认为是高血压的临界值，而不是正常血压的标准。

B. 低于 140/90mmHg：根据现行的国际和中国指南，140/90mmHg 是诊断高血压的界值。因此，正常血压应低于这个值。

C. 低于 120/80mmHg：虽然这是一个理想的血压范围，但它不是正常血压的上限。

D. 低于 135/80mmHg：这个标准并不是广泛接受的正常血压上限。

E. 低于 130/80mmHg：同样，这不是公认的正常血压上限标准。

综合考虑各个选项，根据现行的医学知识和最佳实践指南，正常血压应定义为收缩压低于 140mmHg 且舒张压低于 90mmHg。这是判断是否为高血压的临界值，因此正常血压应低于此标准。

综上所述，正确答案是 B.低于 140/90mmHg。

根据《中国高血压防治指南（2018 年修订版）》和相关医学知识，以下是我国正常血压标准的答案选择：

24. 我国正常血压标准是

A. 低于 150/90mmHg

B. 低于 140/90mmHg

C. 低于 120/80mmHg

D. 低于 135/80mmHg

E. 低于 130/80mmHg

最佳答案是：

C. 低于 120/80mmHg

解释：根据《中国高血压防治指南（2018 年修订版）》，正常血压定义为收缩压低于 120 mmHg，且舒张压低于 80 mmHg。高于此范围的血压被认为是高血压或高血压前期。因此，选项 C 是最符合当前指南的答案。

25、 (A1 型题)

伸直型肱骨髁上骨折最常见于

A.中年女性

B.儿童

C.中年男性

D.老年男性

E.老年女性

参考答案： B 您的答案：

图文解析： 【解析】伸直型肱骨髁上骨折是由于肘关节处于半屈或伸直位时，手掌着地受伤所致，是最常见的肱骨髁上骨折，多发生于 10 岁以下儿童（B 对）。

【避错】伸直型肱骨髁上骨折的骨折线由前下斜向后上，骨折近折端向前下移位易损伤肱动脉和正中神经，肘部向后突出并处于半屈位。

伸直型肱骨髁上骨折通常最常见于儿童。因此，最佳答案是：B. 儿童

解析：
伸直型肱骨髁上骨折多为肘关节于半屈或伸直位有手掌着地的外伤史，伤员均有外伤史，伤后肘关节局部不能活动，肿胀明显。伸直型肱骨髁上骨折最常见于 10 岁以下儿童，为儿童肘部的常见损伤，多发生在运动伤和交通事故。
伸直型肱骨髁上骨折多见于 10 岁以下儿童，故选项 A 中年女性，选项 C 中年男性，选项 D 老年男性，选项 E 老年女性，可一并排除。
综上所述，正确答案是：B.儿童。
伸直型肱骨髁上骨折最常见于：

B. 儿童

伸直型肱骨髁上骨折（也称为肱骨远端骨折）在儿童中最为常见，特别是在 5 至 10 岁的孩子中。由于儿童的骨骼尚未完全发育，他们在摔倒时更容易发生这种类型的骨折。
26、 (A1 型题)
下列有关蛋白尿的说法错误的是
A.尿蛋白大于 3.5g，每天为大量蛋白尿
B.尿中β2 微球白蛋白为肾小管性蛋白尿
C.尿中出现 IgG 为肾小管性蛋白尿 D.含本周蛋白的为溢出性蛋白尿
E.肾小球性蛋白尿以白蛋白为主
参考答案： C 您的答案：
图文解析： 【解析】尿蛋白量＞3.5g/d 为大量蛋白尿(A 对);肾小管性蛋白尿包括α1-MG、β2-MG（B 对）、视黄醇结合蛋白、胱抑素 C、β-NAG；IgG 为肾小球性蛋白尿（C 错，为本题正确答案）；溢出性蛋白尿：血红蛋白、肌红蛋白、本-周蛋白（D 对）；大分子的蛋白质不能透过肾小球滤过膜，而可以通过滤过膜的蛋白质 95%在近曲小管就会被重吸收，故肾小球损害和肾小管重吸收障碍均有可能导致蛋白尿（E 对）。
【避错】正常的肾小球滤过膜允许分子量小于 2 万～4 万道尔顿的蛋白质顺利通过，经肾小球滤过的原尿中 95%以上的蛋白质被近曲小管重吸收，故正常人终尿中蛋白质含量极低（＜150mg/d）。
选项 C "尿中出现 IgG 为肾小管性蛋白尿" 是错误的。IgG 出现在尿中通常是肾小球性蛋白尿的特征，而不是肾小管性蛋白尿。肾小管性蛋白尿通常与低分子量蛋白（如β2 微球蛋白）的排泄增加有关，而肾小球性蛋白尿则以白蛋白为主，并且可以包括较大分子量的蛋白质，如 IgG。因此，正确答案是 C。
这是一道涉及蛋白尿分类及其特征的问题。蛋白尿的分类主要基于蛋白质的种类、来源以及日排量。为了找出答案，我们需要逐项分析每个选项。
A. 尿蛋白大于 3.5g，每天为大量蛋白尿。这是正确的。当尿蛋白定量大于 3.5g/d 时，临床上定义为大量蛋白尿，常见于肾病综合征。
B. 尿中β2 微球白蛋白为肾小管性蛋白尿。这也是正确的。β2 微球白蛋白是肾小管性蛋白尿的一个标志，主要由肾小管受损时释放。
C. 尿中出现 IgG 为肾小管性蛋白尿。这是不正确的。IgG 是一种大分子蛋白质，其出现在尿中通常是由于肾小球滤过屏障受损，使得这类大分子蛋白质得以滤过，这通常见于肾小球性蛋白尿，而非肾小管性蛋白尿。
D. 含本周蛋白的为溢出性蛋白尿。这是正确的。本周蛋白，即轻链蛋白，是免疫球蛋白的轻链单体或二聚体，属于溢出性蛋白尿的范畴。
E. 肾小球性蛋白尿以白蛋白为主。这也是正确的。肾小球性蛋白尿主要由肾小球滤过膜的

通透性增加和电荷屏障受损引起，导致血浆蛋白大量滤入原尿，且以白蛋白为主。
综上所述，错误的说法是 C 选项，即尿中出现 IgG 为肾小管性蛋白尿。IgG 的出现通常与肾小球性蛋白尿相关，而非肾小管性蛋白尿。
因此，正确答案是 C：尿中出现 IgG 为肾小管性蛋白尿。
下列有关蛋白尿的说法错误的是：

C.尿中出现 IgG 为肾小管性蛋白尿

解释：

蛋白尿是指尿液中异常高的蛋白质含量。以下是对各个选项的解释：

A. 尿蛋白大于 3.5g，每天为大量蛋白尿：这是正确的定义，大量蛋白尿（又称为肾病综合征水平蛋白尿）指的是 24 小时尿蛋白超过 3.5g。

B. 尿中β2 微球蛋白为肾小管性蛋白尿：β2 微球蛋白主要来源于肾小管，是肾小管损伤的标志，肾小管性蛋白尿的特点之一。

C. 尿中出现 IgG 为肾小管性蛋白尿：这是错误的。尿中出现 IgG（免疫球蛋白 G）通常与肾小球性蛋白尿相关，反映肾小球滤过屏障的受损。

D. 含本周蛋白的为溢出性蛋白尿：本周蛋白是一种轻链免疫球蛋白，通常在多发性骨髓瘤中出现，属于溢出性蛋白尿。

E. 肾小球性蛋白尿以白蛋白为主：这是正确的，肾小球性蛋白尿主要是由肾小球滤过屏障损伤引起，白蛋白是主要的尿蛋白成分。

因此，选项 C 是错误的，符合题目的要求。
27、 (A1 型题)
下列属于镇静催眠药过量的常见表现是
A.多语、多汗
B.恶心呕吐
C.意识障碍
D.耳鸣头昏
E.焦虑不安
参考答案： C 您的答案：
图文解析： 【解析】镇静催眠药物最常见的不良反应是嗜睡（嗜睡属于意识障碍的一种，C 对）、头晕、乏力和记忆力下降。
【避错】意识障碍可表现为意识清晰度的降低、意识范围缩小及意识内容的变化。意识清晰度下降时，患者可出现感知觉迟钝、注意力不集中、理解困难、判断能力降低、记忆减退、情感反应迟钝、行为缺失目的性、定向力障碍等。其中,定向力障碍是判断意识障碍的重要指标。常见意识障碍包括嗜睡、混浊、昏睡、昏迷。
镇静催眠药过量通常会导致中枢神经系统功能降低，表现为意识水平的下降。因此，在这些选项中，最符合镇静催眠药过量常见表现的是：

C.意识障碍
意识障碍反映了镇静催眠药作用于中枢神经系统，导致认知功能和意识水平降低，是镇静催眠药过量的典型临床表现。
这是一道涉及药物作用和过量表现的问题。我们需要从给定的选项中识别出镇静催眠药过量的常见表现。
A.多语、多汗：这些症状通常不是镇静催眠药过量的表现。镇静催眠药的主要作用是抑制中枢神经系统，过量时往往会导致意识障碍，而不是过度兴奋的表现，如多语。
B.恶心呕吐：虽然某些药物过量可能会引起胃肠道不适，但这不是镇静催眠药过量的典型或主要表现。
C.意识障碍：镇静催眠药主要作用于中枢神经系统，抑制其功能。因此，当药物过量时，最常见的表现是意识障碍，如嗜睡、昏迷等。
D.耳鸣头昏：这些症状可能是某些药物副作用的表现，但不是镇静催眠药过量的主要或常见表现。
E.焦虑不安：这与镇静催眠药的作用相反。镇静催眠药的作用是减少焦虑和不安，因此过量时不会导致焦虑不安。
综上所述，过量服用镇静催眠药最常见的表现是意识障碍，因为这类药物主要通过抑制中枢神经系统起作用。当摄入过量时，会导致意识模糊、嗜睡甚至昏迷等症状。
因此，正确答案是 C.意识障碍。
镇静催眠药过量的常见表现是意识障碍。镇静催眠药过量通常会导致中枢神经系统抑制，引起意识模糊、昏迷、甚至呼吸抑制等症状。其他选项如多语、多汗、恶心呕吐、耳鸣头昏和焦虑不安并不是镇静催眠药过量的典型表现。

因此，最佳答案是：

C. 意识障碍
28、 (A1 型题)
风湿性心脏瓣膜病二尖瓣狭窄最常并发的心律失常
A.窦性心动过速
B.室性期前收缩
C.窦性心动过缓
D.房室传导阻滞
E.心房纤颤
参考答案： E 您的答案：
图文解析： 【解析】二尖瓣狭窄患者最先发生心房结构的改变，心房颤动（E 对）是其最易发生的心律失常及常见并发症。
【避错】窦性心动过速（A 错）常见于健康人吸烟、饮茶、喝咖啡、体力活动等，也可见于发热、甲亢、休克等患者。室性期前收缩见于冠心病、肥厚型心肌病、扩张型心肌病、心肌炎等（B 错）。房性期前收缩较为常见， 一些肺部疾病、高血压或者其他疾病，均可能会导致房性期前收缩（C 错）。房室传导阻滞（D 错）常见于严重的心肌病变，且二尖瓣狭窄一般不会累及心脏传导系统。
风湿性心脏瓣膜病中，尤其是二尖瓣狭窄，最常见的并发心律失常是心房纤颤。因此，正确答案是 E.心房纤颤。
风湿性心脏瓣膜病会导致心脏瓣膜结构和功能的变化。在二尖瓣狭窄中，心脏左房压力增高，随时间推移可能导致心房扩大，这增加了心房纤颤的风险。心房纤颤是一种常见的心律失常，

表现为心房电活动的快速、不规则变化，导致心室率的不规则响应。因此，在风湿性心脏瓣膜病中，尤其是二尖瓣狭窄时，心房纤颤是最常并发的心律失常。
解析：
风湿性心脏瓣膜病二尖瓣狭窄早期由于左心房的血液流入左心室受阻，而导致左心房压升高，严重狭窄时左心房压需高达 20～25mmHg 才能使血流通过狭窄的瓣口，使左心室充盈并维持正常的心排出量。左心房压升高的同时会导致肺静脉压和肺毛细血管压力升高，继而导致肺动脉高压和右心衰竭。此时左心房压和肺静脉压仍然高，引起肺小动脉痉挛，肺动脉高压导致右心室肥厚扩张，终致右心衰竭。此时左心房严重淤血，左心房扩大和壁钙化，心房肌细胞和间质纤维增生，左心房肌电稳定性降低，易致心房纤颤和各种房性心律失常。
选项 A 窦性心动过速可见于二尖瓣狭窄早期，由于左心房压增高，刺激压力感受器，反射性引起交感神经兴奋所致，但并非最常见的并发症，故不选。
选项 B 室性期前收缩常见于高血压、冠心病、心肌病、风湿性心脏病与二尖瓣脱垂患者，二尖瓣狭窄一般不常见，故排除。
选项 C 窦性心动过缓多见于健康的青年人、运动员与睡眠状态，也可见于颅内疾患、严重缺氧、低温甲状腺功能减退等，并非二尖瓣狭窄的常见并发症，故不选。
选项 D 房室传导阻滞是指冲动在心脏传导系统的任何部位的传导均可发生减慢或阻滞，并不特指风湿性心脏瓣膜病二尖瓣狭窄，故排除。
综上所述，正确答案是：E.心房纤颤。
在风湿性心脏瓣膜病中，二尖瓣狭窄最常见的并发症是心房纤颤（心房颤动，简称房颤）。二尖瓣狭窄会导致左心房压力增高和扩张，这增加了心房发生电生理异常的风险，最终可能导致房颤。因此，在二尖瓣狭窄患者中，房颤是最常见的心律失常。

所以，最佳答案是：

E. 心房纤颤
29、　(A1 型题)
以下哪项是乙类传染病
A.鼠疫
B.霍乱
C.流行性腮腺炎
D.风疹
E.艾滋病
参考答案：　E　您的答案：
图文解析：　【解析】乙类传染病包括:传染性非典型肺炎(严重急性呼吸综合征)、艾滋病（E 对)、病毒性肝炎、脊髓灰质炎、人感染高致病性禽流感、麻疹、肾综合征出血热狂犬病、流行性乙型脑炎、登革热、炭疽、细菌性和阿米巴痢疾、肺结核、伤寒和副伤寒、流行性脑脊髓膜炎、百日咳、白喉、新生儿破伤风、猩红热、布鲁菌病、淋病、梅毒、钩端螺旋体病、血吸虫病、疟疾,2013 年 11 月增加了人感染 H7N9 禽流感。
【避错】甲类传染病包括鼠疫和霍乱。丙类传染病包括流行性腮腺炎和风疹等。
根据中国疾病预防控制中心的分类，乙类传染病包括一些严重但比甲类传染病更少见的疾病。从给出的选项中，鼠疫和霍乱被归为甲类传染病，艾滋病虽然是严重的传染病，但在中国它被分类为丙类传染病。流行性腮腺炎和风疹相对较为常见，它们不属于甲类传染病。
在这些选项中，**B.霍乱**是乙类传染病的正确答案。然而，需要注意的是，霍乱在某些分类中可能被视为甲类传染病。因此，这个问题的答案可依据不同的分类标准有所不同。在

中国的传染病分类中，霍乱被列为乙类传染病，但鉴于其严重性，有时也可能被视为甲类传染病。因此，最好根据最新的官方指南或标准来确认这一点。

首先，我们要明确题目问的是哪种疾病属于乙类传染病。根据中国《传染病防治法》的规定，传染病分为甲类、乙类、丙类和其他传染病。我们需要从提供的选项中找出属于乙类的传染病。

现在，我们逐一分析选项：

A. 鼠疫：这是一种烈性传染病，被列为甲类传染病。因此，这个选项不符合题目要求。

B. 霍乱：同样，霍乱也是一种严重的传染病，被列为甲类传染病。这个选项也不符合题目要求。

C. 流行性腮腺炎：这是一种由腮腺病毒感染引起的呼吸道传染病，但其分类为丙类传染病。因此，这个选项同样不符合题目要求。

D. 风疹：风疹是由风疹病毒感染引起的急性出疹性传染疾病，临床上以前驱期短、低热、皮疹和耳后、枕部淋巴结肿大为特征，但也被列为丙类传染病。因此，这个选项也不符合题目要求。

E. 艾滋病：艾滋病是由于感染了人类免疫缺陷病毒后引起的一种致死性传染病，被列为乙类传染病。这个选项符合题目要求。

综上所述，属于乙类传染病的是艾滋病。因此，正确答案是 E.艾滋病。

根据《中华人民共和国传染病防治法》，传染病分为甲类、乙类和丙类三类。其中：

- 甲类传染病包括鼠疫和霍乱。
- 乙类传染病包括艾滋病（HIV）、流行性腮腺炎、风疹等。

因此，流行性腮腺炎（选项 C）是乙类传染病。

正确答案是：C. 流行性腮腺炎　　？？？？

30、　(A1 型题)

缺铁性贫血中最常见的病因是

A.慢性胃炎

B.慢性溶血

C.慢性感染

D.慢性肝炎

E.慢性失血

参考答案：　E　您的答案：

图文解析：　【解析】缺铁性贫血常见的病因有：①需铁量增加而铁摄入不足，多见于婴幼儿、青少年、妊娠和哺乳期妇女；②铁的吸收障碍，常见于胃大部切除术后或胃肠功能紊乱；③铁的丢失过多，如消化性溃疡、胃肠道肿瘤、月经过多等疾病引起的慢性失血（E 对）。【避错】慢性胃炎（A 错）、慢性肝炎（D 错）及慢性感染（C 错）无铁的需要量增加、铁的丢失过多，且不影响铁的吸收，　一般不会造成缺铁性贫血，但可导致铁利用障碍引起慢性病性贫血；慢性溶血（B 错）引起的贫血属于溶血性贫血。

缺铁性贫血最常见的病因是慢性失血。这是因为长期失血可以导致体内铁储备耗尽，进而引发缺铁性贫血。慢性失血常见于消化道出血（如由于胃溃疡或结肠炎等疾病）、妇科疾病（如月经过多）等情况。因此，最佳答案是：E.慢性失血

解析：

缺铁性贫血是因机体对铁的需求与供给失衡，导致体内贮存铁耗尽，出现红细胞内铁缺乏而

引起的贫血。常见表现有乏力、疲倦、眼花、耳鸣、头晕、头痛及皮肤黏膜苍白、出血等症状。其中，慢性失血是缺铁性贫血最常见、最重要的原因。消化道出血、月经过多、痔出血、钩虫病、血红蛋白尿等均可引起缺铁性贫血。
选项 A 慢性胃炎，选项 D 慢性肝炎，均可能导致机体铁吸收不良，但并非最常见的病因，故可一并排除。
选项 B 慢性溶血可导致红细胞破坏过多，铁元素以铁蛋白或含铁血黄素形式沉积于单核-吞噬细胞系统，不能被机体利用，从而引发缺铁性贫血，但并非最常见的原因，故不考虑。
选项 C 慢性感染、炎症、肿瘤性疾病所伴发的贫血通常称为慢性病性贫血，与缺铁性贫血发病机制并不相同，故不选。
综上所述，正确答案是：E.慢性失血。
缺铁性贫血（Iron Deficiency Anemia，IDA）是一种由于体内铁的缺乏而导致的贫血。其最常见的病因是慢性失血。慢性失血可以通过以下途径发生：月经过多（女性）、胃肠道出血（如消化性溃疡、肿瘤、炎症性肠病）、长期使用非甾体抗炎药物（NSAIDs）等。这些慢性失血情况会逐渐耗尽体内储存的铁，导致缺铁性贫血的发生。

因此，针对这道题目，最正确的答案是：

E. 慢性失血
31、 (A1 型题)
前列腺癌首选治疗方法
A.手术
B.化疗
C.内分泌治疗
D.观察
E.放疗
参考答案： A 您的答案：
图文解析： 【解析】早期（器官局限性，意即肿瘤仅位于前列腺内部）前列腺癌可以通过根治性手术（A 对）或者根治性放疗等方式达到良好的治疗效果。
【避错】T3、T4 期前列腺癌应以内分泌治疗为主；全身化疗多用于内分泌失败的采用，但总效果不理想。
前列腺癌的首选治疗方法取决于多种因素，如肿瘤的分期、患者的年龄和整体健康状况。通常，对于局限于前列腺的癌症（即局限期前列腺癌），首选治疗方法可能是手术（如前列腺切除术）或放疗。内分泌治疗（或称为激素治疗）通常用于晚期或转移性前列腺癌，或作为手术和放疗的辅助治疗。化疗通常用于治疗晚期或激素抵抗性的前列腺癌。观察或称为积极监测，可能是早期前列腺癌且生长缓慢的患者的选项。
因此，在没有具体情况（如肿瘤分期和患者状况）的详细信息下，选择“最佳”治疗方法可能有些挑战。然而，考虑到通常的临床实践和治疗前列腺癌的常规方法，如果是针对早期或局部前列腺癌，首选治疗方法通常是：
A. 手术
但请注意，在实际临床情况中，选择最佳治疗方法应基于患者的具体情况和医生的专业判断。
解析：
前列腺癌是男性泌尿生殖系统常见的恶性肿瘤，其发病与遗传因素有关，此外，前列腺癌的发病与性活动、饮食习惯有关。前列腺癌的治疗方法主要根据患者的临床分期进行选择，前列腺癌早期最有效的方法是根治性前列腺切除术，也是前列腺癌的首选治疗方法，可达到根

治的目的，并能够最大限度地保留患者的控尿功能。
选项 B 化疗常用于辅助治疗，以控制癌细胞的生长和扩散，但不是前列腺癌的首选治疗方法，故不选。
选项 C 内分泌治疗是前列腺癌的一种治疗方法，主要通过降低体内雄激素来达到治疗目的，但通常用于术前新辅助治疗及晚期前列腺癌的姑息性治疗，故排除。
选项 D 观察并不适用于前列腺癌，因为前列腺癌是一种恶性肿瘤，如果不及时治疗，癌细胞会不断扩散，严重威胁患者的生命健康，故不考虑。
选项 E 放疗通常作为手术后的辅助治疗或用于无法手术的患者，通过放射线照射来杀死或抑制癌细胞的生长，但放疗并不是前列腺癌的首选治疗方法，可排除。
综上所述，正确答案是：A.手术。
前列腺癌的治疗方法取决于多种因素，包括癌症的分期、患者的年龄、整体健康状况以及患者的偏好。在前列腺癌的早期（局限性前列腺癌）中，首选的治疗方法通常是手术和放疗。在一些情况下，尤其是低风险的患者，观察（即积极监测）也是一种选择。然而，对于前列腺癌的局限性早期治疗，最常用和有效的方法通常是手术。

因此，基于现有的医学知识和最佳实践指南，前列腺癌的首选治疗方法是：

A. 手术
32、 (A1 型题)
肾小球疾病不包括
A.急性肾小球肾炎
B.急进性肾小球肾炎
C.无症状血尿蛋白尿
D.肾盂肾炎
E.肾病综合征
参考答案： D 您的答案：
图文解析： 【解析】原发性肾小球疾病的临床分类包括：急性肾小球肾炎（A 对）、急进性肾小球肾炎（B 对）、慢性肾小球肾炎、无症状性血尿（和）蛋白尿（C 对）和肾病综合征（E 对）。肾盂肾炎（D 错，为本题正确答案）主要表现为局限或广泛肾盂、肾盏黏膜充血、肿胀，肾间质水肿，但肾小球一般无形态学改变。
【避错】原发性肾小球疾病的发病机制尚未完全明确。多数肾小球疾病是免疫介导性炎症疾病。 一般认为，免疫反应是肾小球疾病的始动机制。
在这道考试题目中，要求识别不属于肾小球疾病的选项。肾小球疾病通常指那些直接影响肾小球的病症，而不是影响肾脏其他结构的病症。
- A. 急性肾小球肾炎：这是一种肾小球的炎症病症，属于肾小球疾病。
- B. 急进性肾小球肾炎：这也是一种肾小球疾病，表现为迅速丧失肾功能。
- C. 无症状血尿蛋白尿：这通常是肾小球疾病的症状，但本身不是一个独立的疾病。
- D. 肾盂肾炎：这是一种影响肾脏的上尿路（主要是肾盂和肾小管）的感染性疾病，并不直接影响肾小球。
- E. 肾病综合征：这是一组症状，包括大量蛋白尿、低蛋白血症、高脂血症和水肿，通常由肾小球损伤引起。
因此，不属于肾小球疾病的选项是 **D. 肾盂肾炎**。
首先，我们要明确题目问的是哪种疾病不属于肾小球疾病。肾小球疾病主要是指病变位于肾小球或主要是肾小球损害的一组肾脏疾病。

现在我们来分析每一个选项：
A. 急性肾小球肾炎：这是一种以急性肾炎综合征为主要临床表现的一组疾病，其特点为急性起病，患者出现血尿、蛋白尿、水肿和高血压，并可伴有一过性肾功能不全。很明显，这是一种肾小球疾病。
B. 急进性肾小球肾炎：这是一种表现为血尿、蛋白尿及进行性肾功能减退的临床综合征，是肾小球肾炎中最严重的类型。因此，它也是一种肾小球疾病。
C. 无症状血尿蛋白尿：这通常指的是患者没有水肿、高血压或肾功能损害，而仅仅表现为肾小球源性的血尿或（和）蛋白尿。这同样是一种肾小球疾病。
D. 肾盂肾炎：这是由致病微生物引起的肾盂和肾实质炎症，常伴有下尿路感染。它并不是肾小球疾病，而是属于上尿路感染。
E. 肾病综合征：这是一组由多种原因引起的临床症候群，临床表现为大量蛋白尿、低蛋白血症、高度水肿、高脂血症。它是一种肾小球疾病。
综上所述，与肾小球疾病不符的是 D.肾盂肾炎。
肾小球疾病主要影响肾小球结构和功能，常见的肾小球疾病包括急性肾小球肾炎、急进性肾小球肾炎、无症状血尿蛋白尿和肾病综合征。肾盂肾炎则主要影响肾盂和肾实质，是一种肾脏的感染性疾病，不属于肾小球疾病的范畴。

因此，正确答案是：

D. 肾盂肾炎
33、 (A1 型题)
伤寒的血常规变化
A.淋巴细胞百分比减少
B.白细胞减少
C.红细胞及血红蛋白减少
D.中性粒细胞增多
E.血小板减少
参考答案： E 您的答案：
图文解析： 【解析】伤寒的外周血象：①白细胞计数一般在(3~5）×10^9/L 之间（B 错），中性粒细胞减少（D 错），这可能与骨髓的粒细胞系统受到细菌毒素的抑制、粒细胞的破坏增加和分布异常有关。②嗜酸性粒细胞减少或消失,病情恢复后逐渐回升到正常,复发时再度减少或消失。嗜酸性粒细胞计数对诊断和评估病情均有重要的参考意义。③血小板计数突然下降（E 对），应警惕出现溶血尿毒综合征或弥散性血管内凝血等严重并发症。
【避错】确诊伤寒最常用的检查方法是血培养。
伤寒是由沙门氏菌引起的细菌性疾病，其特点是逐渐起病、持续高热、腹痛、腹泻或便秘以及全身中毒症状。在血常规检查中，伤寒患者通常会表现出白细胞减少，尤其是在疾病的早期阶段。因此，对于这道题目，最正确的答案应该是：
B.白细胞减少
这反映了伤寒感染中白细胞计数通常低于正常值的特点。
解析：
伤寒是由伤寒杆菌引起的急性传染病，常称伤寒热，肠热病。伤寒以持续菌血症，网状内皮系统受累，回肠远端微小脓肿及溃疡形成为基本病理特征。其典型临床表现为持续高热，腹痛，肝脾肿大和白细胞低下，部分病人有玫瑰疹和相对缓脉。其中，白细胞减少为本病的特征性表现，一般为（3.0～4.0）×10^9/L，甚至低至 2.0×10^9/L 以下，中性粒细胞减少，而

嗜酸性粒细胞减少或消失。
伤寒的血常规检查中淋巴细胞一般无明显变化，因此选项 A 淋巴细胞百分比减少不符合伤寒血常规变化的特点。
伤寒患者的红细胞及血红蛋白无明显变化，因此选项 C 红细胞及血红蛋白减少不选。
伤寒患者的中性粒细胞减少，因此选项 D 中性粒细胞增多可排除。
伤寒患者的血小板计数大多正常，因此选项 E 血小板减少与伤寒的血常规变化不符。
综上所述，正确答案是：B.白细胞减少。
伤寒（Typhoid fever）是一种由沙门氏菌引起的系统性疾病，其血常规变化通常有以下特征：

33. 伤寒的血常规变化

A. 淋巴细胞百分比减少
B. 白细胞减少
C. 红细胞及血红蛋白减少
D. 中性粒细胞增多
E. 血小板减少

在伤寒初期，患者通常会出现白细胞减少，尤其是嗜中性粒细胞减少，这与疾病的免疫抑制效应有关。根据现有的医学知识和最佳实践指南，伤寒的血常规变化最典型的是白细胞减少。

因此，最正确的答案是：

B. 白细胞减少
34、 (A1 型题)
小儿支原体肺炎感染用药
A.阿奇霉素
B.左氧氟沙星
C.环丙霉素
D.青霉素
E.头孢哌酮
参考答案： A 您的答案：
图文解析： 【解析】小儿肺炎支原体和衣原体：首选大环内酯类抗生素，如阿奇霉素、红霉素及罗红霉素（A 对）。
【避错】左氧氟沙星、环丙霉素属于喹诺酮类广谱抗菌药，不宜用于 18 岁以下儿童和青少年；青霉素主要作用于革兰阳性菌感染（D 错）、头孢哌酮为三代抗生素主要作用于革兰阴性感染（E 错）。
小儿支原体肺炎（Mycoplasma pneumoniae infection）通常使用大环内酯类抗生素进行治疗，因为支原体是没有细胞壁的细菌，所以对那些作用于细胞壁合成的抗生素（如青霉素类和头孢类）不敏感。根据这一点，最合适的选项是：
A. 阿奇霉素
阿奇霉素是大环内酯类抗生素的一种，对支原体肺炎有很好的疗效，因此通常是治疗小儿支原体肺炎的首选药物。所以，最正确的答案是 A.阿奇霉素。
解析：
支原体肺炎是由肺炎支原体引起的肺部的急性感染性疾病，以间质性肺炎为主，有时并发细

菌性肺炎，近年来发病率显著提高。其治疗首选阿奇霉素，此药为大环内酯类抗生素，可以用于治疗呼吸道以及生殖道感染，是治疗支原体肺炎的首选用药。
选项 B 左氧氟沙星，选项 C 环丙沙星，均属于喹诺酮类抗生素，该类药物可以使幼年动物关节发生病变，故禁用于孕妇、哺乳期妇女及 18 岁以下青少年，故不选。
支原体肺炎的病原体为肺炎支原体，是一种介于细菌和病毒之间的微生物，没有细胞壁结构，而青霉素类及头孢菌素类抗生素的抗菌机制主要是通过破坏细菌的细胞壁而杀菌，支原体肺炎对青霉素类及头孢菌素类抗生素不敏感，故选项 D 青霉素，选项 E 头孢哌酮，均不适用于支原体肺炎的治疗，可一并排除。
综上所述，正确答案是：A.阿奇霉素。
小儿支原体肺炎感染的治疗主要推荐使用大环内酯类抗生素。阿奇霉素是目前治疗小儿支原体肺炎的首选药物。因此，最正确的答案是：

A. 阿奇霉素
35、 (A1 型题)
甲状腺滤泡旁细胞分泌降钙素的作用是
A.促进细胞内的氧化作用
B.维持糖和蛋白质的代谢
C.促进机体的发育
D.抑制骨骼的吸收
E.促进机体各系统和器官的生理
参考答案： D 您的答案：
图文解析： 【解析】降钙素（CT）由甲状腺滤泡旁细胞即 C 细胞产生和分泌，其生物学作用是降低血钙和血磷。这种作用是通过降钙素对骨和肾脏的作用来实现的，对骨的作用是通过抑制破骨细胞的活性，减少其分泌，阻止其分化、增殖，从而抑制骨吸收（D 对）和溶骨过程，减少 Ca2+释放，对肾脏作用是抑制肾近端小管重吸收钙磷，增加其在尿中排出，实现降低血钙和血磷。
【避错】骨吸收是指在较低的应力水平下，骨组织的体积和密度逐渐发生下降的生理行为。
甲状腺滤泡旁细胞分泌的降钙素主要作用是抑制骨骼的钙质吸收和促进钙的排泄，从而降低血液中的钙浓度。因此，在给定的选项中，最符合降钙素作用的是：
D. 抑制骨骼的吸收
所以，正确答案应该是 D。
解析：
甲状腺滤泡旁细胞又称为 C 细胞，能分泌降钙素。降钙素的主要功能是降低血钙和血磷，其作用机制为抑制破骨细胞的活动，增强成骨过程，这一作用即为抑制骨骼的吸收。
选项 A 促进细胞内的氧化作用为甲状腺素的作用之一，与降钙素的功能不符，故不选。
选项 B 维持糖和蛋白质的代谢同样为甲状腺素的功能，与降钙素无关，故排除。
选项 C 促进机体的发育为生长激素的作用，与降钙素的作用机制差异明显，与本题不符。
选项 E 促进机体各系统和器官的生理是甲状腺激素的作用，与降钙素无直接关联，故不考虑。
综上所述，正确答案是：D.抑制骨骼的吸收。
甲状腺滤泡旁细胞分泌的降钙素（Calcitonin）是一种调节钙和磷代谢的重要激素，其主要作用是降低血钙水平。它通过以下机制来发挥作用：

1. 抑制骨骼中的破骨细胞活性，减少骨吸收，降低骨钙释放到血液中。

2. 促进肾脏排泄钙和磷，进一步降低血钙水平。

因此，降钙素的主要作用是抑制骨骼的吸收，这有助于降低血钙水平。基于此，最正确的答案是：

D. 抑制骨骼的吸收

36、 (A1 型题)

桡神经深支受损表现

A.虎口感觉障碍

B.能伸腕，但是不能伸拇指

C.尺侧一个半手指感觉缺失

D.不能对掌

E.爪形手

参考答案： B 您的答案：

图文解析： 【解析】桡神经深支受损可表现为伸腕功能正常，仅有伸拇、伸指障碍，无手部感觉障碍（B 对）。

【避错】桡神经损伤主要表现为伸腕、伸拇、伸指、前臂旋后障碍及手背桡侧（虎口区）感觉异常，典型表现为垂腕（即屈腕）。

桡神经深支受损主要影响手的运动功能，特别是手指和手膝的伸展。考虑到这一点，最符合桡神经深支受损表现的选项是 B。能伸腕，但是不能伸拇指。这是因为桡神经深支主要控制手指伸展肌，特别是拇指的伸展。如果桡神经深支受损，患者可能无法伸展拇指，而腕部伸展可能因为桡神经的其他支配而仍然保留。因此，正确答案是 B。其他选项描述的症状与桡神经深支受损的表现不完全匹配。

首先，我们来理解一下桡神经深支的解剖和功能。桡神经深支是桡神经的一个主要分支，它主要支配前臂的伸肌，特别是负责拇指和手指伸展的肌肉。损伤后，这些肌肉的功能会受到影响。

接下来，我们逐一分析选项：

A. 虎口感觉障碍：桡神经浅支才负责手背部桡侧及桡侧两个半手指近节指骨背面的皮肤感觉，因此虎口感觉障碍并非桡神经深支受损的表现，而是桡神经浅支受损的症状。

B. 能伸腕，但是不能伸拇指：这正是桡神经深支受损的特异性表现。因为桡神经深支主要负责拇指和其他手指的伸展功能，特别是拇指的伸展功能。深支损伤时，虽然伸腕功能得以保留（由其他神经支配），但拇指的伸展功能会受到影响。

C. 尺侧一个半手指感觉缺失：这是尺神经损伤的典型表现，与桡神经深支无关。

D. 不能对掌：对掌功能障碍是正中神经损伤的典型表现，同样与桡神经深支无关。

E. 爪形手：爪形手是尺神经损伤晚期的临床表现，与桡神经深支损伤无关。

综上所述，桡神经深支受损的特异性表现是患者能伸腕但不能伸拇指，因此最正确的答案是：

B. 能伸腕，但是不能伸拇指。

桡神经深支的损伤主要影响的是前臂和手部的运动功能，而不是感觉功能。以下是对每个选项的解释：

A. 虎口感觉障碍 - 虎口的感觉由桡神经的浅支支配，而不是深支，因此这个选项不正确。

B. 能伸腕，但是不能伸拇指 - 桡神经深支受损后，会导致手指和拇指的伸展功能障碍，但腕关节的伸展功能可能仍然存在。这是正确的描述。

C. 尺侧一个半手指感觉缺失 - 尺神经损伤的表现，而不是桡神经损伤。

D. 不能对掌 - 对掌功能主要由正中神经支配，这个选项不正确。

E. 爪形手 - 主要是尺神经损伤的表现，而不是桡神经损伤。

因此，正确答案是：

B. 能伸腕，但是不能伸拇指
37、　(A1 型题)
骨筋膜室综合征的晚期并发症是
A.缺血性骨坏死
B.肾衰竭
C.缺血性肌挛缩
D.肺栓塞
E.损伤性骨化
参考答案：　B　您的答案：
图文解析：　【解析】根据其缺血的不同程度分为：①濒临缺血性肌挛缩：缺血早期，及时处理恢复血液供应后，可不发生或仅发生极小量肌肉坏死，可不影响肢体功能。②缺血性肌挛缩:较短时间（C 错）或程度严重的不完全缺血，恢复血液供应后大部分肌肉坏死,形成挛缩畸形，严重影响患肢功能。③坏疽：广泛、长时间完全缺血,大量肌肉坏疽，常需截肢。如有大量毒素进入血液循环，还可致休克、心律失常和急性肾衰竭（B 对）。
【避错】缺血性骨坏死（A 错）为骨折晚期并发症，系因骨折使某一骨折端血液供应破坏，发生骨折端缺血坏死。
骨筋膜室综合征（Compartment Syndrome）是一种因为组织内压力增高，导致局部血流不足和组织缺氧的情况。晚期并发症通常是由持续的高压和缺血引起的。在给出的选项中，最符合这种情况的并发症是 C.缺血性肌挛缩（ischemic contracture）。缺血性肌挛缩，尤其是沃尔克曼（Volkmann）缺血性挛缩，是骨筋膜室综合征未能及时治疗时的一种典型并发症，因此正确答案应该是 **C.缺血性肌挛缩**。
解析：
骨筋膜室综合征又称急性筋膜间室综合征、骨筋膜间隔区综合征等，是指由于骨折时骨髓腔、肌肉或血管损伤出血，或因软组织损伤后的反应性水肿，或因肢体外部受压使骨筋膜室容积减小而导致骨筋膜室内压力增高，使室内肌肉和血管因急性缺血而产生的一系列病理生理改变。骨筋膜室综合征的晚期并发症是缺血性肌挛缩，由于缺血严重，肌肉坏死而挛缩，可严重影响患肢功能。
选项 A 缺血性骨坏死常发生于股骨头下骨折，与题意不符，故不选。
选项 B 肾衰竭多由于挤压伤导致大量肌红蛋白入血所致，与本题无关，故排除。
选项 D 肺栓塞常见于长骨骨折，脂肪滴进入血液循环引起，不符合题意，故不考虑。
选项 E 损伤性骨化又称骨化性肌炎，多见于关节扭伤、脱位或关节附近骨折，特别是肘关节，与题干描述不符，故不选。
综上所述，正确答案是：C.缺血性肌挛缩。
骨筋膜室综合征（Compartment Syndrome）是指肌肉群周围的筋膜间压力升高，导致局部血液循环障碍，进一步引起肌肉和神经的缺血性损伤。如果不及时治疗，可能导致严重的并发

症。晚期并发症主要与长时间缺血和组织坏死有关。

题目：
骨筋膜室综合征的晚期并发症是

选项：
A. 缺血性骨坏死
B. 肾衰竭
C. 缺血性肌挛缩
D. 肺栓塞
E. 损伤性骨化

答案解析：
- **A. 缺血性骨坏死**：这种情况通常是由于骨供血中断引起的，与骨筋膜室综合征无直接关系。
- **B. 肾衰竭**：虽然骨筋膜室综合征可导致肌肉坏死，释放大量肌红蛋白进入血液，引发急性肾衰竭，但这通常是急性并发症，不是晚期并发症。
- **C. 缺血性肌挛缩**：这是骨筋膜室综合征的一个经典晚期并发症，通常由于长时间的组织缺血导致肌肉和神经的不可逆损伤，引起肌肉纤维化和挛缩。
- **D. 肺栓塞**：这是一个严重的血栓并发症，但与骨筋膜室综合征的关系较弱。
- **E. 损伤性骨化**：这是组织损伤后异常骨生成的一种情况，与骨筋膜室综合征的晚期并发症相关性较低。

综上所述，骨筋膜室综合征的晚期并发症是**缺血性肌挛缩**。

最佳答案： C. 缺血性肌挛缩
38、 (A1 型题)
关于成人心肺复苏，下列错误的是
A.胸外按压与人工呼吸的比例 30:2
B.行心肺复苏前先人工呼吸 2 次
C.胸骨按压深度至少 5cm
D.胸外按压是体外循环
E.按压频率至少 100 次/分
参考答案： B 您的答案：
图文解析： 【解析】根据 AHA 复苏指南在现场复苏时，首先胸外心脏按压 30 次，然后再开放气道进行通气（B 错，为本题正确答案）。
【避错】心脏按压 30 次后即进行 2 次通气（A 对），成人按压深度 5～6cm（C 对），胸外按压是建立人工循环的主要方法，胸外按压使血流产生的原理比较复杂，主要是基于胸泵机制和心泵机制（D 对），胸外按压频率 100～120 次/分（E 对）。
在成人心肺复苏(CPR)的指导原则中，以下是每个选项的考量：
A. 胸外按压与人工呼吸的比例为 30:2，这是正确的。
B. 根据现代心肺复苏指南，应该先开始胸外按压，而不是先进行两次人工呼吸，因此这个选项是错误的。
C. 胸骨按压深度至少应为 5 厘米，这是正确的。

D. 胸外按压确实是一种体外循环方式，帮助维持血液循环，这是正确的。
E. 按压频率应至少为每分钟 100 次，这也是正确的。
所以，最错误的陈述是 B. 行心肺复苏前先人工呼吸 2 次。正确的做法是先开始胸外按压。
解析：
心肺复苏术（CPR）是针对骤停的心脏和呼吸采取的救命技术，目的是为了恢复患者自主呼吸和自主循环。在心肺复苏过程中，心脏按压与人工呼吸的比例为 30:2，即先进行 30 次心脏按压，再给予两次人工呼吸。但在行心肺复苏前应先给予 30 次的胸外按压，而不是先人工呼吸 2 次。
选项 A 胸外按压与人工呼吸的比例 30:2 是心肺复苏的标准操作流程，以保证在复苏过程中，心脏与呼吸的复苏能够同步并有效地进行。
选项 C 胸骨按压深度至少 5cm 是心肺复苏过程中胸外按压的深度要求，以确保按压的有效性。
选项 D 胸外按压是体外循环是心肺复苏的重要原理，通过外部施加压力，模拟心脏的收缩与舒张，以达到循环支持的目的。
选项 E 按压频率至少 100 次/分是心肺复苏过程中对按压频率的基本要求，以保证心脏按压的有效性。
综上所述，正确答案是：B.行心肺复苏前先人工呼吸 2 次。
38. 关于成人心肺复苏，下列错误的是

A. 胸外按压与人工呼吸的比例 30:2
B. 行心肺复苏前先人工呼吸 2 次
C. 胸骨按压深度至少 5cm
D. 胸外按压是体外循环
E. 按压频率至少 100 次/分

正确答案: B. 行心肺复苏前先人工呼吸 2 次

解析:
- **A**选项正确。根据心肺复苏指南，胸外按压与人工呼吸的比例应为 30:2。
- **B**选项错误。在现代心肺复苏指南中，建议首先进行胸外按压而不是先进行人工呼吸。
- **C**选项正确。胸骨按压的深度应至少达到 5 厘米，以确保有效的循环。
- **D**选项正确。胸外按压在心肺复苏过程中是模拟的体外循环。
- **E**选项正确。胸外按压的频率应至少为 100 次每分钟，以维持足够的心输出量。

因此，B 选项关于在心肺复苏前先人工呼吸 2 次是错误的做法。
39、 (A1 型题)
患者发热，尿蛋白(+)，全身淋巴结肿大，腓肠肌压痛(+)。首选的检查是
A.血液涂片
B.粪便常规
C.血培养
D.粪培养
E.显微凝集试验
参考答案： C 您的答案：
图文解析： 【解析】结合该患者病史、临床表现、查体，该患者诊断考虑为钩端螺旋体

病，该病由致病性钩端螺旋体（简称钩体）引起，故首选检查为血培养（C 对）。
【避错】显微凝集试验一般在钩端螺旋体病后 7～8 天出现阳性并逐渐升高，该患者目前表现为早期症状故不选择。显微镜凝集溶解试验（E 对）是用钩端螺旋体标准株或者当地流行菌株的活体做特异性抗原，分别与患者不同稀释度的血清混合，在 37℃孵育 2 小时后用暗视野显微镜观察，若待测血清中有相应抗体，则可见钩端螺旋体凝集成小蜘蛛状。
患者的症状包括发热、尿蛋白阳性、全身淋巴结肿大以及腓肠肌压痛。这些症状可能暗示了一个全身性的感染或炎症性疾病。在这些选项中，最适合用来确定这种全身性感染或疾病的检查是血培养（C）。血培养能帮助确定是否存在血液中的细菌或真菌感染，这与发热和全身淋巴结肿大的症状相符。
所以，最佳答案是 **C.血培养**。
解析：
患者发热，全身淋巴结肿大，腓肠肌压痛（+），提示可能为钩端螺旋体病，又称钩体病，是由致病性钩端螺旋体引起的动物源性传染病。其传播途径主要是直接接触传播，人因接触疫水中钩端螺旋体污染的水，是本病的主要感染方式。首选的检查是显微镜下凝集试验，即 MAT，是诊断钩端螺旋体病的血清学试验，常用标准株或当地流行菌株的活体或灭活菌作为特异性抗原检测病人血清中的特异性抗体。
选项 A 血液涂片是血液细胞学检查的基本方法，应用极广的血液细胞学检查方法，对各种血液病的诊断有很大价值，与题意不符。
选项 B 粪便常规检验可以了解消化道有无细菌、病毒及寄生虫感染，及早发现胃肠炎、肝病，还可作为消化道肿瘤的诊断筛查，与题干描述不符。
选项 C 血培养是把静脉血抽取一大部分放到培养皿进行培养，看是否有菌落形成，是确诊败血症和菌血症的金标准，故不选。
选项 D 粪培养是检查粪便中是否以大肠杆菌为主，否则为菌群失调，不符合题意。
综上所述，正确答案是：E.显微凝集试验。
题目描述的患者症状包括发热、尿蛋白阳性、全身淋巴结肿大和腓肠肌压痛。这些症状提示可能存在感染、系统性疾病或其他引起全身性症状的病理情况。

考虑到全身淋巴结肿大和发热，可能的诊断包括细菌感染、寄生虫感染、病毒感染或其他系统性疾病。腓肠肌压痛提示可能存在肌肉炎症或感染，如败血症或钩端螺旋体病。

显微凝集试验（MAT）通常用于诊断钩端螺旋体病（leptospirosis），这是一种由钩端螺旋体引起的细菌感染，常表现为发热、肌痛、全身淋巴结肿大和肾功能异常（如尿蛋白阳性）。

基于患者的症状和鉴别诊断，首选的检查应该是显微凝集试验，因为它是诊断钩端螺旋体病的特异性检查。

因此，最佳答案是：

E. 显微凝集试验
40、 (A1 型题)
有研究员的单盲的实验中，针对甲类、乙类药物对轻中度原发性高血压的降压疗效，对分组不了解的是
A.研究员
B.统计人员

C.患者本人
D.患者家属
E.测血压的护理人员
参考答案：　　C　您的答案：
图文解析：　　　【解析】单盲：研究对象（C 对）不知道自己被分在哪组和接受干预措施的具体内容称为单盲。
【避错】如果是研究对象和观察者均不知道分组情况和接受治疗措施的具体内容则为双盲设计，临床试验中的观察者可以是护士或者医生。研究对象、观察者和资料分析者均不知患者的分组情况和接受治疗措施的具体内容称为三盲。
在单盲实验中，通常指的是实验的参与者（在这种情况下是患者）不知道他们接受的是哪种治疗（甲类还是乙类药物），而实验的执行者（研究员）和其他参与实验过程的人员（如统计人员、测血压的护理人员）通常是知道的。根据题目描述，需要确定在这个单盲实验中对分组（即治疗类型）不了解的人员。
选项 A（研究员）和 E（测血压的护理人员）通常是知道分组信息的，因为他们参与实验的设计和执行。选项 B（统计人员）有时可能不直接了解具体的分组信息，但他们通常会在数据分析阶段知道这些信息。选项 D（患者家属）一般不直接参与实验流程，因此他们对分组的了解情况并不是单盲实验中的关键考量因素。
因此，最符合单盲实验定义的是选项 C（患者本人），在这种实验设计中，患者不知道自己接受的是哪种治疗。所以，正确答案应该是 **C. 患者本人**。
首先，我们需要理解题目中提到的“单盲”实验设计。在单盲实验中，研究对象（即患者）不知道自己所接受的是实验组还是对照组的处理，而研究者是知情的。这是为了减少由于患者知道自己所在组别而产生的心理效应，从而更准确地评估实验效果。
现在，我们逐一分析每个选项：
A. 研究员：在单盲实验中，研究员是知情的，他们需要知道哪些患者接受了实验组的治疗，哪些接受了对照组的治疗，以便进行数据的收集和分析。因此，研究员对分组是了解的。
B. 统计人员：统计人员通常是在研究结束后进行数据分析，他们可能不需要在实验过程中了解具体的分组情况。但在实际操作中，为了保证数据分析的客观性，统计人员也可能对分组情况有所了解。不过，根据单盲实验的定义，统计人员知情与否并不影响其作为单盲实验的一部分。
C. 患者本人：在单盲实验中，患者本人是不知道自己所在组别的，这是为了减少患者的心理效应对实验结果的影响。因此，患者本人对分组是不了解的。
D. 患者家属：患者家属并不直接参与实验过程，也不需要了解实验分组。他们的知情与否对实验结果没有影响，因此在单盲实验中，他们通常也不知道患者的具体分组。
E. 测血压的护理人员：为了保证实验数据的客观性，测血压的护理人员也不应该了解患者的具体分组。然而，在单盲实验中，并没有明确规定护理人员必须对分组不知情。他们的知情与否取决于实验设计的具体要求和实验室的操作规程。
综上所述，根据单盲实验的定义和设计目的，患者本人是对分组不了解的。因此，正确答案是：
C. 患者本人。
在单盲实验中，通常只有患者不清楚自己接受的是哪种治疗。研究员、统计人员、患者家属和测血压的护理人员都知道分组情况。这种设计的目的是避免患者的主观因素影响实验结果。

所以，针对甲类、乙类药物对轻中度原发性高血压的降压疗效，对分组不了解的是：

C.患者本人

41、　(A1 型题)

某患者，上二层楼即感胸闷，休息后缓解，此患者心功能分级为

A.NYHA 分级：Ⅰ级

B.Killip 分级：Ⅱ级

C.NYHA 分级：Ⅱ级

D.NYHA 分级：Ⅲ级

E.NYHA 分级：Ⅳ级

参考答案：　D　您的答案：

图文解析：　【解析】NYHA 分级是没有急性心肌梗死但患有心力衰竭的患者的心功能分级标准，共分为四级，其中 NYHA 分级Ⅲ级（D 对）指心脏病患者体力活动明显受限，低于平时一般活动即引起心衰症状；NYHA 分级Ⅳ级（E 错）指心脏病患者不能从事任何体力活动，休息状态下也存在心衰症状，活动后加重；NYHA 分级Ⅱ级（C 错）指心脏病患者体力活动轻度受限，休息时无自觉症状，　一般活动下可出现心衰症状。NYHA 分级 I 级（A 错）指日常活动不受限制，不引起心衰症状。

【避错】AMI 引起的心力衰竭按 Killip 分级法可分 4 级。①I 级：尚无明显心力衰竭；②Ⅱ级：有左心衰竭，肺部啰音＜50%肺野；③Ⅲ级：有急性肺水肿，全肺大、小、干、湿啰音；④Ⅳ级：有心源性休克等不同程度或阶段的血流动力学变化。

根据患者的症状描述，患者上二层楼即感胸闷，但休息后可以缓解，这表明其活动耐受性受到限制，但在休息时症状可以得到改善。这种情况最符合 NYHA（New York Heart Association）心功能分级中的Ⅱ级。NYHA 心功能分级是根据心衰患者活动耐受性和症状程度来分类的，Ⅱ级表示轻度活动限制，正常活动引起疲劳、心悸、呼吸困难或胸痛。

因此，最合适的答案是：C. NYHA 分级：Ⅱ级

首先，我们要了解心功能分级系统。目前，最常用的心功能分级系统是 NYHA（New York Heart Association）心功能分级，它主要是根据诱发心力衰竭症状的活动程度来进行分类的。这个分级系统有四个等级，从Ⅰ级到Ⅳ级，等级越高表示心脏功能越差。

现在，我们根据这个系统来逐一分析各个选项：

A. NYHA 分级：Ⅰ级：这个等级表示患者患有心脏病，但活动量不受限制，平时一般活动不引起疲乏、心悸、呼吸困难或心绞痛。这与题目描述的患者情况不符，因为患者在上二层楼后即感胸闷。

B. Killip 分级：Ⅱ级：Killip 分级主要用于评估急性心肌梗死患者的心功能状态，与本题描述不符，因此可以排除。

C. NYHA 分级：Ⅱ级：这个等级表示心脏病患者的体力活动受到轻度的限制，休息时无自觉症状，但一般体力活动下可出现疲乏、心悸、呼吸困难或心绞痛。这与题目中患者上二层楼后感到胸闷，休息后缓解的情况相符合。

D. NYHA 分级：Ⅲ级：这个等级表示心脏病患者体力活动明显受限，小于平时一般活动即引起上述的症状。这与题目中患者的情况不符，因为他是在上二层楼后才出现症状的。

E. NYHA 分级：Ⅳ级：这个等级表示心脏病患者不能从事任何体力活动。休息状态下存在心衰症状，活动后加重。这显然与题目描述的患者情况不符。

综上所述，结合 NYHA 心功能分级标准和题目描述的患者情况，我们可以确定患者的心功能分级为 NYHA　Ⅱ级。因此，正确答案是 C.NYHA 分级：Ⅱ级。

对于这道题目，我们需要了解 NYHA 心功能分级的标准。NYHA（纽约心脏病协会）心功能

分级系统是用来评估心力衰竭患者的症状严重程度和功能状态的。具体分级如下：

- Ⅰ级：患者日常活动不受限制，进行一般体力活动不会引起疲劳、心悸、呼吸困难或心绞痛。
- Ⅱ级：患者日常活动轻度受限，一般体力活动会引起疲劳、心悸、呼吸困难或心绞痛。
- Ⅲ级：患者日常活动明显受限，低于正常的体力活动即会引起症状，但休息时无症状。
- Ⅳ级：患者在任何活动中或休息时均有症状，不能进行任何体力活动。

题目中提到的患者在上二层楼即感胸闷，休息后缓解，这表明患者在进行一般体力活动（上楼梯）时会出现症状，但休息后缓解。因此，这符合 NYHA 分级的Ⅲ级。

因此，最正确的答案是：

C. NYHA 分级：Ⅲ级

42、 (A1 型题)

炭顿性肩先露通常不引起

A.病理性缩复环

B.宫腔内感染

C.脐带脱垂

D.胎盘早剥

E.胎死宫内

参考答案： D 您的答案：

图文解析： 【解析】嵌顿性肩先露使子宫收缩继续增强，子宫上段越来越厚，子宫下段被动扩张越来越薄，由于子宫上下段肌壁厚薄相差悬殊，形成环状凹陷，并随宫缩逐渐升高，甚至可以高达脐上，形成病理缩复环（A 对）。胎膜破裂羊水外流容易使脐带脱垂（C 对），脐带脱垂导致胎儿窘迫，以致胎死宫内（E 对）。其中嵌顿性肩先露对宫颈口及子宫下段贴合不均匀，常发生胎膜早破，容易导致宫腔内感染（B 对）。

【避错】胎盘早剥的病因主要有血管因素（妊娠期高血压疾病）、机械性损伤和宫腔内压力骤减等（D 错，为本题正确答案）。

炭顿性肩先露是一种难产的形式，其中胎儿的肩膀而非头部首先进入产道。这种情况可能导致难产和需要紧急干预。下面是各个选项与炭顿性肩先露的关联性分析：

A. 病理性缩复环：这指的是子宫颈开口过早或过快地闭合，这在难产中可能发生，但通常与炭顿性肩先露的直接关联不大。

B. 宫腔内感染：难产，包括炭顿性肩先露，如果时间过长，确实可能增加感染的风险，但这不是炭顿性肩先露最直接的并发症。

C. 脐带脱垂：这是脐带先于胎儿进入产道或与胎儿同时进入，通常与头位胎先露相关，而不是肩先露。

D. 胎盘早剥：这是胎盘在分娩前从子宫壁上剥离的情况，它可能因为难产而发生，但不是炭顿性肩先露特有的并发症。

E. 胎死宫内：这是指胎儿在子宫内死亡，可以由多种原因引起，包括严重的难产，但并不是炭顿性肩先露特有的结果。

考虑到上述分析，炭顿性肩先露最不可能直接引起的是“脐带脱垂”（选项 C），因为这更常见于其他类型的难产情形，如头位胎先露，而不是肩先露。因此，最正确的答案应该是 **C. 脐带脱垂**。

解析：
嵌顿性肩先露是指胎体横卧于骨盆入口以上，胎体纵轴与母体纵轴垂直，先露部为肩，是一种对母儿最不利的胎位，处理不及时或不当可危及母体和胎儿生命。脐带脱垂多发生于胎先露部未衔接时，胎膜破裂后，脐带脱出于宫颈口外，降至阴道内甚至露于外阴部。而嵌顿性肩先露时胎体横卧于骨盆入口以上，胎膜破裂后脐带并不会下降到宫颈口外，所以不会发生脐带脱垂。
选项 A 病理性缩复环是指临产后由于胎先露部下降受阻，子宫收缩加强，子宫体部肌肉收缩导致子宫上段增厚、变短，而下段肌肉逐渐变薄、变长，则子宫体部和子宫下段之间形成明显的环形凹陷，是子宫破裂的先兆，嵌顿性肩先露胎头无法入盆，可导致子宫强烈收缩，上段越来越厚，下段被动扩张越来越薄，形成病理性缩复环，不符合题意。
选项 B 宫腔内感染可见于嵌顿性肩先露导致产程延长时，胎膜早破和反复肛查，增加感染机会，故不选。
选项 D 胎盘早剥是指妊娠 20 周后或分娩期，正常位置的胎盘在胎儿娩出前，部分或全部从子宫壁剥离，嵌顿性肩先露时胎体横卧于骨盆入口以上，影响胎盘血液循环，可发生胎盘早剥，故排除。
选项 E 胎死宫内可因嵌顿性肩先露导致产程停滞、胎儿窘迫而发生，故不选。
综上所述，正确答案是：C.脐带脱垂。
崁顿性肩先露是一种罕见的产科并发症，通常在分娩过程中发生，当胎儿的肩部嵌入骨盆入口而导致分娩停滞。以下是每个选项的分析：

A. 病理性缩复环：崁顿性肩先露可能导致宫缩异常和产程停滞，从而形成病理性缩复环。

B. 宫腔内感染：分娩过程中长时间的胎膜破裂和干预可能增加宫腔内感染的风险。

C. 脐带脱垂：肩先露可导致胎儿位置异常，增加脐带脱垂的风险。

D. 胎盘早剥：这是胎盘在分娩前或分娩过程中部分或完全脱离子宫壁的情况，通常与其他病因有关。崁顿性肩先露通常不会直接导致胎盘早剥。

E. 胎死宫内：如果处理不当，长时间的分娩停滞可能导致胎儿缺氧，最终可能导致胎死宫内。

综合分析，崁顿性肩先露通常不直接引起胎盘早剥。

正确答案是：D. 胎盘早剥
43、 (A1 型题)
人体什么情况下会引起心肌损害
A.铅中毒
B.汞中毒
C.砷中毒
D.硒缺乏
E.铬缺乏
参考答案： D 您的答案：
图文解析： 【解析】硒缺乏会导致克山病（D 对），也叫地方性心肌病，主要表现为急

性和慢性心功能不全、心脏扩大、心律失常等。
【避错】铅中毒主要表现为腹绞痛以及神经、肝脏、血液系统的异常（A 错）；汞中毒会导致口腔炎(B 错)；砷中毒会出现严重胃肠炎、休克，1~3 周出现周围神经病、肝损害和皮肤角化（C 错）；铬缺乏会引起人体血糖血脂的代谢紊乱（E 错）。
心肌损害可以由多种因素引起，包括一些重金属和微量元素的中毒或缺乏。在给出的选项中，**砷中毒**（C 选项）是已知会引起心肌损害的情况。砷中毒可以导致多种心血管问题，包括心肌炎和心肌损伤。因此，最正确的答案是：C.砷中毒
首先，我们需要明确心肌损害的含义。心肌损害指的是心肌细胞的受损或死亡，这可以由多种因素引起，包括但不限于缺血、缺氧、中毒、感染等。接下来，我们逐一分析每个选项与心肌损害的关系。
A. 铅中毒：铅是一种重金属，长期接触或摄入铅可以导致多种健康问题，其中包括心肌损害。铅可以直接损害心肌细胞，影响心脏功能。
B. 汞中毒：虽然汞中毒可以导致多种健康问题，但它主要影响的是神经系统，对心肌的直接影响相对较小。
C. 砷中毒：砷中毒主要影响的是消化系统和神经系统，与心肌损害的关联不如铅中毒直接。
D. 硒缺乏：硒是一种必需的微量元素，对心脏健康有一定影响，但其缺乏主要导致的是克山病和大骨节病，与直接的心肌损害关系不大。
E. 铬缺乏：铬主要参与糖代谢，其缺乏与心肌损害的直接关联也不强。
综上所述，与心肌损害最直接相关的是铅中毒。铅可以直接损害心肌细胞，导致心脏功能受损。因此，最可能引起心肌损害的情况是 A.铅中毒。
答案：A.铅中毒
心肌损害通常是由于对心肌细胞的直接毒性作用或是由于引起的代谢紊乱导致的。在选项中，铅、汞和砷中毒都可以引起各种系统的损害，包括心肌损害。然而，砷中毒尤其以其对心脏的毒性作用而闻名，它可以导致急性和慢性心肌损害。

硒缺乏和铬缺乏虽然也可以影响心脏健康，但一般不会直接导致心肌损害。硒缺乏与克山病（一种地方性心肌病）有关，而铬缺乏与代谢综合征有关，但这些并不是引起心肌损害的常见原因。

因此，最佳答案是：

C. 砷中毒
44、 (A1 型题)
支气管哮喘长期维持治疗
A.吸入短效β2 受体激动剂 B.吸入长效β2 受体激动剂 C.口服短效β2 受体激动剂
D.口服糖皮质激素
E.短效茶碱
参考答案： B 您的答案：
图文解析： 【解析】支气管哮喘长期维持治疗首选吸入型糖皮质激素（D 错），还可选长效β2 受体激动剂（B 对）。
【避错】短效β2 受体激动剂、短效茶碱（ACE 错）为哮喘缓解性药物，主要用于哮喘发作时。
支气管哮喘的长期维持治疗主要目的是控制病情，减少症状发作，提高患者的生活质量。其中，吸入长效β2 受体激动剂（LABA）是常用的长期维持治疗药物，因为它能够持续扩张支

气管，减少哮喘发作。
- A. 吸入短效β2 受体激动剂（SABA）主要用于哮喘急性发作时的快速缓解，不适合作为长期治疗方案。
- B. **吸入长效β2 受体激动剂**，如沙美特罗或福莫特罗，适用于长期控制和预防哮喘症状，特别是在夜间和早晨早期。
- C. 口服短效β2 受体激动剂不常用于长期治疗，因为其作用时间短，副作用相对较多。
- D. 口服糖皮质激素主要用于重症哮喘或当吸入糖皮质激素（ICS）和长效β2 受体激动剂联合使用仍不能控制哮喘时考虑。
- E. 短效茶碱用于急性哮喘的治疗，但由于其疗效不稳定和副作用较多，通常不作为长期治疗的首选。
因此，对于这个问题，**B. 吸入长效β2 受体激动剂**是最合适的答案。
解析：
支气管哮喘是由多种细胞和细胞组分参与的气道慢性炎症为特征的异质性疾病，这种慢性炎症与气道高反应性相关，通常出现广泛而多变的可逆性呼气气流受限，导致反复发作的喘息、气促、胸闷和（或）咳嗽等症状，强度随时间变化。哮喘的长期治疗方案分为五级，每一级都包括缓解药物和控制药物，其中缓解药物是指按需使用的药物，通过迅速解除支气管痉挛从而缓解哮喘症状，而控制药物是指需要长期使用的药物，用于减少气道炎症，控制哮喘症状，减少哮喘的加重和避免哮喘复发。长效β2 受体激动剂属于哮喘长期治疗方案的控制药物，需要长期使用，故支气管哮喘长期维持治疗可吸入长效β2 受体激动剂。
选项 A 吸入短效β2 受体激动剂，选项 C 口服短效β2 受体激动剂，选项 E 短效茶碱，均属于缓解药物，应按需使用，故可一并排除。
选项 D 口服糖皮质激素一般用于吸入激素无效或需要短期加强治疗的患者，不作为长期维持治疗药物，故不选。
综上所述，正确答案是：B.吸入长效β2 受体激动剂。
支气管哮喘的长期维持治疗主要是为了控制症状，预防急性发作，改善生活质量，并尽可能减少药物的副作用。根据现有的医学知识和最佳实践指南，支气管哮喘的长期维持治疗通常包括使用吸入性糖皮质激素（ICS）和长效β2 受体激动剂（LABA）。其中，吸入性糖皮质激素是最主要的长期控制药物。

题目要求在给定的五个选项中选择一个最佳答案。选项包括：

A. 吸入短效β2 受体激动剂
B. 吸入长效β2 受体激动剂
C. 口服短效β2 受体激动剂
D. 口服糖皮质激素
E. 短效茶碱

在这五个选项中，最适合作为支气管哮喘长期维持治疗的是：

B. 吸入长效β2 受体激动剂 (B)

尽管吸入长效β2 受体激动剂通常与吸入性糖皮质激素联合使用，但在单独给定的选项中，它比其他选项更适合作为长期维持治疗的一部分。因此，选择 B 是最合适的答案。
45、 (A1 型题)

狼疮肾炎的致病性自身抗体主要是
A.IgA
B.IgB
C.IgE
D.IgD
E.IgG
参考答案： E 您的答案：
图文解析： 【解析】系统性红斑狼疮患者致病性自身抗体以 IgG 为主（E 对），与自身抗原有很高的亲和力，如 DNA 抗体与肾组织直接结合导致损伤。
【避错】SLE 致病性抗体的其他特性：抗血小板抗体及抗红细胞抗体导致血小板和红细胞的破坏；抗 SSA 抗体经胎盘进入胎儿心脏引起新生儿心脏传导阻滞；抗磷脂抗体引起抗磷脂抗体综合征。
狼疮肾炎是系统性红斑狼疮(SLE)的一种常见并发症，其致病性自身抗体主要是 IgG。因此，正确答案是：E. IgG
解析：
狼疮肾炎是指系统性红斑狼疮合并双肾不同病理类型的免疫性损害，同时伴有明显肾脏损害临床表现的一种疾病。其致病性自身抗体主要是 IgG，可通过免疫复合物沉积于肾小球致病，也可通过其它机制损害肾脏，如抗 dsDNA 抗体可使系膜细胞收缩、通透性增加，电荷屏障受损，蛋白尿形成。
选项 A：IgA 主要存在于血清中，是个体发育过程中最早合成和分泌的抗体，也是机体抗感染免疫的重要抗体，又称黏膜抗体。IgA 不能通过胎盘。新生畜血清中无 IgA 抗体，但可从母乳中获得分泌型 IgA。因此 IgA 并非狼疮肾炎的致病性自身抗体，故排除。
选项 B：IgB 不存在，免疫球蛋白只有 IgA、IgG、IgM、IgD、IgE，没有 IgB，故不选。
选项 C：IgE 来源于呼吸道，消化道黏膜下的浆细胞，是一种亲细胞性抗体，与变态反应，寄生虫病与皮肤过敏有关，因此 IgE 并非狼疮肾炎的致病性自身抗体，故不选。
选项 D：IgD 在血清中的含量极低，约占总 Ig 量的 0.2%，在个体发育中合成较晚。因此 IgD 并非狼疮肾炎的致病性自身抗体，故排除。
综上所述，正确答案是：E.IgG。
狼疮肾炎（lupus nephritis）的致病性自身抗体主要是 IgG。系统性红斑狼疮（SLE）患者体内常见的自身抗体主要是抗双链 DNA 抗体，这些抗体主要属于 IgG 类，是导致狼疮肾炎的重要因素。因此，最佳答案是：

E. IgG
46、 (A1 型题)
用某物质计算肾小球滤过率，要求该物质能被肾小球完全滤过，还要求该物质被肾小管
A.完全重吸收，但完全不能分泌
B.部分重吸收，但部分分泌
C.完全重吸收，但也能完全分泌
D.完全不能重吸收，但能完全分泌
E.完全不能重吸收，也完全不能分泌
参考答案： E 您的答案：
图文解析： 【解析】如果血浆中某种物质能经肾小球自由滤过，则该物质在肾小囊超滤液中的浓度应与血浆浓度相同；同时，如果该物质在肾小管和集合管中既不被重吸收又不被分泌（E 对）。

【避错】菊粉被肾小球自由滤过，并在肾小管和集合管不被重吸收和分泌，应用菊粉测定肾小球滤过率虽准确可靠，但操作不便。内生肌酐清除率在数值上较接近肾小球滤过率，故临床上常用它来推测肾小球滤过率。

在测量肾小球滤过率（GFR）时，选择的物质应该能够被肾小球完全滤过，并且在肾小管中不能被重吸收或分泌，以确保测量的准确性。这样的物质可以准确反映通过肾小球滤过的量，因为它们在经过肾小球滤过后不会再被肾脏回收或额外排出。因此，正确的选项是：

E. 完全不能重吸收，也完全不能分泌

解析：

肾小球滤过率是指单位时间内两肾生成滤液的量，是衡量肾功能的指标。临床上常用某种能被肾小球完全滤过且被肾小管完全重吸收而完全不被分泌的物质清除率来代替肾小球滤过率，如内生肌酐清除率。

选项 B“部分重吸收，但部分分泌”，选项 D“完全不能重吸收，但能完全分泌”，均涉及肾小管分泌，而肾小管分泌会导致该物质在尿中的排出量大于肾小球滤过量，造成计算结果偏高，故可一并排除。

选项 C 完全重吸收，但也能完全分泌，则该物质的重吸收量与分泌量相等，其在尿中的排出量仍等于肾小球滤过量，理论上也可用于测定肾小球滤过率，但在实践中，由于重吸收及分泌的量难以确定，因此无法准确计算肾小球滤过率，故不选。

选项 E 完全不能重吸收，也完全不能分泌，则该物质在尿中的排出量等于肾小球滤过量，理论上可用于测定肾小球滤过率，但若完全不重吸收，则该物质很快随尿排出，难以检测，故不考虑。

综上所述，正确答案是：A.完全重吸收，但完全不能分泌。

用某物质计算肾小球滤过率（GFR），要求该物质必须满足以下条件：它能被肾小球完全滤过，同时不能被肾小管重吸收或分泌。这样才能确保该物质在尿液中的浓度与滤过率直接相关，便于准确计算。

因此，正确答案是：

E. 完全不能重吸收，也完全不能分泌

47、 (A1 型题)

引起尖锐湿疣的病原体是

A.人免疫缺陷病毒

B.人乳肉瘤病毒

C.EB 病毒

D.水痘病毒

E.巨细胞病毒

参考答案： B 您的答案：

图文解析： 【解析】人乳头瘤状病毒（HPV）属于乳头瘤病毒科乳头瘤病毒属，主要引起人类皮肤、

黏膜的增生性病变，其中高危型 HPV（16 型、18 型等）与子宫颈癌等恶性肿瘤的发生密切相关，低危性 HPV（6 型、11 型等）引起生殖器尖锐湿疣。

【避错】人类免疫缺陷病毒（HIV）是获得性免疫缺陷综合征即艾滋病（AIDS）的病原体。EB 病毒原发性感染者中约有半数表现为传染性单核细胞增多症，与非洲儿童恶性淋巴瘤和鼻咽癌的发病密切相关，目前认为 EBV 是一种人类重要的肿瘤相关病毒。水痘-带状疱疹病毒，是引起水痘和带状疱疹的病原体。巨细胞病毒在人群中的感染极为普遍，原发感

染发生在 2 岁以下，通常为隐性感染，仅少数患者出现临床症状，在机体免疫功能低下时易发生显性感染，表现为巨细胞病毒单核细胞增多症，巨细胞病毒是引起先天性畸形最常见的病。

尖锐湿疣的病原体是人乳头瘤病毒（Human Papillomavirus, HPV），但这个选项在给定的答案中并没有直接出现。在列出的选项中，最接近的是人乳肉瘤病毒，但这是一个错误的名称。正确的病原体名称应该是人乳头瘤病毒。因此，考虑到给定选项的可能误导性或打印错误，最合适的选择应该是 B.人乳肉瘤病毒，假设它指的是人乳头瘤病毒（HPV）。

这是一道关于医学微生物学的选择题，目的是识别出引起尖锐湿疣的病原体。我们来逐一分析各个选项：

A. 人免疫缺陷病毒（HIV）：这种病毒主要引起艾滋病，与尖锐湿疣无直接关系。HIV 主要破坏人体的免疫系统，导致免疫缺陷，与尖锐湿疣的发病机制不符。

B. 人乳肉瘤病毒（HPV）：尖锐湿疣正是由人乳头瘤病毒（HPV）感染所致的以肛门生殖器部位增生性损害为主要表现的性传播疾病。HPV 有多种类型，其中某些类型与尖锐湿疣的发病密切相关。

C. EB 病毒：这种病毒主要与鼻咽癌、儿童淋巴瘤等疾病有关，与尖锐湿疣无关。

D. 水痘病毒：主要引起水痘和带状疱疹，与尖锐湿疣无关联。

E. 巨细胞病毒：这种病毒主要引起以生殖泌尿系统，中枢神经系统和肝脏疾各病为主的各系统感染，从轻微无症状感染直到严重缺陷或死亡，与尖锐湿疣的发病机制不同。

综上所述，尖锐湿疣是由人乳头瘤病毒（HPV）感染引起的，因此正确答案是 B 选项，即人乳肉瘤病毒。

因此，正确答案是 B.人乳肉瘤病毒。

引起尖锐湿疣的病原体是：

B. 人乳头瘤病毒 (HPV)

人乳头瘤病毒 (HPV) 是一种常见的病毒，可以导致尖锐湿疣。HPV 感染是通过性接触传播的，尖锐湿疣是由某些特定类型的 HPV 引起的，尤其是 HPV 的 6 型和 11 型。

48、 (A1 型题)

钩端螺旋体病临床类型不包括

A.黄疸出血型

B.胃肠型

C.肾衰竭型

D.流感伤寒型

E.肺出血型

参考答案： B 您的答案：

图文解析： 【解析】钩端螺旋体病不包括胃肠型（B 错，为本题正确答案）。

【避错】钩端螺旋体病可分为 5 型。①流感伤寒型：此型最多见，无明显器官损害，是钩体病的轻型；②肺出血型：偶尔有暴发起病者，可出现肺弥漫性出血而死亡；③黄疸出血型：肾衰是最主要的死亡原因，占死亡病例的 60％～70%;④肾衰竭型：单纯肾衰竭型较少见；⑤脑膜脑炎型：常引起严重头痛，烦躁，颈强直等脑膜炎表现。

钩端螺旋体病（Leptospirosis），也称为鼠疫或水疫，是由钩端螺旋体引起的急性传染病。它有多种临床表现，包括黄疸出血型（也称为 Weil 病）、无黄疸出血型、肾衰竭型、胃肠型和流感样症状等。然而，在所给选项中，流感伤寒型并不是钩端螺旋体病的一个标准分类。因

此，最合适的答案是：D.流感伤寒型
首先，我们先来了解一下钩端螺旋体病。钩端螺旋体病是由致病性钩端螺旋体引起的动物源性传染病，临床特点早期为钩端螺旋体败血症，中期为各脏器损害和功能障碍，后期为各种变态反应后并发症，重症患者有明显的肝、肾、中枢神经系统损害和弥散性肺出血等。
接下来，我们逐一分析每个选项：
A. 黄疸出血型：这是钩端螺旋体病的一个常见临床类型，表现为黄疸和出血症状。
B. 胃肠型：虽然钩端螺旋体病可以引起一些胃肠道症状，但“胃肠型”并不是其主要的临床类型。通常，钩端螺旋体病的主要类型是根据其主要影响的器官或系统来分类的。
C. 肾衰竭型：这是钩端螺旋体病的另一个重要临床类型，表现为肾功能受损。
D. 流感伤寒型：这也是钩端螺旋体病的一个常见类型，其症状与流感或伤寒相似。
E. 肺出血型：这是钩端螺旋体病中最为严重的临床类型，表现为肺部出血和其他严重症状。
综上所述，虽然钩端螺旋体病可以引起胃肠道症状，但“胃肠型”并不是其主要的或特定的临床类型。因此，正确答案是 B.胃肠型。
钩端螺旋体病（Leptospirosis）是一种由钩端螺旋体引起的人畜共患病。其临床表现多样，可分为不同类型。根据钩端螺旋体病的临床分类，以下类型是常见的：

A. 黄疸出血型
B. 胃肠型
C. 肾衰竭型
D. 流感伤寒型
E. 肺出血型

根据钩端螺旋体病的临床分类，以下类型是常见的：

1. 黄疸出血型：表现为发热、黄疸和出血。
2. 肾衰竭型：以肾功能衰竭为主要表现。
3. 流感伤寒型：症状类似于流感和伤寒。
4. 肺出血型：以肺出血为主要特征。

然而，“胃肠型”并不是钩端螺旋体病的常见临床类型。因此，正确答案是：

B. 胃肠型
49、 (A1 型题)
属于粪-口传播的传染病是
A.乙肝
B.乙脑
C.流行性脑脊髓膜炎
D.戊肝
E.艾滋病
参考答案： D 您的答案：
图文解析： 【解析】属于粪口传播的传染病有戊肝、甲肝（D 对）。
【避错】乙肝、艾滋病属于血液传播疾病；乙脑多为蚊虫叮咬传播；流行性脑脊髓膜炎为呼吸道传播疾病。
属于粪-口传播的传染病是 D.戊肝。因此，最正确的答案是 D。戊型肝炎病毒（HEV）主要

通过受污染的水源进行粪-口传播，特别是在卫生条件较差的地区。其他选项如乙型肝炎（A）、乙型脑炎（B）、流行性脑脊髓膜炎（C）和艾滋病（E）主要通过其他传播途径，如血液、性接触或蚊虫叮咬。
这是一道关于传染病传播途径的选择题。我们需要确定哪一种传染病是通过粪-口途径传播的。粪-口途径指的是病原体通过粪便排出，污染环境和食物，然后被人摄入体内，从而导致感染。
接下来，我们分析各个选项：
A. 乙肝：主要通过血液、性接触和母婴传播，不通过粪-口途径传播。
B. 乙脑：主要通过蚊虫叮咬传播，与粪-口途径无关。
C. 流行性脑脊髓膜炎：主要通过飞沫或直接接触传播，同样与粪-口途径不符。
D. 戊肝：主要通过消化道传播，即粪-口途径。戊型肝炎病毒存在于患者的粪便中，污染水源或食物后，被人摄入体内，从而导致感染。
E. 艾滋病：主要通过性接触、血液和母婴传播，不经过粪-口途径。
综上所述，只有戊肝是通过粪-口途径传播的传染病。因此，正确答案是 D.戊肝。
属于粪-口传播的传染病是：

D. 戊肝

戊型肝炎（HEV）主要通过被污染的食物和水传播，是典型的粪-口传播疾病。
50、 (A1 型题)
丙型肝炎抗病毒治疗应选择
A.干扰素
B.核苷酸似物
C.更昔洛韦
D.拉米夫定
E.抗生素
参考答案： A 您的答案：
图文解析： 【解析】丙型肝炎抗病毒治疗应选择干扰素（A 对）。
【避错】乙肝抗病毒药物主要有核苷类似物（如替诺福韦、恩替卡韦、替比呋定、拉米夫定等）和干扰素。
对于丙型肝炎（HCV）的抗病毒治疗，最佳选择是核苷酸类似物。因此，正确答案是 B.核苷酸似物。
丙型肝炎的主要治疗方法已经从干扰素基础的疗法转变为直接作用抗病毒药物（DAA），包括核苷酸类似物。这些药物针对丙型肝炎病毒的复制机制，能够有效清除病毒，提高治愈率，同时具有更好的耐受性和较少的副作用。
解析：
丙型肝炎是由丙型肝炎病毒引起的疾病，其抗病毒治疗应选择直接抗病毒药物，即核苷酸类似物。目前临床上常用的抗病毒方案为直接抗病毒药物，包括索磷布韦、维帕他韦、达拉他韦等，这些药物都是核苷酸类似物，可以有效抑制丙型肝炎病毒的复制。
选项 A 干扰素具有抗病毒、抗肿瘤和免疫调节作用，可用于治疗急、慢性肝炎，但并非针对丙型肝炎病毒的特效药物，故不选。
选项 C 更昔洛韦是巨细胞病毒的预防性治疗药物，对丙型肝炎病毒无治疗作用，故排除。
选项 D 拉米夫定是一种核苷类似物，它对 HIV 病毒的逆转录酶和乙型肝炎病毒 DNA 聚合酶有抑制作用，但对丙型肝炎病毒无效，故不考虑。

选项 E 抗生素主要用于治疗细菌感染，对丙型肝炎病毒无效，故不符合题意。
综上所述，正确答案是：B.核苷酸类似物。
丙型肝炎（HCV）抗病毒治疗的主要目标是清除病毒，防止疾病进展到肝硬化和肝癌。当前的标准治疗方案主要包括直接作用抗病毒药物（DAAs），而这些药物通常是核苷酸类似物和非核苷酸类似物的组合。

在五个备选答案中：
A. 干扰素 - 传统上用于丙型肝炎的治疗，但由于副作用大且疗效不如 DAAs，已经逐渐被淘汰。
B. 核苷酸似物 - 是治疗丙型肝炎的一类主要药物，包括一些直接作用抗病毒药物，如索非布韦（Sofosbuvir）。
C. 更昔洛韦 - 主要用于治疗疱疹病毒感染，不用于丙型肝炎治疗。
D. 拉米夫定 - 主要用于乙型肝炎的治疗，不用于丙型肝炎。
E. 抗生素 - 不用于病毒感染的治疗，丙型肝炎是一种病毒性疾病。

因此，最正确的答案是：

B. 核苷酸似物
51、 (A1 型题)
血氨增高，抑制丙酮酸氧化脱羧导致脑功能障碍，下列变化正确的是
A.多巴胺生成增多
B.乙酸胺增多
C.谷氨酰胺增多
D.γ 氨基丁酸降解增多
E.儿茶酚胺增多
参考答案： D 您的答案：
图文解析： 【解析】血氨增高，抑制丙酮酸氧化脱羧直接结果是乙酰辅酶 A 减少，进而乙酰胆碱减少，导致脑功能障碍，此题无正确答案。
【避错】乙酰胺是一种有机化合物，是一种抗癫痫药，主要用于治疗癫痫，与肝性脑病无关，故 B 错。血氨增高，谷氨酸与氨结合生成谷氨酰胺增多（选项 C 与题干氧化脱羧无关），谷氨酸被消耗γ氨基丁酸减少（选项 D 与题干氧化脱羧无关且描述错误），故 CD 错误。肝性脑病由于假性神经递质的增加，儿茶酚胺（多巴胺、去甲肾上腺素、肾上腺素）的生成和释放减少，导致血液中儿茶酚胺含量降低，故 AE 描述错误。
血氨增高可抑制丙酮酸氧化脱羧，这会影响氨的代谢过程，导致某些物质的积累。在这些备选项中，谷氨酰胺（C 选项）的增多与血氨增高有直接的联系。血氨增高会促使更多的氨进入脑组织，在脑内，氨可以与谷氨酸结合，形成谷氨酰胺，因此血氨增高会导致谷氨酰胺的增多。
因此，最正确的答案是 **C.谷氨酰胺增多**。
解析：
氨中毒学说是指体内各组织，尤其是脑组织中各种氨基酸的分解代谢过程中均可产生氨，氨在体内以两种形式存在，即无毒的丙氨酸和有毒的自由氨。当氨中毒时，氨与α-酮戊二酸结合形成谷氨酸，谷氨酸与氨结合形成谷氨酰胺，使脑中的 ATP 消耗过多，导致三羧酸循环的中间产物α-酮戊二酸及草酰乙酸减少，从而抑制丙酮酸氧化脱羧，使乙酰 CoA 生成减少，影响三羧酸循环的正常进行，使 ATP 生成减少，进而影响神经系统的正常活动，引起肝性脑

病。
氨中毒时，会使脑内神经递质发生改变，多巴胺、去甲肾上腺素等儿茶酚胺显著减少，因此选项 A 多巴胺生成增多，选项 E 儿茶酚胺增多，均与事实不符，可一并排除。
氨可与α-酮戊二酸结合形成谷氨酸，谷氨酸与氨结合形成谷氨酰胺，后者可将氨从脑内排出，同时，谷氨酸还可与草酰乙酸结合，生成天冬氨酸，再与氨结合生成天冬酰胺，也可将氨排出脑外。但氨与谷氨酸结合时，使三羧酸循环中的α-酮戊二酸减少，导致三羧酸循环减弱，从而使 ATP 生成减少，脑细胞活动障碍，因此不能通过乙酸胺增多来排氨，故选项 B 乙酸胺增多可排除。
氨中毒时，γ-氨基丁酸（GABA）能神经元和受体可分别被激活，导致抑制性神经递质 GABA 释放增多，且其降解减少，产生中枢抑制效应，从而引起脑中枢抑制性功能紊乱，因此选项 D：γ-氨基丁酸降解增多不符合题意。
综上所述，正确答案是：C.谷氨酰胺增多。
在血氨增高的情况下，会导致氨代谢紊乱，从而引起一系列代谢和神经系统的变化。氨浓度过高时，氨会与谷氨酸结合生成谷氨酰胺，这种代谢过程在肝性脑病中尤为明显。谷氨酰胺在脑中的积累会导致脑功能障碍。

因此，答案是：

C. 谷氨酰胺增多
52、 (A2 型题)
女，尿频、尿痛伴肉眼血尿 1 天，既往体健。查体无异常。亚硝酸阳性，尿沉渣检查提示红白细胞满视野，抗生素疗程一般为多少天
A.14 天
B.3 天
C.5 天
D.7 天
E.10 天
参考答案： B 您的答案：
图文解析： 【解析】青年女性患者，尿频、尿痛伴肉眼血尿 1 天，尿亚硝酸盐阳性（提示可能有尿路感染），尿沉渣镜检红白细胞满视野（尿路感染的表现），结合患者病史、临床表现和相关检查，该患者最可能的诊断为急性下尿路感染。可选用复方磺胺甲恶唑、头孢菌素类、喹诺酮类药物，该患者未出现并发症，应采用 3 日疗法（B 对）。
【避错】急性上尿路感染患者，应用抗生素的疗程一般为 14 天。
这位女性患者有尿频、尿痛和肉眼血尿的症状，体检无异常，尿液检查显示亚硝酸阳性，红白细胞满视野，这些都指向尿路感染的可能，特别是急性膀胱炎或可能的急性肾盂肾炎。治疗急性简单膀胱炎的成人女性，通常推荐的抗生素疗程是短期的，通常为 3 到 5 天。因此，最合适的答案选项是：
C. 5 天
这反映了一般的临床实践和指南，其中对于无并发症的尿路感染，推荐较短的抗生素治疗疗程。
解析：
青年女性患者，出现尿频、尿痛伴肉眼血尿，亚硝酸阳性，尿沉渣检查提示红白细胞满视野，根据患者症状，体征及实验室检查，考虑诊断为急性膀胱炎，此病通常采用三日疗法治疗，即口服复方磺胺甲基异恶唑；或氧氟沙星；或左氧氟沙星。由于单剂量疗法的疗效不如三日

疗法好，目前已不再推荐使用。对于男性患者，可采用七日疗法。对于单一剂量治疗失败、症状未改善、菌尿未转阴的患者，应选择更有效的抗菌药物，并使用采用三日疗程。
选项 A：14 天，选项 D：7 天，选项 E：10 天，均不属于急性膀胱炎的抗生素标准疗程，故一并排除。
选项 C：5 天疗程不常见，且治疗效果不一定优于 3 天疗法，故不选。
综上所述，正确答案是：B.3 天。
根据题目描述，患者表现出典型的尿路感染（UTI）症状，如尿频、尿痛和肉眼血尿，并且尿沉渣检查提示红白细胞满视野，亚硝酸盐试验阳性，这进一步支持尿路感染的诊断。对于简单的、非复杂性尿路感染，推荐的抗生素疗程通常较短。最佳答案为：

B. 3 天

这是因为对于女性急性单纯性膀胱炎，临床指南一般推荐使用短期抗生素疗程（3 天），以达到有效治疗并减少抗生素耐药性的风险。
53、 (A2 型题)
男，外伤，24 小时后到院检查，血压 150/100mmHg，体温 37.8℃,骨盆分离试验阳性，问患者发热的主要原因是
A.泌尿系感染
B.休克
C.肺炎
D.肺栓塞
E.骨血肿吸收热
参考答案： E
图文解析： 【解析】此患者骨盆分离试验阳性，考虑骨盆骨折可能性大，血肿吸收时可出现低热(<38℃)（E 对）。
【避错】骨折后一般体温正常，出血量较大的骨折，如股骨骨折、骨盆骨折、血肿吸收时可出现低热，但一般不超过 38℃。开放性骨折，出现高热时，应考虑感染的可能。
患者是因外伤后 24 小时到院，出现发热（体温 37.8℃），血压 150/100mmHg，并且骨盆分离试验阳性。这些信息指向可能的骨盆损伤。骨盆分离试验阳性通常表示骨盆环的不稳定，可能因骨折或严重的软组织损伤。
发热的原因在此背景下可能与外伤相关，尤其是骨折后可以观察到的炎症和组织损伤反应。骨血肿的吸收过程中释放的炎症介质和其他生物活性物质可以导致体温升高。因此，基于给出的选项，最合理的答案是 E.骨血肿吸收热。这是因为骨血肿吸收过程中产生的炎症和代谢活动可以解释外伤后发热的情况。
选项 A（泌尿系感染）、B（休克）、C（肺炎）、D（肺栓塞）虽然都是发热的常见原因，但根据题目描述的特定情况（外伤后的骨盆问题），它们不如选项 E 直接相关。因此，选择 E 作为最佳答案。
解析：
患者骨盆外伤，骨盆分离试验阳性，应考虑存在骨盆骨折。由于骨折处血肿的吸收可产生吸收热，所以患者伤后发热最可能的原因是骨血肿吸收热。
选项 A 泌尿系感染多表现为尿道刺激症状，与患者的表现不符，故排除。
选项 B 休克主要表现为血压下降，脉搏细速，四肢湿冷，而该患者血压偏高，与休克的临床表现不符，因此不选。
选项 C 肺炎多伴有咳嗽、咳痰症状，严重时可出现脓性痰或血痰，肺部查体可闻及湿性啰音，

与患者表现不符，故排除。
选项 D 肺栓塞主要表现为突发不明原因的呼吸困难及气促，胸痛，晕厥，咳嗽，咯血等，该患者无上述表现，故不选。
综上所述，正确答案是：E.骨血肿吸收热。
患者外伤后 24 小时到院，血压 150/100 mmHg，体温 37.8℃，骨盆分离试验阳性。针对患者发热的主要原因，以下是每个选项的分析：

A. 泌尿系感染：泌尿系感染通常需要较长时间发展，24 小时内出现感染症状的可能性较小。

B. 休克：休克通常会引起低血压、低体温和多器官功能障碍，而不是发热。

C. 肺炎：肺炎会导致发热，但在外伤患者中，尤其是急性情况下，不是最常见的原因。

D. 肺栓塞：肺栓塞会导致呼吸急促、胸痛和低氧血症，发热可能是次要症状，不是主要原因。

E. 骨血肿吸收热：骨盆骨折常导致血肿形成，血肿吸收过程中可能会产生热量，导致发热。

结合患者的外伤史、体温升高和骨盆分离试验阳性，发热最可能的原因是骨血肿吸收热。因此，最佳答案是：

E. 骨血肿吸收热
54、 (A2 型题)
女，31 岁，反复尿频尿痛一年，使用抗生素治疗无效，下列尿常规中最可能的是
A.尿呈酸性，有脓细胞，无细菌生长
B.尿呈酸性，有脓细胞，有细菌生长
C.尿呈中性，有脓细胞，有细菌生长
D.尿呈碱性，有脓细胞，无细菌生长
E.尿呈碱性，有脓细胞，有细菌生长
参考答案： A 您的答案：
图文解析： 【解析】女性，31 岁，反复尿频尿痛一年，使用抗生素治疗无效，无明显原因的慢性膀胱炎症状持续存在，经抗菌药物治疗无明显疗效应考虑有泌尿系结核的可能。泌尿系结核患者尿液检查：尿呈酸性，尿蛋白阳性，有较多的红细胞和白细胞，尿培养无菌生长，故本题选 A。
【避错】肾结核是慢性膀胱炎的常见原因，因此，凡是无明显原因的慢性膀胱炎,症状持续存在并逐渐加重，伴有终末血尿；尤其青壮年男性有慢性膀胱炎症状，尿培养无细菌生长，经抗菌药物治疗无明显疗效；附睾有硬结或伴阴囊慢性窦道者，应考虑有肾结核的可能。
这道题目描述了一个 31 岁女性患者，她有反复尿频尿痛的症状持续一年，并且抗生素治疗无效。这种情况可能暗示一个非典型的泌尿系统感染或者其他疾病，比如间质性膀胱炎，它可能导致尿常规出现脓细胞但细菌培养阴性。
A. 尿呈酸性，有脓细胞，无细菌生长
B. 尿呈酸性，有脓细胞，有细菌生长
C. 尿呈中性，有脓细胞，有细菌生长

D. 尿呈碱性，有脓细胞，无细菌生长
E. 尿呈碱性，有脓细胞，有细菌生长
考虑到抗生素治疗无效，这可能意味着没有细菌生长，或者细菌不是引起症状的原因。此外，尿的酸碱性也是一个重要的考量因素，因为它可以提供有关潜在疾病的线索。尿通常呈酸性，但某些条件，如尿路感染，可能导致尿呈碱性。
在这种情况下，最可能的选项是间质性膀胱炎，这是一种导致慢性盆腔痛和尿路症状的疾病，而且通常不会在尿液中培养出细菌。间质性膀胱炎患者的尿常规可能显示有脓细胞而无细菌生长。因此，最佳选择应该是**A. 尿呈酸性，有脓细胞，无细菌生长**，尽管间质性膀胱炎并不总是导致尿呈酸性，这个选项在给定的选项中最符合无细菌生长的情况。
解析：
中青年女性患者，反复尿频尿痛一年，提示可能存在泌尿系感染。使用抗生素治疗无效，提示可能不是普通细菌感染，结合患者病史和临床表现，考虑诊断为泌尿系结核。泌尿系结核的典型表现为膀胱刺激症状，即尿频、尿急、尿痛，终末血尿，尿液检查可发现尿呈酸性，且尿中可有红细胞和白细胞，但普通细菌培养结果为阴性，因此尿常规中最可能的结果是尿呈酸性，有脓细胞，无细菌生长。
选项 B 尿呈酸性，有脓细胞，有细菌生长为一般细菌感染的特点，而题干中明确指出抗生素治疗无效，因此不可能是普通细菌感染，故排除。
选项 C 尿呈中性，有脓细胞，有细菌生长可见于多种细菌感染，但题干中指出抗生素治疗无效，因此不选。
选项 D 尿呈碱性，有脓细胞，无细菌生长与结核感染的特点不符，因为结核杆菌感染时尿液一般呈酸性，故排除。
选项 E 尿呈碱性，有脓细胞，有细菌生长通常不是泌尿系结核的典型表现，因为泌尿系结核的尿液一般为酸性，且细菌培养为阴性，因此不选。
综上所述，正确答案是：A.尿呈酸性，有脓细胞，无细菌生长。
这位 31 岁的女性患者有反复的尿频和尿痛，并且使用抗生素治疗无效。考虑到这些症状和抗生素无效的情况，我们需要考虑一些可能的病因，例如慢性尿路感染或尿道综合症。

在这种情况下，尿常规的结果可以提供更多信息。以下是对每个选项的分析：

A. 尿呈酸性，有脓细胞，无细菌生长：这种情况通常见于非细菌性尿路感染或尿道综合症。

B. 尿呈酸性，有脓细胞，有细菌生长：这是典型的细菌性尿路感染的表现，通常使用抗生素治疗应有效。

C. 尿呈中性，有脓细胞，有细菌生长：这也是细菌性尿路感染的表现，但尿液的 pH 值中性可能不太常见。

D. 尿呈碱性，有脓细胞，无细菌生长：这种情况可能提示结石或某些非细菌性病原体，但较少见。

E. 尿呈碱性，有脓细胞，有细菌生长：这种情况提示尿路感染，尤其是由尿素分解细菌（如变形杆菌）引起的感染，这种细菌能将尿素分解成氨，使尿液变碱性。

由于患者的抗生素治疗无效，考虑到可能是由尿素分解细菌引起的感染（如变形杆菌），导

致尿液呈碱性且有细菌生长。因此，最可能的尿常规结果是：

E. 尿呈碱性，有脓细胞，有细菌生长

这种情况与患者的症状和抗生素无效的治疗史最为符合。
55、 (A2 型题)
男，65 岁，胸痛 3 小时，感胸闷心悸，伴恶心、呕吐、大汗，口服硝酸甘油不缓解，查体：心率 40 次/分，大炮音。冠脉造影示右冠严重堵塞。下列可能出现的心律失常是
A.三度房室传导阻滞
B.二度窦房传导阻滞
C.一度房室传导阻滞
D.一度窦房传导阻滞
E.二度房室传导阻滞
参考答案： A 您的答案：
图文解析： 【解析】三度房室阻滞因房室分离，第一心音强度经常变化，第二心音可呈正常或反常分裂，间或听到响亮亢进的第一心音（大炮音）。此患者心率 40 次/分，大炮音，因此心电图表现最可能是三度房室传导阻滞（A 对）。
【避错】三度房室传导阻滞，阻滞部位可位于房室结、希氏束和双侧束支水平，心室的激动由房室结以下的被动心律产生，即逸搏心律。如出现室性逸搏心律，往往提示发生阻滞的部位较低。由于心房与心室分别由两个不同的起搏点激动，各自保持自身的节律，心电图上表现为：P 波与 QRS 波群毫无关系（PR 间期不固定），心房率快于心室率。
考虑到病人的症状（胸痛、胸闷、心悸、恶心、呕吐、大汗）和体征（心率 40 次/分，大炮音），以及冠脉造影显示的右冠严重堵塞，这些表现指向心脏输出量严重减少和心脏传导系统的受损。大炮音通常与心房和心室的不同步收缩有关，这在三度房室传导阻滞中很常见，因为在这种情况下，心房和心室之间的电气活动完全解耦。
因此，最可能的心律失常是三度房室传导阻滞（A）。三度房室传导阻滞，也称为完全性房室传导阻滞，是心脏传导系统中最严重的阻滞形式，心房和心室之间的电气连接完全丧失，导致心率显著降低，这与患者呈现的症状和体征相符。
解析：
患者老年男性，因胸痛 3 小时就诊，口服硝酸甘油无法缓解，冠脉造影示右冠严重堵塞，根据患者症状，体征及冠脉造影检查，考虑诊断为急性心梗。患者查体心率减慢，出现大炮音，为三度房室传导阻滞的典型体征，结合患者病史和临床表现，该患者最可能出现的心律失常是三度房室传导阻滞。
选项 B 二度窦房传导阻滞，选项 C 一度房室传导阻滞，选项 D 一度窦房传导阻滞，选项 E 二度房室传导阻滞，均可见于心肌梗死，但以上疾病一般无大炮音等体征，故可一并排除。
综上所述，正确答案是：A.三度房室传导阻滞。
根据题目提供的信息，这名 65 岁男性患者出现了急性冠状动脉综合征的典型症状（胸痛、胸闷、心悸、恶心、呕吐、大汗），硝酸甘油无效，心率 40 次/分，听诊有大炮音，并且冠脉造影显示右冠严重堵塞。这些信息提示患者可能出现了严重的心律失常。

在右冠状动脉严重堵塞的情况下，最可能影响右侧心脏的传导系统，特别是供应右心房和房室结的血流。因此，最有可能发生的心律失常是：

A. 三度房室传导阻滞

三度房室传导阻滞（也称为完全性房室传导阻滞）是指心房和心室之间的电信号完全不能传导，导致心房和心室独立跳动。这种情况可能导致心率显著下降，正如题目中提到的心率 40 次/分，而且这种严重的阻滞与右冠状动脉的供血不足密切相关。

因此，最佳答案是：**A. 三度房室传导阻滞**

56、 (A2 型题)

女，70 岁，突发心悸两小时，伴头晕乏力出冷汗，BP80/50mmHg，心脏无扩大，心率 180 次/分，心律绝对不齐，第一心音强弱不等，各瓣膜听诊未闻及病理性杂音，最合

A.临时心脏起搏

B.胺碘酮静注

C.同步电复律

D.美托洛尔静注

E.电除颤

参考答案： C 您的答案：

图文解析： 【解析】老年女性，心率绝对不齐，第一心音强弱不等，考虑房颤。BP80/50mmHg，提示血压下降需要紧急电复律（C 对）。

【避错】对于房颤伴快速心室率、药物治疗无效者，可施行房室结消融或改良术，并同时安置永久起搏器。对于心室率较慢的房颤患者，最长 RR 间期＞5 秒或症状显著者，亦应考虑起搏器治疗（A 错），控制心室率的药物包括 β 受体阻滞剂、钙通道阻滞剂、洋地黄制剂和某些抗心律失常药物（如胺碘酮、决奈达隆）此患者目前血压下降，控制心率不是首要治疗方式（BD 错），电除颤主要适用于心室颤动或快速型心律失常（E 错）。

这位 70 岁女性患者有心悸、头晕、乏力、出冷汗、低血压（BP 80/50 mmHg）和高心率（180 次/分），心律绝对不齐，第一心音强弱不等，这些症状和体征表明她可能经历了一种严重的心律失常。心律绝对不齐可能指示心房颤动。考虑到她的病情紧急（如低血压和高心率），需要立即干预。

选项 C“同步电复律”是在这种情况下首选的治疗方法。同步电复律是一种紧急医疗程序，用于治疗严重的心律失常，如不稳定的心房颤动或心室心动过速，特别是当患者出现血流动力学不稳定（如低血压）时。通过给予心脏精确的电击，来恢复正常的心律。

因此，最合适的答案是：C. 同步电复律

首先，我们要对老年女性的症状进行详细的分析，以便准确诊断她的病情，并选择最合适的治疗方案。

患者老年女性，突发心悸，伴头晕、乏力、出冷汗，这是典型的低血流量症状，可能与心律失常导致的血流动力学不稳定有关。查体发现血压偏低，心率高达 180 次/分，且心律绝对不齐，第一心音强弱不等，这些都是心房颤动的典型表现。结合患者的症状和体征，我们可以初步诊断为心房颤动。

接下来，我们逐一分析选项：

A. 临时心脏起搏：通常用于病态窦房结综合征或高度房室传导阻滞的患者，不适用于房颤。

B. 胺碘酮静注：胺碘酮是广谱抗心律失常药物，可用于转复房颤并维持窦性心律，适用于本例患者。

C. 同步电复律：虽可用于房颤的复律，但在此情况下，由于患者已出现血流动力学不稳定，直接电复律可能带来风险，且在没有充分准备和评估的情况下不宜进行。

D. 美托洛尔静注：主要用于控制心室率，而非复律，且在此血流动力学不稳定的情况下，使用 β 受体阻滞剂可能进一步降低血压，加重病情。

E. 电除颤：通常用于室颤或无脉性室速等致命性心律失常，不适用于房颤。
综上所述，考虑到患者的症状、体征及可能的诊断，最合适的治疗方案是 B.胺碘酮静注，因为它既能转复房颤，又能维持窦性心律，且相对安全。
因此，正确答案是 B.胺碘酮静注。
根据题目中的信息，这位 70 岁的女性患者突发心悸两小时，伴有头晕、乏力、出冷汗，血压为 80/50 mmHg，心率为 180 次/分，心律绝对不齐，第一心音强弱不等，各瓣膜听诊未闻及病理性杂音。

患者表现为急性心律失常（可能是心房颤动或心房扑动）伴有血流动力学不稳定（低血压），根据急性心律失常的处理指南，血流动力学不稳定的心律失常患者应立即进行同步电复律。因此，最佳答案是：

C. 同步电复律
57、 (A2 型题)
男性，27 岁，腹泻、呕吐 1 天。7 月 13 日就诊， 一天前稀水样便 10 次，呕吐 1 次，查体：T37.5℃,P110 次/分，R24 次/分，BP90/52mmHg，WBC8×109/L，N0.78，为快速临床 即进行的检查是
A.血生化检查
B.粪常规及涂片
C.血培养及药敏
D.粪动力及促动试验
E.粪培养及药敏
参考答案： D 您的答案：
图文解析： 【解析】患者腹泻（稀水样便 10 次）、呕吐，血压下降，白细胞、中性粒细胞正常范围（排除细菌感染），考虑霍乱感染可能性大。取发病早期的新鲜粪便或碱性胨水增菌培养 6 小时后，做暗视野显微镜检，可见穿梭状运动的弧菌，即为动力试验阳性，可快速诊断霍乱，故本题选 D。
【避错】霍乱患者腹泻常为首发症状，表现为无痛性剧烈腹泻，不伴有里急后重，速转为米泔样水便或洗肉水样血便。呕吐常发生在腹泻之后，多不伴恶心，呈喷射性呕吐。循环衰竭是严重失水所致的低血容量休克。
这个病例描述了一名 27 岁男性患者，表现有一天的腹泻和呕吐。患者体检发现体温 37.5℃，心率 110 次/分钟，呼吸频率 24 次/分钟，血压 90/52mmHg，白细胞计数 8×10^9/L，中性粒细胞比例 0.78。根据这些症状和体征，需要迅速诊断和处理。
选项分析如下：
- A. 血生化检查，可以用来检查患者的代谢状态和电解质平衡，但对于急性腹泻和呕吐的原因可能不是最直接的诊断手段。
- B. 粪常规及涂片，可用于检查是否有感染性肠炎，是常规的诊断手段之一。
- C. 血培养及药敏，适用于疑似血液感染的情况，虽然患者有发热和心率加快，但没有明显的菌血症症状。
- D. 粪动力及促动试验，更多用于慢性腹泻的诊断。
- E. 粪培养及药敏，适用于疑似感染性腹泻，可以检测特定的病原体并进行药物敏感性测试。
考虑到患者的主要症状是急性腹泻和呕吐，初步判断可能是感染性腹泻。因此，最适合的检查是粪常规及涂片(B)和粪培养及药敏(E)。粪常规及涂片可以快速诊断是否有感染性肠炎，而粪培养及药敏可以确定具体的病原体和选择合适的抗生素。鉴于题目要求选择最佳答案，

且考虑到需要快速临床诊断，我倾向于选择 **E. 粪培养及药敏**，因为它可以提供更具体的病原体信息和治疗指导。
解析：
青年男性患者，腹泻、呕吐 1 天，发病时间为 7 月 13 日，夏季为细菌性痢疾的高发季节，稀水样便、呕吐，低热，心率快，呼吸快，血压低，白细胞正常，中性粒细胞比例增高，根据患者症状，体征，辅助检查结果，考虑诊断为细菌性痢疾。细菌性痢疾为志贺菌感染所致的肠道传染病，为快速确诊，应进行粪便常规及涂片检查，若在粪便中检出巨噬细胞及大量白细胞、红细胞即可确诊。
选项 A 血生化检查主要用于了解肝、肾功能，血糖，血脂，电解质等指标，对于细菌性痢疾的诊断并无帮助，故排除。
选项 C 血培养及药敏，选项 E 粪培养及药敏，二者均可以确定病原体种类并进行药敏试验，为临床选用抗菌药物提供依据，但需要时间较长，故不作为快速诊断的首选。
选项 D 粪动力及促动试验临床上并无此检查，可优先排除。
综上所述，正确答案是：B.粪常规及涂片。
对于该患者，27 岁男性，腹泻和呕吐 1 天，出现了稀水样便，体检显示体温 37.5℃，心率 110 次/分，呼吸频率 24 次/分，血压 90/52mmHg，白细胞计数 8×10^9/L，中性粒细胞比例 0.78。患者有脱水迹象（低血压，心动过速），并且有急性胃肠炎的症状。

在这种情况下，快速临床检查的优先级是确认感染源和处理脱水问题。最直接相关的检查是了解腹泻的原因，特别是是否有细菌感染。因此，快速进行的检查应该是：

B. 粪常规及涂片

通过粪常规及涂片检查，可以迅速评估是否存在细菌感染或者寄生虫，并初步指导抗生素的使用。其他检查如血生化、血培养及药敏和粪培养及药敏虽然也重要，但通常需要更多时间，不是快速临床检查的首选。

所以，最正确的答案是：B. 粪常规及涂片。
58、 (A2 型题)
女，57 岁。左手拇指晨起僵硬伴疼痛 3 年，近半年出现该处的肿胀及活动受限，左手手指及指掌可触及硬性结节，被动活动患指可出现伴疼痛的弹响。左手 X 线无明显异常 最可能是
A.骨关节炎
B.关节内游离
C.类风湿性关节炎
D.风湿性关节炎
E.狭窄性腱鞘炎
参考答案： E 您的答案：
图文解析： 【解析】患者为中老年女性（腱鞘炎好发人群），左拇指晨僵疼痛，屈伸手指时有弹响（狭窄性腱鞘炎典型表现），故该患者最可能的诊断为狭窄性腱鞘炎（E 对）。
【避错】骨关节炎主要是关节的退行性病变，主要是关节疼痛活动后加重，且 X 线有相应表现（A 错），关节腔内游离体的存在会导致关节功能受限、疼痛、关节卡住、肿胀等症状，X 线可见相应表现（B 错）。类风湿性关节炎（C 错）主要表现为反复发作的、对称性的、多发性小关节炎，以近端指间关节、掌指关节最为多见，风湿性关节炎以感染为症

状，常累及大关节，如膝关节、肘关节、肩关节等，不会出现关节畸形，还可表现为关节红肿及疼痛、肠道感染、咽痛、咳嗽、颌下淋巴结肿大等情况，并常与心肌炎、心内膜炎等疾病伴随出现（D 错）。
考虑到病史和症状，最可能的诊断是 E.狭窄性腱鞘炎（也称作触发指）。病人有晨起僵硬和疼痛，这些症状持续三年并且最近六个月有肿胀和活动受限。硬性结节和被动活动时伴随疼痛的弹响是狭窄性腱鞘炎的典型症状。虽然类风湿性关节炎（C 选项）也可能表现为手指的疼痛和僵硬，但触及硬性结节和伴疼痛的弹响更符合狭窄性腱鞘炎的特征。另外，左手 X 线无明显异常，这也可以排除关节病变，如骨关节炎或风湿性关节炎，这些条件通常会在 X 线上显示出明显的异常。因此，E 选项（狭窄性腱鞘炎）是最合适的答案。
首先，我们分析题干：
患者信息：57 岁女性，左手拇指晨起僵硬伴疼痛 3 年。
近半年症状：左手拇指肿胀及活动受限，左手手指及指掌可触及硬性结节。
特殊体征：被动活动患指可出现伴疼痛的弹响。
X 线检查：左手 X 线无明显异常。
接下来，针对每个选项进行逻辑推理：
A. 骨关节炎：骨关节炎主要表现为关节疼痛、僵硬和肿胀，X 线检查常可见关节间隙狭窄、骨赘形成等。但题干中明确提到 X 线无明显异常，所以骨关节炎的可能性不大。
B. 关节内游离：关节内游离体通常是由剥脱性骨软骨炎、骨性关节炎等病变导致的，X 线检查应有异常表现。但题干中指出 X 线无明显异常，因此这一选项也不太可能。
C. 类风湿性关节炎：类风湿性关节炎常表现为多关节的疼痛、肿胀和功能障碍，且 X 线检查通常会有明显的关节改变。但题干描述的症状主要集中在左手拇指，且 X 线检查无异常，所以类风湿性关节炎的可能性较低。
D. 风湿性关节炎：风湿性关节炎常见于儿童，常表现为游走性多关节炎，且可能伴随心脏炎、皮下结节、环形红斑等症状。与题干描述不符，因此可以排除。
E. 狭窄性腱鞘炎：狭窄性腱鞘炎主要表现为手指弹响、疼痛、肿胀和活动受限，特别是在晨起或工作劳累后症状加重。这与题干中描述的“晨起僵硬伴疼痛”和“被动活动患指可出现伴疼痛的弹响”高度一致。
综上所述，结合患者的临床表现和 X 线检查无异常，最可能的诊断是狭窄性腱鞘炎。
因此，正确答案是：E.狭窄性腱鞘炎。
根据题目描述，该患者为 57 岁女性，左手拇指晨起僵硬伴疼痛 3 年，近半年出现肿胀及活动受限，左手手指及指掌可触及硬性结节，被动活动患指可出现伴疼痛的弹响，X 线无明显异常。这些症状提示患者可能患有以下几种疾病：

1. **骨关节炎**：主要表现为关节疼痛、僵硬、活动受限，常见于负重关节（如膝关节、髋关节），X 线常见关节间隙变窄、骨赘形成等特征。
2. **关节内游离**：表现为关节疼痛、活动受限，常伴有关节内异物感，X 线通常可以发现关节内游离体。
3. **类风湿性关节炎**：表现为多发性对称性关节炎，常见于手指、小关节，有晨僵、肿胀、疼痛，X 线可见关节破坏及骨质疏松。
4. **风湿性关节炎**：常见于年轻人，表现为游走性关节炎，伴有发热、心脏受累等，X 线常无特异性表现。
5. **狭窄性腱鞘炎**：也称为扳机指，表现为手指在活动时有弹响和疼痛，尤其是早晨或长时间不活动后，活动受限，X 线一般无异常。

结合该患者的症状，尤其是左手拇指晨起僵硬、疼痛、肿胀及活动受限，手指及指掌可触及硬性结节，被动活动患指可出现伴疼痛的弹响，且 X 线无明显异常，最可能的诊断是狭窄性腱鞘炎。

因此，最正确的答案是：

E. 狭窄性腱鞘炎
59、 (A2 型题)
一男性发热三天，服用扑热息痛治疗后，查血常规显示中心粒细胞中毒现象，白细胞 1.0×10^9/L，红细胞，血小板正常，中性粒细胞绝对值＜0.5×10^9/L，应考虑为什
A.低增生性白血病
B.粒细胞缺乏
C.传染性单核细胞增多症
D.嗜酸细胞增多症
E.骨髓异常增生综合征
参考答案： B 您的答案：
图文解析： 【解析】扑热息痛短期使用不良反应轻，常见恶心和呕吐，偶见皮疹、粒细胞缺乏症（B 对）、贫血、药热和黏膜损害等过敏反应。过量中毒可引起肝损害。长期大量用药，尤其是在肾功能低下者，可出现肾绞痛、急性肾衰竭或慢性肾衰竭（镇痛药性肾病）。【避错】白细胞减少指外周血白细胞绝对计数持续低于 4.0×109/L。中性粒细胞减少指外周血中性粒细胞绝对计数，在成人低于 2.0×109/L，在儿童≥10 岁低于 1.8×109/L 或＜10 岁低于 1.5×109/L；严重者低于 0.5×109/L 时，称为粒细胞缺乏症。
这道题考查的是血液病学知识，特别是对于发热、白细胞减少和中性粒细胞绝对值减少的鉴别诊断。
选项 A，“低增生性白血病”，通常表现为全血细胞减少，但仅凭这些信息难以确诊。
选项 B，“粒细胞缺乏”，这是一个可能的诊断，因为中性粒细胞绝对值＜0.5×10^9/L 表明有明显的粒细胞减少。如果这种减少是由药物（如扑热息痛）引起的，那么可能是药物诱发的中性粒细胞减少症。
选项 C，“传染性单核细胞增多症”，通常会引起白细胞总数的增加，尤其是单核细胞的增加，而不是减少。
选项 D，“嗜酸细胞增多症”，通常表现为嗜酸细胞的增加，与这里描述的情况不符。
选项 E，“骨髓异常增生综合征”，可以引起血细胞减少，但更常见的是多系细胞减少，并且通常会有不同程度的成熟障碍。
综合这些信息，最可能的答案是 B.粒细胞缺乏。因为患者在使用扑热息痛后出现了发热和显著的中性粒细胞减少，这可能是药物诱发的粒细胞缺乏症的表现。
首先，我们先分析题干中的关键信息和数据：
患者男性，发热三天。
服用扑热息痛（一种解热镇痛药）治疗后，血常规出现异常。
中心粒细胞出现中毒现象，这可能意味着存在某种感染或炎症。
白细胞总数明显降低（1.0×10^9/L），但红细胞和血小板数量正常。
中性粒细胞绝对值＜0.5×10^9/L，这是一个关键指标，指向特定的血液学异常。
现在，我们来逐一分析选项：
A. 低增生性白血病：这是一种骨髓增生程度较低的白血病，通常伴有全血细胞减少，包括红细胞、白细胞和血小板。但题干中明确指出红细胞和血小板数量正常，因此这个选项不太

可能。
B. 粒细胞缺乏：这是指中性粒细胞绝对值低于 0.5×10^9/L 的状态，与题干中的描述完全吻合。粒细胞缺乏常由药物（如解热镇痛药、抗生素等）或其他外部因素导致，与患者的用药史和血常规结果相符。
C. 传染性单核细胞增多症：这是一种由 EB 病毒感染引起的疾病，主要表现为发热、淋巴结肿大、咽痛等，血常规检查通常显示淋巴细胞增多，与题干描述不符。
D. 嗜酸细胞增多症：这是一种以嗜酸性粒细胞增多为特征的疾病，与中性粒细胞减少无直接关联。
E. 骨髓异常增生综合征：这是一种起源于造血干细胞的疾病，通常表现为一种或多种血细胞减少，且常伴随有原始细胞的增多，与题干描述不完全吻合。
综上所述，考虑到患者服用了解热镇痛药后出现中性粒细胞减少，且白细胞总数降低，但红细胞和血小板正常，最符合粒细胞缺乏的特征。
因此，正确答案是 B.粒细胞缺乏。
根据题干描述的患者情况：男性发热三天，服用扑热息痛治疗后出现中性粒细胞中毒现象，白细胞显著减少（1.0×10^9/L），中性粒细胞绝对值＜0.5×10^9/L，红细胞和血小板正常。这种表现最可能的诊断是粒细胞缺乏症（中性粒细胞缺乏症）。

粒细胞缺乏症（neutropenia）通常是指中性粒细胞绝对计数（ANC）低于 0.5×10^9/L，患者容易出现感染，尤其是细菌和真菌感染。题目中的血常规结果显示白细胞计数显著降低且中性粒细胞绝对值＜0.5×10^9/L，符合粒细胞缺乏症的诊断标准。

因此，最正确的答案是：

B. 粒细胞缺乏
60、 (A2 型题)
男，45 岁，跑步中跌到，肩部着地，方肩畸形，活动受限，搭肩试验阴性，影像学检查示：肱骨大结节下方骨皮质不连续，向内侧移位，属于肱骨近端骨折 neer 分型
A.neer 一部分骨折
B.neer 二部分骨折
C.neer 三部分骨折
D.neer 四部分骨折
E.其他类型骨折
参考答案： B 您的答案：
图文解析： 【解析】患者外伤史，影像提示：肱骨大结节下方骨皮质不连续，向内侧移位为大结节、外科颈骨折即 neer 三部分骨折（C 对）。
【避错】一部分骨折：肱骨近端骨折，无论骨折线数量是多少，只要未达到上述移位标准，说明骨折部位尚有一定的软组织附着连接，这种骨折为无移位或轻微移位骨折，或称为一部分骨折（A 错）。两部分骨折：仅一个部位发生骨折并且移位者，称之为两部分骨折（B 错），它有 4 种形式，即解剖颈骨折、大结节骨折、小结节骨折或外科颈骨折。四部分骨折（D 错）：当肱骨近端 4 个部分都发生骨折移位时，形成四个分离的骨块，称为四部分骨折，此时肱骨头向外侧脱位，成游离状态，血液供应破坏严重，极易发生缺血坏死。
根据所给信息，患者为 45 岁男性，在跑步中跌倒，出现肩部方肩畸形，活动受限，搭肩试验阴性，影像学检查显示肱骨大结节下方骨皮质不连续且向内侧移位。这些症状和影像学结果指向肱骨近端骨折。

Neer 分型是用来描述肱骨近端骨折的一种分类法，它将肱骨近端骨折分为四个部分：大结节、小结节、肱骨干和肩关节的良性肿瘤。骨折部位的数量决定了分型。
- **Neer 一部分骨折**指的是仅有一个部分断裂，通常是无移位的大结节骨折。
- **Neer 二部分骨折**涉及两个断裂部分，常见的是大结节或小结节与肱骨干的分离。
- **Neer 三部分骨折**包括三个断裂部分，常见的是大结节、小结节和肱骨干均发生断裂。
- **Neer 四部分骨折**指的是四个主要部分均发生了断裂。
题目中描述了肱骨大结节下方骨皮质不连续，向内侧移位，表明至少有一个明确的断裂部分，但未提及其他部位的损伤。这可能指向一个较为简单的骨折类型。因此，最符合描述的是**Neer 二部分骨折**（选项 B），因为它至少涉及到了一个主要的骨折部位及其移位，符合影像学上所见的肱骨大结节下方的骨皮质不连续情况。
解析：
中年男性患者，外伤后导致肩部畸形，活动受限，影像学检查示：肱骨大结节下方骨皮质不连续，向内侧移位，根据 neer 分型标准，若只是一处骨折，属于 neer 一部分骨折，包括外科颈骨折、大结节与小结节骨折、关节盂骨折、解剖颈骨折。本例患者肱骨大结节下方骨皮质不连续，仅涉及一个部位，为 neer 一部分骨折。
选项 B：neer 二部分骨折是指一处骨折且伴随肩关节脱位，或两处骨折但无关节脱位，不符合题意。
选项 C：neer 三部分骨折指两处骨折且伴随肩关节脱位，或三处骨折但无关节脱位，故排除。
选项 D：neer 四部分骨折是指肱骨近端四部分解剖结构均发生骨折，与本例患者的骨折类型不符，因此不选。
选项 E：其他类型骨折在 neer 分型中并无此分类，故排除。
综上所述，正确答案是：A.neer 一部分骨折。
根据题目描述，45 岁男性跑步中跌倒，肩部着地，出现方肩畸形，活动受限，搭肩试验阴性，影像学检查显示肱骨大结节下方骨皮质不连续，向内侧移位。根据这些描述，可以判断这是肱骨近端骨折。Neer 分型系统用于分类肱骨近端骨折，根据骨折的具体部位和移位程度将其分为以下几种：

1. Neer 一部分骨折：骨折不移位或移位不到 1 cm，且角度不超过 45°。
2. Neer 二部分骨折：骨折移位超过 1 cm，或角度超过 45°，通常涉及大结节或小结节。
3. Neer 三部分骨折：两处骨折线，通常涉及大结节、小结节和外科颈。
4. Neer 四部分骨折：三处骨折线，涉及大结节、小结节、外科颈和解剖颈。

根据题干中描述的肱骨大结节下方骨皮质不连续并向内侧移位，这意味着至少有两个部分发生了移位，通常符合 Neer 三部分骨折的定义。

因此，最正确的答案是：

B. neer 二部分骨折
61、 (A2 型题)
男，66 岁。发作性胸痛 1 小时。在问病史过程中突然跌倒，对呼唤和推搡无反应。此时应立即采取的措施是
A.做心脏彩超
B.送抢救室

C.触诊大动脉
D.做心电图
E.查看瞳孔
参考答案：　　C　您的答案：
图文解析：　　【解析】中老年男性患者，发作性胸痛 1 小时（怀疑心肌梗死），意识丧失，呼之不应，应紧急检查心跳呼吸是否存在，故本题选选 C。
【避错】若心跳呼吸停止应立即行心肺复苏。成人心肺复苏的顺序是 C-A-B，C（Circulation） -人工胸外按压：按压频率为 100～120 次/分，成人按压幅度 5～6cm，儿童约 5cm，婴儿约 4cm；A（Airway） -开通气道：先行 30 次胸外按压后，再开通气道；B（Breathing） -人工呼吸：开放气道后，先进行 2 次人工呼吸，每次持续吹气时间 1 秒以上。
这个情况描述了一个 66 岁的男性患者，出现了发作性胸痛并在问诊过程中突然跌倒，对呼唤和推搡无反应。这可能暗示患者出现了心脏停止或严重的循环障碍。
立即采取的措施应该是针对急救和评估患者的生命体征。在这些选项中，最直接和紧急的措施是将患者送往抢救室进行紧急救治，因为这种情况可能需要立即的生命支持和进一步的评估。
因此，最合适的答案是：B.送抢救室。
解析：
老年男性患者，发作性胸痛，提示可能有心绞痛或心肌梗死等心脏疾病，在问病史过程中突然跌倒，对呼唤和推搡无反应，提示可能出现心源性休克，或心脏骤停。此时应首先触诊大动脉，判断是否发生心脏骤停，再采取相应的急救措施。
选项 A 做心脏彩超主要用于检查心脏的结构和运动状态，无法评估患者的意识状态，且不是抢救的首要步骤，故不选。
选项 B 送抢救室应在初步评估患者病情后进行，若患者心脏骤停，应立刻进行心肺复苏，而非直接送抢救室，故排除。
选项 D 做心电图主要用于诊断心律失常，心梗等疾病，该患者已意识丧失，首要任务是判断患者的生命体征，故不考虑。
选项 E 查看瞳孔可评估患者的意识状态，但心脏骤停黄金抢救时间只有 4 分钟，因此应首先通过触诊大动脉的方式，快速判断是否发生心脏骤停，若心脏骤停需立刻进行心肺复苏，若心脏未骤停，再进行其他生命体征的检查，如查看瞳孔，故不选。
综上所述，正确答案是：C.触诊大动脉。
根据题目中的描述，该患者是一名 66 岁的男性，出现了发作性胸痛 1 小时，在问病史过程中突然跌倒，并且对呼唤和推搡无反应。这提示患者可能出现了心脏骤停或其他严重的急症。在这种情况下，最重要的是立即评估患者的生命体征。

在临床急救中，对于突然跌倒且无反应的患者，首要措施是评估有无大动脉搏动，以判断是否存在心脏骤停。因此，正确的答案应该是：

C. 触诊大动脉
62、　(A2 型题)
一男子偶有头痛胸闷，多次自测血压 150/110mmHg 左右，心率 98 次/分，就医按指导控制饮食、运动后仍控制不佳，测血压 150/95mmHg，双侧肾动脉 B 超无特殊。应采取的
A.口服氢氯噻嗪
B.继续观察半年

C.口服比索洛尔
D.症状出现时舌下含服托那普利
E.口服特拉唑嗪
参考答案： C 您的答案：
图文解析： 【解析】中年男性，多次测量血压偏高，考虑高血压，肾脏彩超无特殊考虑原发性高血压，心率偏快选择β受体拮抗剂能抑制心脏β肾上腺素能受体，减慢心率、减弱心肌收缩力、降低血压（C 对）。
【避错】利尿剂类药物可以降血压，但无控制心率作用（A 错）；患者已可确诊高血压需立即治疗，不能仅观察（B 错）；患者血压未到高血压急症，无须舌下含服紧急处理（D 错）；特拉唑嗪为选择性α1 受体阻滞剂，能降低外周血管阻力，对收缩压和舒张压都有降低作用但无控制心率作用（E 错）。
这道题目考察的是高血压的管理和治疗。根据题干信息，这位男子有高血压（150/110 mmHg 降至 150/95 mmHg）和心率偏高（98 次/分），且通过改变生活方式（饮食、运动）后血压仍未得到有效控制。双侧肾动脉 B 超无特殊，说明没有肾源性高血压的迹象。
A 选项的氢氯噻嗪是一种利尿剂，用于治疗高血压，但可能不是首选药物，因为它可能导致血糖和血脂异常，特别是在有心率增快的患者中。
B 选项的继续观察半年，对于已经确认有持续高血压和心率增快的患者来说，并不是一个合适的选择，因为这可能增加心脑血管事件的风险。
C 选项的比索洛尔是一种β阻滞剂，可以降低血压和心率，适用于同时存在高血压和心率增快的患者。
D 选项的托那普利是一种血管紧张素转换酶抑制剂（ACE 抑制剂），通常用于长期管理高血压，而不是仅在症状出现时使用。
E 选项的特拉唑嗪是一种噻嗪类利尿剂，用于治疗高血压，但与氢氯噻嗪相似，可能不是心率增快患者的最佳选择。
综合考虑，最佳选择似乎是 C 选项（口服比索洛尔），因为它可以同时管理高血压和心率增快。不过，实际选择应根据患者具体情况和医生的专业判断。
解析：
患者青年男性，多次自测血压均高，就医控制饮食、运动后血压控制仍不佳，可诊断为高血压病，因患者双侧肾动脉 B 超无特殊，可排除继发性高血压，考虑诊断为原发性高血压。患者就医按指导控制饮食、运动后血压仍控制不佳，应采取药物治疗。患者青年男性，心率偏快，为交感神经张力增高征象，且舒张压高，因此应选择β受体阻滞剂，比索洛尔为选择性β1 受体阻滞剂，可减慢心率，同时降低心输出量，从而降低血压，为首选治疗药物。
选项 A 口服氢氯噻嗪为噻嗪类利尿剂，主要通过排钠，减少细胞外容量，降低外周血管阻力进行降压，适用于单纯收缩期高血压、盐敏感性高血压、合并肥胖或糖尿病、合并心力衰竭的患者，与该患者症状不符，故不选。
患者已出现高血压症状，且经过控制饮食和运动后，血压仍控制不佳，应及时采取药物治疗，因此选项 B 继续观察半年，可排除。
选项 D 症状出现时舌下含服托那普利起效较慢，不适用于高血压急症的治疗，一般用于慢性高血压的维持治疗，故不考虑。
选项 E 口服特拉唑嗪为α受体阻滞剂，因其副作用较多，目前不主张单独使用，故排除。
综上所述，正确答案是：C.口服比索洛尔。
根据题目中提供的信息，该男子偶有头痛胸闷，血压持续在高水平（150/110 mmHg 左右），心率较快（98 次/分），饮食和运动调控无明显效果，且肾动脉 B 超检查未见异常。这些症状和检查结果提示该男子可能患有原发性高血压，需要药物治疗来控制血压。

各选项的分析如下：
- **A. 口服氢氯噻嗪**：氢氯噻嗪是常用的利尿剂，可用于治疗高血压。它通过排出体内多余的钠和水分来降低血压。
- **B. 继续观察半年**：该男子血压已经持续高于正常水平，单靠观察可能会延误治疗时机。
- **C. 口服比索洛尔**：比索洛尔是β受体阻滞剂，可以降低心率和血压。它特别适用于心率较快且有高血压的患者。
- **D. 症状出现时舌下含服托那普利**：托那普利是ACE抑制剂，通常用于长期管理高血压，而不是症状出现时的临时治疗。
- **E. 口服特拉唑嗪**：特拉唑嗪是α受体阻滞剂，主要用于治疗良性前列腺增生症，但也可以用于高血压的治疗。

结合该男子的高血压情况和心率较快的特点，最佳答案是**C. 口服比索洛尔**。比索洛尔不仅可以降低血压，还可以减慢心率，有助于缓解头痛和胸闷等症状。

因此，答案是 **C. 口服比索洛尔**。
63、 (A2 型题)
中年男性，急性广泛前壁心肌梗死入院。第二天，患者气促加重，BP76/50mmHg，P130 次/分，中心静脉压 25cmH2O，双中下肺可闻及湿啰音。治疗选用
A.硝酸甘油
B.呋塞米
C.去甲肾上腺素
D.肾上腺素
E.洋地黄
参考答案： E 您的答案：
图文解析： 【解析】男性患者，急性心梗病史，超 24 小时，血压低（BP76/50mmHg），心率快（P130 次/分），中心静脉压 25cmH2O、双中下肺可闻及湿啰音（提示心功能不全），考虑急性心梗后出现心衰。急性心梗 24 小时内禁用洋地黄，24 小时后出现心功能不全、心衰等可用洋地黄，故本题选 E。
【避错】硝酸甘油主要是扩张血管，应用于心梗（A 错）、患者血压偏低，不考虑使用利尿剂（B 错）；去甲肾上腺素可以增强心肌收缩力同时增加外周阻力，对于改善心脏负荷无明显作用（C 错）；肾上腺素主要增强心肌收缩力同时加快心率，且在心衰时使用容易导致心律失常（D 错）。
中年男性患者出现急性广泛前壁心肌梗死后，第二天气促加重，低血压（BP76/50mmHg），心率加快（P130 次/分），中心静脉压升高（25cmH2O），双中下肺闻及湿啰音，这些症状和体征表明患者可能发展成了心源性休克，伴有肺充血。
治疗选择应该着眼于改善血流动力学状态、提高心脏输出量和降低肺水肿。在这些选项中：
- A. 硝酸甘油可以扩张血管，降低前负荷和后负荷，但在这种情况下可能会因降低血压而加剧休克。
- B. 呋塞米是利尿剂，可用于减轻肺水肿，但不会直接改善血流动力学状态，且在急性期使用时需谨慎，以免导致血压进一步下降和肾功能恶化。
- C. 去甲肾上腺素具有强大的α受体激活作用，可以增加心脏输出量和提高血压，适合治疗心源性休克。
- D. 肾上腺素增加心脏的收缩力和心率，但在心源性休克中常用作对其他治疗无反应时的选

择。
- E. 洋地黄可以增强心脏收缩力，但主要用于治疗心力衰竭和心房颤动，并不适用于急性心源性休克的立即抢救。
考虑到这名患者的情况和需要迅速稳定血流动力学，最佳选择应该是**C. 去甲肾上腺素**。它可以有效提高血压和心脏输出量，适用于治疗由急性心肌梗死引起的心源性休克。
解析：
患者中年男性，因急性广泛前壁心肌梗死入院，现患者出现低血压休克、心率加快、中心静脉压升高和双中下肺湿啰音等症状，考虑诊断为急性心肌梗死后心力衰竭并导致的心源性休克。此时应首选使用血管活性药物维持血压，保证心脏和大脑的血液灌注**。去甲肾上腺素**为强烈的 α 受体激动药，可引起小动脉和小静脉血管收缩，以皮肤、黏膜血管收缩最明显，能迅速升高血压，是抗休克的血管活性药物。
选项 A 硝酸甘油主要用于心绞痛急性发作，也用于降低血压或治疗充血性心力衰竭，但对心源性休克的治疗效果并不明显，故不选。
选项 B 呋塞米为利尿剂，主要用于消除水肿，与该患者当前的治疗目标不符，故排除。
选项 D 肾上腺素常用于过敏性休克的治疗，具有兴奋心肌、升高血压、松弛支气管平滑肌等作用，但因其同时激动 β 1 和 β 2 受体，可使心肌耗氧量增加，引起心律失常，且可加重心肌缺血，一般不用于急性心肌梗死及心源性休克的治疗，故不选。
选项 E 洋地黄为正性肌力药物，可增加心肌耗氧量，心肌梗死 24 小时内应禁用，否则会加重心脏负担，恶化病情，与题意不符。
综上所述，正确答案是：C.去甲肾上腺素。
这位中年男性患者表现出急性广泛前壁心肌梗死后的急性左心衰竭症状，包括气促加重、血压低（BP76/50mmHg）、心率快（P130 次/分）、中心静脉压高（25cmH2O）以及双中下肺湿啰音。这些症状提示患者可能存在心源性休克和急性肺水肿。

针对这种情况，治疗的重点是减轻心脏负担、改善心功能和缓解肺水肿症状。根据临床指南和最佳实践，最适合的治疗选项是：

B. 呋塞米

呋塞米是一种强效利尿剂，可以迅速减轻肺水肿，降低中心静脉压，从而改善呼吸困难。使用呋塞米可以有效缓解患者的症状并稳定病情。

其他选项的分析：
- A. 硝酸甘油：用于缓解心绞痛和降低心脏前负荷，但对于血压过低的患者需谨慎使用。
- C. 去甲肾上腺素：主要用于纠正低血压，但不能直接改善肺水肿症状。
- D. 肾上腺素：主要用于心脏骤停和严重过敏反应的急救，非首选治疗肺水肿。
- E. 洋地黄：用于慢性心衰和心房颤动的控制，不适合急性期的快速干预。

因此，最佳答案是：

B. 呋塞米
64、 (A2 型题)
女，血压 190/110mmHg，B 超提示左肾动脉狭窄 55%，右肾动脉狭窄 50%，导致血压升高的原因是

A.激活交感神经系统
B.激活肾素-血管紧张素-醛固酮系统
C.激活激肽系统
D.血管升压素升高
E.心房利钠肽升高
参考答案：　B　您的答案：
图文解析：　【解析】由于肾动脉狭窄，肾脏血流灌注减少肾脏压力感受器反馈作用会激活肾素-血管紧张素-醛固酮系统，造成血压升高，通过血压升高来促进肾脏灌注（B 对）。
【避错】肾血管性高血压是继发性高血压的第二位原因，系由于单侧或双侧肾动脉主支管腔狭窄，导致肾脏血流灌注降低而引起高血压。肾动脉狭窄的表现包括腹部血管杂音、自发性低血钾和肾功能进行性减退。
此题目考察的是继发性高血压，特别是由肾动脉狭窄引起的。肾动脉狭窄会导致肾脏血流量减少，引发肾脏释放肾素，从而激活肾素-血管紧张素-醛固酮系统（RAAS），增加血管紧张素 II 的生成，最终导致血压升高。因此，导致血压升高的原因是激活肾素-血管紧张素-醛固酮系统。
选项 B“激活肾素-血管紧张素-醛固酮系统”是最合适的答案。
解析：
青年女性患者，血压升高，B 超提示左肾动脉狭窄 55%，右肾动脉狭窄 50%。对于该患者，血压升高最可能的原因是双侧肾动脉狭窄，狭窄的肾动脉可引起肾灌注压下降，从而刺激肾素释放，导致血管紧张素 II 及醛固酮含量增加，进而使血压升高。因此，该患者血压升高的原因是激活肾素-血管紧张素-醛固酮系统。
选项 A 激活交感神经系统可使心率加快，心肌收缩力加强，心输出量增加，血压升高，但一般不会导致如此高的血压，故不选。
选项 C 激活激肽系统可使细动脉扩张、血管通透性增加、支气管平滑肌舒张，并可引起疼痛，不会导致血压升高，故排除。
选项 D 血管升压素升高可使远曲小管和集合管对水的通透性增加，水的重吸收增加，尿液浓缩和稀释功能进一步受到损害，尿量减少，但无升压作用，故不选。
选项 E 心房利钠肽升高可增加肾小球滤过率，抑制肾小管重吸收钠，产生明显的利钠利尿排钾作用，抑制肾素、血管紧张素和醛固酮的分泌，降低血压，与题干描述不符。
综上所述，正确答案是：B.激活肾素-血管紧张素-醛固酮系统。
该题目描述了一名血压高达 190/110mmHg 的女性，B 超提示其左肾动脉狭窄 55%，右肾动脉狭窄 50%。肾动脉狭窄会引起肾脏缺血，进而激活一系列代偿机制来维持肾脏灌注和血压。在这种情况下，最可能导致血压升高的原因是激活肾素-血管紧张素-醛固酮系统（RAAS）。
肾动脉狭窄导致肾脏灌注不足，肾小球旁细胞检测到灌注压降低，进而分泌肾素。肾素催化血管紧张素原生成血管紧张素 I，后者在血管紧张素转换酶（ACE）的作用下转化为血管紧张素 II。血管紧张素 II 是强效的血管收缩剂，还会刺激醛固酮的分泌，进一步增加钠和水的重吸收，导致血容量增加，从而升高血压。
因此，最正确的答案是：B. 激活肾素-血管紧张素-醛固酮系统
65、　(A2 型题)
男，58 岁，突发胸痛 2 小时，呈持续性撕裂样疼痛，向肩背部和腰部放射。既往有高脂血症和高血压 5 年，未规范治疗。查体：左上肢血压 140/85mmHg，右上肢 180/105mmH 次/分，为明确诊断，首选的检查是
A.主动脉 CTA
B.胸部 X 线

C.心电图
D.超声心动图
E.冠状动脉造影
参考答案：　A　您的答案：
图文解析：　【解析】中年男性，突发剧烈疼痛（是主动脉夹层最主要和最常见的临床表现），呈撕裂状，累及肩背部和腰部放射。既往高血压病史（是主动脉夹层最重要的危险因素），查体：左上肢血压 140/85mmHg，右上肢 180/105mmHg（双侧上肢不一致）。综合该患者的临床表现、查体、心电图检查，该患者诊断考虑为主动脉夹层。主动脉夹层首选的检查是：主动脉 CTA（A 对），其敏感性与特异性可达 98%左右，主动脉造影可以显示裂口的部位，明确分支和主动脉受累情况，估测主动脉瓣关闭不全的严重程度。
【避错】超声心动图多用于心脏瓣膜性疾病的诊治（D 错），胸部 X 线可用于观察心脏大小及外形的改变（B 错），心电图主要诊断心梗和心律失常（C 错），冠脉造影主要诊断冠状动脉是否狭窄（E 错）。
考虑到这位 58 岁男性患者突发的持续性胸痛、撕裂样疼痛，以及疼痛放射到肩背部和腰部，结合其高脂血症和高血压病史，首先考虑的诊断是主动脉夹层。这种情况下，左右上肢血压的明显差异也支持这一诊断。因此，为了明确诊断，首选的检查是 **主动脉 CTA（计算机断层扫描血管造影）**。
A. 主动脉 CTA - 此选项是用于确诊主动脉夹层的最佳检查，能够详细显示主动脉的解剖结构和可能存在的夹层。
所以，正确答案是 **A. 主动脉 CTA**。
解析：
患者老年男性，突发胸痛，疼痛剧烈呈撕裂样，并向肩背部和腰部放射，为急性主动脉夹层的典型胸痛表现。患者既往有高脂血症和高血压，查体发现双上肢血压不对称，提示可能存在主动脉夹层，综合患者的病史、症状、体征，该患者考虑诊断为主动脉夹层。为明确诊断，应行主动脉 CTA，其能清楚的显示主动脉腔内的内膜片、内膜破口及所累及的范围等，是诊断主动脉夹层的金标准。
选项 B 胸部 X 线有助于发现动脉钙化影，但对主动脉夹层的诊断价值不大，故不选。
选项 C 心电图主要用于心脏循环系统方面疾病，尤其是心律失常的诊断，对于主动脉夹层的诊断没有帮助，故排除。
选项 D 超声心动图可以识别出真、假腔或内膜裂口下垂的物，但对局限于升主动脉远端和主动脉弓部的病变因受主气道内空气的影响，超声探测可能漏诊，不作为首选检查，故不考虑。
选项 E 冠状动脉造影是诊断冠状动脉粥样硬化性心脏病的一种常用且有效的方法，不用于主动脉夹层的诊断，可排除。
综上所述，正确答案是：A.主动脉 CTA。
根据题干描述，该患者为 58 岁男性，突发胸痛 2 小时，呈持续性撕裂样疼痛，向肩背部和腰部放射，且既往有高脂血症和高血压病史，并且血压存在显著的左右差异。这些症状和体征高度提示主动脉夹层的可能性。

为了明确诊断，最适合的检查是能够详细显示主动脉的形态学变化的检查方法。因此，首选的检查应该是主动脉 CTA（计算机断层扫描血管成像），因为它能够快速、准确地显示主动脉壁的夹层和破裂情况。

因此，最佳答案是：

A.主动脉 CTA

66、 (A2 型题)

男，62 岁，既往陈旧性心肌梗死，PCI 术后 3 年，两年半前开始出现活动后心累，夜间阵发性呼吸困难，间断双下肢水肿，口服利尿药治疗有效。心脏超声示：左室扩大， 段性运动减弱，LVEF35%，近期开始出现阵发性房颤，推荐使用的药物不包括

A.普萘洛尔

B.普罗帕酮

C.地高辛

D.比索洛尔

E.胺碘酮

参考答案： B 您的答案：

图文解析： 【解析】老年男性，既往陈旧性心肌梗死，出现活动后心累，夜间阵发性呼吸困难，间断双下肢水肿，口服利尿药治疗有效（考虑心力衰竭），房颤。心脏超声示左室扩大，左室前壁节段性运动减弱，LVEF35%（考虑心功能不全）。综合考虑此患者为心衰合并房颤，普罗帕酮具有负性肌力作用，可增加中重度心衰患者死亡率，故不考虑使用（B 错，为本题正确答案）。

【避错】β 受体阻滞剂可以控制心率，改善心室重构（AD 对），胺碘酮可以转复心律，致心律失常发生率最低，是目前常用的维持窦性心律药物，特别适用于合并器质性心脏病的患者（E 对），洋地黄类药物可以正性肌力同时减慢心率（C 对）。

这位 62 岁的男性患者有陈旧性心肌梗死的病史，曾进行过经皮冠状动脉介入（PCI）治疗，且自两年半前开始出现心力衰竭的症状，如活动后心累、夜间阵发性呼吸困难和间断的双下肢水肿，而利尿药治疗有效。心脏超声显示左室扩大，段性运动减弱，左室射血分数（LVEF）为 35%，近期出现阵发性房颤。题目要求选择不推荐使用的药物。

在心力衰竭和房颤治疗中，普通使用的药物包括：

- 普萘洛尔（β-受体阻滞剂）
- 普罗帕酮（抗心律失常药）
- 地高辛（心脏糖苷）
- 比索洛尔（β-受体阻滞剂）
- 胺碘酮（抗心律失常药）

根据病例描述，患者有心力衰竭和房颤，因此通常需要使用β-受体阻滞剂（如普萘洛尔或比索洛尔）、抗心律失常药（如普罗帕酮或胺碘酮）和心脏糖苷（如地高辛）。然而，在选择具体药物时，需要考虑病人的整体状况和药物的具体特性。

考虑到这一点，地高辛（选项 C）在治疗心力衰竭和控制房颤方面是有效的，尤其是在降低心率方面。然而，地高辛需要在严密监控下使用，因为它有较窄的治疗窗口和潜在的毒性。但在给定的选项中，没有直接的理由表明地高辛不被推荐。

考虑到上述情况，更可能是其中一个β-受体阻滞剂（普萘洛尔或比索洛尔）或抗心律失常药（普罗帕酮或胺碘酮）不被推荐。在慢性心力衰竭患者中，β-受体阻滞剂被广泛推荐用于减缓病程进展，改善预后。而普罗帕酮和胺碘酮主要用于心律失常的治疗。考虑到胺碘酮的副作用较多，可能在某些心力衰竭患者中不是首选，尤其是在长期使用时可能加重心脏功能不全。

因此，在这些选项中，虽然所有药物在某些情况下都可能适用，选择“不推荐使用”的药物较为复杂。如果患者的心力衰竭状况严重，并且考虑到药物副作用的风险，**胺碘酮（选项 E）**可能不是首选，因为它的长期使用与严重的副作用相关。然而，这需要根据具体的临床情况来决定，因为胺碘酮也是控制房颤的有效药物。

综上所述，最可能的“不推荐使用”的药物是 E.胺
碘酮，主要是基于其潜在的副作用和在特定情况下可能加重心脏状况的风险。
解析：
患者老年男性，既往有心肌梗死及 PCI 手术史，近期活动后心累，夜间阵发性呼吸困难，间断双下肢水肿，超声心动图示左室扩大，左室射血分数降低，提示存在心力衰竭，近期开始出现阵发性房颤，考虑诊断为房颤合并心力衰竭。此时治疗的关键在于控制心室率，恢复窦性心律，预防血栓栓塞。而普萘洛尔为β受体阻滞剂，可阻断心脏的β受体，减慢心率，降低心肌收缩力，减少心输出量，降低血压，一般不用于合并心力衰竭的房颤患者。
选项 B 普罗帕酮，选项 E 胺碘酮，均可用于房颤复律并维持窦性心律，故一并排除。
选项 C 地高辛为洋地黄类药物，可增强心肌收缩力，用于治疗心力衰竭和房颤，符合该患者的治疗原则。
选项 D 比索洛尔是选择性β1 受体阻滞剂，可降低心肌耗氧量，减慢心率，抗心律失常，对于房颤患者有一定疗效。同时对于心力衰竭的患者，比索洛尔从小剂量开始使用，有助于抑制心衰代偿机制中不利的影响，改善心室重塑，因此也适用于该患者。
综上所述，正确答案是：A.普萘洛尔。
这位 62 岁的男性患者既往有陈旧性心肌梗死史，行 PCI 术后 3 年，并且出现心力衰竭的症状，如活动后心累、夜间阵发性呼吸困难和双下肢水肿。心脏超声显示左室扩大，段性运动减弱，LVEF 为 35%，属于心功能不全。近期还出现了阵发性房颤。

对于这种情况的患者，在选择药物时，需要考虑其心功能不全和房颤的治疗。某些药物可能会加重心功能不全，因此不推荐使用。

让我们分析一下各个选项：

A. **普萘洛尔（Propranolol）** - 普萘洛尔是一个非选择性β受体阻滞剂，对心功能不全患者较少使用，因为它可能会加重心力衰竭症状。

B. **普罗帕酮（Propafenone）** - 普罗帕酮是一种抗心律失常药物，对心功能不全患者禁忌，因为它可以导致负性肌力作用和心力衰竭恶化。

C. **地高辛（Digoxin）** - 地高辛常用于心力衰竭和房颤的控制，它可以帮助提高心脏收缩力，并控制心率，是合适的选择。

D. **比索洛尔（Bisoprolol）** - 比索洛尔是选择性β1 受体阻滞剂，适用于心力衰竭患者，可以改善预后，并控制房颤心率。

E. **胺碘酮（Amiodarone）** - 胺碘酮是一种有效的抗心律失常药物，可以用于心功能不全患者的房颤控制。

因此，答案是 **B. 普罗帕酮**，因为它不适合心功能不全的患者，会加重心力衰竭的症状。

最终推荐使用的药物不包括 **B. 普罗帕酮**。
67、 (A2 型题)
患者，女，70 岁。反复双膝关节疼痛 10 年，逐渐加重 2 年，活动时关节有弹响。体检：双

膝关节骨摩擦音(+)，但无明显红肿及压痛。最可能的诊断是
A.骨关节炎
B.类风湿关节炎
C.反应性关节炎
D.强直性脊柱炎
E.系统性红斑狼疮
参考答案： A 您的答案：
图文解析： 【解析】老年女性患者（骨关节炎好发人群），双膝关节疼痛，活动弹响 10 余年，膝关节有骨擦音（骨关节炎常见表现），本例最可能的诊断为骨性关节炎（A 对）。【避错】类风湿性关节炎（B 错）有关节外风湿结节表现；反应性关节炎主要是感染所致，会有关节外感染表现，部分患者会出现红肿（C 错）；强直性脊柱炎（D 错）多见于青年男性，最典型和常见症状为炎性腰背痛；系统性红斑狼疮多为年轻女性，表现有关节疼痛、肿胀、炎症（E 错）。
患者的症状，包括长期的双膝关节疼痛、活动时关节弹响和体检发现的骨摩擦音，但无明显红肿和压痛，最符合骨关节炎（OA）的临床表现。骨关节炎是一种常见的关节疾病，特别是在老年人中，表现为关节软骨逐渐磨损和退化，导致疼痛和功能障碍。
类风湿关节炎（RA）通常表现为关节的疼痛、肿胀和晨僵，而反应性关节炎、强直性脊柱炎和系统性红斑狼疮通常具有其他更特异性的临床特征，这些特征在该病例描述中未提及。因此，最可能的诊断是 **A. 骨关节炎**。
解析：
患者老年女性，反复双膝关节疼痛多年，并逐渐加重，活动时关节有弹响，体检发现双膝关节骨摩擦音阳性，但关节无明显红肿及压痛，结合患者年龄、临床表现及体格检查，考虑骨关节炎可能性大。骨关节炎为一种退行性病变，由于年龄、肥胖、劳损、创伤、关节先天性异常等诸多因素引起关节软骨退化损伤，关节边缘和软骨下骨反应性增生，又称骨关节病、退行性关节炎、老年性关节炎等。
选项 B 类风湿关节炎多见于中年女性，好发于腕、掌指、近端指间关节，多有晨僵，多关节、对称性、持续性肿痛，可有类风湿结节，X 线片改变多有骨质破坏和关节间隙狭窄，与题干中患者临床表现不符，故不选。
选项 C 反应性关节炎多见于青年男性，以下肢大关节受累为多见，非对称性，少关节型，关节炎一般持续 1～3 个月，个别病例可长达半年以上，不符合题意。
选项 D 强直性脊柱炎多见于青年男性，以下腰痛为早期主要症状，有明显的家族聚集发病现象，X 线检查有特征性“竹节样脊柱”，不符合题干中患者表现，故排除。
选项 E 系统性红斑狼疮多见于 20～40 岁育龄女性，临床表现多样，可出现皮疹、关节痛、口腔溃疡、脱发、光过敏等，与该患者症状不符，故不考虑。
综上所述，正确答案是：A.骨关节炎。
根据题目中的描述，患者是一位 70 岁的女性，反复双膝关节疼痛已有 10 年，且近 2 年症状逐渐加重。活动时关节有弹响，并且体检发现双膝关节骨摩擦音（+），但无明显红肿及压痛。这些症状提示可能存在慢性关节疾病。
对于这种情况，最可能的诊断是骨关节炎（OA）。骨关节炎是最常见的关节病，尤其在老年人中常见，表现为关节疼痛、活动时有弹响或摩擦音，病变关节通常无明显红肿及压痛。
因此，最可能的诊断是：A. 骨关节炎
68、 (A2 型题)
化学药品 A 适应证为原发性高血压，现拟用 B 药进行人体生物等效性研究，口服给药剂量 0.5mg，A 药与 B 药的曲线下面积(AUC)分别为 27.2ng/h/ml 和 23.3ng/h/ml，上面关于 生物

利用度描述正确的是
A.A 药与 B 药，不具有生物等效性
B.A 药的绝对生物利用度是 54%
C.B 药的绝对生物利用度是 46%
D.B 药对 A 药的相对生物利用度为 86%
E.A 药对 B 药的相对生物利用度为 86%
参考答案： D 您的答案：
图文解析： 【解析】绝对生物利用度等于血管外给药 AUC/静脉给药 AUC。相对生物利用度等于受试药物 AUC/标准药物 AUC。根据上面两个公式可得 B 药对 A 药的相对生物利用度为 86%，故本题选 D。
【避错】药物的生物利用度指药物以一定的剂量从给药部位吸收进入全身循环的速度和程度。这个参数是决定药物量效关系的首要因素。
生物等效性指的是两种药物在相同剂量下，其体内暴露程度（通常以血药浓度-时间曲线下面积，AUC 来衡量）和时间特性（比如 Cmax，Tmax）相近到足以认为它们是可互换的。相对生物利用度是通过比较测试药物（B 药）与参比药物（A 药）的 AUC 或 Cmax 来计算得出的，公式为（B 药 AUC / A 药 AUC）× 100%。
根据题目所给的数据，A 药的 AUC 是 27.2 ng/h/ml，而 B 药的 AUC 是 23.3 ng/h/ml。要计算 B 药对 A 药的相对生物利用度，我们可以使用以下公式：
$$\text{相对生物利用度} = \left(\frac{23.3}{27.2} \right) \times 100\%$$
计算这个值可以帮助我们确定最正确的答案。
B 药对 A 药的相对生物利用度约为 85.66%。这最接近选项 D，即 B 药对 A 药的相对生物利用度为 86%。因此，正确答案是 D。
这是一道生物等效性评价的问题。首先，我们需要理解生物等效性的定义：若试验制剂和参比制剂的药动学参数的几何均值比值位于 80%～125%之间，即认为两制剂生物等效。
首先，我们逐项分析选项：
A 选项：A 药与 B 药，不具有生物等效性。要确定 A 药和 B 药是否具有生物等效性，我们需要计算两者的 AUC 比值。若比值在 80%～125%之间，则视为生物等效。在此题中，B 药和 A 药的 AUC 比值为 23.3/27.2=0.86，即 86%，落在 80%～125%的范围内，因此 A 药与 B 药具有生物等效性，所以 A 选项错误。
B 选项：A 药的绝对生物利用度是 54%。绝对生物利用度是药物进入体循环的量与给药量的比值，无法从题目中给出的信息直接计算出来，因此 B 选项错误。
C 选项：B 药的绝对生物利用度是 46%。同样，绝对生物利用度不能直接从 AUC 值中计算得出，因此 C 选项错误。
D 选项：B 药对 A 药的相对生物利用度为 86%。相对生物利用度是以某种非静脉给药的制剂（如片剂）为参比制剂获得的结果。在此题中，B 药相对于 A 药的生物利用度计算为（B 药的 AUC/A 药的 AUC）*100%，即（23.3/27.2）*100%=86%，因此 D 选项正确。
E 选项：A 药对 B 药的相对生物利用度为 86%。根据定义，这应该是以 B 药为参比制剂来计算 A 药的生物利用度，即（A 药的 AUC/B 药的 AUC）*100%，这与题目给出的数据不符，所以 E 选项错误。
综上所述，正确答案是 D.B 药对 A 药的相对生物利用度为 86%。
根据题干所述信息和生物等效性研究的原理，回答如下：

生物等效性研究主要关注药物在体内的吸收速度和程度的相似性，通常通过曲线下面积（AUC）来衡量。AUC 代表药物在血液中的总暴露量。

题干给出：
- A 药的 AUC = 27.2 ng・h/mL
- B 药的 AUC = 23.3 ng・h/mL

题目问的是关于生物利用度的描述，重点是 A 药和 B 药的相对生物利用度。

相对生物利用度是指在相同条件下，同剂量的两种药物（或不同剂型）的 AUC 比值，计算公式如下：
$$\text{相对生物利用度} = \frac{\text{B 药的 AUC}}{\text{A 药的 AUC}} \times 100\%$$

根据题目数据：
$$\text{相对生物利用度} = \frac{23.3}{27.2} \times 100\% \approx 85.66\%$$

这与选项 D 中的描述相符，约为 86%。

因此，正确答案是：
D. B 药对 A 药的相对生物利用度为 86%
69、 (A2 型题)
男，39 岁，发热两天，伴畏寒，右上肢剧烈疼痛 1 天，有啮齿动物接触史。查体：T39℃,P110 次／分，R22 次／分，BP120／75mmHg，神志清楚，强迫提问，右侧腋下可触 结，触痛明显，心肺腹未见明显异常，实验室检查：血 WBC12.4×109／L，中性粒 0.86×109／L，淋巴细胞 0.14×109／L。淋巴结穿刺液涂片染色检查可见 G-菌。引起该
A.伤寒杆菌
B.大肠埃希菌
C.奈瑟菌
D.鼠疫耶尔森菌
E.流感嗜血杆菌
参考答案： D 您的答案：
图文解析： 【解析】中青年男性患者，有啮齿动物接触史（鼠疫最主要的传染源），发热、右上肢剧烈疼痛，白细胞增多，右侧腋下可触及肿大淋巴结，淋巴结穿刺液涂片染色检查可见 G-菌（鼠疫耶尔森菌为革兰阴性菌），综合患者病史及临床表现，考虑诊断为鼠疫，致病菌为鼠疫耶尔森菌，故本题选 D。
【避错】鼠疫、霍乱为我国法定的甲类传染病。
这个病例描述的是一位 39 岁的男性，有发热、畏寒、剧烈疼痛和啮齿动物接触史。检查发现右侧腋下淋巴结肿大并触痛，血液检查显示白细胞计数增高，中性粒细胞增多，而淋巴结穿刺液涂片检查发现革兰阴性菌。这些症状和体征结合患者的接触史，最符合的是鼠疫的临床表现。
鼠疫主要由鼠疫耶尔森菌（Yersinia pestis，简称鼠疫杆菌）引起，是一种与啮齿动物接触相关的疾病。鼠疫可以通过被感染的跳蚤传播，或者通过直接接触被感染的动物传播。病人可能会出现突然高热、寒战、头痛、肌肉痛以及相关淋巴结的肿大（腋下、颈部或腹股沟）。血液和淋巴结液体中可以发现鼠疫耶尔森菌。
因此，这个病例的最佳答案是 D.鼠疫耶尔森菌。
首先，我们梳理一下题目的关键信息：

患者中年男性，主要症状有发热、畏寒、右上肢剧烈疼痛，有啮齿动物接触史。查体结果显示体温升高，脉搏、呼吸频率均增快，右侧腋下可触及触痛的结节。实验室检查显示白细胞和中性粒细胞均升高，淋巴结穿刺液涂片染色检查可见 G-菌。
接下来，我们对选项进行逐一分析：
A. 伤寒杆菌：伤寒杆菌是伤寒病的病原体，常称伤寒沙门菌，主要引起肠热症，以持续菌血症，网状内皮系统受累，回肠远端微小脓肿及溃疡形成为基本病理特征，与患者的症状不符。
B. 大肠埃希菌：大肠埃希菌是肠道中的正常栖居菌，但在宿主免疫力下降或细菌侵入肠道外组织器官后，即可成为机会致病菌，引起肠道外感染，但患者无肠道感染表现，故排除。
C. 奈瑟菌：奈瑟菌主要引起人类脑膜炎和败血症等疾病，不会通过啮齿类动物传播，故不选。
D. 鼠疫耶尔森菌：鼠疫耶尔森菌是鼠疫的病原菌，主要通过鼠类和啮齿类动物传播，与人类多种鼠疫相关，包括腺鼠疫、肺鼠疫和败血症型鼠疫等。患者的啮齿动物接触史、发热、畏寒、淋巴结肿痛等症状均符合鼠疫的临床表现，且实验室检查发现 G-菌，与鼠疫耶尔森菌的生物学特性相符。
E. 流感嗜血杆菌：流感嗜血杆菌主要引起婴幼儿肺炎、脑膜炎和成人下呼吸道感染等，与患者的症状不符，故不考虑。
综上所述，正确答案是：D.鼠疫耶尔森菌。
根据题目中给出的症状和实验室检查结果，结合患者有啮齿动物接触史，最可能的致病菌是**D. 鼠疫耶尔森菌**。以下是详细的分析：

1. **症状**：患者出现发热、畏寒、右上肢剧烈疼痛以及右侧腋下可触痛性淋巴结。这些症状与鼠疫的典型表现相符。

2. **体征**：患者发热，体温 39℃，心率加快（P110 次/分），呼吸频率增快（R22 次/分），血压正常。右侧腋下淋巴结肿大且有明显触痛，这也与鼠疫淋巴腺型的特征一致。

3. **实验室检查**：
   - 白细胞计数（WBC）升高，表明有感染。
   - 中性粒细胞比例高（0.86），符合细菌感染的特点。
   - 淋巴细胞比例低（0.14）。
   - 淋巴结穿刺液涂片染色检查可见 G-菌，鼠疫耶尔森菌是一种革兰阴性杆菌。

4. **流行病学史**：患者有啮齿动物接触史，鼠疫的主要传播途径之一是通过受感染的啮齿动物及其跳蚤。

综合以上因素，最可能引起该患者症状的致病菌是鼠疫耶尔森菌。

因此，最佳答案是：
D. 鼠疫耶尔森菌
70、 (A2 型题)
女，66 岁，发热伴尿急、尿频、尿痛 3 天。既往糖尿病病史 10 年。查体：左肾区叩击痛（+），尿沉渣镜检：RBC3~5/HP，WBC 满视野。清洁中段尿细菌定量培养：大肠埃希 106cfu/ml，应首选

A.抗生素治疗 1 周
B.抗生素治疗 2 周
C.不使用抗生素治疗
D.抗生素治疗 3 天
E.单剂量抗生素
参考答案： B 您的答案：
图文解析： 【解析】患者尿频尿急伴发热，左肾叩击痛，尿沉渣镜检：RBC3～5/HP，WBC 满视野。清洁中段尿细菌定量培养：大肠埃希菌 106CFU/ml（肾盂肾炎最常见菌群），诊断为急性肾盂肾炎，抗生素应足量足疗程，治疗 14 天后，如尿菌仍阳性，应该抗生素延长使用 2 周～4 周,故本题选 B。
【避错】（1）病情较轻者：可在门诊口服药物治疗，疗程 10～14 天。常用药物有喹诺酮类（如氧氟沙星 0.2g，2 次 / 日；环丙沙星 0.25g，2 次 / 日或左氧氟沙星）、半合成青霉素类（如阿莫西林 0.5g，3 次 / 日）、头孢菌素类（如头孢呋辛 0.25g，2 次 / 日）等。治疗 14 天后，通常 90%可治愈。如尿菌仍阳性，应参考药敏试验选用有效抗生素继续治疗 4~6 周。（2）严重感染全身中毒症状明显者：需住院治疗，应静脉给药。常用药物，如氨苄西林 1.0～2.0g，每 4 小时一次；头孢噻肟钠 2.0g，每 8 小时一次；头孢曲松钠 1.0～2.0g，每 12 小时一次；左氧氟沙星 0.2g，每 12 小时一次。必要时联合用药。氨基糖苷类抗生素肾毒性大，应慎用。经过上述治疗若好转，可于热退后继续用药 3 天再改为口服抗生素，完成 2 周疗程。治疗 72 小时无好转，应按药敏试验结果更换抗生素，疗程不少于 2 周。经此治疗仍有持续发热者，应注意肾盂肾炎并发症，如肾盂积脓、肾周脓肿、感染中毒症等。
这位 66 岁的女性患者有糖尿病病史，并出现了尿急、尿频和尿痛的症状，左肾区叩击痛阳性，尿沉渣中红细胞和白细胞计数增高，尿细菌培养显示大肠埃希菌（E. coli）的存在，这些都是急性复杂性泌尿系感染，可能为急性肾盂肾炎的表现。在这种情况下，适当的抗生素治疗是必需的。
对于糖尿病患者，尤其是有复杂性泌尿道感染（如急性肾盂肾炎）的患者，建议的抗生素治疗时间通常长于非复杂性泌尿道感染。单剂量或 3 天的治疗对于复杂性感染通常是不够的，因此选项 D 和 E 不是最佳选择。治疗通常需要较长时间，以确保感染完全清除，避免复发或细菌耐药。
因此，对于这种情况，最合适的治疗方案通常是至少 7-14 天的抗生素治疗。考虑到这位患者的情况，选项 A（抗生素治疗 1 周）可能是一个起点，但更常见的做法是进行 2 周的治疗，特别是对于复杂的泌尿道感染或疑似肾盂肾炎的情况。
因此，最正确的答案是 **B. 抗生素治疗 2 周**。
解析：
老年女性患者，发热伴尿急、尿频、尿痛，既往糖尿病病史。查体示左肾区叩击痛，尿沉渣镜检见白细胞满视野，红细胞阳性，清洁中段尿细菌定量培养示大肠埃希菌。根据患者症状、体征、实验室检查，该患者最可能的诊断为急性肾盂肾炎。糖尿病患者的感染一般不易控制，因此急性肾盂肾炎合并糖尿病患者的疗程应适当延长，治疗应首选抗生素治疗 2 周。
选项 A 抗生素治疗 1 周对于糖尿病患者来说疗程过短，可能无法根治，故不选。
该患者已出现急性肾盂肾炎症状，且有糖尿病病史，必须使用抗生素治疗，故选项 C 不使用抗生素治疗可排除。
选项 D 抗生素治疗 3 天，选项 E 单剂量抗生素，疗程过短，对糖尿病患者达不到治疗效果，故一并排除。
综上所述，正确答案是：B.抗生素治疗 2 周。
根据题目提供的病例信息，这位 66 岁的女性患者表现出急性尿路感染的症状，伴有糖尿病

史 10 年。尿液检查显示有红细胞和大量白细胞，并且尿培养显示大肠埃希菌的定量为 10^6 cfu/ml，提示细菌性尿路感染。考虑到患者的年龄、症状和既往病史，尤其是糖尿病，这增加了复杂尿路感染的风险。

对于复杂性尿路感染的治疗，一般推荐使用抗生素治疗更长时间，以确保彻底清除感染，避免复发和并发症。根据临床指南，复杂性尿路感染的抗生素治疗通常建议持续 1-2 周。因此，在此情形下，较为合理的选择是抗生素治疗 2 周。

因此，最佳答案是：

B. 抗生素治疗 2 周
71、 (A2 型题)
女，35 岁，阵发性头痛 2 个月，近一周来间断发生多次癫痫，平日喜食烧烤，查体：神清，颈无抵抗，躯干和四肢可触及多个皮下结节，血 WBC16×109/L，N0.61，L0.20， 颅 CT：脑内可见多个 0.5~1.0cm 结节，最可能的是
A.脑出血
B.癫痫
C.脑肿瘤
D.脑脓肿
E.囊尾蚴病
参考答案： E 您的答案：
图文解析： 【解析】中年女性，阵发性头疼、近一周来间断发生多次癫痫（脑囊尾蚴病皮质型的典型表现），躯干和四肢可触及多个皮下结节（皮下组织和肌肉尾蚴病），平日喜食烧烤，头颅 CT：脑内可见多个 0.5～1.0cm 结节，最可能的诊断是脑囊尾蚴病（E 对）。
【避错】囊尾蚴病，又称囊虫病、猪囊尾蚴病，由猪带绦虫幼虫（囊尾蚴）寄生于人体各组织器官所致的疾病，患囊尾蚴病的原因是进食了猪带绦虫卵。
这个病例描述了一个 35 岁女性患者，有阵发性头痛和近期多次癫痫发作的病史，身体检查发现多个皮下结节，血液检查显示白细胞增多，颅脑 CT 扫描显示多个脑内结节。根据这些症状和检查结果，最可能的诊断是囊尾蚴病（E）。
囊尾蚴病（Neurocysticercosis）是由猪囊尾蚴（Taenia solium）的幼虫形式感染人类中枢神经系统引起的。它可以通过摄入含有猪肉带绦虫卵的食物或水传播。本病例中，患者喜欢吃烧烤，这可能是其感染途径之一。颅脑 CT 发现的多个脑内结节与囊尾蚴病的典型影像学表现相符，这些结节很可能是囊尾蚴的囊肿。此外，癫痫是囊尾蚴病常见的临床表现之一。因此，最符合这个病例描述的答案是 E.囊尾蚴病。
首先，我们来详细分析题干：
青年女性患者，主要的症状是阵发性头痛和间断性癫痫发作。生活习惯上，她喜欢吃烧烤，这可能与寄生虫感染有关。查体结果显示神志清晰，颈无抵抗，但躯干和四肢可触及多个皮下结节。实验室检查发现白细胞计数增高，颅 CT 显示脑内存在多个小结节。
接下来，我们根据题干信息和各个选项进行推理分析：
A. 脑出血：脑出血通常是由于高血压、动脉瘤等原因引起的脑血管破裂。虽然脑出血可能导致头痛，但一般不会引起皮下结节，且颅 CT 表现与题干描述不符。
B. 癫痫：癫痫是一种由脑部神经元异常放电引起的疾病。虽然该患者出现了癫痫症状，但癫痫本身不会引起白细胞升高和皮下结节，故癫痫更像是症状而非根本病因。
C. 脑肿瘤：脑肿瘤虽然可以在颅 CT 上表现为结节，但一般不会导致皮下出现多个结节，且

白细胞计数通常不会因此升高。
D. 脑脓肿：脑脓肿是由于感染引起的脑部局部化脓性炎症。尽管它可能导致头痛和白细胞升高，但通常不会在皮下形成多个结节。
E. 囊尾蚴病：囊尾蚴病是由寄生虫引起的疾病，其中囊尾蚴是猪肉绦虫的幼虫。这种病通常与食用未煮熟的猪肉有关，该患者喜食烧烤，这增加了感染囊尾蚴病的风险。囊尾蚴病可在脑部形成多个结节，并可能引起癫痫发作和皮下结节，同时可能伴有白细胞升高。
综上所述，考虑到患者的临床症状、生活习惯、查体结果和颅 CT 表现，最可能的诊断是囊尾蚴病。
因此，正确答案是 E.囊尾蚴病。
根据题干所述，该患者为 35 岁女性，阵发性头痛 2 个月，近一周来间断发生多次癫痫，查体发现躯干和四肢可触及多个皮下结节，血常规显示白细胞增多，CT 显示脑内多个结节。结合患者喜食烧烤的饮食习惯，最可能的诊断是囊尾蚴病（猪囊虫病）。

猪囊虫病是由于囊尾蚴寄生在人体组织中引起的疾病，常通过食用未煮熟的猪肉或被囊虫卵污染的食物和水传播。囊尾蚴病常表现为皮下结节、头痛和癫痫发作，脑部 CT 可见多个结节。

因此，最可能的诊断是：

E. 囊尾蚴病
72、 (A2 型题)
男，68 岁，胸痛 3 年，位于胸骨后，凌晨发作，数分钟后可自行缓解，发作时心电图提示 V1～V3 导联 ST 段抬高 0.3mv，后复测心电图为正常，该患者为
A.中间综合征
B.初发型心绞痛
C.变异型心绞痛
D.恶化型心绞痛
E.劳累型心绞痛
参考答案： C 您的答案：
图文解析： 【解析】老年男性患者，夜间阵发性胸痛，数分钟自行缓解，心电图 V1～V3 导联 ST 段抬高，考虑为变异性心绞痛。变异型心绞痛也称血管痉挛性心绞痛，特征为静息心绞痛，几乎完全都在静息情况下发生，发病时间多集中在午夜至上午 8 点之间，常伴一过性 ST 段抬高。故本题选 C。
【避错】中间型综合征是指急性有机磷杀虫药中毒所引起的一组以肌无力为突出表现的综合征。多发生在重度有机磷中毒后 24～94 小时，以及复能药用量不足的患者，经治疗胆碱能危象消失，意识清醒或未恢复，在迟发性多发性神经病发生前，突然出现屈颈肌、四肢近端肌无力，还会有部分脑神经支配的肌肉无力，患者会表现为面瘫、呼吸肌麻痹等，最终导致呼吸困难，甚至由于呼吸衰竭而出现死亡（A 错）。初发型劳力性心绞痛（B 错）指在首发症状 1～2 个月内、很轻的体力活动可诱发；恶化型劳力性心绞痛（D 错）是指在相对稳定的劳力性心绞痛基础上心绞痛逐渐增强，两者都有明确的发病诱因如劳动或情绪激动，且心电图胸导联抬高较少见；劳累性心绞痛其特点是疼痛由体力劳累、情绪激动或其他足以增加心肌需氧量的情况所诱发，休息或舌下含用硝酸甘油后迅速消失（E 错）。
该患者的症状描述符合变异型心绞痛（也称普林兹美特尔心绞痛），其特点是在休息时出现胸痛，并伴有 ST 段抬高，这种情况在体力活动时不常见。变异型心绞痛通常与冠状动脉痉

挛有关，并且心电图在发作时显示 ST 段抬高，而在无症状时恢复正常。因此，最佳答案是：
C. 变异型心绞痛
解析：
患者老年男性，胸痛 3 年，凌晨发作，位于胸骨后，数分钟后可自行缓解，为心绞痛常见表现，发作时心电图提示 V1～V3 导联 ST 段抬高 0.3mv，后复测心电图为正常，结合患者症状，体征，辅助检查结果，诊断考虑为变异型心绞痛。变异型心绞痛多发生于休息时，出现一过性的 ST 段动态改变，多伴有一过性的 ST 段抬高，ST 段抬高随着心绞痛的缓解而回到基线。
选项 A 中间综合征是急性有机磷农药中毒引起的，主要表现为肌无力，与题干描述不符，故排除。
选项 B 初发型心绞痛是指患者过去未发生过心绞痛或心肌梗死，而在近 1～2 个月内初次发生的心绞痛，与题干描述不符，故不选。
选项 D 恶化型心绞痛是指原有稳定型心绞痛的患者在相对稳定的劳力性心绞痛基础上心绞痛逐渐增强，即疼痛更剧烈、时间更长或更频繁，按 CCS 分级至少增加Ⅰ级，程度至少 CCS Ⅲ级，与题干描述不符，故不考虑。
选项 E 劳累型心绞痛的特点为阵发性的前胸压榨性疼痛或憋闷感觉，主要位于胸骨后部，常发生于劳力负荷增加时，持续数分钟，休息或用硝酸酯制剂后疼痛消失，与题干描述不符，故不符合。
综上所述，正确答案是：C.变异型心绞痛。
根据题干描述的患者症状和心电图变化，可以分析出最可能的诊断是变异型心绞痛。变异型心绞痛通常在凌晨或休息时发作，持续时间较短（数分钟），并且可以自行缓解。心电图在发作时会出现 ST 段抬高，发作后心电图恢复正常。

因此，最正确的答案是：

C. 变异型心绞痛
73、 (A2 型题)
女，30 岁。月经增多，心肺正常，Hb86g/L，PaO_2 82.3mmHg，$PaCO_2$ 37.6mmHg，pH7.43。血氧容量降低的原因是
A.二氧化碳下降
B.红细胞内 2-3-DPG 下降
C.体温下降
D.pH 下降
E.蛋白下降
参考答案： E 您的答案：
图文解析： 【解析】Hb 氧容量：是指在 100ml 血液中，Hb 所能结合的最大 O_2 量。血氧容量主要受两个因素所影响：血红蛋白含量、Hb 所能结合的最大 O_2 量。该患者 Hb86g/L（贫血，血红蛋白减低），故血氧容量降低的原因是血红蛋白减少（E 对）。
【避错】pH、CO_2、温度、红细胞内 2，3-二磷酸甘油酸主要是影响 Hb 对 O_2 的亲和力，影响氧解离曲线进而影响 PO_2。
本题涉及的是血氧容量的变化。血氧容量是指血液中能够结合的氧的总量，这主要取决于血红蛋白的浓度和血红蛋白的氧结合能力。在这个病例中，女性患者的主要问题是月经增多，导致了血红蛋白（Hb）降低至 86g/L，这是贫血的表现之一。
- A 选项（二氧化碳下降）通常影响血氧的释放，而不是血氧容量。
- B 选项（红细胞内 2-3-DPG 下降）会影响氧和血红蛋白的结合亲和力，从而影响氧的释放，

但不直接影响血氧容量。
- C 选项（体温下降）会影响氧解离曲线的位置，但对血氧容量的影响不大。
- D 选项（pH 下降）同样会影响氧解离曲线，但主要影响氧的释放，而非血氧容量。
- E 选项（蛋白下降）可以指代血红蛋白的降低，因为血红蛋白是血液中的一种蛋白，其下降会直接减少血氧容量。
考虑到上述分析，最合理的答案应该是 **E.蛋白下降**，因为在这种情况下，女性患者的血红蛋白降低（蛋白下降），这直接导致了血氧容量的降低。
解析：
患者青年女性，月经增多，提示存在慢性失血，心肺正常，化验结果提示存在贫血。血氧容量是指在氧分压为 19.95kPa（150mmHg）、二氧化碳分压为 5.32kPa（40mmHg）、温度在 38℃时，100ml 血液中的血红蛋白所能结合的最大氧量。血氧容量的高低可以反映血液携带氧的能力的强弱。血氧容量降低的原因主要有两方面，一是血红蛋白的数量或质量降低，二是红细胞内 2，3-DPG 浓度的下降。由于患者存在贫血，因此血红蛋白的数量和质量降低，同时贫血可导致红细胞内 2，3-DPG 浓度的下降，从而血氧容量下降。
选项 A 二氧化碳下降可刺激外周化学感受器，反射性地引起呼吸中枢兴奋，使呼吸加深加快，增加肺通气，从而使血氧容量上升，与题干矛盾。
选项 C 体温下降，选项 D：pH 下降，均可导致血红蛋白对氧气的亲和力增加，从而使血氧容量上升，与题干描述不符。
选项 E 蛋白下降主要影响血浆胶体渗透压，而与血氧容量关系不大，可排除。
综上所述，正确答案是：B.红细胞内 2-3-DPG 下降。
根据题目提供的信息，该患者是一名 30 岁的女性，月经增多，血红蛋白(Hb)为 86g/L，PaO2 为 82.3mmHg，PaCO2 为 37.6mmHg，pH 为 7.43。首先我们需要分析血氧容量降低的原因。

血氧容量（oxygen capacity）是指血液中能携带的最大氧气量，这通常取决于血红蛋白的浓度和血液的氧合作用能力。血红蛋白浓度降低会直接导致血氧容量降低。

从题目中提供的信息可以看出，患者的血红蛋白值（Hb）明显低于正常范围，这提示可能存在贫血。血红蛋白减少会直接导致血氧容量降低，因为血红蛋白是氧气的主要载体。

题目中的选项与血氧容量降低的关系分析如下：

A. 二氧化碳下降：二氧化碳的变化主要影响血液酸碱平衡，而非直接影响血氧容量。
B. 红细胞内 2-3-DPG 下降：2,3-二磷酸甘油酸(2-3-DPG)的下降会影响血红蛋白与氧气的结合能力，但不会直接减少血红蛋白浓度。
C. 体温下降：体温下降会影响代谢速率和氧气需求，但不会直接影响血红蛋白浓度。
D. pH 下降：pH 下降（酸中毒）会影响氧气释放，但题目中 pH 值为 7.43，处于正常范围，因此不适用。
E. 蛋白下降：全身性蛋白质下降（如低蛋白血症）可能会伴随营养不良，但不会直接影响血红蛋白浓度。

基于上述分析，血氧容量降低的最可能原因是血红蛋白降低。最直接相关的选项是：

E. 蛋白下降

尽管贫血是最直接的因素，而贫血通常与营养相关的蛋白质（如血红蛋白）下降有密切关系。

综上所述，选择答案 **E. 蛋白下降**。

74、 (A2 型题)

男，40 岁，股骨干骨折入院，入院次日突然出现呼吸困难，继而昏迷，广泛皮下出血点，应考虑为

A.脂肪栓塞

B.骨筋膜室综合征

C.骨折端再发严重移位

D.感染

E.大血管破裂

参考答案： A 您的答案：

图文解析： 【解析】股骨干骨折后第 2 天突发呼吸困难，首先考虑脂肪栓塞（A 对）。【避错】脂肪栓塞综合征常发生于成人，是由于骨折处髓腔内血肿张力过大，骨髓被破坏，脂肪滴进入破裂的静脉窦内，可引起肺、脑脂肪栓塞。同时，在肺灌注不良时，肺泡膜细胞产生脂肪酶，使脂肪栓子中的中性脂肪小滴水解成甘油与游离脂肪酸，释放儿茶酚胺，损伤毛细血管壁，使富含蛋白质的液体漏至肺间质和肺泡内，发生肺出血、肺不张和低血氧。临床上出现呼吸功能不全、发绀，胸片显示广泛性肺实变。动脉低血氧可致烦躁不安、嗜睡，甚至昏迷和死亡。

根据描述的症状，包括股骨干骨折后突然出现的呼吸困难、昏迷和广泛的皮下出血点，最有可能的诊断是脂肪栓塞（选项 A）。脂肪栓塞综合症是长骨骨折后的一种严重并发症，尤其是股骨和髓骨折，它可能导致脂肪微粒从骨髓进入血液循环，引起肺部和其他器官的栓塞。所以，最佳答案是 A.脂肪栓塞。

解析：

患者中年男性，股骨干骨折入院，入院次日突然出现呼吸困难，继而昏迷，广泛皮下出血点，考虑患者可能因骨折后骨髓内脂肪滴进入破裂的骨髓血管窦状隙或毛细血管内，并随血流进入肺内血管引起脂肪栓塞，导致呼吸困难，脑栓塞引起昏迷，皮肤、粘膜出血点。

选项 B 骨筋膜室综合征是骨、骨间膜、肌间隔和深筋膜形成的骨筋膜室内的肌肉和血管因急性缺血而产生的一系列早期症候群，多见于前臂和小腿，主要表现为 5P 综合征，即疼痛、苍白、感觉异常、麻痹和无脉，与题干描述不符，故排除。

选项 C 骨折端再发严重移位会有剧烈疼痛，骨折部位会出现明显的畸形，与该患者表现不符，故不选。

选项 D 感染会有局部红肿热痛等炎症表现，该患者无感染表现，可排除。

选项 E 大血管破裂会导致失血性休克等表现，与该患者症状不符，可排除。

综上所述，正确答案是：A.脂肪栓塞。

根据现有医学知识和最佳实践指南，对于 40 岁男性患者因股骨干骨折入院后次日突然出现呼吸困难，继而昏迷，广泛皮下出血点的症状，应考虑为脂肪栓塞。脂肪栓塞综合征（FES）通常发生在长骨骨折后，尤其是股骨和胫骨骨折，常表现为呼吸困难、神经系统症状（如昏迷）和皮下出血点。

因此，最正确的答案是：

A.脂肪栓塞

75、 (A2 型题)

男，34 岁，胃大部切除术后，出现镜面舌，查血常规：WBC3.1×10＾9／L，Hb68g／L，PLT40

×10＾9／L，MCV＞130fl，网织红细胞 0.5％，骨髓细胞学示：骨髓增生活跃 幼浆老”现象，未见病态造血。考虑的诊断是

A.巨幼细胞性贫血

B.缺铁性贫血

C.骨髓增生异常综合征

D.多发性骨髓瘤

E.白血病

参考答案： A 您的答案：

图文解析： 【解析】胃大部切除术（营养性缺血病因）后患者，出现镜面舌，查血常规：WBC3.1×10^9／L，Hb68g／L，PLT40×10＾9／L（三系减少），MCV＞130fl，网织红细胞 0.5％，骨髓细胞学示：骨髓增生活跃，可见“核幼浆老”现象（符合巨幼细胞性贫血骨髓象表现），综合患者病史、检验结果及临床表现，考虑诊断为巨幼细胞性贫血，故本题选 A。

【避错】巨幼红细胞性贫血是核酸合成障碍所致，核酸合成的必需原料是叶酸和维生素 B12。

这位患者的临床表现和实验室检查结果指向了巨幼细胞性贫血（megaloblastic anemia）。关键的线索包括术后镜面舌的出现、低血红蛋白（Hb 68g/L）、大红细胞（MCV > 130 fl）、以及骨髓检查显示的“幼浆老”现象，这些都是巨幼细胞性贫血的典型特征。此外，镜面舌和术后营养不良可能与维生素 B12 或叶酸缺乏有关，这两种营养素缺乏是巨幼细胞性贫血的常见原因。

- A. 巨幼细胞性贫血：对应上述描述的临床表现和检查结果，是最符合的诊断。
- B. 缺铁性贫血：通常会出现小细胞性低色素性贫血，与本例的 MCV 增大不符。
- C. 骨髓增生异常综合征：虽然会导致血细胞减少，但通常不会出现 MCV 明显增大或是“幼浆老”现象。
- D. 多发性骨髓瘤：主要表现为异常免疫球蛋白的增加，与此案例的表现不符。
- E. 白血病：通常会看到血液或骨髓中有异常白细胞的增加，与此病例不符。

因此，最正确的答案是 A. 巨幼细胞性贫血。

解析：

青年男性患者，胃大部切除术后，出现镜面舌，血常规结果显示三系减少，红细胞平均体积增大，提示大细胞性贫血，网织红细胞降低，骨髓细胞学示：骨髓增生活跃，“幼浆老”现象，未见病态造血，结合患者病史、临床表现和相关检查，最可能的诊断是巨幼细胞性贫血。巨幼细胞性贫血是由于叶酸或维生素 B12 缺乏，或某些影响核苷酸代谢的药物导致细胞核脱氧核糖核酸（DNA）合成障碍所引起的贫血，其特点为大细胞性贫血，骨髓中出现巨幼红细胞等造血特点，经维生素 B12 及叶酸治疗有效。

选项 B 缺铁性贫血是由于机体对铁的需求与供给失衡，导致体内贮存铁耗尽，出现红细胞内铁缺乏而引起的贫血，为小细胞低色素性贫血，骨髓象增生活跃，粒红比降低，红细胞系统增生旺盛，以中、晚幼红细胞为主，其体积小、核染色质致密、胞浆少、边缘不整齐，有血红蛋白形成不良表现，即“核老浆幼”的现象，与题意不符，故不选。

选项 C 骨髓增生异常综合征起源于造血干细胞，以血细胞病态造血，高风险向急性髓系白血病转化为特征的难治性血细胞质、量异常的异质性疾病，其血象呈全血细胞减少，骨髓象增生活跃或明显活跃，可见各阶段幼稚细胞，但“幼浆老”现象少见，且题干中已明确指出未见病态造血，故排除。

选项 D 多发性骨髓瘤是恶性浆细胞病，其血象检查中，血红蛋白可降低，血沉加快，骨髓检查可见骨髓瘤细胞，与题干描述不符，故不选。

选项 E 白血病是一类造血干细胞恶性克隆性疾病，克隆性白血病细胞因为增殖失控、分化障

碍、凋亡受阻等机制在骨髓和其他造血组织中大量增殖累积，并浸润其他非造血组织和器官，同时抑制正常造血功能，临床可见不同程度的贫血、出血、感染发热以及肝、脾、淋巴结肿大和骨骼疼痛，与题干描述差异明显，可排除。
综上所述，正确答案是：A.巨幼细胞性贫血。
题目描述的是一名 34 岁的男性，胃大部切除术后出现镜面舌，并伴有以下检查结果：

- 白细胞计数（WBC）：3.1×10^9/L
- 血红蛋白（Hb）：68g/L
- 血小板计数（PLT）：40×10^9/L
- 平均红细胞体积（MCV）：＞130fl
- 网织红细胞：0.5%
- 骨髓细胞学显示：骨髓增生活跃，幼浆老”现象，未见病态造血

这些信息提示了一种贫血类型，特别是伴有 MCV 显著增高（＞130fl）的贫血。我们需要考虑巨幼细胞性贫血的可能性。

分析：

1. **巨幼细胞性贫血**：
   - 胃大部切除术后，患者可能由于内因子缺乏导致维生素 B12 吸收障碍，进而引起巨幼细胞性贫血。
   - 镜面舌是巨幼细胞性贫血的常见表现之一。
   - 血常规中，MCV 显著增高（＞130fl），血红蛋白明显下降，且网织红细胞百分比较低，这些都是巨幼细胞性贫血的典型特征。
   - 骨髓细胞学显示骨髓增生活跃，并有“幼浆老”现象，也是巨幼细胞性贫血的特点之一。

2. **缺铁性贫血**：
   - 一般表现为小细胞低色素性贫血，MCV 通常降低或正常，不符合题干中 MCV＞130fl 的情况。

3. **骨髓增生异常综合征（MDS）**：
   - 虽然 MDS 也可能出现类似的血象变化，但 MCV 显著增高并不是 MDS 的典型特征。

4. **多发性骨髓瘤**：
   - 一般以骨痛、蛋白尿和异常的血清蛋白电泳结果为主要特征，不符合题干中的描述。

5. **白血病**：
   - 白血病通常伴有明显的病态造血现象，骨髓细胞学检查中应有病态造血表现，题干中未见病态造血。

基于以上分析，最符合题干描述的诊断是**巨幼细胞性贫血**。

答案：A. 巨幼细胞性贫血

76、 (A2 型题)
男，28 岁，发热 2 周，最高温度 39.6℃,伴腹痛、腹泻，稀便 3~4 次/天。查体： 一个月前下河游泳，下肢一过性皮疹，肝肋下 2cm，有压痛，脾肋下可触及，WBC15.7×109 E0.4，可选以下哪种药物进行治疗

A.阿苯达唑

B.多西环素

C.吡喹酮

D.左氧氟沙星

E.甲硝唑

参考答案： C 您的答案：

图文解析： 【解析】年轻男性一个月前下河游泳（血吸虫接触史），下肢一过性皮疹，发热 2 周，伴腹痛、腹泻，肝肋下 2cm，有压痛，脾肋下可触及（均为血吸虫临床表现），综合考虑患者诊断为血吸虫病，吡喹酮的毒性小、疗效好、给药方便，为血吸虫病的首选治疗药物（C 对）。

【避错】阿苯达唑能杀灭多种肠道线虫、绦虫和吸虫的成虫及虫卵，不能治疗血吸虫病（A 错）；多西环素首选治疗立克次体感染、支原体感染、以及某些螺旋体感染。四环素类药物还可首选治疗鼠疫、布鲁菌病、霍乱、幽门螺杆菌感染引起的消化性溃疡、肉芽肿鞘杆菌感染引起的腹股沟肉芽肿以及牙龈卟啉单胞菌引起的牙周炎（B 错）；左氧氟沙星为喹诺酮类药物，为广谱抗生素，主要用于呼吸道、泌尿系、消化道感染（D 错）；甲硝唑为厌氧菌感染治疗药物（E 错）。

这道题目描述了一位 28 岁男性患者，有发热、腹痛、腹泻等症状，并提到一个月前有游泳史和下肢皮疹。根据这些信息，我们应该考虑由水源传播的疾病，如寄生虫感染。

A. 阿苯达唑 - 主要用于治疗蛔虫、钩虫、鞭虫、蛲虫等肠道寄生虫感染。

B. 多西环素 - 是一种抗生素，用于治疗多种细菌感染。

C. 吡喹酮 - 主要用于治疗疟疾。

D. 左氧氟沙星 - 是一种广谱抗生素，用于治疗多种细菌感染。

E. 甲硝唑 - 常用于治疗由厌氧菌和原虫引起的感染，如阿米巴病和其他原虫感染。

考虑到患者有游泳史，可能接触了受污染的水源，出现的症状与阿米巴性痢疾相符，其中包括腹痛、腹泻和发热。因此，最合适的药物选择是用于治疗阿米巴性痢疾的甲硝唑（选项 E）。所以，最正确的答案是 E. 甲硝唑。

解析：

患者青年男性，发热、腹痛、腹泻，查体示肝脾大，有河中游泳史，一过性皮疹，实验室检查示白细胞升高，嗜酸性粒细胞增多，结合患者症状、体征及实验室检查，考虑诊断为急性血吸虫病。血吸虫病治疗首选药物是吡喹酮，此药毒性小、疗效好、给药方便、适应症广，可用于各期各型血吸虫病患者。

选项 A 阿苯达唑是一种广谱的驱肠虫药，主要治疗蛔虫、蛲虫、钩虫等寄生虫感染，但对血吸虫无治疗作用，故不选。

选项 B 多西环素是四环素类抗生素，为广谱抑菌剂，高浓度时具杀菌作用，主要用于敏感的革兰氏阳性球菌和阴性杆菌所致的上呼吸道感染、扁桃体炎、胆道感染、淋巴结炎等，对血吸虫感染无效，故排除。

选项 D 左氧氟沙星是喹诺酮类抗生素，主要用于敏感菌所致的呼吸系统感染、泌尿系统感染、生殖系统感染、皮肤软组织感染、肠道感染等，对血吸虫感染没有治疗作用，故不考虑。

选项 E 甲硝唑主要用于厌氧菌的治疗，以及滴虫、阿米巴原虫等的治疗，对血吸虫无效，故不选。

综上所述，正确答案是：C.吡喹酮。
根据题目描述，该患者有发热、腹痛、腹泻和肝脾肿大的症状，同时有下河游泳史和皮疹。结合这些症状和病史，考虑到可能的诊断是血吸虫病（Schistosomiasis）。

血吸虫病的治疗首选药物是吡喹酮。

因此，最正确的答案是：

C. 吡喹酮
77、 (A2 型题)
男，68 岁，发热，头痛腰痛，腹泻 3 天，少尿 300ml/d。查体:体温 38.5℃,心率 116 次/分，呼吸 24 次/分，面部充血，眼睑水肿，软腭有出血点，血常规 Hb134g/L，WBC14 0.84，粪常规及镜检：WBC5/HP，为明确诊断，应当首选的检查是
A.血涂片
B.血培养
C.汉坦病毒抗体
D.钩端螺旋体抗体
E.EB 病毒抗体
参考答案： C 您的答案：
图文解析： 【解析】68 岁男性患者，发热，头痛腰痛，腹泻 3 天（肾综合征出血热发热期全身中毒症状），尿少 1 天、面部充血，眼睑水肿，软腭有出血点（肾综合征出血热毛细血管损害征），实验室检查血 WBC14×109/L（成人正常参考值 4～10×109/L），根据患者的临床表现、个人史和实验室结果，最可能的诊断是肾综合征出血，热汉坦病毒抗体在发病第 2 日即能检出（C 对）。
【避错】肾综合征出血热，又称流行性出血热，以鼠类为主要传染源的一种自然疫源性疾病。本病的主要病理变化是全身小血管和毛细血管广泛性损害，临床上以发热、低血压休克、充血出血和肾损害为主要表现。
这个病例中的患者呈现发热、头痛、腰痛、腹泻、少尿、面部充血、眼睑水肿和软腭出血点等症状。结合体征和实验室检查结果，我们需要考虑多种可能的诊断。
首先，发热和头痛可能指向多种感染性疾病，如细菌、病毒或寄生虫感染。软腭有出血点可能暗示一种血液系统的问题或严重的感染。高白细胞计数表明有感染或炎症的存在。
具体到这个病例，考虑到发热、头痛、腹泻和少尿，以及血常规和粪常规的结果，我们可以排除一些普通的感染，如 EB 病毒感染，因为它通常导致的是淋巴细胞增多，而不是粒细胞。

- A 选项（血涂片）可能用于检测寄生虫感染（如疟疾）或血液疾病。
- B 选项（血培养）是识别血液中细菌或真菌感染的标准方法。
- C 选项（汉坦病毒抗体）用于诊断汉坦病毒感染，这可能导致严重的肾脏问题和出血倾向，符合本病例的一些症状。
- D 选项（钩端螺旋体抗体）用于诊断钩端螺旋体病，通常表现为发热、肌肉痛和可能的肾功能受损。
- E 选项（EB 病毒抗体）用于诊断 EB 病毒感染，通常关联于咽痛、淋巴结肿大、疲劳等症状。
考虑到患者的症状和体征，特别是发热、腹泻、少尿、眼睑水肿和软腭出血点，我倾向于选择 C 选项（汉坦病毒抗体）作为首选检查。汉坦病毒感染可以导致肾综合征出血热，其特点

是发热、出血倾向、肾损害和类似本病例的症状。因此，进行汉坦病毒抗体检测有助于确定这是否为正确诊断。

这是一道关于疾病诊断的问题，需要分析病人的症状、体征和实验室检查结果，以确定最合适的诊断检查。

首先，我们梳理一下题目中的关键信息：

老年男性患者，发热、头痛、腰痛、腹泻 3 天，伴有少尿。

查体示面部充血，眼睑水肿，软腭有出血点。

血常规检查显示血红蛋白（Hb）正常，白细胞（WBC）升高。

粪常规检查及镜检未见明显异常。

接下来，我们分析各选项与题目中患者症状的关联：

A. 血涂片：通常用于检查血液系统疾病，如白血病等，与患者的症状关联不大。

B. 血培养：主要用于诊断细菌感染，但患者无明确的细菌感染迹象。

C. 汉坦病毒抗体：汉坦病毒可引起肾综合征出血热，典型症状包括发热、头痛、腰痛、眼眶痛、恶心、呕吐、胸闷、腹痛、腹泻、全身关节痛等症状，皮肤黏膜三红（脸、颈和上胸部发红），眼结膜充血，重者似酒醉貌，有肾区叩击痛、肝脾肿大、皮下出血点或瘀斑，以及典型的“三痛征”（头痛、腰痛、眼眶痛）和“三红征”（面红、颈红、上胸红），并伴有肾损伤和出血倾向。这些症状与题干中患者的表现高度吻合。

D. 钩端螺旋体抗体：钩端螺旋体病通常表现为发热、酸痛、全身软、眼结膜充血、腓肠肌疼痛、淋巴结肿大等，与患者的症状不完全符合。

E. EB 病毒抗体：EB 病毒感染主要与鼻咽癌、儿童淋巴瘤等病有关，与患者的症状不符。

综合考虑，患者老年男性，发热、头痛、腰痛、腹泻，伴有少尿，查体见面部充血，眼睑水肿，软腭有出血点，这些症状与汉坦病毒感染引起的肾综合征出血热相符。

因此，为了明确诊断，应当首选的检查是 C.汉坦病毒抗体。

根据题干描述，该患者 68 岁，出现发热、头痛、腰痛、腹泻以及少尿等症状，并且查体发现体温升高、心率加快、呼吸急促、面部充血、眼睑水肿和软腭有出血点，这些症状提示可能是感染性疾病。血常规显示白细胞计数升高，这进一步支持感染的可能性。

在这种情况下，我们需要考虑几种可能的感染源：

1. **血涂片**主要用于血液中原虫和某些寄生虫的检查，但对于该患者的症状，特别是软腭出血点和少尿，并不特异。

2. **血培养**是用于检测血液中的细菌或真菌感染，对于确定具体的病原体是有帮助的，但需要时间培养结果才能得出。

3. **汉坦病毒抗体**检测是为了诊断出血热肾综合征（HFRS），其症状包括发热、头痛、肌肉痛、少尿、面部充血、出血点等，和题干描述相符。

4. **钩端螺旋体抗体**检测用于诊断钩端螺旋体病（Weil 病），其症状也包括发热、头痛、肌肉痛、少尿和出血点，但软腭出血点较少见。

5. **EB 病毒抗体**检测主要用于诊断传染性单核细胞增多症（IM），其症状包括发热、咽痛、淋巴结肿大等，但与患者的少尿和软腭出血点不太一致。

结合患者的症状和体征，汉坦病毒感染的可能性较大。因此，最合适的检查应为 **汉坦病毒抗体检测**。

所以，最正确的答案是：
C. 汉坦病毒抗体
78、 (A2 型题)
男，16 岁，参加 1500 米长跑后出现头晕，乏力，尿蛋白(++)，休息 1 天后症状消失，尿蛋白(-)，最可能的情况是
A.分泌性蛋白尿
B.组织性蛋白尿
C.肾小管性蛋白尿
D.功能性蛋白尿
E.溢出性蛋白尿
参考答案： D 您的答案：
图文解析： 【解析】青少年，剧烈运动之后出现蛋白尿（功能性蛋白尿常见诱因），休息后好转（非器质性改变），根据患者病史及检验结果考虑诊断为功能性蛋白尿（D 对）。【避错】功能性蛋白尿：是指因剧烈运动（或劳累）、受寒、发热、精神紧张、交感神经兴奋等所致的暂时性蛋白尿，与肾血管痉挛或充血导致的肾小球毛细血管壁通透性增高有关。多见于青少年，尿蛋白定性不超过（+），定量不超过 500mg/24h。
这个病例描述的是一名 16 岁男孩，在进行 1500 米长跑后出现头晕、乏力和尿蛋白增多的情况，但休息一天后症状和尿蛋白异常消失。根据这些症状和情况，最可能的情况是**功能性蛋白尿**。
功能性蛋白尿是指在某些生理状态下，如剧烈运动、发热、寒冷暴露等情况下，出现的尿蛋白增多，但没有实质性的肾脏病变。这种蛋白尿通常是暂时的，休息或去除诱因后可以恢复正常。在本题中，患者在剧烈运动后出现蛋白尿，休息后症状消失，符合功能性蛋白尿的特点。
因此，正确答案是**D. 功能性蛋白尿**。
解析：
青少年男性患者，运动后出现一过性蛋白尿，休息后蛋白尿消失，结合患者症状和实验室检查，考虑诊断为功能性蛋白尿，其多见于青年人，在剧烈运动、发热、高温、受寒、精神紧张等因素影响下，肾血管痉挛或充血，导致肾小球滤过膜通透性增强而使蛋白大量“漏网”。诱因消除后蛋白尿会消失。
选项 A 分泌性蛋白尿多见于多发性骨髓瘤等疾病，由于骨髓瘤轻链蛋白异常增多，经肾小球滤过后，超过了肾小管重吸收能力而形成的蛋白尿，患者并无骨髓瘤病史，故不选。
选项 B 组织性蛋白尿多见于肾组织的破坏或肾小管分泌的蛋白增多，或某些特殊成分的蛋白异常增多，经肾小球滤过后，超过了肾小管重吸收能力而形成的蛋白尿，题干中并未提及肾脏组织破坏，故排除。
选项 C 肾小管性蛋白尿是由于肾小管结构或功能受损，对正常滤过的小分子蛋白质重吸收障碍，导致蛋白质从尿中排出，常见于肾盂肾炎、间质性肾炎等肾小管损伤性疾病，患者并无肾小管受损证据，故不选。
选项 E 溢出性蛋白尿是由于肾小球滤过、肾小管重吸收均正常，但因血浆中相对分子质量较小或阳性电荷蛋白（如多发性骨髓瘤轻链蛋白、血红蛋白、肌红蛋白等）异常增多，经肾小球滤过，超过肾小管重吸收能力所形成的蛋白尿，常见于多发性骨髓瘤等，患者无相关病史，故排除。

综上所述，正确答案是：D.功能性蛋白尿。
这道题考察的是蛋白尿的类型。根据题干描述的情况：男孩 16 岁，1500 米长跑后出现头晕、乏力，尿蛋白(++)，休息 1 天后症状消失，尿蛋白(-)，最可能的情况是功能性蛋白尿。

功能性蛋白尿是指在某些生理或病理情况下出现的一过性蛋白尿，但肾脏本身没有病变。以下是各类型蛋白尿的简单介绍：

A. **分泌性蛋白尿**：由于肾小管上皮细胞的分泌功能增强而引起的蛋白尿，例如肾小管分泌的 Tamm-Horsfall 蛋白。

B. **组织性蛋白尿**：由于肾小管细胞损伤和坏死，导致肾小管上皮细胞碎片和管型排入尿液中，见于急性肾小管坏死等疾病。

C. **肾小管性蛋白尿**：由于肾小管对蛋白质的重吸收功能障碍，导致小分子蛋白质在尿液中出现，常见于各种肾小管疾病。

D. **功能性蛋白尿**：在一些特殊的生理情况下，如剧烈运动、发热、应激等，肾小球暂时性增透，导致蛋白尿，但肾脏结构和功能正常。休息后可自行消失。

E. **溢出性蛋白尿**：当血浆中某些小分子蛋白（如免疫球蛋白轻链）过多，超过了肾小管的重吸收能力，导致这些小分子蛋白出现在尿液中。常见于多发性骨髓瘤等疾病。

根据题干描述的情况，男孩在剧烈运动后出现的一过性蛋白尿，在休息后自行消失，最符合**功能性蛋白尿**的特征。

因此，正确答案是：

D. **功能性蛋白尿**
79、 (A2 型题)
女，65 岁，糖尿病 12 年，视物模糊 3 月。眼底检查：眼底微血管瘤，少量出血伴棉絮状软性渗出。患者糖尿病视网膜病变分期是
A.2 期
B.4 期
C.3 期
D.1 期
E.5 期
参考答案： C 您的答案：
图文解析： 【解析】老年女性糖尿病患者，行眼底检查：眼底微血管瘤，少量出血伴棉絮状软性渗出，根据分类标准，本题选 C。
【避错】2002 年国际临床分级标准依据散瞳后检眼镜检查，将糖尿病视网膜改变分为两大类、六期。Ⅰ期：微血管瘤、小出血点（D 错）。Ⅱ期：出现硬性渗出（A 错）。Ⅲ期：出现棉絮状软性渗出。Ⅳ期；新生血管形成、玻璃体积血（B 错）。Ⅴ期：纤维血管增殖、玻璃体机化（E 错）。Ⅵ期：牵拉性视网膜脱离、失明。
糖尿病视网膜病变（DR）分为几个不同的阶段，主要根据眼底表现来分类。基于题目描述

中的眼底微血管瘤、出血和棉絮状软性渗出，这些都是糖尿病视网膜病变早期的典型表现。
- 第 1 期（轻微非增殖性糖尿病视网膜病变）：特征是微血管瘤的存在。
- 第 2 期（中度非增殖性糖尿病视网膜病变）：出现更多的微血管瘤、出血和/或黄斑区渗出。
- 第 3 期（重度非增殖性糖尿病视网膜病变）：大量出血和棉絮斑。
- 第 4 期（增殖性糖尿病视网膜病变）：新生血管出现，可能伴有玻璃体出血和视网膜脱离。
- 第 5 期则不属于通常的糖尿病视网膜病变分期体系。

根据题目中的描述，患者的眼底检查显示“眼底微血管瘤，少量出血伴棉絮状软性渗出”，这些症状符合中度非增殖性糖尿病视网膜病变的特点，即第 2 期。因此，最合适的答案是：
A. 2 期
解析：
老年女性患者，糖尿病多年，视物模糊，眼底检查示眼底微血管瘤，少量出血伴棉絮状软性渗出，为糖尿病视网膜病变的典型症状。糖尿病视网膜病变可分为六期，其分期标准如下：
Ⅰ期：视网膜出现微血管瘤和小出血点。
Ⅱ期：出现硬性渗出。
Ⅲ期：出现棉絮状软性渗出。
Ⅳ期：新生血管形成、玻璃体积血。
Ⅴ期：纤维血管增殖、玻璃体机化。
Ⅵ期：牵拉性视网膜脱离、失明。
该患者眼底检查示眼底出现微血管瘤，少量出血伴棉絮状软性渗出，符合糖尿病视网膜病变 3 期的典型表现。
综上所述，正确答案是：C.3 期。
选项 A：2 期表现为出现硬性渗出，该患者为棉絮状软性渗出，故不选。
选项 B：4 期表现为新生血管形成、玻璃体积血，该患者未出现上述症状，故排除。
选项 D：1 期表现为视网膜出现微血管瘤和小出血点，但患者还有棉絮状软性渗出，因此不符合 1 期的诊断，故不选。
选项 E：5 期表现为纤维血管增殖、玻璃体机化，与患者症状不符，故排除。
根据题干描述，患者有糖尿病 12 年，视物模糊 3 个月，眼底检查发现微血管瘤、少量出血和棉絮状软性渗出。这些表现符合糖尿病视网膜病变的特征。糖尿病视网膜病变的分期通常依据的是 ETDRS（Early Treatment Diabetic Retinopathy Study）的标准。

根据 ETDRS 标准，糖尿病视网膜病变分为以下几期：
1. **无明显视网膜病变**（No apparent retinopathy）
2. **轻度非增殖性糖尿病视网膜病变**（Mild NPDR）：仅有微血管瘤。
3. **中度非增殖性糖尿病视网膜病变**（Moderate NPDR）：存在微血管瘤、硬性渗出、棉絮状软性渗出和少量出血。
4. **重度非增殖性糖尿病视网膜病变**（Severe NPDR）：出现广泛的出血、静脉串珠状改变（beading）、视网膜内微血管异常（IRMA）。
5. **增殖性糖尿病视网膜病变**（PDR）：出现新生血管或玻璃体出血。

根据患者的眼底检查结果（眼底微血管瘤、少量出血和棉絮状软性渗出），符合中度非增殖性糖尿病视网膜病变（3 期）的特征。因此，最正确的答案是：

C. 3 期
80、 (A2 型题)

男，高处坠落，腰背挫伤，双下肢弛缓瘫痪，来院急诊。查体：腰部不能活动，双侧腹股沟以下感觉、运动及反射消失。X 线显示胸椎骨折。后其双下肢功能逐渐恢复。 伤可能是

A.脊髓半切综合征

B.脊髓横断损伤

C.脊髓空洞症

D.脊髓震荡

E.马尾综合征

参考答案： D 您的答案：

图文解析： 【解析】患者高处坠落后，下肢感觉障碍（提示可能有脊髓损伤），后逐渐恢复，结合患者病史及临床表现，该患者最可能的诊断为脊髓震荡（D 对）。

【避错】脊髓震荡临床上表现为损伤平面以下感觉、运动及反射完全消失或大部分消失。一般经过数小时至数天，感觉和运动开始恢复，不留任何神经系统后遗症。脊髓半切综合征：为损伤平面以下同侧肢体的运动及深感觉消失，对侧肢体痛觉和温觉消失（A 错）；脊髓横断损伤为髓实质完全性横贯性损害，损伤平面以下的最低位骶段感觉、运动功能完全丧失，包括肛门周围的感觉和肛门括约肌的收缩运动丧失，称为脊髓休克期。2～4 周后逐渐演变成痉挛性瘫痪，表现为肌张力增高，腱反射亢进，并出现病理性锥体束（B 错）；脊髓空洞症一般为慢性、进行性损伤，原因不明，不可恢复（C 错）；马尾综合征患者多表现为疼痛，表现为交替出现了坐骨神经痛，感觉障碍表现为双下肢及会阴部麻木、感觉减弱或消失、大小便失禁（E 错）。

这个病例描述了一个男性患者因高处坠落导致腰背挫伤，出现双下肢弛缓性瘫痪，并伴有腰部活动受限、双侧腹股沟以下感觉、运动及反射消失。X 线显示胸椎骨折，而后双下肢功能逐渐恢复。

这些症状和体征表明患者的脊髓可能受到了影响。考虑到功能的逐渐恢复，完全的脊髓横断损伤(B)不太可能，因为这种情况下患者通常不会自然恢复功能。脊髓半切综合征(A)通常会出现一侧肢体力量减退和另一侧痛温觉丧失，但病例中没有提及这种不对称的体征。脊髓空洞症(C)通常发展较慢，且病例描述的急性事件不符合这种病症。马尾综合征(E)影响的是腰椎以下的神经根，而不是脊髓本身，且通常不会出现在胸椎骨折中。

考虑到上述信息和病例的特点，最合适的诊断是 **D.脊髓震荡**。脊髓震荡是一种因暴力冲击而引起的脊髓功能暂时性障碍，患者可以在几分钟到几周内恢复功能，这与病例描述的情况相符。

解析：

患者高空坠落导致腰背挫伤，查体示双侧腹股沟以下感觉、运动及反射消失，X 线显示胸椎骨折，应考虑为胸椎骨折导致的脊髓损伤。脊髓损伤后双下肢功能逐渐恢复，符合脊髓震荡的特点。脊髓震荡是指脊髓损伤后发生的一种可逆性功能紊乱，与脑震荡相似，是脊髓损伤最轻的一种，其特点为损伤平面以下脊髓功能的迅速、完全恢复，一般认为其恢复时间不超过 24～48 小时。

选项 A 脊髓半切综合征表现为脊髓损伤平面以下同侧肢体的上运动神经元瘫痪和深感觉消失，对侧肢体痛温觉消失，双侧触觉保留的临床综合征，与该患者双侧腹股沟以下感觉、运动及反射消失的症状不符，故不选。

选项 B 脊髓横断损伤表现为损伤平面以下完全瘫痪，各种反射消失，括约肌功能丧失，皮肤感觉丧失，且恢复无望，与题干中患者双下肢功能逐渐恢复不符，故排除。

选项 C 脊髓空洞症是一种慢性、进行性的脊髓变性疾病，其病变特点是脊髓内形成管状空腔以及胶质增生，常好发于颈部脊髓，早期患者的症状比较局限和轻微，晚期症状则表现广泛甚至出现截瘫，不会自行恢复，故不选。

选项 E 马尾综合征是由于腰椎疾病引起的，以腰椎 L3 到尾节神经、神经根或马尾神经受累为主要表现的一种综合征，通常会出现大小便失禁、鞍区麻木、下肢力弱等症状，但一般下肢功能不会逐渐恢复，故排除。
综上所述，正确答案是：D.脊髓震荡。
根据题干描述，该男性高处坠落后腰背部挫伤，出现双下肢弛缓瘫痪，且腰部不能活动，双侧腹股沟以下感觉、运动及反射消失，X 线显示胸椎骨折。之后其双下肢功能逐渐恢复。

从病史和体征来看，初期的双下肢弛缓瘫痪和感觉、运动及反射消失提示脊髓受到影响。之后双下肢功能逐渐恢复，这种表现更符合脊髓震荡的特征。

脊髓震荡（spinal cord concussion）是指脊髓在外力作用下发生短暂的功能障碍，一般不会造成永久性损伤，常见的表现是受伤后出现暂时的脊髓功能丧失，如瘫痪、感觉缺失等，但随后会逐渐恢复。

其他选项解释如下：
- **A. 脊髓半切综合征（Brown-Séquard syndrome）**：多见于脊髓半侧损伤，表现为损伤侧的运动障碍和反射消失，对侧的痛温觉丧失，通常不会完全恢复。
- **B. 脊髓横断损伤（spinal cord transection）**：脊髓完全横断后一般不会完全恢复功能，损伤部位以下会永久性失去运动和感觉功能。
- **C. 脊髓空洞症（syringomyelia）**：多为慢性病程，表现为脊髓内腔隙形成，导致慢性进行性神经功能障碍，通常不会突然发病或完全恢复。
- **E. 马尾综合征（cauda equina syndrome）**：主要表现为马尾神经受压引起的会阴区感觉减退、排尿排便功能障碍和双下肢无力，通常为逐渐发病，不会突然好转。

综上所述，最可能的诊断是：

D. 脊髓震荡
81、 (A2 型题)
男，65 岁，腰痛数年，直腿抬高试验（+），加强试验（-）。查体：左拇指背伸无力。最可能的原因是
A.L4-L5，L5 神经根受压
B.L3-L4，L4 神经根受压
C.L2-L3，L3 神经根受压
D.S1-S2，S2 神经根受压
E.L5-S1，S1 神经根受压
参考答案： A 您的答案：
图文解析： 【解析】患者腰痛，直腿抬高试验（腰椎间盘突出症重要体征），左趾背伸无力，可知该患者受累神经为 L5，结合患者典型症状和体征，此时最可能的诊断是 L4～L5 椎间盘突出（A 对）。
【避错】L5～S1 椎间盘突出对应 S1 神经根受压，表现为踝反射异常、足趾跖屈力减退。
这道题目涉及到判断脊椎神经根受压的情况。考虑到患者主诉为腰痛，直腿抬高试验阳性（+），这通常指示存在下腰部或坐骨神经根受压。查体发现左拇指背伸无力，这一症状涉及到特定的神经功能，即拇指背伸肌，主要由 L5 神经根支配。
结合这些信息，最可能的诊断是 L5 神经根受压。因此，我们需要寻找与 L5 神经根受压相关

的脊椎段。拇指背伸无力暗示 L5 神经功能障碍，因此 A 项（L4-L5，L5 神经根受压）和 E 项（L5-S1，S1 神经根受压）看起来是相关的。但是，由于症状特定指向 L5 神经根，我们可以更具体地指出是在 L4-L5 脊椎段，L5 神经根受压造成的症状。
所以，最佳答案是 A。L4-L5，L5 神经根受压。
解析：
患者老年男性，腰痛数年，查体示左拇指背伸无力，提示桡神经功能障碍。直腿抬高试验阳性，加强试验阴性，可诊断为腰椎间盘突出症。结合患者症状和体征，最可能的原因是 L3-L4，L4 神经根受压。
选项 A：L4-L5，L5 神经根受压主要表现为拇长伸肌肌力减弱，即足拇指背伸肌力减弱，不符合题意。
选项 C：L2-L3，L3 神经根受压主要表现为股四头肌肌力减弱，即伸膝无力，可排除。
选项 D：S1-S2，S2 神经根受压主要表现为腓骨长、短肌肌力减弱，即踝背伸、外翻无力，故不考虑。
选项 E：L5-S1，S1 神经根受压主要表现为踇长屈肌肌力减弱，即足趾屈无力，与题意不符。
综上所述，正确答案是：B.L3-L4，L4 神经根受压。
根据题目描述，65 岁男性患者有腰痛数年，直腿抬高试验（+），加强试验（-），并且查体发现左拇指背伸无力。以下是对各个选项的分析：

- **直腿抬高试验（+）**：通常提示坐骨神经受到牵拉刺激，常见于腰椎间盘突出。
- **加强试验（-）**：表示没有硬脊膜或马尾神经受压。
- **左拇指背伸无力**：提示神经根受压可能性。

拇指背伸无力通常与**L5 神经根**受压相关，因为 L5 神经根支配拇长伸肌。结合腰痛病史和直腿抬高试验阳性，可以推测腰椎间盘突出导致 L5 神经根受压。

因此，最可能的原因是 **L4-L5，L5 神经根受压**，对应选项 A。

最佳答案：
A. L4-L5，L5 神经根受压
82、 (A2 型题)
男，23 岁，高处摔下后右手着地，伤后右前臂畸形，活动受限。查体：右前臂畸形，肿胀，桡骨中下 1/3 交界处可触及骨擦感，腕部可触及异常凸起的尺骨远端，未及尺 可能的诊断是
A.Smith 骨折
B.Monteggia 骨折
C.Colles 骨折
D.Barton 骨折
E.Galeazzi 骨折
参考答案： E 您的答案：
图文解析： 【解析】青年男性，右手臂外伤病史，前臂畸形，肿胀（骨折表现），桡骨中下 1/3 交界处可触及骨擦感，腕部可触及异常凸起的尺骨远端（盖氏（Galeazzi）骨折特征性表现），综合考虑患者为盖氏（Galeazzi）骨折（E 对）。
【避错】尺骨上 1/3 骨干骨折可合并桡骨小头脱位，称为孟氏（Moteggia）（B 错）骨折。伸直型骨折（Colles 骨折）（C 错）多由于腕关节处于背伸位、手掌着地、前臂旋前时受

伤导致，可出现典型畸形姿势，即侧面看呈“银叉”畸形，正面看呈“刺刀样”畸形。史密斯（Smith）骨折是指桡骨远端 2～3 厘米以内的骨折，且骨折远端向掌侧移位或向背侧成角者（A 错）。桡骨远端关节面骨折伴腕关节脱位（Barton 骨折）是在腕背伸、前臂旋前位跌倒，手掌着地，暴力通过腕骨传导，撞击桡骨关节背侧发生骨折，腕关节也随之而向背侧移位。
该患者由于高处摔下导致右前臂畸形，活动受限，且在桡骨中下 1/3 交界处可触及骨擦感，腕部可触及异常凸起的尺骨远端。这些症状符合 Galeazzi 骨折的特点。

Galeazzi 骨折是指桡骨远端骨折伴尺骨远端脱位。而 Monteggia 骨折是尺骨骨折伴桡骨头脱位，Smith 骨折是桡骨远端骨折且向掌侧移位，Colles 骨折是桡骨远端骨折且向背侧移位，Barton 骨折是桡骨远端关节面骨折。因此，根据这位患者的症状和体征，最可能的诊断是 E.Galeazzi 骨折。
这是一道关于骨折类型的鉴别诊断问题。我们需要分析患者的病史、临床表现及查体结果，从而确定最可能的诊断。
首先，我们梳理一下题目中给的关键信息：
青年男性患者，高处摔下后右手着地。
伤后右前臂畸形，活动受限。
查体显示右前臂畸形、肿胀。
桡骨中下 1/3 交界处可触及骨擦感。
腕部可触及异常凸起的尺骨远端。
接下来，我们根据关键信息，分析各个选项的可能性：
A. Smith 骨折：是屈曲型桡骨远端骨折，通常是手背着地引起的损伤，与题干描述不符。
B. Monteggia 骨折：为尺骨上 1/3 骨干骨折合并桡骨头脱位，典型表现为肘后三角关系改变，通常由于肘部外伤导致，与患者情况不符。
C. Colles 骨折：是伸直型桡骨远端骨折，典型表现为“餐叉”样及“枪刺样”畸形，腕部肿胀、压痛明显，但不会出现尺骨远端的异常凸起，因此不符合患者体征。
D. Barton 骨折：是经关节的桡骨远端骨折，虽然也涉及桡骨远端，但主要累及腕关节面，不符合患者前臂畸形和尺骨远端凸起的描述。
E. Galeazzi 骨折：是指桡骨干下 1/3 骨折合并尺骨小头脱位，通常由于摔倒时手掌着地导致。该骨折类型会导致前臂畸形、肿胀，桡骨中下 1/3 交界处可触及骨擦感，腕部可触及异常凸起的尺骨远端，与患者的临床表现和查体结果相符。
综上所述，根据患者的病史、临床表现和查体结果，最可能的诊断是 Galeazzi 骨折。因此，正确答案是 E.Galeazzi 骨折。
综上所述，正确答案是：E.Galeazzi 骨折。
根据题目描述的病史和查体结果，23 岁男性从高处摔下后右手着地，右前臂畸形、肿胀，桡骨中下 1/3 交界处可触及骨擦感，腕部可触及异常凸起的尺骨远端。这些描述符合 Galeazzi 骨折的特点。Galeazzi 骨折是指桡骨干远端 1/3 骨折伴有尺骨远端脱位。

因此，最可能的诊断是：

E. Galeazzi 骨折
83、 (A2 型题)
女，32 岁，怕冷，嗜睡两个月余。查体：脉搏 56 次/分，眼睑水肿，甲状腺弥漫性肿大，质地韧。最可能的甲状腺功能表现是

A.TT3 正常，TT4 正常，TSH 减少
B.TT3 下降，TT4 下降，TSH 增加
C.TT3 增加，TT4 增加，TSH 减少
D.TT3 增加，TT4 增加，TSH 增加
E.TT3 正常，TT4 正常，TSH 增加
参考答案： B 您的答案：
图文解析： 【解析】中青年女性（甲状腺疾病多发人群），怕冷，嗜睡，脉搏 56 次/分，眼睑水肿，甲状腺弥漫性肿大（甲减典型表现），综合症状、体征考虑此患者为甲状腺功能减低。甲状腺功能减退症血清 TSH 增高，TT4 和 FT4 均降低（B 对）。
【避错】TT3 增加，TT4 增加，TSH 减少为甲亢的血清学表现。
这位 32 岁女性患者的症状包括怕冷、嗜睡、脉搏减慢（56 次/分）、眼睑水肿以及甲状腺弥漫性肿大且质地韧。这些临床表现与甲状腺功能减退症相符，这是一种由于甲状腺激素生成不足引起的疾病。
在甲状腺功能减退症中，通常会观察到血清中的三碘甲状腺原氨酸(TT3)和甲状腺素(TT4)水平降低，同时因为反馈机制导致促甲状腺激素(TSH)水平增高。因此，最可能的甲状腺功能表现是 TT3 下降，TT4 下降，TSH 增加。
选项 B（TT3 下降，TT4 下降，TSH 增加）是这种情况的正确答案。
解析：
患者青年女性，怕冷、嗜睡两个月余，查体见脉率缓慢，眼睑水肿，甲状腺弥漫性肿大，质地韧，根据其临床表现及体征，考虑甲状腺功能减退症。甲状腺功能减退症时，甲状腺激素水平下降，对 TSH 负反馈性抑制作用减弱，TSH 水平升高。因此甲状腺功能减退症患者甲状腺功能表现为 TT3 下降，TT4 下降，TSH 增加。
选项 A“TT3 正常，TT4 正常，TSH 减少”；选项 C“TT3 增加，TT4 增加，TSH 减少”；选项 E“TT3 正常，TT4 正常，TSH 增加”；TSH 与 TT3、TT4 的变化趋势相反，故可一并排除。
选项 D：TT3 增加，TT4 增加，TSH 增加不符合甲状腺激素的负反馈调节机制，故不选。
综上所述，正确答案是：B.TT3 下降，TT4 下降，TSH 增加。
根据病人的症状和体征，包括怕冷、嗜睡、脉搏缓慢、眼睑水肿和甲状腺弥漫性肿大，最可能的诊断是甲状腺功能减退（甲减）。

甲减的典型表现是甲状腺激素（TT3 和 TT4）水平降低，而促甲状腺激素（TSH）水平升高，因为垂体会分泌更多的 TSH 来试图刺激甲状腺产生更多的激素。

因此，最可能的甲状腺功能表现是：
B. TT3 下降，TT4 下降，TSH 增加
84、 (A2 型题)
男，22 岁，修理水泵时绞伤右手，查体：右手掌侧可见不规则伤口，出血不止，2~5 指远端皮肤苍白，感觉减退，指间关节屈曲受限，行清创，肌腱神经血管吻合术。术 是
A.患肢制动，弹力绷带固定
B.局部冰敷，预防血肿形成
C.包扎时指噗间隔开，露出指尖
D.石膏固定手指于伸直位
E.鼓励患者早期活动手指
参考答案： C 您的答案：
图文解析： 【解析】患者手部外伤后行肌腱神经血管吻合术，术后包扎时指蹼间隔开，露

出指尖（C
对），便于观察病情，包扎敷料既要压迫止血又不影响肢体血液循环。
【避错】手部外伤患者应将手指固定于功能位，以最大限度保留手部功能。
考题描述了一位 22 岁男性，在修理水泵时右手被绞伤的情况。患者的伤口不规则，有持续出血，且 2 至 5 指远端皮肤苍白，感觉减退，指间关节屈曲受限。经过清创和肌腱神经血管吻合术后，需要考虑术后的恢复措施。
选项分析如下：
- A.患肢制动，弹力绷带固定：这种方法适用于某些骨折或扭伤，需要暂时限制活动，但对于手术后促进血液循环和避免僵硬来说，可能不是最佳选择。
- B.局部冰敷，预防血肿形成：冰敷可以减轻炎症和肿胀，但对于创伤后恢复的长期管理来说，不应该是唯一的措施。
- C.包扎时指噗间隔开，露出指尖：这样做可以避免指尖压迫和促进伤口愈合，但对功能恢复的直接帮助有限。
- D.石膏固定手指于伸直位：这会限制手指活动，可能导致术后僵硬，不利于恢复手指的功能。
- E.鼓励患者早期活动手指：这有助于改善血液循环，防止关节僵硬和肌肉萎缩，是术后康复中常见的建议。
根据上述分析，最佳答案是 **E.鼓励患者早期活动手指**，因为这有助于促进血液循环，防止关节僵硬和肌肉萎缩，加快恢复过程。
解析：
青年男性患者，绞伤右手，查体见右手掌侧不规则伤口，出血不止，2～5 指远端皮肤苍白，感觉减退，指间关节屈曲受限，考虑诊断为右手外伤，已行清创，肌腱神经血管吻合术。术后需局部制动****，弹力绷带固定，以减少吻合处张力，保证吻合口良好愈合。
选项 B 局部冰敷，预防血肿形成虽然有助于减轻肿胀和疼痛，但不是吻合术后的特殊处理，故不选。
选项 C 包扎时指噗间隔开，露出指尖适用于手指的单独包扎，而该患者是手掌受伤，因此不适用，故排除。
术后需保持手指屈曲位以减少肌腱张力，故选项 D 石膏固定手指于伸直位，选项 E 鼓励患者早期活动手指，均不利于吻合口愈合，可一并排除。
综上所述，正确答案是：A.患肢制动，弹力绷带固定。
根据提供的案例，患者右手受到严重伤害，表现为出血不止、远端皮肤苍白、感觉减退和指间关节屈曲受限。在行清创、肌腱神经血管吻合术后，选择最佳的术后处理措施至关重要。以下是对各选项的分析：

A. 患肢制动，弹力绷带固定：这种方法有助于稳定伤肢，减少活动引起的二次损伤和出血。

B. 局部冰敷，预防血肿形成：虽然冰敷可以帮助减少肿胀和血肿，但对于这种复杂的手术后处理不是最优先的选择。

C. 包扎时指噗间隔开，露出指尖：这是为了防止包扎过紧导致的血液循环问题，同时通过露出指尖可以观察指尖的颜色和血液循环情况，是较为重要的措施。

D. 石膏固定手指于伸直位：对于手术后的固定，石膏固定可以提供良好的稳定性，但固定在伸直位可能会限制手指的活动和功能恢复。

E. 鼓励患者早期活动手指：虽然早期活动手指对于防止关节僵硬有帮助，但在初期手术后的恢复期，活动可能会引起出血和损伤，因此不是最优选择。

综合考虑患者的情况和术后处理的原则，**最佳答案是 C. 包扎时指蹼间隔开，露出指尖**。这样可以防止包扎过紧，同时通过观察指尖颜色和血液循环情况来及时发现和处理潜在的问题。

85、 (A2 型题)

女，50 岁，活动后胸闷 1 年，夜间阵发性呼吸困难 4 天。查体：BP130/80mmHg，P2 亢进，心尖部可闻及舒张期隆隆样杂音，该患者最易出现的心律失常是

A.三度房室传导阻滞

B.室上性心动过速

C.心房颤动

D.室性心动过速

E.心房扑动

参考答案： C 您的答案：

图文解析： 【解析】中老年女性，活动后胸闷、夜间阵发性呼吸困难(左心衰竭症状)，P2 亢进，心尖部可闻及舒张期隆隆样杂音（二尖瓣狭窄特征性听诊），综合考虑此患者为二尖瓣狭窄，二尖瓣狭窄最易出现的心律失常为心房颤动（C 对）。

【避错】慢性左心衰的心脏体征：除基础心脏病的固有体征外， 一般均有心脏扩大（单纯舒张性心衰除外）及相对性二尖瓣关闭不全的反流性杂音、肺动脉瓣区第二心音亢进及舒张期奔马律。二尖瓣关闭不全的典型杂音为心尖区全收缩期吹风样杂音。主动脉瓣关闭不全的典型杂音为主动脉瓣区舒张期高调递减叹气样杂音。肺动脉瓣狭窄的典型杂音为胸骨左缘第二肋间响亮的收缩期喷射性杂音。三尖瓣狭窄的典型杂音为胸骨左缘第 4 肋间收缩期前或舒张期滚筒样杂音。

这个病例描述的症状，如活动后胸闷、夜间阵发性呼吸困难以及心尖部可闻及舒张期隆隆样杂音，可能指向心脏瓣膜疾病，特别是二尖瓣病变。P2 亢进指的是第二心音的肺动脉瓣成分增强，这可能是由于左心室末期压力增高导致。这些症状和体征可能与二尖瓣狭窄或者有其他左心室充盈障碍的情况相符。

在这种情况下，最易出现的心律失常是心房颤动（C 选项）。心房颤动在二尖瓣疾病患者中较为常见，特别是在有左房扩大和左心室充盈压力增高的情况下。因此，C 选项（心房颤动）是这题的最佳答案。

解析：

中年女性患者，活动后胸闷，夜间阵发性呼吸困难，查体显示肺动脉第二心音亢进，心尖部可闻及舒张期隆隆样杂音，为二尖瓣狭窄的特征性杂音，结合患者症状及体征，应诊断为二尖瓣狭窄。二尖瓣狭窄患者最常见的心律失常为心房颤动，可能因为左心房压力增高、风湿性心肌炎等导致左心房壁的心肌纤维出现了灶性的肌溶解，心房肌各部位之间出现迷走神经肌电生理不均一性，使各部分心房肌的电兴奋性不稳定，从而诱发房颤。

选项 A 三度房室传导阻滞常见于冠心病、心肌炎、心肌病等疾病，二尖瓣狭窄少见，故不选。

选项 B 室上性心动过速常见于无器质性心脏病者，也可见于风心病、二尖瓣狭窄、冠心病等，但发生率相对较低，故排除。

选项 D 室性心动过速多见于各种器质性心脏病患者，最常见为冠心病，特别是曾有心肌梗死的患者，二尖瓣狭窄少见，故不选。

选项 E 心房扑动多见于器质性心脏病，在房性心动过速与心房颤动之间的过渡型，当心房异位起搏点频率达到 250～350 次/分且呈规则时，引起心房扑动，但发生率较心房颤动低，故排除。
综上所述，正确答案是：C.心房颤动。
根据题目描述，该患者 50 岁，活动后胸闷 1 年，夜间阵发性呼吸困难 4 天，体格检查显示血压 130/80 mmHg，P2 亢进，心尖部可闻及舒张期隆隆样杂音。这些症状和体征提示可能存在心脏功能不全，尤其是左心功能不全。舒张期隆隆样杂音可能提示二尖瓣狭窄。

二尖瓣狭窄常见的并发症之一是心房颤动（房颤），这是因为二尖瓣狭窄导致左心房压力增高，左心房扩大，进而容易引发心房颤动。因此，该患者最易出现的心律失常是：

C. 心房颤动
86、 (A2 型题)
男，18 岁，右踝扭伤 2 小时，右踝肿胀，外踝前方轻压痛，关节稳定性可，X 线未见骨折移位，早期治疗不恰当的是
A.局部按摩
B.休息，减少行走
C.弹力绷带适当固定
D.冷敷
E.右下肢抬高
参考答案： A 您的答案：
图文解析： 【解析】踝部扭伤患者立即按摩（A 错，为本题正确答案）及热敷均会加快患处的血液循环，使肿胀更明显。
【避错】伤后 24 小时内冷敷，以减少局部出血及肿胀，伤后 48 小时应热敷。
早期治疗扭伤踝关节时，应遵循“RICE”原则，即休息（Rest）、冰敷（Ice）、压迫（Compression）和抬高（Elevation）。因此，正确的早期治疗包括休息、减少行走（B）、弹力绷带适当固定（C）、冷敷（D）和受伤肢体抬高（E）。这些措施有助于减少肿胀、疼痛和进一步的组织损伤。
相反，局部按摩（A）可能会加重损伤，因为它可能导致更多的肿胀和出血。在扭伤发生初期，增加局部血流可能会加剧炎症和肿胀，因此不推荐在早期进行按摩。
基于以上信息，最不恰当的早期治疗是**A.局部按摩**。
解析：
青年男性患者，右踝扭伤，右踝肿胀，外踝前方轻压痛，关节稳定性可，X 线未见骨折移位，考虑为单纯踝关节扭伤。单纯踝关节扭伤是临床常见的疾病，在关节及韧带损伤中是发病率最高的疾病。踝关节扭伤后局部软组织因暴力损伤而出血、渗出，导致肿胀和疼痛。此时不宜局部按摩，因按摩会加重局部出血，加重肿胀，不利于软组织的修复。
选项 B 休息，减少行走可减少损伤部位的活动，为软组织的修复提供条件，属于踝关节扭伤的恰当处理。
选项 C 弹力绷带适当固定可起到加压包扎的作用，既能减少出血，又能防止局部再活动，为恰当的处理方式。
选项 D 冷敷可减轻局部充血，缓解肿胀和疼痛，是踝关节扭伤后的恰当处理方式。
选项 E 右下肢抬高可促进下肢血液和淋巴回流，缓解肿胀，对踝关节扭伤后局部软组织的恢复有益。
综上所述，正确答案是：A.局部按摩。

对于右踝扭伤的早期治疗，通常采用急性损伤处理原则，即休息、冰敷、压迫和抬高（RICE原则）。下面逐一分析每个选项：

A. 局部按摩 - 在急性期（受伤后 24 至 48 小时）进行局部按摩是不适当的，因为这可能会增加肿胀和出血，延长恢复时间。

B. 休息，减少行走 - 休息和减少行走有助于防止进一步损伤和促进愈合，是适当的治疗措施。

C. 弹力绷带适当固定 - 弹力绷带固定可以帮助控制肿胀和稳定关节，也是适当的治疗措施。

D. 冷敷 - 冷敷可以减少疼痛和肿胀，是急性期损伤的标准处理方法之一。

E. 右下肢抬高 - 抬高受伤的下肢可以减少肿胀和疼痛，也是适当的治疗措施。

综上所述，不恰当的早期治疗是：
A. 局部按摩
87、 (A2 型题)
男，22 岁。2 周前发热病史，心悸、乏力 3 天。查体：心率 100 次/分，无不齐，心脏超声未见明显异常。最可能的诊断是
A.扩张型心肌病
B.肥厚型心肌病
C.急性病毒性心肌炎
D.冠心病
E.急性心肌梗死
参考答案： C 您的答案：
图文解析： 【解析】患者青年男性（病毒性心肌炎易发人群），发热病史 2 周（前驱感染症状），心悸、乏力，心率 100 次/分，心脏超声未见明显异常，结合患者病史、体征表现，考虑诊断为病毒性心肌炎（C 对）。
【避错】扩张型和肥厚型心肌病可通过超声诊断；冠心病好发于中老年患者，以胸痛及心电图改变可确诊；急性心肌梗死常表现为剧烈且持久的胸骨后疼痛。
患者为 22 岁男性，有 2 周发热病史，近 3 天出现心悸和乏力，心率 100 次/分且齐，心脏超声未见明显异常。根据这些症状和体征，最可能的诊断是 C.急性病毒性心肌炎。
急性病毒性心肌炎常见于年轻患者，可以在病毒感染（如流感病毒、肠道病毒等）后出现心肌损伤和炎症反应。典型症状包括发热、心悸、乏力等。心脏超声可能未显示明显的结构性异常，因为病毒性心肌炎主要是心肌的炎症和功能障碍，而不一定伴有心脏结构的显著变化。
因此，结合这位年轻患者的症状和检查结果，急性病毒性心肌炎是最可能的诊断。
首先，我们要分析患者的病史、症状以及检查结果，从而得出最合适的诊断。
患者青年男性，2 周前有发热病史，这提示患者可能曾有过某种感染，如病毒感染。近 3 天出现心悸和乏力，这是心脏受累的表现。查体显示心率为 100 次/分，虽然稍快但并无不齐，而心脏超声检查未见明显异常。
接下来，我们针对各个选项进行分析：
A. 扩张型心肌病：主要表现为左心室或双心室扩大，伴有心室收缩功能减退。但本题中心

脏超声检查未见明显异常，与此病不符。
B. 肥厚型心肌病：主要特征为心室壁增厚，尤其是室间隔。但此患者的超声并未显示此异常。
C. 急性病毒性心肌炎：多数患者在发病前有病毒感染前驱症状，如发热、全身倦怠等。随后可以有心悸、胸痛、呼吸困难等不适。该患者 2 周前有发热病史，随后出现心悸和乏力，与这一病症高度吻合。
D. 冠心病：多见于中老年，常由冠状动脉粥样硬化导致。此患者为青年，且没有其他冠心病的高危因素，所以此选项不太可能。
E. 急性心肌梗死：通常有心绞痛病史，表现为突发的、持续的、剧烈的胸骨后疼痛。该患者并未提及此类症状。
综上所述，结合患者的病史、症状和检查结果，急性病毒性心肌炎是最合适的诊断。
因此，正确答案是 C.急性病毒性心肌炎。
根据题目描述，患者为 22 岁男性，有 2 周前发热病史，伴有心悸和乏力 3 天，查体显示心率 100 次/分，无不齐，心脏超声未见明显异常。这种情况下，最可能的诊断是急性病毒性心肌炎。

急性病毒性心肌炎通常在病毒感染后出现，并且会表现为发热、心悸、乏力等症状，而超声心动图可能没有明显异常。因此，综合患者的病史和临床表现，最可能的诊断是：

C. 急性病毒性心肌炎
88、 (A2 型题)
女，68 岁，体检时发现血压偏高，但无明显症状，自我感觉良好，未进行规范化治疗。今日早餐后常规散步，突感腹部不适，随到社区医院就诊，初步考虑为胃肠炎，给 治疗。回家途中，突感胸闷，倒地不起，抢救无效死亡。尸检发现心包腔内有血液，约 200ml。该患者最可能的心脏病变是
A.心肌间大量淋巴细胞浸润
B.心肌纤维增粗肥大
C.心肌间可见肉芽肿形成
D.心肌间广泛纤维化
E.左室前壁透壁心梗
参考答案： E 您的答案：
图文解析： 【解析】高血压患者未进行规范化治疗，突感腹部不适，按肠胃炎补液治疗后，突感胸闷，倒地不起（急性心肌梗死的可能），抢救无效死亡。尸检发现心包腔内有血液，约 200ml，综上考虑为急性心肌梗死，故该患者最有可能心脏病变是左室前壁透壁心梗（E 对）。
【避错】心肌间淋巴细胞浸润（A 错）和心肌间可见肉芽肿形成（C 错），为风湿性心肌炎的病理特点。心肌纤维增粗肥大（B 错）为肥厚性心肌病的病理特点。心肌间广泛纤维化（D 错）为慢性缺血性心脏病的病理特点。
这道题目描述了一位 68 岁女性，血压偏高且未进行规范化治疗，突然死亡，尸检发现心包腔内有 200ml 血液。这种情况最有可能是由于急性心梗引起的心包填塞。急性心梗可导致心脏组织的急性缺血和坏死，严重时可导致心壁破裂，血液因此进入心包腔，形成心包填塞，迅速导致患者死亡。
因此，最合适的答案是：
E.左室前壁透壁心梗

这个选项描述了急性心梗导致的透壁性心肌梗死，符合患者急性胸痛、心包腔积血和突然死亡的临床表现。
解析：
患者老年女性，早餐后散步时突感腹部不适，初步诊断为胃肠炎，治疗后回家途中，突感胸闷，倒地不起，抢救无效死亡。尸检发现心包腔内有血液，提示心包积血，结合患者病史和临床表现，该患者最可能的心脏病变是心脏破裂，多见于急性透壁性心肌梗死，心脏破裂常在心梗 1 周内出现，多为心室游离壁破裂，偶为心室间隔破裂造成穿孔或腱索断裂，可导致心包积血引起心脏压塞而猝死。故该患者最可能的心脏病变是左室前壁透壁心梗。
选项 A 心肌间大量淋巴细胞浸润常见于病毒性心肌炎，而该患者为心梗，故不选。
选项 B 心肌纤维增粗肥大是高血压引起的代偿性肥大，不会出现猝死，故排除。
选项 C 心肌间可见肉芽肿形成常见于感染性心内膜炎，而该患者并未提示有感染，故不考虑。
选项 D 心肌间广泛纤维化常见于扩张型心肌病，而该患者并未提示心室扩张，故不符合。
综上所述，正确答案是：E.左室前壁透壁心梗。
根据题目中的描述，68 岁的女性患者在体检时发现血压偏高，但无明显症状，且未进行规范化治疗。她在散步后突感腹部不适，被初步诊断为胃肠炎并进行治疗。在回家途中突感胸闷，倒地不起，最终抢救无效死亡。尸检发现心包腔内有血液，约 200ml。

根据尸检发现心包腔内有血液这一重要线索，结合临床表现，最可能的心脏病变是左室前壁透壁心梗（选项 E）。这种情况可能导致心脏破裂，从而引起心包腔内出血（心包填塞），这是导致患者突然死亡的原因。

答案：E. 左室前壁透壁心梗
89、 (A2 型题)
女，62 岁，左腰痛伴发热 5 天，最高体温 39.2℃,抗生素治疗体温无明显下降，左肾区叩击痛，心率 102 次/分，血压 110/75mmHg。血常规：白细胞 15.3×10^9/L，N0.85，
细胞满视野，RBC3~5/HP。泌尿 CT 平扫：左肾扩张积水，左输尿管上段 1.2cm 结石，下一步治疗措施是
A.体外冲击波碎石
B.输尿管镜取石
C.尿道逆行置管术
D.膀胱输尿管镜取石
E.经皮肾镜取石
参考答案： C 您的答案：
图文解析： 【解析】中老年女性患者，左腰痛伴发热 5 天，抗生素治疗无效，左肾扩张积水，左输尿管上段 1.2cm 结石，综合患者总体情况考虑诊断为左肾结石伴未控制的泌尿系感染（此乃体外冲击波碎石的禁忌证），因此下一步治疗首选尿道逆行置管术（C 对），先把积液排除，控制感染。
【避错】体外冲击波碎石的禁忌证：结石远端尿路梗阻、妊娠、出血性疾病、主动脉或肾动脉瘤、尚未控制的泌尿系感染等。
这个病例描述了一位 62 岁女性，出现左腰痛伴发热，抗生素治疗后体温未明显下降，左肾区叩击痛，血常规显示白细胞升高，CT 平扫显示左肾积水和左输尿管上段结石。这些症状和体征提示左侧肾积水可能是由输尿管结石引起的感染。
A. 体外冲击波碎石（ESWL）适用于小于 2cm 的肾脏和上部输尿管结石，但在有感染和积水的情况下使用可能不安全。

B. 输尿管镜取石可以直接移除结石，并对输尿管造成的阻塞进行处理，适合于输尿管结石，特别是当存在感染和积水时。
C. 尿道逆行置管术主要用于解除尿路梗阻，而不是直接取石。
D. 膀胱输尿管镜取石适用于膀胱或下部输尿管的结石。
E. 经皮肾镜取石适用于较大的肾结石或当其他方法失败时。
考虑到病人的症状、体征和影像学发现，最佳的治疗措施应该是 B. 输尿管镜取石，因为它可以直接移除导致感染和积水的结石，并处理输尿管的阻塞。
解析：
患者老年女性，左输尿管上段结石伴肾积水，合并尿路感染，经抗生素治疗体温无明显下降，表明感染未能有效控制，此时不宜进行有创的手术操作，否则可能导致感染扩散，应先引流积水，控制感染后再处理结石。尿道逆行置管术操作简单，创伤小，可及时引流肾积水，降低肾盂内压力，待感染控制后再二期处理结石。
选项 A 体外冲击波碎石术是通过体外碎石机产生冲击波，由机器聚焦后对准结石，经过多次释放能量而击碎体内的结石，使之随尿液排出体外。该患者合并尿路感染，应先控制感染，否则碎石过程中可能导致感染扩散，故排除。
选项 B 输尿管镜取石，选项 D 膀胱输尿管镜取石，选项 E 经皮肾镜取石，均属于手术治疗，该患者目前有尿路感染征象，不宜手术，否则可能加重感染，应待感染控制后再处理结石，因此一并排除。
综上所述，正确答案是：C.尿道逆行置管术。
这位 62 岁女性患者的临床表现包括左腰痛、发热（体温高达 39.2℃）、抗生素治疗后体温无明显下降、左肾区叩击痛、血常规显示白细胞增高和中性粒细胞比例增高，泌尿 CT 显示左肾扩张积水和左输尿管上段有 1.2cm 结石。这些症状和检查结果提示患者可能存在肾积水和感染，可能是由于输尿管结石引起的上尿路梗阻导致的急性肾盂肾炎。

在这种情况下，最优先的治疗目标是解除梗阻，缓解感染和肾积水。因此，下一步的治疗措施应是尽快解除输尿管梗阻。

选项分析：
A. 体外冲击波碎石：适用于没有急性感染和肾积水的患者，且结石较小。当前不适合，因为患者有急性症状。
B. 输尿管镜取石：直接处理输尿管结石，可以在可视化下取石，适用于急性症状患者。
C. 尿道逆行置管术：可以迅速减轻肾积水和感染，是一种较为保守的应急措施。
D. 膀胱输尿管镜取石：通常用于膀胱和下段输尿管结石，当前结石位于上段。
E. 经皮肾镜取石：适用于较大的肾结石或复杂结石病，但风险和创伤相对较大。

综上所述，考虑到患者的急性症状和当前临床表现，**C. 尿道逆行置管术**是首选，因为它可以迅速缓解梗阻，减轻肾积水和感染症状，然后再根据具体情况选择进一步的取石治疗。

正确答案是：**C. 尿道逆行置管术**
90、 (A2 型题)
男，50 岁，发作性胸痛 1 月，常于凌晨 3 点左右发作，白天活动无特殊不适，Holter 提示胸部导联 ST 段抬高，症状休息后缓解，多次查 cTn 正常，冠脉 CTA 提示前降支轻度狭 期治疗最佳的用药是
A.尼可地尔

B.曲美他嗪
C.美托洛尔
D.卡托普利
E.地尔硫䓬
参考答案：　　A　您的答案：
图文解析：　　【解析】中年男性，发作性胸痛，常于凌晨 3 点左右发作（变异型心绞痛好发于凌晨），白天活动无特殊不适，Holter 提示胸部导联 ST 段抬高，症状休息后缓解，多次查 cTn 正常，冠脉 CTA 提示前降支轻度狭窄，综合考虑患者目前诊断变异型心绞痛。尼可地尔(A 对)是 K+通道激活药，既有激活血管平滑肌细胞膜 K+通道，促进 K+外流，使细胞膜超极化，抑制 Ca2+内流作用，还有释放 NO，增加血管平滑肌细胞内 cGMP 生成的作用。上述两种作用的结果使血管平滑肌松弛，冠脉血管扩张，冠状动脉供血增加和减轻 Ca2+超载对缺血心肌细胞的损害。主要适用于变异型心绞痛和慢性稳定型心绞痛。
该病例描述的是一位 50 岁男性患者，有发作性胸痛，特别是在凌晨 3 点左右，但白天活动时无不适。Holter 监测显示胸部导联 ST 段抬高，休息后症状缓解，血清心肌肌钙蛋白（cTn）检查正常，冠状动脉 CT 血管造影（CTA）显示前降支轻度狭窄。
基于这些信息，这种胸痛的性质和临床表现似乎与变异性心绞痛（普林兹美尔心绞痛）相符，这种心绞痛通常发生在休息时，特别是在夜间或清晨，并且休息和/或硝酸甘油可以缓解症状。变异性心绞痛是由冠状动脉痉挛引起的，与血管的物理阻塞（如在稳定型心绞痛中见到的）不同。
根据这个描述，适当的治疗是使用血管扩张剂来缓解冠状动脉的痉挛。选项中的尼可地尔（A）是一种钙通道阻滞剂，可以用于治疗变异性心绞痛，因为它有助于防止冠状动脉痉挛，扩张血管，从而改善心肌的血液供应。
因此，最合适的答案是 **A. 尼可地尔**。
解析：
中年男性患者，常于凌晨出现发作性胸痛，白天活动无特殊不适，动态心电图提示胸部导联 ST 段抬高，休息后可缓解，多次查心肌肌钙蛋白正常，冠脉 CTA 提示前降支轻度狭窄。结合患者病史及辅助检查，考虑诊断为变异型心绞痛。变异型心绞痛是由于冠状动脉痉挛引起的缺血性心绞痛，其治疗首选钙通道阻滞剂，可扩张冠脉，解除冠脉痉挛，改善心内膜下心肌的供血，其中地尔硫䓬为常用的非二氢吡啶类钙通道阻滞剂，可用于变异型心绞痛的治疗。
选项 A 尼可地尔为钾通道开放剂，与硝酸酯类结构相似，但不属于同一类化合物，适用于各类型心绞痛，尤其适用于存在冠状动脉微血管病变的心绞痛患者，但对冠状动脉痉挛引起的变异型心绞痛效果不如钙通道阻滞剂，故不选。
选项 B 曲美他嗪通过抑制脂肪酸氧化和增加葡萄糖代谢，提高氧的利用效率而治疗心肌缺血，可作为传统治疗不能耐受或无效的稳定性心绞痛的对症治疗，不作为变异型心绞痛的首选治疗，故排除。
选项 C 美托洛尔为β受体阻滞剂，可阻断冠状动脉上面的β受体，导致α受体相对占优势，可诱发冠状动脉痉挛，禁用于变异型心绞痛，故不选。
选项 D 卡托普利为血管紧张素转化酶抑制剂，主要用于高血压和心力衰竭的治疗，对于心绞痛的治疗效果不明确，可排除。
综上所述，正确答案是：E.地尔硫䓬。
根据题目提供的信息，这名 50 岁的男性患者有发作性胸痛，常在凌晨 3 点左右发作，Holter 提示胸部导联 ST 段抬高，症状在休息后缓解，多次查 cTn 正常，冠脉 CTA 提示前降支轻度狭窄。这些信息提示患者可能患有变异型心绞痛（Prinzmetal 心绞痛）。

变异型心绞痛的特征是由于冠状动脉痉挛导致的暂时性心肌缺血，通常在休息时发作，尤其是夜间或凌晨。治疗的主要目标是缓解冠状动脉痉挛，常用的药物包括钙通道阻滞剂和硝酸酯类药物。

在给出的选项中：

A. 尼可地尔（Nicorandil）是一种钾通道开放剂和硝酸酯类药物，具有扩张冠状动脉的作用，常用于治疗变异型心绞痛。

B. 曲美他嗪（Trimetazidine）是一种抗心绞痛药，但主要用于缓解慢性稳定性心绞痛的症状，不是首选的变异型心绞痛治疗药物。

C. 美托洛尔（Metoprolol）是一种β受体阻滞剂，主要用于缓解心绞痛症状，但对于变异型心绞痛，β受体阻滞剂的作用有限，甚至可能加重痉挛。

D. 卡托普利（Captopril）是一种 ACE 抑制剂，主要用于治疗高血压和心力衰竭，对变异型心绞痛的直接效果不明显。

E. 地尔硫䓬（Diltiazem）是一种钙通道阻滞剂，能够有效缓解冠状动脉痉挛，是变异型心绞痛的常用药物之一。

结合上述分析，最适合用于治疗变异型心绞痛的药物是钙通道阻滞剂和硝酸酯类药物。因此，本题的最佳答案是：

E. 地尔硫䓬

91、 (A2 型题)

男，32 岁。体检时心脏听诊发现心尖区收缩期中期喀喇音，闻及 3/6 级收缩中晚期吹风样杂音，X 线显示心影正常。拟诊断为

A.二尖瓣脱垂

B.二尖瓣关闭不全

C.二尖瓣狭窄

D.主动脉关闭不全

E.主动脉狭窄

参考答案： A 您的答案：

图文解析： 【解析】心尖部出现收缩中晚期喀喇音（A 对）提示二尖瓣脱垂。

【避错】三尖瓣关闭不全的杂音特点是胸骨左下缘全收缩期杂音，吸气后杂音增强（B 错）。二尖瓣狭窄（C 错）特征性杂音为心尖区舒张中晚期低调的隆隆样杂音。主动脉关闭不全（D 错）特征杂音为主动脉瓣区舒张期高调叹气样杂音。主动脉狭窄可在胸骨右缘第 2 肋间或者胸骨左缘第 3 肋间，听到收缩期喷射性吹风样杂音，声音粗糙，可以向颈动脉传导（E 错）。

这个案例描述了一名 32 岁男性，在心脏听诊时发现心尖区在收缩期中期有喀喇音，以及在收缩中晚期有 3/6 级吹风样杂音。X 线显示心影正常。

喀喇音通常与瓣膜的异常活动有关，特别是在瓣膜脱垂时会出现。二尖瓣脱垂时，心脏的二尖瓣在收缩期异常突出入左心房，可能产生喀喇音和吹风样杂音。

考虑到这些症状，最符合的诊断是：
A. 二尖瓣脱垂
因此，我选择的答案是 A. 二尖瓣脱垂。
解析：
青年男性患者，体检发现心尖区收缩期中期喀喇音，闻及 3/6 级收缩中晚期吹风样杂音，X 线显示心影正常，结合患者临床表现和相关检查，该患者最可能的诊断为二尖瓣脱垂。
选项 B 二尖瓣关闭不全的典型杂音为心尖区全收缩期吹风样杂音，杂音柔和，常向腋下传导，与题干中杂音描述不符，故排除。
选项 C 二尖瓣狭窄的杂音特点为局限于心尖区的舒张中晚期低调的隆隆样杂音，可伴有舒张期震颤，题干中杂音为收缩期杂音，故不选。
选项 D 主动脉关闭不全的杂音为主动脉瓣区舒张期高调递减型叹气样杂音，可向心尖区传导，与题干中杂音描述差异明显，故不考虑。
选项 E 主动脉狭窄的杂音特点为主动脉瓣区粗糙而响亮的射流性杂音，呈递增-递减型，向颈部传导，在胸骨右缘 1～2 肋间听诊最清楚，杂音特点与题干不符，故不选。
综上所述，正确答案是：A.二尖瓣脱垂。
根据题干描述：

- 32 岁男性，体检时心脏听诊发现心尖区收缩期中期喀喇音。
- 听诊时闻及 3/6 级收缩中晚期吹风样杂音。
- X 线显示心影正常。

这种描述最符合**二尖瓣脱垂**的特征。二尖瓣脱垂的临床表现通常包括心尖区在收缩中期听到喀喇音，以及随后出现的收缩中晚期的吹风样杂音。心影正常也是常见的表现，因为二尖瓣脱垂并不一定会导致心脏的显著增大。

所以，最可能的诊断是：

A. 二尖瓣脱垂
92、 (A2 型题)
女，59 岁。因高热、腹泻静脉点滴庆大霉素治疗，5 天后出现恶心、呕吐、伴少尿。查血白细胞总数及分类正常，尿比重 1.010，蛋白（+），红细胞 0～2/HP，白细胞 3～5 320μmol/L，尿素氮 17mmol/L，尿钠 100mmol/L。该患者肾功能减退最可能的原因是
A.急性肾小管坏死
B.肾前性氮质血症
C.急性肾小球肾炎
D.急进性肾小球肾炎
E.急性间质性肾炎
参考答案： A 您的答案：
图文解析： 【解析】中老年女性，因高热、腹泻应用庆大霉素（肾毒性药物可引起肾小管性急性肾损伤）治疗 5 天后出现少尿、血肌酐 320μmol/L（血肌酐升高），尿素氮 17mmol/L，尿钠 100mmol/L（尿钠小于 20mmol/L 为肾前性急性肾损伤，尿钠大于 40mmol/L 为急性肾小管坏死），故本题选 A。
【避错】肾前性氮质血症是血容量不足或者心功能不全，导致肾血灌注量不足，从而引起肾小球滤过率降低所致，尿钠一般＜20mmol/L（B 错）。急性肾小球肾炎临床特点为血

尿、蛋白尿、水肿和高血压，可伴有一过性肾功能不全；多见于链球菌感染后（C 错）。
急进性肾小球肾炎在急性肾炎综合征上，早期出现少尿或无尿，肾功能快速进展乃至尿毒症期，患者可伴有不同程度贫血（D 错）。急性间质性肾炎常同时具有全身过敏表现，主要见于药物过敏引起的 AIN，可表现为皮疹、发热及外周血嗜酸性白细胞计数增多，部分病例还可有关节痛、淋巴结肿大等（E 错）。
该患者因高热、腹泻接受庆大霉素治疗后出现了恶心、呕吐和少尿的症状，这些都是急性肾损伤的典型表现。考虑到庆大霉素是一种可能导致肾毒性的药物，特别是在剂量大或使用时间较长时，其主要损害机制是引起急性肾小管坏死。血液和尿液检测结果显示尿素氮和血肌酐水平升高，这也支持了急性肾损伤的诊断。
因此，最合适的答案是 A.急性肾小管坏死。
这是一道关于肾功能减退原因的判断题。首先，我们分析患者的病史和检查结果：
患者，女，59 岁，因高热、腹泻接受过庆大霉素治疗。
治疗后出现恶心、呕吐和少尿。
血液检查显示白细胞总数及分类正常，说明没有明显的感染或炎症。
尿液检查显示尿比重降低（1.010），尿蛋白阳性（+），红细胞和白细胞数量均不高。
生化检查显示尿素氮和尿钠水平升高。
接下来，我们根据这些症状和检查结果，分析每个选项的可能性：
A. 急性肾小管坏死：患者接受过庆大霉素治疗，这是已知的肾毒性药物。结合少尿、尿比重降低、尿素氮和尿钠升高，这些都是急性肾小管坏死的典型表现。符合题意。
B. 肾前性氮质血症：通常与血容量不足或心功能不全有关，题干中未提及这些因素。不符合题意。
C. 急性肾小球肾炎：常表现为血尿、蛋白尿、水肿和高血压，而题干中未提及这些症状。不符合题意。
D. 急进性肾小球肾炎：病情发展迅速，早期出现少尿性急性肾衰竭，但题干中未提及相关病史或症状。不符合题意。
E. 急性间质性肾炎：通常与药物过敏有关，表现为肾间质纤维化和肾小管萎缩，题干中未提及过敏史或相关症状。不符合题意。
综上所述，考虑到患者的病史、症状和检查结果，最可能的原因是庆大霉素导致的急性肾小管坏死。
因此，正确答案是：A.急性肾小管坏死。
该患者 59 岁，因高热、腹泻接受静脉点滴庆大霉素治疗后 5 天出现恶心、呕吐、伴少尿，并有以下检查结果：

- 血白细胞总数及分类正常
- 尿比重 1.010
- 尿蛋白（+）
- 尿红细胞 0～2/HP
- 尿白细胞 3～5/HP
- 血肌酐 320μmol/L
- 尿素氮 17mmol/L
- 尿钠 100mmol/L

这些检查结果表明患者存在肾功能减退。以下是对每个选项的分析：

A. **急性肾小管坏死**：庆大霉素是一种氨基糖苷类抗生素，具有肾毒性，可能导致急性肾小管坏死。急性肾小管坏死的典型表现包括尿量减少、血肌酐和尿素氮升高，并且尿钠浓度较高（超过 40mmol/L），尿比重接近 1.010，且可能有少量尿蛋白和尿白细胞。

B. **肾前性氮质血症**：通常由低血容量（如严重腹泻、脱水等）引起。特征包括尿比重增高、尿钠低（通常小于 20mmol/L），与本例患者不符。

C. **急性肾小球肾炎**：特征是血尿、蛋白尿、红细胞管型和水肿等症状。患者的检查结果显示尿红细胞为 0～2/HP，未见明显血尿，不符合急性肾小球肾炎的诊断。

D. **急进性肾小球肾炎**：是一种严重的肾小球疾病，通常会有显著的血尿、蛋白尿和肾功能迅速恶化，临床上常伴有大量红细胞管型，不符合本例患者的表现。

E. **急性间质性肾炎**：通常由药物、感染等引起，可导致尿中白细胞增多，可能伴有尿蛋白、血尿。本例患者用药后出现少尿、恶心、呕吐，尿白细胞增多，但尿中白细胞并不显著，且庆大霉素更常引起急性肾小管坏死。

结合患者的病史（庆大霉素治疗）、症状（少尿、恶心、呕吐）及实验室检查结果（尿比重 1.010，尿钠 100mmol/L，血肌酐和尿素氮升高），最符合的诊断是**急性肾小管坏死**。

因此，最可能的原因是：

A. 急性肾小管坏死
93、 (A2 型题)
男，61 岁，摔倒致左臀部着地 5 小时，左髋部疼痛肿胀，活动障碍，查体：左下肢外旋 60°,Bryant 较健侧缩短 2cm，左侧腹股沟区压痛阳性，左侧大转子叩击痛阳性，考 下列哪种疾病
A.髋关节前脱位
B.股骨干骨折
C.股骨颈骨折
D.股骨转子间骨折
E.髋关节后脱位
参考答案： C 您的答案：
图文解析： 【解析】中老年男性，左臀部摔伤史，左下肢缩短、外旋畸形，左侧腹股沟区压痛阳性，左侧大转子叩击痛阳性，考虑诊断为股骨颈骨折（C 对）。
【避错】髋关节前脱位少见，多发生于交通事故和高处坠落伤，患肢呈外展、外旋和屈曲畸形（A 错）。股骨干骨折的症状往往会有明确的大腿外伤史，如车祸伤，高处坠落伤及挤压伤等，股骨部位出现严重的疼痛，下肢活动功能严重的受限，包括髋关节及膝关节功能受限，大腿部位皮肤青紫色瘀斑，局部可以有明显的肿胀，压痛及叩击痛阳性（B 错）。股骨转子间骨折下肢外旋畸形明显，可达 90° （D 错）。髋关节后脱位表现为患肢短缩，髋关节呈屈曲、内收、内旋畸形（E 错）。
根据描述的症状，患者有左髋部疼痛肿胀，活动障碍，左下肢外旋 60°，Bryant 征表现为患侧比健侧缩短 2cm，左侧腹股沟区压痛阳性，以及左侧大转子叩击痛阳性。这些体征指向髋关节区域的损伤。

A 项的髋关节前脱位通常会导致下肢内旋，而不是外旋。B 项股骨干骨折通常导致严重的活动障碍，可能伴有明显的下肢短缩，但不特定于外旋。D 项的股骨转子间骨折也可能导致下肢外旋，但通常不会有 Bryant 征的明显表现。E 项髋关节后脱位则会导致下肢明显的外旋和短缩，符合 Bryant 征的表现。
综合这些信息，最符合描述的症状是髋关节后脱位，即 E 选项。然而，股骨颈骨折（C 选项）也是老年人摔倒后常见的原因，尤其是在髋部着地的情况下，通常会导致腿部外旋、疼痛和活动障碍。Bryant 征的存在（缩短的下肢）和大转子的叩击痛增加了股骨颈骨折的可能性。因此，最佳答案应该是 C.股骨颈骨折。这类骨折在老年人中较为常见，特别是在跌倒时直接影响到髋部的情况下。
解析：
患者左臀部外伤，左髋部疼痛肿胀，活动障碍，左下肢外旋畸形，Bryant 三角较健侧缩短，提示可能有股骨颈或股骨转子骨折，左侧腹股沟区压痛阳性，左侧大转子叩击痛阳性，结合患者病史、临床表现和查体，可诊断为股骨颈骨折。
选项 A 髋关节前脱位，选项 E 髋关节后脱位，常表现为患肢缩短、内收、内旋或外展外旋畸形，腹股沟处可扪及股骨头，该患者并未出现患肢缩短、内收、内旋或外展外旋畸形等症状，故一并排除。
选项 B 股骨干骨折常表现为骨折部疼痛比较剧烈、压痛、胀肿、畸形和骨擦音等，该患者并未出现压痛、胀肿等症状，故不选。
选项 D 股骨转子间骨折多见于老年人，女性多于男性，转子区出现疼痛，肿胀，瘀斑，下肢不能活动，检查发现转子间压痛，下肢外旋畸形明显，可达 90°，有轴向叩痛，测量可发现下肢缩短，但患者下肢外旋角度为 60°，故排除。
综上所述，正确答案是：C.股骨颈骨折。
根据题目中的描述：

- 患者男性，61 岁，摔倒后左臀部着地。
- 5 小时后出现左髋部疼痛肿胀，活动障碍。
- 查体发现左下肢外旋 60 度，Bryant 三角较健侧缩短 2cm。
- 左侧腹股沟区压痛阳性，左侧大转子叩击痛阳性。

结合这些症状和体征，以下是每种可能的疾病分析：

A. **髋关节前脱位**：髋关节前脱位通常会导致髋部外展和外旋，但并不常见在摔倒后出现髋部肿胀和明显的压痛阳性。

B. **股骨干骨折**：股骨干骨折通常表现为剧烈疼痛、肿胀和畸形，但一般不涉及髋关节及其周围的明显外旋畸形。

C. **股骨颈骨折**：股骨颈骨折是老年人摔倒后的常见骨折类型。患者通常表现为髋部疼痛、肿胀、活动受限，下肢外旋和缩短，且股骨颈骨折常常伴有压痛和叩击痛。

D. **股骨转子间骨折**：股骨转子间骨折会导致髋部疼痛和肿胀，下肢外旋和缩短，但在临床上更常见于股骨颈骨折患者的描述中。

E. **髋关节后脱位**：髋关节后脱位通常表现为髋部内收、内旋而不是外旋，同时伴有髋部

疼痛和活动障碍。

综上所述，题干描述的症状和体征最符合**股骨颈骨折**的特征。

因此，最正确的答案是：

C. **股骨颈骨折**
94、 (A2 型题)
女，30 岁，间断右腹痛 1 周，加重伴尿频、尿急 1 天。查体：右肾区叩击痛阳性，检查：尿 RBC（++），右肾积水，集合系统 1.0cm，考虑尿路结石，结石可能在
A.肾小管和肾盂交界处
B.肾盂输尿管口
C.输尿管下段
D.肾结石
E.膀胱壁内
参考答案： A 您的答案：
图文解析： 【解析】患者女，30 岁，有膀胱刺激征（尿频、尿急 1 天），腹痛、肾区叩击痛、右肾积水、集合系统分离 1.0cm，都提示输尿管或以上部位存在梗阻，综合以上，诊断考虑膀胱壁内（输尿管膀胱移行部）结石。故选择 E。

【避错】尿路结石最常见于以下部位：肾盂输尿管移行处（肾盂输尿管口）、输尿管跨过髂血管位置、输尿管膀胱移行部（膀胱壁内）。肾盂输尿管移行处、输尿管跨过髂血管位一般不出现尿频、尿急膀胱刺激症状，但伴感染时候可出现，患者无相关感染表现，暂不考虑。故 ABCD 错误。
对于这位 30 岁女性患者，间断性右侧腹痛一周，加上尿频和尿急的症状，查体发现右肾区叩击痛阳性，尿液检查显示红细胞（RBC）阳性，且有右肾积水，集合系统显示 1.0cm 的异常，这些迹象表明可能存在尿路结石。
选项 A 至 E 提供了结石可能的位置，基于症状和检查结果，结石可能导致肾盂和输尿管之间的梗阻，引起积水和疼痛。肾结石通常在移动到较窄的输尿管时引起疼痛加剧。因此，结石最有可能位于使尿液流动受阻的位置，从而引起这些症状。
结石在肾盂输尿管口的可能性较高，因为这里是尿液流动的关键狭窄区域，结石卡在这里可以解释积水和疼痛的加剧。因此，最佳答案是 **B.肾盂输尿管口**。
解析：
青年女性患者，间断右腹痛，右肾区叩击痛阳性，检查显示尿红细胞（++），右肾积水，集合系统 1.0cm，考虑尿路结石。尿路结石的典型表现为肾区疼痛伴肋脊角叩击痛，同时尿路结石的患者大多伴有镜下血尿。由于该患者存在右肾积水，提示结石嵌顿在肾盂输尿管口，导致尿液无法从肾盂排入输尿管，从而引起肾积水。
选项 A 肾小管和肾盂交界处结石常无明显症状，大多在体检时发现，且不易引起肾积水，故不选。
选项 C 输尿管下段结石主要症状是疼痛向膀胱会阴部放射，且会伴有明显的膀胱刺激征，与题意不符。
选项 D 肾结石患者大多没有症状，除非肾结石从肾脏掉落到输尿管造成输尿管的尿液阻塞。常见的症状有腰腹部绞痛、恶心、呕吐、烦躁不安、腹胀、血尿等，如果合并尿路感染，也可能出现畏寒发热等现象，与本题不符。

选项 E 膀胱壁内结石的典型症状为排尿突然中断，疼痛放射至远端尿道及阴茎头部，伴排尿困难和膀胱刺激症状，该患者无排尿突然中断症状，故排除。
综上所述，正确答案是：B.肾盂输尿管口。
根据题干的描述，患者 30 岁女性，间断右腹痛 1 周，加重伴尿频、尿急 1 天，体检发现右肾区叩击痛阳性，尿液检查显示红细胞增多，影像检查显示右肾积水，集合系统扩张 1.0cm。综合这些信息，考虑尿路结石。

根据尿路结石的常见部位和症状的描述，最可能的位置是：

B. 肾盂输尿管口

这是因为在肾盂输尿管口处，结石容易导致尿液排出受阻，进而引起肾积水和急性症状如尿频、尿急和肾区叩击痛。

因此，最佳答案是：

B. 肾盂输尿管口
95、 (A2 型题)
女，28 岁，酱油色 3 天。查体：无双侧肾区叩痛，为明确诊断，接下来应做的检查是
A.尿隐血试验
B.尿三杯试验
C.尿沉渣镜检
D.尿常规
E.尿胆原
参考答案： C 您的答案：
图文解析： 【解析】酱油色尿为血尿的一种，多为肌红蛋白尿或血红蛋白尿。血红蛋白尿的出现说明有快速血管内溶血被肾近曲小管上皮细胞重吸收的血红蛋白分解珠蛋白及铁，铁以铁蛋白或含铁血黄素的形式沉积在肾小管上皮细胞中，随上皮细胞脱落由尿液排出，形成含铁血黄素尿，铁离子在酸化的亚铁氰化钾溶液中生成蓝色的亚铁氰化铁（Rous 试验），即普鲁士蓝反应。如尿液中脱落的肾小管上皮细胞有含铁血黄素，显微镜下观察尿沉渣（C 对）肾小管上皮细胞中可有深蓝色物质出现，即为阳性。
【避错】尿隐血反映尿液中存在血红蛋白和肌红蛋白原因很多无特异性（A 错）；尿三杯试验多用于判断泌尿系统出血或炎症的来源和病变部位，用于前尿道、后尿道病变的诊断和鉴别诊断（B 错）；尿常规的检查通常可以查出尿路感染、肾炎、肾性尿糖以及低比重尿无特异性（D 错）；临床上会导致尿胆原检查结果为阳性或强阳性的疾病主要为各类型黄疸，常见的有溶血性黄疸、肝细胞性黄疸等（E 错）。
题目描述了一位 28 岁女性，出现了 3 天的酱油色尿液，但没有双侧肾区叩痛。这种症状通常与血尿有关，其中尿液颜色的改变可能由于血红蛋白、肌红蛋白或其他色素的存在。考虑到这些信息，适当的诊断步骤应旨在确认尿液中的异常成分。
A. 尿隐血试验：用于检测尿液中的红细胞或血红蛋白，对于确定是否存在血尿很有用。
B. 尿三杯试验：用于区分尿路中哪个部分出血，但在初步诊断阶段可能不是首选。
C. 尿沉渣镜检：可以观察到尿液中的红细胞、白细胞、上皮细胞等，有助于诊断，但对于确认尿液颜色改变的原因可能不够具体。
D. 尿常规：包括尿隐血测试，可以提供关于尿液颜色改变的更全面的信息。

E. 尿胆原：主要用于诊断肝脏疾病和胆道疾病，与尿液颜色改变的关系不大。
根据这些信息，最合适的检查是**D. 尿常规**，因为它包括尿隐血试验，并能提供关于尿液成分的广泛信息，有助于确定尿液颜色改变的原因。
解析：
青年女性患者，酱油色尿 3 天，考虑为血红蛋白尿，血红蛋白尿的外观颜色据含血红蛋白的多少而有不同，可呈均匀的浓茶色、葡萄酒色、棕色及酱油色等。因此，为明确诊断，接下来应做的检查是尿沉渣镜检，若尿沉渣镜检发现红细胞，可确诊为血尿。
选项 A 尿隐血试验用于检测尿液中是否含有血红蛋白，以进一步判断是否存在血尿，但无法观察红细胞形态，无法鉴别肾小球源性血尿与非肾小球源性血尿，故不选。
选项 B 尿三杯试验可明确血尿产生的部位和揭示泌尿生殖系统可能存在的疾病，但不能确诊血尿原因，故排除。
选项 D 尿常规可提示尿液的理化性质，但对确定血尿的来源没有帮助，故不选。
选项 E 尿胆原增高常见于肝细胞性黄疸和溶血性疾病等，不符合题意。
综上所述，正确答案是：C.尿沉渣镜检。
根据题目描述，该患者 28 岁，出现酱油色尿液持续 3 天，且体检无双侧肾区叩痛。为了明确诊断，接下来应做的检查是：

首先，我们需要考虑引起酱油色尿液的常见原因，包括血尿、肌红蛋白尿、胆红素尿等。根据临床实践指南和医学知识：

- **尿隐血试验（A 选项）**：用于检测尿液中是否含有血液，但无法提供进一步的信息来明确具体原因。
- **尿三杯试验（B 选项）**：主要用于区分前列腺、膀胱和尿道感染，较少用于鉴别酱油色尿液的原因。
- **尿沉渣镜检（C 选项）**：可观察尿液中的细胞、结晶和其他成分，提供关于血尿或其他成分的信息。
- **尿常规（D 选项）**：综合性检测，可筛查尿液的多种成分，包括红细胞、白细胞、蛋白质、葡萄糖、胆红素等。
- **尿胆原（E 选项）**：主要用于检查胆红素代谢异常，适用于黄疸患者。

考虑到酱油色尿液可能与血尿、肌红蛋白尿或胆红素尿有关，最全面且初步的检查是**尿常规（D 选项）**，因为它能提供多种有价值的信息，有助于进一步明确诊断。

因此，接下来应做的检查是：

D. 尿常规
96、 (A2 型题)
男，50 岁，1 年来心慌胸闷气短，近 1 月来加重，入院后查体：R120 次/分，心音低钝、遥远，Ewart 征阳性，超声提示室间隔不同步，下列最可能的治疗措施是
A.手术治疗
B.心包穿刺术
C.球囊扩张
D.静脉注射呋塞米
E.静脉注射胺碘酮

参考答案：　　B　您的答案：
图文解析：　　【解析】中年男性患者，心慌胸闷气短，心音低钝、遥远，Ewart 征阳性，该患者考虑诊断为心包积液引起心脏压塞。此时应立即采取的措施是心包穿刺抽液（B 对）。【避错】心包积液量大时可于左肩胛骨下出现叩浊音，听诊闻及支气管呼吸音，称心包积液征（Ewart 征），此乃肺组织受压所致，为心包积液特异性体征。
该患者有心慌、胸闷、气短的症状，近期加重，体检发现心率 120 次/分，心音低钝、遥远，Ewart 征阳性，超声显示室间隔不同步。这些症状和体征提示可能存在心包积液或心包压塞，Ewart 征的阳性是心包积液特有的体征之一。
根据所提供的信息和选项，最合适的治疗措施是 **B. 心包穿刺术**。心包穿刺术是用于移除心包内过多液体的程序，这可以迅速减轻心脏的压迫，改善症状。心脏超声的发现以及心音低钝和 Ewart 征的存在，均支持存在心包积液，因此进行心包穿刺术是合理的治疗选择。
解析：
中年男性，心慌胸闷气短 1 年，近 1 月来加重，查体见呼吸急促，心音低钝、遥远，为心包积液的典型表现，Ewart 征阳性是心包积液的特异性体征，超声提示室间隔不同步，根据其临床表现和体征，该患者最可能的诊断为心包积液。心包积液患者如症状严重、心包积液量大，或有心包填塞表现者，可行心包穿刺术，抽出心包腔内积液，降低心包腔内压。
选项 A 手术治疗主要用于治疗缩窄性心包炎，而不是心包积液，故不选。
选项 C 球囊扩张是二尖瓣狭窄的治疗措施，与该患者诊断不符，故排除。
选项 D 静脉注射呋塞米可以利尿以减少血容量，从而降低心包腔内压，但效果不如心包穿刺明显，因此不作为首选。
选项 E 静脉注射胺碘酮是治疗各种室上性与室性快速性心律失常的有效药物，与该患者表现不符，故不选。
综上所述，正确答案是：B.心包穿刺术。
根据题目中的症状和体征，50 岁男性患者出现了一年多的心慌、胸闷、气短，且近一个月来症状加重。查体发现呼吸频率快（R120 次/分），心音低钝、遥远，Ewart 征阳性，超声提示室间隔不同步。

这些临床表现提示可能存在心包积液或心包填塞，Ewart 征阳性也是提示心包积液的一个体征。室间隔不同步可能是由于心包积液引起的心脏压迫所致。基于这些信息，最可能的治疗措施是缓解心包积液所致的症状，解除心脏压迫。因此，心包穿刺术是最可能的治疗措施。

因此，最佳答案是：

B. 心包穿刺术
97、　(A2 型题)
男，72 岁。糖尿病多年，　一直长期服用阿卡波糖，长期胰岛素、瑞格列奈、格列本脲、罗格列酮等药物，空腹血糖 5.2mmol/L，餐后两小时血糖 6.5mmol/L，射血分数 42% 要停药的话，要停哪一种药
A.阿卡波糖
B.长期胰岛素
C.瑞格列奈
D.格列本脲
E.罗格列酮
参考答案：　　E　您的答案：

图文解析：　　　【解析】老年男患者，糖尿病病史多年，空腹血糖 5.2mmol/L，餐后两小时 6.5mmol/L（血糖控制可），射血分数 42％（心功能不全）。心功能不全者禁用罗格列酮（E 对）。

【避错】阿卡波糖肝、肾功能不全者仍应慎用，不宜用于有胃肠功能紊乱者、孕妇、哺乳期妇女和儿童。T1DM 患者不宜单独使用（A 错）；胰岛素禁用于低血糖患者；急性肝炎、肝硬化、溶血性黄疸患者和胰腺炎及肾炎患者禁用（B 错）；瑞格列奈、格列本脲禁用于β细胞功能很差的 T2DM，儿童糖尿病，孕妇、哺乳期妇女，大手术围术期，全胰腺切除术后（CD 错）。

在这个案例中，72 岁的男性病人有长期的糖尿病历史，正在使用多种降糖药物，包括阿卡波糖、长期胰岛素、瑞格列奈、格列本脲和罗格列酮。他的空腹血糖和餐后两小时血糖均处于控制良好的范围，但是他的射血分数为 42%，表明可能存在心功能问题。

在考虑停药时，我们应关注药物的潜在心血管影响。罗格列酮（选项 E）是噻唑烷二酮类（TZD）的成员，已知可能增加心衰风险，尤其是在有心血管病史或心衰风险的患者中。考虑到该患者的射血分数降低，可能指示心衰的风险或存在，应优先考虑停用罗格列酮。

因此，最佳答案是 E.罗格列酮。

解析：

患者老年男性，长期患有糖尿病，目前一直长期服用阿卡波糖，长期胰岛素、瑞格列奈、格列本脲、罗格列酮等药物，空腹血糖 5.2mmol/L，餐后两小时血糖 6.5mmol/L，射血分数 42%。患者目前使用的降糖药物中，格列本脲可加强心肌收缩力，增加心脏负担，易导致心力衰竭，目前患者心功能较差，射血分数降低，应停用格列本脲。

选项 A 阿卡波糖是α-糖苷酶抑制剂，主要在肠道内竞争性抑制葡萄糖甙水解酶，降低多糖及蔗糖分解成葡萄糖，使糖的吸收相应减缓，因此可具有使饭后血糖降低的作用。对心脏无明显影响，故不选。

选项 B 长期胰岛素主要用于 1 型糖尿病、各种严重的糖尿病急性或慢性并发症等，对患者心功能无明显影响，故排除。

选项 C 瑞格列奈通过与胰岛 B 细胞上的磺酰脲受体结合，刺激胰腺释放胰岛素，使血糖水平快速降低，对心功能无明显影响，与题意不符。

选项 E 罗格列酮通过激活过氧化物酶体增殖物激活受体γ起作用，增加靶组织对胰岛素作用的敏感性而降低血糖，对心功能无明显影响，故不考虑。

综上所述，正确答案是：D.格列本脲。

根据题干提供的信息，72 岁的男性患者有糖尿病病史，并且长期使用多种药物来控制血糖水平。患者的空腹血糖为 5.2 mmol/L，餐后两小时血糖为 6.5 mmol/L，这些血糖水平都是在正常范围内。射血分数 42%表明患者有一定程度的心力衰竭。考虑到糖尿病治疗药物的特性以及患者的心功能状态，以下是各药物的分析：

A. **阿卡波糖**：这是一种α-葡萄糖苷酶抑制剂，主要作用于肠道，延缓碳水化合物的吸收，不影响心功能。

B. **长期胰岛素**：胰岛素是糖尿病患者必需的基础治疗药物，对于维持血糖稳定非常重要，不宜停药。

C. **瑞格列奈**：这是一种胰岛素促泌剂，适合于餐时使用，快速作用于餐后血糖控制，不适合长期控制。

D. **格列本脲**：这是一种磺脲类药物，通过刺激胰岛β细胞分泌胰岛素来降低血糖，长期使用有低血糖风险，并且对心功能有不良影响。

E. **罗格列酮**：这是一个噻唑烷二酮类药物，通过提高外周组织对胰岛素的敏感性来降血糖，但已知有增加心力衰竭风险的副作用。

鉴于患者的心力衰竭状况（射血分数 42%），**罗格列酮**（E 选项）可能会加重心力衰竭，应当优先考虑停用。

因此，最正确的答案是：
E. **罗格列酮**
98、 (A2 型题)
女，62 岁，确诊糖尿病多年，胰岛素 30R，早 18 单位，晚 16 单位，餐前半小时给药。晚餐后两小时血糖 6.7mmol/L，晚上感饥饿、头晕，第二天空腹血糖 10.2mmol/L，为了 糖，应该调整的是
A.加用二甲双胍
B.减少晚餐前胰岛素用量
C.增加早餐前胰岛素用量
D.减少晚餐量
E.晚餐后运动
参考答案： B 您的答案：
图文解析： 【解析】中老年糖尿病患者，给予胰岛素治疗后，夜间出现低血糖反应，空腹血糖升高。患者夜间发生低血糖，引起体内胰岛素拮抗激素分泌增加，继而导致反跳性空腹血糖升高即 Somogyi 效应，因此对于本例患者应采取的措施为减少睡前胰岛素用量（B 对），防止夜间低血糖的发生。
【避错】黎明现象为夜间血糖控制良好，也无低血糖的发生，仅于黎明短时间内出现高血糖，可能由于清晨皮质醇、生长激素等分泌增多所致。
这道题目是关于糖尿病患者胰岛素用量的调整。患者晚上感到饥饿和头晕，这可能是因为晚上的胰岛素剂量过高导致夜间血糖过低。但第二天的空腹血糖又偏高，这表明需要调整胰岛素的剂量或给药时间来更好地控制血糖。
选项分析如下：
- A.加用二甲双胍：二甲双胍是一种降糖药，通常用于 2 型糖尿病，对于 1 型糖尿病或依赖胰岛素的糖尿病患者，单独加用二甲双胍可能不足以解决夜间低血糖和早晨高血糖的问题。
- B.减少晚餐前胰岛素用量：这可能会帮助减少晚上的低血糖现象，但不一定能够解决第二天早晨血糖偏高的问题。
- C.增加早餐前胰岛素用量：这可能有助于解决早晨高血糖的问题，但不会解决晚上低血糖的问题。
- D.减少晚餐量：这并不直接解决胰岛素用量不当的问题，且可能导致患者晚上饥饿感更加明显。
- E.晚餐后运动：运动可以帮助降低血糖，但如果患者晚上已经出现低血糖症状，则不建议晚餐后立即进行运动。
考虑到患者晚上的低血糖症状和第二天早晨血糖偏高，合理的调整方案应该既要避免晚上低血糖，也要控制早晨的血糖水平。因此，最佳答案可能是调整胰岛素的给药时间或分配，以确保血糖的稳定。但题目的选项中并没有直接涉及调整给药时间的选项，所以我们需要选择

最接近合理调整的选项。
因此，合理的选择可能是 **B.减少晚餐前胰岛素用量**，以防止晚上低血糖的发生。然后，根据患者的反应进一步调整早餐前的胰岛素用量或进行其他医疗干预。
解析：
老年女性患者，确诊糖尿病多年，使用胰岛素治疗。晚餐后两小时血糖偏低，晚上出现饥饿、头晕等低血糖症状，提示胰岛素用量过多，而第二天空腹血糖又偏高，这可能是因为前一晚的低血糖引起的反跳性高血糖，也称为 Somogyi 效应。因此，应减少晚餐前胰岛素用量，以避免夜间低血糖的发生。
选项 A 加用二甲双胍主要用于单纯饮食控制及体育锻炼治疗无效的 2 型糖尿病，特别是肥胖的 2 型糖尿病，本例患者已经在使用胰岛素，无需加用二甲双胍，故排除。
选项 C 增加早餐前胰岛素用量可能会进一步加重夜间的低血糖情况，进而引发更严重的反跳性高血糖，因此不选。
选项 D 减少晚餐量，选项 E 晚餐后运动，均会加重夜间的饥饿感和低血糖症状，因此可以一并排除。
综上所述，正确答案是：B.减少晚餐前胰岛素用量。
根据题干中提供的患者情况，62 岁女性，糖尿病多年，使用胰岛素 30R，早 18 单位，晚 16 单位，餐前半小时给药。晚餐后两小时血糖 6.7 mmol/L，晚上感饥饿、头晕，第二天空腹血糖 10.2 mmol/L。

考虑到患者晚餐后血糖正常，但夜间出现低血糖症状（饥饿、头晕），而且第二天早上空腹血糖偏高，这表明患者可能在夜间发生了低血糖，导致反弹性高血糖（Somogyi 现象）。

为了改善这种情况，最合适的调整措施是减少晚餐前的胰岛素用量，以避免夜间低血糖的发生。因此，最佳答案是：

B.减少晚餐前胰岛素用量
99、　(A2 型题)
男，18 岁，因“感冒”后自行服用抗菌药物半小时后突然出汗、面色苍白。查体：血压 40/20mmHg，神志不清，面色苍白，脉搏细速，四肢冰冷，发绀，该患者首要治疗
A.多巴胺静滴
B.肾上腺素肌肉注射
C.地塞米松静滴
D.异丙肾上腺素腔内注射
E.吸氧、心电监护
参考答案：　B　您的答案：
图文解析：　【解析】青少年男性患者，自行服用抗菌药物后出现血压 40/20mmHg，神志不清，面色苍白，脉搏细速，四肢冰冷，发绀等一系列休克表现，考虑为过敏性休克，此时应紧急行肾上腺素肌肉注射，故本题选 B。
【避错】过敏性休克表现为小血管扩张和毛细血管通透性增加，出现循环血量降低，血压下降，同时支气管平滑肌痉挛，出现呼吸困难，使用肾上腺素治疗过敏性休克，其激动 α1 受体，收缩小动脉和毛细血管前括约肌，而降低后者的通透性，激动 β1 受体改善心功能出现心肌收缩力增强、心率增快，减少过敏介质释放，扩张冠状动脉，因此可迅速缓解过敏性休克的临床症状，是治疗过敏性休克的首选药物。
这位 18 岁男性患者在服用抗菌药物半小时后突然出汗、面色苍白、血压极低（40/20mmHg）、

神志不清、面色苍白、脉搏细速、四肢冰冷及发绀，这些症状提示可能发生了严重的过敏反应或休克，特别是药物引起的过敏性休克。
考虑到患者的症状和临床表现，首要的治疗目标是迅速恢复血压和改善组织灌注。因此，选项中：
A. 多巴胺静滴：用于治疗休克，但在急性过敏性休克中不是首选。
B. 肾上腺素肌肉注射：是急性过敏反应和过敏性休克的首选治疗，可以迅速改善血流动力学。
C. 地塞米松静滴：用于抗炎和降低免疫反应，在过敏反应治疗中作为辅助治疗。
D. 异丙肾上腺素腔内注射：通常用于哮喘和某些类型的呼吸困难，并不适用于过敏性休克的治疗。
E. 吸氧、心电监护：这是基本的支持治疗，但不足以应对急性过敏性休克的严重性。
因此，最合适的选项是 **B. 肾上腺素肌肉注射**，因为这是对急性过敏性休克的快速和有效治疗。
解析：
青年男性患者，服用抗菌药物半小时后突然出汗、面色苍白。查体见休克血压，神志不清，面色苍白，脉搏细速，四肢冰冷，发绀，根据患者症状及体征，最可能的诊断是过敏性休克。此时应尽快给予肾上腺素肌肉注射，可迅速缓解过敏性休克出现的心脏抑制，支气管痉挛，减少过敏介质释放，扩张冠状动脉，可迅速缓解过敏性休克的临床症状，挽救病人的生命。
选项 A 多巴胺静滴常用于失血性休克、心源性休克等的治疗，对过敏性休克治疗效果不佳，故不选。
选项 C 地塞米松静滴具有抗炎、抗过敏等作用，但起效较慢，常作为抗过敏的辅助用药，不作为首选，故排除。
选项 D 异丙肾上腺素腔内注射主要用于任何部位的房室传导阻滞，但应用于治疗完全性房室传导阻滞时必须安装起搏器或应用起搏电流，不是过敏性休克的首选用药，故不选。
选项 E 吸氧、心电监护是休克的一般辅助治疗措施，但不是首要治疗措施，故排除。
综上所述，正确答案是：B.肾上腺素肌肉注射。
这位 18 岁男性患者在服用抗菌药物后出现了出汗、面色苍白、血压显著下降、神志不清、脉搏细速、四肢冰冷、发绀等症状，考虑可能是过敏性休克（anaphylactic shock）。过敏性休克是一种急性、危及生命的全身过敏反应，治疗的关键是快速恢复血压和维持气道通畅。

在这种情况下，首要治疗措施应该是使用肾上腺素，这是处理过敏性休克的首选药物。

因此，最正确的答案是：

B. 肾上腺素肌肉注射
100、 (A2 型题)
女，40 岁，因甲亢药物治疗 2 年未控制，改用 131I 治疗。131I 治疗 2 天后突感高热、心悸，伴恶心呕吐。查体：T40℃,呼吸急促，大汗淋漓，P160 次/分，律齐。最可能的
A.碘中毒
B.甲状腺危象
C.感染中毒性休克
D.神经中枢系统感染
E.放射性甲状腺炎
参考答案： B 您的答案：

图文解析：　　【解析】中年女性，因甲亢药物治疗 2 年未控制（疾病控制不佳），改用 131I 治疗 2 天（131I 治疗为刺激诱因）；突感高热、心悸，伴恶心呕吐。T40℃,呼吸急促，大汗淋漓，P160 次/分，律齐（甲状腺危象表现）。综合分析该患者最可能的诊断是甲状腺危象（B 对）。

【避错】甲亢危象，是甲状腺毒症急性加重的一个综合征，发生原因与甲状腺激素大量进入循环有关。多发生于较重甲亢未予治疗或治疗不充分的患者。常见诱因有感染、手术、创伤、精神、刺激等。临床表现有：高热或过高热，大汗，心动过速（＞140 次/分），烦躁，焦虑不安，谵妄，恶心，呕吐，腹泻，严重患者可有心衰、休克及昏迷等。

这位患者在接受放射性碘（^131I）治疗后，出现高热、心悸、恶心呕吐、呼吸急促和心率增快的症状。这些症状与甲状腺危象（thyroid storm）相符，甲状腺危象是一种严重的内分泌紧急情况，通常发生在甲亢病人身上，特别是在经历某种应激事件（如感染或放射性治疗）后。因此，最可能的诊断是 B.甲状腺危象。

解析：

中年女性患者，甲亢药物治疗 2 年未控制，131I 治疗甲亢后出现高热、心悸，伴恶心呕吐，查体示高热，呼吸急促，大汗淋漓，心动过速，根据患者的临床表现和体征，该患者最可能的诊断为甲状腺危象。甲状腺危象又称甲亢危象，是甲状腺毒症急性加重的一个综合征，发生原因可能与循环中的甲状腺激素水平增高有关。多发生于较重甲亢未予治疗或治疗不充分的患者，常见诱因为感染、手术、精神刺激等。

选项 A 碘中毒常有误服碘或碘化物病史，主要表现为口中有金属味，流涎，口腔及咽喉部烧灼感、恶心、呕吐、腹痛等，与题干中患者症状不符，故排除。

选项 C 感染中毒性休克常有原发感染灶、迁徙性病灶和败血症的临床表现，严重的感染可能导致脓毒血症，表现为感染性休克、DIC 和多脏器功能衰竭，该患者无感染病史，可排除。

选项 D 神经中枢系统感染常有高热、严重头痛、呕吐、颈项强直、典型的脑膜刺激征阳性等症状，题干中患者无上述表现，故不选。

选项 E 放射性甲状腺炎主要表现为甲状腺局部肿痛，少数患者可出现甲状腺功能亢进症状，但程度一般较轻，题干中患者为甲状腺毒症，症状较重，故不考虑。

综上所述，正确答案是：B.甲状腺危象。

题目描述中，患者是 40 岁的女性，因甲亢药物治疗 2 年未控制，改用 131I 治疗。131I 治疗 2 天后突感高热、心悸，伴恶心呕吐。查体发现体温 40℃，呼吸急促，大汗淋漓，脉搏 160 次/分且律齐。这些症状指向急性严重的全身反应。根据这些症状和背景，最可能的诊断是甲状腺危象。

甲状腺危象是一种危及生命的甲状腺功能亢进的急性加重，常见于甲亢患者在手术、感染、碘放射治疗或其他应激状态后发生。症状包括高热、心悸、恶心呕吐、出汗和快速心率等。

其他选项解释：

- 碘中毒通常不会引起如此剧烈的全身反应。
- 感染中毒性休克和神经中枢系统感染也可能表现为高热和全身症状，但与本例中甲亢患者背景和治疗经过不符。
- 放射性甲状腺炎一般表现为甲状腺局部疼痛，而不是如此剧烈的全身反应。

综上所述，最可能的诊断是 **B. 甲状腺危象**。

101、　(A2 型题)

患者男，2 天前因心梗入院，1 天前突发喘憋和咳粉红色泡沫痰，不能平卧。在心尖部可闻

及 3/6 收缩期杂音。该患者喘憋是由于
A.心肌梗死后综合征
B.乳头肌断裂
C.肺部感染
D.肺栓塞
E.心室膨胀瘤
参考答案：　　B　您的答案：
图文解析：　　【解析】患者心梗后出现喘憋、咳粉红色泡沫痰，心尖部可闻及 3/6 收缩期杂音（乳头肌断裂的听诊杂音），综合分析患者考虑心梗后乳头肌断裂（B 对）。
【避错】心肌梗死后综合征于 MI 后数周至数个月内出现，可反复发生，表现为心包炎、胸膜炎或肺炎，有发热、胸痛等症状（A 错）；肺部感染表现为咳嗽咳痰、伴或不伴胸痛、叩诊肺部可闻及浊音等（C 错）；肺栓塞的临床表现为胸痛、呼吸困难、血压下降，好发于下肢手术，长期制动患者（D 错）；心室壁瘤主要见于左心室，可见左侧心界扩大，心脏搏动范围较广，可有收缩期杂音。瘤内发生附壁血栓时，心音减弱（E 错）。
患者的临床表现（喘憋和咳粉红色泡沫痰，不能平卧，心尖部可闻及 3/6 收缩期杂音）提示急性左心衰竭的特征，这在心脏事件中可能由多种原因引起。考虑到患者有心肌梗死的病史，这些症状可能是心肌梗死后并发症的表现。
A. 心肌梗死后综合征通常表现为发热和心包炎症状，而非急性左心衰竭。
B. 乳头肌断裂会导致急性二尖瓣关闭不全，引起左心衰竭和心尖部的杂音，这与患者的症状相符。
C. 肺部感染通常会有发热、咳嗽、咳痰和呼吸困难，但不太可能导致心尖部杂音。
D. 肺栓塞可导致呼吸困难和胸痛，但不太可能引起心尖部的收缩期杂音。
E. 心室膨胀瘤发生在心肌梗死后，可能导致心室形态和功能的改变，但其表现不典型为急性呼吸困难和心尖部收缩期杂音。
综上所述，最符合患者症状的是 B 选项，乳头肌断裂。因此，患者的喘憋最可能是由于乳头肌断裂引起的。
解析：
患者老年男性，2 天前因心梗入院，1 天前突发喘憋、咳粉红色泡沫痰、不能平卧，为急性左心衰表现，在心尖部可闻及 3/6 级收缩期杂音，结合患者病史、临床表现和体格检查，该患者诊断考虑为心梗后并发乳头肌断裂，导致二尖瓣脱垂并关闭不全，从而引起急性左心衰，出现喘憋。
选项 A 心肌梗死后综合征多于心肌梗死后数周至数月内出现，可反复发生，表现为发热、胸膜炎性胸痛等，与本题患者表现不符，故不选。
选项 C 肺部感染多表现为咳嗽、咳痰等呼吸道症状，该患者无呼吸道症状，可排除。
选项 D 肺栓塞常表现为突发不明原因的呼吸困难及气促，胸痛、晕厥、咳嗽等，与本题不符。
选项 E 心室膨胀瘤又称室壁瘤，指心肌梗死区域出现室壁扩张、变薄、心肌全层坏死，坏死的心肌逐渐被纤维瘢痕组织所替代，病变区薄层的心室壁向外膨出，心脏收缩时丧失活动能力或呈现反常运动，与本题患者症状不符，故不考虑。
综上所述，正确答案是：B.乳头肌断裂。
患者是一位 2 天前因心肌梗死（心梗）入院的男性，1 天前突发喘憋和咳粉红色泡沫痰，不能平卧，并且在心尖部可闻及 3/6 收缩期杂音。根据这些临床表现，最可能的诊断是乳头肌断裂。

乳头肌断裂是心肌梗死的严重并发症之一，常发生在心梗后的数天内。乳头肌断裂会导致二尖瓣急性关闭不全，引起急性左心衰竭，表现为突发的严重呼吸困难（喘憋）、咳粉红色泡沫痰（急性肺水肿）和不能平卧。心尖部的收缩期杂音是由于二尖瓣反流所致。

因此，最可能的答案是：
B. 乳头肌断裂
102、 (A2 型题)
女，近日感乏力、怕冷、面色苍白，每年增重 2kg，甲状腺 2 度肿大,触之疼痛。双下肢水肿，血红蛋白 58g/L，尿常规：红细胞（0~2/HP）。尿蛋白（1~2/HP），未见明显 者诊断为
A.甲状腺功能亢进
B.甲状腺癌
C.桥本甲状腺炎
D.亚急性甲状腺炎
E.甲状腺功能减退
参考答案： D 您的答案：
图文解析： 【解析】患者女性，近日前出现乏力、怕冷、每年增重 2kg（提示甲状腺功能减退症状），甲状腺明显肿大，触之疼痛，有结节（亚急性甲状腺炎明显临床特点）。综合患者病史、症状，先考虑的是亚急性甲状腺炎（D 对）。
【避错】甲状腺功能亢进（A 错）患者呈弥漫性对称性肿大，质软，患者常有心动过速、心悸、食欲亢进，但体重下降；甲状腺癌（B 错）典型的临床表现为甲状腺内发现肿块，早期无明显症状，晚期可产生声音嘶哑、呼吸、吞咽困难和交感神经受压引起 Horner 综合征及侵犯颈丛出现耳、枕、肩等处疼痛和局部淋巴结及远处器官转移等表现。慢性淋巴细胞性甲状腺炎（桥本甲状腺炎）（C 错）患者甲状腺多为双侧对称性、弥漫性肿大，峡部及锥状叶常同时增大，也可单侧性肿大。触诊时，甲状腺质地韧，表面光滑或细沙粒状，也可呈大小不等的结节状， 一般与周围组织无粘连，吞咽运动时可上下移动。甲状腺功能减退（E 错）出现畏寒、乏力、手足肿胀感、嗜睡、记忆力减退、少汗、关节疼痛、体重增加、便秘，但无甲状腺的疼痛。
根据症状描述，患者出现乏力、怕冷、面色苍白、体重增加、甲状腺肿大并伴有疼痛、双下肢水肿以及血红蛋白降低，这些都是甲状腺功能减退的典型表现。甲状腺功能减退时，由于甲状腺激素分泌减少，新陈代谢减慢，导致症状如乏力、怕冷、体重增加等。甲状腺肿大可能是由于甲状腺努力产生更多激素而导致的。血红蛋白下降可能与甲状腺功能减退导致的代谢减慢有关。
选项 A（甲状腺功能亢进）的症状通常与体重减轻、心率增快、焦虑等相符，与本病例描述不符。选项 B（甲状腺癌）虽可能导致甲状腺肿大，但通常不会导致如此多的甲状腺功能减退相关症状。选项 C（桥本甲状腺炎）是一种自身免疫性甲状腺疾病，可以导致甲状腺功能减退，但通常不伴有明显疼痛。选项 D（亚急性甲状腺炎）通常会导致甲状腺肿痛，但伴随的是甲状腺功能的暂时改变，并且症状和进展与本病例描述不完全相符。
综上所述，最符合这些症状的诊断是甲状腺功能减退。因此，正确答案应该是 E.甲状腺功能减退。
解析：
青年女性患者，近日感乏力、怕冷、面色苍白，体重增加，为甲状腺功能减退的代谢减低表现。查体示甲状腺 II 度肿大，触之疼痛，符合亚急性甲状腺炎的特征性表现。双下肢水肿，血红蛋白偏低，为轻度贫血，符合甲状腺功能减退的黏液性水肿和贫血表现。尿常规未见血尿、蛋白尿等异常，可排除肾脏原因造成的水肿。根据患者症状，体征可诊断为亚急性甲状

腺炎伴甲状腺功能减退。
选项 A 甲状腺功能亢进表现为机体代谢亢进和交感神经兴奋，从而引起心悸、出汗、进食和便次增多和体重减少的病症，多数患者还常常同时有突眼、眼睑水肿、视力减退等症状，与题干中患者症状不符，故不选。
选项 B 甲状腺癌常表现为甲状腺内发现肿块，质地硬而固定、表面不平，患者未触及肿块，且甲状腺癌一般不会有触痛，故排除。
选项 C 桥本甲状腺炎表现为甲状腺肿大，多数为弥漫性，少数可为局限性，部分以颜面、四肢肿胀感起病，但甲状腺常无触痛，与题干描述不符，故不考虑。
选项 E 甲状腺功能减退也可出现乏力、怕冷、面色苍白、体重增加等表现，但甲状腺多无触痛，而该患者甲状腺Ⅱ度肿大，触之疼痛，故甲状腺功能减退不能全面解释该患者的所有症状，所以不选。
综上所述，正确答案是：D.亚急性甲状腺炎。
根据题目描述，该女性患者出现乏力、怕冷、面色苍白，每年体重增加 2kg，甲状腺 2 度肿大并且触之疼痛，双下肢水肿，血红蛋白 58g/L，尿常规显示有红细胞和尿蛋白。这些症状和检查结果指向甲状腺功能减退（甲减）。

具体分析如下：

1. **乏力、怕冷、面色苍白**：这些是甲减的典型症状，因为甲减会导致新陈代谢减慢，患者通常会感觉寒冷和疲劳。
2. **每年增重 2kg**：体重增加是甲减的常见症状，由于新陈代谢减慢，体内脂肪容易堆积。
3. **甲状腺 2 度肿大，触之疼痛**：甲状腺功能减退时甲状腺可能会肿大，称为甲状腺肿。
4. **双下肢水肿**：甲减可导致黏液性水肿，表现为四肢和面部的浮肿。
5. **血红蛋白 58g/L**：甲减可导致贫血，这也是乏力和面色苍白的原因之一。
6. **尿常规：红细胞（0~2/HP），尿蛋白（1~2/HP）**：这些可能是由于甲减引起的肾功能异常。

综上所述，最可能的诊断是：
E. 甲状腺功能减退
103、 (A2 型题)
女，27 岁，尿频、尿急、尿痛 2 天，右肾区叩痛阳性，尿沉渣检查，RBC5~10/HP，WBC50~60/HP，最可能的诊断是
A.急性肾小球肾炎
B.急性肾盂肾炎
C.急性膀胱炎
D.急性间质性肾炎
E.急进性肾小球肾炎
参考答案： B 您的答案：
图文解析： 【解析】青年女性患者（急性肾盂肾炎好发人群），尿频尿急尿痛 2 天，右肾区叩痛阳性（急性肾盂肾炎典型临床表现），血尿（+），尿蛋白（+），综合患者的症状、体征、实验室检查，最可能的诊断是急性肾盂肾炎（B 对）。
【避错】急性肾小球肾炎和急进性肾小球肾炎表现为血尿、蛋白尿、水肿和高血压，可伴有一过性肾功能不全（AE 错）；急性间质性肾炎（D 错）患者常有近期用药史，并有皮疹、发热及外周血嗜酸性粒细胞增多等药物过敏表现，伴尿检异常或肾小管及肾小球功能

损害；急性膀胱炎无肾区叩击痛（C 错）。
女性患者出现尿频、尿急、尿痛，并伴有右肾区叩痛阳性，尿沉渣检查显示红细胞（RBC）5~10/HP 和白细胞（WBC）50~60/HP，这些症状和体征最符合急性肾盂肾炎（acute pyelonephritis）的临床表现。
急性肾盂肾炎通常表现为尿频、尿急、尿痛和腰痛，尤其是在肾区叩痛阳性的情况下。尿沉渣检查通常会发现白细胞和红细胞的增加，这与感染相符。因此，最可能的诊断是：B. 急性肾盂肾炎
首先，我们来分析题目中的关键信息和可能的诊断选项。
患者青年女性，主要表现为尿频、尿急、尿痛，这是膀胱刺激征的典型表现，通常与尿路感染有关。此外，右肾区叩痛阳性，这是上尿路感染的体征之一。尿沉渣检查显示红细胞（RBC）轻度升高，白细胞（WBC）显著升高，提示尿路感染。
现在，我们逐一分析各个选项：
A. 急性肾小球肾炎：主要表现为血尿、蛋白尿、水肿和高血压，但一般不会有膀胱刺激征和肾区叩痛，因此不符合该患者的临床表现。
B. 急性肾盂肾炎：这是一种上尿路感染，常表现为发热、腰痛（肾区叩痛阳性）和膀胱刺激征，与该患者的症状相符。
C. 急性膀胱炎：主要表现为膀胱刺激征，但通常不会有肾区叩痛，因此不完全符合患者的表现。
D. 急性间质性肾炎：常表现为寒战、高热、血白细胞增高、核左移等全身感染性症状，以及肾小管性蛋白尿、镜下血尿、白细胞尿等，与该患者症状不完全吻合。
E. 急进性肾小球肾炎：主要表现为血尿、蛋白尿及进行性肾功能减退，与患者的临床表现不符。
综上所述，结合患者的临床表现和实验室检查，最可能的诊断是急性肾盂肾炎。
因此，正确答案是 B. 急性肾盂肾炎。
根据题目描述，27 岁女性出现尿频、尿急、尿痛 2 天，右肾区叩痛阳性，尿沉渣检查显示 RBC 5~10/HP，WBC 50~60/HP。这些症状和检查结果最符合以下诊断：

- 尿频、尿急、尿痛提示泌尿系统感染。
- 右肾区叩痛阳性提示可能涉及肾脏。
- 尿沉渣中有较多白细胞（WBC 50~60/HP），提示感染。

在这几种诊断中：

A. 急性肾小球肾炎通常表现为血尿、蛋白尿，但尿频、尿急、尿痛较少见。
B. 急性肾盂肾炎通常表现为尿频、尿急、尿痛，同时伴有肾区疼痛及明显的尿液中白细胞增多，符合题目描述。
C. 急性膀胱炎会有尿频、尿急、尿痛，但不会有右肾区叩痛阳性。
D. 急性间质性肾炎通常是由药物或其他因素引起的，临床表现与题目不完全符合。
E. 急进性肾小球肾炎是一种严重的肾小球疾病，表现为急性肾功能衰竭、严重蛋白尿和血尿，但不常有尿频、尿急、尿痛。

综合考虑，最可能的诊断是：

B. 急性肾盂肾炎

104、　(A2 型题)
男，48 岁，因急性心梗后入住 ICU，出现心率增快，反复发作室性心动过速和房颤后电复律电除颤抢救成功。形成电风暴的原因是
A.迷走神经兴奋
B.交感神经兴奋
C.副交感系统激动
D.中枢神经系统抑制
E.迷走神经系统抑制
参考答案：　B　您的答案：
图文解析：　【解析】电风暴的发生机制尚未完全明晰，至今认为与下列因素相关：器质性心脏病变是发生电风暴的病理基础，交感神经过度兴奋（B 对）是发生电风暴的促发因素。【避错】室性心律失常风暴系指 24h 内发生≥2～3 次的室性心动过速和/或心室颤动，引起严重血流动力学障碍而需要立即电复律或电除颤等治疗的急性危重性症候群，简称电风暴。
在这个病例中，48 岁男性患者在急性心肌梗死后进入 ICU，并出现心率增快、反复室性心动过速和房颤，随后进行了电复律电除颤抢救。形成电风暴的原因，根据描述的情况，最可能的是交感神经系统的过度激活。
因此，最佳答案是：
B. 交感神经兴奋
交感神经系统的过度激活可以导致心律失常的风险增加，尤其在急性心脏事件（如心肌梗死）后，交感神经系统的兴奋会增加心肌的氧耗，促进异常心电活动，从而可能引发电风暴。
这是一道关于心脏电生理机制的问题。首先，我们需要理解问题中的关键信息和所涉及的医学知识。
理解题干信息：患者中年男性，因急性心梗后入住 ICU，并出现反复发作的室性心动过速和房颤，经电复律电除颤抢救成功。这提示我们，患者的心脏电活动出现了异常，即所谓的“电风暴”。
理解电风暴的含义：心脏电风暴（又称室性心律失常风暴）是指 24 小时内出现≥2～3 次的室性心动过速或心室颤动，需要紧急治疗的临床症候群。这种情况通常与交感神经的过度兴奋和触发活动有关。
分析选项：
A. 迷走神经兴奋：迷走神经主要对心脏产生抑制作用，如果迷走神经兴奋，通常会降低心率，与题干中描述的心率增快和电风暴现象不符。
B. 交感神经兴奋：交感神经兴奋会增加心脏的收缩力和心率，与题干描述的症状相符合。在急性心梗的情况下，交感神经的过度兴奋可能引发电风暴。
C. 副交感系统激动：副交感系统主要指迷走神经，其作用是抑制心脏活动，与题干描述不符。
D. 中枢神经系统抑制：这与心脏的电活动没有直接关系，不会直接导致电风暴。
E. 迷走神经系统抑制：虽然迷走神经抑制可能间接导致心脏活动增加，但它不是导致电风暴的直接原因。
综上所述，交感神经兴奋是导致心率增快和电风暴的主要原因。因此，正确答案是 B.交感神经兴奋。
104. (A2 型题) 男，48 岁，因急性心梗后入住 ICU，出现心率增快，反复发作室性心动过速和房颤后电复律电除颤抢救成功。形成电风暴的原因是

电风暴（electrical storm）是指患者在短时间内反复发生致命性心律失常（如室性心动过速或心室颤动）的状态。急性心梗后，交感神经系统过度激活是导致心脏电活动异常的重要原因之一，容易引起反复发作的室性心动过速和室颤等恶性心律失常。因此，形成电风暴的原因最可能是交感神经兴奋。

所以，最正确的答案是：

B. 交感神经兴奋

105、 (A2 型题)

男孩，7 岁，突发寒战高热，左大腿远端剧痛，左膝关节拒做伸屈活动，查体：局部轻度肿胀，皮温高，深压痛，浮髌试验（-），WBC16×10^9/L，N0.94。早期检查首选

A.局部分层穿刺

B.血常规

C.X 线

D.CT

E.MRI

参考答案： A 您的答案：

图文解析： 【解析】7 岁儿童（急性化脓性骨髓炎好发于儿童和青少年），寒战、高热，白细胞升高，左大腿远端疼痛（化脓性骨髓炎的好发部位，多发于胫骨近端和股骨远端），结合该患者的临床表现和体征，该患者的诊断考虑为急性化脓性骨髓炎。分层穿刺可见脓液及炎性分泌物（A 对）。X 线（C 错）需两周后方有变化。MRI 也具有早期诊断价值，但不作为首选（E 错）。

【避错】凡有下列表现均应想到有急性骨髓炎的可能：①全身中毒症状，高热寒战，局部持续性剧痛，长骨干骺端疼痛剧烈而不愿活动肢体，局部深压痛；②白细胞总数增高，中性粒细胞增高，血培养阳性；③分层穿刺见脓液和炎性分泌物；④X 线平片征象，两周左右方有变化；⑤MRI 检查具有早期诊断价值。

这个问题描述的是一个 7 岁男孩，突然出现寒战、高热、左大腿远端剧痛，以及左膝关节活动受限，伴有局部肿胀和压痛，而且有白细胞增多。这些症状和体征可能提示有感染性关节炎或骨髓炎等炎症性疾病。

在这种情况下，首先需要做的是排除或确认疾病，特别是需要确定是否有骨髓炎或关节炎这样的急性炎症过程。最适合的早期检查方法是选择可以直接观察骨骼和软组织状况的影像学检查。

选项 A 的局部分层穿刺主要用于液体收集和分析，这对于确诊有帮助，但不是最初的检查。选项 B 的血常规是有用的，但它不能直接显示局部的骨骼和软组织状态。选项 C 的 X 线检查可以直观显示骨骼的状况，有助于发现骨骼的异常，如骨折或感染。选项 D 的 CT 可以提供更详细的骨骼和软组织图像，但对于初步检查来说可能过于复杂。选项 E 的 MRI 可以提供非常详细的软组织图像，对于检测骨髓炎或软组织感染特别有用，但它不是最常用的初筛检查手段。

综合考虑，最合适的早期检查选择是 C.X 线，因为它是评估骨骼问题的标准初步检查方法，可以帮助确定是否存在如骨髓炎这样的问题。所以，最佳答案是 C.X 线。

解析：

7 岁患儿，突发寒战高热，左大腿远端剧痛，左膝关节剧痛，拒绝做伸屈活动，提示可能出现骨关节感染性或炎症性疾病。查体见局部轻度肿胀，皮温高，深压痛，实验室检查见血白细胞及中性粒细胞比例显著增高，结合患者病史、临床表现和实验室检查，应高度怀疑该患

儿为急性血源性骨髓炎。为明确诊断，应选择对骨髓水肿敏感的影像学检查，而 MRI 对骨髓内病变敏感，可早期发现局限于骨髓内的炎性浸润，表现为骨髓内异常信号，对急性血源性骨髓炎的早期诊断具有重要价值，因此应作为首选检查。
选项 A 局部分层穿刺为有创检查，可暂不考虑，故不选。
选项 B 血常规检查对于感染性疾病有一定的提示意义，但对确定感染部位价值不大，故排除。
选项 C：X 线，选项 D：CT，均不能发现骨髓内的微小病变，且 X 线检查需发病后 14 天才能发现骨膜反应等阳性征象，对早期诊断价值不大，故可一并排除。
综上所述，正确答案是：E.MRI。
根据题目描述，男孩 7 岁，突发寒战高热，左大腿远端剧痛，左膝关节拒做伸屈活动，局部轻度肿胀，皮温高，深压痛，WBC 升高，N 比例为 0.94。这些症状和体征提示可能存在急性化脓性骨髓炎。

对于急性化脓性骨髓炎，早期诊断非常重要。虽然血常规检查（选项 B）可以提示感染，但它不能提供具体的部位信息。影像学检查对确诊急性骨髓炎更为关键。相比 X 线和 CT，MRI 在检测骨髓炎方面更为敏感，可以更早地显示软组织和骨髓的变化。

因此，早期检查的首选是：

E. MRI
106、 (A2 型题)
男，68 岁。恶心、上腹隐痛、呕吐少许咖啡样液体 2 天。高血压、血脂异常病史 2 年，长期口服阿司匹林 100mg / d。胃镜检查可见胃窦黏膜多发糜烂，表面附着血性黏液。 疗药物是
A.多潘立酮
B.泮托拉唑
C.雷尼替丁
D.枸橼酸铋钾
E.硫糖铝
参考答案： B 您的答案：
图文解析： 【解析】中老年男性患者，长期服用阿司匹林，出现上腹隐痛、呕吐少许咖啡样液体 2 天，胃镜检查可见胃窦黏膜多发糜烂，表面附着血性黏液，综合患者的症状、体征，初步诊断为消化性溃疡，治疗药物首选泮托拉唑（B 对）。
【避错】多潘立酮（A 错）是改善胃肠动力药，适用于十二指肠-胃反流。枸橼酸铋钾（D 错）是胃黏膜保护剂，与两种抗生素联用可以起到杀灭和抑制 Hp 的作用，但治疗消化性溃疡止痛效果较缓慢。硫糖铝（E 错）属于弱碱性抗酸剂，不是治疗消化性溃疡的主要药物。雷尼替丁（C 错）是治疗消化性溃疡的主要药物之一，但疗效不如 PPI。
这道题目涉及的是一位 68 岁男性患者，有恶心、上腹隐痛、呕吐咖啡样液体的症状，有高血压和血脂异常病史，并且长期服用阿司匹林。胃镜检查发现胃窦黏膜多发糜烂，表面附着血性黏液。根据这些信息，患者的症状和胃镜结果指向胃黏膜损伤，可能与长期服用阿司匹林有关。
选项分析如下：
A. 多潘立酮用于治疗恶心呕吐，而这里的主要问题是胃黏膜损伤，所以 A 不是最佳选择。
B. 泮托拉唑是质子泵抑制剂，用于治疗胃溃疡和胃黏膜损伤，能减少胃酸分泌，促进胃黏膜修复。

C. 雷尼替丁是组胺 H2 受体拮抗剂，用于治疗胃溃疡，但由于其副作用和较质子泵抑制剂效果较弱，不是首选。
D. 枸橼酸铋钾用于治疗胃溃疡和胃黏膜损伤，有保护胃黏膜和促进溃疡愈合的作用。
E. 硫糖铝用于治疗胃酸过多和胃溃疡，但主要作用是中和胃酸，不是最佳选择。
鉴于患者的症状和长期服用阿司匹林的背景，最佳选择应是能有效减少胃酸分泌，并促进胃黏膜修复的药物。因此，最佳答案是 **B. 泮托拉唑**，因为它可以减少胃酸分泌，减轻胃黏膜的进一步损伤，并促进已有损伤的修复。
解析：
患者老年男性，长期口服阿司匹林后出现恶心、上腹隐痛、呕吐咖啡样液体，胃镜检查可见胃窦黏膜多发糜烂，表面附着血性黏液，考虑为阿司匹林引起的急性糜烂性出血性胃炎。治疗急性糜烂性出血性胃炎，应首先去除病因，积极治疗原发病和创伤，停用可能引起胃黏膜损伤的药物，同时使用抑制胃酸分泌的药物。选项中泮托拉唑是第三代质子泵抑制剂，能够特异性地抑制壁细胞顶端膜构成的分泌性微管和胞浆内的管状泡上的 H+/K+ATP 酶，引起该酶不可逆性的抑制，从而有效地抑制胃酸的分泌，达到治疗目的。
选项 A 多潘立酮属于促胃肠动力药，主要用于治疗胃食管反流症、功能性消化不良及糖尿病性胃轻瘫，但对胃酸分泌无抑制作用，故不选。
选项 C 雷尼替丁是 H_2受体拮抗剂，也能抑制胃酸分泌，但其抑酸效果较质子泵抑制剂弱，因此不作为首选。
选项 D 枸橼酸铋钾为胃粘膜保护剂，可覆盖在溃疡表面，阻止胃酸、胃蛋白酶侵袭溃疡面，但对胃酸分泌没有抑制作用，故不考虑。
选项 E 硫糖铝同样为胃粘膜保护剂，其机制是在酸性环境下，可解离出硫酸蔗糖复合离子，复合离子聚合成不溶性的带负电荷的胶体，能与溃疡或炎症处带正电荷的蛋白质渗出物相结合，形成一层保护膜，覆盖于病灶表面，阻止胃酸、胃蛋白酶等损害因素进一步侵袭，促进破损粘膜的愈合，但对胃酸分泌无抑制作用，故排除。
综上所述，正确答案是：B.泮托拉唑。
根据患者的病情描述，68 岁的男性，出现恶心、上腹隐痛、呕吐少许咖啡样液体 2 天，有高血压、血脂异常的病史，并长期口服阿司匹林 100mg / d。胃镜检查显示胃窦黏膜多发糜烂，表面附着血性黏液，这提示可能存在阿司匹林诱导的胃黏膜损伤和出血。

对于这种情况下的治疗，应该选择能够有效抑制胃酸分泌并促进胃黏膜愈合的药物。泮托拉唑是一种质子泵抑制剂（PPI），能够强效抑制胃酸分泌，有助于胃黏膜的修复和防止进一步出血。

因此，最适合的治疗药物是：

B. 泮托拉唑
107、 (A2 型题)
女，17 岁，疲劳无力，心烦易怒，怕热多汗，体重下降 5kg，月经量减少，经期缩短，查体：P100 次/分，BP140/70mmHg，手有颤动，甲状腺有弥漫性肿大，无触痛。该患 诊断是
A.糖尿病
B.单纯性甲状腺肿
C.Graves 病
D.自主神经混乱
E.缺铁性贫血

参考答案：　　C　您的答案：
图文解析：　　【解析】青少年女性（甲亢多发人群），心烦易怒，怕热多汗，体重下降，手有颤动，甲状腺有弥漫性肿大，无触痛（甲亢的典型表现），根据患者的症状体征，可诊断为 Graves 病（C 对）。
【避错】单纯性甲状腺肿早期甲状腺弥漫性肿大，腺体表面光滑，质地柔软。后期甲状腺呈不对称结节性肿大，结节大小不一。本病通常表现为甲状腺功能正常的甲状腺肿大，一般无临床症状，仅少数患者可出现甲状腺功能亢进或低下等症状。
根据所描述的症状，如疲劳无力、心烦易怒、怕热多汗、体重下降、月经量减少、经期缩短、心率增快（100 次/分钟）、血压 140/70mmHg、手颤动以及甲状腺的弥漫性肿大且无触痛，最符合的诊断是 Graves 病（C 选项）。Graves 病是一种自身免疫性疾病，会导致甲状腺功能亢进，产生过多的甲状腺激素，引起上述症状。因此，正确答案是 C。Graves 病。
这是一道诊断类问题，我们需要分析患者的症状、体征以及查体结果，从而确定其最可能的诊断。
首先，我们梳理一下题目中的关键信息：
（1）年轻女性患者，出现疲劳无力、心烦易怒、怕热多汗和体重下降的症状。
（2）月经周期和量出现异常，查体发现心率增快，血压升高，手颤动，且甲状腺弥漫性肿大但无触痛。
接下来，我们分析各个选项：
A. 糖尿病：通常表现为多饮、多食、多尿和体重下降，即“三多一少”症状，但一般不会出现甲状腺弥漫性肿大，因此这个选项不符合题意。
B. 单纯性甲状腺肿：主要表现为甲状腺肿大，但通常不会伴随怕热多汗、心烦易怒等症状，故排除。
C. Graves 病：这是一种自身免疫性甲状腺疾病，典型表现为怕热多汗、心烦易怒、心动过速、甲状腺弥漫性肿大等甲状腺功能亢进的症状，与患者的症状体征相符。
D. 自主神经混乱：主要表现为心慌、出汗、烦躁等症状，但不会出现甲状腺弥漫性肿大，故不选。
E. 缺铁性贫血：主要表现为面色苍白、乏力、头晕等症状，与患者的表现不符，可排除。
综上所述，患者最可能的诊断是 Graves 病，这是一种甲状腺功能亢进症，典型表现为怕热多汗、心烦易怒、心动过速、甲状腺弥漫性肿大等。
因此，正确答案是 C. Graves 病。
根据提供的临床表现和查体结果，患者的主要症状包括疲劳无力、心烦易怒、怕热多汗、体重下降、月经量减少和经期缩短。查体发现心率加快（P100 次/分），血压升高（BP140/70mmHg），手有颤动，甲状腺有弥漫性肿大且无触痛。

这些症状和体征与 Graves 病（甲状腺功能亢进症）高度一致。Graves 病是最常见的导致甲状腺功能亢进的原因之一，其特点包括甲状腺肿大、怕热、多汗、体重下降、心率加快、神经系统亢奋（如心烦易怒、手颤）等。

因此，最佳答案是：

C. Graves 病
108、　(A2 型题)
男，22 岁，全身进行性水肿 10 天，既往体健，查体：BP120/80mmHg，颜面及双下肢重度凹陷性水肿，血浆白蛋白 20g/L，血 Cr72μmol/L，血胆固醇 8.6mmol/L，尿蛋白定 沉渣镜

检红细胞 8＜10/HP。下列治疗措施中，错误的是
A.利尿治疗
B.静脉补充大量白蛋白
C.低分子肝素抗凝
D.限制盐摄入
E.根据肾活检病理决定下一步治疗措施
参考答案： B 您的答案：
图文解析： 【解析】青少年男性患者，有大量蛋白尿，尿蛋白大于 3.5g/d、水肿，ALB20g/L（白蛋白正常 40~55g/L）降低，高脂血症，结合患者病史、临床表现及检查，该患者目前最可能的诊断多为肾病综合征。肾病综合征患者应给予正常量的优质蛋白饮食（B 错，为本题正确答案），静脉补充大量蛋白会增加肾小球高滤过，加重蛋白尿并加重病情，故不主张应用。
【避错】肾病综合征的诊断标准是：①大量蛋白尿（＞3.5g/d）；②低白蛋白血症（血清白蛋白小于 30g/L）；③水肿；④高脂血症。其中前两项为诊断的必备条件。
这个病例描述了一名 22 岁男性患者出现全身进行性水肿、血浆白蛋白降低、血胆固醇升高和尿蛋白阳性，这些症状和检查结果暗示可能为肾病综合征。肾病综合征的特点是大量蛋白尿、低蛋白血症、高脂血症和水肿。
根据这些信息，我们可以逐一评估每个治疗选择：
A. 利尿治疗：对于水肿的患者，尤其是那些由低蛋白血症引起的水肿，利尿治疗是合理的。
B. 静脉补充大量白蛋白：在低蛋白血症且严重水肿的情况下，补充白蛋白可以帮助恢复血管内容量，减轻水肿。
C. 低分子肝素抗凝：肾病综合征患者由于尿失凝血因子，有较高的血栓形成风险，因此抗凝治疗在某些情况下是必要的。
D. 限制盐摄入：对于水肿患者，限制钠的摄入是标准治疗的一部分，可以帮助减少水肿。
E. 根据肾活检病理决定下一步治疗措施：这是一个关键步骤，因为它可以确切地诊断出肾脏的损害类型和程度，以及确定最佳的治疗方案。
因此，所有选项看起来都是合理的治疗措施。但考虑到问题要求选择“错误”的治疗措施，我们需要仔细考虑哪一项可能在某些情况下不适宜。在这种情况下，最可能被认为不恰当的是 B 选项“静脉补充大量白蛋白”，因为尽管在某些情况下使用白蛋白可以减轻水肿，但其在治疗肾病综合征中的作用仍有争议，且可能不是首选治疗。有些指南推荐仅在特定情况下（如严重的低蛋白血症）使用白蛋白，而不是作为常规治疗。
综上所述，虽然 B 选项在某些特定情况下是适用的，但考虑到题干中的“错误”要求，B 选项可能是最不适宜的治疗选择。
解析：
患者青年男性，全身进行性水肿 10 天，既往体健，查体示颜面及双下肢重度凹陷性水肿，实验室检查见血浆白蛋白降低，血胆固醇升高，尿蛋白定量升高，尿沉渣镜检红细胞轻度升高，综合患者的病史、临床表现和实验室检查，该患者考虑诊断为肾病综合征。肾病综合征的治疗包括一般治疗，对症治疗，主要治疗即抑制免疫与炎症反应，中医药治疗，防治并发症等。对于低白蛋白血症的治疗，静脉输注白蛋白仅能提高血浆胶体渗透压，输入的蛋白主要在 48 小时内从尿中排出，而且可加重肾小球高滤过及肾小管高代谢，加重蛋白尿并促进肾脏病变进展，因此，静脉补充大量白蛋白这一治疗措施是错误的。
肾病综合征时水肿的治疗原则是限盐、利尿，因此选项 A 利尿治疗，选项 D 限制盐摄入，均属于肾病综合征水肿的常规治疗措施，故排除。
肾病综合征患者容易发生血栓及栓塞并发症，对于这部分患者，可给予选项 C 低分子肝素抗

凝，以预防血栓栓塞性疾病，故不选。
选项 E 根据肾活检病理决定下一步治疗措施是确诊肾病综合征病因的关键步骤，同时也是制定治疗方案的重要参考，是肾病综合征的规范治疗措施，与题意不符。
综上所述，正确答案是：B.静脉补充大量白蛋白。
根据题干描述，患者为 22 岁男性，全身进行性水肿 10 天，伴有重度凹陷性水肿，低血浆白蛋白（20g/L），高血胆固醇（8.6mmol/L），尿蛋白阳性，提示可能是肾病综合征。以下是对各个选项的分析：

A. 利尿治疗：利尿治疗可以帮助减少水肿，是肾病综合征的一部分治疗措施。
B. 静脉补充大量白蛋白：患者有严重的低白蛋白血症，补充白蛋白可以暂时缓解症状，但需要小心，因为大量补充可能导致过度补液和并发症。
C. 低分子肝素抗凝：肾病综合征患者由于低白蛋白血症，容易出现高凝状态，抗凝治疗是合理的。
D. 限制盐摄入：限制盐摄入可以帮助控制水肿，是合理的治疗措施。
E. 根据肾活检病理决定下一步治疗措施：肾活检病理结果是决定进一步治疗的关键依据，是合理的措施。

综上所述，错误的治疗措施是 **B. 静脉补充大量白蛋白**，因为虽然补充白蛋白可以暂时改善症状，但并不是肾病综合征的主要治疗方法，而且大量补充可能导致液体过多和其他并发症。
109、 (A2 型题)
女，54 岁，心悸气短十年，加重伴大咳血一天，查体：颈静脉怒张，双肺可闻及湿啰音，心率 120 次/分，心律不齐，第一心音亢进可听见开瓣音，P2 亢进，心尖部舒张期 音，双下肢轻度水肿，心电图示心房颤动伴快心室率，胸部 X 线片示心影呈梨形心。应首选治疗药物是
A. β 受体拮抗剂
B.利尿剂
C.普罗帕酮
D.美西律
E.西地兰
参考答案： E 您的答案：
图文解析： 【解析】中年女性患者，心悸气短十年，加重伴大咳血一天（二狭的典型临床表现），心律不齐，第一心音亢进可听见开瓣音，P2 亢进，心尖部舒张期隆隆样杂音（二狭患者特异性听诊），心电图示心房颤动伴快心室率，胸部 X 线片示心影呈梨形心（二狭导致的左房增大影），综合患者的症状、体征、辅助检查可诊断为二尖瓣狭窄。对于心房颤动伴快心室率者，应静脉注射洋地黄类药物（E 对）以减慢心室率。对于大咯血患者应取坐位，同时使用镇静剂及利尿剂，以降低肺动脉压，但不是本例中首选治疗举措（B 错）。
【避错】二狭患者风湿热是主要病因，因此治疗时应抗风湿热治疗，长期甚至终生使用苄星青霉素。对于窦性心律患者，如其呼吸困难发生在心率加快时，可使用负性心律药物，如 β 受体拮抗剂（A 错）。
此题目描述了一名 54 岁女性患者，有心悸、气短、咳血、颈静脉怒张、双肺湿啰音、心率加快、心律不齐、心音异常、双下肢轻度水肿、心房颤动伴快心室率以及胸部 X 线片显示的梨形心等症状和体征。这些表现提示可能存在右心功能不全和心房颤动。
选项分析如下：

A. β受体拮抗剂：用于控制心率，对于心房颤动伴快心室率的患者有帮助，但在有明显心衰症状的情况下需谨慎使用。
B. 利尿剂：用于减少水肿，对于心衰患者有利，可以减轻心脏的负担。
C. 普罗帕酮：是一种抗心律失常药物，用于心房颤动的节律控制，但在紧急治疗心衰和控制快速心率方面不是首选。
D. 美西律：也是一种抗心律失常药物，主要用于心室性心律失常，不适用于心房颤动。
E. 西地兰：是强心苷类药物，用于治疗心力衰竭，尤其适用于心房颤动伴心衰的患者。
综合考虑患者的症状和体征，首选治疗应着眼于改善心功能和控制心率。西地兰（E 选项）在治疗心衰和控制心房颤动伴快心室率方面具有双重效果，是合适的选择。因此，最佳答案是 E.西地兰。
解析：
中老年女性患者，心悸气短多年，近期加重伴大咯血，查体示颈静脉怒张，双肺可闻及湿啰音，心率增快，心律不齐，第一心音亢进可听见开瓣音，P2 亢进，双下肢轻度水肿，心电图示心房颤动伴快心室率，胸部 X 线片示心影呈梨形心，综合患者的病史、临床表现、查体及影像学检查，考虑诊断为二尖瓣狭窄并心房颤动。此时首要治疗目的是控制心室率，缓解患者症状，故应首选治疗药物是西地兰，西地兰属于洋地黄类强心药物，通过加强心肌收缩而起作用，具有正性肌力作用、负性频率作用和心脏电生理作用，可用于急性心力衰竭、慢性心衰加重期、快速性心房颤动和心房扑动等。
选项 A：β受体拮抗剂可降低心肌收缩力、自律性、传导性和兴奋性，减慢心率，降低心肌耗氧量，多用于治疗心律失常、心绞痛、心肌梗死、高血压等心血管疾病，与该患者症状不符，故不选。
选项 B：利尿剂主要用于治疗各种水肿，可排除体内多余的水和钠，消除水肿，还可用于降压，与该患者症状不符，故排除。
选项 C：普罗帕酮，选项 D：美西律，均属于广谱高效膜抑制性抗心律失常药，具有膜稳定作用及竞争性β受体阻滞作用，能降低心肌兴奋性，延长动作电位时程及有效不应期，延长传导，可用于预防和治疗室性和室上性异位搏动，室性或室上性心动过速，预激综合征等，还可用于心房扑动或心房颤动，但房颤伴有快心室率时首选西地兰，可一并排除。
综上所述，正确答案是：E.西地兰。
根据提供的病例描述，患者是一名 54 岁的女性，心悸气短已有十年病史，且伴有加重症状和大量咳血。体检发现颈静脉怒张，双肺湿啰音，心率 120 次/分，心律不齐，第一心音亢进，有开瓣音和 P2 亢进，心尖部舒张期音以及双下肢轻度水肿。心电图显示心房颤动伴快心室率，胸部 X 线片示心影呈梨形心。

这名患者的症状和体征提示其可能患有慢性心力衰竭和心房颤动。首要治疗目标是控制心房颤动的快速心室率，从而改善症状和预防进一步的心脏损伤。

选项分析：
- **A. β受体拮抗剂**：可用于心力衰竭和心房颤动伴快心室率的控制。
- **B. 利尿剂**：主要用于缓解心力衰竭引起的水肿，但对心房颤动伴快心室率的控制作用有限。
- **C. 普罗帕酮**：用于心律失常的治疗，但对于有心力衰竭的患者不推荐，因为可能加重心力衰竭。
- **D. 美西律**：用于心律失常的治疗，但其安全性和疗效不如其他药物。
- **E. 西地兰**（地高辛）：可以减慢心房颤动伴快心室率，尤其适用于有心力衰竭的患者。

综合考虑患者的病史、症状和体征，首选治疗药物应当既能控制心房颤动的快速心室率，又能改善心力衰竭症状。β受体拮抗剂和地高辛都适合，但β受体拮抗剂在急性情况下更为常用。

因此，最佳答案是：
A. β受体拮抗剂
110、 (A2 型题)
患者，男，56 岁，既往体健，半年来乏力、面色苍白。血常规示：WBC13×10＾9／L，Hb108g／L，PLT6×10＾9／L，N0.65，L0.23，M0.03。骨髓增生明显活跃，原始细 Auer 小体。考虑诊断为 MDS，为明确 FAB 分型，区别 RA 及 RAS 型应做下列哪项检查
A.铁染色
B.骨髓活检
C.免疫组化检查
D.染色体核型
E.胸片
参考答案： A 您的答案：
图文解析： 【解析】患者考虑诊断为骨髓增生异常综合征，为明确 FAB 分型，区别 RA 及 RAS 型，应行骨髓铁染色（A 对）确定环形铁幼粒细胞比例。
【避错】骨肉瘤（A 错）为恶性骨肿瘤，X 线片可见 Codman 三角或“日光射线”形态，肿瘤边界不清。骨巨细胞瘤（B 错）为交界性肿瘤，X 线片可见骨端偏心性、溶骨性、囊性破坏而无骨膜反应。软骨肉瘤（C 错）是恶性骨肿瘤， X 线片可有云雾状改变，边界不清。骨样骨瘤（E 错）主要症状是疼痛，进行性加重，多数患者服用阿司匹林可缓解症状。
患者的症状和血液检查结果（如 WBC 升高、Hb 降低、PLT 降低）以及骨髓检查发现（如增生活跃，出现 Auer 小体），提示可能为骨髓增生异常综合征（Myelodysplastic Syndromes, MDS）。为了明确 FAB（法国-美国-英国）分型，区别 RA（难治性贫血）及 RAS（难治性贫血伴有环状铁粒幼红细胞）型，需要进行的检查是铁染色（A 选项）。铁染色可以帮助观察骨髓中铁的储存情况，是区分 RA 和 RAS 型 MDS 的重要指标。
因此，最合适的答案是 A.铁染色。
解析：
中老年男性患者，半年来乏力、面色苍白，血常规示全血细胞减少，骨髓增生明显活跃，原始细胞增多，见 Auer 小体，考虑诊断为骨髓增生异常综合征（MDS）。MDS 的 FAB 分型中，RA 为难治性贫血，外周血原始细胞＜1%，骨髓原始细胞＜5%。RAS 为难治性贫血伴原始细胞增多，外周血原始细胞＜5%，骨髓原始细胞在 5%～20%之间。两者的主要区别在于骨髓中原始细胞的比例，故为明确 FAB 分型，区别 RA 及 RAS 型应做骨髓活检，骨髓活检可以了解骨髓中原始细胞的数量，从而进行准确的分型。
选项 A 铁染色主要用于缺铁性贫血和铁粒幼细胞性贫血的诊断，与本题无关。
选项 C 免疫组化检查主要用于肿瘤的诊断和鉴别诊断，与 MDS 的 FAB 分型关系不大，故不选。
选项 D 染色体核型主要用于产前诊断和遗传病诊断，不适用于 MDS 的 FAB 分型，故排除。
选项 E 胸片主要用于检查心肺病变，对 MDS 的 FAB 分型没有帮助，故不考虑。
综上所述，正确答案是：B.骨髓活检。
根据题目中的临床表现和实验室检查结果，患者可能诊断为骨髓增生异常综合征（MDS）。为了进一步明确 FAB 分型，特别是区分难治性贫血（RA）和伴有原始细胞的难治性贫血（RAS），

需要进行下列哪项检查：

答案是 D. 染色体核型。

理由如下：

- FAB 分型的 RA 和 RAS 主要区别在于原始细胞的比例以及是否存在染色体异常。
- 染色体核型检查可以帮助识别特定的染色体异常，这对于明确 MDS 的诊断和分类非常重要。
- 铁染色主要用于检查铁代谢问题，不适用于区分 RA 和 RAS。
- 骨髓活检虽然可以提供骨髓细胞形态和增生情况的信息，但在分型方面不如染色体核型检查具体。
- 免疫组化检查用于鉴定特定的细胞标志物，不适合 MDS 的 FAB 分型。
- 胸片主要用于检查肺部疾病，与此处的诊断无关。

所以，为了明确 MDS 的 FAB 分型，特别是区分 RA 和 RAS 型，应做染色体核型检查。
111、 (A2 型题)
男，22 岁，右膝内侧肿块 5 年，生长缓慢，无明显疼痛，X 线片显示股骨下端内侧干骺端杵状肿块，边缘清楚。应首先考虑的是
A.骨肉瘤
B.骨巨细胞瘤
C.软骨肉瘤
D.骨软骨瘤
E.骨样骨瘤
参考答案： D 您的答案：
图文解析： 【解析】青年男性患者，右膝内侧肿块生长缓慢、无明显疼痛，X 线片示股骨下端内侧干骺端杵状肿块，边缘清楚（骨软骨瘤典型影像学表现），结合患者的症状和影像学表现，应首先考虑的是右股骨骨软骨瘤（D 对）。
【避错】
根据题目描述的病例信息，患者 22 岁，有一个缓慢生长的肿块，存在 5 年无明显疼痛，X 线片显示股骨下端内侧干骺端杵状肿块，边缘清楚。这些特点指向的是良性肿瘤。
A 选项骨肉瘤是一种恶性肿瘤，通常生长迅速，伴有疼痛，不符合这个病例的生长缓慢和无疼痛的特点。B 选项骨巨细胞瘤通常发生在骨的端部，但是其边缘往往不清楚，而且比较少见在股骨下端出现。C 选项软骨肉瘤也是一种恶性肿瘤，通常伴有疼痛和迅速的生长。D 选项骨软骨瘤是良性的，并且通常表现为缓慢生长的肿块，X 线上表现为杵状肿块，边缘清楚，符合题目描述。E 选项骨样骨瘤通常比较小，而且生长极其缓慢，很少形成大的肿块。
因此，根据题目描述和各选项的匹配度，最佳答案应该是 **D.骨软骨瘤**。
解析：
青年男性患者，右膝内侧无痛性肿块多年，X 线片显示股骨下端干骺端杵状肿块，边缘清楚，为骨软骨瘤的典型影像学表现，结合患者临床表现和影像学检查，应首先考虑的诊断是骨软骨瘤。
选项 A 骨肉瘤好发于青少年，常形成梭形的瘤体，疼痛为早期症状，可排除在生长缓慢的肿块之外，故不选。
选项 B 骨巨细胞瘤好发于 20～40 岁，女性略多，好发部位为长骨干骺端和椎体，主要症状

为疼痛和肿胀，与题干描述不符，故不考虑。
选项 C 软骨肉瘤多数发生在 30～60 岁之间，起源于骨膜者多见于头颈及骨盆部，起源于骨髓腔者多见于长管状骨，不符合题意。
选项 E 骨样骨瘤疼痛为主要症状，由轻到重，从间歇性到持续性，从病变局部发展到可伴有神经根放射性痛，不符合题干，故排除。
综上所述，正确答案是：D.骨软骨瘤。
这道题目描述的是一名 22 岁男性，右膝内侧肿块已有 5 年，生长缓慢，无明显疼痛，X 线片显示股骨下端内侧干骺端杵状肿块，边缘清楚。我们需要根据这些信息选择一个最可能的诊断。

考虑到患者的年龄、肿块的生长缓慢、无明显疼痛及 X 线片显示的特点（杵状肿块，边缘清楚），以下是各选项的分析：

A. **骨肉瘤**：骨肉瘤通常表现为快速生长的疼痛性肿块，多见于青少年，X 线片上常见骨质破坏和新骨形成，不太符合本病例的描述。

B. **骨巨细胞瘤**：骨巨细胞瘤多见于 20-40 岁的成人，好发于长骨的骨端，病变常累及关节面，但 X 线片上多见偏心性溶骨性病变，边缘模糊，疼痛较为明显，不太符合本病例的描述。

C. **软骨肉瘤**：软骨肉瘤常见于中老年人，肿瘤生长缓慢，但多有疼痛，X 线片上可见点状或环状钙化，边缘常不清楚，与本病例的描述不完全一致。

D. **骨软骨瘤**：骨软骨瘤是最常见的良性骨肿瘤，多见于青少年，常发生在长骨的干骺端，生长缓慢，X 线片显示为边缘清楚的骨质肿块，本病例的描述与骨软骨瘤非常符合。

E. **骨样骨瘤**：骨样骨瘤多见于青少年，通常表现为剧烈的夜间疼痛，X 线片上可见小的透光区，周围有明显的硬化带，本病例没有疼痛，不符合骨样骨瘤的特点。

综上所述，本病例的描述最符合**骨软骨瘤**的特点，因此正确答案是：

D. 骨软骨瘤
112、 (A2 型题)
男，22 岁，右膝内侧肿块 5 年，生长缓慢，无明显疼痛，X 线片显示股骨下端内侧干骺端杵状肿块，边缘清楚。应首先考虑的是
A.骨肉瘤
B.骨巨细胞瘤
C.软骨肉瘤
D.骨软骨瘤
E.骨样骨瘤
参考答案： D 您的答案：
图文解析： 【解析】青年男性患者，右膝内侧肿块生长缓慢、无明显疼痛，X 线片示股骨下端内侧干骺端杵状肿块，边缘清楚（骨软骨瘤典型影像学表现），结合患者的症状和影像学表现，应首先考虑的是右股骨骨软骨瘤（D 对）。

【避错】骨肉瘤（A 错）为恶性骨肿瘤，X 线片可见 Codman 三角或“日光射线”形态，肿瘤边界不清。骨巨细胞瘤（B 错）为交界性肿瘤，X 线片可见骨端偏心性、溶骨性、囊性破坏而无骨膜反应。软骨肉瘤（C 错）是恶性骨肿瘤， X 线片可有云雾状改变，边界不清。骨样骨瘤（E 错）主要症状是疼痛，进行性加重，多数患者服用阿司匹林可缓解症状。

根据所提供的病例信息，患者 22 岁，出现了右膝内侧肿块，其特点是生长缓慢，无明显疼痛，X 线片显示股骨下端内侧干骺端杵状肿块，边缘清楚。这些特征提示肿块很可能是良性的。

A 选项骨肉瘤，是一种恶性肿瘤，通常生长快速，且疼痛明显，X 线表现为破坏性肿块，与此病例描述不符。

B 选项骨巨细胞瘤，通常发生在长骨的端部，如股骨和胫骨的近端，但其边界不如良性肿瘤清晰。

C 选项软骨肉瘤，是一种恶性肿瘤，生长较快，往往有痛感，并且 X 线片表现出侵袭性特征。

D 选项骨软骨瘤，是一种常见的良性骨肿瘤，生长缓慢，一般无症状，X 线片上呈现为杵状肿块，边缘清晰，与此病例描述相符。

E 选项骨样骨瘤，通常较小，多见于长骨的干骺部，但其特点是引起夜间疼痛，可以被阿司匹林缓解，与本病例描述不完全吻合。

综上所述，最符合这个病例描述的是 D 选项，骨软骨瘤。因此，正确答案应该是 **D. 骨软骨瘤**。

解析：

患者青年男性，右膝内侧肿块多年，无明显疼痛，X 线片示股骨下端内侧干骺端杵状肿块，边缘清楚，结合患者病史、临床表现和影像学检查，应首先考虑骨软骨瘤。骨软骨瘤是一种常见的、软骨源性的良性肿瘤，是位于骨表面的骨性突起物，顶面有软骨帽，中间为髓腔，多发生于青少年，可长期无症状，X 线特征性表现为干骺端从皮质突向软组织的骨性突起。

选项 A 骨肉瘤为最常见的恶性骨肿瘤，好发于青少年，好发部位为长骨干骺端，如股骨远端、胫骨近端和肱骨近端的干骺端，疼痛为早期症状，可伴有局部皮温增高、静脉怒张，X 线可表现为 Codman 三角或呈“日光射线”形态，与题干描述不符，故不选。

选项 B 骨巨细胞瘤为常见的原发性骨肿瘤之一，来源尚不清楚，可能起始于骨髓间充质细胞，以基质细胞和多核巨细胞为主要结构的侵袭性较强的原发性骨肿瘤，X 线片特征性表现为骨端偏心性、溶骨性、囊性破坏而无骨膜反应，病灶呈肥皂泡样改变，与题干描述不符，故排除。

选项 C 软骨肉瘤是常见的恶性骨肿瘤之一，多见于成人和老年人，男性略多见，大多发生在股骨下端、胫骨上端和肱骨上端，症状以钝痛为主，由间歇性逐渐转为持续性，邻近关节者可有关节活动受限，X 线片显示骨质破坏，与题干描述不符，故不选。

选项 E 骨样骨瘤是一种良性成骨性疾患，具有界限清晰的局灶性病灶，一般直径小于 1cm，周围可有硬化骨反应骨膜反应和新生骨产生，X 线特征是有一个圆形或卵圆形的瘤巢，由不同成熟程度的骨质小梁构成，小梁呈放射状排列，周围为硬化骨质，与题干描述不符，故排除。

综上所述，正确答案是：D.骨软骨瘤。

根据题干描述，该患者 22 岁，右膝内侧肿块生长缓慢，已有 5 年历史，无明显疼痛，X 线片显示股骨下端内侧干骺端杵状肿块，边缘清楚。结合这些临床特征，最可能的诊断是骨软骨瘤（D.骨软骨瘤）。

以下是分析过程：

1. **骨肉瘤（A.骨肉瘤）**：这种肿瘤通常发生在青少年或年轻人，病程短且进展迅速，伴随剧烈疼痛，X 线片常显示不规则的骨质破坏和新生骨形成。

2. **骨巨细胞瘤（B.骨巨细胞瘤）**：常见于 20-40 岁之间的成年人，通常位于长骨的骨端，X 线片显示为膨胀性破坏性病变，边缘不清。

3. **软骨肉瘤（C.软骨肉瘤）**：常发生于中老年人，病程较长，X 线片显示软骨样钙化。

4. **骨软骨瘤（D.骨软骨瘤）**：这是最常见的良性骨肿瘤，多见于青少年，病程较长，通常无症状或轻微不适，X 线片显示病变边缘清楚、病灶内可见软骨帽。

5. **骨样骨瘤（E.骨样骨瘤）**：多见于青少年，常发生在长骨干骺端，表现为夜间疼痛，X 线片显示小的硬化病灶，周围有反应性骨质增生。

根据患者的年龄、病程、症状以及 X 线片特征，**骨软骨瘤**是最符合的诊断。

所以，正确答案是 **D.骨软骨瘤**。

113、 (A2 型题)

患者，女，49 岁。颈肩痛半年，向左上肢放射。左上肢肌力下降，手指动作不灵活。颈椎棘突间有压痛，左手拇指感觉减弱。上肢牵拉试验阳性，压头试验阳性。最可能 型是

A.脊髓型

B.神经根型

C.混合型

D.椎动脉型

E.交感神经型

参考答案： B 您的答案：

图文解析： 【解析】患者颈肩痛，并向左上肢放射（神经根型颈椎病早期表现），左上肢肌力下降、手指动作不灵活、左手拇指感觉减弱，上肢牵拉试验阳性、压头试验阳性（神经根型颈椎病主要体征），故该患者最可能的颈椎病类型为神经根型颈椎病（B 对）。

【避错】①脊髓型颈椎病（A 错）常表现为上肢或下肢麻木无力、僵硬、双足踩棉花感，足尖不能离地，触觉障碍、束胸感，双手精细动作笨拙，不能用筷子进食，肌力减退，四肢腱反射活跃或亢进，Hoffmann 征、髌阵挛、踝阵挛及 Babinski 征等阳性。②椎动脉型颈椎病（D 错）主要表现为突发性眩晕而猝倒、偏头痛、听力减退或耳聋、视力障碍等。③交感神经型颈椎病（E 错）主要表现为交感神经受刺激的症状，可为兴奋性也可为抑制性。混④合型颈椎病（C 错）多有两种或多种类型症状同时出现。

这个病例描述的是一名 49 岁女性患者，出现了颈肩痛、向左上肢放射的症状，伴随左上肢肌力下降和手指活动不灵活，颈椎棘突间压痛，左手拇指感觉减弱，以及上肢牵拉试验和压头试验阳性。这些临床表现提示患者可能患有颈椎病。

针对该病例，最可能的诊断是 B.神经根型颈椎病。神经根型颈椎病的特点是颈部和上肢疼痛、感觉减弱、肌力下降，特别是疼痛沿神经根分布放射，手指不灵活也符合神经根受压的表现。压头试验和上肢牵拉试验阳性进一步支持这一诊断，因为这些试验通常用于检查颈部神经根是否受到压迫。

其他选项，如脊髓型、混合型、椎动脉型和交感神经型颈椎病，虽然也是颈椎病的类型，但它们的临床表现与本病例描述不完全吻合。例如，脊髓型颈椎病通常会表现为四肢乏力、步

态不稳等症状，而椎动脉型颈椎病则常见头晕、视力模糊等症状。因此，这些类型与本病例描述的症状不符。
解析：
患者中年女性，颈肩痛半年，向左上肢放射，提示可能有神经根受压。左上肢肌力下降，手指动作不灵活，左手拇指感觉减弱，为神经根受刺激或压迫后出现的根性感觉障碍。颈椎棘突间有压痛，上肢牵拉试验阳性，压头试验阳性，均为神经根型颈椎病的典型表现。结合患者病史、临床表现及体格检查，该患者最可能的诊断是神经根型颈椎病。
选项 A 脊髓型颈椎病表现为上肢或下肢麻木无力、僵硬、双足踩棉花感，足尖不能离地，触觉障碍、束胸感，双手精细动作笨拙，不能用筷子进食，肌力减退，四肢腱反射活跃或亢进，Hoffmann 征、髌阵挛、踝阵挛及 Babinski 征等阳性，与题干描述不符。
选项 C 混合型颈椎病多有两种或多种类型症状同时出现，与患者表现不符，故不选。
选项 D 椎动脉型颈椎病主要表现为突发性眩晕而猝倒、偏头痛、听力减退或耳聋、视力障碍等，与患者的症状描述差异明显。
选项 E 交感神经型颈椎病主要表现为交感神经受刺激的症状，可为兴奋性也可为抑制性，与题干中患者表现不符，故排除。
综上所述，正确答案是：B.神经根型。
根据描述的症状和体征，患者有颈肩痛并放射到左上肢，左上肢肌力下降，手指动作不灵活，颈椎棘突间有压痛，左手拇指感觉减弱，上肢牵拉试验阳性，压头试验阳性。这些症状提示可能存在颈椎病，其中涉及到神经根的压迫。

颈椎病主要分为五种类型：脊髓型、神经根型、混合型、椎动脉型和交感神经型。分析各型的特点：
- 脊髓型颈椎病主要表现为肢体无力、感觉异常、行走不稳，甚至出现大小便障碍。
- 神经根型颈椎病主要表现为单侧或双侧上肢的放射性疼痛、麻木，且与特定神经根支配区域相关。
- 混合型颈椎病表现为以上两种或更多类型症状的混合。
- 椎动脉型颈椎病主要表现为眩晕、恶心、耳鸣等因椎动脉供血不足引起的症状。
- 交感神经型颈椎病主要表现为头痛、视物模糊、心悸等因交感神经功能障碍引起的症状。

结合患者的症状，最符合的是神经根型颈椎病（B）。她的症状包括颈肩痛向左上肢放射、左上肢肌力下降、手指动作不灵活、左手拇指感觉减弱，这些症状典型地提示神经根受到压迫。

因此，最可能的类型是：
B. 神经根型
114、 (A2 型题)
男，64 岁，反复发热伴乏力 1 周，加重 1 天。查体：体温 38.1℃,Hb69g／L，WBC15×109／L，外周血原始细胞 7％，骨髓原始细胞 22％，可见 Auer 小体。诊断为 MDS，其 FAB 为
A.RA
B.RSA
C.CMML
D.RAEB
E.RAEB-t
参考答案： E 您的答案：
图文解析： 【解析】中老年男性患者，反复发热、Hb69g／L（贫血），WBC15×10^9／L，

外周血原始细胞 7％（＞5%），骨髓原始细胞 22％（＞20%，＜30%），可见 Auer 小体，为 RAEB-t 分型（E 对）。

【避错】

这个病例描述的是一位 64 岁男性患者，出现反复发热伴乏力 1 周，体温 38.1℃，血红蛋白 69g／L，白细胞计数 15×109／L，外周血原始细胞 7％，骨髓原始细胞 22％，并可见 Auer 小体。这些临床表现和实验室检查结果符合骨髓增生异常综合征（MDS）的表现，具体到 FAB（法国－美国－英国）分类。

FAB 分类将 MDS 分为以下几类：

- RA（难治性贫血）
- RARS（难治性贫血伴有环状铁粒）
- RAEB（难治性贫血伴有过量的原始细胞）
- RAEB-t（难治性贫血伴有过量的原始细胞，接近急性髓细胞白血病）
- CMML（慢性髓单核细胞白血病）

根据题干中的外周血原始细胞比例 7％和骨髓原始细胞 22％，以及 Auer 小体的出现，我们可以考虑是 RAEB 或 RAEB-t。RAEB 是指骨髓中原始细胞占比在 5%到 20%之间，而 RAEB-t 是指骨髓中原始细胞占比 20%到 30%之间，并且接近急性变。因此，根据骨髓原始细胞 22％的比例，最符合的诊断是 RAEB-t（E 选项）。

解析：

老年男性患者，反复发热伴乏力 1 周，加重 1 天。查体见体温升高，贫血貌，白细胞计数升高，外周血原始细胞升高，骨髓原始细胞明显升高，可见 Auer 小体，为骨髓增生异常综合征（MDS）的典型病理特点，结合患者症状，体征及实验室检查，可诊断为 MDS。MDS 的 FAB 分型中**，RAEB-t（难治性贫血伴原始细胞增多转变型）**的外周血中全血细胞减少，原始细胞大于 5%（但小于 20%），骨髓象中原始细胞大于 20%而小于 30%，或幼粒细胞出现 Auer 小体，符合该患者情况。

选项 A：RA 即难治性贫血，外周血原始细胞＜1%，骨髓原始细胞＜5%，无 Auer 小体，不符合该患者情况，故排除。

选项 B：RAS 即环形铁粒幼细胞难治性贫血，外周血原始细胞＜1%，骨髓原始细胞＜5%，环形铁幼粒细胞＞15%，无 Auer 小体，不符合该患者情况，故不选。

选项 C：CMML 即慢性粒-单核细胞白血病，外周血单核细胞绝对值＞1×10^9/L，骨髓原始细胞 5%～20%，单核细胞增多，无 Auer 小体，不符合该患者情况，故排除。

选项 D：RAEB 即难治性贫血伴原始细胞增多，外周血中全血细胞减少，原始细胞＜5%，骨髓中原始细胞 5%～20%，无 Auer 小体，不符合该患者情况，故不选。

综上所述，正确答案是：E.RAEB-t。

根据患者的症状和实验室检查结果，包括体温、血红蛋白、白细胞计数、外周血和骨髓中的原始细胞比例，以及 Auer 小体的存在，诊断为骨髓增生异常综合征（MDS）。

MDS 的 FAB 分类系统（French-American-British Classification）包括以下几种类型：

1. RA（Refractory Anemia，难治性贫血）
2. RSA（Refractory Anemia with Ring Sideroblasts，难治性贫血伴环形铁粒幼细胞）
3. CMML（Chronic Myelomonocytic Leukemia，慢性粒单核细胞白血病）
4. RAEB（Refractory Anemia with Excess Blasts，难治性贫血伴原始细胞增多）
5. RAEB-t（Refractory Anemia with Excess Blasts in Transformation，转变中的难治性贫血伴原始细胞增多）

患者的骨髓原始细胞比例为22%，外周血原始细胞7%，并可见 Auer 小体，这提示患者处于原始细胞增多的阶段，且有转变为急性白血病的可能。因此，最合适的 FAB 分类应该是 RAEB-t。

正确答案是：E. RAEB-t
115、 (A2 型题)
患者男，55 岁，颈部淋巴结进行性肿大 2 个月，发热 2 周，发病以来体重减轻 14kg。查体：体温 38.7℃,双侧颈部和右腋窝均有数个直径 2～5cm 大小淋巴结，均活动，无压 下未触及。颈部淋巴结活检，可见 R-S 细胞。骨髓涂片中可见淋巴瘤细胞。患者现分期为
A. Ⅰ期
B. Ⅱ期
C.ⅢS 期
D.ⅢE 期
E.ⅣB 期
参考答案： E 您的答案：
图文解析： 【解析】中年男性患者，颈部淋巴结进行性肿大、体重下降 14kg，双侧颈部和右腋窝均有数个淋巴结肿大，颈部淋巴结活检，可见 R-S 细胞，骨髓涂片中可见淋巴瘤细胞，故分期为ⅣB 期（E 对）。
【避错】霍奇金淋巴瘤分为 I~Ⅳ期。
这个病例描述的是一位 55 岁男性患者，有颈部淋巴结进行性肿大 2 个月，伴有发热和体重明显减轻。体检发现双侧颈部和右腋窝有多个大淋巴结，颈部淋巴结活检发现 R-S（里德-斯特恩伯格）细胞，骨髓涂片发现淋巴瘤细胞。这些临床表现提示患者可能患有霍奇金淋巴瘤。
淋巴瘤的分期是根据肿瘤的扩散范围确定的。依据 Ann Arbor 分期系统，淋巴瘤分期如下：
- Ⅰ期：肿瘤局限于一个区域或一个淋巴结区。
- Ⅱ期：肿瘤侵犯两个或更多的淋巴结区，但仅限于膈肌的同一侧。
- Ⅲ期：肿瘤侵犯膈肌两侧的淋巴结区。
- Ⅳ期：广泛的或播散性疾病，可能影响到一个或多个器官或组织以外的淋巴结。
后缀“S”表示脾脏的受累，而“E”表示局部的器官或组织外扩散。
因此，根据该病例的描述，患者有多个区域的淋巴结受累（双侧颈部和右腋窝），且骨髓涂片中可见淋巴瘤细胞，表明病情已经超出了局部淋巴结，涉及到其他器官或组织。这符合Ⅳ期淋巴瘤的特征。但题目中没有明确指出有脾脏受累或其他额外的器官受累的信息，因此最适合的选择应该是Ⅳ期，但具体的Ⅳ期分类（如ⅣA 或ⅣB）未在题目中明确提出。根据常规，没有特殊体征或症状（如明显的全身症状）时，通常默认为ⅣA 期。
然而，考虑到题目给出的选项中没有ⅣA 期，而有ⅣB 期，且患者有体重明显下降这一 B 症状，我们可以考虑选择 E.ⅣB 期作为最佳答案。这反映了疾病的广泛扩散以及伴有的全身症状（如体重下降）。
解析：
中年男性患者，颈部淋巴结进行性肿大，发热，发病以来体重明显减轻。查体见体温升高，双侧颈部和右腋窝淋巴结均肿大，活动，无压痛。颈部淋巴结活检见 R-S 细胞，为霍奇金淋巴瘤特征性病理改变。骨髓涂片中可见淋巴瘤细胞，说明淋巴瘤细胞已侵犯骨髓。根据淋巴瘤的 Ann Arbor 分期标准，弥漫性大 B 细胞淋巴瘤侵犯到骨髓、肝脏、肺等脏器中任一个，即被分期为Ⅳ期，同时患者出现发热大于 38℃、盗汗、半年内体重下降 10%以上任何一种

症状即可分为 B 组，否则为 A 组。本题患者已侵犯骨髓，且有发热、体重明显下降的 B 组症状，故其临床分期为ⅣB 期。
选项 A：Ⅰ期是指肿瘤侵犯单一的淋巴结区或局灶性单个结外器官、部位。本题患者淋巴瘤已侵犯骨髓，与Ⅰ期定义不符。
选项 B：Ⅱ期是指肿瘤侵犯到膈肌同侧的多个淋巴结区，或局灶性单个结外器官及其区域淋巴结，伴或不伴横隔同侧的其他淋巴结区侵犯。本题患者淋巴瘤已侵犯骨髓，与Ⅱ期定义不符。
选项 C：ⅢS 期是指弥漫大 B 细胞淋巴瘤侵犯到横隔上下的淋巴结区，可伴局灶性相关结外器官、脾侵犯或两者均有。S 表示局限期，即一个局灶性结外器官和脾受侵犯。本题患者淋巴瘤已侵犯骨髓，与ⅢS 期定义不符。
选项 D：ⅢE 期 E 表示广泛期，即弥漫性大 B 细胞淋巴瘤广泛侵犯到骨髓、肝脏、肺等脏器。但本题患者有发热大于 38℃、盗汗、半年内体重下降 10%以上的症状，应归为 B 组，而非 E 期。
综上所述，正确答案是：E.ⅣB 期。
根据患者的病情描述和临床检查结果，我们可以进行霍奇金淋巴瘤的分期。

霍奇金淋巴瘤的分期依据 Ann Arbor 分期系统，分为以下几个阶段：

- Ⅰ期：单一淋巴结区或单一器官（淋巴外）。
- Ⅱ期：两个或两个以上淋巴结区位于横隔同一侧（上或下）。
- Ⅲ期：涉及横隔两侧的淋巴结区。
- Ⅳ期：弥漫性或播散性累及一个或多个淋巴外器官。

另外，分期系统还可附加以下标记：
- A：无全身症状。
- B：有以下全身症状之一：不明原因的发热、盗汗、体重减轻超过 10%。
- S：脾脏受累。
- E：局限的淋巴外器官受累。

根据题目中提供的信息：
- 患者有发热和体重减轻，这符合 B 症状。
- 颈部淋巴结活检显示 R-S 细胞，提示霍奇金淋巴瘤。
- 骨髓涂片中可见淋巴瘤细胞，这表示骨髓受累，属于 IV 期。

因此，患者的分期应该是：

E. ⅣB 期

根据上述解释和临床分期系统的应用，选择答案 E. ⅣB 期。
题目材料　　男，58 岁，发热 2 周，查体:体温 38℃,手部皮肤针刺处可见瘀斑，胸骨压痛（＋），心肺未见异常，脾肋下未及，　血常规：Hb86g/L，
WBC12.4×109/L，PLT34×109/L，　骨髓细胞学检查：　增生极度活跃，　胞质内粗大颗粒的早幼粒细胞占
116、　(A3/A4 型题)　　　　题

骨髓检查最可能出现的结果是
A.MPO 阴性
B.CD13 阴性
C.PAS 阳性
D.CD33 阳性
E.NSE 阴性
参考答案： D 您的答案：
图文解析： 【解析】 中老年男性患者，发热，周部皮肤针刺处可见瘀斑，胸骨压痛（+）（白血病浸润表现） ，血常规：Hb86g/L，WBC12.4×109/L，PLT34×109/L（红系、巨核系减少， 白血病细胞增多） ，骨髓细胞学检查：增生极度活跃，胞质内粗大颗粒的早幼粒细胞占 0.75（M3 的典型骨髓象表现） 。考虑最可能诊断为早幼粒细胞白血病，可发现 CD13、CD33 阳性；治疗首选全反式维甲酸。
116 题的回答：骨髓检查最可能出现的结果是 D.CD33 阳性。这种情况常见于急性髓细胞白血病（AML）中，特别是在胞质内有粗大颗粒的早幼粒细胞存在时。
正确答案是：D.CD33 阳性
本题考察的是对骨髓检查相关指标的理解。
选项 A，MPO（髓过氧化物酶）主要存在于中性粒细胞和单核细胞中，是粒细胞和单核细胞的标志之一，在急性髓系白血病中通常为阳性，故排除。
选项 B，CD13 是髓系细胞的标志，在急性髓系白血病中通常为阳性，故不选。
选项 C，PAS（糖原染色）主要用于检测细胞内的糖原和多糖，与急性髓系白血病的诊断关系不大，故排除。
选项 D，CD33 是髓系细胞的特异性标志，在急性髓系白血病中通常为阳性，是诊断急性髓系白血病的重要指标之一，符合题意。
选项 E，NSE（神经元特异性烯醇化酶）是神经元和神经内分泌细胞特有的酸性蛋白酶，与急性髓系白血病的诊断无关，故不选。
问题 116
骨髓检查最可能出现的结果是

A. MPO 阴性
B. CD13 阴性
C. PAS 阳性
D. CD33 阳性
E. NSE 阴性
解答：

该患者骨髓检查显示早幼粒细胞占多，并且临床表现符合急性早幼粒细胞白血病（APL）。APL 中的早幼粒细胞通常 CD33 阳性。故答案为：
D. CD33 阳性
【避错】
117、 (A3/A4 型题) 题
考虑最可能诊断为
A.早幼粒细胞白血病
B.急性髓细胞白血病
C.急性单核细胞白血病

D.急性巨核细胞白血病
E.中枢神经系统白血病
参考答案：　A　您的答案：
图文解析：　【解析】 中老年男性患者，发热，周部皮肤针刺处可见瘀斑，胸骨压痛（+）（白血病浸润表现），血常规：Hb86g/L，WBC12.4×109/L，PLT34×109/L（红系、巨核系减少， 白血病细胞增多），骨髓细胞学检查：增生极度活跃，胞质内粗大颗粒的早幼粒细胞占 0.75（M3 的典型骨髓象表现）。考虑最可能诊断为早幼粒细胞白血病，可发现 CD13、CD33 阳性；治疗首选全反式维甲酸。
【避错】
117 题的回答：考虑最可能的诊断为 B.急性髓细胞白血病（AML）。患者的症状和实验室结果，特别是血常规和骨髓细胞学检查的结果，符合这种诊断。
正确答案是：A.早幼粒细胞白血病
患者中年男性，有发热，查体有胸骨压痛，血常规提示贫血、白细胞升高、血小板减少，骨髓细胞学检查提示增生极度活跃，胞质内含有粗大颗粒的早幼粒细胞显著增加，根据其临床表现、体查、实验室检查，考虑诊断为早幼粒细胞白血病。早幼粒细胞白血病是急性髓细胞白血病的一种特殊类型，被 FAB 协作组定为 AML-M3。
选项 B 急性髓细胞白血病是一组在形态、遗传学、免疫学、临床和预后方面差异较大的异质性肿瘤，包括了从成熟到未成熟阶段的粒细胞或单核细胞的白血病，可分为 M0 到 M7 八个亚型，早幼粒细胞白血病属于其中的一个亚型，与题干描述不符。
选项 C 急性单核细胞白血病是急性髓细胞白血病 M5 型，与题干描述不符，故排除。
选项 D 急性巨核细胞白血病是急性髓细胞白血病 M7 型，与题干描述不符，故不选。
选项 E 中枢神经系统白血病是白血病细胞髓外浸润至蛛网膜或邻近神经组织而产生的临床症状和体征，不是一种独立的白血病类型，故排除。
问题 117
考虑最可能诊断为

A. 早幼粒细胞白血病
B. 急性髓细胞白血病
C. 急性单核细胞白血病
D. 急性巨核细胞白血病
E. 中枢神经系统白血病
解答：

根据临床表现、骨髓细胞学检查结果及早幼粒细胞占多的情况，最可能的诊断为早幼粒细胞白血病（APL）。故答案为：
A. 早幼粒细胞白血病
118、　(A3/A4 型题)　题
治疗用什么
A.全反式维甲酸
B.HA 方案化疗
C.伊马替尼
D.VLDP 方案化疗
E.DA 方案化疗
参考答案：　A　您的答案：

图文解析：　　【解析】 中老年男性患者，发热，周部皮肤针刺处可见瘀斑，胸骨压痛（+）（白血病浸润表现） ，血常规：Hb86g/L，WBC12.4×109/L，PLT34×109/L（红系、巨核系减少， 白血病细胞增多） ，骨髓细胞学检查：增生极度活跃，胞质内粗大颗粒的早幼粒细胞占 0.75（M3 的典型骨髓象表现） 。考虑最可能诊断为早幼粒细胞白血病，可发现 CD13、CD33 阳性；治疗首选全反式维甲酸。

【避错】HA、DA 方案化疗用于急性髓系白血病；伊马替尼用于慢性髓系白血病；VLDP 方案化疗用于急淋白血病（D 错）。

118 题的回答：治疗应选择 E.DA 方案化疗。DA 方案，包括达纳霉素（Daunorubicin）和阿糖胞苷（Cytarabine），是急性髓细胞白血病的常规治疗方案。

正确答案是：A.全反式维甲酸

患者已诊断为早幼粒细胞白血病，其首选治疗为全反式维甲酸（ATRA）+砷剂，因此本题答案为 A。

选项 B，HA 方案（三尖杉酯碱+阿糖胞苷）主要用于急性髓系白血病的治疗，但对早幼粒细胞白血病的治疗效果有限，故不选。

选项 C，伊马替尼主要用于治疗慢性髓系白血病，特别是伴有 BCR-ABL 融合基因的病例，对早幼粒细胞白血病无治疗作用，故排除。

选项 D，VLDP 方案（长春新碱+左旋门冬酰胺酶+柔红霉素+强的松）通常用于治疗急性淋巴细胞白血病，而非早幼粒细胞白血病，故不选。

选项 E，DA 方案（柔红霉素+阿糖胞苷）也常用于急性髓系白血病的治疗，但对早幼粒细胞白血病的治疗效果不如全反式维甲酸显著，故排除。

问题 118

治疗用什么

A. 全反式维甲酸
B. HA 方案化疗
C. 伊马替尼
D. VLDP 方案化疗
E. DA 方案化疗

解答：

急性早幼粒细胞白血病（APL）的标准治疗方案是全反式维甲酸（ATRA）联合化疗。ATRA 能够诱导早幼粒细胞分化。故答案为：

A. 全反式维甲酸

题目材料　　男，22 岁，2 小时前骑自行车不慎摔倒骑跨在车梁上，会阴部肿胀痛，尿道口滴血。查体：生命中稳定，会阴部肿胀，可见瘀斑。急诊用 16　插入尿道膀胱，导出清亮液体。下一步处理的方法男，22 岁，2 小时前骑自行车不慎摔倒骑跨在车梁上，会阴部肿胀痛，尿道口滴血。查体：生命中稳定 肿胀，可见瘀斑。急诊用 16 号导尿管可插入尿道膀胱，导出清亮液体。

119、　(A3/A4 型题)　题

最可能的诊断是

A.尿道球部挫伤
B.尿道膜部损伤
C.尿道阴茎部损伤
D.前列腺损伤

E.膀胱破裂
参考答案：　　A　您的答案：
图文解析：　　【解析】男性前尿道外伤多发生于球部，这段尿道固定在会阴部。会阴部骑跨伤时，将尿道挤向耻骨联合下方，引起尿道球部外伤（故选 A 对）。
【避错】
问题 119：最可能的诊断是什么？
给定的情况描述了一个 22 岁男性自行车事故后出现会阴部肿胀、瘀斑和尿道口滴血的情况。因为可以顺利插入尿道导管并排出清亮的尿液，这表明尿道没有完全撕裂，但有一定程度的损伤。根据描述的受伤机制和临床表现，最可能的诊断是：
A. 尿道球部挫伤 - 尽管可能性存在，但球部尿道受伤通常与更严重的撞击相关。
B. 尿道膜部损伤 - 考虑到伤害的性质和位置，这是最可能的诊断，因为膜部尿道受伤常见于会阴部的外伤。
C. 尿道阴茎部损伤 - 阴茎部尿道损伤通常与直接的阴茎外伤相关。
D. 前列腺损伤 - 前列腺损伤较少见，且通常与更严重的盆腔创伤相关。
E. 膀胱破裂 - 若导尿后尿液清亮，膀胱破裂的可能性较小。
因此，最合理的选择是 B. 尿道膜部损伤。
解析：
青年男性患者，骑自行车不慎摔倒骑跨在车梁上受伤，出现会阴部肿胀痛，尿道口滴血，查体见会阴部肿胀，可见瘀斑，急诊用 16 号导尿管可插入尿道膀胱，导出清亮液体。结合患者病史、临床表现和相关检查，该患者符合尿道球部挫伤的典型表现。
选项 B 尿道膜部损伤多见于骨盆骨折，该患者并无骨盆外伤史，故不选。
选项 C 尿道阴茎部损伤主要表现为局部瘀斑、血肿和尿外渗，该患者未见血肿和尿外渗，故不考虑。
选项 D 前列腺损伤常表现为下腹部疼痛、血尿、排尿困难，该患者未见上述表现，故排除。
选项 E 膀胱破裂主要表现为腹痛、排尿困难、血尿和尿瘘，该患者未见血尿和尿瘘，故不选。
综上所述，正确答案是：A.尿道球部挫伤。
问题 119：最可能的诊断是
患者，22 岁，骑自行车摔倒后会阴部肿胀疼痛，尿道口滴血。查体发现会阴部肿胀，有瘀斑。急诊插入 16 号导尿管，导出清亮液体。这些症状和体征提示了尿道受损的可能性。

诊断分析：

尿道球部挫伤：骑自行车摔倒时会阴部直接撞击容易导致此部位挫伤，常见症状为尿道口出血和会阴部肿胀。
尿道膜部损伤：常见于严重的骨盆骨折或高能量损伤，患者会有会阴部肿胀和尿道出血，但在这种情况下，导尿管通常难以插入。
尿道阴茎部损伤：较为少见，通常发生于外力直接作用于阴茎的情况。
前列腺损伤：这种情况较为少见，且多见于直肠指诊或手术操作后。
膀胱破裂：患者通常会有明显的下腹痛和血尿，导尿管导出的液体通常混有血液。
结合患者的具体病情，最可能的诊断是 A.尿道球部挫伤。
120、　(A3/A4 型题)　题
下一步处理的方法
A.立即手术
B.插尿管 24 小时后手术

C.插尿管 24 小时后拔除，让其自由排尿
D.插尿管，2~3 周拔出
E.插尿管 72 小时后拔出，每周扩张尿道一次
参考答案：　　D　您的答案：
图文解析：　　【解析】该患者为尿道球部挫伤，导尿管插入顺利，可留置导尿管引流 2 周左右（D 对）　。如插入失败，可能有尿道部分裂伤，应即行经会阴尿道修补术，并留置导尿管 2～3 周。
【避错】尿道膜部、尿道阴茎部均为后尿道，多为骨盆骨折导致，常见休克，无会阴部瘀斑表现。
问题 120：下一步处理的方法
对于尿道损伤的管理，关键是防止尿道狭窄的发展，同时允许尿道的恢复。选择处理方法时，要根据尿道损伤的程度和性质来定。考虑到尿道似乎没有完全撕裂（因为可以导尿并排出清亮的尿液），可以采取更保守的治疗方法。选项中的：
A. 立即手术 - 通常保留给完全撕裂或严重尿道损伤。
B. 插尿管 24 小时后手术 - 如果怀疑有复杂的损伤或不能通过保守方法管理，则考虑此方案。
C. 插尿管 24 小时后拔除，让其自由排尿 - 如果尿道损伤较轻，且排尿功能未受影响，可以考虑这种方法。
D. 插尿管，2~3 周拔出 - 对于中度尿道损伤，长期留置尿管可能有助于尿道愈合。
E. 插尿管 72 小时后拔出，每周扩张尿道一次 - 对于有狭窄风险但不需要立即手术的情况，这种方法可能适用。
根据描述的情况和伤害的程度， D. 插尿管，2~3 周拔出 似乎是合适的方法，以便允许尿道充分愈合并减少狭窄的风险。
尿道球部挫伤时，因尿道连续性未破坏，且急诊已用 16 号导尿管插入尿道膀胱，并已导出清亮液体，故此时应插尿管，2~3 周拔出，以观察尿道是否恢复通畅。
选项 A 立即手术适用于尿道断裂等损伤较重的情况，不适用于该患者，故不选。
选项 B 插尿管 24 小时后手术，选项 E 插尿管 72 小时后拔出，每周扩张尿道一次，均不是尿道挫伤的常规处理方法，可一并排除。
选项 C 插尿管 24 小时后拔除，让其自由排尿，因时间过短，此时拔除尿管可能导致尿道未完全恢复，故不考虑。
综上所述，正确答案是：D.插尿管，2~3 周拔出。
问题 120：下一步处理的方法
患者已经成功插入导尿管，并且导出了清亮液体，说明尿道的通畅性较好，没有严重的尿道断裂情况。

处理方法分析：

立即手术：适用于严重的尿道断裂或其他危及生命的损伤情况，当前不适用。
插尿管 24 小时后手术：一般用于观察尿道是否有进一步的损伤迹象，目前不适用。
插尿管 24 小时后拔除，让其自由排尿：可能导致尿道狭窄或其他并发症。
插尿管，2~3 周拔出：这是一种常见的处理尿道球部挫伤的方法，可以让尿道得到充分的恢复时间，减少并发症。
插尿管 72 小时后拔出，每周扩张尿道一次：这种方法适用于尿道狭窄或高风险的患者。
结合患者的病情，最佳的处理方法是 D.插尿管，2~3 周拔出，这可以确保尿道有足够的恢

复时间，避免进一步的损伤或并发症。
题目材料　　　　女，16 岁，心慌、多汗 2 年，体重下降 5kg，大便次数增加，3~4 次/日，不成形，月经 2~3 月一次，量少。查体：P：100 次/分，血压 120/80 突眼，甲状腺Ⅰ度肿大。实验室检查：TT3：8.6nmol/L，TT4：220nmol/L，TSH＜0.002。
121、　(A3/A4 型题)　题
下列哪项有助于诊断
A.TgAb
B.TRAb
C.FT3
D.TPOAb
E.Tg
参考答案：　B　您的答案：
图文解析：　【解析】青少年女性患者（GD 病多发人群），心慌、多汗、体重下降，甲状腺Ⅰ度肿大，TT3：8.6nmol/L，TT4：220nmol/L，TSH＜0.002（TT3、TT4↑，TSH↓)，综合患者症状、体征及实验室检查考虑诊断为弥漫性毒性甲状腺肿。TRAb 已经成为诊断 GD 的第一线指标，未治疗的 GD 患者的阳性率达到 98%。
【避错】60%桥本甲状腺炎 TgAb 阳性，90%桥本甲状腺炎 TPOAb 阳性提示自身免疫；FT3 于血中含量甚微，测定的稳定性不如 TT3、TT4；Tg 无意义。桥本氏甲状腺炎为甲状腺功能减退表现；亚急性甲状腺炎有甲状腺的触痛。
121 题，关于诊断：
这名患者的症状包括心慌、多汗、体重下降、大便次数增加、月经周期延长、甲状腺肿大，并且实验室检查显示 T3 和 T4 升高，TSH 降低。这些都是甲状腺功能亢进症的典型症状。其中，TRAb（甲状腺受体抗体）的检测对于诊断 Graves 病（弥漫性毒性甲状腺肿）特别有用。因此，正确答案是 B.TRAb。
121、有助于诊断的选项是：
B.TRAb
解析：
青年女性患者，有甲亢的典型症状，如心慌、多汗、大便次数增加、月经稀少等，查体出现突眼和甲状腺Ⅰ度肿大，实验室检查示 T3、T4 升高，TSH 明显降低，提示甲状腺功能亢进，综合该患者的病史、查体及实验室检查，考虑诊断为 Graves 病。Graves 病又称弥漫性毒性甲状腺肿，是一种伴甲状腺激素合成增多的器官特异性自身免疫性疾病，患者血清中存在 TSH 受体刺激性抗体，即 TRAb，因此检测患者体内 TRAb 水平有助于诊断。
选项 A：TgAb 即甲状腺球蛋白抗体，主要用于自身免疫性甲状腺疾病的诊断，但不是 Graves 病的特异性抗体，故排除。
选项 C：FT3 即游离三碘甲状腺原氨酸，是诊断甲亢最灵敏的一项指标，但其水平的异常也可见于非甲状腺毒症病因，如急性发热、妊娠等，因此不具有特异性，不能用于 Graves 病的诊断。
选项 D：TPOAb 即抗甲状腺过氧化物酶抗体，与甲状腺组织免疫性损伤密切相关，主要应用于桥本甲状腺炎、原发性甲减等甲状腺疾病的诊断，对 Graves 病无诊断意义。
选项 E：Tg 即甲状腺球蛋白，可作为分化型甲状腺癌的肿瘤标记物，监测其复发和转移，不能用于诊断 Graves 病。
第 121 题：
下列哪项有助于诊断？

A. TgAb
B. TRAb
C. FT3
D. TPOAb
E. Tg

TRAb（甲状腺刺激性抗体）是弥漫性毒性甲状腺肿（Graves 病）的特异性标志，可以帮助诊断。因此，最佳答案是：

B. TRAb
122、 (A3/A4 型题)
该患者所患疾病 题
A.桥本氏甲状腺炎
B.亚急性甲状腺炎
C.弥漫性毒性甲状腺肿
D.单纯性甲状腺肿
E.甲状腺癌
参考答案： C 您的答案：
图文解析： 【解析】青少年女性患者（GD 病多发人群），8.6nmol/L，TT4：220nmol/L，TSH＜0.002 实验室检查考虑诊断为弥漫性毒性甲状腺肿。 患者的阳性率达到 98%。【避错】 心慌、多汗、体重下降， 甲状腺Ⅰ度肿大，TT3：（TT3、TT4↑ ，TSH↓)，综合患者症状、体征及 TRAb 已经成为诊断 GD 的第一线指标，未治疗的 GD
122 题，关于疾病诊断：
根据上述症状和实验室检查结果，患者最有可能患有的是弥漫性毒性甲状腺肿，也就是 Graves 病。所以，正确答案是 C.弥漫性毒性甲状腺肿。
122、该患者所患疾病是：
C.弥漫性毒性甲状腺肿
解析：
青年女性患者，有甲亢的典型症状，如心慌、多汗、大便次数增加、月经稀少等，查体出现突眼和甲状腺Ⅰ度肿大，为 Graves 病的典型体征，实验室检查示 T3、T4 升高，TSH 明显降低，结合患者病史、临床表现和实验室检查，该患者诊断为弥漫性毒性甲状腺肿。
选项 A：桥本甲状腺炎即慢性淋巴细胞性甲状腺炎，是一种以自身甲状腺组织为抗原的慢性自身免疫性疾病，多见于中年女性，好发年龄为 40～50 岁，临床检查可发现甲状腺肿大，质地硬韧，多数患者甲状腺功能正常，但少数可出现甲减症状，与题干中患者症状不符，故排除。
选项 B：亚急性甲状腺炎又称亚急性肉芽肿性甲状腺炎，多见于中年妇女，发病前常有上呼吸道感染史，典型症状以甲状腺肿大、疼痛为主要特征，严重了可以引起全身症状比如发烧、头疼、全身乏力、大便干燥、口干舌燥、心烦等，与患者表现不符，因此不选。
选项 D：单纯性甲状腺肿患者多无明显的临床症状，仅在查体时发现甲状腺肿大，甲状腺功能一般正常，与题干不符，故排除。
选项 E：甲状腺癌早期临床表现不明显，多无自觉症状，随着病情发展可出现颈部肿块、呼吸困难、吞咽困难、声音嘶哑等症状，与患者表现差异明显，所以不选。
第 122 题：
该患者所患疾病是？

A. 桥本氏甲状腺炎
B. 亚急性甲状腺炎
C. 弥漫性毒性甲状腺肿
D. 单纯性甲状腺肿
E. 甲状腺癌

患者表现出甲状腺功能亢进的典型症状，结合实验室检查结果（TT3 和 TT4 升高，TSH 降低），突眼和甲状腺肿大，这些都符合弥漫性毒性甲状腺肿（Graves 病）的临床表现。因此，最佳答案是：

C. 弥漫性毒性甲状腺肿
题目材料　　63 岁男性，进行性右侧睾丸肿大一年，无疼痛，行走不便。查体：睾丸 6×5×4cm，无压痛，右侧睾丸及附睾未触及，透光试验阳性，平卧
123、　(A3/A4 型题)　题
诊断为
A.睾丸鞘膜积液
B.精索鞘膜积液
C.睾丸肿瘤
D.腹股沟斜疝
E.交通性鞘膜积液
参考答案：　　A　您的答案：
图文解析：　　【解析】 中老年男性，进行性无痛性右侧睾丸肿大 1 年，透光试验阳性，平卧后不变，考虑诊断
为睾丸鞘膜积液（ A 对）。
【避错】精索鞘膜积液（B 错）可表现为一个或多个囊肿，呈椭圆形、梭形或哑铃形，沿精索而生长，其下方可扪及正常睾丸、附睾，若牵拉同侧睾丸，可见囊肿随之上下移动。睾丸肿瘤与腹股沟斜疝（CD 错）透光试验均为阴性。交通性鞘膜积液（E 错）透光试验阳性，平卧后消失。
123 题诊断选择：
A. 睾丸鞘膜积液：考虑到无疼痛的肿大和透光试验阳性，睾丸鞘膜积液是一种可能的诊断。
B. 精索鞘膜积液：通常表现为精索区域的可触及肿块。
C. 睾丸肿瘤：通常会导致睾丸硬化，并且透光试验通常为阴性。
D. 腹股沟斜疝：主要表现为腹股沟区域的疝块，通常与睾丸体积变化无关。
E. 交通性鞘膜积液：可能考虑，但通常与睾丸体积不一致。
因此，针对 123 题，最合适的诊断是 A. 睾丸鞘膜积液。
123 题解析：
老年男性患者，进行性右侧睾丸肿大一年，无疼痛，查体示透光试验阳性，平卧时睾丸不消失，结合患者病史、临床表现和体格检查，该患者诊断为睾丸鞘膜积液。
选项 B 精索鞘膜积液表现为腹股沟管内较小的囊性肿块，牵拉同侧睾丸可见肿块移动，不符合题干描述。
选项 C 睾丸肿瘤的典型表现为睾丸肿胀或变硬，透光试验呈阴性，故不选。
选项 D 腹股沟斜疝的疝块常位于腹股沟区，常呈带蒂柄的梨形，并可降至阴囊或大阴唇，透光试验阴性，与题意不符。

选项 E 交通性鞘膜积液的特点是立位时阴囊肿大，但当患者卧位时由于积液流入腹腔，鞘膜囊会明显缩小或消失，阴囊内触及不到睾丸和附睾，透光试验也为阳性，但题干中明确说明平卧时右侧睾丸未消失，故排除。

综上所述，正确答案是：A.睾丸鞘膜积液。

123、(A3/A4 型题)

题：诊断为

A. 睾丸鞘膜积液

B. 精索鞘膜积液

C. 睾丸肿瘤

D. 腹股沟斜疝

E. 交通性鞘膜积液

答案解析：

根据病例描述，患者的右侧睾丸进行性肿大，透光试验阳性。这些特点最常见于鞘膜积液。鞘膜积液是一种在睾丸周围鞘膜腔内积聚液体的病变，通常会导致睾丸肿大且透光试验呈阳性。

因此，最可能的诊断是：

A. 睾丸鞘膜积液

124、 (A3/A4 型题) 题

首选的治疗

A.右侧斜疝修补术

B.右侧睾丸切除术

C.右侧鞘膜突高位结扎术

D.右睾鞘膜睾丸翻转术

E.穿刺抽液

参考答案： D 您的答案：

图文解析： 【解析】 中老年男性，进行性无痛性右侧睾丸肿大 1 年，透光试验阳性，平卧后不变，考虑诊断为睾丸鞘膜积液。成人的睾丸鞘膜积液，如积液量少，无任何症状，不需要手术治疗。积液量多，体积大伴明显的症状，可行睾丸鞘膜切除+翻转术（D 对）。【避错】右侧斜疝修补术为腹股沟斜疝的治疗（A 错）；右侧睾丸切除术用于睾丸肿瘤的治疗（B 错）；右侧鞘膜突高位结扎术为儿童腹股沟斜疝的治疗（C 错）；穿刺抽液为对症治疗（E 错）。

124 题首选治疗选择：

A. 右侧斜疝修补术：适用于腹股沟斜疝，与本病例不符。

B. 右侧睾丸切除术：适用于睾丸肿瘤，而非积液。

C. 右侧鞘膜突高位结扎术：用于治疗精索鞘膜积液或交通性鞘膜积液。

D. 右睾鞘膜睾丸翻转术：通常不是处理睾丸鞘膜积液的首选方法。

E. 穿刺抽液：是处理睾丸鞘膜积液的常见和首选方法，因为它既简单又有效。

因此，对于 124 题，最合适的治疗方法是 E. 穿刺抽液。

124 题解析：

患者已诊断为睾丸鞘膜积液，对于睾丸鞘膜积液，手术是最主要的治疗方法，其目的是在内环处将疝颈做高位结扎，阻断腹水下流，同时行鞘膜翻转术，以避免鞘膜积液的复发。因此，右睾鞘膜睾丸翻转术是该患者的首选治疗方法。

选项 A 右侧斜疝修补术适用于腹股沟疝的患者，与该患者诊断不符，故排除。
选项 B 右侧睾丸切除术主要用于睾丸肿瘤的患者，该患者无肿瘤诊断，故不选。
选项 C 右侧鞘膜突高位结扎术主要适用于小儿交通性鞘膜积液的治疗，与该患者诊断不符，可排除。
选项 E 穿刺抽液是睾丸鞘膜积液的保守治疗方法，但容易复发，不是首选治疗方案，可排除。
综上所述，正确答案是：D.右睾鞘膜睾丸翻转术。
124、(A3/A4 型题)
题：首选的治疗
A. 右侧斜疝修补术
B. 右侧睾丸切除术
C. 右侧鞘膜突高位结扎术
D. 右睾鞘膜睾丸翻转术
E. 穿刺抽液

答案解析：
对于睾丸鞘膜积液的治疗，常见的方法有保守治疗和手术治疗。若积液较少且症状轻微，穿刺抽液可以作为暂时缓解的方法。但对于较大或持续的鞘膜积液，尤其是影响日常活动的情况下，鞘膜突高位结扎术是首选的根治性治疗方法，可以有效阻止液体的继续积聚。

因此，最合适的治疗方法是：
C. 右侧鞘膜突高位结扎术
题目材料　　男，40 岁，水肿，少尿 2 周，血压 160/100mmHg，肌酐 300μmol/L，尿蛋白 2g，镜下血尿 20~30 个，镜下红细胞 8 个，呈新月体征，免疫荧光有
布，
125、　(A3/A4 型题)　题
诊断为
A.急进性肾小球肾炎Ⅰ型
B.急进性肾小球肾炎Ⅱ型
C.急进性肾小球肾炎Ⅲ型
D.肾病综合征
E.急性肾小球肾炎
参考答案：　　A　您的答案：
图文解析：　　【解析】 中年男性患者，水肿，少尿，血压 160/100mmHg（血压升高），肌酐 300μmol/L（肾功能损害），尿蛋白 2g，血尿，呈新月体征（急性型肾炎病理类型），免疫荧光有线型分布(Ⅰ型急性型肾炎免疫病理学类型），诊断为急进性肾小球肾炎Ⅰ型（A 对）。【避错】急进性肾小球肾炎免疫病理学检查是分型的主要依据，Ⅰ型 IgG 及 C3 呈线条状沿肾小球毛细血管壁分布；Ⅱ型 IgG 及 C3 呈颗粒状或团块状沉积于系膜区及毛细血管壁；Ⅲ型肾小球内无或仅有微量免疫沉积物。
对于第一题，急进性肾小球肾炎可分为三型：
Ⅰ型通常与抗肾小球基底膜抗体相关。
Ⅱ型与免疫复合物沉积有关。
Ⅲ型与无免疫复合物沉积的肾小球损伤有关，常见于 ANCA 相关性血管炎。
由于题干中提到“免疫荧光有布”，可能暗示有免疫复合物的沉积或特定免疫标记，更倾向于Ⅱ型急进性肾小球肾炎。但确切的分类还需依据免疫荧光的具体发现，这里我们可以选择

B.急进性肾小球肾炎Ⅱ型 作为最可能的诊断。
答案为 A. 急进性肾小球肾炎Ⅰ型
该患者中年男性，水肿，少尿，高血压，肾功能异常，大量蛋白尿，镜下血尿，镜下红细胞，新月体征，免疫荧光有分布，根据临床表现和实验室检查，诊断考虑为急进性肾小球肾炎。急进性肾小球肾炎是以急性肾炎综合征、肾功能急剧恶化、多在早期出现少尿性急性肾衰竭为临床特征的一组疾病。根据免疫病理可分为三型，Ⅰ型为抗肾小球基底膜抗体型，Ⅱ型为免疫复合物型，Ⅲ型为少免疫沉积物型。该患者新月体征，免疫荧光有分布，考虑诊断为急进性肾小球肾炎Ⅰ型。
问题 125（A3/A4 型题）
诊断为：

A. 急进性肾小球肾炎Ⅰ型
B. 急进性肾小球肾炎Ⅱ型
C. 急进性肾小球肾炎Ⅲ型
D. 肾病综合征
E. 急性肾小球肾炎

分析：
急进性肾小球肾炎（RPGN）是一种快速进展的肾小球肾炎，以迅速发展的肾功能不全为特征，并常伴有血尿和蛋白尿。根据免疫荧光检查结果，急进性肾小球肾炎可分为三型：

Ⅰ型：抗肾小球基底膜抗体相关（如 Goodpasture 综合征）
Ⅱ型：免疫复合物沉积相关（如狼疮性肾炎、IgA 肾病）
Ⅲ型：少免疫复合物沉积（pauci-immune），通常与抗中性粒细胞胞浆抗体（ANCA）相关
根据题干信息，患者的免疫荧光检查有布（通常提示免疫复合物沉积），结合患者的临床表现和实验室检查结果，最可能的诊断是 急进性肾小球肾炎Ⅱ型。

答案：B
126、 (A3/A4 型题) 题
治疗首选
A.血浆置换
B.泼尼松
C.泼尼松+免疫抑制剂
D.血液透析
E.泼尼松+细胞毒药物
参考答案： A 您的答案：
图文解析： 【解析】 中年男性患者，水肿、少尿、血压 160/100mmHg（血压升高），肌酐 300μmol/L（肾功能损害），尿蛋白 2g，血尿，呈新月体征（急性型肾炎病理类型），免疫荧光有线型分布(Ⅰ型急性型肾炎免疫病理学类型），诊断为急进性肾小球肾炎Ⅰ型，首选治疗为血浆置换（A 对）。
【避错】泼尼松及细胞毒药物、免疫抑制剂配合甲泼尼龙冲击用于Ⅱ、Ⅲ型患者。
对于第二题，治疗急进性肾小球肾炎的首选通常包括免疫抑制剂以控制免疫反应，泼尼松是常用的治疗方法，但在许多情况下，尤其是疾病较为严重时，可能需要结合使用免疫抑制剂。因此，C.泼尼松+免疫抑制剂 可能是更合适的选择。

答案为 A. 血浆置换
对于Ⅰ型急进性肾小球肾炎的治疗，强化血浆置换疗法是目前最有效的方法，可以去除循环中的抗肾小球基底膜抗体，减轻肾小球损害，因此治疗首选血浆置换。故本题选 A。选项 B 泼尼松，选项 C 泼尼松+免疫抑制剂，选项 E 泼尼松+细胞毒药物，均适用于Ⅱ、Ⅲ型急进性肾小球肾炎的治疗，故一并排除。选项 D 血液透析仅用于肾功能严重受损时，不是首选，可排除。
问题 126（A3/A4 型题）
治疗首选：

A. 血浆置换
B. 泼尼松
C. 泼尼松+免疫抑制剂
D. 血液透析
E. 泼尼松+细胞毒药物

分析：
急进性肾小球肾炎的治疗需要迅速控制炎症和免疫反应，以防止肾功能进一步恶化。治疗方案通常包括高剂量的糖皮质激素和免疫抑制剂。对于免疫复合物沉积相关的 RPGN（Ⅱ型），通常采用糖皮质激素联合免疫抑制剂。

答案：C
题目材料　　患者胸闷气短数年，2 日前开始出现憋喘、咯血，咳粉红色痰，心电图示房颤，心超显示，左心房内径 56mm，二尖瓣口面积 0.8cm2，成墙垛赘生物，
127、　(A3/A4 型题)　题
考虑是何诊断
A.急性肺栓塞
B.急性心肌梗死
C.支气管扩张
D.扩张型心肌病
E.感染性心内膜炎
参考答案：　E　您的答案：
图文解析：　【解析】 中年男性患者，胸闷气短，2 日前开始出现憋喘、咯血，咳粉红色痰（急性左心衰竭症状），心电图示房颤，左心房内径 56mm，二尖瓣口面积 0.8cm2，成墙垛样改变（二尖瓣狭窄改变），有赘生物（感染性心内膜炎典型心超改变）。考虑诊断为感染性心内膜炎（E 对）。
【避错】急性肺栓塞表现为胸痛、咯血、呼吸困难三联征（A 错）；急性心肌梗死表现为持续性胸痛大于 30 分钟，有典型 ST 段心电图改变（B 错）；支气管扩张表现为咳嗽、咳脓臭痰多年，肺部高分辨 CT 可见双轨征或卷发样阴影（C 错）；扩张型心肌病是一类以左心室或双心室扩大伴收缩功能障碍为特征的心肌病（D 错）。
127 题考虑的是诊断问题。这个病例的特点和心脏超声结果与感染性心内膜炎相符，因为感染性心内膜炎可以引起垛赘生物形成，并可能导致心脏结构如二尖瓣受损。因此，最合适的诊断是：E. 感染性心内膜炎
127 题解析：
患者长期胸闷气短，近期憋喘、咯血，咳粉红色痰，心电图示房颤，心超显示左心房增大，

二尖瓣狭窄且有赘生物，根据患者的临床表现和辅助检查，最可能的诊断是感染性心内膜炎。
选项 A 急性肺栓塞常表现为突发不明原因的呼吸困难及气促，同时伴有胸痛、咳血等，一般无心内膜赘生物，故排除。
选项 B 急性心肌梗死一般有心绞痛病史，主要表现为胸骨后濒死样疼痛等心肌缺血表现，一般不会有憋喘、咯血及咳粉红色痰等症状，与题干描述不符。
选项 C 支气管扩张主要表现为慢性咳嗽、大量浓痰及反复咳血，无心脏相关症状，故不选。
选项 D 扩张型心肌病主要表现为活动时呼吸困难和活动耐量下降，心脏扩大，一般不伴有咯血及咳粉红色痰等症状，故排除。
综上所述，正确答案是：E.感染性心内膜炎。
问题 127 的答案：
考虑是何诊断

A. 急性肺栓塞 - 患者有憋喘、咯血，但没有典型的肺栓塞病史或风险因素。
B. 急性心肌梗死 - 患者无明显胸痛表现，心电图也没有提示。
C. 支气管扩张 - 患者有咯血，但无慢性咳嗽和痰多等支气管扩张的典型表现。
D. 扩张型心肌病 - 患者心脏超声显示的是二尖瓣狭窄，左心房增大，而不是心室的扩张。
E. 感染性心内膜炎 - 患者有赘生物，但没有发热、心脏杂音的变化等感染性心内膜炎的典型表现。

患者有长期的胸闷气短病史、房颤、二尖瓣口严重狭窄和赘生物形成，最符合的是风湿性心脏病导致的二尖瓣狭窄。但是题目中没有明确指出风湿性心脏病，这种情况下，感染性心内膜炎的可能性不能完全排除，因为赘生物可能是由感染导致的。

最佳答案是：E. 感染性心内膜炎
128、 (A3/A4 型题) 题
下列治疗首选的是
A.利尿剂
B.球囊扩张术
C.阿司匹林抗血小板
D.硝酸酯扩冠脉
E.洋地黄
参考答案： E 您的答案：
图文解析： 【解析】 中年男性患者，胸闷气短，2 日前开始出现憋喘、咯血，咳粉红色痰（急性左心衰竭症状），心电图示房颤，左心房内径 56mm，二尖瓣口面积 0.8cm2，成墙垛样改变（二尖瓣狭窄改变），有赘生物（感染性心内膜炎典型心超改变）。考虑诊断为感染性心内膜炎。合并有二尖瓣狭窄伴房颤，药物治疗首选洋地黄（器质性心脏病合并房颤是洋地黄的绝佳适应证）。
【避错】球囊扩张术用于单纯中重度二尖瓣狭窄。
128 题涉及到治疗首选。对于感染性心内膜炎，重要的治疗方式是使用抗生素来控制感染。然而，在提供的选项中，没有直接的抗生素治疗选项。考虑到感染性心内膜炎可能引起的心功能受损和心脏负荷增加，利尿剂可以被用来减轻症状和心脏的负担。因此，在给定的选项中，最合适的治疗首选是：A. 利尿剂
128 题解析：
患者已诊断为感染性心内膜炎，且伴有二尖瓣狭窄，此时首选的治疗方法是球囊扩张术，以

改善二尖瓣狭窄的症状。
选项 A 利尿剂多用于心力衰竭的治疗，该患者未出现心力衰竭，故排除。
选项 C 阿司匹林抗血小板，选项 D 硝酸酯扩冠脉，多用于冠心病的治疗，而该患者并非冠心病，与题意不符。
选项 E 洋地黄是正性肌力药物，多用于伴有快速心房颤动/心房扑动的收缩性心力衰竭，而该患者未出现心力衰竭，故不选。
综上所述，正确答案是：B.球囊扩张术。
问题 128 的答案：
下列治疗首选的是

A. 利尿剂 - 可以缓解症状但不是首选治疗。
B. 球囊扩张术 - 对于二尖瓣狭窄患者是有效的治疗方法，尤其是有症状的严重狭窄。
C. 阿司匹林抗血小板 - 对二尖瓣狭窄和房颤患者可以使用，但不是首选。
D. 硝酸酯扩冠脉 - 主要用于心绞痛，不适用于这种情况。
E. 洋地黄 - 可用于房颤和心衰的控制，但不是根本治疗。

由于患者有严重的二尖瓣狭窄，球囊扩张术可以有效地改善症状和预防血栓形成。

最佳答案是：B. 球囊扩张术
题目材料　　男，42 岁。腹胀伴乏力 2 天。突发胸痛 5 小时。既往高脂血症病史 2 年，未治疗。查体：P68 次/分，BP120/78mmhg，心肺腹未见异常。血 cTnT0.83mg/fl，D-DIMER：0.3g/L，心电图Ⅰ、aVL、V5-V6 导联 ST 段压低 0.2mv。
129、　(A3/A4 型题)
为进一步明确诊断应进行的检查是
A.超声心动图
B.冠状动脉造影
C.肺动脉 CTA
D.主动脉 CTA
E.心脏核磁共振
参考答案：　B　您的答案：
图文解析：　【解析】 中年男性患者，突发胸痛 5 小时（高度怀疑急性心肌梗死），既往高脂血症病史，心电图Ⅰ、aVL、V5-V6 导联 ST 段压低 0.2mv，综合患者症状、病史及心电图表现考虑为急性心肌梗死。冠状动脉造影为诊断冠心病的金标准（B 对）。
【避错】超声心动图为心力衰竭、心脏器质性病变的检查（A 错）；肺动脉 CTA 为肺栓塞的金标准（C 错）；主动脉 CTA 为主动脉狭窄的金标准（D 错）；心脏核磁共振检查较少见（E 错）。
129、
这位 42 岁的男性患者有高脂血症史，未经治疗，并突发胸痛。他的 cTnT 升高（0.83mg/fl）提示有心肌损伤，D-DIMER 略有升高。心电图显示 ST 段压低，这些都是急性冠状动脉综合征的特征。为进一步明确诊断，应选择：
B.冠状动脉造影
这是确定冠状动脉狭窄或闭塞的金标准，有助于诊断急性心肌梗死。
正确答案是：C.肺动脉 CTA
患者突发胸痛，心电图Ⅰ、aVL、V5～V6 导联 ST 段压低，血 cTnT0.83mg/fl，D-dimer：0.3g/L，

考虑患者为肺动脉栓塞症，为进一步明确诊断应进行的检查是肺动脉 CTA，可以直接显示肺动脉内的血栓，表现为肺动脉内的低密度充盈缺损，部分或完全包围在不透光的血流之间，或者呈完全充盈缺损，远端血管不显影，其诊断准确率高，敏感性与特异性均好。
选项 A 超声心动图为非创伤性检查，可反映心脏结构及左心功能，但对肺动脉栓塞诊断价值不大，故不选。
选项 B 冠状动脉造影为诊断冠心病的金标准，不能用于诊断肺动脉栓塞，故排除。
选项 D 主动脉 CTA 主要用于主动脉夹层的诊断，与该患者情况不符，故不考虑。
选项 E 心脏核磁共振为评估心脏结构和功能的金标准，对心肌病的诊断以及心脏肿瘤、心包疾病的诊断具有重要价值，但对肺动脉栓塞诊断意义不大，故不符合题意。
129. 为进一步明确诊断应进行的检查是
患者的主要症状是突发胸痛，结合心电图显示的 ST 段压低和血清心肌肌钙蛋白 T 升高，提示可能存在冠状动脉问题。此外，既往有高脂血症病史，但未治疗，进一步增加了冠状动脉疾病的风险。

考虑到这些因素，最直接的检查应该是冠状动脉造影，因为它能直接显示冠状动脉的阻塞或狭窄情况，有助于明确是否存在冠状动脉粥样硬化性心脏病（例如急性心肌梗死）。

最佳答案: B. 冠状动脉造影
130、 (A3/A4 型题) 题
请问目前考虑什么诊断
A.主动脉夹层
B.急性心肌梗死
C.急性肺动脉梗死
D.急性心肌炎
E.急性心包炎
参考答案： B 您的答案：
图文解析： 【解析】 中年男性患者，突发胸痛 5 小时（高度怀疑急性心肌梗死） ，既往高脂血症病史，心电图Ⅰ 、aVL、V5-V6 导联 ST 段压低 0.2mv，综合患者症状、病史及心电图表现考虑为急性心肌梗死（B 对）。
【避错】主动脉夹层常表现为突发胸部撕裂样疼痛，常有上肢血压增高，两侧肢体血压及脉搏明显不对称（A 错） ；急性肺动脉梗死表现为胸痛、咯血、呼吸困难三联征（C 错） ；急性心肌炎多数于发病前 1～3 周有病毒感染等前驱症状，多数以心律失常为主诉或首见症状（D 错） ；急性心包炎心电图表现为 aVR 导联以外 ST 段广泛抬高（E 错）。
130、考虑到他的 cTnT 升高和心电图改变，最可能的诊断是：B.急性心肌梗死
这种情况下，心电图常见的 ST 段改变和心肌损伤标志物升高，是急性心肌梗死的典型表现。
正确答案是：C.急性肺动脉梗死
患者突发胸痛，心电图Ⅰ、aVL、V5～V6 导联 ST 段压低，血 cTnT0.83mg/fl，D-dimer：0.3g/L，考虑患者为急性肺动脉梗死。肺动脉栓塞时，心电图可出现非特异性的 ST 段和 T 波改变，多数患者有低氧血症，当合并右心衰竭时，可出现相应的临床表现，本题患者心电图导联 ST 段压低，伴有乏力，均为右心衰表现，因此考虑急性肺动脉梗死。
选项 A 主动脉夹层常表现为突发胸背部撕裂样剧痛，伴虚脱表现，两侧肢体动脉血压明显不等，与本题患者症状不符，故不选。
选项 B 急性心肌梗死一般有心绞痛病史，主要表现为胸骨后濒死样疼痛等心肌缺血表现，心电图示心肌梗死的特征性与动态性改变，本题患者无心绞痛表现，故排除。

选项 D 急性心肌炎发病前多有病毒感染前驱症状，如发热、全身倦怠和肌肉酸痛等，与本题无关。
选项 E 急性心包炎主要表现为胸骨后、心前区疼痛，心包摩擦音等，与本题不符。
130. 目前考虑什么诊断
患者的主要症状是胸痛，伴有腹胀和乏力，结合以下临床发现：

心电图：Ⅰ、aVL、V5-V6 导联 ST 段压低
血清心肌肌钙蛋白 T 升高（0.83 mg/L）
这些特征高度提示急性冠状动脉综合征，尤其是急性心肌梗死。心肌梗死通常会导致心电图 ST 段改变和血清心肌标志物（如 cTnT）升高。

最佳答案: B. 急性心肌梗死
题目材料　　女性，20 岁，口干、多饮，多尿半月。每日尿量 7~8L。尿常规提示：血糖：4.8mmol/L，比重：1.007。禁水试验后尿量无明显减少。测量渗 浆渗透压 305mosm/L、尿液渗透压：200mosm/L；静脉注射去胺加压素后，尿量明显减少，测渗透压：血浆：300mosm/L，尿渗透压：550mosm/L。
131、　(A3/A4 型题)　题
考虑以下哪种疾病
A.完全中枢性尿崩症
B.部分中枢性尿崩症
C.完全肾性尿崩症
D.部分肾性尿崩症
E.神经性烦渴
参考答案：　A　您的答案：
图文解析：　　　【解析】青少年女性，　口干、多饮，多尿半月，每日尿量 7～8L 低比重尿（尿崩症的典型症状）　，禁水试验后尿量无明显减少。加压素试验鉴别肾性尿崩症和中枢性尿崩症（中枢性尿崩症注射加压素后尿液渗透压增加，肾性尿崩症则基本无变化）　。静脉注射去氨加压素后，尿量明显减少，血浆渗透压 300mosm/L、尿渗透压 550mosm/L（血浆渗透压基本无变化，尿渗透压增加）考虑中枢性尿崩症，加压素试验中尿液渗透压增加 50%以上，考虑完全中枢性尿崩症（A 对）。
【避错】
对于 131 题，答案应该是 B.部分中枢性尿崩症。患者对去胺加压素有反应，意味着她的肾脏可以对 ADH 起作用，但自身产生的 ADH 可能不足，表明是中枢性尿崩症。由于症状并非完全缺乏 ADH（因为治疗有效），所以更符合部分中枢性尿崩症。
131 题正确答案是：A.完全中枢性尿崩症。
本题考察的是尿崩症的分类及诊断。
选项 A 完全中枢性尿崩症是由于抗利尿激素（ADH）严重缺乏或部分缺乏，导致肾小管重吸收水的功能障碍，从而引起以多尿、烦渴与多饮和低比重尿和低渗尿为特征的一组综合征。该患者存在多尿（每日尿量 7~8L），尿比重低（1.007），禁水试验后尿量无明显减少，符合完全中枢性尿崩症的特征性表现，在静脉注射去胺加压素后，尿量明显减少，尿渗透压明显升高（550mosm/L），进一步验证了该诊断，为正确选项。
选项 B 部分中枢性尿崩症表现为多尿症状较轻，禁水后尿量可以减少，尿比重和尿渗透压可以升高，与题干描述不符。
选项 C 完全肾性尿崩症，选项 D 部分肾性尿崩症，均是由于肾脏病变，导致肾小管对抗利

尿激素不敏感而引起的综合征，其临床特征与中枢性尿崩症相似，对外源性 ADH 无反应或反应减弱，但注射高渗盐水后可使尿量减少，尿比重增加，注射加压素后尿量不减少，尿比重不增加，与题干中患者对加压素反应敏感不符，故一并排除。
选项 E 神经性烦渴是由于精神因素引起烦渴、多饮，从而导致多尿和低比重尿、低渗尿，但这些症状在受到限水时可以得到缓解，与题干中禁水试验后尿量无明显减少不符。
综上所述，正确答案是：A.完全中枢性尿崩症。
患者是一名 20 岁的女性，症状包括口干、多饮、多尿，每日尿量高达 7-8 升，病程为半个月。尿常规提示血糖正常（4.8mmol/L），尿比重低（1.007）。禁水试验后尿量无明显减少，测得血浆渗透压 305mosm/L，尿液渗透压 200mosm/L。静脉注射去氨加压素后，尿量明显减少，测得血浆渗透压 300mosm/L，尿渗透压 550mosm/L。

诊断思路
尿崩症的类型区分：

中枢性尿崩症（Central Diabetes Insipidus）：由垂体或下丘脑的损伤引起，导致抗利尿激素（ADH）分泌不足。
肾性尿崩症（Nephrogenic Diabetes Insipidus）：肾脏对 ADH 反应不足。
神经性烦渴（Primary Polydipsia）：主要由于过度饮水引起。
禁水试验和去氨加压素试验结果：

禁水试验后尿量无明显减少，提示患者不能集中尿液。
静脉注射去氨加压素后，尿量明显减少，尿渗透压显著上升（从 200mosm/L 到 550mosm/L），说明肾脏对 ADH 有反应。
上述结果表明患者可能是中枢性尿崩症，因为在去氨加压素注射后，尿量减少且尿渗透压显著增加。

回答问题
考虑以下哪种疾病
A. 完全中枢性尿崩症
132、 (A3/A4 型题) 题
应首选的药物是
A.氯丙嗪
B.氯磺苯脲
C.氢氯噻嗪
D.去氨加压素
E.卡马西平
参考答案： D 您的答案：
图文解析： 【解析】青少年女性， 口干、多饮，多尿半月，每日尿量 7～8L 低比重尿（尿崩症的典型症状） ，禁水试验后尿量无明显减少。加压素试验鉴别肾性尿崩症和中枢性尿崩症（中枢性尿崩症注射加压素后尿液渗透压增加，肾性尿崩症则基本无变化） 。静脉注射去氨加压素后，尿量明显减少，血浆渗透压 300mosm/L、尿渗透压 550mosm/L（血浆渗透压基本无变化，尿渗透压增加） ，考虑完全性中枢性尿崩症，治疗首选去氨加压素（D 对）。
【避错】氯丙嗪为中枢性多巴胺受体拮抗剂，用于精神分裂者、躁狂症等疾病（A 错） ；

氯磺苯
脲可用于肾性尿崩症，治疗效果较去氨加压素弱（B 错） ；氢氯噻嗪对肾性尿崩症也有效长期服用氢氯噻嗪可能引起低钾、高尿酸血症等(C 错)；卡马西平为抗癫痫药，常用于癫痫单纯或复杂部分性发作、原发型三叉神经痛（E 错）。
对于 132 题，首选的药物应该是 D.去氨加压素，因为这种药物可以用于治疗中枢性尿崩症，它是一种合成的抗利尿激素，可以补充体内缺乏的 ADH，减少尿量，增加尿液浓度。
132 题正确答案是：D.去氨加压素。
本题考察的是尿崩症的治疗。
选项 A 氯丙嗪可抑制垂体分泌催乳素、促性腺激素、生长激素、褪黑素和 ACTH，也可抑制中枢神经系统各个部位，尤其是大脑皮质上某些区具有抗多巴胺受体的功能，可产生镇静作用，但它并不直接作用于肾小管，无法增强水的重吸收，因此不能治疗尿崩症，故排除。
选项 B 氯磺苯脲可刺激抗利尿激素释放并增强抗利尿激素对肾小管的作用，但抗利尿作用不及去氨加压素，仅可用于部分性尿崩症，对于完全中枢性尿崩症效果不佳，故不选。
选项 C 氢氯噻嗪主要抑制远端小管前段和近端小管对氯化钠的重吸收，从而增加远端小管和集合管的 Na^{+}-K^{+}交换，K^{+}分泌增多而发挥利尿作用，与治疗尿崩症的原理相反，与题意不符。
选项 D 去氨加压素为人工合成的抗利尿激素类似物，抗利尿作用强，而无加压作用，不良反应少，为目前治疗尿崩症的首选药物，符合题意。
选项 E 卡马西平是广谱抗癫痫药，主要治疗癫痫、三叉神经痛等疾病，对于尿崩症的治疗没有明显作用，可排除。
综上所述，正确答案是：D.去氨加压素。
应首选的药物是
D. 去氨加压素 (Desmopressin)
题目材料　　男，22 岁，全身进行性水肿 10 天，既往体健。查体：BP120/80mmHg，双下肢及颜面部凹陷性水肿。实验室检查：血浆白蛋白 20g/L；尿蛋白：　尿沉渣检测：RBC：8～10/HP；血 Cr：73μmol/L；血胆固醇：8.2mmol/L。
133、　(A3/A4 型题)　题
该患者可能考虑的诊断是
A.急性肾小球肾炎
B.肾病综合征
C.急性间质性肾炎
D.慢性肾小球肾炎
E.急进性肾小球肾炎
参考答案：　B　您的答案：
图文解析：　【解析】青少年男性患者，有大量蛋白尿，尿蛋白大于 3.5g/d、水肿，ALB20g/L（白蛋白正常 40~55g/L） 降低，高脂血症，结合患者病史、临床表现及检查，该患者目前最可能的诊断多为肾病综合征（B 对）。
【避错】 肾病综合征的诊断标准是：①大量蛋白尿（＞3.5g/d） ；②低白蛋白血症（血清白蛋白小于 30g/L） ；③水肿；④高脂血症。其中前两项为诊断的必备条件。
对于第 133 题，关于患者的可能诊断：
B. 肾病综合征 是最符合描述的诊断，因为患者有大量蛋白尿、低白蛋白血症、水肿和高脂血症。
案例分析
患者男性，22 岁，主要症状是全身进行性水肿 10 天，伴有双下肢及颜面部凹陷性水肿。实

验室检查显示：

血浆白蛋白：20 g/L（正常值：35-55 g/L）
尿蛋白：显著阳性
尿沉渣：RBC 8-10/HP（正常值：0-2/HP）
血清肌酐：73 μmol/L（正常值：44-133 μmol/L）
血胆固醇：8.2 mmol/L（正常值：<5.2 mmol/L）
从以上检查结果可以看出，患者存在低蛋白血症、重度蛋白尿、高胆固醇血症以及浮肿，符合肾病综合征的典型表现。

问题解答
133、该患者可能考虑的诊断是
A. 急性肾小球肾炎
B. 肾病综合征
C. 急性间质性肾炎
D. 慢性肾小球肾炎
E. 急进性肾小球肾炎

答案：B. 肾病综合征
134、 (A3/A4 型题) 题
不应选用的治疗是
A.限盐
B.注射大量白蛋白
C.利尿
D.活检后治疗
E.补液
参考答案： B 您的答案：
图文解析： 【解析】青少年男性患者，有大量蛋白尿，尿蛋白大于 3.5g/d、水肿，ALB20g/L（白蛋白正常 40~55g/L） 降低，高脂血症，结合患者病史、临床表现及检查，该患者目前最可能的诊断多为肾病综合征。肾病综合征患者应给予正常量的优质蛋白饮食（B 错，为本题正确答案） ，静脉补充大量蛋白会增加肾小球高滤过，加重蛋白尿并加重病情，故不主张应用。
【避错】 肾病综合征的诊断标准是：①大量蛋白尿（＞3.5g/d） ；②低白蛋白血症（血清白蛋白小于 30g/L） ；③水肿；④高脂血症。其中前两项为诊断的必备条件。
对于第 134 题，关于不应选用的治疗：
E.补液 通常不推荐用于管理肾病综合征的患者，因为这些患者已经存在水肿，补液可能加重这种情况。
首先，我们分析患者提供的病史和实验室检查结果。
青年男性，全身进行性水肿 10 天，查体发现双下肢及颜面部凹陷性水肿。实验室检查显示：血浆白蛋白明显降低（20g/L），大量蛋白尿（＞3.5g/d），血尿（RBC：8～10/HP），胆固醇明显升高（8.2mmol/L）。根据这些信息，我们可以初步判断患者可能的诊断。
该患者可能考虑的诊断是：
A. 急性肾小球肾炎：虽然可能有血尿和蛋白尿，但血浆白蛋白和胆固醇的水平通常不会如此异常。

B. 肾病综合征：符合患者的所有主要症状，包括大量蛋白尿、低白蛋白血症、高脂血症和水肿。
C. 急性间质性肾炎：主要表现为肾小管功能损害，与患者的症状不符。
D. 慢性肾小球肾炎：通常病程较长，且不一定伴有如此明显的低白蛋白血症和高脂血症。
E. 急进性肾小球肾炎：通常表现为肾功能迅速恶化，与患者的病程和实验室检查不符。
因此，考虑到患者的所有症状，最可能的诊断是肾病综合征。
不应选用的治疗是：
A. 限盐：对于水肿患者，限盐是常规治疗措施，有助于减少水肿。
B. 注射大量白蛋白：在肾病综合征中，由于大量蛋白尿导致的低白蛋白血症，单纯注射白蛋白并不能有效解决根本问题，且可能带来一系列并发症，如过敏反应、容量过载等。
C. 利尿：对于水肿患者，利尿是常规治疗措施，有助于减少水肿。
D. 活检后治疗：为了明确诊断和制定更精确的治疗方案，肾活检是必要的。
E. 补液：在某些情况下，为了维持血流动力学稳定，适当的补液是必要的。
因此，注射大量白蛋白是不应选用的治疗措施。
综上所述，正确答案是：
133. B. 肾病综合征
134. B. 注射大量白蛋白
解题思路
肾病综合征的典型特征包括：大量蛋白尿（通常每天超过 3.5 克）、低白蛋白血症（通常低于 30 g/L）、全身水肿以及高胆固醇血症。这些特征与患者的实验室检查结果完全一致。因此，最可能的诊断是肾病综合征。

134、不应选用的治疗是
A. 限盐
B. 注射大量白蛋白
C. 利尿
D. 活检后治疗
E. 补液

答案：B. 注射大量白蛋白
135、 (B1 型题)
适合 Allis 法治疗的是
A.髋关节后脱位
B.膝关节后脱位
C.肘关节后脱位
D.桡骨小头半脱位
E.肩关节前脱位
参考答案： A 您的答案：
图文解析： 【解析】适合 Allis 法治疗的是髋关节后脱位；肩关节前脱位一般采用局部浸润麻醉，用 Hippocrates 法复位。
【避错】
第 135 题：适合 Allis 法治疗的是
Allis 法通常用于婴儿的髋关节脱位治疗，但在题目的选项中，最接近的是肘关节后脱位。然而，Allis 试验通常不是用来治疗脱位，而是检查婴儿是否有髋关节发育不良。因此，如果是

治疗相关的话，我们可能需要考虑这是一个陷阱问题。通常，Allis 法不用于成人的关节脱位治疗。但如果我们必须从给定的选项中选择，肘关节后脱位可能是最接近的，尽管这并不是一个标准应用。
这是一道关于关节脱位治疗方法的选择题。我们需要根据关节脱位的类型和位置，来判断哪种治疗方法最为合适。
首先，我们来分析第 135 题，即适合 Allis 法治疗的是哪种关节脱位。
A.髋关节后脱位：Allis 法是治疗髋关节后脱位的一种常用手法，该方法通过特定的手法操作来复位脱位的髋关节。
B.膝关节后脱位：膝关节后脱位通常不使用 Allis 法治疗。
C.肘关节后脱位：肘关节后脱位一般通过其他方法复位，而非 Allis 法。
D.桡骨小头半脱位：这种脱位一般通过简单的手法复位即可，不需要使用 Allis 法。
E.肩关节前脱位：肩关节前脱位常采用其他复位方法，如 Hippocrates 手法。
综合以上分析，我们可以确定 Allis 法最适合用于治疗髋关节后脱位，因此第 135 题的答案是 A.髋关节后脱位。
适合 Allis 法治疗的是
A. 髋关节后脱位
解析：
Allis 法主要用于治疗髋关节后脱位，通过病人仰卧位，医生站在病人侧方，将髋关节屈曲成 90 度，双手握住病人的踝部或小腿，进行牵引和旋转来复位。
136、 (B1 型题)
适合用 Hippocrates 手法复位的是
A.髋关节后脱位
B.膝关节后脱位
C.肘关节后脱位
D.桡骨小头半脱位
E.肩关节前脱位
参考答案： E 您的答案：
图文解析： 【解析】适合 Allis 法治疗的是髋关节后脱位；肩关节前脱位一般采用局部浸润麻醉，用 Hippocrates 法复位。
【避错】肘关节后脱位可以采用单人复位法。2%普鲁卡因或 1%利多卡因 10ml 肘关节内麻醉或臂丛麻醉。术者站在患者的前面，提起患者的患肢，环抱术者的腰部，使肘关节置于半屈曲位。以一手握住患者腕部，沿前臂纵轴作持续牵引，另一拇指压住尺骨鹰嘴突，亦沿前臂纵轴方向作持续推挤动作直至复位。桡骨小头半脱位不用麻醉即可进行手法复位。术者一手握住小儿腕部，另一手托住肘部，以拇指压在桡骨头部位，肘关节屈曲至 90°,作轻柔的前臂旋后、旋前活动，反复数次，并用拇指轻轻推压桡骨头即可复位。
第 136 题：适合用 Hippocrates 手法复位的是
Hippocrates 手法主要用于肩关节脱位的复位。这种方法通过对肩关节施加牵引和旋转来复位。因此，对于这个问题，肩关节前脱位是最合适的选项。
接下来，我们分析第 136 题，即适合用 Hippocrates 手法复位的是哪种关节脱位。
A.髋关节后脱位：髋关节后脱位主要采用 Allis 法，而非 Hippocrates 手法。
B.膝关节后脱位：膝关节后脱位不适用 Hippocrates 手法。
C.肘关节后脱位：肘关节后脱位一般通过其他方法复位。
D.桡骨小头半脱位：这种脱位一般通过简单的手法复位，不使用 Hippocrates 手法。
E.肩关节前脱位：Hippocrates 手法是治疗肩关节前脱位的常用方法，通过特定的操作来复位

脱位的肩关节。
综上所述，Hippocrates 手法最适合用于治疗肩关节前脱位，因此第 136 题的答案是 E.肩关节前脱位。
适合用 Hippocrates 手法复位的是
E. 肩关节前脱位
解析：
Hippocrates 手法主要用于肩关节前脱位的复位。病人平躺，医生一手握住病人的手腕，一脚抵在病人的腋窝，通过拉伸手臂与抵压腋窝来复位肩关节。
137、 (B1 型题)
IgA 肾病确诊选择的检查是
A.肾活检
B.肾动脉造影
C.肾 B 超
D.肾 CT
E.肾盂造影
参考答案： A 您的答案：
图文解析： 【解析】IgA 肾病确诊选择肾活检。鉴别急慢性肾衰竭的检查方法首选肾 B 超。
【避错】
137、IgA 肾病确诊选择的检查是
IgA 肾病（IgA 肾病）的确诊通常依赖于肾脏活组织的检查，因为这可以直接观察肾小球中 IgA 沉积。因此，正确答案是：A. 肾活检
首先，对于第 137 题，我们需要确定哪种检查方法最适用于 IgA 肾病的诊断。
选项 A：肾活检是诊断肾脏疾病的金标准，尤其是对于肾小球疾病的诊断和鉴别诊断至关重要。IgA 肾病的确诊依赖于肾活检的组织学检查，因为只有通过病理学检查才能确定肾小球系膜区是否有 IgA 沉积，从而确诊 IgA 肾病。
选项 B：肾动脉造影主要用于检查肾动脉狭窄或阻塞性疾病，与 IgA 肾病的诊断关系不大。
选项 C：肾 B 超主要用于检查肾脏的形态和结构，但不能确诊 IgA 肾病。
选项 D：肾 CT 可以提供肾脏的解剖结构信息，但对 IgA 肾病的诊断价值有限。
选项 E：肾盂造影主要用于检查尿路的形态和功能，对 IgA 肾病的诊断没有直接帮助。
综上所述，对于 IgA 肾病的确诊，最关键的检查方法是肾活检，因此第 137 题的答案是 A.肾活检。
根据医学知识和最佳实践指南，以下是这两道考试题目的正确答案：

IgA 肾病确诊选择的检查是：
A. 肾活检
解释：IgA 肾病的确诊通常需要通过肾活检（肾脏活组织检查）来观察肾小球内沉积的 IgA 和其他免疫复合物。
138、 (B1 型题)
鉴别急慢性肾衰竭的检查方法
A.肾活检
B.肾动脉造影
C.肾 B 超
D.肾 CT

E.肾盂造影

参考答案： C 您的答案：

图文解析： 【解析】IgA 肾病确诊选择肾活检。鉴别急慢性肾衰竭的检查方法首选肾 B 超。

【避错】肾动脉造影能准确显示肾动脉狭窄部位、范围、程度及侧支循环形成情况，是诊断肾动脉狭窄的“金标准”。静脉尿路造影（IVU）可以了解分侧肾功能、病变程度与范围。

138、鉴别急慢性肾衰竭的检查方法

急慢性肾衰竭的鉴别通常不单靠一项检查完成，但肾活检可以提供直接的肾脏组织状态，帮助区分是急性还是慢性损伤。虽然其他影像学检查如肾 B 超、肾 CT 也能提供有用信息，但肾活检在确定肾衰竭的性质方面更为准确。因此，最相关的答案是：A. 肾活检

接下来，我们分析第 138 题，关于鉴别急性和慢性肾衰竭的检查方法。

选项 A：肾活检可以明确肾脏的病理改变，对于鉴别急性和慢性肾衰竭有重要意义。急性肾衰竭的肾脏病理改变通常较轻且可逆，而慢性肾衰竭则有明显的肾小球硬化和肾小管间质纤维化等不可逆改变。

选项 B：肾动脉造影、选项 C：肾 B 超、选项 D：肾 CT、选项 E：肾盂造影，这些检查方法主要用于了解肾脏的形态和结构，对鉴别急性和慢性肾衰竭的帮助有限。

因此，第 138 题的答案是 A.肾活检，因为它是鉴别急性和慢性肾衰竭最直接和有效的方法。

鉴别急慢性肾衰竭的检查方法是：

A. 肾活检

解释：鉴别急性肾衰竭和慢性肾衰竭需要通过肾活检来评估肾脏组织的病理变化，从而确定肾损伤的性质和程度。

139、 (B1 型题)

肠切除术后肠瘘长期禁食的患者可能出现的凝血因子异常是

A.凝血因子Ⅷ和Ⅸ

B.凝血因子Ⅱ和Ⅹ

C.凝血因子Ⅲ和Ⅹ

D.凝血因子Ⅲ和Ⅴ

E.凝血因子Ⅳ和Ⅶ

参考答案： B 您的答案：

图文解析： 【解析】肠切除术后肠瘘长期禁食患者，小肠对脂溶性维生素 ADEK 吸收减少，凝血因子Ⅱ和Ⅹ以及Ⅶ。

139、肠切除术后长期禁食的患者可能会出现维生素 K 缺乏，因为维生素 K 主要通过肠道细菌合成，并且在脂肪中吸收。维生素 K 是合成几种凝血因子（包括凝血因子Ⅱ、Ⅶ、Ⅸ和Ⅹ）所必需的。因此，在这种情况下，凝血因子异常可能涉及Ⅱ和Ⅹ。所以，最合适的选项是 B. 凝血因子Ⅱ和Ⅹ。

问题 139

肠切除术后肠瘘长期禁食的患者可能出现的凝血因子异常是：

正确答案：B. 凝血因子Ⅱ和Ⅹ

解析：
长期禁食会导致维生素 K 缺乏，而维生素 K 是合成凝血因子Ⅱ（凝血酶原）、Ⅶ、Ⅸ和Ⅹ的重要成分。肠切除术后肠瘘患者如果长期禁食，容易导致维生素 K 缺乏，从而影响这些依赖维生素 K 的凝血因子的合成，其中尤以凝血因子Ⅱ和Ⅹ为主要代表。
【避错】
140、 (B1 型题)
血友病患者缺乏的凝血因子是
A.凝血因子Ⅷ和Ⅸ
B.凝血因子Ⅱ和Ⅹ
C.凝血因子Ⅲ和Ⅹ
D.凝血因子Ⅲ和Ⅴ
E.凝血因子Ⅳ和Ⅶ
参考答案： A 您的答案：
图文解析： 【解析】血友病是一组遗传性凝血因子生成障碍引起的出血性疾病，该病的发生是缺少凝血因子Ⅷ和Ⅸ。
【避错】凝血因子Ⅲ为组织因子，凝血因子 V，凝血因子 IV 均为辅因子。
140、血友病是一种遗传性出血性疾病，通常涉及凝血因子的缺乏。血友病 A 是由于凝血因子Ⅷ缺乏引起的，而血友病 B 是由于凝血因子Ⅸ缺乏引起的。因此，对于血友病患者，缺乏的凝血因子是Ⅷ和Ⅸ。所以，对于这个问题，最合适的选项是 A.凝血因子Ⅷ和Ⅸ。
这是一道关于凝血因子的选择题，旨在测试考生对凝血因子及其相关疾病的理解。
首先，我们需要理解各个凝血因子的特点及其在凝血过程中的作用。
139 题分析：
肠切除术后肠瘘长期禁食的患者，由于营养不良，主要影响的是维生素 K 依赖的凝血因子。
维生素 K 依赖的凝血因子包括Ⅱ（凝血酶原）、Ⅶ、Ⅸ和Ⅹ。
在这些因子中，长期禁食主要影响的是凝血因子Ⅱ（凝血酶原）、Ⅶ、Ⅸ和Ⅹ的合成。
但选项中只提到了凝血因子Ⅱ和Ⅹ，因此选择 B.凝血因子Ⅱ和Ⅹ作为最符合题意的答案。
140 题分析：
血友病是一种遗传性凝血障碍性疾病，主要分为血友病 A 和血友病 B。
血友病 A 是凝血因子Ⅷ缺乏所导致的，而血友病 B 则是凝血因子Ⅸ缺乏。
根据题意，血友病患者缺乏的凝血因子应是Ⅷ和Ⅸ，因此选择 A.凝血因子Ⅷ和Ⅸ作为正确答案。
综上所述，139 题的答案是 B.凝血因子Ⅱ和Ⅹ，140 题的答案是 A.凝血因子Ⅷ和Ⅸ。
因此，正确答案是：
B.凝血因子Ⅱ和Ⅹ
A.凝血因子Ⅷ和Ⅸ
问题 140
血友病患者缺乏的凝血因子是：

正确答案：A. 凝血因子Ⅷ和Ⅸ

解析：
血友病是一种遗传性凝血障碍疾病，主要分为血友病 A 和血友病 B。血友病 A 患者缺乏凝血因子Ⅷ，而血友病 B 患者缺乏凝血因子Ⅸ。这两种因子的缺乏都会导致凝血过程受阻，从而引发出血倾向。因此，血友病患者缺乏的凝血因子是凝血因子Ⅷ和Ⅸ。

141、 (B1 型题)
男，35 岁，发热头痛 5 天，于 1 月 5 日就诊，查体：T38.6℃,血压 85/45mmHg，结膜充血，眼睑水肿，腋下可见出血点，WBC17×109/L，PLT55×109/L，尿蛋白（+++），最 源是
A.犬
B.人
C.羊
D.啮齿动物
E.猪
参考答案： D 您的答案：
图文解析： 【解析】青年男性，发热头痛、血压 85/45mmHg（低血压休克期），结膜充血，眼睑水肿，腋下可见出血点（毛细血管损伤症），WBC17×109/L（白细胞升高），PLT55×109/L（血小板降低），尿蛋白（+++），根据患者的临床症状、体征和实验室检查，诊断为肾综合征出血热，传染源是鼠，啮齿动物（D 对）。
【避错】猪（E 错）是流行性乙型脑炎的传染源。
对于第一个问题（141）：
35 岁男性患者出现发热头痛、血压下降、结膜充血、眼睑水肿、皮下出血点、白细胞升高（WBC 17×10^9/L）、血小板降低（PLT 55×10^9/L）和尿蛋白（+++）。
这些症状和体征提示可能是由一种传播自动物的疾病引起的，考虑到病情的急性起病和临床表现，可以考虑汉坦病毒感染，该病毒常通过啮齿动物传播。
因此，第 141 题的答案是 D.啮齿动物。
这是一道传染病学问题，我们需要根据患者的临床表现、实验室检查和流行病学特征来确定可能的传染源。
对于第一题（141 题）：
患者中年男性，发热、结膜充血、眼睑水肿、出血点等，伴有低血压、血小板减少和蛋白尿，这些症状强烈提示流行性出血热。流行性出血热的主要传染源是啮齿动物，如鼠类。啮齿动物携带的病毒可通过其排泄物、分泌物等传播给人类。
题目 141：
男，35 岁，发热头痛 5 天，于 1 月 5 日就诊，查体：T38.6℃,血压 85/45mmHg，结膜充血，眼睑水肿，腋下可见出血点，WBC17×10^9/L，PLT55×10^9/L，尿蛋白（+++）。

这个病例的特征包括发热、低血压、出血点、血小板减少和尿蛋白，这提示有出血热的可能性。在中国，导致出血热的主要病原体是汉坦病毒，而汉坦病毒的主要宿主是啮齿动物。因此，最可能的传染源是 D.啮齿动物。
142、 (B1 型题)
学生，16 岁，发热头痛 4 天，呕吐 2 天后加重伴意识不清 1 天，于 8 月 10 日就诊。体温 39.8℃,血压 140/90mmHg，浅昏迷，颈反抗（+），克氏征及布氏征（+）。白细胞 17.5 N0.75×109/L，脑脊液检查：CSF 透明，压力 200cmH2O，有核细胞 400×106/L，多核细胞 1.5×106/L，蛋白 0.8g/L，糖 3.8mmol/L，氯化物 120mmol/L。最可能的传染源是
A.犬
B.人
C.羊
D.啮齿动物
E.猪
参考答案： E 您的答案：

图文解析：　　【解析】青年患者，高热，头痛，呕吐，伴意识不清（流行性乙型脑炎初期症状），于 8 月 10 日就诊（流行性乙型脑炎夏秋季高发）。浅昏迷，颈反抗（+），克氏征及布氏征（+），血 WBC17.5×109/L（白细胞升高），脑脊液检查压力升高，WBC400×109/L（正常值 0～15×109/L），蛋白 0.8g/L（腰穿正常值 0.2～0.45g/L），糖 3.8mmol/L（腰池正常值 2.5～4.5mmol/L），氯化物 120mmol/L（腰池正常值 120～130mmol/L），根据患者临床表现及实验室检查，该患者最可能的诊断为流行性乙型脑炎，传染源主要是猪（E 对）。
【避错】啮齿动物是肾综合征出血热和鼠疫的传染源。
对于第二个问题（142）：
16 岁学生出现发热头痛、呕吐、意识不清、颈部僵硬、克氏征和布氏征阳性、白细胞增高、脑脊液检查显示细胞计数增加、蛋白质轻微增高。
这些症状和体征表明患者很可能患有细菌性脑膜炎。细菌性脑膜炎的传染源通常是人，通过飞沫传播。
因此，第 142 题的答案是 B.人。
对于第二题（142 题）：
青少年患者，发热、头痛、呕吐、意识障碍，伴有脑膜刺激征阳性，脑脊液检查显示白细胞增多、蛋白轻度升高，这些症状提示乙型脑炎。乙型脑炎主要通过蚊虫叮咬传播，而蚊虫的乙脑病毒主要来源于猪。猪是乙脑病毒的主要储存宿主，因此猪是乙型脑炎的主要传染源。
综上所述，141 题的答案是 D.啮齿动物，142 题的答案是 E.猪。
题目 142：
学生，16 岁，发热头痛 4 天，呕吐 2 天后加重伴意识不清 1 天，于 8 月 10 日就诊。体温 39.8℃,血压 140/90mmHg，浅昏迷，颈反抗（+），克氏征及布氏征（+）。白细胞 17.5 N0.75 ×10^9/L，脑脊液检查：CSF 透明，压力 200cmH2O，有核细胞 400×10^6/L，多核细胞 1.5 ×10^6/L，蛋白 0.8g/L，糖 3.8mmol/L，氯化物 120mmol/L。

这个病例表现出急性脑膜炎的症状，常见的病原体包括细菌和病毒。结合流行病学资料，学生在 8 月发病，提示可能为病毒性脑膜炎，常见传染源为人。因此，最可能的传染源是 B.人。
143、　(B1 型题)
呋塞米的作用是
A.降低心室前负荷
B.降低心室后负荷
C.降低心室前后负荷
D.降低心肌收缩力
E.增加心排血量
参考答案：　A　您的答案：
图文解析：　　【解析】呋塞米是高效能的袢利尿剂，促进 Na+、H2O 的排泄，减少血容量，降低心脏前负荷。
对于第 143 题，呋塞米是一种利尿剂，主要用于治疗水肿和高血压。它通过增加肾脏排钠和水的作用来起效，因此会减少血容量，从而降低心室前负荷和后负荷。因此，最正确的答案是 C.降低心室前后负荷。
这是一道关于药物作用机制的选择题。我们需要准确理解并分析呋塞米和硝普钠的药理作用，然后选出最符合各自药物特性的选项。
首先，我们分析呋塞米的作用。
呋塞米，也被称为速尿，是一种高效能的袢利尿剂。其主要作用是抑制肾小管对钠和氯的重

吸收，从而促进尿液生成和钠、氯、钾等离子的排泄。这种利尿作用可以降低血容量，进而降低心室的前负荷。因此，针对“呋塞米的作用是”这一问题，最符合的选项是 A.降低心室前负荷。

143. 呋塞米的作用是

呋塞米是一种袢利尿剂，它通过抑制肾脏髓袢升支粗段的 Na^{+}-K^{+}-$2Cl^{-}$同向转运体，增加钠、钾、氯及水的排出。它主要用于治疗急性肺水肿、慢性心力衰竭、高血压和各种类型的水肿。因此，它可以减轻心脏的前负荷。

答案：A. 降低心室前负荷

144、 (B1 型题)

硝普钠的作用是

A.降低心室前负荷

B.降低心室后负荷

C.降低心室前后负荷

D.降低心肌收缩力

E.增加心排血量

参考答案： C 您的答案：

图文解析： 【解析】呋塞米是高效能的袢利尿剂，促进 Na+、H2O 的排泄，减少血容量，降低心脏前负荷。硝普钠可降低左心室前后负荷。

【避错】硝普钠是均衡性扩张血管，扩张小动脉，使外周血管阻力即心脏后负荷降低而增加心排血量；扩张小静脉，使回心血量减少，降低左室舒张末压而减轻心脏前负荷，即同时降低左心室前后负荷。

对于第 144 题，硝普钠是一种血管扩张剂，它通过直接作用于血管平滑肌来引起血管扩张，主要降低心室前负荷，但也可能对后负荷有所降低。因此，硝普钠最主要的作用是降低心室前负荷，但鉴于它也可以对心室后负荷产生一定的影响，最合适的答案可能是 C.降低心室前后负荷。然而，通常硝普钠更为人所知的作用是降低心室前负荷，因此如果需要选择一个最主要的作用，答案应该是 A.降低心室前负荷。

硝普钠是一种均衡性扩张血管的药物，能同时直接扩张静脉和动脉，因此它可以同时降低心室的前负荷和后负荷。硝普钠不增加心肌的收缩力，而是通过降低心脏的前后负荷来减轻心脏的工作负担，改善心功能。所以，针对“硝普钠的作用是”这一问题，最符合的选项是 C.降低心室前后负荷。

144. 硝普钠的作用是

硝普钠是一种强效血管扩张剂，通过一氧化氮（NO）释放，直接松弛血管平滑肌，导致动脉和静脉的扩张，因而能同时降低心脏的前负荷和后负荷。它主要用于治疗急性心力衰竭和高血压危象。

答案：C. 降低心室前后负荷

145、 (B1 型题)

女，76 岁，扩张型心肌病，持续房颤，慢性心力衰竭，为增加营养一周前开始每天加饮用牛奶 500ml，3 天前出现下肢水肿及气短，除了限制入量，能够短期控制症状的是

A.呋塞米

B.阿司匹林

C.硝酸甘油

D.普萘洛尔

E.地高辛
参考答案：　　A　您的答案：
图文解析：　　【解析】老年女性，扩张性心肌病，持续房颤，慢性心力衰竭，出现下肢水肿及气短（考虑饮水过多所致），短期内能够控制症状的药物是呋塞米（A 对）。
避错：
【避错】
第 145 题
这个病例描述的是一位老年女性患者，患有扩张型心肌病、持续性房颤和慢性心力衰竭。近期因增加牛奶摄入量后出现下肢水肿和气短。这些症状表明她的心力衰竭状况可能恶化，需要迅速干预以控制症状。在给出的药物选项中：
A. 呋塞米（一种利尿剂），可以迅速帮助减轻体液过载，缓解心力衰竭症状，对于心力衰竭导致的水肿和气短是有效的。
B. 阿司匹林，主要用于抗血小板，此场景不适用。
C. 硝酸甘油，用于缓解心绞痛，通过扩张血管降低心脏前负荷，虽然有助于心力衰竭的管理，但对急性症状控制不如利尿剂直接。
D. 普萘洛尔，β受体阻滞剂，用于控制心率，治疗心衰时需谨慎使用，尤其在血压偏低的情况下。
E. 地高辛，用于增强心脏收缩力和控制心室率，但对于急性水肿和气短的控制不如利尿剂直接。
因此，对于第 145 题，最合适的答案是 A. 呋塞米。
145 题解析：
老年女性患者，有扩张型心肌病、持续房颤和慢性心力衰竭。患者一周前开始每天加饮用牛奶 500ml，3 天前出现下肢水肿及气短，考虑心衰症状恶化，应短期给予利尿剂对症治疗。选项中呋塞米是袢利尿剂，能有效减轻体液潴留，从而短期内迅速缓解心衰症状。
选项 B 阿司匹林是抗血小板聚集药物，对于心衰的治疗并无效果，故排除。
选项 C 硝酸甘油主要用于心绞痛的治疗，也可用于高血压危象和心力衰竭，但其对心衰的治疗作用较弱，起效较慢，不作为心衰的首选治疗方案，故不选。
选项 D 普萘洛尔是β受体阻滞剂，能降低心率，减少心肌耗氧量，改善心肌缺血，减少心绞痛发作，长期应用可显著降低死亡等心血管事件，但在急性心衰时不建议使用，可排除。
选项 E 地高辛是洋地黄类药物，最适合用于有快速心室率的心房颤动并心室扩大伴左心室收缩功能不全者，而题干中患者为房颤，心室率情况未知，因此地高辛并非最佳选项。
综上所述，正确答案是：A.呋塞米。
根据题目的描述和病人的情况，以下是每个问题的最佳答案：

题目描述：76 岁女性，扩张型心肌病，持续房颤，慢性心力衰竭。增加牛奶摄入后出现下肢水肿及气短，除了限制入量，能够短期控制症状的是？
最佳答案：A. 呋塞米
解释：该患者有心力衰竭的症状（如下肢水肿和气短），呋塞米是一种强效的利尿剂，可以迅速减轻液体潴留症状。
146、　(B1 型题)
女，76 岁，扩张型心肌病，持续房颤，给予β受体阻滞剂后血压偏低但心室率仍偏快，应该加用控制心室率的药物是
A.呋塞米
B.阿司匹林

C.硝酸甘油
D.普萘洛尔
E.地高辛
参考答案：　　E　您的答案：
图文解析：　　【解析】给予β受体阻滞剂后血压偏低但心室率仍偏快，应该加用的药物是地高辛（E 对），洋地黄类药物主要用于β受体阻滞剂治疗后仍有症状，尤其用于减慢房颤患者的心室率。
【避错】
第 146 题
描述的是相同的患者，在使用β受体阻滞剂（如普萘洛尔）后，血压偏低但心室率仍然偏快。需要选择一个可以控制心室率但对血压影响较小的药物。在给出的选项中：
A. 呋塞米，利尿剂，在这里不适用。
B. 阿司匹林，抗血小板，不影响心率。
C. 硝酸甘油，主要用于心绞痛，对心率控制作用有限。
D. 普萘洛尔，已经使用且导致血压降低，不适合继续加大剂量。
E. 地高辛，可以控制心室率而对血压影响较小，特别适用于房颤患者的心室率控制。
因此，对于第 146 题，最合适的答案是 E. 地高辛。
146 题解析：
患者老年女性，有扩张型心肌病和持续房颤，在应用β受体阻滞剂后血压偏低，但心室率仍然偏快，应选用对血压影响较小的药物来控制心室率。选项中地高辛是洋地黄类药物，可增强心肌收缩力，用于房颤的治疗可减慢房颤患者的心室率，尤其适合心室率快速的房颤患者，且对血压影响较小，符合题意。
选项 A 呋塞米是利尿剂，主要用于心衰的对症治疗，对心室率的控制没有效果，故不选。
选项 B 阿司匹林是抗血小板聚集药物，对心室率的控制没有帮助，可排除。
选项 C 硝酸甘油主要用于心绞痛的治疗，也可用于高血压危象和心力衰竭，但对心室率的控制作用较弱，不作为首选，因此排除。
选项 D 普萘洛尔是β受体阻滞剂，虽然能降低心率，但由于题干中提到患者在应用β受体阻滞剂后血压偏低，故不宜继续使用普萘洛尔，所以不选。
综上所述，正确答案是：E.地高辛。
题目描述：76 岁女性，扩张型心肌病，持续房颤，给予β受体阻滞剂后血压偏低但心室率仍偏快，应该加用控制心室率的药物是？
最佳答案：E. 地高辛
解释：地高辛是一种强心药和抗心律失常药，可以有效控制房颤患者的心室率，尤其适用于那些血压已经偏低的患者，因为它对血压的影响较小。
147、　(B1 型题)
男，37 岁，血压升高 5 年余，平素血压 160~180/100~120mmHg，未诊治。体型肥胖，有打鼾史。半年前开始在医生的指导下进行低脂饮食控制和药物治疗，厄贝沙坦片每次 两次，托拉塞米 5mg，经过三个月的治疗，血压控制在 150~160/90~100mmHg，该患者为
A.高血压急症
B.继发性高血压
C.顽固性高血压
D.高血压危象
E.恶性高血压
参考答案：　　C　您的答案：

图文解析：　　　【解析】中青年男性，平素血压 160～180/100～120mmHg，未诊治，后服用硝苯地平控释片（钙通道阻滞剂），厄贝沙坦片（血管紧张素 II 受体拮抗剂），托拉塞米（利尿剂），血压控制在 150～160/90～100mmHg（无并发症者，血压目标值 140/90mmHg），根据患者病史，用药方案和血压控制情况，考虑为顽固性高血压（C 对）。
【避错】高血压急症（A 错）最突出的表现为短时间内舒张压＞130mmHg，合并肾损害。继发性高血压（B 错）最常见的是由肾脏疾病引起的高血压，先有肾病，再有高血压。高血压危象（D 错）包括高血压急症及亚急症。病情急剧恶化，同时伴有进行性心、脑、肾、视网膜等重要的靶器官功能不全的表现。恶性高血压（E 错）也称急进性高血压，起病较急骤，典型表现为血压显著升高，舒张压多持续在 130～140mmHg 或更高，并有头痛，视力模糊和视盘水肿等症状，且多在中青年发病。
对于第 147 题，这位 37 岁男性患者有长期未治疗的高血压病史，并伴有肥胖和打鼾症状，可能暗示着睡眠呼吸暂停。他在开始低脂饮食和药物治疗后，血压有所下降，但仍然较高。这表明他的高血压并不容易控制，但没有急性危及生命的症状。因此，最合适的选择是 C. 顽固性高血压，它指的是尽管进行了适当治疗，血压仍难以达到目标水平的情况。
147 题正确答案是：C.顽固性高血压。
患者中年男性，血压升高多年，体型肥胖，有打鼾史，应高度怀疑存在睡眠呼吸暂停低通气综合征，此为顽固性高血压的常见原因。经联合应用足量 3 种不同作用机制的降压药物（包括利尿剂）后，血压仍在目标水平之上，或至少需要 4 种药物才能使血压达标时，称为顽固性高血压（或难治性高血压）。本题中患者体型肥胖，有打鼾史，经低脂饮食控制、厄贝沙坦和托拉塞米治疗后，血压仍高于正常，应考虑为顽固性高血压。
选项 A 高血压急症是指原发性或继发性高血压患者，在某些诱因作用下，血压突然和显著升高（一般超过 180/120mmHg），同时伴有进行性心、脑、肾等重要靶器官功能不全表现，本题患者未出现靶器官功能不全表现，故不选。
选项 B 继发性高血压是指由特定病因引起的高血压，如肾实质性高血压、肾动脉狭窄、原发性醛固酮增多症等，本题中患者无继发性高血压的病因，故不考虑。
选项 D 高血压危象是高血压急症的一种，指血压短时间内急剧升高，同时出现头痛、眩晕、烦躁、恶心、呕吐、心悸、气急及视力模糊等症状，以及伴有动脉痉挛累及靶器官缺血症状，本题患者无上述症状，故排除。
选项 E 恶性高血压是指患者病情急骤发展，舒张压持续≥130mmHg，并有头痛、视力模糊、眼底出血、渗出和乳头水肿，肾脏损害突出，持续蛋白尿、血尿与管型尿，本题患者并未见上述症状，故不选。
第一道题：
患者，37 岁，血压升高 5 年，平素血压在 160180/100120mmHg，体型肥胖，有打鼾史。经过三个月的治疗，血压控制在 150160/90100mmHg。

关键点：

血压升高 5 年，未诊治。
体型肥胖，有打鼾史（提示可能存在阻塞性睡眠呼吸暂停综合征）。
经药物治疗，血压未能降到正常范围内。
答案分析：

A. 高血压急症：　需要立即降低血压以防止靶器官损伤，不符合。
B. 继发性高血压：　由于有肥胖和打鼾史，可能与阻塞性睡眠呼吸暂停相关，但题干并未提

供确切的证据支持继发性高血压。
C. 顽固性高血压： 经过合理的生活方式和药物治疗，血压未能控制在正常范围内，符合顽固性高血压的定义。
D. 高血压危象： 需要立即降低血压，不符合。
E. 恶性高血压： 需要立即处理的高血压，不符合。
最正确的答案是：C. 顽固性高血压
148、 (B1 型题)
男，56 岁，血压升高 3 年，呼吸困难 1 周，加重 1 天。患者 1 周前无明显诱因出现活动时喘憋，1 天前出现呼吸困难，端坐呼吸，伴大汗，有高血压家族史。查体：P：100 次/BP230/150mmHg，双肺可闻及中小水泡音，心率 100 次/分，律齐，各瓣膜区未闻及杂音，双下肢不肿。心电图提示左室肥厚劳损。该患者目前的情况为
A.高血压急症
B.高血压亚急症
C.顽固高血压
D.高血压危象
E.恶性高血压
参考答案： A 您的答案：
图文解析： 【解析】患者有高血压病史，突然发现血压升高，伴有心功能不全的表现，应考虑诊断为高血压急症（A 对）。高血压急症是指原发性或继发性高血压患者，在某些诱因作用下，血压突然和明显升高（一般血压超过 180/120mmHg）并伴有重要靶器官损害。【避错】高血压亚急症（B 错）指血压明显升高，但不伴有重要靶器官损害。恶性高血压（E 错）指舒张压持续≥130mmHg，并有头痛、视力模糊、眼底渗出，尤以肾脏损害突出。顽固高血压（C 错）指使用了三种以上合适剂量的降压药物联合治疗（一般应该包括利尿剂）血压仍未能达到目标水平。高血压危象（D 错）包括高血压急症和亚急症。
对于第 148 题，这位 56 岁的男性患者出现了严重的高血压（230/150mmHg），伴随呼吸困难、端坐呼吸、大汗等症状，这些都是急症情况的迹象。查体发现双肺有中小水泡音，心电图显示左室肥厚劳损，这些都指示有可能的心脏功能受损和肺部液体积聚。因此，最合适的答案是 A.高血压急症，这种情况需要立即治疗，以防止进一步的器官损害。
148 题正确答案是：A.高血压急症。
患者老年男性，血压升高多年，现出现呼吸困难、喘憋、端坐呼吸、大汗，查体见血压升高，双肺可闻及中小水泡音，心电图提示左室肥厚劳损，根据其症状、体征可考虑诊断为高血压急症。高血压急症是指在原发性或继发性高血压患者，在某些诱因作用下，血压突然和明显升高（一般＞180/120mmHg），同时伴有进行性心、脑、肾等重要靶器官功能不全表现。本题患者已出现心功能不全表现，且血压急剧升高，因此诊断为高血压急症。
选项 B 高血压亚急症是指血压显著升高但不伴靶器官损害，本题患者出现明显的心功能不全表现，与题意不符。
选项 C 顽固性高血压是指患者已应用足量且合理联合的 3 种降压药物（包括利尿剂）后，血压仍在目标水平之上，或至少需要 4 种降压药物才能使血压达标，本题中未提及患者用药情况，故不选。
选项 D 高血压危象是高血压急症的一种，指血压短时间内急剧升高，同时出现头痛、眩晕、烦躁、恶心、呕吐、心悸、气急及视力模糊等症状，以及伴有动脉痉挛累及靶器官缺血症状，本题患者并未见上述症状，故排除。
选项 E 恶性高血压是指患者病情急骤发展，舒张压持续≥130mmHg，并有头痛、视力模糊、眼底出血、渗出和乳头水肿，肾脏损害突出，持续蛋白尿、血尿与管型尿，本题患者并未见

上述症状，与题意不符。
第二道题：
患者，56 岁，血压升高 3 年，呼吸困难 1 周，加重 1 天，P：100 次/ BP230/150mmHg，双肺可闻及中小水泡音，心电图提示左室肥厚劳损。

关键点：

血压非常高：230/150 mmHg。
呼吸困难，端坐呼吸，双肺可闻及中小水泡音（提示急性肺水肿）。
心电图提示左室肥厚劳损。
答案分析：

A. 高血压急症： 高血压急症（高血压危象的一部分），包括高血压导致的急性器官损害，符合症状。
B. 高血压亚急症： 高血压亚急症指的是血压显著升高但没有立即的器官损伤，不符合症状。
C. 顽固高血压： 主要指长期治疗未能控制的高血压，不符合症状。
D. 高血压危象： 高血压急症的一部分，指的是急需降压以防止靶器官损伤的情况，符合症状。
E. 恶性高血压： 也可能适用，但急性肺水肿提示急症。
最正确的答案是：A. 高血压急症
149、 (B1 型题)
患者前列腺特异性抗原阴性，经尿道前列腺切除术后病理活检发现前列腺癌病灶，可能的分期是
A.T1 期
B.T2 期
C.T3a 期
D.T3b 期
E.T4 期
参考答案： A 您的答案：
图文解析： 【解析】患者前列腺特异性抗原阴性，经尿道前列腺切除术后病理活检发现前列腺癌病灶，属于 T1 期。
【避错】前列腺癌 TNM 分期，T1 期：仅手术标本中偶发小病灶；T2 期病灶局限在包膜内；T3a 期病灶突破前列腺包膜；T3b 期病灶侵犯精囊；T4 期远处转移到直肠、膀胱颈等。
对于第 149 题，患者前列腺特异性抗原（PSA）阴性，但是经尿道前列腺切除术（TURP）后的病理活检发现前列腺癌。这种情况下，癌症是在无意中发现的，通常是局限在前列腺内的，因此最可能的分期是 T1 期。T1 期指的是肿瘤无法通过肉眼检查或影像学检查发现，仅在前列腺切片或手术中意外发现的癌症。所以，第 149 题的答案应该是 A.T1 期。
解析：
前列腺癌分期主要分为临床分期和病理分期两种。临床分期是根据 DRE、PSA、穿刺活检阳性针数和部位、经直肠超声、骨扫描、MRI 或 CT 检查结果，将前列腺癌分为 T1、T2、T3、T4 期。而病理分期是前列腺癌根治术病理标本检查后进行分期。如前列腺癌未侵犯一叶，为 T2a 期；如已侵犯两叶，但仍局限于前列腺内，则为 T2b 期；如已穿破前列腺一叶或两叶包膜，为 T3a 期；如已侵及精囊，则为 T3b 期；如已侵及除精囊外的其他邻近组织，且癌细胞已穿透前列腺壁，包括侵及膀胱颈、前列腺周围的脂肪组织、肛提肌和盆壁，则为 T4 期。

患者前列腺特异性抗原阴性，经尿道前列腺切除术后病理活检发现前列腺癌病灶，说明癌肿尚局限于前列腺一叶内，为 T1 期。
选项 B：T2 期指癌组织仍局限于前列腺内，但已穿破前列腺一叶或两叶包膜，故不选。
选项 C：T3a 期指癌已侵及一叶前列腺包膜，并穿破一叶或两叶包膜，但仍局限于前列腺内，故排除。
选项 D：T3b 期指癌组织已侵及精囊，但本题中未提及，故不选。
选项 E：T4 期指癌组织已穿透前列腺壁，侵及到前列腺周围邻近组织，但本题中未提及，故排除。
综上所述，正确答案是：A.T1 期。
第 149 题
患者前列腺特异性抗原（PSA）阴性，经尿道前列腺切除术后病理活检发现前列腺癌病灶，可能的分期是：
A.T1 期
B.T2 期
C.T3a 期
D.T3b 期
E.T4 期

经尿道前列腺切除术（TURP）后发现前列腺癌病灶，而患者没有症状或体检发现前列腺肿块，这种情况通常对应于 T1 期前列腺癌。T1 期前列腺癌是指通过手术切除其他组织或切片检查偶然发现的癌症。

答案：A.T1 期
150、 (B1 型题)
前列腺癌根治术后，病理报告突破两叶包膜，未侵犯精囊，最可能的分期是
A.T1 期
B.T2 期
C.T3a 期
D.T3b 期
E.T4 期
参考答案： C 您的答案：
图文解析： 【解析】前列腺癌根治术后，病理报告突破两叶包膜，未侵犯精囊，最可能的分期是 T3a 期。
【避错】
对于第 150 题，根治性前列腺切除术后的病理报告显示肿瘤突破了两叶包膜，但未侵犯精囊。这种情况下，最可能的分期是 T3a 期。T3a 期指的是肿瘤已经突破前列腺包膜，但还未侵犯到精囊。所以，第 150 题的答案应该是 C.T3a 期。
前列腺癌的病理分期中 T3 期分为 T3a 和 T3b 期，其中 T3a 期指癌已侵及一叶前列腺包膜，并穿破一叶或两叶包膜，但仍局限于前列腺内；T3b 期指癌组织已侵及精囊。前列腺癌根治术后，病理报告突破两叶包膜，未侵犯精囊，说明癌组织已穿破一叶或两叶包膜，但仍局限于前列腺内，为 T3a 期。
选项 A：T1 期指癌组织局限于前列腺一叶内，且 PSA 水平升高甚至正常，与本题无关。
选项 B：T2 期指癌组织局限于前列腺内，但已穿破一叶或两叶包膜，可排除。
选项 D：T3b 期指癌组织已侵及精囊，但本题中未提及，可不考虑。

选项 E：T4 期指癌组织已穿透前列腺壁，侵及到前列腺周围邻近组织，但本题中未提及，可排除。
综上所述，正确答案是：C.T3a 期。
第 150 题
前列腺癌根治术后，病理报告突破两叶包膜，未侵犯精囊，最可能的分期是：
A.T1 期
B.T2 期
C.T3a 期
D.T3b 期
E.T4 期

病理报告显示癌症突破前列腺包膜，但未侵犯精囊，这种情况通常对应于 T3a 期前列腺癌。T3a 期是指癌症扩展到前列腺包膜外但没有侵犯精囊。

答案：C.T3a 期

2023 第三单元
1、 (A1 型题)
抑郁自评量表是
A.SDS
B.SAS
C.MHT
D.BDI
E.HAMA
参考答案： A 您的答案：
图文解析： 【解析】抑郁自评量表（SDS）能相当直观地反映患者抑郁的主观感受及严重程度，故本题选 A。
【避错】焦虑自评量表（SAS）测量有无焦虑症状及其严重程度（B 错）；中学生心理健康诊断测验（MHT），又叫中小学生一般性焦虑测验，是适用于我国中小学学生标准化的测验（C 错）；BDI 为贝克抑郁问卷（D 错）；HAMA 为汉密尔顿焦虑量表（E 错）。
抑郁自评量表通常指的是贝克抑郁自评量表（Beck Depression Inventory，BDI）。因此，正确答案是 **D.BDI**。其他选项，如 SDS 指的是自评抑郁量表（Self-Rating Depression Scale），SAS 是自评焦虑量表（Self-Rating Anxiety Scale），而 HAMA 指的是汉密尔顿焦虑量表（Hamilton Anxiety Scale），这些都与抑郁自评量表不是完全一致的。
这是一道关于心理学评估量表的题目。我们需要从提供的选项中找出代表抑郁自评量表的正确缩写。
A. SDS：抑郁自评量表（Self-rating Depression Scale），这是一个常用的自评量表，用于评估个体的抑郁程度。它包含了 20 个项目，每个项目按 1-4 级评分，其中 10 个为正向评分，10

个为反向评分。这是评估抑郁状态的常用工具。
B. SAS：焦虑自评量表（Self-Rating Anxiety Scale），这个量表主要用于评估个体的焦虑程度，而不是抑郁。
C. MHT：多种人格特质问卷（Multiple Human Trait Test），该问卷用于评估多种人格特质，不专门针对抑郁状态。
D. BDI：贝克抑郁量表（Beck Depression Inventory），虽然这个量表也用于评估抑郁，但其缩写并不是 BDI，而是 BDI-II（第二版）。此外，题目中询问的是抑郁自评量表，其标准缩写为 SDS。
E. HAMA：汉密尔顿焦虑量表（Hamilton Anxiety Rating Scale），这个量表主要用于评估焦虑症状，不适用于评估抑郁。
综上所述，抑郁自评量表的正确缩写是 SDS。
因此，正确答案是 A.SDS。
抑郁自评量表是

A. SDS (Self-Rating Depression Scale，自评抑郁量表)

因此，正确答案是 **A. SDS**。
2、 (A1 型题)
为了诊断胃癌患者的临床分期，首选的检查手段是
A.PET-CT
B.腹部增强 CT
C.腹腔镜探查
D.上消化道 X 线钡剂造影
E.内镜超声
参考答案： B 您的答案：
图文解析： 【解析】为了诊断胃癌患者的临床分期，首选的检查手段是腹部增强 CT（B 对）。
【避错】PET-CT（A 错）价格昂贵， 一般不作为首选检查；腹腔镜探查（C 错）是手术治疗方式；上消化道 X 线钡剂造影（D 错）已在很大程度上被内镜检查替代，目前仅用于不愿内镜检查或内镜检查有禁忌者；内镜超声（E 错）是确诊胃癌的主要手段，可通过内镜超声判断肿瘤侵犯深度。
为了诊断胃癌患者的临床分期，最佳的检查手段是内镜超声（E）。内镜超声（EUS）能够详细评估肿瘤的深度和是否侵犯周围结构，对于评估局部淋巴结的情况也非常有用。这使得 EUS 成为胃癌临床分期中首选的检查手段。虽然 PET-CT 和腹部增强 CT 在评估远处转移方面很有价值，但在确定肿瘤的局部侵犯和分期方面，内镜超声更加精确。因此，选择 E（内镜超声）是最合适的。
解析：
胃癌的临床分期诊断需要对肿瘤浸润胃壁的深度、局部淋巴结及远处转移情况进行综合评估。选项中能对胃癌浸润深度、局部淋巴结及邻近器官直接浸润情况进行准确评估的检查是内镜超声，可清晰显示胃壁的各层结构和邻近器官的超声图像，有助于判断胃癌浸润深度、异常肿大的淋巴结以及肿瘤对胃壁的浸润情况，是胃癌 TNM 分期的常规检查手段。
选项 A：PET-CT 多用于发现胃癌的远处转移，在判断肿瘤浸润深度、局部淋巴结情况方面作用有限，故不选。
选项 B：腹部增强 CT 多用于术前临床分期，有助于判断肿瘤的部位、与周围脏器的关系以

及淋巴结转移情况和对疾病分期的判断，但准确度不如内镜超声，故排除。
选项 C：腹腔镜探查是侵入性检查，可直观观察腹腔脏器及淋巴结转移情况，并可在直视下取可疑病灶活检，取得病理的证据，但因其有创性，一般不作首选，故不考虑。
选项 D：上消化道 X 线钡剂造影检查对评价胃癌病变范围、局部胃腔狭窄、胃壁僵硬程度等有一定价值，但无法准确评估胃癌的浸润深度及局部淋巴结情况，故不符合题意。
综上所述，正确答案是：E.内镜超声。
为了诊断胃癌患者的临床分期，首选的检查手段是：

E. 内镜超声

内镜超声（EUS）能够提供胃壁层次的详细信息以及周围淋巴结和器官的状态，对于评估肿瘤的局部浸润和区域淋巴结转移非常重要，因此在胃癌临床分期中具有重要的价值。
3、 (A1 型题)
导致抗利尿激素分泌异常的肺癌类型是
A.肉瘤样癌
B.小细胞癌
C.大细胞癌
D.腺癌
E.鳞癌
参考答案： B 您的答案：
图文解析： 【解析】小细胞肺癌是一种神经内分泌肿瘤，具有内分泌功能，可分泌抗利尿激素（B 对）、儿茶酚胺、组胺等物质。
【避错】肉瘤样癌（A 错）为肺癌的一种，但不常见。大细胞癌（C 错）发生于大支气管，肿块常较大，恶性程度高，生长迅速，转移早而广泛；腺癌（D 错）是女性肺癌最常见的类型；鳞状细胞癌（E 错）为肺癌中最常见的类型。
导致抗利尿激素分泌异常（即综合征）的肺癌类型通常是小细胞肺癌（Small Cell Lung Cancer, SCLC）。因此，最正确的答案是：
B.小细胞癌
小细胞肺癌因其内分泌活性而闻名，可能导致一种称为“适当性抗利尿激素分泌增多症”（SIADH）的状态，其中体内抗利尿激素（ADH）的分泌过多，导致水分重吸收增加和血液稀释性低钠血症。
解析：
肺癌是起源于肺部支气管黏膜或腺体的恶性肿瘤。肺癌按组织学可分为鳞状细胞癌、腺癌、腺鳞癌、小细胞癌、大细胞癌、肉瘤样癌、类癌和唾液腺型癌和和未分类癌。其中，小细胞肺癌患者常伴内分泌异常或类癌综合征，可分泌 5-羟色胺，引起哮喘样支气管痉挛、阵发性心动过速、水样腹泻及皮肤潮红等，也可分泌抗利尿激素，导致低钠血症。
选项 A 肉瘤样癌，选项 C 大细胞癌，选项 D 腺癌，选项 E 鳞癌，均不会分泌抗利尿激素，故可一并排除。
综上所述，正确答案是：B.小细胞癌。
导致抗利尿激素分泌异常的肺癌类型是：

B. 小细胞癌
4、 (A1 型题)
慢性阻塞性肺疾病最核心的特征是

A.长期大量吸烟史
B.气流持续受限
C.受季节相关的症状反复发作
D.慢性咳嗽、咳痰症状
E.小气道功能障碍
参考答案：　B　您的答案：
图文解析：　【解析】慢性阻塞性肺疾病（COPD）简称慢阻肺，其特征是持续存在的呼吸系统症状和气流受限（B 对），通常与显著暴露于有害颗粒或气体引起的气道和（或）肺泡异常有关。
【避错】ACDE 选项均可见于 COPD 患者，但无特征性，故不选。
慢性阻塞性肺疾病（COPD）的最核心特征是气流持续受限。因此，最佳答案是 **B. 气流持续受限**。
虽然长期大量吸烟史（A）、受季节相关的症状反复发作（C）、慢性咳嗽、咳痰症状（D）和小气道功能障碍（E）都与慢性阻塞性肺疾病有关，但这些都不是其最核心的特征。气流持续受限反映了 COPD 的主要病理生理特点，是诊断 COPD 的关键标准。
这是一道关于慢性阻塞性肺疾病（COPD）核心特征的问题。我们需要基于 COPD 的定义和特征来选择最符合题意的选项。
A. 长期大量吸烟史：虽然吸烟是 COPD 的一个重要风险因素，但它不是 COPD 的核心特征，因为 COPD 也可能由其他因素引起，如长期暴露在空气污染或职业性粉尘环境中。
B. 气流持续受限：这是 COPD 的定义性特征。COPD 是一种具有气流受限特征的疾病，气流受限不完全可逆，呈进行性发展。这种气流受限与肺部对有害气体或有害颗粒的异常炎症反应有关。
C. 受季节相关的症状反复发作：虽然 COPD 患者的症状可能因季节变化（如寒冷天气）而加重，但这并非其核心特征。许多呼吸系统疾病都可能表现出季节性变化。
D. 慢性咳嗽、咳痰症状：这些症状虽然常见于 COPD 患者，但并非其核心特征，因为也有其他呼吸系统疾病可能导致这些症状。
E. 小气道功能障碍：小气道功能障碍是 COPD 的病理生理特征之一，但它更多地描述了疾病的机制，而不是其核心特征。气流受限是这一机制导致的直接后果，因此比小气道功能障碍更具代表性。
综上所述，气流持续受限是慢性阻塞性肺疾病（COPD）最核心的特征，因此正确答案是 B：气流持续受限。
慢性阻塞性肺疾病（COPD）的最核心特征是气流受限，这种受限是进行性加重的，并且与气道和肺对有害颗粒或气体的慢性炎症反应有关。虽然其他选项如长期大量吸烟史、慢性咳嗽和咳痰、小气道功能障碍等都与 COPD 密切相关，但核心特征是气流持续受限。

因此，正确答案是：

B. 气流持续受限
5、　(A1 型题)
全身性感染的治疗原则是
A.如为真菌性脓血症，应用全身静脉抗真菌药物，联合广谱抗生素
B.积极寻找原发病灶，尽早行对症治疗
C.待药敏试验结果出来后再选择敏感抗生素
D.早期大量联合

E.应用广谱抗生素
参考答案：　　B　您的答案：
图文解析：　　【解析】全身性感染应用综合性治疗，关键是处理原发感染灶（B 对）。【避错】对于抗菌药物的应用：无培养结果时，根据原发感染灶的性质及早联合应用（C 错），估计有效的两种抗生素，并应用足够剂量（D 错），再根据细菌培养及抗生素敏感试验，调整抗菌药物。对真菌性脓毒症，应尽量停用广谱抗生素（AE 错），或改用必需的窄谱抗生素，并全身应用抗真菌药物。
全身性感染的治疗原则主要包括尽早识别并控制感染源、合理使用抗生素等。因此，从提供的选项中，最符合全身性感染治疗原则的是：
B.积极寻找原发病灶，尽早行对症治疗
这是因为对于全身性感染（如败血症），重要的是尽快识别并控制感染源，同时进行对症支持治疗。虽然在确诊后根据药敏试验选择敏感抗生素（选项 C）是重要的，但不应推迟治疗以等待药敏结果。早期积极干预，包括对症治疗和控制感染源，是提高治愈率和生存率的关键。因此，选项 B 是最符合全身性感染治疗原则的选择。
解析：
全身性感染，是指致病菌侵入宿主血液循环，并在其中生长繁殖、产生毒素而引起严重的全身性感染或中毒症状。其治疗原则是早期、联合、足量、静脉应用抗生素，并根据血培养或病灶分泌物培养结果及时调整用药。
选项 A 如为真菌性脓血症，应用全身静脉抗真菌药物，联合广谱抗生素是抗真菌治疗的原则，并不是全身性感染的治疗原则，故不选。
选项 B 积极寻找原发病灶，尽早行对症治疗是正确的方法，但不是主要的治疗原则，故排除。
选项 C 待药敏试验结果出来后再选择敏感抗生素不符合早期用药的原则，药敏试验需要一定的时间，在等待期间会导致病情延误，故不选。
选项 E 应用广谱抗生素是正确的方法，但单一使用抗生素不能很好的控制感染，需联合用药，故排除。
综上所述，正确答案是：D.早期大量联合。
全身性感染（如败血症）的治疗原则主要包括尽早控制感染源和使用适当的抗生素治疗。最佳的治疗方案通常要求在取得药敏试验结果之前就开始使用经验性抗生素治疗，并且在获得药敏试验结果后再调整抗生素种类。因此，根据当前的医学知识和最佳实践指南，以下是对该题目的答案分析：

A. 如为真菌性脓血症，应用全身静脉抗真菌药物，联合广谱抗生素。
- 这仅适用于真菌性感染，而不是所有全身性感染的治疗原则。

B. 积极寻找原发病灶，尽早行对症治疗。
- 这是正确的原则之一，找到并处理原发感染源非常重要。

C. 待药敏试验结果出来后再选择敏感抗生素。
- 等待药敏试验结果出来后再开始治疗可能会延误病情，不是最佳的初始治疗策略。

D. 早期大量联合。
- 虽然早期联合使用抗生素是正确的，但需要根据具体情况调整用量和种类，“大量”不一定是最佳选择。

E. 应用广谱抗生素。
- 使用广谱抗生素是经验性治疗的常见做法，但应结合其他治疗原则进行综合考虑。

综合考虑，最正确的答案是：

B. 积极寻找原发病灶，尽早行对症治疗。

这符合全身性感染的总体治疗原则，强调了早期干预和对症治疗的重要性。
6、 (A1 型题)
肺动脉高压的诊断标准是
A.静息状态下右心导管测量平均肺动脉压≥25mmHg
B.静息状态下超声心动测量平均肺动脉压≥25mmHg
C.静息状态下右心导管测量平均肺动脉压≥30mmHg
D.静息状态下超声心动测量平均肺动脉压≥30mmHg
E.静息状态下超声心动测量收缩期肺动脉压≥25mmHg
参考答案： A 您的答案：
图文解析： 【解析】肺动脉高压是由多种已知或未知原因引起的肺动脉压异常升高的一种病理生理状态，血流动力学诊断标准为：在海平面、静息状态下，右心导管测量平均肺动脉压（mPAP）≥25mmHg（A 对）。
【避错】静息状态下超声心动测量平均肺动脉压＞50mmHg，结合临床也可诊断肺动脉高压。
肺动脉高压的诊断标准曾经是静息状态下右心导管测量平均肺动脉压≥25mmHg。然而，根据最新的指南，如 2018 年欧洲心脏病学会（ESC）和欧洲呼吸学会（ERS）共同发布的肺动脉高压诊断和治疗指南，这一标准已经被更新。现在的诊断标准是静息状态下右心导管测量平均肺动脉压≥20mmHg。
尽管这个信息没有直接体现在你给出的选项中，但最接近的答案是：
A. 静息状态下右心导管测量平均肺动脉压≥25mmHg
因此，在给定选项中，A 选项曾经是被广泛接受的诊断标准。但请注意，最新指南中的标准已经有所改变。如果这个问题基于最新的医学知识，那么实际上没有一个选项完全准确。如果考试的标准还没有更新，那么 A 选项可能是最合适的答案。
解析：
肺动脉高压是指静息状态下右心导管所测得的平均肺动脉压（mean pulmonary artery pressure，mPAP）≥ 5mmHg，肺毛细血管楔压（Pulmonary capillary wedge pressure，PCWP）≤15mmHg，且需除外引起肺动脉压力升高的呼吸系统疾病、左心疾病、肺血栓栓塞及先天性心脏病等疾病。其中，静息状态下右心导管测量平均肺动脉压≥25mmHg 是诊断肺动脉高压的金标准。
右心导管测量是诊断肺动脉高压的金标准，超声心动图只能作为评估肺动脉高压的筛查方法，其结果仅供参考，故选项 B“静息状态下超声心动测量平均肺动脉压≥25mmHg”，选项 D“静息状态下超声心动测量平均肺动脉压≥30mmHg”，选项 E“静息状态下超声心动测量收缩期肺动脉压≥25mmHg”，可一并排除。
右心导管测得静息状态下平均肺动脉压≥25mmHg 即可诊断为肺动脉高压，而非 30mmHg，故选项 C 静息状态下右心导管测量平均肺动脉压≥30mmHg，可排除。
综上所述，正确答案是：A.静息状态下右心导管测量平均肺动脉压≥25mmHg。
根据现有的医学知识和最佳实践指南，肺动脉高压的诊断标准是通过右心导管测量的静息状

态下的平均肺动脉压。具体标准如下：

A. 静息状态下右心导管测量平均肺动脉压≥25mmHg

因此，最佳答案是：

A. 静息状态下右心导管测量平均肺动脉压≥25mmHg
7、 (A1 型题)
下列关于消化性溃疡出血，说法正确的是
A.生命体征平稳后行胃镜检查
B.口服 PPI
C.药物治疗或胃镜止血无效行手术治疗
D.出血时一般无明显腹痛
E.胃溃疡一般比十二指肠溃疡容易发生
参考答案： C 您的答案：
图文解析： 【解析】消化性溃疡患者消化道大出血，经药物、胃镜及血管介入治疗无效时，应行手术治疗（C 对）。
【避错】单一口服 PPI（B 错）只在消化性溃疡不伴出血时治疗有效，伴出血时均应联合胃镜下治疗（A 错）；部分患者腹痛较为剧烈，有慢性腹痛的患者，出血后腹痛也可减轻（D 错）；十二指肠溃疡比胃溃疡更易出血（E 错）。
关于消化性溃疡出血，正确的说法是：
A. 生命体征平稳后行胃镜检查
这是正确的。在处理消化性溃疡出血时，确保患者生命体征平稳后进行胃镜检查是至关重要的，因为这可以帮助确定出血源，并进行适当的治疗，如内镜下止血。
B. 口服 PPI
虽然口服质子泵抑制剂（PPI）是消化性溃疡治疗的一部分，但在急性出血情况下，更常用的做法是静脉注射 PPI，以更快地控制胃酸分泌。
C. 药物治疗或胃镜止血无效行手术治疗
这也是正确的。当药物治疗和/或胃镜止血措施无效时，可能需要进行手术治疗以控制出血。
D. 出血时一般无明显腹痛
这一说法并不完全正确，因为消化性溃疡出血的患者可能会有腹痛，尤其是在溃疡穿透或穿孔时。
E. 胃溃疡一般比十二指肠溃疡容易发生
这个说法没有充分的依据，因为十二指肠溃疡比胃溃疡更常见，但胃溃疡可能更容易引起出血。
综合考虑，最合适的答案应该是 **C. 药物治疗或胃镜止血无效行手术治疗**，因为它涵盖了消化性溃疡出血治疗的重要方面。
解析：
消化性溃疡出血是消化性溃疡最常见的并发症，一般先采取非手术治疗，主要措施包括输血补液、应用止血药物、内镜止血、抗休克等。若药物治疗或胃镜止血无效时，可考虑手术治疗。
选项 A 生命体征平稳后行胃镜检查，对于消化性溃疡并发出血的患者，急诊胃镜检查应在出血后 24～48 小时内进行，此时胃镜下可见活动性出血，可同时进行内镜下止血治疗，与题意不符。

选项 B 口服 PPI 为消化性溃疡出血的一般治疗措施，属于非手术治疗，并不能作为消化性溃疡出血的特点，故不选。
消化性溃疡患者通常会出现节律性、周期性的上腹疼痛，出血后腹痛可减轻，因此选项 D 出血时一般无明显腹痛，可排除。
十二指肠溃疡比胃溃疡更容易发生穿孔，因此选项 E 胃溃疡一般比十二指肠溃疡容易发生，可排除。
综上所述，正确答案是：C.药物治疗或胃镜止血无效行手术治疗。
关于消化性溃疡出血的正确说法，综合现有的医学知识和最佳实践指南，正确答案是：

C. 药物治疗或胃镜止血无效行手术治疗

解释如下：

- **A. 生命体征平稳后行胃镜检查**：虽然胃镜检查是确诊和治疗消化性溃疡出血的重要手段，但通常在生命体征稳定后尽早进行胃镜检查，以便快速明确出血原因并采取相应治疗。
- **B. 口服 PPI**：质子泵抑制剂（PPI）确实是消化性溃疡的主要药物治疗方法，但在急性出血期通常需要静脉给药，以迅速抑制胃酸分泌。
- **C. 药物治疗或胃镜止血无效行手术治疗**：当药物治疗或内镜下止血无法控制出血时，手术治疗是必要的措施，以避免进一步的并发症和生命危险。
- **D. 出血时一般无明显腹痛**：消化性溃疡出血时患者通常会有上腹痛，特别是在出血量较大时，疼痛可能更加明显。
- **E. 胃溃疡一般比十二指肠溃疡容易发生**：实际上，十二指肠溃疡比胃溃疡更常见，而且十二指肠溃疡的出血风险也较高。
8、 (A1 型题)
与强直性脊柱炎密切相关的遗传基因是
A.HLA-DR2
B.HLA-B27
C.HLA-DR3
D.HLA-DR4
E.HLA-DR5
参考答案： B 您的答案：
图文解析： 【解析】与强直性脊柱炎密切相关的遗传基因是 HLA-B27（B 对）。
【避错】HLA-DR2（A 错）的异常改变可能引起的疾病有系统性红斑狼疮、硬皮病、原发性开角青光眼等。HLA-DR3 抗原（C 错）在重症肌无力患者中检出率较高。HLA-DR4（D 错）与类风湿性关节炎有一定的关联。携带 HLA-DR5（E 错）基因的个体常常与桥本氏甲状腺炎密切相关。
与强直性脊柱炎密切相关的遗传基因是 **B.HLA-B27**。强直性脊柱炎与 HLA-B27 的关联非常明显，大多数患者（约 90%）都是 HLA-B27 阳性。
解析：
强直性脊柱炎是一种主要侵犯脊柱，并可不同程度的累及骶髂关节和周围关节的慢性进行性炎性疾病。本病又名 Marie-strümpell 病、Von Bechterew 病、类风湿性脊柱炎、畸形性脊柱炎、类风湿中心型等，现都称 AS。AS 的特点为腰、颈、胸段脊柱关节和韧带以及骶髂关节的炎症和骨化，髋关节常常受累，其它周围关节也可出现炎症。与强直性脊柱炎密切相关的遗传基因是 HLA-B27。在我国，强直性脊柱炎患者的 HLA-B27 的阳性率高达 90%。

选项 A：HLA-DR2 与类风湿性关节炎有关，与强直性脊柱炎无直接关系，故不选。
选项 C：HLA-DR3 与自身免疫性甲状腺疾病、I 型糖尿病、Addison 病、原发性胆汁性肝硬化等疾病相关，而与强直性脊柱炎无关，故排除。
选项 D：HLA-DR4，选项 E：HLA-DR5，均与强直性脊柱炎无直接关系，目前尚未发现与特定的疾病有明确关联，可一并排除。
综上所述，正确答案是：B.HLA-B27。
与强直性脊柱炎密切相关的遗传基因是：

B. HLA-B27
9、 (A1 型题)
长期内痔患者肛门有肿块脱出，用手不能回纳，为几期
A. Ⅰ期
B. Ⅱ期
C.Ⅲ期
D.Ⅳ期
E. Ⅴ期
参考答案： D 您的答案：
图文解析： 【解析】长期内痔患者肛门有肿块脱出，用手不能回纳，为Ⅳ度（D 对）。【避错】内痔的分度：Ⅰ度：便时带血，便后出血自行停止，无痔脱出；Ⅱ度：常有便血，排便时有痔脱出，便后可自行还纳；Ⅲ度：偶有便血，排便或久站、咳嗽、负重时痔脱出，需用手还纳；Ⅳ度：偶有便血，痔脱出不能还纳或还纳后又脱出。
长期内痔患者肛门有肿块脱出且用手不能回纳，这通常表示该病情已经进展到较严重的阶段。根据内痔的临床分期，肿块不能自行回纳或需要手动推回肛内的通常被分类为Ⅲ期。因此，最正确的答案是 C.Ⅲ期。
解析：
内痔是位于肛管齿状线以上的直肠末端黏膜下血管丛扩张、曲张形成的柔软包块，其实质为曲张的静脉血管团。内痔一般被分为四期，其中，Ⅲ期内痔是指痔核更大，质地较硬，表面微带灰白色，便时痔核脱出肛门外，甚至行走，咳嗽，喷嚏或站立时也会脱出，不能自行回纳，须用手推回，或平卧，热敷后才能回纳，便血不多或不再出血。所以，长期内痔患者肛门有肿块脱出，用手不能回纳，考虑为内痔Ⅲ期。
选项 A：Ⅰ期内痔以便血症状为主，痔核较小，质柔软，痔面鲜红色或青紫色，排便时痔核不脱出肛门外，与题干描述不符。
选项 B：Ⅱ期内痔，便时带血、滴血或喷射状出血，伴有痔核脱出，便后可自行回纳，题干中患者用手不能回纳，故不选。
选项 D：Ⅳ期内痔，痔核脱出不能回纳，嵌顿于外，由于嵌顿时间日久，易形成血栓、坏死、感染，多伴有剧烈的疼痛、发热等全身症状，与题干描述不符，故排除。
内痔分期中无Ⅴ期，故选项 E：Ⅴ期，可优先排除。
综上所述，正确答案是：C.Ⅲ期。
长期内痔患者肛门有肿块脱出，用手不能回纳，为Ⅳ期。

所以，正确答案是：
D. Ⅳ期
10、 (A1 型题)
支气管哮喘早期发作时的血气改变是

A.pH 值上升，PaO2 下降，PaCO2 下降
B.pH 值上升，PaO2 下降，PaCO2 上升
C.pH 值下降，PaO2 下降，PaCO2 下降
D.pH 值下降，PaO2 下降,PaCO2 上升
E.pH 值正常，PaO2 下降，PaCO2 上升
参考答案： A 您的答案：
图文解析： 【解析】支气管哮喘早期：由于过度通气可使 PaCO2 下降，pH 上升，表现为呼吸性碱中毒。
【避错】支气管哮喘后期：缺氧加重，而出现呼吸性酸中毒（CO2 潴留）+代谢性酸中毒（缺氧乳酸增多），提示病情危重。
在支气管哮喘早期发作时，通常会出现呼吸性碱中毒，因为患者会尝试通过增加呼吸频率来减轻呼吸困难，导致二氧化碳排出增多。因此，PaCO2 会下降，导致血液 pH 值上升。另外，由于气道狭窄，氧气交换减少，会导致 PaO2 下降。综上所述，最符合这种情况的选项是：
A.pH 值上升，PaO2 下降，PaCO2 下降
所以，正确答案是 **A**。
解析：
支气管哮喘是由多种细胞和细胞组分参与的气道慢性炎症为特征的异质性疾病，这种慢性炎症与气道高反应性相关，通常出现广泛而多变的可逆性呼气气流受限，导致反复发作的喘息、气促、胸闷或咳嗽等症状，强度随时间变化。支气管哮喘早期发作时，由于气道痉挛，机体缺氧，过度通气，会导致 pH 上升，血氧分压（PaO_2）下降，二氧化碳分压（$PaCO_2$）下降，出现呼吸性碱中毒。
支气管哮喘早期发作时，由于机体缺氧，过度通气，会导致 $PaCO_2$下降，不会出现 $PaCO_2$上升。故选项 B：“pH 值上升，PaO_2下降，$PaCO_2$上升”；选项 D：“pH 值下降，PaO_2下降，$PaCO_2$上升”；选项 E：“pH 值正常，PaO_2下降，$PaCO_2$上升”；可一并排除。
哮喘发作时，由于气道阻塞，呼吸不畅，机体缺氧而过度通气，导致 $PaCO_2$下降，出现呼吸性碱中毒，此时血液 pH 值上升，而非下降，故选项 C：pH 值下降，PaO_2下降，$PaCO_2$下降，可排除。
综上所述，正确答案是：A.pH 值上升，PaO2 下降，PaCO2 下降。
支气管哮喘早期发作时的血气改变主要与急性呼吸道阻塞有关，导致患者出现过度通气（hyperventilation）。这种情况会引起以下变化：

- pH 值上升（由于呼吸性碱中毒）
- PaO2 下降（因为气道阻塞影响氧气交换）
- PaCO2 下降（因为过度通气排出了更多的二氧化碳）

因此，最正确的答案是：

A. pH 值上升，PaO2 下降，PaCO2 下降
11、 (A1 型题)
下列哪项不是急性胰腺炎手术指征
A.直接胆红素进行性升高伴寒战高热
B.合并大出血或假性囊肿
C.急性腹膜炎不能排除其他急腹症
D.血清淀粉酶＞500U/L

E.胰腺及胰周组织坏死继发感染
参考答案： D 您的答案：
图文解析： 【解析】血清淀粉酶升高程度与胰腺炎严重程度不成正比，故血清淀粉酶＞500U/L 不是急性胰腺炎的手术指征（D 错，为本题正确答案）。
【避错】急性胰腺炎手术适应证：①急性腹膜炎不能排除其他急腹症时（C 对）；②伴胆总管下端梗阻或胆道感染者（A 对，直接胆红素的升高说明存在阻塞性黄疸）；③合并肠穿孔、大出血或胰腺假性囊肿（B 对）；④胰腺和胰周坏死组织继发感染（E 对）。
对于急性胰腺炎，手术治疗通常是在特定情况下才考虑的，主要是当有并发症或其他保守治疗无效时。根据提供的选项，我们来分析哪一项不是急性胰腺炎的手术指征：
A. 直接胆红素进行性升高伴寒战高热：这可能指示胆管有问题，如胆管阻塞或感染，可能需要手术干预。
B. 合并大出血或假性囊肿：这些情况可以导致病情恶化，可能需要手术处理。
C. 急性腹膜炎不能排除其他急腹症：急性腹膜炎通常需要手术来确定原因并治疗。
E. 胰腺及胰周组织坏死继发感染：这是急性胰腺炎的严重并发症，通常需要外科手术清除坏死组织。
D. 血清淀粉酶＞500U/L：虽然血清淀粉酶水平的升高是急性胰腺炎的一个重要标志，但它本身并不直接作为手术指征。通常，治疗侧重于保守治疗，包括休息、止痛和液体补充，除非出现并发症或病情加重。
因此，最正确的答案是 **D. 血清淀粉酶＞500U/L**。这项指标虽然是诊断急性胰腺炎的重要依据，但它不直接作为手术治疗的指征。
这是一道关于急性胰腺炎手术指征的选择题。我们需要分析每个选项，确定哪一项不是急性胰腺炎的手术指征。
A. 直接胆红素进行性升高伴寒战高热：这种情况可能提示有胆道梗阻或感染，这是急性胰腺炎中可能需要手术的严重情况之一。
B. 合并大出血或假性囊肿：大出血和假性囊肿是急性胰腺炎的严重并发症，通常需要手术治疗。
C. 急性腹膜炎不能排除其他急腹症：在不能明确诊断的情况下，为了排除其他可能导致急性腹膜炎的疾病，手术探查可能是必要的。
D. 血清淀粉酶＞500U/L：血清淀粉酶升高是急性胰腺炎的典型表现，但单纯的淀粉酶升高并不是手术的直接指征。胰腺炎的严重程度和治疗方式的选择更多地依赖于临床表现和其他实验室及影像学检查。
E. 胰腺及胰周组织坏死继发感染：胰腺及周围组织坏死并继发感染是急性胰腺炎的严重并发症，通常需要手术治疗。
综上所述，单纯的血清淀粉酶升高并不是急性胰腺炎的手术指征。手术决策通常基于疾病的严重程度、并发症的出现以及患者的整体状况。
因此，正确答案是 D.血清淀粉酶＞500U/L。
急性胰腺炎的手术指征主要包括胰腺及胰周组织坏死继发感染、合并大出血或假性囊肿、急性腹膜炎不能排除其他急腹症等。手术指征通常涉及到严重的并发症或无法通过非手术手段控制的情况。我们逐一分析选项：

A. 直接胆红素进行性升高伴寒战高热：直接胆红素升高和寒战高热可能提示胆源性急性胰腺炎，这通常需要进行手术以解除胆道梗阻，但这不是急性胰腺炎本身的手术指征，而是胆源性问题的手术指征。

B. 合并大出血或假性囊肿：假性囊肿和大出血都是急性胰腺炎的严重并发症，需要手术干预。

C. 急性腹膜炎不能排除其他急腹症：急性腹膜炎如果不能排除其他急腹症，需要手术探查来明确诊断并处理。

D. 血清淀粉酶＞500U/L：虽然血清淀粉酶显著升高是急性胰腺炎的标志之一，但单独的高血清淀粉酶并不是手术指征。血清淀粉酶升高只是诊断急性胰腺炎的指标之一。

E. 胰腺及胰周组织坏死继发感染：继发感染是急性胰腺炎的严重并发症，通常需要手术干预。

综上所述，D 项“血清淀粉酶＞500U/L”不是急性胰腺炎的手术指征。

所以，正确答案是：D. 血清淀粉酶＞500U/L。

12、 (A1 型题)

关于判断胃食管反流病患者的症状与病理生理改变的相关检查是

A.胃镜

B.食管测压

C.动态心电图

D.24h 食管 pH 值检测

E.上消化道 X 线钡剂检查

参考答案： A 您的答案：

图文解析： 【解析】胃镜是诊断 RE 最准确的方法，并能判断 RE 的严重程度和有无并发症，结合活检可与其他原因引起的食管炎和其他食管病变（如食管癌等）相鉴别。正常食管黏膜为复层鳞状上皮，胃镜下呈均匀粉红色，当其被化生的柱状上皮代替后呈橘红色（A 对）。

【避错】食管测压、24h 食管 pH 值检测、上消化道 X 线钡剂检查也可用于协助诊断胃食管反流病，但不作为首选。动态心电图一般用于心律失常及短暂心肌缺血的检测。

在胃食管反流病（GERD）的诊断中，关键是确定食管暴露于胃酸的时间和程度，以及食管对酸的反应。虽然胃镜可以直接观察食管和胃的黏膜情况，发现炎症或其他损伤，但它不足以量化酸反流的程度或确立病理生理改变。

食管测压（选项 B）可以评估食管下括约肌的功能和食管蠕动功能，对于评估反流病的病理生理机制很有帮助，但它不直接测量酸反流。

动态心电图（选项 C）用于评估心脏活动，与胃食管反流病的直接诊断无关。

24 小时食管 pH 值检测（选项 D）是评估食管酸暴露时间和酸反流频率的金标准，可以直接测量食管内的 pH 值，以确定酸反流的存在和严重程度。

上消化道 X 线钡剂检查（选项 E）可以显示食管、胃和十二指肠的解剖结构和大的运动异常，但对于酸反流的量化和病理生理改变的评估不够敏感。

因此，最符合判断胃食管反流病患者的症状与病理生理改变的相关检查是**D. 24 小时食管 pH 值检测**。

解析：

胃食管反流病是指胃内容物反流入食管、咽喉、口腔等食管以外的部位所引起的一系列症状和并发症。胃食管反流病的症状包括有胸口灼热感、打嗝、恶心、呕吐、胸口闷痛，以及

胃酸逆流。诊断胃食管反流病最准确的方法是 24h 食管 pH 值检测，可提供食管是否存在过度酸反流的客观证据，是判断胃食管反流病患者的症状与病理生理改变的相关检查，是诊断胃食管反流病重要的辅助检查。
选项 A 胃镜是诊断反流性食管炎最准确的方法，并能判断反流性食管炎的严重程度和有无并发症，结合活检可与其他原因引起的食管炎和其他食管病变作鉴别，但无法判断胃食管反流病患者症状与病理生理改变，故不选。
选项 B 食管测压是反映食管下括约肌功能的指标，当胃食管反流病内科治疗效果不好时，可作为辅助诊断，但不能直接诊断胃食管反流病，故排除。
选项 C 动态心电图是用于心血管系统疾病的检查，与胃食管反流病无关，可排除。
选项 E 上消化道 X 线钡剂检查对评估食管黏膜损伤价值有限，只能提示有无反流的征象，阳性率不高，不能确诊胃食管反流病，与本题不符。
综上所述，正确答案是：D.24h 食管 pH 值检测。
关于判断胃食管反流病患者的症状与病理生理改变的相关检查，以下是各选项的分析：

A. **胃镜**：可以直接观察食管和胃的粘膜状态，对诊断胃食管反流病（GERD）有帮助，但不能直接评估反流的频率和酸度。

B. **食管测压**：可以测量食管的压力和运动功能，评估下食管括约肌的功能，但不是诊断 GERD 的最佳选择。

C. **动态心电图**：用于监测心脏的电活动，与胃食管反流病无关。

D. **24 小时食管 pH 值检测**：是诊断胃食管反流病的金标准。它可以连续监测 24 小时内食管中的酸性反流情况，直接评估反流的频率和酸度，是最直接判断 GERD 症状与病理生理改变相关性的检查。

E. **上消化道 X 线钡剂检查**：可以显示食管和胃的形态学改变，但对诊断反流病的敏感性和特异性较低。

综合以上分析，最正确的答案是：

D. **24 小时食管 pH 值检测**
13、 (A1 型题)
下列属于原发性腹膜炎的是
A.胆管吻合术漏
B.女性生殖道细菌上行感染
C.小肠穿孔
D.胃溃疡穿孔
E.结肠穿孔
参考答案： B 您的答案：
图文解析： 【解析】原发性腹膜炎又称自发性腹膜炎，即腹腔内无原发病灶，其感染途径有：①血行播散；②上行性感染；③直接扩散；④透壁性感染。上行性感染，即来自女性生殖道的细菌，通过输卵管直接向上扩散至腹腔，如淋菌性腹膜炎（B 对）。
【避错】继发性腹膜炎是最常见的腹膜炎。腹腔空腔脏器穿孔、外伤引起的腹壁或内脏破

裂，是急性继发性化脓性腹膜炎最常见的原因。
原发性腹膜炎是指腹膜炎发生在无明显的细菌源性腹部病变的情况下。它通常与血行感染或特定病原体如结核菌的感染有关。相反，继发性腹膜炎通常是由腹部器官的穿孔、撕裂或炎症引起的，这些情况导致细菌直接进入腹腔。
选项 A（胆管吻合术漏）、C（小肠穿孔）、D（胃溃疡穿孔）和 E（结肠穿孔）都可能导致继发性腹膜炎，因为它们涉及腹部器官的破损，从而允许细菌直接进入腹腔。
选项 B（女性生殖道细菌上行感染）通常指的是细菌通过女性生殖道上行至盆腔，可能导致盆腔炎症性疾病，而不是直接引起腹膜炎。然而，在缺乏明显的穿孔或破损的情况下，这种类型的感染可能不会被视为原发性腹膜炎的典型原因。
由于这些选项都与继发性腹膜炎更相关，因此没有一个选项是描述原发性腹膜炎的经典情况。鉴于题目要求选择与原发性腹膜炎相关的情况，但这些选项似乎都指向继发性腹膜炎，可能需要重新考虑这个问题的上下文或是查看是否有其他信息或选项定义原发性腹膜炎的情况。
基于常规医学知识，原发性腹膜炎通常不是由这些条件直接引起的。因此，如果必须从这些选项中选择，并且假设题目可能在询问与原发性腹膜炎最不相关的情况，那么可能需要进一步的澄清。在实际的考试环境中，建议仔细审阅题目和选项，以确保正确理解所问的是什么。
如果是根据上下文选择最有可能与原发性腹膜炎相关的情况，则可能需要考虑选项 B，因为它相对不那么直接与腹部器官的穿孔或破损有关。
这是一道涉及腹膜炎分类的问题。腹膜炎通常分为原发性腹膜炎和继发性腹膜炎两种。我们需要识别哪一种情况属于原发性腹膜炎。
A. 胆管吻合术漏：这是由手术操作导致的腹膜炎，因此属于继发性腹膜炎。

B. 女性生殖道细菌上行感染：这种情况下，细菌通过生殖道上行至腹腔引起的腹膜炎，并非由腹内脏器穿孔或损伤等外部因素导致。这种腹膜炎没有明确的腹腔内病变来源，因此它属于原发性腹膜炎。
C. 小肠穿孔：这是由小肠穿孔引起的腹膜炎，属于继发性腹膜炎。
D. 胃溃疡穿孔：胃溃疡穿孔会导致胃内容物进入腹腔，引发腹膜炎，属于继发性腹膜炎。
E. 结肠穿孔：结肠穿孔同样会导致结肠内容物进入腹腔，引起腹膜炎，也属于继发性腹膜炎。
综上所述，只有选项 B 代表的原发性腹膜炎是由女性生殖道细菌上行感染导致的，没有明确的腹腔内病变来源，符合原发性腹膜炎的定义。
因此，正确答案是 B.女性生殖道细菌上行感染。
原发性腹膜炎（primary peritonitis）是一种没有明显腹内器官病变的腹膜炎，常见于免疫力低下患者、儿童和肝硬化合并腹水的患者。根据这个定义，原发性腹膜炎不是由腹内器官穿孔或外科手术引起的，而是由腹腔内自然存在的细菌感染引起的。

选项分析：
A. 胆管吻合术漏 - 这是腹部外科手术后的并发症，属于继发性腹膜炎。
B. 女性生殖道细菌上行感染 - 这是一种可以导致原发性腹膜炎的情况，因为细菌可以通过生殖道上行至腹腔。
C. 小肠穿孔 - 属于继发性腹膜炎，因为是由小肠破裂引起的。
D. 胃溃疡穿孔 - 属于继发性腹膜炎，因为是由胃的病变引起的。
E. 结肠穿孔 - 属于继发性腹膜炎，因为是由结肠破裂引起的。

根据上述分析，最符合原发性腹膜炎定义的是选项 B：女性生殖道细菌上行感染。

因此，最佳答案是：
B. 女性生殖道细菌上行感染
14、 (A1 型题)
胃十二直肠溃疡穿孔首选检查是
A.立位 X 线腹平片
B.B 超
C.增强 CT
D.胃镜
E.PETCT
参考答案： A 您的答案：
图文解析： 【解析】胃十二直肠溃疡穿孔首选检查是立位 X 线腹平片（A 对），检查可见膈下游离气体。
【避错】B 超（B 错）检查在消化道的应用有限。增强 CT（C 错）检查主要用于检查与血管有关的急腹症、腹内脏器损伤、脏器炎症及腹腔脓肿，用于了解肠梗阻时是否伴有血供障碍。消化性溃疡合并出血时可选胃镜（D 错）。PET-CT（E 错）可早期发现肿瘤，确定性质，可准确对肿瘤进行分期，评价治疗效果。
对于胃十二指肠溃疡穿孔，首选的检查方法是立位 X 线腹平片（选项 A）。这种检查可以发现腹腔内自由气体，是诊断穿孔的关键依据。虽然增强 CT（选项 C）也能用于诊断胃肠道穿孔并提供更多细节信息，但立位 X 线腹平片更为快速和经济，通常是首选方法。因此，最佳答案是 A.立位 X 线腹平片。
解析：
胃十二指肠溃疡穿孔是胃十二指肠溃疡的常见并发症，多由于溃疡不断加深，穿透肌层，浆膜层，最后穿透胃或十二指肠壁而发生穿孔。对于胃十二指肠溃疡穿孔，首选检查是立位 X 线腹平片，典型表现为膈下新月状游离气体影。
选项 B：B 超常用于肝、胆、胰、脾等实质脏器的检查，对空腔脏器穿孔的诊断价值不大，故不选。
选项 C：增强 CT，选项 E：PETCT，均价格昂贵，操作复杂，且对于空腔脏器穿孔的诊断并无特殊意义，因此一并排除。
选项 D：胃镜可直视溃疡部位、病变大小及性质，并可取材活组织检查、确定 HP 感染，还可对部分病例进行镜下止血、息肉切除、肿瘤切除等治疗，但属于有创检查，且无法诊断穿孔，可排除。
综上所述，正确答案是：A.立位 X 线腹平片。
胃十二指肠溃疡穿孔是一种急症，需要快速诊断和治疗。诊断胃十二指肠溃疡穿孔的首选检查应该是能够迅速、准确地显示气体外泄的检查方法。根据现有的医学知识和最佳实践指南，首选检查是：

A.立位 X 线腹平片

立位 X 线腹平片（即立位腹部平片）可以显示膈下游离气体，这是胃十二指肠溃疡穿孔的一个特征性体征。因此，在临床实践中，立位 X 线腹平片通常是首选的初步检查。
15、 (A1 型题)
甲状腺癌颈部淋巴结最小的清扫范围是

A.Ⅵ
B.Ⅱ
C.Ⅲ
D.Ⅳ
E.Ⅴ
参考答案： A 您的答案：
图文解析： 【解析】颈淋巴结清扫的范围目前乃有分歧，但最小范围清扫，既中央区颈淋巴结(Ⅵ)清扫已基本达成共识（A 对）。
【避错】Ⅵ区清扫既清扫了甲状腺癌最易转移的区域，又有助于临床分期、指导治疗、预测颈侧区淋巴结转移的可能性和减少再次手术的并发症。
在甲状腺癌的治疗中，颈部淋巴结清扫的范围根据肿瘤的位置、类型以及淋巴结的扩散情况而定。颈部淋巴结分为几个区域，用罗马数字 I 至 VII 来表示。其中，Ⅵ区通常指的是前颈中央区，这个区域包括了甲状腺周围的淋巴结，是甲状腺癌最常见的淋巴结转移区。
因此，在甲状腺癌的治疗中，最小的清扫范围通常包括Ⅵ区，即前颈中央区的淋巴结，以确保清除可能受累的淋巴结。所以，最正确的答案是 **A.Ⅵ**。
解析：
甲状腺癌是起源于甲状腺滤泡上皮的恶性肿瘤，常表现为无痛性颈部肿块或结节。甲状腺癌的主要病理学类型包括：未分化癌、髓样癌、乳头状癌和未分化癌，恶性程度由高至低为：未分化癌、髓样癌、乳头状癌。甲状腺癌治疗以手术为主，进行甲状腺切除及颈部淋巴结清扫，并根据病理检查结果，决定是否需要碘 131 治疗及 TSH 抑制治疗。颈部淋巴结的分区中，Ⅵ区指颈前区淋巴结，也称为气管周围淋巴结，包括环甲膜淋巴结、气管、甲状腺周围淋巴结、咽后淋巴结等，是甲状腺癌最先转移的区域，因此甲状腺癌颈部淋巴结清扫时，最小的清扫范围是Ⅵ区。
选项 B：Ⅱ区淋巴结指颈内静脉上组淋巴结，上界为颅底，下界为舌骨，为甲状腺癌清扫的第二站，清扫范围大于Ⅵ区，故不选。
选项 C：Ⅲ区淋巴结指颈内静脉中区淋巴结，上界为舌骨，下界为环状软骨下缘，为甲状腺癌清扫的第三站，清扫范围大于Ⅵ区，故排除。
选项 D：Ⅳ区淋巴结指颈内静脉下组淋巴结，上界为环状软骨，下界与锁骨上缘平行，为甲状腺癌清扫的第四站，清扫范围大于Ⅵ区，可排除。
选项 E：Ⅴ区淋巴结指颈后三角淋巴结，包括锁骨上淋巴结，为甲状腺癌清扫的第五站，清扫范围大于Ⅵ区，与题意不符。
综上所述，正确答案是：A.Ⅵ。
甲状腺癌的治疗包括手术切除肿瘤及受累淋巴结。在进行甲状腺癌的颈部淋巴结清扫时，通常会根据癌症的分期、淋巴结受累情况以及手术指南选择合适的清扫范围。

对于甲状腺癌，颈部淋巴结最小的清扫范围通常包括Ⅵ区，也称为中央区或前中央区淋巴结。这一区域位于气管的两侧，包括前气管和气管旁淋巴结，常见于甲状腺癌的早期转移部位。

因此，甲状腺癌颈部淋巴结最小的清扫范围是Ⅵ区。

正确答案是：A.Ⅵ
16、 (A1 型题)
急性上呼吸道感染患者拟行腹腔镜胆囊切除术，手术需延期至急性上呼吸道感染症状恢复后
A.5~7 天

B.14~21 天
C.7~14 天
D.1~3 天
E.3~5 天
参考答案：　　C　您的答案：
图文解析：　　【解析】急性呼吸系统感染者，择期手术应推迟至治愈后 1～2 周（C 对）。【避错】近期有脑卒中病史，择期手术应该至少推迟 2 周，最好 6 周。
对于患有急性上呼吸道感染的患者，在进行非紧急手术，如腹腔镜胆囊切除术之前，通常建议等待直到感染症状完全缓解。这是因为急性上呼吸道感染可能增加麻醉和手术的风险，包括呼吸道并发症和感染扩散的风险。
根据医学指南和最佳实践，手术通常推荐在急性症状完全消退后进行。虽然具体的等待时间可以根据患者的具体情况而变化，但一般建议的时间是在感染症状恢复后的 2 到 4 周内，以确保患者完全康复并减少手术风险。因此，最合适的回答选项是：
B. 14~21 天
这个时间范围为患者提供了充分的恢复时间，减少了急性感染对手术的潜在负面影响。
解析：
急性上呼吸道感染多由病毒感染所致，但部分可由细菌感染所致，也可由混合感染所致。患者常表现为咳嗽、流涕、发热等症状。急性上呼吸道感染时，呼吸道黏膜的防御功能会受到影响，增加了手术中和手术后感染的风险。因此，对于择期手术，一般建议在感染控制、体温恢复正常后 1～2 周再进行手术，以降低手术并发症的风险。
综上所述，正确答案是：B.14~21 天。
选项 A：5~7 天，选项 C：7~14 天，选项 D：1~3 天，选项 E：3~5 天，均属于手术延期时间不足，此时呼吸道黏膜的防御功能可能还未完全恢复，手术并发症的风险仍然较高，故可一并排除。
急性上呼吸道感染患者拟行腹腔镜胆囊切除术，手术需延期至急性上呼吸道感染症状恢复后。急性上呼吸道感染可能会增加麻醉和手术的风险，如导致气道反应增强、分泌物增多、通气不良等，因此通常建议手术延期至感染症状完全消失后再进行。

对于急性上呼吸道感染症状恢复后建议延期的时间，一般认为需要大约 2 周的时间，以确保呼吸道功能完全恢复，减少术中和术后并发症的发生。故正确答案是：

B. 14~21 天
17、　(A1 型题)
目前常用于诊断支气管扩张的检查
A.肺功能
B.支气管镜
C.高分辨 CT
D.胸部 X 线
E.B 超
参考答案：　　C　您的答案：
图文解析：　　【解析】目前常用于诊断支气管扩张的检查为 HRCT（C 对）。HRCT 可在横断面上清楚地显示扩张的支气管。
【避错】其他有助于诊断支气管扩张的辅助检查为：X 线（纵切面上可见双轨征或卷发影）、支气管镜（当支气管扩张呈局灶性且位于段支气管以上时，可发现弹坑样改变）、

肺功能等。B 超检查在呼吸系统的作用有限。
用于诊断支气管扩张的最常用和最有效的检查方法是高分辨率计算机断层扫描（高分辨 CT）。高分辨 CT 可以提供关于气管和支气管壁的详细信息，能够清晰地显示支气管壁的厚度、扩张程度以及是否有其他肺部病变。因此，选择最正确的答案是：C.高分辨 CT
解析：
支气管扩张是由于支气管及其周围肺组织慢性化脓性炎症和纤维化，使支气管壁的肌肉和弹性组织破坏，导致支气管变形及持久扩张。典型的症状有慢性咳嗽、咳大量脓痰和反复咯血。
支气管扩张的诊断依赖于影像学检查，其中高分辨 CT 是支气管扩张诊断的金标准，其对支气管扩张症的确诊率很高，且无创、易重复、易接受。现已成为支气管扩张的主要诊断方法。
选项 A 肺功能检查主要用于慢性阻塞性肺疾病、支气管哮喘等疾病的诊断，对支气管扩张的诊断意义不大，故排除。
选项 B 支气管镜可用于观察病变部位，但因其为创伤性检查，一般不用来诊断支气管扩张，故不选。
选项 D 胸部 X 线检查虽可观察到卷须状的双轨征，但其诊断价值不及高分辨率 CT，故排除。
选项 E 超声检查多用于积液或腹腔实质性脏器的检查，对支气管扩张的诊断意义不大，故不选。
综上所述，正确答案是：C.高分辨 CT。
目前常用于诊断支气管扩张的检查是：

C. 高分辨 CT

高分辨 CT（HRCT）可以详细显示支气管和肺组织的结构变化，是诊断支气管扩张最敏感和特异的检查方法。
18、 (A1 型题)
不属于乳腺癌高危因素的是
A.绝经年龄早
B.产后未哺乳
C.肥胖
D.一级亲属乳腺癌家族史
E.BRCA 基因突变
参考答案： A 您的答案：
图文解析： 【解析】月经初潮年龄早、绝经年龄晚（A 错，为本题正确答案）、不孕及初次足月产的年龄晚与乳腺癌发病均有关。
【避错】产后未哺乳（B 对）、肥胖（C 对）以及一级亲属乳腺癌家族史（D 对）均会增加乳腺癌的患病风险，属于乳腺癌的高危因素。BRCA 基因突变（E 对）会导致乳腺癌、卵巢癌、胰腺癌、前列腺癌等多种癌症类型患癌风险增加。
不属于乳腺癌高危因素的是 A.绝经年龄早。因为晚绝经（绝经年龄晚）而不是早绝经通常被认为是乳腺癌的一个风险因素。晚绝经意味着女性在生命中长时间暴露于雌激素，这可能增加患乳腺癌的风险。相反，早绝经减少了女性体内雌激素的总体暴露时间，因此可能降低乳腺癌的风险。其他选项，如产后未哺乳、肥胖、一级亲属乳腺癌家族史和 BRCA 基因突变，都是已知的乳腺癌高危因素。
首先，我们要明确乳腺癌的高危因素。乳腺癌的高危因素主要包括内源性或外源性雌激素的长期刺激、月经过早来潮（小于 12 岁）或绝经晚（迟于 55 岁）、未生育、晚育（第一胎在 35 岁以后）或生育后不哺乳，乳癌家族史，以及乳腺非典型增生等。

接下来，我们来分析各个选项：
A. 绝经年龄早：这与乳腺癌的高危因素不符。实际上，绝经年龄晚是乳腺癌的高危因素。因此，绝经年龄早不被视为乳腺癌的高危因素。
B. 产后未哺乳：这是乳腺癌的高危因素之一。未哺乳可能导致乳腺组织持续受到雌激素的刺激，从而增加乳腺癌的风险。
C. 肥胖：肥胖也被认为是乳腺癌的高危因素，特别是在绝经后的女性中。肥胖可能导致雌激素水平升高，从而增加乳腺癌的风险。
D. 一级亲属乳腺癌家族史：有明显的乳腺癌家族史是乳腺癌的高危因素之一。如果一级亲属（如母亲，女儿，姐妹）中有乳腺癌患者，那么个体的乳腺癌风险会增加。
E. BRCA 基因突变：BRCA1 和 BRCA2 基因与乳腺癌和卵巢癌的风险增加有关。这些基因的突变可以显著增加乳腺癌的风险。
综上所述，不属于乳腺癌高危因素的是 A.绝经年龄早。
乳腺癌的高危因素包括许多方面，但以下选项中，不属于乳腺癌高危因素的是：

A. 绝经年龄早
B. 产后未哺乳
C. 肥胖
D. 一级亲属乳腺癌家族史
E. BRCA 基因突变

正确答案是：A. 绝经年龄早

解释：乳腺癌的高危因素包括产后未哺乳、肥胖、一级亲属乳腺癌家族史以及 BRCA 基因突变等。而绝经年龄早（相对来说）反而可能减少乳腺癌的风险，因为长时间的雌激素暴露与乳腺癌的发病有关。早绝经意味着雌激素暴露时间较短，可能减少乳腺癌的风险。因此，绝经年龄早不属于乳腺癌的高危因素。
19、 (A1 型题)
重度溃疡性结肠炎首选治疗
A.糖皮质激素
B.硫唑嘌呤
C.英夫利昔单抗
D.环孢素
E.大量抗生素
参考答案： A 您的答案：
图文解析： 【解析】对于重度溃疡性结肠炎，糖皮质激素是首选的治疗药物（A 对）。【避错】溃疡性结肠炎患者首选 5-氨基水杨酸；糖皮质激素用于对 5-氨基水杨酸疗效不佳的轻、中度患者，尤其适用于重度患者；硫唑嘌呤可用于对激素治疗效果不佳或对激素依赖者的维持治疗；环孢素用于重度 UC 静脉使用糖皮质激素治疗无效时，其作为补救治疗；英夫利昔单抗可用于 UC 的诱导缓解及补救治疗。
对于重度溃疡性结肠炎的首选治疗，糖皮质激素（A）通常被推荐作为首线治疗方法。这是因为它们能够迅速减轻炎症和症状。因此，最佳答案是 A.糖皮质激素。
解析：
溃疡性结肠炎是一种慢性非特异性肠道炎症性疾病，主要累及直肠和结肠，多呈反复发作的慢性病程。临床表现为间断性腹泻、黏液脓血便、腹痛及里急后重等。本病治愈难度大，复

发率高，具有较高的癌变率，与结肠癌的发病密切相关。溃疡性结肠炎按严重程度可分为轻度、中度和重度。重度溃疡性结肠炎患者腹泻每日 6 次以上，有明显黏液脓血便，体温＞37.5℃、脉搏＞90 次/分，血红蛋白＜100g/L，血沉＞30mm/h，C-反应蛋白（CRP）升高，血清白蛋白＜30g/L。其治疗应首选糖皮质激素。

选项 B 硫唑嘌呤是免疫抑制药，主要不良反应为骨髓抑制、肝功能损害等，且起效慢，不作为溃疡性结肠炎首选用药，故排除。

选项 C 英夫利昔单抗主要用于对激素治疗效果不佳或对激素依赖的慢性持续型病例，特别适用于对激素无效的重度溃疡性结肠炎患者，但不作为首选，故不选。

选项 D 环孢素为免疫抑制剂，适用于器官移植、骨髓移植的排异反应，难以控制的异位性皮炎、银屑病等自身免疫性疾病，不用于溃疡性结肠炎的治疗，可排除。

选项 E 大量抗生素对于溃疡性结肠炎无效，因为溃疡性结肠炎不是由细菌感染引起的炎症，因此可优先排除。

综上所述，正确答案是：A.糖皮质激素。

重度溃疡性结肠炎的首选治疗通常是糖皮质激素。糖皮质激素可以迅速缓解急性炎症，是重度溃疡性结肠炎的标准初始治疗方法。

因此，最正确的答案是：

A. 糖皮质激素

20、 (A1 型题)

对于明确肺结核是否有传染性最有帮助的是

A.γ-干扰素释放试验

B.痰涂片抗酸染色

C.结核菌素试验

D.胸部 CT

E.抗结核抗体

参考答案： B 您的答案：

图文解析： 【解析】结核病在人群中的传染源主要是结核病患者，即痰直接涂片阳性者（B 对），主要咳嗽、喷嚏、大笑、大声谈话等方式把含有结核分枝杆菌的微滴排到空气中而传播。

【避错】γ-干扰素释放试验（A 错）可以区分结核分枝杆菌自然感染与卡介苗接种和大部分非结核分枝杆菌感染，因此诊断结核感染的特异性明显高于 PPD 试验，但由于成本较高等原因，目前多用于研究评价工作，尚未广泛推行。结核菌素试验（C 错）和抗结核抗体检查（E 错）只能用来鉴别患者是否曾受结核杆菌感染，并不能确定是否为传染源。胸部 CT（D 错）主要能显示各种类型肺结核的特点与性质，有无空洞及进展恶化和吸收好转的变化，并不能明确肺结核是否有传染性。

对于判断肺结核是否有传染性，最有帮助的方法是检查患者是否能够排出含有结核分枝杆菌的痰液。因此，最佳答案是：

B. 痰涂片抗酸染色

痰涂片抗酸染色是一种快速且有效的方法，用于检测痰中是否含有结核分枝杆菌，这是判断肺结核感染是否具有传染性的关键指标。其他选项如γ-干扰素释放试验、结核菌素试验和抗结核抗体检测，主要用于诊断感染的存在，而不是传染性。胸部 CT 则更多用于评估肺部病变的程度和类型。

解析：

肺结核是否具有传染性，主要取决于患者是否排菌，即痰中是否存在结核杆菌。如患者痰中存在结核杆菌，则具有传染性，反之则无传染性。痰涂片抗酸染色可通过检测痰中是否含有抗酸杆菌，来确定患者是否排菌，对于明确肺结核是否有传染性最有帮助。
选项 A：γ-干扰素释放试验，选项 C：结核菌素试验，二者均用于检出结核分枝杆菌的感染，而非检出病菌，故对判断肺结核传染性价值不大，因此可一并排除。
选项 D：胸部 CT 对于肺内病灶的性质、部位、累及范围及与周围组织的关系等观察较准确，但无法确定患者是否排菌，因此不选。
选项 E：抗结核抗体检测阳性说明体内存在抗结核杆菌抗体，表示受试者感染过结核杆菌，但不能判断是否具有传染性，故不考虑。
综上所述，正确答案是：B.痰涂片抗酸染色。
明确肺结核是否有传染性最有帮助的检查是：

B. 痰涂片抗酸染色

原因：痰涂片抗酸染色可以直接检测到结核分枝杆菌（抗酸杆菌）的存在。如果痰涂片抗酸染色阳性，说明患者的痰中有活的结核菌，具有传染性。因此，这是最直接且有效的检测肺结核传染性的方法。
21、 (A1 型题)
肝硬化贲门周围血管断流术不包括哪个血管
A.胃网膜左静脉
B.胃后静脉
C.左膈下静脉
D.冠状静脉
E.胃短静脉
参考答案： A 您的答案：
图文解析： 【解析】肝硬化贲门周围血管断流术不包括胃网膜左静脉（A 错，为本题正确答案）。
【避错】贲门周围血管可分成四组：①冠状静脉（D 对）：包括胃支、食管支及高位食管支等；②胃短静脉（E 对）： 一般为 3～4 支，伴行着胃短动脉，分布于胃底的前后壁，注入脾静脉；③胃后静脉（B 对）：起始于胃底后壁，伴着同名动脉下行，注入脾静；④左膈下静脉（C 对）：可单支或分支进入胃底或食管下段左侧肌层。
肝硬化贲门周围血管断流术（也称为门静脉分流术）旨在减轻门静脉压力和降低食管静脉曲张的风险。在这种手术中，医生会断流一些血管以减少门静脉系统的血流，从而降低门静脉高压。
选项分析如下：
- A. 胃网膜左静脉：通常会在门静脉高压情况下被断流，因为它与门静脉系统相连。
- B. 胃后静脉：也是与门静脉高压相关，可能会被断流。
- C. 左膈下静脉：不直接参与门静脉系统，通常不在此手术的断流范围内。
- D. 冠状静脉：与门静脉高压有关，可能会被断流以减少贲门部的压力。
- E. 胃短静脉：与门静脉高压有关，通常会被断流。
根据上述分析，最不可能被包括在肝硬化贲门周围血管断流术中的血管是 **C. 左膈下静脉**。因此，正确答案应该是 C。
解析：
肝硬化门静脉高压导致的食管胃底静脉曲张破裂大出血是门脉高压症最常见的并发症，其病

死率高、复发出血率高。贲门周围血管离断术是治疗门静脉高压所致食管胃底静脉曲张破裂出血的重要术式，通过离断食管下端和胃近端、贲门周围的反常血流，达到控制食管胃底静脉曲张破裂出血的目的，同时又维持了门静脉向肝血流，从而保证了肝脏的血液供应。胃网膜左静脉走行于胃大弯，沿胃大弯分布并与胃网膜右静脉吻合，注入脾静脉，是脾静脉的属支，不属于贲门周围血管，在贲门周围血管离断术中无需处理。
选项 B 胃后静脉，选项 C 左膈下静脉，选项 D 冠状静脉，选项 E 胃短静脉，均属于贲门周围血管，在行贲门周围血管离断术时需要一并离断，以阻断食管下段和胃底反常血流，减少门静脉高压所致的出血，可一并排除。
综上所述，正确答案是：A.胃网膜左静脉。
肝硬化贲门周围血管断流术（如 Sugiura 手术）是一种针对肝硬化并发食管胃底静脉曲张出血的手术，通过断离贲门周围的静脉血管以减少出血风险。以下是常见的贲门周围血管断流术涉及的血管：

1. 胃网膜左静脉
2. 胃后静脉
3. 冠状静脉
4. 胃短静脉

这些血管都是贲门周围血管断流术中需要处理的血管，而左膈下静脉通常不在该术式中涉及。因此，正确答案是：

C. 左膈下静脉
22、 (A1 型题)
结直肠中最常见的非肿瘤性息肉是
A.增生性息肉
B.错构瘤性息肉
C.化生性息肉
D.炎性息肉
E.腺瘤性息肉
参考答案： A 您的答案：
图文解析： 【解析】结直肠非肿瘤性息肉是指结直肠黏膜表面突出的异常生长的、较少发生癌变的肿物。其主要有幼年性息肉、增生性息肉和炎性息肉三类，其中最常见的非肿瘤性息肉是增生性息肉（A 对）。
【避错】肿瘤性息肉是大肠膜上皮细胞增生的真性肿瘤，其单发者统称为腺瘤，根据其组织学特征和生物学行为的不同又可分为腺管状、绒毛状和混合性 3 类，多发性者常见为家族性腺瘤病。
在结直肠中，最常见的非肿瘤性息肉是增生性息肉。因此，正确答案是 A.增生性息肉。
增生性息肉是结直肠中最常见的非肿瘤性息肉，通常被认为是良性的，很少会发展为癌症。这种类型的息肉通常不需要特别的治疗，但可能需要定期的监测，以确保它们不会发展成更严重的状况。
解析：
结直肠息肉泛指直肠粘膜表面向肠腔突出的隆起性病变。按病理可分为：腺瘤性息肉、错构瘤性息肉、增生性息肉、炎性息肉等。其中增生性息肉又称为化生性息肉，是最常见的一种非肿瘤性息肉，多发生在直肠，中年以后更为常见。

选项 B 错构瘤性息肉虽为非肿瘤性息肉，但不如增生性息肉常见，故排除。
选项 C 化生性息肉属于增生性息肉，不是单独的分类，故不选。
选项 D 炎性息肉又称假性息肉，是由于肠粘膜长期受慢性炎症刺激引起的息肉样肉芽肿，这种息肉多见于溃疡性结肠炎、慢性血吸虫病、阿米巴痢疾及肠结核等病的病变肠道中，属于非肿瘤性息肉，但不如增生性息肉常见，与题意不符。
选项 E 腺瘤性息肉又称肿瘤性息肉，是大肠良性上皮性肿瘤，可发生癌变，不属于非肿瘤性息肉，故排除。
综上所述，正确答案是：A.增生性息肉。
结直肠中最常见的非肿瘤性息肉是：

A. 增生性息肉
23、 (A1 型题)
采取非手术方法治疗急性消化性溃疡穿孔时，错误的是
A.静脉输液
B.胃肠减压
C.糖皮质激素
D.质子泵抑制剂
E.抗生素抗感染
参考答案： C 您的答案：
图文解析： 【解析】急性消化性溃疡穿孔使用糖皮质激素（C 错，为本题正确答案）无明显效果，甚至糖皮质激素会增加胃酸、胃蛋白酶分泌，减少胃黏液分泌，使胃肠黏膜的抵抗力下降，在胃液的侵蚀因素作用下可诱发或加剧胃、十二指肠溃疡以及消化道出血或穿孔。【避错】质子泵抑制剂抑酸作用很强，可使胃内达到无酸水平，多在 2～3 天内控制消化性溃疡症状（D 对）。抗生素抗感染（E 对）、镇静镇痛治疗、胃肠减压（B 对）、静脉输液（A 对）、营养为一般非手术治疗措施，适用于病情轻或病情超过 24h，且腹部体征已减轻或伴有严重心肺等脏器疾患不能耐受手术。
在处理急性消化性溃疡穿孔的非手术治疗中，糖皮质激素（C 选项）通常是不推荐的。急性消化性溃疡穿孔是一种需要紧急处理的临床状况，可能需要外科手术来修补穿孔。非手术治疗主要包括稳定病人的生命体征、抗生素治疗以防止感染、静脉输液维持水电解质平衡和营养支持、以及质子泵抑制剂（PPIs）减少胃酸分泌。
糖皮质激素通常不在急性消化性溃疡穿孔的非手术治疗中使用，因为它们可能抑制免疫反应，延迟溃疡愈合，并增加感染的风险。因此，对于这个问题，错误的答案是 C.糖皮质激素。
解析：
急性消化性溃疡穿孔是消化性溃疡的严重并发症，起病急、变化快、病情重，需要紧急处理。非手术治疗适用于一般情况良好，症状体征较轻的空腹穿孔。在非手术治疗中，糖皮质激素的应用是不适宜的。糖皮质激素虽然可以抑制炎症反应，但同时也可能加重消化道溃疡，增加消化道出血的风险，因此在急性消化性溃疡穿孔的治疗中应避免使用。
选项 A 静脉输液可补充血容量，维持水电解质平衡，是急性消化性溃疡穿孔非手术治疗的基础措施之一，不符合题意。
选项 B 胃肠减压可降低胃肠内压，减少胃肠内容物流入腹腔，有利于穿孔的愈合，是急性消化性溃疡穿孔非手术治疗的重要措施之一，故排除。
选项 D 质子泵抑制剂可抑制胃酸分泌，减少胃酸对胃肠黏膜的刺激，有利于穿孔的愈合，是非手术治疗中的重要药物，故不选。
选项 E 抗生素抗感染可预防和治疗感染，减少并发症的发生，是急性消化性溃疡穿孔非手术

治疗中必不可少的措施，与题意不符。
综上所述，正确答案是：C.糖皮质激素。
采取非手术方法治疗急性消化性溃疡穿孔时，错误的是：

C.糖皮质激素

解释：
急性消化性溃疡穿孔是指胃或十二指肠溃疡穿透胃肠壁引起腹腔内穿孔的急症。非手术治疗主要包括：

- 静脉输液（A）：补充体液和电解质，维持患者的循环稳定。
- 胃肠减压（B）：通过鼻胃管减轻胃肠内容物对穿孔部位的压力，减少进一步的漏出和炎症。
- 质子泵抑制剂（D）：减少胃酸分泌，促进溃疡愈合。
- 抗生素抗感染（E）：预防和治疗腹腔感染。

糖皮质激素（C）不属于急性消化性溃疡穿孔的常规治疗方法，因为它们可能会抑制炎症反应，延缓溃疡愈合，并增加感染的风险。
24、 (A1 型题)
功能性消化不良的定义是
A.症状 12 个月，连续 3 个月
B.症状 6 个月，连续 2 个月
C.症状 12 个月，连续 2 个月
D.症状 6 个月，连续 3 个月
E.症状 12 个月
参考答案： D 您的答案：
图文解析： 【解析】功能性消化不良是指由胃和十二指肠功能紊乱引起的餐后饱胀感、早饱、中上腹痛及中上腹烧灼感等症状，而无器质性疾病的一组临床综合征，是呈持续或反复发作的慢性过程，该病的症状出现至少 6 个月，近 3 个月症状符合其诊断标准（D 对）。【避错】目前我国采用罗马Ⅲ标准的功能性胃肠病命名分类。临床上以功能性消化不良和肠易激综合征多见。
功能性消化不良的定义通常是指症状持续至少 6 个月，并且在最近的 3 个月内有连续的症状。因此，最符合这一定义的选项是 D.症状 6 个月，连续 3 个月。所以正确答案是 D。
解析：
功能性消化不良是指由胃和十二指肠功能紊乱引起的，但没有器质性改变的胃肠道功能性疾病，临床表现主要为餐后饱胀、早饱感、上腹胀痛、嗳气、食欲不振、恶心等。功能性消化不良的诊断需满足以下标准：有上腹痛、上腹灼热感、餐后饱胀和早饱症状之一种或多种，呈持续或反复发作的慢性过程（病程超过 6 个月，近 3 个月症状持续）；同时需排除可解释上述症状的器质性疾病。
综上所述，正确答案是：D.症状 6 个月，连续 3 个月。
功能性消化不良（functional dyspepsia，FD）是一种常见的消化系统疾病，通常根据罗马标准（Rome Criteria）进行定义。根据最新的罗马 IV 标准，功能性消化不良的定义如下：

功能性消化不良的诊断标准包括：
- 在症状首次出现前至少 6 个月开始，并且在最近 3 个月内症状持续存在。

- 主要症状包括上腹部痛、上腹部不适、早饱感或餐后饱胀感。

基于上述信息，最符合罗马 IV 标准的选项是：

D. 症状 6 个月，连续 3 个月

因此，正确答案是：**D. 症状 6 个月，连续 3 个月**

25、 (A1 型题)

哪个部位的溃疡应特别注意卓艾综合征

A.胃窦部

B.幽门部

C.十二指肠球部

D.十二指肠降部

E.回肠部

参考答案： D 您的答案：

图文解析： 【解析】卓艾综合征的溃疡特点为多发、位于不典型部位，例如十二指肠降部（D 对）、对正规抗溃疡药物疗效差等。

【避错】胃溃疡好发于胃角及胃窦小弯部；十二指肠溃疡好发于十二指肠球部；十二指肠球后溃疡容易出现出血并发症。

卓艾综合征（Zollinger-Ellison Syndrome，ZES）是一种由胃泌素瘤（gastrinoma）引起的疾病，这种瘤体通常位于胰腺和十二指肠，导致胃酸分泌过多，从而引起消化性溃疡。在 Zollinger-Ellison 综合征中，溃疡通常发生在十二指肠，尤其是十二指肠球部，但也可能发生在胃或其他肠道部位。因此，当考虑到卓艾综合征时，应特别注意十二指肠球部的溃疡。

选项 C.十二指肠球部是最符合这一描述的选项。所以最正确的答案是 C.十二指肠球部。

解析：

卓艾综合征又称为促胃液素瘤或 Zollinger-Ellison 综合征，是一种以消化道溃疡为主要表现的综合病症。其典型表现为顽固及不典型部位的消化性溃疡及腹泻，溃疡最常见于十二指肠降部和水平部，且常呈多发，顽固难治。

选项 A 胃窦部，选项 B 幽门部，均是消化性溃疡的常见部位，与卓艾综合征无明确关联，可一并排除。

选项 C 十二指肠球部是十二指肠溃疡最常见的好发部位，与卓艾综合征无关，故不选。

选项 E 回肠部并非消化性溃疡的常见发病部位，卓艾综合征中消化道溃疡也罕见于此处，故排除。

综上所述，正确答案是：D.十二指肠降部。

卓艾综合征（Zollinger-Ellison 综合征，ZES）是一种由于胃泌素瘤导致胃泌素分泌过多，从而引起胃酸分泌增加和难治性溃疡的疾病。ZES 的溃疡通常发生在十二指肠的多个部位，并且比常见溃疡更难治疗。

在 ZES 中，溃疡最常见的部位是十二指肠球部，因为这是胃酸最先进入小肠的地方，且十二指肠球部的溃疡最常见。

因此，最正确的答案是：

C. 十二指肠球部

26、 (A1 型题)
关于电烧伤，下列说法正确的是
A.入口处较出口处损伤重
B.损伤范围通常外小内大
C.伤后坏死范围一般不会再扩大
D.局部渗出较一般烧伤轻
E.有明显坏死层面
参考答案： B 您的答案：
图文解析： 【解析】电烧伤时入口处常炭化，形成裂口或洞穴，烧伤常深达肌肉、肌腱、骨骼，损伤范围常外小内大（B 对）。
【避错】电烧伤指由电流通过人体所引起的烧伤，烧伤的主要特点：①出口轻，里面重（A 错），损伤范围外小内大（B 对）；②由于邻近血管的损害，经常出现进行性坏死，伤后坏死范围可扩大数倍（C 错）；③局部渗出较一般烧伤重（D 错）；④没有明显的坏死层面（E 错）。
关于电烧伤的特点，选项 B "损伤范围通常外小内大" 是正确的。电烧伤通常由电流穿过身体引起，可能导致内部组织（如肌肉和血管）受到比皮肤表面更严重的伤害。因此，电烧伤的外部损伤可能看起来比实际的内部损伤轻，所以损伤范围通常是外小内大。因此，最正确的答案是 B。
解析：
电烧伤通常是由于电流通过人体所引起的烧伤，电流通过人体有“入口”和“出口”，入口处较出口处损伤重，入口周围有炭化，形成裂口或洞穴，烧伤常深达肌肉、肌腱、骨周，损伤范围常外小内大。
选项 A 入口处较出口处损伤重，电流从皮肤或黏膜破损处进入体内，在入口处形成电流斑，入口处常有炭化，形成裂口或洞穴，烧伤常深达肌肉、肌腱、骨周，损伤较重，而出口处损伤相对较轻，此选项描述正确，但不是本题最佳答案。
电烧伤的特点是损伤范围通常外小内大，即损伤范围常较受损伤皮肤创面为大，而且伤后坏死范围常随时间增大，数日后才能确定其范围和深度，所以选项 C“伤后坏死范围一般不会再扩大”不符合电烧伤特点，故排除。
选项 D 局部渗出较一般烧伤轻与事实相反，电烧伤不同于一般的烧伤，其特点为“口小底大，外浅内深”，往往深层的组织损伤更为严重，故电烧伤局部渗出常较一般烧伤更为严重，故不选。
电烧伤没有明显的坏死层面，往往伴有周围组织的进行性坏死，有时坏死范围可扩展至肌肉、肌腱、骨骼或更深层组织，因此选项 E 有明显坏死层面与电烧伤特点不符，故不选。
综上所述，正确答案是：B.损伤范围通常外小内大。
关于电烧伤，下列说法正确的是：

B. 损伤范围通常外小内大

解析：
电烧伤的特点之一是损伤范围通常外小内大，这是由于电流通过人体时在体内产生的热量较大，造成深部组织的损伤较为严重，而表面损伤相对较轻。此外，电烧伤的损伤范围在伤后通常会继续扩大，坏死范围也会逐渐增大。因此，选项 B 是正确的。

其他选项解析：

A. 入口处较出口处损伤重：通常电烧伤的入口和出口都可能出现严重的损伤，但具体损伤程度要看电流的路径和电压的高低，一般来说，出口处的损伤可能更严重。
C. 伤后坏死范围一般不会再扩大：电烧伤后的坏死范围会逐渐扩大，因此这说法不正确。
D. 局部渗出较一般烧伤轻：电烧伤的局部渗出可能比一般烧伤更严重，因此这说法不正确。
E. 有明显坏死层面：电烧伤确实会有明显的坏死层面，但这不是最能概括电烧伤特点的正确答案。

综上所述，正确答案是 B。
27、 (A1 型题)
甲状腺结节，纵横比＞1，有丰富血流信号，检查发现降钙素分泌增多，可能为哪种病理类型
A.未分化癌
B.乳头状癌
C.滤泡状癌
D.髓样癌
E.低分化癌
参考答案： D 您的答案：
图文解析： 【解析】甲状腺结节，纵横比＞1，有丰富血流信号，高度怀疑为甲状腺的恶性肿瘤。且该患者降钙素升高，故考虑为髓样癌（D 对）。
【避错】髓样癌来源于 C 细胞，细胞排列呈巢状或囊状，无乳头或滤泡结构，呈未分化状；间质内有淀粉样物沉积，恶性程度中等，可有颈淋巴结侵犯和血行转移。
甲状腺结节的特征，如纵横比大于 1、丰富的血流信号，以及检查发现降钙素分泌增多，通常指向髓样癌。髓样癌是一种由甲状腺的 C 细胞引起的神经内分泌肿瘤，这些细胞负责分泌降钙素。因此，降钙素水平的增加是髓样甲状腺癌的一个重要指标。因此，根据题干描述的特征，最合适的答案是：D. 髓样癌
解析：
甲状腺结节是指在甲状腺内的肿块，可随吞咽动作随甲状腺而上下移动，是临床常见的病症，可由多种病因引起。降钙素即降钙素原，由甲状腺滤泡旁细胞（C 细胞）合成和分泌，主要由降钙素、降钙素基因相关肽和甲状旁腺激素相关蛋白三部分组成。当甲状腺结节且伴有降钙素分泌增多时，最可能的病理类型是甲状腺髓样癌。甲状腺髓样癌来源于滤泡旁降钙素分泌细胞（C 细胞），血清降钙素明显升高常提示髓样癌的存在。
选项 A 未分化癌、选项 B 乳头状癌、选项 C 滤泡状癌、选项 E 低分化癌，均无降钙素升高，可一并排除。
综上所述，正确答案是：D.髓样癌。
题目描述了一种甲状腺结节的特征：纵横比＞1，有丰富血流信号，且降钙素分泌增多。降钙素（Calcitonin）是由甲状腺 C 细胞分泌的激素，升高的降钙素水平常提示髓样癌的存在。结合其他特征，如结节纵横比大于 1 和丰富的血流信号，也支持这一诊断。

所以，最可能的病理类型是：

D. 髓样癌
28、 (A1 型题)
继病因后肝硬化患者的肝功能异常的原因
A.营养不良

B.对激素灭活增加
C.侧支循环形成
D.脾功能亢进
E.腹压升高
参考答案： C 您的答案：
图文解析： 【解析】侧支循环形成（C 对）是继病因之后推动肝功能减退的重要病理生理环节，侧支循环会造成大量门静脉血流绕过肝细胞，致使有毒物质不能代谢与解毒而直接进入体循环，从而使肝功能进一步减退。
【避错】其他选项均为肝硬化的一般临床表现。
肝硬化患者肝功能异常的原因较为复杂，可以包括多个方面的因素。这里的选项中：
- A. 营养不良：营养不良会影响整体健康，但不是肝功能异常的直接原因。
- B. 对激素灭活增加：肝硬化患者的肝脏对荷尔蒙的代谢能力减弱，不是激素灭活增加。
- C. 侧支循环形成：这是肝硬化晚期常见的现象，可以导致门脉高压，但不直接导致肝功能异常。
- D. 脾功能亢进：在肝硬化中，门脉高压可以导致脾功能亢进，这可以间接影响肝功能。
- E. 腹压升高：腹压升高可能是因为腹水增多，这是肝硬化晚期的症状之一，但不是肝功能异常的直接原因。
考虑到肝硬化导致的门脉高压可引起脾功能亢进，进而影响血液成分，如增加红细胞和血小板的破坏，从而影响肝功能，我倾向于选择 **D. 脾功能亢进** 作为答案。脾功能亢进可以引起血细胞减少，进而影响肝功能检测结果，表现为肝功能异常。
解析：
肝硬化是由一种或多种病因长期、反复刺激造成肝脏弥漫性损害，其特点为慢性弥漫性结缔组织增生，肝细胞变性坏死、再生和肝小叶结构损害及假小叶形成。早期肝功能尚可代偿，肝硬化发展到一定程度，超出肝功能的代偿能力，称为肝硬化失代偿期，主要临床表现为肝功能减退和门静脉高压所致的症状和体征。肝功能减退会影响蛋白质的合成，白蛋白合成减少，从而影响血浆胶体渗透压，致使组织液生成过多，而出现营养不良性水肿，因此营养不良是肝功能异常的表现。
选项 B 对激素灭活增加与题意相反，应为对激素的灭活减少，故不选。
选项 C 侧支循环形成，选项 E 腹压升高，均是门静脉高压所导致的临床表现，可一并排除。
选项 D 脾功能亢进主要表现为脾肿大、脾功能亢进为特征，早期以白细胞及血小板减少为主，不是肝功能异常的原因，故不考虑。
综上所述，正确答案是：A.营养不良。
继病因后肝硬化患者的肝功能异常的原因主要包括多种因素，但其中最为直接和常见的原因是脾功能亢进（D.脾功能亢进）。脾功能亢进是由于门静脉高压导致脾脏血流增多，引起脾脏的增大和功能亢进，进而引起血细胞的过度破坏，导致贫血、白细胞减少和血小板减少，这些都会进一步加重肝功能异常。

因此，最正确的答案是：

D. 脾功能亢进
29、 (A1 型题)
食管胃底静脉曲张行手术治疗，需切断的血管不包括
A.胃左静脉
B.胃短静脉

C.胃后静脉
D.左膈下静脉
E.脾静脉
参考答案：　　E　您的答案：
图文解析：　　【解析】食管胃底静脉曲张行手术治疗，需切断的血管不包括大静脉，例如胃网膜右静脉、脾静脉（E 对）。
【避错】贲门周围血管可分成四组：①胃左静脉（A 错），又名冠状静脉：包括胃支、食管支及高位食管支等。②胃短静脉（B 错）：　一般为 3～4 支，伴行着胃短动脉，分布于胃底的前后壁，注入脾静脉。③胃后静脉（C 错）：起始于胃底后壁，伴着同名动脉下行，注入脾静脉。④左膈下静脉（D 错）：可单支或分支进入胃底或食管下段左侧肌层。
在进行食管胃底静脉曲张的手术治疗时，主要目标是减少门脉高压下胃食管区域的压力，从而降低出血的风险。手术中通常需要切断与门脉高压相关的静脉通路，特别是那些直接贡献于食管下段和胃底静脉曲张的静脉。
A. 胃左静脉（left gastric vein）通常会被切断，因为它是食管底部和胃底静脉曲张的主要血液来源之一。
B. 胃短静脉（short gastric veins）也是连接至脾静脉的，参与胃底静脉的血液回流，可能会被切断。
C. 胃后静脉（posterior gastric vein）可能会参与到这一过程中，尤其是如果它们为食管下段或胃底的静脉曲张提供血液供应。
D. 左膈下静脉（left inferior phrenic vein）可能不直接参与到食管下段和胃底的静脉曲张中，因此可能不需要切断。
E. 脾静脉（splenic vein）是主要的血管之一，它通过短胃静脉与胃底静脉曲张相连，通常在门体分流术中被涉及。
根据这些信息，最不可能需要切断的血管是 **D. 左膈下静脉**，因为它不是直接参与食管和胃底静脉曲张的主要血管。
解析：
食管胃底静脉曲张行手术治疗，主要目的是离断食管下段及胃底贲门周围的血管，包括胃左静脉（也称冠状静脉）、胃后静脉、胃短静脉、左膈下静脉及其伴随的同名动脉，同时行脾切除，达到即刻止血的目的。但脾静脉是脾动脉的伴行静脉，行于脾动脉的下方，横过后方左肾和肾静脉的前面，延伸至胰尾，至胰颈后方，向右行横过后方的左肾上腺下极和肾的前面，到达横结肠右曲，再向前汇入门静脉系统。其并不参与食管胃底静脉的构成，且对维持脾脏静脉回流具有重要意义，因此无需离断。
选项 A 胃左静脉又称冠状静脉，是与食管下段静脉丛相吻合的，是手术时需要离断的血管之一。
选项 B 胃短静脉，选项 C 胃后静脉，选项 D 左膈下静脉，均参与食管胃底静脉丛的形成，因此手术时需一并离断，以阻断门奇静脉间的反常血流，达到控制食管胃底静脉曲张破裂出血的目的。
综上所述，正确答案是：E.脾静脉。
食管胃底静脉曲张的手术治疗主要是通过切断供血的静脉来减轻曲张静脉的压力，从而防止出血。以下是几种静脉曲张的手术中常需要切断的血管：

- **胃左静脉**（又称冠状静脉）
- **胃短静脉**
- **胃后静脉**

- **左膈下静脉**

但**脾静脉**通常不是需要切断的血管之一。脾静脉是将血液从脾脏带走并注入门静脉系统的一部分，而不是直接参与食管胃底静脉曲张的供血。

因此，答案是：

E. 脾静脉

30、 (A1 型题)

属于慢性肛裂特点的是

A.有弹性

B.肉芽灰白

C.无瘢痕形成

D.底部浅

E.边缘整齐

参考答案： B 您的答案：

图文解析： 【解析】慢性肛裂的特点是：底深不整齐，质硬，边缘增厚纤维化，肉芽灰白（B 对），有瘢痕形成。

【避错】急性肛裂可见裂口边缘整齐（E 错），底浅（D 错），呈红色并有弹性（A 错），无瘢痕形成（C 错）。

慢性肛裂的特点通常包括边缘硬化、底部可见内肛管肌或纤维组织、并且往往伴有肛乳头增生或肛窦形成。因此，在这些选项中，最符合慢性肛裂特点的是：

B. 肉芽灰白

这个选项指的可能是在肛裂底部看到的肉芽组织，这种灰白色的肉芽组织是由于慢性炎症和愈合过程中形成的。因此，最佳答案应该是 B. 肉芽灰白。

首先，我们来理解一下慢性肛裂的基本概念。肛裂是指肛门皮肤的全层纵行裂开，通常是由于大便干燥或肛门括约肌过度收缩引起的。肛裂可分为急性和慢性两种，其中慢性肛裂通常是由于急性肛裂未得到及时治疗而形成的。

现在，我们来逐一分析各个选项：

A. 有弹性：慢性肛裂由于长期的炎症和愈合过程，组织往往变得硬化，失去弹性。因此，这一选项不符合慢性肛裂的特点。

B. 肉芽灰白：在慢性肛裂中，由于组织的慢性炎症和修复过程，肉芽组织可能呈现出灰白色，这与长期的愈合和瘢痕形成有关。这一选项是符合慢性肛裂特点的。

C. 无瘢痕形成：慢性肛裂由于长期的愈合和再损伤过程，往往会在肛裂周围形成瘢痕组织。因此，这一选项与慢性肛裂的特点不符。

D. 底部浅：慢性肛裂由于长期的慢性炎症和修复，其裂口通常较深，与急性肛裂相比，其底部更深。因此，这一选项不符合慢性肛裂的特点。

E. 边缘整齐：慢性肛裂的边缘往往不规则，有增生和肥厚，不像急性肛裂那样边缘整齐。因此，这一选项也不符合慢性肛裂的特点。

综上所述，符合慢性肛裂特点的是肉芽灰白，这是由于长期的慢性炎症和修复过程所导致的。因此，正确答案是 B。

所以，本题的正确选项是 B.肉芽灰白。

慢性肛裂的特点包括持续时间长、难以愈合、常伴有瘢痕组织和外翻的肛缘。具体的特点如下：

1. 肛裂的边缘常不整齐，有外翻的皮肤皱襞。
2. 底部较深，可见灰白色的肉芽组织。
3. 常伴有瘢痕形成。
4. 一般不会有弹性。

基于上述特点，最正确的答案是：

B. 肉芽灰白

31、 (A1 型题)

桡骨下三分之一骨折合并尺骨小头脱位属于

A.蒙氏骨折

B.盖氏骨折

C.Colles 骨折

D.Smith 骨折

E.尺骨骨折

参考答案： B 您的答案：

图文解析： 【解析】桡骨下三分之一骨折合并尺骨小头脱位属于盖氏骨折。

【避错】尺骨上三分之一骨折合并桡骨头脱位属于蒙氏骨折（A 错）；伸直型骨折（Colles 骨折）（C 错）多由于腕关节处于背伸位、手掌着地、前臂旋前时受伤导致，可出现典型畸形姿势，即侧面看呈“银叉”畸形，正面看呈“刺刀样”畸形，X 线拍片可见骨折远端向桡、背侧移位，近端向掌侧移位；史密斯（Smith）（D 错）骨折是指桡骨远端 2～3cm 以内的骨折，且骨折远端向掌侧移位或向背侧成角。

桡骨下三分之一骨折合并尺骨小头脱位通常被称为蒙氏骨折（Monteggia 骨折）。因此，最正确的答案是：A. 蒙氏骨折

这是一道医学专业知识的问题。首先，我们需要明确各个选项代表的骨折类型，再与题目中描述的桡骨下三分之一骨折合并尺骨小头脱位进行对比。

A. 蒙氏骨折：这种骨折涉及到尺骨上三分之一骨干骨折，同时伴随桡骨头脱位，与题目描述不符。

B. 盖氏骨折：这种骨折指的是桡骨干下三分之一骨折，同时伴随尺骨小头脱位。这与题目中描述的“桡骨下三分之一骨折合并尺骨小头脱位”完全吻合。

C. Colles 骨折：这种骨折是指发生在桡骨远端三厘米以内的骨折，且骨折远端会向桡、背侧移位，近端则向掌侧移位，与题目描述不符。

D. Smith 骨折：这种骨折是屈曲型桡骨远端骨折，或称为反 Colles 骨折，其骨折远端会向掌侧和尺侧移位，与题目描述不符。

E. 尺骨骨折：这一选项仅描述了骨折发生的位置，并未涉及桡骨或尺骨小头脱位的情况，与题目描述不符。

综上所述，桡骨下三分之一骨折合并尺骨小头脱位符合盖氏骨折的定义。

因此，正确答案是 B.盖氏骨折。

桡骨下三分之一骨折合并尺骨小头脱位属于：

B. 盖氏骨折 (Galeazzi fracture)

盖氏骨折是指桡骨远端骨折合并尺骨小头脱位的情况。

32、　(A1 型题)
最常出现 ANCA（中性粒细胞胞质抗体）阳性的肾病是
A.急进性肾小球肾炎
B.急性肾小球肾炎
C.狼疮肾炎
D.急性间质性肾炎
E.急性肾小管坏死
参考答案：　A　您的答案：
图文解析：
最常出现 ANCA（中性粒细胞胞质抗体）阳性的肾病是 A.急进性肾小球肾炎。
急进性肾小球肾炎通常与 ANCA 阳性相关，是一种严重的肾脏疾病，其特征是肾小球的快速损害。ANCA 主要与小血管炎相关，包括急进性肾小球肾炎的两个主要类型：显微镜下多血管炎和坏死性小动脉炎。因此，选择 A 作为最常见的与 ANCA 阳性相关的肾病是最合适的。
解析：
中性粒细胞胞质抗体（ANCA）是一种以中性粒细胞和单核细胞胞质成分为靶抗原的自身抗体，是原发性小血管炎诊断、监测病情活动和预测复发的重要指标，特异性、敏感性均较好。最常出现 ANCA 阳性的肾病是急进性肾小球肾炎。
选项 B 急性肾小球肾炎主要由 β-溶血性链球菌感染后所诱发的免疫反应引起，表现为血尿、蛋白尿、水肿和高血压，不出现 ANCA 阳性，故排除。
选项 C 狼疮肾炎是指系统性红斑狼疮合并双肾不同病理类型的免疫性损害，同时伴有明显肾脏损害临床表现的一种疾病，其发病机制是免疫复合物形成与沉积，不会引起 ANCA 阳性，故不选。
选项 D 急性间质性肾炎是以肾小管间质损害为主的肾脏疾病，主要由多种原因导致肾间质炎性细胞浸润、间质水肿、肾小管受损，肾小球及肾血管一般不受累或受累相对较轻，与免疫复合物沉积有关，与本题不符。
选项 E 急性肾小管坏死主要由缺血和肾毒性物质损害引起，病变主要在近曲小管，与本题无关。
综上所述，正确答案是：A.急进性肾小球肾炎。
32. 最常出现 ANCA（中性粒细胞胞质抗体）阳性的肾病是

A. 急进性肾小球肾炎

急进性肾小球肾炎（RPGN）是最常与 ANCA 阳性相关的肾病。ANCA 相关血管炎（如显微镜下多血管炎和韦格纳肉芽肿病）通常表现为急进性肾小球肾炎，患者可能会出现急性肾功能衰竭，并在肾活检中看到新月体形成。因此，正确答案是 A. 急进性肾小球肾炎。
33、　(A1 型题)
对鉴别是否肾小球源性血尿最有意义的是
A.全程血尿
B.合并尿道刺激征
C.尿潜血阳性
D.肉眼血尿
E.变形红细胞血尿
参考答案：　E　您的答案：
图文解析：　镜检变形红细胞多为肾小球源性的，均一形态正常红细胞为非肾小球源性的。

尿红细胞容积分布曲线，肾小球源性呈非对称性，非肾小球源性呈对称性曲线。
在鉴别是否肾小球源性血尿时，最有意义的是选项 E，变形红细胞血尿。肾小球源性血尿的特点是尿中出现变形红细胞（即异形红细胞），这是由于红细胞经过受损的肾小球时发生形态变化所致。相比之下，非肾小球源性血尿（如下尿路出血）通常呈现非变形的红细胞。因此，检测尿液中的变形红细胞是鉴别肾小球源性血尿的重要指标。
首先，我们来看一下这个问题。题目要求我们鉴别哪种表现对于判断是否为肾小球源性血尿最有意义。
选项 A，全程血尿，指的是从排尿开始到排尿结束，尿液都含有血液。然而，这并不能明确指出血尿的来源，因为除了肾小球源性血尿，其他如输尿管、膀胱或尿道的问题也可能导致全程血尿。
选项 B，合并尿道刺激征，通常与尿路感染相关，并非肾小球疾病的特异性表现。
选项 C，尿潜血阳性，仅表示尿中存在血红蛋白，但并不能明确其来源是肾小球还是其他部位。
选项 D，肉眼血尿，指的是可以通过肉眼直接观察到的血尿。但这同样不能明确血尿的来源。
选项 E，变形红细胞血尿，是指红细胞通过病变的肾小球基底膜时受到挤压和损伤，导致红细胞形态发生改变。这种形态改变的红细胞称为变形红细胞。当尿中出现大量变形红细胞时，通常意味着血尿来源于肾小球，因此这对于鉴别是否为肾小球源性血尿具有重要意义。
综上所述，变形红细胞血尿是鉴别肾小球源性血尿的关键指标。因此，最有意义的选项是 E.变形红细胞血尿。
鉴别是否肾小球源性血尿最有意义的指标是变形红细胞血尿（E）。变形红细胞（dysmorphic red blood cells）在显微镜下呈现不同于正常红细胞的形态，是由于通过受损的肾小球基底膜时受到的物理压力所致。这个特征是肾小球源性血尿的一个重要标志。

因此，答案是：E. 变形红细胞血尿

34、 (A1 型题)

属于激素冲击治疗适应证的是

A.新月体肾炎

B.毛细血管内增生性肾小球肾炎

C.系膜增生性肾小球肾炎

D.局灶节段性肾小球硬化

E.急性间质性肾炎

参考答案： A 您的答案：

图文解析： 新月体肾炎患者及早明确诊断，早期激素强化治疗，预后可得到显著改善。
激素冲击治疗主要用于那些对皮质类固醇治疗有良好反应的疾病。在这些选项中，新月体肾炎（A 选项）是对激素冲击治疗反应良好的肾脏疾病之一。新月体肾炎通常需要积极的免疫抑制治疗，包括高剂量的皮质类固醇（激素冲击治疗），以减少肾脏炎症和避免进一步的肾脏损伤。
因此，最佳答案是 A.新月体肾炎。
这是一道关于肾脏疾病治疗的选择题，我们需要基于各种肾小球疾病的特性和治疗方法来判断哪种疾病适用于激素冲击治疗。
首先，我们来分析各个选项代表的疾病及其治疗方法：
A. 新月体肾炎：这是一种快速进展的肾小球肾炎，通常与抗肾小球基底膜抗体或免疫复合物沉积有关。其特点是肾小球内新月体形成，肾功能迅速恶化。对于这种疾病，早期、强化的免疫抑制治疗是关键，包括大剂量糖皮质激素冲击治疗。

B. 毛细血管内增生性肾小球肾炎：常见于急性肾小球肾炎，主要表现为血尿、蛋白尿、水肿和高血压。其治疗主要是支持治疗和对症治疗，激素冲击治疗不是首选。
C. 系膜增生性肾小球肾炎：这是一种慢性肾小球肾炎，表现为系膜细胞和系膜基质的增多。其治疗包括控制血压、减少蛋白尿等，激素治疗可能有一定效果，但并非首选激素冲击治疗。
D. 局灶节段性肾小球硬化：这是一种慢性肾脏疾病，表现为部分肾小球节段性的硬化和瘢痕形成。治疗主要是控制血压、减少蛋白尿，以及可能的免疫抑制治疗，但不首选激素冲击治疗。
E. 急性间质性肾炎：通常与药物反应、感染或自身免疫性疾病有关。治疗主要是停用相关药物、控制感染或使用免疫抑制剂，但不采用激素冲击治疗。
综上所述，只有新月体肾炎是激素冲击治疗的适应症，因为这种治疗方法可以迅速抑制免疫反应，防止肾功能进一步恶化。
因此，正确答案是 A.新月体肾炎。
对于激素冲击治疗的适应证，新月体肾炎（A.新月体肾炎）是其中之一。新月体肾炎是一种严重的、快速进展的肾小球肾炎，其特征是肾小球的广泛新月体形成。激素冲击治疗在此类疾病中可用于抑制炎症反应，减缓疾病进展。

因此，最正确的答案是：

A. 新月体肾炎
35、 (A1 型题)
急性肾衰竭、高钾血症患者，心率 40 次/分，应首先采取的治疗措施是
A.静脉点滴 5％碳酸氢钠
B.静脉点滴 10％葡萄糖＋胰岛素
C.口服降钾树脂
D.静脉注射 10％葡萄糖酸钙
E.血液透析
参考答案： E 您的答案：
图文解析： 急性肾衰竭少尿期易发生高钾血症，主要表现为心肌收缩力减弱、心音低钝、心率减慢、室性期前收缩、房室传导阻滞、室颤，甚至心跳停搏。本例心率仅 40 次/分，说明已有十分严重的高血钾，引起Ⅲ度房室传导阻滞，因此血液透析是最有效的治疗。ABCD 都是降低血钾的一般性治疗措施。
在这种情况下，患者患有急性肾衰竭和高钾血症，并且心率仅为 40 次/分，这表明患者可能出现了严重的心脏问题，需要立即处理。在高钾血症的紧急处理中，首先要考虑的是稳定心脏膜电位并快速降低血钾水平。
A. 静脉点滴 5%碳酸氢钠用于治疗酸中毒，可以帮助转移体内的钾离子，但它不是直接降低血钾的最快方法。
B. 静脉点滴 10％葡萄糖＋胰岛素可以迅速将血钾转移到细胞内，从而降低血钾水平，这是治疗高钾血症的常用方法，但在心率极低的情况下可能不是首选。
C. 口服降钾树脂是长期控制血钾水平的措施，作用较慢，不适用于紧急情况。
D. 静脉注射 10％葡萄糖酸钙可以快速稳定心脏膜电位，对于心律失常特别是在高钾血症引起的心动过缓情况下，是紧急处理的首选方法。
E. 血液透析是最终治疗高钾血症和急性肾衰竭的方法，但在紧急情况下需要立即采取行动以稳定心脏状况，透析的准备可能需要更多时间。
因此，最合适的首选治疗措施是 D. 静脉注射 10％葡萄糖酸钙，以快速稳定心脏膜电位并防

止心律失常。
解析：
急性肾衰竭患者出现高钾血症时，其处理原则包括：1. 纠正代谢性酸中毒；2. 稳定心肌细胞膜电位；3. 迅速降低血钾浓度；4. 及时处理原发病和恢复肾功能。急性肾衰竭合并高钾血症患者出现心率缓慢时，应高度警惕心脏骤停，因此必须迅速降低血钾浓度，血液透析可迅速清除体内过多的钾离子，是降低血钾的最有效方法。
选项 A 静脉点滴 5%碳酸氢钠可以纠正酸中毒，并促使钾离子向细胞内转移，但降钾作用起效较慢，因此不是首选的治疗方式，故排除。
选项 B 静脉点滴 10%葡萄糖＋胰岛素可以使钾离子转入细胞内，从而降低血清钾浓度，但起效较慢，因此可排除。
选项 C 口服降钾树脂虽然可以迅速降低肠道中的钾离子，从而降低血钾浓度，但起效较血液透析慢，因此不作为首选治疗方式，故不选。
选项 D 静脉注射 10%葡萄糖酸钙可稳定心肌细胞膜电位，但不能降低血钾浓度，故不考虑。
综上所述，正确答案是：E.血液透析。
对于急性肾衰竭伴高钾血症且心率 40 次/分的患者，首先需要迅速纠正高钾血症并保护心脏。最佳答案是：

D. 静脉注射 10%葡萄糖酸钙

理由：葡萄糖酸钙能迅速对抗高钾血症对心肌的抑制作用，稳定心肌细胞膜，防止心脏停搏。这是一种快速的、紧急的治疗措施，可以立即保护心脏功能。其他选项虽然也可以降低血钾水平，但它们的作用较慢或不直接对抗高钾血症对心脏的影响。
36、 (A1 型题)
下述检查对区分上下尿路感染没有意义的是
A.尿白细胞管型
B.B 超示肾盂形态异常
C.膀胱穿刺培养示大肠埃希菌
D.肾区扣痛
E.外周血 WBC 明显升高
参考答案： C 您的答案：
图文解析：
尿路感染最常见的病菌是大肠埃希菌，上下尿路均可有，无法区分。选项 ABDE 可见于上尿路感染，而不可见于下尿路感染，故可鉴别。
为了区分上尿路感染（如肾盂肾炎）和下尿路感染（如膀胱炎），我们需要依赖于那些可以明确指示感染部位的检查。下面是各个选项的分析：
A. 尿白细胞管型：通常出现在上尿路感染（如肾炎）中，可以帮助确定感染部位，因此对区分上下尿路感染有意义。
B. B 超示肾盂形态异常：通过 B 超检查肾盂是否异常，可以帮助判断是否为上尿路的问题，因此对区分上下尿路感染有意义。
C. 膀胱穿刺培养示大肠埃希菌：膀胱穿刺培养能够发现引起下尿路感染的病原体，但对于区分上尿路和下尿路感染来说，不具有特异性，因为大肠埃希菌也可能是上尿路感染的病原体。
D. 肾区扣痛：肾区扣痛是上尿路感染的典型症状，有助于区分上下尿路感染。
E. 外周血 WBC 明显升高：这是全身性的感染反应，不能准确区分上尿路和下尿路感染。

因此，最不能明确区分上下尿路感染的检查是 **E. 外周血 WBC 明显升高**，因为这是一种全身性的反应，不足以区分感染的具体部位。
这道题目要求我们判断哪项检查对于区分上下尿路感染没有意义。为了解决这个问题，我们首先需要了解上下尿路感染的特点及其常见的诊断方法。
A. 尿白细胞管型：白细胞管型表示肾小管内有炎症变化，通常出现在肾盂肾炎等上尿路感染中，因此这对区分上下尿路感染是有意义的。
B. B 超示肾盂形态异常：这通常提示上尿路感染，如肾盂肾炎等，因为下尿路感染一般不会引起肾盂形态的明显变化。所以，这一检查对于区分上下尿路感染同样是有意义的。
C. 膀胱穿刺培养示大肠埃希菌：大肠埃希菌是常见的尿路感染细菌，但它既可以引起上尿路感染也可以引起下尿路感染。因此，仅通过膀胱穿刺培养发现大肠埃希菌并不能明确区分上下尿路感染。
D. 肾区扣痛：这通常是上尿路感染的一个典型症状，如下背部疼痛或肾区扣痛，在肾盂肾炎等上尿路感染中尤为常见。因此，这一症状对于区分上下尿路感染是有帮助的。
E. 外周血 WBC 明显升高：在上尿路感染中，由于炎症的全身性影响，外周血白细胞通常会明显升高。而在下尿路感染中，这种情况较少见。因此，这一检查也有助于区分上下尿路感染。
综上所述，膀胱穿刺培养发现大肠埃希菌既不能明确指示上尿路感染，也不能明确指示下尿路感染，因此它对区分上下尿路感染没有意义。所以，正确答案是 C 选项。

所以，正确答案是 C.膀胱穿刺培养示大肠埃希菌。
下述检查对区分上下尿路感染没有意义的是：

D. 肾区扣痛

解析：

- **尿白细胞管型（A）**：白细胞管型通常提示肾脏的炎症或感染，有助于区分上尿路（肾脏）感染。
- **B 超示肾盂形态异常（B）**：肾盂形态异常可以提示上尿路感染。
- **膀胱穿刺培养示大肠埃希菌（C）**：膀胱穿刺培养直接取样膀胱内的尿液，主要用于下尿路感染的诊断。
- **肾区扣痛（D）**：肾区扣痛虽然可能提示上尿路感染，但它的存在与否并不能有效区分上下尿路感染，其他因素也可能导致肾区疼痛。
- **外周血 WBC 明显升高（E）**：外周血白细胞计数升高可以提示全身炎症反应，但不能特异性区分上下尿路感染。

因此，肾区扣痛虽然可能提示上尿路感染，但并不能有效区分上下尿路感染。
37、 (A1 型题)
口服铁剂治疗有效的缺铁性贫血者，下列化验最先上升的是
A.网织红细胞
B.血红蛋白
C.MCV
D.MCH
E.MCHC

参考答案：　　A　您的答案：
图文解析：　　口服铁剂有效的表现先是外周血网织红细胞增多，高峰在开始服药后 5～10 天，2 周后血红蛋白浓度上升，　一般 2 个月左右恢复正常。铁剂治疗应在血红蛋白恢复正常后至少持续 4~6 个月，待铁蛋白正常后停药。
治疗缺铁性贫血时，最先上升的指标是网织红细胞（A）。口服铁剂治疗后，身体开始制造更多的红细胞，其中网织红细胞是新生红细胞的早期形态，它们的增加标志着骨髓对铁剂的反应和红细胞生成的增加。因此，治疗开始后不久，网织红细胞的计数会首先上升。随后，随着更多成熟红细胞的产生，血红蛋白水平、MCV（平均红细胞体积）、MCH（平均红细胞血红蛋白含量）、MCHC（平均红细胞血红蛋白浓度）等指标也会逐渐改善。所以正确答案是 A。网织红细胞。
解析：
口服铁剂有效的缺铁性贫血患者，骨髓象增生活跃，因此网织红细胞最先上升，一般于服药后 3～4 天上升，7 天左右达高峰，2 周左右网织红细胞逐渐下降，表明治疗有效，血红蛋白逐渐上升。
选项 B 血红蛋白上升一般在治疗两周后开始上升，上升速度较慢，故排除。
选项 C：MCV 即平均红细胞体积，选项 D：MCH 即平均红细胞血红蛋白，选项 E：MCHC 即平均红细胞血红蛋白浓度，三者均用于贫血的形态学分类，其变化较慢，不是最先上升的指标，故可一并排除。
综上所述，正确答案是：A.网织红细胞。
在口服铁剂治疗缺铁性贫血时，最先上升的化验指标是网织红细胞（A）。以下是对选项的解释：

A. **网织红细胞**：网织红细胞是红细胞生成的早期阶段，它们的增加表明骨髓对铁治疗的反应。口服铁剂治疗缺铁性贫血后，通常在 3-7 天内网织红细胞会首先上升。

B. **血红蛋白**：血红蛋白的水平也会随着治疗而逐渐上升，但通常在网织红细胞上升之后，约 2-4 周后开始显著增加。

C. **MCV（平均红细胞体积）**：MCV 是衡量红细胞大小的指标。在缺铁性贫血的早期阶段，MCV 通常较低。随着治疗，MCV 会逐渐恢复正常，但这一变化相对较慢。

D. **MCH（平均红细胞血红蛋白量）**：MCH 反映的是每个红细胞内的平均血红蛋白量。和 MCV 一样，MCH 的恢复也需要较长时间。

E. **MCHC（平均红细胞血红蛋白浓度）**：MCHC 反映的是红细胞中血红蛋白的浓度，变化相对稳定，不是最先上升的指标。

因此，最先上升的是**网织红细胞**。选择 A。
38、　(A1 型题)
下列有关再生障碍性贫血发病机制的检查结果，错误的是
A.血清 IL-2 水平增高
B.血清 TNF 水平增高
C.CD4＋T 细胞增高
D.CD8＋T 细胞增高

E.CD25＋T 细胞增高

参考答案：　　C　您的答案：

图文解析：　　再生障碍性贫血 CD4＋ ：CD8＋比值减低。

再生障碍性贫血（AA）是一种骨髓造血功能减退的疾病，其发病机制与免疫异常有关。具体来说，患者常见的免疫异常包括 T 细胞活性增高和细胞因子水平的改变。因此，血清中的一些免疫调节因子，如 IL-2（白细胞介素 2）和 TNF（肿瘤坏死因子）水平会增高。CD4+T 细胞和 CD25+T 细胞增高也与再生障碍性贫血的免疫异常有关。然而，关于 CD8+T 细胞，其在再生障碍性贫血中的作用并不是总是呈现增高。

所以，在给出的选项中，如果要找到“错误”的描述，需要考虑到哪个选项与再生障碍性贫血的通常表现不一致。虽然 CD8+T 细胞在某些再生障碍性贫血患者中可能增高，但这不是最典型的表现。因此，这个问题可能比较微妙，取决于题目意图和对“错误”的定义。

考虑到上述信息，如果需要选出一个最不符合再生障碍性贫血典型发病机制的选项，我倾向于选择：

D. CD8＋T 细胞增高

这是因为虽然在某些 AA 患者中可能观察到 CD8+T 细胞增高，但这并不是 AA 的典型免疫学特征。然而，需要注意的是，这个选择可能具有争议性，因为在不同的 AA 患者中，CD8+T 细胞的表达可能有所不同。

解析：

再生障碍性贫血简称再障，是一组由多种病因所致的骨髓造血功能衰竭性综合征，以骨髓造血细胞增生减低和外周血全血细胞减少为特征，临床以贫血、出血和感染为主要表现。再生障碍性贫血发病机制的检查结果显示，再生障碍性贫血患者体内活化的 T 淋巴细胞及其分泌的造血负调控因子增加，介导了造血干细胞的损伤和骨髓衰竭。其中 CD25 分子是白细胞介素-2 受体（IL-2R）的 α 链，能特异性地与 IL-2 结合，主要分布在活化的 T 细胞和 NK 细胞，B 细胞、单核-巨噬细胞和少数骨髓瘤细胞表面也有表达。再生障碍性贫血患者 $CD25^{+}$T 细胞数量降低，提示 T 细胞免疫功能下降。

再生障碍性贫血患者体内活化的 T 淋巴细胞及其分泌的造血负调控因子增加，这些造血负调控因子包括 γ 干扰素（IFN-γ）、白细胞介素-2（IL-2）和肿瘤坏死因子（TNF）等，可显著抑制正常人骨髓 CFU-GM 生长，导致骨髓衰竭。因此选项 A 血清 IL-2 水平增高，选项 B 血清 TNF 水平增高，均符合再生障碍性贫血的发病机制。

再生障碍性贫血患者 T 淋巴细胞亚群失衡，T 辅助细胞 I 型（Th1）细胞明显增多，而 T 辅助细胞 II 型（Th2）细胞相对减少，$CD4^{+}/CD8^{+}$ 比例倒置，$CD8^{+}$T 细胞活性增加从而抑制骨髓造血。因此选项 C：$CD4^{+}$T 细胞增高，选项 D：$CD8^{+}$T 细胞增高，均符合再生障碍性贫血的发病机制。

综上所述，正确答案是：E.CD25＋T 细胞增高。

再生障碍性贫血（Aplastic anemia）是一种由骨髓造血干细胞损伤导致的疾病，表现为全血细胞减少。其发病机制涉及免疫介导的干细胞破坏，常见的免疫机制包括 T 细胞和相关细胞因子的异常。

具体来看，各种 T 细胞及细胞因子的异常在再生障碍性贫血中有以下表现：

1. **血清 IL-2 水平增高**：IL-2（白介素-2）是一种重要的细胞因子，可以促进 T 细胞增殖和活化，在再生障碍性贫血患者中，IL-2 水平通常是增高的。

2. **血清 TNF 水平增高**：TNF（肿瘤坏死因子）是一种能够诱导细胞凋亡的细胞因子，其

水平在再生障碍性贫血中也是增高的。

3. **CD4＋T 细胞增高**：CD4＋T 细胞在再生障碍性贫血中的水平并不一定增高，事实上，研究显示其水平通常是正常或降低的。

4. **CD8＋T 细胞增高**：CD8＋T 细胞增高在再生障碍性贫血中较为常见，因为 CD8＋T 细胞能够直接杀伤骨髓造血干细胞。

5. **CD25＋T 细胞增高**：CD25 是 IL-2 受体的 α 链，通常与调节性 T 细胞（Treg）相关，研究表明在再生障碍性贫血患者中，CD25＋T 细胞水平也可能增高。

结合上述分析，选项 C（CD4＋T 细胞增高）在再生障碍性贫血发病机制中的描述是错误的。

因此，答案是：

C. CD4＋T 细胞增高
39、 (A1 型题)
下列可出现原位溶血的疾病是
A.遗传性球形细胞增多症
B.G6PD 缺乏症
C.骨髓增生异常综合征
D.异常血红蛋白病
E.阵发性睡眠性血红蛋白尿
参考答案： C 您的答案：
图文解析： 原位溶血指骨髓内幼红细胞在释入血循环前已经在骨髓内破坏，常见于巨幼红细胞贫血和骨髓增生异常综合征。
原位溶血（intravascular hemolysis）是指在血管内部发生的红细胞破裂和溶解。以下是每个选项的分析：
A. 遗传性球形细胞增多症（Hereditary spherocytosis）通常引起溶血，但主要是通过脾脏过滤机制，而非原位溶血。
B. G6PD 缺乏症可以导致原位溶血，特别是在患者接触到某些药物、感染或其他氧化应激时。
C. 骨髓增生异常综合征（Myelodysplastic syndromes, MDS）主要表现为造血不良，虽然可能有溶血，但不典型为原位溶血。
D. 异常血红蛋白病（Hemoglobinopathies），如镰状细胞贫血，可导致原位溶血。
E. 阵发性睡眠性血红蛋白尿（Paroxysmal nocturnal hemoglobinuria, PNH）是一种罕见疾病，其中红细胞在血管内破裂，导致原位溶血。
考虑到原位溶血的定义和各疾病的特点，最符合原位溶血的疾病是**E. 阵发性睡眠性血红蛋白尿**。PNH 是一种特别以血管内红细胞破裂和溶解为特征的疾病，因此选项 E 是最正确的答案。
解析：
原位溶血也称为无效性红细胞生成或原位溶血性贫血，指骨髓内的幼红细胞在释入血液循环之前已在骨髓内破坏，本质上是一种血管外溶血，常见于骨髓增生异常综合征和巨幼细胞贫血。
选项 A 遗传性球形细胞增多症，选项 B 葡萄糖-6-磷酸脱氢酶（G6PD）缺乏症，选项 D 异常

血红蛋白病，选项 E 阵发性睡眠性血红蛋白尿，以上均属于血管内溶血性疾病，红细胞在血液循环中被破坏，释放游离血红蛋白，与原位溶血的定义不符，故一并排除。
综上所述，正确答案是：C.骨髓增生异常综合征。
下列可出现原位溶血的疾病是

E. 阵发性睡眠性血红蛋白尿

解释：

阵发性睡眠性血红蛋白尿（PNH）是一种获得性、克隆性造血干细胞疾病，特征是红细胞在补体系统的作用下发生溶血。由于 PNH 红细胞缺乏保护性膜蛋白，这些细胞更容易被补体破坏，导致溶血。原位溶血即在血管内发生的溶血，PNH 是其中一种典型的疾病。其他选项如遗传性球形细胞增多症和 G6PD 缺乏症等也可以导致溶血，但主要是发生在血管外（脾脏等），而不是血管内。
40、 (A1 型题)
下列不属于骨髓增生异常综合征骨髓常见病态造血表现的是
A.红系核质发育不平衡
B.粒系核分叶过多
C.粒系细胞颗粒过多
D.粒系细胞颗粒过少
E.见到幼稚型巨核细胞
参考答案： C 您的答案：
图文解析： 病态造血为骨髓异常增生综合征的特征，其表现如下。①红系：比例过大（＞60％）或过少（＜15％），奇数核、核碎裂、核间桥；核质发育不平衡，巨幼样变，成熟红细胞大小、染色不均，RAS 环形铁幼粒细胞＞15%;②粒-单核系：原幼细胞比例增高，核分叶过多或过少，核质发育不平衡，粒系细胞颗粒过少；③巨核系：出现淋巴样小巨核细胞、单圆核小巨核细胞、多圆核小巨核细胞、大单圆核巨核细胞。
在骨髓增生异常综合征（Myelodysplastic Syndromes, MDS）中，骨髓的病态造血表现主要包括：
- 红系核质发育不平衡（A）：指红细胞前体的核与胞浆成熟不同步，是 MDS 的典型表现之一。
- 粒系核分叶过多（B）：粒细胞前体核分叶异常，如过多的核分叶，也是 MDS 的特征之一。
- 粒系细胞颗粒过少（D）：在 MDS 中，粒系细胞可能出现颗粒减少的情况。
- 幼稚型巨核细胞（E）：MDS 中常见巨核细胞系的异常，如幼稚型巨核细胞的出现。
粒系细胞颗粒过多（C）并不是 MDS 的典型病理特征，因此，最佳答案是 C.粒系细胞颗粒过多。
解析：
骨髓增生异常综合征（MDS）是起源于造血干细胞的一组异质性髓系克隆性疾病，特点是髓系细胞分化及发育异常，表现为无效造血、难治性血细胞减少、造血功能衰竭，高风险向急性髓系白血病（AML）转化。MDS 的病态造血包括红细胞、粒细胞及巨核细胞系统的病态造血，粒系细胞颗粒过多不是病态造血的特点。
选项 A 红系核质发育不平衡是 MDS 常见的红系病态造血的特点，即幼红细胞胞质发育落后于胞核，通常胞质少，呈空泡状，核染色质疏松，有核仁。
选项 B 粒系核分叶过多是 MDS 常见的粒系病态造血的特点，可多于 5 叶，常为 6-9 叶，甚

至出现 16 叶。
选项 D 粒系细胞颗粒过少也是 MDS 常见的粒系病态造血的特点，表现为中性粒细胞的胞质颗粒减少，PAS 染色阴性。
选项 E 见到幼稚型巨核细胞是 MDS 常见的巨核系病态造血的特点，可出现淋巴样小巨核细胞、单圆核小巨核细胞、多圆核巨核细胞等。
综上所述，正确答案是：C.粒系细胞颗粒过多。
40、 下列不属于骨髓增生异常综合征骨髓常见病态造血表现的是

A. 红系核质发育不平衡
B. 粒系核分叶过多
C. 粒系细胞颗粒过多
D. 粒系细胞颗粒过少
E. 见到幼稚型巨核细胞

最佳答案：C. 粒系细胞颗粒过多

解析：
骨髓增生异常综合征（MDS）是一组异质性很高的克隆性造血干细胞疾病，其主要特点是骨髓病态造血和无效造血，常见病态造血表现包括：
- 红系核质发育不平衡（A）
- 粒系核分叶过多（B）
- 粒系细胞颗粒过少（D）
- 见到幼稚巨核细胞（E）

而粒系细胞颗粒过多（C）则不是 MDS 的常见病态造血表现。
41、 (A1 型题)
下列检查结果支持 CML 急变期的是
A.血中原粒细胞＞5%
B.血中原粒＋早幼粒细胞＞20%
C.骨髓中原粒细胞＞20%
D.骨髓中原粒细胞＋早幼粒细胞＞40%
E.骨髓活检显示胶原纤维显著增生
参考答案： C 您的答案：
图文解析： 慢性粒细胞白血病（CML）急性变期：骨髓中原粒细胞+早幼粒细胞＞50％，血中原粒+早幼粒细胞＞30％，骨髓中原始细胞或原淋+幼淋＞20％。
在慢性粒细胞性白血病（CML）的急变期，通常会观察到血液或骨髓中原粒细胞和早幼粒细胞（不成熟的粒细胞）的比例显著增加。根据相关医学指南和标准，CML 急变期的诊断标准之一是血液或骨髓中原粒细胞加上早幼粒细胞的比例超过 20%。
因此，根据上述选项，最能支持 CML 急变期的检查结果是：
- **D. 骨髓中原粒细胞＋早幼粒细胞＞40%**
这个选项显示了一个明显高于正常水平的未成熟粒细胞比例，符合 CML 进展到急变期的血液学变化特征。
解析：
慢性髓系白血病（CML）是多能造血干细胞的恶性增殖性疾病，其病程分为慢性期、加速期

和急变期。其中，CML 进入急变期的标准包括外周血中原粒+早幼粒细胞＞30%，骨髓中原粒+早幼粒细胞＞50%，有髓外原始细胞浸润，以及骨髓活检显示原始细胞大量聚集等。其中，骨髓中原粒+早幼粒细胞＞50%，符合 CML 急变期的标准，而选项中**骨髓中原粒细胞＋早幼粒细胞＞40%**与这一标准最为接近。
选项 A 血中原粒细胞＞5%，选项 B 血中原粒＋早幼粒细胞＞20%，均不符合 CML 急变期的标准，故一并排除。
选项 C 骨髓中原粒细胞＞20%为 CML 加速期的标准，不符合题意。
选项 E 骨髓活检显示胶原纤维显著增生是骨髓纤维化的病理特征，不符合 CML 急变期的表现，故不选。
综上所述，正确答案是：D.骨髓中原粒细胞＋早幼粒细胞＞40%。
慢性髓细胞白血病（CML）急变期的诊断标准之一是原粒细胞的比例显著增加。根据目前的医学知识和诊断指南，下列检查结果支持 CML 急变期的是：

C. 骨髓中原粒细胞＞20%

这是因为在 CML 急变期，原粒细胞在骨髓中的比例通常超过 20%。
42、 (A1 型题)
下列属于 T 细胞淋巴瘤的是
A.边缘区淋巴瘤
B.滤泡性淋巴瘤
C.套细胞淋巴瘤
D.间变大细胞淋巴瘤
E.Burkitt 淋巴瘤
参考答案： D 您的答案：
图文解析： 间变大细胞淋巴瘤免疫表型为 T 细胞。
在这些选项中，属于 T 细胞淋巴瘤的是 **D. 间变大细胞淋巴瘤**。边缘区淋巴瘤、滤泡性淋巴瘤、套细胞淋巴瘤以及 Burkitt 淋巴瘤都是 B 细胞起源的淋巴瘤。而间变大细胞淋巴瘤是一种 T 细胞淋巴瘤，它来源于 T 细胞或自然杀伤(NK)细胞。
首先，我们需要明确淋巴瘤的分类。淋巴瘤主要分为霍奇金淋巴瘤和非霍奇金淋巴瘤两大类。在非霍奇金淋巴瘤中，又可以分为 B 细胞淋巴瘤和 T 细胞淋巴瘤。
接下来，我们逐一分析每个选项：
A. 边缘区淋巴瘤：这是一种 B 细胞来源的淋巴瘤，所以不属于 T 细胞淋巴瘤。
B. 滤泡性淋巴瘤：同样是 B 细胞来源的淋巴瘤，也不属于 T 细胞淋巴瘤。
C. 套细胞淋巴瘤：这也是一种 B 细胞淋巴瘤，因此也不符合题目要求的 T 细胞淋巴瘤。
D. 间变大细胞淋巴瘤：这是一种 T 细胞来源的淋巴瘤，符合题目要求。
E. Burkitt 淋巴瘤：这是 B 细胞来源的淋巴瘤，所以也不是 T 细胞淋巴瘤。
综上所述，只有选项 D，即间变大细胞淋巴瘤，是 T 细胞淋巴瘤。
因此，正确答案是 D.间变大细胞淋巴瘤。
下列属于 T 细胞淋巴瘤的是：

D. 间变大细胞淋巴瘤

解析：
- 边缘区淋巴瘤 (A): 属于 B 细胞淋巴瘤。

- 滤泡性淋巴瘤 (B): 属于 B 细胞淋巴瘤。
- 套细胞淋巴瘤 (C): 属于 B 细胞淋巴瘤。
- 间变大细胞淋巴瘤 (D): 属于 T 细胞淋巴瘤。
- Burkitt 淋巴瘤 (E): 属于 B 细胞淋巴瘤。

所以，最佳答案是 D. 间变大细胞淋巴瘤。

43、 (A1 型题)

Henoch 型过敏性紫癜的临床表现中，除皮肤紫癜外，还有

A.关节肿痛

B.便血

C.尿血

D.视网膜出血

E.虹膜炎

参考答案： B 您的答案：

图文解析： 【解析】Henoch 型过敏性紫癜为腹型，表现为恶心，呕吐，呕血，腹泻，便血，其中腹痛最为常见。

Henoch-Schönlein 紫癜（Henoch-Schönlein purpura, HSP）是一种小血管炎，主要影响毛细血管。其临床表现不仅包括皮肤紫癜，还可能涉及关节、肠道和肾脏。因此，除皮肤紫癜外，Henoch-Schönlein 紫癜的典型临床表现包括：

- 关节肿痛（通常影响膝关节和踝关节）
- 腹痛或便血（肠道受累）
- 尿血（肾脏受累）

视网膜出血和虹膜炎并不是 Henoch-Schönlein 紫癜的典型表现。因此，最佳答案是涵盖这种疾病常见额外症状的选项。考虑到上述信息，最合适的答案是：

A.关节肿痛

B.便血

C.尿血

在这些选项中，关节肿痛（A）是 Henoch-Schönlein 紫癜的一个非常典型的额外临床表现。然而，便血（B）和尿血（C）也是很重要的表现，特别是在疾病影响肠道和肾脏时。如果题目要求选择一个最佳答案，我们需要考虑最常见和最具代表性的症状。

由于 Henoch-Schönlein 紫癜最典型的额外临床特征是肾脏受累，表现为尿血，因此正确的答案应该是**C.尿血**。

解析：

过敏性紫癜又称自限性急性出血症，是一种侵犯皮肤和其他器官细小动脉和毛细血管的过敏性血管炎，发病原因可能是病原体感染、某些药物作用、过敏等致使体内形成 IgA 或 IgG 类循环免疫复合物，沉积于真皮上层毛细血管引起血管炎。过敏性紫癜可分为单纯型、腹型、关节型、肾型、混合型等。其中，腹型过敏性紫癜又称为 Henoch 型紫癜，此型患者除皮肤紫癜外，因消化道黏膜及腹膜脏层毛细血管受累，可产生一系列消化道症状及体征，如恶心、呕吐、呕血、腹泻及粘液便、便血等。

选项 A 关节肿痛多见于关节型过敏性紫癜，又称为 Schonlein-Henoch 紫癜，可有关节肿胀、疼痛、压痛及功能障碍等表现，不符合 Henoch 型紫癜的特点，故不选。

选项 C 尿血多见于肾型过敏性紫癜，又称为紫癜性肾炎，可出现血尿、蛋白尿及管型尿，偶见水肿、高血压等症状，不符合 Henoch 型紫癜的特点，故排除。

选项 D 视网膜出血，选项 E 虹膜炎，在过敏性紫癜中少见，且不属于 Henoch 型紫癜的特点，

故不选。
综上所述，正确答案是：B.便血。
Henoch-Schönlein 紫癜（Henoch-Schönlein Purpura, HSP）是一种以皮肤紫癜为主要特征的系统性血管炎，常见于儿童。除皮肤紫癜外，HSP 的临床表现还包括以下几种：

A. 关节肿痛：关节症状是 HSP 的常见表现，患者可能出现关节肿痛，尤其是在膝关节和踝关节。
B. 便血：胃肠道症状是 HSP 的另一个常见表现，患者可能出现腹痛、便血等症状。
C. 尿血：肾脏受累也比较常见，患者可能出现血尿、蛋白尿等表现。

视网膜出血和虹膜炎在 HSP 中较为少见，因此可以排除 D 和 E。

综上所述，Henoch 型过敏性紫癜的临床表现中，除皮肤紫癜外，还有：

A. 关节肿痛
B. 便血
C. 尿血

最佳答案是：
A. 关节肿痛
44、 (A1 型题)
Felty 综合征引起的中性粒细胞减少的最可能机制是
A.生成减少
B.成熟障碍
C.免疫性破坏过多
D.非免疫性破坏过多
E.分布异常
参考答案： C 您的答案：
图文解析：
Felty 综合征是一种罕见的自身免疫性疾病，与类风湿关节炎相关，特征是类风湿关节炎、脾肿大和中性粒细胞减少。中性粒细胞减少主要是由于免疫性破坏，因此最可能的机制是免疫系统对中性粒细胞的过度破坏。
所以，正确答案是：C. 免疫性破坏过多
解析：
Felty 综合征是指类风湿关节炎患者伴有脾大、中性粒细胞减少，有的甚至有贫血和血小板减少，其引起的中性粒细胞减少的最可能机制是免疫性破坏，大量粒细胞被免疫性破坏，导致中性粒细胞减少。
选项 A 生成减少可见于再生障碍性贫血、肿瘤侵犯骨髓、急性白血病、药物或放射线等理化因素及系统性疾病导致的骨髓造血障碍等，与 Felty 综合征发病机制不符，故不选。
选项 B 成熟障碍见于巨幼细胞性贫血、骨髓增生异常综合征，由于叶酸或维生素 B_{12}缺乏，或某些影响核苷酸代谢的药物导致细胞 DNA 合成障碍所致的贫血，不符合 Felty 综合征的发病机制，故排除。
选项 D 非免疫性破坏过多见于感染、炎症或脾功能亢进等情况，与 Felty 综合征发病机制不同，故不选。

选项 E 分布异常可见于异体蛋白反应、内毒素血症，由于中性粒细胞转移至边缘池导致循环池的粒细胞相对减少，但粒细胞总数并不减少，与题干描述不符，故排除。
综上所述，正确答案是：C.免疫性破坏过多。
Felty 综合征 (Felty syndrome) 是一种罕见的疾病，常见于类风湿关节炎患者，主要特征包括脾肿大、中性粒细胞减少和类风湿因子阳性。该病的中性粒细胞减少主要是由于免疫性机制引起的。

根据现有医学知识和最佳实践指南，Felty 综合征引起的中性粒细胞减少最可能的机制是：

C. 免疫性破坏过多
45、 (A1 型题)
不属于阵发性室上性心动过速临床特点的是
A.突发突止
B.心率＞150/分
C.心律绝对规则
D.第一心音强弱不等
E.大部分由折返机制引起
参考答案： D 您的答案：
图文解析： 【解析】该病突发突止，常无器质性病变，常有折返机制，心率 150～250 次/分，节律快而规则。
阵发性室上性心动过速（Paroxysmal Supraventricular Tachycardia，PSVT）的临床特点通常包括突然开始和突然结束（突发突止）、心率通常大于 150 次/分钟、心律规则等。大部分确实是由折返机制引起的。选项 D，“第一心音强弱不等”，通常与心室填充或心脏瓣膜功能障碍相关，并不是阵发性室上性心动过速的典型临床特征。
因此，不属于阵发性室上性心动过速临床特点的是 **D. 第一心音强弱不等**。
首先，我们来了解一下阵发性室上性心动过速（PSVT）的基本特点。PSVT 是一种常见的心律失常，主要由折返机制引发，具有突发突止的特性，心率一般较快，通常超过 150 次/分，心律通常规则。
现在，我们根据这些特点来逐一分析选项：
A. 突发突止：这是阵发性室上性心动过速的一个显著特点，即心动过速的发作和终止都非常突然，所以这个选项是符合阵发性室上性心动过速的特点的。
B. 心率＞150/分：阵发性室上性心动过速时，心率通常会超过 150 次/分，所以这个描述也是准确的。
C. 心律绝对规则：在阵发性室上性心动过速的情况下，心律通常是规则的，这与选项描述相符。
D. 第一心音强弱不等：这一特征更常见于心房颤动，而不是阵发性室上性心动过速。在阵发性室上性心动过速中，心律是规则的，因此第一心音的强度也应该是均匀的，而不是强弱不等。
E. 大部分由折返机制引起：阵发性室上性心动过速确实大多数是由折返机制引起的，这与选项描述一致。
综上所述，不属于阵发性室上性心动过速临床特点的是 D.第一心音强弱不等。这是心房颤动的特征，而非阵发性室上性心动过速的特点。
根据现有医学知识和最佳实践指南，阵发性室上性心动过速（PSVT）的临床特点包括：

A. 突发突止：PSVT 通常突然开始和结束。
B. 心率＞150/分：PSVT 的心率通常超过 150 次每分钟。
C. 心律绝对规则：PSVT 的心律是绝对规则的。
D. 第一心音强弱不等：这不是 PSVT 的特征。PSVT 的第一心音通常是恒定的。
E. 大部分由折返机制引起：大多数 PSVT 是由折返机制引起的。

因此，正确答案是不属于阵发性室上性心动过速临床特点的是：

D. 第一心音强弱不等
46、　(A1 型题)
导致肺水肿最直接的原因是
A.肺动脉压升高
B.右心室压升高
C.肺静脉压升高
D.左心室舒张压升高
E.右心房压升高
参考答案：　　C　您的答案：
图文解析：　　【解析】肺静脉压升高时导致肺毛细血管压升高，直接引起肺间质水肿。左室舒张末压升高，左房压升高等均是通过使肺静脉压升高间接导致肺水肿。
导致肺水肿最直接的原因是肺静脉压升高。肺水肿通常是由于左心功能衰竭导致的，这会引起肺循环的静脉压力增加。当左心室不能有效地将血液泵入体循环时，血液在肺循环中滞留，导致肺静脉压升高。因此，最直接导致肺水肿的是肺静脉压升高。
所以，正确答案是 C.肺静脉压升高。
首先，我们要明确什么是肺水肿。肺水肿是指液体从肺毛细血管异常渗透至肺间质、肺泡，超过了淋巴回流的代偿能力，造成肺血管外液体异常积聚的一种病理状态。
接下来，我们来分析每一个选项与肺水肿的直接关系：
A. 肺动脉压升高：肺动脉压升高主要与肺动脉高压相关，但这不直接导致肺水肿。它可能会影响右心室的功能，但不是肺水肿的直接原因。
B. 右心室压升高：右心室压升高与肺动脉压升高有一定的关联，但同样不是肺水肿的直接原因。
C. 肺静脉压升高：肺静脉压升高意味着肺部血液回流到左心房的阻力增加。当肺静脉压升高到一定程度，液体会渗出到肺泡和肺间质，从而导致肺水肿。因此，这是导致肺水肿的直接原因。
D. 左心室舒张压升高：左心室舒张压升高会影响心脏的舒张功能，但它与肺水肿之间没有直接的因果关系。
E. 右心房压升高：右心房压升高与右心功能有关，但它不直接导致肺水肿。
综上所述，最直接导致肺水肿的原因是肺静脉压升高，因此选择 C. 肺静脉压升高。
导致肺水肿最直接的原因是：

C. 肺静脉压升高

肺水肿是由于液体在肺泡和肺间质内积聚而引起的，最直接的原因是肺静脉压升高。这通常与左心功能不全有关，当左心室不能有效泵出血液时，会导致血液在肺循环中滞留，增加肺静脉的压力，从而导致液体渗出，形成肺水肿。

47、 (A2 型题)
男，14 岁。午餐进食海鱼后，即出现头痛、头晕、胸闷、心跳呼吸加快，伴有眼结膜充血。颜面部及全身潮红，测体温正常，无呕吐、腹泻等症状。患者最可能是
A.河豚中毒
B.肉毒梭菌毒素中毒
C.麻痹性贝类中毒
D.组胺中毒
E.副溶血性弧菌中毒
参考答案： D 您的答案：
图文解析： 【解析】青少年男性患者，在午餐进食海鱼后，出现头痛、头晕、胸闷、心跳呼吸加快，伴有眼结膜充血。颜面部及全身潮红（组胺中毒的过敏表现），故结合患者的诱因及过敏表现，提示为组胺中毒（D 对）。
【避错】河豚中毒后，以多次呕吐的表现为主，有明显的神经症状，但较少见腹痛、腹泻等（A 错）。肉毒梭菌毒素中毒后，起病突然，主要表现为神经系统症状（B 错）。麻痹性贝类中毒（C 错）主要表现为唇、口、舌的感觉异常和麻木，严重者会出现肢体麻木，随意运动障碍，甚至呼吸困难。副溶血性弧菌中毒后，可出现血水样便（E 错）。
该症状表明患者可能经历了一种与食物相关的急性反应。考虑到患者在进食海鱼后出现头痛、头晕、胸闷、心跳呼吸加快和颜面部及全身潮红，但体温正常，没有呕吐或腹泻，这些症状与组胺中毒（也称为鱼类中毒或组胺食物中毒）相符。
组胺中毒是由食用含有高水平组胺的鱼类（如未妥善储存的海鱼）引起的，组胺是在鱼类腐败过程中由细菌作用于组氨酸形成的。患者会出现类似过敏反应的症状，如头痛、头晕、潮红、结膜充血和心跳加快。
因此，最可能的答案是：D. 组胺中毒
解析：
患者青少年男性，进食海鱼后，即出现头痛、头晕、胸闷、心跳呼吸加快，伴有眼结膜充血、颜面部及全身潮红，测体温正常，无呕吐、腹泻等症状，考虑诊断为组胺中毒。组胺中毒通常是因食用不新鲜的鲐鱼、马鲛鱼、金枪鱼、秋刀鱼、青皮红肉鱼等含组氨酸高的鱼类而引起的。组胺是一种生物胺，广泛存在于人体组织之中，当机体受到理化刺激或发生过敏反应时，会引起含有组胺的细胞脱颗粒，导致组胺释放，与靶细胞上的组胺受体结合后就会产生一系列生理反应。当组胺释放后会引起小动脉扩张、毛细血管通透性增加、支气管收缩、痉挛，以及胃肠道平滑肌收缩等生理效应，其症状与荨麻疹、支气管哮喘、过敏性休克类似。
选项 A 河豚中毒表现为口唇感觉异常，之后出现恶心、呕吐、腹痛、腹泻，甚至全身麻痹、瘫痪，严重者出现血压下降、呼吸困难、昏迷、呼吸衰竭而死亡，与题干描述不符，故排除。
选项 B 肉毒梭菌毒素中毒表现为以复视、眼睑下垂、吞咽困难、语言障碍、头颈无力、肌肉麻痹等脑神经损害为主要症状，病死率较高，与患者的症状描述差异明显，故不选。
选项 C 麻痹性贝类中毒主要表现为嘴唇、手和脚趾的麻痹感及恶心、呕吐、腹泻等症状，严重者可导致瘫痪甚至死亡，与题意不符，故排除。
选项 E 副溶血性弧菌中毒表现为上腹部阵发性绞痛，继而腹泻，每天 5～6 次，洗肉水样便，无脓血，无里急后重，部分患者出现呕吐，不符合题干，故不选。
综上所述，正确答案是：D.组胺中毒。
根据题目描述，该男孩在午餐进食海鱼后出现头痛、头晕、胸闷、心跳呼吸加快，伴有眼结膜充血，颜面部及全身潮红，但体温正常，无呕吐、腹泻等症状。这些症状在进食海鱼后立即发生，提示可能是与食物有关的急性中毒反应。

A. 河豚中毒通常会导致口唇麻木、四肢无力、甚至呼吸肌麻痹，但题干未提到这些典型症状。
B. 肉毒梭菌毒素中毒通常表现为对称性神经肌肉麻痹、视物模糊、吞咽困难等，症状出现较为缓慢，也不符合题干所述的急性反应。
C. 麻痹性贝类中毒常见症状为口周及指尖麻木、四肢无力，甚至严重时可导致呼吸困难和瘫痪，但题干描述的症状与之不完全吻合。
D. 组胺中毒（常称为"鲐鱼中毒"）症状包括头痛、头晕、面部潮红、心跳加速、胸闷、眼结膜充血等，与题干中的描述非常吻合。这种中毒是由于摄入了含高浓度组胺的鱼类，组胺是由鱼在保存不当时细菌作用下生成的。
E. 副溶血性弧菌中毒主要表现为急性肠胃炎症状，如呕吐、腹泻、腹痛，通常不会有题干描述的面部及全身潮红等症状。

综上所述，患者最可能是组胺中毒。正确答案是：

D. 组胺中毒
48、 (A2 型题)
女，40 岁，既往体健。突发胸痛，肺压缩 30％，经胸腔穿刺后肺压缩仍为 30％。接下来应怎样治疗
A.胸腔穿刺
B.胸膜固定术
C.胸腔闭式引流
D.手术
E.心包穿刺
参考答案： C 您的答案：
图文解析： 【解析】患者中年女性，突发胸痛，肺压缩 30%，考虑为自发性气胸，经过胸腔穿刺后不能复张，说明胸腔穿刺无效，故不宜再用胸腔穿刺术，且该患者既往体健，接下来应该选择胸腔闭式引流（C 对）。
【避错】胸腔穿刺抽气适用于小量气胸（20%以下）;闭式胸腔引流术的适应证为：①中、大量气胸、开放性气胸、张力性气胸；②经胸腔穿刺术治疗，伤员下肺无法复张者；③需使用机械通气或人工通气的气胸或血气胸者；④拔除胸腔引流管后气胸或血胸复发者；⑤剖胸手术。
针对这个案例，关键信息是患者突发胸痛，肺压缩 30%，经过胸腔穿刺治疗后，肺压缩情况没有改善。基于这些信息，选择最佳的治疗方案应当是 C.胸腔闭式引流。
胸腔闭式引流是治疗肺压缩的常用方法，特别是在胸腔穿刺后如果没有明显改善，表明可能有持续的空气或液体泄漏导致肺压缩。闭式引流可以持续排出胸腔内的空气或液体，帮助肺部重新扩张。其他选项如胸腔穿刺(A)已经尝试过且效果不佳，胸膜固定术(B)和手术(D)通常用于更复杂或特定情况下的治疗，而心包穿刺(E)用于心包积液或心包膜问题，与本案例的情况不符。因此，最适合的选择是 C.胸腔闭式引流。
首先，我们需要理解问题的核心。患者是一位 40 岁的女性，突然发生胸痛，并且肺部压缩了 30%。经过胸腔穿刺后，肺部的压缩程度没有改变，仍为 30%。这意味着简单的胸腔穿刺可能无法有效缓解患者的状况。
现在，我们根据题干信息，结合选项进行逐一分析：
A. 胸腔穿刺：既然已经进行了一次胸腔穿刺，但肺部压缩没有改善，那么再次进行胸腔穿刺可能不会有更好的效果。

B. 胸膜固定术：此手术通常用于治疗复发性气胸或持续漏气，但在此情境下并不是首选。
C. 胸腔闭式引流：对于初次发生的气胸，当简单抽气效果不佳时，胸腔闭式引流是一个有效的治疗方法。它可以持续地将胸腔内的气体引出，从而帮助肺部重新扩张。
D. 手术：对于初次气胸，手术通常不是首选治疗方法，除非其他方法都无效或存在特殊情况。
E. 心包穿刺：这与气胸治疗无关，主要用于心包积液或心脏压塞的治疗。
综上所述，对于该患者，胸腔闭式引流是合适的治疗方法，因为它可以持续地引出胸腔内的气体，帮助肺部恢复。
因此，答案是 C. 胸腔闭式引流。
该题描述了一名 40 岁女性患者，突然出现胸痛，肺压缩 30%，经过胸腔穿刺后肺压缩仍为 30%。这种情况提示可能存在气胸，并且穿刺后肺未能重新膨胀。接下来最适合的治疗方案应该是确保肺重新膨胀并避免再次压缩。根据现有的医学知识和最佳实践指南，以下是各个选项的分析：

A. 胸腔穿刺：已经进行过一次胸腔穿刺，但未能解决问题，因此再次穿刺可能效果不佳。
B. 胸膜固定术：这是较为复杂的手术，一般在其他治疗方法无效时考虑。
C. 胸腔闭式引流：这是处理气胸的常规治疗方法，特别是对于穿刺无效的情况下，可以有效地排除胸腔内的气体，使肺重新膨胀。
D. 手术：通常用于严重或复杂的气胸，或者其他治疗方法无效的情况。
E. 心包穿刺：与当前问题无关，不适用。

在上述选项中，最合适的治疗方案是 **C. 胸腔闭式引流**，因为这是标准的处理方法，可以有效地排除胸腔内的气体，促进肺的重新膨胀。

所以答案是：**C. 胸腔闭式引流**。
49、 (A2 型题)
女，28 岁，孕 28 周，体温 38.5℃。转移性右下腹痛，有腹膜刺激征。下一步处理措施是
A.腹腔镜阑尾切除术
B.引产＋阑尾切除
C.剖宫产＋阑尾切除
D.应用抗生素
E.支持对症治疗
参考答案： A 您的答案：
图文解析： 【解析】患者妊娠期妇女，有发热、转移性右下腹痛、腹膜刺激征阳性，诊断为妊娠期急性阑尾炎， 一旦诊断明确，不用抗生素抗感染等对症保守治疗（DE 错），须急诊行阑尾切除术（A 对）。
【避错】除非有产科急诊指征，原则上仅处理阑尾炎而不同时行剖宫产手术。但以下情况可先行剖宫产再行阑尾切除术：①术中暴露阑尾困难；②阑尾穿孔并发弥漫性腹膜炎，盆腔感染严重，子宫已有感染征象；③近预产期或胎儿基本成熟，已具生存能力（C 错）；胎儿一般情况可，可继续妊娠（B 错）。
这个问题描述的是一位 28 岁孕妇，孕 28 周，出现体温升高和转移性右下腹痛，并有腹膜刺激征。这些症状和体征提示可能是阑尾炎。在孕期，阑尾炎的诊断和处理是比较特殊的，需要考虑到母体和胎儿的安全。
A 选项腹腔镜阑尾切除术是处理非妊娠期阑尾炎的常规手术，但在孕期需要谨慎考虑，特别

是在孕晚期。腹腔镜手术在孕期可行，但需要经验丰富的手术团队，并且要小心操作以避免对胎儿的影响。
B 选项引产＋阑尾切除和 C 选项剖宫产＋阑尾切除通常不是首选，除非有其他迫切的产科指征，因为这会增加早产和其他并发症的风险。
D 选项应用抗生素可能适用于非典型阑尾炎或轻微的症状，但在明确的阑尾炎且存在腹膜刺激征的情况下，仅仅使用抗生素可能不足以解决问题。
E 选项支持对症治疗一般不是针对急性阑尾炎的主要治疗方式。
根据这个情况，最佳的处理措施可能是 A.腹腔镜阑尾切除术。这种方法可以减少对孕妇的干扰，并及时解决阑尾炎的问题。然而，需要在有经验的医疗团队和适当的设备支持下进行。所以，如果条件允许，A 选项（腹腔镜阑尾切除术）是最佳选择。
解析：
青年女性，孕 28 周，体温升高，转移性右下腹痛，腹膜刺激征阳性，考虑妊娠合并急性阑尾炎。妊娠合并急性阑尾炎在妊娠早、中期，可及时进行手术治疗，在妊娠晚期，手术最好同时行剖宫产，若因手术刺激导致早产，其婴儿存活率较剖宫产者为高，但手术时应轻柔，尽量减少对子宫的刺激，术后继续使用广谱抗生素，同时加强术后护理。该患者为孕 28 周，已属于妊娠晚期，应剖宫产同时行阑尾切除术，可一并去除病灶，又可保证胎儿安全。所以下一步处理措施应是剖宫产＋阑尾切除。
选项 A 腹腔镜阑尾切除术虽属于微创手术，但手术过程中需要气腹，腹压增大会对胎儿产生影响，可排除。
引产是指妊娠 12 周后，因母体或胎儿方面的原因，须用人工方法诱发子宫收缩而结束妊娠，本题患者无必须终止妊娠的指征，故选项 B 引产＋阑尾切除，可排除。
选项 D 应用抗生素，选项 E 支持对症治疗，均属于保守治疗措施，仅能起到缓解症状的作用，不能根治，且容易复发，不是首选治疗方案，故一并排除。
综上所述，正确答案是：C.剖宫产＋阑尾切除。
在 28 周孕期出现高热（38.5℃）和右下腹转移性疼痛，并且有腹膜刺激征的女性患者，最可能的诊断是急性阑尾炎。妊娠期急性阑尾炎是一种外科急症，及时处理对于母婴健康至关重要。处理措施应根据妊娠周数、病情严重程度以及胎儿的状况来决定。

在妊娠期的这个阶段（28 周），妊娠仍然相对较早，且如果诊断为急性阑尾炎，外科干预是必要的。抗生素治疗虽然可以暂时缓解症状，但不能从根本上解决问题，而支持对症治疗无法有效处理阑尾炎。因此，对于急性阑尾炎，最合适的处理方式是手术。

腹腔镜阑尾切除术（A）是一种微创手术，适用于妊娠期的急性阑尾炎治疗。相比于开放手术，腹腔镜手术的创伤较小，恢复较快，并且对于胎儿的影响较小。因此，在这种情况下，选择腹腔镜阑尾切除术是最合理的。

引产+阑尾切除（B）或剖宫产+阑尾切除（C）均属于较为激进的治疗措施，不建议在没有明确胎儿或母体危及生命的情况下采用。应用抗生素（D）和支持对症治疗（E）则不足以处理急性阑尾炎的根本问题。

因此，最佳答案是：

A. 腹腔镜阑尾切除术

50、　(A2 型题)

女，54 岁，腹痛腹胀低热 2 月，查体：腹软，脐周压痛。B 超示中等量腹水，腹水 ADA65U/L。下列最有助于诊断的检查是

A.结肠镜

B.γ-干扰素释放试验

C.腹部 CT

D.血沉

E.腹水

参考答案：　　B　您的答案：

图文解析：　　【解析】中年女性患者，腹痛腹胀低热 2 月，查体腹软，脐周压痛，B 超示中等量腹水，腹水 ADA65U/L（结核性腹膜炎常升高）。低热腹痛，腹部柔韧感，根据题意，故诊断为结核性腹膜炎，最有助于诊断的检查是γ-干扰素释放试验（B 对）。

【避错】结肠镜（A 错）主要用于诊断肠结核，结核性腹膜炎需行腹腔镜检查；腹部 CT、B 超、血沉及腹水检查均有助于结核性腹膜炎的诊断，但不如γ-干扰素释放试验有特异性。

这个病例描述了一个 54 岁女性患者，出现腹痛、腹胀和低热持续 2 个月，体检发现脐周压痛，B 超显示有中等量腹水，腹水腺苷脱氨酶(ADA)为 65U/L。腹水 ADA 水平升高可能提示结核性腹膜炎。

在给出的选项中，最有助于诊断这种情况的是：

A. 结肠镜 - 用于检查大肠的情况，通常用于诊断结肠相关的疾病，如大肠癌或炎症性肠病。

B. γ-干扰素释放试验 - 这是一种用于检测结核感染的免疫学测试，可以帮助诊断活动性结核或潜伏性结核感染。

C. 腹部 CT - 可以提供关于腹部器官、包括肠道、腹膜和其他组织的详细图像，有助于检查腹膜炎的迹象。

D. 血沉 - 血液沉降率测试，可用于检测身体中的炎症，但对特定疾病的诊断价值有限。

E. 腹水 - 这可能指的是进行腹水分析，包括生化、细胞和微生物学检查。

考虑到患者腹水 ADA 水平升高，结合临床表现，最可能的诊断是结核性腹膜炎。因此，最有助于诊断的检查是 **B. γ-干扰素释放试验**，因为它有助于确定是否存在结核感染，从而支持结核性腹膜炎的诊断。然而，在临床实践中，通常需要结合腹部影像学（如腹部 CT）和腹水分析来综合评估和诊断。

解析：

中年女性患者，低热 2 月，查体见腹软，脐周压痛，B 超示中等量腹水，腹水腺苷脱氨酶（ADA）65U/L，高度怀疑为结核性腹膜炎。结核性腹膜炎是由结核分枝杆菌引起的慢性弥漫性腹膜感染，任何年龄均可发病，以中青年多见，女性略多于男性，多数起病较缓，但急性发病者亦为数不少，主要症状为倦怠，发热、腹胀和腹痛，亦有畏寒、高热骤然起病者，轻至中度急性腹痛，或钝痛，查体腹部有揉面感，脐周可触及腹部肿块，可出现移动性浊音阳性，结核性腹膜炎腹水为渗出液，以淋巴细胞为主，腹水腺苷脱氨酶活性增高，提示结核性腹膜炎，结核性腹膜炎患者腹水腺苷脱氨酶多大于 40U/L，常用来鉴别腹水的性质。为明确诊断，可进行γ-干扰素释放试验。该实验采用酶联免疫斑点技术或酶联免疫吸附法检测分泌γ干扰素特异性 T 淋巴细胞，用于检测受试者体内是否存在潜伏结核分枝杆菌的感染，其敏感性和特异性明显高于 PPD 试验，且不受卡介苗接种和非结核分枝杆菌的影响，对于该患者**，γ-干扰素释放试验**可帮助明确结核感染的诊断。

选项 A 结肠镜常用于诊断肠道疾病，结核性腹膜炎的诊断价值不大，故不选。

选项 C 腹部 CT 可显示腹腔淋巴结肿大及少量腹水，但不能确诊结核性腹膜炎，可排除。

选项 D 血沉增快可见于多种疾病，如急性炎症、白血病、结缔组织病及结核病的活动期，

不具有特异性，与题意不符。
选项 E 腹水检查虽可帮助诊断，但 γ-干扰素释放试验敏感性及特异性更高，故不考虑。
综上所述，正确答案是：B. γ-干扰素释放试验。
对于一个 54 岁女性患者，症状包括腹痛、腹胀和低热 2 个月，体检发现腹部柔软，脐周压痛。B 超显示中等量腹水，且腹水 ADA（腺苷脱氨酶）值为 65U/L。考虑到这些症状和检查结果，最有可能的诊断是结核性腹膜炎。

为了进一步确诊，最有帮助的检查应该是能够直接检测到结核感染的证据。因此，在以下选项中：

A. 结肠镜：用于检查结肠病变，不是首选。
B. γ-干扰素释放试验：用于检测结核感染，非常有帮助。
C. 腹部 CT：可以显示腹部结构异常，但对于确诊结核感染不如 γ-干扰素释放试验特异。
D. 血沉：虽然可以反映炎症状态，但不特异，意义有限。
E. 腹水：已经检查过了，提供进一步信息有限。

所以，最有助于诊断的检查是：

B. γ-干扰素释放试验
51、 (A2 型题)
男，39 岁，右上腹痛伴发热 1 天，查体：T39.8℃,P105 次/分，BP85/55mmHg，肝区叩诊（+），B 超示胆囊多发结石，胆总管直径 1.6cm，肝总管下段有强回声光团伴声影， 的治疗是
A.急诊行开腹胆囊切除术
B.急诊行胆总管切开，T 管引流术
C.积极抗休克治疗，禁食、抗感染
D.急诊行腹腔镜胆囊切除术
E.积极抗休克治疗，缓解后择期手术
参考答案： B 您的答案：
图文解析： 【解析】中年男性患者，右上腹痛伴发热 1 天，T39.8℃（发热），P105 次/分，BP85/55mmHg（休克），肝区叩诊（+），B 超示胆囊多发高密度团块影（提示有胆囊结石），胆总管直径 1.6cm（正常胆总管直径小于 1cm），肝总管下段有强回声光团伴声影（提示肝总管有结石）。符合急性梗阻性化脓性胆管炎的典型表现，最适宜的治疗是急诊行胆总管切开，T 管引流术（B 对）。
【避错】腹痛、寒战高热、黄疸 Charcot 三联征，伴有休克、神经中枢系统受抑制表现为 Reynolds 五联征。
患者出现的症状和体征表明他可能有急性胆囊炎伴胆石症，并且可能发展成胆管炎或胆管阻塞。根据患者的症状（右上腹痛伴发热）、体征（体温 39.8℃、心率 105 次/分、血压 85/55mmHg）、B 超结果（胆囊多发结石、胆总管直径增大、肝总管下段有强回声光团伴声影），可以判断患者的情况较为严重，需要立即处理。
- A 选项（急诊行开腹胆囊切除术）可能是一个选择，如果考虑到胆囊炎严重且存在并发症。
- B 选项（急诊行胆总管切开，T 管引流术）适用于胆总管结石或严重的胆管炎。
- C 选项（积极抗休克治疗，禁食、抗感染）适用于所有严重腹部感染的初始处理，但可能不足以单独解决问题，尤其是当有明确的手术指征时。
- D 选项（急诊行腹腔镜胆囊切除术）适用于急性胆囊炎，但在患者出现休克和明确胆管问

题时可能需要更多的评估。
- E 选项（积极抗休克治疗，缓解后择期手术）是在控制了急性症状后再进行手术的理想选择。
考虑到患者似乎有休克（低血压）和胆管阻塞的迹象，应首先采取 C 选项（积极抗休克治疗，禁食、抗感染）来稳定患者的状况。随后，根据患者的反应和进一步评估，可能需要手术干预（如 B 或 D 选项）。但在急性期，优先处理休克和感染是关键，因此我倾向于选择 C 选项。
解析：
中年男性患者，右上腹痛伴发热，查体见高热，心率增快，血压降低，出现休克表现。肝区叩诊（+），B 超示胆囊多发结石，胆总管直径增宽，肝总管下段有结石存在。考虑诊断为胆总管结石导致的急性胆管炎，因血压下降出现休克表现，此时应积极抗休克治疗，禁食、抗感染，为手术创造条件。
选项 A 急诊行开腹胆囊切除术，选项 D 急诊行腹腔镜胆囊切除术，均属于胆囊手术，但患者目前诊断为胆总管结石导致的急性胆管炎，且处于休克状态，不能立即行手术治疗，故一并排除。
选项 B 急诊行胆总管切开，T 管引流术虽是治疗胆总管结石的手术之一，但患者目前处于休克状态，需先抗休克治疗，生命体征平稳后再行手术，故不选。
胆总管结石并胆管炎患者，在非手术治疗控制急性炎症期后，根据病因选择合适的手术方法。故选项 E 积极抗休克治疗，缓解后择期手术是合理的处理方式，但不是当前首选的治疗方案，因此不选。
综上所述，正确答案是：C.积极抗休克治疗，禁食、抗感染。
根据题干信息，该患者出现了右上腹痛伴发热 1 天，并且查体显示体温高达 39.8℃，脉搏 105 次/分，血压 85/55mmHg，肝区叩诊阳性。B 超提示胆囊多发结石，胆总管直径 1.6cm，肝总管下段有强回声光团伴声影。这些表现提示患者可能存在急性胆管炎合并胆道梗阻和败血症。

患者的血压低，提示可能存在休克。对于这种情况，首要任务是积极抗休克治疗、禁食、抗感染处理。待患者病情稳定后再考虑进一步的手术治疗。因此，最合适的治疗方案是：

E. 积极抗休克治疗，缓解后择期手术

52、 (A2 型题)
患者胸痛、反酸 3 年，加重伴干咳 3 天，查体未见异常，心电图、胸部 CT 未见异常。患者应使用的药物是
A.硝酸甘油
B.倍氯米松
C.泮托拉唑
D.硝苯地平
E.氢氯噻嗪
参考答案： C 您的答案：
图文解析： 【解析】反酸、烧心为胃食管反流病最常见和典型的症状，反流物刺激食管引起胸痛、刺激气管引起咳嗽，心电图、胸部 CT 未见异常，可排除心肌梗死和肺炎，故诊断为胃食管反流病，首选泮托拉唑抑酸治疗（C 对）。
【避错】硝酸甘油为扩血管药，用于治疗心绞痛（A 错）；倍氯米松为糖皮质激素（B 错）；硝苯地平为二氢吡啶类钙离子通道阻滞剂（D 错）；氢氯噻嗪为排钾型利尿剂（E 错）。

这道题目描述了一个患者长期胸痛和反酸，最近出现干咳，但是体检、心电图和胸部 CT 都未发现异常。根据这些信息，我们可以考虑胸痛和反酸可能与消化系统问题，如胃食管反流病（GERD），有关。

选项分析如下：

A. 硝酸甘油 - 主要用于治疗心绞痛，通过扩张血管来减轻心脏负担。但心电图和胸部 CT 未发现异常，心脏原因的可能性较低。

B. 倍氯米松 - 是一种糖皮质激素，用于治疗炎症和哮喘等。患者没有明显的炎症或哮喘症状。

C. 泮托拉唑 - 是一种质子泵抑制剂，用于治疗胃酸过多、胃食管反流病等。考虑到患者有反酸的症状，这个选项是合理的。

D. 硝苯地平 - 是一种钙通道阻断剂，用于治疗高血压和心绞痛。尽管它可以用来治疗胸痛，但没有证据表明这种胸痛是由心脏问题引起的。

E. 氢氯噻嗪 - 是一种利尿剂，主要用于治疗高血压，并不适用于这种情况。

因此，最合适的答案是 **C. 泮托拉唑**，因为它用于治疗反酸和胃食管反流，与患者的症状相符。

这是一道关于药物选择的问题。我们需要先理解患者的具体病情，再根据患者的主诉和查体结果，以及心电图、胸部 CT 的检查结果来选择最合适的药物。

理解患者背景信息：患者胸痛、反酸 3 年，最近 3 天症状加重并伴有干咳。查体未见异常，心电图和胸部 CT 也未见异常。基于这些信息，我们可以推断患者可能患有胃食管反流病（GERD）。

理解问题核心：我们需要确定在给定选项中哪种药物最适合该患者。

接下来我们分析每个选项：

A. 硝酸甘油：主要用于缓解心绞痛，不适用于胃食管反流病患者。

B. 倍氯米松：这是一种糖皮质激素，主要用于哮喘和慢性阻塞性肺病的治疗，与本例患者的症状不符。

C. 泮托拉唑：这是一种质子泵抑制剂（PPI），能有效抑制胃酸分泌，是治疗胃食管反流病的首选药物。

D. 硝苯地平：主要用于高血压和心绞痛的治疗，与本例患者的症状不符。

E. 氢氯噻嗪：这是一种利尿剂，主要用于高血压和水肿的治疗，同样不适用于本例患者。

综合患者的症状和药物的适应症，我们可以得出结论：患者应使用的药物是 C.泮托拉唑。这是因为泮托拉唑能有效抑制胃酸分泌，是治疗胃食管反流病的首选药物，与患者的症状相符。

根据患者的症状和检查结果，胸痛、反酸伴干咳的情况考虑可能是胃食管反流病（GERD）。胸部 CT 和心电图未见异常，排除了心血管疾病的可能性。治疗 GERD 的一线药物是质子泵抑制剂（PPI），如泮托拉唑。

因此，患者应使用的药物是：

C. 泮托拉唑

53、 (A2 型题)

某一神经科医生在给学生上课时突然晕倒，抽搐，学生手足无措，十几秒后老师跟学生解释他表演癫痫典型症状，学生印象深刻，该记忆方式是

A.有意识记

B.无意识记

C.机械识记

D.理解识记

E.形象识记
参考答案： B 您的答案：
图文解析： 【解析】根据题意，学生是在无意识状态下记忆，故该记忆方式是无意识记（B 对）。
【避错】有意识记是指刻意去记忆某件事或某个概念（A 错）；机械识记为对无意义或缺乏了解的材料，依据其表面特征采用简单重复的方式记忆，例如人名、地名、电话号码等（C 错）；理解识记是指在理解的基础上记忆（D 错）；形象识记是以感知过的客观事物在头脑中再现的具体形象为内容的记忆，它可以帮助我们记住事物的具体形象（E 错）。
在这个案例中，神经科医生通过模拟癫痫的典型症状来教学，使得学生通过观察这一行为过程得到深刻的印象和理解。这种记忆方式侧重于通过直观的体验和形象的场景来加深记忆印象，因此最适合的答案是：
E. 形象识记
形象识记是通过直观、具体的事物或事件来加强记忆的方式，它依赖于视觉或感官体验的形象化，使得学习内容更加生动、易于理解和记忆。在这个情境下，医生的表演让学生通过视觉和情境感受深刻地记住了癫痫的症状，因此形象识记是最佳选项。
解析：
识记是记忆过程的第一个基本环节，是指个体获得知识和经验的过程，它具有选择性的特点。识记的内容和方法对学习材料的选择会发生直接影响。该神经科医生在给学生上课时突然晕倒，抽搐，并在十几秒后解释他表演癫痫典型症状，学生印象深刻，这是通过实践情景中的事物或现象，让学生留下深刻的印象，从而记忆深刻，符合无意识记的特征。无意识记是指没有预定目的，在识记过程中也不需要做一定的意志努力的识记，又称为不随意识记或偶然识记。
选项 A 有意识记是指有预定的识记目的，并运用一定记忆方法的识记，题目中并未提及学生的识记行为是否具有预定目的，故不选。
选项 C 机械识记是指对无意义或缺乏内在联系的材料，主要靠机械重复的方法进行识记，材料本身缺乏意义联系，或学习者不了解材料的意义，不理解其间的内在联系，单靠反复背诵达到识记，题干中并未提及学生反复背诵，故排除。
选项 D 理解识记是识记方法的一种，是在对材料理解的基础上，依据材料的内在联系，并运用已有的知识经验而进行的识记，是主要的识记方法，其基本条件是对材料的理解和进行思维加工。题干中并未提及学生运用已有的知识经验对识记材料进行理解，故不选。
选项 E 形象识记是指在识记过程中，以事物的具体形象为主要的识记特点，材料中并未体现识记过程中是否以事物的具体形象为主要识记特点，故排除。
综上所述，正确答案是：B.无意识记。
答案是：E. 形象识记。

形象识记指的是通过具体形象来记住事物或信息的过程。在这个案例中，医生通过亲自表演癫痫发作的典型症状，使学生对这种情况有了深刻的印象。通过这种具体、生动的表演，学生能够形象地记住癫痫发作的表现，因此这是形象识记的典型例子。
54、 (A2 型题)
男，40 岁。体重 60kg，右上肢肩关节以下右小腿烧伤，深度为浅Ⅱ度至深Ⅱ度，右足部烧伤深度为Ⅱ度。该患者第一个 24 小时的补液量应为
A.2500ml
B.1800ml
C.2000ml

D.3700ml
E.4300ml
参考答案：　　D　您的答案：
图文解析：　　【解析】中年男性患者，右上肢肩关节以下右小腿烧伤，深度为浅Ⅱ度至深Ⅱ度，右足部烧伤深度为Ⅱ度。可计算总烧伤面积 2.5%+3%+3.5%+6.5%+3.5%=19%，体重60kg，可计算出额外补液量 60×19×1.5=1710ml，另加基础需要量 2000ml，故该患者第一个 24 小时的补液量应为 3710ml(D 对)。
【避错】伤后第 1 个 24 小时补液量：成人每 1%Ⅱ度、Ⅲ度烧伤面积每千克体重补充电解质液 1ml 和胶体液 0.5ml（电解质与胶体比例为 2：1），另加基础水分 2000ml。
对于烧伤患者的初始 24 小时补液量，常用的计算公式是：$\text{体重(kg)} \times \text{烧伤体表面积百分比(TBSA\%)} \times 4 \text{ml}$。但是，根据烧伤的程度和范围，TBSA%的确定可能有所不同。
首先，我们需要估计烧伤的体表面积百分比。根据题目描述，患者的右上肢（包括肩部以下到小腿）和右足部受到烧伤，这可以被认为是一个较大的区域。根据“规则的九”，一个成年人的一条完整的臂和手约占 9%，一条腿约占 18%，足部较小，约占 1-2%。因此，如果我们大致估计，这位患者的 TBSA%可能在 10%至 30%之间，具体取决于烧伤的确切范围。
考虑到这位 40 岁男性患者体重为 60kg，如果我们采用一个较中等的 TBSA%估计，比如 20%，那么他的补液量大约是：
$$ 60 \, \text{kg} \times 20 \, \% \times 4 \, \text{ml/kg/\%} = 4800 \, \text{ml} $$
但这个计算结果并不直接符合题目给出的选项，而且这个计算是基于整个 24 小时周期的总补液量，通常第一个 24 小时内给予总量的一半。因此，如果按照 4800ml 的总补液需要量，第一个 24 小时内应给予 2400ml。但由于这个数字也不在选项中，我们可能需要重新考虑TBSA%的估计或是补液方案的细节。
再细看题目描述，可能存在对烧伤面积的更精确计算需要。因此，我们可能需要根据具体的烧伤面积来重新评估补液量。但是，根据给定的选项，最接近 2400ml 的是 2500ml，所以我倾向于选择 A（2500ml）作为最合适的答案。不过，实际的最佳选择还应考虑具体的烧伤评估和医疗指南。
解析：
患者中年男性，体重 60kg，右上肢肩关节以下及右小腿烧伤，深度为浅Ⅱ°至深Ⅱ°，右足部烧伤深度为Ⅱ°。根据中国新九分法，双上肢占 18%，其中双上臂 7%，双前臂 6%，双手 5%，右小腿占体表面积的 6.5%，右足占体表面积的 3.5%，所以患者的烧伤面积为（7%+6%+3%）/2+6.5%+3.5%=16%。根据中国烧伤补液原则，伤后第一个 24 小时，每 1%Ⅱ°、Ⅲ°烧伤面积，每千克体重应补充胶体液 0.5ml 和电解质液 1ml，另外补充水分 2000ml，所以该患者第一个 24 小时的补液量应为 60×（16×1.5）+2000=3700ml。因此该患者第一个 24 小时的补液量应为 3700ml。
综上所述，正确答案是：D.3700ml。
根据烧伤患者的补液量计算公式，通常采用 Parkland 公式。该公式为：

$$ \text{补液量 (ml)} = 4 \times \text{体重 (kg)} \times \text{烧伤面积 (\% TBSA)} $$

这里，TBSA 指的是烧伤的体表面积百分比。

首先，我们需要估算烧伤面积（TBSA）。根据题目描述：
- 右上肢肩关节以下：约 9%

- 右小腿：约 9%
- 右足部：约 3.5%

总共的烧伤面积为：

$$ 9\% + 9\% + 3.5\% = 21.5\% $$

患者体重为 60 kg，因此根据 Parkland 公式：

$$ \text{补液量} = 4 \times 60 \times 21.5 = 5160 \text{ml} $$

但是，补液量通常在第一个 24 小时内给予一半，在第二个 24 小时内给予另一半。因此，患者第一个 24 小时的补液量应为：

$$ \frac{5160 \text{ml}}{2} = 2580 \text{ml} $$

所以，最接近的答案是 A. 2500ml。

因此，答案是：
A. 2500ml

55、 (A2 型题)

男，50 岁。头晕、乏力伴腰痛 3 个月。血常规：Hb72g/L,WBC6.4×109/L，PLT125×109/L，ESR106mm/h，血清蛋白电泳见 M 蛋白带。尿蛋白(+)。骨髓细胞学检查：幼稚浆 腰椎 X 线检查见第 2、3 椎体压缩性骨折。最可能的诊断是

A.反应性浆细胞增多症

B.多发性骨髓瘤

C.慢性肾小球肾炎

D.骨转移癌

E.霍奇金淋巴瘤

参考答案： B 您的答案：

图文解析： 【解析】中年男性患者，头晕、乏力伴腰痛（多发性骨髓瘤常见骨痛，以腰骶部最为常见）3 个月。血常规：Hb72g/L（正常值 120～160g/L，贫血为多发性骨髓瘤常见表现），WBC6.4×109/L，Plt125×109/L（正常值 100～300×109/L），ESR106mm/h（正常值 0~15mm/h），血清蛋白电泳见 M 蛋白带（提示有高黏滞综合征），尿蛋白（+）（提示有肾功能损害）。骨髓细胞学检查：幼稚浆细胞占 0.42。腰椎 X 线检查见第 2、3 椎体压缩性骨折（多发性骨髓瘤易导致病理性骨折），故诊断为多发性骨髓瘤（B 对）。

【避错】反应性浆细胞增多症可由慢性炎症、伤寒、系统性红斑狼疮、肝硬化、转移癌等引起。浆细胞一般不超过 15%且无形态异常，免疫表型为 CD38+、CD56-且不伴有 M 蛋白，IgH 基因重排阴性（A 错）；慢性肾小球肾炎表现为急性肾炎综合征血尿、蛋白尿、水肿、高血压病程大于 3 个月（C 错）；骨转移癌表现为骨痛，无骨髓细胞学表现（D 错）；霍奇金淋巴瘤表现为无痛性进行性淋巴结肿大（E 错）。

这个病例中的关键信息是头晕、乏力、腰痛，血常规显示 Hb 72g/L（贫血），WBC 正常，PLT 稍低，ESR 显著升高，血清蛋白电泳显示 M 蛋白带，尿蛋白阳性，骨髓检查显示幼稚浆细胞，以及 X 线检查显示脊椎压缩性骨折。

这些症状和体征最符合多发性骨髓瘤（Multiple Myeloma, MM）的临床表现，这是一种影响

浆细胞的血液癌症。多发性骨髓瘤的特征包括骨髓中浆细胞的增多，高沉降率（ESR），低血红蛋白（贫血），M 蛋白的出现，和骨质破坏或压缩性骨折。
因此，最可能的诊断是：B. 多发性骨髓瘤
解析：
中年男性患者，出现头晕、乏力伴腰痛症状。血常规结果显示血红蛋白降低，提示贫血，血沉增快，提示存在炎症性疾病或高球蛋白血症等，血清蛋白电泳见 M 蛋白带。尿蛋白阳性，提示肾脏受损。骨髓细胞学检查见幼稚浆细胞，腰椎 X 线检查见第 2、3 椎体压缩性骨折。根据患者的临床表现、实验室检查，可诊断为多发性骨髓瘤。多发性骨髓瘤是一种恶性浆细胞病，其肿瘤细胞起源于骨髓中的浆细胞，而浆细胞是 B 淋巴细胞发育到最终功能阶段的细胞。因此多发性骨髓瘤可以归到 B 淋巴细胞淋巴瘤的范围。多发性骨髓瘤起病徐缓，早期无明显症状，容易被误诊。临床表现多样，主要有贫血、骨痛、肾功能不全、感染、出血、神经症状、高钙血症、淀粉样变等。
选项 A 反应性浆细胞增多症是指一组由多种原因或原发疾病引起的、以骨髓浆细胞增多为特征的继发性疾病，一般无 M 蛋白带，故不选。
选项 C 慢性肾小球肾炎系指蛋白尿、血尿、高血压、水肿为基本临床表现，起病方式各有不同，病情迁延，病变缓慢进展，可有不同程度的肾功能减退，最终发展为慢性肾衰竭的一组肾小球疾病，与题干描述不符，故排除。
选项 D 骨转移癌是指原发于身体其他部位的恶性肿瘤通过各种途径转移至骨骼并在骨内继续生长所形成子肿瘤，患者往往有原发恶性肿瘤的病史，该患者并无相关病史，故不考虑。
选项 E 霍奇金淋巴瘤是淋巴瘤的一种独特类型，为青年人中最常见的恶性肿瘤之一，多发生在 20～40 岁的青年人，由原发于一组淋巴结或淋巴结外的淋巴组织中的淋巴细胞或其前体细胞发生恶性变所致，与题干描述不符，故不选。
综上所述，正确答案是：B.多发性骨髓瘤。
根据提供的病例描述，患者是一名 50 岁的男性，症状包括头晕、乏力和腰痛 3 个月。检查结果如下：

- 血红蛋白 (Hb): 72 g/L（贫血）
- 白细胞计数 (WBC): 6.4×10^9/L（正常）
- 血小板计数 (PLT): 125×10^9/L（轻微减少）
- 红细胞沉降率 (ESR): 106 mm/h（显著升高）
- 血清蛋白电泳：M 蛋白带（提示单克隆免疫球蛋白增多）
- 尿蛋白：阳性（提示蛋白尿）
- 骨髓细胞学检查：幼稚浆细胞增多
- 腰椎 X 线检查：第 2、3 椎体压缩性骨折

综合这些临床特征和检查结果，最可能的诊断是**多发性骨髓瘤**（B）。

理由：
1. **贫血**（Hb 72 g/L）：多发性骨髓瘤常见贫血。
2. **高 ESR**（106 mm/h）：多发性骨髓瘤患者通常有显著的 ESR 升高。
3. **M 蛋白带**：血清蛋白电泳显示 M 蛋白带，提示单克隆免疫球蛋白增多，这是多发性骨髓瘤的特征。
4. **骨痛和骨折**：患者有腰痛和椎体压缩性骨折，多发性骨髓瘤患者常见骨痛和病理性骨折。

5. **尿蛋白**：多发性骨髓瘤可导致肾脏损害，出现蛋白尿。
6. **骨髓细胞学检查**：幼稚浆细胞增多是多发性骨髓瘤的典型表现。

综上所述，最符合患者的临床和检查特征的是多发性骨髓瘤。

正确答案是 **B. 多发性骨髓瘤**。

56、 (A2 型题)

男，44 岁，右下肢清创缝合术后 6 天，发热疼痛 2 天，见伤口处红肿，少量红色液，缝合处张力高，该患者可能是何种感染

A.破伤风

B.厌氧菌

C.溶血性链球菌

D.金葡菌

E.铜绿假单胞菌

参考答案： C 您的答案：

图文解析： 【解析】中年男性患者，右下肢清创缝合术（外科手术史）后 6 天，发热疼痛（提示伤口感染）2 天。见伤口处红肿，少量红色液（溶血性链球菌脓液为淡红色稀薄状），缝合处张力高，最可能感染的细菌是溶血性链球菌（C 对）。

【避错】破伤风多见于伤口较深时，典型症状为肌紧张性收缩的基础上阵发性强烈痉挛（A 错）；厌氧菌临床上多见于口腔感染，也可导致破伤风和气性坏疽（B 错）；铜绿假单胞菌多见于器械操作后感染（E 错）；金黄色葡萄球菌多见于血源性感染（D 错）。

该患者的症状包括发热、疼痛、伤口红肿，以及少量红色液体，这些迹象表明可能发生了伤口感染。缝合处张力高也可能指示感染导致的炎症反应。根据这些症状，我们可以分析选项中的可能病原体：

A. 破伤风：由破伤风梭菌引起，主要症状为肌肉僵硬和痉挛，不太符合患者的表现。

B. 厌氧菌：可能导致严重的感染，但常见的症状包括恶臭的伤口分泌物和气性坏疽，这并非题中描述的情况。

C. 溶血性链球菌：这类细菌可以导致急性炎症反应，常见于皮肤和软组织感染，与患者症状相符。

D. 金葡菌：特别是金黄色葡萄球菌，常引起皮肤和软组织感染，也可能是此类症状的原因。

E. 铜绿假单胞菌：通常与医院相关的感染有关，更常见于免疫系统受损的患者，并且其感染通常与湿润的环境有关，如烧伤或静脉导管的感染。

考虑到症状和常见的皮肤及软组织感染病原体，C 选项“溶血性链球菌”和 D 选项“金葡菌”是比较可能的。但是，由于伤口红肿、发热、疼痛这些症状更典型地与溶血性链球菌相关，因此最可能的答案是 **C. 溶血性链球菌**。

解析：

患者右下肢清创缝合术后出现发热、疼痛，查体见伤口红肿，少量红色液体流出，缝合处张力高，考虑为金黄色葡萄球菌感染，因其能产生血浆凝固酶，可使周围血液或血浆中的纤维蛋白等沉积于细菌表面，阻碍体内吞噬细胞的吞噬，还能保护病菌不受血清中杀菌物质的破坏，常引起侵袭性疾病如皮肤化脓性感染、各种器官及全身感染，产生的症状与本题患者出现的症状相符。

选项 A 破伤风常表现为牙关紧闭、阵发性痉挛、强直性痉挛等，与该患者表现不符，故排除。

选项 B 厌氧菌感染常见于口腔、腹腔、盆腔等部位，且感染部位可有气体产生，与本题患者

症状不符，故不选。
选项 C 溶血性链球菌感染常见于上呼吸道感染、猩红热、风湿热等疾病，与本题无关。
选项 E 铜绿假单胞菌感染常见于皮肤黏膜受损部位，如烧伤、创伤或手术切口等，表现为局部化脓性炎症，脓汁呈淡绿色，与本题中患者伤口少量红色液体流出的表现不符，故不考虑。
综上所述，正确答案是：D.金葡菌。
根据题干描述的症状和体征，患者右下肢清创缝合术后 6 天，出现发热、疼痛、伤口处红肿、少量红色液体渗出、缝合处张力高等症状。这些症状提示伤口有感染的迹象。为了确定可能的感染病原体，我们需要结合临床特点来判断。

以下是每个选项的分析：

A. **破伤风**：破伤风主要表现为肌肉痉挛和张口困难等特征性症状，而非伤口红肿和渗液。

B. **厌氧菌**：厌氧菌感染通常发生在缺氧环境中，表现为坏死组织较多、恶臭味等，虽然可能存在红肿和疼痛，但题干并未提及此类典型表现。

C. **溶血性链球菌**：溶血性链球菌感染可以导致局部红肿、疼痛、脓液渗出，且会引起发热等全身症状，比较符合患者的症状。

D. **金黄色葡萄球菌（金葡菌）**：金葡菌是最常见的伤口感染病原菌之一，感染后常表现为红肿、疼痛、化脓性分泌物，并可引起发热，与题干症状也较为吻合。

E. **铜绿假单胞菌**：这种细菌感染常见于烧伤或免疫力低下患者，伤口会有绿色脓液渗出，但题干中提到的是红色液体，故不太符合。

综合分析，患者清创缝合术后 6 天出现的症状（红肿、疼痛、少量红色液体渗出、缝合处张力高）最符合金黄色葡萄球菌感染的表现。因此，最佳答案是：

D. **金葡菌**
57、 (A2 型题)
女，35 岁，因发热，呕吐头疼 3 天入院，查体：面色潮红，腋下有出血点，尿常规有蛋白尿，酱油色尿液，血常规：WBC23×109/L，异形淋巴细胞 0.12，PLT48×109/L， 可能是
A.流行性脑脊髓膜炎
B.伤寒
C.流行性出血热
D.败血症
E.钩端螺旋体病
参考答案： C 您的答案：
图文解析： 【解析】中年女性患者，因发热，呕吐头疼（发热中毒症状）3 天入院，查体：面色潮红，腋下有出血点（充血、出血征），尿常规有蛋白尿，酱油色尿液（肾损害），血常规：WBC23×109/L，异形淋巴细胞 0.12（肾综合征出血热患者特征性表现），PLT48×109/L，该患者的诊断可能是肾综合征出血热，即流行性出血热（C 对）。
【避错】流行性脑脊髓膜炎为脑膜炎奈瑟球菌引起的急性化脓性脑膜炎，表现为脑膜刺激

征、全身瘀点瘀斑（A 错）；伤寒主要表现为稽留热、肝脾肿大（B 错）；败血症无异形淋巴细胞（D 错）；钩端螺旋体病通常有疫水接触史，显微镜下凝集溶解实验确诊（E 错）。
考虑到这位 35 岁女性患者的临床表现：发热，呕吐，头疼，面色潮红，腋下出血点，尿常规显示有蛋白尿和酱油色尿液，血常规显示白细胞计数(WBC)为 23×10^9/L，异形淋巴细胞 0.12，血小板计数(PLT)为 48×10^9/L，我们可以开始分析每个选项：
A. 流行性脑脊髓膜炎：通常会有高热、剧烈头痛、呕吐和颈项刚硬等症状，但不太可能导致腋下出血点和酱油色尿液。
B. 伤寒：由沙门氏菌引起，症状包括发热、腹痛、头痛和皮疹，但伤寒不太会导致酱油色尿液或出血点。
C. 流行性出血热：病因是汉坦病毒，会导致发热、出血倾向（如腋下出血点）、蛋白尿和酱油色尿液，血常规检查可能显示白细胞增多和血小板减少，与此病例描述吻合。
D. 败血症：虽然可能导致高热、白细胞计数增高和器官功能障碍，但一般不会出现酱油色尿液和局部出血点。
E. 钩端螺旋体病：一种由钩端螺旋体引起的疾病，可导致肾功能受损、黄疸和蛋白尿，但其特征并不完全符合本病例描述。
综合考虑，最符合这位患者症状的是 C 选项，流行性出血热。因此，我会选择 C 作为最佳答案。
首先，我们来分析题干中的关键信息和症状。
患者中年女性，主要症状为发热、呕吐、头疼，已经持续 3 天。查体发现面色潮红，腋下有出血点。实验室检查发现，尿常规有蛋白尿和酱油色尿液，血常规结果显示白细胞显著增高，异形淋巴细胞占比增高，血小板数量降低。
接下来，我们根据这些信息来逐一分析选项：
A. 流行性脑脊髓膜炎：虽然该疾病也伴有发热、头疼等症状，但通常不会出现酱油色尿液和异形淋巴细胞增多，因此可以排除。
B. 伤寒：伤寒主要表现为持续发热、表情淡漠、相对缓脉、玫瑰疹、肝脾大和白细胞减少，与题干描述的症状不符，故排除。
C. 流行性出血热：该疾病是一种以鼠类为主要传染源的自然疫源性疾病，临床上以发热、低血压休克、充血出血和肾损害为主要表现。题干中的面色潮红、腋下出血点、蛋白尿、酱油色尿液（血红蛋白尿，提示血管内溶血）、异形淋巴细胞增多和血小板减少等症状均符合流行性出血热的临床表现。
D. 败血症：虽然败血症也可能引起发热和其他全身症状，但它通常不会导致特异性的出血点或酱油色尿液，因此不符合题干描述。
E. 钩端螺旋体病：这是一种由致病性钩端螺旋体引起的动物源性传染病，主要症状包括发热、酸痛、全身软、眼结膜充血、腓肠肌疼痛、淋巴结肿大等，与题干描述的症状不完全吻合。
综上所述，结合患者的临床症状和实验室检查，最可能的诊断是流行性出血热。
因此，正确答案是 C.流行性出血热。
根据题目提供的病史和检查结果，患者出现发热、呕吐、头疼 3 天，面色潮红，腋下有出血点，尿常规显示蛋白尿和酱油色尿液，血常规显示白细胞总数显著升高和血小板减少，这些症状和体征提示可能的出血性疾病。

结合症状和检查结果，流行性出血热（即肾综合征出血热）是最符合的诊断。流行性出血热通常由鼠传播，特征性表现包括急性发热、出血倾向（如皮肤出血点）、肾脏受损（蛋白尿、酱油色尿液）以及血液学异常（白细胞增多、血小板减少）。

因此，最可能的诊断是：

C. 流行性出血热
58、 (A2 型题)
男，65 岁。间歇性乏力、腹胀 3 年，加重 2 个月。限盐利尿治疗后腹胀无明显缓解。查体：T36.8℃,BP120/80mmHg。巩膜黄染，可见肝掌、蜘蛛痣。腹膨隆，无压痛，肝
移动性浊音阳性。双下肢可见非凹陷性水肿。人血白蛋白 19g/L，血钾 3.7mmol/L，血钠 136mmol/L。接下来最为恰当的治疗措施是
A.腹腔置管持续引流腹腔积液
B.静脉输注白蛋白后利尿
C.继续利尿限盐
D.腹水培养，用敏感抗生素
E.经颈静脉肝内门腔分流术
参考答案： B 您的答案：
图文解析： 【解析】老年男性，间歇性乏力、腹胀，可见肝掌、蜘蛛痣（提示肝功能减退），腹膨隆，无压痛，移动性浊音阳性（提示有腹水）。双下肢可见非凹陷性水肿，人血白蛋白 19g/L（正常范围：35～50g/L，提示低蛋白血症），血钾 3.7mmol/L（正常范围 3.5~5.5mmol/L），血钠 136mmol/L（正常值范围：135～145mmol/L），结合患者临床症状和实验室检查，考虑诊断为肝硬化失代偿期，最重要的治疗是对因治疗，即针对腹水的治疗，已限盐利尿治疗，腹胀无明显缓解，患者低蛋白血症，血浆胶体渗透压下降，毛细血管内液体漏入腹腔或组织间隙，故应静脉输注白蛋白，提升血浆胶体渗透压后利尿（B 对）。【避错】腹腔置管持续引流腹腔积液（A 错）为对症治疗，继续利尿限盐（C 错）为一般治疗；腹水培养，用敏感抗生素（D 错）用于腹膜炎的治疗，该病例无此症状，故不选；经颈静脉肝内门腔分流术（E 错）用于门脉高压所致腹水。
这个病例描述的是一名 65 岁男性患者，有间歇性乏力、腹胀 3 年，近两月加重，且限盐利尿治疗后腹胀无明显缓解。患者体检结果显示巩膜黄染、肝掌和蜘蛛痣，这些都是肝硬化的体征。腹部检查发现腹膨隆，移动性浊音阳性，这表明有腹水。双下肢非凹陷性水肿也符合肝硬化引起的水肿表现。实验室检查显示人血白蛋白低（19g/L），这也是肝功能不全的标志之一。
针对这种情况，选择最恰当的治疗措施需要考虑到腹水的管理和肝功能的支持。选项 A（腹腔置管持续引流腹腔积液）可能适用于紧急情况下腹水大量且迅速积聚导致呼吸困难的情况，但这不是长期管理方案。选项 C（继续利尿限盐）在之前的治疗中未能显著改善症状，可能不够有效。选项 D（腹水培养，用敏感抗生素）通常适用于腹水感染的情况，但目前没有描述感染的迹象。选项 E（经颈静脉肝内门腔分流术）是一种外科手术，用于减轻门脉高压，通常在其他治疗措施无效时考虑。
考虑到患者的低白蛋白血症和腹水，最合适的治疗措施是选项 B，静脉输注白蛋白后利尿。静脉输注白蛋白可以帮助提高血浆白蛋白浓度，改善胶体渗透压，从而有助于控制腹水并减轻水肿。此外，改善血浆白蛋白水平有助于改善患者的总体情况和肝功能。因此，本题的答案是 B。
首先，我们分析题干。
老年男性患者，间歇性乏力、腹胀 3 年，最近 2 个月症状加重。经过限盐利尿治疗后，腹胀并未得到明显缓解。查体发现患者有巩膜黄染、肝掌和蜘蛛痣，这些都是肝功能减退的典型体征。腹部膨隆、肝移动性浊音阳性和双下肢的非凹陷性水肿都提示有腹水。实验室检查发

现人血白蛋白明显降低，这可能与肝功能减退导致的蛋白合成减少有关。血钾和血钠均在正常范围内。
接下来，我们逐一分析选项：
A.腹腔置管持续引流腹腔积液：这种方法虽然可以暂时缓解腹胀，但长期引流可能导致蛋白质和电解质的丢失，不利于患者的长期恢复。
B.静脉输注白蛋白后利尿：由于患者的白蛋白明显降低，这可能导致胶体渗透压下降，从而引起腹水。静脉输注白蛋白可以提高血液的胶体渗透压，从而帮助将组织间的水分吸收入血。随后使用利尿剂可以帮助排出体内多余的水分，从而减轻腹水。
C.继续利尿限盐：考虑到患者之前的限盐利尿治疗并未使腹胀得到明显缓解，因此继续此方法可能效果不佳。
D.腹水培养，用敏感抗生素：题干中并未提及患者有感染的症状或体征，因此使用抗生素可能并不恰当。
E.经颈静脉肝内门腔分流术：此方法通常用于治疗门静脉高压所导致的腹水，但在此情况下，患者的腹水主要是由于肝功能减退和低白蛋白血症导致的，因此该方法可能并不适用。
综上所述，考虑到患者的白蛋白明显降低，最为恰当的治疗措施是静脉输注白蛋白后利尿，以提高血液的胶体渗透压并排出多余的水分。
因此，正确答案是 B.静脉输注白蛋白后利尿。
患者是一位 65 岁的男性，间歇性乏力、腹胀 3 年，加重 2 个月。尽管接受了限盐利尿治疗，腹胀无明显缓解。查体显示黄疸、肝掌、蜘蛛痣、腹膨隆、肝移动性浊音阳性以及双下肢非凹陷性水肿。实验室检查结果包括低白蛋白血症（19g/L），正常血钾（3.7mmol/L）和血钠（136mmol/L）。

考虑到患者的临床表现和实验室结果，最可能的诊断是肝硬化并发腹水。这种情况下，最适当的治疗措施应该是通过提高血浆白蛋白水平来改善腹水，这可以通过静脉输注白蛋白，然后继续利尿治疗来实现。

因此，最为恰当的治疗措施是：

B. 静脉输注白蛋白后利尿
59、　(A2 型题)
男子肛周红肿热痛，查体：T38℃,肛周红肿，有波动感，应选用的种治疗措施是
A.局部理疗缓解
B.抽吸脓肿
C.切开脓肿
D.抗生素治疗
E.温水坐浴
参考答案：　C　您的答案：
图文解析：　【解析】男性患者，肛周红肿，有波动感（直肠肛管周围脓肿典型症状），伴发热（提示有感染），根据患者症状考虑诊断为直肠肛管周围脓肿，首选切开脓肿引流（C对）。
【避错】脓肿切开引流是治疗直肠肛管脓肿的主要方法，　一旦诊断明确，即应切开引流。
这道题目描述的是一个男性患者有肛周红肿、热痛，并伴有波动感，这表明可能存在脓肿。体温　38℃也支持有感染存在。波动感通常意味着脓液积聚，因此需要排脓以减轻症状和治疗感染。

选项分析如下：
- A. 局部理疗缓解：这种方法对于缓解症状可能有一定效果，但不能根本解决脓肿问题。
- B. 抽吸脓肿：这可以暂时减轻症状，但对于大的或深层的脓肿可能不够有效。
- C. 切开脓肿：这是处理脓肿的标准方法，可以有效排出脓液，并且有助于快速缓解症状和治疗感染。
- D. 抗生素治疗：虽然抗生素可以帮助控制感染，但单独使用通常不足以解决已形成的脓肿。
- E. 温水坐浴：这有助于缓解症状和清洁区域，但不足以治疗脓肿。
因此，最佳选择是 C. 切开脓肿，因为这是直接并有效地处理脓肿的方法。
解析：
患者肛周红肿热痛，查体见体温升高，肛周红肿且有波动感，可考虑诊断为肛周脓肿。由于脓肿有波动感，提示脓肿成熟，应及时切开脓肿，并行脓液引流，避免感染进一步扩散。
选项 A 局部理疗缓解，选项 E 温水坐浴，二者均可促进局部血液循环，有助于炎症的消退，可作为辅助治疗措施，但由于该患者脓肿已成熟，单纯理疗和坐浴治疗效果不佳，故不选。
选项 B 抽吸脓肿为姑息性治疗措施，抽吸后脓肿易复发，因此不作为首选，故排除。
选项 D 抗生素治疗为一般性治疗措施，患者已出现全身症状，说明感染已扩散，此时单纯应用抗生素已无法控制感染，故不选。
综上所述，正确答案是：C.切开脓肿。
题目描述了一名男子出现肛周红肿热痛，并且查体时发现有波动感，这提示可能存在脓肿。对于这种情况，最佳的治疗措施是：

C. 切开脓肿

理由如下：
- 肛周脓肿的治疗通常需要外科干预，尤其是当有波动感时，表明脓肿已形成且需要引流。
- 切开引流脓肿可以迅速缓解症状，防止感染扩散，并促进愈合。
- 其他选项如局部理疗、抽吸脓肿、抗生素治疗和温水坐浴可能在某些情况下作为辅助治疗，但不能替代切开引流作为主要治疗方法。

因此，最正确的答案是 C. 切开脓肿。
60、 (A2 型题)
男，40 岁，头痛伴视物模糊 3 个月，在大便中发现虫卵，喜食生肉，此时眼底检查发现视乳头水肿，最可能的诊断是
A.病毒性脑膜炎
B.脑囊虫蚴病
C.结核性脑膜炎
D.脑肿瘤
E.疟疾
参考答案： B 您的答案：
图文解析： 【解析】中年男性患者，喜食生食，头痛伴视物模糊 3 个月，曾在大便中发现虫卵（说明患者有猪带绦虫感染），最可能的诊断是脑囊尾蚴病（B 对）。
【避错】病毒性脑膜炎（A 错）患者可有头痛、恶心、呕吐、轻偏瘫等表现，粪便检查多正常；结核性脑膜炎（C 错）起病缓慢，多于 1～2 周非特异性感染症状后，出现脑膜刺激征；脑肿瘤（D 错）分为良性肿瘤和恶性肿瘤，良性肿瘤生长缓慢，局限在脑内，可引起颅内高压的症状；恶性肿瘤生长迅速，可发生转移，也可引起颅内高压症状，威胁生命，

粪便检查无明显异常；疟疾（E 错）主要为周期性发作的发冷-发热-出汗。
考虑到这位 40 岁男性患者有头痛、视物模糊、眼底检查发现视乳头水肿，并且在大便中发现虫卵，喜食生肉，最可能的诊断是脑囊虫蚴病。因此，正确答案是：
B. 脑囊虫蚴病
脑囊虫蚴病是由摄入含有囊虫蚴的生或半生肉类（通常是猪肉）引起的寄生虫感染。囊虫蚴可以进入脑部，引起神经系统症状，如头痛、视力模糊和视乳头水肿。患者的临床表现和食物史支持这一诊断。
解析：
中年男性患者，头痛伴视物模糊 3 个月，眼底检查发现视乳头水肿，为颅内高压的典型表现，喜食生肉，在大便中发现虫卵，结合患者病史、临床表现和眼底检查，对该患者最可能的诊断为脑囊虫蚴病。
选项 A 病毒性脑膜炎常有前驱感染史，主要表现为发热、反复惊厥发作、不同程度的意识障碍和颅内压增高症状，一般无脑膜刺激征，脑脊液检查外观清亮，白细胞数轻度升高，糖、氯化物和蛋白多无明显变化，与题干描述不符，故排除。
选项 C 结核性脑膜炎起病缓慢，多于 1～2 周非特异性感染症状后，出现脑膜刺激征，脑脊液检查白细胞数明显升高，以淋巴细胞为主，蛋白质明显增高，通常为 1～3g/L，糖及氯化物下降，与本题无关。
选项 D 脑肿瘤起病缓慢，逐渐加重，常有颅内压增高的表现，但无寄生虫感染史，不符合题意。
选项 E 疟疾主要表现为周期性寒战、高热和大汗，热退后可无症状，本题患者无寒战、高热等症状，故不选。
综上所述，正确答案是：B.脑囊虫蚴病。
根据题目描述：40 岁男性，头痛伴视物模糊 3 个月，在大便中发现虫卵，喜食生肉，并且眼底检查发现视乳头水肿，最可能的诊断是脑囊虫蚴病（B）。

解析如下：
1. **头痛和视物模糊**：这些是脑部病变常见的症状。
2. **大便中发现虫卵**：提示可能有寄生虫感染。
3. **喜食生肉**：生肉可能含有囊虫，符合脑囊虫蚴病的感染途径。
4. **视乳头水肿**：这是颅内压增高的表现，脑囊虫蚴病可导致颅内压增高。

综合上述信息，最符合的诊断是 B. 脑囊虫蚴病。
61、 (A2 型题)
患者有脓血便病史，X 线钡剂造影显示结肠多发病变，后症状加重，出现低血钾，诊断考虑为
A.肠结核
B.直肠癌
C.溃疡性结肠炎合并中毒性巨结肠
D.肠易激综合征
E.肠梗阻
参考答案： C 您的答案：
图文解析： 【解析】患者有脓血便病史（溃疡性结肠炎常见症状），X 线钡剂造影显示结肠多发病变（溃疡性结肠炎典型影像学改变），后症状加重，出现低血钾（中毒性巨结肠常见诱因），诊断考虑为溃疡性结肠炎合并中毒性巨结肠（C 对）。

【避错】肠结核主要位于回盲部，多表现为腹泻，有时腹泻便秘交替，大便呈糊样，多无脓血，X 线钡餐可见回盲部充盈呈跳跃征（A 错）；直肠癌好发于中老年人，多表现为血便或粪便隐血阳性，无明显黏液脓血（B 错）；肠易激综合征多表现为腹痛，腹泻，排稀水样黏液便，检查多无异常（D 错）；肠梗阻典型表现为腹痛，腹胀，呕吐，肛门停止排气、排便（E 错）。

这个问题涉及到对病史和诊断考虑的解析。患者有脓血便的病史，这提示可能存在炎症性肠病。X 线钡剂造影显示结肠多发病变，进一步支持了炎症性肠病的可能性。症状加重，出现低血钾，这可能与电解质紊乱有关，后者可以在严重的炎症性肠病中见到。结合这些信息，最符合的诊断是溃疡性结肠炎，可能合并有中毒性巨结肠（这是一种并发症，指的是结肠显著扩张，通常与溃疡性结肠炎有关）。

A. 肠结核可能导致脓血便，但通常影响回肠末端和盲肠，并且钡剂造影表现不会是结肠多发病变。

B. 直肠癌可能导致脓血便，但一般不会导致结肠的多发病变。

D. 肠易激综合征（IBS）通常不会引起脓血便，且 X 线检查多正常。

E. 肠梗阻的临床表现与此病例不符，肠梗阻患者一般不会出现脓血便。

因此，最合适的答案是 **C. 溃疡性结肠炎合并中毒性巨结肠**。

解析：

患者有脓血便病史，X 线钡剂造影显示结肠多发病变，后症状加重，出现低血钾，考虑患者为溃疡性结肠炎合并中毒性巨结肠。溃疡性结肠炎是一种原因不明的，以直肠和结肠黏膜非特异性炎症、溃疡形成为主的病变。中毒性巨结肠是溃疡性结肠炎的严重并发症之一，其 X 线腹部平片可见结肠扩张，结肠袋消失。临床表现为病情急剧恶化，毒血症明显，有脱水与电解质平衡紊乱，出现鼓肠、腹部压痛，肠鸣音消失。

选项 A 肠结核起病缓慢，早期腹痛不明显，多位于右下腹，常有上腹或脐周疼痛，系回盲部病变引起的牵涉痛，常伴有腹泻，但便中多不含粘液及脓血，无里急后重，X 线钡餐激惹征阳性，可排除。

选项 B 直肠癌早期无明显症状，癌肿破溃形成溃疡或感染时才出现症状，直肠刺激症状如便意频繁，排便习惯改变，便前肛门有下坠感、里急后重、排便不尽感，晚期有下腹痛，与题干描述不符，故不选。

选项 D 肠易激综合征是一种以腹痛或腹部不适伴排便习惯改变为特征而无器质性病变的常见功能性肠病，患者以中青年居多，老年人较为少见，腹痛以下腹和左下腹多见，排便或排气后缓解，粪便呈糊状或稀水样，少数可有粘液，但绝无脓血，不符合题意。

选项 E 肠梗阻临床表现有腹痛、呕吐、腹胀及停止自肛门排气排便，钡剂造影显示结肠梗阻部位以上扩张，钡剂无法继续向下流动，故排除。

综上所述，正确答案是：C.溃疡性结肠炎合并中毒性巨结肠。

根据题干描述，患者有脓血便病史，X 线钡剂造影显示结肠多发病变，后症状加重，出现低血钾。考虑以下几个因素：

1. **脓血便**：常见于溃疡性结肠炎。
2. **结肠多发病变**：溃疡性结肠炎可以引起结肠的广泛病变。
3. **低血钾**：可以是中毒性巨结肠的表现，因电解质紊乱导致。

综合以上病史和症状，最符合的诊断是 C. 溃疡性结肠炎合并中毒性巨结肠。

所以最佳答案是：**C. 溃疡性结肠炎合并中毒性巨结肠**。

62、 (A2 型题)
女，50 岁，月经不规则 2 年余，阴道不规则流血 20 天，查体贫血貌，子宫稍大，无压痛，稍软，为明确诊断，应选择的检查是
A.盆腔 CT
B.尿 HCG 测定
C.分段诊刮
D.盆腔 B 超
E.阴道镜检查
参考答案： C 您的答案：
图文解析： 【解析】中年女性患者，月经不规则 2 年余，阴道不规则流血 20 天（约 90%的子宫内膜癌患者出现阴道流血或阴道排液症状），子宫略大（晚期可有子宫明显增大），稍软，应高度怀疑子宫内膜癌，其确诊方法是分段诊刮（C 对），其能刮取子宫内膜和内膜病灶行活组织检查，作出病理检查。
【避错】盆腔 B 超（D 错）可用于早孕的确诊及子宫肌瘤等疾病的诊断；阴道镜检查（E 错）主要用于观察阴道和宫颈，对可疑部位行定位、活检，结合活检方可确诊。尿 hCG 检查（B 错）即尿妊娠试验，临床用于诊断早孕，可用于辅助诊断宫外孕，估计先兆流产和预后，并可协助诊断并随访观察某些恶性肿瘤以及妇女受孕后，但不作为首选检查。盆腔 CT 检查（A 错）能够显示肿瘤的结构特点、周围侵犯及远处转移的情况，多用于妇科肿瘤治疗方案的制定、预后估计、疗效观察及术后复发的诊断，不能作为确定诊断的首选检查。
这位 50 岁女性患者月经不规则已超过 2 年，并且有阴道不规则流血 20 天，体检发现贫血和子宫略大但无压痛，质地稍软。根据这些症状，为了明确诊断，合适的检查应该是那种能够提供子宫及其内膜状况详细信息的检查。
选项 C“分段诊刮”是一种用于评估子宫内膜异常（如不规则出血）的诊断程序。它涉及在视觉引导下从子宫内膜取样，这样可以对取出的组织进行病理学检查。考虑到该患者的年龄、症状（不规则出血和月经不规则）以及体检结果（子宫略大），分段诊刮有助于确定不规则出血的原因，如子宫内膜异常或恶性病变。
因此，最合适的答案是 C.分段诊刮。这项检查可以提供确诊，特别是在考虑到患者可能存在子宫内膜病变的风险时。
首先，我们分析题干。
患者为 50 岁女性，已有月经不规则两年多的历史，最近阴道不规则流血已达 20 天。查体显示贫血貌，子宫稍大、无压痛、稍软。这些信息提示我们，患者可能患有与子宫内膜相关的疾病，特别是考虑到她的年龄和症状，子宫内膜癌的风险增加。
接下来，我们分析各个选项：
A. 盆腔 CT：虽然 CT 可以提供盆腔的详细解剖结构，但它通常不是子宫内膜病变的首选诊断工具。
B. 尿 HCG 测定：这是用于检测怀孕的常用方法，与子宫内膜病变的诊断无直接关联。
C. 分段诊刮：这是一个直接取子宫内膜组织进行检查的方法，能够明确诊断子宫内膜的病变，特别是恶性病变。它可以直接提供组织病理学证据，是诊断子宫内膜癌的金标准。
D. 盆腔 B 超：虽然 B 超可以提供子宫和附件的大致情况，但它不能替代病理诊断，因此不是首选。
E. 阴道镜检查：这主要用于观察阴道和宫颈的病变，并不直接针对子宫内膜。
综合上述分析，为了明确诊断该患者的具体病情，最佳选择是直接取子宫内膜组织进行检查，即进行分段诊刮。这种方法可以提供确切的组织病理学证据，有助于确定是否存在恶性病变。

因此，正确答案是 C.分段诊刮。
为了明确诊断这位 50 岁女性的病情，最合适的检查是 C. 分段诊刮。以下是各选项的解释：

A. 盆腔 CT：盆腔 CT 能提供子宫和附件的影像，但对明确诊断阴道不规则流血的原因，尤其是诊断子宫内膜病变，不如分段诊刮直接有效。

B. 尿 HCG 测定：尿 HCG 测定主要用于检测妊娠情况，对这个 50 岁女性出现的月经不规则和阴道不规则流血不太适用。

C. 分段诊刮：分段诊刮能够采集子宫内膜样本，进行病理学检查，是诊断子宫内膜病变（如子宫内膜增生或癌变）的金标准。

D. 盆腔 B 超：盆腔 B 超可以提供子宫和卵巢的影像学信息，对初步评估有帮助，但不能直接获得病理诊断。

E. 阴道镜检查：阴道镜检查主要用于宫颈及阴道病变的诊断，对子宫内膜病变的诊断帮助不大。

因此，最佳答案是 C. 分段诊刮。
63、 (A2 型题)
女，25 岁，发现右乳肿块两年。无乳房红肿，疼痛，乳头溢液，无发热。查体：双乳外观无明显异常，右乳外上象限可触及大小 2.0cm×1.5cm 肿块，质地中等、光滑、活 楚。应如何治疗
A.局部理疗
B.密切观察
C.针灸治疗
D.手术切除
E.中药逍遥散
参考答案： D 您的答案：
图文解析： 【解析】青年女性（乳腺纤维腺瘤易感人群）发现右乳肿块两年，无乳房红肿，疼痛，乳头溢液，无发热，查体可触及光滑、活动、边界清楚的肿块（乳腺纤维腺瘤典型临床特征），故诊断为乳房纤维腺瘤。手术切除是唯一有效方法（D 对）。
【避错】局部理疗（A 错）、密切观察（B 错）、针灸治疗（C 错）对于乳腺纤维瘤的治疗无效果。中药逍遥散（E 错）具有调和肝脾，疏肝解郁，养血健脾之功效，可用于乳腺囊性增生病。
根据描述，患者 25 岁，有一个两年的乳腺肿块，无其他症状，肿块的特征包括大小为 2.0cm×1.5cm，质地中等、光滑、可移动。考虑到肿块存在的时间长度以及其特点，首先需要排除恶性肿瘤的可能性。
在这种情况下，通常推荐的做法是进行进一步的诊断，如影像学检查（例如乳腺超声或乳腺 X 线摄影）和/或组织活检。然而，这个选择并没有直接出现在选项中。在给定的选项中，最接近正确处理方法的是手术切除（D 选项）。这是因为对于存在时间较长、特征不明的乳腺肿块，需要进行切除以便于组织病理学检查，从而确诊其良性或恶性，并排除乳腺癌的可能。因此，最佳答案是：手术切除
解析：

青年女性患者，发现右乳肿块两年，查体发现右乳外上象限可触及大小 2.0cm×1.5cm 肿块，质地中等、光滑、活动、界限清楚，是乳腺纤维腺瘤的典型临床表现，结合患者病史、临床表现和相关检查，可初步诊断为右侧乳腺纤维腺瘤。乳腺纤维腺瘤虽属良性，但有恶变可能，故一旦发现，应予手术切除。
选项 A 局部理疗，选项 C 针灸治疗，选项 E 中药逍遥散，均为中医辅助治疗方法，对乳腺纤维腺瘤无治疗作用，故可一并排除。
选项 B 密切观察仅适用于肿瘤较小、生长缓慢的患者，该患者肿块已长至 2.0cm×1.5cm，故应手术切除，而非密切观察。
综上所述，正确答案是：D.手术切除。
根据患者的情况描述，25 岁女性，右乳发现肿块两年，无其他症状（如红肿、疼痛、乳头溢液、发热），查体发现右乳外上象限有 2.0cm×1.5cm 肿块，质地中等、光滑、活动。这种情况最可能是良性乳腺肿瘤，如纤维腺瘤。

对于这种乳腺肿块，最佳的治疗方案是手术切除。手术切除不仅可以明确诊断（通过病理检查），还可以防止潜在的恶变风险。其他选项如局部理疗、密切观察、针灸治疗或中药治疗都不能有效解决问题。

因此，最合适的治疗方案是：

D.手术切除
64、 (A2 型题)
男性，34 岁，肛门周围反复疼痛，红肿、流脓 5 个月，查体：肛缘 3cm 处有一直径 1cm 硬结，按压可有脓液流出，沿硬结往里可触及一条索物，延伸至肛管内 3cm，该病诊断
A.皮脂腺囊肿
B.外痔
C.肛管癌
D.肛瘘
E.肛裂
参考答案： D 您的答案：
图文解析： 【解析】青年男性，肛门周围反复疼痛，红肿、流脓（肛瘘的临床表现），肛缘 3cm 处有一硬结，按压可有脓液流出，沿硬结往里可触及一条索物，延伸至肛管内（符合肛瘘的临床特征），结合患者临床表现和特征，故诊断为肛瘘（D 对）。
【避错】皮脂腺囊肿好发于头面部和背部（A 错）；外痔临床表现为肛门肿物突出，如发生血栓，可有剧痛，成为血栓性外痔（B 错）；肛管癌表现为肛门疼痛，瘙痒，无脓液流出（C 错）；肛裂典型表现为疼痛、便秘和出血，肛裂，前哨痔，肛乳头肥大常同时存在（E 错）。
根据所描述的症状，包括肛门周围反复疼痛、红肿、流脓，以及检查发现的硬结、脓液流出和可触及的索物延伸至肛管内，最符合的诊断是肛瘘。肛瘘通常表现为肛门周围的疼痛、红肿、间歇性排脓等症状，且检查时可发现与肛管相通的瘘道。因此，最佳选择应该是：D.肛瘘
解析：
患者青年男性，肛门周围反复疼痛，红肿、流脓 5 个月，查体发现肛缘外硬结，按压有脓液流出，沿硬结可触及一条索物延伸至肛管内，根据患者的病史、临床表现和体查，该患者可诊断为肛瘘。肛瘘是肛门直肠瘘的简称，是发生在肛门直肠周围的脓肿溃破或切口引流的后

遗病变。肛瘘是脓肿后时代，是一个疾病的两个阶段。中医称为肛漏。
选项 A 皮脂腺囊肿俗称“粉瘤”，主要由于皮脂腺排泄管阻塞，皮脂腺囊状上皮被逐渐增多的内容物膨胀所形成的滞留性囊肿，不会出现流脓现象，与题干中患者表现不符，故不选。
选项 B 外痔位于齿线以下，是由痔外静脉丛曲张或肛缘皱襞皮肤发炎、肥大、结缔组织增生或血栓瘀滞而形成的肿块，外痔表面盖以皮肤，可以看见，不能送入肛内，不易病变，以疼痛和有异物感为主要症状，与患者的表现差异明显，故排除。
选项 C 肛管癌早期症状不明显，进展期的临床表现类似直肠下段癌，主要有下列三个方面：大便习惯改变，排粪次数增加，常伴里急后重或排便不尽感；粪便性状改变，粪条变细或变形，常带有黏液或脓血；肛门疼痛，肛门部坠胀，随着癌肿发展，可出现肛门部溃烂、出血、感染等症状，题干中未提及上述症状，故不考虑。
选项 E 肛裂是消化道出口从齿状线到肛缘这段最窄的肛管组织表面裂开，形成小溃疡，方向与肛管纵轴平行，呈梭形或椭圆形，长约 0.5～1.0cm，常引起肛周剧痛，与患者表现不符，故不选。
综上所述，正确答案是：D.肛瘘。
根据题干描述，男性，34 岁，肛门周围反复疼痛、红肿、流脓 5 个月，查体发现肛缘 3cm 处有一直径 1cm 硬结，按压可有脓液流出，沿硬结往里可触及一条索物，延伸至肛管内 3cm。以上表现符合肛瘘的临床特征。

肛瘘（D.肛瘘）通常是由于肛门周围脓肿破溃后形成的，是肛管和皮肤之间的异常通道。其主要症状包括肛门周围的疼痛、红肿、流脓，查体时可触及硬结和脓液。

因此，本题的最佳答案是：

D.肛瘘
65、　(A2 型题)
患者女性，16 岁，甲状腺Ⅲ度肿大，甲状腺摄碘率 2 小时 15%，24 小时 35%，该患者可能的疾病是
A.甲状腺功能减退
B.甲状腺功能亢进
C.桥本甲状腺炎
D.生理性甲状腺肿
E.结节性甲状腺肿
参考答案：　D　您的答案：
图文解析：　【解析】青春期女性，甲状腺Ⅲ度肿大，甲状腺摄碘率 2 小时 15%，24 小时 35%（正常范围内），由于青春期甲状腺激素需求量大，如机体合成的不可以满足生理需要，甲状腺腺体就会出现代偿性增生，肿大，但不伴有功能的改变，故应诊断为生理性甲状腺肿（D 对）。
【避错】结节性甲状腺肿（E 错）可表现为甲减或甲亢症状；甲状腺功能减退（A 错）表现为低代谢症状，实验室检查 T3、T4 减低；甲状腺功能亢进（B 错）T3、T4 升高，131I 摄取（摄碘能力）2 小时＞25%，24 小时＞50%。桥本甲状腺炎（C 错）TPO 抗体，TG 抗体阳性。
这个问题涉及到评估甲状腺功能和肿大的原因。甲状腺摄碘率反映了甲状腺对碘的吸收能力，是评估甲状腺功能的重要指标。
- **甲状腺功能减退**（A）通常伴随着低碘摄取率，但由于甲状腺努力吸收更多碘来产生甲状腺激素，早期可能会出现摄碘率正常或轻微增高。

- **甲状腺功能亢进**（B）是由于甲状腺过度活跃，导致高碘摄取率。
- **桥本甲状腺炎**（C）是一种自身免疫性疾病，最初可能有正常或增加的碘摄取率，但随着病情发展，甲状腺功能会减退。
- **生理性甲状腺肿**（D）通常见于青春期女性，甲状腺功能和摄碘率通常是正常的。
- **结节性甲状腺肿**（E）指的是甲状腺中结节的增生，可以是单个或多个结节，摄碘率可以正常、增高或减低，取决于结节是否功能性活跃。

考虑到这位患者甲状腺III度肿大，摄碘率在 2 小时为 15%，24 小时为 35%，这表明甲状腺有明显的碘摄取但并非过度活跃。这些数据不典型于甲状腺功能亢进（通常会有更高的摄碘率），也不完全符合甲状腺功能减退（摄碘率通常较低）。结节性甲状腺肿可能表现出不同的摄碘率，但通常与结节活性有关。考虑到年龄和症状，桥本甲状腺炎或生理性甲状腺肿的可能性较高。但桥本甲状腺炎更可能在早期表现出正常或略高的摄碘率，随病情进展而减低。因此，最合适的答案可能是**C. 桥本甲状腺炎**，因为它可以在病程早期表现为甲状腺肿大和相对正常或略高的碘摄取率。然而，最终诊断需要结合其他临床信息，如抗甲状腺抗体的水平、甲状腺功能测试（如 TSH、T3、T4 水平）以及可能的甲状腺超声检查。

解析：

青少年女性患者，甲状腺III度肿大，甲状腺摄碘率 2 小时 15%，24 小时 35%。正常人甲状腺摄碘率 2 小时应为 5%～25%，24 小时应为 20%～45%，该患者甲状腺摄碘率在正常范围内，但甲状腺明显肿大，应考虑为单纯性甲状腺肿，亦称为生理性甲状腺肿。

选项 A 甲状腺功能减退时，血清 T3、T4 降低，TSH 升高，甲状腺摄^{131}I 率明显降低，与题干描述不符，故不选。

选项 B 甲状腺功能亢进时，甲状腺摄^{131}I 率增高，且高峰前移，亦与题干描述不符，故排除。

选项 C 桥本甲状腺炎是一种自身免疫性疾病，甲状腺自身抗体常阳性，血清 T3、T4 可降低、正常或增高，TSH 可增高或降低，甲状腺摄^{131}I 率可降低、正常或增高，但题干中并未提及该患者抗体阳性，故不选。

选项 E 结节性甲状腺肿一般不对称，可触及多个或单个结节，结节以外的甲状腺组织无肿大，且结节内常有钙化、囊变和出血，与本例不符，故排除。

综上所述，正确答案是：D.生理性甲状腺肿。

根据题目描述，患者为 16 岁女性，甲状腺III度肿大，甲状腺摄碘率 2 小时为 15%，24 小时为 35%。

分析各个选项：

A. **甲状腺功能减退**：通常表现为甲状腺摄碘率降低，不符合题意。
B. **甲状腺功能亢进**：通常表现为甲状腺摄碘率增高，但题目中的摄碘率并没有明显增高。
C. **桥本甲状腺炎**：多为甲状腺功能减退，并伴有自身抗体，甲状腺摄碘率变化不典型。
D. **生理性甲状腺肿**：常见于青春期和妊娠期等特定生理时期，但摄碘率通常正常或略有变化，不符合题意。
E. **结节性甲状腺肿**：甲状腺可增大，且摄碘率可能不正常。

基于以上分析，最可能的答案是：

E. 结节性甲状腺肿。

66、 (A2 型题)

女，关节痛，蛋白尿，抗 ds-DNA 抗体阳性，补体 C3 下降。下面说法不正确的是

A.病情好转后不可以继续妊娠
B.出现癫痫等脑部表现提示预后不良
C.肾活检可以判断预后
D.可以使用羟氯喹
E.会导致关节畸形
参考答案：　　E　您的答案：
图文解析：　　【解析】青年女性，关节病变，蛋白尿（提示肾脏病变），抗 dsDNA 抗体（+）（狼疮肾炎标记性抗体，活动性最强），补体 C3 下降（提示病情在活动期），患者出现多器官损害现象，结合实验室检查，考虑诊断为狼疮肾炎。SLE 关节损害特点为可恢复的非侵蚀性关节半脱位，无骨质破坏，无畸形（E 错，为本题正确答案）。
【避错】狼疮肾炎患者病情好转后，没有中枢神经系统、肾脏或其他脏器损害者，可以继续妊娠（A 对）；出现癫痫等脑部表现提示预后不良（B 对）；肾活检可以判断预后（C 对）；SLE 的药物治疗主要是糖皮质激素与免疫抑制剂（环磷酰胺）联合治疗，可以使用羟氯喹（D 对）作为背景治疗。
本题描述的是一位女性患者，表现有关节痛、蛋白尿、抗 ds-DNA 抗体阳性和补体 C3 下降。这些症状和实验室结果提示可能为系统性红斑狼疮（SLE）。我们需要找出下面说法中不正确的一个。
A. 病情好转后不可以继续妊娠 — 这个说法是错误的。SLE 患者在病情稳定或好转时，是可以考虑妊娠的，但需要在医生的严格监控下进行。
B. 出现癫痫等脑部表现提示预后不良 — 这是正确的，SLE 若累及中枢神经系统，如出现癫痫，可能提示预后不良。
C. 肾活检可以判断预后 — 这也是正确的，肾活检在 SLE 中是评估肾脏受累程度和预后的重要手段。
D. 可以使用羟氯喹 — 这是正确的，羟氯喹是 SLE 管理中常用的药物之一。
E. 会导致关节畸形 — 虽然 SLE 可以导致关节疼痛和功能受限，但通常不会导致关节畸形，这个选项可能在某些情况下也是错误的。然而，与 A 选项相比，关节畸形不是 SLE 的典型特征，但 SLE 确实可以导致严重的关节问题，包括畸形。
在这些选项中，最不正确的是 A（病情好转后不可以继续妊娠）。因为 SLE 患者在病情控制良好的情况下是可以考虑妊娠的，但需要在医疗团队的监督下仔细规划和管理。因此，正确答案是 A。
解析：
育龄女性患者，抗 ds-DNA 抗体阳性，补体 C3 下降，伴有关节痛和蛋白尿，根据其临床表现和实验室检查，考虑诊断为系统性红斑狼疮。系统性红斑狼疮是一种多发于青年女性的累及多脏器的自身免疫性炎症性结缔组织病，早期、轻型和不典型的病例日渐增多。此病呈缓解与反复发作交替出现，不会导致关节畸形。
系统性红斑狼疮（SLE）好发于育龄女性，患者病情好转后，若无重要脏器损害，病情稳定 1 年或 1 年以上，细胞毒免疫抑制剂如环磷酰胺、甲氨蝶呤或雷公藤停药半年，激素仅需小剂量维持时，可怀孕。因此选项 A 病情好转后不可以继续妊娠说法错误，为本题正确答案。
SLE 患者若出现中枢神经系统损害，出现癫痫等脑部表现，提示预后不良，故选项 B 出现癫痫等脑部表现提示预后不良符合 SLE 的特点。
SLE 患者累及肾脏时称为狼疮性肾炎，病理类型多样，其分型对治疗和预后均有指导意义，肾活检可以判断预后，故选项 C 肾活检可以判断预后，符合 SLE 的特征。
羟氯喹是 SLE 的基础用药，几乎无副作用，且由于该药有抗疟、抗炎、抑制免疫等作用，有利于更好地控制 SLE 活动，保护患者不发生脏器损害，减少激素的用量及不良反应，故选项

D 可以使用羟氯喹，符合 SLE 的治疗原则。
综上所述，正确答案是：E.会导致关节畸形。
这道题目描述了一位女性患者，症状包括关节痛、蛋白尿、抗 ds-DNA 抗体阳性以及补体 C3 下降。这些症状和实验室结果提示系统性红斑狼疮（SLE）的诊断。根据这个诊断，我们来分析选项，找出不正确的说法。

A. 病情好转后不可以继续妊娠 —— 这是不正确的。实际上，系统性红斑狼疮患者在病情得到有效控制（通常指病情稳定至少 6 个月）后是可以考虑妊娠的，但需要密切监控。

B. 出现癫痫等脑部表现提示预后不良 —— 这是正确的。SLE 患者若出现中枢神经系统症状如癫痫，通常预示病情严重，预后较差。

C. 肾活检可以判断预后 —— 这是正确的。SLE 患者的肾损害（狼疮性肾炎）可以通过肾活检来评估，从而判断预后和制定治疗方案。

D. 可以使用羟氯喹 —— 这是正确的。羟氯喹是治疗 SLE 的重要药物之一，有助于控制病情，减少发作频率。

E. 会导致关节畸形 —— 这是不正确的。SLE 尽管可以引起关节痛和关节炎，但一般不会导致像类风湿性关节炎那样的关节畸形。

因此，最不正确的说法是：

E. 会导致关节畸形

总结：选择 E 选项。
67、 (A2 型题)
男性，48 岁，右上腹胀痛 4 月，伴乏力，无恶心、呕吐、发热，自发病以来，尿色稍黄，既往饮白酒 10 年余，约 150~200ml/d。查体：精神差，巩膜轻度黄染，无肝掌、蜘 无异常，腹软，无压痛，肝脾未触及，移动性浊音（-），实验室检查：ALT35、AST25、HBsAg（-），抗 HCV（-），B 超：肝实质回声弥漫密集增强，远场回声明显衰减，
是
A.泼尼松治疗
B.营养支持
C.抗肝纤维化治疗
D.戒酒
E.保肝药物治疗
参考答案： D 您的答案：
图文解析： 【解析】中年男性患者，大量长期饮酒史，乏力、右上腹胀痛，查体及实验室检查提示肝功能尚可，B 超提示脂肪性肝病，综合患者临床表现及检查检验结果，考虑诊断为酒精性肝病。戒酒（D 对）是治疗酒精性肝病患者最重要的措施。
【避错】我国现有的酒精性肝病诊断标准为：有长期饮酒史， 一般超过 5 年，折合酒精量男性≥40g/d，女性≥20g/d；或 2 周内有大量饮酒史，折合酒精量>80g/d。
这个案例描述了一位 48 岁男性患者，长期饮酒，出现右上腹胀痛、乏力，巩膜轻度黄染，

无恶心、呕吐、发热。实验室检查显示肝功能轻度异常，B 超显示肝实质回声弥漫密集增强，远场回声衰减，这些症状和检查结果提示可能的慢性肝病，如酒精性肝病。
对于治疗方案的选择，我们需要考虑患者的主要病因和最有效的干预措施。鉴于患者的长期饮酒史，最关键的干预措施是：
D. 戒酒
戒酒是治疗酒精性肝病的最重要步骤，可以防止肝病进一步恶化，并可能逆转肝损伤。虽然其他选项如营养支持、抗肝纤维化治疗和保肝药物治疗对于肝病患者也是重要的，但在这种情况下，没有比停止损害肝脏的原因更重要的治疗了。因此，选择 D（戒酒）作为最佳答案。
解析：
中年男性患者，长期饮酒史，右上腹胀痛，尿黄，查体见精神差，巩膜轻度黄染，B 超示肝实质回声弥漫密集增强，远场回声明显衰减，考虑诊断为酒精性肝病。酒精性肝病的治疗原则是：戒酒和营养支持，减轻酒精性肝病的严重程度，改善已存在的继发性营养不良和对症治疗。其中戒酒是治疗酒精性肝病的根本，是最重要的治疗措施。
选项 A 泼尼松治疗属于激素治疗，激素会诱发或加剧消化性溃疡，引起血糖、血压升高，增加发生感染的风险，不宜用于治疗酒精性肝病，故排除。
选项 B 营养支持属于酒精性肝病的治疗原则，但不是最重要的措施，最重要的措施是戒酒，故不选。
选项 C 抗肝纤维化治疗可阻止或逆转肝纤维化，但对酒精性肝病的治疗效果欠佳，故不考虑。
选项 E 保肝药物治疗的疗效不确切，且有可能加重肝脏负担，应避免滥用，故不符合题意。
综上所述，正确答案是：D.戒酒。
根据患者的病史和检查结果，这位 48 岁男性有右上腹胀痛和乏力，伴随轻度黄疸，长期饮酒史，并且 B 超显示肝实质回声弥漫密集增强，远场回声明显衰减。这些表现很可能提示患者患有酒精性肝病，特别是酒精性脂肪肝或早期酒精性肝硬化。

从治疗角度出发，酒精性肝病的首要处理措施是戒酒，这可以防止肝脏损伤的进一步恶化，并为肝脏自我修复创造条件。因此，最正确的答案是：

D. 戒酒
68、 (A2 型题)
男，体温 39.1℃,心率 109 次/分，咳嗽咳痰，CT 见液平，入院后予头孢联合喹诺酮抗感染，停药的指征是
A.X 线见病灶吸收
B.咳嗽咳痰症状消失
C.血氧饱和度恢复正常
D.体温恢复正常
E.血常规正常
参考答案： A 您的答案：
图文解析： 【解析】男性患者，体温 39.1℃（提示感染），咳嗽咳痰，CT 见液平（肺脓肿的典型影像学特征），故该患者考虑诊断为肺脓肿，应用抗生素治疗，疗程 6～8 周或胸片恢复正常（A 对）时停药。
【避错】肺炎患者体温正常后 72 小时可停用抗生素。
这个问题关乎抗生素治疗何时停止的决策，一个非常实际的临床问题。治疗的目标是消除感染症状和确保病原体被清除，避免过早停药导致的复发或耐药性发展。考虑到这些因素，虽然体温、血氧饱和度、血常规的恢复都是治疗效果的指标，但仅凭这些指标恢复正常并不一

定意味着感染已经完全得到控制。
在这个案例中，男性患者的 CT 扫描显示液平，这表明有肺部感染。因此，最佳答案应该是选项 A“X 线见病灶吸收”，因为它直接反映了感染部位的改善情况。病灶的吸收意味着感染得到了控制，是一种更直接和可靠的停药指标。所以，正确答案应该是 A。
解析：
患者中年男性，体温升高，心率增快，咳嗽咳痰，CT 见液平，考虑诊断为肺炎合并胸腔积液。该患者入院后予头孢联合喹诺酮抗感染，停药的指征是 X 线见病灶吸收。
选项 B 咳嗽咳痰症状消失仅能代表患者症状改善，但感染是否完全控制需影像学检查辅助诊断，故排除。
选项 C 血氧饱和度恢复正常仅能表示患者目前不存在缺氧，不能代表感染已被控制，故不考虑。
选项 D 体温恢复正常可能是退热药的作用，不能代表感染完全好转，故不选。
选项 E 血常规正常亦不能代表感染已被完全控制，不具有特异性，故排除。
综上所述，正确答案是：A.X 线见病灶吸收。
根据题干提供的信息，该患者存在发热、心率增快、咳嗽咳痰等症状，且 CT 显示液平，提示可能存在细菌性感染。患者接受了头孢联合喹诺酮抗感染治疗。停药指征需要综合考虑临床症状和辅助检查的结果。

在临床实践中，停用抗生素的指征通常包括以下几点：

1. 临床症状显著改善或消失（如发热、咳嗽咳痰症状缓解）。
2. 辅助检查指标恢复正常（如血常规、血氧饱和度等）。
3. 影像学检查提示病灶吸收或明显缩小。

在这道题目中，各选项的分析如下：

A. X 线见病灶吸收：提示病灶显著改善，但不一定意味着感染完全控制。
B. 咳嗽咳痰症状消失：是临床症状改善的重要指标，但单独依赖临床症状可能不够全面。
C. 血氧饱和度恢复正常：也是重要指标，但不能完全反映感染控制的情况。
D. 体温恢复正常：发热消退是感染控制的标志之一，但单独作为停药指征不够全面。
E. 血常规正常：白细胞计数等指标恢复正常是判断感染控制的重要依据之一。

综合考虑，停药的最佳指征应该是病情的综合改善，即不仅体温恢复正常，还应结合其他临床和实验室指标，如咳嗽咳痰症状、血常规等。因此，最合理的选择是：

E. 血常规正常

血常规的正常化通常意味着体内感染已经得到了有效控制，符合临床停药的标准。
69、 (A2 型题)
女，30 岁，阑尾切除术后 6 天，大量进食后面色苍白，烦躁气急，上腹饱胀，呕吐胃内容物，既往消化性溃疡病史 6 年，查体：上腹膨隆，轻压痛，无反跳痛，粪隐血（-） 效的处理措施是
A.针灸理疗
B.肌肉注射新斯的明

C.应用广谱抗生素
D.局部热敷
E.胃肠减压
参考答案： E 您的答案：
图文解析： 【解析】阑尾切除术后 6 天（阑尾炎手术史），大量进食后面色苍白，烦躁气急，上腹饱胀，呕吐胃内容物（术后肠梗阻常见症状），既往消化性溃疡病史 6 年，上腹膨隆，轻压痛，无反跳痛，粪隐血（-），考虑诊断为阑尾炎术后的粘连性肠梗阻，最简单有效的处理措施是胃肠减压（E 对）可缓解腹胀，减轻胃肠压力。
【避错】针灸理疗（A 错），局部热敷（D 错）对上述症状的治疗无效果；应用广谱抗生素（C 错）适用于炎症的预防与治疗。肌肉注射新斯的明（B 错）主要作用是治疗肠道手术后肠功能受限，对肠麻痹有一定的治疗作用。
考虑到这位 30 岁女性术后第 6 天出现面色苍白、烦躁气急、上腹饱胀和呕吐的症状，以及她有长达 6 年的消化性溃疡病史，最合适的处理措施似乎是 E.胃肠减压。这些症状可能指示急性胃扩张或术后胃肠道动力不足，这在阑尾切除术后可能发生。胃肠减压通过插入鼻胃管来缓解胃内容物的压力，减少胃肠膨胀，并预防进一步的并发症。其他选项如针灸理疗、肌肉注射新斯的明、应用广谱抗生素或局部热敷均不直接针对她当前的主要问题。因此，最佳选择是 E.胃肠减压。
解析：
患者阑尾切除术后 6 天，大量进食后面色苍白，烦躁气急，上腹饱胀，呕吐胃内容物，提示可能存在肠梗阻，查体示上腹膨隆，轻压痛，无反跳痛，粪隐血（-），可排除消化道出血性疾病，结合患者病史和临床表现，应考虑为术后肠梗阻。肠梗阻的治疗原则为纠正因肠梗阻所引起的全身生理紊乱和解除梗阻，其中胃肠减压是治疗肠梗阻的重要措施之一，通过胃肠减压吸出胃肠道内的气体和液体，可以减轻腹胀，降低肠腔内压力，减少肠腔内的细菌和毒素，改善肠壁血循环，有利于改善局部病变和全身情况。
选项 A 针灸理疗，选项 D 局部热敷，可一定程度缓解胃肠道痉挛，但无法降低肠腔内压力，对于肠梗阻的治疗效果有限，故可一并排除。
选项 B 肌肉注射新斯的明主要用于重症肌无力，术后腹气胀、尿潴留等，对于肠梗阻的治疗效果并不确切，可排除。
选项 C 应用广谱抗生素主要用于治疗感染性疾病，肠梗阻并非感染性疾病，与题意不符。
综上所述，正确答案是：E.胃肠减压。
在这种情况下，患者在阑尾切除术后 6 天出现面色苍白、烦躁气急、上腹饱胀和呕吐，且有消化性溃疡病史。查体显示上腹膨隆和轻压痛，无反跳痛，粪隐血（-）。这些症状提示可能存在胃肠功能障碍或梗阻，最有效的处理措施应是缓解症状和减轻胃肠道压力。

选项解释：
A. 针灸理疗 - 对于急性症状的处理效果有限，可能不适合。
B. 肌肉注射新斯的明 - 新斯的明可以增强胃肠蠕动，但不一定适用于急性胀气和呕吐的情况，且可能加重症状。
C. 应用广谱抗生素 - 适用于感染性疾病，但当前没有证据支持存在感染。
D. 局部热敷 - 可以缓解轻微的疼痛和不适，但对急性胃肠胀气效果有限。
E. 胃肠减压 - 通过插入鼻胃管，减轻胃肠道的压力，缓解胀气和呕吐，是处理这种症状最有效的措施。

因此，最合适的处理措施是：

E. 胃肠减压
70、 (A2 型题)
女，75 岁，咯血 10 天，每日咯血量约 20ml，既往发作两次，抗感染治疗后好转。患者肺 CT 显示双肺弥漫性支扩，目前首选的治疗是
A.手术治疗
B.止血药物
C.抗感染治疗
D.支气管动脉栓塞
E.垂体后叶素
参考答案： D 您的答案：
图文解析： 【解析】老年女性，CT 提示双肺弥漫性支气管扩张，既往咯血 2 次，抗感染好转，再次咯血，故采用支气管动脉栓塞术（D 对）。
【避错】对反复咯血的患者，经内科治疗（控制感染、口服止血药物、静脉给予垂体后叶素）无效，可考虑介入栓塞治疗或手术治疗。病变局限者可考虑外科手术，否则采用支气管动脉栓塞术治疗。
女性患者，75 岁，有咯血病史且每日咯血量约 20ml，病史中显示她曾因此发作两次并在接受抗感染治疗后有所好转。患者的肺 CT 显示双肺弥漫性支气管扩张症。基于这些信息，我们需要考虑一个治疗方法，既能解决当前的咯血问题，又能针对可能的根本原因，即支气管扩张症。
A. 手术治疗 - 通常考虑于重症病例或当其他治疗无效时。
B. 止血药物 - 对于治疗咯血有效，但不解决根本原因。
C. 抗感染治疗 - 支气管扩张症常伴随感染，考虑到患者既往抗感染治疗后有所好转，这可能是一个合适的选项。
D. 支气管动脉栓塞 - 用于治疗严重或持续咯血，但需考虑到具体情况和风险。
E. 垂体后叶素 - 主要用于治疗尿崩症，与本病例不相关。
综合考虑，最佳答案可能是 **C. 抗感染治疗**，因为患者的病史表明，她对此类治疗有反应，且抗感染治疗可以针对支气管扩张症的潜在感染因素，这符合患者当前的主要症状和诊断。
解析：
患者老年女性，咯血，每日咯血量约 20ml，既往有咯血病史，抗感染治疗后好转，结合患者肺 CT 显示双肺弥漫性支扩，对于该患者，目前应首选抗感染治疗。
选项 A 手术治疗适用于局限性支气管扩张、经充分的内科治疗仍顽固反复发作者，该患者为双肺弥漫性支扩，手术治疗效果不佳，故不选。
患者咯血症状不重，无需使用止血药物，因此选项 B 止血药物，可排除。
选项 D 支气管动脉栓塞主要用于治疗各种原因引起的支气管动脉损伤所造成的咯血，该患者咯血症状不重，经抗感染治疗后可好转，故不作为首选治疗方式。
选项 E 垂体后叶素用于呼吸内科的咯血，尤其是发生大咯血时的治疗，该患者咯血量不大，故不考虑。
综上所述，正确答案是：C.抗感染治疗。
这位 75 岁的女性患者有弥漫性支气管扩张症（支扩）和咯血的病史。目前，她每天咯血约 20 毫升，并且在过去的两次发作中通过抗感染治疗获得好转。基于这些信息，以下是每个选项的分析：

A. **手术治疗**：手术治疗通常用于局限性支扩或反复严重咯血且其他治疗无效的情况。

对于弥漫性支扩，手术不是首选治疗方法。

B. **止血药物**：止血药物可以在短期内减少咯血量，但它们通常不是长期管理弥漫性支扩的最佳选择。

C. **抗感染治疗**：抗感染治疗是支扩的基础治疗，尤其是在急性感染期。然而，这位患者目前的咯血可能并不是由新的感染引起的，而是由于弥漫性支扩本身。因此，虽然抗感染治疗是支扩管理的重要部分，但它可能不是目前最适合控制咯血的首选方法。

D. **支气管动脉栓塞**：支气管动脉栓塞（BAE）是治疗严重和反复咯血的有效方法。它通过栓塞引起咯血的支气管动脉，减少出血，是支扩患者反复咯血的一线治疗选择之一。

E. **垂体后叶素**：垂体后叶素可以暂时减少血流量，帮助控制出血，但它不解决引起咯血的根本原因，通常作为短期紧急措施使用。

综合考虑，**支气管动脉栓塞（D. 支气管动脉栓塞）**是目前控制这位患者咯血的最佳治疗选择，因为它能有效减少出血量，且适用于弥漫性支扩引起的反复咯血情况。

因此，最合适的答案是：

D. 支气管动脉栓塞

71、 (A2 型题)

育龄期女性，产后 4 周， 一直母乳喂养，乳房出现红肿，表面皮温升高，无波动感，以下处理首选

A.切开引流

B.立即停止哺乳

C.立即使用抗生素

D.热敷，按压乳房，促进乳汁排出

E.等待药敏结果出来后再行抗生素治疗

您的答案：D

图文解析： 【解析】育龄期女性，产后 4 周， 一直母乳喂养，乳房出现红肿，表面皮温升高，考虑急性乳腺炎。急性乳腺炎的治疗原则是消除感染、排空乳汁（D 对）。

【避错】切开引流（A 错）为脓肿形成后的处理，该患者乳房无波动感，故不需要做切开引流； 一般不停止哺乳，因停止哺乳，不仅影响婴儿喂养，且提供了乳汁淤积的机会。但患侧乳房应停止哺乳，并以吸乳器吸尽乳汁，促使乳汁通畅排出（B 错）；早期呈蜂窝织炎表现而未形成脓肿之前，应用抗生素可获得良好的效果。因主要病原菌为金黄色葡萄球菌，可不必等待细菌培养的结果，应用青霉素治疗，或用耐青霉素酶的苯唑西林钠，或头孢一代抗生素如头孢拉啶，但不作为首选（CE 错）。

本题描述的是产后 4 周母乳喂养的育龄期女性，出现乳房红肿和表面皮温升高，没有波动感。这种情况最有可能是乳腺炎，特别是考虑到她正在哺乳。

选项 A（切开引流）通常适用于脓肿形成，但题干中没有提到波动感，这表明还没有形成脓肿。选项 B（立即停止哺乳）不是首选，因为哺乳有助于清空乳腺，可能有助于症状缓解。选项 C（立即使用抗生素）是针对乳腺炎的一个常见治疗，但在没有严重感染迹象（如发热、全身病状）的情况下可能不是首选。选项 E（等待药敏结果出来后再行抗生素治疗）在紧急

情况下不是首选，因为可能需要立即干预。
选项 D（热敷，按压乳房，促进乳汁排出）是治疗非脓肿性乳腺炎的首选方法之一。它有助于减轻症状，促进乳汁流通，避免乳腺管堵塞。
因此，最佳答案是 D。
首先，我们来理解题目描述的临床情况。这是一位产后 4 周的育龄期女性，一直进行母乳喂养，现在乳房出现红肿，表面皮温升高，但无波动感。这些症状提示可能是乳腺炎的早期阶段，尚未形成脓肿。
接下来，我们根据题目中的选项进行逐一分析：
A. 切开引流：这一选项通常适用于脓肿形成后。但题目中明确提到"无波动感"，意味着尚未形成脓肿，因此这一选项不适用。
B. 立即停止哺乳：在乳腺炎的早期阶段，停止哺乳并不是首选的处理方法。实际上，继续哺乳有助于乳腺的通畅，从而有助于炎症的消退。
C. 立即使用抗生素：虽然抗生素可以治疗感染，但在没有明确的细菌感染证据之前，不建议盲目使用。
D. 热敷，按压乳房，促进乳汁排出：在乳腺炎的早期阶段，这种方法是非常有效的。热敷可以帮助增加局部的血液循环，而按压乳房和促进乳汁排出有助于消除乳腺的堵塞，从而有助于消退炎症。
E. 等待药敏结果出来后再行抗生素治疗：这一选项虽然看起来更为科学，但在急性乳腺炎的情况下，及时的干预是非常重要的。等待药敏结果可能会延误治疗的最佳时机。
综上所述，考虑到该患者处于乳腺炎的早期阶段，尚未形成脓肿，且需要及时的干预，因此，热敷、按压乳房并促进乳汁排出是首选的处理方法。
因此，正确答案是 D.热敷，按压乳房，促进乳汁排出。
对于育龄期女性，产后 4 周，一直母乳喂养，乳房出现红肿，表面皮温升高，无波动感，考虑乳腺炎的可能性较大。乳腺炎的治疗通常包括抗生素治疗、继续哺乳或使用吸奶器排空乳房，以及适当的对症处理如局部热敷。

根据现有的医学知识和最佳实践指南，首选的处理是：

C.立即使用抗生素

原因如下：
1. **立即使用抗生素**：在乳腺炎的早期阶段，特别是在没有波动感（提示没有脓肿形成）的情况下，立即使用抗生素可以有效控制感染，防止病情恶化。
2. **继续哺乳**：并不需要立即停止哺乳，因为继续哺乳有助于排空乳房，减少乳汁淤积，加速康复。
3. **局部对症处理**：可以采用热敷等对症处理，但这些措施是辅助性的，不能替代抗生素治疗。

因此，最佳答案是：

C.立即使用抗生素
72、 (A2 型题)
男，35，上腹部不适，乏力，消瘦 3 年，查体：Hb80g/L，胃泌素 80pg/ml，消化道造影示胃小弯侧 2.5cm 龛影，胃活动差。考虑诊断是

A.胃溃疡
B.胃息肉
C.复合型溃疡
D.胃癌
E.慢性胃炎
参考答案： D 您的答案：
图文解析： 【解析】中年男性，上腹部不适，乏力，消瘦 3 年（恶性肿瘤患者常见体征），Hb80g/L（贫血），胃泌素 80pg/ml，消化道造影示胃小弯侧 2.5cm 龛影，胃活动差，综合患者情况，考虑诊断为胃癌（D 对）。
【避错】胃溃疡（A 错）多见于餐后腹痛，腹痛可被抑酸或抗酸剂缓解。胃息肉（B 错）多无明显临床表现，偶有便血、腹痛等；复合型溃疡（C 错）指胃和十二指肠均有活动性溃疡，梗阻发生率较高。慢性胃炎（E 错）大多数患者无明显症状，可表现为中上腹不适、饱胀、钝痛等。
此题目是关于一个 35 岁男性患者，主诉上腹部不适、乏力和消瘦 3 年。体检发现血红蛋白(Hb)为 80g/L（偏低），胃泌素水平为 80pg/ml，消化道造影显示胃小弯侧有 2.5cm 的龛影，胃活动差。
考虑到这些信息：
- 低血红蛋白可能指向慢性出血或营养不良。
- 胃泌素水平正常，这有助于区分某些胃部疾病。
- 龛影通常与溃疡或肿瘤性病变相关联，而胃活动差可能暗示病变的刚性和广泛性。
基于这些临床表现，最可能的诊断是胃癌（D 选项）。虽然胃溃疡或其他选项也可能导致上腹部不适或龛影，但结合乏力、消瘦和胃活动差的表现，更应警惕胃癌。胃癌可以导致慢性出血，解释了低血红蛋白的发现，并且胃癌在消化道造影中常表现为刚性的龛影。
因此，最佳答案是 D.胃癌。
这是一道关于胃肠道疾病的诊断问题。我们需要根据患者的病史、查体发现以及影像学结果，综合判断其可能的疾病。
首先，我们梳理一下题目中给的关键信息：
青年男性患者，上腹部不适，乏力，消瘦 3 年。
血红蛋白（Hb）80g/L，低于正常值，提示存在贫血。
胃泌素 80pg/ml，高于正常值，可能与胃酸分泌过多或胃泌素瘤有关。
消化道造影显示胃小弯侧 2.5cm 的龛影，且胃活动差。
接下来，我们逐项分析选项：
A. 胃溃疡：胃溃疡可能导致上腹部不适和消瘦，但胃溃疡的胃泌素水平一般正常，且胃溃疡的龛影一般较小，不会伴有明显的胃活动减弱。
B. 胃息肉：胃息肉一般不会导致长期的上腹部不适和消瘦，且胃息肉在消化道造影上的表现与题干描述不符。
C. 复合型溃疡：复合型溃疡指的是同时有胃溃疡和十二指肠溃疡，但此疾病不会导致明显的胃活动减弱，且胃泌素水平通常正常。
D. 胃癌：胃癌可导致长期上腹部不适、乏力和消瘦，且常伴有胃泌素水平的升高。胃癌在消化道造影上可表现为较大的龛影，且由于癌组织的浸润，胃的活动性通常会减弱。这与题干描述高度一致。
E. 慢性胃炎：慢性胃炎虽然也可导致上腹部不适，但一般不会导致长期的乏力和消瘦，且胃泌素水平和胃的活动性通常正常。
综上所述，结合患者的病史、查体发现以及影像学表现，最可能的诊断是胃癌。因此，答案

是 D.胃癌。
根据患者的临床表现和检查结果，最可能的诊断是：

患者是一名 35 岁的男性，主要症状是上腹部不适、乏力和消瘦，已经持续了 3 年。查体发现血红蛋白（Hb）水平为 80g/L，提示贫血。胃泌素水平为 80pg/ml。消化道造影显示胃小弯侧有 2.5cm 的龛影，且胃活动差。

这种表现综合考虑，最可能的诊断是：

D. 胃癌

理由如下：
1. **上腹部不适、乏力和消瘦**是胃癌的常见症状。
2. **贫血（Hb 80g/L）**可能与胃癌引起的慢性出血或营养不良有关。
3. **胃泌素水平 80pg/ml**虽不特异，但胃癌可导致胃泌素分泌异常。
4. **消化道造影示胃小弯侧 2.5cm 龛影，胃活动差**提示胃壁有异常增厚或肿块，符合胃癌的影像学表现。

以上分析基于现有的医学知识和最佳实践指南，认为胃癌是最有可能的诊断。
73、 (A2 型题)
女，32 岁。急性阑尾炎阑尾切除术后 5 天，体温升高至 39℃,大便频繁伴里急后重，黏液便，量少。查体：切口愈合可，无红肿，未触及明显压痛及里急后重。可能的原
A.溃疡性结肠炎
B.伪膜性肠炎
C.阑尾残株炎
D.盆腔脓肿
E.粪瘘
参考答案： D 您的答案：
图文解析： 【解析】青年女性，阑尾炎切除术后，发热，大便频繁伴里急后重，考虑为盆腔脓肿（D 对），是急性阑尾炎的常见并发症。
【避错】溃疡性结肠炎（A 错）主要表现为腹泻和黏液脓血便，该患者有阑尾炎切除术后史，不考虑溃疡性结肠炎。伪膜性肠炎（B 错）多为近期应用广谱抗菌药物后导致肠道菌群失调，使难辨梭状芽孢杆菌大量繁殖成优势菌群，产生外毒素及侵袭性酶类致病。阑尾残株炎（C 错）是由于阑尾残端保留过长或肠石残留，术后残株可炎症复发，仍表现为阑尾炎的症状。粪瘘（E 错）产生的原因多为阑尾残端单纯结扎，其结扎线脱落等。
考虑到患者术后出现了发热、大便频繁伴有里急后重和黏液便，我们应该考虑那些术后可能引起这些症状的并发症。在提供的选项中，伪膜性肠炎（B）是一种与抗生素治疗相关的肠道感染，其特点是突然发病的水样腹泻、发热、腹痛以及可能的里急后重。考虑到患者是在接受腹部手术后不久出现这些症状，伪膜性肠炎成为一个合理的疑似诊断。
其他选项虽然可能与腹部手术相关，但它们的典型表现与此病例描述不完全符合。因此，最合适的答案是 B.伪膜性肠炎。
解析：
青年女性患者，阑尾切除术后 5 天出现发热，大便频繁伴里急后重，为直肠刺激症状，且排黏液便，量少，切口愈合良好，考虑诊断为伪膜性肠炎。伪膜性肠炎是一种主要发生于结肠

和小肠的急性纤维素渗出性炎症，多系在应用抗生素后导致正常肠道菌群失调，难辨梭状芽胞杆菌大量繁殖，产生毒素而致病，与题干中阑尾切除术后使用抗生素抗感染的病史相符合。
选项 A 溃疡性结肠炎一般起病缓慢，病情轻重不一，症状以腹泻为主，排出含有血、脓和黏液的粪便，常有阵发性结肠痉挛性疼痛，并里急后重，排便后可获缓解，与该患者症状不符，故不选。
选项 C 阑尾残株炎常表现为阑尾炎手术时已行的阑尾切除术后阑尾残端保留过长，或者粪石残留，继续发炎，仍表现为阑尾炎的症状，与该患者表现不符，故排除。
选项 D 盆腔脓肿常表现为弛张型高热，腹膜刺激征更加明显，出现直肠压迫感、排便感及排尿痛等直肠和膀胱刺激症状，并有全身中毒症状，与该患者表现不一致，故不选。
选项 E 粪瘘指各种原因所致的胃肠道与体表或体腔、体腔外出现异常通道，称为内瘘与外瘘，有消化液经瘘管流出，该患者并无瘘管流出，可排除。
综上所述，正确答案是：B.伪膜性肠炎。
根据题干描述的情况，32 岁女性患者在急性阑尾炎阑尾切除术后 5 天，体温升高至 39℃，大便频繁伴有里急后重和黏液便。查体显示切口愈合良好，无红肿，也未触及明显压痛及里急后重。根据这些信息，最可能的诊断是：

B. 伪膜性肠炎

理由如下：
- 术后患者出现高热和频繁的大便，伴有里急后重和黏液便，这些症状提示肠道感染或炎症。
- 伪膜性肠炎（Clostridioides difficile 感染）常见于术后或抗生素使用后，表现为腹泻、里急后重和高热，符合题干描述的症状。
- 溃疡性结肠炎通常是慢性病，不太可能在术后短时间内急性发作。
- 阑尾残株炎和盆腔脓肿也可能导致发热和腹痛，但与题干中描述的频繁大便和黏液便症状不完全匹配。
- 粪瘘一般会导致切口或周围皮肤的感染和排粪，但题干中切口愈合良好，无红肿。

因此，结合患者的症状和病史，最可能的诊断是伪膜性肠炎。
74、 (A2 型题)
患者，女，28 岁，关节疼痛 1 年，易脱发半年就诊。ESR110mm/h，抗“O”正常，Hb86g/L，PLT70×109/L，ANA 呈颗粒型阳性，尿蛋白(+++)，确诊为 SLE，在估计其预后方 项说法不正确
A.不宜妊娠
B.肾脏病变是增殖型表现者，预后不良
C.非缓解期妊娠可加重病情，应避孕
D.反复发作关节炎引起关节畸形和关节周围肌肉萎缩
E.反复癫痫发作及器质性脑病表现者预后不良
参考答案： D 您的答案：
图文解析： 【解析】青年女性，关节疼痛、脱发、血沉升高、贫血、血小板降低、ANA 阳性、尿蛋白阳性，考虑为 SLE。SLE 反复发作关节炎，关节 X 线检查多无关节骨破坏（D 错，为本题正确答案）。
【避错】非缓解期的 SLE 患者容易出现流产、早产和死胎，故应避孕（AC 对）；SLE 出现肾脏病变，多合并狼疮肾炎，提示预后不良（B 对）；反复癫痫发作及器质性脑病表现者为狼疮脑病，提示预后不良（E 对）。

这个题目考察的是系统性红斑狼疮（SLE）的诊断和预后判断。让我们逐一分析每个选项：
A. 不宜妊娠：SLE 患者在活动期不宜妊娠，因为妊娠可能加重疾病活动，但在疾病控制稳定时，妊娠是可能的。因此，这个说法不完全正确，需要根据疾病活动情况具体分析。
B. 肾脏病变是增殖型表现者，预后不良：确实，SLE 患者中的增殖型肾炎与较差的预后相关。
C. 非缓解期妊娠可加重病情，应避孕：这是正确的，SLE 患者在非缓解期妊娠确实可能加重病情，应当避孕。
D. 反复发作关节炎引起关节畸形和关节周围肌肉萎缩：SLE 可以引起关节炎，长期不控制可能导致关节畸形和肌肉萎缩，这是可能的。
E. 反复癫痫发作及器质性脑病表现者预后不良：SLE 引起的神经系统受累，如癫痫发作和器质性脑病，确实与较差的预后相关。
因此，从这些选项中，A 项“不宜妊娠”是最不准确的，因为它没有考虑到疾病的活动状态。在疾病控制稳定的情况下，SLE 患者是可以考虑妊娠的。所以，正确答案应该是 A。
解析：
患者青年女性，关节疼痛、易脱发，ESR 增快，抗“O”正常，中度贫血，血小板计数偏低，ANA 呈颗粒型阳性，尿蛋白阳性，根据症状、体征可确诊为系统性红斑狼疮（SLE）。SLE 患者关节炎主要累及近端指间关节、掌指关节、腕关节、肘关节和膝关节等，常表现为对称性、持续性疼痛，但较少引起关节畸形和关节周围肌肉萎缩。
SLE 好发于育龄女性，妊娠可诱发 SLE 活动，特别是在怀孕的头 3 个月和产后几个月，故选项 A 不宜妊娠，选项 C 非缓解期妊娠可加重病情，应避孕，均符合 SLE 患者的预后情况。
SLE 几乎可以累及每一个系统、组织和器官，其中肾脏损害最常见，可表现为急性肾炎综合征、急进性肾炎综合征、肾病综合征或肾小球肾炎样肾病等，严重者发展为肾功能衰竭，故选项 B 肾脏病变是增殖型表现者，预后不良，符合 SLE 患者的预后特点。
SLE 累及神经系统，又称神经精神狼疮，轻者仅有偏头痛、性格改变、记忆力减退或轻度认知障碍，重者可表现为脑血管意外、昏迷、癫痫持续状态等，因此选项 E 反复癫痫发作及器质性脑病表现者预后不良，是 SLE 患者预后不良的表现。
综上所述，正确答案是：D.反复发作关节炎引起关节畸形和关节周围肌肉萎缩。
题目描述的是一名 28 岁女性患者，患有系统性红斑狼疮（SLE），伴有多个症状和体征，包括关节疼痛、脱发、血沉升高、贫血、血小板减少、ANA 阳性和尿蛋白。这些特征提示疾病活跃且有多系统受累。

对于 SLE 患者的预后评估，以下是选项的分析：

A. **不宜妊娠** - SLE 患者在疾病活动期确实不宜妊娠，因为妊娠可能加重病情，并且对母婴健康有潜在风险。
B. **肾脏病变是增殖型表现者，预后不良** - 增殖型肾炎（如增生性狼疮肾炎）预后较差，需要密切监测和积极治疗。
C. **非缓解期妊娠可加重病情，应避孕** - 在 SLE 活动期妊娠确实可能加重病情，因此建议避孕直至疾病缓解。
D. **反复发作关节炎引起关节畸形和关节周围肌肉萎缩** - SLE 患者的关节炎一般是非侵蚀性的，不像类风湿性关节炎那样导致关节畸形。虽然关节炎可以引起疼痛和功能障碍，但很少导致关节畸形和严重肌肉萎缩。
E. **反复癫痫发作及器质性脑病表现者预后不良** - 中枢神经系统受累，如癫痫发作和器质性脑病，确实预后不良。

综上所述，D 选项的描述不正确，因为 SLE 患者的关节炎通常不会引起关节畸形和严重肌肉萎缩。

所以，正确答案是：

D. 反复发作关节炎引起关节畸形和关节周围肌肉萎缩
75、 (A2 型题)
女，27 岁，确诊系统性红斑狼疮 2 年，妊娠 28 周。 一周前检查：Hb109g/L,PLT209×106/L。患者可以使用的药物是
A.甲氨蝶呤
B.环磷酰胺
C.羟氯喹
D.来氟米特
E.大剂量糖皮质激素
参考答案： C 您的答案：
图文解析： 【解析】育龄期女性，妊娠 28 周，确诊系统性红斑狼疮 2 年，轻度贫血，血小板正常，因羟氯喹对妊娠影响相对较小，可以使用（C 对）。
【避错】甲氨蝶呤为类风湿性关节炎的首选用药（A 错）。环磷酰胺和来氟米特有致畸作用，孕妇不能使用（BD 错）。激素会增加妊娠期糖尿病、高血压及感染风险，不建议长期大剂量使用，应尽快减量至泼尼松≤15mg/d（E 错）。
系统性红斑狼疮（Systemic Lupus Erythematosus, SLE）的治疗需要根据患者的具体情况来定制，尤其是在妊娠期间。在选择药物时，我们需要考虑药物的安全性，特别是对胎儿的影响。
- **甲氨蝶呤（Methotrexate, MTX）**：对妊娠有害，可能导致胎儿畸形，所以孕妇禁用。
- **环磷酰胺（Cyclophosphamide）**：对胎儿有害，可以导致出生缺陷和流产，因此在妊娠期间不推荐使用。
- **羟氯喹（Hydroxychloroquine, HCQ）**：在妊娠期间相对安全，且对 SLE 有治疗效果，是孕妇 SLE 治疗的首选药物之一。
- **来氟米特（Leflunomide）**：孕妇禁用，因为可能导致胎儿畸形。
- **大剂量糖皮质激素**：虽然可以用于 SLE 的治疗，尤其是在活动期，但在妊娠期间应谨慎使用，并尽量控制在最小有效剂量。
因此，最适合这位患者的选项是 **C.羟氯喹**。它既可以有效控制 SLE 的症状，又相对安全地用于妊娠期间。
解析：
患者青年女性，确诊系统性红斑狼疮，现妊娠 28 周。对于妊娠期合并系统性红斑狼疮患者，应选用对胎儿影响小的药物。羟氯喹对妊娠和胎儿无不良影响，且可以控制 SLE 活动，防止 SLE 复发，因此患者可以使用。
选项 A 甲氨蝶呤，选项 B 环磷酰胺，选项 D 来氟米特，均属于免疫抑制剂，有明确致畸作用，孕妇禁用，故一并排除。
选项 E 大剂量糖皮质激素可能对胎儿有影响，只有当收益大于风险时才考虑使用，故不选。
综上所述，正确答案是：C.羟氯喹。
对于一位确诊系统性红斑狼疮（SLE）2 年，妊娠 28 周的女性患者来说，选择适合的药物时需要特别注意其对胎儿的安全性。以下是各选项的分析：

A. **甲氨蝶呤**：是一种抗代谢药物，用于治疗 SLE 和某些癌症。但甲氨蝶呤对胎儿有致畸

作用，妊娠期禁用。

B. **环磷酰胺**：是一种烷化剂，具有免疫抑制作用，但其对胎儿有严重的毒性和致畸作用，妊娠期禁用。

C. **羟氯喹**：是一种抗疟药物，也常用于治疗 SLE。研究表明，羟氯喹在妊娠期是相对安全的，并且对控制 SLE 活动有益。

D. **来氟米特**：是一种免疫抑制剂，具有致畸性，妊娠期禁用。

E. **大剂量糖皮质激素**：虽然糖皮质激素可以用于控制 SLE 的活动，但大剂量使用可能对胎儿产生不利影响，通常在妊娠期间会尽量避免使用大剂量。

因此，综合考虑药物的疗效和对妊娠的安全性，**C. 羟氯喹** 是最合适的选择。

答案：C. 羟氯喹
76、　(A2 型题)
男，70 岁，左腹股沟包块 1 年，包块圆形，大小 5×4cm，平躺消失，按住包块口深环，咳嗽后仍可突出，该包块可能为
A.脂肪瘤
B.腹股沟斜疝
C.腹股沟直疝
D.股疝
E.精索鞘膜积液
参考答案：　C　您的答案：
图文解析：　【解析】老年男性，腹股沟包块圆形，平躺消失，按住包块深环，咳嗽后仍突出，考虑为腹股沟直疝（C 对）。
【避错】脂肪瘤（A 错）为正常脂肪样组织的瘤状物，好发于四肢、躯干，境界清楚，呈分叶状，质软可有假囊性感、无痛。腹股沟斜疝（B 错）好发于儿童及青壮年，疝块呈椭圆形或梨形，可进入阴囊，回纳后按压包块深环，疝块不再突出。股疝（D 错）好发于 40 岁以上的妇女，疝块呈半球形，平卧后疝块不能完全消失。精索鞘膜积液（E 错）肿块较小，在腹股沟管内，牵拉同侧睾丸可见肿块移动。
这个问题描述的是一位 70 岁男性，有左腹股沟包块 1 年。包块圆形，大小 5×4cm，平躺时消失，按住包块口深环，咳嗽后仍可突出。根据这些临床特征，可以排除脂肪瘤（A）和精索鞘膜积液（E），因为这些条件通常不会因体位改变而改变大小，也不会在咳嗽时突出。
在剩下的选项中，腹股沟疝（包括斜疝和直疝）和股疝是与描述相符的常见情况。腹股沟疝通常会在站立或咳嗽时变得更明显，并且可能在躺下时消失。腹股沟斜疝（B）通常是通过内环进入腹股沟管的，而腹股沟直疝（C）是通过腹股沟管的薄弱区直接向前突出的。股疝（D）位于腹股沟下方，通常不会上升到腹股沟管。
由于包块在咳嗽后可以突出，并且在平躺时消失，这更符合腹股沟疝的特点。考虑到病人的年龄和描述的临床表现，最可能的诊断是**腹股沟直疝（C）**。因为在老年男性中，腹股沟直疝比腹股沟斜疝更常见，尤其是在没有先天性成分（如斜疝常见于年轻人）的情况下。
首先，我们分析题干。
老年男性患者，左腹股沟有一包块，圆形，大小为 5×4cm，且在平躺时会消失。关键信息

是，当按住包块口深环并咳嗽时，包块仍可突出。这一特点为我们提供了关于包块的性质的线索。

接下来，我们逐一分析选项：

A.脂肪瘤：脂肪瘤是一种常见的软组织良性肿瘤，由成熟脂肪细胞构成，可发生于身体任何有脂肪的部位。但它不会在平躺时消失，也不会因为按住某个位置而改变其突出状态。故排除。

B.腹股沟斜疝：腹股沟斜疝的疝囊会经过腹股沟管深环突出，并可能进入阴囊。其特点之一是当按住腹股沟管深环并增加腹压（如咳嗽）时，疝块不再突出。但题干中明确提到，按住包块口深环并咳嗽后，包块仍然可以突出，这与腹股沟斜疝的特点不符。故不选择。

C.腹股沟直疝：腹股沟直疝的疝囊经直疝三角区直接由后向前突出，不经过内环，也不进入阴囊。关键的是，当病人站立时，在腹股沟内侧端、耻骨结节上外方会出现一半球形肿块，但当患者平躺后，该肿块多会自行回纳消失。如果用手按住腹股沟管深环并让患者起立并咳嗽，疝块仍可突出，这与题干描述相符。

D.股疝：股疝的疝块往往不大，常在腹股沟韧带下方卵圆窝处表现为一半球形的突起。股疝容易发生嵌顿，且平躺后疝块不能完全回纳消失。与题干描述不符。

E.精索鞘膜积液：这是一种囊肿，多位于睾丸上方或腹股沟管内。其特点是透光试验阳性，即当用手电筒照射时，积液部分会显得透亮。此外，鞘膜积液通常不会因为体位的改变或按压某个部位而消失或突出。与题干描述不符。

综上所述，考虑到患者平躺后包块消失，以及在按住包块口深环并咳嗽后包块仍可突出的特点，最符合腹股沟直疝的表现。因此，正确答案是 C.腹股沟直疝。

根据题目描述，70 岁男性，左腹股沟包块 1 年，包块圆形，大小 5×4cm，平躺消失，按住包块口深环，咳嗽后仍可突出。结合这些症状，以下是对各选项的分析：

A. 脂肪瘤：脂肪瘤一般是皮下柔软、不痛的肿块，不会因为体位变化或咳嗽而消失或突出。

B. 腹股沟斜疝：腹股沟斜疝的特点是包块在站立或咳嗽时出现，平躺时消失。按住深环时，包块在咳嗽时不会再突出。

C. 腹股沟直疝：腹股沟直疝通常发生在腹股沟区中内侧，其特征是在平躺时包块消失，按住深环后，包块在咳嗽时仍可突出。

D. 股疝：股疝通常出现在股骨与腹股沟韧带之间的区域，症状与描述不完全吻合。

E. 精索鞘膜积液：精索鞘膜积液多为阴囊内的液体积聚，通常表现为阴囊肿大，不符合题目中包块位于腹股沟且平躺时消失的描述。

综上所述，最符合题目描述的答案是：

C. 腹股沟直疝

77、 (A2 型题)

男，25 岁，因车祸伤致肝破裂，腹腔出血，失血性休克，经急诊手术后，腹腔出血得到控制，并给予充分补液。但患者中心静脉压 15cmH2O，血压 90/60mmHg，并没有得到 来的处理是

A.继续补液

B.补液试验
C.给予强心剂
D.给予血管扩张剂
E.给予糖皮质激素
参考答案： B 您的答案：
图文解析： 【解析】失血性休克患者，中心静脉压正常、血压低，故应进行补液实验（B对），以此鉴别心功能不全及血容量不足。
考虑到患者的中心静脉压（CVP）为 15 cmH2O，这表明患者已经进行了充分的补液，因为正常 CVP 范围通常在 5-10 cmH2O。患者的血压仍然偏低（90/60 mmHg），暗示可能存在持续的低血容量状态或心功能不足。
选项 A（继续补液）可能不是最佳选择，因为患者的 CVP 已经较高，表明血容量可能已经充足。选项 B（补液试验）通常用于确定是否需要进一步补液，但在高 CVP 的情况下可能不适用。选项 D（给予血管扩张剂）可能会进一步降低血压，所以不是理想的选择。
考虑到这些情况，选项 C（给予强心剂）似乎是最合适的选择。强心剂可以增强心脏收缩力，提高心输出量，从而有助于改善血压。因此，正确答案可能是 **C. 给予强心剂**。
解析：
青年男性患者，肝破裂术后，已给予补液。现患者中心静脉压正常偏高，血压偏低，说明心功能不全或血容量相对过多，心脏不能有效搏出，应给予强心剂以增强心肌收缩力。
选项 A 继续补液适用于血容量严重不足的情况，而该患者中心静脉压正常偏高，表明血容量并非不足，故不选。
选项 B 补液试验主要用于鉴别少尿的原因，与题干不符，故排除。
选项 D 给予血管扩张剂适用于中心静脉压高，血压正常的情况，与题干描述不符，故不选。
选项 E 给予糖皮质激素主要用于严重感染或炎症的治疗，与该患者病情不符，故排除。
综上所述，正确答案是：C.给予强心剂。
在此案例中，患者在车祸导致肝破裂后，出现了腹腔出血和失血性休克。经过急诊手术后，虽然腹腔出血已得到控制，并给予了充分补液，但患者的中心静脉压（CVP）为 15cmH2O，而血压仍然低（90/60 mmHg）。这种情况提示患者可能有容量过多，心功能不全或者其他因素导致的血压低。

在选择答案时需要考虑患者的当前状况：

- 继续补液（A）可能会加重容量负担，导致心衰。
- 补液试验（B）不适合当前已补液充分的情况。
- 给予强心剂（C）可能是合适的选择，因为它有助于增强心脏的收缩功能，改善血压。
- 给予血管扩张剂（D）可能不适合当前情况，因为血压已经偏低，进一步扩张血管会使血压更低。
- 给予糖皮质激素（E）在此情况下没有明确指征。

因此，最佳答案是：
C. 给予强心剂
78、 (A2 型题)
一外伤患者，左大腿被柴刀砍伤，9 小时后送医院急诊，检查：左大腿外侧有 5cm 长，深 1cm 的伤口，边缘尚整齐，伤口内沾有较多泥土。处理宜
A.清创后一期缝合

B.清创后延期缝合
C.酒精消毒后缝合
D.局部应用抗生素
E.肥皂水冲洗
参考答案：　　B　您的答案：
图文解析：　　【解析】开放性伤口常有污染，应行清创术。如果伤口污染较重或处理时间已超过伤后 8~12 小时，但尚未发生明显的感染，皮肤的缝线暂不结扎（B 对），伤口内留置盐水纱条引流。24～48 小时后伤口仍无明显感染者，可将缝线结扎使创缘对合。如果伤口已感染，则取下缝线按感染伤口处理。
【避错】伤后 6~8 小时内清创一般可达到一期愈合；面部伤口即使大于 12 小时也要争取一期缝合。
对于一个外伤患者，特别是伤口较深且有明显污染（如沾有泥土）的情况，首要任务是彻底清创，以防止感染。因此，简单的消毒和缝合通常不够。伤口如果被污染，直接一期缝合（即立即缝合）风险很高，因为它可能促使感染发展。
最佳的处理方法是清创后延期缝合（B 选项）。延期缝合是指在伤口彻底清创（去除污染物和死组织）并在观察一段时间（通常几天）以确保没有感染发生后再进行缝合。这种方法可以减少伤口感染的风险。
所以，对于这个题目，最正确的答案是 **B.清创后延期缝合**。
解析：
患者大腿外侧伤口，深 1cm，伤口内沾有泥土，表明伤口污染较重，送医时受伤时间已达 9 小时，污染伤口超过 8 小时未处理，应按感染伤口处理，即清创后延期缝合。
选项 A 清创后一期缝合适用于伤后 8 小时以内或虽受伤已超过 8～12 小时，但尚未发生明显感染的污染较轻的伤口，与题意不符。
酒精消毒仅适用于完整皮肤的消毒，而该患者已有开放性伤口，故选项 C 酒精消毒后缝合，可排除。
局部应用抗生素不能替代全身治疗，且容易引起过敏反应，故选项 D 局部应用抗生素，可不选。
选项 E 肥皂水冲洗不能达到外科灭菌的要求，仅可作为野外条件下临时处理的措施，故不选。
综上所述，正确答案是：B.清创后延期缝合。
对于这种情况，首先要考虑到伤口感染的风险，因为伤口已经暴露在外 9 小时，并且沾有较多泥土。因此，在处理伤口时，需要注意彻底清创并采取适当的缝合方式，以避免感染。

最适合的处理方案是清创后延期缝合。这样可以先彻底清除伤口内的泥土和坏死组织，降低感染风险，并在确认伤口没有感染后再进行缝合。

因此，最佳答案是：

B. 清创后延期缝合
79、　(A2 型题)
老年男性，消瘦乏力，体重减轻，指检距肛门 8cm 处有一直径 3cm 肿块，结肠镜检查见距离肛门 8~10cm 处约 3cm 菜花样肿块，问此患者首选治疗方法
A.Miles 术
B.Dixon 术
C.Hartmann 术

D.直肠癌局部切除术
E.乙状结肠双腔造口
参考答案： B 您的答案：
图文解析： 【解析】老年男性，消瘦、乏力、体重减轻（癌症典型症状和体征），距肛门 8cm 处有一直径 3cm 肿块，考虑为直肠癌。Dixon 术（经腹直肠癌根治术，B 对）又称作为直肠低位前切术，它是目前应用最多的直肠癌根治术，适用于距离齿状线 5cm 以上（距肛门 7cm 以上）的肿瘤。
【避错】Miles 术（A 错），适用于距离齿状线 5cm 以内（距肛门 7cm 以内）的肿瘤；经腹直肠切除、近端造口、远端封闭手术（Hartmann 手术，C 错），适用于年老体弱等原因不能行 Miles 手术或急性梗阻不宜行 Dixon 手术。直肠癌局部切除术（D 错）适用于 T1 以内的直肠癌。排便困难或肠梗阻，可行乙状结肠双腔造口（E 错）。
本题描述的是一位老年男性患者，出现消瘦、乏力、体重减轻等症状，并通过肛门指检及结肠镜检查发现距肛门 8~10cm 处有约 3cm 的菜花样肿块。根据这些信息，我们可以推断肿块很可能是直肠癌。
现在，我们来看看各个治疗方案：
- **A. Miles 术（前方直肠切除术）**：适用于直肠中、下段癌症，涉及到整个直肠和肛门的切除，通常伴有永久性造口。
- **B. Dixon 术（前切除术）**：适用于上段直肠癌或乙状结肠癌，保留了肛门，不需要造口。
- **C. Hartmann 术**：适用于直肠下段癌症，其中切除病变部分肠段，末端结肠造口，直肠残端闭合，适用于需要急症手术或不适合立即重建肠道连续性的情况。
- **D. 直肠癌局部切除术**：适用于早期直肠癌，特别是癌变较小、较浅且位于直肠下段的情况。
- **E. 乙状结肠双腔造口**：这是一种缓解措施，通常用于急症情况，如肠梗阻或需要减压的情况，而非根治性手术。
考虑到肿块的位置（距离肛门 8~10cm），这表明它位于直肠中或下段。因此，选择 Miles 术（前方直肠切除术）可能是更适合的治疗选择，因为它适用于直肠中下段的癌症，并且是一种根治性手术。
所以，最合适的答案是 **A. Miles 术**。
解析：
老年男性患者，消瘦乏力，体重减轻，为恶性肿瘤常见的恶病质表现，指检距肛门 8cm 处有一直径 3cm 肿块，结肠镜检查见距离肛门 8~10cm 处约 3cm 菜花样肿块，结合患者症状、体征，可诊断为直肠癌。由于该患者癌肿距离肛门 8～10cm，属于低位直肠癌，且癌肿较大，为直径 3cm 的菜花样肿块，有手术指征，应行根治性手术。由于该患者为低位直肠癌，肿块较大，距肛缘小于 7cm，故应选择腹会阴联合直肠癌根治术（Miles 手术）。
选项 B：Dixon 术即经腹直肠癌切除术适用于直肠癌下缘距肛门在 7～11cm 的早期直肠癌，与本题不符。
选项 C：Hartmann 术即经腹直肠癌切除、近端造口、远端封闭手术适用于因全身一般情况差不能耐受 Miles 手术或者急性梗阻不宜行 Dixon 手术的直肠癌病人，与本题患者情况不符。
选项 D：直肠癌局部切除术适用于早期瘤体小、分化程度高的直肠癌，本题患者肿块较大，不宜局部切除。
选项 E：乙状结肠双腔造口仅仅能起到解除梗阻缓解症状的效果，而起不到根治的目的，与题意不符。
综上所述，正确答案是：A.Miles 术。

根据题目描述，该患者是老年男性，消瘦乏力，体重减轻，指检及结肠镜检查发现距肛门 8-10cm 处有一个直径约 3cm 的菜花样肿块，这提示可能为直肠癌。根据直肠癌的治疗原则和当前最佳实践指南，选择合适的治疗方案需要考虑肿瘤的位置、大小、浸润深度及患者的全身情况等因素。

选项分析如下：
- **A. Miles 术**：这种手术是腹会阴联合直肠癌根治术，适用于低位直肠癌（距肛门 5cm 以内），需要切除肛门及其周围组织。
- **B. Dixon 术**：又称为低位前切除术，适用于中低位直肠癌（距肛门 5-10cm），保留肛门功能，是目前较为推荐的手术方式。
- **C. Hartmann 术**：一般用于急性情况或作为姑息性手术，不常作为首选治疗方案。
- **D. 直肠癌局部切除术**：适用于非常早期的、局限于粘膜下层的直肠癌，且无淋巴结转移。
- **E. 乙状结肠双腔造口**：通常作为姑息性手术，主要用于解除肠梗阻等症状。

综上所述，该患者的肿瘤位于距离肛门 8-10cm 处，属于中位直肠癌，Dixon 术（低位前切除术）是最佳选择，因为它可以在根治肿瘤的同时保留肛门功能。

所以，首选治疗方法是 **B. Dixon 术**。
80、 (A2 型题)
男，17 岁。胸痛、气短 2 小时。2 小时前，患者大笑后突发胸痛、气短，有少量咳嗽，无发热。查体：左侧胸廓饱满，左肺叩诊鼓音，左肺呼吸音低，无啰音。心率 101 次/ 示窦性心动过速。最可能的诊断是
A.自发性气胸
B.肺栓塞
C.支气管哮喘急性发作
D.肺气肿
E.急性心肌梗死
参考答案： A 您的答案：
图文解析： 【解析】青年男性，患病前有大笑动作，体征为左侧胸廓饱满，左肺叩诊鼓音，左肺呼吸音低（为自发性气胸典型表现），根据其临床表现和体征，考虑诊断为自发性气胸（A 对）。
【避错】肺栓塞主要表现为突发胸痛、呼吸困难、咯血、晕厥等（B 错）；支气管哮喘急性发作表现为发作性呼吸困难，持续时间为数分钟，发作时可闻及广泛的哮鸣音（C 错）；肺气肿较少出现与青年男性，且体征多为双侧胸廓饱满，叩诊呈清音（D 错）；急性心肌梗死以胸前区剧烈疼痛为首发症状，较少出现一侧胸廓饱满，叩诊呈清音等体征（E 错）。
这道题目描述了一名 17 岁男性患者在大笑后突发胸痛和气短，体检发现左侧胸廓饱满，左肺叩诊呈鼓音，左肺呼吸音降低，无发热和啰音，心率增快。基于这些信息，最可能的诊断是自发性气胸（A 选项）。
自发性气胸通常发生在无明显外伤的情况下，尤其是在年轻人中。典型症状包括突发胸痛和呼吸困难。体检可能发现患侧胸廓饱满，叩诊呈鼓音，呼吸音降低，这与本题描述的临床表现相符。因此，A. 自发性气胸是最可能的诊断。
首先，我们来解析题干。

青年男性患者，突发胸痛、气短，并且是在大笑后出现的。查体显示左侧胸廓饱满，左肺叩诊鼓音，这是气胸的一个典型体征。同时，左肺呼吸音低，没有啰音，心率稍快。

接下来，我们针对每一个选项进行分析：
A. 自发性气胸：考虑到患者大笑后突发症状，左侧胸廓饱满和左肺叩诊鼓音，这些都是自发性气胸的典型症状和体征。青年男性是自发性气胸的好发人群，且常因持重物、屏气、剧烈体力活动等诱发。因此，这一选项高度符合题干描述。
B. 肺栓塞：虽然肺栓塞也可能导致胸痛和气短，但它通常与深静脉血栓有关，并且多见于老年人或长期卧床的患者。题干中并未提及这些风险因素，因此此选项不太可能。
C. 支气管哮喘急性发作：支气管哮喘通常会有反复发作的喘息、气急、胸闷或咳嗽等症状，而且这些症状常在夜间或清晨发作或加剧。题干中并未提及这些症状，因此可以排除。
D. 肺气肿：肺气肿的症状通常包括慢性咳嗽、咳痰、气短或呼吸困难等，并且它是一个慢性疾病，不会因为大笑等突发因素而急性起病。因此，这一选项不符合题干描述。
E. 急性心肌梗死：急性心肌梗死通常表现为持续的、剧烈的胸骨后疼痛，可能伴有恶心、呕吐、大汗等症状。虽然它也可能导致窦性心动过速，但题干中的症状描述和体征与急性心肌梗死不符。
综上所述，考虑到患者的年龄、起病方式、症状和体征，最可能的诊断是自发性气胸。
因此，正确答案是 A.自发性气胸。
根据题目描述，患者是一名 17 岁的男性，胸痛和气短 2 小时。这些症状在大笑后突然发生，并且伴有少量咳嗽，没有发热。体检显示左侧胸廓饱满，左肺叩诊鼓音，左肺呼吸音低，无啰音，心率 101 次/示窦性心动过速。

结合患者的年龄和症状特征，以下是对各选项的分析：

A. **自发性气胸**：这种情况常见于年轻健康的男性，通常表现为突发的单侧胸痛和呼吸困难。体检可以发现患侧胸廓饱满、叩诊呈鼓音、呼吸音降低，这与题目中的描述一致。

B. **肺栓塞**：通常见于有深静脉血栓或其他栓子来源的患者，症状包括突发的呼吸困难、胸痛（可能是胸膜性的）、咯血等。虽然症状可能类似，但题目中没有提到相关的危险因素或典型症状。

C. **支气管哮喘急性发作**：通常表现为呼吸困难、喘息、咳嗽，但不会有明显的胸痛或一侧胸廓饱满的体征。

D. **肺气肿**：通常见于慢性阻塞性肺疾病患者，表现为长期的呼吸困难和慢性咳嗽，急性发作时可能加重，但与题目中的急性、突发症状不一致。

E. **急性心肌梗死**：常见症状包括胸痛（可以放射到其他部位）、气短、出汗等，通常伴有典型的心电图和心肌标志物改变，但患者年仅 17 岁，不太可能是急性心肌梗死的典型发病年龄，且体征也不完全匹配。

综合以上分析，最可能的诊断是 **A. 自发性气胸**。
81、 (A2 型题)
男，33 岁，右上腹胀痛伴寒战、高热 6 小时。查体：T39.9℃,皮肤黏膜无黄染，右上腹压痛，

轻度肌紧张，无明显反跳痛。WBC18×109/L，N0.85，腹部立位平片，右侧 超：肝右后下方可见 8cm 液性暗区。以下最有价值的检查

A.肝核素扫描

B.腹部 CT

C.肝动静脉造影

D.超声下肝穿刺

E.血培养

参考答案： D 您的答案：

图文解析： 【解析】青年男性，右上腹胀痛，寒战高热，B 超显示肝右后下方可见 8cm 液性暗区，可诊断为肝脓肿。此时最具价值的检查应该为超声下肝穿刺（D 对）。

【避错】肝核素扫描（A 错）和肝动静脉造影（C 错） 一般用于肝癌的辅助检查；腹部 CT 虽然对肝脓肿的诊断有辅助价值，但不是最具诊断价值的检查（B 错）；血培养敏感性不及超声下穿刺，有时可为阴性（E 错）。

这个案例描述的是一个 33 岁男性患者出现右上腹胀痛、寒战和高热，体温达到 39.9℃，右上腹有压痛和轻度肌紧张，但无明显反跳痛。患者的白细胞计数升高，超声检查显示肝右后下方有 8cm 的液性暗区。这些临床表现和检查结果可能提示有急性腹部疾病，特别是与肝脏相关的问题。

选项分析如下：

A. 肝核素扫描 - 这是一种评估肝脏血流和功能的检查，对于急性腹痛的诊断不是首选。

B. 腹部 CT - 可以提供详细的腹部结构信息，特别是对于腹腔内器官的炎症、感染或其他病变有很高的诊断价值。

C. 肝动静脉造影 - 主要用于评估肝脏的血管结构，对于本病例的初步诊断可能不是必需的。

D. 超声下肝穿刺 - 可以用于诊断肝脏病变，但在没有更多信息的情况下进行侵入性操作可能不是最佳选择。

E. 血培养 - 对于感染性疾病非常有用，但在确定感染部位之前，可能需要进行更多的成像检查。

综合考虑，最有价值的检查应该是能够提供最多信息以帮助确诊的检查。在这种情况下，**B. 腹部 CT**是最佳选择，因为它可以提供关于腹腔内器官，特别是肝脏的详细信息，有助于诊断肝脏的液性暗区的性质，并指导进一步治疗。

首先，我们要分析患者的症状、体征和已有的检查结果。

患者青年男性，急性起病，出现右上腹胀痛、寒战和高热，这是急性胆囊炎或肝脓肿的典型表现。查体发现体温升高，右上腹有压痛和轻度肌紧张，但无明显反跳痛，白细胞和中性粒细胞均升高，提示存在感染。腹部立位平片和 B 超结果显示肝右后下方有 8cm 的液性暗区，进一步支持肝脓肿的诊断。

接下来，我们分析各个选项：

A. 肝核素扫描：这种检查更多地用于评估肝脏功能和胆道排泄情况，对于确定肝脓肿的诊断价值有限。

B. 腹部 CT：这是一种常用的影像学检查方法，能够清晰地显示肝脏的形态和结构，对肝脓肿的大小、位置和与周围组织的关系都能提供详细的信息。

C. 肝动静脉造影：主要用于显示肝脏的血管结构和血流情况，对于肝脓肿的诊断不是首选。

D. 超声下肝穿刺：虽然肝穿刺可以确诊，但作为一种有创检查，其风险相对较高，不是首选的诊断方法。

E. 血培养：这主要用于确定感染的病原体，虽然对治疗有指导意义，但对于直接诊断肝脓

肿的价值不大。
综合考虑上述信息，腹部 CT 既无创又能提供详细的肝脏形态和结构信息，是诊断肝脓肿的首选检查方法。
因此，最有价值的检查是 B.腹部 CT。
患者为 33 岁男性，右上腹胀痛伴寒战、高热 6 小时，体温 39.9℃，皮肤黏膜无黄染，右上腹压痛，轻度肌紧张，无明显反跳痛。白细胞计数 18×10^9/L，中性粒细胞比例 0.85，腹部超声显示肝右后下方可见 8cm 液性暗区。

根据患者的症状和检查结果，最有可能的诊断是肝脓肿。对于肝脓肿的诊断和进一步评估，以下检查是最有价值的：

B. 腹部 CT

腹部 CT 能够详细显示肝脓肿的大小、位置和数量，同时还能发现其他可能的腹部病变。因此，腹部 CT 是最有价值的检查方法。

正确答案：B. 腹部 CT
82、 (A2 型题)
男，42 岁，腹痛，腹胀伴肛门停止排气排便 2 天。禁食，补液治疗，今晨突发腹痛加剧。既往行阑尾切除术 10 年余。查体：全腹压痛，反跳痛，肌紧张，肠鸣音消失。最 是
A.空气通便
B.手术探查
C.持续性胃肠减压
D.补液
E.禁食
参考答案： B 您的答案：
图文解析： 【解析】中年男性，腹痛、腹胀、肛门停止排气排便（肠梗阻的表现），禁食补液治疗 2 天后腹痛加重（内科治疗无效，考虑手术治疗），有阑尾炎手术史（考虑术后肠粘连造成的粘连性肠梗阻）。查体：全腹压痛，反跳痛，肌紧张，肠鸣音消失。该患者出现腹膜炎表现且经内科治疗无效，考虑绞窄性肠梗阻，应行手术探查（B 对）。
【避错】有下列表现者，应考虑为绞窄性肠梗阻：（1）腹痛发作急骤，初始即为持续性剧烈腹痛；（2）病情发展迅速，早期出现休克；（3）有腹膜炎表现；（4）腹胀不对称，腹部有局部隆起；（5）呕吐出现早而频繁，呕吐物为血性，腹腔穿刺抽出血性液体；（6）X 线检查见孤立扩大的肠袢；（7）经积极的非手术治疗体征无明显改善者。
这道题描述的是一名 42 岁男性患者，出现腹痛、腹胀，已经 2 天没有排气和排便。患者有阑尾切除术的病史，经过禁食和补液治疗后，腹痛加剧。体检发现全腹压痛、反跳痛、肌紧张，且肠鸣音消失。
这些症状和体征表明患者可能发生了肠梗阻或腹腔内急性病变，如肠扭转或肠缺血。在这种情况下，仅靠禁食和补液治疗可能不足以解决问题，需要进一步的诊断和治疗。
选项 B“手术探查”在这种情况下是最合适的选择，因为它允许直接视察腹腔内情况，确定病变的性质，并进行相应的治疗。手术探查可以迅速发现并解决引起症状的原因，如肠梗阻、肠缺血或其他腹腔内问题。
因此，我会选择 B“手术探查”作为这道题目的最佳答案。
首先，我们来分析题干。

患者中年男性，腹痛、腹胀，且肛门停止排气排便已有 2 天，这是肠梗阻的典型症状。经过禁食和补液治疗后，今晨腹痛突然加剧。既往有阑尾切除手术史。查体显示全腹压痛、反跳痛、肌紧张，且肠鸣音消失，这些都是腹膜炎的典型体征。结合患者的病史和体征，我们可以初步诊断为肠梗阻后可能引发的肠绞窄或肠穿孔，导致了急性腹膜炎。
接下来，我们针对每个选项进行分析：
A. 空气通便：在肠梗阻的情况下，使用空气通便可能会加重病情，甚至导致肠穿孔的风险增加，因此这个选项是不合适的。
B. 手术探查：考虑到患者可能的肠绞窄或穿孔情况，手术探查是最直接和有效的方法来确认诊断并进行相应的治疗。这可以防止病情进一步恶化，甚至危及患者生命。
C. 持续性胃肠减压：虽然胃肠减压在肠梗阻的治疗中有一定的作用，可以缓解症状，但在肠绞窄或穿孔的情况下，它不能解决根本问题。
D. 补液：补液对于纠正脱水和电解质失衡是必要的，但它并不能解决肠梗阻或腹膜炎的根本问题。
E. 禁食：禁食可以减少肠道负担，有助于缓解肠梗阻的症状，但在紧急情况下如肠绞窄或穿孔，它不是主要的治疗方法。
综上所述，考虑到患者可能的肠绞窄或肠穿孔，以及急性腹膜炎的体征，最紧迫和必要的措施是进行手术探查以确定病因并进行相应的治疗。
因此，正确答案是 B. 手术探查。
根据患者的症状和体征，最合理的选择是 **B. 手术探查**。以下是分析过程：

患者是一名 42 岁的男性，主要症状包括腹痛、腹胀、肛门停止排气排便 2 天，并且病情在今晨突然加剧。体检发现全腹压痛、反跳痛、肌紧张和肠鸣音消失，这些都是急性腹膜炎的典型表现。

急性腹膜炎通常由腹腔内脏器破裂、感染或缺血导致，需要紧急手术处理。患者的病史显示 10 年前曾行阑尾切除术，可能存在肠粘连或其他并发症，这进一步增加了急性腹膜炎的可能性。突发的腹痛加剧提示可能出现了急性并发症，如肠穿孔或肠梗阻。

因此，综合考虑患者的临床表现和既往病史，最合理的处理方法是紧急手术探查，以明确病因并进行相应的治疗。

其他选项的分析：
- **A. 空气通便**：适用于轻度的肠梗阻，但不适用于急性腹膜炎。
- **C. 持续性胃肠减压**：虽然对肠梗阻有帮助，但无法解决急性腹膜炎或其他严重并发症。
- **D. 补液**：是支持治疗的一部分，但不能解决根本问题。
- **E. 禁食**：同样是支持治疗的一部分，但不能解决根本问题。

因此，最正确的答案是 **B. 手术探查**。
83、 (A2 型题)
女，28 岁，2 周前因接触冷空气出现干咳，喘息，无发热，自服头孢类抗菌药无效。为明确诊断，应进一步做的检查
A.支气管激发试验
B.动脉血气分析
C.特异性变应原检测

D.血嗜酸性粒细胞检测
E.呼出气 NO（FeNO）检测
参考答案：　　A　您的答案：
图文解析：　　【解析】青年女性，两周前因接触冷空气出现干咳、喘息（有过敏原接触史）抗生素治疗无效，考虑为支气管哮喘。支气管激发试验（A 对）对评估气道反应性有重要意义，若 FEV1 下降≥20%，提示存在支气管哮喘。
【避错】动脉血气分析（B 错）主要试验是了解机体呼吸功能和酸碱平衡状态；特异性变应原检测（C 错）对哮喘诊断价值不大；大多数哮喘患者诱导痰液中嗜酸性粒细胞计数增高（＞2.5%），且与哮喘症状相关，但并非血嗜酸性粒细胞检测（D 错）；FeNO 测定（E 错）可以作为评估气道炎症和哮喘控制水平的指标，也可以用于判断吸入激素治疗的反应。
这个案例描述了一位 28 岁女性，因接触冷空气后出现干咳和喘息，没有发热，服用头孢类抗菌药也未见效果。根据病情描述，这种症状可能与哮喘相关。哮喘是一种常见的慢性炎症性气道疾病，其特点是气道对各种刺激的过度反应。考虑到这些症状，特别是在接触冷空气后出现，哮喘的可能性较高。
要明确诊断哮喘，通常需要进行肺功能测试，并可能需要进行特定的诊断测试。在提供的选项中，A 项“支气管激发试验”是一种常用于诊断哮喘的方法，可以检测气道对刺激的反应性。然而，更为常规和无创的初步检测方法是 E 项的“呼出气一氧化氮（FeNO）检测”。FeNO 检测是评估气道炎症，特别是与嗜酸性粒细胞相关炎症的一个非侵入性方法，它在哮喘诊断和管理中具有重要价值。
考虑到这些因素，最佳答案可能是 E 项“呼出气 NO（FeNO）检测”，因为它是一个简单、快速且非侵入性的检测方法，可以帮助诊断哮喘，并且对于监测疾病活动度及指导治疗具有重要价值。
解析：
青年女性患者，2 周前接触冷空气后出现干咳，喘息，无发热，自服头孢类抗菌药无效，该患者考虑诊断为支气管哮喘。支气管哮喘的典型症状为伴有哮鸣音的呼气性呼吸困难，夜间及凌晨发作和加重常是哮喘的特征之一，可在数分钟内发作，经数小时至数天，用支气管舒张剂或自行缓解。该疾病是由多种细胞和细胞组分参与的气道慢性炎症性疾病，慢性炎症与气道高反应性相关，通常出现广泛多变的可逆性气流受限，并引起反复发作性的喘息、气急、胸闷或咳嗽等症状，常在夜间和（或）清晨发作、加剧，多数患者可自行缓解或经治疗后缓解。支气管哮喘需与心源性哮喘、喘息性慢性支气管炎等疾病鉴别，支气管激发试验用以测定气道反应性，常用的吸入激发剂有乙酰胆碱、组胺、甘露醇等，通过依次增加吸入的气道收缩剂的浓度和剂量，观察出现支气管痉挛的剂量和反应，可判断是否存在气道高反应性，对于支气管哮喘的诊断和鉴别诊断具有重要意义。
选项 B 动脉血气分析是指对各种气体、液体中不同类型的气体和酸类物质进行分析的技术过程，可反映肺通气和换气功能，但不能用于支气管哮喘的诊断，故不选。
选项 C 特异性变应原检测用于寻找变应原，为脱敏治疗提供依据，而非用于支气管哮喘的诊断，故排除。
选项 D 血嗜酸性粒细胞检测多用于寄生虫感染及过敏反应的检查，支气管哮喘患者血嗜酸性粒细胞计数可增高，但不具有特异性，不能用于诊断，故不选。
选项 E 呼出气 NO（FeNO）检测可作为气道炎症的生物标志物，FeNO 水平可以反映气道的嗜酸性粒细胞性炎症，但其特异性及敏感性相对较低，因此不作为支气管哮喘的诊断性检查，故排除。
综上所述，正确答案是：A.支气管激发试验。

这道题目描述了一位 28 岁的女性患者，因接触冷空气后出现干咳和喘息，未发热，自服头孢类抗菌药无效。为了明确诊断，应进一步做的检查是什么？

从症状和背景来看，患者可能患有哮喘或过敏性疾病。哮喘通常会表现为干咳和喘息，尤其是在接触某些触发因素（如冷空气）后。

为了确诊哮喘，常用的检查包括支气管激发试验、特异性变应原检测、血嗜酸性粒细胞检测以及呼出气 NO（FeNO）检测。

- **A. 支气管激发试验**：通过吸入特定刺激物（如组胺或甲酰胺）来评估气道反应性，是诊断哮喘的重要方法之一。
- **B. 动脉血气分析**：主要用于评估肺功能和酸碱平衡，通常在严重呼吸困难或慢性阻塞性肺疾病患者中应用。
- **C. 特异性变应原检测**：用于识别过敏原，但并不能直接确诊哮喘。
- **D. 血嗜酸性粒细胞检测**：嗜酸性粒细胞升高常见于哮喘和过敏性疾病，但单独检测嗜酸性粒细胞并不能确诊哮喘。
- **E. 呼出气 NO（FeNO）检测**：呼出气中的一氧化氮水平可以反映气道炎症，常用于哮喘的诊断和监测。

综上所述，为了明确诊断哮喘，最常用的检查方法是支气管激发试验。尽管 FeNO 检测也很有用，但支气管激发试验被认为是更直接的诊断方法。

因此，最佳答案是：

A. 支气管激发试验

84、 (A2 型题)

男，23 岁，右下腹痛，腹泻，消瘦 5 年，既往有肛瘘史，肠镜回示：回盲部充血、水肿，呈鹅卵石样改变，伴多发纵行溃疡，最有可能的是

A.肠结核

B.阿米巴肠炎

C.缺血性肠病

D.克罗恩病

E.溃疡性结肠炎

参考答案： D 您的答案：

图文解析： 【解析】青年男性，右下腹痛、腹泻、消瘦（炎症性肠炎的可能），有肛瘘史，肠镜提示回盲部充血水肿，呈鹅卵石样改变，伴多发纵型溃疡（符合克罗恩病），考虑为克罗恩病（D 对）。

【避错】肠结核（A 错）病变部位通常在回盲部附近，很少累及空肠，节段性改变不如克罗恩病明显，可见腹腔淋巴结中央坏死或钙化等改变。阿米巴肠炎（B 错）病变主要侵犯右侧结肠，也可累及左侧结肠，结肠溃疡较深，边缘潜行。溃疡间的黏膜多正常。缺血性肠病（D 错）主要表现为腹痛、间断便血、肠排空障碍，内镜表现包括黏膜水肿、脆弱，节段性发红，散在的糜烂，纵向溃疡，点状出血和紫色出血结节。溃疡性结肠炎（E 错）肠镜表现为黏膜血管纹理模糊紊乱，或消失充血，病变处见弥漫性糜烂和多发性浅溃疡。

该患者表现为右下腹痛、腹泻、消瘦，且有肛瘘史，肠镜检查显示回盲部充血、水肿，呈鹅

卵石样改变，伴有多发性纵行溃疡。这些临床表现和肠镜所见符合克罗恩病（Crohn's disease）的特征。克罗恩病是一种慢性炎症性肠病，可以影响消化道的任何部分，从口腔到肛门，但最常见于回肠和结肠。鹅卵石样的肠道改变和纵行溃疡是克罗恩病的典型内窥镜发现。
因此，最佳答案是：D. 克罗恩病
解析：
青年男性患者，右下腹痛、腹泻伴消瘦，有肛瘘史，肠镜示回盲部充血、水肿，呈鹅卵石样改变，伴多发纵行溃疡，考虑诊断为克罗恩病。克罗恩病是一种原因不明的肠道炎症性疾病，在胃肠道的任何部位均可发生，但多发于末端回肠和右半结肠。本病和慢性非特异性溃疡性结肠炎统称为炎症性肠病（IBD）。临床表现为腹痛、腹泻、肠梗阻，伴有发热、营养障碍等肠外表现。病程多迁延，反复发作，不易根治。本病又称局限性肠炎、局限性回肠炎、节段性肠炎和肉芽肿性肠炎。
选项 A 肠结核是临床上较为常见的肺外结核病，是因结核杆菌侵犯肠道而引起的慢性感染，其溃疡形态为带状，长径与肠轴垂直，与本题患者溃疡形状不符，故不选。
选项 B 阿米巴肠炎的病变多在回盲部结肠，易复发变为慢性，溃疡较深，边缘潜行，溃疡间的黏膜多属正常，与本题患者症状不符，故不考虑。
选项 C 缺血性肠病主要表现为突然发生的剧烈腹部绞痛，恶心呕吐频繁，腹泻多为血性，部分病人有里急后重感，与本题患者症状不符，故不选。
选项 E 溃疡性结肠炎的病变位于大肠，呈连续性弥漫性分布，与本题无关。
综上所述，正确答案是：D.克罗恩病。
根据题目描述，23 岁的男性患者有右下腹痛、腹泻和消瘦的病史，并且有肛瘘史。肠镜检查显示回盲部充血、水肿，呈鹅卵石样改变，伴多发纵行溃疡。这些症状和检查结果最符合克罗恩病（Crohn's disease）的表现。

克罗恩病的特点包括：
- 回肠末端和回盲部常见病变部位
- 右下腹痛
- 慢性腹泻
- 消瘦
- 肛瘘和其他肛周病变
- 肠镜检查时看到的鹅卵石样改变和纵行溃疡

综上所述，最有可能的诊断是：

D. 克罗恩病
85、 (A2 型题)
2 岁男孩，哭闹、腹痛 18 天，果酱样大便 2 次，查体：右上腹触及“腊肠样”包块，轻微压痛，右下腹空虚，肠鸣音亢进。腹部 B 超：右上腹包块为“靶环征”。最佳诊断
A.急性阑尾炎
B.基因肠系膜淋巴结炎
C.小肠扭转
D.肠套叠
E.肠蛔虫
参考答案： D 您的答案：
图文解析： 【解析】2 岁男童（肠套叠好发于婴幼儿），哭闹、腹痛、果酱样大便，右

上腹触及包块（肠套叠三联征：腹痛、血便、腹部包块），右下腹空虚，肠鸣音亢进，考虑为肠套叠（D 对）。
【避错】急性阑尾炎（A 错）主要表现为右下腹转移性疼痛；急性肠系膜淋巴结炎（B 错）多见于儿童，往往先有上呼吸道感染史，腹部压痛部位偏内侧，超声或 CT 检查发现腹腔淋巴结肿大；小肠扭转（C 错）表现为突然发作剧烈，腹部绞痛常为持续性疼痛阵发性加剧；肠蛔虫（E 错）常突发剑突下钻顶样剧烈绞痛，阵发性加剧，可伴有恶心、呕吐或吐出蛔虫。
此题描述的是一个 2 岁男孩，出现了哭闹、腹痛，并有果酱样大便，体检时在右上腹触及“腊肠样”包块，轻微压痛，右下腹空虚，肠鸣音亢进。腹部 B 超显示“靶环征”，这些都是典型的症状和体征。
选项 D“肠套叠”最符合这些症状。肠套叠是儿童常见的急腹症之一，特别是在婴幼儿期。它是指肠管的一部分滑入相邻的肠段中，导致肠道阻塞和血供不良。肠套叠常常表现为周期性腹痛、腹部包块（腊肠样包块）和“果酱样”血便。B 超显示的“靶环征”也是肠套叠的特征性超声表现。
因此，最佳答案是 D.肠套叠。
解析：
患儿 2 岁男童，因哭闹、腹痛 18 天就诊，排果酱样大便，查体见右上腹触及腊肠样包块、轻微压痛，为肠套叠典型体征，右下腹空虚，肠鸣音亢进，腹部 B 超显示右上腹包块为“靶环征”，为肠套叠典型影像学表现，故考虑诊断为肠套叠。
选项 A 急性阑尾炎典型表现为转移性右下腹痛，查体可有右下腹压痛、反跳痛，腹部 B 超显示阑尾肿胀，直径大于 6mm，该患儿并无转移性右下腹痛及右下腹压痛体征，故排除。
选项 B 基因肠系膜淋巴结炎多见于 7 岁以下小儿，好发于冬春季节，典型表现为发热、腹痛、恶心、呕吐等症状，以右下腹常见，呈阵发性、痉挛性疼痛，腹部 B 超显示肠系膜淋巴结肿大，与题干中患者症状不符，故不选。
选项 C 小肠扭转典型表现为突然发作的剧烈腹部绞痛，多在脐周围，常为持续性疼痛阵发性加重，疼痛可放射至腰背部，呕吐频繁，与该患儿症状差异明显，故不考虑。
选项 E 肠蛔虫表现为反复发作的脐周痛，腹部 B 超检查不会显示“靶环征”，故不选。
综上所述，正确答案是：D.肠套叠。
根据题干描述的症状和体征，考虑到男孩哭闹、腹痛 18 天，果酱样大便 2 次，右上腹触及“腊肠样”包块，右下腹空虚，肠鸣音亢进，以及腹部 B 超显示右上腹包块为“靶环征”，这些特征都非常典型，指向一种常见的儿童急腹症——肠套叠。

肠套叠是一种常见的引起婴幼儿急腹痛的疾病，通常表现为阵发性哭闹、腹痛，果酱样大便，触诊时可以在右上腹摸到“腊肠样”包块，腹部 B 超的“靶环征”是肠套叠的特异性表现。因此，最佳诊断是：

D.肠套叠
86、 (A2 型题)
小儿患者，剧烈咳嗽，肌肉酸痛，宜用下列哪种药物治疗
A.链霉素
B.庆大霉素
C.青霉素
D.阿奇霉素
E.异烟肼

参考答案：　　D　您的答案：

图文解析：　　【解析】剧烈咳嗽为支气管肺炎的典型体征，治疗支原体肺炎，大环内酯类抗生素为首选，如阿奇霉素（D 对）、红霉素、罗红霉素等。

【避错】链霉素、异烟肼（AE 错）用于结核的治疗；庆大霉素（B 错）适用于革兰阴性杆菌感染，如大肠埃希菌、克雷伯菌等；青霉素（C 错）可用于金黄色葡萄球菌肺炎。

在处理小儿剧烈咳嗽和肌肉酸痛的病例时，选择正确的药物非常重要。这些症状可能是由多种原因引起的，如病毒、细菌感染或其他疾病。因此，在没有更多信息（如病因）的情况下，选择最合适的治疗可能具有挑战性。

选项中的药物属于不同类别的抗生素或抗感染药物：

- A. 链霉素：主要用于治疗结核和某些严重的细菌感染。
- B. 庆大霉素：是一种氨基糖苷类抗生素，用于治疗严重的细菌感染。
- C. 青霉素：是一种广泛用于治疗细菌感染的β-内酰胺类抗生素。
- D. 阿奇霉素：是一种大环内酯类抗生素，常用于治疗呼吸道感染、耳部感染等。
- E. 异烟肼：主要用于治疗结核病。

根据题目描述，小儿患者主要症状为剧烈咳嗽和肌肉酸痛，这可能指向呼吸道感染。在这种情况下，选择针对可能的呼吸道感染治疗的药物更为合适。

因此，最佳答案可能是 **D. 阿奇霉素**，因为它是一种常用于治疗各种细菌引起的呼吸道感染的抗生素，如支气管炎、肺炎等。然而，实际选择药物时应根据具体病因和医生的诊断来确定。

解析：

患儿剧烈咳嗽，肌肉酸痛，考虑存在支原体感染，大环内酯类抗生素是治疗支原体肺炎的首选药物，其中阿奇霉素是新一代大环内酯类抗生素，抗菌谱较广，对流感嗜血杆菌、链球菌等革兰阳性菌有效，对支原体、衣原体等非典型病原体也有良好的抗菌作用，常用于支原体肺炎的治疗。

选项 A 链霉素，选项 E 异烟肼，均是抗结核药物，不用于支原体感染的治疗，故可一并排除。

选项 B 庆大霉素属于氨基糖苷类抗生素，主要用于治疗细菌感染，尤其是革兰氏阴性菌引起的感染，不用于治疗支原体感染，故不选。

选项 C 青霉素属于β-内酰胺类抗生素，主要用于革兰阳性球菌、杆菌，革兰阴性球菌及螺旋体所致感染，不用于治疗支原体感染，故排除。

综上所述，正确答案是：D.阿奇霉素。

小儿患者剧烈咳嗽且肌肉酸痛，可能是由细菌感染引起的呼吸道疾病。考虑到常见的病原体和抗生素的适用性，以下是各选项的分析：

A. 链霉素：主要用于结核病的治疗，不适用于常见的呼吸道感染。

B. 庆大霉素：主要用于严重的革兰氏阴性菌感染，有肾毒性和耳毒性，且不常用于呼吸道感染。

C. 青霉素：用于链球菌感染，但对某些呼吸道病原体（如支原体）无效。

D. 阿奇霉素：一种大环内酯类抗生素，对常见的呼吸道病原体（包括支原体、衣原体）有效，且适用于儿童。

E. 异烟肼：用于结核病的治疗，不适用于常见的呼吸道感染。

综合考虑，最适合治疗小儿剧烈咳嗽和肌肉酸痛的药物是阿奇霉素。阿奇霉素覆盖广泛的呼吸道病原体，并且儿童耐受性较好。

因此，最佳答案是：

D. 阿奇霉素
87、 (A2 型题)
男，54 岁，咳嗽伴间断低热半年，胸部 CT 示：右上肺，多发小斑片状高密度影，伴少许空洞，右肺上叶体积减小，最可能诊断是
A.肺癌
B.肺血管炎
C.慢性肺脓肿
D.肺结核
E.肺曲霉菌病
参考答案： D 您的答案：
图文解析： 【解析】中年男性，咳嗽、低热（肺结核经典表现：低热、盗汗、乏力），胸部 CT 示：右上肺，多发小斑片状高密度影伴少许空洞，右上叶体积减小（符合肺结核的 CT 影像，肺结核好发于肺上叶的尖后段、下叶的背段和后基底段，易形成空洞和播散病灶），考虑为肺结核（D 对）。
【避错】肺癌（A 错）主要表现为刺激性干咳、咯血、消瘦，影像表现为，边缘毛糙、可有分叶或切迹的阴影。肺血管炎（B 错）主要表现为发热、乏力、关节痛和皮损，影像学检查能发现血管壁增厚，管腔狭窄等病变。肺脓肿（C 错）起病及中毒症状较重，寒战、高热、咳大量脓臭痰，影像学可见均匀的大片阴影空洞，内常见液平。肺曲霉菌病（E 错）表现为咳嗽，咳痰，咳血发热，胸痛，其影像学表现为空洞内可移动团块，上缘弧形，并与周围形成空气半月征，邻近胸膜可以增厚。
这个病例描述了一个 54 岁男性，有长达半年的咳嗽和间断低热症状，胸部 CT 显示右上肺有多发小斑片状高密度影，伴有少量空洞，以及右肺上叶体积减小。这些症状和影像学特征可能指向几种疾病，但我们需要根据特征来缩小可能性。
A. 肺癌通常会表现为单个肿块，伴随不规则边缘或者肺门淋巴结增大，而非多发小斑片状高密度影，所以这个选项不太可能。
B. 肺血管炎可能导致多发的肺部病变，但通常伴随全身症状如发热、体重下降和全身乏力，并且 CT 上往往显示出弥漫性的肺部损害，不特定于上叶减小或空洞。
C. 慢性肺脓肿可能表现为包含空洞的肺部病变，但这些空洞通常较大，且往往伴有明显的病理壁，不完全符合小斑片状高密度影的描述。
D. 肺结核是一个非常可能的诊断，因为它可以表现为多发小斑片状高密度影，伴有空洞形成，特别是在肺的上叶，还可以导致慢性咳嗽、低热等症状。
E. 肺曲霉菌病通常影响免疫系统受损的个体，表现为肺部的斑片状阴影或空洞，但通常有免疫缺陷的背景，且 CT 表现与描述不完全匹配。
综上所述，根据这些症状和影像学特征，最可能的诊断是 D.肺结核。
首先，我们需要对题目中的关键信息进行解读。
患者，男，54 岁，主要症状是咳嗽和间断性低热持续了半年。这些症状是结核病的典型表现。胸部 CT 显示右上肺有多发小斑片状高密度影和少许空洞，同时右肺上叶体积有所减小。
现在，我们根据这些信息对各选项进行分析：
A. 肺癌：肺癌的症状可能包括咳嗽、胸痛和呼吸困难，但肺癌的 CT 表现通常与题目描述不符，且肺癌不常伴有低热。
B. 肺血管炎：这是一种涉及肺部血管的炎症性疾病。其症状可能包括呼吸困难、咳嗽和胸

痛，但 CT 表现与题目中的描述不匹配。
C. 慢性肺脓肿：这是一种由于感染引起的肺部化脓性炎症。虽然它可能导致空洞形成，但通常伴有高热和其他严重的感染症状，且 CT 表现也与题目描述不完全相符。
D. 肺结核：肺结核是一种由结核分枝杆菌引起的传染病，其典型症状包括长期咳嗽、低热、盗汗和体重减轻。CT 上，肺结核可能表现为多发小斑片状高密度影和空洞，与题目中的描述相符。
E. 肺曲霉菌病：这是一种由曲霉菌引起的肺部感染。其症状可能包括咳嗽、发热和呼吸困难，但 CT 上的表现与题目中的描述不匹配。
综上所述，考虑到患者的症状、低热和 CT 上的特定表现，最可能的诊断是肺结核。
因此，答案是 D.肺结核。
根据题干中的信息，患者 54 岁，咳嗽伴间断低热半年，胸部 CT 显示右上肺多发小斑片状高密度影，伴少许空洞，右肺上叶体积减小。这些症状和影像学表现最可能的诊断是肺结核。

解释如下：
1. **肺癌**：虽然肺癌可以引起咳嗽和胸部 CT 的异常，但通常不会伴有间断性低热和空洞形成，特别是多发小斑片状影像更不像肺癌的表现。
2. **肺血管炎**：肺血管炎常表现为咳嗽和肺部病变，但空洞形成和肺叶体积减小不是其典型特征。
3. **慢性肺脓肿**：慢性肺脓肿也可以表现为空洞病变，但通常伴有明显的脓性痰液和更严重的全身症状。
4. **肺结核**：肺结核最常见的症状是咳嗽和低热，CT 表现为小斑片状高密度影和空洞，同时肺叶体积减小也是肺结核的特征之一。
5. **肺曲霉菌病**：肺曲霉菌病可以有类似的影像学表现，但通常发生在免疫功能低下的患者中，并伴有更严重的全身症状。

综上所述，最可能的诊断是**D. 肺结核**。
88、 (A2 型题)
患者因手术行蛛网膜下腔麻醉后，无不适，意识清晰，切口少许液体渗出，应采取的体位是
A.低半坐位
B.高半坐位
C.15°~30° 头高脚低位
D.下肢抬高 15°~20°,头部和躯干抬高 20°~30°
E.平卧位
参考答案： E 您的答案：
图文解析： 【解析】蛛网膜下腔麻醉后应采取平卧位（E 对），以防止因脑脊液外渗致头痛。
【避错】腹部手术后多取低半坐卧位（A 错）或斜坡卧位，以减少腹壁张力。施行颈胸手术后，多采用高半坐位卧式（B 错），以便于呼吸及有效引流。施行颅脑手术后，如无休克或昏迷，可取 15°~30° 头高脚低斜坡卧位（C 错）。休克患者，应取下肢抬高 15°~20°,头部和躯干抬高 20°~30° （D 错）的特殊体位。
患者在接受蛛网膜下腔麻醉后，若无不适且意识清晰，出现切口少量液体渗出的情况，适宜的体位是为了减少脑脊液压力和促进患者的舒适度。选项 D，“下肢抬高 15°~20°,头部和躯干抬高 20°~30° ”提供了既能促进液体回流，又能保持脑脊液压力适中的体位。这种体位可以帮助减少脑脊液渗漏，并且有助于防止低脑脊液压症状的发生。

因此，最佳选择是 D.下肢抬高 15°~20°,头部和躯干抬高 20°~30°。
解析：
蛛网膜下腔麻醉是腰麻或脊椎麻醉的简称，即将局麻药物经腰椎间隙注入蛛网膜下腔，阻断部分脊神经的传导功能而引起相应支配区域的麻醉作用。蛛网膜下腔麻醉后，为避免脑脊液流失引起的低颅压性头痛，应去枕平卧 6～8 小时，即平卧位。
选项 A 低半坐位适用于心肺功能不全者，因膈肌下降，胸腔容积扩大，利于呼吸，使呼吸困难得到改善，同时腹腔脏器下移，利于健侧代偿，以减轻症状和有利于健肺的通气功能，与题干描述不符，故排除。
选项 B 高半坐位适用于颈、胸手术后或有严重的心肺疾病患者，由于膈肌下移，使胸腔容积扩大，减轻腹腔脏器对心肺的压迫，增加肺活量，有利于气体交换，使呼吸困难得到改善，可排除。
选项 C15°~30°头高脚低位适用于颅内高压时，可降低颅内压，预防脑水肿，不符合题意。
选项 D 下肢抬高 15°~20°，头部和躯干抬高 20°~30°即休克体位，用于休克病人，抬高下肢，有利于静脉血回流，增加心输出量，而头和躯干的抬高有利于呼吸，可排除。
综上所述，正确答案是：E.平卧位。
患者在蛛网膜下腔麻醉后，若切口少许液体渗出，但无不适且意识清晰，此时应采取的体位是：**A.低半坐位**。

低半坐位可以帮助减少蛛网膜下腔麻醉后可能的并发症，如头痛。通过这种体位，能够有效减少脑脊液从脊柱穿刺点的漏出，有助于防止低颅压综合征的发生，同时还能减轻切口液体渗出的影响。

其他选项的解析：
- **B.高半坐位**：通常用于某些特定手术后，但对于蛛网膜下腔麻醉后的病人，不是最佳选择。
- **C.15°~30°头高脚低位**：适用于一些需要减少颅内压的情况，但在蛛网膜下腔麻醉后的常规护理中并不常见。
- **D.下肢抬高 15°~20°,头部和躯干抬高 20°~30°**：这种体位对于某些休克或血流动力学不稳定的患者可能有帮助，但对于本题情况不适用。
- **E.平卧位**：通常在蛛网膜下腔麻醉后最初的短时间内使用，但长时间平卧可能会增加头痛的风险。

综上所述，最佳答案是：**A.低半坐位**。
89、 (A2 型题)
女，55 岁，反复腹痛，乏力，既往有乙肝病史 30 年。查体：神志清，肝肋下 3cm，腹部移动性浊音阴性。实验室：总胆红素 30μmol/L，ALB20g/L，PT19.1s，B 超：肝右前 4cm×3cm，实性，首选的如何治疗措施是
A.化疗
B.动脉栓塞
C.靶向治疗
D.手术
E.无水乙醇注射
参考答案： D 您的答案：
图文解析： 【解析】中老年女性，反复腹痛、乏力，有乙肝病史 30 年，肝脏肿大，总

胆红素升高（提示肝功能异常），ALB 降低，PT19.1s（PT 正常参考值范围：11～13s，凝血酶原时间 PT 超过正常值 3 秒为异常），B 超：肝右前叶 4cm×3cm 实性光团，考虑原发性肝癌。肝癌的治疗性切除术是目前治疗肝癌最有效的方法之一，应首选手术（D 对）。
【避错】肝癌对化疗（A 错）和放疗不敏感。动脉栓塞（B 错）用于治疗不可切除的肝癌或作为肝癌切除术后的辅助治疗。靶向治疗（C 错）主要应用于非小细胞肺癌中的腺癌患者。无水乙醇注射（E 错）主要用于治疗转移复发的肝癌。
根据这位 55 岁女性患者的病史和检查结果，她有 30 年乙肝病史，现在表现为反复腹痛，乏力，肝脏增大（肝肋下 3cm），并且在 B 超检查中发现了肝右前部 4cm×3cm 的实性肿块。实验室结果显示总胆红素 30μmol/L，白蛋白(ALB)20g/L，凝血酶原时间(PT)19.1 秒，这些结果提示肝功能受损和凝血功能不全。
考虑到乙肝长期感染会增加肝癌（尤其是肝细胞癌）的风险，这位患者的情况很可能与肝癌相关。因此，在选择治疗方法时，需要考虑癌症的大小、位置、数量以及患者的整体健康状况。
选项中的治疗方法包括：
- A. 化疗：通常用于晚期或无法手术的癌症治疗。
- B. 动脉栓塞：是一种局部治疗方法，通过堵塞肿瘤的供血来抑制其生长。
- C. 靶向治疗：针对肝癌特定分子或细胞途径的治疗。
- D. 手术：如果肿瘤局限并且患者的整体状况允许，手术可能是首选。
- E. 无水乙醇注射：主要用于小的肿瘤或作为其他治疗方法的辅助。
在这种情况下，首选的治疗措施通常是手术（选项 D），尤其是如果肿瘤没有明显扩散，大小和位置允许手术切除。手术可以提供治愈的机会，尤其是在肿瘤局限于肝脏时。然而，最终的治疗选择应该基于更全面的评估，包括肿瘤的确切性质、分期以及患者的整体健康状况。因此，我会选择 D. 手术作为首选的治疗措施。
解析：
中年女性患者，既往有乙肝病史 30 年，反复腹痛、乏力，为肝癌的典型临床表现，查体见肝脏肿大，实验室检查提示胆红素升高，提示肝功能下降，白蛋白降低，凝血酶原时间延长，均提示肝功能受损，B 超见肝右前有实质性肿块，结合患者既往乙肝病史及 B 超表现，该患者最可能的诊断为原发性肝癌。患者目前神志清楚，无腹水，考虑肝癌早期，对于肝癌早期的患者，应及时手术切除，以防止病情进展恶化。
选项 A 化疗多用于癌症晚期的患者，可延长生存期，该患者目前考虑肝癌早期，应行手术治疗，故不选。
选项 B 动脉栓塞，选项 E 无水乙醇注射，均属于介入治疗，适用于不宜手术的原发性肝癌或术后复发者，不作为肝癌的首选治疗方式，故一并排除。
选项 C 靶向治疗是近年对实体恶性肿瘤的主要治疗手段，可延长生存期，但对早期肝癌患者效果有限，故不选。
综上所述，正确答案是：D.手术。
根据题目中提供的信息，患者是一名 55 岁的女性，反复腹痛、乏力，并有 30 年的乙型肝炎病史。查体显示肝脏肋下 3 厘米，实验室检查显示总胆红素 30μmol/L，ALB 20g/L，PT 19.1 秒，B 超显示肝右前 4cm×3cm 的实性病变。

从这些信息来看，患者可能患有肝癌（肝细胞癌）。对于这种情况，治疗方案需要根据肿瘤的大小、位置、肝功能情况及患者的整体健康状况来决定。

根据患者的情况，肝脏功能较差（低白蛋白、高凝血酶原时间），肿瘤直径为 4cm×3cm，

这提示有可能为肝细胞癌并且可能不适合进行根治性手术。无水乙醇注射（Ethanol injection）是一种微创治疗方法，常用于小型肝癌患者，尤其是在肝功能较差或不适合手术的患者中。

因此，首选的治疗措施应该是：
E.无水乙醇注射
90、　(A2 型题)
男，28 岁，上腹胀痛不适，伴恶心呕吐 2 天，呕吐物为酸臭味的宿食，进食后缓解，常受凉后易发。该症状病因是
A.胃角溃疡
B.食管溃疡
C.十二指肠降部溃疡
D.胃体溃疡
E.十二指肠球部溃疡
参考答案：　E　您的答案：
图文解析：　【解析】青年男性，上腹胀痛不适、恶心呕吐，呕吐物为酸臭宿食（怀疑幽门狭窄或梗阻），饥饿时疼痛，进食后缓解，考虑为十二指肠溃疡怀疑并发幽门狭窄或梗阻。十二指肠球部溃疡（E 对）较易并发幽门梗阻。
【避错】十二指肠球部后壁溃疡更易出血；胃溃疡较十二指肠溃疡更易癌变；溃疡穿孔多发于胃小弯侧或十二指肠球部。
题目描述的是一位 28 岁男性患者，主诉上腹胀痛不适，伴恶心呕吐 2 天，呕吐物为酸臭味的宿食，且进食后症状缓解，经常在受凉后容易发作。根据这些症状，我们可以考虑上消化道溃疡的诊断。
选项 A（胃角溃疡）和 D（胃体溃疡）都是胃溃疡的不同部位，通常会导致进食后疼痛加剧。选项 B（食管溃疡）通常与吞咽痛和反流症状相关，而非特别强调进食后缓解。选项 E（十二指肠球部溃疡）和 C（十二指肠降部溃疡）是十二指肠溃疡的不同部位，这类溃疡常见的特点是餐后疼痛缓解。
根据患者的症状，特别是进食后疼痛缓解这一特点，更符合十二指肠溃疡的临床表现。十二指肠球部是十二指肠的初始部分，而十二指肠降部是其后的部分。鉴于十二指肠球部溃疡更常见，并且这种类型的溃疡在受凉等因素影响后易发，结合患者症状和常见病因学，最佳答案是 E.十二指肠球部溃疡。
解析：
青年男性患者，上腹胀痛不适，伴恶心呕吐，呕吐物为酸臭味的宿食，进食后缓解，常受凉后易发，考虑诊断为幽门梗阻，其最常见于十二指肠球部穿透性溃疡后。十二指肠球部穿透性溃疡后，由于局部炎性水肿及幽门平滑肌痉挛可导致暂时性梗阻，经胃肠减压、输液等保守治疗后可缓解，属炎症水肿性狭窄。随着溃疡的反复发作、局部瘢痕形成和收缩可导致幽门永久性狭窄，此时需外科手术治疗。
选项 A 胃角溃疡，选项 D 胃体溃疡，均多表现为餐后痛，且无幽门梗阻的风险，故可一并排除。
选项 B 食管溃疡常表现为吞咽疼痛和吞咽困难，与题干描述不符，故不选。
选项 C 十二指肠降部溃疡以夜间痛和饥饿痛为主要表现，发生幽门梗阻的风险远低于十二指肠球部，故不考虑。
综上所述，正确答案是：E.十二指肠球部溃疡。
根据题目描述，患者是一位 28 岁的男性，症状包括上腹胀痛不适，伴有恶心呕吐 2 天，呕吐物为酸臭味的宿食，进食后缓解，且常在受凉后易发作。根据这些症状，最佳的诊断是十

二指肠球部溃疡（E）。

解析如下：
- 十二指肠溃疡，尤其是十二指肠球部溃疡，常表现为上腹痛，特别是在进食后 2-3 小时内发作，且进食可以缓解症状。这与患者描述的进食后缓解相符合。
- 呕吐酸臭味的宿食提示胃内容物滞留，这是十二指肠溃疡常见的症状之一。
- 症状在受凉后易发作可能与神经调节和胃肠道功能有关，也常见于十二指肠溃疡患者。

其他选项的解析：
- 胃角溃疡和胃体溃疡的疼痛通常与进食无明显关系，且呕吐宿食的情况较少见。
- 食管溃疡主要表现为吞咽痛、胸骨后疼痛，与本例症状不符。
- 十二指肠降部溃疡较少见，且疼痛特点与十二指肠球部溃疡类似，但更少见呕吐酸臭宿食的情况。

因此，最佳答案是：
E. 十二指肠球部溃疡
91、 (A2 型题)
男，40 岁，腹胀、呕吐 3 天，呕吐物为宿食，既往十二指肠溃疡病史 10 年，为纠正患者可能存在的水电解质代谢紊乱和酸碱平衡失调。应适用的液体是
A.生理盐水
B.5%葡萄糖盐水
C.1.86%乳酸钠溶液+5%葡萄糖盐水
D.10%氯化钾+5%葡萄糖盐水
E.5%碳酸氢钠
参考答案： D 您的答案：
图文解析： 【解析】中年男性，既往十二指肠溃疡病史 10 年，腹胀、呕吐 3 天，可能发生等渗性缺水，应补充平衡液溶液。在纠正缺水后，排钾量会增加血清 K+浓度也因细胞外液量的增加而被稀释降低，血清钾会降低，应注意预防低钾血症的发生，故还应预防性补钾，因此该患者应该补充平衡盐溶液同时补钾（D 对）。
【避错】任何等渗性液体大量丢失所造成的血容量减少，短时间内均属等渗性脱水。临床上常见病因有：消化液急性丧失，如肠外瘘、大量呕吐、腹泻等；体液丧失在软组织内，如腹腔或腹膜后感染、肠梗阻、烧伤等。若胃肠道消化液的持续性丢失则会造成低渗性缺水。
在这个病例中，患者有十二指肠溃疡病史 10 年，出现腹胀和呕吐，呕吐物为宿食，表明可能存在胃排空延迟或梗阻。由于长期呕吐，患者可能发生了电解质失衡和酸碱平衡失调。通常情况下，呕吐会导致体内失去胃酸，引起代谢性碱中毒和电解质失衡，尤其是低钾血症和低氯血症。
对于这种情况，首要的治疗是纠正水电解质和酸碱平衡的紊乱。选择最适合的液体需要考虑患者的具体酸碱平衡和电解质状态。一般来说，如果存在代谢性碱中毒，使用含有氯化物的液体可以帮助纠正碱中毒和电解质失衡。
- A 项的生理盐水（0.9%氯化钠溶液）是一种等渗液，可以用于补充体液和纠正电解质失衡。
- B 项的 5%葡萄糖盐水（5%葡萄糖+0.9%氯化钠溶液）也能补充能量和电解质。
- C 项的 1.86%乳酸钠溶液+5%葡萄糖盐水是一种碱性溶液，一般用于纠正酸中毒。
- D 项的 10%氯化钾+5%葡萄糖盐水用于补充钾，但高浓度的钾盐使用需谨慎，以防过量导

致高钾血症。
- E 项的 5%碳酸氢钠是用于纠正代谢性酸中毒的碱性溶液。
考虑到患者的症状和可能的代谢性碱中毒，最合适的选项是需要补充氯化物帮助纠正碱中毒的液体。因此，A 项生理盐水可能是最适合的选择，因为它可以帮助纠正电解质失衡和轻微的碱中毒。所以，正确答案是 **A. 生理盐水**。
解析：
中年男性患者，既往十二指肠溃疡病史，现出现腹胀、呕吐，呕吐物为宿食，应高度怀疑幽门梗阻。长期反复呕吐可丢失大量胃液，其中含有大量的 H^+ 及 Cl^-，易导致低氯性碱中毒，治疗时需输注含氯溶液和酸性液体以纠正碱中毒，5%葡萄糖盐水既含氯又含钠，既可纠正碱中毒，又可纠正电解质紊乱，为治疗本例患者的理想溶液。
选项 A 生理盐水为等渗氯化钠溶液，纠正碱中毒效果有限，故不选。
选项 C“1.86%乳酸钠溶液+5%葡萄糖盐水”，选项 E“5%碳酸氢钠”，二者均为碱性溶液，会进一步加重碱中毒，故一并排除。
选项 D“10%氯化钾+5%葡萄糖盐水”中钾离子浓度过高，不宜直接输注，且患者并未出现低钾表现，无需额外补钾，故不考虑。
综上所述，正确答案是：B.5%葡萄糖盐水。
根据题目描述，该患者有十二指肠溃疡病史，且目前出现腹胀、呕吐 3 天，呕吐物为宿食，这提示有可能存在胃肠道梗阻的情况。由于长期呕吐，患者可能会出现水、电解质代谢紊乱和酸碱平衡失调。

患者长期呕吐，丢失了大量的氯离子和氢离子，容易出现代谢性碱中毒。因此，纠正代谢性碱中毒和补充氯离子是治疗的重点。

综上所述，最合适的液体是生理盐水（A），因为生理盐水（0.9%的氯化钠溶液）不仅可以补充水分，还能补充丢失的氯离子，从而纠正代谢性碱中毒。

正确答案是：
A. 生理盐水
92、 (A2 型题)
男，42 岁，腹痛，腹胀伴肛门停止排气排便 2 天。禁食，补液治疗，今晨突发腹痛加剧。既往行阑尾切除术 10 年余。查体：全腹压痛，反跳痛，肌紧张，肠鸣音消失。最 是
A.手术探查
B.持续性胃肠减压
C.解痉药物治疗
D.足量抗生素
E.空气灌肠
参考答案： A 您的答案：
图文解析： 【解析】中年男性，腹痛、腹胀、肛门停止排气排便（肠梗阻的表现），禁食补液治疗后腹痛加重（内科治疗无效，考虑手术治疗），有阑尾炎手术史（考虑术后肠粘连造成的粘连性肠梗阻）。查体：全腹压痛，反跳痛，肌紧张，肠鸣音消失。该患者出现腹膜炎表现且经内科治疗无效，考虑绞窄性肠梗阻，应行手术探查（A 对）。
【避错】有下列表现者，应考虑为绞窄性肠梗阻：（1）腹痛发作急骤，初始即为持续性剧烈腹痛；（2）病情发展迅速，早期出现休克；（3）有腹膜炎表现；（4）腹胀不对称，腹部有局部隆起；（5）呕吐出现早而频繁，呕吐物为血性，腹腔穿刺抽出血性液

体；（6）X 线检查见孤立扩大的肠袢；（7）经积极的非手术治疗体征无明显改善者。
这个病例描述了一个 42 岁男性患者，有腹痛、腹胀和停止排气排便 2 天的症状，既往有阑尾切除手术史。目前症状加剧，查体发现全腹压痛、反跳痛、肌紧张，且肠鸣音消失。这些临床表现暗示可能发生了急性肠梗阻或腹膜炎，需要紧急处理。
A 项（手术探查）在急性腹症、特别是怀疑腹膜炎或严重肠梗阻时，当保守治疗无效或情况紧急需要确定诊断并解除梗阻时是必要的。
B 项（持续性胃肠减压）虽然可以缓解症状，但不解决潜在的问题，通常作为手术前准备或辅助治疗手段。
C 项（解痉药物治疗）用于缓解平滑肌痉挛，但在急性腹症中并不适宜作为首选治疗，因为它可能掩盖病情的严重性。
D 项（足量抗生素）虽然对于感染性腹症是重要的，但在未明确感染源和没有手术治疗的情况下，并不能作为唯一的治疗手段。
E 项（空气灌肠）通常用于诊断和治疗轻至中度肠梗阻，但在急性、严重的情况下并不适宜，尤其是在存在腹膜炎或严重梗阻的风险时。
综合考虑，最合适的选择是 A.手术探查，因为患者的症状和体征表明可能存在急需解决的腹内问题，如肠梗阻或腹膜炎，这需要通过手术来诊断和治疗。
首先，我们需要仔细解读患者的病例信息。患者为 42 岁男性，主要症状为腹痛、腹胀，且已经持续 2 天没有排便排气。尽管已经进行了禁食和补液治疗，但今晨腹痛突然加剧。他的腹部查体结果显示全腹压痛、反跳痛、肌紧张和肠鸣音消失，这些都是腹膜刺激征的典型表现。结合既往阑尾切除手术史，我们可以初步怀疑可能存在肠梗阻，且目前已有腹膜炎体征，说明病情可能已经从单纯性肠梗阻发展到绞窄性肠梗阻。
接下来，我们逐一分析选项：
A. 手术探查：对于疑似绞窄性肠梗阻的患者，由于肠道可能已经出现了血运障碍，为避免肠道坏死、穿孔和继发性的腹膜炎，需要紧急手术治疗。这是首选的治疗方案。
B. 持续性胃肠减压：这一措施通常用于单纯性肠梗阻的保守治疗，通过胃肠减压来减轻肠道内的压力。但在绞窄性肠梗阻的情况下，由于存在肠道血运障碍，仅仅进行胃肠减压是不够的。
C. 解痉药物治疗：这类药物主要用于缓解平滑肌痉挛，但在绞窄性肠梗阻的情况下，药物治疗并不能解决根本问题。
D. 足量抗生素：虽然抗生素可以预防或治疗继发性感染，但并不能解决肠梗阻的根本问题。
E. 空气灌肠：这种方法通常用于治疗小儿肠套叠，对于成人绞窄性肠梗阻并不适用。
综上所述，考虑到患者病情可能已经从单纯性肠梗阻发展到绞窄性肠梗阻，且已出现腹膜炎体征，因此应首选手术治疗。所以，正确答案是：A. 手术探查。
这道题目描述了一位 42 岁男性患者，症状包括腹痛、腹胀，并伴有肛门停止排气排便 2 天。患者禁食、补液治疗后今晨突发腹痛加剧。查体发现全腹压痛、反跳痛、肌紧张，肠鸣音消失。这些体征提示可能存在急性腹膜炎，可能由于肠穿孔或肠梗阻导致的。

考虑到患者的既往手术史（10 年前的阑尾切除术），存在肠粘连的可能性，这进一步增加了肠梗阻的可能性。而突发腹痛加剧及全腹压痛、反跳痛和肌紧张等体征非常符合急性腹膜炎的表现。

在这种情况下，最紧急和有效的处理方法是立即进行手术探查（A）。手术探查可以明确病因，进行必要的治疗（如解除肠梗阻、修补肠穿孔等），避免病情进一步恶化。其他选项（如持续性胃肠减压、解痉药物治疗、足量抗生素、空气灌肠）都不能根本解决患者的急性情况，

可能导致延误治疗，增加并发症风险。

因此，最佳答案是：

A. 手术探查

93、 (A2 型题)

患儿，4 岁。发现左上腹包块如拳头大小，质硬，可活动，无压痛，小便正常。应首先考虑

A.左肾积水

B.左肾母细胞瘤

C.脾大

D.胰腺囊肿

E.左肾结核

参考答案： B 您的答案：

图文解析： 【解析】肾母细胞瘤好发于 5 岁以下的儿童，腹部肿块为最常见的症状，表面光滑，中等硬度，无压痛，有一定活动度。

在这个案例中，考虑到患儿年龄（4 岁）和症状（左上腹包块如拳头大小，质硬，可活动，无压痛，小便正常），最可能的诊断是 **B.左肾母细胞瘤**。

解释如下：

- A.左肾积水（Hydronephrosis）通常会伴有疼痛或小便问题。
- B.左肾母细胞瘤（Wilms tumor）是儿童最常见的恶性肾脏肿瘤，其特点是腹部的可触及包块，通常不痛，与此案例相符。
- C.脾大（Splenomegaly）可能因多种原因引起，但通常不表现为一个可活动的硬块。
- D.胰腺囊肿（Pancreatic cyst）在儿童中相对罕见，且通常不表现为硬性可移动包块。
- E.左肾结核（Renal tuberculosis）非常罕见，通常伴有系统性症状，如发热、夜间出汗和体重减轻。

因此，根据提供的信息和临床表现，最可能的诊断是左肾母细胞瘤。

解析：

4 岁患儿，左上腹质硬包块，活动度可，无其他异常表现，结合患者症状和体征，对该患儿最可能的诊断为左肾母细胞瘤。肾母细胞瘤是小儿最常见的恶性肿瘤，多在 5 岁前发病，偶见于大龄儿童及成人，最常见的症状是腹部肿块，且肿块表面光滑、质硬、无压痛，且可随呼吸活动。

选项 A 左肾积水可有腰腹部疼痛、排尿困难、血尿等症状，一般腹部包块不明显，故不选。

选项 C 脾大可见于某些病毒感染、细菌感染、立克次体感染、寄生虫感染等，一般会出现原发病的相应症状，而题干中未提及，故排除。

选项 D 胰腺囊肿多继发于胰腺炎或上腹部外伤后，主要表现为上腹疼痛，饱满感，并可向背部放射，伴恶心、呕吐、食欲下降，与题干描述不符，故不选。

选项 E 左肾结核多见于青壮年，典型表现为膀胱刺激症状，伴终末血尿，可有低热、盗汗等结核中毒症状，一般不会出现腹部包块，故排除。

综上所述，正确答案是：B.左肾母细胞瘤。

根据题目描述，患儿 4 岁，左上腹包块如拳头大小，质硬，可活动，无压痛，小便正常。结合这些临床表现，最可能的诊断是：

B. 左肾母细胞瘤

左肾母细胞瘤（又称为 Wilms 瘤）是儿童中最常见的肾脏恶性肿瘤，常表现为腹部包块。包块通常是无痛的，质硬，可活动，且大小如拳头。由于小便正常，排除泌尿系统的急性病变，如左肾积水或左肾结核。脾大和胰腺囊肿也会引起腹部包块，但结合患儿的年龄和无压痛的特点，左肾母细胞瘤是最可能的诊断。

因此，最佳答案是：

B. 左肾母细胞瘤
94、 (A2 型题)
男性，65 岁。进行性排尿困难已 1 年，不能自行排尿 8 小时，膀胱膨隆，轻压痛。首选的治疗方法应是
A.针灸
B.耻骨上膀胱穿刺
C.导尿并保留导尿管
D.耻骨上膀胱造瘘
E.药物治疗
参考答案： C 您的答案：
图文解析： 【解析】患者可能有前列腺疾病导致膀胱尿潴留，紧急处理是解决膀胱内压力，在病症没有根除前，保留尿管。
在这个病例中，患者是一位 65 岁的男性，表现为进行性排尿困难已有 1 年，最近不能自行排尿 8 小时，伴有膀胱膨隆和轻度压痛。这些症状表明患者可能存在急性尿潴留，这是一种需要立即处理的紧急情况。
对于这种情况，首选的治疗方法是立即解除尿潴留，通常通过导尿来实现。因此，选项 C（导尿并保留导尿管）是最合适的选择。保留导尿管可以暂时缓解尿潴留的问题，并给医生时间来评估患者的情况和计划进一步的治疗策略。其他选项，如针灸、膀胱穿刺、造瘘或单纯的药物治疗，不适用于急性尿潴留的立即缓解。
因此，最佳答案是 **C. 导尿并保留导尿管**。
这是一道涉及泌尿系统疾病的题目。我们需要分析患者的症状，确定疾病的类型，然后选出最合适的治疗方案。
理解题目背景信息：老年男性患者，进行性排尿困难已一年，突然不能自行排尿 8 小时，膀胱膨隆且有轻压痛。这些症状强烈提示良性前列腺增生导致的急性尿潴留。
理解问题的核心：我们需要基于患者的症状，判断哪种治疗方法是首选。
接下来，我们逐一分析每个选项：
A 选项（针灸）：虽然针灸在某些情况下可能对排尿有帮助，但不是急性尿潴留的首选治疗方法。
B 选项（耻骨上膀胱穿刺）：这是一种紧急处理措施，通常用于导尿失败或患者情况危急时，不是首选。
C 选项（导尿并保留导尿管）：对于急性尿潴留，首要任务是迅速有效地排空膀胱。导尿是迅速、简便且有效的方法，同时保留导尿管可以确保膀胱持续排空，避免再次尿潴留。
D 选项（耻骨上膀胱造瘘）：这通常是在导尿失败或患者情况特殊时采用的备选方案，不是首选。
E 选项（药物治疗）：虽然药物治疗对于慢性症状管理可能有效，但在急性尿潴留的情况下，药物治疗起效慢，不能迅速解决问题。
综上所述，考虑到患者需要迅速有效地排空膀胱，同时避免进一步的并发症，导尿并保留导

尿管是最直接、最有效的首选治疗方法。
因此，答案是 C.导尿并保留导尿管。
根据患者的年龄和症状表现，男性，65 岁，有 1 年的进行性排尿困难，现在不能自行排尿 8 小时，膀胱膨隆并有轻压痛，这些症状提示可能是急性尿潴留。急性尿潴留的首要处理措施是立即缓解膀胱的压力，以防止进一步的膀胱和肾脏损伤。

在这种情况下，最合适的首选治疗方法是导尿并保留导尿管，这可以立即缓解膀胱的压力，防止进一步的并发症，并有助于诊断和治疗引起排尿困难的潜在原因。因此，最佳答案是：

C.导尿并保留导尿管
95、　(A2 型题)
血尿患者，膀胱镜见膀胱右侧壁有一 2.5cm×1.0cm 新生物，有蒂，距右输尿管口 3cm，活检诊断移行细胞癌 T1 期。首选的治疗方法是
A.膀胱灌注化疗
B.经尿道膀胱肿瘤电切术
C.膀胱部分切除术
D.膀胱全切除
E.放疗
参考答案：　B　您的答案：
图文解析：　【解析】原则上 Ta、T1 及局限性 T2 期的肿瘤保留膀胱的手术，该患者肿瘤有蒂，则 B 选项较为适合。
在这种情况下，针对血尿患者，其膀胱镜检查显示膀胱右侧壁有一个 2.5cm×1.0cm 的带蒂新生物，且活检诊断为移行细胞癌 T1 期，最适合的治疗方法是选择 B.经尿道膀胱肿瘤电切术。
T1 期移行细胞癌意味着肿瘤仅侵犯膀胱的内层，还没有深入到肌层。在这个阶段，经尿道膀胱肿瘤电切术（TURBT）是首选治疗方法。这种手术通过尿道切除肿瘤，能够准确评估肿瘤的侵袭深度并清除可视肿瘤。这种方法适用于浅层膀胱癌，尤其是 T1 期或更低级别的病变。其他选项如膀胱灌注化疗、膀胱部分切除术、膀胱全切除或放疗通常是对于更高级别或侵袭性更强的肿瘤而考虑的治疗方法。因此，对于这个病例，最合适的选择是 B.经尿道膀胱肿瘤电切术。
解析：
患者血尿，膀胱镜检见膀胱右侧壁带蒂新生物，病理活检诊断为移行细胞癌 T1 期，T1 期指肿瘤侵入上皮下结缔组织，但未达到肌层。Ta、T1 期肿瘤行经尿道膀胱肿瘤切除术（TURBt），是临床最常用的术式，创伤小、恢复快，可以反复进行，不仅可以用于肿瘤的切除，还可以用于了解肿瘤的病理分级、临床分期，以制定下一步的治疗方案。
选项 A 膀胱灌注化疗主要用于原位癌、细胞分化良好的非肌层浸润性膀胱癌的术后辅助治疗以及晚期的肌层浸润性膀胱癌的术前新辅助治疗，不作为首选治疗方式，故不选。
选项 C 膀胱部分切除术适用于 T2、T3 期低级别、局限的膀胱肿瘤或病人不能耐受膀胱全切者，与题意不符。
选项 D 膀胱全切除术适用于多发、反复发作、分化不良、非肌层浸润性膀胱癌或高级别的原位癌以及肌层浸润性膀胱癌，与题干描述不符。
选项 E 放疗效果不及根治性膀胱全切术，仅起解除梗阻、缓解症状的效果，多用于失去根治性手术机会的病人，故排除。
综上所述，正确答案是：B.经尿道膀胱肿瘤电切术。

对于血尿患者，经膀胱镜检查发现膀胱右侧壁有 2.5cm×1.0cm 的新生物，并且活检诊断为移行细胞癌 T1 期（肿瘤仅限于黏膜下层），最佳的治疗方法是经尿道膀胱肿瘤电切术（TURBT）。TURBT 是一种微创手术，可以有效地切除肿瘤，同时保留膀胱功能。

因此，首选的治疗方法是：

B. 经尿道膀胱肿瘤电切术
96、 (A2 型题)
男性，25 岁。骑跨式会阴部外伤后不能自行排尿，会阴部青紫并阴囊血肿，导尿管不能插入膀胱。多为
A.阴囊损伤
B.阴茎部尿道损伤
C.尿道球部损伤
D.后尿道损伤
E.膀胱损伤
参考答案： C 您的答案：
图文解析： 【解析】男性尿道以生殖膈为界分前后尿道，前尿道包括球部和阴茎部，后部包括前列腺部和膜部。会阴骑跨伤时多发生前尿道损伤，将尿道挤向耻骨联合下方，引起球部损伤。
这道题描述的是一个男性因为跨式会阴部外伤后出现了不能自行排尿，会阴部青紫和阴囊血肿，且导尿管不能插入膀胱的情况。这些症状指向了泌尿系统的损伤，特别是尿道的损伤。选项 A“阴囊损伤”虽然可能伴随此类外伤，但仅以阴囊损伤通常不会直接导致无法插入导尿管的问题。选项 E“膀胱损伤”可能导致排尿困难，但在会阴部外伤的情况下，直接导致膀胱损伤而不影响尿道的可能性较小。因此，最可能的损伤位置是尿道，特别是骑跨伤常见的部位。
考虑到不能自行排尿和导尿管无法插入膀胱的症状，以及伤害的机制和位置，最有可能的是尿道受到了损伤。尿道可以分为前尿道和后尿道，其中球部尿道属于前尿道的一部分。由于伤害是由骑跨式会阴部外伤造成的，更有可能是损伤到了较靠前的尿道部分。
因此，最合适的答案是：
B. 阴茎部尿道损伤
这个答案是基于伤害的性质和位置，以及伴随的临床症状来判断的。阴茎部尿道损伤在此类机制下是常见的，而且这种损伤能解释为何导尿管无法正常插入膀胱。
解析：
青年男性患者，会阴部外伤史，不能自行排尿，会阴部青紫并阴囊血肿，导尿管不能插入膀胱，结合患者病史和临床表现，该患者首先考虑尿道球部损伤。男性前尿道外伤多发生于球部，这段尿道固定在会阴部。会阴部骑跨伤时会将尿道挤向耻骨联合下方，而引起球部损伤。
选项 A 阴囊损伤主要表现为阴囊肿胀、疼痛，伴局部淤血、青紫或血肿，严重者伤后即出现会阴部血肿、尿外渗、尿道出血等症状，但患者一般可自行排尿，与题干中患者症状不符，故排除。
选项 B 阴茎部尿道损伤多发生于尿道悬垂部与球部连接处，由于这段尿道不固定，活动度较大，因此受伤机会较小，故不选。
选项 D 后尿道损伤多见于骨盆挤压伤，常合并骨盆骨折，与题意不符，故排除。
选项 E 膀胱损伤的患者多伴有排尿困难、血尿和腹痛，与患者表现不符，可排除。
综上所述，正确答案是：C.尿道球部损伤。

根据题干提供的信息，25 岁男性骑跨式会阴部外伤后出现不能排尿、会阴部青紫和阴囊血肿，导尿管无法插入膀胱，这些症状和体征提示可能的损伤部位应为尿道球部。骑跨式会阴部外伤常见于会阴部撞击，容易造成尿道球部损伤。

因此，最佳答案是：

C. 尿道球部损伤

97、 (A2 型题)

男性，50 岁。因胃癌伴重度贫血入院，既往体健，无输血史。术前化验 Hb56g/L，为纠正贫血。下列最合适的输血治疗是

A.输全血

B.输浓缩红细胞

C.输洗涤红细胞

D.输去白细胞的红细胞

E.输冷冻红细胞

参考答案： B 您的答案：

图文解析： 手术前输入浓缩红细胞可以纠正贫血，低蛋白血症，浓缩红细胞可以提高携氧能力。

在这种情况下，患者有重度贫血（Hb 56g/L），需要输血以提高血红蛋白水平。最合适的输血治疗取决于患者的具体情况和需求，但通常情况下，选择浓缩红细胞（RBC）是因为它们可以有效提高血红蛋白水平，同时减少体积负荷。

- **A. 输全血**：全血包含红细胞、白细胞、血小板和血浆。通常在大量出血需要补充血容量和血细胞成分时使用，对于这位患者来说，全血可能不是最佳选择，因为主要问题是重度贫血。

- **B. 输浓缩红细胞**：浓缩红细胞主要用于治疗贫血，提高血红蛋白和红细胞含量，对于需要增加氧运输能力但不需要额外血浆的患者来说，是最合适的选择。

- **C. 输洗涤红细胞**：洗涤红细胞是去除了大部分血浆和白细胞的红细胞，常用于对血浆蛋白过敏的患者或避免特定免疫反应的情况，不是本例的首选。

- **D. 输去白细胞的红细胞**：去白细胞的红细胞主要用于减少输血相关的免疫反应和传染性问题。虽然比全血更加适合，但不是最优选择。

- **E. 输冷冻红细胞**：冷冻红细胞主要用于长期储存特定血型的红细胞，适用于罕见血型的患者，不适用于此案例。

因此，最合适的选项是 **B. 输浓缩红细胞**。这种类型的输血旨在提高患者的血红蛋白水平，同时避免过多的液体负荷，对于重度贫血患者来说是最佳选择。

解析：

中年男性患者，胃癌伴重度贫血。术前化验提示血红蛋白明显降低，提示小细胞低色素性贫血，即缺铁贫。对于该患者而言，目前急需纠正贫血，因此输浓缩红细胞为最合适的输血治疗。

选项 A 输全血主要用于治疗各种原因导致的大出血，该患者并未出现出血，故排除。

选项 C 输洗涤红细胞主要用于对白细胞凝集素有发热反应者及肾功能不全不能耐受库存血中高钾者，与该患者症状不符，故不选。

选项 D 输去白细胞的红细胞主要用于多次输血后产生白细胞或血小板抗体，引起发热等输血反应的个体，或者预期需要反复输血的患者，如再障、白血病、骨髓增生异常综合征、造血干细胞移植后等患者，以防止产生白细胞抗体，该患者既往无输血史，故排除。

选项 E 输冷冻红细胞解冻时，需加入一定量的氯化钠注射液，以防由于细胞内冰晶融化后水被排出而细胞萎缩，导致红细胞死亡，现已很少应用，可排除。
综上所述，正确答案是：B.输浓缩红细胞。
根据患者的病情和现有的医学指南，考虑到他因胃癌伴重度贫血住院且没有输血史，最适合的输血治疗方案是输浓缩红细胞。浓缩红细胞的输注可以有效提高患者的血红蛋白水平，改善贫血症状，同时避免输全血可能带来的过量液体负荷和其他潜在风险。以下是各选项的解释：

A. 输全血：全血输注包含红细胞和血浆，但对于单纯纠正贫血来说，不需要输注血浆部分，且全血输注的液体负荷较大，不是首选。
B. 输浓缩红细胞：浓缩红细胞是首选，因为它可以有效提高血红蛋白水平，减少不必要的液体负荷。
C. 输洗涤红细胞：主要用于对血浆蛋白过敏或有反复输血反应的患者，不是一般情况下的首选。
D. 输去白细胞的红细胞：主要用于对白细胞有免疫反应的患者或预防输血相关性白细胞反应的情况，也不是常规的首选。
E. 输冷冻红细胞：通常用于罕见血型或需要长期保存血液的情况，非紧急纠正贫血的常规选择。

因此，最适合的选项是：

B. 输浓缩红细胞
98、 (A2 型题)
女性，59 岁。因患乳腺瘤在硬膜外麻醉下施行根治术，出血较多，即输“AB”型红细胞悬液 2 个单位，当输入 20ml 左右时出现畏寒、胸闷，胸背疼痛，呼吸急促，脉搏加 降，立即停止输血，经处理 1 小时体温升至 39℃,排尿一次，呈浓茶样，量少。最可能的输血不良反应是
A.非溶血性发热性输血反应
B.输血相关的急性肺损伤
C.即发性的溶血性输血反应
D.细菌污染反应
E.过敏反应
参考答案： C 您的答案：
图文解析： 排尿一次，呈浓茶样，量少，提示血红蛋白尿，提示溶血反应。
这位 59 岁女性在乳腺瘤根治术中接受输血后，出现了畏寒、胸闷、胸背疼痛、呼吸急促和脉搏加快的症状，随后体温升高至 39℃，并且排尿量少，尿色呈浓茶样。这些症状表明了一种急性且严重的输血反应。根据所描述的症状，尤其是呈浓茶样的尿，这通常指示溶血事件，即红细胞被破坏，释放血红蛋白进入血液循环，最终由肾脏排出。
因此，最可能的输血不良反应是 C.即发性的溶血性输血反应。这种类型的输血反应发生迅速，通常是由于 ABO 血型不匹配导致的，会引起患者出现急性症状，如畏寒、发热、胸痛、背痛、呼吸急促以及尿液颜色的变化。
解析：
患者老年女性，乳腺瘤手术时出血较多，输入红细胞悬液后出现畏寒、胸闷、胸背部疼痛、呼吸急促、脉搏加快，体温升高、尿呈浓茶样等症状，考虑诊断输血相关的急性肺损伤。输

血相关的急性肺损伤是由于供血者血浆中存在白细胞凝集素或人类白细胞抗原特异性抗体所致，当这些异质血浆蛋白进入受血者体内后，与受血者体内的白细胞发生抗原抗体反应，导致白细胞凝集并释放炎性介质，造成肺毛细血管损伤，进而引发急性肺损伤。临床上可出现急性呼吸困难、双侧肺水肿和低氧血症，同时可伴有发热和低血压。

选项 A 非溶血性发热性输血反应是指输血中或输血后引起的体温升高，发热反应是最常见的输血反应，但一般不会出现呼吸困难，故不选。

选项 C 即发性的溶血性输血反应是由于输入血型不合导致的溶血反应，该反应发病急，进展快，表现为输入少量异型血后立即出现沿输血静脉的红肿、疼痛、寒战、高热、呼吸困难、腰背酸痛、心率加快乃至血压下降、休克，随之出现血红蛋白尿和黄疸，严重者可出现少尿、无尿及急性肾衰，与该患者症状不符，故排除。

选项 D 细菌污染反应若污染的细菌毒力较小，数量较少时，可仅有全身感染症状，若污染的细菌数量多、毒力大，则可立即出现内毒素性休克和 DIC，与本题患者症状不符，故不选。

选项 E 过敏反应多发生在输血数分钟后，表现为局限性或全身性瘙痒或荨麻疹，严重者可出现支气管痉挛，呼吸困难，休克，甚至死亡，但不会出现尿色改变，与题意不符。

综上所述，正确答案是：B.输血相关的急性肺损伤。

根据患者的症状描述，最可能的输血不良反应是即发性的溶血性输血反应。该反应的特征包括：

1. 患者在输血过程中或输血后不久出现畏寒、发热。
2. 胸闷、胸背疼痛、呼吸急促。
3. 脉搏变化。
4. 体温升高。
5. 排尿异常（浓茶样尿，提示血红蛋白尿）。

这些症状符合即发性溶血性输血反应的典型表现，因此答案是：

C. 即发性的溶血性输血反应

99、 (A2 型题)

男性，50 岁。近一月来常有刺激性咳嗽，无发热，应用止咳剂及抗生素治疗无效。X 线摄片示：右肺下叶 4cm×3.5cm 块状影。应考虑的疾病是

A.支气管肺炎

B.肺结核

C.肺癌

D.肺脓肿

E.支气管腺瘤

参考答案： C 您的答案：

图文解析： 右肺下叶 4cm×3.5cm 块状影提示占位。“刺激性”咳嗽符合肺癌诊断。

在这种情况下，50 岁男性患者出现持续的刺激性咳嗽，无发热，对止咳剂和抗生素治疗无效，X 线摄片显示右肺下叶有 4cm×3.5cm 的块状影。根据这些症状和 X 线结果，最可能的诊断是肺癌（选项 C）。这是因为：

- 持续性咳嗽和对常规治疗无效提示可能是恶性肿瘤。
- X 线摄片上的块状影通常提示肿瘤存在，尤其是在无发热和感染征象的情况下。
- 肺癌在中老年人中较为常见，特别是在有长期吸烟史的个体中。

虽然支气管肺炎（A）、肺结核（B）、肺脓肿（D）和支气管腺瘤（E）也可以表现为胸部 X

线的块状影，但根据所描述的临床症状和 X 线特征，肺癌是最可能的诊断。因此，正确答案应该是 C.肺癌。
解析：
中老年男性患者，刺激性咳嗽一月余，止咳剂及抗生素治疗无效，影像学检查示右肺下叶块状影，根据其临床表现及影像学特点，应高度怀疑肺癌。
选项 A 支气管肺炎起病急骤或迟缓，多数发病前先有轻度上呼吸道感染，轻者先有流涕、轻咳、低热、纳差，之后突然高热，咳嗽加剧、气促而发病，咳嗽较频，早期为刺激性干咳，极期咳嗽反而减轻，恢复期咳嗽有痰，该患者并无上述表现，故不选。
选项 B 肺结核起病缓慢，病程较长，有低热、倦怠、食欲不振、咳嗽及少量咯血，与题干描述不符，故不考虑。
选项 D 肺脓肿临床特征为高热、咳嗽和咳出大量脓臭痰，胸部 X 线影像显示有一个或多发的含气液平面的空洞，与题干不符，故排除。
选项 E 支气管腺瘤是一种良性肿瘤，生长缓慢，X 线检查可呈现圆形致密阴影或肿瘤引起的支气管阻塞征象，与题意不符，故不选。
综上所述，正确答案是：C.肺癌。
根据题干描述，50 岁男性，近一月来常有刺激性咳嗽，无发热，应用止咳剂及抗生素治疗无效，X 线摄片示右肺下叶 4cm×3.5cm 块状影。

分析如下：
- 支气管肺炎通常伴有发热、咳痰等症状，且 X 线表现为斑片状阴影，持续时间短，抗生素治疗通常有效。
- 肺结核常伴有咳嗽、痰中带血、夜间盗汗、体重减轻等症状，X 线表现为上叶多见，局部斑片或空洞影。
- 肺癌常见于中老年人，症状可以是咳嗽（包括刺激性咳嗽）、痰中带血、胸痛、呼吸困难等，X 线表现为块状影或结节影。
- 肺脓肿通常伴有发热、咳大量脓痰，X 线表现为含气液平面的空腔。
- 支气管腺瘤较少见，症状可以是咳嗽、痰中带血，但 X 线表现多为边界清楚的小结节影。

结合该患者的症状（刺激性咳嗽，无发热，抗生素治疗无效）和影像学表现（右肺下叶 4cm×3.5cm 块状影），最可能的诊断是肺癌。

所以，最正确的答案是：
C. 肺癌
100、 (A2 型题)
男性，65 岁。皮肤巩膜黄染进行性加重 1 个月来诊。自述尿色深黄，大便灰白色。查体：扪诊胆囊无肿大，Murphy 征阴性，腹部未扪及肿块。诊断首先考虑为
A.胰头癌
B.胆总管下端癌
C.乏特壶腹癌
D.肝门部胆管癌
E.十二肠腺癌
参考答案： D 您的答案：
图文解析： 老年男性患者，皮肤巩膜黄染进行性加重，尿色深黄，大便灰白色，查体：扪诊胆囊无肿大，Murphy 征阴性，腹部未扪及肿块，提示病变位于胆管上端，首选考虑为肝

门部胆管癌。
这位 65 岁男性患者出现进行性加重的皮肤及巩膜黄染、尿色深黄、大便灰白色，这些症状表明患者存在梗阻性黄疸。在没有胆囊肿大和腹部肿块的情况下，最可能的原因是胆道的恶性梗阻。
A. 胰头癌通常会引起梗阻性黄疸，因为胰头肿瘤可压迫胆总管。
B. 胆总管下端癌也会引起梗阻性黄疸，但多伴有胆囊肿大（Courvoisier 征阳性）。
C. 乏特壶腹癌（Ampulla of Vater cancer）同样可以引起梗阻性黄疸。
D. 肝门部胆管癌（Klatskin 瘤）会引起梗阻性黄疸，但往往不会导致胆囊显著肿大。
E. 十二肠腺癌一般不是梗阻性黄疸的首要考虑诊断。
考虑到这些因素和症状，最可能的诊断是胰头癌（A），因为它是引起梗阻性黄疸最常见的原因之一，特别是在没有明显胆囊肿大和腹部肿块的情况下。因此，最佳选择是 A.胰头癌。
首先，我们分析题干。
患者老年男性，主要症状为皮肤巩膜黄染进行性加重，尿色深黄，大便灰白色，这些都是典型的梗阻性黄疸表现。胆囊无肿大，Murphy 征阴性，且腹部未扪及肿块，这些体征可以帮助我们进一步缩小诊断范围。
接下来，我们逐项分析选项：
A. 胰头癌：胰头癌可以压迫或浸润胆总管，导致梗阻性黄疸。然而，胰头癌通常在晚期才能导致明显的黄疸，并且往往伴有腹部肿块和胆囊肿大，这与题干描述不符。
B. 胆总管下端癌：胆总管下端癌也会导致梗阻性黄疸，但通常会导致胆囊肿大，因为癌肿阻塞了胆汁流向十二指肠的通路，而胆汁仍然不断分泌到胆囊中，导致胆囊膨胀。这与患者的体征不符。
C. 乏特壶腹癌：乏特壶腹癌同样可以引起梗阻性黄疸，但由于其位置靠近十二指肠乳头，也可能导致胆囊肿大，这同样与患者的临床表现不符。
D. 肝门部胆管癌：肝门部胆管癌位于肝脏内胆管的起始部，这里的阻塞会导致胆汁无法流入胆总管和胆囊，因此胆囊不会肿大。这与患者的临床表现相吻合，即进行性黄疸而无胆囊肿大。
E. 十二指肠腺癌：虽然十二指肠腺癌在晚期可能压迫或浸润胆总管导致黄疸，但其主要症状通常与肠道梗阻或出血有关，且不一定导致胆囊肿大，但这并不是其典型表现，因此可能性相对较小。
综上所述，考虑到患者的主要症状为进行性黄疸，尿色深黄，大便灰白色，且胆囊无肿大，最可能的诊断为肝门部胆管癌。
因此，正确答案是 D.肝门部胆管癌。
根据题干描述，该患者为 65 岁男性，出现了进行性加重的皮肤和巩膜黄染 1 个月，尿色深黄，大便灰白色，这些症状提示患者存在胆汁淤积。查体结果显示胆囊无肿大，Murphy 征阴性，腹部未触及肿块。

考虑到患者的年龄和症状，这些体征提示胆管系统的疾病可能性较大。以下是各个选项的分析：

A. **胰头癌**：胰头癌常导致胆总管梗阻，进而引起黄疸和大便颜色变浅。不过，胰头癌常伴有体重减轻和腹痛，并且有时能够扪及肿块。

B. **胆总管下端癌**：胆总管下端癌会导致胆管完全或部分梗阻，引起进行性加重的黄疸和灰白色大便。该患者的症状和体征符合这一疾病表现。

C. **乏特壶腹癌**：乏特壶腹癌会引起类似于胆总管下端癌的症状，因其位置邻近胆总管下端，会引起梗阻性黄疸、尿色深黄和灰白色大便。

D. **肝门部胆管癌**：肝门部胆管癌通常会引起进行性黄疸，但常常伴有右上腹疼痛，可能会有肝肿大，而此患者没有这些体征。

E. **十二指肠腺癌**：十二指肠腺癌较少引起胆管梗阻性黄疸，主要症状多为消化道症状如腹痛、恶心等，不太符合该患者的表现。

综上所述，结合患者的症状和体征，最可能的诊断是 **乏特壶腹癌** 或 **胆总管下端癌**。但考虑到乏特壶腹癌引起的症状常常更为典型和明显，故最优选择为：

C. **乏特壶腹癌**
101、 (A2 型题)
女性，35 岁。便血并排便不尽感半月就诊，既往有内痔病史。首选的检查方法是
A.大便潜血试验
B.直肠指检
C.直肠镜检
D.结肠镜检
E.钡剂灌肠检查
参考答案： B 您的答案：
图文解析： 首先考虑该患者内痔复发，主要靠肛门直肠指检。
在这种情况下，首选的检查方法应该是结肠镜检（选项 D）。这位 35 岁女性患者有便血和排便不尽的症状，虽然有内痔病史，但是这些症状可能指示更严重的结直肠疾病，如息肉或结直肠癌。结肠镜检可以直接观察结直肠的内部情况，检查是否有病变，如息肉、炎症或肿瘤，并可进行活检。因此，结肠镜检是诊断这类症状最详细且直接的方法。
解析：
青年女性患者，既往有内痔，现出现便血伴排便不尽感，为明确便血原因以及内痔的严重程度，应首选简单、易行的直肠指检。直肠指检可以发现肛门、直肠有无病变和病变的性质，80%以上的直肠癌可以在直肠指检时触及，故直肠指检是简单而重要的临床检查方法，对及早发现直肠癌意义重大。
选项 A 大便潜血试验是测定消化道出血的一种方法，主要用于检验肉眼不可见的少量出血，阳性说明粪便中含有红细胞，但不能确定出血部位，故不选。
选项 C 直肠镜检、选项 D 结肠镜检均可以确定出血部位，但直肠镜检和结肠镜检操作复杂，价格昂贵，不作为首选，故一并排除。
选项 E 钡剂灌肠检查可发现结肠的微小病变，但对直肠病变的诊断价值不大，且操作复杂，不作为首选，故不考虑。
综上所述，正确答案是：B.直肠指检。
根据患者的症状（便血和排便不尽感）以及既往有内痔病史，最首选的检查方法是能够直接观察和评估直肠及下段结肠的情况，以明确出血来源和排便不尽感的原因。

最佳答案是：

D. 结肠镜检

原因如下：

1. **便血和排便不尽感**是可能由结直肠病变引起的症状，如内痔、息肉、炎症性肠病或肿瘤。
2. **结肠镜检**可以全面检查整个结肠和直肠，是最有效的检查方法，能够直接观察病变部位并进行活检，以排除或确诊病变。
3. **直肠指检**虽然是基本的检查，但其范围有限，无法全面评估结肠和直肠的情况。
4. **大便潜血试验**主要用于筛查是否存在隐血，不能确定出血的具体来源。
5. **直肠镜检**仅能检查直肠段，不能全面评估上段结肠病变。
6. **钡剂灌肠检查**虽然能显示肠道结构，但分辨率不如结肠镜检查，且无法进行活检。

因此，对于便血和排便不尽感的患者，尤其是既往有内痔病史，结肠镜检是首选的检查方法。
题目材料　　患者车祸后 4 小时，出现剧烈腹痛，查体：T36.8℃,P89 次/分，BP124/85mmHg，腹部有压痛、反跳痛，血淀粉酶 1200U/L。腹部立位平片未 常。腹部 B 超示右侧腹部少量腹腔积液。
102、　(A3/A4 型题)　题
最可能损伤的器官是
A.肝破裂
B.脾破裂
C.胰腺破裂
D.结肠破裂
E.小肠破裂
参考答案：　C　您的答案：
图文解析：　【解析】患者车祸（方向盘撞击腹部易造成胰腺损伤）后 4 小时，出现剧烈腹痛，查体：T36.8℃ ，P89 次/分，BP124/85mmHg，腹部有压痛、反跳痛（腹膜刺激征），血淀粉酶升高，腹部 B 超示右侧腹部少量腹腔积液。最可能损伤的器官是胰腺（C 对）。【避错】肝破裂为腹部开放性损伤中最常见，常合并右下位肋骨骨折，有出血性休克和腹膜刺激征表现（A 错）；脾破裂为腹部闭合性损伤中最常见，常合并左下位肋骨骨折，有出血性休克表现（B 错）；结肠破裂腹膜炎出现晚但较严重（D 错）；小肠破裂腹膜炎出现早且明显（E 错）。
对于这个案例，患者在车祸后出现剧烈腹痛，体温 36.8℃，心率 89 次/分，血压 124/85mmHg，腹部有压痛和反跳痛，血淀粉酶水平升高至 1200U/L，腹部立位平片未见异常，B 超显示右侧腹部少量腹腔积液。基于这些信息，可以推断以下结论：
最可能损伤的器官是 C.胰腺破裂。这是因为血淀粉酶水平显著升高通常与胰腺损伤相关。虽然其他器官损伤（如肝脾破裂）也可能引起腹痛和腹腔积液，但它们通常不会导致血淀粉酶水平如此显著地升高。
正确答案是：C.胰腺破裂。
本题患者车祸外伤后 4 小时出现剧烈腹痛，查体有腹膜刺激征阳性，实验室检查见血淀粉酶显著升高，提示胰腺组织受损，因此该患者最可能损伤的器官是胰腺。肝破裂、脾破裂通常伴有大量出血，会引起心率增快、血压下降等血容量不足的表现，该患者心率、血压均正常，故可一并排除选项 A 肝破裂，选项 B 脾破裂。而结肠破裂、小肠破裂早期多表现为腹膜炎症状，但淀粉酶多正常，故不考虑选项 D 结肠破裂，选项 E 小肠破裂。

问题 102：最可能损伤的器官
A. 肝破裂：肝破裂通常会导致右上腹痛和大量腹腔积液，但不会导致血淀粉酶显著升高。
B. 脾破裂：脾破裂也会导致腹痛和腹腔积液，但血淀粉酶升高不明显。
C. 胰腺破裂：胰腺破裂可导致血淀粉酶显著升高，并引起剧烈腹痛和腹腔积液。
D. 结肠破裂：结肠破裂可能导致腹痛和腹膜炎症状，但不导致血淀粉酶升高。
E. 小肠破裂：小肠破裂可能导致腹痛和腹膜炎症状，但不导致血淀粉酶升高。
答案：C. 胰腺破裂
103、 (A3/A4 型题) 题
问接下来还要做什么检查是
A.开腹探查
B.诊断性腹腔穿刺
C.腹部 CT
D.腹腔镜探查
E.腹部 B 超
参考答案： C 您的答案：
图文解析： 【解析】患者车祸（方向盘撞击腹部易造成胰腺损伤）后 4 小时，出现剧烈腹痛，查体：T36.8℃ ，P89 次/分，BP124/85mmHg，腹部有压痛、反跳痛（腹膜刺激征），血淀粉酶升高，腹部 B 超示右侧腹部少量腹腔积液。最可能损伤的器官是胰腺。接下来还要做腹部 CT 检查，能显示胰腺轮廓是否整齐及周围有无积血、积液，评估病情（C 对）。
【避错】开腹探查、腹腔镜探查用于失血性休克和明显腹膜刺激征患者，并指导治疗（AD 错）；诊断性腹腔穿刺用于肝脾破裂合并失血性休克患者（B 错）。
对于接下来要进行的检查，选择 C.腹部 CT。CT 扫描是评估腹部创伤的黄金标准，可以提供有关腹腔内器官损伤的详细信息，特别是在怀疑胰腺损伤时。开腹探查通常是在急诊情况下，当有明显的内脏破裂或内出血时进行的。而在这种情况下，首选的是进行 CT 扫描以获得准确的损伤评估。
正确答案是：C.腹部 CT。
患者已有腹部压痛、反跳痛等腹膜刺激症状，血淀粉酶显著升高，怀疑胰腺破裂，为明确诊断应行腹部 CT，其诊断准确率高，不仅可以判断胰腺损伤的程度，还能判断腹腔内脏器及淋巴结转移情况及对疾病分期的判断，较开腹探查、腹腔镜探查而言，属于无创性检查，患者接受度高。诊断性腹腔穿刺为有创检查，患者腹部压痛反跳痛明显，暂不宜行腹腔穿刺，因此可排除选项 B 诊断性腹腔穿刺。而选项 A 开腹探查，选项 D 腹腔镜探查，均为手术探查，不作为首选检查，故一并排除。患者已行腹部 B 超，无需重复，且对胰腺的观察效果不如 CT，故不考虑选项 E 腹部 B 超。
问题 103：接下来还要做什么检查
A. 开腹探查：这是一个侵入性操作，通常在其他检查无法确诊或患者病情危重时进行。
B. 诊断性腹腔穿刺：可以帮助确定腹腔内是否有血液或其他液体，但对具体损伤的器官识别有限。
C. 腹部 CT：能够详细显示腹腔内脏器的结构和损伤情况，特别是对于胰腺这样的深部器官。
D. 腹腔镜探查：虽然可以直接观察腹腔内情况，但属于侵入性检查。
E. 腹部 B 超：已进行过，不足以进一步明确诊断。
答案：C. 腹部 CT
题目材料 女，32 岁，4 小时前进食较多油腻食物导致突发持续上腹痛，伴呕吐进食物。查体：痛苦面容，巩膜无黄染，心肺查体无异常，腹软，左上 反跳痛，心肺查体未见异常，Murphy（-），肠鸣音 2 次/分。

104、 (A3/A4 型题) 题

可考虑诊断为

A.急性胰腺炎

B.急性胆囊炎

C.急性阑尾炎

D.心肌梗死

E.胃溃疡

参考答案： A 您的答案：

图文解析： 【解析】青年女性，进食油腻食物导致突发持续上腹痛，伴呕吐进食物。查体：痛苦面容，腹软，左上腹压痛，murphy 阴性，综合患者病史、临床表现及体征，考虑为急性胰腺炎（A 对）。

【避错】急性胆囊炎（B 错）表现为右上腹或上腹部疼痛、发热及血白细胞增多，墨菲征阳性或扪及右上腹包块。右下腹压痛是急性阑尾炎（C 错）最常见的重要体征。心肌梗死（D 错）表现为胸骨后疼痛，心电图提示 ST 段明显升高或压低。 胃溃疡（E 错）主要表现为餐后胃痛，服用抑酸剂疼痛缓解。

104 题：

A. 急性胰腺炎：考虑到进食油腻食物后发生的上腹部疼痛和呕吐，这是急性胰腺炎的常见病因。

B. 急性胆囊炎：通常伴有右上腹痛，Murphy 征阳性。

C. 急性阑尾炎：通常引起右下腹痛。

D. 心肌梗死：主要表现为胸痛，且心肺查体中未提及与心肌梗死相关的异常。

E. 胃溃疡：可以导致上腹痛，但通常与进食模式有关，而非突发性疼痛。

综合这些信息，最可能的诊断是 A. 急性胰腺炎，因为症状与急性胰腺炎较为吻合。

104 题解析：

患者青年女性，进食油腻食物后突发持续上腹痛，伴呕吐，左上腹压痛、反跳痛，均符合急性胰腺炎的典型表现。故该患者可考虑诊断为急性胰腺炎。

选项 B 急性胆囊炎通常表现为 Murphy 征阳性，即医师用左手掌平放于患者右胸下部，以拇指指腹勾压于右肋下胆囊点处，嘱患者缓慢深吸气，在吸气的过程中发炎的胆囊下移时碰到用力按压的拇指，引起胆囊触痛，如因剧烈疼痛而致吸气终止称 Murphy 征阳性，本题中患者 Murphy 征（-），可排除。

选项 C 急性阑尾炎的典型表现为转移性右下腹痛，本题患者为持续上腹痛，故不考虑。

选项 D 心肌梗死一般有心绞痛病史，主要表现为胸骨后濒死样疼痛等，心电图示心肌梗死的特征性与动态性改变，本题患者心肺查体无异常，且无相关病史，故不选。

选项 E 胃溃疡主要表现为餐后痛，且该患者无胃溃疡相关病史，可优先排除。

综上所述，正确答案是：A.急性胰腺炎。

问题 104：

可考虑诊断为

A.急性胰腺炎

B.急性胆囊炎

C.急性阑尾炎

D.心肌梗死

E.胃溃疡

解析：
病人进食较多油腻食物后突发上腹痛，伴呕吐，并且查体显示上腹反跳痛，这些症状和体征均提示急性胰腺炎的可能性较大。急性胆囊炎患者通常会有 Murphy 征阳性，而此患者 Murphy 征阴性。急性阑尾炎通常表现为右下腹痛。心肌梗死和胃溃疡虽然也可能导致上腹痛，但结合进食油腻食物后的症状，急性胰腺炎的可能性更大。

最佳答案：A. 急性胰腺炎
105、 (A3/A4 型题) 题
为确诊应进行的检查是
A.立位腹平片
B.腹部增强 CT
C.心电图
D.胃镜
E.结肠镜
参考答案： B 您的答案：
图文解析： 【解析】青年女性，进食油腻食物导致突发持续上腹痛，伴呕吐进食物。查体：痛苦面容，腹软，左上腹压痛，murphy 阴性，综合患者病史、临床表现及体征，考虑为急性胰腺炎。腹部增强 CT（B 对）有助于确定胰腺坏死程度，对诊断急性胰腺炎有重要意义。
【避错】立位腹平片（A 错）主要诊断胃肠道穿孔、肠梗阻等疾病。心电图（C 错）主要了解是否存在各种心律失常、心肌缺血/梗死、房室肥大或电解质紊乱等。 胃镜（D 错）是食管、 胃、十二指肠疾病最常用和最准确的检查方法。结肠镜（E 错）则主要用于观察从肛门到回盲瓣的所有结直肠病变。
105 题：
为确诊，应进行的检查可能包括：
A. 立位腹平片：通常用于检查肠梗阻或自由腹腔气体。
B. 腹部增强 CT：对于急性胰腺炎的诊断非常有用，可以显示胰腺的炎症和周围液体积聚。
C. 心电图：用于心脏问题的诊断，此处不适用。
D. 胃镜：用于观察胃黏膜，寻找溃疡或其他病变。
E. 结肠镜：用于检查结肠疾病，此处不适用。
因此，对于这位患者来说，最适合的检查是 B. 腹部增强 CT，以评估胰腺是否存在炎症或其他并发症。
105 题解析：
为了确诊急性胰腺炎，最有价值的检查是腹部增强 CT，其不仅能诊断急性胰腺炎，而且能鉴别是否合并胰腺组织坏死，同时评估胰腺外并发症及严重程度。
选项 A 立位腹平片主要用于消化道穿孔、肠梗阻、腹腔内异物等疾病的诊断，对急性胰腺炎的诊断价值不大，故不选。
选项 C 心电图主要用于心脏相关疾病的诊断，该患者心肺查体无异常，故排除。
选项 D 胃镜是诊断胃部疾病的金标准，本题患者为急性胰腺炎，故不考虑。
选项 E 结肠镜是诊断结肠疾病的金标准，本题患者发病部位为胰腺，与结肠无关，与题意不符。
综上所述，正确答案是：B.腹部增强 CT。
问题 105：
为确诊应进行的检查是

A.立位腹平片
B.腹部增强 CT
C.心电图
D.胃镜
E.结肠镜

解析：
腹部增强 CT 是确诊急性胰腺炎的首选检查方法，可以清楚显示胰腺的炎症和并发症，如胰腺坏死、胰腺周围积液等。立位腹平片主要用于诊断胃肠穿孔，心电图用于排除心脏问题，胃镜和结肠镜则分别用于检查胃和结肠的疾病。

最佳答案：B. 腹部增强 CT
题目材料　　男，35 岁，低热一月余，咳嗽，痰中带血 3 天。2 年前患胸膜炎，经治疗后好转。胸片：左肺不规则阴影，左侧肋膈角变钝。血 WBC8.0×109/ N0.78，ESR30mm/h。
106、　(A3/A4 型题)　题
为明确诊断首选的检查是
A.胸部 CT
B.痰涂片抗酸染色
C.支气管镜
D.γ-干扰素释放试验
E.血清肿瘤标志物
参考答案：　B　您的答案：
图文解析：　【解析】青年男性，低热、咳嗽、痰中带血（肺结核的表现），2 年前患胸膜炎，胸片：左肺不规则阴影，左侧肋膈角变钝（提示为结核性胸膜炎），ESR30mm/h（ESR 正常参考值范围：男性：0～15mm/h 女性：0～20mm/h，血沉增快，可见于结核杆菌感染时，如肺结核、结核性胸膜炎等），综上考虑为结核性胸膜炎。痰涂片抗酸染色（B 对）对诊断结核性胸膜炎是最简单、快速、易行和可靠的方法。
【避错】胸部 CT（A 错）常用于对肺结核的诊断，以及与其他胸部疾病的鉴别诊断。纤维支气管
镜（C 错）常应用于支气管结核和淋巴结支气管瘘的诊断。γ-干扰素释放实验（D 错）由于成本较高等原因，目前多用于研究评价工作，尚未广泛推行。血清肿瘤标记物（E 错）对肺癌的诊断和病情的监测有一定参考价值。
对于第 106 题，为明确诊断首选的检查是：
A. 胸部 CT 可以提供关于肺部阴影性质的详细信息，是评估肺部病变的重要手段，有助于确定病变的性质和范围。
B. 痰涂片抗酸染色主要用于诊断肺结核。
C. 支气管镜检查可以直视下呼吸道并获取组织样本进行病理学分析，适用于肺部病变不明确时。
D. γ-干扰素释放试验主要用于诊断结核感染。
E. 血清肿瘤标志物用于检测可能的肺癌等。
考虑到患者的临床表现和病史，**选项 A（胸部 CT）**可能是首选的检查方法，因为它能提供关于肺部病变的详细信息，有助于明确诊断。
106、解析：

青年男性患者，低热一月余，咳嗽，痰中带血 3 天，为肺结核的典型临床表现，既往有胸膜炎病史。胸片示左肺不规则阴影，左侧肋膈角变钝，实验室检查见血白细胞正常，中性粒细胞偏高，血沉增快，根据患者症状、辅助检查，考虑诊断为肺结核，为明确诊断首选痰涂片抗酸染色，若检查结果阳性，结合患者病史和临床表现，即可确诊为肺结核。

选项 A 胸部 CT 对 X 线胸片发现不了的隐藏在心脏后、脊柱旁沟和在肺尖、锁骨下区的肺部病灶都有很好的显示，但并不能确诊肺结核，故不选。

选项 C 支气管镜适用于做肺叶、段及亚段支气管病变的观察，活检采样，细菌学、细胞学检查，对于肺结核的确诊意义不大，故排除。

选项 D：γ-干扰素释放试验是一种用于结核杆菌感染的体外免疫检测的新方法，其不能用于确诊当前的结核感染，仅起到辅助检查的作用，与题意不符。

选项 E 血清肿瘤标志物主要用于预测直肠癌的预后和监测复发，对肺结核的确诊没有意义，故不考虑。

综上所述，正确答案是：B.痰涂片抗酸染色。

题目 106

为明确诊断首选的检查是

A.胸部 CT

B.痰涂片抗酸染色

C.支气管镜

D.γ-干扰素释放试验

E.血清肿瘤标志物

答案: B. 痰涂片抗酸染色

解析：

患者有低热、咳嗽和痰中带血的症状，同时胸片显示左肺不规则阴影，这些症状和影像学表现提示可能存在肺结核的可能性。痰涂片抗酸染色是诊断肺结核的重要检查，可以直接发现结核杆菌，是确诊结核病的首选方法。因此，痰涂片抗酸染色是明确诊断的首选检查。

107、 (A3/A4 型题)

为明确治疗方案，需重点了解的情况是 题

A.胸膜炎治疗情况

B.家族遗传史

C.青霉素过敏史

D.体重变化情况

E.此前健康体检情况

参考答案： A 您的答案：

图文解析： 【解析】青年男性，低热、咳嗽、痰中带血（肺结核的表现），2 年前患胸膜炎，胸片：左肺不规则阴影，左侧肋膈角变钝（提示为结核性胸膜炎），ESR30mm/h（ESR 正常参考值范围：男性：0～15mm/h 女性：0～20mm/h，血沉增快，可见于结核杆菌感染时，如肺结核、结核性胸膜炎等），综上考虑为结核性胸膜炎。该患者有胸膜炎病史，考虑患者以往胸膜炎治疗不规范而复发，为明确治疗方案则需重点了解的情况为胸膜炎治疗情况（A 对）。

【避错】结核性胸膜炎胸水蛋白含量高，容易引起胸膜粘连，原则上应尽快抽尽胸腔内积液。大量胸水者，每周抽液 2~3 次，首次抽液不超过 700ml， 以后每次抽液不超过 1000ml。

对于第 107 题，为明确治疗方案，需重点了解的情况是：

A. 胸膜炎治疗情况有助于了解患者过往病史和治疗反应。
B. 家族遗传史对于一些遗传性疾病的诊断很重要。
C. 青霉素过敏史对于制定抗生素治疗方案至关重要。
D. 体重变化情况可以反映患者的营养状况和疾病进展。
E. 此前健康体检情况可以提供患者的基线健康状况。
在这种情况下，**选项 A（胸膜炎治疗情况）**对于明确治疗方案可能最为关键，因为了解患者对之前治疗的反应可以帮助制定针对当前症状的治疗计划。
107、解析：
该患者已确诊肺结核，治疗肺结核需用抗结核药物，制定治疗方案需了解患者此前健康体检情况，是否对结核药有耐药以及是否存在其他疾病等情况，以更好地制定治疗方案。
选项 A 胸膜炎治疗情况对制定抗结核治疗方案价值不大，故排除。
选项 B 家族遗传史主要针对一些单基因遗传病，而肺结核不会遗传，与题意不符。
选项 C 青霉素过敏史对抗结核治疗方案的选择影响不大，常用的抗结核药物中不包括青霉素，可排除。
选项 D 体重变化情况对制定治疗方案价值不大，故不选。
综上所述，正确答案是：E.此前健康体检情况。
题目 107
为明确治疗方案，需重点了解的情况是

A.胸膜炎治疗情况
B.家族遗传史
C.青霉素过敏史
D.体重变化情况
E.此前健康体检情况

答案: A. 胸膜炎治疗情况
解析：
患者有胸膜炎病史，并且 2 年前经过治疗后好转。了解他之前胸膜炎的治疗情况对于明确当前症状的病因和制定治疗方案非常重要。如果之前的胸膜炎是由结核引起的，那么患者当前的症状可能是结核复发，需要结合以前的治疗情况制定合适的治疗方案。因此，了解胸膜炎治疗情况是明确治疗方案的重点。
题目材料　　女，44 岁。因右乳腺癌行改良根治术。术后病理：右乳浸润性癌，非特殊型，肿瘤大小 3cm×2cm，组织学III级，ER80%强阳，PR90%强阳，HE ki-67.50%。腋窝淋巴结（4/16），见癌转移，与周围组织有粘连。
108、　(A3/A4 型题)　题
按照 TNM 分期，该患者的分期是
A.T1N1M0
B.T1N2M0
C.T2N1M0
D.T3N2M0
E.T2N2M0
参考答案：　E　您的答案：
图文解析：　【解析】 中年女性患者，因右乳腺癌行改良根治术。肿瘤大小 3cm×2cm（提示为 T2：>2cm，　≤5cm）　，腋窝淋巴结（4/16）（提示为 N2：　同侧腋窝肿大淋巴结彼此

融合， 组织粘连）见癌转移，与周围组织有粘连。全身检查其他器官未见转移（提示为 M0：移） 。按照 TNM 分期，该患者分期 T2N2M0（E 对）。

【避错】乳腺癌 TNM 分期： 癌瘤长径或与周围无远处转

对于问题 108，关于 TNM 分期的选择，我们有以下信息：

肿瘤大小 3cm×2cm，这归入 T2（任何维度大于 2cm 但不超过 5cm 的肿瘤）。

腋窝淋巴结有 4 个受累，显示癌症转移，这归入 N1（1-3 个腋窝淋巴结受累）。

没有提及远处转移，因此 M0（无远处转移）。

基于这些信息，最适合的 TNM 分期为 C.T2N1M0。

108、解析：

中年女性患者，右乳腺癌行改良根治术，术后病理提示肿瘤大小为 3cm×2cm，根据 AJCC 乳腺癌 TNM 分期，该患者原发肿瘤大小为 T2。同时病理提示腋窝淋巴结（4/16）见癌转移，属于 N2。无远处转移为 M0。因此，按照 TNM 分期，该患者的分期是 T2N2M0。

选项 A：T1N1M0 表示原发肿瘤长径小于等于 2cm，同侧腋窝有肿大淋巴结，但可推动，可活动，无远处转移，与题干描述不符。

选项 B：T1N2M0 表示原发肿瘤长径小于等于 2cm，同侧腋窝肿大淋巴结融合，或与周围组织粘连，或同侧胸骨旁有肿大淋巴结，但无远处转移，故不选。

选项 C：T2N1M0 表示原发肿瘤长径大于 2cm 且小于等于 5cm，同侧腋窝有肿大淋巴结，但可推动，可活动，无远处转移，故排除。

选项 D：T3N2M0 表示原发肿瘤大于 5cm，同侧腋窝肿大淋巴结融合，或与周围组织粘连，但无远处转移，与题干描述不符。

综上所述，正确答案是：E.T2N2M0。

108、按照 TNM 分期，该患者的分期是

根据病理结果和 TNM 分期标准：

T：肿瘤大小 3cm × 2cm，根据 TNM 分期标准，应为 T2（肿瘤最大径为 2cm 到 5cm）。

N：腋窝淋巴结（4/16）见癌转移，并与周围组织有粘连，根据 TNM 分期标准，应为 N2（3-4 个淋巴结有癌转移，且与周围组织粘连）。

M：没有远处转移，因此为 M0。

综上，该患者的 TNM 分期应为：T2N2M0。

最佳答案是：

E. T2N2M0

109、 (A3/A4 型题) 题

该患者术后应采取何种综合治疗方案

A.化疗+靶向治疗

B.化疗+内分泌治疗

C.化疗+靶向治疗+内分泌治疗+放疗

D.化疗+放疗+内分泌治疗

E.化疗+靶向治疗+内分泌治疗

参考答案： C 您的答案：

图文解析： 【解析】 中年女性患者，因右乳腺癌行改良根治术。肿瘤大小 3cm×2cm（提示为 T2：癌瘤长径>2cm， ≤5cm） ，腋窝淋巴结（4/16） （提示为 N2： 同侧腋窝肿大淋巴结彼此融合，或与周围组织粘连）见癌转移，与周围组织有粘连。全身检查其他器官未见转移（提示为 M0：无远处转移） 。按照 TNM 分期，该患者分期 T2N2M0。患者右乳腺

癌改良根治术后，ER80%，HER2+，可行治疗为化疗+靶向治疗+内分泌治疗+放疗（C 对）。

【避错】癌基因表皮生长因子受体 2（HER2）有过度表达者，适宜应用术后辅助化疗；乳腺癌细胞中雌激素受体（ER）含量高者，称激素依赖性肿瘤，这些病例对内分泌治疗有效；在保留乳房的乳腺癌手术后，放射治疗是一重要组成部分，应于肿块局部广泛切除后给予适当剂量放射治疗；靶向治疗通过转基因技术制备的曲妥珠单抗对 HER2 过度表达的乳腺癌患者有良好效果，可降低乳腺癌患者术后的复发转移风险，提高无病生存期。

对于第 109 题，患者的肿瘤表达 ER 和 PR 强阳性，Ki-67 标记 50%，表示增殖速度较快。这种情况下，综合治疗方案通常包括内分泌治疗，因为肿瘤是激素受体阳性的。由于 Ki-67 较高，也可能需要化疗来控制快速增长的肿瘤细胞。此外，腋下淋巴结转移可能需要放疗来控制局部病情。因此，适宜的治疗方案可能包括化疗、放疗和内分泌治疗。没有特别提到需要靶向治疗，除非有特定的分子标志物表达，如 HER2 阳性。因此，最合适的答案可能是 D.化疗+放疗+内分泌治疗。但请注意，最终的治疗方案应由具体情况和医生的判断决定。

109、解析：

该患者术后病理提示肿瘤为 ER、PR 强阳性，提示可行内分泌治疗。Ki-67＞14%，提示肿瘤细胞增殖较快，需行化疗。存在 4 枚淋巴结转移，需行放疗。因此该患者术后应采取的综合治疗方案是化疗+放疗+内分泌治疗。

选项 A：化疗+靶向治疗适用于 HER-2 阳性的乳腺癌患者，题干中未提及 HER-2 状态，故不选。

选项 B：化疗+内分泌治疗，选项 E 化疗+靶向治疗+内分泌治疗，均缺乏放疗这一重要辅助治疗措施，放疗是局部治疗手段，可用于根治性治疗和姑息性治疗，该患者已有 4 枚淋巴结转移，放疗必不可少，可一并排除。

选项 C：化疗+靶向治疗+内分泌治疗+放疗包含靶向治疗，而题干中未提及 HER-2 状态，靶向治疗不一定适用，故不考虑。

综上所述，正确答案是：D.化疗+放疗+内分泌治疗。

109、该患者术后应采取何种综合治疗方案

根据患者的病理特点和临床指南，考虑以下几点：

化疗：患者肿瘤组织学分级为III级，表明肿瘤具有较高的恶性度，且淋巴结有癌转移，符合化疗指征。

靶向治疗：虽然题目未明确提到 HER2 状态，但通常在高级别浸润性乳腺癌中，如 HER2 阳性，应考虑加入靶向治疗。

内分泌治疗：患者 ER 和 PR 均为强阳性，符合内分泌治疗的指征。

放疗：有淋巴结转移且与周围组织粘连，应考虑放疗以减少局部复发风险。

综合考虑，最合理的方案是：化疗+靶向治疗+内分泌治疗+放疗。

最佳答案是：

C. 化疗+靶向治疗+内分泌治疗+放疗

题目材料　　男，55 岁，夜间阵发性疼痛，进食后缓解，近 3 个月来加重，门诊收住入院。

110、　(A3/A4 型题)

治疗的手术方式为

A.胃大部切除术

B.胃修补手术

C.迷走神经干切断术

D.选择性迷走神经切断术

E.全胃切除术

参考答案：　　A　您的答案：

图文解析：　　【解析】根据患者情况，考虑诊断为十二指肠溃疡。 胃十二指肠溃疡的主要术式是远端胃大部切除术，也即通常所称的胃大部切除术。手术适应证： 胃十二指肠溃疡保守治疗无效或者并发穿孔、出血、幽门梗阻、癌变者（A 对）。

【避错】 胃修补手术（B 错）适应证于胃或十二指肠溃疡急性穿孔；迷走神经切断术已很少应用（C 错） ；消化性溃疡合并消化道大出血，若经内科治疗后出血仍不止，则可采用胃大部切除，或选择性胃迷走神经切断术（D 错）加行幽门成形术。全胃切除术（E 错）多适用于胃体与胃近端癌。

110、治疗的手术方式为

症状表明可能是消化性溃疡，特别是在饮食后疼痛有所缓解。最常见的治疗方法是选择性迷走神经切断术（D），用于减少胃酸分泌，从而减轻症状。

答案: D. 选择性迷走神经切断术

正确答案是：A.胃大部切除术。

中年男性患者，夜间阵发性疼痛，进食后缓解是十二指肠溃疡的典型表现，考虑诊断为十二指肠溃疡。胃大部切除术是治疗十二指肠溃疡最常用的手术方式，胃大部切除也包括了溃疡病灶的切除，故本题中患者最适合的手术方式是胃大部切除术。选项 B 胃修补手术一般不用于十二指肠溃疡的治疗，故排除。选项 C 迷走神经干切断术，选项 D 选择性迷走神经切断术，现多已弃用，可一并排除。选项 E 全胃切除术主要适用于胃部的恶性肿瘤，对十二指肠溃疡治疗无效，与题意不符。

110、(A3/A4 型题)

治疗的手术方式为：

A.胃大部切除术

理由：患者为 55 岁男性，夜间阵发性疼痛，进食后缓解，症状近 3 个月来加重，符合消化性溃疡的典型表现。胃大部切除术常用于治疗严重的消化性溃疡，特别是当药物治疗无效或溃疡并发症（如出血、穿孔）时。

111、　(A3/A4 型题)　题

手术后，患者胆汁性呕吐，呕吐后腹痛不缓解，属于

A.吻合口瘘

B.输出端梗阻

C.碱性反流性胃炎

D.倾倒综合征

E.十二指肠吻合口破裂

参考答案：　　B　您的答案：

图文解析：　　【解析】根据患者情况，考虑诊断为十二指肠溃疡。 胃十二指肠溃疡的主要术式是远端胃大部切除术，也即通常所称的胃大部切除术。该患者术后出现胆汁性呕吐，呕吐后腹痛不缓解，为术后早期并发症中的输出端梗阻（B 对）。

【避错】吻合口破裂（E 错）或吻合口瘘（A 错） ，多表现为剑突下疼痛，应立即禁食水，胃肠减压并严密观察，出现腹膜炎时手术探查。碱性反流性胃炎（C 错）多出现于术后数月至数年。早期倾倒综合征（D 错）多于进食后半小时出现心悸、出冷汗、乏力、面色苍白等短暂血容量不足的相应表现，并伴有恶心和呕吐、腹部绞痛和腹泻；晚期倾倒综合征发生在禁食后 2～4 小时。主要表现为头晕、面色苍白、出冷汗、乏力、脉搏细数。

111、手术后，患者胆汁性呕吐，呕吐后腹痛不缓解，属于

胆汁性呕吐和呕吐后腹痛不缓解可能指向胆汁返流，常见于胃手术后，这可能导致碱性反流性胃炎（C）。答案: C. 碱性反流性胃炎

正确答案是：C.碱性反流性胃炎。

手术后，患者出现胆汁性呕吐，呕吐后腹痛不缓解，考虑为碱性反流性胃炎。该并发症通常由于术中幽门成形或胃空肠吻合术后，导致胆汁、胰液等十二指肠内容物反流入胃，破坏了胃黏膜的屏障保护作用，从而引起的一系列症状。选项 A 吻合口瘘是指胃肠道手术后，吻合口的地方出现漏口，消化液、肠内容物会从瘘口流出腹腔、腹壁外，与题干描述不符。选项 B 输出端梗阻主要表现为呕吐物含有胆汁的胃内容物，但呕吐后腹痛可稍缓解，故排除。选项 D 倾倒综合征主要表现为进食后出现剑突下不适、心悸、乏力、面色苍白、出冷汗等短暂血容量不足的相应表现，以及恶心、呕吐、腹部绞痛和腹泻等症状，与题干描述不符。选项 E 十二指肠吻合口破裂较少见，主要表现为急性腹膜炎，严重者可出现休克，可排除。

111、(A3/A4 型题)

手术后，患者胆汁性呕吐，呕吐后腹痛不缓解，属于：

C.碱性反流性胃炎

理由：手术后出现胆汁性呕吐，且呕吐后腹痛不缓解，提示胆汁反流进入胃内，导致胃黏膜受损，符合碱性反流性胃炎的临床表现。

112、 (A3/A4 型题) 题

手术后患者有低血糖综合征，此时表现为

A.呕吐物为食物，不含胆汁

B.呕吐物为胆汁，不含食物

C.呕吐物既有胆汁也有食物

D.呕吐物是隔夜宿食

E.无呕吐

参考答案： E 您的答案：

图文解析： 【解析】根据患者情况，考虑诊断为十二指肠溃疡。 胃十二指肠溃疡的主要术式是远端胃大部切除术，也即通常所称的胃大部切除术。术后患者有低血糖综合征，属于晚期倾倒综合征，主要表现为头晕、面色苍白、出冷汗、乏力、脉搏细数。一般无呕吐，故本题选 E。

【避错】呕吐物为食物，不含胆汁为输入袢梗阻（A 错） ；呕吐物为胆汁，不含食物为慢性不完全性输入袢梗阻（B 错） ；呕吐物既有胆汁也有食物为碱性反流性胃炎或输出袢梗阻（C 错）；呕吐物是隔夜宿食多见于幽门狭窄或梗阻（D 错）。

112、手术后患者有低血糖综合征，此时表现为

低血糖综合征常见于胃部手术后，尤其是在胃部大部分切除后。这种情况下，食物过快进入小肠，引起胰岛素过度分泌和随后的低血糖。此时患者可能没有呕吐，因为低血糖综合征的主要表现是与进食后的血糖变化相关的症状。

答案: E. 无呕吐

正确答案是：B.呕吐物为胆汁，不含食物。

手术后患者有低血糖综合征，这是由于胃大部切除术后，食物过快地进入空肠，葡萄糖被过快吸收，血糖呈一过性增高，刺激胰岛素分泌过多，随即发生反应性低血糖。在这种情况下，由于胃大部切除术后胃的容量变小，食物快速进入肠道，而胆汁也可能随食物快速进入肠道，因此呕吐物中可能含有胆汁但不含食物。选项 A“呕吐物为食物，不含胆汁”不符合胃大部切除后的生理改变，故排除。选项 C“呕吐物既有胆汁也有食物”通常不会出现在低血糖综合征的情况下，因为食物和胆汁不会同时停留在胃中，可排除。选项 D“呕吐物是隔夜宿食”

是幽门梗阻的典型表现，与低血糖综合征无关，故不选。选项 E“无呕吐”不符合低血糖综合征时可能出现的症状，因此排除。

112、(A3/A4 型题)

手术后患者有低血糖综合征，此时表现为：

E.无呕吐

理由：低血糖综合征是胃切除术后的一种并发症，主要表现为饭后 2 小时左右出现低血糖症状，如出汗、心悸、乏力、饥饿等。此时患者通常不会有呕吐症状。

题目材料　　女，46 岁，上腹部不适 1 年，加重伴头晕，乏力 2 月，多为进餐前疼痛，既往患有桥本甲状腺炎病史 10 年，胃镜见胃黏膜变薄，黏膜色泽变淡

血管型。

113、　(A3/A4 型题)　题

为明确诊断应该首选检查

A.胃镜

B.13C 呼气试验

C.碘 131 检查

D.腹部 X 线片

E.内因子抗体检查

参考答案：　　E　您的答案：

图文解析:【解析】 中年女性，上腹部不适 1 年，加重伴头晕，乏力（贫血症状）2 月，多为进餐前疼痛（考虑十二指肠溃疡），既往患有桥本甲状腺炎病史 10 年，胃镜见胃体黏膜变薄，黏膜色泽变淡，可透见血管型（符合慢性萎缩性胃炎的典型胃镜表现），综上考虑为 A 型胃炎。血清抗壁细胞抗体、内因子抗体（E 对）及维生素 B12 水平测定，有助于诊断自身免疫性胃炎。

【避错】 胃镜（A 错）及组织学检查是慢性胃炎诊断的关键，仅依靠临床表现不能确诊。13C（B 错）或 14C-尿素呼气试验，是 HP 检测的重要方法之一。131I（C 错）摄取率诊断甲亢的传统方法，目前已经被 sTSH 测定所代替。X 线平片（D 错）仅用于与食管、胃肠道疾病相关的急症检查，包括食管、胃肠道的金属性异物、穿孔和肠梗阻等。

问题 113: 为明确诊断应该首选检查

患者的症状是上腹部不适、头晕、乏力，并且胃镜检查显示胃黏膜变薄，黏膜色泽变淡。考虑到她有桥本甲状腺炎的病史，需要进一步检查可能与此相关的病变。桥本甲状腺炎是一种自身免疫性疾病，可能伴随其他自身免疫性胃炎。

A. 胃镜已经进行过了，显示了胃黏膜的变化。

B. 13C 呼气试验主要用于检测幽门螺杆菌感染，这与当前的临床情况关联不大。

C. 碘 131 检查用于评估甲状腺功能，考虑到患者有桥本甲状腺炎，这可能有助于了解甲状腺的状况，但对确定胃部问题的诊断帮助不大。

D. 腹部 X 线片可以显示一些胃肠道问题，但对于诊断黏膜的具体问题并不是最佳选择。

E. 内因子抗体检查是检测自身免疫性胃炎的重要手段，尤其是与 B12 吸收不良相关的萎缩性胃炎。

因此，对于问题 113，我会选择 E. 内因子抗体检查，因为这项检查有助于诊断自身免疫性胃炎，这与患者的症状和胃镜所见相符。

正确答案：E.内因子抗体检查

该患者上腹部不适，既往患有桥本甲状腺炎，胃镜见胃黏膜变薄，黏膜色泽变淡，血管透见，这些症状强烈提示 A 型胃炎的可能性。A 型胃炎是自身免疫性胃炎，由自身免疫机制形成，

患者体内产生了针对壁细胞或内因子的自身抗体，导致壁细胞总数减少，胃酸分泌降低，内因子减少，进而导致维生素 B12 吸收不良，出现巨幼红细胞性贫血。对于自身免疫性胃炎，内因子抗体及壁细胞抗体的检测是最有价值的实验诊断方法，故首选内因子抗体检查以明确诊断。
选项 A 胃镜虽然可以观察到胃黏膜的变化，但对于自身免疫性胃炎的特异性诊断价值不及内因子抗体检查，故排除。
选项 B：^{13}C 呼气试验主要用于检测幽门螺杆菌感染，不适用于诊断自身免疫性胃炎，因此不选。
选项 C：碘^{131}检查主要用于甲状腺和甲状旁腺疾病的诊断，与自身免疫性胃炎的诊断无关，所以不选。
选项 D：腹部 X 线片对于胃肠道疾病的诊断价值有限，不能明确诊断自身免疫性胃炎，故排除。
113、明确诊断应该首选检查
考虑到患者的症状和胃镜发现，结合其既往桥本甲状腺炎的病史，这位患者可能患有自身免疫性胃炎。为了进一步明确诊断，首选检查应该是评估是否存在内因子抗体（A3/A4 型题）。

正确答案：E. 内因子抗体检查
114、 (A3/A4 型题) 题
患者可能会出现
A.血中胃蛋白酶原增多
B.胃酸分泌增多
C.维生素 B12 缺乏
D.可见 virchow 淋巴结
E.内因子增多
参考答案： C 您的答案：
图文解析： 【解析】 中年女性，上腹部不适 1 年，加重伴头晕，乏力（贫血症状）2 月，多为进餐前疼痛（考虑十二指肠溃疡） ，既往患有桥本甲状腺炎病史 10 年， 胃镜见胃体黏膜变薄，黏膜色泽变淡，可透见血管型（符合慢性萎缩性胃炎的典型胃镜表现） ，综上考虑为 A 型胃炎。A 型胃炎的主要表现为：恶性贫血（即巨幼红细胞贫血，维生素 B12 减少（C 对） ）、 胃蛋白酶原缺乏（A 错） 、 胃酸分泌减少（B 错） 、 内因子缺乏（E 错）。【避错】Virchow 淋巴结常为胃癌、胰腺癌、食管癌、霍奇金淋巴瘤转移的标志。
问题 114: 患者可能会出现
A. 血中胃蛋白酶原增多与胃酸分泌减少有关，但更多见于胃酸分泌正常或增多的情况。
B. 胃酸分泌增多通常与 Zollinger-Ellison 综合征等疾病相关，与本病例描述不符。
C. 维生素 B12 缺乏可能由于自身免疫性胃炎导致萎缩性胃炎，进而影响 B12 的吸收。
D. 可见 virchow 淋巴结是胃癌晚期的体征，与本病例描述不符。
E. 内因子增多与本病例的自身免疫背景和症状不符。
考虑到患者的胃镜结果和自身免疫病史，最可能的情况是萎缩性胃炎导致的维生素 B12 吸收不良。因此，对于问题 114，我会选择 C. 维生素 B12 缺乏。
正确答案：C.维生素 B12 缺乏
解析：
中年女性患者，既往患有桥本甲状腺炎，胃镜见胃黏膜变薄，黏膜色泽变淡，结合患者既往病史、胃镜检查，最可能的诊断为自身免疫性胃炎。自身免疫性胃炎时，体内出现针对壁细胞或内因子的自身抗体，导致胃酸分泌降低，内因子减少，进而导致维生素 B12 吸收不良，

出现巨幼红细胞性贫血，即维生素 B12 缺乏。
选项 A 血中胃蛋白酶原增多多见于胃窦黏膜的 G 细胞分泌促胃液素过多的疾病，如胃泌素瘤，与该患者症状不符，故不选。
自身免疫性胃炎时，体内出现针对壁细胞或内因子的自身抗体，导致胃酸分泌降低，故选项 B 胃酸分泌增多，可排除。
选项 D 可见 Virchow 淋巴结即左锁骨上淋巴结肿大多见于晚期食管癌和胃癌，与题干描述不符，故不选。
自身免疫性胃炎时，体内出现针对壁细胞或内因子的自身抗体，导致胃酸分泌降低，内因子减少，故选项 E 内因子增多，与题意不符。
114、患者可能会出现
自身免疫性胃炎常导致胃黏膜萎缩，内因子缺乏，进而影响维生素 B12 的吸收，导致维生素 B12 缺乏。因此，患者可能会出现以下症状：

正确答案：C. 维生素 B12 缺乏

解题思路
病史和症状分析：

女，46 岁，上腹部不适 1 年，加重伴头晕，乏力 2 月，多为进餐前疼痛。
既往患有桥本甲状腺炎病史 10 年。
胃镜见胃黏膜变薄，黏膜色泽变淡，血管型。
可能的诊断：

自身免疫性胃炎，可能导致内因子缺乏，进而导致维生素 B12 缺乏性贫血。
首选检查：

内因子抗体检查是明确诊断的关键。
可能出现的症状：

维生素 B12 缺乏最可能出现。
这两个问题结合起来，可以得出明确的诊断思路，并选择最佳的检查方法和预期的症状。
题目材料　　男，22 岁，9 天前脚被铁钉刺伤。两天前出现乏力，　伴头痛，　　咀嚼困难，吞咽困难，1 天前出现牙关紧闭，肌肉痉挛，　全身抽搐。
115、　(A3/A4 型题)　　　　　　　题
若疾病继续发展，最威胁生命的是
A.持续呼吸肌痉挛
B.肌肉断裂
C.骨折
D.营养障碍
E.尿潴留
参考答案：　A　您的答案：
图文解析：　【解析】青年男性，9 天前脚被铁钉刺伤（异物刺伤史）　。两天前出现乏力，伴头痛，咀嚼困难，吞咽困难，1 天前出现牙关紧闭，肌肉痉挛，全身抽搐（破伤风典型症状）　，考虑为破伤风。若疾病继续发展，最威胁生命的是持续呼吸肌痉挛（A 对）　，因持

续的呼吸肌和膈肌痉挛，可造成呼吸骤停。
【避错】BCDE 选项也为破伤风的表现，但不如呼吸痉挛紧急。
对于第 115 题，破伤风最威胁生命的并发症是呼吸肌痉挛，因为它可以导致呼吸衰竭。因此，答案是 A.持续呼吸肌痉挛。
A：持续呼吸肌痉挛
解析：
青年男性患者，9 天前脚被铁钉刺伤，后出现乏力、头痛、咀嚼困难、吞咽困难，1 天前出现牙关紧闭、肌肉痉挛、全身抽搐，结合患者病史、症状，应诊断为破伤风。破伤风是由破伤风梭菌侵入人体伤口后，在厌氧环境下生长繁殖，产生嗜神经外毒素而引起全身肌肉强直性痉挛为特点的急性传染病。若疾病继续发展，最威胁生命的是持续呼吸肌痉挛，可因窒息而死亡。
选项 B 肌肉断裂，选项 C 骨折，均可在强烈的肌肉痉挛后出现，但不是最威胁生命的症状，因此可一并排除。
破伤风患者由于不断阵发痉挛，出大汗等，故每日消耗热量和水分丢失较多，易发生营养障碍，因此需要十分重视补充营养和水与电解质平衡的调整，但不是最威胁生命的症状，故选项 D 营养障碍，可排除。
选项 E 尿潴留可在强烈的膀胱括约肌痉挛后出现，但不是最威胁生命的症状，可排除。
题目材料
男，22 岁，9 天前脚被铁钉刺伤。两天前出现乏力，伴头痛，咀嚼困难，吞咽困难，1 天前出现牙关紧闭，肌肉痉挛，全身抽搐。

问题 115
若疾病继续发展，最威胁生命的是：

A. 持续呼吸肌痉挛
B. 肌肉断裂
C. 骨折
D. 营养障碍
E. 尿潴留

答案和解析：
破伤风是一种由破伤风梭菌（Clostridium tetani）引起的急性特异性感染，其毒素（破伤风毒素）主要影响中枢神经系统，导致全身肌肉痉挛。如果疾病继续发展，最威胁生命的是持续呼吸肌痉挛，因为呼吸肌痉挛会导致呼吸困难甚至窒息，这是破伤风患者的主要死亡原因之一。

最佳答案：A. 持续呼吸肌痉挛
116、 (A3/A4 型题) 题
首选治疗为
A.破伤风类毒素
B.破伤风抗毒素
C.青霉素
D.破伤风类毒素+青霉素
E.破伤风抗毒素+青霉素

参考答案：　　E　您的答案：
图文解析：　　【解析】青年男性，9 天前脚被铁钉刺伤（异物刺伤史）　。两天前出现乏力，伴头痛，咀嚼困难，吞咽困难，1 天前出现牙关紧闭，肌肉痉挛，全身抽搐（破伤风典型症状）　，考虑为破伤风。此时首选治疗应选择破伤风抗毒素+青霉素（E 对）。
【避错】破伤风抗毒素（TAT）　，　目的是中和游离的毒素，所以只在早期应用有效，若毒素已与神经组织结合，则难收效。抗生素治疗首选青霉素，剂量为 80 万～100 万 U，肌内注射，每 4～6 小时 1 次，或大剂量静脉滴注，剂量为 200 万～1000 万 U，每日分 2～4 次给药，可抑制破伤风梭菌。
对于第 116 题，破伤风的首选治疗是破伤风抗毒素，用于中和循环中的毒素，防止毒素进一步损害神经系统。同时，通常会使用抗生素，如青霉素，来杀灭残留的肉毒杆菌，防止它们产生更多的毒素。因此，答案是 E.破伤风抗毒素+青霉素。
E：破伤风抗毒素+青霉素
解析：
破伤风患者的首选治疗为尽早皮下注射破伤风抗毒素以中和游离毒素，同时应用青霉素抑制破伤风梭菌，预防和控制感染。
选项 A 破伤风类毒素，选项 D 破伤风类毒素+青霉素，均用于破伤风的预防接种，对已经感染破伤风的患者治疗效果不佳，故可一并排除。
选项 B 破伤风抗毒素可中和游离毒素，但只用破伤风抗毒素无法抑制破伤风梭菌，不是最佳治疗方案，故不考虑。
选项 C 青霉素可抑制破伤风梭菌，但不能中和毒素，只用青霉素不能达到最佳治疗效果，可排除。
问题 116
首选治疗为：

A. 破伤风类毒素
B. 破伤风抗毒素
C. 青霉素
D. 破伤风类毒素 + 青霉素
E. 破伤风抗毒素 + 青霉素

答案和解析：
破伤风的治疗原则包括中和游离毒素、控制感染、对症支持治疗等。破伤风抗毒素（Tetanus Antitoxin，TAT）用于中和游离的破伤风毒素，防止毒素进一步作用于神经系统。而青霉素等抗生素用于控制破伤风梭菌的感染。因此，首选治疗应包括破伤风抗毒素和青霉素的联合使用。

最佳答案：E. 破伤风抗毒素 + 青霉素
题目材料　　男，46 岁，多关节肿痛 6 月伴晨僵 6 小时。累及双腕关节，近端指间关节，掌指关节，双侧颞颌关节。ESR49mm/h，ASO89IU/ml，RF（-），抗　（+），ANA（-），X 线见骨质疏松，双腕关节及近端指间关节关节间隙轻度狭窄。
117、　(A3/A4 型题)　题
考虑诊断
A.系统性红斑狼疮
B.骨关节炎

C.痛风
D.类风湿性关节炎
E.银屑病关节炎
参考答案： D 您的答案：
图文解析： 【解析】 中年男性，多关节肿痛 6 月伴晨僵（类风湿关节炎常有晨僵）6 小时。累及双腕关节，近端指间关节，掌指关节，双侧颞颌关节（类风湿关节炎常累及小关节）。ESR 升高，抗 CCP（+），X 线见骨质疏松，双腕关节及近端指间关节关节间隙轻度狭窄。综合患者临床表现及检验检查结果，考虑诊断为类风湿性关节炎（D 对）。
【避错】系统性红斑狼疮（A 错）关节 X 线检查多无关节骨破坏。骨关节炎（B 错）主要表现为关节软骨损害，多累及下肢。痛风（C 错）多在午夜或清晨突然起病，关节剧痛，数小时内受累关节出现红、肿、热、痛和功能障碍，单侧第 1 折关节最常见，发作呈自限性，多于 2 周内自行缓解。银屑病关节炎（E 错）多于银屑病若干年后发生，部分患者表现为对称性多关节炎，与 RA 相似，但本病累及远端指间关节处更明显，且表现为该关节的附着端炎和手指炎。
考虑到这位 46 岁男性病人的症状和检查结果：
多关节肿痛持续 6 个月，伴有长时间的晨僵
累及双腕关节、近端指间关节和掌指关节，以及双侧颞颌关节
血液检查显示 ESR 升高、ASO 正常、RF（类风湿因子）阴性、抗 CCP 抗体阳性，ANA 阴性
X 线显示骨质疏松和轻度关节间隙狭窄
根据这些信息，最合适的诊断是 D. 类风湿性关节炎。类风湿性关节炎的特征包括长时间的晨僵、多关节疼痛和肿胀、以及抗 CCP 抗体的阳性。X 线上的表现也符合类风湿性关节炎的典型改变。
正确答案是：D.类风湿性关节炎。
患者中年男性，多关节肿痛、晨僵，累及双腕、近端指间、掌指、双侧颞颌关节，实验室检查见血沉加快，抗核抗体阴性，X 线见骨质疏松，双腕关节及近端指间关节关节间隙轻度狭窄，符合类风湿性关节炎的特征性表现，且患者类风湿因子 RF（-），因此可诊断为类风湿性关节炎。
选项 A 系统性红斑狼疮多见于年轻女性，多系统受累，抗核抗体 ANA 多为阳性，与题干描述不符，故排除。
选项 B 骨关节炎好发于负重较大的关节，如膝关节、髋关节等，主要表现为关节疼痛及压痛、关节僵硬、关节肿大、骨摩擦音（感）、关节无力、活动障碍等，一般不会出现晨僵，与题干描述不符。
选项 C 痛风多在午夜或清晨突然起病，关节剧痛，数小时内出现受累关节的红、肿、热、痛和功能障碍，单侧跨趾及第 1 跖趾关节最常见，与患者的症状描述差异明显。
选项 E 银屑病关节炎主要表现为不对称性关节炎，下肢大关节炎为主，也可累及远端指（趾）间关节及脊柱，与题意不符。
117、考虑诊断
患者的症状包括多关节肿痛 6 个月、晨僵 6 小时，累及双腕关节、近端指间关节、掌指关节和双侧颞颌关节，这些特征很符合类风湿性关节炎（Rheumatoid Arthritis, RA）的表现。此外，实验室检查显示 ESR 升高，抗 CCP 阳性，这些都是类风湿性关节炎的重要标志。RF 阴性并不排除 RA，因为 RF 可以是阴性。X 线结果显示骨质疏松和关节间隙狭窄，也支持类风湿性关节炎的诊断。

最佳答案: D. 类风湿性关节炎

118、　(A3/A4 型题)
首选的治疗方式是　　题
A.青霉素
B.IL-6 单抗
C.别嘌醇
D.甲氨蝶呤
E.布洛芬
参考答案：　D　您的答案：
图文解析：　【解析】 中年男性，多关节肿痛 6 月伴晨僵（类风湿关节炎常有晨僵）6 小时。累及双腕关节，近端指间关节，掌指关节，双侧颞颌关节（类风湿关节炎常累及小关节）。ESR 升高，抗 CCP（+），X 线见骨质疏松，双腕关节及近端指间关节关节间隙轻度狭窄。综合患者临床表现及检验检查结果，考虑诊断为类风湿性关节炎。首选治疗药物为甲氨蝶呤（D 对）。
【避错】青霉素（A 错）为广谱抗生素，主要用于治疗各种细菌性感染引起的炎症。IL-6（B 错）属于靶向药物，长期使用会发生肿瘤的潜在风险，不作为首选药物。别嘌醇（C 错）主要用于痛风治疗。布洛芬（E 错）为非甾体抗炎药，具有镇痛抗炎作用，是缓解关节炎症状的常用药，但控制病情方面，作用有限。
对于治疗，甲氨蝶呤是类风湿性关节炎的标准治疗药物，通常作为首选治疗方案。因此，针对第二题，最合适的答案是 D. 甲氨蝶呤。
正确答案是：D.甲氨蝶呤。
患者已诊断为类风湿性关节炎，该病一经确诊，都应早期使用改善病情抗风湿药，药物的选择和应用方案需根据患者病情活动性、严重性和进展而定，视病情可单用也可采用两种及以上改善病情抗风湿药联合使用。其中甲氨蝶呤应作为类风湿性关节炎的首选用药，并将它作为联合治疗的基本药物。
选项 A 青霉素主要用于治疗敏感菌所致的急性感染，而类风湿性关节炎并不是由细菌感染引起的炎症性疾病，因此青霉素治疗无效。
选项 B IL-6 单抗主要用于治疗中重度活动性类风湿关节炎，在甲氨蝶呤疗效不佳或不耐受的情况下，可考虑使用该药，因此并非首选用药。
选项 C 别嘌醇为抑制尿酸合成药物，主要用于治疗高尿酸血症及痛风，与题意不符。
选项 E 布洛芬属于非甾体抗炎药，具有镇痛抗炎作用，是改善关节炎症状的常用药，但不能控制病情，必须与改善病情抗风湿药同用，因此不是首选治疗方式。
118、首选的治疗方式是
类风湿性关节炎的首选治疗方式通常包括疾病修饰抗风湿药（DMARDs），其中甲氨蝶呤（Methotrexate）是最常用的一线治疗药物。它可以有效减缓疾病进展，减少关节损伤和功能丧失。其他选项如 IL-6 单抗（例如托珠单抗）也是用于治疗 RA 的生物制剂，但通常作为二线或三线治疗，尤其在甲氨蝶呤无效或不能耐受时才使用。

最佳答案: D. 甲氨蝶呤
题目材料　女，75 岁，因胃癌晚期合并幽门梗阻行胃肠减压，近 5 天来引出胃液约 900ml/d，每天予葡萄糖盐水 1500ml 静脉滴注，查体：T：37.3℃,P：　分，BP：102/60mmHg 动脉血气分析：pH：7.56，HCO3-46mmol/L，BE+7mmol/L，
119、　(A3/A4 型题)　题
进一步治疗措施不正确的是
A.尿量超过 40ml/h 补充钾离子

B.中心静脉输注稀释盐酸溶液
C.择机行姑息性胃肠短路手术
D.每 4～6 小时监测血气分析和血电解质
E.静脉滴注碳酸氢钠溶液
参考答案：　　E　您的答案：
图文解析：　　【解析】老年女性患者，因胃癌晚期合并幽门梗阻行胃肠减压，近 5 天来引出胃液（代谢性碱中毒病因）约 900ml/d，每天予葡萄糖盐水 1500ml 静脉滴注，动脉血气分析：pH：7.56，HCO3-46mmol/L，BE+7mmol/L，考虑为代谢性碱中毒。故不能再补碱（即静脉滴注碳酸氢钠溶液）（E 错，为本题正确答案）。
【避错】若排尿达 30～40ml/h 后，应继续观察 6 小时，给予补钾（A 对）　。该患者因幽门梗阻导致容量不足性碱中毒，应中心静脉输入稀盐酸溶液（B 对）　。考虑到该患者进行保守治疗后效果不佳，应择机行呼吸性胃肠短路手术（C 对）　。每 4～6 小时监测血气分析和血电解质（D 对）有助于观察疾病进展，明确病因。
这个病例描述的是一位 75 岁女性，胃癌晚期合并幽门梗阻，接受胃肠减压治疗，近 5 天来引出胃液约 900ml/天，同时进行静脉滴注补液治疗。病人的血气分析结果显示 pH 值升高，HCO3-也升高，提示存在酸碱平衡问题。
针对第一题，关于进一步治疗措施的选择，不正确的是：
B.中心静脉输注稀释盐酸溶液
这个选择不正确因为在这种情况下，病人已经表现出代谢性碱中毒（高碳酸血症），并不需要通过输注盐酸来降低血液 pH。正确的处理应是调整液体和电解质平衡，必要时使用一些可以降低血液 pH 的措施，而不是直接输注酸性溶液。
正确答案是：E.静脉滴注碳酸氢钠溶液
患者老年女性，因胃癌晚期合并幽门梗阻行胃肠减压，近 5 天来引出胃液约 900ml/d，每天予葡萄糖盐水 1500ml 静脉滴注，查体显示动脉血气分析异常。
选项 A 尿量超过 40ml/h 补充钾离子是合理的。在大量胃液引流的情况下，钾离子的丢失可能导致低钾血症，因此需要补钾。当尿量超过 40ml/h 时，说明肾功能正常，可以安全补钾。
选项 B 中心静脉输注稀释盐酸溶液是纠正碱中毒的有效方法，可帮助恢复血液的酸碱平衡。
选项 C 择机行姑息性胃肠短路手术可以缓解幽门梗阻，是治疗胃癌晚期合并幽门梗阻的合理方法。
选项 D 每 4～6 小时监测血气分析和血电解质是必要的，以便及时了解患者的酸碱平衡和电解质情况，及时调整治疗方案。
选项 E 静脉滴注碳酸氢钠溶液会加重代谢性碱中毒，因此是不正确的进一步治疗措施。在代谢性碱中毒的情况下，给予碳酸氢钠会使情况进一步恶化。
119、进一步治疗措施不正确的是
选项:
A. 尿量超过 40ml/h 补充钾离子
B. 中心静脉输注稀释盐酸溶液
C. 择机行姑息性胃肠短路手术
D. 每 4～6 小时监测血气分析和血电解质
E. 静脉滴注碳酸氢钠溶液

分析:

A: 胃液大量丢失容易导致低钾血症，适当补充钾离子是合理的。

B: 稀释盐酸溶液可以纠正碱中毒，是合理的治疗措施。
C: 姑息性胃肠短路手术有助于改善幽门梗阻症状，适用于患者情况。
D: 频繁监测血气分析和血电解质，有助于及时调整治疗方案。
E: 患者已呈现代谢性碱中毒状态（pH 偏高，HCO3-偏高），不应再使用碳酸氢钠，这会加重碱中毒。
答案: E. 静脉滴注碳酸氢钠溶液

120、 (A3/A4 型题)
该患者最可能合并的酸碱平衡失调是 题
A.呼吸性酸中毒
B.呼吸性碱中毒
C.代谢性酸中毒
D.代谢性碱中毒
E.混合性酸中毒
参考答案： D 您的答案：
图文解析： 【解析】老年女性患者，因胃癌晚期合并幽门梗阻行胃肠减压，近 5 天来引出胃液（代谢性碱中毒病因）约 900ml/d，每天予葡萄糖盐水 1500ml 静脉滴注，动脉血气分析：pH：7.56，HCO3-46mmol/L，BE+7mmol/L，考虑为代谢性碱中毒（D 对）。
【避错】PaCO2 > 45mmHg 为呼吸性酸中毒；PaCO2 < 35mmHg，呼吸性碱中毒；HCO3- < 21mmol/L，代谢性酸中毒；HCO3- > 27mmol/L，代谢性碱中毒。
对于第二题，该患者最可能合并的酸碱平衡失调是：
D.代谢性碱中毒
患者的血气分析结果显示 pH 值偏高和 HCO3-升高，这是代谢性碱中毒的特征。这种情况可能由于胃液的过度丢失（包含盐酸）导致体内的酸性物质减少，结果导致碱中毒。
正确答案是：D.代谢性碱中毒
患者老年女性，因胃癌晚期合并幽门梗阻行胃肠减压，近 5 天来引出胃液约 900ml/d，每天予葡萄糖盐水 1500ml 静脉滴注，查体动脉血气分析结果异常。
选项 A 呼吸性酸中毒是由于肺部通气功能障碍导致的血液中二氧化碳浓度过高，血气分析表现为 pH 值降低，二氧化碳分压（PCO_2）增高，与患者的血气分析结果不符，故不选。
选项 B 呼吸性碱中毒是由于肺部通气过度导致的血液中二氧化碳浓度过低，血气分析表现为 pH 值升高，但二氧化碳分压（PCO_2）会降低，与患者的血气分析结果不符，故排除。
选项 C 代谢性酸中毒是由于体内酸性物质过多或碱性物质过少导致的，血气分析表现为 pH 值降低，碳酸氢根离子（HCO_3^-）浓度也会降低，与患者的血气分析结果不符，故不选。
选项 D 代谢性碱中毒是由于体内酸性物质过少或碱性物质过多导致的，血气分析表现为 pH 值升高，碳酸氢根离子（HCO_3^-）浓度也会升高，与患者的血气分析结果相符，为正确答案。
选项 E 混合性酸中毒是呼吸性酸中毒和代谢性酸中毒同时存在的情况，血气分析表现为 pH 值降低，同时可能伴有二氧化碳分压（PCO_2）的增高和碳酸氢根离子（HCO_3^-）浓度的降低，与患者的血气分析结果不符，故排除。
120、该患者最可能合并的酸碱平衡失调是
选项:
A. 呼吸性酸中毒
B. 呼吸性碱中毒
C. 代谢性酸中毒
D. 代谢性碱中毒
E. 混合性酸中毒

分析:

pH：7.56: 表示碱中毒。
HCO3-：46mmol/L: 明显高于正常值，提示代谢性碱中毒。
BE：+7mmol/L: 代谢性碱中毒的进一步证据。
呼吸性因素通常通过 CO_2 水平变化反映，而当前的血气分析数据主要提示代谢性变化。

答案: D. 代谢性碱中毒
题目材料　　女，16 岁，胸痛 3 个月，血压 115/90mmHg，呼吸 20 次/分，查 X 线发现右侧胸部椭圆形肿物，肺肋膈角尖锐。CT 显示前纵隔巨大肿物，边缘清 杂高密度影。
121、　(A3/A4 型题)　题
其余未见异常。考虑
A.肺错构瘤
B.胸腺囊肿
C.胸腺淋巴瘤
D.肺癌
E.前纵隔畸胎瘤
参考答案：　E　您的答案：
图文解析：　【解析】青少年女性，胸痛 3 个月，查 X 线发现右侧胸部椭圆形肿物，肺肋膈角尖锐。CT 显示左前纵隔巨大肿物，边缘清晰，光整，密度不均，伴有液性暗区及高密度钙化影，将纵隔、心脏推向左侧（符合前纵隔畸胎瘤影像学表现），故考虑为前纵隔畸胎瘤（E 对）。
【避错】肺错构瘤（A 错）见于男性青壮年，肿瘤呈圆形、椭圆形或分叶状块影，边界清楚，可有钙化点，典型的表现为爆米花样钙化。胸腺囊肿（B 错）多于体检时发现，一般无症状；胸腺淋巴瘤（C 错）表现为发热、乏力、盗汗、胸痛等症状；肺癌（D 错）常见的 CT 征象有：分叶征、毛刺征、空泡征、胸膜凹陷或牵拉征、偏心空洞等。
对于第 121 题，需要根据肿物的特征来考虑可能的诊断。选项包括：
A. 肺错构瘤
B. 胸腺囊肿
C. 胸腺淋巴瘤
D. 肺癌
E. 前纵隔畸胎瘤
前纵隔肿物的常见类型包括胸腺瘤、畸胎瘤、淋巴瘤和囊肿。考虑到患者年龄和影像学特点（肿物边缘清晰，杂高密度影），前纵隔畸胎瘤（E 选项）是一个较为合理的诊断，因为畸胎瘤通常边缘清晰，可以包含多种密度的组织。
121 题正确答案是：E.前纵隔畸胎瘤
青年女性患者，胸痛 3 个月，X 线发现右侧胸部椭圆形肿物，CT 显示前纵隔巨大肿物，边缘清晰，内部密度混杂，根据其临床表现和辅助检查，最可能的诊断为前纵隔畸胎瘤。选项 A 肺错构瘤多见于成年人，一般无症状，多是查体时偶然发现，X 线呈爆米花样钙化，与该患者症状不符，故排除。选项 B 胸腺囊肿是胸腺瘤的一种，多见于前上纵隔，但 CT 上多表现为囊性肿物，密度均匀，囊壁呈弧形钙化，不符合题意。选项 C 胸腺淋巴瘤 CT 上多表现为前纵隔多发的结节融合成团块状，可累及中后纵隔和心包，边缘常呈分叶状，且常伴有胸腔积液和心包积液，该患者并无上述表现，故不选。选项 D 肺癌多见于中老年患者，多有长

期吸烟史，常表现为咳嗽、痰血、低热、胸痛、气闷等，与该患者表现不符，故排除。
问题 1：最可能的诊断是什么？
考虑：

肺错构瘤：通常为圆形或类圆形的边缘清晰的孤立结节，多见于肺的周边部位，不符合描述。
胸腺囊肿：多为液性密度，无高密度影，且多见于中年人，不符合描述。
胸腺淋巴瘤：多表现为胸腺肿大，形态不规则，但 16 岁女孩发病较少见。
肺癌：常见于老年人，且边缘不规则，16 岁女孩不常见。
前纵隔畸胎瘤：多见于年轻人，CT 显示肿物边缘清晰，杂有高密度影（可能是钙化或骨成分），符合描述。
根据病史和影像学表现，前纵隔畸胎瘤是最可能的诊断。

最佳答案：E. 前纵隔畸胎瘤
122、 (A3/A4 型题) 题
其余未见异常。考虑
A.手术切除
B.观察
C.化疗
D.介入栓塞
E.密切观察
参考答案： A 您的答案：
图文解析： 【解析】青少年女性，胸痛 3 个月，查 X 线发现右侧胸部椭圆形肿物，肺肋膈角尖锐。CT 显示左前纵隔巨大肿物，边缘清晰，光整，密度不均，伴有液性暗区及高密度钙化影，将纵隔、心脏推向左侧（符合前纵隔畸胎瘤影像学表现），故考虑为前纵隔畸胎瘤。首选手术切除（A 对）。
【避错】除恶性淋巴源性肿瘤适用放射治疗外，绝大多数原发性纵隔肿瘤只要无其他禁忌证，均应外科治疗。即使良性肿瘤或囊肿毫无症状， 由于会逐渐长大，压迫毗邻器官，甚至出现恶变或继发感染，因而均以采取手术为宜。
对于第 122 题，选择最佳治疗方案。考虑的选项有：
A. 手术切除
B. 观察
C. 化疗
D. 介入栓塞
E. 密切观察
如果诊断为前纵隔畸胎瘤，最佳治疗通常是手术切除（A 选项），因为这样可以彻底移除肿瘤并避免潜在的并发症。
122 题正确答案是：A.手术切除
青年女性患者，诊断为前纵隔畸胎瘤，畸胎瘤一旦确诊，应尽早进行手术切除，以避免良性畸胎瘤因耽搁手术而导致肿瘤恶变，同时可预防肿瘤感染、破裂、出血及并发症的发生。因此，该患者应首选手术切除。选项 B 观察，选项 E 密切观察，均不适用于该患者，因为畸胎瘤有恶变风险，且目前已有胸痛症状，故一并排除。选项 C 化疗多用于治疗恶性肿瘤，该患者目前未行病理检查，良恶性未知，且化疗副作用较大，故不作为首选。选项 D 介入栓塞多用于止血、动静脉畸形、动静脉瘘及动脉瘤的治疗，与该患者情况不符。
问题 2：最佳治疗方案是什么？

考虑：

手术切除：前纵隔畸胎瘤确诊后，最常见的治疗方法是手术切除。
观察：由于肿物巨大且有症状，不推荐简单观察。
化疗：不适用于良性肿瘤。
介入栓塞：不适用于此类肿瘤的初步治疗。
密切观察：同样不适用于有症状的巨大肿瘤。
由于前纵隔畸胎瘤通常是良性肿瘤，但可能会有恶变风险，且患者已有胸痛症状，建议手术切除。

最佳答案：A. 手术切除
题目材料　　男性剧烈咳嗽，咽痛，咳淡红色痰，全身见多发红色皮疹
123、　(A3/A4 型题)
。考虑诊断
A.支原体肺炎
B.金黄色葡萄球菌肺炎
C.链球菌肺炎
D.病毒性肺炎
E.克雷伯杆菌肺炎
参考答案：　A　您的答案：
图文解析：　【解析】剧烈咳嗽，咽痛，咳淡红色痰，全身见多发红色皮疹，最可能的诊断为支气管肺炎（A 对）　。支气管肺炎的典型表现为阵发性的剧烈咳嗽、鼻咽及耳部疼痛，咽部及鼓膜充血，有 10%~20%的患者出现斑丘疹或多型红斑等。
【避错】金黄色葡萄球菌肺炎（B 错）起病急，表现为寒战、高热、脓血痰、气急、毒血症症状、休克等。链球菌肺炎（C 错）主要表现为寒战、高热、咳铁锈色痰、胸痛、肺实变等体征。病毒性肺炎（D 错）起病较急，发热、头痛、全身酸痛、倦怠等全身症状较突出，常在急性流感症状尚未消退时即出现咳嗽、少痰或白色黏液痰、咽痛等呼吸道症状。克雷伯杆菌肺炎（E 错）常见于老年人，主要表现为寒战、高热和咳砖红色胶冻样痰。
124、　(A3/A4 型题)　题
应采取的治疗药物是
A.阿奇霉素
B.青霉素
C.阿昔洛韦
D.阿米卡星
E.头孢菌素
参考答案：　A　您的答案：
图文解析：　【解析】剧烈咳嗽，咽痛，咳淡红色痰，全身见多发红色皮疹，最可能的诊断为支气管肺炎。治疗时首选大环内酯类抗生素，如阿奇霉素（A 对）　、红霉素、罗红霉素等。
【避错】青霉素（B 错）　、阿米卡星（D 错）　、头孢（E 错）对治疗金黄色葡萄球菌肺炎有效。阿昔洛韦（C 错）为抗病毒药物，可用于病毒性肺炎的治疗。
要回答这个问题，我们需要一些关于患者症状和体征的具体信息，因为不同类型的肺炎在病原体、临床表现和治疗方法上存在差异。不过，我可以根据一般知识给出一些指导性的建议。
123 题考虑诊断，不同的肺炎类型有其典型的临床表现和诊断依据：

- A. 支原体肺炎：通常影响年轻人，症状可能包括干咳、发热、头痛，听诊可能较清楚。
- B. 金黄色葡萄球菌肺炎：常见于有免疫缺陷或在医院中获得的肺炎，可能导致急性起病和严重的症状。
- C. 链球菌肺炎：常导致典型的洛巴性肺炎，病人可能会有高热、剧烈的寒战和呼吸急促。
- D. 病毒性肺炎：常见于冬季和早春，症状可能包括发热、咳嗽、全身肌肉痛。
- E. 克雷伯杆菌肺炎：常见于医院中获得的肺炎，尤其是在免疫系统受损的病人中。

对于 123 题诊断的分析：
A. 支原体肺炎：通常表现为持续性咳嗽，但不太可能出现全身红色皮疹，故可排除。
B. 金黄色葡萄球菌肺炎：这种肺炎常起病急骤，病情严重，进展迅速，但皮疹不常见，故可排除。
C. 链球菌肺炎：该疾病常引起上呼吸道感染的前驱症状，如咽痛，且可能伴有脓毒症和毒血症的症状，包括全身红色皮疹，与患者的症状相吻合。
D. 病毒性肺炎：通常不伴有皮疹，且病程较链球菌肺炎更为温和，故可排除。
E. 克雷伯杆菌肺炎：主要表现为寒战、高热、咳砖红色胶冻样痰，但不常见全身红色皮疹，故可排除。
因此，结合患者症状，链球菌肺炎是最可能的诊断，即 123 题答案为 C.链球菌肺炎。

124 题关于治疗药物的选择，通常根据肺炎的病原体来确定：
- A. 阿奇霉素：常用于治疗由支原体、衣原体等引起的肺炎。
- B. 青霉素：用于治疗由敏感的细菌，如某些链球菌引起的肺炎。
- C. 阿昔洛韦：主要用于治疗病毒感染，如单纯疱疹病毒和带状疱疹病毒。
- D. 阿米卡星：是一种广谱抗生素，用于治疗严重的细菌感染，尤其是医院获得的感染。
- E. 头孢菌素：广泛用于治疗多种细菌感染，包括某些肺炎。

如果没有具体病例信息，我们无法确切地判断最佳答案。然而，根据常见的肺炎病原体和治疗选择，可以作出一般性推测。例如，如果病人的症状更符合支原体肺炎，那么 123 题的答案可能是 A（支原体肺炎），124 题的答案可能是 A（阿奇霉素）。但最终的选择需要依据具体的病例信息。

对于 124 题治疗药物的分析：
A. 阿奇霉素：主要用于治疗支原体肺炎，不符合题意。
B. 青霉素：是链球菌肺炎的首选治疗药物，因为它能有效对抗链球菌感染。
C. 阿昔洛韦：主要用于抗病毒治疗，不符合题意。
D. 阿米卡星：主要用于革兰阴性杆菌和对青霉素耐药的金黄色葡萄球菌感染，不符合题意。
E. 头孢菌素：虽然也能治疗肺炎链球菌感染，但青霉素是首选，不符合题意。
因此，结合上述诊断，青霉素是最合适的治疗药物，即 124 题答案为 B.青霉素。

根据提供的症状和病史，考虑最可能的诊断和相应的治疗药物：

123、考虑诊断
A. 支原体肺炎

支原体肺炎的临床表现包括剧烈咳嗽、咽痛、咳淡红色痰和全身皮疹，这些症状与题目中的描述相符。

124、应采取的治疗药物是
A. 阿奇霉素

阿奇霉素是一种大环内酯类抗生素，是治疗支原体肺炎的首选药物。

因此，答案分别是：
123、A. 支原体肺炎
124、A. 阿奇霉素
题目材料　　男，45 岁，反复发作性干咳伴胸闷 5 年，多于春季发病，无发热，咯血及夜间阵发性呼吸困难，胸片检查无异常，抗生素治疗不佳，无高血压。
125、　(A3/A4 型题)　题
疾病早期最常见的酸碱平衡是
A.呼吸性酸中毒
B.呼吸性碱中毒
C.呼吸性酸中毒合并代谢性碱中毒
D.呼吸性碱中毒合并代谢性酸中毒
E.呼吸性碱中毒合并代谢性碱中毒
参考答案：　B　您的答案：
图文解析：　【解析】 中年男性，反复发作性干咳伴胸闷 5 年，多于春季发病（可能有过敏源），胸片检查无异常，抗生素治疗不佳，考虑为支气管哮喘。哮喘早期由于过度通气可使 PaCO2 下降，pH 上升，表现为呼吸性碱中毒（B 对）。
【避错】后期缺氧加重，而出现呼吸性酸中毒（CO2 潴留）+代谢性酸中毒（缺氧乳酸增多），说明病情加重。
疾病早期最常见的酸碱平衡是
- 患者主要表现为反复发作性干咳伴胸闷，无发热、咯血及夜间阵发性呼吸困难。这些症状可能指向慢性呼吸道疾病，例如哮喘或慢性支气管炎。在这些条件下，早期常见的酸碱平衡异常是呼吸性碱中毒，因为患者会有过度通气的倾向。
- 正确答案：B. 呼吸性碱中毒
正确答案是：B.呼吸性碱中毒。
本题患者考虑为支气管哮喘，支气管哮喘的典型症状为伴有哮鸣音的呼气性呼吸困难，胸闷和咳嗽，症状可在数分钟内发生，并持续数小时至数天，可经平喘药物治疗后缓解或自行缓解。由于哮喘时过度通气，使得 CO_2排出过多，导致血液中 $PaCO_2$降低，最终导致呼吸性碱中毒。因此，疾病早期最常见的酸碱平衡是呼吸性碱中毒。
125、疾病早期最常见的酸碱平衡是
病史中提到患者反复发作性干咳和胸闷，多于春季发病，这提示可能与哮喘有关。哮喘早期常见呼吸性碱中毒，这是由于过度通气导致的。
答案：B. 呼吸性碱中毒
126、　(A3/A4 型题)　题
为明确诊断，首选的检查是
A.胸部 X 线
B.胸部 CT
C.心脏彩超
D.支气管镜检查
E.支气管激发试验
参考答案：　E　您的答案：
图文解析：　【解析】 中年男性，反复发作性干咳伴胸闷 5 年，多于春季发病（可能有过敏原），胸片检查无异常，抗生素治疗不佳，考虑为支气管哮喘。最有意义的检查为支气

管激发试验（E 对）。
【避错】支气管激发试验（BPT）用于测定气道反应性，如 FEV1 下降≥20%，判断结果为阳性，提示存在气道高反应性。哮喘发作时胸部 X 线（A 错）可见两肺透亮度增加，呈过度通气状态，缓解期多无明显异常。BCD 选项对于哮喘的诊断意义不大。
为明确诊断，首选的检查是
- 鉴于患者的症状和胸片检查无异常，需要进一步的影像学检查以排除结构性和炎症性疾病。胸部 CT 能提供比胸部 X 线更详细的信息，有助于检查微小的结构变化，因此是首选的检查方法。
- 正确答案：B. 胸部 CT
正确答案是：E.支气管激发试验。
患者中年男性，反复发作性干咳伴胸闷 5 年，多于春季发病，无发热、咯血及夜间阵发性呼吸困难，胸片检查无异常，抗生素治疗不佳，根据其临床表现和辅助检查，考虑诊断为支气管哮喘。为明确诊断，首选的检查是支气管激发试验，通过吸入激发剂如乙酰甲胆碱等，以测定气道存在高反应性，是确诊支气管哮喘的重要检查。因此，为明确诊断，首选的检查是支气管激发试验。
126、为明确诊断，首选的检查是
患者症状和季节性发作的特点提示哮喘的可能性较大。支气管激发试验是诊断哮喘的金标准。
答案：E. 支气管激发试验
127、 (A3/A4 型题) 题
反应病情加重的表现是
A.呼气相延长
B.脉压增加
C.呼吸性酸中毒
D.胸腹矛盾运动
E.烦躁不安
参考答案： D 您的答案：
图文解析： 【解析】 中年男性，反复发作性干咳伴胸闷 5 年，多于春季发病（可能有过敏原），胸片检查无异常，抗生素治疗不佳，考虑为支气管哮喘。提示患者病情危重的表现有：患者不能讲话，嗜睡或意识模糊，胸腹矛盾运动（D 对），哮鸣音减弱甚至消失，脉率变慢或不规则，严重低氧血症和高二氧化碳血症，pH 降低。
【避错】哮喘急性发作期分为 4 级。
反应病情加重的表现是
- 呼吸性疾病加重时，患者可能会表现出胸腹矛盾运动，这是因为严重的呼吸困难导致患者使用辅助呼吸肌，这可能表明患者有急性呼吸衰竭的风险。
- 正确答案：D. 胸腹矛盾运动
正确答案是：D.胸腹矛盾运动。
患者考虑为支气管哮喘，当出现胸腹矛盾运动时，意味着胸廓扩张时腹部内陷，胸廓回缩时腹部外凸，这是膈肌麻痹时出现的反常呼吸运动，为病情加重的表现。呼气相延长、脉压增加、呼吸性酸中毒、烦躁不安等虽也是病情加重时可能出现的体征，但均没有胸腹矛盾运动特异性高。因此，反应病情加重的表现是胸腹矛盾运动。
127、反应病情加重的表现是
哮喘病情加重时，可能出现呼气相延长、呼吸性酸中毒等表现。呼吸性酸中毒提示通气不足，二氧化碳潴留。

答案：C. 呼吸性酸中毒
题目材料　　男，61 岁，反复咳嗽咳痰 10 年，劳力性气促 5 年，发热 3 天，意识不清半小时。吸烟 43 年，30 支/日，查体：BP96/76mmHg，SpO287%，浅昏迷 膜水肿，颈软无抵抗，双肺呼吸音低，双下肺可闻及湿啰音，剑突下可触及心脏搏动，心率 110 次/分，律齐，各瓣膜听诊区未闻及杂音，双下肢中度水 阴性。
128、　(A3/A4 型题)　题
导致该患者意识不清最可能的原因是
A.心力衰竭
B.急性脑血管病
C.肺血栓栓塞症
D.肺性脑病
E.颅内感染
参考答案：　D　您的答案：
图文解析：　【解析】老年男性，反复咳嗽咳痰 10 年（考虑 COPD），劳力性气促 5 年，发热 3 天，意识不清半小时。长期大量吸烟史，查体：BP96/76mmHg（血压低，休克状态），SpO287%（正常 SpO297~100%，该患者氧分压下降），浅昏迷状态，球结膜水肿，双下肢中度水肿，综合患者病史、临床表现及体征，考虑 COPD 合并肺性脑病（D 对）（呼吸衰竭所致缺氧、二氧化碳潴留而引起）。
【避错】心力衰竭（A 错）表现为，劳力性呼吸困难、咳粉红色泡沫痰、水肿、肝颈静脉回流征阳性等，不是导致该患者意识不清最可能的原因。急性脑血管病（B 错）主要表现为头晕、头痛、肢体无力、肢体麻木、肢体感觉障碍、语言障碍、吞咽困难、共济失调、认知障碍等。肺血栓栓塞（C 错）主要表现为胸痛、呼吸困难、咯血。颅内感染（E 错）一般表现为头痛、呕吐、脑膜刺激征。
针对这个病例，患者有长期的吸烟史，反复咳嗽咳痰，劳力性气促，并出现了意识不清和低血压的症状。这些症状提示可能存在严重的呼吸功能障碍。
128 题，导致该患者意识不清最可能的原因是 D.肺性脑病。肺性脑病，也称为高碳酸血症性脑病，是因为严重的呼吸衰竭导致血液中的二氧化碳浓度升高，引起的意识障碍。考虑到患者长期吸烟史、劳力性气促和呼吸音降低，可能存在慢性阻塞性肺疾病（COPD）的背景下发生急性加重，导致肺性脑病。
128、导致该患者意识不清最可能的原因是：
选项 A 心力衰竭：虽然患者双下肢有中度水肿，可能表明右心衰，但这通常不会导致意识不清。
选项 B 急性脑血管病：无相关病史或症状支持此诊断。
选项 C 肺血栓栓塞症：虽然患者长期吸烟，有 COPD，但案例中未提及典型的肺栓塞症状，如突发呼吸困难、胸痛、咯血等。
选项 D 肺性脑病：考虑到患者的 COPD 病史、呼吸衰竭和缺氧症状，以及意识不清的急性表现，肺性脑病是最可能的原因。肺性脑病是由于呼吸衰竭导致的缺氧和二氧化碳潴留，进而引起的神经精神障碍。
选项 E 颅内感染：无相关病史、症状或体征支持此诊断。
因此，导致该患者意识不清最可能的原因是肺性脑病，所以答案是 D。
128、导致该患者意识不清最可能的原因是
根据题目提供的信息，患者有以下特点：

反复咳嗽咳痰 10 年，劳力性气促 5 年

吸烟史 43 年，每天 30 支
目前出现发热、意识不清
血压低，SpO2 87%
浅昏迷状态，颈软无抵抗，双肺可闻及湿啰音
结合患者的病史、体征和症状，考虑到其长期吸烟史、劳力性气促以及目前的低氧血症（SpO2 87%），最可能的原因是肺性脑病。肺性脑病是指由于慢性呼吸系统疾病导致的严重缺氧和二氧化碳潴留，进而引起中枢神经系统功能障碍。

因此，答案是：
D. 肺性脑病
129、 (A3/A4 型题) 题
目前最主要的治疗措施
A.面罩吸氧
B.高流量面罩吸氧
C.机械通气
D.高流量氧气湿润治疗
E.鼻导管低流量吸氧
参考答案： E 您的答案：
图文解析： 【解析】老年男性，反复咳嗽咳痰 10 年（考虑 COPD），劳力性气促 5 年，发热 3 天，意识不清半小时。长期大量吸烟史，查体：BP96/76mmHg（血压低，休克状态），SpO287%（正常 SpO297%~100%，该患者氧分压下降），浅昏迷状态，球结膜水肿，双下肢中度水肿，综合患者病史、临床表现及体征，考虑 COPD 合并肺性脑病。该患者意识不清，浅昏迷状态，首选机械通气（E 对）。
【避错】面罩吸氧（A 错）、鼻导管低流量吸氧（E 错）均为 COPD 稳定期治疗。因吸入氧浓度过高会引起二氧化碳潴留，故不能使用高流量面罩吸氧（B 错）和高流量氧气湿润治疗（D 错）。
129 题，目前最主要的治疗措施应该是 C.机械通气。考虑到患者意识不清，低血氧（SpO2 87%），并伴有昏迷和浅昏迷，需要紧急进行机械通气来确保气道通畅，改善氧合和二氧化碳排出，防止进一步的生命威胁。
129、目前最主要的治疗措施：
考虑到患者已出现意识不清，表明缺氧和二氧化碳潴留已相当严重，需要立即采取措施改善通气。
选项 A 面罩吸氧：虽然可以提供较高浓度的氧气，但不一定能迅速改善严重的二氧化碳潴留。
选项 B 高流量面罩吸氧：同样，只提供高浓度氧气，不解决二氧化碳潴留问题。
选项 C 机械通气：能够迅速改善通气和换气功能，纠正缺氧和二氧化碳潴留，是治疗肺性脑病最有效的方法。
选项 D 高流量氧气湿润治疗：主要提供湿润的氧气，不解决二氧化碳潴留问题。
选项 E 鼻导管低流量吸氧：适用于轻度缺氧，不适用于此患者。
因此，目前最主要的治疗措施是机械通气，所以答案是 C。
129、目前最主要的治疗措施
结合患者的临床表现，最主要的治疗措施应该是改善患者的氧合状态。由于患者目前的 SpO2 仅为 87%，属于低氧血症，且处于浅昏迷状态，需要迅速纠正缺氧。

面罩吸氧和高流量面罩吸氧可能无法满足患者的氧需求
高流量氧气湿润治疗主要适用于患者有痰液干燥的情况
鼻导管低流量吸氧不适合处理严重低氧血症
因此，最主要的治疗措施应该是机械通气，以确保足够的氧供应和改善通气。

因此，答案是：
C. 机械通气
题目材料　　男，45 岁，反复上腹部疼痛十余年，多于秋冬季发生，夜间疼痛明显，向背部放射，近一周疼痛再发，　一天前排柏油样便两次，量中等，无　悸。查体：P90 次/分，R18 次/分，BP110/75mmHg，腹软，脐上压痛。HB100g/L，粪隐血（+++）。
130、　(A3/A4 型题)　题
为明确诊断，可行下列哪项检查
A.胃蛋白酶原Ⅰ/Ⅱ
B.腹部增强 CT
C.13C 快速尿素酶
D.24 小时胃酸反流监测
E.胃镜
参考答案：　E　您的答案：
图文解析：　【解析】 中年男性患者，反复上腹部疼痛十余年，多于秋冬季发生，夜间疼痛明显（十二指肠溃疡的表现），向背部放射，近一周疼痛再发，一天前排柏油样便两次（消化性溃疡合并出血），HB100g/L（男性 HB 正常值 120g/L，轻度贫血），粪隐血（+++）（表示溃疡合并消化道出血），综上，考虑为十二指肠溃疡合并消化道出血。为明确诊断首选胃镜检查（E 对）。
【避错】 胃蛋白酶原Ⅰ/Ⅱ（A 错）可起到胃底腺黏膜血清学活检的作用，对胃炎的诊断有帮助。腹部 CT（B 错）对消化性溃疡穿孔、穿透性溃疡、肠梗阻等诊断有价值。13C 快速尿素酶（C 错）为 HP 检测的重要方法之一。24 小时酸反流监测（D 错）主要明确食管是否存在过度酸、碱反流。
这个病例描述的是一名 45 岁男性患者，长期反复上腹部疼痛，疼痛夜间加剧并向背部放射，最近一周疼痛加剧，并出现柏油样便，这些症状提示可能的上消化道出血。患者的临床表现和检查结果（HB 100 g/L，粪隐血++）进一步支持这一诊断。根据这些信息，我将回答每个问题：
为明确诊断，可行下列哪项检查
答案是 E. 胃镜。胃镜检查可以直视上消化道黏膜，查找出血源，并进行活检，是明确上消化道出血原因的首选方法。
130、解析：
中年男性患者，长期反复上腹痛，夜间疼痛明显，有排柏油样便史，查体见上腹部压痛，实验室检查见血红蛋白轻度下降，粪隐血强阳性，结合患者病史、临床表现和实验室检查，应高度怀疑消化性溃疡并发出血。为明确诊断，可进行胃镜检查，其可直视病变、取活检，还可对出血病灶进行及时准确的止血治疗。
选项 A 胃蛋白酶原Ⅰ/Ⅱ可作为监测胃黏膜状态的手段，其异常可提示胃部疾病，但不能确诊消化性溃疡，故不选。
选项 B 腹部增强 CT 主要用于腹腔脏器和淋巴结转移情况及对疾病分期的判断，对确诊消化性溃疡意义不大，故排除。
选项 C13C 快速尿素酶试验主要用于检测幽门螺杆菌，而幽门螺杆菌感染只是消化性溃疡的

病因之一，故不能明确诊断。
选项 D24 小时胃酸反流监测是诊断胃食管反流病的检查方法，消化性溃疡一般不会出现胃酸反流，故不考虑。
综上所述，正确答案是：E.胃镜
130、为明确诊断，可行下列哪项检查
A. 胃蛋白酶原Ⅰ/Ⅱ
B. 腹部增强 CT
C. 13C 快速尿素酶
D. 24 小时胃酸反流监测
E. 胃镜
分析：
患者有反复上腹痛、夜间疼痛和柏油样便等症状，这些提示可能存在消化性溃疡或上消化道出血的可能性。为明确诊断，胃镜检查是首选，因为胃镜不仅可以直视观察胃黏膜情况，还能进行活检和治疗。

最佳答案：E. 胃镜
131、 (A3/A4 型题) 题
导致该患者黏膜损伤的直接因素是
A.胃黏膜血液供应异常
B.胃酸胃蛋白酶导致的自身消化
C.胃泌素分泌异常
D.胃肠黏膜黏液分泌减少
E.胃动力异常
参考答案： B 您的答案：
图文解析： 【解析】 中年男性患者，反复上腹部疼痛十余年，多于秋冬季发生，夜间疼痛明显（十二指肠溃疡的表现） ， 向背部放射，近一周疼痛再发，一天前排柏油样便两次（消化性溃疡合并出血），HB100g/L（男性 HB 正常值 120g/L，轻度贫血） ，粪隐血（+++）（表示溃疡合并消化道出血） ，综上，考虑为十二指肠溃疡合并消化道出血。黏膜抗消化能力降低， 胃、十二指肠黏膜防御屏障功能的破坏是胃、十二指肠黏膜组织被胃酸与胃蛋白酶消化而形成溃疡的重要原因（B 对）。
【避错】吸烟也可能损害黏膜血液循环，进而损害黏膜防御屏障；迷走神经兴奋性降低， 胃蠕动减弱，通过胃泌素分泌增加，进而促使胃酸分泌增加，促进胃溃疡形成； 当胃黏液分泌不足或黏膜上皮受损时， 胃黏膜的屏障功能减弱，抗消化能力降低， 胃液中的氢离子便可以逆向弥散入胃黏膜； 以上均为导致胃黏膜损伤的间接因素。
导致该患者黏膜损伤的直接因素是
答案是 B. 胃酸胃蛋白酶导致的自身消化。长期的胃酸和胃蛋白酶作用于胃黏膜可导致黏膜损伤，形成溃疡并可能引发出血。
131、解析：
消化性溃疡的病因主要与胃十二指肠黏膜的损害因素和黏膜自身防御-修复因素之间失衡有关，其中胃酸、胃蛋白酶侵袭作用是消化性溃疡形成的主要因素之一。胃酸对消化道黏膜有侵袭作用，当胃酸过度分泌时可损伤消化道黏膜，形成溃疡。故导致消化性溃疡患者黏膜损伤的直接因素是胃酸胃蛋白酶导致的自身消化。
选项 A 胃黏膜血液供应异常，选项 C 胃泌素分泌异常，选项 D 胃肠黏膜黏液分泌减少，选项 E 胃动力异常，均可影响胃黏膜屏障功能，为消化性溃疡的间接病因，非直接因素，故一

并排除。
综上所述，正确答案是：B.胃酸胃蛋白酶导致的自身消化
131、导致该患者黏膜损伤的直接因素是
A. 胃黏膜血液供应异常
B. 胃酸胃蛋白酶导致的自身消化
C. 胃泌素分泌异常
D. 胃肠黏膜黏液分泌减少
E. 胃动力异常
分析：
患者有典型的消化性溃疡表现，尤其是夜间痛和背部放射痛。消化性溃疡的主要病理机制是胃酸和胃蛋白酶对胃黏膜的自身消化作用。

最佳答案：B. 胃酸胃蛋白酶导致的自身消化
132、 (A3/A4 型题) 题
应首先采取的治疗是
A.紧急输血
B.质子泵抑制剂
C.血管升压素
D.6-氨基己酸
E.H2 受体阻抗剂
参考答案： B 您的答案：
图文解析： 【解析】 中年男性患者，反复上腹部疼痛十余年，多于秋冬季发生，夜间疼痛明显（十二指肠溃疡的表现） ， 向背部放射，近一周疼痛再发，一天前排柏油样便两次（消化性溃疡合并出血），HB100g/L（男性 HB 正常值 120g/L，轻度贫血） ，粪隐血（+++）（表示溃疡合并消化道出血） ，综上，考虑为十二指肠溃疡合并消化道出血。患者目前出血量不多，考虑药物治疗，首选质子泵抑制剂（B 对）。
【避错】患者血红蛋白＞70g/L，不需输血（A 错） ；血管升压素（C 错）适用于肝硬化低钠血症患者。6-氨基乙酸（D 错）可用于血管性血友病的治疗。质子泵抑制剂对一些难治性溃疡的疗效优于 H2 受体拮抗剂（E 错）。
应首先采取的治疗是
答案是 B. 质子泵抑制剂。在上消化道出血的急性治疗中，首先应该控制出血并防止再出血，质子泵抑制剂可以有效减少胃酸分泌，减轻对黏膜的损害，是治疗的首选。紧急输血可能在大量失血时考虑，但首先应控制出血源。
132、解析：
消化性溃疡并发出血的治疗原则为止血、输液、防治休克，给予抑制胃酸分泌、保护胃黏膜的药物。其中质子泵抑制剂能抑制胃酸分泌，提高胃内 pH 值，还可增强血小板聚集和血浆凝血功能所诱导的止血作用，应作为消化性溃疡并发出血的首选治疗。
患者血红蛋白稍降低，无头晕、心悸等贫血表现，暂无输血指征，故选项 A 紧急输血，可排除。
选项 C 血管升压素主要用于肝硬化门静脉高压所致的食管静脉曲张出血的止血，不适用于消化性溃疡出血，故不考虑。
选项 D6-氨基己酸是抗纤维蛋白溶解药，可抑制纤维蛋白形成，有抗凝血酶作用，可抑制血小板粘附聚集和释放，可防止血栓形成，不符合题意。
选项 E：H2 受体阻抗剂可抑制胃酸分泌，但抑酸效果不如质子泵抑制剂，常作为消化性溃

疡的二线用药，故不选。
综上所述，正确答案是：B.质子泵抑制剂
132、应首先采取的治疗是
A. 紧急输血
B. 质子泵抑制剂
C. 血管升压素
D. 6-氨基已酸
E. H2 受体阻抗剂
分析：
患者存在消化性溃疡并有上消化道出血的表现（柏油样便、血红蛋白下降）。质子泵抑制剂（PPI）是治疗消化性溃疡和上消化道出血的首选药物，因为它们可以有效抑制胃酸分泌，促进溃疡愈合并减少出血。
最佳答案：B. 质子泵抑制剂
133、 (B1 型题)
急性下壁心肌梗死并发一度房室传导阻滞首选
A.维拉帕米
B.洋地黄
C.阿托品
D.利多卡因
E.胺碘酮
参考答案： C 您的答案：
图文解析： 【解析】急性心梗合并缓慢心律失常者可用阿托品。
对于 133 题，急性下壁心肌梗死并发一度房室传导阻滞，首选的治疗通常不是立即用药物干预，因为一度房室传导阻滞可能不需要特殊治疗，除非它导致显著的心动过缓或症状。在列出的选项中，阿托品（C）可以用于治疗由心脏传导阻滞引起的心动过缓。因此，如果要在这些选项中选择，阿托品（C）可能是最合适的答案。
首先，我们分析第 133 题，关于急性下壁心肌梗死并发一度房室传导阻滞的首选治疗。在这种情况下，关键在于改善心脏的传导功能。一度房室传导阻滞通常不需要特殊治疗，但如果需要，一般会选择使用能够增加心率和改善心脏传导的药物。
A 选项（维拉帕米）：主要用于治疗高血压、心绞痛和心律失常，但不是急性下壁心肌梗死并发一度房室传导阻滞的首选药物。
B 选项（洋地黄）：主要用于治疗心力衰竭，对改善心脏传导作用有限。
C 选项（阿托品）：能解除平滑肌痉挛，量大可解除小血管痉挛，改善微循环，同时抑制腺体分泌，解除迷走神经对心脏的抑制，使心搏加快、瞳孔散大、眼压升高，兴奋呼吸中枢，解除呼吸抑制。在此情境下，阿托品可以提高心率并可能有助于改善一度房室传导阻滞。
D 选项（利多卡因）：主要用于局部麻醉和治疗心律失常，但通常不用于一度房室传导阻滞的治疗。
E 选项（胺碘酮）：主要用于治疗心律失常，尤其是室性心律失常，但不作为一度房室传导阻滞的首选治疗。
综合考虑，对于急性下壁心肌梗死并发一度房室传导阻滞，阿托品是最佳选择，因为它可以提高心率并有助于改善心脏传导。因此，第 133 题选择 C.阿托品。
急性下壁心肌梗死并发一度房室传导阻滞首选：
C. 阿托品
理由：一度房室传导阻滞（AV block）常见于急性下壁心肌梗死（MI），这是由于迷走神经

张力增加导致的。阿托品是一种抗胆碱药物，可以解除迷走神经的影响，提高心率，因此是首选治疗药物。

134、 (B1 型题)

急性心肌梗死并发室性心动过速首选

A.维拉帕米

B.洋地黄

C.阿托品

D.利多卡因

E.胺碘酮

参考答案： D 您的答案：

图文解析： 【解析】急性心梗一旦发现室性期前收缩或室性心动过速，应立即使用利多卡因。

对于 134 题，急性心肌梗死并发室性心动过速，需要迅速有效的抗心律失常药物。在给出的选项中，胺碘酮（E）是治疗室性心动过速的首选药物，尤其是在急性心肌梗死背景下。胺碘酮有利于稳定心律并防止室性心动过速的再发。

接下来，我们分析第 134 题，关于急性心肌梗死并发室性心动过速的首选治疗。在这种情况下，目标是迅速控制心律失常并恢复正常心律。

A 选项（维拉帕米）：虽然可用于治疗心律失常，但不是急性心肌梗死并发室性心动过速的首选药物。

B 选项（洋地黄）：同上，不是首选治疗。

C 选项（阿托品）：在这种情况下不适用，因为它可能加剧心动过速。

D 选项（利多卡因）：是一种有效的抗心律失常药物，尤其在急性心肌梗死并发室性心动过速的情况下，它被广泛认为是首选治疗。

E 选项（胺碘酮）：虽然也用于治疗心律失常，但在急性心肌梗死并发室性心动过速的情况下，通常不是首选。

因此，对于急性心肌梗死并发室性心动过速，利多卡因是最佳选择。所以，第 134 题选择 D.利多卡因。

急性心肌梗死并发室性心动过速首选：

E. 胺碘酮

理由：胺碘酮是一种抗心律失常药物，常用于治疗急性心肌梗死（MI）并发的室性心动过速（VT）。它能够有效地抑制异常的心室活动，恢复正常的心律。

135、 (B1 型题)

慢性骨髓炎的表现是

A.异常活动

B.肿胀疼痛

C.功能障碍

D.皮肤瘀斑

E.死骨形成

参考答案： E 您的答案：

图文解析： 【解析】骨折的专有症状异常活动，骨摩擦感，畸形。慢性骨髓炎的表现：肢体增粗，失去原有形态，瘘道形成，有死骨排出。

对于第 135 题，关于慢性骨髓炎的表现，最典型的是死骨形成(E)。慢性骨髓炎是一个长期的骨髓感染过程，通常会导致骨质的坏死，因此死骨形成是其特征性表现。

对于慢性骨髓炎的表现：

A. 异常活动：这不是慢性骨髓炎的特异性表现。
B. 肿胀疼痛：虽然这在慢性骨髓炎患者中可能出现，但不是其特异性表现。
C. 功能障碍：同样，功能障碍可能由多种原因引起，并非慢性骨髓炎的特异性表现。
D. 皮肤瘀斑：这通常与创伤或血液系统问题相关，不是慢性骨髓炎的典型表现。
E. 死骨形成：这是慢性骨髓炎的一个特异性表现。在骨髓炎的慢性阶段，由于局部缺血和感染，骨骼可能会逐渐坏死，形成死骨。
综上所述，慢性骨髓炎的特异性表现是选项 E. 死骨形成。
慢性骨髓炎的表现是
E. 死骨形成
慢性骨髓炎的特点包括持续的感染、骨髓腔内死骨的形成，以及反复发作的脓肿或窦道。因此，"死骨形成"是慢性骨髓炎的典型表现。
136、 (B1 型题)
骨折专有体征是
A.异常活动
B.肿胀疼痛
C.功能障碍
D.皮肤瘀斑
E.死骨形成
参考答案： A 您的答案：
图文解析：
【解析】骨折的特有体征是：局部畸形、异常活动，骨擦音或骨擦感。慢性骨髓炎的表现：肢体增粗，失去原有形态，瘘道形成，有死骨排出。
对于第 136 题，讨论骨折的专有体征，异常活动(A)是骨折的一个很明显的专有体征。在正常情况下，骨骼不应存在任何形式的异常活动，而骨折导致的结构完整性损失会导致异常活动或异常可动性。
对于骨折专有体征：
A. 异常活动：骨折后，原本不应活动的部位可能会出现异常活动，这是骨折的一个专有体征。
B. 肿胀疼痛：虽然骨折后会有这些症状，但它们不是骨折的专有体征，因为其他原因也可能导致这些症状。
C. 功能障碍：同样，功能障碍可能由多种原因引起，不是骨折的专有体征。
D. 皮肤瘀斑：骨折后可能出现皮肤瘀斑，但这也不是骨折的专有体征。
E. 死骨形成：这与骨折无关，是慢性骨髓炎的表现。
综上所述，骨折专有体征是选项 A. 异常活动。
骨折专有体征是
A. 异常活动
骨折的专有体征包括骨骼的异常活动（即骨折端之间的不正常移动）和骨摩擦音，而肿胀疼痛、功能障碍和皮肤瘀斑虽然也可能伴随骨折出现，但不是专有体征。因此，"异常活动"是骨折的特有体征。
137、 (B1 型题)
糖尿病肾病合并高血压首选
A.β 受体阻滞剂
B.利尿剂
C.α 受体阻滞剂

D.血管紧张素Ⅱ受体阻滞剂
E.钙通道阻滞剂
参考答案： D 您的答案：
图文解析： 【解析】β受体阻滞剂可减慢心率和心收缩力，减低心肌收缩力和耗氧量。血管紧张素Ⅱ受体阻滞剂可降低血压和减轻蛋白尿。
对于第一个问题（糖尿病肾病合并高血压），首选的治疗药物通常是 D.血管紧张素Ⅱ受体阻滞剂（ARBs），因为它们在降低血压的同时还能减缓糖尿病肾病的进展。
对于 137 题，糖尿病肾病合并高血压的首选治疗药物，关键在于选择一种能够针对糖尿病肾病特点的药物。糖尿病肾病的特点包括肾脏损伤和高血压，因此，所选药物需要能够降低血压的同时，对肾脏有保护作用。
分析选项：
A. β受体阻滞剂：虽然能降低血压，但对肾脏的保护作用不明显。
B. 利尿剂：主要用于排水排钠，降低血容量，从而降低血压，但对肾脏的直接保护作用有限。
C. α受体阻滞剂：主要通过阻断血管平滑肌α1受体和直接舒张血管平滑肌作用，使血管扩张，外周阻力降低，血压下降，但对肾脏的保护作用不明显。
D. 血管紧张素Ⅱ受体阻滞剂：这类药物不仅能有效降低血压，还能改善肾脏的血流动力学，减少尿蛋白，对肾脏有明显的保护作用，是糖尿病肾病合并高血压的首选治疗药物。
E. 钙通道阻滞剂：虽然能降低血压，但对肾脏的保护作用不明显。
综上所述，对于糖尿病肾病合并高血压，首选的治疗药物是血管紧张素Ⅱ受体阻滞剂，因此 137 题答案是 D。
138、 (B1 型题)
冠心病稳定型心绞痛合并高血压首选
A.β受体阻滞剂
B.利尿剂
C.α受体阻滞剂
D.血管紧张素Ⅱ受体阻滞剂
E.钙通道阻滞剂
参考答案： A 您的答案：
图文解析： 【解析】β受体阻滞剂可减慢心率和心收缩力，减低心肌收缩力和耗氧量。血管紧张素Ⅱ受体阻滞剂可降低血压和减轻蛋白尿。
对于第二个问题（冠心病稳定型心绞痛合并高血压），首选的治疗药物通常是 A.β受体阻滞剂，因为它们可以减少心脏的氧气需求量，从而减轻心绞痛的症状，同时控制高血压。
接下来分析 138 题，冠心病稳定型心绞痛合并高血压的首选治疗药物。这里的关键在于选择一种能够同时降低血压和改善心绞痛症状的药物。
分析选项：
A. β受体阻滞剂：这类药物能够减慢心率、降低心肌收缩力和心肌耗氧量，从而减少心绞痛发作，同时也能有效降低血压，是冠心病稳定型心绞痛合并高血压的首选治疗药物。
B. 利尿剂、C. α受体阻滞剂、D. 血管紧张素Ⅱ受体阻滞剂、E. 钙通道阻滞剂：虽然这些药物都能降低血压，但对心绞痛的治疗作用不如β受体阻滞剂明显。
因此，对于冠心病稳定型心绞痛合并高血压，首选的治疗药物是β受体阻滞剂，所以 138 题答案是 A。
138. 冠心病稳定型心绞痛合并高血压首选
A. β受体阻滞剂

解释：对于冠心病稳定型心绞痛合并高血压的患者，首选β受体阻滞剂。这类药物不仅能有效控制血压，还能减少心绞痛发作的频率，改善心脏供血，从而降低心血管事件的发生风险。
139、 (B1 型题)
属于纤溶异常的实验室检查是
A.血 vWF 测定
B.PF3 有效性测定
C.血栓素 B2 测定
D.血 PC 测定
E.血 D-二聚体测定
参考答案： E 您的答案：
139 题: 属于纤溶异常的实验室检查是
纤溶系统的异常通常通过监测纤维蛋白溶解的过程来评估，血 D-二聚体的测定可以用来评估纤溶系统的活性。D-二聚体是纤维蛋白溶解后产生的产物，可以指示有纤维蛋白形成和随后的溶解活动，因此它是纤溶异常的指标。
答案：E.血 D-二聚体测定
首先，我们来理解这两个问题的核心。第一个问题是寻找与纤溶异常相关的实验室检查，而第二个问题是关于抗凝异常的实验室检查。
对于纤溶异常，它主要涉及到的是血栓的分解过程。当身体形成血栓后，纤溶系统会将其分解以防止血栓过度增长或堵塞血管。在这个过程中，D-二聚体会被释放出来。因此，血 D-二聚体的测定可以反映纤溶酶的活性，是纤溶异常的一个重要指标。
139、属于纤溶异常的实验室检查是：

A. 血 vWF 测定：Von Willebrand 因子 (vWF) 的测定主要用于诊断 von Willebrand 病及其他与血管性血友病相关的疾病，通常与纤溶异常无关。
B. PF3 有效性测定：血小板因子 3 (PF3) 的测定与纤溶无关，主要与凝血功能相关。
C. 血栓素 B2 测定：血栓素 B2 是血小板活化的标志物，主要与血小板功能和血栓形成有关，不直接反映纤溶状态。
D. 血 PC 测定：蛋白 C (PC) 是一种抗凝蛋白，通常与抗凝功能相关。
E. 血 D-二聚体测定：D-二聚体是纤维蛋白降解产物，其升高提示纤溶活动增加，因此与纤溶异常相关。
正确答案：E. 血 D-二聚体测定
140、 (B1 型题)
属于抗凝异常的实验室检查是
A.血 vWF 测定
B.PF3 有效性测定
C.血栓素 B2 测定
D.血 PC 测定
E.血 D-二聚体测定
参考答案： D 您的答案：
图文解析：
140 题: 属于抗凝异常的实验室检查是
抗凝异常通常涉及血浆中凝血和抗凝因子的测定。血 PC（蛋白 C）测定是评估抗凝系统功能的一个重要指标，因为蛋白 C 是一种具有抗凝和促进纤溶的重要生理活性的蛋白质。当蛋

白 C 水平异常时，可能导致抗凝功能障碍。
答案：D.血 PC 测定
再来看抗凝异常，这与身体的抗凝系统有关，它防止血液过度凝固。在这个系统中，有多种抗凝物质，其中蛋白 C（PC）是一个关键的抗凝因子。当身体缺乏 PC 或 PC 功能异常时，会增加血栓的风险。因此，血 PC 测定是抗凝异常的关键实验室检查。
140、属于抗凝异常的实验室检查是：

A. 血 vWF 测定：如前所述，vWF 测定主要用于诊断 von Willebrand 病，与抗凝功能无关。
B. PF3 有效性测定：如前所述，PF3 主要与凝血功能相关，不是抗凝功能的检查。
C. 血栓素 B2 测定：如前所述，血栓素 B2 与血小板活化和血栓形成相关，不是抗凝功能的检查。
D. 血 PC 测定：蛋白 C 是一种抗凝蛋白，其水平异常可以反映抗凝功能的异常。
E. 血 D-二聚体测定：如前所述，D-二聚体主要用于反映纤溶状态，与抗凝功能无关。
正确答案：D. 血 PC 测定
141、 (B1 型题)
克罗恩病诊断最有意义的检查方法是
A.腹部 B 超
B.腹部 CT
C.粪便潜血
D.胃镜
E.消化道 X 线钡剂造影
参考答案： A 您的答案：
图文解析： 【解析】胃镜可以清楚看出溃疡面、溃疡大小及周围情况。克罗恩病可根据消化道 X 线钡剂造影和结肠镜检查，观察肠管病变跳跃征和阶段性分布。
141、克罗恩病（Crohn's disease）是一种慢性炎症性肠道疾病，它可以影响消化道的任何部分。克罗恩病的诊断通常需要多种检查方法，其中影像学检查尤为重要。消化道 X 线钡剂造影（E 选项）曾是诊断克罗恩病的常规方法之一，因为它可以显示肠道的狭窄、瘘管和其他异常情况。然而，随着技术的进步，腹部 CT（B 选项）因其高分辨率和全面显示肠壁及周围组织的能力，已成为诊断克罗恩病的首选方法。因此，对于克罗恩病诊断最有意义的检查方法，我倾向于选择 B.腹部 CT。
对于 141 题，关于克罗恩病的诊断：
A 选项（腹部 B 超）：虽然 B 超在某些情况下对腹部疾病的诊断有帮助，但对于肠道等空腔脏器的显示效果不佳，不是诊断克罗恩病的首选方法。
B 选项（腹部 CT）：CT 对于腹部脏器和淋巴结的显示有一定优势，但不是克罗恩病诊断的最敏感方法。
C 选项（粪便潜血）：这主要用于消化道出血的检测，不能直接用于诊断克罗恩病。
D 选项（胃镜）：胃镜主要用于检查胃部疾病，对克罗恩病的诊断价值有限。
E 选项（消化道 X 线钡剂造影）：这种方法能清晰显示肠黏膜的微小结构和肠道的狭窄、梗阻等病变，对克罗恩病的诊断具有重要意义。141. E.消化道 X 线钡剂造影
克罗恩病诊断最有意义的检查方法
克罗恩病是一种慢性炎性肠病，常涉及胃肠道的多个部位。对于克罗恩病的诊断，最有意义的检查方法应该能够全面评估胃肠道的病变情况，确定病变的部位、范围和严重程度。以下是对各选项的分析：

A. 腹部 B 超：适用于评估腹部内脏器官，但对胃肠道特别是小肠的评估有限。

B. 腹部 CT：可以提供腹腔内器官和肠道的详细影像，有助于评估克罗恩病的并发症如肠穿孔、脓肿等。

C. 粪便潜血：可以提示肠道出血，但不能确诊克罗恩病。

D. 胃镜：主要用于检查上消化道，如食道、胃和十二指肠，对小肠的评估有限。

E. 消化道 X 线钡剂造影：可以显示消化道的解剖结构和功能状态，但敏感性和特异性不如 CT。

对于克罗恩病，最有意义的检查方法是腹部 CT，因为它可以提供详细的影像信息，有助于全面评估病变范围和严重程度，并发现可能的并发症。因此，答案是：
B. 腹部 CT
142、 (B1 型题)
胃溃疡诊断最有意义的检查方法是
A.腹部 B 超
B.腹部 CT
C.粪便潜血
D.胃镜
E.消化道 X 线钡剂造影
参考答案： D 您的答案：
图文解析： 【解析】胃镜可以清楚看出溃疡面、溃疡大小及周围情况。克罗恩病可根据消化道 X 线钡剂造影和结肠镜检查，观察肠管病变跳跃征和阶段性分布。
142、胃溃疡是一种发生在胃内壁的溃疡性病变。对于胃溃疡的诊断，内镜检查（胃镜，D 选项）是最重要的方法，因为它可以直观地观察到胃壁的溃疡和病变情况，并且能够进行活检来排除恶性病变。因此，对于胃溃疡诊断最有意义的检查方法，我会选择 D.胃镜。
对于 142 题，关于胃溃疡的诊断：
A 选项（腹部 B 超）：如上所述，B 超对空腔脏器的显示效果不佳，不适用于胃溃疡的诊断。
B 选项（腹部 CT）：虽然 CT 能显示腹腔脏器和淋巴结转移情况，但对胃黏膜的直接显示效果有限。
C 选项（粪便潜血）：这只能提示消化道出血，不能明确诊断胃溃疡。
D 选项（胃镜）：胃镜能直观观察胃黏膜的病变，包括胃溃疡的位置、大小和形态，是诊断胃溃疡最直接和有效的方法。
E 选项（消化道 X 线钡剂造影）：虽然能显示胃的形态，但对胃黏膜的微小病变显示效果不如胃镜。
胃溃疡诊断最有意义的检查方法
胃溃疡是一种常见的消化性溃疡病，诊断上需要直观地观察到胃黏膜的损伤情况。以下是对各选项的分析：

A. 腹部 B 超：不适合评估胃内壁的病变。

B. 腹部 CT：可以显示胃的解剖结构，但对胃黏膜的细节表现有限。

C. 粪便潜血：可以提示胃肠道出血，但不能确诊胃溃疡。

D. 胃镜：可以直观地看到胃黏膜，并直接观察到溃疡部位，还可以进行活检和病理学检查，是诊断胃溃疡的金标准。

E. 消化道 X 线钡剂造影：可以显示胃的解剖结构，但不如胃镜直接。

对于胃溃疡，最有意义的检查方法是胃镜，因为它能够直接观察到溃疡部位并进行活检。因此，答案是：

D. 胃镜

143、 (B1 型题)

诊断早期原发性肝癌最有意义的检查是

A.血淀粉酶检测

B.血清甲胎蛋白检测

C.腹部 CT 检查

D.血 CEA

E.血 CRP

参考答案： B 您的答案：

图文解析： 【解析】腹部 CT，尤其增强 CT 是口前诊断胰腺坏死的最佳检查方法，是诊断出血坏死性胰腺炎最有意义的检查。正确答案为 C 而不是 A，因为出血坏死性胰腺炎患者血清淀粉酶可增高、正常甚至低于正常，可见血清淀粉酶测定对出血坏死性胰腺炎的诊断价值并不大。原发性肝癌常表现为血清甲胎蛋白阳性，阳性率约为 68％～74％，为目前诊断早期肝癌最有意义的实验室检查项目。

143、对于诊断早期原发性肝癌，最有意义的检查是 B.血清甲胎蛋白检测。甲胎蛋白（AFP）是肝癌的一个常见标志物，尤其在早期肝癌的诊断中具有重要意义。虽然 AFP 在某些其他情况下也可能升高（如在妊娠或其他肝脏疾病中），但在原发性肝癌的诊断中它是一个关键的指标。

首先，我们来看第一个问题：诊断早期原发性肝癌最有意义的检查是？

选项 A.血淀粉酶检测通常用于评估胰腺功能，特别是在急性胰腺炎的诊断中，与肝癌无直接关系。

选项 B.血清甲胎蛋白检测是诊断原发性肝癌的特异性标志物。甲胎蛋白（AFP）是在胎儿早期由肝脏和卵黄囊合成的一种血清糖蛋白，出生后，AFP 的合成很快受到抑制。当肝细胞或生殖腺胚胎组织发生恶性病变时，有关基因重新被激活，使原来丧失合成 AFP 的细胞又重新开始合成，以致血中 AFP 含量明显升高。因此，原发性肝细胞性肝癌患者血清 AFP 增高，阳性率约为 70%，因此可以作为诊断肝癌的特异性标志物。

选项 C.腹部 CT 检查虽然可以提供肝脏的结构信息，但不如血清甲胎蛋白检测敏感和特异。

选项 D.血 CEA（癌胚抗原）主要作为结直肠癌的肿瘤标志物，与肝癌关系不大。

选项 E.血 CRP（C 反应蛋白）是炎症指标，非特异性，不能用于诊断肝癌。

综上所述，对于第一个问题，诊断早期原发性肝癌最有意义的检查是血清甲胎蛋白检测，因此答案选 B。

诊断早期原发性肝癌最有意义的检查是

答案: B. 血清甲胎蛋白检测

解析: 血清甲胎蛋白（AFP）是诊断早期原发性肝癌最有意义的标志物，特别是对高危人群

进行筛查时。AFP 是一种在胎儿期产生的蛋白质，成人正常情况下其血清水平极低。如果在成人血清中检测到高水平的 AFP，尤其是慢性肝病患者，可能提示存在原发性肝癌。

144、 (B1 型题)

诊断急性出血坏死型胰腺炎最有意义的检查是

A.血淀粉酶检测

B.血清甲胎蛋白检测

C.腹部 CT 检查

D.血 CEA

E.血 CRP

参考答案： C 您的答案：

图文解析： 【解析】腹部 CT，尤其增强 CT 是口前诊断胰腺坏死的最佳检查方法，是诊断出血坏死性胰腺炎最有意义的检查。正确答案为 C 而不是 A，因为出血坏死性胰腺炎患者血清淀粉酶可增高、正常甚至低于正常，可见血清淀粉酶测定对出血坏死性胰腺炎的诊断价值并不大。原发性肝癌常表现为血清甲胎蛋白阳性，阳性率约为 68％～74％，为目前诊断早期肝癌最有意义的实验室检查项目。

144、对于诊断急性出血坏死型胰腺炎，最有意义的检查是 A.血淀粉酶检测。胰腺炎的诊断主要依据是血淀粉酶或脂肪酶的升高，特别是当出现典型的腹痛和影像学上的胰腺异常时。血淀粉酶水平的显著升高通常与急性胰腺炎的诊断相关。腹部 CT 检查也对胰腺炎的诊断有重要价值，特别是在需要评估炎症程度和并发症时。但在初步诊断急性胰腺炎时，血淀粉酶检测是最直接和常用的方法。

接着，我们分析第二个问题：诊断急性出血坏死型胰腺炎最有意义的检查是？

选项 A.血淀粉酶检测是诊断急性胰腺炎的常用方法之一。在急性胰腺炎发病后，血清淀粉酶会在短时间内显著升高，因此是诊断急性出血坏死型胰腺炎的关键指标。

选项 B.血清甲胎蛋白检测如前所述，主要用于诊断肝癌，与胰腺炎无关。

选项 C.腹部 CT 检查虽然能提供胰腺的影像信息，但在急性胰腺炎的早期，血清学标志物的变化往往更为敏感。

选项 D.血 CEA 和选项 E.血 CRP 同样与急性出血坏死型胰腺炎的诊断无直接关系。

因此，对于第二个问题，诊断急性出血坏死型胰腺炎最有意义的检查是血淀粉酶检测，答案选 A。

诊断急性出血坏死型胰腺炎最有意义的检查是

答案: A. 血淀粉酶检测

解析: 血淀粉酶检测是诊断急性胰腺炎的常用方法。急性胰腺炎时，胰腺释放的酶进入血液，导致血淀粉酶水平显著升高。虽然血淀粉酶水平升高并不是急性胰腺炎特有的，但其迅速升高和临床症状结合可以帮助诊断。

145、 (B1 型题)

治疗慢性肾小球肾炎，可引起高血钾的药物是

A. α -受体阻滞剂

B.呋塞米

C.ACEI 类抑制药

D.钙拮抗剂

E. β -受体阻滞剂

参考答案： C 您的答案：

图文解析： 【解析】呋塞米是高效利尿药。ACEI 抑制药类有引起钾离子潴留的作用。

对于题目 145，治疗慢性肾小球肾炎，可能引起高血钾的药物是 C. ACEI 类抑制药。ACE 抑制剂（如便携素、雷米普利等）在治疗慢性肾小球肾炎时，可以减少蛋白尿并保护肾功能，但它们可能导致高血钾，尤其是在肾功能不全的情况下。

第 145 题：治疗慢性肾小球肾炎，可引起高血钾的药物是？

首先，我们分析各个选项与问题的关联性。

A 选项（α-受体阻滞剂）：主要用于治疗血管痉挛性疾病、休克和急性心肌梗死，与引起高血钾无直接关系。

B 选项（呋塞米）：是一种利尿剂，主要用于治疗水肿，其作用是促进钾离子的排泄，因此不会导致高血钾。

C 选项（ACEI 类抑制药）：即血管紧张素转化酶抑制剂，通过抑制血管紧张素转化酶的活性，减少血管紧张素 II 的生成，从而抑制血管收缩和醛固酮的释放。由于醛固酮具有保钠排钾的作用，因此抑制其释放可能导致血钾升高。

D 选项（钙拮抗剂）：主要用于治疗心绞痛和高血压，与血钾水平无直接关系。

E 选项（β-受体阻滞剂）：主要用于治疗心律失常和高血压，与血钾水平无直接关系。

综上所述，与问题最相关的答案是 C 选项（ACEI 类抑制药），因为它可能通过抑制醛固酮的释放而导致血钾升高。

145. 治疗慢性肾小球肾炎，可引起高血钾的药物是

C. ACEI 类抑制药

ACEI（血管紧张素转换酶抑制剂）类药物在治疗慢性肾小球肾炎时，可以通过抑制肾素-血管紧张素-醛固酮系统（RAAS），减少钾的排泄，从而可能引起高血钾。因此，ACEI 类药物如卡托普利、依那普利等都可能引起高血钾。

146、 (B1 型题)

慢性肾小球肾炎，主要原因为水钠潴留的高血压疗治疗首选

A. α-受体阻滞剂

B.呋塞米

C.ACEI 类抑制药

D.钙拮抗剂

E. β-受体阻滞剂

参考答案： B 您的答案：

图文解析： 【解析】呋塞米是高效利尿药。ACEI 抑制药类有引起钾离子潴留的作用。

对于题目 146，慢性肾小球肾炎引起的水钠潴留导致高血压时，首选的治疗药物是 C. ACEI 类抑制药。ACE 抑制剂有助于控制高血压，并且对肾脏有保护作用，减缓肾功能恶化。此外，它们还能减轻肾小球高滤过状态，减少蛋白尿。因此，在慢性肾小球肾炎伴高血压的情况下，ACEI 类药物通常是首选治疗药物。

第 146 题：慢性肾小球肾炎，主要原因为水钠潴留的高血压治疗首选？

同样地，我们再次分析各个选项。

A 选项（α-受体阻滞剂）：对于水钠潴留没有直接的治疗作用。

B 选项（呋塞米）：是一种利尿剂，能有效地促进体内过多的水分和钠离子的排出，从而缓解水钠潴留，是治疗此类高血压的首选药物。

C 选项（ACEI 类抑制药）：虽然也用于治疗高血压，但其主要作用机制不是针对水钠潴留。

D 选项（钙拮抗剂）和 E 选项（β-受体阻滞剂）：同样不是针对水钠潴留的治疗药物。

综上所述，与问题最相关的答案是 B 选项（呋塞米），因为它是利尿剂，能有效地解决水钠潴留问题，从而降低血压。

综上所述，对于 145 题的答案是 C.ACEI 类抑制药，对于 146 题的答案是 B.呋塞米。
146. 慢性肾小球肾炎，主要原因为水钠潴留的高血压治疗首选
B. 呋塞米

呋塞米是一种强效的袢利尿剂，能够通过抑制肾小管对钠和水的重吸收，有效减少水钠潴留，降低血容量，从而降低血压。对于由于水钠潴留引起的高血压，呋塞米是首选药物。
147、 (B1 型题)
高血压性脑出血的常见病变部位在
A.额叶
B.脑干
C.颞叶
D.枕叶
E.基底节
参考答案： E 您的答案：
图文解析：
147、高血压性脑出血的常见病变部位在
答案是 E.基底节。高血压性脑出血常见于脑的基底节区域，这是因为基底节区域的小动脉容易受到高血压影响而发生病变。
这是一道 B1 型选择题，我们需要根据题干和选项，结合医学知识进行分析。
对于 147 题，高血压性脑出血的常见病变部位：
选项 A 额叶，选项 B 脑干，选项 C 颞叶，选项 D 枕叶，虽然都可能出现脑出血，但并不是高血压性脑出血的最常见部位。高血压性脑出血通常是由于长期高血压导致的动脉硬化和微小动脉瘤形成，最终可能因情绪激动、过度脑力或体力劳动等因素导致血压急剧升高，从而引发脑血管破裂。这种出血最常见于基底节区域，特别是豆纹动脉，因为这是大脑中动脉的深穿支，易受高血压的影响。47 题的正确答案是 E.基底节，
高血压性脑出血的常见病变部位在
E. 基底节
148、 (B1 型题)
癫痫复杂部分性发作的病灶多位于
A.额叶
B.脑干
C.颞叶
D.枕叶
E.基底节
参考答案： C 您的答案：
148、癫痫复杂部分性发作的病灶多位于
答案是 C.颞叶。颞叶癫痫是最常见的局部发作性癫痫，特别是复杂部分性发作，病灶通常位于颞叶。
对于 148 题，癫痫复杂部分性发作的病灶多位于：
选项 A 额叶虽然与认知和情感有关，但不是癫痫复杂部分性发作的主要病灶部位。选项 B 脑干主要与生命维持功能相关，如呼吸和心跳，与癫痫病灶关系不大。选项 D 枕叶主要与视觉处理有关，也不是癫痫的主要病灶区域。选项 E 基底节主要与运动控制有关，同样不是癫痫的主要病灶。而选项 C 颞叶与记忆、情感和听觉处理有关，是癫痫复杂部分性发作最常见的病灶部位，特别是在颞叶的内侧结构，如海马体。C.颞叶。

癫痫复杂部分性发作的病灶多位于
C. 颞叶
149、　(B1 型题)
属于选择性 5-羟色胺重吸收抑制剂的是
A.卡马西平
B.碳酸锂
C.氟西汀
D.利培酮
E.阿普唑仑
参考答案：　C　您的答案：
图文解析：　【解析】目前临床上的非经典抗精神病药物是阻断 5-HT 受体，如氯氮平和利培酮。氟西汀又称百忧解，是强效选择性 5-HT 摄取抑制药。
对于第 149 题，选择性 5-羟色胺重吸收抑制剂（SSRIs）主要用于治疗抑郁症。在给出的选项中，C. 氟西汀 是一种典型的选择性 5-羟色胺重吸收抑制剂。
这是一道选择题，我们需要从给定的选项中选择与问题最相关的答案。
首先，我们分析第 149 题，它问的是哪种药物属于选择性 5-羟色胺重吸收抑制剂。
A. 卡马西平：这是一种抗癫痫药，也用作三叉神经痛缓解后的长期预防性用药，并不属于选择性 5-羟色胺重吸收抑制剂。
B. 碳酸锂：这是一种情绪稳定剂，主要用于治疗躁狂症，对躁狂和抑郁交替发作的双相情感性精神障碍有很好的治疗和预防复发的作用，对反复发作的抑郁症也有预防发作作用，同样不属于选择性 5-羟色胺重吸收抑制剂。
C. 氟西汀：这是一种选择性 5-羟色胺再摄取抑制剂（SSRI），它的主要作用是抑制 5-羟色胺在突触间隙的重吸收，从而提高 5-羟色胺在突触间隙的浓度，起到抗抑郁的作用。这是本题的正确答案。
D. 利培酮：这是一种抗精神病药，用于治疗急性和慢性精神分裂症等精神疾病，不属于选择性 5-羟色胺重吸收抑制剂。
E. 阿普唑仑：这是一种苯二氮卓类药物，具有抗焦虑、抗抑郁、镇静、催眠、抗惊厥及肌肉松弛等作用，不属于选择性 5-羟色胺重吸收抑制剂。
综上所述，对于第 149 题，答案是 C. 氟西汀。

属于选择性 5-羟色胺重吸收抑制剂的是
C. 氟西汀
150、　(B1 型题)
属于非典型抗精神病药物的是
A.卡马西平
B.碳酸锂
C.氟西汀
D.利培酮
E.阿普唑仑
参考答案：　D　您的答案：
图文解析：　【解析】目前临床上的非经典抗精神病药物是阻断 5-HT 受体，如氯氮平和利培酮。氟西汀又称百忧解，是强效选择性 5-HT 摄取抑制药。
对于第 150 题，非典型抗精神病药物是一类较新的抗精神病药，与典型抗精神病药相比，它们具有不同的药理特性和副作用谱。在给出的选项中，D. 利培酮 是一种非典型抗精神病药。

接下来，我们分析第 150 题，它问的是哪种药物属于非典型抗精神病药物。
A. 卡马西平、B. 碳酸锂、C. 氟西汀、E. 阿普唑仑：这些药物的作用已在前面描述，它们都不属于非典型抗精神病药物。

D. 利培酮：这是一种非典型抗精神病药物，主要用于治疗精神分裂症和其他精神疾病。这是本题的正确答案。
综上所述，对于第 150 题，答案是 D. 利培酮。
属于非典型抗精神病药物的是
D. 利培酮

2023 第四单元
1、 (A1 型题)
八个月婴儿可出现的体征是
A.颈项强直
B.巴宾斯基阳性
C.克氏征阳性
D.拥抱反射
E.吮吸反射
参考答案： B 您的答案：
图文解析： 【解析】2 岁以下儿童巴宾斯基征阳性亦可为生理现象。
【避错】颈项强直（A 错）见于婴幼儿缺血缺氧性脑病或脑膜炎等。3~4 个月前的婴儿肌张力较高，克氏征可为阳性（C 错）。
拥抱反射（D 错）是一种防御性反射，当新生儿听到声音、受到刺激等情况下，可出现两臂外展伸直，继而屈曲内收到胸前，呈拥抱状的症状。随着年龄增加，神经发育完善，3~6 个月后自行消失。吮吸反射（E 错）是指当乳头或手指触碰新生儿口唇时，新生儿会出现头向触碰方向寻找，并出现口唇及舌的吸吮、蠕动，能够自主进食， 一般在 4~7 个月后消失。
对于八个月大的婴儿，最典型的体征是吮吸反射，选项 E。这是因为吮吸反射是新生儿期到婴儿早期常见的一种原始反射，通常出生后即存在，会在婴儿大约 6 个月大时自然消失。然而，由于这个反射在很多婴儿中可以持续到一岁左右，因此在 8 个月大的婴儿中观察到它并不罕见。
A 项颈项强直，C 项克氏征阳性，和 D 项拥抱反射通常与神经系统的问题相关，不是正常婴儿的典型体征。B 项巴宾斯基阳性反射在婴儿中很常见，但它通常在 12 个月龄以前消失，因此也不是八个月婴儿的典型体征。
综上所述，最合适的答案是 E.吮吸反射。
这是一道关于婴儿生长发育阶段特有体征的选择题。我们需要确定哪种体征是八个月大的婴儿可能出现的。首先，我们分析每个选项代表的体征及其与婴儿年龄的相关性：
A. 颈项强直：这通常不是婴儿期的正常体征，而是病理状态，如脑膜炎等疾病的体征。
B. 巴宾斯基阳性：这是一种病理反射，正常情况下，婴儿在两岁之前巴宾斯基征可为阳性，两岁之后转为阴性，因此，八个月大的婴儿可以出现巴宾斯基阳性体征。
C. 克氏征阳性：这是一种脑膜刺激征，通常与脑膜炎或其他神经系统疾病相关，不是正常婴儿的体征。
D. 拥抱反射：这是新生儿期的原始反射，通常在 3-4 个月后消失，因此在八个月大的婴儿中不会出现。

E. 吮吸反射：这也是新生儿期的原始反射，随着婴儿的成长会逐渐消失，不是八个月大婴儿应有的体征。
综上所述，巴宾斯基阳性是八个月大婴儿可以出现的正常体征，而其他选项要么与病理状态相关，要么是新生儿期的反射，不适合八个月大的婴儿。
因此，正确答案是 B.巴宾斯基阳性。
八个月婴儿可能出现的体征是：

B. 巴宾斯基阳性

解释：巴宾斯基反射（Babinski 反射）是婴儿的一种正常反射，当用硬物划过脚底外侧时，大脚趾上翘，其他脚趾扇形展开。这个反射在婴儿阶段是正常的，通常在 2 岁以内会消失。而颈项强直、克氏征阳性和拥抱反射（Moro 反射）主要见于病理情况下或是新生儿期。而吮吸反射通常在婴儿期较早阶段出现，八个月的婴儿一般已经开始减少了这种反射。
2、 (A1 型题)
关于颅底骨折不正确的是
A.CT 无法显示颅底骨折
B.颅前窝骨折可有“熊猫征”
C.单纯性颅底骨折可保守治疗
D.X 线显示颅内积气
E.可有脑脊液流出
参考答案： A 您的答案：
图文解析： 【解析】颅底骨折可因颅盖骨折延伸至颅底或由颅底骨折延伸到颅盖所致，CT 可以显示颅底骨折。
【避错】“熊猫眼征”为颅前窝骨折累及额骨眶板和筛骨，引起出血流进眶内，眶周皮下及球结合膜下形成瘀血斑。单纯性颅底骨折无脑损伤或颅内血肿， 一般采用非手术治疗。X 线检查可显示颅内积气。颅底骨折患者可出现脑脊液外流，可经耳、鼻、眼睑、口等部位流出。所以不选 BCDE。
关于颅底骨折，以下描述不正确的是：
A. CT 无法显示颅底骨折
CT（计算机断层扫描）是检测颅底骨折的首选影像学方法，因为它能提供颅骨的详细图像，尤其是在骨折较为明显的情况下。因此，选项 A 说 CT 无法显示颅底骨折是不正确的。其他选项描述的情况是可能发生的：颅前窝骨折可有“熊猫征”（眼周出血造成的黑眼圈），单纯性颅底骨折可以保守治疗，X 线可显示颅内积气，且颅底骨折可能导致脑脊液流出。因此，最不正确的选项是 A。
解析：
颅底由前向后的颅前窝、颅中窝和颅后窝呈由高到底的阶梯状排，凹凸不平、有大小不同的骨孔与裂隙容纳颅神经和血管通过。颅底骨折时，常伴发相应的颅神经损伤，如嗅神经、视神经、听神经和面神经的损伤。高分辨率 CT 检查可显示骨折线、气颅、脑肿胀、脑室出血等，对颅底骨折有确诊价值。因此 CT 无法显示颅底骨折这一说法是错误的。
颅前窝骨折时，因出血经前鼻孔流出进入眶内眼睑周围，可呈现紫色，状似熊猫眼，又称“熊猫征”，故选项 B 颅前窝骨折可有“熊猫征”是颅底骨折的临床表现之一。
颅底骨折一般无需特殊治疗，重点在于观察有无脑损伤及处理脑脊液漏、颅神经损伤等合并伤，故选项 C 单纯性颅底骨折可保守治疗符合颅底骨折的治疗原则。
颅底骨折时，X 线摄片检查可显示颅内积气，故选项 D：X 线显示颅内积气符合颅底骨折的

影像学表现。
颅底骨折若伤及鼻旁窦或乳突气房，可伴脑脊液鼻漏或耳漏，选项 E 可有脑脊液流出是颅底骨折的临床表现。
综上所述，正确答案是：A.CT 无法显示颅底骨折。
关于颅底骨折不正确的选项是：

A. CT 无法显示颅底骨折

解释：
颅底骨折通常可以通过 CT 扫描清晰显示，因此选项 A 是不正确的。CT 扫描是诊断颅底骨折的常用影像学检查方法，能够提供详细的骨结构信息，有助于识别骨折部位和范围。
3、 (A1 型题)
下列描述女性生殖系统防御机制不正确的是
A.阴道前后壁紧贴
B.pH＞6.5
C.阴道有自净功能
D.宫颈内口闭合
E.大小阴唇自然合拢
参考答案： B 您的答案：
图文解析： 【解析】阴道 pH 是女性阴道内酸碱度的一个指标，它会影响阴道内的微生物群落和炎症反应。正常阴道 pH 在 3.8~4.4 之间，呈弱酸性。
【避错】女性生殖系统具有多种防御机制，包括阴道前后壁紧贴、阴道自净功能、宫颈内口闭合和大小阴唇自然合拢等。这些机制共同作用，以保护女性生殖健康。阴道前后壁紧贴是女性生殖系统的一种自然状态，它有助于保持阴道内的清洁和干燥，减少感染的风险。阴道自净功能是指阴道内的菌群通过相互作用，维持着酸碱平衡，从而减少感染的风险。宫颈内口闭合也是女性生殖系统的一种自然状态，它有助于防止宫颈内的病原体进入宫腔。大小阴唇自然合拢也是女性生殖系统的一种自我保护机制，它有助于减少外界环境对阴道的污染。所以不选 ACDE。
女性生殖系统的防御机制包括阴道壁的紧贴、阴道酸性环境（pH 值较低）、自净功能、宫颈内口的闭合以及大小阴唇的自然合拢。其中，pH 值的描述尤其关键，因为健康的阴道环境通常具有较低的 pH 值（约 3.8 至 4.5），有助于防止有害细菌的生长。因此，在这些选项中，描述不正确的是：B. pH＞6.5 这是因为健康的阴道 pH 值通常小于 4.5，而不是大于 6.5。因此，B 选项是不正确的描述。
首先，我们要明确这道题目考察的是女性生殖系统的防御机制。女性生殖道因其特殊的解剖结构和生理功能，具有一定的自然防御功能，这些功能可以防御病原体的感染。接下来，我们逐一分析选项：
A. 阴道前后壁紧贴：这是阴道的一种自然状态，有助于防止外部病原体的侵入。当阴道没有被异物（如阴茎或卫生棉条）扩张时，其前后壁是紧贴的，这样可以减少病原体进入的机会。
B. pH＞6.5：正常阴道的 pH 值是≤4.5，这种酸性环境可以抑制其他病原体的生长，称为阴道的自净作用。因此，阴道 pH 值大于 6.5 是不正常的，这会破坏阴道的自然防御机制，使得病原体更容易生长。
C. 阴道有自净功能：这是阴道的一个重要防御机制。阴道内的乳杆菌可使糖原分解为乳酸，从而维持阴道正常的酸性环境。这种酸性环境可以抑制其他病原体的生长，起到防御感染的

作用。
D. 宫颈内口闭合：宫颈是女性生殖系统中一个重要的防御屏障。宫颈内口通常处于闭合状态，这有助于防止上行性感染。
E. 大小阴唇自然合拢：这也是女性生殖系统的一个自然防御机制。大小阴唇的自然合拢状态可以减少外部病原体直接接触阴道口的机会。
综上所述，选项 B（pH＞6.5）是不正确的描述，因为这破坏了阴道的自然酸性环境，降低了其防御功能。
因此，最终答案是 B. pH＞6.5。
女性生殖系统具有多种防御机制，以防止感染和其他问题。以下是对各选项的分析：

A. **阴道前后壁紧贴**：这是正确的。阴道前后壁紧贴有助于防止外来病原体进入。

B. **pH＞6.5**：这是不正确的。健康女性阴道的 pH 值通常在 3.8 到 4.5 之间，酸性环境有助于抑制病原体的生长和繁殖。

C. **阴道有自净功能**：这是正确的。阴道分泌物和正常菌群（如乳酸菌）有助于保持阴道的清洁和防御。

D. **宫颈内口闭合**：这是正确的。宫颈内口的闭合有助于防止病原体进入子宫。

E. **大小阴唇自然合拢**：这是正确的。大小阴唇的自然合拢起到机械屏障的作用，防止外界病原体的侵入。

因此，最正确的答案是：

B. **pH＞6.5**
4、 (A1 型题)
小儿头围测量的方法是
A.沿乳头下缘水平绕胸 1 周
B.沿乳头上缘水平绕胸 1 周
C.眉弓到耳上到枕后结节绕头一周
D.眉间到枕后绕头 1 周
E.眉弓上方最突出处到枕后结节绕头 1 周
参考答案： E 您的答案：
图文解析： 【解析】小儿头围测量的方法是眉弓上方最突出处到枕后结节绕头 1 周。【避错】根据上述解析，排除其他选项。
小儿头围的正确测量方法是选项 C：“眉弓到耳上到枕后结节绕头一周”。这种方法确保了头围的测量围绕了头部的最大周长，通常在耳朵上方和枕骨突出部位之间进行。因此，最佳答案是 C。
解析：
小儿头围的测量，是沿眉弓上方最突出处到枕后结节绕头一周，以皮尺围绕该区域一圈的长度即为头围。头围的增长与脑和颅骨的生长有关，头围测量在 2 岁以内最有价值，头围过小常提示脑发育不良，头围过大或增长过快，则提示脑积水。
选项 A 沿乳头下缘水平绕胸 1 周，选项 B 沿乳头上缘水平绕胸 1 周，均测量的是胸围，与

头围无关，故可一并排除。
选项 C“眉弓到耳上到枕后结节绕头一周”，选项 D“眉间到枕后绕头 1 周”，均不符合头围的标准测量方法，因此可一并排除。
综上所述，正确答案是：E.眉弓上方最突出处到枕后结节绕头 1 周。
小儿头围测量的方法是：

E. 眉弓上方最突出处到枕后结节绕头 1 周
5、 (A1 型题)
鉴别三种非典型苯丙酮尿症的方法是
A.尿三氯化铁试验
B.尿蝶呤图谱分析
C.血浆游离氨基酸分析
D.Guthrie 细菌生长抑制试验
E.DNA 分析
参考答案： B 您的答案：
图文解析： 【解析】非典型苯丙酮尿症是苯丙氨酸羟化酶的辅酶四氢生物蝶呤（BH4）缺乏所致，需测定尿液中蝶呤类物质的水平来鉴别三种非典型苯丙酮尿症，其包括：苯丙氨酸、酪氨酸、苯丙氨酸的羟化代谢产物和苯丙氨酸的旁路代谢产物。
【避错】尿三氯化铁试验用于检查尿液中是否有苯丙氨酸，但不能鉴别三种非典型苯丙酮尿症（A 错）。血浆游离氨基酸分析仅能区分经典型和非经典型苯丙酮尿症（C 错）。Guthrie 细菌生长抑制试验仅能用于新生儿筛查（D 错）。DNA 分析仅能用于基因诊断，不能用于鉴别三种非典型苯丙酮尿症（E 错）。
三种非典型苯丙酮尿症（PKU）的鉴别，通常需要对遗传背景进行详细分析，因为这些形式的 PKU 涉及不同的遗传缺陷。虽然尿三氯化铁试验和 Guthrie 细菌生长抑制试验可以用于初步筛查苯丙酮尿症，但它们并不足以区分非典型 PKU 的不同类型。尿蝶呤图谱分析和血浆游离氨基酸分析有助于了解代谢异常的性质，但在鉴别具体的遗传变异上可能不够具体。
E. DNA 分析是鉴别三种非典型苯丙酮尿症的最佳方法，因为它能够精确识别遗传缺陷，从而确定病因，并区分不同类型的非典型 PKU。通过分析患者的 DNA，可以直接检测到导致苯丙酮尿症的基因变异，从而准确诊断非典型 PKU 的具体类型。因此，最正确的答案是 E. DNA 分析。
解析：
非典型苯丙酮尿症主要包括四氢生物喋呤缺乏症、6-丙酮酰四氢蝶呤合成酶缺乏症和二氢生物蝶呤还原酶缺乏症三种类型。蝶呤是苯丙氨酸、酪氨酸和色氨酸在催化羟化酶反应过程中的辅酶，这些疾病均是由于鸟苷三磷酸环化水合酶、6-丙酮酰四氢蝶呤合成酶或二氢生物蝶呤还原酶等某种酶的缺乏，使四氢生物蝶呤合成或再生障碍，导致苯丙氨酸不能氧化成酪氨酸，只能通过旁路代谢，由苯丙氨酸转氨酶催化生成苯丙酮酸，由尿排出，发生苯丙酮尿症。因此可通过尿蝶呤图谱分析对三种非典型苯丙酮尿症进行鉴别诊断。
选项 A 尿三氯化铁试验，选项 C 血浆游离氨基酸分析，选项 D：Guthrie 细菌生长抑制试验，均属于苯丙酮尿症的筛查试验，只能提示苯丙氨酸羟化酶缺乏，但并不能鉴别三种非典型苯丙酮尿症，故可一并排除。
选项 E：DNA 分析为苯丙酮尿症的产前诊断方法，但同样不能鉴别三种非典型苯丙酮尿症，故不选。
综上所述，正确答案是：B.尿蝶呤图谱分析。
鉴别三种非典型苯丙酮尿症（Phenylketonuria, PKU）的方法需要特异性和准确性。对于这一

问题的备选答案：

A. 尿三氯化铁试验：这是一种用于检测尿中苯丙酮酸的方法，但对非典型苯丙酮尿症的鉴别诊断不够特异。

B. 尿蝶呤图谱分析：这是鉴别非典型苯丙酮尿症的关键方法之一，通过分析尿液中的蝶呤（如生物蝶呤）的代谢物来区分不同类型的 PKU。

C. 血浆游离氨基酸分析：这是一种常用于检测苯丙氨酸水平的方法，对 PKU 的诊断有帮助，但不能特异性地鉴别非典型苯丙酮尿症。

D. Guthrie 细菌生长抑制试验：这是一种早期筛查 PKU 的方法，但对非典型苯丙酮尿症的鉴别没有特异性。

E. DNA 分析：通过基因分析可以鉴定引起非典型苯丙酮尿症的特定基因突变，是一种非常准确的方法，但通常在临床上作为最后的确诊手段使用。

综上所述，鉴别三种非典型苯丙酮尿症最常用的方法是尿蝶呤图谱分析（B），因为它可以通过分析蝶呤代谢物来区分不同类型的苯丙酮尿症。

因此，最佳答案是：

B. 尿蝶呤图谱分析

6、 (A1 型题)

脊髓半切综合征常见于

A.急性髓外硬膜内脓肿

B.急性脊髓炎

C.吉兰巴雷综合征

D.脊髓髓外肿瘤

E.脊髓空洞症

参考答案： D 您的答案：

图文解析： 【解析】脊髓半切综合征常见于脊髓髓外肿瘤，其主要表现为典型的患侧深感觉消失、对侧浅感觉消失，由于失去来自对侧皮层脊髓束的纤维支配，同侧肢体上 1/3 处功能障碍。避错急性髓外硬膜内脓肿（A 错）主要表现为寒战、高热、意识障碍等感染中毒症状，可伴有肌肉痉挛和强迫体位等表现，常伴有患侧浅反射减弱或消失，而深反射亢进，牵张反射亢进，出现病理征及克氏征。急性脊髓炎（B 错）急性起病，常先有脊背部疼痛、胸腹部束带感及下肢麻木感，后迅速出现脊髓受损平面以下运动、感觉及大小便功能障碍。吉兰巴雷综合征（C 错）又称急性炎性脱髓鞘性多发性神经病，是以周围神经和神经根的脱髓鞘病变及小血管炎性细胞浸润为病理特点的自身免疫病，临床表现为多发性神经病、脑脊液中蛋白-细胞分离和电生理改变。脊髓空洞症（E 错）多表现为典型的痛、温觉障碍和深感觉保存的分离性感觉障碍。

【避错】

脊髓半切综合征（Brown-Séquard syndrome）通常是由脊髓的一侧损伤引起的，表现为同侧的运动功能丧失和对侧的痛觉和温度感觉减退。考虑到这一点，最有可能引起脊髓半切综合

征的是那些导致脊髓一侧受损的病症。
选项中：
- A. 急性髓外硬膜内脓肿可能导致脊髓受压，但通常影响脊髓的两侧而不是单侧。
- B. 急性脊髓炎通常影响脊髓的大部分区域，不特异于单侧。
- C. 吉兰巴雷综合征是一种周围神经系统的疾病，不直接影响脊髓。
- D. 脊髓髓外肿瘤可以局部压迫脊髓，导致单侧损伤，因此可能引起脊髓半切综合征。
- E. 脊髓空洞症通常影响脊髓中央部分，虽然可能引起多样化的神经症状，但不特定于造成脊髓半切综合征。

因此，最符合脊髓半切综合征常见病因的选项是 D. 脊髓髓外肿瘤。
解析：
脊髓半切综合征，又称 Brown-Sequard 综合征，指由于外部的压迫和脊髓内部的病变等原因引起脊髓病损等原因引起病损平面以下同侧肢体上运动神经元瘫痪，深感觉消失、精细触觉障碍，血管舒缩功能障碍，对侧肢体痛温觉消失，双侧触觉保留的临床综合征。脊髓半切综合征常见于脊髓髓外肿瘤，髓外肿瘤早期即可产生脊髓压迫症状，导致病损平面以下同侧肢体上运动神经元瘫痪，深感觉消失、精细触觉障碍，对侧肢体痛温觉消失，双侧触觉保留。
选项 A 急性髓外硬膜内脓肿，选项 B 急性脊髓炎，均为脊髓的炎性病变，多为横贯性损害，表现为受损平面以下的各种感觉缺失、上运动神经元性瘫痪及括约肌障碍等，无双侧感觉和运动不对称表现，因此可一并排除。
选项 C 吉兰巴雷综合征多为双侧迟缓性瘫痪，远端重于近端，可有周围性感觉障碍和脑神经损害，脑脊液蛋白-细胞分离现象，与脊髓半切综合征表现不符，故不选。
选项 E 脊髓空洞症主要表现为节段性分离性感觉障碍、运动及反射异常以及皮肤营养障碍等，无双侧感觉和运动不对称表现，故排除。
综上所述，正确答案是：D.脊髓髓外肿瘤。
脊髓半切综合征（Brown-Séquard 综合征）是一种由于脊髓半侧受损导致的综合征，其临床表现通常包括同侧肢体的运动功能丧失和本体感觉丧失，以及对侧肢体的痛温觉丧失。

这种综合征最常见的病因包括脊髓外伤、脊髓肿瘤、脊髓感染以及脊髓的退行性病变。在选项中，最有可能导致脊髓半切综合征的是脊髓髓外肿瘤。

因此，最正确的答案是：

D. 脊髓髓外肿瘤
7、 (A1 型题)
确诊流行性乙型脑炎常检查的抗体是
A.IgM
B.IgG
C.IgE
D.IgC
E.IgB
参考答案： A 您的答案：
图文解析： 【解析】乙脑是虫媒传染，感染后血中出现特异性抗体，而获得免疫。特异性 IgM 抗体（A 对）出现较早（病后 3～4 天），脑脊液中最早在病程第 2 天即可检测到，2 周时达高峰，可作为早期诊断指标。特异性 IgG 抗体（B 错）出现较晚（病后 2 周），用于回顾性诊断和流行病学调查。

【避错】根据上述解析排除 BCDE。
流行性乙型脑炎（也称为日本脑炎）的确诊通常依赖于特异性抗体检测。在这种情况下，检测 IgM 抗体是最重要的，因为 IgM 抗体通常在感染早期出现，并且其存在指示着近期感染。因此，最正确的答案是：A. IgM
首先，我们来理解题目的核心点。题目问的是确诊流行性乙型脑炎时，应该检查哪种类型的抗体。流行性乙型脑炎是一种由乙脑病毒引起的疾病，而人体在感染此类病毒后，会产生不同类型的抗体以对抗病毒。
下来，我们逐一分析每个选项：
A. IgM：在感染流行性乙型脑炎病毒后，机体会首先产生 IgM 抗体。这种抗体通常在感染后的一周内出现，因此它是早期诊断流行性乙型脑炎的敏感指标。
B. IgG：IgG 抗体通常在感染后的一段时间内出现，并且持续时间较长。尽管它也是病毒感染的一个标志，但由于其出现时间相对较晚，不如 IgM 敏感，所以不是早期诊断的首选。
C. IgE：这种抗体主要与过敏反应相关，而不是与病毒感染的诊断直接相关。
D. IgC 和 E. IgB：在医学中，并不存在 IgC 和 IgB 这两种抗体类型，因此这两个选项可以首先被排除。
综上所述，由于 IgM 是机体在感染流行性乙型脑炎病毒后首先产生的抗体，并且出现时间早，敏感度高，因此它是确诊流行性乙型脑炎的首选检查抗体。
因此，正确答案是 A. IgM。
确诊流行性乙型脑炎常检查的抗体是：

A. IgM

流行性乙型脑炎（乙脑）是由乙型脑炎病毒引起的一种病毒性脑炎。IgM 抗体是感染早期产生的抗体，检测 IgM 抗体的存在可以帮助早期诊断乙型脑炎。因此，最正确的答案是 A.IgM。
8、 (A1 型题)
儿童生长发育特点正确的是
A.各个系统生长发育速度一致
B.机体发育不存在个体差异
C.年纪越大，发育越慢
D.婴儿期是体格发育最快的时期
E.1 岁后是体格发育最快的时期
参考答案： D 您的答案：
图文解析： 【解析】儿童生长发育是一个连续的、有阶段性的过程，各系统器官发育的速度不完全一致（A 错）。各年龄段生长发育的速度是相对稳定的，但其生理特征有显著的个体差异（B 错），在各个阶段也存在一定差异。年龄越小，发育越快，近一岁时体格生长速度达到高峰， 一岁后开始减慢（CE 错）。婴儿期（出生到一岁）是体格发育最快的时期（D 对）。
【避错】根据上述解析，排除其他选项。
这个题目的最佳答案是 D，婴儿期是体格发育最快的时期。在儿童的生长发育过程中，婴儿期（出生后的第一年）是体格增长最快的时期，尤其是出生后的前 6 个月。此时期内，婴儿的体重和身高会迅速增加。而且，各个系统的生长发育速度并不一致，体内的各个器官和系统会根据各自的发育规律进行生长。此外，机体发育存在个体差异，即便是同龄的儿童，其生长速率和发育程度也会有所不同。随着年龄的增长，发育速度逐渐减慢，因此选项 C 也部分正确，但比较全面且准确的描述是选项 D。

这是一道关于儿童生长发育特点的选择题。我们需要分析每个选项，找出与儿童生长发育特点相符合的正确答案。
A. 各个系统生长发育速度一致：实际上，儿童的各个系统生长发育速度并不一致。例如，神经系统发育较早，生殖系统发育较晚。因此，这个选项是不正确的。
B. 机体发育不存在个体差异：儿童的生长发育受遗传、营养、环境等多种因素影响，因此会存在个体差异。这个选项也是不正确的。
C. 年纪越大，发育越慢：虽然儿童发育速度会随着年龄增长而逐渐放缓，但不能简单地说年纪越大发育越慢，因为不同阶段的发育速度变化并不完全一致。因此，这个选项表述不够准确。
D. 婴儿期是体格发育最快的时期：婴儿期（从出生到 1 岁）是儿童体格发育最迅速的时期，身高、体重等方面都会有显著的增长。这个选项是正确的。
E. 1 岁后是体格发育最快的时期：实际上，婴儿期（从出生到 1 岁）是体格发育最快的时期，而不是 1 岁后。因此，这个选项是不正确的。
综上所述，正确答案是 D：婴儿期是体格发育最快的时期。这个选项准确地反映了儿童生长发育的一个重要特点。
根据医学知识和最佳实践指南，以下是对题目的解答：

儿童的生长发育特点存在许多独特之处，包括体格发育速度在不同年龄段存在显著差异。尤其是婴儿期（出生到 1 岁）是体格发育最迅速的时期。因此，最正确的答案是：

D.婴儿期是体格发育最快的时期

解释：婴儿期的体重和身高增长速度远高于其他年龄段，例如新生儿在出生后的第一年中，体重通常会增加三倍，身高增长约 25 厘米。这一阶段的迅速发育反映了婴儿对营养和环境刺激的高度敏感性。相比之下，1 岁后虽然仍在持续发育，但速度明显放缓。因此，选项 D 最符合儿童生长发育的特点。
9、 (A1 型题)
脑栓塞最常见的病因是
A.心房颤动
B.动脉粥样硬化
C.高血压
D.糖尿病
E.高脂血症
参考答案： A 您的答案：
图文解析： 【解析】脑栓塞是指各种栓子随血流进入颅内动脉使血管腔急性闭塞或严重狭窄，引起相应供血区脑组织发生缺血坏死及功能障碍的一组临床综合征。脑栓塞按栓子的来源分为心源性、非心源性和来源不明性三大类，其中以心源性脑栓塞最常见，占脑栓塞的 60%~75%，而心源性脑栓塞最常见的病因是心房颤动（A 对）
【避错】动脉粥样硬化（B 错）、高血压（C 错）、糖尿病（D 错）、高脂血症（E 错）均为脑血栓形成最常见的病因。而非脑栓塞的最常见病因。
脑栓塞最常见的病因是心房颤动（A）。心房颤动导致心脏的上房不规则且快速地收缩，从而增加血液在心房内停滞的可能性，这可以导致血栓形成。这些血栓如果脱落，可以随血流进入大脑循环，导致脑栓塞。虽然动脉粥样硬化、高血压、糖尿病和高脂血症也是脑栓塞的重要危险因素，但心房颤动被认为是最常见的直接病因。因此，正确答案是 A.心房颤动。

解析：
脑栓塞是指各种栓子随血流进入颅内动脉使血管腔急性闭塞或严重狭窄，引起相应供血区脑组织发生缺血坏死及功能障碍的一组临床综合征。其中，心源性栓塞是脑栓塞中最常见的原因，约占 60%～75%，栓子在心内膜和瓣膜产生，脱落入脑后致病。其中心房颤动是心源性栓子最常见的原因，约占半数以上。
选项 B 动脉粥样硬化是脑血栓形成最常见的病因，而非脑栓塞，故排除。
选项 C 高血压，选项 D 糖尿病，选项 E 高脂血症，均为动脉粥样硬化的危险因素，但不直接导致脑栓塞，因此可一并排除。
综上所述，正确答案是：A.心房颤动。
脑栓塞最常见的病因是：

A. 心房颤动

心房颤动（AF）是脑栓塞的一个主要风险因素。心房颤动会导致心房内血流滞留，从而形成血栓，这些血栓可能会脱落并进入大脑动脉，导致脑栓塞。因此，选择 A 选项。
10、 (A1 型题)
新生儿坏死性小肠结肠炎最常见的病因是
A.寒冷
B.溶血
C.未及时添加辅食
D.早产
E.双胎
参考答案： D 您的答案：
图文解析： 【解析】新生儿坏死性小肠结肠炎为多种原因导致的，以腹胀、呕吐、血便和低血压为主要表现的危重疾病，早产为常见病因。避错寒冷（A 错）、溶血（B 错）为引起新生儿黄疸的病因。未及时添加辅食（C 错）为生理性贫血的病因。
【避错】寒冷（A 错）可能引起新生儿发热、咳嗽、腹胀症状，不会引起坏死性小肠结肠炎。溶血（B 错）为引起新生儿黄疸的病因。未及时添加辅食（C 错）为生理性贫血的病因。双胎（E 错）不会引起新生儿坏死性小肠结肠炎。
新生儿坏死性小肠结肠炎（Necrotizing Enterocolitis, NEC）最常见的病因是 D.早产。早产儿由于肠道功能不成熟和免疫系统薄弱，更容易发展成坏死性小肠结肠炎。这种情况在早产儿中较为常见，尤其是那些需要肠外营养的极低出生体重儿。因此，正确答案是 D.早产。
解析：
新生儿坏死性小肠结肠炎是一种获得性疾病，是多种原因引起的肠黏膜损害，使之缺血、缺氧，导致小肠、结肠发生弥漫性或局部坏死的一种疾病。新生儿坏死性小肠结肠炎的确切病因及发病机制尚未完全明确，可能与多种致病因素有关，但早产是新生儿坏死性小肠结肠炎发病的一个主要危险因素，因胃肠功能不成熟，胃酸分泌少，胃肠动力差，易出现食物滞留，利于细菌生长；且早产儿本身免疫力较低，胃肠蠕动及消化吸收功能和凝血机制发育尚未完善，肠壁通透性高，屏障功能差，更易于细菌侵入。
选项 A 寒冷可能会引起新生儿体温异常，导致新生儿硬肿症等疾病，但与坏死性小肠结肠炎的发生关系不大，故不选。
选项 B 溶血多见于新生儿溶血性疾病，如新生儿 ABO 溶血，Rh 溶血等，常表现为黄疸、贫血等，与坏死性小肠结肠炎的发病无明显关联，故排除。
选项 C 未及时添加辅食主要影响的是婴儿的营养吸收，可能会引起缺铁性贫血、维生素 D

缺乏性佝偻病等，但不会直接导致坏死性小肠结肠炎，故不考虑。
选项 E 双胎可能使母亲在孕期面临更大的营养需求和心理压力，但与坏死性小肠结肠炎的发生关系不大，故不符合。
综上所述，正确答案是：D.早产。
新生儿坏死性小肠结肠炎（NEC）是新生儿期常见的急性胃肠道疾病，其病因尚不完全明确，但研究表明，多因素共同作用，包括早产、感染、肠道缺血缺氧、肠道菌群失衡等。其中，**早产**是最常见和最重要的危险因素之一。早产儿的胃肠道发育不完全，免疫功能不成熟，更容易发生坏死性小肠结肠炎。

因此，最正确的答案是：

D. 早产
11、 (A1 型题)
儿童阶段发病率及死亡率最高的时期是
A.新生儿期
B.婴儿期
C.幼儿期
D.学龄前期
E.学龄期
参考答案： A 您的答案：
图文解析： 【解析】新生儿各个系统的发育尚不够成熟，自身免疫力尚未形成，对外界环境的变化不能很好地适应，发生各种疾病的概率大大增加，是新生儿期死亡率最高的阶段。【避错】婴儿期（B 错）死亡率仅次于新生儿期，幼儿期（C 错）发病率及死亡率一般低于婴儿期而高于幼儿后期，学龄前期（D 错）发病率及死亡率一般低于幼儿期而高于学龄期，学龄期（E 错）发病率及死亡率最低。
在儿童阶段，发病率及死亡率最高的时期通常是 B.婴儿期。婴儿期指的是出生后的第一个月至一岁之间。这一时期，由于婴儿的免疫系统尚未完全发育，加之营养、环境因素及感染等原因，他们面临较高的健康风险。因此，婴儿期的儿童相对于其他年龄阶段，其发病率和死亡率都要高。
解析：
儿童期包括新生儿期、婴儿期、幼儿期、学龄前期、学龄期和青春期，其中新生儿期是儿童阶段发病率及死亡率最高的时期。这主要是因为新生儿从宫内到宫外环境需要一个适应过程，且新生儿期抗病能力弱，需要更多护理和喂养方面的关注。
选项 B 婴儿期虽然也存在一定的健康风险，但与新生儿期相比，婴儿期的发病率和死亡率相对较低，故不选。
选项 C 幼儿期，选项 D 学龄前期，选项 E 学龄期，随着儿童年龄的增长，免疫系统的逐渐完善，这些时期的发病率和死亡率都相对较低，故一并排除。
综上所述，正确答案是：A.新生儿期。
11. 儿童阶段发病率及死亡率最高的时期是

A. 新生儿期

新生儿期是指婴儿出生后的前 28 天，这一时期婴儿的身体功能尚未完全发育成熟，对环境的适应能力较差，容易受到感染和其他健康问题的影响，因此发病率和死亡率最高。

12、 (A1 型题)
可引起差异性紫癜的是哪种先天性心脏病
A.动脉导管未闭
B.室间隔缺损
C.房间隔缺损
D.法洛四联症
E.肺动脉狭窄
参考答案： A 您的答案：
图文解析： 【解析】动脉导管未闭患者如果导管直径细、分流量小者常无明显症状。导管直径粗、分流量大者常并发充血性心力衰竭,表现为易激惹、气促、乏力、多汗以及喂养困难、发育不良等。当病情发展为严重肺动脉高压且出现右向左分流时,表现为下半身发绀和杵状指/趾,称为“差异性发绀”。
【避错】(1）室间隔缺损（B 错），劳力性呼吸困难：成人、小孩患中、小型室间隔缺损无明显症状，仅在剧烈运动时表现为呼吸急促、劳力性呼吸困难。大型室间隔缺损时，小儿表现出喂养困难、体重不增、发育迟缓等症状。(2）房间隔缺损（C 错）。除较大缺损外，房间隔缺损儿童时期一般无症状，随年龄增长症状逐渐显现，劳力性呼吸困难为主要表现，继之可发生室，上性心律失常，特别是房扑、房颤而使症状加重。有些患者可因右室容量负荷过重而发生右心衰竭。(3）法洛四联症（D 错）主要表现为青紫、蹲踞症状、杵状指和阵发性缺氧发作， 一般无差异性紫癜。(4）肺动脉狭窄（E 错）主要表现为劳累后心悸、气急、乏力、胸闷不适等症状，伴青紫、气短、晕厥等， 一般不会引起差异性紫癜。
差异性紫癜（differential cyanosis）是指下肢与上肢或唇部的氧合血红蛋白水平不同，常见于某些特定的心脏病中。它通常出现在新生儿或早期婴儿，特别是那些有特定类型的先天性心脏病的儿童。
在所给的选项中，法洛四联症（Tetralogy of Fallot）是最可能引起差异性紫癜的先天性心脏病。法洛四联症是一种包括四个心脏结构异常的疾病：室间隔缺损、肺动脉狭窄、右室肥大和主动脉骑跨。这些异常导致血液绕过肺循环，使未充分氧合的血液流入体循环，从而可能导致差异性紫癜。
因此，最正确的答案是：D. 法洛四联症
解析：
差异性紫绀是一种特殊的紫绀现象，即上下肢或上肢、下半身出现不同的紫绀情况，为动脉导管未闭的病理生理改变。在动脉导管未闭的情况下，主动脉的血液会流入肺动脉，导致肺动脉区域含氧量较高的血液与来自右心的静脉血混合，使得原本应该流入上肢的高含氧血液被未完全氧合的血液稀释，上肢皮肤出现轻度青紫，而同时下肢是由腹主动脉供血，血液含氧量仍然较高，因此不出现青紫。故可引起差异性紫癜的先天性心脏病是动脉导管未闭。
选项 B 室间隔缺损，选项 C 房间隔缺损，选项 D 法洛四联症，选项 E 肺动脉狭窄，均不会出现差异性紫绀，可一并排除。
综上所述，正确答案是：A.动脉导管未闭。
差异性紫癜（differential cyanosis）是一种临床表现，主要特征是身体不同部位的皮肤呈现出不同程度的紫绀。它通常与某些先天性心脏病相关，这些疾病会导致心脏和血液循环异常，从而引起全身血氧分布不均。

在上述选项中，**法洛四联症（Tetralogy of Fallot）**是引起差异性紫癜的常见原因。这是一种复杂的先天性心脏病，特征包括室间隔缺损、肺动脉狭窄、主动脉骑跨和右心室肥厚。

这些结构异常导致肺循环和体循环之间的血流混合，特别是在发生严重的右向左分流时，会导致下肢比上肢出现更明显的紫绀现象。

因此，最正确的答案是：

D. 法洛四联症
13、 (A1 型题)
抑郁症的躯体症状群包括
A.情绪低落
B.睡眠障碍
C.思维迟缓
D.动作缓慢
E.自责自罪
参考答案： B 您的答案：
图文解析： 【解析】抑郁症的核心症状包括情绪低落、兴趣缺乏、快感缺失、意志活动减退（自责自罪）、认知功能损害（思维迟缓、动作缓慢），可伴有多种躯体不适症状，如食欲减退、睡眠障碍、体重下降、易疲劳等。选项 A、C、D、E 均不属于躯体症状。【避错】抑郁症有三无（自觉无助、自觉无望、自觉无用）、三自（自罪、自责、自杀）。
在抑郁症的躯体症状群中，虽然多个选项看起来都可能与抑郁症有关，但需要找到最具体的躯体症状。选项 A（情绪低落）、C（思维迟缓）、D（动作缓慢）和 E（自责自罪）更多地描述了心理或行为特征，而不是躯体症状。
B 选项“睡眠障碍”是抑郁症的典型躯体症状，包括入睡困难、夜间频繁醒来或过早醒来。因此，在这里最恰当的选择应该是 **B. 睡眠障碍**。
解析：
抑郁症又称抑郁障碍，以显著而持久的心境低落为主要特征，是心境障碍的主要类型。抑郁症的躯体症状群包括睡眠障碍，以早醒最具特征性。
选项 A 情绪低落，选项 C 思维迟缓，选项 E 自责自罪，均属于抑郁症的心理症状群，而非躯体症状群，可一并排除。
选项 D 动作缓慢多见于抑郁状态严重的患者，此时患者会显得行为迟缓，生活懒散，蓬头垢面，缺乏兴趣和活力，整日无精打采，但这属于精神运动性抑制障碍，不属于躯体症状群，故不选。
综上所述，正确答案是：B.睡眠障碍。
抑郁症的躯体症状群主要包括一些生理和身体上的表现，而不是情绪或认知方面的症状。根据题目提供的选项，以下是对每个选项的分析：

A. 情绪低落 - 这是抑郁症的主要情绪症状，但不属于躯体症状。
B. 睡眠障碍 - 这是抑郁症的常见躯体症状。
C. 思维迟缓 - 这是抑郁症的认知症状，不是躯体症状。
D. 动作缓慢 - 这是抑郁症的躯体症状。
E. 自责自罪 - 这是抑郁症的情感症状，不是躯体症状。

综合以上分析，抑郁症的躯体症状群包括睡眠障碍和动作缓慢。在这道题目中，最佳答案是：

B. 睡眠障碍

14、　(A1 型题)
以下最能提示早期妊娠的是
A.超声发现原始心管搏动
B.宫颈黏液涂片发现羊齿状树叶结晶
C.停经
D.双相体温高温相持续 18 天
E.呕吐
参考答案：　A　您的答案：
图文解析：　【解析】最能提示早期妊娠的是超声发现原始心管搏动（A 对）。
【避错】宫颈黏液涂片发现羊齿状树叶结晶（B 错）是体内雌激素变化的结果，不能提示早期妊娠。停经（C 错）是妊娠最早的症状，但不能提示早期妊娠。双相体温高温相持续 18 天（D 错）是监测有无排卵及黄体功能的一种可靠方法，不是提示早期妊娠的可靠指标。呕吐（E 错）可以是早期妊娠症状，但不是提示早期妊娠的可靠指标。
早期妊娠的诊断可以通过多种方式进行，但在这些选项中，最能明确提示早期妊娠的是 A. 超声发现原始心管搏动。超声检查中发现的原始心管搏动是妊娠大约 5-6 周时可以观察到的现象，是确认早期妊娠的直接和可靠指标。因此，选择 A 作为最正确的答案。其他选项虽然可能与妊娠有关，但它们不如超声检查发现原始心管搏动直接或特异。
解析：
早期妊娠是指妊娠第 14 周以前的阶段，即从妊娠开始到妊娠第 13 周的第 6 天。此阶段正是受精卵向胚胎、胎儿剧烈分化的重要时期，也可以说是胎儿“分化组装成形”的主要时期。早期妊娠的主要症状与体征为停经、早孕反应、乳房和生殖系统的变化，其中最明显的是停经，一旦月经过期 10 日或以上，应疑为妊娠。
选项 A：超声发现原始心管搏动是胚胎存活的标志，此时胚胎已发育到一定时间，并不能提示早期妊娠，故不选。
选项 B：宫颈黏液涂片发现羊齿状树叶结晶只能说明有雌激素影响，在排卵期，妊娠期和月经前均可见到，不具有特异性，故排除。
选项 D：双相体温高温相持续 18 天提示黄体功能良好，但不能提示早期妊娠，故不选。
选项 E：呕吐为早孕反应的一种，但不是每个孕妇都会出现，且早孕反应通常出现在停经 6 周左右，不能提示早期妊娠，故排除。
综上所述，正确答案是：C.停经。
14、(A1 型题)
以下最能提示早期妊娠的是：

A. 超声发现原始心管搏动

解释：超声发现原始心管搏动是最可靠、最明确的早期妊娠指标。这是因为原始心管搏动表明胚胎发育已经开始，并且只有在妊娠的情况下才能观察到这种情况。其他选项，如停经、双相体温高温相持续 18 天、呕吐，虽然也可能提示妊娠，但它们并不是绝对的指标，可能受到其他因素的影响。宫颈黏液涂片发现羊齿状树叶结晶则是一种辅助诊断方法，但也不如超声发现原始心管搏动明确和可靠。
15、　(A1 型题)
子宫肌瘤最常见的变性是
A.囊性变
B.玻璃样变

C.红色样变
D.钙化
E.肉瘤样变
参考答案：　　B　您的答案：
图文解析：　　【解析】子宫肌瘤变性是指子宫肌瘤继发变性，包括玻璃样变、囊性变、红色样变、肉瘤样变及钙化。其中玻璃样变最多见（B 对）。
【避错】根据上述解析，排除其他选项。
子宫肌瘤最常见的变性是红色样变（C 选项）。红色样变（或称为红色变性）通常发生在急性扭转的子宫肌瘤中，由于血液供应突然中断导致肌瘤组织内出血和坏死。这种类型的变性特别在孕妇中比较常见，因为怀孕期间子宫的体积增大，使得肌瘤更容易扭转。因此，最佳答案是 C。
解析：
子宫肌瘤变性是子宫肌瘤失去了原有的典型结构，形成了不同种类的变性。常见的变性包括玻璃样变、囊性变、红色变性、肉瘤样变和钙化。其中玻璃样变是最常见的变性类型，也称为透明变性。
选项 A 囊性变是由玻璃样变继续发展而来的，由肌细胞坏死液化形成，相对少见，故不选。
选项 C 红色样变多见于妊娠期或产褥期，为肌瘤的一种特殊类型的坏死，相对少见，故排除。
选项 D 钙化多见于蒂部细小、血供不足的浆膜下肌瘤，以及绝经后妇女的子宫肌瘤，相对少见，故不选。
选项 E 肉瘤样变较少见，多见于绝经后伴疼痛和出血的患者，与题意不符。
综上所述，正确答案是：B.玻璃样变。
子宫肌瘤的最常见变性是玻璃样变（B. 玻璃样变）。玻璃样变是由于肌瘤组织缺血引起的退行性变，常见于年龄较大的患者。其他变性类型如囊性变、红色样变、钙化和肉瘤样变也可见，但不如玻璃样变常见。
16、　(A1 型题)
下列不属于新生儿特殊生理性改变的是
A.乳房增大
B.马牙
C.假月经
D.生理性黄疸
E.红臀
参考答案：　　E　您的答案：
图文解析：　　【解析】新生儿的常见生理改变有：生理性黄疸（胆红素产生过多且肝脏酶系统发育不完善所致，排除 D）、假月经（阴道出血），排除 C、乳房增大（孕妇雌激素对胎儿影响中断所致，排除 A）、板牙（又称“马牙”，上皮细胞堆积或黏液潴留肿胀所致，排除 B）、螳螂嘴（指两侧颊部出现脂肪垫隆起，对吸奶有利）、新生儿脱发。
【避错】红臀又称新生儿尿布皮炎，是一种常见的皮肤炎症性疾病，常见于接触尿布的部位，皮损开始为轻度潮红、肿胀，逐渐出现丘疹、水疱、糜烂渗出等，边界清楚，可继发细菌或念珠菌感染，出现脓疱或溃疡，是一种病理改变，故本答案为 E。
在新生儿期，特殊的生理性改变通常包括乳房增大、假月经和生理性黄疸，这些都是正常的生理现象。红臀（红色的臀部）也可以在新生儿中观察到，尤其是因为尿布疹或其他皮肤刺激。然而，“马牙”不是通常与新生儿相关的生理性改变。马牙通常指的是某些动物（如马）的特定牙齿，这个词在新生儿生理性改变的语境中似乎不适用。
因此，最正确的答案是 **B.马牙**。

这是一道选择题，要求从给定的选项中选出不属于新生儿特殊生理性改变的一项。
首先，我们来分析每个选项代表的新生儿生理特点：
A. 乳房增大：新生儿出生后，由于母体激素的影响，可能会出现乳房增大的现象，这是一种正常的生理性改变。
B. 马牙：马牙是新生儿期的一种特殊生理状态，通常表现为牙龈上的黄白色小点，对新生儿的健康没有影响，也是一种正常的生理性改变。
C. 假月经：由于母体雌激素的影响，部分新生儿出生后会有阴道流出少许血性分泌物或类似月经的出血，这也是一种正常的生理性改变。
D. 生理性黄疸：新生儿出生后，由于胆红素的代谢特点，往往会出现生理性黄疸，表现为皮肤和巩膜的黄染，这是一种正常的生理过程。
E. 红臀：红臀，也称为尿布疹，通常是由于尿布区域长时间潮湿、摩擦或接触刺激性物质导致的皮肤炎症反应。它并不是新生儿的一种生理性改变，而是一种皮肤问题，与护理和使用尿布的方式密切相关。
综上所述，乳房增大、马牙、假月经和生理性黄疸都是新生儿期正常的生理性改变，而红臀则是一种皮肤问题，不属于新生儿的特殊生理性改变。
因此，正确答案是 E.红臀。
下列不属于新生儿特殊生理性改变的是：

E. 红臀

解析：

- **乳房增大**：新生儿由于母体内激素的影响，出生后可出现乳房增大，属于正常的生理现象。
- **马牙**：新生儿口腔内出现的小白点，通常位于上颚或牙龈，属于正常的生理现象。
- **假月经**：新生儿女孩由于母体激素的作用，可能会在出生后几天内出现少量阴道出血，属于正常的生理现象。
- **生理性黄疸**：新生儿由于肝功能未完全成熟，常在出生后 2-3 天出现黄疸，大多数属于生理性，且可自行消退。

- **红臀**：即尿布疹或尿布皮炎，是由于尿液和粪便对皮肤的刺激导致的皮肤炎症，不属于新生儿的生理性改变，而是病理性改变。
17、 (A1 型题)
有关咳嗽变异型哮喘说法正确的是
A.常在午后发生或加重
B.抗哮喘治疗无效
C.以干咳为主
D.支气管激发试验阴性
E.常发生感染，抗生素治疗有效
参考答案： C 您的答案：
图文解析： 【解析】临床把以咳嗽为唯一症状的不典型哮喘称为咳嗽变异型哮喘，故 C 为正确答案。
【避错】咳嗽变异型哮喘夜间咳嗽较明显，故 A 错；咳嗽变异型哮喘治疗原则与典型哮喘相同，故 B 错；咳嗽变异型哮喘支气管激发试验多为阳性，以咳嗽为唯一症状，无喘息、

气急、胸闷等，发生感染可能性较小，故 DE 错。
咳嗽变异型哮喘（Cough-variant asthma, CVA）是一种特殊类型的哮喘，其主要症状是干咳，特别是在没有其他典型哮喘症状（如喘息或呼吸困难）的情况下。考虑到这些信息，我们可以评估每个选项：
A. 常在午后发生或加重 - 这一描述更多与哮喘的一般症状相关，而不是特指咳嗽变异型哮喘。
B. 抗哮喘治疗无效 - 这不正确，因为咳嗽变异型哮喘通常对抗哮喘治疗（如吸入性皮质类固醇）是敏感的。
C. 以干咳为主 - 这是正确的，咳嗽变异型哮喘的主要症状是持续性的干咳。
D. 支气管激发试验阴性 - 这不正确，因为咳嗽变异型哮喘患者在支气管激发试验中通常呈阳性，显示出支气管高反应性。
E. 常发生感染，抗生素治疗有效 - 这并不准确，咳嗽变异型哮喘不特别倾向于感染，且抗生素治疗对哮喘本身无效。
因此，最正确的答案是 **C. 以干咳为主**。
这是一道关于咳嗽变异型哮喘的问题，需要从给定的选项中选出最符合咳嗽变异型哮喘特点的描述。
A 选项“常在午后发生或加重”：这个描述通常不与咳嗽变异型哮喘直接相关，哮喘的发作并不特定于午后。
B 选项“抗哮喘治疗无效”：这与咳嗽变异型哮喘的治疗原则相悖。实际上，咳嗽变异型哮喘对抗哮喘治疗是敏感的。
C 选项“以干咳为主”：这是咳嗽变异型哮喘的典型症状，患者通常以干咳为主要表现，不伴有喘息和呼吸困难。
D 选项“支气管激发试验阴性”：这是不正确的。在咳嗽变异型哮喘中，支气管激发试验通常是阳性的，因为这是一种诊断哮喘的重要检查方法。
E 选项“常发生感染，抗生素治疗有效”：这与咳嗽变异型哮喘的特点不符。哮喘并不是由感染引起的，因此抗生素治疗通常无效。
综上所述，C 选项“以干咳为主”最符合咳嗽变异型哮喘的特征。
因此，正确答案是 C.以干咳为主。
关于咳嗽变异型哮喘（Cough Variant Asthma， CVA）的说法正确的是：

C. 以干咳为主

解析：
咳嗽变异型哮喘是一种特殊类型的哮喘，其主要表现是长期的干咳而无明显的喘息症状。以下是对其他选项的分析：

A. 常在午后发生或加重：咳嗽变异型哮喘的咳嗽症状通常在夜间或早晨加重，而不是在午后。

B. 抗哮喘治疗无效：抗哮喘治疗对咳嗽变异型哮喘是有效的，尤其是吸入激素和支气管扩张剂。

D. 支气管激发试验阴性：支气管激发试验通常是阳性的，可以用于诊断哮喘及其变异型。

E. 常发生感染，抗生素治疗有效：咳嗽变异型哮喘与感染无关，抗生素治疗无效。

因此，正确答案是 C. 以干咳为主。

18、 (A1 型题)

应行预防性宫颈环扎术的是

A.曾有 3 次以上药物流产史的孕妇

B.曾有 3 次以上人工流产史的孕妇

C.曾有 3 次以上稽留流产史的孕妇

D.曾有 3 次以上足月胎膜早破史的孕妇

E.曾有 3 次以上妊娠中期自然流产史的孕妇

参考答案： E 您的答案：

图文解析： 【解析】对于有孕中期自然流产病史，非孕期宫颈内口可通过 8 号海格式扩张器，可在孕前或孕中进行宫颈环扎手术，故 E 对。

【避错】宫颈环扎术适应证有：宫颈功能不全引起的习惯性流产、早产，前置胎盘，双胎或多胎妊娠，孕中 B 超检查发现宫颈出现进行性缩短，考虑宫颈机能不全。

在预防性宫颈环扎术的背景下，这种手术通常是为了防止宫颈功能不全，导致的早产或流产。考虑到这点，最适合进行预防性宫颈环扎术的情况是在有重复中期流产（通常是无痛性的和无预兆的）的孕妇中。

因此，根据题目所给的选项，最合适的答案是：

E. 曾有 3 次以上妊娠中期自然流产史的孕妇

这是因为多次妊娠中期自然流产可能与宫颈功能不全有关，而宫颈环扎术是防止宫颈过早扩张和随后的流产或早产的一种手段。

解析：

宫颈环扎术是封闭宫颈内口的一种手术，一般算中小手术，需要局部麻醉，手术时间大约半小时左右。宫颈环扎术的目的是修复并建立正常的宫颈内口形态和功能，使妊娠能够维持至足月或胎儿能够存活。手术多于妊娠 14～18 周施行。预防性宫颈环扎术是指对判断为宫颈机能不全者进行环扎术，以预防其发生流产。宫颈机能不全是指在没有宫缩的情况下，子宫颈由于解剖或功能缺陷而不能维持妊娠至足月。有 3 次以上妊娠中期自然流产史的孕妇可考虑宫颈机能不全，可行预防性宫颈环扎术。

选项 A 曾有 3 次以上药物流产史的孕妇，选项 B 曾有 3 次以上人工流产史的孕妇，均属人为终止妊娠，与自然流产无关，不需行宫颈环扎术，可一并排除。

选项 C 曾有 3 次以上稽留流产史的孕妇，应行病因筛查，以确定胎儿死亡原因，与宫颈机能不全关系不大，故不选。

选项 D 曾有 3 次以上足月胎膜早破史的孕妇，应警惕绒毛膜羊膜炎，与宫颈机能无关，故排除。

综上所述，正确答案是：E.曾有 3 次以上妊娠中期自然流产史的孕妇。

预防性宫颈环扎术通常用于预防由于宫颈机能不全导致的流产或早产。宫颈机能不全是一种病症，宫颈在没有收缩的情况下自行扩张，导致妊娠中期或晚期流产或早产。适应症包括曾有多次妊娠中期自然流产或早产史的孕妇。基于这一点，最佳答案是：

E. 曾有 3 次以上妊娠中期自然流产史的孕妇

19、 (A1 型题)

单纯母乳喂养易导致婴儿产生的贫血是

A.缺铁性贫血

B.巨幼红细胞性贫血
C.失血性贫血
D.溶血性贫血
E.再生障碍性贫血
参考答案： A 您的答案：
图文解析： 【解析】婴儿出生后从第六个月开始，单纯的母乳不能满足其生长需要，容易导致缺铁，造成小儿缺铁性贫血，故 A 对。
【避错】叶酸或维生素 B12 的缺乏可导致巨幼红细胞性贫血；失血性贫血多见于因外伤或手术等血液流失过多者；溶血性贫血指红细胞破坏过多,超过了骨髓代偿性造血功能,导致外周血的红细胞减少、血红蛋白下降；再生障碍性贫血是一种多原因引起的骨髓造血衰竭性疾病，主要以造血干细胞质和量的异常以及造血微环境的异常为主。
单纯母乳喂养最常导致婴儿产生的贫血是缺铁性贫血，因为母乳中的铁虽然吸收率高，但总量不足以满足婴儿快速增长的需要。因此，正确答案是：A. 缺铁性贫血
解析：
母乳是婴儿成长最自然、最安全、最完整的天然食物，它含有婴儿成长所需的所有营养和抗体。然而，母乳中的铁含量相对较低，每 100 毫升母乳仅含铁 0.1 毫克，而婴儿每日需要铁 10 毫克左右，因此纯母乳喂养无法满足婴儿对铁的全部需求。长期纯母乳喂养而未及时添加辅食，容易导致婴儿体内铁储备耗竭，从而发生缺铁性贫血。
综上所述，正确答案是：A.缺铁性贫血。
选项 B 巨幼红细胞性贫血主要是由于叶酸或维生素 B12 缺乏所致，这两种营养素在母乳中的含量相对充足，因此不是母乳喂养导致的主要贫血类型，故不选。
选项 C 失血性贫血是由于身体某个部位出血导致的贫血，与喂养方式无关，故排除。
选项 D 溶血性贫血是由于红细胞破坏过多、过快导致的贫血，与喂养方式没有直接关系，故不选。
选项 E 再生障碍性贫血是由于骨髓造血功能衰竭导致的贫血，与喂养方式无关，故排除。
单纯母乳喂养易导致婴儿产生的贫血是：

A. 缺铁性贫血

解析： 单纯母乳喂养的婴儿，特别是在 6 个月后，如果没有及时添加富含铁的辅食，可能会出现铁摄入不足，导致缺铁性贫血。母乳中的铁含量相对较低，且随着婴儿生长，对铁的需求量增加，因此容易出现铁缺乏。缺铁性贫血在婴幼儿中较为常见，而其他类型的贫血（如巨幼红细胞性贫血、失血性贫血、溶血性贫血、再生障碍性贫血）则不常见于单纯母乳喂养的婴儿。
20、 (A1 型题)
闭合性颅盖骨骨折诊断的主要依据是
A.触诊局部有凹陷感
B.出现神经系统损伤体征
C.头皮肿胀有波动感
D.X 线平片
E.有骨擦音
参考答案： D 您的答案：
图文解析： 【解析】对于闭合性颅盖骨折，常行 X 线平片检查明确诊断，故 D 对。
【避错】闭合性颅盖骨折若无明显凹陷且仅为线形骨折时，触诊局部可无凹陷感，未伤及

颅内神经和神经核团时可不出现神经系统损伤体征，头皮肿胀不明显无波动感，可无骨擦音，故 ABCE 错。
闭合性颅盖骨骨折的诊断主要依赖于影像学检查，因此最正确的答案是 D. X 线平片。X 线平片是诊断骨折的常规方法，能够直观显示骨折的位置和程度。虽然其他选项如触诊局部有凹陷感（A）、出现神经系统损伤体征（B）、头皮肿胀有波动感（C）和有骨擦音（E）可能与颅盖骨骨折相关，但这些都不如影像学检查直接和准确。因此，在这些选项中，X 线平片（D）是诊断闭合性颅盖骨骨折的主要依据。
解析：
闭合性颅骨骨折的诊断依赖于影像学检查，其中 X 线平片操作简便易行，价格低廉，常作为颅骨骨折的首选影像学检查方法。X 线平片可以发现颅骨的连续性中断、移位，以及骨折线的走行方向，为颅骨骨折的诊断提供可靠的影像学证据。
颅骨骨折时不一定出现颅骨凹陷，选项 A 触诊局部有凹陷感不是闭合性颅骨骨折的特征性表现，故不选。
选项 B 出现神经系统损伤体征多见于开放性颅脑损伤，闭合性颅骨骨折不常合并神经系统损伤，故排除。
选项 C 头皮肿胀有波动感多见于帽状腱膜下血肿，而颅骨骨折一般无头皮血肿表现，与题意不符。
闭合性颅骨骨折时，由于有头皮覆盖，骨折端不会暴露于外界，因此触诊时不会出现骨擦音，故选项 E 有骨擦音不符合闭合性颅骨骨折的特点，故不考虑。
综上所述，正确答案是：D.X 线平片。
闭合性颅盖骨骨折的诊断主要依靠影像学检查。尽管临床体征如触诊局部有凹陷感、神经系统损伤体征、头皮肿胀有波动感以及有骨擦音都可以提示颅骨骨折，但这些体征都需要影像学检查来进一步确认。因此，X 线平片是诊断闭合性颅盖骨骨折的主要依据。

正确答案是：D. X 线平片
21、 (A1 型题)
深感觉障碍，以下哪种情况容易发生跌倒
A.双手平举上肢平抬
B.闭目
C.单抬腿
D.转颈
E.屈膝
参考答案： B 您的答案：
图文解析： 【解析】深感觉障碍时可出现闭目难立征阳性，表现为患者双足并拢站立时，两手向前平伸，患者闭眼后站立不稳，身体摇摆倾斜欲倒，故 B 对。
【避错】闭目难立征阳性常提示深感觉障碍，是神经系统常见阳性体征，ACDE 与闭目难立征检查无关。
深感觉障碍主要影响对肢体位置和运动的感知，导致平衡和空间定位能力下降，因此在需要精细平衡和空间定位的动作中更容易发生跌倒。考虑到这些因素，选项中闭目（B）是最可能导致跌倒的情况，因为闭目时，人们无法依靠视觉信息来帮助维持平衡，对于存在深感觉障碍的个体来说，这会大大增加跌倒的风险。因此，正确答案是 B.闭目。
解析：
深感觉是指来自肌肉、肌腱、骨膜和关节的本体感觉，包括运动觉、位置觉和震动觉。深感觉障碍提示脊髓后索功能障碍，闭目时，机体无法通过视觉代偿，所以容易发生跌倒。故闭

目时，深感觉障碍患者容易发生跌倒。
选项 A 双手平举上肢平抬，选项 D 转颈，均会干扰平衡觉，但平衡觉属于浅感觉，深感觉障碍患者在进行上述动作时，可通过浅感觉代偿，故不易跌倒，可一并排除。
选项 C 单抬腿主要影响运动觉，但患者可通过位置觉代偿，不易跌倒，故不选。
选项 E 屈膝主要影响位置觉，但患者可通过运动觉代偿，不易跌倒，可排除。
综上所述，正确答案是：B.闭目。
对于深感觉障碍的患者，容易发生跌倒的情况是当他们闭上眼睛时。这是因为深感觉障碍影响了身体对自身位置的感觉，当患者闭上眼睛时，视觉反馈被去除，依赖于深感觉的信息减少，因此更容易失去平衡和跌倒。

所以，最正确的答案是：

B. 闭目
22、 (A1 型题)
合并前列腺增生的帕金森患者不选用的药物是
A.溴隐亭
B.多巴丝肼
C.苯海拉明
D.苯海索
E.金刚烷胺
参考答案： D 您的答案：
图文解析： 【解析】苯海索属于抗胆碱能药，老年患者慎用，闭角型青光眼及前列腺肥大患者禁用，故答案为 D。
【避错】溴隐亭属于多巴胺受体激动剂，直立性低血压和精神症状发生率较高，而症状波动和异动症发生率低；多巴丝肼副作用分为周围性和中枢性两类，前者为恶心、呕吐、低血压、心律失常等，后者有症状波动和异动症、精神症状等；苯海拉明属于抗组胺药，主要用于治疗过敏性疾病、晕动病等；金刚烷胺肾功能不全、癫痫、严重胃溃疡、肝病患者慎用。
合并前列腺增生的帕金森患者不应使用可能加重前列腺症状的药物。在选项中，苯海拉明（C选项）是一种抗组胺药，具有抗胆碱作用，可能导致尿潴留，因此对于前列腺增生的患者来说是不适宜的。因此，最佳答案是 C.苯海拉明。
解析：
前列腺增生是老年男性常见疾病，其病因是由于前列腺的逐渐增大对尿道及膀胱出口产生压迫作用。临床上表现为尿频、尿急、夜间尿次增加和排尿费力，并能导致泌尿系统感染、膀胱结石和血尿等并发症。帕金森为一种常见的神经系统变性疾病，最主要的病理改变是中脑黑质多巴胺能神经元的变性死亡，由此而引起纹状体多巴胺含量显著性减少。复方左旋多巴是治疗帕金森病最基本的药物，该类药物从小剂量开始使用，逐渐增加剂量。其中多巴丝肼为左旋多巴与苄丝肼按比例混合而成的成品，由于苄丝肼为外周脱羧酶抑制剂，可减少左旋多巴在外周的脱羧，增加左旋多巴进入脑内的含量以及减少其外周的副作用，因此该药物相对左旋多巴的副作用有所减少，但多巴丝肼仍可能引起排尿困难等不良反应，加重患者前列腺增生导致的排尿障碍，因此合并前列腺增生的帕金森患者不宜选用。
选项 A 溴隐亭是多巴胺受体激动剂，可直接刺激多巴胺受体位点，其促进释放的催乳素会因为竞争同一受体而更多地结合多巴胺受体，加上 D_2受体阻断剂也有抗催乳素作用，可以减少催乳素的分泌，从而用于治疗或抑制生理性泌乳、高催乳素血症以及与高催乳素血症有

关的生育功能低下、月经紊乱及闭经、溢乳、性腺发育不全、男性女性化乳房等。该药对泌尿系统的影响较小，可用于合并前列腺增生的帕金森患者，故排除。
选项 C 苯海拉明为乙醇胺的衍生物，可与组织中释放出来的组胺竞争效应细胞上的 H1 受体，从而阻止过敏反应的发作，解除组胺的致痉和充血等作用，不会加重患者前列腺增生，故不考虑。
选项 D 苯海索为中枢抗胆碱抗帕金森病药，作用在于选择性阻断纹状体的胆碱能神经通路，而对外周作用较小，从而有利于恢复帕金森病患者脑内多巴胺和乙酰胆碱的平衡，改善患者的帕金森病症状，不会加重患者前列腺增生，与题意不符。
选项 E 金刚烷胺原为抗病毒药，抗帕金森病的机制可能为促进纹状体多巴胺的合成和释放，减少神经细胞对多巴胺的再摄取，并有抗乙酰胆碱作用，从而改善帕金森病患者的症状，对泌尿系统影响较小，可排除。
综上所述，正确答案是：B.多巴丝肼。
合并前列腺增生的帕金森患者应避免使用具有抗胆碱能作用的药物，因为这些药物可能会加重前列腺增生的症状，例如尿潴留。

根据选项：
- **溴隐亭**（Bromocriptine）是一种多巴胺激动剂，用于治疗帕金森病，不具有抗胆碱能作用。
- **多巴丝肼**（Carbidopa-Levodopa）是治疗帕金森病的常用药物，主要作用于多巴胺系统，不具有抗胆碱能作用。
- **苯海拉明**（Diphenhydramine）是一种抗组胺药，具有较强的抗胆碱能作用。
- **苯海索**（Benztropine）是一种抗胆碱能药物，用于治疗帕金森病。
- **金刚烷胺**（Amantadine）主要用于治疗帕金森病，也具有抗胆碱能作用。

在这些药物中，**苯海拉明**和**苯海索**具有抗胆碱能作用。为了避免加重前列腺增生的症状，应该避免使用具有抗胆碱能作用的药物。因此，答案是：

C. 苯海拉明

参考资料:
- 朱红梅, 陈忠明. 实用临床药物手册. 北京：人民卫生出版社, 2018.
- 王林波, 高文英. 神经系统疾病药物治疗学. 北京：科学出版社, 2016. !!!!
23、 (A1 型题)
卵巢上皮性肿瘤一线化疗方案为
A.博来霉素+长春新碱
B.顺铂+长春新碱
C.顺铂+长春新碱+异环磷酰胺
D.顺铂+紫杉醇
E.紫杉醇+卡铂
参考答案： E 您的答案：
图文解析： 【解析】卵巢上皮性肿瘤一线化疗方案被广泛采用的是紫杉醇加铂类，有效率高且毒性低，故 E 对。
【避错】目前卵巢上皮性肿瘤的一线化疗方案，主要有腹腔化疗和静脉化疗 2 种，其中静脉化疗被广泛运用于临床。静脉化疗方案主要包括紫杉醇类加铂类、阿霉素类加紫杉醇类、

阿霉素类加环磷酰胺加铂类、铂类加环磷酰胺。

卵巢上皮性肿瘤的一线化疗方案通常包括铂类药物和紫杉类药物。顺铂（铂类药物之一）和紫杉醇是常用的组合，因此我会选择：

D. 顺铂+紫杉醇

这种组合被广泛用作卵巢上皮性肿瘤的一线治疗，因其有效性和耐受性。然而，在实际情况中，医生可能会根据患者的具体情况调整化疗方案。

解析：

卵巢上皮性肿瘤是指来源于上皮组织的卵巢肿瘤，是常见的妇科肿瘤，占卵巢肿瘤的 50%～70%。其恶性类型占卵巢恶性肿瘤的 85%～90%，多见于中老年妇女，很少发生在青春期前或婴幼儿期。卵巢上皮性肿瘤的一线化疗方案是紫杉醇+卡铂，已成为卵巢上皮性癌初治的标准方案。

选项 A 博来霉素+长春新碱是霍奇金淋巴瘤的首选化疗方案，故不选。

选项 B 顺铂+长春新碱可用于睾丸癌的化疗，但不作为卵巢上皮性肿瘤的一线化疗方案，故排除。

选项 C 顺铂+长春新碱+异环磷酰胺可作为小细胞肺癌、睾丸癌、肉瘤等的化疗方案，但不用于卵巢上皮性肿瘤的化疗，故不选。

选项 D 顺铂+紫杉醇可用于乳腺癌、肺癌等的化疗，但不是卵巢上皮性肿瘤的一线化疗方案，可排除。

综上所述，正确答案是：E.紫杉醇+卡铂。

卵巢上皮性肿瘤一线化疗方案通常为含铂类药物和紫杉类药物的联合方案。根据当前医学指南和临床实践，推荐的标准一线化疗方案为紫杉醇和卡铂的联合治疗。

因此，最正确的答案是：

E. 紫杉醇+卡铂

24、　(A1 型题)

对自己精神状态认识和判断的能力

A.自知力

B.智力

C.领悟力

D.意识

E.定向力

参考答案：　A　您的答案：

图文解析：　【解析】自知力指患者对其自身精神状态的认识和判断的能力，故答案为 A 【避错】智力指人的大脑理解社会、进行日常生活能力的复杂程度。领悟力指理解、领会、感悟事物的能力。意识是人脑对大脑内外表象的觉察。定向力是一个人对时间、地点、人物以及自身状态的认识能力。

这个问题考察的是心理学和精神医学中的一个概念，即个体对自己精神状态的认识和判断能力。正确的答案是 A.自知力。

自知力（insight）是指个体对自身情感、思维和行为的理解和认识能力。在精神健康领域，自知力特别指个体对自己精神状态，包括任何可能的精神疾病或心理问题的认识和理解。一个人的自知力水平可以影响其对治疗的接受程度和治疗效果。因此，在这个选择题中，A 选项“自知力”是最符合题意的答案。

解析：

对自己精神状态的认识和判断能力在精神病学临床上称为自知力，指患者对自己精神状态的认识和判断能力。
选项 B 智力是指生物一般性的精神能力，指人认识、理解客观事物并运用知识、经验等解决问题的能力，包括记忆、观察、想象、思考、判断等，与题意不符，故不选。
选项 C 领悟力是指对事物认识、体会、解悟的能力，并非对自己精神状态的认识和判断能力，故排除。
选项 D 意识是指个体对周围环境及自身状态的感知能力，意识的维持依赖大脑皮质的兴奋，当脑干上行网状激活系统或双侧大脑皮质损害时可导致意识障碍，与题干描述不符，故不考虑。
选项 E 定向力是指一个人自己对时间、地点、人物以及自身状态的认识能力，定向力障碍是意识障碍的一个重要标志，与题干描述不符，可排除。
综上所述，正确答案是：A.自知力。
正确答案是 A.自知力。

自知力是指个体对自身精神状态的认识和判断的能力。它包括对自己存在心理问题的意识，以及对这些问题可能带来的影响的理解。在精神病学评估中，自知力是一个重要的评估指标，用于判断患者对其疾病的理解和应对能力。
25、 (A1 型题)
电镜下子宫内膜有高度分泌活动，腺上皮细胞增大，胞内有分泌颗粒，间质疏松肿大，提示此时子宫内膜处于的月经周期为
A.增生早期
B.增生晚期
C.分泌早期
D.分泌晚期
E.月经前期
参考答案： D 您的答案：
图文解析： 【解析】本题子宫内膜有高度分泌活动，胞内有分泌颗粒，间质疏松肿大，符合分泌晚期表现。避错子宫内膜随卵巢的周期性变化而发生改变，一般分为三期。(1）增生期：相当于卵泡发育成熟阶段，即月经周期的第 5～14 天左右。经期后，在雌激素的作用下，子宫内膜基底层细胞开始增生并修复剥脱处创面，随后继续增生变厚，腺体增多、变宽，血管也增生，间质增生致密。(2）分泌期：约为月经周期的 15～28 天，此期内膜继续增厚，腺体进一步扩大、屈曲，到晚期腺体高度分泌，血管也迅速增长，更加屈曲。间质变疏松并有水肿。(3）月经期：为月经周期第 1～4 天。在内膜功能层形成的散在小血肿，使坏死的内膜剥脱，随血液排出，称之为月经。
【避错】
这道题目描述的是子宫内膜在月经周期中的特定阶段的表现。电镜下观察到的子宫内膜有高度分泌活动，腺上皮细胞增大，胞内有分泌颗粒，间质疏松肿大，这些都是子宫内膜在分泌期的特点。月经周期大致分为增生期和分泌期，分泌期跟随在排卵后，是在黄体期间，这时候子宫内膜为了可能的妊娠进行准备，表现出高度的分泌活动。
根据题干描述，最符合的选项是分泌期。具体到是早期还是晚期，考虑到“高度分泌活动”和“胞内有分泌颗粒”，这些描述更符合分泌早期，但通常分泌颗粒的描述更倾向于分泌的中后期。因此，如果考虑到“间质疏松肿大”，这可能指向分泌期的后期，因为这时子宫内膜为植入做好了充分的准备。
所以，最合适的答案是 **D. 分泌晚期**。

解析：
月经周期主要分为月经期，增生期和分泌期，其中增生期又可分为增生早期、增生中期和增生晚期，分泌期可分为分泌早期、分泌中期和分泌晚期。其中，分泌晚期是月经周期的第24～28 日，此期子宫内膜进一步增厚，表面高低不平，略呈波浪形。上皮下的间质分化为肥大的蜕膜样细胞。内膜腺体开口面向宫腔，有糖原等分泌物溢出，间质更疏松、水肿，进一步疏松。螺旋小动脉迅速增长，超出内膜厚度，也更弯曲，并见许多分支。根据题目描述，此时子宫内膜处于分泌晚期。
选项 A 增生早期为月经周期的第 5～7 日，此期内膜的增生与修复在月经期即已开始，腺上皮细胞呈立方形或低柱状，间质较致密，细胞呈星形，间质中的小动脉较直，壁薄，与题意不符。
选项 B 增生晚期是月经周期的第 11～14 日，此期内膜进一步增厚，表面高低不平，略呈波浪形，腺上皮变为高柱状，增殖明显，细胞界限明显，与题干描述不符。
选项 C 分泌早期是月经周期的第 15～19 日，此期内膜腺体更长，弯曲更明显，腺上皮细胞的核下开始出现含糖原的小泡，称核下空泡，为分泌期的组织学特征，与本题不符。
选项 E 月经前期并不属于月经周期，子宫内膜的周期性变化分为月经期、增生期和分泌期，故不选。
综上所述，正确答案是：D.分泌晚期。
根据子宫内膜在月经周期中的变化，电镜下子宫内膜有高度分泌活动，腺上皮细胞增大，胞内有分泌颗粒，间质疏松肿大，这些特征提示子宫内膜处于分泌期。分泌期进一步分为分泌早期和分泌晚期。

增生期：在增生期，子宫内膜的主要特征是腺体和间质的增生，腺体呈直线状或稍弯曲，腺上皮细胞的分泌活动较低，胞内不会有显著的分泌颗粒。

分泌早期：在分泌早期，腺上皮细胞开始增大，胞内出现分泌颗粒，但间质尚未明显疏松肿大。

分泌晚期：在分泌晚期，腺上皮细胞高度分泌，胞内有大量分泌颗粒，间质疏松且水肿。这些描述与题干中提到的电镜下观察结果最为一致。

月经前期：在月经前期，子宫内膜开始退化，分泌活动减少，间质水肿减轻，准备脱落。

根据上述分析，最符合题干描述的子宫内膜状态是分泌晚期。

所以正确答案是：**D. 分泌晚期**
26、 (A1 型题)
命令性幻听最常见于
A.精神分裂症
B.惊恐障碍
C.躁狂症
D.抑郁症
E.适应障碍
参考答案： A 您的答案：
图文解析： 【解析】命令性幻听是指个人听到命令性语言的一种幻听，多见于精神分裂

症，故 A 对。
【避错】惊恐障碍主要表现为强烈的、反复的惊恐发作，即在短时间内快速出现以心慌、胸闷、出汗等为主的焦虑感、恐惧感、濒死感或失控感。躁狂症以情感高涨、思维奔逸以及言语动作增多为典型症状。抑郁症有兴趣下降或缺乏，“三自”“三无”等典型症状。
适应障碍指因长期存在应激源或困难处境，加上本身人格缺陷，产生的烦恼、抑郁等情感障碍以及适应不良行为和生理功能障碍。
命令性幻听，指的是患者听到声音对其发出命令或指示，这类幻听常常与严重的精神健康问题相关。在列出的选项中，精神分裂症（现在更常被称为精神病性障碍）是最常与命令性幻听相关联的疾病。因此，最正确的答案是：A. 精神分裂症
解析：
命令性幻听是幻听的一种，指幻听的内容为命令患者做某事。这是一种虚幻的听觉，但是这种幻听往往大脑出现一些命令患者的一些命令。比如命令患者跑步、命令患者跳楼、命令患者伤害他人等。命令性幻听多见于精神分裂症，也可见于伴精神病性症状的双相障碍急性发作期，是一种严重的精神病性症状。
选项 B 惊恐障碍是一种突如其来的惊恐体验，表现为严重的窒息感、濒死感和精神失控感，并伴有严重的自主神经功能紊乱症状。惊恐障碍一般不会出现幻觉，因此可排除。
选项 C 躁狂症以情感高涨或易激惹为主要临床表现，伴随精力旺盛、言语增多、活动增多，严重时伴有幻觉、妄想、紧张症状等精神病性症状。躁狂症一般不会有命令性幻听的表现，故不选。
选项 D 抑郁症以显著而持久的心境低落为主要特征，临床表现从闷闷不乐到悲痛欲绝，可有消极自杀企图或行为，甚至发生木僵。抑郁症不会出现命令性幻听，可排除。
选项 E 适应障碍是指在明显的生活改变或环境变化时所产生的短期和轻度的烦恼状态和情绪失调，常有一定程度的行为变化等，但并不出现精神病性症状，与题干描述不符。
综上所述，正确答案是：A.精神分裂症。
命令性幻听（command hallucinations）是一种病理性症状，指病人听到某种声音或指令，要求他们采取某些行为。此症状在精神分裂症患者中最为常见。精神分裂症是一种严重的精神疾病，常表现为幻觉、妄想和思维紊乱等症状。

因此，命令性幻听最常见于精神分裂症。

最佳答案是：
A. 精神分裂症
27、 (A1 型题)
长期大量饮白酒后，突然戒酒多长时间发生震颤谵妄
A.12 小时
B.48 小时
C.6 小时
D.3 小时
E.24 小时
参考答案： B 您的答案：
图文解析： 【解析】酒精戒断后震颤谵妄状态属于重度症状，常开始于停酒后 48~96 小时。【避错】戒酒综合征发生于戒酒或突然减少酒用量的 6~28 小时内。主要表现分为轻度症状、中度症状和重度症状。酒精戒断后震颤谵妄状态具体表现为意识清晰度改变的情况下，出现手、面、舌的粗大震颤，定向力障碍、幻觉和妄想，同时出现癫痫发作和合并躯体症状。

长期大量饮用白酒后，突然戒酒可能会导致震颤谵妄（戒酒综合症，又称酒精戒断综合症），通常在戒酒后 48 小时内最常见。因此，最正确的答案是 B.48 小时。
解析：
震颤谵妄是一种在慢性酒精中毒或长期酒精依赖的基础上骤然减少酒量或停饮后 48 小时左右出现，表现为短暂的、伴有躯体症状的急性意识模糊状态。其典型的三个特征是：意识障碍、行为紊乱、幻觉错觉，并伴有大量出汗、心跳过速、发热、血压升高、瞳孔散大、认知障碍、定向障碍等。长期大量饮白酒后，突然戒酒 48 小时可发生震颤谵妄。
综上所述，正确答案是：B.48 小时。
震颤谵妄（delirium tremens, DT）是长期大量饮酒者在突然戒酒后可能出现的严重戒断症状。它通常发生在戒酒后的一段时间内，具体时间因个体差异有所不同。根据临床实践和相关文献，震颤谵妄最常在戒酒后 48 至 96 小时内发生。

因此，最佳答案是：

B. 48 小时
28、 (A1 型题)
男孩，2 岁，身长 87cm，体重 12.5kg，能双脚跳，会说 2~3 个字的短语，能准确评估该小儿发育情况的是
A.发育正常
B.生长落后
C.超重
D.发育迟缓
E.消瘦
参考答案： A 您的答案：
图文解析： 【解析】一般一岁在 75cm，2 岁在 87cm（男孩身高正常）； 一般一岁时可达 10kg，2 岁在 12.5~13.5kg（体重正常），2 岁左右能双脚跳，会用勺子吃饭（运动发育正常）；会说 2~3 个字的短语（1~2 岁单词阶段，3 岁以后成语阶段，所以语言发育正常），故该男孩可评估为发育正常，故 A 对。
【避错】临床方便的应用：2~6 岁儿童平均身高（cm）=年龄×7+75，1~6 岁体重（kg）=年龄×2+8。
该题目考查的是对 2 岁儿童的发育和生长情况的评估。根据提供的信息，男孩身长 87 厘米，体重 12.5 千克，能够双脚跳，会说 2 到 3 个字的短语。这些信息可以用来判断他的生长发育是否正常。
- 身高和体重：对于 2 岁的儿童，87 厘米的身高和 12.5 千克的体重大致处于正常范围内，但需要具体对照生长曲线图来判断。
- 发育里程碑：2 岁儿童通常能够说出简单的词语和短语，并能进行简单的物理活动如双脚跳。这位男孩能够进行这些活动，说明他的运动和语言发展大致正常。
根据这些信息，最合理的结论是该男孩的发育情况是正常的。因此，最佳答案选项为：A. 发育正常
这是一道关于儿童生长发育评估的问题。我们需要先了解儿童生长发育的标准，再根据题目中给出的信息进行分析判断。
理解背景信息：题目描述了一个 2 岁男孩，给出了他的身高、体重以及某些发育里程碑。
理解问题核心：我们的目标是基于这些信息来准确评估该小儿的发育情况。
现在，我们根据现有医学知识和儿童生长发育的标准来逐一分析选项：

A 选项（发育正常）：
根据题目描述，2 岁男孩身高 87cm，体重 12.5kg，能双脚跳，会说 2-3 个字的短语。参考儿童生长发育标准，这些表现均符合 2 岁儿童的正常发育水平，因此可以判断该儿童的发育是正常的。
B 选项（生长落后）：
题目中并未提及该儿童的生长速度明显低于同龄儿童，且其身长 87cm 和体重 12.5kg 均在正常范围内，因此不能判断为生长落后。
C 选项（超重）：
虽然体重是一个重要的健康指标，但仅凭体重 12.5kg 并不能直接判断该儿童超重。需要结合身高和体重来综合评估，而题目中并未提供足够信息来支持这一判断。
D 选项（发育迟缓）：
根据描述，该儿童能双脚跳，会说 2-3 个字的短语，这些都是 2 岁儿童正常的发育里程碑。因此，不能判断为发育迟缓。
E 选项（消瘦）：
同样，仅凭体重 12.5kg 不能判断该儿童消瘦。需要综合身高和体重来评估，而题目中的信息并不支持这一判断。
综上所述，根据题目描述和儿童生长发育的标准，我们可以确定该儿童的发育是正常的。因此，正确答案是 A.发育正常。
根据现有的医学知识和生长发育标准，一个 2 岁男孩的正常生长发育情况应该包括以下几个方面：

1. **身高**：2 岁男孩的身高范围一般在 85-90 厘米左右。该男孩身高 87 厘米，属于正常范围。
2. **体重**：2 岁男孩的体重一般在 11-14 千克之间。该男孩体重 12.5 千克，属于正常范围。
3. **运动发育**：能双脚跳是 2 岁孩子的正常运动能力。
4. **语言发育**：能说 2-3 个字的短语也是 2 岁孩子的正常语言能力。

综合以上信息，该男孩的生长发育指标均在正常范围内。因此，最正确的答案是：

A. 发育正常
29、 (A1 型题)
法洛四联症不会出现以下哪个体征
A.苍白
B.蹲踞
C.晕厥
D.杵状指（趾）
E.活动耐力下降
参考答案： A 您的答案：
图文解析： 【解析】法洛四联症临床表现常见的有皮肤黏膜青紫，而非苍白（A 错，为本题正确答案）。
【避错】法洛四联症时会出现蹲踞（B 对）喜抱，活动耐力下降（E 对）、杵状指（趾）（D 对），当小儿耗氧或需氧增加时会出现阵发性呼吸困难，严重时会出现晕厥（C 对）、抽搐，甚至死亡。
法洛四联症（Tetralogy of Fallot）是一种先天性心脏病，包括四个特征：右室流出道狭窄、

室间隔缺损、主动脉骑跨和右室肥厚。这种病症会导致血液中的氧含量降低，因此患者可能出现青紫、杵状指（趾）、活动耐力下降、晕厥和蹲踞姿势（用以增加肺血流量和改善氧合）等体征。
- A. 苍白：通常与缺氧相关，但在法洛四联症中，患者更可能出现青紫色的皮肤和黏膜。
- B. 蹲踞：法洛四联症的患者可能采取蹲踞姿势来增加肺血流量和改善氧合。
- C. 晕厥：由于脑部血流减少和氧合不良，法洛四联症患者可能出现晕厥。
- D. 杵状指（趾）：是因长期缺氧而发生的体征，常见于法洛四联症患者。
- E. 活动耐力下降：由于心脏的结构异常和氧合不足，法洛四联症患者通常会表现为活动耐力下降。

因此，在上述选项中，最不可能作为法洛四联症表现的是 A. 苍白，因为这种病通常导致皮肤青紫而不是苍白。所以正确答案是 A. 苍白。
解析：
法洛四联症是一种常见的先天性心脏畸形，其基本病理为室间隔缺损、肺动脉狭窄、主动脉骑跨和右心室肥厚。由于主动脉起跨于两心室之上，主动脉除接受来自左心室的血液外，还直接接受一部分来自右心室的静脉血，因而出现青紫；同时，肺动脉的狭窄使肺循环进行气体交换的血流量减少，更加重了青紫的程度。患者多表现为面色青紫****，而不是苍白。
选项 B 蹲踞为法洛四联症病儿临床上一种特征性姿态、蹲踞可缓解呼吸困难和发绀，与题意不符。
选项 C 晕厥是由于肺动脉瓣狭窄致肺循环阻力增大，右心室排血受阻，收缩压下降，严重者可出现缺氧性发作，意识丧失或抽搐，与法洛四联症的表现相符，故不选。
选项 D 杵状指（趾）常见于慢性缺氧，法洛四联症患者由于慢性缺氧，机体代偿性产生过多的红细胞，导致继发红细胞增多症，致使手指或足趾末端增生、肥厚、呈杵状膨大，符合法洛四联症的体征，故不考虑。
选项 E 活动耐力下降是由于患者长期缺氧，稍微一活动就感到乏力，呼吸费力，不符合题意。
综上所述，正确答案是：A.苍白。
法洛四联症是一种先天性心脏病，常见的体征包括蹲踞、晕厥、杵状指（趾）和活动耐力下降。苍白不属于法洛四联症的典型体征。

因此，正确答案是：

A. 苍白
30、 (A1 型题)
新生儿胆红素生成过多的疾病是
A.新生儿败血症
B.先天性甲减
C.先天性胆道闭锁
D.新生儿窒息
E.胆汁黏膜综合征
参考答案： A 您的答案：
图文解析： 【解析】新生儿败血症可引发溶血，因此新生儿败血症能导致新生儿胆红素生成过多的疾病是新生儿败血症（A 对）。
【避错】先天性甲状腺功能减退症（B 错）由于甲状腺激素分泌减少引起代谢功能低下临床体征有智力落后、生长发育迟缓和生理功能低下。新生儿窒息（D 错）由于缺氧而引起代谢功能低下，当机体代谢功能低下时，胆红素生成会减少而不是增多。先天性胆道闭锁（C 错）

和胆汁黏稠综合征（E 错）都是因胆道梗阻引起的黄疸，并不会引起胆红素量的改变。
题目问的是导致新生儿胆红素生成过多的疾病。其中，先天性胆道闭锁会导致胆汁流动受阻，进而引起胆红素积累和黄疸。因此，正确答案应该是：C. 先天性胆道闭锁
首先，我们来理解一下胆红素生成过多的原因。胆红素的产生主要是由于体内衰老的红细胞被破坏后释放出的血红蛋白转化而来。当红细胞破坏增多，就会导致胆红素生成过多。
现在，我们根据这个原理来逐一分析每个选项：
A. 新生儿败血症：这是一种严重的细菌感染。在感染过程中，大量的红细胞可能被破坏，从而导致胆红素生成过多。这与上述原理相符。
B. 先天性甲减：这是一种由于甲状腺激素分泌不足引起的疾病。它与胆红素的生成没有直接关系。
C. 先天性胆道闭锁：这是一种胆道系统的先天性疾病，主要影响胆汁的排泄，而不是胆红素的生成。
D. 新生儿窒息：这主要是影响氧气供应，与胆红素的生成无直接关系。
E. 胆汁黏膜综合征：这主要影响胆汁的排泄和形成胆栓，同样与胆红素的生成没有直接关系。
综上所述，与胆红素生成过多有直接关系的疾病是新生儿败血症。因此，最正确的答案是：A. 新生儿败血症。
新生儿胆红素生成过多的疾病主要涉及红细胞破坏过多或肝脏处理胆红素的能力受损。根据题目提供的选项，正确答案是 **新生儿败血症**（A）。

新生儿败血症是一种严重的感染性疾病，可以导致红细胞的破坏增加，从而导致胆红素生成过多。其他选项如先天性甲减和先天性胆道闭锁虽然也会引起新生儿黄疸，但它们的机制主要是胆红素代谢和排泄障碍，而非胆红素生成过多。

所以，最佳答案是：

A. 新生儿败血症
31、 (A1 型题)
影响人性格基本形成的关键时期是
A.青年期
B.婴儿期
C.学龄前期
D.幼儿期
E.中年期
参考答案： C 您的答案：
图文解析： 【解析】影响人性格基本形成的关键时期是学龄前期（C 对）。
【避错】围生期是指妊娠满 28 周至出生后 7 天，由于经历环境转换，危险性大，此期为儿童死亡率最高的时期；幼儿期为意外伤害发生率最高的时期。婴儿期指从出生到满 1 周岁以前的一段时期，是生长发育最迅速的时期，也是最短的一个阶段；幼儿期为意外伤害发生率最高的时期；13 周岁~17 周岁为少年（也可称青少年），20 周岁~39 周岁为青年，是人一生的生长发育、身体健康、心理和智力发育的关键时期；中年期， 一般指 40—60 岁,人生的许多重要任务都是在这一时期完成的。
影响人性格基本形成的关键时期通常认为是在早期的发展阶段，尤其是在儿童的早年。因此，最相关的选项可能是：

- A. 青年期
- B. 婴儿期
- C. 学龄前期
- D. 幼儿期
- E. 中年期

其中，婴儿期和幼儿期通常被视为性格形成的关键时期，因为这是个体经历快速心理和情感发展的时期。在这两个阶段，儿童开始建立信任感、独立性以及初步的自我意识。但是，如果要选择一个最为关键的时期，许多心理学理论，如弗洛伊德的精神分析理论和埃里克森的心理社会发展理论，都强调了**幼儿期**（大约 2 到 3 岁）对个体的长远影响，尤其是在性格形成方面。

因此，我认为最佳答案是 **D. 幼儿期**。

解析：

性格是一个人对现实的稳定的态度，以及与这种态度相应的，习惯化了的行为方式中表现出来的人格特征。人的性格并不是一成不变的，但是，一旦形成就比较稳定，影响人性格基本形成的关键时期是学龄前期。学龄前期儿童大脑皮质的兴奋和抑制功能逐渐平衡，皮质对皮下中枢的调节作用逐渐加强，分析综合能力进一步发展，行为的自觉性也逐步形成和发展，其性格特征在学龄前期已基本形成。

选项 A 青年期是性格的成熟和稳定期，而不是形成期，故不选。

选项 B 婴儿期儿童的心理开始发生和发展，已经有了初步的思维活动，但性格尚未形成，故排除。

选项 D 幼儿期是儿童心理发展的重要时期，是语言、思维、个性、社会交往等各方面发展的关键时期，但不是性格基本形成的关键时期，故不考虑。

选项 E 中年期个体的性格基本稳定，一般不会有太大变化，故不选。

综上所述，正确答案是：C.学龄前期。

影响人性格基本形成的关键时期是：

C. 学龄前期

32、 (A1 型题)

与过期妊娠无关的是

A.肥胖

B.宫内发育迟缓

C.羊水过多

D.胎盘功能不良

E.胎儿过度成熟

参考答案： C 您的答案：

图文解析： 【解析】羊水过多与胎膜早破和早产有关，与过期妊娠无关（C 错，为本题正确答案）。

【避错】发生过期妊娠的原因还不明确。引发的可能因素很多，包括肥胖、宫内发育迟缓、胎盘功能不良、胎儿过度成熟、雌孕激素比例失调等。

过期妊娠，也称为过期产或过期分娩，通常指妊娠超过预定的分娩日期，通常是超过 40 周。与过期妊娠相关的因素通常包括胎儿宫内生长迟缓（B）、羊水异常（C）、胎盘功能问题（D），因为这些因素可能导致妊娠延续超过正常期限。肥胖（A）是孕期并发症的一个风险因素，但它本身通常不直接导致过期妊娠。胎儿过度成熟（E）是指胎儿在宫内生长时间过长，实际上与过期妊娠直接相关。因此，与过期妊娠无关的选项是 **A.肥胖**。

解析：
过期妊娠是一种异常妊娠，指妊娠超过 42 周但还未分娩。过期妊娠的胎儿围产病率和死亡率增高，并随妊娠延长而加剧，妊娠 43 周时围产儿死亡率为正常 3 倍。44 周时为正常 5 倍。
过期妊娠可能与以下因素有关：雌、孕激素比例失调：正常妊娠足月分娩时，雌激素增高，孕激素降低。如雌激素不能明显增加，导致孕激素比例升高，抑制前列腺素及缩宫素的作用，从而引发过期妊娠。
胎儿畸形：多见于无脑儿，与胎儿肾上腺皮质激素分泌不足有关。
遗传因素：部分过期妊娠者有家族史，推测该病可能与遗传因素有关。
胎盘硫酸酯酶缺乏：是一种罕见的伴性隐性遗传病，可导致过期妊娠。
子宫收缩刺激反射减弱：部分过期妊娠胎儿较大，可导致头盆不称或胎位不正，胎儿先露部不能与子宫下段及宫颈密切接触，从而减少了反射性子宫收缩，引起过期妊娠。
选项 A 肥胖是妊娠期糖尿病的危险因素，而妊娠期糖尿病会增加巨大儿的发生率，易导致过期妊娠，二者有一定关联。
过期妊娠的胎儿胎盘功能减退，胎儿生长受限，导致胎儿宫内发育迟缓，因此选项 B 宫内发育迟缓与过期妊娠有关。
过期妊娠的胎儿可能继续生长，所以相对于营养过剩的胎儿就会变成巨大儿，且过熟儿颅骨变硬、颅缝较窄，在分娩过程中胎头不易变形，适应产道能力下降，使胎头下降困难，容易出现难产。难产可直接导致胎儿宫内窘迫，此时胎儿经常处于缺氧状态，致使胎儿肛门括约肌松弛，胎粪排出而污染羊水，胎儿吸入污染的羊水后可引起部分肺不张，化学性肺炎及全身感染等，增加了围产儿的发病率和死亡率。且过期妊娠的胎儿，围产儿的死亡率比足月儿高 3-6 倍，其胎盘功能不良的发生率较高，故选项 D 胎盘功能不良符合过期妊娠的特点。
过期妊娠的胎儿，过度成熟，又称为过熟儿，故选项 E 胎儿过度成熟是过期妊娠的表现。
选项 C 羊水过多是胎儿畸形、多胎妊娠、胎盘脐带病变、母儿血型不合、孕妇有合并症等原因导致的**，与过期妊娠无关**。
综上所述，正确答案是：C.羊水过多。
32、与过期妊娠无关的是

A.肥胖
B.宫内发育迟缓
C.羊水过多
D.胎盘功能不良
E.胎儿过度成熟

答案：C.羊水过多

解释：

- **过期妊娠**（妊娠超过 42 周）可能与以下因素有关：
  - **肥胖**（A）：肥胖孕妇的妊娠期延长风险较高。
  - **宫内发育迟缓**（B）：过期妊娠时，胎儿的营养和氧气供给可能受到限制，导致胎儿发育迟缓。
  - **胎盘功能不良**（D）：胎盘功能随着妊娠时间延长可能逐渐衰退，导致胎儿供氧不足。
  - **胎儿过度成熟**（E）：过期妊娠的胎儿可能会表现出过度成熟的体征，如皮肤干燥、指甲长等。

- **羊水过多**（C）：羊水过多通常与妊娠期糖尿病、胎儿畸形、双胎妊娠等因素有关，与过期妊娠无直接关系。
33、　(A1 型题)
不全流产的特征是
A.易发生休克
B.阴道流血
C.腹痛
D.妊娠物完全排出
E.无妊娠物排出
参考答案：　A　您的答案：
图文解析：　【解析】不全流产：难免流产继续发展，部分妊娠物排出宫腔（DE 错），还有部分残留于宫腔内或嵌顿于宫颈口处，或胎儿排出后胎盘滞留宫腔或嵌顿于宫颈口，影响子宫收缩，导致出血，甚至发生休克（A 对）。
【避错】腹痛（C 错）和阴道流血（B 错）是流产的一般性特征，无特异性。
不全流产的特征主要包括阴道流血和腹痛，但其关键特征是妊娠物未能完全排出。因此，正确答案是：
B. 阴道流血
这是因为在不全流产中，妊娠物部分留在子宫内，导致阴道出血和可能伴随的腹痛，但关键是妊娠物没有完全排出。尽管 A（易发生休克）和 C（腹痛）也可能与流产相关，但它们不是不全流产的主要特征。D（妊娠物完全排出）描述的是完全流产，而 E（无妊娠物排出）则与流产的概念不符。
解析：
不全流产是指难免流产继续发展，部分妊娠物排出宫腔，还有部分残留于宫腔内或嵌顿于宫颈口处，或胎儿排出后胎盘滞留宫腔或嵌顿于宫颈口，影响子宫收缩，导致大量出血，甚至发生休克，妇科检查见子宫小于停经周数，阴道流血不止。
选项 A 易发生休克，是难免流产继续发展，导致大量出血时可能出现的表现，不是不全流产的特异性表现，故不选。
选项 C 腹痛在多种流产中均可出现，无特异性，故排除。
选项 D 妊娠物完全排出，选项 E 无妊娠物排出，均为干扰选项，故不选。
综上所述，正确答案是：B.阴道流血。
不全流产的特征是阴道流血和腹痛，但妊娠物未完全排出。因此，最正确的答案是：

B.阴道流血
34、　(A1 型题)
关于受精过程以下说法正确的是
A.卵子排出即可受精
B.精子进入输卵管即可成功受精
C.受精成功即为妊娠的开始
D.整个受精过程需要大概 72 小时
E.受精发生在卵子排出 12 小时内
参考答案：　E　您的答案：
图文解析：　【解析】受精多在排卵后数小时内发生，　一般不超过 12 小时（E 对）。
【避错】卵子（次级卵母细胞）从卵巢排出，经输卵管伞部进入输卵管，在输卵管内与获能

的精子相遇（A 错）。只有发生顶体反应的精子才能次级卵母细胞融合（B 错）。着床后即为妊娠的开始（C 错）。整个受精过程需要大概 24 小时（D 错）。
关于受精过程的正确说法是 E. 受精发生在卵子排出 12 小时内。卵子排出后，其最佳受精能力保持在约 12 至 24 小时内，超过这个时间段，卵子的受精能力迅速下降。因此，受精通常发生在卵子排出后的 12 小时内，而且通常是在输卵管中进行。所以，选项 E 是最正确的答案。
这是一道关于受精过程的题目，我们需要根据现有的医学知识来判断哪个选项是正确的。
首先，我们来分析每个选项：
A. 卵子排出即可受精。这个说法是不准确的。虽然卵子排出是受精的前提，但并不是卵子一排出就能立即受精。它需要与精子结合才能完成受精过程。
B. 精子进入输卵管即可成功受精。这个说法同样不准确。精子进入输卵管只是受精的第一步，它还需要找到并结合卵子才能完成受精。
C. 受精成功即为妊娠的开始。受精成功确实是妊娠的一个重要步骤，但严格来说，妊娠的开始通常被定义为受精卵着床在子宫内膜上，而不仅仅是受精成功。
D. 整个受精过程需要大概 72 小时。这个说法不准确。一般来说，受精过程通常发生在排卵后的 12-24 小时内，而不是 72 小时。
E. 受精发生在卵子排出 12 小时内。这个说法是准确的。正常情况下，受精大多发生在排卵后数小时内，一般不超过 24 小时。当精子与卵子相遇后，首先发生顶体反应，使精子的头、尾分离，并发生透明带反应和卵黄膜的封闭作用，这一系列过程需要一定时间，但通常在卵子排出后的较短时间内（如 12 小时内）完成。
综上所述，正确答案是 E.受精发生在卵子排出 12 小时内。
关于受精过程，以下说法正确的是：

E.受精发生在卵子排出 12 小时内

解释：

- A. 卵子排出即可受精：不完全正确，卵子排出后需要在输卵管与精子相遇才能受精。
- B. 精子进入输卵管即可成功受精：不完全正确，精子需要穿透卵子的外层并与卵细胞结合才能完成受精过程。
- C. 受精成功即为妊娠的开始：不完全正确，受精是妊娠的一个必要步骤，但妊娠的开始通常是指胚胎成功植入子宫内膜。
- D. 整个受精过程需要大概 72 小时：不正确，受精过程通常在数小时内完成。
- E. 受精发生在卵子排出 12 小时内：正确，卵子排出后的受精时间窗通常在 12-24 小时内，超过这个时间卵子就不再具备受精能力。

35、 (A1 型题)
下列关于流行性乙型脑炎病理改变是叙述错误的是
A.神经细胞坏死
B.血管套形成
C.软化灶
D.胶质细胞增生
E.有脓性分泌物
参考答案： E 您的答案：
图文解析： 【解析】流行性乙型脑炎是由乙型脑炎病毒感染引起的急性传染病，其基本

病变有：血管周围淋巴细胞浸润和血管套形成（B 对），神经细胞变性坏死（A 对），软化灶形成（C 对）和胶质细胞增生（D 对）。流行性乙型脑炎由病毒感染引起，无脓性物渗出（E 错，为本题正确答案）。

【避错】蛛网膜下腔有脓性渗出物主要见于流行性脑脊髓膜炎。

流行性乙型脑炎（也称为日本脑炎）主要表现为中枢神经系统的炎症反应。其病理改变通常包括神经细胞坏死、胶质细胞增生、血管周围的淋巴细胞浸润，可能出现血管套形成，以及脑组织的软化灶。根据这些信息：

- A. 神经细胞坏死：这是流行性乙型脑炎的特征性病理改变。
- B. 血管套形成：在病变区周围的小血管周围常见淋巴细胞和浆细胞浸润，形成所谓的血管套。
- C. 软化灶：在严重的病例中，脑组织可能发生坏死，形成软化灶。
- D. 胶质细胞增生：这是脑炎过程中常见的病理改变。

E. 有脓性分泌物：流行性乙型脑炎通常不会引起脑膜的脓性感染，因此不太可能出现脓性分泌物。

因此，描述错误的是 E. 有脓性分泌物。

首先，我们要了解流行性乙型脑炎的基本病理特点。流行性乙型脑炎是由乙型脑炎病毒引起的，其特点是病毒在脑实质细胞内增殖，并导致一系列病理改变。

接下来，我们针对每个选项进行分析：

A. 神经细胞坏死：流行性乙型脑炎病毒在脑实质细胞内增殖，这会导致神经细胞受损甚至坏死。所以，A 选项描述是准确的。

B. 血管套形成：由于流行性乙型脑炎导致的炎症反应，小胶质细胞、少突胶质细胞和淋巴细胞会在血管周围聚集，形成“血管套”。因此，B 选项也是正确的。

C. 软化灶：流行性乙型脑炎会导致脑实质细胞的坏死，进而形成筛网状软化灶。所以，C 选项描述是准确的。

D. 胶质细胞增生：流行性乙型脑炎会导致胶质细胞的增生，特别是在小胶质细胞和少突胶质细胞中。因此，D 选项描述也是正确的。

E. 有脓性分泌物：流行性乙型脑炎是由病毒引起的，而不是细菌。因此，它不会产生脓性分泌物，这与细菌感染有所不同。所以，E 选项描述是不准确的。

综上所述，关于流行性乙型脑炎病理改变的错误叙述是 E 选项：有脓性分泌物。

流行性乙型脑炎（乙脑）是一种由乙型脑炎病毒引起的急性传染病，主要通过蚊虫传播。该病的病理改变主要集中在中枢神经系统，常见病理特征包括神经细胞坏死、血管套形成、软化灶和胶质细胞增生。

各选项解释如下：

- A. 神经细胞坏死：乙脑的主要病理变化之一，是由于病毒感染导致神经细胞的损伤和死亡。
- B. 血管套形成：乙脑感染时，炎症细胞围绕血管形成的“血管套”现象。
- C. 软化灶：乙脑常见的病理特征，是由于神经组织的坏死和液化形成的软化灶。
- D. 胶质细胞增生：由于神经细胞损伤，胶质细胞会增生以修复损伤。
- E. 有脓性分泌物：脓性分泌物一般与细菌性感染相关，而乙脑是病毒感染，通常不产生脓性分泌物。

因此，关于流行性乙型脑炎病理改变的叙述错误的是：

E. 有脓性分泌物

36、　(A1 型题)

短暂性脑缺血发作应用阿司匹林治疗的目的是
A.改善神经功能的缺失
B.保护脑神经
C.增加再灌注
D.预防复发
E.扩张血管
参考答案：　　D　您的答案：
图文解析：　　　【解析】短暂性脑缺血发作（TIA）是由于血流动力学改变、微栓塞等原因导致局部脑或视网膜缺血引起的短暂性神经功能缺失。阿司匹林抗血小板治疗对于非心源性栓塞性 TIA 可以起到抗血小板凝集、防止再次阻塞动脉引起血流动力学障碍的作用，因而可以预防 TIA 的复发（D 对）。
【避错】短暂性脑缺血发作多次复发时应选用华法林。
短暂性脑缺血发作（TIA）的治疗主要目的是防止未来的脑卒中发生。因此，应用阿司匹林治疗短暂性脑缺血发作主要是为了减少再次发作的风险。阿司匹林具有抗血小板聚集作用，可以帮助防止血栓形成，从而减少复发的风险。因此，最正确的答案是：D. 预防复发
这是一道关于短暂性脑缺血发作（TIA）治疗目的的选择题。首先，我们需要明确阿司匹林在 TIA 治疗中的作用，然后分析每个选项与阿司匹林治疗目标的关联度。
阿司匹林是一种抗血小板聚集药物，它在 TIA 治疗中的主要作用是预防血栓形成，从而降低 stroke（中风）的风险。
现在我们来逐一分析各个选项：
A. 改善神经功能的缺失：阿司匹林并不能直接改善已经出现的神经功能缺失，而是通过预防进一步的血栓形成来避免情况恶化。所以 A 选项不正确。
B. 保护脑神经：虽然阿司匹林通过预防中风间接保护了脑神经，但这并不是其直接的治疗目的。因此 B 选项也不准确。
C. 增加再灌注：阿司匹林主要是抗血小板聚集，不直接增加脑组织的再灌注。所以 C 选项与阿司匹林的主要作用不符。
D. 预防复发：阿司匹林通过抑制血小板聚集，降低血栓形成的风险，从而有效预防 TIA 或 stroke 的复发。这与阿司匹林的治疗目的高度相关，因此 D 选项是正确的。
E. 扩张血管：阿司匹林并不具备直接扩张血管的作用，其主要功能是抗血小板聚集。所以 E 选项不正确。
综上所述，阿司匹林在 TIA 治疗中的主要目的是预防复发，因此正确答案是 D 选项：预防复发。
短暂性脑缺血发作（TIA）的治疗目的是预防复发，因为 TIA 是缺血性卒中的高危预兆。因此，应用阿司匹林的主要目的是减少血栓形成，进而预防未来的卒中或再次发生 TIA。

根据现有的医学知识和最佳实践指南，正确答案是：

D. 预防复发
37、　(A1 型题)
不符合面神经炎表现的是
A.患侧额纹消失
B.患侧鼻唇沟变浅
C.患侧不能闭眼
D.患侧不能鼓腮

E.常伴有偏瘫
参考答案：　　E　您的答案：
图文解析：　　【解析】面神经炎多表现为病侧面部表情肌瘫痪，前额皱纹消失、眼裂扩大、鼻唇沟平坦、口角下垂。病侧不能作皱额、蹙眉、闭目、鼓气和噘嘴等动作。不符合面神经炎表现的是偏瘫（E 错，为本题正确答案）。
【避错】面神经是以运动神经为主的混合神经，主要支配面部表情肌和传导舌前 2/3 的味觉及支配舌下腺、下颌下腺和泪腺的分泌。基底核区脑出血常导致偏瘫。
面神经炎，也称为贝尔氏麻痹，主要表现为面部肌肉无力或麻痹。根据这些症状，我们可以分析每个选项：
A. 患侧额纹消失：这是面神经炎的一个典型症状，因为面部表情肌受影响，患者难以皱眉。
B. 患侧鼻唇沟变浅：这也是面神经炎的一个表现，因为面部控制力下降，导致面部的一些纹路变浅。
C. 患侧不能闭眼：这是面神经炎的一个重要症状，称为拉格纳尔征，表明患者无法闭紧患侧眼睛。
D. 患侧不能鼓腮：这同样是面神经炎的症状之一，因为受影响的面部肌肉无法正常工作，导致患者无法鼓腮。
E. 常伴有偏瘫：这个选项不符合面神经炎的典型表现。面神经炎主要影响面部肌肉，而偏瘫是指半边身体的肌肉无力或麻痹，通常是由脑部疾病引起的。
因此，最不符合面神经炎表现的是 **E. 常伴有偏瘫**。
首先，我们逐一审视每个选项，并结合面神经炎（也称为面神经麻痹或面瘫）的典型表现来进行分析。
A. 患侧额纹消失：这是面神经炎的一个典型表现。当面神经受损时，由于面神经支配的额肌无力，会导致患侧额纹消失。
B. 患侧鼻唇沟变浅：这同样是面神经炎的一个表现。由于面神经受损，支配口周肌肉的神经信号减弱，使得患侧鼻唇沟变得不明显。
C. 患侧不能闭眼：面神经炎患者常因眼轮匝肌无力而无法完全闭眼，这也是该病症的一个典型症状。
D. 患侧不能鼓腮：由于面神经受损导致的颊肌无力，患者在进行鼓腮等动作时会感到困难，这是面神经炎的另一个常见症状。
E. 常伴有偏瘫：偏瘫通常与脑血管事件（如中风）相关，而不是面神经炎的典型表现。面神经炎主要影响面部神经和肌肉，通常不会导致身体其他部位的瘫痪。
综上所述，不符合面神经炎表现的是选项 E，即常伴有偏瘫。这一症状与面神经炎的典型表现不符，而更多地与中风等脑血管疾病相关。因此，正确答案是 E。
37. 不符合面神经炎表现的是

A. 患侧额纹消失
B. 患侧鼻唇沟变浅
C. 患侧不能闭眼
D. 患侧不能鼓腮
E. 常伴有偏瘫

答案：E. 常伴有偏瘫

解析：面神经炎（即面瘫）主要表现为患侧面部肌肉的瘫痪，如患侧额纹消失、鼻唇沟变浅、

不能闭眼和不能鼓腮等。然而，面神经炎一般不会伴有偏瘫，因为偏瘫通常是由于脑部病变引起的，而面神经炎是由于面神经的病变引起的。

38、 (A1 型题)

下列妊娠期糖尿病对母儿的影响最可能是

A.胎儿低血糖

B.羊水过少

C.新生儿高血糖

D.巨大儿

E.过期妊娠

参考答案： D 您的答案：

图文解析： 【解析】妊娠期糖尿病对母亲可能会出现酮症酸中毒、引起肾脏疾病这些影响，对胎儿可能产生出现巨大儿（D 对）、引起早产。

【避错】孕妇患有妊娠期糖尿病时，胎儿会长期处于高血糖的状态下，使胰岛β细胞增生，产生较多的胰岛素，合成脂肪，进而引起巨大儿。

妊娠期糖尿病（gestational diabetes mellitus, GDM）对母儿的影响可以多方面考虑，但我们需要找出最可能的影响。选择最正确的答案，我们应当关注妊娠期糖尿病最典型和常见的并发症。

A. 胎儿低血糖 - 这通常发生在出生后，如果母亲患有糖尿病，因为胎儿可能会产生更多的胰岛素来对抗高血糖，出生后可能会导致低血糖。

B. 羊水过少 - 这不是妊娠期糖尿病的典型并发症。

C. 新生儿高血糖 - 通常情况下，妊娠期糖尿病导致的是新生儿低血糖而不是高血糖。

D. 巨大儿 - 这是妊娠期糖尿病最典型的影响之一，因为高血糖环境会促使胎儿生长加速，从而导致出生时体重异常增加。

E. 过期妊娠 - 这并不是妊娠期糖尿病最典型的并发症。

根据上述分析，最可能的影响是巨大儿（D 选项）。因此，对于这个问题，最佳答案是 **D. 巨大儿**。

这是一道涉及妊娠期糖尿病影响的问题。首先，我们来了解一下妊娠期糖尿病的基本知识。妊娠期糖尿病（GDM）指的是在怀孕期间发生的糖尿病，这可能会影响母体和胎儿的健康。接下来，我们针对每个选项进行详细的分析：

A. 胎儿低血糖：虽然妊娠期糖尿病的孕妇可能生出低血糖的胎儿，但这并不是妊娠期糖尿病最直接或最常见的影响。因此，这个选项可能不是最佳答案。

B. 羊水过少：妊娠期糖尿病通常与羊水过多的情况更为关联，而非羊水过少。所以，这个选项不太可能是正确答案。

C. 新生儿高血糖：虽然妊娠期糖尿病可能影响新生儿的血糖水平，但高血糖不是其最直接的影响。此选项也不太可能是正确答案。

D. 巨大儿：妊娠期糖尿病的孕妇，由于血糖控制不佳，胎儿长期处于高血糖状态，容易导致胎儿躯体过度发育，形成巨大儿。这是妊娠期糖尿病的常见并发症之一。

E. 过期妊娠：妊娠期糖尿病与过期妊娠的关联并不强，因此这个选项也不太可能是正确答案。

综合以上分析，妊娠期糖尿病最直接和常见的影响是巨大儿。因此，正确答案是 D.巨大儿。

妊娠期糖尿病对母儿的影响最可能的是：

D. 巨大儿

妊娠期糖尿病最常见的并发症之一是巨大儿（巨大的胎儿），这主要是由于母体的高血糖导致胎儿的胰岛素分泌增加，从而促进胎儿的生长。

39、 (A1 型题)

输卵管疏通术后，发热，宫颈举痛，附件压痛，右侧为重，白细胞升高，可能并发的疾病是

A.盆腔炎

B.子宫剥脱

C.黄体破裂

D.子宫破裂

E.卵巢囊肿

参考答案： A 您的答案：

图文解析： 【解析】输卵管疏通术后，发热，宫颈举痛，附件压痛，右侧为重（盆腔炎的典型体征），白细胞升高，可能并发的疾病是盆腔炎。

【避错】子宫剥脱（B 错）即子宫内膜剥脱，子宫内膜的功能层会随着卵巢周期性分泌雌激素和孕激素,发生周期性内膜剥脱。黄体破裂（C 错）常发生于月经后半期，由于黄体功能不全或外力作用下，出现突然一侧下腹疼痛，阴道无流血或仅有少量流血。子宫破裂（D 错）分为不完全性子宫破裂和完全性子宫破裂，不完全性子宫破裂是子宫肌层部分或全层破裂，但浆膜层完整，宫腔与腹腔不相通，胎儿及其附属物仍在宫腔内；完全性子宫破裂是子宫肌壁全层破裂，宫腔与腹腔相通，产妇突感下腹一阵撕裂样剧痛，子宫收缩骤然停止，无发热，无宫颈举痛及附件压痛。卵巢囊肿蒂扭转（E 错）的典型症状是体位改变后突然发生一侧下腹剧痛，常伴恶心、呕吐甚至休克。

根据题目描述，患者输卵管疏通术后出现发热、宫颈举痛、附件压痛（尤其是右侧较重）和白细胞升高，这些症状和体征提示可能发生了感染和炎症反应。在给出的选项中，盆腔炎（Pelvic Inflammatory Disease, PID）最符合这些临床表现。盆腔炎常见于妇科手术后，如输卵管疏通术，可引起宫颈举痛、附件区压痛及全身炎症反应表现，如发热和白细胞升高。

因此，最可能的并发疾病是：A. 盆腔炎。

解析：

患者输卵管疏通术后，出现发热、宫颈举痛、附件压痛、白细胞升高等表现，考虑并发了盆腔炎。

选项 B 子宫剥脱指的是子宫内膜的周期性脱落，是正常生理现象，不会出现宫颈举痛、附件压痛等症状，故不选。

选项 C 黄体破裂通常发生在排卵后或月经周期之末 1 周，偶可在月经期第 1，2 天发病。部分患者黄体破裂，出血不多，但有突然发作的下腹部疼痛，并由于血液流向腹腔使腹膜受到刺激，引起反应性恶心、呕吐，严重者因此发生休克。该患者无恶心、呕吐等症状，与黄体破裂的症状描述差异明显，故排除。

选项 D 子宫破裂是指妊娠晚期或分娩期子宫体部或子宫下段发生裂开，为产科严重并发症，威胁母儿生命。主要死于出血、感染休克。而该患者为输卵管疏通术后，并无分娩史，故子宫破裂的可能性不大，与题意不符。

选项 E 卵巢囊肿通常无症状，除非囊肿体积较大，但很少出现发热、宫颈举痛等，故不选。

综上所述，正确答案是：A.盆腔炎。

根据题目描述，患者在输卵管疏通术后出现发热、宫颈举痛、附件压痛（右侧为重），并伴有白细胞升高。这个症状提示可能存在感染或炎症。

- **盆腔炎（A）**：输卵管疏通术后，最常见的并发症之一是盆腔炎。这是由于手术过程中细菌可能进入盆腔导致感染。症状包括发热、宫颈举痛、附件压痛和白细胞升高。

- 子宫剥脱（B）：这不是一个常见的术后并发症，并且不符合题目描述的症状。

- 黄体破裂（C）：黄体破裂通常会引起急性腹痛，但不一定伴随发热和白细胞升高。

- 子宫破裂（D）：子宫破裂通常在妊娠或分娩过程中发生，不是输卵管疏通术的常见并发症。

- 卵巢囊肿（E）：卵巢囊肿可能引起腹痛，但不太可能伴随宫颈举痛和发热。

综合考虑患者的症状和术后并发症的常见性，最可能的诊断是：

A. 盆腔炎
40、 (A1 型题)
预防宫颈管套扎的指征是
A.妊娠 24 周
B.妊娠 9 周
C.妊娠 32 周
D.妊娠 12 周
E.妊娠 34 周
参考答案： D 您的答案：
图文解析： 【解析】预防性的宫颈管套扎：适合于以前有过两次以上的怀孕中晚期难免流产，或者在怀孕之前通过做扩宫棒检测已经确定有宫颈机能不全的情况，在孕 12~16 周进行。【避错】根据解析排除其他选项。
宫颈管套扎是一种用于防止早产的手术，通常适用于有早产历史或宫颈异常短的孕妇。进行这项手术的最佳时间是在妊娠中期，早于发生宫颈扩张或早产迹象之前。因此，妊娠 24 周（A 选项）通常被认为是进行宫颈管套扎的合适时间，因为这时候既能有效预防早产，又足够早以避免宫颈扩张的风险增加。所以，正确答案应该是 A.妊娠 24 周。
解析：
宫颈环扎术是封闭宫颈内口的一种手术，一般算中小手术。宫颈环扎术的目的是修复并建立正常的宫颈内口形态和功能，使妊娠能够维持至足月或胎儿能够存活。手术多于妊娠 14～18 周施行，因此预防宫颈管套扎的指征是妊娠 12 周后，此时进行手术，可避免因手术刺激导致流产，又可预防宫颈机能不全的孕妇发生早产。
综上所述，正确答案是：D.妊娠 12 周。
选项 A 妊娠 24 周，选项 C 妊娠 32 周，选项 E 妊娠 34 周，均晚于妊娠 12 周，此时再进行宫颈环扎术，因手术刺激可能导致早产等不良后果，故可一并排除。
选项 B 妊娠 9 周尚属于孕早期，此时胚胎着床尚不稳定，不宜行宫颈环扎术，以免手术刺激导致流产，故不选。
预防宫颈管套扎（即宫颈环扎术）通常用于防止因宫颈机能不全导致的早产或流产。根据现有的医学指南和最佳实践，宫颈环扎术通常在妊娠的早中期进行，特别是在 12 到 24 周之间进行，以预防早产。

选项分析如下：

A. 妊娠 24 周 - 这是一个常见的时间段进行宫颈环扎术。

B. 妊娠 9 周 - 这时间段太早，通常不会进行宫颈环扎术。
C. 妊娠 32 周 - 这时间段太晚，通常不会进行预防性的宫颈环扎术。
D. 妊娠 12 周 - 这是一个合适的时间段进行宫颈环扎术。
E. 妊娠 34 周 - 这时间段太晚，通常不会进行预防性的宫颈环扎术。

在这几项中，最合适的预防宫颈管套扎的指征是 **D. 妊娠 12 周**。

41、 (A1 型题)

有关髋关节结核描述的正确的是

A.多见于儿童

B.双侧发病较多

C.不会形成寒性脓肿

D.“4”字试验阴性

E.髋关节缺血性坏死

参考答案： A 您的答案：

图文解析： 【解析】髋关节结核儿童多见，单侧居多。后期会有寒性脓肿，“4”字试验阳性。

髋关节结核（也称为骨结核）是一种影响骨骼的结核感染，其中髋关节是较常见的受累部位之一。现在我们来分析每个选项：

A. **多见于儿童**：髋关节结核确实可以影响儿童，但也可见于成人。儿童是一个相对常见的群体，因为他们的免疫系统未完全发育。

B. **双侧发病较多**：髋关节结核通常是单侧性的，双侧发生较少。

C. **不会形成寒性脓肿**：这个描述不正确。髋关节结核可以形成寒性脓肿，这是结核感染的特征之一。

D. **“4”字试验阴性**：“4”字试验（也称为 Patrick 试验）用于评估髋关节或骶髂关节的疼痛。在髋关节结核中，这个试验可能是阳性，因为患者可能表现出髋关节的疼痛和受限。

E. **髋关节缺血性坏死**：髋关节结核可能导致关节破坏和关节周围的炎症，但缺血性坏死并不是结核病的特征，而是血液供应受阻的结果。

在这些选项中，**A**（多见于儿童）是最符合髋关节结核描述的。虽然成人也可患病，但儿童更易受到这种感染的影响。因此，最正确的答案是 **A**。

首先，我们逐一分析每个选项：

A. 多见于儿童：髋关节结核确实在儿童中更为常见。这是因为儿童的免疫系统相对较弱，且结核杆菌主要通过呼吸道传播，儿童在接触结核病患者后更容易受感染。

B. 双侧发病较多：实际上，髋关节结核单侧发病较为多见，而不是双侧。

C. 不会形成寒性脓肿：髋关节结核的病变发展可能导致髋关节周围出现脓肿，这些脓肿通常不伴有明显的局部发热或红、热、痛等急性炎症表现，被称为“寒性脓肿”。因此，这个选项是不正确的。

D. “4”字试验阴性：“4”字试验是一个用于检查髋关节疾病的体格检查方法。在髋关节结核的情况下，由于关节活动受限和疼痛，“4”字试验往往呈阳性，而非阴性。

E. 髋关节缺血性坏死：髋关节结核与髋关节缺血性坏死没有直接关系。髋关节缺血性坏死通常与长期使用激素、髋关节外伤等因素有关，而非髋关节结核的直接后果。

综上所述，正确答案是 A，即髋关节结核多见于儿童。这是因为儿童免疫系统相对较弱，且结核杆菌主要通过呼吸道传播，使儿童在接触结核病患者后更容易感染。其他选项均存在错误或与髋关节结核的直接关系不大。

有关髋关节结核描述的正确的是：

A. 多见于儿童

髋关节结核是一种常见的骨关节结核，主要发生在儿童和青少年，故选择 A 选项。其他选项的描述不正确，特此解释如下：

B. 双侧发病较多：髋关节结核通常为单侧发病，双侧发病较为罕见。

C. 不会形成寒性脓肿：髋关节结核常会形成寒性脓肿，这是结核性炎症的一种特征表现。

D. “4”字试验阴性：“4”字试验阳性是髋关节病变的表现之一。

E. 髋关节缺血性坏死：髋关节结核并不会直接导致髋关节缺血性坏死。缺血性坏死通常由其他原因引起，如股骨头的血供障碍。

42、 (A2 型题)

患者，男，近 3 个月来经常思考“人为什么要存在”“历史起源是什么样的”，自己知道思考没有意义，但自己不能控制，为此感到苦恼，坐立不安，主动就诊。患者存 是

A.强迫思维

B.焦虑症状

C.恐惧症状

D.强迫症状

E.惊恐发作

参考答案： A 您的答案：

图文解析： 【解析】强迫思维是以刻板形式反复闯入患者头脑中的观念、表象或冲动思维，它们几乎总是令人痛苦的，内容常常为暴力、猥亵或毫无意义。患者往往试图抵制，但不成功。虽然这些思维并非自愿且令人反感，但患者认为它是属于自己的（A 对）。

【避错】恐惧症发病的症状主要就是过于紧张、害怕，甚至会出现严重的躯体反应，比如心慌、心悸、手抖、出汗，严重时会出现大汗淋漓、呼吸困难、窒息感等等（C 错）。强迫症状往往是为了减轻强迫思维产生的焦虑而不得不采取的行动，明知是不合理的，但不得不做（D 错）。惊恐发作是指一次明确的强烈恐惧、焦虑或不适的发作期，同时伴有多个自主神经症状和认知症状（E 错）。广泛性焦虑障碍是一种以焦虑为主要临床表现的精神障碍，者常常有不明原因的提心吊胆、紧张不安，并有显著的自主神经功能紊乱症状、肌肉紧张及运动性不安（B 错）。

这个问题涉及识别患者表现的精神症状类型。根据题干描述，患者经常思考一些深层次的问题，如“人为什么要存在”、“历史起源是什么样的”，并且虽然他知道这些思考没有意义，但无法控制这些思维，还因此感到苦恼和坐立不安。这些症状符合强迫思维的特点，强迫思维是一种反复出现、不愉快的思想或冲动，个体通常会感到这种思想是无法控制的，并因此感到苦恼。

- A. 强迫思维：指的是反复出现的、难以抗拒的、通常是不愉快的思想、冲动或想象，患者往往认为这些是无意义的，并试图抗拒或摆脱。
- B. 焦虑症状：通常指持续的、过度的、不合理的担忧和紧张，而不是针对特定思想的焦虑。
- C. 恐惧症状： 是对特定物体或情境的强烈恐惧和回避。
- D. 强迫症状：通常指强迫行为（如反复清洗、检查）或强迫思维，这里的描述更偏向于思维层面， 而不是行为。

- E. 惊恐发作：是突然的、强烈的恐惧或不适感，通常伴有躯体症状，如心跳加快、呼吸急促。
根据这些定义，最符合患者描述的是 A.强迫思维。因此，正确答案是 A。
首先，我们仔细解读题干。患者近 3 个月来经常进行一些无意义的哲学性思考，如“人为什么要存在”和“历史起源是什么样的”。关键信息在于，患者自己知道这些思考没有意义，但却不能自我控制，并因此感到苦恼和坐立不安。
现在，我们针对每个选项进行分析：
A. 强迫思维：强迫思维是指反复出现一些想法，明知不必要或不合理，但是无法控制。这与患者的症状高度吻合，即明知思考无意义却无法停止。
B. 焦虑症状：虽然患者表现出苦恼和坐立不安，但这些症状更可能是由强迫思维导致的，而不是单纯的焦虑症状。焦虑症状通常表现为广泛性担忧、紧张和不安，而不特指某种无意义的反复思考。
C. 恐惧症状：恐惧症状通常与特定的对象或情境相关，如恐高症、幽闭恐惧症等。题干中并未提及患者对任何特定事物或情境的恐惧。
D. 强迫症状：强迫症状是一个更广泛的概念，包括强迫思维和强迫行为。虽然患者的症状属于强迫症状的一种，但强迫思维更为具体和准确。
E. 惊恐发作：惊恐发作通常表现为突然的、强烈的恐惧感，伴随生理症状如心跳加速、出汗、呼吸急促等。题干中并未提及这些症状。
综合考虑，患者的症状最符合强迫思维的定义。因此，选择 A 选项“强迫思维”作为最符合题干描述的答案。
综上所述，正确答案是：A.强迫思维。
根据题干描述，患者经常思考“人为什么要存在”“历史起源是什么样的”，尽管他知道这些思考没有意义，但他无法控制这些想法，并且因此感到苦恼，坐立不安。这种情况很可能是强迫思维的表现。

强迫思维（obsessive thoughts）是指患者反复出现一些不由自主、不合逻辑的想法或冲动，尽管患者知道这些想法或冲动是没有意义的，却难以控制，且因此感到痛苦和困扰。

因此，最正确的答案是：

A. 强迫思维
43、 (A2 型题)
男，26 岁，车祸伤左侧头部着地，意识障碍，查体：呼吸深大，右侧瞳孔 2.0mm，左侧瞳孔 4.0mm，视盘水肿，拟行急诊手术，可能的诊断是
A.左侧颞叶钩回疝
B.右侧小脑幕切迹疝
C.枕骨大孔疝
D.左侧小脑扁桃体疝
E.大脑镰疝
参考答案： B 您的答案：
图文解析： 【解析】男性，车祸伤左侧头部着地，意识障碍，呼吸深大，右侧瞳孔 2.0mm，左侧瞳孔 4.0mm（提示左侧瞳孔缩小），视盘水肿（提示颅内压增高），拟行急诊手术，根据该患者的症状、体征，考虑诊断为右侧小脑幕切迹疝（B 对）。
【避错】小脑幕切迹疝 ①颅内压增高的症状：表现为剧烈头痛，与进食无关的频繁呕

吐。头痛程度进行性加重伴烦躁不安。急性脑疝患者视神经盘水肿可无。②瞳孔改变：病初由于病侧动眼神经受刺激导致病侧瞳孔变小，对光反射迟钝，随病情进展病侧动眼神经麻痹，病侧瞳孔逐渐散大，直接和间接对光反射均消失。③运动障碍：表现为病变对侧肢体的肌力减弱或麻痹，病理征阳性。严重时可出现去脑强直发作，这是脑干严重受损的信号。④意识改变。⑤生命体征紊乱：脑干内生命中枢功能紊乱或衰竭，可出现生命体征异常。表现为心率减慢或不规则，血压忽高忽低，呼吸不规则、大汗淋漓或汗闭，面色潮红或苍白。

该病例描述了一个 26 岁男性，经历车祸伤后头部着地，出现意识障碍、呼吸深大，右侧瞳孔 2.0mm，左侧瞳孔 4.0mm，以及视盘水肿。这些症状表明患者可能经历了严重的颅内损伤，导致颅内压增高。

考虑到这些临床表现，特别是瞳孔大小不对称和视盘水肿，可以推测患者可能有颅内疝的情况。颅内疝是指脑组织由于颅内压增高而移位，压迫重要结构。

- A. 左侧颞叶钩回疝，通常会导致瞳孔扩大（通常是同侧），并可能压迫脑干。
- B. 右侧小脑幕切迹疝，这种情况下瞳孔变化可能不明显，但通常伴随意识下降和脑干功能障碍。
- C. 枕骨大孔疝，也称为颅后窝疝，通常会迅速导致生命体征不稳定。
- D. 左侧小脑扁桃体疝，这会导致脑干受压并可能迅速进展为枕骨大孔疝。
- E. 大脑镰疝，这种情况下瞳孔不对称也可能出现，但更多是由于大脑半球间的压迫。

根据描述的症状，特别是左侧瞳孔扩大和意识障碍，最可能的诊断是**左侧颞叶钩回疝**（A）。这种疝通常是由于同侧颅脑损伤引起的，与病人的受伤方式（左侧头部着地）和临床表现相符。

解析：

青年男性患者，车祸伤左侧头部着地后出现意识障碍，查体显示右侧瞳孔正常，左侧瞳孔散大，左侧动眼神经麻痹，双侧视盘水肿，考虑患者颅内高压，结合患者病史、临床表现，该患者最可能的诊断为左侧颞叶钩回疝。颞叶钩回疝又称为小脑幕切迹疝，是由于颅内压增高使颞叶海马回、钩回通过小脑幕切迹被推移至幕下。由于小脑幕切迹与脑干内侧面之间有枕骨小脑幕阻挡，移位的脑组织在小脑幕切迹挤压脑干，使脑干内的网状上行激动系统受累，病人随之陷入昏睡状态；患侧动眼神经受挤压产生患侧瞳孔逐渐散大，直接和间接对光反射消失，并有患侧上睑下垂、眼球外斜；患侧的大脑脚受到挤压造成病变对侧偏瘫，肌张力增高，腱反射亢进，病理征阳性；有时颞叶前部或额叶底部被挤入小脑幕裂孔，可因脑干受压而导致呼吸系统紊乱，突然出现呼吸停止，心跳骤停或死亡。

选项 B 右侧小脑幕切迹疝常表现为右侧动眼神经麻痹，而题干中患者表现为左侧动眼神经麻痹，故排除。

选项 C 枕骨大孔疝，选项 D 左侧小脑扁桃体疝，均为脑疝的一种，但均不会出现动眼神经麻痹的症状，可一并排除。

选项 E 大脑镰疝又称为扣带回疝，极少见，主要表现为剧烈头痛、呕吐和烦躁不安等颅内压增高症状，一般不出现动眼神经麻痹，不符合题意。

综上所述，正确答案是：A.左侧颞叶钩回疝。

根据题目描述，该患者头部受伤后出现意识障碍，呼吸深大，右侧瞳孔 2.0mm，左侧瞳孔 4.0mm，视盘水肿，这些体征提示颅内高压和脑疝。具体分析如下：

1. **瞳孔变化**：一侧瞳孔扩大（左侧 4.0mm），提示同侧颞叶受压或颞叶钩回疝。
2. **意识障碍**：严重的头部损伤可能导致颅内出血和颅内压升高，进而导致意识障碍。
3. **呼吸深大**：可能是由于脑干受压导致的中枢性呼吸变化。

4. **视盘水肿**：提示颅内压增高。

结合这些体征，最可能的诊断是**左侧颞叶钩回疝**，因为钩回疝会导致同侧动眼神经受压，出现同侧瞳孔扩大和对侧瞳孔缩小的表现。左侧钩回疝导致左侧动眼神经受压，使左侧瞳孔扩大。

因此，最佳答案是：

A. 左侧颞叶钩回疝

44、 (A2 型题)

女，26 岁。因车祸撞击头部，头部疼痛 15 小时。醒后出现头晕、呕吐、神志清，眼睑青紫，眼结膜下出血，嗅觉丧失，鼻腔可见淡红色液体流出。最可能的诊断是

A.颅顶骨凹陷性骨折

B.颅底前窝骨折

C.颅底中窝骨折

D.颅底后窝骨折

E.鼻骨骨折

参考答案： B 您的答案：

图文解析： 【解析】青年女性患者，车祸撞击头部，神志清楚，眼睑青紫，眼结膜下出血，嗅觉丧失，鼻腔可见淡红色液体流出，考虑诊断为颅底前窝骨折（B 对）。颅前窝骨折，骨折出血可经前鼻孔流出，或进入眶内在眼睑和球结膜下形成淤血斑，俗称“熊猫眼征”或“眼镜征”。颅中窝骨折，血液和脑脊液可经外耳道流出，形成耳漏。颅后窝骨折，在乳突和枕下部可见皮下淤血（Battle 征）。

【避错】颅中窝骨折（C 错）时，可因出血进入耳内形成耳内出血，并可出现脑脊液耳漏。颅后窝骨折（D 错）可累及咽后壁，咽后壁血肿并可出现软腭麻痹、漏液误咽发生呛咳。鼻骨骨折（E 错）可出现鼻出血、鼻腔堵塞感、头痛和鼻梁偏斜畸形等表现。

这个病例描述的症状，包括头晕、呕吐、眼结膜下出血、嗅觉丧失以及鼻腔流出淡红色液体（可能为脑脊液漏），提示了颅底骨折的可能性。特别是嗅觉丧失和鼻腔流出液体往往与颅底前窝骨折相关，因为前窝骨折会影响嗅神经并可能导致脑脊液从鼻孔流出。

因此，最可能的诊断是：B.颅底前窝骨折

这是一道涉及颅骨骨折类型的诊断问题。首先，我们需要分析患者的病史、症状和体征，以确定最可能的诊断。

病史分析：

患者年轻女性，因车祸撞击头部，出现头痛、头晕、呕吐。

眼睑青紫，眼结膜下出血，嗅觉丧失，鼻腔可见淡红色液体流出。

选项解读：

A.颅顶骨凹陷性骨折：此类型骨折通常会导致头部局部的凹陷，但一般不会导致嗅觉丧失或鼻腔出血。

B.颅底前窝骨折：常见症状包括眼睑和结膜下淤血（熊猫眼征），以及脑脊液鼻漏，符合患者症状。

C.颅底中窝骨折：通常表现为耳后乳突区皮下瘀斑（Battle 征）和脑脊液耳漏，与题干描述不符。

D.颅底后窝骨折：常见症状为乳突和枕下部皮下淤血（枕下区肿胀及皮下瘀斑），不符合患者表现。

E.鼻骨骨折：主要表现为鼻部肿胀、疼痛、鼻出血、鼻梁变形或塌陷，不伴有嗅觉丧失或脑脊液鼻漏。
综合分析：
患者头部外伤后出现眼睑青紫、眼结膜下出血，提示颅底前窝部位可能受损。
嗅觉丧失和鼻腔淡红色液体流出（可能是脑脊液鼻漏）是颅底前窝骨折的典型症状。
综上所述，根据患者的病史、症状和体征，最可能的诊断是颅底前窝骨折。因此，答案是 B.颅底前窝骨折。
根据患者的症状和体征，以下是每个选项的可能性分析：

A. **颅顶骨凹陷性骨折**：通常表现为局部头痛和肿胀，但不会引起眼结膜下出血、嗅觉丧失或鼻腔液体流出。

B. **颅底前窝骨折**：这种骨折可能会导致嗅觉丧失，因为嗅神经通过前颅窝。鼻腔流出淡红色液体可能是脑脊液鼻漏，这也是前颅窝骨折的典型表现。另外，眼睑青紫和眼结膜下出血也是前颅窝骨折的常见体征。

C. **颅底中窝骨折**：这种骨折通常表现为耳部症状，如耳漏、听力丧失和鼓膜出血，不会引起嗅觉丧失或鼻腔液体流出。

D. **颅底后窝骨折**：这种骨折通常会影响脑干和小脑，导致共济失调、眼球震颤等神经系统症状，不会引起嗅觉丧失或鼻腔液体流出。

E. **鼻骨骨折**：虽然会导致鼻出血和眼睑青紫，但不会导致嗅觉丧失和眼结膜下出血，也不太可能引起脑脊液鼻漏。

综上所述，患者的头部撞击后出现的嗅觉丧失、鼻腔淡红色液体流出、眼睑青紫和眼结膜下出血最符合颅底前窝骨折的表现。因此，最可能的诊断是：

B. **颅底前窝骨折**
45、 (A2 型题)
女，26 岁，痛经 3 年逐渐加重，月经量增多，本次行经 10 日，血仍未止，2 年前因不孕做过输卵管通液治疗，子宫较正常略大，后位，活动度差，右侧触及 4cm×4cm×3cm 块，与子宫粘连；左侧附件增厚，双宫骶韧带均触及豆粒大痛性结节，本病首先考虑为
A.陈旧性宫外孕
B.生殖器结核
C.慢性盆腔炎
D.子宫内膜异位症
E.输卵管卵巢囊肿
参考答案： D 您的答案：
图文解析： 【解析】育龄期女性，痛经、月经量增多，2 年前因不孕做过输卵管通液治疗，子宫较正常略大，后位，活动度差，右侧触及 4cm×4cm×3cm 的大韧性包块，与子宫粘连；左侧附件增厚，双宫骶韧带均触及豆粒大痛性结节，本病首先考虑为子宫内膜异位症（D 对）。
【避错】陈旧性宫外孕，表现为输卵管妊娠流产或破裂后，很多患者会出现反复内出血症

状，这样很容易导致盆腔内肿不消散，血肿机化变硬并与周围组织粘连（A 错）。生殖器结核又称结核性盆腔炎，患者可无任何异常症状，常在诊断性刮宫、子宫输卵管造影或腹腔镜手术时发现（B 错）。盆腔炎性疾病主要包括子宫内膜炎、输卵管炎、输卵管卵巢脓肿、盆腔腹膜炎。炎症可局限于一个部位，也可同时累及几个部位，以输卵管炎，输卵管卵巢炎最常见（C 错）。输卵管卵巢囊肿是附件囊肿的一种，多由炎症刺激引起输卵管卵巢囊肿的症状（E 错）。
根据所描述的症状，如痛经逐年加重、月经量增多、子宫略大并后位、附件区增厚以及双宫骶韧带触及痛性结节等，这些特征较为符合子宫内膜异位症（Endometriosis）的临床表现。子宫内膜异位症是一种常见的妇科疾病，其特点是子宫内膜组织存在于子宫以外的部位，常见症状包括痛经、月经不规则、性交痛以及不孕等。此外，与子宫粘连的块状物可能指向内膜异位囊肿的形成。
因此，最符合这位患者症状的诊断是 D.子宫内膜异位症。
首先，我们来仔细解读一下题目的关键信息。患者是一位 26 岁的女性，长期痛经并逐渐加重，月经量增多，并且本次月经已经持续了 10 天还未结束。两年前，她因为不孕问题做过输卵管通液治疗。在检查中，医生发现她的子宫比正常略大，位置后倾，活动度较差。右侧可以触及一个与子宫粘连的肿块，而左侧附件增厚。另外，在双宫骶韧带处都能触及到豆粒大小的痛性结节。
接下来，我们根据这些信息来分析各个选项：
A. 陈旧性宫外孕：通常会有停经后腹痛与阴道流血的症状，并且可能出现腹部包块。但题目中并未提及这些症状，因此这个选项不太可能是正确答案。
B. 生殖器结核：这种疾病通常表现为不孕、月经失调、下腹坠痛以及全身症状如发热、盗汗等。虽然患者有不孕史，但其他结核的典型症状并未提及，因此这个选项也不太可能是正确答案。
C. 慢性盆腔炎：这种疾病主要表现为下腹痛、阴道分泌物增多，并可能出现异常阴道出血等症状。但题目中描述的症状与这些不完全吻合，因此这个选项也不太可能是正确答案。
D. 子宫内膜异位症：这是一种常见的妇科疾病，主要表现为继发性痛经、进行性加重，月经量增多，经期延长或月经淋漓不尽等症状。此外，子宫内膜异位症还可能导致不孕和性交不适。检查时可能会发现子宫后倾固定，一侧或双侧附件处触及囊实性包块，活动差。这些症状和体征与题目中描述的患者情况高度吻合。
E. 输卵管卵巢囊肿：这是一种炎性积液，通常是由盆腔炎性疾病发展而来。虽然患者左侧附件增厚，但没有其他证据表明这是输卵管卵巢囊肿。
综上所述，考虑到患者的痛经、月经量增多、不孕史以及查体发现，最符合的诊断是子宫内膜异位症。
因此，正确答案是 D.子宫内膜异位症。
根据患者的病史和体征，最可能的诊断是子宫内膜异位症（D）。以下是一些支持这个诊断的理由：

1. **痛经逐渐加重**：子宫内膜异位症是痛经常见的原因之一，尤其是当痛经随着时间的推移逐渐加重时。
2. **月经量增多且行经时间延长**：子宫内膜异位症可能导致月经不调，包括月经量增多和行经时间延长。
3. **既往不孕史和输卵管通液治疗**：不孕症与子宫内膜异位症密切相关，且输卵管通液治疗可能提示患者存在盆腔结构异常。
4. **子宫后位、活动度差**：子宫内膜异位症可导致盆腔器官的固定和子宫后位。

5. **触及痛性结节**：双宫骶韧带触及痛性结节是子宫内膜异位症的典型体征之一。

其他选项分析如下：
- **A. 陈旧性宫外孕**：虽然可以有盆腔包块，但一般不会有痛性结节。
- **B. 生殖器结核**：通常会有全身症状，如低热、盗汗、体重减轻等，而患者病史中未提及这些症状。
- **C. 慢性盆腔炎**：虽然也可表现为盆腔疼痛和包块，但通常不会有子宫内膜异位症那样显著的痛经和痛性结节。
- **E. 输卵管卵巢囊肿**：虽然可以解释盆腔包块，但不会解释痛经逐渐加重和痛性结节。

因此，最可能的诊断是子宫内膜异位症（D）。
46、 (A2 型题)
初孕妇，25 岁，妊娠 39 周，腹痛 2 天，查体：BP130／90mmHg，心率 100 次／分，下腹拒按，阴道口可见胎儿上肢，胎心音消失，导尿为淡红色，首选的处理措施是
A.缩宫素引产
B.剖宫产
C.内转胎位术
D.产钳助产
E.静滴地西泮
参考答案： B 您的答案：
图文解析： 【解析】初孕妇，25 岁，妊娠 39 周，腹痛 2 天，下腹拒按（先兆子宫破裂的产妇烦躁不安，呼吸、心率过快，下腹剧痛难忍），阴道口可见胎儿上肢（为忽略性肩先露，可造成梗阻性难产，是先兆子宫破裂的高危因素），胎心音消失（因宫缩过强、过频，胎儿触不清，胎心率加快或减慢或听不清）。导尿呈淡红色（膀胱受压充血，出现排尿困难及血尿），诊断为忽略性肩先露引起的先兆子宫破裂。首先处理措施是立即剖宫产（B 对）。【避错】由于胎儿尚可存活，因此行引产（A 错）不恰当。内倒转后臀牵引（C 错）适用于臀先露。行胎头吸引术与行产钳助产术（D 错）适用于头先露，而本例为肩先露。地西泮用于妊娠期子痫前期（E 错）。
本题描述的是一位处于妊娠晚期的初孕妇，表现为腹痛、高血压、快速心率、下腹部拒按，且有阴道出现胎儿上肢和胎心音消失的情况。这些症状暗示着可能的胎盘早剥或胎儿窘迫，需要紧急处理。
- **A. 缩宫素引产**：通常用于催产或在分娩后减少出血，但在胎儿存活的情况下不是首选，尤其是在胎位异常或胎儿窘迫时。
- **B. 剖宫产**：在胎儿窘迫和非正常分娩进程（如胎儿部分娩出、胎心音消失）情况下，剖宫产是紧急救治胎儿和母亲的最佳选择。
- **C. 内转胎位术**：这是一种手术操作，用于调整胎儿位置以促进阴道分娩，但在胎儿窘迫的情况下不适宜采用。
- **D. 产钳助产**：在头位分娩中可用产钳辅助，但在胎儿部分娩出且紧急情况下不推荐。
- **E. 静滴地西泮**：主要用于镇静，不适用于处理这种紧急妊娠情况。
因此，最合适的处理措施是 **B. 剖宫产**，因为它是在胎儿窘迫和分娩进展不良时的紧急干预措施。
首先，我们分析题干。
这是一位 25 岁的初孕妇，妊娠 39 周，已经足月。她已经腹痛了 2 天，这可能意味着产程已经开始。查体中，她的血压是 130/90mmHg，心率 100 次/分，下腹拒按，这可能提示腹部

有疼痛和紧张。关键信息是，阴道口可以见到胎儿的上肢，而且胎心音消失，这通常不是一个好兆头，可能意味着胎儿已经死亡或者处于非常危险的状态。导尿为淡红色，可能提示有尿血，这进一步表明了产妇可能面临的紧急情况。
接下来，我们逐项分析选项：
A. 缩宫素引产：虽然缩宫素可以促进分娩，但在这个情境中，胎心音已经消失，使用缩宫素引产可能不是最安全的选择。
B. 剖宫产：考虑到胎儿上肢已经在阴道口可见，但胎心音消失，这很可能意味着胎儿处于危险状态。剖宫产可以迅速将胎儿取出，避免进一步的并发症，特别是在胎心音消失的情况下。
C. 内转胎位术：这种方法通常用于胎位不正的情况，但在这个案例中，主要的问题是胎心音消失，所以这不是首选的处理措施。
D. 产钳助产：虽然产钳助产可以帮助分娩，但在胎心音消失的情况下，这种方法可能不是最安全或最有效的。
E. 静滴地西泮：地西泮主要用于抗焦虑和镇静，对于当前的紧急情况并不是首选处理措施。
综上所述，考虑到胎儿上肢已经在阴道口可见但胎心音消失，这可能意味着胎儿处于非常危险的状态，需要立即采取措施。因此，首选的处理措施是进行剖宫产以确保母体和胎儿的安全。
因此，正确答案是 B.剖宫产。
这道题描述了一位初孕妇在妊娠 39 周时出现腹痛并伴有严重的体征（胎儿上肢暴露、胎心音消失、导尿淡红色），提示可能存在胎盘早剥、子宫破裂或脐带脱垂等紧急情况。最佳处理措施应为立即终止妊娠以拯救母亲的生命。因此，首选的处理措施是剖宫产。

因此，正确答案是：

B. 剖宫产
47、 (A2 型题)
男孩，5 岁。持续高热 1 周。皮疹 2 天，查体：T39℃,P128 次 / 分，R36 次 / 分。热病容，双眼结膜充血，口唇干裂，可见草莓舌，皮肤可见弥漫性斑丘疹，颈部左侧可触 淋巴结。心音有力，手足指（趾）硬性水肿。最可能的诊断是
A.川崎病
B.手足口病
C.败血症
D.风湿热
E.猩红热
参考答案： A 您的答案：
图文解析： 【解析】男孩，5 岁。持续高热 1 周（发热 5 天以上）。皮疹 2 天，查体：T39℃,P128 次/分，R36 次 / 分。热病容，双眼结膜充血，口唇干裂，可见草莓舌，皮肤可见弥漫性斑丘疹（皮疹），颈部左侧可触及一直径 2cm 淋巴结（颈部淋巴结肿大）。心音有力，手足指（趾）硬性水肿，综合患者情况，考虑诊断为川崎病（A 对）。
【避错】手足口病（B 错）多发生于 5 岁以下儿童，口腔、手足部位可出现典型皮疹。败血症（C 错）多有寒战，多无手足指（趾）硬性水肿。风湿热（D 错）有 5 个主要表现：游走性多发性关节炎、心肌炎、皮下结节、环形红斑、舞蹈病。猩红热（E 错）疹退后有明显的脱屑，多见杨梅舌。
这个案例描述了一个 5 岁男孩，出现了持续高热、皮疹、双眼结膜充血、口唇干裂、草莓舌、

皮肤弥漫性斑丘疹、颈部淋巴结肿大和手足指硬性水肿等症状。这些症状最符合川崎病的特点。
川崎病是一种影响儿童的疾病，特点是发热超过五天以及多脏器炎症表现，包括结膜炎、唇炎和口腔变化（如草莓舌）、皮疹、手足变化（如硬性水肿）以及颈部淋巴结肿大。
因此，最可能的诊断是：
A. 川崎病
其他选项如手足口病、败血症、风湿热和猩红热虽然也可以导致发热和皮疹，但它们的其他症状与这个案例描述的不完全吻合。
首先，我们来仔细分析题干和选项，以便确定最可能的诊断。
患者是一个 5 岁的男孩，持续高热一周，出现皮疹两天。体检显示高热、心率和呼吸均加快，呈现热病容。关键的临床表现包括双眼结膜充血、口唇干裂、草莓舌、皮肤弥漫性斑丘疹、颈部淋巴结肿大和手足指（趾）硬性水肿。
现在，我们逐一分析选项：
A. 川崎病：川崎病是一种急性发热性出疹性小儿疾病，以全身血管炎为主要病变。其典型临床表现包括持续高热、结膜充血、草莓舌、皮疹等，与题干描述高度一致。此外，手足硬性水肿和膜状脱皮也是川崎病的特征性表现。
B. 手足口病：手足口病主要由肠道病毒引起，常见于 5 岁以下儿童。其症状包括发热、手、足、口腔等部位的皮疹或疱疹。但手足口病通常不伴有结膜充血和草莓舌，且手足硬性水肿不是其典型表现。
C. 败血症：败血症是指致病菌进入血液循环，在其中生长繁殖并产生毒素而引起的全身性感染。虽然败血症可引起高热和皮疹，但它通常不伴有结膜充血、草莓舌或手足硬性水肿。
D. 风湿热：风湿热是一种与 A 组 β 溶血性链球菌感染有关的全身性结缔组织的非化脓性疾病。其主要症状包括关节炎、心脏炎、皮下结节等，与题干描述的症状不符。
E. 猩红热：猩红热是由 A 组溶血性链球菌感染引起的急性呼吸道传染病。其主要症状包括发热、咽峡炎、全身弥漫性鲜红色皮疹等。虽然与题干中的部分症状相似，但猩红热通常不伴有结膜充血和手足硬性水肿。
综上所述，考虑到患者的高热、结膜充血、草莓舌、皮疹和手足硬性水肿等症状，最可能的诊断是川崎病。
因此，正确答案是 A.川崎病。
根据题目提供的病史和查体表现，这个 5 岁男孩的症状包括：

- 持续高热 1 周
- 皮疹 2 天
- 体温 39℃，心率 128 次/分，呼吸 36 次/分
- 热病容
- 双眼结膜充血
- 口唇干裂
- 草莓舌
- 皮肤弥漫性斑丘疹
- 颈部左侧淋巴结肿大
- 心音有力
- 手足指（趾）硬性水肿

这些症状符合川崎病的典型表现。川崎病（Kawasaki disease）是一种儿童常见的急性发热性

疾病，主要表现为高热、皮疹、眼结膜充血、口腔改变（如草莓舌和口唇干裂）、颈部淋巴结肿大以及四肢末端改变（如硬性水肿）。

因此，最可能的诊断是：

A. 川崎病

48、 (A2 型题)

男孩，5 岁。因生长和智力发育落后就诊。查体：身材矮小，头围小，眼距宽，鼻梁低，外耳小，通贯手，心脏听诊有杂音。为明确诊断最合适的检查是

A.染色体核型分析

B.头颅 CT

C.超声心动图检查

D.血 T3、T4

E.智力测定

参考答案： A 您的答案：

图文解析： 【解析】本例患儿生长和智力发育落后，身材矮小，头围小、眼距宽、鼻梁低、外耳小（特殊面容），通贯手（皮纹特点），心脏听诊有杂音（伴发畸形），应诊断为唐氏综合征，为明确诊断最合适的检查是染色体核型分析（A 对）。

【避错】智力测定（E 错）是许多遗传及先天疾病的一般检查，无明确诊断意义。血清 T3、T4 检测（D 错）多用于明确诊断先天性甲状腺疾病。头颅 CT（B 错）用于诊断颅内血肿或肿瘤等。超声心动图检查（C 错）用于诊断小儿先心病。

根据题目描述的症状，包括生长和智力发育落后、身材矮小、头围小、眼距宽、鼻梁低、外耳小、通贯手（指手上的蹼状组织连接），以及心脏听诊有杂音，这些特征指向某种遗传性疾病。这种症状组合让人联想到某种染色体异常，如唐氏综合症，这是一种常见的染色体病，通常与第 21 号染色体的三体状态相关。

最合适的检查方法是**A.染色体核型分析**，因为这种检查可以确诊是否存在染色体异常，进而帮助确定具体的遗传性疾病。其他选项虽然可能有助于评估相关的临床表现（如超声心动图检查心脏问题），但对于明确遗传性疾病的诊断而言，染色体核型分析更为直接和关键。因此，最合适的选择是 A。

解析：

5 岁患儿，生长和智力发育落后，查体身材矮小，头围小，眼距宽，鼻梁低，外耳小，通贯手，心脏听诊有杂音，根据其临床表现和体征，考虑诊断为唐氏综合征。唐氏综合征即 21-三体综合征，又称先天愚型或 Down 综合征，是由多了一条 21 号染色体而导致的疾病。为明确诊断最合适的检查是染色体核型分析，通过检查可明确是否存在 21 号染色体的异常。

选项 B 头颅 CT 主要用于颅脑占位性病变的诊断，如颅内血肿、脑外伤、脑出血、蛛网膜下腔出血、脑梗死、脑肿瘤、脑炎、寄生虫病、退行性病变等，对染色体异常疾病的诊断意义不大，故排除。

选项 C 超声心动图检查可直观显示心脏的结构和运动状态，主要用于诊断心血管疾病，对染色体异常疾病无诊断价值，故不选。

选项 D 血 T3、T4 主要用于甲状腺功能的检测，对染色体异常疾病的诊断没有帮助，故排除。

选项 E 智力测定主要用于儿童智力发育的鉴定以及作为脑器质性损害及退行性病变的参考指标，但对唐氏综合征无诊断意义，故不选。

综上所述，正确答案是：A.染色体核型分析。

根据题目描述，这个 5 岁男孩表现出身材矮小、头围小、眼距宽、鼻梁低、外耳小、通贯手

以及心脏听诊有杂音等特征。为了明确诊断，需要选择一种最合适的检查方法。

这些特征提示可能是某种先天性综合征，最常见的包括唐氏综合征、特纳综合征等。染色体核型分析是用来检测染色体异常的，这对于诊断遗传性疾病非常重要。

综合以上信息，最合适的检查是：

A. 染色体核型分析

49、 (A2 型题)

婴儿，18 个月，逗之不笑，面色蜡黄，有阵发性肢体不规则颤动，经常咳嗽和腹泻，查血象：Hb61g／L，MCV102fl，MCH 升高，MCHC 正常，该患儿最可能缺乏以下哪种物

A.维生素 B12

B.维生素 A

C.铁

D.维生素 C

E.维生素 E

参考答案： A 您的答案：

图文解析： 【解析】婴儿，18 个月，逗之不笑，面色蜡黄，有阵发性肢体不规则颤动（巨幼贫患儿典型临床表现）。血象：Hb61g/L，MCV102fl，MCH 升高，MCHC 正常（提示大细胞性贫血）。根据患儿年龄、临床表现、体征及血常规，最可能的诊断是营养性巨幼细胞性贫血，维生素 B12 缺乏导致（A 对）。

【避错】维生素 A 缺乏的典型表现为皮肤角化过度和干眼症（B 错）。缺铁性贫血患儿面色苍白，可有反甲，异食癖等特有表现，血象为小细胞低色素性贫血（C 错）。维生素 C 缺乏表现为四肢无力、牙龈出血、口臭、维生素 C 缺乏病（D 错）。维生素 E 一般不容易缺乏，更多由于肝脏损伤引起，缺乏维生素 E 表现为轻度的溶血性贫血，MCV 不会增大（E 错）。

这个案例描述了一位 18 个月大的婴儿，表现出不笑、面色蜡黄、阵发性肢体不规则颤动、频繁咳嗽和腹泻。血液检查显示 Hb 61 g/L（贫血），MCV 102 fl（红细胞体积增大），MCH 升高（平均红细胞血红蛋白含量增高），MCHC 正常（平均红细胞血红蛋白浓度正常）。这些症状和血液指标指向了巨幼细胞性贫血。

巨幼细胞性贫血通常由维生素 B12 或叶酸缺乏引起。维生素 B12 缺乏不仅会导致巨幼细胞性贫血，还可能导致神经系统问题，如不规则颤动。而这位婴儿的症状与维生素 B12 缺乏的临床表现相符。

基于这些信息，最可能的答案是：A. 维生素 B12

解析：

18 个月大婴儿，逗之不笑，面色蜡黄，有阵发性肢体不规则颤动，经常咳嗽和腹泻，血象结果提示中度贫血，红细胞体积偏大，结合患儿症状及实验室检查，考虑营养性巨幼红细胞性贫血。巨幼红细胞性贫血主要是由于维生素 B12 和（或）叶酸缺乏所致的一种大细胞性贫血。

选项 B 维生素 A 缺乏主要表现为夜盲、结膜干燥角化、角膜干燥、软化、混浊，甚至穿孔失明、皮肤干燥、脱屑、角化增生、头发干脆易脱落、指甲多纹等，与题干描述不符。

选项 C 铁缺乏主要见于缺铁性贫血，为小细胞低色素性贫血，平均红细胞体积（MCV）、平均红细胞血红蛋白量（MCH）、平均红细胞血红蛋白浓度（MCHC）均降低，该患者为大细胞性贫血，故不选。

选项 D 维生素 C 缺乏可致坏血病，早期症状是性情暴躁、皮肤苍白、消化不良、抵抗力减

弱、倦怠无力、食欲减退、牙龈出血、鼻出血、眼角膜出血、并伴有腹泻和呼吸道感染等，与题干描述差异较大。
选项 E 维生素 E 缺乏主要表现为皮肤粗糙、缺乏弹性、皱纹多、肌肤失润、头发枯黄、易脱落，容易脱屑、色斑加重等早衰表现，与患儿症状不符。
综上所述，正确答案是：A.维生素 B12。
根据题干描述，这名 18 个月大的婴儿有以下症状和体征：

- 不笑
- 面色蜡黄
- 阵发性肢体不规则颤动
- 经常咳嗽和腹泻

血象检查结果：
- 血红蛋白 (Hb) 61 g/L（低于正常范围）
- 平均红细胞体积 (MCV) 102 fl（偏高）
- 平均红细胞血红蛋白含量 (MCH) 升高
- 平均红细胞血红蛋白浓度 (MCHC) 正常

这些症状和血象结果表明该婴儿可能患有巨幼细胞性贫血（megaloblastic anemia），这种贫血常见于维生素 B12 或叶酸缺乏。

选择维生素 B12 缺乏的原因：
1. 面色蜡黄可能是贫血的表现。
2. 巨幼细胞性贫血的特点包括 MCV 增高、MCH 升高、MCHC 正常。
3. 维生素 B12 缺乏常见于素食者或消化道吸收不良的患者。
4. 维生素 B12 缺乏可能会导致神经系统症状，如肢体颤动。

因此，最可能的答案是：

A. 维生素 B12
50、 (A2 型题)
女，9 岁，晨起发现眼睑肿胀，2 周前患咽峡炎。查体：BP170／100mmHg。尿常规：尿蛋白（＋＋），RBC(++)、WBC(++)。肾穿刺活检示肾小球体积增大，囊内腔狭窄，肾 胞和系膜细胞增生。免疫荧光示 IgG 和补体 C3 阳性。脏层上皮细胞和基底膜之间驼峰样电子致密物沉积。该患者最有可能的肾病病理类型是
A.新月体性肾小球肾病
B.毛细血管内增生性肾小球肾病
C.膜增生性肾病
D.微小病变性肾病
E.肾小管间质性肾病
参考答案： B 您的答案：
图文解析： 【解析】9 岁女孩，2 周前患咽峡炎（提示有上感病史）。肾穿刺活检示肾小球体积增大，肾小球内皮细胞和系膜细胞增生。免疫荧光示 IgG 和补体 C3 阳性，脏层上皮细胞和基底膜之间驼峰样电子致密物沉积（肾穿刺提示为急性弥漫性肾小球肾炎典型表现）

（B 对）。
【避错】新月体由肾小球球囊壁层上皮细胞和单核细胞形成，是弥漫性新月体性肾小球肾炎的特征性病变（A 错）。膜性增生性肾小球肾炎，其组织学特点是肾小球基膜增厚、系膜细胞增生和系膜基质增多，增生的系膜细胞突起插入毛细血管袢中，形成车轨状（C 错）。肾小球球囊脏层上皮细胞足突消失是微小病变性肾小球病的特征性病变（D 错）。肾小管和肾间质炎症病变特点是慢性间质性炎症、纤维化和瘢痕形成，主要累及肾间质，很少累及肾小球（E 错）。
这个病例中的患者，9 岁女孩，出现眼睑肿胀，有高血压（BP 170/100 mmHg）、蛋白尿、血尿和白细胞尿，以及肾穿刺活检显示肾小球体积增大，囊内腔狭窄，肾细胞和系膜细胞增生。免疫荧光呈现 IgG 和补体 C3 阳性，电镜下观察到脏层上皮细胞和基底膜之间的驼峰样电子致密物沉积。这些临床和病理特征指向急进性肾炎，特别是以 IgG 和 C3 沉积为特征的类型。在给出的选项中，新月体性肾小球肾病（选项 A）是描述急进性肾炎的一种，特征是肾小球内形成新月体，肾小球细胞和系膜细胞的增生，以及免疫复合物（如 IgG 和 C3）的沉积。鉴于病理报告中提到的驼峰样电子致密物，这是急进性肾炎（尤其是 IgA 肾病）的典型病理特征，但鉴于病理结果中未提到新月体的形成，我们可能需要更仔细地考虑其他类型。
然而，如果没有明确提及新月体，我们也应考虑毛细血管内增生性肾小球肾病（选项 B），这种病变通常见于急性肾炎，如链球菌感染后肾炎，这与病例中的前咽峡炎病史可能相关联。最终，确定最准确的答案需要依据患者的完整病理报告和临床表现，但根据所提供的信息，最有可能的诊断是毛细血管内增生性肾小球肾病（选项 B），因为这种病理类型与患者的病理特征相符，包括免疫复合物沉积和肾小球的增生改变。因此，我倾向于选择 B 作为最可能的答案。
这是一道关于肾病病理类型的问题。我们需要基于患者的临床表现、实验室检查和肾活检结果来判断其肾病的病理类型。
理解背景信息：9 岁女性患者，晨起发现眼睑肿胀，2 周前有咽峡炎病史。查体发现高血压，尿常规检查发现尿蛋白和红细胞、白细胞均阳性。肾穿刺活检显示肾小球体积增大，囊内腔狭窄，肾小球细胞和系膜细胞增生。免疫荧光显示 IgG 和补体 C3 阳性，且脏层上皮细胞和基底膜之间有驼峰样电子致密物沉积。
理解问题的核心：我们需要确定患者肾病的病理类型。
接下来，我们逐一分析选项：
A. 新月体性肾小球肾病：新月体性肾小球肾炎又称为急进性肾小球肾炎，是以血尿、蛋白尿及进行性肾功能不全为表现的一组临床综合征，是肾小球肾炎中最严重的类型，与题干描述不符。
B. 毛细血管内增生性肾小球肾病：这种肾病的病理特点是肾小球细胞和系膜细胞增生，与题干中肾穿刺活检的结果相符合。此外，患者近期有前驱感染史（咽峡炎），出现水肿、高血压、血尿和蛋白尿，这些都是毛细血管内增生性肾小球肾病的典型表现。
C. 膜增生性肾病：这种肾病的病理特点是肾小球基底膜增厚，系膜细胞增生和系膜基质增多，与题干描述不符。
. 微小病变性肾病：这种肾病的病理特点是肾小球基本正常，仅在电镜下可见肾小球脏层上皮细胞足突广泛融合、消失，与题干描述不符。
E. 肾小管间质性肾病：这种肾病的病理特点是肾小管萎缩和肾间质纤维化，与题干描述不符。
综上所述，根据患者的临床表现、实验室检查和肾活检结果，最有可能的肾病病理类型是毛细血管内增生性肾小球肾病。
因此，答案是 B.毛细血管内增生性肾小球肾病。

该患者是一名 9 岁女孩，临床表现为晨起眼睑肿胀，有高血压（BP 170/100 mmHg），尿常规检查显示尿蛋白阳性、红细胞和白细胞增多。肾穿刺活检发现肾小球体积增大，囊内腔狭窄，肾小球细胞和系膜细胞增生，免疫荧光示 IgG 和补体 C3 阳性，并且脏层上皮细胞和基底膜之间有驼峰样电子致密物沉积。这些病理和临床特征提示以下几种肾病类型。

A. 新月体性肾小球肾病：主要特征是新月体形成，一般伴随急进性肾炎综合征，病理表现为新月体形成（>50%肾小球），并没有该患者的典型病理特征。

B. 毛细血管内增生性肾小球肾病：表现为肾小球毛细血管内皮细胞增生和炎症细胞浸润，常见于急性链球菌感染后肾小球肾炎（急性链球菌感染后肾炎），其病理特征与该患者的描述相符。

C. 膜增生性肾病：表现为系膜细胞和系膜基质增生，同时伴有肾小球基底膜增厚和分裂，免疫荧光显示 IgG、C3 阳性沉积，但其病理特征不完全匹配该患者的描述。

D. 微小病变性肾病：常见于儿童，以大蛋白尿为主要特征，光镜下肾小球结构正常，电镜下可见足细胞融合，患者无免疫复合物沉积，明显不符合该患者的病理特征。

E. 肾小管间质性肾病：主要表现为肾小管和间质的损伤和炎症，而非肾小球的病变，不符合该患者的临床和病理特征。

综上所述，最符合该患者的临床和病理特征的肾病病理类型是 B. 毛细血管内增生性肾小球肾病（急性链球菌感染后肾小球肾炎）。

因此，最有可能的肾病病理类型是：

B. 毛细血管内增生性肾小球肾病

51、 (A2 型题)

患者女，G1P0，28 岁，7 月前行人工流产术后闭经，肥胖多毛，行雌孕激素序贯治疗后未有月经，诊断为

A.Turner 综合征

B.闭经泌乳综合征

C.多囊卵巢综合征

D.Asherman 综合征

E.sheehan 综合征

参考答案： D 您的答案：

图文解析： 【解析】人工流产后宫颈或宫腔粘连称为 Asherman 综合征，是由于刮宫时损伤宫颈管黏膜或子宫内膜基底层、肌层所致，临床表现为闭经、月经过少和不育，故 D 对。

【避错】Turner 综合征又称为先天性卵巢发育不全综合征,是由于女性先天缺少一条 X 染色体,从而导致身材矮小，第二性征发育不良或者不发育，但智力正常。闭经泌乳综合征表现为停止哺乳后半年，仍长期持续溢乳，并伴有闭经；或非妊娠却见到乳房分泌乳汁样液体，并有闭经。多囊卵巢综合征是育龄期女性最常见的内分泌疾病，是以生殖障碍、内分泌异常、代谢紊乱和精神问题为特征的一组临床综合征，以无排卵、不孕和肥胖、多毛等

经典临床表现为主。Sheehan 综合征（席汉综合征）指产后大出血导致垂体缺血性坏死引起的垂体前叶功能减退综合征，主要表现为虚弱乏力，没有乳汁分泌，出现闭经或月经量少，月经不规律。
患者的病例提示了闭经（没有月经）、肥胖、多毛等症状，这些都是多囊卵巢综合征（PCOS，Polycystic Ovary Syndrome）的典型特征。多囊卵巢综合征是一种内分泌障碍，常见于育龄女性，表现为月经不规律、过多的男性激素（表现为多毛或者脱发）和多囊卵巢。
- A. Turner 综合征通常在儿童或青春期就被诊断出来，主要特征包括矮小和性腺发育不全。
- B. 闭经泌乳综合征（Sheehan 综合征）通常与分娩后严重出血有关。
- D. Asherman 综合征是子宫内膜下粘连，常由手术引起，如流产手术，但不一定伴有肥胖或多毛。
- E. Sheehan 综合征是垂体梗死，常发生在分娩后出血严重的妇女中。
根据这些信息，最符合描述的是 C.多囊卵巢综合征。因此，最佳答案是 C.多囊卵巢综合征。
解析：
患者为青年女性，7 月前行人工流产术后闭经，肥胖多毛，行雌孕激素序贯治疗后未有月经，考虑诊断为多囊卵巢综合征。多囊卵巢综合征是一种常见的妇科内分泌疾病，在临床上以雄激素过高的临床或生化表现、持续无排卵、卵巢多囊改变为特征，常伴有胰岛素抵抗和肥胖。
选项 A：Turner 综合征又称特纳综合征，是由于全部或部分体细胞中一条 X 染色体完全或部分缺失所致，临床表现为身矮、生殖器与第二性征不发育和躯体的发育异常，不符合题意。
选项 B：闭经泌乳综合征多为垂体肿瘤引起，临床特征为泌乳、闭经、不孕三联征，题干中未提及泌乳症状，故排除。
选项 D：Asherman 综合征即宫腔粘连综合征，多因宫腔操作、感染等导致子宫内膜损伤，从而引起子宫腔闭塞、月经过少或闭经、不孕等，常伴有周期性腹痛、下腹坠痛等，与题干描述不符。
选项 E：sheehan 综合征又称希恩综合征，是由于产后大出血休克，导致垂体尤其是腺垂体促性腺激素分泌细胞缺血坏死，引起腺垂体功能低下而出现的一系列症状，如闭经、无泌乳、性欲减退、毛发脱落等，第二性征衰退，生殖器官萎缩，以及肾上腺皮质、甲状腺功能减退，畏寒、嗜睡、低血压，可伴有严重而局限的眼眶后方疼痛、视野缺损及视力减退、肥胖、皮肤粗糙及色素沉着、神经系统退化、性欲减退、不孕不育、水肿等，不符合题干。
综上所述，正确答案是：C.多囊卵巢综合征。
患者女，28 岁，7 月前行人工流产术后闭经，肥胖多毛，行雌孕激素序贯治疗后未有月经，最可能的诊断是：

C. 多囊卵巢综合征（Polycystic Ovary Syndrome, PCOS）

理由如下：
- 闭经：多囊卵巢综合征常见表现之一。
- 肥胖：多囊卵巢综合征患者中常见。
- 多毛：多囊卵巢综合征由于高雄激素水平引起。

Turner 综合征通常在年轻女孩中表现出原发性闭经和低身高等体征，与本例不符。闭经泌乳综合征和 Sheehan 综合征分别有泌乳和产后大出血病史的特点，与本例不符。Asherman 综合征是宫腔粘连引起的，人工流产后出现，但不伴有肥胖多毛。

综上所述，患者最可能的诊断是 C. 多囊卵巢综合征。

52、 (A2 型题)

女性分娩一子后。怕自己养不活，继而紧张，早醒，体重下降 5kg，该女子可能是

A.焦虑障碍

B.抑郁障碍

C.精神分裂障碍

D.人格障碍

E.心境转换障碍

参考答案： B 您的答案：

图文解析： 【解析】分娩后出现情感障碍且早醒、体重下降，考虑抑郁障碍，故 B 对。【避错】焦虑障碍以广泛、持续性焦虑或反复发作的惊恐不安为主要特征，临床表现为坐立不安、失眠、呼吸紧迫、多汗、皮肤潮红或苍白、心悸等，故 A 错。精神分裂障碍以幻觉、思维障碍、情感障碍、意志和行为障碍、认知障碍为主要表现。人格障碍分为反社会型、偏执型、分裂样型、分裂型、强迫型、自恋型、边缘型、表演型、回避型、依赖型等。心境转换障碍，临床上主要表现为情感高涨或低落，伴有相应的认知和行为改变和有幻觉妄想等精神病性症状。

这个题目描述的情况涉及产后的心理状态，特别是紧张、早醒和体重下降。根据这些症状，最符合的诊断是抑郁障碍。产后抑郁是一种常见的心理健康问题，可以表现为紧张、焦虑、睡眠障碍（如早醒）和体重下降。

A 项的焦虑障碍主要特征是过度的担心和紧张，虽然产后女性可能体验到这些情绪，但根据题干描述的症状，这些并不是唯一或最主要的特征。

C 项的精神分裂障碍涉及思维、感知、情感、行为的广泛障碍，这里没有明显的迹象表明女性有这种障碍。

D 项的人格障碍是指长期的、不适应的行为和思维模式，这些模式通常不是由特定生活事件触发的。

E 项的心境转换障碍，也称为双相情感障碍，涉及情绪高涨和沮丧的极端变化。

因此，最合适的答案是 **B.抑郁障碍**。

首先，我们提取题干中的关键信息：女性分娩后，由于担心养不活孩子而出现的紧张、早醒和体重下降等症状。这些症状可能指向某种心理障碍。

接下来，我们根据所给选项逐一分析：

A. 焦虑障碍：焦虑障碍通常表现为过度担忧、紧张和恐惧。题干中的女性因为担心养不活孩子而出现的紧张和早醒，与焦虑障碍的症状相符。

B. 抑郁障碍：抑郁障碍主要表现为持续的悲伤感、兴趣丧失、疲劳、睡眠障碍等。虽然题干中的女性有早醒和体重下降的症状，但并未明确提及悲伤感或兴趣丧失等典型的抑郁症状。

C. 精神分裂障碍：精神分裂症主要表现为幻觉、妄想、思维混乱等严重精神症状。题干中并未提及这些症状。

D. 人格障碍：人格障碍是指明显偏离正常且根深蒂固的行为方式，具有适应不良的性质。题干中并未提及与人格障碍相关的症状或行为模式。

E. 心境转换障碍：这通常指双相情感障碍，即情绪波动在抑郁和躁狂之间。题干中并未提及躁狂或明显的情绪波动。

综上所述，根据题干描述的症状，最符合的是焦虑障碍，特别是考虑到分娩后的心理压力和担忧可能导致焦虑症状的出现。

因此，正确答案是 A.焦虑障碍。

根据题目中的描述，女性在分娩后出现了紧张、早醒和体重下降的症状，这些症状符合产后

抑郁障碍的表现。产后抑郁是一种在女性分娩后常见的情绪障碍，表现为持续的悲伤、失望、无助感、疲劳、睡眠障碍、食欲不振和体重下降等。

因此，该女子可能是：

B. 抑郁障碍

53、 (A2 型题)

男孩，1 岁 8 个月。发热 6 天，皮疹 2 天。查体:营养发育好，皮肤可见斑丘疹颈部左侧可触及一直径 2cm 淋巴结。双眼球结膜充血，无脓性分泌物。口唇充血水肿，舌乳头突 见硬性水肿，下列治疗措施最合适的是

A.大剂量丙种球蛋白

B.双嘧达莫

C.糖皮质激素

D.抗生素

E.对症治疗

参考答案： A 您的答案：

图文解析： 【解析】患儿发热 6 天，出疹时热更高，皮肤可见斑丘疹，双眼球结膜充血，口唇充血水肿，舌乳头突出，手足可见硬性水肿，根据其临床表现和体征，应诊断为川崎病。川崎病的治疗方案：静脉注射免疫球蛋白量为 1~2/kg,推荐剂量为 2/kg,于 8~12 小时静脉缓慢输入,宜于发病早期(10 天以内)应用,可迅速退热,预防冠状动脉病变发生。应同时合并应用阿司匹林。

【避错】双嘧达莫（B 错）为川崎病的辅助治疗药物。糖皮质激素（C 错）因可促进血栓形成,增加发生冠状动脉病变及冠状动脉瘤的风险,影响冠脉病变修复故不宜单独应用。抗生素（D 错）用于治疗继发感染。对症治疗可以改善症状，但不是最合适的治疗（E 错）。

这个题目描述的症状似乎符合川崎病（Kawasaki disease）的特征，这是一种主要影响儿童的血管炎症状。川崎病的典型表现包括发热、皮疹、颈部淋巴结肿大、结膜充血、口唇充血水肿，以及舌头的变化（草莓舌）。

针对川崎病，最常用的治疗方法是大剂量的静脉注射丙种球蛋白（IVIG）以及口服阿司匹林。因此，在提供的选项中，最合适的治疗措施应该是：

A.大剂量丙种球蛋白

这种治疗可以帮助减少心血管并发症的风险，并改善患儿的整体预后。所以正确答案应该是 A.大剂量丙种球蛋白。

解析：

患儿发热，皮肤可见斑丘疹，颈部淋巴结肿大，双眼球结膜充血，口唇充血水肿，草莓舌，口周可见硬性水肿，根据其临床表现及体征，考虑诊断为川崎病。川崎病为自限性疾病，多数预后良好。治疗的主要目的是控制血管炎症、减少冠脉损伤，防止冠状动脉瘤形成以及血栓性阻塞。川崎病的治疗主要包括阿司匹林联合丙种球蛋白，可降低冠脉瘤的发生率。因此，下列治疗措施最合适的是大剂量丙种球蛋白。

选项 B 双嘧达莫主要用于人工心脏瓣膜置换术后、缺血性心脏病、脑卒中和短暂性脑缺血发作，也可用于心绞痛的预防和治疗，对于川崎病无治疗作用，故不选。

选项 C 糖皮质激素具有抗炎、抗内毒素、抑制免疫、抗休克及增强应激反应等药理作用，但川崎病属于免疫系统疾病，应用糖皮质激素反而可能加重病情，故不考虑。

选项 D 抗生素主要用于治疗由细菌、支原体、衣原体、立克次体、螺旋体、真菌等引起的感染性疾病，川崎病属于免疫系统疾病，而非感染性疾病，使用抗生素并不能达到治疗目的，

故排除。
选项 E 对症治疗是临床常用的治疗方法，但川崎病具有自限性，且该患儿尚未出现任何并发症，因此对症治疗并不是最合适的措施，与题意不符。
综上所述，正确答案是：A.大剂量丙种球蛋白。
根据题目描述，男孩发热 6 天，皮疹 2 天，并伴有淋巴结肿大、结膜充血、口唇水肿和舌乳头突起，这些症状提示可能是川崎病（Kawasaki disease）。川崎病是一种儿童常见的急性发热性疾病，常见于 5 岁以下儿童，主要表现为发热、皮疹、结膜充血、口唇红肿和草莓舌、以及颈部淋巴结肿大。

对于川崎病的治疗，最佳选择是使用大剂量的丙种球蛋白（IVIG）和阿司匹林，以降低冠状动脉病变的风险。

因此，最合适的治疗措施是：

A. 大剂量丙种球蛋白
54、 (A2 型题)
男，62 岁，早晨起床发现右上肢无法抬举、无法独立行走 3 小时，伴言语含糊。发病前晚八点时正常，既往高血压、糖尿病。查体：P90 次/分、R19 次/分、BP160/90mmHg， 含糊，右侧鼻唇沟浅，舌头右偏，右上肢肌力二级，右下肢三级，左侧五级，右侧病理征阳性，右侧偏身针刺下降，血糖 11mmol/L，其余正常，SpO2 99%，头颅 CT 正常，
A.TIA
B.脑血栓形成
C.脑栓塞
D.脊髓炎
E.脑出血
参考答案： B 您的答案：
图文解析： 【解析】患者老年男性，既往高血压、糖尿病病史，晨起突发右上肢无法抬举、无法独立行走，伴言语含糊（左侧大脑中动脉供血区脑血栓形成的症状），查体见右侧鼻唇沟浅，舌头右偏（面神经麻痹症状），右上肢肌力二级，右下肢肌力三级，左侧五级，右侧病理征阳性，右侧偏身针刺下降（偏瘫症状），SpO299%（正常值 95%～99%），头颅 CT 正常，结合患者病史、临床表现和影像学检查，应诊断为左侧大脑中动脉供血区脑血栓形成。
【避错】TIA（A 错）即短暂性脑缺血发作，是由于局部脑或视网膜缺血引起的短暂性神经功能缺损，好发于老年人，发病突然，症状持续时间一般仅数分钟到数小时，过后完全正常，无后遗症状。脑栓塞（C 错）是各种栓子随血流进入颅内动脉使血管腔急性闭塞或严重狭窄，引起相应供血区脑组织发生缺血坏死及功能障碍的一组临床综合征，主要病因为各种心脏栓子脱落，患者多有心脏病病史，发病后病情常比脑血栓形成重，病死率高。脊髓炎（D 错）是由感染、中毒、过敏等因素引起的脊髓炎症，临床以急性起病、进展迅速为主要表现。脑出血（E 错）是脑血管破裂所致的脑内实质性出血，好发于基底节区，最常见于高血压合并细小动脉硬化患者，脑 CT 可见高密度影。
这位 62 岁的男性患者出现的症状包括右上肢无法抬举、行走困难、言语含糊、右侧面部肌肉无力、舌头偏向右侧、右侧肢体肌力减弱、右侧病理征阳性以及右侧偏身针刺感下降。这些症状指向脑血管事件的可能性。患者有高血压和糖尿病病史，这增加了脑血管意外的风险。
A. TIA（短暂性脑缺血发作）的症状类似于中风，但通常在 24 小时内完全恢复，没有持续的脑损伤。

B. 脑血栓形成是由于血管内的血栓阻塞，导致脑部供血减少，可能引起持续的脑组织损伤。
C. 脑栓塞指的是一个血栓（或其他异物）在体内移动并堵塞了供血脑的血管。
D. 脊髓炎涉及的是脊髓的炎症，与本病例描述的症状不符。
E. 脑出血涉及脑内出血，但患者头颅 CT 正常，不支持出血的诊断。
考虑到症状的急性起始、侧面分布的肌力减弱和言语不清，以及患者有脑血管疾病的危险因素，最可能的诊断是脑血栓形成或脑栓塞。鉴于头颅 CT 正常，排除了脑出血和大面积梗塞所致的明显脑实质损伤，这种情况下通常更难区分脑血栓和脑栓塞。但是，脑栓塞通常会导致更突然的症状起始，而脑血栓形成可能伴随逐渐加重的症状。
考虑到这些信息，我倾向于选择 **B. 脑血栓形成** 作为最佳答案，因为症状的逐渐出现和患者的既往病史与这一诊断更为一致。然而，实际临床情况可能需要进一步的诊断工具，如磁共振成像（MRI），来明确具体的病因。
首先，我们分析患者的症状和病史：
老年男性，有高血压和糖尿病史，这两个都是脑血管疾病的危险因素。
早晨起床后发现右上肢无法抬举、无法独立行走，伴言语含糊。这些症状提示可能有神经系统损伤。
查体显示右侧鼻唇沟浅，舌头右偏，右上肢肌力二级，右下肢三级，左侧五级，右侧病理征阳性，右侧偏身针刺觉下降。这些体征符合左侧大脑半球损伤的表现。
头颅 CT 正常，排除了脑出血和其他明显的脑部病变。
现在，我们逐个分析选项：
A. TIA（短暂性脑缺血发作）：通常症状持续时间较短，一般在 24 小时内完全恢复，无后遗症，与该患者情况不符。
B. 脑血栓形成：由于患者有高血压和糖尿病这两个脑血管疾病的危险因素，且症状在早晨起床后发现，可能是夜间血流减慢、血液粘稠度增高导致的血栓形成。这与患者的病史和症状相符。
C. 脑栓塞：通常是由其他部位的栓子脱落随血流进入大脑动脉造成堵塞，起病急骤，但患者病史中未提及可能的栓子来源，且起病并非急骤。
D. 脊髓炎：主要表现为受损平面以下的肢体瘫痪、传导束性感觉障碍和尿便障碍，与该患者症状不符。
E. 脑出血：虽然患者有高血压病史，但头颅 CT 正常，排除了脑出血的可能性。
综上所述，结合患者的病史、症状和查体结果，最可能的诊断是脑血栓形成。
因此，正确答案是 B.脑血栓形成。
根据题干中的信息，62 岁的男性患者早晨起床时发现右上肢无法抬举、无法独立行走 3 小时，伴言语含糊。患者有高血压和糖尿病的病史，查体显示右侧鼻唇沟浅，舌头右偏，右上肢肌力二级，右下肢三级，右侧病理征阳性，右侧偏身针刺感觉减退，血糖为 11mmol/L。头颅 CT 显示正常。

从临床表现来看，患者出现了急性脑血管事件的症状，包括：
- 右侧肢体无力
- 言语含糊
- 舌头右偏
- 右侧病理征阳性

结合头颅 CT 正常，排除了脑出血的可能性。患者的症状在发病后持续了超过 24 小时，排除了 TIA 的可能性。考虑到患者有高血压和糖尿病的病史，这些都是脑血管病的危险因素。

在急性脑血管事件中，脑血栓形成和脑栓塞都是常见原因。脑栓塞通常会有突然发作的特点，而脑血栓形成则可能会在夜间或早晨发作，因为血流缓慢时容易形成血栓。患者早晨起床后症状明显，提示可能是夜间或早晨发作的脑血栓形成。

因此，最可能的诊断是：

B. 脑血栓形成
55、 (A2 型题)
女婴，6 个月，热退后皮疹半天。查体：体温 36.9℃,脉搏 120 次/分，呼吸 36 次/分，精神好，颈部，胸部，上肢可见红色斑丘疹，压之褪色，咽部充血。心、肺、腹及神 常。诊断考虑为
A.幼儿急疹
B.风疹
C.水痘
D.麻疹
E.猩红热
参考答案： A 您的答案：
图文解析： 【解析】本例患儿热退后皮疹，精神好，咽部充血，应诊断为幼儿急疹（A 对）。幼儿急疹主要见于婴幼儿,一般情况好,高热时可有惊厥，耳后枕部淋巴结可肿大,常伴有轻度腹泻。皮疹特点：红色细小密集斑丘疹,头面颈及躯干部多见,四肢较少， 一天出齐,次日即开始消退，无色素沉着、无脱皮。
【避错】风疹（B 错）全身症状轻，耳后、枕后淋巴结肿大并触痛，皮疹多在症状出现后 1~2 天出现，且有面颈部→躯干→ 四肢的先后顺序。水痘（C 错）多为低热，发热第二天出疹，且皮疹由最初的红色斑疹和丘疹变成透明饱满的水泡，皮疹呈向心性。麻疹（D 错）多有咳嗽、畏光、鼻卡他、结膜炎等前驱症状，且皮疹多出现于发热后 3~4 天，疹退后皮肤有糠麸样脱屑并有色素沉着。猩红热（E 错）多为高热，咽痛明显，杨梅舌，环口苍白圈明显等。
这个病例描述了一个 6 个月大女婴，在发热退后出现皮疹半天。根据症状，特别是体温正常、脉搏加快、呼吸频率增加、精神状态良好、颈部、胸部、上肢出现红色斑丘疹且压之褪色，以及咽部充血，可以考虑几种儿童常见的疾病。
选项 A 的幼儿急疹（Roseola infantum），也称为突发性疹或第六病，通常以高热开始，热退后出现皮疹，符合这个病例的描述。幼儿急疹的特点是高热突然消退后出现的皮疹，主要影响年龄较小的儿童。
选项 B 的风疹和选项 D 的麻疹，虽然都是儿童常见的疹病，但它们的皮疹特征和疾病过程与本病例描述不完全相符。风疹的皮疹通常较幼儿急疹更细小，而麻疹则伴有显著的全身症状，如高热、寒战和科普利克斑。
选项 C 的水痘通常以密集的疱疹出现，与本病例描述的斑丘疹不符。
选项 E 的猩红热，特征是咽部感染后出现的猩红色皮疹，通常伴有高热和草莓舌，但这并非本病例的主要表现。
因此，最符合这个病例描述的是 A.幼儿急疹。
解析：
6 个月女婴，热退后出疹，颈部、胸部、上肢可见红色斑丘疹，压之褪色，咽部充血，根据其临床表现和体征，考虑诊断为幼儿急疹。幼儿急疹的特点是突发高热，持续 3～5 天后退

热，随之出现皮疹，皮疹为红色细小密集斑丘疹，以头面颈及躯干部多见，四肢较少，一天出齐，次日即开始消退。
选项 B 风疹全身症状轻，耳后、枕后淋巴结肿大并触痛，皮疹多在症状出现后 1～2 天出现，且有面颈部→躯干→四肢的出疹顺序，与该患儿症状不符，故排除。
选项 C 水痘在发病 24 小时内出现皮疹，皮疹先发于头皮、躯干受压部分，呈向心性分布，与该患儿症状存在差异，故不选。
选项 D 麻疹多于发热后的 3～4 天出疹，且出疹期为发热的高峰期，与题意不符，故不考虑。
选项 E 猩红热的皮疹多在发热 1～2 天出现，且出疹时高热，皮肤弥漫性充血，疹退后伴脱皮，与该患儿症状不同，可排除。
综上所述，正确答案是：A.幼儿急疹。
根据题目提供的信息，女婴 6 个月，热退后皮疹半天，伴有颈部、胸部、上肢的红色斑丘疹，压之褪色，咽部充血，心、肺、腹及神经系统检查正常。以下是各个选项的分析：

A. **幼儿急疹**（又称玫瑰疹）：典型表现为发热 3-5 天后，热退后出现红色斑丘疹，通常见于婴幼儿，皮疹压之褪色，常伴有轻度咽充血。
B. **风疹**：皮疹通常从面部开始，逐渐扩散至全身，伴有轻度发热和淋巴结肿大，但通常不会在热退后突然出现皮疹。
C. **水痘**：皮疹为红色斑疹、丘疹、疱疹、结痂混合存在，分批出现，伴有发热，但不符合热退后皮疹出现的特征。
D. **麻疹**：皮疹从面部开始逐渐扩散至全身，伴有发热、咳嗽、流涕、结膜炎和 Koplik 斑，不符合热退后皮疹出现的特征。
E. **猩红热**：皮疹呈现“鸡皮样”触感，通常伴有高热、草莓舌、咽峡炎，皮疹一般在热退之前出现。

结合上述分析，该女婴的症状最符合 **幼儿急疹** 的典型表现。热退后出现红色斑丘疹，皮疹压之褪色，轻度咽充血，其他系统检查正常。

因此，最正确的答案是：

A. 幼儿急疹
56、 (A2 型题)
8 岁，患重症再生障碍性贫血 1 月，等待造血干细胞移植，需要纠正贫血时宜输注何种血液制品
A.辐照的去白的红细胞
B.去白红细胞
C.悬浮红细胞
D.浓缩红细胞
E.全血
参考答案： A 您的答案：
图文解析： 【解析】重症再生障碍性贫血，拟行异基因造血干细胞移植者应输注辐照后的红细胞。辐照血液的主要功能是预防输血相关性移植物抗宿主病的发生，是指免疫缺损或免疫抑制的患者不能清除输入血液中具有免疫活性的淋巴细胞，使其在体内植活、增殖，将患者的组织器官识别为靶目标进行免疫攻击、破坏的一种致命性输血并发症。血液辐照为目前公认最有效的预防输血相关性移植物抗宿主病发生的方法。

【避错】根据上述解析，排除其他选项。
对于一个 8 岁的重症再生障碍性贫血患儿，等待造血干细胞移植，最佳选择是纠正贫血时输注的血液制品。在这种情况下，应选择辐照的去白的红细胞（选项 A）。这是因为：
1. **辐照处理**：辐照的红细胞可以防止移植物抗宿主病（GVHD），这是在移植前后免疫功能低下的患者中尤为重要的。
2. **去白**：去白细胞有助于减少输血反应的风险，包括对白细胞的过敏反应和病原体的传播。
因此，对于该患儿，最适宜的输血选项是辐照的去白的红细胞。所以正确答案是 **A. 辐照的去白的红细胞**。
解析：
本题患儿已明确诊断为重症再生障碍性贫血，准备行造血干细胞移植术，需要纠正贫血。对于准备进行骨髓移植或其他器官移植的患者，为了避免宿主抗移植物反应，输血前应对血制品进行辐照，以灭活其中具有免疫活性的淋巴细胞，防止移植物抗宿主病的发生。同时为了避免移植物抗宿主反应和发热反应，输血时应选用去除白细胞的血液制品。因此，对于本例患儿，宜输注辐照的去白的红细胞。
选项 B 去白红细胞适用于多次输血后产生白细胞抗体，出现发热反应者，或预期需要反复输血者，以及不能耐受输血发热反应者，但无法避免移植物抗宿主反应，故不选。
选项 C 悬浮红细胞，选项 D 浓缩红细胞，均只去除了大部分血浆，没有去除白细胞，均不能避免移植物抗宿主反应和发热反应，故可一并排除。
选项 E 全血中含有大量的白细胞，更易引起输血反应，故不考虑。
综上所述，正确答案是：A.辐照的去白的红细胞。
在重症再生障碍性贫血患者等待造血干细胞移植期间，需要纠正贫血时宜输注血液制品的选择需要考虑几个因素，如预防移植物抗宿主病（GVHD）以及减少输血相关并发症的风险。

正确答案是：A. 辐照的去白的红细胞

解释：
1. **辐照的去白的红细胞**：辐照可以防止供体淋巴细胞在受体体内增殖，从而预防移植物抗宿主病（GVHD）。去除白细胞有助于减少输血相关的发热反应和传染性疾病的传播风险。这对于免疫功能低下的患者尤其重要，如等待造血干细胞移植的重症再生障碍性贫血患者。

其他选项的解释：
- **去白红细胞**：虽然去除了白细胞，但没有经过辐照，不能有效预防 GVHD。
- **悬浮红细胞**：一般红细胞悬浮液可能含有白细胞，风险较高。
- **浓缩红细胞**：与悬浮红细胞类似，浓缩红细胞可能仍含有白细胞。
- **全血**：全血包含所有血液成分，包括白细胞，风险最高，不推荐用于此类患者。

因此，选择 A 选项即辐照的去白的红细胞，是最合适的。
57、 (A2 型题)
女，28 岁，产后 2 周仍有腹痛，发热，体温 38℃。2 天前阴道流血，约 250ml。查体：宫底耻骨联合上两横指，压痛，宫颈容两指，可见烂肉堵塞。哪项治疗不正确
A.产钳夹除
B.补液支持
C.抗感染治疗

D.给缩宫素
E.负压吸引术
参考答案：　　E　您的答案：
图文解析：　　【解析】该患者产后 2 周仍有腹痛，发热，阴道流血，宫底耻骨联合上两横指（宫底位置高，提示子宫收缩不良），压痛，宫颈容两指（宫颈口未闭，易导致感染），可见烂肉堵塞（有胎盘、胎膜残留），故诊断为晚期产后出血，考虑子宫收缩乏力、胎盘因素、软产道损伤等引起的出血。负压吸引术适用于胎儿娩出后胎盘滞留或部分嵌顿、胎盘粘连而施行人工剥离胎盘术不能奏效者，或者过期流产有严重出血时。本例患者有烂肉堵塞，应考虑为胎盘因素，此时应行产钳夹除（A 对），不可使用负压吸引术（E 错，为本题正确答案）。本例患者失血较多，应给与补液支持（B 对）、抗感染治疗（C 对）预防感染。给缩宫素（D 对）可以促进子宫收缩，使子宫烂肉样组织完全排出，并有利于减少产后出血。
这位 28 岁女性产后 2 周有腹痛和发热，以及阴道出血和宫颈有物体阻塞的症状，暗示可能的产后感染或宫内残留物。选择最不正确的治疗方案需要考虑到她的症状和体征。
- A. 产钳夹除：产钳通常用于助产过程中，用于此情况可能不适合，除非确实有需要移除的大块残留物。
- B. 补液支持：对于因出血或发热导致的可能的体液缺失，补液是适当的。
- C. 抗感染治疗：考虑到发热和可能的感染症状，抗感染治疗是合理的。
- D. 给缩宫素：缩宫素有助于宫缩，清除宫内残留物，减少出血，适用于产后出血或宫内残留物情况。
- E. 负压吸引术：这种方法可以用于清除宫内残留物或血块，适用于此情况。
在这些选项中，最不正确的可能是 A.产钳夹除，因为它主要用于分娩过程中辅助婴儿分娩，而不是用于处理产后出血或感染。对于宫内残留物或烂肉堵塞，通常采用的方法是手术清宫或负压吸引术，而不是使用产钳。因此，选项 A 似乎是最不合适的治疗选择。
解析：
青年女性患者，产后 2 周仍有腹痛、发热，考虑为产褥期感染。患者 2 天前出现阴道流血，查体示宫底位于耻骨联合上两横指，压痛，宫颈容两指，可见烂肉堵塞，考虑诊断为子宫复旧不全合并感染。此时烂肉堵塞，说明存在胎盘或胎膜残留，此时若进行负压吸引术，由于产后时间较长，胎膜或胎盘组织与子宫壁紧密粘连，负压吸引术可能会导致出血较多，甚至损伤子宫内膜，所以负压吸引术为不正确的治疗方法。
选项 A 产钳夹除是指用产钳夹出残留物，是胎盘或胎膜残留时常用的治疗措施，故排除。
选项 B 补液支持治疗，选项 C 抗感染治疗，均是该产妇合并感染时的重要治疗措施，有助于产妇恢复，可一并排除。
选项 D 给缩宫素可促进子宫收缩，有助于止血和子宫恢复，不符合题意。
综上所述，正确答案是：E.负压吸引术。
根据题干描述，28 岁女性产后 2 周仍有腹痛、发热、体温 38℃，伴有阴道流血及宫颈烂肉堵塞，提示可能存在感染或宫内残留物。这种情况下，应采取适当的治疗措施来清除宫内残留物，控制感染并支持患者的恢复。

对于每个选项的分析：

A. **产钳夹除**：产钳夹除可以帮助移除宫颈处的烂肉堵塞，适用于宫颈和阴道内的组织清除。

B. **补液支持**：补液支持是维持患者体液平衡的基本治疗措施，尤其是发热和流血的情况

下。

C. **抗感染治疗**：由于存在感染的症状（发热和腹痛），抗感染治疗是必不可少的。

D. **给缩宫素**：缩宫素用于促进子宫收缩，可以帮助排出宫内残留物，同时减少出血。

E. **负压吸引术**：负压吸引术是一种常用的方法，可以有效地清除宫内残留物。

考虑到这些分析，唯一不合适的治疗是 D. **给缩宫素**。虽然缩宫素可以帮助子宫收缩和减少出血，但在已有明显感染症状时，直接使用缩宫素可能不合适，因为需要优先处理感染和清除残留物。

因此，答案是：**D. 给缩宫素**。
58、　(A2 型题)
女性，35 岁，下腹痛伴恶心 4 小时，无阴道流血，呕吐和发热，平素月经周期正常，末次月经半月前，妇科查体右侧附件区一肿物，5cm，有张力，无触痛。血常规：Hb11 WBC10×109/L，PLT20×109/L，血 HCG(-)，最可能的诊断是
A.急性阑尾炎
B.卵巢囊肿蒂扭转
C.黄体破裂
D.盆腔炎性疾病
E.子宫内膜异位症
参考答案：　B　您的答案：
图文解析：　【解析】青年女性，下腹痛伴恶心 4 小时，无阴道流血，呕吐和发热（提示可能为外科疾病），平素月经周期正常，末次月经半月前（提示此时为黄体期），妇科查体右侧附件区一肿物，5cm，有张力，无触痛（符合卵巢囊肿蒂扭转的典型症状）。血常规：HB110g/L（女性正常值 110～150g/L），WBC10×109/L（正常值 4～10×109/L），PLT20×109/L（正常值 100～300×109/L），血 HCG(-)，最可能的诊断是卵巢囊肿蒂扭转。
【避错】急性阑尾炎（A 错）常表现为转移性右下腹痛及腹膜刺激征，可有不同程度的中毒症状，无停经史，无肿块触及。黄体破裂（C 错）有体位改变诱发，表现为突发性一侧下腹疼痛，可伴有恶心、呕吐等表现。盆腔炎性疾病（D 错）常表现为下腹痛、阴道分泌物增多，腹痛为持续性，活动后或性交后加重，病程长者可有神经衰弱症状。子宫内膜异位症（E 错）典型症状为继发性痛经、进行性加重，无恶心、呕吐等表现。
这个案例描述了一位 35 岁女性患者，出现了下腹痛、恶心、无阴道流血和呕吐，但没有发热。她的月经周期正常，末次月经在半月前。妇科检查发现右侧附件区有一个 5cm 的肿物，这个肿物有张力但无触痛。血液检查显示血红蛋白（Hb）11，白细胞计数（WBC）10×10^9/L，血小板计数（PLT）20×10^9/L，血 HCG 测试为阴性。
基于这些信息，最可能的诊断是**B. 卵巢囊肿蒂扭转**。这是因为患者有一个有张力的肿物在附件区，这通常与卵巢囊肿蒂扭转一致。卵巢囊肿蒂扭转会导致急性腹痛，并且可能伴有恶心和呕吐，但通常不会导致阴道出血或发热，这与病例描述相符。其他选项如急性阑尾炎、黄体破裂、盆腔炎性疾病和子宫内膜异位症在这种情况下较不可能，因为它们的临床表现与此案例描述的不完全一致。
解析：
患者为中年女性，平素月经周期正常，末次月经半月前，现出现下腹痛、恶心，无阴道流血、

呕吐和发热，妇科查体示右侧附件区一肿物，5cm，有张力，无触痛，血常规示血红蛋白正常，白细胞正常，血小板下降，血 HCG 阴性，根据其临床表现和实验室检查，考虑诊断为卵巢囊肿蒂扭转。卵巢囊肿蒂扭转的典型症状是体位的改变后突然发生一侧下腹剧痛，常伴恶心、呕吐甚至休克，系膜扭转引起血管梗阻，导致肿瘤发生坏死、出血、感染或破裂，严重时可能危及生命。
选项 A 急性阑尾炎的典型表现为转移性右下腹痛，该患者无转移性右下腹痛表现，故不选。
选项 C 黄体破裂多发生在月经周期之末 1 周，偶可在月经期第一二天发病，部分患者黄体破裂，出血不多，可自行止血，不留后遗症，但严重者可发生出血性休克，表现为大汗淋漓、头昏头痛、血压下降、四肢冰冷等，如治疗不及时可危及生命，与患者的症状描述差异明显，故排除。
选项 D 盆腔炎性疾病是女性上生殖道感染引起的一组疾病，主要包括子宫内膜炎、输卵管炎、输卵管卵巢脓肿和盆腔腹膜炎，其常见症状为下腹痛、阴道分泌物增多，严重时可出现高热、寒战、头痛等，月经期发病可出现经量增多、经期延长，非月经期发病可有白带增多，与题意不符。
选项 E 子宫内膜异位症是指子宫内膜组织生长在子宫腔与子宫肌层以外的部位，典型症状为下腹痛和痛经、月经异常、不孕、性交不适等，该患者无痛经、月经异常等表现，故不考虑。
综上所述，正确答案是：B.卵巢囊肿蒂扭转。
根据题目中患者的症状和检查结果，最可能的诊断是卵巢囊肿蒂扭转。

以下是对每个选项的分析：

A. 急性阑尾炎：急性阑尾炎常见的症状包括右下腹痛、恶心、呕吐、发热和白细胞增多，但很少会有右侧附件区的肿物。

B. 卵巢囊肿蒂扭转：卵巢囊肿蒂扭转的典型症状包括突然出现的下腹痛，伴有恶心、呕吐，常无发热。妇科查体右侧附件区有一个 5cm 的肿物，有张力但无触痛，符合卵巢囊肿蒂扭转的表现。

C. 黄体破裂：黄体破裂通常会有急性下腹痛和内出血的表现，可能会伴有阴道流血，但无明显肿物。

D. 盆腔炎性疾病：盆腔炎性疾病常见的症状包括下腹痛、发热、白带异常等，妇科查体可能会有压痛，但不一定会有明确的肿物。

E. 子宫内膜异位症：子宫内膜异位症通常表现为慢性盆腔痛、痛经和不孕等，急性发作时不太可能出现类似的肿物。

综上所述，结合患者的症状、妇科查体和辅助检查结果，最可能的诊断是 **B. 卵巢囊肿蒂扭转**。
59、 (A2 型题)
女，30 岁，妊娠 25 周。OGTT：空腹血糖 5mmol/L，1 小时 11.2mmol/L，2 小时 8.2mmol/L。既往体健。母儿可能情况
A.妊娠期高血压
B.妊娠期高血糖

C.滴虫阴道炎
D.羊水过少
E.前置胎盘
参考答案：　　B　您的答案：
图文解析：　　【解析】女，30 岁，妊娠 25 周（妊娠 25 周是确诊糖尿病的重要时期），OGTT：空腹血糖 5mmol/L（正常值 4.4mmol/L～5.1mmol/L），1 小时 11.2mmol/L（正常值小于 8.5mmol/L），2 小时 8.2mmol/L（正常值小于 7.8mmol/L）。患者空腹血糖、OGTT1 小时血糖、OGTT2 小时血糖均超过正常值，确诊为妊娠期糖尿病（B 对）。
【避错】妊娠期高血压（A 错）患者会出现蛋白尿、水肿和高血压。滴虫阴道炎（C 错）主要症状是阴道分泌物增多及外阴瘙痒，间或有灼热、疼痛、性交痛等。羊水过少（D 错）胎儿体表可直接受压，子宫的张力增大，妊娠期高血压的发病率增高。前置胎盘（E 错）会出现无诱因、无痛性反复阴道流血。
根据提供的情况，这位 30 岁的孕妇在妊娠 25 周时进行了口服葡萄糖耐量测试（OGTT），结果显示空腹血糖 5mmol/L，1 小时后为 11.2mmol/L，2 小时后为 8.2mmol/L。根据美国糖尿病协会（ADA）的标准，妊娠期糖耐量测试的诊断标准是：空腹血糖值≥5.1mmol/L，1 小时后≥10.0mmol/L，2 小时后≥8.5mmol/L。本例中，空腹血糖正常，但 1 小时和 2 小时的血糖值超过了上述标准，因此可以诊断为妊娠期糖尿病。
因此，最合适的答案是：B. 妊娠期高血糖
解析：
青年女性患者，OGTT 检查示空腹血糖正常，但餐后 1 小时血糖和餐后 2 小时血糖均偏高，可诊断为妊娠期糖尿病，即妊娠期高血糖。
选项 A 妊娠期高血压是指妊娠与血压升高并存的一组疾病，包括妊娠期高血压、子痫前期、子痫等，常表现为高血压、蛋白尿、水肿等，与题意不符。
选项 C 滴虫阴道炎是由阴道毛滴虫引起的常见阴道炎症，也是常见的性传播疾病，主要症状是阴道分泌物增多及外阴瘙痒，或有灼热、疼痛、性交痛等，与题干描述不符。
选项 D 羊水过少主要与羊水产生减少或羊水外漏增加有关，部分羊水过少原因不明，常见原因有胎儿畸形、胎盘功能不全、药物作用等，该患者并无上述情况，故不选。
选项 E 前置胎盘是指妊娠 28 周后，胎盘下缘毗邻或覆盖宫颈内口，典型症状为妊娠晚期或临产时，发生无诱因、无痛性反复阴道流血，不符合题意。
综上所述，正确答案是：B.妊娠期高血糖。
根据题目描述，该患者 30 岁，妊娠 25 周，OGTT（口服葡萄糖耐量试验）结果显示空腹血糖 5mmol/L，1 小时血糖 11.2mmol/L，2 小时血糖 8.2mmol/L。根据 OGTT 的结果，我们可以判断患者存在血糖升高的情况。

根据国际糖尿病联盟和世界卫生组织的标准，妊娠期糖尿病的诊断标准是：
- 空腹血糖≥5.1 mmol/L 但<7.0 mmol/L
- 或 1 小时血糖≥10.0 mmol/L
- 或 2 小时血糖≥8.5 mmol/L 但<11.1 mmol/L

题目中患者的空腹血糖为 5.0 mmol/L，虽然没有达到妊娠期糖尿病的诊断标准，但 1 小时血糖 11.2 mmol/L 和 2 小时血糖 8.2 mmol/L 超过了正常范围，符合妊娠期糖尿病的诊断标准。因此，母儿的可能情况是妊娠期高血糖。

正确答案是：

B. 妊娠期高血糖
60、　(A2 型题)
男孩，6 岁。水肿，尿少 5 天。查体：T37℃,P82 次/分，R18 次/分，BP100/65mmHg。双下肢凹陷性水肿。实验室检查：人血白蛋白 24g/L，胆固醇 6.3mmol/L，尿 RBC1~2/HP 蛋白定量 1.5g。予泼尼松 2mg/(kg.d)口服三周，查 2 次尿蛋白阴性，接下来的治疗措施
A.继续泼尼松 2mg/(kg•d)，4 周后改为泼尼松 2mg/kg 隔日顿服
B.继续泼尼松 2mg/(kg•d)，5 周后改为泼尼松 2mg/kg 隔日顿服
C.改为泼尼松 2mg/kg 隔日顿服
D.继续泼尼松 2mg/(kg•d)，2 周后改为泼尼松 2mg/kg 隔日顿服
E.停用泼尼松
参考答案：　D　您的答案：
图文解析：　【解析】男孩，6 岁。水肿，尿少 5 天。查体：T37℃,P82 次/分，R18 次/分，BP100/65mmHg。双下肢凹陷性水肿。实验室检查：人血白蛋白 24g/L（人血白蛋白正常值：40～55g/L），24 小时尿蛋白定量 1.5g（尿蛋白定量正常值：≤0.1g/d）。考虑肾病综合征可能性大。9 版儿科学：糖皮质激素中长程疗法可用于各种类型的肾病综合征。先以泼尼松 2mg/(kg.d)最大量 60mg/d，分次服用。若 4 周内尿蛋白转阴,则自转阴后至少巩固 2 周方始减量,以后改为隔日 2mg/kg 早餐后顿服,继续用 4 周,以后每 2~4 周总量减 2.5~5mg,直至停药。疗程必须达 6 个月(中程疗法)。所以选 D。
该题目描述的是一个 6 岁男孩，表现为水肿和尿少，伴有低白蛋白血症和蛋白尿。这些症状和实验室检查结果指向肾病综合征。泼尼松是治疗肾病综合征的常用药物，通过减少炎症和免疫反应来改善症状。
治疗后，孩子的尿蛋白检查两次呈阴性，表明对泼尼松的治疗有良好反应。接下来的治疗步骤通常是减少泼尼松的剂量，以防止副作用，同时维持疗效。
选项 A 和 B 提议继续相同剂量的泼尼松治疗较长时间后再减量，而选项 D 建议较短时间后减量。选项 C 直接建议改为隔日顿服，E 选项建议停用泼尼松。
在肾病综合征的管理中，渐减剂量通常是在症状控制后逐步进行的。因此，直接停用泼尼松（E 选项）通常不被推荐，因为可能会导致病情复发。选项 C 中直接改为隔日顿服可能过于急促，可能不够保守。
考虑到治疗的连贯性和防止病情复发的需要，A 和 B 选项看似都是可行的选择。然而，根据最佳实践，治疗剂量应在病情得到控制后逐步减少，以寻找最小有效剂量并减少副作用。通常建议在症状稳定后逐渐减少剂量，然后过渡到隔日治疗。
因此，选择 B（继续泼尼松 2mg/(kg•d)，5 周后改为泼尼松 2mg/kg 隔日顿服）可能是更为合适的选择，因为它提供了一个更长的稳定期，在完全改变给药频率之前确保病情稳定。
解析：
6 岁患儿，水肿，尿少，查体见双下肢凹陷性水肿，实验室检查见低蛋白血症，高血脂，镜下血尿，大量蛋白尿，符合肾病综合征的特征性表现。该患儿已予泼尼松 2mg/(kg.d)口服三周，查 2 次尿蛋白阴性，说明患儿对糖皮质激素治疗敏感，接下来可将泼尼松改为隔日顿服疗法，即继续泼尼松 2mg/(kg•d)，4 周后改为泼尼松 2mg/kg 隔日顿服，再逐渐减量至停药。选项 B“继续泼尼松 2mg/(kg•d)，5 周后改为泼尼松 2mg/kg 隔日顿服”，选项 D“继续泼尼松 2mg/(kg•d)，2 周后改为泼尼松 2mg/kg 隔日顿服”，均属于糖皮质激素用药时间的错误描述，不符合肾病综合征的糖皮质激素治疗规范，故可一并排除。
由于该患儿对糖皮质激素治疗敏感，应继续服用，直至尿蛋白转阴后两周才能改为隔日顿服，并继续治疗一段时间，故选项 C 改为泼尼松 2mg/kg 隔日顿服，选项 E 停用泼尼松，可一并排除。

综上所述，正确答案是：A.继续泼尼松 2mg/(kg•d)，4 周后改为泼尼松 2mg/kg 隔日顿服。该题目描述了一名 6 岁的男孩，出现水肿和尿少 5 天，查体显示双下肢凹陷性水肿，实验室检查显示低蛋白血症（人血白蛋白 24g/L）和高胆固醇血症（胆固醇 6.3mmol/L），提示患有肾病综合征。泼尼松治疗后尿蛋白阴性，表明病情有所好转。接下来的治疗措施应遵循肾病综合征的治疗原则，即逐步减量。

根据肾病综合征的标准治疗方案，一般是在初期泼尼松治疗取得尿蛋白阴性后，继续全剂量治疗一定时间，然后逐步减量至隔日疗法。因此，正确的答案应为继续全剂量治疗一定时间，然后改为隔日疗法。

最佳答案是：

B. 继续泼尼松 2mg/(kg•d)，5 周后改为泼尼松 2mg/kg 隔日顿服

解释：在泼尼松治疗肾病综合征的过程中，通常在初始治疗后需要维持一段时间的全剂量治疗，然后逐步过渡到隔日疗法。继续全剂量治疗 5 周后再改为隔日顿服，有助于减少复发的风险并确保病情稳定。

61、 (A2 型题)

女，39 岁，G3P2，子宫二度脱垂伴阴道前后壁轻度膨出。妇科检查宫颈长约 6cm，子宫后位正常大小，双附件区未触及肿物，要求手术治疗，首选的手术方式是

A.次全子宫切除术

B.骶韧带缩短术

C.阴道前后壁修补术

D.阴道半封闭术

E.曼氏手术

参考答案： E 您的答案：

图文解析： 【解析】该患者为中年经产妇，子宫二度脱垂伴阴道前后壁轻度膨出，要求手术治疗。对于年轻宫颈延长子宫脱垂患者可行曼氏手术(Manchester 手术)包括阴道前后壁修补、主韧带缩短及宫颈部分切除术。

【避错】次全子宫切除术（A 错）适用于年龄较轻、宫颈较长的子宫脱垂患者。骶韧带缩短术（B 错）适用于仅宫颈外口距处女膜＜4cm，未生育的年轻患者。阴道前后壁修补术（C 错）适用于只有阴道前后壁膨出的患者。阴道半封闭术（D 错）用于年龄较大，无须考虑生育功能的患者。

在这个案例中，考虑到女性 39 岁，G3P2（即三次妊娠，两次分娩），并有子宫二度脱垂伴阴道前后壁轻度膨出的情况，首选的手术方式应该旨在有效地纠正脱垂并减少复发风险。

A 项“次全子宫切除术”主要用于治疗有子宫病变的情况，并不是脱垂的首选治疗方法。

B 项“骶韧带缩短术”是治疗子宫脱垂的一种方法，但它更多用于更严重的脱垂情况。

C 项“阴道前后壁修补术”适用于阴道壁脱垂，可以修复前后壁的膨出，是针对本病例情况的合理选择。

D 项“阴道半封闭术”用于治疗严重的子宫脱垂或阴道脱垂，可能对患者的性生活产生影响，一般不作为首选。

E 项“曼氏手术”是针对阴道脱垂的一种治疗方法，但不是本案例的首选手术。

因此，最合适的答案应该是 C.阴道前后壁修补术，因为这种手术能够直接解决患者的主要问题，即子宫脱垂伴阴道前后壁轻度膨出。

首先，我们先了解并分析该题的关键信息。患者是一位 39 岁的女性，已经有过三次怀孕和两次分娩（G3P2）。她目前的情况是子宫二度脱垂，伴随阴道前后壁轻度膨出。妇科检查结果显示，宫颈长约 6cm，子宫处于后位且大小正常，双附件区未发现肿物。现在，她希望通过手术治疗来改善这一状况。
现在，我们根据这些信息分析各选项的适用性：
A. 次全子宫切除术：这种手术通常用于更严重的子宫疾病，如肌瘤、腺肌症等，但考虑到患者的子宫大小和位置均正常，这一选项可能不是首选。
B. 骶韧带缩短术：该手术旨在缩短和加固子宫的支持结构，但通常不作为子宫脱垂的首选治疗方式。
C. 阴道前后壁修补术：虽然患者存在阴道前后壁轻度膨出，但这并不是主要症状，主要问题是子宫脱垂，因此这一选项可能不够全面。
D. 阴道半封闭术：这种手术主要用于老年妇女，尤其是那些不再需要保留性交功能的患者，不适用于本案例中的年轻女性患者。
E. 曼氏手术：这种手术包括阴道前后壁修补、主韧带缩短及宫颈部分切除术，适用于年龄较轻、宫颈延长的子宫脱垂患者，这与题干中患者的情况相吻合。
综上所述，考虑到患者年轻，子宫大小和位置正常，主要症状是子宫二度脱垂和阴道前后壁轻度膨出，曼氏手术既能解决子宫脱垂的问题，又能处理阴道壁的膨出，因此是首选的手术方式。
因此，正确答案是 E.曼氏手术。
根据题干描述，患者为 39 岁女性，有子宫二度脱垂伴阴道前后壁轻度膨出。要求手术治疗。根据目前的妇科最佳实践指南，对于这种情况，手术的首选方案应考虑既能解决子宫脱垂问题，又能修复阴道前后壁膨出的问题。

各选项解释如下：
A. 次全子宫切除术 - 主要用于治疗子宫病变，不能解决子宫脱垂及阴道前后壁膨出问题。
B. 骶韧带缩短术 - 主要用于提高子宫的支持，但不能解决阴道前后壁膨出问题。
C. 阴道前后壁修补术 - 主要用于修复阴道前后壁膨出，但不能有效解决子宫脱垂问题。
D. 阴道半封闭术 - 主要用于严重子宫脱垂患者，通过减少阴道长度来提供支持，但不适合较年轻的女性，且未解决阴道前后壁膨出。
E. 曼氏手术 - 通过阴道悬吊术来固定子宫，同时修复阴道前后壁膨出，适用于有子宫脱垂和阴道前后壁膨出的情况。

综合考虑患者的情况以及每种手术的适应症，最合适的手术方式是 E. 曼氏手术。它不仅能解决子宫脱垂问题，还能修复阴道前后壁膨出，是最佳选择。

所以答案是：
E. 曼氏手术
62、 (A2 型题)
女，41 岁，反复外阴阴道瘙痒 3 月，既往糖尿病病史 6 年，妇科查体：阴道红肿，分泌物增多，分泌物呈豆腐渣样。最有可能的诊断是
A.外阴阴道假丝酵母菌阴道炎
B.淋病
C.萎缩性阴道炎
D.细菌性阴道炎

E.前庭大腺炎
参考答案：　　A　您的答案：
图文解析：　　【解析】该患者为中年女性，反复外阴阴道瘙痒 3 月（外阴瘙痒为外阴阴道假丝酵母菌阴道炎的常见症状），既往糖尿病病史 6 年（糖尿病是外阴阴道假丝酵母菌阴道炎的易感因素），妇科查体：阴道红肿，分泌物增多，分泌物呈豆腐渣样（分泌物呈豆腐渣样为外阴阴道假丝酵母菌阴道炎的典型表现），根据患者的病史、临床表现，最有可能的诊断是外阴阴道假丝酵母菌阴道炎。
【避错】淋病（B 错）多有淋菌性尿道炎，出现尿道口溢脓，自觉尿痛、尿频、尿急。萎缩性阴道炎（C 错）常见于自然绝经或人工绝经后妇女，表现为外阴灼热不适、瘙痒及阴道分泌物增多，无豆腐渣样分泌物。细菌性阴道炎（D 错）表现为阴道分泌物增多，有鱼腥臭味。前庭大腺炎（E 错）主要症状为一侧大阴唇局部肿胀、灼热感、疼痛明显、行走不便，有时会形成脓肿。
最符合这个病例描述的诊断是 A.外阴阴道假丝酵母菌阴道炎。病人有糖尿病病史，糖尿病患者因血糖控制不良可能导致局部免疫力下降，容易发生念珠菌感染。症状包括外阴阴道瘙痒和红肿，分泌物增多，特别是描述为“豆腐渣样”分泌物，这是念珠菌感染的典型表现。因此，选择 A 作为最可能的诊断。
首先，我们来详细解读一下题目中的关键信息。这是一位 41 岁的女性患者，有长期糖尿病史，反复出现外阴阴道瘙痒，且查体发现阴道红肿，分泌物增多并呈豆腐渣样。
接下来，我们来分析每个选项，以便找出最符合这些症状的诊断：
A. 外阴阴道假丝酵母菌阴道炎：这种阴道炎的典型症状包括外阴瘙痒、灼痛，还可伴有尿频、尿痛及性交痛，阴道分泌物增多，典型的分泌物特征是白色稠厚呈凝乳或豆腐渣样。这些症状与题干描述的患者症状高度吻合。
B. 淋病：淋病主要表现为泌尿生殖系统的化脓性感染，通常会有尿道疼痛、脓性分泌物等症状，与题干描述不符。
C. 萎缩性阴道炎：多见于自然绝经或人工绝经后的妇女，主要症状是外阴灼热不适、瘙痒及阴道分泌物增多，但分泌物通常稀薄，呈淡黄色，感染严重者呈脓血性白带，与患者的症状描述不匹配。
D. 细菌性阴道炎：主要表现为阴道分泌物增多，有鱼腥臭味，尤其性交后加重，可伴有轻度外阴瘙痒或灼热感，分泌物通常呈灰白色、均匀一致、稀薄，与患者的豆腐渣样分泌物不符。
E. 前庭大腺炎：这是一种外阴炎症，主要表现为前庭大腺部位的红、肿、热、痛等症状，与题干描述的症状差异明显。
综合以上分析，我们可以看出，患者的症状与外阴阴道假丝酵母菌阴道炎的典型症状高度一致，特别是阴道分泌物的豆腐渣样特征是该病的一个显著标志。
因此，最有可能的诊断是 A.外阴阴道假丝酵母菌阴道炎。
根据题目描述，这名 41 岁的女性有反复外阴阴道瘙痒 3 个月的病史，并且有 6 年的糖尿病病史。妇科检查显示阴道红肿、分泌物增多，且分泌物呈豆腐渣样。

糖尿病患者容易患上真菌感染，而豆腐渣样分泌物是典型的假丝酵母菌（即念珠菌）感染特征。因此，最有可能的诊断是：

A. 外阴阴道假丝酵母菌阴道炎
63、　(A2 型题)
男孩，两岁半，重度营养不良，近一周出现腹泻，3~8 次/日，稀薄，呈黄色，泡沫较多，

脓性，可见豆渣样细块，可能诊断的疾病是
A.白色念珠菌肠炎
B.致病大肠埃希菌肠炎
C.轮状病毒肠炎
D.金黄色葡萄球菌肠炎
E.耶尔森菌小肠结肠炎
参考答案：　　A　您的答案：
图文解析：　　【解析】该患儿，有腹泻，泡沫较多，脓性，可见豆渣样细块，多为白念珠菌肠炎所致。
该病常并发于其他感染,或肠道菌群失调时。病程迁延,常伴鹅口疮。大便次数增多,黄色稀便,泡沫较多,带黏液,有时可见豆腐渣样细块(菌落)。大便镜检有真菌孢子和菌丝。
【避错】致病大肠埃希细菌肠炎（B 错）多继发于小儿腹泻，表现为发热、腹痛、腹泻、里急后重等。轮状病毒肠炎（C 错）多见于 6～24 个月的婴幼儿，好发于秋冬季，常由于饮食不当和气候变化诱发，典型粪便为水样或蛋花汤样。金黄色球菌肠炎（D 错）:多继发于使用大量抗生素后病和症状常与菌群失调的程度有关,有时继发于慢性疾病的基础上。表现为发热、呕吐、腹泻、不同程度的中毒症状、脱水和电解质紊乱，甚至发生休克。典型大便为暗绿色,量多带黏液，少数为血便。耶尔森菌小肠结肠炎（E 错）临床表现有腹痛、腹泻、发热、呕吐等。发生在冬季和早春,可引起淋巴结肿大,亦可产生肠系膜淋巴结炎,症状可与阑尾炎相似,也可引起咽痛和颈淋巴结炎。
这个考题描述的是一位两岁半男孩，重度营养不良，并出现频繁的黄色泡沫状脓性腹泻，还伴有豆渣样细块。这些症状提示感染性肠炎。我们来逐一分析这些选项：
A. 白色念珠菌肠炎通常会导致白色或奶酪样便便，并且常常伴随免疫系统受损的情况出现，但不太符合脓性腹泻的描述。
B. 致病大肠埃希菌肠炎可能导致血性腹泻，而不是脓性。
C. 轮状病毒肠炎是儿童中最常见的病毒性腹泻原因，但其特征是水样腹泻，通常不包括脓性排泄物。
D. 金黄色葡萄球菌肠炎通常会导致急性腹泻，但与脓性腹泻和豆渣样细块的描述不完全符合。
E. 耶尔森菌小肠结肠炎可以导致脓血性腹泻，并且可能出现在营养不良的儿童中，表现为脓性和有细块的腹泻。
综上所述，最可能的诊断是 E.耶尔森菌小肠结肠炎，因为它与所描述的症状最为吻合。
解析：
患儿两岁半男孩，有重度营养不良病史，近一周出现腹泻，大便 3～8 次/日，稀薄、黄色、泡沫多**，呈脓性**，可见豆渣样细块，考虑诊断为白色念珠菌肠炎。白假丝酵母菌为机会致病菌，主要为内源性传染，可引起皮肤黏膜感染、内脏感染、中枢神经系统感染等，其中内脏感染中最常见的是消化道和肺部感染。消化道感染可引起鹅口疮、舌炎、口角糜烂以及白色念珠菌肠炎，其中白色念珠菌肠炎可有低热、呕吐、腹胀、腹泻等表现，大便为黄色稀水便，泡沫较多，带黏液，可见豆腐渣样细块，大便镜检有真菌孢子和菌丝。
选项 B 致病大肠埃希菌肠炎常表现为高热、呕吐、腹痛、腹泻，水样便，有腥臭味，也可为黏液便、脓血便或血水便，与题干中大便的性状描述差异明显。
选项 C 轮状病毒肠炎常表现为发热、呕吐、腹泻，大便次数及水分多，呈黄色水样或粥样便，无脓性及豆渣样细块，与题干描述不符。
选项 D 金黄色葡萄球菌肠炎典型大便为暗绿色，量多带粘液，少数为血便，不符合题干。
选项 E 耶尔森菌小肠结肠炎典型表现为急性胃肠炎症状、小肠结肠炎，败血症者可有全身感

染中毒症状，还可引起反应性关节炎，与题干描述不符。
综上所述，正确答案是：A.白色念珠菌肠炎。
根据题干描述，患者是两岁半的男孩，重度营养不良，近一周出现腹泻，腹泻次数为 3~8 次/日，稀薄，呈黄色，泡沫较多，脓性，可见豆渣样细块。综合这些症状，最可能的诊断是轮状病毒肠炎（选项 C）。轮状病毒肠炎在婴幼儿中较为常见，通常会引起严重腹泻，排泄物呈黄色、泡沫较多，且往往伴有呕吐和发热。

其他选项的排除理由如下：
A. 白色念珠菌肠炎：多见于免疫功能低下的患者，症状一般不表现为泡沫较多的黄色腹泻。
B. 致病大肠埃希菌肠炎：通常表现为水样或血性腹泻，但泡沫较多的黄色腹泻不常见。
D. 金黄色葡萄球菌肠炎：多见于食物中毒，常表现为急性发作的呕吐和腹泻，粪便不一定呈现描述的特点。
E. 耶尔森菌小肠结肠炎：多见于寒冷季节，症状包括腹痛、发热和血性腹泻，泡沫较多的黄色腹泻不常见。

因此，最可能的诊断是 C. 轮状病毒肠炎。
64、 (A2 型题)
女，24 岁，月经 21~23 天/4~5 天，自然流产 1 次，基础体温双相型，月经第 3 天激素测定未见明显异常，诊断为
A.多囊卵巢综合征
B.黄体功能不全
C.子宫异常内膜脱落
D.无排卵性异常子宫出血
E.闭经溢乳综合征
参考答案： B 您的答案：
图文解析： 【解析】黄体功能不全是指月经周期中有卵泡发育及排卵，但黄体期孕激素分泌不足或黄体过早衰退，导致子宫内膜分泌反应不良和黄体期缩短，引起月经周期缩短。本例患者月经周期缩短，月经第 3 天激素测定未见明显异常，考虑为黄体功能不全。
【避错】多囊卵巢综合征（A 错）在临床上以雄激素过高的临床或生化表现、持续无排卵、卵巢多囊改变为特征，常伴有胰岛素抵抗和肥胖，主要表现为月经失调、雄激素过量和肥胖。子宫异常内膜脱落（C 错）的患者一般无流产史，多数有不孕史，月经第 3 天激素测定可出现异常。该患者激素测定未见明显异常，因此子宫异常内膜脱落的诊断不成立。无排卵性异常子宫出血（D 错）最常见临床表现是子宫不规则出血，表现为月经紊乱，经量增多、经期延长、月经过频、经前点滴出血，诊断性刮宫可发现子宫内膜受雌激素持续作用而无孕激素拮抗，可发生不同程度的增生性改变，少数可呈萎缩性改变。闭经溢乳综合征（E 错）为高催乳素血症的临床表现，闭经泌乳综合征是一种常见的病理生理状态，高催乳素血症时血清催乳素（PRL）升高，常伴有闭经、溢乳及无排卵引起的一系列症状。
该题目提供的信息指向女性患者的生殖健康状况。这位 24 岁的女性月经周期为 21~23 天，持续时间 4~5 天，有一次自然流产的历史，基础体温测量显示双相型，而月经第 3 天的激素水平检查未发现明显异常。基于这些信息，我们需要确定最符合这些临床特点的诊断。
A. 多囊卵巢综合征（PCOS）通常表现为月经不规律、排卵障碍和高雄激素血症。此患者的月经周期较为规律，且无提及雄激素水平异常，因此多囊卵巢综合征可能性较低。
B. 黄体功能不全涉及黄体分泌不足，通常导致月经周期短缩、经期延长或不规则出血。该患者的月经周期和持续时间在正常范围内，而且没有明确的黄体功能不足证据。

C. 子宫异常内膜脱落通常导致不规则的月经出血和可能的不孕，但此患者月经周期和持续时间相对规律，不完全符合此诊断。
D. 无排卵性异常子宫出血表现为无排卵周期导致的不规则出血。然而，该患者的基础体温记录显示有排卵发生（双相型体温曲线），这不符合无排卵性出血。
E. 闭经溢乳综合征（即高泌乳素血症或 Galactorrhea-Amenorrhea Syndrome）主要表现为闭经和乳汁分泌增加。考虑到该患者有规律的月经周期，这个选项也不符合。
综合考虑，虽然没有一个选项完美匹配所有信息，但基于提供的数据，B 选项“黄体功能不全”可能是最符合的诊断，尽管她的月经周期和持续时间在正常范围内，但之前的自然流产和正常的激素水平测试可能暗示了潜在的黄体功能问题。然而，需要注意的是，这种情况在实际诊断中可能需要更多的详细评估和测试。
因此，最合适的选择是 B. 黄体功能不全。
解析：
患者青年女性，月经周期短，自然流产 1 次，基础体温双相型，月经第 3 天激素测定未见明显异常，综合该患者的月经史、流产史、检查，考虑诊断为黄体功能不全。黄体功能不全是指月经周期中有卵泡发育及排卵，但黄体期孕激素分泌不足或黄体过早衰退，导致子宫内膜分泌反应不良和黄体期缩短。临床上以月经周期缩短为主要特征，患者不易受孕或在妊娠早期流产。
选项 A 多囊卵巢综合征多起病于青春期，以雄激素过高的临床或生化表现、持续无排卵、卵巢多囊改变为特征，表现为月经失调、雄激素过量和肥胖，常伴有胰岛素抵抗和代谢综合征，与该患者症状不符，故不选。
选项 C 子宫内膜不规则脱落多见于生育年龄妇女，表现为月经周期正常，但经期延长，长达 9～10 日，且出血量多，该患者月经经期正常，故排除。
选项 D 无排卵性功能失调性子宫出血最常见的症状是子宫不规则出血，表现为月经周期紊乱，经期长短不一，经量不定或增多，甚至大量出血，与题意不符。
选项 E 闭经溢乳综合征临床主要表现为闭经、溢乳、不孕、高催乳素血症及垂体微腺瘤等，与本题无关。
综上所述，正确答案是：B.黄体功能不全。
根据题干信息，这位 24 岁的女性，月经周期为 21~23 天，每次持续 4~5 天，曾自然流产 1 次，基础体温呈双相型，月经第 3 天激素测定未见明显异常。以下是对每个选项的分析：

A. 多囊卵巢综合征：这种情况通常表现为月经稀发、闭经、多毛、肥胖和雄激素过多的症状。题干中没有这些典型的症状，且基础体温呈双相型，表示有排卵。

B. 黄体功能不全：这种情况会导致黄体期缩短，可能表现为月经周期缩短或月经量减少。题干中提到月经周期较短且有自然流产史，这与黄体功能不全较为吻合。

C. 子宫异常内膜脱落：这种情况通常会导致月经不规律或异常出血。题干中未提到这种表现。

D. 无排卵性异常子宫出血：这种情况多见于青春期和围绝经期女性，基础体温通常为单相型。题干中基础体温呈双相型，表示有排卵，不符合无排卵性出血的特点。

E. 闭经溢乳综合征：这种情况主要表现为闭经和溢乳，通常是由于高泌乳素血症引起。题干中没有提到溢乳或闭经，排除。

综合分析，最可能的诊断是：

B. 黄体功能不全
65、 (A2 型题)
男，60 岁。近 3 天夜间行为紊乱，说房间地板上有老鼠、蛇，能看见死人，表情恐怖、紧张，不认识家人，白天较安静，喜卧床，不能回忆夜间行为，能认识家人，头部 C 片状梗死灶，考虑患者处于
A.谵妄状态
B.幻觉妄想状态
C.抑郁状态
D.痴呆状态
E.木僵状态
参考答案： A 您的答案：
图文解析： 【解析】谵妄状态的患者有意识障碍，伴有感知障碍（幻觉、错觉），言语障碍，常有恐怖性幻视，如看到猛兽、鬼神、满屋灰尘等，定向力障碍，感到周围环境犹如陌生而恐怖，患者主客观世界颠倒或出现大量的幻觉、错觉，白天安静，晚上躁动不安。【避错】幻觉妄想状态是指在病态情况下出现病理性妄想，内容离奇、荒谬、脱离现实，甚至具有超自然色彩（B 错）。抑郁状态以显著而持久的情感低落（忧愁、苦闷、唉声叹气、暗自落泪等）为主要表现，伴有兴趣缺乏、快感缺失等，无幻觉、妄想（C 错）。痴呆状态表现为记忆力减退，注意力不能集中，思维迟缓，理解力减退，计算力下降等智能障碍（D 错）。木僵状态表现为不言不语、不动、不食、面无表情、肌肉松弛等（E 错）。幻觉妄想状态是指在病态情况下出现病理性妄想，内容离奇、荒谬、脱离现实，甚至具有超自然色彩（B 错）。抑郁状态以显著而持久的情感低落（忧愁、苦闷、唉声叹气、暗自落泪等）为主要表现，伴有兴趣缺乏、快感缺失等，无幻觉、妄想（C 错）。痴呆状态表现为记忆力减退，注意力不能集中，思维迟缓，理解力减退，计算力下降等智能障碍（D 错）。木僵状态表现为不言不语、不动、不食、面无表情、肌肉松弛等（E 错）。
这个病例描述的是一位 60 岁男性，表现为夜间行为紊乱，出现视觉幻觉（看到老鼠、蛇和死人），表情恐怖、紧张，夜间不认识家人，但白天能较安静，并能认识家人。头部 CT 显示有片状梗死灶。这些症状和临床表现符合谵妄状态（Delirium）的特点。
谵妄状态是一种急性脑功能障碍，常表现为意识水平的波动、认知功能障碍、感知异常（如幻觉）、睡眠-觉醒周期的紊乱等。谵妄通常与脑部病理改变相关，如此病例中的片状梗死灶即可能是诱因之一。
根据这些信息，最合适的答案是：A. 谵妄状态
首先，我们来分析题目给出的关键信息。患者老年男性，近 3 天夜间出现行为紊乱，表现出幻觉（看见不存在的老鼠、蛇、死人），并且表情恐怖、紧张，甚至不认识家人。然而，白天他的行为相对正常，能够认识家人，但不能回忆夜间的异常行为。此外，患者头部 CT 显示存在片状梗死灶。
接下来，我们针对每个选项进行分析：
A. 谵妄状态：谵妄是一种急性、可逆性、广泛性的认知障碍，主要表现为意识障碍，感知觉障碍如幻觉和错觉，以及注意障碍如注意力涣散。此状态通常起病急，病程短暂，病情发展迅速。考虑到患者夜间出现的幻觉、行为紊乱以及不能回忆夜间行为，这与谵妄状态的表现相吻合。
B. 幻觉妄想状态：虽然患者有幻觉表现，但妄想状态通常指的是持续、固定的错误信念，

而患者白天的行为相对正常，能够认识家人，表明其并没有固定的妄想。
C. 抑郁状态：患者并未表现出抑郁的典型症状，如情绪低落、兴趣丧失等。
D. 痴呆状态：痴呆是一个慢性过程，主要表现为记忆力减退、认知能力下降等，通常不会突然出现或消失，与患者的情况不符。
E. 木僵状态：木僵状态表现为不言不动，甚至不吃不喝，而患者在白天能够认识家人并表现出相对正常的行为。
综合以上分析，患者的症状最符合谵妄状态的表现，特别是夜间的幻觉、行为紊乱以及白天的相对正常表现。因此，正确答案是 A.谵妄状态。
根据题干描述的症状，患者在夜间出现行为紊乱、幻觉（看到老鼠、蛇和死人）、紧张恐怖、不认识家人，而在白天较安静，能认识家人且不能回忆夜间的行为。这些症状符合谵妄状态的特征，谵妄状态常表现为急性发作、波动性的意识水平和认知功能障碍，尤其在夜间更为明显。

因此，最可能的诊断是：

A. 谵妄状态
66、 (A2 型题)
女，51 岁，6 周前亲眼目睹丈夫被汽车碾压至死，近 2 周出现恐惧失眠，反复回忆起丈夫去世的场景，不敢进卧室，最有可能的诊断是
A.躯体形式障碍
B.创伤后应激障碍
C.惊恐障碍
D.急性应激障碍
E.广泛性焦虑障碍
参考答案： B 您的答案：
图文解析： 【解析】该患者诊断为创伤后应激障碍，其诊断标准如下：
遭受异乎寻常的创伤性事件（目睹丈夫被汽车碾压致死）；反复重现创伤性体验（反复回忆起丈夫去世的场景）；避免引起痛苦回忆（不敢进卧室）；警觉性提高（恐惧失眠）。因此，该患者最有可能的诊断是创伤后应激障碍。
【避错】躯体形式障碍（A 错）是一种以持久担心或相信各种躯体症状的优势观念为特征的神经症。惊恐障碍（C 错）主要表现为惊恐发作，患者突然出现不可预测的强烈的恐惧感，伴随濒死感或失控感以及严重的自主神经功能失调症状。急性应激障碍（D 错）在遭受异乎寻常的创伤性事件后数分钟至数小时内发病，并且在一个月内缓解。广泛性焦虑障碍（焦虑症）是一种以焦虑为主要表现的精神障碍，患者常有不明原因的提心吊胆、紧张不安，无情感创伤性事件作为致病诱因，与本例不符，故不考虑(E 错)。
这道题目描述了一位 51 岁女性，在 6 周前亲眼目睹丈夫被汽车碾压致死后，近两周出现恐惧、失眠、反复回忆起丈夫去世的场景，并且不敢进卧室的情况。根据这些症状，我们可以分析可能的诊断。
A. 躯体形式障碍（Somatic Symptom Disorder）通常表现为对身体症状的过度关注，与本案例描述的心理症状不太吻合。
B. 创伤后应激障碍（Post-traumatic Stress Disorder, PTSD）是一种在经历或目睹了极度创伤性事件后发展的心理健康状况，表现为反复回忆创伤事件、避免刺激、情绪麻木等症状，与案例描述相符合。
C. 惊恐障碍（Panic Disorder）以突然的、重复的惊恐发作为主要特征，这些发作通常无明

显触发因素，与此案例不完全吻合。
D. 急性应激障碍（Acute Stress Disorder, ASD）通常发生在创伤事件后的一个月内，表现为与 PTSD 类似的症状，但由于这位女性的症状持续了 6 周，因此不太可能是急性应激障碍。
E. 广泛性焦虑障碍（Generalized Anxiety Disorder, GAD）以持续的、广泛的焦虑和担忧为特征，不特定于某一事件或情境，与本案例的特定创伤后反应不完全吻合。
综上所述，最有可能的诊断是 **B. 创伤后应激障碍（PTSD）**。
这是一道医学诊断推理题，我们需要根据题干描述的症状来判断患者的可能疾病。
首先，梳理题目中的关键信息：
51 岁女性患者。
6 周前亲眼目睹丈夫被汽车碾压致死。
近 2 周出现恐惧、失眠，反复回忆起丈夫去世的场景。
不敢进卧室。
接下来，我们根据关键信息，对各选项进行逐一分析：
A. 躯体形式障碍：这是一种以持久地担心或相信各种躯体症状的优势观念为特征的神经症。患者因这些症状反复就医，各种医学检查阴性和医生的解释均不能打消其疑虑。与题干描述不符，可以排除。
B. 创伤后应激障碍（PTSD）：这是一种由于经历或目睹极端威胁或灾难性事件（如战争、自然灾害、严重事故等）所导致的精神障碍。症状可能包括反复出现的与创伤事件相关的噩梦、闪回、触景生情等，以及持续的警觉性增高和回避行为。题干中的患者在丈夫意外去世后出现了与创伤事件相关的恐惧、失眠和反复回忆，这些都是 PTSD 的典型症状。
C. 惊恐障碍：主要表现为突如其来的惊恐体验，伴有濒死感或失控感，以及严重的自主神经功能紊乱症状。题干中未提及这些症状，因此可以排除。
D. 急性应激障碍：指在遭受到急剧、严重的精神创伤性事件后数分钟或数小时内所产生的一过性的精神障碍，一般在数天或一周内缓解，最长不超过 1 个月。题干中患者症状已持续 2 周，且未提及有明显缓解，因此不符合急性应激障碍的特点。
E. 广泛性焦虑障碍：主要表现为不明原因的提心吊胆、紧张不安，与题干描述不符，可以排除。
综上所述，该患者最有可能的诊断是 B.创伤后应激障碍（PTSD）。这是因为患者在经历创伤事件（丈夫去世）后出现了与事件相关的恐惧、失眠和反复回忆，这些都是 PTSD 的典型症状。
因此，正确答案是 B.创伤后应激障碍。
根据描述，这位 51 岁的女性在目睹丈夫被汽车碾压至死后，出现了恐惧、失眠和反复回忆起丈夫去世的场景，不敢进入卧室。这种症状在近 2 周内出现。结合这些信息，最可能的诊断是：

B. 创伤后应激障碍 (PTSD)

创伤后应激障碍的典型症状包括：

- 重复经历创伤事件（如反复回忆或做噩梦）
- 回避与创伤相关的事物或情境（如不敢进卧室）
- 增加的警觉性（如失眠和恐惧）

这种症状通常在经历创伤事件后的一段时间内开始出现，且持续时间超过 1 个月。如果症状

在创伤事件后 4 周内出现并且持续不到 4 周，则更可能是急性应激障碍。然而，由于症状在近 2 周内开始出现，且患者已经经历创伤事件 6 周，所以更符合创伤后应激障碍的诊断。

67、 (A2 型题)

男，25 岁，头痛四个月。加重 3 周，喷射状呕吐，P50 次/分，血压 160/95mmHg，清神志清楚，无双侧视神经乳头状水肿，应该如何处理

A.胸部 X 线+止痛剂

B.心电图+止痛剂

C.颅脑 B 超+甘露醇

D.颅脑 CT/MRI+甘露醇

E.腰椎穿刺+甘露醇

参考答案： D 您的答案：

图文解析： 【解析】患者青年男性，头痛四个月，加重 3 周，喷射状呕吐（颅内压增高的表现），P50 次/分（脉搏徐缓，正常值 60～100 次/分），血压 160/95mmHg（血压增高，正常值 139~90/89～60mmHg），清神志清楚，无双侧视神经乳头状水肿（视网膜中央静脉未受压迫），根据患者的病史、临床表现和体查，患者首先考虑诊断为脑肿瘤。应进行颅脑 CT 或 MRI 检查以明确诊断，颅脑 CT 或 MRI 检查可明确肿瘤的部位、大小、数目、血供以及与周围结构的解剖关系。为减轻肿瘤对脑组织的压迫应行降颅压治疗，20%的甘露醇静脉滴注，故患者应进行的处理为颅脑 CT/MRI+甘露醇。

【避错】胸部 X 线+止痛剂（A 错）主要用于肺部疾病的诊断。心电图+止痛剂（B 错）主要用于心脏疾病的诊断。颅脑 B 超（C 错）主要用于颅内血管疾病的诊断。腰椎穿刺+甘露醇（E 错）主要用于中枢神经系统感染的诊断和治疗。

考虑到这位 25 岁男性患者有长达四个月的头痛病史，近三周内头痛加剧并伴有喷射性呕吐，血压偏高（160/95mmHg），但神志清楚，无双侧视神经乳头状水肿，最合适的处理方式应该是检测可能导致这些症状的原因。

选项 D，即进行颅脑 CT 或 MRI 扫描并给予甘露醇，是最合适的选择。这种做法可以帮助医生评估是否有颅内病变（如肿瘤、出血或肿胀）导致头痛和呕吐，而甘露醇可用于降低颅内压，缓解症状。因此，答案是 D。

解析：

患者青年男性，头痛四月余，近三周加重，伴喷射状呕吐，血压升高，心率降低，应考虑颅内压升高的可能，且患者神志清楚，无双侧视神经乳头水肿，故该患者目前最可能的诊断是颅内压增高症。该疾病一般首选头颅影像学检查，明确是否有颅内占位性病变等，为明确诊断，并缓解颅内高压，该患者应行颅脑 CT/MRI+甘露醇。

选项 A 胸部 X 线+止痛剂中胸部 X 线主要用于胸部外伤、肺部及胸膜疾病等的诊断，与该患者症状无关，故不选。

选项 B 心电图+止痛剂中，心电图主要用于心脏循环系统方面疾病诊断，与题意不符。

选项 C 颅脑 B 超+甘露醇中颅脑 B 超主要用于新生儿颅脑疾病筛查，该患者为青年，故颅脑 B 超不适用。

选项 E 腰椎穿刺+甘露醇中腰椎穿刺为有创检查，主要用于检查脑脊液情况，不能明确是否有颅内占位性病变，与题意不符。

综上所述，正确答案是：D.颅脑 CT/MRI+甘露醇。

根据题目描述，25 岁男性患者头痛四个月，加重三周，伴有喷射状呕吐，提示颅内压增高。患者脉搏 50 次/分，血压 160/95 mmHg，神志清楚，无视神经乳头水肿，这些体征进一步支持颅内病变的可能性。因此，需要进行影像学检查以明确诊断。

选项中，D 项“颅脑 CT/MRI+甘露醇”是最合适的选择。颅脑 CT 或 MRI 可以帮助确定颅内压增高的原因，比如脑肿瘤、脑出血或脑积水等。甘露醇作为一种渗透性利尿剂，可以有效降低颅内压，是治疗颅内压增高的标准措施之一。

因此，正确答案是：

D. 颅脑 CT/MRI+甘露醇
68、 (A2 型题)
男，78 岁，因睡眠差、食欲极度减退、消瘦明显 2 个月入院，患者高血压病史 20 余年，2 年前发现血糖轻度升高，服用降压降糖药后，血糖血压控制良好，患者自主言语减 己去世后老伴无人照顾，住院期间，经常夜间突然自主起床活动，无目的摸床，严重会大喊大叫，不认人，不知道自己身在何处，猜疑子女拿自己值钱的物品，时而发脾 静睡觉，记不得夜间发生的事，患者可能的诊断是
A.谵妄
B.阿尔兹海默症
C.血管性痴呆
D.突发性躁狂
E.精神分裂
参考答案： A 您的答案：
图文解析： 【解析】患者老年男性，因睡眠差、食欲极度减退、消瘦明显 2 个月入院，患者自主言语减少，担忧自己去世后老伴无人照顾，住院期间，经常夜间突然自主起床活动，无目的摸床，严重会大喊大叫，不认人，不知道自己身在何处，猜疑子女拿自己值钱的物品，时而发脾气，白天安静睡觉，记不得夜间发生的事（意识清晰度降低为主的急性认知障碍综合征），应诊断为谵妄（A 对）。
【避错】阿尔茨海默病（AD）（B 错）是一种起病隐匿的进行性发展的神经系统退行性疾病。临床上以记忆障碍、失语、失用、失认、视空间技能损害、执行功能障碍以及人格和行为改变等全面性痴呆表现为特征。血管性痴呆（C 错）是由脑血管病变导致的痴呆。躁狂发作（D）以情感高涨、思维奔逸、活动增多“三高”为临床表现，可伴有夸大观念或妄想、冲动行为等。精神分裂症（E 错）多起病于青壮年，常有感知、思维、情感、行为等多方面的障碍和精神活动的不协调， 一般无意识障碍和明显的智能障碍。
本题描述了一位 78 岁男性患者，有长期高血压病史和轻度血糖升高，表现出睡眠差、食欲减退、消瘦、夜间突然起床活动、不认人、记忆丧失、情绪波动等症状。这些症状表明患者可能有认知功能障碍。
A. 谵妄 - 通常表现为急性起病，意识障碍，症状在一天中变化较大，常见于住院患者。
B. 阿尔兹海默症 - 通常是缓慢进展的认知衰退，以记忆丧失为特征，但不会出现夜间行为极端改变。
C. 血管性痴呆 - 由脑血管疾病引起的认知功能障碍，进展可能快于阿尔兹海默症，症状与脑损伤部位有关。
D. 突发性躁狂 - 突发性躁狂通常表现为过度活跃、冲动或易怒，而不是认知功能丧失。
E. 精神分裂 - 主要特征是思维障碍、幻觉和妄想，与本例描述不完全吻合。
综合患者的年龄、病史以及表现的症状，谵妄（A）是最可能的诊断。因为谵妄可以表现为突然的认知功能改变，包括记忆问题、注意力下降和夜间行为异常，这些都与患者的症状相符。因此，最佳答案是 A.谵妄。
首先，我们分析题干：

患者老年男性，因睡眠差、食欲极度减退、消瘦明显 2 个月入院，同时有高血压和高血糖的慢性病史。住院期间，患者表现出夜间活动增多、无目的行为、大喊大叫、认知能力下降、猜疑和不记得夜间事情等症状。
接下来，我们针对每个选项进行逐一分析：
A. 谵妄：谵妄是一种急性、可逆性、广泛性的认知障碍，尤其是注意力、定向力和感知能力受损。它通常起病急，病程短暂，病情发展迅速，有明显的昼夜节律变化，表现为昼轻夜重。患者的症状（夜间活动增多、无目的行为、大喊大叫、认知能力下降）与谵妄的典型表现相符。
B. 阿尔兹海默症：这是一种起病隐袭、病程缓慢的神经系统退行性疾病，主要表现为渐进性记忆障碍、认知功能障碍、人格改变及语言障碍等神经精神症状。虽然患者的症状包括认知下降，但考虑到病程较短和突然的行为改变，这与阿尔兹海默症的缓慢进展不符。
C. 血管性痴呆：这是由脑血管病变导致的痴呆，通常表现为波动性进展的认知功能障碍、记忆减退、失语、失用、失认等。患者的病史和症状与血管性痴呆的典型表现不完全吻合。
D. 突发性躁狂：这通常表现为情感高涨、活动增多、思维奔逸等。虽然患者有夜间活动增多和喊叫的表现，但其他症状（如认知下降、定向力丧失）与此诊断不符。
E. 精神分裂：精神分裂症是一组病因未明的慢性疾病，多在青壮年缓慢或亚急性起病，临床上往往表现为症状各异的综合征，涉及感知觉、思维、情感和行为等多方面的障碍以及精神活动的不协调。患者多为青壮年，且病程通常较长，与题干描述不符。
综上所述，结合患者的病史和临床表现，最可能的诊断是谵妄。
因此，正确答案是 A.谵妄。
根据题目描述，这位 78 岁的男性患者表现出一系列的精神和认知障碍症状，包括睡眠差、食欲极度减退、消瘦、记忆力减退、定向力障碍（不知道自己身在何处），并伴有猜疑、夜间行为异常和情绪不稳定。这些症状有明显的急性和波动性特征，患者会在夜间突然自主起床活动并发脾气，随后又忘记夜间发生的事。这些特征非常符合谵妄的临床表现。

谵妄（Delirium）是一种急性脑功能紊乱，表现为注意力和认知功能的急性改变，通常是由潜在的医学疾病、药物或其他可逆性因素引起的。它的特征是急性起病、症状波动和夜间症状加重，患者可能表现为定向力障碍、意识混乱、幻觉、错觉、以及情绪不稳定等。

综合上述分析，最可能的诊断是：

A. 谵妄
69、 (A2 型题)
男，25 岁，尿道刺痛流脓 3 天，查体：尿道口红肿，有黄色脓性分泌物，涂片见多核细胞，可找到革兰阴性双球菌，不属于致病原的是
A.内毒素
B.外毒素
C.外膜蛋白
D.菌毛
E.外膜脂多糖
收藏 笔
参考答案： B 您的答案：
图文解析： 【解析】青年男性患者，尿道刺痛流脓 3 天（淋病典型临床表现），查体见尿道口红肿，有黄色脓性分泌物，涂片见多核细胞，可找到革兰阴性双球菌（淋病奈瑟菌主

要革兰阴性双球菌)，结合患者病史、临床表现和实验室检查，该患者最可能的诊断为淋病。淋病奈瑟菌是引起人类泌尿生殖系统黏膜化脓性感染的病原菌，为革兰阴性双球菌，淋病奈瑟菌主要致病物质包括：①侵袭力：淋病奈瑟菌主要致病物质包括：外膜蛋白（C 对)、脂多糖（E 对)、菌毛（D 对）等。②内毒素：内毒素（A 对）是淋病奈瑟菌的次要致病物质，是引起淋病一般症状的主要物质，但不起主要作用，外毒素（B 错，为本题正确答案）不是淋病奈瑟菌的致病物质。

这道题描述的是一个 25 岁男性患者，出现尿道刺痛和流脓症状，体检发现尿道口红肿和黄色脓性分泌物，涂片显示多核细胞和革兰阴性双球菌。这些症状和体征提示了一个典型的性传播感染，特别是淋病的可能性。淋病是由淋病奈瑟菌（Neisseria gonorrhoeae）引起的，这是一种革兰阴性双球菌。

考题询问的是哪一项不属于致病原，我们需要识别出哪个因素通常不被认为是淋病奈瑟菌致病的组成部分。选项中有：

- A. 内毒素
- B. 外毒素
- C. 外膜蛋白
- D. 菌毛
- E. 外膜脂多糖

在这些因素中，内毒素（A 选项）和外膜脂多糖（E 选项）是革兰阴性细菌的典型成分，它们对于细菌的致病性至关重要。外膜蛋白（C 选项）和菌毛（D 选项）同样对细菌的附着和入侵宿主细胞起重要作用。

然而，外毒素（B 选项）通常是由某些细菌产生的，用以直接损害宿主细胞的蛋白质，而淋病奈瑟菌不以产生外毒素作为其主要的致病机制。因此，最不可能属于淋病奈瑟菌致病原的是**B. 外毒素**。

解析：

青年男性患者，尿道刺痛流脓 3 天，查体见尿道口红肿，有黄色脓性分泌物，涂片见多核细胞，可找到革兰阴性双球菌，根据患者的临床表现和实验室检查，该患者可诊断为淋病。淋病的致病菌是淋病奈瑟菌，其致病物质主要包括菌毛、菌毛上的粘附因子、脂寡糖、外膜蛋白以及 IgA1 蛋白酶等，可使淋病奈瑟菌粘附于人类泌尿生殖道粘膜，并在粘膜上定居、繁殖、导致局部急性炎症，出现化脓和感染的症状。而外毒素主要由革兰阳性菌和部分革兰阴性菌产生，并分泌到菌体外的一类毒性蛋白质，淋病奈瑟菌属于革兰阴性菌，但并不产生外毒素。

选项 A 内毒素是革兰阴性菌细胞壁中的一种成分，又称为脂多糖，只有在细菌死亡裂解后才被释放出来，其毒性作用相对较弱，可引起发热、微循环障碍、内毒素休克及播散性血管内凝血等，符合淋病奈瑟菌的致病物质，故不选。

选项 C 外膜蛋白为淋病奈瑟菌的主要致病物质之一，可使淋病奈瑟菌易于粘附到人类泌尿生殖道粘膜上，与题意不符。

选项 D 菌毛可使淋病奈瑟菌与尿道粘膜等组织发生粘附，是淋病奈瑟菌的致病物质，故排除。

选项 E 外膜脂多糖可使淋病奈瑟菌的主要致病物质之一，具有内毒素活性，可使机体致热和具有致死毒性，不符合题意。

综上所述，正确答案是：B.外毒素。

这个案例描述的是一个年轻男性尿道感染的典型症状，根据描述的涂片检查结果，推测是淋病奈瑟菌（Neisseria gonorrhoeae）感染。淋病奈瑟菌是一种革兰阴性双球菌，常见的致病因素包括内毒素（外膜脂多糖），外毒素，外膜蛋白和菌毛。

让我们逐一分析选项：
- A. **内毒素**：内毒素通常指革兰阴性菌细胞壁中的脂多糖（LPS），是淋病奈瑟菌致病因素之一。
- B. **外毒素**：外毒素是细菌分泌的毒性蛋白质或酶，淋病奈瑟菌虽然主要依赖其他致病因素，但也有报告其可能分泌某些外毒素。
- C. **外膜蛋白**：外膜蛋白是革兰阴性菌细胞壁的组成部分，参与致病。
- D. **菌毛**：菌毛有助于细菌黏附在宿主细胞上，是淋病奈瑟菌的重要致病因素。
- E. **外膜脂多糖**：外膜脂多糖（LPS）是革兰阴性菌的重要组成部分，也称为内毒素，是致病因素之一。

综上所述，选项中不属于致病因素的是 **外毒素**。虽然某些革兰阴性菌可以产生外毒素，但淋病奈瑟菌的主要致病机制不包括外毒素。

因此，正确答案是：
B. 外毒素
70、　(A2 型题)
初产妇 28 岁，妊娠 40 周，估计胎儿 3000g，骨盆测量正常，第一产顺利，宫口开 1 小时，S=+2 宫缩 4 次/10 分，羊水清，胎心变异好，偶有早期减速，应采取处置
A.密切观察胎头下降情况
B.会阴侧切加腹压
C.立即剖宫产
D.侧切下胎头吸引
E.产钳助产
参考答案：　A　您的答案：
图文解析：　【解析】青年初产妇，妊娠 40 周（妊娠满 37 周至不满 42 足周期间分娩，为足月产），骨盆测量正常，第一产顺利，宫口开 1 小时（第一产程是指从临产出现规律宫缩至宫口扩张开全（10cm），　一般需要 11～12 小时），S=+2（胎头已下降至坐骨棘平面下 2cm），宫缩 4 次/10 分（正常为 2～3 次/分钟），羊水清，胎心变异好，偶有早期减速（提示胎儿缺氧可能），综上所述，该患者最可能的诊断是正常第一产程，胎儿体重 3000g（正常为 2800~4000g），故应采取的处置是密切观察胎头下降情况（A 对），暂不需行阴道助产术（DE 错）。
【避错】立即剖宫产（C 错）适用于胎儿窘迫、头盆不称等情况。会阴侧切加腹压（B 错）适用于第二产程延长、胎儿窘迫等情况。
这个问题描述了一位初产妇，妊娠期满 40 周，胎儿估计重 3000 克，骨盆测量正常，产程进展顺利，宫口开放速度快（1 小时），胎头下降到 S=+2 位置，宫缩频率为每 10 分钟 4 次，羊水清，胎心率变异好，偶有早期减速现象。这种情况下的处理应侧重于持续监测和评估产妇和胎儿的状态，因为目前没有迹象表明需要紧急干预。
根据描述，产程是正常的，宫缩频率正常，胎心率变异良好，只是偶尔出现早期减速，通常这不是紧急情况，特别是在胎头下降良好且羊水清的情况下。早期减速通常是胎儿头部在产道下降过程中受到压迫的生理反应，如果没有其他不良迹象，可以视为正常的产程一部分。
因此，最佳答案是 A.密切观察胎头下降情况。这意味着继续监测产程的进展、胎心率变异和其他任何可能表明需要干预的迹象。其他选项，如侧切、剖宫产或使用产钳或吸引器，通常在出现严重或持续的胎儿窘迫、产程停滞或骨盆-胎儿不称等情况下才考虑。在这种情况

下，并没有迹象表明需要采取这些更侵入性的干预措施。
解析：
初产妇，妊娠 40 周，骨盆测量正常，胎儿大小正常，宫口已开，S=+2，说明胎头已衔接，产程处于活跃期，宫缩间歇过长，羊水清，胎心变异好，偶有早期减速，此时可通过加强宫缩、密切观察胎头下降情况以判断胎儿是否可以通过正常分娩娩出。
选项 B 会阴侧切加腹压，选项 D 侧切下胎头吸引，选项 E 产钳助产，均是在胎头已达+3 水平，出现胎儿窘迫征象时采用的助产方式，此时胎儿尚未到达此位置，故一并排除。
该产妇胎心变异好，偶有早期减速，产程处于活跃期，可密切观察产程变化，暂无剖宫产指征，因此选项 C 立即剖宫产，不是目前最合适的处理方式，故不选。
综上所述，正确答案是：A.密切观察胎头下降情况。
对于这位 28 岁初产妇，妊娠 40 周，胎儿估计 3000g，骨盆测量正常，第一产程顺利，宫口开全 1 小时，胎儿头部已经下降到 S=+2，宫缩频率为 4 次/10 分钟，羊水清，胎心变异性好，偶有早期减速的情况，应采取的处置是：

A.密切观察胎头下降情况

解析：
- 初产妇的产程通常较长，在胎心率正常且胎头已经下降至 S=+2 的情况下，说明胎儿状态良好，产程进展顺利。
- 宫缩频率为 4 次/10 分钟，属于正常范围，羊水清说明没有胎儿窘迫的表现。
- 早期减速一般是由于宫缩期间胎头受压所致，不是胎儿缺氧的表现，且胎心变异性良好，进一步说明胎儿状态良好。
- 在这种情况下，密切观察胎头下降情况是最合适的选择，以确保产程顺利进行。

因此，最佳答案是：**A.密切观察胎头下降情况**
71、 (A2 型题)
男，14 岁，感冒发热 10 天，体温高达 39.8℃,经医院治疗 3 天后体温恢复，出院后无明显诱因的讲话少，答少问少，在床上大便。查体：T37.9℃,P95 次/分，R20 次/分，BP120/72mmHg。以下诊断最可能是
A.病毒脑炎
B.焦虑障碍
C.躯体感染性精神障碍
D.精神分裂
E.抑郁障碍
参考答案： C 您的答案：
图文解析： 【解析】该患者感冒发热 10 天，体温高达 39.8℃,经医院治疗 3 天后体温恢复，故诊断为躯体感染，即某一种躯体感染性疾病，后出现少言少动少问，在床上大便（意志减退），故最可能诊断为躯体感染性精神障碍。
【避错】病毒性脑炎是由病毒引起的脑实质炎症，可出现发热、头痛、呕吐、惊厥等症状，病情严重时可有意识障碍、精神异常，但发病均较急骤， 一般无前驱期症状，精神症状常在病毒感染的基础上出现，故该患者不能诊断为病毒性脑炎（A 错）。焦虑障碍是一组以焦虑为主要表现的精神障碍，患者常常有不明原因的提心吊胆、紧张不安，与本例不符，故不考虑(B 错)。精神分裂症常起病于成年期，有精神活动的脱离现实性和多方面的“分裂”症状，表现为妄想、幻觉、思维和言语零乱及内向性等， 一般无意识障碍和明显的智能障碍，

病程多迁延。该患者无精神分裂症的典型症状，故不能诊断为精神分裂症（D 错）。抑郁障碍常表现为情绪低落、快感缺失、思维迟缓、意志活动减少、精神运动性迟滞或激越、自责自罪、自杀观念和行为、早醒、食欲减退、体重下降、性欲减退、抑郁心境晨重晚轻的节律改变等，故该患者不能诊断为抑郁障碍（E 错）。

这个病例描述了一个 14 岁男孩，他在感冒发热后出现了讲话减少、答少问少、在床上大便等症状。体温稍高，心率稍快，但血压正常。根据这些症状，我们可以进行一些鉴别诊断：

A. **病毒脑炎**：通常会导致发热、头痛、神经系统症状，如意识障碍、精神症状或抽搐。患者有发热病史和神经系统症状，这可能是一个选项。

B. **焦虑障碍**：通常表现为持续的焦虑和担心，伴随身体症状，如心慌、出汗等。这个病例中没有描述典型的焦虑症状。

C. **躯体感染性精神障碍**：是指在躯体疾病（如感染）的基础上发生的精神障碍，通常与基础疾病密切相关。考虑到孩子之前的高热和当前的神经系统表现，这可能是一个重要的考虑。

D. **精神分裂**：是一种严重的精神障碍，以思维、感知和情感的持续障碍为特征。此病例中缺乏典型的精神分裂症症状。

E. **抑郁障碍**：主要表现为持续的悲伤、绝望或兴趣丧失，但孩子的症状与典型的抑郁症不完全吻合。

考虑到孩子有感冒后的复杂表现，包括高热、讲话和反应减少，以及无明显诱因的行为改变（如床上大便），**躯体感染性精神障碍**（C 选项）可能是最可能的诊断。这种情况可能是由于感染后的身体反应导致的精神状态变化。不过，确诊通常需要进一步的医学评估，包括可能的脑部影像学检查、血液和脑脊液检查等。因此，根据这个案例的描述，最可能的诊断是 C.躯体感染性精神障碍。

这是一道医学诊断问题，我们需要基于患者的病史、症状、体征以及可能的医学知识来推断最可能的诊断。

理解背景信息：14 岁男孩，感冒发热 10 天，体温高达 39.8℃。经医院治疗 3 天后体温恢复，出院后无明显诱因的讲话少，反应迟钝，甚至在床上大便。查体显示体温略高，脉搏和呼吸正常，血压也在正常范围内。

理解问题的核心：我们需要基于这些信息，推断出患者的最可能诊断。

分析选项：

A. 病毒脑炎：尽管患者有病毒感染史，但病毒脑炎通常会伴随明显的神经系统症状，如头痛、恶心、呕吐、脑膜刺激征等，这些症状在题干中并未提及。

B. 焦虑障碍：焦虑障碍主要表现为过度担心、恐惧、紧张和不安，题干中并未提及这些症状。

C. 躯体感染性精神障碍：这是一种由躯体感染引发的精神障碍，通常在感染后出现。考虑到患者有明确的感染史（感冒发热），并且在感染后出现了精神症状（讲话少，答少问少，在床上大便），这个选项符合患者的病史和症状。

D. 精神分裂：精神分裂症主要表现为幻觉、妄想、思维混乱、行为和思想的异常等，题干中并未明确提及这些症状。

E. 抑郁障碍：抑郁障碍主要表现为情绪低落、兴趣丧失、疲劳感、睡眠障碍等，题干中并未明确提及这些症状。

综上所述，考虑到患者有明确的感染史，并且在感染后出现精神症状，最可能的诊断是躯体感染性精神障碍。

因此，正确答案是 C. 躯体感染性精神障碍。

根据题目提供的信息，这名 14 岁的男孩在感冒发热 10 天后，虽然体温恢复正常，但出现了

明显的精神状态改变，如讲话少，答少问少，并且有在床上大便的情况。查体显示体温略微升高，脉搏、呼吸和血压在正常范围内。

综合以上信息，这名男孩很可能是因为急性病毒感染（如感冒）导致了中枢神经系统的影响，表现为精神状态的变化。最可能的诊断是躯体感染性精神障碍。

因此，最佳答案是：

C. 躯体感染性精神障碍

72、 (A2 型题)

女，38 岁，妊娠 18 周，既往生一智力低下儿，后因心脏病夭折，目前需要的检查是

A.NT 检查

B.早期唐筛

C.羊水穿刺

D.超声筛查

E.无创 DNA

参考答案： C 您的答案：

图文解析： 【解析】因为该患者既往生育过智力低下的患儿，此次妊娠属于高龄孕妇（＞35 岁），故此次妊娠属于高危儿，可选择羊膜腔穿刺进行产前诊断。羊水穿刺是产前诊断，是判断胎儿染色体是否异常的金标准，所有产前筛查高风险的孕妇均建议通过该检查来进行胎儿染色体是否异常的诊断。

【避错】NT 检查（A 错）是通过超声检查胎儿颈部透明带厚度的一种检查，通常是产前排除胎儿畸形的常规检查手段。早期唐筛（B 错）是通过抽取孕妇血清，检测妊娠早期二聚体糖蛋白激素、甲胎蛋白、游离雌三醇及抑制素 A 的浓度，并结合孕妇的预产期、体重、年龄和采血时的孕周，计算出唐氏胎儿患病的危险系数的检测方法。超声（D 错）只是筛查，无法判断胎儿染色体异常。无创 DNA（E 错）是抽取孕妇的静脉血，检测孕妇血浆中胎儿来源的游离 DNA 片段，筛查常见的非整倍染色体异常的方法，但检测范围窄，所以在可能存在胎儿其他染色体或基因疾病风险的孕妇、胎儿结构畸形，孕妇本身存在染色体异常等特殊情况下，不宜用该方法。其目前仅用于高危人群的次级筛查。

在这种情况下，考虑到这位女性妊娠 18 周，有智力低下儿童的历史，以及儿童因心脏病去世的情况，选择最合适的检查是很重要的。选项 A 的 NT 检查（颈后透明层检查）通常在妊娠 11-13 周进行，已经错过了这个时间窗口。选项 B 的早期唐筛通常在妊娠的第一学期或早期第二学期进行，对于 18 周的妊娠来说也较晚。

考虑到这位母亲的背景和需要详细检查胎儿的情况，最合适的选项是 C 羊水穿刺。羊水穿刺可以在妊娠第二学期进行，用于检测染色体异常和遗传疾病，这对于有遗传病风险的妊娠来说非常重要。因此，正确答案应该是 C 羊水穿刺。

解析：

患者青年女性，妊娠 18 周，既往生一智力低下儿，后因心脏病夭折，有不良孕产史，属于高危孕妇，再次怀孕时，其胎儿发生遗传性疾病的风险较高，为明确诊断，应行羊水穿刺检查，其是产前诊断的一种方法，一般适合中期妊娠的产前诊断，在超音波导引之下，将一根细长针穿过孕妇的肚皮，子宫壁，进入羊水腔，抽取一些羊水进行检验，以明确胎儿是否存在染色体异常及遗传性疾病等。

选项 A：NT 检查指胎儿颈项透明层检查，常用于产前筛查，可以早期诊断染色体疾病和早期发现多种原因造成的胎儿异常，一般在孕 11 周到 13 周+6 天进行，与题干描述不符。

选项 B：早期唐筛一般在怀孕的 11~14 周进行，主要通过超声检查胎儿颈项透明带的厚度，并结合孕妇年龄和实验室检查值来评估胎儿唐氏综合征的风险，而本例患者已妊娠 18 周，故排除。
选项 D：超声筛查可以发现大部分的胎儿畸形，如无脑畸形、脑积水、开放性脊柱裂、肢体畸形、心脏畸形等，但不能确诊是否存在遗传性疾病，因此可排除。
选项 E：无创 DNA 是指利用新一代的 DNA 测序技术，对母体外周血浆中的游离 DNA 片段进行检测，并从中得到胎儿的遗传信息，从而检测胎儿是否患有染色体异常的检测手段，但只能筛查胎儿染色体非整倍体异常，且其准确性低于羊水穿刺，因此不作为首选检查。
综上所述，正确答案是：C.羊水穿刺。
对于 38 岁的孕妇，妊娠 18 周，既往生一智力低下儿，后因心脏病夭折，目前需要的检查是：

C. 羊水穿刺

解析：
- NT 检查（颈项透明层检查）和早期唐筛（唐氏综合征筛查）通常在妊娠 11-14 周进行，已经错过了最佳时间窗口。
- 超声筛查可以用于评估胎儿的结构异常，但不能直接检测染色体异常。
- 无创 DNA 检测是一种高灵敏度的筛查方法，但羊水穿刺是确诊胎儿染色体异常的金标准。

由于该孕妇年龄较大且有不良生育史，羊水穿刺能够直接检测胎儿的染色体，提供最确凿的诊断信息，因此是最佳选择。
73、 (A2 型题)
男，20 岁，大学生，让其自我介绍时说“我今年 20 岁，今天太阳出来了，是个好日子，红桃 A 是病毒，我女朋友”，此属于
A.思维奔逸
B.思维散漫
C.思维破裂
D.思维不连贯
E.精神分裂
参考答案： C 您的答案：
图文解析： 【解析】该患者让其自我介绍时说“我今年 20 岁，今天太阳出来了，是个好日子，红桃 A 是病毒，我女朋友”，此属于思维破裂（C 对），表现为概念之间的联想断裂，各句含意互不相关，变成语词堆积。
【避错】思维奔逸的患者感到脑子特别灵活，就像机器加了“润滑油”（A 错）。表现为特别健谈，说话滔滔不绝，口若悬河。说话的主题常常从一个概念迅速转移为另一个概念，可因环境改变而突然转换话题。思维散漫的患者联想松弛，内容散漫，缺乏主题（B 错）。思维不连贯的患者表现说话东拉西扯，以至于别人弄不懂患者所要阐述的主题思想是什么（D 错）。精神分裂症主要表现为妄想、幻觉、言语和行为紊乱， 一般无智力障碍（E 错）。
这个案例描述的是一个 20 岁大学生在自我介绍时话题快速跳转，内容之间缺乏逻辑和连贯性。这种表现属于“思维不连贯”。思维不连贯是指个体的语言和思维表达缺乏逻辑性和结构性，表达的想法之间缺少逻辑联系，使得话语难以理解。
因此，最合适的答案是：D. 思维不连贯
这是一道关于精神疾病诊断的问题。我们需要先理解题干中描述的症状，再结合精神疾病的相关知识进行推理判断。

理解背景信息：青年男性患者，自我介绍时言语内容跳跃、不连贯，缺乏逻辑性。
理解问题核心：我们需要基于患者的自我介绍，判断其所表现出的思维障碍类型。
接下来，我们针对每个选项进行详细分析：
A 选项（思维奔逸）：通常表现为特别健谈，说话滔滔不绝，口若悬河，感到脑子特别灵活，就像机器加了“润滑油”一样难以停顿下来，但言语内容多为夸大妄想，与本例不符。
B 选项（思维散漫）：表现为在交谈时，内容散漫，缺乏主题，话题转换缺乏必要的联系，但每个句子本身的结构和语法是正确的，只是句子与句子之间缺乏内在的逻辑关系，这与患者的自我介绍不完全吻合。
C 选项（思维破裂）：表现为患者在意识清楚的情况下，思维联想过程破裂，谈话内容缺乏内在意义上的连贯性和应有的逻辑性，与题干描述的症状较为接近，但仍需与其他选项比较。
D 选项（思维不连贯）：表现为在严重的精神分裂症患者中，言语支离破碎，语句片断之间互不相干，没有逻辑联系，与题干不符。
E 选项（精神分裂）：这不是一种具体的思维障碍，而是一种更广泛的疾病分类，不适用于此处的具体分析。
综合分析，该患者的自我介绍内容跳跃、不连贯，但每个句子本身在语法上并无错误，只是句与句之间缺乏逻辑联系，更符合思维破裂的特征。思维散漫虽然也表现为内容散漫，但每个句子之间通常还有一定的逻辑联系，而本例中患者的言语几乎完全缺乏这种联系。
因此，答案是 C.思维破裂。
根据题干描述，患者的自我介绍中包含了无关紧要的信息和语句之间缺乏逻辑联系，例如“我今年 20 岁，今天太阳出来了，是个好日子，红桃 A 是病毒，我女朋友”。这种情况下的思维特征是思维破裂。

思维破裂是指患者的思维过程缺乏连贯性和逻辑性，表现为说话时各句之间没有逻辑关系，语句前后矛盾，难以理解，是精神分裂症的典型症状之一。

因此，正确答案是：

C. 思维破裂
74、 (A2 型题)
女，32 岁，已婚，G1P0，经量增多 2 年，伴头晕乏力 1 个月，既往月经规律。妇科检查：子宫增大如妊娠 3 个月大小，子宫前壁触及直径约 8cm 突起，质地较硬，最佳处理方
A.子宫肌瘤切除术
B.子宫动脉栓塞术
C.次全子宫切除术
D.药物治疗
E.高能聚焦超声治疗
参考答案： A 您的答案：
图文解析： 【解析】该患者为育龄期女性，经量增多 2 年，伴头晕乏力 1 个月（有导致贫血症状），妇检：子宫增大如妊娠 3 个月大小，子宫前壁触及直径约 8cm 突起（肌瘤位于浆膜下，有突起），质地较硬，应首先考虑子宫肌瘤。患者有经量增多、头晕乏力等症状，说明已引起贫血。患者尚年轻，需保留生育功能，故最佳处理方法是子宫肌瘤切除术。
【避错】子宫动脉栓塞术（B 错）:通过阻断子宫动脉及其分支减少肌瘤的血供,从而延缓肌瘤的生长,缓解症状。但该方法可能引起卵巢功能减退并增加潜在的妊娠并发症的风险,对有生育要求的妇女一般不建议使用。次全子宫切除术（C 错）适用于经量增多、经期延

长和与子宫相关出血症状经保守治疗无效者，不用于有生育要求的妇女。药物治疗（D 错）适用于症状轻、近绝经年龄或全身情况不宜手术者。高能聚焦超声治疗（E 错）非主流治疗方法，主要适用于不适合手术的患者。

这个案例描述的是一位 32 岁女性，婚后首次怀孕（G1P0），有两年经量增多的病史，伴有一个月的头晕和乏力。妇科检查发现子宫增大，相当于妊娠 3 个月的大小，并在子宫前壁触及直径约 8cm 的硬性突起。这些症状和体征最符合子宫肌瘤的诊断。

选项分析如下：

A. 子宫肌瘤切除术（Myomectomy）：适用于希望保留生育能力或子宫的患者，特别是对较大的单个肌瘤或位于子宫壁表面的肌瘤。

B. 子宫动脉栓塞术（Uterine Artery Embolization, UAE）：适用于不希望通过手术治疗的患者，可减少肌瘤大小和症状，但不是所有患者都适合此手术。

C. 次全子宫切除术（Subtotal Hysterectomy）：移除子宫体但保留子宫颈，适用于对保守治疗无反应或有多发肌瘤的患者。这是一种更激进的治疗方式，通常考虑在其他治疗失败或不适用的情况下。

D. 药物治疗：通常用于症状轻微的患者或作为手术前的辅助治疗，以缩小肌瘤大小。

E. 高能聚焦超声治疗（High-intensity Focused Ultrasound, HIFU）：是一种非侵入性治疗方法，适用于特定的患者群体。

鉴于病人的症状、年龄以及保留生育能力的需求，最合适的选择似乎是 A. 子宫肌瘤切除术。这种方法可以去除肌瘤，缓解症状，同时保留子宫和患者的生育能力。因此，我会选择 A. 子宫肌瘤切除术作为最佳处理方案。

首先，我们需要了解患者的具体病情。患者为 32 岁女性，已婚，孕 1 产 0，月经量增多 2 年，并伴有头晕乏力症状 1 个月。妇科检查显示，子宫增大如同妊娠 3 个月大小，且子宫前壁存在一个直径约 8cm 的硬结。这些症状和体征强烈提示患者可能患有子宫肌瘤。

接下来，我们针对各个选项进行分析：

A. 子宫肌瘤切除术：这种方法适用于希望保留生育功能的患者，特别是那些年轻且要求保留子宫的患者。考虑到患者的年龄和未生育的状态，这一选项是合适的。

B. 子宫动脉栓塞术：虽然这也是一种治疗方法，但它通常用于缩小肌瘤体积或缓解症状，并不直接切除肌瘤。

C. 次全子宫切除术：这种方法通常适用于不要求保留生育功能的患者。由于患者尚未生育，这一选项可能不是最佳选择。

D. 药物治疗：药物治疗通常用于缓解症状或作为术前准备，但不作为长期治疗的首选方法。

E. 高能聚焦超声治疗：虽然这是一种非侵入性的治疗方法，但它主要用于缓解症状，而不是直接切除肌瘤。

综合上述分析，考虑到患者年轻且尚未生育，最佳的处理方法是选择能够保留子宫且不影响未来生育能力的方式。因此，子宫肌瘤切除术是最佳选择。

所以，正确答案是 A.子宫肌瘤切除术。

根据提供的病例资料，该 32 岁的女性患者有 2 年的月经量增多病史，并伴有 1 个月的头晕乏力症状。妇科检查显示子宫增大至相当于妊娠 3 个月大小，并在子宫前壁触及直径约 8cm 的突起，质地较硬。综合考虑患者的年龄、症状以及子宫增大的情况，最佳的处理方案应能有效缓解症状并改善患者的生活质量。

在处理子宫肌瘤时，以下是各个选项的简要分析：

A. **子宫肌瘤切除术**：适用于希望保留子宫并有生育需求的患者。它能有效去除肌瘤，

缓解症状，但术后可能复发。

B. **子宫动脉栓塞术**：通过阻断肌瘤供血来缩小肌瘤，适用于希望保留子宫的患者，但可能存在副作用如慢性疼痛和感染。

C. **次全子宫切除术**：适用于不再有生育要求的患者。手术可以彻底解决症状，防止复发。

D. **药物治疗**：通常适用于症状轻微的患者，或作为暂时性措施，药物效果有限且可能需要长期使用。

E. **高能聚焦超声治疗**：一种非侵入性治疗方法，但由于肌瘤较大，可能效果不如手术明显。

在此病例中，患者有显著症状且子宫明显增大，考虑到患者可能还希望保留子宫以维持生育功能，**子宫肌瘤切除术（A 选项）** 是较为适宜的选择。

因此，最佳答案是：
A. **子宫肌瘤切除术**
75、 (A2 型题)
男，48 岁，被木棍打击右颞枕部 12 小时，当时意识丧失，约 3 分钟后意识恢复，无恶心呕吐。查体：神志清，言语流利，双侧瞳孔等大等圆，对光反射灵敏，四肢肌力正 片：右侧枕颞部可见一双凸镜高密度影），最可能的诊断是
A.硬膜下积液
B.硬膜外血肿
C.硬膜下血肿
D.硬膜外脓肿
E.硬膜外积液
参考答案： B 您的答案：
图文解析： 【解析】患者头部外伤后意识丧失，约 3 分钟后意识恢复，脑 CT 检查：右侧枕颞部可见一双凸镜高密度影，即可确诊为硬膜外血肿。硬膜外血肿特点表现为颅骨内板与硬脑膜之间的双凸镜形或弓形高密度影。
【避错】硬膜下血肿（C 错），常有外伤史和原发神经系统损害的病史和体征。 一般表现为伤后即出现意识障碍并进行性加重、偏瘫、头痛、恶心、呕吐等症状。CT 见脑表面与颅骨之间有新月形高密度、混杂密度或等密度影。硬膜外脓肿（D 错）较少见，多有颅骨骨髓炎、中耳炎等慢性化脓性病灶存在病史。硬膜外积液和硬膜下积液 CT 表现应为低密度影（AE 错）。
这个病例描述的是一个 48 岁男性，在头部右侧颞枕部被木棍击中后出现的症状。病人在受伤后立即失去意识，大约 3 分钟后意识恢复，没有恶心和呕吐。体检显示患者意识清晰，言语流利，瞳孔对光反射正常，四肢肌力正常。影像学检查发现右侧枕颞部有高密度影，形状类似双凸镜。
根据描述，最可能的诊断是 B. 硬膜外血肿（epidural hematoma）。硬膜外血肿通常发生在头部外伤后，特别是颅骨骨折导致硬膜外动脉破裂时。典型的 CT 表现为镜面或双凸镜状的高密度影，这与题目中描述的影像学表现相符。另外，患者在伤后短时间内失去意识，然后意识恢复（所谓的“清醒间隙”），也是硬膜外血肿的典型临床特征。
解析：

患者右颞枕部外伤史，当时意识丧失，后意识恢复，提示脑震荡。查体示神志清，言语流利，双侧瞳孔等大等圆，对光反射灵敏，四肢肌力正常，说明患者未出现偏瘫及脑神经损伤表现。CT 片示右侧枕颞部可见一双凸镜高密度影，提示血肿存在。综合患者的病史、临床表现及影像学检查，最可能的诊断是右侧硬膜外血肿。
选项 A 硬膜下积液又称硬膜下水瘤，多为外伤后硬膜下腔出现的脑脊液积聚，CT 表现为新月形低密度影，与该患者 CT 影像不符，故不选。
选项 C 硬膜下血肿是位于硬脑膜和蛛网膜之间的血肿，多由外伤引起，好发于小脑幕上颞顶区，CT 表现为脑表面新月形的高密度、混杂密度或等密度影，该患者 CT 影像表现为双凸镜高密度影，与题意不符。
选项 D 硬膜外脓肿的 CT 表现为颅骨内板与硬脑膜之间的低密度影，增强扫描脓肿壁可以强化，壁呈等密度或稍高密度，脓肿中央为低密度，与该患者 CT 影像不符，故排除。
选项 E 硬膜外积液多为外伤后硬膜下腔出现的脑脊液积聚，CT 表现为新月形低密度影，与该患者 CT 影像不符，可排除。
综上所述，正确答案是：B.硬膜外血肿。
根据题目描述，患者右颞枕部受到木棍打击后出现短暂意识丧失并恢复，CT 片显示右侧枕颞部有一双凸镜高密度影。这些特征提示了硬膜外血肿的可能性。

硬膜外血肿的特点包括：
1. 头部外伤史。
2. 短暂意识丧失后恢复清醒（也称为“清醒期”）。
3. CT 影像上典型的双凸镜形状高密度影。

基于以上分析，最可能的诊断是：

B. 硬膜外血肿
76、　(A2 型题)
足月儿，曾宫内窘迫，羊水 3 度，产钳助产，生后肌张力下降，无自主呼吸，最佳处理
A.气管插管机械通气
B.胸外按压
C.气管插管并吸除胎粪
D.吸氧
E.保暖、摆好体位、清理呼吸道、诱发自主呼吸
参考答案：　C　您的答案：
图文解析：　【解析】该患儿曾宫内窘迫，羊水III度，且产钳助产，应考虑羊水吸入综合征的可能，对于羊水胎粪污染的患儿，应气管插管并吸除胎粪。
【避错】气管插管机械通气（A 错）主要用于出生后没有自主呼吸的患儿，而该患儿应先做通畅气道的处理。胸外按压（B 错）主要用于心跳呼吸停止的患儿。吸氧（D 错）仅为一般处理。保暖、摆好体位、清理呼吸道、诱发自主呼吸（E 错）：不是抢救羊水吸入综合征患儿的最佳方案。
在这个案例中，我们有一个足月儿，经历了宫内窘迫、羊水污染程度为 3 度，并通过产钳助产。生后，婴儿出现了肌张力下降和无自主呼吸的症状。考虑到这些情况，最佳处理应该旨在立即稳定婴儿的呼吸状态并处理任何可能阻碍呼吸的问题。
选项 A（气管插管机械通气）虽然对于需要立即呼吸支持的婴儿是重要的，但在没有首先尝试清理呼吸道的情况下直接进行可能不是最优选择。选项 B（胸外按压）主要用于心跳停止

的情况，并不适用于本案例。选项 C（气管插管并吸除胎粪）考虑到了需要清理呼吸道的重要性，尤其是当有羊水污染时。选项 D（吸氧）可以提供必要的氧气，但并不解决呼吸道阻塞的问题。选项 E（保暖、摆好体位、清理呼吸道、诱发自主呼吸）提供了一系列的步骤，旨在稳定婴儿的状态并促进自主呼吸的恢复。

鉴于这些信息，最佳选择应该是**C. 气管插管并吸除胎粪**，因为这既可以清理阻塞的呼吸道，也可以为婴儿提供必要的呼吸支持。这种方法综合考虑了婴儿的症状和背景情况，旨在解决无自主呼吸的紧急问题。

解析：

足月儿，宫内窘迫史，羊水III度污染，产钳助产史，生后肌张力下降，无自主呼吸，结合病史和临床表现，该患儿应诊断为新生儿窒息。复苏采用国际公认的 ABCDE 复苏方案：A（airway）：清理呼吸道；B（breathing）：建立呼吸；C（circulation）：建立正常循环；D（drugs）：药物治疗；E（evaluation）：评估。前三项最重要，其中 A 是根本，B 是关键，复苏的目的是建立呼吸功能，因此生后应首先进行的是 A（airway）步骤，即保暖、摆好体位、清理呼吸道、诱发自主呼吸。

选项 A 气管插管机械通气，选项 B 胸外按压，均属于 ABCDE 复苏方案中的 C 步骤，是在充分的正压通气 30 秒后心率仍＜60 次/分，且需要进行胸外按压时，才需要采用的处理措施，故不选。

选项 C 气管插管并吸除胎粪是在清理呼吸道时发现羊水胎粪污染且患儿无活力时才需要进行气管插管用胎粪吸引管吸引胎粪，该患儿虽存在宫内窘迫史，羊水 3 度污染，但题干未提及患儿无活力，故不考虑。

选项 D 吸氧是 ABCDE 复苏方案中的 B 步骤，应在清理呼吸道之后，诱发自主呼吸效果不佳或无效后进行，故不符合。

综上所述，正确答案是：E.保暖、摆好体位、清理呼吸道、诱发自主呼吸。

对于这个案例，最重要的是立即处理新生儿呼吸道的阻塞和缺氧问题。宫内窘迫、羊水三度污染、产钳助产等情况提示新生儿可能存在胎粪吸入综合征，因此清理呼吸道是优先考虑的措施。根据新生儿复苏指南，最合适的处理方式是：

C.气管插管并吸除胎粪

77、 (A2 型题)

男婴，5 个月，咳嗽 3 天，喘憋 1 天，查体：体温 37.8℃,P150 次/分，R66 次/分，精神可，鼻翼翕动，口唇轻度发绀，三凹征阳性，双肺呼吸相哮鸣音及少许湿啰音，实验 WBC4.0×109/L，N0.35，L0.65。最可能的诊断是

A.呼吸道合胞病毒性肺炎

B.金黄色葡萄球菌肺炎

C.肺炎链球菌肺炎

D.支原体肺炎

E.腺病毒肺炎

参考答案： A 您的答案：

图文解析： 【解析】患儿体温 37.8 度（低热），P150 次/分，R66 次/分，精神可，鼻翼翕动（轻度缺氧表现），口唇轻度发绀，三凹征阳性（严重缺氧表现），双肺呼吸相哮鸣音及少许湿啰音（符合呼吸道合胞病毒性肺炎的肺部体征），实验室检查血 WBC4.0×109/L（正常值 4~10×109/L），N0.35（中性粒细胞比例正常值 0.5~0.7），L0.65（淋巴细胞比例正常值 0.2~0.4），根据该患儿的临床表现和辅助检查，最可能的诊断是呼吸道合胞病毒性肺炎（A 对）。

【避错】金黄色葡萄球菌肺炎（B 错）起病急、病情严重，进展快，早期可出现循环衰竭；发热时间较长且温度较高；外周血白细胞计数明显升高，以中性粒细胞为主；痰液、脓尿和血培养金黄色葡萄球菌阳性。肺炎链球菌肺炎（C 错）多有上呼吸道感染的前驱症状，起病急骤、高热、寒战，全身肌肉酸痛，患侧胸部疼痛，患侧语颤增强等；可有患侧呼吸运动受限，叩诊浊音，听诊可有支气管呼吸音、湿啰音等体征。支原体肺炎（D 错）的症状主要是乏力、咽痛、头痛、发热、咳嗽、食欲不振、腹泻、肌痛、耳痛等，体格检查可见咽部充血，儿童偶可并发鼓膜炎或中耳炎，颈淋巴结肿大等。腺病毒肺炎（E 错）多见于 6 个月至 2 岁的儿童，中毒症状重，可有麻疹样皮疹；消化系统症状有恶心、呕吐、腹泻等；中枢神经系统症状包括精神萎靡、嗜睡、头痛、惊厥等。

这个男婴表现出的症状包括咳嗽、喘憋、发热、心率和呼吸频率增快、鼻翼翕动、口唇轻度发绀、三凹征阳性以及双肺有哮鸣音和少许湿啰音。实验室检查显示白细胞计数正常，但淋巴细胞比例增高。这些症状和体征提示婴儿可能患有急性病毒性下呼吸道感染。

选项 A 的呼吸道合胞病毒性肺炎（RSV）是婴幼儿中常见的急性呼吸道感染病因，特别是在冬春季节。它可以引起上述症状，尤其是在 5 个月大的婴儿中。RSV 感染通常导致气道炎症、哮鸣音和呼吸困难，与这个案例描述的症状相符。

选项 B 的金黄色葡萄球菌肺炎通常与更严重的细菌感染相关，可能出现较高的发热和白细胞计数增加。

选项 C 的肺炎链球菌肺炎是儿童肺炎的常见原因，但通常会导致更高的白细胞计数。

选项 D 的支原体肺炎更常见于年龄较大的儿童和青少年，且其症状往往比较温和。

选项 E 的腺病毒肺炎可以导致类似的症状，但不如 RSV 感染那么常见于这个年龄段。

综合考虑这些信息和临床表现，最可能的诊断是 A.呼吸道合胞病毒性肺炎。

解析：

患儿，咳嗽，喘憋，低热，查体出现鼻翼翕动，口唇轻度发绀等呼吸困难表现及缺氧症状，三凹征阳性，双肺可闻及哮鸣音及少许湿啰音，实验检查见白细胞正常，淋巴细胞升高，根据其临床表现及实验室检查，对于该患儿最可能的诊断为呼吸道合胞病毒性肺炎。该病常见于婴幼儿，尤以 1 岁以内小儿多见，临床上轻症患者发热、呼吸困难等症状不重，中、重症有较明显的呼吸困难、喘憋、口唇发绀、鼻翼翕动及三凹征，发热可为低、中度热和高热，肺部听诊多有中、细湿啰音。

选项 B 金黄色葡萄球菌肺炎起病急、病情严重、进展快，全身中毒症状明显，发热多呈弛张型，肺部体征出现较早，两肺有散在中、细湿啰音，可发生脓胸、脓气胸和肺大疱，与本题患儿症状不符。

选项 C 肺炎链球菌肺炎发病前常有上呼吸道感染史，咳嗽，咳铁锈色痰，肺部体征可有叩诊浊音、触觉语颤增强并可出现支气管呼吸音，消散期可闻及湿啰音，该患儿并无上述体征，故不选。

选项 D 支原体肺炎起病较缓慢，症状主要有乏力、咽痛、头痛、咳嗽、发热、食欲不振、腹泻、肌痛、耳痛等，肺部体征多不明显，与本题无关。

选项 E 腺病毒肺炎主要表现为患者急起发热，咳嗽，呼吸困难，喘憋，轻症一般 7～11 天体温恢复正常，其他症状也逐渐消失，重症患儿恢复期要 1～6 个月，与该患儿临床表现差异明显。

综上所述，正确答案是：A.呼吸道合胞病毒性肺炎。

根据患者的症状和检查结果，最可能的诊断是呼吸道合胞病毒性肺炎 (Respiratory Syncytial Virus, RSV) 。具体分析如下：

1. **年龄**：患者为 5 个月大的男婴，RSV 感染多见于 6 个月以下的婴儿。

2. **症状**：患者出现咳嗽 3 天，喘憋 1 天，体温 37.8℃，这些症状和 RSV 感染相符。喘憋是 RSV 感染的典型症状。
3. **体征**：体检发现鼻翼翕动，口唇轻度发绀，三凹征阳性，双肺呼吸相哮鸣音及少许湿啰音。这些都是 RSV 感染引起的细支气管炎的特征。
4. **实验室检查**：WBC 4.0×10^9/L，N 0.35，L 0.65。白细胞总数正常或稍低，淋巴细胞比例增加，这也符合 RSV 感染的特点。

相比之下：

- **金黄色葡萄球菌肺炎**和**肺炎链球菌肺炎**：常见于免疫力低下或有基础疾病的儿童，且通常会出现高热和明显的细菌感染特征（如白细胞增高、中性粒细胞比例高）。
- **支原体肺炎**：多见于学龄儿童和青少年，较少见于婴幼儿，且病程较长。
- **腺病毒肺炎**：虽然也可引起类似症状，但腺病毒感染通常会伴有较明显的结膜炎和消化道症状。

综合上述分析，最可能的诊断是 **A. 呼吸道合胞病毒性肺炎**。
78、 (A2 型题)
男，12 岁，头颅 CT 示脑室高密度，脑血管造影示双侧颈内动脉闭塞，颅底异常脑血管网形成，诊断
A.脑动脉瘤
B.脑动静脉畸形
C.脑血管病
D.脑静脉瘤
E.脑烟雾病
参考答案： E 您的答案：
图文解析： 【解析】患儿，颅 CT 示脑室高密度，提示颅内出血，脑血管造影示双侧颈内动脉闭塞，颅底异常脑血管网形成，考虑诊断为烟雾病。烟雾病是一种病因不明的、以双侧颈内动脉末端及大脑前、中动脉起始部动脉内膜缓慢增厚，动脉管腔狭窄以至闭塞为特征，并伴有脑底异常血管网形成的脑血管疾病，异常血管网的主要成分是增生微动脉，因其管壁薄、缺乏外弹力层、管腔小而呈烟雾状，异常血管网在脑血管造影时形似“烟雾”，故得名。【避错】脑动脉瘤（A 错）分为真性动脉瘤、假性动脉瘤和夹层动脉瘤。真性动脉瘤多见于颅内后循环动脉瘤，主要发生于基底动脉分叉处，其次为大脑后动脉及小脑后下动脉。假性动脉瘤：因颈内-后交通支动静脉畸形出血导致形成动-颈内交通动脉瘤。脑动静脉畸形（B 错）主要表现为蛛网膜下腔出血和颅内血肿等神经功能障碍症状。脑血管病（CVD）（C 错）是指由于各种脑血管病变所引起的脑部病变，主要指脑血管破裂出血或血栓形成，引起的以脑部出血性或缺血性损伤症状为主要临床表现的一组疾病。脑静脉瘤（D 错）又称静脉畸形或血管瘤，临床少见。
根据题目描述，12 岁男孩的头颅 CT 显示脑室高密度，脑血管造影显示双侧颈内动脉闭塞以及颅底异常脑血管网形成。这些症状提示了一种特定的血管异常。在给出的选项中，最符合这些临床表现的是 E.脑烟雾病（Moyamoya disease）。
脑烟雾病是一种罕见的血管疾病，其特征是颅内主要动脉的逐渐狭窄，导致血流减少，而周围形成许多细小的血管尝试补偿这种血流不足，这在影像上形成了类似“烟雾”的模糊影像。患者常表现为头痛、短暂性脑缺血发作（TIA）、中风以及神经功能障碍。
因此，对于这个案例，最合适的答案是 E.脑烟雾病。

首先，我们来仔细审视这个问题。题目描述了一个 12 岁的男孩，其头颅 CT 显示脑室高密度，脑血管造影则显示双侧颈内动脉闭塞以及颅底异常脑血管网的形成。基于这些信息，我们需要从提供的选项中选出最可能的诊断。

现在，我们逐个分析选项：

A. 脑动脉瘤：这通常指的是脑动脉壁的囊性膨出，虽然它也可能导致脑血管的异常，但通常不会表现为双侧颈内动脉闭塞和颅底异常脑血管网的形成。因此，这个选项与题目描述的症状不完全吻合。

B. 脑动静脉畸形：这是一种先天性局部脑血管发生学上的变异，但通常不会呈现为脑室高密度或双侧颈内动脉闭塞。所以，这个选项也不太可能是正确答案。

C. 脑血管病：这是一个非常宽泛的术语，涵盖了多种脑血管相关的疾病。然而，它没有具体指出是哪种类型的脑血管病，因此不够具体，不能准确对应题目描述的症状。

D. 脑静脉瘤：这指的是发生在静脉的血管瘤，但它通常不会导致脑室高密度或颈内动脉闭塞，因此这个选项也不太可能是正确答案。

E. 脑烟雾病：这是一种以双侧颈内动脉末端及大脑前动脉、大脑中动脉起始部动脉内膜缓慢增厚，动脉管腔逐渐狭窄以至闭塞，脑底穿通动脉代偿性扩张为特征的疾病。它通常表现为脑室高密度、双侧颈内动脉闭塞以及颅底异常脑血管网的形成，与题目描述的症状高度吻合。

综上所述，考虑到脑室高密度、双侧颈内动脉闭塞以及颅底异常脑血管网的形成等症状，最可能的诊断是脑烟雾病。

因此，正确答案是 E.脑烟雾病。

根据题干描述的男孩头颅 CT 示脑室高密度，脑血管造影示双侧颈内动脉闭塞，颅底异常脑血管网形成的情况，这些症状和影像学特征符合脑烟雾病的典型表现。脑烟雾病（Moyamoya 病）是一种慢性进展性脑血管病，主要特点是双侧颈内动脉闭塞，导致颅底形成异常的血管网。

因此，最正确的答案是：

E. 脑烟雾病

79、 (A2 型题)

患儿，2 岁，发热 39.4℃,3 小时候前突发抽搐一次，后精神良好。拟诊断为

A.病毒性脑炎

B.热性惊厥

C.化脓性脑炎

D.低钙性惊厥

E.中毒性脑病

参考答案： B 您的答案：

图文解析： 【解析】患儿，2 岁，发热 39.4℃（高热惊厥多数发生在 6 月至 3 岁婴幼儿，并且一般在上感体温的最初 24 小时内出现，且大多数在一次热程中仅发作一次），3 小时前突发抽搐一次（表现为全身或局部强直性或阵挛性抽搐），后精神良好（无神经系统异常表现），拟诊断为热性惊厥。

【避错】病毒性脑炎（A 错）会有颅内压增高现象，伴有脑膜刺激征。化脓性脑炎（C 错）会有颅内压增高现象，伴有脑膜刺激征。低钙性惊厥（D 错）多见于 1 岁以内的小儿，常由于维生素 D 缺乏导致血清钙减少，出现神经肌肉兴奋性增高。中毒性脑病（E 错）是以中枢神经系统障碍为主要临床表现的一种急性全身性病变，有原发病表现，如肺炎、菌痢等。

在这个病例中，2 岁的患儿出现发热和突发性抽搐，但之后精神状态良好。这种情况最符合的诊断是热性惊厥。热性惊厥是小儿常见的一种情况，通常发生在 6 个月至 5 岁的儿童中，与体温升高有关，但没有中枢神经系统感染或其他明确的原因。抽搐发生时，儿童通常会出现短暂的意识丧失和肢体抽动，抽搐通常在短时间内自行停止，之后孩子的精神状态会迅速恢复正常。
考虑到这些信息，最佳答案是：B.热性惊厥
解析：
患儿，2 岁，发热，3 小时前突发抽搐一次，后精神良好，最可能的诊断是热性惊厥。热性惊厥是小儿时期最常见的惊厥性疾病，首次发作年龄多于生后 6 个月至 3 岁之间，男孩稍多于女孩，绝大多数 5 岁后不再发作。患儿发热后出现抽搐，热退后精神良好，符合热性惊厥的表现。
选项 A 病毒性脑炎常表现为发热、反复惊厥发作、不同程度的意识障碍和颅内压增高症状，该患儿并未出现意识障碍等症状，故不选。
选项 C 化脓性脑炎是各种化脓性细菌引起的脑膜炎症，部分患者累及脑实质，是儿童，尤其是婴幼儿时期常见的中枢神经系统感染性疾病，临床上以急性发热、惊厥、意识障碍、颅内压增高和脑膜刺激征以及脑脊液脓性改变为特征，与该患儿的症状描述差异明显。
选项 D 低钙性惊厥的患儿多不发热，但有维生素 D 缺乏性佝偻病的症状和体征，该患儿并未见上述症状，故不考虑。
选项 E 中毒性脑病多见于 2～10 岁儿童，患儿有高热、严重头痛、呕吐、烦躁不安和谵妄乃至昏迷，常有惊厥发作，与题意不符。
综上所述，正确答案是：B.热性惊厥。
根据题目描述，患儿 2 岁，发热 39.4℃，3 小时前突发抽搐一次，之后精神良好。以下是各选项的分析：

A. **病毒性脑炎**：病毒性脑炎通常会伴随持续的精神症状，如意识改变、嗜睡、头痛等。题目中提到患儿抽搐后精神良好，不符合病毒性脑炎的特点。

B. **热性惊厥**：热性惊厥常见于 6 个月至 5 岁之间的儿童，多在体温突然升高时发生。惊厥后儿童通常恢复正常，精神状态良好。题目中的情况非常符合热性惊厥的典型特征。

C. **化脓性脑炎**：化脓性脑炎通常会伴有严重的全身症状，如高热、剧烈头痛、呕吐、意识障碍等。患儿抽搐后精神良好，不符合化脓性脑炎的表现。

D. **低钙性惊厥**：低钙性惊厥常见于新生儿和婴儿期，多伴有手足抽搐和其他低钙血症的症状。虽然发热也可能诱发惊厥，但题目中并未提到低钙血症的其他特征。

E. **中毒性脑病**：中毒性脑病通常会有明确的毒物接触史，并伴有神经系统症状，如意识障碍、反复抽搐等。患儿抽搐后精神良好，不符合中毒性脑病的表现。

综合以上分析，最符合题目中患儿情况的诊断是**热性惊厥**。因此，最佳答案是：

B. 热性惊厥
80、　(A2 型题)
男婴，3 月龄，儿童保健门诊体检，足月顺产儿，出生体重 3.2kg，身长 50cm，纯母乳喂养，

查体：体重 6kg，身长 63cm，无乳牙，关于小儿喂养，正确的是
A.继续纯母乳喂养
B.在家庭人的食物的基础，从泥状到碎状食物
C.可以添加米粉，奶瓶喂养
D.可以一次添加 2~3 种辅食
E.混合喂养，为将来停止哺乳做准备
参考答案： A 您的答案：
图文解析： 【解析】出生后前 3 个月体重每月增长 700~800g，前半年身高平均每月增长 2.5cm，小儿理论体重在 5.3kg~5.6kg/身高 57.5cm，该小儿身高、体重都正常，应继续纯母乳喂养。
【避错】1~3 个月：泥状食物，如水果汁、青菜汤、鱼肝油和钙剂。4`6 月婴儿可予泥状食物如：菜泥、水果泥、含铁配方米粉、配方奶（BCE 错）。小儿辅食添加原则为从少到多、从稀到稠、从细到粗、从一种到多种，每引入一种新的食品应适应 2～3 天（D 错）。
本题关于 3 个月大男婴的喂养方式。考虑到婴儿的年龄和目前的情况（足月顺产，体重和身长正常增长，纯母乳喂养），最适合的喂养方法是继续纯母乳喂养。世界卫生组织推荐独自母乳喂养至婴儿 6 个月大。因此，最正确的答案是：A. 继续纯母乳喂养
解析：
3 月龄男婴，足月顺产，纯母乳喂养，体检示发育正常。根据《中国居民膳食指南（2016）》建议，0～6 月龄婴儿应给予纯母乳喂养，满 6 月龄起添加辅食。该婴儿目前 3 个月，故应继续纯母乳喂养。
选项 B 在家庭人的食物的基础，从泥状到碎状食物是在婴儿 7～9 月龄时添加的辅食种类，该患儿目前 3 个月，与题意不符。
米粉为辅食，应在满 6 月龄后添加，故选项 C 可以添加米粉，奶瓶喂养，可排除。
添加辅食的原则为由少到多，由稀到稠，由细到粗，由一种到多种，并在婴儿健康、消化功能正常时添加。故选项 D 可以一次添加 2～3 种辅食，与添加辅食的原则不符，故不选。
对于 6 月龄内婴儿，母乳是其最好的食物，能满足其生长发育所需，故无需混合喂养，为将来停止哺乳做准备，因此选项 E 混合喂养，为将来停止哺乳做准备，可排除。
综上所述，正确答案是：A.继续纯母乳喂养。
对于一个 3 个月大的婴儿，喂养的最佳实践指南建议继续纯母乳喂养。世界卫生组织（WHO）和许多儿科协会推荐婴儿在前 6 个月内进行纯母乳喂养，因为母乳提供了婴儿所需的所有营养，并且有助于增强免疫系统，减少感染的风险。3 个月大的婴儿不需要添加辅食。

因此，最正确的答案是：

A.继续纯母乳喂养
81、 (A2 型题)
男，近年来记忆力渐差，把虚构的事件填补记忆，不认识家人，不辨方向。有时在深夜看到屋里有人影晃动。饮酒 20 年，每天 250ml。最可能的诊断是
A.血管性痴呆
B.震颤谵妄
C.阿尔兹海默病
D.酒精性痴呆
E.科萨科夫综合征
参考答案： E 您的答案：

图文解析：　　【解析】患者记忆力渐差，有虚构和错构（有时在深夜看到屋里有人影晃动），无意地编造经历与情节或远事近移以填补记忆的空白（把虚构的事件填补记忆），定向力障碍（不辨方向），结合长期饮酒史，诊断考虑科萨科夫综合征。故选择 E。
【避错】血管性痴呆有明确脑血管疾病史，不同的血管损伤临床表现不同，故 A 错。震颤谵妄主要见于饮酒者，停用酒精后出现的急性反应，常有不安、幻觉等精神症状，故 B 错。阿尔兹海默病隐匿发病，有记忆减退、定向障碍等症状，但常伴有日常生活不能自理等行为表现，严重影响生活质量，影像学表现可见脑皮质萎缩，故 C 错。需要和酒精性痴呆相鉴别，酒精性痴呆表现为逐渐加重的个性改变，行为失控，记忆力减退，患者无个性改变和行为失控，故 D 错。

这位男性患者的症状包括记忆力逐渐变差，填补记忆的虚构事件，不认识家人，方向感丧失，以及有时在深夜看到屋内有人影晃动。结合他长期大量饮酒的历史（每天 250ml，持续 20 年），最可能的诊断是酒精性痴呆（D）。

酒精性痴呆是长期大量饮酒导致的一种认知功能障碍，表现为记忆力减退、判断力和批判力丧失、人格变化等症状。这与该患者的表现相符。虽然阿尔茨海默病（C）和血管性痴呆（A）也可导致记忆力下降和认知功能障碍，但鉴于患者长期的饮酒史，酒精性痴呆是更可能的诊断。震颤谵妄（B）通常与急性混乱状态相关，而科萨科夫综合征（E）则是由维生素 B1 缺乏导致，主要表现为记忆障碍、共济失调和眼球运动异常。

因此，最合适的答案是 D.酒精性痴呆。

解析：

患者长期饮酒史，近年来记忆力渐差，出现虚构的事件填补记忆障碍，不认识家人，不辨方向，有时在深夜看到屋里有人影晃动，这是明显的近记忆力障碍、定向力障碍和幻觉，结合患者长期饮酒史，考虑患者为酒精性痴呆的可能性大。

选项 A 血管性痴呆指由脑血管病变导致的痴呆，该患者无脑血管病史，可排除。

选项 B 震颤谵妄为酒精戒断反应的一种，典型表现为意识模糊，分不清东南西北，不识亲人，不知时间，有大量的知觉异常，常见形象歪曲而鲜明的恐怖性幻视，如猛兽、毒蛇等，伴随情绪激动，大喊大叫，并有全身肌肉的粗大震颤，发热，大汗淋漓，心跳加快，血压升高，该患者无戒断病史，可排除。

选项 C 阿尔茨海默病是一种起病隐匿的进行性发展的神经系统退行性疾病，临床上以记忆障碍、失语、失用、失认、视空间技能损害、执行功能障碍以及人格和行为改变等全面性痴呆表现为特征，无长期饮酒史，与题干不符。

选项 E 科萨科夫综合征又称健忘综合症，为一种大脑缺乏硫胺（维生素 B1）而引起的精神障碍，主要临床表现包括近记忆严重丧失，时空定向力障碍，尤以近事遗忘、对时间是月、日等不能正确分辨，但即刻回忆保留，停酒后可能有部分恢复，伴随有明显的虚构，该患者无明显的即刻回忆能力，可排除。

综上所述，正确答案是：D.酒精性痴呆。

根据题目描述，这位男性患者具有以下特点：

1. 近年来记忆力渐差。
2. 填补记忆的虚构事件（可能是错构症）。
3. 不认识家人，不辨方向（严重的认知功能障碍）。
4. 深夜看到屋里有人影晃动（幻觉）。
5. 饮酒 20 年，每天 250ml。

综合以上信息，最有可能的诊断是 **E. 科萨科夫综合征**。

科萨科夫综合征（Korsakoff syndrome）是一种由于长期大量饮酒导致的神经系统损害，主要表现为严重的记忆障碍、虚构症（即用虚构的事件填补记忆缺失）、认知障碍以及幻觉。患者通常会经历长期的酒精滥用，导致维生素 B1（硫胺素）缺乏，进而损害脑部结构。

其他选项的分析：
- **A. 血管性痴呆**：通常与脑血管病史相关，表现为渐进性的认知功能下降，但幻觉较少见。
- **B. 震颤谵妄**：通常是急性酒精戒断综合征的一部分，表现为意识模糊、幻觉、震颤等急性症状，但不符合患者长期记忆力渐差的描述。
- **C. 阿尔兹海默病**：通常是逐渐进展的老年痴呆，常见于老年人，但无长期饮酒史的特征。
- **D. 酒精性痴呆**：与长期饮酒有关，但通常不包括虚构症和幻觉，较少见于科萨科夫综合征的特征。

综上所述，最可能的诊断是 **E. 科萨科夫综合征**。
82、　(A2 型题)
男孩，2 岁，纳差，消瘦 2 个月，伴发育倒退，查体:T36.5℃,P120 次/分，R26 次/分，贫血貌，颜面轻度水肿，表情呆滞，皮肤黏膜苍白，心肺未见异常，腹软，肝肋下 4
3cm，无颈抵抗，实验室检查，血 Hb88g/L,RBC1.9×10^12/L,外周血涂片示 RBC 大小不等，以大细胞为多。最可能的诊断是
A.再生障碍性贫血
B.维生素 B12 缺乏所致营养性巨幼细胞性贫血
C.缺铁性贫血
D.地中海贫血
E.叶酸缺乏所致营养性巨幼细胞性贫血
参考答案：　E　您的答案：
图文解析：　【解析】患者中度贫血（Hb60~90g/L），外周血涂片示 RBC 大小不等，以大细胞为多，提示巨幼细胞性贫血。巨幼细胞性贫血分为叶酸缺乏和维生素 B12 缺乏，最常见叶酸缺乏，两者症状区分是维生素 B12 缺乏可引起神经系统症状，如注意力不集中、癫痫样改变等，故 B 错，选择 E。
【避错】再生障碍性贫血临床常见贫血、出血、感染症状，血象三系减少（红细胞、白细胞、血小板），与患者不符，故 A 错。`缺铁性贫血为小细胞低色素性贫血，与患者血涂片不符，故 C 错。地中海贫血轻型常无明显表现，中间型可见中度贫血，重型出生数日即出现贫血、肝脾肿大进行性加重，黄疸，与患者不符，故 D 错。
这个病例描述了一个 2 岁男孩，表现为纳差、消瘦、发育倒退、轻度面部水肿、表情呆滞、皮肤黏膜苍白，体检未发现心肺异常，肝大，血常规检查显示贫血，红细胞(RBC)数量减少，且外周血涂片显示红细胞大小不等，以大细胞为多。这些症状和实验室检查结果指向巨幼细胞性贫血，这是一种红细胞异常大的贫血。
A. 再生障碍性贫血通常表现为骨髓生成功能减退，但需要更多的骨髓检查结果来确诊。
B. 维生素 B12 缺乏所致的营养性巨幼细胞性贫血可能出现在本病例中，因为这种类型的贫血会导致大型红细胞的出现。
C. 缺铁性贫血通常导致小细胞低色素性贫血，表现为小型和色素减少的红细胞，因此这个选项与病例描述不符。

D. 地中海贫血是一种遗传性疾病，导致红细胞生成障碍，但它通常不会导致大细胞的出现。
E. 叶酸缺乏也会导致营养性巨幼细胞性贫血，这与维生素 B12 缺乏有类似的血液学特征。
鉴于上述信息，最可能的诊断是维生素 B12 缺乏所致的营养性巨幼细胞性贫血（B 选项）或叶酸缺乏所致的营养性巨幼细胞性贫血（E 选项）。然而，通常需要更多的实验室测试（如血清维生素 B12 和叶酸水平测定）来区分这两者。考虑到病例中未提及饮食习惯或其他相关症状，可能需要更详细的病史来确定是哪一种。但是，如果只根据提供的信息，我会倾向于选择 B. 维生素 B12 缺乏所致营养性巨幼细胞性贫血，因为它比叶酸缺乏更常见于儿童。
解析：
男性患儿，纳差、消瘦，伴发育倒退。查体显示低热、心率稍快、呼吸正常，有贫血貌和颜面轻度水肿，表情呆滞，皮肤黏膜苍白，肝大，无颈抵抗。实验室检查提示血红蛋白和红细胞均低于正常值，且外周血涂片示红细胞大小不等，以大细胞为主，符合巨幼细胞性贫血的特征。巨幼细胞性贫血的特点是红细胞体积比较大，中性粒细胞核分叶过多，骨髓中的巨幼红细胞出现增生的现象，它又简称为巨幼贫。因缺乏维生素 B12 或（和）叶酸所致，且本例患儿有颜面轻度水肿，为维生素 B12 缺乏的特点，故考虑诊断为维生素 B12 缺乏所致营养性巨幼细胞性贫血。
选项 A 再生障碍性贫血通常表现为全血细胞减少，且多无肝脾肿大，与题干描述不符，故排除。
选项 C 缺铁性贫血为小细胞性贫血，与题干描述的大细胞性贫血不符，故不选。
选项 D 地中海贫血虽为大细胞性贫血，但多有阳性家族史，其特殊面容、生长发育迟缓和骨质改变等症状在幼儿期以后才会逐渐出现，与题干描述不符，故不考虑。
选项 E 叶酸缺乏所致营养性巨幼细胞性贫血虽也可能出现颜面水肿，但相对较少见，维生素 B12 缺乏则常见颜面水肿，故叶酸缺乏相比维生素 B12 缺乏，可能性更低，与题意不符。
综上所述，正确答案是：B.维生素 B12 缺乏所致营养性巨幼细胞性贫血。
根据题目中提供的病史和体检结果，2 岁男孩出现纳差、消瘦、发育倒退，伴有贫血貌、颜面轻度水肿、表情呆滞、皮肤黏膜苍白，实验室检查显示血红蛋白 88g/L，红细胞计数 1.9×10^12/L，外周血涂片显示红细胞大小不等，以大细胞为多。这些信息提示存在严重贫血且红细胞大小不等，多为大细胞。

综合分析，最可能的诊断是：

B. 维生素 B12 缺乏所致营养性巨幼细胞性贫血

理由如下：
1. 患儿贫血貌，颜面轻度水肿，表情呆滞，皮肤黏膜苍白，提示有慢性贫血。
2. 血红蛋白 88g/L 和红细胞计数 1.9×10^12/L，提示中重度贫血。
3. 外周血涂片显示红细胞大小不等，以大细胞为主，提示巨幼细胞性贫血。
4. 维生素 B12 缺乏常见于儿童发育中的营养不良或吸收障碍，而巨幼细胞性贫血是维生素 B12 或叶酸缺乏的典型表现。

其他选项分析：
- A. 再生障碍性贫血：通常表现为全血细胞减少，骨髓检查示造血细胞减少，不符合该患儿的临床表现和实验室检查。
- C. 缺铁性贫血：通常表现为小细胞低色素性贫血，红细胞大小均一，不符合该患儿的大细胞贫血特点。

- D. 地中海贫血：通常为小细胞性贫血，且有家族史及相关地区分布，不符合该患儿的大细胞贫血特点。
- E. 叶酸缺乏所致营养性巨幼细胞性贫血：与维生素 B12 缺乏导致的巨幼细胞性贫血临床表现非常相似，但考虑到年龄和营养状况，维生素 B12 缺乏更为常见。

因此，最可能的诊断是维生素 B12 缺乏所致营养性巨幼细胞性贫血。

83、 (A2 型题)

女，26 岁，妊娠 32 周，1 小时前突发腹痛，阴道少量流血，面苍白，脉搏弱，P120 次/分，BP180/110mmHg，子宫大于妊娠周期，硬如板状，胎心不清。可诊断为

A.先兆早产

B.先兆子宫破裂

C.羊水栓塞

D.胎盘早剥

E.前置胎盘

参考答案： D 您的答案：

图文解析： 【解析】患者孕晚期，突发腹痛，阴道少量流血，影响循环系统（面苍白，脉搏弱，P120 次/分，BP180/110mmHg），结合查体子宫大于妊娠周期，硬如板状，胎心不清提示胎盘早剥，需要防治 DIC、产后出血等并发症，并立即剖宫产，故选择 D。

【避错】先兆流产有不规则宫缩和阴道流血，胎膜完整，故 A 错。先兆子宫破裂一般出现在产程，有腹痛难忍、血尿、病理性缩复环、胎心听不清四大表现，与患者不符，故 B 错。羊水栓塞出现在生产过程中，有呼吸循环衰竭，DIC，多脏器损伤表现，与患者不符，故 C 错。前置胎盘为无诱因无痛性阴道出血，与患者腹痛、循环障碍不符，故 E 错。

这位患者的症状包括突发腹痛，阴道出血，面色苍白，脉搏快速弱，血压升高，子宫大于妊娠周期，硬如板状，胎心不清。这些症状指向的是严重的妊娠并发症。

A 选项“先兆早产”通常会有宫缩，子宫颈的改变，和可能的阴道出血，但不会导致血压升高或子宫硬如板状。

B 选项“先兆子宫破裂”可能会导致急性腹痛和血压下降，但不太可能导致血压升高。

C 选项“羊水栓塞”是妊娠期间罕见但严重的并发症，它会导致急性心肺功能衰竭和凝血功能障碍，但通常不会导致子宫硬如板状。

D 选项“胎盘早剥”是胎盘部分或全部从子宫壁早期剥离，会导致腹痛，阴道出血，子宫硬度增加，胎心异常，和可能的母体循环不稳定，符合这位患者的症状。

E 选项“前置胎盘”通常导致无痛性阴道出血，而不伴随急性腹痛或子宫硬化。

因此，最符合这位患者症状的诊断是 D.胎盘早剥。

首先，让我们仔细分析这个案例。患者是一名 26 岁的女性，妊娠 32 周，突发腹痛，阴道有少量流血，同时面色苍白、脉搏微弱，心率达到 120 次/分，血压显著升高至 180/110mmHg。子宫大于妊娠周期且硬如板状，胎心不清。接下来，我们根据题干和选项进行详细分析：

A. 先兆早产：虽然患者有腹痛和阴道流血的症状，但先兆早产通常不会伴随如此高的血压和板状腹的体征，故该选项不太可能。

B. 先兆子宫破裂：先兆子宫破裂通常表现为下腹剧痛、血尿和胎心率异常等，但一般不会出现板状腹和如此高的血压，因此这个选项也不太符合。

C. 羊水栓塞：羊水栓塞是一种严重的产科并发症，表现为突发的呼吸困难、低血压和出血性休克等。患者的症状虽然严重，但没有呼吸困难或休克的表现，因此这个选项也不太可能。

D. 胎盘早剥：患者的症状包括腹痛、阴道流血、面色苍白、脉搏微弱、高血压和子宫硬如板状，这些都是胎盘早剥的典型表现。特别是在妊娠晚期，高血压和突发的持续性腹痛是胎

盘早剥的重要指征。
E. 前置胎盘：前置胎盘主要表现为妊娠晚期或临产时，发生无诱因、无痛性反复阴道流血，腹部检查子宫软，无压痛，胎心率正常等，与患者的症状不符。
综合以上分析，患者的症状最符合胎盘早剥的诊断。因此，正确答案是 D.胎盘早剥。
根据题目描述，26 岁的孕妇在妊娠 32 周时，突发腹痛，阴道少量出血，面色苍白，脉搏弱（120 次/分），血压高（180/110 mmHg），子宫大于妊娠周期并且硬如板状，胎心不清。

这些症状提示了以下几个关键点：

1. 妊娠 32 周，突发腹痛和阴道出血。
2. 面色苍白，脉搏弱，血压升高，可能提示出血性休克。
3. 子宫大于妊娠周期并硬如板状。
4. 胎心不清。

结合这些症状和体征，最可能的诊断是胎盘早剥（选项 D）。胎盘早剥是指正常位置的胎盘在胎儿娩出前部分或完全从子宫壁剥离，这会导致突发的腹痛、阴道出血、子宫变硬以及胎儿窘迫。

因此，最佳答案是：

D. 胎盘早剥
84、 (A2 型题)
男，16 岁，反复左侧肢体抽搐 1 年，每次发作先由左手抽动，逐渐波及左上肢，并向左肩扩展，最后发展至左面部，每次发作持续 30 秒即可自行缓解。在发作时神志清醒， 痫，则最大可能性为哪种发作类型
A.继发全面性强直阵挛发作
B.肌阵挛发作
C.单纯感觉发作
D.复杂部分发作
E.jackson 发作
参考答案： E 您的答案：
图文解析： 【解析】男患者，16 岁，每次发作先由左手抽动，逐渐波及左上肢，并向左肩扩展，最后发展至左面部，符合 jackson 发作的典型表现（开始为身体某一部位抽搐，随后按一定顺序逐渐向其他部位扩展的一种癫痫发作）故选择 E。
【避错】继发全面性强直阵挛发作，发作时全身抽搐，意识丧失，呼之不应，与本题患者明显不符，故 A 错。肌阵挛发作为身体某个部位突然、快速、有力地抽动，无按顺序传导的特点，故 B 错。单纯部分感觉性发作（单纯体觉发作）常表现肢体麻木感和针刺感，有些特殊感觉性发作可表现视觉性（如闪光或黑蒙）、听觉性、嗅觉性、味觉性和眩晕性（如眩晕感、飘浮感、下沉感）等，患者有明显的癫痫状态和按顺序传导现象，故 C 错。复杂部分发作（精神运动性发作）由颞叶病变引起，起病年龄在 20 岁左右，临床主要表现为意识障碍，也可表现为精神症状和自动症，少数可发展为全身性发作，可有不同程度的记忆障碍，与该患者不符，故 D 错。
在这个病例中，16 岁男孩经历了反复的左侧肢体抽搐，发作从左手开始，逐渐扩展至左上肢、左肩，最后到左面部。发作每次持续大约 30 秒，并且患者在发作期间神志清醒。这种

症状表明抽搐局限于身体的一侧，并且逐步扩散到临近区域，这是 Jackson 发作的典型表现。Jackson 发作，也称为局部运动性癫痫发作，是一种局部起源的癫痫发作，通常影响大脑的运动区。患者在发作时保持意识清醒，且发作往往从身体的一个特定部位开始，逐渐扩展到临近部位。
因此，最符合这个描述的选项是：E. Jackson 发作
解析：
青少年男性患者，反复左侧肢体抽搐 1 年，每次发作先由左手抽动，逐渐波及左上肢，并向左肩扩展，最后发展至左面部，每次发作持续 30 秒即可自行缓解，在发作时神志清醒，根据症状、体征，对于该患者可诊断为 jackson 发作。jackson 发作又称局限性运动性癫痫发作，临床表现为抽搐自手指-腕部-前臂-肘-肩-口角-面部逐渐发展，称为 jackson 发作，是部分性发作中的一种特殊类型。
选项 A 继发全面性强直阵挛发作主要表现为意识丧失、双侧强直后紧跟有阵挛的序列活动，与本题患者症状不符，故排除。
选项 B 肌阵挛发作主要表现为某个肌肉或肌群突然、快速、有力的收缩，引起肢体、躯干或面部快速抽动，与本题患者症状不符，故不选。
选项 C 单纯感觉发作主要表现为身体某一局部发生不自主抽动，或一侧肢体麻木感和针刺感，多发生在口角、舌、手指或足趾，与本题患者症状不符，故不考虑。
选项 D 复杂部分发作主要表现为意识障碍和自动症，与本题患者症状不符，可排除。
综上所述，正确答案是：E.jackson 发作。
根据题目描述，该男孩的症状表现为左侧肢体抽搐，开始于左手，然后逐渐扩展到左上肢、左肩，最终发展到左面部，每次发作持续 30 秒，发作时神志清醒。这些特征符合 Jackson 发作的典型表现。

Jackson 发作（Jacksonian seizure）是一种部分性癫痫发作，从一个身体部位开始，逐渐扩展到同侧身体的其他部位，同时患者在发作过程中通常保持清醒。

因此，最可能的发作类型是：

E. Jackson 发作
85、 (A2 型题)
新生儿，20 天，胎龄 42 周，出生体重 4300g，出生后腹胀，便秘，喂养困难，查体：刺激后反应差，面部和巩膜黄染，心肺未见异常，腹部膨隆，脐疝，四肢肌张力低下， 凉，血常规：Hb122g/L,白细胞 7×109/L，N0.3L0.6，TSH50mU/L，血培养阴性，最可能的诊断是
A.先天性甲状腺功能减退症
B.新生儿缺血缺氧性脑病
C.新生儿溶血病
D.先天性巨结肠
E.新生儿败血症
参考答案： A 您的答案：
图文解析： 【解析】新生儿，有胃肠道麻痹症状（出生后腹胀，便秘，喂养困难），刺激后反应差，腹部膨隆，脐疝，四肢肌张力低下，四肢末梢稍凉，结合 TSH 偏高（正常 3mU/L~12mU/L）、淋巴细胞高（L0.6），诊断考虑先天性甲状腺功能减退症，故选择 A。
【避错】新生儿缺血缺氧性脑病一般有明显宫内窘迫史或产时窒息史，故 A 错。新生儿溶血可有面部和巩膜黄染，生理性黄疸一般仅有消化系统症状，无其他症状；病理性黄疸可

有反应差、厌食、精神差症状，但黄疸症状一般较重，也不符合，故 C 错。先天性巨结肠多有便秘腹胀，发育迟缓，食欲下降， 一般无肌张力下降、反应差、末梢循环差的表现，故 D 错。患者血培养阴性，排除新生儿败血症，故 E 错。

考虑到新生儿的症状，包括出生后腹胀、便秘、喂养困难、面部和巩膜黄染、腹部膨隆、脐疝、四肢肌张力低下，以及检查结果显示 TSH 升高（50mU/L），这些症状和体征指向了甲状腺功能异常。TSH 升高通常表明甲状腺功能减退，因为垂体分泌更多的 TSH 来刺激甲状腺产生甲状腺激素。

结合这些信息，最可能的诊断是：

A. 先天性甲状腺功能减退症

这种情况下，甲状腺功能不足会导致新生儿的代谢减慢，从而出现喂养困难、肌张力低下、黄疸等症状。高 TSH 水平是甲状腺功能减退的典型标志，尤其是在新生儿中。因此，选项 A（先天性甲状腺功能减退症）是最合适的答案。

解析：

新生儿，过期产儿，巨大儿，出生后腹胀，便秘，查体见喂养困难，反应差，面部和巩膜黄染，心肺未见异常，腹部膨隆，脐疝，四肢肌张力低下，皮温凉，结合患者症状，体征，辅助检查结果，对于该患儿最可能的诊断为先天性甲状腺功能减退症。

选项 B 新生儿缺血缺氧性脑病是指在围产期窒息而导致患儿脑的缺氧缺血性损害，临床出现的一系列脑病表现。 这种病不仅严重威胁着新生儿的生命，并且是新生儿期后病残致病中之一，与题干中患者表现不符，故排除。

选项 C 新生儿溶血病是因母子血型不合引起的同族免疫性溶血，使胎儿在母体内发生溶血性贫血，常有水肿，贫血，肝脾肿大等表现，严重者可致死胎，核黄疸，或留有后遗症，题干中未提及血型不合，故不选。

选项 D 先天性巨结肠是由于直肠或结肠远端的肠管持续痉挛，粪便淤滞在近端结肠，使该肠管肥厚，扩张，是小儿常见的先天性肠道畸形，主要表现有胎便排出延迟，顽固性便秘腹胀，与题干不符，故不考虑。

选项 E 新生儿败血症是指新生儿期致病菌经各种途径侵入新生儿血循环，并在其中生长繁殖，产生毒素而造成全身性的感染，主要临床表现有反应差，嗜睡，发热或体温不升，少吃，少哭，少动，体重不增或增长缓慢等症状，与题干不符，故不选。

综上所述，正确答案是：A.先天性甲状腺功能减退症。

根据题干信息，新生儿有以下症状和体征：

- 出生体重 4300g，属于巨大儿。
- 出生后腹胀，便秘，喂养困难。
- 体检发现反应差，面部和巩膜黄染，腹部膨隆，脐疝，四肢肌张力低下，四肢凉。
- 血常规显示 Hb122g/L，白细胞 7×10^9/L，N0.3 L0.6。
- TSH（促甲状腺激素）显著升高，达到 50mU/L。
- 血培养阴性。

这些临床特征指向以下几个重要考虑：

1. **先天性甲状腺功能减退症（A 选项）**：高 TSH、面部黄疸、腹胀、便秘、喂养困难、四肢肌张力低下都是典型表现。先天性甲状腺功能减退症是一种由于甲状腺激素缺乏导致的新生儿代谢紊乱，常见症状包括生长迟缓、精神反应差和便秘等。TSH 显著升高是此病的一个重要诊断指标。

2. **新生儿缺血缺氧性脑病（B 选项）**：主要表现为缺氧后的神经系统症状，但腹胀、便秘等消化系统症状较少见，且 TSH 异常不常见。

3. **新生儿溶血病（C 选项）**：通常表现为黄疸和贫血，血常规中白细胞数目正常且 TSH 不受影响。

4. **先天性巨结肠（D 选项）**：主要表现为便秘和腹胀，但一般不会出现 TSH 显著升高的情况。

5. **新生儿败血症（E 选项）**：可以表现为反应差、腹胀、便秘，但一般伴随全身感染症状如发热或体温不稳定，且血培养通常阳性。

综上所述，结合显著升高的 TSH 和新生儿的临床表现，最可能的诊断是**A. 先天性甲状腺功能减退症**。

86、　(A2 型题)

初产妇，30 岁，孕前体检发现房间隔缺损，现妊娠 26 周，无心慌气短，超声心动图发现房间隔缺损 0.6cm2，EF68%。为发现早期心力衰竭，每周孕检一次，开始的孕周应

A.28 周

B.30 周

C.32 周

D.34 周

E.36 周

参考答案：　C　您的答案：

图文解析：　【解析】患者房间隔缺损，超声心动图发现房间隔缺损 0.6cm2，EF68%，心功能尚在正常范围内，应该从 32 周开始每周一次孕检了解有无心脏负担情况。故选择 C。【避错】孕 32 周时候胎儿明显增大，心脏负担增加，心脏疾患的孕妇应每周孕检一次，了解心脏负担情况，当 EF 值低于 50%提示心脏泵血功能受损，需要及时干预。

对于一位孕前被发现有房间隔缺损的初产妇，定期孕检的目的是为了监测可能出现的心力衰竭等并发症。房间隔缺损（ASD）可能导致左向右分流，随着妊娠进展，心脏的负担增加，所以需要密切监测。本病例中，患者目前没有心慌气短等症状，超声心动图显示房间隔缺损面积为 0.6cm²，射血分数（EF）68%，这意味着她的心脏功能目前保持良好。

通常，孕晚期（第三孕期）是心脏负担增加最显著的时期，这段时间应增加监测频率。考虑到心脏病患者的监测通常应在妊娠中期至晚期开始更加频繁，通常建议从妊娠第 28 周或更早开始进行更密切的监测。

因此，根据上述讨论，我认为最合适的答案是 A.28 周。这样可以确保如果出现早期心力衰竭或其他并发症，可以及时发现并进行干预。

解析：

青年初产妇，孕前已诊断为房间隔缺损，现妊娠 26 周，为发现早期心力衰竭征象，应从妊娠 28 周起，每周检查一次。

选项 B：30 周，选项 C：32 周，选项 E：36 周，开始检查时间均较晚，不能及时发现早期心力衰竭，故一并排除。

选项 D：34 周开始每周孕检主要用于妊娠合并心脏病孕妇评估心功能，而该产妇已明确有房间隔缺损，为及时发现心衰应提早检查，与题意不符。

综上所述，正确答案是：A.28 周。

对于这位 30 岁的初产妇，她在孕前体检时发现有房间隔缺损（ASD），目前妊娠 26 周且无心慌气短的症状，超声心动图显示房间隔缺损为 $0.6cm^2$，射血分数（EF）为 68%。为了早期发现心力衰竭，最佳的每周孕检开始时间应综合考虑其心脏负担和孕期变化。

在这种情况下，推荐在妊娠晚期，即妊娠 28 周左右开始更为频繁的监测，以便及时发现和处理可能的心脏并发症。因此，答案是：

A. 28 周

87、 (A2 型题)

女性，外阴群集鱼子样小丘疹，醋酸白试验阴性，拟诊断为

A.尖锐湿疣

B.假性湿疣

C.皮脂腺异位

D.扁平湿疣

E.生殖器鲍温样丘疹病

参考答案： B 您的答案：

图文解析： 【解析】患者外阴群集鱼子样小丘疹，醋酸白试验阴性，是女性假性湿疣典型表现，故选择 B。

【避错】尖锐湿疣损害初期为细小淡红色丘疹，以后逐渐增大增多，呈乳头样、鸡冠状或菜花样突起，根部常有蒂，且易发生糜烂渗液，触之易出血，皮损裂缝间常有脓性分泌物郁积，致有恶臭，可出现异物感、痛、痒感或性交痛，容易鉴别，故 A 错。皮脂腺异位病变特征为无明显隆起皮肤的粟粒大小扁平丘疹状损害，群集分布，多呈淡黄色或少数为淡白色，直径 1～3mm，故 C 错。扁平湿疣损害为集蔟性红色柔软、扁平的丘疹，可融合成斑片状，无蒂，高低不平，触之坚实，比较平滑），传染性较大，临床上有时压紧的尖锐湿疣团块类似扁平湿疣，用棉签将其拨开即可见明显分开的乳头状疣体，仔细观察即可区别，故 D 错。阴部鲍温样丘疹病呈棕红色到紫色，有少许鳞屑，严重者融合成斑片状，常与尖锐湿疣共存，病变范围小，无溃疡、结痂，不浸润皮下组织，故 E 错。

题目描述的是一名女性患者，外阴出现群集的鱼子样小丘疹，醋酸白试验阴性。根据这些信息，我们可以逐一排除一些选项：

A. 尖锐湿疣：尖锐湿疣通常会在醋酸白试验中呈现白色，因此在醋酸白试验阴性的情况下，尖锐湿疣的可能性较低。

B. 假性湿疣（也称摩擦性角化病）：这是一种常见的非感染性皮肤病，可以呈现为外阴的鱼子样丘疹，通常醋酸白试验为阴性。

C. 皮脂腺异位：这通常指的是皮脂腺在非典型位置的存在，且表现为较小的结节，不太符合鱼子样小丘疹的描述。

D. 扁平湿疣：这种类型的湿疣通常较小、边界清晰，但醋酸白试验结果可能呈现阳性或变化。

E. 生殖器鲍温样丘疹病：这是一种较为罕见的状况，通常呈现为红色或皮肤色的丘疹或斑块，与题干描述的鱼子样丘疹不太吻合。

综上所述，最可能的选择是 B.假性湿疣，因为它的临床表现与题干描述的情况最为接近，而且醋酸白试验通常为阴性。

解析：

女性患者，外阴群集鱼子样小丘疹，醋酸白试验阴性，为假性湿疣的典型表现，因此可诊断

为假性湿疣。假性湿疣是一种发生于女性阴部的颗粒状突起性淡红色丘疹，有时伴轻度瘙痒，病因不明，一般认为是一种与发育异常有关的良性乳头瘤。
选项 A 尖锐湿疣醋酸白试验阳性，故不选。
选项 C 皮脂腺异位症多在青春期后发生，中年人较多见，男性多于女性。该病是由于皮脂腺发育的生理性变型和皮脂腺增生所致。经临床观察，本病不仅仅只发生于口腔黏膜，而且还多见于男性的包皮和龟头、女性的大小阴唇等部位。该患者并无上述表现，故排除。
选项 D 扁平湿疣是二期梅毒的一种表现，好发于外阴、肛周、乳房下等易摩擦浸渍部位，湿性丘疹形如扁豆，表面湿烂，有少量渗液，含大量梅毒螺旋体，传染性强，与本例患者的表现不符，故不选。
选项 E 生殖器鲍温样丘疹病与 HPV 感染有关，典型皮损为多个或单个色素性丘疹，肤色、红褐色，形状不一，大小不等，可呈圆形、椭圆形或不规则形，表面可光亮呈天鹅绒外观，或轻度角化呈疣状，皮损散在分布或群集排列成线状、带状或环状，甚至可融合成斑块状，不符合题意。
综上所述，正确答案是：B.假性湿疣。
根据题干描述，女性外阴出现群集鱼子样小丘疹，且醋酸白试验阴性。我们逐一分析各个选项：

A. 尖锐湿疣：由人乳头瘤病毒（HPV）感染引起，典型特征为菜花状或乳头状的赘生物，醋酸白试验通常为阳性。

B. 假性湿疣：也称为 Vulvar Papillomatosis，通常表现为小的白色或肉色丘疹，醋酸白试验阴性。

C. 皮脂腺异位：皮脂腺异位通常表现为小的黄色或白色丘疹，通常不呈现鱼子样外观。

D. 扁平湿疣：也是由 HPV 引起，通常表现为扁平的丘疹或斑块，醋酸白试验通常为阳性。

E. 生殖器鲍温样丘疹病：这是一种罕见的良性皮肤病，表现为多发的小丘疹，醋酸白试验通常为阴性。

结合题干描述的“鱼子样小丘疹”和“醋酸白试验阴性”，最符合描述的是“假性湿疣”。

因此，最佳答案是：

B. 假性湿疣
88、 (A2 型题)
女，16 岁，进食困难 1 年。过分关注体重，认为自己胖，拼命节食，多吃就催吐，偶有贪食。过去的一年体重从 55kg 下降至 35kg，闭经，自觉精神良好。身高 165cm，无明征。甲状腺功能：FT3：4.16pg/mol，FT4：14.69pg/mol，TSH：14.63IU/ml。可能的诊断是
A.抑郁障碍
B.广泛性焦虑障碍
C.神经性厌食
D.强迫障碍
E.甲状腺功能减退症

参考答案：　　C　您的答案：
图文解析：　　【解析】患者女性，16 岁，过分关注体重,为防止体重增加，拼命节食，多吃就催吐,导致体重下降明显低于正常范围（过去的一年体重从 55kg 下降至 35kg），甚至导致闭经，是神经性厌食的典型表现，故选择 C。
【避错】患者食欲正常，也无抑郁相关症状，故 A 错。患者无焦虑、不安等相关焦虑症状，故 B 错。患者并无过分要求秩序严格和完美、无过分仔细和重复、无过度注意细节而拖延等强迫表现，故 D 错。患者虽有闭经，但甲状腺功能未见特殊异常，症状也不符合甲状腺功能减退症典型表现（表情淡漠、黏液性水肿、反应迟钝、心动过缓、嗜睡、精神差），故 E 错。
这位 16 岁女性患者的主要症状包括进食困难、对体重的过分关注、自我感觉过胖、采取极端节食措施、催吐行为和偶尔的贪食，以及显著的体重下降和闭经。这些临床表现与神经性厌食症的特征相符。神经性厌食症患者常因极度恐惧体重增加和对体型的扭曲感知而进行严格的饮食控制和体重减轻行为。
在提供的甲状腺功能测试中，FT3 和 FT4 水平可能处于正常范围，但 TSH 水平增高，这可以在神经性厌食症患者中观察到，因为他们的身体可能会调节甲状腺功能以减缓新陈代谢率。虽然这种改变也可能与甲状腺功能减退症相似，但根据临床症状，甲状腺功能减退症不太可能是主要问题。
因此，最可能的诊断是 **C. 神经性厌食**。
首先，让我们仔细分析题目中的关键信息来形成清晰的诊断思路。
患者为 16 岁女性，出现进食困难的症状已经持续 1 年。她过分关注自己的体重，认为自己胖，因此拼命节食，并且在吃多了之后会催吐。值得注意的是，患者过去一年体重从 55kg 急剧下降至 35kg，同时出现了闭经的情况。尽管体重显著下降，但患者自觉精神状况良好。此外，甲状腺功能检查结果 FT3 和 FT4 正常，而 TSH 升高。
现在，我们来分析各个选项：
A. 抑郁障碍：虽然厌食和抑郁都是心理疾病，但题目中并未提及患者有明显的情绪低落、兴趣丧失等抑郁症状，因此这个选项可能不是最直接的诊断。
B. 广泛性焦虑障碍：通常表现为持续的、全面的、无明确对象的焦虑，以及自主神经活动亢进等表现。题目中并未提及患者有明显的焦虑症状，因此这个诊断可能不准确。
C. 神经性厌食：患者表现出对体重的过度关注，拼命节食，甚至在吃多了之后会催吐，体重急剧下降，这些都是神经性厌食的典型症状。同时，尽管体重显著下降，患者却自觉精神状况良好，这也符合神经性厌食的心理特征。
D. 强迫障碍：通常表现为反复出现一些想法或行为，明知不必要或不合理，但无法控制。题目中并未提及患者有明显的强迫症状，因此这个诊断可能不准确。
E. 甲状腺功能减退症：虽然患者的 TSH 升高，但 FT3 和 FT4 正常，这与典型的甲状腺功能减退症的甲状腺功能检查结果不符。此外，甲状腺功能减退症通常伴随明显的低代谢症候群，如畏寒、乏力、手足肿胀、嗜睡、记忆力减退等，这些在题目中并未提及。
综合以上分析，患者表现出对体重的过度关注、拼命节食、贪食与催吐行为，以及体重的急剧下降，这些都是神经性厌食的典型症状。同时，尽管体重显著下降，患者却自觉精神状况良好，这也符合神经性厌食的心理特征。
因此，结合患者的症状、体征和实验室检查，最可能的诊断是神经性厌食。所以，正确答案是 C.神经性厌食。
根据题干信息，该患者为 16 岁女性，症状包括进食困难、过分关注体重、拼命节食、催吐、偶尔贪食以及体重显著下降。这些症状符合神经性厌食（Anorexia Nervosa）的表现。此外，患者体重从 55kg 下降至 35kg，导致闭经，并且她自觉精神良好，这进一步支持神经性厌食

的诊断。

甲状腺功能检查结果显示：
- FT3：4.16 pg/mol（正常范围 3.1-6.8 pg/mol）
- FT4：14.69 pg/mol（正常范围 12-22 pmol/L）
- TSH：14.63 IU/ml（正常范围 0.27-4.2 mIU/L）

患者的 TSH 显著升高，而 FT3 和 FT4 在正常范围内，但结合其体重下降和饮食行为异常，更可能是继发于神经性厌食的甲状腺功能异常，而不是原发性甲状腺功能减退症。因此，甲状腺功能减退症（E 选项）不是主要诊断。

综上所述，最可能的诊断是：

C. 神经性厌食
89、 (A2 型题)
患儿，1 岁，活动时喜下蹲及被大人抱，体检发现口唇发绀。下列哪项说法不正确
A.右心房扩大
B.右心室扩大
C.左心室扩大
D.房间隔缺损
E.支气管动脉和肺血管侧支循环建立
参考答案： D 您的答案：
图文解析： 【解析】患儿活动时喜下蹲及被大人抱，体检口唇发绀，诊断考虑法洛四联症。法洛四联症可有右心房增大（选项 A）、右心室扩大（选项 B）、左心室扩大（选项 C），随着病情发展可以出现发绀缓解，因为支气管动脉与肺血管之间形成侧支循环减少了右向左分流（选项 E）。此题选择说法不正确的，故选择 D。
【避错】房间隔缺损的症状随缺损大小而有区别，小型缺损，左向右分流量少者可长期无症状，小儿活动量正常。大型缺损患儿左向右分流量大，症状发生较早，表现为活动后气急、乏力等，生长发育多落后于同龄正常小儿，并易患呼吸道感染、肺炎等内科疾病，很少发生发绀，故说法不正确的是房间隔缺损，此题选择 D。
这道题目描述的是一个 1 岁大的患儿，在活动时会下蹲或喜欢被大人抱，并且体检发现口唇发绀。这些症状提示可能存在先天性心脏病，特别是紫绀型心脏病，如法洛四联症（Tetralogy of Fallot）。法洛四联症包括四个特征：右心室肥大（右心室扩大）、肺动脉瓣狭窄、房间隔缺损和位于室间隔缺损上方的主动脉。患儿活动时下蹲可以增加下肢的血管阻力，有助于增加向肺部的血流量，减少右至左的分流，从而改善缺氧状态。
- A 项“右心房扩大”：虽然法洛四联症中典型的是右心室扩大，但右心房也可能因为负荷增加而扩大。
- B 项“右心室扩大”：这是法洛四联症的一个特征，因此这个陈述是正确的。
- C 项“左心室扩大”：在法洛四联症中，主要是右心室扩大，而左心室通常不会扩大，所以这个选项可能是不正确的。
- D 项“房间隔缺损”：实际上应该是室间隔缺损，不是房间隔缺损，但在紫绀型心脏病中室间隔缺损是常见的。
- E 项“支气管动脉和肺血管侧支循环建立”：在某些紫绀型心脏病，如法洛四联症，可能建立这种侧支循环以改善血氧。

综合考虑，最不正确的说法是 C 项“左心室扩大”，因为在法洛四联症等紫绀型先天性心脏病中，主要是右心室扩大，而不是左心室扩大。
解析：
1 岁患儿，活动时喜下蹲及被大人抱，体检发现口唇发绀，应考虑为法洛四联症。法洛四联症是一种常见的先天性心脏畸形。其基本病理为室间隔缺损、肺动脉狭窄、主动脉骑跨和右心室肥厚。在法洛四联症中，右心室流出道梗阻致右心室压力增高，右心室肥大顺应性降低后，为了维持心排出量，右心室收缩功能代偿性增强，右心室心肌增生，乳头肌及腱索增粗，室上嵴亦增厚，心室可呈局限性或普遍性增厚。左心室位于右心室的左侧，右心室肥厚可使左室相对性缩小，舒张功能受限，心排出量减少。因此左心室不会扩大，反而会相对性缩小。由于右心室流出道梗阻，使右心室压力增高，右心室扩大，进而导致右心房压力增高，右心房代偿性增大，因此选项 A 右心房扩大，选项 B 右心室扩大，均符合法洛四联症的特点。
选项 D 房间隔缺损在法洛四联症中并不常见，但由于法洛四联症是四种畸形同时存在的联合病症，其中包括室间隔缺损，故本题中，房间隔缺损的表述并不完全错误。
选项 E 支气管动脉和肺血管侧支循环建立是机体的一种代偿性反应，当肺动脉主干发生梗阻时，机体为保证肺部血供，会代偿性地形成侧支循环，以保证肺部血液供应。
综上所述，正确答案是：C.左心室扩大。
题目描述了一名 1 岁患儿，活动时喜欢下蹲及被大人抱，体检发现口唇发绀。这提示可能存在心脏方面的疾病，尤其是先天性心脏病。我们需要从给定的选项中选择一个不正确的答案。

选项分析：

A. **右心房扩大**：许多先天性心脏病，例如房间隔缺损或肺动脉高压，会导致右心房扩大。

B. **右心室扩大**：右心室扩大常见于先天性心脏病，尤其是右心流出道梗阻或肺动脉高压等情况。

C. **左心室扩大**：一般情况下，导致口唇发绀的先天性心脏病很少会单独导致左心室扩大。通常，左心室扩大与左心流出道梗阻或大动脉血流量增多有关。

D. **房间隔缺损**：房间隔缺损是常见的先天性心脏病，可能会导致口唇发绀。

E. **支气管动脉和肺血管侧支循环建立**：这种情况一般出现在复杂先天性心脏病中，用于代偿肺血流不足或肺动脉闭锁等情况。

分析患儿表现出的症状及体征，**左心室扩大**通常不与这类心脏病直接相关，尤其是导致发绀的先天性心脏病中，左心室扩大相对不常见。因此，最不正确的选项应是：

C. 左心室扩大
90、 (A2 型题)
男，3 岁，多汗乏力 1 年余，有 2 次肺炎病史，查体：胸骨左缘 2~3 肋间闻及 3/6 级收缩期杂音，肺动脉瓣区第二心音固定分裂，该患儿胸部 X 线心影形态最可能是
A.烧瓶状
B.靴状

C.梨状
D.球状
E.普大型
参考答案：　　C　　您的答案：
图文解析：　　【解析】患儿乏力症状和肺炎病史，结合查体胸骨左缘 2~3 肋间闻及 3/6 级收缩期杂音，肺动脉瓣区第二心音固定分裂，是房间隔缺损杂音典型表现，房间隔缺损胸部 X 线心影最有可能的形态是梨状。梨状心又称二尖瓣型心，多见于二尖瓣病变、房间隔缺损、肺动脉瓣窄、肺动脉高压和肺心病等。故选择 C。
【避错】烧瓶状心是心包积液的特征性表现，故 A 错。靴状心常见于主动脉关闭不全，也可见于主动脉瓣狭窄、高血压性心脏病、法洛四联症，故 B 错。球形心多见于二尖瓣关闭不全、应激性心肌病、心包积液等，故 D 错。普大型心脏常见于心肌炎，全心衰，故 E 错。
这个考题描述了一个 3 岁男孩，有多汗乏力超过一年和两次肺炎病史，查体发现胸骨左缘 2~3 肋间有 3/6 级收缩期杂音，肺动脉瓣区第二心音固定分裂。这些症状和体征提示可能的心脏问题。
根据描述的症状和体征，尤其是收缩期杂音和固定分裂的第二心音，这可能指向先天性心脏病，比如房间隔缺损或室间隔缺损等。心脏 X 线表现通常与特定的心脏病有关：
- A. 烧瓶状：常见于心包积液。
- B. 靴状：与法洛四联症相关，特点是右心室肥大。
- C. 梨状：不是特定心脏疾病的典型表现。
- D. 球状：通常与心房扩大有关，如二尖瓣病变。
- E. 普大型：不特指某种心脏形态改变。
结合症状和典型心脏病的 X 线表现，最符合的描述可能是“靴状”心，因为这是法洛四联症的特征，而该症状群可引起肺动脉瓣区的异常听诊发现和心脏形态改变。然而，病史和体征也可能与其他类型的先天性心脏病相符，但是根据题目给出的选项和信息，选择“靴状”（B）可能是最合适的答案。
解析：
患儿，3 岁男性，多汗乏力 1 年余，有 2 次肺炎病史，胸骨左缘 2~3 肋间闻及 3/6 级收缩期杂音，肺动脉瓣区第二心音固定分裂，符合房间隔缺损的典型听诊特点。房间隔缺损会导致右心室、右心房增大，在胸部 X 线上，轻至中度增大的心脏呈现为“梨形心”，即梨状。
选项 A 烧瓶状心多见于心包积液，其 X 线表现为心影向两侧扩大，下大上小类似烧瓶，与题意不符。
选项 B 靴状心是指左心室增大时胸片上表现为心尖圆隆下移，可见于主动脉瓣狭窄，也可见于主动脉瓣关闭不全，亦称为“主动脉型心”，不符合题意。
选项 D 球状心是二尖瓣关闭不全的 X 线征象，心影呈球形扩张，双侧心室增大圆隆，心胸比率正常或增加，可排除。
选项 E 普大型是指左、右心室增大时，胸片上心影呈烧瓶状，可见于扩张型心肌病，与题干描述不符。
综上所述，正确答案是：C.梨状。
根据题目描述，3 岁男孩有多汗乏力 1 年余和 2 次肺炎病史。查体显示胸骨左缘 2~3 肋间闻及 3/6 级收缩期杂音，肺动脉瓣区第二心音固定分裂。这些症状提示该患儿可能患有房间隔缺损（ASD）。

房间隔缺损的典型体征包括：
- 肺动脉瓣区第二心音固定分裂

- 收缩期杂音

在房间隔缺损的病例中，胸部 X 线片常见的心影形态是“普大型”，因为心脏增大且肺血流增加。

因此，最可能的答案是：

E. 普大型

91、 (A2 型题)

男婴，4 个月，烦躁多汗，冬季出生，足月顺产，母乳喂养，请问可能出现下列哪项情况

A.手足镯

B.颅骨软化

C.方颅

D.鸡胸

E.X、O 型腿

参考答案： B 您的答案：

图文解析： 【解析】男婴，4 个月，烦躁多汗，冬季出生，足月顺产，母乳喂养。考虑患者为维生素 D 缺乏导致的婴幼儿佝偻病。佝偻病为 3 岁以下小儿的常见病，由于维生素 D 缺乏，使体内的钙磷不能正常吸收与利用，从而影响骨骼的生长发育，严重者可导致骨骼畸形。主要症状包括神经症状（易激怒烦躁，夜间哭闹），运动发育迟缓，出牙迟，骨骼改变（颅骨软化、方颅、串珠肋等）。因为婴儿年龄为 4 个月，故应选择（B）颅骨软化。

【避错】题干中的手足镯、颅骨软化、方颅、鸡胸 、X 或 O 型腿皆为佝偻病症状。但颅骨软化主要发生于 3~6 个月患儿，方颅则出现在 8~9 月患儿身上，鸡胸多出现于 6 个月后。

这个题目描述了一个 4 个月大的男婴，烦躁多汗，出生于冬季，足月顺产，母乳喂养。考虑到年龄、症状和喂养方式，我们需要考虑可能与营养不良相关的情况，特别是与钙和维生素 D 的摄入不足相关的情况。

A. 手足镯 - 常见于佝偻病，是因为钙或维生素 D 缺乏导致的骨骼问题。

B. 颅骨软化 - 也是佝偻病的一个特征，由于钙或维生素 D 缺乏导致颅骨变软。

C. 方颅 - 通常是指头骨形状异常，不特定于佝偻病。

D. 鸡胸 - 通常与佝偻病相关，但更多见于稍大的儿童。

E. X、O 型腿 - 也与佝偻病有关，但通常在儿童更大时出现。

考虑到婴儿的年龄和症状，以及冬季出生可能导致的阳光暴露不足（维生素 D 的一个主要来源），最可能的情况是钙和维生素 D 的摄入不足，这可能导致佝偻病。在这种情况下，手足镯和颅骨软化是最可能出现的临床表现，特别是在这个年龄的婴儿中。因此，虽然手足镯（A）和颅骨软化（B）都与佝偻病有关，颅骨软化是比较典型的早期临床表现，所以最合适的答案可能是 B.颅骨软化。

首先，我们需要仔细分析这个问题。问题涉及到一个 4 个月大的男婴，主要症状是烦躁和多汗，且提到是在冬季出生，足月顺产，母乳喂养。这些症状和情况提示我们考虑维生素 D 缺乏性佝偻病的可能性。维生素 D 缺乏性佝偻病在婴儿期较为常见，特别是对于那些日照不足、维生素 D 摄入不足的婴儿。

现在，我们来逐个分析选项：

A. 手足镯：这是维生素 D 缺乏性佝偻病在较大儿童中可能出现的骨骼畸形，但在这个 4 个月大的婴儿中不太可能出现。

B. 颅骨软化：这是维生素 D 缺乏性佝偻病的早期症状，特别是在 3-6 个月的婴儿中常见。

这与题干中描述的症状和婴儿的年龄相符。
C. 方颅：这是维生素 D 缺乏性佝偻病在 7-8 个月大婴儿中常见的症状，对于这个 4 个月大的婴儿来说，出现的可能性较小。
D. 鸡胸：这是佝偻病激期的一种表现，通常出现在 1 岁左右的小儿中，对于 4 个月大的婴儿来说不太可能出现。
E. X、O 型腿：这也是佝偻病的一种骨骼畸形表现，但通常在 1 岁左右出现，不适用于这个 4 个月大的婴儿。
综上所述，考虑到这个婴儿的年龄和症状，最有可能的情况是颅骨软化，这是维生素 D 缺乏性佝偻病在 3-6 个月大婴儿中的典型早期表现。
因此，正确答案是 B.颅骨软化。
根据题干描述，男婴 4 个月，烦躁多汗，冬季出生，足月顺产，母乳喂养，这些症状和情况提示可能是佝偻病（维生素 D 缺乏性佝偻病）。

佝偻病常见于婴幼儿，尤其是在冬季出生的婴儿，由于阳光照射不足，导致维生素 D 缺乏，从而影响钙、磷代谢，进而引起骨骼发育异常。题目中的症状（烦躁多汗）也符合佝偻病的临床表现。

在佝偻病的不同阶段，会出现不同的体征和骨骼变化。4 个月的婴儿在早期可能会出现以下几种典型表现中的一种：

B. 颅骨软化

颅骨软化是早期佝偻病的典型表现，尤其是后脑勺部位，可以出现颅骨软化症状，触摸时感觉柔软。这是由于钙化不完全所致。

其他选项如手足镯、方颅、鸡胸、X、O 型腿等，虽然也是佝偻病的表现，但通常出现在疾病的后期阶段或年龄更大的儿童中。

因此，最有可能的答案是：

B. 颅骨软化
92、 (A2 型题)
男，35 岁，右侧耳后疼痛后，伴口角歪斜，查体：右侧鼻唇沟变浅，右侧额纹消失，Bell 征（+），下列可能的诊断是
A.脑血管病
B.蛛网膜下腔出血
C.面神经炎
D.吉兰-巴雷综合征
E.脑脊膜炎
参考答案： C 您的答案：
图文解析： 【解析】Bell 征又称贝尔氏麻痹或兔眼征，指在面神经麻痹时让患者进行闭眼动作，而患侧眼睑无法完全闭合，露出白色巩膜。Bell 征常见于面神经炎、周围神经疾病导致的周围性面瘫。且患者出现口角歪斜，体格检查见右侧鼻唇沟变浅，右侧额纹消失的面瘫症状，因此考虑患者可能诊断为面神经炎（故 C 正确）。

【避错】脑血管病患者多出现神志问题；蛛网膜下腔出血多出现突然的喷射性呕吐，头痛；吉兰-巴雷综合征表现为急性对称性弛缓性肢体瘫痪；脑脊膜炎会出现颈项强直等脑膜刺激征。

35 岁男性患者出现右侧耳后疼痛，伴有口角歪斜，查体发现右侧鼻唇沟变浅，右侧额纹消失，Bell 征阳性。这些症状和体征指向面部神经受损，特别是面神经。面神经炎，也称为贝尔麻痹，是一种导致面部肌肉暂时性弱化或麻痹的病症，其特点是快速发展的单侧面部肌肉无力或瘫痪。

考虑到这些临床表现，最可能的诊断是 C.面神经炎。其他选项如脑血管病、蛛网膜下腔出血、吉兰-巴雷综合征和脑脊膜炎，虽然可以影响神经功能，但它们的临床表现通常不限于面神经损伤，并且会有其他系统的症状和体征。因此，根据提供的症状和体征，C.面神经炎是最合适的诊断。

解析：

青年男性患者，右侧耳后疼痛后出现口角歪斜，查体示右侧鼻唇沟变浅，右侧额纹消失，Bell 征（+），为面神经麻痹的典型表现，故考虑诊断为面神经炎。

选项 A 脑血管病常见于中老年患者，多有高血压、动脉粥样硬化、糖尿病等脑血管病的危险因素，起病急骤，进展迅速，常有头痛、呕吐等颅内压增高的表现，一般无意识障碍，故可排除。

选项 B 蛛网膜下腔出血起病急骤，一般有明显诱因，如剧烈运动、过度疲劳等，主要表现为头痛、脑膜刺激征和眼部症状，严重时可出现意识障碍，与题干描述不符。

选项 D 吉兰-巴雷综合征主要表现为迟缓性瘫痪，远端重于近端，可有周围性感觉障碍和脑神经损害，与题意不符。

选项 E 脑脊膜炎主要表现为感染症状、脑膜刺激征、颅内压增高、脑实质损害、癫痫发作、瘫痪及颅神经损害症状，不符合题干描述。

综上所述，正确答案是：C.面神经炎。

根据题目中提供的信息，该患者表现出右侧耳后疼痛伴口角歪斜，右侧鼻唇沟变浅，右侧额纹消失，Bell 征（+）。这些症状提示面神经受到影响，最可能的诊断是面神经炎。

面神经炎（Bell's palsy）是一种常见的导致面部肌肉瘫痪的疾病，通常是单侧的。主要症状包括：

- 面部肌肉无力或瘫痪
- 一侧面部表情肌不能正常活动
- 耳后疼痛（有时）
- Bell 征（+）：即在试图闭眼时眼球向上转动，眼睑不能完全闭合。

其他选项的简要解释：

- **脑血管病**：通常伴有其他神经系统体征，可能会涉及多条神经。
- **蛛网膜下腔出血**：表现为剧烈的突发头痛、恶心、呕吐、颈部僵硬等，较少表现为单侧面神经症状。
- **吉兰-巴雷综合征**：通常表现为四肢对称性无力，始于下肢并向上蔓延，不仅仅影响面神经。
- **脑脊膜炎**：通常有发热、头痛、颈部僵硬等全身症状，不局限于单侧面神经。

因此，最可能的诊断是：

C. 面神经炎

93、 (A2 型题)
出生 4 周，妊娠 42 周男婴，出生时烦躁声嘶，喂食情况不好，脐周膨隆，肌张力弱，可能的诊断是
A.缺血缺氧性脑病
B.先天性巨结肠
C.脑出血
D.脑梗死
E.脓毒血症
参考答案： A 您的答案：
图文解析： 【解析】新生儿缺血缺氧性脑病主要是围生期新生儿因缺氧引起的脑部病变，症状多表现为出生前胎心减慢胎动减少，意识障碍，嗜睡迟钝，肌张力改变，伴有频繁的惊厥发作。本题患者出生时烦躁声嘶，喂食情况不好（精神状态差），脐周膨隆，肌张力弱（肌张力减弱的体现）。故考虑诊断为新生儿缺血缺氧性脑病。
【避错】先天性巨结肠患者多表现出腹胀、排泄障碍；脑出血和脑梗死起病急骤，同时应伴有神志改变；脓毒血症应伴有高热发烧的症状。
这个案例描述了一个出生 4 周、妊娠 42 周的男婴，出生时表现为烦躁声嘶、喂食不好、脐周膨隆和肌张力弱。根据这些症状，最可能的诊断是先天性巨结肠（B）。先天性巨结肠（Hirschsprung disease）是一种影响大肠的疾病，导致肠道无法正常推进粪便，从而引起腹胀和喂食困难。脐周膨隆可能是由于肠道扩张引起的，而肌张力弱可能是全身状况不良的体现。因此，选项 B（先天性巨结肠）是这个情景下最合理的诊断。
解析：
患儿，过期产儿，出生时即出现烦躁不安、声嘶，脐周膨隆，肌张力低，结合患儿症状及体征，应诊断为先天性巨结肠。先天性巨结肠是由于直肠或结肠远端的肠管持续痉挛，粪便淤滞在近段结肠，使该肠管肥厚、扩张，是小儿常见的先天性肠道畸形。
选项 A 缺血缺氧性脑病多见于足月儿，常有宫内窘迫史或生后窒息史，该患儿未见窒息史，故不选。
选项 C 脑出血，选项 D 脑梗死，多见于老年人，多有高血压、动脉粥样硬化等基础疾病，新生儿少见，可一并排除。
选项 E 脓毒血症是指化脓性细菌侵入血流后，在其中大量繁殖，并通过血流扩散至宿主体的其他组织或器官，产生新的化脓性病灶，患儿无感染病史，且无化脓性病灶，故排除。
综上所述，正确答案是：B.先天性巨结肠。
根据题目描述，这是一名出生 4 周，妊娠 42 周的男婴，出生时表现为烦躁声嘶，喂食情况不好，脐周膨隆，肌张力弱。这些症状提示我们要考虑以下几个可能的诊断：

- **缺血缺氧性脑病**：通常会有出生窒息史，并伴有意识障碍、抽搐等神经系统症状。
- **先天性巨结肠**：通常表现为新生儿便秘、腹胀，但不太会出现肌张力弱的情况。
- **脑出血**：常见于早产儿，表现为昏迷、惊厥、反应低下等。
- **脑梗死**：新生儿少见，通常伴有偏瘫等局灶性神经症状。
- **脓毒血症**：新生儿脓毒血症可以表现为烦躁、喂食困难、腹胀、肌张力低下等全身症状。

综合考虑，脐周膨隆、肌张力弱、喂食困难及烦躁声嘶这些症状，更符合脓毒血症的表现。因此，最可能的诊断是：

E. 脓毒血症
94、 (A2 型题)
新生儿，15 天，腹胀，3 天未排便。查体：体重 3.2kg，精神欠佳，腹部见静脉曲张，肠鸣音亢进。为明确诊断首选的检查是
A.直肠肛门镜
B.直肠指检
C.直肠肛门肌活检
D.X 线钡剂灌肠
E.立位 X 线平片
参考答案： E 您的答案：
图文解析： 【解析】该新生儿出现便秘腹胀症状，且体格检查见肠鸣音亢进，考虑患者为新生儿急腹症，是否出现新生儿巨结肠可能，为求进一步详细诊断，选择进行腹部平片检查，若平片显示表现为结肠积气、扩张则进行 X 线钡剂灌肠确诊，因此本题选择 E。
【避错】新生儿年纪较小，耐受性较差，应先选择伤害较小的腹部平片检查进行筛选。
这个病例描述了一个 15 天大的新生儿，出现了腹胀和 3 天未排便的症状，体检时发现体重低，精神状态不佳，腹部有静脉曲张和肠鸣音亢进。这些症状和体征可能与先天性肠道问题有关，如先天性巨结肠。
首选的检查方法应该是既能提供解剖结构信息，又能显示肠道功能状态的检查。考虑到患儿年龄和症状，X 线钡剂灌肠（选项 D）是一个合适的选择。这种检查可以显示肠道的形态和功能状态，帮助诊断如巨结肠症等先天性肠道疾病。直肠肛门镜（A）和直肠肛门肌活检（C）虽然也可以提供有用信息，但对于诊断结构性和功能性的问题不如钡剂灌肠全面。直肠指检（B）是一种基本的检查方法，可以发现直肠内的异常，但对于深入的诊断帮助有限。立位 X 线平片（E）可以显示肠道气体分布，但对于确诊具体的结构和功能性疾病也不如钡剂灌肠。
因此，最佳答案是 D.X 线钡剂灌肠。
解析：
新生儿，腹胀，3 天未排便，应高度怀疑肠梗阻或肠套叠等疾病，腹部见静脉曲张提示门脉高压，查体肠鸣音亢进提示肠梗阻的可能性大。肠梗阻时，梗阻近端肠管内会积聚大量气体和液体，X 线下会显示出气液平面，立位 X 线平片可以明确梗阻的部位、程度及可能的性质，是诊断肠梗阻最简单、有效的方法。
选项 A 直肠肛门镜操作复杂，对于肠梗阻的诊断价值不大，故排除。
选项 B 直肠指检对于肠梗阻的诊断没有帮助，故不选。
选项 C 直肠肛门肌活检为有创检查，对于肠梗阻诊断帮助不大，且易加重患儿的痛苦，可排除。
选项 D：X 线钡剂灌肠多用于检查结肠部位的病变，且钡剂灌肠有导致肠梗阻加重的风险，因此肠梗阻患者禁用，故不考虑。
综上所述，正确答案是：E.立位 X 线平片。
根据所提供的新生儿症状（腹胀、未排便、腹部静脉曲张、肠鸣音亢进）和查体结果，最可能的诊断是肠梗阻或先天性巨结肠。为了明确诊断，首选的检查应该是能够显示肠道梗阻部位及其性质的影像学检查。

A. 直肠肛门镜：适用于直肠肛门区病变的检查，但对明确诊断肠梗阻或先天性巨结肠不是首选。

B. 直肠指检：虽然简单，但对明确上消化道或肠道梗阻不是首选。

C. 直肠肛门肌活检：用于确诊先天性巨结肠（Hirschsprung 病），但一般在其他检查（如 X 线钡剂灌肠）提示有问题后才进行。

D. X 线钡剂灌肠：这是明确诊断肠梗阻或先天性巨结肠的首选检查，能够显示肠道的形态和功能变化，是本题的最佳答案。

E. 立位 X 线平片：可用于检测气腹等，但对明确诊断肠梗阻的具体情况不如钡剂灌肠详细。

因此，最佳答案是：

D. X 线钡剂灌肠

95、 (A2 型题)

男，68 岁，吃饭时突感左侧肢体无力，伴视物重影，既往高血压病史。查体：神志清楚，右侧眼球外斜外展，右侧瞳孔散大，双侧额纹正常，左侧鼻唇沟浅，左侧肢体肌 巴宾斯基征阳性，该患者表现符合

A.基底动脉尖综合征

B.大脑脚综合征

C.闭锁综合征

D.霍纳征

E.延髓腹外侧综合征

参考答案： B 您的答案：

图文解析： 【解析】该患者男，68 岁，吃饭时突感左侧肢体无力。查体见神志清楚，右侧眼球外斜外展，右侧瞳孔散大（动眼神经麻痹表现），双侧额纹正常，左侧鼻唇沟浅，左侧肢体肌力 1 级，左侧巴宾斯基征阳性（交叉性偏瘫表现）。大脑脚综合征又称 Weber 综合征是一种神经系统疾病，以交叉性偏瘫和动眼神经麻痹为特征。该患者左侧肢体发病，且出现交叉性偏瘫及动眼神经麻痹，因此该患者应诊断为大脑脚综合征。

【避错】闭塞后导致眼球运动障碍及瞳孔异常、觉醒和行为障碍，可伴有记忆力丧失，对侧偏盲或皮质盲称为基底动脉尖综合征；闭锁综合征对侧痛温觉障碍（脊髓丘脑侧束），与同侧面部感觉障碍构成交叉性感觉障碍；霍纳综合征主要的四个临床表现为：眼裂变小、眼球内陷、瞳孔缩小、面部无汗（四个表现均为病灶同侧）；延髓腹外侧综合征表现为眩晕、恶心、呕吐及吞咽困难及病灶侧共济失调也可出现霍纳综合征。

本题描述的是一个 68 岁男性患者，出现吃饭时左侧肢体无力、视物重影，既往有高血压病史。体检发现右侧眼球外斜外展、右侧瞳孔散大、左侧鼻唇沟浅、左侧肢体肌力减弱及巴宾斯基征阳性。这些症状和体征提示中枢神经系统受累。

A. 基底动脉尖综合征：通常表现为脑干、小脑及部分颅神经功能受损。此案例中的眼球外斜和瞳孔散大可能与颅神经功能障碍有关，但没有提到小脑症状，因此不完全符合。

B. 大脑脚综合征：通常影响大脑脚附近结构，导致对侧偏瘫、脑神经受累等症状。该患者表现出的双侧症状不典型于大脑脚综合征。

C. 闭锁综合征：主要表现为四肢瘫痪、保存意识和眼球运动。此病例中患者有一侧肢体无力，并非全身瘫痪，因此可能不是闭锁综合征。

D. 霍纳综合征：主要表现为瞳孔缩小、眼睑下垂和面部出汗减少。病例中描述的是瞳孔散大，不符合霍纳综合征。

E. 延髓腹外侧综合征（Wallenberg 综合征）：通常由延髓背外侧供血障碍引起，表现为交感神经受损（如 Horner 综合征）、小脑症状、面部感觉障碍等。患者的症状中包括颅神经损害和肢体无力，与延髓受损的表现相符。
综合考虑，患者的症状最符合的是延髓腹外侧综合征（Wallenberg 综合征）。因此，最合适的答案是 E。
首先，我们分析题干：

老年男性患者，既往有高血压病史，吃饭时突然出现左侧肢体无力和视物重影的症状。查体显示神志清楚，右侧眼球外斜外展，右侧瞳孔散大，双侧额纹正常，左侧鼻唇沟浅，左侧肢体肌力异常，巴宾斯基征阳性。
接下来，我们逐项分析选项：
A. 基底动脉尖综合征：这是一种由基底动脉尖部位的血液循环障碍引发的综合征，主要表现为眼球运动障碍、瞳孔异常、觉醒和行为异常，伴有记忆力丧失、对侧偏瘫和偏盲等。该患者的症状（左侧肢体无力、视物重影、右侧眼球外斜外展、右侧瞳孔散大）与此综合征的描述相符。
B. 大脑脚综合征：主要表现为偏瘫，但不会出现眼球外斜和瞳孔散大的症状，与题干描述不符。
C. 闭锁综合征：这是一种严重的脑干损伤，患者虽然意识清楚，但全身瘫痪，不能讲话和吞咽，只能通过瞬目和眼球垂直运动来表达意愿，与题干描述的症状不符。
D. 霍纳征：主要表现为瞳孔缩小、眼球内陷、眼裂变小等，与题干描述的症状不符。
E. 延髓腹外侧综合征：主要表现为眩晕、恶心、呕吐、眼球震颤、同侧肢体共济失调、同侧霍纳征、交叉性感觉障碍等，与题干描述的症状不符。
综上所述，该患者的症状最符合基底动脉尖综合征。
因此，正确答案是 A.基底动脉尖综合征。
根据题干提供的信息，68 岁男性患者在吃饭时突然感到左侧肢体无力，并伴有视物重影。既往有高血压病史。查体发现右侧眼球外斜外展，右侧瞳孔散大，双侧额纹正常，左侧鼻唇沟浅，左侧肢体肌张力低下，巴宾斯基征阳性。

分析各个选项：
- **A. 基底动脉尖综合征**：主要表现为意识改变、双眼凝视麻痹和多发性颅神经麻痹，常伴有四肢瘫痪或偏瘫。
- **B. 大脑脚综合征（Weber 综合征）**：主要表现为中脑病变，通常表现为对侧肢体偏瘫及病灶同侧的动眼神经麻痹。
- **C. 闭锁综合征**：主要表现为四肢瘫痪和失语，但患者意识清楚，可以通过眼球活动进行有限的交流。
- **D. 霍纳征**：表现为同侧眼睑下垂、瞳孔缩小和无汗症，通常由交感神经损伤引起。
- **E. 延髓腹外侧综合征（Wallenberg 综合征）**：表现为病灶同侧的面部痛觉、温觉丧失和对侧的肢体痛觉、温觉丧失，同时伴有小脑症状、吞咽困难、声音嘶哑等。

根据患者的症状：左侧肢体无力，右侧眼球外斜外展，右侧瞳孔散大等特征，符合大脑脚综合征的表现。大脑脚综合征又称为 Weber 综合征，主要由中脑病变引起，表现为病灶对侧的肢体偏瘫及病灶同侧的动眼神经麻痹。

所以，最可能的诊断是：

B. 大脑脚综合征
96、 (A2 型题)
女，40 岁，G4P1，接触性出血，肝肾功能无异常，卵巢功能正常，诊断为
A.子宫内膜癌
B.子宫肌瘤
C.子宫颈炎
D.妊娠
E.葡萄胎
参考答案： C 您的答案：
图文解析： 【解析】子宫颈炎的主要症状包括异常白带分泌物及性交或检查时摩擦出血，即接触性血。本题患者为 40 岁女性，肝肾及卵巢功能均无异常（排除卵巢功能障碍和其他器官障碍），且出现典型接触性出血症状，因此本题患者诊断应为子宫颈炎。
【避错】子宫内膜癌多发生于绝经期妇女；子宫肌瘤表现为阴道不规则出血，与是否接触无关；葡萄胎早期症状同妊娠相似，因此两者可以排除
在这个案例中，40 岁的女性，G4P1（即孕四产一），出现接触性出血，而肝肾功能和卵巢功能均正常。接触性出血通常是指性交、体检或置入宫内器械后发生的出血，这是子宫颈病变的常见症状，尤其是子宫颈炎或子宫颈癌。然而，选项中并没有直接提到子宫颈癌。
A. 子宫内膜癌通常发生在绝经后妇女，主要症状为异常阴道出血。
B. 子宫肌瘤可能导致月经过多或不规则出血，但一般不表现为接触性出血。
C. 子宫颈炎是导致接触性出血的常见原因，尤其是在生育年龄的妇女中。
D. 妊娠通常不会导致接触性出血，除非存在异常情况，如宫外孕或先兆流产。
E. 葡萄胎是一种异常妊娠形式，主要表现为异常阴道出血，但不特定于接触性出血。
根据这些信息，最可能的诊断是**C.子宫颈炎**，因为这是引起接触性出血的常见原因，并且患者的其他器官功能正常。
首先，让我们仔细分析题干并指出关键信息。患者为 40 岁女性，已有四次怀孕经历（G4），并成功分娩一次（P1）。她出现了接触性出血的症状，同时肝肾功能正常，卵巢功能也正常。基于这些信息，我们可以逐项分析选项：
A. 子宫内膜癌：此癌症通常表现为异常子宫出血，但题干中未提及子宫出血，且接触性出血不是子宫内膜癌的典型表现。因此，这个选项可能不是最佳答案。
B. 子宫肌瘤：虽然子宫肌瘤是女性生殖系统中最常见的良性肿瘤，但它通常不引起接触性出血。子宫肌瘤的症状可能包括月经改变、腹部包块、白带增多、腹痛、腰酸、下腹坠胀等，与题干描述不符。
C. 子宫颈炎：这是育龄妇女的常见病，有急性和慢性两种。主要表现为白带增多，呈黏稠的黏液或脓性黏液，有时可伴有血丝或夹有血丝。此外，宫颈炎可能导致接触性出血，这与题干中的描述相符。
D. 妊娠：虽然妊娠是一个可能的选项，但题干中并未提及与妊娠相关的任何症状，如停经、早孕反应等。且接触性出血不是妊娠的典型表现。
E. 葡萄胎：葡萄胎因妊娠后胎盘绒毛滋养细胞增生、间质水肿，而形成大小不一的水泡，水泡间借蒂相连成串，形如葡萄而名之。虽然葡萄胎可能导致阴道出血，但通常不会表现为接触性出血，且题干中未提及葡萄胎的其他典型症状，如停经后阴道流血、子宫异常增大等。
综上所述，考虑到患者接触性出血的症状以及卵巢功能正常等条件，最符合的诊断是子宫颈炎。
因此，正确答案是 C.子宫颈炎。
根据提供的病例描述，这名 40 岁的女性有接触性出血，并且没有肝肾功能异常，卵巢功能

正常。

以下是对选项的逐一分析：

A. **子宫内膜癌**：子宫内膜癌的典型症状包括异常子宫出血，但主要是绝经后妇女常见的疾病，而且不一定是接触性出血。

B. **子宫肌瘤**：子宫肌瘤也可能导致异常出血，但不典型地表现为接触性出血。更常见的症状包括月经过多、月经不规则、腹痛等。

C. **子宫颈炎**：子宫颈炎是子宫颈的炎症，可能导致接触性出血。这是一种比较常见的导致接触性出血的原因。

D. **妊娠**：妊娠的症状包括停经、晨吐等，而接触性出血并不是典型症状。妊娠引起的出血一般是着床出血或其他异常妊娠情况。

E. **葡萄胎**：葡萄胎是妊娠的异常形式，主要表现为停经、阴道出血、妊娠期子宫异常增大等，但接触性出血并不是典型的症状。

综合分析，这名女性 40 岁，有接触性出血，其他功能无异常，最可能的诊断是**子宫颈炎**。

因此，最佳答案是：

C. 子宫颈炎
97、 (A2 型题)
女，31 岁，自然分娩后 1 天，诉下腹部阵发性疼痛。查体：T37.8℃,宫底平脐，质硬，无压痛，阴道少量流血，暗红色，量少于月经量。该患者属于
A.产褥感染
B.正常产褥
C.软产道裂伤
D.产后出血
E.子宫复旧不良
参考答案： B 您的答案：
图文解析： 【解析】产褥期是指产妇全身各器官除乳腺外从胎盘娩出至恢复或接近正常未孕状态所需的时期， 一般规定为 6 周。产后 24 小时内，体温略升高但不超过 38℃,可能与产程长导致过度疲劳，产妇失水或恶露积滞等有关。同时产后血液和坏死脱落的子宫蜕膜等组织经阴道排出，表现为暗红色，称为恶露。因此该患者可诊断为正常产褥。
【避错】产褥感染发热体温应超过 38℃;软产道裂伤及产后出血产生的血液应为鲜红色;子宫复旧不良指产后 6 周子宫仍未能恢复到非孕状态，现产妇仅产后 1 天，无法判断
这道题考察的是产后妇女的临床表现及其诊断。根据题目描述，女性患者在自然分娩后 1 天出现下腹部阵发性疼痛，体温略高（37.8℃），宫底位置正常（平脐），宫体质地硬，无压痛，阴道有少量暗红色流血，但流血量少于月经量。
选项分析如下：

A. 产褥感染通常伴有发热、恶露增多且臭味重，宫缩疼痛等症状。
B. 正常产褥期女性可能会有轻微的下腹疼痛和少量阴道出血，宫缩通常使宫底逐渐下降。
C. 软产道裂伤通常会导致明显的出血，且伴随压痛。
D. 产后出血指的是分娩后过量出血，通常伴有血压下降、心率增快等症状。
E. 子宫复旧不良会导致子宫下降缓慢或不明显，宫体较软，可能伴有出血等问题。
根据患者的症状和体征，宫底位置正常且质硬，流血量较少，主要表现为阵发性下腹疼痛和微热，没有出现明显的异常恶露或严重的出血情况，这些都比较符合正常产褥期的表现。因此，最合适的答案应该是 B.正常产褥。
首先，我们来详细分析题干中的关键信息。患者为 31 岁女性，自然分娩后 1 天，出现下腹部阵发性疼痛。查体结果显示体温略有升高，宫底平脐，质地硬且无压痛，阴道有少量暗红色流血，但量少于月经量。
接下来，我们根据这些信息分析各个选项：
A. 产褥感染：虽然患者体温略有升高，但并未出现产褥感染的其他典型症状，如高热、寒战、阴道脓性分泌物等。此外，宫底无压痛，这与产褥感染的常见体征不符。
B. 正常产褥：患者处于产褥期（产后 6 周内），体温升高可能与分娩过程中的应激反应或轻度脱水有关，但仍处于正常范围内。宫底平脐、质硬且无压痛是产后子宫逐渐收缩复旧的正常表现。阴道流血量少于月经量，也符合产后恶露的正常变化。因此，这些症状均符合正常产褥期的表现。
C. 软产道裂伤：通常会导致产后出血明显增多，但患者阴道流血量少于月经量，且无相关症状描述，因此该选项不符合。
D. 产后出血：患者阴道流血量少于月经量，不符合产后出血的定义。
E. 子宫复旧不良：子宫复旧不良通常表现为子宫底高度高于正常范围，子宫质地较软，并可能出现恶露增多、持续时间长等症状。但患者子宫底高度正常，质地硬，且流血量少，不符合子宫复旧不良的表现。
综上所述，患者的症状最符合正常产褥期的表现。因此，正确答案是：B. 正常产褥期。
根据题干信息，该患者为 31 岁的女性，自然分娩后 1 天，出现下腹部阵发性疼痛。查体结果显示：体温 37.8℃，宫底平脐，质硬，无压痛，阴道少量流血，暗红色，量少于月经量。

我们逐项分析选项：

A. **产褥感染**：产褥感染通常伴有高热（38℃以上），并可能伴有子宫压痛，恶露有臭味等症状。患者体温 37.8℃，未达到高热标准，且无子宫压痛和恶露异常。

B. **正常产褥**：产后 1 天，出现轻度的下腹部阵发性疼痛和少量阴道流血是正常现象，因为子宫在收缩以促进恢复，体温也未达到发热标准。这个描述与患者的症状最为吻合。

C. **软产道裂伤**：通常表现为明显的阴道流血，且多为鲜红色血液。题中患者的流血量少于月经量且为暗红色，不符合软产道裂伤的典型症状。

D. **产后出血**：产后出血一般定义为产后 24 小时内出血量超过 500ml。患者阴道流血量少于月经量，未达到产后出血的标准。

E. **子宫复旧不良**：通常表现为子宫恢复不佳，可能伴有持续性下腹痛和大量阴道流血。患者的症状（少量流血、子宫质硬）不符合子宫复旧不良的典型表现。

综上所述，最符合该患者情况的诊断是：

B. **正常产褥**
98、 (A2 型题)
女，30 岁，结婚 3 年未避孕未孕，伴下腹坠胀。月经 8~9 天/26~28 天，妇科检查：子宫后位，活动度较差，形状不规则，双附件区未触及明显异常。以下检查对诊断价值最没有价值的是
A.盆腔 B 超
B.基础体温测定
C.分段诊刮
D.腹部立位片
E.子宫输卵管碘油造影
参考答案： D 您的答案：
图文解析： 【解析】腹部立位片对于子宫病变检查不明确，影像学上最好使用 B 超或 MRI 对病变部位进行进一步确认。
【避错】基础体温测定除了能诊断患者怀孕状况外，还可诊断患者子宫及卵巢功能；分段诊刮可以排除患者子宫病变；子宫输卵管碘油造影可以确认患者输卵管有无病变。
这个考题考察的是关于不孕症的诊断方法，特别是在评估患者可能的子宫或附件问题时。根据这位女性患者未避孕 3 年未孕和下腹坠胀的症状，以及月经周期和妇科检查的结果，我们需要考虑哪种诊断方法对于确定不孕原因最没有价值。
A. 盆腔 B 超（超声检查）：这是评估子宫和附件（如卵巢）状况的常用方法，对于诊断子宫形态、位置、附件区域的异常有重要价值。
B. 基础体温测定：这是一种评估女性排卵状态的方法，通过监测排卵周期中的体温变化来判断。虽然对于诊断不孕有一定的帮助，但对于子宫形态和结构的评估没有直接价值。
C. 分段诊刮：这是一种子宫内膜组织的取样方法，可以帮助诊断内膜疾病，如子宫内膜异位症等，对诊断不孕的原因有直接价值。
D. 腹部立位片：这是一种 X 线检查方法，主要用于评估腹部器官，如肠道，对于直接评估子宫和附件的状况价值不大。
E. 子宫输卵管碘油造影：这是一种评估输卵管是否通畅的方法，对于诊断不孕尤其是输卵管因素的不孕有重要价值。
综合考虑，选择最没有诊断价值的项目应是 **D.腹部立位片**，因为它主要用于评估腹部的其他器官，对于直接诊断子宫或附件的问题不是最直接有效的方法。
患者青年女性，结婚 3 年未避孕且未孕，这提示可能存在不孕的问题。患者还伴有下腹坠胀的症状。月经周期相对规律，但经期稍长。妇科检查显示子宫后位，活动度较差且形状不规则，而双附件区未触及明显异常。根据这些信息，我们可以合理怀疑可能存在子宫内膜异位症、子宫腺肌症或其他子宫异常。
现在，我们逐一分析各个选项的诊断价值：
A. 盆腔 B 超：对于妇科检查发现子宫形状不规则和活动度差的情况，盆腔 B 超可以进一步观察子宫和附件的形态和结构，有助于发现子宫腺肌症、子宫内膜异位症或其他异常，因此是有价值的。
B. 基础体温测定：这项检查可以间接反映卵巢功能，有助于评估患者的生育能力和月经周期中激素的变化，因此也是有价值的。
C. 分段诊刮：分段诊刮可以通过刮取子宫内膜和内膜病灶进行活组织检查，作出病理诊断，

为临床治疗提供依据，对于诊断子宫内膜病变特别有价值。
D. 腹部立位片：主要用于检查腹部脏器和骨骼的情况，对于评估该患者的不孕和子宫形状不规则等问题几乎没有直接帮助，因此是最没有价值的检查。
E. 子宫输卵管碘油造影：这项检查可以评估输卵管是否通畅，以及子宫腔的形态是否正常，对于不孕症的诊断具有重要价值。
综上所述，对于这位患者的诊断，腹部立位片（选项 D）是最没有价值的检查。
因此，正确答案是 D.腹部立位片。
这是一道关于女性不孕和下腹坠胀的诊断性检查题目。以下是各项检查的解释：

A. 盆腔 B 超：能够检查盆腔内的结构，包括子宫、卵巢及其周围的组织，对发现异常形状的子宫及可能的病变（如子宫肌瘤、卵巢囊肿）非常有帮助。

B. 基础体温测定：用于评估女性排卵功能，通过监测基础体温变化来推断排卵期，是评估不孕症的常用手段之一。

C. 分段诊刮：用于检查子宫内膜，通过刮取子宫内膜组织进行病理检查，能够帮助诊断子宫内膜异常或病变。

D. 腹部立位片：主要用于检查腹部器官的情况，但对评估盆腔内的生殖器官病变价值有限。

E. 子宫输卵管碘油造影：是一种通过注入造影剂来检查子宫和输卵管通畅情况的影像学检查，对评估输卵管通畅性及子宫腔结构有重要价值。

综上所述，对于一个 30 岁结婚 3 年未避孕未孕、伴有下腹坠胀且子宫后位、活动度较差的女性，腹部立位片在这些检查中对诊断价值最没有价值，因为它主要检查腹部脏器，对盆腔内生殖器官的直接检查作用较小。

因此，最没有诊断价值的检查是：

D. 腹部立位片
99、 (A2 型题)
产妇，39 周，胎心正常，先露-2，宫高 35cm，腹围 115cm，出口后矢状径+坐骨结节间径＜15cm，应该怎么处理
A.剖宫产
B.继续观察
C.缩宫素
D.人工破膜
E.引产
参考答案： A 您的答案：
图文解析： 【解析】坐骨结节间径与出口后矢状径之和＞15cm 时，胎头可后移利用出口后三角空隙娩出。若两者之和＜15cm，足月胎儿不能经阴道分娩，应行剖宫产。该产妇孕 39 周，胎心正常，先露-2，宫高 35cm，腹围 115cm，出口后矢状径+坐骨结节间径＜15cm，足月胎儿无法经过孕妇阴道分娩，因此本题答案应为（A）剖宫产。
【避错】剖宫产指征包括：（1）胎儿窘迫；（2）胎位异常；（3）头盆不称；（4）瘢痕子宫；

（5）前置胎盘及前置血管；（6）脐带脱垂；（7）胎盘早剥；（8）妊娠巨大儿者；（9）严重合并症和并发症；（10）产道畸形；（11）妊娠合并肿瘤；（12）外阴疾病；（13）感染性疾病；（14）双胎或多胎妊娠；（15）孕妇要求的剖宫产。
这道题目考查的是产科紧急情况的处理，特别是关于产妇分娩途径的选择。考虑到产妇已经到了 39 周，胎心正常，但出口后矢状径和坐骨结节间径小于 15 厘米，这意味着骨盆可能过窄，不利于顺产。在这种情况下，如果继续顺产，可能会导致难产或胎儿受压。
因此，最佳的处理方式是 A.剖宫产。这样可以避免由于骨盆出口狭窄而引发的分娩难题和可能对胎儿造成的风险。剖宫产是在这种情况下推荐的分娩方式，以保证母婴安全。所以，正确答案是 A.剖宫产。
解析：
该产妇，39 周，胎心正常，先露-2，宫高 35cm，腹围 115cm，出口后矢状径+坐骨结节间径＜15cm，提示骨盆出口平面狭窄，且先露高浮，考虑存在头盆不称，难以经阴道分娩，因此应该选择剖宫产。
选项 B 继续观察不适用于该患者，因已确定存在头盆不称，继续观察可能导致胎儿宫内窘迫，胎死宫内等不良后果，故排除。
选项 C 缩宫素主要用于协调性宫缩乏力、宫口扩张≥3cm、胎心良好、胎位正常、头盆相称者，该产妇存在头盆不称，故使用缩宫素并不能改善分娩情况，反而会增加胎儿窘迫的风险，因此不选。
选项 D 人工破膜主要用于宫口扩张≥3cm、无头盆不称、胎头已衔接而产程延缓者，不适用于头盆不称的患者，故不考虑。
选项 E 引产是指妊娠 12 周后，因母体或胎儿方面的原因，须用人工方法诱发子宫收缩而结束妊娠，不适用于该足月产妇，故不选。
综上所述，正确答案是：A.剖宫产。
这道题涉及到产妇在临产时的处理决策，特别是在胎儿先露、宫高、腹围以及骨盆出口径等参数下的决策。我们来逐一分析这些因素：

- **胎心正常**：这是一个好的迹象，表示胎儿目前没有窘迫。
- **先露-2**：表示胎儿头部尚未完全进入骨盆入口，处于较高的位置。
- **宫高 35cm，腹围 115cm**：这些数值提示胎儿大小可能偏大。
- **出口后矢状径+坐骨结节间径＜15cm**：这表明产妇的骨盆出口径较小，可能影响顺产。

根据以上信息，胎儿的头部位置较高且骨盆出口径较小，提示自然分娩的风险较高。继续观察可能会导致胎儿窘迫或难产的风险增加，而其他选项如缩宫素和人工破膜在这种情况下并不能解决骨盆出口狭窄的问题。

因此，最佳答案应该是采取剖宫产，以确保产妇和胎儿的安全。

答案是：**A. 剖宫产**
100、 (A2 型题)
男童 2 岁，诊断为肾病综合征，激素治疗 3 周后尿蛋白转阴，停药 2 周后复发。该情况一年内出现 3 次。该儿童激素依赖类型是
A.激素依赖型，复发
B.激素敏感型，频复发
C.激素依赖型，频复发

D.激素耐药型，复发
E.激素耐药型，频复发
参考答案：　　C　　您的答案：
图文解析：　　　【解析】根据《儿童激素敏感、复发/依赖肾病综合征诊治循证指南》显示按激素的治疗反应可分以下三型：（1）激素敏感型 NS：以泼尼松足量[2 mg/(kg・d)或 60 mg/(m2・d)]治疗≤4 周尿蛋白转阴者；（2）激素耐药型 NS：以泼尼松足量治疗＞4 周尿蛋白仍阳性者。又可分为初治耐药和迟发耐药。后者指激素治疗 1 次或多次缓解后，再次激素治疗＞4 周尿蛋白仍阳性者；（3）激素依赖型 NS：对激素敏感，但连续两次减量或停药 2 周内复发者。而复发与频复发的定义为，复发：连续 3 天，24 小时尿蛋白定量≥50 mg/kg，或晨尿的尿蛋白/肌酐(mg/mg)≥2.0，或晨尿蛋白由阴性转为(＋＋＋)～(＋＋＋＋)；频复发(FR)：指病程中半年内复发≥2 次，或 1 年内复发≥4 次。因此可以判断该患者为激素依赖型，频复发。
【避错】准确区分复发与频复发区别。
对于这个 2 岁男童的案例，他被诊断为肾病综合征，经过激素治疗后尿蛋白转阴，但在停药后 2 周内复发，这种情况在一年内出现 3 次。根据这个描述，最合适的分类是“激素依赖型，频复发”。在肾病综合征的背景下，激素依赖型是指在激素治疗中呈现良好反应，但在停药后不久发生复发的情况。由于这种情况在一年内发生了多次（频复发），所以最准确的答案是：
C. 激素依赖型，频复发
这种分类反映了患者对激素治疗的依赖性以及复发的频率。
解析：
2 岁男童，诊断为肾病综合征，激素治疗 3 周后尿蛋白转阴，停药 2 周后复发，该情况一年内出现 3 次，为频繁复发，符合激素敏感型，频复发。激素敏感型肾病综合征是指用药 8～12 周内肾病综合征缓解，即连续 3 天，尿蛋白由阳性转为阴性，即尿蛋白＜0.3g/d，或 24 小时尿蛋白定量＜0.3g，或尿蛋白/肌酐（mg/mg）＜0.2；或一周内连续 3 次，尿试纸检查阴性。肾病综合症复发是指连续 3 天，尿蛋白由阴性转为（+++）或（++++），或 24 小时尿蛋白定量≥50mg/kg 或尿蛋白/肌酐（mg/mg）≥2.0；或尿试纸检查由阴性转为（++）及以上，为复发。若半年内复发≥2 次，或一年内复发≥3 次，为频复发。
选项 A“激素依赖型，复发”，选项 C“激素依赖型，频复发”，可一并排除，因为激素依赖型是指对激素敏感，但连续 2 次减量或停药 2 周内复发的肾病综合征，与题意不符。
选项 D“激素耐药型，复发”，选项 E“激素耐药型，频复发”，可一并排除，因为激素耐药型肾病综合征是指激素治疗 8～12 周未缓解，尿蛋白仍呈阳性，该患者尿蛋白已转阴，不符合激素耐药型。
综上所述，正确答案是：B.激素敏感型，频复发。
根据题干描述，2 岁男童被诊断为肾病综合征，激素治疗 3 周后尿蛋白转阴，但停药 2 周后复发，且这种情况在一年内出现了 3 次。以下是对各选项的解释：

A. 激素依赖型，复发：激素依赖型指的是患者在激素治疗期间症状缓解，但一旦减少或停用激素就会复发。单纯复发不考虑频繁复发的次数。

B. 激素敏感型，频复发：激素敏感型指的是患者对激素治疗有效，尿蛋白转阴，频复发指的是一年内复发次数多于 2 次。

C. 激素依赖型，频复发：激素依赖型且频复发指的是患者在激素治疗期间症状缓解，但一

旦减少或停用激素就会复发，并且一年内复发次数多于 2 次。

D. 激素耐药型，复发：激素耐药型指的是患者对激素治疗无反应，复发指的是病情没有得到缓解或再次恶化。

E. 激素耐药型，频复发：激素耐药型且频复发指的是患者对激素治疗无反应，并且病情频繁复发。

根据上述解释和题干所述情况，正确答案是：

C. 激素依赖型，频复发
101、 (A2 型题)
女，孕 40 周，宫高 35cm，腹围 110cm，阴道流血一天，妇科检查：宫口开 1cm，双足先露，接下来应该做的检查是
A.经 B 超外旋转
B.经阴道内旋转
C.自然分娩
D.静点缩宫素
E.剖宫产
参考答案： E 您的答案：
图文解析： 【解析】患者女，孕 40 周，宫高 35cm，腹围 110cm，阴道流血一天，妇科检查见宫口开 1cm，双足先露。胎儿为足先露，胎位异常孕妇需直接行剖宫产，因此选择（E）剖宫产。
【避错】剖宫产指征包括：（1）胎儿窘迫；（2）胎位异常；（3）头盆不称；（4）瘢痕子宫；（5）前置胎盘及前置血管；（6）脐带脱垂（7）胎盘早剥；（8）妊娠巨大儿者；（9）严重合并症和并发症；（10）产道畸形；（11）妊娠合并肿瘤；（12）外阴疾病；（13）感染性疾病；（14）双胎或多胎妊娠；（15）孕妇要求的剖宫产。
这道题目涉及到孕妇在妊娠晚期的处理，特别是在出现异常情况如双足先露和阴道流血时的处理。双足先露是胎位异常的一种，可能导致分娩过程中的并发症，需要特别注意。在此情况下，最先需要考虑的是保证母婴安全。
- A. 经 B 超外旋转：这是在某些胎位异常情况下考虑的，目的是试图将胎儿转至头位，以便自然分娩。但在阴道流血的情况下，进行此操作可能增加风险。
- B. 经阴道内旋转：这个选项通常不是处理胎位异常和阴道流血的首选方法，因为它可能增加子宫撕裂的风险。
- C. 自然分娩：双足先露和阴道流血的情况下，自然分娩可能存在较高的风险，并不是首选处理方法。
- D. 静点缩宫素：这主要用于处理产后出血，而不是分娩前的阴道流血。
- E. 剖宫产：在出现双足先露和阴道流血的情况下，为了保证母婴安全，剖宫产通常是最佳选择。剖宫产可以避免因胎位异常导致的分娩困难和潜在的并发症。
因此，最佳答案是 **E. 剖宫产**。
解析：
青年女性，足月孕妇，阴道流血一天，宫口开 1cm，双足先露，应考虑为足先露，是常见的胎位异常，胎儿双足先于胎头露出可刺激宫颈使宫口迅速扩张，胎膜破裂极易发生脐带脱垂，出现胎儿窘迫甚至死亡。故一经确诊应尽快剖宫产，以保证母婴安全，故接下来应该做的检

查是剖宫产。
选项 A 经 B 超外旋转是检查方法，不是治疗手段，故排除。
选项 B 经阴道内旋转是产科的一种处理方法，但不适用于足先露，足先露时，不可能进行手法复位，故不选。
选项 C 自然分娩适用于头先露的胎位，足先露是剖宫产的绝对指征，不可能进行自然分娩，可排除。
选项 D 静点缩宫素即催产素，主要用于协调性宫缩乏力时的催产和晚期难免流产时的引产，足先露时应尽快剖宫产，故不宜使用催产素，与题意不符。
综上所述，正确答案是：E.剖宫产。
根据你提供的题目信息，我们可以得出以下分析和结论：

患者为足月妊娠（孕 40 周），宫高 35cm，腹围 110cm，阴道流血一天。妇科检查发现宫口开 1cm，胎位为双足先露。

双足先露（也称为双足臀先露）是一种异常的胎位，通常情况下会增加分娩的风险。此时需要考虑最安全的分娩方式。

以下是各选项的分析：

A. 经 B 超外旋转：这种方法是将胎儿从不正常的位置（臀位或横位）旋转到头位（正常位置）。但是，在宫口仅开 1cm 的情况下，这种方法可能不适用。

B. 经阴道内旋转：这种方法也试图改变胎儿的位置，但需要专业技术和条件，且通常在分娩过程中使用，而不是在宫口仅开 1cm 时进行。

C. 自然分娩：由于双足先露会增加母婴的分娩风险，自然分娩并不是最佳选择。

D. 静点缩宫素：缩宫素用于促进宫缩，但在胎位异常的情况下，这可能会增加母婴的风险。

E. 剖宫产：在双足先露情况下，剖宫产是较为安全的选择。它可以避免因胎位异常引起的分娩并发症，保护母婴安全。

综上所述，最佳答案是：

E. 剖宫产
102、 (A2 型题)
女， 一年零 6 个月，发热两天，拒食，T38.6℃,P138 次/分，R38 次/分，颌下淋巴结肿大，咽充血，咽腭弓、腭垂、软腭有数个疱疹，舌面及齿龈有小疱疹，且部分小疱 疡，心肺腹无异常，最可能的病原体是
A.EB 病毒
B.单纯疱疹病毒
C.白色念珠菌
D.腺病毒
E.柯萨奇病毒

参考答案：　　E　您的答案：

图文解析：　　【解析】疱疹性咽峡炎多发生于婴幼儿，患者具有患儿接触史，且伴发高热，咽部充血，出现散在灰白色疱疹，可出现在咽腭弓、腭垂、软腭等处。该患者一年零 6 个月（疱疹性咽峡炎常见发病年龄），发热两天，拒食（疱疹性咽峡炎体征），颌下淋巴结肿大，咽充血，咽腭弓、腭垂、软腭有数个疱疹，舌面及齿龈有小疱疹，且部分小疱疹破溃变成溃疡(疱疹性咽峡炎咽喉处表现)。因此患者可以考虑诊断为疱疹性咽峡炎，该病主要病原体为柯萨奇病毒及肠道病毒 71 型，因此此题正确答案为柯萨奇病毒（E）。

【避错】EB 病毒感染喉咙会出现白色脓样分泌物；单纯性疱疹引起的疱疹性龈口炎主要为群集性小水泡且很快破溃，同时伴有皮肤黏膜的疱疹出现；白色念珠菌感染则引起鹅口疮，表现为奶油状白色的疼痛黏膜斑；由腺病毒感染引起的咽喉炎常伴有咳嗽。

这个病例描述的是一名一岁半的女童，出现了发热、拒食、颌下淋巴结肿大、咽充血，以及咽腭弓、腭垂、软腭和口腔内出现疱疹和小疱疡。这些症状提示可能是由某种病毒感染引起的。

- A. EB 病毒（Epstein-Barr 病毒）通常导致的是传染性单核细胞增多症，主要表现为发热、咽痛、肿大的颈部淋巴结和乏力，但是它不常导致口腔内疱疹。
- B. 单纯疱疹病毒（Herpes simplex virus）通常会在口腔内形成疱疹，尤其是 HSV-1，它经常导致口腔疱疹。
- C. 白色念珠菌（Candida albicans）是一种真菌，通常引起口腔念珠菌病，而非疱疹。
- D. 腺病毒（Adenovirus）主要导致呼吸道感染和结膜炎，并不典型地引起口腔疱疹。
- E. 柯萨奇病毒（Coxsackievirus）是一种引起手足口病的病毒，可以导致口腔疱疹，但这些疱疹通常伴有手和脚的皮疹。

考虑到上述症状和特点，最可能的病原体是**B. 单纯疱疹病毒**，因为这种病毒通常会导致口腔内出现多个疱疹和疱疡。

首先，我们来仔细分析这个案例。

患儿，女，一年零六个月大，主要症状包括发热、拒食，查体发现颌下淋巴结肿大，咽充血，并且在咽腭弓、腭垂、软腭以及舌面和齿龈处发现疱疹。这些症状与体征强烈提示我们考虑疱疹性咽峡炎的可能性。

接下来，我们对选项进行分析：

A. EB 病毒：这种病毒主要与传染性单核细胞增多症有关，与疱疹性咽峡炎的病原体不符。

B. 单纯疱疹病毒：主要导致皮肤黏膜的疱疹，尤其是口唇、生殖器疱疹等，但不特异于咽峡炎。

C. 白色念珠菌：这是一种真菌，与疱疹性咽峡炎无关。

D. 腺病毒：虽然腺病毒可以导致呼吸道感染，但其症状与疱疹性咽峡炎不符。

E. 柯萨奇病毒：这是疱疹性咽峡炎的常见病原体。考虑到患儿的年龄、症状及体征，这一选项与案例描述高度匹配。

综上所述，结合患儿的年龄、发热、拒食、咽部的充血和疱疹等症状，最有可能的病原体是柯萨奇病毒。

因此，正确答案是 E. 柯萨奇病毒。

根据题干描述的症状，患儿发热、拒食、颌下淋巴结肿大、咽充血，且在咽腭弓、腭垂、软腭、舌面及齿龈有疱疹和部分疱疡，这些症状提示为疱疹性口腔炎。综合考虑最可能的病原体是柯萨奇病毒。

柯萨奇病毒（Coxsackie virus）感染常见于儿童，尤其是手足口病和疱疹性咽峡炎，其特征是口腔黏膜和四肢出现疱疹。

因此，最可能的病原体是：

E.柯萨奇病毒
103、 (A2 型题)
女，30 岁，孕 38 周，不规律腹痛 3 小时，阴道流出暗褐色分泌物，诊断为
A.胎盘早剥
B.前置胎盘
C.先兆临产
D.先兆早产
E.临产
参考答案： C 您的答案：
图文解析： 【解析】患者女 30 岁，孕 38 周，不规律腹痛 3 小时，阴道流出暗褐色分泌物。分娩发动前，出现预示不久即将临产的症状，称为先兆临产。在接近分娩时，部分产妇可见阴道有少量的血性分泌物排出，称为见红，是先兆临产的重要症状。因此该患者应被诊断为先兆临产。
【避错】胎盘早剥一般症状为阴道流血且量较大；前置胎盘同样是出现阴道出血症状；早产指自末次月经第 1 日计算，妊娠满 28 周至不足 37 周分娩者为早产。
这位 30 岁孕妇，孕 38 周，出现不规律腹痛和阴道流出暗褐色分泌物。这些症状提示可能的妊娠并发症。在这里，我们可以逐一排除和考虑每个选项的可能性：
A. 胎盘早剥：通常表现为突发的腹痛，可能伴有阴道出血，但通常是鲜红色的。在本案例中，分泌物为暗褐色，可能不是最典型的胎盘早剥表现。
B. 前置胎盘：通常表现为无痛的鲜红色阴道出血。由于本案例中腹痛不规律，并且分泌物颜色不是鲜红，前置胎盘可能性较小。
C. 先兆临产：指的是分娩前的征兆，如不规则宫缩，可能会有阴道分泌物改变，但通常不伴有暗褐色分泌物。
D. 先兆早产：指的是在妊娠 37 周前的分娩征兆，包括宫缩、颈管变化和可能的阴道出血。但考虑到这位妇女已经 38 周，所以先兆早产不适用。
E. 临产：指的是分娩的开始，通常伴随着规律宫缩和阴道分泌物增多。阴道分泌物可能包括血性的“见红”，即暗褐色分泌物，这可能表明宫颈已开始变化并准备分娩。
根据上述分析，最合适的答案似乎是 **E. 临产**，因为 38 周孕龄的不规律腹痛和暗褐色阴道流出物可能是分娩即将开始的迹象。
这是一道关于孕期并发症及产前征兆的题目。我们需要分析孕妇的孕期周数和症状，从而判断其当前状况。
首先，梳理题目中的关键信息：
患者为 30 岁女性，怀孕 38 周。
出现不规律腹痛 3 小时。
阴道流出暗褐色分泌物。
接下来，根据这些信息进行解释和推理：
孕 38 周：属于妊娠晚期，胎儿已足月。
不规律腹痛：这可能是宫缩的表现，但由于腹痛不规律，因此尚未进入正式产程。
阴道流出暗褐色分泌物：这通常是见红的表现，即子宫颈附近的胎膜与宫壁分离，导致毛细血管破裂和少量出血，是临产前的一个重要征兆。
现在，我们对选项进行逐一分析：

A. 胎盘早剥：这是一种紧急情况，通常伴有剧烈腹痛和阴道流血，与题目描述不符。
B. 前置胎盘：前置胎盘是胎盘位于子宫下段，可能导致产前出血，但通常不伴有腹痛，且出血多为鲜红色，与题目描述不符。
C. 先兆临产：是指在正式临产前，孕妇出现一系列预示不久将临产的症状，包括不规律宫缩、胎儿下降感和见红等，与题目描述相符。
D. 先兆早产：这是指妊娠在 28～37 周，孕妇出现早产临产的症状，但宫颈尚未开大 3cm，而该孕妇已经孕 38 周，因此不适用。
E. 临产：是指出现规律宫缩，宫颈管进行性缩短等分娩即将开始的征兆，但题目中描述的是不规律腹痛，故排除。
综上所述，孕妇孕 38 周，不规律腹痛，阴道流出暗褐色分泌物，最可能的诊断是先兆临产。因此，答案是 C.先兆临产。
题目描述中提到患者怀孕 38 周，不规律腹痛 3 小时，阴道流出暗褐色分泌物。根据这些症状，最有可能的诊断是：

A. 胎盘早剥

胎盘早剥（placental abruption）是指胎盘在胎儿娩出前从子宫壁部分或完全剥离，症状包括腹痛、阴道出血（通常为暗红色或暗褐色），这与题目中提到的症状相符。

其他选项解释如下：

B. 前置胎盘：通常会有无痛性亮红色阴道出血，但题目中提到的腹痛和暗褐色分泌物不符合这个诊断。

C. 先兆临产：通常指的是临近分娩时的症状，包括规律性的宫缩和少量阴道出血，但题目中描述的不规律腹痛和暗褐色分泌物不太符合。

D. 先兆早产：虽然腹痛可能与早产有关，但阴道流出暗褐色分泌物更典型的是胎盘早剥。

E. 临产：这意味着分娩过程已经开始，通常伴随规律性的宫缩和子宫颈的改变，不太符合题目中的不规律腹痛。

综上所述，最正确的答案是：

A. 胎盘早剥
104、 (A2 型题)
男，70 岁，走路缓慢伴右上肢摆动消失 2 年，常跌倒，查体：面部呆板，颈肌张力高，右侧肢体肌张力高于左侧，静坐时，右手震颤，四肢肌力 5 级，腱反射减退，双侧 Ba 性，其余神经系统检查无异常，头颅 MRI：皮质内 2 个小的腔隙灶。最可能的诊断是
A.特发性震颤
B.帕金森叠加综合征
C.血管性帕金森综合征
D.多系统萎缩
E.帕金森病

参考答案：　　E　您的答案：
图文解析：　　【解析】帕金森病多发于老年人，主要症状包括静止性震颤，肌肉僵直，运动迟缓，姿势步态障碍，疼痛，感觉异常，吞咽困难言语障碍，典型患者可表现为面具样表现。该 70 岁男患者（帕金森病常见发病年龄），走路缓慢伴右上肢摆动消失 2 年，常跌倒（帕金森病症状：姿势步态障碍伴运动迟缓），查体见面部呆板，颈肌张力高（肌肉僵硬），静坐时，右手震颤（静止性震颤），四肢肌力 5 级，腱反射减退。因此该患者可能诊断为（E）帕金森病。
【避错】特发性震颤多伴有家族史，典型症状是手的节律性外展内收样震颤和屈伸样震颤，且为唯一表现；帕金森叠加综合征为多系统萎缩、进行性核上性麻痹、路易体痴呆及皮质基底节变性的结合；多系统萎缩同时伴有意识障碍，自主神经功能不良，出现尿失禁；血管性帕金森综合征多为急性脑卒中后突然发作，表现为双下肢运动障碍，典型症状为“磁性足反应”。
该病例的描述符合帕金森病（Parkinson's disease, PD）的典型特征。症状包括走路缓慢、一侧肢体的摆动减少、面部表情减少（面部呆板）、肌张力增加（特别是颈部和右侧肢体）、静止性震颤（尤其是在右手）、肌力正常但腱反射减退。这些都是帕金森病的常见临床表现。头颅 MRI 显示的皮质内小的腔隙灶可能是血管性改变，但不足以解释全部症状。
- A 项“特发性震颤”主要表现为双侧对称的震颤，尤其在运动时，不符合本病例的描述。
- B 项“帕金森叠加综合征”和 D 项“多系统萎缩”均指的是有额外的非运动症状或更广泛的系统受累，这在题干中没有明确指出。
- C 项“血管性帕金森综合征”指的是由血管问题引起的帕金森样症状，虽然 MRI 有腔隙性病变，但不典型，且不够支持此诊断。
因此，最可能的诊断是 E 项“帕金森病”。
这是一道诊断推理问题，我们需要从题干中提取关键信息，结合医学知识进行分析。
关键信息整理：
老年男性患者，走路缓慢，右上肢摆动消失，经常跌倒。
查体：面部呆板，颈肌张力高，右侧肢体肌张力高于左侧，静坐时右手震颤，四肢肌力正常，腱反射减退，双侧病理征阳性。
头颅 MRI 显示皮质内有 2 个小的腔隙灶。
接下来，我们逐一分析选项：
A. 特发性震颤：这是一种原因不明的震颤，通常不影响肌张力或运动功能，与题干描述不符。
B. 帕金森叠加综合征：这是一种类似帕金森病的神经系统退行性疾病，但通常伴有其他系统症状，如小脑共济失调、锥体外系症状等，题干中未提及这些症状。
C. 血管性帕金森综合征：这种综合征通常与脑血管病变有关，表现为肌张力增高、运动减少、震颤等症状。考虑到患者年龄、症状（肌张力增高、震颤）以及头颅 MRI 显示的腔隙灶，这一选项与题干描述高度吻合。
D. 多系统萎缩：这是一种神经系统退行性疾病，通常影响多个系统，包括小脑、脑干等，题干中未提及这些症状。
E. 帕金森病：虽然帕金森病也会导致肌张力增高和震颤，但通常不会伴有脑血管病变，如腔隙灶。
综上所述，患者老年男性，走路缓慢伴右上肢摆动消失，经常跌倒，查体显示面部呆板，颈肌张力高，右侧肢体肌张力高于左侧，静坐时右手震颤，头颅 MRI 显示皮质内有小的腔隙灶，最可能的诊断是血管性帕金森综合征。
因此，正确答案是 C. 血管性帕金森综合征。

根据描述，这位 70 岁的男性患者表现出走路缓慢、右上肢摆动消失、常跌倒、面部呆板、颈肌张力高、右侧肢体肌张力高于左侧、静坐时右手震颤等症状。结合查体和 MRI 结果（皮质内有 2 个小的腔隙灶），最可能的诊断是：

C. 血管性帕金森综合征

理由如下：

1. **血管性帕金森综合征**：该综合征常见于老年人，与小血管病变有关。MRI 显示皮质内有小腔隙灶，这支持血管性病变的诊断。患者的帕金森症状，如肌张力高、震颤、面部呆板、走路缓慢、右上肢摆动消失等，也符合血管性帕金森综合征的表现。

2. **特发性震颤**：通常表现为姿势性或意向性震颤，不会出现肌张力高和走路缓慢等症状。

3. **帕金森叠加综合征**：通常指帕金森病与其他神经退行性疾病并存，但患者的 MRI 显示血管性病变，更符合血管性帕金森综合征。

4. **多系统萎缩**：虽然也会出现帕金森样症状，但通常伴有自主神经功能障碍，如直立性低血压、膀胱功能障碍等，且 MRI 通常显示小脑萎缩或脑干萎缩等。

5. **帕金森病**：典型帕金森病 MRI 通常不显示明显病变，而该患者的 MRI 显示有血管性病变，更符合血管性帕金森综合征。

综上所述，最可能的诊断是 **C. 血管性帕金森综合征**。
105、 (A2 型题)
女，30 岁，阴道不规则流血 20 天，咳嗽 5 天。半年前行人工流产，妇科检查：子宫如 40 天妊娠大小，质软，双侧附件区均可触及大小约 5cm 的囊性包块，胸部 X 线片示双侧 球状阴影。首先考虑的诊断是
A.绒癌
B.葡萄胎
C.侵袭性葡萄胎
D.先兆流产
E.稽留流产
参考答案： A 您的答案：
图文解析： 【解析】绒癌多表现于在葡萄胎清宫、足月分娩，自然流产、人工流产、宫外孕后出现不规则阴道流血、咳嗽、抽搐等。主要体现为阴道有紫蓝色结节，子宫增大、宫旁肿块，胸部 X 线摄片可见多个棉团样阴影。该患者女，30 岁（绒癌好发年龄段），半年前行人工流产（绒癌常见诱发病因），阴道不规则流血 20 天，咳嗽 5 天（绒癌常见体征）。妇科检查见子宫如 40 天妊娠大小，质软，双侧附件区均可触及大小约 5cm 的囊性包块（绒癌子宫区影像学表现），胸部 X 线片示双侧中下野多发棉球状阴影（绒癌胸部影像学表现）。因此考虑患者诊断为（A）绒癌。
【避错】葡萄胎表现为停经后的不规则阴道流血，易被误诊为先兆流产。以后逐渐增多，子宫出现异常增大、变软；侵袭性葡萄胎表现为葡萄胎排出 3~4 周后阴道不规则出血；且其他疾病均无胸片为棉球状阴影表现。

在这个案例中，30 岁女性患者出现阴道不规则流血 20 天，咳嗽 5 天，曾在半年前进行人工流产。妇科检查发现子宫类似 40 天妊娠大小，质地柔软，双侧附件区可触及约 5cm 大小的囊性包块，胸部 X 线片显示双侧球状阴影。根据这些临床表现，最可能的诊断是绒癌（A）。绒癌（也称为滋养细胞瘤）是一种高度恶性的妊娠相关肿瘤，通常发生在葡萄胎之后。绒癌的特点包括不规则阴道出血、子宫增大超过妊娠期预期、囊性包块（通常与子宫外转移有关），以及胸部 X 线片上的典型球状阴影，表示远处转移，尤其是肺转移。因此，根据这些症状和临床表现，绒癌是最符合的诊断。

其他选项，如葡萄胎（B）和侵袭性葡萄胎（C），虽然与绒癌相关，但葡萄胎通常不会引起如此严重的症状或远处转移，而侵袭性葡萄胎虽可能有类似的症状，但绒癌的恶性程度更高，且其症状和体征更加显著。先兆流产（D）和稽留流产（E）主要表现为早孕期出血和疼痛，不符合本病例的临床表现。因此，最正确的选择是 A.绒癌。

首先，让我们仔细分析题干中的关键信息，以便更准确地解答这道题。

患者为 30 岁女性，出现阴道不规则流血和咳嗽的症状。半年前曾进行人工流产，现在妇科检查发现子宫增大，双侧附件区有囊性包块，胸部 X 线片还显示双侧球状阴影。这些信息对于确定诊断至关重要。

现在，我们来逐一分析选项：

A. 绒癌：考虑到患者半年前有人工流产史，这是绒癌的一个重要风险因素。绒癌可以发生在流产、足月分娩或葡萄胎之后，且可能伴有阴道不规则流血和肺部转移灶（如 X 线片所示的球状阴影）。这些特点与患者症状相符，因此绒癌是一个合理的诊断考虑。

B. 葡萄胎：虽然葡萄胎也可能导致子宫增大和阴道不规则流血，但它通常不会发生在流产之后，且题干中未提及葡萄状胎块，因此这个选项不太可能。

C. 侵袭性葡萄胎：这是一种恶性肿瘤，但它通常源于葡萄胎而非流产，且题干中未提及与葡萄胎直接相关的信息，因此这个选项也不太可能。

D. 先兆流产：先兆流产通常发生在妊娠早期，伴有腹痛和阴道流血，但题干中提到的是流产后出现的症状，且先兆流产不会伴有肺部阴影，因此这个选项可以排除。

E. 稽留流产：稽留流产指的是胚胎或胎儿在宫内死亡后未能及时自然排出，但题干中明确指出患者半年前进行了人工流产，且目前的症状（如子宫增大、附件包块、肺部阴影）与稽留流产不符，因此这个选项也可以排除。

综上所述，结合患者的病史、症状和影像学表现，最可能的诊断是绒癌。因此，正确答案是：

A. 绒癌。

根据提供的病史和检查结果，30 岁的女性出现了阴道不规则流血，并且有咳嗽的症状。妇科检查发现子宫增大，并且双侧附件区触及囊性包块。此外，胸部 X 线片显示双侧有球状阴影。结合这些信息，最可能的诊断是绒癌（A. 绒癌）。

绒癌（绒毛膜癌）是一种高度恶性且罕见的肿瘤，多发生于生殖器官，尤其是在妊娠后。它常表现为阴道不规则流血，胸部 X 线片的球状阴影提示肺转移，这是绒癌的典型表现之一。患者半年前曾行人工流产，这也是绒癌的危险因素之一。葡萄胎和侵袭性葡萄胎虽有类似表现，但胸部 X 线的球状阴影更加指向绒癌。

因此，最佳答案是：

A. 绒癌

题目材料　　女孩，4 岁，发热咳嗽流涕 4 天，发疹 1 天，体温 39.5℃,心率 130 次／分，呼吸 38 次／分，精神萎靡，头颈部出现红疹，疹间皮肤正常，眼结 分泌物，淋巴结未触及，口腔充血粗糙，心肺正常，肝脾未触及，血红蛋白 115g／L，白细胞 6.5×109／L，中性粒

细胞 0.35，淋巴细胞 0.6，血小板 261

106、 (A3/A4 型题) 题

最可能的诊断是

A.风疹

B.猩红热

C.麻疹

D.幼儿急疹

E.药物疹

参考答案： C 您的答案：

图文解析： 【解析】患者发热 4 天，出疹 1 天，头颈部出现红疹，疹间皮肤正常，眼结膜充血有分泌物，淋巴结未触及，符合麻疹诊断（C 对）。

【避错】风疹是发热第 2 天出疹,并于 1 天内出齐，出疹顺序：面部—颈部—躯干— 四肢（A 错） 。猩红热皮疹于发病后 24 小时迅速出现，出疹顺序：颈部—腋下——腹股沟处，有帕氏线、杨梅舌（B 错） 。幼儿急疹特点是在发热 3～5 天后热度突然下降，皮肤出现玫瑰红色的斑丘疹，皮疹主要见于躯干、颈部和上肢，如无并发症可很快痊愈（D 错） 。药疹是药物通过口服、

外用和注射等途径进入人体而引起的皮肤黏膜炎症的反应。几乎所有的药物都有可能引起皮炎，但最常见的有磺胺类药、解热镇痛药、安眠药类以及青霉素、链霉素等（E 错）。

最可能的诊断是：

风疹（A）通常表现为轻微发热、颈部淋巴结肿大和典型的皮疹。

猩红热（B）通常会有高热、咽痛、草莓舌和细小红疹，特别是在躯干和褶皱部位。

麻疹（C）的特征是高热、科普利克斑、咳嗽、流涕、结膜炎和特征性的红疹。

幼儿急疹（D）多见于幼儿，表现为高热突然退热后出现的皮疹。

药物疹（E）则通常与药物摄入有关，表现为多种皮疹类型。

考虑到上述信息，最符合这些症状的是麻疹（C），因为患者有高热、咳嗽、流涕、精神萎靡、眼结膜分泌物和头颈部红疹。

解析：

患者幼儿女性，发热、咳嗽、流涕 4 天，后出现红疹，为典型的麻疹前驱期表现。查体发现，头颈部出现红疹，疹间皮肤正常，有结膜炎表现，口腔充血粗糙，为典型的麻疹出疹期表现。血常规结果提示存在病毒感染。根据患者临床表现、体征及实验室检查，考虑诊断为麻疹。

选项 A 风疹全身症状轻，耳后、枕后淋巴结肿大并触痛，皮疹多在症状出现后 1～2 天出现，且有面颈部→躯干→四肢的出疹顺序，与题干描述不符。

选项 B 猩红热有发热、咽峡炎、杨梅舌、环口苍白圈等表现，且其皮疹特点是皮肤弥漫性充血，上有密集针尖大小丘疹，全身皮肤均可受累，疹退后伴脱皮，与患者的症状描述差异明显。

选项 D 幼儿急疹特点是热退疹出，且多为红色细小密集斑丘疹，以头面颈及躯干多见，四肢较少，一天出齐，次日即开始消退，与题干中患者症状不符，故排除。

选项 E 药物疹多有近期用药史，且发疹类型多样，但多伴随瘙痒，停药后皮疹多可消退，该患者并未提及近期用药史，与题意不符。

综上所述，正确答案是：C.麻疹。

106. 最可能的诊断是

A. 风疹

B. 猩红热

C. 麻疹

D. 幼儿急疹
E. 药物疹

解析：
根据病史和临床表现，如发热、咳嗽、流涕、发疹（头颈部出现红疹，疹间皮肤正常），眼结膜分泌物，口腔充血粗糙（科氏斑）等特点，最可能的诊断是麻疹。

正确答案：C. 麻疹
107、 (A3/A4 型题) 题
最可能的并发症是
A.肺炎
B.脓气胸
C.肺脓肿
D.心肌炎
E.麻疹脑炎
参考答案： A 您的答案：
图文解析： 【解析】小儿符合麻疹诊断。麻疹最常见的并发症是肺炎（A 对）。
【避错】麻疹患者应隔离至出疹后 5 天，若并发肺炎，则隔离至出疹后 10 天。
最可能的并发症是：
麻疹常见的并发症包括肺炎（A）、耳部感染、腹泻和最严重的并发症之一，麻疹脑炎（E）。考虑到麻疹的严重并发症，**麻疹脑炎（E）**是一个重要的并发症，但需要注意症状的发展才能确定。
解析：
麻疹最常见的并发症是肺炎，该患者为麻疹诊断，故最可能的并发症是肺炎。
选项 B 脓气胸，选项 C 肺脓肿，多见于肺部化脓性细菌感染，麻疹一般不会并发此类疾病，故一并排除。
选项 D 心肌炎虽然可继发于病毒感染之后，但相比肺炎少见，故不选。
选项 E 麻疹脑炎发病率较低，故排除。
综上所述，正确答案是：A.肺炎。
107. 最可能的并发症是
A. 肺炎
B. 脓气胸
C. 肺脓肿
D. 心肌炎
E. 麻疹脑炎

解析：
麻疹的常见并发症包括肺炎和脑炎。结合患儿的症状和病史，最可能的并发症是麻疹脑炎。

正确答案：E. 麻疹脑炎
108、 (A3/A4 型题)
若退热后，头面及躯干皮疹，双肺呼吸音细湿啰音，最佳处理措施是 题
A.隔离治疗
B.抗过敏

C.丙种球蛋白
D.抗生素
E.阿司匹林
参考答案：　A　您的答案：
图文解析：　【解析】麻疹若退热后，头面及躯干皮疹，双肺呼吸音细湿啰音，考虑合并肺炎，最佳处理措施是隔离治疗（A 对）。
【避错】接触麻疹患者 5 天内可注射丙种球蛋白预防发病或减轻症状。麻疹患者不需应用抗生素，若继发细菌感染可给予抗生素。
若退热后，头面及躯干皮疹，双肺呼吸音细湿啰音，最佳处理措施是：
细湿啰音可能表明下呼吸道感染或肺炎，此时**抗生素（D）**治疗是适当的，尤其是在麻疹引发的并发症如细菌性肺炎的情况下。
解析：
患者麻疹诊断明确，若在退热后，出现头面及躯干皮疹，双肺可闻及细湿啰音，应考虑并发肺炎。麻疹并发肺炎常见的致病菌为金黄色葡萄球菌，肺炎链球菌、流感嗜血杆菌等，因此最佳的处理措施是使用针对这些细菌的抗生素。
选项 A 隔离治疗为麻疹的一般治疗措施，但不是最佳处理措施，与题意不符。
选项 B 抗过敏，选项 C 丙种球蛋白，一般用于荨麻疹或过敏性疾病的治疗，该患者并非过敏性疾病，可一并排除。
选项 E 阿司匹林一般不用做抗菌治疗，与本题无关。
综上所述，正确答案是：D.抗生素。
108. 若退热后，头面及躯干皮疹，双肺呼吸音细湿啰音，最佳处理措施是
A. 隔离治疗
B. 抗过敏
C. 丙种球蛋白
D. 抗生素
E. 阿司匹林

解析：
患儿退热后出现细湿啰音，提示可能合并了肺炎，麻疹患者合并细菌感染时需要使用抗生素治疗。

正确答案：D. 抗生素
题目材料　　儿童，7 岁，左膝疼痛 3 天，寒战高热 1 天，查体：T：39.5℃,左侧股骨下段近关节处肿胀疼痛，查体明显压痛，浮髌试验阴性。实验室检查 22×10＾9／L，　N0.86。
109、　(A3/A4 型题)　题
最可能的诊断是
A.骨巨细胞瘤
B.化脓性关节炎
C.骨肉瘤
D.急性血源性骨髓炎
E.膝关节结核
参考答案：　D　您的答案：
图文解析：　【解析】儿童，7 岁（急性血源性骨髓炎多发生于儿童及青少年），左膝疼痛 3 天，寒战高热 1 天，T 39.5℃，左侧股骨下段近关节处肿胀疼痛（好发于长骨干骺端），

查体明显压痛，浮髌试验阴性（排除 B 选项）　。实验室检查：血 WBC 22×10＾9／L（感染征象，排除 E 选项）　，综合患者情况，考虑诊断为急性血源性骨髓炎（D 对）。
【避错】骨巨细胞瘤多见于 20~40 岁女性，典型 X 线表现为病灶部位肥皂泡样骨质改变（A 错）；骨肉瘤，主要症状为局部疼痛、夜间尤重，典型 X 线表现是 Codman 三角或呈日光放射状骨膜反应（C 错）。
109 题：最可能的诊断是
A. 骨巨细胞瘤：这是一种较少见的良性骨肿瘤，通常不会引起急性症状如高热和寒战。
B. 化脓性关节炎：通常表现为关节炎症，但该病例中肿胀在股骨下段，而非关节内，且浮髌试验阴性，不符合化脓性关节炎的典型表现。
C. 骨肉瘤：是一种恶性肿瘤，但它的症状进展通常较慢，不太可能突然出现高热和寒战。
D. 急性血源性骨髓炎：符合高热、寒战、局部骨疼痛和肿胀的症状，且白细胞计数增高，是急性感染的表现。
E. 膝关节结核：虽然关节结核可以引起关节周围疼痛和肿胀，但通常进展缓慢，并伴有慢性症状，不会出现急性高热和寒战。
基于上述分析，最可能的诊断是 D. 急性血源性骨髓炎。
解析：
患儿，左膝疼痛、寒战、高热，查体见高热，左侧股骨下段近关节处肿胀疼痛，明显压痛，浮髌试验阴性，实验室检查见白细胞增高，中性粒细胞比例增高，根据其临床表现及实验室检查，该患者最可能的诊断为急性血源性骨髓炎。
选项 A 骨巨细胞瘤典型表现为疼痛和肿胀，间歇性隐痛，局部压痛及关节活动受限，局部皮温增高，关节肿胀，与题干描述不符，故不选。
选项 B 化脓性关节炎好发于髋关节和膝关节，主要表现为关节局部温度升高、肿胀、疼痛、关节积液，浮髌试验常为阳性，与题干中浮髌试验阴性不符，故排除。
选项 C 骨肉瘤好发于长骨干骺端，尤其是股骨远端和胫骨近端，表现为局部疼痛，多为持续性，逐渐加剧，夜间为甚，伴全身恶病质、发热及贫血等，与该患儿症状差异明显，故不考虑。
选项 E 膝关节结核起病缓慢，有低热、乏力、疲倦、食欲差、消瘦、贫血等全身症状，膝关节周围肿胀，该患儿并无结核病史，且无上述症状，与题意不符。
综上所述，正确答案是：D.急性血源性骨髓炎。
题目 109：最可能的诊断
分析：根据病史和临床表现，孩子出现了高热、局部疼痛和肿胀，实验室检查显示白细胞总数升高和中性粒细胞比例高，这些表现提示有感染存在。以下是选项分析：

A. 骨巨细胞瘤：通常为良性肿瘤，常见于青少年和成年人，表现为渐进性疼痛和肿胀，不太可能导致急性高热和白细胞总数显著升高。
B. 化脓性关节炎：表现为关节的急性炎症、红肿热痛，但浮髌试验阴性提示关节腔积液不明显。
C. 骨肉瘤：恶性骨肿瘤，多见于儿童和青少年，通常为慢性进行性疼痛和肿胀，不太可能引起急性发热和高白细胞计数。
D. 急性血源性骨髓炎：急性发病，表现为高热、局部剧痛、红肿、压痛，白细胞总数和中性粒细胞比例显著升高，符合本例表现。
E. 膝关节结核：慢性病程，表现为长期低热、盗汗、逐渐加重的疼痛和肿胀，不符合急性高热和实验室表现。
基于上述分析，最可能的诊断是 D. 急性血源性骨髓炎。

110、 (A3/A4 型题) 题

治疗首选的抗生素

A.三代头孢

B.氧氟沙星

C.青霉素

D.庆大霉素

E.克林霉素

参考答案： A 您的答案：

图文解析： 【解析】患儿考虑诊断为急性血源性骨髓炎。急性血源性骨髓炎一旦诊断应立即开始足量抗生素治疗，最常见的致病菌是金黄色葡萄球菌，治疗首选头孢类抗生素（A 对）。【避错】全身及局部症状消失后，抗生素仍需连续应用 3~6 周。

110 题：治疗首选的抗生素

A. 三代头孢：对于治疗急性血源性骨髓炎，三代头孢类抗生素（如头孢曲松）是一个好选择，因为它们对抗革兰阳性菌和革兰阴性菌均有良好的覆盖范围。

B. 氧氟沙星：这是一种广谱抗生素，但不是治疗骨髓炎的首选，尤其是在儿童中，因为其对骨骼的潜在影响。

C. 青霉素：虽然对某些感染有效，但针对骨髓炎，它的覆盖范围可能不足够广泛。

D. 庆大霉素：这是一种强力的抗生素，但主要用于复杂的或难治性感染，并且有较高的毒性风险。

E. 克林霉素：可用于治疗骨和关节感染，但不是首选，尤其是在初步治疗急性血源性骨髓炎时。

因此，治疗首选的抗生素应该是 A. 三代头孢。

解析：

针对急性血源性骨髓炎的治疗，需要早期、足量、联合、敏感的抗生素应用，针对该患者情况，首选对金黄色葡萄球菌敏感的抗生素**，青霉素**对金黄色葡萄球菌具有较强的抗菌作用，故首选青霉素。

选项 A 三代头孢对革兰氏阴性杆菌的抗菌效果更强，常用于肺炎克雷伯菌等引起的肺炎，并非金黄色葡萄球菌的首选，故不选。

选项 B 氧氟沙星是喹诺酮类抗生素，常用于肺炎链球菌等引起的呼吸道、泌尿道感染，不是金黄色葡萄球菌的首选，故排除。

选项 D 庆大霉素是一种氨基糖苷类抗生素，对各种革兰阴性细菌及革兰阳性细菌都有良好的抗菌作用，但对金黄色葡萄球菌效果不佳，故不考虑。

选项 E 克林霉素主要用于厌氧菌引起的腹腔和盆腔感染，还可用于敏感的革兰阳性菌引起的呼吸道、关节、皮肤和软组织、骨组织、胆道等感染及败血症、心内膜炎等，与题干不符。

综上所述，正确答案是：C.青霉素。

题目 110：治疗首选的抗生素

分析：对于急性血源性骨髓炎的治疗，广谱抗生素是首选，尤其是覆盖金黄色葡萄球菌（最常见病原菌）的抗生素。以下是选项分析：

A. 三代头孢：如头孢噻肟或头孢曲松，对金黄色葡萄球菌有效，是广谱抗生素的代表，适合首选治疗。

B. 氧氟沙星：属于喹诺酮类抗生素，不推荐用于儿童。

C. 青霉素：虽然对链球菌有效，但对金黄色葡萄球菌效果有限。

D. 庆大霉素：主要用于革兰氏阴性菌感染，毒性较大，单独使用效果有限。

E. 克林霉素：对厌氧菌有效，可作为替代方案，但首选仍为三代头孢。
基于上述分析，治疗首选的抗生素是 A. 三代头孢。
题目材料　　经产妇，32 岁，孕 39 周，估算胎儿体重 3800g，骨盆外测量正常，规律宫缩 15 小时，间歇 8 分钟，持续 30 秒，宫口开 4cm，胎膜已破，LOA，S 图 I 型。
111、　(A3/A4 型题)　题
以下有可能的是
A.子宫协调性乏力
B.子宫不协调性乏力
C.子宫协调性收缩过强
D.子宫不协调性收缩过强
E.膈肌收缩减弱
参考答案：　　A　您的答案：
图文解析：　　【解析】子宫协调性收缩乏力（低张性子宫收缩乏力）子宫收缩具有正常的节律性、对称性和极性，但收缩力弱，宫腔压力低（＜2.0kPa），持续时间短，间歇期长且不规律，宫缩＜2 次/10 分钟。当子宫收缩达极期时，子宫体不隆起和变硬，用手指压宫底部肌壁仍可出现凹陷，产程延长或停滞。本患者为经产妇，32 岁，孕 39 周，估算胎儿体重 3800g，骨盆外测量正常，规律宫缩 15 小时，间歇 8 分钟，持续 30 秒，宫口开 4cm，胎膜已破，LOA，S＝-1，心电图 I 型（孕妇生产情况均良好，产程延长）。因此患者产程延长的时间有可能是（A）子宫协调性乏力。
【避错】子宫不协调性乏力主要表现为下腹部有压痛，胎位触不清，胎心不规律，宫口扩张缓慢或不扩张，胎先露部下降延缓或停滞，产程延长。
首先分析 111 题：
A 项“子宫协调性乏力”指的是宫缩强度不够，可能导致分娩延长。
B 项“子宫不协调性乏力”指的是宫缩强度和频率不规律，亦可能导致分娩延长。
C 项“子宫协调性收缩过强”和 D 项“子宫不协调性收缩过强”都表明宫缩过于强烈。
E 项“膈肌收缩减弱”与分娩过程关系不大。
考虑到宫口开 4cm，规律宫缩已持续 15 小时，但进展不显著，最可能的情况是宫缩强度不足，故 A 项“子宫协调性乏力”可能是正确答案。
根据题目描述，该孕妇规律宫缩 15 小时，间歇 8 分钟，持续 30 秒，宫口开 4cm，这些表现均符合协调性宫缩乏力的特点。现在我们逐项分析选项：
A. 子宫协调性乏力：由于宫缩间歇期较长（8 分钟）且持续时间相对较短（30 秒），这与协调性宫缩乏力的特点相符，即宫缩节律性、对称性和极性均正常，仅是收缩力弱，因此该选项是可能的。
B. 子宫不协调性乏力：这种乏力通常表现为宫缩节律性异常，而题干中描述的是规律宫缩，因此不符合。
C. 子宫协调性收缩过强：这会导致宫缩过于频繁和强烈，与题干描述的宫缩情况不符。
D. 子宫不协调性收缩过强：这通常表现为宫缩节律异常且强烈，与题干描述不符。
E. 膈肌收缩减弱：这与题干描述的宫缩乏力无直接关系，且膈肌收缩情况未在题干中提及。
综上所述，最有可能的答案是 A.子宫协调性乏力。
问题 111: 以下有可能的是
A. 子宫协调性乏力
B. 子宫不协调性乏力
C. 子宫协调性收缩过强
D. 子宫不协调性收缩过强

E. 膈肌收缩减弱
分析：

患者已规律宫缩 15 小时，但宫口只开到 4cm，表明产程进展缓慢。结合宫缩频率（间歇 8 分钟，持续 30 秒），这提示宫缩强度和频率不够，符合“子宫协调性乏力”的特征。

正确答案：A. 子宫协调性乏力
112、 (A3/A4 型题) 题
最恰当的治疗措施是
A.人工破膜
B.静滴缩宫素
C.剖宫产
D.肌注哌替啶
E.肌注地西泮
参考答案： B 您的答案：
图文解析： 【解析】对于子宫协调性乏力的治疗方案可应用缩宫素静脉滴注，主要适用于协调性宫缩乏力、宫口扩张 3cm、胎心良好、胎位正常、头盆相称者。因此本题应该选择静滴缩宫素（B）。
【避错】若发现有头盆不称，估计不能经阴道分娩者，应及时行剖宫产术；若判断无头盆不称和胎位异常，估计能经阴道分娩者，应采取加强宫缩的措施。人工破膜：宫口扩张≥3cm、无头盆不称、胎头已衔接者，可行人工破膜。哌替啶，地西泮主要起到镇静作用，不能增强宫缩。
接着分析 112 题的最恰当治疗措施：
A 项“人工破膜”通常用于加速宫缩，但题干中提到胎膜已破，故此项不适用。
B 项“静滴缩宫素”用于增强宫缩，适用于宫缩乏力的情况。
C 项“剖宫产”是在无法顺产或存在母婴风险时考虑的选项。
D 项“肌注哌替啶”用于镇痛，E 项“肌注地西泮”用于镇静，这两项更多是对症处理，不解决根本问题。
鉴于上述分析，对于 111 题，答案应该是 A“子宫协调性乏力”；对于 112 题，答案应该是 B“静滴缩宫素”，因为它可以增强宫缩力，有助于推进分娩过程。
针对该孕妇的宫缩乏力情况，我们需要选择一个恰当的治疗措施。现在我们分析各个选项：
A. 人工破膜：虽然人工破膜有时用于加强宫缩，但题干中已明确胎膜已破，因此这一选项不适用。
B. 静滴缩宫素：对于协调性宫缩乏力，当宫口扩张≥3cm、胎心良好、胎位正常、头盆相称时，可以静脉滴注缩宫素加强宫缩。题干中的情况符合这些条件，因此这是一个恰当的治疗措施。
C. 剖宫产：在没有其他并发症或紧急情况下，剖宫产不是首选治疗方案。
D. 肌注哌替啶：这通常用于治疗不协调性宫缩乏力，与题干描述的宫缩情况不符。
E. 肌注地西泮：同样多用于治疗不协调性宫缩乏力，不适用于本题中的情况。
综上所述，最恰当的治疗措施是 B.静滴缩宫素。
问题 112: 最恰当的治疗措施是
A. 人工破膜
B. 静滴缩宫素
C. 剖宫产

D. 肌注哌替啶
E. 肌注地西泮
分析：

鉴于子宫协调性乏力，最佳的治疗措施是使用静滴缩宫素，以增强宫缩力量，促进产程进展。其他选项如人工破膜、剖宫产等不适用于这种情况，哌替啶和地西泮主要用于镇痛和镇静，不是治疗子宫乏力的首选。

正确答案：B. 静滴缩宫素
题目材料　　男婴，6 个月，腹泻 6 个月，4~7 次/天，黏稠样便，无呕吐，无发热，食欲尚可，精神尚可，出生体重 3.2kg，现体重 7.1kg，单纯母乳喂养，　脱水样貌，营养发育正常，心肺无异常，腹部肠蠕动，肠鸣音正常。
113、　(A3/A4 型题)　题
考虑的诊断是
A.过敏性腹泻
B.感染性腹泻
C.生理腹泻
D.消化功能紊乱
E.失氯性腹泻
参考答案：　C　您的答案：
图文解析：　【解析】生理性腹泻多见于 6 个月以下的婴儿，其外观虚胖，常有湿疹，出生后不久即腹泻，每天大便次数多，甚至十几次，每次大便量不一定很多，其中含少量水分，一般没有特殊腥臭味。生理性腹泻的婴儿除大便次数增多外，多无其他症状，食欲好，无呕吐，生长发育不受影响，一般添加辅食后即恢复正常。患者为男婴，6 个月，腹泻 6 个月，4~7 次/天（腹泻表现），黏稠样便（大便性状无异常），无呕吐，无发热，食欲精神尚可，体重增长正常，单纯母乳喂养（生理性腹泻一般诱因），体征未见脱水样貌，营养发育正常，心肺无异常，腹部肠蠕动，肠鸣音正常（无明显病理性症状表现）。因此可诊断患儿为（C）生理腹泻。
【避错】过敏性腹泻应伴有其他部位过敏性表现；感染腹泻多伴有发热；消化功能紊乱则患儿生长发育可能受到一定程度影响；失氯性腹泻，　以顽固性腹泻、低氯血症、低钠血症、低钾血症和代谢性碱中毒为主要特征，表现为水样泻。
113 题，考虑的诊断是：
过敏性腹泻 (A)：鉴于婴儿持续的腹泻症状以及单纯母乳喂养的背景，过敏性腹泻成为一个可能的诊断。此情况可能由母乳中的某些食物成分（如牛奶蛋白）引起，导致婴儿出现消化道反应。
感染性腹泻 (B)：通常会伴有发热、呕吐等症状，此案例中未提及这些症状。
生理腹泻 (C)：通常指新生儿期的生理性腹泻，对于 6 个月大的婴儿不太适用。
消化功能紊乱 (D)：可能考虑在内，但通常需要更多证据来支持这一诊断。
失氯性腹泻 (E)：是一种较少见的疾病，通常需要电解质失衡等其他症状来支持诊断。
因此，对于 113 题，最可能的诊断是过敏性腹泻 (A)。
113、正确答案是：C.生理腹泻
患儿 6 个月大，腹泻已持续 6 个月，每天 4~7 次，但除腹泻外，其他情况如食欲、精神均正常，营养发育也未受影响。出生体重 3.2kg，现体重 7.1kg，表明体重增长正常。同时，患儿无呕吐、发热等症状，心肺无异常，肠鸣音也正常。根据患儿的症状和体征，最可能的诊断

是生理性腹泻。生理性腹泻多见于 6 个月以下的婴儿，其外观虚胖，常有湿疹，出生后不久即腹泻，每天大便次数多，甚至十几次，每次大便量不一定很多，其中含少量水分，一般没有特殊腥臭味。生理性腹泻的婴儿除大便次数增多外，多无其他症状，食欲好，无呕吐，生长发育不受影响，添加辅食后，大便即逐渐转为正常。因此，结合患儿情况，考虑诊断为生理腹泻。

113、考虑的诊断是：

男婴，6 个月，腹泻 6 个月，4-7 次/天，黏稠样便，无呕吐，无发热，食欲尚可，精神尚可，体重增长正常（出生体重 3.2kg，现体重 7.1kg），单纯母乳喂养，脱水样貌但营养发育正常，心肺无异常，腹部肠蠕动，肠鸣音正常。这些信息提示婴儿的腹泻情况可能并不是由感染或过敏引起的，而是生理性腹泻或因消化功能不成熟导致的。

答案选择：

C. 生理腹泻

114、 (A3/A4 型题) 题

可行处理方法

A.减少母乳喂养次数，使用蛋白水降解配方奶

B.继续母乳喂养逐步由少到多添加米糊，米糊等辅食

C.使用止泻药

D.使用抗生素

E.停止母乳喂养，使用蛋白水解配方奶

参考答案： B 您的答案：

图文解析： 【解析】患者诊断为生理性腹泻，一般经添加辅食即可好转。

【避错】蛋白水解配方奶多用于适用于乳蛋白过敏患儿，本患儿仅为生理腹泻，不必使用蛋白水解配方奶。

114 题，可行处理方法：

减少母乳喂养次数，使用蛋白水降解配方奶 (A)：如果怀疑过敏性腹泻，可以考虑这种方案，以减少过敏原的摄入。

继续母乳喂养逐步由少到多添加米糊，米糊等辅食 (B)：对于 6 个月大的婴儿，逐渐添加辅食是合适的，但应确保不引入潜在的过敏原。

使用止泻药 (C)：通常不建议在未明确诊断的情况下给婴儿使用止泻药。

使用抗生素 (D)：除非有明确的细菌感染证据，否则不建议使用。

停止母乳喂养，使用蛋白水解配方奶 (E)：这可以是一个选择，如果怀疑是严重的食物过敏。

考虑到这些因素，对于 114 题，如果诊断为过敏性腹泻，则合理的处理方法可能是减少母乳喂养次数，使用蛋白水降解配方奶 (A)。

114、正确答案是：B.继续母乳喂养逐步由少到多添加米糊，米糊等辅食

对于生理性腹泻的患儿，一般不需要特殊治疗，因为生理性腹泻并不是疾病，而是一种正常的生理现象。虽然患儿排便次数较多，但是患儿的精神状态、食欲以及体重增长等均未受到影响。如果盲目给患儿使用止泻药或抗生素，可能会对患儿的肠道微生态造成破坏，反而不利于患儿的健康成长。因此，对于该患儿，可行的处理方法是继续母乳喂养，并逐步由少到多地添加辅食，如米糊等。这样既能够满足患儿的营养需求，又能够逐渐改善患儿的腹泻症状。因此，选项 B 是正确答案。选项 A“减少母乳喂养次数，使用蛋白水降解配方奶”、选项 E“停止母乳喂养，使用蛋白水解配方奶”均不合适，因为母乳是婴儿最好的食物，且该患儿并未出现对母乳不耐受或过敏的情况。选项 C“使用止泻药”和选项 D“使用抗生素”

也不可取，因为该患儿并没有感染或炎症等需要使用药物的病症。
114、可行处理方法：

由于男婴体重增长正常，营养发育正常，说明母乳喂养可以提供足够的营养且对婴儿有益。生理腹泻通常是由于婴儿的消化系统尚未完全成熟导致的，在这种情况下，继续母乳喂养并逐步添加辅食是合理的处理方法。

答案选择：
B. 继续母乳喂养逐步由少到多添加米糊，米糊等辅食
题目材料　　女，38 岁，接触性出血 1 周，月经规律，G3P1,妇科检查：宫颈上唇见 1.0cm ×0.5cm，糜烂样改变，质脆，出血明显，子宫及双附件未触及异 诊宫旁软。
115、　(A3/A4 型题)　题
最有助于确诊的检查是
A.妇科超声检查
B.宫颈染色细胞学检查
C.宫颈活组织检查
D.人乳头状瘤病毒检查
E.阴道分泌物检查
参考答案：　　C　您的答案：
图文解析：　　【解析】患者女，38 岁，接触性出血 1 周，月经规律（激素无异常），体检见宫颈上唇 1.0cm×0.5cm 糜烂样改变，质脆，出血明显（子宫颈病变提示），子宫及双附件未触及异常，三合诊宫旁软（除外子宫颈外其他部位病变）。证明患者病变主要来自子宫颈，为进一步诊断病理性质，因此应取宫颈活组织行病理学检查，因此选择 C。
【避错】子宫及双附件未触及异常，三合诊宫旁软，妇科超声检查意义不大；宫颈染色细胞学检查在细胞组织学的权威性不如病理学诊断；人乳头状瘤病毒检查主要用于诊断 HPV 感染；　阴道分泌物检查不能进一步确认病变性质。
第 115 题
最有助于确诊的检查是宫颈活组织检查。
A. 妇科超声检查：用于观察子宫、附件等结构，但对于宫颈表面的病变不如组织检查直接。
B. 宫颈染色细胞学检查：对筛查宫颈病变有用，但对确诊具体的宫颈病理状态不如活检准确。
C. 宫颈活组织检查：可以直接观察到组织的病理变化，是确诊宫颈病变的金标准。
D. 人乳头状瘤病毒检查：主要用于宫颈癌的风险评估和筛查，而非直接诊断。
E. 阴道分泌物检查：用于检测感染等状况，不适用于宫颈病变的确诊。
因此，最佳答案是 C. 宫颈活组织检查。
115、解析：
青年女性患者，接触性出血，妇科检查见宫颈上唇见糜烂样改变，质脆且易出血，子宫及双附件、宫旁均未见异常，考虑为子宫颈癌。子宫颈癌诊断的“金标准”是宫颈活组织检查。
选项 A 妇科超声检查虽能发现宫颈异常回声肿块，但不能确诊是否为子宫颈癌，可排除。
选项 B 宫颈染色细胞学检查即宫颈细胞学检查或 TCT、LCT，主要用于 CIN 及早期子宫颈癌筛查的基本方法，不能确诊，故不选。
选项 D 人乳头状瘤病毒检查主要用于 CIN 及子宫颈癌发病因素的检查，亦不能确诊，故排除。
选项 E 阴道分泌物检查主要用于女性生殖系统感染、肿瘤的诊断、雌激素水平的判断及 STD 的病原学检查，对于宫颈癌的诊断没有意义，故不考虑。

综上所述，正确答案是：C.宫颈活组织检查。
题目 115：最有助于确诊的检查是

在这种情况下，最有助于确诊宫颈病变的检查是宫颈活组织检查。虽然宫颈染色细胞学检查和人乳头状瘤病毒检查也有助于筛查宫颈病变，但活组织检查能够提供组织学诊断，这是确诊的金标准。

最佳答案：C. 宫颈活组织检查
116、 (A3/A4 型题)
若该患者确诊为鳞状细胞癌，基底膜下浸润深度 2mm，则分期是 题
A. Ⅰ A1
B. Ⅰ A2
C. Ⅱ B1
D. Ⅱ B2
E.ⅢB3
参考答案： A 您的答案：
图文解析： 【解析】宫颈鳞癌分期标准Ⅰ A1：所测量间质浸润深度＜3.0mm； Ⅰ A2：3.0mm ≤所测量间质浸润深度 ≤5.0mm； Ⅰ B1：所测量间质浸润深度＞5.0mm 而最大径线＜=2.0cm； Ⅰ B2：最大径线>2.0cm； Ⅰ B3：最大径线＞2.0cm。因此正确答案应为 A。
【避错】 Ⅰ A 分期浸润深度最大为 5mm， Ⅰ B 则不低于 5mm。
第 116 题
若该患者确诊为鳞状细胞癌，基底膜下浸润深度 2mm，则分期是 IA1。
A. Ⅰ A1：宫颈鳞状细胞癌的分期为 IA1 指的是浸润深度≤3mm 且最大横径≤7mm。
B. Ⅰ A2：浸润深度>3mm 且≤5mm，最大横径≤7mm。
C. Ⅱ B1：更深的浸润或更大的肿瘤，但不适用于此情况。
D. Ⅱ B2：进一步分期的宫颈癌，与本例情况不符。
E. ⅢB3：更高级别的宫颈癌分期，与本例情况不符。
因此，最佳答案是 A. Ⅰ A1。
116、解析：
该患者已确诊为鳞状细胞癌，基底膜下浸润深度 2mm，根据子宫颈癌的临床分期，癌灶局限在子宫颈，即间质浸润深度＜3mm，宽度≤7mm，为Ⅰ A1 期。
选项 B：Ⅰ A2 为间质浸润深度＞3mm 且＜5mm，宽度≤7mm，与题干描述不符。
选项 C：Ⅱ B1，选项 D：Ⅱ B2，均属于Ⅱ B 期，肿瘤超越子宫，但未达骨盆壁或未达阴道下 1/3，不符合题意。
选项 E：ⅢB 期指肿瘤扩展到骨盆壁，和/或肾积水，或肾无功能，与题干描述不符。
综上所述，正确答案是：A. Ⅰ A1。
题目 116：若该患者确诊为鳞状细胞癌，基底膜下浸润深度 2mm，则分期是

根据国际妇产科联盟（FIGO）宫颈癌分期系统，基底膜下浸润深度小于或等于 3mm 的鳞状细胞癌分为Ⅰ A 期。进一步区分为Ⅰ A1 和Ⅰ A2 期：Ⅰ A1 期是基底膜下浸润深度小于或等于 3mm，且水平扩展不超过 7mm； Ⅰ A2 期是基底膜下浸润深度超过 3mm 但不超过 5mm，且水平扩展不超过 7mm。

由于题干中提到的基底膜下浸润深度为 2mm，因此患者的分期为Ⅰ A1 期。

最佳答案：A. Ⅰ A1

题目材料　　女，62 岁，G6P4，绝经 6 年，阴道反复少量流血 4 个月，糖尿病病史 5 年。查体：BP160/105mmHg，身高 155cm，体重 80kg，妇科检查：阴道少 颈光滑，子宫正常大小，双侧附件未见异常。经阴道超声检查：子宫内膜 1.1cm，不规则增厚，其内与相邻肌层内血流丰富。

117、　(A3/A4 型题)　题

与该病无关的因素是

A.多产

B.肥胖

C.高血压

D.绝经延迟

E.糖尿病

参考答案：　A　您的答案：

图文解析：　【解析】该患者 62 岁，G6P4（提示多产），绝经 6 年（提示绝经延后），阴道反复少量流血 4 个月，糖尿病病史 5 年（提示糖尿病史）。血压为 BP160/105mmHg（提示高血压），身高 155cm，体重 80kg（BMI 超标，为超重），主要症状为阴道少量流血，宫颈光滑，子宫正常大小，双侧附件未见异常。阴超检查见子宫内膜 1.1cm，不规则增厚，其内与相邻肌层内血流丰富。子宫内膜增厚病因可因肥胖、高血压、绝经延迟、糖尿病等引起的激素失调导致阴道反复流血，　同多产问题不大。因此选择 A。

【避错】正常绝经时间应为 45~55 岁，本患者绝经时间为 57 岁，属绝经延迟。

117 题：

与该病无关的因素是：

A. 多产

B. 肥胖

C. 高血压

D. 绝经延迟

E. 糖尿病

这里需要确定与子宫内膜癌无关的因素。多产、肥胖、绝经延迟和糖尿病都被认为是子宫内膜癌的风险因素。而高血压与子宫内膜癌的直接关联性较低，因此在这些选项中，C. 高血压 应该是与该病无关的因素。

与该病无关的因素是

答案：D.绝经延迟

解析：患者老年女性，已绝经，出现绝经后阴道流血，超声示子宫内膜增厚，血流丰富，应考虑为子宫内膜癌。子宫内膜癌的发生与多种因素有关，包括无孕酮拮抗的雌激素长期作用下，发生子宫内膜增生症，继而癌变；基因突变如 PTEN 基因失活；林奇综合征等。而绝经是每一个妇女生命进程中必然发生的过程，与子宫内膜癌的发生无关。

选项 A 多产，选项 B 肥胖，选项 C 高血压，选项 E 糖尿病，均与子宫内膜癌发病相关，故一并排除。

117. (A3/A4 型题) 与该病无关的因素是

A. 多产

B. 肥胖

C. 高血压

D. 绝经延迟

E. 糖尿病

答案解析：
这个患者的情况提示可能的子宫内膜病变，如子宫内膜增生或子宫内膜癌。子宫内膜增生和子宫内膜癌的风险因素包括多产、肥胖、高血压、绝经延迟和糖尿病。

与子宫内膜增生或子宫内膜癌无关的因素是：
A. 多产
118、 (A3/A4 型题)
以下适合的手术方法是 题
A.广泛性子宫切除＋双附件子宫切除＋盆腔、腹部动脉淋巴切除术
B.大剂量激素治疗
C.宫腔镜子宫内膜电极切除术
D.筋膜外全子宫切除术＋双附件子宫切除术＋盆腔、腹部动脉淋巴切除术
E.子宫内膜消融术
参考答案： D 您的答案：
图文解析： 【解析】根据病史体征可知，患者有糖尿病史且肥胖，有子宫内膜增厚，患者已 62 岁，绝经已 6 年， 已无生育需求。故应对患者行子宫全切术最佳，筋膜外全子宫切除术适用于一般情况较差，合并其他疾病不能耐受筋膜外全子宫切除术以上手术的患者，因此该患者应该选择（D）筋膜外全子宫切除术＋双附件子宫切除术＋盆腔，腹部动脉淋巴切除术。
【避错】筋膜外全子宫切除术是指接近宫颈分离侧平面，不包括宫颈间质，在宫颈附着处切断子宫骶韧带，切除的阴道壁一厘米左右。广泛性子宫切除指全子宫切除，将子宫主韧带在盆壁在提肛处切除，子宫骶韧带靠近其下外侧附着处切除或保留一厘米的主韧带及骶韧带，以帮助排尿功能的恢复，必须切除阴道上段的三分之一。
118 题：
以下适合的手术方法是：
A. 广泛性子宫切除＋双附件子宫切除＋盆腔、腹部动脉淋巴切除术
B. 大剂量激素治疗
C. 宫腔镜子宫内膜电极切除术
D. 筋膜外全子宫切除术＋双附件子宫切除术＋盆腔、腹部动脉淋巴切除术
E. 子宫内膜消融术
考虑到患者的年龄、症状及超声检查结果，如果确诊为子宫内膜癌，需进行手术治疗。在这些选项中，广泛性子宫切除（包括子宫、附件以及相关淋巴结切除）是较为彻底的治疗方法。然而，选择 A 或 D 取决于癌症的分期和扩散情况。考虑到题目中没有明确的癌症分期信息，但已知子宫内膜明显增厚并且血流丰富，这可能暗示了癌症的存在。D. 筋膜外全子宫切除术＋双附件子宫切除术＋盆腔、腹部动脉淋巴切除术 是一种更彻底的治疗方式，可能适用于高度怀疑或确诊的子宫内膜癌患者，尤其是在考虑到潜在的癌症扩散时。
适合的手术方法是
答案：D.筋膜外全子宫切除术＋双附件子宫切除术＋盆腔、腹部动脉淋巴切除术
解析：患者老年女性，出现绝经后阴道流血，超声示子宫内膜增厚，血流丰富，应考虑为子宫内膜癌。其首选治疗方法为手术治疗，手术目的为手术病理分期，确定病变范围及与预后相关因素，切除病变子宫及其他可能存在的转移病灶，决定术后辅助治疗的选择。该患者为Ⅰ期子宫内膜癌，适合的手术方法为筋膜外全子宫切除加双侧附件切除术，同时加做盆腔和

腹主动脉旁淋巴结清扫。
选项 A 广泛性子宫切除＋双附件子宫切除＋盆腔、腹部动脉淋巴切除术是子宫颈癌的手术方式，与题意不符。
选项 B 大剂量激素治疗，选项 E 子宫内膜消融术，均属于药物保守治疗，主要适用于要求保留生育功能的年轻患者，而该患者已绝经且年龄较大，不适用，故一并排除。
选项 C 宫腔镜子宫内膜电极切除术主要用于治疗功能失调性子宫出血，故不考虑。
118. (A3/A4 型题) 以下适合的手术方法是
A. 广泛性子宫切除＋双附件子宫切除＋盆腔、腹部动脉淋巴切除术
B. 大剂量激素治疗
C. 宫腔镜子宫内膜电极切除术
D. 筋膜外全子宫切除术＋双附件子宫切除术＋盆腔、腹部动脉淋巴切除术
E. 子宫内膜消融术

答案解析：
患者 62 岁，绝经后阴道流血，子宫内膜厚度 1.1cm，不规则增厚，且内膜与肌层血流丰富，这提示可能的子宫内膜癌。根据最佳实践指南，手术治疗应包括切除可能受累的子宫、附件及进行淋巴结清扫以评估病变扩散情况。

适合的手术方法是：
D. 筋膜外全子宫切除术＋双附件子宫切除术＋盆腔、腹部动脉淋巴切除术
题目材料 女，35 岁，反复发作胸闷、心慌半年，再发半小时。平时工作压力大，半年内突发三次胸闷，心慌，呼吸急促，立刻医院急诊就诊，查心电 CT，心肌酶谱，肺功能检查均未见明显异常，吸氧后症状缓解。半小时前再发胸闷，气促，心慌，手掌麻木，无胸痛，无呕吐，查体：T37.5℃,P87 次 次/分，血压 120/70mmHg，紧张面容，听诊未闻及哮鸣音，心律齐，病理征阴性。
119、 (A3/A4 型题) 题
考虑诊断为
A.不稳定心绞痛
B.惊恐障碍
C.广泛焦虑障碍
D.躯体形式障碍
E.支气管哮喘
参考答案： B 您的答案：
图文解析： 【解析】惊恐障碍是一种以反复出现的突如其来的惊恐体验为特征的急性焦虑障碍，典型惊恐发作往往发生在日常活动时，且未见器质性病变。患者女，35 岁，反复发作胸闷、心慌半年，再发半小时。 诱因为工作压力大，半年内突发三次胸闷，心慌，呼吸急促（惊恐障碍症状），立刻医院急诊就诊，查心电图，肺部 CT，心肌酶谱，肺功能检查均未见明显异常（排除器质性病变），吸氧后症状缓解。半小时前再发胸闷，气促，心慌，手掌麻木（惊恐障碍症状），查体未见异常。因此该患者应诊断为（B）惊恐障碍。
【避错】患者心电图及血生化均未出现异常，因此排除不稳定心绞痛；广泛性焦虑障碍是以经常全面的、无明确对象或固定内容的紧张不安及过度为特征。这种焦虑与周围任何特定的情境没有关系；广泛焦虑障碍会体现为无器质性病变的异常皮肤感觉与胃肠道反应；支气管哮喘多有诱因且需要进行支气管诱发实验进行进一步诊断。
119、考虑诊断为：

B. 惊恐障碍
这位患者的症状，包括反复胸闷、心慌、气促和手掌麻木，以及症状的突然发作和缓解，更符合惊恐障碍的表现。心电图、心肌酶谱和肺功能检查未发现异常，支持了这一诊断而非心血管或呼吸系统疾病。
119、正确答案：B.惊恐障碍。
中年女性患者，平时工作压力大，突发胸闷，心慌，呼吸急促，多次查心电、CT、心肌酶谱、肺功能检查均未见明显异常，吸氧后症状缓解，半小时前再发，伴手掌麻木，查体无异常体征，根据其临床表现和辅助检查，考虑诊断为惊恐障碍。惊恐障碍简称惊恐症，是以反复出现显著的心悸，出汗、震颤等自主神经症状，伴以强烈的濒死感或失控感，害怕产生不幸后果的惊恐发作为特征的一种急性焦虑障碍。
选项 A 不稳定心绞痛胸痛性质与稳定型心绞痛相似，为压迫、发闷或紧缩性，但疼痛程度更重，持续时间更长，且胸痛在休息时也可发生，与题意不符。
选项 C 广泛焦虑障碍是以持续的显著紧张不安，伴有自主神经功能兴奋和过分警觉为特征的一种慢性焦虑障碍，题干中未提及过分警觉及自主神经功能兴奋等症状，故不选。
选项 D 躯体形式障碍是一种以持久的担心或相信各种躯体症状的优势观念为特征的神经症，病人因这些症状反复就医，各种医学检查阴性和医生的解释均不能打消其疑虑，与本题患者症状不符。
选项 E 支气管哮喘典型症状为伴有哮鸣音的呼气性呼吸困难，且症状可在数分钟内发生，并持续数小时至数天，可经平喘药物治疗后缓解或自行缓解，夜间及凌晨发作或加重常是哮喘的重要临床特征，该患者无相关表现，故排除。
119、考虑诊断为
B. 惊恐障碍

解释：患者有反复发作的胸闷、心慌、呼吸急促等症状，且每次急诊检查结果均未见明显异常，症状在吸氧后缓解。结合患者紧张的面容和发作时的心慌、手掌麻木等特点，更符合惊恐障碍的表现。
120、 (A3/A4 型题) 题
首选的治疗药物是
A.心境稳定剂
B.扩血管药
C.抗精神病药
D.解痉平喘药
E.抗抑郁药
参考答案： A 您的答案：
图文解析： 【解析】患者诊断为惊恐障碍，首选药物为心境稳定剂，也被称为抗躁狂药，除抗躁狂作用外，对双相情感障碍也有稳定和预防复发作用，故又称情感稳定剂。三大心境稳定剂包括：锂盐、双丙戊酸钠和拉莫三嗪。
【避错】患者需要的是抗焦虑抗躁狂治疗，因此需要心境稳定剂，应避免选择为抗抑郁药物。
120、首选的治疗药物是：
E. 抗抑郁药
惊恐障碍的治疗中，选择性血清素再摄取抑制剂（SSRIs）是首选治疗选项，它属于抗抑郁药。虽然这些药物最初是为治疗抑郁症开发的，但也被证明对惊恐障碍和其他焦虑疾病有效。
120、正确答案：E.抗抑郁药。
惊恐障碍简称惊恐症，是以反复出现显著的心悸，出汗、震颤等自主神经症状，伴以强烈的

濒死感或失控感，害怕产生不幸后果的惊恐发作为特征的一种急性焦虑障碍。治疗时以心理治疗为主，药物治疗为辅，药物主要选择抗抑郁药、苯二氮䓬类、β-肾上腺素能受体阻滞剂，故首选的治疗药物是抗抑郁药。
选项 A 心境稳定剂主要用于治疗躁狂症，是双相情感障碍的基础用药，与该患者症状不符，故不选。
选项 B 扩血管药，选项 D 解痉平喘药，均用于治疗心绞痛及支气管哮喘，与该患者症状不符，可一并排除。
选项 C 抗精神病药主要用于治疗精神分裂症及其他具有精神病性症状的精神障碍，与本题不符。
120、首选的治疗药物是
E. 抗抑郁药

解释：惊恐障碍的首选治疗药物通常是抗抑郁药物，特别是选择性 5-羟色胺再摄取抑制剂（SSRIs），如舍曲林、帕罗西汀等，这类药物在缓解惊恐发作和预防复发方面效果显著。
121、 (A3/A4 型题) 题
药物治疗的时间为
A.1 周
B.1 个月
C.3 个月
D.6 个月
E.1 年
参考答案： D 您的答案：
图文解析： 【解析】心境稳定剂治疗时间 6 个月无效后应进行更换更有效的药物，不推荐两个及两个以上治疗药物进行联用。
【避错】准确记忆药物应用时间。
121、药物治疗的时间为：
D. 6 个月
对于惊恐障碍的治疗，一般推荐至少持续 6 个月的药物治疗。这是为了减少复发的风险，并帮助患者更好地管理和控制其症状。
121、正确答案：B.1 个月。
抗焦虑药起效较慢，往往在用药初期可能出现不良反应，所以用药应从小剂量开始，然后缓慢加量以减少不良反应的发生。在用药 2～4 周后开始起效，所以没有特殊情况不需要换药，一般情况在服用药物 1 个月左右就能有明显的疗效，故药物治疗的时间为 1 个月。
选项 A：1 周时间太短，药物还未起效，故不选。
选项 C：3 个月，选项 D：6 个月，选项 E：1 年，均时间太长，不是最佳治疗时间，故可一并排除。
121、药物治疗的时间为
D. 6 个月

解释：对于惊恐障碍的药物治疗，通常建议至少治疗 6 个月以稳定病情，并防止症状复发。治疗时间过短可能导致症状未完全缓解或易于复发。
题目材料 男，67 岁，患再生障碍性贫血 5 年，通过输血纠正了贫血症状，近两次输血出现了发热反应，但无腰痛，血尿。
122、 (A3/A4 型题)

该患者出现的输血发热反应是
A.过敏反应
B.迟发性溶血性发热
C.急性溶血性发热
D.非溶血性发热
E.受到细菌污染
参考答案：　　D　您的答案：
图文解析：　　【解析】非溶血性发热性输血反应是中较为常见的一种反应。它是指患者在输血中或输血后体温升高≥1℃，并以发热为主要临床表现，且能排除溶血、细菌污染、严重过敏等原因引起发热的一类输血反应。患者男，67 岁，患再生障碍性贫血 5 年，通过输血纠正了贫血症状，近两次输血出现了发热反应（患者有多次输血史，可诱发非溶血性发热），但无腰痛，血尿（排除溶血性发热）。排除细菌污染、过敏及溶血性反应，因此该患者应该诊断为非溶血性发热。
【避错】患者曾有输血史且无其他皮肤表现，因此排除过敏反应；患者无腰痛尿血，因此排除溶血反应；患者发热不明显，且未见血象检查异常，无寒战，因此排除后得此患者可诊断为非溶血性发热。
122 题的答案：
该患者出现的输血发热反应可能是 D.非溶血性发热。非溶血性发热反应是输血最常见的副作用，通常由输血时白细胞、血小板或者血浆中的蛋白质引起，这种发热反应通常不严重，而且患者没有腰痛和血尿的症状，这通常与溶血性反应相关联。
122、解析：
老年男性患者，患有再生障碍性贫血，长期接受输血治疗。近期输血后出现发热反应，但无腰痛、血尿等其他症状。根据临床表现，该患者最可能出现了非溶血性发热。非溶血性发热是最常见的早期输血并发症，发生率可达 2%～10%。多发生于输血开始后 15 分钟～2 小时内。主要表现为畏寒、寒战和高热，体温可上升至 39℃～40℃，同时伴有头痛、出汗、恶心或呕吐。
选项 A 过敏反应多发生在输血数分钟后，也可在输血中或输血后发生，主要表现为皮肤局限性或全身性瘙痒或荨麻疹，严重者可出现支气管痉挛，呼吸困难，胸闷，腹痛，甚至过敏性休克乃至昏迷，死亡，与题意不符。
选项 B 迟发性溶血性发热，选项 C 急性溶血性发热，均会出现溶血表现，如腰背疼痛、血红蛋白尿等，严重者可出现急性肾衰竭和 DIC，与该患者症状不符，故可一并排除。
选项 E 受到细菌污染会出现败血症的表现，该患者并未出现感染征象，故不考虑。122.D.非溶血性发热；
122. 该患者出现的输血发热反应是
A. 过敏反应
B. 迟发性溶血性发热
C. 急性溶血性发热
D. 非溶血性发热
E. 受到细菌污染

正确答案：D. 非溶血性发热

非溶血性发热反应是输血后常见的副作用，通常是由输血过程中受血者对供血者的白细胞或血小板的反应引起的。因为患者没有出现腰痛和血尿，急性和迟发性溶血反应的可能性较小，

而非溶血性发热反应则更为可能。

123、 (A3/A4 型题) 题

该患者出现输血发热反应的原因是

A.存在致热原

B.受血者存在同种白细胞和血小板

C.与共者血型不合

D.血液运输，保存受污染

E.受血者患有溶血性疾病

参考答案： B 您的答案：

图文解析： 【解析】非溶血性发热性输血反应通常与白细胞和/或血小板抗体以及血液保存中产生的细胞因子有关，多发生于输注多人份血小板浓缩液（PCs） 时以及多次输血或多次怀孕的妇女中。因此本题应该选择 B。

【避错】存在致热原及血液运输，保存受污染可以导致感染性发热；与共者血型不合、受血者患有溶血性疾病可发生溶血性反应。

123 题的答案：

该患者出现输血发热反应的原因可能是 B.受血者存在同种白细胞和血小板。这是非溶血性发热反应的常见原因之一，当输血中的白细胞或血小板在受血者体内被识别和摧毁时，可能会引起发热。

123、解析：

关于输血后发热的原因，考虑到患者未出现溶血、过敏或感染的症状，最可能的原因是输血过程中存在致热原，包括药物、生物制品或输血器等被致热原污染，导致患者在输血后出现发热反应。

选项 B 受血者存在同种白细胞和血小板通常与输血后的免疫反应相关，如发热、HLA 抗体引起的无效输血等，但并非直接导致发热，故不选。

选项 C 与供血者血型不合会导致急性溶血反应，但患者并未出现溶血的症状，可排除。

选项 D 血液运输、保存受污染通常会导致败血症等严重感染，但患者并未出现感染征象，不符合题意。

选项 E 受血者患有溶血性疾病通常会导致溶血反应，而患者并未出现溶血的症状，故排除。

123.A.存在致热原。

123. 该患者出现输血发热反应的原因是

A. 存在致热原

B. 受血者存在同种白细胞和血小板

C. 与供者血型不合

D. 血液运输，保存受污染

E. 受血者患有溶血性疾病

正确答案：B. 受血者存在同种白细胞和血小板

题目材料 女，50 岁，反复肢体抽搐 2 年，发作时四肢抽搐，伴意识丧失，数分钟后缓解。4 个月内类似症状出现 3 次。查体：T 36℃,血压：140/90mmH 楚，脑神经检查正常。四肢肌力 5 级，肌反射对称，病理反射阴性。MRI 提示顶叶巨大占位。

124、 (A3/A4 型题) 题

考虑什么

A.脑出血

B.脑脓肿

C.颅内肿瘤
D.静止性震颤
E.癫痫
参考答案：　　C　您的答案：
图文解析：　　【解析】 颅内肿瘤是指生长在颅腔的新生物，可起源于脑、脑膜、神经、血管及脑附件，或由身体的其他组织或脏器转移侵入颅内而形成，大都可产生头痛、颅内高压及局灶性症状。该患者反复肢体抽搐 2 年，发作时四肢抽搐，伴意识丧失，数分钟后缓解（考虑颅脑病变，排除静止性震颤）　。体格检查见 T 36℃ ，血压：　140/90mmHg，神志清楚，脑神经检查正常（脑部神经无异常放电，排除癫痫）　。四肢肌力 5 级，肌反射对称，病理反射阴性（排除脑出血）　。该患者 MRI 发现顶叶有巨大占位（颅内肿瘤影像学表现，提示颅内肿瘤）　，因此考虑为　（C）颅内肿瘤。
【避错】脑出血及脑脓肿应在　MRI　上有相应影像学表现；静止性震颤应表现为肌肉在松弛的情况下出现肢体抖动，睡眠时消失，较有节律；癫痫患者将出现脑神经的异常放电现象。
对于第 124 题，考虑病例的描述和 MRI 结果，最可能的诊断是颅内肿瘤（C 选项）。症状和占位性病变在 MRI 上的发现，提示这可能是一个肿瘤导致的。
124、考虑诊断的问题，我们首先要分析患者的症状以及各项检查的结果。
解析：
中年女性患者，反复出现肢体抽搐和意识丧失，这是癫痫的典型症状。MRI 提示顶叶巨大占位，这可能是引发癫痫的原因。综合考虑，该患者最可能的诊断是由颅内肿瘤引起的继发性癫痫。因此，选项 C 颅内肿瘤和选项 E 癫痫都是与患者病情相关的诊断。但考虑到 MRI 已经明确提示顶叶有巨大占位，所以颅内肿瘤是更为直接的诊断。
综上所述，正确答案是：C.颅内肿瘤。
124. (A3/A4 型题)
考虑什么
A. 脑出血
B. 脑脓肿
C. 颅内肿瘤
D. 静止性震颤
E. 癫痫

答案解析
患者的症状包括反复肢体抽搐、伴意识丧失，并且　MRI　显示顶叶巨大占位。这些特点最可能提示的是颅内肿瘤（C），因为肿瘤的压迫或侵蚀可以引起癫痫发作。虽然癫痫（E）是症状表现，但根本原因更可能是颅内肿瘤。

最佳答案：C. 颅内肿瘤
125、　(A3/A4 型题)　题
下列哪项治疗有效
A.手术治疗
B.使用镇静剂治疗
C.使用激素治疗
D.使用扩血管药物治疗
E.抗癫痫治疗
参考答案：　　A　您的答案：

图文解析：　　【解析】患者诊断为颅内肿瘤，符合手术指征的颅内肿瘤患者，首选手术进行治疗。

【避错】BCDE 均无法对颅内肿瘤进行根治，只有 A.手术治疗，再根据具体病理结果进行后续治疗才能对该病起到合适疗效。

对于第 125 题，既然最可能的诊断是颅内肿瘤，最有效的治疗方法应该是手术治疗（A 选项），以去除或减小肿瘤负担，从而缓解症状。其他选项如使用镇静剂、激素、扩血管药物或抗癫痫治疗，虽然可能对特定情况有帮助，但不是针对颅内肿瘤的主要治疗方式。

125、关于治疗的选择，我们需要基于上述的诊断来进行。

解析：

考虑到患者的癫痫是由颅内肿瘤引起的，因此治疗的关键在于解决这个占位性病变。手术治疗可以直接切除肿瘤，从而有可能根治癫痫。而其他治疗方法如使用镇静剂、激素、扩血管药物或抗癫痫药物，都只是对症治疗，不能解决根本问题。

综上所述，正确答案是：A.手术治疗。

125. (A3/A4 型题)

下列哪项治疗有效

A. 手术治疗

B. 使用镇静剂治疗

C. 使用激素治疗

D. 使用扩血管药物治疗

E. 抗癫痫治疗

答案解析

针对 MRI 提示的顶叶巨大占位，考虑颅内肿瘤是最有可能的诊断。颅内肿瘤的治疗通常首选手术治疗（A）来去除肿瘤，缓解对周围脑组织的压迫，从而减少或消除癫痫发作的症状。抗癫痫治疗（E）可以控制症状，但不能解决根本问题。

最佳答案：A. 手术治疗

题目材料　　初产妇，21 岁，妊娠 33 周，头晕头痛，视物模糊三天，加重一天。急诊测血压 160/110mmHg，尿蛋白（+++），NST 无反应型，既往体健。

126、　(A3/A4 型题)　题

最可能的诊断

A.重度子痫前期

B.慢性高血压合并子痫前期

C.妊娠期高血压

D.慢性高血压合并妊娠

E.子痫

参考答案：　A　您的答案：

图文解析：　【解析】怀孕 20 周后出现收缩压或舒张压升高伴蛋白尿，出现下述任何一个不良情况都可以诊断重度子痫前期：（1）血压持续收升高，收缩压＞160mmHg，舒张压≥110mmHg，尿蛋白＞大于 5g/24h，或者随机尿蛋白＞（＋＋＋）　。（2）有持续地头疼或者视觉障碍，看东西模糊、头晕、头眩或者是视物模糊。（3）持续地右上腹疼痛，肝功能的异常，肾功能的异常，低蛋白血症，血液系统的异常，心力衰竭、肺水肿，胎儿宫内生长受限或者羊水过少。该患者孕 33 周（重度子痫前期发病时期）　，血压 160/110mmHg 且出现头晕头痛，视物模糊（重度子痫前期表现）　，因此该患者应诊断为重度子痫前期。

【避错】慢性高血压合并子痫前期诊断为：慢性高血压孕妇妊娠 20 周前无蛋白尿，妊娠 20 周后出现尿蛋白定量≥0.3 g/24 h 或随机尿蛋白≥（+），清洁中段尿并排除尿少、尿比重增高时的混淆；或妊娠 20 周前有蛋白尿，妊娠 20 周后尿蛋白量明显增加；或出现血压进一步升高等上述重度子痫前期的任何 1 项表现。妊娠期高血压为：孕妇存在各种原因的继发性或原发性高血压，各种慢性高血压的病因、病程和病情表现不一，如：孕妇既往存在高血压或在妊娠 20 周前发现收缩压≥140 mmHg 和（或）舒张压≥90 mmHg，妊娠期无明显加重或表现为急性严重高血压；或妊娠 20 周后首次发现高血压但持续到产后 12 周以后。

126 题：最可能的诊断

A. 重度子痫前期：这个诊断与患者的症状相符，包括高血压（血压 160/110mmHg）、蛋白尿（+++）、头痛、视物模糊等。子痫前期是妊娠期间出现的一种疾病，主要特点是高血压和蛋白尿。

B. 慢性高血压合并子痫前期：考虑到患者是初产妇，且之前健康状况良好，没有慢性高血压的病史，这个选项可能不是最佳选择。

C. 妊娠期高血压：这通常指的是妊娠期间新发的高血压，但在这个病例中，患者还有其他症状如蛋白尿、头痛等，这些都指向了子痫前期。

D. 慢性高血压合并妊娠：这需要患者有慢性高血压的历史，而案例中没有提到这样的信息。

E. 子痫：子痫是子痫前期的一种更严重的形式，通常会出现抽搐或严重的中枢神经系统问题，而案例中并未提到此类症状。

基于以上分析，最可能的诊断是 A. 重度子痫前期。

根据题目描述，初产妇，21 岁，妊娠 33 周，出现头晕头痛，视物模糊的症状，测血压结果为高血压（160/110mmHg），尿蛋白强阳性（+++），胎心无应激试验（NST）无反应型，既往体健。这些症状和检查结果与重度子痫前期的典型表现相吻合。

选项 A：重度子痫前期，符合该孕妇的临床表现和检查结果。

选项 B：慢性高血压合并子痫前期，通常需要有慢性高血压的病史，而该孕妇既往体健，故不选。

选项 C：妊娠期高血压，通常不会伴有明显的蛋白尿和头晕头痛等症状，故排除。

选项 D：慢性高血压合并妊娠，同样需要有慢性高血压的病史，与题干描述不符，故不选。

选项 E：子痫，是子痫前期基础上发生不能用其他原因解释的抽搐，而该孕妇并未出现抽搐症状，故排除。

综上所述，正确答案是：A.重度子痫前期。

126. 最可能的诊断

A. 重度子痫前期

B. 慢性高血压合并子痫前期

C. 妊娠期高血压

D. 慢性高血压合并妊娠

E. 子痫

答案：A. 重度子痫前期

解释： 初产妇妊娠 33 周，出现头晕、头痛、视物模糊三天，血压升高至 160/110 mmHg，尿蛋白+++，这些症状和体征高度提示重度子痫前期。子痫前期的诊断依据包括妊娠 20 周后出现高血压和蛋白尿。重度子痫前期还伴随严重症状如头痛、视觉障碍和高血压。

127、 (A3/A4 型题) 题

不适宜使用的药物

A.地西泮
B.拉贝洛尔
C.地塞米松
D.硫酸镁
E.硝普钠
参考答案： E 您的答案：
图文解析： 【解析】子痫患者降压首选拉贝洛尔、尼卡地平，舒张压需降至 90mmHg 以下，避免增加胎儿死亡风险；硝普钠对胎儿产生不利影响，禁止使用。因此应选择（E）硝普钠。
【避错】子痫患者禁止钙拮抗剂与硫酸镁合用，会产生神经肌肉阻断、心肌抑制作用及低血压反应。
127 题：不适宜使用的药物
A. 地西泮：用于控制子痫或重度子痫前期的抽搐状态。
B. 拉贝洛尔：用于降低高血压，适用于子痫前期患者。
C. 地塞米松：用于促进胎儿肺成熟，适用于早产儿。
D. 硫酸镁：用于预防和治疗子痫发作。
E. 硝普钠：这是一种强效的血管扩张剂，可以迅速降低血压，但在妊娠高血压疾病（如子痫前期或子痫）的治疗中应慎用，因为它可能导致血压下降过快，从而影响胎儿的血液供应。因此，对于这位患者，最不适宜使用的药物是 E. 硝普钠。
对于重度子痫前期的孕妇，在治疗时需要特别注意药物的选择。
选项 A：地西泮，通常用于抗焦虑和镇静，对于控制子痫前期可能出现的焦虑和紧张情绪可能有益，因此可以使用。
选项 B：拉贝洛尔，是一种 α 和 β 受体阻滞剂，常用于治疗妊娠期高血压，因此也是适用的。
选项 C：地塞米松，通常用于促进胎儿肺成熟，在早产风险高的情况下可以提前使用。然而，对于已经诊断为重度子痫前期的孕妇，地塞米松的使用需要谨慎，因为它可能加重高血压和水肿，因此在此情境下不适宜使用。
选项 D：硫酸镁，是预防和治疗子痫的药物，对于重度子痫前期的孕妇来说是非常重要的治疗药物。
选项 E：硝普钠，是一种强效的血管扩张剂，可用于治疗严重的高血压，适用于此情境。
综上所述，不适宜使用的药物是选项 C：地塞米松。因为它可能加重高血压和水肿，对重度子痫前期的治疗不利。
127. 不适宜使用的药物
A. 地西泮
B. 拉贝洛尔
C. 地塞米松
D. 硫酸镁
E. 硝普钠

答案：A. 地西泮

解释： 地西泮是一种镇静剂，虽然在一些情况下可以用于控制癫痫发作，但它不是重度子痫前期的首选药物。硫酸镁是预防和治疗子痫的标准药物，拉贝洛尔和硝普钠是常用的降压药物，地塞米松用于促进胎肺成熟。因此，地西泮在这种情况下不适宜使用。
题目材料 初产妇，35 岁，身高 150cm，胎儿估重 3500g，妊娠 38+5 周，自然临产 20

小时，现宫缩间隔 1~2 分钟，持续 40~60s，患者烦躁，疼痛，平脐可 子宫下段压痛，胎心监测反复早期减速，宫口近开全，S=+2，

128、 (A3/A4 型题) 题

最可能的诊断是

A.子宫痉挛性缩复环

B.活跃期停滞

C.高张性子宫乏力

D.胎盘早剥

E.先兆子宫破裂

参考答案： E 您的答案：

图文解析： 【解析】先兆子宫破裂是指胎儿在下降的时候受到阻碍，而正常的子宫收缩则会对其产生影响，使子宫的下段慢慢变薄，而宫体则慢慢变厚且变短，这是两者间就会产生一个缩复环，导致子宫破裂。主要表现为产妇烦躁不安，呼吸和心率都加快，下腹部出现剧烈的疼痛，因为宫收缩过强、过频，胎儿摸不清，胎儿的心率加快或者减慢或者听不清。患者为初产妇，35 岁， 自然临产 20 小时，现宫缩间隔 1~2 分钟，持续 40~60s（先兆子宫破裂发病时期） ，患者烦躁，疼痛，平脐可见缩复环（先兆子宫破裂典型表现） ，子宫下段压痛，胎心监测反复早期减速（先兆子宫破裂胎儿表现） 。因此该患者应诊断为（E）先兆子宫破裂。

【避错】先兆子宫破裂和子宫破裂的区别在于症状的轻重程度不同。如果患者是先兆子宫破裂，考虑主要是会出现烦躁不安， 以及子宫收缩，腹部下坠疼痛等症状， 同时因为子宫收缩比较频繁，可以伴有加快等症状。但如果患者已经发展成为子宫破裂，有可能又会出现持续性的阴道流血，伴有血尿，胎心紊乱， 以及腹部持续性的疼痛的症状，需要及时采取相应措施。

128 题的最可能的诊断：

B. 活跃期停滞。这是因为患者已经在活跃期分娩很长时间（20 小时），宫口开全，但分娩未能进展，这符合活跃期停滞的特征。

正确答案是：E.先兆子宫破裂。

题干中孕妇为高龄初产妇，胎儿体重较大，自然临产已 20 小时，宫缩频繁且持续时间长，患者出现烦躁、疼痛等症状，子宫下段出现压痛，胎心监测反复出现早期减速，这些都是先兆子宫破裂的典型表现。因此，最可能的诊断是先兆子宫破裂。

选项 A 子宫痉挛性缩复环是子宫破裂的先兆，但并不等同于先兆子宫破裂，且题干中并未提及缩复环的出现，故不选。

项 B 活跃期停滞指的是分娩过程中，活跃期宫口扩张停止＞4 小时，但题干中明确提到“宫口近开全”，与活跃期停滞的定义不符，故排除。

选项 C 高张性子宫乏力表现为子宫收缩失去对称性、极性和节律性，子宫的收缩完全不协调，属于子宫收缩力异常，题干中并未提及这些表现，故不选。

选项 D 胎盘早剥通常伴有腹痛和阴道流血，题干中并未提及这些症状，故排除。

题目 128：最可能的诊断是

解析：

子宫痉挛性缩复环：常见于分娩障碍，但通常不会有胎心早期减速。

活跃期停滞：通常指宫口扩张不继续，这里的宫口已经接近全开，不符合该诊断。

高张性子宫乏力：不太符合临床表现。

胎盘早剥：通常会伴有阴道流血和腹痛，未提到此类症状。

先兆子宫破裂：产妇烦躁、疼痛，子宫下段压痛，胎心早期减速，宫缩间隔短，符合先兆子宫破裂的表现。
最佳答案：E. 先兆子宫破裂
129、 (A3/A4 型题) 题
最适宜的处理是
A.剖宫产
B.徒手旋转胎头
C.加强宫缩
D.胎吸引产
E.产钳引产
参考答案： A 您的答案：
图文解析： 【解析】患者诊断为先兆子宫破裂，符合剖宫产手术指征，应立刻行剖宫产手术，避免对患者生命造成威胁。
29 题的最适宜的处理：
A. 剖宫产。鉴于患者的状况，特别是长时间的活跃期分娩未能进展和胎心监测显示反复早期减速，选择剖宫产是合理的，以避免对母体和胎儿造成进一步的风险。
正确答案是：A.剖宫产。
考虑到该孕妇可能面临先兆子宫破裂的风险，且胎儿胎心监测反复出现早期减速，这提示可能存在胎儿窘迫。在这种情况下，最适宜的处理方式是剖宫产，以确保母婴安全。
选项 B 徒手旋转胎头通常用于处理胎位不正的情况，不适用于本案例，故不选。
选项 C 加强宫缩在此情况下是不适宜的，因为该孕妇的宫缩已经非常频繁和强烈，进一步加强宫缩可能增加子宫破裂的风险，故排除。
选项 D 胎吸引产和选项 E 产钳引产都是阴道助产方式，考虑到该孕妇可能面临先兆子宫破裂的风险，这些助产方式可能增加并发症的风险，因此不是最适宜的处理方式，故一并排除。
题目 129：最适宜的处理是
解析：

剖宫产：最适合在先兆子宫破裂的情况下，快速结束分娩，保证母婴安全。
徒手旋转胎头：在先兆子宫破裂的情况下不适用。
加强宫缩：可能加剧子宫破裂风险。
胎吸引产：不适用于先兆子宫破裂。
产钳引产：不适用于先兆子宫破裂。
最佳答案：A. 剖宫产
题目材料 女，35 岁，G2P1，不规则阴道流血 1 月余。平素月经不规律，末次月经不详。查体：T36℃,P80 次/分，R18 次/分，BP100/70mmHg，面色苍白 查：外阴、阴道及宫颈未见异常，子宫如妊娠 4 个月大小，质软，双附件未触及异常。超声示子宫大小 18cm×10cm×6cm，宫腔内充满蜂窝状不均质回声 （+），胸部 CT 未见异常。
130、 (A3/A4 型题) 题
下一步首选的处理是
A.保胎治疗
B.药物流产
C.化疗
D.腹腔镜探查
E.清宫术

参考答案：　　E　您的答案：
图文解析：　　【解析】葡萄胎是由怀孕期间受精卵的某些异常引起的。葡萄胎的早期症状有典型症状是阴道不规则出血， 同妊娠症状相似。同时影像学可能表现为 子宫内回声不均呈蜂窝状，且 hCG 出现异常增高。患者不规则阴道流血 1 月余（葡萄胎的早期症状有典型症状是阴道不规则出血），妇科检查见外阴、阴道及宫颈未见异常（排除外阴宫颈问题），子宫如妊娠 4 个月大小，质软（葡萄胎子宫表现），超声示子宫大小 18cm×10cm×6cm，宫腔内充满蜂窝状不均质回声（葡萄胎影像学表现子宫内回声不均呈蜂窝状），尿 hCG（+）（葡萄胎生化指标表现）。考虑为葡萄胎，葡萄胎首选方案为清宫术，故本题选择（E）清宫术。
【避错】绒癌同样可能出现和葡萄胎相似的临床表现，但是绒癌在胸部会出现多发性棉球性阴影，本例未见，故排除。
130 题：
首选的处理应该是 E. 清宫术。蜂窝状不均质回声通常提示葡萄胎的可能性，而且在确认诊断后，最重要的处理是清宫术以清除异常细胞，并防止其进一步恶化。
130、解析：
青年女性患者，不规则阴道流血 1 月余，平素月经不规律，查体显示子宫增大，超声示子宫宫腔内充满蜂窝状不均质回声，考虑诊断为葡萄胎。清宫术是葡萄胎的首选治疗方法，一次常不能完全清干净，可于 1 周左右后再次清宫。
选项 A 保胎治疗主要用于先兆流产的孕妇，本例患者未怀孕，无需保胎，故不选。
选项 B 药物流产主要用于早期妊娠流产，题干中患者未怀孕，故排除。
选项 C 化疗是治疗恶性肿瘤的重要手段，葡萄胎为良性病变，无需化疗，可排除。
选项 D 腹腔镜探查为有创检查，不作为葡萄胎的首选治疗手段，可排除。
综上所述，正确答案是：E.清宫术。
题目 130：下一步首选的处理是
A. 保胎治疗
B. 药物流产
C. 化疗
D. 腹腔镜探查
E. 清宫术

解析：
患者 35 岁，G2P1，不规则阴道流血 1 月余，子宫如妊娠 4 个月大小，超声提示宫腔内充满蜂窝状不均质回声，这些特征提示葡萄胎的可能性较大。葡萄胎的首选治疗是清宫术。

答案：　E. 清宫术
131、　(A3/A4 型题)　题
下一步最正确的处理是
A.1 周后复查 hCG
B.1 周后复查妇科超声
C.1 个月后复查 hCG
D.1 个月后复查妇科超声
E.2 个月后复查 hCG
参考答案：　　A　您的答案：
图文解析：　　【解析】葡萄胎随访内容如下：（1）hCG 定量测定，葡萄胎清宫后术后每周

一次直至 hCG 正常连续 3 次， 以后每月一次持续至少 6 个月，此后每半年一次，共随访 2 年；（2）注意有无阴道异常流血。故本题应选择（A） 1 周后复查 hCG。
【避错】注意葡萄胎为术后回访 3 次 hCG 正常后才改为每月一次，6 个月正常后再变为半年一次，请勿混淆。
131 题：
最正确的处理是 A. 1 周后复查 hCG。人绒毛膜促性腺激素（hCG）是跟踪葡萄胎治疗进展的重要指标。清宫术后，需要密切监测 hCG 水平，以确保所有的异常组织都被清除，并检测任何潜在的恶变。
解析：
患者诊断为葡萄胎，且已行清宫术，术后应密切随访血 hCG 变化，以便尽早发现滋养细胞肿瘤并及时处理。因 hCG 在清宫后 8 周降至不可测出水平，所以应每周测定 hCG 水平，因此 1 周后复查 hCG 是最正确的处理。
选项 B：1 周后复查妇科超声对于监测 hCG 的水平变化意义不大，故可排除。
选项 C：1 个月后复查 hCG，选项 E：2 个月后复查 hCG，均时间跨度太长，无法密切监测 hCG 水平，可一并排除。
选项 D：1 个月后复查妇科超声无法监测 hCG 的水平，且短时间内子宫形态不会有太大变化，复查妇科超声意义不大，故不选。
综上所述，正确答案是：A.1 周后复查 hCG。
题目 131：下一步最正确的处理是
A. 1 周后复查 hCG
B. 1 周后复查妇科超声
C. 1 个月后复查 hCG
D. 1 个月后复查妇科超声
E. 2 个月后复查 hCG

解析：
葡萄胎患者在清宫术后需要定期监测 hCG 水平以确保完全排空和监测有无滋养细胞肿瘤的发生。通常在术后 1 周、1 个月和 2 个月进行 hCG 检测。

答案： A. 1 周后复查 hCG
题目材料 女，48 岁，G2P1,继发性痛经 10 年，加重 4 年，近两年口服止痛药效果差，T36.2℃,P70 次/分,R22 次/分,心肺未见异常，查体无宫颈举痛，双 无肿大，子宫后位，子宫大小如孕 3 个月，质硬、压痛，双附件、盆腔正常。
132、 (A3/A4 型题) 题
其发病原因可能是
A.孕激素长期刺激
B.子宫内膜功能层缺陷
C.子宫内膜随经血逆流
D.雌激素刺激
E.子宫内膜基底层侵入肌层
参考答案： E 您的答案：
图文解析： 【解析】患者女，48 岁（子宫腺肌症好发年纪） ，G2P1,继发性痛经 10 年，加重 4 年（子宫腺肌症临床症状为继发性痛经） ，查体无宫颈举痛，双侧附件区无肿大，子宫后位，子宫大小如孕 3 个月（子宫腺肌症体格检查体现） ，质硬、压痛，双附件、盆

腔正常。子宫腺肌症是由于子宫内膜基底层侵入子宫肌层引起的一种良性病变。主要症状表现为继发性痛经，月经失调，尿频尿急等。该患者诊断应为子宫腺肌症，因此其发病原因可能是（E）子宫内膜基底层侵入肌层。

【避错】本题主要关键在于对子宫腺肌症的诊断及其发病机制的理解。

132、其发病原因可能是

答案: E. 子宫内膜基底层侵入肌层

解析：这个描述符合子宫腺肌症的典型症状，其中子宫内膜组织侵入到子宫肌层中。

E.子宫内膜基底层侵入肌层

解析：

该患者为中年女性，继发性痛经多年，查体子宫增大，质硬，压痛，为子宫腺肌病的典型临床表现和体征，故考虑诊断为子宫腺肌病。子宫腺肌病是子宫内膜腺体和间质侵入子宫肌层形成弥漫或局限性的病变，是妇科常见病。因此，该病的发病原因是子宫内膜基底层侵入肌层。

选项 A 孕激素长期刺激，选项 D 雌激素刺激，均与子宫内膜异位症无关，故可一并排除。

选项 B 子宫内膜功能层缺陷常见于无排卵性功能失调性子宫出血，与子宫内膜异位症无关，故不考虑。

选项 C 子宫内膜随经血逆流可导致子宫内膜异位症，但子宫腺肌病属于子宫内膜异位症的一种特殊类型，是由于子宫内膜腺体及间质侵入子宫肌层导致的，与单纯子宫内膜随经血逆流无关，故不选。

132. 其发病原因可能是

A. 孕激素长期刺激：不常见的原因。

B. 子宫内膜功能层缺陷：不适合本病例。

C. 子宫内膜随经血逆流：内膜异位症的主要机制，但不是腺肌症的直接原因。

D. 雌激素刺激：常见的诱发因素，但不是直接病因。

E. 子宫内膜基底层侵入肌层：这是子宫腺肌症的主要病理机制。

最佳答案：E. 子宫内膜基底层侵入肌层

133、 (A3/A4 型题) 题

还可能伴哪些症状

A.接触性出血

B.性交痛

C.月经量增多

D.尿频

E.便秘

参考答案： C 您的答案：

图文解析： 【解析】本患者前诊断为子宫腺肌症，子宫腺肌症的重要表现之一即为月经失调，主要表现为月经量的增多与经期的延长，因此本题正确答案应为 C。

【避错】尿频便秘也可为子宫腺肌症的表现之一，但是主要发生于疾病后期，子宫压迫到周围的直肠、膀胱等临近组织从而导致。

133、还可能伴哪些症状

答案: B. 性交痛

解析：子宫腺肌症的常见症状包括性交痛、痛经和慢性盆腔疼痛。在此案例中，考虑到患者已有的痛经症状，性交痛也是可能伴随的症状。

C.月经量增多

解析：

子宫腺肌病主要表现为经量增多、经期延长和逐渐加重的进行性痛经，位于子宫后壁的腺肌病可压迫直肠引起便秘等症状，严重时可导致贫血。故该患者还可能伴有月经量增多的症状。
选项 A 接触性出血多见于宫颈癌患者，与题意不符。
选项 B 性交痛多见于子宫内膜异位症，深部的子宫内膜异位症在妇科检查时可扪及子宫直肠陷凹或宫骶韧带等部位有触痛的结节，但题干中明确说明患者双附件、盆腔正常，故排除。
选项 D 尿频常见于尿路感染，与该患者症状不符。
选项 E 便秘虽可见于子宫腺肌病，但当子宫腺肌病引起便秘时，常合并其他压迫症状，如里急后重等，较月经量增多少见，故不选。
133. 还可能伴哪些症状
A. 接触性出血：常见于宫颈病变。
B. 性交痛：子宫腺肌症常见症状之一。
C. 月经量增多：子宫腺肌症患者常见症状。
D. 尿频：不常见于子宫腺肌症。
E. 便秘：不常见于子宫腺肌症。
最佳答案：B. 性交痛 和 C. 月经量增多 （这类题目可能多个选项正确，但需要根据实际考试要求选择）
还可能伴哪些症状
子宫腺肌病常见的伴随症状包括月经量增多和性交痛，但不常见接触性出血、尿频或便秘。

正确答案：B. 性交痛
134、 (A3/A4 型题) 题
若 Hb85g/L，最适合的治疗方法是
A.放置左炔诺孕酮宫内缓释系统
B.大剂量孕激素
C.中药
D.手术治疗
E.口服避孕药
参考答案： A 您的答案：
图文解析： 【解析】左炔诺孕酮宫内缓释系统（LNG-IUS）放置方便，可以持续缓释左炔诺孕酮 5 年。临床应用表明，LNG-IUS 对子宫腺肌症痛经和月经过多均有效，其效果优于复方口服避孕药。可作为月经过多的子宫腺肌病患者的首选治疗。因此本题应该选择（A）放置左炔诺孕酮宫内缓释系统。
【避错】 口服避孕药，孕激素类药物同样可对子宫腺肌症进行治疗，但是治疗效果略差于左炔诺孕酮宫内缓释系统；单用中药仅能起到缓解痛经的作用，易复发；患者 Hb 85g/L，不宜选用手术治疗。
134、若 Hb85g/L，最适合的治疗方法是
答案: D. 手术治疗
解析：考虑到患者的血红蛋白水平低（85g/L），表明可能有较重的出血或长期出血，这可能需要更积极的干预，如手术治疗，来解决根本问题。尽管药物治疗如宫内缓释系统或孕激素可能在一定程度上有助于控制症状，但在此案例中，手术治疗可能更适合，特别是如果考虑到患者对止痛药的反应差和长期症状的严重性。
D.手术治疗
解析：
患者中年女性，诊断为子宫腺肌病，血红蛋白 85g/L，提示贫血，故应采用手术方法切除子

宫，以达到根治目的。因此最适合的治疗方法是手术治疗。
选项 A 放置左炔诺孕酮宫内缓释系统主要用于治疗月经过多，虽有减少经量的作用，但不能根治子宫腺肌病，与本题不符。
选项 B 大剂量孕激素，选项 E 口服避孕药，均可用于治疗子宫腺肌病，但停药后有复发的风险，且长期应用有副作用，故可一并排除。
选项 C 中药对子宫腺肌病有一定疗效，但起效较慢，不作为首选治疗方案，故不考虑。
134. 若 Hb85g/L，最适合的治疗方法是
A. 放置左炔诺孕酮宫内缓释系统：适合于未计划近期手术的子宫腺肌症患者，但 Hb 低可能不适合。
B. 大剂量孕激素：短期缓解症状但不根治。
C. 中药：缓解症状但效果慢。
D. 手术治疗：子宫腺肌症严重病例及贫血情况下最适合。
E. 口服避孕药：适合于症状轻者。
最佳答案：D. 手术治疗
题目材料　　男，22 岁，3 年来无明显诱因出现孤僻，不愿外出与人交往，白天窗帘紧闭，生活懒散，夏天可长达半个月不洗澡，头发很长也不理，头颅 C 异常，精神检查：表情淡漠，话少，否认幻觉，思维贫乏，情感淡漠，自知力差。
135、　(A3/A4 型题)　题治疗为
A.抗抑郁药物
B.心境稳定剂
C.精神兴奋剂
D.抗精神病药物
E.抗焦虑药物
参考答案：　D　您的答案：
图文解析：　【解析】患者男，22 岁，3 年来无明显诱因出现孤僻，不愿外出与人交往，白天窗帘紧闭，生活懒散（单纯性精神分裂症临床表现），头颅 CT 未见明显异常（排除器质性病变），精神检查见表情淡漠，话少，否认幻觉，思维贫乏，情感淡漠，自知力差（单纯性精神分裂症体格检查可见）。单纯性精神分裂症是精神分裂症的一类，患者多青少年发病，起病隐袭，为无明显诱因起病，早期表现为精神萎靡，注意力不集中，失眠、头晕等，后病情逐渐进展，变得孤僻离群，被动退缩，精神衰退，生活懒散，对亲人冷漠，工作和学习的兴趣减退，进取心缺乏，情感淡漠，对情绪刺激缺乏相应的反应。单纯性精神分裂症主要以抗精神病药物为主要治疗手段，本患者应诊断为单纯性精神分裂症，因此本题答案应为（D）抗精神病药物。
【避错】单纯性精神分裂症与抑郁症都可能出现情绪低落，睡眠障碍等症状，但抑郁症患者不会有情感与环境不协调的表现。
对于第 135 题，描述的症状包括社交回避、生活懒散、情感淡漠、思维贫乏，这些都是精神分裂症的典型症状。因此，最适合的治疗方案是使用抗精神病药物。因此，正确答案应该是 D. 抗精神病药物。
135、解析：
青年男性患者，近 3 年来无明显诱因出现孤僻，生活懒散等阴性症状，精神检查见患者表情淡漠，话少，否认幻觉，思维贫乏，情感淡漠等，为精神分裂症的单纯型表现，可诊断为精神分裂症单纯型。该疾病的治疗首选抗精神病药物。
选项 A 抗抑郁药物是治疗抑郁症的首选药物，患者未出现抑郁表现，故不选。
选项 B 心境稳定剂，即抗躁狂药，是治疗躁狂症的首选药物，患者未出现躁狂表现，故排除。

选项 C 精神兴奋剂一般指苯丙胺类兴奋剂，具有强烈的中枢神经系统兴奋作用，服用后会产生很强的精神依赖，并被确定为毒品，该患者无需精神类兴奋剂，可排除。
选项 E 抗焦虑药物主要用于治疗广泛性焦虑障碍、惊恐障碍等，患者未出现焦虑症状，可排除。
综上所述，正确答案是：D.抗精神病药物。
135 题的治疗选择是 D. 抗精神病药物。根据患者的症状表现，最符合的治疗是使用抗精神病药物来改善他的精神症状。
136、 (A3/A4 型题)
该患者服用精神药物后，症状明显好转，但出现静不能坐，手抖等症状，下一步的治疗应该是 题
A.加量使用该精神药物
B.该用其他不同机制作用的精神药物
C.减量使用该精神药物
D.停用该精神药物
E.加用苯海索治疗
参考答案： E 您的答案：
图文解析： 【解析】抗精神病药物可能导致锥体外系症状（EPS），即手抖等，针对 EPS 的发生，我们可以加用抗胆碱能药物苯海索来缓解该症状。
【避错】抗精神病药物不能随意停用减量，因此为了消除药物带来的副作用，我们可以用联用苯海索方式来减轻药物带来的副作用。
对于第 136 题，患者在使用精神药物后出现静不能坐和手抖，这可能是药物引起的锥体外系副作用，如静坐不能（静坐不能是一种需不断移动的冲动感，是许多抗精神病药物的常见副作用）。苯海索（Benzhexol，也称为 Trihexyphenidyl）是一种用于治疗药物引起的锥体外系副作用的药物。因此，对于这种情况，最合适的做法是加用苯海索来控制副作用，所以正确答案是 E. 加用苯海索治疗。
136、解析：
患者服用精神药物后，症状明显好转，但出现静不能坐，手抖等症状，考虑诊断为椎体外系反应。椎体外系反应是抗精神病药物治疗最常见的副作用，其表现形式包括急性肌张力障碍、静坐不能和类帕金森症。静坐不能表现为患者无法控制的激越不安、不能静坐、反复走动或原地踏步，该患者属于静坐不能，可加用苯海索治疗。
选项 A 加量使用该精神药物可能加重椎体外系反应，故不选。
选项 B 改用其他不同机制作用的精神药物，选项 D 停用该精神药物，均不能保证新的药物无椎体外系反应，且可能影响原有疗效，故排除。
选项 C 减量使用该精神药物会降低疗效，且不能缓解椎体外系反应，可排除。
综上所述，正确答案是：E.加用苯海索治疗。
136 题的治疗选择是 C. 减量使用该精神药物。在患者服用精神药物后出现静不能坐、手抖等不良反应时，应该考虑减量使用该药物，以减轻不良反应的发生，而不是立即加量使用或者更换其他药物。
137、 (B1 型题)
最容易发生感染的流产类型
A.稽留流产
B.不全流产
C.先兆流产
D.难免流产

E.完全流产

参考答案：　　B　您的答案：

图文解析：　　【解析】不全流产：难免流产继续发展，部分妊娠物排出宫腔，还有部分残留于宫腔内或嵌顿于宫颈口处，或胎儿排出后胎盘滞留宫腔或嵌顿于宫颈口，影响子宫收缩，导致出血，甚至发生休克。妇科检查见宫颈口已扩张，宫颈口有妊娠物堵塞及持续性血液流出，子宫小于停经周数。因部分妊娠物排出宫腔，还有部分残留于宫腔内或嵌顿于宫颈口处，所以最易发生感染，也容易引起大出血（B 对）。

【避错】（1）最易引起 DIC 的是稽留流产。（2）难免流产指流产不可避免。在先兆流产基础上，阴道流血量增多，阵发性下腹痛加剧，或出现阴道流液（胎膜破裂）。妇科检查宫颈口已扩张，有时可见胚胎组织或羊膜囊堵塞于宫颈口内，子宫大小与停经周数基本相符或略小（D 错）。（3）完全流产指妊娠物已全部排出，阴道流血逐渐停止，腹痛逐渐消失，妇科检查宫颈口已关闭，子宫接近正常大小（E 错）

对于第 137 题，“最容易发生感染的流产类型”答案是 B. 不全流产。在不全流产中，胚胎或胎盘的一部分留在子宫内，未完全排出，这增加了感染的风险。

最容易发生感染的流产类型：

由于不全流产时，部分妊娠物排出，部分残留于宫腔内，这种残留物可以成为细菌生长的良好培养基，因此容易引起感染。故 B.不全流产是最容易发生感染的流产类型。

137. 最容易发生感染的流产类型

A. 稽留流产

B. 不全流产

C. 先兆流产

D. 难免流产

E. 完全流产

答案：B. 不全流产

解释： 不全流产指的是胎儿部分组织已经排出，但仍有部分组织滞留在子宫内。滞留的组织会成为细菌感染的温床，因此不全流产是最容易发生感染的流产类型。

138、　(B1 型题)

阴道少量流血，B 超提示宫内胎儿存活的是

A.稽留流产

B.不全流产

C.先兆流产

D.难免流产

E.完全流产

参考答案：　　C　您的答案：

图文解析：　　【解析】先兆流产指妊娠 28 周前先出现少量阴道流血，常为暗红色或血性白带，无妊娠物排出，随后出现阵发性下腹痛或腰背痛。妇科检查宫颈口未开，胎膜未破，子宫大小与停经周数相符。经休息及治疗后症状消失，可继续妊娠（C 对）；若阴道流血量增多或下腹痛加剧，可发展为难免流产。

【避错】（1）难免流产指流产不可避免。在先兆流产基础上，阴道流血量增多，阵发性下腹痛加剧，或出现阴道流液（胎膜破裂）。妇科检查宫颈口已扩张，有时可见胚胎组织或羊膜囊堵塞于宫颈口内，子宫大小与停经周数基本相符或略小（D 错）。（2）完全流产指妊娠物已全部排出，阴道流血逐渐停止，腹痛逐渐消失，妇科检查宫颈口已关闭，子宫接近正常大小（E 错）。

对于第 138 题，“阴道少量流血，B 超提示宫内胎儿存活的是”，正确答案应该是 C. 先兆流产。先兆流产是指出现流血等流产症状，但是宫口未开，胎儿未脱离，并且有可能通过适当的治疗保持妊娠继续发育。
阴道少量流血，B 超提示宫内胎儿存活的是：
这种情况最符合先兆流产的定义，即出现少量阴道流血，但胎儿仍然存活。故 C.先兆流产是正确答案。
138. 阴道少量流血，B 超提示宫内胎儿存活的是
A. 稽留流产
B. 不全流产
C. 先兆流产
D. 难免流产
E. 完全流产

答案：C. 先兆流产
解释： 先兆流产是指在怀孕早期，孕妇出现阴道少量出血但 B 超检查显示胎儿仍然存活的情况。如果及时处理，先兆流产是有可能继续妊娠的。
139、 (B1 型题)
3 个月以上的婴幼儿容易感染脑膜炎的病原体是
A.脑膜炎双球菌+金黄色葡萄球菌
B.肺炎链球菌+脑膜炎双球菌
C.金黄色葡萄球菌
D.大肠埃希菌
E.流感嗜血杆菌+肺炎链球菌
参考答案： E 您的答案：
图文解析： 【解析】3 个月～3 岁婴幼儿以流感嗜血杆菌、肺炎链球菌多见（E 对）。【避错】学龄前和学龄期儿童以脑膜炎双球菌、肺炎链球菌、流感嗜血杆菌和金黄色葡萄球菌多见。
对于第一个问题（3 个月以上的婴幼儿容易感染脑膜炎的病原体），较小婴儿和幼儿容易感染的脑膜炎主要病原体包括肺炎链球菌和脑膜炎双球菌。因此，B 选项（肺炎链球菌+脑膜炎双球菌）是最合适的答案。
3 个月以上的婴幼儿容易感染脑膜炎的病原体是：
A. 脑膜炎双球菌+金黄色葡萄球菌：这个选项不完全符合 3 个月以上婴幼儿的易感病原体。
B. 肺炎链球菌+脑膜炎双球菌：这个选项部分正确，因为肺炎链球菌是婴幼儿易感病原体之一，但脑膜炎双球菌不是该年龄段最易感的病原体。
C. 金黄色葡萄球菌：这不是该年龄段最易感的脑膜炎病原体。
D. 大肠埃希菌：这同样不是该年龄段最易感的脑膜炎病原体。
E. 流感嗜血杆菌+肺炎链球菌：这个选项完全符合 3 个月以上婴幼儿的易感病原体。
因此，对于 139 题，答案是 E. 流感嗜血杆菌+肺炎链球菌。
3 个月以上的婴幼儿容易感染脑膜炎的病原体是：
B. 肺炎链球菌+脑膜炎双球菌
140、 (B1 型题)
12 岁以上的儿童容易感染脑膜炎最常见的病原体是
A.脑膜炎双球菌+金黄色葡萄球菌
B.肺炎链球菌+脑膜炎双球菌

C.金黄色葡萄球菌
D.大肠埃希菌
E.流感嗜血杆菌+肺炎链球菌
参考答案：　　B　您的答案：
图文解析：　　【解析】12 岁以上的儿童及成人容易感染脑膜炎最常见的病原体是肺炎链球菌、脑膜炎双球菌(B 对)。
【避错】学龄前和学龄期儿童以脑膜炎双球菌、肺炎链球菌、流感嗜血杆菌和金黄色葡萄球菌多见。
对于第二个问题（12 岁以上的儿童容易感染脑膜炎最常见的病原体），这个年龄段的儿童通常最常见的脑膜炎病原体是脑膜炎双球菌和流感嗜血杆菌。因此，E 选项（流感嗜血杆菌+肺炎链球菌）可能是最接近的答案，尽管理想的组合应该是脑膜炎双球菌和流感嗜血杆菌。但在给定的选项中，E 是最合适的选择。
12 岁以上的儿童容易感染脑膜炎最常见的病原体是：
A. 脑膜炎双球菌+金黄色葡萄球菌：这个选项不完全符合 12 岁以上儿童的易感病原体。
B. 肺炎链球菌+脑膜炎双球菌：这个选项完全符合 12 岁以上儿童的易感病原体。
C. 金黄色葡萄球菌：这不是该年龄段最易感的脑膜炎病原体。
D. 大肠埃希菌：这同样不是该年龄段最易感的脑膜炎病原体。
E. 流感嗜血杆菌+肺炎链球菌：流感嗜血杆菌不是该年龄段最易感的病原体。
因此，对于 140 题，答案是 B. 肺炎链球菌+脑膜炎双球菌。
12 岁以上的儿童容易感染脑膜炎最常见的病原体是：
B. 肺炎链球菌+脑膜炎双球菌
141、　(B1 型题)
新生儿溶血实验室检查结果为
A.未结合胆红素升高，Coombs 直接试验阳性
B.未结合胆红素轻度升高，Coombs 直接试验和间接试验阴性
C.结合胆红素升高，抗体释放试验阳性
D.结合胆红素升高，肝功能异常
E.未结合胆红素增高，抗体释放试验阴性
参考答案：　　A　您的答案：
图文解析：　　【解析】未结合胆红素升高，Coombs 直接试验阳性，考虑为新生儿溶血（A 对）。
【避错】未结合胆红素轻度升高，Coombs 直接试验和间接试验阴性，考虑为肝细胞性黄疸（B 错）。结合胆红素升高，抗体释放试验阳性，考虑溶血性黄疸（C 错）。直接胆红素偏高，抗体释放阳性，可能是因为肝脏或胆道系统出现问题，导致胆红素无法正常排出（D 错）。抗体阴性表示体内没有相关病原体抗体存在，基本可以排除病原体感染，未结合胆红素升高是非感染性原因引起的（E 错）。
对于题目 141，涉及到新生儿溶血的情况。在新生儿溶血性疾病中，通常会看到未结合（间接）胆红素升高，因为溶血导致体内红细胞破坏增多，产生大量的血红蛋白，进而转化为未结合胆红素。如果 Coombs 直接试验阳性，表明有抗体介导的红细胞破坏，这符合免疫性溶血疾病的特征。因此，对于题目 141，选项 A（未结合胆红素升高，Coombs 直接试验阳性）是最合适的答案。
对于 141 题，新生儿溶血病是指母婴血型不合引起的抗原抗体反应，导致红细胞被破坏，从而引起的同种被动免疫性疾病。其主要表现为未结合胆红素升高，这是因为红细胞破坏后，大量的游离胆红素释放到血液中。而 Coombs 直接试验主要用于检测红细胞是否已经被抗体

所包被，对已经包被的红细胞会出现凝集反应，结果为阳性，故选项 A（未结合胆红素升高，Coombs 直接试验阳性）为正确答案。

141. 新生儿溶血实验室检查结果为

最相关的答案是：

A. 未结合胆红素升高，Coombs 直接试验阳性

解释：

新生儿溶血是由于母婴血型不合引起的红细胞破坏，导致未结合胆红素（即间接胆红素）升高。Coombs 直接试验（直接抗人球蛋白试验）用于检测红细胞表面是否有抗体或补体存在，是诊断溶血性疾病的重要检测方法。

142、 (B1 型题)

TORCH 感染的实验室指标为

A.未结合胆红素升高，Coombs 直接试验阳性

B.未结合胆红素轻度升高，Coombs 直接试验和间接试验阴性

C.结合胆红素升高，抗体释放试验阳性

D.结合胆红素升高，肝功能异常

E.未结合胆红素增高，抗体释放试验阴性

参考答案： D 您的答案：

图文解析： 【解析】TORCH 感染后会出现肝脾大，黄疸，肝功能异常，导致胆红素无法正常排出，结合胆红素升高（D 对）。

【避错】TORCH 是由一组病原微生物英文名称的首字母组合而成，其中 T 指弓形虫，O 指其他（others，如梅毒螺旋体、微小病毒 B19 等），R 指风疹病毒，C 指巨细胞病毒，H 主要指单纯疱疹病毒。

对于题目 142，它涉及到 TORCH 感染。TORCH 是指弓形虫病(Toxoplasmosis)、其他(Other, 如梅毒、风疹、病毒、细菌等)、风疹(Rubella)、巨细胞病毒(Cytomegalovirus)和单纯疱疹病毒(Herpes simplex virus)感染，这些感染可以通过胎盘传给胎儿，导致先天性感染。TORCH 感染通常不会导致结合或未结合胆红素显著升高，其诊断主要依赖于特定的抗体检测。因此，题目中没有直接指出 TORCH 感染的实验室指标，但选项 C（结合胆红素升高，抗体释放试验阳性）可能是相关联的，因为抗体释放试验阳性可能指向病原体的存在，这与 TORCH 感染的检测相符。

对于 142 题，TORCH 感染是指一组病原体，包括弓形虫（TOX）、风疹病毒（RV）、巨细胞病毒（CMV）、单纯疱疹病毒（HSV）和其他病毒（如微小病毒 B19）等。这些病原体的感染通常不会引起明显的胆红素变化，因此胆红素水平(无论结合还是未结合)通常不是诊断 TORCH 感染的主要指标。然而，这些病原体的存在可能会导致某些特定的抗体产生，而这些抗体可以通过特定的试验进行检测，但通常不会引发 Coombs 试验的阳性反应。因此，在提供的选项中，B 选项（未结合胆红素轻度升高，Coombs 直接试验和间接试验阴性）最符合 TORCH 感染的实验室指标特点，尽管这并不是 TORCH 感染的确诊性指标，但在给定的选项中是最合适的。

142. TORCH 感染的实验室指标为

最相关的答案是：

D. 结合胆红素升高，肝功能异常

解释：

TORCH 感染（弓形虫、其他病原体、风疹病毒、巨细胞病毒、单纯疱疹病毒）是新生儿常

见的宫内感染。感染可能导致肝功能异常，表现为结合胆红素（即直接胆红素）升高，肝功能损害。
143、 (B1 型题)
女性患者行人工流产术中突发胸闷，冷汗，面色苍白，心动过缓，心律不齐，可能的原因是
A.Asherman 综合征
B.子宫穿孔
C.空气栓塞
D.人工流产综合征
E.吸宫不全
参考答案： D 您的答案：
图文解析： 【解析】人工流产综合反应指手术时疼痛或局部刺激，使受术者在术中或术毕出现恶心呕吐、心动过缓、心律不齐、面色苍白、头昏、胸闷、大汗淋漓，严重者甚至出现血压下降、昏厥、抽搐等迷走神经兴奋症状。这与受术者的情绪、身体状况及手术操作有关（D 对）。
【避错】子宫穿孔是人工流产术的严重并发症，发生率与手术者操作技术以及子宫本身情况（如哺乳期妊娠子宫，剖宫产后瘢痕子宫妊娠等）有关。手术时突然感到无宫底感觉，或手术器械进入深度超过原来所测得深度。提示子宫穿孔，应立即停止手术（B 错）。Asherman 综合征又称宫腔粘连，是由于人工流产或者其他宫腔操作，损伤子宫内膜而导致的（A 错）。空气栓塞是一种起源于肺的气体栓子阻塞脑血管引起的疾病，通常因在周围压力降低时（如从深水向浅水上升时），膨胀的肺部气体导致肺过度膨胀所致，其常见的特征为疼痛和（或）神经系统症状。空气栓塞是指空气进入血循环至肺，阻塞肺动脉主要通路，引起严重休克（C 错）。吸宫不全指人工流产术后部分妊娠组织物的残留。与操作者技术不熟练或子宫位置异常有关，是人工流产术常见的并发症，表现为手术后阴道流血时间长，血量多或流血停止后再现多量流血症状（E 错）。
对于第 143 题，描述的症状包括胸闷、冷汗、面色苍白、心动过缓和心律不齐，这些症状可能与空气栓塞相关。空气栓塞是人工流产过程中可能发生的严重并发症，可以导致急性心脏和循环功能障碍。因此，最合适的答案是 C.空气栓塞。
首先，我们分析第 143 题。题目描述的是一位女性患者在人工流产术中突然出现的胸闷、冷汗、面色苍白、心动过缓和心律不齐等症状。我们可以根据这些症状逐一分析选项：
A. Asherman 综合征：主要表现为月经过少、闭经、继发性不孕，与题目描述的症状不符，因此可以排除。
B. 子宫穿孔：虽然是一种人工流产的并发症，但主要表现为下腹剧痛，伴有恶心、呕吐、出血等症状，与题目中的心血管症状不符，所以不是最佳选项。
C. 空气栓塞：虽然空气栓塞是一种严重的并发症，但题目中并未提及与空气栓塞直接相关的症状，因此可以排除。
D. 人工流产综合征：主要表现为在人工流产术中或手术结束时出现恶心呕吐、心动过缓、心律不齐、面色苍白、头昏、胸闷、大汗淋漓，严重者甚至出现血压下降、昏厥、抽搐等迷走神经兴奋症状。这与题目描述的症状高度吻合，因此是最合适的选项。
E. 吸宫不全：主要表现为手术后阴道流血时间长，血量多或流血停止后再现多量流血，与题目描述的症状不符，因此不是最佳选项。
综上所述，第 143 题的答案是 D.人工流产综合征。
问题 143
女性患者行人工流产术中突发胸闷，冷汗，面色苍白，心动过缓，心律不齐，可能的原因是：

这些症状提示一种急性、危及生命的情况。胸闷、冷汗、面色苍白、心动过缓和心律不齐都是急性事件的症状，结合人工流产术，可以推测如下：

选项分析：

A. Asherman 综合征：通常不会导致急性症状，是由于宫腔粘连导致的月经异常或不孕。
B. 子宫穿孔：也可能出现急性腹痛和出血，但不太可能直接引起上述心血管症状。
C. 空气栓塞：这是一个急性且严重的并发症，可能引起胸闷、冷汗、心动过缓和心律不齐。
D. 人工流产综合征：也是一个急性并发症，表现为迷走神经反射，导致心动过缓、心律不齐和其他症状。
E. 吸宫不全：通常表现为术后出血，不太可能引起心动过缓、心律不齐。
答案：D. 人工流产综合征
144、 (B1 型题)
人工流产术后阴道持续流血 2 周未净，可能的诊断是
A.Asherman 综合征
B.子宫穿孔
C.空气栓塞
D.人工流产综合征
E.吸宫不全
参考答案： E
图文解析： 【解析】吸宫不全指人工流产术后部分妊娠组织物的残留。与操作者技术不熟练或子宫位置异常有关，是人工流产术常见的并发症。手术后阴道流血时间长，血量多或流血停止后再现多量流血，应考虑为吸宫不全，血或尿 hCG 检测和超声检查有助于诊断（E 对）。
【避错】子宫穿孔是人工流产术的严重并发症。发生率与手术者操作技术以及子宫本身情况（如哺乳期妊娠子宫，剖宫产后瘢痕子宫妊娠等）有关。手术时突然感到无宫底感觉，或手术器械进入深度超过原来所测得深度。提示子宫穿孔，应立即停止手术（B 错）。人工流产综合反应指手术时疼痛或局部刺激，使受术者在术中或术毕出现恶心呕吐、心动过缓、心律不齐、面色苍白、头昏、胸闷、大汗淋漓，严重者甚至出现血压下降、昏厥、抽搐等迷走神经兴奋症状。这与受术者的情绪、身体状况及手术操作有关（D 错）。
Asherman 综合征又称宫腔粘连，是由于人工流产或者其他宫腔操作，损伤子宫内膜而导致的（A 错）。空气栓塞是一种起源于肺的气体栓子阻塞脑血管引起的疾病，通常因在周围压力降低时（如从深水向浅水上升时），膨胀的肺部气体导致肺过度膨胀所致，其常见的特征为疼痛和（或）神经系统症状。空气栓塞是指空气进入血循环至肺，阻塞肺动脉主要通路，引起严重休克（C 错）。
对于第 144 题，阴道持续流血 2 周未净的情况可能与 Asherman 综合征相关。Asherman 综合征是子宫内膜粘连导致的，可能导致月经量减少或无月经，且手术后可能出现持续流血。因此，对于这种情况，最合适的答案是 A.Asherman 综合征。
接着，我们分析第 144 题。题目描述的是人工流产术后阴道持续流血 2 周未净的情况。我们再次根据症状分析选项：
A. Asherman 综合征、B. 子宫穿孔、C. 空气栓塞、D. 人工流产综合征：这些选项的症状与题目描述的阴道持续流血不符，因此可以一并排除。
E. 吸宫不全：主要表现为手术后阴道流血时间长，血量多或流血停止后再现多量流血，与题目描述的症状相符，因此是最佳选项。

综上所述，第 144 题的答案是 E.吸宫不全。
问题 144
人工流产术后阴道持续流血 2 周未净，可能的诊断是：

术后持续出血提示可能有宫腔内残留或其他异常。

选项分析：

A. Asherman 综合征：一般会引起月经减少甚至闭经，而不是持续出血。
B. 子宫穿孔：可能导致急性出血，但不太可能导致术后持续流血 2 周。
C. 空气栓塞：这是一个急性事件，不会导致术后持续出血。
D. 人工流产综合征：通常表现为术中急性反应，而不是术后持续流血。
E. 吸宫不全：术后持续出血最常见的原因是宫腔内有残留物，吸宫不全可能导致术后持续流血。
答案：E. 吸宫不全
145、 (B1 型题)
女，27 岁，孕 30 周，白带增多 6 天，妇科检查：外阴正常，阴道见浆液性脓性分泌物，宫颈红肿，轻度糜烂，Giemsa 染色可见包涵体，该病原体治疗是
A.青霉素
B.克林霉素
C.头孢曲松
D.氧氟沙星
E.阿奇霉素
参考答案： E 您的答案：
图文解析： 【解析】27 岁孕妇，白带增多，阴道见浆液性有脓性分泌物，且分泌物 Giemsa 染色观察可见包涵体，诊断为沙眼衣原体感染，首选阿奇霉素（E 对）。
【避错】孕妇禁用四环素、多西环素和喹诺酮类（氧氟沙星为喹诺酮类）。大环内酯类（阿奇霉素）、头孢类（头孢曲松）、林可霉素类（克林霉素）均不是梅毒的首选药物。
第 145 题中描述的是孕妇患有宫颈炎，伴有浆液性脓性分泌物，Giemsa 染色可见包涵体，这通常暗示了沙眼衣原体（Chlamydia trachomatis）感染。治疗沙眼衣原体感染的药物通常是阿奇霉素或者其他大环内酯类抗生素。因此，对于这个病例，最适合的选择是 E.阿奇霉素。
145 题解析：
患者青年女性，孕 30 周，白带增多，妇科检查见阴道浆液性脓性分泌物，宫颈红肿，轻度糜烂，Giemsa 染色可见包涵体，根据患者临床表现及实验室检查，考虑沙眼衣原体感染可能性大，沙眼衣原体感染的治疗，阿奇霉素为首选，故选用阿奇霉素。
选项 A 青霉素为β-内酰胺类抗生素，主要用于治疗敏感的 G+球菌和杆菌，G-球菌及螺旋体所致的感染，对沙眼衣原体感染效果欠佳，故排除。
选项 B 克林霉素为林可霉素的衍生物，主要针对革兰氏阳性菌和厌氧菌感染的治疗，对沙眼衣原体没有治疗作用，故不选。
选项 C 头孢曲松为第三代头孢菌素类抗生素，对肠杆菌科细菌有强大活性，但对沙眼衣原体感染治疗效果有限，故排除。
选项 D 氧氟沙星属于喹诺酮类抗生素，对沙眼衣原体感染有一定疗效，但疗效不及阿奇霉素，且孕妇及哺乳期妇女禁用，故不选。正确答案是：145.E，
145. (B1 型题)

题目分析：

女，27 岁，孕 30 周，白带增多 6 天。妇科检查显示外阴正常，阴道见浆液性脓性分泌物，宫颈红肿，轻度糜烂，Giemsa 染色可见包涵体。
这种情况提示宫颈炎，Giemsa 染色可见包涵体，常见于衣原体感染。
最佳答案：
E. 阿奇霉素
（阿奇霉素是一种大环内酯类抗生素，对衣原体有良好疗效，尤其适用于孕妇。）
146、 (B1 型题)
女，32 岁，妊娠 12 周，阴道检查发现大阴唇红肿，可见圆形小溃疡，阴道分泌物检查可见活跃的苍白球螺旋体，首选治疗是
A.青霉素
B.克林霉素
C.头孢曲松
D.氧氟沙星
E.阿奇霉素
参考答案： A 您的答案：
图文解析： 【解析】妊娠 12 周，阴道检查发现大阴唇红肿，可见圆形小溃疡，阴道分泌物检查可见活跃的苍白螺旋体，诊断为梅毒，苍白密螺旋体为梅毒的病原体，为原核单细胞生物，具有与细菌相似的细胞壁，对青霉素（青霉素主要通过破坏细菌的细胞壁从而发挥杀菌作用）极为敏感，故孕妇感染苍白密螺旋体首选的治疗药物是青霉素（A 对）。
【避错】孕妇禁用四环素、多西环素和喹诺酮类（氧氟沙星为喹诺酮类）。青霉素、头孢类（头孢曲松）、林可霉素类（克林霉素）不是沙眼衣原体感染治疗药物。
第 146 题中描述的情况提示患者可能患有梅毒，因为苍白球螺旋体是梅毒的病原体。妊娠期梅毒的首选治疗是青霉素，因为它对胎儿安全，并且能有效治疗梅毒。因此，对于这个病例，最适合的选择是 A.青霉素。
146 题解析：
患者青年女性，妊娠 12 周，阴道检查见大阴唇红肿，可见圆形小溃疡，阴道分泌物检查可见活跃的苍白球螺旋体，考虑诊断为梅毒，梅毒的病原体为梅毒螺旋体，梅毒螺旋体对青霉素高度敏感，且至今没有出现对青霉素耐药的梅毒螺旋体，因此青霉素是治疗梅毒的首选药物，故选用青霉素。
选项 B 克林霉素为林可霉素的衍生物，主要针对革兰氏阳性菌和厌氧菌感染的治疗，对梅毒螺旋体感染无效，故排除。
选项 C 头孢曲松为第三代头孢菌素类抗生素，对肠杆菌科细菌有强大活性，但对梅毒螺旋体感染治疗效果有限，故不选。
选项 D 氧氟沙星属于喹诺酮类抗生素，对梅毒螺旋体感染疗效欠佳，且孕妇及哺乳期妇女禁用，故排除。
选项 E 阿奇霉素属于大环内酯类抗生素，可作为青霉素过敏者的替代治疗药物，但疗效不如青霉素，故不选。
综上所述，正确答案是：145.E，146.A。
146. (B1 型题)
题目分析：

女，32 岁，妊娠 12 周，阴道检查发现大阴唇红肿，可见圆形小溃疡，阴道分泌物检查可见

活跃的苍白球螺旋体。
这种情况提示梅毒感染。
最佳答案：
A. 青霉素
（青霉素是治疗梅毒的首选药物，尤其在妊娠期妇女中更为推荐。）
147、 (B1 型题)
能杀灭休眠体的药物是
A.吡喹酮
B.氯喹
C.乙胺嘧啶
D.伯氨喹
E.奎宁
参考答案： D 您的答案：
图文解析： 【解析】伯氨喹能杀灭肝脏中休眠子（D 对），控制疟疾的复发。
【避错】乙胺嘧啶（C 错）能杀灭红细胞外期的子孢子，发挥病因性预防作用。氯喹、奎宁均能杀灭红细胞内期裂殖体，主要用于控制症状发作和预防性抑制疟疾症状发作（BE 错）。吡喹酮（A 错）为广谱抗吸虫药和驱绦虫药，对多种吸虫有强大的杀灭作用，对绦虫感染和囊虫病也有良好效果。
第 147 题：能杀灭休眠体的药物是
答案：A. 吡喹酮。吡喹酮是用于治疗和预防疟疾的药物，特别是对于那些由恶性疟原虫引起的疟疾，它能够杀灭肝脏中的疟原虫休眠体，从而预防疟疾的复发。
能杀灭休眠体的药物是：
首先，我们分析各个选项：
A. 吡喹酮：主要用于治疗血吸虫病，与杀灭疟原虫休眠体无关。
B. 氯喹：主要用于控制疟疾症状，但不能杀灭休眠体。
C. 乙胺嘧啶：属于抗叶酸类药物，能抑制疟原虫的二氢叶酸还原酶，但对已经形成的休眠体无效。
D. 伯氨喹：能杀灭肝脏中休眠子，控制疟疾的复发，并控制疟疾的传播。
E. 奎宁：和氯喹类似，用于治疗疟疾症状，但对休眠体无杀灭作用。
综上所述，正确答案是 D.伯氨喹，因为它能杀灭休眠体，从而控制疟疾的复发和传播。
147、能杀灭休眠体的药物是
根据现有的医学知识和最佳实践指南，伯氨喹（primaquine）是已知的能有效杀灭休眠体的药物。因此，答案是：
D. 伯氨喹
148、 (B1 型题)
控制间日疟发作首选的药物是
A.吡喹酮
B.氯喹
C.乙胺嘧啶
D.伯氨喹
E.奎宁
参考答案： B 您的答案：
图文解析： 【解析】控制间日疟发作的首选药物是氯喹（B 对），该药对各种疟原虫的红细胞内期裂殖体均有较强的杀灭作用，能迅速有效地控制疟疾的临床发作。

【避错】奎宁（E 错）的抗疟作用与氯喹相似，但不良反应较氯喹多，故不宜作为首选药物。伯氨喹（D 错）主要用于控制复发和传播。乙胺嘧啶（C 错）常用于疟疾病因性预防。吡喹酮（A 错）主要用于治疗血吸虫病。
第 148 题：控制间日疟发作首选的药物是
答案：B. 氯喹。氯喹是治疗疟疾的常用药物，特别是对于间日疟（由恶性疟原虫或间日疟原虫引起的疟疾），它能够有效控制症状和治疗急性发作。
控制间日疟发作首选的药物是：
我们再次分析选项：
A. 吡喹酮：不针对疟疾治疗。
B. 氯喹：是控制疟疾症状的首选药物，能有效杀灭红细胞内期裂殖体，主要用于控制症状发作和预防性抑制疟疾症状发作。
C. 乙胺嘧啶：主要用于预防，而非急性发作期治疗。
D. 伯氨喹：主要用于杀灭休眠体，不用于急性发作期。
E. 奎宁：虽可治疗疟疾，但效果通常不如氯喹。
综上所述，正确答案是 B.氯喹，因为它是控制间日疟发作的首选药物。
148、控制间日疟发作首选的药物是
氯喹（chloroquine）是控制疟疾急性发作的常用药物，尤其是对间日疟原虫有效。因此，答案是：
B. 氯喹
149、 (B1 型题)
儿童苯丙酮尿症的确诊检查是
A.血 TSH 测定
B.染色体核型分析 C.尿三氯化铁实验
D.尿蝶呤分析
E.血浆氨基酸分析
参考答案： E 您的答案：
图文解析： 【解析】儿童苯丙酮尿症的确诊检查是血浆氨基酸分析（E 对），即测定血清苯丙氨酸浓度，血清苯丙氨酸浓度的测定主要用于筛查实验阳性患者的确诊。
【避错】】尿三氯化铁试验（C 错）及 2，4—二硝基苯肼试验用于较大儿童苯丙酮尿症的初筛，阳性者需进一步做血苯丙氨酸测定才能确诊。尿蝶呤分析（D 错）主要用于苯丙酮尿症经典型和非经典型的分析鉴别。血 TSH 是甲亢或甲减的确诊方法（A 错）。染色体核型分析是唐氏综合征的确诊方法（B 错）。
对于题目 149，“儿童苯丙酮尿症的确诊检查是”，正确的答案是 E.血浆氨基酸分析。这种检查可以测量血液中的苯丙氨酸浓度，若明显升高则可确诊为苯丙酮尿症。
149 题儿童苯丙酮尿症的确诊检查是：
正确答案应是“E.血浆氨基酸分析”。这个检查可以精确测量血浆中的氨基酸水平，对于确诊苯丙酮尿症至关重要。
149、儿童苯丙酮尿症的确诊检查是
E. 血浆氨基酸分析

解析：苯丙酮尿症（PKU）的确诊需要通过血浆氨基酸分析来检测苯丙氨酸的水平。该病是由于苯丙氨酸羟化酶（PAH）缺乏导致苯丙氨酸代谢障碍，血浆中的苯丙氨酸浓度明显升高。
150、 (B1 型题)
儿童苯丙酮尿症的初筛选用的检查是

A.血 TSH 测定
B.染色体核型分析
C.尿三氯化铁实验
D.尿蝶呤分析
E.血浆氨基酸分析
参考答案：　　C　您的答案：
图文解析：　　【解析】尿三氯化铁试验（C 对）及 2，4—二硝基苯肼试验用于较大儿童苯丙酮尿症的初筛，阳性者需进一步做血浆氨基酸分析才能确诊。
【避错】儿童苯丙酮尿症的确诊检查是血浆氨基酸分析（E 错）。尿蝶呤分析（D 错）主要用于苯丙酮尿症经典型和非经典型的分析鉴别。血 TSH 是甲亢或甲减的确诊方法（A 错）。染色体核型分析是唐氏综合征的确诊方法（B 错）。
对于题目 150，“儿童苯丙酮尿症的初筛选用的检查是”，正确的答案也是 E.血浆氨基酸分析。新生儿筛查通常包括血浆氨基酸的分析，以便早期发现苯丙酮尿症等代谢疾病。
150 题儿童苯丙酮尿症的初筛选用的检查是：
正确答案应是“C.尿三氯化铁实验”。这个检查方法简便、快速且成本低，适合作为初步筛查的方法。
综上所述，对于儿童苯丙酮尿症的确诊检查，应选择血浆氨基酸分析（E），而对于初筛，应选择尿三氯化铁实验（C）。
150、儿童苯丙酮尿症的初筛选用的检查是
C. 尿三氯化铁实验

解析：尿三氯化铁实验是一种快速、简便的初筛方法，通过检测尿中苯丙氨酸的代谢产物（如苯乙酸、苯乙酮酸、苯乙醇）的存在来初步筛查苯丙酮尿症。如果尿液中这些代谢产物与三氯化铁发生化学反应变为绿色，则提示苯丙氨酸代谢异常。
