# Supplementary material for: Large Language Models Evaluation of Medical Licensing Examination Using GPT-4.0, ERNIE Bot 4.0, and GPT-4o
Source: Bioengineering (Basel). 2026 Jan 17;13(1):113. doi: 10.3390/bioengineering13010113 (PMC12837214; doi:10.3390/bioengineering13010113)
Supplement: Supplementary file 1 [file bioengineering-13-00113-s001.zip › English - Version All_Questions_Reference_Answers_and_Model_Responses.pdf]

All questions and each model answers (black characters on the white background is the correct answer, the bottom color font is gpt 4.0 answer, then the bottom yellow font is the 4.0 answer, then the bottom color font is gpt 4 o answer, all color fonts, green is the correct answer, red is the wrong answer)

2023, Unit 1

1. A1 multiple choice questions (there are five alternative answers: A, B, C, D and E. Please choose one of the best answers.）

1. (Type A1 question)

When temporary blood drawing in medical institutions, which of the following is not a requirement for temporary blood drawing in medical institutions

A. The surface antibodies of liver A

B. HBsAg

C. Surface antibodies against hepatitis C

D. HIV antibody

E. Syphilis antibody

Reference answer: A. Your answer:

[Analysis] Temporary blood collection of medical institutions should be able to carry out cross-blood matching, hepatitis B virus surface antigen and hepatitis C

The ability to detect viral antibodies, HIV antibodies and Spi. pallidum antibodies. Therefore, this question is selected A.

[Error] In order to ensure emergency use of blood, medical institutions can temporarily collect blood, but the following conditions must be met:

(1) endangers the patient's life and is in urgent need of blood transfusion; (2) the local blood station cannot provide blood in time and cannot supply other blood in time

Medical institutions regulate blood, but other medical measures cannot replace blood transfusion treatment; (3) have to carry out cross blood matching and type B

Detection ability of hepatitis virus surface antigen, hepatitis C virus antibody, HIV antibody and Treponema pallidum antibody;

(2) Observe with the operating procedures and technical standards of blood collection and supply. Medical institutions shall respond within 10 days after the temporary blood collection

To report to the administrative department of public health of the people's government at or above the county level.

For temporary blood draws at medical facilities, blood tests for a range of infectious diseases are often required to ensure safety and prevent the spread of the disease. Options A, B, C, D, and E mention hepatitis A surface antibodies, hepatitis B surface antigen, hepatitis C surface antibodies, HIV antibodies, and syphilis antibodies, respectively. These tests are usually designed to identify possible infectious diseases to prevent their spread in the medical process.

However, the question is "not a prescribed requirement for a temporary blood draw in the medical institution," meaning that we need to find a test that is not necessarily necessary at the time of a temporary blood draw. Among the options, hepatitis A surface antibodies (option A) are usually not a must for temporary blood draws, because hepatitis A is transmitted more by the oral route than by blood. Therefore, the risk of hepatitis A is lower in blood transmission compared to hepatitis B, hepatitis C, HIV, and syphilis.

Based on this logic, the most correct answer should be A surface antibody of A liver.

analyze:

When the blood is collected temporarily in the medical institutions, the necessary infectious disease examination of the collected blood is required to ensure the blood safety and reduce the risk of disease transmission. The correspondence between the tests items and the diseases involved in the option is as follows:

Option A hepatitis A surface antibody can determine whether the patient is infected with hepatitis A virus or has been vaccinated with hepatitis A vaccine, which is A necessary test for temporary blood collection in medical institutions.

Option B second liver surface antigen is the shell protein of second liver virus, itself does not have infectious, but its appearance often accompanies the existence of second liver virus, it is the sign of infection second liver virus, also be the necessary test item that medical institution collects blood temporarily.

Hepatitis C virus has no surface antibodies, so it cannot detect hepatitis C surface antibodies, usually by testing hepatitis C antibodies to determine the presence of HCV infection. Therefore, option C hepatitis C surface antibody is not a temporary blood drawing test item of medical institutions, which is the correct answer to this question.

Option D: HIV antibody is an important indicator of HIV infection detection, and it is a necessary test item for temporary blood collection in medical institutions.

Option E Syphilis antibody test can help to determine whether there is treponema pallidum infection, which is a necessary test item for temporary blood collection in medical institutions.

In conclusion, the correct answer is: C. Hepatitis C surface antibodies.

Which of the following is not required:

A. The surface antibodies of liver A

B. Hepatitis B surface antigen

C. Surface antibodies against hepatitis C

D. HIV antibody

E. Syphilis antibodies

According to existing medical knowledge and best practice guidelines, medical institutions usually detect hepatitis B surface antigen (HBsAg), hepatitis C antibodies (HCV Ab), HIV antibodies, and syphilis antibodies when taking temporary blood draws, because these are highly infectious agents that require timely screening. Hepatitis A surface antibodies (HAV Ab) are usually not included in routine screening programs for temporary blood draws because it is transmitted mainly transmitted

through the digestive tract rather than blood.

So the answer is:

A. The surface antibodies of liver A

2. (Type A1 question)

The institution with the authority to preserve the physician prescription signature or the relevant signature is

A. The administration of public health where the physician practices

B. The practice institution of registered practitioners

C. Supervision and management department where the physician is registered

D. Provincial health administration department

E. Department of the physician

Reference answer: B. Your answer:

[Analysis] Doctors must sign samples in the registered medical, prevention, health care institutions and special signature for record before issuing the office

square. So this question is selected B.

[To avoid mistakes] Article 5 of the Prescription Management Measures stipulates on the right to prescribe: prescriptions issued by a registered medical assistant practicing physician

It must be valid only after the signature or special signature of the medical practitioner in the practice place. Registered practicing assistant physician in the township,

The medical, preventive and health care institutions in ethnic townships and towns shall obtain the corresponding right to prescribe them at the registered practice place.shakedown period

The prescription of the doctors must be reviewed, signed or added by the medical practitioners in the medical, prevention and health care institutions

It is valid after being stamped with a special seal. Doctors must sign the samples and keep special seals in the registered medical, preventive and health care

institutions

The prescription can only be issued. After a doctor is ordered to suspend his practice, to leave the training period or his practice certificate is revoked,

The prescription right is cancelled immediately.

In China, the preservation of physician prescription signatures or relevant signatures is usually handled by the practice registered of the physician. Therefore, the most appropriate choice should be the practice institution of B. These institutions are responsible for managing the practice activities of physicians, including the preservation of prescription signatures and related documents, to ensure the quality of medical services and compliance with the law. Therefore, my answer is B. the practice of registered practitioners.

First, we need to make it clear that the question is about which institution has the authority to preserve the physician prescription signature or the relevant signature. Next, we can analyze each option one by one:

A. Health administration department of the place of practice: The health administration department is mainly responsible for the supervision and management of public health and medical services. Although they have certain supervision power, they are usually not responsible for the internal management of specific medical institutions, such as the preservation of prescription signatures of doctors.

B. Practicing institution of medical registration: The practicing institution of medical registration is the place where doctors work daily and write prescriptions. According to the relevant regulations, the doctor's prescription signature or relevant signature should be properly kept by the registered practice institution for verification.

C. Supervision and management department of the registration place: This department is mainly responsible for the registration and management of physicians. Although they keep the registration information of physicians, it does not involve the preservation of prescription signature.

D. Provincial health management department: This is a higher level management department, mainly responsible for the health policy and management of the whole province, and does not directly participate in the internal management of medical institutions.

E. Department of the physician: The department is a unit within the hospital. Although physicians work in the department, the preservation of prescription signature or signature is usually carried out by the hospital or practicing institution, rather than a single department.

To sum up, according to the internal management regulations and relevant regulations of medical institutions, the physician prescription signature or relevant signature should be kept by the registered practicing institution. This can ensure the authenticity and traceability of prescriptions, and help to ensure the drug safety and medical quality of patients.

Therefore, the correct answer is B. physician.

Institutions with the authority to preserve physician prescription signatures or relevant signatures based on existing medical knowledge and best practice guidelines are:

F. The practice institution of registered practitioners

3. (Type A1 question)

The prescription validity period may be extended under special circumstances, but the maximum validity period shall not exceed

A.2 days

B.3 days

C.5 days

D.7 days

E.10 days

Reference answer: B. Your answer:

Graphic analysis: [Analysis] The prescription is valid on the day of issuance. If the validity period needs to be extended under special circumstances, the physician who makes the prescription shall indicate the validity

Term, but the maximum period of validity shall not exceed 3 days.

[Avoid mistakes] memory questions, from the "prescription management method".

In China, the validity period of a prescription is usually determined by the relevant medical regulations and policies. The duration of a prescription may vary by region and by case, but in general, the general prescription is usually valid for no more than 7 days. For some special conditions, such as long-term medication prescriptions for people with chronic diseases, but this usually requires special regulation or approval.

In the absence of detailed country or region regulations, it is more reasonable to choose "D.7 days" as the longest expiration period of prescription, because this is the common upper limit of prescription expiration period. However, the practice should be based on the local medical regulations and policies.

Therefore, the most appropriate answer is the D.7 days. However, it is recommended to consult the latest medical regulations and guidelines for the most accurate information.

analyze:

Prescription is an important written document of medical and pharmaceutical preparation, when doctors diagnosis and treatment of patients usually prescribed, and then by pharmacist review, deployment, check, the last in patients with dispensing vouchers, prescription drug validity is generally 3 days, but the prescription in special circumstances need to extend the validity, by the prescription doctor indicate the valid

period, but the longest validity shall not exceed 3 days.

To sum up, the correct answer is: B.3 days.

According to the Prescription Management Measures, under special circumstances, the maximum validity period of a prescription shall not exceed 7 days. So, the correct answer is:

D.8 days

4. (Type A1 question)

The legal qualifications for doctors to prescribe narcotic drugs and category I psychotropic drugs are

A. Qualified to open prescriptions of narcotic drugs and category I psychotropic drugs

B. Have the qualification to open the clinical use of narcotic drugs and class I psychotropic drugs

C. Participate in provincial training courses on narcotic drugs and class I psychotropic drugs

D. Clinical experience in opening narcotic drugs and class I psychotropic drugs

E. Seal cards for narcotic drugs and class I psychotropic drugs

Reference answer: A. Your answer:

[Analysis] A medical institution shall, in accordance with the provisions of the competent department of health under The State Council, conduct training and assessment on the knowledge of the use of narcotic drugs and psychotropic drugs for the medical doctors of the unit, and grant the prescription qualification of narcotic drugs and class I psychotropic drugs if they pass the examination. Therefore, this question is selected A.

[Avoiding errors] Where a medical institution needs to use narcotic drugs and class I psychoactive drugs, it shall go through the people of the city divided into districts where it is located

Approved by the competent health department of the government, seal cards for the purchase and use of narcotic drugs and category I psychotropic drugs are obtained.

In China, doctors must have corresponding legal qualifications for prescribing narcotic drugs and category I psychotropic drugs. This often requires physicians not only relevant clinical experience, but also specific training and officially approved qualifications or certification. The options mentioned "qualified to prescribe narcotic drugs and category I psychotropic drugs", "qualification for clinical use", "attending a training class", "clinical experience" and "obtaining a printing card".

Of these options, option A "qualified to prescribe narcotic drugs, category I psychotropic drugs" points most directly to the statutory requirement, because it indicates that doctors have been officially certified to legally prescribe these drugs. While other options such as training, clinical experience and printing cards may be part of or relevant to obtaining this qualification, it is fundamental that doctors must have formal prescribing qualifications.

Therefore, the correct answer should be A. Qualified to prescribe narcotic drugs and the first class of psychotropic drugs.

analyze:

Narcotic drugs and psychoactive drugs are drugs under special administration by the State. If medical institutions need to use narcotic drugs and class I psychoactive drugs, they shall obtain the approval of the drug regulatory department of narcotic drugs and the city divided into districts. Medical institutions shall, in accordance with the guiding principles for the clinical application of narcotic drugs and psychoactive drugs formulated by the competent department of public health under The State Council, prescribe narcotic drugs and category I psychoactive drugs. The practicing physicians with the right to prescribe narcotic drugs and first class psychotropic drugs shall attend the training on the knowledge and standardized management of narcotic drugs and psychotropic drugs and pass the examination to obtain the right to prescribe narcotic drugs and first class psychotropic drugs.

Option A is qualified to prescribe narcotic drugs and Class I psychotropic drugs;

Option B is qualified for clinical use of narcotic drugs and Class I psychotropic drugs; Option D has clinical experience to open narcotic drugs and Class I psychotropic drugs; the above three options are not accurate, and prescription right qualification or clinical experience does not mean the right to prescribe narcotic drugs and psychotropic drugs, and clinical use qualification is not a necessary condition stipulated by law.

Option C attends the provincial training class of narcotic drugs and psychotropic drugs of the first class, which is not accurate. Participating in the training class is a link to obtain the prescription right, but it is not the only condition, and it also needs to pass the examination, so it is not selected.

To sum up, the correct answer is: E. Printing cards of narcotic drugs and the first class of psychotropic drugs.

In the Chinese medical practitioner examination, the most consistent answer for the legal qualifications required for doctors to prescribe narcotic drugs and the first class of psychotropic drugs is:

A. Qualified to open prescriptions of narcotic drugs and category I psychotropic drugs

According to the relevant laws and regulations in China, doctors need to have the special qualification to open prescriptions for narcotic drugs and category I psychotropic drugs before they can legally prescribe these drugs. This qualification is usually obtained through certification and training from the relevant departments.

5. (Type A1 question)

The following are the cases of bad drugs

A. passing off non-drugs as drugs

B. degenerative

C. The composition of the drug is inconsistent with the ingredients prescribed by

the state

D. The indicated functional indications are beyond the therapeutic scope of the drug

E. Expshelf life

Reference answer: E Your answer:

Graphic analysis: [analysis] the cases of inferior drugs are: (1) the content of drug ingredients does not meet the national drug standards; (2) contaminated

Drugs; (3) drugs that do not indicate or changed their product validity period; (4) drugs that do not indicate or changed their product batch number;

(5) drugs that exceed the validity period; (6) drugs that add preservatives and auxiliary materials without authorization; and (7) other drugs that do not conform to the drug standards

Prospective medicine.

[Avoid mistakes] The cases of counterfeit drugs include: the ingredients of the drug are inconsistent with the ingredients stipulated in the national drug standards; non-drug risk

Charging drugs or impersonate other drugs as such drugs; deteriorated drugs; the indications or functions indicated in the drug

Out of the prescribed scope.

In the Chinese medical practitioner examination, the definition of inferior drugs is those drugs that do not meet the national standards in quality. By this definition, the following cases can be considered as inferior drugs:

A. To impersonate non-drugs as drugs: This situation involves counterfeiting, but it is not directly related to the quality of the drug itself. Therefore, although this is A serious violation, it does not necessarily meet the definition of inferior drugs.

B. Metamorphic: deteriorated drugs are certainly of substandard quality, so they can be considered inferior.

C. The composition of a drug is not consistent with the composition stipulated by the state: this situation directly violates the drug quality standards, so the drug can be considered inferior.

D. The scope of functional indications is beyond the scope of the drug: this involves the issue of drug labeling and advertising, which is not compliant, but does not necessarily mean the quality of the drug itself.

E. Over expiration: Drugs beyond expiration may change in chemical composition and efficacy and will no longer meet the original quality standards, and may, therefore, be considered inferior.

Considering that the definition of inferior drugs is mainly related to the quality of drugs, options B (deteriorated) and C (the composition of the drug is inconsistent with the composition stipulated by the state) and E (beyond the expiration period) all meet the conditions of inferior drugs. However, if we choose the "most correct" answer, we usually look for the option that most directly reflects the quality of drugs. In this case, option C (where the composition of the drug does not match the national composition) may be the most direct case of identifying the drug as inferior, because it directly violates the composition rules of the drug.

Therefore, the most appropriate answer should be C. Drug ingredients are not specified by the state.

This is a question of drug classification, and we need to identify the bad drugs from the given options.

Understanding the definition of inferior drugs is the key to solving this problem. Inferior drugs refer to the drugs whose ingredients do not meet the provisions of the national drug standards. This usually includes the content of drug ingredients that do

not meet the national drug standards, or are contaminated, or does not indicate or change the expiration date, production batch number, and other circumstances that do not meet the drug standards.

Analysis options:

Option A (posing as non-drugs as drugs): This is actually a case of fake drugs, not inferior drugs.

Option B (deteriorated): deteriorated drugs are fake because their properties have changed and may be harmful to human health.

Option C (the composition of the drug): This is also considered as fake because it does not match the prescribed standard.

Option D (the functional indications are beyond the therapeutic range of the drug): This situation may involve false advertising, but does not directly point to inferior drugs.

Option E (beyond the expiration date): This is a case of inferior drugs. Drugs beyond the expiration date may not guarantee their safety and effectiveness, and are therefore considered inferior.

In conclusion, the safety and efficacy of drugs beyond the expiration date may be affected, in line with the definition of inferior drugs.

Therefore, the answer is E. Exexpiration date.

The question is about substandard drugs, and which of the following options is correct.

Analysis options:

-A. Non-drugs posing as drugs: this is fake drugs, not bad drugs.

-B. Metamorphosis: drug deterioration belongs to the category of inferior drugs.

-C. The composition of the drug is not specified by the state: this is fake, not inferior.

-D. The scope of functional indications is beyond the scope of drugs: this belongs to the category of false advertising, not inferior drugs.

-E. Overexpiry date: drug expiry date belongs to the category of inferior drugs.

So, the correct answer is:

F. degenerative

6. (Type A1 question)

Which department issued the epidemic prevention and control issue

A. Provincial grade A A hospital

B. Health administration

C. Provincial epidemic prevention and control center

D. mobile cabin hospital

E. the State Council

Reference answer: E Your answer:

[Analysis] Epidemic prevention and control is issued by the health administration department of The State Council, so E is selected.

[To avoid mistakes] Provincial third-class A hospitals, health management departments, provincial epidemic prevention and control centers, and makeshift hospitals all cooperate with epidemic prevention and control

Implementation of the unit.

Epidemic prevention and control is usually issued by authoritative and responsible official departments. In China, this kind of information is usually released by the national or local health commission or health administration department. So the most appropriate answer is:

F. Health management department

This is a choice question for the epidemic prevention and control information release department. Let's analyze each option:

A. Provincial Grade A hospitals: Grade A hospitals are mainly responsible for medical services, including the diagnosis and treatment of diseases. They are not directly responsible for releasing the epidemic prevention and control information, so they can be excluded.

B. Health authorities: Health authorities are usually responsible for the development and implementation of public health policies, but they are usually not direct publishers of epidemic prevention and control information, especially at the national level.

C. Provincial Epidemic prevention and Control Centers: Although provincial epidemic prevention and control centers are closely related to epidemic prevention and control, they usually implement policies formulated by higher authorities and are not the original publishers of the information.

D. Makeshift hospitals: Makeshift hospitals are mainly used for isolation and treatment of patients and are not responsible for releasing epidemic prevention and control information.

E. The State Council: In China, The State Council is the highest administrative organ, responsible for policy formulation and release at the national level. As a national emergency public health event, the release of epidemic prevention and control is usually carried out by the State Council.

To sum up, The State Council is responsible for the release of epidemic prevention and control information, so the correct answer is E. The State Council.

The relevant policies and guidance on epidemic prevention and control are issued by the health authorities. In China, the main institutions specifically implementing and managing the epidemic prevention and control work are the health commissions at all levels, which are responsible for formulating and issuing prevention and control measures, guidelines and policies. So, the most correct answer is:

F. Health administration

7. (Type A1 question)

To determine the period of detention, quarantine or medical observation for a contact with an infectious disease, mainly based on the infectious disease

A. incubation

B. infective stage

C. Pathogen-carrying period

D. decubation

E. Clinical diagnosis period

Reference answer: A. Your answer:

[Analysis] The incubation period (A pair) is the period from the pathogen invading the human body to the onset of clinical symptoms.infect

The incubation period of disease is not the same, quarantine work can determine the specific situation such as observation population, time,

So as not to miss the inspection.

[Avoid mistakes] The incubation period of each infectious disease has a range (the shortest, the longest), and is a normal distribution, is a quarantine worker

An important basis for the observation and retention of contacts.

When determining the period of detention, quarantine or medical observation of a contact of an infectious disease, the main basis is the incubation period of the infectious disease. The incubation period is the period between the individual's infection and the onset of symptoms. This period is crucial to identify possible infected persons for isolation and observation to prevent further spread of the virus. Therefore, the correct answer is A. latency.

analyze:

A contact of an infectious disease refers to contact with the source of infection and is likely to be infected. The duration of detention, quarantine or medical observation needs to be determined based on the possible duration of infection. The incubation period is the period from the pathogen invading the human body to the beginning of clinical symptoms. Therefore, the period of detention, quarantine or medical observation of infectious disease contacts can be determined according to the incubation period.

Option B transmission period refers to the period when the infected person or patient has the ability to transmit the virus to others, and has nothing to do with the observation period of the infectious disease contact, so it is not selected.

Option C pathogen carrying period refers to that after the clinical symptoms disappear, the patient still retains the pathogen in his body, and the clinical symptoms no longer appear. The pathogen carrier is not infectious, so it is not considered.

Option D recovery period means that when the pathogenesis of the pathogen is weakened or disappeared, and the immune response of the body is gradually strengthened, the pathological changes are gradually reduced, the patient's symptoms gradually improve, and at this time, the infectivity gradually disappears, which has nothing to do with this question.

Option E Clinical diagnosis period is the disease diagnosis and non-infectious disease concept proposed by clinicians based on clinical experience, clinical history, clinical signs, medical imaging, blood biochemistry, immunological examination, cellular and histopathological examination.

In conclusion, the correct answer is: A. Latency period.

Determine the period of check-up, quarantine or medical observation for a contact of an infectious disease, mainly based on the incubation period of the infectious disease. The incubation period is the time from the pathogen to the onset of clinical symptoms. Because although there are no symptoms during the incubation period, but may already be infectious, so to determine the incubation period is an important basis to prevent the spread of infectious diseases.

The correct answer is: A. incubation period

8. (Type A1 questions)

The following class B infectious diseases are those listed below

A. pestis

B. cholera

C. nettle rash

D. Aids

E. angina parotidea

Refer to the answer: D. Your answer:

[Analysis] Class B includes: SARS (severe acute respiratory syndrome), AIDS (D pair), viral

Hepatitis, poliomyelitis, human infection with highly pathogenic avian influenza, measles, renal syndrome hemorrhagic fever, rabies, epidemic type B

Encephalitis, dengue fever, anthrax, bacterial and amoebic dysentery, tuberculosis, typhoid fever and paratyphoid fever, and endemic cerebrospinal membranes

Inflammation, pertussis, diphtheria, neonatal tetanus, scarlet fever, brucellosis, gonorrhea, syphilis, leptospirosis, blood

Trematode, malaria, increased human H7N9 in November 2013.

[Misavoidance] Class A infectious diseases include plague and cholera. Class C infectious diseases include mumps and rubella.

According to the classification of infectious diseases in China, class B infectious diseases include those that are more serious but less contagious or harmful than class A infectious diseases. In the option:

A. Plague is a class A infectious disease.

B. Cholera is also a class A infectious disease.

C. rubella is a class B infectious disease.

D. AIDS is a class B infectious disease.

E. Mumps is not usually classified as a class A / B infectious disease.

Therefore, the most correct answer is D. AIDS.

First, we need to understand the classification system of infectious diseases in China. In China, according to their mode of transmission, 39 statutory infectious diseases are divided into class A infectious diseases, Class B infectious diseases and class C infectious diseases according to their transmission mode, transmission speed, epidemic intensity and the degree of harm to human health, society and economy.

Next, we analyze each option:

A. Plague-plague is a class A infectious disease with a very high fatality rate and infectivity, so it does not meet the requirements of class B infectious disease in the title.

B. Cholera-cholera is also classified as a class A infectious disease, with a very high transmission speed and harm, so it is not a class B infectious disease.

C. rubella-rubella is not in the list of class A or B infectious diseases but is class C infectious diseases and can therefore be excluded.

D. AIDS-AIDS is clearly classified as a class B infectious disease. It is mainly through sexual transmission, blood transmission and mother-to-child transmission, which has serious harm to human health, but the speed and mode of transmission are different from that of class A infectious diseases.

E. Mumps-mumps is also a class C infectious disease, mainly transmitted through droplets, its transmission speed and harmfulness is relatively low.

In conclusion, according to the classification of infectious diseases in China, AIDS is the only disease that meets the definition of class B infectious diseases.

So, the correct answer is D. AIDS.

According to the Law of the Prevention and Treatment of Infectious Diseases of the People's Republic of China, class B infectious diseases include AIDS, tuberculosis, syphilis and viral hepatitis B. The following are the categories of the various infectious diseases:

-Class A infectious diseases: plague, cholera

-Class B infectious diseases: AIDS, mumps, rubella, etc

-Class C infectious diseases: influenza, hand, foot and mouth disease, etc

The topic requires you to choose class B infectious diseases. Among the options, only AIDS (D) is a class B infectious disease.

So, the most correct answer is:

F. Aids

9. (Type A1 question)

The time limit for reporting deaths during the medication is

A.15 hours

B.5 hours

C.3 hours

D. immediately

E.2 hours

Refer to the answer: D. Your answer:

[Analysis] The death caused by the drug in the process of medication should be reported immediately, so this question is selected D.

[Escape errors] Death shall be reported immediately, new and serious adverse reactions shall be reported within 15 days, and general adverse reactions shall be reported within 30 days.

During the medication, this serious adverse event should be reported immediately. Thus, the correct answer is that D. immediately. This timeliness of reporting is essential to prevent further adverse events and to take the necessary measures.

This is a question about the time limit for the reporting of adverse drug events. We need to determine how long this serious adverse event should be reported during the medication.

The key to understanding the problem is to recognize that adverse drug events, especially the events leading to death, are essential for drug regulation and patient safety. Therefore, the timely reporting of such events is of great significance to prevent the recurrence of similar events and to protect the safety of more patients.

Now, let's analyze the various options:

A.16 hours: This is relatively long and it may not be possible to alert drug regulators to take necessary actions.

B. 5 hours: Although shorter than 15 hours, it may not be the most rapid reaction time, especially when dealing with serious adverse drug events that may lead to death.

C. 3 hours: This time is relatively short, but still not the most immediate

response.

D. Immediate: This option emphasizes the urgency of reporting adverse events, especially the need for immediate action to protect patient safety in response to potentially fatal AEs.

E. 2 hours: Although shorter than 3 hours, it is still not "immediate" reporting and may not be applicable for handling the most urgent situations.

In conclusion, when it comes to serious adverse events such as deaths due to drugs, the most important thing is to report them immediately, so that the drug regulator can act as soon as possible to prevent the recurrence of similar events.

Thus, the correct answer is that of D. immediately.

Death during the medication is serious adverse reactions and must be reported immediately to ensure timely treatment and avoidance of further hazards. So, the most correct answer is:

E. immediately

10. (Type A1 questions)

If the medical institution is verified for three years, the hospital is

A. clinic

B. tertiary hospitals

C. Village clinic

D. outpatient department

E. Community Service Center

Reference answer: B. Your answer:

[Analysis] "Regulations on the Management of Medical Institutions" stipulates that the medical institutions with more than 100 beds, its " medical institutions practice

The license shall be checked once every 3 years. More than 100 beds are available in secondary hospitals (100~500) and tertiary hospitals (>

500), so this question is selected for B.

[Avoid mistakes] For medical institutions with less than 100 beds, the Practice License of Medical Institutions shall be verified once a year.

In China, the practice license verification time of medical institutions varies according to the type and level of medical institutions. Generally speaking, larger hospitals, such as tertiary hospitals, have a longer practice license and may be five years. While smaller medical units, such as clinics, village clinics, outpatient departments, and community service centers, may require more frequent verification, such as every three years.

Based on this information, the largest type of medical institution that best meets the triannual verification period may be a clinic, village clinic, outpatient department, or community service center. But in China, tertiary hospitals usually have longer verification cycles, so option B (tertiary hospitals) should not be the correct answer.

Since the question requires the best answer, we need to further determine which type of medical institution has a clear verification period of three years. Generally, relatively small medical facilities such as clinics (option A) or community services (option E) may have shorter license expiration periods, so I tend to choose one of them.

Thus, the most likely answer is the A. clinic or E. community service center. However, the specific answer may depend on the local regulations and policies. Without specific legal texts or policies for reference, determining the exact answer may be somewhat difficult. If one has to be chosen, I may choose the A. Clinic, because it is usually a smaller medical institution that may require more frequent calibration.

analyze:

The verification of the practice license of a medical institution refers to the process in which the administrative department of public health inspects, evaluates and examines the basic conditions and practice conditions of the medical institution according to law, and makes corresponding conclusions according to law. According to the Detailed Rules for the Implementation of the Regulations on the Administration of Medical Institutions, medical institutions shall apply to the registration authority for the verification procedures three months before the expiration of the verification period, and the verification period shall be verified in accordance with the evaluation measures of medical institutions and relevant provisions. Among them, the verification period of more than 100 beds in general hospitals, hospitals of traditional Chinese medicine hospitals, integrated Chinese and western medicine hospitals, ethnic medicine hospitals, specialized hospitals, sanatoriums, rehabilitation hospitals, maternal and child health care hospitals, emergency centers, clinical laboratory centers and specialized disease prevention and treatment institutions is 3 years. The number of beds in tertiary hospitals is generally more than 100, so the verification time of the medical institution is three years.

Option A clinic, option C village clinic, option D outpatient department, and option E community service center are all medical institutions with less than 100 beds, and the verification period should be 1 year, which can be excluded together.

To sum up, the correct answer is: B. tertiary hospitals.

According to China's Regulations on the Administration of Medical Institutions and relevant regulations, the verification time of the practice licenses of medical institutions varies according to different types of medical institutions. The practice license of a tertiary hospital is usually checked once every three years. Therefore, the hospital is:

F. tertiary hospitals

11. (Type A1 questions)

A hospital has carried out the new management of hospital management system,

reception and referral process, psychological consultation, health examination and so on, to ensure the care, attention and fairness of medical staff. The above measures

A. It is conducive to improving the development level of medical science

B. It is conducive to building a harmonious doctor-patient communication

C. Benefit to patient diagnosis and treatment and fair treatment

D. Is conducive to the utilization of medical and health care departments

E. It is conducive to exerting the overall effect of the hospital

Reference answer: E Your answer:

Graphic analysis: [Analysis] A hospital has carried out a series of new management to ensure the care, attention and fairness of medical staff, which is beneficial

To give full play to the overall effect of the hospital.

[Wrong avoidance] option ABCD is not reflected.

This question examines the impact of the reform of hospital management system on hospital operation and medical service. The options include different aspects of the benefits, but you need to choose the answer that best fits the question description. According to the title description, the hospital has carried out the reform of management system, reception and referral process, psychological consultation, health examination and other aspects, with the focus on ensuring the concern, concern and fairness of medical staff, so as to infer the direct effect of these reforms.

A. Help to improving the development level of medical science: Although good management can indirectly promote scientific development, the topic does not directly involve scientific research activities.

B. Benefit to building harmonious doctor-patient communication: This option meets the description of improving hospital management and improving job satisfaction of medical staff, which may directly affect doctor-patient communication.

C. Good for patient treatment and fair treatment: This option is also in line with the topic, especially in the context of emphasizing fairness.

D. Good for the use of health care departments: This option is too specific and not directly related to the management measures described in the title.

E. It is conducive to exerting the overall effect of the hospital: this is a relatively broad option. Although improving the management system can indeed improve the overall efficiency of the hospital, it is not specific enough.

In conclusion, the best answer should be the option that most directly reflects the results of hospital management reform. I tend to choose B. It is conducive to the construction of harmonious doctor-patient communication, because it is directly related to improving hospital management and enhancing the satisfaction of medical staff, and then promoting a harmonious doctor-patient relationship. However, option C also has some relevance, because the reform ensures fairness and may directly affect patient diagnosis and treatment. In this case, option B corresponds more specifically to the reform measures and the expected results described in the title.

analyze:

The hospital has carried out reforms in management system, referral, psychological consultation, health examination and other aspects. These measures not only involve the optimization of medical service process, but also pay attention to the psychology and working status of medical staff, which is obviously a comprehensive hospital management reform. By optimizing the process of receiving and referral, hospitals can improve patients' medical experience and ensure that patients get timely and effective diagnosis and treatment. At the same time, the care, attention and fairness of medical staff can improve the job satisfaction and efficiency of medical staff, so as to play the overall effect of the hospital.

Option A is conducive to improving the development level of medical science, which is usually related to medical research, technological innovation, education and training, and has little to do with the management reform of the hospital, so it is excluded.

Although optimizing the referral process and providing psychological counseling may help to improve doctor-patient communication, the specific measures of doctor-patient communication are not explicitly mentioned in the questions, so option B is conducive to build a harmonious doctor-patient communication is not the best option.

The measures in the question stem pay more attention to hospital management and the care of medical staff, and do not directly make specific reforms to the

diagnosis and treatment and fair treatment of patients. Therefore, option C is conducive to the diagnosis and treatment and fair treatment of patients, and can be excluded.

Although the physical examination may involve the medical and health care departments, the reform measures in the question stem do not particularly emphasize the use of medical and health care departments, so option D is conducive to the use of medical and health care departments, which is inconsistent with the meaning of the question.

To sum up, the correct answer is: E. It is conducive to exerting the overall effect of the hospital.

This topic looks at the effect of hospital management measures. The management system and measures mentioned in the topic, such as hospital management system, reception and referral process, psychological consultation, health examination, etc., aim to care for and pay attention to medical staff while ensuring fairness.

In this context, the most direct and obvious effect of these measures is to promote the harmony of the doctor-patient relationship, because medical staff are more able to treat patients positively after being cared for and treated fairly, thus improving the quality of doctor-patient communication.

So the best answer is:

B It is conducive to building a harmonious doctor-patient communication

12. (Type A1 questions)

If a doctor fails to pass the regular assessment and still fails to pass the assessment again after training, the local health administration department deals with him according to the "Medical Practitioners Law", and the treatment is

A. Cancel registration and withdraw the practicing certificate

B. Suspension of practice activities

C. Revocation of the qualification certificate of medical practitioners

D. Assessment after re-training

E. Lifelong ban

Reference answer: A. Your answer:

[Analysis] The Law on Medical Practitioners stipulates that the health administrative department of the people's government at or above the county level may treat the doctors who fail to pass the assessment

In order to suspend their practice activities for 3 months to 6 months, and to receive training and continuing education, and still fail to pass the examination, it shall be noted

Sell the registration, and withdraw the doctor's practicing certificate.

[Avoting mistakes] The Law on Medical Practitioners stipulates that the administrative departments of public health of the people's governments at or above the county level are responsible for guiding, inspecting and supervising doctors

Teacher assessment work. Institutions or organizations entrusted by the administrative department of public health of the people's government at or above the county level shall follow the practice standards of doctors

Regular professional level, work performance and professional ethics.

When dealing with the failure of the assessment of medical practitioners, the appropriate measures are usually determined according to the specific regulations and circumstances. Given this situation indicates that physicians fail two consecutive examinations, and given the provisions of the Medical Practitioners Act, the most severe measures such as revocation of the medical practitioner license or suspension for life may apply to more serious violations. Usually, if it is still unqualified after re-training, a more strict but limited measure may be chosen, such as suspension of practice activities. Therefore, given these considerations, the most appropriate answer may be:

F. Suspension of practice activities

This not only reflects the punishment of unqualified doctors, but also leaves room for correction, which is easy to be re-assessed after further evaluation and possible training at a certain point in the future.

analyze:

This question examines the unqualified treatment measures of the assessment of medical practitioners.

To sum up, the correct answer is: A. Cancel the registration and withdraw the doctor's practicing certificate.

According to the Law on Medical Practitioners, the administrative department of public health of the people's government at or above the county level may order the doctors to suspend their practice for three to six months and receive training and continuing education. Those who still fail to pass the examination will be cancelled and the practicing certificate will be withdrawn. In this question, if a doctor fails to pass the regular examination, and still fails to pass the examination again after training, his registration should be cancelled and the doctor's practicing certificate should be withdrawn. So option A is the correct answer.

Option B Suspension of practice is a preliminary treatment measure for the doctors who fail the examination, but the question stem describes that the doctor still fails the examination again after training, so the punishment of suspension of practice is no longer applicable and can be excluded.

The Law on Medical Practitioners does not give the health administration department the right to revoke the qualification of medical practitioners, so option C should revoke the qualification of medical practitioners, which does not conform to the provisions of the Law on Medical Practitioners, so it is excluded.

The physician has been trained and failed to pass the examination again. Therefore, option D, the examination after re-training, does not meet the description of the question stem and can be excluded.

The Law on Medical Practitioners does not stipulate the punishment of banning for life, so option E is banned for life, which does not comply with the legal provisions and is inconsistent with this topic.

To sum up, the correct answer is: A. Cancel the registration and withdraw the doctor's practicing certificate.

According to Article 36 of the Law of the People's Republic of China on Medical Practitioners, doctors who fail to pass the regular assessment shall receive training. For those who still fail to pass the second assessment after the training, the administrative department of public health shall cancel their registration or lower their practice categories. According to this regulation, the treatment of the doctor should be to cancel the registration and withdraw the doctor's practicing certificate.

So, the most correct answer is:

A. Cancel registration and withdraw the practicing certificate

13. (Type A1 questions)

In animal experiments, in order to obtain the blood of living mice, the laboratory personnel directly cut off the head of the experimental mice and pressed the residual limbs so that the remaining blood could flow out of the large neck artery. The laboratory staff violated the principle of

A. Give a good living environment for laboratory animals

B. Give adequate life support to the experimental animals

C. To minimize the suffering of the experimental animals

D. Try to use experimental materials instead of live animal experiments

E. Reduction of the unnecessary number of experimental animals

Reference answer: C Your answer:

[Analysis] In this question, the laboratory staff directly cut off the animal's head, and press the limb to get blood, this behavior did not

There is a maximum reduction of the suffering of the experimental animals. Therefore, this question is selected C.

[Misavoidance] The ethical principles that should be followed in animal experiments include: substitution, reduction, optimization and responsibility. Substitution refers to the use of No

Experimental materials of perception to replace live animals, or use lower animals to replace higher animals. The reduction is made in the animals

During the experiment, minimize the use of animals, use a small number of animals to obtain the same amount of test data or use a certain amount

With more trial data. Optimization refers to minimizing non-humane humans when experiments must be performed with animals

Range and extent of sequence effects on animals. Animal welfare means that human beings should avoid causing unnecessary harm to animals, against it

And to prevent cruelty to animals, so that the animals in the state of recreational survival.

The principle violated by the laboratory staff is that C. To minimize the suffering of the laboratory animals. The principle of animal welfare requires that the pain and stress of animals should be minimized in experiments, and humane blood should be obtained, such as needle blood drawing, to reduce the pain of animals. Cutting the animal's head directly is extremely inhumane and is a serious violation of the principle of alleviating the suffering of experimental animals.

This is a multiple-choice question in medical ethics, designed to examine the understanding of the ethical principles of laboratory animals. The title describes the behavior of a laboratory staff directly cutting the head of an experimental mouse in order to obtain blood from living mice. We need to analyze which ethical principles of laboratory animals violate.

First, we analyze the topic and list the important information:

Laboratory personnel to obtain blood from living mice.

Cut off the head of the experimental mouse directly.

Press the residual limb to let the remaining blood flow out.

Then, explain and reason:

The ethical principles of laboratory animals mainly include: providing a good living environment, adequate living security, minimizing the pain of animals, using experimental materials to replace living animal experiments, and reducing the unnecessary number of experimental animals.

In this question, the lab staff obtains blood by directly cutting the head of the mouse, which obviously causes great pain to the animals, contrary to the principle of "minimizing the pain of the experimental animals."

Now let's compare the options to this one:

A. Giving A good living environment for the laboratory animals: The title does not mention the living environment of the laboratory animals, so this option is not consistent with the question description.

B. Give adequate life support to the laboratory animals: Similarly, life support is not mentioned in the topic, so this option is not applicable.

C. Minimize the suffering of laboratory animals: laboratory personnel obtain blood through extreme means, apparently do not follow the principle of reducing animal pain, so this option is in line with the question.

D. Try to use experimental materials instead of live animal experiments: The topic does not mention whether alternative materials were used, so this option does not match the question description.

E. Reduce the unnecessary number of experimental animals: The number of experimental animals is not mentioned in the question, so this option is not applicable.

In conclusion, the laboratory staff cut off the head of the mice directly to obtain

the blood, which violates the principle of "minimizing the suffering of the experimental animals".

Therefore, the correct answer is: C. To minimize the suffering of the experimental animals.

The behavior of the laboratory staff violates the following principles as:

F. To minimize the suffering of the experimental animals

Explanation: In animal experiments, ethical and humanitarian principles require researchers to minimize the pain and discomfort of laboratory animals. Cutting off the mouse's head and pressing on its limb to obtain blood is a method that causes great pain, contrary to the principle of alleviating pain in experimental animals. Therefore, the correct answer is a C.

14. (Type A1 questions)

The following criteria are true for brain death

A. It helps to provide the scientific basis for judging the death of patients

B. The aim is to conserve health resources

C. Necessary requirement for implementing organ donation

D. Cardiopulmonary function must be completely lost

E. The only criterion for judging the death of a patient

Reference answer: A. Your answer:

[Analysis] The direct ethical purpose of the implementation of brain death standards is: ① make the death standard more scientific and guide doctors to correct

Implement resuscitation and rescue, determine accurate time of death and reduce legal disputes; ② Ethics to maintain the dignity of the deceased

(Therefore, this question is selected as A, and BC is wrong). Brain death is a widespread and permanent loss of full function by brain cells

Brain, cerebellum, pons, and medulla oblongata. That is, after the whole brain death, although the heart rate is still alive, but the brain recovery is impossible, the individual death

Has happened and inevitable (D error). Brain death is not the only criterion for determining patient death (E error).

[avoidance] The ethical value and significance of the diagnostic criteria for brain death are mainly in five aspects: scientific and accurate judgment of death and maintenance of the deceased

Dignity behind the death, save social resources, reduce the burden of the family, conducive to the implementation of organ transplantation.

The correct statement about the standard of brain death is A: "To provide the scientific basis for judging the death of patients". Brain death is the complete and irreversible loss of brain function, including the functions of the cerebral cortex and the brainstem. This concept helps the medical and legal communities to provide a clear and scientific basis for judging individual deaths. Although brain death is often associated with organ donation (option C), organ donation is not a necessary requirement for the standard of brain death. Loss of cardiopulmonary function (option D) is the criterion for cardiopulmonary death, not brain death. Thus, option A is the most accurate answer.

This is a multiple-choice question about the criteria for brain death. We need to understand the definition and meaning of brain death, and then analyze each option to find out the criteria for brain death.

Understanding background information: Brain death refers to the complete and irreversible loss of whole brain function, including the brain stem, namely death.

Understand the question core: Analyze each option to find out the claims that meet the criteria for brain death.

Analysis options:

Option A (helps to provide the scientific basis for judging patient death): The brain death standard provides a scientific and objective basis for determining death, which is consistent with the description of option A.

Option B (intended to save health resources): The brain death standard is not set up to save health resources, but to define death more scientifically, so option B is incorrect.

Option C (a necessary requirement for organ donation): Although the brain death criteria are related to organ donation, it is not a necessary requirement for organ donation, so option C is not correct.

Option D (mandatory complete loss of cardiopulmonary function): Brain death criteria do not require a complete loss of cardiopulmonary function, but rather a complete and irreversible loss of total brain function, so option D is incorrect.

Option E (the only criterion for determining patient death): Although brain death is a scientific criterion for death determination, it is not the only criterion, so option E is incorrect.

In conclusion, the answer is that A. helps to provide the scientific basis for judging the death of patients.

15. The following criteria for brain death are true of this statement:

A. It helps to provide the scientific basis for judging the death of patients

analyze:

Brain death refers to the complete and irreversible loss of the function of the brain and brain stem, which is a criterion to judge a person's death. The aim is to provide a scientific basis for medical decision-making. The brain death standard is widely accepted and used in many countries, and its scientific nature and reliability have been confirmed by numerous studies.

- ** A. To provide the scientific basis for judging the death of patients * *: This is true, because the brain death standard provides the scientific basis for judging the death of patients.

- ** B. The goal is to save health resources * *: Although brain death determination can affect the use of health resources, this is not its main purpose.

- ** C. Necessary requirement for organ donation * *: Brain death judgment is usually related to organ donation, but it is not the only or necessary requirement for organ donation.

- ** D. Complete loss of cardiopulmonary function * *: Brain death determination does not require a complete loss of cardiopulmonary function.

- ** E. The only criterion for judging the death of a patient * *: Brain death is a criterion for judging death, but not the only criterion, and cardiac death is also a common criterion.

Therefore, the correct answer is an A.

16. (Type A1 question)

In clinical consultation, doctors should avoid the phenomenon of listless, boundless repeated questions or excessive emotional language, which reflects the ethical requirements of diagnosis and treatment

A. Respect the patients and be impartial

B. Full concentration, proper language

C. Comprehensive, systematic, careful and meticulous

D. dignified ure and warm attitude

E. Listen patiently and guide you correctly

Reference answer: B. Your answer:

[Analysis] In the process of asking for the medical history, doctors should follow the following ethical requirements: dignified manner, enthusiastic attitude; attention

Note, proper language; listen patiently and guide correctly. In this case, doctors avoid listless and repeated questions

The ethical requirement of concentration and proper language, so this question chooses B.

[Avoid] Ethics requirements of physical examination: comprehensive, systematic, careful; caring and considerate, reduce pain; respect for patients,

The heart is selfless.

In clinical consultation, doctors should avoid the phenomenon such as listless, boundless repeated questions or excessive emotional language, which reflects that doctors should concentrate on patients and use appropriate language to communicate. So the best answer should be:

F. Full concentration, proper language

This option emphasizes that doctors should stay focused during the consultation process and use the appropriate language, which helps to establish good communication with patients, thus improving the quality and efficiency of diagnosis and treatment.

analyze:

In clinical consultation, doctors should avoid the phenomenon of listless, boundless repeated questions or excessive emotional language, and require doctors to concentrate on it and have proper language. This is the most basic emotional content of medical ethics and low level of responsibility, and the emotional content of medical ethics and low level of responsibility content.

Option A Respecting patients and impartiality requires medical staff to be serious, serious and meticulous in treating and nursing for patients, respect the personal dignity and autonomy of patients, and at the same time, treat every patient fairly and selflessly, which is inconsistent with the problem.

Option C is comprehensive and systematic, and meticulous means that the doctor should comprehensively and systematically understand the patient's medical history, do not omit any information related to the disease, and the patient's complaint should be carefully analyzed, which is inconsistent with the question.

Option D dignified demeanor and enthusiastic attitude means that the appearance, clothing and posture of the medical staff should reflect the good image of the

professionals, and the medical staff should diagnose and treat the patients enthusiastically, but it is not reflected in the description of the question stem, so it is not selected.

Option E listens patiently. Correct guidance requires the medical staff to listen patiently to the patient's medical history and give correct guidance to the patient's psychological problems, which is inconsistent with the description of the question stem.

To sum up, the correct answer is: B. concentration, proper language.

In clinical consultation, doctors should avoid the phenomenon of listless, boundless repeated questions or excessive emotional language, and the ethical requirements of diagnosis and treatment are * * B. concentration, proper language * *.

** analyze:**

- ** A. Respect for patients, fairness and selflessness * *: Although respect for patients and selflessness are the ethical principles that doctors should follow, they mainly emphasize the fairness and respect of doctors in treating patients, rather than the accuracy of focus and language.

- ** B. Focus and proper language * *: This option emphasizes the need to focus and use proper language, avoid listless and excessive emotional language, and meet the requirements of the questions.

- ** C. Comprehensive, systematic, careful * *: This option emphasizes that doctors should be comprehensive, systematic, careful and meticulous in the process of consultation, but it does not completely match the phenomenon described by the question stem.

- ** D. dignified and enthusiastic attitude * *: This option emphasizes the behavior and attitude of doctors, but it is not direct enough to the language phenomenon of avoiding listlessness and excessive emotion in the topic.

- ** E. Listen patiently and guide correctly * *: This option emphasizes that doctors should listen patiently and guide patients correctly, but it does not exactly

correspond to the phenomenon of "avoiding listless, rambling repeated questions or excessive emotional language" in the question stem.

Therefore, the most consistent answer to the requirements of the question is * * B. concentration, proper language * *.

17. (Type A1 question)

The director of cardiovascular medicine of a hospital was reported by its doctoral student "each install a bracket rebate 10,000 yuan", after the investigation is true, the director violated what principle

A. equitable principle

B. clean

C. Principles of confidentiality

D. principle of optimality

E. Principles of informed consent

Reference answer: B. Your answer:

[Analysis] Medical ethics requires doctors to have basic professional ethics, whether it is conducive to the remission and rehabilitation of patients' diseases; whether it is conducive to the protection and improvement of human survival and the environment; whether it is conducive to prenatal and prenatal care and the health and longevity of the population; whether it is conducive to the development of medical science and social progress. Whether it is conducive to the disease remission and rehabilitation of patients is the primary and supreme standard of medical moral evaluation. The director received kickbacks from patients, which violated the principle of honesty and self-discipline in medical ethics, so we choose B.

[Error avoidance] Informed consent refers to the patient or their family members having the right to know the patient's condition and the prevention and treatment measures taken to medical personnel

The autonomy to decide the choice. The principle of confidentiality means that medical personnel should keep medical secrets in disease prevention and treatment, and should not divulge them at will

The patient's illness, in case of unnecessary harm to the patient. The optimization

principle of clinical diagnosis and treatment ethics

It refers to the careful and careful selection of clinical ethical guidelines to optimize the treatment measures that benefit patients

Capacity mainly includes the best efficacy, safe and harmless, least pain and least cost.

This issue is about medical ethics. Given the options given, the cardiovascular medicine director's acceptance of kickbacks violated the principle of "integrity and self-discipline" in medical ethics. The principle of honesty and self-discipline requires that medical staff should keep honest in professional activities to avoid affecting medical decision-making and patient care. So the most appropriate answer is:

F. clean

This is a question of medical ethics. We need to first understand the background information and the core of the question, then carefully analyze each option, and compare it with the information given in the question.

Background information: The title describes a scene in which the director of the hospital's cardiovascular department was reported to receive kickbacks for installing the stent.

Understanding the core of the problem: We need to judge which ethical principle the director of cardiovascular medicine violates based on this relationship.

Analysis options:

Option A (fairness principle): The fairness principle emphasizes that all patients should be treated fairly and impartial. This principle does not directly address the issue of doctors receiving kickbacks, and can therefore be excluded.

Option B (honesty and self-discipline): Honesty and self-discipline means that medical personnel should adhere to the principle of honesty and self-discipline in medical services, and do not accept any form of kickbacks or benefits. In this case, the director of cardiovascular medicine received kickbacks for the installation of stents, which clearly violated the principle of integrity and self-discipline.

Option C (Confidentiality principle): The confidentiality principle mainly involves the protection of patient information, and it is not directly related to this case, so it can be excluded.

Option D (optimization principle): The optimization principle is to choose the most beneficial treatment plan for the patient in the medical behavior. This principle does not involve the issue of doctors receiving kickbacks, and therefore it can be excluded.

Option E (Informed Consent Principles): The informed and consent in medical behavior. This principle is not directly related to the situation in this question, so it is not considered.

To sum up, the director of cardiovascular medicine in the hospital was reported as "a rebate of 10,000 yuan for each stent installed", which obviously violates the principle of honesty and self-discipline. Therefore, the right answer is B. honesty and self-discipline.

According to the title description, the director of cardiovascular medicine received a rebate of 10,000 yuan for each stent installation, which violated doctors' professional ethics and medical ethics. Specifically, this is a violation of the principle of "honesty and self-discipline".

* * B. Honesty and self-discipline * *

18. (Type A1 question)

Moral judgment is based on the actual effect of action

A. deontology

B. outcome

C. deontology

D. consequential theory

E. Virtue theory

Refer to the answer: D. Your answer:

Graphic analysis: [Analysis] It is the consequence theory, also called teleology or effect theory,

It is an ethical theory that takes the consequences of moral behavior as the final basis for determining moral norms, so we choose D.

[Avoid mistakes] memory knowledge points.

This question examines the different moral theories in ethics. According to the title description, we need to find a theory that takes the actual effects of the action as the basis for moral judgment. The options "B. outcome theory" and "D. consequence theory" are both related to the outcome of action, but the term commonly used in ethics is "consequence theory" (consequentialism), which emphasizes that the outcome of behavior is the most important criterion for judging the morality of behavior. Therefore, the most correct answer is D. The consequence theory.

First of all, we should make it clear that this problem examines the basis of moral evaluation in medical ethics.

Now, let's analyze the options, one by one:

A. Deontology (deonalism): Deontology holds that human behavior should follow moral principles and moral obligations, regardless of the outcome. It focuses on the legitimacy of the behavior, rather than the quality of the results.

B. Results theory: Results theory claims that moral evaluation should be based on the result of the behavior, that is, the quality of the behavior depends on its results. However, in ethics theory, the more common term is "effectiveness theory" rather than "outcome theory". The effect theory holds that the value of morality depends on the result or effect of the behavior, which is consistent with the "actual effect of action as the basis of moral judgment"

in this topic, but the term "effect theory" is not directly used in the option.

C. Duty theory (deactivism): Similar to the moral theory, the obligation theory emphasizes the importance of moral obligations and principles, rather than just the results.

D. Consequence theory: This is a similar concept to the effect theory, but also takes the consequences of behavior as the standard of moral judgment. In ethics, consequence theory and effectiveness theory are often used interchangeably, both referring to result-oriented moral evaluation.

E. Virtue theory: Virtue theory focuses on human character and moral character, rather than the consequences of a single act.

In conclusion, although both "effect theory" and "consequence theory" refer to the result or consequence of behavior as the standard of moral evaluation in ethics, the more accurate term in this topic should be "effect theory". Since the "effect theory" is not directly given in the option, we can choose the "consequence theory" that is closest to its meaning.

Therefore, the correct answer is D. The consequence theory. This option is best fit with the description "based on the actual effect of action".

The theory based on moral judgment on the actual effect of action is the * * result theory * *, also called the consequence theory. This ethical theory holds that the moral value of behavior depends on its consequences or consequences. In other words, the quality of a behavior is judged according to whether the consequences it brings are favorable.

So, the correct answer is:

F. outcome

19. (Type A1 questions)

The following cases require mandatory medical treatment

A. diabetes mellitus

B. Fever in infants and young children

C. senile dementia

D. coronary disease

E. Violent people

Reference answer: E Your answer:

[Analysis] compulsory medical treatment generally includes venereal diseases, drug use, violent mental disorders, serious infectious diseases, etc., so

This question is selected E.

[Wrong avoidance] Compulsory medical treatment, refers to the involuntary compulsory treatment, refers to the country to avoid the public health crisis, through compulsory treatment

The treatment of patients' diseases, to cure the disease, prevent the spread of diseases and safeguard the interests of public health, is mandatory and non-self

The characteristics of willingness and public welfare.

When selecting situations requiring mandatory medical attention, consideration is often those that may present an immediate danger to the patient himself or others. Of these options, the most is:

F. A violent person

Such individuals may constitute harm to themselves or others, and therefore in some cases the law may require a mandatory medical evaluation or treatment to prevent potential harm from occurring. Therefore, option E is the most appropriate answer. Other options such as diabetes, infant fever, senile dementia and coronary heart disease require medical attention, but usually do not require mandatory medical attention.

analyze:

Compulsory medical treatment, refers to the involuntary voluntary compulsory treatment, refers to the state to cure the disease through the treatment of the disease,

prevent the spread of the disease, safeguard the public health interests, at the same time forcing patients to undertake disease treatment system. People who need to be forced to seek medical treatment are generally patients with mental illness. They cannot identify or control their own behaviors, and may harm themselves, endanger the safety of others, disrupt the medical order, and hinder the order of the work of medical personnel. Among the options given, violent people may do harm or harm others, thus requiring mandatory medical treatment.

Option A diabetes, option C senile dementia, option D coronary heart disease, most of these three diseases do not harm themselves or harm others, so they do not belong to the category of compulsory medical treatment, so they are excluded together.

Option B Children with fever generally do not have the ability to harm themselves, and infants do not have the ability to take care of themselves, and often need to be accompanied by parents throughout the whole process, so there is no compulsory medical treatment, so they do not choose.

To sum up, the correct answer is: E. violent people.

The question examines situations requiring mandatory medical treatment in certain cases. Here is an analysis for each option:

A. * * Diabetes * *: Although diabetes is a disease that requires long-term management and treatment, mandatory medical attention is usually not required unless acute complications occur (e. g., diabetic ketoacidosis or hypertonic coma).

B. * * Baby fever * *: Baby fever is a common phenomenon, although it needs close observation and appropriate treatment, but usually does not need compulsory medical treatment.

C. * * Geriatric dementia * *: Alzheimer's disease is a chronic progressive

disease where patients need long-term care and management, but generally does not require mandatory medical treatment.

D. * * Coronary heart disease * *: Patients with coronary heart disease need long-term drug treatment and lifestyle management, and acute coronary syndrome (such as myocardial infarction) needs emergency medical intervention, but usually does not involve mandatory medical treatment.

E. * * Violent people * *: Violent people may pose a threat to themselves or others and need immediate mandatory medical treatment for assessment and management to ensure safety.

In conclusion, * * E. A violent person * * is the only situation that requires mandatory medical treatment.

Therefore, the best answer is * * E * *.

20. (Type A1 question)

The medical mode used in patients with coma and shock is

A. Active-cooperative type

B. Active-participatory type

C. Co-participation type

D. Active-passive type

E. Guiding- -cooperative

Refer to the answer: D. Your answer:

[Analysis] Active-passive mode: this mode is suitable for coma, shock, mental illness patients attack period, severe low intelligence

Patients and infants and other patients who are difficult to express their subjective will (D pair).

[Misavoidance] Guidance cooperation model: This model is for most patients;

joint participation model: This model is for patients

Patients with a certain medical knowledge background or patients with long-term chronic diseases.

For the medical mode of coma and shock patients, the choice should be D. active-passive type. This is because patients in coma or shock are usually unable to participate in their treatment process, thus requiring active treatment and care, while the patient is in a passive state of treatment. Therefore, the most appropriate medical model should be the active-passive type.

analyze:

The doctor-patient relationship is divided into three types: active-passive, guiding-cooperative, and joint participation. Among them, the active-passive type refers to the doctor's active command and the patient's passive obedience, which is suitable for patients who are difficult to express their subjective opinions, such as anesthesia, severe trauma coma patients. Therefore, the medical mode used by coma and shock patients is the active-passive type.

Option A is active-cooperative, and option E is guiding-cooperative, which are both interference items. These two modes do not exist in the doctor-patient relationship, which can be excluded together.

Option B active-participation, option C co-participation, both patients actively participate in their own treatment and discuss treatment plan with doctors. Patients with coma shock cannot express their subjective opinions, so these two modes are not suitable for such patients, so they can be excluded together.

To sum up, the correct answer is: D. Active-passive type.

The medical mode used in coma and shock patients are:

F. Active-passive type

Explanation: In the coma or shock state, the patient cannot actively participate in

or cooperate with the medical decision-making and treatment, so the doctor needs to take measures actively, while the patient is in a passive state of receiving treatment. This medical model emphasizes the initiative and decision-making power of doctors, and is applicable to emergency situations where patients cannot express their wishes or cooperate with treatment.

21. (Type A1 question)

A visitor often goes to the psychological clinic because of conflicts with others. The psychological doctor understands that in the process of communicating with others, he often only considers his own happiness regardless of the "realistic standard". According to the psychoanalysis theory, the visitor occupies a dominant position

A. super-ego

B. id

C. Subject self

D. oneself

E. Object self

Reference answer: B. Your answer:

[Analysis] The instinct, impulse and desire located in the unconscious constitute the original and self, which is the biological face of personality, following the " happy original

Then, so the option B is correct.

[Avoid mistakes] The self is between the present self and the external world, which is the psychological aspect of the personality. The role of the self is to make the individual on the one hand

Be aware of his cognitive ability; on the other hand, he causes the individual to adapt to the reality

"reality principle". Superego is the social aspect of personality, which is the "moralized self", determined by "conscience" and "self-ideal"

Composition, the power of supeto-self is to guide the self, limit the id, and follow the "ideal principle".

In psychoanalytic theory, the personality structure is divided into three parts: the id (id), the ego (ego), and the superego (superego). The id is an innate part, representing an instinctive impulse and desire, and the pursuit of instant gratification,

without regard to reality or social norms. The self is the rational part, trying to meet the needs of the self, while considering both reality and feasibility. Superego represents moral standards and ideals, trying to suppress the impulse of the present self, in line with social and moral norms.

According to the description of the title stem, the client only considers his own happiness in interpersonal communication, regardless of the realistic standards, which is in line with the characteristics of Ben I (id), because Ben I pursues immediate satisfaction, and does not consider the consequences or the needs of others.

Therefore, the correct answer is B. Ben I.

analyze:

In the process of communicating with others, visitors often only consider their own happiness, not the "realistic standard", which is a typical manifestation of "id". In the theory of psychoanalysis, the human psychology contains two main parts, namely, the present self and the self. Later, the concept of superego was added, that is, a positive force to restrain and suppress the self. Among them, I represents a variety of primitive instincts and desires, which act in accordance with the "happy principle", irrational, and blindly pursue satisfaction. In the process of communicating with people, visitors only consider their own happiness, which is the dominant of "id".

Option A represents morality and conscience, under the control of "moral principles", restrain the impulse of the self, forcing me to delay the satisfaction or eliminate the impulse, so do not choose.

Option C The subject self refers to the individual's cognition of their own state of existence, and is the result of the individual's self-evaluation of their social role, which is inconsistent with the description of the question stem, so it is excluded.

Option D self-represents rationality and common sense, under the control of the "realistic principle", and allows the limited performance of the own impulse according to the reality, which is inconsistent with the performance of the visitors, so it is not considered.

Option E The object self refers to the self as a social object, which is a reflection

of social relations, which is recognized and evaluated by others, and is inconsistent with the description of the subject stem, so it is not selected.

To sum up, the correct answer is: B. Ben.

According to psychoanalytic theory, Freud proposed three components of personality: id (Id), ego (Ego), and superego (Superego). The id represents the raw, impulsive desire and instinct, seeking immediate gratification without regard to reality and morality. The ego is the part responsible for realistic perception and behavioral regulation, able to reconcile the ego desires with superego moral standards. Superego represents the internalized social norms and moral standards, and the pursuit of perfection and ideals.

The visitors in the topic often only consider their own happiness, regardless of the "standard of reality", which shows that their behavior is mainly driven by instinct and desire, and lacks realistic and moral constraints. According to this description, the client is the dominant self.

So the correct answer is:

B id

22. (Type A1 questions)

The mental health center of a university evaluates the degree of students at high risk of depression, and the most appropriate way is

A. thematic apperception test

B. Characteristic coping style questionnaire

C. Adaptive behavioral assessment scale

D. The SAS self-assessment scale

E. SDS self-rating scale

Reference answer: E Your answer:

[Analysis] Depression self-evaluation scale (SDS) can quite intuitively reflect the subjective feelings and severity of depression, so this

Topic selection E.

[Wrong avoidance] TAT (subject perception test) (A error) is used for personality measurement. The characteristic coping style questionnaire (B error) is used for the reverse

The positive and negative attitudes and behavioral characteristics of the subjects in the face of difficult setbacks. Adaptive behavior assessment scale (C error) 1

It refers to the Children's Adaptive Behavior Evaluation Scale, which is mostly used for the evaluation test of children's social adaptation behavior. Anxiety self-evaluation

The scale (SAS) measures the presence of anxiety symptoms and its severity (D error).

The most appropriate method is the E. SDS self-evaluation scale.

The SDS (Depression Self-rating Scale) is a common tool used to quantitatively assess the degree of depression. This scale is specifically designed to assess the clinical symptoms of depression, including low mood, decreased interest, decreased energy, and more. It is a self-rating scale, which means that individuals can answer questions according to their own feelings and experiences, which can more directly reflect their psychological state. Therefore, the SDS self-rating scale is a very appropriate choice when assessing quantitative levels of depression in mental health centers for students at high risk for depression.

analyze:

For the quantitative assessment of students at high risk of depression, a scale assessing the degree of depression should be selected. Option involved in the assessment scale each focus, only SDS self-evaluation scale namely depression self-evaluation scale, can directly reflect the subjective feelings of depression and its changes in the treatment, suitable for adults with depression symptoms, especially suitable for find patients with depression, also can be used for psychological counseling clinic and psychiatric outpatient service, to determine the presence of

depressive symptoms, and assess the weight of the degree of depression, especially suitable for the quantitative assessment of depression degree of depression.

Option A, TAT, belongs to the projection personality test. It is mainly used to determine people's needs and the motivation for achievement. It cannot quantitatively assess the degree of depression, so it is not selected.

Option B characteristic coping questionnaire, CSQ, is a self-assessment questionnaire, designed to measure what trait coping methods individuals have, to explain why individuals have different response methods in the face of stress, which has nothing to do with the quantitative assessment of depression, so it is excluded.

Option C Adaptive Behavioral Assessment Scale, abbreviated as ABAS, is mainly used to assess adaptive behaviors in children and adolescents, not for quantitative assessment of depression, so it is not selected.

The DSAS self-rating scale, namely the anxiety self-rating scale, is mainly used to assess the subjective feelings of patient anxiety and its changes in treatment, and cannot be used for quantitative assessment of depression degree, so it is excluded.

In conclusion, the correct answer is: E. SDS self-rating scale.

For this topic, it is important to choose an appropriate scale to quantitatively assess the depression level of students at high risk of depression. Here is a introduction to the options:

A. * * Topic perception test (Thematic Apperception Test, TAT) * *: A projection test that lets subjects tell a story through pictures to reveal the underlying emotion and motivation. It is not specifically used to quantitatively assess the depression.

B. * * Questionnaire (Trait Coping Style Questionnaire) * *: used to assess how individuals respond in the face of stress, non-specific to depression assessment.

C. * * Adaptive Behaviour Assessment Scale (Adaptive Behavior Scale) * *:

Assessment individual adaptive behavior, not specifically used for depression assessment.

D. * * SAS Self-rating Scale (Self-Rating Anxiety Scale, SAS) * *: Self-rating scale developed by Chung for anxiety assessment is not suitable for specific assessment of depression.

E. * * SDS Self-rating Scale (Self-Rating Depression Scale, SDS) * *: A Self-rating scale that was also developed by Chung to assess depression. This scale allows for a quantitative assessment of depression, and is an appropriate tool for depression assessment.

Based on this information, the most appropriate scale for the quantitative assessment of depression among students at high risk of depression is the * * E. SDS self-rating scale * *.

Therefore, the correct answer is: * * E. SDS self-rating scale * *.

23. (Type A1 question)

The most severely damaged organ with acute aflatoxin poisoning is

A. liver

B. kidney

C. spleen

D. oarium

E. cerebrum

Reference answer: A. Your answer:

Graphic analysis: [analysis] Aspergillus flavus widely exists in mildew food. Milwed peanuts, corn and cereals contain the most. Aspergillus flavus

There are many kinds, among which aflatoxin B1 is the most carcinogenic. Aflatoxin B1 is an isocycloaromatic hydrocarbon that is metabolized in the liver to

epoxy

Compound, can cause point mutations in the tumor suppressor gene p53. This toxin can induce hepatocellular carcinoma. So this topic

select A.

[Error avoidance] Hepatitis B virus (HBV) infection leads to chronic injury and regeneration of hepatocytes, which may be given to aflatoxin B1

The mutagenic role provides the conditions. The synergistic effect of HBV infection and aflatoxin B1 may be in the high incidence of liver cancer in China

Important factors causing liver cancer.

Aflatoxin, also known as aflatoxin, is a toxic metabolite produced by Aspergillus flavus. This toxin primarily affects the liver, and thus in the case of acute aflatoxin poisoning, the most severely damaged organ is the liver. Therefore, the correct answer is A. liver.

First, we need to understand the effects of aflatoxin on the human body. Aflatoxin is an strong hepatotoxin that primarily damages the liver. When the body ingests food contaminated with aflatoxin, the toxin is mainly absorbed by the liver, causing liver damage by inhibiting the activity of mixed functional oxidase lines in hepatocytes and damaging other related substances. When aflatoxin is heavily ingested, acute poisoning can occur, with acute hepatitis, hemorrhagic necrosis, hepatocyte steatosis, and bile duct hyperplasia occurring. Although the spleen, kidney, brain and ovary are also the target organs of aflatoxin action, the main damage is still concentrated in the liver.

Now, we analyze each option one by one:

A. Liver: As mentioned above, aflatoxin mainly damages the liver, which can cause acute hepatitis, hemorrhagic necrosis and other serious liver injuries. Therefore, this is a reasonable answer option.

B. Kidney: Although aflatoxin may also have some effect on the kidney, its main damage is the liver, not the kidney.

C. Spleen: Although the spleen is also affected, it is not the main organ damaged by aflatoxin.

D. Ovaries: The ovaries may also be affected by aflatoxin, but they are less affected relative to the liver.

E. The brain: The brain is not the main organ damaged by aflatoxin.

In conclusion, the most severely damaged organ for acute aflatoxin poisoning is the liver. So the correct answer is:

A.liver

Aflatoxin is a toxin produced by Aspergillus flavus, and its main toxic effect is damage to the liver. When acute poisoning, aflatoxin can cause liver cell necrosis, leading to acute hepatitis, jaundice, and even liver failure. Thus, the organ most severely damaged by acute aflatoxin poisoning is the liver.

So, the correct answer is:

A. liver

24. (Type A1 question)

It should not be considered as a factor requiring a comprehensive analysis for the diagnosis of occupational diseases

A. Risk factors for the occupation

B. Auxiliary examination results

C. Contact history of occupational disease risk factors

D. Previous occupational medical history

E. Inincidence of occupational diseases in areas

Reference answer: E Your answer:

[Analysis] The Law of the People's Republic of China on the Prevention and Control of Occupational Diseases for the diagnosis of occupational diseases should be a comprehensive analysis of the following factors:

(1) the patient's occupational history; (2) occupational-disease-inductive contact history and occupational-disease-inductive factors in the workplace; (3) exposure

Bed performance and auxiliary examination results, etc. The incidence of occupational diseases is not considered as a factor for comprehensive analysis, so this topic is selected

F. .

[Avoid mistakes] memory questions.

In the diagnosis of occupational diseases, various factors need to be analyzed comprehensively analyzed, including occupational risk factors, auxiliary examination results, contact history of occupational risk factors, past occupational history, etc. These are all important information points that can help doctors determine whether a person suffers from an occupational disease. However, although the incidence of occupational diseases in the region can provide certain background information, it is not a decisive factor to diagnose whether an individual is sick. Therefore, the factors most unlikely to require a comprehensive analysis for the diagnosis of occupational diseases in individual cases are:

The incidence of occupational diseases in area E

First of all, we should make it clear that the topic requirement is to find out the factors that cannot be used as a comprehensive analysis of the diagnosis of occupational diseases. Next, we analyzed each option one by one.

A. Occupational risk factors: this is an important factor that must be considered when diagnose occupational diseases. Understanding the possible harmful factors in

the patient's occupation is essential to determine the occupational disease.

B. Auxiliary examination results: auxiliary examination, such as blood test, imaging examination, etc., can provide objective evidence about the health status of patients, and is also an indispensable part of the diagnosis of occupational diseases.

C. Contact history of occupational disease risk factors: it is a key link in the diagnosis process to know whether the risk factors that may lead to occupational diseases in the patient.

D. Past occupational medical history: The patient's past occupational medical history, especially that related to the current occupational disease, is very important for both diagnosis and treatment.

E. The incidence of occupational diseases in the region: although the incidence of occupational diseases in the region can provide the common occurrence of occupational diseases within a certain area, it cannot be directly used as the basis to diagnose whether an individual has occupational diseases. The specific situation of each individual should be analyzed according to their occupational risk factor exposure history, clinical manifestations and auxiliary examination results.

In conclusion, although the regional incidence of occupational diseases is helpful to understand the prevalence of occupational diseases in the area, it can not be directly used to diagnose whether an individual has occupational diseases. Therefore, the factor that cannot require comprehensive analysis for the diagnosis of occupational diseases is the incidence of occupational diseases in E. region.

Therefore, the correct answer is the incidence of occupational diseases in the E. area.

Title: Can not be a comprehensive analysis for the diagnosis of occupational diseases

A. Risk factors for the occupation

B. Auxiliary examination results

C. Contact history of occupational disease risk factors

D. Previous occupational medical history

E. Inincidence of occupational diseases in areas

Answer: E. The incidence of occupational diseases in local areas

Analysis: When diagnose occupational disease, it is usually necessary to comprehensively analyze various information, including occupational risk factors, auxiliary examination results, contact history of occupational disease risk factors and previous occupational medical history. However, although the incidence of occupational diseases in the region can be used as a reference, it is not directly used as a factor for diagnosing individual occupational diseases. Therefore, option E is the correct answer.

25. (Type A1 question)

The environmental hazard of HFH chloride is

A. Damage to the natural vegetation

B. Destruction to the ozone layer

C. acid rain

D. Disrupt the ecological balance

E. Generating a greenhouse effect

Reference answer: B. Your answer:

Graphic analysis: [analysis] Chlorofluorocarbons ozone layer destruction is the most serious. It is characterized by strong penetration, can quickly through the atmospheric troposphere, into

Stratosphere, thereby destroying the ozone layer.

[Avoid] Carbon dioxide is a typical greenhouse gas, which strongly absorbs infrared radiation from the ground. Infrared radiation is the main

It is a thermal effect, therefore, this process will warm the atmosphere. Chlorofluorocarbons were commonly used as refrigerants in the past.cause

With its simple production process and low price, CFF is the most effective among the once popular refrigerant "Freon"

The composition of. At that time, refrigerators, freezers, air conditioners, a large number of added CFCs.

In this topic, we want to identify the main environmental hazards to CFCs (Chlorofluorocarbons, the "chlorine fluoride" mentioned in the topic may refer to this substance). The best known environmental impact of CFFC is the destruction of the ozone layer. After they rise to the stratosphere in the atmosphere, they release chlorine atoms due to the effects of solar radiation and cause thinning of the ozone layer, a process known as ozone depletion.

So, the most correct answer is:

F. The destruction of the ozone layer

First, we need to understand the environmental harm of FC. Chlorofluorocarbons is a kind of synthetic organic compounds, often used as refrigerant, foaming agent, etc. Due to its good chemical stability, it will not decompose in the troposphere, and can rise to the stratosphere, and be decomposed under the action of strong ultraviolet light to produce chlorine atoms, which can serve as a catalyst for ozone decomposition and destroy the ozone layer.

Next, we will analyze each option:

A. Damage to natural vegetation: This option does not specifically refer to the influence of chlorofluoride. A variety of environmental pollution and human factors

may lead to the destruction of natural vegetation.

B. Destruction to the ozone layer: As mentioned above, the fluorine hydroxyl chloride can rise to the stratosphere and decompose under the action of ultraviolet light to produce chlorine atoms, which will catalyze the decomposition of ozone, thus destroying the ozone layer. This is the specific environmental impact of HCFH.

C. Acid rain: Acid rain is mainly caused by pollutants such as sulfur dioxide and nitrogen oxides, and has no direct relationship with chlorofluoride.

D. Destroy ecological balance: Although environmental pollution and climate change may ultimately affect ecological balance, this option is too broad to refer specifically to the effects of hydroxyl chloride.

E. Producduce greenhouse effect: greenhouse gases mainly include carbon dioxide, methane, etc., and CFfluoride is not the main greenhouse gas.

In conclusion, the specific harm of CFFC to the environment is the destruction of the ozone layer. So, the best answer is:

F. Destruction to the ozone layer.

Topic 24: The harm to the environment is

Hydroyl (chlorofluorohydrocarbons, CFCs) is a class of compounds containing chlorine, fluorine and carbon, commonly used in refrigerants, foaming agents and detergents. Its main environmental hazard is the destruction of the ozone layer.

Answer: B. destruction to the ozone layer

After the chlorofluorocarbons enter the atmosphere, the chlorine atoms are decomposed under the ultraviolet radiation in the high air, and the chlorine atoms react with ozone, leading to the destruction of the ozone layer. This disruption causes more UV light to reach the Earth's surface, thus causing a range of environmental and health problems. Therefore, the destruction of the ozone layer by CFF is its main environmental hazard.

26. (Type A1 question)

The biggest addiction to smoking is

A. tar

B. CO

C. nicotine

D. CO2

E. NO2

Reference answer: C Your answer:

[Analysis] Nicotine (C pair) is the main substance of tobacco addiction. Because the brain is activated by nicotine for a long time

State, the sensitivity of nicotine decreased, leading to the gradual increase in smoking in most people, and the formation of nicotine dependence over time

Lai is addictive.

[Avoid mistakes] CO, the main component of tar and tobacco smoke, is closely related to the occurrence of lung cancer.

The most addictive component of smoking is C. nicotine. Nicotine, the main addictive substance in tobacco, can quickly enter the brain and stimulate the release of dopamine, creating a sense of pleasure, thus leading to dependence and addiction. Tar, carbon monoxide (CO), carbon dioxide (CO2) and nitrogen dioxide (NO2) are also found in tobacco smoke and are harmful to humans, but they are not the main factors in addiction.

This is a choice question involving tobacco composition and its addiction. We need to identify the components that smoking is most addictive.

Let's analyze the various options:

A. Tar: Tar is the product of tobacco burning and mainly causes human health hazards, but it is not addictive.

B. CO (carbon monoxide): CO is a harmful gas produced when tobacco burns. It has a strong binding ability with hemoglobin, which may lead to hypoxia in the body, but it is not addictive.

C. Nicotine: Nicotine is one of the main components of tobacco, and it is highly addictive. It can combine with the nicotinic receptor in the central nervous system, produce excitation, sedative, pleasure and other effects, and is the key factor leading to smoking addiction.

D. CO2 (carbon dioxide): CO2 is a normal product of respiration and is not addictive to the human body.

E. NO2 (nitrogen dioxide): NO2 is a harmful gas, harmful to the human body, but it is not addictive.

In conclusion, nicotine is the major component leading to smoking addiction. Thus, the correct answer is the C. nicotine.

The most highly addictive components of smoking are these:

F. nicotine

27. (Type A1 question)

In order to improve the rate of maternal hospital delivery in a mountainous area, a county established a green channel for maternal hospital delivery, this measure

belongs to

A. Adjust the direction of health services

B. Strengthening of community action

C. Create a supportive environment

D. Develop personal health skills

E. Develop public policies for health promotion

Reference answer: E Your answer:

[Analysis] The areas of health promotion activities include: developing public policies that can promote health, creating supportive environments, strengthening community actions, developing individual skills, and redirecting health services. It clearly requires relevant departments to establish and implement health promotion policies, whose purpose is to make it easier for people to make more healthy choices. In this case, the hospital established a green channel for maternal hospital delivery belongs to this item, so this question E is selected.

[Avoid] Creating supporting environment refers to creating a safe, comfortable, satisfactory and pleasant working and living environment; strengthening community action

It aging community strength and use community resources to enhance self-help and social support; developing personal skills is by providing fitness

Health information and education help people improve their ability to make health decisions; adjust the direction of health services refers to the health sector

Bed treatment services, and should also provide preventive and health promotion services.

The issue is about the classification of public health interventions. The establishment of the green channel for hospital delivery aims to increase the rate of hospital delivery in a mountainous area. Such measures usually involve improving visit conditions, providing convenience and supporting environment, and make women more receptive to professional delivery services. Therefore, the most appropriate option should be "C. Create a supporting environment". This option refers to the promotion of health behaviors and the use of services by providing favorable conditions and resources, such as easy transportation, financial support, and healthcare resources.

analyze:

A county has established a green channel for maternal hospital delivery, aiming to improve the accessibility of maternal hospital delivery, reduce the risk of delivery, and improve the health level of mother and child through policy means. This measure reflects the government's attention and investment to public health, and belongs to the formulation of public policies to promote health.

Option A The adjustment of the direction of health services mainly refers to shifting the focus of health services from treatment to prevention, from urban to rural areas, and from the upper level to the grassroots, which is not consistent with the topic, so it is not chosen.

Option B Strengthening community action mainly refers to community organizations improving the health awareness and health behavior of community residents through self-management and participatory research, which is not directly related to the establishment of the green channel for maternal hospital delivery, so it is excluded.

Option C creating supportive environment refers to creating a healthy environment from social, economic, cultural and other aspects. Although it is related to the green channel of maternal hospital delivery, it is not the most directly related option, so it is not selected.

Option D Developing personal health skills refers to improving personal health awareness and skills through health education, health promotion and other ways. Although important, it is not directly related to the establishment of the green channel for maternal hospital delivery, so it is excluded.

To sum up, the correct answer is: E. Develop public policies to promote health.

28. In order to improve the rate of maternal hospital delivery in a mountainous area, a county established a green channel for maternal hospital delivery, which belongs to

A. Adjust the direction of health services

B. Strengthening of community action

C. Create a supportive environment

D. Develop personal health skills

E. Develop public policies for health promotion

* * Answer: C. Create a supportive environment * *

The establishment of the green channel for maternal hospital delivery is to support maternal hospital delivery by providing convenient medical services, so as to improve the rate of hospital delivery. Such measures belong to provide convenient conditions and resources, and create an environment conducive to healthy behavior, so option C "creating a supportive environment" is the most suitable answer.

29. (Type A1 question)

The country selects the AIDS epidemic in a certain area, and conducts a unified survey method and method for the total population in the region, which the statistical method belongs to

A. Passive monitoring

B. Surveillance of cause of death

C. Symptom monitoring

D. Sentinel monitoring

E. Behavioral monitoring

Refer to the answer: D. Your answer:

[Analysis] Sentinel monitoring refers to a clearer understanding of a specific public health problem, according to the disease, health status or

Distribution characteristics of risk factors, a number of representative areas and (or) populations are selected as monitoring points, according to the unified regulations

Collect the data (D pair).

[To avoid mistakes] In the process of testing, the lower unit reported the monitoring data according to the routine, and the higher testing unit passively accepted, said

For passive monitoring (A wrong); higher monitoring unit to organize investigation or require lower testing unit to collect strictly in accordance with the regulations

Set of data, called active monitoring. Cause of death monitoring (B error) can understand the mortality and death of various diseases (including injuries) in the population

Due to the distribution, grasp the health level of the population, and determine the main cause of death and the focus of disease prevention and treatment in different periods. Symptom monitoring (C is wrong)

Also known as syndromic monitoring or syndrome monitoring, it is especially suitable for some new diseases, diseases of unknown etiology, clinically still

Disease without a definite diagnostic method. Behavioral monitoring (E error) is applicable to the behavioral monitoring of a disease, such as AIDS

Monitoring of intravenous injection behavior and sexual behavior in AIDS patients.

The topic examines the different types of epidemiological surveillance."Passive monitoring", "cause of death monitoring", "symptom monitoring", "sentinel monitoring" and "behavioral monitoring" in the options are all different methods for monitoring public health problems, especially infectious diseases.

A. Passive surveillance refers to the surveillance method that relies on medical institutions to report cases in their daily work.

B. Cause of death monitoring is focused on collecting data on causes of death.

C. Symptom monitoring usually focuses on the occurrence of specific symptoms to detect the disease outbreak.

D. Sentinel surveillance refers to purposeful, systematic disease surveillance at a specific site or institution to represent the health status of the entire population or a specific group.

E. Behavioral monitoring focuses on the way people behave and their impact on health.

The title describes a unified national survey of AIDS prevalence within a certain range, which involves purposeful and systematic monitoring of a specific population,

most in line with the definition of "sentinel surveillance". So the best answer is:

F. Sentinel surveillance

analyze:

Public health surveillance includes infectious disease surveillance and non-infectious disease surveillance, among which infectious disease surveillance is the main body of disease surveillance. Infectious disease surveillance is a long-term and continuous collection, verification and analysis of the dynamic distribution and influencing factors of diseases, and the timely reporting and feedback of information, so as to take timely intervention measures. Its types include hospital-based monitoring, laboratory monitoring, sentinel surveillance, and population survey. In the problem, the country selects the AIDS epidemic in a certain area, and conducts a unified way and method for the total population in the region, which is a systematic and continuous surveillance for specific diseases, and the statistical method belongs to sentinel surveillance.

Option A Passive monitoring refers to the data reported by the subordinate units according to the routine, and the superior units passively accept it. It does not belong to the specific monitoring for specific diseases and populations, so it is not selected.

Option B Cause of death monitoring refers to the monitoring of the cause of death in a region or population. The question stem describes the monitoring of AIDS as a specific disease, which is not consistent with the meaning of the question.

Option C symptom monitoring is a part of the monitoring system, which refers to the monitoring of specific symptoms, but the problem is the monitoring of AIDS epidemic, not just AIDS symptoms, so excluded.

Option E Behavioral monitoring is usually used to study the behavioral patterns of specific populations, rather than the prevalence of specific diseases, which does not fit to the topic.

In conclusion, the correct answer is: D. sentinel monitoring.

The country selects the AIDS epidemic in a certain area, and conducts a unified method and method survey method for the total population in the region. The statistical method belongs to:

G. Sentinel surveillance

Sentinel surveillance refers to the systematic and continuous collection, analysis, and interpretation of data on certain diseases or health-related events in specific locations and populations in order to detect and control disease transmission in a timely manner. This method is commonly used for the surveillance of infectious diseases, including AIDS. Sentinel surveillance can understand the prevalence of disease by conducting regular surveys in certain high-risk areas or in specific populations.

30. (Type A1 question)

Which data is most suitable for collection

A. attack

B. case rate

C. attack rate

D. death rate

E. secondary attack rate

Reference answer: B. Your answer:

Graphic analysis: [analysis] the current situation survey is also known as cross-sectional survey, prevalence survey, etc. At a certain time, there is a certain disease (including

Old and new cases) as a percentage of the total population. It is mostly used for census. So choose B.

[Fault avoidance] incidence = (number of new cases / number of the population exposed in the same period) k (k= 100%, 1000 ‰, or

100000 / 10 0000), so A wrong. The ence rate is used to measure disease frequency in a small range and short time, and is often used in disease outbreaks

Or popular investigation, so C wrong. Mortality is the frequency of a single disease dying due to the disease, so D is wrong. The renewal rate refers to one

After the first case occurring in 11 families, wards, dormitories, nurseries and kindergarten groups occurs, the disease is the shortest and the most

Cases appearing between the long incubation period are called continuation cases, so E is wrong.

The question asks which data is the best place to collect when conducting the current situation survey. Current situation survey usually refers to a cross-sectional study of health status in a certain population at a given point in time. Therefore, the key is to determine which rates best reflect the health of the study population at a given point in time.

A. Incidence rate: the number of new cases within a certain period of time, usually used to reflect the occurrence of the disease.

B. Prevalence: refers to the presence of a disease in a certain population of people at a specific time point or period, reflecting the sum of old and new cases.

C. Disease rate: This is not a commonly used epidemiological term and may be a synonym for prevalence.

D. Mortality rate: the ratio of the number of deaths from certain causes to the total population over a certain period of time.

E. Renewal rate: usually the proportion of new, minor, symptoms or diseases developed in a population with a preexisting disease.

For current situation surveys, we usually focus on the presence of the disease in a population at a specific point in time, so the prevalence (B) is the most suitable data to collect, because it can provide information on the prevalence of the disease in a population at a certain point in time. So the most correct answer is the B. prevalence.

First, we need to make it clear that this is a question about the choice of epidemiological survey methods. The question asks which kind of data is most suitable for the survey, which requires us to understand the characteristics of the survey and the concept of various rates.

A Current situation survey, also known as a cross-sectional study, is a survey of the health status, related factors or diseases of a specific population at a specific point in time. Its purpose is to collect information on the population health status and related factors at a certain time section, and to describe the distribution characteristics of the data.

Next, we will analyze each option:

A. Incidence: indicates the frequency of new cases of a certain population within A certain period of time. This is a period index, which is usually used for disease monitoring and describe the distribution of disease, and is not suitable for current situation investigation.

B. Prevalence: indicates the ratio of the number of people in a certain population at a certain point to the number of people at that certain point. This is a time point index, which is very suitable for the current situation survey, and can reflect the number of patients in the population.

C. Disease rate: similar to the incidence, it is also an indicator of new cases, but it is suitable for small measurement of disease frequency in a short period of time, and is not suitable for current situation investigation.

D. Mortality rate: indicates the ratio of the number of deaths due to a certain disease in a certain period to the number of patients suffering from a certain disease in the same period. It is mainly used to measure the severity of the disease and the medical treatment and diagnosis level of the hospital, and is not applicable to the current situation investigation.

E. Renewal rate: refers to the percentage of susceptible contacts of an infectious disease with the incidence between the shortest incubation period and the longest

incubation period in the total number of all susceptible contacts, which is more used for the study of disease transmission and infection control, and is not suitable for current situation investigation.

In conclusion, prevalence is the best indicator of the purpose and characteristics of the survey, because it represents the proportion of people suffering from a certain disease at a certain point in time, which is suitable to describe the health status of the population at a certain point in time.

Therefore, the correct answer is the B. prevalence.

Current situation survey (cross-sectional study) is a survey of a specific population at a specific point in time. The main purpose is to understand the distribution of certain specific health indicators in that population. Therefore, the current situation survey is most suitable to collect data on * * prevalence * *. Prevalence refers to the proportion of the total number of existing cases of a certain disease or health state in a given population at a given point in time.

The correct answer is: * * B. Prevalence * *.

31. (Type A1 question)

Here's what secondary prevention is

A. To relieve the pain in patients with angina pectoris

B. Diabetic patients by blood glucose screening

C. Monitoring of harmful gases in the environment

D. Develop health education

E. Smokers start quitting smoking

Reference answer: B. Your answer:

[Analysis] Secondary prevention includes early detection, early diagnosis and early treatment, in which early detection of diseases can be achieved through general

use

Examination, screening (B pair), regular health examination, key project examination of high-risk groups and the establishment of specialized outpatient clinics, etc.

[Wrong avoidance] Primary prevention is a measure for healthy individuals; tertiary prevention is timely and effective for sick patients

Treatment measures to terminate the development of the disease and prevent its deterioration.

Secondary prevention aims to diagnose and treat at the initial stages of the disease to stop its progression and mitigate its effects. It aims to detect conditions early and intervene to reduce the severity of the disease and avoid potential health problems.

Options A (angina pain relief) and E (smokers start quitting smoking) are closer to primary prevention or treatment, not secondary prevention. Options C (monitoring harmful gases in the environment) and D (conducting health education) are more like primary prevention because they are designed to prevent disease.

Option B (screening patients with diabetes through blood glucose) is secondary prevention because it is a screening measure aimed at early detection of the disease and diabetes even before symptoms appear, allowing early treatment and management to prevent the disease from deteriorating.

Therefore, the most correct answer is B. blood glucose screening.

This is a multiple-choice question that requires us to analyze the definition of "secondary prevention" and find out the answers from the options provided that meet the definition of "secondary prevention".

Understanding secondary prevention: Secondary prevention refers to intervention at the early stage of the disease to stop or slow the development of the

disease and reduce the occurrence of complications and sequelae. This usually includes early diagnosis and appropriate treatment.

Analysis options:

Option A (relieve pain in patients): This is more like A treatment for a diagnosed disease, falling under the category of tertiary prevention, that is, taking measures to prevent the deterioration of the disease and complications.

Option B (screening for diabetes by blood glucose): This meets the definition of secondary prevention. Through screening, diagnosis and treatment can be performed in the early stages of diabetes to prevent the deterioration and the occurrence of complications.

Option C (monitoring harmful gases in the environment): This is the primary prevention, which is used for the whole group to prevent the occurrence of disease.

Option D (conducting health education): Health education is mainly to improve the public's health awareness and knowledge level, which belongs to the category of primary prevention.

Option E (smokers start quitting): This is also primary prevention, which prevents disease through lifestyle changes.

In conclusion, only option B (in patients with diabetes by blood glucose screening) meets the definition of secondary prevention, namely diagnosis and intervention at the early stages of the disease to prevent exacerbations and the onset of the complications.

Therefore, the answer is B. diabetes patients through blood glucose screening.

Secondary prevention refers to the prevention of further disease development or the occurrence of complications through screening, early diagnosis and early treatment in the early stage of the disease.

In the above options:

A. relieve pain in patients-this is a treatment, not a preventive measure.

B. Screening for diabetes by blood glucose-this is early screening and diagnosis of diabetes and belongs to secondary prevention.

C. Monitoring of harmful gases in the environment-which is the content of primary prevention, with the aim of reducing the exposure to harmful factors.

D. Health education- -which is the content of primary prevention, aiming to improve the health level of the population and prevent diseases.

E. Smokers start quitting smoking-this is the content of primary prevention, aiming to prevent the occurrence of smoking-related diseases.

So, the most correct answer is:

F. Diabetic patients by blood glucose screening

32. (Type A1 questions)

A patient uses immunosuppressants, and the most likely infection in a hospital is

A. Infection with bacteria

B. ectogenous infection

C. cross infection

D. Transfusion infection

E. Infection in epidemic areas

Reference answer: C Your answer:

[Analysis] The occurrence of hospital infection can be caused by endogenous resident bacteria in the skin, nasal cavity, digestive tract and other parts, but also by the medical environment, medical equipment and medical equipment, visitors, medical personnel to patients, also known as cross-infection (C pair).[Error avoidance] carrier infection (A error) refers to the latent pathogen bacteria in normal healthy people. Once the host immune function is reduced, the pathogen can reproduce and

cause disease. Exogenous infection (B error) is an infection caused by a pathogen originating from outside the host. Infection in an epidemic area (E error) means that the pathogen exists in the body or body surface of the source of infection and is transmitted to others through different transmission routes.

Long-term use of immunosuppressants by patients increases the risk of infection, especially in the hospital setting. With a suppressed immune system, patients have weakened resistance to various pathogens. In this case, the most common one is a hospital-acquired infection, also known as a nosocomial infection.

In the option:

A. carrier infection usually refers to an infection caused by pre-existing microorganisms within an individual.

B. Exogenous infection refers to an infection originating from the external environment.

C. Cross-infection refers to the transmission from one patient or its environment to another patient.

D. Transfusion infection is one transmitted by blood transfusion.

E. Infection in an epidemic area refers to an infection within or associated with a specific epidemic area.

Considering that these patients are more susceptible to infection in the hospital environment, especially when the immune system is suppressed, the risk of cross-infection (C. cross-infection) is high, because microorganisms can be transmitted in hospitals through various routes, such as improper hand hygiene, medical equipment or environmental surfaces. Thus, the most likely answer is the C. cross-infection.

analyze:

Patients with long-term use of immunosuppressants are immunocompromised and susceptible to infection. Exogenous infection refers to the infection caused by the pathogen coming from the patient and invading the body through different ways. For immunocompromised patients, there are a large number of microorganisms in the

hospital environment, which are easy to invade the body and cause exogenous infection.

Option A carrier infection refers to the infection caused by the carrier bacteria, the carrier is generally asymptomatic, but can not be regularly discharged, become the source of infection, but it is not the most common infection of immunosuppressant users, so it is not selected.

Option C Cross-infection refers to the mutual infection between patients within a medical unit, which has little to do with immunosuppressant users, so it is not considered.

Option D Transfusion infection is infection caused by blood transfusion or blood products, with no history of blood transfusion, so excluded.

Option E Epidemic area infection refers to when the patient is infected in the epidemic area of an infectious disease. There is no mention of the patient in the epidemic area, so it is not selected.

In conclusion, the correct answer is: B. Exogenous infection.

For patients with chronic immunosuppressants, the most likely type of infection is * * exogenous infection * * (option B). Immunosuppressants can weaken the patient's immune system, making it more susceptible to pathogens coming from the external environment. Thus, such patients are most vulnerable to infection by external pathogens in the hospital setting. The options are explained as follows:

A. * * Band infection * *: Usually the patient itself carries the infection when the immunity drops, but not as common as exogenous infection.

B. * * Exogenous infection * *: It means that the pathogen comes from the external environment and enters the human body through air, contact and other channels, which is very common in immunosuppressed patients, because their immune system cannot effectively resist the external pathogens.

C. * * Cross infection * *: refers to infection transmitted between medical staff, instruments or other patients. Although it is also common in the hospital environment, the main factor is from exogenous pathogens.

D. * * Transfusion infection * *: It is transmitted by blood transfusion, generally with a low incidence of strict transfusion screening.

E. * * Infection in epidemic areas * *: refers to the infection in epidemic areas, which is directly related to the environment of the patient and is not as common as exogenous infection.

Therefore, the answer is * * B. Exogenous infection * *.

33. (Type A1 questions)

For one patient, considering vitamin B12 deficiency anemia, which of the following is correct

A. intravenous infusion of folic acid if folic acid absorption is impaired

B. A prolonged infusion of vitamin B12 if vitamin B12 is malabsorbed

C. Folic acid should not be taken orally together with the antioxidant vitamin C

D. Vitamin B12 cannot be taken orally together because it affects the absorption of folic acid

E. Because folic acid promotes the synthesis of vitamin B12, take folic acid first

Reference answer: B. Your answer:

[Analysis] The body's ability to store vitamin B12 is limited. If vitamin B12 is malabsorbed, the time of vitamin B12 is prolonged to improve the storage of vitamin B12 in the body, so B is selected.

[Error avoidance] folic acid absorption disorder can be injected with folic acid (but not as maintenance therapy, unless it is a disease of intestinal malabsorption

People), do not drip, so A wrong. Folic acid and vitamin C are taken at the same time can promote absorption, so it can be oral at the same time, so C

wrong. Folic acid can be taken orally along with vitamin B12, with no effect on

absorption, so D is wrong. Folic acid is a water-soluble group B vitamin

element, through the action of dihydrofolate reductase and vitamin B12, forms tetrahydrofolate, the latter with multiple one-carbon units (including

CH3, CH2, CHO, etc.) combined into tetrahydrofolate coenzymes, delivering one carbon unit, involved in many important reactions and nuclei in the body

The synthesis of acids and amino acids, rather than promoting vitamin B12 synthesis, so E is wrong.

For the treatment of vitamin B12 deficiency anemia, the correct approach is that if vitamin B12 is poorly absorbed, vitamin B12 should be given by intramuscular injection to ensure that the patient can directly absorb and utilize the vitamin. Therefore, option B (prolonged vitamin B12 infusion if vitamin B12 is malabsorbed) is correct. This is because the malabsorption of vitamin B12 often needs to be supplemented by giving vitamin B12 directly, rather than relying on the oral route, which can bypass the absorption disorders of the gastrointestinal tract.

Other options such as A, C, D and E are not standard practice for treatment of vitamin B12 deficiency anemia or are based on misunderstanding. In particular, option E promoting vitamin B12 synthesis is inaccurate; in fact, excess folic acid can mask the hematologic manifestations of vitamin B12 deficiency, but does not promote vitamin B12 synthesis. Thus, the most correct answer is the B.

analyze:

Vitamin B12 deficiency anemia, also known as pernicious anemia, is caused by malabsorption of vitamin B12 caused by the deficiency of internal factors. The treatment principle is to supplement vitamin B12. If there is vitamin B12 malabsorption, the course of vitamin B12 should be extended, that is, if vitamin B12 absorption is malabsorbed, the duration of vitamin B12 should be prolonged.

Folic acid absorption disorder, can take oral folic acid, so option A if folic acid absorption disorder, intravenous folic acid, can be excluded.

Folic acid and vitamin C can be taken together, there is no interaction between the two and will not affect human health, so option C folic acid can not be taken orally with the antioxidant vitamin C, can be excluded.

There is no mutual effect between folic acid and vitamin B12, so they can be

taken together, so option D "Vitamin B12 cannot be taken orally together because it affects the absorption of folic acid", option E "because folic acid should take large amounts of folic acid because it promotes the synthesis of vitamin B12", which can be excluded together.

In conclusion, the correct answer is: B. If vitamin B12 is malabsorbed, the muscle infusion of vitamin B12 is prolonged.

The treatment of vitamin B12 deficiency anemia is mainly vitamin B12 supplementation. Based on existing medical knowledge and best practice guidelines, the following is an analysis of each options:

A. * * If intravenous folate absorption disorder * * -Treatment of folic acid absorption disorder does include intravenous folic acid infusion, but this is not directly related to the direct treatment of vitamin B12 deficiency anemia.

B. * * Vitamin B12 infusion of vitamin B12 * * -Vitamin B12 malabsorption (e. g., patients with pernicious anemia) usually requires long-term intramuscular infusion of vitamin B12, initially daily, and then gradually decreases in frequency. This option is correct.

C. * * Folate cannot be taken orally together with the antioxidant vitamin C * * -Folate and vitamin C can be taken orally together, and there is no evidence that both cannot be taken together.

D. * * Because vitamin B12 affects the absorption of folic acid, * * -Vitamin B12 and folic acid can be taken together in the body, and there is no evidence that vitamin B12 affects the absorption of folic acid.

E. * * Because folic acid promotes the synthesis of vitamin B12, large oral doses of folic acid * * -folic acid will not promote the synthesis of vitamin B12. Vitamin B12 needs to be obtained through diet or supplement, in different ways and ways.

So, the correct answer is:

* * B. Extension of vitamin B12 infusion if vitamin B12 is malabsorbed * *

34. (Type A1 question)

The trophic arteries of the femoral head were not included

A. arteriae circumflexa ilium profunda

B. The ascending branch of the femoral shaft trophic artery

C. Branch of the lateral wound femoral artery

D. Small concave artery

E. Branch of the medial spiral femoral artery

Reference answer: A. Your answer:

[Analysis] The blood supply source of femoral head is concave artery, ascending branch of femoral shaft nourishing artery and internal and lateral femoral arteries.

(A wrong, is the correct answer to this question)

[Avoid mistakes] The medial femoral artery can be divided into lateral epiphyseal artery, superior metaphyseal artery, inferior metaphyseal artery, including the epiphyseal

The lateral artery supplies the blood circulation from 2 / 3 to 4 / 5 areas of the femoral head, and is the main source of blood supply to the femoral head.

The main blood supply of the femoral head comes from the medial femoral artery, the branches of the lateral femoral artery and the ascending branch of the nourishing artery of the femoral shaft. The deep iliac arteries supply mainly to the soft tissue around the hip and not directly to the femoral head. Therefore, among these options, excluding the femoral head trophic arteries are:

A. arteriae circumflexa ilium profunda

So the correct answer is an A.

analyze:

The femoral head is the upper end of the femur, spherical, toward the interior, and forms the hip joint with the acetabulum. The blood supply to the femoral head are foveal artery, ascending branch of the femoral backbone trophic artery and internal and lateral femoral arteries. The deep iliac artery produced by the iliopsoas artery mainly nourishes the iliac bone, which does not supply blood directly to the femoral head.

Option B the ascending branch of the femoral backbone nourishing artery, C the lateral femoral artery branch, D the minor concave artery, and E the medial femoral artery branch, which are vegetative arteries of the femoral head, can be excluded together.

In conclusion, the correct answer is: A. the deep iliac artery.

35. The trophic arteries of the femoral head are not included

A. arteriae circumflexa ilium profunda

B. The ascending branch of the femoral shaft trophic artery

C. Branch of the lateral wound femoral artery

D. That of the foveal artery

E. Branch of the medial spiral femoral artery

The correct answer is A. the deep iliac artery.

Explanation: The main vegetative arteries of the femoral head include the medial femoral artery, foveal artery, ascending branch of the femoral shaft trophic artery and branches of the lateral femoral artery. The deep iliac artery was not involved in the blood supply to the femoral head and was therefore not included.

36. (Type A1 question)

One day, when the doctor made a ward round, he found a patient with pale face, wet limbs, cold sweat, accelerated pulse, restlessness, and difficulty breathing. The patient was immediately in shock. The process of this mental activity belongs to doctors

A. sense perception

B. consciousness

C. imagine

D. thought

E. personality

Refer to the answer: D. Your answer:

Graphic analysis: [analysis] thinking is the human brain indirectly summarizes the reflection of objective things. Human thinking is a psychological process in which the general and essential characteristics and regularity of things are understood on the basis of perceptual knowledge with the help of concepts, representations and actions. By observing the objective performance of the patient, the doctor realized that the patient is in a state of shock. This process belongs to thinking, so this question chooses D.

[Avoid mistakes] Psychological phenomena are divided into psychological processes and personality characteristics. Psychological processes include cognitive process, emotional process and will

rule. Cognitive processes include feeling, perception, memory, thinking, imagination, etc.

The topic is a judgment about the process of mental activity. According to the title description, the doctor quickly judged the patient as shock by observing the

external symptoms (pale face, cold limbs, cold sweat, faster pulse, restlessness, and breathing difficulties) during ward rounds. This process is accomplished through the receipt and interpretation of patient symptoms, focusing on the integration and understanding of information to make a judgment.

Among these options, feeling (A) mainly refers to the process of receiving external information through the senses; perception (B) refers to the process of processing and explaining the information of perception, forming the understanding of things; imagination (C) refers to the process of constructing the image of things in the brain, usually without direct connection with reality observation; thinking (D) refers to the psychological process of processing, analyzing, integrating, judging, judging, or solving the information; and personality (E) refers to the sum of individual psychological characteristics, which is not directly related to the mental activity process of this topic.

Therefore, the most consistent description is "perception" (B), because the doctor needs to explain and process the observed symptoms to determine that the patient is in shock. So the correct answer should be B. perception.

First, let's analyze this problem carefully. The title describes a doctor's judgment that the patient is in shock by observing the specific symptoms of the patient (pale face, wet and cold limbs, etc.). This process involves the physician's perception and understanding of the patient's symptoms and drawing conclusions from it.

Now, we analyze each option one by one:

A. Feeling: Feeling is A reflection of the individual characteristics of objective things, such as seeing colors, hearing sounds, etc. In this case, the doctor did feel the patient's symptoms, but feeling alone was not enough to form a shock judgment.

B. Perception: Perception is the integration and interpretation of sensory

information, which is higher than sensation. Although doctors are integrating patient symptoms, perception itself does not contain in-depth analysis and judgment of information.

C. Imagination: Imagination is the process of processing and transforming the existing representations in the mind and forming a new image. This option has little to do with the doctor's judgment process.

D. Thinking: Thinking is the human brain's understanding of the nature of objective things and the internal connection between things. In this case, the doctor quickly judged the patient in shock by observing the symptoms and using his medical knowledge. This process involves in-depth analysis and reasoning, which is the embodiment of thinking ability.

E. Personality: Personality is a general term for a person's psychological quality, which has little to do with this specific judgment process.

To sum up, doctors quickly make the judgment of patients' shock by observing the various symptoms of patients and combining with their own medical knowledge. This process mainly reflects the doctor's thinking ability, namely option D. In this process, the doctor not only senses the patient's symptoms, but also makes an in-depth analysis and judgment of these symptoms, which cannot be achieved by simple perception or perception.

Therefore, the right answer is D. Thinking.

According to the psychological activity process of the doctor described in the title, this activity involves the comprehensive judgment and analysis of the patient's symptoms, and making a diagnosis, which belongs to the thinking process. So, the most correct answer is:

F. thought

37. (Type A1 question)

The examinations to determine the site, depth, and severity of the urethral injury were

A. catheter

B. ultrasonic examination

C. urethra CT

D. retrctrograde urethrography

E. ureteroscope

Refer to the answer: D. Your answer:

Graphic analysis: [analysis] retrograde urethrography can show the site and degree of urethral trauma (9th edition of Surgery) (D pair).

[Misavoidance] While urethral trauma can be diagnostic, the integrity and continuity of the urethra can only be understood (A

wrong). Ultrasound is mainly used in urology to determine the nature of renal mass, stones and hydronephrosis, and to determine residual urine and measurement

Prostate volume, check the scrotum, testis, epididymis, etc. (B wrong). CT is mainly used in urology to identify the renal sac

Mass and renal substantial lesions, determine the extent and extent of renal injury and the diagnosis and stage of the tumor (C error). There was no ureteroscopy

Method depth of damage (E wrong).

The most accurate method to determine the site, depth, and severity of the urethral injury is to perform retrograde urethrography (Retrograde Urethrography, RUG). This examination is able to clearly show the contour of the urethra, helping to determine the specific location and extent of the injury. Therefore, the best option is for D. retrograde urethrography.

analyze:

Urethral injury is the most common injury of the urinary system and is more common in men. In ically, the male urethra is bounded by the urinary diaphragm, which is divided into anterior and posterior segments. The anterior urethra includes the bulb and the penis, and the posterior urethra includes the prostate and membrane.

Anterior urethra injury mostly occurs in the ball, and the tube diameter of this urethra is large, which is a fixed part of the urethra, while the membrane urethra crosses the urine diaphragm, and the position is relatively fixed, so traumatic urethral rupture is easy to occur here. The examination to determine the site, depth, and severity of the urethral injury is retrograde urethrography, which clearly shows the site, extent, and the extent of urinary extravasation.

Option A urinary catheter, which is mainly used for preoperative and postoperative catheterization or for exploring whether the urethra is continuous and complete after urethral injury, but the site, depth and severity of urethral injury cannot be judged, so it is not selected.

Option B ultrasound examination can detect urinary extravasation and peribladder hematoma, but it is difficult to detect posterior urethral injury, so it cannot be used as an examination method to determine the site, depth and severity of urethral injury, so it is excluded.

Option C Urethral CT can show pelvic fracture, but it cannot clearly show the site of urethral injury, and it is expensive, so it is not selected.

Option E ureteroscope is mainly used for the diagnosis and treatment of renal pelvis and ureteral diseases, and it has little diagnostic value for urethral injury, so it is excluded.

In conclusion, the correct answer is: D. Retrograde urethrography.

38. The examination to determine the site, depth, and severity of the urethral injury is

The best answer is:

D. Retrograde urethrography

Retrograde urethrography is the standard test for assessing urethral injury and

can detail the site, depth and severity of the urethra. This examination method clearly shows the morphology and damage of the urethra by retrograde injection of contrast medium and X-ray shooting.

39. (Type A1 question)

The stones formed in the alkaline urine are

A. calculus urate

B. oxalate stones

C. cystine calculus

D. phosphate calculus

E. xanthic calculus

Refer to the answer: D. Your answer:

[Analysis] alkaline urine is conducive to the formation of phosphate; oxalic acid stone, uric acid stone, cystine stone and xanthine stone are easily formed in acidic urine (D pair).

The most frequently formed stone in alkaline urine is the phosphate stone. Therefore, the correct answer is the D. phosphate stones. Phosphate stones mainly include calcium phosphate and magnesium ammonium phosphate stones, which are more likely to form in an alkaline environment. Other types of stones, such as uric acid and cystine stones, more often form in acidic urine.

analyze:

The type of stone that is easily formed in alkaline urine is phosphate stone. Because its solubility is low in alkaline urine, it is easy to precipitate to form stones.

Option A Uric acid stones are mainly formed in acid urine, so they are not chosen.

Option B oxalate stone, option C cystine stone, option E xanthine stone, these three stones have little to do with urine acidity and can be formed in acidic or alkaline urine, but they are not easier to form in alkaline urine than in other pH environments, so they are excluded together.

In conclusion, the correct answer is: D. phosphate stones.

Stones formed in alkaline urine are usually phosphate stones. So the best answer

is:

F. phosphate calculus

40. (Type A1 question)

In the case of chronic renal failure, the abnormal calcium and phosphorus metabolism is

A. Normal blood phosphorus and elevated blood calcium

B. Reduced blood phosphorus and increased blood calcium

C. Blood phosphorus and blood calcium

D. Higher blood phosphorus and decreased blood calcium

E. Higher blood phosphorus and higher blood calcium

Refer to the answer: D. Your answer:

Graphic analysis: [Analysis] In the early stage of chronic renal failure, blood calcium and blood phosphorus can still remain within the normal range. As the disease progressed, the kidney reduced phosphorus and developed hyperphosphatemia and hypocalcemia (D). Hypocalcemia is mainly related to insufficient calcium intake, active vitamin D deficiency, hyperphosphatemia, and metabolic acidosis.(9th Edition of Internal Medicine)

In the case of chronic renal failure, increased blood phosphorus and decreased blood calcium usually occur. This is because the renal regulation of calcium is impaired, resulting in decreased blood calcium. In addition, the reduced activation of vitamin D by the kidney also affects the calcium absorption and metabolism, further reducing the blood calcium level. Therefore, the best answer is that D. Blood phosphorus increases and blood calcium decreases.

analyze:

In chronic renal failure, due to the damage of the nephron, the ability of the kidney to excrete fixed acid is weakened or lost, leading to the excretion of acid metabolites, causing acid poisoning, and can lead to renal tubular acidosis. In order to maintain the acid-base balance of body fluids, the body will mobilize bone calcium to neutralize the acid metabolites, leading to the decrease of blood calcium. Meanwhile, due to the secretion of H in the renal tubules, H binding to HPO_4^{2} into H_2PO_4 is

reduced, resulting in increased reabsorption of HPO $_4^2$ and increased blood phosphorus. Therefore, the abnormal calcium and phosphorus metabolism caused by chronic renal failure is usually increased blood phosphorus and decreased blood calcium.

Option A "normal blood phosphorus, the blood calcium increased", option B "blood phosphorus decreased, blood calcium increased", option E "blood phosphorus increased, blood calcium increased", and chronic renal failure, to maintain fluid acid-base balance, will mobilize bone calcium to neutralize acid metabolites, cause blood calcium decreased, therefore can be excluded.

Option C decreased blood phosphorus and blood calcium can be seen in patients with hypoparathyroidism, which does not conform to the pathophysiology of chronic renal failure, so it is not selected.

In conclusion, the correct answer is: D. Blood phosphorus increased, blood calcium decreased.

In chronic renal failure (CKD), phosphorus excretion decreases due to decreased renal function. At the same time, the ability of the kidney to synthesize active vitamin D (1,25-dihydroxyvitamin D) has decreased, resulting in reduced calcium absorption and lower blood calcium levels. This condition stimulates the parathyroid gland to produce more parathyroid hormone (PTH) in an attempt to increase blood calcium levels through bone absorption, but usually leads in a high phosphorus and low calcium state.

So the correct answer is:

F. Higher blood phosphorus and decreased blood calcium

41. (Type A1 question)

Neural mechanism of hepatic encephalopathy resulting from gastrointestinal bleeding

A. Enhanced phenylethylamine metabolism

B. The enhanced glutamine metabolism

C. Increased acetylcholine synthesis

D. Manganese crosses the blood-brain barrier into the brain

E. γ -aminobutyric acid decomposition

Reference answer: B. Your answer:

[Analysis] The dysfunction of the central nervous system is mainly metabolic or functional, which is the result of the comprehensive effect of a variety of disease factors. At present, the mainstream theory is mainly related to the increase of blood ammonia. In normal, blood ammonia content is small, and a small amount of ammonia in brain tissue forms glutamate with α -ketoglutaric acid under the action of glutamate dehydrogenase. Glutamate, with the participation of glutamyl synthetase, ATP and Mg2 +, was then combined with ammonia to form glutamine. Glutamine is a form of ammonia transport that occurs in the liver and kidney by glutaminase breaks into glutamate and ammonia. Ammonia forms urea through the ornithine cycle in the liver, and NH4 + formed in the kidney is excreted by the kidney. When blood ammonia increases, blood ammonia increases the ammonia through the blood-brain barrier into the brain tissue, and then damages the function of the central nervous system. So this question is selected B.

An important neurological mechanism of hepatic encephalopathy resulting from gastrointestinal bleeding is due to elevated blood ammonia levels. Ammonia is a key factor in the pathogenesis of hepatic encephalopathy, as liver damage cannot effectively convert ammonia to urea, leading to the accumulation of ammonia in the blood. However, in the given option, ammonia is not directly mentioned, so we need to consider the option that is most relevant to ammonia metabolism.

A. Enhanced phenylethylamine metabolism

B. The enhanced glutamine metabolism

C. Increased acetylcholine synthesis

D. Manganese passes through the blood-brain barrier and passes into the brain

E. Increased decomposition of γ -aminobutyric acid (GABA)

Of these options, option B "enhanced glutamine metabolism" is most associated with the metabolism of ammonia. In hepatic encephalopathy, due to the accumulation of ammonia, glutamate in the brain binds with ammonia to form glutamine, leading to

enhanced metabolism of glutamine. This process helps to reduce the toxicity of ammonia, but at the same time leads to an unbalanced brain metabolism, which in turn promotes the development of hepatic encephalopathy.

Thus, the most appropriate answer is the enhanced B. glutamine metabolism.

analyze:

Hepatic encephalopathy caused by gastrointestinal bleeding is mostly due to digestive tract bleeding, a large number of blood protein digestion products are decomposed by bacteria in the intestinal tract, producing a large amount of ammonia, causing ammonia poisoning, leading to hepatic encephalopathy. The digestive tract hemorrhage caused by neural mechanism of hepatic encephalopathy is the digestive tract bleeding, intestinal bacteria decomposition of blood, produce a lot of ammonia, ammonia into the blood and brain barrier into the brain tissue, cause brain edema, ammonia in the brain and α -ketoglutaric acid, form glutamate, which combined with ammonia to generate glutamine, and consume a lot of ATP and other coenzymes, cause brain cell dysfunction, lead to hepatic encephalopathy.

Option A Enhanced phenyllethylamine metabolism was not associated with the occurrence of hepatic encephalopathy, so it was excluded.

The increased synthesis of C acetylcholine is related to the pathogenesis of myasthenia gravis, and has no direct relationship with hepatic encephalopathy, so it is not selected.

Manganese does not cross the blood-brain barrier under normal circumstances, and can only enter the brain tissue when the liver function is impaired, but this is not the neural mechanism of hepatic encephalopathy caused by gastrointestinal bleeding, so option D manganese through the blood-brain barrier into the brain, does not conform to the question.

Option E: increased γ -aminobutyric acid breakdown was not associated with hepatic encephalopathy, but in fact, the inhibitory neurotransmitter γ -aminobutyric acid synthesis was increased during hepatic encephalopathy, so it was not chosen.

In conclusion, the correct answer is: B. Glutamine metabolism enhancement.

The neural mechanisms underlying hepatic encephalopathy due to gastrointestinal bleeding are mainly related to the disorder of ammonia metabolism

and the accumulation of toxic substances. Hepatic encephalopathy (HE) is the decline of liver detoxification function caused by liver failure or portal hypertension, which makes toxic substances (such as ammonia) accumulate in the blood and enter the brain, leading to neurological dysfunction.

During gastrointestinal bleeding, a large amount of hemoglobin decomposes to produce ammonia, which the liver fails to effectively detoxify, which crosses the blood-brain barrier to enter the brain, further affecting the nervous system. The enhanced metabolism of glutamine is a key mechanism, because glutamine is the product of ammonia and glutamate, and too much ammonia causes increased glutamine levels in the brain, leading to neurotoxicity.

So, the most appropriate answer is:

F. The enhanced glutamine metabolism

42. (Type A1 question)

Venous blood supply that is not in the danger triangle is

A. venae angularis

B. venae ophthalmica superior

C. venae ophthalmica inferior

D. venae temporales superficiales

E. venae faciei profunda

Refer to the answer: D. Your answer:

[Analysis] The danger triangle is a triangle area formed by the connection between the mouth on both sides and the nose root. The facial vein communicates through the inner canthal vein, ocular vein (the eye vein is: the central retinal vein, upper ocular vein, present vein, and eye vein) and the cavernous sinus, and also through the facial deep vein and wing plexus and the cavernous sinus. Therefore, ABCE is right, and D is wrong.

In the medical field, the danger triangle usually refers to the triangular area of the

face extending from the bridge of the nose to the sides of the corners of the mouth. The veins in this area have direct or indirect venous connections to the intracranial venous sinus, so infection potentially potential intracranial transmission through these venous channels, causing serious complications.

A. The medial canthus vein is the venous blood supply in the dangerous triangle.

B. The superior ocular vein is also the venous blood supply in the dangerous triangle.

C. Current veins are also the venous blood supply in the dangerous triangle.

D. The superficial temporal vein does not directly belong to the venous blood supply in the dangerous triangle, which is located in the temporal region.

E. Deep facial veins are the venous blood supply in the dangerous triangle.

Therefore, the venous blood supply that is not in the danger triangle is the D. superficial temporal vein. So, the correct answer is the D. superficial temporal vein.

analyze:

Dangerous triangle usually refers to the two sides of the mouth to nose root connection formed by the triangle area, due to the facial vein without venous flap, this area infection may through the inner canthus vein, eye vein into the intracranial cavernous venous sinus, cause suppurative cavernous venous sinus, eyes and the surrounding tissue of redness and induration, accompanied by pain and tenderness, and chills, fever, headache, etc., even severe cases occur sepsis, toxemia, life-threatening. Therefore, avoid the facial "dangerous triangle" pus point, do not squeeze, scratch squeeze or pick, so as to avoid the spread of inflammation, causing more serious complications. The venous blood supply in the facial "dangerous triangle" includes the anterior vein, deep facial vein, medial canthus vein, superior ocular vein and present vein, etc. The superficial temporal vein belongs to the branch of the external jugular vein and does not belong to the venous blood supply in the dangerous triangle.

Option A medial canthal vein, B superior ocular vein, C present vein, and E lateral deep vein, both are venous blood supply in the dangerous triangle, and therefore can be excluded together.

To sum up, the correct answer is: D. The superficial temporal vein.

In human anatomy, the danger triangle of the face refers to the area between the inner canthus and the mouth corner, where the venous blood supply is connected to the intracranial venous system, and if the infection may cause severe intracranial infection. The veins within the danger triangle include the medial canthus veins, superior ocular veins, and deep facial veins. The superficial temporal vein is not within the venous blood supply range in the dangerous triangle.

So the correct answer is:

F. venae temporales superficiales

43. (Type A1 question)

In the aortic and pulmonary artery flow, the anatomical structures involved are

A. sinnses obliquus pericardii

B. Anterior inferior pericardial sinus

C. sinuses venarum cavarum

D. coronary sinus

E. sinuses transversus pericardii

Reference answer: E Your answer:

Graphic analysis: [Analysis] The transverse pericardial sinus is the gap between the pericardial cavity in the ascending aorta, the pulmonary artery trunk and the superior vena cava, and the anterior wall of the left atrium. When careful direct surgery to block the blood flow of the aorta and pulmonary artery, the large artery can be clamped from the front and back through this sinus. Therefore, this question is selected E.

The anatomical structure usually involved in clamp aortic and pulmonary artery flow in open cardiac surgery is the transverse pericardial sinus. Because the transverse pericardial sinus is located in the posterior aspect of the aorta and pulmonary artery and is an important anatomical marker, this area often needs to be identified during surgery to avoid damage to adjacent structures. Therefore, the most appropriate option is the E. pericardial transverse sinus.

analyze:

In open cardiac surgery, in order to temporarily block the aorta and pulmonary arteries, it is necessary to clamp the aorta and pulmonary arteries. The transverse pericardial sinus is the space between the pericardial cavity in the aorta, the ascending artery and the superior vena cava, and the anterior wall of the left atrium. It is the only way to block the blood flow of the aorta and pulmonary artery during cardiac surgery.

Option A The oblique pericardial sinus is located in the pericardial space between the left posterior atrium, left and right pulmonary veins, the inferior vena cava and the posterior pericardial wall. The operation needs to block the return of the inferior vena cava to pass through this gap, not with the meaning of the question.

Option B The anterior inferior pericardial sinus is located in the anterior lower part of the anterior pericardial wall, posterior to the sternal stem, and the pericardial cavity anterior to the phrenic nerve. Pericardial puncture is unrelated to clamp aorta, and pulmonary artery flow, so it is excluded.

Option C The vena cava sinus is located between the upper and inferior vena cava mouth, and is the lowest position in the pericardial cavity. When the body is upright, fluid in the pericardial cavity will first accumulate here, which is inconsistent with the dry description.

Option D The coronary sinus is open in the right atrium, collecting venous blood from the heart and being injected into the right atrium. This structure is not involved in the operation, so it is not selected.

In conclusion, the correct answer is: E. The transverse pericardial sinus.

In direct cardiac surgery, when clamp aortic and pulmonary artery flow is required, the anatomy involved is the transverse pericardial sinus (transverse pericardial sinus). The transverse pericardial sinus, located between the ascending aorta and the pulmonary artery, is an important anatomical marker to easily clamp these large vessels during surgery. So, the correct answer is:

F. sinuses transversus pericardii

44. (Type A1 questions)

The ligament that maintains the uterine anteversion is

A. fallopian arch

B. alae vespertilionis

C. cardinal ligament of uterus

D. ovarian proper ligament

E. ligamenta infundibulo-pelvinum

Reference answer: A. Your answer:

[Analysis] The uterine ligament consists of 4 ligaments, namely: uterine main ligament, uterine broad ligament, uterine round ligament, and sacral uterine ligament. The broad ligament can prevent lateral displacement of the uterus and maintain the median uterus. The uterine ligament mainly maintains uterine tilt. The main ligament of the uterus positions the cervix to prevent the uterus from prolapse. The uterosacral ligament indirectly maintains the uterine forward tilt position. Therefore, this question is selected A.

The ligament that maintains the anteversion is the uterine ligament. So the correct answer is A. uterine ligament. The uterine ligament is a pair of structures that originate on both sides of the uterus and extend through the pelvis to the inner thigh, helping to maintain the position and forward tilt of the uterus.

analyze:

There are 4 pairs of uterine ligaments, including the round ligament, broad ligament, main ligament and uterine sacral ligament. The uterine round ligament starts from the front of the uterine horn and slightly below the proximal end of the fallopian tube, leans forward near the superficial ring of the inguinal canal, and stops at the front of the labia labum via the inguinal canal. The round ligament plays an important role in maintaining the anterior uterine inclination.

Option B The broad uterine ligament is a double peritoneal wrinkle formed by the peritoneum covering the anterior and posterior uterine wall extending from the lateral edge of the uterus to both sides of the pelvic wall. Its function is to fix the cervix and keep the uterus in the center of the pelvic cavity, so it is not selected.

Option C The main uterine ligament is a transverse fiber bundle formed by the thickening of the lower broad ligament, also known as the transverse cervical ligament. It is the main structure to fix the cervical position and prevent uterine prolapse. It has nothing to do with the maintenance of uterine forward tilt, so it is excluded.

Option D The innate ligament of the ovary is formed by the thickening of the broad ligament between the medial ovary and the uterine horn. Its function is to connect the ovary and the uterus and has no effect on the position of the uterus, so it is not considered.

Option E The ovarian suspension ligament is a structure formed by the broad ligament near the arteriovein of the ovary. which passes through the ovarian arterial vein, which is the symbol of finding the ovarian arterial vein during the operation. It has no effect on the position of the uterus and can be excluded.

To sum up, the correct answer is: A. The uterine circle ligament.

The ligaments that maintain the uterine anteversion are:

A. fallopian arch

The role of the uterine ligament is to keep it in the normal anatomical position by fixing the forward position of the uterus. Therefore, in this question, the most correct answer is the A. uterine circle ligament.

45. (Type A1 questions)

The main mechanism of insulin reduction in insulin is

A. Promoting gluconeogenesis

B. Promote the absorption of glucose by muscle, fat, and protein

C. Inhibition of glucose absorption by the proximal renal tubules by the brain

D. Inhibiting the synthesis of hepatic glycogen and muscle glycogen suppresses the breakdown of glucose

Reference answer: B. Your answer:

[Analysis] The mechanism of insulin reducing blood sugar is mainly the effect of insulin on glucose metabolism, promoting the uptake and utilization of glucose by peripheral tissue cells (B pair), promoting the synthesis of muscle glycogen and liver glycogen (D wrong), and inhibiting gluconeogenesis (A wrong), so as to reduce blood sugar.

Insulin is an important hormone that regulates blood glucose levels, which reduces blood glucose concentrations through several mechanisms. Depending on the options provided, the main mechanism of action of insulin is to promote the absorption of glucose by muscle, fat, and protein. So, the most correct answer is:

E. Promote the absorption of glucose by muscle, fat, and protein

This reflects how insulin helps cells absorb glucose from the blood and use it as energy, thereby lowering blood glucose levels.

analyze:

Insulin is secreted by pancreatic B cells and has protein hormones that lower blood sugar. The main mechanism of insulin hypoglycemic is to promote the absorption of glucose by muscle, fat and protein, thus reducing blood sugar levels.

Option A promotes gluconeogenesis, option D inhibits the synthesis of liver glycogen and muscle glycogen and inhibits the decomposition of glucose, which is the action mechanism of glucagon, which is opposite to the effect of insulin, so it can be excluded together.

Insulin reduces blood glucose by promoting glucose uptake and utilization by muscle and adipose tissue, rather than by inhibiting cerebral glucose absorption by the proximal renal tubules. Therefore, option C inhibits the absorption of glucose by the proximal renal tubules, which is inconsistent with the question.

In conclusion, the correct answer is: B. Promote the absorption of glucose by muscle, fat, and protein.

The main mechanism of insulin hypoglycemic mechanism is to promote the absorption of glucose by muscle, fat and protein. Insulin is a hormone secreted by the β -cells of the pancreas, and its main effect is to reduce blood sugar levels. It achieves this effect by promoting cellular glucose absorption and utilization, inhibiting glucose production in the liver, and promoting the synthesis of hepatic glycogen and muscle glycogen. So, the most correct answer is:

F. Promote the absorption of glucose by muscle, fat, and protein

46. (Type A1 questions)

Small doses that can increase the renal artery blood flow are available

A. adrenalin

B. arterenol

C. isoprenaline

D. dopamine

E. aminophylline

Refer to the answer: D. Your answer:

[Analysis] Small dose of dopamine can increase the blood flow of the renal artery, mainly because it suppresses the activity of Na + -K + -ATP enzyme in the near convoluted tubule and reduces the reabsorption of sodium, D on. Adrinephrine is mainly used for cardiopulmonary resuscitation and the rescue of anaphylactic shock (A wrong). Norrepinephrine has a strong α -adrenergic excitation effect, is a very effective pressor drug, for normal or low blood volume shock body, norepinephrine will reduce renal blood flow and urine volume (B wrong). Isoproterenol excites the β 2 receptor of vascular smooth muscle (mainly skeletal muscle, liver, coronary vessels) for vasodilation (C

wrong). The main clinical effect of aminophylline is to relieve bronchospasm (E error).

The physiological effects of the drug needs to be considered when selecting drugs that increase renal artery blood flow. Adrenaline, noradrenaline, and isoproterenol act mainly on α and β receptors in the heart and blood vessels, while dopamine and aminophylline have different mechanisms of action.

Adrinephrine (A) acts primarily on α and β receptors and leading to vasoconstriction, which affects renal artery blood flow.

Norerepinephrine (B) has a strong effect on α receptors, and can also cause vasoconstriction, which is not conducive to increasing renal artery blood flow.

Isoproterenol (C) is a cardiac agent that mainly acts on the β 1 receptor in the heart and has little direct effect on renal artery blood flow.

Dopamine (D) acts mainly on dopamine receptors at low doses, which can increase renal blood flow, and is one of the drugs used to treat acute renal insufficiency.

Aminophylline (E) is mainly used for the treatment of asthma, which increases

the airway opening by relaxing the smooth muscle, and has little direct effect on the renal artery blood flow.

Therefore, the best answer is D. dopamine because it increases renal blood flow at small doses.

analyze:

We mainly investigated the effect of vasoactive drugs on renal artery blood flow. Small doses of dopamine can excite the dopamine receptors (D_1 receptors) in the kidney, dilate, increase renal blood flow, increase glomerular filtration rate, increase urine volume, and have the effect of sodium diuretic. Therefore, small doses of dopamine can increase the renal artery blood flow.

Option A epinephrine can bind to α and β adrenergic receptors to produce strong, rapid and transient excited α and β effects. The excitation of β_1 receptors in the heart can enhance cardiac contractility, accelerate heart rate, increase myocardial oxygen consumption, and increase renal artery blood flow is not obvious, so it is excluded.

Option B norepinephrine can bind to myocardial β_1 adrenergic receptor, thus enhancing cardiac contractility and accelerating heart rate. It can also combine with vascular smooth muscle α receptor, which is inconsistent with the problem.

Option C Isoproterenol is a β receptor agonist, mainly used for bronchial asthma and atrioventricular block, with no significant effect on renal artery blood flow, so it is not selected.

Option E aminophylline is a phosphodiesterase inhibitor, increased intracellular cAMP levels and dilate bronchchi, less effect on renal artery blood flow, can be excluded.

In summary, the correct answer is: D. dopamine.

Small doses that can increase the renal artery blood flow are:

F. dopamine

** analyze **:

Dopamine is a dopamine receptor agonist that can cause dilation of renal vessels. Small doses of dopamine act mainly on the dopamine receptors (D1 receptor), which can dilate the renal arteries and increase renal blood flow. Thus, dopamine can be used at small doses to treat acute renal failure or other conditions that require increased renal blood flow. Other options such as epinephrine, norepinephrine and isoproterenol act mainly on α and β adrenoceptors, usually causing vasoconstriction and cardiostimulation effects and are not appropriate for increasing renal blood flow. However, aminophylline increases the level of intracellular cAMP mainly by inhibiting phosphodiesterase, which is mainly used for the treatment of asthma and chronic obstructive pulmonary disease.

47. (Type A1 question)

The primary cells responsible for HIV infection are

A. DC

B. T cell

C. CD4 T cells

D. B leukomonocyte

E. CD8 T cells

Reference answer: C Your answer:

[Analysis] CD4 + T lymphocytes are the most important target cells of HIV infection. After HIV infection of human body, there is a progressive reduction of CD4 + T lymphocytes, inversion of CD4 + / CD8 + T lymphocyte ratio, impaired cellular immune function, and C pair.

The main cells infected by HIV (human immunodeficiency virus) are CD4 T cells. Thus, the most correct answer is the C. CD4 T cells.

analyze:

HIV, the human immunodeficiency virus, is a retrovirus causing defects in the human immune system. HIV mainly invades the human body's immune system, including CD4 T cells, macrophages, and dendritic cells. However, after HIV infects the human body, it will selectively adsorb to the CD4 receptor of the target cells, and enter the host cells with the help of the auxiliary receptor. It will mainly infect CD4 T cells, and replicate and expand in it, eventually leading to the rupture and death of CD4 T cells.

Option A Dendritic cells are also the target cells of HIV invasion, but they are not the main object of infection, so they are not selected.

Option B: T cells, option D: B lymphocytes, option E: CD8 T cells, are not the main HIV infection object, so they can be excluded together.

In conclusion, the correct answer is: C.CD4 T cells.

The primary target cell for HIV (human immunodeficiency virus) infection is CD4 T cells. These cells are a very important part of the immune system, and the HIV virus enters them by binding CD4 receptors and reproduces them, leading to the destruction of the immune system. So, the most correct answer is:

F. CD4 T cells

48. (Type A1 question)

Mainly the cells infected with HIV were not included

A. Kupffer's cell

B. T cell

C. mononuclear macrophage

D. B leukomonocyte

E. glia cells

Reference answer: A. Your answer:

Graphic resolution: [parse] after HIV invaded the human body, under the action of direct cytotoxicity and immunopathology, mainly CD4 + lymphocytes of a variety of cells (CD8 + T cells, monocytes-macrophages, B lymphocytes, lymphocytes, NK cells, small glial cells, etc.) function damage and a large number of damage, lead to the body cell immune deficiency, prompted the body with a kinds of serious opportunistic infection and tumor. Therefore, select A, excluding Kopffer cells.

HIV (human immunodeficiency virus) mainly infects cells in the immune system, especially cells with CD4 receptors. Consider the following cell types:

A. Kupffer cells-These are macrophages in the liver that, and although they can be infected by HIV, are not the primary target cells for HIV.

B. T cells-HIV predominantly infect CD4 + T cells, a key link in the HIV life cycle.

C. Monocytic macrophages-HIV can also infect mononuclear macrophages, which are important factors for viral transmission and persistence.

D. B lymphocytes-Although B cells are not the primary target of HIV, they can be affected, especially in the advanced stages of HIV infection.

E. Glial cells-HIV can infect cells of the central nervous system, including microglia, but glial cells (such as astrocytes and oligodendrocytes) are not the primary target cells of HIV.

Thus, the cells least likely to be mainly infected by HIV are E. glia.

First, we need to understand which cells HIV or human immunodeficiency virus mainly infects. HIV mainly binds to CD4 molecules on the cell surface and enters the cell interior, and then reproduces its own RNA and protein using the cell biosynthetic system and asseminto new viral particles. Next, we analyze each option one by one:

A. Kupfer cells: Kupfer cells are macrophages in the liver, whose main function is to remove harmful substances from the blood. HIV does not primarily infect these cells, and although macrophages can be infected by HIV, Kupffer cells, as specific types of macrophages, are not the primary target of HIV infection.

B. T cells: HIV replication and spreads mainly through infection of CD4 + T cells. These cells play a key role in the immune system and are the main target of HIV attack.

C. Mononuclear macrophages: These cells are also part of the immune system and can be infected by HIV. Although they are not a primary target of HIV, they can still be exploited by viruses for replication.

D. B lymphocytes: Although B lymphocytes are not the primary target of HIV infection, they can also be infected by HIV in some cases.

E. Glial cells: HIV can invade the nervous system in the late stage and infect glial cells and neurons, leading to AIDS, dementia syndrome and other neurological diseases.

In conclusion, HIV does not primarily infect Kupffer cells, although these cells could theoretically be infected. Therefore, the most appropriate answer is the A. Kupffer cell.

Which of the following cells does not include the cells infected by HIV? As we know, HIV (human immunodeficiency virus) mainly attacks and destroys the human immune system, especially the following cells: T cells (especially CD4 + T cells), mononuclear macrophages, and glial cells. Kupffer cells are macrophages in the liver and may also be infected by HIV. However, B lymphocytes are not the primary target cells for HIV.

So, the most correct answer is:

F. B leukomonocyte

49. (Type A1 question)

AIDS late lymph node pathological changes are

A. lymph foilicie hyperplasia

B. paracocortical areas

C. Sinus histiocytic sis

D. The disappearance of the lymphocytes

E. Narrowing of the parapracortical areas

Refer to the answer: D. Your answer:

Graphic analysis: [analysis] changes in lymphoid tissue in AIDS patients: (1) early, lymphadenectasis; Microscopic obvious hyperplasia of lymphoid follicles (active germinal center, "All over the sky star" phenomenon), More plasma cells in the medulla; EM visualization of HIV particles located within the germinal center, Mainly focused on the follicular dendritic cells, It can also appear in macrophages and CD4 + T cells; (2) With the development of the lesions, The reduction or disappearance of lymphocytes in the outer layer of the follicles, Small blood vessel proliferation, Germinal centers are divided by scattered pieces; Progressive reduction of CD4 + T cells in paraneortical areas, Instead, with plasma cell infiltration; (3) Late stage, The lymph nodes are barren, Almost disappeared lymphocytes, Only a few macrophages and plasma cells remained. Therefore, this question is chosen D.

The nonode pathological changes in the advanced stages of AIDS are characterized by the disappearance of lymphocytes. AIDS is caused by the human

immunodeficiency virus (HIV), which destroys the body's immune system, especially the CD4 + T cells. As the disease progresses, the lymphocyte numbers will decrease significantly, especially in the lymph nodes.

So, the most correct answer is:

F. The disappearance of the lymphocytes

analyze:

The main characteristics of the pathological changes in advanced AIDS are: the lymph nodes are barren and the lymphocytes disappear. This is because HIV invades CD4 T lymphocytes, leading to cellular immune deficiency, thus secondary opportunistic infections and malignant tumors, resulting in the destruction of lymph node structure and a large reduction of lymphocytes.

Option A Lymphoid follicular hyperplasia is A pathological feature of follicular lymphoma, which has nothing to do with AIDS, so it is not selected.

Option B paracortical hyperplasia and paracortical narrowing of option E can be seen in the early stage of AIDS, but will not appear in the late stage of AIDS and can be excluded together.

Option C sinus histiocytic hyperplasia is a pathological characteristic of Rosa-Dorfman disease (RDD) rather not AIDS, so it is not considered.

In conclusion, the correct answer is: D. The lymphocytes disappear.

AIDS (AIDS) is a chronic infectious disease caused by human immunodeficiency virus (HIV). Its late stage often shows the severe destruction of the immune system, leading to the occurrence of a variety of opportunistic infections and tumors. Pathological changes in the lymph nodes in the advanced stage of AIDS have characteristic manifestations.

In the early and middle stages of AIDS, lymph nodes usually develop lymphoid follicle hyperplasia and hyperplasia of paracocortical areas. However, in the late stages of AIDS, the structure of the lymph nodes is significantly altered due to further failure of the immune system. Specifically, the significant reduction or even disappearance of lymphocytes in the lymph nodes, leading to atrophy and regression

of lymphoid tissue.

Therefore, the most correct answer for the pathological changes in late AIDS is:

G. The disappearance of the lymphocytes

50. (Type A1 question)

The pathological features of the breast fibroadenoma are

A. Comconsists of proliferplastic fibrous stroma and glands, which are squeezed by fibers

B. Cancer cells infiltrate in fibers

C. A painful solitary mass

D. Fibroadenoma occurs in the upper quadrant of the breast and is round or oval

E. The most common malignancy of the female breast

Reference answer: A. Your answer:

[Analysis] Fibroadenoma is the most common benign breast tumor (E error) in women, which is composed of two mixture of glandular epithelium and fibrous tissue and can occur at all ages, more commonly in women aged 15 to 35. It is mainly a painless breast lump (C wrong), which occurs in the upper quadrant of the breast. The clinical palpation is mostly oval, rubber-like lump, with clear boundary (D wrong) and good range of motion. General ly is solid, well-defined oval nodules, gray-brown, bulging, lobated, often visible fissure like cavity (A pair).

Breast fibroadenoma is a common benign breast tumor whose pathological features mainly include proliferplastic fibrous and glandular tissue. These proliferative glands are extruded by fibrous tissue and often present a cleft-like structure. So, the most correct answer to this question is:

A.It consists of proliferating fibrous stroma and glands, which are squeezed by the fibers

analyze:

Breast fibroadenoma is the most common benign tumor of the breast, composed of epithelial and interstitial tissue. Its pathological features are composed of

proliferating fibrous stroma and glands, which are squeezed by fibers and slit.

Option B The infiltration of cancer cells is a pathological feature of invasive lobular carcinoma and is unrelated to breast fibroadenoma, so it is not selected.

Most of the breast fibroadenomas are painless masses, only 14% have mild pain, so the option C painful solitary mass is not the pathological feature of breast fibroadenoma, so it is excluded.

Breast fibroadenoma is good to occur in the upper quadrant of the breast, so option D breast Fibroadenoma is good to occur in the upper quadrant of the breast, which is round or oval, and can be excluded.

Option E The most common malignancy of the female breast is invasive ductal carcinoma, not a breast fibroadenoma, so it is not chosen.

In conclusion, the correct answer is: A. It consists of the hyperplasia of the fiber stroma and the glands, and the hyperplasia of the glands are squeezed by the fibers and appear like cracks.

51. The pathological features of breast fibroadenoma are

A. Comconsists of proliferplastic fibrous stroma and glands, which are squeezed by fibers

* * Answer: A * *

Explanation: Breast fibroadenoma is a benign tumor composed of proliferating fibrous stroma and glands. Its pathological feature is characterized by gland hyperplasia and a cleft arrangement squeezed by fibrous tissue. This structure distinguishes distinguished from other lesions such as the invasive features of breast cancer and painful masses.

52. (Type A1 question)

The most common metastatic pathway for pancreatic tail cancer is

A. Translymphatic metastasis to the retroperitoneal lymph node

B. transferred to the lumbar spine

C. Transfer to the liver via the portal vein

D. Direct spread was transferred to the peritoneum

E. After hematogenous transfer to the liver

Reference answer: E Your answer:

[Analysis] The most common metastatic site of pancreatic tail cancer is the liver, and the pancreatic blood flow comes back to the liver through the portal vein. Therefore, this question is selected E.

The most common route of metastasis in pancreatic tail cancer is the hematogenous metastasis to the liver. Therefore, the best answer is E. Hemogenous transfer to the liver. Pancreatic cancer (including cancers of the tail of the pancreas) is usually transmitted through the blood circulation, with the liver being the most common site of distant metastasis.

analyze:

Pancreatic cancer has a high degree of malignancy and is prone to metastasis. The main metastasis routes include lymphatic metastasis, hematogenous metastasis, direct invasion, metastasis along the nerve periphery, etc. Lymphatic metastasis of pancreatic cancer occurs earlier, and its metastasis route is usually through lymphatic vessels, first to the peritoneum and then to the supraclavicular lymph nodes. The most common metastasis in pancreatic tail cancer is lymphatic metastasis to the retroperitoneal lymph node.

Lymphatic metastasis of pancreatic tail cancer is the main metastatic route, while hematogenous metastasis is relatively rare, so option B "blood transfer to lumbar spine" and option E "blood transfer to liver" can be excluded together.

Option C Metastasis to the liver through the portal vein is common in hepatocellular carcinoma, not the metastasis route of pancreatic tail cancer, so it is not chosen.

Advanced pancreatic cancer can directly invade the adjacent lower end of the common bile duct, portal vein, inferior vena cava and superior mesenteric arteriovein, etc., etc. However, advanced pancreatic tail cancer does not often spread directly to the peritoneum, so option D is directly spread to the peritoneum, which does not conform to the characteristics of pancreatic tail cancer metastasis, so it is excluded.

In conclusion, the correct answer is: A. Translymphatic metastasis to the retroperitoneal lymph node.

The most common route of metastasis in pancreatic tail cancer (pancreatic tail cancer) is by blood transfer to the liver (hepatic metastasis via hematogenous spread). So the best answer is:

F. Transfer to the liver via the portal vein

53. (Type A1 question)

The main exudate of lobular pneumonia is

A. cellulose

B. seriflux

C. neutrophile granulocyte

D. leukomonocyte

E. eosinophile granulocyte

Reference answer: C Your answer:

[Analysis] Lobular pneumonia mainly uses bronchioles as the center, lung tissue and neutrophil invasion of lung tissue

In dominant, C vs.

Lobular pneumonia (also known as bronchopneumonia) is usually characterized by exudates in the bronchi and alveoli, which are mainly composed of neutrophils. Therefore, for the question, " What is the main exudate of lobular pneumonia?"", The best answer is:

F. neutrophile granulocyte

analyze:

Lobular pneumonia is mainly caused by suppurative bacteria, with a focal acute suppurative inflammation with the pulmonary lobule as the lesion unit. The lesion begins in the bronchus and spreads to the alveoli surrounding it. Lobular pneumonia is more common in children and the elderly and the infirm. Clinically, fever, cough and expectoration are the main symptoms. Wet rales sound in the lungs on auscultation,

and focal shadows of different sizes are visible on X-ray chest film. Total peripheral blood leukocytes and neutrophil numbers increased. The lesions of lobular pneumonia are characterized by the spread of some purulent inflammatory lesions centered on the bronchioles within the lung tissue. In severe cases, the lesions fuse with each other or even involve the whole lung, forming streptococcus pneumoniae pneumonia. Microscopically, the main exudate in the lesion was the neutrophils.

Option A cellulose was mainly seen in lobar pneumonia, inconsistent with lobular pneumonia, so it was excluded.

Option B serous fluid is the main exudate of serous inflammation, and lobular pneumonia is suppurative inflammation, so it is not selected.

Option D lymphocytes are mostly found in chronic inflammation and viral infection, and are the main exudate of non-lobular pneumonia, which can be excluded.

Option E Eosinophils are mostly found in parasitic infection and IgE-mediated inflammatory response, and the main exudate of non-lobular pneumonia is inconsistent with the idea.

In conclusion, the correct answer is: C. Neutrophils.

Lobular pneumonia (also known as bronchopneumonia) is mainly caused by bacterial infections and is characterized by focal inflammation of the alveoli and peribronchial tissues. Its main exudate is usually neutrophils, due to the fact that neutrophils are one of the most important defense cells of the body in response to bacterial infections.

So the answer is:

G. neutrophile granulocyte

54. (Type A1 question)

Molecules capable of presenting antigenic peptides to human CD8 + T cells are

A. B7

B. CD1

C. HLA-2

D. HLA-1

E. CD4

Refer to the answer: D. Your answer:

[Analysis] HLA, also known as MHC, the main histocompatibility complex, is a protein molecule present on the surface of antigen-presenting cells responsible for antigen presentation. After binding to the antigen, HLA-1 can be presented to the cell surface and delivered to CD8 + T cells, and then activates CD8 + cells, so D vs.

In humans, the antigens recognized and responded by CD8 + T cells are presented by major histocompatibility complex (MHC) class I molecules. HLA-1 (human leukocyte antigen-1) is a class of MHC class I molecules that in this context is responsible for the presentation of antigen peptides to CD8 + T cells. So the best answer is:

F. HLA-1

analyze:

Antigenic peptides are short peptides presented by MHC molecules recognized by T cell receptors. MHC-class I molecules can bind to endogenous antigen peptides presented to CD8 cytotoxic T cells and MHC-class molecules can bind to exogenous antigen peptides presented to CD4 helper T cells. Among them, MHC-class I molecules are also known as HLA-class I molecules in humans, including HLA-A, HLA-B, and HLA-C. Thus, the molecule capable of presenting an antigenic peptide to human CD8 + T cells is HLA-1.

Option A: B7 is a synergistic stimulating molecule that plays an important role in autoimmune monitoring, humoral immune response and transplantation response, but does not participate in the presentation of antigen peptide, so it is not selected.

Option B: CD1 is a transmembrane glycoprotein, similar in structure to MHC-class I molecules, which can present lipid antigen to T cells, but does not present antigen peptide to CD8 + T cells, so it is excluded.

Option C: HLA-2 is not a typing of MHC molecules, is an interference term and can be preferentially excluded.

Option E: CD4 is a marker molecule on the surface of T cells, which can be expressed on the surface of some T cells, thymocytes and some macrophages. It does not participate in the presentation of antigen peptides, which is inconsistent with the idea.

In conclusion, the correct answer is: D. HLA-1.

The correct answer is the D. HLA-1.

Explanation: CD8 + T cells recognize antigenic peptides by binding to major histocompatibility complex class I molecules (MHC I, also known as HLA-I). The HLA-1 molecule is a class MHC I molecule that presents antigen peptides to CD8 + T cells, while HLA-2 (class MHC II) is a molecule that presents antigen peptides to CD4 + T cells. Thus, the molecule capable of presenting an antigenic peptide to human CD8 + T cells is HLA-1.

55. (Type A1 question)

The site of the histamine synthesis

A. clasmatoblast

B. endothelial cells

C. red blood cell

D. white blood cell

E. macrophage

Reference answer: A. Your answer:

Text analysis: [analysis] histamine is mainly synthesized by mast cells, A pair. The main function of the vascular endothelium is the barrier function, while the vascular endothelium has an important endocrine function, which can synthesize and release a variety of endothelium-derived vasoactive factors (B error). The main effects of macrophages are to phagocytose debris, damage cells, dead cells and apoptotic cells, and inhibit the production of proinflammatory cytokines by macrophages, E error.

[fault avoidance]

Histamine is a biogenic amine that is synthesized and stored mainly in vivo by mast cells and alkalophiles. Therefore, the most correct answer to the "synthesis site of histamine" in the question is:

A.clasmatoblast

analyze:

Histamine is widely present in human tissues and has a powerful pro-inflammatory effect. Under trauma, inflammation and other stimuli, histamine decarboxylase in mast cells will be activated, catalyzing the decarboxylation of histamine and producing histamine. So the site of histamine synthesis is in mast cells.

Option B endothelial cells, C red blood cells, D white blood cells, and E macrophages, which do not produce histamine and can be excluded together.

In conclusion, the correct answer is: A. mast cells.

Histamine (Histamine) is an important bioactive amine that plays an important role in allergic reactions and immune reactions. It is mainly synthesized and stored by mast cells and basophils. Here is an analysis of each option:

A. * * Mast cells * *: Mast cells are the main cell type that synthesize and release histamine, especially during allergic reactions and inflammation.

B. Endothelial cells: The main function of endothelial cells is to constitute the inner wall of blood vessels, not the main synthesis site of histamine.

C. Red blood cells: The main function of red blood cells is to transport oxygen and carbon dioxide and do not synthesize histamine.

D. White blood cells: Leukocyte cells include many types of cells, such as neutrophils and lymphocytes. Although they play a role in the immune response, they are not the main synthesis sites of histamine.

E. Macrophages: The main function of macrophages is to swallow up and remove pathogens and cell debris from the body, and it is not the main site of histamine synthesis.

In conclusion, the main site of histamine synthesis is the mast cells. So, the most correct answer is:

A. clasmatoblast

56. (Type A1 questions)

What belongs to the central immune organ is that

A. marrow

B. epityphlon

C. lymph gland

D. spleen

E. tonsil

Reference answer: A. Your answer:

[Analysis] central immune organs: bone marrow and thymus. Peripheral immune organs: spleen, lymph nodes, tonsils, and small intestine collection

Lymph nodes, appendix, etc. Therefore, this question A is right.

[fault avoidance]

Central immune organs are those responsible for generating and / or mature immune cells. According to this definition, bone marrow belongs to the central immune organ because it is the origin of all types of blood cells, including immune cells (e. g., B cells and T cells). So the correct answer is:

A.marrow

This is a choice question about the composition of the immune system, which requires us to accurately distinguish between central immune organs and peripheral immune organs.

First, let's comb through the key information in the topic: the topic requires to identify the options that belong to the central immune organs.

Next, we analyzed each option:

A. Bone marrow: Bone marrow is the place where all kinds of blood cells and immune cells occur and mature, and it is an important central immune organ of the body. It can produce a variety of immune cells and deliver them to peripheral immune organs, thus meeting the requirements.

B. Appenddix: The appendix is a tubular organ rich in lymphoid tissue, but it mainly serves as a peripheral immune organ, which is different from the central immune organ, so it is excluded.

C. Lymph nodes: Lymph nodes are important peripheral immune organs, which filter lymph fluid and participate in immune reactions, but they do not belong to the central immune organs, so they are not selected.

D. Spleen: The spleen is also a peripheral immune organ, whose main function is to filter blood and remove pathogens, which does not meet the definition of a central immune organ, so it is excluded.

E. Tonsils: tonsils also belong to the peripheral immune organ, which is an important lymphoid tissue in the throat, but do not belong to the central immune organ, so it is not selected.

In conclusion, the bone marrow is the central immune organ responsible for the production and export of immune cells. Therefore, the correct answer is A. bone marrow.

Those belonging to the central immune organs are:

A. marrow

explain:

The central immune organ is the organ that produces and develops immune cells, mainly including the bone marrow and thymus. The bone marrow is the main site of blood and immune cell generation, and therefore belongs to the central immune organ. The appendix, lymph nodes, spleen, and tonsils all belong to the peripheral immune organs, and the main function is to perform an immune response.

57. (Type A1 question)

The form of the most common viruses belonging to humans and animals is

A. Ball-shape or ball-like shape

B. Brick

C. linear

D. Tadpole shape

E. Bullet

Reference answer: A. Your answer:

[Analysis] The basic morphology of the virus is ball (adenovirus), rod (tobacco mosaic virus), tadpole shape. There are also bullet shape, filamentous, brick shape and other forms. The most common zoonosis caused by the virus is epidemic encephalitis, which belongs to ball virus, A pair.

The most common co-occurring human and animal viruses are usually spherical or spherical, so the correct answer is A. spherical or spherical. This form of virus includes many respiratory viruses and some enteric viruses that can spread between humans and animals.

analyze:

The morphology of virus particles is diverse, the common morphology is spherical, rod, tadpole, brick, silk, etc. Among them, spherical or spherical is the most common form of virome, and the most common virus such as influenza virus and rabies virus are spherical or spherical.

Option B brick virus, such as poxvirus, mainly infects animals and infects fewer cases in humans, so it is not selected.

Option C line viruses, such as tobacco mosaic virus, infecting only plants, not animals and humans, was excluded.

Option D Tadpole-shaped viruses, such as bacteriophages, mainly infect bacteria and have little to do with human and animal virus infections, so they are not selected.

Option E rhabdovirus, such as rabies virus, measles virus, is spherical or spherical, rather than elastic, so excluded.

In conclusion, the correct answer is: A. spherical or spherical shape.

The forms of the most common viruses shared between humans and animals are:

A. Ball-shape or ball-like shape

This form of virus includes many common viruses transmitted from animals to humans, such as influenza virus, coronavirus and so on. So the answer is:

A. Ball-shape or ball-like shape

58. (Type A1 question)

Are not characteristic of M. avium

A. Cell wall with phosphoic acid

B. opportunistic pathogens

C. Poor effect of drug treatment

D. Fast growth rate

E. Positive staining for acid-fast staining

Reference answer: A. Your answer:

[Analysis] Mycobacterium avium is the most common opportunistic pathogen, and non-tuberculosis mycobacteria is very similar to the two pathogen phenotypes, fast growth rate, and poor drug treatment effect, positive acid resistance staining, so it is difficult to distinguish from tuberculosis in the early stage, BCDE is the characteristics of Mycobacterium avium. The bacterial wall of this bacterium had neither phosphoic acid from gram-positive bacteria nor lipopolysaccharide from gram-negative bacteria. This question is selected A.

The characteristics of Mycobacterium avium (Mycobacterium avium complex, MAC) mainly include:

A. Cell wall has phosphoic acid: This is A common feature of the genus Mycobacterium. The cell wall contains phosphoic acid, which helps bacteria to resist the invasion of drugs such as antibiotics.

B. Opportunistic pathogens: MAC is an opportunistic pathogen that usually causes disease in individuals with compromised immunity.

C. Poor effect of drug treatment: Due to the particularity of cell wall structure,

the effect of drug treatment on MAC is not good, and the treatment process is more complex and long-term.

D. Fast growth rate: This option is incorrect. Indeed, mycobacteria usually grow slower, especially the MAC, which grow and reproduce slower than many other bacteria.

E. acid-resistant positive: Mycobacteria, including MAC, are acid-resistant positive, which means they are able to maintain dye during acid-resistant staining, an important feature in the diagnosis of this type of bacterial infections.

Therefore, the option least characteristic of M. avium is * * D. Rapid growth rate * *.

analyze:

Mycobacterium avium belongs to one of non-tuberculous mycobacteria, which have neither phosphoic acid nor abundant lipids in their cell wall, and thus differs from the virulence and pathogenicity of M. tuberculosis.

Option B Opportunistic pathogens refer to some microorganisms that are usually not pathogenic and only pathogenic under specific conditions. Mycobacterium avium is opportunistic pathogens and can be excluded.

After mycobacterium avium infection, there is no specific and efficient antibacterial drugs, so the treatment effect is poor, so the poor treatment effect of option C is the characteristics of Mycobacterium avium, which is inconsistent with the meaning of the question.

M. avium grows faster than other non-tuberculous mycobacteria, so option D is a characteristic of M. avium and can be excluded.

Option E Positive acid staining is characteristic of mycobacteria, so it is not considered.

In conclusion, the correct answer is: A. Cell wall has phosphoic acid.

Title: Not characteristic of Mycobacterium avium

A. Cell wall with phosphoic acid

B. opportunistic pathogens

C. Poor effect of drug treatment

D. Fast growth rate

E. Positive staining for acid-fast staining

Mycobacterium Mycobacterium avium complex (MAC) is an opportunistic pathogen commonly found in immunocompromised patients, such as AIDS patients. Their cell walls contain large amounts of lipids, stain positive for acid resistance, and have generally poor effects of drug treatment. However, they grow slower, which is different from some other fast-growing mycobacteria. Therefore, option D "grow faster" is incorrect.

The correct answer is: D. Fast growth rate

[fault avoidance]

59. (Type A1 question)

The hormone most commonly secreted by ovarian sex cord stromal cells is

A. progestational hormone

B. estrogen

C. HCG

D. LH

E. FSH

Reference answer: B. Your answer:

Graphic analysis: [analysis] ovarian sex cord interstitial cells most often secrete estrogen. Progestogen is a steroid hormone secreted by the luteal cells of the ovary. HCG is a glycoprotein secreted by the trophoblast cells of the placenta. Luteinizing hormone (LH) is a hormone produced by the pituitary gland. Follicular stimulating hormone (FSH) is a glycoprotein gonadotropin synthesized and secreted by basophils of the human anterior pituitary gland. So this question is selected B.

The most commonly secreted hormone by ovarian sex cord stromal cells is estrogen. Therefore, the correct answer is the B. oestrogen. The ovary consists of multiple cell types, including sex cord stromal cells, which are mainly responsible for

the synthesis and secretion of estrogen, especially estradiol. Estrogen plays an important role in the female reproductive system and in multiple other physiological processes.

analyze:

Ovarian sex cord stromal cells, a type of ovarian tumors, originating from the sex cord and stromal tissue in the primitive gonads, which develop into different cell types in men and women, respectively, and form different tumors. In women, the hormone most commonly secreted by the ovarian strocord cells is estrogen.

Option A progesterone is mainly secreted by luteal body cells, rather than ovarian sex cord stromal cells, so it is not selected.

Option C: HCG, option D: LH, option E: FSH, are secreted by the gland, rather than ovarian cord stromal cells, so they can be excluded together.

To sum up, the correct answer is: B. Oestrogen.

Ovarian sex cord stromal cells mainly include follicular membrane cells and granulosa cells, which play important endocrine functions in the ovary. The most commonly secreted hormone by ovarian sex cord stromal cells is estrogen.

So, the most correct answer is:

F. estrogen

60. (Type A1 question)

FSH in the cells acting on the testis are

A. spermatogonium

B. Sertoli's cells

C. mesenchymal cells

D. androgone

E. spermatocyte

Reference answer: B. Your answer:

[Analysis] After FSH acts on supporting cells and binds to their specific receptor, the supporting cells produce androgen binding protein, which can bind with testosterone and transport it to the seminiferous tubules to maintain the concentration of testosterone in the lumen and promote the growth and development of spermatogenic epithelial cells at all levels. Therefore, this question B is right.

FSH (follicle-stimulating hormone) functions mainly in the supporting cells (also known as Sertoli cells) in the testis, which are responsible for maintaining the appropriate environment for spermatogenesis. So, the most correct answer is:

F. Sertoli's cells

analyze:

Follicular hormone (FSH) is a gonadotropin secreted by the pituitary gland. It mainly acts on the supporting cells of the testis, namely Sertoli cells, to stimulate its synthesis and secretion of androgen binding proteins and promote spermatogenesis and maturation. Thus the cells that FSH acts on the testis are Sertoli cells.

Option A spermatogonia are the original cells of sperm and the stem cells that form sperm. Their development is influenced by gonadotropin-releasing hormone (GnRH). FSH does not directly act on such cells, so it is excluded.

Option C Interstitial cells, also known as Leydig cells, can synthesize and secrete androgens and are affected by luteinizing hormone (LH), rather than FSH, so it is not chosen.

Option D spermatogenic cells include spermatogonia, primary spermatocytes, secondary spermatocytes, spermatocytes and sperm at different stages of spermatogenesis, which is affected by a combination of multiple hormones. FSH does not directly act on such cells, so it is not considered.

Option E spermatocyte is a stage of the development of spermatogenic cells, which is also subject to the joint action of multiple hormones. FSH does not directly act on such cells and can be excluded.

In summary, the correct answer is: B. Support cells.

The main role of FSH (follicle-stimulating hormone) in the male reproductive system is to stimulate the supporting cells (Sertoli cells) in the testis and promote spermatogenesis and testicular function. Thus, the cells in which FSH acts on the

testis are Sertoli cells.

The correct answer is: B. Support cells

61. (Type A1 question)

The peculiar motor form of the small intestine is

A. tonic contraction

B. segmentation movement

C. mass movement

D. segmentation movement

E. wriggle

Reference answer: B. Your answer:

Graphic analysis: [analysis] The movement of the small intestine mainly includes tension contraction, segmented movement and peristalsis. Secmental movement is a unique form of the small intestine (B pair). Section movement is a kind of rhythmic contraction and diastolic movement based on the circular muscle of the intestinal wall, which can repeatedly divide food into many segments. Its significance is: (1) fully mix the chyme and digestive fluid to facilitate chemical digestion; (2) make the chyme in close contact with the intestinal wall and create good conditions for absorption; (3) squeeze the intestinal wall, which is conducive to the reflux of blood and lymphatic fluid.

The characteristic form of movement of the small intestine is the B. segmented movement. Section movement is a mode of movement in the small bowel, whose purpose is to mix and advance food through the contraction and relaxation of the muscles to facilitate digestion and absorption. This movement segments and stirs the intestinal contents, facilitating full contact with the digestive juice. Therefore, the correct answer is the B. segmental motion.

This is a medical problem that examines the form of small bowel movement. First, we need to understand the motor characteristics of the small bowel and select the characteristic motor forms of the small bowel from the options provided.

First, we analyze the forms of motion represented by each option:

A. Tonic contraction: This is a common basic contraction in the smooth muscle

of the digestive tract, not specific to the small intestine.

B. Section movement: this is a unique movement form of the small intestine. It adopts the rhythmic contraction and diastolic movement based on the cyclic muscle, which makes the contents of the small intestine continuously divided, which is conducive to the full mixing of chyme and digestive juice, and promotes the return of blood lymph in the intestinal wall.

C. Group movement: This is a propulsion movement of the large intestine, and it is not unique to the small intestine.

D. Segmental movement: This form of movement does not refer to the small intestine, and is not specific to it.

E. Peristle: This is a common form of movement in the digestive tract, which pushes the contents toward the anus, which is not specific to the small intestine.

In conclusion, the characteristic motor form of the small intestine is segmented motion, which facilitates the full mixing of digesta with digestive juice while promoting blood and lymphatic reflux in the small intestinal wall.

Therefore, the answer is B. segmented motion.

B

62. (Type A1 question)

Cells that have no endocrine function are

A. The pancreatic ductal cells

B. adrenal medullary cells

C. Thyroid cells

D. Parathyroid cells

E. G cells of the gastric mucosa

Reference answer: A. Your answer:

[Analysis] Adrenal medulla cells mainly secrete adrenaline and norepinephrine, and the follicular cells in the thyroid produce two main thyroid hormones, tetraiodothyronine (thyroxine, T4) and triiodothyronine (T3). The main role of the parathyroid gland is the synthesis and secretion of PTH. The gastric mucosal G cells

mainly secrete gastrin.to sum up,

This question is selected A.

Cells that are not endocrine-competent are A. pancreatic ductal cells.

The pancreas consists of two parts: the exocrine part and the endocrine part. Pancreatic ductal cells belong to the exocrine fraction, which function to transport digestive enzymes to the small intestine. These cells do not themselves have an endocrine function, that is, they do not secrete hormones into the blood. Instead, cells in the endocrine component (e. g. islet cells) are responsible for the production and secretion of hormones such as insulin and glucagon.

The cells in the other options have endocrine functions:

-B. Adrenaline and noradrenaline secretion by adrenal medullary cells.

-C. Thyroid hormone secretion by thyroid cells.

-D. Parathyroid hormone secretion by parathyroid cells.

-E. Gastrin secretion by gastric mucosal G cells.

First, we need to clarify what the endocrine function is. Endocrine refers to some cells or tissues in the body can secrete hormones, to regulate various physiological activities of the body through blood. Next, we analyze each option:

A. Pancreatic ductal cells: pancreatic ductal cells are mainly exocrine cells that are responsible for delivering pancreatic fluid to the small intestine to aid digestion. These cells do not have any endocrine functions.

B. Adrenal medullary cells: These cells secrete epinephaline and norepinephrine, two hormones that play a key role in regulating heartbeat, blood pressure and stress responses. Thus, these cells have endocrine functions.

C. Thyroid cells: Thyroid cells are capable of producing thyroid hormones, such as T3 and T4, which have important effects on the body's metabolic rate, protein synthesis, and many other physiological functions. So, these cells also have endocrine functions.

D. Parathyroid cells: Parathyroid cells are able to secrete parathyroid hormone, and this hormone plays an important role in the regulation of blood calcium and blood phosphorus levels. This shows the endocrine function of these cells.

E. G cells of the gastric mucosa: The G cells in the gastric mucosa can secrete gastrin, a hormone that stimulates the secretion of the gastric acid. Thus, these cells also have an endocrine function.

In conclusion, the cells that do not have endocrine function are pancreatic ductal cells because they are mainly exocrine cells responsible for the delivery of pancreatic juice rather than the secretion of hormones. So, the correct answer is A. pancreatic duct cells.

Cells that have no endocrine function are:

A. pancreatic ductal cells

Explanation: The pancreatic ductal cells are mainly responsible for secreted digestive enzymes into the small intestine, rather than hormones, so they are not endocrine. The cells in the other options have endocrine functions: adrenal medulla cells secrete epinephrine and norepinephrine, thyroid cells secrete thyroid hormone, parathyroid cells secrete parathyroid hormone, and gastric mucosal G cells secrete gastrin.

63. (Type A1 question)

Anatomical shunting is most likely seen in

A. obstructive emphysema

B. chronic bronchitis

C. bronchial asthma

D. bronchiectasia

E. pulmonary embolism

Refer to the answer: D. Your answer:

[Analysis] alveolar ventilation dysfunction includes diffusion disorder, alveolar ventilation and blood flow ratio imbalance, anatomical shunt. In physiological conditions, a portion of the venous blood in the lung flows directly into the pulmonary artery, called an anatomical shunt. Under pathological conditions (such as

bronchiectasis, etc.), bronchial vascular dilation and arteriovenous short-circuit opening in the lung increase anatomical shunt and abnormal venous blood doping, leading to respiratory failure. The blood is completely free of gas exchange process, so it is called true shunt. During pulmonary consolidation and atelectasis, the diseased alveoli completely lost their ventilatory function, but

There is still blood flow, and the blood flows through the artery without gas exchange, which is a functional shunt. To sum up, this question is selected

F. .

Anatomical shunt is a process in which blood flows directly from the left side of the heart and bypassing gas exchange in the lungs. This condition usually occurs in the presence of certain structural cardiac or pulmonary abnormalities. Given the disease in the option, pulmonary embolism (E) is most likely to cause anatomical shunting. Pulmonary embolism can cause blood flow to bypass obstructed areas, causing blood that has not exchange gas from the lungs to flow directly into the systemic circulation.

Therefore, the correct answer is the E. pulmonary embolism.

analyze:

Anatomical shunt refers to the direct oxygenation of venous blood in the pulmonary artery into the pulmonary vein, resulting in a reduction of the partial oxygen pressure, which is a special case of ventilation / flow ratio. It is common in pulmonary arteriovenous fistula, that is, the venous blood in the pulmonary artery flows directly into the pulmonary vein without oxygenation, resulting in reduced blood oxygen partial pressure, which is a kind of congenital heart disease. During pulmonary embolism, embolus obstruction of the pulmonary artery and its branches causes an increase in blood flow resistance, forming pulmonary hypertension, increasing the right heart load, and even right heart failure. Part of the blood flows directly into the pulmonary veins without oxygenation, forming anatomical shunt, resulting in hypoxemia.

Option A obstructive emphysema, option B chronic bronchitis, option C bronchial asthma, are persistent airflow limited, with the development of the disease, lung tissue elasticity, alveolar enlargement, residual volume, cause lung ventilation dysfunction, this part of the alveolar flow without gas exchange, formed the functional shunt, does not belong to the anatomical shunt, therefore ruled out.

Option D Bronchiectasis is due to chronic suppurative inflammation and fibrosis of the bronchial tissue and its surrounding lung tissue, which destroys the muscle and elastic tissue of the bronchial wall, resulting in bronchial deformation and lasting dilatation. Typical symptoms include chronic cough, large purulent sputum and repeated hemoptysis, and no anatomical shunt, so it is not selected.

In conclusion, the correct answer is: E. Pulmonary embolism.

Anatomical shunt (anatomic shunt) is the portion of blood flow in the lungs that does not flow through the alveoli. This condition is usually seen in certain specific pulmonary pathological states. Let's analyze the options one by one:

A. * * Obstructive emphysema * *: Obstructive emphysema is mainly caused by the destruction of the alveolar wall and airway obstruction, which results in reduced gas exchange efficiency but not necessarily an anatomical shunt.

B. * * Chronic bronchitis * *: Chronic bronchitis mainly shows inflammation and increased secretions of the airways, and is less likely to cause anatomical shunting.

C. * * Bronchial asthma * *: Bronchial asthma is wheezing and dyspnea due to reversible obstruction of the airways, and again does not involve anatomical shunting.

D. * * Bronchiectasis * *: Bronchiectasis is caused by permanent dilatation and inflammation of the airways and, although causing pulmonary infection and retention of secretions, does not necessarily form anatomical shunting.

E. * * Pulmonary embolism * *: Pulmonary embolism is when the pulmonary artery or its branch is embolized, resulting in a part of the lung tissue to lose its blood supply. Since blood flow is blocked, blood in the blocked area does not participate in gas exchange, which can lead to anatomical shunting.

Therefore, an anatomical shunt is most likely to be seen in * * E. pulmonary embolism * *.

64. (Type A1 question)

The fastest conduction velocity in cardiac electrical activity is

A. sinus node cells

B. atrial muscle cell

C. The cardiac ventricular myocytes

D. Aschoff's node

E. PF

Reference answer: E Your answer:

[Analysis] The action potential generated by Purkinje cell excitation is fast response action potential. Stage 0 depolarization is caused by Na + influx. Due to the high density of INa channel on the cell membrane of Purkinje cell, the depolarization rate in stage 0 is fast, so the conduction rate of this cell is the fastest. Therefore, this question is selected E.

The fastest conduction velocity in the electrical activity of the heart is * * E. Purkinje fiber * *.

Purkinje fibers (Purkinje fibers) are part of the inner heart that transmit electrical pulses to ventricular myocytes, allowing the ventricles to contract quickly and harmoniously. Purkinje fibers conduct very fast, which helps the ventricle to effectively pump blood through the body. Thus, of the options given, Purkinje fibers are the fastest conduction velocity.

First, we need to understand the conduction velocity of the different parts of the electrical activity of the heart. The conduction velocity of electrical activity in the heart depends on the type and characteristics of cardiomycells.

Now let's analyze it item by item:

A. Sinus node cells: This is the pacing point of electrical activity in the heart, but its conduction velocity is not the fastest. Sinus node cells are mainly responsible for the generation of electrical signals in the heart rather than rapid conduction.

B. Atrial muscle cells: Although atrial muscle cells are able to conduct electrical signals, their conduction velocity is not the fastest. Atrial myocytes are mainly responsible for atrial contraction.

C. Ventricular myocytes: ventricular myocytes are responsible for the contraction of the ventricles, although they also have a certain electrical signal conduction capacity, but they are not the fastest.

D. AV node: The AV node plays a key role in the conduction of cardiac electrical signals, but its conduction speed is also not the fastest.

E. Purkinje fibers: Purkinje fibers are the fastest conduction part of the heart

conduction system. These specialized cardiomyocytes are capable of rapidly transmitting electrical signals from the atrium to the ventricle, ensuring a synchronous contraction of the heart.

In conclusion, the fastest conduction velocity in electrical cardiac activity is in Purkinje fibers.

Therefore, the correct answer is the E. Purkinje fiber.

The fastest conduction velocity in cardiac electrical activity is

F. PF

Purkinje fibers are part of the cardiac conduction system, which are located in the ventricle and are responsible for rapidly conducting electrical signals to ensure that the ventricular muscles can contract in unison. Purkinje fibers have significantly higher conduction velocity than other cardiac structures and are thus the fastest conducting part of the heart during electrical activity.

65. (Type A1 questions)

In the heart or blood vessels in vivo, the blood clots or some solid mass formed in the blood is

A. embolus

B. thrombus

C. embolism

D. extravasated blood

E. cruor

Reference answer: B. Your answer:

[Analysis] In the living heart and blood blood coagulation or some blood in the formation of the formation of solid mass process, called thrombosis, the formation of solid mass is called thrombosis. So this question is selected B.

In the heart or blood vessels in vivo, the blood clots or some solid masses formed in the blood are called blood clots. Therefore, the correct answer is the B. thrombus.

- ** A. embolus * *: Usually refers to substances that fall off and migrate through the blood stream, which can be gas, liquid or solid, which can cause embolism in other points in the body.

- ** B. Thrombus * *: A solid mass of blood coagulation inside the vessel, which is usually attached to the vessel wall.

- ** C. Embolization * *: It is the obstruction of blood vessels caused by emboli, which may be thrombus, but also other substances.

- ** D. Silting * *: refers to the blood flow slow or stagnation, resulting in blood accumulation in a certain part of the phenomenon.

- ** E. Coagulation * *: refers to the transition of blood from liquid to coagulation, which is part of hemostasis.

analyze:

Blood coagulation in the heart or blood vessels, or the formation of the blood points stick to each other, the process of forming a solid mass is called thrombosis, the formation of a solid mass is called thrombosis.

Option A embolus refers to the abnormal substance that blocks blood vessels in the circulating blood, which can be solid, liquid or gas, which is inconsistent with the description of the question stem, so it is not selected.

Option C embolization refers to the phenomenon that abnormal substances insoluble in the blood appear in the circulating blood and flow with the blood, thus blocking the lumen of the blood vessel, which is quite different from the description of the problem stem.

Option D congestion refers to that the blood is deposited in the venules and capillaries due to the obstruction of venous blood return, which is obviously different from the description of the trunk.

Option E Coagulation is an important link of physiological hemostasis, a process of blood from a flowing liquid state to a mobile gel state. It is a part of physiological hemostasis, and the problem describes a pathological process, so it is excluded.

To sum up, the correct answer is: B. thrombosis.

In the heart or blood vessels in which blood clots or some solid mass blocks formed in the blood are called thrombosis.

So, the most correct answer is:

F. thrombus

66. (Type A1 questions)

Of the fat-soluble vitamins are

A. vitamin PP

B. vitamin B

C. vitamin C

D. vitamin A

E. Vitamin B12

Refer to the answer: D. Your answer:

[Analysis] Fat-soluble vitamins refer to vitamins soluble in fat and non-polar organic solvents (such as benzene, ether and chloroform, etc.), including vitamin A, vitamin D, vitamin E, vitamin K, etc. Therefore, this question is selected A. Water-soluble vitamins are soluble in water. After entering the body, they will be absorbed by the small intestine and transported to the required parts of the body. The rest will be excreted with the excrement, mainly B vitamins and vitamin C.

Fat-soluble vitamins include vitamins A, D, E, and K. According to this information, the fat-soluble vitamin is option D, vitamin A. So the correct answer is:

F. vitamin A

This is a question about vitamin classification. We need to identify which vitamin is fat-soluble. Vitamins can be divided into two categories according to their solubility: fat-soluble vitamins and water-soluble vitamins.

Next, we analyze each option one by one:

A. Vitamin PP (niacin, nicotinamide): This is a water-soluble vitamin, also known as vitamin B3, which is not fat-soluble.

B. Vitamin B: Most members of the vitamin B group, such as vitamin B1, B2, B6, etc., are water-soluble.

C. Vitamin C: Vitamin C is a typical water-soluble vitamin that has an

antioxidant effect.

D. Vitamin A: Vitamin A is A fat-soluble vitamin that plays an important role in visual function and cell growth regulation in the body.

E. Vitamin B12: Although vitamin B12 contains structurally lipid-soluble parts (i. e., alkyl chains), it is usually classified as a water-soluble vitamin because of its high solubility in aqueous solution.

In conclusion, the fat-soluble vitamins are vitamin A. Therefore, the correct answer is the D. vitamin A.

The following fat-soluble vitamins are:

F. vitamin A

67. (Type A1 questions)

In genetic engineering, the role of the plasmid is often

A. donor

B. The vector of the target gene

C. was used as the target gene

D. receptor

E. Toolase

Reference answer: B. Your answer:

[Analysis] Plasmid is a common small circular DNA in bacteria, which is distinguished from the large circular DNA, among which genes can be expressed. Plasmids are often used in genetic engineering as a vector to carry the target gene, pair B.

In genetic engineering, plasmids most commonly function as carriers of target genes, as they can carry foreign DNA into the host cell. Thus, the most correct answer is the vector of the B. target gene.

First, let's understand the key concepts in the title. This question questions the role of plasmids in genetic engineering. Plasmids are small circular DNA molecules in bacteria, and they are often used as vectors in genetic engineering because they can

readily accept and carry foreign DNA.

Next, we analyze the options one by one:

A. Donor: In genetic engineering, a donor usually refers to the original organism or DNA fragment that provides the target gene, and the plasmid is not a donor, it is a vector used to carry these genes. Therefore, the A option is incorrect.

B. Vectors of the target genes: Plasmids are often used as vectors carrying the target gene in genetic engineering. They can be engineered to contain specific target genes and subsequently imported into recipient cells. This is the major role of plasmids in genetic engineering. Therefore, option B is correct.

C. As a target gene: The plasmid itself does not act as a target gene. They are tools used to carry and transfer the target gene, not the target gene itself. Therefore, the C option is incorrect.

D. Receptors: In genetic engineering, a receptor is usually a cell or organism that receives foreign DNA, rather than a plasmid. Plasmids are vectors that carry genes, not receptors that accept genes. Therefore, the D option is incorrect.

E. Tool enzymes: Tool enzymes are usually enzymes used in genetic engineering to cut, link, or modify DNA, such as restriction enzymes, DNA ligases, etc. The plasmid is not an enzyme, but a vector of the DNA. Therefore, the E option is incorrect.

In conclusion, the main role of plasmids in genetic engineering is to serve as carriers of target genes for carrying and transfer genes into the recipient cells. Therefore, the correct answer is the vector of the B. target gene.

In genetic engineering, the role of the plasmid is often

F. The vector of the target gene

Plasmids are circular DNA molecules commonly used for genetic engineering that can carry foreign genes and introduce them into host cells. As a vector, the plasmid can help the gene replication and expression of the target gene in the host cell, so option B is the correct answer.

68. (Type A1 question)

The gout-associated metabolic pathways are

A. The purine nucleotide metabolism

B. pentose phosphate metabolism

C. amino acid metabolism

D. Pyrimidine nucleotide metabolism

E. Triglyceride metabolism

Reference answer: A. Your answer:

[Analysis] Gout is a kind of recurrent inflammatory disease caused by increased purine bioanabolism, excessive uric acid production or poor excretion of uric acid, and the deposition of uric acid crystals in the joint synovial membrane, bursa, cartilage and other tissues. So I choose A. The pentose phosphate pathway is a mode of oxidative decomposition of glucose. Protein catabolism is first hydrolyzed into amino acids under the catalysis of enzymes, and then each amino acid is catabolized, or transformed into other substances, or participates in the synthesis of new proteins. Thus, amino acid metabolism is the central content of protein catabolism. The catabolism of pyrimidine nucleotides is the removal of phosphate and ribose to produce the pyrimidine base, which is degraded in the liver, and the degradation products are easily soluble in water.

Gout is a disease caused by elevated uric acid levels in the body. whose root cause is abnormal metabolism of purines. Thus, the metabolic pathway associated with gout is purine nucleotide metabolism. So the correct answer is:

A.metabolism of purine nucleotide

analyze:

Gout is caused by single sodium urate deposition crystal correlation joint disease, and purine nucleotide metabolism disorder and (or) uric acid excretion caused by hyperuricemia directly related, refers to acute characteristic arthritis and chronic tophi disease, mainly including acute arthritis, tophi formation, tophi chronic arthritis, urate nephropathy and uric acid urinary calculi, severe can appear joint disability and renal insufficiency. Gout is often accompanied by abdominal obesity, hyperlipidemia, hypertension, type 2 diabetes mellitus and cardiovascular disease.

Option B pentose phosphate metabolism, option C amino acid metabolism,

option D pyrimidine nucleotide metabolism, and option E triglyceride metabolism were not significantly associated with the occurrence of gout and can be excluded together.

In conclusion, the correct answer is: A. purine nucleotide metabolism.

Gout is a disease caused by the deposition of urate crystals in the joints and tissues. Uric acid is an end product of purine metabolism, and therefore the relevant metabolic pathway for gout is purine nucleotide metabolism.

The correct answer is:

A. The purine nucleotide metabolism

69. (Type A1 question)

The following are the essential amino acids for the human body

A. glycocoll

B. Phe

C. arginine

D. Ser

E. proline

Reference answer: B. Your answer:

[Analysis] There are 9 kinds of essential amino acids: lysine, tryptophan, phenylalanine, methionine, threonine, isoleucine, leucine, valine, histidine. So this question is selected B.

In the human body essential amino acids, the body can not be synthesized, must be taken through the diet. By this definition, the essential amino acids in the option are:

F. Phe

Therefore, the correct answer is the B. phenylalanine. Other amino acids, such as glycine, arginine, serine and proline, can be synthesized by the body, so they are not

classified as essential amino acids.

This is a biochemical problem, and we need to identify which one is the essential amino acid from a given option.

First, we need to clarify what is an essential amino acid. Essential amino acids refer to the amino acids that cannot be synthesized by themselves or cannot meet the needs of the human body and must be taken from food.

Now we will analyze each option:

A. Glycine: Glycine is A non-essential amino acid that can be synthesized by the human body by itself, so it does not meet the requirements of the question.

B. Phenylalanine: Phenylalanine is one of the essential amino acids that cannot be synthesized by the human body and must be obtained from food. This meets the title requirements.

C. Arginine: Although arginine is considered a semi-essential amino acid in some cases, it is often considered nonessential in adults because the body is able to synthesize sufficient amounts.

D. Serine: Serine is a non-essential amino acid that can be synthesized by the human body.

E. Proline: Proline is also a non-essential amino acid that can be synthesized by the human body.

In conclusion, only option B. phenylalanine is an essential amino acid that the human body cannot synthesize by itself and must be obtained from food.

Therefore, the correct answer is the B. phenylalanine.

The following essential amino acids for the human body are:

F. Phe

70. (Type A1 questions)

The molecules that increase their concentrations in the blood during ketoacidosis are

A. glycerin trilaurate

B. lactic acid

C. acetylformic acid

D. purine trione

E. acetacetic acid

Reference answer: E Your answer:

[Analysis] Under various causes of diabetes patients, due to severe insulin deficiency, hormones such as glucagon, catecholamine, growth hormone, and adrenal corticoid hormone have more significant effects on metabolism, which accelerates the decomposition of fat. Fatty acids in the liver by β -oxidation of a large number of ketone bodies (including acetylacetic acid, β -hydroxybutyrate and ketone), when the ketone body production is greater than tissue utilization and renal excretion, can make blood and urine ketone body rise, called ketosis. Acetacetic acid and β -hydroxybutyric acid consume a large amount of alkali reserves in the body, which can easily lead to metabolic acidosis, so it is called ketoacidosis.to sum up,

This question is selected E.

The molecule with increased concentrations in the blood during ketoacidosis is E. acetoacetate. Ketoacidosis is a pathological state in which ketone bodies (mainly acetoacetate and β -hydroxybutyrate) accumulate in the blood due to accelerated glycogen depletion in the body. Thus, the concentration of acetoacetate would increase in this case.

analyze:

Ketone bodies mainly include acetoacetate, β -hydroxybutyrate, and acetone. Ketoacidosis timing insulin absolute or relative insufficiency, liver glycogen increased, glycolysis, lead to fatty acid β -oxidation, thus generate a large number of acetyl CoA, acetyl CoA accumulation promoted the generation of ketone body, ketone body lead to syndrome and ketonuria, at the same time due to the extrahepatic tissue using ketone body, increased acetyl acetate concentration in the blood, condition further deterioration, then ketoacidosis.

Option A triglycerides generally do not change significantly during ketoacidosis, so they are not chosen.

Option B Lactate during hypoxia or strenuous exercise, due to enhanced anaerobic glycolysis, which is unrelated to ketoacidosis, was excluded.

Option C pyruvate is an intermediate of glycolysis, and under normal circumstances pyruvate can be converted into the TCA cycle during ketoacidosis, so it

is not considered.

Option D Uric acid is the end product of purine metabolism. When uric acid production in the body is excessive and (or) excreted too little, it can lead to hyperuricemia, which is unrelated to ketoacidosis, so it is not selected.

In conclusion, the correct answer is: E. Acetyloacetic acid.

In Diabetic Ketoacidosis (DKA), the body is unable to use glucose as a main source of energy and instead generates energy through fat decomposition. Fatty acids produced by lipolysis are converted to ketone bodies in the liver, which include acetoacetate, β -hydroxybutyrate, and acetone. The generation and accumulation of these ketone bodies leads to elevated ketone body concentrations in the blood, which causes ketoacidosis.

Therefore, the molecule with increased concentration in the blood during ketoacidosis is the ketone body, which includes acetylacetic acid.

The answer is: E. Acetacetic acid

71. (Type A1 questions)

The respiratory chain inhibitor rotenone can inhibit electron transport, which is inhibited by

A. complexus I

B. complexus II

C. complexus III

D. complexus IV

E. complexus V

Reference answer: A. Your answer:

[Analysis] Rotenone is an inhibitor of mitochondrial electron transport chain complex I, which induced the apoptosis of cells by promoting the production of mitochondrial reactive oxygen species. So A to.

Rotenone is a known inhibitor of the respiratory chain that acts by inhibiting

complex I (NADH dehydrogenase) in the electron transport chain within the mitochondria. Thus, it blocks the process of electron delivery from NADH to coenzyme Q, thereby inhibiting the oxidative phosphorylation of the mitochondria and ATP production.

So, the correct answer is * * A. complex I * *.

analyze:

The respiratory chain, also known as the oxidative respiratory chain or electron transport chain, is a combination of hydrogen delivery reactions and electron delivery reactions located on the inner mitochondrial membrane arranged in a certain order to deliver pairs of hydrogen atoms from metabolites to oxygen, creating water and ATP. The key enzymes or molecules involved in this process are called the complex, where complex I, the NADH-Q reductase, functions to deliver electrons from NADH to ubiquinone while pumping protons from the mitochondrial matrix into the intermembrane space. Rotenone is a respiratory chain inhibitor, and the main object of its inhibitory effect is complex I.

In conclusion, the correct answer is: A. Complex I.

Option B complex is succinate-Q reductase, which functions to deliver electrons from succinate to ubiquinone while pumping protons from the mitochondrial matrix into the intermembrane space. Its specific inhibitors are rust rust spirit, powder rust ning, malonate acid and other substances, rotenone can not inhibit its function, so it is not selected.

Option C complex, or Q-cytochrome c reductase, is a large protein complex on the inner mitochondrial membrane that transmits electrons from the prototype ubiquinone to cytochrome c. Its specific inhibitor was antimycin A and other substances, and rotenone did not inhibit it, so it was excluded.

Option D complex, namely cytochrome c oxidase, is the terminal complex of electron transport in the respiratory chain of the mitochondrial membrane. It is the final link of electron transport in the oxidative respiratory chain, which can transmit electrons from cytochrome c to oxygen molecules. Its specific inhibitors are cyanide, hydrogen sulfide, sodium azide and other substances, and rotenone is not its inhibitor, so it is not considered.

Option E complex V, namely ATP synthase, is an enzyme complex on the mitochondrial inner membrane. It can use the proton electrochemical gradient to

catalyze the phosphorylation of ADP to generate ATP. Its inhibitor is oligomycin and other substances, and rotenone has no inhibitory effect on it, which can be excluded.

The respiratory chain inhibitor rotenone (rotenone) is a commonly used respiratory chain inhibitor that suppresses complex I (NADH dehydrogenase) in the electron transport chain. By inhibiting complex I, rotenone prevents the electrons generated by NADH from entering the respiratory chain, thereby inhibiting ATP production.

So the correct answer is:

A. complexus I

72. (Type A1 question)

The nucleic acid molecules that store and transmit the genetic information are

A. DNA

B. mRNA

C. tRNA

D. rRNA

E. hnRNA

Reference answer: A. Your answer:

[Analysis] DNA is the main material basis for the storage, replication and transmission of genetic information, so A is chosen. RNA also plays an important role in the process of protein synthesis, in which transfer ribonucleic acid, or tRNA, plays the role of carrying and transferring activated amino acids; messenger ribonucleic acid, or mRNA, is the template for protein synthesis; and ribonucleic acid, or rRNA, is the main place of cell protein synthesis. Nuclear heterogeneous RNA, namely hnRNA, is a precursor of mRNA and becomes mRNA.

The nucleic acid molecules that store and transmit genetic information is DNA. Thus, the most correct answer is the A. DNA.

DNA (deoxyribonucleic acid) is responsible for storing genetic information in the organism and transmitting it to future generations as cells divide. Other options

such as mRNA (messenger ribonucleic acid), tRNA (transport ribonucleic acid), rRNA (ribosomal nucleic acid) and hnRNA (heteronuclear ribonucleic acid) are all related to the processing and translation of genetic information, but are not the main genetic information storage media.

analyze:

Nucleic acids include DNA and RNA, and DNA is deoxyribonucleoic acid, and RNA is ribonucleoic acid. DNA is the carrier of genetic information, which is the main genetic material, a large molecular polymer composed of deoxynucleotides. In the DNA molecular structure, two single strands of deoxynucleotides are coiled around a common central axis, forming a double helical structure. With the function of storing and transmitting genetic information, the nucleic acid molecules that store and transmit genetic information are DNA.

Option B: mRNA is the messenger RNA, which is a direct template to guide protein biosynthesis and does not store genetic information, so it is not selected.

Option C: tRNA is transportRNA, its main function is to carry amino acids into the ribosome and synthesize proteins under the guidance of mRNA, It does not transmit genetic information, so it is excluded.

Option D: rRNA is ribosomal RNA, whose main function is to form a ribosome with ribosome ribosomal proteins. As a site for protein synthesis, it is not consistent with the problem stem.

Option E: hnRNA, or heterogeneous nuclear RNA, is the precursor of mRNA. The direct product of transcription formation in eukaryotes needs to be processed and modified to become mRNA. It does not store genetic information and can be excluded.

In summary, the correct answer is: A.DNA.

The nucleic acid molecules that store and transmit the genetic information are

A. DNA

73. (Type A1 question)

Is not characteristic of protein denaturation

A. Increased viscosity

B. With decreased solubility

C. Enhanced crystallization capacity

D. is more susceptible to hydrolysis by the enzyme

E. Reduced biological activity

Reference answer: C Your answer:

[Analysis] Protein denaturation refers to the destruction of certain physical and chemical factors, leading to the change of its physicochemical properties and biological properties, such as reduced solubility, increased viscosity, loss of crystallization ability, loss of biological activity, easy to be proteolysis by proteases. Denaturation of proteins mainly causes disulfide bond and non-covalent bond destruction, without any change in molecular weight.

In this problem, we need to determine which term is not a characteristic of protein denaturation. Protein denaturation usually means that the 3 D structure of a protein is changed, but its primary structure, namely the amino acid sequence, remains unchanged. Such structural changes usually result in changes in certain physical and chemical properties of the protein, such as reduced solubility, reduced biological activity, and increased sensitivity to thermal or other factors. Based on this information, we can analyze the various options:

A. Increased viscosity – proteins after denaturation may cause increased viscosity due to structural changes.

B. Decreased solubility – this is a common result of protein denaturation, as changes in its three-dimensional structure may affect its solubility in water.

C. enhanced crystallization ability-Usually, the crystallization ability of a denatured protein is reduced because its regular structure is destroyed.

D. More easily hydrolysis by enzymes-denatured proteins may be more accessible or more difficult to hydrolysis by enzymes, depending on how their structural changes affect the site of action of the enzyme.

E. Reduced biological activity-which is often a direct consequence of protein denaturation, since its activity often depends on its specific three-dimensional structure.

Therefore, not part of the protein denaturation feature is * * C. crystallization

ability enhancement * *, because denaturation usually impairs the crystallization ability of the protein, rather than enhancing it.

analyze:

Protein denaturation refers to the destruction of the spatial structure of the protein under some physical and chemical factors, resulting in altered physicochemical properties and loss of biological activity. After protein denaturation, its physicochemical properties and biological properties are changed, such as the solubility decreases, the viscosity increases, the crystallization ability disappears, the biological activity disappears, and it is easy to be hydrolyzed by proteases. Therefore, the crystallization ability will not increase, but will disappear.

Option A viscosity increase is due to the protein denaturation of the protein, the hydration layer inside the protein molecule is destroyed, the polypeptide chain extension is loose, more easy to form a network structure, so that the solution viscosity increases, in line with the characteristics of protein denaturation.

Option B reduced solubility is due to the exposure of hydrophobic groups originally buried within the molecule of the protein after denaturation, which reduces the protein solubility, consistent with the characteristics of protein denaturation.

Option D is more susceptible to enzymatic hydrolysis is due to the protein denaturation, its specific spatial structure is destroyed, peptide bonds are more exposed and thus more susceptible to enzymatic hydrolysis, consistent with the characteristics of protein denaturation.

Option E decreased bioactivity is due to the disrupted protein denaturation of its specific spatial structure and the disappearance of biological activity, consistent with the characteristics of protein denaturation.

To sum up, the correct answer is: C. Enhance the crystallization ability.

74. does not belong to the characteristics of protein denaturation is

A. Increased viscosity

B. With decreased solubility

C. Enhanced crystallization capacity

D. is more susceptible to hydrolysis by the enzyme

E. Reduced biological activity

Protein denaturation means a protein molecule undergoing structural changes for loss of its native conformation and function. Degeneration usually leads to the following characteristics:

-Decreased solubility (B): The denatured proteins usually aggregate, resulting in a decrease in solubility.

-More likely to be hydrolysis by enzymes (D): Denaturation makes the protein structure loose and easier to be decomposed by proteases.

-Decreased bioactivity (E): proteins usually lose their biological function due to structural changes.

-Increased viscosity (A): The denatured protein molecules may form larger aggregates, leading to increased viscosity in the solution.

However, the enhanced crystallization capacity (C) is not a typical feature of protein denaturation. Denaturation usually causes the protein to lose its ordered structure and does not easily crystallize. Therefore, the answer is: C. increased crystallization ability

75. (Type A1 question)

Wet gangrene often occurs in the region

A. Brain, spleen, and liver

B. Brain, intestine, and uterus

C. Lung, intestine, and uterus

D. Lung, intestine, and liver

E. Lung, intestine, and uterus

Reference answer: E Your answer:

Graphic analysis: [Analysis] Wet gangrene is a kind of gangrene, which mostly occurs in the internal organs (intestines, uterus, lung, etc.) connected with the outside world, and also can be seen in the limbs (accompanied by congestion and edema). Common wet gangrene has gangrene appendicitis, intestinal gangrene, lung gangrene and postpartum gangrene endometritis. Therefore, this question is selected E.

Wet gangrene (wet gangrene) is the accumulation of fluid in necrotic tissue due

to bacterial infection. This type of gangrene usually occurs at the end of the organs or limb in the body, especially when blood flow is blocked. Of the given options, we should consider those organs that are vulnerable to poor blood flow and that are prone to bacterial infection.

Option A (brain, spleen, liver), B (brain, intestine, uterus), and D (lung, intestine, liver) contain some organs that are susceptible to infection, but wet gangrene tends to occur in areas with significant infection and poor blood flow, such as the intestine. Therefore, we can exclude the option of include brain and spleen, because these organs are not the most common sites of wet gangrene.

Option C (lung, intestine, uterus) and E (lung, intestine, uterus) look similar, but option E is actually repetitive and may be an input error. Given that wet gangrene is common at the end of the limbs as well as in the internal organs, especially those prone to blood flow disorders and bacterial infections, the intestine is a common site. However, there is no choice pointing directly to the limbs in these options, and we can only choose from the organs given.

Therefore, the most appropriate option seems to be D (lung, intestine, liver), as these sites are more likely to the brain and spleen, especially considering the blood flow problems and infection tendency of the intestine. However, this type of gangrene is usually more related to the ends of the limbs, but based on the option given by the title, D appears to be the closest answer.

There is a problem with the topic!

analyze:

Wet gangrene is a kind of gangrene, refers to tissue necrosis because of more water, spoilage bacteria infection is serious, local obvious swelling, dark green or dirty black. Because spoilage bacteria decompose protein, produce odor, fecal odor, etc., resulting in foul odor. Wet gangrene often occurs in the lung, intestine, uterus, appendix, gallbladder and other organs, and can also occur in the limbs with blocked arteries but unimpeded venous return.

Option A "brain, spleen, liver", option B "brain, intestine, uterus", option D "lung, intestine, liver", all have brain in them. After brain tissue necrosis, it generally belongs to coagulation necrosis, rather than wet gangrene, so it is excluded together.

Option D The liver in lung, intestine and liver belongs to parenchymal organs, and the organs after necrosis are mostly coagulation necrosis, rather than wet

gangrene, so it is not selected.

To sum up, the correct answer is: E. Lung, intestine, and uterus.

Wet gangrene (Wet gangrene) is usually caused by bacterial infections. It is most common in organs and tissues with high water content, especially those that are susceptible to infection. Common sites of wet gangrene include the intestine, lung, and uterus.

Therefore, according to existing medical knowledge and best practice guidelines, wet gangrene often occurs in the lungs, intestine, and uterus.

The correct answer is: C. The lungs, the intestines, and the uterus

76. (Type A2 questions)

Male, 47 years old. She had a history of chronic hepatitis B for more than 10 years, and had dull pain and discomfort in the right upper abdomen 1 month ago. Physical examination: right abdominal bulging, palpable hard, uneven surface of the mass, mobile turbidity positive, abdominal effusion.yes

A. hepatic echinococcosis

B. primary carcinoma of liver

C. hepatic cyst

D. hepatapostema

E. hepatic hemangioma

Reference answer: B. Your answer:

Graphic analysis: [analysis] hepatitis, liver cirrhosis, liver cancer trilogy. The old blood ascites and uneven surface of the mass suggest malignant tumor.

This case describes a 47-year-old man with a history of chronic hepatitis B with dull pain in the right upper abdomen and a firm, superficial mass in the right abdomen with positive mobile turbidity and hemorrhagic abdominal fluid accumulation. Based on these symptoms, we can identify alternative answers one by one:

A. Liver hydatidosis: Although it can cause a liver mass, it is generally

accompanied by a specific epidemiological history, and the mass usually does not cause bloody ascites.

B. Primary liver cancer (hepatocellular carcinoma): it is relatively common in patients with chronic hepatitis B, which can cause a hard mass, uneven surface, and may be accompanied by bloody ascites, in line with the case description.

C. Liver cyst: usually a benign lesion with a smooth surface of the mass that is unlikely to cause bloody ascites.

D. Liver abscess: Although it can cause abdominal pain and lumps, the lump is usually soft, and the symptoms will have obvious acute inflammation, such as fever, chills, etc.

E. Hepatic hemangioma: usually an asymptomatic benign tumor that rarely causes bloody ascites or abdominal pain.

Therefore, based on the case description and related symptoms, the most appropriate answer is B. primary liver cancer.

This is a problem involving the diagnosis of the disease that requires identifying the most likely disease based on the patient's history, symptoms, and signs.

First, let's review the key information given in the topic:

The patient was a middle-aged male with a history of chronic hepatitis B for more than 10 years.

1 month ago, he developed dull pain in the right upper abdomen.

Physical examination revealed a right abdominal, palpable and palpable lump.

The abdominal effusion was bloody and positive, suggesting significant ascites.

Next, we analyzed the corresponding diseases and their characteristics:

A. Hepatic hydatidosis: This is a parasitic disease presenting mainly as an abdominal mass but usually without ascites and a history inconsistent with the patient's description.

B. Primary liver cancer: closely related to the history of hepatitis B, often manifested as pain in the liver area, large liver, hard texture, and possible ascites. The patient's medical history, symptoms, and signs correspond with this.

C. Liver cyst: usually asymptomatic and is unlikely to cause ascites and pain unless the cyst is very large.

D. Liver abscess: the main manifestations are chills, high fever, liver pain and large liver, but not bloody ascites and hard mass.

E. Hepatic hemangioma: This is a benign tumor that is usually asymptomatic, and is unlikely to cause pain and ascites unless the hemangioma is very large or ruptured.

In conclusion, combining a history of hepatitis B, dull pain in the right upper abdomen, abdominal bulge, stiff and bumpy mass, and bloody ascites, the most likely diagnosis was primary liver cancer.

The correct answer is B. primary liver cancer.

According to the title description, the patient was a 47-year-old patient with a history of chronic hepatitis B for more than 10 years, and developed dull pain and discomfort in the right upper abdomen 1 month ago. Physical examination found that the right abdominal bulging, palpable hard texture, uneven surface mass, mobile turbidity positive, and bloody abdominal effusion. These symptoms and signs are highly suggestive of primary liver cancer.

Patients with chronic hepatitis B are at high risk of primary liver cancer, and the mass characteristics and bloody abdominal effusion described in the title are also common manifestations of primary liver cancer.

Therefore, the most likely diagnosis is: B. primary liver cancer

77. (Type A2 questions)

A male, 52 years old, had type 2 diabetes mellitus with BMI of 32 kg/m2 and waist circumference of 90cm. The following exercises were suitable for the man

A. weight lifting

B. swim

C. rope skipping

D. tug-of-war

E. long-distance race

Reference answer: B. Your answer:

[Analysis] In middle-aged and elderly male patients, with BMI value of 32 kg/m2, obesity, which is most suitable for low intensity exercise with low joint

pressure, such as swimming (B pair).

[To avoid mistakes] Weight lifting, tug-of-war and other antagonistic sports are more violent, not suitable for this patient. And rope skipping, long-distance running on the lower limb large joints

The pressure caused is greater, so it is not suitable.

Considering that the 52-year-old type 2 diabetic's BMI of 32 kg/m2 indicates that he is obese, the appropriate exercise regimen should be low to moderate intensity, low impact, helping to improve glycemic control and weight loss. Based on this information, the most appropriate option is:

B swim

Swimming is a whole body exercise with little impact on joints, suitable for people with obesity and type 2 diabetes, and can help improve cardiopulmonary function, control blood sugar and lose weight. Compared with the other options, swimming is a low-impact exercise, suitable for patients with higher BMI, and it is effective in controlling blood sugar levels and reducing body weight, so it is the best option.

analyze:

A middle-aged male patient, diagnosed with type 2 diabetes mellitus, body mass index (BMI) indicating obesity and large waist circumference. Regular exercise in diabetic patients can reduce blood sugar and blood lipid, increase energy consumption, reduce insulin resistance, reduce the risk of diabetic cardiovascular lesions and microvascular complications, and improve the overall health level. When choosing exercise mode, moderate and low intensity aerobic exercise, such as swimming, is particularly suitable for patients who cannot carry weight or walk due to complications.

Option A weight lifting, option C rope skipping, option D tug-of-war, option E long-distance running, are all high-intensity exercise, which is not suitable for the patient. Tug-of-war also requires teamwork and is not suitable for patients alone.

To sum up, the correct answer is: B. Swimming.

For a 52-year-old man with type 2 diabetes and a BMI of 32 kg/m^2, the proper exercise style should be safe, sustainable and can help control blood glucose levels

and body weight. The following is an analysis of each movement mode:

- ** Weightlifting * *: Although lifting can increase muscle mass and strength, there may be too much risk for diabetics, especially those who start exercise, especially cardiovascular burden.

- ** Swimming * *: Swimming is a low impact, efficient whole body exercise, conducive to heart and lung health, reduce weight, and less pressure on the joints. For patients with diabetes, it is a very suitable way of exercise.

- ** Skipping * *: Skipping is a kind of high-intensity aerobic exercise. Although it is conducive to cardiopulmonary function, it has a large impact on joints and is not easy to adhere to for a long time, which may not be suitable for 52-year-old and large weight diabepatients.

- ** Tug-of-war * *: Tug-of-war is a high-intensity short-term exercise, and participants need explosive force and large physical strength, which is not recommended for diabetic patients.

- ** Long-distance running * *: Long-distance running can effectively improve the cardiopulmonary function and metabolic rate, but for patients with large weight of diabetes, long running may cause great pressure on the knee joint and other joints, easy to cause damage.

To sum up, the sports mode suitable for this man is * * B. swimming * *.

78. (Type A2 questions)

Female, 32 years old, afraid of cold, drowsiness for more than two months. Physical examination: pulse 56 times / minute, eyelid edema, diffuse enlarged thyroid, tough texture. The most likely manifestation of thyroid function is

A. Normal TT 3, normal TT4, and reduced TSH

B. TT3 decreased, TT4 decreased, and TSH increased

C. Increased TT 3, increased TT4, and decreased TSH

D. TT3, TT4, and TSH

E. Normal TT 3, normal TT4, and increased TSH

Reference answer: B. Your answer:

[Analysis] middle-aged female patients, afraid of cold, drowsiness, eyelid edema, diffuse thyroid enlargement, all belong to the typical manifestations of hypothyroidism, diagnosed as hypothyroidism. An increased serum TSH and decreased both TT4 and FT4. Serum TT3 and FT3 were normal in early stage and decreased in late stage. So this question is selected B.

[Wrong avoidance] Primary hypothyroidism due to the thyroid gland itself lesions, causing the reduction of thyroid secretion of thyroid hormone, for the lower

Negative feedback inhibition was diminished in thalamus and pituitary gland, and TSH secretion was increased. TSH changed more than thyroid hormone (TT4, FT4)

sensitive.

This 32-year-old female presented with symptoms of cold, drowsiness, reduced pulse (56 beats / minute), eyelid edema and diffuse thyroid enlargement, which are typical of hypothyroid function (hypothyroidism). At low thyroid function, the levels of thyroid hormone (TT3 and TT4) decrease, while the levels of thyroid stimulating hormone (TSH) increase because the hypothalamic-pituitary-thyroid axis attempts to stimulate the thyroid to produce more thyroid hormone by increasing TSH.

Based on this information, the most likely manifestations of thyroid function are:

The-TT3 is decreased

The-TT4 was decreased

-TSH increase

Thus, the most correct answer is a decrease in B.TT3, a decrease in TT4, and an increase in TSH.

analyze:

The patient was a young woman, afraid of cold, drowsiness for more than two months, with bradycardia, eyelid edema, diffuse thyroid enlargement and tough texture, and considered as hypothyroidism. In hypothyroidism, thyroid hormone secretion is insufficient, and the hypothalamus and pituitary gland will increase the secretion of TSH to promote the secretion of thyroid hormone, so the serum TSH level increases. At the same time, the production of thyroid hormones including TT_3 and TT_4 is reduced, so in hypothyroidism, the most likely thyroid function is

decreased TT3, decreased TT4, and increased TSH.

In hypothyroidism, serum TT_3 and TT_4 levels decreased and TSH levels increased. Therefore, option A "normal TT3, normal TT4, decreased TSH", option C "increased TT3, increased TT4, decreased TSH", option D "increased TT3, increased TT4, increased TSH", option E "normal TT3, normal TT4, increased TSH", all of which are inconsistent with thyroid function during hypothyroidism and can be excluded together.

In conclusion, the correct answer is: B.TT3 decreases, TT4 decreases, and TSH increases.

The 32-year-old female patient presented with cold, drowsiness, and physical examination revealed a slow pulse (56 beats / minute), eyelid edema, and diffuse thyroid enlargement. These symptoms and signs suggest that she may have hypothyroidism (hypothyroidism).

When hypothyroidism, the secretion of thyroid hormone is reduced, leading to the body metabolism, manifested as fear of cold, drowsiness, slow pulse and other symptoms. As the thyroid hormone (TT3 and TT4) levels decrease, the negative feedback effect decreases, and thyroid stimulating hormone (TSH) secretion increases to stimulate the thyroid to produce more hormones.

Thus, the most likely manifestations of thyroid function are:

F. TT3 decreases, TT4 decreases, and TSH increases

79. (Type A2 question)

Female, 30 years old, 34 weeks of pregnancy, sudden persistent abdominal pain with the genital bleeding for 2 hours, body examination: body temperature 36.2℃, P120 times / points, R: 26 times / points, blood pressure: 80 / 60 mmHg, consciousness, limbs, eyeball and limbs multiple stasis check: Hb70g/L,RBC:2.7×1012/L,Plt:85×109/L,Fg:1.48g/L, D-dimer: 10.8mg/L, peripheral blood with split cells, B ultrasound display between pelvic cavity and endometrium has unclear low density echo shadow, the effect is not correct

A. Microthrombus causes reduced reduced effort

B. Increased kallikdyase leads in reduced peripheral resistance

C. Complement activation leads to reduced peripheral resistance

D. Schizyytosis leading to coronary artery constriction

E. Less preload causes decreased cardiac discharge

Refer to the answer: D. Your answer:

[Analysis] The patient was 34 weeks pregnant, with persistent abdominal pain with genital bleeding for 2 hours, hypovolemic shock with circulatory disorder, and diffuse intravascular hemolysis (DIC) was considered for diagnosis. There are cracks in the center, the central pale area is flat, quite like the open mouth or fish mouth, visible in DIC, but does not lead to coronary artery contraction, coronary artery contraction is mainly caused by microthrombosis, this question is the incorrect effect on blood pressure, so choose D.

[Error avoidance] common microthrombosis in DIC results in reduced return effort (option A), which leads to reduced preload leading to cardiac output

Amount reduction (option E). DIC can directly or indirectly activate the kinin system and complement system, so that kinin and complement generation increase

Many, kinin can relax the microartery and capillary anterior sphincter, increase the capillary permeability, and reduce the peripheral resistance (optional

Item B). Complement degranulates mast cells and, by releasing histamine, exerts a similar role as kinin to reducing peripheral resistance

Less (option C).

The topic is about a pregnant woman's emergency situation during pregnancy, examining the understanding of the relevant clinical manifestations and the physiological mechanisms behind it. Based on the stem information, the woman developed severe symptoms, including persistent abdominal pain, pudendal bleeding, significant anemia (Hb 70g / L), thrombocytopenia, coagulopathy (low fibrinogen and high D-dimer), and the presence of cleft cells, suggestive of possible severe DIC (disseminated intravascular coagulation) and thrombotic microangiopathy.

The option is as correct:

A. Microthrombosis causes reduced blood volume: may be true because DIC and microvascular thrombosis reduce effective circulating blood volume.

B. Increased kallikdyase causes reduced peripheral resistance: probably true

because activation of the kinin system can cause vasodilation, thereby reducing peripheral resistance.

C. Complement activation leads to reduced peripheral resistance: Perhaps correctly, complement activation can cause vasodilation in an inflammatory response.

D. Histocytosis leading to coronary constriction: This option seems unreasonable, because the presence of cleft cells is mainly a sign of mechanical destruction of red blood cells and microangiopathy, and does not directly lead to coronary constriction.

E. Reduced preload leads to reduced cardiac discharge bleeding: probably true, because anemia and reduced blood volume can reduce the preload of the heart, thus reducing cardiac output.

Thus, option D "schistocytosis causes coronary constriction" is improperly described, which is inconsistent with the appearance of cleft cells and the associated pathophysiological mechanisms. So the most correct answer should be a D.

analyze:

Patients with young women, pregnancy sudden abdominal pain with vaginal bleeding, physical examination shock blood pressure, limbs wet cold, eyeball and limbs multiple ecticymosis, prompt may appear DIC, laboratory examination see low hemoglobin, suggesting anemia, low platelet, low fibrinogen, D-dimer is high, all conform to the laboratory diagnosis of DIC, and peripheral blood split cells, B ultrasound showed after amniotic hematoma, therefore can be diagnosed as placental abruption with DIC.

Option A Microthrombosis leads to the reduction of blood effort is due to the formation of A large number of microthrombosis by DIC, leading to systemic microcirculation disorders, tissue ischemia and hypoxia, thus reducing the reduction of blood effort, in line with the impact of DIC on the body.

Option B increased peripheral resistance is reduced in the process of DIC, kinin release enzyme is activated, into kinin release enzyme, the latter catalytic bradykinin into bradykinin and lysintokinin, can make fine artery dilation, increased vascular permeability, bronchial smooth muscle contraction, and can cause pain, so can reduce peripheral resistance.

Option C complement activation is due to the activation of the complement system during DIC, producing anaphylaxins C3a and C5a, which can release histamine from mast cells or basophils, cause vascular dilation, increase vascular

permeability, and reduce peripheral resistance.

Option D chistocytosis leads to coronary artery contraction, because coronary artery contraction is mainly related to the excitation of the sympathetic-adrenal medulla system, but no direct relationship with cleft cells, and chistocytosis is mainly due to DIC red blood cell damage.

Option E is due to the formation of microthrombosis during the DIC process, which leads to the reduction of return effort, that is, the preload of the heart, which reduces the cardiac discharge.

In conclusion, the correct answer is: D. Histocysis leads to coronary artery constriction.

This topic describes a woman at 34 weeks of gestation with sudden persistent abdominal pain with pudendal bleeding when her vital signs were unstable, low blood pressure, decreased hemoglobin and platelets, significantly elevated D-dimer, and laceration in peripheral blood. Based on this information, it can be speculated that the patient may have developed placental abruption with disseminated intravascular coagulation (DIC). We need to choose the incorrect influencing factors.

Let's analyze the options, item by item:

A. * * Microthrombosis decreases return effort * * -This is true. DIC can cause microthrombosis, which reduces the amount of return effort.

B. * * Increasing kallikrekase results in reduced peripheral resistance * * -this is true. Increased kallikrein causes vascular dilation and reduced peripheral resistance.

C. * * Complement activation leads to a reduction in peripheral resistance * * -this is true. Activation of the complement system leads to vascular dilatation and reduced peripheral resistance.

D. * * Bistocytosis leading to coronary artery constriction * * -This is incorrect. The increase in cleft cells is mainly due to the destruction of red blood cells and does not directly lead to coronary artery constriction.

E. * * Reduced preload causes decreased cardiac bleeding * * -this is true. Less

preload causes less cardiac discharge due to bleeding and microthrombosis.

Considering the above analysis, option D "schistocysis causes coronary artery constriction" is not correct, so the correct answer is:

F. Schizyytosis leading to coronary artery constriction

80. (Type A2 question)

Male, 45 years old, spinal cord thoracic damage due to a car accident. After 2 months, the most likely voiding disturbance was

A. frequency of urinatior

B. diuresis

C. incontinentia urinae

D. Bladder neck spasm

E. No tension in the urinary bladder

Reference answer: C Your answer:

[Analysis] After 2 to 4 weeks of spinal cord injury, the reflex activity gradually recovered and changed into central paralysis, with increased muscle tone, hyperreflexia, positive pathological symptoms and reflex urination (C pair).

[Error avoidance] The acute phase of spinal cord injury often involves spinal shock, including below-plane flaccid paralysis and muscle tone

Reduced tendon reflex, weakened tendon reflexes, negative pathological reflexes and urinary retention.

After SCI, especially in the thoracic segment, patients may develop different voiding dysfunction depending on the extent and location of the injury. SCI often leads to dysregulation of the lower urinary tract, especially in the acute and subacute stages. The initial phase (acute phase) usually presents as spinal shock, at which the bladder may be without an inability to urinate autonomously. But over time, especially after 2 months, the situation may change, and patients may develop different symptoms.

In this case, the most likely urination disturbance is * * no tension bladder (E) *

*. The bladder is due to the lack of neural control resulting in the inability of the bladder muscles to contract normally, causing difficulty or unable to urinate independently. However, it should be noted that over time and at the site of injury, patient symptoms may shift to other types of voiding disturbance, such as the neurogenic bladder, with possible urinary frequency, incontinence or bladder neck spasm.

Therefore, according to the title description, the most likely urination disturbance after 2 months is * * no tension bladder (E) * *.

analyze:

The patient was a middle-aged male with spinal thoracic damage due to a car accident. Different sites of spinal cord damage, patients can have different types of bladder dysfunction. Spinal cord urination center is located in the lumbosacral segment, when spinal cord injury in thoracic or above, urination process will not be controlled by the spinal cord low urination center, urination process will be controlled by the brain stem and cerebral cortex tissue, no tension bladder, namely bladder afferent nerve damage, bladder filling afferent signal cannot be perceived, spinal cord urination reflex central activity disorder, bladder detrusor muscle contraction weakness, lead to a lot of residual urine, urinary retention.

Option A urinary frequency is the main manifestation of bladder irritation, which is mostly seen in urinary tract infection and renal tuberculosis, so it is excluded.

Option B polyuria is more common in diabetes, diabetes insipidus, etc., and has nothing to do with spinal cord injury, so it is not chosen.

Option C urinary incontinence is divided into true urinary incontinence, pseudourinary incontinence and stress urinary incontinence, most of which are related to bladder sphincter damage, nerve dysfunction and labor injury, but the patient did not damage the lumbosacral segment, so there will be no urinary incontinence, so it is not considered.

Option D bladder neck spasm is more common in acute bacterial cystitis, so it is not chosen.

To sum up, the correct answer is: E. No tension of the bladder.

For this topic, we need to consider the effect of spinal cord injury on voiding

function. Injury to the thoracic segment of the spinal cord may lead to disruption of neural control, which subsequently affects the function of the bladder.

After SCI, voiding disturbance usually depends on the site and extent of the injury. Thoracic spinal cord injury often leads to bladder dysfunction, specifically as urinary incontinence or no tension bladder. Two months later, the patient is most likely to have autonomic dysfunction, causing the bladder to contract normally or urinate.

In conclusion, the most likely voiding disorders are:

F. No tension in the urinary bladder

81. (Type A2 questions)

A 55-year-old man with cervical lymph node dissection for thyroid cancer showed left shoulder droop, limited upper lifting of the left upper limb, and possible nerve damage after surgery

A. nervus phrenicus

B. vagus

C. accessorius

D. nervi occipitalis minor

E. great auricular nerve

Reference answer: C Your answer:

[Analysis] When one side of the accessory nerve is damaged, it will lead to the ipsilateral trapezius muscle and sternocleidomastoid muscle dysfunction, resulting in muscle paralysis, accompanied by muscle atrophy. At this time, it is weak when turning the neck to the opposite side, and accompanied by the same side shoulder droop, unable to shrug the shoulders. In the long run, it is also prone to the corresponding muscle atrophy. After muscle atrophy, scapular displacement is easy to occur, which makes the brachial plexus nerve pulled, resulting in the upper limb of the affected side cannot be lifted, the limb abduction function is limited, and

contracture and deformity will appear in the later stage. Therefore, this question is selected C.

[To avoid errors] Phrenic nerve injury can cause diaphragmatic paralysis, limited spontaneous breathing, and diaphragmatic spasm. When vagal nerve injury can occur

Hovoice, cough, etc. Occipital skin sensory dysfunction when the small occipital nerve is damaged. Oear nerve injury can appear aurskin

Skin numbness.

This patient developed left shoulder droop and left upper limb lift limitation after cervical lymph node dissection for thyroid cancer, these symptoms indicating possible damage to the accessory nerve. The accessory nerve (Accessory nerve, also known as the XI cranial nerve) mainly innervates the trapezius and sternocleidomastoid muscles, and its function is closely related to the movement of the shoulder. Thus, common manifestations after accessory nerve injury include shoulder weakness, sagging, and restricted upper limb elevation.

The phrenic nerve (Phrenic nerve) in option A mainly controls the diaphragm and is related to breathing, rather than directly affecting the movement of the shoulder or upper limbs.

The vagus nerve (Vagus nerve) of option B affects visceral function, including the heart, lungs, and digestive system, and does not directly control shoulder or upper limb movements.

The small occipital nerve (Lesser occipital nerve) in option D is mainly related to the sensation of the scalp.

The large ear nerve (Great auricular nerve) in option E affects the sensation of the ear and face.

Therefore, the most consistent for this symptom is C. accessory nerve.

This is a question of medical knowledge. We need to first understand the background information and the core of the question, and then carefully analyze each option, and compare it with the information given in the question.

Background information: First, the topic described a 55-year-old male patient with drooping left shoulder and limited upper lift of the left upper limb. We need to judge the possible damage by surgery according on these symptoms.

At the core of the problem: We need to find out the nerves that may be damaged

after the operation.

Analysis options:

Option A (phrenic nerve): The phrenic nerve is mainly responsible for the movement of the diaphragm. After injury, it will lead to abnormal diaphragm function and dyspnea and other symptoms, which are inconsistent with the symptoms described in the topic.

Option B (vagus nerve): The vagus nerve is mainly responsible for the movement and sensation of multiple organs such as the heart, lung and gastrointestinal tract. It may lead to corresponding organ dysfunction after injury, but will not show symptoms of motor movement in the left upper limb.

B option (accessory nerve): accessory nerve main dominant sternocleidomastoid and trapezius muscle, the sternocleidomastoid and trapezius muscle contraction, after injury can cause sternocleidomastoid muscle and trapezius muscle paralysis, with lateral sterocleidomastoid and trapezius muscle atrophy, patients to the affected side turn head weakness, ipsilateral sternocleidomastoid muscle atrophy, ipsilateral sterocleidomastoid muscle and trapezius muscle paralysis, thus leading to left shoulder ptosis, left upper limb lift limited, this is consistent with the symptoms described in the title.

C Option (small occipital nerve): the small occipital nerve is mainly responsible for the sensation of the occipital part, and the occipital paresthesia will occur after injury, which is not consistent with the symptoms described in the topic.

D Option (large ear nerve): The large ear nerve is mainly responsible for the sensation of the back of the auricle. After injury, there will be sensory abnormalities in the corresponding area, which is not consistent with the symptoms described in the topic.

In conclusion, the nerves that may be damaged after surgery are C. accessory nerves.

For the situation described in the topic, that is, left shoulder droop and left upper limb lifting limitation after cervical lymph node dissection for thyroid cancer, the most likely nerve damage is the accessory nerve (Accessory nerve). The accessory nerve innerates the trapezius muscle, and the upper limb, so damage to the accessory nerve can cause the sagging shoulder and limited upper lift of the upper limb.

So the correct answer is: C. accessory nerve

82. (Type A2 question)

A 50-year-old woman with abdominal pain and distension, a semicircular mass under the inguinal ligament, pain in touch, the medial tissue structure of the mass was

A. arcus iliopectineus

B. femoral vein

C. fallopian arch

D. arcus cruralis

E. lacunar ligament

Reference answer: E Your answer:

[Analysis] The patient is a 50-year-old woman with obstruction symptoms (abdominal pain, abdominal distension to stop exhaust and defecation), a semicircular mass can be touched under the inguinal ligament, pain of touch, femoral hernia is considered for diagnosis. Femoral hernia An extraventral hernia protruding through the femoral ring, femoral tube, and oval socket to the root of the thigh. The femoral tube consists of four walls and two veins: the internal wall is the lacunar ligament (also known as the socket ligament); the lateral wall is the fibrous septum beside the femoral vein of the femoral sheath (also the femoral vein); the anterior wall is the inguinal ligament; the posterior wall is the pubic comb ligament (the Cooper ligament); and the two are saphenous vein holes of the femoral rim and the femoral side. Therefore, the tissue structure of the mass is considered as lacunar ligament

select E.

[Misavoidance] The iliopubarch is a structure that separates the space between the inguinal ligament and the hip bone, with one end to the inguinal ligament and the other end

The iliopubeminence attached to the hip bone, unrelated to femoral hernia, is A wrong. The femoral vein is the lateral wall of the femoral hernia, so the B is wrong. The uterus is round and tough

Belt is the maintenance of uterine forward tilt function, so C wrong. The inguinal

ligament is the anterior wall of the femoral hernia, so D is wrong.

This case describes a 50-year-old female patient with abdominal pain, abdominal distension, cessation of exhaust defecation and palpable a semicircular painful mass under the inguinal ligament. These symptoms and signs suggest the possibility of an inguinal hernia. In women, inguinal hernia may contain the uterine ligament.

The various structures in the option are located in different anatomical locations, in which the uterine ligament is one of the structures leading to the female inguinal canal, especially in the case of inguinal hernia, where the uterine uterine ligament may highlight and form a mass.

So, the most appropriate answer is:

B fallopian arch

analyze:

The middle-aged female of the patient stopped venting and defecation due to abdominal pain and abdominal distension, and a semicircular mass could be touched under the inguinal ligament, which was considered as femoral hernia. The hernia sac of the femoral hernia is protruding through the femoral ring and through the femoral canal to the oval socket, showing a half spherical protrusion in the oval socket below the inguinal ligament. The hernia mass sometimes does not completely disappear. Because there are many ligaments outside the hernia sac, the medial tissue structure is the lacunal ligament, also known as the rapine ligament, which is the fibrous thickening part of the medial end of the inguinal ligament and the internal edge of the femoral ring.

Option A The ilipubic arch is a marker to judge femoral hernia and oblique hernia. It is surrounded by the lower margin of the internal oblique muscle and the transverse ventrinal muscle within the inguinal ligament, which is located in the lateral side of the femoral hernia, so it is not selected.

Option B femoral vein was located lateral to the femoral hernia and was therefore excluded.

Option C The round uterine ligament starts from the front of the uterine horn and slightly below the proximal end of the fallopian tube, and walks on the anterolateral

side under the cover of the anterior lobe of the broad ligament. After reaching the lateral walls of the pelvis on both sides, the inguinal tube stops at the front end of the labia major, adjacent to the femoral hernia, but not its medial structure, so it is excluded.

Option D The inguinal ligament is a ligament-like structure formed by the thickening of the aponeurosis at the outer edge of the pubic crest, located lateral to the femoral hernia, so it is not selected.

In conclusion, the correct answer is: E. The lacunar ligament.

According to the description, the patient was a 50-year-old woman, with abdominal pain, abdominal distension, stopped exhaust and defecation, and a semicircular mass under the inguinal ligament with pain. This description fits with the characteristics of an inguinal hernia.

The question asked is what is the tissue structure on the medial side of the mass. Anatomically, the anatomy of the inguinal hernia is well defined, and the medial aspect of the mass is usually the femoral vein. Therefore, the most likely answer is: B. femoral vein

83. (Type A2 questions)

Female, 32 years old. Double lower limb edema, arthralgia, oral ulcer. Blood routine: Hb100g / L, urinary protein (+ + +), anti-dsDNA (+), ANCA 1:1000, the most relevant immune for this patient is

A. IgA

B. IgE

C. immune complex

D. Immunomolecules

E. IgD

Reference answer: C. Your answer:

Graphic analysis: [Analysis] Patients with double lower limb edema,

arthralgia, oral ulcer, laboratory tests: dsDNA (+) (is the diagnosis of SLE, specific antibody), ANCA (+), urinary protein (+ + +) (suggestive of systemic lupus erythematosus), so according to the clinical manifestations and laboratory examination, the patient was diagnosed as systemic lupus erythematosus disease. Anti-dsDNA antibody (C correct): it is a specific antibody for SLE and a marker antibody for SLE; in the active phase of SLE, the titer of anti-dsDNA antibody is closely related to disease activity, increasing anti-dsDNA titer, indicating the high risk of recurrence and more strict monitoring.

[Avoid] RF (+) and anti-CCP antibody (+) common in rheumatoid arthritis; ANA (+), anti-SSA (+) and anti-SSB (+) in Sren's syndrome.

The symptoms of this case, including bilateral lower extremity edema, arthralgia, and laboratory findings (Hb 100g / L, urinary protein + + +, anti-dsDNA positive, ANCA 1:1000) point to systemic lupus erythematosus (Systemic Lupus Erythematosus, SLE) or associated autoimmune disease.

In SLE, the most typical immunological feature is the production of autoantibodies against different autoantigens, especially anti-double-stranded DNA (dsDNA) antibodies, which have been confirmed positive in laboratory tests. An important feature of SLE is the formation and deposition of immune complexes that can trigger inflammation and damage in multiple organs and tissues throughout the body.

Thus, the most relevant immunization to this patient is the C. immune complex.

analyze:

The young female patient, with double lower limb edema, arthralgia, oral ulcer, decreased hemoglobin, massive proteinuria, anti-dsDNA positive, ANCA titer significantly increased, was considered for the diagnosis of systemic lupus erythematosus. The disease is an autoimmune inflammatory connective tissue disease involving multiple organs more frequently in young women, with increasing early, mild and atypical cases. The pathogenesis is the formation and deposition of immune complexes, which causes a series of immune responses, leading to tissue damage. So, the most relevant immune to this patient is the immune complex.

Option A: IgA nephropathy is the most common primary glomerular disease. It refers to the primary glomerular disease with IgA or IgA deposition in the mesangial area, with or without other immunoglobulin deposition in the mesangial area of the glomerulus, and the diagnosis was inconsistent with that patient and was excluded.

Option B: IgE refers to an antibody of the human body, which exists in the blood, is a cytophilic antibody, which is related to skin allergy, and does not agree with this case, so it is not selected.

Option D: Immune molecules are a variety of molecules involved in immune response and immune regulation, including cytokines, antibodies, complement, MHC molecules, etc. They are active substances playing an important role in the immune system. Their concept is too broad and not the best option, so it is not chosen.

Option E: IgD antibody is the transformation of human B lymphocytes into plasma cells under antigen stimulation, which produces antibodies that can specifically bind to the corresponding antigens. IgD accounts for only 0.6% of serum immunoglobulins. IgD, produced by B lymphocytes converted into plasma cells upon antigen stimulation, may be related to the initiation of antibodies production by B cells and to some hypersensitivity, but does little to this patient and is therefore excluded.

In conclusion, the correct answer is: C. immune complexes.

The title described a 32-year-old female patient with symptoms including bilateral lower limb edema, arthralgia, and oral ulcers. Laboratory tests showed anemia (Hb 100g / L), urinary protein (+ + +), positive anti-dsDNA antibody (+), and ANCA titer of 1:1000. These signs and laboratory results suggest the possibility of systemic lupus erythematosus (SLE).

Systemic lupus erythematosus is an autoimmune disease, often characterized as arthralgia, rash, oral ulcers, and nephritis (urinary protein). Positive anti-dsDNA antibody is one of the important diagnostic markers of SLE, and ANCA is usually associated with other autoimmune diseases such as anti-neutrophil cytoplasmic antibody-associated vasculitis, but is also seen in SLE. The pathological mechanism of SLE usually involves the formation and deposition of immune complexes that can cause inflammation and damage in sites such as glomeruli, leading to the corresponding clinical symptoms.

Therefore, the most relevant immunfor this patient are:

F. immune complex

The deposition of immune complexes is an important link in the pathological mechanism of SLE and can explain the clinical manifestations and laboratory findings of this patient.

84. (Type A2 question)

In summer, when construction workers work on the construction site, they suddenly have dizziness and headache, no sweat after sweating, dry skin, body temperature over 40°C, blood pressure 88 / 60mmHg, accompanied by consciousness disorder, the patient may be diagnosed as

A. shock

B. heat cramps

C. heat apoplexy

D. heat prostration

E. heatstroke

Reference answer: C. Your answer:

Graphic analysis: [Analysis] When working in high temperature in summer, the body temperature exceeds 40°C, the blood pressure is 88 / 60mmHg, accompanied by consciousness disorder, the diagnosis was made as heat sickness, so this question is C.

[Error avoidance] Heat spasm has no obvious temperature increase, no mental disorder; heat failure is more common in the elderly, children and patients with chronic diseases, the temperature increase mostly exceeds 40°C, no mental disorder.

The question describes a construction worker working in a hot summer environment, with symptoms of dizziness, headache, excessive sweating, dry skin, body temperature over 40°C, decreased blood pressure and disturbance of consciousness. These symptoms indicate that the patient may have suffered from a severe heat-related illness.

The option analysis is performed as follows:

-A. Shock: Although the blood pressure drop may be related to shock, there are

many causes of shock, and the situation and symptoms described here are more consistent with heat-related diseases.

-B. Heat spasm: usually muscle spasm due to heavy sweating and electrolyte loss, but not high fever or disturbance of consciousness.

-C. Heat sickness: It is the most serious heat-related disease in high temperature environments, characterized by a sharp increase in body temperature (usually more than 40°C), central nervous system dysfunction (e. g. disorder of consciousness), and dry skin without sweating. This fits very well with the symptoms described in the title.

-D. Heat failure: It is caused by insufficient water and salt, manifested by heavy sweating, weakness, fatigue, and a slight increase in body temperature, but usually not as sharply as heat sickness.

-E. Heat stroke: This is a general term for a variety of health problems due to high temperatures, but in the medical classification, more specific conditions (such as heat failure or heat sickness) are diagnosed separately.

In conclusion, the most consistent diagnosis for this condition is * * C. thermosis * *. HST is an emergency medical condition that requires immediate treatment to reduce high body temperature and address a potential life threat.

analyze:

In summer, the patient was working in the construction site with sudden dizziness and headache, no sweat after heavy sweating, dry skin, body temperature over 40°C, blood pressure decreased, accompanied by consciousness disorder, which was considered as heat radiation disease. Heat stroke is the most serious type of heat stroke, mainly manifested as high fever, no sweat, consciousness disorder, and multiple organ function damage.

Option A shock is the body suffered strong pathogenic factors, due to effective circulating blood volume, tissue blood perfusion extensive, sustained, significantly reduced, poor systemic microcirculation function, vital organs of life syndrome, but generally will not have high fever, no sweat, dry skin, etc.

Option B heat spasm is a phenomenon of heat stroke. In the dry and hot environment conditions, work, excessive sweating, with sweat discharge a lot of NaCl, the occurrence of limb and abdominal wall muscle spasm phenomenon, the patient's body temperature does not rise, does not meet the meaning of the question.

Option D Heat failure is mostly due to excessive sodium loss due to excessive sweating and insufficient blood volume, manifested as dizziness, headache, nausea, vomiting, palpitation and blood pressure drop, but generally there will be no high fever, no sweat, dry skin and other manifestations, which can be excluded.

Option E Heat stroke refers to the acute comprehensive syndrome mainly characterized by cardiovascular and central nervous system dysfunction due to central dysfunction of thermoregulation or sweat gland failure, and excessive loss of water and electrolytes, in the environment of high temperature or humidity and low wind. The symptoms are mild and do not meet the meaning of the question.

To sum up, the correct answer is: C. Heat radiation disease.

According to the title description, the patient suddenly developed dizziness, headache, no sweat after heavy sweating, dry skin, body temperature over 40℃, blood pressure 88 / 60 mmHg, accompanied by consciousness disorder. These symptoms are highly suggestive of heat sickness.

Heat emission sickness (Heat Stroke) is a serious heat-related disease that usually occurs in high-temperature environments. Typical manifestations include high fever (body temperature usually exceeds 40℃), abnormal central nervous system function (such as consciousness disorders, epilepsy, etc.), and dry heat and no skin sweating (in typical heat sickness, motor heat sickness may still have sweating). If not managed, HST may lead to multiple organ failure and death.

Other option analysis:

- ** A. Shock * *: The patient's blood pressure is low, but the diagnosis of shock needs to combine more clinical manifestations and causes, and the more prominent ones in the problem stem are high fever and consciousness disorders.

- ** B. Heat spasm * *: The main manifestation is muscle spasm, usually the body temperature does not reach 40℃, and there is no obvious disorder of consciousness.

- ** D. Heat failure * *: also known as heat failure syndrome, mainly manifested

as a lot of sweating, weakness, fatigue, dizziness, etc., but there will be no obvious high fever and consciousness disorders.

- ** E. Heat stroke * *: It is a broad term that can include heat spasm, heat failure and heat sickness, but the specific symptoms in the problem stem are more consistent with the characteristics of heat sickness.

So, the most appropriate answer is:

* * C. Heat sickness * *

85. (Type A2 question)

A chemical factory worker had nausea, vomiting, tremor and other emergency hospitalization in an accident. Physical examination: skin mucosa was cherry red, with bitter almond taste, suspected to be acute occupational poisoning, most likely

A. carbon monoxide

B. hepatic gas

C. arsenic hydride

D. aniline

E. prussiate

Reference answer: E. Your answer:

Graphic analysis: [Analysis] When cyanide poisoning, the breath smell has a bitter almond taste (E pair).

[Avoid mistakes] Hydrogen sulfide poisoning is upper respiratory mucosa irritation, palpitations, pulmonary edema, coma, etc., hydrogen arsenic, hemoglobinuria, anemia, severe acute renal failure; benzene is mainly the anesthetic effect on the central nervous system.

The symptoms described by the title, including nausea, vomiting, tremor, cherry red skin mucosa, and a bitter almond taste in exhaled breath, were most consistent with the clinical manifestations of cyanide poisoning. Cyanide poisoning is an emergency that requires immediate treatment because it rapidly leads to a blockade of cellular respiration and tissue hypoxia.

The answer is E. cyanide.

analyze:

The patient's chemical factory workers developed nausea, vomiting and tremor due to occupational exposure. Physical examination showed that the skin mucosa was cherry red, with bitter almond taste in exhaled breath. According to the patient's occupation, clinical manifestations and signs, it was suspected to be cyanide poisoning. Cyanide poisoning mainly by respiratory tract inhalation, skin absorption or eating, etc., lead to tissue hypoxia and asphyxia, its clinical manifestations of fatigue, dizziness, headache, nausea, after tonic and paroxysmal convulsions, until the Angle arch, blood pressure, urine, incontinence, often with brain edema and respiratory failure, its unique performance for mucous membrane and skin is bright red, breath has bitter almond taste.

Option A Carbon monoxide poisoning often shows cherry red lips, but no special smell, which is not consistent with the description of the question stem, so it is excluded.

Option B hydrogen sulfide poisoning often has photophobia, tears, eye tingling, foreign body sensation and other symptoms, and the smell of smelly eggs in the breath is not consistent with the description of the question, so it is not selected.

Option C Hydrogen arsenide poisoning is mainly manifested as different degrees of acute hemolysis and kidney damage, with no special smell in the exhaled breath, which is inconsistent with the description of the title stem and can be excluded.

Option D aniline poisoning mainly damages the blood system, causing hemolytic anemia and liver and kidney damage. The skin can be pale green purple, but it will not exhale bitter almond taste, so it is not selected.

In conclusion, the correct answer is: E. cyanide.

According to the title description, the workers in the chemical factory suffered from nausea, vomiting, tremor and other symptoms in the accident. The physical examination found that the skin mucosa was cherry red, and there was bitter almond taste in the breath. These characteristics suggested that the possibility of acute poisoning was very high.

Combining these symptoms and signs, the most likely cause is cyanide poisoning.

The typical features of cyanide poisoning include:

- ** Cherry red skin mucosa * *: Cyanide by inhibiting cytochrome oxidase, leading to tissue hypoxia, despite the high oxygen content in the blood, but the tissue can not use oxygen, resulting in the appearance of cherry red skin mucosa.

- ** There is bitter almond taste in the breath * *: This is a typical odor characteristic of cyanide poisoning.

Characteristics of the other options:

-Carbon monoxide (A): It usually causes headaches, dizziness, fatigue, disturbance of consciousness, cherry-red skin, but no bitter almond taste.

-Hydrogen sulfide (B): rotten egg flavor, mainly respiratory irritation.

-Hydrogen arsenide (C): it usually presents as hemolytic anemia, jaundice, and hemoglobinuria.

-Aniline (D): can cause methemoglobinemia, resulting in blue or gray skin and mucosa.

In summary, the answer that most fits the description is:

B prussiate

86. (Type A2 questions)

Male, 18 years old, suddenly sweating and pale after half an hour after taking antibiotics for himself due to "cold". Physical examination: blood pressure 40 / 20mmHg, confused, pale, fine pulse, cold limbs, cyanosis, the patient primary treatment

A. Still drops of dopamine

B. Epinephrine was injected intramuscularly

C. Quiet drops of dexamethasone vs

D. Intramuscular injection of isoproterenol

E. Oxygen inhalation and ecg monitoring

Reference answer: B. Your answer:

[Analysis] Juvenile male patients with blood pressure of 40 / 20mmHg, unconscious, pale, fine pulse, cold limbs, cyanosis and other series of shock manifestations, were considered as anaphylactic shock. At this time, emergency intramuscular injection should be performed, so B.

[Avoid] Anaphylactic shock showed dilated small blood vessels and increased capillary permeability, decreased circulating blood volume and blood pressure

Desperline, simultaneous bronchial smooth muscle spasm, developed dyspnea, the use of adrenaline for anaphylactic shock, its excitement

α 1 receptor, constricting arterioles and anterior capillary sphincter, while decreasing the permeability of the latter and agonistic β 1 receptors improve cardiac work

It can enhance myocardial contractility, increase heart rate, reduce the release of allergic mediators, and expand coronary arteries, so it can be quickly relieved

The clinical symptom of anaphylactic shock is the first choice for the treatment of anaphylactic shock.

The 18-year-old man experienced sudden sweating, pallor, decreased blood pressure (40 / 20 mmHg), confusion, fine pulse, cold limbs, and cyanosis, which suggested a severe anaphylactic reaction or shock, especially drug-induced anaphylactic shock.

The primary treatment should be the immediate stabilization of the patient's hemodynamic status and improved oxygenation. Given the severity of his symptoms, the preferred treatment should be epinephrine, as it can raise blood pressure quickly and relieve allergic reactions. Epinephrine is usually administered by intramuscular injection to ensure rapid absorption and onset of action, especially in first-aid situations.

Therefore, the most appropriate option is: B. Epinephrine intramuscular injection.

analyze:

Juvenile male patients, who developed hypotension, confusion, pale face, fine pulse, cold limbs, cyanosis and other shock symptoms, were considered for anaphylactic shock. Adrenaline can excite α and β receptors, thus enhancing myocardial contractility, increase cardiac output, accelerate heart rate, contract blood vessels, relax bronchial smooth muscle, is the first choice for the treatment of anaphylactic shock. Therefore, patients at this time they should choose adrenaline intramuscular injection.

Option A dopamine hydrodrip is mainly used for shock syndrome caused by myocardial infarction, trauma, endotoxin sepsis and other conditions, but not as the first choice of anaphylactic shock, so it is excluded.

Option C Dexamethasone is a commonly used anti-allergy drug, but it has a slow effect and is generally not the first choice for anaphylactic shock, so it is not selected.

Option D Intramuscular injection of isoproterenol as a β receptor agonist, which can enhance myocardial contractility, accelerate heart rate, dilate peripheral vessels and bronchi, but has no effect on α receptor, so the pressure boost effect is not obvious, so it is not considered.

Option E Oxygen inhalation and ecg monitoring are the general adjuvant treatment measures for shock patients, not the primary treatment measures, so they do not meet the meaning of the question.

In conclusion, the correct answer is: B. Epinephrine intramuscular injection.

This patient developed an acute allergic reaction after taking the antimicrobial medication, showing symptoms of shock. Shock is a life-threatening emergency that must be addressed quickly. According to existing medical guidelines and best practice, the preferred treatment for anaphylactic shock (shock from anaphylactic reaction) is that of epinephrine injection.

In this case, the most correct answer is:

F. Epinephrine was injected intramuscularly

Adrinephrine can rapidly contract blood vessels, increase cardiac output, reverse

the shock state, and reduce the severity of allergic reactions. Although dopamine and isoproterenol also have some pressor effects, they are not the drug of choice for anaphylactic shock. Dexamethasone is a glucocorticoid that contributes to the treatment of allergic reactions, but is not the drug of choice for first aid. Oxygen inhalation and ECG monitoring are supportive care, but are unable to rapidly reverse the shock.

87. (Type A2 questions)

After the patient is admitted to the hospital, the special situation requires emergency treatment. The patient cannot be approved and there is no contact person or family members present, so the tube bed physician needs to handle it and carry out it after the approval

A. Ward Director

B. Department head

C. Superior physician

D. Hospital supervision and administration department

E. Hospital Head

Reference answer: E Your answer:

Graphic analysis: [Analysis] During operation, special examination or special treatment, the patient shall obtain the consent of the patient, obtain the consent and signature of the family member of the patient or related person; obtain the consent and signature of the patient; without the family member or related person, or the treating physician shall propose the medical treatment plan after the approval of the responsible person (E pair) of the medical institution or the authorized medical institution responsible person.

[Avoid mistakes] memory questions.

In an emergency, if the patient is unable to give consent and no contact or family member is present, handling this condition usually requires following the hospital's emergency medical intervention policy. Although this may vary between national and hospital policies, often in an emergency setting, approval from a superior physician or hospital head is required for treatment, especially when it comes to potentially life-threatening emergencies.

In China, emergency medical services laws usually stipulate that doctors are

responsible to take the necessary treatment measures when a patient's life is in danger and timely consent is not available. In the absence of explicit provision, superior physicians usually have the power to make such urgent decisions.

Considering these factors, the best answer may be:

F. The superior physician

This is because in an emergency situation, the superior physician is usually responsible for making prompt medical decisions, especially where there is no other authorized person to contact.

analyze:

"Regulations on the administration of medical institutions", in an emergency to save dying patients with emergency medical measures cause adverse consequences do not belong to the medical accident, medical institutions and medical personnel in medical activities, must strictly abide by the medical and health management laws, administrative regulations, department rules and medical nursing norms, routine, abide by the medical service professional ethics. Doctors shall abide by laws, regulations and technical operation norms in their practice activities. For the emergency of the patient, emergency treatment is required. If the patient cannot be approved and no relevant contact person or family members are present, the bed management physician shall implement the corresponding medical treatment measures after the approval of the hospital leader or the authorized responsible personnel.

To sum up, the correct answer is: E. Hospital head.

When the patient has special circumstances after admission, requiring emergency treatment and the patient consent cannot be obtained, and no relevant contact person or family members are present, emergency treatment is usually required after the approval of the superior physician according to Chinese medical regulations and best practice guidelines. This is because superior physicians often have more experience and authority to quickly make accurate decisions needed to ensure patient safety and health. So the answer is:

G. The superior physician

88. (Type A2 questions)

Male, 65 years old. Diarrhea for one week and palpitations for 2 days. He had a previous history of hypertension and atrial fibrillation. Oral warfarin, perindopril, nitrate esters, and digoxin treatment. Emergency electrocardiogram: frequent preventricular contraction, short array of ventricular tachycardia. If the cause of the change, which test should be chosen

A. Determination of digoxin concentration

B. coagulation time test

C. D-dimer assay

D. Demarkers of myocardial necrosis

E. Brain sodium peptide

Reference answer: A. Your answer:

Text analysis: [analysis] elderly male patients, oral warfarin, perindopril, nitrate ester, digoxin therapy. Diarrhea 1 week, palpitations for two days, emergency electrocardiogram: frequent ventricular contraction (digitalis poisoning, the most important manifestation is all kinds of arrhythmia, to the ventricular contraction is common), short array ventricular tachycardia, according to the patient examination and clinical manifestations, digoxin concentration determination, so this question choose A.

[Error avoidance] The gastrointestinal manifestations of digitalis poisoning such as nausea, vomiting, and neurological symptoms such as blurred vision, yellow vision,

Green vision, disorientation disorder, consciousness disorder and so on are relatively rare.

In this case, the patient had a history of hypertension and atrial fibrillation along with warfarin (an anticoagulant), perindopril (BP lowering agent), nitrate esters (for treatment of angina) and digoxin (for cardiac function problems). The emergency ECG showed frequent preventricular contraction and short-array ventricular tachycardia, which may indicate digoxin toxicity, especially considering the patient has a history of diarrhea, which may cause an electrolyte imbalance and increase the risk of digoxin toxicity.

Therefore, the correct answer should be * * A. digoxigenin concentration

determination * *. This examination allowed for the presence of digoxin toxicity, the most likely cause after considering the patient's medical history and clinical presentation. Other options may be relevant in some cases, but the determination of digoxin concentration is the most direct and relevant examination for this particular case and the patient's symptoms.

analyze:

An elderly male patient with one week diarrhea, palpitations for 2 days, ECG showed frequent preventricular contractions and short array ventricular tachycardia. Due to the patient's long-term oral digoxin, diarrhea for a week, suspected of digoxin poisoning. The safety range of digoxin is narrow, and the effective amount and poisoning volume are very close. The blood concentration should be monitored when used, so the measurement of digoxin concentration should be done.

Option B coagulation time measurement is mainly used to monitor the anticoagulant effect of warfarin, which is inconsistent with the meaning of the question.

Option C: D-dimer measurement can be used as an auxiliary diagnosis of hyperfibrinolysis and thrombosis, and it does not reflect digoxin poisoning, so it is not selected.

Option D: Cardiac necrosis markers were used to diagnose myocardial infarction. The patient did not have MI, so it was excluded.

Option E: Brain natriuretic peptide is mainly used for the diagnosis of heart failure. This patient did not have heart failure, so it is not considered.

In conclusion, the correct answer is: A. Degoxigenin concentration determination.

According to the title description, the patient was 65-year-old with a history of hypertension and atrial fibrillation and was on warfarin, perindopril, nitrate esters and digoxin. There was diarrhea for a week and palpitations for 2 days, and the emergency electrocardiogram showed frequent preventricular contractions and a short array of ventricular tachycardia.

In this case, it needs to be considered whether the patient's medication has

contributed to the worsening of the current symptoms. In particular, digoxin is a drug used to treat heart failure and atrial fibrillation, but its therapeutic window is narrow and prone to poisoning. The manifestations of digoxin poisoning include gastrointestinal symptoms (e. g. diarrhea) and cardiac symptoms (e. g. ventricular arrhythmia).

Therefore, the most appropriate examination is * * A. digoxigenin concentration determination * *.

This will help to determine whether the patient's digoxin level is too high to cause the current symptom changes. Other options such as coagulation time determination, D-dimer determination, and myocardial necrosis markers determination, while useful in some clinical situations, in this specific case, digoxin concentration determination is the most direct and relevant examination.

Answer: * * A. Determination of digoxin concentration * *

89. (Type A2 question)

Male, 76 years old. Urinary frequency, urgency, nocturia increased, dysuria for two months, the lesion involvement of the structure is

A. alveus urogenitalis

B. The prostatic peripheral zone

C. The prostatic fibrous muscular stroma

D. Central zone of the prostate gland

E. Prostate transitional zone

Reference answer: E Your answer:

Graphic analysis: [analysis] elderly male patients, urinary frequency, urgency, nocturia increased, dysuria for two months, diagnosed as benign prostatic hyperplasia. Prostatic hyperplasia is mainly found in the periurethral transitional band (E pair).

[Avoid mistakes] Prostate cancer is mainly adenocarcinoma, often in the peripheral zone.

A 76-year-old man presented with symptoms of frequency, urgency, increased nocturia, and the most likely involved prostate structure being the prostate transitional zone. These symptoms are usually associated with benign prostatic hyperplasia (BPH), which occurs mainly in the transitional zone of the prostate. So the best answer is:

B Prostate migration area

First of all, we need to understand the main symptoms of the patient: elderly men, with urinary frequency, urgency, increased nocturia, and symptoms of dysuria, which all point to prostatic hyperplasia.

Now, let's analyze the options, one by one:

A. Prostatic capsule: This is not the main area involved in prostatic hyperplasia, so it is excluded.

B. Peripheral prostate area: Although this is also a part of the prostate, it is not the main involvement area of prostatic hyperplasia, so it is not selected.

C. Prostate fibromuscular matrix: although this part is related to the function and structure of the prostate, it is not the main lesion area of prostatic hyperplasia, so it is excluded.

D. Central prostate area: Similarly, this area is not the main lesion site of prostatic hyperplasia, so it is not selected.

E. Prostate migration area: This is the most frequently involved area of prostatic hyperplasia. The enlarged prostate tissue is mainly derived from the hyperplasia of the gland tissue and the stromal tissue in the transitional zone of the prostate. This hyperplasia can compress the urethra, causing symptoms such as frequent urination, urgency, increased nocturia and dysuria.

In conclusion, the structure involved in this patient was most likely the prostate transitional zone.

Therefore, the correct answer is the E. prostate migration zone.

This title describes a 76-year-old male with symptoms including frequency, urgency, increased nocturia and dysuria. These symptoms suggest the possible presence of the prostate-associated lesions. The prostate areas most likely to be involved based on the clinical presentation are:

A. alveus urogenitalis

B. The prostatic peripheral zone

C. The prostatic fibrous muscular stroma

D. Central zone of the prostate gland

E. Prostate transitional zone

Combining the patient's symptoms and a common area of prostate lesions, the prostate migration area is a common site of prostatic hyperplasia (BPH), and BPH usually causes these urinary symptoms. Therefore, the most likely answer is: E. prostate migration

90. (Type A2 questions)

At term, 12h after birth, sclera appeared for 6h, poor consciousness. The mother had a history of abortion, type B blood, Rh negative, his father type A blood, Rh positive, blood test: Hb142g / L, the antibody related to the disease of the child was

A. IgM

B. IgE

C. IgG

D. IgD

E. IgA

Reference answer: C Your answer:

Graphic analysis: [analysis] term children, 12h after birth, sclera yellow 6h, the mother has A history of abortion type B blood, Rh negative, his father type A blood, Rh positive, considered for newborn hemolysis, blood type Bh mother for blood transfusion, abortion or delivery after Rh + red blood cells stimulation, can produce anti-Rh 1gG antibody. Therefore, this question is selected C.

[Avoid mistakes] The natural antibody of ABO blood group system is IgM, and blood transfusion with inconsistent blood group can lead to serious hemolytic reaction

This exam question involves neonatal hemolytic disease, especially caused by blood group and Rh incompatibility. In this case, the mother is Rh negative and the father is Rh positive, possibly causing the fetus to be Rh positive. If the mother produces antibodies against Rh-positive red blood cells, these antibodies can cross the placenta and attack the red blood cells of the fetus, causing hemolysis.

For newborns, jaundice (such as the yellow sclera of the dye in question) and poor consciousness occur shortly after birth, possibly due to hyperbilirubinemia caused by hemolytic disease. Maternal Rh-negative with paternal Rh-positive blood group mismatch increases this possibility.

The type of antibody that can cross the placenta and cause hemolysis in newborns is IgG. Thus, the relevant antibody most likely causing this neonatal disease is IgG.

So, the correct answer is the C. IgG.

analyze:

In term children, sclera yellow dye 12 hours after birth, should be considered as neonatal hemolytic disease. The mother is type B blood, Rh negative, has a history of abortion, the father is type A blood, Rh positive, according to the blood group of both parents, it can be inferred that the blood group of the child may be type A, Rh positive, so it can cause the production of immune Rh antibody in the mother, pregnancy again through the placenta into the fetal body can cause hemolysis. Because immune Rh antibodies belong to IgG type antibodies and have a small molecular weight, they can enter the fetus through the placenta to cause hemolysis.

Option A: IgM has a large molecular weight, cannot cross the placenta, and will not cause hemolytic disease to the fetus, so it is excluded.

Option B: IgE is related to the body's antiparasitic immunity and skin allergy, which is not involved in the occurrence of hemolytic disease in newborns, and is therefore excluded.

Option D: The function of IgD is unknown, which may be related to the initiation of antibodies in B cells, and some hypersensitivity, which is inconsistent.

Option E: IgA mainly exists in milk, saliva, tears, as well as the mucosal surface of the respiratory tract, digestive tract and urogenital tract, participating in the mucosal immunity of the body, which is inconsistent with this topic.

In conclusion, the correct answer is: C. IgG.

This problem describes a full-term child with jaundice 12 hours after birth with a history of maternal miscarriage with blood type B Rh negative and a father type A Rh positive. Combining this information, the newborn is likely to have neonatal

hemolysis (a form of neonatal jaundice), an immune response due to maternal and infant incompatibility, especially Rh incompatibility.

In the case of Rh incompatibility, the mother's Rh negative blood may have an immune response to the fetal Rh positive blood, generate antibodies against fetal red blood cells, which enter the placenta and cause the destruction of fetal red blood cells, causing hemolysis and jaundice. This class of antibodies is of the IgG type, because IgG is the only antibody that can cross the placental barrier.

So the correct answer is:

F. IgG

91. (Type A2 question)

A researcher obtained data on the prevalence of type 2 diabetes from 1998 to 2017 in a northern city of Our city. Which statistical chart should be used to describe the prevalence and time of type 2 diabetes in the city over the past 20 years

A. diagram

B. locus

C. bar chart

D. histogram

E. scatter diagram

Reference answer: A. Your answer:

[Analysis] The line diagram (A pair) is to use the rise and fall of the line segments to express the continuous change of the index (variable), which is applicable to describe the change trend of one variable with another variable.

[Avoid mistakes] In the circle diagram (B error), the total area of the circle is 100% to represent the whole of things, and the fan area in the circle is used

To represent the proportion of the whole. Bar chart (C wrong) is also known as bar chart, with the length of the equal width straight bar to represent the phase

The numerical magnitude of the mutually independent statistical indicators and

the contrasting relations between them. Histogram (D error) is represented by straight rectangular area

The sum of the frequencies and each rectangular area represents the sum of the frequencies. It is mainly used to represent the frequency distribution of continuous variables.come loose

The dot plot (E error) shows the straight line or curve relationship between the two indicators.

To describe the trend in the prevalence and time of type 2 diabetes over the past 20 years, line charts (option A) are best used. The line graph is able to clearly show the trend of data over time and is ideal for expressing continuous data changes, such as prevalence over time. Although circle chart, histogram, histogram and scatter plot can show data distribution or proportion, they are not as intuitive and effective as line chart to show trend changes over a long time span. Therefore, the best answer is the A. line chart.

This is a multiple-choice question about data visualization. The most appropriate statistical map of the data should be selected according to the characteristics of the data and the display requirements. Let's start with analyzing the key information in the topic:

First, to understand the background and requirements of the topic: The researcher obtained 20-year prevalence data of type 2 diabetes in a city, aiming to show the trend of prevalence over time.

Next, we analyzed the applicability of each option:

A. Line diagram: Line diagram is the fluctuation of line segments to represent the continuous change of variables. It is suitable to describe the change trend of one variable with another variable, and is usually used to describe the change trend of statistical indicators over time.

B. Circle diagram: The circle diagram is mainly used to represent the proportion of the various parts of things, and is not applicable to display the time series data.

C. straight bars: straight bars are mainly used to represent the numerical size of independent statistical indicators and the comparative relationship between them, which is not suitable for showing continuous change trends.

D. Histogram: Histogram is mainly used to represent the frequency distribution of continuous variables, and is not applicable in this case.

E. Scatter chart: Scatter chart is mainly used to show the relationship between two indicators. Although it can show data points, it is not intuitive to show the changing trend as line chart.

Combined with the requirements of the title, it is the changing trend of type 2 diabetes, and the line chart is best used to show this continuity.

To sum up, the correct answer is: A. Line chart.

To describe the trend of the prevalence and time of type 2 diabetes in a city over the past 20 years, the most suitable statistical chart is the line chart. Line grams can clearly show trends over time, helping researchers to observe an increase or decrease in prevalence.

Correct answer: A. Line diagram

92. (Type A2 question)

To study the relationship between age X and mean weight Y (kg) in children aged 2 to 7 years can be expressed as Y=7 + 2X, then the following is true

A. The mean body weight of children aged 0 years was 7kg

B. The mean body weight of children aged 11 years was 29kg

C. The mean body weight of the 5-year-old children was 17kg

D.6 years 7kg more than 5 years

E. 2kg increase from 1 to 2 years

Reference answer: C Your answer:

[Analysis] The relationship between 2-7 years (A, B, E wrong, 0,1 and 11 years) and average age weight Y (kg) can be expressed as Y=7 + 2X, according to the formula, the average weight of 5 years old children is 17kg, and the average weight of 6 years old children is 19kg (D wrong), so C.

[Fault avoidance] Note that the value range of X in Eq.

According to the given linear equation Y = 7 + 2X, where X represents the age and Y represents the mean weight (kg), we can calculate the mean weight of children of different ages. By replacing the value of X, we can find out which option is correct.

-For option A, when X=0, Y=7 + 2 * 0=7, so that the mean weight of children aged 0 years is 7kg, which appears to be correct.

-For option B, when X=11, Y=7 + 2 * 11=29, so the average weight of 11-year-olds is 29kg, which is also true.

-For the C option, when X=5, Y=7 + 2 * 5=17, so the average weight of 5 years old children is 17kg, which is also true.

-For option D, comparing the difference in weight between children aged 6 and 5, Y=7 + 2 * 6=19 at 6, Y=17 at 5,2kg more than 5, not 7kg, so this option is incorrect.

-For option E, the increased weight from age 1 to 2 years is 2 * (2-1) =2kg, so this option is also correct.

Of these options, A, B, C and E are correct, but the questions requires the best answer. Since this is a mathematical calculation of linear relations, and all of these answers can be calculated directly from a given equation without an explicit "best" answer, we need to find one that is most reasonable in the actual situation.

Considering the reality, the average weight of 7kg (option A) seems the most reasonable, because it is based on the baseline set by the equation. Thus, while multiple options are mathematically correct, option A is probably the most appropriate answer to the question.

This is an inference question about the relationship between children's age and average body weight.

First, we need to understand the formula given in the title: Y = 7 + 2X, where Y represents the average weight and X represents the age of the child. This formula suggests a linear relationship between children's average weight and their age, specifically by 2kg and a base weight (i. e., weight at age 0) of 7kg.

Now, we analyze each option one by one:

A. The average weight of children aged 0 years is 7kg: according to the formula Y = 7 + 2X, when X=0, Y=7. This means that the average weight of 0 years is 7kg. This option is correct.

B. The average body weight of 11-year-old children is 29kg: substitute X=11 into the formula to obtain Y = 7 + 2 * 11 = 29. However, the topic clearly points out that the scope of research is children aged 2 to 7, so 11 is not within the scope of

research. Although this option is mathematically correct, it does not match the meaning of the question, so it is wrong.

C. The average body weight of 5-year-old children is 17kg: substitute X=5 into the formula to obtain Y = 7 + 2 * 5 = 17. This fits the question meaning, but compared to the correct answer, A, it is not the direct answer to the question.

D. 7kg more average weight than 5 years: According to the formula, the difference in weight between 6 and 5 years should be 2kg (because 2kg for each additional year) rather than 7kg. This option is wrong.

E. 2kg increase from 1 to 2 years: This option is correct because the weight gain is 2kg for each additional year, according to the formula. But compared to the correct answer A, it is not the direct answer to this question.

In conclusion, the correct answer is A: the average weight of children at age 0 is 7kg.

According to the question stem, the relationship between age X and mean weight Y (kg) of children aged 2 to 7 years is given, which can be expressed by formula Y=7 + 2X.

Let's analyze it option by option:

A. The mean body weight of children aged 0 years was 7kg

The study requires the weight relationship between children aged 2 and 7 years, and children aged 0 years are not included in the study. The assumption of this option is not within the scope of the question stem, so it does not fit the question meaning.

B. The mean body weight of children aged 11 years was 29kg

Studying only children aged 2 to 7 years, age 11 is beyond the scope of the study to calculate their weight with a given formula, so this option is incorrect.

C. The mean body weight of the 5-year-old children was 17kg

Enter the formula Y=7 + 2X, X=5, i. e., Y=7 + 25=7 + 10=17kg. This is the

correct calculation result.

D. 6 years 7kg more than 5 years

The body weight was calculated in 6-year-old children: Y=7 + 26=7 + 12=19kg.

The body weight was calculated in 5-year-old children: Y=7 + 25=7 + 10=17kg.

6 years more than 5 years: 19kg-17kg = 2kg. So this option is wrong.

E. 2kg increase from 1 to 2 years

The research range given by the subject stem is 2 to 7 years old, and it does not involve the 1-year-old situation. This option is beyond the scope of the question, so it does not fit the question.

To sum up, the correct answer is:

D. The mean body weight of the 5-year-old children was 17kg

93. (Type A2 question)

To analyze the prevalence of hookworm in A and B, it is known that the prevalence of men is higher than that of women, men and more men than women, more women in B than men, today to compare the prevalence of hookworm in A and B, the following statement is correct

A. Standardize the sex first before counting

B. cannot be compared

C. Comparison made by sex

D. chi-square test

E. Z checkout

Reference answer: A. Your answer:

[Analysis] When comparing the prevalence, incidence, mortality and other data of two different groups, in order to eliminate the influence of its internal composition (such as age, gender, length of service, length of illness, severity of the disease) on the rate, the standardized rate (A pair) can be used.

[Fault avoidance] The main purpose of χ 2 test is to infer whether there is any

difference between two or more overall rate or composition ratio, Z test, χ 2

Inspection are only some inspection methods, unable to eliminate the error caused by its internal composition.

The correct answer is an A. To compare the prevalence of hookworm disease in both A and B, one should consider the differences in gender composition, as the prevalence is known to be higher in men than in women. There are more males than women and more females than men, and this difference in sex ratio can affect the direct comparison of prevalence. Therefore, gender should be standardized first to eliminate the effect of gender structure differences on the prevalence comparison, before calculating and comparing the standardized prevalence in both places. This allows for a more fair and accurate comparison of hookworm prevalence in both places.

analyze:

This question needs to compare the prevalence of hookworm in A and B, but the ratio of men and women in A and B is not consistent, so the sex ratio of the two places should be adjusted first, that is, the prevalence should be calculated before standardizing the gender, so as to eliminate the different effects of gender.

If the prevalence of hookworm is directly compared between A and B, the results will be inaccurate due to the inconsistent male and female ratio between A and B, so option B cannot be compared.

The comparison of option C by gender can only yield the difference in prevalence between different gender, but not the difference in prevalence between A and B, so it is not selected.

Option D chi-square test is used to infer whether there are differences between two or more population rates or composition ratios, independent of this problem.

The Z-test for option E is suitable for comparison between two groups in large samples and is not suitable for comparison of prevalence, so it is not considered.

To sum up, the correct answer is: A. Standardize gender before calculating.

To compare the prevalence of hookworm disease in A and B, it is known that there are more men than women in B, and the prevalence of men is higher than women, which will make the direct comparison of the prevalence results in the two

places distorted. For more accurate comparison results, sex should be standardized before calculation. So, the most correct answer is:

A. Standardize the sex first before counting

94. (Type A2 questions)

The survey of 500 patients with Salmonella infections in one region best reflects the average duration of Salmonella in the region

A. average

B. geometrical mean

C. median

D. P75

E. standard error

Reference answer: C Your answer:

[Analysis] The median is the middle number of a set of data arranged in size order, which is usually used to represent the "median" level of a set of data. Because the Salmonella infection situation is graded data, and the median number reflects the central trend of a set of data, the best indicator of the average situation of Salmonella infection in the region is the median number (C pair).

[Misavoidance] The average is the sum of a set of numbers divided by the number of the groups, which is usually used to represent the "average" water of a set of data

Ping (A wrong). Geometric means are the n degree root of the product of a set of numbers and are usually used to represent the " mean change of a set of data

Rate " (B is wrong). P75 represents the 75th percentile, the value of the 75th percentile of the data in the order of size, and is usually used to represent the "upper edge" level of a set of data (D error). The standard deviation indicates the degree of dispersion of a set of data, usually

Used to represent the "fluctuation" situation of a set of data (E error).

In this case, for the best representation of the average length of Salmonella infection in the region, the most appropriate statistic is the median (option C). The median is the value between datasets, and the median more accurately reflects the

central trend than the mean when the data distribution is asymmetrical or when outliers are included. The duration of Salmonella infection may be extremely variable by individual differences, so that the median is a more robust metric in this case, and it is not affected by extreme values. So, the correct answer is C. median.

analyze:

In this question, we want to find a statistic that most accurately reflects the average length of Salmonella infection in a patient. Given that data for infection duration may present a skewed distribution, especially when extreme values exist, arithmetic means may not accurately reflect the situation in most patients. The median is the number in the middle after arranging a set of data from small to large. In a skewed distribution, the median better represents the case for most of the data because it is not affected by extreme values. Thus, for the skewed distribution data of Salmonella infection length, the median is the statistic that most reflects the length of infection in most patients.

To sum up, the correct answer is: C. Median.

Option A mean, the arithmetic mean, is suitable when the data is normally or approximately normally distributed, and is susceptible to extreme values. However, the duration of Salmonella infection in this question may present A skewed distribution, so it is not selected.

Option B The geometric mean is usually used to reflect the average level of a set of log-transformed data that is normally distributed, or to calculate the average growth rate, and therefore is not considered.

Option DP75 is a percentile, indicating that 75% of the data is less than or equal to this value, but it does not fully reflect the average level of the overall data, so it is excluded.

Option E standard deviation is used to measure the dispersion of the data rather than the mean level, so it is not selected.

In statistics, there are many indicators used to describe the central trends in a set of data. For infection in Salmonella infected patients, it is very important to choose appropriate central trend indicators if the average duration is to be reflected.

1. * * Mean (A) * *: The arithmetic mean, which is the sum of all data values divided by the number of data. It is greatly affected by the extreme values, and if the data distribution is biased or outliers exist, the average may be pulled too high or too low.

2. * * Geometric mean (B) * *: Geometric mean applies to the data of product relations, such as growth rate. It is not commonly used in the length of Salmonella infection because temporal data are generally not presented in a multiplicative relationship.

3. * * Median (C) * *: Median is the value in the middle after arranging the data in order of size. If the data is biased or has extreme values, the median is not affected by extreme values as the mean. Thus, the median is more representative of the central trend of a set of skewed data.

4. * * P75 (D) * *: The 75th percentile, indicating that the 75% of the data value is less than or equal to that value. It is mainly used to describe the distribution of the data, rather than a central trend.

In conclusion, the median (C) * * that reflects the average duration of Salmonella infection in a region best represents the trend in the data set and is not affected by extreme values.

So the best answer is:

**C. median **

95. (Type A2 question)

A total of 87 patients were collected for the efficacy of a urinary tract infection, of which 43 were cured, 17 were effective, 6 improved and 21 were ineffective. The information belongs to

A. Data on continuous variables

B. Data on orderly and multi-categorical variables

C. Data on disordered multiple categorical variables

D. quantitative data

E. qualitative data

Reference answer: B. Your answer:

[Analysis] Grade data, also known as orderly multi-classification data (B pair), is that the observation results of variables are qualitative, but there are differences in degree or order between various categories (attributes), such as the treatment effect of drugs is classified according to "effective, effective, improved, and invalid".

[Wrong avoidance] The qualitative data shows the quality characteristics of the research object, which are manifested as incompatible categories. Quantitative data is change

The observation results of quantity are numerical type to illustrate the quantitative characteristics of the research object, which is that it can be measured by numerical size

The level of different characteristics of the observation units, generally have units of measurement.

In this case of UTI drug efficacy testing, we saw that the efficacy of patients was divided into four categories: "cured", "significant", "improved" and "ineffective". These categories are sequential, from 'cure' to 'ineffective' indicating a gradual reduction in efficacy. Thus, this data type is classified according to different levels of efficacy, and these classifications are ordered.

The option is resolved as follows:

A. Continuous variable data refers to numerical data that can be subdivided indefinitely, such as height, weight, which is not applicable here.

B. Orderly multi-classification variable data means that the data is divided into several orderly categories, which is applicable to this case.

C. Disordered multi-classification variable data refers to that the data is divided into several categories without internal order, such as blood type, occupation, etc., which is not applicable here.

D. Quantitative data are numerical data that can be quantified, usually continuous or countable, and are not applicable here.

E. Qualitative data refers to the classified data that cannot be expressed in numerical form. Although the data in this case is classified, it more specifically belongs to the orderly classification.

Therefore, the most appropriate answer is * * B. orderly multi-classification variable data * *.

This is a question about data type identification. First of all, we need to understand the data given in the topic and its classification, and then combine the definition of each option to determine which type of data is most consistent with the description of the title.

Understanding background information: The title describes a study on the drug treatment effects of urinary tract infections, involving 87 patients, whose treatment effects were classified as curative, significant, improved, and ineffective.

Understand the core of the problem: We need to choose the most appropriate type from a given option based on the characteristics of these data.

Now, we analyze each option one by one:

A. Data of continuous variables: This refers to the variables that can take any real value, such as height, weight, etc. The data in this question are categorical, not continuous values, so option A does not fit.

B. Orderly multi-classification variable data: This refers to multiple categories of variables and a certain sequential relationship between these categories. The treatment effect (cure, effective, improvement, ineffective) does have a sequence relationship from good to poor, so option B seems reasonable.

C. Disordered multi-classification variables: there is no clear order relationship between categories, such as blood group (A, B, O, AB). The classification in this question is orderly, so option C is not correct.

D. Quantitative data: This refers to quantifiable data, such as continuous variables such as height and weight. Although the treatment effect can be quantified (e. g. cure =4, significant =3, significant, improvement =2, ineffective =1), the original data itself is not in quantity, so the D option is not fit.

E. Qualitative data: refers to the data that describes the nature and cannot perform mathematical operations. Although the treatment effect can be quantified by transformation, its nature is still descriptive in nature, and the title does not give specific quantitative values, so it can be regarded as qualitative data.

Taken together, although the treatment effect seems to be orderly (cure, effective, improved, ineffective), in this problem, we are more concerned with the "qualitative" difference of the treatment effect, rather than the difference of "quantity". That is, we focus on whether the patient is cured, whether it is effective, rather than the "amount" of the treatment effect. Therefore, the data is more consistent with the characteristics of the qualitative data.

Therefore, the most correct answer is E. qualitative data.

According to the title description, the results of the drug efficacy test include "cure", "effective", "improved" and "ineffective". Such data are characterized by ordered categorical variables, that is, these categories can be ranked but not measured quantitatively. So, the most correct answer is:

B Data on orderly and multi-categorical variables

96. (Type A2 questions)

A burn patient came to the outpatient clinic to change the medicine, questioned the doctor's professional ability, and said why the two drugs were not used together. The doctor patiently explained that one of the drugs would weaken the efficacy of the other drug, and could not be used together. The process reflects that doctors' medication is

A. Strictly observe laws and regulations

B. Save money and distribute it fairly

C. Reasonable compatibility, timely adjustment

D. Rational drug use and careful observation

E. Treat the right medicine for the people

Reference answer: C Your answer:

[Analysis] The doctor explained to the burn patient why the two drugs could not

be used together. This process reflects the reasonable compatibility in the process of medication and the principle of timely adjustment, so this question chooses C.

[To avoid mistakes] The medication principle of epilepsy patients should be: medication by type, single medicine, early medication, small amount of start, long-term treatment

cure.

This question examines the principles and considerations of doctors in the process of medication. The doctor explained that two drugs cannot be used together because one of them reduces the efficacy of the other, which reflects the principles of proper compatibility and timely adjustment. So the correct answer should be:

B Reasonable compatibility, timely adjustment

This option emphasizes the rational selection and adjustment of medication based on their knowledge of drug interactions to ensure the effectiveness and safety of the treatment.

This is a choice of medical ethics and the principles of medication. We need to first understand the background information and the core of the question, and then carefully analyze each option, and compare it with the information given in the question.

Understanding background information: The doctor explained to the patient that two drugs cannot be used simultaneously because one drug weakens the other drug.

At the heart of the understanding problem: We need to determine which option best reflects this medication principle.

Analysis options:

A. Strict compliance with regulations: Although doctors should abide by relevant regulations in the course of practice, the doctor's explanation in this topic does not directly reflect this point.

B. Cost saving and fair distribution: This option involves the allocation and economic considerations of medical resources, and is not directly related to the situation in the topic.

C. Reasonable compatibility and timely adjustment: This option directly corresponds to the situation in the topic. The doctor explained why the two drugs cannot be used at the same time, that is, there is an interaction between the drugs, and

reasonable compatibility is needed.

D. Rational drug use and careful observation: Although rational drug use is an important principle for doctors to use drugs, the focus of this topic is on the interaction and compatibility between drugs, rather than simply rational drug use and observation.

E. Treatment for people: This option emphasizes the treatment for individual differences, and is not directly related to the drug compatibility problem in the topic.

To sum up, the doctor's explanation directly reflects the need to pay attention to the reasonable compatibility of drugs in the process of medication and avoid the adverse interaction between drugs. Therefore, option C "reasonable compatibility, timely adjustment" is the choice most consistent with the description of the title.

Therefore, the answer is C. Reasonable compatibility, timely adjustment.

The title describes a doctor explaining to a patient why two drugs cannot be used together, because one drug weakens the efficacy of the other. This reflects that doctors follow the principle of reasonable compatibility and timely adjustment in the process of medication. Reasonable compatibility refers to the interaction between drugs to ensure the safety and effectiveness of drug combinations and to avoid adverse reactions or reduced efficacy. Timely adjustment refers to the adjustment of the treatment plan according to the specific situation of the patient to achieve the best treatment effect.

Therefore, the most correct answer is: C. Reasonable compatibility, timely adjustment

97. (Type A2 questions)

One old man because of bad teeth, often with rice porridge, porridge as the main food, also often eat meat, eggs, but often constipation, consider the constipation of the elderly

A. Eating too few vegetables

B. is mainly cereal

C. Eating too little of meat

D. Excessive consumption of soy products

E. Pasta as the staple food

Reference answer: A. Your answer:

[Analysis] The patient had a simple diet, lack of crude fiber, reduced the volume of stool, increased the viscosity, slow movement in the intestine, excessive absorption of water and constipation. Therefore, this question is selected A.

[Avoid mistakes] Constipation refers to the reduction of defecation times, difficulty in defecation and dry stool. Normal people defecate 1~2 times or 1

~2 days defecation once, constipation patients defecate less than 3 times a week, and defecation effort, fecal induration, less quantity.

There may be many reasons for constipation, but according to the dry information, the old man mainly eats rice porridge, porridge, and often eats meat and eggs. Without mentioning vegetable intake, it can be speculated that vegetable intake may be insufficient. Vegetables are rich in fiber, which is very important to promote intestinal peristalsis and prevent constipation. Therefore, the most likely reason is the underconsumption of vegetables.

Option A "Eating too few vegetables" is the best reason for the old man's constipation, so the correct answer should be A.

analyze:

The eating habits of elderly patients to rice porridge, porridge and other staple food, also often eat meat, eggs, but often constipation. Analysis of the patient's diet, staple food is given priority to with cereals, protein source is given priority to with meat and eggs, lack of vegetables rich in dietary fiber, and dietary fiber is hydrophilic, can absorb water, make the food residue expansion and form lubricating gel, easy to promote feces, and dietary fiber can stimulate the intestinal wall, speed up the intestinal peristalsis, so less dietary fiber intake is the main cause of patients with constipation.

Option B is mainly cereal, while option E is pasta as the staple food, which can provide the carbohydrates needed by the body, and has a certain stimulating effect on the intestinal tract, which can promote defecation, It is not the cause of constipation, so it can be excluded together.

Option C Eating too little meat will generally lead to insufficient protein intake, will not lead to constipation, so do not choose.

Soybean products mostly contain oligosaccharides and other substances, the

volume of these substances combined with water can expand, can cause stimulation to the intestinal tract, accelerate intestinal peristalsis, so as to relieve constipation, option D Eating too much soy products will not cause constipation, but also help defecation, so eliminate.

In conclusion, the correct answer is: A. Eating too little on vegetables.

In this topic, an old man often eats rice porridge and porridge as staple, but also eats meat and eggs, but often constipation. To find out the cause of constipation, we need to consider his eating habits.

According to the title, the elderly's diet contains less vegetables, and vegetables are an important source of dietary fiber, fiber helps promote intestinal peristalsis and prevent constipation. Therefore, the main cause of constipation may be due to insufficient vegetable intake.

So, the most likely answer is:

A. Eating too few vegetables

98. (Type A2 questions)

A doctor screened 1,160 people in an area, and found a total of 140 patients with diabetes. They were tested for fasting blood glucose at 6.1. The results were as follows, asking what was the positive test rate was

A. 27/85

B. 87/89

C. 31/112

D. 124/140

E. 58/85

Refer to the answer: D. Your answer:

[Analysis] For the fasting blood glucose test of 140 diabetic patients, the fasting blood glucose of 6.1 mmol/L was positive, and the test result was positive: 124, negative: 16, so the positive test rate of this standard was 124 / 140, so we choose D.

[Error avoidance] Sensitivity, also known as sensitivity or true positive rate, refers to the positive test to be evaluated in cases confirmed by gold standards

The percentage of the people in the population. It can reflect the ability of the test to be evaluated to correctly judge the actual sick case as a certain disease.

Specialty, also known as true negative rate, refers to the percentage of non-cases to be judged negative by the gold standard

compare. It can reflect the ability of the test to be evaluated to correctly judge the actual research subjects without a disease as without a disease.

In this issue, we need to determine the positive test rate, namely the number of detected fasting glucose 6.1 divided by the number of diabetic patients with total fasting glucose testing.

The given information does not mention how many people had fasting blood glucose 6.1, except that a total of 140 diabetic patients were found and tested for fasting blood glucose. Generally, the positive test rate is the proportion of positive test results in all test subjects, so we should calculate the number of positive results divided by the total number of people tested (140 patients with diabetes).

The numbers in the option should represent a combination of different positive test results and the total number of tests. The most reasonable speculation here is that the number of positives should be close to 140, as this is the total number of diabetic patients tested.

Thus, the closest option to the 140 positives is D. 124 / 140. This indicates a positive fasting glucose measurement in 124 diabetic patients, with a total of 140 diabetic patients tested.

Therefore, the positive test rate should be 124 / 140, and choose D as the answer.

This is a question about diabetes screening that requires us to calculate the positive rate of fasting blood glucose testing.

Background information: This question involved screening 1,160 people for diabetes, and found 140 patients with diabetes. Next, these diabetic patients were tested positive for fasting glucose 6.1.

Understand the core of the problem: We need to calculate the positive detection rate based on this data.

Analysis options:

A. 27 / 85: This ratio does not match the data provided in the title.

B. 87 / 89: Similarly, this ratio does not match the data in the title.

C. 31 / 122: This ratio also does not fit the data in the title.

B 124 / 140: According to the title description, fasting glucose 6.1 is considered positive, and the number of clear fasting glucose 6.1 was 124. The total number of tests was 140, so the positive test rate should be 124 / 140.

E. 58 / 85: This ratio does not match the data in the title.

In conclusion, the positive rate of fasting glucose testing should be the number of people who tested positive divided by the total number of tests, namely 124 / 140.

Therefore, the correct answer is D.124 / 140.

To calculate the positive test rate, we need to understand the definition: the positive test rate is the rate of positive results in the screening results. In this question, we focus on the positive results of the fasting blood glucose test.

From the title, we know that 1,160 people were screened and 140 people were found to have diabetes. These diabetic patients were tested positive for fasting glucose and fasting glucose 6.1.

Therefore, we need to find the following proportions:

\[\ Text {positive detection rate} = \ fic {\ text {number of positive results}} {{text {total number of tests}} \]

Among the options provided by the question, only the denominator of option D is 140 (total number of tests), so we only need to see if the number of positive results

is 124.

The answer is:

\[D. 124/140 \]

So the positive test rate is:

\[\frac{124}{140} = 0.8857 \]

To proximately 88.57%.

The correct answer is D. 124 / 140.

99. (Type A2 question)

A hospital selected 117 pregnant women to study whether the injection of pregnant women with oxytocin had on neonatal jaundice. Among them, 60 pregnant women gave drug treatment, and 57 pregnant women did not give drug treatment to observe whether the newborn developed neonatal jaundice, thus associated with neonatal jaundice. This method of study is considered as follows

A. case-control study

B. cohort study

C. Research on current conditions

D. experimental research

E. cross-sectional study

Refer to the answer: D. Your answer:

[Analysis] Clinical trial (D pair) randomly divides the research subjects from the same population into experimental group and control group. The experimental group

and the control group does not give the experimental factor, and then prospectively follow up the outcome of each group and compare the degree of difference so as to judge the effect of experimental factors.

[Wrong avoidance] A case-control study (A error) is to select people with and without a specific disease as the case group and, respectively

Control group, investigate the proportion or level of past exposure to some or some suspected risk factors, by comparing groups

The difference in the proportion or level of exposure, judging whether the exposure factors are associated with the disease studied and the degree of association

An observational study approach. A cohort study (B error) involves having a well-defined population exposed to a suspicious cause

Su or exposure was divided into different subgroups, and the outcomes of each group were tracked and compared to determine the exposure factors and outcomes

An observational study method of whether and not the degree of association. The current condition studies and cross-sectional studies (CE error) are

The most widely used method in descriptive studies. It is the application of census or sampling survey methods to collect a specific time,

Data on disease, health status and related factors in specific populations, and describe the distribution characteristics of the data.

This question examines the identification of different types of study designs. Case – control studies (A) are often retrospective, comparing past exposures between people with a disease (or outcome) and those who do not. The cohort study (B) was prospective or retrospective, tracking two groups (exposed and non-exposed) populations to see if they develop a disease or outcome. A current situation study (C) is a cross-sectional study of the health status or certain variables in a specific population at a certain point in time. The experimental study (D) involves the study design of an intervention, such as a randomized controlled trial. Horizontal research (E) is another version of the current situation study, which is also conducted at a certain point in time.

According to the information, the researchers selected pregnant women, one group received oxytocin, and the other group did not, to compare the incidence of neonatal jaundice. There is a clear intervention (oxytocin injection), and neonatal outcomes are observed prospectively in both groups of pregnant women. This fits

with the characteristics of the experimental studies.

Therefore, the correct answer is * * D. Experimental study * *.

First, we briefly review the key information in the topic stem: this is a study of the effects of neonatal jaundice after oxytocin injection in pregnant women. In the study, 117 pregnant women were divided into two groups, one group given medication (oxytocin) and the other group given without medication. The aim was to observe whether the newborn developed jaundice and to explore the association with oxytocin.

Next, we analyze the options:

A. Case-control studies: This method is usually used to determine whether exposure factors are associated with the disease studied and the degree of association. It is usually a comparison of the proportion of people with and without a disease with past exposure to a possible risk factor to analyze and infer the association of that factor with the disease. The study described by the title stem is not retrospective based on existing cases, so option A is incorrect.

B. Cohort study: Cohort study is an observational study method that divides a well-defined population into different subgroups according to whether they are exposed to a suspicious factor or exposure degree, tracks the outcome of each group and compares its differences, so as to determine whether there is a causal association and the degree of association between exposure factors and outcome. The study grouped the pregnant women according to whether to give drug treatment (oxytocin) and tracked the jaundice of the newborns, which matched the definition of the cohort study.

C. Current situation research: This method is the method of census or sampling survey to collect certain data, and describe the distribution characteristics of the data. It is usually used to understand the disease or health condition at a certain time, rather than to infer causality. The research described by the stem clearly involves the inquiry of causality, so option C is not correct.

D. Experimental research: Although there are Settings of experimental group and control group in the question stem, experimental research usually involves the strict control of experimental conditions and the random allocation of experimental subjects. The title stem did not explicitly mention the key elements of the experimental design, so it is not entirely certain that this is an experimental study.

E. Horizontal research: This usually refers to the research method of psychological experimental research or investigation of subjects in different age groups within the same time, which is inconsistent with the research described by the title stem.

In conclusion, according to the study design and purpose of the title description, the most consistent is the cohort study. This approach can prospectively observe the health status of different groups (exposed and non-exposed groups) over a period of time and determine the causal relationship between exposure factors and disease.

Therefore, the correct answer is the B. cohort study.

According to the title description, this research method belongs to the * * experimental study * *.

The explanation is as follows:

- ** Experimental study * *: The investigator intervened with some variables and observed the results. In this topic, the investigator observed the effect on neonatal jaundice by intervention (giving oxytocin).

- ** Case-control study * *: Individuals with a certain disease (case group) are compared with individuals who do not have the disease (control group) to look for possible pathogenic factors. The study is usually retrospective.

- ** Cohort study * *: A group of individuals (cohort) without a certain disease was divided into two groups according to whether they were exposed to a pathogenic factor, and then the onset of the two groups was observed prospectively.

- ** Current situation study * *: Observe a certain group of people at a certain point in time to understand the status quo or distribution of a certain disease.

- ** Horizontal research * *: Observation of a certain population at a specific point in time is usually used to describe the distribution of disease or health-related factors at a certain point, similar to current situation studies.

The description in the title subgroups 117 pregnant women with or without oxytocin and observes the occurrence of neonatal jaundice, a typical experimental study design. So, the correct answer is:

B experimental research

100. (Type A2 question)

An investigator conducted a randomized single-blind trial to compare the antihypertensive efficacy of A and B drugs in patients with mild to moderate essential hypertension. In this experiment, the most likely situation of the trial group was not known

A. Experimental designer

And B. Statistical analysts

B. Patients receiving the treatment

C. Physician who administered the treatment

D. Nurses who measured the blood pressure

Reference answer: C Your answer:

[Analysis] The single-blind study subjects (C pair) did not know which group they were assigned to and the specific content of the intervention was called single-blind.

[Error avoidance] If neither the study subject nor the observer know the grouping situation and the specific content of the treatment measures, it is double-blind

DESIGN The observer in a clinical trial can be a nurse or a doctor. The study subjects, observers and data analysts did not know

The grouping of patients and the specific content of the treatment measures are called triple blind.

In a randomized single-blind trial, we usually keep the group blinded to ensure

the objectivity and impartiality of the experiment. A single-blind experiment means that one of the parties (usually the participants themselves) does not know whether they have received experimental or control treatment. Therefore, the patients most likely unaware of the trial grouping were the patients receiving treatment.

Option C (for the treated patient) is the most correct answer.

analyze:

Randomized single-blind trial refers to the study subjects do not know which group they are assigned to, and the researchers know the grouping. Single-blind is mainly to avoid the possible effects of subjective factors of the research subjects. According to the topic, the antihypertensive efficacy of A drugs and B drugs in patients with mild to moderate essential hypertension was compared. In this trial, a randomized single-blind study method was adopted, and the treated patients were most probably not aware of the group status.

In the randomized single-blind trial, the investigator is aware of the grouping, so the designer of option A, option B, and the doctor who performs the treatment in option D will know the grouping and can be excluded.

Option E The nurse who measured blood pressure was the researcher and also knew the grouping in the single-blind trial, so she was not chosen.

In conclusion, the correct answer is: C. Patients receiving treatment.

In randomized single-blind trials, it is usually the patients who are not aware of the trial grouping. This design was designed to prevent patient expectations or bias from influencing trial results. Patients do not know whether they have received A or B drugs, thus avoiding the impact of psychological factors on the results.

Therefore, the most likely unaware of the trial grouping is:

E. Patients receiving the treatment

101. (Type A2 questions)

The purpose of the case-control study data is

A. Controls the selection offset

B. Control the information offset

C. Control for confounding bias

D. Control for recall bias

E. Control for no-response bias

Reference answer: C Your answer:

Graphic analysis: [analysis] stratified analysis is to divide the research population into different layers according to a certain feature or factor, and then analyze the association between exposure and disease in each layer respectively. The factors used for stratification are possible confounding factors. Through stratification analysis, the factors can be judged whether the factor plays a confounding role. If the confounding role plays, it can be controlled by stratification analysis, so the purpose of stratification analysis is to control the confounding bias (C pair).

[Wrong avoidance] common selection bias: (1) the admission rate bias, (2) the detection of syndrome bias, and (3) the current sick cases-new cases

Bias, (4) no response bias, (5) susceptibility bias; common information bias: (1) recall bias, (2) reporting

Reporting bias, (3) exposure suspicion bias, (4) diagnostic suspicion bias, (5) measurement bias, and (6) publication bias.

The primary aim of the stratified analysis in case-control studies is to control for confounding bias. Therefore, the correct answer is C. Control for confounding bias.confounding bias occurs in observational studies and when one or more additional variables are simultaneously associated with the exposure and outcome of the study, this may affect the results causing erroneous associations. Stratified analysis is a statistical method in which these confounders can be considered in the analysis, thereby reducing their impact on the study results.

First, let's parse the topic and point out the key information. This question asks about the purpose of a stratified analysis in a case-control study.

Next, each option is analyzed one by one:

A. Control selection offset: Selection offset is mainly due to the deviation caused by the selection methods or criteria of the research objects. Stratified analysis in case-control studies were not designed to control for selection shift.

B. Control information offset: information offset is mainly caused by errors in measurement or information collection. The stratified analysis was primarily used to control for confounders rather than information offset.

C. Control for confounding bias: confounding bias is due to interference by other factors, which deviates the study results from the truth. The stratified analysis is precisely by stratifying study subjects by potential confounders to control for the influence of these confounders on the results. Therefore, this option is correct.

D. Control for recall bias: recall bias is mainly due to the subject's error in recalling information. Although case-control studies may be subject by recall bias, stratified analyses were not designed to control for this bias.

E. Control for non-response bias: non-response bias is a bias caused to some of the subjects not responding to the survey or study. The stratified analysis was not related to this issue.

In conclusion, the main objective of the stratified analysis in case-control studies is to control for confounding bias, namely, option C. This approach more accurately evaluates the relationship between exposure factors and disease by stratifying study subjects by potential confounding factors (e. g., age, gender, etc.).

Therefore, the correct answer is C. Control for confounding bias.

A case-control study is an observational study design, often used to explore the association between disease and underlying risk factors. Stratified analysis is a statistical method used to control for the effect of confounding variables on study outcomes.

confounding bias (confounding factors) is the presence of one or more factors associated with both exposure and outcome, which may confound the true relationship between exposure and outcome. Through stratified analysis, the relationship between exposure and outcome can be analyzed separately at each levels, thereby controlling the influence of these confounding factors.

Therefore, the purpose of the stratified analysis of the case-control study data is

to:

B Control for confounding bias

102. (Type A2 questions)

A scholar wants to use the method of case-control study to explore the influencing factors of coronary heart disease, and the most inappropriate thing is to choose cases

A. Try to choose new cases

B. Try to select cases diagnosed by common diagnostic criteria at home and abroad

C. Try to include cases from different hospitals

D. Select community-derived cases as far as possible

E. Try to select cases from large hospitals

Reference answer: E Your answer:

[Analysis] Due to the use of hospital patients as cases and controls, patients to hospitals, hospitals to patients, cases are not random samples of all patients, so the choice to carry out hospital-based case-control research is easy to produce admission rate bias. Patients derived from large hospitals have more severe symptoms, so it is the most inappropriate to choose, so this question chooses E.

[Avoid] Option C in this question is a confusing option, coronary heart disease is a chronic long-term disease, community cases have mild to severe cases, but not

Critical and severe cases, so it can be used as a research object.

In case-control studies, investigators usually aim to compare people with a disease (cases) and those without it (controls) to identify risk factors for the disease. Case selection should be considered with multiple factors to ensure the validity and representativeness of the study.

Option A refers to the selection of new cases as far as possible, which is often appropriate because it reduces recall bias and selection bias and more accurately reflects the current condition and risk factors.

Option B refers to selecting cases diagnosed by common diagnostic criteria at home and abroad, which is also appropriate because the use of standardized

diagnostic criteria helps to ensure the accuracy of cases and the comparability of study results.

Option C means to select cases from different hospitals as much as possible, which can increase the diversity and representativeness of the study sample and thus improve the extrapolation of the findings.

Option D is the selection of community-derived cases as far as possible, which helps to ensure that the study sample is not limited to specific healthcare facilities, thus increasing the representativeness of the sample.

Option E is to select cases from large hospitals as far as possible, which may lead to selection bias because cases in large hospitals may not represent the general population, especially if the study focuses on the status of the whole community or population.

Therefore, the most inappropriate choice was E, selecting cases from large hospitals as far as possible, as this may introduce selection bias, limiting the representativeness and generalizability of the study.

analyze:

A case-control study, also known as a retrospective study, compares the percentage of exposure to a possible risk factor between a patient and no control patient to analyze whether these factors are associated with the disease. Is one of the most basic and important types of studies in analyzing epidemiological methods. When selecting cases, if cases are selected from large hospitals as far as possible, selection bias may be introduced, because cases in large hospitals may be more serious and complex, and may include cases referred from small hospitals, so they are not representative and cannot reflect the overall situation of patients with coronary heart disease.

Option A: Choosing new cases as closely as possible can reduce the recall bias and ensure the authenticity of the results, which is the ideal choice for case selection.

Option B Try to select the confirmed cases with common domestic and foreign diagnostic criteria to ensure the diagnostic accuracy of the cases and make the study results more reliable.

Option C to select cases from different hospitals as far as possible could expand the sample source and make the findings more representative.

Option D selecting community-derived cases as far as possible can reflect the

real situation of CHD in grassroots patients, increasing the external validity of the study.

To sum up, the correct answer is: E. Try to select cases from large hospitals.

A case-control study is a retrospective study that identifies risk factors for disease by comparing the characteristics of a case group (individuals with disease) and a control group (individuals without disease). In selecting selection, the following are some considerations:

- ** New cases (option A) * *: Choosing new cases helps to reduce selection bias, as new cases are more representative of current disease characteristics and risk factors.

- ** Diagnostic Criteria (option B) * *: Cases confirmed using diagnostic criteria common at home and abroad ensure the homogeneity and diagnostic accuracy of cases, which helps to improve the internal validity of the study.

- ** Inclusion of cases from different hospitals (C option) * *: Selection of cases from different hospitals can increase the representativeness of the sample and reduce the bias due to a single hospital characteristics.

- ** Community-derived cases (option D) * *: Community-derived cases are generally better representative of the general population, thus improving the external validity of the findings.

- ** Cases selected by large hospitals (option E) * *: Choosing cases from large hospitals whenever possible may lead to selection bias, because patients in large hospitals are often seriously ill and do not well represent the disease situation of the general population.

In conclusion, in case-control studies, the most inappropriate case selection is * * E. To select cases from large hospitals as far as possible.

So, the answer is up to this:

* * E. Try to select cases from a large hospital * *

103. (Type A2 questions)

A city in 2006 to primary and secondary schools vision health survey, found that primary and middle school students eyesight health is not good. The primary and secondary schools in the city were divided into experimental group and control group. The experimental group carried out eye health care publicity and stopped bad eye behavior, and the results were analyzed after a period. The study is for

A. experimental research

B. Community intervention experiment

C. cohort study

D. case-control study

E. Individual experiments

Reference answer: A. Your answer:

Graphic analysis: [analysis] experimental study (A pair) divides the random subjects from the same population into experimental group and control group, the experimental group gives experimental factor, the control group does not give the factor, and then prospectively follow up the outcome of each group and compare the degree of difference to judge the effect of experimental factors.

[Error avoidance] The case-control study is to select the population with and without a specific disease as the case group and the control group, respectively,

Investigate the proportion or level of past exposure to certain or certain suspected risk factors by comparing exposures between groups

Differdifference in proportion or level, an observation of whether the exposure factor is associated with the disease studied and the degree of association

Methods of sexual research. A cohort study divides a well-defined population into whether exposed to a suspicious factor or exposure

Different subgroups tracked the outcome of each group and compared their differences to determine whether there is any association and correlation between exposure factors and outcome

An observational study of the degree of association.

This title describes a visual health survey of primary and middle school students,

and then divides the students into experimental group and control group. The experimental group took intervention measures to promote eye health care and stop bad eye behavior. The key point here is a clear intervention (eye care presentation and suppression of bad eye behavior) and a control group, which has characterized the intervention study.

Option A 'Experimental research' usually refers to studies conducted under tightly controlled conditions, either in a laboratory or community setting, but this option is too broad.

Option B 'Community Intervention Experiment' refers to an intervention implemented at the community level to observe its effect on certain health outcomes, consistent with the context described in the title.

The C option 'Cohort study' is usually observational studies following two or more groups to compare their exposure factors and health outcomes with no proactive interventions.

Option D "Case-control study" is usually retrospective, comparing exposure between individuals with (cases) of disease and those without disease (controls).

The E option 'individual experiment' does not provide sufficient information in this context and does not fit the description of the whole group intervention.

In conclusion, the most appropriate answer is the B. Community intervention experiment, as the study implemented a clear intervention in a community (school) environment and set up a control group for comparison.

First, let's look at the experimental design described in the title. In this study, the researchers first found the problem of poor visual health of primary and middle school students, and then divided the city's primary and middle school students in the group, experimental group and control group. The experimental group received eye health care and bad eye behavior. The core of this design is to implement a certain intervention (eye care preaching and bad eye behavior suppression) for a part of the people (experimental group) in a specific community (primary and secondary schools), and then observe the effect of this intervention.

Next, we analyze the options one by one:

A. Experimental research: Although the study involves experimental grouping,

the term "experimental research" is relatively broad and not enough to accurately describe the characteristics of the study.

B. Community intervention experiment: This option is highly consistent with the title description. The study was conducted in a specific community (primary and secondary school), with a specific intervention (eye care presentation and bad eye behavior suppression), and then evaluating the effect of this intervention. This is a typical experimental design for a community intervention.

C. Cohort studies: Cohort studies often involve long-term follow-up of specific populations to observe the relationship between certain exposure factors (e. g., smoking, eating habits, etc.) and health outcomes (e. g., disease incidence). The study design in this topic is inconsistent with this one.

D. Case-control studies: This study design is usually used to assess whether a certain exposure factor is associated with a certain disease. It involves selecting individuals already sick (case group) and not sick (control group) and then retrospectively comparing their exposure history. The design in this topic is not such a study.

E. Individual experiments: This option emphasizes experiments at the individual level, while the research in this topic is conducted at the community (school) level.

In conclusion, the study best fits the definition of "community intervention experiment". In such experiments, the researcher implements a specific intervention for a subset of the population within a specific community, and then evaluates the impact of the intervention on the health of the population.

Therefore, the correct answer is the B. Community intervention experiment.

According to the title description, the study involved grouping and intervening for primary and secondary school students (eye care presentation and bad eye behavior suppression), and then analyzing the results. The characteristics of this study design fit the definition of experimental research as it includes intervention and contrast groups. Here is an analysis of the options:

A. Experimental research: It is indeed an experimental study, because it includes interventions and an experimental control group.

B. Community intervention experiment: Although this study involves the community, its specific intervention object is students, so it is more suitable to be called experimental research.

C. Cohort studies: Cohort studies usually involve long-term follow-up of a group of individuals without a specific intervention, not eligible here.

D. Case-control study: This study design to compare past exposures in sick and disease-free individuals is not as described here.

E. Individual experiments: Individual experiments are an intervention study of a single individual, where the subject is a group.

So, the most appropriate answer is:

A. experimental research

104. (Type A2 questions)

In the United States, 300 healthy elderly male employees were selected and followed up for 10 years to conclude that type A personality was the cause of cardiovascular disease

A. perspective study

B. retrospective study

C. Investigational study

D. cohort study

E. observational study

Reference answer: A. Your answer:

Graphic analysis: [Analysis] Prospective research is based on the present to track the future research method, which can make up for the defects of retrospective research. The study of 300 healthy elderly male employees for 10 years is A prospective study, so this question A is chosen.

[Error avoidance] Retrospective research is based on the present and goes back to the past. Experimental studies will be derived from the same population

The study subjects are randomly divided into experimental group and control

group, the experimental factor, the control group does not give this factor, and then before

To follow up the outcome of each group and compare the degree of difference to determine the effect of experimental factors. Cohort study is what will be one

The well-defined populations were divided into different subgroups according to whether they were exposed to a suspicious factor or exposure, and the outcome of each group was tracked

An observational study that compares the association and the association between the exposure factors and the outcome

law. Observational research is the application of census or sampling survey methods to collect a specific time of population disease, health conditions

And related factors of information, and the distribution characteristics of the data.

This topic describes a study in which 300 healthy elderly male employees were followed for 10 years to determine the association between type A personality and cardiovascular disease. The study approach involved tracking changes in a given group over time without mention of any intervention. This type of study design is usually classified as a cohort study.

A cohort study is an observational study method used to study the association between exposure and outcome. It involves selecting a group of people who are not sick but are exposed to a factor (type A personality in this case) and tracking them over time to observe and document the occurrence of outcomes (e. g., cardiovascular disease).

Therefore, the most appropriate answer is: D. Cohort study

analyze:

In this topic, the United States in the "behavior study" selected 300 healthy elderly male employees, follow-up 10 years concluded that type A personality is the cause of cardiovascular disease, the study group according to whether exposure type A personality characteristics, after 10 years of prospective observation, compare the incidence of cardiovascular disease between the two groups, thus concluded that type A personality is the cause of cardiovascular disease, this research method is called prospective cohort study. Prospective cohort study refers to an observational study method that groups people without a certain disease according to whether they are

exposed to a suspicious factor or exposure degree, tracks and observes their respective outcomes and compares their differences, so as to determine whether there is a causal association and degree of association between exposure factors and outcome.

Option B The retrospective study is based on the present, looking back to the past study, and is inconsistent with the research method described, so it is not selected.

Option C experimental study is a research method for researchers to reasonably control or create certain conditions according to the research purpose, artificially change the research object, so as to verify the hypothesis and explore the causality of educational phenomenon. It is inconsistent with the description of the question stem, so it is excluded.

Option D Cohort study is an observational study method of dividing a specific population into subgroups by exposure, tracking the occurrence of outcomes (such as disease) between the groups and comparing the difference of the incidence of cause and effect between the outcome, including prospective cohort and historic cohort studies. Prospective cohort study is a kind of cohort study, and the option expression is not accurate enough, so it is not selected.

Option E Observational studies means that researchers objectively observe and record the behavior or status information of research subjects without any intervention measures, so as to understand the distribution characteristics of diseases, health status and related factors in the population, without giving any intervention measures, which is inconsistent with the description of the subject stem, so it is not selected.

In conclusion, the correct answer is: A. prospective study.

The description of this problem involves the long-term follow-up of a group of healthy elderly male employees to draw research conclusions about the relationship between type A personality and cardiovascular disease. According to the topic content, this is a long-term follow-up observation study.

The key points mentioned in the title stem are:

1 The study population was composed of 300 healthy elderly male employees.

2 The study method is to follow-up for 10 years.

3 The study concluded that type A personality is A cause of cardiovascular disease.

With this information, we can analyze the various options:

- ** Prospective study * *: This research method is to collect data from now and follow up observation in the future.

- ** Retrospective study * *: This research method is to review the past data or cases, for analysis.

- ** Pilot study * *: This research method is to conduct intervention trials by setting up trials and control groups.

- ** Cohort study * *: This is a type of prospective study involving long-term follow-up of a specific group of population (cohort).

- ** Observational study * *: This is a non-intervention, only observational study approach that can include both prospective and retrospective studies.

Option A (prospective study) and D (cohort study) both appear to fit the description, but the more accurate answers are:

B * * Cohort Study * *

Because the subject stem describes a long-term follow-up of a specific group of people to observe their health changes and draw conclusions, this is exactly the definition of a cohort study.

105. (Type A2 questions)

Male, 40 years old, 1 week after a business trip to eat street stalls, fever, sclera mild yellow dye, AST, ALT increased, HAVIgM (+), HBsAg (+), for the pathogen of the patient is correct

A. prone to recessive infection

And B. Mother-to-child transmission is possible

B. It can cause a secondary viremia

C. is a DNA virus

D. nonenveloped virus

Reference answer: A. Your answer:

[Analysis] Anti-HAVIgM positivity is the specific index of early diagnosis of hepatitis A. Blood HBsAg (+) indicates asymptomatic carriers or patients with chronic hepatitis B, and the history of unclean food consumption and clinical manifestations of viral hepatitis A. Hepatitis A virus is the genus Hepatoviruses in the family Small RNA Viridae. After human infection with HAV, most people show subclinical or recessive infection, and only a few people present with acute hepatitis A. Can recover completely commonly, do not turn for chronic hepatitis, also have no chronic carrier.

[Error avoidance] Anti-HAV-IgG positive is a marker of previous HAV infection or immunization, which can exist for a long time and cannot be used as a

The diagnostic basis of the liver.

The patient's symptoms included fever, mild yellow sclera, elevated AST and ALT, positive HAV IgM (hepatitis A virus immunoglobulin M) and positive HBsAg (hepatitis B surface antigen). Positive HAV IgM means that the patient had A recent hepatitis A virus infection, while HBsAg positive indicates the presence of hepatitis B virus infection.

-A. Easy to recessive infection: more common in hepatitis B virus (HBV).

-B. Mother-to-child transmission: This is characteristic of hepatitis B virus and can be transmitted from mother to child.

-C. Can cause secondary viremia: this is not A common feature in the hepatitis A virus.

-D. Is A DNA virus: Hepatitis B virus is a DNA virus, while hepatitis A virus is an RNA virus.

-E. Unenveloped virus: Hepatitis A virus is an unenveloped RNA virus.

Given patients the patients of both hepatitis A and hepatitis B. However, based on the information and options provided, we prefer the characteristics of hepatitis B virus because it is the key pathogen in the topic (especially HBsAg positive). Therefore, the most suitable answer option is * * B. Mother-to-child transmission can

be transmitted * *, as this is a clear feature of hepatitis B virus.

analyze:

The middle-aged male, with fever, mild yellow dye, AST, ALT increased, HAVIgM positive, HBsAg positive, was considered for diagnosis of hepatitis A with hepatitis B. Hepatitis B is caused by hepatitis B virus, hepatitis B virus is DNA virus, can cause acute or chronic hepatitis B after infecting human body, chronic hepatitis is easy to develop for liver cirrhosis, liver cancer.

Option A is prone to recessive infection of hepatitis A virus, hepatitis A virus is an RNA virus, mainly fecal-oral transmission, the population is generally susceptible, can obtain lasting immunity after infection, recessive infection is very common, does not meet the meaning of the question.

Option B The pathogen that can be mother-to-child transmission is HIV. Although hepatitis B virus can be mother-to-child transmission, it is less likely than mother-to-child transmission of AIDS, so it is excluded.

Option C can cause secondary viremia is dengue virus, hepatitis B virus does not have this characteristic, so it is not selected.

Option E Unenveloped virus is poliovirus, whereas hepatitis B virus is enveloped virus and it can therefore be preferentially excluded first.

In summary, the correct answer is: D. A DNA virus.

According to the information, the 40-year-old male patient developed fever and mild yellow sclera, increased AST and ALT, and HAV IgM (+) and HBsAg (+) after eating the food stalls. These suggest that the patient was coinfected with hepatitis A virus (HAV) and hepatitis B virus (HBV).

Let's analyze the various options:

A. Easy to recessive infection: Hepatitis A virus (HAV) is usually A dominant infection, that is, patients will appear obvious symptoms, and recessive infection is not common.

B. Mother-to-child transmission: Hepatitis B virus (HBV) can be transmitted

from mother to child.

C. Can cause secondary viremia: secondary viremia is generally associated with chronic viral infection, and hepatitis A virus (HAV) is usually an acute self-limiting disease that does not cause secondary viremia.

D. Is a DNA virus: Hepatitis B virus (HBV) is a DNA virus, while hepatitis A virus (HAV) is an RNA virus.

E. Unenveloped virus: Hepatitis A virus (HAV) is unenveloped virus, while hepatitis B virus (HBV) is an enveloped virus.

Combining the HAV IgM (+) and HBsAg (+) of the patient, we need to select a feature suitable for these two viruses.

The correct answer is:

And B. Mother-to-child transmission is possible

Because the hepatitis B virus (HBV) can be transmitted from mother to child.

100, (Type A2 questions)

A 28-year-old female presented with pruritus of the vulva for 1 week without fever. Gynecological examination: the vulva is visible tofu residue-like secretions. Pathogen characteristics causing the disease are not included

A. Can be grown in sand weak medium

B. for a unicellular microorganism

C. Can cause deep tissue and organ damage

D. for the opportunistic pathogens

E. for the Gram stain-negative bacteria

Reference answer: E Your answer:

Graphic and text analysis: [analysis] young women, vulva pruritus for 1 week, no fever. Gynecological examination can see bean curd-like secretions, the patient's most likely diagnosis of vulvovaginal Candidosis, Candida is gram-positive bacteria, so this question E.

[Fault avoidance] C. albicans grew well on common agar, blood agar and sand retention medium (pair A), with single fine

Cell microorganisms (B pair), can infect the skin, mucosa, viscera (C pair,) vulvovaginal candidosis is mainly internal

Origin infection, Candida as an opportunistic pathogen (D pair), in addition to the vagina, can also be parasitic in the human mouth, intestine

Dow, this part of the Candida bacteria can infect each other, also can be transmitted directly through sexual intercourse.

This title described a 28-year-old female patient complaining of pruritus in the vulva for 1 week. A gynecological examination found tofu residue-like secretions in the vulva. Based on these symptoms, the most likely pathogen is Candida albicans (Candida albicans), a common agent of Candida infection.

For the characteristics of the pathogen, we can analyze:

A. C. albicans can grow in sand weak medium, so the A option is the correct feature.

B. C. albicans is a unicellular fungus, so the B option is also correct.

C. albicans usually causes infections of the skin and mucosa, causing less damage to deep tissues or organs, but can cause deep tissue damage in cases of compromised immunity. Thus, the C option is correct to some extent.

D. C. albicans is an opportunistic pathogen that usually causes infection during normal flora imbalance or decreased immunity, so the D option is correct.

E. C. albicans is Gram-positive, so the E option description does not match the characteristics of C. albicans.

According to the above analysis, the correct answer should be * * E. Gram stain negative bacteria * *, because the pathogen causing the disease, Candida albicans, is actually Gram stain positive.

analyze:

In young female patients, vulva pruritus, vulva visible tofu residue-like secretions, the most likely diagnosis is vulvovaginal candidosis, the pathogen is Candida albicans, a kind of fungi. However, the cell wall of fungi contains chitin and β -glucan, which is easily colored on Gram staining, so Candida leucopus is Gram-positive.

Candida albicans is a fungus, which can grow in sand-protected weak medium, so option A can grow in sand-protected weak medium belongs to the characteristics of this bacterium.

Candida albicans is a fungus, which is a eukaryote with real nuclei and organelles and belong to unicellular microorganisms, so option B is a unicellular microorganism.

In addition to invading the skin and mucosa, Candida albicans can also cause acute and chronic visceral infection, so option C can cause deep tissue and organ damage in line with its characteristics.

Candida albicans is an opportunistic pathogen, mainly endogenous infection. In addition to parasitic the vagina as an opportunistic pathogen, it can also be parasitic in the oral cavity and intestinal tract. Once the conditions are appropriate, it can cause infection, so option D as an opportunistic pathogen is its characteristic.

In conclusion, the correct answer is: E. Gram-negative bacteria.

According to the case information provided by the Gan, the patient was a 28-year-old woman with pruritus in the vulva for 1 week and no fever. Gynecological examination found tofu-like secretions in the vulva. Based on these symptoms and signs, the most likely diagnosis is * * Candida vulvovaginalis infection * * (i. e., vaginal candidiasis).

Candida is a common yeast infection, especially common in women, and the typical symptoms include vulva pruritus and tofu residue-like leucorrhea.

Now we will analyze each alternative answer:

A. * * Can grow in sand weak medium * *: correct. Candida species can grow in

sand weak medium, a medium commonly used to culture fungi.

B. * * for unicellular microbe * *: correct. Candida species are unicellular microorganisms, belonging to a species of fungi.

C. * * Can cause deep tissue and organ damage * *: error. Although Candida may cause systemic invasive infections in immunocompromised patients usually usually only superficial infections such as skin and mucosal infections in immunocompetent patients. Deep tissue and organ damage is not typical features.

D. * * for opportunistic pathogens * *: Correct. Candida usually exists as an opportunistic pathogen, usually parasitic on the human mucous membrane and skin, and causes infection when the human immunity drops or the local environment changes.

E. * * for Gram stain-negative bacteria * *: error. Candida species are gram stain-positive bacteria.

Therefore, * * the most correct answer is that C. can cause deep tissue and organ damage * *.

101. (Type A2 questions)

A male found his wife cheating, the next day the lower limbs can not walk, and appeared epilepsy symptoms, lower limbs weakness, examination nervous system negative, with Minnesota personality test, the patient belongs to

A. hypo

B. paranoia

C. depression

D. hysteria

E. schizophrenia

Refer to the answer: D. Your answer:

Graphic analysis: [analytical] hysteria refers to a kind of separation symptoms (partial or complete loss of self identity and memory of the past) and conversion symptoms (in insoluble problems and conflict of unhappy mood, in the form of somatic symptoms) of mental disorders, including hysteria conversion symptoms including hysteria blindness, paralysis, hysterical loss, hysterical convulsions. Therefore, this question is chosen D.

[Avoid wrong] hysteria is also called hysteria, separation disorder. Hysteria sex epilepsy, the preferred psychotherapy method is hypnotic suggestion therapy

law. Hychondria (A wrong) Patients have unfounded belief that they have a serious physical or incurable disease.vain hope

An order (B error) is one or more non-grotesque delusions without any other psychotic symptoms

(C wrong) It can be summarized as low mood, slow thinking, and decreased will activity. Schizophrenia (E wrong) generally has hallucinations, delusion

Thinking, agitation behavior, social failure, lack of interest, loss of willpower and other symptoms.

This title describes a presentation of physical symptoms in a patient after experiencing emotional trauma, such as weakness and inability to walk in both lower limbs, and with symptoms of epilepsy, but no abnormalities were detected by neurological examination. In this case, the symptoms are likely to be caused by psychological causes. Based on the title description and options, the most appropriate answer is D. hysteria.

Hysteria, also known as conversion disorder, is a mental illness in which the patient exhibits symptoms related to the nervous system, such as paralysis, difficulty walking or seizures, but these symptoms cannot be explained by medical examination. Such symptoms are often associated with psychological factors, such as occurring after experiencing extreme stress or emotional trauma. Therefore, according to the situation and symptoms described in the title, choosing D. hysteria is the most appropriate choice.

analyze:

The middle-aged male patient, after discovering his wife cheating, suddenly could not walk in both lower limbs, and appeared symptoms similar to epilepsy,

weakness of both lower limbs, neurological physical examination showed no abnormalities, Minnesota personality test can assist the diagnosis, according to the patient's symptoms, physical examination, the most likely diagnosis of the patient is hysteria. Hysteria is the mental disorder caused by mental factor, such as life event, inner conflict, hint or self-suggestion, act on the individual of easy disease, the main performance has separation symptom and conversion symptom two kinds. Separation refers to a complete or partial mismatch between perceptions of past experiences and today's environment and self-identity. Conversion refers to the emotional response caused by mental stimulation, followed by the physical symptoms. Once the physical symptoms appear, the emotional reaction will fade or disappear. The patient developed bilateral lower limb weakness and seizure-like symptoms, which were the conversion symptoms of hysteria.

Option A is an excessive or discommensurate concern about the physical condition, mainly manifested by the perception that he has some or more serious progressive physical disorders. The patient did not think he was ill, and was not selected.

Option B paranoia, also known as paranoid mental disorder, is a mental illness that usually involves a distorted perception of reality, believing that they are persecuted, cheated or hurt, and the patient does not show such symptoms, so it is not selected.

Option C Depression is characterized by significant and persistent low mood, clinical manifestations from depression to grief, negative suicide attempts or behaviors, or even wood rigidity, inconsistent with the symptoms of the patient's symptoms, so it is excluded.

Option E Schizophrenia is a group of chronic diseases with unknown etiology, most of which are slow or subacute onset in young adults. Clinically, it often presents as syndromes with different symptoms, involving sensory perception, thinking, emotional and behavioral disorders, as well as the incoordination of mental activities. The patient did not see the above symptoms, so it was not considered.

In summary, the correct answer is: D. Hysteria.

According to the title description, the patient found that his wife was cheating and developed symptoms of both lower limbs, epilepsy and weakness the next day,

but the neurological examination was unabnormal. This condition suggests that the symptoms may be caused by the psychological factors.

The Minnesota Personality Test (MMPI) is a psychological test used to assess an individual's mental health status and personality traits. The symptoms described in the topic appeared suddenly and were related to a psychological traumatic event (wife cheating), and the neurological examination was not abnormal, considering that it may be caused by psychological disorders.

Based on this information, the best answer is:

B Hysteria (also called conversion disorder)

Hysteria is a psychological disorder, often manifested as sudden neurological symptoms that cannot be explained by medical examination, such as paralysis, seizures, often associated with psychological stress or traumatic events.

102. (Type A2 questions)

A 52-year-old male with repeated upper abdominal discomfort for many years was diagnosed as gastric ulcer by gastroscopy, but the improvement was not obvious after treatment with anti-ulcer drugs. After the doctor asked, the symptoms of the pressure or the symptoms increased. The treatment given was

A. Power-induced shock therapy

B. psychotherapy

C. Giving anti-schizophrenia drug therapy

D. Treatment with sedative drugs

E. Increase of anti-ulcer drugs

Reference answer: B. Your answer:

[Analysis] Stomach ulcer belongs to the category of psychosomatic diseases, the implementation of psychological intervention for psychosomatic diseases should

focus on the elimination of psychosocial stimuli, the elimination of psychological causes and the elimination of biological symptoms as the main goal. The main principles are mental and physical treatment, but for specific cases, they mainly include: for patients with acute onset and severe physical symptoms, physical symptomatic treatment, supplemented by psychological treatment; for psychological symptoms, mainly somatic symptoms, implement the conventional physical treatment.

So this question is selected B.

[Avoid mistakes] Psychosomatic disease refers to the body organic disease where psychosocial factors play an important role in the occurrence and development of the disease

Disease and somatic functional dysfunction.(1) Cardisomatic diseases of the cardiovascular system include coronary atherosclerotic heart disease and array

Primary tachycardia, arrhythmia, essential hypertension, essential hypotension, Raynaud's disease, etc.; (2) respiratory disease

Cardisomatic diseases include bronchial asthma, excessive ventilation syndrome, neurological cough, etc.; (3) psychobody of the digestive system

Diseases include gastric and duodenal ulcers, anorexia nervosa, neuropathic vomiting, ulcerative colitis, and irritable bowel syndrome

; (4) psychosomatic diseases of the skin system include neurodermatitis, alopecia areata, psoriasis, hyperhidrosis, chronic urticaria,

Eczema et al.

For this 52-year-old male patient, who had a prolonged history of recurrent upper abdominal discomfort, gastroscopy indicated gastric ulcer but showed little response to antiulcer medication. Patients also reported increasing symptoms when stressful or stressful, which suggests a possible influence of psychological or mental stress factors.

Of the options, * * B. Psychological therapy * * may be the most appropriate option. This is because patients' symptoms are related to psychological stress, and psychotherapy can help patients learn how to manage stress and emotions, which may reduce symptoms. Although increasing the dose of anti-ulcer drugs (option E) is also a possible option, psychological treatment may be more justified given the poor response to the initial treatment.

Electrical shock therapy, anti-schizophrenia pharmacotherapy, and sedative medication are commonly used for more severe psychiatric disorders, and these treatments do not appear to be preferred for the situation described in this case. Therefore, the most suitable option is B. psychotherapy.

First, we analyze the question stem.

Middle-aged male patient, with long-term epigastric discomfort, was diagnosed with gastric ulcer. The key message is that increased patient symptoms are associated with high stress or stressful mood, suggesting that psychological factors may have played an important role in the disease process.

Next, we analyze the options one by one:

A. Power shock therapy: This is a therapy that stimulates the brain to cause temporary consciousness and memory loss. It is usually used to treat certain mental disorders, such as depression, schizophrenia and bipolar disorder. In this case, the patient's main problem was gastric ulcer, and the psychological factor was the aggravating factor rather than the main cause, so electric shock treatment was not applicable.

B. Psychotherapy: Given that the aggravation of the patient's symptoms is related to psychological stress, psychotherapy may be an effective treatment. Psychotherapy can help patients learn how to cope with stress and reduce anxiety and tension, which may help reduce the symptoms of gastric ulcers.

C. Anti-schizophrenia medication: The patient was not diagnosed with schizophrenia, so the use of anti-schizophrenia drugs is not reasonable.

D. Sedatives: Although sedatives may help relieve tension and anxiety, they are usually not a long-term solution and may have side effects. Moreover, they do not directly target the treatment of gastric ulcers.

E. Increasing anti-ulcer drugs: Although anti-ulcer drugs are the main treatment of gastric ulcers, in this case, the aggravation of symptoms is mainly related to psychological stress, so increasing the dosage of drugs alone may not be the most effective solution.

In conclusion, psychotherapy may be the most appropriate treatment for the most related aggravating symptoms of patients and psychological stress. Psychotherapy can help patients learn more effective ways to cope with stress that may reduce the symptoms of gastric ulcers.

Therefore, the correct answer is B. psychotherapy.

According to the title description, the 52-year-old man repeatedly had upper abdominal discomfort for many years, and proposed gastric ulcer by gastroscopy, and the treatment effect of anti-ulcer drugs was not obvious. At the same time, the patient reported that the pressure or stress, indicating that his gastric ulcer may be related to psychological factors. Therefore, the most suitable treatment regimen should consider the effects of psychological factors.

A. Power shock therapy is mainly used for severe mental disorders or refractory depression, not in this condition.

B. Psychotherapy can help patients cope with stress and emotional tension, which may relieve the symptoms of gastric ulcer associated with this.

C. Antischizophrenia medication is not suitable for the symptoms in this patient.

D. Sedative medication can temporarily relieve anxiety, but it is not the best option to solve the fundamental problems caused by psychological factors.

E. Increasing the dose of anti-ulcer drugs may not solve the problem in this case and may cause side effects.

Based on the above analysis, the most suitable treatment plan is * * B. Psychological therapy * *.

103. (Type A2 questions)

A doctor received a patient, looked at the CT results and test results, and combined with the patient's symptoms, gave the patient with lung cancer conclusion. This thought process belongs to

A. convergent thinking

B. logical thinking

C. divergent thinking

D. innovative thinking

E. abstract thinking

Reference answer: A. Your answer:

[Analysis] Aggregation thinking refers to a directional and organized way of thinking that generates logical conclusions from known information and seeks correct answers from ready-made data. In this question, the doctor gives the diagnosis of lung cancer according to the examination and test results and the symptoms, which belongs to the aggregation thinking (A pair).

[Avoid wrong] Divergent thinking (C wrong), also known as radiation thinking, radiation thinking, diffusion thinking or different thinking, refers to the brain

A thinking mode presented in a diffuse state of thinking. Abstract thinking (BE error), also known as the word thinking or logical thinking

Dimension refers to the process of using words to judge, reason and draw conclusions. Abstract thinking reflects reality mediated by words.bring forth new ideas

Thinking (D wrong) is the reorganization of the existing knowledge and experience, to produce a novel, unprecedented, creative imagination to participate in

The thinking of.

The topic examines how doctors diagnose a disease using different information (CT results, laboratory results, and the patient's symptoms). In this case, doctors must extract relevant information from various examination results and then apply their medical knowledge and experience to synthesize this information to draw diagnostic conclusions.

The option analysis is performed as follows:

-A. Aggregate thinking: It refers to the process of bringing different information, data or facts together to form A whole. In medical diagnosis, converging thinking may involve the combined consideration of different examination findings and clinical manifestations.

-B. Logical thinking: refers to using the logical method of thinking and reasoning to draw reasonable conclusions. In the medical field, logical thinking is used to infer a possible illness based on the patient's symptoms and examination findings.

-C. Divergent thinking: It is a way of thinking designed to generate creative ideas by exploring many possible solutions. In this context, it may not be the best fit, as physicians need to draw definitive conclusions based on specific evidence.

-D. Innovative thinking: It involves creating new ideas or concepts. In the diagnostic process, although innovation is important, it is more applied in therapeutic methods or when solving problems.

-E. Abstract thinking: it is the ability to understand complex concepts and think without specific information. It is not the most directly related mode of thinking in the diagnostic process.

Taken together, the best way to describe the thinking process of diagnodiseases by analyzing CT results, test results and patient symptoms is aggregate thinking (option A). Therefore, the best answer to this question should be A. aggregate thinking.

First, we analyze the topic and list the important information. This question describes the doctor giving the conclusion that the patient has lung cancer after combining the CT results, the test results and the patient's symptoms.

Next, we need to analyze each option to determine which thought process is best consistent with the title description:

A. Aggregated thinking: This way of thinking means to draw a centralized conclusion through logical reasoning and comprehensive analysis, starting from the known information. This is consistent with the process of combining the results and the patient's symptoms.

B. Logical thinking: Although the reasoning process of doctors contains logical components, logical thinking focuses more on the rigor and formalization of reasoning, rather than just integrating information to draw conclusions.

C. Divergent thinking: This way of thinking emphasizes the generation of a variety of different ideas or solutions from a central point. This is inconsistent with the process of drawing a single conclusion after integrating the information.

D. Innovative thinking: This way of thinking focuses on generating novel, unique ideas or solutions. No innovative or novel elements are reflected in the title.

E. Abstract thinking: This way of thinking emphasizes the extraction of its common essential characteristics from concrete things. Although the doctor's diagnosis process contains certain abstract thinking, the focus of the title description is on integrating information to draw conclusions rather than abstracting common features.

To sum up, the thinking process of doctors is most consistent with the definition

of aggregate thinking, that is, starting from a variety of information and examination results, and drawing a centralized conclusion after comprehensive analysis.

Therefore, the right answer is A. aggregate thinking.

In this case, the doctor concluded that the patient's lung cancer by reviewing the patient's CT results and testing results, combined with the patient's symptoms. This process involves a comprehensive analysis of various information and reasoning to draw conclusions. This thinking process is most consistent with * * logical thinking * *.

* * Logical thinking * * refers to the process of drawing conclusions through reasoning and analysis based on known facts and evidence. In medical diagnosis, doctors often use logical thinking to synthesize the results of various examinations and tests, combine the patient's symptoms, and finally make a diagnosis.

So the correct answer is:

B logical thinking

104. (Type A2 questions)

The staff investigated the health situation of the children in a village, and found that the artificially fed babies in the village recently had diarrhea, vomiting and cyanosis. The examination found that the children had increased methemoglobin in their blood, considering the reason is

A. nitrite poisoning

B. methyl mercury intoxication

C. Drinking water is high in chlorine content

D. Burning of inferior coal

E. The bottle material contains bisphenol

Reference answer: A. Your answer:

[Analysis] artificially fed infants (suspected of citing unclean water) recently developed diarrhea, vomiting and cyanosis (the first symptoms of nitrite poisoning), the examination found that the methemoglobin in children's blood was increased (indicating nitrite poisoning), considering the diagnosis of nitrite poisoning, so this question choose A.

[Avoid mistakes] Nitrite mostly exists in pickled pickles, meat, unclean well water and spoiled vegetables.

In this case, diarrhea, vomiting, and cyanosis, combined with increased methemoglobin (methemoglobin) in the blood, were most likely due to nitrite poisoning. Nitrite poisoning can cause the oxidation of blood iron ions from + 2 valence to + 3 valence, forming methemoglobin and reducing the oxygen-carrying capacity of the blood, thus causing symptoms such as diarrhea, vomiting and cyanosis. This intoxication is usually associated with the ingestion of water or foods containing higher amounts of nitrite.

Therefore, the best answer is A. nitrite poisoning.

analyze:

Artificial fed children in the village recently developed diarrhea, vomiting and cyanosis. Meanwhile, the examination found increased methemoglobin in the blood, which is a typical manifestation of nitrite poisoning. Nitrite poisoning is due to eating nitrite or drinking nitrite content high well water or pickled vegetables caused by poisoning, can also be for gastrointestinal dysfunction, gastrointestinal nitrate reducing bacteria reproduction, feed into the vegetables rich in nitrate, nitrate in the gut is reducing bacteria into nitrite, causing poisoning. Nitrite can oxidize the normal oxygen-carrying low-iron hemoglobin in the blood to methemoglobin, thus losing the oxygen-carrying capacity and causing tissue hypoxia and cyanosis.

Option B Methylmercury poisoning is common in people with long-term contact with mercury operations. Clinically, psychiatric-neurological abnormalities, gingivitis and tremor are the main symptoms, which are not consistent with the description of the problem stem, so it is not selected.

Option C High chlorine in drinking water can cause diarrhea and other symptoms, but it cannot explain the increase of methemoglobin, so it was excluded.

Option D Burning inferior coal may cause carbon monoxide poisoning, but it will not increase methemoglobin in the blood, not in line with the question.

Option E bottle material containing bisphenol may affect the health of infants, but it is not directly related to methemoglobin increase, so it is not chosen.

In conclusion, the correct answer is: A. nitrite poisoning.

According to the information provided in the topic, the artificially fed infant had diarrhea, vomiting and cyanosis, and the examination found increased methemoglobin in the blood, considering nitrite poisoning.

Nitrite poisoning leads to methemoglobinemia (Methemoglobinemia), which increases methemoglobin in the blood, and then leads to tissue hypoxia, presenting with cyanosis (blue skin and mucous membranes). Artificial feeding of infants may sometimes consume nitrite through contaminated water sources or food.

So the most likely answer is:

A. nitrite poisoning

105. (Type A2 questions)

Wang mou and his friends out dinner, point the Yangzhou meat, tuna, pickles, green beans and mushroom food, eat about ten minutes wang mou and his friends are headache, chest tightness, heart rate, general discomfort, head and neck chest burning and tingling, one of the friends asthma attacks, breathing difficulties, analysis of the main reason is

A. muscarinism

B. histamine poisoning

C. Cyanoside type of food poisoning

D. kidney bean poisoning

E. nitrite poisoning

Reference answer: B. Your answer:

[Analysis] After taking green skin red meat fish, headache, chest tightness,

accelerated heart rate, general discomfort, head, neck and chest flushing, accompanied by burning and tingling, considering the diagnosis of histamine poisoning, so this question chose B.

[Avoid mistakes] When ingesting the green skin and red meat fish (rich in histidine) in Marine fish, such as fish, mackerel, autumn swordfish, sardine

Fish, mackerel, bamboo pod fish, tuna and other fish can cause histamine poisoning.

The question describes a series of symptoms of a group of people after eating Yangzhou food meat, tuna, pickles, green beans, and mushroom food, such as headache, chest tightness, increased heart rate, general discomfort, burning and tingling, as well as asthma attacks and dyspnea in one of the friends. Based on these symptoms, we need to consider which food-induced poisoning may have contributed to these reactions.

Option A is muscarinosis, often associated with consumption of wild mushrooms, but symptoms of mushroom poisoning are more related to gastrointestinal and neurological symptoms such as nausea, vomiting, abdominal pain, visual distortions and hallucinations.

Option B is histamine poisoning, which is usually associated with the consumption of stale seafood (such as tuna), and can cause headache, chest tightness, increased heart rate, general discomfort and other symptoms, which matches the symptoms described in this case.

Option C is cyanoside food poisoning. Although some foods contain cyanoside, the toxic symptoms mainly include dizziness, headache, dyspnea, blood pressure drop, etc., which is not completely consistent with this case.

Option D is green bean poisoning, which may contain if undercooked phytohemagglutinin, which can cause symptoms such as abdominal pain and vomiting, but is unlikely to lead to this widespread neurological and respiratory symptoms.

Option E is nitrite poisoning, which can cause some severe symptoms but is more related to the intake of improperly processed processed foods and typically causes a reduced ability to transport oxygen from the blood.

In conclusion, the most consistent for these symptoms is option B, histamine poisoning, especially related to the consumption of potentially inadequate fresh tuna.

analyze:

Wang and his friends went out to dinner and ordered a variety of food. For about ten minutes after dinner, they had headache, chest tightness, accelerated heart rate, general discomfort, burning and tingling in the head, neck and chest. One of his friends had an asthma attack and breathing difficulties. A possible histamine poisoning was considered. Histamine poisoning is characterized by rapid onset, mild symptoms, rapid recovery, and little death. Main manifestations for skin flushing, conjunctival congestion, like drunk, dizziness, headache, heartbeat, chest tightness and shortness of breath, some patients appear conjunctival congestion, eyelid edema, pharyngeal pain, dysphagia, even, urticaria, vomiting, diarrhea, etc., heavy person can appear bronchospasm, dilated pupils, difficulty breathing, blood pressure drop.

Option A muscarinic mainly for parasympathetic nerve endings excessive excitement, similar muscarinic like symptoms, characterized by smooth muscle spasm and gland secretion, such as nausea, vomiting, abdominal pain, more sweat, pupil, heart rate, salivation, incontinence, severe cases can appear pulmonary edema, dyspnea, coma, convulsions, etc., does not accord with the idea.

Option C cyanoside class food poisoning refers to the cyanoside plant poisoning, such as cassava poisoning, after eating can be released under the action of gastric acid, lead to poisoning, the main symptoms of nerve and digestive tract damage, nausea, vomiting, abdominal pain, diarrhea, some dizziness, headache, mouth bitter almond, pupil, severe coma, finally died because of respiratory paralysis and cardiac stop, with the topic dry description.

Option D green bean poisoning is the poisoning caused by the consumption of green beans, green beans contain toxic protein and saponin, if not cooked thoroughly, people will cause poisoning after 1 to 5 hours after eating, mild headache and dizziness, severe nausea, vomiting, abdominal pain, which is inconsistent with the dry problem.

Option E Nitrite poisoning often shows cyanosis caused by tissue hypoxia. When possible, the quantitative test of methemoglobin in blood and that of nitrite in surplus food can be done, which is inconsistent with the performance of Wang and his friends, so it is excluded.

To sum up, the correct answer is: B. histamine poisoning.

According to the symptoms of Wang and his friends, including headache, chest tightness, accelerated heart rate, general discomfort, burning and tingling in the head, neck and chest, and one of the friends had asthma attacks and breathing difficulties, these symptoms are very typical of the manifestations of histamine poisoning. Histamine poisoning is common in eating foods high in histamine, such as tuna and kimchi. Especially in cases of improper food handling or preservation, the histamine content may be significantly increased.

Therefore, the most likely answer is: B. histamine poisoning

106. (Type A2 question)

In 2017, a municipal health bureau created the selection of "advanced departments", and selected a total of 30 "excellent advanced departments". In this good environment, all hospital departments strive to be "advanced departments", and the psychological promoting factor reflected in this case is

A. Raise public opinion and educate people by the environment

B. Case analysis, by reasoning

C. Technical clearance, strength first

D. Take the code first, and be moving in shape

E. Knowledge education, to educate people

Refer to the answer: D. Your answer:

Graphic analysis: [Analysis] Create the selection of advanced departments, in order to set up a typical, the first, to the shape of moving, so that the hospital departments in a good atmosphere are striving to do "advanced departments", so this question selected D.

[Avoid avoid mistakes] common sense questions.

In this case, as hospital departments are striving to become "advanced departments", which reflects a psychological promoting factor, that is, encouraging and motivating others by creating excellent models or examples. This approach motivates other people or groups by demonstrating the standards or achievements expected. So the most appropriate answer is:

B. Take the code first, and be moving in shape

This option means infecting and motivating people by setting typical examples that strive to become similar "advanced departments."

This is a multiple choice question involving medical psychology and health pedagogy, designed to analyze psychological promoters in a practical case.

First of all, we should understand the background of the topic: a city health bureau selected "advanced departments", and successfully selected 30 "excellent advanced departments". This activity has stimulated the enthusiasm of each hospital department to be the "advanced department".

Now, let's analyze the options, one by one:

A. Public opinion suppression and education by environment: Although the case mentioned that the selection activity has created a good environment, it does not clearly reflect the static effect of public opinion, so this option may not be the most directly relevant.

B. Case analysis: The case does not mention of motivating or persuading others through case analysis, so this option does not meet the meaning of the question.

C. Technical clearance, strength first: Although technical strength may be very important for the selection of "advanced departments", technical factors are not particularly emphasized in the case, so this option is not the most directly related.

D. Take Canon first, with shape moving: This option is highly related to the case. The 30 "excellent advanced departments" have actually become role models (models), and their performance has stimulated the desire of other departments to follow suit. This is the embodiment of "Canon first", which influences and inspires others by establishing examples.

E. Knowledge education, moral education: The case does not explicitly mention to cultivate moral character through knowledge education, so this option is not consistent with the meaning of the question.

To sum up, the answer is D. Canon first, and moving in shape. This option most directly reflects the psychological promoting factor of the hospital departments striving to be "advanced departments", that is, to influence and motivate other departments by setting up excellent models.

According to the description in the title, a city health bureau selected "advanced

departments", creating a good environment, encourage the hospital departments to strive to be "advanced departments". This practice makes use of the power of example and the typical demonstration effect to encourage other departments to improve themselves, and strive to become an "advanced department".

Therefore, the psychological promoting factors reflected in this case are:

C Take the code first, and be moving in shape

This way by setting up advanced models, with practical actions and achievements to encourage and infect others, to strive to be advanced, to create a good environment for competition and development.

107. (Type A2 questions)

Male, 11 years old, was clinically diagnosed with brain death due to a car accident. The patient's parents were extremely sad, but accepted the idea of organ donation and asked to meet the recipient and consider them relatives. The organ donor doctor insisted on refusing to meet, and the patient's parents offered him. In this case, the medical ethics embodied is

A. The dignity and protection of the donor

B. Patients' health interests come first

C. Free and non-commercial

D. informed consent

E. maintain secrecy

Reference answer: E Your answer:

[Analysis] Organ donor doctors insist on refusing the meeting between donor and recipient reflects the principle of confidentiality of human organ transplantation (E pair).

[To avoid mistakes] The ethical principles of human organ transplantation in China: (1) the principle of patient interests first, (2) voluntary, free and forbidden

Principles of stop trading, (2) principle of informed consent, (4) principle of

respecting and protecting the donor, (5) principle of confidentiality, (6) public

Peace, fairness and openness.

The medical ethical principle embodied in this case is A. the dignity and protection of the donor. The situation here revolves around the issue of organ donation for a brain-dead patient, emphasizing the protection of the dignity of the patient, i. e. (the donor). Although the patient's parents wanted to see and establish contact with the organ recipient, the physician refused this request to protect the donor's privacy and dignity and prevent possible social and psychological problems. Moreover, it involves the special protection of minor donors and ensuring that their dignity is not violated, even after the end of their life. Therefore, option A is the most appropriate answer to this context.

This is a matter of medical ethics. We first need to understand and analyze the key information in the topic, and then combine this information to make logical reasoning about the given options.

Critical Information Analysis:

An 11-year-old boy was brain dead in a car accident.

The patient's parents, sad, accepted the idea of organ donation.

The patient's parents asked to meet the recipient and wished to relative with the recipient.

Organ donor physicians declined their request to meet with the recipient.

Option analysis:

A. The dignity and protection of the donor: Although the dignity and protection of the donor are important ethical principles, in this case, the issue focuses more on protecting the anonymity of the recipient and the anonymity of the donation process than directly on the dignity of the donor. Thus, this option may not be the most directly relevant.

B. Patient health interests first: This principle mainly focuses on the health interests of patients. In this case, although organ donation was made to save the lives of other patients, the core of the problem was the ethical issues in the donation process rather than a direct focus on the health benefits of patients. Thus, this option may not be the most relevant.

C. Free and non-commercial: This principle emphasizes that organ donation

should be free and non-commercial. However, the focus of this case is on protecting the anonymity of the donation process and protecting the identity of the donor parties, which is not directly related to the compensation and non-commercialization.

D. Informed consent: Although informed consent is an important principle in medical ethics, in this case, the crux of this question is not whether informed consent was obtained, but about privacy and anonymity in the donation process.

E. Confidentiality: In this case, the organ donor physician refused the patient's request to meet the recipient, most likely to maintain the privacy and anonymity of both parties. This is to protect both donor and recipient from unnecessary stress and interference and ensure the impartiality and objectivity of the donation process. Thus, this option is highly correlated to the context in the case.

In conclusion, the most consistent with the medical ethical principles of this case is option E: confidentiality. This principle embodies the protection of the privacy of both donor and recipient, as well as ensuring the impartiality and objectivity of the organ donation process.

This topic describes a case involving organ donation in brain death involving multiple medical ethical principles. The title requires the selection of one that best reflects the ethical principles in the case.

In the title, the patient's parents asked to meet the recipient and consider him a relative, and the organ donor physician refused the request. The reasons for rejection may involve the protection of the donor's privacy and the privacy of the recipient, which is closely related to the principle of confidentiality (E. confidentiality) in medical ethics. The confidentiality principle emphasizes the protection of patient privacy and information security in the doctor-patient relationship.

In conclusion, the medical ethical principles best embodied in this case are:

D maintain secrecy

108. (Type A2 questions)

Children were born breastfeeding, without added complementary food, the recent loss of appetite, pale. Physical examination: P125 times / min, 3cm under liver rib, 1cm under spleen rib, hemoglobin 87g / L. Peripheral blood smear: enlarged central light staining area. No wait, mainly small. The most likely type of amino acid lacking in infants is

A. tryptophan

B. glycocoll

C. Thr

D. Leu

E. Ile

Reference answer: A. Your answer:

Graphic analysis: [analysis] pediatric patients have anemia symptoms. Tryptophan is an essential amino acid that is a precursor for the synthesis of serotonin, which has important roles in the regulation of mood, sleep, and appetite. Because breastfed infants are usually unable to obtain enough tryptophan from their mother's diet, they are prone to symptoms such as growth retardation and loss of appetite. In addition, isoleucine (option E) and glycine (option B) levels may also be affected in infants, but they are less affected relative to tryptophan.

[fault avoidance]

This question examines the amino acid deficiency associated with infant malnutrition. The baby showed decreased appetite, pallor, physical examination of anemia (hemoglobin 87g / L) and mild enlargement of liver and spleen, and peripheral blood smear showed enlargement of the central light staining area, which were all manifestations of anemia. Anemia may be associated with deficiencies of multiple nutrients, including iron, vitamin B12, folic acid, and certain amino acids.

Considering that infants are exclusively breastfed without complementary foods, a lack of essential amino acids is most likely, although breast milk, as an ideal food for infants, may not meet the need for certain nutrients without complementary foods. Among the options given, leucine (Leucine) and isoleucine (Isoleucine) are branched-chain amino acids that are very important for child growth and development.

Leucine is an essential amino acid that the human body cannot synthesize on its own and must be obtained through food. Leucine is important for protein synthesis,

muscle repair, and blood glucose regulation. The requirement for leucine is particularly important in infants and children during the stages of rapid growth and development.

According to this information, the most likely amino acid deficiency should be one closely related to growth and development. Among these options, D. leucine (Leucine) is a more reasonable choice because it is an important essential amino acid and plays a key role in the growth and development of children. Therefore, it is reasonable to choose D. leucine as the answer.

analyze:

Children breastfed, without adding complementary food, recently decreased appetite, pale. Physical examination showed hepatosplenomegaly, increased heart rate, a significant decrease in hemoglobin, and anemia. The peripheral blood smear showed an enlarged central hemogram area, variable cell size, and the diagnosis of nutritional iron deficiency anemia was considered. In iron deficiency anemia, the synthesis of hemoglobin decreases, and the raw materials of hemoglobin include iron and globin, and the synthesis of globin is amino acid, especially glycine, which is the essential amino acid for globin synthesis. Therefore, this child is most likely to lack glycine in vivo.

A tryptophan, C threonine, D leucine and E isoleucine, are essential amino acids, but they are not essential in the synthesis of globin, so they can be excluded together.

To sum up, the correct answer is: B. glycine.

This topic described a breast-fed child without complementary food, recent loss of appetite and pale face, physical examination found fast heart rate, hepatosplenomegaly, decreased hemoglobin, peripheral blood smear showed an expansion of the central light staining area, indicating anemia. Combined with the above symptoms and signs, what is the most likely amino acid type that the baby will lack?

Considering that breastfed children do not have added complementary foods, it is likely that some essential nutrients are lacking, including iron, vitamin D, vitamin B12, etc. The low hemoglobin and the enlarged central light staining area of the

peripheral blood smear mentioned in the title stem are the typical manifestations of iron deficiency anemia. The main cause of iron deficiency anemia is iron deficiency rather than a specific amino acid.

However, if the topic requires us to choose an amino acid, we can take into account the metabolic and nutritional characteristics of the amino acids in the body. For infants, essential amino acids are essential because the body cannot synthesize these amino acids and must be taken through the diet. Tryptophan (A), glycine (B), threonine (C), leucine (D), and isoleucine (E) are all amino acids required by the human body, but given the amino acid composition of breast milk, infants are less likely to lack tryptophan because breast milk contains sufficient tryptophan.

Based on this reasoning, the most likely type of amino acid deficiency in breastfed infants without the addition of complementary food is threonine (C). Because threonine is an essential amino acid and is in high demand in infants with rapid growth periods.

So, the most correct answer is:

B Thr

109. (Type A2 question)

A 67-year-old man was bitten by a silver ring snake one hour ago, followed by limb weakness, breathing difficulties, and drooping eyelids. After physical examination, two tooth marks in the skin, the size of the needle tip, and the acetylcholine at the nerve-muscle joint changed

A. Diffusion blocked in the gap

B. blocked binding to the receptor

C. Synthesis is blocked

D. Release blocked

E. Degradation is blocked

Reference answer: B. Your answer:

[Analysis] silver ring snake has α -, β -bungarotoxin, two neurotoxins, one is the postsynaptic neurotoxin or α -neurotoxin, this toxin competitive with the acetylcholine receptor at the neuromuscular joint, block neurotransmitter conduction (therefore B); the other is presynaptic neurotoxin or β -neurotoxin, which directly acts on the presynaptic membrane of motor nerve, blocking the release of acetylcholine, make the skeletal muscle lose its contraction function and paralysis.

[fault avoidance]

Silver-ring snake bites can cause acetylcholine changes at the nerve-muscle joint, which often involves a blocked release of neurotransmitter material. The toxin mainly affects nerve-muscle joints and blocks the transmission of nerve impulses to muscles, leading to symptoms such as muscle weakness, dyspnea and eyelid ptosis. This toxin hinders the normal release of acetylcholine into the synaptic cleft of the nerve-muscle junction, thus affecting the normal muscle contraction.

Therefore, the most appropriate answer to this question is: D. Release is blocked

analyze:

In an elderly male patient, he developed limb weakness, breathing difficulties and eyelid ptosis. After physical examination, there were two tooth marks in the skin and the size of the needle tip, which could be diagnosed as silver ring snake bite. The snake toxin mainly damages the nervous system, causing neuromuscular paralysis and respiratory paralysis, which is a neurotoxin. After the bite of the silver ring snake, its neurotoxin acts on the acetylcholine receptor at the nerve-muscle joint, resulting in the binding of acetylcholine and the receptor, thus blocking the nerve conduction of the nerve-muscle joint and causing skeletal muscle flaccid paralysis.

The dispersion of acetylcholine at the nerve-muscle joint in the gap is not affected by accotoxin, so the diffusion in the gap is excluded.

Arnodotoxin does not affect the synthesis of acetylcholine, so the blocked synthesis of option C is not consistent with the question.

The toxin has no effect on the release of acetylcholine, so option D release is not considered.

The toxin affects the acetylcholine receptor and does not affect the degradation of acetylcholine, so option E degradation can be excluded.

In conclusion, the correct answer is: B. Receptor binding is blocked.

According to the title, the 67-year-old man was bitten by a silver-ring snake and

later developed symptoms of weakness, difficulty breathing, and drooping eyelids. Silver ring venom is mainly a neurotoxin that affects acetylcholine function in the nerve-muscle joints.

By blocking the binding of acetylcholine to the receptor, producing symptoms similar to myasthenia gravis. Therefore, the associated acetylcholine changes at the nerve-muscle joint should be:

B blocked binding to the receptor

110. (Type A2 questions)

The patient, 50 years old, had a low-fat diet due to hyperlipidemia, and his visual perception decreased recently, so which of the following foods should be added

A. bread

B. urad

C. Daucus carota

D. Benincasa hispida

E. apple

Reference answer: C Your answer:

Graphic analysis: [analysis] low fat diet visual perception decline, more considered for the lack of vitamin A, so should supplement the food containing vitamin A, such as carrots and animal liver, so this question choose C.

[Avoid mistakes] Vitamin A deficiency causes night blindness. Toasis is caused by vitamin PP deficiency; beriberi is developed

Vitamin B1 deficiency; vitamin C deficiency is caused by vitamin C deficiency; rickets is caused by vitamin D deficiency.

This topic involves decreased visual perception, which may be related to A lack of vitamin A, because vitamin A is very important for vision, especially in low-light environments. Vitamin A deficiency can lead to night blindness and reduced visual perception.

Among the food options given, carrots are the well-known source of vitamin A

because they are rich in β -carotene, which the body can convert into vitamin A. Therefore, carrot supplementation is beneficial for a patient who may have had an insufficient vitamin A intake due to a low-fat diet.

So, the most correct answer is * * C. carrot * *.

analyze:

The middle-aged male, on a low-fat diet due to hyperlipidemia, recently decreased his visual perception, considering the night blindness caused by vitamin A deficiency. Vitamin A deficiency disease, also called toad, because of vitamin A deficiency caused by eye and skin lesions of systemic disease, in 1~4 years old children, the earliest symptom is poor, eye conjunctiva and dry cornea, later development for corneal softening and dry skin and hair follicle keratinization, so also called night blindness, dry eye disease, corneal softening. When patients with hyperlipidemia eat a low-fat diet, if the intake of fat food is excessively restricted, it will affect the absorption of fat-soluble vitamin A, resulting in the manifestation of vitamin A deficiency. Therefore, the food rich in vitamin A can be appropriately increased in the diet, such as carrots, which is rich in β -carotene. After intake of human digestive organs, it can be converted into vitamin A, which is the most safe to supplement vitamin A products (simple supplement chemical synthesis of vitamin A, excessive will cause people).

Option A bread, option B mung bean, option D wax gourd, option E apple, the vitamin A content of the above food is low, which has limited effect on the decrease of visual perception of the patient, so it can be excluded together.

To sum up, the correct answer is: C. carrot.

According to the information in the topic, the patient had a low-fat diet due to hyperlipidemia, and recently his visual perception decreased. This case, given the possible nutrient deficiencies, especially with vision-related nutrients.

Carrots are rich in vitamin A (in the form of β -carotene), and vitamin A is very important for maintaining normal visual function. Deficiency of vitamin A may cause night blindness and other vision problems. Therefore, supplementary foods rich in vitamin A are recommended.

The correct answer is:

C Daucus carota

111. (Type A2 questions)

Female, 70 years old, vomiting for two days, 5~6 times a day, diarrhea a day, more than 10 times a day, urine volume decreased for 1 day, physical examination: 36.5℃, heart rate 110 times / minute, pulse 26 times / minute, blood pressure 85 / 60mmHg. The expression was indifferent, and the oral mucosa was pale, at 300ml / 24h. Heart rate 110 beats / min, heart rhythm, the cause in blood pressure

A. The excited vascular motor system

B. Reduction in effective circulating blood volume

C. Increased catecholamine secretion

D. Microscopic vein dilatation

E. Reduction of return effort

Reference answer: B. Your answer:

[Analysis] Elderly women, frequent vomiting, diarrhea (a large amount of body fluid loss), urine volume decreased for 1 day, heart rate 110 times / min, pulse 26 times / min, blood pressure 85 / 60mmHg, indifference, indicating the existence of shock. Therefore, the reason for oliguria is the loss of large body fluids caused by the reduction of effective circulating blood volume, so this question is B.

[Error avoidance] Shock is the pathology of the body's effective blood volume reduction, insufficient tissue perfusion, cell metabolism dysfunction and impaired function

Cheng, whose fundamental change is insufficient tissue perfusion.

This 70-year-old female patient presented with vomiting, diarrhea, decreased urine output, increased heart rate (110 beats / min), decreased blood pressure (85 / 60mmHg), and pallor of the oral mucosa. These clinical manifestations suggest that there may be a fluid loss in this patient, resulting in decreased blood volume. The decrease in blood pressure may be due to a reduced effective circulating blood volume. Therefore, in this case, the most likely reason is a decrease in effective circulating blood volume. Therefore, choose B. effective circulating blood volume reduction as

the best answer.

First of all, we analyze the question stem:

The main symptoms of elderly female patients were vomiting, diarrhea, and reduced urine volume. Physical examination showed normal temperature, but the heart rate was fast, blood pressure was low, in the range of shock and blood pressure, and the symptoms of indifferent expression and pale oral mucosa. These information suggests that patients may have hypovolemic shock due to vomiting and diarrhea.

Next, we analyze the options one by one:

A. Excitation of the vasomotor system: Although the state of the vasomotor system may affect blood pressure, in this situation, the excitation of the vasomotor system is insufficient due to the large loss of body fluids to maintain normal blood pressure, so it is not selected.

B. Reduce effective circulating blood volume: Considering the patient's vomiting, diarrhea, and reduced urine volume, this is likely to lead to significant loss of fluid, thus reducing effective circulating blood volume and thus affecting blood pressure. This is a plausible explanation.

C. Increased catecholamine secretion: The increased secretion of catecholamines is usually associated with a stress response, but it is not the direct cause of decreased blood pressure, especially in the case of a large loss of body fluid, so it is excluded.

D. Microvein dilatation: Although dilation and contraction of blood vessels can affect blood pressure, it is not the main cause of blood pressure drop in this context, which is inconsistent with the problem stem description.

E. Reduced blood effort: Although fluid loss may lead to reduced blood effort, it is more a result of reduced effective circulating blood volume than a direct cause of altered blood pressure, which can be excluded.

In conclusion, the most plausible explanation is that the large loss of body fluids due to vomiting and diarrhea, which leads to a significant reduction in effective circulating blood volume, which is the main cause of the decrease in blood pressure.

Therefore, the correct answer is B. The effective circulating blood volume reduction.

The problem describes a 70-year-old female patient who developed vomiting and

diarrhea associated with decreased urine output, hypotension, and increased heart rate. These symptoms indicate that the patient may be in a state of shock, especially hypovolemic shock due to loss of body fluids.

Here is an analysis of the options:

A. Vasomotor excitation: This condition generally leads to increased blood pressure, not lower blood pressure.

B. Reduce the effective circulating blood volume: Vomiting and diarrhea can lead to the loss of body fluids, thus reducing the effective circulating blood volume and leading to hypotension.

C. Increased catecholamine secretion: An increase in catecholamines causes increased heart rate and vasoconstriction, usually causing increased blood pressure.

D. Microvein dilatation: Microvein dilatation causes blood accumulation in the periphery, which may lead to a drop in blood pressure, but is not the main cause of this acute fluid loss.

E. Reduced blood effort volume: Although reduced blood effort can lead to decreased blood pressure, the main reason here is the loss of body fluids caused by vomiting and diarrhea, which directly leads to a decrease in effective circulating blood volume.

Overall, the most likely answer is:

B Reduction in effective circulating blood volume

112. (Type A2 questions)

A doctor did not tell the patient the relevant matters for attention. After the local county health department knew about it, he ordered him to rectify. If he did not rectify and continued to violate the relevant regulations of vaccination, the local health management department stopped the relevant regulations of his license, and the time when he stopped his practice was

A. Over 1 year and more than 15 months

B. Above 1 year and below 18 months

C. Under 6 months

D. Under 3 months

E. Above 6 months and below 12 months

Reference answer: E Your answer:

[Analysis] The Law on Medical Practitioners stipulates that if a doctor violates the administrative rules and regulations of public health in his practice activities and causes serious consequences, the health administrative department of the people's government at or above the county level shall give him a warning or order him to suspend the practicing activities for more than six months and more than one year (E pair).

[To avoid mistakes] A doctor who, in violation of the provisions of this Law, commits any of the following acts shall be protected by the people's government at or above the county level

The administrative department shall give a warning or order to suspend its practice activities for less than six months but less than one year; if the circumstances are serious, the practice shall be revoked

Certificate; constitute a crime, the case shall be investigated for criminal responsibility according to law: ① Violation of health administrative rules and regulations or technical operation norms, causing

Serious consequences; ② Unresponsible delay of rescue and treatment of emergency patients, causing serious consequences; ③ causing medical treatment

Liability for the accident; ④ Having signed diagnosis, treatment, epidemiological and other certification documents without personal diagnosis and investigation

Life and death certificates; ⑤ concealing, forging or destroying medical documents and relevant materials without authorization; ⑥ used unused

Drugs, disinfection agents and medical devices approved for use; ⑦ do to use narcotic drugs and toxic drugs for medical use as required

Products, psychotropic substances and radioactive drugs; ⑧ experimental clinical treatment on patients without the consent of the patients or their family members;

⑨ Lege patients' privacy and causing serious consequences; ⑩ takes advantage of his position to solicit or illegally accept patients or seek property

His illegitimate interests; (11) the occurrence of natural disasters, epidemics of infectious diseases, sudden accidents of major casualties and other serious threats

In an emergency threatening the life and health of the people, it does not obey the administrative department of public health; (12) medical accidents or discovery occur

Failure to report the epidemic situation of infectious diseases or abnormal death of patients in accordance with the provisions.

When dealing with permission to practice for a physician who violates vaccination regulations, the severity and duration of violations by that physician is usually considered. Since the title description mentioned that the doctor continued to violate the vaccination regulations without rectification, this indicates that the violations were repeated and not corrected. In China's relevant laws and regulations, the suspension of practice permits is usually longer for such repeated violations.

Although the specific suspension time may vary according to the actual situation and local policies, a longer suspension period is justified given the seriousness and repeatability of the violation. Therefore, options A and B look more appropriate. However, without specific legal provisions as a reference, I prefer a medium-length suspension period to reflect serious violations but also leave some room for adjustment.

Based on the above analysis, I will choose * * E. Over 6 months and less than 12 months * * as the most appropriate answer. This option reflects the serious handling of physician violations, while considering the flexibility in possible laws and regulations. However, specific answers answer needs to be determined according to local laws and regulations.

analyze:

According to the Vaccine Administration Law, before implementing vaccination, medical and health personnel should strictly check the information of the recipients, check the vaccines, syringes and needles, and inform the recipients or their guardians of the varieties, effects, contraindications, adverse reactions and precautions in accordance with the requirements of the vaccination standards. If the applicant fails to inform the recipient or his guardian, the competent health department of the people's government at or above the county level shall order it to correct and give a warning; if the applicant refuses to correct, the responsible unit shall be fined between 5,000 yuan and 50,000 yuan, 0 and the medical and health personnel directly responsible shall be fined between 3,000 yuan or ordered to suspend practice activities for not less than 6 months but not more than one year.

To sum up, the correct answer is: E. Over 6 months and less than 12 months.

According to Article 37 of the Law of the People's Republic of China on Medical Practitioners, if a doctor violates the administrative rules and regulations of public health, and the circumstances are serious, the public health administrative department may order him to suspend his practice activities for not less than six months and not more than one year. So the correct answer is:

C Above 6 months and below 12 months

113. (Type A2 questions)

Infants, 7 months old, are vaccinated as planned, and the following are still unvaccinated

A. The MMR vaccine

B. Polio vaccine

C. hepatitis B vaccine

D. DPT vaccine

E. Bacille Calmette-Guerin

Reference answer: A. Your answer:

Graphic analysis: [Analysis] The first type of MR vaccine is 18 to 24 months

old (A pair).

[Avoid mistakes] The time of hepatitis B vaccination is birth, 1 month and 6 months of age. BCG is occurs at birth. Measles vaccine at the beginning

It's August old. The DPT vaccine was administered at 3,4, and 5 months of age.

For 7-month-old infants, according to the routine vaccination schedule:

-A. MMR vaccine (measles, mumps, and rubella vaccine), usually at 12 months of age.

-B. Polio vaccine, which usually begins within the first few months of birth, and completes multiple intensification during infancy.

-C. Hepatitis B vaccine, usually at birth, one month and six months of age.

-D. DPT vaccine (pertussis, diphtheria, tetanus vaccine), usually within 2,4, or 6 months after birth.

-E. BCG vaccine (for tuberculosis), in China, is usually vaccinated shortly after birth.

According to the above information, 7-month-old infants should have already received the polio vaccine, hepatitis B vaccine, DPT vaccine and BCG vaccine. Therefore, the vaccine that has not yet been vaccinated is * * MMR vaccine * *.

Option * * A. MMR vaccine * * is the correct answer.

This is a question about the infant vaccination program. First, we need to understand that in China, there is a clear schedule for infant vaccination, which is when to vaccinate according to the age. Next, we will analyze the options one by one from this background information to determine which vaccine is still unvaccinated in 7-month-old infants.

A. MMR vaccine: According to the Chinese vaccination plan, MMR vaccine (MMR, including measles, mumps and rubella vaccine) is usually administered at 18 months of age. So, for a 7-month-old baby, the vaccine is not yet available.

B. Polio vaccine: This vaccine is usually administered at 2,3, and 4 months of age, so a 7-month-old baby should have been vaccinated.

C. Hepatitis B vaccine: Hepatitis B vaccine is administered at birth, 1 month and 6 months, so 7-month-old babies should have been vaccinated.

D. DPT vaccine: This vaccine is administered at 3,4 and 5 months old, so 7-month-old babies should have been vaccinated.

E. BCG vaccine: The BCG vaccine is usually vaccinated at birth to prevent tuberculosis, so 7-month-old babies should have been vaccinated.

In conclusion, for a 7-month-old infant, MR vaccine is unvaccinated because it is usually administered at 18 months.

Therefore, the correct answer is the A. MMR vaccine.

The infant vaccination schedule is arranged according to the Immunization Procedures and Instructions for Children under the National Immunization Program of China. Under this procedure, 7-month-old infants usually have already received the following vaccines:

1. * * Hepatitis B vaccine * * (vaccination at birth, 1 month, 6 months old)

2. * * BCG vaccine * * (inoculation at birth)

3. * * Polio vaccine * * (2,3,4 months vaccination)

4. * * DPT vaccine * * (3,4,5 months vaccination)

But 7-month-old babies are do not received the following:

5. * * MMR vaccine * * (measles, mumps, rubella combination vaccine, usually started at 8 months of age)

So the correct answer is:

A. The MMR vaccine

114. (Type A2 questions)

Patient, female, is 16 years old. Recently depressed mood, and secretly hidden a large bottle of sleeping pills. After the parents found out, they were sent to a mental hospital. The receiving doctor diagnosed him as depression and recommended hospitalization, but his parents did not agree. This is what is taken

A. voluntary hospitalization

B. Commitment

C. Inspection and evaluation

D. medical appraisal

E. Re-diagnosis

Reference answer: C Your answer:

[Analysis] If the patient has his own behavior, the guardian requires consent before hospitalization. The patient in this topic already has a plan to harm himself, but it has not been implemented, and the guardian refuses to be hospitalized, so the decision should be made after further examination and evaluation (C pair).

[Wrong avoidance] Compulsory medical treatment, refers to the involuntary compulsory treatment, refers to the country to avoid the public health crisis, through compulsory treatment

The treatment of patients' diseases, to cure the disease, prevent the spread of diseases and safeguard the interests of public health, is mandatory and non-self

The characteristics of willingness and public welfare. They generally include venereal diseases, drug use, violent mental disorders, and serious infectious diseases

class.

For this situation, option C "check assessment" may be the most appropriate option. Although the receiving physician recommends hospitalization, further evaluation is often required to determine the patient's mental state and treatment needs if the patient or their family disagrees. Such assessments can help decide whether other measures such as forced hospitalization or other interventions are needed. Therefore, the choice of C is the appropriate clinical operation performed in this setting.

analyze:

The young female patient was diagnosed as depression, and the doctor suggested

hospitalization, and the parents did not agree, indicating that the patient did not go through the hospitalization procedures at this time, so the patient was not hospitalized at this time, but was in the state of outpatient examination and evaluation.

Option A Voluntary hospitalization refers to the situation in which the patient has complete self-awareness and voluntarily asks to be hospitalized. However, the question stem clearly states that the patient's parents do not agree to hospitalization, so it is excluded.

Option B forced hospitalization means that the patient's self-knowledge is incomplete. With the consent of the guardian, the hospital enforces the patient for hospitalization, but the parents do not agree to the hospitalization, so it is not selected.

Since the patient did not go through the hospitalization procedures at this time and was in the outpatient stage, option D for medical identification and option E for rediagnosis could be excluded together.

To sum up, the correct answer is: C. Check and evaluate.

According to the information, the 16-year-old woman was depressed recently and had a lot of sleeping pills, a sign of suicide risk. She was diagnosed with depression and recommended hospitalization. However, the parents did not consent to the hospitalization. In this case, the medical institution has the right to take compulsory measures to protect the patient if the patient is obviously suicidal or poses a danger to themselves or others.

So the best answer is:

B. Commitment

115. (Type A2 questions)

Female, 30 years old. Every time after drinking milk, abdominal pain, abdominal distension, diarrhea symptoms, suspected to be the lack of some enzyme, after examination found that some enzyme activity is reduced, in order to reduce the symptoms, it can be recommended to choose which kind of milk

A. Compound milk

B. dried skimmed milk

C. leben

D. modified milk powder

E. evaporated milk

Reference answer: C Your answer:

[Analysis] Every time after drinking milk, abdominal pain, abdominal distension, diarrhea symptoms, considering the lack of lactase, resulting in milk lactose can not be decomposed. You can drink yogurt in moderation, because the lactic acid bacteria in yogurt itself will produce a large number of lactase, which can help the body to better digest lactose, so this question is selected C.

[Avoid mistakes] Yogurt is made of fresh milk and lactobacillus fermentation, and its clot is fine and high acidity, which is conducive to digestion and absorption. Sweet refining

Milk is the fresh milk by vacuum concentration or other methods to remove most of the water, concentrated to the original volume of 25%~40% of the milk

Product, then add 40% sugar canned, sugar content is too high.

The 30-year-old woman presented with symptoms of abdominal pain, bloating, and diarrhea, often associated with lactose intolerance, due to reduced or lack of lactase activity. Lactase is an enzyme responsible for the breakdown of lactose into glucose and galactose. If the body lacks lactase, undigested lactose enters the large intestine and will be fermented by bacteria, producing gases and other substances that causing abdominal pain, bloating and diarrhea.

In order to reduce the symptoms, you can choose yogurt, because the lactic acid produced by lactic acid bacteria during the fermentation process will help break down the lactose, thus reducing the amount of lactose into the large intestine and reducing the symptoms of lactose intolerance. Therefore, the best option is: C. yogurt

First, we need to identify the key point of the problem: this 30-year-old woman developed abdominal pain, bloating and diarrhea after drinking milk, which is usually caused by lactose intolerance. Lactose intolerance is due to the lack of lactase in the body to break down the lactose in milk, which causes these symptoms.

Next, we will analyze each option:

A. Compound milk: it usually contains a variety of nutrients, but it does not

solve the problem of lactose intolerance, because lactose still exists.

B. skim milk powder: Although the fat in the milk is removed, the lactose content is not reduced, so it is not suitable for patients with lactose intolerance.

C. Yogurt: In the process of making yogurt, lactic acid bacteria will decompose most of the lactose in milk into lactic acid, thus significantly reducing the lactose content. Therefore, yogurt is a good choice for patients with lactose intolerance and can alleviate or avoid the symptoms of lactose intolerance.

D. Mixed milk powder: This milk powder may be processed, but if it is not specifically low in lactose or not lactose free, then it is not a good option for patients with lactose intolerance.

E. Light condensed milk: light condensed milk is also a milk product, and the lactose content is not significantly reduced, so it is not suitable for patients with lactose intolerance.

In conclusion, yogurt is the best choice for patients with lactose intolerance, because it significantly reduces the lactose content and is able to reduce the symptoms caused by lactose intolerance.

Therefore, the correct answer is C. yogurt.

The patient developed abdominal pain, abdominal distension and diarrhea after each milk drink, which was suspected to be caused by lactose intolerance. Lactose intolerance is caused by the lack of lactase activity in the body to effectively decompose lactose. For this case, milk products with low lactose content or lactose treatment can be selected to reduce the symptoms.

Here is an analysis of each milk type:

A. Compound milk: It contains many ingredients, but not necessarily is low in lactose.

B. skim milk powder: skim milk powder mainly removes fat, but the lactose content is still high.

C. Yogurt: The lactose in yogurt is partially decomposed into lactic acid during the fermentation process, with low lactose content, so it is suitable for lactose

intolerant patients.

D. Make milk powder: the ingredients are complex, but not necessarily low in lactose.

E. Light condensed milk: It is a concentrated milk with a high lactose content.

Therefore, the most suitable choice for lactose intolerance patients is yogurt, because yogurt lactose has been partially decomposed, can reduce the symptoms.

The correct answer is: C. yogurt

116. (Type A2 questions)

A 62-year-old woman had chest tightness for 1 month with oliguria for 3 days. Previous history of hypertension and diabetic nephropathy. Serum sodium 138 mmol/L, potassium 7.8 mmol/L, and T wave high tip of ECG. The mechanism leading to the ECG changes is that responsible for the ventricular myocytes

A. Sdown of Ca^{2} ion influx in phase 0

B. Accelerated influx of Ca^{2} ions in phase 2

C. Accelerated K-ion outflow in phase 3

D. K ion outflow is slowed in phase 4

E. Acceleration of the Na ion influx in phase 0

Reference answer: C Your answer:

Text analysis: [analysis] middle-aged and elderly female patients, previous history of hypertension and diabetic nephropathy, blood potassium 7.8 mmol/L (more than 5.5 mmol/L), electrocardiogram T wave high point, considered for diagnosis of hyperkalemia. In the case of increased blood potassium concentration, the difference between the potassium ion concentration inside and outside the cell decreases, the potassium ion balance decreases, and the threshold potential decreases, resulting in increasing the permeability of the cell membrane to the potassium ion, resulting in the shortening of the action potential time course. At the same time, the cardiomyocyte stage 3 repolarization speed is accelerated, which causes the high tip of the T wave and the shortened QT interval in the electrocardiogram performance. Therefore, this question is selected C.

[Avoid mistakes] In hyperkalemia, mild muscle tremor, paresthesia in hands and feet, limb weakness, tendon reflexes loss or disappearance,

Even delayed paralysis appears. Hyperkalemia can cause sinus bradycardia, atrioventricular block, or tachyarrhythmia

Often, the most dangerous one is ventricular fibrillation or cardiac arrest. Hyperkalemia often has abnormal electrocardiogram changes, early changes for T wave high

However, sharp, Q-T interval shortened, QRS wave broadening accompanied with amplitude decreased, and P wave amplitude decreased and gradually disappeared.

This issue concerns the mechanism of ECG changes, especially in a patient with hypertension, diabetic nephropathy and hyperkalemia (potassium 7.8 mmol/L). The ECG shows a high T wave, which is usually associated with hyperkalemia. In the action potential of cardiac myocytes, the ion flow properties of each phase have a direct effect on the waveform of the ECG. In particular, the flow of potassium ions is crucial in the third phase of the action potential (repolarization phase).

Options A, B, and E discuss the flow of calcium and sodium ions, which are more important in stages 0 and 2 of the ventricular myocyte action potential, which are not key factors in hyperkalemia affecting T wave morphology.

Option C mentions the acceleration of phase 3 K ion outflow, an important part of repolarization, but not accelerated in hyperkalemia; instead, it slows down by the increased extracellular potassium concentration.

The slowing of K ion outflow in stage 4 indicated by option D is consistent with the ECG T wave due by hyperkalemia. Because in hyperkalemia, the increase of extracellular potassium concentration slows down the outflow of potassium ions from ventricular myocytes, which leads to the prolonged duration of the action potential, eventually showing the high tip of the T wave on the electrocardiogram.

Therefore, the correct answer is that D: K ion outflow slows down in phase 4.

analyze:

An elderly female patient with a previous history of diabetic nephropathy had significantly elevated serum potassium and a high pointed ECG T wave, a typical ECG manifestation of hyperkalemia. During hyperkalemia, the extracellular K concentration increases and K outflow decreases, which slows down the automatic

depolarization of ventricular myocytes in stage 4, that is, K outflow slows down, thus affecting the self-discipline of cardiomyocytes and leading to changes in ECG.

Option A: Stage 0 Ca^{2} ion influx slowing was mainly seen in hypocalcemia and was unrelated to hyperkalemia, so it was not chosen.

Option B: acceleration of Ca^{2} ion influx, option C: acceleration of K ion outflow, will shorten the action potential time of ventricular myocytes, which is not consistent with the performance of electrocardiogram during hyperkalemia, so it is excluded.

Option E: Accelerated Na ion influx in stage 0 is a manifestation of stimulated cardiomyocyte excitation and is not directly related to hyperkalemia, so it is not considered.

In conclusion, the correct answer is: K ion outflow slows down in the D.4 stage.

According to the information in the problem, the 62-year-old female patient had chest tightness of 1 month, worsened with oliguria in the past 3 days, and had a history of hypertension and diabetic nephropathy. Laboratory tests showed serum potassium 7.8 mmol/L and ECG showed a T wave high tip. This suggests that the patient has hyperkalemia, which often causes characteristic changes on the ECG.

The effect of hyperkalemia on ECG is mainly reflected in the following aspects:

1. * * T wave high tip * *: This is an early characteristic manifestation of hyperkalemia and usually occurs when serum potassium exceeds 5.5 mmol / L.

2. P-wave disappearance or amplitude reduction.

3. The QRS wave group broadening.

4. A lower heart rate can eventually lead to cardiac arrest.

The high T-wave tip caused by hyperkalemia is mainly due to the repolarization process of ventricular myocytes (i. e., stage 3 in the electrocardiogram). The specific mechanism is as follows:

Hyperkalemia increases the extracellular potassium ion concentration, leading to

increased K ion efflux during repolarization. Since repolarization (phase 3) is dominated by K ion efflux, elevated serum potassium accelerates this process, leading to T-wave high tips.

Therefore, the ECG changes in the test stem patients are due to the accelerated * * 3-stage K ion efflux * * in the ventricular myocytes. The correct answer is:

C. Accelerated K-ion outflow in phase 3

117. (Type A2 question)

Female, 26 years old. Multidipsia, polyuria, hunger, weight loss for 6 months, examination: fasting blood glucose 14.2mmol/L, HbAlc8.6%, fasting serum insulin 7U / L (reference value 10 ~ 20 mU / L), urine sugar (+ + +). This patient may be changed in vivo and is

A. An increase in the TCA cycle

B. Increased glycogen synthesis

C. Tissue reduced with sugar

D. Increased decomposition of glucose

E. Reduction of gluconeogenesis

Reference answer: C Your answer:

[Analysis] Young female patients, polydipsia, polyuria, hunger, weight loss for 6 months (typical clinical manifestations of diabetes), combined with examination indicated increased blood glucose (fasting blood glucose 14.2mmol/L), insulin deficiency (HbAlc8.6%, fasting serum insulin 7U / L), the diagnosis was considered as type 1 diabetes. Type 1 diabetes is mainly due to the inability of the pancreas to secrete enough insulin, glucose in the cytoplasm cannot enter the mitochondria for oxidative decomposition, and glucose decomposition decreases (D wrong), resulting in the reduction of the tricarboxylic acid cycle substrate, thus affecting the tricarboxylic acid cycle (A wrong). Also leads to a decrease in glycogen synthesis

Less, increased decomposition (B wrong). Muscle and adipose tissue were reduced with sugar (C pair). Increased gluconeogenesis (E is wrong). So this question

select C.

This 26-year-old female presented symptoms of polydipsia, polyuria, hunger and body weight loss with hyperglycemia (fasting glucose 14.2mmol/L) and hyperglycated hemoglobin (HbAlc8.6%) with low fasting serum insulin levels (7U / L, reference 10 to 20 mU / L) and urinary glucose (+ + +). These clinical findings and laboratory findings fit with the diagnosis of diabetes.

In the diabetic state, due to insufficient insulin action or weakened cellular response to insulin, resulting in reduced intracellular glucose utilization and increased blood glucose levels. In addition, because cells feel an energy deficit, they replenish energy by increasing gluconeogenesis (synthesis of glucose from non-carbohydrate precursors) and fatty acid oxidation, rather than by increasing glycogen synthesis. Therefore, an increase in glycogen synthesis does not occur in this case. At the same time, with reduced glucose utilization in diabetics, the process of glucose decomposition in the body is actually increased in an attempt to replenish energy.

Based on the above analysis, the most appropriate option is the increased decomposition of D. glucose. This reflects reduced cellular glucose utilization resulting from insulin deficiency in diabetes, while the body attempts to meet energy needs by increasing glucose breakdown.

First, let's review the key information in the topic:

(1) In young female patients, the main symptoms were polydipsia, polyuria, hunger and weight loss, which lasted for 6 months.

(2) Fasting blood glucose 14.2mmol/L (normal value should be less than 6.1 mmol/L), HbA1c 8.6% (normal reference value is 4%~6%), these two indicators show that patients have poor blood glucose control, and the possibility of diabetes is great.

(3) Fasting serum insulin is 7U / L, which is 10 ~ 20 mU / L lower than the reference value, indicating low insulin levels in the patient.

(4) Urinary sugar (+ + +), indicating that the glucose content in the urine is high, consistent with the hyperglycemia state.

Next, we analyze the options:

A. The TCA cycle increases: Although the TCA cycle is an important link in glucose aerobic oxidation, the topic stem information does not mention any biochemical index changes directly related to this, so it is impossible to determine whether the TCA cycle is increased.

B. Increased glycogen synthesis: In the case of insulin deficiency, glycogen synthesis is usually reduced because insulin is the main hormone that promotes glycogen synthesis. The patient's fasting serum insulin was below normal, so the possibility of increased glycogen synthesis is unlikely.

C. Reduction in tissue sugar use: Due to low insulin levels, the uptake and utilization of glucose by tissue cells will decrease, which is consistent with the typical pathophysiological process of diabetes mellitus. During insulin deficiency, glucose uptake by cells is blocked, resulting in reduced tissue sugars.

D. Increased glucose decomposition: There is no information directly related to the decomposition of glucose in the test stem, and in the state of hyperglycemia, glucose decomposition usually does not increase, but may decrease due to insulin deficiency.

E. Reduced gluconeogenesis: gluconeogenesis is an important route of glucose production in the body, especially during starvation or prolonged fasting. In the case of diabetes, gluconeogenesis may actually increase because the body needs to try to maintain blood glucose levels.

In conclusion, considering the patient hyperglycemia status and low insulin levels, the most plausible explanation is reduced glucose uptake and utilization by tissue cells, leading to decreased glucose use in tissue. This is a direct consequence of insulin deficiency in a diabetic state.

Therefore, the correct answer is C. tissue sugar reduction.

According to the information provided by the topic, the patient was a 26-year-old woman with symptoms of polydipsia, polyuria, hunger and weight loss, and the examination results showed fasting blood glucose 14.2mmol/L, HbA1c 8.6%, fasting serum insulin 7U / L (reference value 10 ~ 20 mU / L) and urine glucose (+ + +). These findings suggest that this patient may have diabetes, especially type 1 diabetes or late type 2 diabetes, showing marked hyperglycemia and insulin deficiency.

In diabetic patients, glucose cannot be used effectively due to insulin deficiency or insulin obstruction, the following may occur:

- ** The TCA cycle increases * *: Otherwise, diabetic glucose cannot be used effectively, and the TCA cycle activity may be reduced.

- ** Increased glycogen synthesis * *: No, glycogen synthesis is reduced in diabetic patients because the liver and muscle cells fail to use the glucose efficiently.

- ** Sugar reduction * *: Compliance, tissue glucose utilization is significantly reduced due to insulin deficiency or resistance.

- ** Increased glucose breakdown * *: No, although high glucose levels will not increase due to limited insulin action.

- ** Reduction of gluconeogenesis * *: Otherwise, liver gluconeogenesis in diabetic patients increased instead, leading to a further increase in blood sugar.

To sum up, the most likely answer is:

C. * * Tissue is reduced with sugar * *

118. (Type A2 questions)

Male, 15 years old, sudden fatigue, nausea, vomiting, weariness and greasy food, school more than 30 people have the same symptoms, no jaundice, 1cm under the liver ribs, tenderness. Most likely

A. Typhoid fever and paratyphoid fever

B. hepatitis A

C. cholera

D. hepatitis B

E. E hepatitis

Reference answer: B. Your answer:

[Analysis] The school group has sudden fatigue, nausea, vomiting, greasy food, no jaundice (in the course of hepatitis A, the most infectious stage is early jaundice), according to the symptoms and physical examination is most likely to be diagnosed as hepatitis A (B pair).

[Avoid mistakes] The common clinical manifestations of typhoid fever are persistent fever, relatively slow pulse, rose rash, liver and spleen large. Typical

clinical of cholera

The manifestation is that cholera enterotoxin causes intestinal spasm, which then causes intestinal spasm, with a large number of watery stools and severe vomiting, among which

Diarrhea is characterized by no fever, no urgency, most of which are not accompanied by abdominal pain, and consciously relaxed after defecation.

This case describes a 15-year-old boy and more than 30 people in his school with sudden fatigue, nausea, vomiting and weariness of greasy food, all typical of acute hepatitis. Jaundice was not mentioned in the case, but mentioned hepatic tenderness at 1cm, which may indicate hepatic involvement.

Depending on the symptoms and the simultaneous occurrence of similar symptoms in the school, the most likely is viral hepatitis, especially the type of transmission by diet. Hepatitis A is transmitted through mouth and feces, often related to the hygiene of food or water, and may spread rapidly in groups, consistent with the simultaneous occurrence of symptoms in many people.

Therefore, the most likely answer is * * B. Hepatitis A * *. Hepatitis A usually causes acute hepatitis symptoms, such as fatigue, nausea, vomiting and loss of appetite, especially tired of greasy food, and does not necessarily accompany jaundice. Furthermore, it often bursts under dense residential conditions, such as schools, which matches the situation described by the title.

First of all, we extracted the key information: the patient was a 15-year-old man, whose main symptoms were sudden fatigue, nausea, vomiting, and weariness of greasy food. In addition, more than 30 people from the same school have the same symptoms, which is an important epidemiological clue that may point to food poisoning or some infectious disease. Physical examination revealed 1cm of the liver with tenderness but no jaundice.

Next, we analyzed the corresponding disease characteristics of each option:

A. Typhoid fever and paratyphoid fever: The main manifestations were persistent fever, indifferent expression, relatively slow pulse, rose rash, liver and spleen size, and leukopenia, which were not consistent with the symptoms of the patient.

B. Hepatitis A: mainly transmitted through the fecal-oral route and often occurs by consumption of contaminated food or water. Its incubation period is about 2 to 6 weeks, with an average of 4 weeks. Clinical manifestations are divided into acute

jaundice type and no jaundice type, the latter is more common. Acute phase often manifested as fever, fatigue, nausea, vomiting, greasy food and other symptoms, highly consistent with the description of the stem.

C. Cholera: The main manifestations are severe diarrhea, vomiting, dehydration, muscle spasm, and, in severe cases, circulatory failure and acute kidney failure, which are not consistent with the symptoms of the patient.

D. Hepatitis B: mainly through blood, sexual contact, mother to child transmission, generally not through food or water transmission, and the incubation period is relatively long, an average of 3 months, so it can be excluded.

E. Hepatitis E: Although it is also transmitted through the fecal-oral route, its incubation period is about 2 to 9 weeks, with an average of 6 weeks, and its clinical manifestations are similar to those of hepatitis A, but compared with hepatitis A, the outbreak of hepatitis E is relatively rare.

Based on the above information, considering that more than 30 patients in the school have the same symptoms and the clinical manifestations of the patient, the most likely diagnosis is hepatitis A. Therefore, the correct answer is the B. hepatitis A.

According to the information provided in the question, the 15-year-old boy and more than 30 people in the school have sudden fatigue, nausea, vomiting, greasy food and other symptoms, and no jaundice, 1cm under the liver ribs, tenderness. These symptoms and signs point to a possible infectious disease that, combined with the collective onset of the patient and the surrounding population, the most likely diagnosis is hepatitis A.

Hepatitis A is usually transmitted through the fecal-oral route, has a strong infectivity, common in schools, kindergartens and other collective places. Symptoms include fatigue, nausea, vomiting, loss of appetite, weariness of greasy food, etc. Although jaundice may occur in some patients, it is not present in all cases.

So the most likely answer is:

F. hepatitis A

119. (Type A2 questions)

The patient, male, was 38 years old. Fever with body pain for 7 days, physical examination: T39.6℃, P95 times / min, R31 times / min, inguinal lymph node enlargement, gastrocnemius tenderness (+), urine routine: urine protein (+ + +), urine sediment microscopy: WBC (+), RB step examination is

A. blood smear

B. stool for routine

C. blood culture

D. coproculture

E. Microagglutination assay

Reference answer: E Your answer:

Graphic analysis: [analysis] male, 38 years old (mainly young adults, Male is more common than female), Fever with body pain for 7 days (systemic infection poisoning), And enlarged inguinal lymph nodes, Gastrocnemius tenderness (typical clinical manifestation of leptosis), Combined with the patient's clinical history, clinical findings, and physical examination, This patient was considered for the diagnosis as leptospirosis, The disease is caused by pathogenic Leptospira (short), Microscopic agglutination test (E pair) is a specific antigen with leptospira standard strain or a living local circulating strain, Mixed mixed with serum at different dilutions of patients, Observed by dark-field microscopy after 2 hours of incubation at 37℃, If there is a corresponding antibody in the serum to be tested, Lptospira agglutates into a small spider shape.

[Error avoidance] Fertilizer reaching test is a kind of test tube agglutination reaction with known Typhi O and H antigen and paratyphoid rod A and B

F. antigen, with the serum to be tested as a tube or microplate to determine the presence of corresponding antibodies in the serum, as

Reference for the diagnosis of typhoid fever and paratyphoid fever. It was used to assist in the diagnosis of Rickettsia disease, a strain of Proteus O

Antigens share common antigens with typhus rickettsia and scrub typhus, so the O antigen from these strains can be used instead of ricker

Secondary body antigens were cross-agglutinated with the patient's serum to detect the corresponding antibodies in the patient's serum.

This 38-year-old male patient had symptoms of fever, general soreness, enlarged groin lymphadenopathy, gastrocnemius tenderness, positive urinary protein and a small number of white blood cells and red blood cells appearing in urinary sediment. These symptoms may point to a variety of causes, including infections (bacteria, viruses, parasites, etc.), inflammatory diseases, or other systemic diseases.

Based on these symptoms and signs, especially high fever, enlarged inguinal lymph nodes, and positive urinary protein, an important consideration is the possible infectious disease. Therefore, we should look for tests that can aid in the diagnosis of infectious diseases.

A. Blood smears: They can be used to check for malaria parasites or other blood parasites.

B. fecal routine: for testing for intestinal infections, such as bacterial, viral or parasitic infections.

C. Blood culture: Can detect bloodstream infections to help determine the type of infection and the bacteria or fungi that cause the infection.

D. Faeces culture: used to confirm specific enteric pathogens.

E. Microagglutination test: used to test for certain bacterial infections, such as leptospirosis (e. g. plague).

Considering the patient's symptoms, such as fever, inguinal lymphadenopathy, and positive urine protein, the microagglutination test (E) may be an effective method for diagnosing leptospirosis (e. g., regression to fever or plague). However, given the comprehensiveness and severity of the symptoms, performing a blood culture (C) to detect possible bloodstream infections seems a more direct and urgent need, as this can indicate a systemic infection that requires prompt recognition and treatment.

Therefore, I would choose * * C. blood culture * * as the next step to identify the cause of the infection and provide guidance for treatment.

analyze:

Young male patients, fever, body aches, high fever, heart rate, breathing, inguinal lymph node enlargement * *, gastrocnemius muscle tenderness * *, laboratory examination see strong positive urine protein, urine white ocyte positive, red blood positive, according to the patient's history, clinical manifestations, physical examination and laboratory examination, considering the diagnosis of leptospirosis.

Leptospirosis is an animal-borne infectious disease caused by pathogenic leptospira, which can be confirmed by microscopic agglutination test. It is a serological method to detect the presence of specific antibodies in serum, and is the most commonly used diagnostic method in China.

Option A blood smear is mainly used for examining parasites, which is of little significance for the diagnosis of leptospira infection, so it is excluded.

Option B fecal routine, option D fecal culture, which is generally used for the diagnosis of infectious diseases of the digestive tract, while leptospirosis is mainly infected by contact with bacterial wild animals and livestock, so it can be excluded together.

Option C blood culture is mainly used for the diagnosis of bacterial infectious diseases, but the in vitro culture of leptospirosis is more difficult and is rarely carried out in China, so it is not considered.

In conclusion, the correct answer is: E. Microagglutination test.

According to the patient's symptoms and signs, the 38-year-old male patient had fever associated with body pain for 7 days. Physical examination revealed high fever, rapid heart rate, increased respiratory rate, inguinal lymph node enlargement, gastrocnemius tenderness, and routine urine showed increased urinary protein and positive white blood cells. These symptoms suggest the possibility of a severe infection.

Considering these symptoms, especially high fever and enlarged inguinal lymph nodes, the possibility of bacterial infection is greater. Microagglutination tests (MAT) are usually used to diagnose leptospirosis (leptosis), while blood cultures help to detect bacteria in the blood.

The best answer should be:

G. blood culture

Blood cultures can help detect the presence of bacteria in the blood and identify the source of the infection.

120, (Type A2 questions)

Female, 16 years old, traveled to remote areas with the tour group. After dinner, she had abdominal pain, diarrhea and diarrhea 4 times a day. After treatment in the local health center, she improved after the sun under the hot sun. What may be the medicine

A. ceftazidime

B. gentamicin

C. berberine hydrochloride

D. Ofloxacin

E. praziquantel

Refer to the answer: D. Your answer:

Graphic analysis: [Analysis] After taking light-sensitive drugs, the skin appeared erythema and itching after the sun under the hot sun the next day. Common light-sensitive drugs are quinolones (D pair), hydrochlorothiazide, chlorpromazine, tetracycline, etc.

[To avoid mistakes] Gentamicin belongs to aminoglycosides (ototoxic, renal toxic, muscle toxicity, allergy), and praziquantel is anti-schistosoma.

This question describes a 16-year-old woman who experienced abdominal pain and diarrhea during the travel trip. These symptoms may be related to the treatment she received.

Based on the symptoms and situations described, we can screen these drugs one by one:

-A. Ceftazidime: This is an antibiotic that is mainly used to treat bacterial infections. It usually does not cause sun-induced sensitivity.

-B. gentamicin: also an antibiotic that is used mainly for the treatment of severe bacterial infections and is also unlikely to cause sun sensitivity.

-C. Oberberine hydrochloride: used to treat various diseases, including malaria and certain skin diseases, but usually does not cause sun sensitivity.

-D. ofloxacin: This is a broad-spectrum antibiotic, fluoroquinolones, which may

lead to light sensitivity that makes the skin more sensitive to sunlight and may cause erythema and itching in the sun.

-E. praziquantel: used to treat parasitic infections, such as malaria, is usually not associated with sun sensitivity.

In conclusion, the most likely drug is * * D. ofloxacin * *, as it is known to potentially elicit a photosensitivity response.

analyze:

The adolescent female patient developed abdominal pain and diarrhea after traveling, which was diagnosed as traveler diarrhea, after treatment, improved, erythema and pruritus appeared on the skin after sunburn the next day, and the diagnosis was made as drug photosensitive reaction was considered. According to the patient's medical history and clinical manifestations, the drug used for diarrhea may be berberine hydrochloride. After taking this drug, sunlight may occur, leading to skin erythema and itching.

Option A ceftazidime, option B gentamicin, option D ofloxacin, are antibiotics, can be used for the treatment of traveler diarrhea, but generally do not cause photosensitive reaction, so excluded.

Option E praziquantel is the first choice for the treatment of schistosomiasis, and the patient has no signs of schistosomiasis infection, so it was not chosen.

In conclusion, the correct answer is: C. berberine hydrochloride. According to the title description, the woman developed symptoms of skin erythema and pruritus after taking the drug during the trip, which appeared after sunlight, suggesting a possible photosensitivity reaction to the drug. Many antimicrobial agents may cause photosensitivity, but ofloxacin (D. ofloxacin) is one of the most common drugs to cause photosensitivity.

Therefore, the most likely drug is:

H. Ofloxacin

121. (Type A2 questions)

Female, 45 years old, increased menstruation, extended for 2 years, Palpitations, dizziness for 2 months, physical examination: Uterine size 14 weeks, multiple fibroids, the largest 876cm, blood routine: Hb 71 g / L, WBC 2.451012 / L, PaO282.3mm,pH7.43. Consistent with the blood oxygen change in this patient is

A. Reduction in arterial oxygen saturation

B. The arterial blood partial oxygen pressure was decreased

C. Reduction in the arterial blood oxygen content

D. Arviovenous oxygen partial pressure difference increases

E. Left shift shift from the curve

Reference answer: B. Your answer:

[Analysis] The patient had increased menstrual volume, lengthening, palpitations, dizziness for 2 months (common clinical manifestations of anemia), uterine size like 14 weeks of pregnancy, multiple uterine fibroids, Hb71g/L,WBC2.45×1012/L,PaO282.3mmHg (decreased blood oxygen and partial pressure, B pair), based on the patient's history and clinical manifestations, consider the diagnosis of anemia and uterine fibroids.

[Avoid avoidance] PaO 2 (blood oxygen partial pressure) refers to the tension produced by physically dissolved oxygen molecules in arterial blood, reflecting the hypoxia sensitivity of the body

Sense index, mainly to judge whether the body hypoxia and its degree. Arterial blood oxygen partial pressure test, mainly used for hypoxia or oxygen poisoning

Diagnosis of sexual disease, a normal value of 95 to 100 mmHg. Blood oxygen saturation (SaO 2) is the percentage of the volume of bound oxyhemoglobin (HbO 2) in the blood to the total bound hemoglobin volume, that is, the concentration of blood oxygen in the blood, which represents the oxygen carrying capacity of red blood cells.

The 45-year-old woman had multiple uterine fibroids, increased menstrual volume, palpitations, dizziness, and routine blood tests showed low hemoglobin (Hb 71g / L), which indicated that she may have anemia. Anemia can affect blood oxygen carrying capacity, so we need to consider which option best fits this situation.

A. Arterial oxygen saturation decline-Blood oxygen saturation is the extent to which hemoglobin in the blood binds to oxygen. Although anemia reduces the total

amount of hemoglobin in the blood, reducing the total oxygen carried, it does not necessarily affect saturation unless there is also poor oxygenation.

B. arterial oxygen partial pressure drop-this refers to the partial pressure of oxygen in the blood, related to the gas exchange capacity of the lung.20 This patient's PaO 2 was 82.3mmHg, which was within the normal range and so not the optimal option.

C. decreased arterial oxygen content-this seems a reasonable option because anemia reduces the total hemoglobin and in turn reduces the blood oxygen content.

D. Increasing arteriovenous oxygen partial pressure difference- -which usually reflects the oxygen demand and uptake of the tissue, and is not closely related to the situation in this case.

E. Left shift from the curve – this indicates an increased affinity of hemoglobin for oxygen, not directly related to the anemia itself.

In conclusion, the most consistent for this patient's symptoms and examination results was option C, with decreased arterial oxygen content. Therefore, the answer should be a C.

First, we analyze the question stem.

The patient was a middle-aged female, long-term menstrual volume increased and prolonged, physical examination found abnormal uterine enlargement, diagnosed as multiple uterine fibroids. The blood routine results showed moderate anemia, and the blood oxygen partial pressure (PaO 2) was 82.3mmHg, within the normal range (the normal value was 80-100mmHg).

Next, we analyzed the options item by item:

A. Decin arterial oxygen saturation: Blood oxygen saturation is usually directly related to blood oxygen partial pressure. Because the patient's blood oxygen partial pressure is in the normal range, the blood oxygen saturation should also be normal, so option A is not correct.

B. Arterial blood oxygen partial pressure drop: It has been clearly indicated that the patient's blood oxygen partial pressure is 82.3mmHg, which is within the normal range, so option B is not correct.

C. Arterial blood oxygen content decreased: arterial blood oxygen content refers to the actual oxygen amount of hemoglobin (Hb) per 100ml of blood. Since the patient had significantly lower hemoglobin (Hb 71g / L, 110-150g / L for normal

women), the total amount of oxygen in the blood decreased, although normal partial oxygen pressure decreased. Therefore, the option C is correct.

D. Arteriovenous oxygen differential increases: arteriovenous oxygen differential usually reflects the use of oxygen by tissues. The venous blood oxygen partial pressure is not mentioned in the topic stem, and the anemia mainly affects the arterial blood oxygen content, rather than the arteriovenous oxygen partial pressure difference, so the D option is not correct.

E. Left shift of the oxygen separation curve: The oxygen separation curve describes the relationship between blood oxygen partial pressure and blood oxygen saturation. In the anaemic case, the blood oxygen-carrying capacity decreases due to decreased hemoglobin, but this does not directly cause a left shift of oxygen from the curve. Indeed, in the anaemic case, due to decreased hemoglobin, it may result in relatively lower oxygen saturation at the same partial pressure, manifested by a right shift of oxygen from the curve. Therefore, the E option is incorrect.

In conclusion, the correct answer is: C. Arterial blood oxygen content has decreased.

For this topic, we need to carefully analyze the patient's situation and the meaning of each option.

A 45-year-old woman, had a history of increased and prolonged periods and experienced palpitations and dizziness in the last 2 months. Physical examination found that the uterus, such as the size of 14 weeks of gestation, had multiple uterine fibroids, the largest fibroids was 876cm. Blood routine showed Hb 71g / L, indicating severe anemia, and WBC 2.4510 ^ 9 / L, suggesting possible leukopenia. Arterial blood gas analysis showed that PaO2 82.3mmHg, pH 7.43.

Based on the above conditions, the patient has severe anemia (Hb 71g / L), which may cause palpitations, dizziness and other symptoms. Anemia can cause a decrease in arterial oxygen levels because hemoglobin is the main carrier of oxygen. When the hemoglobin level is reduced, the blood oxygen content decreases even when the arterial blood oxygen partial pressure is normal. Therefore, the options best

matching the blood oxygen changes in this patient are:

B Reduction in the arterial blood oxygen content

Subject materials, male, 28 years old. Before marriage to a certain health care institution for pre-marital examination. After examination, the medical and health care institutions issued a medical pre-marital examination certificate, which listed the patient's current disease, and his girlfriend, causing the man's strong dissatisfaction.

122, (Type A3 / A4 questions) subject

The disease that should not be listed on the certificate list is

A. Diseases that do not medically recommend marriage

B. Specific infectious diseases during the infectious period

C. Mental illness in the disease

D. Genetic diseases with severe effects on fertility

E. Self-limiting infectious diseases

Reference answer: E Your answer:

[Analysis] Pre-marital medical examination includes the examination of the following diseases: (1) serious genetic diseases: due to the congenital formation of genetic factors, patients all or part of the ability to live independently, offspring reproduction risk is high, medical considered unsuitable for reproductive diseases (A, D wrong).(2) Designated infectious diseases: AIDS, gonorrhea, syphilis and other infectious diseases stipulated in the Law of the People's Republic of China to affect marriage and childbirth.(3) Related psychosis: schizophrenia, manic-depressive psychosis, and other severe psychosis (C wrong).(4) Other diseases related to marriage and childbearing, such as important organ diseases and reproductive system diseases. After pre-marital medical examination, the physician shall propose a designated infectious disease within the infectious period or the relevant mental illness within the onset period

Medical opinion; both men and women preparing to marry should postpone the marriage, B wrong. To sum up, this question chooses E.

[fault avoidance]

122、The question was asked about diseases that should not be listed on the proof

list. The key here is to consider the information on which disease is not necessary to be disclosed during the premarital examination, as it may not affect individual health or marital life in the long term. By this criterion, E. Self-limiting infectious diseases are the most appropriate option, as self-limiting diseases are usually self-resolving and require no special treatment or may not affect the long-term health of both parties.

The correct answer is: A. Diseases diseases diseases is not recommended.

According to the Marriage Law, pre-marital examinations should be checked for the presence of serious genetic diseases, designated infectious diseases and related psychoillness. Self-limiting diseases usually do not require special treatment and can recover by themselves, so there is no need to list them specifically in the pre-marital examination certificate. The diseases that do not recommend marriage in medicine are not a clear medical classification, and may contain many diseases that do not affect marriage, so they should not be listed on the pre-marital examination certificate. Therefore, option A "Medical diseases that do not recommend marriage" should not be listed on the pre-marital examination certificate.

122. For diseases that should not be listed on the certificate list, the

A. Diseases that do not medically recommend marriage

B. Specific infectious diseases during the infectious period

C. Mental illness in the disease

D. Genetic diseases with severe effects on fertility

E. Self-limiting infectious diseases

Correct answer: E. Self-limiting infectious diseases

analyze:

Self-limiting infectious disease is a kind of disease that can be self-limiting and will heal naturally. It generally does not constitute a long-term threat or marital obstacle, and should not be listed on the pre-marital examination certificate sheet.

123, (A3 / A4 type question) subject

From the perspective of medical psychology, the man's unmet need is

A. Survival needs

B. Need for communication

C. The need for acceptance

D. Need for respect

E. Self-actualizing needs

Refer to the answer: D. Your answer:

Graphic analysis: [Analysis] When doctors put forward medical opinions such as "not suitable for marriage", "not suitable for childbirth" and "postponed for marriage", they should fully respect the wishes of the service object, patiently and carefully explain the scientific truth, and give key explanations to the possible consequences. D to.

[fault avoidance]

123、The question examines the knowledge of medical psychology. In this context, a man is strongly dissatisfied with the health care institution disclosing his health condition to his girlfriend, which may violate his need for D. respect. The needs of respect include self-esteem, status, recognition and the needs of others, and the man here may feel his status and self-esteem threatened.

The correct answer is: D. The need for respect

During the pre-marital check-up, his illness was listed and told to his girlfriend, causing his strong dissatisfaction. From the perspective of medical psychology, this dissatisfaction mainly stems from his feeling that his privacy rights and dignity have been violated, that is, his need for respect is not met. Respect needs include pride in one's abilities and achievements, and the pursuit of independence, freedom and dignity. Therefore, the D option "need for respect" is correct.

123. From the perspective of medical psychology, the man felt that the unmet need was

A. Demand for survival

B. Need for communication

C. The need for acceptance

D. Need for respect

E. Self-actualizing needs

Correct answer: D. Need for respect

analyze:

In that case, a man was dissatisfied and angry about the disclosure of his personal health information, mainly because his privacy and dignity were not respected, which falls under the category of respect needs.

124, (A3 / A4 type question) subject

In this case, the primary physician moral obligation violated by the receiving physician is

A. Health education for the patients

B. Protection of patient privacy

C. Follow the laws of medical science

D. Standard writing of medical documents

E. Compliance with the operating specifications

Reference answer: B. Your answer:

Graphic analysis: [Analysis] The pre-marital medical examination form should be properly kept confidential for personal privacy. The question of this question is mainly to emphasize that the doctor informed the girlfriend without the patient's consent to the disease, so this question B is more appropriate.

[fault avoidance]

124. The question asks about the moral obligations of the main physician violated by the receiving physician. In this case, the doctor failed to protect the patient's privacy information and disclosed the personal health status to a third party without consent, so the violation was B. to protect the patient's privacy. This is an important part of physician ethics and requires physicians to protect patients' privacy and confidential information.

The correct answer is: B. Protect patients' privacy

In the case, the receiving physician informed the girlfriend of his illness without

his consent, violating the patient's privacy rights. Protecting patients 'privacy is one of the important moral obligations of doctors. Doctors should respect patients' privacy and confidentiality rights, and should not disclose patients' personal information and health status without reason. Therefore, the primary physician ethical obligation violated by the attending physician is the protection of patient privacy. Other options, such as health education for patients, following medical science laws, writing medical documents, and following operational practices, are also the ethics that physicians should follow, but are not directly involved in this case.

124. In this case, the primary physician ethical obligation violated by the receiving physician was

A. Health education for the patients

B. Protection of patient privacy

C. Follow the laws of medical science

D. Standard writing of medical documents

E. Compliance with the operating specifications

F. Protect the privacy of patients

analyze:

According to the Law on Medical Practitioners and relevant medical ethical requirements, physicians should protect patients' privacy rights. In this case, the failure of the receiving physician to protect the privacy of the patient led to the disclosure of the patient's disease information, which caused dissatisfaction and the violation of the moral obligation of the physician to protect the privacy of the patient.

In a city, 206 adult men and 201 adult women were randomly selected to understand their HBsAg carrying status. Among them, 33 men were positive, the positive rate was 16.02%, the positive rate was 10.94%, the known positive rate of men in the province was 17.3%, and the positive rate was $P > 0.05$, according to the standard of $\alpha = 0.05$.

125, (A3 / A4 type question) subject

If the carrier rate of HBsAg in the city is $P > 0.05$, the difference between the

sample rate and men in the province depends on

A. Sample number is too large

B. Effects of the systematic error

C. Effect of the calculation error

D. The overall difference

E. Effect of sampling error

Reference answer: E Your answer:

Graphic analysis: [analysis] P> 0.05, that is, there is no significant difference between the two, the city is the same as the whole province. The large sample size gives the estimator closer to the overall parameter, the larger the sample size, the smaller the error, A error. Systematic error is the error caused by the measurement method itself, according to the question stem and the current medical level, the determination of hepatitis B should not have too much error, so exclude B option. There is no calculation error, similar statistical error, also known as statistical data error, is the gap between the actual statistical data results and the corresponding objective true value, in the statistics of hepatitis B patients is not easy to appear obvious error, C wrong. The overall difference, leading to the city and the province's statistics are the same, this logic is wrong, D wrong. The results of the city are obtained by random selection, which is prone to sampling error, so question E is chosen.

[fault avoidance]

The correct answer is an E. In the statistical analysis, a P-value greater than 0.05 indicates that the differences observed in the sample may be due to sampling error rather than true differences between populations. Here, the P-value was greater than 0.05 more than province-wide male HBsAg positive carriers, indicating that the difference between the two rates may simply be due to sampling error.

I. Effect of sampling error

analyze:

If the carrier rate of HBsAg in men in the city, P> HBsAg is 0.05, it indicates that there is no statistical difference between the two. While the difference between the sample rate and the overall rate is often due to the sampling error, that is, the sample is not perfectly representative of the population. This error is random and

decreases with increasing sample size, but could not be completely eliminated.

125. If the carrier rate of HBsAg in the city is P> 0.05 compared with the carrier rate of HBsAg in the province, the difference between the sample rate of men depends on the difference

A. Sample number is too large

B. Effects of the systematic error

C. Effect of the calculation error

D. Overall difference

E. Effect of sampling error

Answer: E. The effect of sampling error

Analysis: When P> 0.05, it indicates that there is insufficient statistical evidence to show that there is a significant difference between the male HBsAg carriers in the city and the male HBsAg carriers in the province. This difference may be due to sampling error rather than systematic error, calculation error or actual differences in the population.

126, (A3 / A4 type question)

The following conclusion is correct subject

A. Statistically significant differences in gender carriage rates between men and women

B. Male carriage rate> that in females

C. Male carriage rate <in females

D. Male carriage rate = female

E. Carrying rates cannot be considered different between men and women

Reference answer: E Your answer:

Graphic analysis: [Analysis] According to the question stem, compare the P> 0.05, indicating that the difference is not statistically significant, A wrong. Since there

is no significant difference between men and women, there is no claim that male carrying rate is higher than women and men are lower than women, and BCD is wrong. To sum up, this question chooses E.

[fault avoidance]

The correct answer is an E. With P-values greater than 0.05, we cannot reject the null hypothesis, meaning that there is insufficient evidence of a statistically significant difference in HBsAg carriage rates between men and women, and therefore different male-female carriage rates cannot be considered.

E. Carrying rates cannot be considered different between men and women

analyze:

"P> 0.05", which shows that the difference in HBsAg carriage rate between men and women is not statistically significant, so we should not consider the difference between men and women.

126. The following conclusions are correct

A. Statistically significant differences in gender carriage rates between men and women

B. Male carriage rate> that in females

C. Male carriage rate <in females

D. Male carriage rate = female

E. Carrying rates cannot be considered different between men and women

Answer: E. Differdifference between men and women

Analysis: The topic mentioned the comparison of gender carriage rates, P> 0.05, indicating that there is not statistically sufficient evidence for a significant difference between male and female carriage rates. Therefore, the correct conclusion is that the carriage rate is not yet different between men and women.

127, (Type A3 / A4 questions) subject

To compare the two rates with the Z-test, the following statement is true

A. The larger the P, the more reasonable there is that the two overall rates are different

B. The smaller the P, the smaller the difference between the two samples

C. The smaller the P, the more reason there is that the two overall rates are different

The larger D.P, the more reasonable there is that the two sample rates are different

D. The smaller the P, the more reasonable it is to suggest that the two sample rates are different

Reference answer: C Your answer:

[Analysis] Z test is a hypothesis test. In hypothesis test, P value represents the probability of current evidence or stronger evidence when the null hypothesis H0 is correct. The smaller the P-value, the stronger the evidence provided by the sample, the more favorable the original hypothesis, and the more supported the alternative hypothesis. In this question, the alternative hypothesis should be that the overall rate of men and women is different, so this question chooses C. Note that the other option is to compare the sample rate, comparing the rate ratio of the sample, the significance of the hypothesis test is to estimate the rate of the whole population through the randomly drawn sample rate, so BDE error.

[fault avoidance]

The correct answer is a C. When comparing two ratios using the Z-test, a smaller P-value (usually less than 0.05) indicates that the difference between the two samples is statistically significant, so it is more reasonable to assume that the two overall rates are not identical.

E. The smaller the P, the more reason there is that the two overall rates are different

analyze:

The Z-test is a statistical method used to compare two proportions or rates. In this test, the smaller the P value means the smaller the probability of the observed data appearing assuming that two population rates are identical, thus the more reason it is to reject this hypothesis, namely that the two population rates are different. So option C "the smaller P, the more reasonable the two overall rates are different" is correct. However, a larger P-value indicates that the higher the probability of the observed

data appearing when assuming that the two population rates are identical, so we cannot reject this hypothesis. So, the A, B, D, and E options are all wrong.

127. Using the Z-test to compare the two rates, the following statement is true

A. The larger the P, the more reasonable there is that the two overall rates are different

B. The smaller the P, the smaller the difference between the two samples

C. The smaller the P, the more reason there is that the two overall rates are different

The larger D. P, the more reasonable there is that the two sample rates are different

D. The smaller the P, the more reasonable it is to suggest that the two sample rates are different

E. Answer: The smaller the P, the more reason there is that the two overall rates are different

Analysis: In the Z-test, the smaller the P-value, the more significant the difference between the sample rates, so there is a better reason to think that the two overall rates are not the same.

Title material male, 22 years old, 3 years no obvious cause solitary, don't want to go out with people, curtains closed during the day, lazy life, summer can be up to half a month not take a bath, hair hair, see obvious abnormal, mental check: expression, less words, deny illusion, poor thinking, emotional indifference, self-knowledge is poor.

128, (A3 / A4 type question) subject

This patient was considered for this diagnosis of

A. schizophrenia

B. generalized anxiety

C. autism

D. depression

E. Social avoidant personality disorder

Reference answer: A. Your answer:

[Analysis] This difficult problem is the differential diagnosis of schizophrenia and depression, the main differentiation point is that schizophrenia patients can appear mental activities and the environment is obviously not coordinated, and depression patients will not. In this case, the patient with closed curtains during the day and not taking a bath for up to half a month in summer belongs to the disharmony between mental activities and environment, so it is considered schizophrenia rather than depression. Most patients with schizophrenia start in the prime of life, around the age of 25. Simple schizophrenia clinical manifestation of poor thinking, emotional indifference, or will decline "negative symptoms" is given priority to, early can be characterized as similar neurasthenia symptoms, such as depression, attention, dizziness, insomnia, etc., then gradually withdrawn, lazy, interest, emotional indifference and behavior, and cannot adapt to the needs of society, but no delusion, hallucinations and other obvious "positive symptoms". Disease duration was at least 2 years. This patient had consistent clinical findings, and A vs. Extensive anxiety is continuous significant nervous, accompanied by autonomic nervous function and excessive vigilance characterized by a chronic anxiety disorder, patients often have characteristic appearance, such as facial distortion, frown, posture, and fidgeting, and even trembling, skin pale, palms, feet and armpit sweat. B inconformity. Autism starts in infancy, and C is wrong. Evidant personality disorder is characterized by avoidance of social situations or interactions involving rejection, criticism or humiliation risk, incompatible with the present patient, E wrong.

128: The symptoms are consistent with the characteristics of schizophrenia, such as social withdrawal, emotional indifference, poor self-knowledge, etc. Therefore, the most appropriate diagnosis is: A. schizophrenia

analyze:

Young male patients, in recent years, no obvious inducement to appear solitary, do not want to go out to communicate with people, lazy life, mental examination found indifferent expression, less words, denied hallucinations, poor thinking, emotional indifference, poor self-knowledge, according to its symptoms and signs, consider the diagnosis of schizophrenia. Schizophrenia is a kind of severe psychosis with unknown etiology, which is usually slow or subacute in young adults. It is often

manifested as a syndrome with different symptoms, involving sensory perception, thinking, emotional and behavioral disorders, as well as the incoordination of mental activities. Patients are generally aware and their intelligence is basically normal, but some patients will have the impairment of cognitive function in the course of the disease.

Option B generalized anxiety is a chronic anxiety disorder characterized by continuous significant nervousness, accompanied by autonomic function excitation and excessive vigilance. It is not consistent with the symptoms of this patient, so it is not selected.

Option C Autism occurs in infancy, mainly characterized by different degrees of speech development disorders, interpersonal disorders, narrow interest and rigid behavior, which are inconsistent with the age and symptoms of the patients in this question, so they were excluded.

Option D Depression is characterized by significant and persistent low mood, clinical manifestations from depression to grief, negative suicide attempts or behavior, or even wood rigidity, has nothing to do with this problem.

Option E Social avoidant personality disorder, also known as evasive personality, is characterized by behavioral withdrawal, psychological inferiority, and more avoidance attitude or inability to cope with challenges, which is inconsistent with this topic.

In conclusion, the correct answer is: A. schizophrenia.

diagnostic analysis:

Schizophrenia (A): in line with the solitary, unwilling to communicate, emotional indifference, poor thinking, poor self-knowledge and other manifestations, the most in line with the patient's symptoms.

Generalized anxiety (B): Generally manifested as constant tension and concern that does not match the main manifestations of the patient in this case.

Autism (C): mostly occurs in childhood, mainly manifested as social communication and communication disorders, not fully consistent with the performance of this adult patient.

Depression (D): Although there are symptoms of lazy life and emotional

indifference, but poor thinking, poor self-knowledge and other more inclined to schizophrenia.

Social avoidant personality disorder (E): mainly manifested as extreme social fear and avoidance, but not necessarily accompanied by emotional symptoms such as emotional apathy and poor thinking.

Therefore, the best diagnosis was A. schizophrenia.

129, (Type A3 / A4 questions) subject

The drug that the treatment should choose is

A. antidepressant drugs

B. A mood stabilizer

C. incitantia

D. antipsychotic drug

E. anxiolytic drugs

Refer to the answer: D. Your answer:

[Analysis] For the drug treatment of schizophrenia, antipsychotic drugs should be selected, D pair.

129: Antipsychotic drugs often require the treatment of schizophrenia because they help control hallucinations, delusions, and confusion in thinking and behavior. So, the right drug is: D. Antipsychotics

129, analysis:

Since this patient was considered for schizophrenia, the medication that should be chosen for treatment was antipsychotic. Such drugs can effectively control the psychopathological symptoms of schizophrenia and significantly improve the quality of life and prognosis of patients.

Option A antidepressants were mainly used to treat depression, so they were excluded.

Option B mood stabilizer, or anti-manic drug, is mainly used to treat mania, which is not consistent with this question.

Option C Psychostimulant is not a professional medical term. In psychiatry, "stimulant" is usually used to describe certain drugs that can stimulate the central nervous system, but these drugs are usually not used to treat schizophrenia, so they

are not selected.

Option E Antianxiety drugs are mainly used to treat generalized anxiety and panic disorder, which do not meet the meaning of the question.

To sum up, the correct answer is: D. Antipsychotics.

Treatment options:

Antidepressant drugs (A): mainly used to treat depression, not suitable for this patient.

Mood stabilizer (B): used for bipolar disorder, not suitable for this patient.

Psychostimulant (C): used for treating attention deficit hyperactivity disorder (ADHD), etc., not suitable for this patient.

Antipsychotic (D): Suitable for patients with schizophrenia and effectively control symptoms.

Antimolytic drugs (E): used for anxiety disorders, not suitable for this patient.

Thus, the best drug of choice was D. antipsychotics.

130, (Type A3 / A4 questions)

After taking psychotropic drugs, the symptoms were significantly improved, but there were symptoms such as static cannot sit, hand shaking, and the next step of treatment should be subject

A. Excessive use of this psychotropic medication

B. The psychotropic drugs with other different mechanisms

C. Reduced use of this psychotropic drug

D. Discontinuation of this psychotropic medication

E. Treatment with phenenhisol

Reference answer: E Your answer:

Graphic analysis: [analysis] The symptoms of patients unable to sit and hand shaking belong to extrapyramidal reaction. There are five different forms of extrapyramidal reactions that are common, mainly: acute dystonia, sedentary inability, pharmacogenetic Parkinson's syndrome, cleft lip syndrome, and delayed dyskinesia. Benyisol is often used in combination with antipsychotic drugs to improve or even

prevent extrapyramidal reactions caused by such drugs, E vs.

130: Symptoms described, such as static sitting and hand shaking, may be drug side effects, especially extrapyramidal side effects caused by antipsychotic drugs. Marine (Benzhexol) is commonly used to treat this side effect. Therefore, the next step should be: E

130. Analysis:

After taking psychotropic drugs, the symptoms are significantly improved, but there are symptoms such as static sitting and hand shaking, which is the external vertebral system reaction caused by the drug, so the next treatment should be added. Benyisol is a central antichololine anti-parkinsonian drug, clinically used for Parkinson's disease, Parkinson's syndrome, can also be used for extrapyramidal diseases caused by drugs.

Option A The use of this psychotropic drug may aggravate the side effects of patients, so it is not selected.

Option B switching to other psychotropic drugs with different mechanisms does not exactly address the current extrapyramidal response problem and may cause other uncertain side effects that can be ruled out.

Option C reduces the use of this psychotropic drug may affect the treatment effect and does not meet the question.

Option D Discontinuation of this psychotropic drug may cause the patient's condition to relapse, which is inconsistent with this question.

In conclusion, the correct answer is: E.

Handling of drug side effects:

After taking psychotropic drugs, the symptoms improved, but there were side effects such as static sitting and hand shaking, which may be drug-induced extrapyramidal reaction (such as static sitting and tremor).

Increasing dosage of this psychotropic drug (A): unsuitable and may aggravate side effects.

Replace psychotropic drugs with different mechanisms (B): there is some rationality, but the balance between efficacy and side effects should be considered.

Reduced use of this psychotropic drug (C): reduces side effects and may be a reasonable choice.

Discontinuation of this psychotropic drug (D): not recommended, which may lead to recurrence of symptoms.

Additional therapy (E): It is an anticholinagent that relieves extrapyramidal responses.

Therefore, the best treatment regimen is E. Alphenhyline therapy.

Male, 68 years old, was hospitalized for acute myocardial infarction. After 3 weeks, the patient had all other symptoms except for the slight discomfort after the anterior heart area, and the laboratory tests were negative, and the treating doctor simply discharged him in advance. One month later, the patient presented to the emergency department of the hospital with angina and died of ventricular fibrillation. The patient's family thought that the patient's death was related to the last doctor's advice to leave the hospital, and a dispute with the hospital

131, (A3 / A4 type question) subject

The hospital has the conditions for frozen storage, and the legal maximum time limit for the autopsy is

A. 48 hours after patient death

F. Treatment of the patients at 3 days after their death

And C. 7 days after the patient's death

D. 48 hours after the signature of the patient's close relatives

E. 3 days after the signature of the patient's close relatives

Reference answer: C Your answer:

Graphic analysis: [Analysis] Referring to article 18 of the "Regulations on Handling Medical Accidents", if the patient dies, the doctor and patient parties cannot determine the cause of death or have objections to the cause of death, the autopsy should be conducted within 48 hours after the death of the patient. If the body is frozen, it can be extended to 7 days, C.

As for the legal maximum time limit for the freezing of corpses, according to the law I know, the options do not fully meet the standard laws of any country, because the time limit of autopsy varies by national or regional law. However, however, autopsy should be performed as early as possible to avoid further changes in the body

that affect the autopsy results. If an option has to be chosen, I tend to choose B. 3 days after the patient died because this period is relatively reasonable for the family to make decisions and make the necessary legal and medical preparation.

131. The correct answer is: C. 7 days after patient death

According to the Regulations on the Handling of Medical Accidents, if the patient cannot determine the cause of death or disagree with the cause of death, the autopsy should be conducted within 48 hours after the death of the patient; if the body is frozen, it can be extended to 7 days. Therefore, the hospital has the conditions for frozen storage, and the legal maximum time limit for autopsy is 7 days after the death of the patient's death, and option C is correct.

131. Hospitals are qualified for frozen storage, and the maximum legal time limit for autopsy is

A. 48 hours after patient death

G. Treatment of the patients at 3 days after their death

And C. 7 days after the patient's death

D. 48 hours after the signature of the patient's close relatives

E. 3 days after the signature of the patient's close relatives

Best answer: C. 7 days after patient death

The legal maximum time limit for autopsy is usually prescribed as within 7 days of the patient's death. This is done to ensure the timeliness and accuracy of the autopsy.

132, (A3 / A4 type question) subject

Family behavior belongs to

A. Type H behavior

B. Type D behavior

C. Type X behavior

D. Type B behavior

E. Type A behavior

Reference answer: E Your answer:

Graphic analysis: [Analysis] Type A behavior is mainly extremely competitive, aggressive, impatient, impatient, and hostile, usually dissatisfied with some core aspects of life, extremely competitive. Options A conform to. Type B behaviors are less competitive and less hostile. Type-D behavior is mainly reflected in pessimism.

With regard to the types of family behavior, these options appear to point to specific patterns or types of behavior, but these types are not widely endorsed or well-defined medical or psychological terms. If it is based on conventional understanding, there may not be enough information to determine the type of family's behavior. Therefore, there is no clear answer, but if you have to choose, I would tend to choose type C. X behavior, here assuming that type X behavior refers to a particular or undefined type of behavior.

The correct answer is: C. X type behavior

X-type behavior mode is an "exchange type" behavior mode in response to social pressure. It means that in social competition, some people will take a kind of utilization, resistance, regulatory behavior or exchange to deal with conflict. The patient's family had a dispute with the hospital due to the patient's death and the last doctor's advice to discharge her, which reflects that the patient's family took a rebellious behavior to deal with the conflict and belongs to the X-type behavior mode, so option C is correct.

The A-type behavior mode shows A high desire for achievement, full of challenge and competitive spirit, prone to unprovoked hostility, competitive, impatience, time urgency, etc., which is not consistent with the meaning of the question, so option A can be excluded.

Type B behavior is A personality trait opposite to type A behavior. It is loose and aloof from worldly worldly, which is inconsistent with this question, so option B can be excluded.

Type D behavior is withdrawn behavior, which is silent, withdrawn, extremely shy, not good at social, stubborn, emotional instability. This kind of people are prone to cardiovascular diseases and malignant tumors, and can be accompanied by

abnormal psychology, which has nothing to do with this question, so option D can be excluded.

There is no H pattern, so option E can be excluded first.

132. Family behavior belongs to

A. Type H behavior

B. Type D behavior

C. Type X behavior

D. Type B behavior

E. Type A behavior

Best answer: Type B. D type behavior

Type D behavior usually refers to the type of behavior in which emotions are easily agitated, susceptible to stress, and may therefore adopt extreme behavior. In this case, the patient's family has a dispute with the hospital because of the doctor's decision, which belongs to type D behavior.

133, (A3 / A4 type question)

The patient's family members disputed the decision of the attending physician's request to discharge early at that time, believing that the behavior caused the death of the patient. Which of the following statements is in line with medical ethics subject

A. The attending doctor should fully communicate with the patient's family members to explain the reasons and status of the early discharge request at that time

B. The patient's family has no right to question the attending doctor's decision at that time

C. The hospital should conduct an accountability investigation by the attending physician

D. Patients can make trouble in the hospital

E. The attending physician is responsible for the death of the patient

Reference answer: A. Your answer:

[Analysis] Patients and their families have the right to question the decision of the attending doctor, but making trouble in the hospital is a violation of the relevant laws and regulations. If the medical staff of the hospital is at fault in the diagnosis and treatment of the patient, the hospital shall assume certain responsibility for the death of the patient. Because this case has not been determined whether the doctor and the hospital are at fault. In conclusion, the BCDE is wrong.

For the third question, what is in line with medical ethics is A. The attending doctor should fully communicate with the patient's family to explain the reasons and status of the early discharge at that time. This embodies the principles of transparency and communication in medical ethics, ensuring that patients and their families understand treatment decisions and discharge decisions, while helping in avoiding misunderstanding and mistrust.

133. The correct answer is: A. The attending doctor should fully communicate with the patient's family members to explain the reasons and status of the request for early discharge

From the perspective of medical ethics, the attending doctors should choose to communicate honestly with the objections of the patient's families and explain the medical reasons for early discharge and the specific situation of the patient, which can help eliminate misunderstanding and maintain the harmonious doctor-patient relationship. Thus, option A meets the requirements of medical ethics.

Option B "The patient's family has no right to question the attending doctor's decision at that time" violates the patient's right of informed consent, and the patient and his family have the right to question the doctor's medical decision, so it is excluded.

Option C "The hospital should conduct accountability investigation by the attending doctor" is too arbitrary. Direct accountability investigation without sufficient communication and understanding of the situation does not conform to the principle of justice, so it is not selected.

Option D "Patients can make trouble in the hospital" obviously violates the requirements of social order and medical environment. It not only fails to solve the problem, but also may cause more chaos and harm, which can be excluded.

Option E The statement that "the attending doctor should be responsible for the

death of the patient" is too absolute. Whether the patient's death is directly caused by the doctor's decision can only be determined by professional medical malpractice identification, which cannot be simply attributed to the doctor, so it does not meet the question.

133. The patient's family disputed the decision of the attending physician's request to discharge early at that time, believing that the behavior caused the death of the patient. which of the following statements is in line with medical ethics

A. The attending doctor should fully communicate with the patient's family to explain the reasons and status of the early discharge at that time

B. The patient's family has no right to question the attending doctor's decision at that time

C. The hospital should conduct an accountability investigation by the attending physician

D. Patients can make trouble in the hospital

E. The attending physician should be responsible for the death of the patient

Best answer: A. The attending doctor should fully communicate with the patient's family to explain the reason and the condition of the early discharge at that time

Patients with closed abdominal injury were accompanied by shock, and no coagulation was extracted by abdominal puncture.

134, (A3 / A4 type question)

The most important treatment link that should be used before the surgery is this one

A. keep quiet

B. relieve pain

C. Prevention and treatment of shock

D. gastrointestinal decompression

E. Application of antibiotics

Reference answer: C Your answer:

Graphic and text analysis: [analysis] abdominal puncture extraction without coagulation suggests intra-abdominal bleeding, organ rupture. The injured with visceral injury are easy to have shock, so the prevention and treatment of shock is an important part of the treatment. In patients with progressive abdominal bleeding, the bleeding was rapidly dissected.

Question 134 focuses on the primary treatment process that should be used before surgery is performed. The options given include keeping quiet, relieving pain, preventing shock, gastrointestinal decompression and application of antibiotics. In this case, the most urgent thing is to handle shock, because shock is a life-threatening state and requires immediate management to maintain circulation and tissue perfusion. Therefore, the correct answer is C. Prevention against shock.

The most important treatments that should be used before the surgery are:

For this problem, considering that the patient is already in shock and that the abdominal puncture does not clot, this usually means intra-abdominal bleeding. In this case, the most important treatment link should be the rapid and effective management of the shock state.

A. Keep quiet-Although it is important for patients to be quiet, it is not a primary treatment of shock.

B. Pain relief-analgesia is important, but it is not the main link in the management of shock.

C. Prevention of shock- -this is the most critical. Shock is an emergency situation that requires prompt management to ensure the patient's life safety.

D. Gastrointestinal decompression-Although probably necessary in some cases of abdominal injury, it is not the primary treatment for shock.

E. Application of antibiotics- -which is used primarily to prevent infection and is not the main treatment of shock.

So the answer is: C. Prevent shock

For patients with closed abdominal injury with shock and abdominal puncture, the following is the analysis and selection of the best answer:

134, (A3 / A4 type question)

The most important treatments that should be used before the surgery are:

H. Prevention and treatment of shock

Shock is a life-threatening emergency and must be managed immediately to maintain the patient's vital signs and organ perfusion. While quiet (A), pain relief (B), gastrointestinal decompression (D), and application of antibiotics (E), although important in some cases, the first primary step to be taken in the face of shock is to prevent shock.

135, (Type A3 / A4 questions)　　subject

The therapeutic principle that should be taken is that

A. Blood transfusion and anti-shock with pressor drugs

B. Anti-shock to wait for surgery after blood pressure improves

C. First antishock and then surgery

D. Anti-shock and simultaneous surgery

E. No surgery with a lot of hemostatic drugs

Refer to the answer: D. Your answer:

Graphic and text analysis: [analysis] abdominal puncture extraction without coagulation suggests intra-abdominal bleeding, organ rupture. The injured with visceral injury are easy to have shock, so the prevention and treatment of shock is an important part of the treatment. In patients with progressive abdominal bleeding, the bleeding was rapidly dissected.

The 135 questions focused on the therapeutic principles that should be adopted. The options given include blood transfusion and anti-shock with anti-shock, anti-shock surgery after blood pressure improves, anti-shock and then surgery, anti-shock surgery at the same time, non-surgery with a large number of hemostatic drugs. In patients with an abdominal closed injury accompanying shock, the priority is to stabilize the patient's hemodynamic status, but also can not delay surgical treatment because the internal organs may be impaired. Therefore, anti-shock therapy (e. g., fluid transfusion, booster drugs) and surgical treatment should be performed

simultaneously to control internal bleeding and address the source of injury. So, the correct answer is D. Anti-simultaneous surgery.

The therapeutic principles that should be taken are follows:

In managing patients with abdominal closure injuries associated with shock, the treatment principles need to take comprehensive consideration of the overall condition.

A. rehydration fluid transfusion and antishock with pressor drugs-which is only part of the treatment of shock but does not involve the management of abdominal injuries.

B. Antishock for surgery after blood pressure improves-which may delay treatment of abdominal injuries.

C. Anti-shock before surgery-with B option, which may cause treatment delay.

D. Anti-shock simultaneous surgery-this is a comprehensive treatment that addresses both shock and abdominal injuries.

E. No surgery with a lot of hemostatic agents-relying on hemostatic agents alone without surgery may not be sufficient to manage abdominal injuries.

Taken together, the most appropriate treatment principle is both anti-shock therapy and surgery. Therefore, the answer is: D. Anti-shock and simultaneous surgery

135, (Type A3 / A4 questions)

The therapeutic principles that should be taken are follows:

I. Anti-shock and simultaneous surgery

In patients with a closed abdominal injury in this condition, immediate surgical control of the bleeding source is required for intraperitoneal bleeding, while anti-shock therapy is required to maintain blood pressure and organ perfusion. Therefore, the optimal treatment principle is anti-shock simultaneous surgery (D).

Patient, female, 28 years old, 34 weeks pregnant.10 days ago, he began to feel fatigue and poor appetite. In the past 5 days, vomiting, yellow sclera, and was hospitalized. His blood pressure was 135 / 90mmHg, ALT35176 μmol/L, and urinary protein (-).

136, (A3 / A4 type question) subject

The first selected check is

A. CBC

B. alkaline phosphatase

C. cholalic acid

D. Antibodies to the hepatitis virus antigen, item 7

E. blood sugar

Refer to the answer: D. Your answer:

[Analysis] any period of pregnancy may be infected by hepatitis virus infection, among which hepatitis B virus infection is the most common. Clinical manifestations: digestive system symptoms that cannot be explained by pregnancy reaction or other reasons, such as appetite loss, liver pain, fatigue, abdominal distension; some patients may have yellow skin mucosa and dark urine. Serum bilirubin was mostly above 17 μmol/L. The ALT and AST were mostly within the normal range.

136 The first test is that, considering the patient's symptoms and pregnancy, we need to exclude the most urgent situation and may have a significant impact on the mother and baby. Complete blood count in item A can help assess infection or hematological problems; item B elevated alkaline phosphatase may point to liver or biliary disease; item C bile acid test is specific for the diagnosis of pregnancy-related liver disease; item D 7 for hepatitis; item E blood glucose is also important for the diabetes status of pregnant women. Here, considering the jaundice and abnormal liver function, the examination of C bile acid is more critical for the diagnosis of pregnancy-related liver disease, so the choice of C is the most appropriate.

The correct answer is: D. Hepatitis virus antigen antibody seven.

Young female patient, pregnant 34 weeks old, appear digestive tract symptoms and jaundice, ALT rise, prompt liver function is impaired, should be excluded in the first place viral hepatitis, therefore the first choice examination is hepatitis virus antigen antibody 7, in order to clear whether it is viral hepatitis and hepatitis type. Option A complete blood count, although routine, was not diagnostic for hepatitis, so it was excluded. Option B Alkaline phosphatase is mainly used in the diagnosis and differential diagnosis of skeletal and hepatobiliary diseases, especially jaundice, but it is not the first choice for the diagnosis of hepatitis, so it is not selected. Option C cholic acid is mainly used for the diagnosis of intrahepatic cholestasis during pregnancy. The patient in this question had no pruritus, so it was excluded. Option E

blood glucose is a routine examination, but it has no diagnostic value for hepatitis, so it is not selected.

136, the first selected check is

A. Complete blood cell count

B. alkaline phosphatase

C. cholalic acid

D. Seven items for antibodies to hepatitis virus antigen

E. blood sugar

analyze:

Complete blood count: used to screen for anemia or infection, but not the first test.

Alkaline phosphatase: used for hepatobiliary disease, but not specific disease.

Cholic acid: Intrahepatic cholestasis (ICP) during pregnancy can cause cholic acid elevation, but it is not the preferred test.

Hepatitis virus antigen antibody seven: used for screening for viral hepatitis.

Blood glucose: screen for diabetes or gestational diabetes.

Because the patient showed jaundice and abnormal liver function, combined with the specific situation of pregnancy, the possibility of viral hepatitis was considered first, so D. hepatitis virus antigen antibody seven was selected as the first test.

Answer: D. Hepatitis virus antigen antibody seven

137, (type A3 / A4 questions) subject

The best diagnosis is

A. pregnancy fatty liver

B. Intrahepatic cholestasis of pregnancy

C. hypertensive liver damage in pregnancy

D. medicamentous liver lesion

E. In pregnancy complicated with severe hepatitis

Reference answer: E Your answer:

Graphic analysis: You may be infected by the hepatitis virus at any period of pregnancy, and hepatitis B virus infection is the most common. Clinical manifestations: digestive system symptoms that cannot be explained by pregnancy reaction or other reasons, such as appetite loss, liver pain, fatigue, abdominal distension; some patients may have yellow skin mucosa and dark urine. Serum bilirubin was mostly above 17 μmol/L. The ALT and AST were mostly within the normal range.

137 The best diagnosis is that, according to the clinical manifestations of the patient, liver diseases related to pregnancy should be considered, including fatty liver of pregnancy, intrahepatic cholestasis in pregnancy, hypertensive liver damage in pregnancy, drug-induced liver damage, and complicated with severe hepatitis in pregnancy. Among them, fatty liver of pregnancy usually shows severe liver function impairment and coagulation dysfunction; intrahepatic cholestasis in pregnancy is mainly manifested as pruritus and jaundice; pregnancy hypertensive liver damage with hypertension and proteinuria; drug-induced liver damage; pregnancy with severe hepatitis shows hepatitis symptoms. Considering the clinical presentation and laboratory indicators in the present case, especially consciousness disturbance and significant jaundice, most likely was A. gestational fatty liver.

The correct answer is: E. pregnancy with severe hepatitis.

Young female patients, 34 weeks pregnant, 10 days ago began to feel weak, poor appetite, nearly 5 days worse, with vomiting, sclera yellow, prompt jaundice, should be highly suspected viral hepatitis, and patients with low consciousness, ALT and bilirubin, according to the clinical manifestations and laboratory examination, the patient is the most likely diagnosis of pregnancy with severe hepatitis. Option A Fatty liver of pregnancy is often manifested as persistent nausea, vomiting, fatigue, upper abdominal pain or headache. Junterus appears after a few days to 1 week and has progressive deepening, which is inconsistent with the symptoms of the patients in this question, so it is not selected. Option B Intrahepatic cholestasis in pregnancy is characterized by the skin pruritus and high values of cholic acid. Patients in this question had no pruritus, and no bile acid values were found, so it was excluded. Option C pregnancy hypertension liver damage should have hypertension, proteinuria and edema and other pregnancy-induced hypertension manifestations, this patient has normal blood pressure, no proteinuria and edema manifestations, can be excluded.

Option D Drug-induced liver damage should be a medication history, not mentioned in this question, so it is not chosen.

137, and the best diagnosis is made

A. pregnancy fatty liver

B. Intrahepatic cholestasis of pregnancy

C. hypertensive liver damage in pregnancy

D. Drug-induced hepatic damage

E. In pregnancy complicated with severe hepatitis

analyze:

Gestational fatty liver: it usually occurs in the third trimester, presenting with fatigue, jaundice, and abnormal liver function, but ALT is usually significantly elevated.

Intrahepatic cholestasis in pregnancy (ICP): mainly pruritus and increased bilirubin, but mildly increased ALT.

Pregnancy hypertension liver damage: common in pregnancy hypertension disease, ALT is usually significantly increased.

Drug-induced hepatic damage: Drug history needs to be defined, not appropriate for the patient.

Pregnancy with severe hepatitis: severe fatigue, jaundice, significantly abnormal liver function, and ALT was usually elevated, but the ALT was only 35 U / L.

The patient's symptoms and laboratory findings (ALT 35 U / L, total bilirubin 176 μmol/L) were more consistent with the diagnosis of intrahepatic cholestasis in pregnancy (ICP).

Answer: B. intrahepatic cholestasis of pregnancy

A 30-year-old male patient with a disease duration of 4 months. Before headache onset, admission showed left limb weakness and vomiting. Physical examination on admission: clear awareness, fundus optic nerve papilloedema, muscle strength level of left upper and lower limb, leaping, pathological signs (+).

138, (A3 / A4 type question) subject

The diagnosis of this patient was a

A. cerebral infarction

B. hematencephalon

C. Subarachnoid gap hemorrhage

D. encephaledema

E. intracranial hypertension

Reference answer: E Your answer:

[Analysis] Typical manifestations of intracranial hypertension: headache, vomiting, optic nerve papilloedema. The cause of intracranial hypertension should be found out as soon as possible to fundamentally solve the problem of intracranial hypertension. CT is the preferred examination for the diagnosis of intracranial lesions.

Analysis of 38 questions:

The medical history of this 30-year-old male patient included headache, left limb weakness, vomiting, clarity, fundus optic papilledema, left muscle grade and leaping pathology, which indicated possible neurological damage and increased intracranial pressure. The direct sign of intracranial hypertension is papillary edema of the fundus optic nerve. Thus, the diagnosis most consistent with these clinical findings is the E. intracranial hypertension.

Topic 138 Analysis:

The middle-aged male patient had a long course of disease, accompanied by headache and vomiting, physical examination of the fundus optic nerve papilledema, decreased muscle strength of the left upper and lower limbs, and positive pathological signs. These symptoms and signs indicated intracranial hypertension. The three main diseases of intracranial hypertension are headache, vomiting and optic papilloedema.

Option A Cerebral infarction usually has an acute onset, most of which have no obvious symptoms of increased intracranial pressure, such as headache, vomiting and papilloedema, mainly manifested as three partial symptoms (hemiplegia, hemianopia, partial sensory disorder), which is not consistent with the description of the problem stem.

Option B Cerebral hemorrhage usually has a significant history of hypertension, sudden onset, mostly in emotional excitement or activity, which is inconsistent with

the description of the problem stem.

Option C subarachnoid gap hemorrhage starts sharply, mostly caused by aneurysm rupture, abnormal cerebrovascular rupture and other reasons, often accompanied by obvious headache, meningeal irritation and eye symptoms, which is inconsistent with the performance of patients in the problem stem.

Option D Brain edema usually refers to a pathological phenomenon of increased water and brain volume in brain tissue. It is a pathological state accompanied by a variety of diseases. It does not specifically refer to a certain disease and generally does not appear alone, so it is not selected.

Question 138: The patient's diagnosis is

A. cerebral infarction

B. hematencephalon

C. Subarachnoid gap hemorrhage

D. encephaledema

E. intracranial hypertension

analyse:

The patient had symptoms such as headache, vomiting, left limb weakness, and a physical examination found papilloedema of the fundus optic nerve, which indicated increased intracranial pressure. Long course of disease (4 months), and positive pathological signs, these characteristics suggest possible intracranial space-occupying lesions. Taken together, the most likely diagnosis is intracranial hypertension.

Best answer: E. intracranial hypertension

139, (A3 / A4 type question) subject

The tests to be used are

A. X-ray cranial radiograph

B. electroencephalogram

C. brain angiography

D. CT

E. ECT

Refer to the answer: D. Your answer:

[Analysis] Typical manifestations of intracranial hypertension: headache, vomiting, optic nerve papilloedema. The cause of intracranial hypertension should be found out as soon as possible to fundamentally solve the problem of intracranial hypertension. CT is the preferred examination for the diagnosis of intracranial lesions.

Analysis of questions 139:19:

For the etiology diagnosis of intracranial hypertension, imaging examination is required to view the internal structure of the brain. CT scan is a common and rapid method to diagnose intracranial hemorrhage, tumor, and cerebral edema. Therefore, the most appropriate method of examination is the D. CT.

Topic 139 Analysis:

Considering that the patient's diagnosis was intracranial hypertension, further examination is required in order to clarify its etiology.

Option A: X-ray skull radiograph is mainly used for the diagnosis of skull lesions, which is not helpful for the diagnosis of the etiology of intracranial hypertension, so it is not selected.

Option B: EEG is mainly used for the diagnosis of epilepsy and other diseases, and has limited value for the etiology of intracranial hypertension, so it is excluded.

Option C: Although cerebral angiography can show the morphology and structure of cerebral blood vessels, it is not the preferred examination method for intracranial hypertension, so it is not chosen.

Option D: CT scan can clearly show the morphology and size of the brain parenchyma, ventricles and cisternae, etc. It is the preferred imaging examination method for the diagnosis of intracranial hypertension and its etiology, so it is the correct answer.

Option E: ECT (emission single-photon computed tomography) is mainly used for the examination of thyroid and bone tumors, and has little to do with the diagnosis of intracranial hypertension, so it is excluded.

Question 139: The inspection is

A. X-ray skull film

B. electroencephalogram

C. brain angiography

D. CT

E. ECT

analyse:

To further clarify the diagnosis, CT can quickly and effectively show intracranial space-occupying lesions, cerebral edema, and intracerebral hemorrhage. Therefore, CT is the preferred examination method.

Best answer: D. CT

140. (Type B1 question)

The process in which granulation tissue replaces necrotic tissue, thrombus, and exudates is called

A. metaplasia

B. organization

C. become divided

D. revive

E. hyperplasia

Reference answer: B. Your answer:

Graphic analysis:

The process in which granulation tissue replaces necrotic tissue, thrombus, and exudates is called D. regeneration.

-This description is consistent with the role of granulation tissue in the healing process, usually present in the late stages of inflammation, where granulation tissue formation is part of the repair process, but more accurate to say "replacing" necrotic tissue in a process called repair or hyperplasia. Therefore, there may be a little ambiguity here that needs to be considered according to the actual situation. It is also

reasonable to choose E. hyperplasia if the formation and function of granulation tissue are considered.

The correct answer is: B. Mechanized

analyze:

The process by which granulation tissue replaces necrotic tissue, thrombus, and exudates is called mechanization. Mechanization refers to the process in which new granulation tissue grows into and replaces necrotic tissue, thrombus, pus, and foreign body after tissue and cell damage. Granulation tissue plays a key role in the mechanical process, which can be gradually transformed into scar tissue, so that the damaged tissue can be repaired.

Option A metaplasia is A process in which one differentiated mature cell type is replaced by another differentiated mature cell type, inconsistent vs.

Option C differentiation refers to the process in which cells of the same origin gradually produce cell groups with different morphological, structural and functional characteristics, which can be excluded.

Option D regeneration refers to the process of repairing and restoring the defect with the same cells around the body during the damage, which is inconsistent with the description of the question stem.

Option E hyperplasia refers to the phenomenon of active cells, resulting in increased number of cells in the tissue or organ, which can be excluded.

140. (Type B1 question)

The process by which granulation tissue replaces necrotic tissue, thrombus, and exudate is called:

B organization

141. (Type B1 question)

The process of repair after tissue injury by dividing the proliferation of its adjacent healthy cells is called

A. metaplasia

B. organization

C. become divided

D. revive

E. hyperplasia

Refer to the answer: D. Your answer:

[Analysis] Regeneration refers to the process of repair by surrounding cells after the damage of tissues and cells.

The process of repairing tissue damage by its adjacent healthy cells is called D. regeneration.

-Regeneration is the process by which the cells of the tissue divide the hyperplasia to restore the normal structure and function after injury.

141, The correct answer is: D. regeneration

analyze:

The process of repair after tissue damage by dividing the proliferation of its adjacent healthy cells is called regeneration. Regeneration is the process in which the body repairs and recovers the defect with the surrounding similar cells after injury. In this question, the key word is the healthy cell division hyperplasia ", which happens to be the definition of regeneration.

Option A metaplasia is the process in which one differentiated mature cell type is replaced by another differentiated mature cell type and can be excluded.

Option B refers to the process in which new granulation tissue grows into and replaces necrotic tissue, thrombus, pus, foreign body, etc., after tissue and cell damage, which does not conform to the meaning of the question.

Option C differentiation refers to the process in which cells of the same origin gradually produce cell groups with different morphological structure and functional characteristics, so it is not considered.

Option E hyperplasia refers to the phenomenon that active cells lead to the number of cells in tissues or organs, so it is not chosen.

141. (Type B1 question)

The process of repair after tissue injury by dividing the proliferation of their adjacent healthy cells is called:

F. revive

142. (Type B1 question)

The process of transformation of the differentiated mature tissue of the same

germ layer into another mature tissue is called

A. metaplasia

B. organization

C. become divided

D. revive

E. hyperplasia

Reference answer: A. Your answer:

Graphic analysis:

The process of transforming the differentiated mature tissue of the same germ layer into another mature tissue is called B. mechanization.

-This description is similar to the concept of transformation and is often used to describe a mature tissue type into another. But in medical terms, the term "mechanical chemistry" is usually used to describe the process of tissue such as thrombus turning into fibrous tissue during the pathological process. Therefore, more context may need to be considered if it to be accurately matched. If the problem refers to a change in the type of the normal tissue, this may be "differentiation" (C), but this usually does not occur in the mature tissue.

The correct answer is: A. Chemistry

analyze:

The process of transformation of the differentiated mature tissue of the same germ layer into another mature tissue is called metaplasia.metaplasia is a pathological process transforming a differentiated mature tissue into another mature tissue that is reversible, usually appearing only in cell types with more active division and proliferative capacity.

Option B mechanization refers to the process of new granulation tissue growing into and replacing necrotic tissue, thrombus, pus, foreign body, etc., after tissue and cell damage, which is not consistent with the meaning of the question.

Option C differentiation refers to the process in which cells of the same origin gradually produce cell groups with different morphological, structural and functional characteristics, which can be excluded.

Option D regeneration refers to the process of repairing and restoring the defect with the surrounding similar cells when the body is damaged, which does not conform

to the meaning of the question.

Option E hyperplasia refers to the phenomenon of increased number of cells in a tissue or organ, so it is not considered.

142. (Type-B1 question)

The process of transforming the differentiated mature tissue of the same germ layer into another mature tissue is called:

A. metaplasia

143. (Type B1 question)

Immature type of teratoma belongs to

A. borderline tumor

B. early carcinoma

C. benign tumour

D. therioma

E. precancerous lesion

Refer to the answer: D. Your answer:

Graphic analysis:

143, the immature type of teratoma belongs to

Answer: D. malignant tumor

Explanation: Immature teratomas are malignant tumors because they contain immature, embryo-like cells and have a high risk of deterioration.

143, the immature type of teratoma belongs to

F. therioma

Explanation: Immature teratomas contain undifferentiated cellular components with malignant potential that usually require further treatment and monitoring.

144. (Type B1 question)

Gastrointestinal cancer infiltrating only the mucosal layer and submucosa

A. borderline tumor

B. early carcinoma

C. benign tumour

D. therioma

E. precancerous lesion

Reference answer: B. Your answer:

Graphic analysis:

144, Gastrointestinal cancer that only infiltrates the mucosal layer and submucosa

Answer: B. Early-stage cancer

Explanation: Gastrointestinal cancer that only infiltrates the mucosal layer and submucosa is called early cancer because it has not yet penetrated the muscle layer or spread to distant tissue.

144, Gastrointestinal cancer that only infiltrates the mucosal layer and submucosa

F. early carcinoma

Explanation: Early cancer means that cancer cells are limited to the mucosal layer and submucosa, and do not invade deeper tissues and organs. Early detection and treatment may improve the cure rates.

145. (Type B1 question)

Straight and colonic familial multiple adenomatous polyps belong to

A. borderline tumor

B. early carcinoma

C. benign tumour

D. therioma

E. precancerous lesion

Reference answer: E Your answer:

Graphic analysis:

145, straight, colon familial multiple adenomatous polyps belong to

Answer: E. precancerous lesions

Explanation: Familial adenomatous polyposis (FAP) is a genetic condition involving the formation of multiple adenomatous polyps in the straight and the colon. These polyps themselves are benign, but if left untreated, have a high risk of malignant becoming colorectal cancer. Therefore, they are regarded as precancerous lesions.

First, let's understand the meaning of each option:

A. Borderline tumor: This is A tumor with tissue morphology and biological behavior between benign and malignant behavior.

B. Early cancer: It usually refers to the early stages of cancer, usually with small lesions and no metastases.

C. Benign tumors: slow-growing tumors that are not capable of invasion and metastasis.

D. Malignant tumor: refers to tumors with invasive and metastatic ability, which is more harmful to health.

E. Premalignant lesions: a lesion or disease underlying cancer.

Next, we analyze each problem one by one:

Immature teratas are usually aggressive and metastatic, so it is a malignant tumor. So the answer is D. malignant tumor.

For gastrointestinal cancer that only infiltrates the mucosal layer and submucosa, this is usually considered the early stage of cancer, so the answer is B. early cancer.

Straight and colonic familial multiple adenomatous polyps, although benign in itself, are usually regarded as a precancerous lesion due to its high malignant potential. So the answer is E. precancerous lesions.

In conclusion, the answer is: 143. D. malignant tumors 144. B. early cancer 145. E. premalignant lesions

145, straight, colon familial multiple adenomatous polyps belong to

G. precancerous lesion

Explanation: Familial adenomatous polyposis (FAP) is a genetic disease

characterized by the formation of a large number of adenomatous polyps in the colon and rectum. Untreated, these polyps are highly likely to evolve into colorectal cancer and are therefore considered precancerous lesions.

146. (Type B1 question)

It indicates an insufficient blood volume

A. Central venous pressure was very low and urine volume was high

B. Low central venous pressure and low urine volume

C. Low central venous pressure and high urine output

D. Very high central venous pressure and high urine output

E. Very high central venous pressure and low urine volume

Reference answer: B. Your answer:

Graphic analysis:

146, indicating insufficient blood volume

Answer: B. Low central venous pressure, low urine volume

Explanation: When the blood volume is insufficient, the central venous pressure usually drops because the circulating blood volume decreases. At the same time, urine volume is reduced due to reduced blood volume.

146, indicating insufficient blood volume.

Answer: B. Low central venous pressure, low urine volume

Central venous pressure represents the change of right atrium or thoracic vena cava pressure, which can reflect the relationship between systemic blood volume and right heart function, and is one of the main indicators of clinical hemodynamic observation. Low central venous pressure usually indicates insufficient blood volume, and low urine volume further confirms insufficient blood volume and insufficient renal perfusion. Therefore, the low central venous pressure and low urine volume suggest insufficient blood volume.

146, indicating insufficient blood volume

F. Low central venous pressure and low urine volume

Explanation: When the blood volume is low, the central venous pressure (CVP)

is low, resulting in insufficient renal perfusion and reduced urine volume.

147. (Type B1 question)

It indicates that the fluid volume has been fully replenished

A. Central venous pressure was very low and urine volume was high

B. Low central venous pressure and low urine volume

C. Low central venous pressure and high urine output

D. Very high central venous pressure and high urine output

E. Very high central venous pressure and low urine volume

Refer to the answer: D. Your answer:

Graphic analysis:

147, indicating that the liquid volume has been fully replenished

Answer: E. High central venous pressure, low urine volume

Explanation: This option is slightly complicated, but if excessive fluid supplementation causes a significant increase in central venous pressure, there may be a temporary decrease in the volume of urine. This condition can occur in patients with excessively rapid infusion or cardiac insufficiency. However, under normal conditions, the urine output should increase after adequate fluid supplementation. Therefore, this issue may be examining the case of excessive fluid supplementation.

147, indicating that the liquid volume has been fully replenished.

Answer: D. High central venous pressure, high urine volume

The size of the central venous pressure depends on the relationship between the cardiac ejection capacity and the amount of venous return effort. Central venous pressure is very high, indicating sufficient blood volume and high urine volume indicating sufficient renal perfusion and good renal function. Therefore, the central venous pressure is very high, and the high urine volume can indicate that the fluid volume has been replenished with sufficient amount.

147, indicating that the liquid volume has been fully replenished

G. Very high central venous pressure and high urine output

Explanation: When the fluid volume is replenished, the central venous pressure

increases, and the kidneys are well infused and the urine volume increases.

148. (Type B1 question)

There may be a cardiac dysfunction present

A. Central venous pressure was very low and urine volume was high

B. Low central venous pressure and low urine volume

C. Low central venous pressure and high urine output

D. Very high central venous pressure and high urine output

E. Very high central venous pressure and low urine volume

Reference answer: E Your answer:

Graphic analysis:

148, There may be a cardiac dysfunction

Answer: E. High central venous pressure, low urine volume

Explanation: During cardiac insufficiency, the heart's ability to pump blood decreases, leading to increased venous return and increased central venous pressure. Also, the decreased renal perfusion, which may lead to decreased urine volume. This situation reflects the results of the interaction of cardiac dysfunction and renal function.

148, There may be a cardiac dysfunction.

Answer: E. High central venous pressure, low urine volume

The size of the central venous pressure depends on the relationship between the cardiac ejection capacity and the amount of venous return effort. If the central venous pressure is high but the urine volume is low, this may mean that despite adequate blood volume, the cardiac ejection function may be limited, resulting in a renal insufficient perfusion and reduced urine volume. This condition may suggest a cardiac insufficiency. Therefore, central venous pressure is high and low urine volume may indicate the presence of cardiac insufficiency.

148, There may be a cardiac dysfunction

H. Very high central venous pressure and low urine volume

Explanation: During cardiac insufficiency, the heart cannot effectively pump

blood, resulting in blocked venous reflux and increased central venous pressure, but due to insufficient renal perfusion, the urine volume is reduced.

149. (Type B1 question)

Hypoglycemia syndrome after gastric surgery

A. Vomesis is for food, without bile

B. The vomit is bile, without food

C. The vomit has both bile and food

D. The vomit is sour and has a bad food

E. No vomiting

Reference answer: E Your answer:

Graphic analysis:

For postoperative hypoglycemia syndrome (question 149), common symptoms include early postprandial fullness, abdominal pain, tremor, sweating, palpitation, etc., rather than a special emphasis on the characteristics of vomiting. But if chosen in a given option, symptoms associated with hypoglycemia, such as manifestations of hunger or indigestion, can be considered. Therefore, I prefer D (vomit smells sour, has good food) because it may be related to the food entering the small intestine too quickly and it being absorbed quickly, leading to a drop in blood sugar levels.

149 Postgastric hypoglycemia syndrome

Postgastric hypoglycemia syndrome is common after gastrectomy. Because the gastric contents can enter the small intestine quickly after eating, excessive insulin secretion occurs and symptoms of hypoglycemia appear. Vomiting in this case is not usually the main symptom, so E. No vomiting is the most likely answer.

150, (Type B1 question)

Output loop obstruction after gastric surgery

A. Vomesis is for food, without bile

B. The vomit is bile, without food

C. The vomit has both bile and food

D. The vomit is sour and has a bad food

E. No vomiting

Reference answer: C Your answer:

For postoperative output loop obstruction (question 150), vomiting is a common symptom, especially when food does not pass normally through the small intestinal interface. Output loop obstruction usually results in vomit containing undigested food and gastric acid. Therefore, I tend to choose A (vomit is food, no bile) because the output loop obstruction usually blocks before the bile is discharged into the gut, resulting in the vomit mainly food rather than bile.

This is a question about the different complications and their manifestations after stomach surgery. We need to choose the most consistent description of the vomit according to the characteristics of each complication.

First, we analyze the postoperative hypoglycemic hypoglycemia. In this case, the gastric emptying is too fast, resulting in the rapid absorption of glucose and the excessive secretion of insulin, followed by reactive hypoglycemia. This does not usually lead to vomiting, so the key is to identify which symptoms are least associated to the syndrome.

For postoperative gastric output loop obstruction, it usually occurs after a major gastrectomy for a major postoperative anastomosis. In this case, due to the output loop obstruction, the bile and digestive juice such as pancreatic juice accumulate in the proximal end of the obstruction, and the food residue can not pass through. Therefore, when vomiting occurs, the vomit will contain both bile and food.

Now, let's analyze the options one by one:

Post-gastric hypoglycemia syndrome:

A. The vomit is food without bile-this is more related with the obstruction of the input loop.

B. vomit is bile without food-this does not consistent with the presentation of hypoglycemic syndrome.

C. The vomit has both bile and food-also inconsistent.

D. The vomit is sour and has good food- -which is usually associated with pyloric obstruction.

E. No vomiting-Given that the hypoglycemic syndrome is not directly associated

with vomiting, this is the most appropriate option.

Therefore, for question 149, the most appropriate choice is the E. no vomiting.

Postoperative gastric loop of output obstruction:

A. Vomus as food without bile-not consistent with the manifestation of output loop obstruction.

B. Vis bile without food-this is more like a manifestation of input loop obstruction.

C. The vomit has both bile and food- -which is typical of output loop obstruction, as both bile and food accumulate in the proximal end of the obstruction.

D. Svomit is vomit with good food-inconsistent with output loop obstruction.

E. No vomiting-apparently not the case, as output loop obstruction usually causes vomiting.

Therefore, for the 150 questions, the most appropriate choice is that C. vomit has both bile and food.

In conclusion, the correct answer is: 149. E. no vomiting; 150. C. the vomit has both bile and food.

150. Postoperative gastric output loop obstruction

Postoperative gastric output loop obstruction is the mechanical obstruction of the output loop after gastric surgery. At this point, the vomit usually contains bile without food because food fails to reach the small bowel through the site of obstruction. The most appropriate answer is that B. vomit is bile, no food.

2023 Unit 2

1. (Type A1 question)

Cells that have no endocrine function are

A. The pancreatic ductal cells

B. interstitial glands

C. Parathyroid master cells

D. adrenal mededullary cells

E. G cells of the gastric mucosa

Reference answer: A. Your answer:

[Analysis] Pancreatic duct cells secrete pancreatic fluid, which belongs to the exocrine function of the pancreas and does not have endocrine function (A wrong, is the correct answer to this question).

[To avoid mistakes] Adrenal medullary hormones secrete catecholamines, parathyroid main cells secrete parathyroid hormone, G of the gastric mucosa

Cells secrete gastrin, and testicular Leydig cells secrete androgens.

In this question, we need to identify which type of cells has no endocrine function. Endocrine cells often involve the process of secreting hormones in the blood. Let's look at each option:

-A. Pancreatic ductal cells: These cells participate in the delivery of pancreatic fluid, rather than directly in the production of endocrine hormones.

-B. Leydig testicular cells: These cells secrete testosterone and belong to endocrine cells.

-C. Parathyroid main cells: they secrete parathyroid hormone and have endocrine function.

-D. Adrenal medulla cells: They secrete adrenaline and norepinephrine, and they also have endocrine functions.

-E. The G cells of the gastric mucosa: they secrete gastrin and have an endocrine function.

Therefore, the most correct answer is * * A. pancreatic ductal cells * *, as they are mainly responsible for the transmission of pancreatic fluid to the small intestine and are not directly involved in the production of endocrine hormones.

First, we need to clarify that endocrine cells are cells that can synthesize and secrete bioactive substances and deliver them to other organs or cells through blood or tissue fluid. These bioactive substances are often known as hormones.

Now, let's look at each option:

A. Pancreatic ductal cells: The main function of pancreatic ductal cells is to

deliver pancreatic fluid, and they do not have the ability to secrete hormones, so they do not have the endocrine function.

B. Testicular stromal cells: These cells secrete androgens, which are an endocrine function.

C. Parathyroid master cells: These cells can secrete parathyroid hormone, which is also an endocrine function.

D. Adrenal medulla cells: These cells can secrete adrenaline and norepinephrine, apparently with endocrine functions.

E. G cells of the gastric mucosa: these cells secrete gastrin, which also has an endocrine function.

In conclusion, only the pancreatic ductal cells do not have an endocrine function. Therefore, the answer is the A. pancreatic ductal cells.

This topic looks at the endocrine function of different cells. Here is an analysis of each option:

A. * * Pancreatic ductal cells * *: The main function is to secrete bicarbonate (HCO $_3$) into the pancreatic duct to help neutralize gastric acid, not with endocrine function.

B. * * Testicular stromal cells * *: Also known as Leydig cells, they secrete androgens (such as testosterone) and have endocrine function.

C. * * Parathyroid main cell * *: Secreting parathyroid hormone (PTH), with endocrine function.

D. * * Adrenal medulla cells * *: Secreting adrenaline and norepinephrine, with endocrine function.

E. * * G cells of gastric mucosa * *: they secrete gastrin and have endocrine function.

To sum up, the answer is:

A. * * Pancreatic duct cells * *

2. (Type A1 question)

Adverse effects of phenytoin were not included

A. Gingival damage

B. incoordination

C. Kidney damage

D. anaphylaxis

E. anemia

Reference answer: C Your answer:

[Analysis] Common adverse reactions of phenytoin sodium are: ataxia, irritability, gingival hyperplasia, anemia, allergic reaction, liver toxicity, etc.; rare adverse reactions: jaundice, vitamin D and calcium metabolism disorder, fracture, bone abnormalities, etc. This drug is metabolized through the liver, so it generally does not cause kidney damage, so this question is selected for C.

[Error] Phenytoin is an antiepileptic as well as an anticonvulsant. For the treatment of systemic tonic-clonic seizures, complex

Heterogeneous seizures (mental mental seizures, temporal lobe seizures), simple partial seizures (localized seizures) and seizures

Continued state.

Phenytoin is a commonly used antidiphtheria drug, whose adverse effects may include gingival hyperplasia (gingival damage), ataxia, allergic reactions, and anemia. However, renal impairment is not a common adverse effect of phenytoin sodium. Therefore, the most correct answer is: C. Kidney damage

First, let's take a detailed look at phenytoin and its possible adverse reactions.

Phenytoin is an antiepileptic and antiarrhythmic drug that can trigger a variety of

adverse effects. Among them, gingival hyperplasia is one of its common adverse effects, which may be related to the direct stimulation of gingival tissue by drugs. In addition, phenytoin sodium may also affect the nervous system, causing adverse reactions such as vertigo, tremor, diplopia, ataxia and so on. In a few cases, phenytoin may also cause allergic reactions, such as skin rash, fever, and exfoliative dermatitis.

Now, we analyze the options based analyze the options:

A. Gingival damage: This is a known adverse effect of phenytoin sodium, so this option is not correct.

B. Ataxia: As mentioned above, phenytoin may affect the nervous system and lead to adverse effects such as ataxia, so this option is also incorrect.

C. Renal damage: Although phenytoin sodium is mainly metabolized through the liver, it may also cause some damage to the kidney, especially during long-term use or in large doses. Therefore, this option is also incorrect.

D. Allergic reaction: As mentioned earlier, phenytoin may cause an allergic reaction, so this option is not correct.

E. Anemia: No adverse effects of phenytoin are clearly reported in the existing medical literature. Although long-term use of phenytoin may affect the hematopoietic system, this effect usually presents as leukocyte or thrombocytopenia rather than anemia.

In conclusion, the adverse effects of phenytoin do not include anemia. Therefore, the correct answer is the E. anemia.

Phenytoin is a widely used antiepileptic drug whose common adverse effects include gingival damage (A), ataxia (B), allergic reaction (D), and anemia (E). However, renal impairment (C) is not a common adverse effect of phenytoin sodium.

So the correct answer is:

F. Kidney damage

3. (Type A1 question)

The indicator for storing iron is

A. serum iron

B. serum ferritin

C. Transferrin saturation

D. myoglobin iron

E. Intraerythrocytic iron

Reference answer: B. Your answer:

Graphic analysis: [Analysis] Under normal physiological conditions, serum ferritin measurement (B pair) can sensitively reflect the amount of iron stored in the body.

[Error] Serum iron measurement and transferrin saturation measurement reflect the iron content in plasma; iron and muscle red

Protein iron is the functional iron in the body.

Storage of iron is mainly reflected by serum ferritin, which is the predominant form of stored iron in the body. Therefore, the most correct answer is: B. serum ferritin

First, we need to make it clear that the question is the indicator of iron storage. Storage iron refers to the iron stored in the body, which is not directly involved in hemoglobin synthesis or other physiological activities, but rather exists as an iron reserve in the body.

Next, we analyze each option one by one:

A. Serum iron: This mainly reflects the amount of iron bound to transferrin in the blood, but more reflects the iron available in the blood than the iron stored.

B. Serum ferritin: Ferritin is the main form of iron stored in the body. The concentration of serum ferritin can directly reflect the iron storage situation in the body and is a good indicator of iron storage.

C. Transferrin saturation: This is the ratio of the concentration of transferrin in serum to the maximum binding capacity of transferrin in serum. It reflects the carrying capacity of transferrin and the degree of iron ion binding in the blood, and does not directly represent the stored iron.

D. Myoglobin iron: Myoglobin is mainly found in the heart muscle, and skeletal

muscle. Although its iron content is related to some diseases, it does not directly reflect the state of iron stored in the body.

E. Iron in erythrocytes: This mainly reflects the content of iron in red blood cells, which is related to the synthesis and function of hemoglobin, rather than iron storage.

Combining the above analysis, we can determine that serum ferritin is a direct indicator reflecting the status of iron storage in vivo.

Therefore, the correct answer is the B. serum ferritin.

The indicators for iron storage are:

F. serum ferritin

4. (Type A1 question)

For NSAIDs use in chronic injuries of the motor system, the following is correct

A. Dindexacin and aspirin can be combined to reduce liver function impairment

B. NSAID oral liquid may be used for focal superficial lesions

C. COX-1 can be used to reduce the impairment of gastrointestinal function

D. NSAIDs can be used in a variety of ways to enhance the efficacy

E. Short-term minimum effective drug dose

Reference answer: E Your answer:

[Analysis] Non-steroidal anti-inflammatory drugs are commonly used drugs in the treatment of chronic injuries of the motor system. The following points should be noted: ① short-term medication (E pair); ② localized topical NSAIDs (B error), ③ selective cyclooxidase 2 (COX-2) inhibitor (C error), ④ renal insufficiency; ⑤ simple, structural and nitrogen free drugs to reduce the effect on liver function, and aspirin (A error); ⑥ NSAIDs should be used alone (D error), combined anti-inflammatory and analgesic effect will not increase but double the side effects of the drug.

[Avoid avoidance] The causes of chronic injury of motor system are complex, common causes are: ① Local tissue disease caused by systemic disease

Rational tension and spasm; ② Local vasospasm due to ambient temperature, decreased circulation supply and local metabolites

Accumulation; ③ long-term, repeated, continuous repetition of the same posture,

work, learning and professional movements, beyond the human body local

The compensatory ability, causing tissue damage and not timely repair; ④ operation is unskilled, inattention, posture

Incorrect potential, causing local abnormal stress; ⑤ abnormal body physiological structure or posture, uneven stress distribution; ⑥ acute

Improper rehabilitation after injury leads to chronic injury.

Non-steroidal anti-inflammatory drugs (NSAIDs) are a common treatment for chronic injuries of the motor system. Choosing the best answer requires considering the mechanism of NSAID action, side effects, and principles of clinical use.

A. Dindexacin and aspirin are both NSAIDs, and the combination of both may increase the risk of gastrointestinal bleeding and does not reduce liver function impairment.

B. Focal superficial lesions may not require oral NSAIDs, and topical preparations may be used.

C. COX-1 inhibitors (e. g., aspirin) may increase gastrointestinal function impairment, rather than mitigate it.

Multiple combinations of D. NSAIDs are not recommended because it increases the risk of side effects, especially in the kidneys and gastrointestinal tract.

D. Use of the lowest effective dose of NSAIDs and short-term use is a recommended practice to reduce side effects and improve safety.

Therefore, the best answer is E, the short-term use of the lowest effective drug dose for the treatment of chronic motor system injuries. This practice can minimize side effects, particularly on the gastrointestinal tract and the kidney.

analyze:

Non-steroidal anti-inflammatory drugs are commonly used as antipyretic, analgesic and anti-inflammatory drugs in clinical practice. They mainly play a role by inhibiting the synthesis of prostaglandins, and have antipyretic, analgesic, anti-inflammatory, anti-rheumatic, anticoagulation and other effects. However, when using, the minimum effective drug dose should be used in the short term to avoid the side effects caused by long-term heavy medication.

Option A To reduce liver function impairment, the statement that the combination of indomexacin and aspirin is wrong, because NSAIDs such as aspirin

are metabolized in the liver, and the use of two or more NSAIDs will increase the incidence of adverse reactions and increase liver toxicity, thus excluded.

There are many dosage forms of NSAIDs, among which latex, paste, patch and external dressing are mainly suitable for local medication, especially for superficial chronic pain in segments, so option B focal superficial lesions can use NSAIDs oral liquid, which can be excluded.

NSAIDs anti-inflammatory drugs by inhibiting COX, thus reducing the synthesis of PG, COX-1 is normal tissue cells used to synthesis for the body beneficial prostacyclin necessary, so long-term use of COX-1 inhibitors may have obvious damage to the digestive tract, such as induced gastric ulcer, gastric bleeding, gastric perforation, so option C in order to reduce gastrointestinal function damage can use COX-1, does not conform to the question.

Since adverse effects between NSAIDs can be additive and simultaneous use of two or more NSAIDs can significantly increase the incidence of adverse effects, option D NSAIDs can be used in multiple combinations to enhance the efficacy and can be excluded.

In conclusion, the correct answer is: E. Short-term minimum effective drug dose.

For the use of NSAIDs in chronic motor system injury, the following statement is correct:

E. Short-term minimum effective drug dose

Explanation: Non-steroidal anti-inflammatory drugs (NSAIDs) are often used to relieve pain and inflammation in chronic injuries of the motor system. However, the use of these drugs requires caution to minimize side effects. The usual recommendation is to use the lowest effective dose as short as possible to reduce potential damage to organs such as the gastrointestinal tract, liver and kidney. The combination of multiple NSAIDs may increase the risk of adverse effects and is therefore not recommended.

5. (Type A1 question)

Use of antiplatelet drugs for PCI surgery for acute myocardial infarction

A. aspirin 300mg + clopidogrel 450mg

B. aspirin 100mg + clopidogrel 300mg

C. aspirin 100mg + clopidogrel 600mg

D. aspirin 300mg + clopidogrel 300mg

E. Aspirin 300mg + clopidogrel 600mg

Reference answer: E Your answer:

Graphic analysis: [analysis] STEMI patients without contraindications should immediately take enterocoated aspirin loading dose of 150~300mg (BC

Wrong), followed by 75 ~ 100 mg/d. Clopidogrel may be used when ticagrelor is not available or contraindications

600mg loading dose (E pair), only at 300mg (D wrong).

[Error avoidance] Compared with metal bare stents, patients with coronary heart disease are preferred to implant drug-eluting stents, in order to control the symptoms of ischemia

Reduced the occurrence of myocardial infarction and death, requiring aspirin and clopidogrel antiplatelet therapy for at least 12

A month.

Non-steroidal anti-inflammatory drugs (NSAIDs) are a common treatment for chronic injuries of the motor system. Choosing the best answer requires considering the mechanism of NSAID action, side effects, and principles of clinical use.

A. Dindexacin and aspirin are both NSAIDs, and the combination of both may increase the risk of gastrointestinal bleeding and does not reduce liver function impairment.

B. Focal superficial lesions may not require oral NSAIDs, and topical preparations may be used.

C. COX-1 inhibitors (e. g., aspirin) may increase gastrointestinal function impairment, rather than mitigate it.

Multiple combinations of D. NSAIDs are not recommended because it increases the risk of side effects, especially in the kidneys and gastrointestinal tract.

D. Use of the lowest effective dose of NSAIDs and short-term use is a

recommended practice to reduce side effects and improve safety.

Therefore, the best answer is E, the short-term use of the lowest effective drug dose for the treatment of chronic motor system injuries. This practice can minimize side effects, particularly on the gastrointestinal tract and the kidney.

analyze:

Acute myocardial infarction is a potentially life-threatening acute condition that damages the heart with a result of a lack of blood supply. PCI surgery, or percutaneous coronary intervention, refers to the cardiac catheterization technique to dredge the narrow or even occluded coronary artery lumen, thus improving the myocardial blood perfusion. After PCI, dual antiplatelet therapy, namely aspirin combined with clopidogrel, is aspirin 300mg chewing + clopidogrel 300mg orally.

Aspirin is an anti-platelet aggregation drug, which can inhibit the aggregation of platelets on atherosclerotic plaques, facilitate the release of endogenous fibrinolytic active substances and inhibit thrombosis, and is the cornerstone of the prevention of cardiovascular and cerebrovascular diseases. Clopidogrel is an ADP receptor antagonist that inhibits platelet binding to the ADP receptor, thereby reducing platelet aggregation. The routine dosage of clopidogrel is 75mg daily, but for patients undergoing PCI, load 300mg, so option A "aspirin 300mg + 450mg clopidogrel"; option C "Aspirin 100mg + clopidogrel 600mg"; option E "Aspirin 300mg + clopidogrel 600mg"; may be excluded.

The amount of aspirin for antiplatelet aggregation is generally 100mg per day, but for patients with acute myocardial infarction undergoing PCI, 300mg load should be administered, so option B "aspirin 100mg + clopidogrel 300mg" can be excluded.

In conclusion, the correct answer is: D. aspirin 300mg + clopidogrel 300mg.

In percutaneous coronary intervention (PCI) in patients with acute myocardial infarction (AMI), the recommended antiplatelet regimen is to rapidly inhibit platelet aggregation and prevent thrombosis. According to current medical guidelines and best practices, the dosage and usage of aspirin and clopidogrel are as follows:

- ** Aspirin * *: A loading dose of 300mg is generally used in the acute phase.

- ** Clopidogrel * *: The loading dose of 600mg is recommended in the acute phase.

Therefore, the most appropriate option is:

B aspirin 300mg + clopidogrel 600mg

6. (Type A1 question)

Hypertension with bilateral renal artery stenosis, which of the following drugs is prohibited

A. Valsartan

B. metoprolol

C. Del sulphuric acid

D. digitalis

E. furosemide

Reference answer: A. Your answer:

[Analysis] Angiotensin-converting enzyme inhibitors and angiotensin receptor antagonists, such as captopril, losartan, valsartan, etc., so this question is selected A.

[Eravoidance] ACEI and ARB drugs are also prohibited in women with hyperkalemia and pregnancy. Adverse reactions of ACEclass I drugs are stimuli

Sexual dry cough and angioedema, ARB directly related to drug adverse reactions, generally do not cause irritant dry cough.

Patients with hypertension with bilateral renal artery stenosis need to take careful carefully during treatment to avoid further damage to renal function. Among these options, furosemide (option E) is a diuretic, usually used for the treatment of hypertension, but it increases the renal burden, especially in the case of renal artery stenosis, which may lead to further deterioration of renal function.

However, more critical is the choice of C, diltidium (magnesium sulfate), which is usually prohibited in the case of hypertension with bilateral renal artery stenosis because it may lead to reduced renal blood flow and exacerbate renal ischemia. But diltiur is mainly used clinically in the treatment of severe heart disease and sedation in some cases, but not commonly used in the treatment of hypertension.

In fact, the more appropriate drugs are angiotensin-converting enzyme inhibitors

(ACEI) or angiotensin receptor antagonists (ARB), such as valsartan (option A), which are usually banned in patients with bilateral renal artery stenosis because they reduce the amount of blood produced by the kidneys and may lead to acute renal failure. Therefore, in this context, the most appropriate answer should be * * A. Valsartan * *.

analyze:

Hypertension with bilateral renal artery stenosis is a special type of hypertension that considers the effect of antihypertensive drugs on renal blood flow. In patients with bilateral renal artery stenosis, the perfusion of renal blood flow has been reduced. If angiotensin receptor blocker is used, the small artery can dilate, the pressure of glomerular capsule decreases, filtration pressure decreases, and glomerular filtration rate decreases, thus aggravating renal damage and even renal failure. Therefore, valsartan is contraindicated in patients with hypertension associated with bilateral renal artery stenosis. Valsartan belongs to an angiotensin receptor blocker.

Option B Metoprolol is a β receptor blocker, which mainly achieves antihypertensive effect by inhibiting central and peripheral RAAS, inhibiting myocardial contractility and reducing cardiac output. It has no effect on kidney function and can be used in patients with renal artery stenosis, so it is excluded.

Option C diltiur belongs to calcium channel blockers, mainly by blocking extracellular calcium through voltage-dependent L calcium channel into vascular smooth muscle cells, weaken the excitation contraction coupling, reduce resistance vasoconstriction reaction, so as to achieve antihypertensive effect, no effect on kidney function, can be used in patients with renal artery stenosis, so not choose.

Option D digitalis is a strong heart tonic drug, mainly used in patients with heart failure, can enhance myocardial contractility, no effect on kidney function, can be used in patients with renal artery stenosis, unrelated to this question.

Option E furosemide is a diuretic, which mainly reduces blood volume and peripheral vascular resistance. It has no effect on kidney function and can be used in patients with renal artery stenosis, so it is not considered.

In conclusion, the correct answer is: A. Valsartan.

The banned drug prescribed in patients with hypertension associated with bilateral renal artery stenosis is valsartan. Valsartan is an angiotensin receptor antagonist (ARB), which lowers blood pressure by inhibiting the effects of

angiotensin. As angiotensin plays an important role in maintaining renal perfusion pressure and glomerular filtration rate, the use of ARB may cause further deterioration of renal function in patients with bilateral renal artery stenosis.

So, the correct answer is:

A. Valsartan

7. (Type A1 question)

The most unlikely clinical presentation in acute leukemia patients is

A. coilonychia

B. be green about the gills

C. gingival dleeding

D. Spleen enlargement

E. The intermittent hematuria

Reference answer: A. Your answer:

[Analysis] Keynail is the performance of iron deficiency anemia (A wrong, is the correct answer to this question).

[To avoid mistakes] AL onset onset is different. Urgent person can suddenly high fever, similar to "cold", can also be serious bleeding.slow

Slow often pale (B), skin purpura, menorrhagia or after tooth extraction bleeding difficult to stop (C) and medical treatment was sent

present. Hemorrhage as the early manifestation of nearly 40%, bleeding can occur in all parts of the body (E pair), skin pechiae, ecchymosis,

Epistaxis, gingival bleeding, and menorrhagia are more common. The proliferative infiltrate of leukemic cells showed lymph nodes and hepatosplenomegaly (D

Right), joint and bone pain, etc.

Acute leukemia is a type of cancer that develops rapidly and mainly affects the bone marrow and blood. Its symptoms may vary by the type of leukemia and other factors, but usually include fatigue, fever, bleeding tendency (e. g., gingival bleeding),

and normal cytopenia in the bone marrow space occupied by leukemic cells.

-A. Sponail: usually associated with iron deficiency anemia, not common in acute leukemia.

-B. Pallor: It may occur in patients with acute leukemia, because leukemia can reduce red blood cells and cause anemia.

-C. Gingival bleeding: common in acute leukemia because thrombocytopenia.

-D. Spleen enlargement: It may occur in acute leukemia, especially in certain subtypes, such as acute myeloid leukemia.

-E. Intermittent hematuria: Although acute leukemia may cause a systemic bleeding tendency, intermittent hematuria is not a typical symptom.

Considering the above information, the most unlikely clinical presentation is A. tabpoate as it is usually associated with other pathological states, particularly with iron deficiency anemia rather than acute leukemia.

analyze:

Acute leukemia is a class of clonal malignant diseases with abnormal hematopoietic stem cells. Leukemic cells in the clone lose the ability to further differentiate and arrest at different stages of cell development, where hyperplasia accumulate in the bone marrow and other hematopoietic tissues and infiltrate other organs and tissues, while normal hematopoiesis is inhibited. Symptoms include anemia, bleeding, infection, and infiltration of leukemia cells.

Option A spoon nail is A kind of nail deformity, the performance is nail thinning, central depression and all around the bulge, spoon shape, common in iron deficiency anemia, onychitis and other diseases, and leukemia has no direct relationship, for the correct answer.

Option B Pallor is the manifestation of anemia, leukemia cell proliferation will inhibit the bone marrow generation of normal blood cells, reduced erythrocytoiesis can cause anemia, pale and other symptoms, is a common manifestation of leukemia patients.

Option C Gingival bleeding may be caused by reduced platelet count or coagulation dysfunction. The abnormal leukemia cell proliferation in the bone marrow during leukemia affects the normal blood cell generation, and decreased platelet count or dysfunction, thus causing bleeding, which is a common manifestation in leukemia patients.

Option D Splenic enlargement is due to reactive spleen proliferation or infiltration of leukemic cells, consistent with the clinical manifestation of leukemia.

Option E Intermittent hematuria may be caused by infiltration of the urinary system by leukemic cells and is a common symptom in leukemia patients.

To sum up, the correct answer is: A. Keynail.

The most unlikely clinical manifestations in patients with acute leukemia are:

A. coilonychia

Common clinical manifestations of acute leukemia include anemia (causing paleness), bleeding (e. g., bleeding gums), an enlarged spleen, and various infections and bleeding symptoms (e. g., intermittent hematuria). However, the spoon nail (with a sunken nail shape) is usually associated with chronic anemia, especially iron deficiency anemia, rather than a typical manifestation of acute leukemia. Therefore, the most unlikely clinical presentation in patients with acute leukemia is tabate.

8. (Type A1 questions)

The typical presentation of the blood routine in malaria patients is

A. Elevated leukocytes

B. Elevated granulocytes

C. Elevated reticulocytes

D. Red blood cells and decreased hemoglobin

E. thrombocytopenia

Refer to the answer: D. Your answer:

[Analysis] Repeated attacks of malaria patients cause a large number of red blood cell destruction, which can make patients with different degrees of anemia (D pair) and splenomegaly. Reticulocytes increased (C wrong) and platelets slightly decreased (E wrong), but neither is typical. The total number of white blood cells can be slightly increased at the beginning, after the normal or slightly lower (AB wrong).

[Misavoidance] The typical symptoms of malaria are sudden chills, high fever and heavy sweating. Chiller often lasts for 20 minutes to an hour.follow

The body temperature rises rapidly, usually to more than 40℃, accompanied by headache, body aches, fatigue, but conscious. Fever is often sustained

2~6 Hours. Then they began to sweat a lot, and the body temperature plummeted, lasting for 30 minutes to 1 hour. At this point, the patient's consciousness is obvious

Better, but often feel weak, dry mouth. There is a certain interval between the two episodes of various malaria. Interval very early in the disease course

The period may be irregular, but it gradually becomes regular after several attacks. The intermittent period of P. vivax and P. ovale is approximately 48 hours, three

Manax malaria is about 72 hours. Malparum falciparum ranges from 36 to 48 hours.

Blood blood blood findings in malaria patients are reductions of red blood cells and hemoglobin. Malaria is caused by parasitic infection, mainly affecting red blood cells, leading to hemolytic anemia. Therefore, the best answer is: D. Less red blood cells and hemoglobin

analyze:

Malaria is an insect-borne infection caused by the bite of Anopheles mosquitoes or feeding into the blood of people with Plasmodium. There are four species of Plasmodium parasites, Plasmodium vivax, Plasmodium sinensis, Plasmodium falciparum and Plasmodium ovale. Different Plasmodium cause P. vivax, P. malax, P. falciparum and P. ovale. Malaria is mainly manifested as periodic and regular attacks, systemic chills, fever, sweating, long-term multiple attacks, can cause anemia and splenomegaly. Therefore, the typical blood routine expression of malaria patients is reduced red blood cells and hemoglobin.

Option A elevated leukelevation is common in acute bacterial infection, severe tissue injury, massive bleeding, poisoning and leukemia, and is not directly related to malaria, so it is not selected.

Option B granulocyte elevation is commonly seen in infections, physical or chemical stimuli, immune diseases, and blood system diseases, not typical blood routine manifestations of malaria, so it is not considered.

Option C reticulocyte elevation was mainly seen in hemolytic anemia or acute blood loss, not the typical blood routine manifestation of malaria, so it did not consistent.

Option E Thrombocytopenia is commonly seen in platelet generation disorders, increased platelet destruction or consumption, abnormal platelet distribution, and is not a typical blood routine manifestation of malaria, so it is excluded.

In conclusion, the correct answer is: D. Less red blood cells and hemoglobin.

Malaria is a disease caused by Plasmodium infection and is common in tropical and subtropical regions. Its clinical manifestations include periodic attacks of high fever, chills, headache, fatigue and other symptoms. Malaria has a significant effect on the blood system, and typical changes in the blood routine can provide clues to the diagnosis.

Routine blood tests in malaria patients will usually reveal that:

-Red blood cells and hemoglobin decreased: because of the Plasmodium parasite in the red blood cells, causing red blood cell destruction, leading to anemia.

-Thrombocytopenia: This is one of the common hematologic changes in malaria.

Based on the above knowledge, the typical manifestation of blood routine in malaria patients is:

F. Red blood cells and decreased hemoglobin

9. (Type A1 question)

A clavicle fracture does not require surgery

A. 2 / 3 pairs of the middle clavicle

B Distal clavicle associated with injury to the coracoid ligament

C Can not tolerate the bandage

D three hours after the open injury

E Nonunion of old fractures

Reference answer: A. Your answer:

[Analysis] It is generally believed that 80%~90% of the middle clavicle fracture

(A pair) can be treated by non-surgical methods, that is, manual reduction, horizontal "8" bandage fixed.

[Avoid mistakes] Colvicular fracture in the following conditions, open reduction and internal fixation can be considered: ① Patients can not tolerate the "8" strain

Pain with fixation (C error); ② reset before displacement, affecting appearance; ③ with nerve and vascular damage; ④ openness

Fracture (D error); ⑤ old fracture non-union (E error); ⑥ external end fracture, combined with coracal lock ligament rupture (B error)

For the treatment of clavicle fractures, non-surgical treatment is usually indicated for undisplaced or slightly displaced midclavicle fractures. Therefore, option A "Middle clavicle 2 / 3 alignment" (i. e. middle clavicle fracture with good fracture end alignment) usually does not require surgical treatment. This is because most middle clavicle fractures can be treated by conservative treatment, such as using octaband or clavicle band for fixation, to achieve good healing.

The conditions mentioned in options B, C, D and E usually require further evaluation or treatment and may include surgery. Especially when accompanied with coracoid ligament injury, inability to tolerate conservative fixation methods, open fracture, or fracture nonunion, surgical treatment should usually be considered. In conclusion, the best answer is A. Middle clavicle 2 / 3 pairs.

First, we analyzed each option one by one:

A. 2 / 3 pairs of the middle clavicle: most of the clavicle fractures occur at the junction of the clavicle. If the fracture end of 2 / 3 of the middle clavicle is well positioned, it indicates that the position and stability of the fracture are relatively good. In this case, surgical treatment is usually not required, and healing can be achieved by conservative treatment such as eight-character bandage.

F Distal clavicle associated with coracoid ligament injury: When the distal clavicle fracture is associated with coracoid ligament injury, this usually means that the fracture is more severe and may affect the stability of the acromioclavicular joint. In this case, surgery may be necessary to ensure the proper function and stability of the joint.

G Unable to tolerate the bandage: For those patients who can not tolerate the bandage, conservative treatment may not be the best choice. At this point, surgery may be a better solution to ensure the correct alignment and stability of the fracture.

H Three hours after open injury: Open fractures usually require thorough debridement and fixation due to a high risk of infection. Therefore, surgical treatment is usually necessary in this setting.

I Nonunion of old fractures: For old fractures, surgery may be necessary to promote fracture healing.

In conclusion, surgical treatment is not required only when 2 / 3 of the middle clavicle is well positioned. The conditions described in the other options will often require surgical intervention.

Therefore, the correct answer is: A. Middle clavicle 2 / 3 in pairs.

The cases where a clavicle fracture does not require surgery are:

A. 2 / 3 pairs of the middle clavicle

Fractures of the middle clavicle can usually be managed by conservative treatment such as a bandage without the need for surgery. Other options suggest the need for surgical intervention, such as B (with coracoid ligament injury), C (bandage is not tolerated), D (open injury), and E (old fracture nonunion).

10. (Type A1 questions)

Laboratory tests useful to confirm the diagnosis of PNH are

A. Bone marrow cytology

B. reticulocyte detection

C. A peripheral blood smear

D. FLAER detection in blood granulocytes

E. Red BC permetic fragility assay

Refer to the answer: D. Your answer:

Graphic analysis: [Analysis] Flow cytometry detection of fluorescent labeled variants (FLAER) in peripheral blood granocytes and monocytes (D pair) can distinguish GPI protein positive and negative cells. At present, FLAER is generally used for the detection of nucleated cells, which cannot evaluate the red blood cell PNH clone. It is a new method for PNH detection, which is more sensitive and

specific, especially for the detection of tiny PNH clones, and is not affected by transfusion and hemolysis.

[Error avoidance] Paroxysmal sleep hemoglobinuria (PNH) is an acquired hematopoietic stem cell gene mutation

Erythrocytic membrane-deficient chronic endovascular hemolysis disease. Due to increased sensitivity to complement, in the acidified serum

(pH 6.6 to 6.8), after 37°C incubation, easy hemolysis, the Ham test (acid hemoltest) (+), this test is

Specific diagnostic basis of paroxysmal sleep hemoglobinuria.

The most useful laboratory test for confirming PNH (paroxysmal nocturnal hemoglobinuria) is the D. Blood granulocyte FLAER test. FLAER (Fluorescence-labeled aerosol antigen deletion probe) is a method for detecting the absence of specific proteins on the surface of blood cells, which is typical in PNH. The FLAER assay can accurately identify blood cells lacking the GPI (glycosylphosphatidylinositol) ankyrin, which is a key feature of PNH. Therefore, the most correct answer is the D. blood granulocyte FLAER test.

analyze:

Paroxysmal sleep hemoglobinuria (PNH) is a acquired acquired red cell membrane defective hemolytic disease caused by acquired hematopoietic stem cells, is a classic intravascular hemolytic disease. The main feature of the disease is complement-sensitive erythrocytosis, which is characterized by more than 15% erythrocyte lysis in acidified serum hemolysis test (Ham test), or more than 5% erythrocyte lysis in sugar water test, or positive hemolysis test for snake venom factor. The exact diagnosis depends on flow cytometry detection of blood granulocytic FLAER, FLAER is fluorescently labeled aeromonas water (Aerolysin) variant, can specifically bind with glycosylated phosphatidylinositol (GPI) on the cell membrane, on the flow cytes, GPI and monocytes.

Option A bone marrow cytology is to determine the degree of hyperplasia of nucleated cells. According to the ratio of mature red blood cells and nucleated cells, the degree of bone marrow hyperplasia can be divided into five grades: hyperactive hyperplasia, hyperactive hyperplasia, active hyperplasia, reduced hyperplasia, and extremely reduced hyperplasia, so PNH cannot be confirmed, so it is excluded.

Option B reticulocyte test is mainly used to reflect the generating function of

bone marrow red blood cells, which is an auxiliary examination of a variety of anaemic diseases. reticulocyte increase is commonly seen in hemolytic anemia, acute hemorrhagic anemia, iron deficiency anemia, etc., while reticulocyte reduction is common in aplastic anemia, and PNH cannot be confirmed, so it is not selected.

Option C Peripheral blood smear is to take a drop of peripheral blood to make an appropriate push film. After Giemsa staining, the patient is examined with microscope oil microscope and diagnosed according to various blood cells and abnormal cell morphological characteristics. This method can only provide cell morphological information, and PNH cannot be confirmed, so it is not considered.

Option E red cell permeability fragility test is the determination of red cell resistance to various concentrations of low permeability solution, in salt water, water through the cell membrane, make the red blood cells gradually expansion, destruction of red cell permeability mainly depends on the ratio of surface area and volume of red blood cells, surface area and small volume of permeability salt solution resistance is larger, otherwise the resistance is small, has nothing to do with the diagnosis of PNH, can be ruled out.

In conclusion, the correct answer is: D. Blood granuyte FLAER detection.

Type A1 multiple-choice questions ask you to choose the best answer. For the diagnosis of PNH (paroxysmal sleep hemoglobinuria), the following is an analysis of each option:

A. Bone marrow cytology: Bone marrow cytology can be used to exclude other hematological diseases, but can not directly confirm PNH.

B. reticulocyte test: reticulocyte test can show hemolytic status, but not specific examination, can not confirm PNH.

C. Peripheral blood smear: Peripheral blood smear can detect hemolysis characteristics, but can not specifically confirm PNH.

D. FLAER test in blood granulocytes: The FLAER (fluorescently labeled amine

acetate) test is a highly specific laboratory test that detects the lack of GPI anchor protein on the PNH cell membrane, and is the first test for the diagnosis of PNH.

E. Red cell penetration fragility test: This test is mainly used to detect hereditary spherocytosis, independent of PNH.

In conclusion, the laboratory tests most conducive to confirm the diagnosis of PNH are:

F. FLAER detection in blood granulocytes

11. (Type A1 questions)

The trophic arteries of the femoral head were not included

A. arteriae circumflexa ilium profunda

B. The ascending branch of the femoral shaft trophic artery

C. Branch of the lateral wound femoral artery

D. Small concave artery

E. Branch of the medial spiral femoral artery

Reference answer: A. Your answer:

[Analysis] The blood supply source of femoral head is concave artery, ascending branch of femoral shaft nourishing artery and internal and lateral femoral arteries.(A wrong, is the correct answer to this question)

[Avoid mistakes] The medial femoral artery can be divided into lateral epiphyseal artery, superior metaphyseal artery, inferior metaphyseal artery, including the epiphyseal

The lateral artery supplies the blood circulation from 2 / 3 to 4 / 5 areas of the femoral head, and is the main source of blood supply to the femoral head.

The main blood supply to the femoral head comes from the artery of the femoral ligament (mainly the lateral artery beside the hip) and the vascular network surrounding the femoral neck, which consists of branches of the medial and the lateral femoral artery. The femoral shaft nourishing artery mainly supplies the bone marrow

and bone mass of the femoral shaft and does not directly supply the femoral head. Therefore, the most appropriate choice is: B. femoral backbone nourishing artery ascending branch

analyze:

The femoral head is located at the upper end of the femur, spherical, toward the upper, and associated with the acetabum. A small concave in the center of the head, the ligament is the attachment of the femoral head, and the ligament has the artery that nourishes the femoral head. The trophic arteries of the femoral head are mainly from the ascending branch of the femoral shaft trophic artery and the branches of the internal and lateral arteries.

Option A The deep iliac artery is A branch of the internal and external iliac artery, mainly distributed around the hip joint, supplying the adjacent muscles and skin * *, and not directly supplying the femoral head * *, which is the correct answer.

The ascending branch of the B femoral shaft is the vegetative artery of the femoral shaft, and some of its branches will rise to the femoral head and supply blood to it.

Option C lateral femoral artery branch, and option E medial femoral artery branch, which belong to the branch of the spiral femoral artery, rise along the base of the femoral neck, enter the femoral head, and supply blood to it.

Option D The concave artery is the concave artery of the femoral head that provides nutrition to the femoral head.

In conclusion, the correct answer is: A. the deep iliac artery.

The vegetative arteries of the femoral head include multiple sources, but these do not include the deep spiral iliac arteries. The main trophic arteries of the femoral head are the medial femoral artery, lateral femoral artery, foveal artery and ascending branch of the femoral shaft trophic artery.

So the correct answer is:

A. arteriae circumflexa ilium profunda

12. (Type A1 questions)

Drug for the treatment of supraventricular tachycardia

A. amiodarone

B. lidocaine

C. adenosine

D. metoprolol

E. verapamil

Reference answer: C Your answer:

[Analysis] Drug therapy is the most commonly used and effective method to terminate the onset of tachycardia. Preferred adenosine with rapid onset of action (C pair).

[Fault avoidance] When adenosine is ineffective, static verapamil (E wrong) can be used instead, and other optional drugs include β -blockers

(D wrong), digitalis, propafenone. The effect of amiodarone (A wrong) belongs to the class of prolonging the action potential,

It is a broad-spectrum antiarrhythmic drug, which blocks potassium channels, inhibits potassium ion efflux, and significantly extends APD and ERP. Lidocaine main

Be used for ventricular tachycardia (B error).

The treatment of supraventricular tachycardia (SVT, Supraventricular Tachycardia) is usually preferred with adenosine (Adenosine) because it rapidly stops most of the supraventricular tachycardia. The effect of adenosine is very transient and usually does not lead to persistent hypotension or other serious side effects. Therefore, the best answer to this question is to be C. adenosine.

analyze:

Supraventricular tachycardia, or simply supraventricular tachycardia. It mainly includes paroxysmal supraventricular tachycardia, disciplined atrial tachycardia and non-paroxysmal borderline tachycardia. Paroxysmal epiventricular velocity is a paroxysmal fast and regular ectopic heart rhythm. It is characterized by a sudden cessation of sudden seizures. During the attack, the patient felt the heart beat very fast, as if to jump out like, very uncomfortable. Its preferred therapeutic agent is adenosine. It is mainly used for the treatment of supraventricular tachycardia and is often used as the preferred drug due to its rapid onset of action. However, adenosine half-life is

very short, so its action time is very short, adenosine static injection almost immediately after the administration of drugs, usually need to switch to other drugs or electrical cardioversion.

Option A Amiodarone is A broad-spectrum antiarrhythmic drug that can be used for various supraventricular and ventricular tachyarrhythmia, including atrial flutter and fibrillation, and preexcitation syndrome; hypertrophic cardiomyopathy, ventricular arrhythmia after myocardial infarction, prevention of ventricular arrhythmia after resuscitation, is not the preferred treatment for supraventricular tachycardia, so it is excluded.

Option B lidocaine, option D metoprolol, option E verapamil, are all broad-spectrum antiarrhythmic drugs that can treat various supraventricular and ventricular tachyarrhythmias, but are not the preferred treatment for supraventricular tachycardia, so they were excluded.

To sum up, the correct answer is: C. adenosine.

The preferred drug for supraventricular tachycardia (Supraventricular Tachycardia, SVT) is adenosine (Adenosine). Adenosine is an effective and rapid antiarrhythmic drug commonly used in the acute management of supraventricular tachycardia to terminate the tachycardia attack by briefly blocking the conduction of the atrioventricular node.

So, the most correct answer is:

F. adenosine

13. (Type A1 questions)

The presentation of the hypertensive fundus lesion stage is

A. retinal venous crossing

B. papilloedema

C. The increased reflection of the retinal arteries

D. Floc exudation of the retina

E. Tinner-induced thinning of the retinal arteries

Reference answer: B. Your answer:

Graphic analysis: [analysis] Keith-Wagener fundus classification method is currently used: grade I: retinal artery thinning, reflective enhancement (CE wrong); grade: retinal artery stenosis, arteriovenous cross compression (A wrong); grade: fundus hemorrhage and cotton exudation (D wrong) on the basis of the above lesions; grade: optic disc edema (B pair).

[Avoid mistakes] memory questions.

Hypertensive fundus lesions can be divided into different stages, where the fourth stage is the most severe and usually features include optic disc edema. Therefore, for this question:

14. The presentation of the hypertensive fundus lesion stage is

A. retinal venous crossing

B. papilloedema

C. The increased reflection of the retinal arteries

D. Floc exudation of the retina

E. Tinner-induced thinning of the retinal arteries

The best answer is * * B. optic disc edema * *. Optic disc edema is a common presentation and this stage of fundus changes may be accompanied by a severe decrease in visual acuity.

analyze:

Hypertensive fundus lesions are divided into four levels, among which, the grade is the most severe grade of hypertensive fundus lesions. Its manifestations is papilledema, retinal hemorrhage, exudation accompanied by papilloedema, may lead to serious consequences such as blindness, namely optic disc edema.

Option A Retinal venous crossing is hypertensive fundus lesion grade, with retinal artery hardening, arterial thinning, enhanced reflection, and venous cross compression, which is not consistent with the meaning of the question.

Option C The reflective enhancement of retinal arteries is hypertensive fundus lesions grade I, functional stenosis or mild sclerosis of retinal arteries, and generally mild narrowing of retinal arteries, which is inconsistent with this question.

Option D retinal cotton wool exudation was hypertensive fundus lesion grade, retinal artery obvious hardening, retinal hemorrhage, exudation and cotton wool spots, retinal edema, but papilloedema was ignored, which was not consistent with the dry description.

Option E retinal artery thinning to hypertensive fundus lesions grade I, functional stenosis or mild sclerosis of retinal arteries, and generally mild narrowing of retinal arteries, which can be excluded.

In conclusion, the correct answer is: B. optic disc edema.

Hypertensive fundus lesions can be divided into four stages according to the different fundus changes, with the fourth stage showing optic disc edema. So the correct answer is:

F. papilloedema

15. (Type A1 questions)

Clinical manifestations of typhoid fever were not included

A. Bleeding skin rash

B. splenauxe

C. relative infrequent pulse

D. apathy

E. Continuous high fever

Reference answer: A. Your answer:

[Analysis] The characteristic clinical manifestations of typhoid fever (polar phase) include: persistent fever (E pair); symptoms of neurological poisoning, patients with indifference (D pair); relatively slow pulse (C pair); rose rash; digestive tract symptoms; hepatosplenomegaly (B pair). The hemorrhagic rash is the clinical manifestation of typhus (A wrong, is the correct answer to this question).

[Error avoidance] Complications of typhoid fever include intestinal bleeding, intestinal perforation, toxic hepatitis, toxic myocarditis, and others, such as pneumonia,

thrombophlebitis. Enteric bleeding was the most common, and intestinal perforation was the most severe.

Typhid fever is an acute infectious disease caused by Salmonella typhimurium (Salmonella Typhi). Its clinical manifestations include persistent high fever, headache, abdominal pain, constipation or diarrhea, splenic size, and relatively slow veins. Inathy may also be one of the symptoms, especially in severe cases. However, hemorrhagic rashes are not a typical clinical manifestation of typhoid fever, and although in some cases a rash (e. g. rose rash) may occur in typhoid patients, they are usually not hemorrhagic.

Therefore, according to the question, choose the option of not including the clinical manifestations of typhoid fever, the answer is * * A. hemorrhagic rash * *.

analyze:

Typhoid fever is an acute infectious disease caused by typhoid fever bacteria, often called typhoid fever, enteric fever. Typhi is characterized by persistent bacteremia, involvement of the reticular endothelial system, and minimal abscess and ulcer formation in the distal ileum. The incubation period of typhoid fever is 10 – 14 days. Its symptoms include high fever up to 39° to 40°C; other symptoms include abdominal pain, severe diarrhea, headache, rose spots in the body, and intestinal bleeding or perforation is the most serious complication. Hemorrhagic rash can be seen in a variety of infectious and non-infectious diseases, such as measles, rubella, scarlet fever, typhoid fever and paratyphi, typhus, herpes simplex, herpes simplex, erysipelas, insect bite dermatitis, drug dermatitis, hand, foot and mouth disease, dengue fever, etc., which is not characteristic clinical manifestations of typhoid fever.

The pathology of typhoid is characterized by hyperplastic reactions of the mononuclear-phagocytic system, lymphoid hyperplasia, swelling, necrosis, and ulcer formation in the lower ileum, hence the spleen B.

The clinical manifestations of typhoid fever with continuous bacteremia, reticulate endothelial system involvement, the distal ileal small abscess and ulcer formation as the basic pathological features, its typical clinical manifestations for high fever, special poisoning face, relatively slow pulse, skin, rose rash, liver and spleen, surrounding the total number of white blood cells, eosinophils disappear, bone marrow with typhoid cells. Therefore, option C is relatively slow pulse, option D has indifferent expression, and option E has continuous high fever, all of which are clinical manifestations of typhoid fever.

In conclusion, the correct answer is: A. hemorrhagic rash.

Typhid fever is a systemic infectious disease caused by Salmonella, and its

typical clinical manifestations include:

- ** Persistent high fever * *: Patients usually experience high fever for several weeks, with a temperature often between 39 and 40 degrees Celsius.

- ** Splenic enlargement * *: Splenic enlargement is a common sign of typhoid fever and usually occurs within one to two weeks of disease onset.

- ** Relatively slow pulse * *: In the case of high fever, the pulse is relatively slow, which is one of the characteristic manifestations of typhoid fever.

- ** Inindifferent expression * *: Patients often show listless and indifferent expression.

However, * * haemorrhagic rash * * is not a typical manifestation of typhoid fever. Although patients with typhoid fever may develop a rose rash, a small pink maculopapular rash, a hemorrhagic rash is uncommon.

So, the most correct answer is:

A. Bleeding skin rash

16. (Type A1 question)

Painless gross hematuria is the main clinical manifestation

A. tumor of bladder

B. nephrophthisis

C. renal cyst

D. Upper urinary tract calculi

E. nephropyelitis

Reference answer: A. Your answer:

[Analysis] The main clinical manifestation of bladder tumor is hematuria (A pair), hematuria is the most common symptom of bladder cancer, about 85% of patients show intermittent painless macroscopic hematuria.

[Avoid error] The typical manifestations of renal tuberculosis (B error) are frequency of urination, urgency, urination pain with terminal hematuria, and patients often have low fever and theft

Sweat and other symptoms of tuberculosis poisoning. Simple renal cysts generally have no symptoms and only appear when the cyst presses the surrounding tissue

Symptoms, such as lumbar and abdominal pain, abdominal mass, hematuria, etc. (C wrong). The typical manifestation of upper urinary calculi (D wrong) is the waist

Pain, hematuria or microscopic hematuria, if there is a stone stuck in the ureter may appear costal ridge angle colic or urethral obstruction.nephropyelitis (E

Wrong) often have high fever, chills, low back pain and frequent urination, urgent urination, urination pain bladder irritation symptoms.

Painless gross hematuria is a condition in which blood can be seen in the urine without pain. The symptoms are most common in the bladder tumours. So, the most appropriate answer to this question is:

A. tumor of bladder

Although upper urinary tract stones, renal tuberculosis, renal cysts, and pyelonephritis may all cause hematuria, they are usually accompanied by pain or other symptoms. Bladder tumors are more likely to cause painless gross hematuria. Therefore, the choice of A as the best answer was based on current medical knowledge and best practice guidelines.

First of all, let's analyze the topic first. The title asked us to identify which disease with painless gross hematuria as the main clinical manifestation.

Next, we analyze the options one by one:

A. Bladder tumors: The typical manifestations of bladder tumors include intermittent, fully painless macroscopic hematuria. This corresponds with the symptoms described by the title.

B. Renal tuberculosis: The typical manifestation of renal tuberculosis is bladder irritation symptoms, such as frequent urination, urgency, urination pain, and may be accompanied by terminal hematuria. In addition, patients may also have symptoms of tuberculosis poisoning, such as low fever and night sweats. This is inconsistent with the description of a painless gross hematuria.

C. Renal cysts: renal cysts generally do not have obvious clinical manifestations, unless the cyst is very large and compresses the surrounding tissue, the symptoms may appear. But the symptoms in this case are not consistent with the painless gross hematuria.

D. Upper urinary tract stones: The main symptoms of upper urinary tract stones are pain and hematuria. The pain can be dull, dull, bulging or colic, which is inconsistent with the description of painless hematuria.

E. Pelelonephritis: pyelonephritis is mainly manifested as systemic symptoms such as fever, chills and headache, as well as bladder irritation symptoms such as frequent urination, urgent urination and urination pain. Some patients may have hematuria, but it is usually accompanied by pain, which does not meet the description of painless hematuria.

In conclusion, only option A bladder tumors met the main clinical manifestations of painless macroscopic hematuria as described in the title.

Therefore, the correct answer is the A. bladder tumor.

The most likely disease with painless gross hematuria as the main clinical manifestation is the bladder tumors. Painless gross hematuria is often an important manifestation of urourinary tumors, especially bladder cancer. Other options, such as renal tuberculosis, renal cysts, upper urinary tract stones, and pyelonephritis, are often associated with pain or other symptoms.

So, the most correct answer is:

A. tumor of bladder

17. (Type A1 question)

The main treatment modality for Addison-disease is

A. glucocorticoid replacement therapy

B. Saleralocorticoid replacement therapy

C. operative treatment

D. symptomatic treatment

E. Intravenous infusion of glucocorticoids

Reference answer: A. Your answer:

[Analysis] Addison disease (primary chronic adrenocortical dysfunction) treatment includes glucocorticoid replacement therapy, salt and mineralocorticoid replacement therapy, adrenal crisis treatment (including fluid rehydration, anti-infection and other symptomatic treatment), surgery or other stress treatment. Among them, glucocorticoid replacement therapy is the main treatment method (A pair). Most patients can achieve satisfactory results after adequate salt intake, and some patients need to add salinocorticoid when they still have symptoms.

[Avoid avoidance] The most characteristic of primary chronic adrenocortical dysfunction is the deepening of skin pigment, exposed area

Friction, areola, scar and other places are particularly obvious, mucosal pigmentation is seen in the gums, tongue, buccal mucosa and other places.

Addison Disease (Atherton's disease) is mainly characterized by adrenal cortical dysfunction, leading to a decrease in glucocorticoid and minerocorticoid levels. Therefore, its main treatment is to supplement these hormones to replace the body's inability to produce sufficient amounts of hormones.

-A. glucocorticoid replacement therapy

-B. Saleralocorticoid replacement therapy

In fact, treatment for Addison disease usually requires alternative treatment with both glucocorticoids (e. g., cortisol) and mineralocorticosteroids (e. g., flurhydrocortisone). However, if a "primary" treatment modality must be selected from these options, it is generally considered * * mineralocorticoid replacement therapy * *, as this treatment is essential to maintain electrolyte balance and prevent shock. So, the correct answer is most likely to be * * B. mineralocorticoid replacement therapy * *. However, in practice, patients often require two types of hormone replacement.

First, we need to understand Addison's disease, also known as primary chronic adrenocortical hypofunction. This is a disease due to the destruction of the vast majority of the bilateral adrenal glands, and the most common cause of it is autoimmune adrenalitis. The main manifestation of this disease is cortisol and aldosterone deficiency.

Now, we do a detailed analysis for each option:

A. Glucocorticoid replacement therapy: As cortisol levels decrease in patients with Addison disease, glucocorticoid replacement therapy can effectively supplement this deficiency and thus relieve symptoms. This is a long-term treatment designed to simulate the body's natural cortisol secretion.

B. Mineralocorticoid replacement therapy: Although aldosterone is also minerocorticoid, it is usually not the main treatment in the treatment of Addison disease. The main role of mineralocorticoids is to regulate water-salt metabolism, while glucocorticoids have a broader physiological role.

C. Surgical treatment: For Addison disease, surgical treatment is not the main treatment method. Because it is mainly caused by autoimmune diseases, surgery does not solve the problem.

D. Symptomatic treatment: Although symptomatic treatment is necessary in some cases, it does not address the root cause of Addison's disease, namely the deficiency of cortisol and aldosterone.

E. Intravenous corticosteroids: This is usually used for emergency treatment of acute adrenal crisis rather than conventional treatment for Addison's disease.

Considering the above analysis, the main treatment method for Addison disease is glucocorticoid replacement therapy, which aims to mimic the natural cortisol secretion and thus relieve the symptoms.

Therefore, the correct answer is A. glucocorticoid replacement therapy.

The primary treatment modality for Addison-disease is glucocorticoid replacement therapy.

The best answer is: * * A. glucocorticoid replacement therapy * *

18. (Type A1 question)

Small doses that can increase the renal artery blood flow are available

A. adrenalin

B. arterenol

C. isoprenaline

D. dopamine

E. aminophylline

Refer to the answer: D. Your answer:

[Analysis] Dopamine (D pair) acts on D1 receptors at a low concentration, dilating the renal blood vessels, increasing the renal blood flow, and increasing the glomerular filtration rate. Meanwhile, dopamine has sodium exclusion diuretic effect, which may be the direct effect of dopamine on renal tubular D1 receptor. Large doses of α receptors stimulating the renal vessels can significantly contract the renal vessels.

[Avoid avoidance] Adrenaline in the kidney mainly excited α receptor on vascular smooth muscle, vasoconstriction (A error); norkidney

Adenotor α 1 receptor, mainly small arteries and venules (B wrong); isoproterenaline

Pharmacological effects usually include excited β 1, β 2 receptors, and relaxation of bronchial smooth muscle (C wrong); aminophylline can increase renal blood flow

Volume and glomerular filtration rate inhibit the reabsorption of sodium and water by renal tubules, so it has the effect of strong heart diuresis, but it has no dose area

Don't (E wrong).

Small doses of dopamine have the effect of increasing renal arterial blood flow by stimulating dopamine receptors. Thus, among these options, the most appropriate answer is the D. dopamine. Dopamine can dilate renal vessels and increase blood flow to the kidneys at low doses.

First, let's analyze each option one by one:

A. Adrenaline: it mainly acts on the cardiac muscle, vascular smooth muscle, bronchial smooth muscle, etc. Adrephrine increases the heart contraction and contracts small blood vessels in the skin, viscera, and kidneys, but slightly expands blood vessels in skeletal muscle and liver. Therefore, epinephrine is not the best choice.

B. Nrepinephrine: This drug contracts the arterioles and venules of the body, only the coronary vessels. It mainly affects the α 1 receptor of vascular smooth muscle, constricting blood vessels and thus raising hypertension. Therefore,

norepinephrine is not the best option to increase the renal artery blood flow.

C. Isoproterenol: This drug mainly agonizes β receptors, with strong agonistic effects on both β 1 and β 2 receptors, and has little effect on α receptors. Its main function is to enhance myocardial contractility, accelerate heart rate, accelerate conduction, and increase cardiac output and myocardial oxygen consumption. However, it mainly dilates the arterioles, but this dilation is not specific to the renal artery, so it is not the best answer.

D. Dopamine: At small doses, dopamine mainly stimulates dopamine receptors, expanding the kidney and mesenteric vessels, increasing renal blood flow and glomerular filtration rate, as well as urine volume and sodium excretion. This feature is exactly in line with the "small doses can increase renal artery blood flow" described in the title.

E. Aminophylline: This drug is mainly used to relieve asthma, it can directly relax the bronchial smooth muscle, while also can dilate the coronary arteries and peripheral blood vessels. But it does not specifically increase the blood flow to the renal artery.

In conclusion, it is dopamine that small doses can increase renal artery blood flow. So the best answer is: D. dopamine.

With available medical knowledge and best practice guidelines, a small dose that increases renal artery blood flow is dopamine (D).

Dopamine has different effects at different doses:

-Small dose (0.5-2 μ g / kg/min): mainly on dopamine receptors to increase renal blood flow and urine volume.

-Moderate dose (2-10 μ g / kg/min): Mainly on β 1-adrenergic receptors to increase cardiac contractility and cardiac output.

-High dose (> 10 μ g / kg/min): it mainly acts on α -adrenergic receptors, leading to vasoconstriction and increased blood pressure.

Thus, the correct answer is the D. dopamine.

19. (Type A1 questions)

The most commonly used indicator to evaluate the glomerular filtration rate is

A. blood urea

B. serum creatinine

C. inulin clearance

D. Endogenous creatinine clearance

E. eGFR

Refer to the answer: D. Your answer:

Graphic analysis: [analysis] endogenous creatinine clearance is close to the glomerular filtration rate, so it is commonly used to predict glomerular filtration

Over-rate (D pair).

[Avoid mistakes] Blood urea (A wrong) is all filtered by the glomerulus, 30%~40% is reabsorbed by the renal tubules, and the renal tubules also secrete less

Volume urea, not used to evaluate the glomerular filtration rate. Blood creatinine (B wrong) can be affected by protein intake and intestinal absorption function

Sound, can not accurately evaluate the glomerular filtration rate. Inulin is freely filtered by the glomerulus and not reaspirated in the tubules and collecting ducts

Collection and secretion, using inulin to determine the glomerular filtration rate is accurate and reliable, but the operation is inconvenient (C wrong). The eGFR was done with the serum

Adding the creatinine value into the formula is not accurate (E error).

When evaluating the glomerular filtration rate (GFR), the most commonly used indicator is the E. eGFR (to estimate the glomerular filtration rate). The eGFR was calculated from blood creatinine levels, age, sex, and race, and was used to estimate the filtering function of the kidney. Although other options, such as blood creatinine and endogenic creatinine clearance, are also associated to renal function, eGFR is the most commonly used clinically indicator to assess glomerular filtration rate. Thus, the most correct answer is the E. eGFR.

analyze:

Glomerular filtration rate (GFR) refers to the amount of filtrate produced by the two kidneys per unit of time, and is a measure of renal function. The most commonly used index to evaluate the glomerular filtration rate is eGFR, that is, the estimate of

the glomerular filtration rate, which refers to the glomerular filtration rate calculated based on the blood creatinine detection value, age, sex, weight, and CKD-EPI formula. This method is convenient to calculate and is of great significance for the evaluation of renal function.

Although option A blood urea can also reflect renal function, urea can be affected by many extra-renal factors, such as high-protein diet, gastrointestinal bleeding, dehydration and the use of glucocorticoids, tetracycline and other drugs, so it is not as accurate as blood creatinine and can be excluded.

Although option B blood creatinine has a certain correlation with glomerular filtration rate, and the measurement method is simple, it is influenced by various factors such as age, sex, shape, dietary structure, diet, muscle consumption, creatinine distribution volume and abnormal tubular secretion, so it cannot be solely dependent on blood creatinine concentration to evaluate renal function, which can be excluded.

Option C inulin clearance is the clearance capacity of both kidneys equivalent to several ml of plasma within 1 minute. Inulin clearance rate is completely equal to glomerular filtration rate, which is the most ideal method to determine glomerular filtration rate. However, due to the determination method is complex, expensive, and inulin is an exogenous substance, which requires continuous constant velocity injection, which is not suitable for conventional application and can be excluded.

Option D The endogenous creatinine clearance test is complicated, requiring 24-hour urine collection and blood collection for blood creatinine and urinary creatinine concentration measurement, and creatinine clearance calculation, which cannot be used for the diagnosis of acute kidney injury, and can be excluded clinically.

To sum up, the correct answer is: E.eGFR.

The most commonly used indicator to evaluate the glomerular filtration rate is

F. Endogenous creatinine clearance

20. (Type A1 question)

The typical signs of a massive pericardial effusion are

A. distention of jugular vein

B. The heart is far away

C. Griesinger-Kussmaul sign

D. hypopiesia

E. cyanosis of lips

Reference answer: C Your answer:

Text analysis: [Analysis] The clinical characteristics of pericardial effusion are Beck's triad: hypotension, low heart tone, jugular vein irritability. A large amount of pericardial effusion can reduce the systolic blood pressure, but the diastolic blood pressure does not change much, so the pulse pressure becomes smaller. According to the degree of cardiac compression, pulse can be weakened or strange pulse (C pair), which is a typical sign of pericardial effusion, and jugular vein irritability and hypotension can also be seen in many diseases such as heart failure.

[Error avoidance] Ewart sign is pericardial effusion sign, which refers to the occurrence of percussion turbidity under the left scapula and auscultation

Tracheal breath sounds, which are the signs of pericardial effusion.

Large pericardial effusion may cause compression of the heart, affecting its normal function, thus triggering a range of clinical symptoms and signs. Based on this information, you can screen the options one by one:

A. Jugular vein irritation: Pericardial effusion can cause obstruction of heart reflux, which leads to jugular vein irritation. However, this is not the most typical sign of a massive pericardial effusion.

B. Heart sound is distant: a large amount of pericardial effusion will make the heart far away from the chest wall, causing the heart sound to appear distant on auscultation. This is a typical sign of a massive pericardial effusion.

C. Qi pulse: Qi pulse is a drop in blood pressure of more than 10 mm Hg during inspiration, which is common in cardiac tamponade, but is not a typical sign of a large pericardial effusion.

D. Hypotension: Severe pericardial effusion may cause cardiac tamponade, which can lead to hypotension, but this is not the most specific sign.

E. Cyanosis of the mouth: This is a sign of poor oxygenation, and although severe pericardial effusion may cause insufficient circulation to cause cyanosis, this is not the most typical sign.

Therefore, the most typical sign is B. heart distant.

analyze:

Pericardial effusion refers to the accumulation of excessive fluid in the pericardial cavity, including liquid, serous fibrin, bloody, purulent, etc. Normal pericardial cavity contains 25~30ml of liquid, and any reason increases the amount of liquid in the pericardial cavity, beyond 50ml, that is, the pericardial effusion appears. Pericardial effusion lasts for more than several months and constitutes a chronic pericardial effusion. When the pericardial effusion is large, it can cause a series of clinical manifestations, such as dyspnea, jugular vein irritability, etc. Among the signs of pericardial effusion, strange pulse has the most diagnostic value. Strange pulse refers to the obvious pulse weakening or disappearance during inspiration, which is caused by the decrease of blood volume of left ventricular stroke, and is an important sign of pericardial effusion.

Option A jugular vein irritability can be seen in pericardial effusion, but also in right heart failure, vasoconstriction pericarditis and other diseases, it is not specific, so it is not selected.

Option B Heart sound is far visible in a large amount of pericardial effusion, but also in dilated cardiomyopathy, severe heart failure and other diseases, so it is excluded.

Option D hypotension can be seen in pericardial effusion, but also in massive bleeding, acute myocardial infarction, severe trauma and other diseases, which does not meet the question.

Option E Cyanosis can be seen in the pericardial effusion, but also in chronic obstructive pulmonary disease, pulmonary thromboembolism and other diseases, so it is not selected.

To sum up, the correct answer is: C. Qi mai.

Among the typical signs of a large amount of pericardial effusion, * * * * * * * * * * * * * * * * are possible, but the most typical sign is * * strange pulse * *.

Qi pulse refers to the decrease of arterial systolic pressure of more than 10 mmHg during inhalation, which is due to the compression of a large pericardial effusion on the heart, which affects the filling and exhaust function of the heart during inhalation. Thus, while other options may also appear in patients with massive pericardial effusion, Qipulse is the most specific sign.

So the best answer is:

F. Griesinger-Kussmaul sign

21. (Type A1 question)

The cause of massive hemoptysis in mitral valve stenosis is

A. bronchial vein

B. The rupture of the pulmonary capillaries

C. rupture uptured bronchial artery

D. Pulmonary congestion

E. pulmonary embolism

Reference answer: A. Your answer:

[Analysis] The sudden massive hemoptysis in rheumatic heart disease patients is caused by severe mitral stenosis, sudden increase in left atrial pressure, increased pulmonary venous pressure, and bronchial vein rupture (A pair) bleeding.

[Avoid error] Pulmonary capillary rupture (B error) can lead to blood, blood sputum or pink bubbles in sputum in patients with mitral stenosis

foaming phlegm. Bronchial artery rupture is common in bronchiectasis (C error). Pulmonary congestion is usually caused by left heart failure and is often a powder

Red foam sputum, for capillary rupture (D wrong). The causes of pulmonary embolism and hemorrhage are blood stasis, lung tissue and edema, and alveoli

Wall, or bronchial intimal capillaries rupture, bronchial submucosal bronchial varicose veins rupture, blood into the alveoli or

Bronchus, finally cough out through the trachea (E wrong).

Mitral stenosis usually leads to increased pressure in the left atrium, which subsequently causes pulmonary congestion. When the pulmonary vein pressure increases, it may lead to increase the pressure of the pulmonary capillaries, resulting in pulmonary capillary rupture and hemoptysis. Therefore, of these options, the most consistent cause for mitral stenosis causing massive hemoptysis is * * B. Pulmonary capillary rupture * *.

analyze:

When mitral valve stenosis, left atrial pressure increases, pulmonary venous blood reflux obstacle, lead to increased pulmonary venous pressure, can cause bronchial venular rupture bleeding, the performance is sudden large amount of blood, called mitral valve stenosis large hemoptysis. Therefore, the cause of mitral stenosis is the rupture of the bronchial vein.

Option B Pulmonary capillary rupture is mostly seen in pulmonary congestion and edema caused by incomplete mitral valve closure. Due to the high posterior venular pressure of the capillary and the enhanced permeability of hypoxia in the tube wall, the fluid seeps out of the capillaries and enters the alveoli, causing pulmonary edema rather than massive hemoptysis, so it is not selected.

Option C bronchial artery rupture can lead to massive hemoptysis, but mitral stenosis generally does not involve the bronchial artery, so bronchial artery rupture is not the cause of mitral stenosis and massive hemoptysis, so it is excluded.

Option D Pulmonary congestion is caused by increased left atrial pressure caused by mitral stenosis, but does not lead to massive hemoptysis and can be excluded.

Option E Common symptoms of pulmonary embolism include unexplained dyspnea and shortness of breath, especially after activity, chest pain, syncope, cough, hemoptysis, etc., but the amount of hemoptysis is generally not large, so it is not the cause of mitral stenosis and large hemoptysis, and can be excluded.

In conclusion, the correct answer is: A. bronchial vein rupture.

The main cause of massive hemoptysis caused by mitral stenosis is increased pulmonary vein pressure, which has ruptured the bronchial vein. So, the most correct answer is:

A. bronchial vein

22. (Type A1 questions)

To confirm hyperparathyroidism, which of the following tests can be used

A. neck CT

B. mediastinum CT

C. MIBI Nuclide scan

D. Neck ultrasound

E. neck MRI

Reference answer: C Your answer:

Graphic analysis: [Analysis] Location diagnosis of hyperparathyroid: After qualitative diagnosis, neck ultrasound examination, radionuclide examination such as MIBI scan, neck and mediastinal CT scan are still needed, among which MIBI parathyroid imaging has extremely high specificity.

[Avoid mistakes] The changes of blood calcium, blood phosphorus and urine calcium are high blood calcium, low blood phosphorus and high urine calcium.

The most common method to confirm hyperparathyroidism is the MIBI nuclide scan, as this examination is able to pinpoint the glands with hyperparathyroidism. So, the correct answer is: C. MIBI Nuclide scan

First, we need to understand what hyperparathyroidism is. Hyperparathyroidism is the excessive secretion of parathyroid hormone, which is usually due to some abnormality of the parathyroid gland, such as hyperplasia, adenoma or adenocarcinoma. To diagnose the disease, we need to find tests that can directly show the parathyroid function and structure.

Now, let's look at the options one by one:

A. Neck CT: Although CT can provide good anatomical structure information, it is not very specific for parathyroid glands and has large radiation, which is not the preferred examination method.

B. Mediastinal CT: This examination focuses more on the structure and disease of the chest, and has little to do with the parathyroid gland.

C. MIBI Nuclide scanning: MIBI (methoxyisobutyl isonitrile) is a tumor-friendly imaging agent that can be used to diagnose hyperparathyroidism, especially when a tumor or hyperplasia of the parathyroid gland is suspected. MIBI can be ingested by hyperfunctioning parathyroid tissue and thus can be clearly displayed in the scan, providing strong evidence for a confirmed diagnosis.

D. Neck ultrasound: Although ultrasound can reveal some structures of the neck, it has limited specific diagnostic value for the parathyroid gland.

E. Neck MRI: MRI can provide more detailed anatomical information, but also for parathyroid specificity, the price is relatively high, and the operation is more

complex.

In conclusion, MIBI nuclide scan is the method of choice for confirming the diagnosis of hyperparathyroidism for its ability to specifically display hyperfunctional parathyroid tissue.

Thus, the correct answer is the C. MIBI nuclide scan.

The best test for confirming the diagnosis of hyperparathyroidism (hyperparathyroidism, PTH) is the * * C. MIBI Nuclide scan * *.

The diagnosis of hyperparathyroidism usually relies on multiple means of examination, including serum calcium level, PTH level, etc. Imaging examination plays a key role in locating the abnormal parathyroid glands. Here is an analysis of the options:

A. * * Neck CT * *: Cervical CT can be used to look for anatomical abnormalities in the parathyroid gland, but is weak for the assessment of functionality.

B. * * Mediastinal CT * *: Mediastinal CT is not commonly used in the initial diagnosis of hyperparathyroidism unless there is ectopic parathyroid tissue requiring localization.

C. * * MIBI Nuclide scan * *: MIBI (methoxyisobutyl isopronitrile) Nuclide scan is the preferred method for the diagnosis of hyperparathyroidism. It utilizes the uptake of radioisotope-labeled compounds in the parathyroid gland and evaluates the functional status of the parathyroid gland.

D. * * Cervical ultrasound * *: neck ultrasound can be used for initial screening of parathyroid glands, but its detection rate of small or ectopic parathyroid lesions is low.

E. * * Cervical MRI * *: Cervical MRI is superior in soft tissue contrast, but is less effective than MIBI nuclear scan for functional assessment of parathyroid glands.

Therefore, MIBI nuclide scan is the best choice for confirming hyperparathyroidism.

23. (Type A1 question)

Diseases requiring plasmapheresis are

A. acute nephritis

B. Acute renal tubular necrosis

C. Acute interstitial nephritis

D. lupus nephritis

E. ANCA-associated vasculitis with pulmonary hemorrhage

Reference answer: E Your answer:

Graphic resolution: [analytical] ANCA associated vasculitis with pulmonary hemorrhage (E) is due to a large number of anti-GBM (glomerular basement membrane) antibodies and ANCA (anti-neutrophil cytosolic antibody) on the kidney and lung, lead to acute crescent type glomerulonephritis and pulmonary hemorrhage, need emergency treatment, plasma exchange can quickly clear the serum antibodies, alleviate disease progress.

[Error avoidance] Plasma exchange refers to the application of plasma exchange machine to separate plasma and blood cells, discard plasma to equal the amount of normal people

The plasma (or plasma albumin) and the patient's blood cells are re-imported into the body and repeated several times until the serum antibody (e. g. anti

GBM antibody, ANCA) turned negative or the condition improved. Acute nephritis (A wrong) is mainly bed rest, symptomatic treatment and anti-infection treatment, and only dialysis treatment for acute renal failure; acute tubular necrosis (B wrong) is mainly correcting the reversible cause as soon as possible, maintaining internal environment stability, nutritional support, preventing complications and renal replacement treatment; acute interstitium

Sex nephritis (C wrong) generally to stop the renal damage drugs, immunosuppressive therapy, hemodialysis therapy; lupus nephritis

(D wrong) Treatment with hormone and cytotoxic drugs, none of the above

diseases need plasmapheresis treatment.

Among these options, the disease requiring plasmapheresis is E. ANCA-associated vasculitis with pulmonary hemorrhage. ANCA-associated vasculitis (anti-neutrophil cytoplasmic antibody-associated vasculitis) is an autoimmune disease that causes vasculitis and tissue damage. In cases associated with severe pulmonary hemorrhage, plasmapheresis can be used to rapidly remove circulating ANCA antibodies and inflammatory mediators, reduce the condition, and improve survival. Therefore, the best answer is the E. ANCA-associated vasculitis with pulmonary hemorrhage.

analyze:

Plasmapheresis is a blood purification therapy that removes large molecules from the blood. The basic process is to separate the patient's blood into plasma and cellular components through the plasma separator, by removing the pathogenic plasma or selectively remove some pathogenic factors in the plasma, and then transfusion the formed and required albumin or fresh plasma, to achieve the purpose of treatment. Indications for plasmapheresis include acute glomerulonephritis, myasthenia gravis crisis, acute Guillain Barre syndrome, etc. Among them, acute glomerulonephritis is associated with autoimmunity, and anti-glomerular basement membrane antibodies exist in the patient, so plasmapheresis therapy can be used to clear the pathogenic antibodies * *. Lupus nephritis * * is an autoimmune disease, there are a variety of autoantibodies in the body, plasma exchange can remove pathogenic antibodies, so as to alleviate the disease.

Option A acute nephritis, option B acute tubular necrosis, which are non-immune kidney disease, and plasmapheresis is ineffective, so it can be excluded together.

Option C Acute interstitial nephritis is mostly related to drug allergy, allergy drugs are stopped, and glucocorticoids are quickly recovered, without plasma exchange, so it is not selected.

Option E: ANCA-associated vasculitis with pulmonary hemorrhage is an autoimmune disease, but rapid onset, rapid progress, multiple impact therapy combined with immunosuppressants, plasmapheresis is not the first choice, so it is excluded.

In conclusion, the correct answer is: D. lupus nephritis.

Diseases requiring plasmapheresis are:

F. ANCA-associated vasculitis with pulmonary hemorrhage

ANCA-associated vasculitis with pulmonary hemorrhage is a disease requiring plasmapheresis. Plasmapheresis in such diseases, thereby reducing the severity of the immune response.

24. (Type A1 question)

Stones formed in the alkaline urine

A. calculus urate

B. oxalate stones

C. cystine calculus

D. phosphate calculus

E. xanthic calculus

Refer to the answer: D. Your answer:

[Analysis] Easy ammonium phosphate and phosphate precipitation in alkaline urine (D pair). Uric acid, oxalic acid, xanthine stone and cystine crystals are easily formed in acidic urine.

[To avoid mistakes] In urinary stones, calcium-containing stones are the most common, which can be developed on the X-ray plain film, called positive stones, such as calcium oxalate

Stone stones, calcium phosphate stones, calcium carbonate stones, mixed stones, etc. Negative stones do not contain calcium salt, can pass through X-ray, and do not develop on X-ray flat film, such as pure uric acid stones, cystine stones, etc.

The most easily formed in alkaline urine is phosphate stones, because in alkaline environment, phosphate can easily precipitate to form stones. Therefore, the most correct answer is: D. phosphate stones

First of all, we need to make it clear that this is a problem about urinary stones, involving which urine environment of different types of stones are easy to form. The formation mechanism of urinary stones is complex, influenced by various factors such as urine pH and composition.

Now, we analyze each option one by one:

A. Uric acid stones: These stones usually form in the acidic urine, because the solubility of uric acid is reduced in the acidic environment, and it is easy to precipitate and form crystals. Therefore, this option does not match the alkaline urine environment required by the topic.

B. Oxaloate stones: The formation of oxalate stones has little to do with the acidity of urine, and is more affected by the dietary oxalic acid content and other storigenic factors. So, this option is not the best answer either.

C. Cystine stone: cystine stones are mainly related to genetic factors and have little relationship with the acidity of urine. Therefore, this option also does not meet the topic requirements.

D. Phosphate stones: These stones have reduced solubility in alkaline urine and tend to crystallize. This is consistent with the alkaline urine environment mentioned in the title, and it is a reasonable answer.

E. Xanthine stones: xanthine stones are relatively rare, and their formation is mainly related to genetic factors or the lack of some enzymes, but is not directly related to the acidity of urine.

In conclusion, the stones that are easier to form in alkaline urine are phosphate stones. Therefore, the correct answer is the D. phosphate stones.

The stones formed in alkaline urine are mainly phosphate stones.

So the best answer is:

F. phosphate calculus

25. (Type A1 question)

The standard of normal blood pressure in China is

A. is below 150 / 90mmHg

B. is below 140 / 90mmHg

C. is below 120 / 80mmHg

D. is below 135 / 80mmHg

E. is below 130 / 80mmHg

Reference answer: C Your answer:

Graphic analysis: [analysis] normal blood pressure systolic blood pressure <120mmHg, diastolic blood pressure <80mmHg (C pair).

[Error avoidance] Hypertension was defined as a systolic blood pressure of 140mmHg and (or) diastolic blood pressure in the clinic without the use of antihypertensive drugs

≥90mmHg。Normal high BP systolic blood pressure was 120 to 129 mmHg, and diastolic blood pressure was 80 to 89 mmHg.

Normotensive criteria are usually defined as less than 120 / 80mmHg. However, slightly higher BP values can sometimes be accepted depending on different clinical guidelines and context. The upper limit of options A and B is high and is usually seen as the defining criteria for hypertension. The D and E options provide lower BP criteria but were still higher than routinely defined normotensive. Therefore, based on the most commonly accepted medical criteria, * * C. below 120 / 80mmHg * * should be the most accurate answer.

This is a test question about the normal blood pressure criteria. First, we need to define the normal range of blood pressure, which is an important indicator of assessing cardiovascular health.

Next, we analyze each option:

A. Below 150 / 90mmHg: This criterion is usually considered a cut-off for hypertension, not normoblood pressure.

B. Below 140 / 90mmHg: 140 / 90mmHg is the cut-off value for the diagnosis of hypertension according to the current international and Chinese guidelines. Therefore, normotensive should be below this value.

C. Below 120 / 80mmHg: Although this is an ideal blood pressure range, it is not the upper limit of normal blood pressure.

D. Below 135 / 80mmHg: This criterion is not the widely accepted upper limit of normal blood pressure.

E. Below 130 / 80mmHg: Again, this is not the accepted upper limit for normal blood pressure.

Considering the options together, normoBP should be defined as SBP below

140mmHg and DBP below 90mmHg per current medical knowledge and best practice guidelines. This is the cut-off value to determine whether hypertension, so normal blood pressure should be lower than this standard.

In conclusion, the correct answer is that B. is below 140 / 90mmHg.

According to the Guidelines for the Prevention and Treatment of Hypertension in China (2018 Revision) and relevant medical knowledge, the following are the answers to the standard of normal blood pressure in China:

26. The standard for normal BP in China is

A. is below 150 / 90mmHg

B. below 140 / 90mmHg

C. is below 120 / 80mmHg

D. is below 135 / 80mmHg

E. is below 130 / 80mmHg

The best answer is:

F. is below 120 / 80mmHg

Explanation: According to the Chinese Guidelines for the Prevention and Treatment of Hypertension (revised 2018), normal blood pressure was defined as systolic BP below 120 mmHg and diastolic BP below 80 mmHg. Blood pressure above this range was considered hypertension or prehypertension. Therefore, option C is the answer that most conforms to the current guidelines.

27. (Type A1 question)

Straextended supraocondylar humeral fractures are most common in

A. Middle-aged women

B. children

C. Middle-aged men

D. Older men

E. Older women

Reference answer: B. Your answer:

[Analysis] The extended supracdylar fracture of the humerus is caused by the palm landing injury when the elbow is in half flexion or extended position. It is the most common supracondylar humerus fracture, which mostly occurs in children under 10 years old (B pair).

[Avoid mistakes] The fracture line of extended supracondylar humeral fracture is inclined from forward and backward upward, and the proximal end of the fracture is displaced forward and lower and easy to damage the brachius

The tery and median nerve, the elbow protruding protruding backward and in a-flexion position.

Straended supracondylar humerus fractures are usually most common in children. So the best answer is: B. Children

analyze:

The extended supraocondylar humeral fractures are mostly a history of trauma to the elbow joint in half flexion or extension. All the patients have a history of injury. The elbow joint cannot move locally after the injury and the swelling is obvious. Straightening-type supraocondylar humerus fracture, the most common occurrence in children under 10 years of age, is a common elbow injury in children, mostly occurring in sports injuries and traffic accidents.

Stratended supraocondylar humerus fractures are more common in children under 10 years of age, so option A middle-aged women, option C middle-aged men, option D elderly men, and option E elderly women, can be excluded together.

In conclusion, the correct answer is: B. Children.

Stratended supraocondylar humeral fractures are most common in:

F. children

Straended supraocondylar fractures (also known as distal humerus fractures) are most common in children, especially in those ages 5 to 10. Since children's bones are not fully developed, they are more prone to this type of fracture when falls.

28. (Type A1 question)

The following statement of proteinuria is wrong

A. Urinary protein was greater than 3.5g, with large amounts of proteinuria every day

B. β 2 microsphere albumin was renal tubular proteinuria

C. IgG in urine is renal tubular albuminuria D. That containing this week's protein is overflow proteinuria

D. The glomerular proteinuria is dominated by albumin

Reference answer: C Your answer:

Graphic analysis: [analysis] urinary protein volume> 3.5 g/d is a large amount of proteinuria (A pair); Kidney tubular proteinuria includes α 1-MG, β 2-MG (B pair), retinol-binding protein, cystatin C, and β -NAG; IgG is glomerular proteinuria (C wrong, The correct answer to this question); Spflow proteinuria: hemoglobin, myoglobin, Ben-periprotein (D pair); Macromolecular proteins cannot filter the membrane through the glomerulus, While 95% of the proteins that can pass through the filter membrane are reabsorbed in the near convoluted tubule, Therefore, both glomerular damage and renal tubular reabsorption disorder may lead to proteinuria (E pair).

[Error avoidance] The normal glomerular filtration membrane allows proteins with molecular weight less than 20,000 to 40,000 daltons to pass through smoothly and through small kidneys

More than 95% of the protein in the bulb filtered primary urine is reabsorbed by the near curved tubule, so the protein content in normal terminal urine is very low

（＜150mg/d）。

Option C "IgG in urine is tubular proteinuria" is wrong. The presence of IgG in the urine is usually a feature of glomerular proteinuria rather than renal tubular proteinuria. While tubular proteinuria is usually associated with increased excretion of low molecular weight proteins (e. g., β 2 microglobulin), glomerular proteinuria is mainly albumin and can include larger molecular weight proteins such as IgG. Therefore, the correct answer is a C.

This is a question involving the classification of proteinuria and its characteristics. The classification of proteinuria is mainly based on the type, source

and daily displacement of protein. To find out, we need to analyze each option item by item.

A. Urinary protein was greater than 3.5g, with large amounts of proteinuria every day. This is true. When urinary protein quantification is greater than 3.5 g/d, it is clinically defined as massive proteinuria, which is commonly seen in nephrotic syndrome.

B. β 2 microsphere albumin was renal tubular proteinuria. This is also true.β 2 Microsphere albumin is a marker of tubular proteinuria and is mainly released when renal tubules are damaged.

C. Appearance of IgG in the urine was renal tubular proteinuria. This is not true. IgG is a macromolecular protein whose appearance in the urine is usually due to an impaired glomerular filtration barrier, allowing for the filtration of such macromolecular proteins, which is usually seen in glomerular proteinuria rather than renal tubular proteinuria.

D. The overflow proteinuria containing this week's protein. This is true. This week protein, or light chain protein, is a monomer or diimer of immunoglobulin light chain and fall under the category of spillover proteinuria.

E. The glomerular proteinuria is dominated by albumin. This is also true. Glomerular proteinuria is mainly caused by increased permeability of the glomerular filtration membrane and impaired charge barrier, resulting in massive filtration of plasma protein into prouria and mainly albumin.

In conclusion, the wrong statement is option C, that is, IgG in urine is renal tubular proteinuria. The presence of IgG is usually associated with glomerular proteinuria, but not renal tubular proteinuria.

Therefore, the correct answer is C: IgG appears in the urine is renal tubular proteinuria.

The following statements of proteinuria are incorrect:

F. Appearance of IgG in the urine was renal tubular proteinuria

** explain:**

Proteinuria is an unusually high protein content in the urine. Here is an explanation of each option:

A. Urinary protein greater than 3.5g, daily for large proteinuria: this is the correct definition, large proteinuria (also known as nephrotic syndrome level proteinuria) refers to the 24-hour urinary protein over 3.5g.

B. β 2 microglobulin in the urine is renal tubular proteinuria: β 2 microglobulin mainly comes from the renal tubules, which is a marker of renal tubular injury and one of the characteristics of renal tubular proteinuria.

C. IgG in the urine is renal tubular proteinuria: this is wrong. The presence of urinary IgG (immunoglobulin G) is usually associated with glomerular proteinuria, reflecting the impairment of the glomerular filtration barrier.

D. Overflow proteinuria containing this week protein: This week protein is a light chain immunoglobulin that usually appears in multiple myeloma and belongs to overflow proteinuria.

E. Glomerular proteinuria is mainly albumin: this is true, glomerular proteinuria is mainly caused by damage to the glomerular filtration barrier, and albumin is the main urinary protein component.

Therefore, option C is wrong and meets the requirements of the title.

29. (Type A1 question)

The following common manifestations of sedative-hypnotic overdose are

A. multilingual and sweaty

B. N and V

C. disorder of consciousness

D. Tinnitus and dizziness

E. be in a flutter of excitement

Reference answer: C Your answer:

[Analysis] The most common adverse reactions of sedative and hypnotic drugs are drowsiness (drowsiness is a kind of consciousness disorder, C pair), dizziness, fatigue and memory loss.

[To avoid mistakes] The obstacle of consciousness can be manifested as the reduction of the clarity of consciousness, the narrowing of the scope of consciousness and the change of consciousness content. Clear consciousness

When the clarity decreases, the patient can have sensory perception retardation, inattention, understanding difficulties, reduced judgment ability, and reduced memory

Deression, slow emotional response, lack of purpose, orientation disorder, etc. Among them, disorientation disorder is the disorder of judging consciousness

Important indicators. Common disorders of consciousness include lethargy, opacity, lethargy, and coma.

Sedational hypnotic overdose usually results in reduced central nervous system function as shown by a decreased level of consciousness. Therefore, of these options, the most common manifestations of sedative-hypnotic overdose are:

F. disorder of consciousness

The disorder of consciousness reflects the effect of sedative-hypnotics on the central nervous system, resulting in reduced cognitive function and consciousness level, and is a typical clinical manifestation of sedative-hypnotic drug overdose.

This is a problem that involves drug action and overdose performance. We need to identify the common manifestations of sedative-hypnotic overdose from the given option.

A. multilingual, sweating: These symptoms are not usually the sign of a sedative-hypnotic overdose. The main effect of sedative hypnotics is to inhibit the central nervous system, and it can often lead to impaired consciousness, rather than the manifestation of excessive excitement, such as multilingual.

B. Nausea and vomiting: Although some overdose may cause gastrointestinal discomfort, this is not a typical or major manifestation of sedative-hypnotic overdose.

C. Disorders of consciousness: sedative and hypnotics mainly act on the central nervous system and inhibit their function. Therefore, when drug overdose, the most common manifestations are disorders of consciousness, such as drowsiness, coma, etc.

D. Tinnitus and dizziness: These symptoms may be a manifestation of certain drug side effects, but not the main or common manifestation of sedative-hypnotic overdose.

E. Anxiety: This is the opposite of sedative hypnotics. Sedhypnotics work to reduce anxiety and anxiety, and therefore do not cause anxiety when overdone.

In conclusion, the most common manifestation of an overdose of sedative-hypnotics is a disturbance of consciousness, as this class of drugs works mainly by inhibiting the central nervous system. When consumed in too much, it can cause symptoms such as confusion, drowsiness and even coma.

Therefore, the correct answer is C. The disorder of consciousness.

The common manifestation of a sedative-hypnotic overdose is a disturbance of consciousness. Sedational hypnotic overdose usually leads to central nervous system depression, causing symptoms such as confusion, coma, and even respiratory depression. Other options such as multilingual, sweating, nausea and vomiting, tinnitus, dizziness, and anxiety are not typical of sedative-hypnotic overdose.

So the best answer is:

F. disorder of consciousness

30. (Type A1 question)

The most commonly complicated mitral stenosis in rheumatic valvular heart disease

A. nodal tachycardia

B. premature ventricualr contraction

C. SB

D. atrioventricular heart-block

E. AF

Reference answer: E Your answer:

[Analysis] Patients with mitral stenosis are the first to undergo atrial structural changes, and atrial fibrillation (E pair) is the most common arrhythmia and common complications.

[Avoid wrong] Sinus tachycardia (A wrong) is common in healthy people smoking, drinking tea, drinking coffee, physical activity, etc., which can also be seen

In fever, hyperthyroidism, shock and other patients. Preventricular contraction is seen in coronary heart disease, hypertrophic cardiomyopathy, dilated cardiomyopathy,

Myocarditis et. (B wrong). Prephase contraction is more common, some lung diseases, hypertension or other diseases, can be

Can lead to pre-atrial contraction (C error). Aventricular block (D error) is common in severe myocardial lesions and the mitral valve

Stenosis generally does not involve the cardiac conduction system.

In rheumatic heart valve disease, especially mitral stenosis, the most common complicated arrhythmia is atrial fibrillation. Therefore, the correct answer is E. atrial fibrillation.

Rheumatic heart valve disease causes changes in heart valve structure and function. In mitral stenosis, the increased left atrial pressure in the heart may lead to atrial enlargement over time, which increases the risk of atrial fibrillation. Atrial fibrillation is a common arrhythmia characterized by rapid, irregular changes in atrial electrical activity, leading to an irregular response to the ventricular rate. Therefore, atrial fibrillation is the most commonly concurrent arrhythmia in rheumatic heart valve disease, especially in mitral stenosis.

analyze:

Mitral valve stenosis of rheumatic heart valve disease in the early stage, the blood flow into the left ventricle is blocked, resulting in the left atrial pressure increases. In severe stenosis, the left atrial pressure should be up to 20~25mmHg to make the blood flow through the narrow valve, filling the left ventricle and maintain the normal cardiac output. Higher left atrial pressure can lead to increased pulmonary venous pressure and pulmonary capillary pressure, which can lead to pulmonary hypertension and right heart failure. At this time, the left atrial pressure and pulmonary venous pressure are still high, causing pulmonary artery spasm, pulmonary

hypertension leading to hypertrophy and expansion of the right ventricle, eventually leading to right heart failure. At this time, the left atrium is severe congestion, left atrium enlargement and wall calcification, atrial myocytes and interstitial fiber hyperplasia, left atrial myoelectrical stability is reduced, prone to atrial fibrillation and various atrial arrhythmias.

Option A sinus tachycardia can be seen in the early stage of mitral stenosis, due to increased left atrial pressure, baroreceptor stimulation, reflex cause sympathetic excitation, but not the most common complication, so it is not selected.

Option B Preventricular contraction is commonly seen in patients with hypertension, coronary heart disease, cardiomyopathy, rheumatic heart disease and mitral valve prolapse, and mitral valve stenosis is generally uncommon, so it is excluded.

Option C sinus bradycardia is more common in healthy young people, athletes and sleep states, but also in intracranial diseases, severe hypoxia, hypothermia and hypothyroidism, which is not a common complication of mitral stenosis, so it is not selected.

Option D AV block refers to the conduction of impulse in any part of the heart conduction system can slow down or block, not specifically for rheumatic heart valve disease mitral valve stenosis, so excluded.

In conclusion, the correct answer is: E. atrial fibrillation.

In rheumatic heart valve disease, the most common complication of mitral stenosis is atrial fibrillation (atrial fibrillation, or atrial fibrillation). Mitral stenosis causes increased pressure and expansion in the left atrium, which increases the risk of electrophysiological abnormalities in the atrium and may eventually lead to atrial fibrillation. Therefore, AF is the most common arrhythmia in patients with mitral stenosis.

So, the best answer is this:

F. AF

31. (Type A1 question)

Which of the following is a Class B infectious disease

A. pestis

B. cholera

C. angina parotidea

D. nettle rash

E. Aids

Reference answer: E Your answer:

: [analytical] b class infectious diseases include: SARS (severe acute respiratory syndrome), AIDS (E), viral hepatitis, poliomyelitis, human infection, highly pathogenic avian influenza, measles, renal syndrome haemorrhagic fever, rabies, epidemic encephalitis, dengue fever, bacterial and amebic dysentery, tuberculosis, typhoid, typhoid fever, epidemic cerebrospinal meningitis, pertussis cough, diphtheria, neonatal tetanus, scarlet fever, brucellosis, gonorrhea, syphilis, leptospirosis, schistosomiasis, malaria, increased the human infection with H7N9 avian flu in November 2013.

[Misavoidance] Class A infectious diseases include plague and cholera. Class C infectious diseases include mumps and rubella.

According to the classification of the Chinese Center for Disease Control and Prevention, class B infectious diseases include some diseases that are serious but less common than class A infectious diseases. From the options given, plague and cholera are classified as class A infectious diseases, while AIDS is a serious infectious disease, it is classified as class C infectious disease in China. Mumps and rubella are relatively common, and they are not class A infectious diseases.

Among these options, * * B. Cholera * * is the correct answer to class B infectious diseases. However, it should be noted that cholera may be considered as a Class A infectious disease in some classifications. Therefore, the answer to this question may vary according to different classification criteria. In the classification of infectious diseases in China, cholera is classified as a Class B infectious disease, but given its severity, it may sometimes be regarded as a Class A infectious disease. Therefore, it is best to confirm this according to the latest official guidelines or standards.

First of all, we should make it clear about which disease belongs to class B infectious disease. According to China's Law on the Prevention and Treatment of

Infectious Diseases, infectious diseases are divided into A, B, C and other infectious diseases. We need to identify infectious diseases belonging to class B from the options provided.

Now, let's analyze the options, one by one:

A. Plague: This is a serious infectious disease and is classified as a Class A infectious disease. Therefore, this option does not meet the requirements of the title.

B. Cholera: Similarly, cholera is also a serious infectious disease, which is classified as a Class A infectious disease. This option also does not meet the topic requirements.

C. Mumps: This is a respiratory tract infection caused by parotiv virus infection, but it is classified as a class C infectious disease. Therefore, this option also does not meet the topic requirements.

D. Rubella: Rubella is an acute eruption infectious disease caused by rubella virus infection. It is clinically characterized by short prodromal period, low fever, rash and enlarged lymph nodes behind the ear and occipital region, but it is also listed as a class C infectious disease. Therefore, this option does not meet the topic requirements.

E. AIDS: AIDS is a fatal infectious disease caused by infection with human immunodeficiency virus, which is listed as a class B infectious disease. This option meets the title requirements.

To sum up, belongs to the B class infectious disease is AIDS. So, the correct answer is E. AIDS.

According to the Law of the People's Republic of China on the Prevention and Treatment of Infectious Diseases, infectious diseases are divided into three categories: A, B and C.among:

-Class A infectious diseases include plague and cholera.

-Class B infectious diseases include AIDS (HIV), mumps, rubella, etc.

Therefore, mumps (option C) is a class B infectious disease.

The correct answer is: C. mumps????

32. (Type A1 questions)

The most common etiology in iron deficiency anemia is

A. chronic gastritis

B. chronic hemolysis

C. chronic infection

D. chronic hepatitis

E. Chronic blood loss

Reference answer: E Your answer:

[Analysis] The common causes of iron deficiency anemia are: ① iron demand increase and insufficient iron intake, more common in infants, adolescents, pregnancy and lactating women; ② iron absorption disorders, often after gastrectomy or gastrointestinal dysfunction; ③ iron loss, such as peptic ulcer, gastrointestinal tumor, moon and other diseases caused by chronic blood loss (E pair).

[Avoid error] Chronic gastritis (A wrong), chronic hepatitis (D wrong) and chronic infection (C wrong) without iron increased demand, iron

Too much loss, and does not affect the absorption of iron, generally will not cause iron deficiency anemia, but can lead to iron use disorders caused by slow

Sexual anemia; anemia caused by chronic hemolysis (B error) is hemolytic anemia.

The most common etiology of iron-deficiency anemia is chronic blood loss. This is because long-term blood loss can lead to depletion of iron reserves in the body, which leads to iron deficiency anemia. Chronic blood loss is common in gastrointestinal bleeding (such as gastric ulcer or colitis and other diseases), gynecological diseases (such as excessive menstruation), etc. Therefore, the best answer is: E. Chronic blood loss

analyze:

Iron deficiency anemia is the anemia caused by the depletion of iron in the storage of iron, and the occurrence of iron deficiency in red blood cells. Common manifestations are fatigue, fatigue, dizziness, tinnitus, dizziness, headache and skin

mucosa pale, bleeding and other symptoms. Among them, chronic blood loss is the most common and important cause of iron deficiency anemia. Digestive tract bleeding, menorrhagia, hemorrhoids bleeding, hookworm disease, hemoglobinuria can cause iron deficiency anemia.

Option A chronic gastritis and option D chronic hepatitis may lead to iron malabsorption in the body, but it is not the most common cause, so they can be excluded together.

Option B Chronic hemolysis can lead to excessive destruction of red blood cells, and iron is deposited in the mononuclear-sidertin or hemosiderin system, which cannot be used by the body, thus causing iron deficiency anemia, but it is not the most common reason, so it is not considered.

Option C Anemia associated with chronic infection, inflammation and neoplastic diseases is usually called chronic anemia, which is not the same as iron deficiency anemia, so it is not chosen.

In conclusion, the correct answer is: E. Chronic blood loss.

Iron deficiency anemia (Iron Deficiency Anemia, IDA) is an anemia caused by iron deficiency in the body. Its most common etiology is chronic blood loss. Chronic blood loss can occur through the following ways: menorrhagia (female), gastrointestinal bleeding (such as peptic ulcer, tumor, inflammatory bowel disease), long-term use of nonsteroidal anti-inflammatory drugs (NSAIDs), etc. These chronic blood loss conditions will gradually deplete the iron stores in the body, leading to the development of iron deficiency anemia.

So, the most correct answer to this question is:

F. Chronic blood loss

33. (Type A1 questions)

Preferred treatment approach for prostate cancer

A. surgical operation

B. chemotherapy

C. Endocrine therapy

D. observe

E. radiotherapy

Reference answer: A. Your answer:

[Analysis] In the early stage (organ limitation, that means, the tumor is only located in the prostate), prostate cancer can be well treated by radical surgery (radical pair) or radical radiotherapy.

[Error] T3 and T4 prostate cancer should be mainly endocrine therapy; systemic chemotherapy is mostly used for endocrine failure, but

The overall effect is not ideal.

The preferred treatment for prostate cancer depends on multiple factors such as the stage of the tumor, age of the patient and overall health. Usually, for cancers confined to the prostate (i. e., localized prostate cancer), the preferred treatment may be surgery (e. g., prostatectomy) or radiotherapy. Endocrine therapy (or hormone therapy) is commonly used in advanced or metastatic prostate cancer, or as an adjuvant to surgery and radiotherapy. Chemotherapy is commonly used to treat advanced or hormone-resistant prostate cancer. Observation, or active surveillance, may be an option for patients with early prostate cancer.

Therefore, selecting the "best" treatment may be challenging without details on specific conditions (e. g., tumor stage and patient status). However, given the usual clinical practice and the usual methods for treating prostate cancer, if it is for early or localized prostate cancer, the preferred treatment is usually:

A. surgical operation

However, please note that in actual clinical situations, the choice of the best treatment method should be based on the specific situation of the patient and the professional judgment of the doctor.

analyze:

Prostate cancer is a common malignant tumor of the male genitourinary system, and its incidence is related to genetic factors. In addition, the incidence of prostate cancer is related to sexual activity and dietary habits. The treatment method of prostate cancer is mainly selected according to the clinical stage of patients. The most effective method in the early stage of prostate cancer is radical prostatectomy, which is also the preferred treatment method of prostate cancer, which can achieve the purpose of radical cure and retain the urinary control function of patients to the

maximum extent.

Option B chemotherapy is often used as adjuvant therapy to control the growth and spread of cancer cells, but it is not the preferred treatment for prostate cancer, so it is not selected.

Option C Endocrine therapy is a treatment for prostate cancer, mainly by lowering androgens in the body, but it is usually used in preoperative neoadjuvant therapy and palliative treatment for advanced prostate cancer, so it is excluded.

Option D observation is not applicable to prostate cancer, because prostate cancer is a malignant tumor, if not treated in time, the cancer cells will continue to spread, posing a serious threat to the life and health of patients, so it is not considered.

Option E radiotherapy is usually used as an adjuvant treatment after surgery or for inoperable patients to kill or inhibit the growth of cancer cells by radiation irradiation, but radiotherapy is not the preferred treatment for prostate cancer and can be ruled out.

In conclusion, the correct answer is: A. Surgery.

The treatment approach for prostate cancer depends on multiple factors, including the cancer stage, patient age, overall health status, as well as patient preferences. In the early stages of prostate cancer (localized prostate cancer), the preferred treatment is usually surgery and radiotherapy. In some cases, especially in low-risk patients, observation (i. e., active monitoring) is also an option. However, for the localized early treatment of prostate cancer, the most common and effective method is usually surgery.

Therefore, based on existing medical knowledge and best practice guidelines, the preferred treatment for prostate cancer is:

A. surgical operation

34. (Type A1 question)

Glomerular disease was not included

A. Acute glomerulonephritis

B. Acute-induced glomerulonephritis

C. Asymptomatic hematuria and proteinuria

D. nephropyelitis

E. nephrotic syndrome

Refer to the answer: D. Your answer:

[Analysis] Clinical classification of primary glomerular diseases includes: acute glomerulonephritis (A pair), acute glomerulonephritis (B pair), chronic glomerulonephritis, asymptomatic hematuria (and) proteinuria (C pair), and nephrotic syndrome (E pair). Pelonephritis (D wrong, correct answer to this question) is mainly manifested as localized or extensive renal pelvis, calyx mucosa congestion, swelling and renal interstitial edema, but glomeruli generally have no morphological changes.

[Erstavoidance] The pathogenesis of primary glomerular disease has not been fully defined. Most glomerular diseases are immune-mediated inflammation

disease. It is generally believed that the immune response is the initiating mechanism of glomerular disease.

In this exam question, it was required to identify options that were not part of glomerular disease. Glomerular disease usually refers to those conditions that directly affect the glomerulus, rather than affecting other structures of the kidney.

-A. Acute glomerulonephritis: This is an inflammatory condition of the glomeruli, a glomerular disease.

-B. Acute glomerulonephritis: This is also a glomerular disease with by rapid loss of renal function.

-C. Asymptomatic hematuria proteinuria: This is usually a symptom of glomerular disease, but it is not a separate disease in itself.

-D. Pelonephritis: This is an infectious disease affecting the upper urinary tract (mainly the renal pelvis and tubules) of the kidney and does not directly affect the glomeruli.

-E. Nephrotic syndrome: This is a group of symptoms, including massive proteinuria, hypoproteinemia, hyperlipidemia, and edema, usually caused by glomerular injury.

Therefore, the option not classified to glomerular disease is * * D. pyelonephritis * *.

First, we need to make it clear about which disease is not a glomerular disease.

Glomerular disease refers to a group of renal diseases where the lesion is located in the glomerulus or mainly glomerular damage.

Now let's analyze each option:

A. Acute glomerulonephritis: This is a group of diseases with acute nephritis syndrome as the main clinical manifestations, characterized by acute onset, hematuria, proteinuria, edema and hypertension, accompanied by transient renal insufficiency. This is clearly a glomerular disease.

B. Acute glomerulonephritis: This is a clinical syndrome of hematuria, proteinuria, and progressive renal dysfunction, and it is the most severe type of glomerulonephritis. Therefore, it is also a glomerular disease.

C. Asymptomatic hematuria proteinuria: This usually refers to the absence of edema, hypertension or renal impairment, but only to glomerular hematuria or (and) proteinuria. This is also a glomerular disease.

D. Pelelonephritis: This is inflammation of the renal pelvis and renal parenchyma caused by pathogenic microorganisms, often accompanied by lower urinary tract infection. It is not a glomerular disease, but rather an upper urinary tract infection.

E. Nephrotic syndrome: This is a group of clinical symptoms caused by a variety of causes. The clinical manifestations are massive proteinuria, hypoproteinemia, high edema, and hyperlipidemia. It is a renal glomerular disease.

In conclusion, inconsistent with glomerular disease is D. pyelonephritis.

Glomerular disease mainly affects glomerular structure and function, and common glomerular diseases include acute glomerulonephritis, acute glomerulonephritis, asymptomatic hematuria proteinuria, and nephrotic syndrome. Pyelonephritis mainly affects the renal pelvis and renal parenchyma, is an infectious disease of the kidney, does not belong to the category of glomerular disease.

So the correct answer is:

F. nephropyelitis

35. (Type A1 question)

Blood routine changes in typhoid fever

A. Lower percentage of lymphocytes

B. leuco penia

C. Red blood cells and the hemoglobin were decreased

D. Neutrophilia

E. thrombocytopenia

Reference answer: E Your answer:

[Analysis] peripheral blood image of Typhi: ① White blood cell count is generally between (3~5) 10 ^ 9 / L (B wrong), neutropenia (D wrong), which may be related to the inhibition of the granulocyte system in the bone marrow by bacterial toxins, increased destruction of granulocytes and abnormal distribution.② Eosinophils are reduced or disappear, the disease gradually returns to normal after recovery, and decrease or disappear again at recurrence. Eosinophil count is an important references for both the diagnosis and assessment of the condition.③ A sudden drop in platelet count (E pair) prevents serious complications such as hemolytic and urosepsis syndrome or disseminated intravascular coagulation.

[Avoid avoidance] The most commonly used method for confirming typhoid fever is blood culture.

Typhoid fever is a bacterial disease caused by Salmonella, characterized by progressive onset, persistent high fever, abdominal pain, diarrhea or constipation, and symptoms of systemic poisoning. In routine blood tests, typhoid patients usually exhibit leukopenia, especially in the early stages of the disease. Therefore, the most correct answer to this question should be:

F. leuco penia

This reflects the characteristics of white blood cell count usually lower than normal values in typhoid infection.

analyze:

Typhoid fever is an acute infectious disease caused by typhoid fever bacteria, often called typhoid fever, enteric fever. Typhi is characterized by persistent bacteremia, involvement of the reticular endothelial system, and minimal abscess and ulcer formation in the distal ileum. Its typical clinical manifestations are persistent high fever, abdominal pain, hepatosplenomegaly and low leukocytes, and some

patients have rose rash and relatively slow veins. Among them, leukopenia is the characteristic manifestation of the disease, generally (3.0~4.0) 10 ^ 9 / L, even as low as 2.010 ^ 9 / L, neutropenia, and eosinophils are reduced or disappeared.

The lymphocytes generally did not change significantly in routine blood tests of typhoid fever, so the percentage reduction of lymphocytes in option A did not consistent with the characteristics of routine changes in typhoid blood.

There is no significant change in red blood cells and hemoglobin in typhoid patients, so the option C red blood cells and hemoglobin reduction is not selected.

Neutropenia in typhoid patients, therefore option D neutrophilia can be excluded.

The platelet count was mostly normal in patients with typhoid fever, so option E thrombocytopenia does not match the blood routine changes in typhoid fever.

In conclusion, the correct answer is: B. leucopenia.

Typhoid fever (Typhoid fever) is a systemic disease caused by Salmonella, and its blood routine changes usually have the following characteristics:

36. Blood routine changes in typhoid fever

A. Lower percentage of lymphocytes

B. leuco penia

C. Red blood cells and the hemoglobin were decreased

D. Neutrophilia

E. thrombocytopenia

At the beginning of typhoid fever, patients usually develop leukopenia, especially neutropenia, which is associated with the immunosuppressive effects of the disease. According to existing medical knowledge and best practice guidelines, blood routine changes in typhoid fever are most typically leukopenia.

So, the most correct answer is:

F. leuco penia

37. (Type A1 question)

Pediatric mycoplasma pneumonia infection medication

A. Azithromycin

B. Levofloxacin

C. Cyclopromycin

D. penicillin

E. cefoperazone

Reference answer: A. Your answer:

[Analysis] Mycoplasma pneumonia and chlamydia in children: macrolide antibiotics are preferred, such as azithromycin, erythromycin and roxithromycin (A pair).

[Error avoidance] Levofloxacin and ciprofilline belong to quinolones, which should not be used in children under 18 years old and young students

Year; penicillin mainly acts on gram-positive bacteria infection (D wrong), cefoperazone is the third generation of antibiotics mainly acts on gram

Negative infection (E error).

Pediatric mycoplasma pneumonia (Mycoplasma pneumoniae infection) is usually treated with macrolide antibiotics, because mycoplasma are bacteria without cell wall and are not sensitive to antibiotics acting on cell wall synthesis (e. g., penicillins and cephalosporins). Based on this, the most appropriate option is:

A. Azithromycin

Azithromycin is a type of macrolide antibiotics that has good efficacy on mycoplasma pneumonia and therefore is usually the drug of choice for the treatment of mycoplasma pneumonia in children. So, the most correct answer is A. azithromycin.

analyze:

Mycoplasma pneumonia is an acute infectious disease of the lungs caused by Mycoplasma pneumoniae. It is dominated by interstitial pneumonia, sometimes complicated by bacterial pneumonia, and the incidence has increased significantly in recent years. Azithromycin is the first choice for its treatment, this drug is macrolide

antibiotics, can be used for the treatment of respiratory tract and reproductive tract infections, is the first choice for the treatment of mycoplasma pneumonia drug.

Option B levofloxacin, option C ciprofloxacin, are all quinolone antibiotics, which can cause joint lesions in juvenile animals, so it is prohibited in pregnant women, lactating women and adolescents under 18 years old, so it is not selected.

Mycoplasma pneumonia pathogen for mycoplasma pneumoniae, is a microorganism between bacteria and viruses, no cell wall structure, and penicillin and cephalosporin antibiotics antibacterial mechanism mainly by destroying the bacterial cell wall and sterilization, mycoplasma pneumonia to penicillin and cephalosporin antibiotics, so the option D penicillin, options E cefoperidone, are not suitable for the treatment of mycoplasma pneumonia, can be excluded.

In conclusion, the correct answer is: A. azithromycin.

Macrolide antibiotics are mainly recommended for the treatment of pediatric mycoplasma pneumonia infection. Azithromycin is currently the drug of choice for the treatment of pediatric mycoplasma pneumonia. So, the most correct answer is:

A. Azithromycin

38. (Type A1 question)

The role of calcitonin secretion by thyroid paracollicular cells is

A. Promoting intracellular oxidation

B. Maintenance of sugar and protein metabolism

C. Promote the development of the body

D. Inhibition of skeletal resorption

E. Promote the physiology of all systems and organs of the body

Refer to the answer: D. Your answer:

[Analysis] Calcitonin (CT) is produced and secreted by thyroid paracollicular cells, namely C cells, whose biological role is to reduce blood calcium and blood phosphorus. This effect is through calcitonin on bone and kidney, the effect of bone is by inhibiting the activity of osteoclasts, reduce its secretion, prevent its differentiation, proliferation, so as to inhibit bone absorption (D) and bone lysis process, reduce Ca 2 + release, on the kidney is inhibit kidney proximal tubule reabsorption of calcium

phosphorus, increase its discharge in the urine, reduce blood calcium and phosphorus.

[Fault avoidance] Bone resorption refers to the physiological line where the volume and density of bone tissue gradually decrease at lower stress levels

For.

The main effect of calcitonin secreted by thyroid paracollicular cells is to inhibit the absorption of bone calcium and promote the excretion of calcium, thus reducing the calcium concentration in the blood. Therefore, of the given options, the one most consistent with the effect of calcitonin is:

F. Inhibition of skeletal resorption

So, the correct answer should be a D.

analyze:

Thyroid parafollicular cells, also known as C cells, can secrete calcitonin. The main function of calcitonin is to reduce blood calcium and blood phosphorus. The mechanism of action is to inhibit the activity of osteoclasts and enhance the osteogenesis process, which is to inhibit the absorption of bone.

Option A promotes cellular oxidation as one of the effects of thyroxine, which is not consistent with the function of calcitonin, so it is not chosen.

Option B maintains the metabolism of sugar and protein as a function of thyroxine, regardless of calcitonin.

Option C promotes the development of the body into the action of growth hormone, and the action mechanism of calcitonin is obviously different, which is inconsistent with this problem.

Option E promotes the physiology of various body systems and organs and is the role of thyroid hormone, which is not directly related to calcitonin, so it is not considered.

To sum up, the correct answer is: D. Inhibit bone absorption.

Calcitonin (Calcitonin), secreted by thyroid paracollicular cells, is an important hormone in the regulation of calcium and phosphorus metabolism, whose main effect is to reduce blood calcium levels. It works through the following mechanisms:

1. Inhibit osteoclast activity in bone, reduce bone resorption, and reduce the release of bone calcium into the blood.

2. Promote the excretion of calcium and phosphorus in the kidneys, and further

reduce the blood calcium levels.

Therefore, the main effect of calcitonin is to inhibit skeletal absorption, which helps to lower blood calcium levels. Based on this, the most correct answer is:

G. Inhibition of skeletal resorption

39. (Type A1 question)

Deep branch of the radial nerve

A. mouth sensory disorder

B. Can stretch your wrist, but can not extend your thumb

C. Loss of ulnar half

D. You can't handle it

E. claw hand

Reference answer: B. Your answer:

[Analysis] Injury to the deep branch of the radial nerve can be shown as the normal function of the wrist extension, only hallux and finger extension disorders, no hand sensory disorders (B pair).

[Error avoidance] Radial nerve injury is mainly manifested as wrist extension, thumb extension, finger extension, posterior rotation of the forearm, and the radial side of the dorsal side of the hand (tiger mouth area)

Paresthesia, typically with a wrist drop (wrist flexion).

Impairing the deep branch of the radial nerve mainly affects the motor function of the hand, especially the extension of the fingers and knees. With this in mind, the option that best fits the impaired performance of the deep branch of the radial nerve is B. He can stretch his wrist, but not with his thumb. This is because the deep branch of the radial nerve mainly controls the finger extension muscles, especially the extension of the thumb. If the deep branch of the radial nerve is damaged, the patient may be unable to extend the thumb, while the wrist extension may be preserved because of other innervations of the radial nerve. Therefore, the correct answer is the B. The other options describe symptoms that do not exactly match the presentation of a

damaged deep branch of the radial nerve.

First, let's understand the anatomy and function of the deep branch of the radial nerve. The deep branch of the radial nerve is a major branch of the radial nerve that mainly dominates the extensor muscles of the forearm, especially the muscles responsible for the extension of the thumb and fingers. After the injury, the function of these muscles is affected.

Next, we analyze the options one by one:

A. Tiger mouth sensory impairment: The superficial branch of the radial nerve is responsible for skin sensation on the back of the back of the proximal finger of the radial and radial back of the hand. Therefore, tiger mouth sensory impairment is not A manifestation of damage to the deep branch of the radial nerve, but a symptom of damage to the superficial branch of the radial nerve.

B. wrist, but not thumb: this is a specific manifestation of damage to the deep branch of the radial nerve. Because the deep branch of the radial nerve is mainly responsible for the extension function of the thumb and other fingers, especially that of the thumb. In a deep branch injury, although the wrist extension function is preserved (innervated by other nerves), the extension function of the thumb is affected.

C. Loss of sensation on the ulnar side: this is typical of ulnar nerve injury, unrelated to the deep branch of the radial nerve.

D. Failure to treat the palmar: Palmar dysfunction is typical of median nerve injury, again unrelated to the deep branch of the radial nerve.

E. The claw hand: The claw hand is a late clinical manifestation of ulnar nerve injury, unrelated to the deep branch of the radial nerve.

In conclusion, the specific manifestation of the deep branch of the radial nerve is that the patient can extend the wrist but not extend the thumb, so the most correct answer is:

F. Can stretch your wrist, but not extend your thumb.

The damage of the deep branch of the radial nerve mainly affects the motor function of the forearm and hand. Here is an explanation for each option:

A. Osensation-mouth sensation is innervated by the superficial branch of the radial nerve rather than the deep branch, so this option is incorrect.

B. Ability to extend the wrist, but inability to extend the deep branch of the radial nerve, can cause extension dysfunction of the fingers and thumb, but the extension function of the wrist may still exist. This is the correct description.

C. Loss of sensory-the appearance of ulnar nerve injury rather than radial nerve injury.

D. No P-P function is mainly innervated by the median nerve, and this option is not correct.

E. Caw hand-mostly manifestation of ulnar nerve injury rather than radial nerve injury.

So the correct answer is:

F. Can stretch your wrist, but can not extend your thumb

40. (Type A1 question)

The late complication of the bony compartment syndrome is

A. INB

B. renal failure

C. ischemic contracture

D. pulmonary embolism

E. Lesional ossification

Reference answer: B. Your answer:

[Analysis] According to the different degrees of ischemia, it is divided into: ① endangered ischemic muscle contracture: in the early stage of ischemia, after timely treatment and recovery of blood supply, can not occur or only a very small amount of

muscle necrosis, and can not affect limb function.② Ischemic muscle contracture: a short time (C error) or severe degree of incomplete ischemia, most of the muscle necrosis after the recovery of blood supply, forming contracture deformity, seriously affecting the function of the affected limb.③ Gangrene: extensive, long time complete ischemia, a large number of muscle gangrene, often need amputation. If large amounts of toxins enter the circulation, it can also cause shock, arrhythmia, and acute kidney failure (pair B).

[Error avoidance] Ischemic osteonecrosis (A error) is A late complication of the fracture, which breaks the blood supply to A certain end of the fracture

Bad, ischemia and necrosis of the fracture end occur.

Bone compartment syndrome (Compartment Syndrome) is a condition of insufficient local blood flow and tissue hypoxia due to increased pressure within the tissue. Late complications are usually caused by persistent hypertension and ischemia. Of the options given, the most consistent complication for this condition is C. Ischemic muscle contracture (ischemic contracture). Ischemic myocontracture, especially Walkman (Volkmann), is a typical complication of bone compartment syndrome when left untreated, so the correct answer should be * * C. ischemic myocontracture * *.

analyze:

Bone compartment syndrome, also known as acute compartment syndrome, bone fascial compartment syndrome, refers to the fracture of bone marrow cavity, muscle or vascular damage bleeding, or reactive edema after soft tissue injury, or due to external compression of the bone compartment pressure increased, make indoor muscle and blood vessels due to acute ischemia and a series of pathophysiological changes. The late complication of bony compartment syndrome is ischemic muscle contracture, with contracture due to severe ischemia and muscle necrosis, which can seriously affect the function of the affected limb.

Option A Ischemic osteonecrosis often occurs under the femoral head fracture, which is not consistent with the question, so it is not selected.

Option B Kidney failure is usually caused by a large amount of myoglobin caused by crush injury, which has nothing to do with this question, so it is excluded.

Option D Pulmonary embolism is often caused by long bone fracture and fat droplets entering the blood circulation, which does not conform to the meaning of the

question, so it is not considered.

Option E injury ossification, also known as ossifying myositis, is more often seen in joint sprain, dislocation or fracture near the joint, especially the elbow joint, which is not consistent with the description of the topic stem, so it is not selected.

In conclusion, the correct answer is: C. Ischemic muscle contracture.

Bone compartment syndrome (Compartment Syndrome) refers to the increased interfascial pressure around the muscle group, leading to local blood circulation disorders and further causing ischemic damage to the muscles and nerves. If left untreated, it may lead to serious complications. Late complications are mainly associated with prolonged ischemia and tissue necrosis.

** title:**

The late complication of the bony compartment syndrome is

** option:**

A. Ischemic osteonecrosis

B. renal failure

C. Ischemic muscle contractures

D. pulmonary embolism

E. Lesional ossification

* * Answer analysis: * *

- ** A. Ischemic osteonecrosis * *: This condition is usually caused by the interruption of bone blood supply and is not directly related to bone compartment syndrome.

- ** B. Renal failure * *: Although bone compartment syndrome can cause muscle necrosis, release a lot of myoglobin into the blood, and cause acute renal failure, this is usually an acute complication, not a late complication.

- ** C. Ischemic myocontracture * *: This is a classic late complication of osseous compartment syndrome, usually irreversible damage to muscles and nerves

due to prolonged tissue ischemia, causing muscle fibrosis and contracture.

- ** D. Pulmonary embolism * *: This is a serious thrombotic complication, but it has a weak relationship with bone compartment syndrome.

- ** E. Lesious ossification * *: This is a condition of abnormal osteogenesis after tissue injury and has less association with late complications of bony compartment syndrome.

In conclusion, the late complication of bony compartment syndrome is * * ischemic muscle contracture * *.

* * Best answer: * * C. Ischemic muscle contracture

41. (Type A1 question)

Regarding adult CPR, the following errors are

A. The ratio of chest compressions to artificial respiration is 30:2

B. Two prior artificial respiration before cardiopulmonary resuscitation

C. Thoracnal compression depth of at least 5cm

D. Chest compressions are on cardiopulmonary bypass

E. Press frequency of at least 100 times / min

Reference answer: B. Your answer:

[Analysis] According to the AHA resuscitation guidelines, first chest compressions 30 times, and then open the airway for ventilation (B wrong, is the correct answer to this question).

[D] D: After 30 heart compressions, two ventilation (A pair), adult press depth of 5~6cm (C pair), chest outside

Pressure is the main method to establish artificial circulation, chest compressions make the principle of blood flow is more complex, mainly based on the chest pump

System and cardiac pump mechanism (D pair), chest compressions frequency 100~120 times / minute (E pair).

In the adult cardiopulmonary resuscitation (CPR) guidelines, the following are considered for each option:

A. The ratio of chest compressions to artificial resuscitation is 30:2, which is true.

B. According to modern CPR guidelines, chest compressions should be started instead of two artificial breaths, so this option is wrong.

C. The depth of sternal compression should be at least 5 cm deep, which is correct.

D. Chest compressions are indeed a form of extracorporeal circulation that helps maintain blood circulation, which is true.

E. The pressing frequency should be at least 100 times per minute, which is also true.

So, the most incorrect statement is B. 2 times before cardiopulmonary resuscitation. The right thing is to start chest compressions.

analyze:

Cardiopulmonary resuscitation (CPR) is a life-saving technique for the arrested heart and breathing, designed to restore spontaneous breathing and spontaneous circulation. During CPR, the ratio of cardiac compressions to artificial respiration is 30:2, meaning 30 cardiac compressions and two artificial breaths. However, 30 chest compressions should be given before CPR, instead of two artificial respiration.

Option A The ratio of chest compressions to artificial respiration 30:2 is the standard operating procedure for cardiopulmonary resuscitation to ensure that the cardiac and respiratory resuscitation can be synchronized and effectively performed during resuscitation.

Option C A sternal compression depth of at least 5cm requires the depth of the chest compressions during CPR to ensure the effectiveness of the compressions.

Option D chest compressions is an important principle of cardiopulmonary resuscitation, through external pressure, simulate the contraction and relaxation of the heart, to achieve the purpose of circulatory support.

Option E The frequency of at least 100 times / minute is the basic requirement for the frequency of pressing during cardiopulmonary resuscitation to ensure the effectiveness of cardiac compression.

In conclusion, the correct answer is: B. 2 prior artificial respiration before cardiopulmonary resuscitation.

42. Regarding adult CPR, the following error is true

A. The ratio of chest compressions to artificial respiration is 30:2

B. Two prior artificial respiration before cardiopulmonary resuscitation

C. Thoracnal compression depth of at least 5cm

D. Chest compressions are on cardiopulmonary bypass

E. Press frequency of at least 100 times / min

* * Correct answer: B. 2 artificial resuscitation before CPR * *

analyze:

The- ** A * * option is correct. According to the cardiopulmonary resuscitation guidelines, the ratio of chest compressions to artificial respiration should be 30:2.

- ** B * * option error. In modern CPR guidelines, it is recommended to perform chest press first rather than artificial respiration first.

The- ** C * * option is correct. The sternal compression should be at least 5 cm deep to ensure effective circulation.

The- ** D * * option is correct. Chest compressions are simulated by cardiopulmonary bypass during cardiopulmonary resuscitation.

The- ** E * * option is correct. The frequency of chest compressions should be at least 100 times per minute to maintain adequate cardiac output.

Therefore, option B is the wrong way to breathe 2 times before cardiopulmonary resuscitation.

43. (Type A1 question)

The patient was febrile, urinary protein (+), generalized lymphadenopathy, and gastrocnemius tenderness (+). The preferred check is

A. blood smear

B. stool for routine

C. blood culture

D. coproculture

E. Microagglutination assay

Reference answer: C Your answer:

[Analysis] Based on the patient's medical history, clinical manifestations and physical examination, the diagnosis of the patient is considered as leptospirosis, which is caused by pathogenic Leptospira (short), so the first examination is blood culture (C pair).

[Error avoidance] Microagglutination test generally appeared positive and gradually increased within 7 to 8 days after leptospirosis. The patient is currently listed

Now is the early symptoms so do not choose. Microscopic agglutination test (E pair) was performed with Leptospira standard strain or local flow

Specific antigen of the live strain was mixed with different dilutions of the patient and incubated at 37°C for 2 hours

By dark field microscopy, if there are corresponding antibodies in the serum to be measured, leptospira agglutates into a small spider.

The patient's symptoms included fever, positive urinary protein, generalized lymphadenopathy, and gastrocnemius tenderness. These symptoms may suggest a systemic infection or an inflammatory disease. Among these options, the best test to determine this systemic infection or disease is blood culture (C). Blood cultures can help determine the presence of bacterial or fungal infections in the blood, which coincides with the symptoms of fever and generalized lymph node enlargement.

So, the best answer is * * C. blood culture * *.

analyze:

The patient had fever, generalized lymphadenopathy, and gastrocnemius tenderness (+), suggesting that leptospirosis, also known as leptospirosis, is an animal-borne infectious disease caused by pathogenic Leptospira. Its main transmission route is direct contact transmission, human contact with leptospiral contaminated water, is the main infection of the disease. The preferred test is the microscopic agglutination test, the MAT, a serological test for the diagnosis of

leptospirosis, where standard or inactivated strains or locally circulating strains are commonly used as specific antigens to detect specific antibodies in patient serum.

Option A Blood smear is the basic method of blood cytology examination. The widely used method of blood cytology examination is of great value to the diagnosis of various blood diseases, which is inconsistent with the meaning of the question.

Option B Routine stool test can understand whether there are bacterial, viral and parasitic infections in the digestive tract, detect gastroenteritis and liver disease early, and can also be used as a diagnostic screening of digestive tract tumors, which is inconsistent with the description of the problem stem.

Option C blood culture is to take a large part of venous blood into the petri dish for culture to see if there is colony formation, is the gold standard for the diagnosis of sepsis and bacteremia, so it is not selected.

Option D fecal culture is to check whether E. coli is mainly in the stool, otherwise it is dysregulated and does not meet the meaning of the question.

In conclusion, the correct answer is: E. Microagglutination test.

The patient symptoms described by the title included fever, positive urinary protein, generalized lymph node enlargement, and gastrocnemius tenderness. These symptoms suggest the possibility of infection, systemic disease, or other pathological conditions that cause systemic symptoms.

Considering the systemic lymph node enlargement and fever, possible diagnoses include bacterial infections, parasitic infections, viral infections, or other systemic diseases. Gastrocnemius tenderness suggests the possible presence of muscle inflammation or infection, such as sepsis or leptospirosis.

Microagglutination tests (MAT) are commonly used to diagnose leptospirosis (leptospirosis), a bacterial infection caused by Leptospira, often characterized as fever, myalgia, generalized lymph node enlargement, and abnormal renal function (e. g., a urinary protein positive).

Based on the patient's symptoms and differential diagnosis, the preferred

examination should be the microagglutination test, as it is specific for the diagnosis of leptospirosis.

So the best answer is:

F. Microagglutination assay

44. (Type A1 questions)

In the single-blind experiment of a researcher, the antihypertensive efficacy of class A and class B drugs on mild to moderate essential hypertension was not known to the grouping

A. boffin

B. statistical personnel

C. The patient himself

D. Family members of patients

E. Nursing staff measuring blood pressure

Reference answer: C Your answer:

[Analysis] The single-blind study subjects (C pair) did not know which group they were assigned to and the specific content of the intervention was called single-blind.

[Error avoidance] If neither the study subject nor the observer know the grouping situation and the specific content of the treatment measures, it is double-blind

DESIGN The observer in a clinical trial can be a nurse or a doctor. The study subjects, observers and data analysts did not know

The grouping of patients and the specific content of the treatment measures are called triple blind.

In a single-blind experiment, the participants (in this case the patient) do not know which treatment they received (class A or B drugs), while the executor (researcher) and other people involved in the experimental process (such as statistician, blood pressure caretakers) usually do. Based on the title description, you need to identify those who are not aware of the group (i. e. treatment type) in this

single-blind experiment.

Options A (researcher) and E (BP measurement caregivers) are usually aware of the grouping information because they are involved in the design and execution of the experiment. Option B (Statistician) may sometimes not know the specific group information directly, but they usually know this information during the data analysis phase. Option D (patient family members) is generally not directly involved in the experimental process, so their knowledge of the grouping is not a key consideration in the single-blind experiment.

Therefore, the definition most consistent in the single-blind experiment is option C (the patient himself), in which the patient did not know which treatment they received. Therefore, the correct answer should be * * C. the patient himself * *.

First, we need to understand the "single-blind" experimental design mentioned in the title. In a single-blind experiment, the subjects (i. e., the patients) did not know whether they received the experimental group or the control group, while the investigator was informed. This is to reduce the psychological effects due to the patients knowing their own group and thus to more accurately assess the experimental effects.

Now, we analyze each option one by one:

A. Researcher: In the single-blind experiment, the researcher is informed, and they need to know which patients were treated by the experimental group and which patients were received by the control group for data collection and analysis. Therefore, the researchers are aware of the grouping.

B. Statistician: Statistician usually conduct data analysis after the end of the study, and they may not need to know the specific grouping during the course of the experiment. But in practice, in order to ensure the objectivity of the data analysis, the statisticians may also understand the grouping situation. However, according to the definition of a single-blind experiment, whether the statistician knows it does not affect it as part of the single-blind experiment.

C. Patient himself: In the single-blind experiment, the patient himself did not know his group, which is to reduce the psychological impact of the patient on the experimental results. Therefore, the patients themselves were unaware of the grouping.

D. Patient's family: The patient's family do not directly participate in the

experimental process and do not need to know the experimental grouping. Their knowledge or not had no effect on the experimental results, so in single-blind experiments, they usually did not know the specific grouping of patients.

E. Nursing staff measuring blood pressure: In order to ensure the objectivity of the experimental data, the nursing staff measuring blood pressure should not know the specific grouping of patients. However, in the single-blind experiments, it is not specified that caregivers must be blinded to the grouping. Their knowledge depends on the specific requirements of the experimental design and the operating procedures of the laboratory.

In conclusion, according to the definition and design purpose of the single-blind experiment. So, the correct answer is:

F. The patient himself.

In single-blind experiments, usually only patients do not know which treatment they receive. Researlows, statisticians, patient families, and caregivers measuring the BP were aware of the grouping. The purpose of this design is to avoid patient subjective factors affecting the experimental results.

Therefore, as for the antihypertensive efficacy of class A and class B drugs on mild to moderate essential hypertension, what is not known about the grouping is:

G. The patient himself

45. (Type A1 questions)

A patient felt chest tightness on the second floor, which was relieved after rest. The patient's cardiac function was classified as

A. NYHA grade: Grade I

B. Killip Grade: Grade

C. NYHA classification: Grade

D. NYHA classification: Grade

E. NYHA classification: Grade

Refer to the answer: D. Your answer:

[Analysis] NYHA classification is the cardiac function of patients with heart failure, which is divided into four levels. NYHA classification (D pair) indicates heart disease patients with limited physical activity; NYHA classification (E wrong) means that patients with heart disease cannot have heart failure symptoms at rest, aggravated after activity; NYHA classification (C wrong) indicates that heart disease patients have mild limited physical activity in heart disease, no conscious symptoms at rest, and heart failure symptoms in general activity. NYHA grade I (A error) means that daily activities are not restricted and do not cause symptoms of heart failure.

[Error avoidance] Heart failure caused by AMI can be divided into 4 grades according to the Killip classification method.① Grade I: No significant heart failure; ②

Grade I: left heart failure, lung rales <50% lung field; Grade ③: acute pulmonary edema, whole lung large, small, dry, wet

Sound; grade ④: hemodynamic changes of different degrees or stages such as cardiogenic shock.

According to the patient's symptoms, the patient felt chest tightness on the second floor, but it could be relieved after rest, which indicates that the activity tolerance is limited, but the symptoms can be improved at rest. This condition is most consistent with the NYHA (New York Heart Association) cardiac function classification. The NYHA cardiac function classification is classified according to the activity tolerance and degree of symptoms in patients with heart failure, and the grade indicates mild activity restriction, and normal activity causes fatigue, palpitations, dyspnea, or chest pain.

Therefore, the most appropriate answer is: C. NYHA grade: grade

First, we need to understand the grading system of cardiac function. At present, the most commonly used cardiac function grading system is NYHA (New York Heart Association) cardiac function classification, which is mainly classified according to the degree of activity that induce heart failure symptoms. This grading system has four grades, from I to grade, with higher grades indicating worse cardiac function.

Now, we follow this system to analyze each option individually:

A. NYHA Grade: I: This level indicates that the patient has heart disease, but the activity is not limited, and the usual activity does not cause fatigue, palpitations, dyspnea or angina. This is not consistent with the description of the patient in the

topic, because the patient felt chest tightness after the second floor.

B. Killip Grade: Grade: Killip Grade is mainly used to evaluate the cardiac function status of patients with acute myocardial infarction, which is inconsistent with the description of this question, so it can be excluded.

C. NYHA Grade: Grade: This level indicates that patients with heart disease are mildly limited in physical activity and have no voluntary symptoms during rest, but fatigue, palpitations, dyspnea or angina under general physical activity. This is in line with the situation that the patient felt chest tightness after climbing the second floor and was relieved after rest.

D. NYHA grade: Grade: This level indicates that the physical activity of cardiac patients is significantly limited, less than the usual activity that causes the above symptoms. This is not consistent with the situation of the patient in the title, because he developed symptoms after climbing the second floor.

E. NYHA Grade: Level: This level indicates that patients with heart disease cannot perform any physical activity. Symptoms of heart failure were present at rest, which worsened after activity. This is clearly not consistent with the patient situation described in the title.

In conclusion, combining the NYHA cardiac function grading criteria and the patient condition described by the title, we can determine the patients' cardiac function grade as grade NYHA II. Therefore, the correct answer is the C. NYHA grade: grade.

For this topic, we need to understand the criteria of NYHA heart function classification. The NYHA (New York Heart Association) cardiac function grading system is used to assess symptom severity and functional status in patients with heart failure. The specific grades are given as follows:

-Level I: Daily activities are not restricted, and general physical activities do not cause fatigue, palpitations, dyspnea, or angina pectoris.

Grade- II: Patients with mild limitation of daily activities, general physical activity will cause fatigue, palpitations, dyspnea or angina pectoris.

Grade- III: Patients' daily activities are significantly limited, below the normal

physical activity will cause symptoms, but no symptoms at rest.

Grade- IV: Patients are symptomatic during any activity or at rest and should not perform any physical activity.

The patient mentioned in the topic felt chest tightness on the upper second floor and was relieved after rest, which indicates that the patient experienced symptoms during general physical activity (going up the stairs), but was relieved after rest. Therefore, this fits within the grade of the NYHA classification.

So, the most correct answer is:

B NYHA classification: Grade

46. (Type A1 questions)

Kandton shoulder loss does not usually cause

A. Pathological constriction of the compound ring

B. intrauterine infection

C. omphaloproptosis

D. placental abruption

E. dead fetus in uterus

Refer to the answer: D. Your answer:

[Analysis] incarcerated shoulder first makes the uterine contraction continue to strengthen, the upper uterine section is getting thicker and thicker, the passive expansion of the lower part is getting thinner and thinner, due to the thickness of the upper and lower parts of the uterus, the formation of a ring depression, and gradually increased with the contractions, and even up to the umbilical, forming the pathology of retraction ring (A pair). The rupture of fetal membrane amniotic fluid outflow is easy to cause the umbilical cord prolapse (C pair), and the umbilical cord prolapse leads to fetal distress, resulting in fetal death in utero (E pair). Among them, incarcerated shoulder exposure is uneven to the cervix and lower uterine segments, and premature rupture of fetal membrane often occurs, which is easy to lead to

intrauterine infection (B pair).

[Error avoidance] The main causes of placental abruption are vascular factors (hypertensive disease in pregnancy), mechanical injury and intrauterine pressure

Force drop, etc. (D wrong, is the correct answer to this question).

Kandton shoulder first is a form of dystocia in which the shoulder, not the head, first enters the birth canal. This condition may lead to dystocia and the need for urgent intervention. The following is an correlation analysis of each options and KKshoulder shoulder shoulder:

A. Pathological retraction: This refers to the premature or rapid closure of the cervical opening, which may occur in dystocia but is usually not directly associated with hallow shoulder exposure.

B. Intrauterine infection: dystocia, including first shoulder exposure, may indeed increase the risk of infection if too long, but this is not the most direct complication of first shoulder exposure.

C. Umbilical cord prolapse: This is the entry of the umbilical cord into the birth canal before the fetus or at the same time as the fetus, usually associated with the head position, rather than the shoulder first.

D. Placental abruption: This is the case of the placenta being removed from the uterine wall before delivery, which may occur due to dystocia but is not a unique complication of low shoulder manifestation.

E. Fetal death in utero: This refers to the death of the fetus in utero and can be caused by a variety of causes, including severe dystocia, but not the result of hallow shoulder exposure.

Considering the above analysis, the least likely direct cause is "cord prolapse" (option C), as this is more common in other types of obstructed labor, such as head position rather than shoulder first. Therefore, the most correct answer should be * * C. umbilical cord prolapse * *.

analyze:

Emcerated shoulder first exposed refers to the fetal body lying above the entrance of the pelvis, the longitudinal axis of the fetal body is perpendicular to the longitudinal axis of the mother, the first exposed is the shoulder, is the most adverse fetal position for the mother and child, not timely or improper treatment can endanger the life of the mother and the fetus. Umbilical cord prolapse occurs when the fetal

dew is not connected. After the fetal membrane rupture, the umbilical cord prolapse outside the cervix, falls to the vagina and even exposed in the vulva. When the incarcerated shoulder is exposed, the fetal body lies above the entrance of the pelvis, and the umbilical cord will not fall outside the cervix after the rupture of the fetal membrane, so the umbilical cord prolapse will not occur.

Option A pathological shrink complex ring refers to the fetus first dew drop, uterine contraction, uterine body muscle contraction causes the upper uterine section thickening, shorter, and the lower muscle gradually thinning, longer, the uterine body and the lower uterine form obvious annular depression, is the precursor of uterine rupture, embedded shoulder cannot into the basin, can lead to strong uterine contraction, more and more thick, lower passive expansion more and more thin, form pathological shrink complex ring, does not conform to the topic.

Option B Intrauterine infection can be seen in incarcerated shoulder exposure, premature rupture of membranes and repeated anal examination increase the chance of infection, so it is not selected.

Option D Placental abruption refers to that after 20 weeks of gestation or during childbirth, part or all of the normal placenta is dissected from the uterine wall before the fetus, and the fetal body of the incarcerated shoulder lies above the entrance of the pelvis, affecting the blood circulation of the placenta, and placental abruption can occur, so it is excluded.

Option E fetal death can occur due to labor stagnation and fetal distress, so it is not selected.

In conclusion, the correct answer is: C. Cord cord prolapse.

Kandon shoulder loss is a rare obstetric complication that usually occurs during labor when the fetal shoulder is embedded in the pelvic entrance. Here is an analysis of each option:

A. Pathological retraction ring: The first shoulder dew may lead to abnormal contractions and labor stagnation, thus forming A pathological retraction ring.

B. Intrauterine infection: prolonged rupture of membranes and intervention during delivery may increase the risk of intrauterine infection.

C. Umbilical cord prolapse: Shoulder exposure can lead to abnormal fetal position and increase the risk of umbilical cord prolapse.

D. Placental abruption: This is a condition where the placenta is partially or completely detached from the uterine wall before or during delivery, usually related to other causes. Klower shoulder exposure usually does not directly lead to placental abruption.

E. Fetal death in utero: If not handled properly, prolonged delivery stagnation may lead to fetal hypoxia, which may eventually lead to fetal death in utero.

Comprehensive analysis, low shoulder exposure usually does not directly cause placental abruption.

The correct answer is: D. placental abruption

47. (Type A1 question)

When can the human body can cause myocardial damage

A. plumbism

B. mercury poisoning

C. arseniasis

D. selenium deficiency

E. Chromium deficiency

Refer to the answer: D. Your answer:

[Analysis] Selenium deficiency will lead to Keshan disease (D pair), also known as endemic cardiomyopathy, mainly manifested as acute and chronic cardiac insufficiency, cardiac enlargement, arrhythmia and so on.

[Avoid mistakes] Lead poisoning is mainly manifested by abdominal cramps and

nervous, liver and blood system abnormalities (A mistake); mercury poisoning can be

Cause stomatitis (B error); arsenic poisoning, severe gastroenteritis, shock, peripheral neuropathy and liver damage at 1 to 3 weeks

Skin keratosis (C wrong); chromium deficiency causes metabolic disorders in blood glucose (E wrong).

Myocardial damage can be caused by several factors, including the poisoning or deficiency of some heavy metals and trace elements. Of the options given, * * arsenism * * (option C) is a condition known to cause myocardial damage. Arsenic poisoning can cause a variety of cardiovascular problems, including myocarditis and myocardial damage. So the most correct answer is: C. arsenic poisoning

First, we need to clarify the meaning of myocardial damage. Myocardial damage refers to the damage or death of cardiomyocytes, which can be caused by various factors, including but not limited to ischemia, hypoxia, poisoning, infection, etc. Next, we analyzed each option for myocardial damage one by one.

A. Lead poisoning: Lead is a heavy metal, and chronic exposure or ingestion can lead to a variety of health problems, including myocardial damage. Lead can directly damage cardiomyocytes and affect cardiac function.

B. Mercury poisoning: Although mercury poisoning can lead to a variety of health problems, it mainly affects the nervous system and has a relatively little direct impact on the heart muscle.

C. Arsenic poisoning: Arsenic poisoning mainly affects the digestive system and nervous system, and is not as directly related to myocardial damage than lead poisoning.

D. Selenium deficiency: Selenium is an essential trace element, which has a certain impact on heart health, but its deficiency mainly leads to Keshan disease and Kashin-Beck disease, which has little relationship with direct myocardial damage.

E. Chromium deficiency: Chromium is mainly involved in glucose metabolism, and its lack is not a direct association with myocardial damage.

In conclusion, the most directly related to myocardial damage is lead poisoning. Lead can directly damage cardiomyocytes, leading to impaired cardiac function. Therefore, the most likely case to cause myocardial damage is A. lead poisoning.

Answer: A. Lead poisoning

Myocardial damage is usually caused by direct toxic effects on cardiomyocytes or by metabolic disorders. Among the options, lead, mercury, and arsenic poisoning can all cause damage to a variety of systems, including myocardial damage. However, arsenpoisoning is particularly known for its toxic effects on the heart, which can cause acute and chronic myocardial damage.

Although selenium deficiency and chromium deficiency can also affect heart health, they generally do not directly cause myocardial damage. Selenium deficiency is associated with Keshan disease (an endemic cardiomyopathy), and chromium deficiency is associated with metabolic syndrome, but these are not a common cause of myocardial damage.

So the best answer is:

F. arseniasis

48. (Type A1 question)

Long-term maintenance treatment for bronchial asthma

A. Inhaled short-acting β 2 receptor agonist B. inhaled long-acting β 2 receptor agonist C. oral short-acting β 2 receptor agonist

B. Oral corticosteroids

C. Short-acting theophylline

Reference answer: B. Your answer:

[Analysis] Long-term maintenance treatment of bronchial asthma preferred inhaled glucocorticoids (D wrong), but also can choose a long-acting β 2 receptor agonist (B pair).

[Misavoidance] short-acting β 2 receptor agonists and short-acting theophylline (ACE error) are asthma relief drugs, mainly used for asthma attacks

time.

The main purpose of long-term maintenance treatment of bronchial asthma is to control the condition, reduce the onset of symptoms, and improve the quality of life of

patients. Among them, an inhaled long-acting β 2 receptor agonist (LABA) is a commonly used long-term maintenance therapy because it can continuously dilate the bronchi and reduce asthma attacks.

-A. Inhaled short-acting β 2 receptor agonists (SABA) is mainly used for rapid remission of acute asthma attacks and is not suitable as A long-term treatment option.

-B. * * Inhalation of long-acting β 2 receptor agonists * *, such as salmeterol or formoterol, is suitable for the long-term control and prevention of asthma symptoms, especially at night and early in the morning.

-C. Oral short-acting β 2 receptor agonists are not commonly used for long-term treatment because of their short duration of action and relatively many side effects.

-D. Oral corticosteroids are mainly used in severe asthma or are considered when combined with inhaled corticosteroids (ICS) and long-acting β 2 receptor agonists still fail to control asthma.

-E. Short-acting theophylline is used in the treatment of acute asthma, but it is usually not used as the preferred choice for long-term treatment due to its unstable efficacy and more side effects.

Therefore, for this question, * * B. inhalation of long-acting β 2 receptor agonists * * is the most appropriate answer.

analyze:

Bronchial asthma is characterized by a variety of cells and cell components involved in airway chronic inflammation of heterogeneous disease, the chronic inflammation associated with airway hyperreactivity, usually appear wide and variable reversible expiratory airflow limitation, lead to repeated episodes of wheezing, shortness of breath, chest tightness and (or) cough symptoms, intensity changes over time. Long-term treatment of asthma is divided into five levels, each level includes relief drugs and control drugs, relief drugs refers to the use of drugs, by quickly relieve bronchospasm to relieve asthma symptoms, and control drugs refers to need long-term use of drugs, used to reduce airway inflammation, control asthma symptoms, reduce asthma aggravation and avoid asthma recurrence. Long-acting β 2 receptor agonists belong to the control drugs of long-term treatment of asthma, need long-term use, so long-term maintenance treatment of bronchial asthma can be inhaled long-acting β 2 receptor agonists.

Option A inhalation short-acting β 2 receptor agonist, option C oral short-acting

β 2 receptor agonist, and option E short-acting theophylline, both are relief drugs and should be used as needed, so they can be excluded together.

Option D Oral corticosteroids are generally used in patients with ineffective inhaled hormones or who need short-term intensive therapy. They are not used as long-term maintenance therapy, so they are not selected.

In conclusion, the correct answer is: B. inhalation of long-acting β 2 receptor agonists.

Long-term maintenance treatment of bronchial asthma is mainly designed to control the symptoms, prevent acute attacks, improve the quality of life, and minimize the side effects of drugs. Based on existing medical knowledge and best practice guidelines, long-term maintenance treatment of bronchial asthma usually includes the use of inhaled corticosteroids (ICS) and long-acting β 2 receptor agonists (LABA). Among them, inhaled corticosteroids are the most important long-term control drugs.

The questions require choosing one of the best answers among the given five options. Options include:

A. Inhalation of the short-acting β 2-receptor agonists

B. Inhalation of long-acting β 2-receptor agonists

C. Oral of short-acting β 2 receptor agonists

D. Oral corticosteroids

E. Short-acting theophylline

Of these five options, the best candidates for the long-term maintenance treatment of bronchial asthma are:

F. Inhalation of long-acting β 2-receptor agonists (B)

Although inhaled long-acting β 2 receptor agonists are commonly used in

combination with inhaled corticosteroids, it is more appropriate as part of long-term maintenance therapy than other options when given alone. Therefore, choosing B is the most appropriate answer.

49. (Type A1 question)

Pathogenic autoantibodies of lupus nephritis are mainly

A. IgA

B. IgB

C. IgE

D. IgD

E. IgG

Reference answer: E Your answer:

[Analysis] The pathogenic autoantibodies in patients with SLE are mainly IgG (E pair), which have high affinity with autoantigens, such as the direct binding of DNA antibodies and renal tissue, resulting in injury.

[Error avoidance] Other characteristics of SLE pathogenic antibodies: anti-platelet antibodies and anti-red blood cell antibodies lead to platelets and red blood cells

Disruption of; anti-SSA antibodies transplacenta into the fetal heart cause neonatal heart block; antiphospholipid antibodies cause anti-phosphorus

Lipid-antibody syndrome.

Lupus nephritis is a common complication of systemic lupus erythematosus (SLE), whose pathogenic autoantibodies are mainly IgG. So the correct answer is: E. IgG

analyze:

Lupus nephritis is a disease of systemic lupus erythematosus combined with the immune damage of different pathological types of both kidneys, along with the clinical manifestations of obvious renal damage. Its pathogenic autoantibodies are mainly IgG, which can be deposited in the glomerulus by immune complexes, or damage the kidney by other mechanisms. For example, anti-dsDNA antibodies can shrink the mesangial cells, increase their permeability, damage the charge barrier, and form albuminuria.

Option A: IgA is mainly present in serum. It is the earliest antibody synthesized and secreted in the process of individual development. It is also an important antibody for anti-infection immunity, also known as mucosal antibody. IgA fails to cross the placenta. IgA antibodies are absent in newborn animal serum, but secretory IgA can be obtained from human milk. Therefore, IgA was not a pathogenic autoantibody to lupus nephritis and was excluded.

Option B: IgB is not present, immunoglobulin only IgA, IgG, IgM, IgD, IgE, no IgB, so it is not selected.

Option C: IgE comes from plasma cells under the mucosa of respiratory tract. It is a cytophilic antibody that is related to allergic reaction and parasitic disease and skin allergy. Therefore, IgE is not a pathogenic autoantibody of lupus nephritis, so it is not selected.

Option D: IgD is extremely low in serum, representing about 0.2% of the total Ig amount, and is synthesized late in ontogeny. IgD, IgD is not a pathogenic autoantibody of lupus nephritis and is excluded.

In conclusion, the correct answer is: E. IgG.

The pathogenic autoantibodies of lupus nephritis (lupus nephritis) are mainly IgG. The common autoantibodies in patients with systemic lupus erythematosus (SLE) are mainly anti-double-stranded DNA antibodies. These antibodies mainly belong to the IgG class and are an important factor leading to lupus nephritis. So the best answer is:

F. IgG

50. (Type A1 question)

Calculate the glomerular filtration rate with a substance, which requires the substance to be completely filtered by the glomerulus, and also requires the substance to be filtered by the renal tubules

A. Complete reabsorbed, but not secretory at all

B. Parally reabsorbed but partially secreted

C. Fully reabsorbed, but is also fully secreted

D. is not completely reabsorbed, but can be fully secreted

E. Not reabsorbed at all, and not secreted at all

Reference answer: E Your answer:

[Analysis] If a substance in plasma can be freely filtered through the glomerulus, the concentration of the substance in the renal capsule should be the same as in the plasma concentration; meanwhile, if the substance is neither reabsorbed nor secreted in the renal tubules and collecting duct (E pair).

[Avoid mistakes] Inulin is freely filtered by the glomerulus, and is not reabsorbed and secreted in the renal tubules and the collecting duct, as measured by inulin

The glomerular filtration rate is accurate and reliable, but the operation is inconvenient. Internal creatinine clearance is numerically closer to the glomerular filtration rate,

Therefore, it is commonly used clinically to predict the glomerular filtration rate.

When measuring the glomerular filtration rate (GFR), the selected substance should be completely filtered by the glomerulus and should not be reabsorbed or secreted in the renal tubules to ensure the accuracy of the measurement. Such substances can accurately reflect the amount of filtration through the glomerulus because they are not recovered or excreted extra by the kidney after the glomerular filtration. So, the correct options are:

F. Not reabsorbed at all, and not secreted at all

analyze:

Glomerular filtration rate refers to the amount of filtrate produced by both kidneys per unit of time, which is an indicator of renal function. Clinically, in place of the glomerular filtration rate, it is completely filtered by the glomerulus and completely reabsorbed by the glomerulus, such as endophytic creatinine clearance.

Option B "partially reabsorbed but partially secreted", option D "cannot be reabsorbed at all, but can be completely secreted", both involving tubular secretion, which causes the discharge of the substance in the urine greater than the excess glomerular filter, resulting in high calculation results, so it can be excluded together.

Option C completely reabsorption, but also can be completely secreted, the material reabsorption and secretion amount, equal in the output in the urine is still equal to the glomerular filtration, theoretically can also be used to determine the glomerular filtration rate, but in practice, due to the amount of reabsorption and

secretion is difficult to determine, so unable to accurately calculate the glomerular filtration rate, so do not choose.

Option E cannot be reabsorbed at all and cannot be secreted at all, so the amount of this substance in the urine is equal to excessive glomerular filtration, which can theoretically be used to determine the glomerular filtration rate. However, if it is not reabsorbed at all, the substance is quickly excreted in the urine and is difficult to detect, so it is not considered.

To sum up, the correct answer is: A. Complete reabsorption, but not secretion at all.

Calculating the glomerular filtration rate (GFR) with a substance must meet the following conditions: it can be completely filtered by the glomerulus and can not be reabsorbed or secreted by the renal tubules. In this way, the concentration of the substance in the urine is directly related to the filtration rate to facilitate accurate calculation.

So the correct answer is:

G. Not reabsorbed at all, and not secreted at all

51. (Type A1 question)

The pathogen that causes condyloma acuminatum is

A. Human immunodeficiency virus

B. human milk sarcoma virus

C. EB virus

D. chickenpox virus

E. cytomegalovirus

Reference answer: B. Your answer:

[Analysis] Human papillomavirus (HPV) belongs to the papillomavirus genus of the papilloma virus family, which mainly causes human skin

Hyperplastic mucosal lesions, in which the high-risk HPV type (type 16, type 18, etc.) and the occurrence of cervical cancer and other malignant tumors are closely

related

Related, low-risk HPV (type 6, type 11, etc.) causes genital condyloma acuminatum.

[Error avoidance] Human Immunodeficiency virus (HIV) is the etiological agent of acquired immunodeficiency syndrome, known as AIDS (AIDS)

body. About half of those primary infected with EB virus present with infectious mononucleosis, associated with malignant lymph in African children

Tumor and the onset of NPC are closely related, and EBV is currently recognized as an important tumor-associated virus in humans. Chicken pox-banded blister

Rubella virus, is the causative agent of varicella and herpes zoster. CMV infection in the population is extremely common, primary sensation

Dyeing occurs under 2 years of age, usually is a recessive infection, only a few patients appear clinical symptoms, when the body's immune function is low

It is prone to dominant infection, manifested as cytomegalovirus monocytosis, and cytomegalovirus is the most commonly causing congenital malformations

See the disease.

The causative agent of condyloma is human papillomavirus (Human Papillomavirus, HPV), but this option does not appear directly in the given answer. Of the options listed, the closest is the human milk sarcoma virus, but this is an incorrect name. The correct pathogen name should be the HPV. Therefore, given the possible misleading or miserror of a given option, the most appropriate option should be B. human mammary asarcoma virus, assuming it refers to human papillomavirus (HPV).

This is a choice question about medical microbiology to identify the pathogen that causes condyloma acuminatum. Let's analyze each option one by one:

A. Human immunodeficiency virus (HIV): This virus mainly causes AIDS and has no direct relationship with condyloma acuminatum. HIV mainly destroys the body's immune system, leading to immune deficiency, which is inconsistent with the pathogenesis of condyloma acuminatum.

B. Human lacosarcoma virus (HPV): condyloma acuminatum is a sexually transmitted disease caused by human papillomavirus (HPV) infection with proliferative damage of the anogenital site. There are many types of HPV, some of

which are closely related to the onset of condyloma acuminatum.

C. EB virus: this virus is mainly related to nasopharyngeal cancer, childhood lymphoma and other diseases, not with condyloma acuminata.

D. Varicella virus: mainly causes varicella and herpes zoster, with condyloma acuminatum.

E. Cytomegalovirus: This virus mainly causes infection of each system with genitourinary system, central nervous system and liver diseases, from mild asymptomatic infection to severe defects or death, which is different from the pathogenesis of condyloma acuminatum.

In conclusion, condyloma acuminatum is caused by human papillomavirus (HPV) infection, so the correct answer is option B, namely human milk sarcoma virus.

Therefore, the correct answer is B. human phasarcoma virus.

The pathogens that cause condyloma acuminatum are:

F. Human papillomavirus (HPV)

Human papillomavirus (HPV) is a common virus that can cause condyloma acuminatum. HPV infection is transmitted by sexual contact, and condyloma acuminata is caused by some specific types of HPV, especially the HPV types 6 and 11.

52. (Type A1 question)

Leptospirosis clinical type is not included

A. icterohemorrhagic form

B. gastrointestinal type

C. Renal failure type

D. Influenza typhoid type

E. Pulmonary bleeding type

Reference answer: B. Your answer:

[Analysis] Leptospirosis does not include gastrointestinal type (B is wrong, which is the correct answer to this question).

[Erstavoidance] Leptospirosis can be divided into type 5.① Influenza typhoid type: this type is the most common, no obvious organ damage, is the hook

Mild form of body disease; ② Pulmonary hemorrhage type: death from diffuse pulmonary hemorrhage; ③ jaundice hemorrhage

Type: renal failure is the most important cause of death, accounting for 60%~70% of death cases; ④ renal failure type: simple renal failure type is relatively rare; ⑤ meningoencephalitis type: often cause severe headache, irritability, cervical rigidity and other meningitis manifestations.

Leptospirosis (Leptospirosis), also known as plague or water epidemic, is an acute infectious disease caused by Leptospira. It has a variety of clinical manifestations, including jaundice blood type (also known as Weil disease), no jaundice blood type type, renal failure, gastrointestinal type, and flu-like symptoms. However, among the options given, influenza typhoid type is not a standard classification of leptospirosis. Therefore, the most appropriate answer is: D. influenza typhoid type

First, let's learn about leptospirosis. Leptospirosis is an animal-derived infectious disease caused by pathogenic leptospirosis. Its clinical characteristics are leptospiral sepsis in the early stage, the damage and dysfunction of all organs in the middle stage, and the complications after various metamorphosis in the later stage. Severe patients have obvious liver, kidney, central nervous system damage and disseminated pulmonary hemorrhage.

Next, we analyze each option one by one:

A. Jaundice and bleeding blood type: This is A common clinical type of leptospirosis, presenting with jaundice and bleeding symptoms.

B. Gastrointestinal type: Although leptospirosis can cause some gastrointestinal symptoms, the "gastrointestinal type" is not its main clinical type. Usually, the main types of leptospirosis are classified according to the organs or systems of their main effects.

C. Renal failure type: This is another important clinical type of leptospirosis, manifested by impaired renal function.

D. Influenza and typhoid fever: This is also a common type of leptospirosis, with symptoms similar to influenza or typhoid fever.

E. Pulmonary hemorrhage type: This is the most severe clinical type of

leptospirosis, presenting with pulmonary hemorrhage and other severe symptoms.

In conclusion, although leptospirosis can cause gastrointestinal symptoms, the "gastrointestinal type" is not its primary or specific clinical type. Therefore, the correct answer is the B. gastrointestinal type.

Leptospirosis (Leptospirosis) is a zoonosis caused by Leptospira. Its clinical manifestations are diverse and can be divided into different types. According to the clinical classification of leptospirosis, the following types are common:

A. icterohemorrhagic form

B. gastrointestinal type

C. Renal failure type

D. Influenza typhoid type

E. Pulmonary hemorrhage type

According to the clinical classification of leptospirosis, the following types are common:

1. Jaundice and blood type: manifested as fever, jaundice and bleeding.
2. Renal failure type: renal failure is the main manifestation.
3. Influenza typhoid type: symptoms are similar to influenza and typhoid.
4. Pulmonary hemorrhage type: pulmonary hemorrhage is the main feature.

However, "gastrointestinal type" is not a common clinical type of leptospirosis. So the correct answer is:

F. gastrointestinal type

53. (Type A1 question)

Infectious diseases belonging to fecal-oral transmission are

A. hepatitis B

B. epidemic encephalitis B

C. Epidemic cerebrospinal meningitis

D. The liver

E. Aids

Refer to the answer: D. Your answer:

Graphic analysis: [Analysis] Infectious diseases of fecal-oral transmission include hepatitis E and hepatitis A (D pair).

[Avoid] Hepatitis B and AIDS are blood-borne diseases; JE is mostly transmitted by mosquito bites; epidemic cerebrospinal meningitis is

Respiratory transmission diseases.

A faecal-to-oral infectious disease is D. hepatitis E. Thus, the most correct answer is the D. Hepatitis E virus (HEV) is mainly fecal-oral transmitted through contaminated water sources, especially in areas with poor sanitation. Other options such as hepatitis B (A), Japanese encephalitis (B), epidemic cerebrospinal meningitis (C), and AIDS (E) are mainly used through other routes of transmission, such as blood, sexual contact, or mosquito bites.

This is a choice question about the transmission route of infectious diseases. We need to determine which infectious disease is transmitted by the fecal-oral route. The fecal-oral route refers to the excretion of pathogens through feces, polluting the environment and food, and then being ingested into the body, thus leading to infection.

Next, we analyze each option:

A. Hepatitis B: mainly transmitted through blood, sexual contact and mother-to-child transmission, not through the fecal-oral route.

B. JE: mainly transmitted by mosquito bites, unrelated of the fecal-oral route.

C. Epidemic cerebrospinal meningitis: mainly transmitted by droplets or direct contact, also inconsistent with the fecal-oral route.

D. Hepatitis E: mainly transmitted through the digestive tract, namely the fecal-oral route. HEV is present in a patient's feces, is contaminated with water or food, and is ingested, leading to infection.

E. AIDS: mainly through sexual contact, blood, and mother-to-child transmission, not through the fecal-oral route.

In conclusion, only HEE is an infectious disease transmitted by the faecal-oral route. Therefore, the correct answer is D. hepatitis E.

Infectious diseases belonging to fecal-oral transmission are:

B The liver

Hepatitis E (HEV) is mainly transmitted through contaminated food and water, and is a typical fecal-oral transmitted disease.

54. (Type A1 question)

Hepatitis C antiviral therapy should be selected

A. interferon

B. Nucleotide analog material

C. Ganciclovir

D. Lamivudine

E. antibiotic

Reference answer: A. Your answer:

Graphic analysis: [Analysis] Interferon (A pair) should be selected for the antiviral treatment of hepatitis C.

[Avoid] Hepatitis B antiviral drugs mainly include nucleoside analogues (such as tenofovir, entecavir, tibifuidine, lamif

F.) and interferon.

The best antiviral option for hepatitis C (HCV is a nucleotide analogue). Therefore, the correct answer is the B. nucleotide analog object.

The mainstay of treatment of hepatitis C has shifted from interferon-based therapy to direct-acting antiviral drugs (DAA), including nucleotide analogs. These drugs target the replication mechanism of HCV, effectively removing the virus and improving the cure rate while having better tolerance and fewer side effects.

analyze:

Hepatitis C is a disease caused by hepatitis C virus, and direct antiviral drugs should be selected, namely nucleotide analogues. At present, the commonly used clinical antiviral programs are direct antiviral drugs, including sofosbuvir, vipathivir, tarativir, etc. These drugs are nucleotide analogs, which can effectively inhibit the replication of hepatitis C virus.

Option A interferon has antiviral, anti-tumor and immunomodulatory effects, and can be used for the treatment of acute and chronic hepatitis, but it is not a specific drug for hepatitis C virus, so it is not selected.

Option C ganciclovir is a prophylactic treatment for CMV and has no therapeutic effect on HCV, so it was excluded.

Option D lamivudine is a nucleoside analogue that inhibited HIV reverse transcriptase and HIV hepatitis B virus DNA polymerase, but not for hepatitis C virus, and is not considered.

Option E Antibiotic is mainly used to treat bacterial infection, which is ineffective for hepatitis C virus, so it does not meet the question.

In conclusion, the correct answer is: B. nucleotide analogues.

The primary goal of antiviral therapy for hepatitis C (HCV) is to clear the virus and prevent disease progression to cirrhosis and liver cancer. Current standard treatment options mainly include direct-acting antiviral drugs (DAAs), which are usually a combination of nucleotide analogues and non-nucleotide analogues.

Of the five alternative answers:

A. IFon, traditionally used in the treatment of hepatitis C, has been phased out due to high side effects and less efficacy than DAAs.

B. Nucleotide compounds-are a major class of drugs for the treatment of hepatitis C, including some directly acting antivirals, such as sofebuvir (Sofosbuvir).

C. Ganciclovir-primarily for herpesvirus infections, not for hepatitis C treatment.

D. Lamivudine- -mainly for the treatment of hepatitis B, not for hepatitis C.

E. Antibiotics-Not used for the treatment of viral infections, hepatitis C is a viral disease.

So, the most correct answer is:

F. Nucleotide analog material

55. (Type A1 questions)

Increased blood ammonia, inhibition of pyruvate oxidative decarboxylation leads to brain dysfunction, the following changes are correct

A. Increased dopamine production

B. More amine acetate

C. Glutamine was increased

D. γ aminobutyric acid degradation

E. Cased catecholamines

Refer to the answer: D. Your answer:

[Analysis] Blood ammonia increase, inhibition of pyruvate oxidation and decarboxylation is the direct result of acetyl-CoA reduction, and then reduced acetylcholine, resulting in brain dysfunction, there is no correct answer to this question.

[Error avoidance] Acetamide is an organic compound, is an anti-epileptic drug, mainly used in the treatment of epilepsy, and without hepatic encephalopathy

Pass, so B wrong. Blood ammonia increased, glutamate and ammonia combined to produce glutamine increased (option C and dry oxidation and decarboxylation no

Guan), glutamate is reduced by γ aminobutyric acid (option D is unrelated to oxidation decarboxylation and miscorrectly), so CD is wrong

mistake. Hepatic encephalopathy due to increased pseudoneurotransmitters, catecholamines (dopamine, norepinephrine, epinephrine)

The generation and release of is reduced, resulting in a reduction of catecholamines in the blood, so the AE is misdescribed.

Higher blood ammonia can inhibit the oxidation and decarboxylation of pyruvate, which will affect the metabolic process of ammonia and lead to the accumulation of certain substances. In these options, the increase in glutamine (option C) was directly

associated with increased blood ammonia. Increased blood ammonia will cause more ammonia to enter the brain tissue. In the brain, ammonia can bind to glutamate to form glutamine, so increased blood ammonia will lead to an increase in glutamine.

Therefore, the most correct answer is * * C. glutamine increase * *.

analyze:

Ammonia poisoning refers to that ammonia can be produced in the catabolism of various amino acids in the body, especially in the brain tissue. Ammonia exists in two forms in the body, namely non-toxic alanine and toxic free ammonia. When ammonia poisoning, ammonia and α -ketoglutaric acid to form glutamate, glutamate and ammonia to form glutamine, make the ATP consumption in the brain, lead to tricA cycle intermediates α -ketoglutaric acid and oxaloacetate, thus inhibiting pyruvate oxidation decarboxylation, reduce acetyl CoA generation, affect the normal tricarboxylic acid cycle, reduce ATP generation, and affect the normal activity of the nervous system, cause hepatic encephalopathy.

When ammonia poisoning will change the neurotransmitters in the brain, and catecholamines such as dopamine and norepinephrine are significantly reduced. Therefore, option A dopamine and E catecholamines are increased, which are inconsistent with the facts and can be excluded together.

Ammonia can combine with α -ketoglutarate to form glutamate, glutamate is combined with ammonia to form glutamine, the latter can expel ammonia from the brain, at the same time, glutamate can also combine with oxaloacetic acid to form aspartate, and then combine with ammonia to form asparagine, which can also expel ammonia from the brain. However, when ammonia is combined with glutamate, the α -ketoglutarate acid in the TCA cycle is reduced, leading to the weakening of the TCA cycle, resulting in the decrease of ATP generation and brain cell activity disorder. Therefore, the ammonia cannot be discharged by the increase of acetate amine, so the increase of B acetate amine can be excluded.

When ammonia poisoning, γ -aminobutyric acid (GABA) ergic neurons and receptors can be activated respectively, leading to the release of inhibitory neurotransmitter GABA, and its degradation decreases, producing central inhibitory effect, thus causing the inhibitory dysfunction of the brain center, so option D: γ -aminobutyric acid degradation is not consistent with the question.

To sum up, the correct answer is: C. glutamine increase.

In the case of increased blood ammonia, it can lead to ammonia metabolism disorders, which causes a series of metabolic and nervous system changes. When the ammonia concentration is too high, ammonia binds to glutamate to produce glutamine, and this metabolic process is particularly evident in hepatic encephalopathy. The accumulation of glutamine in the brain leads to brain dysfunction.

So the answer is:

F. Glutamine was increased

56. (Type A2 questions)

Female, frequent urination, urination pain with gross hematuria for 1 day, past physical health. There was no abnormality in physical examination. Nitrite positive, urine sediment examination suggests that red and white blood cells full field, the course of antibiotics is generally how many days

A.14 days

B.3 days

C.5 days

D.7 days

E.10 days

Reference answer: B. Your answer:

Graphic analysis: [analytical] young female patients, urinary frequency, urine pain with gross hematuria 1 day, a positive urinary nitrite (hint may have urinary tract infection), urine sediment microscopic red and white blood cells (manifestation of urinary tract infection), combined with patient history, clinical manifestations and related examination, the patient with the most likely diagnosis of acute lower urinary tract infection. Compound sulfamethoxazole, cephalosporins and quinolones can be selected. The patient has no complications, so the 3-day therapy should be used (with B pair).

[Avoid avoidance] In patients with acute upper urinary tract infection, the course of antibiotics is generally 14 days.

This female patient had symptoms of frequent urination, urinary pain and gross

hematuria, no abnormalities, urine examination, positive nitrite and full view of red and white blood cells, which all pointed to the possibility of urinary tract infection, especially acute cystitis or possibly acute pyelonephritis. For adult women with acute simple cystitis, the usually recommended course of antibiotics is short-term, usually 3 to 5 days. Therefore, the most appropriate answer option is:

C.6 days

This reflects general clinical practice and guidelines in which shorter courses of antibiotic therapy are recommended for uncomplicated urinary tract infections.

analyze:

In young female patients, frequent urination, urinary pain with gross hematuria, positive nitrite, urine sediment examination indicated red blood cells with full view. According to the patient's symptoms, signs and laboratory tests, the diagnosis of acute cystitis was considered, which was usually treated with three-day therapy, namely oral compound sulfa methylisoxazole; or ofloxacin; or levofloxacin. Because single-dose therapy is not as effective as three-day therapy, it is no longer recommended. For male patients, a seven-day therapy is available. For patients with failed single dose treatment, no improvement in symptoms, and no change of bacteriuria, more effective antimicrobial drugs should be selected and used for a three-day course of treatment.

Option A: 14 days, option D: 7 days, and option E: 10 days, which were not the standard course of antibiotics for acute cystitis, and were excluded.

Option C: The 5-day treatment course is uncommon, and the treatment effect is not necessarily better than the 3-day therapy, so it is not chosen.

To sum up, the correct answer is: B.3 days.

According to the title description, the patient showed typical symptoms of urinary tract infection (UTI), such as urinary frequency, urinary pain and gross hematuria, and urine sediment examination indicated full view of red and white blood cells and positive nitrite test, which further supported the diagnosis of urinary tract infection. For simple, uncomplicated urinary tract infections, the recommended antibiotic courses are usually shorter. The best answer is:

B.4 days

This is because clinical guidelines generally recommend short courses of antibiotics (3 days) to achieve effective treatment and reduce the risk of antibiotic resistance.

57. (Type A2 question)

Male, trauma, to the hospital 24 hours later, blood pressure 150 / 100mmHg, body temperature 37.8℃, pelvic separation test positive, asked the main reason for fever is

A. urinary infection

B. shock

C. pneumonia

D. pulmonary embolism

E. Bone of absorption the bone heat

Reference answer: E

Graphic analysis: [analysis] This patient has a positive pelvic separation test, considering the possibility of pelvic fracture, low fever (<38℃) can occur during hematoma absorption (E pair).

[To avoid mistakes] After fracture, the body temperature is generally normal, and the fracture with a large amount of bleeding, such as femoral fracture, pelvic fracture, and hematoma absorption

Low heat may occur, but generally does not exceed 38℃. Open fracture, when presenting with high fever, the possibility of infection should be considered.

The patient presented to the hospital 24 hours after trauma, developed fever (temperature 37.8℃), blood pressure 150 / 100mmHg, and positive pelvic separation test. This information points to a possible pelvic lesion. A positive pelvic separation test usually indicates an instability of the pelvic ring, possibly due to a fracture or severe soft tissue injury.

The cause of fever in this context may be related to trauma, especially the inflammation and tissue injury response that can be observed after the fracture. Inflammatory mediators and other bioactive substances released during resorption of bone hematoma can lead to elevated body temperature. Therefore, based on the options given, the most reasonable answer is E. bone hematoma absorption fever. This is because the inflammatory and metabolic activities generated during bone hematoma

resorption may explain the post-traumatic fever situation.

Options A (urinary tract infection), B (shock), C (pneumonia), and D (pulmonary embolism) are all common causes of fever, but they are not as directly related as option E based on the specific condition described (pelvic problems after trauma). Therefore, E was chosen as the best answer.

analyze:

The patient had a pelvic trauma and a positive pelvic separation test, and the presence of a pelvic fracture should be considered. Because the absorption of the hematoma at the fracture can produce absorption heat, the most likely cause of the fever after the injury is the bone hematoma.

Option A urinary tract infections usually showed urethral irritation symptoms, which was inconsistent with the patient's performance, so it was excluded.

Option B shock mainly showed decreased blood pressure, fine pulse, and wet and cold limbs, while the patient had high blood pressure, which was inconsistent with the clinical manifestations of shock, so it was not selected.

Option C pneumonia is usually accompanied by cough and sputum symptoms, severe purulent or blood sputum, lung examination and wet rales, not consistent with the patient's performance, so it is excluded.

Option D Pulmonary embolism was mainly manifested as sudden unexplained dyspnea and shortness of breath, chest pain, syncope, cough, hemoptysis, etc. The patient did not have the above manifestations, so it was not selected.

In conclusion, the correct answer is: E. Bone hematoma absorbs heat.

The patient presented to the hospital 24 hours after trauma, blood pressure was 150 / 100 mmHg, temperature was 37.8°C, and pelvic separation test was positive. For the main cause of fever in patients, the following is an analysis for each option:

A. urinary tract infections: Urotract infections usually take a longer time to develop and are less likely to have symptoms of infection within 24 hours.

B. Shock: Shock usually causes hypotension, hypothermia, and multiorgan dysfunction rather than fever.

C. Pneumonia: Pneumonia can cause fever, but it is not the most common cause in patients with trauma, especially in the acute setting.

D. Pulmonary embolism: Pulmonary embolism leads to shortness of breath, chest pain, and hypoxemia, and fever may be a secondary symptom, not the primary cause.

E. Bone sorption fever: pelvic fractures often lead to the formation of hematoma, which may produce heat during the absorption process, leading to fever.

Combined with the patient's history of trauma, elevated temperature and positive pelvic separation test, the most likely cause of fever was bone hematoma absorption fever. So the best answer is:

F. Bone of absorption the bone heat

58. (Type A2 question)

A 31-year-old woman responded with antibiotics for one year, most likely in the following urine routine

A. Urine was acidic, with purulent cells and no bacterial growth

B. Urine is acidic, with purulent cells and bacterial growth

C. Urine was neutral, with purulent cells and bacterial growth

D. Urine was alkaline, with purulent cells and no bacterial growth

E. Urine is alkaline, with purulent cells and bacterial growth

Reference answer: A. Your answer:

[Analysis] female, 31 years old, repeated frequent urination pain for one year, antibiotic treatment is ineffective, no obvious cause of chronic cystitis symptoms persist, no obvious effect after antimicrobial treatment should consider the possibility of urinary tuberculosis. Urine examination of patients with urinary tuberculosis: urine is acidic, urine protein is positive, there are more red blood cells and white blood cells,

urine culture sterile growth, so this question is selected A.

[Avoid avoidance] Renal tuberculosis is a common cause of chronic cystitis, therefore, all no obvious cause of chronic cystitis, symptoms

Continued presence and gradually aggravated, with terminal hematuria; especially young men with chronic cystitis symptoms, urine culture without bacteria

Long, no obvious effect with antimicrobial treatment; epididymis with desicle or with scrotal chronic sinus, renal tuberculosis

ability.

This title describes a 31-year-old female patient who had recurrent urinary pain for one year and who failed to receive antibiotic treatment. This condition may suggest an atypical urinary infection or other disease, such as interstitial cystitis, which may lead to routine urinary purulent cells but negative bacterial culture.

A. Urine was acidic, with purulent cells and no bacterial growth

B. Urine is acidic, with purulent cells and bacterial growth

C. Urine was neutral, with purulent cells and bacterial growth

D. Urine was alkaline, with purulent cells and no bacterial growth

E. Urine is alkaline, with purulent cells and bacterial growth

Given that antibiotic treatment was ineffective, this could mean that no bacteria grew, or that the bacteria were not what cause the symptoms. In addition, the acidity and alkalinity of the urine is also an important consideration, because it can provide clues about the underlying disease. Urine is usually acidic, but certain conditions, such as urinary tract infections, may lead to an alkaline urine.

In this scenario, the most likely option is interstitial cystitis, a disease that causes chronic pelvic pain and urinary tract symptoms, and usually does not develop bacteria in the urine. Urinary routine in patients with interstitial cystitis may show purulent cells without bacterial growth. Therefore, the best choice should be * * A. acidic urine, with purulent cells, and no bacterial growth * *, although interstitial cystitis does not always cause acidic urine, this option is best consistent with the absence of bacterial growth in the given option.

analyze:

Young and middle-aged female patients, repeated frequent urination pain for a year, suggesting the possibility of urinary tract infection. The failure of antibiotic

treatment suggests that it may not be a common bacterial infection, considering the patient's history and clinical manifestations. The typical manifestations of urinary tract tuberculosis are bladder stimulation symptoms, that is, frequent urination, urgent urination, urination pain, terminal hematuria, urine examination can find the urine acid, and there can be red blood cells and white blood cells in the urine, but the common bacterial culture results are negative, so the most likely result of the urine routine is urine acid, purulent cells, no bacterial growth.

Option B urine is acidic, with pus cells, and bacterial growth is characterized by a general bacterial infection, while the problem stem clearly points out that antibiotic treatment is ineffective, so it is impossible to be a common bacterial infection, so it is excluded.

Option C urine is neutral, with pus cells, and bacterial growth can be seen in a variety of bacterial infections, but the problem stem indicates that antibiotic treatment is ineffective, so it is not selected.

Option D The urine was alkaline, with purulent cells, and no bacterial growth was inconsistent with the characteristics of tuberculosis infection, because the urine was generally acidic when tuberculosis infection, so it was excluded.

Option E The urine is alkaline, has purulent cells, and bacterial growth is usually not typical of urinary tuberculosis, because urinary tuberculosis urine is generally acidic, and the bacterial culture is negative, so it is not selected.

In conclusion, the correct answer is: A. The urine is acidic, with purulent cells, and no bacterial growth.

This 31-year-old female had recurrent frequency and pain with antibiotics. Considering these symptoms and the ineffectiveness of antibiotics, we need to consider some possible etiologies, such as a chronic urinary tract infection or urethral syndrome.

In this case, the results of the urine routine could be more informative. Here is an analysis of each option:

A. Urine is acidic, with purulent cells, and no bacterial growth: this condition is usually seen in non-bacterial urinary tract infections or urethral syndrome.

B. Urine is acidic, with purulent cells and bacterial growth: this is a typical manifestation of bacterial urinary tract infection, and treatment should usually be effective with antibiotics.

C. Urine is neutral, with purulent cells and bacterial growth: this is also a manifestation of bacterial urinary tract infections, but the pH neutrality in urine may be less common.

D. Urine is alkaline, with purulent cells, and no bacterial growth: this condition may indicate stones or some non-bacterial pathogens, but less commonly.

E. Urine is alkaline, purulent cells, and bacterial growth: this situation suggests urinary tract infection, especially the infection caused by urea breakdown bacteria (such as proteus), which can decompose urea into ammonia and make urine alkaline.

Because the patient's antibiotic treatment was ineffective, considering the infection (e. g. proteus), it resulted in alkaline urine and bacterial growth. Therefore, the most likely routine result of urine is:

B Urine is alkaline, with purulent cells and bacterial growth

This condition was most consistent with the patient's symptoms and a history of ineffective antibiotics.

59. (Type A2 question)

Male, 65 years old, with chest pain for 3 hours, chest tightness and palpitations, accompanied by nausea, vomiting, excessive sweating, oral nitroglycerin did not relieve, physical examination: heart rate 40 times / minute, cannon tone. Coronary angiography showed a severe blockage of the right crown. The following possible cardiac arrhythmias are among these

A. third-degree atrioventricular block

B. Second-degree sinus conduction block

C. One-degree atrioventricular block

D. One-degree sinus conduction block

E. Second-degree atrioventricular block

Reference answer: A. Your answer:

[Analysis] the third degree atrioventricular block due to the atriochamber separation, the intensity of the first heart sound often changes, the second heart sound can be normal or abnormal split, or hear the loud and loud first heart sound (cannon sound). The patient had a heart rate of 40 beats / min and A cannon tone, so the ECG performance was most likely A third-degree atrioventricular block (pair A).

[Error avoidance] Third degree atrioventricular block, block site can be located in the atrioventricular node, tract bundle and bilateral bundle branch level, ventricular shock

Movement is produced by the passive heart rhythm below the atrioventricular node, that is, the escape heart rhythm. If appear ventricular escape stroke cardiac rhythm, often suggest to produce block

The site is lower. Because the atrium and ventricle are excited by two different pacing points, each maintains its own rhythm, ECG

On the figure, the P wave has no relationship with the QRS wave group (the PR interval is not fixed), and the atrial rate is faster than the ventricular rate.

Considering the patient's symptoms (chest pain, chest tightness, palpitations, nausea, vomiting, sweating) and signs (heart rate 40 beats, cannon tone), and the severe blockage of the right crown indicated by coronary angiography, these findings point to a severe reduction in cardiac output and damage to the cardiac conduction system. Cannon sounds are often associated with asynchronous contraction of the atrium and ventricles, which is common in third-degree AV block with completely decoupled electrical activity between the atrium and ventricle.

Thus, the most likely arrhythmia is a third-degree atrioventricular block (A). Three-degree AV block, also known as complete AV block, is the most severe form of block in the cardiac conduction system, with a complete loss of the electrical connection between the atrium and ventricles leading to a significant decrease in heart

rate, consistent with the symptoms and signs presented by the patient.

analyze:

The patient was an elderly male, due to chest pain for 3 hours, oral nitroglycerin could not relieve, coronary angiography showed severe blockage of the right crown, according to the patient's symptoms, signs and coronary angiography examination, the diagnosis of acute myocardial infarction was considered. The patient's heart rate slowed down and the cannon tone appeared, which was a typical sign of third-degree atrioventricular block. Combined with the patient's medical history and clinical manifestations, the most likely arrhythmia in this patient was third-degree atrioventricular block.

Option B second degree sinus block, option C one degree atrioventricular block, option D first degree sinus block, option E second degree atrioventricular block, which can be seen in myocardial infarction, but the above diseases generally have no signs such as cannon tone, so they can be excluded together.

In conclusion, the correct answer is: A. Third-degree atrioventricular block.

According to the information provided by the topic, the 65-year-old man presented with typical symptoms of acute coronary syndrome (chest pain, chest tightness, palpitations, nausea, vomiting, sweating), ineffective nitroglycerin, heart rate 40 times / minute, cannon sound on auscultation, and coronary angiography showed severe blockage of the right crown. These information suggested that the patient may have developed a serious arrhythmia.

In the case of severe blockage of the right coronary artery, it may most likely affect the conduction system of the right heart, especially the blood flow supplying the right atrium and AV node. Therefore, the most likely cardiac arrhythmia to occur is:

* * A. 3-degree atrioventricular block * *

Three-degree atrioventricular block (also known as complete atrioventricular block) refers to the complete failure of electrical signals between the atrium and

ventricles, leading to the independent beating of the atrium and ventricles. This condition may lead to a significant decrease in heart rate, as mentioned in the topic at 40 beats / min, and this severe block is strongly associated with insufficient blood supply to the right coronary artery.

Therefore, the best answer is: * * A. Third-degree atrioventricular block * *

60. (Type A2 question)

Female, 70 years old, sudden palpitations for two hours, accompanied by dizziness and fatigue, cold sweat, BP80 / 50mmHg, no heart enlargement, heart rate 180 times / min, absolute irregular heart rhythm, the first heart sound strength, the valve auscultation and pathological murmur, the most close

A. Temporary cardiac pacing

B. Quiet injection of amiodarone vs

C. synchronized cardioversion

D. Mettoprolol note

E. electric defibrillation

Reference answer: C Your answer:

Graphic analysis: [analysis] elderly women, heart rate is absolutely uneven, the strength of the first heart sound, consider atrial fibrillation. BP80 / 50mmHg, indicating that the blood pressure drop required urgent electrical cardioversion (C pair).

[Error avoidance] For atrial fibrillation with rapid ventricular rate and ineffective drug treatment, atrioventricular node ablation or modification can be performed, and at the same time

Place for a permanent pacemaker. For AF patients with a slower ventricular rate, the longest RR interval of> 5 seconds or significant symptoms should also be tested

Considering pacemaker therapy (A error), the drugs that control the ventricular rate include β -blockers, calcium channel blockers, and digitalis production

Agents and certain antiarrhythmic drugs (e. g., amiodarone, dronedarone) This patient currently has decreased blood pressure, and heart rate control is not the first

To treat patients (BD error), electrical defibrillation is mainly suitable for

ventricular fibrillation or rapid arrhythmia (E error).

This 70-year-old woman had palpitations, dizziness, fatigue, cold sweat, hypotension (BP 80 / 50 mmHg) and high heart rate (180 beats / min), absolute irregular rhythm and varying first heart sounds, indicating that she may have experienced a severe arrhythmia. Absolutely irregular heart rhythm may indicate atrial fibrillation. Given her disease urgency (such as hypotension and high heart rate), immediate intervention was required.

Option C "Synchronous electrical cardioversion" is the preferred treatment in this setting. Synchronous electrocardioversion is an emergency medical procedure used to treat severe arrhythmias such as unstable atrial fibrillation or ventricular tachycardia, especially when the patient develops hemodynamic instability (e. g., hypotension). To restore normal heart rhythm by giving the heart.

Therefore, the most appropriate answer is: C. synchronous electrical repetition

First, we will conduct a detailed analysis of the elderly woman's symptoms in order to accurately diagnose her condition and choose the most appropriate treatment option.

The patient was an elderly female with sudden palpitations, dizziness, fatigue, and cold sweat, which are typical symptoms of low blood flow and may be related to hemodynamic instability caused by arrhythmia. Physical examination revealed low blood pressure, heart rate as high as 180 beats per minute, absolute irregular rhythm, and varying first heart sound, which are typical manifestations of atrial fibrillation. Combined with the patient's symptoms and signs, we can make a preliminary diagnosis of atrial fibrillation.

Next, we analyze the options one by one:

A. Temporary cardiac pacing: usually used in patients with morbid sinus syndrome or high atrioventricular block, not for atrial fibrillation.

B. Amiodarone intravenous injection: Amiodarone is a broad-spectrum antiarrhythmic drug used for restoring atrial fibrillation and maintaining sinus rhythm in this patient.

C. Synchronous electrocardioversion: Although it can be used for AF cardioversion, in this case, direct electrical cardioversion may pose a risk due to the patient's hemodynamic instability, and should not be performed without adequate preparation and evaluation.

D. Mettoprolol static note: Mainly used to control ventricular rate, not cardioversion, and in this case of hemodynamic instability, the use of β -blockers may further reduce blood pressure and aggravate the condition.

E. Electrodefibrillation: usually used for fatal arrhythmias such as ventricular fibrillation or pulseless ventricular tachycardia, not for atrial fibrillation.

In conclusion, considering the patient's symptoms, signs and possible diagnosis, the most appropriate treatment option is B. amidarone static infusion, because it both overturns AF and maintains sinus rhythm and is relatively safe.

Therefore, the correct answer is the B. amiodarone static infusion.

According to the information in the topic, the 70-year-old female patient had sudden palpitations for two hours, accompanied by dizziness, fatigue, cold sweat, blood pressure of 80 / 50 mmHg, heart rate of 180 beats / minute, absolute uneven heart rhythm, first heart sound, and pathological murmur.

Patients present with acute arrhythmia (possibly atrial fibrillation or atrial flutter) with hemodynamic instability (hypotension), according to the management guidelines for acute arrhythmia. So the best answer is:

F. synchronized cardioversion

61. (Type A2 questions)

A 27-year-old male presented with 1 day of diarrhea and vomiting. On July 13, the watery stool 10 times, vomiting 1, physical examination: T37.5℃, P110 / min, R24 / min, BP90 / 52mmHg, WBC 8109 / L, N0.78, which was a rapid clinical examination

A. Blood biochemical examination

B. fecal routine and smear

C. Blood culture and drug sensitivity

D. Faecal motility and actuation test

E. fecal culture and drug susceptibility

Refer to the answer: D. Your answer:

Graphic analysis: [analysis] The patient had diarrhea (ten rare watery stools), vomiting, decreased blood pressure, white blood cells and normal range of neutrophils (excluding bacterial infection), considering the possibility of cholera infection. After 6 hours of culture of fresh feces or alkaline water bacteria in the early stage, do dark field microscope, visible shuttle moving vibrio, that is, positive power test, can quickly diagnose cholera, so this question chose D.

[Avoid mistakes] Diarrhea in cholera patients is often the first symptom, manifested as painless severe diarrhea, not accompanied by urgent weight, quickly turn to

Rice swill sample water stool or wash meat water sample blood stool. Vomiting often occurs after diarrhea, often with nausea, with jet vomiting.recurrence

Failure is a hypovolemic shock caused by severe water loss.

This case describes a 27-year-old male patient presenting with a day of diarrhea and vomiting. Physical examination revealed the patient with body temperature 37.5℃, heart rate 110 beats / min, respiratory rate 24 beats / min, blood pressure 90 / 52mmHg, white blood cell count 810 ^ 9 / L, and neutrophil ratio 0.78. Based on these symptoms and signs, prompt diagnosis and management are required.

The option analysis is performed as follows:

-A. Blood biochemical tests can be used to check the metabolic status and electrolyte balance of patients, but may not be the most direct diagnosis for the causes of acute diarrhea and vomiting.

-B. fecal routine and smear, which can be used to check for infectious enteritis, is one of the routine diagnostic methods.

-C. Blood culture and drug sensitivity, suitable for suspected blood infection, although the patient has fever and increased heart rate, but no obvious symptoms of bacteremia.

-D. Faecal motility and actuation test, more used for the diagnosis of chronic diarrhea.

-E. Fecal culture and drug sensitivity, suitable for suspected infectious diarrhea, which can detect specific pathogens and perform drug sensitivity testing.

Considering that the main symptoms were acute diarrhea and vomiting, the initial judgment may be infectious diarrhea. Therefore, the most suitable examination are routine and smear (B) and fecal culture and drug sensitivity (E).fecal routine and

smear can rapidly diagnose infectious enteritis, while fecal culture and susceptibility can identify the specific pathogen and choose the appropriate antibiotics. Given the requirement for the best answer and the need for rapid clinical diagnosis, I tend to choose * * E. fecal culture and susceptibility * * because it can provide more specific pathogen information and treatment guidance.

analyze:

Young male patients, diarrhea, vomiting 1 day, onset time for July 13, summer season for bacterial diarrhea, watery, vomiting, low heat, heart rate, fast breathing, low blood pressure, normal white blood cells, increased neutrophils, according to the patient symptoms, signs, auxiliary examination results, considering the diagnosis of bacterial dysentery. Bacterillary dysentery is an intestinal infectious disease caused by Shigella infection. For rapid diagnosis, stool routine and smear examination should be carried out. If macrophages and a large number of white blood cells and red blood cells are detected in stool, it can be confirmed.

Option A Blood biochemical examination is mainly used to understand the liver and kidney function, blood glucose, blood lipid, electrolytes and other indicators, which is not helpful for the diagnosis of bacterial dysentery, so it is excluded.

Option C blood culture and drug sensitivity, option E fecal culture and drug sensitivity, both can determine the pathogen type and conduct drug sensitivity test, to provide a basis for the clinical selection of antibiotics, but it takes a long time, so it is not regarded as the first choice for rapid diagnosis.

Option D Faecal motility and actuation test are not available clinically and can be excluded preferentially.

To sum up, the correct answer is: B. fecal routine and smear.

For this patient, a 27-year-old man with 1 day of diarrhea and vomiting developed thin watery stool, and physical examination showed body temperature of 37.5°C, heart rate of 110 beats / min, respiratory rate of 24 beats / min, blood pressure of 90 / 52mmHg, white blood cell count of 810 ^ 9 / L, and neutrophil ratio of 0.78. The patient had signs of dehydration (hypotension, tachycardia) and symptoms of acute gastroenteritis.

In this case, the priority of rapid clinical examination is to confirm the source of infection and address the dehydration problem. The most directly related examination

is to understand the cause of diarrhea, especially the presence of bacterial infection. Therefore, the quick examination should be:

B fecal routine and smear

Through routine fecal tests and smear tests, the presence of bacterial infections or parasites can be quickly assessed, and the use of antibiotics is initially guided. Other tests such as blood biochemistry, blood culture and drug sensitivity and fecal culture and drug sensitivity, while also important, often require more time and are not the first choice for rapid clinical examination.

So, the most correct answer is: B. fecal routine and smear.

62. (Type A2 question)

Female, 57 years old. The left thumb is stiff with pain for 3 years. In the past half a year, the swelling and limited activity in the place, the left fingers and the palm can touch hard nodules, and the passive affected finger can sound with pain. The absence of significant abnormalities in the left X-ray is most likely

A. osteoarthritis

B. intra-articular detachment

C. Rheumatoid arthritis

D. rheumatic arthritis

E. tendinitis stenosans

Reference answer: E Your answer:

[Analysis] The patient is middle-aged and elderly women (people with tenosynovitis), left thumb morning stiffness pain, flexion and extension fingers (typical manifestations of stenosis tenosynovitis), so the most likely diagnosis of the patient is stenosis tenosynovitis (E pair).

[Avoid mistakes] Osteoarthritis is mainly a degenerative lesion of the joint, mainly is the joint pain after the activity of the aggravation, and the X-ray has the corresponding

Performance (A error), the presence of the free body in the joint cavity will lead to limited joint function, pain, joint jam, swelling and other diseases

Form, X-ray visible corresponding performance (B error). Rheumatoid arthritis (C wrong) is mainly manifested by recurrent, symmetrical attacks

Multiple small arthritis, to the proximal interphalangeal joint, metacarpophalangeal joint is the most common, rheumatoid arthritis to infection

Form, often involve large joints, such as knee joint, elbow joint, shoulder joint, will not appear joint deformity, and can also be shown as a joint

Red swelling and pain, intestinal infection, sore throat, cough, subjaw lymph node enlargement and other conditions, and often with myocarditis, endocarditis

And other diseases appear together (D wrong).

Given the history and symptoms, the most likely diagnosis is E. tenosynovitis (also known as trigger finger). The patient had morning stiffness and pain that lasted for three years and had swelling and limited mobility in the last six months. Hard nodules and accompanying painful bounce sounds during passive activity are typical symptoms of stenotic tenosynovitis. Although rheumatoid arthritis (option C) may also present as pain and stiffness of the fingers, touching a hard nodule and bouncing with pain are more consistent with the characteristics of stenosis tenosynovitis. In addition, there are no obvious abnormalities in the left hand X-ray, which can also exclude joint lesions, such as osteoarthritis or rheumatoid arthritis, these conditions that usually show obvious abnormalities in the X-ray. Therefore, option E (stenotic tenosynovitis) is the most appropriate answer.

First of all, we analyze the question stem:

Patient information: 57-year-old female with left thumb stiffness and pain for 3 years.

Symptoms in nearly half a year: swelling and limited movement of the left thumb, left fingers and palms of the fingers can touch hard nodules.

Special signs: passive movement can sound with pain.

X-ray inspection: the left hand X-ray has no obvious abnormality.

Next, do the logical reasoning for each option:

A. Osteoarthritis: Osteoarthritis is mainly manifested as joint pain, stiffness and swelling. The joint space narrowing and osteophyte formation can often be seen in

X-ray examination. However, it is clearly mentioned that the X-ray has no obvious abnormalities, so the possibility of osteoarthritis is unlikely.

B. Intraarticular free: intra-articular free body is usually caused by exfoliative osteochondrotis, osteoarthritis and other lesions, and X-ray examination should be abnormal. However, the question indicates that the X-ray has no obvious abnormality, so this option is unlikely.

C. Rheumatoid arthritis: Rheumatoid arthritis often presents with pain, swelling, and dysfunction of multiple joints, and the X-ray examination usually has significant joint changes. However, the symptoms described by the problem stem are mainly concentrated in the left thumb, and the X-ray examination is not abnormal, so the possibility of rheumatoid arthritis is low.

D. Rheumatoid arthritis: Rheumatoid arthritis is common in children, often manifested as wandering polyarthritis, and may be accompanied by carditis, subcutaneous nodules, annular erythema and other symptoms. It is not consistent with the description of the title stem, so it can be excluded.

E. Stenotic tenosynovitis: stenotic tenosynovitis is mainly manifested by finger ringing, pain, swelling, and limited movement, especially in the morning or after work fatigue. This is highly consistent with the description in the title stem of "morning stiffness with pain" and "passive activity affected finger can appear with pain".

In conclusion, combining the clinical presentation and the X-ray examination with no abnormal findings, the most likely diagnosis is stenotic tenosynovitis.

Therefore, the correct answer is: E. tenotic tenosynovitis.

According to the title description, the patient is a 57-year-old female with morning stiffness of the left thumb with pain for 3 years, swelling and limited activity in the past half a year, hard nodules in the left fingers and palms of the fingers can be touched, passive fingers can snap with pain, and the X-ray has no obvious abnormal. These symptoms suggest that the patient may have the following diseases:

1. * * Osteoarthritis * *: The main manifestations are joint pain, stiffness, limited movement, common in weight-bearing joints (such as knee joint, hip joint), X-ray common joint space narrowing, osteophyte formation and other characteristics.

2. * * Intraarticular free * *: joint pain, limited movement, often accompanied by

intraarticular foreign body sensation, X-ray can usually be found intraarticular free body.

3. * * Rheumatoid arthritis * *: multiple symmetrical arthritis, common in fingers, small joints, morning stiffness, swelling, pain, X-ray visible joint destruction and osteoporosis.

4. * * Rheumatoid arthritis * *: common in young people, the performance of wandering arthritis, accompanied by fever, heart involvement, etc., X-ray often has no specific performance.

5. * * tenotic tenosynovitis * *: Also known as trigger finger, especially in the morning or after a long time of inactive, activity is limited, X-ray is generally not abnormal.

Combined with the symptoms of the patient's symptoms, especially the stiffness, pain, swelling and limited movement of the left thumb in the morning, passive fingers with pain, and no obvious abnormalities in X-ray, the most likely diagnosis is stricture tenosynovitis.

So, the most correct answer is:

F. Narrowing tenosynovitis

63. (Type A2 questions)

A male with fever for three days and after paracetamol treatment, routine blood examination showed the phenomenon of central myelocyte poisoning, white blood cells 1.010 ^ 9 / L, red blood cells, normal platelets, absolute value of neutrophils <0.510 ^ 9 / L, which should be considered

A. hypoproliferative leukemia

B. agranulocytosis

C. Infectious mononucleosis

D. Acocytosis

E. Myelodysplastic syndrome

Reference answer: B. Your answer:

[Analysis] Paracetamol short-term use of mild adverse reactions, common nausea and vomiting, occasionally rash, agranulocyte deficiency (B pair), anemia, fever and mucosal damage and other allergic reactions. Excessive poisoning can cause liver damage. Long-term heavy drug use, especially in patients with low renal function, may develop renal colic, acute renal failure, or chronic renal failure (analgesic nephropathy).

[Error avoidance] leukopenia refers to the absolute leukocyte count of peripheral blood that was consistently lower than 4.0109 / L. Outside of neutropenia

Absolute peripheral blood neutrophil count, below 2.0109 / L in adults and below 1.810 9 / L in children aged 10 years or <10

Years than 1.5109 / L; severe cases less than 0.5109 / L, called granulocytosis.

This question examines hematological knowledge, especially for the differential diagnosis of fever, leukopenia and absolute neutrophils.

Option A, "hypoproliferative leukemia", which usually presents with pancytopenia, but is difficult to diagnose with this information alone.

Option B, 'granulocyte deficiency', which is a possible diagnosis, as absolute neutrophil values of <0.510 ^ 9 / L indicate a marked granulocytopenia. If this reduction is caused by drugs (e. g., paracetamol), then it may be drug-induced neutropenia.

Option C, "infectious mononucleosis", usually causes an increase in the total number of leukocytes, especially in monocytes, rather than decreasing.

Option D, "oncocytosis", which typically presents as an increase in oncocytes, is inconsistent with the situation described here.

Option E, "myelodysplastic syndrome", which can cause cytopenia but more commonly multilineage cytoreduction and often has varying degrees of maturation impairment.

Combining this information, the most likely answer is B. granulocyte deficiency. Because the patient developed fever and significant neutropenia after paracetamol administration, this may be a manifestation of drug-induced granulocytosis.

First, let's analyze the key information and data in the question stem:

The patient was male, with fever for three days.

After taking paracetamol (an antipyretic and analgesic drug), the blood routine was abnormal.

Central trophils are poisoning, which may indicate some kind of infection or inflammation.

The total number of leukocytes was significantly decreased (1.010 ^ 9 / L), but the numbers of red blood cells and platelets were normal.

The absolute neutrophil value was <0.510 ^ 9 / L, which is a key indicator pointing to specific hematological abnormalities.

Now, let's analyze the options one by one:

A. Hypoproliferative leukemia: This is a less myeloproliferative leukemia, usually associated with pancytopenia, including red blood cells, white blood cells, and platelets. But it is clear that red blood cells and platelets are normal, so this option is unlikely.

B. Granulocyte deficiency: This refers to the absolute value of neutrophils being lower than 0.510 ^ 9 / L, which corresponds exactly to the description in the title stem. Granulocyte deficiency is often caused by drugs (such as antipyretic analgesics, antibiotics, etc.) or other external factors, consistent with the patient's medication history and blood routine results.

C. Infectious mononucleosis: this is a disease caused by EB virus infection, mainly manifested as fever, enlarged lymph nodes, sore throat, etc., and routine blood examination usually shows increased lymphocytes, which is inconsistent with the description of the title stem.

D. Oncocytosis: This is a disease characterized by eosinophilia, with no direct association with neutropenia.

E. Myelodysplastic syndrome: This is a disease originating from hematopoietic stem cells, usually reduced by one or more blood cells, and is often accompanied by an increase in original cells, which does not completely coincide with the description.

In conclusion, considering that the patient had neutropenia and decreased total leukocyte count, but normal erythrocytes and platelets, it most fits the characteristics of granulocyte deficiency.

Thus, the correct answer is a B. granulocyte deficiency.

According to the patient described by the stem: men had fever for three days,

neutrophil poisoning after paracetamol treatment, significantly decreased leukocytes (1.010 ^ 9 / L), absolute neutrophils <0.510 ^ 9 / L, and normal red blood cells and platelets. The most likely diagnosis of this manifestation is granulocytosis (neutrophilic deficiency).

Granulocytosis (neutropenia) usually refers to the absolute neutrophil count (ANC) lower than 0.510 ^ 9 / L, and patients are prone to infection, especially bacterial and fungal infections. The blood routine results in the topic showed a significant decrease in white blood cell count and the absolute value of neutrophils <0.510 ^ 9 / L, which met the diagnostic criteria for granulocytosis.

So, the most correct answer is:

B agranulocytosis

64. (Type A2 question)

A 45-year-old male, fell during running, landed on the shoulder, square shoulder deformity, limited movement, negative shoulder test. Imaging examination showed that the bone cortex below the large humerus was not continuous, medially displaced, belonging to the proximal humerus fracture neer classification

A. neer part of the fracture

B. neer bipartite fracture

C. neer for a tripartite fracture

D. neer four-part fracture

E. Other types of fractures

Reference answer: B. Your answer:

[Analysis] The patient's previous history of trauma, which suggested that the bone cortex was discontinuous under the greater tuberosity of the humerus, which shifted medially to the large tuberosity and surgical neck fracture, that is, neer triple fracture (C pair).

[Error avoidance] Part of the fracture: the proximal humerus fracture, regardless

of the number of fracture lines, as long as the above displacement mark is not reached

Accurate, indicating that there is some soft tissue attachment connection at the fracture site, this fracture is not displaced or slightly displaced fracture, or said

Is a partial fracture (A wrong). Two parts of the fracture: only one site of the fracture and displacement, called the two parts of the bone

Fold (B error), it has 4 forms, namely anatomical neck fracture, large tuberosity fracture, small tuberosity fracture or surgical neck fracture.four-part

Frfracture (D error): When all four parts of the proximal humerus are displaced, four separate bone blocks are called four

Fracture, at this time the humeral head lateral dislocation, into a free state, blood supply damage is seriously serious, easy to occur ischemia necrosis.

According to the information given, the patient was a 45-year-old man who fell during running, developed square shoulder deformity in the shoulder, limited movement, negative shoulder test, and imaging examination showed discontinuous and medial displaced bone cortex below the greater tuberosity of the humerus. These symptoms and radiographic findings point to the proximal humeral fracture.

The Neer classification is a classification used to describe proximal humeral fractures. It divides proximal humerus fractures into four parts: large nodules, small nodules, benign tumors of the humeral trunk and the shoulder joint. The number of fracture sites determines the classification.

- ** A portion of the Neer fracture * * refers to only one partial fracture, usually without displacement.

- ** Neer binary fracture * * involves two broken parts, the common being the separation of the large or small tuberosity from the humeral trunk.

- ** Neer tripartite fracture * * includes three broken parts, commonly broken in the large tuberosity, small tuberosity and humeral shaft.

- ** A Neer four-part fracture * * refers to all four major parts having fractures.

The title describes cortical discontinuity below the greater tuberculus, with medially displacement, indicating at least one definite fracture part, but no mention of damage elsewhere. This may point to a relatively simple fracture type. Therefore, the most consistent description is * * Neer bipartite fracture * * (option B), as it involves

at least one major fracture site and its displacement, consistent with the discontinuity of the great tuberosity of the humerus.

analyze:

Middle-aged male patients, after trauma cause shoulder deformity, limited activity, imaging examination: the humerus nodule cortex discontinuous, medial displacement, according to the nuclear classification standard, if only a fracture, belong to the new part of the fracture, including surgical neck fracture, large nodules and small nodules fracture, glenoid fracture, anatomical neck fracture. In this case, the bone cortex was discontinuous below the greater tuberosity of the humerus, involving only one site, which was part of the neer fracture.

Option B: neer bipartite fracture is one fracture with shoulder dislocation, or two fractures without joint dislocation, which does not meet the meaning.

Option C: The neer tripartite fracture refers to two fractures with associated shoulder dislocation, or three fractures without joint dislocation.

Option D: neer quadruple fracture refers to the four anatomy of the proximal humerus, which is not consistent with the type of fracture in the present patient, so it is not selected.

Option E: Other types of fractures were not classified in the neer classification and were excluded.

To sum up, the correct answer is: A. nuclear part of the fracture.

According to the title description, a 45-year-old man fell and landed on the shoulder, developed square shoulder deformity, limited movement, and negative shoulder test. Imaging examination showed that the bone cortex below the macrotuberosity of the humerus was medially displaced. From these descriptions, it can be judged that this was a proximal humerus fracture. The Neer classification system is used to classify proximal humerus fractures according to their specific site and degree of displacement:

1. Part of the fracture of Neer: The fracture is not displaced or displaced by less than 1 cm, and the angle does not exceed 45°.

2. Neer bipartite fracture: fracture displacement more than 1 cm, or angle more than 45°, usually involving large or small nodules.

3. Neer tripartite fractures: two fracture lines, usually involving large nodules, small nodules, and surgical neck.

4. Neer four-part fracture: three fracture lines, involving large tuberosity, small nodule, surgical neck and anatomical neck.

According to the discontinuous and medial displacement of the bone cortex below the humeral greater tuberosity described in the title stem, this means that at least two parts were displaced, usually meeting the definition of a Neer tripartite fracture.

So, the most correct answer is:

B neer bipartite fracture

65. (Type A2 questions)

Male, 66 years old. Epodic chest pain for 1 h. He fell suddenly during his history and did not respond to calling and shoving. The measures that should be taken immediately now are this

A. Heart color ultrasound

B. Send it to the rescue room

C. Palpation of the great artery

D. Do electrocardiogram

E. View pupils

Reference answer: C Your answer:

Text analysis: [analysis] middle-aged and elderly male patients, onset chest pain for 1 hour (suspected myocardial infarction), loss of consciousness, call should not be, should be urgent to check whether the existence of heartbeat breathing, so this question optional C.

[Avoid mistakes] If the heart beat and breathing stop, CPR should be performed immediately. The order of CPR in adults is C-A-B, C

(Circulation) -Artificial chest compressions: the pressing frequency is 100~120

times / minute, and the pressing amplitude of adults is 5~6cm

About 5cm for children and 4cm for infants; A (Airway) -airway opening: 30 chest compressions before airway opening; B

(Breathing) -Artificial respiration: After the opening of the airway, first two artificial respiration, each lasting for 1 second

falling-rising tone.

This condition describes a 66-year-old male patient who developed paroxysmal chest pain and a sudden fall during the consultation, unresponsive to calling and pushing. This may imply that the patient had a cardiac arrest or severe circulatory disturbance.

The immediate measures taken should be targeting first aid and assessing the patient's vital signs. Among these options, the most immediate and urgent measure is to transport the patient to the emergency room for emergency treatment, as this condition may require immediate life support and further assessment.

Therefore, the most appropriate answer is: B. Send to the emergency room.

analyze:

An elderly male patient with paroxysmal chest pain, suggestive of possible cardiac disease such as angina or myocardial infarction, a sudden fall during the history, and no response to calling and pushing, suggesting the possibility of cardiogenic shock, or cardiac arrest. At this time, the main artery should be palpated first, to determine whether the cardiac arrest has occurred, and then take the corresponding emergency measures.

Option A for cardiac color ultrasound is mainly used to check the structure and movement state of the heart, can not assess the state of consciousness of the patient, and is not the first step of rescue, so it is not selected.

Option B to be sent to the emergency room should be performed after the initial evaluation of the patient's condition. If the patient has a cardiac arrest, CPR should be performed immediately, rather than directly sent to the emergency room, so it is excluded.

Option D ECG is mainly used for the diagnosis of arrhythmia, myocardial infarction and other diseases. The patient has lost consciousness, and the primary task is to judge the patient's vital signs of the patient, so it is not considered.

Option E check the pupil can assess the state of consciousness, but cardiac arrest gold rescue time only 4 minutes, so should first through the way of palpation artery, quickly determine whether cardiac arrest, if cardiac arrest need immediately cardiopulmonary resuscitation, if the heart did not stop, then other vital signs, such as check the pupil, so do not choose.

To sum up, the correct answer is: C. Palpation of the large artery.

According to the description in the title, the patient was a 66-year-old male who developed paroxysmal chest pain for 1 hour, suddenly fell during the history, and did not respond to calling and shoving. This suggests that the patient may have had a cardiac arrest or any other severe emergency. In this case, it is most important to assess the patient's vital signs immediately.

In clinical first aid, for patients with a sudden fall and no response, the primary measure is to assess the presence of large artery beats to determine the presence of cardiac arrest. So the correct answer should be:

F. Palpation of the great artery

66. (Type A2 question)

A man occasionally had headache and chest tightness. His self-measured blood pressure was about 150 / 110mmHg for many times, and his heart rate was 98 times / minute. After medical treatment, diet and exercise were not good, his blood pressure was measured at 150 / 95mmHg, and bilateral renal artery B ultrasound was not special. Should take

A. Oral hydrochlorothiazide

B. Continue to observe for half a year

C. Oral bisoprolol

D. Taking tolapril under the tongue when the symptoms appear

E. Oral terazosin

Reference answer: C Your answer:

[Analysis] middle-aged men, multiple measurements of high blood pressure, considering hypertension, kidney color ultrasound without special consideration of

essential hypertension, heart rate fast selection of β receptor antagonist can inhibit the heart β adrenergic receptor, slow heart rate, weaken myocardial contractility, reduce blood pressure (C pair).

[Error avoidance] Diuretics can lower blood pressure, but have no heart rate control (A error); patients have confirmed hypertension

Immediate treatment, not only observation (B error); hypertensive pressure did not required emergency treatment (D

Wrong); Trazosin is a selective α 1-receptor blocker that reduces peripheral vascular resistance to both systolic and diastolic blood pressure

Reduced effect but no controlled heart rate effect (E error).

This topic examines the management and treatment of hypertension. According to the information, the man had high blood pressure (150 / 110 mmHg to 150 / 95 mmHg) and high heart rate (98 beats / min), and his blood pressure was not effectively controlled after lifestyle changes (diet, exercise). Bilateral renal artery B ultrasound was not special, indicating that there was no sign of nephrogenic hypertension.

Hydrochlorothiazide in option A is A diuretic for treating hypertension, but may not be the drug of choice because it may cause blood glucose and dyslipidemia, especially in patients with increased heart rate gain.

Further observation of option B is not an appropriate option for patients who have confirmed persistent hypertension and increased heart rate, as this may increase the risk of cardiovascular and cerebrovascular events.

Bisoprolol with option C is a β blocker that lowers blood pressure and heart rate and is suitable for patients with both hypertension and increased heart rate increase.

Topapril with option D is an angiotensin-converting enzyme inhibitor (ACE inhibitor) that is commonly used in the long-term management of hypertension rather than only when symptoms occur.

Trazosin with option E is a thiazide diuretic used to treat hypertension but, similar to hydrochlorothiazide, may not be the best option for patients with a higher heart rate increase.

Taken together, the best option seems to be option C (oral bisoprolol), because it can manage both hypertension and heart rate growth. However, the actual choice should be based on the specific situation of the patient and the doctor's professional

judgment.

analyze:

The patient was a young male with high multiple self-test blood pressure, poor blood pressure control after medical treatment, diet control and exercise. He could be diagnosed as hypertension. Because the patient had no special bilateral renal artery B ultrasound, secondary hypertension could be excluded, and the diagnosis of essential hypertension was considered. Patients seek medical treatment according to the guidance to control diet, blood pressure is still not well controlled after exercise, should be treated with drugs. The patient had a fast heart rate, a sign of increased sympathetic tension and high diastolic pressure, so β -blocker should be selected, and bisolol is a selective β 1-receptor blocker, which can slow heart rate and reduce cardiac output, thus reducing blood pressure, and is the first choice of treatment.

Option A Oral hydrochlorothiazide is A thiazide diuretic, which mainly reduces sodium, reducing extracellular volume and reducing peripheral vascular resistance. It is suitable for patients with simple systolic hypertension, salt-sensitive hypertension, obesity or diabetes and heart failure, which is not consistent with the symptoms of this patient, so it is not selected.

The patient has developed symptoms of hypertension, and after controlled diet and exercise, the blood pressure is still poorly controlled, so drug treatment should be taken in time. Therefore, option B should be observed for half a year, which can be ruled out.

Option D: Toranapril is not suitable for the treatment of acute hypertension, and is generally used for the maintenance treatment of chronic hypertension, so it is not considered.

Option E Oral terrazosin is a α -blocker and is not recommended alone because of its many side effects, so it is excluded.

In conclusion, the correct answer is: C. Oral bisoprolol.

According to the information provided in the topic, the man occasionally had headache and chest tightness, blood pressure remained at high levels (150 / 110 mmHg), fast heart rate (98 times / min), diet and exercise regulation had no obvious effect, and renal artery B-ultrasound examination showed no abnormalities. These symptoms and findings suggested that the man may have essential hypertension and required medication to control blood pressure.

Each option is analyzed as follows:

- ** A. Oral hydrochlorothiazide * *: hydrochlorothiazide is A commonly used diuretic and can be used to treat hypertension. It lowers blood pressure by draining excess sodium and water from the body.

- ** B. Continue observation for half a year * *: The man's blood pressure has been higher than normal, observation alone may delay the treatment opportunity.

- ** C. Oral bisoprolol * *: Bisoprolol is a β -blocker that reduces heart rate and blood pressure. It is especially suitable for patients with a fast heart rate and have hypertension.

- ** D. Taking tolapril under the tongue when symptoms appear * *: Tolapril is an ACE inhibitor and is usually used for long-term management of hypertension rather than temporary treatment at the onset of symptoms.

- ** E. Oral terrazosin * *: Trazosin is a α receptor blocker, mainly used for the treatment of benign prostatic hyperplasia, but can also be used in the treatment of hypertension.

Combined with the man's hypertension and the characteristics of a fast heart rate, the best answer is * * C. oral bisoprolol * *. Bisoprolol not only lowers blood pressure, but also slows the heart rate and helps relieve symptoms such as headaches and chest tightness.

Therefore, the answer is * * C. oral bisoprolol * *.

67. (Type A2 question)

A middle-aged male with acute extensive anterior wall MI was admitted. The next day, the patient had increased BP76 / 50mmHg, P130 beats / min, central venous pressure of 25cmH2O in both middle and lower lungs. Treatment selection

A. devil's brew

B. furosemide

C. arterenol

D. adrenalin

E. digitalis

Reference answer: E Your answer:

[Analysis] Male patients, history of acute myocardial infarction, more than 24 hours, low blood pressure (BP76 / 50mmHg), fast heart rate (P130 times / min), central venous pressure of 25cmH2O, double middle and lower lungs and wet rales (indicating cardiac insufficiency), considered heart failure after acute myocardial infarction. Gitalis is prohibited within 24 hours of acute myocardial infarction, and cardiac dysfunction and heart failure are available after 24 hours, so this question is selected E.

[Avoid mistakes] Nitroglycerin is mainly dilating blood vessels, used in myocardial infarction (A wrong), the patient's blood pressure is low, do not consider the use of profit

Urine (B wrong); norepinephrine enhances myocardial contractility and increases peripheral resistance to improve cardiac load

No significant effect (C error); adrenaline mainly increases myocardial contractility while increasing heart rate, and is easy to use in heart failure

Lead to cardiac arrhythmia (D wrong).

A middle-aged male patient with acute extensive anterior wall myocardial infarction aggravated the following day, hypotension (BP76 / 50mmHg), increased heart rate (P130 beats / min), increased central venous pressure (25cmH2O), double lower lung sounds and wet rales, both signs and signs indicate that the patient may develop cardiogenic shock with pulmonary congestion.

Treatment options should aim at improving hemodynamic status, increasing cardiac output and reducing pulmonary edema. Of these options:

-A. Nitroglycerin can dilate blood vessels and reduce preload and afterload, but in this case, the shock may be exacerbated by lowering blood pressure.

-B. Furosemide is a diuretic that can be used to reduce pulmonary edema but does not directly improve the hemodynamic state and should be used in the acute phase to avoid further decline in blood pressure and deterioration of renal function.

-C. Nrepinephrine has potent α receptor activation effects to increase cardiac output and increase blood pressure, suitable for treating cardiogenic shock.

-D. Adrinephrine increases cardiac contractility and heart rate, but is commonly

used in cardiogenic shock as an option when unresponsive to other treatments.

-E. Digitalis can enhance cardiac contractility, but it is mainly used for the treatment of heart failure and atrial fibrillation, and is not suitable for the immediate rescue of acute cardiogenic shock.

Given this patient's condition and the need for rapid hemodynamic stabilization, the best option should be * * C. norepinephrine * *. It is effective in increasing blood pressure and cardiac output and is suitable for the treatment of cardiogenic shock caused by acute myocardial infarction.

analyze:

The middle-aged male was admitted for acute extensive anterior wall myocardial infarction. Now the patient had symptoms of hypotensive shock, accelerated heart rate, increased central venous pressure and double middle and lower lung wet rales, which was diagnosed as heart failure after acute myocardial infarction. At this time, vasoactive drugs should be preferred to maintain blood pressure and ensure blood perfusion in the heart and brain * *. Norerepinephrine * * is a strong α receptor agonist, which can cause vasoconstriction of arterioles and venules, and the skin and mucosal vasoconstriction is the most obvious, which can quickly rise hypertension. It is a vasoactive drug against shock.

Option A nitroglycerin is mainly used for acute attack of angina pectoris, also used for reducing blood pressure or treatment of congestive heart failure, but the treatment effect on cardiogenic shock is not obvious, so it is not selected.

Option B furosemide was a diuretic, mainly used to eliminate edema, inconsistent with the patient's current treatment target, and was excluded.

Option D epinephrine is commonly used in the treatment of anaphylactic shock, with excited myocardial, hypertension, relaxation bronchial smooth muscle, but because of its excited β 1 and β 2 receptors, can increase myocardial oxygen consumption, cause arrhythmia, and can aggravate myocardial ischemia, generally not used for the treatment of acute myocardial infarction and cardiogenic shock, so do not choose.

Option E digitalis is an inotropic drug, which can increase myocardial oxygen consumption, myocardial infarction should be banned within 24 hours, otherwise it will increase the burden of the heart, worsen the condition, and is inconsistent with the question.

In conclusion, the correct answer is: C. norepinephrine.

This middle-aged male patient presented symptoms of acute left heart failure after acute extensive anterior wall myocardial infarction, including increased aggravation, low blood pressure (BP76 / 50mmHg), fast heart rate (P130 beats / min), high central venous pressure (25cmH2O), and double middle and lower lung wet rales. These symptoms suggest that the patient may have had cardiogenic shock and acute pulmonary edema.

For this situation, treatment focuses on reducing the heart burden, improving cardiac function and relieving pulmonary edema symptoms. According to the clinical guidelines and best practices, the most appropriate treatment options are:

B furosemide

Furosemide is a potent diuretic that rapidly reduces pulmonary edema and lowers central venous pressure, thereby improving dyspnea. The use of furosemide can effectively relieve the patient's symptoms and stabilize the condition.

* * Analysis of other options: * *

-A. Nitroglycerin: used to relieve angina and reduce cardiac preload, but carefully in patients with low blood pressure.

-C. Nrepinephrine: mainly used to correct hypotension, but not directly improve the symptoms of pulmonary edema.

-D. Adrenaline: mainly used for cardiac arrest and severe allergic reactions, not preferred treatment of pulmonary edema.

-E. Digitalis: For the control of chronic heart failure and atrial fibrillation, it is not suitable for rapid intervention in the acute phase.

So the best answer is:

B furosemide

68. (Type A2 question)

Female, blood pressure 190 / 110mmHg, B ultrasound suggested 55% left renal artery stenosis and 50% right renal artery stenosis, the cause of increased blood pressure

A. Activation of the sympathetic nervous system

B. Activation of the renin-angiotensin-aldosterone system

C. Activations of the kinin system

D. Elevated vasopressin

E. Elevated atrial natriuretic peptide

Reference answer: B. Your answer:

[Analysis] Due to renal artery stenosis, renal blood perfusion reduces renal baroreceptor feedback will activate the renin-angiotensin-aldosterone system, resulting in increased blood pressure, and promote renal perfusion through increased blood pressure (B pair).

[Error avoidance] renovascular hypertension is the second cause of secondary hypertension, due to the unilateral or bilateral main branches of the renal artery

Lumen stenosis, resulting in decreased renal blood perfusion and causing hypertension. The presentation of renal artery stenosis include abdominal vascular murmur, since

Primary hypokalemia and progressive renal function.

This topic examines secondary hypertension, especially caused by renal artery stenosis. Renal artery stenosis causes reduced blood flow to the kidney and triggers the release of renin, which activates the renin-angiotensin-aldosterone system (RAAS), increases the production of angiotensin II, and ultimately leads to increased blood pressure. Therefore, the cause of the elevated blood pressure is the activation of the renin-angiotensin-aldosterone system.

Option B "Activate the renin-angiotensin-aldosterone system" is the most appropriate answer.

analyze:

In a young female patient, blood pressure was elevated, and B ultrasound

suggested 55% stenosis of the left renal artery and 50% stenosis of the right renal artery. For this patient, the most likely cause of elevated BP was bilateral renal artery stenosis, which caused decreased renal perfusion pressure, thus stimulating the release of renin, resulting in increased angiotensin and aldosterone content, and then increased blood pressure. Therefore, the elevated blood pressure in this patient was caused by the activation of the renin-angiotensin-aldosterone system.

Option A Activation of the sympathetic nervous system can increase heart rate, increases cardiac contractility, increases cardiac output, and increases blood pressure, but generally does not lead to such A high blood pressure, so it is not chosen.

Option C activates the kinin system to dilate the fine arteries, increased vascular permeability, bronchial smooth muscle relaxation, and causes pain, without raising blood pressure.

Option D The increase of vasopressin can increase the permeability of the distal convoluted tubules and the collecting duct to water, increase the reabsorption of water, further damage the urine concentration and dilution function, and reduce the urine volume, but there is no pressor effect, so it is not selected.

Option E elevated atrial natriuretic peptide can increase glomerular filtration rate, inhibit renal tubular reabsorption of sodium, produce significant natriuretic diuretic potassium discharge effect, inhibit the secretion of renin, angiotensin and aldosterone, and reduce blood pressure, inconsistent with the dry description.

In conclusion, the correct answer is: B. To activate the renin-angiotensin-aldosterone system.

The title described a woman with a blood pressure of 190 / 110mmHg, whose B ultrasound indicated 55% stenosis of the left renal artery and 50% stenosis of the right renal artery. Renal artery stenosis causes renal ischemia, which in turn activates a series of compensatory mechanisms to maintain renal perfusion and blood pressure.

In this case, the most likely cause of increased blood pressure is the activation of the renin-angiotensin-aldosterone system (RAAS). Renal artery stenosis leads to renal hypoperfusion, with decreased perfusion pressure detected by paragaglomerular cells, and subsequently renin secretion. Renin catalyzes the generation of angiotensinogen to angiotensin I, which is converted to angiotensin II in the presence of an angiotensin-converting enzyme (ACE). Angiotensin II is a potent vasoconstrictor that also stimulates the secretion of aldosterone, further increasing the reabsorption of sodium and water, leading to an increase in blood volume and thus a high blood

pressure.

Therefore, the most correct answer is: B. To activate the renin-angiotensin-aldosterone system

69. (Type A2 question)

A 58-year-old male with sudden chest pain for 2 hours showed persistent tear-like pain radiating to the shoulder, back and waist. She had had hyperlipidemia and hypertension for 5 years without standardized treatment. Physical examination: the blood pressure of the left upper limb is 140 / 85mmHg, and 180 / 105mmH times / minute of the right upper limb. For a clear diagnosis, the preferred examination is

A. aorta CTA

B. Chest X-ray

C. electrocardiogram

D. echocardiogram

E. coronary arteriongraphy

Reference answer: A. Your answer:

[Analysis] middle-aged men, sudden severe pain (is the most important and the most common clinical manifestation of aortic dissection), tearing, involving the shoulder, back and waist radiation. Previous history of hypertension (the most important risk factor for aortic dissection), physical examination: blood pressure 140 / 85mmHg in left upper limb, 180 / 105mmHg in right upper limb (inconsistency in both upper limbs). Based on the clinical manifestations, physical examination and electrocardiogram examination, the patient was considered as aortic dissection. The preferred examination for aortic dissection is: aortic CTA (for A pair), whose sensitivity and specificity can reach about 98%, aortogram can show the site of the cleft, define branch and aortic involvement, and estimate the severity of aortic valve insufficiency.

[Fault avoidance] Echocardiography is mostly used for the diagnosis and treatment of heart valve disease (D error), and chest X-ray can be used to observe the size of the heart

And appearance change (B error), electrocardiogram mainly diagnosed myocardial infarction and arrhythmia (C wrong), coronary angiography mainly diagnosed coronary form

Whether the artery is narrow (E is wrong).

Considering the 58-year-old male patient with sudden persistent chest pain, tear-like pain, and pain radiating to the shoulder, back and waist, combined with his history of hyperlipidemia and hypertension, the first diagnosis considered was aortic dissection. In this case, the apparent difference in left and right upper limb blood pressure also supports this diagnosis. Therefore, to clarify the diagnosis, the preferred examination is * * aortic CTA (computed tomography angiography) * *.

A. Aortic CTA-This option is the best test for the diagnosis of aortic dissection, detailed aortic anatomy and possible dissection.

So, the correct answer is * * A. aortic CTA * *.

analyze:

The patient is an elderly male patient with sudden chest pain, severe tearing pain, and radiation to the shoulder, back and waist, which is the typical chest pain manifestation of acute aortic dissection. The patient had previous hyperlipidemia and hypertension, and physical examination revealed asymmetric blood pressure in both upper limbs, suggesting the possibility of aortic dissection. Based on the patient's medical history, symptoms and signs, the patient was considered for the diagnosis of aortic dissection. In order to clarify the diagnosis, aortic CTA should be performed, which can clearly show the intima in the aortic cavity and the extent involved, and is the gold standard for the diagnosis of aortic dissection.

Option B chest X-ray helps to detect arterial calcifications, but it has little diagnostic value for aortic dissection, so it was not chosen.

Option C ECG is mainly used for the diagnosis of cardiac and circulatory system diseases, especially the arrhythmia, which is not helpful for the diagnosis of aortic dissection, so it is excluded.

Option D echocardiography can identify true, false lumen or intimal prolapse, but the lesions confined to the distal ascending aorta and the upper arch and the air in the main airway are not considered.

Option E coronary angiography is a common and effective method for the diagnosis of coronary atherosclerotic heart disease, which is not used for the diagnosis of aortic dissection and can be ruled out.

In conclusion, the correct answer is: A. aortic CTA.

According to the description, the patient was a 58-year-old man with sudden

chest pain for 2 hours, sustained tear pain, radiation to the shoulder, back and waist, and a previous history of hyperlipidemia and hypertension, and a significant left-right difference in blood pressure. These symptoms and signs highly suggest the possibility of aortic dissection.

To clarify the diagnosis, the most suitable examination is the examination method that can detail the morphological changes of the aorta. Therefore, the preferred examination should be the aortic CTA (computed tomography vascular imaging), because it can quickly and accurately demonstrate the dissection and rupture of the aortic wall.

So the best answer is:

A. aorta CTA

70. (Type A2 question)

A 62-year-old male with previously old myocardial infarction, 3 years after PCI, two and a half years ago, nocturnal paroxysmal dyspnea, intermittent bilateral lower extremity edema, and effective oral diuretic therapy. Cardiac ultrasound showed: LV enlargement, decreased segmental motion, LVEF35%, recent onset of paroxysmal atrial fibrillation, recommended drugs excluded

A. inderal

B. propafenone

C. digoxin

D. bisoprolol

E. amiodarone

Reference answer: B. Your answer:

[Analysis] Elderly men, previous old myocardial infarction, active cardiac fatigue, nocturnal paroxysmal dyspnea, intermittent double lower extremity edema, effective oral diuretic treatment (considering heart failure), atrial fibrillation. Cardiac ultrasound showed LV enlargement, decreased segmental motion of the LV anterior wall, LVEF35% (considering cardiac insufficiency). Considering that this patient is heart failure with atrial fibrillation, propafenone has negative inotropic effect, which

can increase the mortality of patients with moderate and severe heart failure, so it is not considered (B wrong, is the correct answer to this question).

[Error avoidance] β blockers can control heart rate and improve ventricular remodeling (AD pair), and amiodarone can cardiovert, causing

With the lowest incidence of arrhythmia, it is commonly used to maintain sinus rhythm, especially for combining organic heart disease

Patient (E), digitalis can reduce positive muscle strength and slow heart rate (C).

This 62-year-old man had a history of old myocardial infarction, percutaneous coronary intervention (PCI), and heart failure involvement such as cardiac exertion, nocturnal paroxysmal dyspnea and intermittent lower extremity edema, while diuretic therapy was effective. Cardiac ultrasound showed an enlarged LV, reduced segmental motion, left ventricular ejection fraction (LVEF) of 35%, and recent paroxysmal atrial fibrillation. The topic asked for choosing drugs that are not recommended.

In the treatment of heart failure and atrial fibrillation, the commonly used drugs include:

-Propranolol (β -beta-blocker)

-Propatenone (antiarrhythmic drug)

-Digoxin (cardiac glycoside)

-Bisoprolol (β -blocker)

-Amiodarone (antiarrhythmic drug)

According to the case, the patient had heart failure and atrial fibrillation, so β -blockers (e. g., propranolol or bexolol), antiarrhythmic drugs (e. g., propafenone or amiodarone), and cardiac glycosides (such as digoxin) were usually required. However, the overall condition of the patient and the specific characteristics of the drug need to be considered when choosing specific drugs.

With this in mind, digoxin (option C) is effective in treating heart failure and controlling AF, especially in reducing heart rate. However, digoxin needs to be used under close monitoring because it has a narrow therapeutic window and potential toxicity. But there is no direct reason that digoxin is not recommended.

Given the above, it is more likely that one of the β -blockers (propranolol or bisoprolol) or antiarrhythmic drugs (propafenone or amiodarone) is not recommended. In patients with chronic heart failure, β -blockers are widely recommended to slow

down the course of the disease and improve the prognosis. Compafenone and amiodarone are mainly used for the treatment of cardiac arrhythmias. Considering the high side effects of amiodarone, it may not be preferred in some patients with heart failure, especially during long-term use.

Therefore, among these options, while all drugs may be applicable in some cases, choosing drugs "not recommended" is complicated. If the patient's heart failure condition is severe and the risk of drug side effects is considered, * * Amiodarone (option E) * * may not be preferred because its long-term use is associated with serious side effects. However, this needs to be decided according to the specific clinical circumstances, as amiodarone is also an effective drug to control AF.

In conclusion, the most likely "not recommended" drug is E. amine

iodarone, mainly based on its potential side effects and the risk of potentially aggravating cardiac conditions in specific situations.

analyze:

The patient was an elderly male with a history of myocardial infarction and PCI surgery, recent activity after heart tired, nocturnal paroxysmal dyspnea, intermittent double lower limb edema, echocardiography showed left ventricular enlargement, left ventricular ejection fraction decreased, suggesting heart failure, recent onset of paroxysmal atrial fibrillation, considering the diagnosis of atrial fibrillation combined with heart failure. At this point, the key to treatment is to control the ventricular rate, restore sinus rhythm, and prevent thromboembolism. Propranolol is a β -blocker that can block β receptors in the heart, slow down heart rate, cardiac contractility, cardiac output and blood pressure, and is generally not used in patients with atrial fibrillation with heart failure.

Option B propafenone, option E amiodarone, can be used for AF fibrillation and maintain sinus rhythm, so were excluded.

Option C digoxin is a digitalis drug that enhances myocardial contractility for the treatment of heart failure and atrial fibrillation, in line with the treatment principles of this patient.

Option D bisoprolol is a selective β 1-blocker that reduces myocardial oxygen consumption, slows heart rate and antiarrhythmia, and has certain efficacy in patients with atrial fibrillation. Meanwhile, in patients with heart failure, bisoprolol is started at small doses, which helps to inhibit the adverse effects in the compensatory

mechanism of heart failure and improve the ventricular remodeling, so it is also applicable for this patient.

In conclusion, the correct answer is: A. Propranolol.

This 62-year-old man had a previous history of old myocardial infarction 3 years after PCI and developed symptoms of heart failure such as postmobilization heart involvement, nocturnal paroxysmal dyspnea and edema of both lower limbs. Cardiac ultrasound showed an enlarged LV with diminished segmental motion and an LVEF of 35%, indicating cardiac insufficiency. Recent paroxysmal atrial fibrillation.

For patients with this condition, the treatment of cardiac insufficiency and atrial fibrillation needs to be considered when choosing drugs. Certain drugs may aggravate cardiac insufficiency and are not recommended.

Let's analyze the various options:

A. * * Propranolol (Propranolol) * * -Propranolol is a non-selective β receptor blocker that is less frequently used in patients with cardiac dysfunction because it may aggravate symptoms of heart failure.

B. * * Proafenone (Propafenone) * * -Proafenone is an antiarrhythmic drug that is contraindicated in patients with cardiac dysfunction because it can cause negative inotropic effects and deterioration of heart failure.

C. * * Digoxin (Digoxin) * * -digoxin is commonly used in the control of heart failure and atrial fibrillation. It can help improve cardiac contractility and control the heart rate, which is an appropriate choice.

D. * * Bisoprolol (Bisoprolol) * * -Bisoprolol is a selective β 1-blocker in patients with heart failure, improving outcomes and controlling the heart rate of atrial

fibrillation.

E. * * Amiodarone (Amiodarone) * * -Amiodarone is an effective antiarrhythmic drug that can be used to control atrial fibrillation in patients with cardiac insufficiency.

Therefore, the answer is * * B. propafenone * *, because it is not suitable for patients with cardiac insufficiency and can aggravate the symptoms of heart failure.

The final-recommended drug for use does not include * * B. propafenone * *.

71. (Type A2 question)

Patient, female, is 70 years old. Repeated knee pain for 10 years, gradually aggravated for 2 years, and the joint sounded during activity. Physical examination: bone friction sound (+) in both knees, but no obvious swelling and tenderness. The most likely diagnosis is the

A. osteoarthritis

B. rheumatoid arthritis

C. adjuvant arthritis

D. ankylosing spondylitis

E. systemic lupus erythematosus

Reference answer: A. Your answer:

[Analysis] elderly female patients (people with good osteoarthritis), both knee pain, activity bounce for more than 10 years, knee bone rubbing (common manifestations of osteoarthritis), the most likely diagnosis of this case is osteoarthritis (A pair).

[Avoid wrong] Rheumatoid arthritis (B wrong) has extra-articular rheumatic nodules; reactive arthritis is mainly infection

Therefore, there will be extra-articular infection, some patients will appear redness (C wrong); ankylosing spondylitis (D wrong) is more common in green

Male years, the most typical and common symptoms is inflammatory back pain;

systemic lupus erythematosus is mostly young women with joint pain

Pain, swelling, and inflammation (E is wrong).

The patient's symptoms, including long-term bilateral knee pain, joint ringing and bone friction sounds found by physical examination, but without obvious swelling and tenderness, were most consistent with the clinical manifestations of osteoarthritis (OA). Osteoarthritis is a common joint disease, especially in the elderly, manifested by gradual wear and degeneration of articular cartilage, leading to pain and functional impairment.

Rheumatoid arthritis (RA) usually presents with pain, swelling, and morning stiffness in the joint, while reactive arthritis, ankylosing spondylitis, and systemic lupus erythematosus usually have other, more specific clinical features not mentioned in this case description.

Therefore, the most likely diagnosis is * * A. osteoarthritis * *.

analyze:

The elderly female of the patient had repeated pain in both knees for many years, and gradually worsened, and the joints were active. The physical examination found that the bone friction sound was positive in both knee joints, but there was no obvious swelling and tenderness in the joints. Combined with the age, clinical manifestations and physical examination of the patient, the possibility of osteoarthritis was considered. Osteoarthritis is a degenerative disease, due to age, obesity, strain, trauma, joint congenital abnormalities and many other factors cause articular cartilage degradation damage, joint edge and subchondral bone reactive hyperplasia, also known as osteoarthrosis, degenerative arthritis, senile arthritis, etc.

Option B rheumatoid arthritis is more common in middle-aged women, better in the wrist, metacarpal fingers, proximal interphalangeal joints, morning stiffness, multiple joints, symmetry, persistent swelling and pain, rheumatoid nodules, X-ray changes more bone destruction and joint space stenosis, is not consistent with the clinical manifestations of patients in the stem, so it is not selected.

Option C reactive arthritis is more common in young men, with the involvement of large joints of lower limbs, asymmetric, less joint type, arthritis generally lasts for 1 to 3 months, and some cases can last more than half a year, which does not meet the meaning of the question.

Option D Ankylosing spondylitis is more common in young men, the following

low back pain is the main early symptoms, there are obvious familial aggregation phenomenon, X-ray examination has characteristic "bamboo like spine", which does not conform to the performance of the patients in the problem stem, so it is excluded.

Option E Systemic lupus erythematosus is most common in women of childbearing age between 20 and 40 years old. The clinical manifestations are diverse, including rash, arthralgia, oral ulcer, alopecia, light allergy, etc., which is not consistent with the symptoms of the patient, so it is not considered.

In conclusion, the correct answer is: A. osteoarthritis.

According to the description in the title, the patient is a 70-year-old woman with recurrent double knee pain for 10 years, and the symptoms have gradually worsened in the past 2 years. During the movement, the joints sounded, and the physical examination found bone friction sounds (+) in both knee joints, but no obvious swelling and tenderness. These symptoms suggest the possible presence of a chronic joint disease.

For this condition, the most likely diagnosis is osteoarthritis (OA). Osteoarthritis is the most common joint disease, especially in the elderly, which is manifested as joint pain, movement with sound or friction sound, and diseased joints usually without obvious swelling and tenderness.

Therefore, the most likely diagnosis is: A. osteoarthritis

72. (Type A2 question)

Chemical drug A is indicated to be essential hypertension, and it is proposed to use drug B for human bioequivalence study. The oral dose is 0.5mg, and the area under the curve (AUC) of drug A and drug B is 27.2ng/h/ml and 23.3ng/h/ml, respectively. The above description of bioavailability is correct

A.A and B, not bioequivalent

The absolute bioavailability of the B.A drug was 54%

The absolute bioavailability of the C.B drug was 46%

D. The relative bioavailability of drug B for drug A is 86%

E. The relative bioavailability of drug A to drug B is 86%

Refer to the answer: D. Your answer:

Graphic analysis: [Analysis] Absolute bioavailability is equal to extravascular AUC / intravenous AUC. Relative bioavailability is equal to the test drug AUC /

standard drug AUC. According to the above two formulas, the relative bioavailability of drug B to drug A is 86%, so we choose D.

[Error avoidance] The bioavailability of a drug refers to the speed and process at which the drug is absorbed from the administered site into the systemic circulation

linear measure. This parameter is the first factor in determining the drug-dosage-effect relationship.

Bioequivalence is that the exposure of two drugs (usually measured by the area under the plasma concentration-time curve, AUC) and temporal characteristics (e. g., Cmax, Tmax) are similar enough to be interchangeable. Relative bioavailability was calculated by comparing the AUC or Cmax of the test drug (drug B) to the reference drug (drug A) as (drug B AUC / drug A AUC).

According to the data given in the topic, the AUC of drug A was 27.2 ng / h / ml, while the AUC of drug B was 23.3 ng / h / ml. To calculate the relative bioavailability of drug B to drug A, we can use the following formula:

\[\ Text {relative bioavailability} = \ left (\ frac {23.3} {27.2} \ right) \ times 100\%\]

Calculating this value can help us to identify the most correct answer.

The relative bioavailability of drug B to drug A was approximately 85.66%. This is closest to option D, where the relative bioavailability of drug B to drug A is 86%. Therefore, the correct answer is the D.

This is a question of bioequivalence evaluation. First, we need to understand the definition of bioequivalence: if the geometric mean ratio of the pharmacokinetic parameters of the test preparation and the reference preparation is between 80% and 125%.

First, we analyze the options item by item:

Option A: drugs A and B, which are not bioequivalent. To determine whether drugs A and B are bioequivalent, we need to calculate the AUC ratio of both. If the ratio is between 80% and 125%, it is considered as bioequivalent. In this question, the AUC ratio of drug B and drug A is 23.3/27.2=0.86, that is, 86%, which falls within the range of 80% to 125%. Therefore, the drug A and drug B are bioequivalent, so option A is wrong.

Option B: The absolute bioavailability of drug A is 54%. Absolute bioavailability is the ratio of the amount of drug entering the systemic circulation to the amount

administered, which cannot be calculated directly from the information given in the topic, so option B is incorrect.

Option C: The absolute bioavailability of drug B is 46%. Similarly, the absolute bioavailability cannot be calculated directly from the AUC values, so the C option is incorrect.

Option D: The relative bioavailability of drug B to drug A is 86%. Relative bioavailability is obtained with a non-intravenous formulation (e. g., tablet) as a reference formulation. In this question, the bioavailability of drug B relative to drug A is calculated as (AUC of drug B) * 100%, i. e. (23.3/27.2) * 100%=86%, so the D option is correct.

Option E: The relative bioavailability of drug A to drug B is 86%. By definition, this should be used to calculate the bioavailability of drug A, i. e. (AUC / AUC of drug B) * 100%, which is inconsistent with the data given in the title, so the E option is wrong.

In conclusion, the correct answer is that the relative bioavailability of drug D.B to drug A is 86%.

Based on the described information and the principles of the bioequivalence study, the answers are as follows:

Bioequivalence studies focus on the similarity in the rate and degree of drug absorption in the body, usually measured by the area under the curve (AUC). The AUC represents the total exposure of the drug in the blood.

The question is as follows:

-AUC of drug A = 27.2 ng · h / mL

AUC = 23.3 ng · h / mL

The question is about a description of bioavailability, focusing on the relative bioavailability of drugs A and B.

Relative bioavailability is the AUC ratio of two drugs (or different dosage forms) at the same dose under the same conditions, calculated using the following formula:

\[\ Text {relative bioavailability} = \ frac {\ text {B drug AUC}} {\ text {A drug AUC}} \ times 100\% \]

According to the title data:

\[\ Text {Relative bioavailability} = \ frac {23.3} {27.2} \ times 100 \% \ approx 85.66 \% \]

This matches the description in option D, at approximately 86%.

So the correct answer is:

D. The relative bioavailability of drug B for drug A is 86%

73. (Type A2 questions)

A 39-year-old male with fever with chills and severe right upper limb pain for 1 day had a history of rodent exposure. Physical examination: T39℃, P110 / min, R22 / min, BP120 / 75mmHg, conscious, forced questioning, right armpit touch, obvious tenderness, cardiopulmonary abdomen, laboratory examination: blood WBC 12.4109 / L, neutroils 0.86109 / L, lymphocytes 0.14109 / L. G-bacteria were detected by smear staining of lymph nodes. Cause the

A. Bacillus typhi

B. E.coli

C. Neisseria

D. Yersinia pestis

E. Haemophilus influenzae

Refer to the answer: D. Your answer:

Graphic parsing: [analytical] young and middle-aged male patients, rodent contact history (plague is the main source of infection), fever, right upper limb severe pain, leukocytosis, the right armpit palpable swollen lymph nodes, lymph node puncture fluid smear examination visible G-bacteria (yersinia plague for

gram-negative bacteria), comprehensive patient history and clinical performance, considering the diagnosis of plague, pathogenic bacteria for yersinia plague, so this topic D.

[Avoid mistakes] Plague and cholera are legal class A infectious diseases in China.

This case describes a 39-year-old man with a history of fever, chills, severe pain, and rodent exposure. The examination found the right axillary lymph node enlargement and tenderness, the blood examination showed increased white blood cell count and neutrophil increase, and the lymph node puncture fluid smear examination found gram-negative bacteria. These symptoms and signs, combined with the patient's contact history, are most consistent with the clinical manifestations of the plague.

Plague is mainly caused by Yersinia (Yersinia pestis. pestis) and is a disease associated with rodent contact. Plague can be transmitted through infected fleas, or through direct contact with infected animals. Patients may experience sudden high fever, chills, headache, muscle pain, and enlargement of the associated lymph nodes (armpit, neck, or groin). Yersinia pestis can be found in the blood and lymph node fluid.

Therefore, the best answer to this case is D. Yersinia pestis.

First, let's review the key information about the topic:

The patient was a middle-aged male with major symptoms of fever, chills, severe pain in the right upper limb, and a history of rodent exposure. Physical examination results showed increased body temperature, increased pulse and respiratory rate, and palpable tender nodules under the right armpit. Laboratory tests showed both leukocytes and neutrophils, and G-bacteria were visualized on smear staining of lymph nodes.

Next, we analyze the options one by one:

A. Typhi: Typhi is the pathogen of typhoid fever, often called Salmonella Typhi, mainly causing enteric fever, continuous bacteremia, reticular endothelial system involvement, and the formation of small abscess and ulcer in the distal ileum are the basic pathological features, which are not consistent with the symptoms of patients.

B. Escherichia coli: Escherichia coli is a normal resident bacteria in the intestinal tract, but after the host immunity drops or the bacteria invade the intestinal tissues and organs, the bacteria can become an opportunistic pathogen, causing extra-intestinal

infection, but the patient has no intestinal infection, so it is excluded.

C. Neisseria: Neisseria mainly causes human meningitis and sepsis, not transmitted through rodents, so it is not selected.

D. Yersinia pestis: Yersinia pestis is a pathogen of plague, mainly transmitted through rodents and rodents, and is associated with a variety of human plague, including bubonic plague, pneumonic plague and septicemic plague. The patient's rodent contact history, fever, chills, lymph node swelling and pain and other symptoms were all consistent with the clinical manifestations of plague, and laboratory tests found G-bacteria, consistent with the biological characteristics of Yersinia pestis.

E. Haemophilus influenzae: Haemophilus influenzae mainly causes infant pneumonia, meningitis and adult lower respiratory tract infection, which is not consistent with the symptoms of patients, so it is not considered.

In conclusion, the correct answer is: D. Yersinia pestis.

Based on the symptoms and laboratory findings given in the title, and the patient's history of rodent exposure, the most likely pathogenic pathogen was * * D. Yersinia pestis * *. Here is a detailed analysis:

1. * * Symptoms * *: The patient developed fever, chills, severe pain in the right upper limb, and palpable lymph nodes under the right armpit. These symptoms correspond with the typical presentation of plague.

2. * * Signs * *: The patient was febrile with body temperature of 39℃, heart rate increased (P110 beats / min), respiratory rate increased (R22 beats / min), and normal blood pressure. The right subaxillary lymph node was enlarged and had significant tenderness, which is also consistent with the characteristics of the plague lymphadenotype.

3. * * Laboratory examination * *:

-White blood cell count (WBC) increased infection.

-High proportion of neutrophils (0.86), consistent with the characteristics of

bacterial infection.

-Low proportion of lymphocytes (0.14).

-Nopuncture fluid smear stain showed G-bacteria, Yersinia pestis is a Gram-negative bacilli.

4. * * Epidemiological history * *: The patient had a history of rodent exposure, and one of the main routes of plague is through infected rodents and their fleas.

Considering these factors, the most likely pathogen to cause the patient's symptoms is Yersinia pestis.

So the best answer is:

* * D. Yersinia pestis * *

74. (Type A2 questions)

A 66-year-old woman had fever with urgency, frequency and pain for 3 days. Past medical history of diabetes mellitus for 10 years. Physical examination: percussion pain (+) in the left kidney area, urine sediment microscopy: RBC 3 ~ 5 / HP, WBC full view. Clean mid-course urinary bacterial quantitative culture: intestinal 106 cfu / ml, should be preferred

A. antibiotic treatment for 1 week

B. Antibiotics for treatment for 2 weeks

C. was treated without antibiotics

D. Antibiotics for treatment for 3 days

E. Single dose of antibiotics

Reference answer: B. Your answer:

[Analysis] The patient had frequent urination, fever, left kidney percussion pain, urine sediment microscopy: RBC 3 ~ 5 / HP, WBC full view. Quantitative culture of clean middle urinary bacteria: Escherichia coli 106 CFU / ml (the most common flora of pyelonephritis), diagnosed as acute pyelonephritis, antibiotics should be sufficient treatment, 14 days of treatment, if the urine bacteria is still positive, antibiotics should

be extended for 2 to 4 weeks, so this question is B.

[To avoid mistakes] (1) mild disease: can be treated in the outpatient service, the course of 10~14 days. Commonly used drugs are quinolones

Class (e. g., ofloxacin 0.2g, 2 times / day; ciprofloxacin 0.25g, 2 times / day or levofloxacin), semisthetic penicillium

Vegetarian (e. g. amoxicillin 0.5g, 3 times / day), cephalosporins (e. g. cefuroxime 0.25g, 2 times / day), etc.rule

After 14 days of therapy, it is usually 90% curative. If the urine bacteria is still positive, effective antibiotics should be used to continue treatment 4~

6 Weeks.(2) Severe infection with obvious symptoms of systemic poisoning: need hospitalization, should be administered intravenous drugs. Commonly used drugs, such as ampicyl

Cillin 1.0~2.0g, every 4 hours; cefotaxime sodium 2.0g, every 8 hours; ceftriaxone sodium 1.0~2.0g,

Once every 12 hours; levofloxacin 0.2g, once every 12 hours. Combination combination if necessary. Aminoglycoside antibiotics in the kidney

It should be used with caution. If the above treatment is better, oral antibiotics can be changed for 3 days after the heat withdrawal

It becomes a 2-week course. If there is no improvement in treatment for 72 hours, antibiotics should be changed according to the results of drug susceptibility test, and the course of treatment should not be less than 2 weeks. After this treatment, there is still persistent fever, attention should be paid to the complications of pyelonephritis, such as renal pus, perirenal abscess, infectious poisoning, etc.

The 66-year-old female patient with a history of diabetes, and the symptoms of urgency, frequency and urine pain, left kidney area percussion pain positive, urine sediment in red blood cells and white blood cell count, urine bacteria culture shows the presence of Escherichia coli (E. coli), these are acute complex urinary tract infection, may be the manifestation of acute pyelonephritis. In this case, appropriate antibiotic treatment is required.

In patients with diabetes, especially those with complicated urinary tract infections (e. g., acute pyelonephritis), the recommended antibiotic treatment is usually longer than non-complicated urinary tract infections. A single dose or 3 days of treatment is usually insufficient for complex infections, and therefore options D

and E are not optimal. Treatment usually takes longer times to ensure complete clearance of the infection and to avoid recurrence or bacterial resistance.

Therefore, the most appropriate treatment regimen for this condition is usually at least 7-14 days. Given this patient's condition, option A (1 week of antibiotics) may be a starting point, but it is more common practice to perform 2 weeks of treatment, especially for complicated urinary tract infection or suspected pyelonephritis.

Therefore, the most correct answer is * * B. Antibiotic treatment for 2 weeks * *.

analyze:

Elderly female patient with fever with urgency, frequency and pain, past history of diabetes. Physical examination showed percussion pain in the left kidney area, urine sediment microscopy showed white blood cells full view, positive red blood cells, clean middle quantitative culture of urinary bacteria showed Escherichia coli. Based on the patient's symptoms, signs, and laboratory tests, the most likely diagnosis of this patient was acute pyelonephritis. Infection in diabetic patients is generally not easy to control, so the course of treatment in patients with acute pyelonephritis combined with diabetes should be appropriately extended, and antibiotics should be preferred for 2 weeks.

Option A Antibiotic treatment for 1 week for diabetic patients is too short, it may not be cured, so it is not chosen.

The patient had symptoms of acute pyelonephritis and a history of diabetes mellitus and must be treated with antibiotics, so option C without antibiotic therapy was excluded.

Option D antibiotic treatment for 3 days, option E single dose of antibiotic, the course was too short to achieve the treatment effect for diabetic patients, so it was excluded.

In conclusion, the correct answer is: B. Antibiotics for 2 weeks.

Based on the case information provided by the title, the 66-year-old woman presented with symptoms of an acute urinary tract infection with a 10-year history of diabetes. Urine examination showed red blood cells and large white blood cells, and urine cultures showed a quantification of Escherichia coli at 10 ^ 6 cfu / ml, suggesting a bacterial urinary tract infection. This increases the risk of complicated urinary tract infections given the patient's age, symptoms and past medical history, especially diabetes.

For the treatment of complicated urinary tract infections, antibiotic therapy is generally recommended for longer periods to ensure complete clearance of the infection and to avoid recurrence and complications. According to clinical guidelines, antibiotic therapy for complicated urinary tract infections is usually recommended for 1 to 2 weeks. Therefore, in this case, the more reasonable choice is the antibiotic treatment for 2 weeks.

So the best answer is:

B Antibiotics for treatment for 2 weeks

75. (Type A2 questions)

Female, 35 years old, paroxysmal headache for 2 months, intermittent seizures in the past week, daily eating barbecue, physical examination, neck resistance, trunk and limbs can touch multiple subcutaneous nodules, blood WBC 16109 / L, N0.61, L0.20, cranial CT: multiple 0.5~1.0cm nodules in the brain, most likely

A. hematencephalon

B. epilepsia

C. brain tumor

D. brain abscess

E. cysticercosis

Reference answer: E Your answer:

Graphic analysis: [analytical] middle-aged women, paroxysmal headache, nearly a week to intermittent epilepsy (brain cysticercus cortical typical), trunk and limbs can touch multiple subcutaneous nodules (subcutaneous tissue and muscle cercariosis), usually like barbecue, head CT: brain multiple 0.5~1.0cm nodules, the most likely diagnosis of brain cysticercosis (E).

[Avoid mistakes] cysticercosis, also known as cysticercosis, pig cysticercosis, by the tapeworm larvae (cysticercaria) parasitic in the human body

Diseases caused by tissues and organs, the cause of cysticercosis is the feeding of taenia worm eggs.

This case describes a 35-year-old female with a history of paroxysmal headache and multiple recent seizures with multiple subcutaneous nodules on physical examination, blood examination showing leukocytosis and craniocerebral CT scan showing multiple intracerebral nodules. Based on these symptoms and findings, the most likely diagnosis is cysticercosis (E).

Cysticercosis (Neurocysticercosis) is caused by infection of the human CNS in the larval form of cysticeriae (Taenia solium). It can be transmitted by ingestion of food or water containing pork taenia eggs. In this case, the patient likes to eat barbecue, which may be one of its routes of infection. Multiple intracerebral nodules identified by brain CT correspond with the typical imaging findings of cysticercosis, which are most likely cysts of cysticeriae. In addition, epilepsy is one of the common clinical manifestations of cysticercosis.

Therefore, the answer best fitting with the description of this case is E. cysticercosis.

First, let's analyze the questions in detail:

In young female patients, the main symptoms are paroxysmal headache and intermittent seizures. Living habits, she likes to eat barbecue, which may be related to parasitic infection. Physical examination showed clarity and no resistance to the neck, but multiple subcutaneous nodules were palpable on the trunk and extremities. Laboratory examination revealed an increased white blood cell count, and cranial CT showed the presence of multiple small nodules in the brain.

Next, we conducted the inference analysis according to the question stem information and each option:

A. Cerebral hemorrhage: Cerebral hemorrhage is usually caused by cerebrovascular rupture caused by hypertension, aneurysm and other reasons. Although cerebral hemorrhage may cause headache, it generally does not cause subcutaneous nodules, and the cranial CT findings do not match the description of the problem stem.

B. Epilepsy: Epilepsy is a disease caused by the abnormal discharge of neurons in the brain. Although the patient developed symptoms of epilepsy, the epilepsy itself did not cause elevated leukocytes and subcutaneous nodules, so epilepsy is more like a symptom than the underlying cause.

C. Brain tumors: Although brain tumors can appear as nodules on cranial CT,

they generally do not lead to multiple nodules under the skin, and the white blood cell count does not usually increase.

D. Brain abscess: A brain abscess is a local suppurative inflammation of the brain caused by an infection. Although it may cause headache and elevated leukocytes, it usually does not form multiple nodules under the skin.

E. Cysticercosis: Cysticercosis is a disease caused by parasites, in which cysticercariae are the larvae of the pork tapeworm. The disease is usually associated with the consumption of undercooked pork, and the patient likes to eat barbecue, which increases the risk of cysticercosis. Cysticercosis can form multiple nodules in the brain and may cause seizures and subcutaneous nodules, along with possibly elevated leukocytes.

In conclusion, considering the clinical symptoms, lifestyle habits, physical findings and cranial CT findings, the most likely diagnosis is cysticercosis.

Thus, the correct answer is that for E. cysticercosis.

According to the problem, the patient was a 35-year-old woman with paroxysmal headache for 2 months and had intermittent seizures in the past week. Physical examination found multiple subcutaneous nodules palpable on the trunk and limbs, blood routine showed leukocytosis, and CT showed multiple nodules in the brain. Combined with the patient's barbecue diet, the most likely diagnosis is cysticercosis (porcine cysticercosis).

Porcine cysticercosis is a disease caused by cysticercus parasitism in human tissues and is often transmitted by consumption of undercooked pork or food and water contaminated by clastocystis eggs. Cysticercosis often presents with subcutaneous nodules, headache, and seizures, with multiple nodules seen on brain CT.

Therefore, the most likely diagnosis is:

F. cysticercosis

76. (Type A2 questions)

A 68-year-old man with 3-year chest pain, located after the sternum, onset in the early morning and resolved spontaneously after a few minutes. The electrocardiogram at the onset suggested that ST elevation from V 1 to V 3 was 0.3 mv, and the retest electrocardiogram was normal

A. intermediate syndrome

B. initial angina

C. variant angina pectoris

D. progressive angina pectoris

E. angina of effort

Reference answer: C Your answer:

Graphic analysis: [analysis] elderly male patients with nocturnal paroxysmal chest pain, several minutes of spontaneous relief, electrocardiogram V1~V3 lead ST segment elevation, considered as variable angina pectoris. Variant angina, also known as vasospastic angina, is characterized by resting angina, almost entirely in the resting condition, the onset time is mostly concentrated between midnight and 8 a. m., often accompanied by transient ST segment elevation. Therefore, this question is selected C.

[Erstavoidance] Intermediate type syndrome refers to a group of muscle weakness caused by acute organohorus insecticide poisoning

Joint levy. Mainly occurred in the patients 24 to 94 hours after severe organophosphorus poisoning, and the patients with insufficient medicinal amount of compound energy, were treated with choline

Can crisis disappeared, conscious or not recovered, before the late onset of polyneuropathy, the sudden appearance of flexor neck muscles, limbs

Proximal muscle weakness, there will also be part of the brain nerve innervation of the muscle weakness, patients will show facial paralysis, respiratory muscle paralysis, the most

This eventually leads to difficulty breathing and even death due to respiratory failure (A fault). Primary labor angina (B error)

Mild physical activity within 1 to 2 months; aggravated exertional angina (D wrong)

On the basis of stable labor angina, the angina gradually increased, both have

clear causes such as labor or emotional shock

Movement and ECG thoracic lead elevation is rare; exertive angina is characterized by pain from physical fatigue, emotional or emotional

He was induced by conditions sufficient to increase myocardial oxygen demand and rapidly disappeared after rest or sublingual nitroglycerin (E error).

The patient was described with variant angina (also known as metel angina), characterized by chest pain at rest with ST elevation, which is uncommon during physical activity. Variant angina is usually associated with coronary artery spasm and ECG shows ST elevation during the episode and normalized in the absence of symptoms. So the best answer is:

C.variant angina pectoris

analyze:

The patient was an elderly male with chest pain for 3 years, onset in the early morning, located in the sternum, can relieve itself after a few minutes, which was a common manifestation of angina. During the attack, the electrocardiogram suggested the V1~V3 lead ST elevation 0.3 mv, and the electrocardiogram was normal, combined with the patient's symptoms, signs and auxiliary examination results, the diagnosis was considered as variant angina. Variant angina pectoris most often occurs at rest, with transient ST segment dynamic changes, often with transient ST segment elevation, and ST segment elevation returning to baseline with the relief of angina pectoris.

Option A intermediate syndrome is caused by acute organophosphorus pesticide poisoning, mainly manifested as muscle weakness, which was not consistent with the description of the problem stem, so it was excluded.

Option B primary angina refers that the patient has not had angina pectoris or myocardial infarction in the past, but the first angina pectoris in nearly 1~2 months, which is inconsistent with the description of the problem, so it is not selected.

Option D worsened angina refers to the original stable angina in patients with relatively stable labor angina, that is, the pain is more intense, longer or more frequent, at least increased grade I, at least CCS, which is inconsistent with the description of the title stem, so it is not considered.

Option E fatigue angina is characterized by paroxysmal anterior chest pressing pain or stuffy feeling, mainly located in the back of the sternum, often occurs when

the labor load increases, lasting for several minutes, rest or with nitrate preparation after the pain disappeared, not with the dry description, so it does not meet.

In conclusion, the correct answer is: C. Variant angina pectoris.

Based on the patient symptoms and ECG changes described by the problem stem, the most likely diagnosis of variant angina pectoris can be analyzed. Variant angina usually occurs in the early hours or at rest, is of a short duration (minutes) and resolves spontaneously. The electrocardiogram will have ST segment elevation during the attack, and the electrocardiogram will return to normal after the attack.

So, the most correct answer is:

C. Variant angina pectoris

77. (Type A2 question)

Female, 30 years old. With normal menstruation, Hb 86 g / L, PaO2 82.3mmHg, PaCO2 37.6mmHg, pH7.43. The reason for the decreased blood oxygen volume is that

A. Carbon dioxide is decreased

B. Decreased 2-3-DPG within erythrocytes

C. loss of body temperature

D. pH descend

E. Protein decreased

Reference answer: E Your answer:

[Analysis] Hb oxygen capacity: refers to the maximum amount of O2 that Hb can bind in 100ml of blood. Blood oxygen volume is mainly affected by two factors: hemoglobin content and the maximum amount of O2 that Hb can bind to. The patient had Hb 86 g / L (anemia, decreased hemoglobin decreased), so the decreased oxygen volume was due to decreased hemoglobin (E pair).

[Error avoidance] pH, CO2, temperature, 2 in erythrocytes, 3-diphosphoglycerate mainly affect the affinity of Hb to O2, affect the oxygen dissociation curve and then affect PO2.

This question involves the changes in blood oxygen volume. Blood oxygen volume is the total amount of oxygen bound in the blood, which depends mainly on the concentration of hemoglobin and the oxygen binding capacity of hemoglobin. In this case, the main problem of the female patient was increased menstruation, resulting in a decrease in hemoglobin (Hb) to 86g / L, one of the manifestations of anemia.

-Option A (CO_2 drop) usually affects blood oxygen release rather than blood oxygen volume.

Option-B (2-3-DPG decrease in erythrocytes) affects the binding affinity of oxygen and hemoglobin, thereby affecting oxygen release, but not directly affecting blood oxygen volume.

The-C option (temperature drop) affects the position of the oxygen dissociation curve, but has little effect on the blood oxygen volume.

The-D option (pH drop) also affects the oxygen dissociation curve, but mainly affects oxygen release rather than blood oxygen volume.

The-E option (protein drop) can refer to the decrease of hemoglobin, because hemoglobin is a protein in the blood, and its decrease directly reduces the blood oxygen volume.

Considering the above analysis, the most reasonable answer should be * * E. protein decreased * *, because in this case, the female patient'hemoglobin decreases (protein decreased), which directly leads to the decrease in blood oxygen volume.

analyze:

The patient was a young woman with increased menstruation, suggesting chronic blood loss, normal heart and lung, and laboratory results suggesting anemia. Blood oxygen volume refers to the maximum amount of oxygen bound by 100ml of blood at an oxygen partial pressure of 19.95kPa (150mmHg), CO_2 of 5.32 kPa (40mmHg) and a temperature of 38°C. The level of blood oxygen volume can reflect the ability of the blood to carry oxygen. There are two main reasons for the decrease in blood oxygen volume, one is the decreased quantity or quality of hemoglobin, and the decrease of the 2,3-DPG concentration inside the red blood cells. Due to the presence of anemia in the patient, the quantity and quality of hemoglobin are reduced, and anemia can lead to a decrease in 2,3-DPG concentration, and thus blood oxygen volume.

Option A carbon dioxide drop can stimulate the peripheral chemoreceptors, reflexively cause the excitement of the respiratory center, accelerate the breathing, increase lung ventilation, so that the blood oxygen volume rise, and conflict with the problem stem.

Option C body temperature drops, and option D: pH drops, which can lead to increase the affinity of hemoglobin for oxygen, thus increasing blood oxygen volume, which is inconsistent with the dry description.

Option E protein decrease mainly affects the plasma colloidal osmolarity pressure, which has little relationship to the blood oxygen volume and can be excluded.

In conclusion, the correct answer is: B. 2-3-DPG decreased in the internal erythroid cells.

According to the information provided in the topic, the patient was a 30-year-old woman with increased menstruation, hemoglobin (Hb) 86g / L, PaO 282.3mmHg, PaCO237.6mmHg and pH 7.43. First, we need to analyze the reasons for the reduced blood oxygen volume.

Oxygen volume (oxygen capacity) is the maximum amount of oxygen that can be carried in the blood, usually depending on the concentration of hemoglobin and the oxygen cooperation capacity of the blood. Lower hemoglobin concentration will directly lead to decreased blood oxygen volume.

According to the information provided in the topic, the hemoglobin value (Hb) was significantly lower than the normal range, which suggests the possibility of anemia. Reducing hemoglobin will directly lead to decreased blood oxygen volume, because hemoglobin is the main carrier of oxygen.

The relationship between the options and the decrease of blood oxygen volume was analyzed as follows:

A. Reduction in carbon dioxide: Changes in carbon dioxide mainly affect

acid-base balance in blood, rather than directly affecting blood oxygen volume.

B. A decrease of 2-3-DPG in red blood cells: The decrease in 2,3-diphosphoglycerate (2-3-DPG) affects the ability of hemoglobin to bind to oxygen, but does not directly reduce the hemoglobin concentration.

C. Decline in body temperature: Decline in body temperature will affect metabolic rate and oxygen demand, but will not directly affect hemoglobin concentration.

D. pH drop: The pH drop (acidosis) will affect the oxygen release, but the pH value in the title is 7.43, which is in the normal range, so it is not applicable.

E. Protein decline: Systemic protein decline (e. g., hypoproteinemia) may accompany malnutrition, but it does not directly affect the hemoglobin concentration.

Based on the above analysis, the most likely reason for the decreased blood oxygen volume is the decreased hemoglobin. The most directly related options are:

* * E. Protein drop * *

Although anemia is the most direct factor, anemia is often closely related to a decline in nutrition-related protein (such as hemoglobin).

In conclusion, the choice answer * * E. protein drop * *.

78. (Type A2 question)

Male, 40 years old, was admitted with femoral shaft fracture, the next day of sudden dyspnea, then coma, extensive subcutaneous bleeding points, which should be considered

A. fat embolism

B. Bone-compartment syndrome

C. Severe redisplacement of the fracture end

D. infect

E. The rupture of the large blood vessels

Reference answer: A. Your answer:

Graphic analysis: [analysis] the second day after femoral shaft fracture sudden dyspnea, first consider fat embolism (A pair).

[Avoid mistakes] Fat embolism syndrome often occurs in adults, which is due to the hematoma tension in the fracture cavity, and the bone marrow is broken

Bad, fat droplets into the ruptured venous sinus, can cause pulmonary and cerebral fat embolism. Meanwhile, in poor lung perfusion, the alveoli

Membrane cells produce lipase that hydrolyzes small neutral fat droplets in fat emboli into glycerol and free fatty acids, releasing catechol

Amine, damage the capillary wall, make the protein-rich fluid leak into the pulmonary interstitium and alveoli, pulmonary hemorrhage, atelectasis and occur

Low blood oxygen. Clinically, respiratory insufficiency, cyanosis, and chest X-ray showed generalized pulmonary consolidation. Arterial hypoxia can cause irritability

Restless, drowsiness, and even coma and death.

Based on the described symptoms, including sudden dyspnea, coma, and extensive subcutaneous bleeding sites after a femoral shaft fracture, the most likely diagnosis was fat embolism (option A). Fat embolism syndrome is a serious complication after long bone fractures, especially the femur and pulp, which may cause fat particles from the bone marrow into the blood circulation, causing embolization of the lungs and other organs. So, the best answer is A. fat embolism.

analyze:

Patients with middle-aged male, femoral shaft fracture admission, admission the next day suddenly dyspnea, then coma, extensive subcutaneous bleeding point, consider patients may enter the bone marrow after the fracture of bone marrow vascular sinusoidal gap or capillaries, and with blood flow into the pulmonary vessels cause fat embolism, cause the breathing difficulties, cerebral embolism cause coma, skin, mucosal bleeding point.

Option B bone compartment syndrome is bone, intersseous membrane, muscle interval and deep fascia of the muscles and blood vessels caused by acute ischemia and a series of early syndrome, more in the forearm and calf, mainly for 5P syndrome,

namely pain, pale, paresthesia, paralysis and pulse, inconsistent with the dry description, so excluded.

Option C: Severe redisplacement of the fracture end will have severe pain, and obvious deformity will appear at the fracture site, which is inconsistent with the performance of the patient, so it is not selected.

Option D infection will have inflammatory manifestations such as local redness, heat and pain, and the patient has no infection manifestations and can be ruled out.

Option E Large vessel rupture can cause hemorrhagic shock and other manifestations, which are not consistent with the patient's symptoms and can be excluded.

In conclusion, the correct answer is: A. fat embolism.

According to existing medical knowledge and best practice guidelines, fat embolism should be considered for a 40-year-old male patient hospitalized for femoral shaft fracture. Fat embolism syndrome (FES) usually occurs after long bone fractures, especially in the femur and tibia, often presenting as dyspnea, neurological symptoms (e. g., coma), and subcutaneous bleeding sites.

So, the most correct answer is:

A. fat embolism

79. (Type A2 questions)

Male, 34 years old, after large gastrectomy, microscopic tongue, routine blood examination: WBC 3.110 ^ 9 / L, Hb 68 g / L, PLT 4010 ^ 9 / L, MCV> 130 fl, reticulocyte 0.5%, bone marrow cytology: active hyperplasia and aging " phenomenon, no pathological hematopoiesis. The diagnosis considered is

A. megaloblastic anemia

B. hypoferric anemia

C. Myelodysplastic syndrome

D. Bozzolo's disease

E. leukemia

Reference answer: A. Your answer:

[Analysis] After major gastrectomy (nutritional ischemia etiology), the patient

had endoscopic tongue and routine blood examination appeared: WBC 3.110 ^ 9 / L, Hb 68 g / L, PLT 4010 ^ 9 / L (three series reduction), MCV> 130 fl, reticulocyte 0.5%, bone marrow cytology showed active bone marrow hyperplasia, and the phenomenon of "nuclear and plasma aging" (consistent with the bone marrow appearance of megaloblastic anemia

Now), based on the patient's medical history, test results and clinical manifestations, considering the diagnosis of megaloblastic anemia, so this question is selected A.

[Avoid mistakes] Melanoblastic anemia is caused by the disorder of nucleic acid synthesis, and the essential raw materials for nucleic acid synthesis are folic acid and vitamins

B12.

This patient's clinical findings and laboratory findings pointed to megaloblastic anemia (megaloblastic anemia). Key clues include the appearance of postoperative mirror tongue, low hemoglobin (Hb 68g / L), large red blood cells (MCV> 130 fl), and "young plasma aging" as shown by bone marrow examination, which are typical of megaloblastic anemia. Furthermore, endoscopic tongue and postoperative malnutrition may be associated with vitamin B12 or folate deficiency, a common cause of megaloblastic anemia.

-A. Megaloblastic anemia: the clinical findings and findings described above.

-B. Iron deficiency anemia: Microcellular hypochromic anemia usually occurs, inconsistent with the MCV enlargement in this case.

-C. Myelodysplastic syndrome: Although it causes cytopenia, it usually does not show significant MCV enlargement or "old aging".

-D. Multiple myeloma: mainly an increase in abnormal immunoglobulin, inconsistent with the findings in this case.

-E. Leukemia: An increase in abnormal white blood cells in the blood or bone marrow is usually seen, inconsistent with this case.

Thus, the most correct answer is A. megaloblastic anaemia.

analyze:

Young male patients, after large gastrectomy, a mirror tongue, blood routine results show three series, red blood cells increase, mean prompt macrocytic anemia,

decreased reticulocytes, bone marrow cytology: bone marrow hyperplasia active, "young pulp old" phenomenon, no pathological hematopoiesis, combined with patient history, clinical manifestations and related examination, the most likely diagnosis is giant young cell anemia. Megaloblastic anemia is anemia caused by deficiency of folic acid or vitamin B12, or some drugs affecting the metabolism of nucleotides that cause the synthesis of nuclear deoxyribonucleic acid (DNA). It is characterized by macellastic anemia, hematopoietic characteristics in the bone marrow, and is effective in treatment with vitamin B12 and folic acid.

Option B iron deficiency anemia is due to the body's demand for iron and supply imbalance, lead to storage iron storage, red blood cell iron lack of anemia, for small cell hypochromic anemia, bone marrow like hyperplasia, red, decreased, red blood cell system hyperplasia, give priority to with middle and late young red blood cells, its small size, dense nuclear chromatin, less cytoplasm, edge, poor hemoglobin form bad performance, namely the phenomenon of "nuclear old pulp young", does not accord with the idea, so do not choose.

Option C myelodysplastic syndrome originated from hematopoietic stem cells, with blood cells pathological hematopoietic, high risk to acute myeloid leukemia characterized by refractory blood cytoplasm, abnormal amount of heterogeneous disease, the blood in whole blood cells, bone marrow hyperplasia active or active, visible stage naive cells, but "young pulp old" phenomenon is rare, and dry has clearly did not see pathological hematopoiesis, so excluded.

Option D Multiple myeloma is malignant plasma cell disease. In the blood examination, hemoglobin can be reduced and blood sedimentation is accelerated. The myeloma cells can be seen in the bone marrow examination, which is not consistent with the description of the main stem, so it is not selected.

Option E leukemia is a kind of hematopoietic stem cell malignant clonal disease, clonal leukemia cells because of proliferation out of control, differentiation disorder, apoptosis mechanism proliferation in the bone marrow and other hematopoietic tissue, and infiltrate other hematopoietic tissues and organs, at the same time inhibit normal hematopoiesis, clinical visible different degree of anemia, bleeding, infection, fever and liver, spleen, lymph node enlargement and bone pain, and dry difference, can be ruled out.

In conclusion, the correct answer is: A. megaloblastic anemia.

The topic describes a 34-year-old male with a mirror tongue after a major

gastrectomy, along with the following findings:

-White blood cell count (WBC): 3.110 ^ 9 / L

-Hemoglobin (Hb): 68g / L

-Platelet count (PLT): 4010 ^ 9 / L

-Mean red blood cell volume (MCV):> 130 fl

-reticulocytes: 0.5%

-Bone marrow cytology showed active bone marrow hyperplasia, young and old " phenomenon, no pathological hematopoiesis

These information suggested a type of anemia, particularly the anemia with a significantly higher MCV (> 130 fl). We need to consider the possibility of megalyocytic anemia.

** analyse:**

1. * * Megaloblastic anemia * *:

-After a partial gastrectomy, patients may have a vitamin B12 absorption disorder due to internal factor deficiency, which may then cause megaloblastic anemia.

-The ular tongue is one of the common manifestations of megaloblastic anemia.

-Significant higher MCV (> 130 fl), a significant decrease in hemoglobin, and a lower percentage of reticulocytes, which are typical features of megaloblastic anemia.

-Bone marrow cytology shows active myeloid hyperplasia, and has a "young plasma aging" phenomenon, which is also one of the characteristics of megaloblastic anemia.

2. * * Iron deficiency anemia * *:

-Generally presenting as small cellular hypochromic anemia, MCV is usually reduced or normal, not consistent with the MCV> 130 fl in the problem stem.

3. * * Myelodysplastic Syndrome (MDS) * *:

-Although similar hemochanges may occur in MDS, significantly higher MCV is not a typical feature of MDS.

4. * * Multiple myeloma * *:

-Generally, bone pain, proteinuria and abnormal serum protein electrophoresis results are the main characteristics, which do not conform to the description in the title stem.

5. * * leukemia * *:

-Leukemia is usually accompanied by obvious pathological hematopoiesis phenomenon, there should be pathological hematopoiesis in bone marrow cytology, and no pathological hematopoiesis is seen in the problem stem.

Based on the above analysis, the diagnosis most consistent with the description of the problem stem is * * megaloblastic anemia * *.

* * Answer: A. megaloblastic anemia * *

80. (Type A2 question)

A 28-year-old male with fever for 2 weeks and maximum temperature of 39.6℃, accompanied by abdominal pain, diarrhea and loose stools 3 to 4 times per day. Physical examination: one month ago, swimming in the river, transient rash, 2cm under the liver ribs, tenderness, palpable under the spleen ribs, WBC 15.7109 E0.4, which of the following drugs was available for treatment

A. albendazole

B. deoxytetracycline

C. praziquantel

D. Levofloxacin

E. metronidazole

Reference answer: C Your answer:

Graphic analysis: [analytical] young men a month before swimming (schistosomiasis contact history), lower limb transient rash, fever for 2 weeks, with abdominal pain, diarrhea, liver ribs 2cm, have tenderness, spleen ribs palpable (both schistosomiasis clinical manifestations), considering the patients with diagnosis of schistosomiasis, praziquantel small toxicity, good curative effect, convenient administration, for the preferred treatment of schistosomiasis (C).

[Avoid mistakes] Albendazole can kill a variety of intestinal nematodes, tapeworms and fluke adults and eggs, can not treat schistosomiasis

(A wrong); doxycycline is the preferred treatment for rickettsial infections, mycoplasma infections, and some spirochete infections.achromycin

Drugs can also be preferred to treat plague, brucellosis, cholera, Helicobacter pylori infection caused by peptic ulcer, granuloma

inguinal granuloma caused by sphingomyces infection and periodontitis caused by Porphyromonas gingivalis (B error); levofloxacin

Quinolone, a broad-spectrum antibiotic, mainly used for respiratory tract, urinary tract, digestive tract infections (D wrong); methyl nitrate

The azole is the treatment of anaerobic bacteria infection (E error).

This title describes a 28-year-old male patient with fever, abdominal pain, and diarrhea, and mentions a history of swimming and a rash in the lower limbs a month earlier. In the light of this information, we should consider diseases transmitted by water sources, such as parasitic infections.

A. Albendazole-It is mainly used to treat intestinal parasitic infections such as roundworm, hookworm, whip worm and worm.

B. Dxycycline-is an antibiotic used to treat a variety of bacterial infections.

C. praziquantel-which is mainly used to treat malaria.

D. Levofloxacin-is a broad-spectrum antibiotic used for the treatment of multiple bacterial infections.

E. Metronidazole- -is commonly used to treat infections caused by anaerobic bacteria and protozoa, such as amoebiasis and other protozoan infections.

Considering that the patient had a swimming history and may have been exposed to contaminated water sources, the symptoms presented coincided with amoebic dysentery, which included abdominal pain, diarrhea, and fever. Therefore, the most appropriate drug choice is metronidazole for the treatment of amebic dysentery (option E). So, the most correct answer is the E. metronidazole.

analyze:

The patient was a young man with fever, abdominal pain, diarrhea, liver and spleen, a history of river swimming, transient rash, laboratory examination showed elevated white blood cells and eosinophilia. Combined with the patient's symptoms, signs and laboratory tests, the patient was diagnosed as acute schistosomiasis was considered. The first choice for the treatment of schistosomiasis is praziquantel, which has little toxicity, good efficacy, convenient administration and wide indications, and can be used in patients with various types of schistosomiasis in each stage.

Option A Albendazole is A broad-spectrum insecticide drug, which mainly treats roundworm, worm, hookworm and other parasitic infections, but has no therapeutic effect on schistosomiasis, so it is not selected.

Option B doxycycline is a tetracycline antibiotic, which is a broad-spectrum bacteriostatic agent and has bactericidal effect at high concentration. It is mainly used for upper respiratory tract infection, tonsillitis, biliary tract infection, lymphadenitis and other infection caused by sensitive gram-positive cocci and negative bacteria, and is not effective for schistosome infection, so it is excluded.

Option D Levofloxacin is a quinolone antibiotic, which is mainly used for respiratory system infection, urinary system infection, reproductive system infection, skin and soft tissue infection, and intestinal infection caused by sensitive bacteria. It has no therapeutic effect on schistosome infection, so it is not considered.

Option E metronidazole is mainly used for the treatment of anaerobic bacteria, as well as the treatment of trichhomonas, amoeba, etc., so it is not selected.

In conclusion, the correct answer is: C. praziquantel.

According to the title description, the patient had symptoms of fever, abdominal pain, diarrhea, and hepatosplenomegaly, along with a history of river swimming and a

rash. Considering these symptoms and medical history, the possible diagnosis is schistosomiasis (Schistosomiasis).

The first choice for the treatment of schistosomiasis is praziquantel.

So, the most correct answer is:

B praziquantel

81. (Type A2 questions)

Male, 68-year-old, with fever, headache, and low back pain, diarrhea for 3 days, and oliguria for 300 ml/d. Physical examination: body temperature 38.5℃, heart rate 116 times / min, breathing 24 times / min, facial congestion, eyelid edema, soft palate bleeding point, normal blood routine Hb134g / L, WBC14 0.84, fecal routine and microscopic examination: WBC 5 / HP, for a clear diagnosis, the preferred examination is

A. blood film

B. blood culture

C. antibodies to Hantavirus

D. Leptospira antibody

E. EB viral antibody

Reference answer: C Your answer:

Graphic analysis: [analytical] 68-year-old male patient, fever, headache, low back pain, diarrhea 3 days (renal syndrome hemorrhagic fever fever systemic poisoning symptoms), urine 1 days, facial congestion, eyelid edema, soft palate bleeding point (renal syndrome hemorrhagic fever capillary damage), laboratory examination blood WBC 14109 / L (adult normal reference value 4~10109 / L), according to the patient's clinical manifestations, personal history and laboratory results, the most likely diagnosis is renal syndrome bleeding, hot virus antibody in the second day can be detected (C pair).

[Error avoidance] renal syndrome hemorrhagic fever, also known as epidemic

hemorrhagic fever, is a natural epidemic disease with rodents as the main source of infection

illness. The main pathological changes of this disease are the systemic damage of small vessels and capillaries, clinical fever and hypotension

F. congested bleeding and renal damage are the main manifestations.

The patient in this case presented symptoms of fever, headache, low back pain, diarrhea, oliguria, facial congestion, eyelid edema and bleeding points in the soft palate. Combining the signs and laboratory results, we need to consider multiple possible diagnoses.

First, fever and headache may point to a variety of infectious diseases, such as bacterial, viral, or parasitic infections. A bleeding point in the soft palate may suggest a hematologic problem or a serious infection. A high leukocyte count indicates the presence of an infection or inflammation.

Specifically in this case, considering fever, headache, diarrhea and oliguria, and the results of blood routine and fecal routine, we can exclude some common infections, such as EB virus infection, as it usually causes lymphocytosis rather than granulocytes.

-A option (blood smear) may be used to detect parasitic infections (e. g. malaria) or blood diseases.

The-B option (blood culture) is the standard method for identifying bacterial or fungal infections in the blood.

Option-C (hantavirus antibody) was used for the diagnosis of hantavirus infection, which may cause severe renal problems and bleeding tendency, consistent with some of the symptoms in this case.

The-D option (Leptospira antibody) is used to diagnose leptospirosis, usually presenting with fever, muscle pain, and possibly impaired renal function.

The-E option (EB virus antibody) is used to diagnose EB virus infection, usually associated with symptoms such as sore throat, enlarged lymph nodes, and fatigue.

Considering the patient's symptoms and signs, especially fever, diarrhea, oliguria, eyelid edema, and soft palate bleeding points, I tend to choose option C (hantavirus antibody) as the preferred examination. Hantavirus infection can cause hemorrhagic

fever in renal syndrome, characterized by fever, bleeding tendency, renal impairment, and symptoms similar to this case. Therefore, performing hantavirus antibody testing is help to determine whether this is the correct diagnosis.

This is a question about the diagnosis of the disease that requires the analysis of the patient's symptoms, signs, and laboratory findings to determine the most appropriate diagnostic workup.

First, let's review the key information in the topic:

Elderly male patient, fever, headache, lumbago, diarrhea for 3 days, with oliguria.

Physical examination showed facial congestion, eyelid oedema, and bleeding spots in the soft palate.

Routine blood tests showed normal hemoglobin (Hb) and elevated white blood cells (WBC).

Routine fecal examination and microscopic examination showed no obvious abnormalities.

Next, we analyzed the association of each option with the patient's symptoms in the topic:

A. Blood smear: Usually used to check for hematological disorders, such as leukemia, and it has little association with the patient's symptoms.

B. Blood culture: mainly used for the diagnosis of bacterial infection, but the patient has no clear signs of bacterial infection.

C. Hantavirus antibody: hantavirus can cause renal syndrome hemorrhagic fever, typical symptoms include fever, headache, pain, orbital pain, nausea, nausea, vomiting, chest tightness, abdominal pain, diarrhea, systemic joint pain, skin mucosa three red (face, neck and upper chest red), eye conjunctival congestion, heavy like drunk, kidney area percussion pain, liver and spleen enlargement, subcutaneous bleeding or ecchymosis, and typical "three pain" (headache, pain, orbital pain) and "three red sign" (red, red, chest red), accompanied by kidney injury and bleeding tendency. These symptoms were highly consistent with the patient's performance in the problem stem.

D. Leptospira antibody: Leptospirosis usually presents as fever, pain, soft body, conjunctival congestion, gastrocnemius pain, lymph node enlargement, etc., which is not completely consistent with the symptoms of the patients.

E. EB virus antibody: EB virus infection is mainly related to nasopharyngeal cancer, childhood lymphoma and other diseases, which is not consistent with the symptoms of patients.

Taken together, the patient was an elderly male with fever, headache, low back pain, diarrhea, oliguria, congestion in the body department, eyelid edema, and bleeding points in the soft palate, these symptoms consistent with the hemorrhagic fever of renal syndrome caused by hantavirus infection.

Therefore, in order to clarify the diagnosis, the preferred test should be the C. hantavirus antibody.

According to the description, the 68-year-old patient, developed fever, headache, low back pain, diarrhea and oliguria, and physical examination found elevated temperature, increased heart rate, shortness of breath, rapid, facial congestion, eyelid edema and soft palate bleeding points, these symptoms may be infectious disease. Blood routine showed an elevated white blood cell count, which further supports the possibility of infection.

In this case, we need to consider several possible sources of infection:

1. * * Blood smear * * is mainly used for the examination of primary worms and some parasites in blood, but it is not specific for the symptoms of this patient, especially the soft palate and oliguria.

2. * * Blood culture * * is used to detect bacterial or fungal infections in the blood, which is helpful to determine specific pathogens, but it takes time to culture the results.

3. * * Hantavirus antibody * * test is to make the diagnosis of hemorrhagic fever renal syndrome (HFRS), whose symptoms include fever, headache, muscle pain, oliguria, facial congestion, bleeding points, etc., consistent with the description of the problem stem.

4. * * Leptospira antibody * * test is used to diagnose leptospirosis (Weil

disease), whose symptoms also include fever, headache, muscle pain, oliguria and bleeding points, but the soft palate are rare.

5. * * EB virus antibody * * test is mainly used for the diagnosis of infectious mononucleosis (IM), its symptoms include fever, sore throat, lymph node enlargement, etc., but it is not consistent with the patient's oliguria and soft palate bleeding points.

Combined with the symptoms and signs of the patient, the possibility of hantavirus infection is greater. Therefore, the most appropriate test should be the * * Hantavirus antibody detection * *.

So, the most correct answer is:

F. antibodies to Hantavirus

82. (Type A2 question)

Male, 16 years old, developed dizziness, fatigue, urinary protein (+ +) after the 1500 m run, symptoms disappeared after 1 day of rest, urine protein (-), most likely

A. excretory proteinuria

B. histological proteinuria

C. The renal tubular proteinuria

D. functional proteinuria

E. overflow proteinuria

Refer to the answer: D. Your answer:

[Analysis] In teenagers, proteinuria (a common cause of functional proteinuria) appeared after strenuous exercise, and improved after rest (non-organic changes). According to the medical history and test results of the patient's diagnosis of functional proteinuria (D test).

[Avoid mistakes] Functional proteinuria: refers to strenuous exercise (or fatigue), cold, fever, mental tension, sympathetic nerve

Temporary proteinuria caused by excitement, and increased permeability of the glomerular capillary wall caused by renal vasospasm or congestion

close. More common in adolescents, urinary protein qualitative not more than (+), quantitative not more than 500mg / 24h.

This case describes a 16-year-old boy who developed dizziness, fatigue and increased urinary protein after the 1500 m run, but the symptoms and abnormal urinary protein disappeared after a day of rest. Based on these symptoms and conditions, the most likely scenario is * * functional proteinuria * *.

Functional albuminuria refers to the increase of urinary protein in some physiological states, such as strenuous exercise, fever, and cold exposure, but without substantial renal lesions. This proteinuria is usually temporary and can normalize after rest or removal of triggers. In this topic, the patient developed proteinuria after intense exercise, and the symptoms disappeared after rest, consistent with the characteristics of functional proteinuria.

Therefore, the correct answer is * * D. functional albuminuria * *.

analyze:

Young male patients, exercise after transient proteinuria, proteinuria disappear after rest, combined with symptoms and laboratory examination, considering the diagnosis of functional proteinuria, more in young people, in strenuous exercise, fever, high temperature, cold, nervous under the influence of factors, renal vasospasm or congestion, lead to enhanced glomerular filtration membrane permeability and make a large number of protein "leakage". Proteinuria will disappear after the cause is eliminated.

Option A secretory proteinuria is mostly seen in multiple myeloma and other diseases. Due to the abnormal increase of light chain protein in myeloma, proteinuria is formed beyond the renal tubular reabsorption capacity after glomerular filtration. The patient has no history of myeloma, so he was not selected.

Option B histological proteinuria is mostly seen in the destruction of renal tissue or the increase of protein secreted by renal tubules, or the abnormal increase of protein of some special components. After glomerular filtration, the proteinuria formed by the renal tubular reabsorption capacity of the kidney tissue, so it is excluded.

Option C renal tubular proteinuria is due to renal tubular structure or function damage, the normal filtration of small molecule protein reabsorption disorder, resulting in protein discharge from the urine, it is common in pyelonephritis,

interstitial nephritis and other renal tubular damage diseases, patients have no evidence of renal tubular damage, so it is not selected.

Option E overflow proteinuria is due to glomerular filtration, tubular reabsorption are normal, but because of the relatively small molecular mass in plasma or positive charge protein (such as multiple myeloma light chain protein, hemoglobin, myoglobin, etc.) abnormal increase, by glomerular filtration, more than tubular reabsorption ability of proteinuria, common in multiple myeloma, patients with no related history, so excluded.

To sum up, the correct answer is: D. Functional proteinuria.

This question examines the type of proteinuria. According to the situation described by the problem: the boy was 16 years old, with dizziness, fatigue, urinary protein (+ +), symptoms disappeared after 1 day of rest, urine protein (-), the most likely case is functional proteinuria.

* * Functional proteinuria * * is transient proteinuria seen in some physiological or pathological situations but without lesions in the kidney itself. The following is a brief introduction of each type of proteinuria:

A. * * Secretory proteinuria * *: Prominuria due to the enhanced secretory function of renal tubular epithelial cells, such as the Tamm-Horsfall protein secreted by the renal tubules.

B. * * Histoproteinuria * *: Due to tubular cell injury and necrosis, tubular epithelial debris and tubular cells are discharged into urine, which is seen in acute tubular necrosis and other diseases.

C. * * Tubular proteinuria * *: Due to the dysfunction of the reabsorption of protein by the renal tubules, the appearance of small molecules appears in urine, which is common in various renal tubular diseases.

D. * * Functional albuminuria * *: In some special physiological circumstances, such as strenuous exercise, fever, stress, etc., the glomerulus temporarily increases, leading in proteinuria, but the kidney structure and function are normal. It can disappear itself after rest.

E. * * Spillover proteinuria * *: When some small molecule proteins (such as immunoglobulin light chains) are too much in the plasma, they exceed the reabsorption capacity of the renal tubules, causing these small molecule proteins to appear in the urine. Common in multiple myeloma and other diseases.

According to the situation described by the problem stem, the transient proteinuria that appeared in boys after strenuous exercise disappeared spontaneously after rest, which is most consistent with the characteristics of * * functional proteinuria * *.

So the correct answer is:

F. * * Functional albuminuria * *

83. (Type A2 question)

Female, 65 years old, diabetes mellitus for 12 years, blurred vision in March. Fundus examination: fundus microhemangioma, a small amount of bleeding with cotton wool soft exudation. The patient's diabetic retinopathy stage was

Phase A.2

F. Phase 4

G. Phase 3

H. Phase 1

E.5 phase

Reference answer: C Your answer:

[Analysis] Elderly female diabetic patients, fundus examination: fundus

microhemangioma, a small amount of bleeding with cotton wool soft exudation, according to the classification criteria, this question is selected C.

[Eravoid avoidance] In 2002, the diabetic retinal changes were divided into two major categories

Class, six. Stage I: microhemangioma, small bleeding point (D wrong). Period: hard exudation (A wrong). Adventitia period: out

Now cotton culent soft exudation. Stage; neovascularization, vitreous blood accumulation (B error). Stage V: fibrovascular proliferation,

Glass body machine (E wrong). Period: traction retinal detachment, blindness.

Diabetic retinopathy (DR) is divided into several different stages, mainly according to the fundus findings. Based on the fundus microhemangioma, bleeding and cotton soft exudation, these are typical of the early stages of diabetic retinopathy.

-Stage 1 (mild non-proliferative diabetic retinopathy): characterized by the presence of microhemangioma.

-Stage 2 (moderate non-proliferative diabetic retinopathy): More microangiomas, bleeding, and / or exudation from the macular area.

-Stage 3 (severe non-proliferative diabetic retinopathy): massive bleeding and cotton wool spots.

-Stage 4 (proliferative diabetic retinopathy): neovascularization occurs, with possible vitreous haemorrhage and retinal detachment.

-Stage 5 does not belong to the usual staging system of diabetic retinopathy.

According to the description in the title, the patient's fundus examination showed "fundus microhemangioma, a small amount of bleeding with cotton wool soft exudation", and these symptoms fit the characteristics of moderate non-proliferative diabetic retinopathy, namely stage 2. So the most appropriate answer is:

A. Phase 2

analyze:

The elderly female patient with diabetes for many years, blurred vision, fundus microhemangioma on fundus examination, a small amount of bleeding with cotton wool soft exudation, is a typical symptom of diabetic retinopathy. Diabetic retinopathy can be divided into six stages, and the staging criteria are as follows:

Stage I: Microangioma and small bleeding sites appear in the retina.

Period: appear hard exudation.

Period: appear cotton flocculent soft exudation.

Stage: neovascularization, vitreous blood accumulation.

Stage V: fibrovascular proliferation, vitreous mechanization.

Period: traction retinal detachment, blindness.

Fundus examination of this patient showed microhemangioma in the fundus with a small amount of bleeding with cotton wool soft exudation, consistent with the typical presentation of stage 3 diabetic retinopathy.

To sum up, the correct answer is: the C.3 issue.

Option A: Stage 2, the patient is cotton soft exudation, so it was not selected.

Option B: Stage 4 showed neovascularization and vitreous hemorrhage, and the patient did not show these symptoms, so it was excluded.

Option D: Stage 1 showed microhemangioma and small bleeding points in the retina, but the patient also had cotton wool soft exudation, so it did not meet the diagnosis of stage 1, so it was not selected.

Option E: Stage 5 showed fibrovascular proliferation and vitrification, which did not match the patient's symptoms, so it was excluded.

According to the description, the patient had diabetes for 12 years and blurred vision for 3 months. fundus examination revealed microhemangioma, small bleeding, and cotton soft exudation. These findings fit the features of diabetic retinopathy. Stage of diabetic retinopathy is usually based on the ETDRS (Early Treatment Diabetic Retinopathy Study) criteria.

According to the ETDRS criteria, diabetic retinopathy is divided into the following stages:

1. * * No significant retinopathy * * (No apparent retinopathy)

2. * * Mild non-proliferative diabetic retinopathy * * (Mild NPDR): only microangioma.

3. * * Moderate non-proliferative diabetic retinopathy * * (Moderate NPDR): the presence of microhemangioma, rigid exudation, cotton wool soft exudation and a small amount of bleeding.

4. * * Severe non-proliferative diabetic retinopathy * * (Severe NPDR): extensive bleeding, venous bead changes (beading), intraretinal microvascular abnormalities (IRMA).

5. * * Proliferative diabetic retinopathy * * (PDR): neovascularization or vitreous hemorrhage occurred.

Based on the patient's fundus examination findings (fundus microhemangioma, minimal bleeding, and cotton wool soft exudation), the characteristics of moderate non-proliferative diabetic retinopathy (stage 3) were consistent. So, the most correct answer is:

B. Phase 3

80. (Type A2 questions)

Male, falling from high place, back contusion, lower limbs relaxation paralysis, to the hospital emergency. Physical examination: the waist can not move, bilateral groin below the sensation, movement and reflex disappeared. X-ray shows a thoracic vertebral fracture. Later, the function of both lower limbs was gradually recovered. The injury may be

A. spinal cord hemection syndrome

B. spinal cord injury

C. myelosyringosis

D. spinal concussion

E. syndrome of cauda equina

Refer to the answer: D. Your answer:

[Analysis] After the patient fell from height, lower limb sensory impairment (suggesting the possibility of spinal cord injury), and gradually recovered. Combined with the patient's history and clinical manifestations, the most likely diagnosis of the patient is spinal cord concussion (D pair).

[Error avoidance] Spinal cord concussion clinically shows the complete or most disappearance of sensory, motor and reflexes below the plane of injury.

Generally after a few hours to several days, sensory and movement began to recover, not leaving any neurological sequelae. Spinal cord hemi-cut synthesis

Sign: the disappearance of movement and deep sensation of the ipsilateral limb below the injury plane, and the disappearance of pain and temperature of the contralateral limb (A wrong);

The spinal cord transection injury is a complete transverse damage of the pulp parenchyma, with the sensory and motor functions of the lowest sacral segment below the plane of the injury

Total loss, including the sensation and loss of the anal sphincter, is called the spinal shock phase. After 2~4 weeks

It gradually evolved into spastic paralysis, manifested by increased muscle tone, hyperreflexia, and pathological pyramidal bundles (B

Wrong); syringomyelia is generally chronic, progressive injury of unknown cause, not recovery (C wrong); cauda equina syndrome

Most patients presented with pain, alternating with sciatica, and sensory impairment with bilateral lower limbs and perineal anesthesia

Wood, feeling weakened or disappear, incontinence (E wrong).

This case describes a male patient with low back contusion due to a high fall and flaccid paralysis of both lower limbs with limited lumbar movement and loss of bilateral subgroin sensation, movement, and reflexes. The X-ray showed a thoracic vertebral fracture with a gradual functional recovery of the latter two lower limbs.

These symptoms and signs indicate that the patient's spinal cord may have been affected. Given the gradual recovery of function, a complete spinal cord transection injury (B) is unlikely, because patients usually do not regain function naturally. Spinal cord hemisection syndrome (A) usually shows loss of strength on one side and loss of pain and temperature on the other side, but this asymmetric sign was not mentioned in the case.syringomyelia (C) usually develops slowly and an acute events not match the condition. Cauda equina syndrome (E) affects the nerve roots below the lumbar spine, not the spinal cord itself, and usually does not occur in thoracic fractures.

Considering the above information and the characteristics of the case, the most appropriate diagnosis is * * D. spinal cord concussion * *. Spinal cord concussion is a temporary impairment of spinal cord function caused by a violent shock, and patients can regain function within minutes to weeks, consistent with the case description.

analyze:

The patient suffered from lumbar back contusion, physical examination showed loss of bilateral below groin sensation, motor and reflexes, and X-ray showed thoracic spine fracture, which should be considered as spinal cord injury caused by thoracic spine fracture. The function of both lower limbs gradually recovered after SCI, consistent with the characteristics of spinal cord concussion. Spinal cord concussion refers to a reversible functional disorder that occurs after spinal cord injury. Similar to concussion, it is the lightest type of spinal cord injury. It is characterized by rapid and complete recovery of spinal cord function below the injury plane, and it is generally believed that its recovery time does not exceed 24 to 48 hours.

Option A spinal cord hemiresection syndrome showed paralysis of upper motor neurons and deep sensation of the ipsilateral limb below the plane of SCI, loss of nociception of the contralateral limb, and clinical syndrome of bilateral tactile preservation, which was inconsistent with the disappearance of bilateral sensory, motor and reflex symptoms in the patient, so it was not selected.

Option B spinal cord transection injury showed complete paralysis below the injury plane, loss of various reflections, loss of sphincter function, loss of skin sensation, and hope of recovery, which was not consistent with the gradual recovery of lower limb function of the patient in the problem stem, so it was excluded.

Option C syringomyelia is a chronic and progressive spinal cord degeneration disease, which is characterized by the formation of tubular cavity and glial hyperplasia in the spinal cord, often occurs in the cervical spinal cord, the symptoms in the early stage of patients are limited and mild, late symptoms are widespread and even paraplegia, will not recover by itself, so it is not selected.

Option E cauda equina syndrome is caused by lumbar disease, with the lumbar L3 to caudal nerve, nerve root or cauda equina involvement as the main syndrome, usually appear incontinence, sellar numbness, lower limb weakness and other symptoms, but the general lower limb function will not gradually recover, so it is excluded.

In conclusion, the correct answer is: D. Spinal cord concussion.

According to the description of the problem, the man fell back back contusion, relaxation paralysis of both lower limbs, and the waist can not move, bilateral groin sensation, movement and reflex disappeared, X-ray showed thoracic vertebra fracture. After that, his lower limb function gradually recovered.

From the medical history and signs, the initial relaxation paralysis of both lower limbs and the loss of sensory, motor and reflexes suggested that the spinal cord was affected. After that, the function of both lower limbs gradually recovered, which is more in line with the characteristics of spinal cord concussion.

Spinal cord concussion (spinal cord concussion) refers to the transient dysfunction of the spinal cord under the action of external force, which generally does not cause permanent damage. The common manifestation is the temporary loss of spinal cord function after injury, such as paralysis, sensory loss, etc., but then gradually recover.

Other options are explained as follows:

- ** A. Spinal cord half resection syndrome (Brown-Sequard syndrome) * *: It is often seen in the spinal cord half side injury, manifested by the loss of movement disorders and reflexes on the injured side, and the loss of pain perception on the contralateral side, usually not complete recovery.

- ** B. Spinal cord transection injury (spinal cord transection) * *: Complete transection of the spinal cord generally does not fully restore function, and causes a permanent loss of motor and sensory function below the injury site.

- ** C. syringomyelia (syringomyelia) * *: Most are chronic disease, showing the formation of internal spaces in the spinal cord, leading to chronic progressive neurological dysfunction, usually without sudden onset or complete recovery.

- ** E. Cauda equina syndrome (cauda equina syndrome) * *: The main manifestations are the perineal sensation, dysfunction of the defecation and weakness of the lower limbs, usually gradually without sudden improvement.

In conclusion, the most likely diagnosis is:

* * D. Spinal cord concussion * *

81. (Type A2 questions)

Male, 65 years old, with lumbar pain for several years, straight leg elevation test (+), strengthening test (-). Physical examination: the left thumb back extension is weak. The most likely reason is that

A. L4-L5, L5 nerve root compression

B. L3-L4, L4 nerve root compression

C. L2-L3, L3 nerve root compression

D. S1-S2, S2 nerve root compression

E. L5-S1, S1 nerve root compression

Reference answer: A. Your answer:

[Analysis] The patient's low back pain, straight leg elevation test (an important sign of lumbar disc herniation), and dorsal weakness of left toe, it is known that the patient involved nerve is L5, combined with the typical symptoms and signs, the most likely diagnosis at this time is L4~L5 disc herniation (A pair).

[Error avoidance] L5~S1 intervertebral disc herniation corresponds to the compression of the S1 nerve root, which is manifested by abnormal ankle reflex and decreased plantar flexion of the toes.

The topic involves judging the compression of the spinal nerve roots. Considering that the patient complained of low back pain with a positive straight leg elevation test (+), this usually indicates the presence of lower lumbar or sciatic nerve root compression. Physical examination revealed weakness in the dorsal extension of the left thumb, a symptom that involves a specific neurological function, the dorsal extensor of the thumb, innervated by the L5 nerve root.

Combined with this information, the most likely diagnosis is L5 nerve root compression. Therefore, we need to look for the spinal segments associated with L5 nerve root compression. Dorsal weakness of the thumb suggests L5 nerve dysfunction, so items A (L4-L5, L5 nerve root compression) and E (L5-S1, S1 nerve root compression) appear related. However, because the symptoms specifically point to the L5 nerve root, we can more specifically indicate the symptoms caused by the compression of the L5 nerve root in the L4-L5 spinal segment.

So, the best answer is an A. L4-L5, L5 nerve roots with compression.

analyze:

The patient was an elderly male with lumbar pain for several years, and physical

examination showed weakness of the dorsal extension of the left thumb, suggesting radial nerve dysfunction. Straight leg elevation test is positive, strengthening test is negative, can be diagnosed as lumbar disc herniation. Combined with the patient's symptoms and signs, the most likely cause is the L3-L4, L4 nerve roots compression.

Option A: L4-L5, L5 nerve root compression is mainly manifested by the weakened muscle strength of the extensor pollicis long muscle, that is, the weakened muscle strength of the dorsal extensor of the thumb, which does not meet the meaning of the question.

Option C: L2-L3, L3 nerve root compression is mainly manifested by weakened quadriceps muscle strength, that is, extended knee weakness, which can be excluded.

Option D: S1-S2, S2 nerve root compression is mainly manifested as weakened muscle strength of fibula length and short muscle, that is, ankle dorsal extension and valgus weakness, so it is not considered.

Option E: L5-S1, S1 nerve root compression is mainly manifested by the weakened muscle strength of the hallux long flexor muscle, that is, foot plantarflexion weakness, which is inconsistent with the meaning of the question.

In conclusion, the correct answer is: B.L3-L4, L4 nerve root compression.

According to the title description, a 65-year-old male patient had low back pain for several years, straight leg elevation test (+), booster test (-), and physical examination revealed weakness of the back of the left thumb. Here is an analysis of each option:

- ** Straight leg elevation test (+) * *: usually indicates that the sciatic nerve is pulled stimulation, common in lumbar disc herniation.

- ** Enhancement test (-) * *: indicates no dura or cauda equina compression.

- ** Weak dorsal extension of the left thumb * *: indicating the possibility of nerve root compression.

Dorsal extension weakness of the thumb is usually associated with * * L5 nerve root * * compression, because the L5 nerve root innervates the extensor pollicis longus. Combined with a history of low back pain and a positive straight leg elevation

test, it can be speculated that lumbar disc herniation compressed the L5 nerve root.

Therefore, the most likely reason is * * L4-L5, L5 nerve root compression * *, corresponding to option A.

Best answer:

A. L4-L5, L5 nerve root compression

82. (Type A2 questions)

Male, 23 years old, right hand landing, right forearm deformity after injury, limited movement. Physical examination: the right forearm deformity, swelling, the middle and lower junction of the radius, the wrist can touch the abnormal bulge of the distal ulna, the possible diagnosis of the ruler is

A. Smith for the fracture

B. Monteggia for fracture

C. Colles. for a fracture

D. Barton. for the fracture

E. Galeazzi. for a fracture

Reference answer: E Your answer:

[Analysis: A young man with a history of right arm trauma, forearm deformity, swelling (fracture manifestations), bone rubbing sensation at the junction of the middle and lower part of the radius, and the wrist of the distal ulna (characteristic manifestation of Gage (Galeazzi) fracture), the patient was Gai (Galeazzi) fracture (E pair).

[Error avoidance] 1 / 3 of the upper ulna backbone fracture can be combined with the small dislocation of the radial head, called Meng (Moteggia) (B wrong) fracture.

Straight fracture (Colles fracture) (C wrong) due to the wrist joint in the back extension, palm landing, forearm rotation before

Injury jurinjury, can appear typical deformity posture, that is, the side is "silver fork" deformity, the front is "bayonet like" deformity.history

A Mith (Smith) fracture is a fracture within 2 to 3 cm of the distal radius with a distal volar or dorsal displacement

Angner (A wrong). The articular surface fracture of distal radius with wrist dislocation (Barton fracture) is in wrist extension and forearm rotation

Front fall, the palm of the ground, violence through the wrist bone conduction, hit the radial joint dorsal fracture, the wrist joint also followed

To the dorsal shift.

The patient had a deformity of the right forearm due to a high fall, limited movement, and a palpable bone rub at the junction of the middle and lower radius, and the abnormally raised distal ulna at the wrist. These symptoms fit the characteristics of a Galeazzi fracture.

Galeazzi A fracture is a fracture of the distal radius associated with a dislocation of the distal ulna. While Monteggia fracture is an ulnar fracture with radial head dislocation, Smith fracture is a distal radius fracture and displacement to the volar side, Colles fracture is a distal radius fracture and dorsal displacement, and Barton fracture is a distal radius articular surface fracture. Therefore, based on this patient's symptoms and signs, the most likely diagnosis is an E.Galeazzi fracture.

This is a question about the differential diagnosis of fracture types. We need to analyze the patient's medical history, clinical manifestations, and physical examination results to determine the most likely diagnosis.

First, let's comb through the key information given in the topic:

The young male patient fell with his right hand on the ground.

The right forearm was deformed and movement was restricted.

Physical examination revealed a deformity and swelling of the right forearm.

Bone rubbing can be reached at the middle and lower 1 / 3 of the radius.

The distal is distal at the wrist.

Next, we analyze the possibilities of each option based on the key information:

A. Smith fracture: A flexion distal radius fracture, usually caused by the back of the hand, not with the trunk description.

B. Monteggia fracture: 1 / 3 shaft fracture of the upper ulna with radial head

dislocation, usually manifested by the triangular change of the posterior elbow, usually caused by elbow injury, inconsistent with the patient's condition.

C. Colles fracture: it is a straight distal radius fracture, typically "meal fork" and "spear prick" deformity, wrist swelling and tenderness, but no abnormal bulge of the distal ulna, so it does not meet the patient's signs.

D. Barton fracture: a transarticular fracture of the distal radius, which also involves the distal radius, but mainly involves the wrist surface, which does not meet the description of the forearm deformity and the bulge of the distal ulna.

E. Galeazzi fracture: a 1 / 3 fracture of the lower radial shaft with dislocation of the ulna head, usually caused by landing on the palm during a fall. This fracture type can cause deformity and swelling of the forearm, bone rubbing at the middle and lower half of the radius, and an abnormally raised distal end of the ulna at the wrist, consistent with the patient's clinical findings and physical findings.

In conclusion, the most likely diagnosis is Galeazzi fracture based on patient history, clinical presentation and physical findings. Therefore, the correct answer is the E.Galeazzi fracture.

To sum up, the correct answer is: E.Galeazzi fracture.

According to the medical history and physical examination results described by the title, a 23-year-old man fell on the ground, the right forearm was deformed and swollen, the bone rub was felt at the junction of the middle and lower radius, and the abnormally raised distal ulna was reached at the wrist. These descriptions are fit with the characteristics of the Galeazzi fracture. Galeazzi A fracture refers to 1 / 3 fractures of the distal radius shaft with a dislocation of the distal ulna.

Therefore, the most likely diagnosis is:

I. Galeazzi. for a fracture

83. (Type A2 question)

Female, 32 years old, afraid of cold, drowsiness for more than two months. Physical examination: pulse 56 times / minute, eyelid edema, diffuse enlarged thyroid, tough texture. The most likely manifestation of thyroid function is

A. Normal TT 3, normal TT4, and reduced TSH

B. TT3 decreased, TT4 decreased, and TSH increased

C. Increased TT 3, increased TT4, and decreased TSH

D. TT3, TT4, and TSH

E. Normal TT 3, normal TT4, and increased TSH

Reference answer: B. Your answer:

[Analysis] Young and middle-aged women (people with multiple thyroid diseases), afraid of cold, drowsiness, pulse 56 times / minute, eyelid edema, diffuse thyroid enlargement (typical manifestations of hypothyroidism), comprehensive symptoms and signs considering this patient is reduced thyroid function. Higher serum TSH and decreased TT4 and FT4 (B pair).

[Error avoidance] TT3 increased, TT4 increased, and TSH decreased as serological manifestations of hyperthyroidism.

The 32-year-old woman experienced cold fear, drowsiness, reduced pulse (56 beats / min), eyelid edema, and diffuse thyroid enlargement and tough texture. These clinical manifestations are consistent with hypothyroidism, a disease caused by insufficient thyroid hormone production.

In hypothyroidism, lower levels of triiodothyronine (TT3) and thyroxine (TT4) in the serum are commonly observed, along with increased levels of thyroid-stimulating hormone (TSH) due to feedback mechanisms. Thus, the most likely manifestations of thyroid function are decreased TT3, decreased TT4, and increased TSH.

Option B (TT3 decreased, TT4, TSH increased) is the correct answer to this situation.

analyze:

The patient was a young woman, afraid of cold and drowsiness for more than two months, with slow pulse rate, eyelid edema, diffuse thyroid enlargement and tough texture. According to her clinical manifestations and signs, hypothyroidism was considered. In hypothyroidism, thyroid hormone levels decrease, and the negative feedback inhibition of TSH is weakened, and TSH levels increase. Therefore, the thyroid function of hypothyroidism patients shows decreased TT3, decreased TT4, and increased TSH.

Option A "TT3 normal, TT4 normal, decreased TSH"; option C "TT3 increased, TT4 increased, decreased TSH"; option E "increased TT3 normal, normal TT4, TSH"; TSH has opposite trends to TT3 and TT4, which can be excluded together.

Option D: increased TT3, TT4, and TSH do not comply with the negative feedback regulation mechanism of thyroid hormone, so it is not selected.

In conclusion, the correct answer is: B.TT3 decreases, TT4 decreases, and TSH increases.

Based on the patient's symptoms and signs, including cold fear, drowsiness, slow pulse, eyelid edema, and diffuse thyroid enlargement, the most likely diagnosis is hypothyroidism (hypothyroidism).

Typical manifestations of hypothyroidism are lower levels of thyroid hormone (TT3 and TT4) and increased thyroid stimulating hormone (TSH), because the pituitary produces more TSH in an attempt to stimulate the thyroid to produce more hormones.

Thus, the most likely manifestations of thyroid function are:

F. TT3 decreased, TT4 decreased, and TSH increased

84. (Type A2 questions)

Male, 22 years old, twisted his right hand while repairing the water pump, physical examination: irregular wound on the right palm, bleeding, 2~5 distal skin pale, decreased sensation, limited interdigital joint flexion, debridement, tendon and neurovascular anastomosis. The art is

A. Brake of affected limb and elastic bandage

B. Local ice compress to prevent hematoma formation

C. Ffingers fingers fingers fingers fingers the tips

D. Plaster fixed finger in straightening

E. Patients are encouraged to move their fingers early on the stage

Reference answer: C Your answer:

Graphic analysis: [Analysis] The patient underwent tendon neurovascular

anastomosis after the hand trauma, and the finger webbed interval was open and the fingertips were exposed (C

Yes), easy to observe the condition, dressing dressing should compress hemostasis and do not affect the blood circulation of the limbs.

[Error avoidance] Patients with hand trauma should fix their fingers in the functional position to maximize the preservation of hand function.

The exam described a 22-year-old man who twisted his right hand while repairing a pump. The patient had irregular wounds, persistent bleeding, and pale skin distal to the 2 to 5 digits, hypoesthesia, and limited interphalangeal joint flexion. After debridement and tenoneurovascular anastomosis, postoperative recovery measures need to be considered.

The option analysis is performed as follows:

-A. Affected limb braking, elastic bandage fixation: This method is suitable for certain fractures or sprains and requires temporary movement restriction, but it may not be the best choice for promoting blood circulation and avoiding stiffness after surgery.

-B. Local ice compress to prevent hematoma formation: Ice compress can reduce inflammation and swelling, but it should not be the only measure for the long-term management of post-traumatic recovery.

-C. Open the fingertips: This can avoid fingertip compression and promote wound healing, but the direct help to functional recovery is limited.

-D. Fixed the finger in a straight position: this will limit the finger movement and may lead to postoperative stiffness, which is not conducive to the recovery of finger function.

-E. Encouraging patients to move their fingers early: This helps improve blood circulation, prevent joint stiffness and muscle atrophy, and is a common recommendation in postoperative rehabilitation.

According to the above analysis, the best answer is * * E. Encourage the patient to move the fingers early * *, because it helps to promote blood circulation, prevent joint stiffness and muscle atrophy, and speed up the recovery process.

analyze:

In a young male patient, twisted the right hand, with irregular wound on the right

palm, excessive bleeding, 2 to 5 distal skin pale, hyposensation, limited interdigital joint flexion, the diagnosis was considered as right hand trauma, debridement and tendon neurovascular anastomosis was performed. The postoperative local braking * * * * and the elastic bandage fixation are required to reduce the tension of the anastomosis and ensure the good healing of the anastomosis.

Option B is local ice to prevent hematoma formation. Although it can help reduce swelling and pain, it is not a special treatment after anastomosis, so it is not chosen.

In Option C, the finger is open for a separate dressing of the finger, while the patient had a palm injury, so it was not applicable, so it was excluded.

The finger flexion position is required to reduce the tendon tension, so option D plaster places the finger in the straight position. Option E encourages patients to move the finger early, which is not conducive to anastomotic healing and can be excluded together.

To sum up, the correct answer is: A. affected limb braking, elastic bandage fixation.

According to the case provided, the patient suffered severe injury to the right hand, presenting with excessive bleeding, pale distal skin, hypoesthesia and limited flexion of the interphalangeal joint. After debridement, the best postoperative treatment is crucial. Here is an analysis of the options:

A. braking of the affected limb, elastic bandage fixation: This method helps stabilize the injured limb and reduce secondary damage and bleeding caused by activity.

B. Local ice compress to prevent hematoma formation: Although ice compress can help reduce swelling and hematoma, it is not the top priority for this complex post-surgical treatment.

C. When dressing, the fingertips are exposed: this is to prevent the blood circulation problems caused by excessive dressing. At the same time, you can observe

the color of the fingertips and the blood circulation situation by exposing the fingertips, which is a more important measure.

D. plaster fixation in the straight position: For postoperative fixation, plaster fixation can provide good stability, but fixation in the extended position may limit finger movement and functional recovery.

E. Encourage patients to move their fingers early: Although early finger movement is helpful to prevent joint stiffness, movement may cause bleeding and injury during the recovery period after the initial surgery, and therefore is not the optimal choice.

Considering the patient's condition and the principles of postoperative treatment, the best answer * * is C. Open the finger when dressing, exposing the fingertips * *. This prevents bandages from being tight, and addresses potential problems by observing the fingertip color and blood circulation.

85. (Type A2 question)

A 50-year-old female had chest tightness for 1 year after activity and nighttime paroxysmal dyspnea for 4 days. Physical examination: BP130 / 80mmHg, P2 hyperactivity, apeical smell and diastolic rumble murmur, the most common arrhythmia in this patient

A. third-degree atrioventricular block

B. Supraventricular tachycardia

C. auricular fibrillation

D. ventricular tachycardia

E. atrial flutter

Reference answer: C Your answer:

Graphic analysis: [analytical] middle-aged and old women, activity after chest tightness, night paroxysmal dyspnea (left heart failure symptoms), P2 hyperactivity, apex can smell and diastolic rumble murmur (characteristic mitral stenosis

auscultation), considering the patients for mitral stenosis, mitral stenosis is the most likely to arrhythmia for atrial fibrillation (C).

[Avoid error] Heart signs of chronic left heart failure: in addition to the inherent signs of underlying heart disease, there are usually heart enlargement (simple

Except for diastolic heart failure) and regurgitation murmur of relative mitral insufficiency, second heart tone hyperactivity and relaxation in the pulmonary valve area

Zhang Qi Ben horse law. The typical murmur of mitral insufficiency is a full systolic wind-like murmur in the apical area. Aortic valve is closed not

The full typical murmur is a high-profile diastolic sigh murmur in the aortic valve area. The typical murmur of pulmonary valve stenosis is the sternum

A loud systolic jet murmur in the second intercostal region of the left margin. The typical murmur of tricuspid stenosis is intercostal constriction of the left margin of the sternum

Pre-period or diastolic drum-like murmur.

Symptoms described in this case, such as post-active chest tightness, nocturnal paroxysmal dyspnea, and audible and diastolic rumble murmur at the apex, may point to heart valvular disease, particularly mitral valve lesions. P2 hyperactivity refers to the enhancement of the pulmonary valve component of the second heart tone, which may be due to the increased pressure in the late left ventricular phase. These symptoms and signs may correspond with mitral stenosis or other left ventricular filling disorders.

In this case, the most common arrhythmia is atrial fibrillation (option C). Atrial fibrillation is more common in patients with mitral valve disease, especially in the presence of left atrial enlargement and increased left ventricular filling pressure. Therefore, option C (atrial fibrillation) is the best answer to this question.

analyze:

In a middle-aged female patient, chest tightness after activity, nocturnal paroxysmal dyspnea, physical examination showed hypersecond heart tone of pulmonary artery, and diastolic rumble murmur at the apex, which was the characteristic murmur of mitral stenosis. Combined with the symptoms and signs of the patient, she should be diagnosed as mitral stenosis. Mitral valve stenosis patients the most common arrhythmia for atrial fibrillation, may because of increased left

atrial pressure, rheumatic myocarditis cause left atrial wall of the focal myolysis, atrial muscle parts between vagus nerve muscle electrophysiological heterogeneity, make each part of the atrial muscle electrical excitability is not stable, causing atrial fibrillation.

Option A third degree atrioventricular block is common in coronary heart disease, myocarditis, cardiomyopathy and other diseases, mitral stenosis is rare, so it is not selected.

Option B Supraventricular tachycardia is common in patients without organic heart disease, but also can be seen in wind heart disease, mitral stenosis, coronary heart disease, etc., but the incidence is relatively low, so it is excluded.

Option D ventricular tachycardia is more common in patients with various organic heart disease, the most common is coronary heart disease, especially in patients with myocardial infarction, mitral stenosis is rare, so it is not selected.

Option E Atrial flutter is more common in organic heart disease. In the transition type between atrial tachycardia and atrial fibrillation, when the frequency of atrial ectopic pacing reaches 250~350 times / minute and is regular, the atrial flutter is caused, but the incidence is lower than atrial fibrillation, so it was excluded.

To sum up, the correct answer is: C. atrial fibrillation.

According to the title description, the patient was a 50-year-old patient with chest tightness for 1 year after activity, and nighttime paroxysmal dyspnea for 4 days. Physical examination showed blood pressure of 130 / 80 mmHg, P2 hyperactive, and diastolic rumble murmur at the apex. These symptoms and signs suggest the possibility of cardiac insufficiency, especially left cardiac insufficiency. Diastolic rumble murmur may indicate mitral stenosis.

One of the common complications of mitral stenosis is atrial fibrillation (atrial fibrillation), because mitral stenosis causes increased left atrial pressure and left atrial enlargement, which can easily lead to atrial fibrillation. Therefore, the most common arrhythmias in this patient are:

B auricular fibrillation

86. (Type A2 question)

Male, 18 years old, right ankle sprain for 2 hours, right ankle swelling, mild tenderness in front of external malleolus, joint stability, no fracture displacement of X-ray, early treatment is inappropriate

A. Local massage

B. Rest, and reduce walking

C. The elastic bandage is fixed appropriately

D. cold compress

E. Right lower limb elevation

Reference answer: A. Your answer:

Graphic analysis: [Analysis] Patients with ankle sprain immediately massage (A wrong, is the correct answer to this question) and hot compress will accelerate the blood circulation of the affected area, making the swelling more obvious.

[Avoid mistakes] Cold compress within 24 hours after injury to reduce local bleeding and swelling. hot compress should be applied 48 hours after injury.

Early treatment of the ankle sprain should follow the "RICE" principle, namely rest (Rest), ice (Ice), compression (Compression), and elevation (Elevation). Therefore, correct early treatment includes rest, reduced walking (B), proper elastic bandage fixation (C), cold compress (D), and injured limb elevation (E). These measures can help to reduce the swelling, pain, and further tissue damage.

Conversely, local massage (A) may aggravate the injury as it may cause more swelling and bleeding. Increase in local blood flow may increase the inflammation and swelling, so early massage is not recommended.

Based on the above information, the most inappropriate early treatment is * * A. local massage * *.

analyze:

In a young male patient, right ankle sprain, right ankle swelling, mild tenderness before lateral malleolus, stable joint stability, no fracture displacement of X-ray, which was considered as simple ankle sprain. Ankle sprain alone is a common clinical disease, with the highest incidence in joint and ligament injuries. The ankle sprain causes bleeding and exudation due to violent injury, resulting in swelling and pain. At this time, it is not suitable for local massage, because massage will aggravate local bleeding and swelling, which is not conducive to the repair of soft tissue.

Option B: Rest, reducing walking can reduce the activity of the injury site, and provide conditions for soft tissue repair, which belongs to the appropriate treatment of ankle sprain.

Option C The elastic bandage properly fixed can play the role of pressure bandage, which can not only reduce bleeding, but also prevent local reactivity, as an appropriate treatment.

Option D cold compress can reduce local congestion, relieve swelling and pain, and is the appropriate way to handle it after ankle sprain.

Option E Right lower limb elevation promotes blood and lymphatic return of the lower limbs, relieves swelling, and improves local soft tissue recovery after ankle sprain.

To sum up, the correct answer is: A. Local massage.

For the early treatment of the right ankle sprain, the acute injury management principle is usually used, namely, rest, ice compress, compression, and elevation (RICE principle). Each option is analyzed below:

A. Local massage-Local massage in the acute phase (24 to 48 hours after injury) is inappropriate, as this may increase swelling and bleeding and prolong recovery time.

B. Rest, reduce walking-Rest and reduce walking help prevent further damage and promote healing and are appropriate therapeutic measures.

C. Proper fixation of the elastic bandage-The elastic tension bandage fixation can help control the swelling and stabilize the joint, and is also an appropriate treatment.

D. Cold compress-cold compress can reduce pain and swelling, and is one of the standard treatments of acute phase injury.

E. Right lower limb elevation-Elevation The injured lower limb reduces swelling and pain and is also an appropriate treatment.

In conclusion, the inappropriate early treatment is:

A. Local massage

87. (Type A2 question)

Male, 22 years old. History of fever 2 weeks ago, palpitations and fatigue for 3 days. Physical examination: heart rate 100 times / minute, all together, cardiac ultrasound showed no obvious abnormalities. The most likely diagnosis is the

A. dilated cardiomyopathy

B. hypertrophic cardiomyopathy

C. Acute viral myocarditis

D. coronary disease

E. acute myocardial infarction

Reference answer: C Your answer:

[Analysis] The patient was a young male (people prone to viral myocarditis) with a 2-week history of fever (prodromal infection symptoms), palpitations, fatigue, heart rate 100 times / min, no obvious abnormalities in cardiac ultrasound, combined with the patient's history and signs, considering the diagnosis of viral myocarditis (C pair).

[Avoid] dilated and hypertrophic cardiomyopathy can be diagnosed by ultrasound; coronary heart disease in middle-aged and elderly patients with chest pain and heart

Electrographic changes are confirmed; acute myocardial infarction often presents with severe and persistent retrosternal pain.

A 22-year-old man with a 2-week history of fever developed palpitations and fatigue in the last 3 days, heart rate 100 beats / minute, and no significant abnormalities on cardiac ultrasound. Based on these symptoms and signs, the most likely diagnosis is a C. acute viral myocarditis.

Acute viral myocarditis is common in young patients, which can cause myocardial injury and inflammation after viral infection (such as influenza virus, enterovirus, etc.). Typical symptoms include fever, palpitations, fatigue, etc. Cardiac

ultrasound may not show significant structural abnormalities because viral myocarditis is mainly inflammation and dysfunction of the myocardium, not necessarily with significant changes in cardiac structure. Therefore, combining the symptoms and examination results of this young patient, acute viral myocarditis is the most likely diagnosis.

First, we need to analyze the patient's medical history, symptoms, and examination results, so as to obtain the most appropriate diagnosis.

The patient was a young male with a history of fever 2 weeks ago, which suggests that the patient may have had some infection, such as a viral infection. Palpitations and fatigue in the last 3 days, which is the manifestation of cardiac involvement. Physical examination showed that the heart rate was 100 beats per minute, which was slightly faster but all together, while the cardiac ultrasound examination showed no obvious abnormalities.

Next, we analyzed for each option:

A. Dilated cardiomyopathy: an enlarged left or double ventricle with decreased ventricular systolic function. However, the cardiac ultrasound examination in this question did not see obvious abnormalities, which is inconsistent with this disease.

B. Hypertrophic cardiomyopathy: The main feature is ventricular wall thickening, especially the ventricular septum. However, the ultrasound of this patient did not show this abnormality.

C. Acute viral myocarditis: most patients have prodromal symptoms of viral infection, such as fever and systemic burnout, before the onset. Then you can have palpitations, chest pain, dyspnea and other discomfort. This patient had a history of fever 2 weeks earlier, followed by palpitations and fatigue, which highly coincided with this condition.

D. Coronary heart disease: more common in middle-aged and old age, often caused by coronary atherosclerosis. This patient was young and had no other risk factors for CHD, so this option is unlikely.

E. Acute myocardial infarction: usually with a history of angina pectoris, presenting with sudden, persistent, and severe retrosternal pain. The patient did not mention such symptoms.

In conclusion, combining the patient history, symptoms and examination results, acute viral myocarditis is the most appropriate diagnosis.

Therefore, the correct answer is the C. acute viral myocarditis.

According to the title description, the patient was a 22-year-old man with a history of fever 2 weeks ago, with palpitations and fatigue for 3 days. Physical examination showed a heart rate of 100 times / min, and no obvious abnormalities in cardiac ultrasound. In this case, the most likely diagnosis is an acute viral myocarditis.

* * Acute viral myocarditis * * Usually occurs after viral infection and shows symptoms of fever, palpitations, fatigue, while echocardiography may not be significantly abnormal. Therefore, considering the patient's medical history and clinical presentation, the most likely diagnosis is:

B Acute viral myocarditis

88. (Type A2 questions)

A 68-year-old female, found high blood pressure during physical examination, but had no obvious symptoms, felt good about herself and had no standardized treatment. After breakfast today, I took a routine walk and felt abdominal discomfort. I went to the community hospital for treatment. On the way home, suddenly feel chest tightness, fell to the ground, rescue invalid death. Necropsy revealed blood in the pericardial space, about 200ml. The most likely cardiac lesion in this patient was

A. Mass lymphocyte infiltration between the myocardium

B. Cardiac muscle fiber thickening and hypertrophy

C. Granuloma formation was seen between the myocardium

D. Extensive fibrosis

E. Left ventricular anterior wall transmMI

Reference answer: E Your answer:

[Analysis] Hypertension patients did not undergo standardized treatment, sudden abdominal discomfort, according to gastroenteritis rehydration treatment, sudden feeling of chest tightness, can not afford to fall to the ground (the possibility of acute myocardial infarction), rescue ineffective death. The autopsy found blood in the pericardial cavity, about 200ml, which was considered acute myocardial infarction, so the patient was most likely to have left ventricular anterior wall wall myocardial

infarction (E pair).

[Avofrom errors] Intermyocardial lymphocyte infiltration (A wrong) and myocardial granuloma formation (C wrong), for rheumatic myocarditis

Pathological characteristics. And hypertrophy (B error) is the pathological characteristic of hypertrophic cardiomyopathy. Extensive fibrosis between the myocardium

(D error) is a pathological feature of chronic ischemic heart disease.

The title described a 68-year-old woman with high blood pressure and no standardized treatment, and an autopsy found 200ml of blood in the pericardial cavity. This condition is most likely due to a cardiac tamponade due to an acute myocardial infarction. Acute myocardial infarction can lead to acute ischemia and necrosis of heart tissue, serious can lead to heart wall rupture, blood will therefore enter the pericardial cavity, forming cardiac tamponade, quickly lead to the death of patients.

So the most appropriate answer is:

B Left ventricular anterior wall transmMI

This option describes a transmural myocardial infarction due to an acute myocardial infarction, consistent with the clinical manifestations of acute chest pain, pericardial hemorrhage, and sudden death.

analyze:

The elderly woman of the patient suddenly felt abdominal discomfort when walking after breakfast, and was initially diagnosed as gastroenteritis. On the way home after treatment, she suddenly felt chest tightness, fell to the ground, and died after rescue. Blood autopsy found pericardial cavity, prompt pericardial accumulation, combined with patient history and clinical manifestations, the patients with the most likely heart disease is heart rupture, more common in acute wall myocardial infarction, heart rupture in myocardial infarction within 1 week, more ventricular free wall rupture, occasionally for ventricular interval rupture perforation or tendon rupture, can cause pericardium caused cardiac compression and sudden death. Therefore, the most likely cardiac attack is left ventricular anterior wall wall myocardial infarction.

Option A Large lymphocytic infiltration between the myocardium is common in viral myocarditis, and the patient was myocardial infarction, so it was not selected.

Option B cardiac fiber hypertrophy is compensatory hypertrophy caused by hypertension without sudden death, so it is excluded.

Option C myocardial granuloma formation was common in infectious endocarditis, and this patient did not indicate infection, so it was not considered.

Option D Extensive myocardial fibrosis was common in dilated cardiomyopathy, and this patient did not indicate ventricular dilation, so it was not consistent.

In conclusion, the correct answer is: E. Left ventricular anterior wall transmural myocardial infarction.

According to the description in the title, the 68-year-old woman was found to have high blood pressure during the physical examination, but had no obvious symptoms and no standardized treatment. She felt sudden abdominal discomfort after walking, and was initially diagnosed with gastroenteritis and treated. On the way home, he suddenly felt chest tightness, fell to the ground, and finally died after rescue. Necropsy revealed blood in the pericardial space, about 200ml.

Based to the important clue of blood in the pericardial cavity, combined with the clinical manifestations, the most likely cardiac lesion is LV anterior wall wall MI (option E). This condition may cause heart rupture, resulting in pericardial hemorrhage (tamponade), resulting to sudden death.

* * Answer: E. Left ventricular anterior wall wall myocardial infarction * *

89. (Type A2 question)

A 62-year-old female with left lumbago with fever for 5 days, the highest body temperature of 39.2°C, no significant decrease with antibiotic treatment, percussion pain in the left kidney area, heart rate 102 beats / min and blood pressure 110 / 75mmHg. Blood routine: white blood cells 15.310 ^ 9 / L, N0.85,

Cells with full view and RBC 3 to 5 / HP. Plain urinary CT scan: the left kidney has dilated hydronephrosis, 1.2cm stone in the upper left ureter, and the next treatment step is

A. extracorporeal shock wave lithotripsy

B. stone removal by ureteroscopy

C. urethral retrograde catheterization

D. Stone removal by cystoureteroscopy

E. Stone removal by percutaneous nephroscopy

Reference answer: C Your answer:

Graphic parsing: [analytical] middle-aged and old female patients, left back pain with fever for 5 days, antibiotic treatment is ineffective, left kidney expansion, left ureter 1.2cm stones, comprehensive overall diagnosis of patients with left kidney stones with uncontrolled urinary tract infection (this is in vitro shock wave lithotripsy contraindications), so the next step treatment choice urethra retrograde catheterization (C), the effusion out, control infection.

[Error avoidance] Contraindications for extracorporeal shock wave lithotripsy: stone distal urinary tract obstruction, pregnancy, hemorrhagic disease, aorta or kidney

Aneurysm, uncontrolled urinary tract infection, etc.

This case describes a 62-year-old woman with left low back pain with fever, no significant decrease in body temperature after antibiotic treatment, percussion pain in the left renal area, elevated leukocytes, and left hydronephrosis and left upper ureteric calculi on plain CT scan. These symptoms and signs suggest that the left hydronephrosis may be an infection caused by ureteric calculi.

A. Extracorporeal shock wave lithotripsy (ESWL) is suitable for renal and upper ureteral stones of less than 2cm, but may not be safe for use in the presence of infection and hydropathy.

B. The ureteroscopic lithotomy allows for the direct removal of the stone and the management of the obstruction caused by the ureter, which is suitable for ureteral stones, especially when infection and water accumulation are present.

C. urethral retrograde catheterization is mainly used to relieve urinary tract obstruction rather than direct stone removal.

D. Bicouretertomy is suitable for stones in the bladder or lower ureter.

E. Percutaneous nephrolithotomy is suitable for larger renal stones or when other methods fail.

Considering the patient's symptoms, signs and radiographic findings, the best treatment should be B. ureteroscopy, as it can directly remove the stones that cause infection and water accumulation and manage the obstruction of the ureter.

analyze:

The elderly woman, the upper left ureteral stone with hydronephrosis, combined

with urinary tract infection, the temperature did not drop significantly after antibiotic treatment, indicating that the infection could not be effectively controlled, invasive surgery should not be carried out at this time, otherwise it may lead to the spread of infection. The water should be drained first, and the stone was treated after controlling the infection. Retrograde urethra catheterization is simple to operate and has little trauma. It can drain the hydronephrosis in time, reduce the pressure in the renal pelvis, and treat the stones in the second stage after infection control.

Option A extracorporeal shock wave lithotripsy is A shock wave generated by an external lithotripsy machine, which focuses on the stones by the machine, and releases energy for many times to smash the stones in the body from the urine. The patient with urinary tract infection should control the infection first, otherwise the spread of infection may lead to the process of lithotripsy, so it is excluded.

Option B ureteroscopy, option D bladder ureteroscopy, option E percutaneous nephroscopy, are surgical treatment. The patient currently has signs of urinary tract infection and is not suitable for surgery, otherwise the infection may aggravate, and stones should be treated after infection control, so they are excluded.

In conclusion, the correct answer is: C. urethral retrograde catheterization.

The clinical manifestations of this 62-year-old female patient included left low back pain, fever (temperature up to 39.2℃), no significant decrease in body temperature after antibiotic treatment, percussion pain in the left kidney area, increased blood routine, and urinary CT, dilated left kidney and 1.2cm stones in the upper left ureter. These symptoms and examination findings suggest that the patient may have hydronephrosis and infection, possibly from acute pyelonephritis due to obstruction of the upper urinary tract due to ureteric calculi.

In this case, the most priority treatment goal is to relieve the obstruction and relieve the infection and hydronephrosis. Therefore, the next therapeutic step should be to remove the ureteral obstruction as soon as possible.

Option analysis:

A. extracorporeal shock wave lithotripsy: suitable for patients without acute infection and hydronephrosis with small stones. Currently unsuitable because the

patient has acute symptoms.

B. Stone extraction by ureteroscope: Direct treatment of ureteral stones can be taken under visualization, which is suitable for patients with acute symptoms.

C. Urethral retrograde catheterization: it can quickly reduce hydronephrosis and infection, which is a more conservative emergency measure.

D. Bladder vesicoureter ureteroscopy: usually used for bladder and lower ureteral stones, currently located in the upper segment.

E. Percutaneous nephrolithotomy: suitable for large kidney stones or complex stone disease, but with relatively high risk and trauma.

In conclusion, considering the acute symptoms and current clinical manifestations, * * C. urethral retrograde catheterization * * is preferred because it can quickly relieve obstruction, reduce hydronephrosis and infection symptoms, and then choose further stone treatment according to the specific situation.

The correct answer is: * * C. urethral retrograde catheterization * *

90. (Type A2 questions)

Male, 50-year-old, with paroxysmal chest pain for January, often at around 3 am, without special discomfort in daytime activities, Holter indicated ST segment elevation of chest guide, relief of symptoms after rest, normal cTn examination, coronary CTA suggested that the best medication for mild narrow treatment of anterior descending artery was

A. nicorandil

B. trimetazidine

C. metoprolol

D. captopril

E. Del sulphuric acid

Reference answer: A. Your answer:

Graphic resolution: [analytical] middle-aged men, onset of chest pain, often at about 3 o'clock in the morning (variant angina good in the morning), daytime

activity without special discomfort, Holter prompt chest lead ST segment elevation, symptoms after rest, repeatedly check cTn is normal, coronary CTA prompt before descending mild stenosis, considering the current diagnosis of variant angina. Niccodil (A pair) is A K + channel activation drug, which activates K + channel of vascular smooth muscle cell membrane, promote K + outflow, hyperpolarize cell membrane, inhibit Ca 2 + influx, and release NO and increase the generation of cGMP in vascular smooth muscle cells. The results of the above two effects relax vascular smooth muscle, coronary vasodilation, increased coronary blood supply and alleviate the damage to ischemic cardiomyocytes by Ca 2 + overload. It is mainly used for variant angina pectoris and chronic stable angina pectoris.

This case describes a 50-year-old man with incident chest pain, particularly around 3 am, but without discomfort during daytime activity. Holter Surveillance showed ST elevation of the chest lead, symptoms resolved after rest, serum myocardial troponin (cTn) examination was normal, and coronary CT angiography (CTA) showed mild stenosis of the anterior descending artery.

Based on this information, the nature and clinical presentation of this chest pain appear to be consistent with variable angina (angina), which usually occurs at rest, especially at night or early in the morning, and can be relieved by rest and / or nitroglycerin. Variable angina is caused by coronary artery spasm, different from the physical obstruction of blood vessels (as seen in stable angina).

According to this description, the appropriate treatment is the use of vasodilators to relieve the spasm of the coronary artery. Nicodil (A) is A calcium channel blocker that can be used to treat variable angina because it helps prevent coronary artery spasm and dilate blood vessels, thereby improving the blood supply to the heart muscle.

Therefore, the most appropriate answer is * * A. Nicole * *.

analyze:

Middle-aged male patients often had paroxysmal chest pain in the early morning without special discomfort during daytime activities. Holter electrocardiogram suggested ST elevation of the chest lead, which can be relieved after rest. Cardiac troponin was normal for many times, and coronary CTA suggested mild stenosis of the anterior descending branch. Combined with the patient's medical history and auxiliary examination, the diagnosis of variant angina pectoris was considered. Variant angina is ischemic angina caused by coronary artery spasm, and calcium

channel blocker is the first choice for treatment, which can expand the coronary artery, relieve coronary spasm and improve the blood supply of the subendocardium. Diertiur is a commonly used non-dihydropyridine calcium channel blocker, which can be used in the treatment of variant angina.

Option A nicoredil is A potassium channel open agent, similar to nitrate ester structure, but does not belong to the same class of compounds, suitable for all types of angina pectoris, especially for patients with coronary microvascular lesions, but the effect of variant angina pectoris caused by coronary artery spasm is not as good as calcium channel blockers, so it is not chosen.

Option B Trimetazidine treatment of myocardial ischemia by inhibiting fatty acid oxidation, increasing glucose metabolism and improving the utilization efficiency of oxygen can be used as a symptomatic treatment for stable angina that cannot be tolerated or ineffective in traditional treatment, and not as the preferred treatment for variant angina, so it is excluded.

Option C metoprolol is a β receptor blocker, which can block the β receptor above the coronary artery, resulting in the relative dominance of α receptors, can induce coronary artery spasm, not variant angina, so it is not chosen.

Option D captopril is an angiotensin converting enzyme inhibitor, which is mainly used in the treatment of hypertension and heart failure. The treatment effect of angina pectoris is not clear and can be ruled out.

To sum up, the correct answer is: E. Disulphur.

According to the information provided by the topic, the 50-year-old man had paroxysmal chest pain, often occurring around 3 am. Holter indicated ST elevation of the chest guide, symptoms were relieved after rest, repeated cTn was normal, and coronary CTA suggested mild stenosis of the anterior descending branch. These information suggest that the patient may have variant angina (Prinzmetal angina).

Variant angina is characterized by temporary myocardial ischemia due to coronary artery spasm, usually occurring at rest, especially at night or early morning. The primary goal of treatment is to of coronary spasm and commonly used drugs include calcium channel blockers and nitrate drugs.

In the options given:

A. Niccodil (Nicorandil) is a potassium channel open agent and nitrate drug that dilates the coronary arteries and is often used in the treatment of variant angina.

B. Trimetazidine (Trimetazidine) is an anti-angina drug, but it is mainly used to relieve the symptoms of chronic stable angina, and is not the preferred treatment for variant angina.

C. Metoprolol (Metoprolol) is a β blocker that is mainly used to relieve angina symptoms, but for variant angina, β blockers have limited effects and may even aggravate spasticity.

D. Captopril (Captopril) is an ACE inhibitor, mainly used for the treatment of hypertension and heart failure, with no direct effect on variant angina pectoris.

E. Diltifide (Diltiazem) is a calcium channel blocker that can effectively relieve coronary artery spasm and is one of the commonly used drugs for variant angina.

Combined with the above analysis, the most suitable drugs for the treatment of variant angina are calcium channel blockers and nitrate ester drugs. So, the best answer to this question is:

F. Disulphur

91. (Type A2 questions)

Male, 32 years old. During physical examination, cardiac auscultation found the noise in the middle contraction of the apical area, and the noise in the middle and late contraction of grade 3 / 6. X-ray showed normal heart shadow. The proposed

diagnosis was

A. mitral valve prolapse

B. mitral valve insufficiency

C. mitral stenosis

D. Aortic closure insufficiency

E. aorta angusta

Reference answer: A. Your answer:

Graphic analysis: [analysis] Apical contraction in the middle and late kala sound (A pair) indicates mitral valve prolapse.

[Misavoidance] The murmur of incomplete tricuspid valve closure is characterized by a total systolic murmur at the left lower margin of the sternum, and the murmur is enhanced after inhalation (B

wrong). The characteristic murmur of mitral stenosis (C fault) is a low-key rumble murmur in the middle and late diastolic period in the apical area. Aortic clearance

The insufficiency (D error) characteristic murmur is a diastolic sigh murmur in the aortic valve area. Aortic stenosis may be in the right margin of the sternum

The second intercostal or the left margin of the third intercostal, hear the systolic jet air noise, the sound is rough, can be to the carotid artery

Conducting (E-error).

This case describes a 32-year-old male with a hala tone in the apical area during cardiac auscultation and a grade 3 / 6 wind-like murmur in the middle to late contraction. The X-ray shows a normal heart shadow.

Karala is usually associated with abnormal activity of the valve, especially during valve prolapse. During mitral valve prolapse, the mitral valve of the heart is abnormally prominent into the left atrium during systole, which may produce a hara and wind-like murmur.

Considering these symptoms, the most consistent diagnosis is:

A. mitral valve prolapse

Therefore, the answer I choose is A. mitral valve prolapse.

analyze:

In a young male patient, the sound in the middle systolic systole of the apical area was found, and the murmur in the middle and late systolic phase of grade 3 / 6 was detected. The X-ray showed normal heart shadow. Combined with the patient's clinical manifestations and related examinations, the most likely diagnosis of the patient was mitral valve prolapse.

Option B The typical murmur of incomplete mitral valve is the full systolic murmur in the apical area. The murmur is soft and often transmitted to the underarm, which is not consistent with the murmur in the main stem, so it was excluded.

Option C The murmur of mitral stenosis is characterized by low-key rumble murmur in the middle and late diastolic confined to the apical area, accompanied by diastolic tremor, and the murmur is systolic murmur, so it is not selected.

Option D is a high-profile diastolic sigh-like murmur in the aortic valve area, which can be transmitted to the apical area. It is significantly different from the murmur in the main stem, so it is not considered.

Option E The noise of aortic stenosis is characterized by rough and loud jet noise in the aortic valve area, with increasing-decreasing type and conduction to the neck, and the auscultation is clearer between the right margin of the sternum, and the characteristics of the noise are not consistent with the problem stem, so it is not selected.

In conclusion, the correct answer is: A. Mitral valve prolapse.

According to the title stem description:

-A 32-year-old male with halala in midsystole on cardiac auscultation during physical examination.

-Middle and late interval on auscultation.

- -The X-ray shows a normal heart shadow.

This description is most consistent with the characteristics of a * * mitral valve prolapse * *. Clinical manifestations of mitral valve prolapse usually include the hearing of sounds in the apical region in the middle contraction, and the subsequent wind-like murmur in the middle and late contraction. Normal heart shadow is also a

common finding because mitral valve prolapse does not necessarily lead to significant enlargement of the heart.

So, the most likely diagnosis is:

A. mitral valve prolapse

92. (Type A2 questions)

Female, 59 years old. Due to high fever and diarrhea, nausea, vomiting and oliguria appeared 5 days later. The total number and classification of blood leukocytes were normal, urine specific gravity 1.010, protein (+), red blood cells 0~2 / HP, white blood cells 3~5 320 μmol/L, urea nitrogen 17 mmol/L, and urinary sodium 100 mmol/L. The most likely cause of hyporenal function in this patient was

A. Acute renal tubular necrosis

B. Presenric azacemia

C. Acute glomerulonephritis

D. Acute-induced glomerulonephritis

E. Acute interstitial nephritis

Reference answer: A. Your answer:

Text analysis: [analytical] elderly women, because of high fever, diarrhea application gentamicin (renal toxicity drugs can cause tubular acute kidney injury) treatment after 5 days oliguria, blood creatinine 320 μmol/L (blood creatinine), urea nitrogen 17 mmol/L, urinary sodium 100 mmol/L (urinary sodium less than 20 mmol/L for prerenal acute kidney injury, urinary sodium greater than 40 mmol/L for acute renal tubular necrosis), so this question A.

[Avoid mistakes] Anterior renal azotemia is caused by insufficient blood volume or cardiac insufficiency, which leads to insufficient renal blood perfusion

Due to the reduced glomerular filtration rate, urinary sodium is generally <20 mmol/L (B wrong). Acute glomerulonephritis is clinically characterized by blood

Urine, proteinuria, edema and hypertension, associated with transient renal insufficiency; more often after streptococcal infection (C error).

Acute glomerulonephritis in acute nephritis syndrome, early oliguria or anuria, rapid progression of renal function and even urination

During the disease period, patients may have different degrees of anemia (D error). Acute interstitial nephritis often has both systemic allergies, mainly

For AIN caused by drug allergy, it may appear as rash, fever and increased eosinophilic leukocyte count in peripheral blood

Cases may also have arthralgia, lymph node enlargement (E wrong).

The patient developed symptoms of nausea, vomiting, and oliguria after receiving gentamicin for high fever, and diarrhea, which are typical of acute kidney injury. Considering that gentamicin is a drug that may cause nephrotoxicity, especially at large doses or longer duration, the main mechanism of damage is causing acute tubular necrosis. Blood and urine tests showing increased levels of BUN and blood creatinine also supported the diagnosis of acute kidney injury.

Therefore, the most appropriate answer is A. acute tubular necrosis.

This is a judgment question about the causes of renal dysfunction. First, we analyzed the patient's medical history and examination results:

A 59-year-old female patient was treated with gentamicin for high fever and diarrhea.

Treatment developed nausea, vomiting, and oliguria.

Blood examination showed a normal total white blood cell count and classification, indicating that there was no obvious infection or inflammation.

Urine examination showed a decrease in urine proportion (1.010), positive urine protein (+), and a low number of red blood cells and white blood cells.

Biochemical examination showed elevated BUN and urinary sodium levels.

Next, we analyzed the possibility of each option based on these symptoms and examination results:

A. Acute tubular necrosis: The patient had been treated with gentamicin, a known nephrotoxic agent. Combined with oliguria, decreased urine specific gravity, and increased BUN and urinary sodium, these are typical of acute tubular necrosis. In line with the meaning of the question.

B. Prenal azoperaemia: usually associated with blood volume insufficiency or cardiac insufficiency, which is not mentioned in the title stem. Not consistent with the

meaning of the question.

C. Acute glomerulonephritis: often manifested as hematuria, proteinuria, edema and hypertension, and these symptoms are not mentioned in the title trunk. Not consistent with the meaning of the question.

D. Acute glomerulonephritis: The disease developed rapidly with early onset of oliguric acute renal failure, but no medical history or symptoms were mentioned in the problem. Not consistent with the meaning of the question.

E. Acute interstitial nephritis: usually associated with drug allergy, renal interstitial fibrosis and tubular atrophy, no history of allergy or symptoms related. Not consistent with the meaning of the question.

In conclusion, considering the patient's medical history, symptoms, and examination results, the most likely cause was acute tubular necrosis due to gentamicin.

Therefore, the correct answer is: A. acute tubular necrosis.

The 59-year-old patient developed nausea, vomiting, and oliguria 5 days after receiving intravenous drip gentamicin for high fever and diarrhea, and had the following findings:

-Total number and classification of blood leukocytes were normal

-Urine specific gravity of 1.010

-Urine protein (+)

-2 to 2 / HP

-Urinary leukocytes 3 to 5 / HP

-Blood creatinine at 320 μ mol / L

-BUN: 17mmol / L

-100mmol / L

These findings indicated that the patient had decreased renal function. Here is an analysis of each option:

A. * * Acute tubular necrosis * *: Gentamicin is an aminoglycoside antibiotic

with nephrotoxicity that may cause acute tubular necrosis. Typical manifestations of acute tubular necrosis include decreased urine volume, increased blood creatinine and blood urea nitrogen, higher urinary sodium concentration (over 40 mmol/L), nearly 1.010, and possible amounts of urinary protein and leukocytes.

B. * * Prenal azogenemia * *: usually caused by hypovolemia (such as severe diarrhea, dehydration, etc.). Features include increased urine specific gravity and low urine sodium (usually less than 20 mmol/L), inconsistent with the patient.

C. * * Acute glomerulonephritis * *: It is characterized by hematuria, proteinuria, erythrocyte tubular pattern, and edema. The patient's examination results showed urine red blood cells from 0 to 2 / HP and no obvious hematuria, which did not consistent with the diagnosis of acute glomerulonephritis.

D. * * Acute glomerulonephritis * *: It is a severe glomerular disease with significant hematuria, proteinuria and rapid deterioration of renal function. It is often accompanied by a large number of red blood cell tube types, which does not accord with the manifestations of this patient.

E. * * Acute interstitial nephritis * *: usually caused by drugs, infection, etc., can lead to leukocytosis in urine, possibly accompanied by urinary protein, hematuria. In this case, oliguria, nausea, vomiting, and leukocytosis, but urinary leukocytes were not significant, and gentamicin more often caused acute tubular necrosis.

Combined with the patient's medical history (gentamicin treatment), symptoms (oliguria, nausea, vomiting) and laboratory results (urine specific gravity 1.010, urine sodium 100 mmol/L, blood creatinine and urea nitrogen increased), the most consistent diagnosis was * * acute tubular necrosis * *.

Therefore, the most likely reasons are:

A. Acute renal tubular necrosis

93. (Type A2 question)

Male, 61-year-old, fell left hip landing for 5 hours, pain and swelling, mobility disorder of left hip, physical examination: external rotation of left lower limb 60°, Bryant shortening of 2cm, left inguinal area tenderness positive, left greater trochanter percussion pain positive, which of the following diseases

A. anterior dislocation of hip joint

B. fracture of femoral shaft

C. transcervical fracture

D. Intertrochanteric femoral fracture

E. The posterior dislocation of the hip joint

Reference answer: C Your answer:

[Analysis] In middle-aged and elderly men, a history of left hip fall, left lower limb shortening, external rotation deformity, left groin area tenderness positive, left greater trochanter percussion pain positive, considering the diagnosis of femoral neck fracture (C pair).

[Error avoidance] Hip anterior dislocation is rare, which mostly occurs in traffic accidents and high falling injuries, and the affected limbs show abduction, external rotation and flexion

Malformation (A is wrong). The symptoms of femoral shaft fracture often have a clear history of thigh trauma, such as car accident injury, falling injury and

Squeeze injury, severe pain in the femur, severe limitation of lower limb mobility, including hip and knee joint work

Can be limited, the thigh skin green purple ecchymosis, local can have obvious swelling, tenderness and percussion pain positive (B

wrong). External deformity of intertrochanteric fractures up to 90° (D wrong). The posterior dislocation of the hip appeared as the affected limb

Shorting, flexion, adduction, and internal rotation deformity of the hip joint (E error).

According to the described symptoms, the patient had pain and swelling of the left hip, impaired mobility, external rotation of the left lower limb, Bryant sign: 2cm

shortening of the affected side than the healthy side, positive tenderness in the left inguinal area, and positive percussion pain in the left great trochanter. These signs point to the damage in the hip region.

Anterior dislocation of the hip in item A usually causes internal rotation of the lower extremities rather than external rotation. B fractures of the femoral shaft usually result in severe mobility disturbances that may be associated with significant shortening of the lower limbs but not specific to external rotation. The femoral intertrochanteric fracture in item D may also lead to external rotation of the lower limbs, but there usually is no obvious manifestation of Bryant's sign. E posterior dislocation of the hip will lead to obvious external rotation and shortening of the lower limbs, in line with Bryant's sign.

Combined with this information, the most consistently described symptom is the posterior dislocation of the hip, the E option. However, femoral neck fractures (option C) are also a common cause of falls in older elderly, especially with hip landing, which usually leads to external leg rotation, pain and mobility disorders. The presence of Bryant sign (shortened lower limbs) and percussion pain of the greater trochanter increased the possibility of femoral neck fracture.

Therefore, the best answer should be a C. femoral neck fracture. Such fractures are more common in the elderly, especially if the condition directly affects the hip when falling.

analyze:

Patients with left hip trauma, left hip pain and swelling, activity disorder, left lower limb external rotation deformity, Bryant triangle side shortening, may have femoral neck or femoral trochanteric fracture, left inguinal area tenderness positive, left greater trochanter percussion pain positive, combined with the patient history, clinical manifestations and physical examination, can be diagnosed as femoral neck fracture.

Option A hip anterior dislocation, option E hip posterior dislocation, often manifested as affected limb shortening, adduction, internal rotation or external rotation deformity, the femoral head can be palpable in the groin, the patient did not have symptoms of affected limb shortening, adduction, internal rotation or external rotation deformity, so it was excluded.

Option B femoral shaft fracture is often manifested as severe fracture pain, tenderness, swelling, deformity and bone rubbing, etc. The patient did not appear

tenderness, swelling and other symptoms, so it is not selected.

Option D femoral intertrochanteric fracture is more common in the elderly, more women than men, the trochanter area pain, swelling, ecchymosis, lower limbs can not move, examination found intertrochanteric tenderness, lower limb external rotation deformity is obvious, up to 90°, axial percussion pain, measurement can find lower limb shortening, but the patient lower limb external rotation Angle is 60°, so excluded.

To sum up, the correct answer is: C. femoral neck fracture.

According to the description in the title:

-The patient was a 61-year-old male, landing on the left hip after the fall.

-5 hours later she developed painful swelling of the left hip and impaired mobility.

-Physical examination revealed that the left lower limb was in 60 degrees of external rotation, and the Bryant triangle was shortened by 2cm from the healthy side.

-Positive tenderness in the left inguinal area and positive percussion pain in the left greater trochanter.

Combining these symptoms and symptoms, the following is an analysis of each possible disease:

A. * * Anterior hip dislocation * *: Anterior hip dislocation usually causes hip abduction and external rotation, but it does not often cause hip swelling and significant positive tenderness after a fall.

B. * * Femoral shaft fractures * *: Femoral shaft fractures usually present with severe pain, swelling and deformity, but generally do not involve significant external rotation deformity in and around the hip joint.

C. * * Femoral neck fracture * *: Femoral neck fracture is a common type of fracture after the elderly fall. Patients usually present with hip pain, swelling, limited mobility, external rotation and shortening of the lower limbs, and femoral neck fractures are often associated with tenderness and percussion pain.

D. * * Intertrochanteric femoral fractures * *: Intertrochanteric femoral fractures cause hip pain and swelling, external rotation and shortening of the lower limbs, but are more commonly described clinically in patients with femoral neck fractures.

E. * * posterior hip dislocation * *: posterior hip dislocation usually presents as hip adduction, internal rotation rather than external rotation, associated with hip pain and mobility disorders.

In conclusion, the symptoms and signs described by the problem stem are most consistent with the characteristics of * * femoral neck fracture * *.

So, the most correct answer is:

F. * * femoral neck fracture * *

94. (Type A2 question)

A 30-year-old female with intermittent right abdominal pain for 1 week was aggravated with frequent urination and urgency for 1 day. Physical examination: positive percussion pain in the right kidney area, examination: urine RBC (+ +), right hydronephrosis, aggregate system 1.0cm, considering urinary stones, stones may be in

A. Tubule and pyeloid junction

B. The ureteral orifice of the renal pelvis

C. Lower ureter segment

D. kidney stone

E. Inside the bladder wall

Reference answer: A. Your answer:

[Analysis] The patient was female, 30 years old, with bladder stimulation symptoms (frequent urination and urgency for 1 day), abdominal pain, percussion pain in renal area, right hydronephrosis, 1.0cm separation of aggregate system, all suggesting obstruction of the ureter or above. Overall, the stones in the bladder wall (ureteral bladder migration) were considered for diagnosis. So choose E.

[Avoid mistakes] Urinary tract stones are most common in the following places: the migration of the ureter (the ureter mouth), the ureter crossing

Location of iliac vessels, migration of the ureteric bladder (within the bladder wall). The migration of the ureter across the iliac vessel

Generally, there are no symptoms of frequent urination and urgent urination and bladder irritation, but it can appear when accompanied by infection, and the patients have no related infection performance, temporarily not

think over. So the ABCD error.

In this 30-year-old female patient with a week of intermittent right abdominal pain, frequent urination and urgency, physical examination was positive for percussion pain in the right kidney area, urine examination showed positive red blood cells (RBC), and right hydronephrosis, and a 1.0cm abnormality of possible urinary calculi.

Options A to E provide the possible location of the stone which, based on symptoms and findings, may cause obstruction between the renal pelvis and ureter, causing fluid and pain. Kidney stones usually cause increased pain when moving to the narrower ureter. Therefore, stones are most likely located at locations that block the flow of urine, thus causing these symptoms.

The possibility of stones in the ureteral mouth is higher, because it is the key narrow area of urine flow, and stone stuck here can explain the aggravation of water and pain. Therefore, the best answer is * * B. The ureteral orifice * *.

analyze:

Young female patient with intermittent right abdominal pain, positive percussion pain in the right kidney area, examination showed urinary red blood cells (+ +), right hydronephrosis, aggregate system 1.0cm, considering urinary calculi. The typical

manifestation of urinary stones is pain in the kidney area with percussion pain, and most patients with urinary stones have microscopic hematuria. Because the patient had right hydronephrosis, the stone was embedded in the mouth of the ureter, causing urine from the renal pelvis into the ureter, thus causing hydronephrosis.

Option A renal tubule and pyeloid junction stones often have no obvious symptoms, most of them are found during physical examination, and they are not easy to cause hydronephrosis, so it is not selected.

Option C The main symptom of lower ureteral stones is pain radiating to the perineal region of the bladder, and it will be accompanied by obvious bladder irritation, which is inconsistent with the question.

Option D Patients with kidney stones are mostly asymptomatic unless the stones drops from the kidney to the ureter causes urine obstruction of the ureter. Common symptoms include lumbar and abdominal cramps, nausea, vomiting, restlessness, abdominal distension, hematuria, etc. If combined with urinary tract infection, there may also be chills and fever and other phenomena, which is inconsistent with this question.

Option E The typical symptoms of stones in the bladder wall were sudden interruption of urination, pain radiating to the distal urethra and penile head, associated with dysuria and bladder irritation. The patient had no sudden interruption of urination, so it was excluded.

In conclusion, the correct answer is: B. The ureteral orifice.

According to the description of the problem, a 30-year-old female patient with intermittent right abdominal pain for 1 week, aggravated by frequency and urgency for 1 day. Physical examination found positive in the right kidney area percussion pain, urine examination showed increased erythrocysis, imaging examination showed right kidney rosis, and the aggregate system dilated 1.0cm. Combining this information, urinary stones were considered.

Based on the description of common sites and symptoms of urinary stones, the most likely location are:

B The ureteral orifice of the renal pelvis

This is because at the mouth of the renal pelvis, stones easily lead to the obstruction of urine discharge, which then causes hydronephrosis and acute symptoms such as frequent urination, urgency and percussion pain in the renal area.

So the best answer is:

C The ureteral orifice of the renal pelvis

95. (Type A2 questions)

Female, 28 years old, soy sauce color for 3 days. Physical examination: no bilateral renal area percussion pain, in order to make a clear diagnosis, the next examination should be done is

A. urine for occult blood test

B. three-glass test

C. Microscopy of urinary sediment

D. routine urine test

E. urobilinogen

Reference answer: C Your answer:

[Analysis] soy sauce color urine is a kind of hematuria, mostly for myoglobinuria or hemoglobinuria. The emergence of hemoglobinuria has rapid vascular hemolysis by renal tubule epithelial cells reabsorption of hemoglobin decomposition globin and iron, iron in the form of iron tin or hemosiderin deposits in renal tubular epithelial cells, with epithelial cells shed by the urine, form hemosiderin urine, iron ions in acidified potassium ferrocyanide solution generated blue iron ferrocyanide (Royce test), the Prussian blue reaction. If the renal tubular epithelial cells shed in urine have hemosiderin, urinary sediment (C pair) in renal tubular epithelial cells, that is, positive.

[Avoid error] Urine occult blood reflects the presence of hemoglobin and myoglobin in urine for many reasons without specificity (A error); three cups of urine were taken

The als are mostly used to determine the source and site of bleeding or inflammation in the urinary system, and are used for the diagnosis of lesions in the

anterior and posterior urethra

And differential diagnosis (B error); routine urine tests usually detect urinary tract infection, nephritis, renal urinary glucose, and low proportion

No specificity in urine (D wrong); clinically, the diseases that will lead to positive or strongly positive urobiliary test results are mainly of various types

Jaundice, common have hemolytic jaundice, liver cell sex jaundice to wait (E is wrong).

The title describes a 28-year-old female who developed 3 days of soy-colored urine but no bilateral percussion pain in the kidney region. This symptom is often associated with hematuria, where changes in the urine color may be due to the presence of hemoglobin, myoglobin, or other pigments. Considering this information, appropriate diagnostic steps should be designed to confirm abnormal components in urine.

A. Urinary occult blood test: useful for detecting red blood cells or hemoglobin in urine, useful in determining the presence of hematuria.

B. three-cup test: to distinguish which part of the urinary tract bleed, but may not be preferred at the initial diagnostic stage.

C. Microscopy of urinary sediment: red blood cells, white blood cells, epithelial cells can be observed in urine, which is helpful for diagnosis, but it may not be specific enough to confirm the reasons for the change of urine color.

D. Urine routine: including a urine occult blood test, which can provide more comprehensive information about color changes in urine.

E. Galluria: It is mainly used for the diagnosis of liver diseases and biliary tract diseases, and it has little to do with urine color change.

According to this information, the most appropriate test is * * D. urine routine * * because it includes a urine occult blood test and provides extensive information on the composition of urine, helping to determine the cause of urine color change.

analyze:

For young female patients, soy sauce color urine for 3 days is considered as hemoglobinuria. The appearance color of hemoglobinuria is different according to the amount of hemoglobin, which can be uniform strong tea, wine, brown and soy sauce color. Therefore, in order to make a clear diagnosis, the next examination that should

be done is urine sediment microscopy. If urine sediment microscopy finds red blood cells, it can be diagnosed as hematuria.

Option A urine occult blood test is used to test whether the urine contains hemoglobin to further determine the presence of hematuria, but it cannot observe the red blood cell morphology and identify glomerular hematuria and non-glomerular hematuria, so it is not selected.

Option B urine three-cup test can identify the site of hematuria production and reveal the possible disease of the genitourinary system, but could not confirm the cause of hematuria, so it was excluded.

Option D urine routine can indicate the physicochemical properties of urine, but it is not helpful to determine the source of hematuria, so it is not chosen.

Option E is common in hepatocellular jaundice and hemolytic diseases, which does not meet the meaning of the question.

In conclusion, the correct answer is: C. Microscopy of urinary sediment.

According to the title description, the 28-year-old patient developed soy sauce-colored urine for 3 days, and the physical examination did not have bilateral renal percussion pain. To clarify the diagnosis, the next tests are:

First, we need to consider the common causes of soy sauce-colored urine, including hematuria, myoglobinuria, bilirubinuria, etc. According to the clinical practice guidelines and medical knowledge:

- ** Urinary occult blood test (option A) * *: Is used to test the urine for blood, but no further information can be provided to clarify the specific cause.

- ** The three-cup urine test (option B) * *: It is used mainly to distinguish between prostate, bladder and urinary tract infections, and it is less used to identify the causes of soy sauce-colored urine.

- ** Microscopy (option C) * *: cells, crystals and other components in urine to provide information about hematuria or other components.

- ** Routine urine (D option) * *: Comprehensive test, can screen for a variety of urine components, including red blood cells, white blood cells, protein, glucose,

bilirubin, etc.

- ** Chinogen (option E) * *: It is mainly used for examining abnormal bilirubin metabolism and is suitable for patients with jaundice.

Considering that soy sauce urine may be related to hematuria, myoglobinuria or bilirubinuria, the most comprehensive and preliminary examination is * * urine routine (option D) * *, as it provides multiple valuable information to further clarify the diagnosis.

Therefore, the next inspection to be made is to:

* * D. Urine routine * *

96. (Type A2 questions)

Male, 50 years old, 1 years of palpitation and chest shortness of breath, aggravated in recent one month, physical examination after admission: R120 times / minute, low heart sound, distant, positive Ewart sign, ultrasound suggested that the ventricular interval is not synchronized, the following most likely treatment measures are

A. operative treatment

B. paracentesis pericardii

C. Expansion of the balloon

D. intravenous furosemide

E. Amtravenous amiodarone

Reference answer: B. Your answer:

[Analysis] A middle-aged male patient, palpitation, chest tightness, low heart sound, distant, Ewart sign positive, the patient was considered diagnosed as pericardial effusion caused by cardiac pressure plug. The first measure that should be taken immediately at this time is the pericardiocentesis solution (B pair).

[Avoid mistakes] When the pericardial effusion volume is large, it can appear under the left scapula, auscultation and bronchial respiratory sound, weighing the

pericardial accumulation

The fluid sign (Ewart sign), caused by pulmonary tissue compression, is a specific sign of pericardial effusion.

The patient had symptoms of palpitation, chest tightness and shortness of breath, which were recently aggravated. The physical examination found the heart rate of 120 beats / minute, low and distant heart sound, positive Ewart sign, and ultrasound. These symptoms and signs suggest the possibility of pericardial effusion or pericardial compression, and a positive Ewart's sign is one of the signs unique to pericardial effusion.

Based on the information and options provided, the most appropriate treatment is * * B. pericardiocentesis * *. Pericardiocentesis is a procedure used to remove excessive fluid in the pericardium, which can quickly reduce the compression of the heart and improve the symptoms. The finding of cardiac ultrasound and the presence of low heart sounds and Ewart's sign all support the presence of pericardial effusion, so pericardiocentesis is a reasonable treatment option.

analyze:

Middle-aged men, flustered chest tightness short 1 year, nearly months to aggravate, physical examination see shortness of breath, low, distant, for the typical manifestation of pericardial effusion, positive Ewart sign is specific signs of pericardial effusion, ultrasound suggests ventricular interval is not synchronized, according to its clinical manifestations and signs, the patient's most likely diagnosis is pericardial effusion. For patients with pericardial effusion with serious symptoms, large pericardial effusion, or pericardial tamponade, pericardiocentesis is feasible to extract the pericardial effusion to reduce the intrapericardial pressure.

Option A Surgical treatment is mainly used to treat constriction pericarditis rather than pericardial effusion, so it is not chosen.

Option C balloon dilatation was a treatment for mitral stenosis, so the diagnosis was excluded.

Option D intravenous furosemide was diuretic to reduce blood volume and thus intrapericardial pressure, but was less effective than pericardiocentesis and therefore not preferred.

Option E Intravenous amiodarone is an effective treatment for various supraventricular and ventricular tachyarrhythmias, which did not match the

performance of the patient's performance, so it was not selected.

In conclusion, the correct answer is: B. Pericardiocentesis.

According to the symptoms and signs in the topic, the 50-year-old male patient developed palpitation, chest tightness and shortness of breath for more than a year, and the symptoms worsened for nearly a month. Physical examination revealed fast respiratory rate (R120 beats / min), low heart sounds, distant, positive Ewart sign, and ultrasound indicating synchronized ventricular interval.

These clinical findings suggest the possibility of a pericardial effusion or tamponade, and a positive Ewart sign was also a sign of a pericardial effusion. Septal asynchrony may be due to cardiac compression due to the pericardial effusion. Based on this information, the most likely therapeutic measure is to relieve the symptoms caused by the pericardial effusion and relieve the cardiac compression. Therefore, pericardiocentesis is the most likely therapeutic measure.

So the best answer is:

F. paracentesis pericardii

97. (Type A2 questions)

Male, 72 years old. Diabetes for many years, has been taking acarbose for a long time, long-term insulin, repaglinide, gliburide, rosiglitazone and other drugs, fasting blood glucose 5.2 mmol/L, two hours after the meal blood glucose 6.5 mmol/L, ejection fraction 42% to stop the drug, which drug should be stopped

A. acarbose

B. long-term insulin

C. Repaglinide

D. glibenclamide

E. rosiglitazone

Reference answer: E Your answer:

Text analysis: [Analysis] An elderly male patient with a history of diabetes for

many years, fasting blood glucose 5.2 mmol/L, 6.5 mmol/L two hours after a meal (blood glucose control), ejection fraction 42% (cardiac insufficiency). Rosiglitazone (E pair) is prohibited for cardiac dysfunction.

[To avoid mistakes] Acarbose liver and kidney insufficiency should still be used with caution, should not be used for people with gastrointestinal disorders, pregnant women, breastfeeding

Period of women and children. T1DM should not be used alone (A error); insulin is prohibited in patients with hypoglycemia; acute hepatitis

Patients with cirrhosis, hemolytic jaundice and patients with pancreatitis and nephritis (B error); reglilinide

β T2DM with very poor cell function, childhood diabetes, pregnant and lactating women, major surgery, total pancreatectomy

After this (the CD is wrong).

In this case, a 72-year-old male patient with a long history of diabetes and was using a variety of hypoglycemic agents, including acarbose, long-term insulin, repaglinide, glibenclamide and rosiglitazone. His fasting blood glucose and two-hour postprandial blood glucose were in the well-controlled range, but his ejection fraction was 42%, indicating possible problems with cardiac function.

When considering discontinuation, we should focus on the potential cardiovascular effects of the drug. Rosiglitazone (option E) is a member of the thiazolidinedione (TZD) and is known to potentially increase the risk of heart failure, especially in patients with a history of cardiovascular disease or risk of heart failure. Given the reduced ejection fraction in this patient that may indicate the risk or presence of heart failure, discontinuation of rosiglitazone should be preferred.

Therefore, the best answer is for E. rosiglitazone.

analyze:

The patient is an elderly man with long-term diabetes, who has been taking acarbose, long-term insulin, repaglinide, gliburide, rosiglitazone and other drugs, fasting blood glucose 5.2 mmol/L, two hours postprandial blood glucose 6.5 mmol/L, and ejection fraction of 42%. Among the hypoglycemic drugs currently used by patients, glyburide can strengthen the myocardial contractility, increase the heart burden, and easily lead to heart failure. At present, the patient has poor cardiac function and reduced ejection fraction, so glyburide should be stopped.

Option A acarbose is A α -glycosidase inhibitor, which mainly inhibits glucoside hydrolase in the intestinal tract, reduces the decomposition of polysaccharide and sucrose into glucose, and slows down the absorption of sugar accordingly, so it can have the effect of reducing blood sugar after meals. No obvious effect on the heart, so do not choose.

Option B Long-term insulin is mainly used for type 1 diabetes, various severe acute or chronic complications of diabetes, and has no obvious effect on the patient's cardiac function, so it is excluded.

Option C repaglinide binds to the sulfonylurea receptor on the pancreatic islet B cells, stimulates the pancreas to release insulin, and rapidly reduces the blood glucose level, with no obvious effect on cardiac function, which is not consistent with the question.

Option E rosiglitazone acts by activating the peroxisome proliferator-activated receptor γ, increasing the sensitivity of the target tissue to insulin action and reducing blood glucose, with no obvious effect on cardiac function, so it is not considered.

In conclusion, the correct answer is: D. glibenclamide.

According to the information provided, the 72-year-old man had a history of diabetes and had long used multiple drugs to control his blood sugar levels. The patient's fasting glucose was 5.2 mmol/L and two-hour postprandial glucose was 6.5 mmol / L, and these glucose levels were within the normal range. Ejection fraction of 42% indicated that the patient had some degree of heart failure. Considering the characteristics of diabetes treatment drugs and the cardiac function status of the patient, the following is an analysis of each drug:

A. * * Acarbose * *: This is a α -glucosidase inhibitor, which mainly acts on the intestine and delays the absorption of carbohydrates and does not affect cardiac function.

B. * * Long-term insulin * *: Insulin is an essential basic treatment drug for diabetic patients. It is very important to maintain blood glucose stability, and the medication should not be stopped.

C. * * Rupaglinide * *: This is an insulin secretion promoter, suitable for meal

use, quickly acting on postprandial blood glucose control, and is not suitable for long-term control.

D. * * glibenclamide * *: This is a sulfonylurea that reduces blood glucose by stimulating insulin secretion from islet β cells. Long-term use has a risk of hypoglycemia and has adverse effects on cardiac function.

E. * * Rosiglitazone * *: This is a thiazolidinedione that lowers blood glucose by increasing insulin sensitivity in peripheral tissues, but is known to have side effects that increase the risk of heart failure.

Given the patient's heart failure status (ejection fraction 42%), * * Rosiglitazone * * (option E) may aggravate heart failure and discontinuation should be preferred.

So, the most correct answer is:

F. * * Rosiglitazone * * *

98. (Type A2 questions)

Female, 62 year old, diagnosed with diabetes for many years, insulin 30R, 18 units early, 16 units later, administered half an hour before the meal. Two hours after dinner, blood glucose is 6.7 mmol/L, hunger and dizziness at night, the next day, fasting blood glucose is 10.2mmol/L, for sugar, should be adjusted

A. Add metformin

B. Reduce the amount of insulin used before the dinner meal

C. Increase the amount of insulin consumption before breakfast

D. Reduce the amount of dinner meals

E. Exercise after dinner

Reference answer: B. Your answer:

[Analysis] Middle-aged and elderly diabetic patients, after insulin treatment, hypoglycemia at night and fasting blood glucose increased. Patients have

hypoglycemia at night, which causes the increase of insulin antagonistic hormone secretion in the body, and then leads to the increase of rebound fasting blood glucose, i. e., Somogyi effect. Therefore, measures should be taken for this patient to reduce the amount of insulin before bed (B pair) to prevent the occurrence of hypoglycemia at night.

[To avoid mistakes] The phenomenon at dawn is that blood sugar is well controlled at night, and there is no hypoglycemia, and only high blood occurs in a short time at dawn

Sugar, may be due to cortisol, growth hormone secretion in the morning.

The topic is about the adjustment of insulin dosage in diabetes patients. Patients feel hungry and dizzy at night, which may be due to the high insulin dose at night. However, the fasting glucose on the next day was high, which indicates that the dose of insulin or administration time should be adjusted to better control blood glucose.

The option analysis is performed as follows:

-A. Add metformin: Metformin is a hypoglycemic agent commonly used in type 2 diabetes, and metformin alone may not be enough to address nocturnal hypoglycemia and morning hyperglycemia in type 1 diabetes or insulin-dependent diabetes.

-B. Reduce insulin use before dinner: This may help to reduce hypoglycemia at night, but it does not necessarily solve the problem of high blood sugar in the next morning.

-C. Increase insulin dosage before breakfast: This may help solve the problem of morning hyperglycemia, but not the evening hypoglycemia.

-D. Reduce the amount of dinner: This does not directly address the problem of inappropriate insulin consumption, and may lead to more pronounced hunger at night.

-E. Exercise after dinner: Exercise can help lower blood sugar, but if patients already experience hypoglycemia at night, it is not recommended to exercise immediately after dinner.

Considering the symptoms of hypoglycemia at night and the high blood sugar in the next morning, a reasonable adjustment plan should avoid hypoglycemia in the evening and control the blood sugar level in the morning. Therefore, the best answer may be to adjust the duration of administration or allocation of insulin to ensure blood glucose stability. However, the option does not directly adjust the timing of

administration, so we need to choose the option closest to the reasonable adjustment.

Therefore, a reasonable choice may be * * B. to reduce the amount of insulin used before dinner * *, in order to prevent the occurrence of hypoglycemia at night. Then, the amount of insulin before breakfast was further adjusted according to the patient's response or other medical interventions were performed.

analyze:

Elderly female patients, diagnosed with diabetes for many years, are treated with insulin. Two hours after dinner, blood glucose was low, and hypoglycemia symptoms such as hunger and dizziness occurred at night, indicating the dosage of insulin, and the fasting blood glucose on the next day was high. This may be because of the rebound hyperglycemia caused by hypoglycemia the previous night, also known as Somogyi effect. Therefore, the amount of insulin used before the evening dinner should be reduced to avoid the occurrence of nocturnal hypoglycemia.

Option A plus metformin is mainly used for type 2 diabetes, which is ineffective in dietary control and physical exercise, especially obese type 2 diabetes. This patient was already using insulin without adding metformin, so it was excluded.

Option C Increasing the amount of insulin before breakfast may further aggravate the hypoglycemia at night, which may cause more severe rebound hyperglycemia, so it is not selected.

Option D reduces the amount of dinner, and option E does exercise after dinner, which will aggravate hunger and hypoglycemia symptoms at night, so it can be excluded together.

To sum up, the correct answer is: B. Reduce the amount of insulin used before dinner.

According to the patient provided in the question stem, a 62-year-old woman with diabetes for many years, was on insulin 30R, 18 units early, 16 units later, and given half an hour before the meal. Two hours after dinner, blood glucose was 6.7 mmol/L, he felt hungry and dizzy at night, and the next day, fasting blood glucose was 10.2 mmol / L.

Considering that the patient had normal blood glucose after dinner, but nighttime hypoglycemia symptoms (hunger, dizziness), and high fasting blood glucose in the

next morning, this indicates that the patient may have experienced hypoglycemia at night, leading to rebound hyperglycemia (Somogyi phenomenon).

To improve this situation, the most appropriate adjustment measure is to reduce the amount of insulin used before dinner to avoid the occurrence of nocturnal hypoglycemia. So the best answer is:

F. Reduce the amount of insulin used before the dinner meal

99. (Type A2 questions)

Male, 18 years old, suddenly sweating and pale after half an hour after taking antibiotics for himself due to "cold". Physical examination: blood pressure 40 / 20mmHg, confused, pale, fine pulse, cold limbs, cyanosis, the patient primary treatment

A. Still drops of dopamine

B. Epinephrine was injected intramuscularly

C. Quiet drops of dexamethasone vs

D. Intravaluminal injection of isoproterenol

E. Oxygen inhalation and ecg monitoring

Reference answer: B. Your answer:

[Analysis] Juvenile male patients with blood pressure of 40 / 20mmHg, unconscious, pale, fine pulse, cold limbs, cyanosis and other series of shock manifestations, were considered anaphylactic shock. At this time, emergency intramuscular injection should be performed. For this question, choose B.

[Avoid] Anaphylactic shock showed dilated small blood vessels and increased capillary permeability, decreased circulating blood volume and blood pressure

Desperline, simultaneous bronchial smooth muscle spasm, developed dyspnea, the use of adrenaline for anaphylactic shock, its excitement

α 1 receptor, constricting arterioles and anterior capillary sphincter, while decreasing the permeability of the latter and agonistic β 1 receptors improve cardiac work

It can show enhanced myocardial contractility, increased heart rate, reduce the release of allergic mediators, and dilate the coronary arteries, so it can be quickly relieved

The clinical symptom of anaphylactic shock is the first choice for the treatment of anaphylactic shock.

The 18-year-old man with sudden sweating, pale face, very low blood pressure (40 / 20mmHg), confusion, pale face, fine pulse, cold limbs, and cyanosis after half an hour suggested severe anaphylactic reaction or shock, especially drug-induced anaphylactic shock.

Considering the patient's symptoms and clinical manifestations, the primary treatment goal is to rapidly restore blood pressure and improve tissue perfusion. Therefore, in the options of:

A. dopamine dopamine: for shock but not preferred in acute anaphylactic shock.

B. Epinephrine intramuscular injection: it is the preferred treatment for acute allergic reaction and anaphylactic shock, which can rapidly improve hemodynamics.

C. Dexamethasone static drip: used for anti-inflammatory and reduced immune response, as adjuvant therapy in anaphylaxis therapy.

D. Intracinal injection of isoproterenol: usually used for asthma and some types of dyspnea, not for the treatment of anaphylactic shock.

E. Oxygen inhalation and ecg monitoring: This is basic supportive care, but not enough to deal with the severity of acute anaphylactic shock.

Therefore, the most appropriate option is * * B. epinephrine intramuscular injection * *, as this is a rapid and effective treatment for acute anaphylactic shock.

analyze:

The young male patient suddenly sweating and became pale half an hour after taking antimicrobial drugs. Physical examination showed shock, blood pressure, confusion, pale face, fine pulse, cold limbs, cyanosis, according to the patient's symptoms and signs, the most likely diagnosis was anaphylactic shock. At this time, epinephrine should be given intramuscularly as soon as possible, which can quickly relieve the cardiac depression of anaphylactic shock, bronchospasm, reduce the release of allergic mediators, dilate the coronary artery, can quickly relieve the clinical symptoms of anaphylactic shock, and save the patient's life.

Option A dopamine hydrodrip is often used in the treatment of hemorrhagic shock and cardiogenic shock, which is not effective for anaphylactic shock, so it is not selected.

Option C Dexamethasone static drip has anti-inflammatory and anti-allergy effects, but the effect is slow. It is often used as an adjuvant to anti-allergy, and is not the first choice, so it is excluded.

Option D Intravaluminal injection of isoproterenol is mainly used for atrioventricular block at any site, but a pacemaker or pacing current is not the preferred drug for anaphylactic shock, so it is not selected.

Option E Oxygen inhalation and ecg monitoring were the general adjuvant treatment measures for shock, but not the primary treatment measures, so they were excluded.

In conclusion, the correct answer is: B. Epinephrine intramuscular injection.

The 18-year-old man developed signs of sweating, pale complexion, significantly decreased blood pressure, confusion, fine pulse, cold limbs, cyanosis after taking antimicrobial drugs, considered as anaphylactic shock (anaphylactic shock). Anaphylactic shock is an acute, life-threatening systemic allergic reaction, and the key to treatment is rapid recovery of blood pressure and maintenance of airway patency.

In this case, the primary therapeutic measure should be the use of epinephrine, the drug of choice for handling anaphylactic shock.

So, the most correct answer is:

F. Epinephrine was injected intramuscularly

100, (Type A2 questions)

A 40-year-old female was switched to 131I for 2 years of uncontrolled hyperthyroidism. After 2 days of 131I treatment, he felt high fever and palpitations, along with nausea and vomiting. Physical examination: T40°C, shortness of breath, sweating, P160 times / min, rhythm.most probable

A. iodism

B. thyroid crisis

C. infectious toxic shock

D. NeuroCNS infection

E. Radiothyroiditis

Reference answer: B. Your answer:

[Analysis] middle-aged women, due to hyperthyroidism drug treatment for 2 years (poor disease control), switched to 131I for 2 days (131I as a stimulus cause); sudden high fever, palpitations, accompanied by nausea and vomiting. T40℃, shortness of breath, sweating, P160 times / min, rhythm (thyroid crisis manifestation). Comprehensive analysis of the most likely diagnosis of this patient is a thyroid crisis

(B to).

[Avoid error] Hyperthyroidism crisis is a syndrome of acute aggravation of thyrotoxicosis, with the cause of a large number of thyroid hormones

Into the circulation. Most occur in patients with untreated or inadequate treatment of severe hyperthyroidism. Common triggers are infection, surgery,

Trauma, spirit, stimulation, etc. Clinical manifestations include: high fever or too high fever, excessive sweating, tachycardia (> 140 times / minute), and annoyance

Manic, anxiety, delusion, nausea, vomiting, diarrhea, severe patients can have heart failure, shock and coma.

This patient, treated with radioiodine (^ 131I), developed symptoms of high fever, palpitations, nausea and vomiting, tachypnea, and increased heart rate. These symptoms are consistent with thyroid crisis (thyroid storm), a serious endocrine emergency, usually occurring in patients with hyperthyroidism, especially after experiencing a certain stress event (e. g., infection or radiation therapy). Thus, the most likely diagnosis is a B. thyroid crisis.

analyze:

The middle-aged female patient, hyperthyroidism treatment for 2 years, 131I showed high fever, palpitations, accompanied by nausea and vomiting, physical examination showed high fever, shortness of breath, sweating and tachycardia. According to the clinical manifestations and signs, the patient was most likely diagnosed as thyroid crisis. Thyroid crisis, also known as hyperthyroidism crisis, is a

syndrome of acute aggravation of thyroid toxicosis, which may be related to the increased levels of circulating thyroid hormones. It mostly occurs in patients with severe hyperthyroidism not treated or undertreated, and the common triggers are infection, surgery, mental stimulation, etc.

Option A iodine poisoning often has A history of taking iodine or iodide, mainly manifested as metal taste in the mouth, salivation, mouth and throat, burning feeling, nausea, vomiting, abdominal pain, etc., do not consistent with the symptoms of the patients in the dry problem, so it was excluded.

Option C Infection Poisulent shock often has clinical manifestations of primary infection, migratory lesions and sepsis. Severe infection may lead to sepsis as septic shock, DIC and multiple organ failure. This patient has no history of infection and can be excluded.

Option D nervous central system infection often has high fever, severe headache, vomiting, neck rigidity, typical positive meningeal stimulation and other symptoms, the patient does not have the above manifestations, so it is not selected.

Option E Radiactive thyroiditis is mainly manifested as local swelling and pain of the thyroid gland. A few patients can have symptoms of hyperthyroidism, but the degree is generally mild. The patients have thyrotoxicosis with severe symptoms, so it is not considered.

To sum up, the correct answer is: B. thyroid crisis.

In the title description, the patient was a 40-year-old female, switched to 131I for hyperthyroidism for 2 years. After 2 days of 131I, he felt high fever and palpitations, along with nausea and vomiting. Physical examination found that the body temperature was 40℃, shortness of breath, sweating, pulse 160 times / minute and rhythm. These symptoms point to an acute and severe systemic response. Based on these symptoms and background, the most likely diagnosis is a thyroid crisis.

Thyroid crisis is an acute life-threatening aggravation of hyperthyroidism that commonly occurs in patients with hyperthyroidism after surgery, infection, iodine radiotherapy or other stress states. Symptoms include high fever, palpitations, nausea and vomiting, sweating, and a rapid heart rate.

Other option explanations:

-Iodine poisoning usually does not cause such a dramatic systemic reaction.

-Infection toxic shock and nervous central system infection may also show high fever and systemic symptoms, but they are not consistent with the background and treatment of hyperthyroidism patients in this case.

-Radiactive thyroiditis generally presents as local pain in the thyroid rather than such a violent systemic reaction.

In conclusion, the most likely diagnosis is * * B. thyroid crisis * *.

101. (Type A2 questions)

The patient was male, hospitalized for myocardial infarction 2 days ago, suddenly suffocated and coughed pink foam sputum 1 day ago, and could not lie supine. A 3 / 6 systolic murmur can be heard in the apex. The patient was due to the wheezing

A. Post-myocardial infarction syndrome

B. rupture of papillary muscles

C. pulmonary infection

D. pulmonary embolism

E. cardiac aneurysm

Reference answer: B. Your answer:

Graphic analysis: [analysis] After myocardial infarction, the patient appeared gasping, cough pink foam sputum, apex and 3 / 6 systolic murmur (auscultation murmur of papillary muscle rupture), comprehensive analysis of the patient considered papillary muscle rupture after myocardial infarction (B pair).

[Error avoidance] Postmyocardial infarction syndrome appears within a few weeks to several months after MI, and can occur repeatedly, manifested as pericarditis and chest

Membranitis or pneumonia, fever, chest pain (A error); pulmonary infection, cough, sputum, with or without chest pain,

Lung tapping and cloudy sounds (C error); the clinical manifestations of pulmonary embolism are chest pain, dyspnea, decreased blood pressure, and good

onset

In lower limb surgery, long-term braking patients (D wrong); ventricular wall tumor is mainly seen in the left ventricle, with the left heart boundary enlarged and the heart visible

Dirty beat range is wide, can have systolic murmur. When mural thrombus occurs in the tumor, the heart sound is weakened (E error).

The patient's clinical manifestations (wheezing and coughing up pink foam sputum, not supine, apical and 3 / 6 systolic murmur) suggest features of acute left heart failure, which may be caused by multiple causes in cardiac events. Given the patient's history of MI, these symptoms may be manifestations of post-MI complications.

A. Post-myocardial infarction syndrome usually presents with symptoms of fever and pericarditis rather than acute left heart failure.

B. Papillary muscle rupture causes acute mitral insufficiency, causing left heart failure and a murmur in the apex, consistent with the patient's symptoms.

C. Pulmonary infections usually have fever, cough, expectoration, and dyspnea, but are unlikely to cause an apical murmur.

D. Pulmonary embolism can cause dyspnea and chest pain, but is unlikely to cause a systolic murmur in the apex.

E. Ventricular dilatoma occurs after myocardial infarction and may lead to changes in ventricular morphology and function, but its presentation is atypical of acute dyspnea and apical systolic murmur.

In conclusion, the most consistent with the patient's symptoms is option B, papillary muscle rupture. Therefore, the patient's wheezing is most likely caused by the papillary muscle rupture.

analyze:

Patients with elderly men, 2 days ago for myocardial infarction hospital, 1 day ago sudden asthma, cough pink foam sputum, cannot supine, for acute left heart failure, in the apex can smell and grade 3 / 6 systolic noise, combined with patient history, clinical manifestations and physical examination, the patient diagnosis for myocardial infarction after concurrent papillary rupture, mitral valve prolapse and closed, causing acute left heart failure, asthma.

Option A after myocardial infarction syndrome appears more than within weeks to months after myocardial infarction, and can occur repeatedly, manifested as fever, pleuritic chest pain, etc., which is not consistent with the performance of this question, so it is not selected.

Option C Pulmonary infection usually showed respiratory symptoms such as cough and expectoration, and the patient had no respiratory symptoms and could be ruled out.

Option D Pulmonary embolism often presents as sudden unexplained dyspnea and shortness of breath, chest pain, syncope, cough, etc., which is inconsistent with this question.

Option E ventricular dilator, also known as ventricular tumor, refers to the myocardial infarction area wall expansion, thinning, myocardial necrosis, necrosis of myocardial gradually replaced by fibrous scar tissue, lesion area thin layer of ventricular wall outward, heart contraction loss of activity or abnormal movement, inconsistent with this problem symptoms, so do not consider.

In conclusion, the correct answer is: B. papillary muscle rupture.

The patient was a man admitted to myocardial infarction (myocardial infarction) 2 days ago, wheezing and pink foam sputum 1 day ago, unable to lie supine and could hear 3 / 6 systolic murmur at the apex. Based on these clinical findings, the most likely diagnosis is a papillary muscle rupture.

Papillary muscle rupture is one of the serious complications of myocardial infarction, which often occurs within several days after myocardial infarction. Papapillary muscle rupture can lead to acute insufficiency of the mitral valve, acute left heart failure, manifested as severe dyspnea (wheezing), pink foam sputum (acute pulmonary edema), and inability to recumbent. The systolic murmur in the apex is due to mitral regurgitation.

So the most likely answer is:

B rupture of papillary muscles

102. (Type A2 questions)

Female, recently feel weak, afraid of cold, pale, annual weight gain 2kg, thyroid 2 degrees enlargement, touch the pain. Edema of both lower limbs, hemoglobin 58g / L, urine routine: red blood cells (0~2 / HP). Urinary protein (1 to 2 / HP), no obvious diagnosis

A. Hyperthyroidism

B. cancerous goiter

C. Hashimoto's thyroditis

D. sub-acute thyroiditis

E. Hypothyroidism

Refer to the answer: D. Your answer:

Text analysis: [Analysis] Female patients, recently appeared fatigue, fear of cold, and weight gain of 2kg per year (indicating symptoms of hypothyroidism), thyroid gland, obvious enlargement, and pain of touch, and nodules (obvious clinical features of subacute thyroiditis). Based on the patient's medical history and symptoms, subacute thyroiditis (D pair) was considered first.

[Avoid error] Patients with hyperthyroidism (A error) show diffuse symmetrical enlargement, soft quality, patients often have tachycardia,

Palpitation, hyperappetite, but weight loss; thyroid cancer (B error) typical mass in the thyroid,

Early stage has no obvious symptoms, late stage can produce hoarseness, breathing, dysphagia and sympathetic compression to cause Horner synthesis

Symptoms and invasion of the cervical plexus showed pain in the ear, pillow, shoulder and local lymph node and distant organ metastasis. Chronic lymphatics

Patients with cytoplastic thyroiditis (Hashimoto's thyroiditis) (C wrong) mostly have bilateral symmetrical, diffuse enlargement and isthmus

And tapered leaves often increase at the same time, can also be unilateral enlargement. On palpation, the thyroid gland is tough, smooth or granular,

Can also be the size of the nodular, generally with the surrounding tissue without adhesion, swallowing movement can be moved up and down.thyroid function

Decreased (E error) appeared chills, fatigue, swelling of the hands and feet, drowsiness, memory loss, less sweat, joint pain, weight

Increases, constipation, but no thyroid pain.

According to the symptom description, the patient developed fatigue, cold, pale face, weight gain, thyroid enlargement with pain, lower limb edema and decreased hemoglobin reduction, which are typical manifestations of hypothyroidism. In hypothyroidism, due to the reduction of thyroid hormone secretion, the metabolism slows down, leading to symptoms such as fatigue, fear of cold, weight gain and so on. Goiter may result from the thyroid gland trying to produce more hormones. Falling hemoglobin may be related to a slower metabolism due to hypothyroidism.

The symptoms of option A (hyperthyroidism) are usually consistent with weight loss, heart rate increase, anxiety, etc., with the case description. Option B (thyroid cancer) may cause thyroid enlargement, but it does not usually cause so many hypothyroidism-related symptoms. Option C (Hashimoto's thyroiditis) is an autoimmune thyroid disease that can cause hypothyroidism, but usually without significant pain. Option D (subacute thyroiditis) usually causes swollen thyroid pain, but is accompanied by temporary changes in thyroid function, and the symptoms and progression do not completely match those described in this case.

In conclusion, the diagnosis most consistent with these symptoms is hypothyroidism. Therefore, the correct answer should be the one E. hypothyroidism.

analyze:

Young female patients, recently feel fatigue, afraid of cold, pale, weight gain, for the metabolic reduction performance of hypothyroidism. Physical examination showed thyroid degree enlargement and pain of touch, which was consistent with the characteristic manifestations of subacute thyroiditis. Double lower limb edema, low hemoglobin, mild anemia, consistent with the myinous oedema and anemia of hypothyroidism. No abnormalities of hematuria and proteinuria can be observed, and edema caused by kidney can be excluded. Based on the patient's symptoms, the physical signs can be diagnosed as subacute thyroiditis with hypothyroidism.

Option A Hyperthyroidism is manifested as the hyperactivity of metabolism and sympathetic excitation, which causes palpitations, sweating, eating and increased defecation and weight loss. Most patients also often have eye protrusion, eyelid edema, vision loss and other symptoms, which are not consistent with the symptoms of the patients in the problem stem, so it is not selected.

Option B Thyroid cancer is often manifested as a mass found in the thyroid gland, with hard and fixed texture and uneven surface. The patient did not touch the mass,

and thyroid cancer generally does not have tenderness, so it is excluded.

Option C Hashimoto thyroiditis shows goiter, most of which are diffuse, and a few may be limited. Some are affected by face and limb swelling, but the thyroid is often not tender, which is not consistent with the description of the title stem, so it is not considered.

Option E Hypothyroidism can also appear fatigue, fear of cold, pale face, weight gain and other manifestations, but no thyroid tenderness, and the patient's thyroid degree is enlarged and touch pain, so hypothyroidism cannot fully explain all the symptoms of the patient, so it is not selected.

In conclusion, the correct answer is: D. Subacute thyroiditis.

According to the title description, the female patient developed fatigue, afraid of cold, pale face, annual weight gain of 2kg, 2 degree thyroid enlargement and touch pain, lower limb edema, hemoglobin 58g / L, and routine urine showed red blood cells and urinary protein. These symptoms and examination findings point to hypothyroidism (hypothyroidism).

The specific analysis is provided as follows:

1. * * Weak, cold, pale * *: These are typical symptoms of hypothyroidism, because hypothyroidism can slow down metabolism, patients usually feel cold and tired.

2. * * 2kg annual weight gain * *: Weight gain is a common symptom of hypothyroidism, due to slow metabolism, body fat is easy to accumulate.

3. * * Thyroid 2 degrees enlargement, touch pain * *: hypothyroidism thyroid may be enlarged, called goiter.

4. * * * *: Methhypothyroidism can lead to mucinous edema, manifested as edema of limbs and face.

5. * * hemoglobin 58g / L * *: Hypothyroidism can cause anemia, which is also one of the reasons for fatigue and pale face.

6. * * Urinary routine: red blood cells (0~2 / HP), urinary protein (1~2 / HP) * *: These may be due to abnormal renal function in hypothyroidism.

In conclusion, the most likely diagnosis is:

C Hypothyroidism

103. (Type A2 questions)

A 27-year-old female with frequent urination, urgency and urinary pain for 2 days, positive percussion pain in the right kidney area, urine sediment examination, RBC 5 ~ 10 / HP, and WBC 50 ~ 60 / HP, the most likely diagnosis was

A. Acute glomerulonephritis

B. acute pyelonephritis

C. acute cystitis

D. Acute interstitial nephritis

E. Acute-progressive glomerulonephritis

Reference answer: B. Your answer:

Graphic resolution: [parse] young female patients (acute pyelonephritis), frequent urination pain 2 days, the right kidney area percussion pain positive (typical clinical manifestations of acute pyelonephritis), hematuria (+), urine protein (+), comprehensive patients with symptoms, signs, laboratory tests, the most likely diagnosis is acute pyelonephritis (B).

[Avoid mistakes] Acute glomerulonephritis and acute glomerulonephritis show hematuria, proteinuria, edema and hypertension, which can be accompanied

One transient renal insufficiency (AE error); acute interstitial nephritis (D error) often has recent medication history and skin

Rash, fever, peripheral blood eosinophilia and other drug allergies, accompanied by abnormal urine test or renal tubular and glomerular function

Damage; acute cystitis without renal tapping pain (C wrong).

The female patient had frequent urination, urgency, urination pain, accompanied by positive percussion pain in the right kidney area. Urinary sediment examination showed red blood cells (RBC) 5~10 / HP and white blood cells (WBC) 50~60 / HP, and these signs and signs were most consistent with the clinical manifestations of acute pyelonephritis (acute pyelonephritis).

Acute pyelonephritis usually presents with urinary frequency, urgency, urine pain, and low back pain, especially in cases of positive tapping pain in the renal area.

Urinary sediment examination usually reveals an increase in white blood cells and red blood cells, consistent with the infection. Therefore, the most likely diagnosis is: B. acute pyelonephritis

First, we analyze the key information and possible diagnostic options.

The patient was a young female, mainly presenting with frequency, urgency and pain, which are typical of bladder irritation signs and are usually associated with urinary tract infection. Moreover, the right renal area was positive for percussion pain, one of the signs of upper urinary tract infection. Urinary sediment examination showed slightly elevated red blood cells (RBC) and significantly elevated white blood cells (WBC), suggesting urinary tract infection.

Now, we analyze each option one by one:

A. Acute glomerulonephritis: the main manifestations are hematuria, proteinuria, edema and hypertension, but generally there will be no bladder irritation and percussion pain in the renal area, so it does not meet the clinical manifestations of the patient.

B. Acute pyelonephritis: This is an upper urinary tract infection, often characterized by fever, low back pain (positive percussion pain in the renal region), and bladder irritation signs, consistent with the patient's symptoms.

C. Acute cystitis: the main manifestation is the bladder irritation sign, but there is usually no renal percussion pain, so it is not fully consistent with the patient's performance.

D. Acute interstitial nephritis: often manifested as systemic infectious symptoms, including chills, high fever, increased blood leukocytes, and left nuclear migration, as well as renal tubular proteinuria, microscopic hematuria, leukocyuria, etc., which are not completely consistent with the symptoms of the patient.

E. Acute glomerulonephritis: the main manifestations are hematuria, proteinuria and progressive renal dysfunction, which are inconsistent with the clinical manifestations of the patient.

In conclusion, combining the clinical presentation and laboratory examination, the most likely diagnosis is acute pyelonephritis.

Therefore, the correct answer is the B. acute pyelonephritis.

According to the title description, a 27-year-old woman had frequent urination, urgency and urination pain for 2 days, with positive percussion pain in the right

kidney area. The urine sediment examination showed RBC 5 ~ 10 / HP and WBC 50~60 / HP. These symptoms and test findings best match the following diagnoses:

-Frequency, urgency and pain indicate urinary infection.

-Positive percussion pain in the right kidney region suggests possible renal involvement.

-More leukocytes (WBC 50~60 / HP) indicate infection.

In these several diagnoses:

A. Acute glomerulonephritis usually presents as hematuria and proteinuria, but urinary frequency, urgency and urine pain are less common.

B. Acute pyelonephritis usually presents with urinary frequency, urinary urgency, and urine pain, along with pain in the renal area and significant leukocytosis in the urine, consistent with the title description.

C. Acute cystitis will have frequent urination, urgent urination, urination pain, but no positive percussion pain in the right kidney region.

D. Acute interstitial nephritis is usually caused by drugs or other factors, and the clinical manifestations is not completely consistent with the topic.

E. Acute glomerulonephritis is a severe glomerular disease characterized by acute renal failure, severe proteinuria, and hematuria, but not often frequent urination, urgency, or painful urination.

Taken together, the most likely diagnosis is:

B Acute pyelonephritis

104. (Type A2 questions)

A 48-year-old male was admitted to ICU due to acute myocardial infarction, with rapid heart rate increase, repeated ventricular tachycardia and atrial fibrillation.

The cause of the electric storm is that

A. vagal excitation

B. Sympathetic nerve excitation

C. Excited parasympathetic system

D. Central nervous system inhibition

E. Vagagal nervous system inhibition

Reference answer: B. Your answer:

[Analysis] The occurrence mechanism of electric storm has not been fully clear, and it is believed to be related to the following factors: organic heart disease is the pathological basis of electric storm, sympathetic excessive excitability (B pair) is the driving factor of electric storm.

[Erstavoidance] Ventricular arrhythmia storm refers to 2 to 3 episodes of ventricular tachycardia and / or ventricular fibrillation within 24h

Severe hemodynamic disorders requiring immediate electrical cardioversion or electrical defibrillation, or acute critical syndrome

sudden and violent.

In this case, a 48-year-old man entered the ICU after an acute myocardial infarction with increased heart rate, recurrent ventricular tachycardia and atrial fibrillation, followed by electrical cardioversion and electrical defibrillation. The cause of the electrical storm, depending on the situation described, is most likely overactivation of the sympathetic nervous system.

So the best answer is:

B Sympathetic nerve excitation

Excessive activation of the sympathetic nervous system can lead to an increased risk of arrhythmias, especially after acute cardiac events (such as myocardial infarction). The excitation of the sympathetic nervous system can increase the oxygen consumption of the myocardium and promote abnormal cardiac electrical activity, which may trigger an electrical storm.

This is a question about the electrophysiological mechanisms of the heart. First, we need to understand the key information in the question and the medical knowledge involved.

Information: The middle-aged male, admitted to ICU after acute myocardial infarction, and developed recurrent ventricular tachycardia and atrial fibrillation, and was successfully rescued by electrical cardioversion and electrical defibrillation. This suggests us that the patient's electrical activity of the heart was abnormal, the so-called "electric storm".

Understand the meaning of electric storm: cardiac electric storm (also known as ventricular arrhythmia storm) refers to the clinical syndrome of ventricular tachycardia or ventricular fibrillation occurring 2 to 3 times within 24 hours, requiring emergency treatment. This condition is often associated with sympathetic hyperexcitability and triggering activity.

Analysis options:

A. Vagagal nerve excitation: The vagal nerve mainly has an inhibitory effect on the heart. If the vagal nerve is excited, it usually lowers the heart rate, which is inconsistent with the heart rate increase and electric storm described in the question stem.

B. Sympathetic excitation: Sympathetic excitation increases the contractility and heart rate of the heart, consistent with the symptoms described by the trunk. In the case of an acute myocardial infarction, excessive excitation of the sympathetic nerves may trigger an electrical storm.

C. Parasympathetic system excitement: Parasympathetic system mainly refers to the vagus nerve, and its function is to inhibit heart activity, which is inconsistent with the description of the title stem.

D. Central nervous system inhibition: This is not directly related to the electrical activity of the heart and does not directly lead to electrical storms.

E. Vagal nervous system inhibition: Although vagal inhibition may indirectly lead to increased cardiac activity, it is not the direct cause of the electrical storm.

In conclusion, sympathetic excitation is the main cause of the heart rate increase and electrical storm. Therefore, the correct answer is B. sympathetic excitation.

104. (Type A2) male, 48 years old, admitted to ICU after acute myocardial infarction, heart rate increased, repeated ventricular tachycardia and atrial fibrillation. The cause of the electric storm is that

Electrical storm (electrical storm) is a patient with recurrent fatal arrhythmia (e. g., ventricular tachycardia or ventricular fibrillation) in a short period of time. After acute myocardial infarction, excessive activation of the sympathetic nervous system is one of the important causes of abnormal electrical activity of the heart, which is easy to cause recurrent malignant arrhythmias such as ventricular tachycardia and ventricular fibrillation. Therefore, the cause of the electrical storm is most likely to be sympathetic excitation.

So, the most correct answer is:

F. Sympathetic nerve excitation

105. (Type A2 questions)

A 7-year-old boy, with sudden chills and high fever, severe distal left knee pain, flexion of left knee, physical examination: mild local swelling, high skin temperature, deep tenderness, floating patellar test (-), WBC 1610 ^ 9 / L, N0.94. Early examination is preferred

A. Local layered puncture

B. routine blood test

C. X line

D. CT

E. MRI

Reference answer: A. Your answer:

Graphic resolution: [resolution] 7 years old children (acute suppurative osteomyelitis in children and adolescents), chills, high fever, white blood cells, left distal thigh pain (suppurative osteomyelitis, often in the proximal tibia and distal femur), combined with the patient's clinical manifestations and signs, the patient diagnosis of acute suppurative osteomyelitis. The layered puncture showed pus and inflammatory secretions (A pair). The X-ray (C is wrong) takes two weeks to change. MRI is also of early diagnostic value, but it is not the preferred option (E error).

[Avoid mistakes] All the following manifestations should be thought of the possibility of acute osteomyelitis: ① systemic poisoning symptoms, high fever chills,

local

Persistent pain, long shaft seal pain and reluctant to move the limb, local deep tenderness; ② total white blood cells increased, medium

Gonulocytes increased, positive blood culture; ③ layered puncture for pus and inflammatory secretions; ④ X-ray plain film signs, two weeks left

Change on the right side; ⑤ MRI test is valuable for early diagnosis.

This question describes a 7-year-old boy with a sudden onset of chills, high fever, severe pain in the distal left thigh, and limited movement of the left knee joint with localized swelling and tenderness, and leukocytosis. These symptoms and signs may suggest an inflammatory disease such as infectious arthritis or osteomyelitis.

In this case, the first thing to be done is to exclude or confirm the disease, and especially to determine whether there is an acute inflammatory process such as osteomyelitis or arthritis. The most suitable early examination method is the choice of imaging examination that allows the direct observation of bone and soft tissue conditions.

The local layered puncture of option A was mainly used for fluid collection and analysis, which is helpful for confirming the diagnosis, but not for the initial examination. Option B's blood routine is useful, but it does not directly show the local bone and soft tissue status. The X-ray of option C can visually show the condition of the bone and help detect bone abnormalities, such as fractures or infections. CT of option D may provide more detailed images of bone and soft tissue, but may be overly complicated for preliminary examination. The MRI of option E can provide very detailed soft tissue images and is particularly useful for detecting osteomyelitis or soft tissue infection, but it is not the most commonly used means of primary screening.

Taken together, the most appropriate early examination option is the C.X-ray, as it is the standard preliminary examination method for assessing skeletal problems and can help determine whether a problem such as osteomyelitis exists. So, the best answer is the C.X-ray.

analyze:

A 7-year-old child with sudden chills and high fever, severe pain in the distal left thigh, and severe pain in the left knee joint refused to do extension and flexion activities, suggesting the possibility of infectious or inflammatory diseases. Physical examination showed local mild swelling, high skin temperature, deep tenderness, and

laboratory examination showed that the proportion of blood leukocytes and neutrophils was significantly increased. Combined with the patient's medical history, clinical manifestations and laboratory examination, the child should be highly suspected of acute blood-borne osteomyelitis. In order to clarify the diagnosis, imaging examination sensitive to bone marrow edema should be selected, while MRI is sensitive to intrablary lesions, which can detect early inflammatory infiltrate confined to the bone marrow, showing abnormal signal within the bone marrow, which is of great value for the early diagnosis of acute bloody osteomyelitis, so it should be used as the first choice.

Option A partial layered puncture is an invasive examination, but it is not considered temporarily, so it is not selected.

Option B Blood routine examination has some implications for infectious diseases, but it is of little value to determine the infection site, so it is excluded.

Option C: X-ray, option D: CT, no small lesions in the bone marrow can be found, and positive signs such as periosteal reaction can only be found 14 days after the onset of X-ray examination, which is of little value for early diagnosis, so it can be ruled out together.

In summary, the correct answer is: E.MRI.

According to the title, the boy was 7 years old with sudden chills and high fever, severe pain in the distal thigh of the left knee, refused to do flexion activities in the left knee, with mild local swelling, high skin temperature, deep tenderness, increased WBC, and N ratio of 0.94. These symptoms and signs suggest the possibility of acute suppurative osteomyelitis.

For acute suppurative osteomyelitis, the early diagnosis is very important. Although routine blood tests (option B) may indicate infection, it cannot provide specific site information. Imaging examination is more critical to diagnose acute osteomyelitis. MRI is more sensitive to detecting osteomyelitis and can show earlier changes in soft tissue and bone marrow.

Therefore, the first choice for the early examination is:

B MRI

106. (Type A2 question)

Male, 68 years old. Nausea, dull upper abdominal pain, vomiting of a little coffee-like liquid for 2 days. A history of hypertension and dyslipidemia for 2 years, and long-term oral aspirin 100 mg/d. Gastroscopy showed multiple erosion of the antral mucosa and bloody mucus attached to the surface. The cure is

A. domperidone

B. pantoprazole

C. ranitidine

D. Colloidal Bismuth Subcitrate

E. sucralfate

Reference answer: B. Your answer:

Graphic resolution: [analytical] middle-aged and old male patients, long-term taking aspirin, upper abdominal dull pain, vomiting a little coffee liquid 2 days, gastroscopy visible gastric sinus mucosa multiple erosion, surface adhesion hemorrhagic mucus, comprehensive patients with symptoms and signs, preliminary diagnosis of peptic ulcer, treatment preferred pantoprazole (B).

[Error avoidance] Domperidone (A wrong) is a drug to improve gastrointestinal motility, suitable for duodenal-gastric reflux. Colloidal Bismuth Subcitrate (D

Wrong) is a gastric mucosal protective agent, combined with two antibiotics can kill and inhibit Hp, but the treatment of peptic ulceration

The analgesic effect is relatively slow. Aluminum (E wrong) belongs to weak alkaline antacid, is not the main treatment of peptic ulcer

thing. Ranitidine (C wrong) is one of the main drugs for peptic ulcer treatment, but it is not as effective as PPI.

The topic involved a 68-year-old male patient with symptoms of nausea, dull pain in the upper abdomen, vomiting of coffee-like fluid, a history of hypertension and dyslipidemia, and long-term aspirin use. Gastroscopy revealed multiple erosion of the gastric sinus mucosa and bloody mucus attached to the surface. According to these information, the patient's symptoms and gastroscopy findings point to gastric mucosa damage that may be related to long-term aspirin administration.

The option analysis is performed as follows:

A. domperidone is used for the treatment of nausea and vomiting, and the main

problem here is the gastric mucosa damage, so A is not the best choice.

B. Pantoprazole is a proton pump inhibitor, which is used in the treatment of gastric ulcers and gastric mucosal damage, which can reduce gastric acid secretion and promote gastric mucosal repair.

C. Ranitidine is a histamine H2 receptor antagonist used in the treatment of gastric ulcers but is not preferred due to its side effects and weaker efficacy than a proton pump inhibitor.

D. Bismuth potassium citrate is used to treat gastric ulcer and gastric mucosal damage, which has the effect of protecting gastric mucosa and promoting ulcer healing.

E. Aluminum sulfate is used to treat excessive gastric acid and gastric ulcer, but the main effect is to neutralize gastric acid, not the best choice.

Given the patient's symptoms and the background of long-term aspirin use, the best choice should be drugs effective in reducing gastric acid secretion and promoting gastric mucosal repair. Therefore, the best answer is * * B. pantoprazole * *, because it reduces gastric acid secretion, reduces further damage to the gastric mucosa, and promotes the repair of existing damage.

analyze:

An elderly male patient presented with nausea, dull pain in the upper abdomen and vomiting coffee-like fluid after long-term oral administration of aspirin. On gastroscopy, he showed multiple erosion of antral mucosa and bloody mucus on the surface, which was considered as acute erosive hemorrhagic gastritis caused by aspirin. In the treatment of acute erosive hemorrhagic gastritis, the cause should be removed first, the primary disease and trauma should be actively treated, the drugs that may cause gastric mucosa damage should be stopped, and the drugs that inhibit gastric acid secretion should be used. In the option, pantoprazole is the third generation proton pump inhibitor, which can specifically inhibit the H + / K + ATP enzyme on the secreted microtubules formed by the apical membrane and the tubular bubble in the cytoplasm, causing the irreversible inhibition of the enzyme, thus effectively inhibiting the secretion of gastric acid and achieving the purpose of therapeutic treatment.

Option A domperidone is A gastrointestinal motility drug, which is mainly used for the treatment of gastroesophageal reflux disease, functional dyspepsia and diabetic

gastroparesis, but it has no inhibitory effect on gastric acid secretion, so it is not selected.

Option C ranitidine is an H_2 receptor antagonist that also suppresses gastric acid secretion, but has less acid suppression than proton pump inhibitors, and therefore is not preferred.

Option D Bismuth potassium citrate is a gastric mucosal protective agent, which can cover the surface of the ulcer to prevent gastric acid and pepsin from invading the ulcer surface, but it has no inhibitory effect on gastric acid secretion, so it is not considered.

Option E aluminum also for gastric mucosa protective agent, its mechanism is in the acidic environment, can dissociate sulfate sucrose compound ions, composite ion polymerization into insoluble negatively charged colloid, can with ulcer or inflammation with positive charge protein exudation, form a layer of protective film, covering the lesion surface, prevent gastric acid, pepsin damage factors further invasion, promote the healing of damaged mucosa, but no inhibition of gastric acid secretion, so out.

To sum up, the correct answer is: B. Pantoprazole.

According to the patient's condition description, a 68-year-old man presented with nausea, dull pain in the upper abdomen, vomiting of a little coffee-like fluid for 2 days, a history of hypertension and dyslipidemia, and a long-term oral aspirin of 100 mg/d. Gastroscopy showed multiple erosion of the antral mucosa and surface adhesion of bloody mucus, which suggested possible aspirin-induced gastric mucosal damage and hemorrhage.

For the treatment in this case, drugs that can effectively inhibit gastric acid secretion and promote gastric mucosal healing should be selected. Pantoprazole is a proton pump inhibitor (PPI) that effectively suppresses gastric acid secretion, helps repair the gastric mucosa and prevent further bleeding.

Therefore, the most suitable therapeutic drugs are:

B pantoprazole

107. (Type A2 questions)

Female, 17 years old, tired, weak, upset and irritable, afraid of heat and sweating, weight loss of 5kg, reduced menstrual volume, shortened menstruation, physical examination: P100 times / min, BP140 / 70mmHg, hand fibrillation, thyroid diffuse thyroid enlargement, no tenderness. The diagnosis is

A. diabetes mellitus

B. A simple goiter

C. Graves Disease

D. Autonomic nerve confusion

E. hypoferric anemia

Reference answer: C Your answer:

[Analysis] adolescent women (people with hyperthyroidism), upset, afraid of heat and sweating, weight loss, hand fibrillation, thyroid diffuse swelling, no tenderness (typical manifestations of hyperthyroidism), according to the symptoms and signs of patients, can be diagnosed as Graves disease (C pair).

[Avoid mistakes] Simple goiter has diffuse thyroid enlargement in the early stage, with smooth gland surface and soft texture. Later thyroid

They showed asymmetric nodular enlargement, and the nodules varied in size. This disease usually presents as an enlarged thyroid gland with normal thyroid function, one

Like no clinical symptoms, only a few patients can appear hyperthyroidism or low symptoms.

According to the symptoms described symptoms, such as fatigue, weakness, irritability, sweating, weight loss, decreased menstruation, shortened menstruation, increased heart rate (100 beats / min), blood pressure 140 / 70mmHg, hand fibrillation and diffuse enlargement and no tenderness of thyroid, the most consistent diagnosis was Graves disease (option C). Graves Disease is an autoimmune disease that can cause hyperthyroidism and produce excessive thyroid hormones, causing these symptoms. Therefore, the correct answer is a C. Graves illness.

This is a diagnostic problem, and we need to analyze the patient's symptoms, signs, and physical examination results to determine the most likely diagnosis.

First, let's review the key information in the topic:

(1) Young female patients have symptoms of fatigue, weakness, irritability, fear of heat, sweating and weight loss.

(2) Abnormal menstrual cycle and volume, physical examination found increased heart rate, elevated blood pressure, hand fibrillation, and diffuse thyroid enlargement but no tenderness.

Next, we analyze each option:

A. Diabetes: usually manifested as polydipsia, hyperdiagia, polyuria and weight loss, that is, "three more and one less" symptoms, but generally does not appear diffuse thyroid enlargement, so this option does not meet the question.

B. Simple goiter: the main manifestation is goiter, but usually not accompanied by fear of heat, sweating, irritability and other symptoms, so it is excluded.

C. Graves's disease: This is an autoimmune thyroid disease, typically showing symptoms of hyperthyroidism, such as fear of heat, excessive sweating, irritability, tachycardia, and diffuse thyroid enlargement, which is consistent with the symptoms and signs of the patient.

D. Autonomic nerve disorder: the main manifestations are palpitation, sweating, irritability and other symptoms, but not diffuse thyroid enlargement, so it is not selected.

E. Iron deficiency anemia: the main manifestations are pale face, fatigue, dizziness and other symptoms, which are inconsistent with the patient's performance, and can be excluded.

In conclusion, the most likely diagnosis of patients is Graves disease, a form of hyperthyroidism, usually characterized as fear of heat, irritability, tachycardia, and diffuse enlargement of the thyroid.

Thus, the correct answer is the C. Graves disease.

Based on the clinical manifestations and physical examination results provided, the main symptoms include fatigue, weakness, irritability, heat fear and excessive sweating, body weight loss, decreased menstrual volume, and shortened menstrual period. Physical examination revealed increased heart rate (P100 beats / min), increased blood pressure (BP140 / 70mmHg), hand fibrillation, diffuse thyroid enlargement and no tenderness.

These symptoms and signs are highly consistent with Graves's disease (hyperthyroidism). Graves Disease is one of the most common causes of hyperthyroidism, including goiter, heat fear, sweating, weight loss, increased heart rate, neurological hyperactivity (such as irritability, hand fibrillation) and so on.

So the best answer is:

C Graves Disease

108. (Type A2 questions)

Male, 22 years old, with progressive systemic edema for 10 days, previous physical examination: BP120 / 80mmHg, severe depressed edema in face and lower limbs, plasma albumin 20g / L, blood Cr72 μmol/L, blood cholesterol 8.6 mmol/L, 8 <10 / HP. Of the following treatments, this is wrong

A. diuretic therapy

B. intravenous supplementation of albumin

C. LMWH for anticoagulation

D. Limit the salt intake

E. Decisions on the next treatment measures according to the pathology of the renal biopsy

Reference answer: B. Your answer:

[Analysis] adolescent male patients with a large amount of proteinuria, urinary protein greater than 3.5 g/d, edema, ALB20g / L (normal albumin of 40~55g / L) decreased, hyperlipidemia, combined with the patient's history, clinical manifestations and examination, the most likely diagnosis of the patient is nephrotic syndrome. Patients with nephrotic syndrome should be given a normal amount of high quality protein diet (B wrong, the correct answer to this question), intravenous supplementation of a large amount of protein will increase glomerular hyperfiltration, aggravate proteinuria and aggravate the disease, so it is not recommended.

[Error avoidance] The diagnostic criteria for nephrotic syndrome are: ① massive proteinuria (> 3.5 g/d); ② hypoalbuminemia (serum

Albumin was less than 30g / L); ③ oedema; ④ hyperlipidemia. The first two are

prerequisites for diagnosis.

This case describes a 22-year-old male patient presenting with systemic progressive oedema, decreased plasma albumin, elevated blood cholesterol and positive urinary protein, symptoms and examination findings suggestive of possible nephrotic syndrome. Nephrotic syndrome is characterized by massive proteinuria, hypoproteinemia, hyperlipidemia, and oedema.

With this information, we can evaluate each treatment option one by one:

A. Diuretic therapy: It is reasonable for patients with edema, especially those caused by hypoproteinemia.

B. intravenous albumin supplementation: In cases of hypoproteinemia and severe edema, albumin supplementation can help restore the vascular content and reduce edema.

C. Low molecular weight heparin anticoagulation: Patients with nephrotic syndrome have a high risk of thrombosis due to urinary coagulation factor, so anticoagulation therapy is necessary in some cases.

D. Limit salt intake: For patients with edema, sodium restriction is part of standard treatment and can help reduce edema.

E. Determine the next treatment step based on kidney biopsy pathology: This is a critical step, because it can diagnose exactly the type and extent of renal damage and determine the best treatment options.

Thus, all options appear as reasonable therapeutic measures. But given the choice of "wrong" treatment, we need to carefully consider which may not be appropriate in some cases. In this case, the most likely considered inappropriate is the B option "intravenous supplementation of massive albumin", because although albumin can reduce edema in some cases, its role in the treatment of nephrotic syndrome is still controversial and may not be the preferred treatment. Some guidelines recommend the use of albumin only in specific situations (e. g., severe hypoproteinemia) rather than as conventional treatment.

In conclusion, although the option B is applicable in some specific situations, the B option may be the least appropriate treatment option, considering the "error" requirement in the title stem.

analyze:

Patient young men with progressive systemic edema for 10 days, previous body

health, physical examination and double lower limb severe pitting edema, laboratory examination to reduce plasma albumin, blood cholesterol, quantitative urine protein, urine sediment microscopy, the comprehensive history, clinical manifestations and laboratory examination, the patient consider the diagnosis of nephrotic syndrome. The treatment of nephrotic syndrome includes general treatment, symptomatic treatment, main treatment namely inhibiting immune and inflammatory reaction, traditional Chinese medicine treatment, prevention and treatment of complications, etc. For the treatment of low albuminemia, intravenous infusion of albumin can only improve plasma colloidal osmotic pressure, input protein mainly in 48 hours from the urine, and can aggravate glomerular hyperfiltration and tubular high metabolism, aggravating proteinuria and promote the progress of kidney lesions, therefore, intravenous supplement a lot of albumin this treatment is wrong.

The treatment principle of edema in nephrotic syndrome is salt restriction and diuresis, so option A diuretic treatment, option D limits salt intake, which are the conventional treatment of edema in nephrotic syndrome edema, so they are excluded.

Patients with nephrotic syndrome are prone to thrombosis and embolic complications. For these patients, option C low molecular weight heparin can be given to prevent thromboembolic disease, so it is not selected.

Option E deciding the next treatment step according to the pathology of renal biopsy is a key step to confirm the cause of nephrotic syndrome, and also an important reference to develop treatment plans. It is a standard treatment measure for nephrotic syndrome, which is inconsistent with the topic.

In conclusion, the correct answer is: B. intravenous supplementation of massive albumin.

According to the problem stem description, the patient was a 22-year-old man with progressive systemic edema for 10 days, with severe depressed edema, low plasma albumin (20g / L), high blood cholesterol (8.6 mmol/L), and positive urinary protein, suggesting possible nephrotic syndrome. Here is an analysis of each option:

A. Diuresis therapy: Diuresis therapy can help reduce edema and is part of the treatment of nephrotic syndrome.

B. Intravenous massive albumin supplementation: The patient has severe hypoalbuminemia, and albumin supplementation can temporarily relieve the

symptoms, but caution should be taken, as heavy supplementation may lead to excessive rehydration and complications.

C. Low molecular weight heparin anticoagulation: Patients with nephrotic syndrome are prone to hypercoagulant state due to hypoalbuminemia, and anticoagulation therapy is reasonable.

D. Limit salt intake: limiting salt intake can help control edema and is a reasonable treatment.

E. Determine the next therapeutic measures according to the pathology of renal biopsy: the pathological results of renal biopsy are the key basis for determining further treatment and are a reasonable measure.

In conclusion, the wrong treatment measure is * * B. intravenous supplementation of massive albumin * *, because although albumin supplementation temporarily improves symptoms, it is not the main treatment for NS, and extensive supplementation may lead to excess fluid and other complications.

109. (Type A2 question)

Female, 54 years old, palpitations shortness of breath for ten years, aggravated with large hemoptysis one day, physical examination: jugular vein irritability, double lung can smell and wet rales, heart rate 120 times / minute, arrhythmia, the first heart tone can hear open flap, P2 hyperactivity, apex diastolic tone, double lower limb mild edema, electrocardiogram showed atrial fibrillation with fast ventricular rate, chest X-ray heart shadow pear-shaped heart. The therapeutic agent that should be preferred is

A. β receptor antagonist

B. diuretic

C. propafenone

D. mexiletine

E. cedilanid

Reference answer: E Your answer:

Graphic resolution: [analytical] middle-aged female patients, palpitations shortness of breath for ten years, aggravated with large hemoptysis day (two narrow

typical clinical manifestations), arrhythmia, the first heart sounds can hear flap, P2 hyperactivity, apex diastolic rumble murmur (two narrow patients specific auscultation), electrocardiogram showed atrial fibrillation with fast ventricular rate, chest X-ray shows heart shadow pear-shaped heart (two narrow left atrial enlargement), comprehensive patient symptoms, signs, auxiliary examination can be diagnosed as mitral stenosis. For patients with atrial fibrillation and fast ventricular rate, digitalis drugs (with E pair) should be injected intravenously to slow down the ventricular rate. For patients with massive hemoptysis, use sedative agents and diuretics to reduce pulmonary artery pressure, but it is not the preferred treatment in this case (B error).

[Avoid] Rheumatic fever is the main cause, so anti-rheumatic fever should be treated, long-term or even lifelong use of benzyl

Astropenicillin. In patients with sinus rhythm, if their dyspnea occurs at increased heart rate,

Such as the β receptor antagonist (A wrong).

This topic describes a 54-year-old female patient with symptoms and signs of palpitations, shortness of breath, hemoptysis, jugular irritability, wet rales, accelerated heart rate, arrhythmia, abnormal heart sounds, mild edema of both lower limbs, atrial fibrillation with fast ventricular rate, and pear-shaped heart shown by chest X-ray. These manifestations suggest the possibility of right cardiac dysfunction and atrial fibrillation.

The option analysis is performed as follows:

A. β receptor antagonist: helpful for heart rate control in patients with atrial fibrillation with fast ventricular rate, but with caution in cases of significant symptoms of heart failure.

B. Diuretics: used to reduce edema, beneficial for patients with heart failure, and can reduce the burden on the heart.

C. propafenone: an antiarrhythmic drug used for rhythm control of atrial fibrillation, but not preferred for emergency treatment of heart failure and rapid heart rate control.

D. Mesc: also an antiarrhythmic drug, mainly used for ventricular arrhythmias, not for atrial fibrillation.

E. Sidl: a drug used in the treatment of heart failure, especially in patients with

atrial fibrillation and heart failure.

Considering the patient's symptoms and symptoms, the preferred treatment should aim at improving cardiac function and controlling heart rate. Silidl (E option) is a suitable choice in the treatment of heart failure and control of atrial fibrillation with fast ventricular rate. Therefore, the best answer is E. Silland.

analyze:

Middle-aged and old female patients, palpitations, shortness of breath for many years, the recent aggravation with massive hemoptysis, physical examination shows jugular vein irritability, double lungs can smell and wet rales, heart rate, arrhythmia, the first heart tone can hear open flap, P2 hyperactivity, double lower limb mild edema, electrocardiogram atrial fibrillation with fast ventricular rate, chest X-ray shows pear shaped heart, comprehensive history, clinical manifestations, physical examination and imaging examination, considering the diagnosis of mitral stenosis and atrial fibrillation. Primary treatment purpose is to control the ventricular rate, relieve patients with symptoms, so the preferred treatment is sildil, siltill belongs to digitalis, strong heart drugs, by strengthening myocardial contraction, with positive muscle force, negative frequency and cardiac electrophysiological effect, can be used for acute heart failure, chronic heart failure, rapid atrial fibrillation and atrial flutter, etc.

Option A: β Receptor antagonist can reduce myocardial contractility, self-discipline, conductivity and excitability, slow down heart rate, and reduce myocardial oxygen consumption. It is mostly used to treat arrhythmia, angina pectoris, myocardial infarction, hypertension and other cardiovascular diseases, which are not consistent with the symptoms of the patient, so it is not selected.

Option B: Diuretics are mainly used for the treatment of various edema, which can eliminate excess water and sodium in the body, eliminate edema, and can also be used for blood pressure, which is not consistent with the symptoms of the patient, so it is excluded.

Option C: propatenone, option D: beauty, all belong to the broad spectrum efficient membrane inhibitory antiarrhythmic, membrane stability and competitive β receptor block, can reduce myocardial excitability, prolong the action potential duration and effective refractory period, prolong conduction, can be used for prevention and treatment of ventricular and ventricular ectopic beats, ventricular or ventricular tachycardia, preexcitation syndrome, can also be used for atrial flutter or

atrial fibrillation, but atrial fibrillation with fast ventricular rate preferred l, can be excluded.

To sum up, the correct answer is: E. Silland.

According to the case description provided, the patient was a 54-year-old woman with a history of shortness of breath for ten years with aggravating symptoms and massive hemoptysis. Physical examination revealed jugular irritability, wet rales in both lungs, heart rate 120 beats / min, arrhythmia, first heart hyperactivity, open flap and P2 hyperactivity, apical diastolic tone and mild edema of both lower limbs. The ECG showed atrial fibrillation with fast ventricular rate, and the chest X-ray showed a pear-shaped heart.

The patient's symptoms and signs suggested the possibility of chronic heart failure and atrial fibrillation. The primary treatment goal is to control the rapid ventricular rate of atrial fibrillation, thereby improving symptoms and preventing further cardiac injury.

Option analysis:

- ** A. β receptor antagonist * *: It can be used for the control of heart failure and atrial fibrillation with fast ventricular rate.

- ** B. Diuretics * *: It is mainly used to relieve edema caused by heart failure, but it has limited control of atrial fibrillation with fast ventricular rate.

- ** C. propafenone * *: For the treatment of arrhythmias, but not recommended for patients with heart failure because it may aggravate heart failure.

- ** D. Mercil * *: For the treatment of arrhythmia, but it is less safe and effective than other drugs.

- ** E. Sydney * * (digoxin): It slows down atrial fibrillation with fast ventricular rate, especially in patients with heart failure.

Considering the patient's history, symptoms, and signs, the preferred treatment should both control the rapid ventricular rate of atrial fibrillation and improve the symptoms of heart failure. Both β -receptor antagonists and digoxin are suitable, but β

-receptor antagonists are more commonly used in acute situations.

So the best answer is:

A. β receptor antagonists

110. (Type A2 questions)

The patient, male, 56 years old, previously healthy, weak and pale for half a year. Blood routine showed: WBC1310 ^ 9 / L, Hb108g / L, PLT 610 ^ 9 / L, N0.65, L0.23, and M0.03. Myeloplasia was clearly active with primitive fine Auer bodies. Considering the diagnosis of MDS, in order to clarify the FAB classification, which of the following tests should be performed to distinguish RA and RAS types

A. Iron staining

B. bone marrow biopsy

C. Immunohistochemical examination

D. Chromosome karyotype

E. rabat

Reference answer: A. Your answer:

[Analysis] The patient should be diagnosed as myelodysplastic syndrome. In order to clarify the FAB classification and distinguish RA from RAS type, bone marrow iron staining (A pair) should be performed to determine the proportion of ring iron granuloclasts.

[Error avoidance] Osteosarcoma (A error) is A malignant bone tumor, and the Codman triangle or "daylight ray" morphology can be seen on the X-ray ray, and the tumor boundary is unclear. Giant cell tumor of bone (B wrong) is a borderline tumor with eccentric, osteolytic and cystic destruction on the X-ray plate without periosteal reaction. Chondrosarcoma (C wrong) is a malignant bone tumor whose X-ray can have cloud changes with unclear boundaries. The main symptom of osteoid osteoma (E error) is pain, progressive aggravation, with aspirin relieved in most patients.

The patient's symptoms and blood findings (e. g. increased WBC, decreased Hb, decreased PLT) and bone marrow findings (e. g., active hyperplasia, Auer bodies) suggested a possible myelodysplastic syndrome (Myelodysplastic Syndromes, MDS). To define FAB (France-USA-UK) typing and to distinguish RA (refractory anemia)

from RAS (refractory anemia with ring young cells), iron staining (option A). Iron staining can help observe iron storage in the bone marrow and is an important indicator to distinguish RA from RAS type MDS.

Therefore, the most appropriate answer is the A. iron staining.

analyze:

Middle-aged and elderly male patients were weak and pale in the past half a year, and blood routine showed pancytopenia, significantly active myeloid hyperplasia and increased primitive cellularity, and Auer body, which was considered as myelodysplastic syndrome (MDS). In the FAB classification of MDS, RA was refractory anemia with <1% peripheral blood blast cells and <5% bone marrow blast cells. The RAS was refractory anemia with increased blast cells, <5% peripheral blood blast cells, and bone marrow blast cells between 5% and 20%. The main difference between the two is the proportion of primitive cells in the bone marrow, so in order to clarify the FAB typing, RA and RAS types should be differentiated from bone marrow biopsy. Bone marrow biopsy can understand the number of primitive cells in the bone marrow, so as to carry out accurate typing.

Option A iron staining is mainly used for the diagnosis of iron deficiency anemia and cuocytic anemia, unrelated to this problem.

Option C Immunohistochemical examination is mainly used for the diagnosis and differential diagnosis of tumors, and it has little relationship with the FAB typing of MDS, so it is not chosen.

Option D chromosome karyotype was mainly used for prenatal diagnosis and genetic disease diagnosis, and was not suitable for FAB typing of MDS, so it was excluded.

Option E chest X-ray is mainly used to examine cardiopulmonary lesions and is not helpful in the FAB typing of MDS, so it is not considered.

In conclusion, the correct answer is: B. Bone marrow biopsy.

Based on the clinical findings and laboratory results in the title, the patient may be diagnosed with myelodysplastic syndrome (MDS). To further define the FAB classification, especially to differentiate refractory anemia (RA) from refractory anemia (RAS) with primitive cells:

The answer is the D. chromosome karyotype.

The reasons are as follows:

The main difference between RA and RAS by-FAB typing is the proportion of primitive cells and the presence of chromosomal abnormalities.

-Chromosome karyotyping can help to identify specific chromosomal abnormalities, which is important to clarify the diagnosis and classification of MDS.

-Iron staining is mainly used to examine iron metabolism problems and is not suitable to distinguish between RA and RAS.

-Bone marrow biopsy, while providing information on the morphology and hyperplasia of bone marrow cells, is not as specific in subtyping as chromosomal karyotyping.

-Immunohistochemical examination was used to identify specific cellular markers not suitable for FAB typing in MDS.

-Chest radiograph is mainly used to check for lung disease and is not related to the diagnosis here.

Therefore, in order to clarify the FAB typing of MDS, especially to distinguish between RA and RAS types, the chromosome karyotype examination should be done.

111. (Type A2 questions)

A 22-year-old male with a 5-year medial mass in the right knee showed slow growth without significant pain and an X-ray showed a clubbing mass in the medial metaphysis of the inferior femur with clear margins. It should be considered first

A. osteosarcoma

B. giant cell tumor of bone

C. chondroma sarcomatosum

D. osteochondroma

E. osteoidosteoma

Refer to the answer: D. Your answer:

Graphic resolution: [analytical] young male patients, the right knee medial mass slow growth, no obvious pain, X-ray shows the inferior femoral medial metaphysis pestle mass, clear edge (osteochondroma typical imaging findings), combined with patient symptoms and imaging performance, should be first considered is the right femoral osteochondroma (D).

[fault avoidance]

According to the case information described by the title, the 22-year-old patient had a slowly growing mass with no significant pain for 5 years and X-ray showed the medial metaphysis of the inferior femur with clear margins. These characteristics point to the benign tumors.

Option A The osteosarcoma is a malignancy that usually grows rapidly with pain, not consistent with the slow-growing and pain-free characteristics of this case. Option B Giant cell tumors of the bone usually occur at the end of the bone, but the margins are often unclear and are relatively rare at the lower end of the femur. Option C Chondrosarcoma is also a malignant tumor, usually associated with pain and rapid growth. Option D The osteochondroma is benign and usually presents as a slowly growing mass with a clubbing mass on the X-line, consistent with the title description. Option E Osteoid osteomas are usually small and grow extremely slowly, rarely forming large masses.

Therefore, based on the title description and the matching degree of each option, the best answer should be * * D. osteochondroma * *.

analyze:

In a young male patient with a painless mass inside the right knee for many years, X-ray showed the epiphyseal of the lower femoral mass with clear margins, which was a typical imaging manifestation of osteochondroma. Combined with the clinical manifestations and imaging examination, the first diagnosis was osteochondroma.

Option A osteosarcoma occurs in teenagers, often form A spindle-shaped tumor body, pain is an early symptom, can be excluded from the slow-growing mass, so it is not selected.

Option B giant cell tumor of bone occurs from 20 to 40 years old, slightly more women, good places for the long shaft epiphysis and vertebral body, the main symptoms are pain and swelling, which is not consistent with the description of the

problem, so it is not considered.

Option C chondrosarcoma mostly occurs between 30 and 60 years old, originating from the periosteum in the head and neck and pelvis, and originating from the bone marrow cavity in the long tubular bone, which does not meet the meaning of the question.

Option E Osteoid osteoma pain is the main symptom, from light to severe, from intermittent to persistent, from the local development of the lesion to can be accompanied by nerve root radiation pain, not consistent with the problem dry, so it is excluded.

In conclusion, the correct answer is: D. osteochondroma.

This title describes a 22-year-old male with a medial mass in the right knee for 5 years, slow growth and no significant pain, and an X-ray showing a clubbing mass in the inferior femur with clear margins. We need to choose the most probable diagnosis based on this information.

Considering the patient's age, slow growth of the mass, no significant pain, and the characteristics of the X-ray display (clubbing mass, clear margins), the following is an analysis of the options:

A. * * Osteosarcoma * *: Osteosarcoma usually presents as a rapidly growing painful mass, more common in adolescents, with common bone destruction and new bone formation on X-ray, which is not quite consistent with the description of this case.

B. * * Giant cell tumor of bone * *: Giant cell tumor of bone is mostly common in adults 20-40 years old, which occurs at the bony end of the long bone, and the lesions often involve the articular surface. However, more eccentric osteolytic lesions are seen on the X-ray film, with blurred margins and pain is more obvious, which does not accord with the description of this case.

C. * * Chondrosarcoma * *: Chondrosarcoma is common in middle-aged and

elderly people, and tumors grow slowly, but often have pain. Point or annular calcification is visible on X-ray, and margins are often unclear, which is not completely consistent with the description of this case.

D. * * Osteochondroma * *: Osteochondroma is the most common benign bone tumor, mostly common in teenagers, often occurs in the metaphysis of long bones, slow growth, and X-ray shows a clear margin. The description of this case is very consistent with osteochondroma.

E. * * Osteoid osteoma * *: Osteoid osteoma is more common in adolescents, usually presenting as severe nocturnal pain, with small light transmittance area visible on X-ray and obvious sclerotic band. This case has no pain, which does not conform to the characteristics of osteoid osteoma.

In conclusion, the description of this case is most consistent with the characteristics of * * osteochondroma * *, so the correct answer is:

D osteochondroma

112. (Type A2 questions)

A 22-year-old male with a 5-year medial mass in the right knee showed slow growth without significant pain and an X-ray showed a clubbing mass in the medial metaphysis of the inferior femur with clear margins. It should be considered first

A. osteosarcoma

B. giant cell tumor of bone

C. chondroma sarcomatosum

D. osteochondroma

E. osteoidosteoma

Refer to the answer: D. Your answer:

Graphic resolution: [analytical] young male patients, the right knee medial mass

slow growth, no obvious pain, X-ray shows the inferior femoral medial metaphysis pestle mass, clear edge (osteochondroma typical imaging findings), combined with patient symptoms and imaging performance, should be first considered is the right femoral osteochondroma (D).

[Error avoidance] Osteosarcoma (A error) is A malignant bone tumor, and the Codman triangle or "daylight ray" morphology can be seen on the X-ray ray, and the tumor boundary is unclear. Giant cell tumor of bone (B wrong) is a borderline tumor with eccentric, osteolytic and cystic destruction on the X-ray plate without periosteal reaction. Chondrosarcoma (C wrong) is a malignant bone tumor whose X-ray can have cloud changes with unclear boundaries. The main symptom of osteoid osteoma (E error) is pain, progressive aggravation, with aspirin relieved in most patients.

According to the case information provided, the patient was 22-year-old and developed a medial mass in the right knee characterized by slow growth and no significant pain with an X-ray showing the medial metaphysis clubbing mass of the inferior femur with clear margins. These features suggested that the mass is most likely benign.

Option A Osteosarcoma, a malignant tumor that usually grows rapidly and has significant pain, with the X-ray presenting as a destructive mass, inconsistent with this case description.

Option B The giant cell tumor of the bone, usually occurring at the end of the long bone, such as the proximal femur and tibia, but is less well defined than benign tumors.

Option C chondrosarcoma, a malignant tumor, grows faster, tends to feel painful, and the X-rays show invasive features.

B Option osteochondroma, a common benign bone tumor with slow growth and generally asymptomatic, presented as a clubbing mass on the X-ray, consistent with this case description.

Option E The osteoid osteoma, usually smaller, is more common in the metaphysis of the long bone, but is characterized by causing nocturnal pain that can be relieved by aspirin and does not completely coincide with the present case description.

In conclusion, the most consistent with this case description is the D option, osteochondroma. Therefore, the correct answer should be * * D. osteochondroma * *.

analyze:

The patient was a young male with a medial mass in the right knee for many years, no obvious pain, X-ray, pestle of the lower femur with clear margins, combined with the patient's history, clinical manifestations and imaging examination, osteochondroma should be considered first. Osteochondroma is a common, chondrogenic benign tumor, is located in the bone surface of the bony process, the top surface has a cartilage cap, the middle of the pulp cavity, mostly in teenagers, can be long-term asymptomatic, X-ray characteristic performance of the metaphyseal from the cortical process to the soft tissue bony process.

Option A osteosarcoma as the most common malignant bone tumor, good in teenagers, good parts for long backbone metaphysis, such as distal femur, proximal tibia and proximal humeral metaphysis, pain for early symptoms, can be accompanied by increased local skin temperature, venous irritability, X ray can be characterized as Codman triangle or "daylight ray" form, inconsistent with the topic dry description, so do not choose.

Option B bone giant cell tumor is one of the common primary bone tumor, the source is not clear, may start in the bone marrow mesenchymal cells, with stromal cells and multinucleated giant cells as the main structure of aggressive primary bone tumor, X-ray characteristic performance of eccentric bone end, osteolysis, cystic destruction without periosteal reaction, lesions are soap bubble change, inconsistent with the dry description, so excluded.

Option C chondrosarcoma is one of the common malignant bone tumor, more in adults and the elderly, men slightly more, mostly occurred in the lower end of the femur, tibia and upper humerus, symptoms is given priority to with dull pain, from intermittent gradually to persistent, adjacent joint activity is limited, X-ray shows bone destruction, inconsistent with the dry description, so do not choose.

Option E osteoid osteoma is a benign kind of osteogenic diseases, with clear focal lesions, generally less than 1cm in diameter, can have bone sclerosis surrounding periosteum reaction and new bone, X-ray characteristic is a round or oval tumor nest, composed of different maturity of bone, trabecular radial arrangement, surrounding for hardening bone, with the dry description, so excluded.

In conclusion, the correct answer is: D. osteochondroma.

According to the description of the problem stem, the 22-year-old patient had a slow-growing medial mass in the right knee for 5 years and had no significant pain.

The X-ray showed a clubbing mass in the medial metaphysis of the lower femur with clear margins. Combining these clinical features, the most likely diagnosis is osteochondroma (D. osteochondroma).

The following is the analysis process:

1. * * Osteosarcoma (A. Osteosarcoma) * *: This tumor usually occurs in teenagers or young people, with a short course and rapid progression, accompanied by severe pain, and the X-ray often shows irregular bone destruction and new bone formation.

2. * * Giant cell tumor of bone (B. Giant cell tumor of bone) * *: Common in adults aged 20-40, usually located at the end of the long bone, the X-ray shows expansive destructive lesions with indistinct margins.

3. * * Chondrosarcoma (C. chondrosarcoma) * *: Often occurs in middle-aged and elderly people with a long course of disease, and X-ray shows chondroid calcification.

4. * * Osteochondroma (D. Ostechondroma) * *: This is the most common benign bone tumor, mostly seen in adolescents with a long course of disease, usually asymptomatic or slight discomfort. X-ray shows the clear margin and cartilage cap in the lesion.

5. * * Osteoid osteoma (E. osteoid osteoma) * *: More common in teenagers, often occur in the long shaft of the epiphysis, manifested as nighttime pain, X-ray shows small sclerosing lesions, surrounding reactive osteoid hyperplasia.

According to the patient's age, course of disease, symptoms, and X-ray

characteristics, * * osteochondroma * * is the most consistent diagnosis.

So, the correct answer is * * D. osteochondroma * *.

113. (Type A2 questions)

Patient, female, 49 years old. Collar and shoulder pain for half a year, radiating to the left upper limb. The left muscle strength decreased in the upper limb and the finger movements were inflexible. There was tenderness between the cervical spinprocesses and decreased sensation in the left thumb. The upper limb pull test was positive and the head test was positive. The most likely type is

A. myeloid form

B. Neural root type

C. mixed type

D. Vertebral artery type

E. The sympathetic nerve type

Reference answer: B. Your answer:

Graphic resolution: [resolution] patients with neck and shoulder pain, and the left upper limb radiation (early nerve root type cervical spondylosis), left upper limb muscle strength, finger movements, the left thumb feeling, upper limb positive pull test, pressure head test positive (nerve root type of cervical spondylosis main signs), so the patient with the most likely type of cervical spondylosis for nerve root type of cervical spondylosis (B).

[To avoid mistakes] ① Cervical spondylotic myelopathy (A wrong) is often manifested as numbness and weakness of the upper or lower limbs, stiffness, foot stepping cotton feeling,

Foot toe can not be off the ground, tactile disorders, chest feeling, both hands fine movements clumsy, can not eat with chopsticks, muscle strength loss, four

Limlimb tendon reflexes were active or hyperactive and positive for Hoffmann, patellar clonus, ankle clonus and Babinski signs.② vertebral artery type neck

Vertebral disease (D wrong) is mainly manifested as sudden vertigo and cataplexy, migraine, hearing loss or deafness, visual impairment, etc.③

Sympathetic type of cervical spondylosis (E fault) is mainly manifested as the

symptoms of sympathetic nerve stimulation, which can be either excitability or inhibition

nature. Mixed ④ type of cervical spondylosis (C wrong) has two or more types of symptoms at the same time.

This case describes a 49-year-old female patient with neck and shoulder pain, radiation to the left upper limb, along with decreased left upper limb muscle strength and inflexible finger mobility, cervical interspinous tenderness, reduced left thumb sensation, and positive upper limb pull test and pressure head test. These clinical findings suggest that the patient may have had a cervical spondylosis.

For this case, the most likely diagnosis was B. radiculopathy. Cervical radiculopathy is characterized by neck and upper limb pain, reduced sensation, decreased muscle strength, especially the pain along the nerve root distribution radiation, finger imflexibility is also in line with the performance of nerve root compression. A positive pressure head test and upper limb pull test further support this diagnosis, as these tests are commonly used to check whether the nerve roots in the neck are compressed.

Other options, such as spinal, mixed, vertebral artery and sympathetic type cervical spondylosis, although also the type of cervical spondylosis, their clinical manifestations did not completely coincide with this case description. For example, cervical spondylotic myelopathy usually shows symptoms such as weakness of limbs and gait instability, while cervical spondylotic spondylosis is common symptoms such as dizziness and blurred vision. Therefore, these types do not match the symptoms described in this case.

analyze:

The patient was a middle-aged woman with neck and shoulder pain for half a year, radiating to the left upper limb, suggesting possible nerve root compression. Falling muscle strength of the left upper limb, inflexible finger movements, and weakened sensation of the left thumb, which is a root sensory disorder after stimulation or compression of the nerve root. There is tenderness between cervical spinprocesses, positive upper limb pull test and positive head test, which are typical manifestations of cervical radiculopathy. Combined with the patient's medical history, clinical manifestations, and physical examination, the most likely diagnosis of the patient was cervical radiculopathy.

Option A cervical spondylosis for upper limb or lower limb numbness, stiffness,

feet on cotton feeling, toe can not off the ground, touch disorder, chest, both hands fine action clumsy, can not eat with chopsticks, muscle strength, limb tendon reflex active or hyperactivity, Hoffmann, patellar clonus, ankle clonus and Babinski positive, inconsistent with the problem dry description.

Option C Mixed cervical spondylosis has two or more types of symptoms at the same time, inconsistent with the patient's performance, so it is not selected.

Option D vertebral artery cervical spondylosis is mainly manifested by sudden vertigo, migraine, hearing loss or deafness, visual impairment, etc., which are obviously different from the symptom description of patients.

Option E sympathetic type cervical spondylosis mainly shows the symptoms of sympathetic nerve stimulation, which can be excitable or inhibitory, and is inconsistent with the performance of patients in the problem stem, so it is excluded.

To sum up, the correct answer is: B. radiculata.

According to the described symptoms and signs, the patient had neck and shoulder pain radiating to left upper limb, decreased muscle strength of left upper limb, inflexible finger movements, tenderness between cervical spine processes, weakened sensation of left thumb, positive upper limb pull test and positive compression head test. These symptoms suggest the possibility of cervical spondylosis, which involves the compression of the nerve roots.

Cervical spondylosis is mainly divided into five types: spinal cord type, nerve root type, mixed type, vertebral artery type and sympathetic nerve type. Analyze the characteristics of each type:

-Cervical spondylotic myelopathy mainly presents as limb weakness, paresthesia, unstable walking, and even bowel and feces disorders.

-Cervical radiculopathy mainly shows radiation pain and numbness in unilateral or bilateral upper limbs, and is associated with specific nerve root innervated areas.

-Mixed cervical spondylosis presents as a mixture of the above two or more types of symptoms.

-Cervical spondylosis is mainly manifested as dizziness, nausea, tinnitus and other symptoms caused by insufficient blood supply to the vertebral artery.

-Sympathetic cervical spondylosis is mainly manifested as headache, blurred

vision, palpitations and other symptoms caused by sympathetic dysfunction.

Combined with the patient's symptoms, the most consistent is the cervical radiculopathy (B). Her symptoms included neck and shoulder pain radiating to the left upper limb, decreased muscle strength of the left upper limb, inflexible finger movements, and diminished sensation of the left thumb, which typically suggested compression of the nerve roots.

Therefore, the most likely type is:

F. radiculata

114. (Type A2 questions)

Male, 64 years old, recurrent fever with fatigue for 1 week, aggravated for 1 day. Physical examination: body temperature 38.1°C, Hb 69 g / L, WBC 15109 / L, 7% peripheral blood cells, 22% bone marrow cells, and Auer bodies. The diagnosis was MDS, and its FAB was

A. RA

B. RSA

C. CMML

D. RAEB

E. RAEB-t

Reference answer: E Your answer:

[Analysis] In middle-aged and elderly male patients, recurrent fever, Hb 69 g / L (anemia), WBC 1510 ^ 9 / L, peripheral blood cells 7% (> 5%), bone marrow cells 22% (> 20%, <30%), Auer body, RAEB-t type (E pair).

[fault avoidance]

This case describes a 64-year-old male presenting with recurrent fever with fatigue for 1 week, temperature 38.1°C, hemoglobin 69g / L, white ocyte count 15109 / L, peripheral blood cell 7%, 22% marrow cell, and visible Auer bodies. These clinical findings and laboratory findings correspond with the myelodysplastic syndrome (MDS) findings in the FAB (France-US-UK) classification.

The FAB classification divides the MDS into the following categories:

-RA (refractory anemia)

-RARS (refractory anemia with ring iron iron)

-RAEB (refractory anemia with excess primary cells)

-RAEB-t (refractory anemia with excess blast cells close to acute myeloid leukemia)

-CMML (chronic myeloid monocytic leukemia)

Based on the proportion of peripheral blood primitive cells in the problem stem, 7% and 22% bone marrow primitive cells, and the appearance of Auer bodies, we can consider either RAEB or RAEB-t. RAEB refers to the marrow between 5% and 20%, while RAEB-t refers to the blast between 20% and 30% in the marrow and close to acute change. Therefore, according on the percentage of 22%, the most consistent diagnosis is RAEB-t (E option).

analyze:

The elderly male patient had recurrent fever with fatigue for 1 week and aggravated for 1 day. Physical examination showed increased body temperature, anemia, increased white blood cell count, increased peripheral blood blast cells, and significantly increased bone marrow blast cells, and showed the Auer body, which is a typical pathological feature of myelodysplastic syndrome (MDS). Combined with patient symptoms, signs and laboratory tests, it can be diagnosed as MDS. The FAB typing of MDS * *, RAEB-t (refractory anemia with transition) * *, primary blood cells greater than 5% (but less than 20%), primary cells greater than 20% and less than 30%, or Auer bodies, according to the condition of this patient.

Option A: RA, refractory anemia, <1% peripheral blood cells, <5% bone marrow cells, no Auer body, did not fit the patient, so it was excluded.

Option B: RAS, ring siderocyte refractory anemia, peripheral blood blast cells <1%, bone marrow blast cells <5%, ring iron granuloclasts> 15%, no Auer body, did not conform to the condition of the patient's condition, so it was not selected.

Option C: CMML is chronic granular-monocyte leukemia, absolute value of peripheral blood monocytes> 110 ^{9} / L, 5%~20% bone marrow primitive cells, increased monocytes, no Auer body, which did not conform to the condition of the patient, so it was excluded.

Option D: RAEB is refractory anemia with procytosis, pancytopenia in peripheral blood, <5% primitive cells, 5%~20% primitive cells in bone marrow, no Auer body, which did not conform to the condition of the patient, so it was not selected.

In summary, the correct answer is: E. RAEB-t.

Based on patient symptoms and on the results of laboratory tests, including body temperature, hemoglobin, white blood cell count, the proportion of primitive cells in the peripheral blood and bone marrow, and the presence of Auer bodies.

The FAB classification system for MDS (French-American-British Classification) includes the following types:

1. RA (Refractory Anemia, refractory anemia)

2. RSA (Refractory Anemia with Ring Sideroblasts, refractory anemia with annular derderocytes)

3. CMML (Chronic Myelomonocytic Leukemia, chronic monocytic leukemia)

4. RAEB (Refractory Anemia with Excess Blasts, refractory anemia with protocytosis)

5. RAEB-t (Refractory Anemia with Excess Blasts in Transformation, transition refractory anemia with increased blast)

The proportion of bone marrow primitive cells was 22% and 7%, and Auer bodies were visible, which suggests that the patient was in the stage of increased primitive cells and had the possibility of transition to acute leukemia. Therefore, the most appropriate FAB classification should be the RAEB-t.

The correct answer is: E. RAEB-t

115. (Type A2 questions)

A 55-year-old male patient with progressive cervical lymph node enlargement for 2 months, fever for 2 weeks and weight loss of 14kg since disease onset. Physical

examination: the body temperature was 38.7℃, and there were several lymph nodes of 2~5cm in both neck and right axilla, both of which were active and not touched without pressure. For cervical lymph node biopsy, R-S cells were seen. Lymphoma cells were seen in the bone marrow smear. The patient is now in staging

A. I designated time

B. II designated time

C. IIIS designated time

D. IIIE designated time

E. IVB designated time

Reference answer: E Your answer:

[Analysis] A middle-aged male patient with progressive cervical lymph nodes, weight loss of 14kg, several lymph nodes in both neck and right armpit, cervical lymph node biopsy, R-S cells, lymphoma cells in bone marrow smear, so the stage was stage B (E pair).

[Avoid avoidance] Hodgkin's lymphoma is divided into stage I~.

This case describes a 55-year-old male patient with progressive enlargement of the cervical lymph nodes for 2 months, associated with fever and significant weight loss. Physical examination revealed multiple large lymph nodes in the neck and right axilla, cervical lymph node biopsy revealed R-S (Reed-Sternberg) cells and lymphoma cells in a bone marrow smear. These clinical findings suggest that the patient may have had Hodgkin's lymphoma.

The stage of the lymphoma was determined based on the extent of the tumor spread. According to the Ann Arbor staging system, the lymphoma staging is as follows:

-Stage I: The tumor is confined to one region or a lymph node region.

Phase- II: The tumor invaded two or more lymph node areas, but was limited to the same side of the diaphragm.

Stage- III: The tumor invaded the lymph node areas on both sides of the diaphragm.

Phase- IV: extensive or disseminated disease that may affect lymph nodes other than one or more organs or tissues.

The suffix "S" indicates involvement of the spleen, while "E" indicates local

spread outside the organ or tissue.

Thus, according to the case, the patient had multiple areas of lymph node involvement (bilateral neck and right axillary) and lymphoma cells were visible in the bone marrow smear, indicating that the disease was beyond the local lymph nodes and involved other organs or tissues. This fits with the characteristics of stage lymphoma. However, there is no information that spleen involvement or other additional organ involvement is clearly indicated in the title, so the most suitable choice should be the period, but the specific period classification (such as A or B) is not clearly stated in the title. As usual, when there are no specific signs or symptoms (e. g. obvious systemic symptoms).

However, considering that the problem has no stage A and stage B, and the patient has a significant B symptom of weight loss, we can consider choosing stage E. B as the best answer. This reflects the widespread spread of the disease and the associated systemic symptoms (e. g., weight loss).

analyze:

Middle-aged male patients, progressive cervical lymph node enlargement, fever, and significant weight loss since the onset. Physical examination showed elevated body temperature, and both neck and right axillary lymph nodes were enlarged, active and without tenderness. The cervical lymph node biopsy showed R-S cells, which showed characteristic pathological changes of Hodgkin's lymphoma. Lymphoma cells were seen in the bone marrow smear, indicating that the lymphoma cells had invaded the bone marrow. According to the Ann Arbor staging criteria of lymphoma, diffuse large B cell lymphoma invasion into any of the bone marrow, liver, lung and other organs, namely the stage, and patients with fever more than 38℃, night sweats, weight loss of more than 10% in half A year can be divided into group B, otherwise group A. The patient in this question had bone marrow invasion and had group B symptoms with fever and significant weight loss, so his clinical stage was stage B.

Option A: Stage I refers to the tumor invasion of a single lymph node area or focal single extranodal organs or sites. The patient's lymphoma had invaded the bone marrow and did not match the definition of stage I.

Option B: Phase refers to tumor invasion into multiple lymph node areas on the ipsilateral side of the diaphragm, or focal single extranodal organs and their regional lymph nodes, with or without other nodal areas on the same side of the transverse septum. The patient's lymphoma has invaded the bone marrow and does not consistent

with the definition of period.

Option C: S phase is the invasion of diffuse large B cell lymphoma into the transverse lymph node area, associated with focal associated extranodal organs, splenic invasion, or both. S represents the limitation period, where a focal extranodal organ and spleen are invaded. The patient's lymphoma had invaded the bone marrow and did not match the definition of S phase.

Option D: Stage E E indicates extensive stage, where diffuse large B cell lymphoma is widely invaded into the bone marrow, liver, lung and other organs. However, the patients in this question with symptoms of fever greater than 38℃, night sweats and weight loss of more than 10% within half a year should be classified as group B rather than stage E.

In summary, the correct answer is: E. B stage.

Based on the patient's condition description and clinical examination results, we can stage Hodgkin's lymphoma.

The staging of Hodgkin lymphoma is based on the Ann Arbor staging system:

-Stage I: a single lymph node area or a single organ (extra-lymphoid).

Phase- II: Two or more lymph node areas are on the same side of the transverse (upper or lower).

Phase- III: involving the lymph node areas on both sides of the transverse septum.

Phase- IV: Diffuse or disseminated involvement of one or more extramymphatic organs.

In addition, the following markers can be attached to the staging system:

-A: No systemic symptoms.

-B: One of the following systemic symptoms: unexplained fever, night sweats, and weight loss of more than 10%.

-S: Spleen involvement.

-E: localized extra-lymphoid organ involvement.

Based on the information provided in the topic:

-The patient had fever and weight loss, which fit the B symptoms.

-Neck lymph node biopsy showed R-S cells, suggesting Hodgkin's lymphoma.

-Lymphoma cells are seen in the bone marrow smear, which indicates bone marrow involvement and is stage IV.

Therefore, the patient's staging should be:

C IVB designated time

Based on the above interpretation and the application of the clinical staging system, the answer was selected for stage E. B.

Male, 58 years old, fever for 2 weeks, physical examination: 38℃, ecchymosis on the acupuncture spot of the hand skin, sternum tenderness (+), no abnormal cardiopulmonary, not under the spleen ribs, Blood routine: Hb 86 g / L,

WBC12.4×109/L，PLT34×109/L， bone marrow cytomorphologic examination:polar hyperplasia, Promyelocytes with bulky granules in the cytoplasm

116, (A3 / A4 type question) subject

The most likely outcome of a bone marrow examination is that

A.MPO negative

And B.CD13 was negative

G. PAS positive

H. CD33 was positive

I. NSE negative

Refer to the answer: D. Your answer:

[Analysis] Middle-aged and elderly male patients with fever, ecchymosis, sternum tenderness (+) (leukemic manifestations of leukemia infiltration), blood routine: Hb 86 g / L, WBC 12.4109 / L, 109 / L, PLT 34109 / L (erythroid, megakaryus decreased, increased leukemia cells), bone marrow cytology examination: hyperactive hyperplasia, promyelocytes of large particles in the cytoplasm accounted for 0.75 (typical myeloid manifestation of M3). Considering the most likely diagnosis of promyelocytic leukemia, CD13 and CD33 positive; all-trans retinoic acid is preferred.

Answer to question 116: The most likely result of a bone marrow examination is D.CD33 positive. This condition is common in acute myeloid leukemia (AML), especially in the presence of promyelocytes with large inner granules in the cytoplasm.

The correct answer is: D.CD33 positive

This question examines the understanding of the indicators related to the bone marrow examination.

Option A, MPO (myeloperoxidase) is mainly found in neutrophils and monocytes, which is one of the hallmarks of granulocytes and monocytes, and is usually positive in acute myeloid leukemia, so it is excluded.

Option B, CD13 is a marker of myeloid cells and is usually positive in acute myeloid leukemia, so it is not chosen.

Option C, PAS (glycogen staining) is mainly used to detect intracellular glycogen and polysaccharides, and it has little to do with the diagnosis of acute myeloid leukemia, so it is excluded.

Option D, CD33 is a specific marker of myeloid cells, which is usually positive in acute myeloid leukemia, and is one of the important indicators in the diagnosis of acute myeloid leukemia, consistent with the question.

Option E, NSE (neuron-specific enolase) is an acid protease unique to neurons and neuroendocrine cells, and is not related to the diagnosis of acute myeloid leukemia, so it is not selected.

Question 116

The most likely outcome of a bone marrow examination is that

A.MPO negative

And B. CD13 was negative

C PAS positive

D CD33 was positive

E NSE negative

answer:

Bone marrow examination of the patient showed a proportion of promyelocytes and clinical findings were consistent with acute promyelocytic leukemia (APL). Promyelocytes in APL are usually positive for CD33. So the answer is:

C CD33 was positive

[fault avoidance]

117. (Type A3 / A4 questions) subject

Consider the most likely diagnosis of

A. Promyelocytic leukemia

B. acute myeloid leukemia

C. Acute monocytic leukemia

D. Acute megakaryocytic leukemia

E. Central nervous system leukemia

Reference answer: A. Your answer:

[Analysis] Middle-aged and elderly male patients with fever, ecchymosis, sternum tenderness (+) (leukemic manifestations of leukemia infiltration), blood routine: Hb 86 g / L, WBC 12.4109 / L, 109 / L, PLT 34109 / L (erythroid, megakaryus decreased, increased leukemia cells), bone marrow cytology examination: hyperactive hyperplasia, promyelocytes of large particles in the cytoplasm accounted for 0.75 (typical myeloid manifestation of M3). Considering the most likely diagnosis of promyelocytic leukemia, CD13 and CD33 positive; all-trans retinoic acid is preferred.

[fault avoidance]

Answer to question 117: Consider the most likely diagnosis of B. Acute myeloid

leukemia (AML). The patients' symptoms and laboratory results, especially the results of routine blood tests and bone marrow cytology, were consistent with this diagnosis.

The correct answer is: A. Promyelocytic leukemia

Patients with middle-aged men, fever, sternum tenderness, blood routine, anemia, white blood cells, thrombocytopenia, bone marrow cytology suggests hyperplasia hyperactive, cytoplasm contains large particles of early myelocytes cells increased significantly, according to its clinical manifestations, physical examination, laboratory examination, considering the diagnosis of early myelocytic leukemia. Promyelocytic leukemia is a specific type of acute myeloid leukemia designated as AML-M3 by the FAB Collaborative.

Option B acute myeloid leukemia is a group of differences in morphology, genetics, immunology, clinical and prognosis of heterogeneous tumors, including from mature to immature stage of granulocytes or monocyte leukemia, can be divided into M0 to M7 eight subtypes, promyelocytic leukemia belongs to one of the subtype, inconsistent with the dry description.

Option C Acute monocytic leukemia is acute myeloid leukemia M5 type, which does not match the description of the title stem, so it was excluded.

Option D Acute megakaryocytic leukemia is acute myeloid leukemia M7 type, which is not consistent with the description of the title stem, so it is not selected.

Option E CNS leukemia is a clinical symptom and sign produced by the extramedullary infiltration of leukemic cells into the arachnoid or adjacent nerve tissue. It is not an independent type of leukemia, so it is excluded.

Question 117

Consider the most likely diagnosis of

A. Promyelocytic leukemia

B. acute myeloid leukemia

C. Acute monocytic leukemia

D. Acute megakaryocytic leukemia

E. Central nervous system leukemia

answer:

Based on the clinical findings and early myelocyte findings, the most likely diagnosis is promyelocytic leukemia (APL). So the answer is:

A. Promyelocytic leukemia

118, (Type A3 / A4 questions) subject

What to use for treatment

A. RA

B. HA regimen for chemotherapy

C. Imatinib

D. VLDP regimen chemotherapy

E. DA regimen of chemotherapy

Reference answer: A. Your answer:

[Analysis] Middle-aged and elderly male patients with fever, ecchymosis, sternum tenderness (+) (leukemic manifestations of leukemia infiltration), blood routine: Hb 86 g / L, WBC 12.4109 / L, 109 / L, PLT 34109 / L (erythroid, megakaryus decreased, increased leukemia cells), bone marrow cytology examination: hyperactive hyperplasia, promyelocytes of large particles in the cytoplasm accounted for 0.75 (typical myeloid manifestation of M3). Considering the most likely diagnosis of promyelocytic leukemia, CD13 and CD33 positive; all-trans retinoic acid is preferred.

[Misavoidance] HA and DA chemotherapy were used for acute myeloid leukemia; imatinib for chronic myeloid leukemia; and VLDP chemotherapy for acute lymphocyte leukemia (D error).

Answer to question 118: Treatment should choose the E.DA regimen for chemotherapy. The DA regimen, including danamycin (Daunorubicin) and cytarabine (Cytarabine), is the usual treatment regimen for AML.

The correct answer is: A. All-trans-retinoic acid

The patient has been diagnosed with promyelocytic leukemia, whose preferred treatment is all-trans-retinoic acid (ATRA) + arsenic agent, so the answer to this question is A.

Option B, HA regimen (three acute acute alkali + cytarabine) is mainly used for the treatment of acute myeloid leukemia, but the treatment effect on promyelocytic leukemia is limited, so it is not selected.

Option C, Imatinib was mainly used to treat chronic myeloid leukemia, especially in cases with the BCR-ABL fusion gene, and had no therapeutic effect on promyelocytic leukemia, so it was excluded.

Option D, VLDP regimen (vincristine + L-asparaginase + daunorubicin + prednisone) is usually used for acute lymphocytic leukemia rather than promyelocytic leukemia, so it is not chosen.

Option E, DA regimen (daunorubicin + cytarabine) was also commonly used in the treatment of acute myeloid leukemia, but the treatment of promyelocytic leukemia was not as significant as all-trans retinoic acid, so it was excluded.

Question 118

What to use for treatment

A. All-trans-retinoic acid

B. HA regimen for chemotherapy

C. imatinib

D. VLDP regimen chemotherapy

E. DA regimen of chemotherapy

answer:

The standard treatment regimen for acute promyelocytic leukemia (APL) is all-trans-retinoic acid (ATRA) combined with chemotherapy. ATRA is able to induce promyelocytic differentiation. So the answer is:

A. All-trans-retinoic acid

Male, 22 years old, accidentally fell down while riding a bicycle 2 hours ago and rode on the car beam, with perineal swelling and pain, and dripping blood at the urethral opening. Physical examination: stable in life, perineal swelling, visible ecchymosis.16 and clear fluid was exported. The next step, male, 22 years old, 2 hours ago riding a bicycle accidentally fell down on the car beam, perineal swelling and pain, the urethral opening dripping blood. Physical examination: stable swelling in life, visible ecchymosis. An emergency 16-gauge catheter can be inserted into the urethral bladder to produce clear fluid.

119, (Type A3 / A4 questions) subject

The most likely diagnosis is the

A. Ball contusion of the urethra

B. Injury to the membrane part of the urethra

C. urethral penile injury

D. prostate injury

E. cystorrhexis

Reference answer: A. Your answer:

Graphic analysis: [Analysis] Male anterior urethral trauma mostly occurs in the ball part, and this section of the urethra is fixed in the perineum. When perineal riding cross injury, squeeze the urethra below the pubis joint, causing trauma to the urethral bulb (so choose A pair).

[fault avoidance]

Question 119: What is the most likely diagnosis?

The given condition describes a 22-year-old male presenting with perineal swelling, ecchymosis, and blood drops at the urethral opening after a bicycle accident. Because the urethral catheter can be inserted smoothly and the clear urine drained, this indicates that the urethra is not completely torn, but with some degree of damage. Based on the described mechanism of injury and clinical presentation, the most likely diagnosis is:

A. urethral bulbar contusion-Although the possibility exists, bulbar urethral injuries are usually associated with more severe impact.

B. urethral injuries-Considering the nature and location of the injury, this is the most likely diagnosis as urethral injuries are common in trauma to the perineum.

C. Urethral penile injury-penal injury is usually associated with direct penile trauma.

D. Prostate injury-prostate injury is less common and is often associated with more severe pelvic trauma.

E. Bladder rupture-if urine is clear after catheterization.

Therefore, the most reasonable choice is B.

analyze:

A young male patient accidentally fell down on a bicycle and was injured on the beam of the car, with perineal swelling and pain, blood drops at the urethral opening, physical examination, and visible ecchymosis. Emergency No.16 catheter could be inserted into the urethral bladder to produce a clear liquid. Combined with the patient's history, clinical findings and related examinations, the patient was consistent with the typical findings of urethra bulb trauma.

Option B urethral membrane injury is more seen in pelvic fractures. This patient has no history of pelvic trauma, so it was not selected.

Option C The urethral penile injury mainly showed local ecchymosis, hematoma and urinary extravasation, and no hematoma and urinary extravasation occurred in this patient, so it was not considered.

Option D Prostate injury was often manifested as lower abdominal pain, hematuria, and dysuria, which this patient did not see, so it was excluded.

Option E Bladder rupture mainly showed abdominal pain, dysuria, hematuria, and urinary fistula. This patient had no hematuria and urinary fistula, so it was not selected.

In conclusion, the correct answer is: A. contusion of the urethral bulb.

Question 119: The most likely diagnosis is

The patient, a 22-year-old, had painful perineal swelling and bleeding at the urethral opening after falling by bicycle. Physical examination revealed perineal swelling with ecchymosis. A 16-gauge catheter was inserted emergency to export clear fluid. These symptoms and signs suggest the possibility of an impaired urethra.

diagnostic analysis:

Urethral ball contusion: the direct impact of the perineum can easily lead to contusion in this area, and the common symptoms are urethral opening bleeding and perineal swelling.

Urethral membrane injury: common in severe pelvic fractures or high-energy injuries, patients will have perineal swelling and urethral bleeding, but in this case, the catheter is usually difficult to insert.

Urethral penile injury: relatively rare, usually when external forces directly act on the penis.

Prostate injury: This condition is rare and is often seen after digital rectal examination or surgical procedures.

Bladder rupture: Patients usually have significant lower abdominal pain and hematuria, and the catheter-derived fluid is usually mixed with blood.

Combined with the specific condition, the most likely diagnosis is A. bulbar contusion.

120, (Type A3 / A4 questions)   subject

The next step in the process

A. Surgery immediately

B. Surgery 24 h

C. The inserted catheter was removed 24 hours later and allowed to urinate freely

D. The urinary catheter was inserted and removed at 2 to 3 weeks

E. Uter removed removed 72 hours and dilated once a week

Refer to the answer: D. Your answer:

[Analysis] The patient had contusion of the ball of the urethra, the catheter was inserted smoothly, and the catheter could be drained for about 2 weeks (D pair). If the insertion fails, there may be partial urethra laceration, including perineal urethra repair and urinary catheter for 2 to 3 weeks.

[To avoid mistakes] The urethra membrane and the penis are the posterior urethra, which are mostly caused by pelvic fractures, common shock, and no ecchymosis in the perineum.

Question 120: The next step

For the management of urethral injury, the key is to prevent the development of urethral strictures while allowing the recovery of the urethra. When choosing the treatment method, according to the degree and nature of the urethral injury. Given that the urethra does not appear to be completely torn (because it can catheterization and shed clear urine), a more conservative approach could be taken. In the option:

A. Immediate surgery-usually reserved for a complete tear or severe urethral

injury.

B. Surgery 24 h after catheter insertion-Consider this protocol if a complex injury is suspected or cannot be managed by conservative methods.

C. Remove the catheter after 24 hours to urinate freely-this method can be considered if the urethra injury is mild and the voiding function is not affected.

D. Catheal insertion, 2 to 3 weeks extraction-For moderate urethral injury, long-term indwelling catheter may contribute to urethral healing.

E. Pull out after 72 hours and the urethra once a week-This approach may be applicable for cases at risk of stenosis but not requiring immediate surgery.

Depending on the situation described, and the extent of injury, D. insertion, 2 to 3 weeks extraction seems to be the appropriate method in order to allow the urethra to heal adequately and reduce the risk of stricture.

When the ball of the urethra was bruised, the continuity of the urethra was not destroyed, and the 16 catheter had been inserted into the emergency bladder, and the clear liquid has been exported. Therefore, the urethra should be inserted and pulled out for 2~3 weeks to observe whether the urethra was restored.

Option A immediate surgery is suitable for severe injuries such as urethral fracture, not for this patient, so it is not selected.

Option B is surgery 24 h, option E is pulled out 72 h and expand the urethra once a week, which is not the conventional treatment of urethral contusion and can be excluded together.

Option C, remove the catheter after 24 hours and let them urinate freely. Due to the short time, the catheter removal may lead to incomplete recovery of the urethra, so it is not considered.

In conclusion, the correct answer is: D. inserted catheter, 2 to 3 weeks to pull out.

Question 120: The next step

The patient had successfully inserted the catheter and exported clear fluid, indicating that the urethra had good patency with no serious urethral fracture.

Treatment method analysis:

Immediate surgery: Suitable for severe urethral breaks or other life-threatening injuries, not currently applicable.

Surgery after 24 hours of catheter insertion: generally used to observe the urethra for further signs of injury and is not applicable.

The catheter is removed 24 hours to urinate freely: it may lead to urethral stricture or other complications.

Catheal insertion, 2~3 weeks extraction: this is a common method of dealing with the urethra bulb contusion, which can make the urethra get sufficient recovery time and reduce complications.

Pull out the catheter after 72 hours and expand the urethra once a week: This method is suitable for patients with high urethral stricture or high risk.

Combining the patient's condition, the best management is D. catheterization, extracted at 2 to 3 weeks, which ensures sufficient recovery time for the urethra to avoid further injuries or complications.

Female, 16 years old, palpitation, sweating for 2 years, weight loss of 5kg, stool times increased, 3~4 times / day, unformed, menstruation 2~3 months once, less quantity. Physical examination: P: 100 times / minute, blood pressure of 120 / 80 protruding eyes, thyroid degree I enlargement. Laboratory tests: TT3:8.6nmol/L, TT4:220 n mol/L, TSH <0.002.

121, (Type A3 / A4 questions) subject

Which of the following items facilitates the diagnosis

A. TgAb

B. TRAb

C. FT3

D. TPOAb

E. Tg

Reference answer: B. Your answer:

[Analysis] adolescent female patients (people with GD disease), palpitation, sweating, weight loss, thyroid I enlargement, TT3:8.6nmol/L, TT4:220 n mol/L, TSH <0.002 (TT3, TT 4, TSH), patients were diagnosed as diffuse toxic goiter based on symptoms, signs and laboratory examination. TRAb has become the first line indicator in the diagnosis of GD, with a positive rate of 98% in untreated GD patients.

[Error] 60% Hashimoto's thyroiditis TgAb positive, and 90% Hashimoto's thyroiditis TPOAb positive indicates autoimmunity; FT3 is very little in blood, and the determination was not as stable as TT3 and TT4; Tg is meaningless. Hashimoto's thyroiditis is a sign of hypothyroidism; subacute thyroiditis has thyroid tenderness.

Question 121, about the diagnosis:

Symptoms in this patient included palpitation, hyperhidrosis, weight loss, increased stool count, prolonged menstrual cycles, goitre, and laboratory tests showed elevated T3 and T4 and decreased TSH. These are the typical symptoms of hyperthyroidism. Among them, TRAb (thyroid receptor antibody) testing is particularly useful for the diagnosis of Graves's disease (diffuse toxic goiter). Thus, the correct answer is the B. TRAb.

121. The helpful options for the diagnosis are:

B TRAb

analyze:

Young female patients, the typical symptoms of hyperthyroidism, such as flustered, sweating, stool frequency, menstruation, etc, body examination and thyroid I enlargement, laboratory examination showed T3, T4 rise, TSH significantly reduced, suggest hyperthyroidism, comprehensive the patient history, physical examination and laboratory examination, considering the diagnosis of Graves disease. Graves Disease, also known as diffuse toxic goiter, is an organ-specific autoimmune disease with increased synthesis of thyroid hormone. TSH receptor stimulating antibodies, namely TRAb, are present in the serum of patients, so the detection of TRAb levels in the patients is helpful for diagnosis.

Option A: TgAb, namely thyroglobulin antibody, is mainly used in the diagnosis of autoimmune thyroid disease, but it is not a specific antibody for Graves disease, so it is excluded.

Option C: FT3, or free triiodothyronine, is the most sensitive indicator for the diagnosis of hyperthyroidism. However, its abnormal level can also be seen in the causes of non-thyrotoxicosis, such as acute fever, pregnancy, etc., so it is not specific and cannot be used for the diagnosis of Graves disease.

Option D: TPOAb, namely anti-thyroid peroxidase antibody, is closely related to the immune damage of thyroid tissue. It is mainly used in the diagnosis of Hashimoto's thyroiditis, primary hypothyroidism and other thyroid diseases, and has

no diagnostic significance for Graves disease.

Option E: Tg, or thyroglobulin, can be used as a tumor marker for differentiated thyroid cancer to monitor its recurrence and metastasis, and can not be used for the diagnosis of Graves disease.

Question 121:

Which of the following facilitates the diagnosis?

A. TgAb

B. TRAb

C. FT3

D. TPOAb

E. Tg

TRAb (thyroid irritant antibody) is a specific marker of diffuse toxic goiter (Graves disease) and can help in the diagnosis. So the best answer is:

B. TRAb

122, (Type A3 / A4 questions)

The patient's disease subject

A. Hashimoto's thyroiditis

B. sub-acute thyroiditis

C. Diffuse toxic goiter

D. Simple goiter

E. cancerous goiter

Reference answer: C Your answer:

Graphic and text analysis: [analysis] adolescent female patients (people with GD disease),

8.6 n mol/L, TT4:220 n mol/L, TSH <0.002 Laboratory examination was

considered for the diagnosis of diffuse toxic goiter. The positive rate of the patients reached 98%.[fault avoidance]Fluitation, hyperhidrosis, weight loss, enlarged thyroid I, TT3: (TT3, TT4, TSH), comprehensive patient symptoms, signs and TRAb have become the first line index of diagnosis of GD, untreated GD

Question 122, about disease diagnosis:

Based on the above symptoms and laboratory results, patients are most likely to have a diffuse toxic goiter, or Graves's disease. So, the correct answer is C. diffuse toxic goiter.

122. The patient's disease is:

C. Diffuse toxic goiter

analyze:

Young female patients, the typical symptoms of hyperthyroidism, such as flustered, sweating, stool frequency, menstruation, rare, body examination and thyroid I enlargement, for the typical signs of Graves disease, laboratory tests showed T3, T4, TSH is significantly reduced, combined with patient history, clinical manifestations and laboratory examination, the patient diagnosed as diffuse toxic goiter.

Option A: Hashimoto thyroiditis is chronic lymphocytic thyroiditis, is A kind of thyroid tissue as antigen of chronic autoimmune disease, more in middle-aged women, good age is 40~50 years old, clinical examination can find goiter, texture hard tough, most patients with normal thyroid function, but A few can appear hypothyroidism symptoms, inconsistent with the problem in dry symptoms, so excluded.

Option B: subacute thyroiditis also known as subacute granulomatous thyroiditis, more in middle-aged women, often before the history of upper respiratory tract infection, typical symptoms of goiter, pain as the main characteristics, serious can cause systemic symptoms such as fever, headache, fatigue, dry stool, dry mouth, upset, etc., does not accord with patients, so do not choose.

Option D: Most patients with simple goiter had no obvious clinical symptoms. The goiter was found only during physical examination, and the thyroid function was generally normal, which was not consistent with the problem stem, so it was excluded.

Option E: The early clinical manifestations of thyroid cancer are not obvious, and there are no self-conscious symptoms. With the development of the disease, neck mass, dyspnea, dysphagia, hoarseness and other symptoms can appear, which are

significantly different from the patients, so it is not selected.

Question 122:

Is the patient's disease?

A. Hashimoto's thyroiditis

B. sub-acute thyroiditis

C. Diffuse toxic goiter

D. Simple goiter

E. cancerous goiter

The patient presented typical symptoms of hyperthyroidism, combined with laboratory findings (increased TT3 and TT4, decreased TSH), protrusion and thyroid enlargement, all consistent with the clinical presentation of diffuse toxic goiter (Graves's disease). So the best answer is:

G. Diffuse toxic goiter

A 63-year-old man with progressive right testicular enlargement for one year, without pain and no walking problems. Physical examination: testis 654cm, no tenderness, right testis and epididymis not palpated, positive transmittance test, supine

123, (A3 / A4 type question) subject

Diagnostic as

A. hydrocele testis

B. funicular hydrocele

C. orchioncus

D. indirect inguinal hernia

E. Traffic hydrocele

Reference answer: A. Your answer:

[Analysis] middle-aged and elderly men, progressive painless right testicular

enlargement for 1 year, positive light test positive, unchanged after supine, considering diagnosis

Is the testicular hydrops (pair A).

[Avoid wrong] The effusion (B wrong) can be manifested as one or more cysts, oval, spindle or dumbbell shaped, growing along the spermatic cord, and the normal testis and epididymis can be palpbelow. If the testis on the same side, the cyst can move up and down. Testicular tumors and oblique inguinal hernia (CD error) were negative. Traffic hydrocele (E wrong) transmittance test was positive and disappeared after lying down.

123:

A. Testicular hydrocele: Hydrocele is a possible diagnosis.

B. Hydroeffusion: usually presenting as a palpable mass in the spermatic cord area.

C. Testicular tumor: It usually causes testicular sclerosis, and the light transmittance test is usually negative.

D. Ininguinal hernia: a hernia mass in the inguinal region, usually independent of testicular volume changes.

E. Traffic hydroe: possible considered but usually inconsistent with testicular volume.

Therefore, for question 123, the most appropriate diagnosis is the A. testicular hydrocele.

123 Question Analysis:

In an elderly male patient, progressive right testicular enlargement for one year, no pain, positive body examination showed light transmittance test, and the testis did not disappear when supine. Combined with the patient's medical history, clinical manifestations and physical examination, the patient was diagnosed as testicular fluid effusion.

Option B hydroeffusion appeared as a smaller cystic mass in the inguinal canal, with the mass moving in the ipsilateral testis, which did not conform to the trunk description.

Option C testicular tumors usually showed testicular swelling or stiffness and a negative light transmittance test, so it was not selected.

Option D The hernia mass of oblique inguinal hernia is often located in the groin area, often pear-shaped with a pedicle, and can be reduced to the scrotum or labia majora, negative transmittance test, inconsistent with the question.

Option E Traffic hydroeffusion is characterized by enlargement of the scrotum at position, but when the patient lies and the effusion flows into the abdominal cavity, the capsule will significantly shrink or disappear, and the testis and epididymis can not be touched in the scrotum. The transmittance test was also positive, but the topic stem clearly indicates that the right testis did not disappear when supine, so it was excluded.

In conclusion, the correct answer is: A. Testicular hydrocele.

123, (A3 / A4 type question)

Question: Diagnosis is

A. Testicular hydrocele

B. Hydroeffusion of spercord sheath

C. orchioncus

D. indirect inguinal hernia

E. Traffic hydrocele

Answer analysis:

According to the case description, the patient had enlarged right testis with a positive transmittance test. These features are most common in the hydrocele. Hydrocele is a lesion that accumulates fluid in the sheath membrane space around the testis and usually results in testicular enlargement and a positive light transmittance test.

Therefore, the most likely diagnosis is:

A. Testicular hydrocele

124, (A3 / A4 type question) subject

The preferred treatment

A. Right-sided oblique hernia repair

B. Right orchiectomy

C. High-level ligation of the right sheath process

D. Right testicular of testicular right testicular lip

E. Score extraction fluid

Refer to the answer: D. Your answer:

[Analysis] In middle-aged and elderly men, progressive painless right testicular enlargement for 1 year, positive light transmittance test, unchanged after supine, was considered to be diagnosed as testicular hydrocele. Adult testicular hydrops, such as low hydrops, do not have any symptoms and do not require surgical treatment. High fluid volume, large volume with obvious symptoms, feasible testicular sheath resection + turnover (D pair).

[Error avoidance] Right oblique hernia repair is the treatment of inguinal hernia (A error); right orchiectomy is used for the treatment of testicular tumor (B error); high ligation of the right sheath is the treatment of pediatric inguinal hernia (C error); and puncture solution is symptomatic treatment (E error).

124:

A. Right oblique hernia repair: suitable for oblique inguinal hernia, inconsistent with this case.

B. Right orchiectomy: for testicular tumors, than fluid.

C. High ligation of the right sheath process: for the treatment of hydrocele or fluid.

D. Right testicular sheath testicular reversal: usually not the preferred method for handling testicular hydrocele.

E. Punsolution: It is a common and preferred method for handling testicular hydrops because it is simple and effective.

Therefore, for question 124, the most appropriate treatment is E. aspiration.

124 Question Analysis:

The patient has been diagnosed as testicular hydrocele. For testicular hydrocele, surgery is the main treatment method. The purpose is to tie the hernia neck at the internal ring, and flip the sheath to avoid the recurrence of hydrocele. Therefore, right testicular sheath testicular turnover is the preferred treatment for this patient.

Option A Right oblique hernia repair was indicated for patients with inguinal hernia not at diagnosis and was excluded.

Option B Right orchiectomy is mainly used for patients with testicular tumor, who have no tumor diagnosis, so it is not selected.

Option C: High ligation of the right sheath process is mainly suitable for the treatment of pediatric traffic hydrocele, which is inconsistent with the diagnosis of this patient and can be excluded.

Option E aspiration is a conservative treatment for testicular hydrocele, but it is prone to relapse and is not the preferred treatment option and can be ruled out.

In conclusion, the correct answer is: D.

124, (A3 / A4 type question)

Title: Preferred treatment

A. Right-sided oblique hernia repair

B. Right orchiectomy

C. High-level ligation of the right sheath process

D. Right testicular of testicular right testicular lip

E. Tsolution

Answer analysis:

For the treatment of testicular hydrocele, the common methods are conservative treatment and surgical treatment. If the effusion is small and the symptoms are mild, the puncture solution can be used as a temporary relief method. However, for large or continuous hydrocele, especially when affecting daily activities, high ligation of the sheath process is the preferred radical treatment, which can effectively prevent the continued accumulation of fluid.

Therefore, the most appropriate treatment method is considered to be:

B. High-level ligation of the right sheath process

Male, 40 years old, edema, oliguria for 2 weeks, blood pressure 160 / 100mmHg, creatinine 300 μmol/L, urine protein 2g, microscopic hematuria from 20 to 30,8

microscopic red blood cells, crescent signs, immunofluorescence

cloth,

125, (A3 / A4 type question) subject

Diagnostic as

A. Acute-induced glomerulonephritis type I

B. Urgent glomerulonephritis subtype

C. Urgent glomerulonephritis type

D. nephrotic syndrome

E. Acute glomerulonephritis

Reference answer: A. Your answer:

[Analysis] middle-aged male patients, edema, oliguria, blood pressure 160 / 100mmHg (elevated blood pressure), creatinine 300 μ mol / L (renal function impairment), urinary protein 2g, hematuria, crescent signs (acute nephritis pathological type), linear distribution (type I acute nephritis pathology type), diagnosed as acute glomerulonephritis type I (A pair).[Error] Immunopathological examination of acute glomerulonephritis is the main basis for classification. Type I IgG and C3 are distributed along the glomerular capillary wall; type IgG and C3 are granular or mass deposited in the mesangial area and capillary wall; no or only trace immune deposits in the glomerulus.

For the first question, acute glomerulonephritis can be divided into three types:

Type I is usually associated with anti-glomerular basement membrane antibodies.

Type is associated with immune complex deposition.

Type 1 is associated with glomerular injury without immune complex deposition and is commonly seen in ANCA-associated vasculitis.

Because the title stem mentioned that "immunofluorescence has cloth", it may imply the deposition of immune complexes or specific immune labeling, which prefers acute glomerulonephritis. However, the exact classification should be based on the specific findings of immunofluorescence, where we can choose B. emergency glomerulonephritis type as the most likely diagnosis.

The answer is A. acute glomerulonephritis type I

The patient was a middle-aged male with edema, oliguria, hypertension,

abnormal renal function, massive proteinuria, microscopic hematuria, microscopic red blood cells, crescent signs, immunofluorescence, according to clinical manifestations and laboratory examination, and considered as acute glomerulonephritis. Acute glomerulonephritis is a group of diseases characterized by acute nephritis syndrome, rapid deterioration of renal function, and the occurrence of oliguric acute renal failure in the early stage. According to the immunopathology, it can be divided into three types, type I is anti-glomerular basement membrane antibody type, type is immune complex type, and type is less immune sediment type. The patient had crescent signs and immunofluorescence distribution, which was considered for the diagnosis of acute glomerulonephritis type I.

Question 125 (type A3 / A4)

The diagnosis is:

A. Acute-induced glomerulonephritis type I

B. Urgent glomerulonephritis subtype

C. Urgent glomerulonephritis type

D. nephrotic syndrome

E. Acute glomerulonephritis

analyse:

Acute glomerulonephritis (RPGN) is a rapidly progressive glomerulonephritis characterized by rapidly developing renal dysfunction, often accompanied by hematuria and proteinuria. According to the results of immunofluorescence examination, acute glomerulonephritis can be classified into three types:

Type I: anti-glomerular basement membrane antibody associated (e. g. Goodpasture syndrome)

Type: Immune complex deposition-related (e. g., lupus nephritis, IgA nephropathy)

Type: less immune complex deposition (pauci-immune), usually associated with

anti-neutrophil cytoplasmic antibodies (ANCA)

According to the stem information, the patient's immunofluorescence test was cloth (usually indicating immunological complex deposition), combined with the patient's clinical findings and laboratory findings, the most likely diagnosis was acute glomerulonephritis.

answer:B

126, (A3 / A4 type question) subject

Treatment preferred

A. plasmapheresis

B. metacortandracin

C. prednisone + immunosuppressive agent

D. hemodialysis

E. Prednisone + cytotoxic drugs

Reference answer: A. Your answer:

[Analysis] middle-aged male patients, edema, oliguria, blood pressure 160 / 100mmHg (elevated blood pressure), creatinine 300 μ mol / L (renal impairment), urinary protein 2g, hematuria, crescent signs (acute nephritis pathological type), immunofluorescence linear distribution type (type I acute nephritis immunopathology type), diagnosed as acute glomerulonephritis type I, preferred treatment is plasma exchange (A pair).

[Error avoidance] Prednisone and cytotoxic drugs, immunosuppressants combined with methylprednisolone shock are used in patients with type 1.

For the second question, the preferred treatment for acute glomerulonephritis usually includes immunosuppressants to control the immune response. Prednisone is a common treatment, but in many cases, especially with severe disease, a combination of immunosuppressants may be required. Therefore, C. prednisone + immunosuppressants may be a more appropriate choice.

The answer is A. plasma exchange

For the treatment of type I acute glomerulonephritis, intensive plasma exchange therapy is currently the most effective method, which can remove circulating

anti-glomerular basement membrane antibodies and reduce glomerular damage, so plasma exchange is the first choice for treatment. Therefore, this question is selected A. Option B prednisone, option C prednisone + immunosuppressant, and option E prednisone + cytotoxic agent, were suitable for the treatment of acute glomerulonephritis, so they were excluded. Option D Hemodialysis is only used for severely impaired renal function and is not preferred and can be excluded.

Question 126 (Type A3 / A4 questions)

Treatment preferred:

A. plasmapheresis

B. metacortandracin

C. prednisone + immunosuppressive agent

D. hemodialysis

E. Prednisone + cytotoxic drugs

analyse:

Treatment of acute glomerulonephritis requires rapid control of inflammation and immune responses to prevent further deterioration of renal function. Treatment options often include high doses of corticosteroids and immunosuppressive agents. For RPGN (type), corticosteroids combined with immunosuppressive agents are usually used.

answer:C

The patient with chest tightness for several years, 2 years ago began to appear asthma, hemoptysis, cough pink sputum, electrocardiogram showed atrial fibrillation, heart ultrasound showed, left atrial diameter of 56mm, mitral valve area of 0.8cm2, wall stack vegetation,

127, (Type A3 / A4 questions) subject

Consider what diagnosis is

A. acute pulmonary embolism

B. acute myocardial infarction

C. bronchiectasia

D. dilated cardiomyopathy

E. Infective endocarditis

Reference answer: E Your answer:

Graphic resolution: [analytical] middle-aged male patients, chest shortness, 2 recently began to suppress asthma, hemoptysis, cough pink sputum (acute left heart failure symptoms), electrocardiogram showed atrial fibrillation, left atrial diameter 56mm, mitral valve area 0.8cm2, into wall stack sample change (mitral valve stenosis change), have vegetation (infectious endocarditis typical heart super change). The diagnosis of infective endocarditis was considered (E pair).

[Avoid wrong] Acute pulmonary embolism shows triad of chest pain, hemoptysis, and dyspnea (A wrong); acute myocardial infarction shows persistent chest pain greater than 30 minutes, with typical ECG changes in ST segment (B wrong); bronchiectasis shows cough, pus and sputum for many years, high-resolution CT double or curly shadow (C wrong); dilated cardiomyopathy is a cardiomyopathy characterized by left ventricle or double ventricle enlargement with systolic dysfunction (D wrong).

127 is the diagnostic question. The characteristics of this case and the cardiac ultrasound findings correspond with infective endocarditis because it can cause compound vegetation formation and may cause damage to cardiac structures such as the mitral valve. Therefore, the most appropriate diagnosis is: E. infective endocarditis

127 Question Analysis:

The patient had long-term chest tightness and short breath, recent wheezing, hemoptysis, cough pink sputum, electrocardiogram showed atrial fibrillation, left atrial enlargement, mitral stenosis and vegetation. According to the patient's clinical manifestations and auxiliary examination, the most likely diagnosis was infective endocarditis.

Option A Acute pulmonary embolism is often manifested as sudden unexplained dyspnea and shortness of breath, accompanied by chest pain, hemoptysis, etc., generally no endocardial vegetation, so it was excluded.

Option B Acute myocardial infarction generally has a history of angina pectoris,

which is mainly manifested as myocardial ischemia such as retrosternal fatal pain. Generally, there are no symptoms such as wheezing, hemoptysis and cough of pink sputum, which is inconsistent with the description of the title stem.

Option C bronchiectasis mainly shows chronic cough, heavy sputum and repeated hemoptysis, and no heart-related symptoms, so it is not selected.

Option D dilated cardiomyopathy is mainly manifested as active dyspnea and decreased activity tolerance, heart enlargement, generally not accompanied by hemoptysis and cough pink sputum and other symptoms, so it is excluded.

In conclusion, the correct answer is: E. infective endocarditis.

Answer to Question 127:

Consider what diagnosis is

A. Acute pulmonary embolism-The patient had wheezing, hemoptysis, but no typical history of pulmonary embolism or risk factors.

B. Acute myocardial infarction-The patient had no significant chest pain findings, and no electrocardiogram was indicated.

C. Bronchiectasis-The patient had hemoptysis but no typical findings of bronchiectasis such as chronic cough and polysputum.

D. Dilated cardiomyopathy- -The patient's cardiac ultrasound showed a mitral stenosis with left atrial enlargement rather than an expansion of the ventricle.

E. Infective endocarditis-The patient has vegetations but no typical manifestations of infective endocarditis such as fever, changes in the heart murmur.

The patient had a long history of short chest tightness, atrial fibrillation, severe mitral valve stenosis, and vegetation formation, most consistent with mitral valve stenosis due to rheumatic heart disease. However, rheumatic heart disease is not specified in the title. In this case, the possibility of infective endocarditis cannot be completely excluded, because the vegetation may be caused by the infection.

The best answer is: E. infective endocarditis

128, (A3 / A4 type question) subject

The following treatments are preferred

A. diuretic

B. The balloon dilatation procedure

C. The antiplatelet of aspirin

D. Nitrate was used for coronary artery enlargement

E. digitalis

Reference answer: E Your answer:

Graphic resolution: [analytical] middle-aged male patients, chest shortness, 2 recently began to suppress asthma, hemoptysis, cough pink sputum (acute left heart failure symptoms), electrocardiogram showed atrial fibrillation, left atrial diameter 56mm, mitral valve area 0.8cm2, into wall stack sample change (mitral valve stenosis change), have vegetation (infectious endocarditis typical heart super change). A diagnosis of infective endocarditis was considered. With mitral valve stenosis and atrial fibrillation, digitalis is preferred for medical treatment (organic heart disease combined with atrial fibrillation is an excellent indication for digitalis).

[Fault avoidance] Balloon expansion is used for simple moderate to severe mitral stenosis.

128 It involves treatment preference. For infective endocarditis, an important treatment modality is the use of antibiotics to control the infection. However, among the options provided, there is no direct antibiotic treatment option. Given the possible impaired cardiac function and increased cardiac load caused by infective endocarditis, diuretics can be used to reduce the symptoms and the burden on the heart. Therefore, of the given options, the most appropriate treatment is preferred: A. diuretics

128 Question Analysis:

The patient had been diagnosed with infective endocarditis with mitral stenosis, and at this time the preferred treatment was balloon dilatation to improve the symptoms of mitral stenosis.

Option A diuretics were mostly used in the treatment of heart failure. The patient did not have heart failure, so it was excluded.

Option C aspirin antiplatelet, option D nitrate expanded coronary artery, mostly used in the treatment of coronary heart disease, and the patient is not coronary heart

disease, is not with the meaning of the question.

Option E Digitalis is an inotropic drug used for systolic heart failure with rapid atrial fibrillation / atrial flutter, but the patient did not have heart failure, so it was not selected.

In conclusion, the correct answer is: B. balloon dilatation.

Answer to Question 128:

The following treatments are preferred

A. Diuretics-which provides symptomatic relief but is not the preferred treatment.

B. Balloon dilatation-An effective treatment for patients with mitral stenosis, especially for symptomatic severe stenosis.

C. Aspirin antiplatelet-available in patients with mitral stenosis and atrial fibrillation but not preferred.

D. Nitrate coronary enlargement-mainly used for angina pectoris, not suitable for this case.

E. Digitalis-can be used for control of AF and heart failure but not fundamental treatment.

Because the patient has severe mitral stenosis, balloon dilatation is effective in improving symptoms and preventing thrombosis.

The best answer is: B. Balloon distension

Male, 42 years old. Abdominal distension was associated with fatigue for 2 days. Sudden chest pain for 5 hours. Previous history of hyperlipidemia for 2 years, untreated. Physical examination: P68 times / minute, BP120 / 78 mmHg, no abnormality in heart, lung and abdomen. Blood cTnT0.83mg/fl,D-DIMER:0.3g/L, ECG I, aVL, V5-V6 lead ST segment depression of 0.2 mv.

129, (Type A3 / A4 questions)

The examination that should be performed for a further definite diagnosis is

A. echocardiogram

B. coronary arteriongraphy

C. pulmonary artery CTA

D. aorta CTA

E. Cardiac MRI

Reference answer: B. Your answer:

[Analysis] middle-aged male patient, sudden chest pain for 5 hours (highly suspected of acute myocardial infarction), previous history of hyperlipidemia, electrocardiogram I, aVL, V5-V6 lead ST segment depression 0.2 mv, comprehensive patient symptoms, medical history and electrocardiogram performance were considered as acute myocardial infarction. Coronary angiography was the gold standard for the diagnosis of coronary heart disease (B pair).

[Error] Echocardiography is the examination of heart failure and cardiac organic lesions (A error); pulmonary artery CTA is the gold standard for pulmonary embolism (C error); aortic CTA is the gold standard for aortic stenosis (D error); and cardiac MRI is rare (E error).

129、

This 42-year-old male patient had a history of hyperlipidemia, untreated, and sudden chest pain. His elevated cTnT (0.83mg / fl) suggested myocardial injury and a slight increase in D-DIMER. The ECG showed ST segment depression, which are characteristic of the acute coronary syndrome. To further clarify the diagnosis, it should be selected:

B coronary arteriongraphy

This is the gold standard for determining coronary artery stenosis or occlusion that facilitates the diagnosis of acute myocardial infarction.

The correct answer is: C. Pulmonary artery CTA

Patients with sudden chest pain, electrocardiogram I, aVL, V5~V6 lead ST segment depression, blood cTnT0.83mg/fl,D-dimer:0.3g/L, considering the patient for pulmonary embolism, for further clear diagnosis should be examined is pulmonary artery CTA, can directly show the pulmonary artery thrombosis, pulmonary artery low density filling defect, partially or completely surrounded between opaque blood flow, or completely filling defect, distal vessels is not visible, the diagnostic accuracy is

high, sensitivity and specificity are good.

Option A echocardiography is A non-traumatic examination, which can reflect the cardiac structure and left heart function, but it has little diagnostic value for the diagnosis of pulmonary embolism, so it is not selected.

Option B coronary angiography was the gold standard for the diagnosis of coronary heart disease and could not be used for the diagnosis of pulmonary embolism, so it was excluded.

Option D Aortic CTA is mainly used for the diagnosis of aortic dissection and is not consistent with the patient, so it was not considered.

Option E cardiac MRI is the gold standard for evaluating the structure and function of the heart. It is of great value in the diagnosis of cardiomyopathy and cardiac tumor and pericardial disease, but it is of little significance to the diagnosis of pulmonary artery embolism, so it does not meet the meaning of the question.

129 The examination that should be performed for a further definite diagnosis is

The main symptom of the patient was sudden chest pain, combined with ECG ST depression and elevated serum cardiac troponin T, suggesting possible coronary problems. In addition, a previous history of hyperlipidemia, but untreated, further increased the risk of coronary artery disease.

Taking these factors into account, the most direct examination should be coronary angiography, because it directly shows the obstruction or stenosis of the coronary artery, helping to determine the presence of coronary atherosclerotic heart disease (e. g., acute myocardial infarction).

Best answer: B. coronary angiography

130, (Type A3 / A4 questions) subject

What diagnosis is being considered at present

A. dissection of aorta

B. acute myocardial infarction

C. Acute pulmonary artery infarction

D. acute myocarditis

E. acute pericarditis

Reference answer: B. Your answer:

[Analysis] middle-aged male patient, sudden chest pain for 5 hours (highly suspected of acute myocardial infarction), previous history of hyperlipidemia, electrocardiogram I, aVL, V5-V6 lead ST segment depression 0.2 mv, comprehensive patient symptoms, medical history and electrocardiogram performance is considered for acute myocardial infarction (B pair).

[Error] Aortic dissection is often manifested by sudden chest tear pain, often increased blood pressure of upper limbs, significant asymmetry of blood pressure and pulse on both limbs (A wrong); acute pulmonary infarction shows the triad of chest pain, hemoptysis, and dyspnea (C wrong); acute myocarditis has prodromal symptoms such as viral infection 1 to 3 weeks before the onset, most of which is arrhythmia or first symptom (D wrong); acute pericarditis electrocardiogram shows extensive elevation of ST segment beyond aVR lead (E wrong).

130, considering his elevated cTnT and ECG changes, the most likely diagnosis was: B. acute myocardial infarction

In this case, the electrocardiogram, the common ST segment changes and elevated myocardial injury markers, are the typical manifestations of acute myocardial infarction.

The correct answer is: C. Acute pulmonary artery infarction

The patient had sudden chest pain, ECG I, aVL, V 5 to V 6 lead ST depression, blood cTnT0.83mg/fl,D-dimer:0.3g/L, and was considered for acute pulmonary infarction. When pulmonary artery embolism, electrocardiogram can appear non-specific ST segment and T wave changes, most patients have hypoxemia, and when combined with right heart failure, corresponding clinical manifestations can appear. In patients with electrocardiogram lead ST segment depression, accompanied by weakness, all of which are right heart failure, so acute pulmonary infarction is considered.

Option A Aortic dissection is often manifested as sudden chest and back tear pain, with collapse, and arterial blood pressure on both limbs, which is not consistent with the symptoms of patients in this question, so it is not selected.

Option B Acute myocardial infarction generally has a history of angina pectoris,

which is mainly manifested as myocardial ischemia such as retrosternal agonal pain. ECG showed the characteristic and dynamic changes of myocardial infarction. The patient in this question had no angina pectoris, so it was excluded.

Option D Before the onset of acute myocarditis, there are more prodromal symptoms, such as fever, systemic burnout and muscle soreness, which have nothing to do with this question.

Option E Acute pericarditis is mainly manifested as retrosternal, precardiac area pain, pericardial friction sound, etc., which is inconsistent with this question.

130. What diagnoses are currently being considered

The main symptoms were chest pain with abdominal distension and fatigue, combined with the following clinical findings:

ECG: ST segment depression in lead I, aVL, and V5-V6

Serum myocardial troponin T increase (0.83 mg/L)

These features are highly suggestive of an acute coronary syndrome, especially of an acute myocardial infarction. Myocardial infarction usually causes ECG ST changes and elevated serum myocardial markers (such as cTnT).

The best answer: B. Acute myocardial infarction

Female, 20 years old, dry mouth, polydipsia, more urine for half a month. The daily urine volume was 7 to 8 L. Urinary routine prompt: blood glucose: 4.8 mmol/L, specific gravity: 1.007. There was no significant decrease in urine volume after the no-water test.305 mosm / L and urine osmotic pressure: 200 mosm / L; after intravenous vasopressin, urine volume decreased significantly; osmotic pressure: plasma: 300 mosm / L, and urine osmolality: 550 mosm / L.

131, (A3 / A4 type question) subject

Consider which of the following diseases

A. complete central diabetes insipidus

B. The presence of a partial central diabetes insipidus

C. Complete renal diabetes insipidus

D. partial renal diabetes insipidus

E. Neurosia

Reference answer: A. Your answer:

[Analysis] adolescent women, dry mouth, polydipsia, more urine for half a month, daily urine volume 7~8L low specific urine (typical symptoms of diabetes insipidus), no significant reduction after the water ban test. The vasopressin test identified renal diabetes insipidus and central diabetes insipidus (urine osmolarity increased after vasopressin injection without renal diabetes insipidus). After intravenous injection of desmopressin, urine volume was significantly reduced, with plasma osmotic pressure of 300 most m / L and urine osmotic pressure of 550 ost m / L (plasma osmotic pressure basically unchanged, urinary osmotic pressure increased) considering central diabetes insipidus, urine osmotic pressure increased by more than 50% in vasopressin test, and complete central diabetes insipidus (A pair) was considered.

[fault avoidance]

For question 131, the answer should be B. partial central diabetes insipidus. The patient responded to demaminated vasopressin, meaning that her kidneys could act on ADH, but the self-produced ADH may be insufficient, indicating a central diabetes insipidus. Since the symptoms are not completely lacking in ADH (because the treatment is effective), they are more consistent with some central diabetes insipidus.

A. Complete central diabetes insipidus.

This question examines the classification and diagnosis of diabetes insipidus.

Option A Complete central diabetes insipidus is due to A severe or partial deficiency of antidiuretic hormone (ADH), which causes dysfunction of renal tubular reabsorption of water, resulting in a group of syndromes characterized by polyuria, polydipsia and polydipsia and low specific gravity urine and hypotonolar urine. The patient had polyuria (daily urine volume of 7~8L), low urine proportion (1.007), no significant reduction after water prohibition test, which met the characteristic manifestation of complete central diabetes insipidus. After intravenous injection of demaminated vasopressin, the urine volume was significantly reduced and the urine osmotic pressure was significantly increased (550 mosm / L), which further verified the diagnosis and was the correct option.

Option B central diabetes insipidus shows mild polyuria, the urine volume can be

reduced after water prohibition, and the urine specific gravity and urine osmotic pressure can be increased, which is not consistent with the description of the problem stem.

Option C complete renal diabetes insipidus, option D part renal diabetes insipidus, are due to renal lesions, lead to tubular against diuretic hormone insensitive syndrome, its clinical characteristics similar to central diabetes insipidus, no response to exogenous ADH or weakened, but can reduce the urine volume, urine proportion increase, after injection vasopressin does not reduce the urine volume, urine proportion does not increase, with dry patients sensitive to vasopressin response, so excluded.

Option E Neurogenic hypodipsia is due to dysphoria and polydipsia caused by mental factors, which leads to polyuria, low specific gravity urine and low permeability urine. However, these symptoms can be alleviated by water restriction, which is not consistent with the significant reduction of urine volume after the water restriction test.

In conclusion, the correct answer is: A. complete central diabetes insipidus.

The patient was a 20-year-old woman with symptoms including dry mouth, polydipsia and polyuria with daily urine volume up to 7-8 liters and a course of half a month. Urinary routine indicated normal blood glucose (4.8 mmol/L) and low urine proportion (1.007). After the water-off test, the plasma osmotic pressure was 305 mosm / L and the urine osmolality was 200 mosm / L. After intravenous injection of desmopressin, urine volume was significantly reduced, measuring plasma osmolarity pressure of 300 mosm / L and urinary osmotic pressure of 550 mosm / L.

Diagnostic ideas

Type differentiation of diabetes insipidus:

Central diabetes insipidus (Central Diabetes Insipidus): caused by injury to the pituitary or hypothalamus, resulting in insufficient secretion of antidiuretic hormone (ADH).

Renal diabetes insipidus (Nephrogenic Diabetes Insipidus): inadequate response of the kidney to ADH.

Neuroprodirexia (Primary Polydipsia): mainly caused by excessive drinking water.

Results of water prohibition test and desmopressin test:

There was no significant reduction in urine volume after the water prohibition test, indicating that the patient could not concentrate the urine.

After intravenous injection of desmopressin, urine volume was significantly reduced and urine osmolality increased significantly (from 200 mosin / L to 550 mosin / L), indicating a kidney response to ADH.

The above results suggest that the patient may be central diabetes insipidus with decreased urine volume and significant increase in urinary osmolality pressure after desmopressin injection.

question answering

Consider which of the following diseases

A. complete central diabetes insipidus

132, (A3 / A4 type question) subject

The drug that should be preferred is

A. chlorpromazine

B. Chlorosulphonylurea

C. DCT

D. desmopressin

E. carbamazepine

Refer to the answer: D. Your answer:

[Analysis] adolescent women, dry mouth, polydipsia, polyurine for half a month, daily urine volume 7~8L low specific urine (typical symptoms of diabetes insipidus), no significant reduction after the water ban test. The vasopressin test identified renal diabetes insipidus and central diabetes insipidus (urinary osmolarity increased after vasopressin injection, essentially unchanged in renal diabetes insipidus). After intravenous injection of desmopressin, urine volume was significantly reduced, with

plasma osmolality of 300 mosm / L and urine osmotic pressure of 550 mosm / L (plasma osmotic pressure and urine osmotic pressure increased almost unchanged). Considering complete central diabetes insipidus, desmopressin was preferred (D pair) for treatment.

[Error avoidance] Chlorpromazine is A central dopamine receptor antagonist for schizophrenia, mania and other diseases (A wrong); chlorosulfobenzene

Urea may be used for renal diabetes insipidus, less effective than desmopressin (B wrong); hydrochlorothiazide is also effective for renal diabetes insipidus. Long-term use of hydrochlorothiazide may cause hypokalemia, hyperuricemia (C wrong); carbamazepine is an antiepileptic drug, often used for simple or complex partial seizures, original trigeminal neuralgia (E wrong).

For question 132, the preferred drug should be D. desmopressin because this drug can be used to treat central diabetes insipidus, a synthetic antidiuretic hormone that supplements ADH deficiency in the body, reduces urine volume and increases urine concentration.

The correct answer to the question 132 is: D. Desmopressin.

This question examines the treatment of diabetes insipidus.

Option A chlorpromazine can inhibit pituitary secretion prolactin, gonadotropin, growth hormone, melatonin and ACTH, also can inhibit the various parts of the central nervous system, especially some areas on the cerebral cortex has the function of the dopamine receptor, can produce sedative effect, but it is not directly act on the renal tubules, cannot enhance the reabsorption of water, so cannot treat diabetes insipidus, so excluded.

Option B chlorosulfodiphlurea can stimulate the release of antidiuretic hormone and enhance the effect of antidiuretic hormone on renal tubules, but the antidiuretic effect is less than desmopressin, which can only be used for partial diabetes insipidus, not good for complete central diabetes insipidus, so it is not selected.

Option C hydrochlorothiazide mainly inhibits the reabsorption of sodium chloride in the distal tubule and proximal tubule, thus increasing the Na-K exchange of the distal tubule and the collecting duct, increasing K secretion and play a diuretic effect, which is contrary to the principle of treating diabetes insipidus and inconsistent with the problem.

Option D Desmopressin is a synthetic antidiuretic hormone analogue, with

strong antidiuretic effect, but no pressure effect, and few adverse reactions. It is the first choice for the treatment of diabetes insipidus, which meets the meaning of the question.

Option E carbamazepine is a broad-spectrum antiepileptic drug, which mainly treats epilepsy, trigeminal neuralgia and other diseases, but has no obvious effect in the treatment of diabetes insipidus and can be ruled out.

In conclusion, the correct answer is: D. Desmopressin.

The drug that should be preferred is

H. Desmopressin (Desmopressin)

Male, 22 years old, with progressive systemic edema for 10 days, previously healthy. Physical examination: BP120 / 80mmHg, depressed edema of both lower limbs and face. Laboratory test: plasma albumin 20g / L; urine protein: urine sediment test: RBC: 8~10 / HP; blood Cr: 73 μmol/L; blood cholesterol: 8.2 mmol/L.

133, (A3 / A4 type question) subject

The diagnosis that may be considered in this patient is that of

A. Acute glomerulonephritis

B. nephrotic syndrome

C. Acute interstitial nephritis

D. Chronic glomerulonephritis

E. Acute-progressive glomerulonephritis

Reference answer: B. Your answer:

[Analysis] adolescent male patients with a large amount of proteinuria, urinary protein greater than 3.5 g/d, edema, ALB20g / L (normal albumin of 40~55g / L) decreased, hyperlipidemia, combined with the patient's history, clinical manifestations and examination, the most likely diagnosis of the patient is nephrotic syndrome (B pair).

[Error avoidance] The diagnostic criteria for nephrotic syndrome were: ① massive proteinuria (> 3.5 g/d); ② hypoalbuminemia (serum albumin less than 30g / L); ③ edema; ④ hyperlipidemia. The first two are prerequisites for diagnosis.

For question 133, regarding the possible diagnosis of the patient:

F. Nephrotic syndrome was the diagnosis most consistent with the description

because the patient had massive proteinuria, hypoalbuminemia, edema, and hyperlipidemia.

case analysis

A 22-year-old male patient was characterized by progressive systemic edema for 10 days with depressed edema in both lower limbs and face. Laboratory tests show:

Plasma albumin: 20 g / L (normal: 35-55 g / L)

Urinary protein: significantly positive

Urinary sediment: RBC 8-10 / HP (normal: 0-2 / HP)

Serum creatinine: 73 μmol/L (normal: 44-133 μ mol / L)

Blood cholesterol: 8.2 mmol/L (normal value: <5.2 mmol / L)

It can be seen from the above examination results that the patient had hypoproteinemia, severe proteinuria, hypercholesterolemia and edema, which was consistent with the typical manifestations of nephrotic syndrome.

trouble shooting

133, the possible diagnosis for this patient is

A. Acute glomerulonephritis

B. nephrotic syndrome

C. Acute interstitial nephritis

D. Chronic glomerulonephritis

E. Acute-progressive glomerulonephritis

Answer: B., nephrotic syndrome

134, (A3 / A4 type question) subject

Treatment that should not be selected for use is

A. salt restriction

B. A massive injection of albumin

C. diuresis

D. Post-biopsy for treatment

E. fluid infusion

Reference answer: B. Your answer:

[Analysis] adolescent male patients with a large amount of proteinuria, urinary protein greater than 3.5 g/d, edema, ALB20g / L (normal albumin of 40~55g / L) decreased, hyperlipidemia, combined with the patient's history, clinical manifestations and examination, the most likely diagnosis of the patient is nephrotic syndrome. Patients with nephrotic syndrome should be given a normal amount of high quality protein diet (B wrong, the correct answer to this question), intravenous supplementation of a large amount of protein will increase glomerular hyperfiltration, aggravate proteinuria and aggravate the disease, so it is not recommended.

[Error avoidance] The diagnostic criteria for nephrotic syndrome were: ① massive proteinuria (> 3.5 g/d); ② hypoalbuminemia (serum albumin less than 30g / L); ③ edema; ④ hyperlipidemia. The first two are prerequisites for diagnosis.

For question 134, for treatments that should not be used instead:

I. Fluhydration is usually not recommended for the management of patients with nephrotic syndrome, where edema is already present and may aggravate this condition.

First, we analyzed the medical history and laboratory findings provided by the patients.

In a young man with progressive systemic edema for 10 days, physical examination revealed bilateral lower limbs and facial sunken edema. Laboratory tests showed a significant decrease in plasma albumin (20g / L), massive albuminuria (> 3.5 g/d), hematuria (RBC: 8 to 10 / HP), and a significant increase in cholesterol (8.2 mmol/L). Based on this information, we can preliminarily judge the possible diagnosis of the patient.

The possible diagnosis considered in this patient are:

A. Acute glomerulonephritis: Although hematuria and albuminuria may occur, the levels of plasma albumin and cholesterol may not usually be so abnormal.

B. Nephrotic syndrome: It fits all of the main symptoms of the patient, including massive proteinuria, hypoalbuminemia, hyperlipidemia, and oedema.

C. Acute interstitial nephritis: the main manifestation is renal tubular function impairment, which is inconsistent with the patient's symptoms.

D. Chronic glomerulonephritis: usually with a long course and not necessarily accompanied by such significant hypoalbuminemia and hyperlipidemia.

E. Acute glomerulonephritis: usually a rapid deterioration of renal function inconsistent with the patient's course and laboratory tests.

Therefore, considering all the symptoms of the patient, the most likely diagnosis is nephrotic syndrome.

Treatment that should not be selected are:

A. Salt restriction: For patients with edema, salt restriction is a routine treatment measure and helps to reduce edema.

B. Injection of massive albumin: in nephrotic syndrome, due to hypoalbuminemia caused by massive proteinuria, albumin injection alone can not effectively solve the fundamental problem, and may bring a series of complications, such as allergic reaction, volume overload, etc.

C. Diuresis: For patients with edema, diuresis is a routine treatment to help reduce edema.

D. Post-biopsy therapy: Kidney biopsy is necessary to clarify a diagnosis and develop a more precise treatment plan.

E. rehydration: In some cases, proper rehydration is necessary in order to maintain hemodynamic stability.

Therefore, the injection of massive albumin is not an optional treatment.

To sum up, the correct answer is:

133. B. nephrotic syndrome

134 B. injection of albumin

ideas of solving a problem

Typical features of nephrotic syndrome include massive proteinuria (usually more than 3.5 grams per day), hypoalbuminemia (usually less than 30 g / L), systemic edema, and hypercholesterolemia. These characteristics were completely consistent with the patient's laboratory findings. Therefore, the most likely diagnosis is the nephrotic syndrome.

134. Treatment that should not be used selectively is

A. salt restriction

B. A massive injection of albumin

C. diuresis

D. Post-biopsy for treatment

E. fluid infusion

Answer: B. Large amount of injection of albumin

135. (Type B1 question)

Suitable for the Allis method of treatment is

A. Post dislocation of hip dislocation

B. posterior knee dislocation

C. The posterior dislocation of the elbow joint

D. Subluxation of the small radial head

E. anterior dislocation of shoulder

Reference answer: A. Your answer:

Graphic analysis: [Analysis] Allis method is suitable for posterior hip dislocation; anterior shoulder dislocation generally uses local infiltration anesthesia and reduction by Hippocrates method.

[fault avoidance]

Question 135: Suitable for the Allis method of treatment is

The Allis method is commonly used for the treatment of hip dislocation in infants, but in the options for the topic, the closest approach is the posterior elbow dislocation. However, the Allis trial is not usually used to treat dislocation but to examine infants for hip dysplasia. Therefore, we may need to consider that this is a trap issue. Usually, the Allis method is not used for the treatment of joint dislocation in adults. But if we have to choose from the given option, the posterior elbow dislocation may be the closest, although this is not a standard application.

This is a choice question about the treatment of joint dislocation. We need to determine which treatment is the most appropriate according to the type and location

of joint dislocation.

First, we will analyze question 135, which is which joint dislocation is suitable for Allis treatment.

A. posterior dislocation of the hip joint: The Allis method is A common technique for the treatment of posterior dislocation of the hip joint. This method reduces the dislocated hip joint through specific manipulation.

B. Rear knee dislocation: Rear knee dislocation is usually not treated with the Allis method.

C. Postposterior dislocation of elbow: posterior dislocation of elbow is usually reduced by other methods than Allis method.

D. Subluxation of the radial head: This dislocation is generally achieved by simple manipulation without the Allis method.

E. Anterior shoulder dislocation: anterior shoulder dislocation is often used by other reduction methods, such as Hippocrates maneuver.

Considering the above analysis, we can determine that the Allis method is most suitable for treating posterior hip dislocation, so the answer to question 135 is A. posterior hip dislocation.

Suitable for the Allis method of treatment is

A. Post dislocation of hip dislocation

analyze:

Allis method is mainly used to treat posterior dislocation of the hip. Through the patient's supine position, the doctor stands on the patient's side, bends the hip to 90 degrees, holds the patient's ankle or calf with both hands, and performs traction and rotation reduction.

136. (Type B1 question)

Suitable for Hippocrates technique reduction is

A. Post dislocation of hip dislocation

B. posterior knee dislocation

C. The posterior dislocation of the elbow joint

D. Subluxation of the small radial head

E. anterior dislocation of shoulder

Reference answer: E Your answer:

[Analysis] Allis method for posterior hip dislocation; anterior shoulder dislocation generally using local infiltration anesthesia, Hippocrates method reduction.

[Avoid mistakes] elbow dislocation can be used single reduction method.2% procaine or 1% lidocaine in 10ml elbow 1% or brachial plexus anesthesia. The surgeon stands in front of the patient, raises the patient's affected limb, and surrounds the surgeon's waist, so that the elbow joint is placed in half

flexed position. Hold the patient's wrist in one hand, make continuous traction along the longitudinal axis of the forearm, and the other thumb presses on the ulnar beak tip, also along

Keep the longitudinal axis of the forearm until reset. Subluxation of the radial head can be performed without anesthesia.art

The person holds the child's wrist in one hand, holds the elbow in the other hand, presses the thumb on the radial head, and makes the elbow flexion to 90°

Gentle forearm rotation, before rotation activity, repeated several times, and the thumb gently push the radial head can be reset.

Question 136: suitable for Hippocrates technique reduction is

Hippocrates The technique is mainly used for the reduction of the shoulder joint dislocation. This method is reset by applying traction and rotation to the shoulder joint. Therefore, an anterior shoulder dislocation is the most appropriate option for this issue.

Next, we analyzed question 136, which is which kind of joint dislocation is suitable for Hippocrates manipulation reduction.

A. Postposterior hip dislocation: Postposterior hip dislocation mainly uses Allis method rather than Hippocrates.

B. Rear knee dislocation: posterior knee dislocation is not suitable for Hippocrates maneuver.

C. Postdislocation of elbow: Postdislocation of elbow is generally reduced by other methods.

D. Subluxation of the radial head: This dislocation is generally reduced by simple manipulation without Hippocrates technique.

E. Anterior shoulder dislocation: Hippocrates maneuver is a common method in the treatment of anterior shoulder dislocation to reducing the shoulder dislocation through specific operations.

In conclusion, the Hippocrates maneuver is most suitable for the treatment of anterior shoulder dislocation, so the answer to question 136 is E. Shoulder anterior dislocation.

Suitable for Hippocrates technique reduction is

B. Front dislocation of the shoulder joint

analyze:

Hippocrates The maneuver is mainly used for the reduction of anterior shoulder dislocation. The patient lies flat, and the doctor holds the patient's wrist in one hand, lays one foot against the patient's armpit, and repositions the shoulder joint by stretching the arm and compressing the armpit.

137. (Type B1 question)

The examination of choice for the diagnosis of IgA nephropathy is

A. renal biopsy

B. renoarteriography

C. Kidney B ultrasound

D. kidney CT

E. pyelography

Reference answer: A. Your answer:

Graphic analysis: [analysis] IgA nephropathy diagnosis of kidney biopsy. Renal B-ultrasound is the first test method for identifying acute and chronic renal failure.

[fault avoidance]

137, the examination for the diagnosis of IgA nephropathy is

The diagnosis of IgA nephropathy (IgA nephropathy) usually depends on the examination of the kidney biopsy, as this allows for the direct observation of IgA deposits in the glomeruli. Therefore, the correct answer is: A. Kidney biopsy

First, for question 137, we need to determine which examination method is most suitable for the diagnosis of IgA nephropathy.

Option A: Renal biopsy is the gold standard for the diagnosis of kidney disease,

which is especially important for the diagnosis and differential diagnosis of glomerular disease. The diagnosis of IgA nephropathy depends on the histological examination of renal biopsy, because only pathological examination can confirm the diagnosis of IgA nephropathy.

Option B: Renal arteriography is mainly used to examine renal artery stenosis or obstructive disease, and it has little relationship with the diagnosis of IgA nephropathy.

Option C: Renal B ultrasound is mainly used to examine the morphology and structure of the kidney, but cannot confirm IgA nephropathy.

Option D: Renal CT can provide anatomical information of the kidney, but has limited diagnostic value for IgA nephropathy.

Option E: Pelography is mainly used to examine the morphology and function of the urinary tract and is not directly helpful in the diagnosis of IgA nephropathy.

In conclusion, the most critical method for the diagnosis of IgA kidney disease is kidney biopsy, so the answer to question 137 is A. kidney biopsy.

According to the medical knowledge and best practice guidelines, the following are the correct answers to the two test questions:

The tests for the diagnosis of IgA nephropathy are:

A. renal biopsy

Explanation: The diagnosis of IgA nephropathy usually requires a renal biopsy (kidney biopsy) to observe the glomerular deposition of IgA and other immune complexes.

138. (Type B1 question)

Examination method for identifying acute and chronic renal failure

A. renal biopsy

B. renoarteriography

C. Kidney B ultrasound

D. kidney CT

E. pyelography

Reference answer: C Your answer:

Graphic analysis: [analysis] IgA nephropathy diagnosis of kidney biopsy. Renal B-ultrasound is the first test method for identifying acute and chronic renal failure.

[Error avoidance] Renal artery angiography can accurately show the location, extent, extent of renal artery stenosis and collateral circulation formation of renal artery, which is a diagnosis

The "gold standard" for severed renal artery stenosis. Intravenous urography (IVU) can understand the lateral renal function, lesion degree and pattern

Around.

138. Examination method to identify acute and chronic kidney failure

The differentiation between acute and chronic renal failure is usually not accomplished by a single examination, but renal biopsy can provide direct tissue status of the kidney, helping to distinguish between acute or chronic injury. Although other imaging tests such as renal B ultrasound and renal CT can also provide useful information, renal biopsy is more accurate in determining the nature of renal failure. Therefore, the most relevant answer is: A. Kidney biopsy

Next, we analyzed question 138, regarding the differentiation between acute and chronic renal failure.

Option A: Renal biopsy can identify the pathological changes of the kidney, which is important to distinguish between acute and chronic renal failure. The renal pathological changes in acute renal failure are usually mild and reversible, while chronic renal failure has obvious irreversible changes such as glomerulosclerosis and tubulointerstitial fibrosis.

Option B: renal arteriography, Option C: renal B ultrasound, Option D: renal CT, option E: pyelography. These tests are mainly used to understand the morphology and structure of the kidney and have limited help in identifying acute and chronic renal failure.

Therefore, the answer to question 138 is A. kidney biopsy, because it is the most direct and effective method to distinguish between acute and chronic kidney failure.

The tests for identifying acute and chronic renal failure are:

A. renal biopsy

Explanation: Identification of acute and chronic renal failure requires renal

biopsy to assess pathological changes in kidney tissue to determine the nature and extent of kidney injury.

139. (Type B1 question)

The possible abnormality of coagulation factors in patients with prolonged fasting of intestinal fistula after intestinal resection is

A. Clotting factors and

B. Coagulation factors and the X

C. Coagulation factors and the X

D. Coagulation factors and V

E. Coagulation factors and

Reference answer: B. Your answer:

Graphic analysis: [Analysis] Patients with long-term fasting of intestinal fistula after intestinal resection have reduced small intestinal absorption of fat-soluble vitamin ADEK, coagulation factors and X and.

139、Patients with prolonged fasting after bowel resection may develop vitamin K deficiency because vitamin K is mainly synthesized by gut bacteria and is absorbed in fat. Vitamin K is required for the synthesis of several coagulation factors (including coagulation factors,, and X). Thus, in this case, coagulation factor abnormalities may be involved and X. So, the most appropriate options are B. clotting factor and X.

Question 139

The possible abnormalities of coagulation factors in patients with prolonged fasting of intestinal fistula after intestinal resection are:

Correct answer: B. coagulation factor and X

analyze:

Chronic fasting leads to vitamin K deficiency, an important component in the synthesis of coagulation factors (prothrombin),, and X. If long-term fasting, it is easy to lead to vitamin K deficiency, thus affecting the synthesis of these vitamin K-dependent coagulation factors, with coagulation factors and X as the main representatives.

[fault avoidance]

140. (Type B1 question)

The coagulation factors lacking in hemophilia patients are

A. Clotting factors and

B. Coagulation factors and the X

C. Coagulation factors and the X

D. Coagulation factors and V

E. Coagulation factors and

Reference answer: A. Your answer:

Graphic analysis: [Analysis] Hemophilia is a group of hemorrhagic diseases caused by the generation of genetic coagulation factors, the occurrence of which is the lack of coagulation factors and.

[Avoid avoidance] coagulation factor is tissue factor, coagulation factor V, and coagulation factor IV are all cofactors.

140、Hemophilia is a hereditary hemorrhagic disease that often involves a deficiency of clotting factors. Hemophilia A is caused by blood coagulation factor deficiency, while hemophilia B is caused by blood coagulation factor deficiency. Therefore, for hemophilia patients, the deficient coagulation factor is and. So, the most appropriate option for this problem is the A. coagulation factors and.

This is a multiple choice question on coagulation factors, designed to test candidates' understanding of coagulation factors and their related diseases.

First, we need to understand the characteristics of the individual coagulation factors and their role in the coagulation process.

Analysis of questions 139:19:

Patients with long-term fasting of intestinal fistula after intestinal resection, due to malnutrition, mainly affect the vitamin K-dependent coagulation factors.

Vitamin K-dependent coagulation factors include (prothrombin),, and X.

Among these factors, long-term fasting mainly affects the synthesis of coagulation factors (prothrombin),, and X.

However, only the coagulation factors and X were mentioned in the options, so B. The coagulation factors and X were selected as the best answers to the question.

Analysis of the following 140 questions:

Hemophilia is a genetic coagulopathy disorder that is mainly divided into hemophilia A and hemophilia B.

Hemophilia A is caused by the lack of a coagulation factor, while hemophilia B is a coagulation factor deficiency.

According to the question, the coagulation factor lacking in hemophilia patients should be and, so A. coagulation factor was chosen as the correct answer.

In summary, the answers to questions 139 were B. clotting factors and X, and the answers to questions 140 were A. clotting factors and.

So the correct answer is:

B. Coagulation factors and the X

A. Clotting factors and

Question 140

The clotting factors lacking in hemophilia patients are:

Correct answer: A. Clotting factors and

analyze:

Hemophilia is a genetic coagulation disorder that is mainly divided into hemophilia A and hemophilia B. Patients with hemophilia A lack a coagulation factor, while hemophilia B patients do not. Deficiency of both factors causes an obstruction of the coagulation process, which triggers a bleeding tendency. Therefore, the coagulation factors lacking in hemophilia patients are the coagulation factors and.

141. (Type B1 question)

Male, 35 years old, with fever and headache for 5 days, presented on January 5,

physical examination: T38.6℃, blood pressure 85 / 45mmHg, conjunctival congestion, eyelid edema, visible bleeding point under the axilla, WBC 17109 / L, PLT 55109 / L, urinary protein (+ + +), most importantly

A. dog

B. human being

C. sheep

D. rodent

E. hog

Refer to the answer: D. Your answer:

[Analysis] Young male, fever, headache, 85 / 45mmHg (hypotensive shock), conjunctival congestion, eyelid edema, axillary bleeding point (capillary injury), WBC 17109 / L (elevated white blood cells), PLT 55109 / L (platelet reduction), urinary protein (+ + +), according on clinical symptoms, signs and laboratory tests, diagnosed as hemorrhagic fever with renal syndrome, rats, rodents (D pair).

[Avoid error] Pig (E wrong) is the source of infection of epidemic Japanese encephalitis.

For the first question (141):

A 35-year-old male patient presented with fever, headache, decreased blood pressure, conjunctival congestion, eyelid edema, subcutaneous bleeding sites, elevated leukocytes (WBC 1710 ^ 9 / L), platelet reduction (PLT 5510 ^ 9 / L), and urinary protein (+ + +).

These symptoms and signs suggest that it may be caused by a disease transmitted from the animal, and given the acute onset and clinical presentation of the condition, hantavirus infection, which is often transmitted through rodents.

Therefore, the answer to question 141 is found to be D. rodentium.

This is an infectious disease problem, and we need to identify the possible source of infection based on the clinical manifestations, laboratory tests and epidemiological characteristics.

For question 1 (question 141):

The patient was a middle-aged male with fever, conjunctival congestion, eyelid edema, and bleeding sites, accompanied by hypotension, thrombocytopenia, and proteinuria, symptoms strongly suggestive of epidemic hemorrhagic fever. The main

source of epidemic hemorrhagic fever is rodents such as rodents. The virus carried by rodents can be transmitted to humans through their excreta, secretions, etc.

Topic 141:

A 35-year-old man with fever and headache for 5 days was examined on January 5: T38.6℃, blood pressure 85 / 45mmHg, conjunctival congestion, eyelid edema, bleeding points under the axilla, WBC 1710 ^ 9 / L, PLT 5510 ^ 9 / L, urinary protein (+ + +).

The characteristics of this case included fever, hypotension, bleeding points, thrombocytopenia, and urinary protein, which suggest the possibility of hemorrhagic fever. In China, the main pathogen causing hemorrhagic fever is hantavirus, while the main host of hantavirus is rodents. Thus, the most likely source of infection is for D. rodentium.

142. (Type B1 question)

Student, 16 years old, with fever and headache for 4 days, aggravated by vomiting and unconsciousness for 1 day after 2 days, was seen on August 10. Body temperature 39.8℃, blood pressure 140 / 90mmHg, shallow coma, neck resistance (+), Kirschliner's sign and Brinell's sign (+). Leukocytes 17.5 N0.75109 / L, CSF examination: CSF transparent, pressure 200cmH2O, nucleated cells 400106 / L, multinucleated cells 1.5106 / L, protein 0.8g / L, sugar 3.8 mmol/L, and chloride 120 mmol/L. The most likely source of infection is

A. dog

B. human being

C. sheep

D. rodent

E. hog

Reference answer: E Your answer:

[Analysis] Young patients, with high fever, headache, vomiting, and unconsciousness (initial symptoms of Japanese encephalitis), were treated on August 10 (high incidence of Japanese encephalitis in summer and autumn). Shallow coma, neck resistance (+), Kirschner's sign and Burner's sign (+), blood WBC 17.5109 / L (white blood cells increased), cerebrospinal fluid examination pressure increased,

WBC400109 / L (normal value 0~15109 / L), protein 0.8g / L (waist wear normal value 0.2~0.45g/L), sugar 3.8 mmol/L (lumbar pool normal

Value 2.5~4.5mmol/L), chloride 120 mmol/L (lumbar pool normal value 120 to 130 mmol/L), according to the patient clinically

Performance and laboratory examination, the most likely diagnosis of the patient was epidemic Japanese encephalitis, and the source of infection was mainly pigs (E pair).

[Error] Rodents are the source of hemorrhagic fever and plague in renal syndrome.

For the second question (142):

The 16-year-old student developed fever, headache, vomiting, unconsciousness, neck stiffness, positive Kirzi and Bruster's sign, increased leukocytes, cerebrospinal fluid examination showed increased cell count and slightly increased protein.

These symptoms and signs indicate that the patient is very likely to have bacterial meningitis. The source of bacterial meningitis is usually human, transmitted by droplets.

Therefore, the answer to question 142 is the B. human.

For the second question (question 142):

Adolescent patients, fever, headache, vomiting, consciousness disorders, accompanied by positive meningeal irritation, cerebrospinal fluid examination showed leukocytosis and mild elevated protein, these symptoms suggest Japanese encephalitis. Japanese encephalitis is mainly transmitted by mosquito bites, where JEV mainly comes from pigs. Pigs are the main reservoir of JEV, so pigs are the main source of infection of Japanese encephalitis.

In summary, questions 141 answered D. rodents and 142 answered E. pig.

Topic 142:

Student, 16 years old, with fever and headache for 4 days, aggravated by vomiting and unconsciousness for 1 day after 2 days, was seen on August 10. Body temperature 39.8℃, blood pressure 140 / 90mmHg, shallow coma, neck resistance (+), Kirschner's sign and Bruster's sign (+). Leukocytes 17.5 N0.7510 ^ 9 / L, CSF examination: CSF clear, pressure 200cmH2O, nucleated cells 40010 ^ 6 / L, multinucleated cells 1.510 ^ 6 / L, protein 0.8g / L, sugar 3.8 mmol/L, and chloride

120 mmol/L.

This case presented with symptoms of acute meningitis, with common pathogens including bacteria and viruses. Combined with epidemiological data, students in August, suggesting that may be viral meningitis, the common source of infection is human. Thus, the most likely source of infection is in B. humans.

143. (Type B1 question)

The role of furosemide is

A. Reduction of the ventricular preload

B. Reduce the ventricular afterload

C. Reduce the anterior and posterior ventricular load

D. Reducing the myocardial contractility

E. Increase in cardiac output

Reference answer: A. Your answer:

[Analysis] Furosemide is a high-efficiency loop diuretic, promoting the excretion of Na + and H2O, reducing blood volume, and reducing cardiac preload.

For problem 143, furosemide is a diuretic that is mainly used to treat oedema and hypertension. It acts by increasing the effects of renal sodium excretion and water, and therefore reduces blood volume, thereby reducing ventricular preload and afterload. Therefore, the most correct answer is the C. Reducing the anterior and posterior ventricular load.

This is a choice question about the mechanism of action. We need to accurately understand and analyze the pharmacological effects of furosemide and sodium nitroprusside, and then select the options that best match the characteristics of the respective drugs.

First, we analyzed the effects of furosemide.

Furosemide, also known as furosemide, is a highly potent loop diuretic. Its main effect is to inhibit the reabsorption of sodium and chloride by renal tubules, thus promoting urine production and the excretion of sodium, chloride and potassium plasma. This diuretic effect can decrease the blood volume, and subsequently reduce the preload in the ventricles. Therefore, the best option for the role of furosemide is A.

Reduce ventricular preload.

143 The role of furosemide is

Furosemide is a loop diuretic that increases the excretion of sodium, potassium, chlorine and water by inhibiting the Na-K-2 Cl isotropic transporter in the thick segment of the ascending branch of the kidney loop. It is mainly used for the treatment of acute pulmonary oedema, chronic heart failure, hypertension, and various types of oedema. Thus, it can reduce the preload in the heart.

Answer: A. Reduce the ventricular preload

144. (Type B1 question)

The effect of sodium nitroprusside is that

A. Reduction of the ventricular preload

B. Reduce the ventricular afterload

C. Reduce the anterior and posterior ventricular load

D. Reducing the myocardial contractility

E. Increase in cardiac output

Reference answer: C Your answer:

[Analysis] Furosemide is a high-efficiency loop diuretic, promoting the excretion of Na + and H2O, reducing blood volume, and reducing cardiac preload. Sodium nitroprusside reduces the anterior and posterior load of the left ventricle.

[Avoid mistakes] Nitroprusside is a balanced dilate blood vessels, dilate small arteries, so that peripheral vascular resistance is the heart afterload reduced and increase

Add cardiac output; dilate small veins, reduce return blood volume, reduce left ventricular end-diastolic pressure and reduce cardiac preload, that is

Reduce the left ventricular anterior and posterior load.

For problem 144, sodium nitroprusside is a vasodilator that causes vasodilation by direct action on vascular smooth muscle, mainly reducing the ventricular preload but may also reduce the afterload. Therefore, the most important effect of sodium nitroprusside is to reduce the ventricular preload, but given that it can also have some effect on the ventricular afterload, the most appropriate answer may be that C. reduces

the ventricular afterload. However, generally sodium noprusside is better known to reduce ventricular preload, so if the most important effect is needed, the answer should be A. Reducing ventricular preload.

Sodium nitroprusside is a balanced drug that dilates blood vessels and directly dilates the veins and arteries simultaneously, so it can reduce both the preload and afterload of the ventricles. Sodium nitroprusside does not increase the contractility of the heart muscle, but reduces the work burden of the heart and improves the heart function by reducing the anterior and posterior load of the heart. Therefore, for the question of "the role of sodium nitroprusside is", the most consistent option is for C. to reduce the ventricular anterior and posterior load.

144. The effect of sodium nitroprusside is that of

Sodium nitroprusside is a potent vasodilator that releases nitric oxide (NO) to directly relax the vascular smooth muscle, leading to the expansion of arteries and veins, thus reducing both the preload and afterload of the heart. It is mainly used for the treatment of acute heart failure and hypertensive crisis.

Answer: C. Reduce the ventricular anterior and posterior load

145. (Type B1 question)

A 76-year-old woman with dilated cardiomyopathy, persistent atrial fibrillation, chronic heart failure, started drinking 500ml of milk every day one week before increasing nutrition, and developed lower limb edema and shortness of breath 3 days ago. In addition to limiting the amount, the symptoms could be controlled in a short time

A. furosemide

B. aspirin

C. devil's brew

D. inderal

E. digoxin

Reference answer: A. Your answer:

[Analysis] Elderly women, dilated cardiomyopathy, persistent atrial fibrillation, chronic heart failure, lower limb edema and shortness of breath (considering too much

water), the drug that can control the symptoms in the short term is furosemide (A pair).

fault avoidance:

[fault avoidance]

Question 145

This case describes an elderly female patient with dilated cardiomyopathy, persistent atrial fibrillation, and chronic heart failure. Recent lower limb edema and shortness of breath due to increased milk intake. These symptoms indicate that her heart failure condition may worsen and require prompt intervention to control the symptoms. In the drug options given:

A. Furosemide (a diuretic) that quickly helps to relieve fluid overload and symptoms of heart failure, and is effective for oedema and shortness of breath caused by heart failure.

B. Aspirin, mainly used for antiplatelet purposes, is not applicable in this scenario.

C. Nitroglycerin, used for angina relief, reduces cardiac preload by dilating blood vessels and, while helpful in the management of heart failure, can less directly control acute symptoms than diuretics.

D. Propranolol, a β blocker, for heart rate control and caution for heart failure, especially with low blood pressure.

E. Digoxin, used to enhance cardiac contractility and control ventricular rate, but less directly diuretic for acute edema and shortness of breath.

Therefore, for question 145, the most appropriate answer is A. furosemide.

145 Question Analysis:

Elderly female patient, with dilated cardiomyopathy, persistent atrial fibrillation, and chronic heart failure. The patient began to drink 500ml of milk a day a week ago, and developed lower limb edema and shortness of breath 3 days ago. Considering the worsening symptoms of heart failure, diuretics should be given short-term symptomatic treatment. Option, furosemide is a loop diuretic that can effectively reduce fluid retention and quickly relieve symptoms of heart failure in the short term.

Option B aspirin is an antiplatelet aggregation drug and is not effective in the treatment of heart failure, so it is excluded.

Option C nitroglycerin is mainly used for the treatment of angina pectoris, can also be used for hypertension crisis and heart failure, but its therapeutic effect on heart failure is weak, the effect is slow, is not the preferred treatment of heart failure, so it is not selected.

Option D Propranolol is a β -blocker, which can reduce heart rate, reduce myocardial oxygen consumption, improve myocardial ischemia, reduce angina attacks, and long-term application can significantly reduce cardiovascular events such as death, but it is not recommended in acute heart failure and can be ruled out.

Option E digoxin is a digitalis drug, which is most suitable for atrial fibrillation with rapid ventricular rate and left ventricular systolic dysfunction, while patients with atrial fibrillation have unknown ventricular rate, so digoxin is not the best option.

In conclusion, the correct answer is: A. Furosemide.

Based on the description of the question and the patient, here are the best answers to each question:

Description: A 76-year-old female with dilated cardiomyopathy, persistent atrial fibrillation, and chronic heart failure. Lower extremity edema and shortness of breath after increasing milk intake, in addition to limited intake, can short-term control symptoms?

Best answer: A. Furosemide

Explanation: The patient had symptoms of heart failure (such as lower extremity edema and shortness of breath), and furosemide was a powerful diuretic that quickly reduced fluid retention symptoms.

146. (Type B1 question)

A 76-year-old woman with dilated cardiomyopathy, persistent atrial fibrillation, low blood pressure after β blocker but still fast ventricular rate should be added to control ventricular rate

A. furosemide

B. aspirin

C. devil's brew

D. inderal

E. digoxin

Reference answer: E Your answer:

[Analysis] Blood pressure is low after administration of β -blockers, but the ventricular rate is still fast. The drug that should be added is digoxin (E pair), and digitalis are mainly used after β -blockers, especially to slow down the ventricular rate in patients with atrial fibrillation.

[fault avoidance]

Question 146

Description for the same patients with low BP but still faster ventricular rate after administration of β blockers (e. g. propranolol). Need drug that can control ventricular rate but has less effect on blood pressure. In the options given:

A. Furosemide, a diuretic, is not applicable here.

B. Aspirin, an antiplatelet agent, which does not affect the heart rate.

C. Nitroglycerin, mainly used for angina pectoris, has limited effect on heart rate control.

D. Propranolol, already used and resulting in lower blood pressure, is not suitable for further dose escalation.

E. Digoxin, which can control the ventricular rate with little effect on blood pressure, is especially suitable for the ventricular rate control in patients with atrial fibrillation.

Therefore, for question 146, the most appropriate answer is E. digoxin.

146 Question Analysis:

The elderly female patient with dilated cardiomyopathy and persistent atrial fibrillation had low blood pressure after the application of β -blocker, but the ventricular rate was still too fast, so the drugs with less influence on blood pressure should be selected to control the ventricular rate. Option, digoxin is a digitalis drug, which can enhance myocardial contractility, and the treatment of atrial fibrillation can slow down the ventricular rate of patients with atrial fibrillation, especially for patients with rapid ventricular rate, and has little impact on blood pressure, which is in line with the question.

Option A Furosemide is A diuretic, mainly used for the symptomatic treatment of heart failure, and has no effect on the control of the ventricular rate, so it is not

selected.

Option B aspirin is an antiplatelet aggregation drug that is not helpful to the control of the ventricular rate and can be ruled out.

Option C nitroglycerin is mainly used for the treatment of angina, but also for hypertensive crisis and heart failure, but the control of ventricular rate is weak and is not preferred and therefore excluded.

Option D Propranolol is a β blocker, although it can reduce the heart rate, it is not appropriate due to the low treatment, so it is not chosen.

To sum up, the correct answer is: E. Digoxin.

Description: A 76-year-old woman with dilated cardiomyopathy, persistent atrial fibrillation, low blood pressure after β blocker but still fast ventricular rate, should be added to control the ventricular rate?

Best answer: E. Digoxin

Explanation: Digoxin is a cardiotonic and antiarrhythmic drug that effectively controls the ventricular rate in patients with atrial fibrillation, especially for those with already low blood pressure, because it has less impact on blood pressure.

147. (Type B1 question)

Male, 37 years old, with increased blood pressure for more than 5 years, plain blood pressure of 160~180 / 100~120mmHg, without diagnosis and treatment. Was obese, with a history of snoring. Half a year ago, low-fat diet control and drug treatment began under the guidance of doctors. Irbesartan tablets were taken twice and torasemil 5mg. After three months of treatment, blood pressure was controlled at 150~160 / 90~100mmHg, and the patient was

A. Hypertensive emergency

B. secondary hypertension

C. In treatment-resistant hypertension

D. hypertensive crisis

E. malignant hypertension

Reference answer: C Your answer:

[Analysis] Young and middle-aged men, plain blood pressure 160-180 / 100~120mmHg, without treatment, after taking nifedipine controlled release tablets

(calcium channel blocker), irbesartan tablets (angiotensin receptor antagonist), tolasemide (diuretic), blood pressure was controlled at 150-160 / 160 / 90~100mmHg (uncomplicated, blood pressure target value 140 / 90mmHg), according to the patient's medical history, medication regimen and blood pressure control, considered as refractory hypertension (C pair).

[Avoid avoidance] The most prominent manifestation of hypertension (A mistake) is diastolic blood pressure 130mmHg in A short time, combined with renal damage.continue

Primary hypertension (B wrong) is the most common hypertension caused by kidney disease, first with kidney disease, then hypertension. High blood

Pressure crisis (D wrong) include hypertensive emergencies and sub-emergencies. The condition deteriorated dramatically, accompanied by a progressive heart, brain, kidney, and

The manifestation of important target organ dysfunction, such as the retina. Malignant hypertension (E wrong) is also known as acute hypertension, the onset of disease

Sudden, typical manifestations are a significant increase in blood pressure, diastolic blood pressure mostly continues at 130 to 140 mmHg or higher, and headache, blurred vision and optic disc edema and other symptoms, and mostly in young and middle-aged people.

For question 147, this 37-year-old man had a long history of untreated hypertension with obesity and snoring symptoms, possibly suggestive of sleep apnea. He had decreased but still higher blood pressure after starting a low-fat diet and medication. This suggests that his hypertension was not easily controlled but without acute life-threatening symptoms. Therefore, the most appropriate option is C. resistant hypertension, which refers to the difficulty of BP to reach target levels despite appropriate treatment.

C. Resistant hypertension.

A middle-aged male patient with elevated blood pressure for many years, an obese patient with a history of snoring, should be highly suspected of having sleep apnea hypopnea syndrome, which is a common cause of resistant hypertension. After the combination of three sufficient amounts of antihypertensive drugs (including diuretics) with different mechanisms of action, blood pressure is still above the target

level, or when at least four drugs are needed to meet the standard, it is called resistant hypertension (or refractory hypertension). In this question, the patient was obese, had a history of snoring, and his blood pressure was still higher than normal after low-fat diet control, irbesartan and torasemil, which should be considered as resistant hypertension.

Option A Hypertension emergency refers to patients with primary or secondary hypertension, whose blood pressure increases suddenly and significantly (generally over 180 / 120mmHg), accompanied by progressive heart, brain, kidney and other important target organ dysfunction. Patients do not show target organ insufficiency, so it is not selected.

Option B Secondary hypertension refers to hypertension caused by specific causes, such as renal substantial hypertension, renal artery stenosis, primary aldosteronism, etc. The patient in this question does not have the cause of secondary hypertension, so it is not considered.

Option D Hypertension crisis is a kind of hypertension emergency, which refers to a sharp increase in blood pressure in a short period of time, headache, dizziness, irritability, nausea, vomiting, palpitation, shortness and blurred vision, as well as arterial spasm and ischemia in target organ symptoms, so it were excluded.

Option E Malignant hypertension refers to the sudden development of the patient's condition, diastolic blood pressure lasting 130mmHg, headache, blurred vision, fundus hemorrhage, exudation and papilloedema, prominent renal damage, persistent proteinuria, hematuria and tubular uria. The patient did not see the above symptoms, so he is not selected.

The first question:

The 37-year-old patient, with elevated blood pressure for 5 years, and plain BP at 160180 / 100120mmHg, was obese with a history of snoring. After three months of treatment, the blood pressure was controlled at 150160 / 90100mmHg.

key point:

Blood pressure increased for 5 years without medical treatment.

Obobese with history of snoring (suggesting the possibility of obstructive sleep

apnea syndrome).

After medication, the blood pressure failed to fall to the normal range.

Answer analysis:

A. Hypertension emergency: requiring immediate blood pressure reduction to prevent target organ damage, not consistent.

B. Secondary hypertension: It may be associated with obstructive sleep apnea due to a history of obesity and snoring, but it does not provide conclusive evidence for secondary hypertension.

C. Treatment with resistant hypertension: After a reasonable lifestyle and drug treatment, the blood pressure is not controlled within the normal range, which meets the definition of resistant hypertension.

D. Hypertension crisis: requiring immediate blood pressure reduction, not consistent.

E. Malignant hypertension: hypertension requiring immediate management, not consistent with it.

The most correct answer is: C. resistant hypertension

148. (Type B1 question)

A 56-year-old male with elevated blood pressure for 3 years and dyspnea for 1 week and aggravated for 1 day. The patient had no wheezing during activity 1 week ago, developed dyspnea, orthopnea, with sweating, and a family history of hypertension. Physical examination: P: 100 times / BP230 / 150mmHg, small and medium blisters in both lungs, heart rate 100 times / minute, rhythm, no noise in all valves, no swelling in both lower limbs. The ECG suggested left ventricular hypertrophy and strain. The current condition of this patient was

A. Hypertensive emergency

B. hypertensive suburgency

C. of resistant hypertension

D. hypertensive crisis

E. malignant hypertension

Reference answer: A. Your answer:

[Analysis] The patient with a history of hypertension, suddenly found elevated blood pressure, accompanied by cardiac insufficiency, should be considered as hypertensive emergency (A pair). Hypertensive emergency refers to patients with primary or secondary hypertension, with a sudden and significant increase in blood pressure (generally over 180 / 120mmHg) associated with important target organ damage.

[Misavoidance] Hypertension subemergency (B fault) refers to a significant increase in blood pressure, but without damage to important target organs.malignant hypertension

(E error) refers to the diastolic pressure of 130mmHg, headache, blurred vision, fundus exudation, especially prominent renal damage.

Infracant hypertension (C wrong) refers to the combination of more than three appropriate doses of antihypertensive drugs (generally should include diuresis

Agent) The blood pressure still missed its target level. Hypertensive crisis (D wrong) includes hypertensive emergency and subemergency.

For question 148, the 56-year-old man developed severe hypertension (230 / 150mmHg), associated with dyspnea, orthopnea, and sweating, which were signs of acute conditions. Physical examination revealed small blister sounds in both lungs, and the electrocardiogram showed left ventricular hypertrophy and strain, which are all indicative of possible impaired heart function and fluid accumulation in the lungs. Therefore, the most appropriate answer is A. hypertensive emergency, a condition that requires immediate treatment to prevent further organ damage.

148 The correct answer is: A. Hypertension emergency.

The patient was an elderly male with elevated blood pressure for many years, with dyspnea, asthma, orthopnea, sweating, physical examination, elevated blood pressure, small and small blisters in both lungs, electrocardiogram indicating left ventricular hypertrophy and strain, according to his symptoms and signs. Hypertensive emergency refers to the sudden and significant increase in blood pressure (generally> 180 / 120mmHg) under the action of some causes, accompanied by progressive dysfunction of important target organs such as heart, brain and kidney. The patient in this question had cardiac insufficiency and blood pressure increased sharply, so he was diagnosed as hypertensive urgency.

Option B Hypertension sub-emergency refers to a significant increase in blood

pressure without target organ damage. The patient in this question has obvious cardiac insufficiency, which is inconsistent with the meaning of the question.

Option C resistant hypertension refers to that the patient has applied above 3 antihypertensive drugs (including diuretics) before the blood pressure is still above the target level, or at least 4 antihypertensive drugs are needed to meet the blood pressure. The drug situation of the patient is not mentioned in this question, so it is not selected.

Option D Hypertension crisis is a kind of hypertension emergency, which refers to a sharp increase in blood pressure in a short period of time, including headache, dizziness, irritability, nausea, vomiting, palpitations, shortness of breath and blurred vision, as well as arterial spasm and target organ ischemia symptoms. Patients did not see the above symptoms, so they were excluded.

Option E Malignant hypertension refers to the patient's sudden development, diastolic blood pressure lasting 130mmHg, headache, blurred vision, fundus hemorrhage, exudation and papilloedema, prominent renal damage, persistent proteinuria, hematuria and tubular uria. The patient did not see the above symptoms, which is inconsistent with the meaning of the question.

The second question:

The 56-year-old patient had elevated blood pressure for 3 years, dyspnea for 1 week, aggravated for 1 day, P: 100 times / BP230 / 150mmHg, audible in both lungs and medium-sized blister sounds, and electrocardiogram suggested left ventricular hypertrophy and strain.

key point:

Blood pressure is very high: 230 / 150 mmHg.

Dyspnea, orthopnea, and small blisters in both lungs (suggesting acute pulmonary edema).

The ECG suggested a left ventricular hypertrophy and strain.

Answer analysis:

A. Hypertensive emergency: A hypertensive emergency (a part of the hypertensive crisis), including the acute organ damage caused by hypertension, which meets the symptoms.

B. Hypertension suburgency: Hypertension suburgency refers to a significant increase in blood pressure without immediate organ damage that does not meet the symptoms.

C. Stubborn hypertension: mainly refers to hypertension that cannot be controlled by long-term treatment and does not meet the symptoms.

D. Hypertension crisis: a part of hypertension emergency, which refers to the urgent need for blood pressure reduction to prevent target organ damage, which meets the symptoms.

E. Malignant hypertension: It may also apply, but acute pulmonary edema suggests urgency.

The most correct answer is: A. Hypertension and emergency

149. (Type B1 question)

The patient was prostate-specific antigen negative and pathological biopsy revealed prostate cancer lesions after transurethral prostatectomy, the possible staging being

A. T1 stage

B. T2 stage

C. T3a phase

D. T3b phase

E. T4 phase

Reference answer: A. Your answer:

Graphic analysis: [analysis] The patient was negative for prostate specific antigen, and prostate cancer was found by pathological biopsy after transurethral resection of the prostate gland, which belongs to T1 stage.

[Error] TNM stage of prostate cancer, T1: only occasional small lesions in surgical specimens; T2 lesions confined in the capsule;

T3a lesions breach the prostate capsule; T3b lesions invade the seminal vesicle; T4 distant metastasis to the rectum and bladder neck.

For question 149, the patient was negative for prostate-specific antigen (PSA), but found prostate cancer on pathological biopsy after transurethral resection of the prostate (TURP). In this case, cancer is found detected and usually confined within the prostate, and therefore the most likely stage is T1. T1 is a cancer where a tumor cannot be detected by visual inspection or imaging, and is only accidentally detected in prostate sections or during surgery. So, the answer to question 149 should be the A.T1 issue.

analyze:

Prostate cancer stage is mainly divided into clinical stages and pathological stages. Clinical staging is to classify prostate cancer into T1, T2, T3 and T4 based on the results of DRE, PSA, number of positive needle and site of needle biopsy, transrectal ultrasound, bone scan, and classification of T 1, T 2, T 3 or CT findings. The pathological stage is staged after the pathological specimen examination of radical prostate cancer. If prostate cancer does not invade one lobe, T2a stage; if it has invaded, but still confined to the prostate, it is T2b stage; if it has pierced the prostate lobe or capsule, T3a stage; if it has invaded the seminal vesicle, it is T3b stage; if it has invaded and other adjacent tissues except the seminal vesicle, and the cancer cells have penetrated the prostate wall, including bladder neck, levator muscle and pelvic wall, then T4 stage. The patient was negative for prostate specific antigen, and pathological biopsy after transurethral prostatectomy, indicating that the tumor was confined within the prostate lobe and was stage T1.

Option B: In T2, the cancer tissue is still confined to the prostate, but it has pierced the capsule of the prostate, so it is not selected.

Option C: T3a index carcinoma has invaded the prostate capsule of one lobe and perforated the capsule of one or two leaves, but was still confined to the prostate, so it was excluded.

Option D: T3b index cancer tissue has invaded the seminal vesicle, but it is not mentioned in this question, so it is not selected.

Option E: T4 stage cancer tissue has penetrated the prostate wall and invaded the adjacent tissue around the prostate gland, but it was not mentioned in this topic, so it was excluded.

To sum up, the correct answer is: A.T1 issue.

Question 149

The patient was negative for prostate-specific antigen (PSA), and the pathological biopsy revealed prostate cancer lesions after transurethral resection of the prostate, with the possible stages:

A. T1 stage

B. T2 stage

C. T3a phase

D. T3b phase

E. T4 phase

Prostate cancer is detected after transurethral resection of the prostate (TURP) with no symptoms or a physical detected prostate mass, which usually corresponds to T1 prostate cancer. T1 prostate cancer is a cancer detected incidentally by surgical removal of other tissue or sections.

Answer: A.T1 issue

150, (Type B1 question)

After radical prostate cancer surgery, the pathological report broke through the capsule and did not invade the seminal vesicle, the most likely stage was

A. T1 stage

B. T2 stage

C. T3a phase

D. T3b phase

E. T4 phase

Reference answer: C Your answer:

Graphic analysis: [Analysis] After radical prostate cancer, the pathological report broke through the two capsule and did not invade the seminal vesicle, and the most likely stage was T3a.

[fault avoidance]

For question 150, the pathology report after radical prostatectomy showed that

the tumor breakthrough the capsule but did not invade the seminal vesicle. In this case, the most likely staging is stage T3a. T3a refers to the tumor has broken through the prostate capsule, but has not invaded the seminal vesicle. So, the answer to question 150 should be in issue C.T3a.

In the pathological stage of prostate cancer, T3 is divided into T3a and T3b. T3a has invaded the prostate capsule and perforates one or both leaves, but still confined in the prostate; T3b has invaded the seminal vesicle. After radical prostatectomy, the pathological report broke through the two leaf capsule and did not invade the seminal vesicle, indicating that the cancer tissue had penetrated one or two leaf capsule, but was still confined to the prostate, with T3a stage.

Option A: T1 index cancer tissue is confined to the prostate lobe, and PSA levels are increased or even normal, unrelated to this question.

Option B: T2 Cancer tissue is confined to the prostate, but has perforated one or two leaflets and can be excluded.

Option D: T3b cancer tissue has invaded the seminal vesicle, but not mentioned in this question, may not be considered.

Option E: T4: The cancer tissue has penetrated the prostate wall and invaded the adjacent tissue around the prostate, but it is not mentioned in this topic and can be excluded.

To sum up, the correct answer is: C.T3a stage.

Question 150

After radical prostate cancer, the pathological report broke through the capsule without invading the seminal vesicle, and the most likely stage was:

A. T1 stage

B. T2 stage

C. T3a phase

D. T3b phase

E. T4 phase

Pathological reports show that the cancer did breach the prostate capsule but did not invade the seminal vesicle, a condition that usually corresponds to stage T3a

prostate cancer. The T3a stage is a cancer extending beyond the prostate capsule but not invading the seminal vesicle.

Answer: C.T3a period

2023 Unit 3

1. (Type A1 question)

The depression self-rating scale is a

A. SDS

B. SAS

C. MHT

D. BDI

E. HAMA

Reference answer: A. Your answer:

[Analysis] Depression self-evaluation scale (SDS) can quite intuitively reflect the subjective feeling and severity of depression in patients, so this question is selected A.

[Misavoidance] Anxiety Self-assessment Scale (SAS) measures the presence of anxiety symptoms and their severity (B error); mental health of middle school students

Diagnostic test (MHT), also known as the general anxiety test for primary and secondary school students, is applicable to the standardized test for primary and

secondary school students in China

Test (C error); BDI is Beck Depression questionnaire (D error); HAMA is Hamilton Anxiety Scale (E error).

The self-rating scale for depression usually refers to the Beck self-rating scale for depression (Beck Depression Inventory, BDI). Therefore, the correct answer is * * D. BDI * *. Other options, such as the SDS is the self-rated depression Scale (Self-Rating Depression Scale), the SAS is the self-rated anxiety Scale (Self-Rating Anxiety Scale), and the HAMA refers to the Hamilton Anxiety Scale (Hamilton Anxiety Scale), which are not entirely consistent with the self-rated depression scale.

This is a topic about the psychological assessment scale. We need to find the correct abbreviations representing the self-rating scale for depression from the options provided.

A. SDS: The Depression Self-rating Scale (Self-rating Depression Scale), a commonly used self-rating scale used to assess the degree of depression in individuals. It contains 20 items, each scored by level 1-4, with 10 positive and 10 reverse scores. This is a common tool to assessing depressive status.

B. SAS: Anxiety Self-Scale (Self-Rating Anxiety Scale), which is used to assess individual anxiety rather than depression.

C. MHT: Multiple Personality Traits Questionnaire (Multiple Human Trait Test), which is used to assess multiple personality traits and not specifically for depressive status.

D. BDI: The Beck Depression Scale (Beck Depression Inventory), which is also used to assess depression, is abbreviated not as BDI but as BDI-II (second edition). In addition, the depression self-rating scale, which is abbreviated as SDS.

E. HAMA: The Hamilton Anxiety Scale (Hamilton Anxiety Rating Scale), which is mainly used to assess anxiety symptoms, not for depression.

In conclusion, the correct abbreviation of the depression self-rating scale is the SDS.

Therefore, the correct answer is the A. SDS.

The depression self-rating scale is a

A. SDS (Self-Rating Depression Scale, Self-rated Depression Scale)

Therefore, the correct answer is * * A. SDS * *.

2. (Type A1 question)

To diagnose the clinical staging of gastric cancer patients, the preferred means of examination is

A. PET-CT

B. Abdominal-enhanced CT

C. laparoscopic approach

D. Upper gastrointestinal X-ray barium contrast

E. endoscopic ultrasonography

Reference answer: B. Your answer:

[Analysis] In order to diagnose the clinical stage of gastric cancer patients, the preferred means of examination is abdominal enhanced CT (B pair).

[Wrong avoidance] PET-CT (A error) is expensive and generally not the preferred test; laparoscopic exploration (C error) is surgical treatment

Method; Upper gastrointestinal X-ray barium contrast (D error) has been largely replaced by endoscopy and is currently only used for internal use

With scopy or endoscopy; endoscopic ultrasound (E error) is the main means of confirming gastric cancer and can be confirmed by endoscopic ultrasound

Determine the depth of tumor invasion.

In order to diagnose the clinical staging of gastric cancer patients, the best examination method is endoscopic ultrasound (E). Endoscopic ultrasound (EUS) enables a detailed assessment of the tumor depth and invasion of surrounding structures, and is also very useful for the assessment of local lymph nodes. This makes EUS the preferred means of examination in the clinical staging of gastric cancer. Although PET-CT and abdominal-enhanced CT are valuable in assessing distant metastasis, endoscopic ultrasound is more precise in determining local invasion and staging of tumors. Therefore, the choice of E (endoscopic ultrasound) is the most appropriate.

analyze:

The clinical stage diagnosis of GC requires a comprehensive evaluation of the depth of tumor infiltration into the gastric wall, local lymph nodes and distant

metastasis. Options to gastric cancer infiltration depth, local lymph nodes and adjacent organs directly assessment is endoscopic ultrasound, can clearly show the structure of the stomach wall and ultrasound images of adjacent organs, help to determine gastric cancer infiltration depth, abnormal enlarged lymph nodes and tumor infiltration of the stomach wall, is a routine examination means of gastric cancer TNM stage.

Option A: PET-CT is mostly used to detect distant metastasis of gastric cancer, and has limited role in judging the depth of tumor invasion and local lymph nodes, so it is not selected.

Option B: Abdominal enhanced CT is mostly used for preoperative clinical staging, which is helpful to determine the location of the tumor, the relationship with the surrounding organs, lymph node metastasis and the disease stage, but the accuracy is not as good as endoscopic ultrasound, so it was excluded.

Option C: Laparoscopic exploration is an invasive examination, which can directly observe the situation of abdominal organs and lymph nodes metastasis, and the biopsy of suspicious lesions can be taken under direct vision to obtain pathological evidence, but because of its invasive, it is generally not the first choice, so it is not considered.

Option D: X-ray barium contrast examination of the upper digestive tract is of certain value in evaluating the scope of gastric cancer, local gastric cavity stenosis and gastric wall stiffness, but it cannot accurately assess the depth of invasion and local lymph nodes of gastric cancer, so it does not conform to the question.

In conclusion, the correct answer is: E. endoscopic ultrasound.

To diagnose the clinical staging of gastric cancer patients, the preferred examination methods are:

B endoscopic ultrasonography

Endoscopic ultrasound (EUS) can provide detailed information of the gastric wall hierarchy and the status of the surrounding lymph nodes and organs, which is very important for the evaluation of local tumor infiltration and regional lymph node metastasis, so it is of important value in the clinical staging of gastric cancer.

2. (Type A1 question)

The type of lung cancer that causes abnormal vasopressin secretion is

A. carcinoma sareomatodes

B. small cell carcinoma

C. large cell carcinoma

D. adenocarcinoma

E. SC

Reference answer: B. Your answer:

[Analysis] Small cell lung cancer is a kind of neuroendocrine tumor, with endocrine function, can secrete antidiuretic hormone (B pair), catecholamines, histamine and other substances.

[Away] Sarcomatoid cancer (A wrong) is A type of lung cancer, but not common. Large cell carcinoma (C wrong) occurs in the large bronchus,

Often large, highly malignant, rapid growth, early and extensive metastasis; adenocarcinoma (D wrong) is the most common lung cancer in women

Type; Squamous cell carcinoma (E error) is the most common type in lung cancer.

The type of lung cancer that causes abnormal antidiuretic hormone secretion (i. e., the syndrome) is usually small cell lung cancer (Small Cell Lung Cancer, SCLC). So, the most correct answer is:

F. small cell carcinoma

Small cell lung cancer is known for its endocrine activity, which may lead to a state called "increased secretion of appropriate antidiuretic hormone" (SIADH), in which excessive secretion of antidiuretic hormone (ADH) in the body leads to increased water reabsorption and hemodilution hyponatremia.

analyze:

Lung cancer is a malignant tumor originating from the bronchial mucosa or glands of the lungs. Lung cancer can be classified by histology into squamous cell carcinoma, adenocarcinoma, adenosquamous carcinoma, small cell carcinoma, large cell carcinoma, sarcomatoid carcinoma, carcinoid and salivary gland type carcinoma and unclassified carcinoma. Among them, patients with small cell lung cancer are

often accompanied by endocrine abnormalities or carcinoid syndrome, which can secrete serotonin, causing asthma-like bronchospasm, paroxysmal tachycardia, watery diarrhea and skin flushing, etc., and can also secrete antidiuretic hormone, leading to hyponatremia.

Option A sarcomatoid carcinoma, C large cell carcinoma, D adenocarcinoma, and E squamous cell carcinoma, which do not secrete antidiuretic hormone, so it can be excluded together.

To sum up, the correct answer is: B. Small cell carcinoma.

The types of lung cancer that cause abnormal antidiuretic hormone secretion are:

G. small cell carcinoma

3. (Type A1 question)

The most central feature of chronic obstructive pulmonary disease is that

A. Long-term history of heavy smoking

B. Continuously limited airflow

C. Repeated episodes of season-related symptoms

D. Chronic cough and sputum symptoms

E. Small airway dysfunction

Reference answer: B. Your answer:

Graphic analysis: [Analysis] Chronic Obstructive Pulmonary Disease (COPD), or COPD, is characterized by persistent respiratory symptoms and airflow limitation (pair B), usually associated with airway and (or) alveolar abnormalities caused by significant exposure to harmful particles or gases.

[Wrong avoidance] ACDE option can be seen in COPD patients, but no characteristic, so it is not selected.

The most core feature of chronic obstructive pulmonary disease (COPD) is persistent airflow limitation. Therefore, the best answer is * * B. Continuously limited airflow * *.

Although A long-term history of heavy smoking (A), recurrent episodes of

season-related symptoms (C), chronic cough, sputum symptoms (D), and small airway dysfunction (E) are all associated with chronic obstructive pulmonary disease, these are not their core features. The stent airflow limitation reflects the main pathophysiological features of COPD and is a key criterion for the diagnosis of COPD.

This is a question about the core features of chronic obstructive pulmonary disease (COPD). We need to choose the options that best fit the question based on the definition and characteristics of COPD.

A. Long-term history of heavy smoking: Although smoking is an important risk factor for COPD, it is not a core feature of COPD, because COPD may also be caused by other factors, such as long-term exposure to air pollution or occupational dust.

B. Persistent airflow limitation: This is a defining feature of COPD. COPD is a disease with characteristics of airflow limitation, which is not fully reversible and progressive. This airflow limitation is associated with an abnormal inflammatory response of the lungs to harmful gases or harmful particles.

C. Recurrent episodes of season-related symptoms: Although COPD symptoms may be exacerbated by seasonal changes (e. g., cold weather), this is not a core feature. Many respiratory diseases may exhibit seasonal variation.

D. Chronic cough and sputum symptoms: these symptoms are common in COPD patients, but they are not their core characteristics because there are other respiratory diseases that may cause these symptoms.

E. Small airway dysfunction: Small airway dysfunction is one of the pathophysiological features of COPD, but it tells more about the mechanism of the disease than its core features. Airflow limitation is a direct consequence of this mechanism and is therefore more representative than small airway dysfunction.

In conclusion, persistent airflow limitation is the core feature of chronic obstructive pulmonary disease (COPD), so the correct answer is B: persistent airflow limitation.

The most central feature of chronic obstructive pulmonary disease (COPD) is airflow limitation, which is progressively aggravated, and is associated with chronic inflammatory responses of the airways and lungs to harmful particles or gases. Although other options such as long-term heavy smoking history, chronic cough and expectoration, and small airway dysfunction are closely related with COPD, the core

feature is persistent airflow limitation.

So the correct answer is:

F. Continuously limited airflow

4. (Type A1 question)

The principle of treatment of systemic infection is

A. For fungal sepsis, systemic intravenous antifungal drugs were used, combined with broad-spectrum antibiotics

B. Actively seek for primary lesions and conduct symptomatic treatment as early as possible

C. Choose sensitive antibiotics after the results of drug susceptibility test

D. Early mass association

E. Application of broad-spectrum antibiotics

Reference answer: B. Your answer:

Graphic analysis: [analysis] systemic infection application of comprehensive treatment, the key is to deal with the primary infection focus (B pair).

[Error avoidance] For the application of antimicrobial drugs: when there is no culture result, the early combined application according to the nature of the primary infection focus (C

Wrong), two antibiotics and sufficient dose (D wrong) applied according to bacterial culture and antibiotic sensitivity

Trial, adjusting antimicrobial agents. For fungal sepsis, stop broad spectrum antibiotics (AE wrong) or use necessary

Narrow-spectrum antibiotics, and the systemic application of antifungal agents.

The treatment principles of systemic infection mainly include early identification and control of the source of infection and rational use of antibiotics. Therefore, from the options provided, the most consistent with the principles of systemic infection treatment are:

F. Actively seek for primary lesions and conduct symptomatic treatment as early as possible

This is because for systemic infections (such as sepsis), it is important to identify and control the source of the infection as soon as possible, along with symptomatic supportive treatment. Although it is important to select sensitive antibiotics based on susceptibility testing (option C) after diagnosis, treatment should not be delayed to wait for susceptibility results. Early active intervention, including symptomatic treatment and control of the infectious agent, is key to improving cure rates and survival. Therefore, option B is the choice most consistent with the principles of systemic infection treatment.

analyze:

Systemic infection refers to the pathogenic bacteria invade the host blood circulation, and grow and reproduce, produce toxins and cause serious systemic infection or poisoning symptoms. The treatment principle is early, combined, sufficient amount, intravenous application of antibiotics, and timely adjustment of medication according to the results of blood culture or lesion secretion culture.

Option A, such as fungal sepsis, the application of systemic intravenous antifungal drugs, combined with broad-spectrum antibiotics is the principle of antifungal treatment, is not the treatment principle of systemic infection, so it is not selected.

Option B actively seeks for the primary lesion, and early symptomatic treatment is the right method, but it is not the main treatment principle, so it is excluded.

Option C Choosing sensitive antibiotics after the results of drug susceptibility test does not conform to the principle of early medication. Drug susceptibility test needs a certain amount of time, which will lead to disease delay during the waiting period, so it is not selected.

Option E Application of broad-spectrum antibiotics is the correct method, but a single use of antibiotics can not well control the infection, so the combination of drugs is needed, so it is excluded.

To sum up, the correct answer is: D. The early mass association.

The principles of treatment for systemic infections (e. g. sepsis) include early control of the source of the infection and appropriate antibiotic treatment. The optimal treatment regimen usually requires the initiation of empirical antibiotic therapy before

susceptibility test results are obtained and the adjustment of antibiotic species after susceptibility test results are obtained. Therefore, based on current medical knowledge and best practice guidelines, here is an analysis of the answers to the topic:

A. For fungal sepsis, systemic intravenous antifungal drugs were used, combined with broad-spectrum antibiotics.

-This applies only to fungal infections, not the principles of treatment for all systemic infections.

B. Actively seek for primary lesions and conduct symptomatic treatment as early as possible.

-This is one of the right principles, and it is very important to find and process the primary infection source.

C. Choose sensitive antibiotics after the results of drug susceptibility test.

-Waiting for treatment results may delay the condition and is not the optimal initial treatment strategy.

D. Early mass association.

-Although the combination of antibiotics is correct early on, the dosage and type need to be adjusted according to the specific situation, and a "large amount" is not necessarily the best choice.

E. Application of broad-spectrum antibiotics.

-The use of broad-spectrum antibiotics is a common practice in empiric therapy, but should be considered comprehensively in conjunction with other treatment principles.

Overall, the most correct answer is:

* * B. Actively seek for primary lesions and conduct symptomatic treatment as soon as possible.**

This is in line with the overall treatment principles of systemic infections, emphasizing the importance of early intervention and symptomatic treatment.

5. (Type A1 question)

The diagnostic criteria for pulmonary arterial hypertension are

A. Mean pulmonary artery pressure of 25mmHg measured by right heart catheter in resting state

B. Resting-state echocardiography measurements of a mean pulmonary arterial pressure of 25mmHg

C. Mean pulmonary artery pressure of 30mmHg was measured by the right heart catheter in the resting state

D. Resting-state echocardiographic measurements of a mean pulmonary arterial pressure of 30mmHg

E. Echocardiography measurements of systolic pulmonary artery pressure of 25mmHg at resting state

Reference answer: A. Your answer:

[Analysis] Pulmonary artery hypertension is A pathophysiological state of abnormal increase of pulmonary artery pressure caused by various known or unknown causes. The diagnostic criteria of hemodynamics are: mean pulmonary artery pressure (mPAP) measured by right pulmonary heart catheter at sea level and resting state (A pair).

[Error avoidance] The mean pulmonary artery pressure of resting state echocardiography is> 50mmHg, which can also be diagnosed in clinical practice

press.

The diagnostic criteria for pulmonary hypertension was once a mean pulmonary

artery pressure of 25mmHg measured by a right heart catheter in the resting state. However, this standard has been updated according to the latest guidelines, such as the Diagnosis and Treatment of Pulmonary Hypertension jointly published by the European Society of Cardiology (ESC) and the European Respiratory Society (ERS). The diagnostic criterion now is a mean pulmonary artery pressure of 20mmHg measured by the right heart catheter in the resting state.

Although this message is not directly reflected in the options you give, the closest answer is:

A. Mean pulmonary artery pressure of 25mmHg measured by right heart catheter in resting state

Thus, the A option was once a widely accepted diagnostic criterion in a given option. But note that the standards in the latest guidelines have changed. If this question is based on the latest medical knowledge, then actually none of the options are completely accurate. If the test criteria have not been updated, option A may be the most appropriate answer.

analyze:

Pulmonary artery hypertension refers to the mean pulmonary artery pressure (mean pulmonary artery pressure, mPAP) of 5mmHg and pulmonary capillary wedge pressure (Pulmonary capillary wedge pressure, 15mmHg of PCWP) measured by the right heart catheter in resting state, except for respiratory disease, left heart disease, pulmonary thromboembolism and congenital heart disease that cause increased pulmonary artery pressure. Among them, the mean pulmonary artery pressure of 25mmHg measured by the right heart catheter in the resting state is the gold standard for the diagnosis of pulmonary hypertension.

Right heart catheter measurement is the gold standard for the diagnosis of pulmonary hypertension, echocardiography can only be used as a screening method to assess pulmonary hypertension, the results are for reference only, so option B "echocardiography in resting state mean pulmonary pressure 25mmHg", option D "echocardiography in resting state 30mmHg", option E "echocardiography in resting state systolic 25mmHg", which can be excluded.

The mean pulmonary artery pressure in the right heart catheter is diagnosed as pulmonary hypertension instead of 30mmHg, so option C is the right heart catheter in the resting state, which can be excluded.

In conclusion, the correct answer is: A. The mean pulmonary artery pressure measured by the right heart catheter is 25mmHg.

According to existing medical knowledge and best practice guidelines, the diagnostic criteria for pulmonary hypertension is mean pulmonary artery pressure in the resting state as measured by a right heart catheter. The specific criteria are provided as follows:

A. Mean pulmonary artery pressure of 25mmHg measured by right heart catheter in resting state

So the best answer is:

A. Mean pulmonary artery pressure of 25mmHg measured by right heart catheter in resting state

6. (Type A1 question)

The following is true for peptic ulcer bleeding

A. Gastroscopy was performed after the stable vital signs

B. profess to be convinced PPI

C. Drug treatment or gastroscopy failed to achieve hemostasis

D. No obvious abdominal pain during bleeding

E. Gastric ulcers are generally more common than duodenal ulcers

Reference answer: C Your answer:

[Analysis] When digestive ulcer patients with gastrointestinal bleeding, after drugs, gastroscopy and vascular intervention treatment is ineffective, should undergo surgical treatment (C pair).

[Avoid] A single oral PPI (B error) is only effective in the treatment of peptic ulcer without bleeding, which should be combined with the stomach

Microscopic treatment (A error); some patients have severe abdominal pain, and

chronic abdominal pain can also be reduced after bleeding (D

Wrong); duodenal ulcer is more bleeding than gastric ulcer (E wrong).

The correct statement about peptic ulcer bleeding is that:

A. Gastroscopy was performed after the stable vital signs

This is true. In dealing with peptic ulcer bleeding, it is vital to perform gastroscopy after ensuring the patient's vital signs are stable, as this can help identify the source of the bleeding and perform appropriate treatment such as endoscopic hemostasis.

B. profess to be convinced PPI

Although oral proton pump inhibitors (PPI) are part of the treatment of peptic ulcer disease, intravenous PPI is more commonly used to control gastric acid secretion more quickly.

C. Drug treatment or gastroscopy failed to achieve hemostasis

This is also true. When medication and / or endoscopic hemostasis is ineffective, surgical treatment may be required to control the bleeding.

D. No obvious abdominal pain during bleeding

This is not entirely true because patients with bleeding peptic ulcers may have abdominal pain, especially when the ulcer penetrates or punches it.

E. Gastric ulcers are generally more common than duodenal ulcers

This claim is not well founded because duodenal ulcers are more common than gastric ulcers, but gastric ulcers may be more likely to cause bleeding.

Overall, the most appropriate answer should be * * C. Drug therapy or gastroscopy failed to perform surgical treatment * *, because it covers important aspects of the treatment of peptic ulcer bleeding.

analyze:

Digestive ulcer bleeding is the most common complication of peptic ulcer. Generally, non-surgical treatment is taken first. The main measures include blood transfusion, application of hemostatic drugs, endoscopic hemostasis, anti-shock, etc. If drug therapy or gastroscopy for hemostasis is ineffective, surgery may be considered.

Option A should perform gastroscopy after stable vital signs. For patients with

peptic ulcer and bleeding, emergency gastroscopy should be performed within 24 to 48 hours after bleeding. At this time, active bleeding can be seen under gastroscopy, and endoscopic hemostasis treatment can be performed simultaneously, which is inconsistent with the question.

Option B Oral PPI is a general treatment measure for peptic ulcer bleeding, which is a non-surgical treatment and cannot be used as a characteristic of peptic ulcer bleeding, so it is not selected.

Patients with peptic ulcer usually have rhythmic and periodic upper abdominal pain, and abdominal pain can be reduced after bleeding, so option D generally has no obvious abdominal pain when bleeding, which can be excluded.

Duodenal ulcers are more likely to cause perforation than gastric ulcers, so option E gastric ulcers are generally more likely to occur than duodenal ulcers and can be excluded.

In conclusion, the correct answer is: C. Drug treatment or gastroscopy hemostasis ineffective surgical treatment.

The correct statement of peptic ulcer bleeding, combined with the existing medical knowledge and best practice guidelines, the correct answer is:

F. Drug treatment or gastroscopy failed to achieve hemostasis

The explanation is as follows:

- ** A. Gastroscopy after stable vital signs * *: Although gastroscopy is an important means to diagnose and treat bleeding peptic ulcer, gastroscopy is usually performed as soon as possible after vital signs are stable, so as to quickly identify the cause of bleeding and take corresponding treatment.

- ** B. Oral PPI * *: Proton pump inhibitors (PPI) are indeed the main drug treatment for peptic ulcer disease, but they are usually administered intravenously during acute bleeding periods to rapidly inhibit gastric acid secretion.

- ** C. Drug therapy or gastroscopy is ineffective for surgical hemostasis * *: When drug therapy or endoscopic hemostasis cannot control bleeding, surgical

treatment is necessary to avoid further complications and life hazards.

- ** D. Generally no obvious abdominal pain when bleeding * *: Patients with peptic ulcer bleeding usually have upper abdominal pain, especially when the bleeding amount is large, the pain may be more obvious.

- ** E. Gastric ulcers are generally likely to occur than duodenal ulcers * *: In fact, duodenal ulcers are more common than gastric ulcers, and they have a higher risk of bleeding.

7. (Type A1 questions)

The es closely associated with ankylosing spondylitis are

A. HLA-DR2

B. HLA-B27

C. HLA-DR3

D. HLA-DR4

E. HLA-DR5

Reference answer: B. Your answer:

Graphic analysis: [Analysis] The genetic gene closely related to ankylosing spondylitis is HLA-B27 (B pair).

[Wrong avoidance] Abnormal changes of HLA-DR2 (A error) may cause systemic lupus erythematosus, scleroderma, and primary diseases

Open-angle glaucoma et al. HLA-DR3 antigen (C error) has a high detection rate in patients with myasthenia gravis. HLA-DR4 (D error) is somewhat associated with rheumatoid arthritis. Individuals carrying the HLA-DR5 (E error) gene are often closely associated with Hashimoto's thyroiditis.

The genetic gene closely related to ankylosing spondylitis is * * B. HLA-B27 * *. The association between ankylosing spondylitis and HLA-B27 is very obvious, and most patients (about 90%) are HLA-B27 positive.

analyze:

Ankylosing spondylitis is a chronic progressive inflammatory disease that mainly invades the spine and can involve different degrees of the sacroiliac joints and surrounding joints. This disease is also known as Marie-strumpell disease, Von

Bechterew disease, rheumatoid spondylitis, dysmorphic spondylitis, rheumatoid central type, etc., are now called AS. AS is characterized by inflammation and ossification of the lumbar, cervical and thoracic spinal joints and ligaments, as well as the sacroiliac joints. The hip joint is often involved, and other surrounding joints can also have inflammation. The genetic gene closely related to ankylosing spondylitis is HLA-B27. In China, the positive rate of HLA-B27 in patients with ankylosing spondylitis is as high as 90%.

Option A: HLA-DR2 is associated with rheumatoid arthritis and has no direct relationship with ankylosing spondylitis, so it is not chosen.

Option C: HLA-DR3 was associated with autoimmune thyroid disease, type I diabetes, Addison disease, primary biliary cirrhosis, but not with ankylosing spondylitis, so it was excluded.

Option D: HLA-DR4, option E: HLA-DR5, are not directly related to ankylosing spondylitis, and have found no clear association with specific diseases, which can be excluded together.

In conclusion, the correct answer is: B. HLA-B27.

The genes closely associated with ankylosing spondylitis are:

F. HLA-B27

8. (Type A1 question)

Long-term internal hemorrhoids patients have anal mass prolapse, can not return with the hand, for a few periods

A. I designated time

B. II designated time

C. III designated time

D. IV designated time

E. V designated time

Refer to the answer: D. Your answer:

Graphic and text analysis: [analysis] long-term internal hemorrhoids patients with anal mass prolapse, used by hand can not return, for the degree (D pair).

[To avoid mistakes] internal hemorrhoids degree: I degree: stool with blood, stool bleeding to stop, no hemorrhoids prolapse; degree: often stool

Blood, defecation when hemorrhoids out, after defecation can be returned; degree: occasional blood in the stool, defecation or long standing, cough, weight-bearing hemorrhoids

Exit, need to return by hand; degree: occasional blood in the stool, hemorrhoids withdrawal can not return or return after the withdrawal.

Patients with long-term internal hemorrhoids have an anal mass prolapse and can not return with the hand, which usually indicates that the disease has progressed to a more serious stage. According to the clinical stage of internal hemorrhoids, the mass that cannot return by itself or requires manual push back into the anus are usually classified as periods. Therefore, the most correct answer is the C. period.

analyze:

Internal hemorrhoids is a soft mass formed by the dilated and varicose submucosal vascular plexus at the end of the rectum above the dentate line of the anal canal. Its essence is actually a varicose vein vascular group. Internal hemorrhoids is generally divided into four stages, among them, the period of hemorrhoids is larger, hard texture, surface with gray, hemorrhoids outside the anus, even walking, cough, sneeze or stand will take out, cannot back, must push back, or supine, hot compress to return, blood is not much or no longer bleeding. Therefore, long-term internal hemorrhoids patients have anal mass prolapse, with the hands can not return, consider the internal hemorrhoids period.

Option A: Stage I hemorrhoids mainly blood symptoms, small hemorrhoids, soft, hemorrhoids bright red or green purple, defecation when the hemorrhoids does not take out of the anus, not consistent with the description of the dry.

Option B: internal hemorrhoids, with blood, blood or jet bleeding, accompanied by hemorrhoids prolapse, can return after the stool, the patient can not return with their hands, so they do not choose.

Option D: during the period of hemorrhoids, hemorrhoids prolapse can not be returned, embedded in the embedded time, easy to form thrombosis, necrosis, infection, often accompanied by severe pain, fever and other systemic symptoms, and the description of the dry, so excluded.

There is no stage V in the internal hemorrhoids stage, so option E: stage V, which

can be excluded preferentially.

To sum up, the correct answer is: C. Period.

Long-term internal hemorrhoids patients have anal mass prolapse, can not return with the hand, for the period.

So, the correct answer is:

F. IV designated time

9. (Type A1 questions)

Blood gas changes during the early onset of bronchial asthma are

A. pH value increased, PaO 2 decreased, and PaCO2 decreased

B. pH values increased, PaO 2 decreased, and PaCO2 increased

C. pH, PaO 2 and PaCO2

D. pH values decreased, PaO 2 decreased, and PaCO2 increased

E. pH values were normal, PaO 2 decreased and PaCO2 increased

Reference answer: A. Your answer:

[Analysis] Early bronchial asthma: because hyperventilation can reduce PaCO 2, pH rise, manifested as respiratory alkalosis.

[Avoid mistakes] Late stage of bronchial asthma: hypoxia was aggravated, and respiratory acidosis (CO2 retention) + metabolic acidosis occurred

(Hypoxia and lactic acid increased), indicating a critical condition.

During early episodes of bronchial asthma, respiratory alkalosis usually occurs as patients will try to reduce breathing difficulties by increasing respiratory rate, leading in increased carbon dioxide excretion. Therefore, PaCO2 will decrease, causing higher blood pH values. In addition, reduced oxygen exchange, due to airway stenosis, leads to a decrease in PaO 2. In summary, the best options for this scenario are:

A.pH value increased, PaO 2 decreased, and PaCO2 decreased

So, the correct answer is * * A * *.

analyze:

Bronchial asthma is a heterogeneous disease characterized by chronic inflammation of the airway involving a variety of cells and cell components. This chronic inflammation is related with airway hyperreactivity, and usually appears extensive and variable reversible expiratory airflow limitation, leading to recurrent symptoms such as wheezing, shortness of breath, chest tightness or cough, and the intensity changes over time. In the early onset of bronchial asthma, due to airway spasm, body hypoxia, hyperventilation, will lead to pH rise, decrease of blood oxygen partial pressure (PaO_2), decrease of carbon dioxide partial pressure ($PaCO_2$), and respiratory alkalosis.

In the early onset of bronchial asthma, due to body hypoxia and hyperventilation, it will lead to a decline in $PaCO_2$, with no increase in $PaCO_2$. Therefore, option B: "pH rises, PaO_2 down, $PaCO_2$ up"; option D: "pH drops, PaO_2 down, $PaCO_2$ up"; option E: "Normal pH value, PaO_2 down, $PaCO_2$ up"; can be excluded together.

During asthma attack, due to airway obstruction, poor breathing and hyperventilation, the decline of $PaCO_2$ and respiratory alkalosis occurred. At this time, blood pH value increased, rather than decreased, so option C: pH value decreased, PaO_2 decreased, and $PaCO_2$ decreased, which can be excluded.

In conclusion, the correct answer is: A.pH value rises, PaO 2 drops, and PaCO2 decreases.

Blood gas changes in the early onset of bronchial asthma are mainly related to acute airway obstruction, leading to hyperventilation (hyperventilation). This condition causes the following changes:

-pH rise (due to respiratory alkalosis)

-PaO 2 decreased (because airway obstruction affects oxygen exchange)

-PaCO 2 decreased (because hyperventilation emits more carbon dioxide)

So, the most correct answer is:

A. pH values increased, PaO 2 decreased, and PaCO 2 decreased

10. (Type A1 questions)

Which of the following is not a surgical indication for acute pancreatitis

A. Progressive elevation of direct bilirubin associated with chills and high fever

B. complicated with massive hemorrhage or pseudocyst

C. Acute peritonitis cannot be excluded

D. Serum amylase is> 500U / L

E. Secondary infection of the pancreas and peripancreatic tissue by necrosis

Refer to the answer: D. Your answer:

[Analysis] The increase of serum amylase is not proportional to the severity of pancreatitis, so serum amylase> 500U / L is not a surgical indication for acute pancreatitis (D wrong, is the correct answer to this question).

[Avoid errors] surgical indications for acute pancreatitis: ① acute peritonitis cannot be excluded from other acute abdomen (C pair); ② with gallbladder

Main duct obstruction or biliary tract infection (in A pair, elevation of direct bilirubin indicates obstructive jaundice); ③ with bowel

Perforation, massive bleeding, or pancreatic pseudocyst (pair B); ④ Secondary infection of pancreatic and peripancreatic necrotic tissue (pair E).

For acute pancreatitis, surgical treatment is usually considered in specific situations, mainly when there are complications or other conservative treatment is ineffective. Based on the options provided, we will analyze which one is not the surgical indication for acute pancreatitis:

A. Direct progressive elevation of bilirubin with chills and high fever: This may indicate problematic bile duct, such as bile duct obstruction or infection, and may require surgical intervention.

B. Combined with massive bleeding or pseudocyst: These conditions can worsen and may require surgery.

C. Acute peritonitis cannot be excluded from other acute abdomen: Acute peritonitis usually requires surgery to determine the cause and treat it.

D. Secondary infection of the pancreas and peripancreatic tissue necrosis: This is a serious complication of acute pancreatitis that usually requires surgical removal of necrotic tissue.

E. Serum amylase> 500U / L: Although an increased serum amylase level is an important marker of acute pancreatitis, it is not itself a direct indication for surgery. Often, treatment focuses on conservative management, including rest, analgesia, and fluid supplementation, unless complications or exacerbations occur.

Therefore, the most correct answer is * * D. Serum amylase> 500U / L * *. Although this indicator is an important basis for the diagnosis of acute pancreatitis, it is not directly used as an indication for surgical treatment.

This is a multiple-choice question about the surgical indications for acute pancreatitis. We need to analyze each option to determine which one is not the surgical indication for acute pancreatitis.

A. Direct progressive elevation of bilirubin with chills and high fever: This condition may indicate biliary obstruction or infection, which is one of the serious conditions where surgery may be required in acute pancreatitis.

B. Combined massive bleeding or pseudocyst: Major bleeding and pseudocysts are serious complications of acute pancreatitis that usually require surgical treatment.

C. Acute peritonitis cannot exclude other acute abdomen: where a clear diagnosis cannot be made, surgical exploration may be necessary in order to exclude other diseases that may lead to acute peritonitis.

D. Serum amylase> 500U / L: elevated serum amylase is a typical presentation of acute pancreatitis, but elevated amylase alone is not a direct indication for surgery. The severity of pancreatitis and the choice of treatment modality depend more on the clinical manifestations and other laboratory and imaging tests.

E. Pancreatic and peripancreatic tissue necrosis secondary infection: necrosis of the pancreas and surrounding tissue with secondary infection is a serious complication of acute pancreatitis that usually requires surgical treatment.

In conclusion, a simple elevation of serum amylase is not a surgical indication for acute pancreatitis. Surgical decisions are usually based on the severity of the disease, the emergence of complications, and the overall condition of the patient.

Therefore, the correct answer is that D. Serum amylase> 500U / L.

The surgical indications for acute pancreatitis mainly include secondary infection of the pancreas and peripancreatic tissue necrosis, complicated with massive bleeding or pseudocyst, and acute peritonitis, other acute abdomen cannot be excluded. Surgical indications often involve serious complications or conditions that cannot be

controlled by non-surgical means. We analyze the options one by one:

A. Progressive elevation of direct bilirubin with chills fever: direct bilirubin elevation and high fever may indicate biliary acute pancreatitis, which usually requires surgery to remove biliary obstruction, but this is not a surgical indication for acute pancreatitis itself, but for biliary problems.

B. Major hemorrhage or pseudocyst: Both pseudocyst and massive hemorrhage are serious complications of acute pancreatitis that require surgical intervention.

C. Acute peritonitis cannot be ruled out for other acute abdomen: Acute peritonitis If other acute abdomen cannot be excluded, surgical exploration is required to make a clear diagnosis and management.

D. Serum amylase> 500U / L: Although significantly elevated serum amylase is one of the hallmarks of acute pancreatitis, high serum amylase alone is not an indication for surgery. Elevated serum amylase is only one of the indicators of acute pancreatitis.

E. Secondary infection of the pancreas and peripancreatic tissue necrosis: Secondary infection is a serious complication of acute pancreatitis and usually requires surgical intervention.

In conclusion, item D "serum amylase> 500U / L" is not a surgical indication for acute pancreatitis.

So, the correct answer is: D. serum amylase> 500U / L.

11. (Type A1 question)

The examination of the correlation between symptoms and pathophysiological changes in patients with gastroesophageal reflux disease is

A. gastroscope

B. esophageal manometry

C. DCG

D.24h esophageal pH value detection

D. Barium X-ray examination of the upper gastrointestinal tract

Reference answer: A. Your answer:

[Analysis] Gastroscopy is the most accurate method for the diagnosis of RE, and can judge the severity and complications of RE. Combined with biopsy, it can be differentiated from esophagitis and other esophageal lesions (such as esophageal cancer, etc.). Normal esophageal mucosa is stratified squamous epithelium, which is uniformly pink under gastroscopy, and appears orange when replaced by metaplastic columnar epithelium (A pair).

[Error avoidance] esophageal pressure measurement, 24h esophageal pH value detection, upper gastrointestinal X-ray barium examination can also be used to assist in the diagnosis of gastroesophageal reflux disease, but not as the first choice. Holter electrocardiogram is generally used for the detection of cardiac arrhythmias and transient myocardial ischemia.

In the diagnosis of gastroesophageal reflux disease (GERD), it is critical to determine the duration and extent of oesophageal exposure to stomach acid, and the oesophageal response to acid. Although gastroscopy allows direct observation of the mucosal conditions of the esophagus and stomach to detect inflammation or other damage, it is not sufficient to quantify the extent of acid reflux or to establish pathophysiological alterations.

Esophageal manometry (option B) can evaluate the function of the lower esophageal sphincter, and esophageal peristalsis function, which is helpful in assessing the pathophysiology of reflux disease, but it does not directly measure acid reflux.

The Holter electrocardiogram (option C) was used to assess cardiac activity not associated to the direct diagnosis of gastroesophageal reflux disease.

The 24-hour esophageal pH test (option D) is the gold standard for assessing the

duration of esophageal acid exposure and acid reflux frequency, allowing a direct measurement of pH within the esophagus to determine the presence and severity of acid reflux.

Upper gastrointestinal X-ray barium examination (option E) can reveal anatomical and large motor abnormalities in the esophagus, stomach and duodenum, but is not sensitive enough for quantification of acid reflux and assessment of pathophysiological changes.

Therefore, the most consistent examination for judging the correlation between symptoms and pathophysiological changes in patients with gastroesophageal reflux disease is * * D. 24-hour esophageal pH value detection * *.

analyze:

GERD refers to a series of symptoms and complications caused by reflux of gastric contents into areas other than the esophagus, throat and mouth. Symptoms of gastroesophageal reflux disease include a burning chest sensation, hiccups, nausea, vomiting, chest pain, and acid reflux. The most accurate method for the diagnosis of gastroesophageal reflux disease is the 24h esophageal pH test, which can provide objective evidence of excessive acid reflux in the esophagus. It is a related examination to judge the symptoms and pathophysiological changes of patients with gastroesophageal reflux disease, and is an important auxiliary examination for the diagnosis of gastroesophageal reflux disease.

Option A Gastroscopy is the most accurate method to diagnose reflux esophagitis, and can judge the severity and complications of reflux esophagitis. Combined with biopsy can distinguish esophagitis and other esophageal lesions caused by other causes, but it cannot judge the symptoms and pathophysiological changes of patients with gastroesophageal reflux disease, so it is not selected.

Option B esophageal pressure measurement is an index reflecting the function of the lower esophageal sphincter. When the medical treatment effect of gastroesophageal reflux disease is not good, it can be used as an auxiliary diagnosis, but gastroesophageal reflux disease cannot be directly diagnosed, so it is ruled out.

Option C Holter is used for examination of cardiovascular system disease and is unrelated to gastroesophageal reflux disease and can be ruled out.

Option E upper gastrointestinal X-ray barium examination has limited value for evaluating esophageal mucosal damage, which can only indicate whether there are

signs of reflux. The positive rate is not high, and gastroesophageal reflux disease cannot be confirmed, which is inconsistent with this question.

In conclusion, the correct answer is: D.24h esophageal pH value detection.

Regarding the correlation of symptoms and pathophysiological changes in patients with gastroesophageal reflux disease, the following is an analysis of the options:

A. * * Gastroscopy * *: The mucosal status of the esophagus and stomach can be directly observed, which is helpful in the diagnosis of gastroesophageal reflux disease (GERD), but the frequency and acidity of reflux cannot be directly assessed.

B. * * Esophageal manometry * *: It can measure the pressure and motor function of the esophagus and evaluate the function of the lower esophageal sphincter, but it is not the best choice for diagnostic GERD.

C. * * Holter * *: To monitor electrical activity of the heart, not associated to gastroesophageal reflux disease.

D. * * 24-hour esophageal pH value detection * *: It is the gold standard for the diagnosis of gastroesophageal reflux disease. It can continuously monitor the acid reflux in the esophagus within 24 hours, directly assess the frequency and acidity of the reflux, and is the most direct examination to determine the correlation between GERD symptoms and pathophysiological changes.

E. * * Upper gastrointestinal X-ray barium examination * *: It can show morphological changes in the esophagus and stomach, but it has low sensitivity and specificity for the diagnosis of reflux disease.

Considering the above analysis, the most correct answer is:

F. * * 24-hour esophageal pH detection * *

12. (Type A1 questions)

The following are for primary peritonitis

A. Choledochal anastomosis leakage

B. Up-line infection of bacteria in the female genital tract

C. enteric perforation

D. Perforated gastric ulcer

E. perforation of colon

Reference answer: B. Your answer:

[Analysis] Primary peritonitis, also known as spontaneous peritonitis, that is, there is no primary lesion in the abdominal cavity, the infection route is: ① hematogenous dissemination; ② ascending infection; ③ direct spread; ④ transmural infection. An ascending infection, or bacteria from the female reproductive tract, spreads directly upward through the fallopian tube to the abdominal cavity, such as gonoperitonitis (pair B).

[Error avoidance] Secondary peritonitis is the most common peritonitis. Organ perforation of abdominal cavity, abdominal wall or internal rupture caused by trauma

Schfts, is the most common cause of acute secondary suppurative peritonitis.

Primary peritonitis refers to peritonitis occurring in the absence of significant abdominal lesions of bacterial origin. It is usually associated with hematogenous infections or infection of specific pathogens such as M. tuberculosis. In contrast, secondary peritonitis is usually caused by perforation, tearing or inflammation of the abdominal organs, conditions leading cause direct entry of bacteria into the abdominal cavity.

Options A (bile duct anastomosis leakage), C (small bowel perforation), D (gastric ulcer perforation), and E (colonic perforation) may all cause secondary peritonitis as they involve breakage of the abdominal organs that allow bacteria directly into the abdominal cavity.

Option B (ascending infection of bacteria in the female reproductive tract) usually refers to the bacteria moving up through the female genital tract to the pelvic cavity, which may cause pelvic inflammatory diseases rather than direct peritonitis.

However, in the absence of significant perforation or breakage, this type of infection may not be considered as a typical cause of primary peritonitis.

As these options are all more relevant to secondary peritonitis, no option is to describe the classical condition of primary peritonitis. Given that the topic requires selecting conditions related to primary peritonitis, but these options seem to point to secondary peritonitis, you may need to reconsider the context of this issue or to see if there is other information or options defining the primary peritonitis.

Based on conventional medical knowledge, primary peritonitis is usually not usually directly caused by these conditions. Therefore, further clarification if it must be selected from these options, and with the assumption that the topic may be asked about the least relevant situation to primary peritonitis, may be required. In the practical exam environment, it is recommended to carefully review the questions and options to ensure a correct understanding of what is being asked. If the context selection is most likely associated with primary peritonitis, option B may need to be considered as it is relatively less directly associated with perforation or breakage of the abdominal organ.

This is a problem involving the classification of peritonitis. Peritonitis is usually divided into primary peritonitis and secondary peritonitis. We need to identify which condition belongs to the primary peritonitis.

A. Boledochal anastomosis leakage: This is peritonitis caused by surgical operation, so it is secondary peritonitis.

B. Upinfection of bacteria in the female reproductive tract: In this case, peritonitis caused by bacteria through the reproductive tract to the abdominal cavity is not caused by external factors such as perforation or injury of abdominal organs. This peritonitis has no clear source of intraperitoneal lesions and therefore it belongs to primary peritonitis.

C. Small bowel perforation: This is a peritonitis caused by a small bowel perforation, which belongs to secondary peritonitis.

D. Perforated gastric ulcer: perforation of gastric ulcer will cause gastric contents to enter the abdominal cavity, causing peritonitis, which belongs to secondary peritonitis.

E. Colon perforation: Colon perforation can also cause the colon contents into

the abdominal cavity, causing peritonitis, which is also secondary peritonitis.

In conclusion, only the primary peritonitis represented by option B resulted from ascending infection of bacteria in the female genital tract without a clear source of intraabdominal lesions, fitting the definition of primary peritonitis.

Therefore, the correct answer is upward infection of bacteria in the female genital tract.

Primary peritonitis (primary peritonitis) is a form of peritonitis without significant intra-abdominal organ lesions and is commonly seen in patients with low immunity, children, and patients with cirrhosis with ascites. By this definition, primary peritonitis is not caused by perforation of the intraabdominal organ or surgical procedure but by a bacterial infection that naturally exists in the abdominal cavity.

Option analysis:

A. Choledochal anastomosis leakage — A complication after abdominal surgery and is secondary to peritonitis.

B. Up-infection of bacteria in the female genital tract-a condition that can cause primary peritonitis because bacteria can travel up through the genital tract to the abdominal cavity.

C. Small bowel perforation-belongs to secondary peritonitis because it is caused by a rupture of the small intestine.

D. Perforated gastric ulcer-which is secondary to peritonitis because it is caused by a lesion in the stomach.

E. Colon perforation-It is secondary peritonitis because it is caused by colon rupture.

Based on the above analysis, the most consistent for the definition of primary peritonitis is option B: bacterial ascending infection in the female genital tract.

So the best answer is:

F. Up-line infection of bacteria in the female genital tract

13. (Type A1 questions)

The first examination for perforation of gastrodorectal ulcer is

A. Vertical X-ray

B. B exceed

C. strengthen CT

D. gastroscope

E. PETCT

Reference answer: A. Your answer:

[Analysis] The preferred examination for twelve rectal ulcer perforation is vertical X-ray abdominal plain film (A pair), the examination can see free gas under the diaphragm.

[Avoid avoidance] The application of B ultrasound (B error) examination in digestive tract is limited. Enhanced CT (C error) examination is mainly used to check the blood vessels

Related acute abdomen, intra-abdominal organ injury, organ inflammation and abdominal abscess are used to understand whether the blood supply obstruction is accompanied by intestinal obstruction

hinder. Peptic peptic ulcer with bleeding, choose gastroscope (D wrong). PET-CT (E error) provides early tumor detection with certainty

Quality, it can accurately stage the tumor and evaluate the treatment effect.

For gastroduodenal ulcer perforation, the preferred examination method is the vertical X-ray abdominal plain film (option A). This examination can find the free gas in the abdominal cavity, and it is the key basis for the diagnosis of perforation. Although enhanced CT (option C) can also be used to diagnose gastrointestinal perforation and provide more details, vertical X-ray is more rapid and economical and is usually the preferred method. Therefore, the best answer is A. vertical X-ray.

analyze:

Perforation of gastroduodenal ulcer is a common complication of gastroduodenal ulcer, mostly due to the deepening of the ulcer, through the muscle layer, serosa layer,

and finally through the stomach or duodenal wall and perforation. For gastroduodenal ulcer perforation, the preferred examination is the vertical X-ray abdominal plain film, which typically shows a crescent-shaped free gas shadow under the diaphragm.

Option B: B ultrasound is commonly used for the examination of liver, gallbladder, pancreas and spleen, and has little diagnostic value for perforation of empty organs, so it is not selected.

Option C: enhanced CT, option E: PETCT, which is expensive, complicated to operate, and has no special significance for the diagnosis of cavity and organ perforation, so it is excluded together.

Option D: Gastroscope can directly look at the ulcer site, the size and nature of the lesion, and use biopsy to determine HP infection. Some cases can also treat the patients, such as microscopic hemostasis, polyp resection and tumor resection, but it is invasive examination, and perforation cannot be diagnosed, which can be ruled out.

To sum up, the correct answer is: A. vertical X-ray abdominal flat film.

Perforated gastroduodenal ulcer is an emergency that requires rapid diagnosis and treatment. The preferred test for the diagnosis of gastroduodenal ulcer perforation should be a rapid and accurate indication of gas leakage. Based on the existing medical knowledge and best practice guidelines, the preferred examination is:

* * A. Vertical X-ray abdominal plain film * *

Vertical X-ray abdominal plain film (namely vertical abdominal plain film) can show the free gas under the diaphragm, which is a characteristic sign of gastroduodenal ulcer perforation. Therefore, in clinical practice, vertical X-ray is usually the preferred preliminary examination.

14. (Type A1 question)

The smallest extent of dissection of the cervical lymph nodes in thyroid cancer was

A. VI

B. II

C. III

D. IV

E. V

Reference answer: A. Your answer:

[Analysis] The scope of cervical lymph node dissection is different at present, but the minimum scope of dissection, both the central area of cervical lymph node () dissection has basically reached A consensus (A pair).

[Wrong avoidance] Area cleaning not only cleans the area where thyroid cancer is most likely to metastasize, but also contributes to clinical staging, guiding treatment and prognosis

Measure the possibility of lymph node metastasis in the lateral cervical area and reduce the complications of reoperation.

In the treatment of thyroid cancer, the extent of cervical lymph node dissection depends on the location, type of tumor, and the spread of lymph nodes. The cervical lymph nodes are divided into several regions, indicated by Roman numerals I to VII. Among them, the area usually refers to the central area of the anterior neck, which includes the lymph nodes around the thyroid gland, and is the most common lymph node metastasis area for thyroid cancer.

Therefore, in the treatment of thyroid cancer, the minimum dissection range usually includes areas, the lymph nodes in the central area of the anterior neck, to ensure the clearance of the lymph nodes that may be involved. So, the most correct answer is * * A. * *.

analyze:

Thyroid cancer is a malignant tumor originating from the thyroid follicular epithelium and often presenting as a painless neck mass or nodule. The main pathological types of thyroid cancer include: undifferentiated carcinoma, medullary carcinoma, papillary carcinoma and undifferentiated carcinoma, and the malignant degree from high to low is: undifferentiated carcinoma, medullary carcinoma, papillary carcinoma. The treatment of thyroid cancer is mainly surgery, with thyroidectomy and cervical lymph node dissection, and whether to determine the need for iodine-131 treatment and TSH inhibition therapy according to the pathological examination results. In the division of cervical lymph nodes, the area refers to the anterior cervical lymph nodes, also known as peritracheal lymph nodes, including the

cyclic thyroid membrane lymph nodes, trachea, perithyroid lymph nodes, retropharyngeal lymph nodes, which are the first metastasis of thyroid cancer. Therefore, when the cervical lymph node dissection of thyroid cancer, the smallest dissection area is the area.

Option B: Regional lymph nodes The upper group of internal jugular vein, the upper boundary is the skull base, the lower boundary is the hyoid bone, which is the second stop of thyroid cancer dissection. The dissection range is greater than the area, so it is not selected.

Option C: Regional lymph node The middle zone lymph node of the internal jugular vein, the upper boundary is the hyoid bone, and the lower boundary is the lower edge of the annular cartilage, which is the third stop of thyroid cancer dissection. The dissection range is greater than the area, so it is excluded.

Option D: Regional lymph nodes in the lower group of internal jugular vein, the upper boundary is annular cartilage, the lower boundary is parallel to the upper clavicle, which is the fourth stop of thyroid cancer dissection. The dissection range is greater than the area, and can be excluded.

Option E: The posterior cervical triangular lymph node in area V, including the supraclavicular lymph node, is the fifth stop of thyroid cancer dissection. The dissection range is greater than the area, which is inconsistent with the meaning of the question.

To sum up, the correct answer is: A.

Treatment of thyroid cancer includes surgical resection of the tumor and the involved lymph nodes. When performing cervical lymph node dissection for thyroid cancer, the appropriate dissection range is usually selected based on the stage of the cancer, lymph node involvement, and surgical guidelines.

For thyroid cancer, the smallest dissection area of cervical lymph nodes usually includes areas, also known as central or pre-central zone lymph nodes. This region is located on both sides of the trachea, including the anterior and paracheal lymph nodes and is common in early metastatic sites of thyroid cancer.

Therefore, the smallest dissection of thyroid cancer is the area.

The correct answer is: A

15. (Type A1 question)

Patients with acute upper respiratory tract infection are planned to undergo laparoscopic cholecystectomy, and the operation should be postponed until the symptoms of acute upper respiratory tract infection recover

A. 5~7 days

B. for 14 to 21 days

C. From 7 to 14 days

D. 1~3 days

E. 3~5 days

Reference answer: C Your answer:

[Analysis] For acute respiratory infected persons, elective surgery should be postponed to 1 to 2 weeks after treatment (C pair).

[Avoid] With a recent history of stroke, elective surgery should be delayed for at least 2 weeks, preferably 6 weeks.

In patients with acute upper respiratory tract infection, it is usually recommended to wait until complete resolution of infection symptoms before non-emergency surgery such as laparoscopic cholecystectomy. This is because acute upper respiratory tract infections may increase the risk of anesthesia and surgery, including the risk of respiratory complications and the spread of the infection.

According to medical guidelines and best practice, surgery is usually recommended after complete resolution of acute symptoms. Although the specific waiting time can vary depending on the patient, the generally recommended time is not within 2 to 4 weeks after recovery of symptoms of infection to ensure complete recovery and reduce the risk of surgery. Therefore, the most appropriate answer option is:

F. for 14 to 21 days

This time frame provides adequate recovery time for patients, reducing the potential negative impact of acute infection on surgery.

analyze:

Acute upper respiratory tract infections are mostly caused by viral infections, but some can be caused by bacterial infections or mixed infections. Patients often present with cough, runny nose, fever and other symptoms. In acute upper respiratory tract infection, the defense function of the respiratory mucosa is affected, increasing the risk of infection during and after surgery. Therefore, for elective surgery, it is generally recommended to perform additional surgery 1 to 2 weeks after infection control and normal body temperature recovery to reduce the risk of surgical complications.

To sum up, the correct answer is: B.14~21 days.

Option A: 5~7 days, option C: 7~14 days, option D: 1~3 days, and option E: 3~5 days, all of which are insufficient surgery delay time. At this time, the defense function of respiratory mucosa may not be fully recovered, and the risk of surgical complications is still high, so it can be excluded.

Patients with acute upper respiratory tract infection are planned to undergo laparoscopic cholecystectomy, and the operation should be postponed until the symptoms of acute upper respiratory tract infection recover. Acute upper respiratory tract infection may increase the risk of anesthesia and surgery, such as increased airway response, increased secretions, and poor ventilation, so it is usually recommended to postpone the surgery until the symptoms of infection have completely disappeared.

For the recommended delay after the recovery of symptoms of acute upper respiratory tract infection, it is generally believed to take approximately 2 weeks to ensure the complete recovery of respiratory function and reduce the occurrence of intraoperative and postoperative complications. So the correct answer is:

G. for 14 to 21 days

16. (Type A1 question)

It is currently commonly used in the examination for the diagnosis of bronchiectasis

A. lung function

B. bronchoscope

C. high resolution CT

D. Chest X-ray

E. B exceed

Reference answer: C Your answer:

Graphic analysis: [analysis] The examination commonly used in the diagnosis of bronchiectasis is HRCT (C pair). HRCT can clearly show dilated bronchi in cross sections.

[Avoid] Other auxiliary examinations conducive to the diagnosis of bronchiectasis are: X-ray (double track sign or curly hair can be seen on the longitudinal section

Shadow), bronchoscope (if dilatation of the bronchial is focal and above the segment)

Lung function, etc. The B-ultrasound examination has a limited role in the respiratory system.

The most common and effective test method for diagnostic bronchiectasis is high resolution computed tomography (high resolution CT). Highly resolved CT can provide detailed information about the tracheal and bronchial walls, clearly showing the thickness, dilatation and the presence of other lung lesions. Therefore, the most correct answer is: C. High-resolution CT

analyze:

Bronchiectasis is due to chronic suppurative inflammation and fibrosis of the bronchus and its surrounding lung tissue, which destroys the muscle and elastic tissues of the bronchial wall, resulting in bronchial deformation and lasting dilatation. Typical symptoms include chronic cough, large cough of purulent sputum and recurrent hemoptysis. The diagnosis of bronchiectasis relies on imaging examination, in which high-resolution CT is the gold standard for the diagnosis of bronchiectasis, which has a high rate of diagnosis of bronchiectasis, and is non-invasive, reproducible and easily acceptable. It has now become the main diagnostic method of bronchiectasis.

Option A lung function test is mainly used for the diagnosis of chronic obstructive pulmonary disease, bronchial asthma and other diseases, which has little

significance for the diagnosis of bronchiectasis, so it is excluded.

Option B bronchoscopy can be used to observe the lesion site, but because it is a traumatic examination, it is generally not used to diagnose bronchiectasis, so it is not selected.

Option D Chest X-ray showed a double-track sign, but its diagnostic value was less than high-resolution CT, so it was excluded.

Option E Ultrasound examination is mostly used for fluid accumulation or the examination of substantial abdominal cavity organs, which is of little significance to the diagnosis of bronchiectasis, so it is not selected.

In summary, the correct answer is: C. High-resolution CT.

Current tests commonly used to diagnose bronchiectasis are:

F. high resolution CT

High resolution CT (HRCT) can detail the structural changes in bronchial and lung tissue, which is the most sensitive and specific examination method to diagnose bronchiectasis.

17. (Type A1 question)

Not a risk factor for breast cancer

A. Early age of menopause

B. No postpartum lactation

C. fat

D. Family history of breast cancer in first-degree relatives

E. BRCA gene mutations

Reference answer: A. Your answer:

[analysis] Early menarche age, late age of menopause (A wrong, is the correct answer to this question), infertility and late age of first term birth are related to the onset of breast cancer.

[Misavoidance] Postpartum unlactation (B pair), obesity (C pair) and family

history of breast cancer in first-degree relatives (D pair) all increase

The risk of breast cancer is a high-risk factor of breast cancer. Mutations in the BRCA gene (E pair) can cause breast cancer and ovaries

Cancer, pancreatic cancer, prostate cancer, and many other cancer types have an increased cancer risk.

Is not a risk factor for breast cancer is A. The early age of menopause. Because late menopause (late age of menopause) rather than early menopause is generally considered as a risk factor for breast cancer. Late menopause means that women are exposed to estrogen in life, which may increase the risk of breast cancer. Conversely, early menopause reduces the overall exposure time of estrogen in women and thus may potentially reduce the risk of breast cancer. Other options, such as postpartum unlactation, obesity, family history of breast cancer in first-degree relatives and BRCA gene mutations, are known high-risk factors for breast cancer.

First, we need to identify the high-risk factors for breast cancer. The high risk factors for breast cancer mainly include long-term stimulation of endogenous or exogenous estrogen, premature menstruation (less than 12 years old) or late menopause (later than 55 years old), no birth, late childbearing (after 35 years old) or no lactation after birth, family history of breast cancer, and breast dysplasia.

Next, we will analyze each option:

A. Early age of menopause: This is inconsistent with high-risk factors for breast cancer. Indeed, late age at menopause is a high-risk factor for breast cancer. Therefore, early age at menopause is not regarded as a high-risk factor for breast cancer.

B. postpartum not breastfeeding: this is one of the high risk factors for breast cancer. Unlactation may lead to sustained estrogen stimulation of breast tissue, which increases the risk of breast cancer.

C. Obesity: Obesity is also recognized as a high-risk factor for breast cancer, especially in postmenopausal women. Obesity may lead to elevated estrogen levels that may increase the risk of breast cancer.

D. Family history of breast cancer in first-degree relatives: A significant family history of breast cancer is one of the high-risk factors for breast cancer. If a first-degree relative (e. g., mother, daughter, sister) has breast cancer patients, the individual risk of breast cancer increases.

E. BRCA gene mutations: The BRCA 1 and BRCA 2 genes are associated with

an increased risk of breast cancer and ovarian cancer. Mutations in these genes can significantly increase breast cancer risk.

In conclusion, not being A high-risk factor for breast cancer is the early age of A. menopause.

The risk factors for breast cancer include many aspects, but the following options are not the risk factors:

A. Early age of menopause

B. No postpartum lactation

C. fat

D. Family history of breast cancer in first-degree relatives

E. BRCA gene mutations

The correct answer is: A. Early age of menopause

Explanation: High risk factors for breast cancer include postpartum lactation, obesity, family history of breast cancer in first-degree relatives, and BRCA gene mutation. Early age at menopause (relatively speaking) may actually reduce the risk of breast cancer, because prolonged oestrogen exposure is associated with breast cancer onset. Early menopause means a shorter exposure to estrogen and may reduce the risk of breast cancer. Therefore, the early age of menopause is not a high-risk factor for breast cancer.

18. (Type A1 question)

Treatment treatment for severe ulcerative colitis

A. glucocorticoid

B. azathioprine

C. infliximab

D. ciclosporin

E. Large number of antibiotics

Reference answer: A. Your answer:

[Analysis] For severe ulcerative colitis, glucocorticoid is the preferred treatment drug (A pair).

[Error] 5-aminosalicylic acid is preferred in patients with ulcerative colitis; glucocorticoids are ineffective against 5-aminosalicylic acid

Mild and moderate patients, especially for severe patients; azathioprine can be used for poor hormone therapy or hormone adherence

Maintenance treatment in patients; cyclosporin as salvage treatment when intravenous glucocorticoid therapy for severe UC failed;

Infliximab can be used to induce remission and salvage therapy in UC.

As the preferred treatment for severe ulcerative colitis, glucocorticoid (A) is usually recommended as the first line treatment. This is because they are able to rapidly reduce the inflammation and symptoms. Therefore, the best answer is A. glucocorticoids.

analyze:

Ulcerative colitis is a chronic non-specific intestinal inflammatory disease, mainly involving the rectum and colon, and often has a recurrent chronic course. The clinical manifestations are intermittent diarrhea, mucus, pus and blood stool, abdominal pain and severe emergency, etc. This disease is difficult to cure, high recurrence rate, has a high cancer rate, and is closely related to the incidence of colon cancer. Ulcerative colitis can be classified as mild, moderate or severe by severity. Patients with severe ulcerative colitis had diarrhea more than 6 times a day, with obvious mucinous pus and bloody stools, body temperature> 37.5℃, pulse> 90 times / min, hemoglobin <100g / L, sedimentation> 30mm / h, increased C-reactive protein (CRP), and serum albumin <30g / L. Its treatment should be preferred by glucocorticoids.

Option B azathioprine is an immunosuppressive drug, the main adverse reactions are bone marrow suppression, liver function damage, and the effect is slow, so it is not the first choice for ulcerative colitis, so it is excluded.

Option C infliximab is mainly used in chronic persistent cases with poor hormone therapy or hormone dependence. It is especially suitable for patients with severe ulcerative colitis that is not respond to hormones, but it is not preferred, so it is

not selected.

Option D cyclosporin is an immunosuppressant, which is suitable for the rejection of organ transplantation and bone marrow transplantation, and autoimmune diseases such as ectopic dermatitis and psoriasis that are difficult to control, which are not used for the treatment of ulcerative colitis and can be excluded.

Option E Large numbers of antibiotics are ineffective for ulcerative colitis because it is not an inflammation caused by a bacterial infection and can be therefore excluded preferentially.

In conclusion, the correct answer is: A. corticoids.

The preferred treatment for severe ulcerative colitis is usually corticosteroids. Glucocorticoids rapidly relieve acute inflammation and are the standard initial treatment for severe ulcerative colitis.

So, the most correct answer is:

A. glucocorticoid

19. (Type A1 question)

Most helpful in determining whether tuberculosis is infectious is

A. γ -interferon release assay

B. Acid-fast staining of the sputum smear

C. tubercalin test

D. chest CT

E. Anti-TB antibodies

Reference answer: B. Your answer:

[Analysis] Tuberculosis in the population is mainly tuberculosis patients, that is, sputum direct smear positive (B pair), mainly cough, sneeze, laugh, loud talk and other ways to contain mycobacterium tuberculosis micro droplets into the air and spread.

[Wrong avoidance] γ -interferon release test (A error) can distinguish natural

infection of Mycobacterium tuberculosis from BCG vaccination and most parts

By non-tuberculous mycobacteria infection, so the specificity of the diagnosis of TB infection is significantly higher than the PPD test, but due to the higher cost

For other reasons, it is mostly used for research and evaluation and has not been widely implemented. Tuberculin test (C error) and anti-TB antibodies

Examination (E error) can only be used to identify if the patient has been infected with tuberculosis and is not the source of infection.chest

CT (D wrong) can mainly show the characteristics and properties of various types of tuberculosis, whether there is no cavity and progression deterioration and absorption improvement

Change, and cannot be clear whether pulmonary tuberculosis is infectious.

The most helpful way to determine whether pulmonary tuberculosis is contagious is to check whether the patient is able to discharge sputum containing Mycobacterium tuberculosis. So the best answer is:

F. Acid-fast staining of the sputum smear

Acid-fast stain of sputum smear is a rapid and effective method for detecting whether sputum contains Mycobacterium tuberculosis, which is a key indicator to determine whether tuberculosis infection is infectious. Other options such as γ -interferon release assay, tuberculin assay and anti-TB antibody detection are mainly used to diagnose the presence of infection rather than infectivity. Chest CT is more often used to assess the extent and type of lung lesions.

analyze:

Whether tuberculosis is infectious, mainly depends on whether the patient is discharged bacteria, namely whether tuberculosis bacilli exists in the sputum. If the patient has tuberculosis bacilli in the sputum, it is infectious, and otherwise, it is not contagious. Sputum smear acid resistant stain can be tested by the sputum contains acid resistant bacilli, to determine whether the patient discharged bacteria, to determine whether tuberculosis is infectious most helpful.

Option A: γ -interferon release test, option C: tuberculin test, both are used to detect mycobacterium tuberculosis infection, rather than bacteria, so it has little infectious value for determining tuberculosis, so it can be excluded together.

Option D: Chest CT is accurate for the nature, location, involvement and

relationship of the lesions in the lung, but it is impossible to determine whether the patient is discharged, so it is not selected.

Option E: Positive anti-TB antibody test indicates anti-TB bacterium antibody in the body, indicating that the subject has been infected with TB bacteria, but it cannot judge whether it is infectious, so it is not considered.

In conclusion, the correct answer is: B. sputum smear of acid-fast staining.

The most helpful tests to determine whether tuberculosis is infectious are:

G. Acid-fast staining of the sputum smear

Reason: The presence of Mycobacterium tuberculosis (acid fast bacillus) can be directly detected by sputum smear acid fast staining. If the sputum smear is positive for acid resistance staining, it means that the patient has live tuberculosis bacteria in the sputum, which is infectious. Therefore, this is the most direct and effective method to detect the infectivity of pulmonary tuberculosis.

20. (Type A1 question)

Cirpercardiac cardiac cardiac ation does not include any vessel

A. venae gastroepiploica sinistra

B. vena gastrica posterior

C. Left inferior diaphragmatic vein

D. coronary vein

E. short gastric veins

Reference answer: A. Your answer:

[Analysis] cirrhosis pericardiac vascular bypass does not include the left vein of gastric omenentum (A wrong, is the correct answer to this question).

[Avoid mistakes] The blood vessels around the cardia can be divided into four groups: ① coronary vein (D pair): including gastric branch, esophageal branch and high esophagus

Branch, etc.; ② A short gastric vein (E pair): generally 3~4 branches,

accompanied by a short gastric artery, distributed in the anterior and posterior walls of the gastric fundus, injection

Into the splenic vein; ③ posterior gastric vein (in B pair): starting from the posterior wall of the gastric fundus, with the same artery descending, injected into the spleen static; ④ left

Lower diaphragmatic vein (C pair): a single branch or branch into the gastric fundus or the lower esophagus.

Pericardiac cardiac bypass (also known as portal shunt) is designed to reduce portal pressure and reduce the risk of esophageal varices. In this procedure, the doctor cuts off some blood vessels to reduce blood flow to the portal system, thus reducing portal hypertension.

The option analysis is performed as follows:

-A. Left vein of the gastric omenum: usually cut off in the case of portal hypertension because it is connected to the portal system.

-B. Intragastric vein: also associated with portal hypertension and may be disconnected.

-C. Left inferior diaphragmatic vein: not directly involved in the portal system and usually not within the flow range of this procedure.

-D. Coronary vein: associated with portal hypertension and may be disconnected to reduce pressure in the cardia.

-E. Short gastric vein: associated with portal hypertension.

Based on the above analysis, the vessel least likely to be included in the pericardiac vascular rupture in cirrhosis is * * C. left inferior diaphragmatic vein * *. Therefore, the correct answer should be a C.

analyze:

Major bleeding of ruptured esophageal and gastric varices caused by portal hypertension of liver cirrhosis is the most common complication of portal hypertension, with a high mortality rate and a high recurrent bleeding rate. Around cardiac vascular dissection is the treatment of portal hypertension caused by esophageal gastric varices rupture bleeding important operation, through the lower esophagus and gastric proximal, cardiac abnormal blood flow, to control the purpose of esophageal gastric varices rupture bleeding, while maintaining the portal vein to

the liver blood flow, so as to ensure the blood supply of the liver. The left vein of the gastric omentum travels in the great gastric bend, distributed along the great gastric curvature and anastomosis with the right vein of the gastric omentum, and is injected into the splenic vein. It is a branch of the splenic vein, does not belong to the pericardia vessels, and is not treated in the pericardia dissection.

Option B posterior gastric vein, option C left inferior diaphragmatic vein, option D coronary vein, option E short gastric vein, which belong to pericardiac vessels. During the pericardiac vascular dissection, they needs to be removed together to block abnormal blood flow in the lower esophagus and gastric fundus and reduce bleeding caused by portal hypertension, which can be excluded together.

In conclusion, the correct answer is: A. Left vein of the gastric omentum.

Pericardiac vascular disconnection (such as Sugiura surgery) is a procedure for bleeding from the esophageal and gastric varices of cirrhosis that reduces the risk of bleeding by breaking the venous vessels around the cardia. The following are the common pericardiac vascular vessels:

1. Left vein of the gastric omentum
2. and that of the retrogastric vein
3. Coronary veins
4. Short gastric veins

These vessels are all vessels to be treated in the pericardiac vascular bypass, and the left inferior diaphragmatic vein is usually not involved in this procedure. So, the correct answer is:

F. Left inferior diaphragmatic vein

21. (Type A1 questions)

The most common non-neoplastic polyps in the colorectal population are

A. hyperplastic polyp

B. Hamartomatous polyp

C. metaplastic polyp

D. inflammatory polyp

E. adenomatous polyp

Reference answer: A. Your answer:

[Analysis] Colorectal non-neoplastic polyps refer to the abnormal growth of the surface of the colorectal mucosa. There are mainly juvenile polyps, hyperplastic polyps and inflammatory polyps, among which the most common non-neoplastic polyps is hyperplastic polyps (pair A).

[Error avoidance] neoplastic polyps are true tumors of epithelial epithelial cells in the large intestine, and their single tumors are collectively referred to as adenoma, according to their group

The different weaving characteristics and biological behavior can be divided into glandular tubular, villous and mixed 3 categories, multiple people are common for the home

Familial adenomatosis.

In the colorectal population, the most common non-neoplastic polyps are hyperplastic polyps. Therefore, the correct answer is the A. hyperplastic polyps.

Hyperplastic polyps are the most common non-neoplastic polyps in the colorectum, which are generally considered benign and rarely develop cancer. This type of polyps usually do not require specific treatment, but may require regular monitoring to ensure that they do not develop into a more severe condition.

analyze:

Colorectal polyps generally refer to bulging lesions protruding from the surface of the rectum lining into the intestinal lumen. According to the pathology, it can be divided into: adenomatous polyps, hamartomatous polyps, hyperplastic polyps, inflammatory polyps, etc. Among them, hyperplastic polyps, also known as metaplastic polyps, are the most common non-neoplastic polyps, which mostly occur in the rectum and are more common after middle age.

Option B hamartomatous polyps, although non-neoplastic polyps, were not as common as hyperplastic polyps, so they were excluded.

Option C metaplastic polyps belong to hyperplastic polyps, which are not

classified separately, so they are not selected.

Option D inflammatory polyp, also known as false polyp, is due to the intestinal mucosa caused by chronic inflammation of polyp granuloma, the polyp more in ulcerative colitis, chronic schistosomiasis, amoeba dysentery and intestinal tuberculosis disease of intestinal lesions, belong to the neoplastic polyps, but not common, with the problem.

Option E Adenomatous polyp, also known as neoplastic polyp, is a benign epithelial tumor of the large intestine, which can become cancerous and does not belong to non-neoplastic polyp, so it is excluded.

In conclusion, the correct answer is: A. Hyperplastic polyps.

The most common non-neoplastic polyps in the colorectal population are:

A. hyperplastic polyp

22. (Type A1 question)

Non-surgical approach for acute peptic ulcer perforation is wrong

A. V.T

B. gastrointestinal decompression

C. glucocorticoid

D. proton pump inhibitor

E. Antibiotics against infection

Reference answer: C Your answer:

Graphic resolution: [analytical] acute peptic ulcer perforation using glucocorticoid (C wrong, for the correct answer) no obvious effect, even glucocorticoid will increase gastric acid, pepsin secretion, reduce gastric mucus secretion, make the resistance of gastrointestinal mucosa, under the erosion of gastric fluid can induce or aggravate the stomach, duodenal ulcer and gastrointestinal bleeding or perforation.

[Erstors avoidance] Proton pump inhibitors have a strong acid inhibitory effect, which can reach the acid-free level in the stomach and control digestion within 2 to 3 days

Ulcer symptoms (D pair). Antibiotics against infection (E), sedation and analgesic treatment, gastrointestinal decompression (B), and intravenous infusion

(A pair), nutrition is A general non-surgical treatment measure, suitable for mild disease or disease for more than 24h, and the abdominal signs have been reduced

Or accompanied by severe heart and lung and other organ diseases can not tolerate surgery.

In the non-surgical treatment of an acute peptic ulcer perforation, corticosteroids (option C) are usually not recommended. Acute peptic ulcer perforation is a clinical condition that requires urgent management and may require surgical procedures to repair the perforation. Non-surgical treatment mainly includes stabilization of the patient's vital signs, antibiotic treatment to prevent infection, maintenance of water and electrolyte balance and nutritional support with intravenous fluids, and reduction of gastric acid secretion by proton pump inhibitors (PPIs).

Glucocorticoids are generally not commonly used in the nonsurgical treatment of acute peptic ulcer perforation, as they may suppress immune responses, delay ulcer healing, and increase the risk of infection. Therefore, the wrong answer to this question is C. glucocorticoids.

analyze:

Acute perforation of peptic ulcer is a serious complication of peptic ulcer, with acute onset, rapid change and severe condition, which requires urgent treatment. Non-surgical treatment is indicated for fasting perforations with good general condition and mild symptoms and signs. In the non-surgical treatment, the application of glucocorticoids is not appropriate. Although corticosteroids can inhibit the inflammatory response, they may also aggravate digestive tract ulcers and increase the risk of gastrointestinal bleeding, so they should be avoided in the treatment of acute peptic ulcer perforation.

Option A intravenous infusion can supplement blood volume and maintain water and electrolyte balance. It is one of the basic measures for non-surgical treatment of acute peptic ulcer perforation and does not meet the requirements.

Option B Gastrointestinal decompression can reduce gastrointestinal pressure, reduce gastrointestinal content into the abdominal cavity, which is conducive to the healing of perforation. It is one of the important measures for non-surgical treatment of acute peptic ulcer perforation, so it is excluded.

Option D proton pump inhibitor can inhibit gastric acid secretion, reduce the stimulation of gastric acid on gastrointestinal mucosa, and is conducive to the healing of perforation. It is an important drug in non-surgical treatment, so it is not selected.

Option E Antibiotic against infection can prevent and treat infection and reduce the occurrence of complications. It is an essential measure in the non-surgical treatment of acute peptic ulcer perforation, which is inconsistent with the problem.

In conclusion, the correct answer is: C. corticoids.

In non-surgical treatment for acute peptic ulcer perforation, the mistake is:

F. glucocorticoid

explain:

Acute peptic ulcer perforation is an acute in where the gastric or duodenal ulcer penetrates the gastrointestinal wall and causes intra-abdominal perforation. Non-surgical treatment mainly includes:

-Intravenous fluids (A): Supplement of body fluids and electrolytes to maintain stable patient circulation.

-Gastrointestinal decompression (B): reduce the pressure of the gastrointestinal contents on the perforation site through the nasogastric tube, reducing further leakage and inflammation.

-Proton pump inhibitor (D): Reduce gastric acid secretion and promote ulcer healing.

-Antibiotic anti-infection (E): prevention and treatment of abdominal cavity infection.

Glucocorticoids (C) are not a conventional treatment for acute peptic ulcer perforation because they may inhibit the inflammatory response, delay ulcer healing, and increase the risk of infection.

23. (Type A1 question)

Functional dyspepsia is defined as a defined condition

A. Symptoms for 12 months for 3 consecutive months

B. Symptoms for 6 months for 2 consecutive months

C. Symptoms for 12 months for 2 consecutive months

D. Symptoms for 6 months for 3 months

E. Symptoms for 12 months

Refer to the answer: D. Your answer:

Graphic analysis: [analytical] functional dyspepsia refers to by stomach and duodenal dysfunction caused after filling, early full, upper abdominal pain and burning in upper abdomen symptoms, and a group of clinical syndrome, no organic disease, is continuous or recurrent chronic process, the symptoms of the disease appear at least 6 months, nearly 3 months symptoms meet its diagnostic criteria (D).

[Error avoidance] At present, the Roman standard of functional gastrointestinal disease naming classification is adopted in China. Clinically with functional dyspepsia and

IBS is more common.

Functional dyspepsia is usually defined as symptoms lasting for at least 6 months and with continuous symptoms in the last 3 months. Thus, the most appropriate option for this definition is D. symptoms for 6 months and 3 consecutive months. So the correct answer is a D.

analyze:

Functional dyspepsia refers to the gastrointestinal functional diseases caused by gastric and duodenal dysfunction, but without organic changes. The clinical manifestations are mainly fullness after the meal, early satiety, upper abdominal distension pain, belching, loss of appetite, nausea, etc. The diagnosis of functional dyspepsia should meet the following criteria: one or more symptoms of upper abdominal pain, upper abdominal burning, postprandial fullness and early fullness, with a persistent or recurrent chronic course (more than 6 months, symptoms lasting nearly 3 months); and organic diseases that explain these symptoms.

In conclusion, the correct answer is: D. Symptoms for 6 months, for 3

consecutive months.

Functional dyspepsia (functional dyspepsia, FD) is a common disorder of the digestive system and is usually defined by the Rome Standard (Rome Criteria). According to the latest Rome IV standard, functional dyspepsia is defined as follows:

* * Diagnostic criteria for functional dyspepsia include: * *

-Beginning at least 6 months before the first presentation, and symptoms persist within the last 3 months.

-Major symptoms include upper abdominal pain, epigastric discomfort, early satiety, or post-meal fullness.

Based on the above information, the most appropriate options to the Rome IV criteria are:

F. Symptoms for 6 months for 3 months

Therefore, the correct answer is: * * D. Symptoms for 6 months, 3 consecutive months * *

24. (Type A1 question)

Which area of the ulcer should pay special attention to Zhuo ai syndrome

A. Gastrosinuses

B. pylorus

C. duodenal bulb

D. descendant duodenum

E. The ileum

Refer to the answer: D. Your answer:

[Analysis] The ulcers of Zhuoai syndrome are characterized by multiple cases,

located in atypical sites, such as the descending part of the duodenum (D pair), and poor efficacy of regular anti-ulcer drugs.

[Avoid error] gastric ulcer in the gastric corner and small bend of the gastric sinus; duodenal ulcer in the duodenal bulb; duodenum

Retrobulbar ulcers are prone to bleeding complications.

Zollinger-Ellison Syndrome (ZES) is a disease caused by gastrinoma (gastrinoma), which is usually located in the pancreas and duodenum, leading to excessive secretion of gastric acid and thus peptic ulcers. In Zollinger-Ellison syndrome, ulcers usually occur in the duodenum, especially in the duodenal bulb, but may also occur in the stomach or other intestinal sites. Therefore, special attention should be given to ulcers in the duodenal bulb when considering Zhuoai syndrome.

Option C. The duodenal bulb is the best fit for this description. So the most correct answer is C. duodenbulb.

analyze:

Zhuoai syndrome, also known as gastrinoma or Zollinger-Ellison syndrome, is a comprehensive disease with gastrointestinal ulcer as the main manifestation. Its typical manifestations are stubborn and atypical parts of peptic ulcer and diarrhea, ulcers are most common in the descending and horizontal parts of the duodenum, and often multiple, refractory to treat.

Option A gastric gastric antrum, option B gastric pylorus, are all common sites of peptic ulcer, which have no clear association with Zhuoai syndrome, and can be excluded together.

Option C The duodenal bulb is the most common site of duodenal ulcer and has nothing to do with Zhuoai syndrome, so it is not selected.

Option E The ileum is not a common site of peptic ulcer, and gastrointestinal ulcer in Zhuoai syndrome is also rare here, so it is excluded.

In conclusion, the correct answer is: D. Lower duodenum.

Zhuoai syndrome (Zollinger-Ellison syndrome, ZES) is a disease of gastrin hypersecretion due to gastrinoma, resulting in increased gastric acid secretion and refractory ulcers. Ulcers in ZES usually occur in multiple parts of the duodenum and are more difficult to treat than common ulcers.

In ZES, the most common site of ulcers is the duodenal bulb, because this is the first place where gastric acid enters the small intestine, and ulcers in the duodenal bulb are the most common.

So, the most correct answer is:

F. The duodenal bulb

25. (Type A1 question)

Regarding electrical burns, the following statement is correct

A. The entrance is more seriously damaged than at the exit

B. The damage range is usually small outside and large inside

C. The scope of necrosis after injury is generally not expanded again

D. Local exudation is less severe than general burns

E. There is of necrosis

Reference answer: B. Your answer:

[Analysis] When electric burn, the entrance is often carbonized, forming a crack or cave, the burn is often as deep as muscle, tendon, bone, and the damage range is often small outside and large inside (B pair).

[To avoid mistakes] Electric burn refers to the burn caused by the electric current through the human body, the main characteristics of the burn: ① light exit, heavy inside

(A wrong), small outside the damage range and large (B pair); ② progressive necrosis and injury

Later necrosis range can be expanded several times (C wrong); ③ local exudation is heavier than general burn (D wrong); ④ has no obvious necrotic layer

Face (E wrong).

Regarding the characteristics of electrical burns, option B "The damage range is usually small outside and large inside" is correct. Electrical burns are usually caused by an electrical current across the body and may cause more serious damage to

internal tissues (such as muscles and blood vessels) than to the skin surface. Therefore, external injuries in electrical burns may appear less severe than actual internal injuries, so the damage range is usually small outside and large inside. Thus, the most correct answer is the B.

analyze:

Electric burn is usually caused by the current through the human body, the current through the human body has "entrance" and "exit", the entrance is more damaged than the exit, the entrance around the carbonization, forming cracks or cave, burn often deep muscle, tendon, bone, the damage range is often small outside and large inside.

Option A heavy damage at the exit from the entrance, the current into the body, from the skin or mucosal damage to the entrance, the entrance often carbonization, form cracks or cave, burn often deep to muscle, tendon, bone, damage is heavier, and the outlet damage is relatively light, this option is correct, but not the best answer.

The characteristic of electric burns are that the damage range is usually small outside and large inside, that is, the damage range is often larger than the injured skin wound, and the scope of necrosis after injury often increases with time, and its scope and depth can be determined after a few days. Therefore, option C "The scope of necrosis after injury is generally not expanded" does not conform to the characteristics of electric burns, so it is excluded.

Option D Local exudation is lighter than general burns contrary to the fact that electric burns are different from general burns, characterized by "small mouth and large bottom, shallow outside and deep inside", deep tissue damage is often more serious, so the local exudation of electric burns is often more serious than general burns, so it is not selected.

Electrical burns have no obvious necrosis level, often accompanied by progressive necrosis of surrounding tissues. Sometimes, the scope of necrosis can be extended to muscle, tendon, bone or deeper tissues. Therefore, option E has obvious necrosis level not consistent with the characteristics of electrical burns, so it is not selected.

In conclusion, the correct answer is: B. The damage range is usually small outside and large inside.

Regarding electrical burns, the following statement is true:

F. The damage range is usually small outside and large inside

** analyze:**

One of the characteristics of electric burns is that the damage range is usually small on the outside and large on the inside, which is due to the large heat generated in the body when the current passes through the human body, resulting in more serious damage to the deep tissue, while the surface damage is relatively light. In addition, the damage range of electrical burns usually continues to expand after the injury, and the range of necrosis will gradually increase. Thus, option B is correct.

Other option resolution:

A. The entrance is more seriously damaged than the exit: usually, serious damage may occur at the entrance and exit of electrical burns, but the specific degree of damage depends on the current path and voltage. Generally speaking, the damage at the exit may be more serious.

G. The range of necrosis after injury generally does not expand: the scope of necrosis will gradually expand after electrical burns, so this is not true.

H. Local exudation is lighter than general burns: local exudation from electrical burns may be more serious than general burns, so this is incorrect.

I. There is obvious necrosis: electrical burns do have obvious necrosis, but this is not the right answer to summarize the characteristics of electrical burns.

To sum up, the correct answer is that of B.

26. (Type A1 question)

Thyroid nodules, with an aspect ratio> 1, had rich blood flow signals, and examination found increased calcitonin secretion, which possible pathological type

A. undifferentiated carcinoma

B. papillary carcinoma

C. Filtal carcinoma

D. cephaloma

E. Hypodifferentiated carcinomas

Refer to the answer: D. Your answer:

[Analysis] Thyroid nodules, aspect ratio> 1, rich blood flow signal, highly suspected of thyroid malignant tumor. The patient had elevated calcitonin, hence considered medullary carcinoma (pair D).

[Error avoidance] Medullary carcinoma is derived from C cells, with nests or cysts, without nipple or follicular structure, and is undifferentiated

Form; amyloid deposits with moderate malignancy, cervical lymph node invasion and hematogenous metastasis.

Characteristics of thyroid nodules, such as an aspect ratio greater than 1, abundant blood flow signals, and examination of increased calcitonin secretion, usually point to medullary carcinoma. Medullary carcinoma is a neuroendocrine tumor caused by the C cells of the thyroid gland that are responsible for the secretion of calcitonin. Therefore, the increased calcitonin level is an important indicator of medullary thyroid cancer. Therefore, according to the characteristics of the question stem description, the most appropriate answer is: D. medullary cancer

analyze:

Thyroid nodule refers to the mass in the thyroid gland, which can move up and down with the thyroid gland with swallowing action. It is a common clinical disease and can be caused by a variety of causes. Calcitonin is procalcitonin, which is synthesized and secreted by thyroid paracollicular cells (C cells), mainly composed of calcitonin, calcitonin gene-related peptide and parathyroid hormone-related protein. When thyroid nodules are associated with increased calcitonin secretion, the most likely pathological type is medullary thyroid carcinoma. Medullary thyroid carcinoma is derived from paracollicular calcitonin-secreting cells (C cells), and a significant increase in serum calcitonin often indicates the presence of medullary carcinoma.

Option A undifferentiated carcinoma, B papillary carcinoma, C follicular carcinoma, and E poorly differentiated carcinoma had no elevated calcitonin and could be excluded together.

In conclusion, the correct answer is: D. medullary carcinoma.

The title describes the characteristics of a thyroid nodule: an aspect ratio of> 1, rich blood flow signal, and increased calcitonin secretion. Calcitonin (Calcitonin) is a

hormone secreted by thyroid C cells, and elevated levels of calcitonin often indicate the presence of medullary carcinoma. Combined with other features such as nodular aspect ratio greater than 1 and abundant flow signals also supports this diagnosis.

So, the most likely pathological type is:

F. cephaloma

27. (Type A1 question)

The cause of abnormal liver function in patients with liver cirrhosis after the etiology

A. innutrition

B. Increases in the antihormone inactivation

C. Llateral cycle formation

D. hypersplenia

E. Elevated abdominal pressure

Reference answer: C Your answer:

[Analysis] The formation of collateral circulation (C pair) is an important pathophysiological link to promote liver function decline after the cause. Lateral circulation will cause a large number of portal vein blood flow to bypass liver cells, resulting in toxic substances can not be metabolized and detoxification and directly enter the systemic circulation, so as to further decrease liver function.

[Error] Other options are the general clinical manifestations of cirrhosis.

The causes of abnormal liver function in patients with cirrhosis are more complex and can include multiple factors. Of the options available here:

-A. Malnutrition: Malnutrition affects overall health, but it is not the direct cause of abnormal liver function.

-B. Increased hormone inactivation: a less cirrhotic liver ability to metabolize hormones, not increased hormone inactivation.

-C. Formation of collateral circulation: This is a common phenomenon in

advanced stages of cirrhosis, which can lead to portal hypertension, but does not directly lead to abnormal liver function.

-D. Hypersplenism: In liver cirrhosis, portal hypertension can lead to hypersplenism, which can indirectly affect liver function.

-E. Elevated abdominal pressure: increased abdominal pressure may be due to increased ascites, which is one of the advanced symptoms of liver cirrhosis, but not the direct cause of abnormal liver function.

Considering that portal hypertension caused by cirrhosis can cause hypersplenism, which then affects blood components, such as increasing the destruction of red blood cells and platelets, thus affecting liver function, I tend to choose * * D. hypersplenism * * as the answer. Hypersplenism can cause blood cytopenia, and then affect the results of liver function test, manifested as abnormal liver function.

analyze:

Liver cirrhosis is a diffuse damage to the liver caused by long-term and repeated stimulation of one or more causes, which is characterized by chronic diffuse connective tissue hyperplasia, hepatocyte degeneration and necrosis, regeneration, damage of hepatic lobule structure, and the formation of false lobules. Early liver function can be compensated, and cirrhosis develops to a certain extent, beyond the compensatory ability of liver function, which is called the decompensation period of cirrhosis. The main clinical manifestations are the symptoms and signs caused by liver function decline and portal hypertension. Liver function decline will affect the synthesis of protein, albumin synthesis is reduced, thus affecting the plasma colloid osmotic pressure, resulting in excessive production of tissue fluid, and the occurrence of malnutrition and edema, so malnutrition is the performance of abnormal liver function.

Option B increased hormone inactivation is opposite to the question, but should decrease hormone inactivation, so it is not selected.

Option C collateral circulation formation, option E abdominal pressure increase, are the clinical manifestations of portal hypertension, can be excluded together.

Option D Hypersplenism was mainly characterized by splenomegaly and hypersplenism, and mainly by early leukocyte and thrombocytopenia, which was not the cause of abnormal liver function, so it was not considered.

To sum up, the correct answer is: A. malnutrition.

The causes of abnormal liver function in patients with liver cirrhosis mainly include many factors, but the most direct and common cause is hypersplenism (D. hypersplenism). Hyperhyplenism is due to increased blood flow in the spleen caused by portal hypertension, which causes the enlargement and hyperfunction of the spleen, and then causes excessive destruction of blood cells, leading to anemia, leukopenia and thrombocytopenia, which will further aggravate the abnormal liver function.

So, the most correct answer is:

F. hypersplenia

28. (Type A1 question)

Esophageal and gastric fundus varices were treated surgically, and the blood vessels to be cut were not included

A. venae gastrica sinistra

B. short gastric veins

C. vena gastrica posterior

D. Left inferior diaphragmatic vein

E. splenic vein

Reference answer: E Your answer:

[Analysis] For surgical treatment of esophageal and gastric varices, the vessels to be cut do not include large veins, such as the right vein of gastric omentum and splenic vein (E pair).

[Avoid mistakes] The blood vessels around the cardia can be divided into four groups: ① left gastric vein (A wrong), also known as coronary vein: including gastric branch and food

Tube branch and high esophageal branch, etc.② Gastric short vein (B wrong): generally 3~4 branches, accompanied by a short gastric artery, distributed in the stomach

Front and posterior walls of the floor, were injected into the splenic vein.③ Pogastric vein (C wrong): starts from the posterior wall of the gastric fundus, with the same artery,

The splenic vein was injected.④ Left inferior diaphragmatic vein (D wrong): single or branch into the gastric fundus or the left muscle layer of the lower esophagus.

In performing surgical treatment of esophagogastric varices, the main goal is to reduce the pressure in the gastroesophageal area under portal hypertension and thus reduce the risk of bleeding. Surgery usually requires cutting of venous access associated with portal hypertension, especially those that directly contribute to the lower esophageal segment and gastric fundus varices.

A. The left gastric vein (left gastric vein) is usually cut off because it is one of the main sources of blood for the base of the esophagus and fundus gastric varices.

B. The short gastric vein (short gastric veins) is also connected to the splenic vein, which is involved in the blood reflux of the bottom gastric vein, and may be cut off.

C. The retrogastric veins (posterior gastric vein) may be involved in this process, especially if they provide a blood supply to the lower esophagus or fundus of the stomach.

D. The left inferior diaphragmatic vein (left inferior phrenic vein) may not be directly involved in the varices of the lower esophagus and the stomach, and may not be cut.

E. The splenic vein (splenic vein) is one of the main blood vessels, which is connected to the gastric fundus varicose veins through a short gastric vein, and is usually involved in the portal body shunt operation.

According to this information, the most unlikely vessel to be cut is * * D. left subdiaphragmatic vein * * as it is not the primary vessel directly involved in the esophageal and gastric floor varices.

analyze:

The main purpose of surgical treatment of esophageal and gastric varices is to remove the blood vessels around the lower esophagus and the gastric cardia, including the left gastric vein (also known as coronary vein), the posterior gastric vein, the short gastric vein, the left inferior diaphragmatic vein and the accompanying artery of the same name, and to perform splenectomy to achieve the purpose of immediate

hemostasis. However, the splenic vein is the companion vein of the splenic artery, under the splenic artery, across the front of the posterior left kidney and renal vein, extending to the tail of the pancreas, to the back of the pancreas, to the front of the posterior left adrenal gland and the kidney, to the right curvature of the transverse colon, and then forward into the portal vein system. It is not involved in the composition of esophageal and gastric fundus vein, and it is important for the maintenance of splenic vein reflux, so there is no need to dissociation.

Option A The left gastric vein, also known as the coronary vein, is consistent with the venous plexus of the lower esophagus, and is one of the blood vessels that need to be severed during surgery.

Option B short gastric vein, option C retrogastric vein, option D left inferior diaphragmatic vein, all involved in the formation of esophagogastric fundus venous plexus, so the operation should be removed together to block the abnormal blood flow between the portal vein, to achieve the purpose of controlling the ruptured bleeding of esophagogastric fundus varices.

To sum up, the correct answer is: E. Splenic vein.

Surgical treatment of esophageal and gastric fundus varices mainly reduces the pressure of the varicose veins by cutting off the feeding veins, thus preventing bleeding. The following are the vessels often cut in surgery for varicose veins:

- ** Left gastric vein * * (also known as coronary vein)

- ** Short gastric vein * *

- ** Retrogastric vein * *

- ** Left inferior phrenic vein * *

But * * The splenic vein * * is usually not one of the vessels that needs to be cut. The splenic vein is part of removing blood from the spleen and feeding into the portal system rather than being directly involved in the blood supply to the oesophageal-gastric fundus varices.

So the answer is:

F. splenic vein

29. (Type A1 questions)

The characteristic of chronic anal fissure is

A. elastic

B. The granulation is gray

C. without scar formation

D. The bottom is shallow

E. The edges are neat

Reference answer: B. Your answer:

[Analysis] Chronic anal fissure is characterized by irregular deep bottom, hard mass, edge thickening, fibrosis, granulation gray (B pair), and scar formation.

[Avoid error] Acute anal fissure shows the fissure edge neat (E wrong), shallow bottom (D wrong), red and elastic (A wrong),

No scar formation (C error).

Chronic anal fissure features usually include marginal sclerosis, internal anal muscle or fibrous tissue at the bottom, and often with anal papillary hyperplasia or anal sinus formation. Therefore, among these options, the best characteristics of chronic anal fissure are:

F. granulation gray

This option may refer to the possible granulation tissue seen at the base of the anal fissure, with this gray-white granulation tissue formed during chronic inflammation and healing. Therefore, the best answer should be B. granulation gray.

First, let's understand the basic concept of chronic anal fissure. Anal fissure is the whole-layer longitudinal fissure of the anal skin, usually caused by dry stool or excessive contraction of the anal sphincter. Anal fissure can be divided into acute and chronic two types, in which chronic anal fissure is usually formed because the acute anal fissure is not treated in time.

Now, one by one:

A. Resilience: Chronic anal fissure often harden and loses elasticity due to long-term inflammation and healing processes. Therefore, this option does not fit with the characteristics of chronic anal fissure.

B. Granulation gray: In chronic anal fissure, the granulation tissue may appear gray-white due to the chronic inflammation and repair process of the tissue, which is associated with long-term healing and scar formation. This option is consistent with the characteristics of a chronic anal fissure.

C. No scarring formation: In chronic anal fissure, due to the long-term healing and reinjury process, scar tissue is often formed around the anal fissure. Therefore, this option is inconsistent with the characteristics of a chronic anal fissure.

D. Shallow bottom: Chronic anal fissure Due to chronic chronic inflammation and repair, the fissure is usually deep and deeper at the bottom than acute anal fissure. Therefore, this option does not fit with the characteristics of chronic anal fissure.

E. Neat edges: The edges of chronic anal fissure are often irregular, with hyperplasia and hyperplasia, not as neat as acute anal fissure. Therefore, this option is also not consistent with the characteristics of a chronic anal fissure.

In conclusion, consistent with the characteristics of chronic anal fissure is granulation gray, which is due to the long-term chronic inflammation and repair process. Therefore, the correct answer is the B.

So, the correct option for this question is B. granulation gray.

Characteristics of chronic anal fissure include long duration, difficulty in healing, often with scar tissue and anal margin with valgus. The specific features are shown as follows:

1. The edge of the anal fissure is often not neat, and there are valgus skin wrinkles.

2. The bottom is deeper, showing gray-white granulation tissue.

3. It is often accompanied by scar formation.

4. There is generally no elasticity.

Based on the above characteristics, the most correct answer is:

F. granulation gray

30. (Type A1 questions)

The lower third fracture of the radius combined with the small head dislocation of the ulna belongs to

A. Mont's fracture

B. Gai's fracture

C. Colles. for a fracture

D. Smith for the fracture

E. ulnar fracture

Reference answer: B. Your answer:

[Analysis] The lower third fracture of the radius combined with the dislocation of the ulna belongs to Gycher's fracture.

[Misavoidance] One third fracture of the ulna combined with radial head dislocation is Montessori fracture (A error); extended fracture

(Colles fracture) (C wrong) mostly due to the wrist joint in the back extension, palm landing, forearm rotation injury, but

The typical deformity posture, that is, the side is "silver fork" deformity, the front is "bayonet" deformity, X-ray visible

The fracture is displaced distal to the radius and dorsal side and proximal to the volar side; and a Smith (Smith) (D error) fracture is the distal radius

Fractures within 2 to 3 cm with distal volar or angulated dorsal.

A fracture of the lower third of the radius with dislocation of the ulnhead is commonly known as Montessori fracture (Monteggia fracture). Therefore, the most correct answer is: A. Montessori fracture

This is a question of medical expertise. First, we need to identify the fracture type represented by each option, and then compare it with the fracture of the radius with the dislocation of the ulna.

A. Monchsori fracture: This fracture involves a shaft fracture of the upper third of the ulna, accompanied by a dislocation of the radial head, inconsistent with the title description.

B. Gai fracture: This fracture refers to a fracture of the lower third of the radial shaft, accompanied by a dislocation of the ulna head. This coincides with the "fracture of the lower third of the radius with the dislocation of the ulna" described in the title.

C. Colles fracture: This fracture refers to the fracture occurring within three centimeters of the distal radius, and the distal end of the fracture will shift to the radius and dorsal side, and the proximal part is shifted to the volar side, which is inconsistent with the description of the title.

D. Smith fracture: This fracture is a flexion distal radius fracture, or reverse Colles fracture, with the distal end of the fracture leading to the volar and ulnar displacement, inconsistent with the title description.

E. Ulnar fracture: This option only describes the location where the fracture occurred and does not involve the dislocation of the radius or ulna small head, which is inconsistent with the title description.

In conclusion, the lower third of the radius fracture with ulna dislocation fits the definition of Gaycher fracture.

Therefore, the correct answer is B. Gaius fracture.

The lower third fracture of the radius combined with the small head dislocation of the ulna belongs to:

B Gayi fracture (Galeazzi fracture)

Gage's fracture is a fracture of the distal radius with a dislocation of the ulna.

31. (Type A1 question)

The most common ANCA (neutrophil cytosolic antibody) positive nephropathy is

A. aggressive glomerulonephritis

B. Acute glomerulonephritis

C. lupus nephritis

D. Acute interstitial nephritis

E. Acute renal tubular necrosis

Reference answer: A. Your answer:

Graphic analysis:

The most common occurrence of ANCA (neutrophil cytosolic antibody) -positive nephropathy is A. acute glomerulonephritis.

Acute glomerulonephritis, usually associated with a positive ANCA, is a severe renal disease characterized by a rapid impairment of the glomerulus. ANCA is mainly associated with small vasculitis, including two major types of acute glomerulonephritis: microscopic polyvasculitis and necrotizing small arteritis. Therefore, the choice of A as the most common nephropathy associated with ANCA positivity is the most appropriate.

analyze:

Neutrophil cytoplasmic antibody (ANCA) is a kind of autoantibody with the cytosolic components of neutrophils and monocytes as the target antigen. It is an important indicator for the diagnosis, monitoring of disease activity and predicting recurrence of primary small vasculitis, with good specificity and sensitivity. The most commonly observed positive ANCA nephropathy is acute glomerulonephritis.

Option B Acute glomerulonephritis was mainly caused by the immune response induced after β -hemolytic streptococcus infection, showing hematuria, proteinuria, edema and hypertension, without positive ANCA, so excluded.

Option C lupus nephritis refers to a disease of systemic lupus erythematosus with different pathological damage of both kidneys, accompanied by clinical manifestations of obvious renal damage. Its pathogenesis is the formation and deposition of immune complexes, and will not cause ANCA positive, so it is not selected.

Option D Acute interstitial nephritis is a kidney disease with by tubular interstitial damage. It mainly leads to inflammatory cell infiltration of interstitial kidney, interstitial edema, and damaged renal tubules. The glomerulus and renal blood vessels are generally not involved or relatively mild, which is related to the deposition of immune complex, which is inconsistent with this question.

Option E Acute tubular necrosis is mainly caused by ischemia and nephrotoxic substance damage, and the lesions are mainly in the proximal convoluted tubules, unrelated to this problem.

In conclusion, the correct answer is: A. Acute glomerulonephritis.

32. The most common ANCA (neutrophil cytosolic antibody) positive nephropathy is

A. aggressive glomerulonephritis

Acute glomerulonephritis (RPGN) is the kidney disease most commonly associated with ANCA positivity. ANCA-associated vasculitis (such as microscopic polyvasculitis and Wegener's granulomatosis) usually presents as acute glomerulonephritis, where patients may develop acute renal failure and crescent formation is seen on renal biopsy. Therefore, the correct answer is A. acute glomerulonephritis.

33. (Type A1 question)

The most significant thing for identifying whether glomerular hematuria is

A. total hematuria

B. Combined urethral stimulation sign

C. positive urinary occult blood

D. gross hematuria

E. Deformed red blood cell hematuria

Reference answer: E Your answer:

Graphic and text analysis: microscopic deformed red blood cells were mostly glomerular, and all normal red blood cells were non-glomerular. Urinary red blood cell volume distribution curve, glomerular origin showed asymmetry, non-glomerular origin showed symmetry curve.

In identifying glomerular hematuria, the most meaningful is option E, erythroid

hematuria. Glomerular hematuria is characterized by the appearance of deformed red blood cells (i. e., heteromorphic red blood cells) in the urine, which is due to morphological changes when the red blood cells pass through the damaged glomeruli. In contrast, non-glomerular hematuria (following tract below) usually presents non-deformed red blood cells. Therefore, the detection of deformed red blood cells in urine is an important indicator to identify glomerular-derived hematuria.

First, let's take a look at this problem. The topic asked us to identify which performance is the most meaningful way to determine whether it is glomerular hematuria.

Option A, full hematuria, where urine contains blood from the beginning of urination to the end of urination. However, this does not clearly indicate the source of hematuria, because besides glomerulatogenous hematuria, problems other, such as ureter, bladder or urethra may also lead to full hematuria.

Option B, combined with the urethral irritation sign, usually associated with urinary tract infection, is not a specific manifestation of glomerular disease.

Option C, positive urinary occult blood, only indicates the presence of hemoglobin in the urine, but does not determine whether the source of the glomerulus or elsewhere.

Option D, macroscopic hematuria, refers to the hematuria that can be observed directly by the naked eye. But this also does not define the source of the hematuria.

Option E, deformed erythrocyte hematuria, means that the red blood cells are squeezed and damaged when passing through the diseased glomerular basement membrane, resulting in changes in the red blood cell morphology. Such morphological altered red blood cells are known as deformed red blood cells. When a large number of deformed red blood cells appear in the urine, it usually means that hematuria originates from the glomerulus, so it is important to identify whether it is glomerulogenous hematuria.

In conclusion, deformed erythroid hematuria is a key indicator to identify glomerular-origin hematuria. Thus, the most meaningful option is E. metamorphosis erythrocyte hematuria.

The most significant indicator to identify glomerular hematuria is erythroid hematuria (E). Deformed red blood cells (dysmorphic red blood cells) show a microscopic morphology different from normal red blood cells due to the physical

pressure exerted when passing through the damaged glomerular basement membrane. This feature is an important marker of glomerular homogenous hematuria.

Therefore, the answer is: E. deformed red cell hematuria

34. (Type A1 question)

It belongs to the indication for hormone shock therapy

A. Cilla nephritis

B. Intravapillary proliferative glomerulonephritis

C. Mesangial proliferative glomerulonephritis

D. Focal segmental glomerulosclerosis

E. Acute interstitial nephritis

Reference answer: A. Your answer:

Graphic analysis: early diagnosis, early hormone intensive therapy, the prognosis can be significantly improved.

Hormonal shock therapy is mainly used for those diseases that respond well to corticosteroid therapy. Among these options, crescentic nephritis (option A) is one of the kidney diseases that respond well to hormonal shock therapy. The crescentic nephritis usually requires aggressive immunosuppressive therapy, including high-dose corticosteroids (hormonal shock therapy), to reduce kidney inflammation and avoid further kidney damage.

Therefore, the best answer is A. crescophytic nephritis.

This is a choice of kidney disease therapy, and we need to determine which disease is suitable for hormonal impact therapy based on the properties and treatments of various glomerular diseases.

First, we will analyze the diseases represented by each option and their treatments:

A. crescent nephritis: This is a rapidly progressive glomerulonephritis, usually associated with the deposition of anti-glomerular basement membrane antibodies or immune complexes. It is characterized by formation of intrtragerular crescents and rapid deterioration of renal function. For this disease, early, intensive

immunosuppressive therapy is critical, including high-dose glucocorticoid shock therapy.

B. Intravillary proliferative glomerulonephritis: common in acute glomerulonephritis, mainly for hematuria, proteinuria, edema and hypertension. Its treatment is mainly supportive therapy and symptomatic treatment, and hormonal shock therapy is not the first choice.

C. Mesangial proliferative glomerulonephritis: This is a chronic glomerulonephritis, manifested with an increase in mesangial cells and mesangial matrix. Its treatment includes blood pressure control, proteinuria reduction, etc. Hormone therapy may have some effect, but not hormone shock therapy.

D. Focal segmental glomerulosclerosis: This is a chronic kidney disease associated with segmental sclerosis and scarring of some glomeruli. Treatment mainly involves blood pressure control, proteinuria reduction, and possibly immunosuppressive therapy, but hormonal shock therapy is not preferred.

E. Acute interstitial nephritis: usually associated with drug response, infection, or autoimmune disease. Treatment mainly involves withdrawal of relevant drugs, infection control or use of immunosuppressive agents but not hormonal shock therapy.

In conclusion, only crescentonephritis is the indication for hormonal shock therapy because this treatment can rapidly suppress the immune response and prevent further deterioration of renal function.

Therefore, the correct answer is A. crescentus nephritis.

Crescent nephritis (A. crescent nephritis) is one of the indicated indications for hormonal shock therapy. Crenonephritis is a severe, rapidly progressive glomerulonephritis characterized by extensive crescentia formation of the glomerulus. Hormonal shock therapy can be used in such diseases to suppress the inflammatory response and slow down disease progression.

So, the most correct answer is:

A. scilla nephritis

35. (Type A1 question)

Patients with acute renal failure and hyperkalemia, heart rate 40 times / minute, the first treatment measures should be taken is

A. An intravenous drip of 5% sodium bicarbonate

B. An intravenous drip of 10% glucose + insulin

C. Oral potassium-reducing resin

D. IV 10% calcium gluconate

E. hemodialysis

Reference answer: E Your answer:

Graphic analysis: acute renal failure, mainly manifested as weakened myocardial contractility, low heart tone, slow heart rate, ventricular contraction, atrioventricular block, ventricular fibrillation, and even cardiac arrest. In this case, the heart rate was only 40 times / min, indicating that there was a very severe hyperkalemia, causing atrioventricular block, so hemodialysis is the most effective treatment. ABCD is a general treatment measure to reduce serum potassium.

In this case, the patient had acute renal failure and hyperkalemia and had a heart rate of only 40 beats / min, suggesting that the patient may have developed severe heart problems that required immediate management. In the urgent management of hyperkalemia, the first consideration is to stabilize the cardiac membrane potential and rapidly reduce blood potassium levels.

A. intravenous 5% sodium bicarbonate used to treat acidosis can help transfer potassium ions in the body, but it is not the quickest way to directly reduce blood potassium.

B. An intravenous drip of 10% glucose + insulin can rapidly transfer blood potassium into the cell, which is commonly used for hyperkalemia, but may not be preferred in cases of extremely low heart rate.

C. Oral potassium lowering resin is a long-term control of blood potassium level, and is not suitable for emergency.

D. Intravenous injection of 10% calcium gluconate rapidly stabilizes the cardiac membrane potential and is the preferred method for emergency management for arrhythmias especially in cases of bradycardia due to hyperkalemia.

E. Hemodialysis is the definitive treatment for hyperkalemia and acute renal failure, but it requires immediate action to stabilize the cardiac condition in

emergencies, and preparation for dialysis may take more time.

Therefore, the most appropriate preferred treatment is D. intravenous 10% calcium gluconate to rapidly stabilize the cardiac membrane potential and prevent arrhythmias.

analyze:

When patients with acute renal failure develop hyperkalemia, the treatment principles include: 1. Correction of metabolic acidosis; 2. stabilization of myocardial cell membrane potential; 3. Rapid reduction of blood potassium concentration; 4. Timely treatment of primary disease and recovery of renal function. When patients with acute kidney failure combined with hyperkalemia have slow heart rate, they should be highly alert to cardiac arrest, so the blood potassium concentration must be quickly reduced. Hemodialysis can quickly remove excessive potassium ions in the body, which is the most effective way to reduce blood potassium.

Option A intravenous drip 5% sodium bicarbonate can correct acidosis and promote potassium ion transfer to the cell, but potassium reduction is slower, so it is not the preferred treatment.

Option B intravenous drip 10% glucose + insulin can transfer potassium ions into cells, thus reducing serum potassium concentration, but acts more slowly and therefore can be excluded.

Option C Although oral potassium lowering resin can quickly reduce the potassium ions in the intestine, thus reducing the blood potassium concentration, the effect is slower than hemodialysis, so it is not used as the preferred treatment, so it is not selected.

Option D Intravenous injection of 10% calcium gluconate stabilized the cardiomyocyte membrane potential but did not reduce the blood potassium concentration, so it was not considered.

In conclusion, the correct answer is: E. hemodialysis.

In patients with acute renal failure with hyperkalemia and a heart rate of 40 beats / min, they first need to quickly correct the hyperkalemia and protect the heart. The best answer is:

F. IV 10% calcium gluconate

Rationale: Calcium gluconate can quickly combat the inhibitory effect of hyperkalemia on the myocardium, stabilize the myocardial cell membrane, and prevent cardiac arrest. This is a rapid, urgent treatment measure that immediately protects against cardiac function. Other options, while also reducing blood potassium levels, work slower or do not directly combat the effects of hyperkalemia on the heart.

36. (Type A1 question)

The following tests are not meaningful in distinguishing between the upper and lower urinary tract infections

A. Urinary leukocyte tube type

B. B shows abnormal renal pelvis morphology

C. Culture culture culture culture showed E hia culture

D. Knockout pain in kidney area

E. The WBC was significantly increased in the peripheral blood

Reference answer: C Your answer:

Graphic analysis:

The most common pathogen of urinary tract infection is Escherichia coli, which is available in the upper and lower urinary tract and cannot be distinguished. Option ABDE is visible in the upper urine

Road infection, but can not be seen in the lower urinary tract infection, so it can be identified.

To distinguish between upper urinary tract infections (e. g., pyelonephritis) and lower urinary tract infections (e. g., cystitis), we need to rely on those tests that can clearly indicate the site of infection. Here is an analysis of each option:

A. Urinary leukocyte tube type: usually seen in upper urinary tract infections (e. g. nephritis), it can help determine the site of infection, thus making sense to distinguish upper and lower urinary tract infections.

B. B ultrasound shows abnormal renal pelvis morphology: whether the examination of abnormal renal pelvis by B ultrasound can help to determine whether it is a problem of the upper urinary tract, so it is meaningful to distinguish the upper and lower urinary tract infections.

C. Bladder puncture culture shows Escherichia coli: bladder puncture culture can detect the pathogen causing lower urinary tract infection, but is not specific for distinguishing upper urinary tract and lower urinary tract infection, because Escherichia coli may also be the causative agent of upper urinary tract infection.

D. Bucpain in the renal area: buckle pain in the renal area is a typical symptom of upper urinary tract infection and helps to distinguish upper and lower urinary tract infections.

E. WBC is significantly increased in peripheral blood: this is a systemic response to infection and does not accurately distinguish between upper and lower urinary tract infections.

Therefore, the examination that cannot clearly distinguish between upper and lower urinary tract infections is * * E. significantly elevated WBC in peripheral blood * *, as this is a systemic response and is not sufficient to distinguish the specific site of infection.

This title asked us to judge which test is not meaningful in distinguishing between upper and lower LUIs. To solve this problem, we first need to understand the characteristics of the upper and lower urinary tract infections and their common diagnostic methods.

A. Urinary leukocyte duct type: Leukocyte duct type indicates inflammatory changes in the renal tubules, which usually occur in upper urinary tract infections such as pyelonephritis, so it is meaningful to distinguish between upper and lower urinary tract infections.

B. B shows abnormal morphology of the renal pelvis: this usually suggests upper urinary tract infection, such as pyelonephritis, because lower urinary tract infection generally does not cause significant changes in renal pelvis morphology. So, this test is also meaningful to distinguish between the upper and lower urinary tract infections.

C. Bladder puncture culture: Escherichia coli is a common urinary tract infection bacterium, but it can cause both upper and lower urinary tract infections. Therefore, the finding of E. coli by bladder puncture culture alone does not clearly distinguish between upper and lower urinary tract infections.

D. Bucpain in the renal region: This is usually a typical symptom of upper urinary tract infections, following back pain or buckle pain in the renal region, especially in upper urinary tract infections such as pyelonephritis. Therefore, this

symptom is helpful in distinguishing between the upper and lower urinary tract infections.

E. The peripheral blood WBC increases significantly: In upper urinary tract infections, the peripheral blood leukocytes usually are significantly higher due to the systemic effects of inflammation. In lower urinary tract infections, this condition is less common. Therefore, this examination also helps to differentiate between the upper and lower urinary tract infections.

In conclusion, bladder puncture culture found that E. coli could neither clear upper nor lower urinary tract infection, so it was not meaningful for distinguishing between upper and lower urinary tract infections. So, the correct answer is option C.

So, the correct answer is C. bladder bladder culture of E. coli.

The following tests are meaningless in distinguishing between upper and lower urinary tract infections:

F. Bucpain in the renal region

** analyze:**

- ** Urinary leukocyte tube type (A) * *: Leukocyte tube type usually indicates inflammation or infection in the kidney, which helps to differentiate the upper urinary tract (kidney) infection.

- ** B shows abnormal morphology of renal pelvis (B) * *: Abnormal morphology of renal pelvis can indicate upper urinary tract infection.

- ** Bladder puncture culture shows Escherichia coli (C) * *: The bladder puncture culture directly samples the urine in the bladder, mainly for the diagnosis of lower urinary tract infection.

- ** Buckle pain in the renal area (D) * *: Although buckle pain in the renal area may indicate an upper urinary tract infection, its presence or not can not effectively distinguish the upper and lower urinary tract infections, and other factors may also

cause pain in the renal area.

- ** Significant increase in WBC in peripheral blood (E) * *: Higher peripheral blood leukocyte count can indicate a systemic inflammatory response, but can not specifically distinguish the upper and lower urinary tract infections.

Therefore, although buckle pain in the renal region may indicate upper urinary tract infection, it does not effectively distinguish between upper and lower urinary tract infections.

37. (Type A1 question)

Oral iron treatment for effective iron deficiency anemia, the following tests are the first to rise

A. granulofilocyte

B. hemoglobin

C. MCV

D. MCH

E. MCHC

Reference answer: A. Your answer:

Graphic analysis: the effective performance of oral iron agent first increased reticulocyte in peripheral blood, peak 5~10 days after the start of the medication, hemoglobin concentration increased after 2 weeks, and generally returned to normal in about 2 months. Iron therapy should last for at least 4 to 6 months after hemoglobin returns to normal, and the medication should be stopped after ferritin is normal.

When treating iron deficiency anemia, the first indicator to rise is reticulocytes (A). After oral iron treatment, the body begins to make more red blood cells, of which reticulocytes are the early morphology of newborn red blood cells, and their increase marks the bone marrow response to iron and an increase in erythropoiesis. Therefore, the reticulocyte count rises first shortly after treatment initiation. Subsequently, with the production of more mature red blood cells, hemoglobin levels, MCV (mean hemoglobin volume), MCH (mean BC hemoglobin content), and MCHC (average hemoglobin hemoglobin concentration) will gradually improve. So the correct answer is an A.granulofilocyte.

analyze:

The patients with iron deficiency anemia with effective oral iron agent have active bone marrow hyperplasia, so reticulocytes rise first, generally 3 to 4 days after taking the drug, reaching the peak in about 7 days, and reticulocytes gradually decline in about 2 weeks, indicating that the treatment is effective and hemoglobin is gradually rising.

Option B hemoglobin rise generally starts to rise after two weeks of treatment, so it is excluded.

Option C: MCV is the mean red cell volume, option D: MCH is the mean red cell hemoglobin, and option E: MCC is the mean red cell hemoglobin concentration. All of the three are used for the morphological classification of anemia. The change is slow and not the first index to rise, so they can be excluded together.

To sum up, the correct answer is: A. reticulocytes.

In oral iron treatment for iron deficiency anemia, the first rising test index is the reticulocyte (A). Here is an explanation of the options:

A. * * reticulocytes * *: The reticulocytes are the early stages of erythropoiesis and their increase indicates a bone marrow response to iron treatment.reticulocytes usually rise first within 3-7 days after oral iron treatment for iron deficiency anemia.

B. * * Hb * *: The level of hemoglobin also increases gradually along with treatment, but usually increases significantly after about 2-4 weeks.

C. * * MCV (mean red blood cell volume) * *: MCV is a measure of red blood cell size. MCV is usually low in the early stages of iron deficiency anemia. The MCV gradually returns to normal with treatment, but this change is relatively slow.

D. * * MCH (mean red blood cell hemoglobin amount) * *: MCH reflects the mean amount of hemoglobin within each red blood cell. Like MCV, the recovery of MCH also requires a longer time.

E. * * MCHC (average red blood cell hemoglobin concentration) * *: MCHC reflects the concentration of hemoglobin in red blood cells, and the change is relatively stable and is not the first indicator to rise.

Therefore, the first to rise is the * * reticulocyte * *.select A.

38. (Type A1 question)

The following results of the pathogenesis of aplastic anemia were wrong

A. Higher serum levels of IL-2

B. Higher serum TNF levels

C. Increased growth in CD 4 + T cells

D. Increased growth in CD 8 + T cells

E. Increased growth in CD 25 + T cells

Reference answer: C Your answer:

Aplastic anemia: CD4 +: CD8 + ratio decreased.

Aplastic anemia (AA) is a disease of bone marrow hematopoietic decline, and its pathogenesis is related to immune abnormalities. Specifically, common immune abnormalities in patients include increased T cell activity and altered cytokine levels. Therefore, the serum levels of some immune regulatory factors, such as IL-2 (interleukin 2) and TNF (tumor necrosis factor), are increased. Higher CD4 + T cells and CD25 + T cells are also associated with immune abnormalities in aplastic anemia. However, with regard to CD8 + T cells, their role in aplastic anemia is not always elevated.

So, in the options given, if the "wrong" description is to be found, you need to consider which option is inconsistent with the usual manifestation of aplastic anemia. Although CD8 + T cells may be increased in some patients with aplastic anemia, this is not the most typical manifestation. Therefore, the question may be more subtle, depending on the topic intent and the definition of "error".

Considering the above information, if you need to choose the option least fitting the typical pathogenesis of aplastic anemia, I prefer to choose:

F. Increased growth in CD 8 + T cells

This is because, although elevated CD8 + T cells may be observed in some AA patients, this is not a typical immunological feature of AA. However, it should be noted that this choice may be controversial because the expression of CD8 + T cells may vary in different AA patients.

analyze:

Aplastic anemia, referred to as aplastic anemia, is a group of bone marrow hematopoietic failure syndromes caused by a variety of causes, characterized by the decrease of bone marrow hematopoietic cell hyperplasia and peripheral blood whole blood cells, clinical anemia, bleeding and infection as the main manifestations. The results of the pathogenesis of aplastic anemia showed that the activated T lymphocytes and their secreted negative regulators of hematopoiesis were increased in aplastic anemia patients, mediating the damage of hematopoietic stem cells and bone marrow failure. The CD25 molecule is the α chain of interleukin-2 receptor (IL-2R), which can specifically bind to IL-2, mainly distributed in activated T cells and NK cells, and also expressed on the surface of B cells, monocyte-macrophages and a few myeloma cells. The decreased number of CD25 T cells in aplastic anemia patients suggests a decreased T-cell immune function.

The activated T lymphocytes and their secreted negative hematopoietic regulators are increased in patients with aplastic anemia, including γ interferon (IFN-γ), interleukin-2 (IL-2) and tumor necrosis factor (TNF), which can significantly inhibit the CFU-GM growth of bone marrow in normal people, leading to bone marrow failure. Therefore, increased serum IL-2 levels and increased serum TNF levels in option B are consistent with the pathogenesis of aplastic anemia.

In patients with aplastic anemia, T helper cell type I (Th 1) cells, T helper cell type (Th 2) cells, inverted CD4 / CD8 ratio, and increased activity of CD8 T cells to inhibit bone marrow hematopoiesis. Therefore, option C: CD4 T cells and option D: CD8 T cells are consistent with the pathogenesis of aplastic anemia.

In conclusion, the correct answer is: E.CD25 + T cells are increased.

Aplastic anemia (Aplastic anemia) is a disease caused by bone marrow hematopoietic stem cell damage, showing pancytopenia. Its pathogenesis involves immune-mediated destruction of stem cells, and common immune mechanisms include abnormalities of T cells and related cytokines.

Specifically, the abnormalities of various T cells and cytokines have the following manifestations in aplastic anemia:

1. * * Increased serum IL-2 levels * *: IL-2 (interleukin-2) is an important cytokine that promotes T cell proliferation and activation, and IL-2 levels are usually increased in patients with aplastic anemia.

2. * * Increased serum TNF levels * *: TNF (tumor necrosis factor) is a cytokine that can induce cell apoptosis, and its levels are also increased in aplastic anemia.

3. * * Increased CD4 + T cells * *: The levels of CD4 + T cells in these patients are not necessarily increased in aplastic anemia, in fact, studies have shown that their levels are usually normal or decreased.

4. * * CD8 + T cells increased * *: CD8 + T cells increased are common in aplastic anemia, because CD8 + T cells can directly kill bone marrow hematopoietic stem cells.

5. * * Increased CD25 + T cells * *: CD25 is the α -chain of the IL-2 receptor and is usually associated with regulatory T cells (Treg), and studies have shown that CD25 + T cell levels may also be increased in patients with aplastic anemia.

Combined with the above analysis, option C (CD4 + T cell increase) is wrong in the pathogenesis of aplastic anemia.

So the answer is:

G. Increased growth in CD 4 + T cells

39. (Type A1 question)

The following diseases that may develop hemolysis in situ are

A. Inherited spherocytosis

B. G6PD deficiency

C. Myelodysplastic syndrome

D. Abnormal hemoglobinopathy

E. Paroxysmal sleep-induced hemoglobinuria

Reference answer: C Your answer:

Orthotopic hemolysis means that the young red blood cells in the bone marrow have been destroyed in the bone marrow before being released into the blood circulation, which is commonly seen in megaloblastic erythroid anemia and myelodysplastic syndrome.

In situ hemolysis (intravascular hemolysis) is the rupture and dissolution of red blood cells that occurs inside the blood vessel. Here is an analysis of each option:

A. Hereditary spherocytosis (Hereditary spherocytosis) usually causes hemolysis, but mainly through the spleen filtration mechanism rather than in situ hemolysis.

B. G6PD deficiency can cause in situ hemolysis, especially when patients are exposed to certain drugs, infections, or other oxidative stress.

C. Myelodysplastic syndrome (Myelodysplastic syndromes, MDS) is characterized by poor hematopoiesis and, although possible hemolysis, atypical in situ hemolysis.

D. Abnormal hemoglobinopathies (Hemoglobinopathies), such as sickle cell anemia, can cause hemolysis.

E. Paroxysmal sleep hemoglobinuria (Paroxysmal nocturnal hemoglobinuria, PNH) is a rare disease in which red blood cells rupture in blood vessels, causing in situ hemolysis.

Considering the definition of in situ hemolysis and the characteristics of each

disease, the disease most consistent with in situ hemolysis is * * E. paroxysmal sleep hemoglobinuria * *. PNH is a disease particularly characterized by rupture and lysis of intravascular red blood cells, so option E is the most correct answer.

analyze:

In situ hemolysis, also known as ineffective erythropoiesis or in situ hemolytic anemia, means that the young red blood cells in the bone marrow are destroyed in the bone marrow before they are released into the blood circulation, and is essentially an extravascular hemolysis, commonly seen in myelodysplastic syndrome and megaloblastic anemia.

Option A hereditary spherocytosis, option B glucose-6-phosphate dehydrogenase (G6PD) deficiency, option D abnormal hemoglobinopathy, option E paroxysmal sleep hemoglobinuria, above all belong to the endovascular hemolytic disease, red blood cells destroyed in blood circulation, release free hemoglobin, inconsistent with the definition of in situ hemolysis, therefore are excluded.

In conclusion, the correct answer is: C. myelodysplastic syndrome.

The following diseases that may develop hemolysis in situ are

F. Paroxysmal sleep-induced hemoglobinuria

explain:

Paroxysmal sleep hemoglobinuria (PNH) is an acquired, clonal hematopoietic stem cell disease characterized by hemolysis of red blood cells under the action of the complement system. Due to the lack of protective membrane proteins in PNH RBCs, these cells are more susceptible to complement destruction, leading to hemolysis. In situ hemolysis is hemolysis that occurs in blood vessels, and PNH is one of the typical diseases. Other options such as hereditary spherocytosis and G6PD deficiency can also lead to hemolysis, but occur mainly extraascular (spleen, etc.) rather than intravascular.

40. (Type A1 questions)

The following are not the common morbid hematopoietic manifestations of myelodysplastic syndrome

A. Unbalanced erythroid nucleoplasm development

B. Excessive leaf segmentation of the granular nuclei

C. Excessive granules of granulocytic cells

D. Too few granulocycells

E. Observation of naive megakaryocytes

Reference answer: C. Your answer:

Graphic analysis: Morbid hematopoiesis is characteristic of a myelodysplastic syndrome that presents as follows.① Red lineage: too proportion (> 60%) or too little (<15%), odd nuclei, nuclear fragmentation, internuclear bridge; unbalanced nuclear development, large size, uneven size and staining of mature red blood cells, RAS ring iron larvae> 15%; ② -monocyte: increased proportion of primary cells, too little nuclear differentiation, unbalanced nuclear development, too few granulocytic cell granules; ③ megakaryocytes: lymphoid, single, multiple and small megakaryocytes.

In the myelodysplastic syndrome (Myelodysplastic Syndromes, MDS), the pathological hematopoietic manifestations of the bone marrow mainly include:

-Erythroid nucleoplasmic development imbalance (A): the nucleus and cytoplasm maturation, which is one of the typical manifestations of MDS.

-Excessive nuclear segmentation (B): abnormal nuclear segmentation of granulocyte precursors, such as excessive nuclear segmentation, is also one of the features of MDS.

-Too few granuloloid cells (D): granuloid cells may be reduced in MDS.

-Naive megakaryocytes (E): Common megakaryocyte lineage abnormalities in MDS, such as the appearance of naive megakaryocytes.

Excessive granulosa (C) is not a typical pathological feature of MDS, so the best answer is excessive C. granulosa.

analyze:

Myelodysplastic syndrome (MDS) is a group of heterogeneous myeloid clonal diseases originating from hematopoietic stem cells, characterized by abnormal differentiation and development of myeloid cells, showing ineffective hematopoiesis, refractory hemocytopenia, hematopoietic failure, and high risk of transformation to

acute myeloid leukemia (AML). Normal hematopoiesis of MDS includes the pathological hematopoiesis of erythrocyte, granulocyte and megakaryocytic system. Excessive granulocytic cell granules is not a characteristic of pathological hematopoiesis.

Option A erythroid nucleocytoplasmic development is A common characteristic of erythroid pathological hematopoiesis in MDS, that is, juvenile erythrocyte cytoplasmic development lags behind the nucleus, usually with less cytoplasm, vacuoles, loose nuclear chromatin, and nucleoli.

Option B is a common feature of MDS, more than 5, often 6-9, or even 16.

Option D Too few granulocytic granules is also a common feature of granulocytic hematopoiesis in MDS, showing reduced cytoplasmic granules of neutrophils and negative PAS staining.

Option E Seeing ve megakaryocytes is a common feature of megakaryocytes, with lymphoid small megakaryocytes, mononuclear small megakaryocytes, polypolonuclear megakaryocytes, etc.

In conclusion, the correct answer is: C. Excessive granulocyte granules.

41. the following does not belong to the bone marrow of myelodysplastic syndrome

A. Unbalanced erythroid nucleoplasmic development

B. Excessive leaf segmentation of the granular nuclei

C. Excessive granules of granulocytic cells

D. Too few granulocycells

E. Observation of naive megakaryocytes

Best answer: C. Excessive granulocytes

analyze:

Myelodysplastic syndromes (MDS) is a group of highly heterogeneous clonal hematopoietic stem cell diseases, which are mainly characterized by bone marrow pathological hematopoiesis and ineffective hematopoiesis. Common pathological hematopoietic manifestations include:

-Erythroid nucleocytoplasmic development imbalance (A)

-Excessive-of nuclear overation (B)

-Pargrangrangrangrangrancells (D)

-See naive megakaryocytes (E)

Excessive granulocylet (C) is not a common pathological hematopoietic manifestation of MDS.

42. (Type A1 questions)

The following examination results support the period of CML emergency change

A. Blood protogranulocytes> 5%

B. Blood granules + promyelocytes> 20%

C. Bone marrow with primary granulocytes of> 20%

D. Bone marrow grana + promyelocytes> 40%

E. Bone marrow biopsy showed significant proliferation of collagen fibers

Reference answer: C. Your answer:

Graphic analysis: Acute stage of chronic myelogenous leukemia (CML): bone marrow granulocytes + promyelocytes> 50%, blood granules + promyelocytes> 30%, primitive cells or primitive lymph + larvae> 20%.

During the onset of chronic myelogenous leukemia (CML), a significant increase in the proportion of promyelocytes and progranuytes (immature granulocytes) is usually observed. According to relevant medical guidelines and criteria, one of the diagnostic criteria for CML acute change is the proportion of progranulocytes in blood or bone marrow plus promyelocytes over 20%.

Therefore, according to the above options, the inspection results that can best support the CML emergency change period are:

- ** D. Bone marrow promyelocytes + promyelocytes> 40%**

This option shows a significantly higher than normal proportion of immature granulocytes, consistent with hematological changes in CML progression to acute change.

analyze:

Chronic myeloid leukemia (CML) is a malignant proliferative disease of multipotent hematopoietic stem cells, and its course of disease is divided into chronic stage, accelerated stage and acute change stage. Among them, the criteria for CML to enter the stage of acute change include peripheral blood plasma + promyelocytes> 30%, bone marrow plasma +> 50% promyelocytes, extramedullary primitive cell infiltration, and large accumulation of primitive cells shown by bone marrow biopsy. Among them, bone marrow cells + promyelocytes> 50%, meeting the criteria of CML acute change, while the option * * bone marrow granulocyocytes + promyelocytes> 40% * * is the closest to this standard.

Option A blood granulocytes> 5% and blood option B did not meet the criteria for CML acute change, so they were excluded together.

Option C Bone marrow granulocytes> 20% was the criterion for CML acceleration phase and did not meet the meaning.

Option E Bone marrow biopsy showed that significant hyperplasia of collagen fibers was a pathological feature of myelofibrosis, which did not conform to the manifestation of CML emergency change, so it was not selected.

In conclusion, the correct answer is: D. Bone marrow granuytes + promyelocytes> 40%.

One of the diagnostic criteria for acute change in chronic myeloid leukemia (CML) is a significant increase in the proportion of protogranulocytes. With current medical knowledge and diagnostic guidelines, the following findings support the acute change of CML:

F. Bone marrow with primary granulocytes of> 20%

This is because the proportion of granulocytes in the bone marrow usually

exceeds 20% during the onset of CML change.

43. (Type A1 questions)

The following are those for T cell lymphoma

A. Marginal zone lymphoma

B. follicular lymphoma

C. lymphoma mantle cell

D. Intermediate-large cell lymphoma

And E.Burkitt's lymphoma

Reference answer: D. Your answer:

Graphic analysis: The immunophenotype of intervariable large cell lymphoma is T cells.

Among these options, belonging to T cell lymphoma is * * D. large cell lymphoma * *. Marginal zone lymphoma, follicular lymphoma, mantle cell lymphoma, and Burkitt lymphoma are all lymphomas of B cell origin. While intervariable large cell lymphoma is a T-cell lymphoma derived from T cells or natural killer (NK) cells.

First, we need to clarify the classification of lymphoma. Lymphoma is mainly divided into two categories: Hodgkin's lymphoma and non-Hodgkin's lymphoma. In non-Hodgkin's lymphoma, it can be divided into B cell lymphoma and T cell lymphoma.

Next, we analyze each option one by one:

A. Marginal zone lymphoma: This is a B-cell-derived lymphoma, so it is not a T-cell lymphoma.

B. Follicular lymphoma: Also a B-cell-derived lymphoma, but not a T-cell lymphoma either.

C. Pulling cell lymphoma: This is also a B-cell lymphoma, so a T-cell lymphoma that does not meet the requirements of the title.

D. Intergenic large cell lymphoma: This is a T cell-derived lymphoma that meets the requirements.

E. Burkitt lymphoma: This is a B-cell-derived lymphoma, so it is not a T-cell

lymphoma.

In conclusion, only option D, intervariable large cell lymphoma, is T cell lymphoma.

Therefore, the correct answer is D. large cell lymphoma.

The following T cell lymphoma are:

F. Intermediate-large cell lymphoma

analyze:

-Marginal zone lymphoma (A): a B-cell lymphoma.

-Follicular lymphoma (B): a B-cell lymphoma.

-Pulacket cell lymphoma (C): B cell lymphoma.

-Intermediate large cell lymphoma (D): T cell lymphoma.

-Burkitt lymphoma (E): a B-cell lymphoma.

So, the best answer is D. interlarge cell lymphoma.

44. (Type A1 question)

In the clinical manifestations of type Henoch allergic purpura, in addition to the skin purpura, there are also

A. Joint swelling and pain

B. hematochezia

C. hematuria

D. retinal hemorrhage

E. iritis

Reference answer: B. Your answer:

[Analysis] Type Henoch allergic purpura is abdominal type, manifested as nausea, vomiting, hematemesis, diarrhea, hematochezia, among which abdominal

pain is the most common.

Henoch-Schonlein Purpura (Henoch-Schonlein purpura, HSP) is a small vasculitis that mainly affects capillaries. Its clinical manifestations include not only cutaneous purpura, but also the joints, intestines, and kidneys. Therefore, besides cutaneous purpura, the typical clinical manifestations of Henoch-Schonlein purpura include:

-Joint pain (usually affecting knee and ankle)

-Abdominal pain or hematochezia (intestinal involvement)

-Urinary blood (kidney involvement)

Retinal hemorrhage and iritis are not typical manifestations of Henoch-Schonlein purpura. Therefore, the best answer is the option that covers the additional symptoms common to this disease. Considering the above information, the most appropriate answer is:

A. Joint swelling and pain

B. hematochezia

C. hematuria

Among these options, joint swelling and pain (A) is a very typical additional clinical manifestation of Henoch-Schonlein purpura. However, hematochezia (B) and urinary blood (C) are also important manifestations, especially when the disease affects the gut and kidneys. If the question asks to choose the best answer, we need to consider the most common and representative symptoms.

Since the most typical additional clinical feature of Henoch-Schonlein purpura is renal involvement, presenting as urinary blood, the correct answer should be * * C. urinary blood * *.

analyze:

Allergic purpura, also known as self-limited acute blood, is a invasion of the skin and other organs small arteries and capillary allergic vasculitis, cause may be pathogen infection, some drugs, allergy cause the formation of IgA or IgG circulating immune complex, deposition in the upper dermal capillaries cause vasculitis. Allergic purpura can be divided into simple type, abdominal type, joint type, renal type, mixed type, etc. Among them, abdominal allergic purpura is also known as Henoch purpura. In addition to skin purpura, this type of patients because of digestive tract mucosa and

peritoneal dirty layer capillary involvement, can produce a series of gastrointestinal symptoms and signs, such as nausea, vomiting, hematemesis, diarrhea and mucous stool, hematochezia, etc.

Option A joint swelling and pain is more common in joint type allergic purpura, also known as Schonlein-Henoch purpura, can have joint swelling, pain, tenderness and dysfunction, does not conform to the characteristics of Henoch type purpura, so it is not selected.

Option C urine blood is more common in renal type allergic purpura, also known as purpura nephritis, can appear hematuria, proteinuria and tubular urine, occasionally see edema, hypertension and other symptoms, do not conform to the characteristics of Henoch type purpura, so excluded.

Option D retinal hemorrhage, option E iritis, which is rare in allergic purpura, and does not belong to the characteristics of type Henoch purpura, so it is not selected.

In summary, the correct answer is: B. Blood in the stool.

Henoch-Schonlein purpura (Henoch-Schonlein Purpura, HSP) is a systemic vasculitis mainly characterized by cutaneous purpura, and is commonly seen in children. In addition to cutaneous purpura, the clinical manifestations of HSP include the following:

A. Joint swelling and pain: Joint symptoms are common manifestations of HSP, and patients may experience joint swelling and pain, especially in the knee and ankle joints.

B. Blood in chezia: Gastrointestinal symptoms are another common manifestation of HSP, patients may have abdominal pain, blood in chezia and other symptoms.

C. Urine blood: kidney involvement is also relatively common, and patients may have hematuria, proteinuria and other manifestations.

Retinal haemorrhage and iriditis are rare in HSP to excluding D and E.

In conclusion, in the clinical manifestations of type Henoch allergic purpura, in

addition to skin purpura, there are also:

A. of swollen and painful joints

B. hematochezia

C. hematuria

The best answer is:

A. of swollen and painful joints

45. (Type A1 question)

The most likely mechanism of neutropenia caused by Felty syndrome is

A. Reduced generation

B. dysmaturity

C. Excessive immune destruction

D. Excessive non-immune destruction

E. Abnormal distribution

Reference answer: C Your answer:

Graphic analysis:

Fty syndrome is a rare autoimmune disorder associated with rheumatoid arthritis characterized by rheumatoid arthritis, splenomegaly, and neutropenia. Neutropenia is primarily due to immune destruction, and thus the most likely mechanism is excessive destruction of neutrophils by the immune system.

So, the correct answer is: C. Excessive immune damage

analyze:

Fty syndrome refers to patients with rheumatoid arthritis with spleen, neutropenia, and some even anemia and thrombocytopenia. The most likely mechanism of neutropenia is immune destruction, and a large number of granulocytes are destroyed by immune destruction, leading to neutropenia.

Option A generation reduction can be seen in aplastic anemia, tumor bone

marrow invasion, acute leukemia, physical and chemical factors such as drugs or radiation, and bone marrow hematopoietic disorders caused by systemic diseases, which are not consistent with the pathogenesis of Felty syndrome, so it is not selected.

Option B maturation disorder is seen in megaloblastic anemia, myelodysplastic syndrome, anemia caused by cellular DNA synthesis disorder due to folic acid or vitamin B_{12} deficiency, or some drugs that affect nucleotide metabolism, which does not comply with the pathogenesis of Felty syndrome, so it was excluded.

Option D Non-immune destruction is overseen in infection, inflammation or hypersplenism, which is different from the pathogenesis of Felty syndrome, so it is not selected.

The abnormal distribution of option E can be seen in allogeneic protein response and endotoxemia. Due to the transfer of neutrophils to the marginal pool and relatively fewer neutrophils, the total number of granulocytes was not reduced, which was not consistent with the title stem description, so it was excluded.

In conclusion, the correct answer is: C. Excessive immune destruction.

Fty syndrome (Felty syndrome) is a rare disease commonly seen in patients with rheumatoid arthritis, with major features including splenomegaly, neutropenia and positive rheumatoid factor. Neutropenia in this disease is primarily due to immunological mechanisms.

With available medical knowledge and best practice guidelines, the most likely mechanism of neutropenia caused by Felty syndrome is:

F. Excessive immune destruction

46. (Type A1 question)

Are not a clinical characteristic of paroxysmal supraventricular tachycardia

A. Sudden sudden stop

B. Heart rate> 150 / min

C. Absolute rule of heart rhythm

D. The first heart sound strength varies

E. Most are caused by a reentrant mechanism

Refer to the answer: D. Your answer:

[Analysis] The disease is sudden sudden stop, often without organic lesions, often return mechanism, heart rate 150~250 times / minute, rhythm is fast and regular.

The clinical characteristics of paroxysmal supraventricular tachycardia (Paroxysmal Supraventricular Tachycardia, PSVT) usually include sudden onset and sudden end (sudden arrest), heart rate is usually greater than 150 beats per minute, and heart rhythm rule. Most of it is indeed caused by a reentrant mechanism. Option D, "varying first heart tone strength", is usually associated with ventricular filling or heart valve dysfunction and is not a typical clinical feature of paroxysmal supraventricular tachycardia.

Therefore, the clinical characteristics of paroxysmal supraventricular tachycardia are * * D. The strength of the first heart sound varies from * *.

First, let's take a look at the basic characteristics of paroxysmal supraventricular tachycardia (PSVT). PSVT is a common arrhythmia, mainly caused by the reentrant mechanism, which has the characteristics of sudden arrest. The heart rate is generally fast, usually more than 150 beats / minute, and the heart rhythm is usually regular.

Now, we analyze the options based on these features:

A. Sudden arrest: This is a significant feature of paroxysmal supraventricular tachycardia, where the onset and termination of tachycardia are very sudden, so this option is consistent with the characteristics of paroxysmal supraventricular tachycardia.

B. Heart rate> 150 / min: During paroxysmal supraventricular tachycardia, the heart rate usually exceeds 150 beats / min, so this description is also accurate.

C. Absolute rule of heart rhythm: In the case of paroxysmal supraventricular tachycardia, the heart rhythm is usually regular, which matches the description of the option.

D. First heart sounds vary: This feature is more common in atrial fibrillation than in paroxysmal supraventricular tachycardia. In paroxysmal supraventricular tachycardia, the heart rhythm is regular, so the intensity of the first heart sound should also be uniform, rather than unequal strength.

E. Most are caused by a reentrant mechanism: paroxysmal supraventricular tachycardia is indeed mostly caused by a reentrant mechanism, consistent with the

option description.

In conclusion, the clinical characteristics that do not belong to the paroxysmal supraventricular tachycardia are the strength of D. first heart sounds. This is characteristic of atrial fibrillation, but not of paroxysmal supraventricular tachycardia.

With available medical knowledge and best practice guidelines, the clinical characteristics of paroxysmal supraventricular tachycardia (PSVT) include:

A. Sudden: PSVT usually starts and ends suddenly.

B. Heart rate> 150 / min: PSVT heart rate usually exceeds 150 beats per minute.

C. Absolute rule of heart rhythm: The heart rhythm of PSVT is the absolute rule.

D. The first heart sound varies: this is not the characteristic of PSVT. The first heart tone of the PSVT is usually constant.

E. mostly by reentrant mechanism: most PSVT is caused by reentrant mechanism.

Therefore, the correct answer is not that the clinical characteristics of paroxysmal supraventricular tachycardia are:

F. The first heart sound strength varies

47. (Type A1 question)

The most direct cause of pulmonary oedema is that

A. increased pulmonary artery pressure

B. An elevated right ventricular pressure

C. Raised pulmonary venous pressure

D. Raised left ventricular diastolic blood pressure

E. Raised right atrial pressure

Reference answer: C Your answer:

[Analysis] the increase of pulmonary venous pressure leads to the increase of

pulmonary capillary pressure, which directly causes pulmonary interstitial edema. Raised left ventricular end-diastolic pressure and raised left atrial pressure all indirectly lead to pulmonary edema by increasing pulmonary venous pressure.

The most direct cause of pulmonary oedema is the elevated pulmonary venous pressure. Pulmonary edema usually results from left heart failure, which causes increased venous pressure in the pulmonary circulation. When the left ventricle cannot effectively pump blood into the systemic circulation, the blood is trapped in the pulmonary circulation, resulting in increased pulmonary venous pressure. Therefore, the most direct cause of pulmonary oedema is the elevated pulmonary venous pressure.

So, the correct answer is that C. pulmonary venous pressure increases.

First, we need to make it clear about what a pulmonary edema is. Pulmonary edema refers to a pathological state in which abnormal penetration of fluid from pulmonary capillaries into the lung interstitium and alveoli, exceeding the compensatory capacity of lymphatic reflux, resulting in abnormal accumulation of pulmonary extravascular fluid.

Next, we analyze the direct relationship between each option and pulmonary edema:

A. Raised pulmonary artery pressure: increased pulmonary artery pressure is mainly related to pulmonary hypertension, but this does not directly lead to pulmonary edema. It may affect the RV function, but is not a direct cause of pulmonary edema.

B. Raised right ventricular pressure: increased right ventricular pressure is associated with increased pulmonary artery pressure, but it is also not the direct cause of pulmonary edema.

C. Raised pulmonary venous pressure: Higher pulmonary venous pressure means increased resistance to blood return from the lungs to the left atrium. When pulmonary venous pressure rises to a certain level, fluid seeps into the alveoli and lung interstitium, resulting in pulmonary edema. Therefore, this is the direct cause of the pulmonary oedema.

D. Raised left ventricular diastolic pressure: increased left ventricular diastolic pressure can affect the diastolic function of the heart, but there is no direct causal relationship between it and pulmonary edema.

E. Raised right atrial pressure: increased right atrial pressure is related to right heart function, but it does not directly lead to pulmonary edema.

In conclusion, the most direct cause of pulmonary edema is increased pulmonary venous pressure, so C.

The most direct causes of pulmonary oedema are:

F. Raised pulmonary venous pressure

Pulmonary edema is caused by the accumulation of fluid in the alveoli and pulmonary interstitium, most directly caused by elevated pulmonary venous pressure. This is usually associated with left heart dysfunction, and when the left ventricle fails to pump blood effectively, it causes blood retention in the pulmonary circulation and increasing pressure in the pulmonary veins, resulting in fluid exudation and pulmonary edema.

48. (Type A2 question)

Male, 14 years old. After eating sea fish for lunch, there is headache, dizziness, chest tightness, rapid heartbeat and breathing, accompanied by eye conjunctival congestion. Face and body flushing, temperature is normal, no vomiting, diarrhea and other symptoms. Patients are most likely

A. fugu poisoning

B. Clostridium botulinum toxin poisoning

C. paralytic shellfish poisoning

D. histamine poisoning

E. Vibrio parahaemolyticus poisoning

Refer to the answer: D. Your answer:

[Analysis] adolescent male patients, after eating sea fish at lunch, have headache, dizziness, chest tightness, rapid heartbeat and breathing, accompanied by conjunctival congestion. Face and systemic flushing (allergic manifestations of histamine poisoning), so combined with the inducement and allergic manifestations of the patient, it suggests that histamine poisoning (D pair).

[To avoid mistakes] After puffer fish poisoning, mainly vomiting, with obvious neurological symptoms, but less common abdominal pain and diarrhea

et al. (A wrong). Clostridium botulinum toxin poisoning, sudden onset, mainly for neurological symptoms (B error). Paralysis

Shellfish poisoning (C wrong) is mainly manifested as paresthesia and numbness of lips, mouth and tongue, and in severe cases, limb numbness will follow

Italian motor disorders, and even breathing difficulties. After vibrio parahaemolyticus poisoning, blood-watery stools (E wrong) may occur.

The symptom indicates that the patient may have experienced an acute food-related response. Considering that the patient experienced headache, dizziness, chest tightness, rapid heartbeat, face and general flushing, but normal body temperature, no vomiting or diarrhea, these symptoms were consistent with histamine poisoning (also known as fish poisoning or histamine food poisoning).

Histamine poisoning is caused by the consumption of fish containing high levels of histamine (such as sea fish not properly stored), formed by bacteria acting on histidine during fish corruption. Patients develop symptoms similar to anaphylaxis such as headache, dizziness, flushing, conjunctival congestion and rapid heartbeat.

Therefore, the most likely answer is: D. histamine poisoning

analyze:

After eating sea fish, the patient had headache, dizziness, chest tightness, rapid heartbeat and breathing, accompanied by conjunctival congestion, face and whole body flushing, normal temperature, no vomiting, diarrhea and other symptoms, and was diagnosed as histamine poisoning. Histamine poisoning is usually caused by the consumption of fish with high histidine content, such as mackerel, mackerel, tuna, autumn swordfish, green skin and red meat fish. Histamine is a kind of biological amine, which is widely found in human tissues. When the body is subjected to physical and chemical stimulation or allergic reaction, it will cause the degranulation of cells containing histamine, leading to the release of histamine, and a series of physiological reactions after binding with the histamine receptor on the target cells. When histamine is released, it will cause physiological effects such as arteriole expansion, increased capillary permeability, bronchoconstriction, spasm, and contraction of gastrointestinal smooth muscle, whose symptoms are similar to urticaria, bronchial asthma, and anaphylactic shock.

Option A Fugu poisoning was characterized by lip paresthesia, followed by nausea, vomiting, abdominal pain, diarrhea, and even general paralysis and paralysis. In severe cases, blood pressure decreased, breathing difficulties, coma, respiratory failure and death, which was not consistent with the description of the title stem, so it was excluded.

Option B Clostridium botulinum toxin poisoning is characterized by diplopia, eyelid ptosis, dysphagia, language impairment, head and neck weakness, muscle paralysis and other cranial nerve damage, with high fatality rate, obviously different from the symptom description of patients, so it was not selected.

Option C paralytic shellfish poisoning is mainly manifested as paralysis of lips, hands and toes, nausea, vomiting, diarrhea and other symptoms, serious cases can lead to paralysis or even death, not with the title, so excluded.

Option E Vibrio parahaemolyticus poisoning showed paroxysmal colic in the upper abdomen, followed by diarrhea, 5~6 times a day, washing meat with watery stool, no pus, no blood, and no urgent weight. Some patients had vomiting, which did not meet the dry problem, so it was not selected.

To sum up, the correct answer is: D. histamine poisoning.

According to the title description, the boy had headache, dizziness, chest tightness, rapid heartbeat and breathing after eating sea fish at lunch, accompanied by conjunctival congestion, face and body flushing, but the body temperature was normal, no vomiting, diarrhea and other symptoms. These symptoms occurred immediately after eating the marine fish, suggesting a possible acute food-related toxic reaction.

A. Fugu poisoning usually causes numbness in the lips, weakness in the limbs, or even respiratory muscle paralysis, but the problem does not mention these typical symptoms.

B. Clostridium botulinum toxin poisoning usually presents as symmetrical neuromuscular paralysis, blurred vision, dysphagia, etc., and the symptoms appear relatively slowly, and do not meet the acute reaction described in the problem stem.

C. Common symptoms of paralytic shellfish poisoning are numbness of perioral and fingertips, limb weakness, and even severe cases can lead to dyspnea and paralysis, but the symptoms described by the title stem are not completely consistent with it.

D. Symptoms of histamine poisoning (often known as "mackerel poisoning") include headache, dizziness, facial flushing, rapid heartbeat, chest tightness, and conjunctival congestion, which are very consistent with the description in the problem stem. This poisoning is due to the ingestion of fish containing a high concentration of histamine, which is produced by the fish under the action of preserved bacteria.

E. Vibrio parahaemolyticus poisoning is characterized by acute gastroenteritis, such as vomiting, diarrhea, abdominal pain, usually without facial and systemic flushing described by the problem.

In conclusion, patients are most likely histamine poisoning. The correct answer is:

F. histamine poisoning

49. (Type A2 question)

Female, 40 years old, previously physical health. Sudden chest pain, lung compression was 30%, and lung compression remained 30% after thoracic puncture. What should be treated next

A. thoracentesis

B. pleurodesis

C. closed drainage of thoracic cavity

D. surgical operation

E. paracentesis pericardii

Reference answer: C Your answer:

[Analysis] The middle-aged woman, sudden chest pain, lung compression of 30%, considered to be spontaneous pneumothorax, after chest puncture, indicating that chest puncture is ineffective, so thoracocentesis should not be used, and the patient was previously healthy, the next chest closed drainage should be selected (C pair).

[Error avoidance] Chest puncture gas extraction is suitable for small amount pneumothorax (less than 20%); the indication of closed chest drainage is: ①,

Mass pneumothorax, open pneumothorax, and tension pneumothorax; ② After thoracentesis, no lower lung recurrence of the injured patient; ③ required

Pneumothorax or hemopneumothorax using mechanical or artificial ventilation; ④ Patients with pneumothorax or hemothorax recurrence after removal of thoracic drainage tube; ⑤

Caesarean section.

In this case, the key information was that the patient had sudden chest pain, lung compression of 30%, and the lung compression did not improve after thoracacentesis. Based on this information, the best treatment option should be C. closed chest drainage.

Closed chest drainage is commonly used in the treatment of lung compression, especially if no significant improvement after thoracocentesis, indicating that there may be persistent air or fluid leakage leading to lung compression. Closed drainage continuously removes air or fluid from the chest, helping the lungs to expand again. Other options such as thoracentesis (A) have been tried and ineffective, pleurodesis (B) and surgery (D) are usually used for more complex or specific conditions, and pericardiocentesis (E) for pericardial effusion or pericardial problems is not the case in this case. Therefore, the most suitable option is the C. thoracic closed cavity drainage.

First, we need to understand the core of the problem. The patient was a 40-year-old woman with sudden chest pain and a 30% lung compression. After thoracentesis, the degree of compression of the lungs remained unchanged and remained 30%. This means that a simple thoracentesis may not be effective in relieving the patient's condition.

Now, we will analyze the questions one by one according to the stem information and combined with the options:

A. Thoracentesis: Since one thoracic puncture has been performed, but lung compression has not improved, A repeat thoracacentesis may not work better.

B. Pleurodesis: This procedure is usually used to treat recurrent pneumothorax or persistent air leakage, but is not preferred in this setting.

C. Closed chest drainage: For primary pneumothorax, closed chest drainage is an effective treatment when simple extraction is not effective. It can continuously draw out the gas in the chest, helping the lungs to expand again.

D. Surgery: For primary pneumothorax, surgery is not usually the preferred treatment, unless other methods are ineffective or exceptional.

E. Pericardiocentesis: This is not related to pneumothorax treatment and is mainly used for the treatment of pericardial effusion or cardiac tamponade.

In conclusion, for this patient, closed chest drainage is an appropriate treatment as it consistently draws gas in the chest and aids lung recovery.

Therefore, the answer is C. closed chest drainage.

The question describes a 40-year-old female patient with sudden chest pain and lung compression of 30% and still 30% after thoracic puncture. This condition suggests the possible presence of pneumothorax and the lung failed to reexpand after puncture. The next most suitable treatment option should be to ensure that the lung is reinflated and avoid recompression. Based on existing medical knowledge and best practice guidelines, the following is an analysis of each options:

A. Thoracentesis: It has been performed once but failed to resolve the problem, so it may not be effective again.

B. Pleurodesis: This is a more complex operation, which is generally considered when other treatments are ineffective.

C. Closed chest drainage: this is a conventional treatment for the treatment of pneumothorax, especially in the case of puncture is ineffective, which can effectively eliminate the gas in the chest and expand the lung again.

D. Surgery: Usually used in severe or complex pneumothorax, or where other treatments are ineffective.

E. Pericarocentesis: unrelated to the current problem, not applicable.

Of the above options, the most appropriate treatment option is * * C. closed chest drainage * *, as this is the standard treatment that can effectively exclude gas in the chest and promote the reexpansion of the lung.

So the answer is: * * C. Closed chest drainage * *.

50. (Type A2 question)

Female, 28 years old, 28 weeks pregnant, with a body temperature of 38.5℃. Metastatic right lower abdominal pain with signs of peritoneal irritation. The next step is this

A. A laparoscopic appendectomy

B. labor induction + appendectomy

C. Cesarean section + appendectomy

D. Application of antibiotics

E. Supporting the symptomatic treatment

Reference answer: A. Your answer:

[Analysis] Pregnant women with fever, metastatic right lower abdominal pain, and positive peritoneal stimulation signs were diagnosed as acute appendicitis during pregnancy. Once the diagnosis is clear, no antibiotics, anti-infection and other symptomatic conservative treatment (DE error), emergency appendectomy (A pair).

[To avoid mistakes] Unless there is obstetric emergency, in principle, only appendicitis is treated without cesarean section. But the following situation

Appenddectomy can be performed first: ① intraoperative appendix exposure difficulties; ② appendix perforation with diffuse peritonitis, pelvis

Luminal infection is serious, uterine signs of infection; ③ near the due date or fetal basic maturity, has a viability (C wrong);

The fetus is generally OK, can continue to conceive (B wrong).

This question describes a 28-year-old pregnant woman at 28 weeks of gestation, presenting with elevated body temperature and metastatic right lower abdominal pain with signs of peritoneal irritation. These symptoms and signs suggest a possible form of appendicitis. During pregnancy, the diagnosis and management of appendicitis is relatively special, and we need to consider the safety of the mother and the fetus.

Option A laparoscopic appendectomy is A routine procedure for non-gestational appendicitis, but should be carefully considered carefully during pregnancy, especially in the third trimester. Laparoscopic surgery is feasible during pregnancy but requires an experienced surgical team and careful carefully to avoid effects on the fetus.

Option B labor induction + appendectomy and C option cesarean section + appendectomy are usually not preferred unless there are other urgent obstetric indications as this would increase the risk of preterm birth and other complications.

Option D applying antibiotics may be suitable for atypical appendicitis or mild symptoms, but in cases of clear appendicitis and the presence of the signs of the peritoneal irritation, antibiotics alone may not be sufficient to solve the problem.

Option E supports that symptomatic treatment is not generally the primary treatment modality for acute appendicitis.

Based on this situation, the optimal approach may be A. laparoscopic appendectomy. This method can reduce the interference with pregnant women and solve the problem of appendicitis timely. However, it needs to be supported with an experienced medical team and appropriate equipment. Therefore, if conditions permit, option A (laparoscopic appendectomy) is the best option.

analyze:

A young female, 28 weeks pregnant, with elevated body temperature, metastatic right lower abdominal pain, and positive peritoneal irritation sign, considering pregnancy with acute appendicitis. Pregnancy with acute appendicitis in early and middle pregnancy, can be timely surgery, in the third trimester, surgery best cesarean section at the same time, if caused by surgical stimulation, the baby survival rate is cesarean section is high, but surgery should be gentle, try to reduce the stimulation of the uterus, continue to use broad spectrum antibiotics, after at the same time strengthen postoperative care. The patient is 28 weeks pregnant, already belongs to the third trimester, appendectomy should be performed at the same time, can remove the lesion together, and can ensure the safety of the fetus. So the next step should be cesarean section + appendectomy.

Option A Laparoscopic appendectomy is minimally invasive surgery, but pneumoperitoneum is needed during the operation, and increased abdominal pressure will have an impact on the fetus, which can be excluded.

Induction of labor refers to the end of pregnancy after 12 weeks of gestation, due to maternal or fetal reasons, and must be induced to induce uterine contraction. Patients in this question have no indication for termination of pregnancy, so option B induction of labor + appendectomy can be excluded.

Option D applies antibiotics, and option E supports symptomatic treatment,

which are conservative treatment measures, which can only play a role in relieving symptoms, can not be cured, and is easy to relapse, so it is not the preferred treatment plan, so it is excluded together.

In conclusion, the correct answer is: C. cesarean section + appendectomy.

Women with high fever (38.5°C) and metastatic pain in the right lower abdomen and the most likely diagnosis of acute appendicitis. Acute appendicitis during pregnancy is a surgical emergency, and timely management is crucial for the maternal and infant health. Treatment measures should be based on the number of gestational weeks, the severity of the condition and the condition of the fetus.

At this stage of gestation (28 weeks), pregnancy is still relatively early, and surgical intervention is necessary if acute appendicitis is diagnosed. Although antibiotic therapy can temporarily relieve symptoms, it can not fundamentally solve the problem, and supporting symptomatic treatment can not effectively handle appendicitis. Therefore, for acute appendicitis, the most appropriate management method is surgery.

Laparoscopic appendectomy (A) is A minimally invasive procedure suitable for the treatment of acute appendicitis during pregnancy. Compared to open surgery, laparoscopic surgery is less traumatic, has faster recovery, and has less impact on the fetus. Therefore, choosing a laparoscopic appendectomy is the most reasonable option in this case.

Labor induction + appendectomy (B) or cesarean section + appendectomy (C) are more aggressive treatment measures and are not recommended in the absence of clear fetal or maternal life-threatening conditions. The application of antibiotics (D) and supportive symptomatic treatment (E) is not sufficient to address the underlying problem of acute appendicitis.

So the best answer is:

A. A laparoscopic appendectomy

51. (Type A2 questions)

A 54-year-old female with abdominal pain and low fever for 2 months, physical examination: soft abdomen and periumbilical tenderness. B ultrasound showed a moderate amount of ascites, ascites ADA65U / L. The following tests most helpful for the diagnosis are

A. colonoscope

B. γ -interferon release assay

C. belly CT

D. erythrocyte sedimentation rate

E. ascites

Reference answer: B. Your answer:

[Analysis] middle-aged female patients with abdominal pain, abdominal distension and low fever for 2 months, soft abdominal examination, periumbilical tenderness, B ultrasound showed moderate amount of ascites, ascites ADA65U / L (tubercular peritonitis often increased). Low fever abdominal pain, abdominal flexibility, according to the meaning of the question, so the diagnosis of tubercular peritonitis, the most helpful examination for the diagnosis is the γ -interferon release test (B pair).

[Fault avoidance] Colonoscopy (A error) is mainly used for the diagnosis of intestinal tuberculosis, and tuberculous peritonitis requires laparoscopy; abdominal CT and B

Ultra, sedimentation and ascites were all helpful in the diagnosis of tuberculous peritonitis, but they were not as specific as γ -interferon release test

nature.

This case describes a 54-year-old female patient with abdominal pain, abdominal distension and low fever for 2 months, physical examination revealed periumbilical tenderness, B ultrasound showed a moderate amount of ascites, ascites adenosine deaminase (ADA) of 65U / L. Elevated ADA levels of ascites may indicate

tuberculous peritonitis.

Of the options given, the most helpful to diagnose this condition is:

A. Colonoscopy-used to examine the condition of the large intestine, often used to diagnose colon-related diseases, such as colorectal cancer or inflammatory bowel disease.

B. γ -interferon release assay-which is an immunological test used to detect TB infection that can aid in the diagnosis of active or latent TB infection.

C. Abdominal CT-can provide detailed images of the abdominal organs, including the intestine, peritoneum and other tissues, helping in the examination for signs of peritonitis.

D. Blood sedimentation-blood sedimentation rate test, which can be used to detect inflammation in the body, but has limited diagnostic value for specific diseases.

E. Ascites-This may refer to performing an ascites analysis, including biochemical, cellular, and microbiological examination.

Considering the patient's elevated level of ascites ADA, combined with the clinical presentation, the most likely diagnosis was tuberculous peritonitis. Therefore, the workup most helpful for diagnosis is * * B. γ -interferon release test * *, as it helps to determine the presence of TB infection and thus supports the diagnosis of tuberculous peritonitis. However, in clinical practice, a combination of abdominal imaging (e. g., abdominal CT) and ascites analysis is usually required for a comprehensive evaluation and diagnosis.

analyze:

A middle-aged female patient with low fever of 2 months, with soft abdomen, periumbilical tenderness, B ultrasound showed moderate amount of ascites, ascites adenosine deaminase (ADA) 65U / L, highly suspected of tuberculous peritonitis. Tuberculous peritonitis is a chronic diffuse peritoneal infection caused by Mycobacterium tuberculosis, It can occur at any age, To see more than young and middle-aged people, Slightly more females than males, Most patients had a slower onset, But there are also a lot of acute cases, The main symptoms are burnout, Fever, abdominal distension, and abdominal pain, There are also chills, high fever suddenly onset, Mild to moderate acute abdominal pain, Or dull pain, Physical examination of the abdomen has a kneading feeling, Periumbilical palpable abdominal mass, Mobile turbidity positive, Tuberculous peritonitis ascites as exudate, Predominantly with the

lymphocytes, Increased adenosine deaminase activity in ascites fluid, Suggested that tuberculous peritonitis, Patients with tuberculous peritonitis with ascites adenosine deaminase more than 40U / L, Often used to identify the properties of ascites. For a definite diagnosis, a γ -interferon release test can be performed. This experiment used enzyme-linked immunospot technology or enzyme-linked immunosorbent assay to detect γ interferon-specific T lymphocytes for the presence of latent M. tuberculosis infection in the subject, whose sensitivity and specificity were significantly higher than that of PPD test, and it was not affected by BCG vaccination and non-tuberculous mycobacteria. For this patient * *, γ -interferon release test * * could help to clarify the diagnosis of TB infection.

Option A Coloscope is often used to diagnose intestinal diseases. The diagnosis value of tuberculosis peritonitis is not great, so it is not selected.

Option C abdominal CT shows enlarged abdominal lymph nodes and a small amount of ascites, but can not confirm tuberculous peritonitis and can be ruled out.

Option D blood rapid increase can be seen in a variety of diseases, such as acute inflammation, leukemia, connective tissue disease and tuberculosis, is not specific, inconsistent with the meaning of the question.

Option E ascites test can help in the diagnosis, but the γ -interferon release test has higher sensitivity and specificity, so it is not considered.

In conclusion, the correct answer is: B. γ -interferon release assay.

In a 54-year-old female, symptoms included abdominal pain, abdominal distension and low fever for 2 months, a physical examination revealed a soft abdomen and periumbilical tenderness. B ultrasound a moderate amount of ascites with an ADA (adenosine deaminase) value of 65U / L. Considering these symptoms and examination findings, the most likely diagnosis is tuberculous peritonitis.

For further confirmation, the most helpful test should be able to directly detect evidence of TB infection. Therefore, in the following options:

A. Colonoscopy: Examination for colon lesions is not preferred.

B. γ -interferon release assay: used for detecting TB infection, very helpful.

C. Abdominal CT: It can show structural abdominal abnormalities, but it is less specific than the γ -interferon release test for confirmed TB infection.

D. Blood destruction: Although it can reflect the inflammatory state, it is not specific and has limited significance.

E. Ascites: It has been checked, and further information is limited.

So, the most helpful tests for the diagnosis are:

* * B. γ -interferon release assay * *

52. (Type A2 questions)

Male, 39 years old, right upper abdominal pain with fever for 1 day, physical examination: T39.8℃, P105 times / minute, BP85 / 55mmHg, hepatic area percussion (+), B ultrasound gallbladder multiple stones, common bile duct diameter 1.6cm, the lower segment of the liver duct, the treatment is

A. Open cholecystectomy was performed

B. Common bile duct incision and T tube drainage were performed emergency

C. Active anti-shock therapy, fasting, and anti-infection

D. Emergency department for laparoscopic cholecystectomy

E. Active anti-shock therapy, and elective surgery after remission

Reference answer: B. Your answer:

[Analysis] A middle-aged male patient with right upper abdominal pain with fever for 1 day, T39.8℃ (fever), P105 times / min, BP85 / 55mmHg (shock), hepatic percussion (+), B-ultrasound gallbladder (indicating gallbladder node

Stone), the diameter of the common bile duct is 1.6cm (normal common bile duct diameter is less than 1cm), and the lower segment of the common liver duct is strong

(Suggestive of a stone in the common hepatic duct). Consistent with the typical presentation of acute obstructive suppurative cholangitis, the most appropriate treatment is in the emergency department

Common bile duct incision and T tube drainage (B pair) were performed.

[To avoid mistakes] The Charcot triad of abdominal pain, chills, high fever and

jaundice, accompanied by shock and nervous central system suppression

Reynolds Five joint signs.

The patient developed symptoms and signs indicating that he may have acute cholecystitis with cholelithiasis and may develop cholangitis or bile duct obstruction. According to the patient's symptoms (right upper abdominal pain with fever), signs (body temperature 39.8℃, heart rate 105 times / min, blood pressure 85 / 55mmHg), B ultrasound results (multiple gallbladder stones, increased diameter of common bile duct, strong echo light mass with acoustic shadow in the lower segment of the common liver duct), the patient can be judged to be more serious and needs immediate treatment.

-Option A (emergency open cholecystectomy) may be an option if cholecystitis is severe and complications are considered.

-B option (emergency choledochotomy, T tube drainage) is indicated for cholecholithiasis or severe cholangitis.

Option-C (active anti-shock therapy, fasting, anti-infection) is appropriate for all initial management of severe abdominal infections, but may not be sufficient to resolve problems alone, especially if there is a clear indication for surgery.

-The D option (emergency laparoscopic cholecystectomy) is appropriate for acute cholecystitis, but more evaluation may be required when patients develop shock and definite bile duct problems.

The-E option (active anti-shock therapy, elective surgery after remission) is ideal for surgery after controlling for acute symptoms.

Considering that the patient appears to have signs of shock (hypotension) and bile duct obstruction, option C (active anti-shock therapy, fasting, anti-infection) should be taken first to stabilize the patient's condition. Subsequently, surgical intervention (e. g., option B or D) may be required. But in the acute phase, prioritizing shock and infection is key, so I tend to choose the C option.

analyze:

A middle-aged male patient with right upper abdominal pain with fever, high fever after physical examination, increased heart rate, decreased blood pressure, and shock manifestations. In the percussion (+) in the liver area, B ultrasound showed multiple gallbladder stones, widened the diameter of the common bile duct, and there were stones in the lower segment of the common hepatic duct. Considering the

diagnosis of acute cholangitis caused by choledogithiasis, and shock manifestations due to decreased blood pressure. At this time, active anti-shock treatment, fasting and anti-infection should be used to create conditions for surgery.

Option A emergency open cholecystectomy, and option D emergency laparoscopic cholecystectomy, which were gallbladder surgery, but the patient was currently diagnosed as acute cholangitis caused by common bile duct stones and was in shock state, and could not be treated immediately, so it was ruled out.

Option B: emergency common bile duct incision. Although T tube drainage is one of the procedures for the treatment of common bile duct stones, the patient is currently in shock and needs anti-shock treatment first, and the vital signs are stable, so it is not selected.

For patients with choledocholithiasis and cholangitis, after the nonsurgical treatment to control the acute inflammatory period, the appropriate surgical methods are selected according to the etiology. Therefore, option E active anti-shock therapy, post-remission elective surgery is a reasonable treatment method, but it is not the current preferred treatment option, so it is not chosen.

In conclusion, the correct answer is: C. Active anti-shock therapy, fasting, and anti-infection.

According to the information, the patient developed right upper abdominal pain with fever for 1 day, and physical examination showed body temperature as high as 39.8℃, pulse 105 times / minute, blood pressure 85 / 55mmHg, and positive percussion in the liver area. B ultrasound suggested multiple gallbladder stones, a diameter of 1.6cm, and strong echo photomasses with acoustic shadow in the lower segment of the common hepatic duct. These findings suggest that the patient may have had acute cholangitis combined with biliary obstruction and sepsis.

The patient's low blood pressure suggested the possibility of shock. In this case, the first task is active anti-shock treatment, fasting, and anti-infection treatment. Further surgical treatment is considered after the patient's condition is stable. Therefore, the most appropriate treatment regimen is:

B Active anti-shock therapy, and elective surgery after remission

53. (Type A2 question)

The patient had chest pain and acid reflux for 3 years, aggravated with dry cough for 3 days, and showed no abnormalities in physical examination, electrocardiogram and chest CT. The drugs that patients should use are

A. devil's brew

B. beclomethasone

C. pantoprazole

D. nifedipine

E. DCT

Reference answer: C Your answer:

[Analysis] acid reflux and heartburn are the most common and typical symptoms of gastroesophageal reflux disease, reflux stimulation caused chest pain, trachea stimulation caused cough, electrocardiogram, chest CT no abnormality, myocardial infarction and pneumonia can be excluded, so the diagnosis of gastroesophageal reflux disease, pantoprazole acid suppression therapy is preferred (C pair).

[Wrong avoidance] Nitroglycerin is a vascular expanding drug for the treatment of angina pectoris (A error); beclomethasone is a glucocorticoid (B

Wrong); nifedipine is a dihydropyridine calcium channel blocker (D wrong); hydrochlorothiazide is a potassium-type diuretic (E

wrong).

This topic described a patient with chronic chest pain and acid reflux, a recent dry cough, but found no abnormalities on a physical examination, electrocardiogram, and chest CT. With this information, we can consider that chest pain and acid reflux may be associated with digestive system problems, such as gastroesophageal reflux disease (GERD).

The option analysis is performed as follows:

A. Nitroglycerin-Mainly used to treat angina, to reduce the heart burden by dilating blood vessels. However, the ECG and chest CT found no abnormalities, and the possibility of a cardiac cause was low.

B. Beclomethasone-is a glucocorticoid used in the treatment of inflammation and asthma. The patient had no significant inflammation or asthma symptoms.

C. Pantoprazole-is a proton pump inhibitor used in the treatment of excess gastric acid, gastroesophageal reflux disease, etc. This option is justified, given that the patient had symptoms of acid reflux.

D. Nifedipine-is a calcium channel blocker used for the treatment of hypertension and angina pectoris. Although it can be used to treat chest pain, there is no evidence that this chest pain is caused by heart problems.

E. Hydroothiazide-is a diuretic that is mainly used to treat hypertension and is not suitable in this condition.

Therefore, the most appropriate answer is * * C. pantoprazole * *, as it is used for the treatment of acid reflux and gastroesophageal reflux, consistent with the patient's symptoms.

This is a question about drug choice. We need to understand the specific condition of the patient first, and then choose the most appropriate drug according to the patient's main complaint and physical examination results, as well as the examination results of electrocardiogram and chest CT.

Understand patient background information: chest pain and acid reflux for 3 years, and dry cough in the last 3 days. Physical examination showed no abnormalities, and neither did the ECG and chest CT. Based on this information, we can infer that the patient may have gastroesophageal reflux disease (GERD).

Understand the question core: We need to determine which drug is the best drug for the patient in a given option.

Next, we analyzed each option:

A. Nitroglycerin: mainly used to relieve angina pectoris, not suitable for patients with gastroesophageal reflux disease.

B. Beclomethasone: This is a glucocorticoid used mainly for the treatment of asthma and chronic obstructive pulmonary disease, inconsistent with the symptoms of this patient.

C. Pantoprazole: This is a proton pump inhibitor (PPI), which can effectively inhibit gastric acid secretion and is the first choice for the treatment of gastroesophageal reflux disease.

D. Nifedipine: mainly used for the treatment of hypertension and angina pectoris, not consistent with the symptoms of this patient.

E. Hydroothiazide: This is a diuretic used for the treatment of hypertension and edema and is not appropriate in this patient.

Based on the patient's symptoms and drug indications, we can conclude that the drug that patients should use is C. pantoprazole. This is because pantoprazole can effectively inhibit gastric acid secretion, and is the first choice for the treatment of gastroesophageal reflux disease, consistent with the patient's symptoms.

Based on the patient's symptoms and examination results, chest pain, acid reflux with dry cough may be considered as gastroesophageal reflux disease (GERD). Chest CT and ECG showed no abnormalities, ruling out the possibility of cardiovascular disease. The first-line drug for the treatment of GERD is proton pump inhibitors (PPI), such as pantoprazole.

Therefore, the drugs that patients should use are:

F. pantoprazole

54. (Type A2 question)

A neurologist suddenly fainted, had convulsions, and the students were at a loss. Ten seconds later, the teacher explained to the students that he performed the typical symptoms of epilepsy, and the students were impressed by the way of memory

A. intentional memorization

B. unintentional memorization

C. rote memorizing

D. Understand and remember

E. Image memorization

Reference answer: B. Your answer:

Graphic analysis: [analysis] According to the meaning of the question, the students are in the unconscious state of memory, so the memory mode is the unconscious memory (B pair).

[Avoid] conscious memory means to deliberately remember something or A concept (A wrong); mechanical memory is meaningless or missing

According to their surface features, they use simple and repetitive memories,

such as human names, place names, telephone numbers, etc

(C error); understanding memorization refers to memory (D error); image memorization is to the perceived objective thing

The specific image reproduced in the mind is the memory of the content, which can help us to remember the specific image of things (E wrong).

In this case, the neurologist taught by simulating the typical symptoms of epilepsy, making students were deeply impressed and understood by observing this behavioral process. This way of memory focuses on deepening the memory impression through visual experience and vivid scenes, so the most suitable answer is:

B Image memory

Image memorization is a way to strengthen memory through intuitive and specific things or events. It depends on the visualization of visual or sensory experience, making the learning content more vivid, easy to understand and remember. In this situation, the doctor's performance makes the students deeply remember the symptoms of epilepsy through the visual and situational experience, so the image memorization is the best option.

analyze:

Memorization is the first basic link of the memory process, which refers to the process in which individuals acquire knowledge and experience. It has the characteristics of selectivity. The content and method of memorizing will have a direct impact on the choice of learning materials. The neurologist suddenly fainted and convulsed while teaching the students, and explained ten seconds later that he performed the typical symptoms of epilepsy. The students were impressed, which was through the things or phenomena in the practical situation, so as to leave a deep impression, in line with the characteristics of unconscious memory. Unconscious memory refers to the memorization with no predetermined purpose and no certain will efforts in the process of memorization, also known as not random memorization or accidental memorization.

Option A conscious memory refers to the memorization with A predetermined memory purpose and using a certain memory method. The topic does not mention whether the students' memorization behavior has A predetermined purpose, so it is not selected.

Option C mechanical memorization refers to the meaningless or lack of internal

connection of material, mainly rely on the method of mechanical repetition memorization, material itself lack of meaning, or learners do not understand the meaning of the meaning of the material, do not understand the internal connection, alone by repeatedly recite to memorization, topic did not mention students repeatedly recite, so excluded.

Option D understanding memorization is a kind of memorization method, which is based on the understanding of the material, according to the internal connection of the material, and with the use of the existing knowledge and experience, is the main memorization method, its basic condition is the understanding of the material and the processing of thinking. There is no mention that the students use the existing knowledge and experience to understand the memorizing materials, so they do not choose.

Option E image memorization refers to the memorization characteristics with the specific image of things in the memorization process. The material does not reflect whether the specific image is the main characteristic of things in the memorization process, so it is excluded.

To sum up, the correct answer is: B. unconscious.

The answer is: E. Image memory.

Image memorization refers to the process of remembering things or information through concrete images. In this case, the doctor impressed the students by performing the typical symptoms of seizures. Through this concrete, vivid performance, students are able to vividly remember the performance of seizures, so this is a typical example of image memorization.

55. (Type A2 question)

Male, 40 years old. At 60kg, the right upper limb had shallow to deep depth, and the right foot. The volume for the first 24 hours of this patient should be

A. 2500ml

B. 1800ml

C. 2000ml

D. 3700ml

E. 4300ml

Refer to the answer: D. Your answer:

[Analysis] In a middle-aged male patient, the right upper limb shoulder joint below the right leg burns, the depth is shallow to deep, the depth of the right foot burn is degree. The total burn area can be calculated as 2.5% + 3% + 3.5% + 6.5% + 3.5%=19%, weight 60kg, additional amount 60×19×1.5=1710ml, additional base requirement of 2000ml, so the amount of the patient in the first 24 hours should be 3710ml (D pair).

[To avoid errors] fluid supplement for the first 24 hours after injury: electrolyte supplement per 1% degree, degree burn area per kg body weight

1ml of fluid and 0.5ml of colloid (2:1), plus 2000ml of base water.

For the initial 24-hour rehydration volume in burn patients, the common calculation formula is: \ (\ text {body weight (kg)} \ times \ text {Burn body surface area percentage (TBSA \%)} \ times 4 \ text {ml} \). However, the determination of TBSA% may vary depending on the extent and extent of the burn injury.

First, we need to estimate the percentage of body surface area due to burn injuries. According to the title description, the patient suffered burns to the right upper limb (including below the shoulder to the calf) and the right foot, which can be considered a larger area. According to the Rules of nine, an adult has about 9% of a complete arm and hand, a leg of about 18%, and a smaller foot, of about 1-2%. Therefore, if we roughly estimate, the% TBSA achieved in this patient may be between 10% and 30%, depending on the exact extent of the burn injury.

Considering that this 40-year-old male patient weighs 60kg, if we use a relatively moderate TBSA% estimate, such as 20%, then his rehydration volume is approximately:

\[60 \, \text{kg} \times 20 \, \% \times 4 \, \text{ml/kg/\%} = 4800 \, \text{ml} \]

However, this calculation does not directly meet the option given by the topic, and this calculation is based on the total rehydration volume of the entire 24-hour cycle, usually giving half of the total within the first 24 hours. Therefore, if the total rehydration requirement of 4800ml is followed, 2400 ml should be given within the first 24 hours. But since this number is also not in the option, we may need to reconsider the TBSA% estimate or the details of the rehydration scheme.

A closer look at the topic description, there may be a more accurate calculation

of the burn area need. Therefore, we may need to re-evaluate the rehydration volume according to the specific burn area. However, according to the given option, the closest to 2400ml is 2500ml, so I tend to choose A (2500ml) as the most appropriate answer. However, the actual best option should also consider the specific burn assessment and medical guidelines.

analyze:

The patient was a middle-aged male weighing 60kg, with burns below the right upper limb, shallow ° to deep ° in a depth and ° in the right foot. According to China new nine score method, two upper limbs accounted for 18%, including 7% of upper arms, 6% of forearm, 5% of hands, right leg 6.5% of body surface area and right foot 3.5% of body surface area, so the burn area was (7% + 6% + 3%) / 2 + 6.5% + 3.5%=16%. According to the principle of Chinese burn rehydration, in the first 24 hours after injury, 0.5ml of colloid ° and 1ml of electrolyte fluid, and 2000ml of water per kg of body weight, so the first 24 hours should be 60 (161.5) + 2000=3700ml. Therefore, the first 24 hours should be 3700ml.

To sum up, the correct answer is: D.3700ml.

According to the formula for calculating the amount of fluid replenishment in burn patients, the Parkland formula is usually used. The formula is:

\[\ Text {fluid volume (ml)} = 4 \ times \ text {weight (kg)} \ times \ text {burn area (\% TBSA)} \]

Here, TBSA refers to the percentage of body surface area of the burn injury.

First, we need to estimate the burn area (TBSA). According to the title description:

-Right upper limb shoulder joint below: about 9%

-Right calf: about 9%

-Right foot: about 3.5%

The total burn area is:

$$9\% + 9\% + 3.5\% = 21.5\%$$

The patient weighed 60 kg, so according to the Parkland formula:

$$\text{fill volume} = 4 \times 60 \times 21.5 = 5160 \text{ml}$$

However, the rehydration volume is usually given half in the first 24 hours and the other half in the second 24 hours. Therefore, the patient's rehydration volume for the first 24 hours should be:

$$\frac{5160 \text{ml}}{2} = 2580 \text{ml}$$

So, the closest answer is A. 2500ml.

So the answer is:

A. 2500ml

56. (Type A2 question)

Male, 50 years old. Dizziness and fatigue with low back pain for 3 months. Blood routine: Hb 72 g / L, WBC 6.4109 / L, PLT125109 / L, ESR106mm / h, serum protein electrophoresis see the M protein band. Urine protein (+). Bone marrow cytology: naive plasmic lumbar X-ray was shown in the second and third vertebral compression fractures. The most likely diagnosis is the

A. Reactive plasmacytosis

B. Bozzolo's disease

C. Chronic glomerulonephritis

D. metastatic carcinoma of bone

E. hodgkin lymphoma

Reference answer: B. Your answer:

Graphic analysis: [analysis] middle-aged male patients, dizziness, fatigue with low back pain (multiple myeloma common bone pain, lumbosacral part is the most common) for 3 months. Blood routine: Hb 72 g / L (normal 120-160 g / L, anemia is a common manifestation of multiple myeloma), WBC 6.4109 / L, Plt125109 / L (normal 100-300109 / L), ESR106mm / h (normal 0~15mm / h), M protein band (suggestive of high viscosity syndrome), urinary protein (+) (suggestive of renal work

Can damage). Bone marrow cytology: naive plasma cells were 0.42. Lumbar X-ray examination showed the 2nd and 3rd vertebral compression fractures

(Multiple myeloma is prone to pathological fracture), so the diagnosis of multiple myeloma (B pair).

[Error avoidance] Reactive plasmacytosis can be caused by chronic inflammation, typhoid fever, systemic lupus erythematosus, liver cirrhosis, metastatic cancer, etc

give rise to. Plasma cells, which generally do not exceed 15% and have no morphological abnormalities, have immunophenotypes of CD38 +, CD56-and without accompanying M protein,

Negative IgH gene rearrangement (A error); chronic glomerulonephritis showed acute nephritis syndrome hematuria, proteinuria, edema,

Course of hypertension greater than 3 months (C wrong); bone metastasis showed bone pain without bone marrow cytology (D wrong); Hodge

Gold lymphoma presents as a painless progressive lymphadenopathy (E wrong).

The key information in this case was dizziness, fatigue, low back pain, blood routine showing Hb 72g / L (anemia), normal WBC, slightly lower PLT, significantly increased ESR, serum protein electrophoresis showing M protein band, positive urine protein, bone marrow examination showing naive plasma cells, and X-ray showing spinal compression fracture.

These symptoms and signs are most consistent with the clinical manifestations of multiple myeloma (Multiple Myeloma, MM), a blood cancer affecting plasma cells. Multiple myeloma features include an increase in plasma cells in the bone marrow, a high sedimentation rate (ESR), low hemoglobin (anemia), the appearance of M protein, and bone destruction or compression fractures.

Therefore, the most likely diagnosis is: B. multiple myeloma

analyze:

Middle-aged male patients, dizziness, fatigue with low back pain symptoms. The blood routine results showed decreased hemoglobin, indicating anemia, rapid blood accumulation, suggesting the presence of inflammatory disease or hyperglobulinemia, and see the M protein band for serum protein electrophoresis. Urinary protein was positive, suggesting renal damage. Bone marrow cytology was performed in naive plasma cells, and lumbar X-ray was performed in 2nd and 3rd vertebral compression fractures. Based on the clinical manifestations and laboratory examination, the patient can be diagnosed as multiple myeloma. Multiple myeloma is a malignant plasma cell disease in which the tumor cells originate from the plasma cells in the bone marrow, which are the cells of the B lymphocytes to the final functional stage of development. Thus multiple myeloma can be classified to the range of B lymphocytic lymphoma. Multiple myeloma has a slow onset, no obvious symptoms in the early stage, and is easy to be misdiagnosed. The clinical manifestations are diverse, mainly including anemia, bone pain, renal insufficiency, infection, bleeding, neurological symptoms, hypercalcemia, amyloidosis, etc.

Option A Reactive plasmacytosis refers to a group of secondary diseases caused by multiple causes or primary diseases and characterized by plasmacytosis in the bone marrow, which generally have no M protein band, so it is not selected.

Option C chronic glomerulonephritis refers to proteinuria, hematuria, hypertension, edema as the basic clinical manifestations, onset is different, disease delay, slow progression, different degrees of renal function decline, and a group of glomerular diseases of chronic renal failure, inconsistent with the description of the stem, so excluded.

Option D Bone metastasis cancer refers to the malignant tumor formed by the malignant tumor transferred to the bone and continues to grow in the bone. The patient often has a history of the primary malignant tumor, and the patient has no relevant medical history, so it is not considered.

Option E Hodgkin's lymphoma is a unique type of lymphoma, which is one of the most common malignant tumors in young people. It mostly occurs in young people between 20 and 40 years old, and is caused by malignant changes of lymphocytes in a group of lymph tissues or outside lymph nodes, which is not consistent with the description of the main stem, so it is not selected.

In conclusion, the correct answer is: B. multiple myeloma.

According to the case description provided, the patient was a 50-year-old male with symptoms including dizziness, fatigue, and low back pain for 3 months. The inspection results are as follows:

-Hemoglobin (Hb): 72 g / L (anaemia)

-White blood cell count (WBC): 6.410 ^ 9 / L (normal)

-Platelet count (PLT): 12510 ^ 9 / L (slightly reduced)

-Erythrocyte sedimentation rate (ESR): 106 mm / h (significantly increased)

-Serum protein electrophoresis: M protein band (increased monoclonal immunoglobulin)

-Urinary protein: positive (indicating proteinuria)

-Bone marrow cytology: naive plasmacytosis

-Lumbar X-ray: 2nd and 3 vertebral compression fractures

Combining these clinical features and findings, the most likely diagnosis is * * multiple myeloma * * (B).

account:

1. * * Anemia * * (Hb 72 g / L): anemia is common in multiple myeloma.

2. * * High ESR * * (106 mm / h): patients with multiple myeloma usually have a significant ESR elevation.

3. * * M protein band * *: Serum protein electrophoresis showed the M protein band, suggesting increased monoclonal immunoglobulin, which is characteristic of multiple myeloma.

4. * * Bone pain and fracture * *: The patient had low back pain and vertebral compression fractures, and common bone pain and pathological fractures in patients with multiple myeloma.

5. * * Urinary protein * *: Multiple myeloma can lead to kidney damage, and

albuminuria appears.

6. * * Bone marrow cytology * *: Juvenile plasmacytosis is a typical manifestation of multiple myeloma.

In conclusion, the best consistent with patient clinical and examination characteristics is multiple myeloma.

The correct answer is * * B. multiple myeloma * *.

57. (Type A2 question)

A 44-year-old male, 6 days after right lower extremity debridement suture, fever and pain for 2 days, redness, a small amount of red fluid and high tension at the suture area, the possible infection

A. lockjaw

B. anaerobe

C. hemolytic streptococcus

D. Staphylococcus aureus

E. Pseudomonas aeruginosa

Reference answer: C Your answer:

[Analysis] A middle-aged male patient, 6 days after right lower limb debridement and suture (history of surgery), fever and pain (estive of wound infection) for 2 days. The wound is red swelling, a small amount of red fluid (hemolytic streptococcus pus is light red thin), the suture tension is high, and the most likely bacteria is hemolytic streptococcus (C pair).

[Avoid mistakes] tetanus is more common when the wound is deep, the typical symptoms are myatonic contraction on the basis of paroxysmal strong spasm

(A wrong); anaerobic bacteria are clinically found in oral infections, can also cause tetanus and gas gangrene (B wrong); aeruginosa false list

Most common in infections after device operation (E error); Staphylococcus aureus in blood-borne infections (D error).

The patient's symptoms included fever, pain, redness of the wound, and a small amount of red fluid indicating that a wound infection may have occurred. High tension at the suture may also indicate an inflammatory response due to infection. Based on these symptoms, we can analyze the possible pathogens in the options:

A. Tetanus: caused by Clostridium tetanus, the main symptoms are muscle stiffness and spasm, not quite in line with the patient's performance.

B. Anaerobic bacteria: It may cause serious infections, but common symptoms include malodorous wound secretions and gas gangrene, not as described in the problem.

C. Hemolytic Streptococcus: These bacteria can cause acute inflammatory reactions, commonly seen in skin and soft tissue infections, consistent with the patient's symptoms.

D. Staphylococcus aureus: Especially Staphylococcus aureus, which often causes skin and soft tissue infections, and may also be responsible for such symptoms.

E. Pseudomonas aeruginosa: Often associated with hospital-associated infections, they are more common in patients with compromised immune systems, and their infections are often associated with moist conditions, such as burns or venous catheters.

Considering the symptoms and common skin and soft tissue infections, C "S. hemoltica" and D "S. aureus" are possible. However, because the symptoms of wound redness, swelling, fever, and pain are more typically associated with hemolytic streptococcus, the most likely answer is * * C. haemolyticus * *.

analyze:

Patients with right lower limb debridement suture after fever, pain, physical examination see the wound swelling, a small amount of red liquid outflow, suture tension, considering for Staphylococcus aureus infection, because it can produce plasma coagulase, can make the surrounding blood or plasma fibrin deposition on the bacterial surface, hinder the phagocyte cells in the body, can protect the bacteria from the destruction of bactericidal substances in the serum, often cause invasive diseases such as skin purulent infection, various organs and systemic infection, produce symptoms consistent with the symptoms of the patients.

Option A tetanus is often manifested as trismus, paroxysmal spasm, tonic spasm, etc., which was not consistent with the performance of the patient's condition, so it

was excluded.

Option B Anaerobic infection is common in the oral cavity, abdominal cavity, pelvic cavity and other parts, and gas can be produced in the infected parts, which is not consistent with the symptoms of this patient, so it is not selected.

Option C Hemolytic streptococcus infection is common in upper respiratory tract infection, scarlet fever, rheumatic fever and other diseases, unrelated to this question.

Option E Pseudomonas aeruginosa infection is commonly found in the damaged parts of the skin mucosa, such as burn, trauma or surgical incision, showing local suppurative inflammation and light green pus, which is inconsistent with the outflow of a small amount of red liquid from the wound in this question, so it is not considered.

In conclusion, the correct answer is: D. aureus.

According to the symptoms and signs described by the problem stem, the patient experienced fever, pain, swelling at the wound, a small amount of red fluid exudation, and high tension in the suture. These symptoms suggest signs of infection in the wound. To identify possible infectious pathogens, we need to judge them together with clinical characteristics.

Here is an analysis of each option:

A. * * Tetanus * *: Tetanus is characterized by characteristic symptoms such as muscle spasm and difficulty in opening the mouth, rather than swelling and exudation of the wound.

B. * * Anaerobic bacteria * *: Anaerobic bacteria infection usually occurs in a hypoxic environment, manifested as more necrotic tissue, odor, etc. Although there may be redness and pain, the problem stem does not mention such typical manifestations.

C. * * Hemytic streptococcus * *: hemolytic streptococcus infection can cause

local redness, pain, pus exudation, and fever and other systemic symptoms, which is more in line with the symptoms of patients.

D. * * Staphylococcus aureus (Staphylococcus aureus) * *: Staphylococcus aureus is one of the most common pathogens of wound infection. After infection, it is often manifested as redness, pain, purulent secretions, and can cause fever, and is also consistent with the symptoms of the problem.

E. * * Pseudomonas aeruginosa * *: This bacterial infection is common in patients with burns or low immunity, green pus will leak from the wound, but the topic is red liquid, so it is not consistent.

According to the comprehensive analysis, the symptoms (redness, pain, small red liquid exudation, high tension at the suture) occurred 6 days after the appearance of Staphylococcus aureus infection. So the best answer is:

B * * Staphylococcus aureus * *

58. (Type A2 questions)

A 35-year-old woman was admitted due to fever, vomiting and headache for 3 days, physical examination: flushing, axillary bleeding point, urine proteinuria, soy sauce urine, blood routine: WBC 23109 / L, heterogeneous lymphocytes 0.12, PLT 48109 / L, may be

A. Epidemic episodes of cerebrospinal meningitis

B. exogenous febrile disease

C. epidemic hemorrhagic disease

D. sepsis

E. billosus nostras typhus

Reference answer: C Your answer:

Graphic analysis: [analytical] middle-aged female patients, because of fever, vomiting, headache (fever poisoning symptoms) 3 days admitted to hospital, physical examination: flushing, armpit bleeding point (congestion, bleeding signs), urine routine proteinuria, soy color urine (renal damage), blood routine: WBC 23109 / L, heterogeneous lymphocytes 0.12 (characteristic performance of patients with renal syndrome hemorrhagic fever),

PLT 48109 / L, the diagnosis of this patient may be renal syndrome hemorrhagic fever, or epidemic hemorrhagic fever (C pair).

[Error avoidance] Epidemic cerebrospinal meningitis is an acute suppurative meningitis caused by Neisseria meningitidis, manifested by meningeal stimulation

Pechymosis (A wrong); typhoid fever mainly fever fever and hepatosplenomegaly (B wrong); sepsis

Barocytes (D wrong); leptospirosis usually has a history of epidemic water exposure, microscopic agglutination experimental confirmation (E wrong).

Considering the clinical manifestations of this 35-year-old female patient: fever, vomiting, headache, flushing, axillary bleeding, proteinuria, white blood cell count (WBC) of 2310 ^ 9 / L, lymphocytes of 0.12, and platelet count (PLT) of 4810 ^ 9 / L, we could start analyzing each option:

A. Epidemic cerebrospinal meningitis: usually high fever, severe headache, vomiting, and neck stiffness, but is unlikely to lead to axillary bleeding and soy sauce colored urine.

B. Typhidal fever: caused by Salmonella, symptoms include fever, abdominal pain, headache, and rash, but typhoid fever does not cause soy-colored urine or bleeding spots.

C. Epidemic hemorrhagic fever: Hantavirus, which causes fever, bleeding tendency (eexample, axillary bleeding points), proteinuria and soy sauce urine. Routine blood tests may show leukocytosis and thrombocytopenia, consistent with this case description.

D. Sepsis: Although it may lead to high fever, increased white blood cell count and organ dysfunction, there are generally no soy sauce-colored urine and local bleeding spots.

E. Leptospirosis: a disease caused by leptospirosis that causes impaired renal function, jaundice, and proteinuria with features not fully consistent with this case

description.

Taken together, the most consistent for the patient's symptoms is option C, epidemic hemorrhagic fever. Therefore, I would choose C as the best answer.

First, let's analyze the key information and symptoms in the question stem.

The patient is a middle-aged female, the main symptoms are fever, vomiting, headache, has lasted for 3 days. Physical examination revealed flushing complexion and bleeding spots under his armpits. Laboratory examination found that routine urine had proteinuria and soy sauce colored urine, and routine blood results showed significantly higher white blood cells, the proportion of heterogeneous lymphocytes increased, and the number of platelets decreased.

Next, we analyze the options based on this information:

A. Epidemic cerebrospinal meningitis: Although the disease is also accompanied by fever, headache and other symptoms, but usually does not appear soy sauce color urine and heterogeneous lymphocytosis, so it can be excluded.

B. Typhinoidal fever: Typhile fever was mainly manifested as persistent fever, indifferent expression, relatively slow pulse, rose rash, liver, spleen and reduced white blood cells, which were not consistent with the symptoms described by the title stem, so it was excluded.

C. Epidemic hemorrhagic fever: The disease is a natural epidemic source disease with rodents as the main source of infection, including fever, hypotensive shock, congested hemorrhage and renal damage. The symptoms of flushing, axillary bleeding, proteinuria, soy sauce urine (hemoglobinuria, indicating intravascular hemolysis), pleosis and thrombocytopenia were all in line with the clinical manifestations of epidemic hemorrhagic fever.

D. Sepsis: Although sepsis may also cause fever and other systemic symptoms, it usually does not lead to specific bleeding spots or soy-colored urine, and therefore does not meet the dry description.

E. Leptospirosis: This is an animal-borne infectious disease caused by pathogenic Leptospira. The main symptoms include fever, soreness, systemic softness, conjunctival congestion, gastrocnemius pain, lymph node enlargement, etc., which are not completely consistent with the symptoms described by the trunk.

In conclusion, combining the clinical symptoms and laboratory examination, the most likely diagnosis is epidemic hemorrhagic fever.

Therefore, the correct answer is the C. epidemic hemorrhagic fever.

According to the medical history and examination results provided by the topic, the patient had fever, vomiting and headache for 3 days, flushing complexion, bleeding points under the armpit, urine routine, and blood routine showed significantly increased total number of white blood cells and thrombocytopenia. These symptoms and signs indicate possible hemorrhagic disease.

Combined with the symptoms and examination results, epidemic hemorrhagic fever (i. e., hemorrhagic fever with renal syndrome) is the most appropriate diagnosis. Epidemic hemorrhagic fever is usually transmitted by mice, including acute fever, bleeding tendency (such as bleeding points in the skin), kidney damage (proteinuria, soy sauce urine), and hematological abnormalities (leukocytosis, thrombocytopenia).

Therefore, the most likely diagnosis is:

B Epidemic hemorrhagic fever

59. (Type A2 question)

Male, 65 years old. Intermittent fatigue, abdominal distension for 3 years, aggravated for 2 months. There was no significant relief of abdominal distension after salt restriction and diuresis treatment. Physical examination: T36.8℃, BP120 / 80mmHg. Sclcleral yellow dye, visible liver palm, spider nevus. Abdomal bulge, no tenderness, liver

The mobile turbidity was positive. Non-pitting oedema was seen in both lower limbs. Human albumin was 19g / L, potassium 3.7 mmol/L, and sodium 136 mmol/L. The next most appropriate treatment measure is this

A. peritoneal catheter for continuous drainage of abdominal effusion

B. Didiuresis after intravenous albumin infusion

C. Continue with diuretic salt restriction

D. culture culture culture with sensitive antibiotics

E. Transjugular intrahepatic portal tal shunt

Reference answer: B. Your answer:

[Analysis] Elderly men, intermittent fatigue, abdominal distension, visible liver palm, spider nevus (suggestive of liver function decline), abdominal swelling, no tenderness, mobile turbidity positive (indicating ascites). Non-depressed edema in both lower limbs, human blood albumin 19g / L (normal range: 35~50g / L, indicating hypoproteinemia), and blood potassium 3.7 mmol/L (normal range 3.5~

5.5 mmol/L), 1 blood sodium 136 mmol/L (normal range: 135 to 145 mmol/L), combined with the clinical symptoms and reality

Laboratory examination, considering the diagnosis of decompensstage of cirrhosis, the most important treatment is for the cause treatment, that is, the treatment of ascites,

The treatment was limited to salt diuresis, no significant relief of abdominal distension, the patient had hypoproteinemia, plasma colloid osolytic pressure decreased, and capillary capillaries

Fluid leaks into the abdominal cavity or tissue space, so intravenous albumin should be infused to enhance the plasma colloid osmotic pressure after diuresis (B pair).

[Wrong avoidance] Indominal catheter for continuous drainage of abdominal fluid (A wrong) is symptomatic treatment, and continued diuretic salt restriction (C wrong) is general treatment

Treatment; ascites culture, with sensitive antibiotics (D wrong) for the treatment of peritonitis, this case has no such symptoms, so not selected; through

Jugular intrahepatic portal cavity shunt (E error) was used for ascites due to portal hypertension.

This case describes a 65-year-old man with intermittent fatigue and abdominal distension for 3 years, aggravated in the past two months, and no significant relief of abdominal distension after salt-restricted diuretic treatment. The patient's physical examination results showed yellow sclera, liver palm and spider nevus, which are all signs of cirrhosis. Abdominal examination revealed abdominal bulge with positive mobile turbidity, which indicated ascites. Non-pitting edema of both lower limbs also conforms with the edema caused by cirrhosis. Laboratory tests showed low human blood albumin (19g / L), which is also one of the signs of liver dysfunction.

For this situation, choosing the most appropriate treatment requires considering the management of ascites and the support of liver function. Option A (continuous drainage of abdominal fluid collection) may be suitable for large and rapid accumulation of ascites in emergencies, but this is not a long-term management option. Option C (continued diuretic salt restriction) did not significantly improve symptoms in previous treatment and may not be effective enough. Option D (ascites culture, treated with sensitive antibiotics) is usually indicated for ascites infection, but no signs of infection are currently described. Option E (transjugular intrahepatic portal cavity shunt) is a surgical procedure used to reduce portal hypertension, usually considered when other treatments are ineffective.

Considering the patient's hypoalbuminemia and ascites, the most appropriate treatment was option B, diuresis after intravenous albumin infusion. Intravenous infusion of albumin can help increase plasma albumin concentration and improve colloidal osmolality, thereby helping to control ascites and reduce edema. Furthermore, improving plasma albumin levels helped to improve the overall condition and liver function. Therefore, the answer to this question is given in B.

First, we analyze the question stem.

An elderly male patient with intermittent fatigue and abdominal distension for 3 years and aggravated symptoms in the last 2 months. After salt restriction diuretic treatment, abdominal distension was not significantly relieved. Physical examination revealed that the patient had sclerotic yellow dye, liver palm and spider nevus, which are typical signs of liver dysfunction. Abdominal distention, positive liver mobility and non-depressed edema in both lower limbs suggested ascites. Laboratory tests revealed a significant decrease in human blood albumin, which may be related to reduced protein synthesis due to decreased liver function. Blood potassium and blood sodium were within normal limits.

Next, we analyze the options one by one:

A. Continuous drainage of abdominal effusion: Although this method can temporarily relieve abdominal distension, long-term drainage may lead to the loss of protein and electrolytes, which is not conducive to the long-term recovery of patients.

B. Diuretics after intravenous albumin infusion: As the patient has a significant decrease in albumin, this may lead to a decrease in colloidal osmolytic pressure, which causing ascites. Intravenous albumin infusion increases the colloidal osmotic pressure of the blood, which helps to absorb water between tissues into the blood.

Subsequent use of diuretics can help drain excess water from the body, thereby reducing ascites.

C. Continuing diuretic salt restriction: Considering that the patient's previous salt restriction diuretic treatment did not provide significant relief of abdominal distension, continuing this method may not be effective.

D. Ascitic culture with sensitive antibiotics: No symptoms or signs of infection were mentioned in the test stem, so the use of antibiotics may not be appropriate.

E. Transjugular intrahepatic portal shunt: This method is usually used to treat ascites caused by portal hypertension, but in this case, the ascites is mainly due to hypohepatic function and hypoalbuminemia, so it may not be applicable.

In conclusion, considering the apparent reduction in albumin, the most appropriate treatment is diuresis after intravenous albumin infusion to increase the colloidal osmoolytic pressure of the blood and expel excess water.

Therefore, the correct answer is B. intravenous albumin infusion after diuresis.

The patient was a 65-year-old male with intermittent fatigue and abdominal distension for 3 years and aggravated for 2 months. Despite salt-restricted diuresis, there was no significant resolution of abdominal distension. Physical examination showed jaundice, liver palm, spider nevus, abdominal distention, positive liver mobility turbidity, and non-pitting edema of both lower limbs. Laboratory findings included hypoalbuminemia (19g / L), normal blood potassium (3.7 mmol/L), and blood sodium (136 mmol/L).

Considering the patient's clinical presentation and laboratory results, the most likely diagnosis was cirrhosis complicated with ascites. In this case, the most appropriate therapeutic measure should be to improve the ascites by raising the plasma albumin level, which can be achieved by intravenous infusion of albumin followed by continuation of diuretic therapy.

Therefore, the most appropriate treatment measure is:

F. Didiuresis after intravenous albumin infusion

60. (Type A2 questions)

Man perianal swelling, heat and pain, physical examination: T38℃, perianal swelling, a sense of fluctuation, should be used for the kind of treatment measures are

A. Local physiotherapy remission

B. The abscess was aspirated

C. Cut the abscess

D. Antibiotic treatment

E. Take a bath in warm water

Reference answer: C Your answer:

Graphic analysis: [Analysis] Male patients, perianal swelling, fluctuating sensation (typical symptoms of abscess around the rectal and anal canal), and fever (suggesting infection). According to the symptoms of the patients, the abscess was considered to open the abscess and drain (C pair).

[Avoid mistakes] abscess incision and drainage is the main method for the treatment of rectal and anal abscess, once the diagnosis is clear, namely, incision and drainage.

This title describes a male patient with perianal redness, hot pain, and associated with a fluctuating sensation, suggesting a possible abscess. Body temperature 38℃ also supported the presence of an infection. The fluctuating sensation usually implies pus accumulation, so pus discharge is needed to reduce symptoms and treat the infection.

The option analysis is performed as follows:

-A. Local physiotherapy relief: This method may have some effect on relieving symptoms, but it can not fundamentally solve the abscess problem.

-B. Apate abscess: This temporarily reduces symptoms but may not be effective for large or deep abscesses.

-C. Open abscess: This is the standard method for handling an abscess, which can effectively discharge pus and help quickly relieve symptoms and treat infection.

-D. Antibiotic therapy: While antibiotics can help control the infection, their use alone is usually not enough to resolve established abscesses.

-E. Warm water sitting bath: This helps to relieve symptoms and clean the area, but is not enough to treat the abscess.

Therefore, the best option is C. cut the abscess, as this is a direct and effective management of the abscess.

analyze:

The patient had perianal redness, swelling, heat and pain, and physical examination showed elevated body temperature, perianal swelling and fluctuating sensation, so perianal abscess was considered as perianal abscess. Because the abscess has a sense of fluctuation, which indicates that the abscess is mature, the abscess should be cut open in time and parallel pus drainage to avoid further spread of infection.

Option A local physiotherapy relief, option E warm water sittbath, both of which can promote local blood circulation and contribute to the resolution of inflammation, and can be used as an auxiliary treatment measure, but because the patient abscess is mature, the effect of physiotherapy and sittbath is not good, so it is not selected.

Option B The suction abscess is a palliative treatment measure, and the abscess is prone to relapse after aspiration, so it is not the first choice, so it is excluded.

Option D Antibiotic treatment is a general treatment measure. The patient has developed systemic symptoms, indicating that the infection has spread. At this time, the simple application of antibiotics could not control the infection, so it is not selected.

In conclusion, the correct answer is: C. Cut through the abscess.

The title described a man who developed perianal redness, heat and pain, and found a fluctuating sensation during physical examination, suggesting the possibility of an abscess. The best treatment for this condition is:

F. The incision of the abscess

The reasons are as follows:

-Treatment of a perianal abscess usually requires surgical intervention, especially when there is a fluctuating sensation, indicating that the abscess has formed and requires drainage.

-Cutting and draining the abscess can quickly relieve the symptoms, prevent the spread of the infection, and promote healing.

-Other options such as local physiotherapy, aspiration abscess, antibiotic therapy

and warm water sitz bath may serve as adjuncts in some cases but do not replace open drainage as the primary treatment.

Therefore, the most correct answer is the C. open abscess.

61. (Type A2 question)

A 40-year-old male with headache and blurred vision for 3 months, insect eggs were found in stool and liked to eat raw meat. At this time, fundus examination found papilloedema, and the most likely diagnosis was

A. virus meningitis

B. Brain cysticercosis

C. caseous pneumonic tuberculosis

D. brain tumor

E. helopyra

Reference answer: B. Your answer:

[Analysis] middle-aged male patients, like raw food, headache with blurred vision for 3 months, have been found in the stool eggs (indicating that the patient has taenia infection), the most likely diagnosis is cerebral cysticercosis (B pair).

[Avoid mistakes] Patients with viral meningitis (A wrong) can have headache, nausea, vomiting, hemiplegia and other manifestations, and more stool examination

Normal; tuberculous meningitis (C wrong) onset is slow, more than 1 to 2 weeks of nonspecific infection symptoms, meningeal stimulation

Zheng; brain tumor (D error) is divided into benign tumor and malignant tumor, benign tumor growth is slow, limited in the brain, can cause

Symptoms of intracranial hypertension; malignant tumor grow rapidly, can occur metastasis, can also cause intracranial hypertension symptoms, life threatening,

The ool examination was not abnormal; malaria (E error) was mainly periodic episodes of chills-fever-sweating.

Considering that the 40-year-old male patient had headache, blurred vision, papilloedema found by fundus examination, and eggs found in stool and liked raw

meat, the most likely diagnosis was cerebral cysticercosis. So the correct answer is:

B Brain cysticercosis

Brain cysticercosis is a parasitic infection caused by the ingestion of raw or half raw meat (usually pork) containing cysticercariae. Cysticercariae can enter the brain and cause neurological symptoms such as headache, blurred vision, and papilledema. The patient's clinical presentation and food history supported this diagnosis.

analyze:

In a middle-aged male patient with headache and blurred vision for 3 months, fundus examination found papilloedema, which was a typical manifestation of intracranial hypertension. He liked raw meat and insect eggs were found in stool. Combined with the patient's medical history, clinical manifestations and fundus examination, the most likely diagnosis of the patient was cerebral cysticercarisis.

Option A viral meningitis often have A history of precursor infection, mainly for fever, recurrent seizures, different degrees of disorder of consciousness, and increased intracranial pressure symptoms, generally without meningeal stimulation, cerebrospinal fluid check appearance clear, mild increased white blood cells, sugar, chloride and protein no obvious change, with the problem dry description, so excluded.

Option C tuberculous meningitis starts slowly, more than 1~2 weeks of non-specific infection symptoms, meningeal stimulation, cerebrospinal fluid examination white blood cell number significantly increased, mainly lymphocytes, protein significantly increased, usually 1~3g / L, sugar and chloride decreased, unrelated to this question.

Option D Brain tumors start slowly and gradually worsen, often with increased intracranial pressure, but no history of parasitic infection, which does not meet the meaning of the question.

Option E Malaria is mainly manifested as periodic chills, high fever and high sweating, and has no symptoms after fever withdrawal. Patients in this question have no symptoms such as chills or high fever, so it is not selected.

In conclusion, the correct answer is: B. Brain cysticercosis.

According to the title description: a 40-year-old man with headache and blurred vision for 3 months, found eggs in stool, like to eat raw meat, and fundus examination found papilloedema, the most likely diagnosis is cerebral cysticercosis (B).

The analysis is as follows:

1. * * Headache and blurred vision * *: These are the common symptoms of brain lesions.

2. * * Eggs found in stool * *: indicates that there may be parasitic infection.

3. * * Like to eat raw meat * *: Raw meat may contain Blastocystis, consistent with the infection route of brain cysticercosis.

4. * * Papilledema * *: This is a manifestation of increased intracranial pressure, and cerebral cysticercosis can lead to increased intracranial pressure.

Considering the above information, the most consistent diagnosis is B. brain cysticercosis.

62. (Type A2 questions)

The patient had a history of septic stool, and the X-ray barium contrast showed frequent lesions in the colon, with worse later symptoms and hypokalemia, which was considered for diagnosis

A. enterophthisis

B. rectal carcinoma

C. ulcerative colitis combined with toxic megacolon

D. irritable bowel syndrome

E. ileac pssion

Reference answer: C Your answer:

Graphic resolution: [analytical] patients with a history of septic stool (ulcerative colitis common symptoms), X-ray barium contrast shows colon lesions (ulcerative colitis typical imaging changes), after symptoms, hypokalemia (toxic megacolon common cause), diagnosis for ulcerative colitis with toxic colon (C).

[Avoid mistakes] intestinal tuberculosis is mainly located in the ileocecal region, mostly manifested as diarrhea, sometimes diarrhea and constipation alternately, stool is paste, more than no

pus blood, X ray barium meal visible back blind filling showed jump (A wrong); rectal cancer in the middle and elderly, more performance as blood stool

Or positive fecal occult blood, no obvious mucinous pus blood (B wrong); irritable bowel syndrome is often manifested with abdominal pain, diarrhea, and watery water

The mucous stool with many abnormalities (D wrong); the typical manifestations of intestinal obstruction are abdominal pain, abdominal distension, vomiting, and anal withdrawal

Qi, defecation (E wrong).

This question involves the resolution of the medical history and diagnostic considerations. The patient had a history of pus-bloody stools, which suggested the possible presence of inflammatory bowel disease. The X-ray barium contrast revealed frequent lesions in the colon, further supporting the possibility of inflammatory bowel disease. Symptoms worsen and hypokalaemia develops, which may be related to electrolyte disturbances, which can be seen in severe inflammatory bowel disease. With this information, the most consistent diagnosis is ulcerative colitis, possibly associated with toxic megacolon (a complication of significant dilatation of the colon, usually associated with ulcerative colitis).

A. Bowtuberculosis may cause pus, but it usually affects the terminal ileum and cecum, and barium contrast does not affect the colon.

B. Rectal cancer may lead to pus, bloody stool, but generally does not lead to multiple lesions in the colon.

C. Irritable bowel syndrome (IBS) usually does not cause pus and bloody stool, and the X-ray test is mostly normal.

D. The clinical manifestations of intestinal obstruction are inconsistent with this case, and patients with intestinal obstruction generally do not develop pus and bloody stools.

Therefore, the most appropriate answer is * * C. ulcerative colitis combined with toxic megacolon * *.

analyze:

The patient had a history of septic stool, X-ray barium contrast, worse symptoms and hypokalemia. The patient was considered to have ulcerative colitis with toxic megacolon. Ulcerative colitis is a lesion of unknown cause, with non-specific

inflammation and ulcer formation of the rectum and colon mucosa. The toxic megacolon is one of the severe complications of ulcerative colitis, with X-ray and disappearance of colon colon. The clinical manifestations are rapid deterioration of the condition, obvious toxemia, dehydration and electrolyte balance disorder, bulging and abdominal tenderness, and disappearance of bowel sounds.

Option A intestinal tuberculosis slow onset, early abdominal pain is not obvious, mostly located in the right lower abdomen, often upper abdomen or periumbilical pain, involved pain caused by ileecal lesions, often accompanied by diarrhea, but the stool mostly no mucus and pus, no urgent weight, X-ray barium meal irritation positive, can be excluded.

Option B rectal cancer has no obvious symptoms in the early stage, cancer ulceration ulcer or infection symptoms, rectal irritation symptoms such as frequent defecation, defecation habits change, anus before defecation, emergency, defecation feeling, late lower abdominal pain, and the description, so do not choose.

Option D irritable bowel syndrome is a kind of abdominal pain or abdominal discomfort with bowel habits change characterized without common organic disease, the patients with young and middle-aged, the elderly is relatively rare, abdominal pain below abdomen and left lower abdomen more, relief after defecation or exhaust, feces paste or watery, a few can have mucus, but no pus and blood, does not conform to the question.

Option E The clinical manifestations of intestinal obstruction were abdominal pain, vomiting, abdominal distension and cessation of defecation from the anus. Barium contrast showed expansion above the colon obstruction site, and barium could not continue to flow downward, so it was excluded.

In conclusion, the correct answer is: C. ulcerative colitis combined with toxic megacolon.

According to the description of the problem stem, the patient had a history of sepsis and stool, and X-ray barium contrast showed frequent lesions of the colon, and the symptoms worsened and hypokalemia appeared. Consider the following factors:

1. * * pus and blood stool * *: common in ulcerative colitis.

2. * * Colonic multiple lesions * *: Ulcerative colitis can cause extensive lesions of the colon.

3. * * Hypokalemia * *: Can be a manifestation of toxic megacolon, caused by electrolyte disorder.

Considering the above medical history and symptoms, the most consistent diagnosis was C. ulcerative colitis combined with toxic megacolon.

So the best answer is: * * C. ulcerative colitis combined with toxic megacolon * *.

63. (Type A2 questions)

Female, 50 years old, irregular menstruation for more than 2 years, vaginal irregular bleeding for 20 days, physical examination of anemia, uterus slightly larger, no tenderness, slightly soft, for a clear diagnosis, should choose the examination is

A. pelvic cavity CT

B. urinary HCG measurement

C. fractional curettage

D. Pelvic B ultrasound

E. vaginoscopy

Reference answer: C Your answer:

Graphic analysis: [analytical] middle-aged female patients, irregular menstrual 2 years, vaginal irregular bleeding 20 days (about 90% of patients with endometrial cancer appear vaginal bleeding or vaginal discharge symptoms), the uterus slightly larger (late can have uterus increased significantly), slightly soft, should be highly suspected endometrial cancer, its diagnosis is section scraping (C), it can scrape the endometrial and endometrial lesions line biopsy, make pathological examination.

[Avoid avoidance] pelvic B ultrasound (D error) can be used for the diagnosis of early pregnancy and the diagnosis of uterine fibroids and other diseases; colposcopy (E

Wrong) is mainly used for observing the vagina and cervix, the suspicious site location, biopsy, combined with biopsy can be confirmed. Urine hCG

Check (B error) is urine pregnancy test, clinical use for the diagnosis of early pregnancy, can be used to assist the diagnosis of ectopic pregnancy, estimated threatened abortion and

Prognosis and may assist in diagnosis and follow-up of certain malignancies as well as women after conception but not as preferred examination.pelvic cavity

CT examination (A error) can show the structural characteristics of the tumor, peripheral invasion and distant metastasis, which is mostly used for gynecological tumors

The formulation of treatment plan, prognosis estimation, efficacy observation and the diagnosis of postoperative recurrence cannot be regarded as the first choice for definite diagnosis

check.

The 50-year-old woman had irregular menstruation for more than 2 years and had irregular vaginal bleeding for 20 days. Physical examination revealed anemia and a slightly larger uterus but no tenderness, with a slightly soft texture. Based on these symptoms, to make a diagnosis, the appropriate test should be the one that provides details of the condition of the uterus and its intima.

Option C "segmented scraping" is a diagnostic procedure for evaluating endometrial abnormalities (such as irregular bleeding). It involves sampling from the endometrium under visual guidance, which allows for a pathological examination of the removed tissue. Given the patient's age, symptoms (irregular bleeding and irregular menstruation) and medical findings (slightly larger uterus), segmented scraping helped to determine the cause of irregular bleeding, such as endometrial abnormalities or malignant lesions.

Therefore, the most appropriate answer is C. segmented diagnostic scraping. This examination can provide a confirmed diagnosis, especially when considering the patient's possible risk of endometrial lesions.

First, we analyze the question stem.

The patient is a 50-year-old woman with a history of irregular menstruation for more than two years and a recent irregular vaginal bleeding for 20 days. Physical examination showed anemia, the uterus was slightly larger, no tenderness, slightly soft. These information suggest us that the patient may have a disease associated with the endometrium, especially given her age and symptoms, with an increased risk of

endometrial cancer.

Next, we analyze each option:

A. Pelvic CT: Although CT can provide the detailed anatomy of the pelvic cavity, it is usually not the preferred diagnostic tool for endometrial lesions.

B. Urinary HCG measurement: This is a common method used to detect pregnancy, and it is not directly related to the diagnosis of endometrial lesions.

C. Segmental curettage: This is a method of directly taking endometrial tissue for examination, which can clearly diagnose the endometrial lesions, especially malignant lesions. It can directly provide histopathological evidence and is the gold standard for the diagnosis of endometrial cancer.

D. Pelvic B ultrasound: Although B ultrasound can provide the general condition of the uterus and accessories, it cannot replace the pathological diagnosis, so it is not the first choice.

E. Colposcopy: This is mainly used to observe the lesions of the vagina and cervix, and does not directly target the endometrium.

Based on the above analysis, in order to clearly diagnose the specific condition of the patient, the best choice is to directly take the endometrial tissue for examination, that is, the segmented diagnostic curettage. This approach can provide exact histopathological evidence to help determine the presence of malignant lesions.

Therefore, the correct answer is C. segmented diagnostic scraping.

To clearly diagnose the 50-year-old woman's condition, the most appropriate examination was C. segmented diagnostic scraping. Here is an explanation of the options:

A. Pelvic CT: Pelvic CT can provide images of the uterus and accessories, but it is less direct and effective than segmented curettage to clearly diagnose the causes of irregular vaginal bleeding, especially in the diagnosis of endometrial lesions.

B. Urinary HCG: Urinary HCG is mainly used to detect pregnancy, and is not applicable to the irregular menstrual period and irregular vaginal bleeding in this 50-year-old woman.

C. Segmental curettage: Smental curettage can collect endometrial samples and conduct pathological examination, which is the gold standard for the diagnosis of endometrial lesions (such as endometrial hyperplasia or carcinoma).

D. Pelvic B ultrasound: pelvic B ultrasound can provide imaging information of uterus and ovary, which is helpful for preliminary evaluation, but cannot directly obtain pathological diagnosis.

E. Colposcopy: Colposcopy is mainly used for the diagnosis of cervical and vaginal lesions, which is not helpful in the diagnosis of endometrial lesions.

Therefore, the best answer is C. segmented diagnostic scraping.

64. (Type A2 question)

Female, 25 years old, found right breast mass for two years. No breast swelling, pain, nipple discharge, no fever. Physical examination: the appearance of double breasts had no obvious abnormality, and the size 2.0cm×1.5cm mass could be palpable in the outer upper quadrant of the right breast, with medium, smooth and live texture. How to treat it

A. Local physiotherapy

B. Watch closely

C. acupunture therapy

D. exairesis

E. Chinese medicine

Refer to the answer: D. Your answer:

[Analysis] A young woman (breast fibroadenoma susceptible population) found the right breast mass for two years, no breast swelling, pain, nipple overflow, no fever, physical examination can touch smooth, active, well-defined mass (typical clinical

characteristics of breast fibroadenoma), so the diagnosis of breast fibroadadenoma. Surgical resection is the only effective method (D pair).

[Avoid mistakes] Local physical therapy (A wrong), close observation (B wrong), acupuncture treatment (C wrong) for the treatment of breast fibroma

all-or-none. Traditional Chinese medicine Xiaoyao powder (E wrong) has the effect of harmonizing liver and spleen, dredging liver and relieving depression, nourishing blood and invigorating the spleen, and can be used in mammary gland sac

Sexual hyperplasia.

According to the description, the patient was 25-year-old and had a two-year breast mass without other symptoms, and features of the mass included size 2.0cm×1.5cm with medium, smooth, mobile texture. Considering the length of the presence of the mass and its characteristics, the possibility of malignancy needs to be excluded first.

In this cases, it is often recommended to perform further diagnosis such as imaging (e. g. breast ultrasound or breast X-ray) and / or tissue biopsy. However, this choice does not appear directly in the option. Of the given option, the closest approach to correct management is the surgical resection (option D). This is because for long-lived, unidentified breast masses, resection is required for histopathological examination to confirm a benign or malignant disease and to rule out breast cancer. Therefore, the best answer is: surgical resection

analyze:

In a young female patient, the right breast mass was found for two years, and physical examination found a palpable 2.0cm×1.5cm mass in the upper quadrant of the right breast, with medium texture, smooth, active and clear boundaries, which was a typical clinical manifestation of breast fibroadenoma. Combined with the patient's medical history, clinical manifestations and related examinations, the right breast fibroadenoma was initially diagnosed. Although mammary gland fibroadenoma is benign, but have evil change possible, so once found, should be surgically removed.

Option A local physiotherapy, option C acupuncture treatment, and option E Traditional Chinese medicine Xiaoyao Powder, are all adjuvant treatment methods of traditional Chinese medicine, which have no therapeutic effect on breast fibroadenoma, so they can be excluded together.

Option B Close observation is only for patients with small and slow-growing

tumors, whose mass has grown to 2.0cm×1.5cm, and therefore should be removed surgically, rather than under close observation.

In conclusion, the correct answer is: D. Surgical resection.

According to the patient's description, a 25-year-old woman with a mass in her right breast for two years, no other symptoms (such as redness, swelling, pain, nipple discharge, fever), and physical examination revealed a 2.0cm×1.5cm mass in the outer upper quadrant of the right breast, with moderate, smooth texture and active. This condition is most likely expected for benign breast tumors, such as fibroadenoma.

The best treatment option for this breast mass is surgical resection. Surgical resection can not only make a clear diagnosis (through pathological examination), but also prevent the potential risk of malignancy. Other options such as local physiotherapy, close observation, acupuncture treatment or traditional Chinese medicine treatment are not effective in solving the problem.

Therefore, the most appropriate treatment regimen is:

F. exairesis

65. (Type A2 question)

Male, 34 years old, repeated pain around the anus, redness, swelling and pus for 5 months, physical examination: a diameter of 1cm induration 3cm at the anal margin, press can have pus flowing out, a cord along the induration, extended to 3cm in the anal canal, the diagnosis of the disease

A. sebaceous cyst

B. external hemorrhoid

C. carcinoma of anal canal

D. archosyrinx

E. anal fissure

Refer to the answer: D. Your answer:

Graphic analysis: [partic] young men, repeated pain around the anus, redness, pus (clinical manifestations of anal fistula), anal edge 3cm with a knot, pressure can have pus outflow, along the knot into can touch a cord, extends to the anal canal (in line with the clinical characteristics of anal fistula), combined with the clinical manifestations and characteristics of patients, so the diagnosis of anal fistula (D).

[Avoid mistakes] sebaceous cysts occur on the head, face and back (A wrong); the clinical manifestations of external hemorrhoids are prominent anal masses, such as hair

Raw thrombosis, can have severe pain, become thrombotic external hemorrhoids (B wrong); anal canal cancer performance for anal pain, itching, no pus flow

Out (C wrong); anal fissure typically shows pain, constipation, and bleeding, anal fissure, sentinel hemorrhoids, and anal nipple hypertrophy are often present

(E wrong).

Based on the symptoms described here, including recurrent pain around the anus, redness, pus, and examination detected induration, pus outflow, and palpable cord extending into the anal canal, the most consistent diagnosis was the anal fistula. Anal fistula usually presents as pain, redness, intermittent discharge and other symptoms around the anus, and the fistula connected with the anal canal. Therefore, the best choice should be: D. anal fistula

analyze:

The patient was a young man with recurrent pain around the anus, redness and pus for 5 months, and pus outflow. Along the induration, a cord of the patient was extended to the anal canal. According to the patient's medical history, clinical manifestations and physical examination, the patient could be diagnosed as anal fistula. Anal fistula is the abbreviation of anorectal fistula, is occurring around the anorectal abscess rupture or incision drainage after the residual lesion. Anal fistula is a post-abscess era and is the two stages of a disease. Traditional Chinese medicine is called anal leakage.

Option A sebaceous gland cyst commonly known as "powder", mainly due to the obstruction of the sebaceous gland excretion duct, sebaceous gland cystic epithelium by the expansion of the content, will not appear pus phenomenon, and is not consistent with the performance of the patients in the dry, so it is not selected.

Option B external hemorrhoids below the tooth line, is by hemorrhoids external

varicose or anal edge wrinkle skin inflammation, hypertrophy, connective tissue hyperplasia or thrombus stasis and the formation of mass, external hemorrhoids surface cover with skin, you can see, cannot into the anus, not easy to change, with pain and foreign body sensation as the main symptoms, with the patient's obvious difference, so excluded.

Option C early symptoms of anal canal cancer are not obvious, the progressive clinical manifestations are similar to the lower stage of the rectal cancer, mainly have the following three aspects: stool habit change, the number of fecal discharge increased, often accompanied by urgent or defecation feeling; fecal traits change, fecal strip thin or deformation, often with mucus or pus blood; anal pain, anal swelling, with the development of cancer, anal ulceration, bleeding, infection and other symptoms, the problem is not mentioned the above symptoms, so it is not considered.

Option E anal fissure is the fissure of the narrowest anal canal from the tooth line to the anal canal, forming a small ulcer. The direction is parallel to the longitudinal axis of the anal canal, spindle or oval, about 0.5~1.0cm long, often causing perianal pain, which is inconsistent with the performance of patients, so it is not selected.

In conclusion, the correct answer is: D. anal fistula.

According to the description of the stem, male, 34 years old, repeated pain, swelling, pus around the anus for 5 months, physical examination found a diameter of 1cm 3cm induration, press can have pus out, along the induration can be touched a cord, extended to the anal canal 3cm. The above findings accord with the clinical features of the anal fistula.

Anal fistula (D. anal fistula) is usually formed by the rupture of an abscess around the anus, and is an abnormal passage between the anal canal and the skin. The main symptoms include pain, redness, pus around the anus, and stiffness and pus during physical examination.

So, the best answer to this question is:

F. archosyrinx

66. (Type A2 question)

The patient was 16 years old, thyroid enlargement, thyroid iodine photography rate 2 hours 15%, 24 hours 35%, whose possible disease was

A. hypothyroidism

B. Hyperthyroidism

C. Hashimoto's thyroditis

D. Phyologic goiter

E. nodular goiter

Refer to the answer: D. Your answer:

Graphic analysis: [analytical] adolescent women, thyroid enlargement, thyroid iodine rate 15% 2 hours, 24 hours 35% (within the normal range), due to the demand of thyroid hormone puberty, such as the body synthesis can not meet the physiological needs, thyroid glands will appear compensatory hyperplasia, enlargement, but not functional change, therefore should be diagnosed as physiological goiter (D).

[Avoid mistakes]: nodular goiter (E wrong) can show hypothyroidism or hyperthyroidism; hypothyroidism (A wrong)

For low metabolic symptoms, laboratory tests decreased T3 and T4; hyperthyroidism (B wrong) T3 and T4 increased, and 131I uptake

(Iodine photography capacity) 2 hours> 25%, 24 hours> 50%. Hashimoto's thyroiditis (C wrong) TPO antibody, TG antibody positive.

This issue involves the assessment of the thyroid function and the cause of the enlargement. The thyroid iodine consumption rate reflects the ability of the thyroid to absorb iodine and is an important indicator to assess thyroid function.

- ** Hypothyroidism * * (A) is usually accompanied by low iodine uptake rates, but as the thyroid tries to absorb more iodine to produce thyroid hormones, early iodine uptake may be normal or slightly increased.

- ** Hyperthyroidism * * (B) is due to thyroid hyperactivity, leading to a high iodine uptake rate.

- ** Hashimoto's thyroiditis * * (C) is an autoimmune disease that may initially have normal or increased iodine uptake rates, but the thyroid function decreases as the disease progresses.

- ** Physiological goiter * * (D) is usually seen in adolescent women, and thyroid function and iodine intake rate are usually normal.

- ** Nodular goiter * * (E) refers to the hyperplasia of nodules in the thyroid gland, which can be a single or multiple nodules. The iodine intake rate can be normal, increased or reduced, depending on whether the nodules are functional and active.

Considering this patient, the iodine intake rate was 15% at 2 hours and 35% at 24 hours, suggesting that the thyroid had significant iodine uptake but not overactivity. These data are not typical for hyperthyroidism (usually with higher iodine consumption rates) and are not fully consistent with hypothyroidism (usually lower iodine consumption rates). Nodular goiter may exhibit different rates of iodine consumption, but is usually associated with nodular activity. Hashimoto had the higher likelihood of thyroiditis or physiological goiter, given the age and symptoms. But Hashimoto's thyroiditis is more likely to exhibit normal or slightly higher iodine uptake early on, which decreases with disease progression.

Therefore, the most appropriate answer may be * * C. Hashimoto's thyroiditis * *, as it can manifest with goitosis and relatively normal or slightly higher iodine uptake rates early in the course of the disease. However, the final diagnosis requires combining other clinical information, such as the levels of anti-thyroid antibodies, thyroid function tests (e. g., TSH, T3, T4 levels), and possible thyroid ultrasonography.

analyze:

In adolescent female patients, thyroid iodine photography rate was 2 hours 15% and 35% 24 hours. The normal thyroid iodine consumption rate should be 5%~25% for 2 hours and 20%~45% in 24 hours. The thyroid iodine consumption rate is within the normal range, but the thyroid is obviously enlarged, which should be considered as simple goiter, also known as physiological goiter.

Option A In hypothyroidism, serum T3 and T4 decreased, TSH increased, and thyroid photography ^{131}I rate was significantly reduced, which was not consistent with the description of the title stem, so it was not selected.

Option B In hyperthyroidism, thyroid photography ^{131}I rate increased, and the peak was forward, which was also inconsistent with the description of the question stem, so it was excluded.

Option C Hashimoto thyroiditis is an autoimmune disease, thyroid

autoantibodies are often positive, serum T3, T4 can be decreased, normal or increased, TSH can be increased or decreased, thyroid ^{131}I rate can be decreased, normal or increased, but the patient is not mentioned positive in the stem, so it is not selected.

Option E Nodular goiter is generally asymmetric and can reach multiple or single nodules. The thyroid tissue outside the nodules is not enlarged, and calcification, cyst and hemorrhage are common in the nodules, which is not consistent with this case, so it was excluded.

In conclusion, the correct answer is: D. physical goiter.

According to the title description, the patient was a 16-year-old female with an enlarged thyroid iodine photography rate of 15% in 2 hours and 35% in 24 hours.

Analyze the individual options:

A. * * Hypothyroidism * *: It usually shows a reduced thyroid iodine intake rate, which does not meet the question.

B. * * Hyperthyroidism * *: usually the thyroid iodine rate increases, but the iodine rate in the topic is not significantly higher.

C. * * Hashimoto's thyroiditis * *: mostly hypothyroidism, accompanied by autoantibodies, and changes in thyroid iodine photography rate were atypical.

D. * * Physiological goiter * *: It is common in specific physiological periods such as puberty and pregnancy, but the iodine consumption rate is usually normal or slightly changed, which does not meet the meaning of the question.

E. * * Nodular goiter * *: The thyroid gland can be enlarged, and the iodine intake rate may not be normal.

Based on the above analysis, the most likely answer is:

* * E. nodular goiter * *.

67. (Type A2 questions)

Female, arthralgia, proteinuria, positive anti-ds-DNA antibody and decreased complement C3. The following statement is incorrect

A. The pregnancy can not continue after the condition improves

B. The appearance of brain manifestations such as epilepsy suggests a poor prognosis

C. Renal biopsy determines the prognosis

D. Hydroxychloroquine can be used

E. It can cause a joint deformity

Reference answer: E Your answer:

Graphic resolution: [analytical] young women, joint lesions, proteinuria (renal lesions), anti-dsDNA antibody (+) (lupus nephritis marker antibody, activity), complement C3 drop (prompt in active), patients with multiple organ damage, combined with laboratory examination, considering the diagnosis of lupus nephritis. SLE joint damage is characterized by recoverable non-erosive joint subluxation, no bone destruction, and no deformity (E error, correct answer to this question).

[Avoid mistakes] lupus nephritis patients improve, no central nervous system, kidney or other organs damage, can follow

Continuing pregnancy (pair A); brain findings such as epilepsy suggest poor prognosis (pair B); and renal biopsy (C

Right); Drug therapy for SLE is mainly a combination of glucocorticoids and immunosuppressants (cyclophosphamide) and can be used

Hydroxychloroquine (pair D) was used as the background therapy.

This topic describes a female patient presenting with arthralgia, albuminuria, a positive anti-ds-DNA antibody, and a decrease in complement C3. These symptoms and laboratory findings suggest the possibility of systemic lupus erythematosus (SLE). We need to find out the one of the following statements that is incorrect.

A. The pregnancy should not continue after the condition improves- -this statement is wrong. Patients with SLE can be considered for pregnancy when their disease is stable or improving, but it needs to be conducted under strict monitoring by their doctor.

B. The occurrence of brain manifestations such as epilepsy suggests a poor prognosis- -this is true, if SLE involving the central nervous system, if epilepsy

appears, may indicate a poor prognosis.

C. Renal biopsy can determine the prognosis — which is also true, and renal biopsy is an important means to assess the degree of renal involvement and prognosis in SLE.

D. Hydroxychloroquine can be used- -which is correct, one of the drugs commonly used in the management of SLE.

E. Cause joint deformities- -While SLE can cause joint pain and limited function, it usually does not cause joint deformities, and this option may also be wrong in some cases. However, in contrast to option A, joint deformities are not typical of SLE, but SLE can indeed cause severe joint problems, including deformities.

Of these options, the most incorrect is A (pregnancy can not continue after the condition improves). Because patients with SLE can be considered for pregnancy in well-controlled conditions, but need careful planning and management under the supervision of the medical team. Therefore, the correct answer is an A.

analyze:

Female patient of childbearing age, with positive anti-ds-DNA antibody, decreased complement C3, with arthralgia and proteinuria, was considered for the diagnosis of systemic lupus erythematosus based on her clinical presentation and laboratory investigations. Systemic lupus erythematosus is an autoimmune inflammatory connective tissue disease involving multiple organs more frequently in young women, with increasing early, mild and atypical cases. The disease is relieved with repeated episodes and does not lead to joint deformity.

Systemic lupus erythematosus (SLE) occurs in women of childbearing age, the patient's condition improves, if no important organ damage, stable for 1 year or more than 1 year, cytotoxic immunosuppressive agents such as cyclophosphamide, methotrexate or tripterydii stopped for half a year, hormone only maintained in small doses, can pregnancy. Therefore, option A can not continue the pregnancy after the condition is better. The wrong statement is the correct answer to this question.

If patients with SLE have central nervous system damage, epilepsy and other brain manifestations, which indicates poor prognosis, so option B, epilepsy and other brain manifestations suggest poor prognosis is in line with the characteristics of SLE.

SLE patients with kidney involvement is called lupus nephritis, with various pathological types, and its classification has guiding significance for both treatment

and prognosis. Kidney biopsy can judge the prognosis, so option C renal biopsy can determine the prognosis, which is in line with the characteristics of SLE.

Hydroxxychloroquine is the basic drug of SLE with almost no side effects. Moreover, because the drug has anti-malaria, anti-inflammatory and immune suppression, it is conducive to better control of SLE activities, protect patients from organ damage, and reduce the dosage and adverse reactions of hormones. Therefore, option D can use hydroxychloroquine, which is in line with the treatment principles of SLE.

In conclusion, the correct answer is: E. It causes joint deformity.

This title described a female patient with symptoms including arthralgia, proteinuria, positive anti-ds-DNA antibodies, and a decrease in complement C3. These symptoms and laboratory results suggest a diagnosis of systemic lupus erythematosus (SLE). Based on this diagnosis, we will analyze the options and find out the incorrect statement.

A. It is not possible to continue pregnancy after improvement. ——. This is incorrect. In fact, pregnancy in patients with SLE can be considered after their condition is effectively controlled (usually stable for at least 6 months), but it requires close monitoring.

B. The appearance of brain manifestations such as epilepsy suggesting poor prognosis —— This is correct. SLE patients with central nervous system symptoms such as epilepsy usually indicate severe disease and have a poor prognosis.

C. Renal biopsy can determine the prognosis of ——. This is true. Renal damage (lupus nephritis) in SLE patients can be assessed by renal biopsy to determine the prognosis and develop treatment options.

D. Can use hydroxychloroquine —— which is correct. Hydroxychloroquine is one of the important drugs for treating SLE, which helps to control the condition and reduces the frequency of attacks.

E. Can cause a joint deformity —— This is incorrect. Although SLE can cause arthralgia and arthritis, it generally does not cause joint deformities like rheumatoid arthritis.

So, the most incorrect statement is that:

F. It can cause a joint deformity

Summary: Select the E option.

68. (Type A2 question)

A 48-year-old man with right upper abdominal distension for 4 months, accompanied by fatigue, no nausea, vomiting or fever. Since the onset, his urine was slightly yellow and he drank liquor for more than 10 years, about 150 ~ 200 ml/d. Physical examination: poor spirit, mild yellow sclera, no liver palm, no abnormality, soft abdomen, no tenderness, liver and spleen, mobile turbidity (-), laboratory examination: ALT 35, AST 25, HBsAg (-), anti-HCV (-), B ultrasound: liver parenchyma echo diffuse dense enhancement, far field echo attenuation,

yes

A. prednisone for treatment

B. nutritional support

C. Anti-hepatic fibrosis therapy

D. abstinence

E. Liver-sparing drugs

Refer to the answer: D. Your answer:

[Analysis] middle-aged male patients, a large number of long-term drinking history, fatigue, right upper abdominal pain, physical examination and laboratory examination indicate that liver function is ok, B ultrasound suggests fatty liver disease,

based on the clinical manifestations and examination results of the patient, considering the diagnosis of alcoholic liver disease. Alcohol abstinence (D pair) is the most important measure to treat patients with alcoholic liver disease.

[To avoid mistakes] The existing diagnostic criteria for alcoholic liver disease in China are: long-term drinking history, generally more than 5 years, equivalent to the amount of alcohol

40 g/d men and 20 g/d women; or history of alcohol for 2 weeks,> 80 g/d.

This case describes a 48-year-old male patient with chronic alcohol consumption, right upper abdominal pain, fatigue, mild yellow sclera, no nausea, vomiting, or fever. Laboratory tests showed mild abnormalities of liver function, B ultrasound showed diffuse dense enhancement of liver parenchyma and attenuation of far-field echo, and these symptoms and findings suggest possible chronic liver disease, such as alcoholic liver disease.

For the choice of treatment options, we need to consider the main etiology of the patient and the most effective interventions. Given the patient's long history of drinking, the most critical interventions are:

B abstinence

Alcohol withdrawal is the most important step in the treatment of alcoholic liver disease, which prevents further deterioration of liver disease and may reverse liver injury. While other options such as nutritional support, anti-fibrotic therapy, and liver-sparing drugs are also important for patients with liver disease, there is no more important treatment than stopping the liver. Therefore, choose D (abstinence) as the best answer.

analyze:

A middle-aged male patient with a long-term history of alcohol consumption, right upper abdominal distension, yellow urine, poor spirit after physical examination, mild yellow sclera, B ultrasound showed diffuse dense enhancement of liver parenchymal echo and obvious attenuation of far field echo, was considered to be diagnosed as alcoholic liver disease. The treatment principles of alcoholic liver disease are: abstinence and nutritional support, reduce the severity of alcoholic liver disease, improve the existing secondary malnutrition and symptomatic treatment. Among them, alcohol abstinence is the fundamental treatment of alcoholic liver disease, which is the most important treatment measure.

Option A prednisone therapy is hormone therapy, which will induce or aggravate peptic ulcer, cause increased blood sugar and blood pressure, and increase the risk of infection. It should not be used for the treatment of alcoholic liver disease, so it is excluded.

Option B nutritional support belongs to the treatment principle of alcoholic liver disease, but it is not the most important measure, the most important measure is to quit alcohol, so it is not chosen.

Option C anti-hepatic fibrosis therapy prevents or reverses liver fibrosis, but the poor treatment of alcoholic liver disease is not considered.

Option E The efficacy of liver protection medication is not exact, and it may increase the burden of the liver, so the abuse should be avoided, so it does not meet the meaning of the question.

To sum up, the correct answer is: D. Alcohol abstinence.

According to the patient's medical history and examination findings, this 48-year-old man had right upper abdominal pain and fatigue, accompanied by mild jaundice, long history of alcohol consumption, and B ultrasound showed diffuse dense enhancement of hepatic parenchymal echo and marked attenuation of far-field echo. These manifestations are likely to suggest an alcoholic liver disease, particularly alcoholic fatty liver disease or early alcoholic cirrhosis.

From a therapeutic perspective, the primary treatment measure of alcoholic liver disease is alcohol abstinence, which can prevent further deterioration of liver damage and create conditions for the liver to repair itself. So, the most correct answer is:

B abstinence

69. (Type A2 question)

Male, with body temperature 39.1℃, heart rate 109 beats / min, cough and sputum, CT, cin combined with quinolone against infection, the indication for drug withdrawal is

A. X-ray is shown in the lesion absorption

B. Symptoms of cough and sputum disappeared

C. Blood oxygen saturation returned to normal

D. Body temperature has returned to normal

E. Blood routine was normal

Reference answer: A. Your answer:

[Analysis] The male patient with body temperature was 39.1℃ (estive of infection), cough and sputum, CT leveling (typical imaging characteristics of lung abscess), so the patient was diagnosed as lung abscess and treated with antibiotics, and the drug was stopped during the course of 6 to 8 weeks or when the chest X-ray returned to normal (A pair).

[Avoid mistakes] Antibiotics can be stopped 72 hours after normal body temperature.

This question concerns the decision of when antibiotic treatment is stopped, a very practical clinical question. The goal of treatment is to eliminate infection symptoms and ensure that the pathogen is cleared and to avoid relapse or development of resistance due to premature withdrawal. Considering these factors, although body temperature, blood oxygen saturation, and blood routine recovery are all indicators of treatment effect, restoring these indicators alone does not necessarily mean that the infection is already under complete control.

In this case, a CT scan of the male patient showed a fluid level, which indicated a pulmonary infection. Therefore, the best answer should be option A "X-ray see lesion absorption", as it directly reflects the improvement at the site of infection. The absorption of the lesions means that the infection is controlled and is a more direct and reliable indicator of drug withdrawal. So, the correct answer should be an A.

analyze:

The patient was a middle-aged male with elevated body temperature, increased heart rate, cough and sputum, and fluid leveling on CT, which was considered as pneumonia combined with pleural effusion. After admission, the patient was combined with quinolone against infection, and the indication for withdrawal was X-ray for lesion absorption.

Option B The disappearance of cough and sputum symptoms can only represent the improvement of the patient's symptoms, but whether the infection is completely controlled requires imaging auxiliary diagnosis, so it was excluded.

Option C oxygen saturation can only indicate that the patient does not have hypoxia and does not mean that the infection has been controlled, so it is not considered.

Option D The return of normal body temperature may be the effect of antipyretic, which does not represent the complete improvement of infection, so it is not selected.

Option E Normal blood routine does not mean that the infection is completely controlled and is not specific, so it is excluded.

In conclusion, the correct answer is: A.X-ray see lesion absorption.

According to the information provided by the problem stem, the patient had symptoms such as fever, increased heart rate, cough and sputum, and the CT showed fluid level, indicating the possibility of bacterial infection. The patient received anti-infective treatment with cephalosporin plus quinolone. The indications for discontinuation requires comprehensive consideration of clinical symptoms and the results of ancillary examinations.

In clinical practice, the indications for antibiotic discontinuation usually include the following:

1. Clinical symptoms significantly improved or disappeared (such as fever, cough and sputum symptoms relieved).

2. The auxiliary examination index is returned to normal (such as blood routine, blood oxygen saturation, etc.).

3. Imaging examination indicates the absorption or significant shrinkage of the lesions.

In this topic, the options are analysed as follows:

A. X-ray shows lesion absorption: indicating significant improvement, but not necessarily complete control of infection.

B. Disappearance of cough and sputum symptoms: it is an important indicator of

the improvement of clinical symptoms, but relying on clinical symptoms alone may not be comprehensive enough.

C. Oxygen saturation returns to normal: it is also an important indicator, but it does not fully reflect infection control.

D. Body temperature returns to normal: regression of fever is one of the signs of infection control, but not comprehensive enough as an indication for drug withdrawal.

E. Normal blood routine: the normalization of white blood cell count and other indicators is one of the important bases for judging infection control.

Overall, the best indication for drug withdrawal should be the comprehensive improvement of the condition, that is, not only the return of body temperature to normal, but also other clinical and laboratory indicators, such as cough, sputum symptoms, and blood routine. Therefore, the most reasonable choice is to:

* * E. Normal blood routine * *

Normalization of blood routine usually means that the in vivo infection has been effectively controlled, meeting the criteria for clinical discontinuation.

70. (Type A2 questions)

Female, 30 years old, 6 days after appendectomy, after a lot of eating, pale, irritability, abdominal fullness, stomach content, vomiting, previous history of peptic ulcer for 6 years, physical examination: upper abdominal swelling, mild tenderness, no rebound pain, fecal blood (-) effective treatment measures are

A. Acupuncture physiotherapy

B. intramuscularly injected neostigmine

C. Using broad-spectrum antibiotics

D. Local hot compress

E. gastrointestinal decompression

Reference answer: E Your answer:

Graphic analysis: [analytical] 6 days after appendectomy (appendicitis surgery), a lot of eating pale, irritable, upper fullness, vomiting stomach content (postoperative intestinal obstruction common symptoms), previous peptic ulcer history for 6 years, upper abdominal swelling, mild tenderness, no rebound pain, fecal blood (-), considering the diagnosis of appendicitis postoperative adhesion intestinal obstruction, the most simple and effective treatment measures is gastrointestinal decompression (E) can relieve abdominal distension, reduce gastrointestinal pressure.

[Avoid] Acupuncture therapy (A wrong), local hot compress (D wrong) has no effect on the treatment of these symptoms; broad-spectrum antibiotics

(C is wrong) It is suitable for the prevention and treatment of inflammation. The main effect of neomuscle injection (B error) is to treat intestinal surgery

Intestinal function is limited, which has a certain therapeutic effect on intestinal paralysis.

Considering the 30-year-old woman with symptoms of paleness, irritability, upper abdominal fullness and vomiting, and a history of peptic ulcer for up to 6 years, the most appropriate management measure seemed to be E. gastrointestinal decompression. These symptoms may indicate an acute gastric dilatation or postoperative gastrointestinal undermotility, which may occur after appendectomy. Gastrointestinal decompression relieves the pressure of the gastric contents by inserting a nasogastric tube, reduces gastrointestinal distension, and prevents further complications. Other options such as acupuncture therapy, intramuscular neostigmine, broad-spectrum antibiotics, or topical hot compress did not directly address her current main problem. Therefore, the best choice is for E. gastrointestinal decompression.

analyze:

Patients with 6 days after appendectomy, a lot of eating pale, irritable, upper stomach, vomiting stomach content, prompt may exist intestinal obstruction, examination shows upper abdominal bulge, mild tenderness, no rebound pain, fecal blood (-), can exclude gastrointestinal hemorrhagic disease, combined with patient history and clinical manifestations, should be considered for postoperative intestinal obstruction. Principles of intestinal obstruction to correct the systemic physiological disorders caused by intestinal obstruction and relieve obstruction, including gastrointestinal decompression is one of the important measures for the treatment of intestinal obstruction, through gastrointestinal decompression absorb the gas and fluid

in the gastrointestinal tract, can reduce abdominal distension, reduce the bacteria and toxins, improve the intestinal wall blood circulation, is beneficial to improve local lesions and systemic conditions.

Option A acupuncture and physiotherapy, option D local hot compress can relieve gastrointestinal spasm to A certain extent, but it cannot reduce the pressure in the intestinal lumen. The treatment effect of intestinal obstruction is limited, so it can be excluded together.

Option B Intramuscular injection of neostigmine is mainly used for myasthenia gravis, postoperative abdominal distension, urinary retention, etc. The treatment effect of intestinal obstruction is not exact and can be ruled out.

Option C broad-spectrum antibiotics are mainly used for the treatment of infectious diseases, intestinal obstruction is not infectious disease, inconsistent with the topic.

In conclusion, the correct answer is: E. gastrointestinal decompression.

In this condition, the patient developed pallor, irritability, upper abdominal fullness and vomiting and a history of peptic ulcer 6 days after appendectomy. Physical examination showed upper abdominal swelling and mild tenderness, without rebound pain, and fecal hidden blood (-). These symptoms suggest the possibility of gastrointestinal dysfunction or obstruction, and the most effective management measures should be to relieve the symptoms and reduce the gastrointestinal stress.

Option explanation:

A. Acupuncture physiotherapy-Management of acute symptoms has a limited effect and may not be appropriate.

B. An intramuscularly injection of neostigmine-neostigmine can enhance gastrointestinal peristalsis, but may not necessarily apply to acute flatulence and vomiting, and may aggravate symptoms.

C. Application of broad-spectrum antibiotics-suitable for infectious diseases, but there is no current evidence to support the presence of infection.

D. Topical hot compress-relieves mild pain and discomfort but has limited effect on acute flatulence.

E. Gastrointestinal decompression-Reducing the pressure of the gastrointestinal

tract by inserting a nasogastric tube, is the most effective measure to deal with this symptom.

Therefore, the most appropriate treatment measure is:

F. gastrointestinal decompression

71. (Type A2 question)

A 75-year-old female, with hemoptysis for 10 days, had two previous episodes and improved after anti-infective treatment. Pulmonary CT shows diffuse branch expansion in both lungs and the preferred treatment is

A. operative treatment

B. Hemostatic drugs

C. anti-infective therapy

D. The bronchial artery embolization

E. hypophysin

Refer to the answer: D. Your answer:

[Analysis] In elderly women, CT indicated diffuse bronchodilectasis in both lungs, hemoptysis twice before, anti-infection improved, hemoptysis again, so bronchial artery embolization was used (D pair).

[Avoid mistakes] For patients with repeated hemoptysis, after internal medicine treatment (infection control, oral hemostatic drugs, intravenous administration of the posterior pituitary gland

Su) is ineffective, interventional embolization treatment or surgical treatment can be considered. Surgical operation may be considered, otherwise the branch gas is used

Tubular artery embolization for treatment.

The 75-year-old woman with a history of about 20ml daily hemoptysis history showed two episodes and improvement after anti-infective treatment. The patient's pulmonary CT showed diffuse bronchiectasis in both lungs. Based on this information, we need to consider a treatment that addresses both the current hemoptysis problem and addresses the possible underlying cause, namely bronchiectasis.

A. Surgical treatment- -usually considered in severe cases or when other treatments are ineffective.

B. Hemostatic medication-effective for treating hemoptysis, but not addressing the root cause.

C. Anti-infective therapy-Bronchiectasis is often accompanied by infection, which may be an appropriate option considering that the patient has improved after previous anti-infective treatment.

D. Bronchial artery embolization-used to treat severe or persistent hemoptysis with specific circumstances and risks.

E. Retropituitary-mainly used for the treatment of diabetes insipidus, not relevant to this case.

Taken together, the best answer may be * * C. anti-infective therapy * * because the patient's history indicates that she responded to such treatment and that anti-infective therapy could target potential infectious factors in bronchiectasis, consistent with the patient's current primary symptoms and diagnosis.

analyze:

The patient was an elderly female patient with daily hemoptysis of about 20ml, previous history of hemoptysis, and improved after anti-infection treatment. Combined with pulmonary CT, he showed diffuse branch expansion in both lungs. For this patient, anti-infection treatment should be preferred at present.

Option A Surgical treatment is suitable for localized bronchiectasis, and it is still stubborn and repeated after adequate medical treatment. The patient is diffuse branch expansion in both lungs, and the surgical treatment effect is not good, so it is not selected.

The patient has no severe symptoms of hemoptysis and does not need to use hemostatic drugs, so option B hemostatic drugs can be excluded.

Option D Bronchial artery embolization is mainly used for the treatment of hemoptysis caused by various causes. The patient with hemoptysis is not severe and can be improved after anti-infection treatment, so it is not used as the preferred treatment.

Option E posterior pituitary element is used for hemoptysis in respiratory medicine, especially in the event of massive hemoptysis. The patient is not large, so it is not considered.

In conclusion, the correct answer is: C. Anti-infection treatment.

This 75-year-old female had a history of diffuse bronchiectasis (enlargement) and hemoptysis. Currently, she has hemoptysis of about 20 ml per day and has been improved with anti-infective treatment in the past two episodes. Based on this information, the following is an analysis of each option:

A. * * Surgical treatment * *: Surgical treatment is usually used in localized expansion or recurrent severe hemoptysis and other treatments are ineffective. For diffuse branch expansion, surgery is not the preferred treatment.

B. * * Hemostatic drugs * *: Hemostatic drugs can reduce the amount of hemoptysis in the short term, but they are usually not the best choice for the long-term management of diffuse branch expansion.

C. * * Anti-infective treatment * *: Anti-infective treatment is the basic treatment of expansion, especially in the acute infection period. However, this patient's current hemoptysis may not be caused by a new infection but due to diffuse expansion itself. Therefore, although anti-infective therapy is an important part of expansion management, it may not be the most preferred method for current control of hemoptysis.

D. * * Bronchial artery embolization * *: Bronchial artery embolization (BAE) is an effective treatment for severe and recurrent hemoptysis. It causes hemoptysis in the bronchial artery through embolization, reduces bleeding, and is one of the first-line treatment options for repeated hemoptysis in patients with branch expansion.

E. * * Retropituitary * *: Retropituitary can temporarily reduce blood flow and help control bleeding, but it does not address the root cause of hemoptysis and is usually used as a short-term emergency measure.

In general consideration, * * bronchial artery embolization (D. bronchial artery

embolization) * * is currently the best treatment option for controlling hemoptysis in this patient, because it can effectively reduce the amount of bleeding and is suitable for repeated hemoptysis caused by diffuse branch enlargement.

So the most appropriate answer is:

B The bronchial artery embolization

72. (Type A2 questions)

Women of childbearing age, 4 weeks postpartum, have been breastfeeding, breast swelling, increased surface skin temperature, no sense of fluctuation, the following treatment is preferred

A. incision and drainage

B. Stop breastfeeding immediately

C. Use of antibiotics immediately

D. Heat compress, press on the breast to promote milk discharge

E. Wait for the drug susceptibility results before antibiotic treatment

Your answer: D

[Analysis] Women of childbearing age, 4 weeks postpartum, has been breastfeeding, breast swelling, elevated surface skin temperature, consider acute mastitis. The treatment principle of acute mastitis is to eliminate the infection and empty the milk (D pair).

[Error avoidance] The incision and drainage (A wrong) is the treatment after the formation of the abscess. The patient's breast has no sense of fluctuation, so there is no need to do the incision

Drainage; generally do not stop breastfeeding, because stop breastfeeding, not only affect infant feeding, but also provides the opportunity of milk deposition.only

The breast on the affected side should stop breastfeeding and suck with breast pump to facilitate smooth discharge (B wrong); early honeycomb

The application of antibiotics can achieve good results before the inflammation

of abscess formation. Because the main pathogen is staphylococcus aureus

Bacteria, may not have to wait for the results of the bacterial culture, apply penicillin treatment, or with penicillinase-resistant oxacillin sodium, or head

Generation generation antibiotics such as ceffrazidime, but not preferred (CE wrong).

This topic describes the 4 weeks of postpartum breastfeeding childbearing age women, breast swelling and increased surface skin temperature, no sense of fluctuation. This condition is most likely mastitis, especially given that she is breastfeeding.

Option A (incision and drainage) is usually suitable for abscess formation, but there is no mention of volatility in the trunk, indicating that an abscess has not yet formed. Option B (stop breastfeeding immediately) is not preferred because breastfeeding helps empty the breast and may help relieve symptoms. Option C (immediate antibiotics) is a common treatment for mastitis, but may not be preferred in the absence of severe signs of infection (e. g., fever, systemic symptoms). Option E (antibiotics pending the susceptibility results) is not preferred in an emergency because immediate intervention may be required.

Option D (hot compress, breast press, promote milk discharge) is one of the preferred treatments for non-abscess mastitis. It helps reduce symptoms, promote milk circulation and avoid mammary duct blockage.

Therefore, the best answer is the D.

First, we will understand the clinical situation described by the title. This is a 4 weeks of postpartum childbearing age woman, has been breastfeeding, now the breast is red and swollen, surface skin temperature increased, but no sense of fluctuation. These symptoms suggest a possible early stage of mastitis where an abscess has not yet formed.

Next, we analyze it one by one according to the options in the topic:

A. incision and drainage: This option is usually applied after the abscess has formed. However, the clear mention of "no volatility" in the title means that an abscess has not yet formed, so this option does not apply.

B. immediate cessation of lactation: In the early stages of mastitis, cessation of lactation is not the preferred treatment. In fact, continued breastfeeding helps to smooth the mammary gland, thus contributing to the resolution of inflammation.

C. Immediate use of antibiotics: Although antibiotics can treat infections, they are not recommended until clear evidence of bacterial infection.

D. Hot compress, press on the breast, and promote milk discharge: in the early stages of mastitis, this method is very effective. Hot compress can help increase local blood circulation, while pressing the breast and promoting milk discharge can help eliminate the blockage, thus helping to resolve inflammation.

E. Waiting for the susceptibility results before antibiotic treatment: While this option may seem more scientific, timely intervention is very important in the case of acute mastitis. Waiting for drug susceptibility results may delay the optimal timing of treatment.

In conclusion, considering that the patient was in the early stage of mastitis, no abscess had not formed and required timely intervention, hot compress, breast pressing and promoting milk discharge were the preferred treatment.

Therefore, the correct answer is D. Hot compress, press the breast to promote milk discharge.

For women of childbearing age, 4 weeks postpartum, always breastfeeding, breast swelling, increased surface skin temperature, no sense of fluctuation, considering the possibility of mastitis. Treatment of mastitis usually involves antibiotic treatment, continued breastfeeding or emptying the breast by a breast pump, and appropriate symptomatic management such as topical hot compress.

Based on existing medical knowledge and best practice guidelines, the preferred treatment is:

F. Use of antibiotics immediately

The reasons are as follows:

1. * * Immediate use of antibiotics * *: In the early stages of mastitis, especially in the absence of fluctuating sensation (suggesting no abscess formation), the immediate use of antibiotics can effectively control the infection and prevent the deterioration of the disease.

2. * * Continuing * *: It is not necessary to stop breastfeeding immediately, because continuing breastfeeding helps to empty the breast, reduce milk deposition, and accelerate recovery.

3. * * Local symptomatic treatment * *: symptomatic treatment such as hot compress can be used, but these measures are adjuvant and cannot replace antibiotic treatment.

So the best answer is:

G. Use of antibiotics immediately

73. (Type A2 questions)

Male, 35, upper abdominal discomfort, weakness, wasting for 3 years, physical examination: Hb 80 g / L, gastrin 80 pg/ml, gastrointestinal angiography showed 2.5cm side of gastric small curvature, poor gastric activity. Consider that the diagnosis is

A. gastric ulcer

B. polyp of stomach

C. Complex ulcer

D. gastric cancer

E. chronic gastritis

Refer to the answer: D. Your answer:

[Analysis] middle-aged male, upper abdominal discomfort, fatigue, wasting for 3 years (common signs of malignant tumors), Hb 80 g / L (anemia), gastrin 80 pg/ml, digestive tract angiography showed 2.5cm niche of gastric curvature, poor gastric activity, the patient, considered the diagnosis of gastric cancer (D pair).

[Avoid wrong] Gastric ulcer (A wrong) is more common in postprandial abdominal pain, abdominal pain can be relieved by acid suppression or antacid agents. Gastric polyps (B wrong) many

No obvious clinical manifestations, occasionally hematochezia, abdominal pain,

etc.; compound ulcer (C wrong) refers to active ulceration in the stomach and duodenum

The incidence of obstruction is higher. Chronic gastritis (E wrong) most patients have no obvious symptoms, can be manifested as upper abdominal discomfort,

Full of distension, dull pain, etc.

The topic was about a 35-year-old male patient complaining of upper abdominal discomfort, fatigue and wasting for 3 years. Physical examination found that hemoglobin (Hb) was 80g / L (low) and gastrin level was 80 pg/ml. Gastrointestinal angiography showed a 2.5cm niche on the small curvature of the stomach with poor gastric activity.

Consider this information:

-Low hemoglobin may point to chronic bleeding or malnutrition.

-Normal gastrin levels, which helps to distinguish between certain stomach diseases.

-The es is usually associated with ulcerative or neoplastic lesions, while poor gastric activity may suggest rigidity and universality of the lesion.

Based on these clinical findings, the most likely diagnosis is gastric cancer (option D). Although gastric ulcer or other options may also cause epigastric discomfort or niche, fatigue, wasting, and poor gastric activity and gastric cancer. Gastric cancer can lead to chronic bleeding, explaining the finding of low hemoglobin levels, and gastric cancer often appears as a rigid niche in gastrointestinal angiography.

Therefore, the best answer is D. gastric cancer.

This is a diagnostic question about gastrointestinal diseases. We need to comprehensively judge the possible diseases of patients based on their medical history, physical findings and imaging results.

First, let's comb through the key information given in the topic:

Young male patient, with upper abdominal discomfort, fatigue, and wasting for 3 years.

Hemoglobin (Hb) was 80g / L, below normal values, indicating the presence of anemia.

Gastrin 80 pg/ml, above normal values, may be related to gastric acid

hypersecretion or gastrinoma.

Gastrointestinal angiography showed a 2.5cm niche shadow on the small curvature of the stomach with poor gastric activity.

Next, we analyzed the options item by item:

A. Gastric ulcer: Gastric ulcer may lead to upper abdominal discomfort and wasting, but the gastrin level of gastric ulcer is generally normal, and the niche of gastric ulcer is generally small, with no significant weakened gastric activity.

B. gastric polyps: gastric polyps generally do not cause long-term upper abdominal discomfort and wasting, and the performance of gastric polyps on gastrointestinal angiography is not consistent with the description of the main stem.

C. Complex ulcer: Complex ulcer refers to both gastric ulcer and duodenal ulcer, but this disease does not lead to a significant weakening of gastric activity, and gastrin levels are usually normal.

D. Gastric cancer: gastric cancer can lead to long-term upper abdominal discomfort, fatigue and wasting, often accompanied by increased gastrin levels. Gastric cancer can show a large niche on gastrointestinal angiography, and gastric activity is usually weakened due to the infiltration of cancerous tissue. This is highly consistent with the title stem description.

E. Chronic gastritis: Although chronic gastritis can also lead to upper abdominal discomfort, it generally does not lead to long-term fatigue and emaciation, and gastrin levels and gastric activity are usually normal.

In conclusion, combining the patient's history, physical findings, and imaging findings, the most likely diagnosis is gastric cancer. Therefore, the answer is D. gastric cancer.

Based on the patient's clinical presentation and examination results, the most likely diagnosis is:

The patient was a 35-year-old male with major symptoms of upper abdominal discomfort, weakness and wasting, which had been ongoing for 3 years. Physical examination revealed a hemoglobin (Hb) level of 80g / L, indicating anemia. The gastrin level was 80 pg/ml. Gastrointestinal angiography showed a 2.5cm niche on the small curvature of the stomach with poor gastric activity.

Overall, the most likely diagnosis is:

* * D. gastric cancer * *

The reasons are as follows:

1. * * Upper abdominal discomfort, weakness and wasting * * are common symptoms of gastric cancer.

2. * * Anemia (Hb 80g / L) * * may be associated with chronic bleeding or malnutrition caused by gastric cancer.

3. * * Gastridin level of 80 pg/ml * * is not specific, but gastric cancer can lead to abnormal gastrin secretion.

4. * * Gastrointestinal angiography showed a 2.5cm niche in the small curvature of the stomach, and poor gastric activity * * indicated abnormal thickening or mass in the gastric wall, which is consistent with the imaging findings of gastric cancer.

The above analysis based on existing medical knowledge and best practice guidelines identified gastric cancer as the most likely diagnosis.

74. (Type A2 question)

Female, 32 years old. Five days after appendectomy for acute appendicitis, the body temperature increased to 39℃, with frequent stool and severe stool, mucous stool and low amount. Physical examination: the incision can heal, no swelling, no obvious tenderness and urgency. Possible original

A. colitis gravis

B. pseudomembranous enteritis

C. Anendal inflammation

D. pelvic abscess

E. fecal fistula

Refer to the answer: D. Your answer:

Text analysis: [analysis] young women, after appendicitis resection, fever, frequent stool with severe emergency, considered as pelvic abscess (D pair), is a common complication of acute appendicitis.

[Avoid mistakes] Ulcerative colitis (A mistake) was mainly manifested as diarrhea, mucous pus and bloody stools, and the patient had appendectomy

History, regardless of ulcerative colitis. Pseudomembranous enteritis (B error) is mostly intestinal bacteria caused by the recent application of broad-spectrum antimicrobial drugs

Group disorder, so that clostridium difficile blooms into a dominant bacterial flora, producing exotoxins and invasive enzymes.epityphlon

Residual strain inflammation (C error) is due to the residual retention of the appendix or residual intestinal stone, postoperative residual strain can be inflammation recurrence, still manifested as the appendix

Symptoms of tailitis. The causes of fecal fistula (E error) are mostly the simple ligation of the appendix stump and the shedding of the ligation line.

Considering that the patient has developed postoperative fever, frequent stools with tenesmus and mucous stools, we should consider those complications that may cause these symptoms postoperatively. Among the options provided, pseudomembrane enteritis (B) is an intestinal infection associated with antibiotic therapy, characterized by a sudden onset of watery diarrhea, fever, abdominal pain, and possibly immediate urgency. Given that the patient was experiencing these symptoms shortly after undergoing abdominal surgery, pseudomembranous enteritis became a reasonable suspected diagnosis.

Although other options may be associated with abdominal surgery, their typical findings do not fully consistent with this case description. Therefore, the most appropriate answer is B. pseudombranous enteritis.

analyze:

In a young female patient, she had fever within 5 days after appendectomy, frequent stool and severe urgency, symptoms of rectal irritation, mucous stool discharge, small amount, and good incision healing, so the diagnosis of pseudomembranous enteritis was considered. Pseudomembranic enteritis is a kind of acute cellulose exudative inflammation mainly occurring in the colon and small

intestine. Most of them lead to normal intestinal flora imbalance after the application of antibiotics, clostridium difficile reproduction, producing toxins and causing disease, which is consistent with the history of using antibiotics against infection after appendectomy in the trunk.

Option A Ulcerative colitis generally starts slowly, the severity of the disease is different, the symptoms are mainly diarrhea, discharge containing blood, pus and mucus feces, often paroxysmal colonic spastic pain, and severe emergency, can be relieved after defecation, which is not consistent with the symptoms of the patient's symptoms, so it is not selected.

Option C Appendal appendicitis often shows appendicitis surgery after the preservation of the appendix stump is too long, or residual faecite, continued inflammation, still shows the symptoms of appendicitis, which is not consistent with the performance of the patient, so it was excluded.

Option D pelvic abscess is often manifested as relaxation type of high fever, peritoneal stimulation is more obvious, rectal and bladder stimulation symptoms such as rectal pressure, defecation feeling and urination pain, and systemic poisoning symptoms, which is not consistent with the performance of the patient, so it is not selected.

Option E fecal fistula refers to the abnormal passage of the gastrointestinal tract and body surface or body cavity and body cavity caused by various reasons, which is called internal fistula and external fistula. The digestive fluid flows out through the fistula, but the patient has no fistula outflow and can be excluded.

In conclusion, the correct answer is: B. pseudomembranous enteritis.

According to the situation described by the problem stem, a 32-year-old female patient had her body temperature increased to 39℃ 5 days after acute appendicitis, with frequent stool. Physical examination showed good healing of the incision, no redness, and no significant tenderness and severe pain. Based on this information, the most likely diagnosis is:

F. pseudomembranous enteritis

The reasons are as follows:

-Postoperative patients develop high fever and frequent stool, with tenesmus and

mucinous stools, and these symptoms suggest intestinal infection or inflammation.

-Pseudomembranous enteritis (Clostridioides difficile infection) is commonly seen after surgery or after antibiotics, manifested as diarrhea, emergency and high fever, which meet the symptoms described by the problem stem.

-Ulcerative colitis is usually chronic and is unlikely to be acute in a short postoperative period.

-Appendiceal stump inflammation and pelvic abscess may also cause fever and abdominal pain, but do not exactly match the frequent stool and mucus stool symptoms described in the problem trunk.

-Faecal fistula generally leads to infection and fecal discharge of the incision or surrounding skin, but the incision is healed well without redness and swelling.

Therefore, combined with the patient's symptoms and medical history, the most likely diagnosis is pseudomembranous enteritis.

75. (Type A2 question)

The patient, female, 28 years old, was presented with joint pain for 1 year and easy hair loss for six months. ESR110mm / h, normal anti- "O", Hb 86 g / L, PLT 70109 / L, ANA granular positive, urine protein (+ + +), diagnosed as SLE, incorrect in the estimation of the prognosis

A. No pregnancy

B. Kidney lesions are the proliferative patients with a poor prognosis

C. Non-remission pregnancy can aggravate the condition, and contraception should be used

D. Recurrent arthritis causes joint deformities and periarticular muscle atrophy

E. Poor prognosis of recurrent seizures and organic encephalopathy

Refer to the answer: D. Your answer:

[Analysis] Young women, joint pain, alopecia, elevated blood sedimentation, anemia, platelet reduction, ANA positive, urine protein positive, considered as SLE. SLE recurrent arthritis, joint X-ray examination mostly without joint bone damage (D wrong, is the correct answer to this question).

[Fault avoidance] Non-remission SLE patients are prone to abortion, premature birth and stillbirth, so contraception (AC pair); SLE appears kidney

Dirty lesions, often combined with lupus nephritis, suggesting a poor prognosis (B pair); recurrent seizures and organic encephalopathy manifestations are

Lupus encephalopathy, suggesting a poor prognosis (E pair).

This topic examines the diagnosis and prognosis of systemic lupus erythematosus (SLE). Let's analyze each option one by one:

A. pregnancy: Patients with SLE should not be pregnant during active period, because pregnancy may aggravate disease activity, but pregnancy is possible when the disease control is stable. Therefore, this statement is not completely correct and needs to be analyzed according to the disease activity.

B. Kidney lesions are proliferative manifestations, with a poor prognosis: indeed, proliferative nephritis in SLE patients is associated with a worse prognosis.

C. Non-remission pregnancy can aggravate the condition, should be contraception: this is true, SLE patients in non-remission pregnancy may indeed aggravate the condition, should be contraception.

D. Recurrent arthritis causes joint deformity and periarticular muscle atrophy: SLE can cause arthritis, and long-term uncontrolled arthritis may lead to joint deformity and muscle atrophy, which is possible.

E. Poor prognosis in patients with recurrent seizures and organic encephalopathy: Neurological involvement caused by SLE, such as seizures and organic encephalopathy, is indeed associated with a poorer prognosis.

Therefore, from these options, item A "unfavorable for pregnancy" is the least accurate because it does not consider the activity status of the disease. In the case of stable disease control, SLE patients can be considered for pregnancy. So, the correct answer should be an A.

analyze:

The patient was a young woman with joint pain, easy hair loss, rapid ESR, normal anti- "O", moderate anemia, low platelet count, ANA granular positive, positive urine protein, and could be diagnosed as systemic lupus erythematosus (SLE) according to symptoms and signs. Arthritis in SLE patients mainly involves the proximal interphalangeal, metacarpophalangeal, wrist, elbow, and knee joints, often showing symmetry, persistent pain, but less causing joint deformity and periarticular

muscle atrophy.

SLE occurs in women of childbearing age, and pregnancy can induce SLE activity, especially in the first 3 months of pregnancy and a few months postpartum, so option A is not suitable for pregnancy, and option C non-remission pregnancy can aggravate the condition, which is in line with the prognosis of SLE patients.

SLE can involve almost every system, tissues and organs, including kidney damage is the most common, can be manifested as acute nephritis syndrome, acute nephritis syndrome, nephrotic syndrome or glomerulonephritis like nephropathy, severe development into renal failure, so option B kidney disease is proliferative type, poor prognosis, accord with the prognosis of patients with SLE.

SLE involving the nervous system, also known as neuropsychiatric lupus, light only migraine, personality change, memory loss or mild cognitive impairment, heavy can be cerebrovascular accident, coma, epileptic status, etc., so options E repeated seizures and organic encephalopathy of poor prognosis, is the performance of SLE patients with poor prognosis.

In conclusion, the correct answer is: D. Repeated arthritis causes joint deformities and atrophy around the articular muscles.

The topic describes a 28-year-old female patient with systemic lupus erythematosus (SLE) with multiple symptoms and signs, including joint pain, alopecia, elevated sedimentation, anemia, thrombocytopenia, positive ANA, and urinary protein. These features suggest active disease and multisystem involvement.

For the outcome assessment of SLE patients, the following is an analysis of the options:

A. * * Unsuitable for pregnancy * * -SLE patients are indeed not suitable for pregnancy during the disease activity period, because pregnancy may aggravate the disease and has potential risks to the health of the mother and the child.

B. * * Kidney lesions are proliferative manifestations with poor prognosis * * -Proliferative nephritis (such as proliferative lupus nephritis) has a poor prognosis and requires close monitoring and active treatment.

C. * * Non-remission pregnancy can worsen the condition, and should be

contraception * * -A pregnancy during active SLE may indeed worsen the condition, so contraception is recommended until the disease resolves.

D. * * Recurrent arthritis causes joint deformities and periarticular muscle atrophy * * -Arthritis in patients with SLE is generally non-erosive and does not cause joint deformities like rheumatoid arthritis. Although arthritis can cause pain and dysfunction, it rarely causes joint deformities and severe muscle atrophy.

E. * * Poor prognosis of recurrent seizures and organic encephalopathy * * -Central nervous system involvement, such as seizures and organic encephalopathy, indeed has a poor prognosis.

In conclusion, the D option is not described correctly, because arthritis in SLE patients does not usually cause joint deformity and severe muscle atrophy.

So, the correct answer is:

* * D. Recurrent arthritis causes joint deformity and periarticular muscle atrophy * *

76. (Type A2 question)

A 27-year-old female was diagnosed with systemic lupus erythematosus for 2 years and was 28 weeks gestation. Check up one week ago: Hb109g / L, PLT209106 / L. The drugs that the patients can use are

A. methotrexate

B. cyclophosphamide

C. hydroxychloroquine

D. leflunomide

E. High-dose glucocorticoids

Reference answer: C Your answer:

[Analysis] Women of childbearing age, 28 weeks gestation, diagnosed with systemic lupus erythematosus for 2 years, mild anemia, normal platelets, because

hydroxychloroquine has a relatively small effect on pregnancy, can be used (C pair).

[Misavoidance] Methotrexate is the drug of choice for rheumatoid arthritis (A error). Teratogenic of cyclophosphamide and leflunomeres

Use, pregnant women can not use (BD wrong). Hormones can increase the risk of gestational diabetes, hypertension and infection, and long-term risk is not recommended

For high dose, it should be reduced to prednisone 15 mg/d as soon as possible (E wrong).

Treatment of systemic lupus erythematosus (Systemic Lupus Erythematosus, SLE) needs to be tailored to the patient context, especially during pregnancy. When choosing drugs, we need to consider the safety of the drug, especially the effect on the fetus.

- ** Methotrexate (Methotrexate, MTX) * *: It is harmful to pregnancy and may lead to fetal malformation, so pregnant women are prohibited.

- ** Cyclophosphamide (Cyclophosphamide) * *: It is harmful to the fetus and can cause birth defects and miscarriage, and it is therefore not recommended during pregnancy.

- ** Hydroxychloroquine (Hydroxychloroquine, HCQ) * *: It is relatively safe during pregnancy and has a therapeutic effect on SLE, and is one of the first choice drugs for the treatment of SLE in pregnant women.

- ** Leflunomide (Leflunomide) * *: Pregnant women are prohibited because it may cause fetal abnormalities.

- ** High-dose corticosteroids * *: Although they can be used for the treatment of SLE, especially during the active period, they should be used carefully during pregnancy and controlled at the minimum effective dose.

Therefore, the most suitable option for this patient is * * C. hydroxychloroquine * *. It can both effectively control the symptoms of SLE and use it relatively safely during pregnancy.

analyze:

The patient was a young female, diagnosed with systemic lupus erythematosus, now at 28 weeks' gestation. For pregnant patients with systemic lupus erythematosus, drugs with small effects on the fetus should be selected. Hydroxychloroquine has no

adverse effects on pregnancy and fetus, and can control SLE activity and prevent SLE recurrence, so patients can use it.

Option A methotrexate, option B cyclophosphamide, and option D leflunomide, which are immunosuppressants with clear teratogenic effects, were banned in pregnant women, so they were excluded.

Option E High-dose glucocorticoids may have an effect on the fetus and are only considered when the benefits outweigh the risks, so it is not chosen.

In conclusion, the correct answer is: C. Hydroxychloroquine.

For a female patient diagnosed with systemic lupus erythematosus (SLE) for 2 years and at 28 weeks' gestation, special attention should be paid to the safety of the fetus when choosing the appropriate drug. Here is an analysis of the options:

A. * * Methotrexate * *: It is an antimetabolizing drug used for the treatment of SLE and certain cancers. However, methotrexate has a teratogenic effect on the fetus and is prohibited during pregnancy.

B. * * Cyclophosphamide * *: It is an alkylating agent with immunosuppressive effects, but it has serious toxicity and teratogenic effects on the fetus, and it is prohibited during pregnancy.

C. * * Hydroxychloroquine * *: It is an antimalarial drug that is also commonly used in the treatment of SLE. Studies have shown that hydroxychloroquine is relatively safe during pregnancy and is beneficial in controlling SLE activity.

D. * * Lflunomide * *: It is an immunosuppressant with teratogenicity and prohibited during pregnancy.

E. * * High-dose glucocorticoids * *: Although corticosteroids can be used to control the activity of SLE, the fetus and is often avoided as much as possible during pregnancy.

Therefore, considering the efficacy of the drug and the safety of pregnancy, * * C. hydroxychloroquine * * is the most appropriate choice.

* * Answer: C. Hydroxychloroquine * *

77. (Type A2 question)

Male, 70 years old, left groin package for 1 year, round package, 54cm in size, lying flat disappeared, hold the deep ring of the lump, it can still highlight after coughing, the lump may be

A. lipoma

B. indirect inguinal hernia

C. direct inguinal hernia

D. femoral hernia

E. funicular hydrocele

Reference answer: C Your answer:

[Analysis] The elderly men, inguinal package round, lying flat disappeared, hold the deep ring of the lump, cough still prominent, considered for inguinal hernia (C pair).

[Avoid error] lipoma (A wrong) is the tumor of normal fat tissue, good hair in the limbs, trunk, clear realm, presented

Lfoliation, soft can have false sac sexy, painless. Obmic inguinal hernia (B wrong) in children and young adults, the hernia mass is oval

Round or pear-shaped, can enter the scrotum, after pressing the mass deep ring, the hernia block is no longer protruding. Femoral hernia (D wrong) good at 40

In women over 20, the hernia mass is hemispherical and does not completely disappear after recsupine. Perhydroe (E wrong) mass

Small, in the inguinal canal, pulling the ipsilateral testicular visible mass movement.

This question describes a 70-year-old male with left inguin mass for 1 year. The

package block is round, 54cm in size, it will disappear when lying flat, press down the deep ring of the package block mouth, and it can still highlight after coughing. Based on these clinical features, lipoma (A) and hydroeffusion (E) can be excluded because these conditions usually do not change in size due to position and do not highlight during coughing.

Of the remaining options, the inguinal hernia (including oblique and straight hernia) and the femoral hernia were common conditions consistent with the description. Inguinal hernia can often become more pronounced during standing or coughing and may disappear while lying down. An oblique inguinal hernia (B) usually passes into the inguinal canal through the internal ring, while a direct inguinal hernia (C) projects directly forward through the weak area of the inguinal canal. The femoral hernia (D) is located below the groin and usually does not rise to the inguinal canal.

Since the mass can highlight after coughing and disappear while lying flat, this is more consistent with the characteristics of an inguinal hernia. Considering the patient's age and the described clinical presentation, the most likely diagnosis was * * straight inguinal hernia (C) * *. Because in older men, direct inguinal hernia is more common than oblique inguinal hernia, especially in the absence of congenital components (e. g., oblique hernia is common in young people).

First, we analyze the question stem.

In an elderly male patient, there is a left groin, round, 54cm in size, and will disappear when lying flat. The key message is that the lump can still be prominent when holding the deep ring and coughing. This feature provides us with clues about the nature of the parcel block.

Next, we analyze the options one by one:

A. Lipoma: lipoma is a common benign soft tissue tumor consisting of mature fat cells and can occur in any fatty part of the body. But it does not disappear while lying flat, nor does it change its prominence by holding down a position. So exclude.

B. oblique inguinal hernia: The hernia sac of an oblique inguinal hernia will project through the deep ring of the inguinal canal and may enter the scrotum. One of the characteristics is that when the groinal canal is held down and the abdominal pressure is increased (such as cough), the hernia mass is no longer protruding. However, it is clearly mentioned in the title stem that the deep ring of the lump can still highlight after the cough, which is not consistent with the characteristics of

oblique inguinal hernia. So don't choose.

C. Inguinal direct hernia: The hernia sac of the direct inguinal hernia projects directly through the triangular region of the straight hernia, not through the internal ring or into the scrotum. Critically, when the patient is standing, half of the medial mass appears at the medial end of the pubic tubercle, but when the patient lies flat, the mass will disappear spontaneously. If the hand holds down the inguinal canal deep ring and causes the patient to rise and cough, the hernia mass can still highlight, which matches the trunk description.

D. Femoral hernia: The mass of femoral hernia is often small, with a half-spherical protrusion in the ovale fossa below the inguinal ligament. Femoral hernia is prone to incarceration, and the hernia mass can not completely disappear after lying back. Not consistent with the description of the title stem.

E. This is a cyst located above the testis or in the inguinal canal. It is characterized by a positive light transmittance test, that is, when illuminated with a flashlight, the effusion part will appear bright. Furthermore, hydrocele usually does not disappear or protrude due to a change in position or pressing on a site. Not consistent with the description of the title stem.

In conclusion, the appearance of the inguinal hernia occurred considering the appearance of the mass and the appearance of the inguinal hernia. Therefore, the correct answer is C. inguinal hernia.

According to the title description, a 70-year-old male, left groin package for 1 year, round mass, 54cm in size, lying flat disappeared, holding the deep ring of the lump, can still highlight after coughing. Combined with these symptoms, the following is an analysis of the various options:

A. Lipoma: Lipoma is generally a soft, painless lump under the skin, and will not disappear or highlight due to changes in position or cough.

B. Inguinal oblique hernia: Inguinal oblique hernia is characterized by a mass appearing when standing or coughing and disappearing when lying flat. When holding the deep ring, the mass will not protrout when coughing.

C. Direct inguinal hernia: Direct inguinal hernia usually occurs in the middle and medial part of the inguinal area, which is characterized by the disappearance of the mass when lying flat, and after holding the deep ring, the mass can still highlight when coughing.

D. Femoral hernia: Femoral hernia usually occurs in the area between the femur and the inguinal ligament, and the symptoms do not coincide completely with the description.

E. Hydroeffusion: Hydroeffusion is mostly fluid accumulation in the scrotum, usually manifested as scrotal enlargement, which does not meet the description of the mass in the groin and disappearing when lying flat.

To sum up, the most consistent answer to the question description is:

F. direct inguinal hernia

78. (Type A2 question)

A 25-year-old male suffered from a liver rupture, abdominal bleeding, and hemorrhagic shock. After emergency surgery, the abdominal bleeding was controlled and adequate rehydration was given. However, the patient's central venous pressure was 15cmH2O, and the blood pressure was 90 / 60mmHg, and it was not treated here

A. Continue rehydration

B. Replication test

C. Giving a heart booster

D. Giving a vasodilator

E. Administration of corticosteroids

Reference answer: B. Your answer:

Graphic analysis: [analysis] In patients with hemorrhagic shock, with normal central venous pressure and low blood pressure, so fluid rehydration experiment (B pair) should be carried out to identify cardiac insufficiency and blood volume insufficiency.

Considering that the patient's central venous pressure (CVP) is 15 cmH2O, this indicates that the patient has undergone adequate rehydration because the normal CVP range is usually 5-10 cmH2O. The patient's blood pressure remained low (90 / 60 mmHg), suggesting a persistent hypovolemic state or inadequate cardiac function.

Option A (continued rehydration) may not be optimal because the patient had already high CVP, indicating that the blood volume may have been adequate. Option B (rehydration test) is usually used to determine if further rehydration is required, but may not be applicable at high CVP. Option D (vasodilator given) may further lower blood pressure, so it is not ideal.

Given these conditions, option C (booster) seems to be the most appropriate option. Cardiac agents can help improve cardiac contractility and increase cardiac output, thereby improving blood pressure. Therefore, the correct answer may be * * C. Giving a heart booster * *.

analyze:

The young male patient, after hepatic rupture, had been given fluid rehydration. At present, the central venous pressure is normally high and the blood pressure is low, indicating cardiac insufficiency or relatively excessive blood volume, the heart can not be effectively beat, and should be given to enhance the myocardial contractility.

Option A is suitable for severe blood volume insufficiency, and the central venous pressure is normally high, indicating that the blood volume is not insufficient, so it is not selected.

Option B fluid rehydration test is mainly used to identify the causes of oliguria, which is not consistent with the problem stem, so it is excluded.

Option D: Vasodilator administration is suitable for high central venous pressure and normal blood pressure, which is not consistent with the problem stem description, so it is not selected.

Option E provided corticosteroids for severe infection or inflammation, which were not consistent with the patient's condition, and were excluded.

To sum up, the correct answer is: C. Give a heart booster.

In this case, the patient suffered from abdominal bleeding and hemorrhagic shock after a liver rupture caused by a car crash. After emergency surgery, although abdominal bleeding was controlled and adequate rehydration was given, the patient's central venous pressure (CVP) was 15cmH2O while blood pressure was still low (90 / 60 mmHg). This condition suggests that patients may have excess volume, cardiac insufficiency or other factors.

The current condition of the patient needs to be considered when selecting the answer:

-Continued rehydration (A) may increase the volume burden and lead to heart failure.

-The rehydration test (B) is not suitable for the current adequate rehydration situation.

-Giving a heart agent (C) may be appropriate because it helps to enhance the systolic function of the heart and improve blood pressure.

-Vasodilator (D) may not be appropriate for the current situation because blood pressure is already low and further dilation will lower blood pressure.

-Administration of corticosteroids (E) has no clear indication in this case.

So the best answer is:

B Giving a heart booster

79. (Type A2 question)

A trauma patient, the left thigh was cut by a firewood knife, and was sent to the hospital for emergency treatment 9 hours later. The examination: the outer wound of the left thigh was 5cm long and 1cm deep, the edge was still neat, and the wound was stained with more soil. Deal with appropriate

A. Primary suture after debridement

B. deferred suture after debridement

C. Suture after alcohol disinfection

D. Local antibiotic application

E. Wash out with soapy water

Reference answer: B. Your answer:

Graphic analysis: [analysis] Open wounds are often contaminated, and debridement should be performed. If the wound pollution is heavy or the treatment time has exceeded 8~12 hours after the injury, but no obvious infection has occurred, the suture of the skin is not ligated temporarily (B pair), and the saline gauze in the wound for drainage. After 24 to 48 hours, the wound is still no obvious infection, and the suture can be ligated to match the wound margin. If the wound is infected, remove the suture as the infected wound.

[Avoid] Primary healing can be achieved within 6 to 8 hours after injury; even facial wound should be greater than 12 hours

Phase suture.

For a trauma patient, especially those with a deep wound and significant contamination (such as soil), the first priority is thorough debridement to prevent infection. Therefore, simple disinfection and suturing are usually inadequate. If the wound is contaminated, the risk of direct primary closure (i. e. immediate closure) is high because it may prompt the infection.

The best treatment is to delay the suture after debridement (option B). A tended suture is performed after the wound has been thoroughly cleaned (removing contaminants and dead tissue) and observed for a period of time (usually days) to ensure that no infection has occurred. This approach can reduce the risk of wound infection.

Therefore, the most correct answer for this question is * * B. Late suture after debridement * *.

analyze:

The wound on the lateral thigh of the patient was 1cm deep and stained with soil, indicating that the wound was seriously polluted. The injury time had reached 9 hours, and the contaminated wound was not treated for more than 8 hours, which should be treated as the infected wound, that is, delayed suture after debridement.

Option A the first phase after debridement is suitable for less than 8 hours after

injury or although the injury has been more than 8 to 12 hours, but no obvious infection of less contaminated wound, which is inconsistent with the question.

Alcohol disinfection is only suitable for disinfection of intact skin, and the patient has an open wound, so option C alcohol disinfection after disinfection can be excluded.

Topical application of antibiotics cannot replace systemic treatment, and it is easy to cause allergic reactions, so option D topical application of antibiotics is not optional.

Option E Soap water flushing cannot meet the requirements of surgical sterilization, and can only be used as a temporary treatment measure under field conditions, so it is not selected.

To sum up, the correct answer is: B. Postdelay suture after debridement.

In this case, the risk of wound infection should be considered first, because the wound has been exposed for 9 hours and has more dirt. Therefore, thorough debridement and appropriate suturing should be noted to avoid infection.

The most suitable treatment scheme is the delayed suture after debridement. This completely removes soil and necrotic tissue from the wound, reduces the risk of infection, and sutures after confirming that the wound is not infected.

So the best answer is:

B deferred suture after debridement

80. (Type A2 question)

Elderly men, wasting and fatigue, weight loss, refers to a 3cm diameter mass 8cm from the anus, colonoscopy see 8~10cm from the anus, the patient preferred treatment method

A. Miles art

B. Dixon art

C. Hartmann for surgery

D. Local resection of rectal cancer

E. Double elostomy in the sigmoid colon

Reference answer: B. Your answer:

[Analysis] Elderly men, wasting, fatigue, weight loss (typical symptoms and signs of cancer), a 3cm diameter mass 8cm away from the anus, considered for rectal cancer. Dixon (radical abdominal rectal cancer, B), also known as low anterior rectal resection, is the most used radical rectal cancer, which is suitable for tumors more than 5cm away from the dentate line (more than 7cm away from the anus).

[Fault avoidance] Miles (A fault) is suitable for tumors within 5cm from the dentate line (within 7cm from the anus); transabdominal rectal resection, proximal stoma, distal closure surgery (Hartmann surgery, C fault), which is suitable for elderly and frail reasons

Miles surgery or acute obstruction is not suitable for Dixon surgery. Local resection of rectal cancer (D wrong) is suitable for straight within T1

intestinal cancer. Difficult defecation or bowel obstruction, flexible sigmoid double ostomy (E wrong).

This topic describes an elderly male patient, wasting, fatigue, weight loss and other symptoms, and through the anal finger examination and colonoscopy found about 3cm 8 to 10 cm from the anus of the vegetable pattern mass. From this information, we can infer that the mass was most likely a rectal cancer.

Now, we will look at the various treatment options:

- ** A. Miles (anterior rectal resection) * *: Suitable for middle and lower rectal cancers involving the resection of the entire rectum and anus, usually with a permanent stoma.

- ** B. Dixon surgery (anterior resection) * *: Suitable for upper rectal cancer or sigmoid colon cancer, with the anus preserved and without the need for a stoma.

- ** C. Hartmann * *: Suitable for lower rectal cancer, where resection of the diseased bowel, terminal colostomy, closure of rectal stump, suitable for emergency surgery or not suitable for immediate reconstruction of intestinal continuity.

- ** D. Local resection of rectal cancer * *: Suitable for early rectal cancer, especially where the cancer is small, shallow and located in the lower section of the

rectum.

- ** E. sigmoid double ostomy * *: This is a relief, usually used in acute conditions, such as intestinal obstruction or conditions requiring decompression, rather than radical surgery.

Considering the location of the mass (8 to 10 cm from the anus), this indicates that it was located in the middle or lower segment of the rectum. Therefore, the choice of Miles (anterior rectal resection) may be a more suitable treatment option because it is suitable for cancer in the middle and lower rectal segments and is a radical procedure.

So, the best answer is * * A. Miles * *.

analyze:

The elderly male patient, wasting, fatigue, weight loss, is a common cachexia manifestation of malignant tumors. There is a 3cm diameter mass 8cm away from the anus, and a colonoscopy vegetable pattern mass is about 3cm 8~10cm away from the anus. Combined with the symptoms and signs of the patient, it can be diagnosed as rectal cancer. Because the patient's swelling is 8~10cm away from the anus, it belongs to low rectal cancer, and the cancer is large, with a diameter of 3cm, and radical surgery should be performed for surgery. Because the patient had low rectal cancer with a large mass and less than 7cm from the anal margin, the radical resection of abdominal perineum combined with rectal cancer (Miles surgery) should be selected.

Option B: Dixon resection of transabdominal rectal cancer is suitable for early rectal cancer where the lower edge of rectal cancer is 7~11cm from the anus, which is inconsistent with this question.

Option C: Hartmann transabdominal rectal cancer resection, proximal stoma and distal closure surgery is suitable for patients with rectal cancer who cannot tolerate Miles surgery due to poor general condition or acute obstruction unsuitable for Dixon surgery, which is inconsistent with the condition of this question.

Option D: Local resection of rectal cancer is suitable for early rectal cancer with small tumor body and high differentiation. The patient has a large mass and is not suitable for local resection.

Option E: The sigmoid double-lumen stomy can only relieve obstruction and relieve symptoms, without the purpose of radical cure, which is inconsistent with the meaning of the question.

In summary, the correct answer is: A. Miles.

According to the title description, the patient is an elderly male, wasting and fatigue, weight loss, digital examination and colonoscopy found a diameter of about 3cm at 8-10cm from the anus, which suggested that it may be rectal cancer. According to the treatment principles of rectal cancer and current best practice guidelines, choosing an appropriate treatment regimen requires considering factors such as tumor location, size, depth of invasion, and the systemic condition of the patient.

The option analysis is performed as follows:

- ** A. Miles * *: This surgery is a radical resection of abdominal and perineal combined rectal cancer. It is suitable for low rectal cancer (within 5cm from the anus), and requires removal of the anus and surrounding tissues.

- ** B. Dixon * *: Also known as low anterior resection, it is suitable for middle and low rectal cancer (5-10cm away from the anus), preserving the anal function, which is the recommended operation method at present.

- ** C. Hartmann * *: Generally used in acute situations or as a palliative procedure and not often as the preferred treatment.

- ** D. Local resection of rectal cancer * *: Suitable for very early, rectal cancer confined to the submucosa without lymph node metastasis.

- ** E. Double cavity stoma of sigmoid colon * *: Usually as a palliative procedure, it is mainly used to relieve symptoms such as intestinal obstruction.

In conclusion, the patient's tumor is located 8-10cm away from the anus, which belongs to the median rectal cancer. Dixon surgery (low anterior resection) is the best choice, because it can preserve the anal function while eradicating the tumor.

Therefore, the preferred treatment method is * * B. Dixon surgery * *.

81. (Type A2 questions)

Male, 17 years old. Chest pain, shortness of breath for 2 hours. Two hours ago,

the patient had sudden chest pain, shortness of breath and a small cough without fever. Physical examination: the left chest was full, the left lung percussion, and the left lung breathing was low with no rales. Heart rate was 101 beats / index sinus tachycardia. The most likely diagnosis is the

A. therapeutic pneumothorax

B. pulmonary embolism

C. Acute attack of bronchial asthma

D. emphysema pulmonum

E. acute myocardial infarction

Reference answer: A. Your answer:

[Analysis] A young man had laughing movements before the disease, whose signs were full in the left chest, percussion in the left lung, and low respiratory sound in the left lung (typical manifestation of spontaneous pneumothorax). According to the clinical manifestations and signs, the diagnosis was considered as spontaneous pneumothorax (A pair).

[Avoid] Pulmonary embolism is mainly manifested by sudden chest pain, dyspnea, hemoptysis, syncope, etc. (B wrong); acute bronchial asthma

The episodes presented as episodic dyspnoea, lasting for several minutes and widely extensive ble during the episode (C

Wrong); emphysema is less common than young men, and the signs are mostly bilateral thoracic fullness, percussion is clear (D wrong); urgent

In sexual myocardial infarction, severe pain in the chest area was the first symptom, with less chest fullness and clear signs on percussion

(E wrong).

This topic described a 17-year-old male patient with sudden chest pain and shortness of breath after laughing. Physical examination found that the left chest was full, left lung percussion, decreased respiratory sound in the left lung, no fever and rales, and increased heart rate. Based on this information, the most likely diagnosis is a spontaneous pneumothorax (option A).

Spontaneous pneumothorax usually occurs in the absence of significant trauma, especially in young adults. Typical symptoms include sudden chest pain and difficulty breathing. Physical examination may find the full chest of the affected side, the

percussion sound, and the reduced breathing sound, which is consistent with the clinical manifestations described in this question. Therefore, A. spontaneous pneumothorax is the most likely diagnosis.

First, let's go through the question stem.

Young male patients, sudden chest pain, shortness of breath, and appeared after laughing. Physical examination showed a full left thorax and percussion of the left lung, which is a typical sign of pneumothorax. At the same time, the left lung respiratory sound is low, with no rales, and the heart rate is slightly faster.

Next, we analyzed for each option:

A. Spontaneous pneumothorax: Considering the sudden symptoms after laughter, the left chest and the left lung percussion, these are the typical symptoms and signs of spontaneous pneumothorax. Young men are good people of spontaneous pneumothorax, and often induced by holding heavy objects, breath, strenuous physical activity. Therefore, this option is highly consistent with the problem stem description.

B. Pulmonary embolism: Although pulmonary embolism may also cause chest pain and shortness of breath, it is usually associated with deep vein thrombosis, and is more common in the elderly or long-term bedridden patients. These risk factors are not mentioned in the title stem, so this option is unlikely.

C. Acute attacks of bronchial asthma: Bronchial asthma usually has recurrent wheezing, shortness of breath, chest tightness, or cough, which often develop or worsen at night or early in the morning. These symptoms were not mentioned in the title stem, so they can be excluded.

D. Emphysema: Symptoms of emphysema usually include chronic cough, expectoration, shortness of breath, or dyspnea, and it is a chronic disease, not acute onset due to sudden factors such as laughter. Therefore, this option does not conform to the problem stem description.

E. Acute myocardial infarction: Acute myocardial infarction usually presents with persistent, severe retrosternal pain that may be accompanied by nausea, vomiting, excessive sweating and other symptoms. Although it may also cause sinus tachycardia, the symptoms and signs in the trunk do not consistent with acute myocardial infarction.

In conclusion, considering the patient's age, mode of onset, symptoms, and signs, the most likely diagnosis is spontaneous pneumothorax.

Therefore, the correct answer is the A. spontaneous pneumothorax.

According to the title description, the patient was a 17-year-old male with chest pain and shortness of breath for 2 hours. These symptoms occurred suddenly after laughter and were accompanied by a small cough without fever. Physical examination showed full left thorax, percussion percussion in the left lung, low respiratory tone and no rales in the left lung, and heart rate of 101 beats / sinus tachycardia.

The following is the analysis of each option:

A. * * Spontaneous pneumothorax * *: This condition is common in young and healthy men, usually presenting with sudden unilateral chest pain and dyspnea. The physical examination can find the full chest of the affected side, percussion sound and reduced breathing sound, which is consistent with the description in the topic.

B. * * Pulmonary embolism * *: It is usually seen in patients with deep vein thrombosis or other embolic sources, and symptoms include sudden dyspnea, chest pain (possibly pleural), hemoptysis, etc. Although the symptoms may be similar, no associated risk factors or typical symptoms were mentioned in the title.

C. * * Acute onset of bronchial asthma * *: Usually with dyspnea, wheezing, cough, but no significant chest pain or signs of thoracic fullness on one side.

D. * * Emphysema * *: Usually seen in patients with chronic obstructive pulmonary disease, manifested as long-term dyspnea and chronic cough, which may worsen in acute onset, but is not consistent with the acute and sudden symptoms in the topic.

E. * * Acute myocardial infarction * *: Common symptoms include chest pain (which can radiate to other parts), shortness of breath, sweating, etc., usually accompanied by typical electrocardiogram and myocardial marker changes, but the patient is only 17 years old, which is unlikely to be the typical onset age of acute myocardial infarction, and the signs are not completely matched.

Based on the above analysis, the most likely diagnosis is * * A. spontaneous pneumothorax * *.

82. (Type A2 questions)

Male, 33 years old, right upper abdominal distension and pain with chills and high fever for 6 hours. Physical examination: T39.9℃, no yellow skin mucosa, right upper abdomen tenderness, mild muscle tension, no obvious rebound pain. WBC 18109 / L, N0.85, abdominal positioning, right ultrasound: 8cm liquid dark area below the right posterior liver. The following are among the most valuable checks

A. Hepatnuclide scan

B. belly CT

C. Liver arteriovenous angiography

D. Liver puncture under ultrasound

E. blood culture

Refer to the answer: D. Your answer:

[Analysis] A young male, right upper abdominal distension and pain, chills and high fever, B ultrasound showed 8cm liquid dark area of the liver, can be diagnosed as liver abscess. The most valuable test at this time should be ultrasound liver puncture (D pair).

[Fault avoidance] Hepnuclide scan (A error) and hepatic arteriovenous angiography (C error) are generally used for auxiliary examination of liver cancer; although abdominal CT has auxiliary value in the diagnosis of liver abscess, it is not the most diagnostic examination (B error); blood culture sensitivity is not

Ultrasound puncture can sometimes be negative (E error).

This case describes a 33-year-old male patient with right upper abdominal

distension, chills, and high fever with a body temperature of 39.9°C, tenderness and mild muscle tension in the right upper abdomen, but no significant rebound pain. The patient had an elevated white blood cell count and ultrasonography revealed an 8cm liquid dark area in the lower right of the liver. These clinical findings and examination findings may suggest acute abdominal disease, especially problems related to the liver.

The option analysis is performed as follows:

A. Hepatannolin scan-This is an examination to assess hepatic blood flow and function and is not preferred for the diagnosis of acute abdominal pain.

B. Abdominal CT-can provide detailed information about the abdominal structure, especially for inflammation, infection, or other lesions in the abdominal organs.

C. Liver arteriovenous angiography-mainly used to evaluate the vascular structure of the liver and may not be required for the initial diagnosis of this case.

D. Liver puncture under ultrasound- -can be used to diagnose liver lesions, but invasive procedures without more information may not be the best option.

E. Blood culture- -Very useful for infectious diseases, but more imaging tests may be required before identifying the site of infection.

Taken together, the most valuable test should be the one that can provide the most information to help confirm the diagnosis. In this case, * * B. abdominal CT * * is the best choice because it can provide detailed information about the intra-abdominal organs, especially the liver, help to diagnose the nature of the liquid dark zone of the liver and guide further treatment.

First, we want to analyze the patient's symptoms, signs and existing examination results.

The patient was a young male with acute onset of right upper distension, chills and high fever, typical of acute cholecystitis or liver abscess. Physical examination revealed elevated body temperature, tenderness and mild muscle tension in the right upper abdomen, but no significant rebound pain, and elevated leukocytes and neutrophils, suggesting the presence of infection. The abdominal vertical plain film and B ultrasound results showed an 8cm liquid dark area in the lower right posterior part of the liver, which further supported the diagnosis of liver abscess.

Next, we analyze each option:

A. Hepanuclein scan: This test is more used to assess liver function and biliary excretion, and has limited value in determining the diagnosis of liver abscess.

B. Abdominal CT: This is a common imaging method, which can clearly show the morphology and structure of the liver, and provide detailed information on the size, location of the liver abscess and the relationship to the surrounding tissue.

C. Liver arteriovenous angiography: mainly used to show the vascular structure and blood flow of the liver, and is not the first choice for the diagnosis of liver abscess.

D. Liver puncture under ultrasound: Although liver puncture can be confirmed, as an invasive examination, it is relatively risky and is not the preferred diagnostic method.

E. Blood culture: This is mainly used to determine the pathogen of infection, although it is instructive for treatment, it has little value for direct diagnosis of liver abscess.

Considering the above information comprehensively, abdominal CT is both non-invasive and provides detailed morphological and structural information of the liver, which is the preferred examination method for the diagnosis of liver abscess.

Thus, the most valuable examination is the B. abdominal CT.

The patient was a 33-year-old man with right upper abdominal distension with chills and high fever for 6 hours, body temperature of 39.9°C, no yellow skin mucosa, right upper abdominal tenderness, mild muscle tension and no obvious rebound pain. The white blood cell count was 1810 ^ 9 / L and the neutrophil ratio was 0.85, and abdominal ultrasound showed an 8cm fluid dark zone in the lower right posterior liver.

Based on the patient's symptoms and examination findings, the most likely diagnosis is a liver abscess. For the diagnosis and further evaluation of the liver abscess, the following examinations are the most valuable:

F. belly CT

Abdominal CT was able to detail the size, location and number of liver abscesses

while also detecting other possible abdominal lesions. Therefore, abdominal CT is the most valuable method of examination.

Correct answer: B. CT of the abdomen

83. (Type A2 questions)

A 42-year-old male had abdominal pain with anal withdrawal for 2 days. Fasting, rehydration treatment, sudden abdominal pain worsened this morning. Previous appendectomy for more than 10 years. Physical examination: whole abdominal tenderness, rebound pain, muscle tension, bowel sounds disappeared. The most is

A. Air laxative

B. operations research

C. Continuous gastrointestinal decompression

D. fluid infusion

E. jejunitas

Reference answer: B. Your answer:

[Analysis] middle-aged men, abdominal pain, abdominal distension, anal stop exhaust defecation (manifestations of intestinal obstruction), abdominal pain aggravated 2 days after fasting rehydration treatment (medical treatment is ineffective, consider surgical treatment), a history of appendicitis surgery (considering adhesive intestinal obstruction caused by postoperative intestinal adhesion). Physical examination: whole abdominal tenderness, rebound pain, muscle tension, bowel sounds disappeared. The patient developed peritonitis without medical treatment. Considering strangulated intestinal obstruction, surgical exploration should be performed (B pair).

[Avoid mistakes] The following manifestations should be considered as strangulation intestinal obstruction: (1) abdominal pain onset, the initial is persistent

Severe abdominal pain; (2) the rapid development of the disease, early shock; (3) peritonitis; (4) the wrong abdominal distension

Said, there was a local bulge in the abdomen; (5) vomiting appeared early and frequently, vomiting was bloody, and blood fluid was extracted by abdominal

puncture

Body; (6) X-ray; (7) aggressive non-operative signs.

This problem describes a 42-year-old male patient with abdominal pain, distension and defecation for 2 days. The patient had a history of appendectomy with exacerbated abdominal pain after fasting and rehydration therapy. Physical examination found total abdominal tenderness, rebound pain, muscle tension, and bowel sounds disappeared.

These symptoms and signs indicate that the patient may have developed intestinal obstruction or acute intra-abdominal lesions such as volvulus or bowel ischemia. In this case, fasting and rehydration therapy alone may not be enough to solve the problem and will require further diagnosis and treatment.

Option B "Surgical exploration" is the most appropriate option in this setting as it allows direct inspection of the intra-abdominal condition, determining the nature of the lesion, and performing treatment accordingly. Surgical exploration can quickly detect and resolve the causes of symptoms, such as intestinal obstruction, intestinal ischemia, or other intra-abdominal problems.

Therefore, I would choose B "surgical exploration" as the best answer to this question.

First, let's analyze the questions.

The patient is a middle-aged male with abdominal pain, abdominal distension, and anal stop exhaust defecation for 2 days, which is a typical symptom of intestinal obstruction. After fasting and rehydration treatment, the abdominal pain suddenly worsened this morning. Previous history of appendectomy. Physical examination showed total abdominal tenderness, rebound pain, muscle tension, and the disappearance of bowel sounds, which are all typical signs of peritonitis. Combined with the patient's history and signs, we can make a preliminary diagnosis of intestinal strangulation or intestinal perforation caused after intestinal obstruction, leading to acute peritonitis.

Next, we analyzed for each option:

A. Air defecation: In the case of intestinal obstruction, the use of air defecation may aggravate the condition and even lead to an increased risk of intestinal perforation, so this option is not appropriate.

B. Surgical exploration: Considering the possible intestinal strangulation or

perforation, surgical exploration is the most direct and effective method to confirm the diagnosis and conduct the corresponding treatment. This can prevent further deterioration and even endanger the patient's life.

C. Persistent gastrointestinal decompression: Although gastrointestinal decompression has a certain role in the treatment of intestinal obstruction and can relieve the symptoms, it can not solve the fundamental problem in the case of intestinal strangulation or perforation.

D. Rehydration: Rehydration is necessary to correct dehydration and electrolyte imbalance, but it does not address the fundamental problems of intestinal obstruction or peritonitis.

E. Fasting: Fasting can reduce the intestinal burden and help relieve the symptoms of intestinal obstruction, but it is not the primary treatment in emergency situations such as intestinal strangulation or perforation.

In conclusion, considering the patients' possible bowel strangulation or bowel perforation, and the signs of acute peritonitis, the most urgent and necessary measure is to perform surgical exploration to determine the cause and treat it accordingly.

Therefore, the correct answer is the B. surgical exploration.

Based on the patient's symptoms and signs, the most reasonable choice is * * B. surgical exploration * *. The following is the analysis process:

The patient was a 42-year-old man with major symptoms including abdominal pain, abdominal distension, anal cessation of exhaust and defecation for 2 days, and the condition suddenly worsened this morning. Physical examination revealed total abdominal tenderness, rebound pain, muscle tension, and loss of bowel sounds, which are typical of acute peritonitis.

Acute peritonitis is usually caused by rupture of intra-abdominal organs, infection or ischemia and requires urgent surgical management. The patient's medical history showed an appendectomy 10 years ago, with possible intestinal adhesions or other complications, which further increased the possibility of acute peritonitis. Sudden aggravation of the abdominal pain suggests possible acute complications, such as intestinal perforation or bowel obstruction.

Therefore, considering the clinical manifestations and past medical history, the most reasonable treatment is emergency surgical exploration to clarify the cause and conduct corresponding treatment.

Analysis of the other options:

- ** A. Air defecation * *: Suitable for mild intestinal obstruction, but not for acute peritonitis.

- ** C. Persistent gastrointestinal decompression * *: Although helpful for intestinal obstruction, it is unable to resolve acute peritonitis or other serious complications.

- ** D. rehydration * *: Is part of supportive care, but does not solve the fundamental problem.

- ** E. Fasting * *: Also is part of supportive care, but it does not solve the fundamental problem.

Therefore, the most correct answer is * * B. Surgical exploration * *.

84. (Type A2 question)

A 28-year-old female, developed dry cough and wheezing due to cold air 2 weeks ago, and self-administered cephalosporins were ineffective. To clarify the diagnosis, further examination should be done

A. Bronchial challenge test

B. arterial blood gas analysis

C. Specific allergen detection

D. Blood eosinophil detection

E. Air NO (FeNO) test

Reference answer: A. Your answer:

[Analysis] Young women, two weeks ago due to cold air contact with dry cough,

wheezing (allergen contact history) antibiotic treatment is ineffective, considered as bronchial asthma. The bronchial challenge test (A pair) was important for assessing airway responsiveness, and a 20% decrease in FEV 1 indicates the presence of bronchial asthma.

[Fault avoidance] Arterial blood gas analysis (B error) The main test is to understand the body respiratory function and acid-base balance state; specific changes

Aggen testing (C error) had little value for asthma diagnosis; most asthma patients induced increased eosinophil counts in sputum

High (> 2.5%) and associated with asthma symptoms, but not a blood eosinophil test (D error); FeNO assay (E

Wrong) can be used as an indicator to assess the level of airway inflammation and asthma control, and can also be used to judge the reverse of inhaled hormone therapy

answer.

This case describes a 28-year-old woman who developed dry cough and wheezing after exposure to cold air, no fever and no effect with cephalosporins. Based on the condition description, this symptom may be associated with asthma. Asthma is a common chronic inflammatory airway disease characterized by an excessive airway response to various stimuli. Considering these symptoms, especially after exposure to cold air, the likelihood of asthma is higher.

A definitive diagnosis of asthma usually requires pulmonary function tests, and specific diagnostic tests may be required. Among the options provided, item A "bronchial provocation test" is A method commonly used to diagnose asthma and can detect airway responsiveness to stimuli. However, the more routine and non-invasive primary test method is the "exhaled air nitric oxide (FeNO) test" in item E. The FeNO test is a non-invasive method to evaluate airway inflammation, especially the inflammation associated with eosinophils, and it has great value in the diagnosis and management of asthma.

Given these factors, the best answer may be item E "exhaled breath NO (FeNO) test" because it is a simple, rapid and non-invasive test that can help diagnose asthma and is of great value for monitoring disease activity and guiding treatment.

analyze:

The young female patient, who developed dry cough, wheezing and no fever

after cold air exposure 2 weeks ago, was ineffective in self-administered cephalosporin antibiotics, and the patient was considered for the diagnosis of bronchial asthma. The typical symptoms of bronchial asthma are expiratory dyspnea with wheezing. Night and early morning attacks and aggravation are often one of the characteristics of asthma. It can attack in a few minutes, after several hours to several days, with bronchodilators or spontaneous relief. The disease is caused by a variety of cells and cell components involved in airway chronic inflammatory diseases, chronic inflammation associated with airway hyperreactivity, usually appear widely variable reversible airflow limitation, and cause recurrent wheezing, shortness of breath, chest tightness or cough symptoms, often at night and (or) in the morning attack, aggravation, most patients can relieve or after treatment. Bronchial asthma and cardiac asthma, asthma chronic bronchitis and other diseases, bronchial excitation test to determine airway responsiveness, commonly used inhalation stimulating agents have acetylcholine, histamine, mannitol, etc., by increasing the dose and dose of inhaled airway constrictants, observe the dose and response of bronchospasm, can judge whether the existence of airway hyperresponsiveness, for the diagnosis and differential diagnosis of bronchial asthma is of great significance.

Option B Arterial blood gas analysis refers to the technical process of analyzing different types of gases and acids in various gases and liquids, which can reflect lung ventilation and ventilation function, but cannot be used for the diagnosis of bronchial asthma, so it is not selected.

Option C-specific allergen test was used to search for allergens and provide the basis for desensitization treatment, rather than for the diagnosis of bronchial asthma, so it was excluded.

Option D Blood eosinophil test is mostly used for the examination of parasitic infection and anaphylaxis. The blood eosinophil count in bronchial asthma patients can be increased, but it is not specific and cannot be used for diagnosis, so it is not selected.

Option E exhaled breath NO (FeNO) test can be used as a biomarker of airway inflammation. FeNO level can reflect the eosinophilic inflammation of the airway, but its specificity and sensitivity are relatively low, so it is not a diagnostic test of bronchial asthma, so it is excluded.

In conclusion, the correct answer is: A. Bronchial provocation test.

This topic describes a 28-year-old female patient who developed dry cough and

wheezing after cold air and failed to self-administered cephalosporins. What further tests should be done in order to clarify the diagnosis?

From symptoms and background, patients may have asthma or allergic disease. Asthma usually presents as a dry cough and wheezing, especially after exposure to certain triggers (e. g., cold air).

To diagnose asthma, common tests include the bronchial challenge test, specific allergen test, blood eosinophil test, and exhaled breath NO (FeNO) test.

- ** A. Bronchial challenge test * *: assessing airway responsiveness by inhalation of specific stimuli (such as histamine or formamide) is one of the most important ways to diagnose asthma.

- ** B. Arterial blood gas analysis * *: It is mainly used to assess lung function and acid-base balance, and it is usually used in patients with severe dyspnea or chronic obstructive pulmonary disease.

- ** C. Specific allergen test * *: used to identify allergens, but can not directly confirm asthma.

- ** D. Blood eosinophil test * *: elevated eosinophils are common in asthma and allergic diseases, but eosinophils alone does not confirm asthma.

- ** E. Detection of exhaled breath NO (FeNO) * *: The level of nitric oxide found in exhaled breath can reflect airway inflammation and is often used in the diagnosis and monitoring of asthma.

In conclusion, in order to clarify the diagnosis of asthma, the most common examination method is the bronchial provocation test. Although the FeNO test is also useful, the bronchial provocation test is considered a more direct diagnostic approach.

So the best answer is:

* * A. Bronchial provocation test * *

85. (Type A2 questions)

A 23-year-old man with right lower abdominal pain, diarrhea, emaciation for 5 years, previous history of anal fistula, colonoscopy findings: congestion and edema in the ileocecum, cobblestone-like changes with multiple longitudinal ulcers, most likely

A. enterophthisis

B. amoebic enteritis

C. ischemic enteropathy

D. Crohn's disease

E. colitis gravis

Refer to the answer: D. Your answer:

[Analysis] A young male with right lower abdominal pain, diarrhea, emaciation (possibility of inflammatory enteritis), history of anal fistula, colonoscopy suggesting ileocecal congestion and edema, cobblestone changes, with multiple longitudinal ulcers (consistent with Crohn's disease), was considered to be Crohn's disease (D pair).

[Avoid avoidance] The intestinal tuberculosis (A wrong) lesion is usually near the ileocaeca, rarely involving the jejunum, and segmental changes are inferior to gram

Rohn's disease was obvious, and changes such as central necrosis or calcification of the abdominal lymph nodes were seen. Amoebic enteritis (B wrong) lesions of the main invasion

The right colon, which can also involve the left colon, with deep ulcers and deep margins. The mucosa between the ulcers was more normal.ischemic

Enteropathy (D error) mainly abdominal pain, intermittent hematochezia, intestinal emptying disorder, endoscopic manifestations including mucosal edema, fragility,

Segmental redness, scattered erosion, longitudinal ulceration, punctate hemorrhage, and purple bleeding nodules. Ulcerative colitis (E is wrong)

Colenoscopy showed blurred and disordered mucosal vascular texture, or disappeared congestion, and showed diffuse erosion and multiple superficial ulcers in the lesion.

This patient presented with right lower abdominal pain, diarrhea, wasting, and a history of anal fistula, and colonoscopy showed congestion and edema in the ileocecum, cobblestone-like changes with multiple longitudinal ulcers. These clinical findings and colonoscopy correspond with the characteristics of Crohn's disease (Crohn's disease). Crohn's disease is a chronic inflammatory bowel disease that can affect any part of the digestive tract, from the mouth to the anus, but is most common in the ileum and colon. Peboid intestinal changes and longitudinal ulcers are typical endoscopic findings in Crohn's disease.

So, the best answer is: D. Crohn's disease

analyze:

A young male patient with right lower abdominal pain, diarrhea with wasting, a history of anal fistula, colonoscopy showing congestion and edema in ileocecal cobblestone changes with multiple longitudinal ulcers, was considered for diagnosis of Crohn's disease. Crohn's disease is an intestinal inflammatory disease of unknown cause that can occur anywhere in the gastrointestinal tract but more frequently in the terminal ileum and right colon. This disease and chronic non-specific ulcerative colitis are collectively known as inflammatory bowel disease (IBD). The clinical manifestations are abdominal pain, diarrhea, and intestinal obstruction, with parenteral manifestations such as fever and nutritional disorders. The course of the disease is more prolonged, repeated attacks, not easy to have a radical cure. This disease is also known as localized enteritis, localized ileitis, segmental enteritis, and granulomatous enteritis.

Option A intestinal tuberculosis is A clinically common external pulmonary tuberculosis, which is A chronic infection caused by tuberculosis bacilli. Its ulcer shape is ribbon, and its long diameter is perpendicular to the intestinal axis, which is inconsistent with the ulcer shape of the patient, so it is not selected.

Option B The lesions of amoeba enteritis are mostly in the ileocecal colon, which is easy to relapse to chronic, deep ulcer, edge margin, and the mucosa between ulcers is mostly normal, which is not consistent with the symptoms of this question, so it is not considered.

Option C Ischemic intestinal disease is mainly manifested as sudden severe

abdominal colic, frequent nausea and vomiting, and mostly bloody diarrhea. Some patients have urgent urgency, which is not consistent with the symptoms of the patients in this question, so it is not selected.

Option E The lesions of ulcerative colitis are located in the large intestine and showed a continuous diffuse distribution, unrelated to this problem.

To sum up, the correct answer is: D. Crohn's disease.

According to the title description, a 23-year-old man had a history of right lower abdominal pain, diarrhea and wasting, and a history of anal fistula. Enteroscopy showed congestion and edema in the ileocecum with cobblestone-like changes with multiple longitudinal ulcers. These symptoms and findings most consistent with the presentation of Crohn's disease (Crohn's disease).

The characteristics of Crohn's disease include:

-Common lesions in the terminal ileum and ileocaeca

-Right lower abdominal pain

-chronic diarrhea

-become thin

-Anal fistula and other perianal lesions

-Peoid changes and longitudinal ulcers seen during colonoscopy

In conclusion, the most likely diagnosis is:

B Crohn's disease

86. (Type A2 question)

A 2-year-old boy with crying and abdominal pain for 18 days, with 2 jam-like stools. After physical examination, the right upper abdomen touched the "salami" package, slight tenderness, empty right lower abdomen, and loud bowel sounds. Abdominal B-ultrasound: the right upper abdominal mass is the "target ring sign".excellent diagnostics

A. acute appendicitis

B. Gene of mesenteric lymphadenitis

C. enteric volvulus

D. intestinal intussusception

E. Enteroroundworm

Refer to the answer: D. Your answer:

[Analysis] 2-year-old boy (intussusception in infants), crying, abdominal pain, jam stool, right upper abdominal touch mass (triad of intussusception: abdominal pain, bloody stool, abdominal mass), right lower abdomen emptiness, hyperbowel sound, considered as intussusception (D pair).

[Avoid wrong] Acute appendicitis (A wrong) mainly shows metastatic pain in the right lower abdomen; acute mesenteric lymphadenitis (B wrong)

It is more common in children, often with a history of upper respiratory tract infection, the abdominal tenderness site is medial, ultrasound or CT examination found abdominal lymph

Kt enlargement; small bowel volvulus (C wrong) as sudden onset severe, abdominal colic often persistent pain paroxysmal

Drama; intestinal roundworm (E wrong) often sudden and severe colic, can be accompanied by nausea, vomiting or vomiting

Out of the roundworm.

This question describes a 2-year-old boy who developed crying, abdominal pain and jam-like stool. During physical examination, the "sausage oid" package in the right upper abdomen with slight tenderness, empty right lower abdomen, and high bowel sounds. Abdominal B ultrasound shows a "target ring sign", which are typical symptoms and signs.

Option D "Intussusception" best fits these symptoms. Intussusception is one of the common acute abdomen diseases in children, especially in early and young children. It is a part of the intestinal tube sliding into adjacent intestinal segments, resulting in intestinal obstruction and poor blood supply. Intussusception often presents as periodic abdominal pain, abdominal lumps (salsalid lumps) and "jam-like" bloody stools. The "target ring sign" displayed by B ultrasound is also a characteristic ultrasound manifestation of intussusception.

Therefore, the best answer is D. intussusception.

analyze:

Children with 2 years old boy, because of crying, abdominal pain 18 days, jam stool, physical examination see right upper abdomen touch wax mass, mild tenderness, typical signs for intussusception, right lower abdomen emptiness, bowel voice hyperactivity, abdominal B ultrasound display right upper abdomen mass for the "target ring", for intussusception typical imaging performance, so considering the diagnosis of intussusception.

Option A Acute appendicitis was characterized by metastatic right lower abdominal pain, which could include right lower abdominal tenderness and rebound pain after physical examination. abdominal B ultrasound showed appendix swelling with A diameter greater than 6mm. The child had no metastatic right lower abdominal pain and signs of right lower abdominal tenderness, so it was excluded.

Option B gene mesenteric lymphadenitis is mostly common in children under 7 years old, usually in winter and spring, the typical symptoms are fever, abdominal pain, nausea, vomiting and other symptoms, common in the right lower abdomen, paroxysmal, spasmodipain, abdominal B ultrasound shows mesenteric lymph node enlargement, not with the symptoms of the patients, so it is not selected.

Option C Small bowel volvulus is typically manifested as a sudden onset of severe abdominal colic, mostly around the umbilicus, often persistent pain paroxysmal aggravation, pain can radiate to the back, frequent vomiting, and the symptoms of this child, so it is not considered.

Option E The intestinal roundworm shows repeated episodes of periumbilical pain, and the abdominal B-ultrasound examination will not show the "target ring sign", so it is not selected.

To sum up, the correct answer is: D. Intussusception.

According to the problem of the symptoms and signs, considering the boy crying, abdominal pain 18 days, jam sample stool 2 times, right upper abdomen hit "sausage sample" block, right lower abdomen empty, bowel sounds, and abdominal B ultrasound show right upper abdomen mass for "target ring", these characteristics are very typical, pointing to a common children acute abdomen —— intussusception.

Intussusception is a common disease causing acute abdominal pain in infants. It is usually manifested as paroxysmal crying, abdominal pain and jam-like stool.

During palpation, "sausage id" package can be felt in the right upper abdomen, and the "target ring sign" of abdominal B-ultrasound is a specific manifestation of intussusception. Therefore, the best diagnosis is:

B intestinal intussusception

87. (Type A2 question)

Pediatric patients, severe cough, muscle pain, should be treated with which one of the following drugs

A. streptomycin

B. gentamicin

C. penicillin

D. Azithromycin

E. INH

Refer to the answer: D. Your answer:

[Analysis] Severe cough is a typical sign of bronchobronchial pneumonia, and for the treatment of mycoplasma pneumonia, macrolide antibiotics are the first choice, such as azithromycin (D pair), erythromycin, roxithromycin, etc.

[Misavoidance] Streptomycin, isoniazid (AE wrong) for the treatment of TB; gentamicin (B wrong) for Gram-negative rods

Bacterial infections, such as Escherichia coli, Klebsiella bacteria; penicillin (C wrong) can be used in Staphylococcus aureus pneumonia.

When managing pediatric cases of severe cough and muscle soreness, it is very important to choose the right medication. These symptoms may be caused by multiple causes, such as viruses, bacterial infections, or other diseases. Therefore, selecting the most appropriate treatment without any more information (such as the etiology) can be challenging.

The drugs in the option belong to different classes of antibiotics or anti-infective drugs:

-A. Streptomycin: mainly used for the treatment of tuberculosis and some serious bacterial infections.

-B. gentamicin: It is an aminoglycoside antibiotic used for the treatment of serious bacterial infections.

-C. Penicillin: a β -lactam antibiotic widely used in the treatment of bacterial infections.

-D. Azithromycin: It is a macrolide antibiotic, often used in the treatment of respiratory tract infections, ear infections, etc.

-E. isoniazid: mainly used for the treatment of tuberculosis.

According to the title description, the main symptoms of pediatric patients are severe cough and muscle soreness, which may point to respiratory infection. In this case, it is more appropriate to choose drugs for possible respiratory TI treatment.

Therefore, the best answer may be * * D. azithromycin * *, because it is an antibiotic commonly used in the treatment of respiratory tract infections caused by various bacteria, such as bronchitis, pneumonia, etc. However, the actual selection of drugs should be determined based on the specific etiology and the doctor's diagnosis.

analyze:

Children with severe cough, muscle soreness, considering the presence of mycoplasma infection, macrolide antibiotics is the first choice for the treatment of mycoplasma pneumonia, including azithromycin is a new generation of macrolide antibiotics, antibacterial spectrum, effective for haemophilus influenzae, streptococcus, gram-positive bacteria, for mycoplasma, chlamydia and other atypical pathogens also have good antibacterial effect, commonly used in the treatment of mycoplasma pneumonia.

Option A streptomycin, option E isoniazid, are antitubercular drugs, not used for the treatment of mycoplasma infection, so they can be excluded together.

Option B Gentamicin belongs to aminoglycoside antibiotics, mainly used for the treatment of bacterial infections, especially those caused by gram-negative bacteria, and is not used for the treatment of mycoplasma infection, so it is not selected.

Option C penicillin belongs to β -lactam antibiotics, mainly used for infections caused by gram-positive cocci, bacilli, gram-negative cocci and spirochetes, not used for the treatment of mycoplasma infection, so it is excluded.

In conclusion, the correct answer is: D. azithromycin.

Pediatric patients with severe cough and muscle soreness, may be a respiratory

disease caused by bacterial infections. Given the applicability of common pathogens and antibiotics, the following is an analysis of the options:

A. Streptomycin: mainly used for the treatment of tuberculosis, not for common respiratory tract infections.

B. Gentamicin: mainly used for severe Gram-negative bacterial infections, with nephrotoxicity and ototoxicity, and not commonly used in respiratory tract infections.

C. Penicillin: used for streptococcal infections, but not for some respiratory pathogens (for example, mycoplasma).

D. Azithromycin: a macrolide antibiotic effective against common respiratory pathogens (including mycoplasma, chlamydia) and suitable for children.

E. Isoniazid: for the treatment of tuberculosis, not for common respiratory infections.

Taken together, the most suitable drug for severe cough and muscle soreness is azithromycin. Azithromycin covers extensive respiratory pathogens and is well tolerated in children.

So the best answer is:

F. Azithromycin

88. (Type A2 question)

Male, 54 years old, with cough with intermittent low fever for half a year, chest CT showed: right upper lung, multiple small plaque-like high density shadow, with a little cavity, reduced volume of the upper lobe of the right lung, the most likely diagnosis is

A. carcinoma of the lungs

B. pulmonary vasculitis

C. chronic pulmonary abscess

D. tuberculosis

E. pulmonary aspergillosis

Refer to the answer: D. Your answer:

Graphic analysis: [analytical] middle-aged men, cough, low heat (tuberculosis classic performance: low heat, night sweats, fatigue), chest CT: right upper lung, multiple small plaque high density shadow with a little hollow, right upper lobe volume reduction (accord with tuberculosis CT imaging, tuberculosis in the tip of the lung, lower back and basal segment, easy to form hollow and spread lesions), consider for tuberculosis (D).

[Avoid wrong] lung cancer (A wrong) is mainly manifested as irritant dry cough, hemoptysis, emaciation, the image performance is, rough edge, but

Shading with leaf segmentation or notch. Pulmonary vasculitis (B error) is mainly characterized by fever, fatigue, arthralgia and skin lesions, imaging

Examination can find blood vessel wall thickening, lumen stenosis and other lesions. Pulmonary abscess (C wrong) onset and poisoning symptoms, chills,

High fever, cough a large amount of pus, smelly sputum, imaging can be seen uniform large shadow cavity, common fluid level inside.pulmonary aspergillosis (E

Wrong): cough, sputum, hemoptysis, fever, chest pain, and a mobile mass in the cavity, upper edge arc

Form, and form with the surrounding air half-moon sign, adjacent to the pleura can be thickened.

This case describes a 54-year-old man with cough and intermittent hypothermia symptoms, chest CT showing multiple patchy dense shadows in the right upper lung with a small void, and a decrease in the volume of the right upper lobe. These symptoms and imaging features may point to several diseases, but we need to narrow down the possibility based on the features.

A. Lung cancer usually presents as A single mass, associated with irregular margins or enlarged hilar lymph nodes, rather than multiple small plaque, high-density shadows, so this option is unlikely.

B. Pulmonary vasculitis may cause multiple pulmonary lesions, but it is usually accompanied by systemic symptoms such as fever, weight loss, and generalized

weakness, and often shows diffuse pulmonary damage on CT, not specific to upper lobe reduction or emptiness.

C. Chronic lung abscesses may present as pulmonary lesions containing vovities, but these are usually large and often accompanied by distinct pathological walls, not exactly meeting the description of small patchy high-density shadows.

D. Tuberculosis is a very likely diagnosis because it can present as multiple small patchy high-density shadows, with void formation, especially in the upper lobe of the lung, and can also cause symptoms such as chronic cough, low fever, etc.

E. Pulmonary aspergillosis usually affects individuals with a compromised immune system, showing patchy shadows or holes in the lungs, but often with a background of immunodeficiency, and CT findings do not exactly match the description.

In conclusion, based on these symptoms and imaging features, the most likely diagnosis is D. tuberculosis.

First, we need to read the key information in the topic.

The patient, male, 54 years old, whose main symptoms were cough and intermittent low fever persisted for half a year. These symptoms are typical of TB. Chest CT showed multiple small patches and a little holes in the right upper lung, along with a decrease in the volume of the right upper lobe.

Now, we analyze the options based on this information:

A. Lung cancer: Symptoms of lung cancer may include cough, chest pain, and dyspnea, but the CT findings of lung cancer are usually not consistent with the title description, and lung cancer is not often accompanied by low fever.

B. Pulmonary vasculitis: This is an inflammatory disease involving the blood vessels in the lungs. The symptoms may include dyspnea, cough, and chest pain, but the CT findings do not match the description in the title.

C. Chronic lung abscess: This is a suppurative inflammation of the lungs due to infection. Although it may cause cavity formation, it is usually accompanied by high fever and other severe symptoms of infection, and the CT findings are not completely consistent with the title description.

D. Tuberculosis: Tuberculosis is an infectious disease caused by Mycobacterium tuberculosis. Typical symptoms include long-term cough, low fever, night sweats, and weight loss. On CT, tuberculosis may appear as multiple patchy high density shadow

and cavity, consistent with the description in the title.

E. Pulmonary aspergillosis: This is a pulmonary infection caused by Aspergillus species. The symptoms may include cough, fever, and dyspnea, but the performance on CT did not match the description in the title.

In conclusion, considering the patient's symptoms, low fever, and specific findings on CT, the most likely diagnosis is pulmonary tuberculosis.

Therefore, the answer is D. tuberculosis.

According to the information in the problem stem, the 54-year-old patient had a cough with intermittent low fever for half a year. Chest CT showed multiple small plaque-like high-density shadows in the right upper lung, with a little cavity, and reduced volume in the upper lobe of the right lung. The most likely diagnosis of these symptoms and imaging findings is pulmonary tuberculosis.

The explanation is as follows:

1. * * Lung cancer * *: Although lung cancer can cause cough and abnormal chest CT, it is usually not accompanied by intermittent low fever and cavity formation, especially multiple small patchy images are less like the manifestations of lung cancer.

2. * * Pulmonary vasculitis * *: Pulmonary vasculitis often presents as cough and lung lesions, but cavity formation and reduced lobe volume are not typical features.

3. * * Chronic lung abscess * *: Chronic lung abscess can also appear as empty lesions, but usually with obvious purulent sputum and more severe systemic symptoms.

4. * * Tuberculosis * *: The most common symptoms of tuberculosis are cough and low fever, CT manifestations are small plaque high density shadow and cavity, while the reduced lobe volume is also one of the characteristics of tuberculosis.

5. * * Pulmonary aspergillosis * *: Pulmonary aspergillosis can have similar imaging findings, but usually occur in immunocompromised patients with more severe systemic symptoms.

In conclusion, the most likely diagnosis is * * D. tuberculosis * *.

89. (Type A2 questions)

After subarachnoid anesthesia due to surgery, there is no discomfort, clear consciousness, a little fluid exudation of the incision, the position that should be taken is

A. Low half seat

B. High half seat

C.15°~30° head, high and low foot

C. Lower extremities were elevated 15 ° to 20 ° and head and trunk 20 ° to 30 °

D. horizontal position

Reference answer: E Your answer:

[Analysis] After subarachnoid anesthesia, the supine position (E pair) should be taken to prevent headache caused by cerebrospinal fluid extravasation.

[Avoid mistakes] After abdominal surgery, take the low semi-sitting position (A wrong) or slope position to reduce the abdominal wall tension. Carry out neck, chest and hand

After surgery, the high half sitting position (B wrong) is used to facilitate breathing and effective drainage. After craniocerebral surgery, if there is no rest

gram or coma, desirable 15°~30° head high low slope position (C wrong). Shock patients, the lower limbs should be raised 15°~

20°, head and trunk raised 20 ° (30 ° of D wrong).

After receiving subarachnoid anesthesia, if there is no discomfort and clear consciousness, there is a small amount of fluid exudation incision, the appropriate position is to reduce the cerebrospinal fluid pressure and promote the comfort of the patient. Option D, "Lower limb elevation 15 ° to 20 ° and head and trunk elevation 20 ° to 30 °" provides a position that can both promote fluid reflux and maintain moderate cerebrospinal fluid pressure. This position can help reduce CSF leakage and help prevent the occurrence of low CSF pressure symptoms.

Therefore, the best choice is D. lower limb elevation 15 ° to 20 ° and head and trunk elevation 20 ° to 30 °.

analyze:

Subarachnoid anesthesia is short for lumbar anesthesia or spinal anesthesia, that

is, local anesthesia drugs are injected into the subarachnoid space through the lumbar space to block the conduction function of some spinal nerves and cause the anesthetic effect of the corresponding innervated area. After subarachnoid anesthesia, in order to avoid low cranial pressure headache caused by cerebrospinal fluid loss, the pillow should be placed supine for 6~8 hours, that is, the supine position.

Option A low half sitting position is suitable for patients with cardiopulmonary insufficiency. Due to the decrease of the diaphragm and the expansion of the chest volume, it is conducive to breathing and the improvement of dyspnea. At the same time, the abdominal organs are moved down, which is conducive to the healthy side compensation, to reduce the symptoms and the ventilation function conducive to the lung health, which is inconsistent with the description of the problem stem, so it is excluded.

Option B is suitable for patients after neck and chest surgery or with severe cardiopulmonary diseases. Due to the downward movement of the diaphragm, the volume of the chest cavity is expanded, which reduces the compression of abdominal organs, increases lung capacity, is conducive to gas exchange, improves dyspnea, and can be eliminated.

Option C15°~30° head and high foot low is suitable for intracranial hypertension, can reduce intracranial pressure, prevent cerebral edema, does not meet the meaning of the question.

Option D Lower limb elevation 15°~20°, head and trunk elevation 20°~30°, i. e., for shock patients, raise lower limbs, conducive to venous blood return and increase cardiac output, while head and trunk elevation is conducive to breathing, which can be excluded.

In conclusion, the correct answer is: E. The supine position.

After subarachnoid anesthesia, if the incision is A little fluid exudation, but no discomfort and clear consciousness, the body position should be taken at this time is: * * A. Low half sitting position * *.

The low half sitting position can help reduce possible complications after subarachnoid anesthesia, such as headache. Through this position, it can effectively reduce the leakage of cerebrospinal fluid from the spinal puncture point, help prevent the occurrence of low cranial pressure syndrome, and also reduce the effects of

incision fluid exudation.

Resolution of the other options:

- ** B. High-half sitting position * *: It is usually used after certain specific operations, but it is not the best choice for patients after subarachnoid anesthesia.

- ** C.15°~30° head, high and low * *: Suitable for some situations requiring reduction of intracranial pressure, but it is not common in routine care following subarachnoid anesthesia.

- ** D. Lower limb elevation 15 ° to 20 °, head and trunk elevation 20 ° to 30 ° * *: This position may be helpful in some patients with shock or hemodynamic instability, but not applicable to this case.

- ** E. Recumbent position * *: Usually used for an initial short period after subarachnoid anesthesia, but prolonged supine may increase the risk of headache.

To sum up, the best answer is: * * A. Low half seat * *.

90. (Type A2 questions)

A 55-year-old female had recurrent abdominal pain, fatigue and a previous history of hepatitis B for 30 years. Physical examination: clear, 3cm under the liver rib, negative abdominal mobility turbidity. Laboratory: total bilirubin 30μmol/L,ALB20g/L,PT19.1s, B ultrasound: hepatic right anterior 4cm 3cm, solid, the preferred treatment measure is

A. chemotherapy

B. arterial embolism

C. targeted therapy

D. surgical operation

E. For absolute ethanol injection

Refer to the answer: D. Your answer:

Graphic resolution: [analytical] middle-aged and old women, repeated abdominal pain, fatigue, hepatitis b history of 30 years, liver enlargement, total bilirubin

(suggesting abnormal liver function), ALB, PT19.1s (PT normal reference range: 11~13s, prothrombin time PT more than normal value of 3 seconds is abnormal), B ultrasound: right anterior lobe 4cm 3cm solid light mass, considering primary liver cancer. Therapeutic resection of liver cancer is one of the most effective methods for the treatment of liver cancer, and surgery should be preferred (D pair).

[Wrong avoidance] Liver cancer is not sensitive to chemotherapy (A error) and radiotherapy. Arterial embolization (B error) for the treatment of unresectable liver cancer or

As an adjuvant therapy after liver CC resection. Targeted therapy (C error) is mainly used in adenocarcinoma patients in non-small cell lung cancer

A person. Anhydroethanol injection (E wrong) is mainly used for the treatment of metastasis and recurrent liver cancer.

According to the medical history and examination results of the 55-year-old female patient, she had a 30-year history of hepatitis B and now presented with recurrent abdominal pain, fatigue, liver enlargement (3cm below the liver), and a solid mass of 4cm 3cm in the right anterior part of the liver in the B-ultrasound examination. Laboratory results showed total bilirubin 30 μmol/L, albumin (ALB) 20g / L, prothrombin time (PT) 19.1 seconds, which indicated impaired liver function and coagulation.

Given that long-term infection with hepatitis B increases the risk of liver cancer (especially hepatocellular carcinoma), this patient's condition is likely to be associated with liver cancer. Therefore, the size, location, number and the overall health of patients need to be considered when choosing treatment.

Treatment in the options include:

-A. Chemotherapy: Usually used for advanced or inoperable cancer treatment.

-B. Arterial embolization: It is a local treatment that suppresses the growth of a tumor by blocking its blood supply.

-C. Targeted therapy: therapy targeting specific molecular or cellular pathways in Hcancer.

-D. Surgery: Surgery may be preferred if the tumor is limited and the patient's overall condition permits.

-E. absolute ethanol injection: mainly used in small tumors or as an adjunct to other treatments.

In this case, the preferred treatment is usually surgery (option D), especially if the tumor is not significantly spread, the size and location allow for surgical resection. Surgery can provide a chance of cure, especially when the tumor is confined to the liver. However, the final treatment option should be based on a more comprehensive evaluation including the exact nature of the tumor, staging as well as the overall health of the patient. Therefore, I would choose D. surgery as the preferred treatment measure.

analyze:

Middle-aged female patients, have a history of hepatitis B for 30 years, repeated abdominal pain, fatigue, for the typical clinical manifestations of liver cancer, see liver enlargement, laboratory examination suggest bilirubin, decreased liver function, albumin, prothrombin time extension, are impaired liver function, B super see liver right have substantial mass, combined with previous history of hepatitis B and B ultrasound performance, the patient with the most likely diagnosis of primary liver cancer. At present, the patient is conscious and has no ascites, considering the early stage of liver cancer. For patients in the early stage of liver cancer, the surgical resection should be carried out in time to prevent the progression of the disease.

Option A Chemotherapy is mostly used in patients with advanced cancer, which can prolong the survival period. At present, this patient is considered in the early stage of liver cancer and should be treated with surgery, so it is not selected.

Option B arterial embolization and option E absolute ethanol injection are all interventional therapy, suitable for primary liver cancer unsuitable for surgery or postoperative recurrence, not as the preferred treatment for liver cancer, so they are excluded.

Option C targeted therapy is the main treatment for solid malignant tumors in recent years, which can prolong survival, but has limited effect on patients with early liver cancer, so it is not selected.

In conclusion, the correct answer is: D. Surgery.

Based on the information provided in the title, the patient was a 55-year-old woman with recurrent abdominal pain, fatigue, and a 30-year history of hepatitis B. Physical examination showed 3cm below the ribs of liver, laboratory examination showed total bilirubin 30 µmol/L, ALB 20g / L, PT 19.1 seconds, and B ultrasound showed solid right anterior 4cm 3 cm of liver.

From this information, the patient may have liver cancer (hepatocellular carcinoma). In this case, the treatment plan needs to be decided based on the size, location of the tumor, liver function and the overall health of the patient.

Based on the patient's condition, the liver function was poor (low albumin, hyperprothrombin time) and the tumor diameter was 4cm 3cm, which suggests that hepatocellular carcinoma is possible and may not be suitable for radical surgery. Anhydrous ethanol injection (Ethanol injection) is a minimally invasive treatment, often used in patients with small liver cancer, especially in patients with poor liver function or not suitable for surgery.

Therefore, the preferred treatment measures should be:

B For absolute ethanol injection

91. (Type A2 questions)

Male, 28 years old, upper abdominal distension, pain and discomfort, accompanied by nausea and vomiting for 2 days, vomiting is a sour and bad night food, relieved after eating, often after catching cold. The etiology of this symptom is

A. Gastric horn ulcer

B. esophageal ulcer

C. Downdescending duodenal ulcer

D. Gastric ulcer

E. Global duodenal ulcer

Reference answer: E Your answer:

Text analysis: [analysis] young men, upper abdominal distension pain and discomfort, nausea and vomiting, vomiting for acid and smelly night food (suspected pyloric stenosis or obstruction), pain when hunger, relief after eating, considered for duodenal ulcer suspected complicated with pyloric stenosis or obstruction. Duodenal bulb ulcer (E pair) is more prone to be complicated with pyloric obstruction.

[Avoid mistakes] posterior wall ulcer of duodenal bulb is more prone to bleeding; gastric ulcer is more cancerous than duodenal ulcer; ulcer is perforated

From the small curved side of the stomach or the duodenal bulb.

The title describes a 28-year-old male patient who complained of upper abdominal distension, pain and discomfort, accompanied by nausea and vomiting for 2 days. The vomit was a sour and smelly night food, and the symptoms were relieved after eating, and often easy to attack after catching cold. Based on these symptoms, we can consider the diagnosis of an upper gastrointestinal ulcer.

Options A (gastric angle ulcer) and D (gastric body ulcer) are both different parts of the gastric ulcer and usually lead to increased pain after eating. Option B (esophageal ulcer) is usually associated with dysphagia and reflux symptoms, rather than a particular emphasis on relief after eating. Option E (bulb duodenal ulcer) and C (descending duodenal ulcer) are different parts of duodenal ulcer that are commonly characterized by postprandial pain relief.

According to the patient's symptoms, especially the pain relief after eating, it is more consistent with the clinical manifestations of duodenal ulcer. The duodenal bulb is the initial part of the duodenum, while the descending duodenum is the subsequent part. Given that duodenal bulb ulcers are more common and that this type of ulcer is prone to be affected by factors such as cold, the best answer is E.

analyze:

Young male patients, upper abdominal distension pain and discomfort, accompanied by nausea and vomiting, vomiting for the sour smell of common food, relieved after eating, often catch cold prone, considering the diagnosis of pyloric obstruction, its most common after the penetrating ulcer of the duodenal bulb. After penetrating ulcer of duodenal bulb, local inflammatory edema and pyloric smooth muscle spasm can lead to temporary obstruction, which can be relieved after conservative treatment such as gastrointestinal decompression and infusion, which is an inflammatory edema stenosis. With the repeated onset of ulcers, local scar formation and contraction can lead to permanent stenosis of the pylorus, then requiring surgical treatment.

Option A gastric ulcer and option D gastric ulcer, which usually show postprandial pain and have no risk of pyloric obstruction, can be excluded together.

Option B esophageal ulcer often presents as swallowing pain and dysphagia, which is not consistent with the description of the problem stem, so it is not selected.

Option C descending duodenal ulcer was characterized by nocturnal pain and

hunger pain, and the risk of pyloric obstruction was much lower than the duodenal bulb, so it was not considered.

In conclusion, the correct answer is: E. duodenal bulb ulcer.

According to the title description, the patient is a 28-year-old man with symptoms including upper abdominal distension, pain and discomfort, accompanied by nausea and vomiting for 2 days. The vomit is a sour and smelly night food, which is relieved after eating, and often attacks after catching cold. Based on these symptoms, the best diagnosis is a global duodenal bulb ulcer (E).

The analysis is as follows:

-Duodenal ulcers, especially duodenal bulb ulcers, often appear as upper abdominal pain, especially within 2-3 hours after eating, and eating can relieve the symptoms. This is consistent with the postfeeding remission described by the patient.

-The retention of gastric contents, which is one of the common symptoms of duodenal ulcer.

-Symptoms onset after cold may be related to neuromodulation and gastrointestinal function, and are also common in patients with duodenal ulcers.

Resolution of the other options:

-Pain in gastric horn ulcers and gastric body ulcers is usually not significantly associated with eating, and vomiting is less common.

-The esophageal ulcer mainly shows dysphagia and retrosternal pain, which is inconsistent with the symptoms of this case.

-Duodenal descending ulcers are less common, and the pain characteristics are similar to duodenal bulb ulcers, but less often vomiting.

So the best answer is:

F. Global duodenal ulcer

92. (Type A2 questions)

A 40-year-old man with 3 days of abdominal distension and vomiting, vomit was a 10-year history of duodenal ulcer to correct the possible disorder of water and electrolyte metabolism and acid-base balance. The liquid that should be applicable is

A. normal saline

B.5% glucose in saline

C.1.86% sodium lactate solution + 5% dextrose saline

D.10% KCl + 5% glucose saline

E.5% sodium bicarbonate

Refer to the answer: D. Your answer:

[Analysis] middle-aged men, the previous history of duodenal ulcer for 10 years, abdominal distension, vomiting for 3 days, may occur isotonic water shortage, should supplement the equilibrium fluid solution. After correcting the lack of water, the potassium discharge will increase and the serum K + concentration is also diluted and reduced due to the increase of extracellular fluid volume, and the serum potassium will be reduced. Attention should be paid to the prevention of hypokalemia, so preventive potassium supplementation should be added, so the patient should be supplemented with balanced salt solution and potassium supplementation (D pair).

[To avoid errors] Any blood volume reduction caused by the massive loss of isotonic fluid is isotonic dehydration in a short time.clinical

The common causes are: acute loss of digestive juice, such as intestinal fistula, massive vomiting, diarrhea; loss of body fluids in soft tissue,

Such as abdominal cavity or retroperitoneal infection, intestinal obstruction, burns, etc. Persistent loss of gastrointestinal digestive fluid will cause hypotonic deficiency

water.

In this case, the patient had a 10-year history of duodenal ulcer with abdominal distension and vomiting, indicating possible delayed gastric emptying or obstruction. Due to long-term vomiting, patients may have had electrolyte imbalance and acid-base balance imbalance. Usually, vomiting can cause the loss of gastric acid in the body, causing metabolic alkalosis and electrolyte imbalance, especially hypokalemia and hypochlorolaemia.

For this situation, the first treatment is to correct the disorder of the

water-electrolyte and acid-base balance. Choosing the most suitable fluid requires considering the patient's specific acid-base balance and electrolyte status. In general, in the presence of metabolic alkalosis, using fluids containing chloride can help correct the alkalosis and electrolyte imbalance.

-Saline (0.9% sodium chloride solution) is an isotonic solution that can be used to supplement body fluid and correct electrolyte imbalance.

-B The 5% glucose saline (5% glucose + 0.9% sodium chloride solution) also provides energy and electrolytes.

Item-C The 1.86% sodium lactate solution + 5% dextrose saline solution is an alkaline solution that is generally used to correct acidosis.

10% potassium chloride + 5% glucose saline in item D is used to supplement potassium, but high concentrations of potassium should be used to prevent hyperkalemia.

The 5% sodium bicarbonate in item-E is the alkaline solution used to correct the metabolic acidosis.

Considering the patient's symptoms and possible metabolic alkalosis, the most appropriate option is the need for chloride supplementation to help correct the fluid for the alkalosis. Therefore, item A saline may be the most suitable choice because it can help to correct the electrolyte imbalance and minor alkalosis. So, the correct answer is * * A. saline * *.

analyze:

A middle-aged male patient, with a previous history of duodenal ulcer, now has abdominal distension, vomiting, vomit as chronic food, and pyloric obstruction should be highly suspected. Long-term repeated vomiting can lose a lot of gastric juice, which contains a lot of H and Cl $^{-}$, easy to lead to low chloride alkali poisoning, treatment need infusion chlorine solution and acid liquid to correct alkalosis, 5% glucose saline both chlorine and sodium, can correct alkalosis, and correct electrolyte disorder, is the ideal solution for the treatment of this patient.

Option A normal saline is isotonic sodium chloride solution, and the effect of correcting alkalosis is limited, so it is not chosen.

Option C "1.86% sodium lactate solution + 5% dextrose saline", option E "5% sodium bicarbonate", both of which are alkaline solutions, which would further aggravate alkalosis, so they were excluded together.

Option D The potassium ion concentration in "10% potassium chloride + 5% glucose saline" is too high for direct infusion, and the patient did not show low potassium performance, without additional potassium supplementation, so it was not considered.

In conclusion, the correct answer is: B.5% glucose saline.

According to the title description, the patient had a history of duodenal ulcer, and currently had abdominal distension and vomiting for 3 days. The vomit was a chronic food, which suggests the possibility of gastrointestinal obstruction. Due to long-term vomiting, patients may have disordered water, electrolyte metabolism and disordered acid-base balance.

The patient vomited for a long time, lost a large number of chloride ions and hydrogen ions, prone to metabolic alkalosis. Therefore, correction of metabolic alkalosis and chloride supplementation are the focus of treatment.

In conclusion, the most appropriate liquid is saline (A), because saline (0.9% sodium chloride solution) supplements not only the water, but also the lost chloride ions to correct metabolic alkalosis.

The correct answer is:

A. normal saline

93. (Type A2 questions)

A 42-year-old male had abdominal pain with anal withdrawal for 2 days. Fasting, rehydration treatment, sudden abdominal pain worsened this morning. Previous appendectomy for more than 10 years. Physical examination: whole abdominal tenderness, rebound pain, muscle tension, bowel sounds disappeared. The most is

A. operations research

B. Continuous gastrointestinal decompression

C. spasmolytic medication

D. An adequate amount of antibiotics

E. air enema

Reference answer: A. Your answer:

[Analysis] middle-aged men, abdominal pain, abdominal distension, anal stop exhaust defecation (manifestations of intestinal obstruction), abdominal pain after fasting fluid treatment (medical treatment is ineffective, considering surgical treatment), have a history of appendicitis surgery (considering adhesive intestinal obstruction caused by postoperative intestinal adhesion). Physical examination: whole abdominal tenderness, rebound pain, muscle tension, bowel sounds disappeared. The patient developed peritonitis without medical treatment. Considering the strangulated intestinal obstruction, surgical exploration should be performed (A pair).

[Avoid mistakes] The following manifestations should be considered as strangulation intestinal obstruction: (1) abdominal pain onset, the initial is persistent

Severe abdominal pain; (2) the rapid development of the disease, early shock; (3) peritonitis; (4) the wrong abdominal distension

Said, there was a local bulge in the abdomen; (5) vomiting appeared early and frequently, vomiting was bloody, and blood fluid was extracted by abdominal puncture

Body; (6) X-ray; (7) aggressive non-operative signs.

This case describes a 42-year-old male patient with abdominal pain, abdominal distension and cessation of exhaust defecation for 2 days with a previous history of surgical appendectomy. At present, the symptoms are aggravated, and the physical examination found that the total abdominal tenderness, rebound pain, muscle tension, and the bowel sounds disappeared. These clinical manifestations imply that acute intestinal obstruction or peritonitis may have occurred and requires urgent management.

Item A (surgical exploration) is necessary in an acute abdomen, especially suspected peritonitis or severe intestinal obstruction, when conservative treatment is ineffective or urgent to establish the diagnosis and removal of the obstruction.

Item B (persistent gastrointestinal decompression) relieves symptoms, but it does not address the underlying problems and is usually used as a pre-operative preparation or adjunctive treatment.

Item C (spasmodic drug therapy) is used to relieve smooth muscle spasm, but it

is not appropriate as the preferred treatment in the acute abdomen because it may mask the severity of the condition.

Item D (adequate antibiotics), although important for infectious abdominal disease, can not be used as the only treatment in the absence of a clear infection source and surgical treatment.

Item E (air enema) is usually used for the diagnosis and treatment of mild to moderate intestinal obstruction, but it is not appropriate in acute, severe cases, especially when there is a risk of peritonitis or severe obstruction.

Taken together, the most appropriate option is A. surgical exploration, as the patient's symptoms and signs indicate the possibility of urgent intra-abdominal problems such as intestinal obstruction or peritonitis, which need to be diagnosed and treated by surgery.

First, we need to carefully interpret the patient's case information. The patient was a 42-year-old man with major symptoms of abdominal pain and abdominal distension, which had been present for 2 days without defecation and exhaust. Despite fasting and fluid rehydration therapy, the abdominal pain suddenly worsened this morning. His abdominal physical examination revealed total abdominal tenderness, rebound pain, muscle tension, and loss of bowel sounds, which are typical of peritoneal irritation signs. Combined with the previous history of appendectomy, we can initially suspect that there may be intestinal obstruction, and the current signs of peritonitis, indicating that the disease may have progressed from simple intestinal obstruction to strangulated intestinal obstruction.

Next, we analyze the options one by one:

A. Surgical exploration: For patients with suspected strangulation intestinal obstruction, emergency surgical treatment is needed to avoid intestinal necrosis, perforation and secondary peritonitis, as intestinal blood disorders may already occur. This is the preferred treatment option.

B. Persistent gastrointestinal decompression: This measure is usually used in the conservative treatment of simple intestinal obstruction, reducing the intestinal pressure through gastrointestinal decompression. However, in the case of strangulation intestinal obstruction, it is not enough to simply perform gastrointestinal decompression due to the presence of intestinal blood disorders.

C. Sspasmolytic drug therapy: This kind of drugs are mainly used to relieve

smooth muscle spasm, but in the case of strangulated intestinal obstruction, drug therapy does not solve the fundamental problem.

D. Enough antibiotics: Although antibiotics may prevent or treat secondary infections, they do not solve the fundamental problem of intestinal obstruction.

E. Air enema: This method is usually used for the treatment of pediatric intussusception and is not suitable for adult strangulation ileus.

In conclusion, surgical treatment should be preferred given that the patient's condition may have progressed from simple to strangulated ileus and had signs of peritonitis. So, the correct answer is: A. Surgical exploration.

This title describes a 42-year-old male patient with symptoms including abdominal pain, abdominal distension associated with anal stop defecation for 2 days. The patient had a sudden abdominal pain aggravated this morning after fasting and rehydration treatment. Physical examination revealed total abdominal tenderness, rebound pain, muscle tension, and disappeared bowel sounds. These signs suggest the possible presence of acute peritonitis, possibly due to bowel perforation or bowel obstruction.

Considering the patient's previous surgical history (appendectomy 10 years ago), the possibility of intestinal adhesion existed, which further increased the possibility of bowel obstruction. And the sudden abdominal pain and total abdominal tenderness, rebound pain and muscle tension and other signs are very consistent with the performance of acute peritonitis.

In this case, the most urgent and effective management method is the immediate surgical exploration (A). Surgical exploration can identify the cause and conduct necessary treatment (such as removal of intestinal obstruction, repair of intestinal perforation, etc.) to avoid further deterioration of the condition. Other options (such as continuous gastrointestinal decompression, spasmolytic drug therapy, adequate antibiotics, and air enema) do not fundamentally solve the patient's acute condition, which may lead to delayed treatment and increase the risk of complications.

So the best answer is:

A. operations research

94. (Type A2 question)

Children, 4 years old. The left upper abdomen was found as the size of a fist, hard, movable, no tender, and normal urination. First consideration should be given

A. Left hydronephrosis

B. Left nephron cell tumor

C. splenauxe

D. pancreatic cyst

E. nephrophthisis sinistro

Reference answer: B. Your answer:

[Analysis] Wilms cell tumor occurs in children under 5 years old, abdominal mass is the most common symptom, smooth surface, medium hardness, no tenderness, and a certain degree of motion.

In this case, considering the age of the child (4 years) and symptoms (left upper abdomen like fist size, hard, active, no tenderness, normal urine), the most likely diagnosis was * * B. left nephms tumor * *.

The explanation is as follows:

-A. The left hydronephrosis (Hydronephrosis) is usually associated with pain or urination problems.

-B. Left Wilms Blastoma (Wilms tumor) is the most common malignant renal tumor in children, characterized by a palpable mass of the abdomen, usually painless, consistent with this case.

-C. Splspleen (Splenomegaly) may be caused by various reasons, but usually does not appear as an moable hard block.

-D. Pancreatic cysts (Pancreatic cyst) are relatively rare in children and usually do not present as a rigid mobile mass.

-E. Left renal tuberculosis (Renal tuberculosis) is very rare and is usually associated with systemic symptoms, such as fever, nocturnal sweating, and weight loss.

Therefore, based on the information provided and the clinical presentation, the

most likely diagnosis is a left nephroblastoma.

analyze:

A 4-year-old child with a left upper abdominal mass and no other abnormal findings, combined with the patient's symptoms and signs, made the most likely diagnosis of the child was left Wilms tumor. Wilms cell tumor is the most common malignant tumor in children, most of which occur before 5 years old, and occasionally seen in older children and adults. The most common symptom is abdominal mass, and the surface of the mass is smooth, hard, no tenderness, and can be active with breathing.

Option A left hydronephrosis can have abdominal pain, dysuria, hematuria and other symptoms, and the general abdominal mass is not obvious, so it is not chosen.

Option C spleen size can be seen in some virus infection, bacterial infection, rickettsia infection, parasitic infection, etc., generally the corresponding symptoms of the original disease will appear, but not mentioned in the question stem, so it is excluded.

Option D Pancreatic cysts are often secondary to pancreatitis or upper abdominal trauma, mainly manifested as upper abdominal pain, fullness, and can radiate to the back, accompanied by nausea, vomiting, decreased appetite, and not consistent with the description of the problem, so it is not selected.

Option E left kidney tuberculosis is more common in young adults, the typical manifestations of bladder stimulation symptoms, accompanied by terminal hematuria, low fever, night sweats and other tuberculosis poisoning symptoms, generally will not appear abdominal mass, so excluded.

In conclusion, the correct answer is: B. Left nephroblastoma.

According to the title description, the child was 4 years old, and the left upper abdomen mass was about the size of a fist, hard, active, with no tenderness, and normal urination. Combined with these clinical findings, the most likely diagnosis is:

B Left nephron cell tumor

Left Wilms 'tumor (also known as Wilms' tumor) is the most common renal

malignancy in children, often presenting as an abdominal mass. The masses are usually painless, hard, movable, and of the size of a fist. Acute urinary lesions such as left hydronephrosis or left renal tuberculosis were excluded due to normal urination. Splenomegalis and pancreatic cysts also cause abdominal mass, but combined with the age of the child and the characteristics of no tenderness, left nephroblastoma is the most likely diagnosis.

So the best answer is:

C Left nephron cell tumor

95. (Type A2 question)

Male sex, aged 65 years old. Progressive dysuria has been difficult for 1 year, unable to urinate spontaneously for 8 hours, bladder swelling, and mild tenderness. The preferred treatment approach should be

A. acupuncture and moxibustion

B. Suprapubic bladder puncture

C. catheterization and keeping the catheter

D. Suprapubic cystostomy

E. medication

Reference answer: C Your answer:

[Analysis] Patients may have prostate disease leading to urinary bladder retention, emergency treatment is to solve the pressure in the bladder, before the disease is not eradicated, retain the urinary catheter.

In this case, the patient was a 65-year-old man who presented with progressive dysuria for 1 year and had recently been unable to urinate spontaneously for 8 hours with cystococele and mild tenderness. These symptoms indicate that the patient may have acute urinary retention, an emergency that requires immediate management.

For this condition, the preferred treatment is the immediate removal of urinary retention, which is usually achieved by catheterization. Therefore, option C (catheterization and catheter retention) is the most appropriate option. The catheter

preservation can temporarily alleviate the problem of urinary retention and give the physician time to evaluate the patient's condition and plan further treatment strategies. Other options, such as acupuncture, bladder puncture, fistula, or medication alone, are not suitable for the immediate remission of acute urinary retention.

Therefore, the best answer is * * C. catheterization and keep the catheter * *.

This is a topic involving urinary system diseases. We need to analyze the patient's symptoms, determine the type of disease, and then choose the most appropriate treatment plan.

Background information: The elderly male patient, who had progressive dysuria for one year, suddenly failed to urinate for 8 hours, cystoele and mild tenderness. These symptoms strongly suggest acute urinary retention due to benign prostatic hyperplasia.

At the heart of understanding the problem: We need to determine which treatment is preferred based on the patient's symptoms.

Next, we analyze each option one by one:

Option A (Acupuncture): Although acupuncture may be helpful for urination in some cases, it is not the preferred treatment for acute urinary retention.

Option B (suprapuic bladder puncture): This is an emergency treatment that is usually used for failed catheterization failure or when the patient is in critical condition.

Option C (catheterization and catheter preservation): For acute urinary retention, the first priority is to empty the bladder quickly and effectively. Urinary catheterization is a rapid, simple and effective method, while preserving the urinary catheter ensures continuous bladder emptying and avoids reurinary retention.

Option D (Suprapupubic cystostomy): This is usually an alternative when catheterization fails or the patient is exceptional, and is not preferred.

Option E (medication): While medication may be effective for chronic symptom management, it is slow in acute urinary retention and cannot resolve problems quickly.

In conclusion, considering the patients need to empty the bladder quickly and effectively, the catheterization and catheter preservation are the most direct and effective preferred treatment while avoiding further complications.

Therefore, the answer is to perform C. catheterization and preserve the catheter.

Based on the patient's age and symptom presentation, male, 65 years old, with 1 year progressive vosuria, now unable to urinate spontaneously for 8 hours, cystoele with mild tenderness, these symptoms are suggestive of possible acute urinary retention. The primary management measure for acute urinary retention is the immediate stress relief of the bladder to prevent further bladder and kidney damage.

In this case, the most appropriate preferred treatment is catheterization and catheter preservation, which can immediately relieve pressure on the bladder, prevent further complications and aid in the diagnosis and treatment of the underlying causes of dysuria. So the best answer is:

F. catheterization and keeping the catheter

96. (Type A2 questions)

In patients with hematuria, there was a 2.5cm×1.0cm new organism on the right wall of the bladder, pedicle, 3cm from the right ureter orifice, and biopsy for diagnosis of T1 transitional cell carcinoma. The preferred treatment approach is the

A. urinary bladder perfusion with chemotherapy

B. Transurethral resection of urinary bladder tumors

C. A partial cystectomy

D. Total total bladder resection

E. radiotherapy

Reference answer: B. Your answer:

Graphic analysis: [Analysis] In principle, Ta, T1 and localized T2 tumor bladder sparing surgery, the patient's tumor pedicle, the B option is more suitable.

In this case, for patients with hematuria, the cystoscopy showed a 2.5cm×1.0cm led new organism on the right wall of the bladder, and the biopsy was diagnosed as transitional cell carcinoma T1. The most suitable treatment was to choose B. Transurethral resection of bladder tumor.

T1 transitional cell carcinoma means that the tumor invaded only the inner layer of the bladder and not yet deep into the myometrium. At this stage, transurethral resection of bladder tumors (TURBT) is the preferred treatment. This procedure enables accurate assessment of the depth of invasion and removal of the visible tumor. This method is suitable for superficial bladder cancer, especially for T1 stage or lower grade lesions. Other options such as bladder perfusion chemotherapy, partial cystectomy, total cystectomy, or radiotherapy are often treatments considered for higher-grade or more aggressive tumors. Therefore, for this case, the most appropriate option is B. transurethral bladder tumor electroresection.

analyze:

The patient had hematuria, and a new tissue on the right side of the bladder. Pathpathological biopsy diagnosed the transitional cell carcinoma stage T1, T1, when the tumor invaded the subepithelial connective tissue, but did not reach the muscle layer. Transurethral bladder tumor resection (TURBt) for Ta and T1 tumors is the most commonly used clinical operation. It has small trauma and fast recovery, and can be repeated repeatedly. It can not only be used for tumor resection, but also to understand the pathological grade and clinical stage of the tumor, so as to formulate the next treatment plan.

Option A Bladder perfusion chemotherapy is mainly used for postoperative adjuvant therapy for carcinoma in situ, well-differentiated non-muscle-invasive bladder cancer and preoperative neoadjuvant therapy for advanced myo-invasive bladder cancer, which is not used as the preferred treatment, so it is not selected.

Option C Partial cystectomy is suitable for low-grade, localized bladder tumors in T2 and T3 stages, or for patients who cannot tolerate total bladder resection, inconsistent with the question.

Option D Total cystectomy is suitable for multiple, recurrent, poorly differentiated, non-muscle-invasive or high grade carcinoma in situ, and myoinvasive bladder cancer, inconsistent with the topic stem description.

Option E radiotherapy was not as effective as radical total bladder resection, and only had the effect of relieving obstruction and relieving symptoms. It was mostly used for patients who lost the opportunity of radical surgery, so it was excluded.

In conclusion, the correct answer is: B. Transurethral bladder tumor electroresection.

In patients with hematuria, cystoscopy revealed a new organism of 2.5cm×1.0cm on the right wall of the bladder, and the biopsy diagnosis of transitional cell carcinoma T1 (tumor is limited to the submucosa). The best treatment was transurethral resection of bladder tumor (TURBT). TURBT Is a minimally invasive procedure that effectively removes the tumor while preserving bladder function.

Therefore, the preferred treatment approach is to:

F. Transurethral resection of urinary bladder tumors

97. (Type A2 questions)

Male sex, aged 25 years old. After riding straddle perineal trauma, you can not urinate by yourself, perineal blue and scrotal hematoma, catheter can not be inserted into the bladder.mainly

A. scrotal injury

B. urethral injury in the penis

C. Globbar injury of the urethra

D. The posterior urethral injury

E. bladder injury

Reference answer: C Your answer:

[Analysis] The male urethra takes the genital diaphragm as the boundary before and after the urethra, the anterior urethra includes the bulb and the penis, and the posterior part includes the prostate and the membrane. During perineal riding cross injury, the urethra is squeezed under the pubis joint, causing ball injury.

This question describes a man who is unable to urinate, perineal blue and scrotal hematoma, and a catheter that cannot be inserted into the bladder. These symptoms point to the damage in the urinary system, especially in the urethra.

Option A "scrotal injury" may accompany such trauma, scrotal injury alone usually does not directly lead to the problem of failure to insert the catheter. Option E "bladder injury" may cause dysuria, but in the case of perineal trauma, it is less likely to directly cause bladder injury without affecting the urethra. Thus, the most likely

location of injury is the urethra, especially where riding stradinjuries are common.

Given the symptoms of the inability to urinate spontaneously and the ininsertion of the catheter into the bladder, and the mechanism and location of the injury, it is most likely that the urethra was injured. The urethra can be divided into the anterior and posterior urethra, where the bulbar urethra is part of the anterior urethra. Since the injury is caused by the riding-span perineal trauma, it is more likely to be the damage to the more anterior part of the urethra.

So the most appropriate answer is:

F. urethral injury in the penis

This answer is judged based on the nature and location of the injury, and the accompanying clinical symptoms. The urethra injury to the penis is common under such mechanisms, and the damage can explain why the catheter fails to insert properly into the bladder.

analyze:

The young male patient, with a history of perineal trauma, was unable to urinate by himself, the perineal blue and scrotal hematoma, and the catheter could not be inserted into the bladder. Combined with the patient's medical history and clinical manifestations, the patient first considered the urethral ball injury. Male anterior urethral trauma mostly occurs in the ball part, and this segment of the urethra is fixed in the perineum. During the perineal riding injury, the urethra is pushed below the pubis joint, causing ball injury.

Option A scrotal injury is mainly manifested as scrotal swelling and pain, accompanied by local congestion, violet or hematoma. After severe injury, perineal hematoma, urinary extravasation, urethral bleeding and other symptoms appear, but patients can generally urinate by themselves, which is not consistent with the symptoms of the patients in the main problem, so it is excluded.

Option B The urethral injury of the penis mostly occurs at the junction between the urethral overhang and the ball. Because the urethra is not fixed and has a large range of activity, the chance of injury is small, so it is not selected.

After option D, urethral injury is more seen in pelvic crush injury, often combined with pelvic fracture, inconsistent with the meaning of the question, so it is excluded.

Option E Patients with bladder injury often have dysuria, hematuria and

abdominal pain, which are inconsistent with patient performance and can be excluded.

In conclusion, the correct answer is: C. urethral bulb injury.

According to the information provided by the problem stem, a 25-year-old man riding trans-perineum was unable to urinate, perineal cyanum and scrotal hematoma, and the catheter could not be inserted into the bladder. These symptoms and signs suggest that the possible injury site should be the urethral bulb. Riding span perineal trauma is common in the perineal impact, easy to cause urethral ball injury.

So the best answer is:

G. Globbar injury of the urethra

98. (Type A2 questions)

Male sex, aged 50 years old. He was admitted for gastric cancer with severe anemia and had no history of blood transfusion. Hb 56 g / L was tested to correct anemia. The following most appropriate transfusion therapy are

A. Whole blood transfusion

B. Confusion of concentrated erythrocytes

C. Wash throwashed washed transwashed

D. Red blood cells with to leukocytes

E. Frozen red blood cells fused

Reference answer: B. Your answer:

Input of concentrated red blood cells before surgery can correct anaemia, hypoproteinemia, and concentrated red blood cells can improve the oxygen-carrying capacity.

In this case, the patient had severe anemia (Hb 56g / L) and required a blood transfusion to improve the hemoglobin level. The most appropriate transfusion treatment depends on the patient details and needs, but usually, concentrated red blood cells (RBC) are chosen because they are effective in increasing hemoglobin levels while reducing the volume load.

- ** A. Whole blood transfusion * *: Whole blood contains red blood cells, white blood cells, platelets, and plasma. Usually used when massive bleeding requires supplementary blood volume and blood cell components, whole blood may not be optimal for this patient because the main problem is severe anemia.

- ** B. concentrated red blood cells * *: concentrated red blood cells are mainly used to treat anemia and improve hemoglobin and red blood cell content, which is the most appropriate choice for patients who need increased oxygen transport capacity but do not need additional plasma.

- ** C. Washing red blood cells * *: Washing red blood cells are red blood cells that remove most plasma and white blood cells. They are often used in patients with allergy to plasma proteins or avoid specific immune reactions, and are not the first choice in this case.

- ** D. Red blood cells fused * *: The red blood cells are mainly used to reduce transfusion-related immune reactions and infectious problems. Although more suitable than whole blood, it is not the optimal choice.

- ** E. Frozen red blood cells * *: Frozen red blood cells are mainly used for long-term storage of specific red blood cells. They are suitable for patients with rare blood types, not in this case.

Therefore, the most appropriate option is * * B. Confusion of concentrated red blood cells * *. This type of blood transfusion aims to improve patient hemoglobin levels while avoiding excessive fluid loading and is the best option for patients with severe anaemia.

analyze:

Middle-aged male patient with gastric cancer with severe anemia. Preoperative tests indicated a significant decrease in hemoglobin, suggesting small cellular hypochromic anemia, or iron deficiency. For this patient, it is urgently needed to correct the anemia, so the concentrated red blood cells are the most appropriate transfusion therapy.

Option A: Whole blood transfusion was mainly used for the treatment of massive bleeding caused by various causes. The patient did not have bleeding, so it was excluded.

Option C transfusion and washing red blood cells are mainly used for febrile reactions to leukocyte lectin and renal insufficiency cannot tolerate high potassium in

the blood stock, which is not consistent with the symptoms of the patient, so it is not selected.

Option D transfusion to white blood cell red blood cells are mainly used for white blood cells or platelet after blood transfusion antibodies, cause fever blood transfusion reaction, or expected need repeated blood transfusion patients, such as barrier, leukemia, myelodysplastic syndrome, hematopoietic stem cell transplantation, patients, to prevent white blood cell antibodies, the patient has no blood transfusion, so excluded.

Option E When thawing frozen red blood cells, a certain amount of sodium chloride injection should be added to prevent the atrophy of cells due to the melting of ice crystals in the cells, resulting in the death of red blood cells, which is rarely used and can be excluded.

In conclusion, the correct answer is: B. concentrated red blood cells.

According to the patient's condition and existing medical guidelines, considering that he was hospitalized for gastric cancer with severe anemia and had no history of transfusion, the most suitable transfusion treatment regimen was transfusion of concentrated red blood cells. Infusion of concentrated red blood cells can be effective in increasing patient hemoglobin levels and improving anemia symptoms, while avoiding excessive fluid load and other potential risks that may be caused by whole blood transfusion. Here is an explanation of the options:

A. Whole blood transfusion: whole blood transfusion contains red blood cells and plasma, but for the correction of anemia alone, plasma transfusion is not required, and the fluid load of whole blood transfusion is large, which is not the first choice.

B. Transconcentrated red blood cells: concentrated red blood cell is preferred because it can effectively increase hemoglobin levels and reduce unnecessary fluid load.

C. Washing red blood cells: mainly used in patients allergic to plasma proteins or with repeated transfusion reactions, not the first choice in general.

D. Red blood cells fused: mainly used for patients with immune responses to white blood cells or to prevent transfusion-related white blood cell reactions, and is not routinely preferred.

E. Frozen red blood cells: usually used for rare blood types or conditions requiring long-term blood preservation, a routine choice for non-emergency correction of anemia.

Therefore, the most suitable option is:

F. Confusion of concentrated erythrocytes

99. (Type A2 questions)

Female, age 59. Because of breast tumor under epidural anesthesia, more bleeding, that is, "AB" red blood cell suspension 2 units, when input 20ml, chills, chest tightness, chest and back pain, shortness of breath, pulse down, immediately stop blood transfusion, after 1 hour, the body temperature rose to 39℃, urination once, a strong tea like, less quantity. The most likely adverse transfusion reaction was

A. non-hemolytic febrile transfusion reaction

B. blood transfusion-related acute lung injury

C. A spontaneous, hemolytic transfusion reaction

D. The bacterial contamination reaction

E. anaphylaxis

Reference answer: C Your answer:

Graphic and text analysis: urination once, strong tea like, less amount, indicating hemoglobinuria, indicating hemolytic reaction.

The 59-year-old woman received blood transfusion during radical mastectomy and developed chills, chest tightness, chest and back pain, shortness of breath and accelerated pulse, followed by a body temperature rise to 39℃, low urine output and strong tea-like urine. These symptoms indicate an acute and severe transfusion reaction. Based on the described symptoms, especially strong tea-like urine, this is usually indicative of hemolytic events where the red cells are destroyed, releasing hemoglobin into the circulation and ultimately excreted by the kidneys.

Therefore, the most likely adverse transfusion reaction is the C. serie hemolytic transfusion reaction. This type of transfusion reaction occurs rapidly, usually due to an ABO mismatch, causing acute symptoms such as chills, fever, chest pain, back pain,

shortness of breath, and changes in the color of the urine.

analyze:

The elderly female of the patient had more bleeding during mammatoma surgery, including chills, chest tightness, chest and back pain, rapid breath, accelerated pulse, elevated body temperature and strong tea-like urine, so the diagnosis of acute lung injury related to blood transfusion was considered. Blood transfusion related acute lung injury is due to the blood donors in plasma or human leukocyte antigen specific antibodies, when these heterogeneous plasma protein into the blood body, with the blood leukocyte antigen antibody reaction, lead to leukocyte agglutination and release of inflammatory media, cause lung capillary damage, and cause acute lung injury. Clinically, acute dyspnea, bilateral pulmonary edema and hypoxemia can be accompanied by fever and hypotension.

Option A Non-hemolytic febrile transfusion reaction refers to the increase of body temperature caused during or after transfusion. Fever reaction is the most common transfusion reaction, but generally does not appear dyspnea, so it is not selected.

Option C namely sexual hemolytic blood transfusion reaction is due to input blood incompatibility caused by hemolysis reaction, the reaction onset urgent, rapid progress, appear immediately after input a small amount of heterogeneous blood vein, swelling, pain, chills, high fever, dyspnea, back pain, heart rate and blood pressure drop, shock, then hemoglobin urine and jaundice, severe cases can appear oliguria, anuria and acute renal failure, with the patient's symptoms, so excluded.

Option D Bacterial contamination reaction If the contaminated bacteria are less virulent and the number is small, only systemic infection symptoms can be found. If the number of contaminated bacteria is large and virulence, endotoxic shock and DIC can appear immediately, which is inconsistent with the symptoms of this patient, so it is not selected.

Option E allergic reaction occurs a few minutes after blood transfusion, manifested as localized or generalized pruritus or urticaria. In severe cases, bronchospasm, dyspnea, shock, or even death, but no urine color change, which is inconsistent with the question.

In conclusion, the correct answer is: B. Blood transfusion-related acute lung injury.

According on the patient's symptom description, the most likely adverse transfusion reaction was an immediate hemolytic transfusion reaction. The characteristics of the reaction include:

1. Patients develop chills and fever during or shortly after blood transfusion.

2. Chest tightness, chest and back pain, and shortness of breath.

3. Pulse change.

4. Temperature rises.

5. Abnormal urination (strong tea-like urine, suggesting hemoglobinuria).

These symptoms match the typical manifestation of an immediate hemolytic transfusion reaction, so the answer is:

F. A spontaneous hemolytic transfusion reaction

100. (Type A2 questions)

Male sex, aged 50 years old. In recent months, often irritating cough, no fever, the application of cough agent and antibiotic treatment is ineffective. X-ray ray: 4cm×3.5cm block shadow of the lower lobe of the right lung. The diseases that should be considered are

A. alveobronchiolitis

B. tuberculosis

C. carcinoma of the lungs

D. pulmonary abscess

E. bronchial adenoma

Reference answer: C Your answer:

Graphic analysis: 4cm×3.5cm block shadow of the lower lobe of the right lung indicates occupancy."Iritant" cough fits the diagnosis of lung cancer.

In this case, a 50-year-old man presented with a persistent irritating cough without fever that was refractory to cough suppressants and antibiotic therapy, and an

X-ray ray showed a massive shadow of 4cm×3.5cm in the lower lobe of the right lung. Based on these symptoms and the X-ray results, the most likely diagnosis is lung cancer (option C).this is because:

-Persistent cough and failure to conventional treatment suggest possible malignancy.

-Block shadows on X-ray photography usually indicate the presence of tumors, especially in the absence of signs of fever and infection.

-Lung cancer is more common in middle-aged and older adults, especially in individuals with a long smoking history.

Although bronchopneumonia (A), tuberculosis (B), lung abscess (D), and bronchial adenoma (E) can also show A massive chest X-ray shadow, lung cancer is the most likely diagnosis based on the described clinical symptoms and X-ray features described. Therefore, the correct answer should be C. lung cancer.

analyze:

Middle-aged and elderly male patients with irritant cough for more than a month, cough suppressive agent and antibiotic treatment was ineffective, imaging examination showed a massive shadow of the lower lobe of the right lung, according to their clinical manifestations and imaging characteristics, lung cancer should be highly suspected.

Option A bronchial pneumonia onset sudden or slow, most before the first mild upper respiratory tract infection, light first runny nose, light cough, low heat, difference, after suddenly high fever, cough, shortness of breath and disease, cough is frequent, early for excitant dry cough, extreme cough but reduce, convalescent cough with phlegm, the patient without the above performance, so do not choose.

Option B Tuberculosis has a slow onset, a long course of disease, low fever, burnout, loss of appetite, cough and a small amount of hemoptysis, not consistent with the description of the stem, so it is not considered.

Option D Lung abscess was clinically characterized by high fever, cough and a large amount of pus and smelly sputum. Chest X-ray imaging showed one or multiple holes with air-liquid plane, which was not consistent with the dry stem, so it was excluded.

Option E bronchial adenoma is a benign tumor, slow growth, and the X-ray examination can show a round dense shadow or signs of bronchial obstruction caused

by the tumor, which is not consistent with the title, so it is not selected.

In conclusion, the correct answer is: C. Lung cancer.

According to the description of the stem, a 50-year-old man had irritating cough and no fever in the past month, cough agent and antibiotics. The X-ray film showed 4cm×3.5cm block shadow of the lower lobe of the right lung.

The analysis is as follows:

-Bronchopneumonia is usually accompanied by symptoms such as fever and productive cough, and the X-ray shows a patchy shadow with a short duration, and antibiotic treatment is usually effective.

-Tuberculosis is often accompanied by cough, sputum with blood, night sweats, weight loss and other symptoms, X-ray performance is the upper lobe, local plaque or hollow shadow.

-Lung cancer is common in middle-aged and elderly people. The symptoms can be cough (including irritant cough), blood in sputum, chest pain, dyspnea, etc. X-ray shows massive shadow or nodular shadow.

-Pulmonary abscess is usually accompanied by fever, large cough of purulent sputum, and X-ray appears as a cavity with air-fluid plane.

-Bronchial adenoma is less rare, and the symptoms can be cough and blood in sputum, but X-ray is mostly well-demarcred small nodules.

Combined with the patient's symptoms (irritant cough, no fever, ineffective antibiotic therapy) and imaging findings (4cm×3.5cm block of the lower lobe of the right lung), the most likely diagnosis was lung cancer.

So, the most correct answer is:

F. carcinoma of the lungs

100, (Type A2 questions)

Male sex, aged 65 years old. Progressive aggravation of skin scleral yellowing occurred for 1 month. Self-reported urine color dark yellow, stool gray white.

Physical examination: there was no gallbladder enlargement, Murphy sign was negative, and no palpable mass in the abdomen. Diagnosis was first considered as a diagnosis

A. carcinoma of head of pancreas

B. carcinoma of the lower end of the common bile duct

C. Cancer of the ampulla of Bte

D. hilar hilar cholangiocarcinoma

E. Duodenum adenocarcinoma

Refer to the answer: D. Your answer:

Graphic analysis: In elderly male patients, skin scleral yellow dye was progressive, dark yellow urine, gray stool, physical examination: the gallbladder was not enlarged, Murphy sign was negative, and the abdominal mass was not palpable, indicating that the lesion was located at the upper end of the bile duct, and the hilar cholangiocarcinoma was the first choice.

The 65-year-old man developed progressive aggravated yellow skin and sclera, dark yellow urine, and gray-white stool, which indicated obstructive jaundice. In the absence of an enlarged gallbladder and an abdominal mass, the most likely cause is the malignant obstruction of the biliary tract.

A. Pancreatic head cancer usually causes obstructive jaundice because tumors of the pancreatic head can compress the common bile duct.

B. Cancer of the lower end of the common bile duct can also cause obstructive jaundice, but more often with an enlarged gallbladder (positive Courvoisier sign).

C. Ampullary cancer (Ampulla of Vater cancer) can also cause obstructive jaundice.

D. Porlar cholangiocarcinoma (Klatskin tumor) can cause obstructive jaundice but often not significant enlargement of the gallbladder.

E. Duodenal adenocarcinoma is generally not considered as a primary consideration for the diagnosis of obstructive jaundice.

Considering these factors and symptoms, the most likely diagnosis is pancreatic head cancer (A), as it is one of the most common causes of obstructive jaundice, especially in the absence of a significantly enlarged gall bladder and abdominal mass. Therefore, the best choice is A. pancreatic head cancer.

First, we analyze the question stem.

The patient was an elderly male, and the main symptoms were progressive aggravation of skin scleral yellow dye, dark yellow urine, and gray-white stool, which are typical manifestations of obstructive jaundice. There was no gallbladder enlargement, a negative Murphy sign, and no palpable mass in the abdomen. These signs can help us to further narrow the diagnosis.

Next, we analyzed the options item by item:

A. Pancreatic head cancer: Pancreatic head cancer can compress or infiltrate the common bile duct, resulting in obstructive jaundice. However, pancreatic head cancer usually results in obvious jaundice, and is often associated with an abdominal mass and an enlarged gallbladder, which is not consistent with the title trunk description.

B. Cancer of the lower end of the choleduct: Cancer of the lower end of the cholechal also causes obstructive jaundice, but usually causes gallbladder enlargement because the tumor blocks the flow of the bile to the duodenum, and the bile is still secreted into the gallbladder, leading to gallbladder swelling. This did not match the patient's physical signs.

C. Ampullary cancer: Ampullary cancer can also cause obstructive jaundice, but due to its location close to the duodenal papilla, which is also inconsistent with the clinical manifestations of the patient.

D. hilar cholangiocarcinoma: hilar cholangiocarcinoma is located at the beginning of the bile duct in the liver, where the obstruction will cause the bile unable to flow into the common bile duct and gallbladder, so the gallbladder will not expand. This coincided with the patient's clinical presentation, namely, progressive jaundice without gallbladder enlargement.

E. Adenocarcinoma of the duodenum: Although duodenal adenocarcinoma may compress or infiltrate the common bile duct to cause jaundice, its main symptoms are usually associated with intestinal obstruction or bleeding and do not necessarily lead to gallbladder enlargement, this is not a typical manifestation and is relatively unlikely.

In conclusion, considering that the main symptoms were progressive jaundice, dark yellow urine, gray white stool, and absence of enlarged gallbladder, the most likely diagnosis was hilar cholangiocarcinoma.

Therefore, the correct answer is D. hilar cholangiocarcinoma.

According to the problem stem description, the patient was a 65-year-old man with progressive aggravating skin and scleral yellow staining for 1 month, dark yellow urine and gray-white stool, and these symptoms suggested the presence of cholestasis in the patient. Physical examination showed no enlargement of the gallbladder, a negative Murphy sign, and no palpable mass in the abdomen.

Considering the patient's age and symptoms, these signs suggest a greater possibility of disease in the bile duct system. Here is an analysis of each option:

A. * * Pancreatic head cancer * *: Pancreatic head cancer often leads to obstruction of the common bile duct, leading in jaundice and lighter stool. However, pancreatic head cancer is often accompanied by weight loss and abdominal pain, and sometimes has a palpable mass.

B. * * Cancer of the lower end of the choleduct * *: Cancer of the lower end of the common bile duct can cause complete or partial obstruction of the bile duct, causing progressive jaundice and gray-white stool. The patient's symptoms and signs were consistent with this disease presentation.

C. * * Ampullary cancer * *: ampullary cancer can cause symptoms similar to cancer at the lower end of the choleary duct, which is adjacent to the lower end of the common bile duct, and can cause obstructive jaundice, dark yellow urine and gray-white stools.

D. * * Porlar cholangiocarcinoma * *: Porlar cholangiocarcinoma usually causes progressive jaundice, but often with right upper abdominal pain and may have hepatomegaly, and this patient does not have these signs.

E. * * Duodenal adenocarcinoma * *: Duodenal adenocarcinoma causes less biliary obstructive jaundice, and the main symptoms are mostly digestive tract

symptoms such as abdominal pain, nausea, which is not in line with the performance of the patient.

In conclusion, combined with the patient's symptoms and signs, the most likely diagnosis is * * of the ampullary carcinoma * * or * * carcinoma of the lower end of the common bile duct * *. However, considering that the symptoms caused by ampullary cancer are often more typical and obvious, the optimal choice is:

F. * * Bte ampulla cancer * *

101. (Type A2 questions)

Female, aged 35 years old. Blood in the stool side by side will not feel half a month doctor, previously have a history of internal hemorrhoids. The preferred method of examination is the

A. fecal occult blood test

B. digital rectal examination

C. Rectoscopy

D. Colonoscopy

E. Barium enema examination

Reference answer: B. Your answer:

Graphic analysis: first consider the patient internal hemorrhoids recurrence, mainly rely on the anorectal digital examination.

In this case, the preferred test method should be a colonoscopy (option D). The 35-year-old woman had symptoms of hematochezia and endless defecation that, despite a history of internal hemorrhoids, may indicate more severe colorectal disease, such as polyps or colorectal cancer. Colonoscopy directly examines the interior of the colorectal tract, checks for lesions, such as polyps, inflammation, or tumors, and a biopsy can be performed. Therefore, colonoscopy is the most detailed and direct method to diagnose such symptoms.

analyze:

Young female patients, previously internal hemorrhoids, now blood in the stool

with defecation feeling, in order to clarify the cause of blood in chezia and the severity of internal hemorrhoids, simple, easy rectal examination should be preferred. Finger rectal examination can find that the anus and rectum have lesions and the nature of lesions, more than 80% of rectal cancer can be touched in the digital rectal examination, so the digital rectal examination is a simple and important clinical examination method, which is of great significance to the early detection of rectal cancer.

Option A fecal occult blood test is A method to determine gastrointestinal bleeding, which is mainly used to test the small amount of bleeding invisible to the naked eye. The positive result indicates that the stool contains red blood cells, but the bleeding site cannot be determined, so it is not selected.

Option C rectal microscopy and option D colonoscopy can determine the bleeding site, but rectal microscopy and colonoscopy are complicated and expensive, and are not the first choice, so they are excluded together.

Option E barium enema examination can find small lesions of the colon, but the diagnosis of little value for rectal lesions, and the operation is complicated, not as the first choice, so it is not considered.

In conclusion, the correct answer is: B. digital rectal examination.

According to the patient's symptoms (blood in stool and defecation) and previous history of internal hemorrhoids, the most preferred examination method is to be able to directly observe and evaluate the rectum and the lower colon, in order to identify the source of bleeding and the cause of defecation.

The best answer is:

* * D. Colonoscopy * *

The reasons are as follows:

1. * * Blood in the stool and endless defecation * * are symptoms that may be

caused by colorectal lesions, such as internal hemorrhoids, polyps, inflammatory bowel disease, or tumors.

2. * * Colonoscopy * * can comprehensively examine the whole colon and rectum, is the most effective examination method, can directly observe the lesion site and biopsy, to exclude or confirm the lesion.

3. * * Digital rectal examination * * is a basic examination, its scope is limited to comprehensively assess the condition of the colon and rectum.

4. * * Fecal occult blood test * * It is mainly used to screen for the presence of occult blood, and the specific source of bleeding cannot be determined.

5. * * Rectal microscopy * * can only examine the rectal segment, can not fully evaluate the upper segment of the colon lesions.

6. * * Barium enema examination * * can show intestinal structure, but the resolution is not good to colonoscopy, and biopsy cannot be performed.

Therefore, colonoscopy is the preferred method for patients with stool and defecation, especially with a history of internal hemorrhoids.

Four hours after the accident, the patient had severe abdominal pain, physical examination: T36.8℃, P89 times / min, BP124 / 85mmHg, abdominal tenderness, rebound pain, blood amylase 1200U / L. Abdominal vertical flat film is not usual. Abdominal B ultrasound showed a small abdominal fluid accumulation in the right abdomen.

102. (Type A3 / A4 questions) subject

The most likely damaged organs are

A. hepatic rupture

B. lienal rupture

C. pancreatic rupture

D. rupture of colon

E. Breakdown of small intestine

Reference answer: C Your answer:

[Analysis] 4 hours after the car accident (the steering wheel hit the abdomen is easy to cause pancreatic injury), severe abdominal pain, physical examination:

T36.8°C, P89 times / minute, BP124 / 85mmHg, abdominal tenderness, rebound pain (peritoneal irritation), elevated blood amylase, abdominal B-ultrasound showed a small amount of abdominal effusion in the right abdomen. The most likely damaged organ is the pancreas (C pair).

[Avoid wrong] Liver rupture is the most common in open abdominal injury, often combined with right lower rib fracture, hemorrhagic shock and peritoneal irritation (A wrong); splenic rupture is the most common in the abdominal closed injury, often combined with left lower rib fracture, hemorrhagic shock (B wrong); colon rupture peritonitis appeared late but more serious (D wrong); small bowel rupture peritonitis appeared early and obvious (E wrong).

In this case, the patient experienced severe abdominal pain after the accident, with body temperature 36.8°C, heart rate 89 beats / min, blood pressure 124 / 85mmHg, abdominal tenderness and rebound pain, blood amylase level increased to 1200U / L, no abnormal abdominal vertical plain film, and B-ultrasound showed a small amount of abdominal effusion in the right abdomen. Based on this information, the following conclusions can be inferred:

The organ most likely to be injured was C. pancreas rupture. This is because significantly elevated blood amylase levels are usually associated with pancreatic injury. Although other organ injuries (e. g., liver and spleen rupture) may also cause abdominal pain and abdominal effusion, they usually do not result in such significant increases in blood amylase levels.

The correct answer is: C. A tured pancreas.

The patient had severe abdominal pain 4 hours after the accident trauma, positive peritoneal stimulation signs after physical examination, and a significant elevation of blood amylase, indicating that the pancreatic tissue was damaged, so the most likely damaged organ of the patient was the pancreas. Liver rupture and spleen rupture are usually accompanied by massive bleeding, which will cause insufficient heart rate increase, blood pressure drop and other blood volume. The patient's heart rate and blood pressure are normal, so option A liver rupture and option B spleen rupture can be excluded. However, colon rupture and small bowel rupture are mostly manifested as peritonitis, but amylase is normal, so option D colon rupture, and option E small bowel rupture is not considered.

Question 102: Organ most likely to be damaged

A. Liver rupture: Liver rupture usually causes right upper abdominal pain and

large abdominal effusion but not a significant increase in blood amylase.

B. Splenic rupture: splenic rupture also causes abdominal pain and abdominal effusion, but blood amylase is not elevated.

C. Pancreatic rupture: Pancreatic rupture can lead to a significant increase in blood amylase and severe abdominal pain and abdominal effusion.

D. Colon rupture: Colonic rupture may cause abdominal pain and symptoms of peritonitis, but not elevated blood amylase.

E. Small bowel rupture: Small bowel rupture may cause abdominal pain and peritonitis symptoms, but not elevated blood amylase.

Answer: C. A tured pancreas

103, (Type A3 / A4 questions) subject

Ask what to do next

A. Open abdominal exploration

B. Diagnostic abdominal cavity puncture

C. belly CT

D. laparoscopic approach

E. Abdominal B-ultrasound

Reference answer: C Your answer:

[Analysis] 4 hours after the car accident (the steering wheel hit the abdomen is easy to cause pancreatic injury), severe abdominal pain, physical examination: T36.8℃, P89 times / minute, BP124 / 85mmHg, abdominal tenderness, rebound pain (peritoneal irritation), elevated blood amylase, abdominal B-ultrasound showed a small amount of abdominal effusion in the right abdomen. The most likely damaged organ is the pancreas. Next, we should also do abdominal CT examination, which can show whether the contour of the pancreas is neat and whether there is blood and fluid accumulation around it, and evaluate the condition (C pair).

[Fault avoidance] Open abdominal exploration and laparoscopic exploration are used in patients with hemorrhagic shock and obvious peritoneal irritation signs, and to guide the treatment (AD

Wrong); diagnostic abdominal puncture for use in patients with liver and spleen rupture with hemorrhagic shock (B wrong).

For the following examination to be performed, a C. abdominal CT was performed. A CT scan is the gold standard for assessing abdominal trauma and can provide detailed information about intra-abdominal organ damage, especially when pancreatic injury is suspected. Open abdominal exploration is usually performed in emergency cases when there is significant visceral rupture or internal bleeding. While in this case, a CT scan is preferred to obtain accurate injury assessment.

The correct answer is: C. abdominal CT.

Patients with abdominal tenderness, rebound pain, blood amylase significantly increased, suspected pancreatic rupture, for clear diagnosis should be abdominal CT, the diagnostic accuracy is high, not only can determine the degree of pancreatic damage, also can judge the abdominal organs and lymph node metastasis and the judgment of the disease stage, is open, laparoscopic exploration, belongs to the non-invasive examination, patients with high acceptance. Diagnostic abdominal puncture was invasive examination, and the patient had obvious abdominal tenderness and rebound pain, which was not suitable for abdominal puncture. Therefore, option B diagnostic abdominal puncture can be excluded. Option A open abdominal exploration and option D laparoscopic exploration, which are surgical exploration, not as the preferred examination, so they were excluded. The patient had undergone abdominal B-ultrasound without any repetition, and the observation effect on the pancreas was not as good as CT, so the option E abdominal B-ultrasound was not considered.

Question 103: What's the one more check to do next

A. Open abdominal exploration: This is an invasive procedure, usually performed when other tests are not confirmed or when the patient is critically ill.

B. Diagnostic abdominal puncture: Can help determine whether there is blood or other fluid in the abdominal cavity, but there is limited organ identification of specific damage.

C. Abdominal CT: can detail the structure and damage of the abdominal organs, especially for deep organs like the pancreas.

D. Laparoscopic exploration: Although the abdominal cavity can be directly observed, it is an invasive examination.

E. Abdominal B ultrasound: has been performed, not enough to further clarify the diagnosis.

Answer: C. abdominal CT

Female, 32 years old, eat more greasy food for 4 hours led to sudden persistent upper abdominal pain, accompanied by vomiting into food. Physical examination: painful face, sclera without yellow dye, cardiopulmonary physical examination without abnormal, soft abdomen, upper left rebound pain, cardiopulmonary physical examination without abnormal, Murphy (-), bowel sound 2 times / minute.

104, (A3 / A4 type question) subject

A diagnosis may be considered

A. acute pancreatitis

B. acute cholecystitis

C. acute appendicitis

D. myocardial infarction

E. gastric ulcer

Reference answer: A. Your answer:

[Analysis] Young women, eating greasy food led to sudden and persistent upper abdominal pain, accompanied by vomiting into food. Physical examination: painful face, soft abdomen, left upper abdomen tenderness, negative murphy, comprehensive patient history, clinical manifestations and signs, considered as acute pancreatitis (A pair).

[Avoid wrong] Acute cholecystitis (B wrong) showed right upper or upper abdominal pain, fever and blood leukocytosis, Murphy's sign positive or palpable the right upper abdominal mass. Right lower quadrant tenderness is the most common important sign of acute appendicitis (C error). Myocardial infarction (D error) showed retrosternal pain and ECG indicating significantly elevated or depressed ST segment. Gastric ulcer (E error) is mainly manifested as postprandial stomachache, and pain relief with acid inhibitor.

104 Questions:

A. Acute pancreatitis: This is a common cause of acute pancreatitis, considering the upper abdominal pain and vomiting occurring after eating greasy food.

B. Acute cholecystitis: usually associated with right upper abdominal pain and positive Murphy sign.

C. Acute appendicitis: usually causing right lower abdominal pain.

D. Myocardial infarction: chest pain and no mention of myocardial infarction related abnormalities.

E. Gastric ulcer: It can cause epigastric pain but is usually associated with eating pattern rather than sudden pain.

Combined with this information, the most likely diagnosis was A. acute pancreatitis, as the symptoms coincided with acute pancreatitis.

104 Question analysis:

The patient was a young female who had sudden upper abdominal pain with vomiting, left upper abdominal tenderness and rebound pain, which were consistent with the typical manifestations of acute pancreatitis. Therefore, the patient may be considered for the diagnosis of acute pancreatitis.

Option B acute cholecystitis usually show Murphy sign positive, namely the doctor with the left palm flat in the right chest, with thumb finger abdominal pressure in the right costal gallbladder point, told the patient slowly deep inspiration, in the process of inhaling inflamed gallbladder down when press the thumb, cause gallbladder pain, such as severe pain caused by inhaling termination said Murphy sign positive, patients Murphy sign (-), can be excluded.

Option C The typical manifestation of acute appendicitis is metastatic right lower abdominal pain, which is persistent upper abdominal pain, so it is not considered.

Option D Myocardial infarction generally has a history of angina pectoris, mainly manifested as retrosternal fatal pain, etc. ECG showed the characteristic and dynamic changes of myocardial infarction. The patient in this question had no abnormal cardiopulmonary examination and no relevant medical history, so it was not selected.

Option E gastric ulcer mainly showed postprandial pain, and the patient has no history of gastric ulcer, which can be excluded preferentially.

In conclusion, the correct answer is: A. Acute pancreatitis.

Question # 104:

A diagnosis may be considered

A. acute pancreatitis

B. acute cholecystitis

C. acute appendicitis

D. myocardial infarction

E. gastric ulcer

analyze:

After eating more greasy food, the patient suddenly had epigastric pain, accompanied by vomiting, and physical examination showed upper abdominal rebound pain. These symptoms and signs suggested a greater possibility of acute pancreatitis. Patients with acute cholecystitis usually have a positive Murphy sign, while this patient has a negative Murphy sign. Acute appendicitis usually presents with right lower abdominal pain. Although myocardial infarction and gastric ulcer may also cause upper abdominal pain, combined with symptoms after eating greasy food makes a greater possibility of acute pancreatitis.

Best answer: A. Acute pancreatitis

105, (Type A3 / A4 questions) subject

The test for diagnosis is

A. Vertical horizontal abdominal film

B. Abdominal-enhanced CT

C. electrocardiogram

D. gastroscope

E. colonoscope

Reference answer: B. Your answer:

[Analysis] Young women, eating greasy food led to sudden and persistent upper abdominal pain, accompanied by vomiting into food. Physical examination: painful face, soft abdomen, left upper abdomen tenderness, negative murphy, comprehensive patient history, clinical manifestations and signs, considered as acute pancreatitis. Enhanced abdominal CT (pair B) helps to determine the degree of pancreatic necrosis and is important for the diagnosis of acute pancreatitis.

[Error avoidance] Vertical abdominal plain film (A wrong) mainly diagnoses gastrointestinal perforation, intestinal obstruction and other diseases. ECG (C error) mainly to understand whether there are various arrhythmias, myocardial ischemia / infarction, atrioventricular hypertrophy or electrolyte disorders. Gastroscopy (D wrong) is the most commonly used and accurate examination method for esophageal, gastric and duodenal diseases. Colonoscopy (E error) is mainly used to observe all colorectal lesions from the anus to the ileocecal valve.

105 Questions:

For a confirmed diagnosis, the tests to be performed may include:

A. Vertical abdominal film: usually used for intestinal obstruction or free abdominal gas.

B. Abdominal enhanced CT: Very useful for the diagnosis of acute pancreatitis, showing inflammation of the pancreas and the accumulation of fluid around the pancreas.

C. ECG: used for the diagnosis of cardiac problems, not applicable here.

D. Gastroscopy: To the gastric mucosa for ulcers or other lesions.

E. Colonoscopy: used for examining colon diseases, not applicable here.

Therefore, the most suitable examination for this patient is B. abdominal enhanced CT to assess the presence of inflammation or other complications in the pancreas.

105 Question analysis:

In order to diagnose acute pancreatitis, the most valuable test is abdominal enhanced CT, which can not only diagnose acute pancreatitis, but also identify whether combined with pancreatic tissue necrosis, and evaluate extrapancreatic complications and severity.

Option A Vertical abdominal plain film is mainly used for the diagnosis of digestive tract perforation, intestinal obstruction, intraabdominal foreign body and other diseases, which is of little value for the diagnosis of acute pancreatitis, so it is not selected.

Option C ECG was mainly used for the diagnosis of heart-related diseases, and the patient had no abnormal cardiopulmonary examination, so it was excluded.

Option D Gastroscopy is the gold standard for the diagnosis of gastric diseases.

This patient is acute pancreatitis, so it is not considered.

Option E colonoscopy is the gold standard for the diagnosis of colon disease. The onset site of the patient is the pancreas, which is unrelated to the colon and inconsistent with the meaning of the question.

In conclusion, the correct answer is: B. abdominal abdominal CT.

Question 105:

The test for diagnosis is

A. Vertical horizontal abdominal film

B. Abdominal-enhanced CT

C. electrocardiogram

D. gastroscope

E. colonoscope

analyze:

Enhanced abdominal CT is the preferred examination method for the diagnosis of acute pancreatitis, which can clearly show the inflammation and complications of the pancreas, such as pancreatic necrosis, peripancreatic effusion, etc. Vertical horizontal abdominal films are mainly used to diagnose gastrointestinal perforation, electrocardiogram is used to exclude heart problems, and gastroscopy and colonoscopy are used to check the stomach and colon diseases, respectively.

Best answer: B. abdominal abdominal CT

Male, 35 years old, low fever for more than a month, cough, sputum with blood for 3 days. He developed pleuritis 2 years ago and improved after treatment. Chest radiograph: irregular shadow of left lung, left rib diaphragm angle becomes dull. Blood WBC 8.0109 / N0.78, ESR30mm / h.

106, (A3 / A4 type question) subject

The preferred examination for a definite diagnosis is

A. chest CT

B. Acid-fast staining of the sputum smear

C. bronchoscope

D. γ -interferon release assay

E. Serum tumor markers

Reference answer: B. Your answer:

Text analysis: [analytical] young men, low fever, cough, sputum with blood (tuberculosis), 2 years ago with pleisy, chest radiograph: left lung irregular shadow, left rib diaphragm angle blunt (hint for tuberculous pleuritis), ESR30mm / h (ESR normal reference value range: male: 0~15mm / h women: 0~20mm / h, blood increased faster, seen in tuberculosis bacterium infection, such as tuberculosis, tuberculous pleurisy, etc.), overall consideration for tuberculous pleuritis. Acid-fast staining of sputum smear (B pair) is the simplest, rapid, easy and reliable method to diagnose tuberculous pleuritis.

[Wrong avoidance] Chest CT (A error) is often used in the diagnosis of tuberculosis, as well as the differential diagnosis from other chest diseases. Fibrobronchial

Microscopy (C error) is often used in the diagnosis of bronchial tuberculosis and lymph nodes. Due to the high cost, the γ -interferon release experiment (D error) is currently mostly used for research and evaluation, and has not been widely implemented. Serum tumor markers (E error) are of certain reference value for the diagnosis and monitoring of lung cancer.

For question 106, the preferred test for a definite diagnosis is:

A. Chest CT can provide detailed information on the nature of lung shadows, is an important means to evaluating lung lesions and helps to determine the nature and extent of the lesions.

B. Antiacid stain of sputum smear is mainly used to diagnose pulmonary tuberculosis.

C. Bronchoscopy can directly view the lower respiratory tract and obtain tissue samples for pathological analysis, suitable when lung lesions are unclear.

D. The γ -interferon release assay is mainly used for the diagnosis of TB infection.

E. Serum tumor markers for the detection of possible lung cancer, etc.

Given the patient's clinical presentation and medical history, * * option A (chest CT) * * may be the preferred examination method because it provides detailed information about the lung lesion that facilitates the definite diagnosis.

106, analysis:

The young male patient, with low fever for more than a month, cough and blood in sputum for 3 days, was a typical clinical manifestation of pulmonary tuberculosis with a previous history of pleurisy. Chest left lung irregular shadow, the left rib diaphragm Angle blunt, laboratory examination see normal blood leukocytes, neutrophhil high, faster, according to the patient symptoms, auxiliary examination, considering the diagnosis of tuberculosis, for clear diagnosis preferred sputum smear acid stain, if the examination result is positive, combined with patient history and clinical manifestations, can be diagnosed as tuberculosis.

Option A chest CT has A good display of the lung lesions hidden in the heart, the spinal groove and the lung tip and subclavian area that can not be found by X-ray chest film, but it does not confirm tuberculosis, so it is not selected.

Option C bronchoscopy is suitable for the observation of lung lobe, segment and subsegment bronchial lesions, biopsy sampling, bacteriology, cytology examination, with little significance for the diagnosis of tuberculosis, so it is excluded.

Option D: The γ -interferon release assay is a new method for in vitro immunodetection of TB infection. It cannot be used to confirm the current TB infection and only provides auxiliary examination, which is not consistent with the meaning of the question.

Option E serum tumor markers are mainly used to predict the prognosis of rectal cancer and monitor recurrence, and have no significance for the diagnosis of tuberculosis, so they are not considered.

In conclusion, the correct answer is: B. sputum smear of acid-fast staining.

Title 106

The preferred examination for a definite diagnosis is

A. chest CT

B. Acid-fast staining of the sputum smear

C. bronchoscope

D. γ -interferon release assay

E. Serum tumor markers

Answer: B. acid-fast staining of sputum smear

analyze:

The patient had symptoms of low fever, cough and blood in the sputum, while the chest X-ray showed irregular shading of the left lung, and these symptoms and imaging findings suggested the possibility of pulmonary tuberculosis. Sputum smear acid stain is an important examination for the diagnosis of tuberculosis, can directly find tuberculosis, is the preferred method to diagnose tuberculosis. Therefore, the sputum smear acid-fast stain is the preferred examination for a definitive diagnosis.

107, (A3 / A4 type question)

In order to clarify the treatment plan, focus on the situation subject

A. Treatment of the pleurisy

And B. Family genetic history

B. History of penicillin allergy

C. Body weight changes

D. Previous health checkup status

Reference answer: A. Your answer:

Text analysis: [analytical] young men, low fever, cough, sputum with blood (tuberculosis), 2 years ago with pleisy, chest radiograph: left lung irregular shadow, left rib diaphragm angle blunt (hint for tuberculous pleuritis), ESR30mm / h (ESR normal reference value range: male: 0~15mm / h women: 0~20mm / h, blood increased faster, seen in tuberculosis bacterium infection, such as tuberculosis, tuberculous pleurisy, etc.), overall consideration for tuberculous pleuritis. The patient had A history of pleuritis. Considering that the previous treatment of pleuritis was not standardized, in order to clarify the treatment plan, we need to focus on the treatment of pleuritis (A pair).

[Avoid mistakes] tuberculous pleurisy has high pleural hydroprotein content, which is easy to cause pleural adhesion. In principle, the pleural effusion should be

drained as soon as possible. For a large amount of pleural effusion, pump 2~3 times a week, the first solution should not exceed 700ml, and each solution should not exceed 1000ml.

For question 107, in order to clarify the treatment plan, the focus is:

A. Treatment of pleurisy helps to understand the patient's past medical history and treatment response.

B. Family genetic history is important for the diagnosis of some genetic diseases.

C. A history of penicillin allergy is crucial for the development of antibiotic therapy options.

D. Body weight changes can reflect the patient's nutritional status and disease progression.

E. Previous health checks can provide the baseline health status of the patient.

In this case, * * option A (Pleuritis treatment situation) * * may be most critical to clarifying the treatment regimen, as understanding the patient's response to the previous treatment can help develop a treatment plan for the current symptoms.

107. Analysis:

The patient has been diagnosed with tuberculosis, and anti-tuberculosis drugs are needed to treat tuberculosis. To develop the treatment plan, we should know the patient's previous physical examination, whether there is resistance to tuberculosis drugs and whether there are other diseases, so as to make a better treatment plan.

Option A The treatment of pleurisy is of little value to the formulation of an anti-tuberculosis treatment plan, so it is excluded.

Option B family genetic history is mainly for some single gene genetic diseases, and tuberculosis will not be inherited, inconsistent with the question.

Option C The allergic history of penicillin has little influence on the choice of anti-tuberculosis treatment regimen, and penicillin is not included in the commonly used anti-tuberculosis drugs and can be excluded.

Option D Weight change has little value for treatment formulation, so it is not chosen.

To sum up, the correct answer is: E. Previous health examination.

Title 107

In order to clarify the treatment plan, focus on the situation

A. Treatment of the pleurisy

And B. Family genetic history

B. History of penicillin allergy

C. Body weight changes

D. Previous health checkup status

Answer: A. Treatment of pleuritis

analyze:

The patient had a history of pleurisy and had improved after treatment 2 years earlier. Understanding the management of his previous pleurisy is important to clarify the etiology of the current symptoms and to develop treatment options. If the previous pleurisy is caused by tuberculosis, then the patient's current symptoms may be tuberculosis recurrence, and the appropriate treatment plan needs to be developed in combination with the previous treatment situation. Therefore, understanding the treatment situation of pleuritis is the focus of a clear treatment plan.

Female, 44 years old. Modified radical resection was performed for right breast cancer. Postoperative pathology: invasive carcinoma of the right breast, non-special type, tumor size 3cm 2cm, histological grade, ER80%, PR90%, HEki-67.50%. Axillary lymph nodes (4 / 16), with cancer metastasis and adhesion to the surrounding tissue.

108, (Type A3 / A4 questions)　　subject

Following TNM staging, the staging of this patient is

A. T1N1M0

B. T1N2M0

C. T2N1M0

D. T3N2M0

E. T2N2M0

Reference answer: E Your answer:

[Analysis] A middle-aged female patient, due to right breast cancer underwent modified radical resection. Tumor size was 3cm 2cm (T2:> 2cm, 5cm), axillary lymph nodes (4 / 16) (N2: ipsilateral axillary lymph nodes fusion with each other and tissue adhesion), cancer metastasis and adhesion with surrounding tissues. Systemic examination of other organs showed no metastasis (indicated M0: shift). Following TNM stage, the patient was T2N2M0 (E pair).

[Misavoidance] TNM stage of breast cancer: Long diameter or no distant rotation with the surrounding area

For question 108, we have the following information on the selection of the TNM stage:

Tumor size was 3cm 2cm, which falls into T2 (any tumor with a dimension greater than 2cm but not more than 5cm).

Four axillary lymph nodes were involved, showing cancer metastasis, which falls to N1 (1-3 axillary lymph nodes were involved).

No mention of distant metastasis, hence M0 (no distant metastasis).

Based on this information, the most suitable TNM staging is C.T2N1M0.

108, analysis:

The middle-aged female patient underwent modified radical resection for right breast cancer, and the postoperative pathology suggested that the tumor size was 3cm 2cm. According to the TNM stage of AJCC breast cancer, the primary tumor size of this patient was T2. Meanwhile, the pathology suggested that the axillary lymph nodes (4 / 16) showed cancer metastasis, which belongs to N2. No distant metastases were M0. Therefore, according to the TNM staging, the stage of this patient was T2N2M0.

Option A: T1N1M0 means that the long diameter of the primary tumor is less than or equal to 2cm, and there are enlarged lymph nodes in the ipsilateral axilla, but propulsion, movable, and no distant metastasis, which is inconsistent with the description of the title stem.

Option B: T1N2M0 indicates that the primary tumor is less than or equal to 2cm, enlarged lymph node fusion, or adhesion to surrounding tissues, or enlarged lymph node in the lateral sternal side, but no distant metastasis, so it is not selected.

Option C: T2N1M0 indicates that the primary tumor is greater than 2cm and less than 5cm, and has enlarged lymph nodes in the ipsilateral armpit, but propulsion,

movable, and no distant metastasis, so it was excluded.

Option D: T3N2M0 indicates that the primary tumor is greater than 5cm, enlarged lymph node fusion, or adhesion to surrounding tissue with but without distant metastasis, inconsistent with the title stem description.

In summary, the correct answer is: E.T2N2M0.

108, according to the TNM staging, the staging of this patient is

According to the pathological findings and the TNM staging criteria:

T: Tumor size 3cm 2cm, expected to be T2 according to TNM staging criteria (maximum tumor diameter 2cm to 5cm).

axillary lymph nodes (4 / 16) with cancer metastasis and adhesion with surrounding tissues, as per TNM staging criteria: N2 (3-4 lymph nodes with adhesion to surrounding tissues).

no distant metastasis, and therefore, M0.

In conclusion, the TNM stage of this patient should be: T2N2M0.

The best answer is:

B T2N2M0

109, (Type A3 / A4 questions) subject

What comprehensive treatment plan should be taken for this patient after surgery

A. chemotherapy + targeted therapy

B. chemotherapy + endocrine therapy

C. Chemotherapy + targeted therapy + endocrine therapy + radiotherapy

D. chemotherapy + radiotherapy + endocrine therapy

E. Chemotherapy + targeted therapy + endocrine therapy

Reference answer: C Your answer:

[Analysis] A middle-aged female patient, due to right breast cancer underwent modified radical resection. Tumor size was 3cm 2cm (T2: long diameter> 2cm, 5cm),

axillary lymph nodes (4 / 16) (N2: ipsilateral enlarged axillary lymph nodes fused with each other or adhesion with surrounding tissues), cancer metastasis and adhesion with surrounding tissues. Systemic examination of other organs showed no metastasis (indicated by M0: no distant metastasis). Following TNM staging, this patient was staged as T2N2M0. After the modified radical resection of right breast cancer, ER80%, HER 2 +, and the feasible therapy was chemotherapy + targeted therapy + endocrine therapy + radiotherapy (C pair).

[Error avoidance] Oncogene epidermal growth factor receptor 2 (HER 2) overexpression, suitable for postoperative adjuvant chemotherapy; patients with high estrogen receptor (ER) content in breast cancer cells, called hormone-dependent tumors, these cases are effective for endocrine therapy; after breast cancer surgery, radiotherapy is an important part and should be given after local extensive resection of the mass; trastuzumab prepared by targeted therapy has good effect on patients with HER 2 overexpression, reducing the risk of postoperative recurrence and metastasis of breast cancer patients and improving the disease-free survival period.

For problem 109, the tumor was strongly positive for ER and PR, with Ki-67 marking 50%, indicating faster proliferation. In this case, the comprehensive treatment regimen usually includes endocrine therapy because the tumor is hormone receptor positive. Since Ki-67 is higher, chemotherapy may also be required to control the rapidly growing tumor cells. In addition, axillary lymph node metastases may require radiotherapy to control the local condition. Therefore, the appropriate treatment options may include chemotherapy, radiotherapy, and endocrine therapy. Not specifically mentioned the need for targeted therapy unless specific molecular markers are expressed, such as HER 2 positivity. Therefore, the most appropriate answer may be D. Chemotherapy + radiotherapy + endocrine therapy. Note, however, that the final treatment plan should be determined by the specific circumstances and the judgment of the doctor.

109, analysis:

The postoperative pathology of this patient indicated strongly positive ER and PR, which indicated feasible endocrine therapy. Ki-67> 14%, suggesting that tumor cells proliferate rapidly and require chemotherapy. Four lymph node metastases were present, and radiotherapy was required. Therefore, the comprehensive treatment plan for this patient after surgery is chemotherapy + radiotherapy + endocrine therapy.

Option A: Chemotherapy + targeted therapy is suitable for HER-2-positive breast

cancer patients. HER-2 status is not mentioned in the title stem, so it is not selected.

Option B: Chemotherapy + endocrine therapy, option E therapy + targeted therapy + endocrine therapy, lacks an important adjuvant therapy. Radiotherapy therapy is a local treatment and can be used for radical therapy and palliative treatment. The patient has 4 lymph node metastases, which is indispensable and can be excluded together.

Option C: Chemotherapy + targeted therapy + endocrine therapy + radiotherapy includes targeted therapy, but HER-2 status is not mentioned in the title stem, so targeted therapy is not necessarily applicable, so it is not considered.

In conclusion, the correct answer is: D. chemotherapy + radiotherapy + endocrine therapy.

109. What comprehensive treatment plan should be taken after surgery

According to the patient's pathological characteristics and clinical guidelines, the following points are considered:

Chemotherapy: The histological grade of the tumor, indicating high malignancy and cancer metastasis in lymph nodes, conforms to the indication for chemotherapy.

Targeted therapy: Although HER 2 status is not explicitly mentioned in the title, targeted therapy should be considered in high-grade invasive breast cancer, such as HER 2 positive.

Endocrine therapy: The patient had strongly positive ER and PR, consistent with the indications of endocrine therapy.

Radiotherapy: With lymph node metastases and adhesion to the surrounding tissues, radiotherapy should be considered to reduce the risk of local recurrence.

Comprehensive consideration, the most reasonable scheme is: chemotherapy + targeted therapy + endocrine therapy + radiotherapy.

The best answer is:

B Chemotherapy + targeted therapy + endocrine therapy + radiotherapy

Male, 55 years old, with paroxysmal pain at night, relieved after eating, aggravated in nearly 3 months, and was admitted in the outpatient department.

110, (A3 / A4 type question)

The surgical mode of treatment is

A. subtotal gastrectomy

B. gastric repair surgery

C. Vagus xotomy

D. Selective vagotomy

E. total gastrectomy

Reference answer: A. Your answer:

Graphic analysis: [analysis] According to the patient's situation, consider the diagnosis of duodenal ulcer. The main procedure of gastroduodenal ulcer is a distal gastrectomy, commonly known as a partial gastrectomy. Surgical indications: ineffective conservative treatment of gastroduodenal ulcer or complicated by perforation, bleeding, pyloric obstruction, and cancer (A pair).

[Wrong avoidance] Gastric repair surgery (B wrong) is indicated for acute perforation of gastric or duodenal ulcer; vagotomy is rarely used (C wrong); peptic ulcer combined with massive gastrointestinal bleeding, if bleeding continues after medical treatment, gastric resection or selective gastric vagotomy (D wrong) pyloric plasty. Total gastrectomy (E error) is mostly suitable for gastric body and proximal gastric cancer.

110. The surgical mode of treatment is

Symptoms indicate a possible peptic ulcer, especially when pain relief after the diet. The most common treatment is selective vagotomy (D), which is used to reduce gastric acid secretion and thus reduce symptoms.

Answer: D. Selective vagotomy

The correct answer is: A. major gastrectomy.

A middle-aged male patient with nocturnal paroxysmal pain and relief after eating is a typical manifestation of duodenal ulcer. Subtotal gastrectomy is the most commonly used surgical method for the treatment of duodenal ulcer. Major gastrectomy also includes the resection of ulcer disease, so the most suitable surgical method for patients in this question is major gastrectomy. Option B Gastric repair surgery is generally not used for the treatment of duodenal ulcer, so it is excluded. Option C vagotomy, option D selective vagotomy, is now mostly abandoned and can

be excluded together. Option E total gastrectomy is mainly suitable for malignant tumors of the stomach, ineffective treatment of duodenal ulcer and not consistent with the problem.

110, (A3 / A4 type question)

The surgical mode of treatment is:

A. subtotal gastrectomy

Rationale: The patient was a 55-year-old man with paroxysmal pain at night, relieved after eating and worsened for nearly 3 months, consistent with the typical manifestations of peptic ulcer. Massive gastrectomy is often used to treat severe peptic ulcers, especially when drug treatment is ineffective or ulcer complications (e. g., bleeding, perforation).

111. (Type A3 / A4 questions)subject

After the operation, the patient had bilious vomiting, and the abdominal pain did not relieve after vomiting, belonging to

A. anastomotic fistula

B. Output-end obstruction

C. alkaline reflux gastritis

D. dumping syndrome

E. The rupture of the duodenal anastomosis

Reference answer: B. Your answer:

Graphic analysis: [analysis] According to the patient's situation, consider the diagnosis of duodenal ulcer. The main procedure of a gastroduodenal ulcer is a distal partial gastrectomy, also commonly known as a large partial gastrectomy. The patient developed postoperative biliary vomiting, and the abdominal pain did not relieve after vomiting, which was the output end obstruction in the early postoperative complications (B pair).

[Avoid mistakes] Astomotic rupture (E wrong) or anastomotic fistula (A wrong), mostly manifested as pain under the xiphoid process, should immediately fast water, gastrointestinal decompression and close observation, peritonitis when surgical exploration. Alkaline reflux gastritis (C wrong) mostly occurs in the months to years

after surgery. Early dumping syndrome (D wrong) is more than the corresponding manifestations of palpitations, cold sweat, fatigue and pallor half an hour after eating, accompanied by nausea and vomiting, abdominal cramps and diarrhea; late dumping syndrome occurs 2 to 4 hours after fasting. The main performance is dizziness, pale complexion, cold sweat, fatigue, pulse count.

111. After the operation, the patient had bilious vomiting, and the abdominal pain did not relieve after vomiting, which

Failure of abdominal pain after biliary vomiting and vomiting may point to bile reflux, commonly seen after gastric surgery, which may lead to alkaline reflux gastritis (C). Answer: C. alkaline reflux gastritis

The correct answer is: C. Alkaline reflux gastritis.

After the operation, the patient developed bilious vomiting, and the abdominal pain did not relieve after vomiting, considering alkaline reflux gastritis. This complication is usually caused by the reflux of duodenal contents such as bile and pancreatic fluid into the stomach after intraoperative pyloric plasty or gastrojejunostomy, which destroys the barrier protective effect of the gastric mucosa. Option A Astomotic fistula refers to the leakage of the anastomosis after gastrointestinal surgery. The digestive fluid and intestinal contents will flow out of the abdominal cavity and abdominal wall from the fistula mouth, which is not consistent with the description of the topic stem. The obstruction at the output end of option B mainly showed that the vomit contained the gastric content of bile, but the abdominal pain could be slightly relieved after vomiting, so it was excluded. Option D Dumping syndrome is mainly manifested as the corresponding symptoms of short blood volume insufficiency such as subxiphoid discomfort, palpitations, fatigue, pale face, cold sweat, as well as nausea, vomiting, abdominal cramps and diarrhea, which are not consistent with the description of the problem. Option E duodenal anastomosis rupture is relatively rare, mainly manifested as acute peritonitis, severe shock, can be excluded.

111. (Type A3 / A4 questions)

After the operation, the patient had bilious vomiting, and the abdominal pain did not relieve after vomiting, belonging to:

C. alkaline reflux gastritis

Reason: biliary vomiting occurred after surgery, and the abdominal pain did not relieve after vomiting, indicating that bile reflux entered the stomach, leading to damage to the gastric mucosa, which is in line with the clinical manifestations of alkaline reflux gastritis.

112, (A3 / A4 type question) subject

After the surgery the patient had hypoglycemic syndrome, which at this time showed as

A. vomit is food, no bile

B. The vomit is bile and contains no food

C. The vomit has both bile and food

D. Vomus is a overnight night

E. No vomiting

Reference answer: E Your answer:

Graphic analysis: [analysis] According to the patient's situation, consider the diagnosis of duodenal ulcer. The main procedure of gastroduodenal ulcer is a distal gastrectomy, commonly known as a partial gastrectomy. The postoperative patients had hypoglycemia syndrome, which belongs to the advanced dumping syndrome, mainly manifested as dizziness, pale face, cold sweat, fatigue, and fine pulse. Generally no vomiting, so this question choose E.

[Avoid error] vomit is food, no bile is input loop obstruction (A wrong); vomit is bile, no food is chronic incomplete input loop obstruction (B wrong); vomit has both bile and food is alkaline reflux gastritis or output loop obstruction (C wrong); vomit is pyloric stenosis or obstruction (D error).

112. After the operation, the patient had hypoglycemia syndrome, which appeared at this time

Hypoglycaemic syndrome is common after gastric surgery, especially after most removal of the stomach. In this case, food enters the small intestine too quickly, causing excessive insulin secretion and subsequent hypoglycemia. The patient may not have vomiting at this time because the main manifestations of hypoglycemic syndrome are symptoms associated with glycemic changes after eating.

Answer: E. No vomiting

The correct answer is: B. The vomit is bile, without food.

After the operation, the patient had hypoglycemia syndrome, which was due to the food entering the jejunum too quickly after the large gastrectomy, glucose was absorbed too quickly, and the blood sugar increased briefly, which stimulated excessive insulin secretion, and then reactive hypoglycemia occurred. In this case, because the volume of the stomach after large gastrectomy decreases, food quickly enters the intestines, and bile may also quickly enter the intestines with food, so the vomit may contain bile but not food. Option A "vomit is food, no bile" does not conform to the physiological changes after the large gastric resection, so it was excluded. Option C "vomit has both bile and food" does not usually occur in the case of hypoglycemia syndrome because food and bile do not stay in the stomach and can be excluded. Option D "vomit is overnight night food" is a typical manifestation of pyloric obstruction, unrelated to hypoglycemia syndrome, so it is not chosen. Option E "no vomiting" does not meet possible symptoms in hypoglycemia syndrome and was therefore excluded.

112, (A3 / A4 type question)

After the operation, the patient had hypoglycemic syndrome, which at this time showed:

E. No vomiting

Reason: Hypoglycemia syndrome is a complication after gastrectomy, which is mainly manifested as hypoglycemia symptoms occurring about 2 hours after meals, such as sweating, palpitations, fatigue, hunger, etc. Patients usually do not experience vomiting at this time.

Female, 46 years old, with upper abdominal discomfort for 1 year, aggravated by dizziness and fatigue for 2 months, most of them were pain before meals, previous history of Hashimoto thyroiditis for 10 years, and thinning of gastric mucosa and light color in gastroscopy

Vascular type.

113, (A3 / A4 type question) subject

Examination should be preferred for a clear diagnosis

A. gastroscope

B.13C breath test

B. iodine 131 examination

C. X-ray

D. Internal factor antibody examination

Reference answer: E Your answer:

Graphic analysis: [analytical] middle-aged women, upper abdominal discomfort 1 year, aggravated with dizziness, fatigue (anemia) in February, more pain before eating (consider duodenal ulcer), previously with Hashimoto thyroiditis history of 10 years, gastroscope see gastric mucosa thinning, mucosa color pale, can see vascular type (conform to the typical gastroscope performance of chronic atrophic gastritis), overall consideration for type A gastritis. Measurement of serum parietal cell antibodies, internal factor antibodies (E pair) and vitamin B12 levels helps in the diagnosis of autoimmune gastritis.

[Error avoidance] Gastroscopy (A error) and histological examination are the key to the diagnosis of chronic gastritis, which cannot be confirmed only by relying on clinical manifestations.

13C (B error) or 14C-urea breath test, is one of the important methods of HP detection.131I (C error) uptake rate diagnosis

The traditional method of hyperthyroidism has been replaced by sTSH measurement. Plain X-ray (D wrong) is only used for emergency examinations related to esophageal and gastrointestinal diseases, including metallic foreign bodies in the esophagus and gastrointestinal tract, perforation, and intestinal obstruction.

Question 113: Examination should be preferred to clarify the diagnosis

The patient's symptoms were upper abdominal discomfort, dizziness, fatigue, and gastroscopy showed thin gastric mucosa and pale color. Given her history of Hashimoto's thyroiditis, further examination of possible lesions might be required. Hashimoto's thyroiditis is an autoimmune disease that may accompany other autoimmune gastritis.

A. Gastroscopy has been performed, showing changes in the gastric mucosa.

The B. The 13 C breath test is mainly used to detect H. pylori infection, which has little relevance to the current clinical situation.

B. Iodine 131 examination was used to assess thyroid function, considering that the patient had Hashimoto's thyroiditis, which may help to understand the condition of

the thyroid, but not much to determine the diagnosis of stomach problems.

C. Abdominal X-ray film can show some gastrointestinal problems, but it is not optimal for diagnosing specific problems in the mucosa.

D. Internal factor antibody examination is an important means to detect autoimmune gastritis, especially in the atrophic gastritis associated with B12 malabsorption.

Therefore, for question 113, I would choose the E. Internal factor antibody test, because this test is helpful to diagnose autoimmune gastritis, which is consistent with the patient's symptoms and gastroscopy.

Correct answer: E. Internal factor antibody examination

The patient had discomfort in the upper abdomen and previously had Hashimoto's thyroiditis, with thin gastric mucosa, light mucosal color and blood vessels visible in the gastroscope. These symptoms strongly suggested the possibility of type A gastritis. Type A gastritis is autoimmune gastritis, which is formed by autoimmune mechanism, and autoantibodies against parietal cells or internal factors are produced in the patient's body, resulting in A decrease in the total number of parietal cells, gastric acid secretion and internal factors, which leads to malabsorption of vitamin B12 and giant erythrocytic anemia. For autoimmune gastritis, the detection of internal factor antibody and parietal cell antibody is the most valuable experimental diagnosis method, so the internal factor antibody examination is preferred to make a clear diagnosis.

Option A Although the changes of gastric mucosa can be observed, the specific diagnostic value of autoimmune gastritis is not as good as the internal factor antibody examination, so it was excluded.

Option B: ^{13}C breath test is mainly used to detect H. pylori infection, and it is not suitable for the diagnosis of autoimmune gastritis, so it is not selected.

Option C: iodine ^{131} examination is mainly used for the diagnosis of thyroid and parathyroid diseases, and is unrelated to the diagnosis of autoimmune gastritis, so it is not selected.

Option D: Abdominal X-ray film has limited diagnostic value for gastrointestinal diseases, and cannot clearly diagnose autoimmune gastritis, so it is excluded.

113, examination should be preferred for a clear diagnosis

Considering the patient's symptoms and gastroscopy findings, combined with his

previous history of Hashimoto's thyroiditis, this patient may have had autoimmune gastritis. To further clarify the diagnosis, the preferred test should be to assess the presence of internal factor antibodies (A3 / A4).

Correct answer: E., internal factor antibody test

114, (A3 / A4 type question) subject

Patients may appear

A. More pepsinogen was found in the blood

B. Increased gastric acid secretion

C. Vitamin B12 deficiency

D. virchow lymph nodes are visible

E. Increased internal factors

Reference answer: C Your answer:

Graphic analysis: [analytical] middle-aged women, upper abdominal discomfort 1 year, aggravated with dizziness, fatigue (anemia) in February, more pain before eating (consider duodenal ulcer), previously with Hashimoto thyroiditis history of 10 years, gastroscope see gastric mucosa thinning, mucosa color pale, can see vascular type (conform to the typical gastroscope performance of chronic atrophic gastritis), overall consideration for type A gastritis. The main manifestations of type A gastritis are: pernicious anemia (i. e., giant erythroid anemia, vitamin B12 reduction (C pair)), pepsinogen deficiency (A error), reduced gastric acid secretion (B error), and internal factor deficiency (E error).

[Error avoidance] Virchow Lymph nodes are often the markers of gastric cancer, pancreatic cancer, esophageal cancer and Hodgkin's lymphoma metastasis.

Question 114: Patients may occur

A. Increased pepsinogen in blood is related to decreased gastric acid secretion, but more often in normal or increased gastric acid secretion.

B. Increased gastric acid secretion is usually associated with diseases such as Zollinger-Ellison syndrome, inconsistent with this case description.

C. Vitamin B12 deficiency may lead to atrophic gastritis due to autoimmune gastritis, which subsequently affects the absorption of B12.

D. virchow lymph nodes are advanced signs of gastric cancer, inconsistent with this case description.

E. Increased endofactors not with the autoimmune background and symptoms of the present case.

Considering the patient's gastroscopy findings and a history of autoimmune disease, the most likely scenario was vitamin B12 malabsorption due to atrophic gastritis. Therefore, for question 114, I would choose C. vitamin B12 deficiency.

Correct answer: C. Vitamin B12 deficiency

analyze:

The middle-aged female patient had Hashimoto's thyroiditis before, with gastric mucosa thinning and light mucosal color in gastroscopy. Combined with the patient's past medical history and gastroscopy, the most likely diagnosis was autoimmune gastritis. In autoimmune gastritis, autoantibodies against parietal cells or internal factors appear in the body, leading to a decrease in gastric acid secretion and internal factors, which leads to malabsorption of vitamin B12 and megaloblastic erythrocytic anemia, that is, vitamin B12 deficiency.

Option A More pepsinogen in blood is more seen in the disease secreted by G cells in antral mucosa, such as gastrinoma, which is not consistent with the symptoms of this patient, so it is not selected.

In autoimmune gastritis, autoantibodies against parietal cells or internal factors appear in the body, resulting in decreased gastric acid secretion, so option B has increased gastric acid secretion, which can be excluded.

Option D It can be seen that the Virchow lymph node, that is, the left supraclavicular lymph node enlargement, is mostly seen in advanced esophageal cancer and gastric cancer, which is not consistent with the description of the title stem, so it is not selected.

In autoimmune gastritis, autoantibodies against parietal cells or internal factors appear in the body, resulting in the decrease of gastric acid secretion and internal factors, so the number of internal factors in option E increases, which is inconsistent with the meaning of the question.

114. Patients may appear

Autoimmune gastritis often leads to gastric mucosal atrophy and lack of internal factors, which in turn affects the absorption of vitamin B12, leading to vitamin B12

deficiency. Therefore, the patient may experience the following symptoms:

Correct answer: C. Vitamin B12 deficiency

ideas of solving a problem

Medical history and symptom analysis:

Female, 46 year old, with upper abdominal discomfort for 1 year, aggravated by dizziness and fatigue, mostly pain before meals.

He had a previous history of Hashimoto's thyroiditis for 10 years.

Gastroscope saw gastric mucosa thinning, mucosa color becomes light, vascular type.

Possible diagnoses:

Autoimmune gastritis may lead to internal factor deficiency, which may lead to vitamin B12 deficiency anemia.

First choice check:

Examination of internal factor antibody is crucial to clarify the diagnosis.

Possible symptoms:

Vitamin B12 deficiency is most likely to occur.

These two questions are combined to give a clear diagnostic idea and select the best examination method and expected symptoms.

Male, 22 years old, was stabbed in the foot 9 days ago. With weakness, two days ago, With headache, Difficulties chewing, swallowing, trismus 1 day ago, muscle cramps, General convulsions.

115. (Type A3 / A4 questions) subject

If the disease continues to develop, the most life-threatening thing is this

A. Persistent respiratory muscle spasm

B. myorrhexis

C. cataclasis

D. alogotrophy

E. retention of urine

Reference answer: A. Your answer:

[Analysis] young male, 9 days before the foot was nail stab (history of foreign body stab). Two days ago, fatigue, headache, difficulty chewing, difficulty swallowing, trismus, muscle spasm, and systemic convulsions (typical symptoms of tetanus), considered as tetanus. If the disease continues to develop, the most life-threatening thing is persistent respiratory muscle spasm (A pair), which can cause respiratory arrest due to persistent respiratory muscle and diaphragm spasm.

[Misavoidance] BCDE option is also a manifestation of tetanus, but not as urgent as respiratory spasm.

For Problem 115, the most life-threatening complication of tetanus is respiratory muscle spasm, as it can cause respiratory failure. Therefore, the answer is A. persistent respiratory muscle spasm.

A: Continuous respiratory muscle spasm

analyze:

The young male patient was stabbed in the foot by iron nails 9 days, followed by fatigue, headache, difficulty in chewing and difficulty in swallowing. Ttrago, muscle spasm and systemic convulsions. Combined with the patient's medical history and symptoms, he should be diagnosed as tetanus. Tetanus is an acute infectious disease characterized by clostridium tetanus invading human wounds and growing and reproducing in an anaerobic environment, producing neurotropic exotoxin and causing muscle tonic spasm in the whole body. If the disease continues to develop, the most life-threatening condition is persistent respiratory muscle spasm, which can be caused by asphyxiation.

Option B muscle fracture, option C fracture, both can occur after intense muscle

spasm, but are not the most life-threatening symptoms, so they can be excluded together.

Tetanus patients due to continuous spasm, sweating, etc., so the daily consumption of heat and water loss is more, easy to occur nutritional disorders. Therefore, it is necessary to pay great attention to the supplement of nutrition and the adjustment of water and electrolyte balance, but not the most life-threatening symptoms, so option D nutritional disorders, can be eliminated.

Option E Urinary retention can present after intense bladder sphincter spasm but is not the most life-threatening symptom and can be ruled out.

Title material

Male, 22 years old, 9 days before the foot by the nails. Two days ago, fatigue, headache, difficulty in chewing, swallowing, and trismus, muscle spasm and generalized convulsions 1 day ago.

Question 115

If the disease continues, the most life-threatening thing is:

A. Persistent respiratory muscle spasm

B. myorrhexis

C. cataclasis

D. alogotrophy

E. retention of urine

Answers and parsing:

Tetanus is an acute specific infection caused by Clostridium tetanus (Clostridium tetani), whose toxin (tetanus toxin) mainly affects the central nervous system, leading to muscle spasm throughout the body. If the disease continues to develop, the most life-threatening factor is persistent respiratory muscle spasm, which can cause dyspnea and even asphyxia, which is one of the leading causes of death in patients with tetanus.

Best answer: A., Continuous respiratory muscle spasm

116, (A3 / A4 type question) subject

The preferred treatment is

A. tetanus toxoid

B. antitetanicum sera

C. penicillin

D. tetanus toxoid + penicillin

E. Tetanus antitoxin + penicillin

Reference answer: E Your answer:

[Analysis] young male, 9 days before the foot was nail stab (history of foreign body stab). Two days ago, fatigue, headache, difficulty chewing, difficulty swallowing, trismus, muscle spasm, and systemic convulsions (typical symptoms of tetanus), considered as tetanus. At this time, the preferred treatment should be tetanus antitoxin + penicillin (E pair).

[Error avoidance] tetanus antitoxin (TAT) aims to neutralize the free toxin, so it is only effective in early application. If the toxin has been combined with neural tissue, it is difficult to work. Penicillin is preferred for antibiotic treatment, the dose of 800,000 to 1 million U, intramuscular injection, every 4 to 6 hours, or high dose intravenous infusion, the dose of 2 million to 10 million U, 2 to 4 times a day, can inhibit Clostridium C. tetanus.

For question 116, the preferred treatment for tetanus is the tetanus antitoxin, which is used to neutralize the circulating toxins and prevent the toxins from causing further damage to the nervous system. At the same time, antibiotics such as penicillin are usually used to kill residual carbotulinum and prevent them from producing more toxins. Therefore, the answer is E. tetanus antitoxin + penicillin.

B: tetanus antitoxin + penicillin

analyze:

The preferred treatment for tetanus patients is the early subcutaneous injection of tetanus antitoxin to neutralize the free toxin, and the application of penicillin to inhibit C. tetanus and prevent and control the infection.

Option A tetanus toxoid, option D tetanus toxoid + penicillin, are used for tetanus vaccination, with poor treatment effect for patients already infected with tetanus, so

they can be excluded together.

Option B tetanus antitoxin can neutralize the free toxin, but tetanus antitoxin alone cannot inhibit C. tetanus and is not the best treatment option, so it is not considered.

Option C penicillin can inhibit Clostridium tetani, but it does not neutralize the toxin, and using penicillin alone can not achieve the best treatment effect, which can be excluded.

Question 116

The preferred treatment is:

A. tetanus toxoid

B. tetanus antitoxin

C. penicillin

D. tetanus toxoid + penicillin

E. Tetanus antitoxin + penicillin

Answers and parsing:

The treatment principles of tetanus include neutralization of free toxin, infection control, symptomatic supportive treatment, etc. Tetanus antitoxin (Tetanus Antitoxin, TAT) is used to neutralize the free tetanus toxin to prevent further toxin action on the nervous system. Antibiotics such as penicillin are used to control infection by C. tetani. Therefore, the preferred treatment should include a combination of tetanus antitoxin and penicillin.

Best answer: E. tetanus antitoxin + penicillin

Male, 46 years old, multiple joint swelling and pain in June with morning stiffness for 6 hours. Biwrist involvement, proximal interphalangeal joints, metacarpophalangeal joints, and bilateral temporomandibular joint. ESR49mm / h, ASO89IU / ml, RF (-), anti (+), ANA (-), X-ray showed osteoporosis, mild wrist and proximal interphalangeal joint space.

117. (Type A3 / A4 questions) subject

Consider the diagnosis

A. systemic lupus erythematosus

B. osteoarthritis

C. arthrolithiasis

D. rheumatoid arthritis

E. psoriatic arthritis

Refer to the answer: D. Your answer:

Graphic analysis: [analysis] middle-aged men, multiple joint swelling and pain in June with morning stiffness (rheumatoid arthritis often morning stiffness) 6 hours. Involinvolves both wrist joints, proximal interphalangeal joint, metacarpophalangeal joint, and bilateral temporomandibular joints (rheumatoid arthritis). Elevated ESR, anti-CCP (+), X-ray osteoporosis, mild wrist and proximal interphalangeal joints. Based on the clinical manifestations and examination results, the diagnosis of rheumatoid arthritis (pair D) was considered.

[Avoid mistakes] Systemic lupus erythematosus (A wrong) joint X-ray examination without joint bone destruction. Osteoarthritis (B error) is mainly manifested as articular cartilage damage, often involving the lower limbs. Gout (C fault) usually occurs at midnight or early in the morning, sudden onset, severe joint pain, redness, swelling, heat, pain and dysfunction of the affected joints within a few hours, the unilateral first broken joint is the most common, the attack is self-limited, and resolves spontaneously more than within 2 weeks. Psoriatic arthritis (E error) occurs more than psoriasis several years later, and some patients show symmetrical polyarthritis, similar to RA, but the disease is more pronounced in the distal interphalangeal joint, and is manifested as appressoritis and fingeritis of the joint.

Considering the symptoms and findings of the 46-year-old male patient:

Multiple joint swelling and pain persisted for 6 months, associated with prolonged morning stiffness

Involinvolved both wrist, proximal interphalangeal and metacarangeal joints, and bilateral temporomandibular joints

Blood tests showed elevated ESR, normal ASO, negative RF (rheumatoid factor), positive anti-CCP antibody, and negative ANA

The X-ray showed osteoporosis and mild joint space stenosis

Based on this information, the most appropriate diagnosis is that of D. rheumatoid arthritis. Features of rheumatoid arthritis include prolonged morning stiffness, pain and swelling in multiple joints, and positivity for anti-CCP antibodies. The X-ray findings also fit with the typical changes in rheumatoid arthritis.

The correct answer is: D. Rheumatoid arthritis.

Patients with middle-aged men, more joint swelling pain, morning stiffness, involving double wrist, proximal fingers, fingers, bilateral temporomandibular joint, laboratory examination see blood sink, anti-nuclear antibody negative, X ray see osteoporosis, double wrist joint and proximal finger joint mild narrowing, accord with the characteristic performance of rheumatoid arthritis, and patients with rheumatoid factor RF (-), therefore can be diagnosed as rheumatoid arthritis.

Option A Systemic lupus erythematosus was more common in young women, with multiple systemic involvement, and mostly positive antinuclear antibody ANA, which was inconsistent with the description of the title stem, so it was excluded.

Option B Osteoarthritis occurs in the joints with large weight bearing, such as knee joint, hip joint, etc., mainly manifested as joint pain and tenderness, joint stiffness, joint enlargement, bone friction sound (sensation), joint weakness, mobility disorder, etc., generally will not appear morning stiffness, which is inconsistent with the description of the topic stem.

Option C Gout usually starts suddenly at midnight or early in the morning, with severe joint pain, redness, swelling, heat, pain and dysfunction of the affected joints appear within a few hours. Unilateral hallux and first metatarsophaleal joint are the most common, which is significantly different from the symptom description of patients.

Option E Psoriatic arthritis is mainly manifested as asymmetric arthritis, mainly large arthritis of the lower limbs, which can also involve the joints and spine between the distal finger (toe), which is inconsistent with the problem.

117, considering the diagnosis

The patient's symptoms include multiple joint pain for 6 months, 6 hours of morning stiffness, involving both wrists, proximal interphalangeal, metacarangeal and bilateral temporomandibular joints, features well consistent with the findings of rheumatoid arthritis (Rheumatoid Arthritis, RA). Moreover, laboratory tests showed

elevated ESR and positive anti-CCP, which are all important markers of rheumatoid arthritis. Negative RF does not exclude RA, because RF can be negative. The X-ray results showed osteoporosis and joint space narrowing and also supported the diagnosis of rheumatoid arthritis.

Best answer: D. Rheumatoid arthritis

118. (Type A3 / A4 questions)

The preferred treatment modality is the subject

A. penicillin

B. IL-6 mAb

C. allopurinol

D. methotrexate

E. ibuprofen

Refer to the answer: D. Your answer:

Graphic analysis: [analysis] middle-aged men, multiple joint swelling and pain in June with morning stiffness (rheumatoid arthritis often morning stiffness) 6 hours. Involinvolves both wrist joints, proximal interphalangeal joint, metacarpophalangeal joint, and bilateral temporomandibular joints (rheumatoid arthritis). Elevated ESR, anti-CCP (+), X-ray showed osteoporosis, and mild narrowing of the joint space in both wrist and proximal interphalangeal joints. Based on the clinical manifestations and examination results of the patient, the diagnosis of rheumatoid arthritis was considered. The preferred treatment agent is methotrexate (D pair).

[Misavoidance] Penicillin (A wrong) is A broad-spectrum antibiotic, mainly used for the treatment of inflammation caused by various bacterial infections.IL-6（B

Wrong) belongs to targeted drugs, long-term use of the potential risk of tumor, not as the preferred drug. Allopurinol (C is wrong) is mainly used in the treatment of gout. Ibuprofen (E wrong) is a non-steroidal anti-inflammatory drug, with analgesic and anti-inflammatory effect, is a common drug to relieve arthritis symptoms, but the effect of the control of the disease is limited.

For treatment, methotrexate is the standard treatment agent for rheumatoid arthritis and is usually used as the preferred treatment option. Therefore, for the

second question, the most appropriate answer is D. methotrexate.

The correct answer is: D. methotrexate.

The patient has been diagnosed with rheumatoid arthritis, and once the disease is confirmed, it should be used early to improve the disease. The choice and application plan of the drugs should be based on the activity, severity and progression of the patient, and two or more drugs can be used to improve the condition. Among them, methotrexate should be the first choice for rheumatoid arthritis and used as an essential drug for combination therapy.

Option A penicillin is mainly used to treat acute infections caused by sensitive bacteria, while rheumatoid arthritis is not an inflammatory disease caused by bacterial infection, so penicillin treatment is ineffective.

Option BIL-6 mAb is mainly used for the treatment of moderate to severe active rheumatoid arthritis, and the drug can be considered in the case of poor efficacy or intolerance of methotrexate, so it is not the preferred medication.

Option C allopurinol is a drug to inhibit uric acid synthesis, which is mainly used to treat hyperuricemia and gout, which is inconsistent with the idea.

Option E ibuprofen is a non-steroidal anti-inflammatory drug, with analgesic and anti-inflammatory effect, is a common drug to improve the symptoms of arthritis, but can not control the condition, must be used with anti-rheumatic drugs to improve the condition, so it is not the preferred treatment.

118, the preferred treatment method is

The preferred treatment option for rheumatoid arthritis usually includes disease-modifying anti-rheumatic drugs (DMARDs), of which methotrexate (Methotrexate) is the most commonly used first-line treatment drug. It can effectively slow down disease progression and reduce joint damage and loss of function. Other options such as IL-6 mab (e. g. tozumab) are also biological agents for the treatment of RA, but are usually used as second-or third-line therapy, especially when methotrexate is ineffective or not tolerated.

The best answer: D. Methotrexate

A 75-year-old woman underwent gastrointestinal decompression due to advanced gastric cancer complicated with pyloric obstruction. In the past 5 days,

about 900 ml/d, 1500ml intravenous infusion, physical examination: T: 37.3°C, P: points, BP: 102 / 60mmHg arterial blood gas analysis: pH: 7.56, HCO3-46 mmol/L, BE + 7mmol / L,

119, (Type A3 / A4 questions) subject

Further treatment is not correct

A. Uroutput over 40ml / h with potassium supplementation

B. Central intravenous infusion of the diluted HCl solution

C. Select the palliative gastrointestinal short-circuit surgery

D. Blood gas analysis and blood electrolytes were monitored every 4 to 6 hours

E. IV infusion of sodium bicarbonate solution

Reference answer: E Your answer:

[Analysis] The elderly female patient underwent gastrointestinal decompression due to advanced gastric cancer combined with pyloric obstruction. The gastric juice (the cause of metabolic alkalosis) was about 900 ml/d, 1500ml of glucose saline every day, arterial blood gas analysis: pH: 7.56, HCO3-46 mmol/L, BE + 7 mmol/L, considered as metabolic alkalosis. Therefore, alkali can not be supplemented (i. e., intravenous sodium bicarbonate solution) (E wrong, is the correct answer to this question).

[Avoid mistakes] If urination reaches 30~40ml / h, you should observe for 6 hours and give potassium supplementation (A pair). The patient had volume-volume alkalosis due to pyloric obstruction, and a dilute hydrochloric acid solution (pair B) was input to the central vein. Considering the poor effect of conservative treatment, respiratory gastrointestinal short circuit surgery should be performed (C pair). Monitoring of blood gas analysis and blood electrolytes (in D pair) every 4 to 6 hours helps to observe the disease progression and identify the cause.

This case describes a 75-year-old woman with advanced gastric cancer complicated with pyloric obstruction, treated with gastrointestinal decompression for nearly 5 days to extract about 900ml / day, along with intravenous rehydration therapy. The results of blood gas analysis showed increased pH and HCO3-, suggesting acid-base balance problems.

For the first question, the choice of further treatment measures is incorrect:

B Central intravenous infusion of the diluted HCl solution

This choice is incorrect because in this case the patient already exhibits metabolic alkalosis (hypercapnia) and a hydrochloride infusion is not required to lower blood pH. The correct treatment should be to adjust the fluid and electrolyte balance, and to use some measures that can reduce the blood pH if necessary, rather than using a direct infusion of the acidic solution.

The correct answer is: E. intravenous infusion of sodium bicarbonate solution

The elderly female of the patient underwent gastrointestinal decompression for advanced gastric cancer and pyloric obstruction. In the past 5 days, gastric juice was about 900 ml/d, and 1500ml of glucose saline was injected every day. Physical examination showed abnormal arterial blood gas analysis.

Option A urine output over 40ml / h for potassium ion supplementation is justified. In the case of massive gastric fluid drainage, loss of potassium ions may lead to hypokalemia and therefore require potassium replacement. When the urine volume exceeds 40ml / h, it indicates that the renal function is normal and it can safely replenish potassium.

Option B Central intravenous infusion of diluted hydrochloric acid solution is an effective way to correct alkalosis and can help restore the acid-base balance of the blood.

Option C palliative gastrointestinal short-circuit surgery can relieve pyloric obstruction and is a reasonable method to treat advanced gastric cancer combined with pyloric obstruction.

Option D It is necessary to monitor blood gas analysis and blood electrolytes every 4 to 6 hours, in order to timely understand the patient's acid-base balance and electrolytes, and timely adjust the treatment plan.

Option E intravenous infusion of sodium bicarbonate solution aggravates metabolic alkalosis and is therefore incorrect for further treatment. In the case of metabolic alkalosis, the administration of sodium bicarbonate can further worsen the situation.

119. Further treatment measures are incorrect

option:

A. Uroutput over 40ml / h with potassium supplementation

B. Central intravenous infusion of the diluted HCl solution

C. Select the palliative gastrointestinal short-circuit surgery

D. Blood gas analysis and blood electrolytes were monitored every 4 to 6 hours

E. IV infusion of sodium bicarbonate solution

analyse:

A: Large loss of gastric juice can easily lead to hypokalemia, so it is reasonable to supplement potassium ions appropriately.

B: Diluted hydrochloric acid solution can correct alkalosis and is a reasonable treatment measure.

C: Palliative gastrointestinal short-circuit surgery can help improve the pyloric obstruction symptoms and is suitable for patients.

D: Frequent monitoring of blood gas analysis and blood electrolytes facilitates the timely adjustment of the treatment schedule.

E: The patient has had metabolic alkalosis (pH high, HCO3-high), and sodium bicarbonate should not be used, which will aggravate alkalosis.

Answer: E. intravenous infusion of sodium bicarbonate solution

120, (Type A3 / A4 questions)

The most likely combination of acid-base balance imbalance in this patient is subject

A. respiratory acidosis

B. respiratory alkalosis

C. metabolic acidosis

D. metabolic alkalosis

E. mixed acidosis

Refer to the answer: D. Your answer:

[Analysis] For elderly female patients, due to advanced gastric cancer combined with pyloric obstruction, gastrointestinal decompression, nearly 5 days to introduce gastric fluid (cause of metabolic alkalosis) about 900 ml/d, glucose saline 1500ml

intravenous infusion every day, arterial blood gas analysis: pH: 7.56, HCO3-46 mmol/L, BE + 7 mmol/L, considered as metabolic alkalosis (D pair).

[Avoid] PaCO245mmHg is respiratory acidosis; PaCO235mmHg, respiratory alkalosis; HCO3-

21 mmol/L, Metabolic acidosis; HCO3-27 mmol/L, metabolic alkalosis.

For the second question, the most likely acid-base imbalance is:

C metabolic alkalosis

The results of blood gas analysis showed high pH and elevated HCO3-, which is characteristic of metabolic alkalosis. This condition may be less acidic due to excessive loss of gastric juice (including hydrochloric acid), resulting in alkalosis.

The correct answer is: D. metabolic alkalosis

The elderly female of the patient underwent gastrointestinal decompression for advanced gastric cancer and pyloric obstruction. In the past 5 days, gastric juice was about 900 ml/d, and 1500ml of glucose saline was given every day. The analysis results were abnormal.

Option A Respiratory acidosis is due to the high concentration of carbon dioxide in the blood caused by pulmonary ventilation dysfunction. The blood gas analysis shows decreased pH value and increased partial pressure of carbon dioxide (PCO_2), which is not consistent with the results of the blood gas analysis of the patient, so it is not selected.

Option B Respiratory alkalosis is due to the low carbon dioxide concentration in the blood caused by excessive pulmonary ventilation. The blood gas analysis showed that the pH value was increased, but the partial pressure of carbon dioxide (PCO_2) would decrease, which was inconsistent with the results of the blood gas analysis of the patient, so it was excluded.

Option C Metabolic acidosis is caused by excessive acid or too little alkaline substances in the body. The blood gas analysis shows that the pH value decreases, and the concentration of bicarbonate ion (HCO_3) also decreases, which is inconsistent with the blood gas analysis results of the patient, so it is not selected.

Option D Metabolic alkalosis is caused by too little acid or too many alkaline substances in the body. The blood gas analysis shows increased pH value, and the concentration of bicarbonate ion (HCO_3) also increases, which is consistent with the blood gas analysis results and is the correct answer.

Option E Mixed acidosis was the combination of respiratory acidosis and metabolic acidosis. Blood gas analysis showed a decrease of pH, which may be accompanied by an increase of partial pressure of carbon dioxide (PCO_2) and a decrease of the concentration of bicarbonate ion (HCO_3), which was inconsistent with the patient's blood gas analysis results, so it was excluded.

120, the most likely acid-base balance disorder is

option:

A. Respiratory acidosis

B. Respiratory alkalosis

C. Metabolic acidosis

D. The metabolic alkalosis

E. Mixed acidosis

analyse:

PH: 7.56: indicates alkalosis.

HCO3-: 46 mmol/L: significantly higher than the normal value, indicating metabolic alkalosis.

BE: + 7 mmol/L: further evidence of metabolic alkalosis.

Respiratory factors are usually reflected by changes in CO2 levels, while the current blood gas analysis data mainly suggest metabolic changes.

Answer: D. metabolic alkalosis

Female, 16 years old, with chest pain for 3 months, blood pressure 115 / 90mmHg, breathing 20 times / minute, X-ray examination found the right chest oval mass, sharp lung rib and diaphragm angle. CT showed a large mass of the anterior mediastinum with a dense marginal clearance.

121, (A3 / A4 type question) subject

The rest showed no abnormalities.think over

A. hamartoma of lung

B. cyst of thymus

C. Thymic lymphoma

D. carcinoma of the lungs

E. The anterior mediastinal teratoma

Reference answer: E Your answer:

Graphic analysis: [analysis] adolescent women, chest pain for 3 months, X-ray examination found the right chest oval mass, lung rib diaphragm horn sharp. CT showed a huge mass in the left anterior mediastinum with clear margins, optical integration and uneven density, with liquid dark area and high density calcifications, pushing the mediastinum and heart to the left (in line with the imaging findings of anterior mediastinal teratoma), which was considered as anterior mediastinal teratoma (E pair).

[Error avoidance]: Pulmonary hamartoma (A error) is seen in young men, with round, oval or lobulated tumors, with clear boundaries and calcification points, typically popcorn-like calcification. Thymic cyst (B wrong) is more found than during physical examination, usually asymptomatic; thymic lymphoma (C wrong) shows fever, fatigue, night sweats, chest pain and other symptoms; common CT signs of lung cancer (D wrong) include leaf segmentation, burr, vacuolation, pleural depression or pull, eccentric cavity, etc.

For question 121, a possible diagnosis needs to be considered based on the characteristics of the mass. Options include:

A. hamartoma of lung

B. cyst of thymus

C. Thymic lymphoma

D. carcinoma of the lungs

E. The anterior mediastinal teratoma

Common types of anterior mediastinal masses include thymomomas, teratomas, lymphomas, and cysts. Considering the patient's age and imaging characteristics (clear mass margins, miscellaneous high density shadow), anterior mediastinal teratoma (option E) is a more reasonable diagnosis because teratoma is usually clear margins and can contain multiple densities of tissue.

The correct answer to question 121 is: E. anterior mediastinal teratoma

In a young female patient with 3-month chest pain, right chest oval mass found on X-ray, CT showed huge anterior mediastinal mass with clear margins and mixed internal density, and the most likely diagnosis was anterior mediastinal teratoma based on her clinical presentation and ancillary examination. Option A lung hamartoma is more common in adults, generally asymptomatic, most of which was found accidentally during physical examination. X-ray showed popcorn-like calcification, which was not consistent with the symptoms of the patient's symptoms, so it was excluded. Option B Thymic cyst is a kind of thymoma, which is mostly seen in the anterior and superior mediastinum, but it is mostly cystic mass on CT, with uniform density and arc calcification of the cystic wall, which does not meet the meaning of the question. Option C thymic lymphoma CT mostly showed multiple nodules of the anterior mediastinum fused into clumps, which can involve the middle and posterior mediastinum and pericardium. The margins are often lobulated, and often accompanied by pleural effusion and pericardial effusion. This patient did not have the above manifestations, so it was not selected. Option D Lung cancer is more common in middle-aged and elderly patients, and often have a long-term smoking history, often manifested as cough, phlegm, blood, low fever, chest pain, tightness, which is not consistent with the patient's performance, so it was excluded.

Question 1: What is the most likely diagnosis?

think over:

Pulmonary hamartoma: usually circular or circular marginal clear solitary nodules, mostly seen in the peripheral parts of the lung, do not meet the description.

Thymic cyst: mostly fluid density, no high-density shadow, and more common in middle-aged people, do not conform to the description.

Thymic lymphoma: more manifestations of thymic enlargement, irregular morphology, but 16-year-old girls are less common.

Lung cancer: common in the elderly and with irregular margins, uncommon in 16-year-old girls.

Anterior mediastinal teratoma: mostly seen in young people, CT showed clear mass margins, mixed with high density shadow (possibly calcification or bone component), meeting the description.

Based on the history and imaging findings, anterior mediastinal teratoma is the most likely diagnosis.

Best answer: E. Anterior mediastinal teratoma

122, (Type A3 / A4 questions) subject

The rest showed no abnormalities.think over

A. exairesis

B. observe

C. chemotherapy

D. Interventional embolization

E. Watch closely

Reference answer: A. Your answer:

Graphic analysis: [analysis] adolescent women, chest pain for 3 months, X-ray examination found the right chest oval mass, lung rib diaphragm horn sharp. CT showed a huge mass in the left anterior mediastinum with clear margins, optical integration and uneven density, with liquid dark area and high density calcifications, pushing the mediastinum and heart to the left (in line with the imaging findings of anterior mediastinal teratoma), so anterior mediastinal teratoma was considered. Surgical resection (A pair) is preferred.

[Error avoidance] In addition to malignant lymphoid tumors suitable for radiotherapy, the vast majority of primary mediastinal tumors should be treated surgically as long as there is no other contraindication. Even if the benign tumor or cyst is asymptomatic, because it will gradually grow up, compress the adjacent organs, or even appear malignant changes or secondary infection, so surgery is appropriate.

For question 122, choose the best treatment option. The options considered are:

A. exairesis

B. observe

C. chemotherapy

D. was used for interventional embolization

E. Close observation

With a diagnosis of anterior mediastinal teratoma, the best treatment is usually surgical resection (option A), as this allows for the complete removal of the tumor and avoids potential complications.

122 The correct answer is: A. Surgical resection

Young female patients are diagnosed as anterior mediastinal teratoma. Once the teratoma is diagnosed, surgery should be removed as soon as possible to avoid benign malignant tumor changes caused by the delay of surgery, and to prevent the occurrence of tumor infection, rupture, bleeding and complications. Therefore, surgical resection should be preferred for this patient. Option B observation and option E close observation were not suitable for this patient, excluding teratoma because it was at risk of malignant change and currently had symptoms of chest pain. Option C chemotherapy is mostly used for the treatment of malignant tumors. The patient has not undergone pathological examination at present, and the benign and malignant conditions are unknown, and the chemotherapy has large side effects, so it is not the first choice. Option D Interventional embolization is mostly used in the treatment of hemostasis, arteriovenous malformation, arteriovenous fistula and aneurysm, which is inconsistent with the situation of this patient.

Question 2: What is the best treatment option?

think over:

Surgical resection: After the diagnosis of anterior mediastinal teratoma, the most common treatment is surgical resection.

Observation: Because the mass is large and symptomatic, simple observation is not recommended.

Chemotherapy: Not suitable for benign tumors.

Interventional embolization: It is not suitable for the initial treatment of such tumors.

Close observation: Also not suitable for symptomatic giant tumors.

Since anterior mediastinal teratoma are usually benign but may be at risk of malignancy and the patient already has chest pain, surgical resection is recommended.

Best answer: A. Surgical resection

Male severe cough, sore throat, cough light red sputum, see multiple red rashes throughout the body

123, (A3 / A4 type question)

. Consider the diagnosis

A. Eaton agent pneumonia

B. Staphylococcus aureus pneumonia

C. streptococcus pneumonia

D. virus pneumonia

E. Klebsiella pneumonia

Reference answer: A. Your answer:

[Analysis] Severe cough, sore throat, light red sputum, multiple red rash throughout the body, the most likely diagnosis of bronchopneumonia (A pair). The typical manifestations of bronchopneumonia are paroxysmal severe cough, nasopharyngeal and ear pain, pharyngeal and eardrum congestion, 10%~20% of patients appear maculopapular rash or multitype erythema.

[Avoid mistakes] Staphylococcus aureus pneumonia (B wrong) onset rapidly, manifested as chills, high fever, pus, blood and phlegm, shortness of breath, toxemia symptoms, shock, etc. Streptococcal pneumonia (C wrong) is mainly manifested by chills, high fever, rust, sputum, chest pain, lung consolidation and other signs. Viral pneumonia (D wrong) has a rapid onset, fever, headache, body pain, burnout and other systemic symptoms are more prominent, often acute influenza symptoms that cough, less phlegm or white mucus phlegm, sore throat and other respiratory symptoms have not subsided. Klebsiella pneumonia (E wrong) is common in the elderly, mainly manifested as chills, high fever and cough brick red glue jelly sputum.

124, (A3 / A4 type question) subject

The therapeutic drugs that should be taken are

A. Azithromycin

B. penicillin

C. acyclovir

D. amikacin

E. cephalosperin N

Reference answer: A. Your answer:

[Analysis] Severe cough, sore throat, cough light red sputum, multiple red rash throughout the body, the most likely diagnosis is bronchopneumonia. Macrolide antibiotics are preferred for treatment, such as azithromycin (A pair), erythromycin, roxithromycin, etc.

[Error avoidance] Penicillin (B wrong), amikacin (D wrong) and cephalosporin (E wrong) are effective in the treatment of Staphylococcus aureus pneumonia. Acyclovir (C-wrong) is an antiviral drug that can be used in the treatment of viral pneumonia.

To answer this question, we need some specific information about the patient's symptoms and signs, as different types of pneumonia differ in pathogens, clinical manifestations and treatment methods. However, I can give some instructional advice based on general knowledge.

123. Different pneumonia types have their typical clinical manifestations and diagnostic basis:

-A. Mycoplasma pneumonia: usually affects young people, symptoms may include dry cough, fever, headache, which may be clear by auscultation.

-B. Staphylococcus aureus pneumonia: common is immunocompromised or acquired in a hospital and may lead to acute onset and severe symptoms.

-C. Streptococcal pneumonia: often leading to typical loboba pneumonia, patients may have high fever, severe chills and shortness of breath.

-D. Viral pneumonia: Common in winter and early spring, and symptoms may include fever, cough, and systemic muscle pain.

-E. Klebsiella pneumonia: pneumonia is commonly acquired in hospitals, especially in patients with a compromised immune system.

Analysis of the diagnosis of the following 123 questions:

A. Mycoplasma pneumonia: usually a persistent cough, but is unlikely to have a systemic red rash, so it can be excluded.

B. Staphylococcus aureus pneumonia: This pneumonia often has a rapid onset, severe disease and rapid progression, but the rash is not common, so it can be excluded.

C. Streptococcal pneumonia: The disease often causes prodromal symptoms of upper respiratory tract infection, such as sore throat, and may have symptoms of sepsis and toxaemia, including a red rash throughout the body, consistent with the patient's symptoms.

D. Viral pneumonia: usually without skin rash and with a more mild course than streptococcal pneumonia, so it can be excluded.

E. Klebsiella pneumonia: the main manifestations are chills, high fever, cough brick red jelly sputum, but the systemic red rash is not common, so it can be excluded.

Therefore, combined with the patient's symptoms, streptococcal pneumonia was the most likely diagnosis, namely, the answer to question 123 was C. streptococcal pneumonia.

124 The choice of medication is usually determined based on the agent of pneumonia:

-A. azithromycin: commonly used for the treatment of pneumonia caused by mycoplasma, chlamydia, etc.

-B. Penicillin: used to treat pneumonia caused by sensitive bacteria, such as some streptococci.

-C. Acyclovir: mainly used for the treatment of viral infections, such as herpes simplex virus and herpes zoster virus.

-D. Amikacin: It is a broad-spectrum antibiotic used to treat severe bacterial infections, especially hospital-acquired infections.

-E. Cephalins: widely used to treat various bacterial infections, including certain pneumonia.

Without specific case information, we cannot accurately determine the best answer. However, based on the common pneumonia pathogens and treatment options, general speculation can be made. For example, if the patient's symptoms are more consistent with mycoplasma pneumonia, then the answer to question 123 may be A (mycoplasma pneumonia), and the answer to question 124 may be A (azithromycin). But the final selection is based on the specific case information.

Analysis of the question 124 therapeutic drugs:

A. Azithromycin: mainly used for the treatment of mycoplasma pneumonia, not meet the question.

B. Penicillin: It is the preferred treatment for streptococcal pneumonia because it is effective against streptococcal infection.

C. Acyclovir: mainly used for antiviral treatment, does not meet the question.

D. Amikacin: Mainly used for gram-negative bacilli and penicillin-resistant Staphylococcus aureus infection, not within the question.

E. Cephalosporin: Although it can also treat streptococcus pneumoniae infection, penicillin is the first choice and does not meet the question.

Therefore, combined with the above diagnosis, penicillin is the most appropriate therapeutic drug, namely, the answer to question 124 is B. penicillin.

Based on the symptoms and medical history provided, consider the most likely diagnosis and the corresponding therapeutic agent:

123, considering the diagnosis

A. Eaton agent pneumonia

The clinical manifestations of mycoplasma pneumonia include severe cough, sore throat, cough with reddish sputum, and systemic rash, which are consistent with the description in the title.

124, should take the therapeutic drugs are

A. Azithromycin

Azithromycin is a macrolide antibiotic and is the first choice for the treatment of mycoplasma pneumonia.

Therefore, the answers are respectively:

123, A. Mycoplasma pneumonia

124, and A. azithromycin

Male, 45 years old, recurrent dry cough with chest tightness for 5 years, more than spring onset, no fever, hemoptysis and nocturnal paroxysmal dyspnea, no abnormal chest X-ray examination, poor antibiotic therapy, no hypertension.

125, (A3 / A4 type question) subject

The most common acid-base balance in the early stages of the disease is

A. respiratory acidosis

B. respiratory alkalosis

C. Respiratory acidosis combined with metabolic alkalosis

D. Respiratory alkalosis combined with metabolic acidosis

E. Respiratory alkalosis combined with metabolic alkalosis

Reference answer: B. Your answer:

[Analysis] middle-aged men, recurrent dry cough with chest tightness for 5 years, more than spring disease (may have allergens), chest radiograph examination without abnormal, antibiotic treatment is not good, considered for bronchial asthma. Early asthma hyperventilation decreased PaCO2 and pH with respiratory alkalosis (B pair).

[To avoid errors] In the later stage, hypoxia was aggravated, and respiratory acidosis (CO2 retention) + metabolic acidosis (increased hypoxia and lactic acid) appeared,

It indicated that the condition had worsened.

The most common acid-base balance in the early stages of the disease is

-The patient mainly showed recurrent dry cough with chest tightness, no fever, hemoptysis and nocturnal paroxysmal dyspnea. These symptoms may point to chronic respiratory diseases, for example, asthma or chronic bronchitis. Under these conditions, the early common abnormal acid-base balance is respiratory alkalosis, as patients will have a tendency to hyperventilation.

-Correct answer: B. Respiratory alkalosis

The correct answer is: B. Respiratory alkalosis.

The patient is considered bronchial asthma. The typical symptoms of bronchial asthma are expiratory dyspnea with wheezing, chest tightness and cough. Symptoms can occur within a few minutes and last for several hours to several days, and can be relieved or spontaneously after treatment with counterasthma drugs. Due to

hyperventilation in asthma, the CO_2 is overdischarged, leading to lower $PaCO_2$ in the blood, and eventually leading to respiratory alkalosis. Thus, the most common acid-base balance in the early stages of the disease is the respiratory alkalosis.

125, the most common acid-base balance in the early stage of the disease is

The medical history mentioned recurrent dry cough and chest tightness, more than in spring, which suggested that it may be related to asthma. Respiratory alkalosis is common in the early stages of asthma, which is due to hyperventilation.

Answer: B. Respiratory alkalosis

126, (A3 / A4 type question) subject

For a definite diagnosis, the preferred examination is

A. Chest X-ray

B. chest CT

C. Heart color ultrasound

D. bronchoscopy

E. Bronchial challenge test

Reference answer: E Your answer:

[Analysis] middle-aged men, recurrent dry cough with chest tightness for 5 years, more than spring disease (may have allergens), chest radiograph examination without abnormal, antibiotic treatment is not good, considered for bronchial asthma. The most meaningful test was the bronchial challenge test (E pair).

[Fault avoidance] Bronchial challenge test (BPT) is used to determine airway responsiveness. For example, if FEV 1 decreases by 20%, the judgment result is positive, indicating the existence of airway hyperresponsiveness. In the chest X-ray (A wrong), the brightness of the two lungs increased, and it was in A state of excessive ventilation, and there was no obvious abnormality in the remission period. The BCD option has little significance for the diagnosis of asthma.

For a definite diagnosis, the preferred examination is

-Given the patient's symptoms and chest X-ray, further imaging is required to exclude structural and inflammatory disease. Chest CT provides more detailed information than chest X-rays, helps to examine small structural changes, and therefore is therefore the preferred method of examination.

-Correct answer: B. Chest CT

The correct answer is: E. Bronchial challenge test.

The patient was a middle-aged man with recurrent dry cough with chest tightness for 5 years, more than in spring, no fever, hemoptysis and nocturnal paroxysmal dyspnea, no abnormal chest radiograph, and poor antibiotic treatment. According to his clinical manifestations and auxiliary examination, the diagnosis of bronchial asthma was considered. In order to clarify the diagnosis, the preferred examination is the bronchial provocation test, which determines the airway hyperresponsiveness by inhalation of provocation agents such as acetylmethylcholine, and is an important examination for the diagnosis of bronchial asthma. Therefore, for a definitive diagnosis, the preferred examination is the bronchial provocation test.

126, for a clear diagnosis, the preferred examination is

The characteristics of patient symptoms and seasonal attacks suggest a greater possibility of asthma. The bronchial challenge test is the gold standard for diagnosing asthma.

Answer: E. Bronchial challenge test

127, (Type A3 / A4 questions) subject

The manifestation of the reaction condition aggravation is

A. Exiratory phase extension

B. Increased pulse pressure

C. respiratory acidosis

D. Chest-abdominal paradoxical movements

E. have the fidgets

Refer to the answer: D. Your answer:

[Analysis] middle-aged men, recurrent dry cough with chest tightness for 5 years, more than spring disease (may have allergens), chest radiograph examination without abnormal, antibiotic treatment is not good, considered for bronchial asthma. It is suggested that the patient's critical manifestations include: inability to speak, drowsiness or confusion, chest and abdominal contradictory movement (D pair), decreased or even disappeared, slow or irregular pulse rate, severe hypoxemia and hypercapnoxemia, and decreased pH.

[Avoid mistakes] The acute onset of asthma is divided into 4 levels.

The manifestation of the reaction condition aggravation is

-When respiratory disease is aggravated, the patient may exhibit chest-abdominal paradoxical movements because severe dyspnea leads to the use of assisted respiratory muscles, which may indicate that the patient is at risk for acute respiratory failure.

-Correct answer: D. Chest and abdominal conflict movements

The correct answer is: D. Chest and abdominal contradictory movement.

The patient is considered for bronchial asthma. When the chest and abdominal paradoxical movement occurs, it means that the abdomen is trapped when the chest expands, and the abdomen is convex when the chest retract. This is the abnormal respiratory movement of diaphragm paralysis, which is the manifestation of aggravation of the condition. Proexpiratory phase, increased pulse pressure, respiratory acidosis, and irritability are also signs that may appear when the condition aggravates, but they do not have high specificity of chest and abdominal contradictory movement. Therefore, the manifestation of the reaction disease aggravation is the chest and abdominal paradoxical exercise.

127, The aggravation of the reaction condition is

When asthma disease aggravates, there may be breath phase extension, respiratory acidosis and other manifestations. Respiratory acidosis suggested hypoventilation and carbon dioxide retention.

Answer: C. Respiratory acidosis

Male, 61 years old, repeated cough and sputum for 10 years, labor shortness of breath for 5 years, fever for 3 days, unclear consciousness for half an hour. Smoking for 43 years, 30 cigarettes / day, physical examination: BP96 / 76mmHg, SpO287%, shallow coma membrane edema, soft neck, no-resistance, low breath in both lungs, heard and wet rales in both lungs, palpable heart beat under the xiphoid, heart rate 110 times / minute, rhythm, unheard noise in the auscultation area of each valve, moderate water negative in both lower limbs.

128, (A3 / A4 type question) subject

The most likely reason why the patient was involved in unconsciousness was

A. heart failure

B. acute cerebrovascular disease

C. pulmonary thromboembolism

D. pulmonary encephalopathy

E. intracranial infeion

Refer to the answer: D. Your answer:

[Analysis] Elderly men, repeated cough and sputum for 10 years (considering COPD), labor shortness of breath for 5 years, fever for 3 days, unclear consciousness for half an hour. Long-term history of heavy smoking, physical examination: BP96 / 76mmHg (low blood pressure, shock state), SpO287% (normal SpO297~100%, the patient oxygen pressure drop), shallow coma, conjunctiva edema, moderate edema, comprehensive patient history, clinical manifestations and signs, considering COPD combined with pulmonary encephalopathy (D) (hypoxia, carbon dioxide retention caused by respiratory failure).

[Avoid failure] Heart failure (A failure) shows exertional dyspnea, pink foam sputum, edema, liver and jugular venous reflux sign positive, etc., which is not the most likely cause of the patient's unconsciousness. Acute cerebrovascular disease (B fault) is mainly manifested as dizziness, headache, limb weakness, limb numbness, limb sensory impairment, language impairment, dysphagia, ataxia, cognitive impairment, etc. Pulmonary thromboembolism (C error) is mainly manifested as chest pain, dyspnea, and hemoptysis. Intracranial infection (E error) usually shows headache, vomiting, and meningeal irritation.

In this case, the patient had a long history of smoking, repeated cough and expectoration, exertional shortness of breath, and developed symptoms of unconsciousness and hypotension. These symptoms suggest the potential for severe respiratory dysfunction.

128, The most likely cause of the patient's confusion is D. pneumonic encephalopathy. Pulmonary encephalopathy, also known as hypercapnic encephalopathy, is a disorder of consciousness caused by severe respiratory failure resulting in an elevated concentration of carbon dioxide in the blood. Considering the patient's chronic smoking history, exertional wheezing, and reduced respiratory sounds, an acute exacerbation may occur in the context of chronic obstructive pulmonary disease (COPD), leading to pulmonary encephalopathy.

128. The most likely reasons for this patient are:

Option A Heart failure: Although the patient has moderate edema in both lower

limbs, which may indicate right heart failure, this does not usually lead to unconsciousness.

Option B Acute cerebrovascular disease: no relevant history or symptoms supporting this diagnosis.

Option C Pulmonary thromboembolism: Although the patient was a chronic smoker and had COPD, there were no mention of typical pulmonary embolism symptoms, such as sudden dyspnea, chest pain, hemoptysis, etc.

Option D Pulmonary encephalopathy: Pulmonary encephalopathy was the most likely cause, considering the patient's history of COPD, the symptoms of respiratory failure and hypoxia, and the acute presentation of unconsciousness. Pulmonary encephalopathy is a neuropsychiatric disorder caused by hypoxia and carbon dioxide retention due to respiratory failure.

Option E Intracranial infection: There is no relevant medical history, symptoms, or signs to support this diagnosis.

Therefore, the most likely cause of confusion in this patient was pulmonary encephalopathy, so the answer is D.

128. The most likely reason for the patient is the confusion

Based on the information provided by the topic, the patient has the following characteristics:

Repeated cough cough sputum for 10 years, labor shortness of breath for 5 years

Smoking history for 43 years, 30 cigarettes per day

At present, there is fever, unconsciousness

Low blood pressure, at SpO2 87%

Shallow coma state, soft neck without resistance, both lungs can smell and wet rales

Combined with the patient's medical history, signs, and symptoms, considering his long-term smoking history, exertional shortness of breath, and current hypoxemia (SpO2 87%), the most likely cause was pulmonary encephalopathy. Pulmonary encephalopathy refers to the severe hypoxia and carbon dioxide retention caused by chronic respiratory diseases, which then leads to central nervous system dysfunction.

So the answer is:

G. pulmonary encephalopathy

129, (Type A3 / A4 questions) subject

The most important treatment measures at present

A. Oxygen absorption in mask

B. High-flow mask for oxygen inhalation

C. mechanical ventilation

D. High-flow oxygen-wetting treatment

E. Low-flow oxygen inhalation through the nasal catheter

Reference answer: E Your answer:

[Analysis] Elderly men, repeated cough and sputum for 10 years (considering COPD), labor shortness of breath for 5 years, fever for 3 days, unclear consciousness for half an hour. Long-term history of heavy smoking, physical examination: BP96 / 76mmHg (low blood pressure, shock state), SpO287% (normal SpO297%~100%, the patient's oxygen partial pressure decreased), shallow coma, conjunctiva edema, moderate edema of both lower limbs, comprehensive medical history, clinical manifestations and signs of the patient, considering COPD with pulmonary encephalopathy. The patient was unconscious and in a shallow coma, and mechanical ventilation was preferred (E pair).

[Error avoidance] Mask oxygen inhalation (A error) and low flow oxygen inhalation of nasal catheter (E error) are all stable COPD treatment. Because high inhaled oxygen concentration can cause carbon dioxide retention, high flow mask for oxygen inhalation (B wrong) and high flow oxygen wetting treatment (D wrong) cannot be used.

129, the main treatment should be C. mechanical ventilation. Considering the patient had unconsciousness, low blood oxygen (SpO_2 87%), and associated coma and shallow coma, emergency mechanical ventilation was needed to ensure airway patency, improve oxygenation and carbon dioxide discharge, and prevent further life threats.

129. The important treatment measures:

Considering that the patient had developed unconsciousness, indicating that hypoxia and carbon dioxide retention are quite severe, and immediate measures to

improve ventilation are needed.

Option A mask for oxygen: While providing higher concentrations of oxygen, it may not quickly improve severe carbon dioxide retention.

Option B High flow mask oxygen inhalation: Similarly, provides only high concentration oxygen and does not address the carbon dioxide retention problem.

Option C Mechanical ventilation: It can quickly improve ventilation and ventilation function, correct hypoxia and carbon dioxide retention, and is the most effective treatment for pulmonary encephalopathy.

Option D High flow oxygen wetting treatment: It mainly provides moist oxygen and does not solve the problem of carbon dioxide retention.

Option E nasal catheter low flow oxygen: suitable for mild hypoxia, not for this patient.

Therefore, the most important treatment measure is mechanical ventilation, so the answer is C.

129, the most important treatment measures

Combined with the clinical manifestations of the patient, the main therapeutic measure should be to improve the oxygenation status of the patient. As the patient's current SpO 2 was only 87%, hypoxemic and in a shallow coma, hypoxia needed to be corrected quickly.

Mask oxygen inhalation and high-flow mask oxygen inhalation may not meet the patient's oxygen needs

High-flow oxygen wetting therapy is mainly suitable for patients with dry sputum

Low-flow oxygen inhalation in the nasal catheter is not suitable for the management of severe hypoxemia

Therefore, the most important therapeutic measure should be mechanical ventilation to ensure adequate oxygen supply and improve ventilation.

So the answer is:

F. mechanical ventilation

Male, 45 years old, repeated upper abdominal pain for more than ten years, more than autumn and winter, night pain is obvious, radiation to the back, nearly a week of pain again, the front row of asphalt sample twice a day, medium amount, no fear. Physical examination: P90 / min, R18 / min, BP110 / 75mmHg, soft abdomen, supraoumbilical tenderness. HB100g / L, fecal occult blood (+ + +).

130, (Type A3 / A4 questions) subject

To clarify the diagnosis, which of the following tests is feasible

A. Pepsinogen I /

B. Abdominal-enhanced CT

C.13C rapid urease

D.24-hour acid reflux monitoring

H. gastroscope

Reference answer: E Your answer:

Graphic analysis: [analytical] middle-aged male patients, repeated abdominal pain for more than ten years, more than autumn and winter, night pain (duodenal ulcer), radiation to the back, nearly a week, the front asphalt twice a day (peptic ulcer bleeding), HB100g / L (male HB normal value 120g / L, mild anemia), fecal blood (+ + +) (ulcer with gastrointestinal bleeding), in conclusion, consider for duodenal ulcer with gastrointestinal bleeding. Gastroscopy (E pair) is preferred for a definite diagnosis.

[Error avoidance] Gepepsin I / (A error) can play A role in serological biopsy of gastric fundus gland mucosa, which is helpful for the diagnosis of gastritis. Abdominal CT (B wrong) is valuable for the diagnosis of peptic ulcer perforation, penetrating ulcer and intestinal obstruction.13C rapid urease (C error) is one of the important methods for HP detection.24-hour acid reflux monitoring (D error) mainly clarified whether excessive acid and alkali reflux exist in the esophagus.

This case describes a 45-year-old male patient with prolonged recurrent upper abdominal pain, increased pain at night and radiation to the back, the latest week, and tarred stools, symptoms suggestive of possible upper gastrointestinal bleeding. This diagnosis was further supported by her clinical findings and examination findings (HB 100 g / L, fecal hidden blood + +). Based on this information, I will answer each

question:

To clarify the diagnosis, which of the following tests is feasible

The answer is E. gastroscope. Gastroscopy can directly view the upper gastrointestinal mucosa, find the source of bleeding, and biopsy, which is the preferred method to clarify the cause of upper gastrointestinal bleeding.

130. Analysis:

Middle-aged male patients with long-term recurrent upper abdominal pain, obvious pain at night, a history of asphalt discharge, physical examination, upper abdominal tenderness, mild decrease in hemoglobin in laboratory examination, and strong positive fecal occult blood. Combined with the patient's medical history, clinical manifestations and laboratory examination, peptic ulcer complicated with bleeding should be highly suspected. In order to clarify the diagnosis, gastroscopy can be carried out, which can directly look at the lesion, take a biopsy, and also conduct timely and accurate hemostatic treatment for the bleeding lesion.

Option A pepsinogen I / can be used as a means to monitor the state of gastric mucosa. Its abnormality can indicate gastric disease, but it cannot confirm peptic ulcer, so it is not selected.

Option B Abdominal enhanced CT is mainly used for abdominal organs and lymph node metastasis and the judgment of the disease stage, which is of little significance to the diagnosis of peptic ulcer, so it is excluded.

Option C13C rapid urease test is mainly used to detect Helicobacter pylori, and Helicobacter pylori infection is only one of the causes of peptic ulcer, so it cannot be clearly diagnosed.

Option D24-hour acid reflux monitoring is the examination method for the diagnosis of gastroesophageal reflux disease, peptic ulcer generally does not appear acid reflux, so it is not considered.

In conclusion, the correct answer is: E. gastroscope

130, which of the following tests is feasible for a clear diagnosis

A. Pepsinogen I /

B. Abdominal-enhanced CT

C. 13C rapid urease

D. 24-hour acid reflux monitoring

C. gastroscope

analyse:

The patient had symptoms such as recurrent epigastric pain, nocturnal pain and taryuan stools, which suggested the possibility of peptic ulcer or upper gastrointestinal bleeding. To clarify the diagnosis, gastroscopy is the first choice, because gastroscopy can not only directly observe the gastric mucosa conditions, but also perform biopsy and treatment.

The best answer: E. gastroscope

131, (A3 / A4 type question) subject

The direct factor leading to the mucosal damage in this patient is that

A. Abnormal blood supply to the gastric mucosa

B. Autodigestion caused by stomach acid and pepsin

C. Abnormal gastrin secretion

D. Reduced mucus secretion in the gastrointestinal mucosa

E. Abnormal gastric motility

Reference answer: B. Your answer:

Graphic analysis: [analytical] middle-aged male patients, repeated abdominal pain for more than ten years, more than autumn and winter, night pain (duodenal ulcer), radiation to the back, nearly a week, the front asphalt twice a day (peptic ulcer bleeding), HB100g / L (male HB normal value 120g / L, mild anemia), fecal blood (+ + +) (ulcer with gastrointestinal bleeding), in conclusion, consider for duodenal ulcer with gastrointestinal bleeding. The mucosal antidigestive ability is reduced, and the destruction of the gastric and duodenal mucosal defense barrier function is an important reason for the digestion of gastric and duodenal mucosal tissues by gastric acid and pepsin to form ulcers (B pair).

[Avoid mistakes] Smoking may also damage the mucosal blood circulation, and then damage the mucosal defense barrier; vagal nerve excitability, gastric peristalsis, gastric in secretion, and gastric acid secretion; when insufficient gastric mucus secretion or mucosal epithelial damage, the barrier function of the gastric mucosa is weakened, and the hydrogen ions in the gastric juice can reverse diffuse into the

gastric mucosa; the above are indirect factors leading to gastric mucosa damage.

The direct factor contributing to the mucosal damage in this patient is that

The answer is self-digestion caused by B. gastric acid pepsin. Long-term gastric acid and pepsin action on the gastric mucosa can cause mucosal damage, form ulcers and may cause bleeding.

131. Analysis:

The etiology of peptic ulcer is mainly related to the imbalance between the damage factors of gastroduodenal mucosa and the defense-repair factors of mucosal itself, among which the invasion of gastric acid and pepsin is one of the main factors in the formation of peptic ulcer. Gastric acid has an invasive effect on the digestive tract mucosa. When gastric acid is excessively secreted, it can damage the digestive tract mucosa and form ulcers. Therefore, the direct factor causing mucosal damage in peptic ulcer patients is the self-digestion caused by gastric acid pepsin.

Option A abnormal blood supply to gastric mucosa, option C abnormal gastrin secretion, option D decreased gastrointestinal mucosal mucus secretion, option E abnormal gastric motility, which can affect the gastric mucosal barrier function, which is an indirect cause of peptic ulcer, so it is excluded together.

In conclusion, the correct answer is: B. Self-digestion caused by gastric acid pepsin

131, the direct factor leading to the mucosal damage in this patient is

A. Abnormal blood supply to the gastric mucosa

B. Autodigestion caused by stomach acid and pepsin

C. Abnormal gastrin secretion

D. Reduced mucus secretion in the gastrointestinal mucosa

E. Abnormal gastric motility

analyse:

The patient had typical manifestations of peptic ulcer, especially nocturnal pain and back radiating pain. The main pathological mechanism of peptic ulcer is the autodigestive effect of gastric acid and pepsin on the gastric mucosa.

Best answer: B. Self-digestion caused by gastric acid pepsin

132, (A3 / A4 type question) subject

The treatment that should be taken first is that of

A. Emergency blood transfusion

B. proton pump inhibitor

C. antidiuretic hormone

D.6-aminocaproic acid

D. H2 receptor impedance agent

Reference answer: B. Your answer:

Graphic analysis: [analytical] middle-aged male patients, repeated abdominal pain for more than ten years, more than autumn and winter, night pain (duodenal ulcer), radiation to the back, nearly a week, the front asphalt twice a day (peptic ulcer bleeding), HB100g / L (male HB normal value 120g / L, mild anemia), fecal blood (+ + +) (ulcer with gastrointestinal bleeding), in conclusion, consider for duodenal ulcer with gastrointestinal bleeding. The patient has little blood loss, preferred proton pump inhibitor (B pair).

[Error avoidance] Patients with hemoglobin> 70g / L without blood transfusion (A wrong); vasopressor (C wrong) for patients with hyponatremia in cirrhosis.6-aminoacetic acid (D wrong) can be used in the treatment of von Willebrand. Proton pump inhibitors benefit some refractory ulcers better than H2 receptor antagonists (E error).

The treatment that should be taken first is that of

The answer is that B. proton pump inhibitors. In the acute treatment of upper gastrointestinal bleeding, bleeding should be controlled and rebleeding prevented. Proton pump inhibitors can effectively reduce gastric acid secretion and reduce the damage to the mucosa, which is the first choice for treatment. Emergency blood transfusion may be considered during massive blood loss, but the bleeding source should be controlled first.

132. Analysis:

The treatment principles of peptic ulcer complicated with bleeding are hemostasis, infusion, prevention and treatment of shock, and drugs to inhibit gastric acid secretion and protect gastric mucosa. The proton pump inhibitor can inhibit gastric acid secretion, increase gastric pH value, and enhance the hemostasis induced

by platelet aggregation and plasma coagulation function, which should be used as the preferred treatment for peptic ulcer complicated with bleeding.

The patient had slightly decreased hemoglobin, no anemia such as dizziness, palpitations, and no indication for blood transfusion, so option A emergency blood transfusion can be excluded.

Option C vasopressor is mainly used for hemostasis of esophageal varices bleeding caused by portal hypertension of liver cirrhosis, and is not suitable for peptic ulcer bleeding, so it is not considered.

Option D6-aminohexanic acid is an antifibrinolytic drug, which can inhibit fibrin formation, has antithrombin effect, can inhibit platelet adhesion aggregation and release, and can prevent thrombosis, which does not meet the question.

Option E: H2 receptor impedance can inhibit gastric acid secretion, but the acid suppression effect is not as good as proton pump inhibitor. It is often used as a second-line drug for peptic ulcer, so it is not selected.

In conclusion, the correct answer is: B. proton pump inhibitors

132, should take the first treatment is

A. Emergency blood transfusion

B. Proton-pump inhibitors

C. antidiuretic hormone

D. 6-aminocaproic acid

D. H2 receptor impedance agent

analyse:

The patient had peptic ulcer and signs of upper gastrointestinal bleeding (tarm stool, hemoglobin drop). Proton pump inhibitors (PPI) are the drugs of choice for peptic ulcers and upper gastrointestinal bleeding because they are effective in inhibiting gastric acid secretion, promoting ulcer healing and reducing bleeding.

Best answer: B. Proton pump inhibitors

133. (Type B1 question)

Acute inferior myocardial infarction complicated with one-degree atrioventricular block is preferred

A. verapamil

B. digitalis

C. atropine

D. lidocaine

E. amiodarone

Reference answer: C Your answer:

Graphic analysis: [analysis] acute myocardial infarction combined with slow arrhythmia can use atropine.

For question 133, acute inferior myocardial infarction complicated by one-degree AV block, the preferred treatment is usually not immediate pharmacological intervention because one-degree AV block may not require specific treatment unless it causes significant bradycardia or symptoms. Among the options listed, atropine (C) can be used to treat the bradycardia caused by a heart block. Therefore, atropine (C) may be the most appropriate answer.

First, we analyzed question 133, the preferred treatment for acute inferior myocardial infarction complicated by one-degree atrioventricular block. In this case, the key is in improving the conduction function of the heart. Once atrioventricular block usually does not require specific treatment, but if needed, use drugs that increase heart rate and improve heart conduction.

Option A (Verapamil): mainly for hypertension, angina and arrhythmia, but not the drug of choice for acute inferior myocardial infarction with primary atrioventricular block.

Option B (digitalis): Mainly used to treat heart failure and has limited effect on improving heart conduction.

Option C (atropine): can relieve smooth muscle spasm, large amount can relieve small vasospasm, improve microcirculation, inhibit the secretion of glands, relieve the inhibition of vagus nerve on the heart, accelerate the stroke, dilated pupils, increase intraocular pressure, stimulate the respiratory center, and relieve respiratory depression. In this context, atropine increases heart rate and may help to improve one-degree atrioventricular block.

Option D (lidocaine): Mainly used for local anesthesia and treatment of arrhythmias, but usually not used for one-degree atrioventricular block treatment.

Option E (amiodarone): It is mainly used for treating cardiac arrhythmias,

especially ventricular arrhythmia, but not as the preferred treatment for one-degree atrioventricular block.

Taken together, atropine is the best option for acute inferior myocardial infarction complicated by one-degree atrioventricular block, because it increases heart rate and helps improve heart conduction. Therefore, C. atropine was chosen for question 133.

Acute inferior myocardial infarction complicated with one-degree atrioventricular block is preferred:

F. atropine

Rationale: Once atrioventricular block (AV block) is commonly seen in acute inferior myocardial infarction (MI) due to increased vagal tone. Atropine is an anticholindrug that relieves the vagus nerve and increases heart rate and is therefore the preferred therapeutic agent.

134. (Type B1 question)

Acute myocardial infarction complicated by ventricular tachycardia is preferred

A. verapamil

B. digitalis

C. atropine

D. lidocaine

E. amiodarone

Refer to the answer: D. Your answer:

[Analysis] Once an acute myocardial infarction is found with preventricular contraction or ventricular tachycardia, lidocaine should be used immediately.

For question 134, an acute myocardial infarction complicated by ventricular tachycardia requires rapid and effective antiarrhythmic drugs. Among the options given, amiodarone (E) is the drug of choice for the treatment of ventricular tachycardia, especially in the context of acute myocardial infarction. Amiodarone is beneficial for stabilizing the heart rhythm and preventing the recurrence of ventricular tachycardia.

Next, we analyzed question 134, regarding the preferred treatment for acute myocardial infarction complicated by ventricular tachycardia. In this case, the goal is to rapidly control the arrhythmia and restore the normal heart rhythm.

Option A (Verapamil): Although available for arrhythmia treatment, it is not the drug of choice for acute myocardial infarction complicated with ventricular tachycardia.

Option B (digitalis): Again, not the preferred treatment.

Option C (atropine): not applicable in this case because it may exacerbate tachycardia.

Option D (lidocaine): It is an effective antiarrhythmic drug, especially in the setting of acute myocardial infarction, which is widely accepted as the preferred treatment for ventricular tachycardia.

Option E (amiodarone): Although also used for treating arrhythmias, it is usually not preferred in cases of acute myocardial infarction complicated by ventricular tachycardia.

Therefore, lidocaine is the best choice. So, question 134 chooses D. Lidocaine.

Acute myocardial infarction complicated by ventricular tachycardia is preferred:

G. amiodarone

Rationale: Amiodarone is an antiarrhythmic drug that is commonly used for the treatment of ventricular tachycardia (VT) associated with acute myocardial infarction (MI). It is able to effectively inhibit abnormal ventricular activity and restoring normal heart rhythm.

135. (Type B1 question)

The presentation of chronic osteomyelitis is

A. abnormal movement

B. Swelling and pain

C. dysfunction

D. Skin bruises

E. sequestration

Reference answer: E Your answer:

Graphic analysis: [analysis] the proprietary symptoms of fracture abnormal activity, bone friction feeling, deformity. Symptoms of chronic osteomyelitis: limb thickening, loss of the original morphology, fistula formation, and discharge of dead bones.

For question 135, the most typical manifestation of chronic osteomyelitis is dead bone formation (E). Chronic osteomyelitis is a long-term bone marrow infection process that usually leads to necrosis of bone bones, so dead bone formation is its characteristic manifestation.

For the presentation of chronic osteomyelitis:

A. Abnormal activity: This is not a specific manifestation of chronic osteomyelitis.

B. Swelling pain: Although this may occur in patients with chronic osteomyelitis, it is not specific.

C. Dysfunction: Similarly, dysfunction may be caused by multiple causes, not specific manifestations of chronic osteomyelitis.

D. Skin ecchymosis: This is usually associated with trauma or hematological problems and is not typical of chronic osteomyelitis.

E. Dead bone formation: this is a specific manifestation of chronic osteomyelitis. During the chronic phase of osteomyelitis, bones may become necrotic due to ischemia and infection.

In conclusion, the specific manifestation of chronic osteomyelitis is option E. Dead bone formation.

The presentation of chronic osteomyelitis is

G. sequestration

Characteristics of chronic osteomyelitis include persistent infection, formation of dead bone in the bone marrow cavity, and recurrent episodes of abscesses or sinus tract. Thus, "dead bone formation" is a typical manifestation of chronic osteomyelitis.

136. (Type B1 question)

The fractures' proprietary signs are

A. abnormal movement

B. Swelling and pain

C. dysfunction

D. Skin bruises

E. sequestration

Reference answer: A. Your answer:

Graphic analysis:

[Analysis] The unique signs of fracture are: local deformity, abnormal activity, bone rubbing sound or bone rubbing sensation. The table of chronic osteomyelitis

Now: limb thickening, loss of the original form, fistula formation, dead bone discharge.

For question 136, discussing the proprietary signs of the fracture, abnormal activity (A) is a very distinct proprietary sign of the fracture. Under normal circumstances, there should not be any form of abnormal activity, and the loss of structural integrity due to the fracture can lead to abnormal activity or abnormal mobility.

For proprietary signs of fracture:

A. Abnormal activity: After the fracture, abnormal activity may occur in the area that should not be active, which is an exclusive sign of the fracture.

B. Swelling and pain: Although these symptoms may occur after a fracture, they are not exclusive signs of a fracture, because other causes may also cause these symptoms.

C. Dysfunction: Similarly, dysfunction may be caused by a variety of causes, and is not a proprietary sign of fracture.

D. ecchymosis: ecchymosis may occur after the fracture, but this is not a exclusive sign of the fracture.

E. Dead bone formation: This is unrelated to fracture and is a manifestation of chronic osteomyelitis.

In conclusion, the fracture proprietary signs are option A. Abnormal activity.

The fractures' proprietary signs are

A. abnormal movement

Proprietary signs of a fracture include abnormal activity of the bone (i. e., abnormal movement between the fracture ends) and bone friction sounds, while swelling, pain, dysfunction, and skin ecchymosis, although they may also accompany the fracture, are not proprietary signs. Therefore, "abnormal activity" is a characteristic sign of a fracture.

137. (Type B1 question)

Diabetic nephropathy combined with hypertension is preferred

A. β -blockers

B. diuretic

C. α -blockers

D. Angiotensin receptor blockers

E. Ca2+ channel blocker

Refer to the answer: D. Your answer:

[Analysis] β blockers can slow heart rate and cardiac contractility, reduce myocardial contractility and oxygen consumption. Angiotensin receptor blockers reduce blood pressure and reduce proteinuria.

For the first problem (diabetic nephropathy with hypertension), the preferred treatment is usually D. angiotensin receptor blockers (ARBs), because they reduce blood pressure and slow the progression of diabetic nephropathy.

For question 137, the first choice for diabetic nephropathy combined with hypertension, the key is to choose a drug that can target the characteristics of diabetic nephropathy. The characteristics of diabetic nephropathy include kidney injury and hypertension, so the selected drugs need to be able to lower blood pressure while protect the kidney.

Analysis options:

A. β -blockers: They lower blood pressure, but not significant kidney protection.

B. Diuretics: mainly used to drain sodium, reduce blood volume, thus reducing blood pressure, but the direct protective effect on the kidney is limited.

C. α -blockers: mainly by blocking vascular smooth muscle α 1 receptor and direct relaxation of vascular smooth muscle, vascular dilation, peripheral resistance and blood pressure, but the protective effect on the kidney is not obvious.

D. Angiotensin receptor blockers: this kind of drugs can not only effectively reduce blood pressure, but also improve the hemodynamics of the kidney, reduce urinary protein, have obvious protective effect on the kidney, is the first choice for the treatment of diabetic nephropathy combined with hypertension.

E. Calcium channel blockers: Although they reduce blood pressure, they are not significantly kidney protective.

In conclusion, for diabetic nephropathy combined with hypertension, the preferred therapeutic agent is the angiotensin receptor blocker, so the answer to

question 137 is D.

138. (Type B1 question)

Coronary heart disease stable angina pectoris combined with hypertension is preferred

A. β -blockers

B. diuretic

C. α -blockers

D. Angiotensin receptor blockers

E. Ca^{2+} channel blocker

Reference answer: A. Your answer:

[Analysis] β blockers can slow heart rate and cardiac contractility, reduce myocardial contractility and oxygen consumption. Angiotensin receptor blockers reduce blood pressure and reduce proteinuria.

For the second problem (coronary heart disease stable angina with hypertension), the preferred treatment is usually A. β blockers, because they can reduce the oxygen requirement of the heart, thus reducing the symptoms of angina, while controlling hypertension.

Next, question 138, the preferred treatment for stable angina combined with hypertension. The key here is to choose a drug that simultaneously reduces blood pressure and improves angina symptoms.

Analysis options:

A. β blockers: this kind of drugs can slow down heart rate, reduce myocardial contractility and myocardial oxygen consumption, thus reducing the attack of angina pectoris, but also can effectively reduce blood pressure, is the preferred treatment of coronary heart disease stable angina combined with hypertension.

B. Diuretics, C. α -blockers, D. angiotensin receptor blockers, E. Calcium channel blockers: Although these drugs reduce blood pressure, they are not as effective for angina as β -blockers.

Therefore, for coronary heart disease stable angina combined with hypertension, the preferred treatment drug is β receptor blocker, so the answer to question 138 is A.

138. Stable angina pectoris of coronary heart disease combined with

hypertension is preferred

A. β -blockers

Explanation: β blockers are preferred for patients with stable angina with hypertension. These drugs can not only effectively control blood pressure, but also reduce the frequency of angina attacks and improve the blood supply to the heart, thus reducing the risk of cardiovascular events.

139. (Type B1 question)

Laboratory tests belonging to the fibrinolytic abnormalities are

A. Blood vWF determination

B. PF3 effectiveness determination

C. Thromxane B2 measurement

D. Blood for the PC assay

E. Blood D-dimer assay

Reference answer: E Your answer:

Question 139: The laboratory examination belonging to the fibrinolysis abnormality is

Abnormalities in the fibrinolytic system are usually assessed by monitoring the process of fibrinolysis, and determination of blood D-dimer can be used to assess the activity of the fibrinolytic system. The D-dimer is a product produced after fibrinolysis and can be indicative of fibrin formation and subsequent solubilization activity, so it is an indicator of abnormal fibrinolysis.

Answer: E. Blood D-dimer determination

First, we will understand the core of these two issues. The first was looking for laboratory tests associated with abnormal fibrinolysis, while the second was laboratory tests regarding anticoagulation abnormalities.

For fibrinolysis abnormality, it mainly involves the decomposition process of thrombosis. When the body forms a thrombus, the fibrinolytic system breaks it down to prevent it from growing excessively or clogging blood vessels. In this process, the D-dimer is released. Therefore, the determination of blood D-dimers can reflect the activity of plasmin and is an important indicator of abnormal fibrinolysis.

139. The laboratory tests of abnormal fibrinolysis are:

A. Measurement of blood vWF: The determination of factor Von Willebrand (vWF) is mainly used to diagnose von Willebrand disease and other diseases related to von Willebrand disease, usually not associated with fibrinolytic abnormalities.

B. Measurement of PF 3 effectiveness: Measurement of platelet factor 3 (PF 3) is not correlated with fibrinolysis, but mainly with coagulation function.

C. Measurement of thromboxane B2: thromboxane B2 is a marker of platelet activation, which is mainly related to platelet function and thrombosis, and does not directly reflect the fibrinolytic state.

D. Blood PC measurement: Protein C (PC) is an anticoagulant protein that is usually associated with the anticoagulant function.

E. Blood D-dimer measurement: D-dimer is a product of fibrin degradation, indicating increased fibrinolysis activity, and it is therefore associated with abnormal fibrinolysis.

Correct answer: E. Blood D-dimer determination

140. (Type B1 question)

Laboratory tests that belong to the anticoagulation abnormalities are

A. Blood vWF determination

B. PF3 effectiveness determination

C. Thromxane B2 measurement

D. Blood for the PC assay

E. Blood D-dimer assay

Refer to the answer: D. Your answer:

Graphic analysis:

140: The laboratory examination of anticoagulant abnormalities is

Anticoagulation abnormalities usually involve the determination of coagulation and anticoagulant factors in plasma. Blood PC (protein C) measurement is an important indicator to evaluate the function of the anticoagulation system, because protein C is a protein with an important physiological activity of anticoagulation and

promoting fibrinolysis. When protein C levels are abnormal, it may lead to anticoagulation dysfunction.

Answer: D. Blood PC determination

Then at the anticoagulant abnormalities, which are related to the body's anticoagulant system, which prevents blood from clotting. In this system, there are several anticoagulants, among which protein C (PC) is a key anticoagulant factor. When the body lacks PC or has abnormalities of PC function, it increases the risk of thrombosis. Therefore, blood PC measurement is a key laboratory test for anticoagulant abnormalities.

140. The laboratory tests belonging to the anticoagulant abnormalities are:

A. Blood vWF measurement: As previously mentioned, vWF measurement is mainly used to diagnose von Willebrand disease and is not related to anticoagulant function.

B. PF 3 effectiveness measurement: As previously mentioned, PF 3 is mainly related to coagulation function, not an examination of anticoagulant function.

C. Thromxane B2 measurement: As previously described, thromboxane B2 is associated with platelet activation and thrombosis and is not an examination of anticoagulant function.

D. Blood PC measurement: Protein C is an anticoagulant protein, whose abnormal levels can reflect the abnormal anticoagulant function.

E. Blood D-dimer measurement: As mentioned above, the D-dimer is mainly used to reflect the fibrinolytic state and is independent of the anticoagulant function.

Correct answer: D. Blood PC determination

141. (Type B1 question)

The most meaningful test method for Crohn's disease diagnosis is

A. Abdominal B ultrasound

B. belly CT

C. Fecal latent blood

D. gastroscope

E. Digestive tract X-ray barium contrast

Reference answer: A. Your answer:

Graphic analysis: [analysis] gastroscope can clearly see the ulcer surface, ulcer size and surrounding situation. Crohn's disease can be observed by X-ray barium contrast and colonoscopy of the digestive tract and periodic distribution of intestinal lesions.

141、Crohn's disease (Crohn's disease) is a chronic inflammatory intestinal disease that can affect any part of the digestive tract. The diagnosis of Crohn's disease usually requires multiple methods of examination, where imaging is particularly important. Barium X-ray contrast (E option) was once one of the routine methods to diagnose Crohn's disease because it shows stenosis, fistula, and other abnormalities in the gut. However, with technological advances, abdominal CT (option B) has become the method of choice for the diagnosis of Crohn's disease because of its high resolution and its ability to comprehensively display the bowel wall and surrounding tissues. Therefore, I tend to choose B. abdominal CT for the most meaningful examination method for the diagnosis of Crohn's disease.

For question 141, regarding the diagnosis of Crohn's disease:

Option A (abdominal B-ultrasound): Although B-ultrasound is helpful for the diagnosis of abdominal diseases in some cases, it is not effective for the display of intestinal tract and other empty cavity organs, and is not the preferred method to diagnose Crohn's disease.

Option B (Abdominal CT Abdominal): CT has some advantages for the display of abdominal organs and lymph nodes, but is not the most sensitive method for the diagnosis of Crohn's disease.

Option C (fecal occult blood): This is mainly used for detecting gastrointestinal bleeding, and is not directly used to diagnose Crohn's disease.

Option D (Gastroscopy): Gastroscopy is mainly used to check gastric diseases, and has limited value for the diagnosis of Crohn's disease.

Option E barium contrast (X-ray of digestive tract): This method can clearly show the tiny structure of intestinal mucosa and stenosis and obstruction of intestinal tract, which is of great significance for the diagnosis of Crohn's disease.141. E. X-ray barium contrast of the digestive tract

The most meaningful examination method for the diagnosis of Crohn's disease

Crohn's disease is a chronic inflammatory bowel disease that often involves

multiple sites of the gastrointestinal tract. For the diagnosis of Crohn's disease, the most meaningful examination method should enable a comprehensive assessment of the lesions in the gastrointestinal tract and determine the site, extent, and severity of the lesions. Here is an analysis of the options:

A. Abdominal B ultrasound: suitable for the evaluation of abdominal internal organs, but limited assessment of the gastrointestinal tract, especially the small intestine.

B. Abdominal CT: It can provide detailed images of the intraabdominal organs and intestines, and help to evaluate the complications of Crohn's disease, such as intestinal perforation, abscess, etc.

C. Fecal occult blood: intestinal bleeding but not Crohn's disease.

D. Gastroscopy: mainly used for examining the upper digestive tract, such as the esophagus, stomach and duodenum, with limited assessment of the small intestine.

E. Barium X-ray contrast: it can show the anatomical structure and functional status of the digestive tract, but the sensitivity and specificity are inferior to CT.

For Crohn's disease, the most meaningful examination method is abdominal CT, as it can provide detailed imaging information, facilitate a comprehensive assessment of the extent and severity of the lesion and detect possible complications. So the answer is:

B. belly CT

142. (Type B1 question)

The most meaningful test for diagnose gastric ulcers is

A. Abdominal B ultrasound

B. belly CT

C. Fecal latent blood

D. gastroscope

E. Digestive tract X-ray barium contrast

Refer to the answer: D. Your answer:

Graphic analysis: [analysis] gastroscope can clearly see the ulcer surface, ulcer size and surrounding situation. Crohn's disease can be observed by X-ray barium contrast and colonoscopy of the digestive tract and periodic distribution of intestinal lesions.

142、Gastric ulcer is an ulcerative lesion occurring in the lining of the stomach. For the diagnosis of gastric ulcer, endoscopy (gastroscopy, D option) is the most important method, because it can intuitively observe the ulcers and lesions of the gastric wall, and it is able to perform a biopsy to exclude malignant lesions. Therefore, I choose the most meaningful examination method for gastric ulcer diagnosis, D. Gastroscopy.

For question 142, about the diagnosis of gastric ulcer:

Option A (abdominal B ultrasound): As mentioned above, B ultrasound is not effective for the display of cavity organs and is not suitable for the diagnosis of gastric ulcer.

Option B (Abdominal CT): Although CT can show abdominal viscera and lymph node metastasis, the direct visualization of gastric mucosa is limited.

Option C (fecal occult blood): This only indicates gastrointestinal bleeding, not a clear diagnosis of gastric ulcer.

Option D (gastroscopy): Gastroscopy can directly observe the lesions of gastric mucosa, including the location, size and morphology of gastric ulcer, which is the most direct and effective method to diagnose gastric ulcer.

Option E (X-ray barium contrast of the digestive tract): Although it can show the shape of the stomach, it is not as effective as small lesions of the gastric mucosa than gastroscopy.

The most meaningful examination method for the diagnosis of gastric ulcer

Gastric ulcer is a common peptic ulcer disease, requiring intuitive observation of the damage of gastric mucosa. Here is an analysis of the options:

A. Abdominal B ultrasound: not suitable for patients to evaluate the lesions in the gastric wall.

B. Abdominal CT: can show the anatomy of the stomach, but show limited details of the gastric mucosa.

C. Fecal occult blood: can indicate gastrointestinal bleeding, but can not confirm gastric ulcer.

D. Gastroscopy: the gastric mucosa can be visually seen, and the ulcer site can be directly observed. biopsy and pathological examination can also be performed, which is the gold standard for the diagnosis of gastric ulcer.

E. X-ray barium contrast of the digestive tract: it can show the anatomy of the stomach, but not as directly as gastroscopy.

For gastric ulcer, the most meaningful examination method is gastroscopy, because it is able to directly observe the ulcer site and perform a biopsy. So the answer is:

C. gastroscope

143. (Type B1 question)

The most meaningful work-up to diagnose early primary HCC is

A. Blood amylase test

B. Serum alpha-fetoprotein detection

C. A CT examination of the abdomen

D. blood CEA

E. blood CRP

Reference answer: B. Your answer:

[Analysis] Abdominal CT, especially enhanced CT, is the best examination method for preoral diagnosis of pancreatic necrosis, and it is the most meaningful examination for the diagnosis of hemorrhagic necrotizing pancreatitis. The correct answer is C rather than A, because serum amylase in patients with hemorrhagic necrotizing pancreatitis can be increased, normal or even below normal, indicating that serum amylase measurement is not of great diagnostic value for hemorrhagic necrotizing pancreatitis. Primary liver cancer is often positive for serum alpha-fetoprotein, and the positive rate is about 68%~74%, which is the most meaningful laboratory test for the diagnosis of early liver cancer.

143、For the diagnosis of early stages of primary liver cancer, the most meaningful test is the B. serum alpha-fetoprotein test. Al-fetoprotein (AFP) is a common marker of liver cancer, which is especially important in the diagnosis of early liver cancer. Although AFP may also be elevated in some other conditions (such as in pregnancy or other liver diseases), it is a key index in the diagnosis of primary liver cancer.

First, let's look at the first question: is the most meaningful diagnosis of early primary liver cancer?

Option A. Blood amylase testing is commonly used to assess pancreatic function, especially in the diagnosis of acute pancreatitis, with no direct relation to liver cancer.

Option B. Serum alpha-fetoprotein test is a specific marker for the diagnosis of primary liver cancer. AFP-fetoprotein (AFP) is a serum glycoprotein synthesized from the liver and yolk sac in the early fetal stage. AFP synthesis is rapidly inhibited after birth. When liver cells or gonad embryonic tissues undergo malignant lesions, the relevant genes are reactivated, so that the cells that had lost AFP synthesis can start synthesis again, so that the content of AFP in the blood is significantly increased. Therefore, the serum AFP of patients with primary hepatocellular carcinoma is increased with a positive rate of about 70%, which can be used as a specific marker for the diagnosis of HCC.

Option C. Abdominal CT examination, while providing structural information about the liver, is not as sensitive and specific as serum alpha-fetoprotein detection.

Option D. Blood CEA (carcinoembryonic antigen) is mainly used as a tumor marker for colorectal cancer and has little to do with liver cancer.

Option E. Blood CRP (C-reactive protein) is an indicator of inflammation that is not specific and cannot be used for the diagnosis of liver cancer.

In conclusion, for the first question, the most meaningful test for the diagnosis of early primary liver cancer is the serum alpha-fetoprotein test, so the answer is selected in B.

The most meaningful work-up to diagnose early primary HCC is

Answer: B. Serum alpha-fetoprotein test

Serum alpha-fetoprotein (AFP) is the most meaningful marker for the diagnosis of early primary liver cancer, especially when screening high-risk groups. AFP is a protein produced during the fetal stage and has very low serum levels in adults. If high levels of AFP are detected in adult serum, especially in patients with chronic liver disease, it may indicate the presence of primary liver cancer.

144. (Type B1 question)

The most meaningful test for the diagnosis of acute bleeding and necrotizing pancreatitis is

A. Blood amylase test

B. Serum alpha-fetoprotein detection

C. A CT examination of the abdomen

D. blood CEA

E. blood CRP

Reference answer: C Your answer:

[Analysis] Abdominal CT, especially enhanced CT, is the best examination method for preoral diagnosis of pancreatic necrosis, and it is the most meaningful examination for the diagnosis of hemorrhagic necrotizing pancreatitis. The correct answer is C rather than A, because serum amylase in patients with hemorrhagic necrotizing pancreatitis can be increased, normal or even below normal, indicating that serum amylase measurement is not of great diagnostic value for hemorrhagic necrotizing pancreatitis. Primary liver cancer is often positive for serum alpha-fetoprotein, and the positive rate is about 68%~74%, which is the most meaningful laboratory test for the diagnosis of early liver cancer.

144、The most meaningful test for the diagnosis of acute bleeding and necrotizing pancreatitis is the A. blood amylase test. The diagnosis of pancreatitis is mainly based on the elevation of blood amylase or lipase, especially when typical abdominal pain and radiographic pancreatic abnormalities occur. A significant

increase in blood amylase levels is usually associated with the diagnosis of acute pancreatitis. Abdominal CT examination is also of great value for the diagnosis of pancreatitis, especially when needed to assess the degree of inflammation and complications. But in the initial diagnosis of acute pancreatitis, blood amylase test is the most direct and common method.

Next, we analyze the second question: what is the most meaningful test for the diagnosis of acute bleeding and necrotizing pancreatitis?

Option A. Blood amylase test is one of the methods commonly used in the diagnosis of acute pancreatitis. After the onset of acute pancreatitis, serum amylase can be significantly increased in a short period of time, so it is a key indicator in the diagnosis of acute bleeding necrotizing pancreatitis.

Option B. Serum alpha fetoprotein testing was performed as described and mainly for diagnosis of liver cancer, independent of pancreatitis.

Option C. Abdominal CT, while providing imaging information of the pancreas, tends to have more sensitive changes in serological markers in the early stages of acute pancreatitis.

Option D. Blood CEA and option E. Blood CRP is also not directly related to the diagnosis of acute bleeding and necrotizing pancreatitis.

Therefore, for the second question, the most meaningful test for the diagnosis of acute hemorrhagic necrotic pancreatitis is the blood amylase test, and the answer is selected in A.

The most meaningful test for the diagnosis of acute bleeding and necrotizing pancreatitis is

Answer: A. Blood amylase test

Analysis: Blood amylase test is a common method to diagnose acute pancreatitis. In acute pancreatitis, enzymes released from the pancreas enter the blood, resulting in a significant increase in blood amylase levels. Although elevated blood amylase levels are not specific to acute pancreatitis, the combination of its rapid elevation and clinical symptoms can aid in the diagnosis.

145. (Type B1 question)

The treatment of chronic glomerulonephritis, can cause hyperkalemia drugs is

A. α -beta-blockers

B. furosemide

C. ACE class I inhibitors

D. calcium antagonist

E. β -beta-blockers

Reference answer: C Your answer:

[Analysis] furosemide is a highly effective diuretic. The ACEI inhibitor has the effect of causing potassium ion retention.

For title 145, the treatment for chronic glomerulonephritis that may cause hyperkalemia is C. ACE class I inhibitors. ACE inhibitors (such as portable, ramipril) reduce proteinuria and protect renal function in the treatment of chronic glomerulonephritis, but they may cause hyperkalemia, especially in the case of renal insufficiency.

Question 145: to treat chronic glomerulonephritis, is the drug that can cause high blood potassium?

First, we analyze the relevance of each option to the problem.

Option A (α -blocker): mainly used for the treatment of vasospastic disease, shock and acute myocardial infarction, not directly related to causing hyperkalemia.

Option B (furosemide): A diuretic mainly used for the treatment of edema that is used to promote the excretion of potassium ions and therefore does not cause hyperserum potassium.

Option C (ACEI class I inhibitors): angiotensin-converting enzyme inhibitors, which reduce the production of angiotensin by inhibiting the activity of angiotensin-converting enzyme, thereby inhibiting vasoconstriction and the release of aldosterone. Since aldosterone has sodium protection and potassium rejection effect, inhibition of its release may lead to increased blood potassium.

Option D (calcium antagonist): mainly used to treat angina and hypertension with no direct relationship with blood potassium level.

The E option (β -blocker): mainly used for treating arrhythmias and hypertension with no direct relationship with serum potassium level.

In conclusion, the most relevant answer to the question is option C (ACE class I inhibitor), because it may lead to increased serum potassium by inhibiting the release

of aldosterone.

145. Drugs for treating chronic glomerulonephritis that can cause hyperkalemia are

C. ACE class I inhibitors

ACEI (angiotensin converting enzyme inhibitor) drugs that treat chronic glomerulonephritis can reduce potassium excretion by inhibiting the renin-angiotensin-aldosterone system (RAAS), which may cause hyperkalemia. Therefore, ACEs class I drugs such as captopril and enalapril may cause hyperkalemia.

146. (Type B1 question)

Chronic glomerulonephritis, the main cause is the water and sodium retention of hypertension therapy preferred

A. α -beta-blockers

B. furosemide

C. ACE class I inhibitors

D. calcium antagonist

E. β -beta-blockers

Reference answer: B. Your answer:

[Analysis] furosemide is a highly effective diuretic. The ACEI inhibitor has the effect of causing potassium ion retention.

For title 146, when water and sodium retention from chronic glomerulonephritis causes hypertension, the preferred treatment agent is C. ACE class I inhibitors. ACE inhibitors help control hypertension and have a protective effect on the kidney and slow the deterioration of renal function. In addition, they can also reduce the glomerular hyperfiltration status and reduce proteinuria. Therefore, ACE class I is usually the preferred treatment in the setting of chronic glomerulonephritis with hypertension.

Question 146: chronic glomerulonephritis, the main reason for water and sodium retention of hypertension treatment first choice?

Similarly, we analyze the individual options again.

Option A (α -blocker): There is no direct therapeutic effect on water and sodium retention.

Option B (furosemide): It is a diuretic that effectively promotes the discharge of excessive water and sodium ions in the body, thereby relieving water and sodium retention, and is the first choice for this type of hypertension.

Option C (ACEclass I inhibitor): Although it is also used to treat hypertension, the main mechanism of action is not water or sodium retention.

Option D (calcium antagonist) and option E (β -blocker): again not for water or sodium retention.

In conclusion, the most relevant answer to the question is option B (furosemide), because it is a diuretic that effectively solves the problem of water and sodium retention, thus reducing blood pressure.

In conclusion, the answer to questions 145 was C. ACE class I inhibitors, and the answer to questions 146 was B. furosemide.

146. Chronic glomerulonephritis is mainly caused by the preferred treatment of hypertension with water and sodium retention

B. furosemide

Furosemide is a potent loop diuretic that effectively reduces blood sodium retention and reduces blood volume by inhibiting the reabsorption of sodium and water by renal tubules, thus lowering blood pressure. For hypertension due to water and sodium retention, the furosemide is the preferred drug.

147. (Type B1 question)

The common lesions of hypertensive intracerebral hemorrhage are in

A. frontal lobe

B. brainstem

C. lobi temporalis

D. occipital lobe

E. basal ganglia

Reference answer: E Your answer:

Graphic analysis:

147. The common lesions of hypertensive cerebral hemorrhage are in place

The answer is E. basal section. Hypertensive intracerebral hemorrhage is commonly found in the basal ganglia region of the brain because the arterioles in the basal ganglia region are susceptible to hypertension.

This is a B1 type multiple choice question, we need to analyze according to the questions and options, combined with medical knowledge.

For question 147, the common lesions of hypertensive intracerebral hemorrhage:

Option A frontal lobe, B brainstem, C temporal lobe, and D occipital lobe, although all possible with cerebral hemorrhage, are not the most common sites of hypertensive intracerebral hemorrhage. Hypertensive cerebral hemorrhage is usually due to arteriosclerosis and small aneurysm formation caused by long-term hypertension, which may eventually increase blood pressure sharply due to emotional excitement, excessive mental or physical labor, leading to cerebrovascular rupture. This bleeding is most common in the basal ganglia region, especially the legume artery, as it is a deep perforator of the middle cerebral artery and is susceptible to hypertension. The correct answer to question 47 is E. basal section,

The common lesions of hypertensive intracerebral hemorrhage are in

C. basal ganglia

148. (Type B1 question)

The lesions of complex partial seizures are mostly located

A. frontal lobe

B. brainstem

C. lobi temporalis

D. occipital lobe

E. basal ganglia

Reference answer: C Your answer:

148, the lesions of complex partial seizures are mostly located

The answer is the C. the temporal lobe. Temporal lobe epilepsy is the most common local seizure epilepsy, especially complex partial seizures, usually located in the temporal lobe.

For question 148, most of the complex partial seizures were located in:

Option A frontal lobe, although related with cognition and emotion, is not the main focal site for complex partial onset of seizures. Option B The brainstem is mainly related to life-sustaining functions, such as breathing and heartbeat, and not to epileptic focus. Option D The occipital lobe is mainly related to visual processing and is not the main focal area of epilepsy. Option E basal segment is mainly related to motor control and is also not the main focus of epilepsy. While option C temporal lobe, associated with memory, emotional and auditory processing, is the most common focal site for complex partial seizures of epilepsy, especially in medial structures of the temporal lobe, such as the hippocampus. C. lobi temporalis.

The lesions of complex partial seizures are mostly located

F. lobi temporalis

149. (Type B1 question)

A selective serotonin reabsorption inhibitor is

A. carbamazepine

B. lithium carbonate

C. fluoxetine

D. Risperidone

E. alprazolam

Reference answer: C Your answer:

[Analysis] Current clinical non-classical antipsychotic drugs block 5-HT receptors, such as clozapine and risperidone. Fluoxetine, also known as Prozac, is a potent and selective 5-HT uptake inhibitor.

For issue 149, selective serotonin reabsorption inhibitors (SSRIs) are used primarily used for the treatment of depression. Among the options given, C. fluoxetine is a typical selective serotonin reabsorption inhibitor.

This is a multiple-choice question, and we need to choose the most relevant answer to the question from the given option.

First, we analyze question # 149, which asks, which drug belongs to a selective serotonin reabsorption inhibitor.

A. carbamazepine: This is an antiepileptic drug, also used as a long-term

prophylactic drug for the relief of trigeminal neuralgia, and is not a selective serotonin reabsorption inhibitor.

B. Lithium carbonate: This is a mood stabilizer, mainly used for the treatment of mania. It has a good treatment and prevention of bipolar disorder alternating between mania and depression, and also protects against recurrent depression, and also is not a selective serotonin reabsorption inhibitor.

C. Fluoxetine: This is a selective serotonin reuptake inhibitor (SSRI), whose main effect is to inhibit the reabsorption of serotonin in the synaptic cleft, thus increasing the concentration of serotonin in the synaptic cleft and playing an antidepressant effect. This is the correct answer to this question.

D. Risperidone: It is an antipsychotic used to treat mental disorders, such as acute and chronic schizophrenia, and is not a selective serotonin reabsorption inhibitor.

E. Alprazolam: This is a benzodiazepine with anxiolytic, depression, antidepressant, sedative, hypnotic, anticonvulsant and muscle relaxation, not a selective serotonin reabsorption inhibitor.

In conclusion, for question 149, the answer is C. fluoxetine.

A selective serotonin reabsorption inhibitor is

B fluoxetine

150, (Type B1 question)

Are classified to the atypical antipsychotics

A. carbamazepine

B. lithium carbonate

C. fluoxetine

D. Risperidone

E. alprazolam

Refer to the answer: D. Your answer:

[Analysis] Current clinical non-classical antipsychotic drugs block 5-HT receptors, such as clozapine and risperidone. Fluoxetine, also known as Prozac, is a potent and selective 5-HT uptake inhibitor.

For question 150, atypical antipsychotics are a newer class of antipsychotics with different pharmacological properties and side effect profile compared to typical antipsychotics. Among the options given, D. risperidone is an atypical antipsychotic. Next, we analyzed question 150, which asked which drug belongs to atypical antipsychotics.

A. carbamazepine, B. lithium carbonate, C. fluoxetine, E. alprazolam: The effects of these drugs have been described previously and, none of them are atypical antipsychotics.

C Risperidone: This is an atypical antipsychotic drug used mainly to treat schizophrenia and other psychiatric disorders. This is the correct answer to this question.

In conclusion, for question 150, the answer is D. risperidone.

Are classified to the atypical antipsychotics

G. Risperidone

2023, Unit 4

1. (Type A1 question)

The possible physical signs of infants at eight months of age are

A. neck rigidity

And B. Babinski was positive

B. Positive K-cruzi sign

C. embrace reflex

D. sucking reflex

Reference answer: B. Your answer:

[Analysis] Children under 2 years of age Babinski sign positive can also be a physiological phenomenon.

[Avoid error] Cervical rigidity (A error) is seen in infant ischemic hypoxic encephalopathy or meningitis, etc. Inftone before 3 to 4 months of age

High force, k-zi sign can be positive (C error).

The hug reflex (D error) is a defensive reflex that can occur when the newborn hears sounds, receives stimulation, and so on

Arm extension straight, then flexion inside the chest, a hug-like symptoms. Neuroneuroneurodevelopment with age,

It disappeared spontaneously after 3 to 6 months. The sucking reflex (E error) refers to the onset when the nipple or finger touches the newborn's lip

Now the head to touch the direction of the search, and the lips and tongue sucking, peristalsis, can eat independently, generally in 4~7 months later

disappear.

For eight-month-old infants, the most typical sign is the sucking reflex, option E. This is because the sucking reflex is a common primitive reflex from the neonatal period to early infancy, usually present after birth and will naturally disappears when the baby is about 6 months of age. However, since this reflex can last until around a year in many infants, it is not uncommon to be observed in infants at 8 months of age.

A stiff, C positive, and D hug reflex are usually associated with neurological problems and are not typical signs in normal infants. Item B Babinski positive reflex is common in infants, but it usually disappears before 12 months of age and is therefore not a typical sign in an eight-month infant.

In summary, the most appropriate answer is the E. sucking reflex.

This is a multiple-choice question about the characteristic signs of the infant's growth and development stage. We need to determine which signs are possible in eight-month-old infants. First, we analyzed the signs represented by each option and their correlation with infant age:

A. Cervical rigidity: This is usually not A normal sign of infancy, but A pathological condition, such as diseases such as meningitis.

B. Babinski positive: This is a pathological reflex, which normally has a positive Babinski sign before the age of two and turns negative after the age of two, so infants eight months old can present with Babinski positive signs.

C. Positive cruzi sign: This is a meningeal stimulus, usually associated with meningitis or other neurological diseases, and is not a sign in normal infants.

D. Embrace reflex: This is the original reflex of the neonatal period that usually disappears after 3-4 months and therefore does not appear in eight-month-old infants.

E. Sucking reflex: This is also the original reflex of the neonatal period, which gradually disappears as the baby grows up, not the physical sign of an eight-month-old baby.

In conclusion, Babinski positivity is a normal sign that can occur in eight-month-old infants, while other options are either associated with a pathological state or a reflex in the neonatal period and are not suitable for eight-month-old infants.

Therefore, the correct answer is a B. Babinski positive test.

The possible signs in an eight-month infant are:

And B. Babinski was positive

Explanation: The Babinski reflex (Babinski reflex) is a normal reflex in infants. When a hard object runs across the outside of the foot, the big toe turns up and the other toes fan out. This reflex is normal in the infancy phase and usually disappears within 2 years of age. Cervical rigidity, positive K-Kirschner sign, and hug reflex (Moro reflex) are mainly seen in pathological conditions or in the neonatal period. The sucking reflex usually appears earlier in infancy, and eight-month-old infants generally begin to reduce it.

2. (Type A1 question)

Incorrect thing about skull base fractures is

A. CT was unable to show a skull base fracture

B. anterior fossa fracture can have "panda sign"

C. Simple skull base fractures can be treated conservatively

D. The X-line shows the intracranial gas accumulation

E. With possible cerebrospinal fluid outflow

Reference answer: A. Your answer:

[Analysis] The skull base fracture can be caused by the extension of the skull base fracture or from the skull base fracture extending to the skull cover, and CT can show the skull base fracture.

[Avoid] "Panda eye sign" is a fracture of anterior cranial fossa involving the

frontal orbital plate and ethmoid bone, causing blood flow into the orbit and periorbital subcutaneous dermis

And the formation of blood spots under the membrane. Simple skull base fractures have no brain injury or intracranial hematoma and are generally treated nonoperically.

The X-ray examination can show the intracranial gas accumulation. Cranull base fracture patients can appear cerebrospinal fluid outflow, through the ear, nose, eyelids, mouth and other parts

The outflows. So do not choose the BCDE.

Regarding skull base fractures, the following description is incorrect:

A. CT was unable to show a skull base fracture

CT (computed tomography) is the preferred imaging method for detecting skull base fractures because it provides detailed images of the skull, especially when fractures are obvious. Therefore, option A is incorrect to say that CT is unable to show skull base fractures. Other options are possible: anterior fossa fracture can have "panda sign" (dark circles of the eye caused by periocular bleeding), simple skull base fracture can be treated conservatively, X-ray can show intracranial air accumulation, and skull base fracture may cause cerebrospinal fluid outflow. Thus, the most incorrect option is the A.

analyze:

The skull base is arranged from the anterior to the anterior fossa to the posterior, uneven, with different size bone pores and cracks to accommodate the passage of cranial nerves and blood vessels. Cranull base fracture, often accompanied by corresponding cranial nerve injury, such as olfactory nerve, optic nerve, auditory nerve and facial nerve injury. High-resolution CT examination can show fracture line, gas skull, brain swelling, intraventricular hemorrhage, etc., which has a diagnostic value for skull base fracture. Thus the claim that a CT could not show a skull base fracture is wrong.

When the anterior fossa is fractured, the bleeding flows out into the orbital eyelid through the anterior nose, which can appear purple, like the panda eye, also known as "panda sign", so option B anterior fossa fracture can have "panda sign", which is one of the clinical manifestations of skull base fracture.

Cranial base fracture generally does not require special treatment, the focus is to

observe whether there is brain injury and treatment of cerebrospinal fluid leakage, cranial nerve injury and other combined injuries, so option C simple skull base fracture can be treated conservatively in line with the treatment principle of skull base fracture.

When skull base fracture, X-ray photographic examination can show intracranial air accumulation, so option D: X-ray shows that intracranial air accumulation is consistent with the imaging findings of skull base fracture.

If the skull base fracture is injured to the paranasal sinus or mastoid atrium, it can be accompanied by cerebrospinal fluid rhinorrhea or ear leakage. In Option E, cerebrospinal fluid outflow is the clinical manifestation of the skull base fracture.

In conclusion, the correct answer is: A.CT cannot show A skull base fracture.

The incorrect options for a skull base fracture are:

A. CT was unable to show a skull base fracture

explain:

Skull base fractures can usually be clearly visualized by CT scan, so option A is incorrect. CT scan is a common imaging method for the diagnosis of skull base fractures, which can provide detailed bone structure information and help to identify the fracture site and range.

3. (Type A1 question)

The following describes the defense mechanism of the female reproductive system

A. the anterior and posterior walls of the vagina

B. pH＞6.5

C. The vagina has a self-purification function

D. Enof the cervical closure

E. Large and small labia to close naturally

Reference answer: B. Your answer:

[Analysis] Vaginal pH is an indicator of the female pH in the vagina, which will

affect the microbial community and inflammatory response in the vagina. Normal vaginal pH was between 3.8 and 4.4 and was weakly acidic.

[Error avoidance] The female reproductive system has a variety of defense mechanisms, including the anterior and posterior vaginal wall close, vaginal self-purification function, and the cervix

Oral closure and small labia naturally closed, etc. Together these mechanisms work together to protect female reproductive health. The anterior and posterior walls of the vagina are tight

It is a natural state of the female reproductive system that helps keep the vagina clean and dry, reducing the wind of infection

a place difficult of access. Vaginal self-purification function means that the bacterial flora in the vagina maintain the acid-base balance through interaction, thus reducing the wind of infection

a place difficult of access. Entraoral closure is also a natural state of the female reproductive system that helps prevent the entry of pathogens within the cervix

uterine cavity. The natural closure of the labia Yin is also a self-protection mechanism of the female reproductive system, which helps to reduce the external environment

The contamination of the vagina. So choose no ACDE.

The defense mechanisms of the female reproductive system include the tightness of the vaginal wall, the acidic environment of the vagina (low pH), self-purification function, the closure of the internal cervix and the natural closure of the large and small labia vulvora. Among them, the description of the pH value is particularly critical, because a healthy vaginal environment usually has a low pH value (about 3.8 to 4.5), helping to prevent the growth of harmful bacteria. Therefore, in these options, the incorrect description is: B. pH> 6.5 This is because the healthy vaginal pH value is usually less than 4.5, rather than greater than 6.5. Thus, option B is incorrectly described.

First, we need to make it clear that this topic examines the defense mechanism of the female reproductive system. Because of its special anatomical structure and physiological functions, the female reproductive tract has certain natural defense functions, which can protect against the infection of pathogens. Next, we analyze the options one by one:

A. The anterior and posterior walls of the vagina: This is A natural state of the vagina that helps prevent the invasion of external pathogens. When the vagina is not dilated by a foreign body (such as the penis or tampon), the front and posterior walls are close, which reduces the chance of pathogen entry.

B. pH> 6.5: The pH of a normal vagina is 4.5, an acidic environment that can inhibit the growth of other pathogens, called the self-purification of the vagina. Therefore, a vaginal pH greater than 6.5 is abnormal, which can destroy the natural defense mechanism of the vagina and make pathogens easier to grow.

C. Vaginal self-purification function: this is an important defense mechanism of the vagina. Lactobacillus in the vagina can decompose glycogen into lactic acid, thus maintaining the normal acidic environment of the vagina. This acidic environment can inhibit the growth of other pathogens and act as a defense against infection.

D. Closing of the inner cervix: The cervix is an important defensive barrier in the female reproductive system. The inner uterine cervix is usually in a closed state, which helps to prevent an ascending infection.

E. Labia sum closes naturally: this is also a natural defense mechanism of the female reproductive system. The natural closing state of the large labia reduces the chance of direct contact with the vaginal opening.

In conclusion, Option B (pH> 6.5) is improperly described because it destroys the natural acidic environment of the vagina and reduces its defense function.

Thus, the final answer is that B. pH> 6.5.

The female reproductive system has multiple defense mechanisms to prevent infections and other problems. Here is an analysis of the options:

A. * * The anterior and posterior walls of the vagina are close to it * *: This is true. The closeness of the anterior and posterior walls of the vagina helps to prevent the entry of foreign pathogens.

B. * * pH> 6.5 * *: This is not true. The vaginal pH value of healthy women is usually between 3.8 and 4.5, and the acidic environment helps to inhibit the growth and reproduction of pathogens.

C. * * Vaginal has a self-purification function * *: This is true. Vaginal secretions and normal flora (e. g., lactic acid bacteria) help to keep the vagina clean and defensive.

D. * * the cervix closed * *: This is true. The closure of the endocervical opening helps prevent the pathogen from entering the uterus.

E. * * The labia small shade closes naturally * *: This is true. The natural closure of the large and small labia Yin acts as a mechanical barrier to prevent the invasion of external pathogens.

So, the most correct answer is:

F. **pH＞6.5**

4. (Type A1 question)

The method of pediatric head circumference measurement is

A. Around the thorax horizontally along the lower margin of the papilla for 1 week

B. circled the thorax horizontally along the upper margin of the papilla for 1 week

C. The brow arch to the ear to the posterior occipital tubercle around the head

D. Around the head from the brow to the pillow for 1 week

E. The most prominent point above the eyebrow arch to the posterior occipital tubercle around the head for 1 week

Reference answer: E Your answer:

Graphic analysis: [analysis] The measurement method of pediatric head circumference is the most prominent place above the eyebrow arch to the posterior occipital nodule around the head for 1 week.

[Error avoidance] According to the above analysis, exclude other options.

The correct measurement method of children's head circumference is option C: "the eyebrow arch to the ear to the posterior occipital nodule around the head". This method ensures that the head circumference is measured around the maximum circumference of the head, usually between just above the ear and the site of the occipital protrusion. Therefore, the best answer is the C.

analyze:

The measurement of children's head circumference is along the most prominent point on the top of the eyebrow arch to the posterior occipital nodule around the head, and the length of a circle around the area with a leather ruler is the head circumference. The growth of head circumference is related to the growth of brain and skull. The measurement of head circumference is the most valuable within 2 years. A small head circumference often indicates brain dysplasia, and the head circumference is too large or grows too fast, indicating hydrocephalus.

Option A circles the chest horizontally along the lower edge of the nipple for 1 week, and option B circles the chest horizontally along the upper edge of the nipple for 1 week, which measures chest circumference, regardless of head circumference, so it can be excluded together.

Option C "eyebrow arch to ear to pillow nodule around the head", option D "eyebrow to pillow around the head for 1 week", which do not meet the standard measurement method of head circumference, so they can be excluded together.

To sum up, the correct answer is: E. The most prominent point above the eyebrow arch to the posterior occipital nodule around the head for 1 week.

The method of pediatric head circumference measurement is:

H. The most prominent point above the eyebrow arch to the posterior occipital tubercle around the head for 1 week

5. (Type A1 question)

The identification of the three atypical phenylketonuria is

A. Urinary ferric chloride test

B. urinary pterin profile analysis

C. Analysis of plasma-free amino acids

D. Guthrie bacterial growth inhibition assay

E. DNA analyse

Reference answer: B. Your answer:

Graphic analysis: [analytical] atypical phenylketonuria is phenylalanine hydroxylase coenzyme tetrahydropterin (BH4) lack, need to determine the level of pterin in the urine to identify three atypical phenylketonuria, including: phenylalanine, tyrosine, phenylalanine hydroxylation metabolites and phenylalanine bypass metabolites.

[Error avoidance] The urinary ferric chloride test was used to check the urine for phenylalanine, but could not identify the three atypical phenylacetones

Urinary (A wrong). Analysis of plasma free amino acids can only distinguish between classic and nonclassic phenylketonuria (C error). Guthrie Bacterial growth inhibition test is only available for newborn screening (D error). DNA analysis is available only for genetic diagnosis and not for identifying the three atypical phenylketonuria (E error).

The identification of three atypical phenylketonuria (PKU) usually requires a detailed analysis of the genetic background, as these forms of PKU involve different genetic defects. Although the urinary ferric chloride test and the Guthrie bacterial growth inhibition test can be used for initial screening for phenylketonuria, they are not sufficient to distinguish between the different types of atypical PKU. Urpterin mapping and plasma free amino acids help to understand the nature of metabolic abnormalities but may not be specific enough in identifying specific genetic variants.

F. DNA analysis is the best method to identify three atypical phenylketonuria because it is able to precisely identify genetic defects to determine the etiology, and distinguish different types of atypical PKU. By analyzing the patient's DNA, the genetic variants leading to phenylketonuria can be directly detected to accurately diagnose the specific types of atypical PKU. Thus, the most correct answer is the E. DNA analysis.

analyze:

Atypical phenylketonuria mainly includes three types: tetrahydrobiopterin deficiency, 6-acetonyltetrahydroopterate synthase deficiency and dihydrobiopterin reductase deficiency.pterin is phenylalanine, tyrosine and tryptophan in the process of catalytic hydroxylase reaction coenzyme, these diseases are due to guanosine

triphosphate cyclic hydroatase, 6-pyrusyl tetrahydrogenopterin synthase or dihydrogen biopterin reductase enzyme deficiency, make tetrahydrobiopterin synthesis or regeneration disorder, phenylalanine can not oxidation to tyrosine, only through bypass metabolism, by phenylalanine transaminase catalysis of phenylpyruvate, excreted by urine, phenylketonuria. Therefore, the differential diagnosis of three atypical phenylketonuria can be made by uropterin mapping analysis.

Option A urinary ferrichloride test, option C plasma free amino acid analysis, and option D: Guthrie bacterial growth inhibition test, all screening test for phenylketonuria, can only indicate phenylalanine hydroxylase deficiency, but can not identify three atypical phenylketonuria, so they can be excluded together.

Option E: DNA analysis is a prenatal diagnosis method for phenylketonuria, but it also does not identify the three atypical phenylketonuria, so it is not chosen.

In conclusion, the correct answer is: B. urinary pterin atlas analysis.

The identification of three atypical phenylketonuria (Phenylketonuria, PKU) requires specificity and accuracy. An alternative answer to this question:

A. Urinary ferric chloride test: This is a method for detecting phenylpyruvate in the urine, but the differential diagnosis of atypical phenylketonuria is not specific enough.

B. Urinary pterin mapping: This is one of the key methods to identify atypical phenylketonuria, distinguishing different types of PKU by analyzing metabolites of pterin (e. g., biopterin).

C. Analysis of plasma free amino acids: This is a method commonly used to detect phenylalanine levels, which is helpful in the diagnosis of PKU, but can not specifically identify atypical phenylketonuria.

D. Guthrie Bacterial growth inhibition test: This is an early screening method for PKU, but it is not specific for the identification of atypical phenylketonuria.

E. DNA analysis: The identification of specific gene mutations causing atypical phenylketonuria is a very accurate method, but it is usually used clinically as a final means of diagnosis.

In conclusion, the most commonly used method to identify three atypical phenylketonuria is uropterin mapping analysis (B), as it can distinguish different types of phenylketonuria by analyzing pterin metabolites.

So the best answer is:

F. urinary pteropterin profile analysis

6. (Type A1 question)

Spinal cord hemisection syndrome is common in

A. Acute extrtramedullary intradural abscess

B. avute myelitis

C. Guilam Barre syndrome

D. Extramedullary tumor of the spinal cord

E. myelosyringosis

Refer to the answer: D. Your answer:

[Analysis] Spinal cord half resection syndrome is commonly seen in extramedullary tumors of the spinal cord, which is mainly manifested as the typical disappearance of deep sensation on the affected side and the disappearance of the contralateral superficial sensation, due to the loss of fiber innervation from the contralateral cortex spinal tract, and dysfunction in 1 / 3 of the ipsilateral limb. Avoiding acute extrtramedullary intradural abscess (A wrong) is mainly manifested as chills, high fever, consciousness disorder and other infection poisoning symptoms, can be accompanied by muscle spasm and forced position, often accompanied by the affected side superficial reflex weakened or disappear, and deep hyperactive, hyperreflexia, pathological signs and Kirschner's sign. Acute onset of acute myelitis (B error), often with dorsal pain, chest and abdomen band and numbness of the lower

limbs, and then the movement below the plane of spinal cord damage. Giranbare syndrome (C wrong), also known as acute inflammatory demyelinating polyneuropathy, is the detachment of peripheral nerves and nerve roots

Myelin lesions and inflammatory cell infiltration of small blood vessels are autoimmune diseases with pathological characteristics, with clinical manifestations of polyneuropathy and brain

Protein-cell separation and electrophysiological alterations in the spinal fluid.syringomyelia (E error) usually typical pain, temperature disturbance and

Separative sensory disorder of deep sensory preservation.

[fault avoidance]

Spinal cord half-resection syndrome (Brown-Sequard syndrome) is usually caused by an injury to one side of the spinal cord, manifested by loss of ipsilateral motor function and loss of pain and temperature on the contralateral side. With this in mind, the most likely cause of this syndrome are those that cause damage to one side of the spinal cord.

In the option:

-A. Acute extrtramedullary intradural abscess may cause compression of the cord but usually affects both sides of the cord rather than unilateral.

-B. Acute myelitis usually affects most areas of the spinal cord and is not specifically unilateral.

-C. Guilare Barry syndrome is a disease of the peripheral nervous system that does not directly affect the spinal cord.

-D. Extramedullary tumors of the spinal cord can locally compress the spinal cord, causing unilateral injury and thus may cause spinal cord hemisection syndrome.

-E. Syringingomyelia usually affects the central part of the spinal cord, although may cause diverse neurological symptoms but is not specific to causing spinal cord hemisection syndrome.

Thus, the option most consistent with the common etiology of spinal hemiresection syndrome is D. extramedullary tumors of the spinal cord.

analyze:

Spinal cord cutting syndrome, also known as Brown-Sequard syndrome, refers to the external compression and internal lesions caused by spinal cord damage under the

plane below the limb motor neurons paralysis, deep sensation disappear, fine touch disorder, vasomotor dysfunction, contralateral limb tenderness disappear, bilateral touch retention clinical syndrome. Spinal cord half resection syndrome is commonly seen in spinal cord extramedullary tumors, and spinal cord compression symptoms of extramedulullary tumors can occur in the early stage, leading in paralysis of motor neurons on the ipsilateral limb below the plane of the lesion, loss of deep sensation, loss of fine tactile sensation, loss of pain and temperature in the contralateral limb, and preservation of bilateral tactile sensation.

Option A acute extrtramedullary intramural abscess, option B acute myelitis, both inflammatory lesions of the spinal cord, mostly transverse damage, manifested by various sensory loss below the damaged plane, upper motor neuron paralysis and sphincter disorder, without bilateral sensory and motor asymmetry, so can be excluded together.

Option C Guilan barre syndrome is mostly bilateral delayed paralysis, distal is more than proximal, can have peripheral sensory disorders and cerebral nerve damage, cerebrospinal fluid protein-cell separation phenomenon, inconsistent with spinal hemiresection syndrome performance, so it is not selected.

Option E syringomyelia mainly showed segmental separation sensory impairment, motor and reflex abnormalities, and skin nutritional disorders, without bilateral sensory and motor asymmetry, so it was excluded.

In conclusion, the correct answer is: D. Examedullary tumor of the spinal cord.

Spinal cord half resection syndrome (Brown-Sequard syndrome) is a syndrome caused by damage to the spinal cord, whose clinical manifestations usually include loss of motor function and proprioception in the ipsilateral limb, and loss of nociception in the contralateral limb.

The most common etiologies of this syndrome include spinal cord trauma, spinal cord tumors, spinal cord infections as well as degenerative lesions of the spinal cord. Among the options, the most likely cause of spinal hemiresection syndrome is extramedullary tumor of the spinal cord.

So, the most correct answer is:

F. Extramedullary tumor of the spinal cord

7. (Type A1 question)

The antibody commonly tested for confirmed epidemic Japanese encephalitis is

A. IgM

B. IgG

C. IgE

D. IgC

E. IgB

Reference answer: A. Your answer:

Graphic analysis: [analysis] JE is insect media infection, specific antibodies appear in the blood after infection, and obtain immunity. Specific IgM antibodies (A pair) appeared earlier (3 to 4 days after the disease), which could be detected on the second day of the course of the disease, and reached the peak at 2 weeks, which can be used as an early diagnosis index. Specific IgG antibodies (B error) appeared late (2 weeks later) and were used for retrospective diagnosis and epidemiological investigation.

[Fault avoidance] BCDE was excluded according to the above analysis.

The diagnosis of epidemic Japanese encephalitis (also known as Japanese encephalitis) usually depends on specific antibody testing. In this case, detection of IgM antibodies is most important because IgM antibodies usually appear early in infection and its presence is indicative of recent infection. Therefore, the most correct answer is: A. IgM

First, let's understand the core point of the topic. The question is which type of antibody should be checked when epidemic Japanese encephalitis is diagnosed. Epidemic Japanese encephalitis is a disease caused by JEV, and when the human body is infected with this virus, it produces different types of antibodies to fight the virus.

Down, we analyze each option one by one:

A. IgM: After infection with the epidemic Japanese encephalitis virus, the body will first produce IgM antibodies. This antibody usually appears within a week of infection, so it is a sensitive indicator for early diagnosis of epidemic encephalitis.

B. IgG: IgG antibodies are usually present over a period after infection and for a longer duration. Although it is also a hallmark of viral infection, it is not the first

choice for early diagnosis because of its relatively late onset and less sensitive than IgM.

C. IgE: This antibody is mainly associated with allergic reactions, not directly to the diagnosis of viral infection.

D. IgC and E. IgB: In medicine, there are no IgC or IgB antibodies, so these two options can be excluded first.

In conclusion, since IgM is the first antibody produced by the body after infection with Japanese encephalitis virus, and appears early and has high sensitivity, it is the first choice of test antibody for the diagnosis of Japanese encephalitis B.

Therefore, the correct answer is the A. IgM.

The antibodies commonly tested for confirmed epidemic Japanese encephalitis are:

A. IgM

Epidemic Japanese encephalitis (JE) is a type of viral encephalitis caused by Japanese encephalitis virus. IgM antibodies are antibodies produced early on of infection, and detecting the presence of IgM antibodies can help in the early diagnosis of Japanese encephalitis. Thus, the most correct answer is the A. IgM.

8. (Type A1 questions)

Children's growth and development characteristics are right is

A. Consistent growth and development rates across the various systems

B. There are no individual differences in body development

C. Older age and slower development

D. Infant life is the fastest period of physical development

E. The fastest physical development occurs after the age of 1 year

Refer to the answer: D. Your answer:

[Analysis] Children's growth and development is a continuous, phased process, the speed of organ development of each system is not completely consistent (A wrong). The rate of growth and development in each age group is relatively stable, but

there are significant individual differences in its physiological characteristics (B error), and there are some differences in each stage. The younger the age, the faster the development, the peak physical growth rate in the last one year, and began to slow down after one year (CE error). Infancy (birth to one year) is the period of fastest physical development (D pair).

[Error avoidance] According to the above analysis, exclude other options.

The best answer to this question is D, and infancy is the fastest period of physical development. During children's growth and development, infancy (the first year of life) is the fastest physical growth period, especially in the first 6 months after birth. During this period, the baby's weight and height will increase rapidly. Moreover, the growth and development rate of each system is not consistent, and each organ and system in the body will grow according to their own developmental rules. In addition, there are individual differences in body development, and even children of the same age will have different growth rate and development degree. Development slows down with age, so that option C is also partially correct, but a more comprehensive and accurate description is option D.

This is a multiple-choice question about children's growth and development characteristics. We need to analyze each option to find the correct answer that corresponds with the child's growth and development characteristics.

A. The consistent growth and development rate of various systems: in fact, the growth and development rate of children's various systems is not consistent. For example, the nervous system develops earlier and the reproductive system develops later. Therefore, this option is incorrect.

B. There are no individual differences in body development: the growth and development of children are affected by various factors, such as genetics, nutrition, environment and other factors, so there will be individual differences. This option is also incorrect.

C. Older age, slower development: Although child development slows down with age, it cannot be simply said that older development is slower, because development speed does not change completely at different stages. Therefore, this option is not accurate enough.

D. Infancy is the fastest period of physical development: infancy (from birth to 1 year old) is the period of the most rapid physical development of children, with significant increases in height, weight and other aspects. This option is correct.

E. After the age of 1 is the fastest period of physical development: in fact, infancy (from birth to age 1 year) is the fastest period of physical development, not after the age of 1 year. Therefore, this option is incorrect.

In conclusion, the correct answer is D: infancy is the fastest period of physical development. This option accurately reflects an important characteristic of child growth and development.

According to the medical knowledge and best practice guidelines, the following are the answers to the questions:

There are many unique features of children's growth and development, including significant differences in physical development speed at different ages. Especially infancy (birth to 1 year) is the most rapid physical development. So, the most correct answer is:

F. Infant life is the fastest period of physical development

Explanation: Weight and height grow much faster in infancy than at other ages, such as newborns in the first year of life, who usually triple in weight and grow about 25 cm in height. The rapid development of this stage reflects the high sensitivity of infants to nutritional and environmental stimuli. In contrast, after 1 year, it slowed significantly. Therefore, option D is best consistent with the characteristics of child growth and development.

9. (Type A1 question)

The most common cause of cerebral embolism is the

A. auricular fibrillation

B. atherosclerosis

C. hypertension

D. diabetes mellitus

E. hyperlipemia

Reference answer: A. Your answer:

[Analysis] Brain embolism refers to a group of clinical syndromes in which various emboli enter the intracranial artery with the blood flow for acute occlusion or severe stenosis of the vascular lumen, resulting in ischemic necrosis and dysfunction of the brain tissue in the corresponding blood supply area. According to the source of embolus, cerebral embolism can be divided into three categories: cardiac origin, non-cardiac origin and unknown origin. Among them, cardiogenic cerebral embolism is the most common, accounting for 60%~75% of cerebral embolism, and the most common cause of cardiogenic cerebral embolism is atrial fibrillation (A pair)

[Avoid mistakes] Atherosclerosis (B wrong), hypertension (C wrong), diabetes (D wrong), hyperlipidemia (E wrong)

The most common etiology of cerebral thrombosis. And not the most common cause of cerebral embolism.

The most common cause of cerebral embolism is atrial fibrillation (A). Atrial fibrillation causes an irregular and rapid contraction of the upper chamber of the heart, thereby increasing the possibility of blood stagnation within the atrium, which can lead to thrombosis. If these clots fall off, they can enter the brain circulation with the blood flow, leading to cerebral embolism. Although atherosclerosis, hypertension, diabetes, and hyperlipidemia are also important risk factors for cerebral embolism, AF is considered the most common direct etiology. Therefore, the correct answer is A. atrial fibrillation.

analyze:

Cerebral embolism refers to a group of clinical syndromes in which various emboli enter the intracranial artery with the blood flow for acute occlusion or severe stenosis of the vascular lumen, causing ischemia, necrosis and dysfunction of the brain tissue in the corresponding blood supply area. Among them, cardiac embolism is the most common cause of cerebral embolism, accounting for about 60%~75%. emboli are produced in the endocardium and valve, falling off into the brain and cause disease. Among them, atrial fibrillation is the most common cause of cardiac embolus, accounting for more than half.

Option B Atherosclerosis was the most common cause of cerebral thrombosis, not cerebral embolism, so it was excluded.

Option C hypertension, option D diabetes, and E hyperlipidemia, which are all risk factors for atherosclerosis, but do not directly lead to cerebral embolism, and therefore can be excluded together.

In conclusion, the correct answer is: A. atrial fibrillation.

The most common causes of cerebral embolism are:

A. auricular fibrillation

Atrial fibrillation (AF) is a major risk factor for cerebral embolism. Atrial fibrillation can cause trapped blood flow in the atrium, creating blood clots that can fall off and enter the cerebral artery, leading to a cerebral embolism. Therefore, select the A option.

10. (Type A1 questions)

The most common etiology of neonatal necrotizing enterocolitis is

A. cold

B. hematolysis

C. No supplementary food was added in time

D. premature birth

E. twins

Refer to the answer: D. Your answer:

[Analysis] Neonatal necrotic enterocolitis is caused by a variety of reasons, with abdominal distension, vomiting, blood stool and hypotension as the main manifestations of critical disease, premature birth is a common cause. Avoid wrong cold (A wrong), hemolysis (B wrong) are the causes of neonatal jaundice. Not timely add supplementary food (C wrong) is the cause of physiological anemia.

[Avoid error] Cold (A wrong) may cause neonatal fever, cough, abdominal distension symptoms, and will not cause necrotizing small bowel colon

scorching. Hemolysis (B error) is the cause of neonatal jaundice. Did not add complementary food (C wrong) for physiological anemia

follow. Twins (E wrong) do not cause necrotizing enterocolitis in the newborn.

The most common cause of neonatal necrotizing enterocolitis (Necrotizing Enterocolitis, NEC) is D. preterm birth. Premature infants are more likely to develop necrotizing enterocolitis due to immature intestinal function and weak immune system.

This condition is more common in preterm infants, especially those with very low birth weight who require parenteral nutrition. Therefore, the correct answer is D. prematurity.

analyze:

Neonatal necrotizing enterocolitis is an acquired disease, which is a variety of causes of intestinal mucosa damage, ischemia, hypoxia, leading to diffuse or local necrosis of the small intestine and colon. The exact etiology and pathogenesis of neonatal necrotizing enterocolitis have not been fully defined, which may be related to a variety of pathogenic factors, but premature birth is a major risk factor of neonatal necrotizing enterocolitis. Due to immature gastrointestinal function, less gastric acid secretion, poor gastrointestinal power, easy food retention, conducive to bacterial growth; Moreover, premature immunity, gastrointestinal peristalsis, digestive and absorption function and coagulation mechanism are not perfect, high intestinal wall permeability, poor barrier function, more prone to bacterial invasion.

Option A Cold may cause abnormal neonatal body temperature, leading to neonatal hard swelling and other diseases, but it has little to do with the occurrence of necrotizing enterocolitis, so it is not selected.

Option B hemolysis is more common in neonatal hemolytic diseases, such as neonatal ABO hemolysis, Rh hemolysis, which are often manifested as jaundice, anemia, etc., and is not significantly associated with the onset of necrotizing enterocolitis, so it is excluded.

Option C does not timely add complementary food mainly affects the nutrition absorption of infants, which may cause iron deficiency anemia, vitamin D deficiency rickets, etc., but will not directly lead to necrotizing enterocolitis, so it is not considered.

Option E twins may make the mother face greater nutritional needs and psychological pressure during pregnancy, but it is not related to the occurrence of necrotizing enterocolitis, so it does not conform.

To sum up, the correct answer is: D. Prematurity.

Neonatal necrotizing enterocolitis (NEC) is a common acute gastrointestinal disease in the neonatal period, and its etiology is not fully clear, but studies show that multiple factors work together, including prematurity, infection, intestinal ischemia and hypoxia, intestinal flora and microflora imbalance. Among them, * * preterm

birth * * is one of the most common and important risk factors. Premature infants have incomplete gastrointestinal tract development, immature immune function, and are more prone to necrotizing enterocolitis.

So, the most correct answer is:

F. premature birth

11. (Type A1 questions)

The period of the highest childhood morbidity and mortality is

A. neonatal period

B. infancy

C. infancy

D. pre-school age

E. school age

Reference answer: A. Your answer:

[Analysis] The development of each neonatal system is not mature enough, the immunity has not yet formed, the changes in the external environment can not be well adapted, the probability of various diseases greatly increased, is the highest mortality stage in the neonatal period.

[Wrong avoidance] The mortality rate of infancy (B error) is second only to the neonatal period, and the morbidity and mortality rate of early childhood (C error) are generally lower than that

The infancy is higher than that of late childhood, the morbidity and mortality of preschool age (D wrong) are generally lower than that of early childhood and higher than that of school age, and the morbidity and mortality of school age (E wrong) are the lowest.

In childhood, the highest morbidity and mortality rate is usually in B. infancy. Infinfancy is between the first month of birth and one year of life. During this period, infants were at high health risk due to their immune system was not fully developed, coupled with nutrition, environmental factors and infections. Therefore, children in

infancy have higher morbidity and mortality relative to other ages.

analyze:

Childhood includes the neonatal period, infant period, early childhood period, preschool age period, school age period and adolescence, among which the neonatal period is the highest morbidity and mortality period in childhood. This is mainly because the newborn needs an adaptation process from intrauterine to extrauterine environment, and has weak disease resistance in the neonatal period, which requires more attention to care and feeding.

Option B Although there are certain health risks in infancy, the morbidity and mortality in infancy are relatively low compared with the neonatal period, so it is not chosen.

Option C early childhood, option D preschool age, option E school age, with the age of children and the gradual improvement of the immune system, the morbidity and mortality of these periods are relatively low, so they were excluded.

In conclusion, the correct answer is: A. neonatal period.

12. The period of the highest morbidity and mortality in childhood is

A. neonatal period

The neonatal period refers to the first 28 days after birth, when the baby's physical function is not fully mature, poor adaptability to the environment, and vulnerable to infections and other health problems, so the morbidity and mortality are the highest.

13. (Type A1 question)

What kind of congenital heart disease that can cause differential purpura

A. P fistula or window

B. interventricular septal defect

C. ASD

D. tetralogy of Fallot

E. pulmonary stenosis

Reference answer: A. Your answer:

Text analysis: [analysis] Patients with patent ductus arteriosus with thin diameter and small flow often have no obvious symptoms. Patients with thick catheter diameter and large flow rate are often complicated with congestive heart failure, which is manifested as irritability, shortness of breath, fatigue, sweating, feeding difficulties, and dysplasia, etc. When the disease develops severe pulmonary hypertension and a right-to-left shunt occurs, it shows lower body cyanosis and clubbing finger / toe, called "differential cyanosis"."

[Avoid error] (1) ventricular septal defect (B error), exertional dyspnea: moderate and small ventricular septal defect in adults and children

There are no obvious symptoms, only as shortness of breath and exertional dyspnea during strenuous exercise. When a large ventricular septal defect, the small

Children show symptoms such as feeding difficulties, no weight gain, and developmental delay.(2) atrial septal defect (C error). In addition to the larger defect

Externally, atrial septal defect is generally asymptomatic in childhood, and the symptoms gradually appear with age, with exertional dyspnea as the main factor

Performance, then can occur ventricular, superior arrhythmia, especially atrial flutter, atrial fibrillation and the symptoms aggravated. Some patients can be right

Right heart failure due from ventricular overload.(3) Tetralogy of Fallot (D wrong) is mainly manifested as blue, squatting symptoms,

Clubbing finger and paroxysmal hypoxia attack, generally not differentiated purpura.(4) Pulmonary artery stenosis (E error) is mainly manifested as fatigue

After palpitations, shortness of breath, fatigue, chest tightness and other symptoms, accompanied by blue, shortness of breath, syncope, etc., generally will not cause differential purple

purplish or white patches on the skin.

Differential purpura (differential cyanosis) refers to the different oxyhemoglobin level of the lower limbs and the upper limbs or lips, which is common in some specific heart diseases. It is usually present in newborns or early infants, especially those children with specific types of congenital heart disease.

Of the options given, tetralogy of Fallot (Tetralogy of Fallot) is the most likely congenital heart disease to cause differential purpura. Tetralogy of Fallot is a disease

involving four structural cardiac abnormalities: ventricular septal defect, pulmonary stenosis, right ventricular hypertrophy, and aortic riding span. These abnormalities cause blood to bypass the pulmonary circulation, bringing inadequately oxygenated blood into the systemic circulation and thus potentially leading to differential purpura.

Therefore, the most correct answer is: D. Tetralogy of Fallot

analyze:

Differential cyanosis is a special cyanosis phenomenon, that is, different cyanosis occurs in the upper and lower limbs, which is the pathophysiological change of patent ductus arteriosus. In the case of patent ductus arteriosus, aortic blood will flow into the pulmonary artery, lead to pulmonary artery area high oxygen content of blood and venous blood from the right heart, makes should have flowed into the upper limb high oxygen containing blood is incomplete oxygenation, upper limb skin mild blue, and while the lower limb is by the abdominal aorta blood, blood oxygen content is still high, so do not blue. Therefore, the congenital heart disease that can cause differential purpura is the patent ductus arteriosus.

Option B ventricular septal defect, option atrial septal defect, option C, option D Fallot tetralogy, option E pulmonary stenosis will have no differential cyanosis and can be excluded.

In conclusion, the correct answer is: A. patent ductus arteriosus.

Differential purpura (differential cyanosis) is a clinical manifestation that is characterized by different degrees of cyanosis of the skin in different parts of the body. It is often associated with certain congenital heart diseases that cause abnormalities in the heart and blood circulation, causing cause blood oxygen distribution throughout the body.

Among the above options, * * Tetralogy of Fallot (Tetralogy of Fallot) * * is a common cause of differential purpura. This is a complex congenital heart disease characterized by ventricular septal defects, pulmonary stenosis, aortic riding span, and right ventricular hypertrophy. These structural abnormalities lead to the mixing of blood flow between the pulmonary and systemic circulation, especially during severe right-to-left shunting, leading to a more pronounced cyanosis in the lower extremities than in the upper limbs.

So, the most correct answer is:

F. tetralogy of Fallot

14. (Type A1 questions)

The somatic symptom clusters of depression are included

A. hip

B. dyssomnia

C. retardation of thinking

D. bradypragia

E. Self-blame

Reference answer: B. Your answer:

[Analysis] The core symptoms of depression include depression, lack of interest, anhedonia, loss of will activity (self-blame), cognitive function impairment (slow thinking, slow movement), can be accompanied by a variety of physical discomfort symptoms, such as loss of appetite, sleep disorders, weight loss, easy fatigue and so on. Options A, C, D, and E were not somatic symptoms.[Avoid mistakes] depression has three no (conscious helpless, conscious hopeless, conscious useless), three self (since sin, self-blame, suicide).

In the somatic symptom cluster of depression, although multiple options may seem to be related to depression, the most specific somatic symptoms need to be found. Options A (depressed), C (slow thinking), D (slow movement), and E (self-blame) describe more psychological or behavioral characteristics than somatic symptoms.

Option B "Sleep disturbance" is typical somatic symptoms of depression, including difficulty falling asleep, frequent waking at night or early. Therefore, the most appropriate choice here should be * * B. Sleep disorder * *.

analyze:

Depression, also known as depression disorder, is characterized by significant and persistent low mood, and is the main type of mood disorder. The somatic symptom cluster of depression includes sleep disorders, which are most characterized by early waking.

Option A is depressed, option C is slow, and option E is self-blame, all belong to

the psychological symptom group of depression, rather than the physical symptom group, which can be excluded together.

Option D Slow movement is more common in patients with severe depression. At this time, patients will appear slow behavior, lazy life, unscale, lack of interest and vitality, and listless all day, but this belongs to psychomotor inhibition disorder, does not belong to the physical symptom group, so it is not selected.

In conclusion, the correct answer is: B. Sleep disorders.

The somatic symptom cluster of depression mainly includes some physiological and physical manifestations, rather than emotional or cognitive symptoms. Based on the options provided for the topic, here is an analysis of each option:

A. Low mood- -This is the major mood symptom of depression, but it is not a somatic symptom.

B. Sleep disorders- -This is a common somatic symptom of depression.

C. Thinking retardation- -This is a cognitive symptom of depression, not a somatic symptom.

D. Slow movement- -This is a somatic symptom of depression.

E. Self-blame- -This is an emotional symptom of depression, not a somatic symptom.

Based on the above analysis, the somatic symptom group of depression includes sleep disturbance and slow movement. In this question, the best answer is:

F. dyssomnia

15. (Type A1 questions)

The following would most suggest an early pregnancy

A. Primordial heart tube beat was detected by ultrasound

B. Cervical cervical smear smear found found of in in cervical cervical cervical smear

C. menelipsis

D. Body temperature lasts for 18 days

E. vomit

Reference answer: A. Your answer:

Graphic analysis: [analysis] the most possible indication of early pregnancy is ultrasound discovery of the original heart tube pulsation (A pair).

[Avoid mistakes] Cervical mucus smear found sheep tooth-like leaf crystallization (B wrong) is the result of estrogen changes in the body, can not be suggested

early pregnancy. Menopause (C error) is the earliest symptom of pregnancy, but it does not indicate an early pregnancy. Biphasic body temperature continues

16. days (D error) is a reliable method to monitor for ovulation and luteal function, and is not a reliable indicator of early pregnancy. Vomiting (E error) can be a symptom of early pregnancy, but it is not a reliable indicator of early pregnancy.

The diagnosis of early pregnancy can be made in many ways, but of these options, the most clear indication of early pregnancy is the A. ultrasound finding of primitive heart tube beats. The original cardiac tube beat seen on ultrasound is a phenomenon that can be observed at approximately 5-6 weeks of gestation and is a direct and reliable indicator for confirmation of early pregnancy. Therefore, A was chosen as the most correct answer. Other options, while potentially related to pregnancy, are less direct or specific than ultrasound.

analyze:

Early pregnancy refers to the stage before the 14th week of pregnancy, from the beginning of pregnancy to the 13th week of gestation. This stage is an important period for the vigorous differentiation of the fertilized egg to the embryo and the fetus, and can also be said to be the main period of fetal "differentiation, assembly and formation". The main symptoms and signs of early pregnancy are menopause, early pregnancy reaction, changes in the breast and reproductive system, among which the most obvious is menopause. Once the menstrual period is 10 days or above, it should be suspected as pregnancy.

Option A: Ultrasound found that the original heart tube beat is a sign of embryo survival. At this time, the embryo has developed to a certain time, and can not indicate early pregnancy, so it is not selected.

Option B: The crystallization of sheep tooth leaves found in cervical mucus

smear can only indicate the influence of estrogen, which can be seen during ovulation, pregnancy and before menstruation, and is not specific, so it is excluded.

Option D: Biphasic body temperature temperature for 18 days indicated good luteal function, but could not indicate early pregnancy, so it was not selected.

Option E: Vomiting is a kind of early pregnancy reaction, but not every pregnant woman appears, and the early pregnancy reaction usually occurs in about 6 weeks of menopause, which does not indicate the early pregnancy, so it is excluded.

To sum up, the correct answer is: C. menopause.

17. (Type A1 questions)

The following would best suggest an early pregnancy, if any, is:

A. Primordial heart tube beat was detected by ultrasound

* * Explanation * *: Ultrasound detection of primitive heart tube pulsation is the most reliable and definitive indicator of early pregnancy. This is because primitive heart tube pulsation indicates that embryonic development has begun and is only observed in the case of pregnancy. Other options, such as menopause, biphasic body temperature lasting for 18 days, and vomiting, may also indicate pregnancy, but they are not absolute indicators and may be affected by other factors. The detection of sheep tooth leaf crystallization in cervical mucus smear is an auxiliary diagnostic method, but it is not as clear and reliable as the ultrasound detection of the original heart tube beat.

18. (Type A1 questions)

The most common degeneration of uterine fibroids is

A. cystis degeneration

B. hyalinization

C. Red morphism

D. calcification

E. Sarcoma-like changes

Reference answer: B. Your answer:

Graphic analysis: [Analysis] Uterine fibroid degeneration refers to the secondary degeneration of uterine fibroids, including vitaline, cystic, red, sarcoma and calcification. The most common glass changes are seen (B pair).

[Error avoidance] According to the above analysis, exclude other options.

The most common degeneration of uterine fibroids is a red color change (option C). Red degeneration (or red degeneration) usually occurs in acutely reversed uterine fibroids, causing bleeding and necrosis in the fibroid tissue due to a sudden interruption of the blood supply. This type of degeneration is particularly common in pregnant women because the increased size of the uterus during pregnancy makes fibroids more likely to twist. Therefore, the best answer is the C.

analyze:

Uterine fibroids degeneration is a typical example of uterine fibroids losing their original structure, forming different types of degeneration. Common degeneration includes hyaloid, cystic change, red degeneration, sarcomosis, and calcification. Among them, glass osis is the most common type of degeneration, also known as transparent degeneration.

Option A cystic change is developed from glass change, formed by muscle cell necrosis, relatively rare, so it is not selected.

Option C red pattern is more common in pregnancy or puerperium, which is a special type of necrosis of fibroids, relatively rare, so it is excluded.

Option D calcification is more common in subserosal fibroids with small pedicle and insufficient blood supply, and uterine fibroids in postmenopausal women, relatively rare, so it is not selected.

Option E sarcomatoid change is rare in postmenopausal patients with pain and bleeding, inconsistent with the problem.

To sum up, the correct answer is: B. Glass change.

The most common degeneration of uterine fibroids is hyaline (B. hyaline). Vitaly is degeneration due to myomic tissue ischemia and is commonly seen in older patients. Other degeneration types such as cystic, erythroid, calcification and sarcoma were also visible, but not as common as hyaloid.

19. (Type A1 question)

The following ones that do not belong to the special physiological changes of

newborns are

A. barymastia

B. gingival cyst of mucous gland in the newborn

C. pseudomenstruation

D. physiologic icterus

E. Red buttock

Reference answer: E Your answer:

Graphic parse: [analytical] newborn common physiological changes: physiological jaundice (excessive bilirubin and liver enzyme system, exclude D), false menstruation (vaginal bleeding), eliminate C, breast enlargement (pregnant women estrogen on fetal effects caused by the interruption, eliminate A), plate teeth (also known as "teeth", epithelial cell accumulation or mucus retention swelling, exclude B), mantis mouth (refers to the cheek fat pad uplift, beneficial for milk), neonatal hair loss.

[Avoid mistakes] Red buttock, also known as neonatal diaper dermatitis, is a common skin inflammatory disease, common in the contact part of diapers

Position, skin lesions began to be mild flushing, swelling, gradually appear papules, blisters, erosion exudation, clear boundary, can be secondary

Bacterial or Candida infection, pustules or ulcers, is a pathological change, so the answer is E.

In the neonatal period, special physiological changes usually include breast enlargement, false menstruation, and physiological jaundice, which are all normal physiological phenomena. Red buttocks (red buttocks) can also be observed in newborns, especially because of diaper rash or other skin irritation. However, "horse tooth" is not the physiological change usually associated with newborns. Horse teeth usually refer to the specific teeth of certain animals (such as horses), a term that does not seem to apply in the context of physiological changes in the newborn.

Therefore, the most correct answer is * * B. Ma * *.

This is a multiple choice question that requires selecting from a given option that does not belong to the special physiological change of the newborn.

First, we will analyze the neonatal physiological characteristics represented by each option:

A. Breast enlargement: After the birth of A newborn, due to the influence of maternal hormones, the phenomenon of breast enlargement may occur, which is A normal physiological change.

B. Horse tooth: Horse tooth is a special physiological state in the neonatal period. It is usually manifested as small yellow and white spots on the gums, which has no impact on the health of the newborn and is also a normal physiological change.

C. False menstruation: Due to the influence of maternal estrogen, some newborns will have a little vaginal bloody secretions or bleeding similar to menstruation after birth, which is also a normal physiological change.

D. Physiological jaundice: after the newborn is born, because of the metabolic characteristic of bilirubin, often can appear physiological jaundice, the performance is the yellow dye of skin and sclera, this is a kind of normal physiological process.

E. Red buttock: Red buttock, also known as diaper rash, is usually a skin inflammation caused by prolonged moisture, friction, or exposure to irritating substances in the diaper area. It is not a physiological change in the newborn, but a skin problem that is closely related to the way the care and diapers are used.

In conclusion, breast enlargement, horse teeth, false menstruation and physiological jaundice are all normal physiological changes in the neonatal period, while red buttock is a kind of skin problem and does not belong to the special physiological changes of newborns.

Therefore, the correct answer is E. red hip.

The following do not belong to the newborn special physiological changes are:

F. Red buttock

analyze:

- ** Breast enlargement * *: In the newborn, due to the influence of hormones in the mother, breast enlargement can appear after birth, which belongs to a normal physiological phenomenon.

- ** Horse teeth * *: Small white spots appearing in the mouth of newborns,

usually located in the palate or gingiva, is a normal physiological phenomenon.

- ** False menstruation * *: newborn girls due to the action of maternal hormones, may appear a small amount of vaginal bleeding within a few days after birth, belongs to a normal physiological phenomenon.

- ** Physiological jaundice * *: newborn is not complete because of liver function mature, often appear 2-3 days after birth jaundice, most belong to physiological, and can subside spontaneously.

- ** Red buttock * *: That is, diaper rash or diaper dermatitis, is the skin inflammation caused by the skin, does not belong to the physiological change of the newborn, but pathological change.

20. (Type A1 question)

What is true about cough variant asthma

A. Often occur or worsen in the afternoon

B. ineffective anti-asthma therapy

C. was dominated by dry cough

D. Bronchial challenge test was negative

E. Infections often occur, and the antibiotic treatment is effective

Reference answer: C Your answer:

[Analysis] Clinical atypical asthma with cough as the only symptom is called cough variant asthma, so C is the correct answer.

[Avoid error] Cough variant asthma cough is obvious at night, so A wrong; treatment principles of cough variant asthma and typical asthma

Similarly, therefore B wrong; cough variant asthma bronchial challenge test is mostly positive, with cough as the only symptom, no wheezing

Qi shortness, chest tightness, etc., the possibility of infection is small, so DE is wrong.

Cough-variant asthma (CVA) is a specific type of asthma characterized by dry cough, especially in the absence of other typical asthma symptoms (e. g. wheezing or dyspnea). Considering this information, we can evaluate each option:

A. often occurs or worsen in the afternoon – this description is more associated with general symptoms of asthma than specifically to cough variant asthma.

B. Anti-asthma therapy is ineffective-This is not true because cough variant asthma is usually sensitive to antiasthma therapy (e. g., inhaled corticosteroids).

C. Predominantly dry cough-this is true that the main symptom of cough variant asthma is persistent dry cough.

D. Bronchial challenge test negative-this is not correct because cough variant asthma patients are usually positive in bronchial challenge test showing bronchial hyperresponsiveness.

E. Infections often occur, antibiotic therapy is effective-this is not accurate, cough variant asthma is not particularly prone to infection, and antibiotic therapy is not effective in asthma itself.

Therefore, the most correct answer is * * C. Dry cough is mainly used * *.

This is a question about cough variant asthma that requires a description from the given option that best fits the characteristics of cough variant asthma.

Option A "Often occur or aggravated in the afternoon": This description is usually not directly related to cough variant asthma, asthma attacks are not specific to the afternoon.

Option B "No anti-asthma therapy": this goes against the treatment principles of cough variant asthma. In fact, cough-variant asthma is sensitive to anti-asthma therapy.

F. Option: "mostly dry cough": This is a typical symptom of cough variant asthma, and patients usually show mainly dry cough, without wheezing and dyspnea.

G. option "Negative bronchial challenge test": this is not correct. In cough variant asthma, the bronchial provocation test is usually positive because it is an important examination for the diagnosis of asthma.

H. option "common infection, antibiotic treatment is effective": this is not consistent with the characteristics of cough variant asthma. Asthma is not caused by an infection, and so antibiotic treatment is usually ineffective.

In conclusion, the C option "mainly dry cough" most closely fits the characteristics of cough variant asthma.

Therefore, the correct answer is C. mainly dry cough.

Regarding cough variant asthma (Cough Variant Asthma, CVA):

I. was dominated by dry cough

analyze:

Cough-variant asthma is a special type of asthma that is mainly characterized by a long-term dry cough without obvious wheezing symptoms. Here is an analysis of the other options:

A. Often occurs or aggravates in the afternoon: cough variant asthma usually worsen at night or in the morning rather than in the afternoon.

B. Anti-asthma therapy is ineffective: Anti-asthma therapy is effective in cough-variant asthma, especially with inhaled hormones and bronchodilators.

J. Endobronchial challenge test is negative: The bronchial provocation test is usually positive and can be used to diagnose asthma and its variants.

K. Infection often occurs, and antibiotic therapy is effective: cough variant asthma is not associated with infection, and antibiotic therapy is ineffective.

Therefore, the correct answer is C. mainly dry cough.

21. (Type A1 questions)

Prophylactic cervical cerclage should be performed is

A. Pregnant women with a history of more than three medical abortions

B. Pregnant women with a history of more than three induced abortions

C. Pregnant women who have had a history of more than 3 times of miscarriage

D. Pregnant women with a history of PROM more than 3 times

E. Pregnant women with a history of more than three spontaneous abortion in the second trimester of pregnancy

Reference answer: E Your answer:

[Analysis] For the history of spontaneous abortion in the middle of pregnancy, non-pregnancy cervical opening can be through the No.8 sea format dilator, can be performed before pregnancy or in pregnancy, so E on.

[Avoid avoidance] The indications for cervical ligation include: habitual abortion, premature birth, placenta previa, and twins

Or multiple pregnancy, pregnancy B ultrasound examination found that the progressive shortening of the cervix, considering cervical insufficiency.

In the context of prophylactic cervical cerclage, this procedure is usually designed to prevent cervical insufficiency, resulting in preterm birth or miscarriage. With this in mind, the most suitable scenario for prophylactic cervical cerclage is in pregnant women with repeat intermediate abortion (usually painless and unthreatened).

Therefore, based on the options given in the question, the most appropriate answer is:

F. Pregnant women with a history of more than three spontaneous abortion in the second trimester of pregnancy

This is because multiple second trimester spontaneous abortions may be associated with cervical insufficiency, and cervical cerclage is a means of preventing premature cervical dilatation and subsequent miscarriage or preterm birth.

analyze:

Cervical ligation is a kind of surgery to close the cervical opening, which is generally small and medium surgery, requiring local anesthesia, and the operation time is about half an hour. The purpose of cervical cerclage is to repair and establish the normal morphology and function of the intracervix so that pregnancy can be maintained until term or the fetus can survive. Surgery was performed more often than at 14 to 18 weeks of gestation. Prophylactic cervical cerclage refers to cerclage judged to be cervical insufficiency to prevent abortion. Cervical insufficiency is the inability of the cervix to maintain pregnancy to term due to anatomical or functional defects. Pregnant women with more than 3 history of spontaneous abortion in the

second trimester of pregnancy can consider cervical insufficiency, feasible preventive cervical ligation.

Option A for pregnant women with A history of more than 3 medical abortion, and option B with A history of more than 3 induced abortion are all artificial termination of pregnancy, which has nothing to do with spontaneous abortion, without cervical cerclage, which can be excluded together.

Option C Pregnant women who have had a history of miscarriage for more than 3 times should be screened to determine the cause of fetal death, which has little to do with cervical insufficiency, so it is not selected.

Option D Pregnant women with a history of premature rupture of membranes at term for more than 3 times should be alert to chorioamnionitis, which has nothing to do with cervical function, so they is excluded.

In conclusion, the correct answer is: E. Pregnant women who have had more than three times of spontaneous abortion in the second trimester of pregnancy.

Prophylactic cervical ring ligation is usually used to prevent miscarriage or preterm birth due to cervical insufficiency. Cervical insufficiency is a condition where the cervix expands spontaneously without contraction, leading to a second or third trimester miscarriage or premature birth. Indications included pregnant women who had a history of multiple spontaneous abortion or preterm birth. Based on this, the best answer is:

G. Pregnant women with a history of more than three spontaneous abortion in the second trimester of pregnancy

22. (Type A1 question)

Breast-feeding alone easily leads to anemia in infants

A. hypoferric anemia

B. megaloblastic anemia

C. Blood loss anemia

D. erythronoclastic anemia

E. Aplastic anemia

Reference answer: A. Your answer:

[Analysis] After the birth of the baby from the sixth month, simple breast milk can not meet its growth needs, easy to lead to iron deficiency, resulting in iron deficiency anemia in children, so A pair.

[Avoid errors] Deficiency of folic acid or vitamin B12 can lead to giant erythroid anemia; hemorrhagic anemia is more often due to trauma or

Patients with excessive blood loss through surgery; hemolytic anemia refers to excessive destruction of red blood cells that exceeds the compensatory hematopoietic function of bone marrow

Reducdecreased red blood cells, hemoglobin; aplastic anemia is a cause of bone marrow hematopoietic failure

Sexual diseases are mainly abnormalities in the cytoplasm and quantity of hematopoietic stem cells and the hematopoietic microenvironment.

The anemia most often caused by breastfeeding alone is iron deficiency anemia, because the iron in breast milk, while high, is not large enough to meet the rapid growth of infants. Therefore, the correct answer is: A. iron deficiency anemia

analyze:

Breast milk is the most natural, safest and most complete natural food for infant growth. It contains all the nutrients and antibodies needed for infants to grow. However, the iron content in breast milk is relatively low, containing only 0.1 mg of iron per 100 ml of breast milk, while infants need about 10 mg of iron per day, so exclusive breastfeeding does not meet the full demand for iron. Long-term exclusive breastfeeding without timely addition of complementary food is easy to lead to the depletion of iron reserves in infants, resulting in iron deficiency anemia.

To sum up, the correct answer is: A. iron deficiency anemia.

Option B Meggaloblastic anemia is mainly due to deficiency of folic acid or vitamin B12. These two nutrients are relatively abundant in breast milk, so they are not the main type of anemia caused by breastfeeding, so it is not selected.

Option C Hemagic anemia is anemia caused by bleeding in a part of the body, unrelated to feeding method, and was excluded.

Option D Hemolytic anemia is the anemia caused by excessive and excessive red blood cell destruction, which is not directly related to the feeding method, so it is not selected.

Option E aplastic anemia was anemia due to bone marrow hematopoietic failure, independent of feeding mode, and was excluded.

Anemia prone to breastfeeding alone is:

A. hypoferric anemia

* * Analysis: * * Simply breastfed infants, especially after 6 months, if there is no timely addition of iron-rich complementary food, there may be insufficient iron intake, leading to iron deficiency anemia. Iron in breast milk is relatively low, and the demand for iron is increased as infants grow, making it prone to iron deficiency. Iron deficiency anemia is common in infants, while other types of anemia (such as megalthrocyte anemia, hemorrhagic anemia, hemolytic anemia, aplastic anemia) are not common in breastfed infants.

23. (Type A1 question)

The main basis for the diagnosis of a closed skull cover bone fracture is that

A. Local depression on palpation

B. showed signs of neurological damage

C. A fluctuating sense of scalp swelling

D. X line flat sheet

E. Bone erasure

Refer to the answer: D. Your answer:

Graphic analysis: [analysis] For closed skull cover fracture, X-ray plain examination for clear diagnosis, so D pair.

[Avoid error] If there is no obvious depression and only linear fracture, there can be no depression in the local palpation, without injury

Intracranial nerve and nerve nucleus can not appear signs of nervous system damage, scalp swelling is not obvious, no fluctuation feeling, but no bone rub

Sound, so the ABCE is wrong.

The diagnosis of closed skull cover bone fracture mainly depends on imaging examination, so the most correct answer is D. X-ray plain film. Plain X-ray is a

routine method for the diagnosis of fracture, which can visually show the location and degree of fracture. While other options such as local depression (A) on palpation, signs of neurological damage (B), fluctuating scalp swelling (C), and bone rubbing (E) may be associated with skull fractures, these are less direct and accurate than imaging. Therefore, among these options, flat X-ray (D) is the main basis for the diagnosis of closed skull skull fractures.

analyze:

The diagnosis of closed skull fracture relies on imaging examination, in which X-ray plain film is easy to operate and cheap, and is often used as the preferred imaging examination method for skull fracture. Plain X-ray can detect the continuity interruption and displacement of the skull, as well as the walking direction of the fracture line, and provide reliable imaging evidence for the diagnosis of skull fracture.

Skull depression may not occur in skull fracture. Option A is not A characteristic manifestation of closed skull fracture, so it is not chosen.

Option B shows more signs of neurological injury in open craniocerebral injury, and closed skull fracture is not often combined with neurological injury, so it is excluded.

Option C Scalp swelling is more seen in cap subaponeurotic hematoma, while skull fracture generally shows no scalp hematoma, which is inconsistent with the question.

In closed skull fracture, due to scalp coverage, the fracture end is not exposed to the outside world, so there will be no bone rub during palpation, so option E does not meet the characteristics of closed skull fracture, so it is not considered.

To sum up, the correct answer is: D.X-ray flat slice.

The diagnosis of closed skull cover bone fractures relies mainly on imaging examination. Although clinical signs such as local pitting, signs of neurological damage, fluctuating scalp swelling, and bone rubbing can suggest skull fracture, these signs require further confirmation by imaging. Therefore, flat X-ray film is the main basis for the diagnosis of closed skull cover bone fracture.

The correct answer is: D. X-ray flat sheet

24. (Type A1 questions)

Deep sensory disorder, which of the following conditions is prone to fall

A. Lift upper and upper limbs with both hands

B. eye closure

C. Lift your legs alone

D. Turn the neck

E. go down on one's knees

Reference answer: B. Your answer:

[Analysis] deep sensory disorder can appear closed eyes difficult to stand positive, the patient with the feet standing together, hands forward flat, the patient closed his eyes after standing unstable, the body swing inclined to fall, so B.

[To avoid mistakes] Closed eyes are difficult to establish a positive sign often indicates deep sensory disorder, which is a common positive sign of the nervous system, and ACDE is difficult to stand with closed eyes

It has nothing to do with the examination.

Deep sensory impairment mainly affects the perception of limb position and movement, resulting in decreased balance and spatial positioning ability, and thus is more prone to fall during movements requiring fine balance and spatial positioning. Given these factors, closed eyes (B) is the option most likely condition to fall, because people cannot rely on visual information to help maintain balance, which greatly increases the risk of falling in individuals with deep sensory disorders. Therefore, the correct answer is the B. Closed eyes.

analyze:

Deep sensation refers to proprioception from muscles, tendons, periosteum, and joints, including movement perception, location perception, and vibrations. Deep sensory disorder suggests dysfunction of the spinal cord. When eyes are closed, the body cannot compensate through vision, so it is easy to fall. Therefore, when closing the eyes, the patients with deep sensory disorder are prone to fall.

Option A raises both hands and upper limbs flat, and option D turns the neck, which will interfere with the balance sense, but the balance sense belongs to shallow sensation. Patients with deep sensory disorder can compensate for the above movements, so it is not easy to fall, which can be excluded together.

Option C single leg lift mainly affects motion perception, but patients can

compensate through location perception and are not easy to fall, so they is not selected.

Option E knee flexion mainly affects location perception, but patients can be compensated by motion perception, not easy to fall, can be excluded.

To sum up, the correct answer is: B. Close your eyes.

For patients with deep sensory disorder, the situation prone to falls is when they close their eyes. This is because deep sensory disorder affects the body's perception of its position, and when the patient closes his eyes, visual feedback is removed, reducing information dependent on deep sensation, and thus more prone to lose balance and fall.

So, the most correct answer is:

* * B. Closed-looking eyes * *

25. (Type A1 questions)

Parkinson's patients with prostatic hyperplasia are not choose

A. bromocriptine

B. Benserazide

C. diphenhydramine

D. trihexylphenidyl

E. amantadine

Refer to the answer: D. Your answer:

[Analysis] benzene is an anticholinergic drug, elderly patients with caution, angle closure glaucoma and prostatic hypertrophy patients are prohibited, so the answer is D.

[Error avoidance] Bromocriptine is a dopamine receptor agonist, with a higher incidence of orthostatic hypotension and psychiatric symptoms, but a symptom wave

The incidence of motility and abnormal disorder is low; the side effects are divided into peripheral and central, the former is nausea, vomiting and low

Blood pressure, arrhythmia, etc., the latter has symptom fluctuation, mental

symptoms; diphenhydramine belongs to antihistamine, main

Should be used to treat allergic diseases, motion sickness, etc.; amantadine renal insufficiency, epilepsy, severe gastric ulcer, liver disease patients

cautiously use.

PD patients with prostatic hyperplasia should not be given drugs that may worsen prostatic symptoms. Among the options, diphenhydramine (option C) is an antihistamine with anticholine effects that may cause urinary retention and therefore is not appropriate for patients with prostatic hyperplasia. Therefore, the best answer is for C. diphenhydramine.

analyze:

Prostatic hyperplasia is a common disease in elderly men, its cause is due to the gradual enlargement of the prostate on the urethra and bladder outlet compression effect. Clinically, it shows frequency of urination, urgency, increased nocturnal urination and laborious urination, and can lead to complications such as urinary system infections, bladder stones and hematuria. Parkinson's disease is a common neurological degeneration disease, and the most important pathological change is the degenerative death of dopaminergic neurons in the midbrain substantia nigra, resulting in a significant reduction in striatal dopamine content. Compound levodopa is the most basic drug for Parkinson's disease. It is used in small doses and gradually increases the dose. Of dobasezine for levodopa and benzopine mixed proportional to the finished product, because benzopine for peripheral decarboxylase inhibitor, can reduce levodopa in peripheral decarboxylation, increase the content of levodopa into the brain and reduce the peripheral side effects, so the drug relative to levodopa side effects, but dopa hydrazine may still cause dysuria, aggravating prostate hyperplasia caused by urination disorders, so patients with prostatic hyperplasia of Parkinson's patients should not be selected.

Option A bromocryptine is dopamine receptor agonist, can directly stimulate the dopamine receptor site, promote the release of prolactin because compete the same receptor and more dopamine receptor, plus D_2 receptor blockers also has anti-prolactin effect, can reduce the secretion of prolactin, thus used to treat or inhibit physiological lactation, high prolactinemia, and high prolactitinemia low fertility, menstrual disorders, and amenorrhea, lactation, gonadal insufficiency, male female breast, etc. The drug had little effect on the urinary system and could be used in Parkinson's patients with prostatic hyperplasia, so it was excluded.

Option C diphenhydramine is a derivative of ethanolamine, which can compete with histamine released in tissues for the H1 receptors on effector cells, thus preventing the onset of anaphylaxis, relieving the spasmodic and congestion effects of histamine, and will not aggravate prostatic hyperplasia in patients, so it is not considered.

Option D benzene for central anti-choline against Parkinson's drugs, effect is selective block the striatal cholinergic nerve pathway, and smaller peripheral effect, to restore Parkinson's disease brain dopamine and acetylcholine balance, improve Parkinson's disease symptoms, will not aggravate patients with prostatic hyperplasia, is inconsistent with the idea.

Option E Amanadine is an antiviral drug. The mechanism of anti-Parkinson's disease may promote the synthesis and release of striatal dopamine, reduce the reuptake of dopamine by nerve cells, and have anti-acetylcholine effect, thus improving the symptoms of Parkinson's disease patients, which has little impact on the urinary system and can be excluded.

In conclusion, the correct answer is: B. dobasalazine.

Parkin patients with prostatic hyperplasia should be avoided with drugs with anticholinergic effects as these may aggravate symptoms of prostatic hyperplasia, such as urinary retention.

According to the option:

- ** Bromocriptine * * (Bromocriptine) is a dopamine agonist used in the treatment of Parkinson's disease and does not have anticholinergic effects.

- ** Dopastalazine * * (Carbidopa-Levodopa) is a commonly used drug in the treatment of Parkinson's disease, which mainly acts on the dopamine system and does not have anticholinergic effects.

- ** Diphenhyydramine * * (Diphenhydramine) is an antihistamine with a strong anticholinergic effect.

- ** Marine * * (Benztropine) is an anticholinergic drug used to treat Parkinson's disease.

- ** Amantadine * * (Amantadine) is mainly used for the treatment of Parkinson's disease, and it also has anticholinergic effects.

Among these drugs, * * diphenhydramine * * and * * phenyl* * have anticholinergic effects. To avoid aggravating the symptoms of prostatic hyperplasia, drugs with anticholinergic effects should be avoided. So the answer is:

* * C. Diphenhydramine * *

* * References * * *:

-Zhu Hongmei, Chen Zhongming. Manual of practical clinical medicines. Beijing: People's Health Publishing House, 2018.

-Wang Linbo, Gao Wenying. Pharmacotherapeutics for neurologic disorders. Beijing: Science Press, 2016.!!!!

26. (Type A1 question)

The first-line chemotherapy regimen for ovarian epithelial tumors was

A. bleomycin + vincristine

B. Cisplatin + vincristine

C. Cisplatin + vincristine + ifosfamide

D. Cisplatin + paclitaxel

E. Paclitaxel plus carboplatin

Reference answer: E Your answer:

[Analysis] The first-line chemotherapy regimen for ovarian epithelial tumor is widely used as paclitaxel plus platinum, which has high effective rate and low toxicity, so E pair.

[Error avoidance] At present, the first-line chemotherapy regimen for ovarian epithelial tumors mainly includes intraperitoneal chemotherapy and intravenous chemotherapy, among which static

Pulse chemotherapy is widely used in clinical practice. Intravenous chemotherapy regimen mainly includes paclitaxel plus platinum, doxorubicin plus paclitaxel, doxorubicin plus cyclophosphamide plus platinum, and platinum plus cyclophosphamide.

First-line chemotherapy regimens for ovarian epithelial tumors often include

platinum and taxane drugs. Cisplatin (one of the platinum drugs) and paclitaxel are commonly used combinations, so I will choose:

F. Cisplatin + paclitaxel

This combination is widely used as a first-line treatment for ovarian epithelial tumors due to its effectiveness and tolerability. However, in practice, the doctor may adjust the chemotherapy regimen according to the patient.

analyze:

Ovarian epithelial tumor refers to the ovarian tumor derived from the epithelial tissue, which is a common gynecological tumor, accounting for 50%~70% of the ovarian tumors. Its malignant type accounts for 85%~90% of ovarian malignant tumor, and is more common in middle-aged and elderly women, and rarely occur in the period before puberty or infancy. The first-line chemotherapy regimen for ovarian epithelial tumors is paclitaxel + carboplatin, which has become the standard regimen for primary treatment of ovarian epithelial cancer.

Option A bleomycin + vincristine is the preferred chemotherapy regimen for Hodgkin lymphoma, so it is not chosen.

Option B Cisplatin + vincristine could be used for chemotherapy for testicular cancer, but not as a first-line chemotherapy regimen for ovarian epithelial tumors, so it was excluded.

Option C cisplatin + vincristine + ifosfamide can be used as chemotherapy for small cell lung cancer, testicular cancer, sarcoma, etc., but not for chemotherapy for ovarian epithelial tumors, so it is not selected.

Option D cisplatin + paclitaxel can be used for chemotherapy for breast cancer and lung cancer, but it is not the first-line chemotherapy regimen for ovarian epithelial tumors, which can be ruled out.

In conclusion, the correct answer is: E. paclitaxel + carboplatin.

First-line chemotherapy regimen for ovarian epithelial tumors is usually a combination of platinum-containing drugs and taxane drugs. According to current medical guidelines and clinical practice, the recommended standard first-line chemotherapy regimen is a combination of paclitaxel and carboplatin.

So, the most correct answer is:

G. Paclitaxel plus carboplatin

27. (Type A1 question)

The ability to know and judge one's own mental state

A. insight

B. intelligence

C. perception

D. be conscious of

E. directive force

Reference answer: A. Your answer:

[Analysis] Self-knowledge refers to the patient's ability to understand and judge their own mental state, so the answer is A

[Escape from mistakes] Intelligence refers to the complexity of the human brain's ability to understand society and conduct daily life. Understanding refers to understanding and understanding

The ability to know and feel things. Consciousness is the perception of the appearance of the brain inside and outside the brain. Directional force is a person to the time, the ground

Points, the character and their own state.

This question examines a concept in psychology and psychiatry, that is, an individual's ability to recognize and judge their own mental state. The right answer is A. Self-knowledge.

Self-knowledge (insight) refers to an individual's ability to understand and recognize their own emotions, thinking and behavior. In the field of mental health, self-knowledge specifically refers to the individual's knowledge and understanding of his or her own mental state, including any possible mental illness or psychological problems. A person's level of self-awareness can influence their acceptance of treatment and treatment effectiveness. Therefore, in this multiple-choice question, option A "self-knowledge" is the most appropriate answer to the question.

analyze:

The ability to know and judge their own mental state is called self-knowledge in psychiatry clinically, which refers to the patient's ability to know and judge their own

mental state.

Option B Intelligence refers to the general spiritual ability of biology, which refers to the ability to understand and understand objective things and use knowledge and experience to solve problems, including memory, observation, imagination, thinking, judgment, etc., which is not consistent with the meaning of the question, so it is not selected.

Option C comprehension refers to the ability to understand, experience and understand things, and is not the ability to understand and judge one's own mental state, so it is excluded.

Option D consciousness refers to the individual's perception ability to the surrounding environment and its own state. The maintenance of consciousness depends on the excitation of the cerebral cortex. When the brain stem ascending reticular activation system or bilateral cerebral cortex damage can lead to consciousness disorders, which is inconsistent with the description of the question stem, so it is not considered.

Option E orientation refers to a person's ability to understand the time, place, people and their own state. Direction disorder is an important sign of consciousness disorder, which is inconsistent with the description of the question stem and can be excluded.

To sum up, the correct answer is: A. Self-knowledge.

The correct answer is A. Self-knowledge.

Self-knowledge refers to the individual's ability to recognize and judge his or her own mental state. It includes awareness of their own psychological problems and an understanding of the impact that these problems may have. In psychiatric assessment, self-knowledge is an important assessment indicator used to judge a patient's ability to understand and cope with their illness.

28. (Type A1 question)

Under electron microscope, the endometrium has high secretory activity, enlarged glandular epithelial cells, secretory granules in the cell, and enlarged interstitium, suggesting that the endometrium is in the menstrual cycle at this time

A. Early hyperplasia

B. Late hyperplasia

C. Early secretion

D. Late secretion

E. premenstrua

Refer to the answer: D. Your answer:

[Analysis] This endometrium has a high secretory activity, secretory granules in the cell, interstitial loose and enlargement, in line with the late secretion performance. The wrong-avoidance endometrium changes with the periodic changes of the ovaries, which is generally divided into three stages.(1) Hyperplasia period: equivalent to the mature stage of follicle development, that is, about the 5th to 14th days of the menstrual cycle. After the menstrual period, under the action of estrogen, the cells in the basal layer of the endometrium began to hyperplasia and repair the peeling wound, and then continue to hyperplasia and thicken, glands increase, widen, blood vessels also hyperplasia, and the stromal hyperplasia is dense.(2) Secretion period: about 15 to 28 days of the menstrual cycle, and the intima continues to increase during this period

Thick, the gland is further expanded, flexion, to the late gland is highly secreted, the blood vessels also rapidly grow, more flexion. Between qualitative change

Looose and edema.(3) Menstruation period: it is the first to fourth day of the menstrual cycle. The scattered small hematoma formed in the inner membrane functional layer, make the necrotic inner membrane peeling, along with the blood discharge, called menstruation.

[fault avoidance]

This title describes the presentation of the endometrium at specific stages of the menstrual cycle. The high secretory endometrium observed by electron microscopy, enlarged glandular epithelial cells, secretory granules in the cell, and enlarged interstitium, which are the characteristics of the endometrium during the secretory period. The menstrual cycle is roughly divided into hyperplasia period and secretory period, followed by ovulation, during the corpus luteum, at which time the endometrium prepares for a possible pregnancy and shows a high degree of secretory activity.

According to the question stem description, the most consistent option is the secretion period. As to whether it is early or late, considering "highly secretory

activity" and "intracellular secretory granules", these descriptions are more consistent with early secretory stages, but often secretory granules are more inclined to mid-late secretion. Therefore, if considered, this may point to the late secretory phase, when the endometrium is well prepared for implantation.

So, the most appropriate answer is * * D. Late late * *.

analyze:

Menstrual cycle is mainly divided into menstrual period, hyperplasia period and secretion period, among which hyperplasia period can be divided into early hyperplasia, middle hyperplasia and late hyperplasia, secretion period can be divided into early secretion, middle secretion and late secretion. Among them, the late secretion is the 24th ~ 28 days of menstrual cycle, the endometrium is further thickened, the surface is uneven, slightly wavy. The stroma under the epithelium is differentiated into hypertrophic decidual-like cells. The opening of the inner membrane gland faces the uterine cavity, and the secretions such as glycogen overflow, and the stroma is more loose, edema, and further loose. The spiral arterioles grew rapidly beyond intimal thickness and were also more curved and seen in many branches. According to the title description, the endometrium is now in a late stage of secretion.

Option A is the early stage of the 5th to 7th days of the menstrual cycle, when the hyperplasia and repair of the intima begin. The glandular epithelial cells are square or low column, the stroma is dense, the cells are star, the arterioles in the stroma are straight and the wall is thin, which is inconsistent with the question.

Option B The late stage of hyperplasia is the 11th to 14th day of the menstrual cycle. During this period, the inner membrane is further thickened, the surface is uneven, slightly wavy, and the glandular epithelium becomes high columnar, with obvious proliferation and obvious cell boundaries, which is inconsistent with the description of the topic stem.

The early stage of option C secretion is the 15th to 19th day of the menstrual cycle. At this stage, the endometrial glands are longer and more curved, and glycogen-containing vesicles begin to appear under the nucleus of glandular epithelial cells, called subnucleolar vacuoles, which are the histological feature of the secretory period, which is not consistent with this question.

Option E Early menstruation does not belong to the menstrual cycle. The periodic changes of the endometrium are divided into menstrual period, hyperplasia

period and secretory period, so it is not selected.

To sum up, the correct answer is: D. Late secretion.

According to the changes of the endometrium in the menstrual cycle, the endometrium has high secretory activity, enlarged glandular epithelial cells, secretory granules in the cell, and enlarged interstitium, which suggest that the endometrium is in the secretory stage. The secretory phase is further divided into early secretion and late secretion stages.

* * Hyperplastic period * *: In the hyperplasia period, the main characteristics of the endometrium is the hyperplasia of glands and stroma, the glands are straight or slightly curved, the secretory activity of glandular epithelial cells is low, and there will be no significant secretory granules in the cell.

* * Early secretion * *: In the early secretion, glandular epithelial cells begin to increase, and secretory granules appear in the cell, but the stroma is not significantly loose and enlarged.

* * Late secretion * *: In the late secretion, glandular epithelial cells are highly secreted, there are a large number of secretory granules in the cell, loose stroma and edema. These descriptions are most consistent with the EM observations mentioned in the stem.

* * Early menstruation * *: In the early menstrual period, the endometrium began to degenerate, secretion activity reduced, interstitial edema reduced, ready to fall off.

According to the above analysis, the endometrial state best matching the description of the title stem is late secretion.

So the correct answer is: * * D. Late secretion * *

29. (Type A1 question)

Commandering auditory hallucinations are most common in

A. schizophrenia

B. panic disorder

C. vesania

D. depression

E. Adjustment disorder

Reference answer: A. Your answer:

Graphic analysis: [analysis] command hearing refers to the hearing of command language, which is more seen in schizophrenia, so A pair.

[Avoid mistakes] Panic disorder is mainly manifested as strong, repeated panic attacks, that is, in a short period of time to panic,

Chest tightness, sweating and other main anxiety, fear, sense of death or out of control. Mania is driven by high emotion and high thinking

And increased speech and movements are typical symptoms. Depression has interest decline or lack, "three self", "three no" and other typical symptoms.

Adaptation disorder refers to the annoyance and depression caused by long-term stressors or difficult situations, plus their own personality defects

Disorders as well as maladaptive behavior and physiological dysfunction.

Ordering auditory hallucinations, in which a patient gives him or her commands or instructions, are often associated with serious mental health problems. Among the options listed, schizophrenia (now more commonly known as psychotic disorder) is the disorder most commonly associated with imperative auditory hallucinations. So the right answer is: A. schizophrenia

analyze:

Ordering auditory hallucinations is a kind of auditory hallucinations, refers to auditory hallucinations content to command the patient to do something. It's a kind of illusory hearing, but this hearing often commands the brain. Such as ordering patients to run, ordering patients to jump off buildings, and ordering patients to harm others. Ordering auditory hallucinations are more often seen in schizophrenia, but also in the

acute onset of bipolar disorder with psychotic symptoms, which is a severe psychotic symptom.

Option B Panic disorder is a sudden experience of panic, presenting with severe feelings of suffocation, dying and loss of mind, along with severe symptoms of autonomic dysfunction. Panic disorder generally does not have hallucinations and therefore can be excluded.

Option C Mania with emotional upsurge or irritability as the main clinical manifestations, accompanied by exuberant energy, increased speech, increased activity, severe accompanied by hallucinations, delusions, tension symptoms and other psychotic symptoms. Mania generally does not have command auditory hallucinations, so do not choose.

Option D Depression is characterized by significant and persistent low mood, with clinical manifestations ranging from depression to grief, negative suicide attempts or behaviors, or even wooden rigidity. Depression does not appear command sex auditory hallucinations, can be ruled out.

Option E adaptation disorder refers to the short-term and mild annoyance state and emotional disorder caused by obvious life changes or environmental changes. There are often a certain degree of behavioral changes, but no psychotic symptoms, which is inconsistent with the description of the question stem.

In conclusion, the correct answer is: A. schizophrenia.

Ordering auditory hallucinations (command hallucinations) is a pathological symptom in which a patient hears a voice or instruction asking them to take certain behavior. This symptom is most common in patients with schizophrenia. Schizophrenia is a serious mental illness, often presenting with symptoms such as hallucinations, delusions, and disorders of thinking.

Thus, imperative auditory hallucinations occur most frequently in schizophrenia.

The best answer is:

A. schizophrenia

30. (Type A1 question)

After drinking a large amount of liquor for a long time, how long the tremor

A.12 hours

B.48 hours

C.6 hours

D.3 hours

E.24 hours

Reference answer: B. Your answer:

[Analysis] The tremor delusion state after alcohol withdrawal belongs to severe symptoms, often starting from 48 to 96 hours after drinking.[Misavoidance] abstinence syndrome occurs within 6 to 28 hours of abstinence or sudden reduction in alcohol consumption. The main manifestations are divided into mild symptoms, moderate symptoms, and severe symptoms. The tremor state after alcohol withdrawal is specifically manifested in the case of changed consciousness, the hand, face, tongue thick tremor, orientation dysfunction, hallucinations and delusions, and seizures and physical symptoms.

Sudden abstinence after a prolonged period of drinking may lead to tremor (abstinence syndrome, also known as alcohol withdrawal syndrome), usually most common within 48 hours of abstinence. Thus, the most correct answer is the B.48 h.

analyze:

Tremor is an acute form of chronic alcoholism or long-term alcohol dependence or cessation. Its three typical characteristics are: consciousness disorder, behavior disorder, hallucination illusion, accompanied by a lot of sweating, tachycardia, fever, elevated blood pressure, dilated pupil, cognitive impairment, disorientation and so on. After drinking a large amount of liquor for a long time, suddenly quit drinking for 48 hours can occur tremor.

To sum up, the correct answer is: B.48 hours.

Tremor (delirium tremens, DT) is a severe withdrawal symptom that may occur after a sudden withdrawal. It usually occurs during a period of time after abstinence, and the specific time varies depending on individual differences. According to clinical practice and related literature, tremor delusion most often occurs within 48 to 96 hours after abstinence.

So the best answer is:

I. for 48 hours

28. (Type A1 question)

The boy, 2 years old, 87cm long, weight 12.5kg, can jump on both feet, can say 2~3 words of phrases, can accurately assess the development of the child is

A. Normal development

B. Backgrowth

C. overload

D. hypoevolutism

E. become thin

Reference answer: A. Your answer:

[Analysis] Generally, 75cm, 2,2,87cm (normal height); usually, 2,12.5~13.5kg (normal weight), 2 years old, eat with a spoon (normal development); 2~3 words (1~2 years old, idiom stage after 3 years old, so the language development is normal), so the boy can be assessed as normal development, so A pair.

[Error avoidance] Clinically convenient application: average height (cm) = age 7 + 75 in children aged 2 to 6 years, weight (kg) in 1 to 6 years = years

Endovirus 2 + 8.

The topic examines the assessment of the development and growth of a 2-year-old child. According to the information, the boy is 87 centimeters long and weighs 12.5 kilograms, and can jump on both feet and speak phrases of two to three words. This information can be used to determine whether his growth and development are normal.

-Height and weight: For 2-year-old children, the height of 87 cm and 12.5 kg are roughly within the normal range, but need to be determined by the growth curve.

-Developmental milestones: Two-year-olds are usually able to say simple words and phrases, and can perform simple physical activities such as jumping on their feet. The boy's ability to perform these activities shows that his movement and language development were roughly normal.

Based on this information, the most reasonable conclusion is that the boy's

development was normal. Therefore, the best answer option is: A. Normal development

This is a question about the assessment of growth and development in children. We need to first understand the standards of children's growth and development, and then analyze and judge according to the information given in the topic.

Understanding background information: The title describes a 2-year-old boy with his height, weight, and certain developmental milestones.

Understand the question core: Our goal is to accurately assess the development of the child based on this information.

Now, we analyze the options based on existing medical knowledge and criteria for child growth and development:

Option A (normal development):

According to the title, the 2-year-old boy is 87cm tall, weighs 12.5kg, can jump on both feet and speak 2-3 word phrases. According to the child growth and development standards, these manifestations are in line with the normal development level of the 2-year-old child, so it can be judged that the development of the child is normal.

Option B (growth growth):

There is no mention in the title that the growth rate of the child was significantly lower than that of children of the same age, and his length of 87cm and his weight of 12.5kg were within the normal range, so it could not be judged to be backward.

Option C (overweight):

Although body weight is an important health indicator, body weight 12.5kg alone does not directly judge the child to be overweight. A combination of height and weight needs to be assessed, and the topic did not provide enough information to support this judgment.

Option D (developmental delay):

According to the description, the child can jump on both feet and speak 2-3-word phrases, which are normal developmental milestones for a 2-year-old child. Therefore, it cannot be judged as mentally retarded.

J. option (wasting):

Similarly, weight 12.5kg alone cannot be judged that the child is thin. Combined

height and weight were needed, and the information in the topic did not support this judgment.

In conclusion, according to the title description and the criteria of the child's growth and development, we can determine that the child's development is normal. Therefore, the correct answer is A. normal development.

According to the existing medical knowledge and growth and development standards, the normal growth and development situation of a 2-year-old boy should include the following aspects:

1. * * Height * *: The height range of 2-year-old boys is generally about 85-90 cm. The boy was 87 cm tall and was within the normal range.

2. * * Weight * *: Two-year-old boys usually weigh between 11-14 kg. The boy weighed 12.5 kg, within the normal range.

3. * * Sports development * *: Ability to jump on both feet is the normal exercise ability of 2-year-old children.

4. * * Language development * *: The phrase that can speak 2-3 words is also the normal language ability of 2-year-old children.

Based on the above information, the boy's growth and development indicators were within the normal range. So, the most correct answer is:

A. The development was normal

29. (Type A1 question)

Tetralogy of Fallot does not show any of the following signs

A. pallor

B. squatting

C. syncope

D. Clubbing of the finger (toe)

E. Activity endurance is decreased

Reference answer: A. Your answer:

[Analysis] The clinical manifestations of Fallot tetralogy are common skin mucosa blue, rather than pale (A wrong, is the correct answer to this question).

[Avoid mistakes] Tetralogy of Fallot will squat (B), decreased endurance (E), clubbing (toe) (D), when children with oxygen consumption or oxygen demand increases, there will be paroxysmal dyspnea, severe syncope (C), convulsions, and even death.

Tetralogy of Fallot (Tetralogy of Fallot) is a congenital heart disease with four features: right ventricular outflow stenosis, ventricular septal defect, aortic span, and right ventricular hypertrophy. The condition causes lower oxygen levels in the blood, so patients may develop blue fingers, clubbing (toes), decreased endurance, syncope, and squatting (to increase pulmonary blood flow and improve oxygenation).

-A. Pallor: usually associated with hypoxia, but in tetralogy of Fallot, patients are more likely to have green purple skin and mucosa.

-B. Squat: Patients with tetralogy of Fallot may adopt a squatting position to increase pulmonary blood flow and improve oxygenation.

-C. Syncope: syncope may occur in patients with tetralogy of Fallot due to reduced cerebral blood flow and poor oxygenation.

-D. Clubbing (toe): It is a sign of prolonged hypoxia that is commonly seen in patients with tetralogy of Fallot.

-E. Decreased mobility endurance: Patients with tetralogy of Fallot usually present with decreased mobility endurance due to structural abnormalities and inadequate oxygenation in the heart.

Thus, of the above options, the least likely to manifest as a tetralogy of Fallot is A. pallor, as the disease usually causes blue skin rather than paleness. So the correct answer is A. pale.

analyze:

Tetralogy of Fallot is a common congenital cardiac malformation whose underlying pathology is the ventricular septal defect, pulmonary artery stenosis, aortic riding span, and right ventricular hypertrophy. Since the aorta extends above the left ventricles, the aorta receives the venous blood from the left ventricle and the venous blood from the left ventricle, and the stenosis of the pulmonary artery reduces the blood flow of the pulmonary circulation for gas exchange, even too much. Most

patients are blue * * * * rather than pale.

Option B squatting is a clinical characteristic posture of Fallot patients, squatting can relieve dyspnea and cyanosis, which is not consistent with the problem.

Option C syncope is due to increased pulmonary circulation resistance, obstruction of right ventricular output, and decrease in systolic pressure. In severe cases, hypoxia attack, loss of consciousness or convulsions may occur, which is consistent with the manifestations of tetralogy of Fallot, so it is not selected.

Option D clubbing finger (toe) is common in chronic hypoxia. Due to chronic hypoxia in patients with tetralogy of Fallot, the body compensates to produce excessive red blood cells, resulting in secondary polycytosis, resulting in hyperplasia, hypertrophy and clubbing enlargement of fingers or digits, which meets the signs of tetralogy of Fallot, so it is not considered.

Option E activity endurance decline is due to long-term hypoxia, a little activity will feel fatigue, breathing effort, do not meet the meaning of the question.

To sum up, the correct answer is: A. pale.

Tetralogy of Fallot is a congenital heart disease, and common signs include squatting, syncope, finger clubbing (toe), and decreased movement endurance. Pallor is not a typical sign of tetralogy of Fallot.

So the correct answer is:

A. pallor

30. (Type A1 questions)

The disease with excessive neonatal bilirubin production is

A. neonatal septicemia

And B. Congenital hypothyroidism

B. congenital biliary atresia

C. asphyxia neonatorum

D. Bile mucosa syndrome

Reference answer: A. Your answer:

[Analysis] Neonatal sepsis can cause hemolysis, so neonatal sepsis can lead to neonatal bilirubin production of excessive disease is neonatal sepsis (A pair).

[Avoid errors] Congenital hypothyroidism (B error) causes low metabolic function due to reduced secretion of thyroid hormone. Clinical signs include mental retardation, growth retardation and low physiological function. Neonatal asphyxia (D error) causes low metabolic function due to hypoxia. When the body's metabolic function is low, bilirubin production will decrease rather than increase. Congenital biliary atresia (C wrong) and bile viscosity syndrome (E wrong) are both jaundice caused by biliary obstruction and do not cause changes in bilirubin content.

The question is a disease that causes excess bilirubin production in newborns. Among them, congenital biliary atresia can lead to blocked bile flow, and then cause bilirubin accumulation and jaundice. Therefore, the correct answer should be: C. congenital biliary atresia

First, let's understand the causes of excessive bilirubin production. Bilirubin production is mainly due to the conversion of hemoglobin released after the destruction of the aging red blood cells in the body. When red blood cell destruction increases, it leads to excessive bilirubin production.

Now, we analyze each option one by one by this principle:

A. Neonatal sepsis: This is A serious bacterial infection. During the course of infection, a large number of red blood cells may be destroyed, resulting in excessive bilirubin production. This is consistent with the above principle.

B. Congenital hypothyroidism: This is a disease caused by insufficient secretion of thyroid hormones. It is not directly related to the bilirubin production.

C. Congenital biliary atresia: This is a congenital disease of the biliary system that mainly affects bile excretion, rather than bilirubin production.

D. Neonatal asphyxia: This mainly affects the oxygen supply and has no direct relationship with the production of bilirubin.

E. Bile mucosa syndrome: this mainly affects the excretion of bile and the formation of bile plugs, and also is not directly related to the production of bilirubin.

In conclusion, the disease with a direct relationship with excessive bilirubin production is neonatal sepsis. So, the most correct answer is:

A.neonatal septicemia.

Diseases of hyperbilirubin in newborns mainly involve excessive destruction of red blood cells or impaired ability of the liver to handle bilirubin. According to the option provided in the question, the correct answer is * * neonatal sepsis * * (A).

Neonatal sepsis is a serious infectious disease that can lead to increased destruction of red blood cells, resulting in excessive bilirubin production. Other options such as congenital hypothyroidism and congenital biliary atresia can also cause neonatal jaundice, but their mechanism is mainly bilirubin metabolism and excretion disorders, rather than excessive bilirubin production.

So, the best answer is this:

A. Neonatal sepsis

31. (Type A1 questions)

The key period that affects the basic formation of people's character is

A. adolescence

B. infancy

C. pre-school age

D. infancy

E. middle age

Reference answer: C Your answer:

[Analysis] The key period affecting the basic formation of people's character is the preschool period (C pair).

[To avoid mistakes] the perinatal period refers to the full 28 weeks of pregnancy to 7 days after birth, due to the experience of environmental change, high risk, this period is a child

The highest period of child mortality; early childhood is the highest incidence of accidental injury. Infant age is from birth to one year of age

The previous period is the most rapid growth and development, and the shortest period; early childhood is accidental injury

The period with the highest birth rate; 13 to 17 are teenagers (also known as teenagers), and 20 to 39 are young people

A critical period of lifetime growth and development, physical health, mental and intellectual development; middle age, generally 40-60 years old

Many of the important tasks of life were accomplished during this period.

The critical period that affects the basic formation of human character is usually considered to be in the early developmental stages, especially in the early years of children. Therefore, the most relevant option may be:

-A. adolescence

-B. infancy

-C. Preschool age

-D. infancy

-E. middle age

Among them, infancy and early childhood are often seen as critical periods for character formation, as this is a period when individuals experience rapid psychological and emotional development. In both stages, children begin to build trust, independence, and a preliminary sense of self. However, if the most critical period is chosen, many psychological theories, such as Freud's psychoanalysis and Eriksson's psychosocial development, emphasize the long-term impact of * * early childhood * * (about 2 to 3 years old) on individuals, especially in terms of character formation.

Therefore, I think the best answer is * * D. Early childhood * *.

analyze:

Character is a person's stable attitude towards the reality, and the personality characteristics shown in the habitual behavior mode corresponding to this attitude. People's character is not invariable, but, once formed, it is relatively stable, and the key period affecting the basic formation of people's character is the preschool period. The excitation and inhibitory functions of the cerebral cortex of preschool children are gradually balanced, the regulatory effect of the cortex on the subcutaneous center is gradually strengthened, the comprehensive ability of analysis is further developed, the consciousness of behavior is gradually formed and developed, and its personality characteristics have been basically formed in the preschool period.

Option A Youth is the maturity and stability of character, rather than the

formative period, so it is not chosen.

Option B The psychology of infancy began to occur and develop, with initial thinking activities, but personality has not yet been formed, so it is excluded.

Option D Early childhood is an important period for children's psychological development, and a key period for the development of language, thinking, personality, social communication and other aspects, but it is not a key period for the basic formation of character, so it is not considered.

Option E The personality of the middle-age individuals is basically stable, and generally they will not change too much, so they do not choose.

In conclusion, the correct answer is: C. Preschool period.

The key period that affects the basic formation of people's character is:

B pre-school age

32. (Type A1 question)

Independent of the expired pregnancy is

A. fat

B. intrauterine growth retardation

C. hydramnion

D. Poor placental function

E. Excessive maturity of the fetus

Reference answer: C Your answer:

[Analysis] Hyhydramnios is related to premature rupture of membranes and premature birth, and has nothing to do with expired pregnancy (C wrong, is the correct answer to this question).

[Avoid error] The reason for expired pregnancy is not clear. There are many possible factors involved, including obesity and late intrauterine development

Slow, poor placental function, fetal overmaturity, estrogen progesterone ratio imbalance, etc.

Expired pregnancy, also known as expired or expired delivery, usually refers to pregnancy beyond the scheduled date of delivery, usually more than 40 weeks.

Factors associated with expired pregnancy usually include fetal intrauterine growth retardation (B), amniotic fluid abnormalities (C), and placental function problems (D), as these factors may lead to pregnancy continuation beyond the normal duration. Obesity (A) is A risk factor for complications in pregnancy, but it itself does not usually directly lead to an expired pregnancy. Excessive fetal maturity (E) refers to the long fetal growth time in utero, which is actually directly related to expired pregnancy. Therefore, the option unrelated to the expired pregnancy is * * A. obesity * *.

analyze:

An expired pregnancy is an abnormal pregnancy, which is more than 42 weeks of gestation but has not yet been delivered. The rate of fetal perinatal disease and mortality in expired pregnancy is increased, which increases with prolonged pregnancy, and the perinatal mortality rate is 3 times more common than normal at 43 weeks of gestation. Five times more normal at 44 weeks. Expired pregnancy may be related to the following factors: female and progesterone ratio imbalance: normal pregnancy at term delivery, estrogen increased, progesterone decreased. If estrogen can not be significantly increased, leading to the proportion of progesterone increased, inhibit the effect of prostaglandin and oxytocin, thus causing expired pregnancy.

Fetal malformation: more common in encephalless children, and fetal adrenal hormone secretion deficiency.

Genetic factors: Some expired pregnancies have a family history, and it is speculated that the disease may be related to genetic factors.

Placental sulfatase deficiency: It is a rare associated recessive disorder that leads to expired pregnancy.

Uterine contraction stimulation reflex is weakened: some expired pregnancy fetus is larger, can lead to the head basin is not called or the fetal position is not straight, the fetal dew can not be in close contact with the lower uterine segment and cervix, thus reducing the reflex uterine contraction, causing expired pregnancy.

Option A Obesity is A risk factor for gestational diabetes, which increases the incidence of macrosomia and easily leads to expired pregnancy, which is partly related.

Ffetal placental function and fetal growth restriction lead to fetal intrauterine growth retardation, so option B intrauterine growth retardation is related to expired

pregnancy.

The fetus of expired pregnancy may continue to grow, so compared with the fetus of excess nutrition will become macrosomia, and the skull hardening, cranial suture is narrow, in the process of delivery, the fetal head is not easy to deformation, adapt to the decline of the birth canal, make the fetal head decline difficult, prone to dystocia. Pararia can directly lead to fetal intrauterine distress, at this time the fetus is often in a state of hypoxia, resulting in fetal anal sphincter relaxation, meconium discharge and pollution of amniotic fluid, fetal inhalation of contaminated amniotic fluid can cause some lung atelectasis, chemical pneumonia and systemic infection, increase the morbidity and mortality of perinatal children. Moreover, the mortality rate of perinatal fetus is 3-6 times higher than that of term infants, and the incidence of poor placental function is higher. Therefore, option D poor placental function is in line with the characteristics of expired pregnancy.

Exdue pregnancy fetus, excessive maturity, also known as overripe children, so option E fetal excessive maturity is the performance of expired pregnancy.

Option C polyhydramnios is caused by fetal malformation, multiple pregnancy, placenta umbilical cord lesions, maternal and child blood type incompatibility, maternal complications and other reasons, and has nothing to do with expired pregnancy * *.

To sum up, the correct answer is: C. Excessive amniotic fluid.

33. unrelated to expired pregnancy

A. fat

B. intrauterine growth retardation

C. hydramnion

D. Poor placental function

E. Excessive maturity of the fetus

* * Answer: C. Too much fluid * *

** explain **:

- ** Exdue pregnancy * * (over 42 weeks gestation) may be related to the following factors:

- ** Obesity * * (A): Obese pregnant women are a higher risk of prolonged pregnancy.

- ** Intrauterine growth retardation * * (B): During expired pregnancy, the fetal nutrition and oxygen supply may be limited, leading to fetal growth retardation.

- ** Poor placental function * * (D): Placental function may gradually decline with the extension of pregnancy, resulting to insufficient oxygen supply to the fetus.

- ** Excessive fetal maturity * * (E): The expired pregnant fetus may show signs of excessive maturity, such as dry skin, long nails, and so on.

- ** Hydramnios * * (C): Hyhydramnios is usually related to gestational diabetes, fetal malformation, twin pregnancy and other factors, and no direct relationship with expired pregnancy.

34. (Type A1 question)

Intotal abortion are characterized by

A. prone to shock

B. colporrhagia

C. abdominal pain

D. Complete discharge of the pregnancy

E. No gestational discharge

Reference answer: A. Your answer:

[Analysis] incomplete abortion: abortion is inevitable to continue to develop, some pregnancies discharge uterine cavity (DE wrong), and some residual in the uterine cavity or incarcerated in the cervix, or fetal discharge placenta retention uterine cavity or incarcerated in the cervix, affecting uterine contraction, leading to bleeding, and even shock (A pair).

[Avoid mistakes] Abdominal pain (C wrong) and vaginal bleeding (B wrong) are

the general characteristics of abortion, without specificity.

The characteristics of incomplete abortion mainly include vaginal bleeding and abdominal pain, but the key feature is the failure of the pregnancy. So, the correct answer is:

B. colporrhagia

This is because in incomplete abortion, the pregnancy part remains in the uterus, leading to vaginal bleeding and possibly accompanying abdominal pain, but the key is that the pregnancy is not completely excreted. Although A (prone to shock) and C (abdominal pain) may also be associated with miscarriage, they are not the main features of incomplete miscarriage. D (complete discharge of pregnancy) describes a complete abortion, while E (no discharge of pregnancy) is inconsistent with the concept of abortion.

analyze:

Incomplete abortion refers to the inevitable abortion continue to develop, part of pregnancy discharge uterine cavity, and some residue in the uterine cavity or embedded in the cervix, or fetal discharge placenta stranded uterine cavity or embedded in the cervix uterine cavity, affect the uterine contraction, lead to a lot of bleeding, and even shock, examination of department of gynaecology see uterus less than menopause, vaginal bleeding.

Option A is prone to shock, which is the possible manifestation of inevitable abortion, leading to massive bleeding. It is not the specific manifestation of incomplete abortion, so it is not selected.

Option C Abdominal pain occurred in a variety of abortions without specificity, so it was excluded.

Option D Complete pregnancy, and option E no pregnancy, are interference options, so it is not selected.

To sum up, the correct answer is: B. vaginal bleeding.

Intotal abortion was characterized by vaginal bleeding and abdominal pain, but the pregnancy was not completely expelled. So, the most correct answer is:

C. colporrhagia

34. (Type A1 question)

The following statement is true of the fertilization process

A. Eggs can be fertilized when discharged

B. Sperm enters the fallopian tube

C. Successful fertilization is the beginning of pregnancy

D. The entire fertilization process takes about 72 hours

E. Fertilization occurs within 12 hours of egg discharge

Reference answer: E Your answer:

Graphic analysis: [analysis] fertilization mostly occurs within a few hours after ovulation, generally not more than 12 hours (E pair).

[Avoid error] The egg (secondary oocyte) is discharged from the ovary, enters the fallopian tube through the umbrella part of the fallopian tube, and meets the capacitated sperm in the fallopian tube (A wrong). Only acrosome reactive sperm can fuse with secondary oocytes (B error). After implantation is the beginning of pregnancy (C wrong). The entire fertilization process takes about 24 hours (D is wrong).

The correct statement about the fertilization process is that E. fertilization occurs within 12 hours of egg discharge. After the egg is discharged, its optimal fertilization capacity remains within about 12 to 24 hours, beyond which the egg is rapidly ishes. Therefore, fertilization usually occurs within 12 hours after the egg is excreted, and is usually performed in the fallopian tubes. So, option E is the most correct answer.

This is a topic about the fertilization process, and we need to judge which option is correct based on the existing medical knowledge.

First, we will analyze each option:

A. Eggs can be fertilized when discharged. This statement is inaccurate. Although egg discharge is a prerequisite for fertilization, it is not performed immediately when the egg is discharged. It needs to associate with the sperm to complete the fertilization process.

B. Sperm enters the fallopian tube. The statement is equally inaccurate. The entry of sperm into the fallopian tube is only the first step in fertilization, and it also needs to find and bind the egg to complete fertilization.

C. Successful fertilization is the beginning of pregnancy. Fertilization success is indeed an important step in pregnancy, but strictly speaking, the beginning of

pregnancy is often defined as implantation of the fertilized egg on the endometrium and not just fertilization success.

D. The entire fertilization process takes about 72 hours. This statement is not accurate. In general, the fertilization process usually occurs within the first 12-24 hours after ovulation, rather than within the first 72 hours.

E. Fertilization occurs within 12 hours of egg discharge. This statement is accurate. Under normal circumstances, fertilization mostly occurs within a few hours after ovulation, generally not more than 24 hours. When the sperm meets the egg, the acrosome reaction occurs first, so that the head and tail of the sperm are separated, and the zona pellucida reaction and the sealing of the yolk membrane occur. This series of processes takes a certain time, but it is usually completed in a short time after the egg is discharged (e. g., within 12 hours).

In conclusion, the correct answer is that E. fertilization occurs within 12 hours of egg discharge.

Regarding the fertilization process, the following statement is correct:

D. Fertilization occurs within 12 hours of egg discharge

explain:

-A. Eggs can be fertilized after the discharge: not completely correct, the egg needs to meet in the fallopian tube and sperm to be fertilized.

-B. Sperm entering the fallopian tube: not completely correct, sperm needs to penetrate the outer layer of the egg and combine with the egg to complete the fertilization process.

-C. Successful fertilization is the beginning of pregnancy: not completely correct, fertilization is a necessary step in pregnancy, but the beginning of pregnancy usually refers to the successful implantation of the embryo into the endometrium.

-D. The entire fertilization process takes about 72 hours: incorrect, and the fertilization process is usually completed in a few hours.

-E. Fertilization occurs within 12 hours of the discharge of the egg: correctly, the

fertilization time window after the discharge of the egg is usually within 12-24 hours, after which time the egg is no longer able to fertilize.

35. (Type A1 question)

The following information about the pathological changes of epidemic Japanese encephalitis is wrong

A. Necrosis of the nerve cells

B. Blood vessel cuff formation

C. Softening the stove

D. gliocyte proliferation

E. With a purulent discharge

Reference answer: E Your answer:

[Analysis] Epidemic Japanese encephalitis is an acute infectious disease caused by Japanese encephalitis virus infection. Its basic lesions include: perivascular lymphocyte infiltration and formation of vascular sleeve (B pair), degeneration and necrosis of nerve cells (A pair), formation of softening focus (C pair) and glial hyperplasia (D pair). Epidemic Japanese encephalitis is caused by viral infection, no purulent exudate (E wrong, is the correct answer to this question).

[Avoid avoidance] Pative exudate in the subarachnoid space is mainly found in epidemic cerebrospinal meningitis.

Epidemic Japanese encephalitis (also known as Japanese encephalitis) is mainly manifested as the inflammatory reaction of the central nervous system. Its pathological changes usually include nerve cell necrosis, gliosis, perivascular lymphocyte infiltration, possible vascular cuff formation, and a softening focus of brain tissue. Based on this information:

-A. Nerve cell necrosis: This is A characteristic pathological change of epidemic Japanese encephalitis.

-B. Formation of vessel sleeves: Common infiltration of lymphocytes and plasma cells around small blood vessels around the diseased area forms the so-called vessel sleeves.

-C. Focus of softening: In severe cases, the brain tissue may develop necrosis to form a softening focus.

-D. Gliosis: This is a common pathological change during encephalitis.

F. Pyogenic discharge: Epidemic Japanese encephalitis B does not usually cause a purulent infection of the meninges, so a purulent discharge is unlikely to occur.

Thus, the misdescribed is E. with purulent discharge.

First, we need to understand the basic pathological characteristics of epidemic Japanese encephalitis. Epidemic Japanese encephalitis is caused by Japanese encephalitis virus, characterized by proliferation of the virus in the brain parenchyma and a series of pathological changes.

Next, we analyzed for each option:

A. Nerve cell necrosis: Epidemic Japanese encephalitis virus proliferates in the cells of the brain parenchyma, which can lead to damage or even necrosis of nerve cells. So, the option A description is accurate.

B. Vascular cuff formation: Due to the inflammatory reaction caused by epidemic Japanese encephalitis, microglia, oligodendrocytes and lymphocytes will accumulate around the blood vessels, forming a "vascular cuff". Therefore, option B is also correct.

C. Softening focus: epidemic Japanese encephalitis can lead to necrosis of brain parenchymal cells, and then the formation of screen mesh softening focus. So, the C option description is accurate.

D. Gliosis: Epidemic Japanese encephalitis causes glial proliferation, especially in microglia and oligodendrocytes. Therefore, the D option description is also correct.

E. Pogenic secretions: Japanese encephalitis is caused by viruses, not bacteria. Thus, it does not produce purulent secretions, which is somewhat different from bacterial infection. So, the E option description is inaccurate.

In conclusion, the erroneous narrative of pathological changes in epidemic Japanese encephalitis is option E: purulent discharge.

Epidemic Japanese encephalitis (JE) is an acute infectious disease caused by Japanese encephalitis virus, mainly transmitted by mosquitoes. The pathological changes of the disease are mainly concentrated in the central nervous system, and the common pathological features include nerve cell necrosis, vascular cuff formation, softening focus, and gliosis.

The options are explained as follows:

-A. Nerve cell necrosis: One of the main pathological changes in JE is the damage and death of nerve cells caused by viral infection.

-B. Formation of vascular covers: the phenomenon of "vascular sleeves" formed by inflammatory cells around blood vessels during JE infection.

-C. The softening focus: the common pathological feature of JE is caused by the necrosis and liquefaction of nerve tissue.

-D. Gliosis: Due to nerve cell damage, glia hyperplasia to repair the damage.

-E. Having purulent secretions: purulent secretions are generally associated with bacterial infections, while JE is a viral infection that usually does not produce purulent secretions.

Therefore, the narrative of the pathological changes of epidemic Japanese encephalitis is wrong:

F. With a purulent discharge

36. (Type A1 question)

The purpose of transient ischemic attack using aspirin therapy is

A. Loss of improved neurological function

B. Protect the cranial nerves

C. increased reperfusion

D. Prevention of recurrence

E. Expansion of blood vessels

Refer to the answer: D. Your answer:

[Analysis] Transient ischemic attack (TIA) is the loss of transient nerve function caused by local cerebral or retinal ischemia due to hemodynamic changes, microembolism and other reasons. Aspirin antiplatelet therapy can play a role in non-cardiac embolic TIA, prevent antiplatelet agglutination and cause hemodynamic disorders, and thus prevent the recurrence of TIA (D pair).

[Error avoidance] Warfarin should be used for multiple recurrence of transient ischemic attack.

The main purpose of the treatment of transient ischemic attack (TIA) is to

prevent future stroke development. Therefore, the application of aspirin for transient ischemic attacks is primarily intended to reduce the risk of recurrence. Aspirin has an antiplatelet aggregation effect and can help prevent thrombosis, thereby reducing the risk of recurrence. Therefore, the most correct answer is: D. Prevent recurrence

This is a choice question for the treatment of transient ischemic attack (TIA). First, we need to clarify the role of aspirin in TIA treatment, and then analyze the association degree of each option with the therapeutic target of aspirin.

Aspirin is an antiplatelet aggregation drug, whose main role in TIA therapy is to prevent thrombosis, thus reducing the risk of stroke (stroke).

Now let's analyze the options one by one:

A. Loss of improved neurological function: Aspirin does not directly improve the absent neurological function already present, but rather avoids deterioration by preventing further thrombosis. So the option A is incorrect.

B. Protection of cranial nerves: Although aspirin indirectly protects cranial nerves by preventing stroke, this is not its direct therapeutic purpose. So the option B is also inaccurate.

C. Increased reperfusion: Aspirin is mainly antiplatelet aggregation and does not directly increase reperfusion of brain tissue. So option C is not consistent with the main role of aspirin.

D. Prevention of relapse: Aspirin effectively prevents the recurrence of TIA or stroke by inhibiting platelet aggregation and reducing the risk of thrombosis. This is highly relevant with the therapeutic purpose of aspirin, so the D option is correct.

E. Dilatation: Aspirin does not have direct dilatation, its main function is anti-platelet aggregation. So the E option is incorrect.

In conclusion, the main purpose of aspirin in the treatment of TIA is to prevent relapse, so the correct answer is option D: to prevent relapse.

The goal of transient ischemic attack (TIA) is to prevent recurrence, as TIA is a high risk warning of ischemic stroke. Therefore, the main purpose of the aspirin application is to reduce thrombosis, and then prevent future stroke or recurrence of TIA.

Based on existing medical knowledge and best practice guidelines, the correct

answer is:

F. Prevention of relapse

37. (Type A1 question)

Does not match the appearance of facial neuritis is

A. Loss of the affected lateral frontal lines

B. Shallow nasolabial groove on the affected side

C. Do not close your eyes on the affected side

D. No bulging of gills on the affected side

E. It is often accompanied by hemiplegia

Reference answer: E Your answer:

[Analysis] facial neuritis is mostly manifested by lateral facial muscle paralysis, forehead wrinkles disappear, eye fissure expansion, nasolabial groove flat, and drooping mouth. The sick side can not make wrinkle forehead, frown, close eyes, puff and pout and other movements. Do not meet the performance of facial neuritis is hemiplegia (E wrong, for the correct answer).

[Wrong avoidance] The facial nerve is a mixed nerve dominated by the motor nerve, which mainly governs the facial expression muscle and the taste of the first 2 / 3 of the conduction tongue

Sense and governs the secretion of sublingual, submandibular and lacrimal glands. Cerebral hemorrhage in the basal nucleus region often leads to hemiplegia.

Facial neuritis, also known as Bell's palsy, presents mainly with weakness or paralysis of the facial muscles. Based on these symptoms, we can analyze each option:

A. Disappearance of lateral frontal lines: This is a typical symptom of facial neuritis, because the facial expression muscles are affected.

B. Shallow nasolabial groove on the affected side: This is also a manifestation of facial neuritis, as facial control decreases, leading to some shallow facial lines.

C. Failure to close the eyes on the affected side: This is an important symptom of facial neuritis, called ragnar sign, indicating that the patient cannot close the eyes on the affected side.

D. No bulge gills on the affected side: This is also one of the symptoms of facial neuritis, because the affected facial muscles do not work properly, making the patient unable to bulge gills.

E. Common with hemiplegia: This option does not match the typical appearance of facial neuritis. Facial neuritis mainly affects the facial muscles, and hemiplegia refers to the muscle weakness or paralysis of one half of the body, usually caused by brain diseases.

Therefore, the most incompatible manifestation of facial neuritis is * * E. often accompanied by hemiplegia * *.

First, we examined each option one by one and analyzed it with the typical manifestations of facial neuritis (also known as facial nerve palsy or facial paralysis).

A. Disappearance of the affected lateral frontal pattern: This is A typical manifestation of facial neuritis. When the facial nerve is damaged, the facial nerve can lead to the disappearance of the lateral frontal lines.

B. Shallow nasolabial groove on the affected side: this is also a manifestation of facial neuritis. With damaged facial nerve, the nerve signals innervating the periororal muscles are weakened, making the nasolabial groove on the affected side less obvious.

C. Failure to close the eyes on the affected side: Patients with facial neuritis are often unable to completely close their eyes due to the weakness of the orbicularis oculi muscle, which is also a typical symptom of this disease.

D. No gills on the affected side: Patients can feel difficult to perform facial nerve weakness, which is another common symptom of facial neuritis.

E. Frequent hemiplegia: Hemiplegia is usually associated with cerebrovascular events (e. g., stroke), rather than a typical manifestation of facial neuritis. Facial neuritis mainly affects facial nerves and muscles and usually does not lead to paralysis in other parts of the body.

In conclusion, what does not show in facial neuritis is option E, which is often associated with hemiparesis. This symptom is inconsistent with the typical manifestation of facial neuritis and is more associated with cerebrovascular diseases such as stroke. Therefore, the correct answer is the E.

38. It does not meet the appearance of facial neuritis

A. Loss of the affected lateral frontal lines

B. Shallow nasolabial groove on the affected side

C. Do not close your eyes on the affected side

D. No bulging of gills on the affected side

E. It is often accompanied by hemiplegia

Answer: E., often accompanied by hemiplegia

Analysis: facial neuritis (facial paralysis) mainly manifested as the paralysis of the affected side muscles, such as the disappearance of the affected forehead lines, nasolabial groove shallow, can not close the eyes and can not bulge the gills. However, facial neuritis is generally not usually associated with hemiparesis, because hemiplegia is usually caused by brain lesions, and facial neuritis is caused by lesions of the facial nerve.

39. (Type A1 question)

The following effects of gestational diabetes on mothers and children are most likely

A. The fetal hypoglycemia

B. hypamnion

C. neonatal hyperglycemia

D. giant baby

E. prolonged pregnancy

Refer to the answer: D. Your answer:

[Analysis] Gestational diabetes may cause ketoacidosis, kidney disease, produce macrosomia (D pair), and cause premature birth.

[Avoid mistakes] When pregnant women suffer from gestational diabetes, the fetus will be in a state of hyperglycemia for a long time, making the islet β cells increase

Raw, produce more insulin, synthesize fat, and then cause macrosomia.

The effects of gestational diabetes mellitus (gestational diabetes mellitus, GDM) on mothers and children can be considered in many ways, but we need to find out the

most likely effects. In choosing the most correct answer, we should focus on the most typical and common complications of gestational diabetes mellitus.

A. Fetal hypoglycemia-This usually occurs after birth, if the mother has diabetes, because the fetus may produce more insulin to fight hyperglycemia, which may cause hypoglycemia after birth.

B. oligohydramnios-This is not a typical complication of gestational diabetes mellitus.

C. Neonatal hyperglycemia-Usually, gestational diabetes leads to neonatal hypoglycemia rather than hyperglycemia.

D. Macrosomia-This is one of the most typical effects of gestational diabetes, as a hyperglycemic environment accelerates fetal growth, resulting in abnormal weight gain at birth.

E. Overdue pregnancy-This is not the most typical complication of gestational diabetes mellitus.

Based on the above analysis, the most likely effect is macrosomia (option D). Therefore, the best answer to this question is * * D. Giant son * *.

This is a problem involving the effects of gestational diabetes mellitus. First, let's take a look at the basics of gestational diabetes. Gestational diabetes mellitus (GDM) is the diabetes mellitus that occurs during pregnancy, which may affect the health of both the mother and the fetus.

Next, we performed a detailed analysis for each option:

A. Fetal hypoglycemia: Although pregnant women with gestational diabetes may have a fetus with hypoglycemia, this is not the most direct or common effect of gestational diabetes. Therefore, this option may not be the best answer.

B. oligohydramnios: Gestational diabetes is usually more associated with polyhydramnios than oligohydramnios. So, this option is unlikely to be the correct answer.

C. Neonatal hyperglycemia: Although gestational diabetes may affect blood glucose levels in newborns, hyperglycemia is not its most direct effect. This option is also unlikely to be the correct answer.

D. Macrosomia: Pregnant women with gestational diabetes, due to poor blood glucose control, the fetus is in a state of high blood glucose, which is easy to lead to

excessive development of the fetal body and form macrosomia. This is one of the common complications of gestational diabetes mellitus.

E. expired pregnancy: Gestational diabetes is not strongly associated with expired pregnancy, so this option is unlikely to be the correct answer.

Based on the above analysis, the most direct and common effect of gestational diabetes is macrosomia. Therefore, the correct answer is D. macrosomia.

The most likely effects of gestational diabetes on mothers and children are:

F. giant baby

One of the most common complications of gestational diabetes is macrosomia (giant fetus), which is mainly due to increased fetal insulin secretion due to maternal hyperglycemia, which promotes fetal growth.

40. (Type A1 question)

After tubal dredging, fever, cervical pain, accessory tenderness, the right side, elevated white blood cells, possible concurrent disease is

A. pelvic inflammation

B. Uterine peeling

C. rupture of corpus luteum

D. hysterorrhexis

E. oophoritic cyst

Reference answer: A. Your answer:

[Analysis] After tubal dredging, fever, cervical pain, accessory tenderness, the right side is severe (typical signs of pelvic inflammation), white blood cells increased, may be complicated disease is pelvic inflammation.

[Avoid error] uterine stripping (B wrong) is endometrial stripping, the functional layer of the endometrium will periodically secrete the female along with the ovary

Hormones and progesterone, and periodic intima stripping occurs. Luteal rupture (C error) often occurs in the second half of menstruation, due to luteal function

Can be incomplete or external force, sudden side of the lower abdominal pain, no

bleeding or only a small amount of bleeding.hysterorrhexis

(D wrong) is divided into incomplete uterine rupture and complete uterine rupture, incomplete uterine rupture is the myometrium part or

The whole layer breaks, but the serous layer is intact, the uterine cavity and the abdominal cavity do not communicate, the fetus and its appendages are still in the uterine cavity; complete uterus

Rupture is the rupture of the whole layer of the uterine muscle wall, uterine cavity and abdominal cavity, maternal sudden feeling of the lower abdomen a tear like severe pain, uterine contraction suddenly

However stopped, no fever, no cervical pain and accessory tenderness. The typical symptom of ovarian cyst torsion (E wrong) is the sudden pain in the lower abdomen of one side, often accompanied by nausea, vomiting and even shock.

According to the title description, the patient developed fever, cervical pain, adnexal tenderness (especially heavy on the right side) and elevated white blood cells after the fallopian tube dredging operation, and these symptoms and signs suggested a possible infection and inflammatory reaction. Of the options given, pelvic inflammatory disease (Pelvic Inflammatory Disease, PID) most closely fits these clinical findings. Pelvic inflammation is common after gynecological surgery, such as fallopian dredging, which can cause cervical pain, tenderness in the accessory area and systemic inflammatory reaction, such as fever and elevated white blood cells.

Therefore, the most likely concurrent disease is: A. pelvic inflammation.

analyze:

After the tubal dredging operation, the patient appeared fever, cervical pain, adnexal tenderness, elevated white blood cells and other manifestations, considered concurrent pelvic inflammation.

Option B uterine stripping refers to the periodic shedding of the endometrium, which is a normal physiological phenomenon, and will not appear cervical pain, accessory tenderness and other symptoms, so it is not selected.

Option C corpus luteum rupture usually occurs after ovulation or at the last 1 week of the menstrual cycle, and can occasionally occur on menstrual days 1 and 2. Some patients had luteal rupture and not much bleeding, but there was a sudden onset of lower abdominal pain, and the peritoneum was stimulated due to the blood flow to the abdominal cavity, causing reactive nausea and vomiting, and shock in severe cases.

The patient had no symptoms such as nausea and vomiting, and was significantly different from the symptom description of luteal rupture, so it was excluded.

Option D Uterine rupture refers to the dehiscence of the uterine body or lower uterine segment during the third trimester or childbirth, which is a serious obstetric complication and threatens the life of the mother and child. He died mainly from bleeding and septic shock. However, the patient had no history of delivery, and there was no history of childbirth, so the possibility of uterine rupture was small, which was inconsistent with the question.

Option E Ovarian cysts are usually asymptomatic, unless the cyst volume is large, but rarely appear fever, cervical pain, etc., so it is not selected.

To sum up, the correct answer is: A. pelvic inflammation.

According to the title description, the patient developed fever, cervical pain, adnexal tenderness (heavy right side) and elevated white blood cells after fallopian dredging. This symptom suggests the possibility of infection or inflammation.

- ** Pelvic inflammation (A) * *: After the fallopian tube dredging surgery, one of the most common complications is pelvic inflammation. This is due to the possible entry of bacteria during the operation into the pelvic cavity. Symptoms include fever, cervical lifting, adnexal tenderness and elevated leukocytes.

-Exfoliation (B): This is not a common postoperative complication and does not meet the symptoms described in the title.

-Lutetis rupture (C): luteal rupture usually causes acute abdominal pain, but not necessarily accompanied by fever and elevated white blood cells.

-Uterine rupture (D): Uterine rupture usually occurs during pregnancy or delivery and is not a common complication of tubal dredging.

-Ovarian cyst (E): Ovarian cysts may cause abdominal pain, but are unlikely to

be accompanied by cervical pain and fever.

Considering the patient symptoms and the prevalence of postoperative complications, the most likely diagnosis is:

* * A. Pelvic inflammation * *

41. (Type A1 question)

The indication for the prevention of cervical tube ligation is

And A., at 24 weeks of gestation

B. 9 weeks gestation

E. and at 32 weeks of gestation

F. gestation at 12 weeks of gestation

G. At 34 weeks of gestation

Refer to the answer: D. Your answer:

Graphic analysis: [Analysis] Preventive cervical tube puncture: suitable for more than two previous pregnancy in the middle and third trimester of inevitable abortion, or before pregnancy through the uterine bar test has been determined to have cervical insufficiency, in 12 to 16 weeks of pregnancy.

[Wrong avoidance] Exclude other options based on the resolution.

Cervical tube cuff is a procedure used to prevent preterm birth and is usually indicated for pregnant women with a history of preterm birth or an abnormal cervix. The best time to perform this procedure is in the second trimester, well before the onset of cervical dilatation or signs of prematurity. Therefore, 24 weeks of gestation (option A) is usually considered an appropriate time for cervical tube ligation because it is effective in preventing preterm birth and early enough to avoid an increased risk of cervical dilation. So, the correct answer should be A. 24 weeks of gestation.

analyze:

Cervical cerclage is a closure of the cervical opening, generally calculate small and medium surgery. The purpose of cervical cerclage is to repair and establish a normal morphology and function of the intracervix, so that pregnancy can be

maintained until term or the fetus can survive. Surgery is more than 14 to 18 weeks of pregnancy, so the indication for preventing cervical tube ligation is after 12 weeks of pregnancy, at this time of surgery, can avoid abortion caused by surgical stimulation, and can prevent premature birth in pregnant women with cervical insufficiency.

In conclusion, the correct answer is: D. 12 weeks of gestation.

Option A 24 weeks gestation, option C 32 weeks gestation, option E 34 weeks gestation, all later than 12 weeks gestation, then cervical ligation, because surgical stimulation may cause premature birth and other adverse consequences, so can be excluded.

Option B 9 weeks of gestation still belongs to the early pregnancy, at this time the embryo implantation is not stable, it is not suitable for cervical ring ligation, to avoid surgical stimulation caused by abortion, so it is not selected.

Prevention of cervical tube ligation (i. e. cervical ligation) is usually used to prevent premature birth or miscarriage due to cervical insufficiency. According to existing medical guidelines and best practices, cervical cerclage is usually performed in the early second trimester of gestation, especially between 12 and 24 weeks, to prevent preterm birth.

The option analysis is performed as follows:

A. 24 weeks of gestation-This is a common time period for performing cervical cerclage.

B. 9 weeks of gestation-This period is too early for cervical cerclage.

C. 32 weeks gestation-This period is too late to undergo preventive cervical cerclage.

D. 12 weeks of gestation-This is an appropriate time period for cervical cervical ligation.

E. 34 weeks of gestation-This period is too late to prevent preventive cervical cerclage.

Among these items, the most appropriate indication for preventing cervical tube

ligation is * * D. 12 weeks of gestation * *.

41. (Type A1 questions)

The correct description of hip tuberculosis is true

A. More common in children

B. More bilateral cases

C. No cold abscess formation

D. The "4" word test is negative

E. Ischemic necrosis of the hip joint

Reference answer: A. Your answer:

Graphic analysis: [analysis] hip tuberculosis children are more common, mostly unilateral. Later there will be cold abscess, "4" test positive.

Hip tuberculosis (also known as bone tuberculosis) is a tuberculosis infection that affects the bone, and the hip joint is one of the more common sites involved. Now we'll analyze each option:

A. * * More common in children * *: Hip tuberculosis can indeed affect children, but it is also seen in adults. Children are a relatively common group because their immune system is not fully developed.

B. * * * *: Tuberculosis of the hip is usually unilateral and occurs less bilaterally.

C. * * Does not form a cold abscess * *: This description is incorrect. Hip tuberculosis can form a cold abscess, which is one of the characteristic features of TB infection.

D. * * "4" test negative * *: "4" test (also known as Patrick test) is used to assess pain in the hip or sacroiliac joint. In hip tuberculosis, this test may be positive because the patient may exhibit pain and limitation in the hip.

E. * * Ischemic necrosis of the hip * *: Tuberculosis of the hip may cause joint destruction and inflammation around the joint, but ischemic necrosis is not a feature of tuberculosis but a result of a blocked blood supply.

Of these options, * * A * * (mostly in children) is the most well described for the hip TB. Although adults can also become disease, children are more susceptible to this infection. So, the most correct answer is * * A * *.

First, we analyzed each option one by one:

A. More common in children: Hip tuberculosis is indeed more common in children. This is because children have a relatively weak immune system, and TB bacilli are mainly transmitted through the respiratory tract, and children are more likely to be infected after exposure to TB patients.

B. Bilateral incidence: in fact, unilateral incidence of hip tuberculosis is more common, rather than bilateral.

C. No cold abscess: the development of lesions in the hip tuberculosis may lead to abscess around the hip, these abscesses are usually not accompanied by significant local fever or acute inflammation such as red, heat, pain, known as "cold abscess". Therefore, this option is incorrect.

D. Negative "4" test: The "4" test is a physical examination method for hip disease. In the case of hip tuberculosis, the "4" word test is often positive rather than negative due to limited joint motion and pain.

E. Ischemic necrosis of the hip: There is no direct relationship between tuberculosis and ischemic necrosis of the hip. Ischemic necrosis of the hip is often associated with factors such as prolonged hormonal use, hip trauma and so on, rather than a direct consequence of hip tuberculosis.

In conclusion, the correct answer is A, that is, hip tuberculosis is more common in children. This is because children have a relatively weak immune system, and TB bacilli are mainly transmitted through the respiratory tract, making children more susceptible to infection after exposure to TB patients. All other options had errors or are not directly related to hip tuberculosis.

The correct description of hip TB is:

A. More common in children

Hip tuberculosis is A common bone and joint tuberculosis, mainly occurring in children and adolescents, so option A is chosen. Other options are not described and are explained as follows:

B. Bilateral incidence: tuberculosis of the hip is usually unilateral, and bilateral

incidence is rare.

C. No cold abscess formation: the tuberculosis of the hip joint often forms a cold abscess, which is a characteristic manifestation of tuberculous inflammation.

D. Negative "4" test: A positive "4" test is one of the manifestations of hip lesions.

E. Ischemic necrosis of the hip: Tuberculosis of the hip does not directly cause ischemic necrosis of the hip. Ischemic necrosis is usually caused by other causes, such as the blood supply disorder of the femoral head.

42. (Type A2 question)

In the past three months, he often thinks about "why people exist" and "what is the origin of history". He knows that thinking is meaningless, but he can not control it. Therefore, he feels distressed, fidgeting and takes the initiative to see a doctor. Patient storage is

A. Forced thinking

B. Anxiety symptoms

C. Fear symptoms

D. Forced symptoms

E. panic attack

Reference answer: A. Your answer:

[Analysis] Forced thinking is the idea, appearance or impulsive thinking in the minds of patients in a rigid form. They are almost always painful, and the content is often violent, obscene or meaningless. Patients often try to resist, but are unsuccessful. Although these thoughts are not voluntary and offensive, patients consider it belongs (A pair).

[Avoid mistakes] The main symptoms of phobia are too nervous, afraid, and even serious physical reactions, such as

Fluitation, palpitations, hand shaking, sweating, serious will appear sweating,

breathing difficulties, suffocation and so on (C wrong). Compulsive symptoms are often actions that you have to take to reduce the anxiety caused by compulsive thinking, knowing that it is unreasonable, but that you have to do it (D wrong). Panic attack is a clearly defined episode of intense fear, anxiety, or discomfort associated with multiple autonomic and cognitive symptoms (E error). Generalized anxiety disorder is a kind of mental disorder with anxiety as the main clinical manifestation. People often have unexplained anxiety, nervousness, and significant symptoms of autonomic dysfunction, muscle tension and motor restlessness (B error).

This question involves identifying the type of psychiatric symptoms the patient presents. According to the description, the patient often thinks about some deep questions, such as "why do people exist" and "what is the origin of history", and although he knows that these thoughts are meaningless, he could not control these thinking, and therefore felt distressed and restless. These symptoms fit the characteristics of obsessive thinking, a recurring, unpleasant thought or impulse, which the individual often feels uncontrollable and thus distressed.

-A. Forced thinking: refers to recurrent, irresistible, often unpleasant thoughts, impulse or imagination, which patients tend to regard as meaningless and try to resist or get rid of them.

-B. Anxiety symptoms: usually refers to persistent, excessive, unreasonable worry and tension, rather than anxiety for a specific mind.

-C. Fear symptoms: intense fear and avoidance of a specific object or situation.

-D. Compulsive symptoms: usually refers to compulsive behavior (e. g., repeated cleaning, checking), or compulsive thinking, which is described more on the thinking level than behavior.

-E. Panic attack: a sudden, intense sense of fear or discomfort, usually accompanied by somatic symptoms, such as a rapid heartbeat and shortness of breath.

By these definitions, the most appropriate to the patient description is A. forced thinking. Therefore, the correct answer is an A.

First, let's read the question stem carefully. In the past 3 months, patients often have some meaningless philosophical thinking, such as "why do people exist" and "what is the historical origin". The key message is that the patients themselves know that these thoughts are meaningless, but they cannot control themselves, and therefore feel distressed and restless.

Now, we analyze it for each option:

A. Forced thinking: Forced thinking refers to repeated thoughts that are unnecessary or unreasonable, but cannot be controlled. This is highly consistent with the patient's symptoms, that is, knowing that thinking is meaningless but can not stop.

B. Anxiety symptoms: Although patients show distress and restlessness, these symptoms are more likely to be caused by obsessive thinking, rather than simply anxiety symptoms. Anxiety symptoms are often manifested as generalized worry, nervousness, and restlessness, not specifically some kind of meaningless reflection.

C. Fear symptoms: Fear symptoms are usually related to specific objects or situations, such as acrophobia, claustrophobia, etc. There was no mention of the patient's fear of any particular thing or situation.

D. Obsessive symptoms: Compulsive symptoms are a broader concept that includes compulsive thinking and compulsive behavior. Although the patient's symptoms are one of compulsive symptoms, compulsive thinking is more specific and accurate.

E. Panic attacks: Panic attacks usually appear as a sudden, strong sense of fear, along with physical symptoms such as a rapid heart rate, sweating, shortness of breath, etc. The symptoms were not mentioned in the title stem.

Taken together, the patient's symptoms most fit the definition of compulsive thinking. Therefore, option A "forced thinking" was chosen as the answer that best fits the question stem description.

To sum up, the correct answer is: A. Forced thinking.

According to the title stem description, the patient often thinks about "why should people exist" and "what is the historical origin of it?". Although he knew that these thoughts were meaningless, he could not control these thoughts, and therefore felt upset and restless. This situation is most likely a manifestation of forced thinking.

* * Forced thinking * * (obsessive thoughts) refers to the repeated occurrence of some involuntary, illogical thoughts or impulses, although the patient knows that these thoughts or impulses are meaningless, but difficult to control, and therefore feel pain and distress.

So, the most correct answer is:

A. Forced thinking

43. (Type A2 question)

Male, 26 years old, car accident injury left head on the ground, consciousness disorder, physical examination: deep breathing, right pupil 2.0mm, left pupil 4.0mm, optic disc edema, proposed emergency surgery, the possible diagnosis is

A. left temporal lobe

B. A notch hernia of the right cerebellum

C. cerebellar tonsillar hernia

D. The left side of the cerebellar tonsillar hernia

E. Brain sickle hernia

Reference answer: B. Your answer:

Graphic analysis: [analytical] male, car accident injury left head landing, consciousness disorders, breathing, the right pupil of 2.0mm, the left pupil of 4.0mm (prompt left pupil narrowing), optic disc edema (suggesting increased intracranial pressure), emergency surgery, according to the patient's symptoms and signs, considering the diagnosis for the right small brain screen notch hernia (B).

[Error avoidance] cerebellar curtain notch hernia ① Symptoms of increased intracranial pressure: severe headache, frequent vomiting unrelated to eating

spit. The degree of headache was progressive aggravation with irritability. Optic disc edema is absent in patients with acute cerebral hernia.② Pupil change: disease

At the beginning, the pupil on the diseased side becomes smaller, and the light reflex is dull, along with the progression of the disease

Paralysis, the sick side pupil gradually dilated, and both direct and indirect light reflection disappeared.③ Movement disorders: manifested as the opposite limb of the lesion

The muscle strength of the body is weakened or paralytic, and the pathological signs are positive. In severe cases, this is a letter of severe damage to the brain stem

Number.④ consciousness change.⑤ Vital signs disorder: in the brainstem life central function disorder or failure, can appear different vital signs

ordinary. The performance is a slow or irregular heart rate, blood pressure

suddenly high and low, irregular breathing, sweating or sweat closed, complexion flushing

Or pale.

This case describes a 26-year-old man with a head landing after a crash injury, deep breathing, right pupil of 2.0mm, left pupil of 4.0mm, and optic disc edema. These symptoms suggest that the patient may have experienced severe intracranial injury, resulting in increased intracranial pressure.

Considering these clinical manifestations, especially the pupil-size asymmetry and optic disc edema, it can be speculated that the patient may have an intracranial hernia. Intracranial hernia refers to the displacement of brain tissue due to increased intracranial pressure and the compression of important structures.

-A. hernia of the left temporal lobe, usually causing dilated pupils (usually ipsilateral) and possible compression of the brainstem.

-B. Hernia of the right cerebellar tract, in which pupil changes may not be significant but are usually accompanied by decreased consciousness and brainstem dysfunction.

-C. The foramen occipital hernia, also known as posterior fossa hernia, usually rapidly leads to instability of vital signs.

-D. Tonsillar hernia of the left cerebellum, which can cause compression of the brain stem and may progress rapidly to a foramen magnum hernia.

-E. Sickle cerebral herniation, in which pupil asymmetry may also occur, but more due to interhemispheric compression.

Based on the described symptoms, especially left dilated pupils and impaired consciousness, the most likely diagnosis was * * left temporal hook lobe hernia * * (A). This hernia is usually caused by ipsilateral brain injury, consistent with the way the patient's injury (landing on the left head) and clinical presentation.

analyze:

The young male patient developed consciousness disorder after landing on the left head of the accident injury. Physical examination showed normal pupil on the right side, dilated pupil, left oculomotor nerve palsy, and bilateral optic disc edema. Considering the intracranial hypertension, combined with the patient's history and clinical manifestations, the patient was most likely diagnosed as left temporal lobe hook hernia. Temporal lobe hook hernia, also known as cerebellar tentorial hernia, is

due to the increased intracranial pressure that pushes the temporal hippocampus gyrus and the hook gyrus through the cerebellar tentorial notch. Due to occipital cerebellorial block between the cerebellum notch and the inner side of the brainstem, The shifted brain tissue in the cerebellar screen notch squeezing the brain stem, Involving the network-like ascending agonist system in the brain stem, The patient then falls into a drowsy state; The extrusion of the affected eye nerve produces the gradual dispersion of the affected eye pupil, Direct and indirect responses to the light reflection disappear, And have the affected side of ptosis, external eye deviation; The foot of the affected brain caused the lesion contralateral hemiplegia, hypermyotonia, tendon hyperreflexia, Positive pathological signs; Sometimes the anterior temporal lobe or the underfrontal lobe is squeezed into the cerebellar hiatus, Can cause respiratory disorders due to brain stem compression, Sudden onset of respiratory arrest, Cardiac arrest or death.

Option B The right cerebellar tentorial herniation often showed right oculomotor palsy, while the patient in the topic trunk showed left oculomotor palsy, so it was excluded.

Option C foramen occipital hernia and option D left cerebellar tonsillar hernia, are a kind of cerebral hernia, but they do not show symptoms of eye nerve paralysis, which can be excluded together.

Option E sickle cerebral hernia, also known as cingulate hernia, is rarely seen and mainly manifested as symptoms of increased intracranial pressure such as severe headache, vomiting and irritability. Generally, there is no eye movement paralysis, which does not meet the meaning of the question.

In conclusion, the correct answer is: A. left temporal lobe hook hernia.

According to the title description, the patient developed consciousness disturbance after head injury, deep breathing, 2.0mm on the right and 4.0mm on the left, and optic disc edema. These signs suggested intracranial hypertension and brain herniation. The specific analysis is provided as follows:

1. * * Pupil change * *: One side of dilated pupil (4.0mm left), suggesting ipsilateral temporal lobe compression or temporal lobe hook hernia.

2. * * Loss of consciousness * *: Severe head injury may lead to intracranial hemorrhage and increased intracranial pressure, which can lead to impairment of

consciousness.

3. * * Deep breathing * *: It may be central breathing changes caused by brain stem compression.

4. * * Optic disc edema * *: indicates increased intracranial pressure.

Combining these signs, the most likely diagnosis was * * left temporal lobe hernia * * because it causes ipsilateral oculotor compression and signs of ipsilateral pupil enlargement and contralateral pupil constriction. The hernia of the left oculomotor nerve and dilated the left pupil.

So the best answer is:

A. left temporal lobe

44. (Type A2 questions)

Female, 26 years old. Accident hit the head and suffered head pain for 15 hours. After waking up, dizziness, vomiting, confusion, eyelid blue, eye subconjunctival hemorrhage, loss of anosmia, nasal cavity visible reddish liquid outflow. The most likely diagnosis is the

A. A recessed fracture of the parietal bone

B. skull base anterior fossa fracture

C. Fracture of the skull base middle fossa

D. Skial base fracture of the posterior fossa

E. fracture of nasal bone

Reference answer: B. Your answer:

[Analysis] Young female patients, car accident hit the head, conscious, eyelid blue, subconjunctival bleeding, loss of smell, visible reddish liquid outflow of the nasal cavity, considering the diagnosis of the anterior fossa fracture (B pair). Anterior fossa fracture, fracture bleeding can flow through the anterior nostrils, or into the orbital eyelid and the conjunctiva to form congestion, commonly known as "panda eye sign" or "glasses sign". Cranial fossa fracture, blood and cerebrospinal fluid can

flow through the external auditory canal, forming auricular. The posterior cranial fossa was fractured with subcutaneous congestion in the mastoid process and lower occipital region (Battle sign).

[Avoid error] When the skull fossa fracture (C wrong), it can enter the ear due to bleeding into the ear, and the cerebrospinal fluid ear can occur

leak. Posterior fossa fracture (D wrong) can involve the posterior wall of the pharynx, retropharyngeal wall hematoma and soft palate paralysis, leakage of pharynx choking

cough. Nasal bone fracture (E error) can cause nosebleed, nasal congestion, headache and deviated bridge of the nose.

The symptoms described in this case, including dizziness, vomiting, subconjunctival hemorrhage, anosmia, and nasal outflow of reddish fluid (possibly cerebrospinal fluid leak), suggest the possibility of skull base fracture. In particular, anosmia and nasal fluid outflow are often associated with fractures of the skull base anterior fossa, which can affect the olfactory nerve and may cause the outflow of cerebrospinal fluid from the nostrils.

Therefore, the most likely diagnosis is: B. Base fracture of the anterior fossa

This is a diagnostic problem involving the type of skull fracture. First, we need to analyze the patient's history, symptoms and signs to determine the most likely diagnosis.

Medical history analysis:

The patient was a young woman who hit the head with headache, dizziness and vomiting.

Bluish eyelids, subconjunctival hemorrhage, loss of anosmia, and pale reddish fluid outflow from the nasal cavity.

Option interpretation:

A. Epietal bone depression fracture: This type of fracture usually leads to local depression of the head, but generally does not lead to anosmia or nasal bleeding.

B. Anterior skull base and anterior fossa fracture: Common symptoms include eyelid and subconjunctival congestion (panda eye sign), and cerebrospinal fluid rhinorrhea, which meets the patient's symptoms.

C. Fracture of the middle fossa of the skull base: usually manifested as

subcutaneous ecchymosis (Battle sign) in the retromastoid area and cerebrospinal fluid aurleakage, which is not consistent with the description of the main stem.

D. Base base posterior fossa fracture: common symptoms are mastoid and inferior occipitum (swelling and subcutaneous ecchymosis in the suboccipital area), which do not meet the patient's performance.

E. Nasal bone fracture: mainly nasal swelling, pain, nosebleed, deformation or collapse of the bridge of the nose, without loss of anosmia or cerebrospinal fluid rhinorrhea.

aggregate analysis:

The patient had blue eyelid and subconjunctival hemorrhage after head trauma, suggesting that the anterior fossa may be damaged.

Loss of anosmia and reddish outflow of the nasal cavity (possibly cerebrospinal fluid rhinorrhea) are typical symptoms of fractures of the anterior fossa.

In conclusion, based on the patient's history, symptoms and signs, the most likely diagnosis is a fracture of the anterior skull base fossa. Therefore, the answer is the B. skull base anterior fossa fracture.

Here is the likelihood analysis for each option, based on the patient's symptoms and signs:

A. * * sunken fracture of the parietal bone * *: Usually presents as local headache and swelling, but does not cause subconjunctival hemorrhage, loss of anosmia or fluid outflow from the nasal cavity.

B. * * Fracture of the skull base anterior fossa * *: This fracture may cause a loss of anosmia because the olfactory nerve passes through the anterior fossa. The nasal outflow of pale red fluid may be a cerebrospinal fluid rhinorrhea, which is also typical of anterior fossa fractures. In addition, eyelid blue and ocular subconjunctival hemorrhage are also common signs of anterior fossa fractures.

C. * * * *: This fracture usually presents as ear symptoms, such as aurleakage, hearing loss, and tympanic hemorrhage, and does not cause loss of smell or nasal fluid

outflow.

D. * * Fracture of the posterior fossa * *: This fracture usually affects the brain stem and cerebellum, leading to neurological symptoms such as ataxia and nystagmus, without causing loss of smell or nasal fluid outflow.

E. * * Nasal bone fracture * *: Although it can cause nosebleed and eyelid blue, it does not cause loss of anosmia and subconjunctival bleeding, and is unlikely to cause cerebrospinal fluid rhinorrhea.

In conclusion, the presence of anosmia, rasceria, azulence, and subconjunctival hemorrhage in the eye were most consistent with the findings of anterior fossa fracture. Therefore, the most likely diagnosis is:

F. * * Fracture of the anterior fossa * *

45. (Type A2 question)

Female, 26 years old, dysmenorrhea 3 years, menstruation increased, the 10 days, blood is still not stopped, 2 years ago for infertility done fluid treatment, the uterus is slightly larger, posterior, poor mobility, right touch 4cm 4cm 3cm, and uterine adhesion; the left attachment thickening, bilateral uterine sacral ligament touch bean big painful nodules, this disease is first considered as

A. old ectopic pregnancy

B. genital tuberculosis

C. chronic pelvic inflammatory disease

D. endometriosis

E. Tubal ovarian cyst

Refer to the answer: D. Your answer:

[Analysis] childbearing age women, dysmenorrhea, menstrual volume increased, 2 years ago for infertility done fallopian tube fluid treatment, the uterus is normal

slightly larger, posterior, poor mobility, right touch of 4cm 4cm 3cm mass, uterine adhesion; left attachment thickening, bilateral uterine sacral ligament touch bean grain big painful nodules, this disease is first considered as endometriosis (D pair).

[Avoid mistakes] old ectopic pregnancy, the performance of tubal pregnancy abortion or rupture, many patients will appear repeated internal bleeding

Form, it is easy to lead to the pelvic swelling does not dissipate, hematoma machine hardening and adhesion with the surrounding tissue (A wrong).genital organ

Tuberculosis is also known as tuberculosis pelvic inflammation, patients can not have any abnormal symptoms, often in the diagnostic curettage, uterosalpingography or abdomen

Found during endoscopic surgery (B error). Pelvic inflammatory diseases mainly include endometritis, salpingitis, fallopian tube and ovarian pus

Mass, pelvic peritonitis. Inflammation can be limited to one site, but it can also involve several sites at the same time, to salpingitis, fallopian tube

Ovariitis is the most common (C wrong). Tubal ovarian cyst is a kind of adnexal cyst, mostly caused by tubal eggs by inflammatory stimulation

Symptoms of a nest cyst (E-error).

According to the described symptoms, such as annual aggravation of dysmenorrhea, increasing menstrual volume, slightly large and posterior uterus, thickening of the adnexa area, and painful nodules of the bilateral sacral ligament, these characteristics are more consistent with the clinical manifestations of endometriosis (Endometriosis). Endometriosis is a common gynecological disease characterized by the presence of endometrial tissue outside of the uterus, and common symptoms include dysmenorrhea, irregular menstrual periods, painful sexual intercourse, and infertility. Furthermore, masses adhered to the uterus may point to the formation of endometriotic cysts.

Thus, the diagnosis most consistent with this patient's symptoms was that of D. endometriosis.

First, let's read the key information about the topic. The patient is a 26-year-old woman, long-term dysmenorrhea and gradually aggravated, menstruation increased, and this menstruation has lasted for 10 days. Two years ago, she had tubal fluid treatment for infertility. During the examination, the doctor found that her uterus was slightly larger than normal, positioned backward, and less active. A mass adhering to

the uterus can be reached on the right side, while the left adnexa is thickened. In addition, painful nodules can be reached in the bilateral sacral ligament.

Next, we analyze the various options based on this information:

A. Old ectopic pregnancy: There is usually postmenopausal abdominal pain and vaginal bleeding, and A possible abdominal mass. These symptoms were not mentioned in the questions, so the option is unlikely to be the correct answer.

B. Genital tuberculosis: This disease usually presents with infertility, menstrual disorders, lower abdominal falling pain, and systemic symptoms such as fever and night sweats. Although the patient had a history of infertility, other typical symptoms of TB were not mentioned, and therefore this option is also unlikely to be the correct answer.

C. Chronic pelvic inflammation: this disease is mainly manifested by lower abdominal pain, increased vaginal secretions, and possible abnormal vaginal bleeding and other symptoms. But the symptoms described in the questions do not exactly match these, so this option is unlikely to be the correct answer.

D. Endometriosis: This is a common gynecological disease, mainly manifested as secondary dysmenorrhea, progressive aggravation, increased menstrual volume, prolonged menstruation or endless menstrual symptoms. Moreover, endometriosis may also cause infertility and sexual intercourse discomfort. On examination, posterior tilt may be fixed, one or two attachments, and poor activity. These symptoms and signs were highly compatible with the patient condition described in the title.

E. Tubal ovarian cyst: This is an inflammatory fluid, usually developed from a pelvic inflammatory disease. Although the patient had a left adnexal thickening, there was no other evidence that this was a tubal-ovarian cyst.

In conclusion, considering the patient's dysmenorrhea, increased menstrual volume, infertility history, and physical examination findings, the most consistent diagnosis is endometriosis.

Therefore, the correct answer is D. endometriosis.

Based on the patient's history and signs, the most likely diagnosis is endometriosis (D). Here are some reasons to support this diagnosis:

1. * * * *: Endometriosis is one of the common causes of dysmenorrhea, especially when dysmenorrhea gradually increases over time.

2. * * Increased menstrual volume and prolonged menstrual time * *: Endometriosis may lead to irregular menstruation, including increased menstrual volume and prolonged menstrual time.

3. * * Previous history of infertility and tubal fluid treatment * *: Infertility is closely related to endometriosis, and tubal fluid treatment may indicate pelvic structural abnormalities in the patient.

4. * * posterior uterus and poor mobility * *: Endometriosis can lead to the fixation of pelvic organs and posterior uterus.

5. * * Pain nodules * *: Pain nodules in the bilateral sacral ligament is one of the typical signs of endometriosis.

Other options are analyzed as follows:

- ** A. Old ectopic pregnancy * *: Although there can be pelvic mass, there will be no painful nodules.

- ** B. Genital tuberculosis * *: There are usually systemic symptoms, such as low fever, night sweats, and weight loss, which are not mentioned in the patient's medical history.

- ** C. Chronic pelvic inflammation * *: Although it can also appear as pelvic pain and mass, there are usually no significant dysmenorrhea and painful nodules like endometriosis.

- ** E. Tubal ovarian cyst * *: Although the pelvic mass, it does not explain the gradual worsening of dysmenorrhea and painful nodules.

Therefore, the most likely diagnosis is endometriosis (D).

46. (Type A2 question)

Primary pregnant woman, 25 years old, 39 weeks of gestation, abdominal pain for 2 days, physical examination: BP130 / 90mmHg, heart rate 100 times / min, lower abdominal refusal, fetal upper limb visible in the vaginal mouth, fetal heart sound disappeared, catheterization was light red, the preferred treatment measure is

A. Dioxytocin for labor induction

B. C-sect

C. internal version

D. Clip midwifery

E. Still-dropping diazepam

Reference answer: B. Your answer:

Text parse: [analytical] pregnant women, 25,39 weeks of gestation, abdominal pain 2 days, lower abdomen refused to press (aura of uterine rupture maternal agitated, breathing, heart rate, too fast, lower abdominal pain), vaginal mouth visible fetal upper limb (to ignore the shoulder first, can cause obstructed labor, is a high risk factor of aura uterine rupture), fetal heart sound disappear (because of contractions too strong, too frequency, fetal touch is not clear, fetal heart rate accelerated or slow or hear). The catheterization was reddish (bladder pressure and congestion, dysuria and hematuria), and was diagnosed as threatened uterine rupture caused by neglected shoulder exposure. The first treatment is an immediate cesarean section (B pair).

[Avoid avoidance] Because the fetus is alive, it is inappropriate to induce labor (A error). Internal backward hip traction (C error) is suitable for

breech presentation. Tire head suction and forceps midwifery (D error) are suitable for head first exposure, while in this case, shoulder first exposure.diazepam

For preeclampsia during pregnancy (E error).

This question describes a pregnant woman in the third trimester, with abdominal pain, hypertension, rapid heart rate, lower abdominal refusal, and vaginal loss of fetal upper limb and fetal heart sounds. These symptoms imply a possible placental abruption or fetal distress requiring urgent management.

- ** A. Dioxytocin induction * *: Usually used for oxytocin or for reducing bleeding after delivery, but is not preferred in the case of fetal survival, especially in fetal position abnormalities or fetal distress.

- ** B. Cesarean section * *: In the case of fetal distress and abnormal delivery process (such as partial fetal delivery, fetal heart sound disappearance), cesarean section is the best choice for emergency treatment of the fetus and the mother.

- ** C. Transfer * *: This is a surgical procedure to adjust the fetal position to facilitate vaginal delivery, but is not suitable in cases of fetal distress.

- ** D. forceps midwifery * *: forceps can be used in head delivery, but not recommended in partial fetal delivery and in emergency.

- ** E. Static diazepam * *: mainly used for sedation, not for managing this emergency pregnancy.

Therefore, the most appropriate management is * * B. cesarean section * *, as it is an urgent intervention in the event of fetal distress and poor labor progression.

First, we analyze the question stem.

This is a 25-year-old pregnant woman, 39 weeks gestation and already full term. She had abdominal pain for 2 days, which may mean that labor had begun. On physical examination, her blood pressure was 130 / 90mmHg, heart rate 100 beats / min, and refusal in the lower abdomen, which may indicate pain and tension in the abdomen. The key message is that the upper limbs of the fetus can be seen in the vaginal mouth and the fetal heart sound disappears, which is usually not a good sign that the fetus is dead or in a very dangerous state. The catheterization is reddish, which may indicate urinary blood, which further indicates the emergency the mother may face.

Next, we analyzed the options item by item:

A. Oxytocin induction: Although oxytocin can promote labor, in this situation, the fetal heart sound has disappeared, and using oxyoxytocin induction may not be the safest option.

B. Cesarean section: Considering that the upper limbs of the fetus are already visible in the vaginal mouth, but the fetal heart sound disappears, it is likely to mean that the fetus is in a dangerous state. Cesarean section can quickly remove the fetus and avoid further complications, especially if the fetal heart sounds disappear.

C. Internal fetal transfer: This method is usually used in cases of abnormal fetal position, but in this case, the main problem is the disappearance of fetal heart sound, so this is not the preferred treatment measure.

D. forceps midwifery: Although forceps midwifery can help delivery, this method may not be the safest or most effective when the fetal heart sound disappears.

E. Still-drop diazepam: Diazepam is mainly used for anxiolysis and sedation, and is not the preferred treatment for the current emergency.

In conclusion, considering that the fetal upper limbs are already visible in the vaginal mouth but the fetal heart sounds disappear, this may mean that the fetus is in a very dangerous state and that immediate measures are needed. Therefore, the preferred treatment measure is to perform a cesarean section to ensure both maternal

and fetal safety.

Therefore, the correct answer is the B. cesarean section.

This problem describes a primary pregnant woman who developed abdominal pain with severe signs at 39 weeks of gestation with symptoms (fetal upper limb exposure, fetal heart tone loss, light red catheterization), suggesting possible emergencies such as placental abruption, uterine rupture or umbilical cord prolapse. The best treatment should be the immediate termination of the pregnancy to save the mother's life. Therefore, the preferred treatment measure is the cesarean section.

So the correct answer is:

F. C-sect

47. (Type A2 question)

Boy, 5 years old. High fever continued for 1 week. Rash for 2 days, physical examination: T39℃, P128 times / min, R36 times / min. Heat volume, conjunctival congestion in both eyes, dry and cracked lips, strawberry tongue, diffuse maculopapular rash, and lymph nodes on the left side of the neck. Strong heart sound, hand and foot finger (toe) hard oedema. The most likely diagnosis is the

A. Kawasaki disease

B. hand-foot-and-mouth disease

C. sepsis

D. acute articular rheumatism

E. scarlatina

Reference answer: A. Your answer:

Text analysis: [analysis] boy, 5 years old. Continuous high fever for 1 week (fever for more than 5 days). Rash for 2 days, physical examination: T39℃, P128 times / min, R36 times / min. Heat volume, conjunctival congestion in both eyes, dry lips, strawberry tongue, diffuse maculopapular rash (rash) on the skin, and a 2cm diameter lymph node (cervical lymph node enlargement) on the left side of the neck. Strong heart sound, hard edema of hand and foot finger (toe), considering the patient's condition, considering the diagnosis of Kawasaki disease (A pair).

[Avoid mistake] Hand, foot and mouth disease (B mistake) mostly occurs in children under 5 years old, and typical rashes can occur in the oral cavity, hands and feet.sepsis

Disease (C wrong) more chills, more without hand and foot fingers (toe) hard edema. Rheumatic fever (D wrong) has 5 main manifestations: wandering

Sexual polyarthritis, myocarditis, subcutaneous nodules, annular erythema, chorea. Scarlet fever (E error) rash is obvious after the withdrawal

Desquamation, more see red bayberry tongue.

This case describes a 5-year-old boy with persistent high fever, rash, conjunctival congestion in both eyes, dry lips, strawberry tongue, diffuse maculopapular rash, swollen neck lymph nodes, and rigid edema of the hand, foot and fingers. These symptoms are most consistent with the characteristics of Kawasaki disease.

Kawasaki disease is a disease affecting children characterized by fever for more than five days and multiple organ inflammation, including conjunctivitis, cheilitis and oral changes (e. g., strawberry tongue), rash, hand and foot changes (e. g., rigid edema), and enlarged cervical lymph nodes.

Therefore, the most likely diagnosis is:

A. Kawasaki disease

Other options such as HFMD, sepsis, rheumatic fever, and scarlet fever can also cause fever and rash, but their other symptoms do not completely match those described in this case.

First, we will carefully analyze the problem stem and options in order to determine the most likely diagnosis.

The patient was a 5-year-old boy with persistent high fever for a week and a rash for two days. Physical examination showed high fever, heart rate and breathing, showing heat capacity. Key clinical manifestations include conjunctival congestion in both eyes, dry lips, strawberry tongue, diffuse maculopapular rash in the skin, enlarged cervical lymph nodes and rigid edema of the hand and foot fingers (toes).

Now, let's analyze the options, one by one:

A. Kawasaki disease: Kawasaki disease is an acute febrile eruption of pediatric disease, with systemic vasculitis as the main lesion. Its typical clinical manifestations include persistent high fever, conjunctival congestion, strawberry tongue, rash, etc.,

which are highly consistent with the description of the topic trunk. In addition, rigid foot edema and membranous peeling are also characteristic manifestations of Kawasaki disease.

B. HFMD: HFMD is mainly caused by enteric viruses and is common in children under 5 years old. Symptoms include fever, rash or herpes in the hands, feet, mouth, or other areas. However, HFMD is usually not accompanied by conjunctival congestion and strawberry tongue, and rigid edema of hands and feet is not its typical manifestation.

C. Sepsis: Septicemia is a systemic infection caused by pathogenic bacteria that enter the blood circulation, grow and multiply and produce toxins. Although sepsis can cause high fever and rash, it is usually not accompanied by conjunctival congestion, strawberry tongue, or rigid edema of hands and feet.

D. Rheumatic fever: Rheumatic fever is a non-suppurative disease of systemic connective tissue associated with group A β hemolytic streptococcus infection. Its main symptoms include arthritis, carditis, subcutaneous nodules, etc., with the symptoms described.

E. Scarlet fever: Scarlet fever is an acute respiratory tract infection caused by group A hemolytic streptococcus infection. Its main symptoms include fever, pharyngitis, and a diffuse bright red skin rash all over the body. Although similar to some symptoms in the problem stem, scarlet fever may not usually be accompanied by conjunctival congestion and rigid edema of the hands and feet.

In conclusion, considering the symptoms of high fever, conjunctival congestion, strawberry tongue, rash and rigid edema, the most likely diagnosis was Kawasaki disease.

Therefore, the correct answer is the A. Kawasaki disease.

Based on the medical history and physical examination findings provided in the topic, the symptoms of the 5-year-old boy include:

-Continuous high fever for 1 week

-Rashes for 2 days

-Body temperature 39°C, heart rate 128 beats / min and 36 breaths / min

-Heat disease capacity

-Conjuncval congestion in both eyes

-Dry and cracked lips

-raspberry tongue

-Diffuse maculopapular rash in the skin

-enlarged lymph nodes on the left side of the neck

-Heart sound powerful

-Hard edema of fingers (toe)

These symptoms are typical of Kawasaki disease. Kawasaki disease (Kawasaki disease) is a common acute febrile illness in children, characterized by high fever, rash, conjunctival congestion, oral changes (such as strawberry tongue and cleft lips), enlarged cervical lymph nodes, and changes at the ends of the limbs (such as hard edema).

Therefore, the most likely diagnosis is:

A. Kawasaki disease

48. (Type A2 question)

Boy, 5 years old. Due to poor growth and intellectual development. Physical examination: short stature, small head circumference, wide eye distance, low nose bridge, small external ear, pass through the hand, heart auscultation murmur. The most appropriate test for a definitive diagnosis is the one

A. Chromosome karyotyping analysis

B. head CT

C. Echocardiography

D. Blood, T3 and T4

E. Intelligence determination

Reference answer: A. Your answer:

Graphic analysis: [analytical] this case children with backward growth and

intellectual development, short stature, small head circumference, wide eye distance, low nose, small outer ear (special face), through the hand (skin features), heart auscultation murmur (with deformity), should be diagnosed as down syndrome, for clear diagnosis of the most appropriate examination is chromosome karyotype analysis (A).

[Avoid avoidance] Intelligence determination (E error) is a general examination of many genetic and congenital diseases, with no clear diagnostic significance. Serum T3 and T4 tests (D error) are mostly used to clearly diagnose congenital thyroid diseases. Cranial CT (B error) is used to diagnose intracranial hematoma or tumor, etc. Echocardiography examination (C error) is used for the diagnosis of congenital heart disease in children.

Symptoms described by the title include poor growth and mental development, short stature, small head circumference, wide eye distance, low nose bridge, small external ear, webbed tissue through the hand (the finger), and a murmur on cardiac auscultation, which point to a genetic disease. This combination of symptoms is reminiscent of a certain chromosomal abnormality such as Down syndrome, a common chromosomal disorder that is often associated with the tromic status of chromosome 21.

The most appropriate test is * * A. chromosome karyotyping * *, because this test can confirm the presence of chromosomal abnormalities, which in turn can help identify specific genetic diseases. While other options may be useful for assessing the relevant clinical manifestations (e. g., echocardiography for cardiac problems), chromosome karyotyping is more straightforward and critical for defining the diagnosis of genetic disease. Therefore, the most appropriate choice is the A.

analyze:

A 5-year-old child with backward growth and intellectual development, short physical examination, small head circumference, wide eye distance, low nose bridge, small external ear, complete hand, and murmur on cardiac auscultation was considered as Down syndrome according to his clinical manifestations and signs. Down's syndrome, or trisomy 21 syndrome, also known as congenital fool or Down syndrome, is a disease caused by an extra chromosome 21. The most appropriate test for a definite diagnosis is chromosome karyotyping, which allows for the presence of abnormalities on chromosome 21.

Option B head CT is mainly used for the diagnosis of brain space-occupying

lesions, such as intracranial hematoma, brain trauma, cerebral hemorrhage, subarachnoid hemorrhage, cerebral infarction, brain tumor, encephalitis, parasitic disease, degenerative lesions, etc., which is of little significance to the diagnosis of chromosomal abnormalities, so it is excluded.

Option C Echocardiography can intuitively show the structure and motor state of the heart. It is mainly used for the diagnosis of cardiovascular diseases, and has no diagnostic value for chromosomal abnormalities, so it is not selected.

Option D Blood T3 and T4 are mainly used for the detection of thyroid function, which are not helpful for the diagnosis of chromosomal abnormalities, so they are excluded.

Option E Intelligence measurement is mainly used for the identification of children's intellectual development and as a reference index of brain organic damage and degenerative lesions, but it has no diagnostic significance for Down syndrome, so it is not selected.

In conclusion, the correct answer is: A. chromosome karyotyping.

According to the title description, the 5-year-old boy showed short stature, small head circumference, wide eye distance, low nose bridge, small external ear, complete hands and cardiac auscultation murmur. To clarify the diagnosis, the most appropriate examination method is needed to be selected.

These features suggest that it may be some congenital syndrome, most commonly including Down syndrome, Turner syndrome, etc. Chromosome karyotyping is used to detect chromosomal abnormalities, which are important to diagnose genetic diseases.

Considering the above information, the most appropriate inspection is:

A. Chromosome karyotyping analysis

49. (Type A2 question)

Baby, 18 months old, funny, sallow, paroxysmal irregular fibrillation, frequent cough and diarrhea, blood examination: Hb 61 g / L, MCV102fl, elevated MCH,

normal MCHC, the child is most likely to lack any of the following

A. Vitamin B12

B. vitamin A

C. iron

D. vitamin C

E. vitamin E

Reference answer: A. Your answer:

Graphic analysis: [analysis] baby, 18 months, not funny, sallow complexion, paroxysmal limb irregular fibrillation (typical clinical manifestations of huge, young and poor children). Hematology: Hb 61 g / L, MCV102fl, elevated MCH, normal MCHC (estive of macrocytic anemia). Based on the age, clinical manifestations, signs, and blood routine, the most likely diagnosis was nutritional megaloblastic anemia, caused by vitamin B12 deficiency (an A pair).

[Wrong avoidance] The typical manifestations of vitamin A deficiency are skin hyperkeratosis and dry eye disease (B error). Children with iron deficiency anemia have a pale complexion

Pale, can have anti-armour, pica and other unique manifestations, blood image for small cell hypochromic anemia (C wrong). Vitamin C deficiency

It showed weakness of the limbs, bleeding gums, halitosis, and vitamin C deficiency (D wrong). Vitamin E is generally not prone to deficiency,

More due to liver damage, vitamin E deficiency shows mild hemolytic anemia, and MCV does not increase (E error).

This case describes an 18-month-old infant showing non-smiling, sallow complexion, paroxysmal irregular limb fibrillation, frequent cough and diarrhea. Blood tests showed Hb 61 g / L (anemia), MCV 102 fl (increased red blood cell volume), increased MCH (increased mean red cell hemoglobin), and normal MCHC (normal mean red cell hemoglobin concentration). These symptoms and hematological indicators point to megaloblastic anemia.

Meggaloblastic anemia is usually caused by vitamin B12 or folate deficiency. Vitamin B12 deficiency can not only cause megaloblastic anemia, but may also cause neurological problems, such as irregular fibrillation. And the infant's symptoms match the clinical picture of vitamin B12 deficiency.

Based on this information, the most likely answer is: A. Vitamin B12

analyze:

The 18-month-old baby was not funny, sallow, with paroxysmal irregular limb fibrillation, frequent cough and diarrhea, blood results indicated moderate anemia, and red blood cell volume was large. Combined with the symptoms and laboratory examination, nutritional giant erythroid anemia was considered. Meloblastic anemia is a macrocytic anemia due to vitamin B12 and (or) folate deficiency.

Option B Vitamin A deficiency is mainly manifested as night blindness, conjunctiva dry keratosis, the cornea is dry, softening, opacity, and even perforation blindness, skin dry, desquamation, keratosis hyperplasia, hair simply easy to fall off, nail lines, which is inconsistent with the description of dry.

Option C iron deficiency was mainly seen in iron deficiency anemia, which was small cell hypochromic anemia, and the mean red blood cell volume (MCV), mean red blood cell hemoglobin amount (MCH), and mean red blood cell hemoglobin concentration (MCHC) were all decreased. The patient was macrocytic anemia, so it was not selected.

Option D Vitamin C deficiency can cause scurvy. The early symptoms are irritability, pale skin, indigestion, weakened resistance, burnout, loss of appetite, bleeding gums, epistaxis, corneal bleeding, accompanied by diarrhea and respiratory tract infection, which are quite different from the description of the problem.

Option E Vitamin E deficiency is mainly manifested as rough skin, lack of elasticity, wrinkles, skin loss, yellow hair, easy to fall off, easy desquamation, color aggravation and other premature aging manifestations, which is inconsistent with the symptoms of children.

In summary, the correct answer is: A. Vitamin B12.

According to the title stem description, the 18-month-old baby has the following symptoms and signs:

-Don't laugh

-The complexion is sallow

-Irregular paroxysmal limb fibrillation

-Frequent cough and diarrhea

Blood image test results:

-Hemoglobin (Hb) 61 g / L (below normal range)

-Mean red cell volume (MCV) 102 fl (high)

-Higher mean red cell hemoglobin content (MCH)

-Mean red blood cell hemoglobin concentration (MCHC) was normal

These symptoms and bloody findings suggest that the infant may have megaloblastic anemia (megaloblastic anemia), which is commonly seen in vitamin B12 or folic acid deficiency.

* * Reasons for choosing a vitamin B12 deficiency: * *

1. A sallow complexion may be a sign of anemia.

2. Characteristics of megaloblastic anemia include increased MCV, increased MCH, and normal MCHC.

3. Vitamin B12 deficiency is common in vegetarians or patients with digestive tract malabsorption.

4. Vitamin B12 deficiency may lead to neurological symptoms, such as limb fibrillation.

So the most likely answer is:

* * A. Vitamin B12 * *

50. (Type A2 questions)

Female, 9 year old, found eyelid swelling in the morning and had pharyngitis 2 weeks ago. Physical examination: BP170 / 100mmHg. Urine routine: urine protein (+ +), RBC (+ +), WBC (+ +). Renal needle biopsy showed enlarged glomerular volume, narrow capsular lumen, and hyperplasia of renal cells and mesangial cells.

Immunofluorescence showed complement positivity for IgG and complement C3. Hump-like electron compact deposition between dirty layer epithelial cells and the basement membrane. The most likely type of renal pathology in this patient was

A. crescent-induced glomerular nephropathy

B. Inapcapillary proliferative glomerular nephropathy

C. Membranous proliferative nephropathy

D. Microscopic lesional nephropathy

E. Tubulointerstitial nephropathy

Reference answer: B. Your answer:

[Analysis] A 9-year-old girl suffered from pharyngitis 2 weeks ago (indicating a history of infection). Renal needle biopsy showed enlarged glomerular volume and hyperplasia of glomerular endothelial and mesangial cells. Immunofluorescence showed positive IgG and complement C3, and hump-like electron compacments between the dirty epithelium and the basement membrane (renal puncture suggested typical findings of acute diffuse glomerulonephritis) (the B pair).

[Avoid mistakes] The crescent body is formed by the epithelial cells and monocytes of the glomerular balloon, which is a diffuse crescent glomerular kidney

Characteristic lesions of inflammation (A wrong). Membranous proliferative glomerulonephritis is histologically characterized by the thickening and lineage of the glomerular basement membrane

Membrane cell proliferation and mesangial matrix are increased, and hyperplastic mesangial cell processes are inserted into the capillary loop to form a car rail (C

wrong). The disappearance of foot cells in the dirty layer of glomerular balloon is a characteristic lesion of micropathological glomerular disease (D error).

The renal tubules and renal interstitial inflammatory lesions are characterized by chronic interstitial inflammation, fibrosis, and scarring, mainly involving the renal interstitium,

Less involved glomeruli (E wrong).

The patient in this case, a 9-year-old girl, developed eyelid swelling, hypertension (BP 170 / 100 mmHg), albuminuria, hematuria and leukocyuria, and renal biopsy showed increased glomerular volume, narrowing of the capsular space,

and hyperplasia of renal and mesangial cells. Immunofluorescence was positive for IgG and complement C3, and hump-like electron-dense deposits between the dirty layer epithelial cells and the basement membrane were observed by electron microscopy. These clinical and pathological features point to acute nephritis, particularly the types characterized by IgG and C3 deposits.

Among the options given, crescentic glomerular nephropathy (option A) is a description of acute nephritis characterized by the formation of the crescent body within the glomerulus, hyperplasia of glomerular and mesangial cells, and deposition of immune complexes (e. g., IgG and C3). Given the hump-like electron compacts mentioned in the pathology report, which is typical of acute nephritis (especially IgA nephropathy), given the absence of crescent not mentioned in the pathology results, we may need to consider other types more carefully.

However, without explicit mention of crescentia, we should also consider intracapillary proliferative glomerular nephropathy (option B), this lesion is usually seen in acute nephritis, such as poststreptococcal infection, which may be associated with a history of anterior pharyngitis in the case.

Ultimately, determining the most accurate answer is based on the patient's complete pathology report and clinical presentation, but based on the information provided, the most likely diagnosis is incapillary proliferative glomerular nephropathy (option B), as this type of pathology is consistent with the patient's pathological characteristics, including immune complex deposition and changes in glomerular hyperplasia. Therefore, I tend to choose B as the most likely answer.

This is a question about the pathological type of nephropathy. We need to judge the pathological type of their nephropathy based on their clinical manifestations, laboratory tests and kidney biopsy results.

Understanding background information: A 9-year-old female patient with eyelid swelling in the morning and a history of pharyngitis 2 weeks ago. Physical examination revealed hypertension, and routine urine examination was positive for urine protein and red blood cells and white blood cells. Renal needle biopsy showed enlarged glomerular volume, narrowing of the capsular lumen, and proliferation of glomerular and mesangial cells. Immunofluorescence was positive for IgG and complement C3 and hump-like electron compact deposits between the dirty layer epithelial cells and the basement membrane.

Understand the core of the problem: We need to determine the pathological type

of kidney disease.

Next, we analyze the options one by one:

A. Lunar glomerular nephropathy: crescent glomerulonephritis, also known as acute glomerulonephritis, is a group of clinical syndrome manifested by hematuria, proteinuria and progressive renal insufficiency. It is the most serious type of glomerulonephritis and is inconsistent with the description of the subject stem.

B. Capcapillary proliferative glomerular nephropathy: The pathological feature of this nephropathy is characterized by hyperplasia of glomerular and mesangial cells, which is consistent with the results of kidney puncture biopsy. In addition, the patient had a recent history of prodromal infection (pharyngitis), edema, hypertension, hematuria, and proteinuria, which are typical of intracapillary proliferative glomerular nephropathy.

C. Membranous proliferative nephropathy: The pathological characteristics of this nephropathy is characterized by thickening of the glomerular basement membrane, hyperplasia of mesangial cells and increased mesangial matrix, which is inconsistent with the description of the trunk.

. Micropathological nephropathy: the pathological characteristic of this nephropathy is that the glomerulus is basically normal, and only the foot cells of the dirty layer can be widely fused and disappeared, which is inconsistent with the description of the problem stem.

D. Tubulointerstitial nephropathy: The pathological nature of this nephropathy is characterized by tubular atrophy and renal interstitial fibrosis, which is inconsistent with the description of the title trunk.

In conclusion, based on the patient's clinical presentation, laboratory tests and renal biopsy results, the most likely pathological type of nephropathy is intracapillary proliferative glomerular nephropathy.

Therefore, the answer is the B. Capillary endoproliferative glomerular nephropathy.

The patient was a 9-year-old girl with morning eyelid swelling, hypertension (BP 170 / 100 mmHg), and a routine urine examination showed positive urine protein, red blood cells and leukocytosis. Renal needle biopsy revealed enlarged glomerular volume, narrow cystic lumen, hyperplasia of glomerular and mesangial cells, immunofluorescence positive for IgG and complement C3, and hump-like electron

dense deposits between the dirty layer epithelial cells and the basement membrane. These pathological and clinical features suggest several types of nephropathy.

A. Crescent systemic glomerular nephropathy: the main feature is the formation of crescent body, generally accompanied by acute nephritis syndrome, pathological manifestations of crescent body formation (> 50% glomeruli), and does not have the typical pathological features of the patient.

B. Capcapillary proliferative glomerular nephropathy: glomerular capillary endothelial cell hyperplasia and inflammatory cell infiltration, commonly seen in glomerulonephritis after acute streptococcal infection (nephritis after acute streptococcal infection), and its pathological characteristics are consistent with the description of this patient.

C. Membranous proliferative nephropathy: hyperplasia of mesangial cells and mesangial matrix, accompanied by thickening and division of the glomerular basement membrane, immunofluorescence showed positive deposition of IgG and C3, but the pathological features did not fully match the description of the patient.

D. Micropathological nephropathy: common in children, characterized by large proteinuria, normal glomerular structure under light microscope, podocyte fusion under electron microscope, and the patient had no immune complex deposition, which obviously did not conform to the pathological characteristics of the patient.

E. Tubulointerstitial nephropathy: it mainly shows the injury and inflammation of the renal tubules and interstitium, rather than the glomerular lesions, which does not conform to the clinical and pathological characteristics of the patient.

In conclusion, the nephropathpathology most consistent with the clinical and pathological characteristics of the patient was B. intravascular proliferative

glomerular nephropathy (glomerulonephritis after acute streptococcal infection).

Therefore, the most likely pathological type of nephropathy is:

F. Inapcapillary proliferative glomerular nephropathy

51. (Type A2 questions)

The patient, G1P0,28 years old, amenorrhea after induced abortion in July, obese and hairy, after sequential treatment of estrogen progesterone, was diagnosed as

And A.Turner syndrome

H. amenorrhea and lactation syndrome

I. polycystic ovary syndrome

J. Asherman syndrome

And E.sheehan's syndrome

Refer to the answer: D. Your answer:

[Analysis] After induced abortion, cervical or uterine adhesion is called Asherman syndrome, which is caused by damage to the cervical canal mucosa or endometrial basal layer and muscle layer during curettage. The clinical manifestations are amenorrhea, less menagia and infertility, so D pair.

[Avoid mistakes] Turner syndrome, also known as congenital ovarian hypoplasia syndrome, is due to the lack of a female X staining

Body, resulting in short stature, secondary sexual sign dysplasia or dysplasia, but normal intelligence. Medamenorrhea lactation syndrome table

Now for half a year after stopping breastfeeding, still long-term continuous galactorrhea, accompanied by amenorrhea; or non-pregnant but seen breast secretion milk-like liquid

Body, and have amenorrhea. Polycystic ovary syndrome is the most common endocrine disease in women of childbearing age, which is reproductive disorders

A group of clinical syndromes characterized by urinary abnormalities, metabolic disorders and psychiatric problems, with anovulation, infertility and obesity, hirsutism, etc

Classical clinical findings were predominant. Sheehan Syndrome (Xihan syndrome) refers to the pituitary ischemic necrosis caused by postpartum hemorrhage

The anterior pituitary function syndrome, mainly manifested by weakness, no milk secretion, amenorrhea or menstrual volume

Less, irregular menstruation.

The patient's case suggests amenorrhea (no menstruation), obesity, and hypertism, which are typical of polycystic ovary syndrome (PCOS, Polycystic Ovary Syndrome). Polycystic ovary syndrome is an endocrine disorder common in women of childbearing age, characterized by irregular menstruation, excessive male hormones (manifested as hairy or alopecia) and polycystic ovaries.

-A. Turner syndrome is usually diagnosed either in childhood or adolescence, and the main features include short stature and gonadal hypoplasia.

-B. Menenorrhoea syndrome (Sheehan syndrome) is usually associated with severe bleeding after delivery.

-D. Asherman syndrome is a subendometrial adhesion, often caused by surgery, such as abortion, but not necessarily associated with obesity or hypertrichosis.

-E. Sheehan syndrome is a pituitary infarction, which often occurs in women with severe hemorrhage after delivery.

With this information, the most consistent description is C. polycystic ticovary S. Therefore, the best answer is C. polycystic cystic ovary S.

analyze:

The patient was a young woman who had amenorrhea after induced abortion in July, obese and hairy. After sequential treatment of estrogen progesterone, she was diagnosed as polycystic ovary syndrome. Polycystic ovary syndrome is a common gynecological endocrine disease clinically characterized by clinical or biochemical manifestations of excessive androgen levels, persistent anovulation, and ovarian polycystic ovarian changes, often accompanied by insulin resistance and obesity.

Option A: Turner syndrome, also known as Turner's syndrome, is caused by the complete or partial deletion of an X chromosome in all or part of the somatic cells. Its clinical manifestations are short growth, genital and secondary sexual signs and physical development abnormalities, which do not conform to the meaning of the question.

Option B: amenorrhea lactation syndrome is mostly caused by pituitary tumors, with the clinical features of lactation, amenorrhea and infertility. Lactation symptoms were not mentioned in the title stem, so it was excluded.

Option D: Asherman syndrome is uterine adhesion syndrome, which usually leads to uterine operation and infection, resulting in uterine cavity occlusion, little menorrhagia, amenorrhea, infertility, etc., often accompanied by periodic abdominal pain, lower abdominal pain, etc., which is inconsistent with the description of the problem.

Option E: sheehan syndrome, also known as Sheehan syndrome, Is due to a massive postpartum haemorrhagic shock, Leading to ischemia and necrosis of the pituitary, especially the adenopituitary gonadotropin-secreting cells, A series of symptoms that cause hypopituitarism, Such as amenorrhea, no lactation, decreased libido, hair loss, etc., Secondary sexual sign decline, And genital atrophy, And the adrenal cortex, hypothyroidism, chills, drowsiness, hypotension, Can be accompanied by severe and localized posterior orbital pain, visual field defect and vision loss, obesity, rough skin and hyperpigmentation, neurological degeneration, decreased libido, infertility, edema, etc., Does not conform to the question stem.

In conclusion, the correct answer is: C. polycystic ovary syndrome.

The 28-year-old female patient had amenorrhea in July after induced abortion, obese and hairy, and no menstruation after sequential treatment of estrogen progesterone. The most likely diagnosis was:

B Polycystic ovary syndrome (Polycystic Ovary Syndrome, PCOS)

The reasons are as follows:

-Amenor: one of the common manifestations of polycystic ovary syndrome.

-Obesity: commonly seen in patients with PCOS.

-Polyhairy: polycystic ovary syndrome due to high androgen levels.

Turner Syndrome usually shows signs such as primary amenorrhea and low height in young girls, which is inconsistent with this case. Closed lactation syndrome

and Sheehan syndrome had a history of lactation and postpartum hemorrhage, respectively, which is inconsistent with this case. Asherman Syndrome is caused by intrauterine adhesions, appear after induced abortion, but not with obesity hairy.

In conclusion, the most likely diagnosis for the patient was C. PCOS.

52. (Type A2 questions)

After a woman gives birth to a child. Afraid of their own not live, then nervous, early wake up, weight loss 5kg, the woman may be

A. Anxiety disorder

B. Depressive disorder

C. Schizic disorder

D. personality disturbance

E. Mood-switching disorder

Reference answer: B. Your answer:

[Analysis] after childbirth and early wake up, weight loss, considering depression disorder, so B.

[Error avoidance] Anxiety disorders are characterized by widespread, persistent anxiety or recurrent episodes of panic, and are clinically manifested as sitting

Inurgeting, insomnia, breathing urgency, sweating, skin flushing or pale, palpitations, etc., so A wrong. Schizophrenic disorder to the unreal

Perception, thinking disorders, emotional disorders, will and behavior disorders, cognitive disorders are the main manifestations. Personality disorders are classified as antisocial

Type, paranoid, split, split, split, forced, narcissistic, borderline, expressive, avoidant, dependent

class. Mood conversion disorder, clinically mainly manifested as high or low emotion, accompanied by the corresponding cognitive and behavioral changes and have

Delusions, such as psychotic symptoms.

The situation described in this topic involves postpartum psychological states,

especially stress, early awakening and weight loss. Based on these symptoms, the most consistent diagnosis was a depressive disorder. Postpartum depression is a common mental health problem that can be manifested as stress, anxiety, sleep disturbance (e. g., early waking), and weight loss.

The main characteristics of anxiety disorders in item A are excessive worry and nervousness, and although postpartum women may experience these emotions, these are not the only or most important characteristics according to the symptoms described in the problem stem.

Schizophrenic disorder in item C involves a wide range of disorders of thinking, perception, emotion, and behavior, and here there is no obvious indication that women have this disorder.

Personality disorders in term D are long-term, maladaptive behavioral and thought patterns that are often not commonly triggered by specific life events.

Mood switching disorder in item E, also known as bipolar disorder, involves extreme changes in elevated mood and depression.

Therefore, the most appropriate answer is * * B. depressive disorder * *.

First, we extract the key information from the problem stem: stress, early waking up and weight loss after childbirth. These symptoms may point to some kind of psychological disorder.

Next, we analyze each one according to the given options:

A. Anxiety disorders: Anxiety disorders are usually manifested as excessive worry, tension, and fear. The stress and early waking up of women who worry about raising their children are consistent with the symptoms of anxiety disorder.

B. Depression disorder: Depression disorder is mainly manifested as persistent sadness, loss of interest, fatigue, sleep disorders, etc. Although the women in the list had symptoms of early awakening and weight loss, there were no specific mentions of typical depressive symptoms such as sadness or loss of interest.

C. Schizophrenia disorder: Schizophrenia is mainly manifested as hallucinations, delusions, confusion and other serious mental symptoms. These symptoms were not mentioned in the title stem.

D. Personality disorder: Personality disorder refers to the obvious deviation from normal and deep-rooted behavior, with maladaptation. There was no mention of the symptoms or behavior patterns related to personality disorder.

E. Mood switching disorder: This usually refers to bipolar disorder, in which mood swings are seen between depression and mania. There was no mention of mania or obvious mood swings.

In conclusion, the symptoms described by the title stem are most consistent with anxiety disorders, especially considering that the psychological stress and worries after childbirth may lead to the appearance of anxiety symptoms.

Therefore, the correct answer is A. Anxiety disorder.

According to the description in the title, women experienced symptoms of nervousness, early waking, and weight loss after delivery, which coincided with the manifestation of postpartum depressive disorder. Postpartum depression is a common mood disorder in women after childbirth, manifested by persistent sadness, disappointment, helplessness, fatigue, sleep disturbance, loss of appetite and weight loss.

Therefore, the woman may be:

F. Depressive disorder

53. (Type A2 question)

Boy, aged 1 year and 8 months. Fever was noted for 6 days, and the rash occurred for 2 days. Physical examination: good nutritional development, the skin visible maculopapular lymph node on the left side of the neck. The conjunctiva was congested in both eyes with no purulent discharge. Lip congestion and edema, tongue papilla sudden see hard edema, the following treatment measures are the most appropriate

A. High-dose gamma-globulin

B. dipyridamole

C. glucocorticoid

D. antibiotic

E. symptomatic treatment

Reference answer: A. Your answer:

[Analysis] The child had fever for 6 days, and the fever was higher when the rash was out, including maculopapular rash, conjunctival congestion, lip congestion and edema, prominent tongue papilla, and hard edema on hands and feet. According to its clinical manifestations and signs, he should be diagnosed as Kawasaki disease. Treatment plan for Kawasaki disease: the amount of intravenous immunoglobulin is 1~2 / kg, the recommended dose is 2 / kg, slow venous input in 8 to 12 hours, should be applied in the early stage (within 10 days), which can quickly reduce fever and prevent the occurrence of coronary artery disease. The application shall be combined simultaneously

aspirin.

[Wrong avoidance] Dipyridamole (B error) is an adjuvant therapy for Kawasaki disease. Glucocorticoid hormone (C error) can promote thrombus shape

It increases the risk of coronary artery lesions and coronary aneurysm, and affects the repair of coronary artery lesions, so it should not be used alone.pntibiosis

element (D wrong) is used to treat secondary infections. Symptomatic treatment can improve symptoms, but is not the most appropriate treatment (E error).

The symptoms described in this topic seem to fit the characteristics of Kawasaki disease (Kawasaki disease), a form of vasculitis that primarily affects children. Typical manifestations of Kawasaki disease include fever, rash, enlarged lymph nodes in the neck, conjunctival congestion, lip congestion and edema, and changes in the tongue (strawberry tongue).

The most commonly used treatments for Kawasaki disease are high-dose administration of intravenous gamma globulin (IVIG) and oral aspirin. Therefore, of the options provided, the most appropriate treatment measures should be:

A. High-dose gamma-globulin

This treatment could help reduce the risk of cardiovascular complications and improve the overall outcomes of the child. So the correct answer should be A. High-dose Proglobulin.

analyze:

The child had fever, maculopapular rash, cervical lymph nodes, conjunctival congestion, oral edema, strawberry tongue and hard edema around the mouth. According to the clinical manifestations and signs, the diagnosis of Kawasaki disease was considered. Kawasaki disease is a self-limiting disease, and most patients have a

good prognosis. The main aim of treatment is to control vascular inflammation, reduce coronary damage, and prevent coronary aneurysm formation as well as thrombotic obstruction. The treatment of Kawasaki disease mainly includes aspirin combined with gamma globulin, which reduces the incidence of coronary aneurysm. Therefore, the following treatments are most appropriate for high-dose gamma globulin.

Option B dipyridamole is mainly used for artificial heart valve replacement, ischemic heart disease, stroke and transient ischemic attack. It can also be used for the prevention and treatment of angina pectoris. It has no therapeutic effect on Kawasaki disease, so it is not selected.

Option C glucocorticoids have pharmacological effects such as anti-inflammatory, anti-endotoxin, immune suppression, anti-shock and enhanced stress response, but Kawasaki disease is an immune system disease, and the application of glucocorticoids may aggravate the disease, so it is not considered.

Option D Antibiotics are mainly used for the treatment of infectious diseases caused by bacteria, mycoplasma, chlamydia, rickettsia, spiroplasma, fungi, etc. Kawasaki disease is an immune system disease, rather than an infectious disease, and the use of antibiotics can not achieve the purpose of treatment, so it is excluded.

Option E Symptomatic treatment is a common treatment method in clinical practice, but Kawasaki disease is self-limited, and the child has not had any complications, so symptomatic treatment is not the most appropriate measure, inconsistent with the meaning of the question.

In conclusion, the correct answer is: A. High-dose gamma globulin.

According to the title, the boy had a fever for 6 days and a rash for 2 days, accompanied by lymph node enlargement, conjunctival congestion, lip edema and papilla of the tongue, which suggested suggest Kawasaki disease (Kawasaki disease). Kawasaki disease is a common acute febrile illness in children, which is common in children under 5 years of age, mainly manifested as fever, rash, conjunctiva, congestion, red lips and strawberry tongue, and enlarged lymph nodes in the neck.

As a treatment for Kawasaki disease, the best option is to use high doses of gamma globulin (IVIG) and aspirin to reduce the risk of coronary artery disease.

Therefore, the most appropriate treatment measure is:

A. High-dose gamma-globulin

54. (Type A2 question)

Male, 62 years old, got up in the morning to found that the right upper limb could not lift, could not walk independently for 3 hours, accompanied by vague speech. Normal at 8 PM before the onset, previous hypertension and diabetes. Physical examination: P90 times / minute, R19 times / minute, BP160 / 90mmHg, vague, shallow right nasorabial groove, right tongue, right upper limb muscle strength grade two, right lower limb grade 3, left grade five, positive pathological symptoms, right side acupuncture decreased, blood glucose 11 mmol/L, the rest was normal, SpO2 99%, head CT was normal,

A. TIA

B. cerebral thrombosis

C. cerebral embolism

D. myelitis

E. hematencephalon

Reference answer: B. Your answer:

Graphic analysis: [analysis] the patient's elderly male, Past medical history of hypertension and diabetes mellitus, In the morning, the right upper limb cannot lift or walk independently, With vague speech (symptoms of cerebral thrombosis in the feeding area of the left middle cerebral artery), Physical examination revealed the right nasolabial groove, Right deviation of the tongue (facial nerve palsy symptoms), Second level of muscle strength of the right upper limb, Third muscle strength of the right lower limb, Left Side Level 5, Positive right pathological sign, Right bias needle drop (hemiplegia symptoms), SpO299% (95% to 99% of normal values), Cranial CT was normal, Combined with patient history, clinical findings and imaging, Cerebral thrombosis in the feeding area of the left middle cerebral artery should be diagnosed.

[Wrong avoidance] TIA (A error) is A transient ischemic attack, which is a transient nerve caused by local cerebral or retinal ischemia

Functional defect, good onset in the elderly, sudden onset, the duration of the symptoms is generally only a few minutes to a few hours, after the completely positive

Often, without sequelae symptoms. Cerebral embolism (C error) is an acute occlusion or strictness of the vascular lumen by various emboli entering the intracranial artery

A group of clinical syndromes that cause ischemia necrosis and dysfunction of the brain tissue in the corresponding blood supply area, the main cause

Various heart emboli fall off, patients have a history of heart disease, the disease is often heavier than cerebral thrombosis, the fatality rate is high.spine

Myullitis (D wrong) is spinal cord inflammation caused by infection, poisoning, allergy and other factors, with acute clinical onset and rapid progression

For the main performance. Cerebral hemorrhage (E wrong) is the substantial hemorrhage in the brain caused by cerebrovascular rupture, which occurs in the basal ganglia, the most

It is commonly seen in hypertensive patients with fine arteriosclerosis, and high-density shadow can be seen on cerebral CT.

The 62-year-old man presented with symptoms including inability to lift the right upper limb, difficulty walking, slurred speech, right facial muscle weakness, tongue bias to the right side, reduced muscle strength in the right limb, positive pathological signs on the right side, and decreased bias needling on the right side. These symptoms point to the possibility of a cerebrovascular event. The patient had a history of hypertension and diabetes, which increased the risk of cerebrovascular accidents.

A. TIA (transient ischemic attack) has symptoms similar to stroke, but usually fully completely within 24 hours without sustained brain damage.

B. Cerebral thrombosis is due to thrombus obstruction in the blood vessels, which causes reduced blood supply to the brain and may cause continuous brain tissue damage.

C. Brain embolism is when a blood clot (or other foreign body) moves through the body and blocks the blood vessels that supply the brain.

D. Myelitis involves inflammation of the spinal cord, inconsistent with the symptoms described in this case.

E. ICH involved intracerebral hemorrhage, but the patient had normal skull CT and did not support the diagnosis of hemorrhage.

Given the acute onset of symptoms, reduced lateral distribution of muscle

strength and poor speech, and the patient having risk factors for cerebrovascular disease, the most likely diagnosis is cerebral thrombosis or cerebral embolism. Given the normal head CT, excluding significant cerebral parenchymal injury caused by cerebral hemorrhage and massive infarction, it is usually more difficult to distinguish between cerebral thrombosis and cerebral embolism. However, cerebral embolism usually leads to a more sudden onset of symptoms, and cerebral thrombosis may be accompanied by gradually increasing symptoms.

Considering this information, I prefer to choose * * B. cerebral thrombosis * * as the best answer, because the gradual onset of symptoms and the patient's past medical history are more consistent with this diagnosis. However, actual clinical conditions may require further diagnostic tools, such as magnetic resonance imaging (MRI), to clarify the specific etiology.

First, we analyzed the patients' symptoms and medical history:

Older male, with a history of hypertension and diabetes, both of which are risk factors for cerebrovascular disease.

After getting up in the morning, I found that the right upper limb could not lift and could not walk independently, accompanied by vague speech. These symptoms suggest a possible neurological impairment.

Physical examination showed shallow right nasolabial groove, right deviation of tongue, second muscle strength of right upper limb, third grade of right lower limb and left grade five, with positive right pathology and decreased needle prick perception of right side. These signs were consistent with findings of left hemispheric lesion.

Cranial CT was normal, excluding cerebral hemorrhage and other significant brain lesions.

Now, we'll analyze the options one by one:

A. TIA (transient ischemic attack): Usually the symptoms were of short duration, generally fully recovered within 24 hours without sequelae, inconsistent with the patient's condition.

B. Cerebral thrombosis: Because the patient has hypertension and diabetes, two risk factors for cerebrovascular diseases, and the symptoms are found after getting up in the morning, the thrombosis may be caused by slow blood flow at night and increased blood viscosity. This corresponds with the patient's history and symptoms.

C. Cerebral embolism: It is usually blocked by the shedding of emboli from other parts into the cerebral artery, with sudden onset, but the possible source of emboli is not mentioned in the patient's medical history, and the onset is not sudden.

D. Myelitis: The main manifestations are limb paralysis below the damaged plane, conduction tract sensory disorder and urinary stool disorder, which are not consistent with the symptoms of the patient.

E. Cerebral hemorrhage: Although the patient had a history of hypertension, the head CT was normal, ruling out the possibility of cerebral hemorrhage.

In conclusion, combining the patient history, symptoms and physical findings, the most likely diagnosis is cerebral thrombosis.

Therefore, the correct answer is the formation of B. cerebral thrombosis.

According to the information in the question stem, the 62-year-old male patient got up in the morning and was unable to lift the right upper limb and walk independently for 3 hours, with vague speech. The patient had a history of hypertension and diabetes mellitus, and physical examination showed superficial right nasolabial groove, right tongue, secondary muscle strength in the right upper limb, grade III of the right lower limb, positive pathological signs on the right side, decreased acupuncture on the right side, and blood glucose of 11 mmol/L. Cranial CT showed normal findings.

From the clinical manifestations, the patient experienced symptoms of acute cerebrovascular events, including:

-Right-side limb weakness

-Verbal ambiguity

-The tongue to the right

-Positive for the right-sided pathological sign

Combined with a normal head CT, it ruled out the possibility of cerebral hemorrhage. The patient's symptoms persisted over 24 hours after disease onset, ruling out the possibility of TIA. Considering that the patient had a history of hypertension and diabetes, these were all risk factors for cerebrovascular disease.

In acute cerebrovascular events, both cerebral thrombosis and cerebral embolism are the common causes. Cerebral embolism is usually characterized by sudden onset, while cerebral thrombosis may occur at night or in the morning, due to the easy formation of thrombosis when the blood flow is slow. The patient got up in the morning, suggesting that cerebral thrombosis may occur overnight or in the morning.

Therefore, the most likely diagnosis is:

B cerebral thrombosis

55. (Type A2 question)

Female girl, 6 months old, with a rash for half a day. Physical examination: body temperature 36.9℃, pulse 120 times / min, breathing 36 times / min, good spirit, neck, chest, upper limbs visible red macular papules, pressure fading, pharynx congestion. Heart, lung, abdomen and god often. The diagnosis was considered as

A. Dukes' disease

B. nettle rash

C. crystalli

D. hives

E. scarlatina

Reference answer: A. Your answer:

[Analysis] In this case, the child with fever and rash, good spirit, pharyngeal congestion, should be diagnosed as acute rash (A pair). Children with acute rash is mainly seen in infants, the general situation is good, high fever can have convulsions, occipital lymph nodes behind the ear can be enlarged, often accompanied by mild diarrhea. Characteristics of rash: red small dense maculapule, head, neck and body cadres more common, less limbs, one day, the next day began to subside, no pigmentation, no peeling.

[Avoid errors] Rubella (B error) has mild systemic symptoms, swollen lymph nodes behind the ear and pillow and tenderness, and rash usually after the symptoms

appear

1~2 days appear, and have the order of face, neck and trunk limbs. Water pox (C wrong) is mostly low heat, fever the next day out

Rash, and the rash from the initial red rash and papules to transparent and plump blisters, the rash is concentric. Measles (D wrong)

There are cough, photophobia, snaphobia, conjunctivitis and other prodrome symptoms, and the rash appears 3 to 4 days after fever, rash after the skin

Skin has bran-like desquamation and has pigmentation. Scarlet fever (E wrong) is mostly high fever, sore throat is obvious, red bayberry tongue, pale ring mouth

The circle is obvious, etc.

This case describes a 6-month-old baby girl who developed a rash for half a day after fever withdrawal. According to the symptoms, especially normal body temperature, increased pulse, increased respiratory rate, good mental status, neck, chest, upper limb red maculopapular rash and fading of the pressure, and pharyngeal congestion, several common diseases in children can be considered.

Option A The infantile acute rash (Roseola infantum), also known as sudden rash or sixth disease, usually begins with high fever and has a rash after fever withdrawal, as described in line with this case. Acute rash in young children is characterized by a rash after the sudden resolution of high fever, mainly affecting younger children.

Rubella in option B and measles in option D, although both are common eruptions in children, their rash characteristics and disease course are not completely compatible with those described in this case. The rubella rash is usually smaller than that in young children, while measles is associated with significant systemic symptoms such as high fever, chills, and coplick spots.

The chickenpox in option C usually appears as dense herpes, inconsistent with the maculopapular rash described in this case.

Scarlet fever in option E, characterized by a scarlet rash following pharyngeal infection, usually with high fever and strawberry tongue, but this was not the main presentation in this case.

Therefore, the most consistent description in this case is A. infantile acute rash.

analyze:

A 6-month-old girl with a rash after fever withdrawal, red maculopapular rash in

the neck, chest and upper limbs, fading of pressure, and pharyngeal congestion. According to her clinical manifestations and signs, she was diagnosed as acute rash. Children's acute rash is characterized by sudden high fever, lasting 3~5 days of backward fever, followed by rash, rash is red small and dense macule, head, neck and body cadres more common, less limbs, one day, the next day began to subside.

Option B rubella has mild systemic symptoms, posterior ear and posterior pillow lymph node enlargement and tenderness, the rash usually appeared 1 to 2 days after the onset of symptoms, and the face, neck, trunk and limbs, which was inconsistent with the symptoms of the child, so it was excluded.

Option C Varicella showed rash within 24 hours of onset. The rash first affected the compressed part of the scalp and trunk, and showed concentric distribution, which was different from the symptoms of this child, so it was not selected.

Option D Measles is more rash than 3~4 days after fever, and the eruption period is the peak of fever, which is not consistent with the meaning of the question, so it is not considered.

Option E Scarlet fever rash usually occurs in 1 to 2 days of fever, and high fever when the rash occurs, diffuse skin congestion, rash and peeling, different symptoms from this child, which can be excluded.

To sum up, the correct answer is: A. Baby acute rash.

According to the information provided by the topic, the baby was 6 months old, with a rash for half a day, accompanied by red maculopapular rash in the neck, chest and upper limbs, fading of pressure, pharyngeal congestion, and normal heart, lung, abdominal and neurological examination. Here is an analysis of each option:

A. * * Children acute rash * * (also known as rose rash): the typical manifestation is fever 3-5 days, fever red maculopapular rash after regression, usually seen in infants, rash pressure fade, often accompanied by mild pharyngeal congestion.

B. * * Rubella * *: The rash usually starts on the face and spreads throughout the body, with mild fever and enlarged lymph nodes, but usually does not appear suddenly after the fever has subsided.

C. * * Varicella * *: The rash is a mixture of red rash, papules, herpes and scab, which appears in batches, accompanied by fever, but does not meet the characteristics

of the rash after heat withdrawal.

D. * * Measles * *: The rash gradually spread from the face to the whole body from the beginning, accompanied by fever, cough, runny nose, conjunctivitis and Koplik spots, which did not meet the characteristics of the rash after fever withdrawal.

E. * * Scarlet fever * *: The rash is "chicken skin-like" tactile, usually accompanied by high fever, strawberry tongue, and pharyngitis, and the rash usually appears before the fever fades.

Combined with the above analysis, the symptoms of the baby girl are most consistent with the typical manifestations of * * infant rash * *. After fever withdrawal, red maculopapular rash, faded rash pressure, mild pharyngeal congestion, and other systemic tests were normal.

So, the most correct answer is:

A. Dukes' disease

56. (Type A2 question)

5. years old, suffering from severe aplastic anemia for January, waiting for hematopoietic stem cell transplant, what blood products should be infused when the need to correct the anemia

A. Irradiated white-removing red blood cells

B. to to red blood Cs

C. The suspended red blood cells

D. Concentrate the red blood cells

E. whole blood

Reference answer: A. Your answer:

Graphic analysis: [analysis] severe aplastic anemia, the proposed allogeneic hematopoietic stem cell transplant patients should be infused with irradiated red blood cells. Irradiation of the main function of blood is to prevent the occurrence of graft-versus-host disease, refers to the immune defect or immune suppression patients

cannot clear input blood immunoactive lymphocytes, make it in the body plant, proliferation, the patient's tissues and organs identified as the target for immune attack, destruction of a lethal complication of blood transfusion. Irradiation of blood is currently recognized as the most effective method to prevent the occurrence of transfusion-related GVHD.

[Error avoidance] According to the above analysis, exclude other options.

For an 8-year-old child with severe aplastic anemia, waiting for hematopoietic stem cell transplantation, the best choice is to correct blood products to correct anemia. In this case, irradiated deached RBCs should be selected (option A).this is because:

1. * * Irradiation treatment * *: Irradiated red blood cells can prevent graft-versus-host disease (GVHD), which is particularly important in immunocompromised patients before and after transplantation.

2. * * Diswhitening * *: Eliminating white blood cells helps reduce the risk of transfusion reactions, including allergic reactions to white blood cells and the spread of pathogens.

Therefore, for this child, the most appropriate transfusion option is irradiated white red blood cells. So the correct answer is * * A. irradiated white red blood cells * *.

analyze:

The child has been clearly diagnosed as severe aplastic anemia, and is ready for hematopoietic stem cell transplantation, which requires correction of anemia. For patients preparing for bone marrow transplantation or other organ transplantation, to avoid host anti-versus-graft responses, blood products should be irradiated before blood transfusion to inactivate immunoactive lymphocytes and prevent the occurrence of graft-versus-host disease. At the same time, in order to avoid graft-versus-host response and fever reaction, blood products that remove white blood cells should be used during blood transfusion. Therefore, for this child, irradiated white red blood cells should be infused.

Option B White red blood cells is suitable for those who produce leukocyte antibodies after multiple transfusions, fever, or who are expected to need repeated transfusions, and those who cannot tolerate blood transfusion, but cannot avoid graft-versus-host reaction, so it is not selected.

Option C suspended red blood cells, option D concentrated red blood cells, only

removed most of the plasma, no white blood cells, can not avoid graft-versus-host reaction and fever reaction, so it can be excluded together.

Option E Whole blood contains a large number of white blood cells, which is more likely to cause a transfusion reaction, so it is not considered.

In conclusion, the correct answer is: A. irradiated white-removed red blood cells.

The choice of blood product transfusion for anemia in severe aplastic anemia requires several considerations, such as prevention of graft-versus-host disease (GVHD) and reducing the risk of transfusion-related complications.

* * The correct answer is: A. irradiated white red blood cells * *

explain:

1. * * Irradiated dewhitening red blood cells * *: Irradiation prevents donor lymphocytes from proliferating in the recipient, thus preventing graft-versus-host disease (GVHD). Removal of white blood cells helps to reduce transfusion-related fever reactions and the risk of transmission of infectious diseases. This is particularly important for immunocompromised patients, such as patients with severe aplastic anemia awaiting hematopoietic stem cell transplantation.

Explanation of the other options:

- ** White blood cells * *: Although white blood cells are removed, but not through irradiation, can not effectively prevent GVHD.

- ** Suspension of red blood cells * *: the general red blood cell suspension may contain white blood cells, the risk is high.

- ** Concentrated red blood cells * *: Like suspended red blood cells, concentrated red blood cells may still contain white blood cells.

- ** Whole blood * *: Whole blood contains all blood components, including white blood cells, which is at the highest risk and is not recommended for such patients.

Therefore, the choice of option A, the irradiated red blood cells, is the most

appropriate.

57. (Type A2 questions)

A 28-year-old female still had abdominal pain, fever and body temperature of 38°C at 2 weeks postpartum. Vaginal bleeding 2 days ago, approximately 250ml. Physical examination: the pubis combined with two horizontal fingers, tenderness, cervical two fingers, visible rotten meat blockage. Which treatment is not correct

A. Clip clip

B. Replication support

C. anti-infective therapy

D. Give colistin

E. Negative pressure suction technique

Reference answer: E Your answer:

Graphic resolution: [resolution] the patient postpartum 2 weeks still have abdominal pain, fever, vaginal bleeding, hysteris pubis combined on two horizontal finger (high, suggest poor uterine contraction), tenderness, cervical volume two fingers (open cervix, lead to infection), visible rotten meat blockage (placenta, membrane residue), so the diagnosis of late postpartum hemorrhage, considering the uterine contraction, placenta, soft birth canal injury caused by bleeding. Negative pressure suction is suitable for the placenta retention or partially embedded after delivery, placenta adhesion and artificial dissection placenta can not work, or expired abortion has serious bleeding. In this case, the patient had rotten meat blockage and took the examination

Consider for placental factors, at this time should be removed by forceps clamp (A pair), do not use negative pressure suction (E wrong, correct answer for this question

table). In this case, the patient had more blood loss, so the rehydration support (B pair) and anti-infection treatment (C pair) should be given to prevent infection. To shrink

Palcin (D pair) can promote uterine contraction, make the uterine rotten meat tissue completely discharged, and help to reduce postpartum bleeding.

This 28-year-old woman had abdominal pain and fever 2 weeks postpartum, as well as vaginal bleeding and object obstruction of the cervix, suggesting a possible

postpartum infection or intrauterine residue. Choosing the most incorrect treatment regimen requires considering her symptoms and signs.

-A. forceps clamp: forceps are usually used during midwifery and may not be suitable for this condition unless there is indeed a large residue to be removed.

-B. rehydration support: rehydration is appropriate for possible fluid loss due to bleeding or fever.

-C. Anti-infective therapy: Anti-infective therapy is reasonable considering fever and possible infectious symptoms.

-D. Oxytocin: Oxytocin helps with contractions, removes intrauterine residues, and reduces bleeding, which is suitable for postpartum bleeding or intrauterine residues.

-E. Negative pressure suction: This method can be used to remove intrauterine residues or blood clots.

Of these options, perhaps the most incorrect is the A. forceps clamp, as it is mainly used to assist infant delivery during labor rather than for the management of postpartum hemorrhage or infection. For intrauterine residue or rotten meat blockage, the usual method is surgical clearance or negative pressure suction, rather than using forceps. Thus, option A seems to be the least appropriate treatment option.

analyze:

The young female patient still had abdominal pain and fever after 2 weeks postpartum, which was considered as puerperium infection. The patient had vaginal bleeding 2 days ago. Physical examination showed that the uterine floor was located in the two transverse fingers of the pubic union, tenderness, cervical volume and visible rotten meat blockage. Diagnosis was considered as uterine involution and coinfection. At this time, the blockage of rotten meat indicates that there is a placenta or fetal membrane residue. At this time, if the negative pressure suction is performed, due to the long postpartum time, the fetal membrane or placenta tissue is closely adhered to the uterine wall, the negative pressure suction may lead to more bleeding, or even damage the endometrium, so the negative pressure suction is an incorrect treatment method.

Option A clamp refers to the clamp to remove the residue, which is A common treatment when the placenta or fetal membrane remains, so it is excluded.

Option B rehydration supportive treatment and option C anti-infection treatment

are all important treatment measures when the puerpera is co-infected, which can help the maternal recovery and can be excluded together.

Option D to oxytocin can promote uterine contraction, facilitate hemostasis and uterine recovery, and does not meet the question.

To sum up, the correct answer is: E. Negative pressure suction technique.

According to the description, the 28-year-old woman still had abdominal pain, fever, body temperature 38℃ 2 weeks postpartum, accompanied by vaginal bleeding and cervical rotten meat blockage, suggesting the possibility of infection or intrauterine residue. In this case, appropriate therapeutic measures should be taken to remove the intrauterine residue, control the infection and support the recovery of the patients.

For each option of analysis:

A. * * Remove * *: The clamp can help remove the rotten meat blockage at the cervix, which is suitable for tissue removal in the cervix and vagina.

B. * * rehydration support * *: rehydration support is a basic treatment measure to maintain fluid balance in patients, especially in cases of fever and bleeding.

C. * * Anti-infective treatment * *: Anti-infective treatment is essential due to the symptoms of infection (fever and abdominal pain).

D. * * Diactocin * *: Diactocin is used to promote uterine contractions and can help expel intrauterine residues while reducing bleeding.

E. * * Negative pressure suction * *: Negative pressure suction is a common method that can effectively remove intrauterine residues.

Considering these analyses, the only inappropriate treatment was with D. * *

given to oxytocin * *. Although chectocin can aid uterine contraction and reduce bleeding, direct use of chectocin may be inappropriate in cases of already apparent infection due to the need for preferential management of infection and removal of residue.

So the answer is: * * D. Give oxytocin * *.

58. (Type A2 question)

A 35-year-old woman with lower abdominal pain and nausea for 4 hours, no vaginal bleeding, vomiting and fever, normal menstrual cycle, half a month before the last menstruation, a mass in the right adnexal area, 5cm, with tension and no tenderness. Blood routine: Hb11 WBC10109 / L, PLT 20109 / L, blood HCG (-), the most likely diagnosis is

A. acute appendicitis

B. Dle torsion of the ovarian cyst

C. rupture of corpus luteum

D. pelvic inflammatory disease

E. endometriosis

Reference answer: B. Your answer:

Graphic analysis: [analytical] young women, lower abdominal pain with nausea for 4 hours, no vaginal bleeding, vomiting and fever (suggest may be surgical disease), normal menstrual cycle, last menstrual half a month (prompt for luteal period), gynecological examination right accessory area of a mass, 5cm, tension, no tenderness (in line with the typical symptoms of ovarian cyst torsion). Blood routine: HB110g / L

(Normal 110-150 g / L), WBC 10109 / L (normal 4~10109 / L), PLT 20109 / L (normal 100 to 300109 / L), blood HCG (-), the most likely diagnosis was torsion of ovarian cyst.

[Avoid mistakes] Acute appendicitis (A mistake) is often manifested as metastatic right lower abdominal pain and peritoneal irritation, which can be in different degrees

Poxic symptoms, no history of menopause, no mass touch. Luteal rupture (C error) is induced by position change, manifested by the sudden side

Lower abdominal pain, can be accompanied by nausea, vomiting and other manifestations. Pelvic inflammatory disease (D wrong) often presents as lower abdominal pain, vaginal secretion

More increase, abdominal pain is persistent, after activity or after sexual intercourse, the course of the elderly can have neurasthenia symptoms. The endometrium is different

The typical symptoms of disorder (E error) are secondary dysmenorrhea, progressive aggravation, no nausea, vomiting and other manifestations.

This case describes a 35-year-old female patient presenting with lower abdominal pain, nausea, no vaginal bleeding and vomiting, but no fever. Her menstrual cycle was normal, with her last menstrual period occurring about half a month ago. Gynecologic examination revealed a 5-cm mass in the right adnexal area, which had tension but no tenderness. Blood test showed hemoglobin (Hb) 11, white blood cell count (WBC) 1010 ^ 9 / L, platelet count (PLT) 2010 ^ 9 / L, and blood HCG test was negative.

Based on this information, the most likely diagnosis is * * B. ovarian cyst pedictorsion * *. This is because the patient had a mass with tension in the adnexal area, which is usually consistent with the pedicle torsion of the ovarian cyst. Torsion of an ovarian cyst causes acute abdominal pain and may accompany nausea and vomiting, but usually does not cause vaginal bleeding or fever, consistent with the case description. Other options such as acute appendicitis, corpus luteal rupture, pelvic inflammatory disease and endometriosis are less likely in this setting because their clinical manifestations are not completely consistent with those described in this case.

analyze:

Patients for middle-aged women, plain with normal menstrual cycle, last menstrual half month ago, now appear lower abdominal pain, nausea, no vaginal bleeding, vomiting and fever, a gynecological physical examination shows the right accessory area, 5cm, tension, no tenderness, blood routine shows normal hemoglobin, normal white blood cells, platelet decline, blood HCG negative, according to its clinical manifestations and laboratory examination, considering the diagnosis of ovarian cyst torsion. The typical symptom of ovarian cyst pedictorsion is the sudden pain of the lower abdomen of one side after the change of position, often accompanied by nausea, vomiting and even shock. Mesangial torsion causes blood

vessel obstruction, leading to necrosis, bleeding, infection or rupture of the tumor, which may be life-threatening in serious cases.

Option A The typical manifestation of acute appendicitis was metastatic right lower abdominal pain, and the patient had no metastatic right lower abdominal pain, so it was not selected.

Option C luteal rupture occurs at the end of the menstrual cycle of 1 week, occasionally in the first two days of menstruation, some patients with luteal rupture, bleeding, can stop bleeding, no sequelae, but severe hemorrhagic shock can occur, characterized by sweating, dizziness, headache, blood pressure, cold limbs, such as treatment is not life-threatening, and the patient's symptom description difference is obvious, so excluded.

Option D pelvic inflammatory disease is a group of diseases caused by female reproductive tract infection, mainly including endometritis, salpingitis, fallopian tube, ovarian abscess and pelvic peritonitis, its common symptoms of lower abdominal pain, vaginal secretions, severe fever, chills, headache, menstrual period, menstrual onset can increase, does not accord with the idea.

Option E Endometriosis refers to the growth of endometrial tissue in the uterine cavity and myometrium. The typical symptoms are lower abdominal pain and dysmenorrhea, menstrual abnormalities, infertility, sexual intercourse discomfort, etc. The patient has no dysmenorrhea, menstrual abnormalities and other manifestations, so it is not considered.

In conclusion, the correct answer is: B. torsion of ovarian cyst.

Based on the patient's symptoms and the results of the examination, the most likely diagnosis is torsion of the ovarian cyst.

Here is an analysis of each option:

A. Acute appendicitis: Common symptoms of acute appendicitis include right lower abdominal pain, nausea, vomiting, fever, and leukocytosis, but rarely a mass in the right adnexal area.

B. Detorsion of ovarian cyst: Typical symptoms of detorsion of ovarian cyst include sudden onset of lower abdominal pain, associated with nausea and vomiting,

often without fever. There was a 5cm mass in the right adnexal area with tension but no tenderness, consistent with the appearance of torsion of the ovarian cyst.

C. Luteal rupture: corpus luteal rupture usually has acute lower abdominal pain and internal bleeding, which may be accompanied by vaginal bleeding, but no obvious mass.

D. Pelvic inflammatory diseases: Common symptoms of pelvic inflammatory diseases include lower abdominal pain, fever, abnormal leucorrhea, etc. gynecological physical examination may have tenderness, but there may not be definite masses.

E. Endometriosis: Endometriosis usually presents with chronic pelvic pain, dysmenorrhea and infertility, and similar masses are unlikely to occur during acute attacks.

In conclusion, combining the patient's symptoms, gynecological physical examination and ancillary findings, the most likely diagnosis is * * B. ovarian cyst pedictorsion * *.

59. (Type A2 questions)

Female, 30 years old, at 25 weeks' gestation. OGTT: fasting blood glucose 5 mmol/L, 1 hour 11.2mmol/L, 2 hours 8.2 mmol/L. In the past health. Mother and son may be the case

A. Hypertension in pregnancy

B. Hyperglycemia during pregnancy

C. trichomonal vaginitis

D. hypamnion

E. placenta praevia

Reference answer: B. Your answer:

[Analysis] female, 30 years old, 25 weeks of gestation (25 weeks of gestation is

an important period for the diagnosis of diabetes), OGTT: fasting blood glucose 5 mmol/L (normal value 4.4mmol/L~5.1mmol/L), 1 hour 11.2mmol/L (normal value less than 8.5 mmol/L), 2 hours 8.2 mmol/L (normal value less than 7.8 mmol/L). The patient had fasting blood glucose and OGTT for 1 hour blood

Sugar and OGTT exceeded normal values for 2 hours, confirming gestational diabetes (pair B).

[Avoid avoidance] Patients with gestational hypertension (A error) will develop proteinuria, edema and hypertension. Trichomonas vaginitis (C wrong) master

To the symptoms are increased vaginal secretion and vulva pruritus, occasionally burning, pain, sexual pain, etc. Too much hydramnios (D wrong)

The fetal body surface can be directly compressed, the tension of the uterus increases, and the incidence of gestational hypertension increases. Placenta previa (E wrong)

There will be no inducement, painless recurrent vaginal bleeding.

As provided, the 30-year-old woman had an oral glucose tolerance test (OGTT) at 25 weeks of gestation with fasting glucose of 5 mmol/L, 11.2mmol/L after 1 hour and 8.2 mmol/L after 2 hours. According to the American Diabetes Association (ADA) criteria, the diagnostic criteria for glucose tolerance tests during pregnancy are: fasting blood glucose values of 5.1 mmol/L, 10.0mmol/L after 1 hour, and 8.5 mmol/L after 2 hours. In this case, fasting glucose was normal but the 1 and 2 hour glucose values exceeded the above criteria, allowing a diagnosis of gestational diabetes.

Therefore, the most appropriate answer is: B. Hyperglycemia during pregnancy

analyze:

In young female patients, OGTT examination showed normal fasting blood glucose, but high blood glucose at 1 hour after meals and 2 hour after meal, which could be diagnosed as gestational diabetes, that is, hyperglycemia during pregnancy.

Option A Pregnancy hypertension refers to A group of diseases with pregnancy and elevated blood pressure, including pregnancy hypertension, preeclampsia, eclampsia, etc., often manifested as hypertension, proteinuria, edema, etc., inconsistent with the meaning of the question.

Option C Trichomomonas vaginitis is a common vaginal inflammation caused by trichomonas vagina, is also a common sexually transmitted disease, the main symptoms are increased vaginal secretions and vulva pruritus, or burning, pain, sexual

pain, etc., is not consistent with the description of the problem.

Option D oligohydramnios is mainly related to the decrease of amniotic fluid production or the increase of amniotic fluid leakage. Part of the oligohydramnios is unknown, common causes are fetal malformation, placental insufficiency, drug effect, etc. The patient does not have the above situation, so it is not selected.

Option E Placenta previa means that after 28 weeks of gestation, the lower edge of the placenta is adjacent to or covering the cervix. The typical symptoms are in the third trimester or before labor, painless repeated vaginal bleeding, which does not meet the meaning of the question.

In conclusion, the correct answer is: B. Hyperglycemia during pregnancy.

According to the title description, the patient was 30 years old at 25 weeks of gestation. OGTT (oral glucose tolerance test) results showed fasting glucose 5 mmol/L, 1 hour glucose 11.2mmol/L and 2 hour glucose 8.2 mmol/L. According to the results of OGTT, we can judge that the patient has elevated blood glucose.

According to the criteria of the International Diabetes Federation and the World Health Organization, the diagnostic criteria for gestational diabetes are:

-Fasting blood glucose was 5.1 mmol/L but <7.0 mmol / L

-Or 1-hour blood glucose of 10.0 mmol / L

-Or 2-hour blood glucose of 8.5 mmol/L but <11.1 mmol / L

The fasting blood glucose of the patient in the title was 5.0 mmol/L. Although it did not meet the diagnostic criteria for gestational diabetes, the 1-hour blood glucose 11.2 mmol/L and 2-hour blood glucose 8.2 mmol/L exceeded the normal range, which met the diagnostic criteria for gestational diabetes. Therefore, the possible condition of the mother and child is hyperglycemia during pregnancy.

The correct answer is:

F. Hyperglycemia during pregnancy

60. (Type A2 question)

Boy, 6 years old. Oedema, urine less than 5 days. Physical examination: T37℃, P82 / min, R18 / min, BP100 / 65mmHg. Pitting oedema of both lower limbs. Laboratory examination: human blood albumin 24g / L, cholesterol 6.3 mmol/L, urinary RBC 1 ~ 2 / HP protein quantification 1.5g. Prednisone 2mg/(kg.d) oral for three weeks, two urine protein negative, the next treatment measures

A. Prednisone 2mg / (kgd) was continued and changed to prednisone 2mg / kg on alternate days after 4 weeks

B. Prednisone 2mg / (kgd) was continued and changed to prednisone 2mg / kg on alternate days after 5 weeks

C. Change to prednisone 2mg / kg alternate day clothing

D. Continue prednisone 2mg / (kgd) and change to prednisone 2mg / kg 2 weeks later

E. Prednisone was continued

Refer to the answer: D. Your answer:

Text analysis: [analysis] boy, 6 years old. Oedema, urine less than 5 days. Physical examination: T37℃, P82 / min, R18 times / min, BP100 / 65mmHg. Pitting oedema of both lower limbs. Laboratory examination: human blood albumin 24g / L (human blood albumin normal: 40~55g / L), 24-hour urine protein: 1.5g (urine protein: normal: 0.1 g/d). Consider the high possibility of nephrotic syndrome.9 edition of Pediatrics: Long-range therapy in glucocorticoids can be used in various types of nephrotic syndrome. Prednisone 2mg/(kg.d) at a maximum of 60 mg/d, taken in doses. If the urinary protein turns negative within 4 weeks, the amount will be reduced by at least 2 weeks after the conversion, changed to 2mg / kg after breakfast on the other day, and continued for 4 weeks. The total amount will be reduced by 2.5~5mg every 2 to 4 weeks until the drug is stopped. The course of treatment must be 6 months (medium-range therapy). So choose D.

The title describes a 6-year-old boy presenting with oedema and oliguria, with hypoalbuminemia and proteinuria. These symptoms and the laboratory findings point to the nephrotic syndrome. Prednisone is a commonly used drug for the treatment of nephrotic syndrome and improves symptoms by reducing inflammation and immune response.

After treatment, the child's urine protein examination was negative twice, indicating a good response to treatment with prednisone. The next treatment step is

usually reducing the dose of prednisone to prevent side effects while maintaining efficacy.

Options A and B propose to continue the same dose of prednisone after a longer reduction, while option D recommends a reduction after a shorter time. Option C directly recommends changing alternate day change, and option E recommends stopping prednisone.

In the management of nephrotic syndrome, the dose reduction is usually gradual after symptom control. Therefore, direct discontinuation of prednisone (option E) is usually not recommended for possible disease recurrence. Option C may be too rapid and may not be conservative enough.

Given the coherence of treatment and the need to prevent disease relapse, both options A and B seem to be viable options. However, according to best practice, the therapeutic dose should be gradually reduced after the condition is controlled to find the minimum effective dose and reduce side effects. Progressive dose reduction is usually recommended after symptom stabilization and then transition to alternate-day treatment.

Therefore, the choice of B (continued prednisone at 2mg / (kg d) and changed to prednisone at 2mg / kg on alternate days after 5 weeks) may be more appropriate as it provides a longer stabilization period that ensures stable disease before completely changing the frequency of dosing.

analyze:

A 6-year-old child with edema, oliguria, physical examination showed depressed edema of both lower limbs, and laboratory examination showed hypoproteinaemia, hyperlipidemia, microscopic hematuria, and massive proteinuria, consistent with the characteristic manifestations of nephrotic syndrome. The child has been given prednisone 2mg/(kg.d) oral administration for three weeks, negative urine protein negative, indicating that the child is sensitive to glucocorticoid therapy, then prednisone can be changed to alternate day therapy, that is, prednisone 2mg / (kgd), 4 weeks later to prednisone 2mg / kg alternate day, and then gradually reduced to withdrawal.

Option B "Continue Prednisone 2mg / (kg d), take prednisone 2mg / kg for alternate days after 5 weeks", option D "Continue Prednisone 2mg / (kg d), 2 weeks to prednisone 2mg / kg for alternate days", glucocorticoid medication, do not meet the requirements of glucocorticoid treatment for nephrotic syndrome, so can be excluded.

Because the child is sensitive to glucocorticoid therapy, he should continue to take it until two weeks after the urinary protein change, and continue treatment for a period of time, so option C to prednisone 2mg / kg for alternate day, option E stop prednisone and can be excluded.

In conclusion, the correct answer is: A. Continue Prednisone 2mg / (kg d) and change to prednisone 2mg / kg after 4 weeks.

The topic described a 6-year-old boy who developed edema and oliguria for 5 days, physical examination showed depressed edema in both lower limbs, and laboratory tests showed hypoproteinemia (human albumin 24g / L) and hypercholesterolemia (cholesterol 6.3 mmol/L), suggestive of nephrotic syndrome. Urinary protein was negative after prednisone treatment, indicating some improvement. The following treatment measures should follow the treatment principle of nephrotic syndrome, that is, the gradual reduction.

According to the standard treatment plan of nephrotic syndrome, generally in the initial treatment of prednisone achieved urinary protein negative, continue full dose treatment for a certain time, and then gradually reduced to the alternate day therapy. Therefore, the correct answer should be to continue the full dose treatment for a certain period of time and then change to alternate day therapy.

The best answer is:

B Prednisone 2mg / (kgd) was continued and changed to prednisone 2mg / kg on alternate days after 5 weeks

Explanation: In the treatment of prednisone for nephrotic syndrome, a full dose of therapy is usually maintained for a period of time after the initial treatment, followed by a gradual transition to alternate day therapy. Continuing the full dose for 5 weeks before switching to an alternate day can help to reduce the risk of recurrence and ensure stable disease.

61. (Type A2 questions)

A 39-year-old female, G3P2, second uterine prolapse with mild distension of the anterior and posterior vaginal walls. For gynecological examination, the cervix is about 6cm long, the posterior uterus is normal in size, and the mass is not touched in the double accessory area, so surgical treatment is required. The preferred surgical method is

A. Subtotal hysterectomy

B. sacral ligament shortening

C. Repair of the anterior and posterior vaginal walls

D. vaginal closure

E. Manchester operation

Reference answer: E Your answer:

[Analysis] The patient was a middle-aged menstrual woman, with second uterine prolapse with mild swelling of the anterior and posterior vaginal walls, and surgical treatment was required. Mann surgery (Manchester surgery) is feasible in young patients with prolonged uterine prolapse, including anterior and posterior vaginal wall repair, major ligament shortening, and partial cervical resection.

[Error avoidance] Subtotal hysterectomy (A error) is suitable for younger patients with uterine prolapse with longer cervix. Sacral ligament shortening (B error) is suitable for young patients <4cm. Anterior vaginal wall repair (C error) is indicated for patients with only anterior vaginal wall expansion. Vaginal semi-closure (D wrong) is used in older patients without considering reproductive function.

In this case, considering the woman of 39 years old, G3P2 (i. e., three pregnancies, two deliveries), and second uterine prolapse with mild bulging of the anterior and posterior vaginal walls, the preferred surgical approach should be designed to effectively correct the prolapse and reduce the risk of recurrence.

Item A "subtotal hysterectomy" is mainly used for the treatment of uterine lesions, and is not the preferred treatment for prolapse.

Item B "sacral ligament shortening" is a treatment for uterine prolapse, but it is more used for more severe prolapse conditions.

Item C "anterior and posterior vaginal wall repair" is suitable for vaginal wall prolapse and can repair the swelling of the anterior and posterior walls, which is a reasonable choice for the situation of this case.

Item D "vaginal semi-closure" is used to treat severe uterine prolapse or vaginal prolapse, which may have an impact on the sexual life of patients, and is generally not taken as the first choice.

Item E "Man's surgery" is a treatment for vaginal prolapse, but is not the preferred procedure in this case.

Therefore, the most appropriate answer should be C. anterior and posterior vaginal wall repair, as this procedure can directly solve the main problem of the patient, namely uterine prolapse with mild swelling of the anterior and posterior vaginal wall.

First, we first understand and analyze the key information of the question. The patient is a 39-year-old woman who had had three pregnancies and two deliveries (G3P2). Her current condition was a second uterine prolapse with mild distension of the anterior and posterior vaginal walls. The gynecological examination results showed that the cervix was about 6cm long, the uterus was in the posterior position and of normal size, and no mass was found in the double accessory area. Now, she hopes to improve the condition with surgery.

Now, we analyze the applicability of the options using these information:

A. Subtotal hysterectomy: This procedure is usually used for more severe uterine diseases, such as myoma and adenomyosis, but this option may not be preferred given the normal size and position of the uterus.

B. Sacral ligament shortening: This procedure is designed to shorten and reinforce the supporting structure of the uterus, but is not used as the treatment of choice for uterine prolapse.

C. anterior and posterior vaginal wall repair: Although the patient has mild swelling of the anterior and posterior walls of the vagina, this is not the main symptom. the main problem is uterine prolapse, so this option may not be comprehensive.

D. Vaginal semi-closure: This procedure is mainly used in elderly women, especially those who no longer need preserved intercourse, and is not used in young female patients in this case.

E. Mann's surgery: This procedure includes repair of the anterior and posterior vaginal walls, with shortening of the main ligament and partial cervical resection. It is suitable for younger patients with prolonged uterine prolapse, which coincides with

the situation of patients in the trunk.

In conclusion, considering that the patient is young, the uterine size and position are normal, and the main symptoms are second uterine prolapse and mild swelling of the anterior and posterior vaginal walls, Man's surgery can solve both the problem of uterine prolapse and the swelling of the vaginal wall, so it is the preferred surgical method.

Therefore, the correct answer is E. Man's surgery.

According to the description, the patient was a 39-year-old woman with second uterine prolapse with mild swelling of the anterior and posterior vaginal walls. Surgical treatment was requested. According to the current gynecological best practice guidelines, the preferred option for this condition should consider both the solution of uterine prolapse and the repair of anterior and posterior vaginal wall bulging.

The options are explained as follows:

A. Subtotal hysterectomy-It is mainly used to treat uterine lesions and does not solve the problems of uterine prolapse and anterior and posterior vaginal wall swelling.

B. Sacral ligament shortening-mainly used to improve the support of the uterus, but does not solve the problem of anterior and posterior vaginal wall swelling.

C. Front and posterior vaginal wall repair-mainly used for the repair of anterior and posterior vaginal wall swelling, but can not effectively solve the problem of uterine prolapse.

D. Vaginal semi-closure-mainly used in patients with severe uterine prolapse, providing support by reducing vaginal length but not suitable for younger women and with unresolved anterior and posterior vaginal wall distension.

E. Mann's surgery-Immobilize the uterus by vaginal suspension while repairing anterior and posterior vaginal bulging, suitable for cases with uterine prolapse and anterior and posterior vaginal bulging.

Considering the patient's condition and the indications for each procedure, the most appropriate procedure is E. mann. It can not only solve the problem of uterine

prolapse, but also repair the anterior and posterior walls of the vaginal swelling, is the best choice.

So the answer is:

B Manchester operation

62. (Type A2 questions)

Female, 41 years old, repeated vulvovaginal pruritus for 3 months, past history of diabetes for 6 years, gynecological physical examination: vaginal redness, increased secretions, secretions were tofu residue like. The most likely diagnosis is the

A. vulvovinalis vaginitis

B. clap

C. atrophic vaginitis

D. Bacterial vaginitis

E. bartholinitis

Reference answer: A. Your answer:

Graphic resolution: [analytical] the patient for middle-aged women, repeated vulovaginal itching march (vulva itching for vulvovinalis vaginitis common symptoms), past history of diabetes (diabetes is vulvovagalis vaginitis susceptibility factors), gynecological physical examination: vaginal redness, secretions, secretions are tofu residue (secretions are tofu residue for vaginal yeast vaginitis typical manifestation), according to the patient's history, clinical manifestations, the most likely diagnosis is vulvovaginalis silk yeast vaginosis.

[To avoid mistakes] gonorrhea (B wrong) more gonorrhea urethritis, urethral mouth overflow pus, conscious urination pain, frequent urination, urgent urination.decline

Reduction vaginitis (C wrong) is common in natural menopause or artificial postmenopausal women, manifested as vulva burning discomfort, pruritus and Yin

Road secretions increased, no tofu residue-like secretions. Bacterial vaginitis (D wrong) is shown by increased vaginal discharge and fish

The smell. The main symptoms of vestibular macroadenitis (E wrong) are local

swelling, burning sensation of one labia majora, obvious pain and walking

Inconvenient, and sometimes can form an abscess.

The diagnosis most consistent with this case description was A. vulvovinalis vaginitis. Patients have a history of diabetes, diabetic patients may lead to poor blood glucose control, prone to Candida infection. Symptoms include itching and redness in the vulvovagina, and increased secretions, especially when described as "tofu residue-like" secretions, which are typical of Candida infection. Therefore, A was chosen as the most likely diagnosis.

First, let's go into the key information in the title. This is a 41-year-old female patient with a long-term history of diabetes, repeated vulvovaginal pruritus, and physical examination found vaginal redness, increased secretions and tofu residue sample.

Next, we analyze each option to find the diagnosis that best fits these symptoms:

A. vulvovaginal vaginitis: the typical symptoms of this vaginitis include vulva itching, burning pain, can also be accompanied by frequent urination, urine pain and sexual pain, increased vaginal secretions, typical secretions are characterized by white thick thick curd or tofu residue. These symptoms highly coincide with the patient symptoms described by the stem.

B. Gonorrhoea: Gonorrhea is mainly manifested as suppurative infection of the urogenital system, usually with urethral pain, purulent secretions and other symptoms, which is not consistent with the description of the title stem.

C. Atrophic vaginitis: it is more common in women with natural menopause or artificial menopause. The main symptoms are vulva burning discomfort, itching and increased vaginal secretions, but the secretions are usually thin, pale yellow, and the severe infection is pus and blood leucorrhea, which does not match the symptom description of the patient.

D. Bacterial vaginitis: the main manifestations are increased vaginal secretions, fishy smell, especially after sexual intercourse, accompanied by mild vulva itching or burning, secretions are usually gray-white, uniform, thin, not consistent with the patient's tofu residue secretions.

E. Vestibule adenitis: This is a kind of vulval inflammation, mainly manifested as red, swelling, heat, pain and other symptoms of the vestibular gland, which are obviously different from the symptoms described by the main stem.

Based on the above analysis, we can see that the patient's symptoms are highly consistent with the typical symptoms of vulvovinalis vaginitis, especially the bean curd residue-like characteristics of vaginal secretions is a significant marker of the disease.

Therefore, the most likely diagnosis is A. vulvovovinalis vaginitis.

According to the title description, the 41-year-old woman had a 3-month history of recurrent vulvovaginal pruritus and a 6-year history of diabetes. Gynecological examination showed vaginal redness, increased secretions, and secretions were tofu residue.

Diabetic patients are prone to fungal infections, and tofu residue-like secretions are typical of Candida (Candida) infection. Therefore, the most likely diagnosis is:

A. vulvovinalis vaginitis

63. (Type A2 question)

The boy, two and a half years old, severe malnutrition, diarrhea in the past week, 3~8 times / day, thin, yellow, more foam, purulent, visible bean residue like fine block, may diagnose the disease is

A. Candida albicans and his enteritis

B. Pathogenic Escherichia coli enteritis

C. rotavirus enteritis

D. Staphylococcus aureus enteritis

E. Yersinia enterocolitis

Reference answer: A. Your answer:

Analysis: [Analysis] The child, with diarrhea, more foam, purulent, visible bean residue like fine block, mostly caused by Candida albicans enteritis.

The disease is often complicated by other infections, or when the intestinal flora is dysregulated. The course of the disease is prolonged, often accompanied by oral thrush. The number of stool increased, yellow thin stool, more foam, with mucus, sometimes visible tofu residue like thin block (bacterial colonies). The stool

microscopy showed fungal spores and hyphae.

[Avoid mistakes] Disease-causing large intestinal Ai Xi bacterial enteritis (B wrong) is mostly secondary to pediatric diarrhea, manifested as fever, abdominal pain, diarrhea, severe emergency, etc. Rotavirus enteritis (C wrong) is more common in 6~24 months of infants, good hair in autumn and winter, often due to improper diet and climate change induced, typical feces for water or egg flower soup.coccus aureus enteritis (D error): often secondary to the use of numerous antibiotics and symptoms are often associated with the degree of dysregulation, sometimes secondary to chronic diseases. Symptoms include fever, vomiting, diarrhea, varying degrees of poisoning symptoms, dehydration and electrolyte disorders, and even shock. Typical stool is dark green, more amount with mucus, a few for blood stool. Yersinia enterocolitis (E wrong) clinical manifestations of abdominal pain, diarrhea, fever, vomiting, etc. Occurs in winter and early spring, can cause lymph node enlargement, also can produce mesenteric lymphadenitis, symptoms can be similar to appendicitis, also can cause pharyngeal pain and cervical lymphadenitis.

This question describes a two-and-a-half-year-old boy, severely malnourished, with frequent yellow foamy purulent diarrhea, along with thin bean residue-like lumps. These symptoms suggest infectious enteritis. Let's analyze these options one by one:

A. Candida albicans enteritis usually causes white or cheese-like poop and often occurs with an impaired immune system, but less closely fits the description of septic diarrhea.

B. Pathogenic Escherichia coli enteritis may cause bloody diarrhea rather than purulent sex.

C. Rotavirus enteritis is the most common cause of viral diarrhea in children, but is characterized by watery diarrhea, which usually does not include purulent excreta.

D. S. aureus enteritis usually causes acute diarrhea, but it is not exactly consistent with the description of purulent diarrhea and bean residue-like thin mass.

E. Yersinia enterocolitis can cause septic diarrhea and may occur in malnourished children, presenting as purulent and diarrhea with fine masses.

In conclusion, the most likely diagnosis is E. Yersinia enterocolitis, as it fits best with the symptoms described.

analyze:

The two-and-a-half-year-old boy with a history of severe malnutrition, diarrhea

in nearly a week, stool 3~8 times / day, thin, yellow, foam * *, purulent * *, visible bean residue like fine block, considering the diagnosis of Candida albicans enteritis. Candida albicans is an opportunistic pathogen, mainly endogenous infection, can cause skin and mucosal infection, visceral infection, central nervous system infection, etc., among which the most common visceral infection is digestive tract and lung infection. Digestive tract infection can cause thrush, glossitis, mouth erosion and Candida albicans enteritis, of which alitians enteritis can have low fever, vomiting, abdominal distension, diarrhea and other manifestations, stool for yellow watery stool, foam, with mucus, visible tofu residue like thin block, stool microscopy of fungal spores and hyphae.

Option B pathogenic Escherichia coli enteritis is often manifested as high fever, vomiting, abdominal pain, diarrhea, watery stool, smelly, but also mucus stool, pus, bloody stool or bloody stool, which is obviously different from the description of stool in the dry problem.

Option C rotavirus enteritis often showed fever, vomiting, diarrhea, stool frequency and water, yellow water or porridge, no purulent and bean residue thin block, not consistent with the description of the stem.

Option D Staphylococcus aureus enteritis typical stool is dark green, the amount with mucus, a few for blood stool, do not conform to the dry.

Option E Yersinia enterocolitis is acute gastroenteritis symptoms, enterocolitis, septicemia can have systemic infection poisoning symptoms, can also cause reactive arthritis, with the description of the stem.

In conclusion, the correct answer is: A. Candida albicans enteritis.

According to the description of the problem, the patient was a two-and-a-half-year-old boy with severe malnutrition and diarrhea in the past week. The number of diarrhea was 3~8 times / day, thin, yellow, more foam, purulent nature, and thin bean dregs. Combining these symptoms, the most likely diagnosis is rotavirus enteritis (option C). Rotavirus enteritis is more common in infants and young children, usually causing severe diarrhea, yellow excrement, more foam, and often accompanied by vomiting and fever.

The other options are excluded for the following reasons:

A. Candida albicans enteritis: more common in immunocompromised patients,

the symptoms are generally not manifested as more foamy yellow diarrhea.

B. Pathogenic Escherichia coli enteritis: it usually presents as watery or bloody diarrhea, but more foamy yellow diarrhea is less common.

C. Staphylococcus aureus enteritis: more common in food poisoning, often manifested as acute vomiting and diarrhea, feces may not necessarily present the described characteristics.

D. Yersinia enterocolitis: more common in the cold season, symptoms include abdominal pain, fever and bloody diarrhea, more foam yellow diarrhea is less common.

Therefore, the most likely diagnosis is C. rotavirus enteritis.

64. (Type A2 question)

Female, 24 years old, 21 to 23 days / 4 to 5 days, 1 spontaneous abortion, basal body temperature bipolar, no obvious hormone abnormalities on the third day of menstruation, was diagnosed as

A. polycystic ovary syndrome

B. luteal phase defect

C. abnormal endometrium

D. No ovulatory abnormal uterine bleeding

E. Malgalorrhoea syndrome

Reference answer: B. Your answer:

[Analysis] Luteal insufficiency refers to the development of follicles and ovulation in the menstrual cycle, but insufficient progesterone secretion or premature decline of the luteal phase, leading to poor endometrial secretion reaction and shortening of the luteal phase, causing shortening of the menstrual cycle. In this case, the menstrual cycle was shortened, and no obvious abnormalities were found on the 3rd day of menstruation, which was considered as luteal insufficiency.

[Avoid mistakes] Polycystic ovary syndrome (A mistake) is clinically characterized by clinical or biochemical manifestations of excessive androgen, continuous anovulation, and polycystic ovarian changes, often accompanied by insulin resistance and obesity, mainly manifested as menstrual disorders, androgen

excess and obesity. Patients with abnormal endometrial shedding (C wrong) generally have no history of abortion, most of them have a history of infertility, and the hormone measurement can appear abnormal on the third day of menstruation. No obvious abnormalities were found in this patient, so the diagnosis of abnormal endometrial shedding was not established. No ovulatory abnormal uterine bleeding (D wrong) is the most common clinical manifestation of uterine irregular bleeding, characterized by menstrual disorders, menstrual volume, menstrual frequency, menstrual drip, diagnostic curettage can be found endometrial sustained effect by estrogen and no progesterone antagonism, can occur different degrees of proliferative change, a few can be atrophic changes. Menstracorrhoea syndrome (E wrong) is the clinical manifestation of hyperprolactinemia. amenorrhea syndrome is a common pathophysiological state, and serum prolactin (PRL) is elevated in hyperprolactitinemia, often accompanied by a series of symptoms caused by amenorrhea, galactorrhea and anovulation.

The topic provides information pointing to the reproductive health status of female patients. The 24-year-old woman had a menstrual cycle of 21 to 23 days for 4 to 5 days, a history of a spontaneous abortion, and basal body temperature measurements showed bipolar patterns, while examination of the third day of menstruation revealed no significant abnormalities. Based on this information, we need to identify the diagnosis that best fits these clinical characteristics.

A. Polycystic ovary syndrome (PCOS) usually presents with irregular menstruation, ovulation, and hyperandrogenism. This patient had a more regular menstrual cycle and no mention of abnormal androgen levels, so the possibility of polycystic ovary syndrome was low.

B. Luteal insufficiency involves insufficient luteal secretion, usually leading to shortened menstrual cycles, prolonged menstruation, or irregular bleeding. Her menstrual cycle and duration were within normal limits and there was no clear evidence of inadequate luteal function.

C. Abnormal endometrium shedding usually leads to irregular menstrual bleeding and possible infertility, but the menstrual cycle and duration of this patient are relatively regular and do not fully consistent with this diagnosis.

D. Unovulatory abnormal uterine bleeding presents as irregular bleeding due to the anovulatory cycle. However, the patient's basal body temperature record showed ovulation (biphasic body temperature curve), which did not consistent with

anovulatory bleeding.

E. Menacorrhotosis syndrome (i. e. hyperprolactinemia or Galactorrhea-Amenorrhea Syndrome) is mainly characterized by amenorrhea and increased milk secretion. Given this patient's regular menstrual cycle, this option also did not fit.

Taken together, although no option perfectly matches all the information, based on the data provided, option B "luteal insufficiency" may be the most consistent diagnosis, and although her menstrual cycle and duration are within the normal range, previous spontaneous abortion and normal hormone level tests may suggest potential luteal function problems. However, it should be noted that this condition may require more detailed evaluation and testing in the actual diagnosis.

Therefore, the most appropriate choice is the B. luteal insufficiency.

analyze:

The patient was a young woman with short menstrual cycle, 1 spontaneous abortion, and bipolar basic body temperature. No obvious abnormalities were found on the third day of menstruation. Based on the menstrual history, abortion history and examination of the patient, the patient was diagnosed as luteal insufficiency. Luteal insufficiency refers to the development of follicles and ovulation in the menstrual cycle, but insufficient progesterone secretion or premature progesterone decline in the luteal period leads to poor endometrial secretion reaction and shortened luteal phase. Clinically, the shortened menstrual cycle is the main feature, and patients are not easy to conceive or abortion in early pregnancy.

Option A polycystic ovary syndrome starts from puberty, characterized by clinical or biochemical manifestations of excessive androgen, continuous anovulation, polycystic ovarian changes, menstrual disorders, androgen excess and obesity, often accompanied by insulin resistance and metabolic syndrome, which are inconsistent with the symptoms of the patient, so it is not selected.

Option C irregular shedding of endometrium was more common in women of childbearing age, showing normal menstrual cycle but prolonged menstrual period for 9 to 10 days, and the amount of bleeding was large. The patient had normal menstrual period, so it was excluded.

Option D Unovulatory dysfunctional uterine bleeding is irregular uterine bleeding, which is manifested as menstrual cycle disorder, varying menstrual length,

variable or increased menstruation, or even massive bleeding, inconsistent with the meaning of the question.

Option E Menstractorrhoea syndrome is mainly characterized by amenorrhea, galactorrhea, infertility, hyperprolactinemia and pituitary microadenoma, unrelated to this question.

In conclusion, the correct answer is: B. luteal insufficiency.

According to the information, the 24-year-old woman, menstrual cycle for 21 to 23 days, each lasting 4 to 5 days, had 1 spontaneous abortion, basal body temperature was bipolar, and no obvious hormone abnormalities on the third day of menstruation. Here is an analysis of each option:

A. Polycystic ovary syndrome: This condition is usually symptoms of loose menstruation, amenorrhea, hypertrichotism, obesity and excessive androgens. There are no of these typical symptoms in the question stem, and the basal body temperature is biphasic, indicating ovulation.

B. Luteal insufficiency: This condition can lead to a shortened luteal phase, which may show a shortened menstrual cycle or reduced menstrual volume. It is mentioned that the menstrual cycle is short and there is a history of spontaneous abortion, which is consistent with the corpus luteal function.

C. Abnormal endometrial shedding of the uterus: This condition usually leads to irregular menstruation or abnormal bleeding. This performance is not mentioned in the question stem.

D. No ovulatory abnormal uterine bleeding: This condition is more common in adolescent and perimenopausal women, and the basal body temperature is usually monophasic type. The basal body temperature in the problem stem is biphasic, indicating ovulation, which does not conform to the characteristics of anovulatory bleeding.

E. Menaperrhoea syndrome: This condition is mainly manifested by amenorrhea

and galactorrhea, usually caused by hyperprolactinemia. There is no mention of lactation or amenorrhea, excluded.

Combined analysis, the most likely diagnosis is:

F. luteal insufficiency

65. (Type A2 question)

Male, 60 years old. Nearly 3 days of night behavior disorder, said the floor of the room mice, snakes, can see the dead, expression terror, nervous, do not know the family, quiet during the day, like to bed, can not recall the night behavior, can know the family, head C slice infarction, consider the patient in

A. delirious state

B. hallucinatory paranoid state

C. depressive state

D. Dementia status

E. stuporous state

Reference answer: A. Your answer:

Graphic resolution: [analytical] false state patients conscious disorder, accompanied by perceptual disorders (illusion, illusion), speech disorders, often terror vision, such as see the beast, ghosts, house dust, orientation, feel the surrounding environment is strange and terror, the subjective and objective world reversed or a lot of illusion, illusion, quiet during the day, restless at night.

[Avoid error] illusion delusion state refers to the pathological delusion under pathological circumstances, the content is bizarre, absurd, divorced from reality, and even with supernatural color (B wrong). The depressed state is mainly characterized by significant and lasting emotional depression (sadness, depression, sighing, crying, etc.), accompanied by lack of interest, anhedonia, etc., no illusion, delusion (C error). Dementia state is manifested by memory loss, inattention, slow thinking, decreased comprehension, decreased computing power and other intelligent disorders (D error). The stiff state is silent, motionless, eating, expressionless, muscle relaxation, etc. (E wrong). The illusion delusion state refers to the pathological delusion in the

pathological circumstances, the content is bizarre, absurd, divorced from reality, and even has a supernatural color (B wrong). The depressed state is mainly characterized by significant and lasting emotional depression (sadness, depression, sighing, crying, etc.), accompanied by lack of interest, anhedonia, etc., no illusion, delusion (C error). Dementia state is manifested by memory loss, inattention, slow thinking, decreased comprehension, decreased computing power and other intelligent disorders (D error). The stiff state is silent, motionless, eating, expressionless, muscle relaxation, etc. (E wrong).

This case describes a 60-year-old man who showed disordered behavior at night, visual hallucinations (seeing rats, mice, snakes and dead people), looked horrible and nervous, did not know his family at night, but was quiet during the day and got to know his family. A CT of the head showed a lamellipodial infarction. These symptoms and clinical manifestations are consistent with the characteristics of the state (Delirium).

False state is a kind of acute brain dysfunction, often manifested as the level of consciousness fluctuations, cognitive dysfunction, perception abnormalities (such as hallucinations), sleep-wake cycle disorder, etc. It is usually associated with pathological changes in the brain, so flachy infarction in cases may be one of the causes.

Based on this information, the most appropriate answer is: A. False state

First, let's analyze the key information given by the topic. The patient was an elderly male, with a night behavior disorder in the past 3 days, showing hallucinations (seeing no mice, snakes, dead people), and the expression was horrible, nervous, and even did not know the family. However, he behaved relatively normally during the day, being able to meet family members but not recall abnormal behavior at night. Furthermore, CT of the patient's head had revealed the presence of flachy infarct.

Next, we analyzed for each option:

A. False state: delusion is an acute, reversible, widespread cognitive impairment, mainly manifested as disorders of consciousness, sensory disorders such as hallucinations and illusions, and attention disorders such as lax attention. This state is usually acute, the course of the disease is short, and the disease develops rapidly. Considering the nocturnal hallucinations, behavioral disorders, and the inability to recall nocturnal behavior, this coincided with the performance of the delusion state.

B. Delusional state: Although the patient has hallucination, delusional state

usually refers to persistent and fixed wrong beliefs, while the patient's behavior during the day is relatively normal and he can know his family members, indicating that there is no fixed delusion.

C. Depression status: The patient did not show the typical symptoms of depression, such as low mood and loss of interest.

D. Dementia state: Dementia is a chronic process, mainly manifested as memory loss, cognitive decline, etc., usually will not suddenly appear or disappear, inconsistent with the patient's situation.

E. Digory state: Digory state is motionless, or even does not eat or drink, while the patient can know the family during the day and show relatively normal behavior during the day.

Based on the above analysis, the patient's symptoms are most consistent with the performance of the false state, especially in the night hallucinations, behavioral disorders and relatively normal performance during the day. Therefore, the correct answer is A. false state.

According to the symptoms described by the problem, the patient had behavior disorder, hallucinations (see rats, snakes and dead), nervous terror, do not know the family, and quiet during the day, can know the family and can not recall the behavior at night. These symptoms are consistent with the characteristics of the delusion state, which often presents as acute onset, fluctuating levels of consciousness, and cognitive dysfunction, especially at night.

Therefore, the most likely diagnosis is:

A. delirious state

66. (Type A2 questions)

The 51-year-old woman witnessed her husband being run over by a car to his death six weeks ago. In the past two weeks, she experienced fear and insomnia, repeatedly recalled the scene of her husband's death, and was afraid to enter the bedroom. The most likely diagnosis was

A. somatoform disorders

B. Posttraumatic stress disorder

C. panic disorder

D. Acute stress disorder

E. Generalized anxiety disorder

Reference answer: B. Your answer:

[Analysis] The patient was diagnosed as post-traumatic stress disorder, and the diagnostic criteria were as follows:

Unusually traumatic event (witnessing a husband being crushed to death by a car); repeated traumatic experience (repeated return

Remember the death of her husband); avoid painful memories (go to the bedroom); increased alertness (fear, insomnia). Therefore, the most likely diagnosis for this patient was PTSD.

[Error avoidance] Somform disorder (A error) is characterized by the advantage of lasting worry or belief in various physical symptoms

Neurosis. Panic disorder (C error) is mainly panic attack, sudden unpredictable fear

Feeling, accompanied by a sense of dying or loss of control and severe autonomic dysfunction symptoms. Acute stress disorder (D wrong) is suffering

It occurs within minutes to hours after an unusual traumatic event and resolves within a month. Widespread anxiety disorder

Anxiety disorder (anxiety disorder) is a kind of mental disorder as the main performance of anxiety, patients often have unknown reasons for fear, tension

Unease, no emotional traumatic event as a cause, not consistent with this case, so not considered (E wrong).

The title describes a 51-year-old woman who was terrified, had insomnia, had repeated memories of her husband's death and was scared to death by a car six weeks ago. Based on these symptoms, we can analyze the possible diagnoses.

A. Somatoform disorder (Somatic Symptom Disorder) usually shows excessive concern for physical symptoms, which does not coincide with the psychological symptoms described in this case.

B. Post-traumatic Stress Disorder (PTSD) is a mental health condition that

develops after experiencing or witnessing an extremely traumatic event, manifested as repeated recall of traumatic events, the avoidance of irritation, emotional numbness and other symptoms, consistent with the case description.

C. Panic disorder (Panic Disorder) is characterized by sudden, repetitive panic attacks, which usually have no obvious triggers, and are not completely consistent with this case.

D. Acute stress disorder (Acute Stress Disorder, ASD) usually occurs within a month of a traumatic event and shows symptoms similar to PTSD, but because this woman's symptoms persist for 6 weeks, it is unlikely to be an acute stress disorder.

E. Generalized anxiety disorder (Generalized Anxiety Disorder, GAD) is characterized by persistent, widespread anxiety and concern, not specific to an event or situation, and does not coincide with the specific post-traumatic response in this case.

In conclusion, the most likely diagnosis is * * B. Posttraumatic stress disorder (PTSD) * *.

This is a medical diagnostic reasoning problem, and we need to judge the patient's possible disease according to the symptoms described by the problem stem.

First, comb through the key information in the topic:

A 51-year-old female patient presented here.

She saw her husband being run over to death by a car six weeks ago.

In the past two weeks, fear, insomnia, repeatedly recalled the death of her husband.

Dare not into the bedroom.

Next, we analyzed the options one by one based on the key information:

A. Somatoform disorder: This is a neurosis characterized by persistent concern for or belief in the superiority of various somatic symptoms. Patients repeatedly seek medical treatment for these symptoms, various negative medical tests and the doctor's explanation can not dispel their doubts. Do not agree with the description of the question stem, you can be excluded.

B. Post-traumatic stress disorder (PTSD): This is a mental disorder caused by experiencing or witnessing extreme threats or catastrophic events (such as war, natural disasters, serious accidents, etc.). Symptoms may include recurrent nightmares

related to traumatic events, flashbacks, and situational situations, as well as persistent increased alertness and avoidance behavior. The patient in the exercise experienced fear, insomnia, and repeated memories related to the unexpected death of her husband, which are typical of PTSD.

C. Panic disorder: mainly manifested as a sudden panic experience, accompanied by a sense of near death or loss of control, and severe symptoms of autonomic dysfunction. These symptoms were not mentioned in the title stem, so they can be excluded.

D. Acute stress disorder: refers to a transient mental disorder occurring within minutes or hours after suffering a sharp, severe traumatic event, generally relieving within a few days or a week, and lasting no more than 1 month. The symptoms have been persistent for 2 weeks and have not mentioned significant remission, so it does not meet the characteristics of acute stress disorder.

E. Generalized anxiety disorder: the main performance is unexplained fear, nervous, is not consistent with the description of the question stem, can be excluded.

In conclusion, the most likely diagnosis for this patient is B. post-traumatic stress disorder (PTSD). This is because the patient experienced event-related fear, insomnia, and repeated recall after a traumatic event (the death of her husband), which are typical of PTSD.

Therefore, the correct answer is the B. Posttraumatic stress disorder.

According to the description, the 51-year-old woman was afraid to enter her bedroom after witnessing her husband being crushed by a car. This symptom appears in almost 2 weeks. Combined with this information, the most likely diagnosis is:

B Post-traumatic stress disorder (PTSD)

The typical symptoms of PTSD include:

-Repeated traumatic events (such as repeated recall or nightmares)

-Avoid traumatic things or situations (such as not into the bedroom)

-Increased alertness (e. g., insomnia and fear)

The symptoms usually begin to appear within a period after experiencing a traumatic event and lasts for more than 1 month. If the symptoms appear within 4 weeks of the traumatic event and last less than 4 weeks, it is more likely to be an acute stress disorder. However, as symptoms started to appear within almost 2 weeks and the 6 weeks the patient had been experiencing a traumatic event.

67. (Type A2 question)

Male, 25 years old, with headache for four months. Exgravuated for 3 weeks, jet vomiting, P50 times / min, blood pressure 160 / 95mmHg, clear mind, without bilateral optic nerve papillary edema, how should be handled

A. Chest X-ray + analgesics

B. ECG + analgesics

C. Cranial B super + mannitol

D. Cranial brain CT / MRI + mannitol

E. Lumbar puncture + mannitol

Refer to the answer: D. Your answer:

Graphic analysis: [analysis] Young male, headache for four months, aggravated for 3 weeks, jet vomiting (manifestation of increased intracranial pressure), P50 times / minute (slow pulse, normal value of 60~100 times / minute), blood pressure of 160 / 95mmHg (increased blood pressure, normal value of 139~90 / 89~60mmHg), clear mind, no bilateral optic nerve papillary edema (central retinal vein is not compressed), according to the patient's medical history, clinical manifestations and physical examination, the patient was first considered for the diagnosis of brain tumor. CT or MRI should be performed to confirm the diagnosis, and CT or MRI should determine the location, size, number, blood supply, and anatomical relationship with the surrounding structures. To reduce the compression of the tumor, the skull pressure should be reduced, 20% intravenous mannitol,

Therefore, the patient should be treated with the brain CT / MRI + mannitol.

[Avoid] Chest X-ray + analgesic (A error) is mainly used for the diagnosis of lung diseases. ECG + analgesic (B wrong) mainly

For the diagnosis of cardiac disease. Cranial B-ultrasound (C error) is mainly used for the diagnosis of intracranial vascular diseases. Lumbar puncture + mannitol

(E error) is mainly used for the diagnosis and treatment of central nervous system infections.

Considering that the 25-year-old man had a four-month history of headache, aggravated with jet vomiting in the past three weeks, and high blood pressure (160 / 95mmHg), but conscious without bilateral optic nerve papillary edema, the most appropriate treatment should be to detect the possible cause of these symptoms.

Option D, with a brain CT or MRI scan and given a mannitol, is the most appropriate option. This practice can help doctors assess if intracranial lesions (such as tumors, bleeding, or swelling) cause headache and vomiting, while mannitol can be used to reduce intracranial pressure and relieve symptoms. Therefore, the answer is that the D.

analyze:

The patient was a young man with headache for more than four months and aggravated in nearly three weeks, accompanied by jet vomiting, increased blood pressure and decreased heart rate. The possibility of increased intracranial pressure should be considered, and the patient was conscious and without bilateral optic nerve papillary edema. Therefore, currently the most likely diagnosis of the patient is increased intracranial pressure. Cranial imaging examination is generally preferred for this disease to determine whether there are intracranial space-occupying lesions. In order to clarify the diagnosis and relieve intracranial hypertension, the patient should undergo craniocerebral CT / MRI + mannitol.

Option A chest X-ray + analgesic chest X-ray is mainly used for the diagnosis of chest trauma, lung and pleural diseases, but is unrelated to the patient's symptoms, so it is not selected.

Option B electrocardiogram + analgesics, electrocardiogram is mainly used for the diagnosis of diseases in the cardiac circulatory system, which is inconsistent with the meaning of the question.

Option C craniocerebral B ultrasound + mannitol in craniocerebral B ultrasound is mainly used for neonatal craniocerebral disease screening. This patient is a young man, so craniocerebral B ultrasound is not applicable.

Option E lumbar puncture + mannitol lumbar puncture is invasive examination, mainly used to check the cerebrospinal fluid, cannot be clear whether there is intracranial space occupying lesions, inconsistent with the meaning of the question.

In conclusion, the correct answer is: D. Brain CT / MRI + mannitol.

According to the title description, a 25-year-old male patient had a headache for four months and aggravated for three weeks, accompanied by jet vomiting, suggesting increased intracranial pressure. The patient had a pulse of 50 beats / min, a blood pressure of 160 / 95 mmHg, conscious, and no optic nerve papilloedema, and these signs further supported the possibility of intracranial lesions. Therefore, an imaging examination is needed to clarify the diagnosis.

In the options, item D "brain CT / MRI + mannitol" is the most appropriate choice. Brain CT or MRI can help determine the causes of increased intracranial pressure, such as brain tumors, cerebral hemorrhage, or hydrocephalus. As a permeable diuretic, mannitol can effectively reduce intracranial pressure and is one of the standard measures for the treatment of increased intracranial pressure.

So the correct answer is:

F. Cranial brain CT / MRI + mannitol

68. (Type A2 question)

Male, 78 years old, because of poor sleep, extreme loss of appetite, thin obvious 2 months in hospital, patients with hypertension history more than 20 years, 2 years ago found slightly elevated blood sugar, take blood sugar, blood pressure after good control, patients autonomous speech loss after his wife no care, during the hospital, often suddenly get up at night, aimless bed, serious will yell, don't know where you are, suspicious children take their valuable items, sometimes hair spleen static sleep, remember what happened at night, patients may diagnosis is

A. deliration

And B., for Alzheimer's disease

B. vascular dementia

C. Sudden mania

D. split personality

Reference answer: A. Your answer:

Graphic resolution: [resolution] patients elderly men, because of poor sleep, appetite, thin obvious 2 months admission, patients autonomous speech, worry after his wife no care, during hospitalization, often suddenly get up at night, aimless touch bed, serious Shouting, not recognized, don't know where you are, suspicious children take their valuable items, sometimes temper, quiet sleep during the day, remember what happened at night (reduced clarity is mainly acute cognitive impairment syndrome), should be diagnosed as false (A).

[Fault avoidance] Alzheimer's disease (AD) (B error) is a neurological degenerative disease with insidious onset and progressive development. Clinically, it is characterized by generalized dementia including memory impairment, aphasia, aphasia, agpsia, impairment of visual spatial skills, executive dysfunction, and personality and behavior changes. Vascular dementia (C wrong) is a dementia caused by cerebrovascular disease. Manic episode (D) with high emotion, thinking, increased activity "three high" as clinical manifestations, can be accompanied by exaggerated ideas or delusions, impulsive behavior, etc. Schizophrenia (E wrong) many diseases in young adults, often perception, thinking, emotion, behavior and other disorders and mental activities of the incoordination, general unconscious disorders and obvious intelligence disorders.

This question describes a 78-year-old male patient with a long history of hypertension and mild elevated blood glucose, showing poor sleep, loss of appetite, wasting, sudden waking up at night, human rejection, memory loss, and mood swings. These symptoms suggest that the patient may have a cognitive dysfunction.

A. False-usually presents with acute onset, disturbance of consciousness, symptoms vary greatly throughout the day and are common in hospitalized patients.

B. Alzheimer's disease-usually slowly progressive cognitive decline characterized by memory loss but without extreme alteration of nocturnal behavior.

C. Vascular dementia- -cognitive dysfunction caused by cerebrovascular disease that may progress faster than Alzheimer's, with symptoms related to the site of brain injury.

D. Sudden mania-burst mania usually presents as hyperactivity, impulse or irritability, rather than loss of cognitive function.

E. Schizia-The main features are thought disorders, hallucinations, and delusions,

not exactly exactly with the description in this case.

Considering the patient's age, history and symptoms, false (A) is the most likely diagnosis. Because delusion can manifest as sudden changes in cognitive function, including memory problems, decreased attention, and nocturnal behavioral abnormalities, all consistent with the patient's symptoms. So the best answer is A. false.

First of all, we analyze the question stem:

The elderly male patient was admitted for 2 months due to poor sleep, extreme appetite loss, and significant wasting, and had a chronic history of hypertension and hyperglycemia. During hospitalization, patients showed symptoms of increased nocturnal activity, aimless behavior, yelling, cognitive decline, suspicion, and not remembering nocturnal events.

Next, we analyzed each option one by one:

A. delusion: delusion is an acute, reversible, generalized cognitive impairment, especially impaired attention, orientation and perception. It usually has a rapid onset, a short course of disease, rapid development, and obvious circadian rhythm changes, manifested as light day and heavy night. The symptoms (increased nighttime activity, aimless behavior, Shouting behavior, cognitive decline) were consistent with the typical manifestation of delusion.

B. Alzheimer's disease: This is a neurological degenerative disease with dull onset and slow course. It is mainly manifested as neuropsychiatric symptoms such as progressive memory impairment, cognitive dysfunction, personality change and language disorders. Although the patient's symptoms included cognitive decline, this is inconsistent with the slow progression of Alzheimer's disease, given the shorter disease course and abrupt behavioral changes.

C. Vascular dementia: This is dementia caused by cerebrovascular disease, usually manifested as fluctuating and progressive cognitive dysfunction, memory loss, aphasia, loss of use, disrecognition, etc. The patient's history and symptoms did not coincide coincide with the typical presentation of vascular dementia.

D. Sudden mania: This is usually manifested as emotional upsurge, increased activity, progressive thinking, etc. Although the patient had increased nocturnal activity and yelling, other symptoms (e. g. cognitive decline, loss of directional force) did not consistent with this diagnosis.

E. Schizophrenia: Schizophrenia is a group of chronic diseases with unknown etiology, most of which are slow or subacute onset in young adults and often manifested as syndromes with different symptoms, involving sensory perception, thinking, emotional and behavioral disorders, as well as the incoordination of mental activities. Most of the patients are young adults, and the course of the disease is usually long, which is inconsistent with the description of the problem stem.

In conclusion, combining the patient's history and clinical presentation, the most likely diagnosis is false.

Therefore, the correct answer is A. false.

According to the title, the 78-year-old male showed a range of mental and cognitive impairment, including poor sleep, extreme loss of appetite, wasting, memory loss, orientation dysfunction (not knowing where he was), and suspicion, nocturnal behavior and emotional instability. These symptoms have obvious acute and fluctuating characteristics, and patients will suddenly wake up at night and lose their temper, and then forget what happened during the night. These features are very consistent with the false clinical presentation.

Delusions (Delirium) is an acute brain disorder characterized by acute changes in attention and cognitive function, usually caused by underlying medical diseases, drugs, or other reversible factors. It is characterized by acute onset, symptom fluctuations, and nighttime symptoms, and patients may present with disorientation, confusion, hallucinations, illusions, and emotional instability.

Combining the above analysis, the most likely diagnosis is:

A. deliration

69. (Type A2 questions)

Male, 25 years old, urethral tingling pus for 3 days, physical examination: urethral opening red, yellow purulent secretions, smear see multinucleated cells, can find gram-negative diococcus, do not belong to the pathogen is

A. endotoxin

B. exotoxin

C. outer membrane protein

D. pili

E. Outer membrane LPS

collect pen

Reference answer: B. Your answer:

Graphic resolution: [analytical] young male patients, urethral tingling pus 3 days (typical clinical manifestations), physical examination see urethral opening redness, yellow purulent secretions, smear see multinuclear cells, can find gram negative double coccus (neisseria gonorrhoea mainly gram negative double cocci), combined with patient history, clinical manifestations and laboratory examination, the patient the most likely diagnosis of gonorrhea. Neisseria gonorrhoeae is the pathogen causing mucosal suppurative infection of human urogenital system, a gram-negative double coccus, the main pathogenic substances of Neisseria gonorrhoeae include: ① Invasion: Neisseria gonorrhoeae main pathogenic substances include: outer membrane protein (C pair), lipopolysaccharide (E pair), pilus (D pair), etc.② Endotoxin: Endotoxin (pair A) is a secondary pathogenic substance of Neisseria gonorrhoeae,

It is the main substance that causes the general symptoms of gonorrhea, but it does not have the main effect. Exotoxin (B wrong, correct answer for this question) is not the pathogenic substance of Neisseria gonorrhea.

This question describes a 25-year-old male patient with urethral tingling and pus, physical examination revealed redness and yellow purulent discharge, smear showing multinucleated cells and gram-negative diococci. These symptoms and signs suggest the possibility of a typical STI, particularly the gonorrhea. Gonorrhoea is caused by Neisseria gonorrhoeae (Neisseria gonorrhoeae), a Gram-negative double-coccus bacterium.

The exam questions ask which item is not causative, and we need to identify which factors are not generally considered components of N. Neseria gonorrhoeae. The options are:

-A. endotoxin

-B. exotoxin

-C. Outer membrane proteins

-D. pili

-E. Outer membrane LPS

Among these factors, endotoxin (option A) and outer membrane lipopolysaccharide (option E) are typical components of gram-negative bacteria that are essential for bacterial pathogenicity. Outer membrane proteins (option C) and pili (option D) also play an important role in bacterial attachment and invasion of host cells.

However, exotoxins (option B) are usually produced by certain bacteria to directly damage proteins from host cells, while N. Neseria gonorrhoeae does not produce exotoxins as its main pathogenic mechanism. Thus, the most least likely to belong to the Neisseria gonorrhoeae pathogen is * * B. exotoxin * *.

analyze:

In a young male patient, urethral tingling and pus for 3 days, physical examination, yellow purulent discharge, multinuclear cells in the smear, gram-negative dicoccus could be found. According to the patient's clinical manifestations and laboratory examination, the patient could be diagnosed as gonorrhea. The pathogenic bacteria of gonorrhea is neisseria gonorrhoeae, its pathogenic substances mainly include on the pilus, the adhesion factor, lipid oligosaccharide, outer membrane protein and IgA 1 protease, etc., can make N. gonorrhoeae adhere to the human urogenital tract mucosa, and settlement on the mucous membrane, reproduction, lead to local acute inflammation, suppuration and infection symptoms. Exotoxins are mainly produced by gram-positive bacteria and some gram-negative bacteria, and secreted into a class of toxic proteins of bacteria. Neisseria gonorrhoeae belongs to gram-negative bacteria, but it does not produce exotoxins.

Option A endotoxin is A component of the cell wall of gram-negative bacteria, also known as lipopolysaccharide, is only released after bacterial death lysis, its toxic effect is relatively weak, can cause fever, microcirculation disorders, endotoxin shock and disseminated intravascular coagulation, etc., meet the pathogenic substances of neisseria gonorrhoeae, so do not choose.

Option C outer membrane protein is one of the main pathogenic substances of Neisseria gonorrhoeae, which can make neisseria gonorrhoeae easy to adhere to the mucosa of the human urogenital tract, which is not consistent with the idea.

Option D can adhesion Neisseria gonorrhoeae to tissues such as urethral mucosa, which is the pathogenic substance of Neisseria gonorrhoeae, so it is excluded.

Option E Outer membrane lipopolysaccharide can make one of the main pathogenic substances of Neisseria gonorrhoeae, with endotoxin activity, can make the body heat and lethal toxicity, do not conform to the meaning of the question.

In conclusion, the correct answer is: B. exotoxin.

This case describes a typical symptom of urethral infection in a young man, a Neisseria gonorrhoeae infection based on the described smear findings (Neisseria gonorrhoeae). Neisseria gonorrhoeae is a Gram-negative double-coccus, and common pathogenic factors include endotoxin (outer membrane lipopolysaccharide), exotoxin, outer membrane proteins, and pili.

Let's analyze the options one by one:

-A. * * Endotoxin * *: Endotoxin usually refers to lipopolysaccharide (LPS) in the cell wall of gram-negative bacteria, and is one of the pathogenic factors of Neisseria gonorrhoeae.

-B. * * * *: Exotoxins are toxic proteins or enzymes secreted by bacteria. Although Neisseria gonorrhoeae is mainly dependent on other pathogenic factors, there are also reports that they may secrete some exotoxins.

-C. * * Outer membrane protein * *: Outer membrane proteins are components of the cell wall of Gram-negative bacteria and are involved in pathogenesis.

-D. * * hairs * *: The hairs contribute to bacteria adhere to host cells and are an important pathogenic factor in Neisseria gonorrhoeae.

-E. * * Outer membrane lipopolysaccharide * *: Outer membrane lipopolysaccharide (LPS) is an important component of gram-negative bacteria, also known as endotoxin, which is one of the pathogenic factors.

In conclusion, among the options that do not belong to the pathogenic factors are * * exotoxin * *. Although certain Gram-negative bacteria can produce exotoxins, the main pathogenic mechanism of Neisseria gonorrhoeae does not include exotoxins.

So the correct answer is:

* * B. exotoxin * *

70. (Type A2 question)

The primipara is 28 years old, 40 weeks pregnant, estimated fetus 3000g, pelvis measurement is normal, the first birth is smooth, uterine opening for 1 hour, S= + 2 contractions 4 times / 10 points, amniotic fluid, good fetal heart mutation, occasionally early deceleration, should be treated

A. Closely observe the fetal head decline

B. lateral perineal incision plus abdominal pressure

C. performed by immediate cesarean section

D. Lateral cut fetal head suction

E. Clip midwifery

Reference answer: A. Your answer:

Graphic analysis: [analysis] Young primipara, 40 weeks gestation (delivery during 37 weeks gestation to less than 42 foot weeks, For full-term production), Pelpelvic measurements were normal, The first production is smooth, 1 hour of uterine opening (the first stage of labor is from regular contractions to uterine opening (10cm), Generally takes 11 to 12 hours), S= + 2 (the fetal head has dropped to 2cm below the sciatic spine plane), 4 contractions / 10 points (normal 2-3 times / min), Clear the amniotic fluid, Fetal heart rate mutation is good, Occasionally, early deceleration (suggesting possible fetal hypoxia), in summary, The most likely diagnosis in this patient is the normal first stage of labor, Fetal weight: 3000g (normal: 2800 to 4000 g), Therefore, the treatment that should be taken is to closely observe the decline of the fetal head (A pair), Vaginal midwifery is not required temporarily (DE error).

[Avoid wrong] immediate cesarean section (C wrong) is suitable for fetal distress, head basin and other conditions. Perperineal incision plus abdominal pressure (B wrong)

Suitable for the second extension of labor, fetal distress and other conditions.

This problem describes a primipara with 40 weeks of gestation, an estimated weight of 3000 grams, normal pelvis, smooth pelvic measurement, rapid uterine opening (1 hour), fetal head drop to S= + 2 position, frequency of contractions 4 times every 10 minutes, amniotic fluid, good fetal heart rate variation, and occasionally

early deceleration. Management in this case should focus on continuous monitoring and assessment of maternal and fetal status, as there is currently no indication of the need for urgent intervention.

According to the description, the course of labor is normal, the frequency of contractions is normal, the fetal heart rate is well variable, only occasionally early deceleration, which is not usually an emergency, especially in the case of good fetal head decline and amniotic fluid serum. Early deceleration is usually a physiological reaction to the compression of the fetal head during the descending birth canal, and can be regarded as part of the normal labor process if there are no other adverse signs.

Therefore, the best answer is that A. Closely observe the fetal head decline. This means continued monitoring of progression of labor, fetal heart rate variation and any other signs that may indicate the need for intervention. Other options, such as lateral incision, cesarean section or use of forceps or aspirator, are usually considered in the presence of severe or persistent fetal distress, stalled labour, or pelvic-fetal unweighing. In this case, there is no indication that these more invasive interventions are needed.

analyze:

Primigravida women, 40 weeks gestation, pelvis is normal, normal fetal size, uterine mouth is open, S= + 2, head has cohesion, labor in active period, contractions interval too long, amniotic fluid, fetal heart mutation, occasionally early deceleration, now by strengthening contractions, closely observe fetal head drop to determine whether the fetus can through normal childbirth.

Option B perineal lateral cutting plus abdominal pressure, option D lateral cutting fetal head suction, option E forceps midwifery, which is the midwifery mode used when the fetal head has reached + 3 level and the signs of fetal distress appear. At this time, the fetus has not yet reached this position, so it is excluded.

The maternal fetal heart mutation is good, occasionally early deceleration, the labor process is in the active period, the labor process can be closely observed, there is no indication for cesarean section, so option C immediate cesarean section, is not the most appropriate treatment at present, so it is not selected.

To sum up, the correct answer is: A. Closely observe the decline of the fetal head.

For this 28-year-old first mother, 40 weeks of gestation, the fetus is estimated

3000g, the pelvis measurement is normal, the first stage of labor is smooth, uterine opening for 1 hour, the fetal head has dropped to S= + 2, the frequency of contractions is 4 times / 10 minutes, amniotic fluid, fetal heart variability is good, occasionally early deceleration, should be taken is:

A. Closely observe the fetal head decline

** analyze **:

-primiparous labor is usually long, and the fetal heart rate is normal and the head has decreased to S= + 2, indicating that the fetus is in good condition and the labor is progressing smoothly.

-The traction frequency was 4 times / 10 min, which was in the normal range and amniotic fluid indicated no manifestation of fetal distress.

-Early deceleration is generally caused by the fetal head compression during contractions, not a manifestation of fetal hypoxia, and good fetal heart variability, which further indicates that the fetus is in good condition.

-In this case, close observation of the fetal head drop is the most appropriate choice to ensure the smooth progress of labor.

Therefore, the best answer is: * * A. Closely observe the decline of the fetal head * *

71. (Type A2 questions)

Male, 14 years old, with cold and fever for 10 days, with a temperature as high as 39.8℃. After 3 days of hospital treatment, he made less speech without obvious inducement, answered less questions, and defecate in bed. Physical examination: T37.9℃, P95 times / min, R20 times / min, BP120 / 72mmHg. The following diagnoses are most likely to be the following

A. Viral encephalitis

B. Anxiety disorder

C. Somatic infectious psychiatric disorder

D. split personality

E. Depressive disorder

Reference answer: C Your answer:

[Analysis] The patient had a cold and fever for 10 days, the body temperature was as high as 39.8℃, after hospital treatment, the body temperature was diagnosed as physical infection, that is, a physical infectious disease, less move less ask, stool in bed (will decreased), so it is most likely to be diagnosed as physical infectious mental disorder.

[Avoid] viral encephalitis is caused by virus brain substance inflammation, can appear fever, headache, vomiting, convulsions, serious conscious disorder, mental abnormalities, but the onset is sudden, generally no precursor symptoms, mental symptoms often appear on the basis of virus infection, so the patient can not be diagnosed with viral encephalitis (wrong). Anxiety disorder is a group of mental disorders with anxiety as the main performance. Patients often have unexplained fear and nervousness, which is inconsistent with this case, so it is not considered (B wrong). Schizophrenia often starts in adulthood, has the mental activity divorced from reality and various "split" symptoms, manifested as delusion, illusion, thinking and speech confusion and introversion, generally unconscious disorders and obvious intelligent disorders, the course of the disease is delayed. The patient had no typical symptoms of schizophrenia, so he could not be diagnosed as schizophrenia (D wrong). Depression disorder often shows low mood, anhedonia, slow thinking, reduced activity, psychomotor retardation or agitation, remorse, suicide, and behavior, early awakening, loss of appetite, weight loss, sexual desire, depression mood morning heavy late light rhythm change, etc., so the patient can not be diagnosed as depression disorder (E wrong).

This case describes a 14-year-old boy who developed symptoms of less speech, less answers, and taking a stool in bed after a cold and fever. With a slightly higher body temperature and a slightly faster heart rate, but the blood pressure was normal. Based on these symptoms, we can make some differential diagnoses:

A. * * Viral encephalitis * *: It usually causes fever, headache, and neurological symptoms, such as impaired consciousness, psychiatric symptoms, or tics. The patient had a history of fever and neurological symptoms, which may be an option.

B. * * Anxiety disorder * *: Usually manifested as persistent anxiety and worry, accompanied by physical symptoms, such as palpitation, sweating, etc. No typical

symptoms of anxiety were described in this case.

C. * * Somatic infectious mental disorder * *: refers to the mental disorder occurring on the basis of physical disease (such as infection), usually closely related to the underlying disease. This may be an important consideration considering the child's prior high fever and current neurological manifestations.

D. * * Schizia * *: It is a serious mental disorder characterized by persistent disorders of thinking, perception, and emotion. Typical schizophrenia symptoms were absent in this case.

E. * * Depression disorder * *: Mainly persistent sadness, despair, or loss of interest, but the child's symptoms do not completely coincide with typical depression.

Given the complex manifestations of a cold, including high fever, reduced speech and response, and behavioral changes without significant triggers (e. g., stool in bed), * * Physical infectious mental disorder * * (option C) may be the most likely diagnosis. This condition may be due to changes in mental state due to the physical response following infection. However, a diagnosis often requires further medical evaluation, including possible brain imaging tests, blood and cerebrospinal fluid tests. Thus, based on the description of this case, the most likely diagnosis is C. somatoinfectious psychiatric disorder.

This is a medical diagnostic problem, and we need to infer the most likely diagnosis based on the patient's history, symptoms, signs, and possible medical knowledge.

Understanding background information: a 14-year-old boy with a cold and fever for 10 days and a body temperature as high as 39.8℃. After 3 days of hospital treatment, the temperature recovered, after discharge without obvious inducement speech less, slow reaction, and even in bed stool. Physical examination showed a slightly higher temperature, normal pulse and breathing, and blood pressure within the normal range.

At the heart of the problem: We need to infer the most likely diagnosis based on this information.

Analysis options:

A. Viral encephalitis: Although the patient has a history of viral infection, viral encephalitis is usually accompanied by obvious neurological symptoms, such as headache, nausea, vomiting, and meningeal irritation, which are not mentioned in the

problem stem.

B. Anxiety disorder: Anxiety disorders are mainly manifested as excessive worry, fear, tension and restlessness, which are not mentioned in the problem stem.

C. Somatic infectious mental disorders: This is a mental disorder caused by a physical infection, usually occurring after an infection. Given that the patient had a clear history of infection (cold and fever) and developed psychiatric symptoms after infection (less speech, less answers, less stool in bed), this option fits the patient's medical history and symptoms.

D. Schizophrenia: schizophrenia is mainly manifested as hallucinations, delusions, mental confusion, behavior and thought abnormalities, and these symptoms are not explicitly mentioned in the question stem.

E. Depression disorder: Depression disorder is mainly manifested as low mood, loss of interest, fatigue, sleep disorders, etc., and these symptoms are not explicitly mentioned in the problem stem.

In conclusion, considering that the patient had a clear history of infection and developed psychiatric symptoms after the infection, the most likely diagnosis was a somatic infectious psychiatric disorder.

Therefore, the correct answer is C. somatic infectious psychiatric disorders.

According to the information provided by the topic, the 14-year-old boy, 10 days after a cold and fever, but had a significant change in mental state, such as less speech, less questions, and stool in bed. Physical examination showed a slight increase in body temperature with pulse, breath and blood pressure within normal limits.

Combined, the boy is likely that an acute viral infection (such as a cold) caused the effects of the central nervous system, manifested by a change in mental state. The most likely diagnosis is a somatic infectious psychiatric disorder.

So the best answer is:

F. Somatic infectious psychiatric disorder

72. (Type A2 questions)

Female, 38 years old, pregnant 18 weeks old, previously a mental retardation, later due to heart disease, the current examination is

A. NT check up

B. Early Tang sieve

C. amniocentesis

D. Ultrasound screening

E. Non-invasive DNA

Reference answer: C Your answer:

[Analysis] Because the patient has previously given birth to a child with low intelligence, this pregnancy belongs to the elderly pregnant woman (> 35 years old), so this pregnancy is a high-risk child, and amniocentesis can be selected for prenatal diagnosis. Amniocentesis is the prenatal diagnosis, which is the gold standard to determine whether the fetal chromosome is abnormal. All pregnant women at high risk of prenatal screening are recommended to diagnose whether the fetal chromosome is abnormal through this examination.

[Wrong avoidance] NT examination (A error) is A kind of examination to check the thickness of the zona pellucidia of the fetal neck through ultrasound, which is usually a routine examination means to exclude fetal malformation before delivery. Early Tang screening (B error) is A detection method to calculate the concentration of dimeric glycoprotein hormone, alpha-fetoprotein, free estriol and inhibin A in early pregnancy, combined with the expected date of the pregnant woman, weight, age and gestational age during blood collection. Ultrasound (D error) is only a screening test to determine fetal chromosomal abnormalities. Non-invasive DNA (E wrong) is extract pregnant women of venous blood, detect pregnant women plasma fetal source of free DNA fragments, screening of common anechromosome abnormalities method, but the detection range is narrow, so there may be other fetal chromosome or genetic disease risk of pregnant women, fetal structure, pregnant women itself has special circumstances such as chromosome abnormalities, should not use this method. It is currently used for secondary screening in high-risk groups.

In this case, it is important to choose the most appropriate test, given that the woman was 18 weeks gestation, had a history of a mentally retarded child, and the condition where the child had died of heart disease. NT for option A (the posterior cervical transparent layer) is usually performed at 11-13 weeks of gestation and has

missed this time window. The early Tang screen of option B is usually performed in the first or early second semester of gestation and is also later for an 18-week gestation.

Given the mother's background and the need to detail the fetus, the most appropriate option is C amniocentesis. Amniocentesis can be performed in the second semester of pregnancy and is used for detecting chromosomal abnormalities and genetic disorders, which are very important for pregnancies at risk of genetic disorders. Therefore, the correct answer should be the C amniocentesis.

analyze:

Patients with young women, 18 weeks gestation, had a mental retardation, after heart disease died, have adverse pregnancy history, belong to the high risk of pregnant women, pregnant again, the fetal risk of genetic diseases, for the purpose, should be a clear diagnosis, amniotic fluid puncture examination, it is a method of prenatal diagnosis, generally suitable for the second pregnancy prenatal diagnosis, under the ultrasound guide, a slender needle through the belly of pregnant women's belly, uterine wall, into the amniotic fluid cavity, extract some amniotic fluid test, to clarify whether the fetus has chromosomal abnormalities and genetic diseases, etc.

Option A: NT examination refers to the transparent layer examination of fetal neck, which is often used in prenatal screening. It can allow early diagnosis of chromosomal diseases and early detection of fetal abnormalities caused by various causes. It is generally conducted in 11 to 13 weeks + 6 days, which is inconsistent with the description of the question.

Option B: Early Tang screening is usually performed at 11 to 14 weeks of pregnancy. The risk of fetal Down syndrome was assessed by ultrasound for the thickness of the fetal neck, the maternal age and laboratory tests. But this patient was 18 weeks pregnant, so it was excluded.

Option D: Ultrasound screening can detect most of the fetal malformations, such as anencephaly, hydrocephalus, open spina bifida, limb malformation, heart malformation, etc., but can not confirm the existence of genetic diseases, so it can be excluded.

Option E: non-invasive DNA refers to the use of a new generation of DNA sequencing technology, the maternal peripheral plasma free DNA fragments, and get the fetal genetic information, to detect whether the fetus has chromosome abnormalities detection means, but only screen the fetal chromosome aneuploidy, and

its accuracy is lower than amniotic fluid puncture, so not as the first choice.

In conclusion, the correct answer is: C. Amniocentesis.

For a 38-year-old pregnant woman, at 18 weeks of gestation, previously had a mental retardation child, and later died of heart disease, the current tests required are:

F. amniocentesis

analyze:

-NT tests (the transparency of the neck) and early Tang screening (Down syndrome screening) are usually performed at 11-14 weeks of gestation and have missed the optimal time window.

-Ultrasound screening can be used to assess structural abnormalities in the fetus, but does not directly detect chromosomal abnormalities.

-Non-invasive DNA testing is a highly sensitive screening method, but amniocentesis is the gold standard for confirming the diagnosis of fetal chromosomal abnormalities.

Due to the older age and adverse fertility history, amniocentesis can directly detect the chromosomes of the fetus and provide the most conclusive diagnostic information, which is therefore the best choice.

73. (Type A2 question)

Male, 20 years old, college student, let him introduce himself to say, "I am 20 years old this year, today the sun came out, it is A good day, red peach A is A virus, my girlfriend", this belongs to

A. flight of ideas

B. loosening of association

C. split of thought

D. incoherence of thought

E. split personality

Reference answer: C Your answer:

[Analysis] The patient introduced himself said "I am 20 years old this year, today the sun came out, is A good day, peach A is A virus, my girlfriend", this belongs to the thinking breakdown (C to), the performance of the concept of the association fracture, the meaning of each other, into the accumulation of words.

[Avoid mistakes] thinking patients feel particularly flexible, like A machine with "lubricating oil" (A wrong). As a particularly talkative, eloquent, eloquent. The subject of speech often quickly shifts from one concept to another, which can be suddenly changed due to environmental changes. Disthinking patients have loose association, loose content and lack of theme (B wrong). Patients with incoherent thinking talk so much so that others do not understand the topic that the patient wants to explain (D wrong). Schizophrenia is mainly manifested as delusions, hallucinations, speech and behavior disorders, generally no mental retardation (E error).

This case describes a 20-year-old college student who introduces himself to topics jumping quickly, lacking logic and coherence between them. This manifestation belongs to the term "mental incoherence". Incoherence refers to the lack of logic and structure in individual language and thinking expression, and the lack of logical connection between expressed ideas, which makes discourse difficult to understand.

Therefore, the most appropriate answer is: D. Incoherence

This is a question about the diagnosis of mental illness. We need to first understand the symptoms described in the problem stem, and then combined with the relevant knowledge of mental illness for reasoning and judgment.

Understanding background information: young male patients, leaping and incoherent, lack of logic.

Understand the core of the problem: We need to judge the type of mental disorder that patients show based on their self-introduction.

Next, we performed a detailed analysis for each option:

Option A (thinking running): usually manifested as particularly talkative, eloquent, feel the brain is particularly flexible, just like A machine with "lubricating oil" is difficult to stop, but the speech content is mostly exaggerated delusion, which is inconsistent with this case.

Option B (loose thinking): In the conversation, the content is loose, lack of

theme, and the topic transformation lacks the necessary connection, but the structure and grammar of each sentence itself are correct, but there is a lack of internal logical relationship between sentences and sentences, which is not completely consistent with the patient's self-introduction.

Option C (thinking breakdown): it shows that the thinking and association process is broken with clear consciousness, the content of the conversation lacks the coherence and logic in the internal sense, and is close to the symptoms described by the subject stem, but it still needs to be compared with other options.

"D" option (incoherent thinking): in severe schizophrenia patients, the speech is fragmented, the sentences are irrelevant, no logical connection, and inconsistent with the problem.

Option E (Schizophrenia): This is not a specific thinking disorder, but a broader classification of the disease, not suitable for the specific analysis here.

According to the comprehensive analysis, the patient's self-introduction content is leaping and incoherent, but each sentence itself has no error in grammar, but the lack of logical connection between sentences, which is more in line with the characteristics of broken thinking. Although loose thinking is also manifested as loose content, there is usually a certain logical connection between each sentence, and in this case, the patient's speech almost completely lacks this connection.

Therefore, the answer is C. mind breakdown.

According to the description, the patient's self-introduction contains a lack of logical connection between irrelevant information and statements, such as "I am 20 years old, today the sun comes out, it is A good day, peach A is A virus, my girlfriend". The characteristic of thinking in this case is mental breakdown.

The breakdown of thinking refers to the lack of coherence and logic in the thinking process of patients, which shows that there is no logical relationship between the sentences when speaking, and the sentences are inconsistent and difficult to understand, which is one of the typical symptoms of schizophrenia.

So the correct answer is:

F. split of thought

74. (Type A2 question)

Female, 32 years old, married, G1P0, increased menstruation for 2 years, with dizziness and fatigue for 1 month, previous regular menstruation. Gynecological examination: uterine enlargement such as the size of 3 months of pregnancy, the anterior uterine wall touched a diameter of about 8cm protrusion, texture is hard, the best treatment

A. myomectomy

B. uterine artery embolization

C. Subtotal hysterectomy

D. medication

E. High-energy focused ultrasound therapy

Reference answer: A. Your answer:

Graphic resolution: [resolution] the patients for women of childbearing age, after 2 years, with dizziness fatigue 1 month (have caused anemia symptoms), women: uterine enlargement such as pregnancy 3 months size, the anterior wall touch diameter of about 8cm protrusion (fibroids is located in the subserosa, protrusion), texture is hard, should first consider uterine fibroids. The patient has increased volume, dizziness and other symptoms, indicating that has caused anemia. The patient is still young and needs to preserve the reproductive function, so the best management method is uterine myomectomy.

[Error avoidance] Uterine artery embolization (B error): reduce the blood supply of fibroids by blocking the uterine artery and its branches, thus delaying

Growth of fibroids, relieve symptoms. But this method may cause ovarian hypofunction and increase the risk of potential pregnancy complications,

It is generally not recommended for women with fertility requirements. Subtotal hysterectomy (C error) is suitable for increased menstrual volume and prolonged menstruation

Long and uterine-related bleeding symptoms are not responding to conservative treatment, not used in women with fertility requirements. Drug therapy (D wrong) is suitable for those with mild symptoms, near-menopausal age or general conditions unsuitable for surgery. High-energy focused ultrasound therapy (E error) is not a mainstream treatment method, mainly suitable for patients who are not suitable for

surgery.

This case describes a 32-year-old woman with a first married pregnancy (G1P0) with a two-year history of increased menstruation and a month of dizziness and fatigue. Gynecological examination revealed an enlarged uterus, equivalent to the size of 3 months of gestation, and touched a rigid protrusion of about 8cm in the anterior wall in diameter of the uterus. These symptoms and signs are most consistent with the diagnosis of uterine fibroids.

The option analysis is performed as follows:

A. Myomectomy (Myomectomy): indicated for patients wishing to preserve fertility or the uterus, especially for large single fibroids or fibroids located on the surface of the uterine wall.

B. Uterine artery embolization (Uterine Artery Embolization, UAE): Suitable for patients who do not want to be treated by surgery, reducing fibroids size and symptoms, but not all patients are suitable for this procedure.

C. Subtotal hysterectomy (Subtotal Hysterectomy): Removing the uterine body but preserving the cervix for patients who do not respond to conservative treatment or have multiple fibroids. This is a more aggressive treatment modality and is usually considered where other treatments fail or are not applicable.

D. Medical therapy: usually used in patients with mild symptoms or as an adjunct before surgery to reduce the size of fibroids.

E. High-energy focused ultrasound therapy (High-intensity Focused Ultrasound, HIFU): It is a non-invasive treatment method, suitable for a specific patient population.

Given the patient's symptoms, age, and the need for fertility preservation, the most appropriate option seems to be an A. myomectomy. This method can remove fibroids and relieve symptoms while preserving the uterus and patient fertility. Therefore, I would choose A. myomectomy as the best option.

First, we need to know the specific condition of the patient. The patient is a 32-year-old woman, married, pregnant at 1 and 0, increased menstruation for 2 years, with dizziness and fatigue symptoms for 1 month. Gynecological examination showed that the uterus was enlarged like the size of 3 months of gestation, and there was an induration of about 8cm in diameter in the anterior wall of the uterus. These symptoms and signs strongly suggest that the patient may have had uterine fibroids.

Next, we analyzed for each option:

A. Myomectomy: This method is suitable for patients who want to preserve fertility, especially those who are young and require uterine preservation. This option was appropriate given the patient's age and unfertile status.

B. Uterine artery embolization: Although this is also a treatment, it is usually used to reduce the volume of the fibroids or to relieve the symptoms, and does not directly remove the fibroids.

C. Subtotal hysterectomy: This method is usually suitable for patients who do not require fertility preservation. As the patient is not yet fertile, this option may not be the best option.

D. Medical therapy: Medical therapy is usually used to provide symptom relief or as preoperative preparation, but not as the preferred method for long-term treatment.

E. High-energy focused ultrasound therapy: Although this is a non-invasive treatment, it is primarily used to relieve symptoms rather than direct removal of fibroids.

Considering the above analysis, considering that the patient is young and not yet fertile, the best treatment is to choose a way that can preserve the uterus without affecting future fertility. Therefore, myomectomy is the best choice.

So, the correct answer is A. myomectomy.

According to the case data provided, the 32-year-old woman had a 2-year history of increased menstrual volume with 1 month of dizziness and fatigue. Gynecological examination showed an enlarged uterus to the size of the equivalent of 3 months of pregnancy, and touched a protrusion of about 8cm in diameter in the anterior uterine wall, with a hard texture. Considering the patient's age, symptoms, and uterine enlargement, the best treatment plan should effectively relieve the symptoms and improve the patient's quality of life.

When managing uterine fibroids, the following is a brief analysis of each option:

A. * * Myomectomy * *: Suitable for patients who want to preserve the uterus

and need fertility. It is effective in removing fibroids and relieving symptoms, but it may recur after surgery.

B. * * Uterine artery embolization * *: Reducing fibroids by blocking the blood supply to fibroids is suitable for patients who want to preserve the uterus, but there may be side effects such as chronic pain and infection.

C. * * Subtotal hysterectomy * *: For patients who no longer have fertility requirements. Surgery can completely solve the symptoms and prevent recurrence.

D. * * medication * *: Usually for patients with mild symptoms, or as a temporary measure, with limited effect and may require long-term use.

E. * * High-energy focused ultrasound therapy * *: A non-invasive treatment, but large fibroids are probably not as effective as surgery.

In this case, the patient had significant symptoms and the uterus was significantly enlarged, considering that the patient might also wish to preserve the uterus to maintain the fertility function, * * myomectomy (option A) * * was the more appropriate choice.

So the best answer is:

A. * * Myomectomy * *

75. (Type A2 question)

A 48-year-old male was struck to the right temporal occipital for 12 hours, at that time, loss of consciousness, about 3 minutes later, without nausea and vomiting. Physical examination: clear, fluent speech, bilateral pupils and other large eyes, sensitive to light reflex, muscle strength of the limbs: a pair of convex lens can be seen in the right occipital and temporal areas), the most likely diagnosis is

A. subdural collection of fluid

B. epidural hematoma

C. Subdural hematoma

D. epidural abscess

E. Epidural effusion

Reference answer: B. Your answer:

[Analysis] The patient lost consciousness after head trauma, and recovered after about 3 minutes. Brain CT examination: a pair of convex mirrors in the right occipitotemporal part, which could be confirmed as epidural hematoma. Epidural hematoma is characterized by a double convex mirror or a high bow density shadow between the inner skull plate and the dura mater.

[Avoid mistakes] Subdural hematoma (C wrong), often have a history of trauma and the medical history and signs of primary nervous system damage. The general performance is the disorder of consciousness and progressive aggravation, hemiplegia, headache, nausea, vomiting and other symptoms after the injury. On CT, there was a crescent density, confounding density or equal density between the brain surface and the skull. Epidural abscess (D wrong) is rare, often with a history of chronic suppurative lesions such as skull osteomyelitis and otitis media. Epidural and epidural subdural CT should be low density (AE error).

This case describes the symptoms of a 48-year-old man after a stick shot on the right temporoccipital part of the head. The patient lost consciousness immediately after the injury and consciousness consciousness about 3 minutes without nausea and vomiting. Physical examination showed that the patient had clear consciousness, fluent speech, normal pupil to light reflex, and normal muscle strength in the limbs. Imaging examination revealed high-density shadows in the right occipitotemporal region, similar in shape to a double convex lens.

According to the description, the most likely diagnosis was a B. epidural hematoma (epidural hematoma). Epidural hematoma usually occurs after head trauma, especially when skull fractures cause rupture of the epidural artery. The typical CT shows a specular or double-convex, high-density shadow, which is consistent with the imaging findings described in the title. In addition, patients lose consciousness for a short time after the injury, and then regain consciousness (the so-called "awake space"), which is also a typical clinical feature of epidural hematoma.

analyze:

The patient had a history of right temporal occipital trauma, with loss of consciousness and later consciousness suggestive of concussion. Physical examination showed clear mind, fluent speech, bilateral pupils and other large equal eyes, sensitive to light reflex, and normal muscle strength of the limbs, indicating that the patient did not have hemiplegia or cranial nerve injury. The CT film showed a pair of convex lens in the right occipitotemporal region, indicating the presence of hematoma. Based on the patient's history, clinical manifestations and imaging examination, the most likely diagnosis was a right epidural hematoma.

Option A subdural effusion, also known as dural water tumor, is mostly the accumulation of cerebrospinal fluid in the subdural space after trauma, and CT shows A crescent-shaped low-density shadow, which is inconsistent with the CT image of the patient, so it is not selected.

Option C subdural hematoma is a hematoma located between the dura and the arachnoid, mostly caused by trauma and occurs in the superior temporoparietal region of the cerebellum. CT shows high density, mixed density or equal density shadow of the brain surface. The CT image of this patient shows high density shadow of double convex lens, which is inconsistent with the question.

Option D The CT of epidural abscess showed low density shadow between the internal skull plate and the dura mater. The enhanced scan abscess wall could be strengthened, and the wall was equal density or slightly high density, and the center of the abscess is low density, which was not consistent with the CT image of the patient, so it was excluded.

Option E epidural fluid is mostly the accumulation of cerebrospinal fluid in the subdural space after trauma, and the CT showed a low-density crescent shadow, which was inconsistent with the CT image of the patient and can be excluded.

To sum up, the correct answer is: B. epidural hematoma.

According to the title description, the patient had a transient loss of consciousness and recovery in the right temporal occipitotemporal region after a stick blow, and the CT film showed a pair of convex lens in the right occipitotemporal side. These features suggest the possibility of an epidural hematoma.

The characteristics of the epidural hematoma include:

1. History of head trauma.

2. Recovery after a brief loss of consciousness (also known as the "waking period").

3. Typical double convex mirror shape on CT image.

Based on the above analysis, the most likely diagnosis is:

B epidural hematoma

76. (Type A2 question)

At term, Zeng intrauterine distress, 3 degrees of amniotic fluid, forceps midwifery, muscle tone decreased after birth, no spontaneous breathing, optimal treatment

A. Mechanical ventilation for endotracheal intubation

B. external chest compression

C. Tracheal intubation and meconium aspiration

D. oxygen uptake

E. Keep warm, position your body, clean up the respiratory tract, and induce spontaneous breathing

Reference answer: C Your answer:

[Analysis] The child had intrauterine distress, amniotic fluid, and forceps midwifery, the possibility of amniotic fluid inhalation syndrome should be considered, for the child with meconium pollution, should be endotracheal intubation and meconium suction.

[Error avoidance] Mechanical ventilation of endotracheal intubation (A error) is mainly used for children who have no spontaneous breathing after birth, and the children should be treated with smooth airway first. Chest compressions (B errors) are mainly used in children with cardiac and respiratory arrest. Oxygen inhalation (D is wrong) is a normal treatment only. Keeping warm, positioning, cleaning the respiratory tract, and inducing spontaneous breathing (E wrong): it is not the best plan to rescue children with amniotic fluid inhalation syndrome.

In this case, we had a full-term child who experienced intrauterine distress, sheep water contamination degree of 3 degrees, and midwifery through forceps. After birth, the baby developed symptoms of decreased muscle tone and no spontaneous breathing. Given these conditions, optimal management should be designed to immediately stabilize the infant's breathing status and address any problems that may hinder breathing.

Option A (mechanical ventilation for endotracheal intubation), although important for infants requiring immediate respiratory support, it may not be optimal to proceed directly without a first attempt to clear the airway. Option B (chest compressions) is mainly used for cardiac arrest and is not applicable in this case. Option C (endotracheal intubation and meconium suction) considers the importance of the need to clean up the respiratory tract, especially when there is sheep water contamination. Option D (oxygen) provides the necessary oxygen but does not address airway obstruction. Option E (keep warm, positioned, clear the airways, and induce spontaneous breathing) provides a series of steps designed to stabilize the baby's state and promote recovery of spontaneous breathing.

Given this information, the best option should be * * C. endotracheal intubation and suction of meconium * *, as this can both clear the obstructed respiratory tract and provide the necessary respiratory support for the baby. This approach combines the baby's symptoms and background conditions, and aims to address the urgent problem of no spontaneous breathing.

analyze:

Full-term infants, history of intrauterine distress, amniotic fluid contamination, history of forceps midwifery, decreased muscle tone and no spontaneous breathing, combined with medical history and clinical manifestations, the child should be diagnosed with neonatal asphyxia. The recovery uses the internationally recognized ABCDE resuscitation protocol: A (airway): clearing the airway; B (breathing): establish respiration; C (circulation): establish normal circulation; D (drugs): drug treatment; E (evaluation): evaluation. The first three are the most important, among which A is fundamental and B is the key. The purpose of resuscitation is to establish respiratory function. Therefore, the A (airway) step should be conducted first, that is, keep warm, set the body position, clean the respiratory tract, and induce spontaneous breathing.

Option A mechanical ventilation for endotracheal intubation and option B chest

compressions, step C in ABCDE resuscitation protocol, is the heart rate is still <60 times / minutes after 30 seconds of full positive pressure ventilation, and chest compressions are required, so not selected.

Option C endotracheal intubation and meconium removal were found during cleaning the respiratory tract and invitality. Although the child had a history of intrauterine distress and 3 degrees of amniotic fluid pollution, the question did not mention the child's invitality, so it was not considered.

Option D Oxygen inhalation is step B in ABCDE resuscitation protocol, which should be performed after clearing the respiratory tract or inducing poor or ineffective spontaneous breathing, so it does not consistent.

To sum up, the correct answer is: E. Keep warm, position, clean the respiratory tract, and induce spontaneous breathing.

For this case, the most important thing is the immediate management of the obstruction and hypoxia of the respiratory tract of the newborn. Intrauterine distress, three-degree contamination of amniotic fluid, forceps midwifery and other conditions suggest that newborns may have meconium aspiration syndrome, so cleaning the respiratory tract is a priority. According to the neonatal resuscitation guidelines, the most appropriate approach is:

F. Tracheal intubation and meconium aspiration

77. (Type A2 question)

Male infant, 5 months, cough for 3 days, wheezing for 1 day, physical examination: body temperature 37.8°C, P150 / min, R66 / min, mental, nose, mild cyanosis, positive, respiratory sounds and a little wet rales, experiment WBC 4.0109 / L, N0.35, L0.65. The most likely diagnosis is the

A. Respiratory syncytial virus pneumonia

B. Staphylococcus aureus pneumonia

C. S. pneumoniae pneumonia

D. Eaton agent pneumonia

E. adenovirus pneumonia

Reference answer: A. Your answer:

[Analysis] The child's body temperature was 37.8 degrees (low fever), P150 times / min, R66 times / minute, mental, nose (mild hypoxia), mild cyanosis, positive (severe hypoxia), respiratory phase and a little wet rales (pulmonary signs of respiratory syncytial viral pneumonia), laboratory tests of blood WBC 4.0109 / L (normal 4~10109 / L), N0.35 (neutrophil ratio normal value 0.5~0.7), L0.65 (normal lymphocyte ratio

2. 2~0.4), based on the clinical presentation and ancillary examination, the most likely diagnosis was respiratory syncytial viral pneumonia (pair A).

[Avoid error] Staphylococcus aureus pneumonia (B wrong) acute onset, serious disease, rapid progression, early circulatory failure; long fever time and high temperature; peripheral blood leukocyte count significantly increased, mainly neutrophils; sputum, pyuria and blood culture positive for Staphylococcus aureus. Streptococcus pneumoniae pneumonia (C fault) has predrome symptoms of upper respiratory tract infection, sudden onset, high fever, chills, muscle pain, chest pain on the affected side, increased voice tremor on the affected side; limited breathing movement on the affected side, cloudy percussion, bronchial breathing sound and wet rales on auscultation. The symptoms of mycoplasma pneumonia (D wrong) are mainly fatigue, sore throat, headache, fever, cough, loss of appetite, diarrhea, myalgia, earache, etc. On physical examination, children can be complicated with tympanitis or otitis media, cervical lymph neck enlargement, etc. Adenovirus pneumonia (E wrong) is usually common in children aged 6 months to 2 years, with severe poisoning symptoms including measles rash; digestive symptoms including nausea, vomiting, diarrhea; central nervous system symptoms including listlessness, lethargy, headache, convulsions, etc.

The boy showed cough, wheezing, fever, increased heart rate and respiratory rate, alar twitching, mild cyanosis of the lips, positive three concave symptoms, and a few wet rales in both lungs. Laboratory tests showed a normal white blood cell count, but an increased proportion of lymphocytes. These symptoms and signs suggest that the infant may have an acute viral lower respiratory tract infection.

Respiratory syncytial virus pneumonia (RSV) is A common cause of acute respiratory infections in infants and young children, especially in winter and spring. It can cause the above symptoms, especially in 5-month-old infants. RSV infection usually causes airway inflammation, wheezing, and dyspnea, consistent with the symptoms described in this case.

S. aureus pneumonia in option B is usually associated with more severe bacterial infections, possibly with a higher fever and increased white blood cell count.

Option C of S. pneumoniae pneumonia is a common cause of childhood pneumonia, but usually results in a higher leukocyte count.

Mycoplasma pneumonia in option D is more common in older children and adolescents, and its symptoms tend to be mild.

Adenovirus pneumonia in option E can cause similar symptoms, but is less common in this age group as RSV infection.

Considering these information and clinical presentation together, the most likely diagnosis is A. respiratory syncytial viral pneumonia.

analyze:

Children, cough, asthma, low heat, body appear nose, lips mild cyanosis dyspnea and hypoxia symptoms, three concave sign positive, double lung can smell and wheezing and a little wet rales, experimental examination see normal leukocytes, elevated lymphocytes, according to its clinical manifestations and laboratory examination, for the child with the most likely diagnosis of respiratory syncytial virus pneumonia. The disease is common in infants, especially in children within 1 year old. Clinical mild patients with fever, dyspnea and other symptoms are not severe, moderate and severe patients have obvious breathing difficulties, asthma, mouth cyanosis, nose movement and three concave symptoms, fever can be low, moderate heat and high fever, lung auscultation has more medium and fine wet rales.

Option B Staphylococcus aureus pneumonia has acute onset, serious disease, rapid progression, systemic poisoning symptoms are obvious, fever is more relaxed, pulmonary signs appear early, two lungs have scattered, fine wet rales, empyema, purulent pneumothorax and bullae can occur, which is inconsistent with the symptoms of children in this question.

F. often had the history of upper respiratory tract infection before the onset of Streptococcus pneumonia, cough, rust sputum, lung signs, percussion, tactile tremor and bronchial breathing sounds, and wet rales heard in the dissipation period. The child did not have the above signs, so it was not selected.

Option D Mycoplasma pneumonia starts relatively slowly, and the symptoms mainly include fatigue, sore throat, headache, cough, fever, loss of appetite, diarrhea, myalgia, earache, etc., and the pulmonary signs are not obvious, which has nothing to

do with this question.

Option E Adenovirus pneumonia is mainly manifested as acute fever, cough, difficulty breathing, and asthma. In mild cases, the body temperature returns to normal for 7 to 11 days, and other symptoms gradually disappear. The recovery period of severe children is 1 to 6 months, which is significantly different from the clinical manifestations of this child.

In conclusion, the correct answer is: A. Respiratory syncytial virus pneumonia.

Based on the patient's symptoms and examination results, the most likely diagnosis is respiratory syncytial viral pneumonia (Respiratory Syncytial Virus, RSV). The specific analysis is provided as follows:

1. * * Age * *: The patient was a 5-month-old male infant, and RSV infection was mostly common in infants under 6 months.

2. * * Symptoms * *: The patient coughed for 3 days, gasped for 1 day, and his body temperature was 37.8°C. These symptoms were consistent with RSV infection. Gasping is a typical symptom of RSV infection.

3. * * Signs * *: Physical examination found nose itching, mild cyanosis of the lips, positive three concave symptoms, respiratory wheezing and a little wet rales. These are all characteristic of bronchiolitis caused by RSV infection.

4. * * Laboratory examination * *: WBC 4.010 ^{9} / L, N 0.35, L 0.65. The total number of leukocytes was normal or slightly lower and the proportion of lymphocytes increased, which also fits with the characteristics of RSV infection.

by comparison:

- ** Staphylococcus aureus pneumonia * * and * * Streptococcus pneumoniae pneumonia * *: common in children with low immunity or basic diseases, and usually appear high fever and obvious bacterial infection characteristics (such as increased white blood cells, high proportion of neutrophils).

- ** Mycoplasma pneumonia * *: It is more common in school-age children and adolescents, less rarely in infants, and has a long course of disease.

- ** Adenovirus pneumonia * *: Although similar symptoms can be caused, adenovirus infection is usually accompanied by more obvious conjunctivitis and gastrointestinal symptoms.

Combining the above analysis, the most likely diagnosis is * * A. respiratory syncytial viral pneumonia * *.

78. (Type A2 question)

A 12-year-old male with head CT showed high density of cerebral ventricles, cerebral angiography showed bilateral internal carotid artery occlusion and abnormal cerebrovascular network formation at the skull base, and was diagnosed

A. cerebral aneurysm

B. cerebral arteriovenous malformation

C. cerebrovascular disease

D. Cerebral vein tumor

E. Brain smoke disease

Reference answer: E Your answer:

[Analysis] In the child, cranial CT showed high density of the ventricles, indicating intracranial hemorrhage, cerebral angiography showed bilateral occlusion of the internal carotid artery, and abnormal cerebrovascular network formation at the skull base, so the diagnosis of moyamoya disease was considered. Moyamoya disease is a kind of unknown etiology, at the end of the bilateral internal carotid artery and the brain, the middle artery starting arterial intima slow thickening, arterial lumen stenosis and occlusion, accompanied by abnormal network disease of cerebrovascular network, the main component of abnormal vascular network is hyperplasia, because of its thin wall, lack of external elastic layer, small lumen and smoke, abnormal vascular network in cerebral angiography shape like "smoke", hence the name.

[Fault avoidance] Cerebral aneurysm (A error) is classified into true aneurysm, pseudoaneurysm and dissecting aneurysm. True aneurysms are mostly seen in the intracranial posterior circulation aneurysms, mainly at the bifurcation of the basilar artery, followed by the posterior cerebral artery and the posterior inferior cerebellar artery. Pseudoaneurysm: intra-cervical artery-to-cervical artery aneurysm. Cerebral arteriovenous malformation (B error) is mainly manifested as subarachnoid

hemorrhage and intracranial hematoma and other neurological dysfunction. Cerebrovascular disease (CVD) (C error) refers to the brain lesions caused by various cerebrovascular lesions, mainly refers to the cerebrovascular rupture, bleeding or thrombosis, caused by the symptoms of hemorrhagic or ischemic injury to the brain as the main clinical manifestations. Cerebral venous tumor (D wrong), also known as venous malformation or hemangioma, is rare in clinical practice.

According to the title description, the head CT of a 12-year-old boy showed a high density of the ventricles, and cerebral angiography showed bilateral internal carotid artery occlusion and abnormal cerebrovascular network formation at the skull base. These symptoms suggest a specific vascular abnormality. Among the options presented, the most consistent for these clinical manifestations is E. Brain moyamoya disease (Moyamoya disease).

Brain MMD is a rare vascular disease characterized by a gradual narrowing of the main intracranial arteries, resulting in reduced blood flow, and the formation of many tiny surrounding blood vessels attempts to compensate for this insufficient blood flow, which creates a similar "smoke" fuzzy image on the image. Patients often present with headache, transient ischemic attack (TIA), stroke, and neurological dysfunction.

Therefore, for this case, the most appropriate answer is the E. brain moyamoya disease.

First, we look closely at this issue. The title describes a 12-year-old boy whose cranial CT showed a high density of the ventricles, and cerebral angiography showed bilateral internal carotid artery occlusion and the formation of an abnormal cerebral vascular network at the skull base. Based on this information, we need to select the most likely diagnoses from the options provided.

Now, we'll analyze the options one by one:

A. Cerebral aneurysm: This usually refers to the cystic enlargement of the cerebral artery wall, and although it may also lead to cerebrovascular abnormalities, it usually does not present as an occlusion of the bilateral internal carotid artery and the formation of an abnormal cerebral vascular network at the skull base. Therefore, this option does not exactly coincide with the symptoms described by the title.

B. Cerebral arteriovenous malformation: This is a congenital pathological variant of local cerebral vasculogenesis, but usually does not present as a ventricular high density or bilateral occlusion of the internal carotid artery. So, this option is also

unlikely to be the right answer.

C. Cerebrovascular disease: This is a very broad term, covering a wide variety of cerebrovascular-related diseases. However, it does not specify which type of cerebrovascular disease, and therefore is not specific enough to accurately correspond to the symptoms described by the title.

D. Cerebral vein: This refers to a hemangioma that occurs in a vein, but it does not usually cause high density of the ventricles or occlusion of the internal carotid artery, so this option is unlikely to be the right answer.

E. Moyamoya disease: This is a disease characterized by slow thickening of the intima of the artery at the end of the bilateral internal carotid artery and the anterior cerebral artery and the beginning of the middle cerebral artery, gradual narrowing and occlusion of the arterial lumen, and compensatory expansion of the perforating artery. It usually shows a high density of the ventricles, bilateral internal carotid artery occlusion, and the formation of an abnormal cerebrovascular network at the skull base, which is highly consistent with the symptoms described by the title.

In conclusion, considering the high density of the ventricles, bilateral internal carotid artery occlusion, and the formation of an abnormal cerebral vascular network in the skull base, the most likely diagnosis is BMMD.

Therefore, the correct answer is the E. Brain moyamoya disease.

According to the description, head CT showed high density of ventricles, cerebral angiography showed bilateral internal carotid artery occlusion, and abnormal cerebrovascular network formation at the skull base. These symptoms and imaging characteristics are consistent with the typical manifestations of MMD. Brain moyamoya disease (Moyamoya disease) is a chronic progressive cerebrovascular disease characterized by bilateral occlusion of the internal carotid artery, resulting in the formation of abnormal vascular networks at the skull base.

So, the most correct answer is:

B Brain smoke disease

79. (Type A2 question)

The child, 2 years old, with fever 39.4℃, had sudden convulsions 3 hours ago and good spirits. The proposed diagnosis was

A. viral encephalitis

B. febrile convulsion

C. suppurative encephalitis

D. hypocalcium convulsions

E. toxic encephalopathy

Reference answer: B. Your answer:

Graphic analysis: [analytical] children, 2 years old, fever 39.4℃ (febrile convulsion mostly in 6 to 3 years old infants, and generally in the first 24 hours, and most in a heat course only once), 3 hours before a sudden convulsions (systemic or local tonic or clonic convulsions), after good spirit (no abnormal nervous system), proposed diagnosis of thermal convulsion.

[Avoid error] Viral encephalitis (A error) will have increased intracranial pressure, accompanied by meningeal stimulation. Pyogenic encephalitis (C wrong) will have increased intracranial pressure phenomenon, accompanied by meningeal irritation signs. Low calcium convulsions (D wrong) is more common in children under 1 year old, often by

Yu Vitamin D deficiency leads to a decrease in serum calcium and increased neuromuscular excitability. Toxic encephalopathy (E error) is central

Neurological disorders are the main clinical manifestations of an acute systemic lesions of the primary disease, such as pneumonia, dysentery, etc.

In this case, the 2-year-old child developed fever and sudden convulsions, but was later in a good mental state. The most consistent diagnosis for this condition is a febrile convulsion. Thermal convulsion is a common condition in pediatric children, usually occurs in children aged 6 months to 5 years, and is associated with hyperthermia, but without CNS infections or other defined causes. When convulsions occur, children usually have a brief loss of consciousness and limb twitch, convulsions usually stop in a short period of time, after which the child's mental state will quickly return to normal.

Given this information, the best answer is: B. Thermal convulsions

analyze:

The child, 2 years old, febrile, had a sudden convulsion 3 h ago, later mentally well, and the most likely diagnosis was febrile convulsion. Thermal convulsion is the most common convulsive disease in childhood. The first onset is more than between 6 months and 3 years of life, boys are slightly more than girls, and the vast majority of them no longer attack after the 5 years of age. The child developed convulsions after fever, and had good spirits, which was consistent with the manifestations of febrile convulsions.

Option A viral encephalitis is often manifested as fever, recurrent seizures, different degrees of consciousness disorder and increased intracranial pressure. The child does not have symptoms such as consciousness disorder, so it is not selected.

Option C suppurative encephalitis is a variety of suppurative bacteria caused by meningeal inflammation, some patients with brain parenchyma, is children, especially infants common central nervous system infectious diseases, clinical with acute fever, convulsions, consciousness disorders, increased intracranial pressure and meningeal stimulation and cerebrospinal fluid purulent change, with the symptoms of the children.

Option D Children with low calcium convulsions did not have fever, but had symptoms and signs of vitamin D deficiency rickets, which did not see the above symptoms, so it was not considered.

Option E toxic encephalopathy is mostly common in children aged 2 to 10 years old. Children have high fever, severe headache, vomiting, restlessness, delusion and even coma, often convulsion, inconsistent with the meaning of the question.

In conclusion, the correct answer is: B. Thermal convulsions.

According to the title description, the child was 2 years old with a fever of 39.4℃. He had a convulsions 3 hours ago and was in good spirits. Here is an analysis of the options:

A. * * Viral encephalitis * *: Viral encephalitis is usually accompanied by persistent mental symptoms, such as changes of consciousness, lethargy, headache, etc. The topic mentioned that the children after convulsions of good spirit, does not conform to the characteristics of viral encephalitis.

B. * * Thermal convulsion * *: Thermal convulsion is common in children between 6 months and 5 years of age, mostly when a sudden rise in body temperature occurs. After convulsions and are in good mental condition. The situation in the topic fits very well with the typical characteristics of febrile convulsions.

C. * * Pative encephalitis * *: Posogenic encephalitis is usually accompanied by severe systemic symptoms, such as high fever, severe headache, vomiting, consciousness disorders, etc. The child had good spirits after convulsions, which was not consistent with the performance of suppurative encephalitis.

D. * * Low calcium convulsions * *: Low calcium convulsions are common in newborns and infancy, often accompanied by hand and foot convulsions and other symptoms of hypocalcemia. Although fever may also induce convulsions, other features of hypocalcemia were not mentioned in the title.

E. * * Toxic encephalopathy * *: Toxic encephalopathy usually has a clear history of toxic exposure, accompanied by neurological symptoms, such as disorders of consciousness and recurrent convulsions. The child was in good spirits after convulsions, which was not consistent with the manifestations of toxic encephalopathy.

Based on the above analysis, the most consistent with the diagnosis of the children in the problem is * * febrile convulsion * *. So the best answer is:

F. febrile convulsion

80. (Type A2 questions)

Male infant, 3 months of age, physical examination in child health outpatient clinic, natural term, birth weight 3.2kg, 50cm long, exclusive breastfeeding, physical examination: weight 6kg, 63cm long, without deciduous teeth, about pediatric feeding, it is correct

A. continued with exclusive breastfeeding

B. On the basis of household food, from muddy to broken food

C. Rice noodles can be added and bottle fed

D. 2 to 3 kinds of complementary food can be added at a time

D. Mixed feeding, in preparation for the future cessation of lactation

Reference answer: A. Your answer:

[Analysis] In the first three months of birth, the weight increased by 700~800g per month, and the average height in the first half of the year increased by 2.5cm. The theoretical weight of children is 5.3kg~5.6kg/ height 57.5cm, the height and weight are normal, should continue exclusive breastfeeding.

[Avoid mistakes] 1~3 months: muddy food, such as fruit juice, green vegetable soup, cod liver oil and calcium. Infants: 4 ` 6 months

Things such as: vegetable puree, fruit puree, iron-containing formula rice noodles, formula milk (BCE wrong). The principle of adding complementary food in children is from less to more, from thin to thick, from thin to thick, from one to many kinds, each introduction of a new food should be adapted to 2~3 days (D wrong).

This topic is about the feeding method of a 3-month-old baby boy. Considering the baby's age and current conditions (full-term vaginal birth, normal increase in weight and length, exclusive breastfeeding), the most suitable feeding method is to continue exclusive breastfeeding. The World Health Organization recommends breastfeeding alone until the baby is 6 months old. Therefore, the most correct answer is: A. Continue exclusive breastfeeding

analyze:

A 3-month-old male infant with vaginal delivery at term, exclusively breastfeeding, showed normal development by physical examination. According to the Dietary Guidelines for Chinese Residents (2016), infants aged from 0 to 6 months should be exclusively breastfed, and complementary food should be added from the age of 6 months. The baby is currently 3 months old and should continue exclusive breastfeeding.

Option B is based on the type of family food, from muddy to broken food at 7 to 9 months of age, and the child is currently 3 months old, which does not match the question.

Rice noodles as a complementary food, should be added after 6 months of age, so option C can add rice noodles, bottle feeding, can be excluded.

The principle of adding complementary food is from less to more, from thin to thick, from thin to coarse, from one to many kinds, and it is added when the baby is healthy and the digestive function is normal. Therefore, option D can add 2 to 3 kinds of complementary food at a time, which is not consistent with the principle of adding complementary food, so it is not selected.

For infants within 6 months of age, breast milk is the best food and can meet their needs of growth and development, so mixed feeding is not necessary to prepare for future cessation of breastfeeding, so option E mixed feeding, in preparation for future cessation of breastfeeding, can be excluded.

To sum up, the correct answer is: A. continue exclusive breastfeeding.

For a 3-month-old infant, the best practice guidelines for feeding recommend continued exclusive breastfeeding. The World Health Organization (WHO) and many associations of paediatric children recommend exclusive breastfeeding for the first 6 months because breast milk provides all the nutrients the baby needs and helps strengthen the immune system and reduce the risk of infection.3-month-old infants do not need to add complementary foods.

So, the most correct answer is:

A. continued with exclusive breastfeeding

81. (Type A2 questions)

Male, in recent years, the memory is getting worse, the fictional events to fill the memory, do not know the family, do not distinguish the direction. Sometimes in the middle of the night I see a figure shaking in the room.250ml daily for 20 years. The most likely diagnosis is the

A. vascular dementia

B. enomania

C. Alzheimer's disease

D. alcoholic dementia

And E. Corsakov syndrome

Reference answer: E Your answer:

Graphic resolution: [resolution] patients with memory, fiction and error (sometimes in the middle of the night to see the house figure shaking), inadvertently fabricated experience and the plot or far close to fill the blank of memory (the fictional events fill memory), orientation disorder (direction), combined with long-term drinking history, diagnosis considering osakov syndrome. So choose E.

[Error avoidance] Vascular dementia has A clear history of cerebrovascular disease, and different clinical manifestations of vascular injury are different, so A is wrong.tremble

False is mainly seen in drinkers, the acute reaction after stopping alcohol, often uneasy, hallucinations and other mental symptoms, so B wrong. Alzheimer's disease has insidious onset, with symptoms such as memory loss and disorientation, but often accompanied by behaviors such as inability to take care of themselves in daily life, which seriously affects the quality of life. The imaging manifestations show cerebral cortical atrophy, so C is wrong. It needs to be differentiated from alcoholic dementia. Alcohol dementia is gradually increasing personality change, behavior out of control, memory loss, no personality change and behavior out of control, so D is wrong.

The male patient's symptoms include a deteriorating memory, filling in fictitious memories, not knowing family, loss of direction, and sometimes seeing figures moving in the room late at night. Combined with his long history of heavy alcohol consumption (250ml per day for 20 years), the most likely diagnosis was alcoholic dementia (D).

Alcoholic dementia is a cognitive dysfunction caused by long-term heavy drinking, such as memory loss, loss of judgment and critical power, and personality changes. This was consistent with the patient's presentation. Although Alzheimer's disease (C) and vascular dementia (A) can also lead to memory loss and cognitive impairment, alcoholic dementia is a more likely diagnosis given their long history of alcohol use. Tremor shock (B) is usually associated with acute confusion, while Kosakov syndrome (E) is caused by vitamin B1 deficiency, mainly characterized by memory impairment, ataxia, and abnormal eye movements.

Therefore, the most appropriate answer is the D. alcoholic dementia.

analyze:

Patients with long-term drinking history, memory in recent years, fictional events to fill memory disorders, don't know the family, no direction, sometimes in the night to see the house, this is obvious near memory impairment, orientation disorder and hallucinations, combined with long-term drinking history, considering the possibility of patients with alcoholic dementia.

Option A Vascular dementia refers to dementia caused by cerebrovascular disease, and this patient has no history of cerebrovascular disease and can be excluded.

Option B tremor for alcohol withdrawal reaction, a typical performance is fuzzy, confused southeast northwest, unknown relatives, do not know time, there are a lot of abnormal perception, common image distortion and sharp terror vision, such as beasts, snakes, with emotional, yelling, and have body muscle thick tremor, fever, sweating, faster heart rate, elevated blood pressure, the patient has no withdrawal history, can be ruled out.

Option C Alzheimer's disease is a progressive neurological degenerative disease with insidious onset. It is characterized by memory impairment, aphasia, loss of use, agnsia, visual spatial skills impairment, executive dysfunction, and personality and behavior changes, and no long history of drinking, which is inconsistent with the problem.

Option E division sarakov syndrome, also called amnesia syndrome, for a lack of thiamine (vitamin B1) caused by brain mental disorders, main clinical manifestations including severe loss of memory, spatial and temporal orientation disorder, especially recently forgotten, the time is month, day can not correctly resolved, but immediate recall, stop drinking may have partial recovery, with obvious fiction, the patient with no obvious immediate recall ability, can be ruled out.

In conclusion, the correct answer is: D. alcoholic dementia.

According to the title description, this male patient has the following characteristics:

1. Memory has deteriorated in recent years.

2. Fill in memory fictional events (possibly haerism).

3. Do not know the family members, do not identify the direction (severe

cognitive dysfunction).

4. Late a figure shaking in the house (illusion).

5. Drink alcohol at 250ml per day for 20 years.

Based on the above information, the most likely diagnosis is * * E. Korsakov syndrome * *.

* * Kosakov syndrome * * (Korsakoff syndrome) is a type of neurological damage caused by long-term heavy drinking, mainly manifested by severe memory impairment, fiction (i. e., filling up memory loss with fictitious events), cognitive impairment, and hallucinations. Patients often experience long-term alcohol abuse, resulting in vitamin B1 (thiamine) deficiency, which damages brain structures.

Analysis of the other options:

- ** A. Vascular dementia * *: Usually associated with a history of cerebrovascular disease, there is a progressive cognitive decline, but hallucinations are less common.

- ** B. Tremor delusion * *: It is usually part of acute alcohol withdrawal syndrome, which shows acute symptoms such as confusion, hallucinations, and tremor, but it does not meet the description of long-term memory deterioration.

- ** C. Alzheimer's disease * *: Usually a progressive Alzheimer's disease, common in the elderly, but not characterized by a long history of alcohol consumption.

- ** D. Alcoholic dementia * *: Associated with chronic alcohol consumption, but usually does not include fiction and hallucinations, and is less common than features of Kosakov syndrome.

In conclusion, the most likely diagnosis is * * E. Cossakov syndrome * *.

82. (Type A2 questions)

The boy, 2 years old, na poor, wasting for 2 months, accompanied by

developmental regression, physical examination: T36.5°C, P120 times / min, R26 times / min, anemia, mild facial edema, dull expression, pale skin mucosa, no abnormal heart and lung, soft abdomen, subcostal liver, 4

5. cm, no neck resistance, laboratory tests, blood Hb 88 g / L, RBC 1.910 ^ 12 / L, peripheral blood smear showed variable RBC size, with large cells. The most likely diagnosis is the

A. aplastic anemia

B. Nutritional megaloblastic anemia caused by vitamin B12 deficiency

C. hypoferric anemia

D. Cooley's anemia

E. Nutritional megalocytic anemia due by folic acid deficiency

Reference answer: E Your answer:

Graphic analysis: [analysis] Patients with moderate anemia (Hb 60 ~ 90 g / L), peripheral blood smear showed different RBC size, with large cells, indicating megaloblastic anemia. Meggaloblastic anemia is divided into folic acid deficiency and vitamin B12 deficiency, the most common folic acid deficiency. The difference between the two symptoms is vitamin B12 deficiency can cause neurological symptoms, such as inattention, epileptiform changes, so B is wrong, choose E.

[Error avoidance] aplastic anemia common clinical anemia, bleeding, infection symptoms, blood elephant three line reduction (red blood cells, white blood cells, platelets), not consistent with the patient, so A wrong.` Iron deficiency anemia is small cell hypochromic anemia, which is inconsistent with the patient's blood smear, so C is wrong. Thalassemia mild often no obvious performance, intermediate type visible moderate anemia, severe anemia that a few days of birth, liver and spleen splenomegaly progressive aggravation, jaundice, not consistent with the patient, so D wrong.

This case describes a 2-year-old boy, performance by poor, thin, developmental regression, mild facial edema, expression, pale skin mucosa, physical examination was not found cardiopulmonary abnormalities, liver, blood routine examination showed anemia, red blood cells (RBC) number reduction, and peripheral blood smear shows the size of red blood cells, with large cells. These symptoms and laboratory findings point to megaloblastic anemia, an anemia with abnormally large red blood cells.

A. Aplastic anemia usually presents with hypomyelopoiesis but more marrow findings are needed to confirm the diagnosis.

B. Nutritional megaloblastic anemia due to vitamin B12 deficiency may appear in the present case because this type of anemia leads to the appearance of large red blood cells.

C. Iron deficiency anemia usually results in small cell hypochromic anemia presenting as small and less pigmented red blood cells, so this option does not consistent with the case description.

D. Thalassemia is a genetic disorder that causes impaired erythropoiesis, but it usually does not lead to the appearance of large cells.

E. Folate deficiency also causes nutritional megaloblastic anemia, which has a similar hematologic profile to vitamin B12 deficiency.

Given the information presented above, the most likely diagnosis is either nutritional megaloblastic anemia due to vitamin B12 deficiency (option B) or nutritional megaloblastic anemia due to folic acid deficiency (option E). However, more laboratory tests (such as serum vitamin B12 and folate levels determination) are often needed to distinguish between the two. Given that dietary habits or other associated symptoms were not mentioned in the case, a more detailed history may be needed to determine which one. However, if only based on the information provided, I would prefer to choose B. vitamin B12 deficiency because it is more common in children than folic acid deficiency.

analyze:

Male children, poor tolerance, wasting, accompanied by developmental regression. Physical examination showed low fever, slightly faster heart rate, normal breathing, mild edema in anemia and face, dull expression, pale skin mucosa, large liver, and no neck resistance. Laboratory tests indicated that both hemoglobin and red blood cells were lower than normal values, and the peripheral blood smear showed different sizes of red blood cells, mainly large cells, meeting the characteristics of megaloblastic anemia. The characteristic of galoblastic anemia is the large size of red blood cells, excessive segmentation of neutrophil nucleus, and the phenomenon of hyperplasia of giant red blood cells in the bone marrow, which is also referred to as giant poor. Due to the deficiency of vitamin B12 or (and) folic acid, and the mild facial edema in this child, which was characteristic of vitamin B12 deficiency, the diagnosis of nutritional megalyocytic anemia caused by vitamin B12 deficiency was

considered.

Option A Aplastic anemia usually showed pancytopenia, and usually had no hepatosplenomegaly, which was inconsistent with the title stem description, so it was excluded.

Option C iron deficiency anemia is microcytic anemia, which is not consistent with the macrocytic anemia described in the title stem, so it is not selected.

Option D thalassemia is macrocytic anemia, but it has a positive family history. Its symptoms such as special appearance, growth retardation and bone changes will gradually appear after early childhood, which is not consistent with the description of the subject stem, so it is not considered.

Option E nutritional megaloblastic anemia caused by folic acid deficiency may also appear facial edema, it is relatively rare, while vitamin B12 deficiency is common facial edema, so folic acid deficiency is less likely than vitamin B12 deficiency, which is not consistent with the question.

In conclusion, the correct answer is: B. nutritional megaloblastic anemia caused by vitamin B12 deficiency.

According to the medical history and physical examination results provided in the topic, the 2-year-old boy had poor tolerance, wasting, developmental regression, accompanied by anemia, mild edema, dull expression, pale skin mucosa, laboratory examination showed hemoglobin 88g / L, red blood cell count 1.910 ^ 12 / L, peripheral blood smear showed different red blood cells, with large cells. These information suggests the presence of severe anemia and variable sizes of red blood cells, mostly large cells.

Combined analysis, the most likely diagnosis is:

F. Nutritional megaloblastic anemia caused by vitamin B12 deficiency

The reasons are as follows:

1. The child was anemia, with mild facial edema, dull expression and pale skin mucosa, suggesting chronic anemia.

2. Hemoglobin 88g / L and red blood cell count were 1.910 ^ 12 / L, indicating moderate to severe anemia.

3. The peripheral blood smear showed different size of red blood cells, mainly large cells, suggesting megaloblastic anemia.

4. Vitamin B12 deficiency is common in the malnutrition or absorption disorders in developing children, and megaloblastic anemia is a typical manifestation of vitamin B12 or folate deficiency.

Other option analysis:

-A. Aplastic anemia: usually presenting as pancytopenia, bone marrow examination shows hematopoietic cell reduction, not consistent to the clinical manifestations and laboratory tests of the child.

-C. Iron deficiency anemia: usually manifested as small cell hypochromic anemia, with the same red blood cell size, which does not meet the characteristics of macellular anemia in this child.

-D. Thalassemia: usually microcytic anemia with a family history and related regional distribution, which does not meet the characteristics of macrocytic anemia in the child.

-E. Nutritional megaloblastic anemia due to folic acid deficiency: very similar to the clinical manifestations of megaloblastic anaemia due to vitamin B12 deficiency, but vitamin B12 deficiency is more common given the age and nutritional status.

Therefore, the most likely diagnosis is nutritional megalyocytic anemia due to vitamin B12 deficiency.

83. (Type A2 question)

Female, 26 years old, 32 weeks gestation, sudden abdominal pain, small vaginal bleeding, pale face, weak pulse, P120 beats / min, BP180 / 110mmHg, uterus greater than pregnancy cycle, hard as plate, and unclear fetal heart. Can be diagnosed as

A. Threatened premature labor

B. threatened rupture of uterus

C. amniotic embolism

D. placental abruption

E. placenta praevia

Refer to the answer: D. Your answer:

Graphic parse: [analysis] patients with late pregnancy, sudden abdominal pain, a small amount of vaginal bleeding, affect the circulation system (the pale face, weak pulse, P120 times / points, BP180 / 110mmHg), combined with physical uterus is greater than pregnancy cycle, hard as plate, fetal heart unclear prompt placental abruption, need the prevention and treatment of DIC, postpartum bleeding complications, and immediately cesarean section, so choose D.

[To avoid mistakes] threatened abortion has irregular contractions and vaginal bleeding, fetal membrane integrity, so A wrong. The threatened uterine rupture generally appears in the process of labor, there are abdominal pain, hematuria, pathological shrinkage ring, fetal heart can not hear four manifestations, inconsistent with the patient, so B wrong. Amniotic fluid embolism appeared in the production process, with respiratory and circulatory failure, DIC, and multiple organ damage performance, which is not consistent with the patient, so the C is wrong. Placenta previa is painless vaginal bleeding without inducement, which is not consistent with abdominal pain and circulatory disorders, so E is wrong.

This patient's symptoms include sudden abdominal pain, vaginal bleeding, pale complexion, rapidly weak pulse, elevated blood pressure, a uterus greater than the pregnancy cycle, hard as a plate, and indistinct fetal heart. These symptoms point to serious pregnancy complications.

Option A "threatened premature birth" usually has contractions, changes in the cervix, and possible vaginal bleeding, but does not cause elevated blood pressure or a rigid uterus.

Option B The threatened uterine rupture may cause acute abdominal pain and decreased blood pressure, but is unlikely to cause elevated blood pressure.

Option C "amniotic fluid embolism" is a rare but serious complication during pregnancy that causes acute cardiopulmonary failure and coagulopathy but usually does not result in a rigid plate uterus.

Option D, "Placental abruption" is a partial or complete early dissection of the placenta from the uterine wall that causes abdominal pain, vaginal bleeding, increased uterine stiffness, abnormal fetal heart rate, and possibly unstable maternal circulation,

consistent with this patient's symptoms.

The E option "placenta previa" usually results in painless vaginal bleeding without accompanying acute abdominal pain or uterine sclerosis.

Thus, the diagnosis most consistent with this patient's symptoms was D. placental abruption.

First, let's analyze the case carefully. The patient was a 26-year-old woman at 32 weeks gestation with sudden abdominal pain, minimal vaginal bleeding, pale face, weak pulse, heart rate of 120 beats / min and a significant increase in blood pressure to 180 / 110mmHg. The uterus is larger than the pregnancy cycle and hard as plate, fetal heart is unclear. Next, we make a detailed analysis according to the question stem and options:

A. Seminal prematurity: Although patients have symptoms of abdominal pain and vaginal bleeding, threatened prematurity is usually not accompanied by such high blood pressure and plain abdominal signs, so this option is unlikely.

B. threatened uterine rupture: threatened uterine rupture is usually manifested as severe pain in the lower abdomen, hematuria and abnormal fetal heart rate, but generally does not appear plate abdomen and such high blood pressure, so this option is not very consistent.

C. Amniotic fluid embolism: Amniotic fluid embolism is a serious obstetric complication manifested by sudden dyspnea, hypotension, and hemorrhagic shock. The patient's symptoms, although severe, have no signs of dyspnea or shock, so this option is also unlikely.

D. Placental abruption: Symptoms include abdominal pain, vaginal bleeding, pale complexion, weak pulse, high blood pressure and a stiff uterus, which are typical of placental abruption. Especially in the third trimester, hypertension and sudden persistent abdominal pain are important indications for placental abruption.

E. Placenta previa: placenta previa is mainly manifested in the third trimester or before labor, with no inducement, painless repeated vaginal bleeding, soft uterine abdominal examination, no tenderness, and normal fetal heart rate, which is inconsistent with the patient's symptoms.

Based on the above analysis, the patient's symptoms were most consistent with the diagnosis of placental abruption. Therefore, the correct answer is D. placental abruption.

According to the title, the 26-year-old pregnant woman had sudden abdominal pain, minimal vaginal bleeding, pale complexion, weak pulse (120 times / minute), high blood pressure (180 / 110 mmHg), uterus greater than the pregnancy cycle and hard as plate, fetal heart rate.

These symptoms suggest the following key points:

1. Sudden abdominal pain and vaginal bleeding at 32 weeks of gestation.

2. Pallor, weak pulse, and elevated blood pressure may indicate hemorrhagic shock.

3. The uterus is larger than the pregnancy cycle and is as hard as a plate.

4. Unclear fetal heart rate.

Combining these symptoms and signs, the most likely diagnosis is placental abruption (option D). Placental abruption is the removal of the placenta partially or completely from the uterine wall before delivery of the fetus, which leads to sudden abdominal pain, vaginal bleeding, hardening of the uterus, and fetal distress.

So the best answer is:

G. placental abruption

84. (Type A2 questions)

Male, 16 years old, repeated left limb twitching for 1 year, each episode first by the left hand tic, gradually spread to the left upper limb, and expanded to the left shoulder, finally developed to the left face, each attack lasted 30 seconds can relieve itself. At the onset of attack, epilepsy, the greatest possibility is which attack type

A. Secondary to generalized tonic-clonic seizures

B. myoclonic seizure

C. Simple sensory episodes

D. complex partial seizure

E. jackson at the onset of the

Reference answer: E Your answer:

Graphic analysis: [analytical] male patients, 16 years old, each attack by the left hand first, gradually spread to the left upper limb, and extended to the left shoulder, finally developed to the left face, conform to the typical performance of jackson attacks (start to a part of the body convulsions, then in a certain order gradually extended to other parts of a seizure) so choose E.

[Avoid mistakes] secondary generalized tonic clonic seizure, seizure of systemic convulsions, loss of consciousness, call should not be, with the patient in this question

Obviously A discrepancy, so A is wrong. Myoclonus is a sudden, rapid and powerful part of the body, without sequential conduction

Characteristics, so B wrong. Simple partial sensory attack (simple physical perception attack) often show limb numbness and acupuncture feeling, there are

Some special sensory attacks can show visual (such as flash or dark), auditory, olfactory, taste and vertigo

(Such as vertigo feeling, floating feeling, sinking feeling, etc.), patients have obvious epilepsy state and sequential conduction phenomenon, so C wrong.

Complex partial attacks (psychomotor attacks) are caused by temporal lobe lesions, and the onset age is about 20 years old, and the main clinical manifestations

For consciousness disorders, it can also be manifested as mental symptoms and automation, and a few can develop into systemic attacks, which may have different degrees

Memory impairment, not consistent with the patient, so D is wrong.

In this case, the 16-year-old boy experienced recurrent left limb twitching, and the onset started with the left hand and gradually extended to the left upper limb, left shoulder, and finally to the left face. The episode lasted approximately 30 seconds, and the patient was conscious during the episode. This symptom indicates that convulsions are confined to one side of the body and spread to adjacent areas, typical of Jackson episodes.

Jackson Attack, also known as local motor seizures, is a seizure of local origin that usually affects the motor area of the brain. Patients remain conscious during the

attack, and the attack often begins in a specific part of the body and gradually extends to adjacent areas.

Therefore, the best option for this description is: E. Jackson attack

analyze:

Adolescent male patients, repeated left limb twitching for 1 year, each attack first by the left hand, gradually spread to the left upper limb, and expanded to the left shoulder, and finally developed to the left face, each attack lasts 30 seconds can relieve spontaneously, in the attack of conscious, according to the symptoms and signs, signs, for the patient can be diagnosed as jackson attack.jackson Attack is also known as localized motor seizures, the clinical manifestations of convulsions from the finger-wrist-forearm-elbow-shoulder-mouth-face gradually developed, known as jackson seizure, is a special type of partial attack.

Option A secondary generalized tonic-clonic seizures were mainly manifested by loss of consciousness and clonic sequence activity after bilateral tonic, which was not consistent with the symptoms of the patient, so it was excluded.

Option B Myoclonic seizure is mainly manifested as sudden, rapid and powerful contraction of a muscle or muscle group, causing rapid twitching of limbs, trunk or face, which is not consistent with the symptoms of this question, so it is not selected.

Option C is mainly manifested as involuntary itching of a certain part of the body, or numbness and acupuncture on one side of the limb, mostly in the mouth, tongue, fingers or toes, which is not consistent with the symptoms of patients in this question, so it is not considered.

Option D The episode of complex parts is mainly manifested as consciousness disorder and automation, which are not consistent with the symptoms of patients in this question and can be excluded.

To sum up, the correct answer is: E.jackson attack.

According to the title, the boy's symptoms were left limb twitching, starting in the left hand, then gradually extended to the left upper limb and left shoulder, and eventually to the left face, with each episode lasting 30 seconds. These features are consistent with the typical presentation of Jackson episodes.

* * Jackson seizure * * (Jacksonian seizure) is a partial seizure starting from one

body site and gradually extending to other parts of the ipsilateral body, while the patient usually remains awake during the attack.

Therefore, the most likely type of episode is:

H. Jackson at the onset of the

85. (Type A2 questions)

Newborn, 20 days, gestational age 42 weeks, birth weight 4300g, postnatal abdominal distension, constipation, feeding difficulties, physical examination: poor response after stimulation, facial and sclera yellow dye, cardiopulmonary abnormalities, abdominal bulge, umbilical hernia, low muscle tension of limbs, cold, blood routine: Hb122g / L, 7×109/L,N0.3L0.6, TSH50mU / L, negative blood culture, the most likely diagnosis is

A. congenital hypothyroidism

B. Neonatal ischemic and hypoxic encephalopathy

C. hemolytic disease of newborn

D. congenital dilatation of colon

E. neonatal septicemia

Reference answer: A. Your answer:

Graphgraphic: [analytical] newborn, have the symptoms of gastrointestinal paralysis (after birth abdominal distension, constipation, feeding difficulties), poor response after stimulation, abdominal bulge, umbilical hernia, limbs muscle tension, limbs slightly cool, combined with high TSH (normal 3 mU / L ~ 12 mU / L), high lymphocytes (L0.6), diagnosis considering congenital hypothyroidism, so choose A.

[To avoid mistakes] Neonatal ischemic hypoxic encephalopathy generally has A significant history of intrauterine distress or labor asphyxia, so A is wrong. Neonatal dissolution

Blood can have facial and sclera yellow dye, physiological jaundice generally only digestive system symptoms, no other symptoms; pathological jaundice can be

Have reaction poor, anorexia, spirit poor symptom, but jaundice symptom is

commonly heavier, also do not accord with, reason C is wrong.congenital dilatation of colon

More than constipation, abdominal distension, developmental delay, decreased appetite, generally no muscle tone decline, poor response, poor peripheral circulation performance,

So D wrong. The patient's blood culture was negative and neonatal sepsis was excluded, so E was wrong.

Considering the symptoms of the newborn, including postnatal abdominal distension, constipation, feeding difficulties, facial and scleral yellowing, abdominal distention, umbilical hernia, limb hypotonia, and examination findings showing elevated TSH (50 mU / L), these symptoms and signs pointed to abnormal thyroid function. Elevated TSH usually indicates hypothyroidism because the pituitary gland secrete more TSH to stimulate the thyroid to produce thyroid hormone.

Combined with this information, the most likely diagnosis is:

A. congenital hypothyroidism

In this case, insufficient thyroid function can lead to a slower metabolism in the newborn, resulting in symptoms such as feeding difficulties, muscle hypotonia, and jaundice. High TSH levels are a typical hallmark of hypothyroidism, especially in neonates. Therefore, option A (congenital hypothyroidism) is the most appropriate answer.

analyze:

Newborn, expired birth, macrosomia, abdominal distension after birth, constipation, examination feeding difficulties, poor response, face and sclera yellow dye, cardiopulmonary abnormalities, abdominal bulge, umbilical hernia, limb muscle tension, low skin temperature, combined with symptoms, signs, auxiliary examination results, for the child with the most likely diagnosis of congenital hypothyroidism.

Option B Neonatal ischemia-hypoxic encephalopathy refers to a series of clinical encephalopathy manifestations caused to the hypoxic-ischemic damage of the brain caused by perinatal asphyxia. This disease is not only a serious threat to the life of the newborn, but also one of the residual diseases after the neonatal period, and the performance of the patient in the problem stem, so it is excluded.

Option C neonatal hemolysis disease is the same group caused by mother and child blood type incompatibility, so that the fetus has hemolytic anemia in the mother,

often edema, anemia, liver and spleen splenomegaly and other manifestations, serious cases can kill fetal, nuclear jaundice, or have sequelae, no blood type incompatibility in the problem stem, so it is not selected.

Option D Congenital megacolon is due to continuous spasm of the rectum or the distal colon, and fecal stasis in the proximal colon, which makes the intestine hypertrophy and expansion. It is a common congenital intestinal malformation in children. The main manifestations are delayed fetal stool discharge, intractable constipation and abdominal distension, which is inconsistent with the dry problem, so it is not considered.

Option E neonatal septicemia refers to the neonatal pathogenic bacteria through various ways into neonatal blood circulation, and growth in reproduction, produce toxins and cause systemic infection, the main clinical manifestations have poor response, drowsiness, fever or temperature does not rise, eat less, cry less, less, weight or slow growth symptoms, does not tally with the problem dry, so do not choose.

In conclusion, the correct answer is: A. congenital hypothyroidism.

According to the question information, the newborn has the following symptoms and signs:

-Birth weight of 4300g, belongs to macrosomia.

-Postnatal abdominal distention, constipation, and feeding difficulties.

-Physical examination found poor response, yellow face and sclera, abdominal bulge, umbilical hernia, low muscle tone in the limbs, and cool limbs.

-Blood routine showed Hb122g / L, 710 ^ 9 / L and N0.3 L0.6.

-TSH (thyroid-stimulating hormone) was significantly increased to 50 mU / L.

-Blood culture was negative.

These clinical features point to several important considerations:

1. * * Congenital hypothyroidism (option A) * *: High TSH, facial jaundice, abdominal distension, constipation, feeding difficulties, and muscular hypotonia in the

limbs are all typical manifestations. Congenital hypothyroidism is a neonatal metabolic disorder due to thyroid hormone deficiency. Common symptoms include growth retardation, poor mental response, and constipation. Significantly elevated TSH is an important diagnostic index of this disease.

2. * * Neonatal ischemia and hypoxic encephalopathy (option B) * *: Mainly neurological symptoms after hypoxia, but digestive system symptoms such as abdominal distension and constipation are rare, and TSH abnormalities are not common.

3. * * Hemolysis (C option) * *: Usually jaundice and anemia, normal leukocyte count and TSH is not affected.

4. * * Congenitolon (option D) * *: Mainly constipation and abdominal distension, but generally no significant increase in TSH.

5. * * Neonatal sepsis (E) * *: It can be manifested as poor response, abdominal distension, constipation, but generally accompanied by systemic infection symptoms such as fever or unstable body temperature, and blood culture is usually positive.

In conclusion, combining the significantly elevated TSH and the clinical presentation of the newborn, the most likely diagnosis is * * A. congenital hypothyroidism * *.

86. (Type A2 question)

primipara, 30 years old, found atrial septal defect in prepregnancy physical examination, now 26 weeks of gestation, without palpitation and shortness of breath, echocardiography found atrial septal defect 0.6cm2, EF68%. In order to detect early heart failure, a pregnancy test once a week, the beginning of the gestational week should be

A.28 weeks

B.30 weeks

C.32 weeks

D.34 weeks

E.36 weeks

Reference answer: C Your answer:

[Analysis] The patient atrial septal defect, echocardiography found that the atrial septal defect 0.6cm2, EF68%, cardiac function is still within the normal range, should be weekly from 32 weeks to know whether there is cardiac burden. So choose C.

[Avoid mistakes] at 32 weeks of pregnancy, the fetus increased significantly, the burden of the heart increased, and pregnant women with heart diseases should have a pregnancy test once a week

The cardiac burden situation, when the EF value is less than 50%, the heart pumping function is impaired, requiring timely intervention.

For a primipara who is found to have atrial septal defects before pregnancy, the purpose of regular pregnancy tests is to monitor possible complications such as heart failure. Atrial septal defect (ASD) may cause left-to-right shunting, and the burden of the heart increases as pregnancy progresses, so close monitoring is required. In this case, the patient has no symptoms such as palpitation and shortness of breath, and the echocardiography shows an atrial septal defect area of $0.6cm^2$ and ejection fraction (EF) of 68%, which means that her heart function is currently maintained well.

Usually, the third trimester (third trimester) is the most significant increase in the heart burden, and the frequency of monitoring should be increased. Considering that monitoring of patients with heart disease should usually start more frequently in the second to third trimester, closer monitoring starting from the 28th week or earlier is usually recommended.

Therefore, based on the above discussion, I think the most appropriate answer is A.28 weeks. This ensures that early heart failure or other complications can be detected and intervened.

analyze:

Young primipara, before pregnancy has been diagnosed as atrial septal defect, now 26 weeks of gestation, to find early signs of heart failure, should be examined weekly from 28 weeks of gestation.

Option B: 30 weeks, option C: 32 weeks, option E: 36 weeks, the examination started late, and early heart failure cannot be detected in time, so it was excluded.

Option D: Weekly pregnancy test from 34 weeks is mainly used to evaluate cardiac function in pregnant women with heart disease, and the woman has a clear atrial septal defect. In order to detect heart failure, it should be checked in advance, which is inconsistent with the question.

To sum up, the correct answer is: A.28 weeks.

For this 30-year-old pripara, who had an atrial septal defect (ASD) during pre-pregnancy physical examination at 26 weeks of gestation, echocardiography showed an atrial septal defect of $0.6cm^2$ and an ejection fraction (EF) of 68%. For early detection of heart failure, the optimal start time of weekly pregnancy tests should consider both heart burden and changes during pregnancy.

In this case, more frequent monitoring is recommended to start in the third trimester, around 28 weeks of gestation, for timely detection and management of possible cardiac complications. So the answer is:

A.29 weeks

87. (Type A2 question)

Female, vulvar cluster of roe-like small papules, negative acetate white test, to be diagnosed

A. cauliflower excrescence

B. pseudo-condyloma

C. sebaceous gland aberrance

D. condyloma

E. Genital bowvan-like papular disease

Reference answer: B. Your answer:

Graphic analysis: [analysis] The patient vulvar cluster of roe-like small papules, acetic acid white test was negative, which is a typical manifestation of false condyloma in women, so B was selected.

[To avoid mistakes] condyloma damage at the beginning of small light red papules, after gradually increased, nipple, comb or

Cish pattern protrusions, root often pedicle, and easy to produce erosion exudate, touch easy bleeding, skin lesions cracks often between purulent secretions

Dstasis, resulting in odor, can appear foreign body sensation, pain, itching or sexual pain, easy to identify, so A wrong. Ectopic disease of sebaceous glands

It is characterized by flat damage of millet size without obvious bulge skin, cluster distribution, mostly light yellow or a few light

White, 1~3mm in diameter, so the C is wrong. The damage of flat condyloma is a soft red, flat papules, which can fuse into spots

Patky, pedicless, uneven, solid touch, relatively smooth), infectious, clinical sometimes tight sharp wet

The wart mass is similar to flat wet warts, with a cotton swab will be visible apart from the papillary warts, carefully observe the area

Don't, so D wrong. Paudrivan-like papular disease is brown red to purple, with a few scales, severe fusion into patchy, often

Coexistence with condyloma acuminatum, the lesion range is small, no ulcer, scab, no infiltration of subcutaneous tissue, so E wrong.

The title describes a female patient with a cluster of roe-like papules in the vulva and a negative acetate white test. Based on this information, we can exclude some options, one by one:

A. Condyloma acuminatum: Condyloma acuminatum usually appears white in the acetate white test, so in the case of negative acetate white test, the possibility of condyloma sharp water is low.

B. Pseudocondyloma (also known as frictional keratosis): This is a common non-infectious skin disease, can present as a vulva roe-like papules, usually acetate white test is negative.

C. Apia: This usually refers to the presence of sebaceous glands in atypical locations and present as smaller nodules, less consistent with the description of roe-like papules.

D. Flat condyloma: This type of condyloma is usually small and well-defined, but the white acetate test results may be positive or variable.

E. Genital balvan-like papules: This is a rare condition, usually presents as red or skin-colored papules or plaques that do not coincide with the roe-like papules described in the trunk.

In conclusion, the most likely option is B. pseudolatoma because its clinical presentation is closest to the situation described in the title stem and the acetate white test is usually negative.

analyze:

Female patients, vulvar cluster of roe-like papules, negative acetate white test, is the typical manifestation of pseudocondyloma, so can be diagnosed as pseudocondyloma. Pseudocondyloma is a type of granular pale reddish papule occurring in the female vagina, sometimes with mild pruritus of unknown etiology, generally considered to be a benign papilloma associated with developmental abnormalities.

Option A is positive for acetate white test, so it is not chosen.

Option C sebaceous ectopic occurs after puberty, more middle-aged people, more men than women. The disease is due to the physiological variation of sebaceous gland development and sebaceous gland hyperplasia. Through clinical observation, the disease not only occurs in the oral mucosa, but also more common in the male foreskin and glans, female small labia and other parts. This patient did not appear as described above, so it was excluded.

Option D flat condyloma is a manifestation of secondary syphilis, good in the vulva, perianal, breast and other easy to rub impregnated parts, wet papules shape like lentils, wet surface, a small amount of exudate, containing a lot of syphilis treponema, strong infectivity, with the performance of this patient, so it is not selected.

Option E genital temperature-like papule associated with HPV infection, typical skin lesions for multiple or single pigment papules, skin color, reddish brown, different shape, size, round, oval or irregular shape, the surface can be bright velvet appearance, or mild keratosis warts, skin lesions scattered distribution or cluster arranged into linear, ribbon or ring, and even can fuse into patches, does not conform to the question.

To sum up, the correct answer is: B. False condyloma.

According to the description, the female vulvar had a cluster of fish-like papules, and the acetate white test was negative. We analyze each option one by one:

A. Condyloma acuminatum: caused by human papillomavirus (HPV) infection, the typical characteristics of cauliflower or papillary vegetation, acetate white test is usually positive.

B. Pseudocondyloma: also known as Vulvar Papillomatosis, usually presenting as small white or fleshy papules with a negative acetate white test.

C. Setic ectopia: ectopia usually presents as small yellow or white papules and usually do not have a roe-like appearance.

D. Flat condyloma: also caused by HPV, usually manifested as flat papules or plaques, acetate white test is usually positive.

E. Genital Bowen-like papule disease: This is a rare benign skin disease presenting with multiple small papules, and the white acetate test is usually negative.

Combined with the dry description of "fish roe-like small papules" and "acetic acid white test negative", the most consistent with the description is "pseudocondyloma".

So the best answer is:

F. pseudo-condyloma

88. (Type A2 questions)

Female, 16 years old, had difficulty eating for 1 year. Too much attention to weight, think oneself fat, desperately diet, eat more on vomiting, occasionally gluttony. In the past year, my body weight decreased from 55kg to 35kg, with amenorrhea and good spirit. Height 165cm, without

go on a journey. Thyroid function: FT3:4.16pg/mol,FT4:14.69pg/mol,TSH:14.63IU/ml. The possible diagnosis is

A. Depressive disorder

B. pervasive anxiety disorder

C. hysteric apepsia

D. obsessive-compulsive disorder

E. Hypothyroidism

Reference answer: C Your answer:

[Analysis] Patient female, 16 years old, too much attention to weight, in order to prevent weight gain, desperately diet, eat more vomiting, resulting in weight loss is significantly lower than the normal range (in the past year weight dropped from 55kg to 35kg), and even lead to amenorrhea, is a typical performance of anorexia nervation, so choose C.

[Avoid avoidance] The patient has normal appetite and no depression related symptoms, so A is wrong. Patients have no anxiety, anxiety and other related anxiety disorders

So B is wrong. The patient did not require excessive strict order and perfection, without excessive care and repetition, without too much attention to details

Procrastination and other forced performance, so that D is wrong. Although the patient had amenorrhea, there were no special abnormalities in thyroid function and the symptoms did not consistent with A

Typical manifestations of hypogonadism (indifference, mucinous oedema, slow response, bradycardia, drowsiness, mental spirit

Poor), so the E is wrong.

The main symptoms of this 16-year-old female patient included feeding difficulties, excessive weight concerns, self-perceived overweight, extreme dieting, emetic behavior and occasional bustiness, and significant weight loss and amenorrhea. These clinical manifestations correspond with the characteristics of anorexia nervosa. Patients with anorexia nervosa often undergo strict dietary control and weight loss behaviors due to extreme fear of weight gain and a distorted perception of body size.

In the provided thyroid function tests, FT3 and FT4 levels may be in the normal range, but with increased TSH levels, which can be observed in patients with anorexia

nervosa because their bodies may regulate thyroid function to slow the metabolic rate. Although this change may also be similar to hypothyroidism, hypothyroidism is unlikely to be the main problem according on the clinical symptoms.

Therefore, the most likely diagnosis is * * C. anorexia nervosa * *.

First, let's carefully analyze the key information in the topic to form a clear diagnostic idea.

The patient was a 16-year-old woman with symptoms of eating difficulties for 1 year. She cared too much about her weight and thought she was fat, so she went on a diet and vomited after eating too much. Notably, the patient's body weight has decreased sharply from 55kg to 35kg in the past year, accompanied by amenorrhea. Despite significant weight loss, the patient felt a good mental condition. Furthermore, thyroid function tests showed normal FT3 and FT4, with elevated TSH.

Now, let's analyze the various options:

A. Depression disorder: Although anorexia and depression are both psychological disorders, the topic does not mention that the patient has significant depressive symptoms such as low mood and loss of interest, so this option may not be the most direct diagnosis.

B. Generalized anxiety disorder: usually manifested as persistent, comprehensive, anxiety without definite subjects, as well as autonomic hyperactivity. The title did not mention that the patient had significant anxiety symptoms, so the diagnosis may not be accurate.

C. Anorexia: Patients show excessive attention to weight, dieting, even vomiting after eating more, and sharp weight loss, which are typical symptoms of anorexia nervosa. At the same time, despite the significant weight loss, the patient was in good mental condition, which is also in line with the psychological characteristics of anorexia nervosa.

D. Compulsive disorder: usually manifested as repeated thoughts or behaviors, knowing unnecessary or unreasonable, but unable to control. The title did not mention that the patient had significant compulsive symptoms, so the diagnosis may not be accurate.

E. Hypothyroidism: Although the patient had elevated TSH, FT3 and FT4 were normal, which was inconsistent with the thyroid function test results of typical hypothyroidism. In addition, hypothyroidism is usually accompanied by significant

hypometabolic symptoms, such as chills, fatigue, swelling of the hands and feet, drowsiness, and memory loss, which are not mentioned in the topic.

Based on the above analysis, the patients showed excessive attention to body weight, desperate dieting, gluttony and emetic behavior, and a sharp loss of body weight, which are the typical symptoms of anorexia nervosa. At the same time, despite the significant weight loss, the patient was in good mental condition, which is also in line with the psychological characteristics of anorexia nervosa.

Therefore, combined with patient symptoms, signs and laboratory tests, the most likely diagnosis is anorexia nervosa. So, the correct answer is C. Anoxia nervosa.

According to the problem stem information, the patient was a 16-year-old woman, whose symptoms included difficulty eating, excessive weight attention, desperate dieting, vomiting, occasional gluttony, and significant weight loss. These symptoms are consistent with the appearance of anorexia nerneria (Anorexia Nervosa). Furthermore, the patient's weight decreased from 55kg to 35kg, resulting in amenorrhea, and she consciously mentally spirits, which further supported the diagnosis of anorexia nervosa.

Thyroid function test results showed that:

-FT3:4.16 pg / mol (normal range 3.1-6.8 pg / mol)

-FT4:14.69 pg / mol (normal range of 12-22 pmol / L)

-TSH: 14.63 IU / ml (Normal range of 0.27-4.2 mIU / L)

The patient's significantly elevated TSH, while FT3 and FT4 were within the normal range, but combined with their body weight loss and abnormal eating behavior, was more likely an abnormal thyroid function secondary to anorexoxia rather than primary hypothyroidism. Therefore, hypothyroidism (option E) is not the primary diagnosis.

In conclusion, the most likely diagnosis is:

F. hysteric apepsia

89. (Type A2 question)

The child, 1 year old, squatted down and was held by adults, and found cyanosis by physical examination. Which of the following statements is incorrect

A. Right atrium enlargement

B. right ventricular dilatation

C. left ventricular enlargement

D. ASD

E. Establishment of collateral circulation in the bronchial arteries and pulmonary vessels

Refer to the answer: D. Your answer:

[Analysis] Children squat and be held by adults, physical examination of cyanosis, diagnosis of tetralogy of Fallot. Paralogy of Fallot may have right atrial enlargement (option A), right ventricle (option B), and left ventricular enlargement (option C), and cyanosis as the disease develops because the formation of collateral circulation between the bronchial artery and pulmonary vessels reduces right to left shunt (option E). This question choice is not correct, so choose D.

[Error avoidance] The symptoms of atrial septal defect vary with the size of the defect, small defect, left to right with small flow can be absent for a long time

Symptoms, normal activity. Children with large defects have large left to right split flow and early symptoms, manifested as post-activity qi

Urgent, fatigue, etc., growth and development behind the same age of normal children, and prone to respiratory tract infection, pneumonia and other internal diseases, very

Less cyanosis, so the incorrect statement is the atrial septal defect, this question chose D.

The question describes a 1-year-old child who squats or likes to be held by adults, and finds cyanosis of the mouth. These symptoms suggest the possibility of congenital heart disease, particularly cyanotic heart disease, such as tetralogy of Fallot (Tetralogy of Fallot). The tetralogy of Fallot consists of four characteristics: right ventricular hypertrophy (right ventricular enlargement), pulmonary valve stenosis, atrial septal defect, and the aorta located above the ventricular septal defect. Squatting

can increase the vascular resistance of the lower limbs, help to increase the blood flow to the lungs, reduce the right to the left shunt, thus improving the state of hypoxia.

-A "Right atrium enlargement": Although right ventricular enlargement is typical in tetralogy of Fallot, the right atrium may also expand due to increased load.

-Item B "Right ventricle enlargement": This is a feature of tetralogy of Fallot, so this statement is correct.

-C "Left ventricle enlargement": In tetralogy of Fallot, it is mainly right ventricular enlargement, while the left ventricle is usually not enlarged, so this option may be incorrect.

-D "atrial septal defect": it should actually be a ventricular septal defect, not an atrial septal defect, but it is common in cyanotic heart disease.

-E "Establishment of collateral circulation in bronchial arteries and pulmonary vessels": In some cyanotic heart diseases, such as quadlot of Fallot, this collateral circulation may be established to improve blood oxygen.

Taken together, the most incorrect statement is "left ventricle" in item C, because in cyanotic congenital heart disease such as tetralogy of Fallot, enlargement, not left ventricle.

analyze:

A 1-year-old child who was happy about squatting and being held by adults, with cyanosis found by physical examination, should be considered as tetralogy of Fallot. Tetralogy of Fallot is a common congenital heart malformation. The underlying pathologies are ventricular septal defect, pulmonary stenosis, aortic riding span, and right ventricular hypertrophy. In tetralogy of Fallot, right ventricular outflow tract obstruction increases, and right ventricular hypertrophy compliance decreases. In order to maintain cardiac output, the right ventricular systolic function is compensatory enhanced, the right ventricular myocardial hyperplasia, the papillary muscle and tendinae thickening, the upper ventricular ridge is thickened, and the ventricle can be localized or generalized. The left ventricle is located on the left side of the right ventricle, and the right ventricular hypertrophy can reduce the LV relativity, restrict the diastolic function, and reduce the cardiac output. So the left ventricle will not expand, but will relatively shrink.

Due to the obstruction of the right ventricular outflow tract, the pressure on the right ventricle increases and the right ventricle expands, which leads to the increased

pressure on the right atrium and the right atrium enlargement and option B are in line with the characteristics of tetralogy of Fallot.

Option D Atrial septal defect is not common in tetralogy of Fallot, but because tetralogy of Fallot is a combination of four malformations, including ventricular septal defect, the expression of atrial septal defect in this topic is not completely wrong.

Option E The establishment of collateral circulation of bronchial artery and pulmonary vessels is a compensatory response of the body. When the main pulmonary artery is obstructed, the body will guarantee the blood supply to the lungs to form collateral circulation to ensure the blood supply to the lungs.

In conclusion, the correct answer is: C. Left ventricular enlargement.

The title describes a 1-year-old child, who likes to squat and be held by adults, and the physical examination found cyanosis of the mouth. This suggests that there may be cardiac disease, especially congenital heart disease. We need to choose an incorrect answer from a given option.

Option analysis:

A. * * Right atrial enlargement * *: Many congenital heart diseases, such as an atrial septal defect or pulmonary hypertension, can lead to enlargement of the right atrium.

B. * * Right ventricle enlargement * *: Right ventricle enlargement is common in congenital heart disease, especially in right heart outflow tract obstruction or pulmonary hypertension.

C. * * Left ventricle enlargement * *: Generally, congenital heart disease that causes cyanosis of the mouth rarely causes an enlarged left ventricle alone. Usually, left ventricular enlargement is associated with left cardiac outflow tract obstruction or increased blood flow to the large arteries.

D. * * Atrial septal defect * *: Atrial septal defect is a common congenital heart disease that may cause cyanosis of the mouth.

E. * * Establishment of collateral circulation of bronchial arteries and pulmonary vessels * *: This condition generally occurs in complex congenital heart disease and is used to compensate for insufficient pulmonary blood flow or pulmonary atresia.

Analysis of the symptoms and signs of the child, * * left ventricle enlargement * * is usually not directly related to this type of heart disease, especially in the congenital heart disease that causes cyanosis, left ventricle enlargement is relatively uncommon. Therefore, the most incorrect option should be:

* * C. Left ventricle enlargement * *

90. (Type A2 questions)

A 3-year-old male, with more than 1 year, had 2 history of pneumonia, physical examination: 2-3 intercostal rib and grade 3 / 6 systolic murmur, the second heart sound in the pulmonary valve area was fixed division, the chest X-ray heart shadow was the most likely

A. Bottle-like

B. Boot

C. Pear-shaped

D. spherality

E. General size

Reference answer: C Your answer:

[Analysis] Symptoms of fatigue and history of pneumonia, combined with 2~3 interrib smell of the left margin of the sternum and grade 3 / 6 systolic murmur, the second heart sound in the pulmonary valve area, is the typical manifestations of atrial septal defect murmur, and the most likely form of chest X-ray heart shadow of atrial septal defect is piriform. Piriform heart, also known as mitral valve heart, is mostly seen in mitral valve lesions, atrial septal defect, pulmonary artery stenosis, pulmonary

hypertension and cor pulmonale disease. So choose C.

[Avoid mistakes] Flask-shaped heart is the characteristic performance of pericardial effusion, so A is wrong. Boot heart is common in aortic insufficiency, also

It can be seen in aortic stenosis, hypertensive heart disease, tetralogy of Fallot, so B is wrong. Spherocentrids are more common in mitral valve closure

Inwhole, stress cardiomyopathy, pericardial effusion, etc., so D wrong. General large heart is common in myocarditis, total heart failure, so E is wrong.

This exam described a 3-year-old boy with a history of sweating for more than one year and two pneumonia, physical examination revealed grade 3 / 6 systolic murmur in the intercostal left margin of the sternal bone 2 to 3, and fixed division of the second heart sound in the pulmonary valve area. These symptoms and signs suggest possible heart problems.

Based on the described symptoms and signs, especially the systolic murmur and fixed split second heart sounds, this may point to congenital heart disease, such as atrial septal defect or ventricular septal defect, etc. Cardiac X-ray findings are often associated with a specific heart disease:

-A. Flask shape: common in pericardial effusion.

-B. Boot form: associated with tetralogy of Fallot, characterized by right ventricular hypertrophy.

-C. piriform: not typical of a specific heart disease.

-D. Sphule: usually associated with atrial enlargement, such as mitral valve lesions.

-E. General size: not a particular heart morphology change.

Combining the symptoms and the X-ray findings of typical heart disease, the most consistent description may be the "boot" heart, as it is characteristic of tetralogy of Fallot, and this symptom group can cause abnormal auscultation findings in the pulmonary valve area and altered cardiac morphology. However, the history and signs may also correspond to other types of congenital heart disease, but based on the options and information given in the topic, choosing "boots" (B) may be the most appropriate answer.

analyze:

A 3-year-old man with sweating for more than 1 year, had two history of

pneumonia, 2-3 interrib stal and grade 3 / 6 systolic murmur, and the second heart sound in the pulmonary valve area, which met the typical auscultation characteristics of atrial septal defect. Atrial septal defect can cause enlargement of the right ventricle and right atrium, and on the chest X-ray, the light to moderately enlarged heart appears as a "pear-shaped heart", namely piriform.

Option A flask-shaped heart is mostly seen in pericardial effusion. The X-ray shows the heart shadow expanding to both sides, and the bottom is similar to the flask, which is inconsistent with the meaning of the question.

Option B Boot heart refers to the enlargement of the left ventricle, which can be seen in the aortic valve stenosis, and also visible in the aortic valve insufficiency, also known as the "aortic heart", which does not meet the meaning of the question.

Option D The spheroid is an X-ray sign of mitral insufficiency, with a spherical dilated heart shadow, bilateral ventricular enlargement, and a normal or increased cardiothoracic ratio that can be ruled out.

Option E large size means that when the left and right ventricles are enlarged, the heart shadow on the chest film is flask-shaped, which can be seen in dilated cardiomyopathy, which is inconsistent with the description of the main stem.

In summary, the correct answer is: C. piriform.

According to the title description, the 3-year-old boy had a history of sweating and fatigue for more than 1 year and two times of pneumonia. Physical examination showed 2~3 intercostal smell of the left margin and grade 3 / 6 systolic murmur, and fixed division of the second heart sound in the pulmonary valve area. These symptoms suggest that the child may have an atrial septal defect (ASD).

Typical signs of an atrial septal defect include:

-Fixed division of the second heart tone in the pulmonary valve area

-systolic accentuated

In the case of atrial septal defect, the common chest X-ray heart shadow pattern is "generally large" because of the enlarged heart and increased pulmonary blood flow.

So the most likely answer is:

B General size

91. (Type A2 questions)

Baby boy, 4 months, irritable and sweaty, born in winter, full-term natural birth, breastfeeding, which of the following conditions may occur

A. Hand and foot bracelets

B. craniomalacia

C. caput quadratum

D. pigeon breast

E. X and O legs

Reference answer: B. Your answer:

[analysis] Male boy, 4 months, irritable sweating, born in winter, term natural birth, breastfeeding. Consider the patient for vitamin D deficiency. Rickets is a common disease in children under 3 years of age. Due to the lack of vitamin D, the calcium and phosphorus in the body can not be properly absorbed and used normally, thus affecting the growth and development of bones, and severe cases can lead to bone deformities. The main symptoms include neurological symptoms (irritability, crying at night), delayed motor development, delayed teething, bone changes (cranomalacia, square skull, bead ribs, etc.). Because the baby is 4 months old, (B) calomalacia should be chosen.

[Avoid mistakes] hand and foot bracelets, calomalacia, square skull, chicken chest, X or O legs are all symptoms of rickets. But the skull

Softening mainly occurs in children aged 3 to 6 months, while the square head appears in children aged 8 to September, and the chicken breast appears mostly 6 months later.

This title describes a 4-month-old boy, irritable, born in winter, delivered at term, and breastfed. Considering age, symptoms and feeding practices, we need to consider conditions that may be associated with malnutrition, especially with inadequate intake of calcium and vitamin D.

A. Hand-foot bracelets-commonly seen in rickets, resulting from bone problems due to calcium or vitamin D deficiency.

B. Cranomalacia-also a characteristic of rickets due to calcium or vitamin D deficiency.

C. skull-usually the skull and is not specific to rickets.

D. chick thorax-usually associated with rickets but more often in slightly older children.

E. X, O legs-also associated with rickets but usually present when children are larger.

Given the age and symptoms of the baby, and the possible inadequate sun exposure (a major source of vitamin D) from winter birth, the most likely case is an inadequate intake of calcium and vitamin D, which can lead to rickets. In this case, hand and foot bracelets and calomalacia are the most likely clinical manifestations, especially in infants at this age. Therefore, although hand-foot bracelets (A) and calomalacia (B) are associated with rickets, calvaromalacia is A typical early clinical manifestation, so the most appropriate answer may be B. calomalacia.

First, we need to analyze this issue carefully. The problem involved a 4-month-old boy whose main symptoms were irritability and sweating, which was mentioned in winter, full term and breastfeeding. These symptoms and conditions prompted us to consider the possibility of vitamin D deficiency rickets. Vitamin D deficiency rickets is common in infancy, especially in infants with insufficient sunlight and insufficient vitamin D intake.

Now, let's analyze the options one by one:

A. Hand and foot bracelets: This is a possible bone deformity of vitamin D deficiency rickets in older children, but it is unlikely in this 4-month-old baby.

B. Cranomalacia: This is an early symptom of vitamin D deficiency rickets, especially common in infants aged 3 to 6 months of age. This matched the symptoms described in the title stem and the age of the baby.

C. Square head: This is a common symptom of vitamin D deficiency rickets in 7-to 8-month-old infants, and is less likely in this 4-month-old infant.

D. Chicken breast: This is a manifestation of the aggressive period of rickets, which usually occurs in children around 1 year old, and is unlikely for 4-month-old infants.

E. X, O legs: This is also a skeletal malformation of rickets, but it usually occurs around the age of 1 and is not suitable for this 4-month-old baby.

In conclusion, considering the age and symptoms of this infant, the most likely scenario is calomalacia, a typical early manifestation of vitamin D deficiency rickets in infants 3 – 6 months of age.

Therefore, the correct answer is B. calomalacia.

According to the description of the problem stem, the male baby 4 months, irritable sweating, winter born, term natural birth, breastfeeding, these symptoms and conditions may be rickets (vitamin D deficiency rickets).

Rickets is common in infants and young children, especially those born in winter. Due to insufficient sunlight exposure, vitamin D deficiency, which affects calcium and phosphorus metabolism, and then causes abnormal bone development. The symptoms in the topic (irritability and sweating) are also consistent with the clinical manifestations of rickets.

There are different signs and bone changes in different stages of rickets. Infants at 4 months may have one of the following typical manifestations early on:

F. craniomalacia

Cranomalacia is a typical manifestation of early rickets, especially in the back of the head, which can show symptoms of cranomalacia and feel soft when touched. This is due to the incomplete calcification.

Other options such as hand and foot bracelets, square skull, chicken breasts, X, and O legs, although also manifestations of rickets, usually occur in later stages of the disease or in older children.

So the most likely answer is:

G. craniomalacia

92. (Type A2 questions)

A 35-year-old male with right retroear pain, physical examination: right nasolabial groove shallow, right frontal grain loss, Bell's sign (+), the following possible diagnosis is

A. cerebrovascular disease

And B. Subarachnoid hemorrhage

B. facial neuritis

C. Guiland-Barre syndrome

D. cerebrospinal meningitis

Reference answer: C Your answer:

[Analysis] Bell's sign, also known as Bell's palsy or rabbit eye sign, refers to the patient in the facial nerve palsy, and the affected lateral eyelid can not be completely closed, exposing the white sclera. Bell's sign is common in the peripheral facial paralysis caused by facial neuritis and peripheral nerve diseases. In addition, the patient showed the askew of the mouth, the right nasolabial groove was shallow, and the right frontal lines disappeared. Therefore, the patient may be diagnosed as facial neuritis (so C is correct).

[To avoid mistakes] Most cerebrovascular disease patients have mental problems; subarachnoid hemorrhage has sudden jet vomiting and head

Pain; Gililan-Barre syndrome shows acute symmetrical tric cid paralysis; cerebrospinal spinal with with spinal brain

Membrane stimulation.

A 35-year-old man developed right retroauricular pain with mouth askew, physical examination revealed superficial right nasolabial groove, absent right frontal lines, and positive Bell sign. These symptoms and signs point to the facial nerve damage, especially the facial nerve. Facial neuritis, also known as Bell palsy, is a condition that causes temporary weakening or paralysis of the facial muscles characterized by a rapidly developing weakness or paralysis of unilateral facial

muscles.

Considering these clinical findings, the most likely diagnosis is C. facuritis. Other options such as cerebrovascular disease, subarachnoid hemorrhage, Gililan-Barre syndrome, and cerebrospinal meningitis, although they can affect neurological function, their clinical manifestations are usually not limited to facial nerve damage and will have symptoms and signs of other systems. Therefore, C. facuritis, based on the symptoms and signs provided, is the most appropriate diagnosis.

analyze:

In a young male patient, the mouth was askew after right retroear pain, physical examination showed superficial change in the right nasolabial groove, and the right frontal lines disappeared. Bell's sign (+), which was a typical manifestation of facial nerve palsy, so the diagnosis of facial neuritis was considered.

Option A Cerebrovascular disease is common in middle-aged and elderly patients, and there are many risk factors of cerebrovascular disease such as hypertension, atherosclerosis, diabetes, sudden onset, rapid progress, often headache, vomiting and other increased intracranial pressure, generally unconscious disorders, so it can be eliminated.

Option B subarachnoid hemorrhage onset is acute, generally with obvious triggers, such as strenuous exercise, excessive fatigue, mainly manifested as headache, meningeal irritation and eye symptoms, serious consciousness disorders can occur, inconsistent with the description of the problem.

Option D Guilan-Barre syndrome mainly presents as delayed paralysis, distal more severe than proximal, can have peripheral sensory disorders and cranial nerve damage, inconsistent with the question.

Option E Encephalingitis is mainly manifested as infection symptoms, meningeal irritation, increased intracranial pressure, brain parenchymal damage, seizures, paralysis and cranial nerve damage, which does not meet the description of the problem.

In conclusion, the correct answer is: C. facial neuritis.

According to the information provided in the topic, the patient showed right posterior ear pain with skew of the mouth corner, shallow right nasolabial groove, disappeared right frontal lines, and Bell's sign (+). These symptoms suggest that the

facial nerve was affected, and the most likely diagnosis is facial neuritis.

* * Facial neuritis * * (Bell's palsy) is a common condition that causes paralysis of the facial muscles, usually unilateral. Main symptoms include:

-Facial muscle weakness or paralysis

-One side of the facial expression muscle does not move properly

-Post-ear pain (sometimes)

-Bell sign (+): that is, when the eye turns upward when trying to close the eyes, the eyelid cannot be completely closed.

Brief explanation of the other options:

- ** Cerebrovascular disease * *: It is usually accompanied by other neurological signs and may involve multiple nerves.

- ** Subarachnoid hemorrhage * *: severe sudden headache, nausea, vomiting, neck stiffness, etc., less unilateral facial nerve symptoms.

- ** Gililan-Barre syndrome * *: It usually presents as symmetrical weakness of the limbs, starting in the lower limbs and spreading upward, not only affecting the facial nerve.

- ** * *: usually fever, headache, neck stiffness and other systemic symptoms, not limited to the unilateral facial nerve.

Therefore, the most likely diagnosis is:

* * C. facial neuritis * *

93. (Type A2 question)

A male infant, 4 weeks old and 42 weeks gestation, was irritable and hoarse, with poor feeding, periumbilical bulge, and the possible diagnosis was

A. Ischaemic, hypoxic encephalopathy

B. congenital dilatation of colon

C. hematencephalon

D. cerebral infarction

E. Pyemia

Reference answer: A. Your answer:

[Analysis] Neonatal ischemic hypoxic encephalopathy is mainly caused by hypoxia, the symptoms are mostly manifested by the fetal heart rate before birth, consciousness disorders, dull drowsiness, muscle tone changes, accompanied by frequent convulsion seizures. The patient was born irritable and hoarse, poor feeding condition (poor mental state), periumbilical ocele, weak muscle tone (the reflection of weakened muscle tone). Therefore, the diagnosis of neonatal ischemic and hypoxic encephalopathy was considered.

[To avoid errors] Patients with congenital megacolon often show abdominal distension and excretion disorders; cerebral hemorrhage and cerebral infarction should occur suddenly, but at the same time

With ed change; sepsis should be accompanied by high fever.

This case describes a male infant born 4 weeks old and 42 weeks gestation, poor feeding, periumbilical ocele and weak muscle tone. Based on these symptoms, the most likely diagnosis is congenital megacolon (B). Congenital megacolon (Hirschsprung disease) is a disease that affects the large intestine and causes the intestine to advance feces normally, causing bloating and feeding difficulties. Periomphalocele may be due to intestinal expansion, while weak muscle tone may be a manifestation of a poor systemic condition. Therefore, option B (congenital megacolon) is the most reasonable diagnosis in this scenario.

analyze:

Children, expired birth, born irritability, hoarseness, periumbilical swelling, low muscle tone, combined with the symptoms and signs of the children, should be diagnosed as congenital megacolon. Congenital megacolon is due to the continuous spasm of the distal intestine of the rectum or colon, and fecal stasis in the proximal colon, making the intestine hypertrophic and dilated, which is a common congenital intestinal malformation in children.

Option A Ischemic and hypoxic encephalopathy was more common in full-term infants, often with A history of intrauterine distress or postnatal asphyxia. This child had no history of asphyxia, so it was not selected.

Option C cerebral hemorrhage, option D cerebral infarction, more common in

the elderly, more hypertension, atherosclerosis and other basic diseases, the newborn is rare, can be excluded together.

Option E Sepsis refers to the suppurative bacteria invading the blood flow, reproducing in large numbers, and spreading to other tissues or organs of the host through the blood flow, producing new suppurative lesions. The child has no history of infection and no suppurative lesions, so it is excluded.

In conclusion, the correct answer is: B. congenital megacolon.

According to the title description, this is a 4-week baby boy born at 42 weeks of gestation, born with irritable hoarseness, poor feeding, periumbilical swelling, and weak muscle tone. These symptoms suggest to consider several possible diagnoses:

- ** Ischemic and hypoxic encephalopathy * *: usually have a history of birth asphyxia, accompanied by consciousness disorders, convulsions and other neurological symptoms.

- ** Congenital megacolon * *: usually manifested as neonatal constipation, abdominal distension, but not very weak muscle tension.

- ** Intracerebral hemorrhage * *: common in premature infants, manifested as coma, convulsions, low response, etc.

- ** Cerebral infarction * *: Neonates are rare, usually accompanied by focal neurological symptoms such as hemiplegia.

- ** Sepsis * *: Neonatal sepsis can be manifested as irritability, feeding difficulties, abdominal distension, muscle hypotonia and other systemic symptoms.

Taken together, the symptoms of periumbilical ocele, muscle weakness, feeding difficulties, and irritability and hoarseness are more consistent with the manifestations of sepsis. Therefore, the most likely diagnosis is:

B Pyemia

94. (Type A2 question)

Newborn, 15 days, abdominal distension and 3 days without defecation. Physical

examination: weight 3.2kg, poor spirit, abdominal varicose veins, hyperbowel sounds. The preferred examination for a definite diagnosis is

A. rectoanoscopy

B. digital rectal examination

C. Rectoanal muscle biopsy

D. X-ray barium enema

E. Vertical X-ray flat

Reference answer: E Your answer:

Graphic analysis: [resolution] the newborn constipation abdominal distension symptoms, and physical examination see bowel noise, consider patients with neonatal acute abdomen, whether neonatal megacolon may, for further detailed diagnosis, choose abdominal examination, if flat show colon gas, expansion is X-ray barium enema diagnosis, so this choose E.

[To avoid avoidance] Newates are young and have poor tolerance, so plain abdominal examination with less damage should be selected for screening.

This case describes a 15-day-old newborn with abdominal distension and 3 days of no bowel movements, low weight, poor mental status, varicose veins and hypersounds in the abdomen. These symptoms and signs may be related to congenital intestinal problems, such as the congenital megacolon.

The preferred method should be tests that provide both anatomical information and show the functional status of the gut. Considering the age and symptoms of the child, an X-ray barium enema (option D) is an appropriate option. Such examinations can show the morphological and functional status of the intestine, helping in the diagnosis of congenital intestinal diseases such as megacolon. Rectoscopy (A) and rectoanal muscle biopsy (C), while also providing useful information, are not as comprehensive as barium enema for diagnostic structural and functional problems. A digital rectal examination (B) is a basic method that detects abnormalities in the rectum but has limited aid for in-depth diagnosis. Vertical X-ray plain film (E) can show the distribution of intestinal gas, but it is also inferior to barium enema for the diagnosis of specific structural and functional diseases.

Therefore, the best answer is the D.X-ray barium enema.

analyze:

Newborns, abdominal distension, 3 days without defecation, should be highly suspected of intestinal obstruction or intussusception and other diseases, abdominal varicose veins indicates portal hypertension, physical examination of hyperbowel indicates the possibility of intestinal obstruction. During intestinal obstruction, a large amount of gas and liquid will accumulate in the proximal intestine of the obstruction, and the air and liquid plane will show under the X line. The vertical X-ray plain film can identify the site, degree and possible nature of the obstruction, which is the most simple and effective method to diagnose intestinal obstruction.

Option A Rectoanoscopy was complicated and had little diagnostic value for intestinal obstruction, so it was excluded.

Option B digital rectal examination is not helpful for the diagnosis of intestinal obstruction, so it is not chosen.

Option C rectoanal muscle biopsy is invasive examination, which is not helpful for the diagnosis of intestinal obstruction, and is easy to aggravate the pain of the child, which can be ruled out.

Option D: X-ray barium enema is mostly used to examine lesions in the colon, and barium enema is at risk of aggravating intestinal obstruction, so patients with intestinal obstruction are prohibited, so it is not considered.

To sum up, the correct answer is: E. Vertical X-ray flat slice.

Based on the neonatal symptoms provided (abdominal distension, no bowel movement, abdominal varices, hyperbowel sounds) and physical findings, the most likely diagnosis is intestinal obstruction or congenital megacolon. To clarify the diagnosis, the preferred examination should be the imaging examination that can show the site of the intestinal obstruction and its nature.

A. Rectanoscopy: suitable for the examination of lesions in the rectal and anal region, but is not preferred for A clear diagnosis of intestinal obstruction or congenital megacolon.

B. Digital rectal examination: Although simple, it is not preferred for clear upper gastrointestinal or intestinal obstruction.

C. Rectal and anal muscle biopsy: used to confirm the diagnosis of congenital megacolon (Hirschsprung disease), but generally performed only after other tests (such as X-ray barium enema) indicate a problem.

D. X-ray barium enema: this is the first choice for the clear diagnosis of intestinal obstruction or congenital megacolon, which can show the morphological and functional changes of the intestine, and is the best answer to this question.

E. Vertical X-ray plain film: it can be used to detect pneumoperitoneum, but the specific situation of clear diagnosis of intestinal obstruction is not as detailed as barium enema.

So the best answer is:

F. X-ray barium enema

95. (Type A2 questions)

A 68-year-old man experienced left limb weakness with a history of hypertension. Physical examination: conscious, oblique abduction of the right eyeball, right dilated pupil, normal bilateral frontal lines, shallow left nasolabial groove, and positive left limb muscle. The patient's performance was consistent

A. basilar apx syndrome

B. peduncular syndrome

C. apallic syndrome

D. Horner's sign

E. The ventrolateral medulla oblongata syndrome

Reference answer: B. Your answer:

[Analysis] The patient, a 68-year-old male, felt left limb weakness during eating. After physical examination, the right eye was dilated, dilated pupil (oculomotor nerve

palsy), normal bilateral frontal lines, shallow left nasolabial sulcus, left limb muscle strength grade 1, and positive left Babinski sign (crossed hemiplegia). Brain foot syndrome, also known as Weber syndrome, is a neurological disorder characterized by crossover hemiparesis and oculomotor nerve palsy. The patient had a left limb onset and developed crossover hemiparesis and oculomotor nerve palsy, so the patient should be diagnosed with cerebral foot syndrome.

[Avoid mistakes] after occlusion leads to eye movement disorders and pupil abnormalities, arousal and behavior disorders, can be accompanied by memory loss, right

Lateral hemianopia or cortical blindness is called basilar tip syndrome; atresia syndrome contralateral hyperthermia disturbance (lateral spinothalamus tract),

Cross-sensory disorder; the four main clinical manifestations of Horner syndrome are: ocular fission

Small, enophthalmos, dilated pupils, facial no perspiration (all four manifestations are ipsilateral to the lesion); ventrolateral medulla syndrome

Horner syndrome can also occur with vertigo, nausea, vomiting and dysphagia and focal ataxia.

This question describes a 68-year-old male patient with left limb weakness, physical weight, and a previous history of hypertension. Physical examination revealed external oblique abduction of the right eye, dilated pupil on the right side, superficial left nasolabial groove, weakened muscle strength in the left limb and positive Babinski sign. These symptoms and signs suggest a central nervous system involvement.

A. Basilar tip syndrome: usually impaired function in the brainstem, cerebellum and some of the cranial nerves. Ectropia and dilated mydriasis in this case may be related to cranial nerve dysfunction, but the cerebellar symptoms were not mentioned and therefore not fully consistent.

B. Brain foot syndrome: usually affects the structure near the brain foot, resulting in contralateral hemiplegia, cranial nerve involvement and other symptoms. This patient presented bilateral symptoms atypical of cerebral foot syndrome.

C. Acresia syndrome: the main manifestations are quadriplegia, preservation of consciousness, and eye movements. In this case, the patient was weak on one side, not paralyzed, and therefore may not be atresia syndrome.

D. Horner's syndrome: It mainly presents with reduced pupils, drooping eyelids, and reduced facial sweating. The pupil described in the case was dilated and not consistent with Horner syndrome.

E. ventrolateral medulla syndrome (Wallenberg syndrome): usually caused by the dorsolateral blood supply of the medulla, manifested as sympathetic damage (such as Horner syndrome), cerebellar symptoms, facial sensory disorders, etc. The patient's symptoms included cranial nerve damage and limb weakness, consistent with impaired manifestations of the medulla oblongata.

In combination, the patient's symptoms are most consistent with the ventrolateral medulla extension syndrome (Wallenberg syndrome). Therefore, the most appropriate answer is the E.

First of all, we analyze the question stem:

An elderly male patient with a history of hypertension suddenly developed symptoms of left limb weakness and visual weight during eating. Physical examination showed clarity, external oblique abduction of the right eye, dilated pupil on the right side, normal bilateral frontal lines, superficial left nasolabial groove, abnormal muscle strength of the left limb, and positive Babinski sign.

Next, we analyzed the options item by item:

A. Basilar artery tip syndrome: This is A syndrome caused by blood circulation disorders at the tip of the basilar artery, mainly manifested as eye movement disorder, abnormal pupil, arousal and behavioral abnormalities, accompanied by memory loss, lateral hemiplegia and hemianopia. The patient's symptoms (left limb weakness, visual weight, external oblique abduction of the right eye, right dilated pupil) matched the description of this syndrome.

B. Brain foot syndrome: the main manifestation is hemiplegia, but there will not be the symptoms of external eye deviation and dilated pupils, which is inconsistent with the description of the problem stem.

C. Acresia syndrome: This is a severe brain stem injury. Although the patient is aware, he is paralyzed, unable to talk and swallow, and can only express his wishes through instantaneous and vertical eye movements, which is not consistent with the symptoms described by the problem stem.

D. Horner's sign: mainly manifested as pupil narrowing, eye prolapse, small eye fission, etc., which is not consistent with the symptoms described by the problem stem.

E. ventrolateral medulla oblongata syndrome: the main manifestations are vertigo, nausea, vomiting, nystagmus, ipsilateral limb ataxia, ipsilateral Horner's sign, cross sensory disorder, etc., which are not consistent with the symptoms described by the stem.

In conclusion, the patient's symptoms were most consistent with basilar apx syndrome.

Therefore, the correct answer is A. basilar apx syndrome.

According to the information provided by the subject stem, a 68-year-old male patient suddenly felt left limb weakness during eating. She had a previous medical history of hypertension. Physical examination revealed external oblique abduction of the right globe, dilated right pupil, normal bilateral frontal lines, superficial left nasolabial groove, low muscle tone of the left limb, and a positive Babinski sign.

Analyze the individual options:

- ** A. Basilar artery tip syndrome * *: Changes of consciousness, binocular gaze palsy, and multiple cranial nerve palsy, often with tetraplegia or hemiparesis.

- ** B. Brain foot syndrome (Weber syndrome) * *: Midbrain lesions, usually contralateral limb hemiplegia and oculomotor palsy on the same side of the lesion.

- ** C. Closed lock syndrome * *: Mainly, quadriplegia and aphasia, but the patient is conscious and can have limited communication through eyeball activities.

- ** D. Horner's sign * *: ipsilateral ptosis, dilated pupils and anhidrosis, usually caused by sympathetic damage.

- ** E. ventrolateral medulla oblongata syndrome (Wallenberg syndrome) * *: manifested as loss of facial pain and temperature on the same side of the lesion and loss of limb pain and temperature on the contralateral side, accompanied by cerebellar symptoms, dysphagia, hoarseness, etc.

According to the patient's symptoms: left limb weakness, external oblique abduction of the right eyeball, right dilated pupil and other characteristics, in line with the performance of cerebral foot syndrome. Cerebral foot syndrome, also known as

Weber syndrome, is mainly caused by midbrain lesions, which is manifested as limb hemiplegia on the opposite side of the lesion and oculomotor nerve palsy on the same side of the lesion.

So, the most likely diagnosis is:

* * B. cerebral foot syndrome * *

96. (Type A2 questions)

A 40-year-old female with G4P1 with contact bleeding, no abnormal liver and kidney function and normal ovarian function was diagnosed

A. carcinoma of endometrium

B. fibroid

C. cervicitis

D. encyesis

E. acephalocystis racemosa

Reference answer: C Your answer:

[Analysis] The main symptoms of cervicitis include abnormal leucorrhea secretions and friction bleeding during sexual intercourse or examination, that is, contact blood. The patient was a 40-year-old female with no abnormal liver and ovarian function (excluding ovarian dysfunction and other organ disorders), and typical contact bleeding symptoms, so the patient should be diagnosed as cervicitis.

[Avoid mistakes] Endometrial cancer mostly occurs in menopausal women; uterine fibroids show irregular vaginal bleeding, and whether contact with

No; early symptoms of hydatidiform mole are similar to pregnancy, so both can be excluded

In this case, a 40-year-old woman, G4P1 (four births), had contact bleeding with normal liver and kidney functions and ovarian functions. Contact bleeding usually refers to bleeding occurring after sexual intercourse, physical examination, or placement of intrauterine devices, which is a common symptom of cervical lesions, especially cervicitis or cervical cancer. However, cervical cancer is not directly mentioned in the option.

A. Endometrial cancer usually occurs in postmenopausal women, and the main

symptoms are abnormal vaginal bleeding.

B. Uterine fibroids may cause menorrhagia or irregular bleeding, but generally do not present as contact bleeding.

C. Cervicitis is a common cause of contact bleeding, especially in women of reproductive age.

D. Pregnancy usually does not result in contact bleeding unless abnormal conditions such as ectopic pregnancy or threatened abortion exist.

E. Hydatidiform mole is an abnormal form of pregnancy presenting mainly with abnormal vaginal bleeding but not specific to contact bleeding.

Based on this information, the most likely diagnosis is * * C. cervicitis * * as it is a common cause of contact bleeding and the patient has normal functioning of other organs.

First, let's analyze the questions carefully and point out the key information. The patient was a 40-year-old woman with four pregnancies (G4) and one successful delivery (P1). She developed symptoms of contact bleeding, along with normal liver and kidney function and normal ovarian function. Based on this information, we can analyze the options item by item:

A. Endometrial cancer: This cancer usually presents with abnormal uterine bleeding, but uterine bleeding is not mentioned in the trunk, and contact bleeding is not a typical manifestation of endometrial cancer. Therefore, this option may not be the best answer.

B. Uterine fibroids: Although uterine fibroids are the most common benign tumors in the female reproductive system, they do not usually cause contact bleeding. Symptoms of uterine fibroids may include menstrual changes, abdominal mass, increased leucorrhea, abdominal pain, waist acid, lower abdomen swelling, etc., which is inconsistent with the description of the problem stem.

C. Cervicitis: This is a common disease in women of childbearing age, both acute and chronic. The main performance is leucorrhea increase, showing viscous mucus or purulent mucus, sometimes accompanied by bloodshot or sandwiched with bloodshot. Furthermore, cervicitis may cause contact bleeding, which matches the description in the title trunk.

D. Pregnancy: Although pregnancy is a possible option, there is no mention of any symptoms related to pregnancy, such as menopause and early pregnancy reaction.

And contact bleeding is not typical of pregnancy.

E. Hydatidiform mole: hydatidiform mole because of pregnancy placental villus trophoblast hyperplasia, interstitial edema, and the formation of different sizes of blisters, blisters between the pedicle connected into a string, shaped like grapes. Although the hydatidiform mole may cause vaginal bleeding, it usually does not present as contact bleeding, and other typical symptoms of the hydatidiform mole are not mentioned in the problem stem, such as vaginal bleeding after menopause and abnormal enlargement of the uterus.

In conclusion, considering the symptoms of contact bleeding in the patient and the conditions such as normal ovarian function, the most consistent diagnosis is cervicitis.

Therefore, the correct answer is the C. cervicitis.

According to the case description provided, the 40-year-old woman had contact bleeding and had no abnormal liver and kidney function and normal ovarian function.

Here is an individual analysis of the options:

A. * * Endometrial cancer * *: The typical symptoms of endometrial cancer include abnormal uterine bleeding, but mainly common in postmenopausal women, and not necessarily contact bleeding.

B. * * Uterine fibroids * *: Uterine fibroids may also cause abnormal bleeding, but they do not typically present as contact bleeding. More common symptoms include menorrhagia, irregular menstruation, abdominal pain, etc.

C. * * Cervicitis * *: Cervicitis is an inflammation of the cervix that may lead to contact bleeding. This is a more common cause of contact bleeding.

D. * * Pregnancy * *: Symptoms of pregnancy include menopause, morning sickness, and contact bleeding is not typical symptoms. Bleeding caused by

pregnancy is usually implantation bleeding or other abnormal pregnancy conditions.

E. * * Hydatidiform mole * *: Hydatidiform mole is an abnormal form of pregnancy, mainly manifested as menopause, vaginal bleeding, and abnormal enlargement of the uterus during pregnancy, but contact bleeding is not a typical symptom.

Combined analysis, the woman was 40-year-old with contact bleeding and no other functional abnormalities, and the most likely diagnosis was * * cervicitis * *.

So the best answer is:

B cervicitis

97. (Type A2 questions)

A 31-year-old female, 1 day after spontaneous delivery, complained of paroxysmal pain in the lower abdomen. Physical examination: T37.8℃, uterine fundus umbilical level, hard quality, no tenderness, a small amount of vaginal bleeding, dark red, less than menstrual volume. This patient belongs to

A. puerperal infection

B. normal puerperium

C. Soft birth canal laceration

D. postpartum hemorrhage

E. Poor uterine recovery

Reference answer: B. Your answer:

[Analysis] puerperium refers to the maternal body organs except the breast from the placenta to recover or close to the normal not pregnant state required for the period, generally stipulated for 6 weeks. Within 24 hours after delivery, the body temperature is slightly increased but not more than 38℃, which may be related to excessive fatigue caused by the long labor process, maternal water loss or lochia

stagnation. At the same time, postpartum blood and necrotic uterine decidua and other tissues are discharged through the vagina, manifested as dark red, called lochia. Therefore, the patient can be diagnosed as normal puerper.

[Avoid mistakes] Fever temperature should exceed 38°C; blood produced by soft canal laceration and postpartum hemorrhage should be bright red;

Poor uterine involution refers to the failure to recover to the non-pregnant state after 6 weeks postpartum, and the present puerpera is only 1 day postpartum, which cannot be judged

This question examines the clinical manifestations and their diagnosis of postpartum women. According to the title description, female patients had paroxysmal pain in the lower abdomen one day after natural delivery, with slightly higher body temperature (37.8°C), normal uterine fundus position (flat umbilical cord), hard uterine constitution, no tenderness, and a small amount of dark red vaginal bleeding, but the amount of bleeding was less than menstrual volume.

The option analysis is performed as follows:

A. Puerperal infection is usually accompanied by fever, increased lochia, heavy odor, painful contractions and other symptoms.

B. Normal puerperium women may have mild lower abdominal pain and a little vaginal bleeding, and the contractions usually gradually decrease the uterine floor.

C. Soft birth canal laceration usually causes significant bleeding, with accompanying tenderness.

D. Postpartum hemorrhage refers to excessive hemorrhage after delivery, usually accompanied by symptoms such as decreased blood pressure and increased heart rate.

E. Poor uterine involution will lead to slow or insignificant uterine decline, soft uterine body, and may be accompanied by bleeding and other problems.

According to the symptoms and signs of the patient, the position of the uterine fundus is normal and hard, and the amount of bleeding is less, mainly manifested as paroxysmal lower abdominal pain and microheat, with no obvious abnormal lochia or serious bleeding, which are more in line with the performance of normal puerperium. Therefore, the most appropriate answer should be B. normal puerperal.

First, we will analyze the key information in the problem stem in detail. A 31-year-old female presented 1 day after spontaneous delivery. The physical examination showed that the body temperature was slightly increased, the bottom of

the uterus was flat, the texture was hard and no tenderness, and there was a small amount of dark red bleeding in the vagina, but the amount was less than the menstrual volume.

Next, we analyze the individual options based on these information:

A. Puerperal infection: Although the patient's body temperature increased slightly, there were no other typical symptoms of puerperal infection, such as high fever, chills, vaginal purulent discharge, etc. Moreover, there was no tenderness, which did not consistent with the common signs of puerperal infection.

B. Normal puerperal pressure: The patient is in the puerperium (within 6 weeks postpartum), and the body temperature increase may be related to the stress reaction or mild dehydration during delivery, but still within the normal range. The uterine floor, hard quality and no tenderness are the normal manifestations of postpartum uterine contraction and aging. The amount of vaginal bleeding is less than the menstrual volume, but also in line with the normal changes of postpartum lochia. Therefore, these symptoms are consistent with the normal puerperium performance.

C. Soft birth canal laceration: usually leads to a significant increase in postpartum bleeding, but the vaginal bleeding is less than menstrual volume, and there is no relevant symptoms described, so this option does not meet.

D. Postpartum hemorrhage: Patient's vaginal bleeding is less than menstrual bleeding, which does not meet the definition of postpartum hemorrhage.

E. Poor uterine involution: Poor uterine involution usually shows the uterine fundus height above the normal range, soft uterine texture, and increased lochia, long duration and other symptoms. However, the base of the uterus was normal, the texture was hard, and the amount of bleeding was less, which did not accord with the poor performance of uterine recovery.

In conclusion, the patient's symptoms were most consistent with the normal puerperium presentation. Therefore, the correct answer is: B. Normal puerperium.

According to the problem stem information, the patient was a 31-year-old woman with paroxysmal lower abdominal pain 1 day after natural delivery. The physical examination results showed that the body temperature was 37.8℃, the uterine bottom, hard, no tenderness, a small amount of vaginal bleeding, dark red, less than menstrual volume.

We analyze the item-by-item options:

A. * * puerperal infection * *: puerperal infection is usually accompanied by high fever (above 38℃), and may be accompanied by uterine tenderness, choria and other symptoms. The patient's temperature was 37.8℃, not meeting the standard for high fever, and there was no uterine tenderness and lochia abnormalities.

B. * * Normal puerperal * *: One day after delivery, mild low abdominal paroxysmal pain and a small amount of vaginal bleeding were normal, because the uterus was contracted to promote recovery, and the body temperature did not meet the fever standard. This description fits best with the patient's symptoms.

C. * * Soft birth canal laceration * *: usually manifested as obvious vaginal bleeding, and mostly bright red blood. The amount of bleeding in the patient is less than the menstrual amount and is dark red, which does not meet the typical symptoms of laceration in the soft birth canal.

D. * * Postpartum hemorrhage * *: Postpartum hemorrhage is generally defined as the amount of hemorrhage exceeding 500ml within 24 hours postpartum. The patient had vaginal bleeding volume less than menstrual volume and did not meet the criteria for postpartum bleeding.

E. * * Poor uterine recovery * *: Usually with poor uterine recovery, possibly persistent lower abdominal pain and massive vaginal bleeding. The patient's symptoms (a small amount of bleeding, hard uterus) do not accord with the typical manifestations of poor uterine rejuvenation.

In conclusion, the diagnosis most consistent with the patient's condition is:

B * * Normal puerperal labor * *

98. (Type A2 question)

Female, 30 years old, married for 3 years without contraception without pregnancy, accompanied by lower abdominal drop distension. Menstrual 8~9 days / 26~28 days, gynecological examination: posterior uterus, poor mobility, irregular shape, no obvious abnormal touch in the double accessory area. The following tests are the least valuable to the diagnostic value

A. Pelvic B ultrasound

B. examination of basal body temperature

C. fractional curettage

D. Positional abdominal film

E. Opiodol contrast

Refer to the answer: D. Your answer:

Graphic analysis: [analysis] abdominal vertical film is not clear for uterine lesions, and it is best to use B ultrasound or MRI to further confirm the lesion site on imaging.

[Avoid avoidance] Basic temperature measurement can not only diagnose pregnancy status, but also diagnose uterine and ovarian function; segmentation

Medical curettage eliminates the uterus; lipiodol angiography can confirm the lesion.

This exam question examines the diagnostic methods of infertility, especially when evaluating possible uterine or adnexal problems. Based on the symptoms of this female patient without 3 years without pregnancy and lower abdomen, and the results of menstrual cycle and gynecological examinations, we need to consider which diagnostic method is the least valuable in determining the cause of infertility.

A. Pelvic B ultrasound (ultrasound): This is a common method to evaluate the condition of the uterus and adnexa (such as ovary), and is of great value for the diagnosis of uterine morphology, location, and adnexal area abnormalities.

B. Basal body temperature measurement: This is a method to assess the ovulation status of women, judging by monitoring the change in body temperature during the ovulation cycle. Although helpful for the diagnosis of infertility, it has no direct value for the assessment of uterine morphology and structure.

C. Segmental curettage: This is a sampling method of endometrial tissue, which

can help diagnose endometrial diseases, such as endometriosis, and has direct value in diagnose the causes of infertility.

D. Abdominal positioning film: This is an X-ray method, mainly used to evaluate abdominal organs, such as the intestines, and is of little value for direct assessment of the condition of the uterus and adnexa.

E. Opiodol angiography: This is a method to evaluate the patency of the fallopian tube, which is of great value for the diagnosis of infertility, especially for the fallopian tube factors.

Taken together, the choice of the least diagnostic value should be * * D. * *, as it is mainly used to evaluate other organs in the abdomen and is not the most direct and effective method for direct diagnosis of the uterus or adnexa.

The patient was a young woman who had been married for 3 years and was not pregnant, which suggested the possible problem of infertility. The patient also has symptoms of lower abdomen. The menstrual cycle is relatively regular, but the menstrual period is slightly longer. Gynecological examination showed posterior uterus, poor mobility and irregular shape, and no obvious abnormality in the double accessory area. With this information, it is reasonable to suspect that endometriosis, adenomyosis, or other uterine abnormalities may exist.

Now, we analyze the diagnostic value of each option one by one:

A. Pelvic B ultrasound: In the case of irregular shape and poor mobility of the uterus found in gynecological examination, pelvic B ultrasound can further observe the morphology and structure of the uterus and appendages, and help to find adenomyosis, endometriosis or other abnormalities, so it is valuable.

B. Basal thermography: This test can indirectly reflect ovarian function, help to assess fertility and hormonal changes in the menstrual cycle, and is therefore valuable.

C. Segmental curettage: segmental curettage can scrape the endometrial and endometrial lesions to make pathological diagnosis, provide a basis for clinical treatment, and is particularly valuable for the diagnosis of endometrial lesions.

D. Abdominal positioning film: it is mainly used to examine the abdominal organs and bones, and has almost no direct help to evaluate the infertility and irregular uterine shape of the patient, so it is the most worthless examination.

E. Opiodol angiography: This examination can assess whether the patency of the fallopian tube is smooth, and whether the morphology of the uterine cavity is normal,

and is of great value in the diagnosis of infertility.

In conclusion, the abdominal positioning film (option D) is the least valuable test for the diagnosis of this patient.

Therefore, the correct answer is D. abdominal.

This is a diagnostic examination topic about female infertility and lower abdomen swelling. Here are the explanations of the inspections:

A. Pelvic B-ultrasound: the ability to examine the structure of the pelvic cavity, including the uterus, ovary and its surrounding tissues, is very helpful for the discovery of abnormal shape of the uterus and possible lesions (such as uterine fibroids, ovarian cysts).

B. Basal body temperature measurement: it is used to evaluate the ovulation function of women, and to infer the ovulation period by monitoring the changes of basal body temperature, which is one of the common means to evaluate infertility.

C. Segmental curettage: used to examine the endometrium, by scraping endometrial tissue for pathological examination, can help diagnose endometrial abnormalities or lesions.

D. Abdominal positioning position: mainly used to examine abdominal organs, but of limited value to the evaluation of genital lesions in the pelvic cavity.

E. Lipiodol angiography: it is an imaging examination to examine the patency of the uterus and fallopian tube, which is of great value for evaluating the patency of the fallopian tube and the structure of the uterine cavity.

In conclusion, for a 30-year-old woman who has been married for 3 years without contraception, accompanied by lower abdominal swelling and poor mobility, abdominal positioning film has the least diagnostic value in these examinations, because it mainly examines the abdominal organs and has little effect on the direct

examination of the reproductive organs in the pelvic cavity.

Therefore, the most valuable diagnostic test is:

* * D. Abdominal positioning tablet * *

99. (Type A2 question)

Maternal, 39 weeks, normal fetal heart rate, first dew-2, uterine height 35cm, abdominal circumference 115cm, after the exit sagittal diameter + sciatic tuberosity diameter <15cm, how should be dealt with

A. C-sect

B. Continue to observe

C. oxytocin

D. Artificial membrane breaking

E. induced labour

Reference answer: A. Your answer:

[Analysis] When the sum of the diameter between the sciatic tuberosity and the sagittal diameter after the exit is> 15cm, the fetal head can be moved back and delivered through the triangular space after the exit. If the sum of the two is <15cm, the term fetus cannot be delivered vaginally, and should undergo cesarean section. The maternal pregnancy is 39 weeks old, the fetal heart rate is normal, first dew-2, uterine height 35cm, abdominal circumference 115cm, after the exit sagittal diameter + sciatic nodule diameter <15cm, the full-term fetus can not deliver vaginally through the pregnant woman, so the answer to this question should be (A) cesarean section.

[Misavoidance] Indications for cesarean section include: (1) fetal distress; (2) abnormal fetal position; (3); (4) scar uterus; (5) placenta previa; (6) umbilical cord prolapse; (7) placental abruption; (8) pregnant macrosomia; (9) severe complications; (10) birth canal malformation; (11) pregnancy with tumors; (12) vulvar disease; (13) infectious diseases; (14) twin or multiple pregnancy; (15) cesarean section required by pregnant women.

This topic examines the management of obstetric emergencies, especially regarding the choice of maternal delivery routes. Considering that the puerpera has

reached 39 weeks, the fetal heart rate is normal, but the sagittal diameter and sciatic diameter after exit is less than 15 cm, which means that the pelvis may be too narrow and not conducive to vaginal birth. In this case, if vaginal labor continues, it may lead to dystocia or fetal compression.

Therefore, the best treatment is for A. cesarean section. This can avoid labor difficulties and possible risks to the fetus due to the narrow pelvic outlet. Cesarean section is the recommended mode of delivery in this case to ensure the safety of the mother and infant. So, the correct answer is A. cesarean section.

analyze:

The puerpera, 39 weeks, normal fetal heart rate, first dew-2, uterine height 35cm, abdominal circumference 115cm, after the exit sagittal diameter + sciatic tuberosity diameter <15cm, indicating that the pelvic exit plane is narrow, and first high floating, considering the existence of the head and pelvis is not weighed, it is difficult to vaginal delivery, so cesarean section should be chosen.

Option B continued observation is not suitable for the patient, because of the presence of head basin, continued observation may lead to fetal distress, fetal death and other adverse consequences.

Option C Diactocin is mainly used for those with weak coordination contractions, uterine mouth dilation of 3cm, good fetal heart, normal fetal position, and the head basin of the head basin, so the use of oxytocin will not improve the delivery situation, but will increase the risk of fetal distress, so it is not selected.

Option D Artificial film breaking is mainly used for patients with 3cm uterine expansion, no head basin, fetal head connected and delayed labor. It is not suitable for patients with head basin, so it is not considered.

Option E induction of labor refers to the pregnancy, due to maternal or fetal reasons, the pregnancy is not suitable for the full-term woman, so it is not selected.

In conclusion, the correct answer is: A. cesarean section.

This problem involves the maternal processing decision during labor, especially under the parameters such as fetal first exposure, uterine height, abdominal circumference and pelvic out diameter. Let's analyze these factors one by one:

- ** Fetal heart rate * *: This is a good sign that the fetus is not in distress.

- ** Xian-2 * *: It means that the fetal head has not fully entered the entrance of the pelvis and is in a higher position.

- ** Uterine height 35cm, abdominal circumference 115cm * *: These values suggest that the fetal size may be large.

- ** Post-exit sagittal diameter + sciatic tuberosity diameter <15cm * *: This indicates that the pelvic diameter of the pelvis is small, which may affect vaginal birth.

According to the above information, the fetus has a higher head position and a smaller pelvic diameter, suggesting a higher risk of natural delivery. Continued observation may lead to an increased risk of fetal distress or dystocia, and other options such as oxytocin and artificial membrane rupture do not address the problem of pelvic outlet stenosis in this case.

Therefore, the best answer should be to take a cesarean section to ensure both maternal and fetal safety.

The answer is: * * A. cesarean section * *

100, (Type A2 questions)

The 2-year-old boy was diagnosed with nephrotic syndrome, and urinary protein turned negative after 3 weeks of hormone therapy, and relapsed after 2 weeks of withdrawal. The situation occurs three times in a year. This child is of a hormone-dependent type

A. hormone-dependent type, relapse

B. Hormone-sensitive type, with frequent recurrence

C. Horone-dependent type with frequent recurrence

D. Hormone-resistant type, with relapse

E. Hormone-resistant type, with frequent recurrence

Reference answer: C Your answer:

[Analysis] according to the evidence-based guidelines for the diagnosis and

treatment of hormone-sensitive, relapse / dependent nephrotic syndrome in children according to the treatment of the hormone can be divided into the following three types: (1) hormone sensitive NS: prednisone foot dose [2 mg / (kg · d) or 60 mg / (m2 · d)] for 4 weeks; (2) hormone resistant NS: prednisone foot dose for> 4 weeks. It can also be divided into initial drug resistance and late drug resistance. The latter refers to those who are still positive for urinary protein for> 4 weeks after one or more remission of hormone therapy; (3) hormone-dependent NS: those who are sensitive to hormone but relapse within 2 weeks of two consecutive reduction or withdrawal. While recurrence and frequent recurrence were defined as recurrence: 3 consecutive days, 24 hours urine protein quantification 50 mg / kg, or morning urine protein / creatinine (mg / mg) 2.0, or morning urine protein from negative to (+ + +) ~ (+ + + +); frequent recurrence

(FR): refers to 2 recurrence within half a year, or 4 recurrence within 1 years. Therefore, the patient can be judged to be hormone-dependent

Type, frequency recurrence.

[Aavoid avoidance] accurately distinguish between recurrence and frequency recurrence.

In the case of a 2-year-old boy, who was diagnosed with nephrotic syndrome, urinary protein turned after hormone therapy, but recurred within 2 weeks of withdrawal, three times in a year. According to this description, the most appropriate classification is "hormone-dependent type, frequent recurrence". In the context of nephrotic syndrome, the hormone-dependent type is a condition that presents a good response to hormonal therapy but develops a relapse shortly after withdrawal. Since this situation has occurred multiple times in a year (frequent recurrence), the most accurate answer is:

F. Horone-dependent type with frequent recurrence

This classification reflects the patient dependence on hormonal therapy as well as the frequency of relapse.

analyze:

A 2-year-old boy was diagnosed with nephrotic syndrome. Urinary protein turned negative after 3 weeks of hormone treatment, and relapsed after 2 weeks of drug withdrawal. This situation occurred 3 times in a year, which was frequent recurrence, consistent with hormone sensitive type and frequent recurrence.

Hormone-sensitive nephrotic syndrome refers to the remission of nephrotic syndrome within 8 to 12 weeks of medication, that is, 3 consecutive days, urine protein from positive to negative, that is, urine protein <0.3 g/d, or 24-hour urine protein quantification <0.3g, or urine protein / creatinine (mg / mg) <0.2; or three consecutive times within a week, negative urine dipstick examination. Recurrence of nephrotic syndrome refers to urinary protein from negative to (+ + +) or (+ + + +), or 24-hour urinary protein quantification 50mg / kg or urinary protein / creatinine (mg / mg) 2.0; or urine dipstick examination from negative to (+ +) or above. If 2 times within half a year, or 3 times within a year, frequency recurrence.

Option A "hormone-dependent, relapse", option C "hormone-dependent, frequent relapse", can be excluded together, because hormone-dependent type refers to the nephrotic syndrome that is sensitive to hormone but recurred within 2 weeks of two consecutive reduction or withdrawal, which does not match the meaning of the question.

Option D "hormone resistance type, relapse", option E "hormone resistance type, frequent relapse", can be excluded together, because hormone drug-resistant nephrotic syndrome refers to hormone treatment for 8 to 12 weeks, and the urinary protein is still positive. The urinary protein of the patient has turned negative, and does not conform to the hormone resistance type.

In conclusion, the correct answer is: B. hormone-sensitive type, frequent recurrence.

According to the description, the 2-year-old boy was diagnosed with nephrotic syndrome, and the urinary protein turned negative after 3 weeks of hormone treatment, but relapsed after 2 weeks of withdrawal, and this condition occurred three times in a year. Here is an explanation of the options:

A. Hormone-dependent type, relapse: Hormone-dependent type refers to the patient whose symptoms resolve during hormone therapy, but who will relapse once the hormone is reduced or stopped. Recurrence alone occurred irrespective of the number of frequent relapses.

B. Hormone sensitive type, frequent recurrence: Hormone sensitive type refers to the patient's effective in hormone therapy, urinary protein turns negative, and frequent

recurrence refers to the number of recurrence more than 2 times within a year.

C. Hormone-dependent, frequent relapse: Hormone-dependent and frequent relapse refers to the remission of symptoms during hormone therapy, but relapse once hormones are reduced or withdrawn, and relapse more than twice times a year.

D. Hormone resistance type, relapse: Hormone resistance type refers to the patient's nonresponse to hormone therapy, relapse refers to the disease is not in remission or deteriorate again.

E. Hormone resistance type, frequent recurrence: hormone resistance type and frequent recurrence refers to the patient's nonresponse to hormone therapy, and the frequent recurrence of the disease.

Based on the above explanation and the question, the correct answer is:

* * C. hormone-dependent type, frequent recurrence * *

101. (Type A2 questions)

Female, pregnant 40 weeks old, uterine height 35cm, abdominal circumference 110cm, vaginal bleeding one day, gynecological examination: uterine opening 1cm, feet first, the next examination should be done is

A. External rotation in B

B. Intravaginal rotation

C. natural labor

D. Oxytocin

E. C-sect

Reference answer: E Your answer:

[Analysis] The patient is female, 40 weeks pregnant, uterine height 35cm,

abdominal circumference 110cm, vaginal bleeding one day, gynecological examination see the uterine opening 1cm, both feet first exposed. The fetus is exposed first, and pregnant women with abnormal fetal position need to perform direct cesarean section, so (E) cesarean section is selected.

[Misavoidance] Indications for cesarean section include: (1) fetal distress; (2) abnormal fetal position; (3) head; (4) scar uterus; (5) placenta previa; (6) umbilical cord prolapse (7) placental abruption; (8) macrosomia; (9) severe complications and complications; (10) birth canal malformation; (11) pregnancy with tumors; (12) vulvar disease; (13) infectious disease; (14) twin or multiple pregnancy; (15) cesarean section required by pregnant women.

This topic involves the management of pregnant women in the third trimester, especially in the presence of abnormal conditions such as double foot first exposure and vaginal bleeding. Bipedal preexposure is a kind of abnormal fetal position that may lead to complications during delivery and requires special attention. In this case, the first thing to be considered is to ensure the safety of the mother and the child.

-A. External rotation by B ultrasound: This is considered in some abnormal fetal position conditions, and the purpose is to try to transfer the fetus to the head position for natural delivery. However, in the case of vaginal bleeding, performing this procedure may increase the risk.

-B. Intravaginal rotation: This option is typicnot usually the preferred method for handling abnormal fetal position and vaginal bleeding because it may increase the risk of uterine tears.

-C. Natural delivery: High risk of both foot exposure and vaginal bleeding, and is not the preferred treatment.

-D. Dioxytocin: This is mainly used to handle postpartum bleeding, rather than vaginal bleeding before delivery.

-E. Cesarean section: In the case of first foot exposure and vaginal bleeding, cesarean section is usually the best choice to ensure the safety of the mother and the baby. Cesarean section can avoid labor difficulties and potential complications due to abnormal fetal position.

Therefore, the best answer is * * E. cesarean section * *.

analyze:

Young women, full-term pregnant women, vaginal bleeding one day, uterine

mouth open 1cm, feet first exposed, should be considered for the foot first exposed, is a common fetal position abnormality, fetal feet before the fetal head exposed can stimulate the cervix to make the uterine mouth rapid expansion, fetal membrane rupture is easy to occur umbilical cord prolapse, fetal distress and even death. Therefore, once diagnosed, cesarean section should be delivered as soon as possible to ensure the safety of mother and infant, so the next examination should be done is cesarean section.

Option A by super B external rotation is the examination method, not the treatment method, so it is excluded.

Option B transvaginal rotation is a treatment method of obstetrics, but it is not suitable for manual reduction when the foot is first to expose, so it is not selected.

Option C Natural delivery is suitable for the fetal position of head first exposure, which is the absolute indication of cesarean section, so it is impossible to have natural delivery and can be excluded.

Option D static point oxytocin, mainly used for coordination contractions of oxytocin and late abortion induction, sufficient exposure should be cesarean section as soon as possible, so it is not appropriate to use oxytocin, inconsistent with the topic.

To sum up, the correct answer is: E. cesarean section.

Based on the topic information you provide, we can draw the following analysis and conclusions:

The patient was full-term pregnant (40 weeks of gestation), with a uterine height of 35cm, an abdominal circumference of 110cm, and vaginal bleeding for one day. Gynecological examination found that the uterine mouth opened 1cm, fetal position for the foot first exposed.

Double foot exposure (also known as double foot-hip exposure) is an abnormal fetal position that usually increases the risk of delivery. The safest mode of delivery needs to be considered at this point.

Here is an analysis of the options:

A. By B super external rotation: This method is to rotate the fetus away from the abnormal position (breech or transverse position) to the head position (normal position). However, this method may not be applicable when the uterine opening is only 1cm open.

B. Intravaginal rotation: This method also attempts to change the position of the fetus, but requires professional techniques and conditions and is usually used during labor, not than only 1cm of the uterine opening.

C. Natural delivery: Since double exposure is increasing the risk of delivery, natural delivery is not the best choice.

D. Diyotocin: Diactocin is used to promote contractions, but this may increase the risk of abnormal fetal position.

E. Cesarean section: Cesarean section is the preferred choice. It can avoid the delivery complications caused by abnormal fetal position and protect the safety of mother and baby.

In summary, the best answer is:

B C-sect

102. (Type A2 question)

Female, one year and 6 months, fever for two days, food refusal, T38.6℃, P138 times / minutes, R38 times / minutes, submandibular lymph node enlargement, pharyngeal congestion, pharyngeal palate arch, palatal pituitary, soft palate with several herpes, lingual surface and gums, and some small blisters, heart, lung and abdomen without abnormal, the most likely pathogen is

A. EB virus

B. herpes simplex virus

C. albicans Saccharomyces

D. adenovirus

E. Coxsackie virus

Reference answer: E Your answer:

[Analysis] Herpetic pharyngitis mostly occurs in infants, patients with a contact history of children, and accompanied by high fever, pharyngeal congestion, scattered gray and white herpes, can appear in the palatal arch, palatal pituitary, soft palate and other places. The patients with one year and 6 months (herpetic pharyngitis common age), fever for two days, cold (herpes pharyngitis signs), lymph node enlargement, pharyngeal congestion, pharyngeal palate arch, palate, soft palate has several herpes, tongue face and gums have small herpes, and some small herpes rupture into ulcer (herpetic pharyngitis throat performance). Therefore, patients can consider the diagnosis of herpetic pharyngitis, the main pathogen of the disease is

Coxsackievius and enterovirus 71, so the correct answer to this question is coxsackievius (E).

[Avoid mistakes] EB virus will cause white purulent discharge in the throat; mainly herpes gingival stomatitis caused by herpes simplex

Cluster small blisters and quickly rupture, along with herpes appearance of skin mucosa; Candida albicans infection causes thrush, manifested as creamy white, painful mucous membrane spots; pharyngitis caused by adenovirus infection is often accompanied by cough.

This case describes a one-and-a-half year girl with fever, food refusal, lymph node enlargement, pharyngeal congestion, and herpes and blisters in the pharyngeal arch, palatal gland, soft palate and oral cavity. These symptoms suggest that they may be caused by a certain viral infection.

-A. EB virus (Epstein-Barr virus) usually causes infectious mononucleosis, mainly manifested by fever, sore throat, enlarged neck lymph nodes, and fatigue, but it does not often lead to intraoral herpes.

-B. Herpes simplex virus (Herpes simplex virus) usually forms herpes in the mouth, especially HSV-1, which often causes herpes in the mouth.

-C. Candida albicans (Candida albicans) is a fungus that usually causes oral candidiasis, not herpes.

-D. Adenovirus (Adenovirus) mainly causes respiratory tract infections and conjunctivitis, and does not typically cause oral herpes.

-E. Coxsackievirus (Coxsackievirus) is a virus that causes hand, foot and mouth disease and can cause herpes in the mouth, but these herpes are usually accompanied by rashes on the hands and feet.

Considering the above symptoms and characteristics, the most likely pathogen is * * B. herpes simplex virus * *, as the virus usually causes multiple herpes and blisters in the mouth.

First, let's analyze the case carefully.

The child, female, one year and six months old, the main symptoms include fever and food refusal, physical examination revealed enlarged submandibular lymph nodes, pharyngeal congestion, and herpes found in the pharyngeal and palatal arch, palatal prolapse, soft palate, as well as the lingual surface and gums. These symptoms and signs strongly suggest us to consider the possibility of herpetic pharyngitis.

Next, we analyzed the options:

A. EB virus: This virus is mainly associated with infectious mononucleosis and is inconsistent with the pathogen of herpetic pharyngitis.

B. Herpes simplex virus: it mainly leads to herpes in the skin mucosa, especially lips, genital herpes, but not specific to pharyngitis.

C. Candida albicans: This is a fungus not associated with herpetic pharyngitis.

D. Adenovirus: Although adenovirus can cause respiratory tract infections, its symptoms are not consistent with herpetic pharyngitis.

E. Coxsackivirus: This is a common agent of herpetic pharyngitis. This option was highly matched with the case description, given the age, symptoms, and signs of the child.

In conclusion, combined with the age, fever, food refusal, pharyngeal congestion and herpes, the most likely pathogen is coxsackievvirus.

Therefore, the correct answer is the E. coxsackius viruses.

According to the symptoms described by the problem stem, the child had fever, food refusal, enlarged submandibular lymph nodes, pharyngeal congestion, and

herpes and some blisters in the pharyngeal and palate arch, palate, soft palate, lingual face and gums. These symptoms suggested herpetic stomatitis. The most likely pathogen is Coxsackievirus.

Coxsackievirus (Coxsackie virus) infections are common in children, especially hand, foot and mouth disease and herpetic pharyngitis, characterized by herpes in the oral mucosa and limbs.

Thus, the most likely pathogen is:

G. Coxsackie virus

103. (Type A2 questions)

A 30-year-old female, 38 weeks pregnant, with irregular abdominal pain and dark brown discharge from the vagina, was diagnosed

A. placental abruption

B. placenta praevia

C. threatened labor

D. Threatened premature labor

E. about to give birth

Reference answer: C Your answer:

[Analysis] The patient is 30 years old female, 38 weeks pregnant, irregular abdominal pain for 3 hours, vaginal outflow of dark brown secretions. Before the start of childbirth, the symptoms that indicate labor soon, called threatened labor. When close to delivery, some women can see a small amount of vaginal secretions discharge, called red, is an important symptom of threatened labor. Therefore, this patient should be diagnosed with imminent labor.

[Avoid] The general symptom of placental abruption is vaginal bleeding and large amount; placenta previa also shows vaginal bleeding symptoms; early

Birth refers to the first day of the last menstruation, after 28 weeks to less than 37 weeks of gestation for premature birth.

The 30-year-old woman, 38 weeks pregnant, developed irregular abdominal pain and dark brown discharge from her vagina. These symptoms suggest possible pregnancy complications. Here, we can exclude and consider the possibility of each option one by one:

A. Placental abruption: It usually presents with sudden abdominal pain that may have vaginal bleeding, but is usually in bright red. In the present case, the secretion was dark brown and may not be the most typical manifestation of placental abruption.

B. Placenta previa: It usually presents as painless, bright red vaginal bleeding. Given the irregular abdominal pain in this case and the secretions were not bright red, placenta previa was less likely.

C. Menoringer of labor: refers to the signs before labor, such as irregular contractions, there may be vaginal secretion changes, but usually without dark brown secretions.

D. Seminal preterm birth: the signs of delivery before 37 weeks of gestation, including contractions, cervical canal changes, and possible vaginal bleeding. But considering that the woman was already 38 weeks old, a threatened premature birth is not applicable.

E. Labor: refers to the beginning of labor, usually accompanied by regular contractions and increased vaginal discharge. Vaginal discharge may include a bloody "see red", or dark brown discharge, which may indicate that the cervix has begun to change and prepare for delivery.

Based on the above analysis, the most appropriate answer seems to be * * E. imminent labor * *, as irregular abdominal pain and dark brown vaginal outflow at 38 weeks of gestational age may be signs that the delivery is about to begin.

This is a topic about pregnancy complications and prenatal signs. We need to analyze the number of weeks of pregnancy and their symptoms to determine their current condition.

First, comb through the key information in the topic:

The patient was a 30-year-old woman at 38 weeks' gestation.

He had irregular abdominal pain for 3 hours.

Dark brown discharge flows from the vagina.

Next, explain and reason from this information:

38 weeks: belongs to the third trimester, the fetus has term.

Irregular abdominal pain: This may be a sign of contractions, but because of irregular abdominal pain, it has not yet entered the formal labor.

Vaginal outflow of dark brown secretions: this is usually a red performance, that is, the membranes near the cervix and the uterine wall separated, resulting in capillary rupture and a small amount of bleeding, is an important sign before labor.

Now, we analyze the options one by one:

A. Placental abruption: This is an emergency, usually accompanied by severe abdominal pain and vaginal bleeding, not described with the title.

B. Placenta previa: placenta previa is the placenta located in the lower part of the uterus, which may lead to prenatal bleeding, but usually without abdominal pain, and the bleeding is mostly bright red, which is inconsistent with the description of the title.

C. threatened labor: refers to the occurrence of a series of symptoms before the formal labor, including irregular contractions, fetal decline and red, which is consistent with the description of the title.

D. Seminal premature birth: this refers to the pregnancy at 28 to 37 weeks, the symptoms of premature birth, but the cervix has not opened 3cm larger, and the pregnant woman has been 38 weeks pregnant, so it is not applicable.

E. Labor: refers to the occurrence of regular contractions, progressive cervical canal shortening and other signs of labor is about to begin, but the title describes irregular abdominal pain, so it is excluded.

In conclusion, pregnant women are 38 weeks pregnant, irregular abdominal pain, vaginal outflow of dark brown secretions, the most likely diagnosis is threatened labor.

Therefore, the answer is C. threatened labor.

The title description mentioned that the patient was 38 weeks pregnant, with irregular abdominal pain for 3 hours, and dark brown secretions flowed from the vagina. Based on these symptoms, the most likely diagnosis is:

A. placental abruption

Placental abruption (placental abruption) is when the placenta is partially or completely dissected from the uterine wall before delivery of the fetus, and symptoms include abdominal pain, vaginal bleeding (usually dark red or dark brown), consistent with the symptoms mentioned in the title.

Other options are explained as follows:

B. Placenta previa: There is usually painless bright red vaginal bleeding, but the abdominal pain and dark brown secretions mentioned in the topic do not fit this diagnosis.

C. Menoringer labor: usually refers to symptoms near delivery, including regular contractions and a small amount of vaginal bleeding, but the irregular abdominal pain and dark brown secretions described in the title do not consistent.

D. threatened term birth: Although abdominal pain may be associated with preterm birth, dark brown discharge from the vagina is more typical of placental abruption.

E. Labor: This means that the labor process has begun, usually accompanied by regular contractions and changes in the cervix, not quite consistent with the irregular abdominal pain in the problem.

In summary, the most correct answer is:

A. placental abruption

104. (Type A2 questions)

Male, 70 years old, walking slowly with right upper limb swing disappear 2

years, often fall, physical examination: facial dull, neck muscle tension, right limb muscle tension is higher than the left, static, right hand tremor, limb muscle strength level 5, tendon reflexes, bilateral Ba, the rest of the neurological examination without abnormalities, head MRI: two small lacuna in the cortex. The most likely diagnosis is the

A. essential tremor

And B. Parkinson's overlay syndrome

B. Vascular Parkinson's syndrome

C. MSA

D. agitans paralysis

Reference answer: E Your answer:

[Analysis] Parkinson's disease is more common in the elderly, the main symptoms include static tremor, muscle rigidity, bradykinesia, postural gait disorder, pain, paresthesia, dysphagia and speech disorders, typical patients can be shown as mask-like manifestations. The 70-year-old male patient (age of common onset of Parkinson's disease), slow walking with right upper limb swing disappeared for 2 years, frequent falling (Parkinson's disease symptoms: postural gait disorder with bradykinesia), rigid physical examination, high cervical muscle tone (muscle stiffness), and sedentary

When, the right hand tremor (static tremor), limb muscle strength level 5, tendon reflexes decreased. Therefore, this patient may be diagnosed as (E)

agitans paralysis.

[Error] essential tremor is usually accompanied by family history, and its typical symptoms are the only manifestations; Parkinson syndrome is a combination of multiple system atrophy, progressive supruclear palsy, Lewy body dementia and cortical basal ganglia degeneration; multiple system atrophy is accompanied by consciousness disorder, poor autonomic function, and urinary incontinence; vascular Parkinson syndrome is mostly sudden onset after acute stroke, showing movement disorder of both lower limbs, and the typical symptoms are "magnetic foot reaction".

This case is described typical of Parkinson's disease (PD). Symptoms include slow walking, reduced swing on one side of the limb, reduced facial expression (facial rigidity), increased muscle tone (especially in the neck and right limb), static tremor (especially in the right hand), and normal muscle strength but decreased tendon

reflexes. These are the most common clinical manifestations of PD. The small intracortical lacunar foci revealed by cranial MRI may be vascular changes but not sufficient to explain the full symptoms.

-Item A "essential tremor" mainly shows bilateral symmetrical tremor, especially during exercise, which does not meet the description of this case.

-Item B "Parkinson's superposition syndrome" and item D "multisystem atrophy" both refer to having additional non-motor symptoms or broader systemic involvement, which is not specified in the problem stem.

-C "Vascular Parkinson's syndrome" refers to Parkinson-like symptoms caused by vascular problems, although MRI has lacunar lesions, it is atypical and is not enough to support this diagnosis.

Therefore, the most likely diagnosis is item E "Parkinson's disease".

This is a diagnostic reasoning problem, and we need to extract key information from the question stem and analyze it combined with medical knowledge.

Key information arrangement:

An elderly male patient, walking slowly, loss of right upper limb swings and frequent falls.

Physical examination: facial rigidity, high cervical muscle tone, muscle tension of the right limb was higher than that of the left side, right hand tremor when sitting, muscle strength of the limbs was normal, tendon reflex was decreased, and bilateral pathological signs were positive.

Cranial MRI revealed 2 small lacunar foci within the cortex.

Next, we analyze the options one by one:

A. essential tremor: This is an unexplained tremor that usually does not affect muscle tone or motor function and is not consistent with the title stem description.

B. Parkinson's superposition syndrome: This is a neurodegenerative disease similar to Parkinson's disease, but is usually accompanied by other systemic symptoms, such as cerebellar ataxia, extrapyramidal symptoms, which are not mentioned in the problem stem.

C. Vascular Parkinson's syndrome: This syndrome is usually associated with cerebrovascular disease, manifested by increased muscle tone, reduced movement, tremor and other symptoms. Considering the patient's age, symptoms (increased

muscle tone, tremor), and the skull MRI.

D. Multisystem atrophy: This is a degenerative disease of the nervous system, usually affecting multiple systems, including the cerebellum, brain stem, etc., which are not mentioned in the stem.

E. Parkinson's disease: Although Parkinson's disease also causes increased muscle tone and tremor, it is usually not accompanied by cerebrovascular lesions, such as lacunar lesions.

In conclusion, the elderly male patient, slow walking with right upper limb swing disappeared, often falls, physical examination showed facial rigidity, high cervical muscle tone, the right limb muscle tone is higher than that of the left side, the right hand tremor while sitting, head MRI showed a small lacuna focus in the cortex, the most likely diagnosis is vascular Parkinson's syndrome.

Therefore, the correct answer is C. vascular Parkinson's syndrome.

According to the description, the 70-year-old male patient showed slow walking, loss of swing in the right upper extremities, frequent falls, facial rigidity, high cervical muscle tone, higher muscle tone in the right limb than the left side, and tremor of the right hand while sitting. Combined with the physical examination and MRI results (2 small lacunar foci in the cortex), the most likely diagnosis is:

* * C. vascular Parkinson's syndrome * *

The reasons are as follows:

1. * * Vascular Parkinson's syndrome * *: This syndrome is common in the elderly and is associated with small vessel lesions. MRI showed small lacunar foci within the cortex, which supports the diagnosis of vascular lesions. The patient's Parkinson's symptoms, such as high muscle tension, tremor, facial rigidity, slow walking, and disappearance of right upper limb swing, are also consistent with the manifestations of vascular Parkinson's syndrome.

2. * * Sential tremor * *: Usually postural or intentional tremor, without symptoms such as high muscle tone and slow walking.

3. * * Parkinson's superposition syndrome * *: Parkinson's disease coexist with other neurodegenerative diseases, but the patient's MRI shows vascular lesions, which is more consistent with vascular Parkinson's syndrome.

4. * * Multisystem atrophy * *: Although Parkinson-like symptoms also occur, they are usually accompanied by autonomic dysfunction, such as orthostatic hypotension, bladder dysfunction, and MRI usually shows cerebellar atrophy or brainstem atrophy.

5. * * Parkinson's disease * *: Typical Parkinson's disease MRI usually does not show obvious lesions, while the MRI of this patient shows vascular lesions, which is more consistent with vascular Parkinson's syndrome.

In conclusion, the most likely diagnosis is * * C. vascular Parkinson's syndrome * *.

105. (Type A2 questions)

Female, 30 years old, had irregular vaginal bleeding for 20 days and cough for 5 days. Artificial abortion half a year ago, gynecological examination: the size, soft, cystic mass about 5cm in bilateral accessory areas, chest X-ray showed bilateral spherical shadow. The first diagnosis considered is the

A. chorioepithelioma

B. acephalocystis racemosa

C. invasive mole

D. threatened abortion

E. missed abortion

Reference answer: A. Your answer:

[Analysis] Choriocarcinoma is mostly manifested in hydatidiform fetal palace, term delivery, spontaneous abortion, induced abortion, ectopic pregnancy after irregular vaginal bleeding, cough, convulsions, etc. It is mainly reflected in purple-blue vaginal nodules, uterine enlargement, paraintrauterine mass, and multiple cotton-like shadows in chest X-ray film. The patient was 30 years old (good age of veloriocarcinoma), with induced abortion (common cause of choriocarcinoma), irregular vaginal bleeding for 20 days, cough for 5 days (common signs of veloriocarcinoma). On gynecological examination, the uterus was the size of 40-day pregnancy, soft, cystic mass of about 5cm in size (imaging findings in veloriocarcinoma) could be reached in bilateral adnexa, and chest X-ray showed multiple cotton ball shadows in bilateral middle and lower fields (chest imaging findings of veloriocarcinoma). Patients were therefore considered for diagnosis of (A) caveocarcinoma.

[Avoid mistakes] The hydatidiform mole shows irregular vaginal bleeding after menopause, which is easy to be misdiagnosed as threatened abortion. Later, abnormal enlargement and softening; invasive hydatidiform mole showed irregular vaginal bleeding 3 to 4 weeks; and no chest radiographs showed cotton globular shadow.

In this case, a 30-year-old female patient had irregular vaginal bleeding for 20 days and a cough for 5 days, and had had an induced abortion six months ago. Gynecologic examination revealed that the uterus was similar to the size of a 40-day pregnancy with soft texture, a cystic mass of about 5cm size palpable in the bilateral adnexal area, and chest X-ray showed bilateral globular shadow. Based on these clinical findings, the most likely diagnosis is choriocarcinoma (A).

Ploriocarcinoma (also known as trophoblastic tumor) is a highly malignant pregnancy-associated tumor that usually occurs after a hydatidiform mole. Choriocarcinoma features include irregular vaginal bleeding, uterine enlargement beyond pregnancy expectations, cystic mass (usually associated with extrauterine metastases), and typical globular shadows on the chest X-ray, indicating distant metastases, especially lung metastases. Therefore, based on these symptoms and clinical manifestations, choriocarcinoma is the most compatible diagnosis.

Other options, such as hydatidiform (B) and invasive hydatidiform (C), although associated with choriocarcinoma, usually do not cause such severe symptoms or distant metastasis, while invasive hydatidiform mole may have similar symptoms, but choriocarcinoma is more malignant and has more significant symptoms and signs.

The threatened abortion (D) and retained abortion (E) are mainly manifested as bleeding and pain during early pregnancy, which do not consistent with the clinical manifestations of this case. Therefore, the most correct choice is A. pomenocarcinoma.

First, let's carefully analyze the key information in the problem stem, in order to answer the problem more accurately.

The patient was a 30-year-old woman with symptoms of irregular vaginal bleeding and a cough. Half a year ago, induced abortion, now gynecological examination found uterine enlargement, cystic mass in the bilateral accessory area, chest X-ray also showed bilateral spherical shadow. This information is essential to determine the diagnosis.

Now, let's analyze the options, one by one:

A. Ploriocarcinoma: This is an important risk factor for choriocarcinoma, considering that the patient's history of induced abortion six months ago. Ploriocarcinoma can occur after miscarriage, term delivery, or mole and may have irregular vaginal bleeding and lung metastases (as in X-ray). These characteristics are consistent with the patient's symptoms, so that choriocarcinoma is a reasonable diagnostic consideration.

B. Hydatidiform mole: Although hydatidiform mole may also cause uterine enlargement and irregular vaginal bleeding, it does not usually occur after miscarriage, and the hydatidiform mole is not mentioned in the stem, so this option is unlikely.

C. Invasive hydatidiform mole: This is a malignant tumor, but it usually originates from the hydatidiform mole, not abortion, and no information directly related to the mole is mentioned in the title stem, so this option is unlikely.

D. Seminal abortion: Seminal abortion usually occurs in the first trimester of pregnancy, accompanied by abdominal pain and vaginal bleeding, but the symptoms mentioned after abortion, and threatened abortion is not accompanied by shadows of the lungs, so this option can be excluded.

E. The abortion: the abortion refers to the failure of the embryo or fetus to discharge naturally in time after the death in utero, but the problem is clearly pointed out that the patient had an induced abortion six months ago, and the current symptoms (such as uterine enlargement, attachment mass, lung shadow) are inconsistent with the abortion, so this option can also be excluded.

In conclusion, combining the patient's history, symptoms and imaging findings, the most likely diagnosis was villonocarcinoma. So, the correct answer is:

A. chorioepithelioma.

Based on the medical history and examination results provided, the 30-year-old woman developed irregular vaginal bleeding and symptoms of cough. Gynecological examination revealed uterine enlargement and cystic mass involvement in the bilateral adnexa area. Furthermore, the chest X-ray showed bilateral globular shadows. Combined with this information, the most likely diagnosis is velonocarcinoma (A. velonocarcinoma).

Meloriocarcinoma (choriocarcinoma) is a highly malignant and rare tumor that often occurs in the reproductive organs, especially after pregnancy. It often presents as irregular vaginal bleeding, and the globular shadow of the chest X-ray indicates lung metastasis, which is one of the typical manifestations of choriocarcinoma. The patient had an induced abortion six months ago, which is also one of the risk factors for choriocarcinoma. Although hydatidiform mole and invasive hydatidiform mole have similar manifestations, the spherical shadow of chest X-ray is more pointed to choriocarcinoma.

So the best answer is:

A. chorioepithelioma

Girl, 4 years old, fever cough and runny nose 4 days, rash 1 day, temperature 39.5°C, heart rate 130 times / min, breathing 38 times / min, depression, head and neck rash, normal skin, eye node secretions, lymph nodes not touched, rough oral congestion, normal cardiopulmonary, liver and spleen not touched, hemoglobin 115g / L, white blood cells 6.5109 / L, neutrophils 0.35, lymphocytes 0.6, platelet 261

106, (Type A3 / A4 questions) subject

The most likely diagnosis is the

A. nettle rash

B. scarlatina

C. hives

D. Dukes' disease

E. drug rash

Reference answer: C Your answer:

[Analysis] The patient had fever for 4 days and rash for 1 day, red rash on the head and neck, the skin was normal, the conjunctiva was congested with secretions, and the lymph nodes were not touched, which met the diagnosis of measles (C pair).

[Avoid mistakes] Rubella is an eruption on the second day of fever, and come out within 1 day, the order of eruption: face-neck-trunk-limbs (A

wrong). Scarlet heat rash appeared rapidly 24 hours after the onset, the order of eruption: neck-axillary —— groin, Pa line, bayberry tongue (B wrong). Acute rash is characterized by a sudden drop in heat after 3~5 days of fever, and rose red maculopapular rash on the skin. The rash is mainly seen in the trunk, neck and upper limbs, and can be cured quickly and without complications (D wrong). Drug rash is drugs by oral,

Skin and mucosal inflammation caused by external use and injection. Almost all drugs may cause dermatitis, but the most common are sulfonamides, antipyretic analgesics, sleeping pills, penicillin, streptomycin, etc. (E wrong).

The most likely diagnosis is:

* * Rubella (A) * * Usually presents with mild fever, enlarged cervical lymph nodes, and a typical rash.

* * Scarlet fever (B) * * usually has high fever, sore throat, strawberry tongue, and tiny rashes, especially in the trunk and folds.

* * Measles (C) * * is characterized by high fever, coplick spots, cough, runny nose, conjunctivitis and characteristic red rash.

* * Children rash (D) * * Most common in children, manifested as rash after sudden fever of high fever.

* * Drug rash (E) * * is usually associated with drug intake in a variety of rash types.

Considering the above information, the most consistent for these symptoms is measles (C) because the patient had high fever, cough, runny nose, listlessness, conjunctival discharge and head and neck rash.

analyze:

The patient was infantile female with fever, cough and runny nose for 4 days, and then developed red rash, which was typical prodromal measles. Physical examination found that the head and neck appeared rash, normal skin, conjunctivitis, rough oral congestion, typical of measles rash stage. The blood routine results indicated the presence of a viral infection. Measles diagnosis was considered based on the patient's clinical presentation, signs and laboratory tests.

Option A Rubella has mild systemic symptoms, posterior and posterior lymph nodes enlargement and tenderness, the rash usually appeared 1 to 2 days after the onset of symptoms, and the eruption order of the face, neck, trunk and limbs, which is not consistent with the description of the trunk.

Option B Scarlet fever has fever, pharyngitis, bayberry tongue, pale ring and other manifestations, and the rash is characterized by diffuse skin congestion, dense papules of needle size, and the skin of the whole body, accompanied by peeling, which is obviously different from the symptom description of patients.

Option D Children's acute rash is characterized by hot rash, and mostly red small and dense maculopropapular rash, head, face, neck and trunk, less limbs, one day, the next day began to subside, which is not consistent with the symptoms of the patients in the stem, so it is excluded.

Option E drug rash had recent medication history and various rash types, but it was accompanied by itching, and the rash can subside after drug withdrawal. The patient did not mention recent medication history, which was not consistent with the question.

In conclusion, the correct answer is: C. measles.

106 The most likely diagnosis is a diagnosis

A. nettle rash

B. scarlatina

C. hives

D. Dukes' disease

E. drug rash

analyze:

According to the medical history and clinical manifestations, such as fever, cough, runny nose, rash (head and neck rash, normal skin), ocular conjunctival secretions, rough oral congestion (Ke's spot) and other characteristics, the most likely diagnosis is measles.

Correct answer: C. measles

107, (A3 / A4 type question) subject

The most likely complication is that

A. pneumonia

B. pyopneumothorax

C. pulmonary abscess

D. carditis

E. measles encephalitis

Reference answer: A. Your answer:

Graphic analysis: [analysis] children accord with the diagnosis of measles. The most common complication of measles is pneumonia (pair A).

[Avoid mistakes] Measles patients should be isolated until 5 days after the eruption, and if complicated with pneumonia, they should be isolated until 10 days after the eruption.

The most likely complications are:

Common complications of measles include pneumonia (A), ear infection, diarrhea, and one of the most serious complications, measles encephalitis (E).

Considering the serious complications of measles, * * measles encephalitis (E) * * is an important complication, but it requires attention to the development of symptoms to be determined.

analyze:

The most common complication of measles is pneumonia, which is diagnosed with measles, so the most likely complication is pneumonia.

Option B pypneumothorax, option C lung abscess, mostly common in the lung suppurative bacterial infection, measles is generally not complicated with such

diseases, so it is excluded.

Option D myocarditis can be secondary to viral infection, but it is less common than pneumonia, so it is not chosen.

Option E had a low incidence of measles and encephalitis, so it was excluded.

In conclusion, the correct answer is: A. pneumonia.

107 The most likely complication is that

A. pneumonia

B. pyopneumothorax

C. pulmonary abscess

D. carditis

E. measles encephalitis

analyze:

Common complications of measles include pneumonia and encephalitis. Combined with the child's symptoms and medical history, the most likely complication is measles encephalitis.

Correct answer: E. Measles and encephalitis

108, (Type A3 / A4 questions)

If the fever, the head and trunk rash, both lungs breathing sound fine wet rales, the best treatment measure is subject

A. Isolation treatment

B. antianaphylaxis

C. gamma globulin

D. antibiotic

E. aspirin

Reference answer: A. Your answer:

[Analysis] If measles subsided after fever, rash on the head and trunk, breathing in both lungs is fine and wet rales, considering pneumonia, the best treatment measure

is isolation treatment (A pair).

[Avoid mistakes] Patients with measles can be injected within 5 days to prevent the onset or reduce symptoms. Patients with measles do not need antibiotics, and antibiotics can be given if secondary bacterial infection.

If the fever decreases, the head and trunk are rash, and the breathing sound of both lungs is fine and wet. The best treatment measures are:

Fine wet rales may indicate a lower respiratory tract infection or pneumonia, when * * antibiotic (D) * * treatment is appropriate, especially in the case of measles-induced complications such as bacterial pneumonia.

analyze:

The patient has a clear diagnosis of measles. If there is a rash on the head and trunk, which can be heard in both lungs, pneumonia should be considered. The common pathogenic bacteria of measles and pneumonia are Staphylococcus aureus, Streptococcus pneumoniae, haemophilus influenzae, etc., so the best treatment measure is to use antibiotics against these bacteria.

Option A isolation is A general treatment for measles, but not the best treatment and is inconsistent with the topic.

Option B is anti-allergy, and option C gamma globulin is generally used for the treatment of urticaria or allergic diseases. The patient is not an allergic disease and can be excluded together.

Option E aspirin generally does not do antimicrobial treatment, unrelated to this question.

To sum up, the correct answer is: D. antibiotics.

108. If the fever has subsided, the head and trunk rash, both lungs breathing sound fine wet rales, the best treatment measure is

A. Treatment in isolation

B. antianaphylaxis

C. gamma globulin

D. antibiotic

E. aspirin

analyze:

After fever, the child appeared fine wet rales, indicating that it may be combined with pneumonia, and measles patients with bacterial infection.

Correct answer: D. antibiotics

The child, 7 years old, left knee pain for 3 days, chills and high fever for 1 day, physical examination: T: 39.5℃, swelling and pain near the joint of the left lower femur, physical examination was obvious tenderness, and floating patellar test was negative. Laboratory examination was 2210 ^ 9 / L, N0.86.

109, (Type A3 / A4 questions) subject

The most likely diagnosis is the

A. giant cell tumor of bone

B. suppurative arthritis

C. osteosarcoma

D. Acute hematogenous osteomyelitis

E. tuberculosis of knee joint

Refer to the answer: D. Your answer:

[Analysis] Children, 7 years old (acute blood-borne osteomyelitis mostly occurs in children and adolescents), left knee pain for 3 days, chills and high fever for 1 day, T 39.5℃, swelling and pain near the joint of the left inferior femur (in the long shaft), obvious tenderness, negative floating patellar test (excluding option B). Laboratory tests: blood WBC 2210 ^ 9 / L (signs of infection, excluded option E), considering the diagnosis of acute bloody osteomyelitis (pair D).

[Error avoidance] Bone giant cell tumor is mostly seen in women between 20 and 40 years old, and typical X-ray is soap-like bone change at the lesion (A error); osteosarcoma, the main symptoms are local pain, especially heavy at night, and typical X-ray is Codman triangle or solar radial periosteal reaction (C error).

Question 109: The most likely diagnosis is

A. Giant cell tumor of bone: This is a less common benign bone tumor that usually does not cause acute symptoms such as high fever and chills.

B. Pogenic arthritis: usually joint inflammation, but the swelling in this case was

in the lower segment of the femur, not intraarticular, and a negative floating patella test, not consistent with the typical findings of purulent arthritis.

C. Osteosarcoma: It is a malignancy, but its symptoms usually progress more slowly, and a sudden onset of high fever and chills is unlikely.

D. Acute bloodborne osteomyelitis: meet the symptoms of high fever, chills, local bone pain and swelling, and increased white blood cell count, is the manifestation of acute infection.

E. Knee tuberculosis: Although joint tuberculosis can cause pain and swelling around the joint, it usually progresses slowly with chronic symptoms and no acute high fever and chills.

Based on the above analysis, the most likely diagnosis was D. acute hematogenous osteomyelitis.

analyze:

Children, left knee pain, chills, high fever, physical examination see high fever, left femur near the lower joint swelling pain, obvious tenderness, floating patellar test negative, laboratory examination see increased white blood cells, higher proportion of neutrophils, according to its clinical manifestations and laboratory examination, the patient with the most likely diagnosis of acute blood osteomyelitis.

Option A Giant cell tumor of bone usually showed pain and swelling, intermittent dull pain, local tenderness and limited joint movement, and increased local skin temperature and joint swelling, which did not consistent with the description of the problem stem, so it was not selected.

Option B purulent arthritis occurs in the hip joint and knee joint, mainly manifested as local joint temperature increase, swelling, pain, and joint effusion. The floating patella test is often positive, which is inconsistent with the negative floating patellar test in the main stem, so it is excluded.

Option C osteosarcoma occurs at the epiphysis of the long shaft, especially the distal femur and the proximal tibia. It shows local pain, which is mostly persistent and gradually aggravated, especially at night, accompanied by systemic cachexia, fever and anemia, which is significantly different from the symptoms of the child, so it is not considered.

Option E Knee tuberculosis onset is slow, low fever, fatigue, fatigue, poor appetite, wasting, anemia and other systemic symptoms, swelling around the knee

joint, the child has no history of tuberculosis, and no above symptoms, inconsistent with the meaning of the question.

In conclusion, the correct answer is: D. Acute bloodborne osteomyelitis.

Topic 109: Most likely diagnosis

Analysis: Based on the history and clinical manifestations, the child developed high fever, local pain and swelling, and laboratory tests showed elevated total white blood cells and a high proportion of neutrophils, which indicated the presence of infection. The following is the option analysis:

A. Giant cell tumor of the bone: usually A benign tumor, common in adolescents and adults, with progressive pain and swelling that is unlikely to lead to acute high fever and a significant increase in total white blood cells.

B. Pogenic arthritis: acute inflammation of the joint, redness, heat and pain, but the negative floating patella test indicates that fluid accumulation in the joint cavity is not obvious.

C. Osteosarcoma: Malignant bone tumors, more often in children and adolescents, usually chronic progressive pain and swelling, unlikely to cause acute fever and high white blood cell count.

D. Acute blood-borne osteomyelitis: acute onset, manifested as high fever, local pain, swelling, tenderness, significantly increased total proportion of white blood cells and neutrophils, consistent with the manifestations of this case.

E. Knee tuberculosis: chronic course, manifested by long-term low fever, night sweats, gradually increasing pain and swelling, not consistent with acute high fever and laboratory manifestations.

Based on the above analysis, the most likely diagnosis was that of D. acute hematogenous osteomyelitis.

110, (A3 / A4 type question) subject

The preferred antibiotics for treatment

A. Three generations of cephalosporin

B. Ofloxacin

C. penicillin

D. gentamicin

E. clindamycin

Reference answer: A. Your answer:

Graphic analysis: [Analysis] The child was considered to as acute osteomyelitis. Acute blood antibiotic treatment should be started immediately, once diagnosed. The most common pathogenic bacterium is Staphylococcus aureus, and cephalosporin antibiotics are preferred for treatment (an A pair).

[Error avoidance] After the disappearance of systemic and local symptoms, antibiotics still need to be applied continuously for 3 to 6 weeks.

Question 110: The preferred antibiotic for treatment

A. Three generation cephalosporin: Three generation cephalosporin antibiotics (such as ceftriaxone) are a good choice for treating acute bloody osteomyelitis because they have good coverage against both Gram-positive and Gram-negative bacteria.

B. ofloxacin: This is a broad-spectrum antibiotic, but it is not the first choice for the treatment of osteomyelitis, especially in children, because of its potential effects on bone.

C. Penicillin: Although effective for some infections, its coverage may not be wide enough for osteomyelitis.

D. Gentamicin: This is a powerful antibiotic, but it is mainly used for complex or refractory infections, and has a high risk of toxicity.

E. Clindamycin: Use for bone and joint infections, but not preferred, especially in the initial treatment of acute bloody osteomyelitis.

Therefore, the preferred antibiotic for treatment should be A. Cephalum.

analyze:

For the treatment of acute blood-borne osteomyelitis, early, sufficient, combined and sensitive antibiotics are needed. For the patient's situation, the first antibiotic sensitive to Staphylococcus aureus is preferred, and penicillin * * has a strong antibacterial effect on Staphylococcus aureus, so penicillin is preferred.

Option A generation cephalosporin has A stronger antibacterial effect on Gram-negative bacilli. It is often used in pneumonia caused by Klebsiella pneumoniae. It is not the first choice for Staphylococcus aureus, so it is not selected.

Option B ofloxacin is a quinolone antibiotic, often used in respiratory tract and urinary tract infections caused by streptococcus pneumoniae, and is not the first choice for Staphylococcus aureus, so it is excluded.

Option D Gentamicin is an aminoglycoside antibiotic, which has good antibacterial effect on various gram negative bacteria and gram positive bacteria, but not good effect on Staphylococcus aureus, so it is not considered.

Option E clindamycin is mainly used for abdominal and pelvic infections caused by anaerobic bacteria, but also can be used for sensitive respiratory tract, joints, skin and soft tissue, bone tissue, biliary tract infection and sepsis, endocarditis, etc., which is not consistent with the problem.

In conclusion, the correct answer is: C. penicillin.

Topic 110: Antibiotics preferred for treatment

Analysis: For the treatment of acute blood-borne osteomyelitis, broad-spectrum antibiotics are preferred, especially those covering Staphylococcus aureus (the most common pathogen). The following is the option analysis:

A. Three-generation cephalosporin: such as cefotaxime or ceftriaxone, which is effective against Staphylococcus aureus, is representative of broad-spectrum antibiotics and suitable for preferred treatment.

B. ofloxacin: a quinolone antibiotic that is not recommended in children.

C. Penicillin: Although effective against streptococcus, it has limited effect against Staphylococcus aureus.

D. Gentamicin: mainly used for Gram-negative bacteria infection, high toxicity, limited effect alone.

E. Clindamycin: effective against anaerobic bacteria and can be used as an alternative, but it is still a three-generation cephalosporin.

Based on the above analysis, the preferred antibiotic for treatment is A. Cephalosporium.

Maternal, 32 years old, 39 weeks pregnant, estimated fetal weight 3800g, normal pelvic measurement, regular contractions 15 hours, 8 minutes, 30 seconds, uterine opening 4cm, broken membranes, LOA, S Figure type I.

111. (Type A3 / A4 questions)subject

The following is possible

A. Lack of uterine coordination

B. Weak uterine discoordination

C. Excessive contraction of uterine coordination

D. Excessive uterine incoordination tcontraction

E. Reduced diaphragm contraction

Reference answer: A. Your answer:

[Analysis] Lack of uterine coordination contraction (hypotonic uterine contraction weakness) uterine contraction has normal rhythm, symmetry and polarity, but weak contraction force, low uterine pressure (<2.0 kPa), short duration, long and irregular interval, contractions <2 times / 10 minutes. When the uterine contraction reaches the polar stage, the uterine body does not bulge and harden, with fingers pressing the bottom of the uterine muscle wall can still appear depression, prolonged labor or stagnation. The patient was 32 years old, 39 weeks pregnant, estimated fetal weight of 3800g, normal pelvic measurement, regular contractions for 15 hours, intermittent for 8 minutes for 30 seconds, 4cm, broken membranes, LOA, S= -1, electrocardiogram type I (pregnant women have good production, prolonged labor). Therefore, the patient's prolonged labor period may be (A) poor uterine coordination.

[To avoid mistakes] Lack of uterine disharmony is mainly manifested as tenderness in the lower abdomen, unclear fetal position, irregular fetal heart, slow or no dilation, delayed decline of the fetus or stagnation, and prolonged labor process.

First, analyze the question 111:

Item A "weak uterine coordination" refers to the lack of intensity of contractions, which may lead to prolonged labor.

Item B "lack of uterine disharmony" refers to the irregular intensity and frequency of contractions, which may also lead to prolonged labor.

B. "strong uterine contraction" and D "strong uterine contraction" both indicate that contractions are too strong.

Item E "weakened diaphragmatic contraction" has little to do with the delivery process.

Considering that the uterine opening is 4cm, the regular contractions have lasted

for 15 hours, but the progress is not significant, the most likely case is the lack of contractions, so "weak uterine coordination" may be the correct answer.

According to the description of the title, the pregnant woman regularly contractions for 15 hours, intermittent for 8 minutes, lasting 30 seconds, and opened for 4cm. These manifestations are in line with the characteristics of weak coordination contractions. Now we'll analyze the options item by item:

A. Lack of uterine coordination: Given A long interval (8 minutes) and a relatively short duration (30 seconds), this is consistent with the weakness of coordination contractions, that is, rhythmicity, symmetry, and polarity are normal, with only weak contractility.

B. Lack of uterine discoordination: this fatigue is usually manifested as a rhythmic abnormality of contractions, and the problem described in the stem is regular contractions, so it does not conform.

C. Excessive uterine coordination contraction: this can lead to too frequent and strong contractions, inconsistent with the contractions described by the title stem.

D. Excessive uterine contraction: this is usually manifested as abnormal and strong contractions rhythm, inconsistent with the description of the topic stem.

E. Reduced diaphragm contraction: this is not directly related to the weakness of contractions described in the stem, and the diaphragm contraction was not mentioned in the stem.

In conclusion, the most likely answer is A. Lack of uterine coordination.

Question 111: The following is possible

A. Lack of uterine coordination

B. Weak uterine discoordination

C. Excessive contraction of uterine coordination

D. Excessive uterine incoordination tcontraction

E. Reduced diaphragm contraction

analyse:

The patient had regular contractions for 15 hours, but the uterine opening was only open to 4cm, indicating a slow progression of labor. Combined with the

frequency of contractions (8 minutes intermittent, for 30 seconds), this indicates that the intensity and frequency of contractions are not enough, in line with the characteristics of "weak uterine coordination".

Correct answer: A. Lack of uterine coordination

112, (A3 / A4 type question) subject

The most appropriate treatment measure is this

A. Artificial membrane breaking

B. Ditilloxytocin

C. C-sect

D. intramuscular pepersine

E. intramuscular diazepam

Reference answer: B. Your answer:

[Analysis] For the treatment of uterine coordination can be applied oxycotocin intravenous infusion, mainly suitable for coordination of uterine contraction weakness, uterine mouth dilation 3cm, good fetal heart, normal fetal position, head and basin commensurate. Therefore, we should choose static drop oxytocin (B).

[To avoid mistakes] If the head basin is not weighed, it is estimated that can not vaginal delivery, should be timely cesarean section; if no head basin is not weighed and abnormal fetal position, estimated that vaginal delivery, should be taken to strengthen contractions. Artificial membrane breaking: uterine mouth expansion of 3cm, no head basin, fetal head has been connected, feasible artificial membrane breaking. Piperidine, diazepam is mainly sedative and cannot enhance contractions.

Then analyze the most appropriate treatment measures in the 112 questions:

Item A "artificial film" is usually used to accelerate contractions, but the question stem mentioned that the fetal film has been broken, so this item is not applicable.

Item B "still drop oxytocin" is used to enhance contractions and is suitable for cases of weak contractions.

Item C "cesarean section" is the option considered when a vaginal birth is impossible or when there is a maternal and infant risk.

Item D "intramuscular pethidine" for analgesia and item E "intramuscular

diazepam" for sedation. These two items are more symptomatic treatment and do not solve the fundamental problem.

Given the above analysis, the answer to 111 should be A "weak uterine coordination" and to 112 should be B "totocin" because it enhances contractions and helps advance the labor process.

In view of the pregnant woman's uterine contraction weakness, we need to choose a proper treatment measure. Now we analyze the various options:

A. Artificial film breaking: Although artificial film breaking is sometimes used to strengthen contractions, it has been clear that the fetal membrane has been broken in the problem stem, so this option is not applicable.

B. Oxytocin: For coordinated contractions, when the uterine mouth expands 3cm, the fetal heart is good, the fetal position is normal, and the head and basin are commensurate, oxytocin can be infused to strengthen the contractions. The situation in the title stem meets these conditions, so it is an appropriate treatment measure.

C. Cesarean section: Cesarean section is not the preferred treatment option in the absence of other complications or emergencies.

D. intramuscular piperidine: This is usually used to treat discordant contractions, inconsistent with the contractions described by the title stem.

E. Incular diazepam: also used for the treatment of incongruous contractions, not for the situation in this question.

In conclusion, the most appropriate treatment is B. paroxytocin.

Question 112: The most appropriate treatment measure is this

A. Artificial membrane rupture

B. Still-drop drop oxytocin

C. C-sect

D. intramuscular pepersine

E. intramuscular diazepam

analyse:

Given the lack of uterine coordination, the best treatment is the use of oxytocin to enhance uterine actions and promote the progression of labor. Other options such as

artificial membrane rupture and cesarean section are not applicable in this situation, while pethidine and diazepam are mainly used for analgesia and sedation and are not preferred for the treatment of uterine fatigue.

Correct answer: B. Oxytocin

Boy, 6 months, diarrhea 6 months, 4~7 times / day, sticky stool, no vomiting, no fever, fair appetite, fair spirit, birth weight 3.2kg, present weight 7.1kg, simple breastfeeding, dehydrated appearance, normal nutritional development, no abnormal heart and lung, abdominal bowel peristalsis, normal bowel sound.

113. (Type A3 / A4 questions) subject

The diagnosis considered is

A. Allergic diarrhea

B. infectious diarrhea

C. Physiological diarrhea

D. disorders of digestion

E. Chlorine-loss diarrhea

Reference answer: C Your answer:

[Analysis] physiological diarrhea is more common in infants under 6 months, its appearance is fat, often eczema, diarrhea soon after birth is diarrhea, the number of stool every day, or even more than a dozen times, each time the amount of stool is not necessarily a lot, which contains a small amount of water, generally no special smell. Infants with physiological diarrhea in addition to the number of stool increased, more than no other symptoms, good appetite, no vomiting, growth and development is not affected, generally add complementary food is restored to normal. Patients for a boy, 6 months, diarrhea 6 months, 4~7 times / day (diarrhea), sticky stool (stool traits without abnormal), no vomiting, no fever, appetite spirit, normal weight gain, simple breastfeeding (physiological diarrhea general cause), signs without dehydration appearance, normal nutrition, cardiopulmonary abnormalities, abdominal peristalsis, normal sounds (no obvious pathological symptoms). Therefore, the children can be diagnosed as (C) physiological diarrhea.

[Avoid] Allergic diarrhea should be accompanied by allergic manifestations in other parts; infectious diarrhea is often accompanied by fever; the growth and

development of the child may be affected to some extent; chlorine loss diarrhea with intractable diarrhea, hyponachanemia, hyponatraemia, hypokalemia and metabolic alkalosis, mainly characterized by watery diarrhea.

In Question 113, the diagnosis considered is:

Allergic diarrhea (A): Given the persistent symptoms of infant diarrhea and the background of breastfeeding alone, allergic diarrhea becomes a possible diagnosis. This condition may be caused by certain food components in breast milk (such as milk protein), leading to digestive tract reactions in the infant.

Infectious diarrhea (B): usually fever, and vomiting, which were not mentioned in this case.

Physiological diarrhea (C): usually physiological diarrhea in the neonatal period and is not suitable for 6-month-old infants.

Digestive disorders (D): may be taken into account, but more evidence is often needed to support this diagnosis.

Chlorine-loss diarrhea (E) is a less common disease that usually requires other symptoms such as electrolyte imbalance to support the diagnosis.

Therefore, for question 113, the most likely diagnosis is allergic diarrhea (A).

The correct answer is: C. Physiological diarrhea

The child was 6 months old, and the diarrhea had lasted for 6 months, 4~7 times a day, but except for diarrhea, other conditions such as appetite and spirit were normal, and the nutritional development was not affected. Birth weight was 3.2kg and present weight was 7.1kg, indicating normal weight gain. At the same time, the child had no vomiting, fever and other symptoms, no heart and lung abnormalities, and bowel sounds were normal. Based on the symptoms and signs of the child, the most likely diagnosis is physiological diarrhea. Physiological diarrhea is more common in the baby below 6 months, its appearance is fat, often eczema, soon after birth is diarrhea, the number of stool every day, even more than a dozen times, each time the amount of stool is not necessarily a lot, which contains a small amount of water, generally no special smell. Infants with physiological diarrhea in addition to the number of stool increased, more than no other symptoms, good appetite, no vomiting, growth and development are not affected, after adding complementary food, stool is gradually become normal. Therefore, combined with the condition of the child, the diagnosis of physiological diarrhea was considered.

113, The diagnosis considered are:

Male infant, 6 months, diarrhea for 6 months, 4-7 times / day, sticky stool, no vomiting, no fever, fair appetite, fair spirit, normal weight gain (birth weight 3.2kg, now weight 7.1kg), simple breastfeeding, dehydrated appearance but normal nutritional development, no abnormal heart and lung, abdominal bowel peristalsis, normal bowel sounds. These information suggests that the diarrhea situation in infants may not be caused by infection or allergy, but rather by physiological diarrhea or immature digestive function.

Answer selection:

B. Physical diarrhea

114, (A3 / A4 type question) subject

Possible treatment method

A. Reduce the frequency of breastfeeding and use protein water to degrade formula milk

B. Continue to breastfeeding gradually from less to more add rice paste, rice paste and other complementary food

C. Use of an antidiarrheal medication

D. Use of antibiotics

E. Discontinue breastfeeding and use a proteolytic formula

Reference answer: B. Your answer:

Graphic analysis: [Analysis] The patient was diagnosed with physiological diarrhea, which can generally be improved by adding complementary food.

[Error avoidance] Proteolytic formula is mostly used for children with milk protein allergy. This child is only physiological diarrhea, and there is no need to use proteolytic formula.

Question 114 questions, feasible treatment method:

Reduce the frequency of breastfeeding and use protein water degradation formula (A): If allergic diarrhea is suspected, consider this regimen to reduce allergen

intake.

Continue breastfeeding gradually from less to more add rice paste, rice paste and other complementary food (B): for 6-month-old infants, gradually add complementary food is appropriate, but should ensure that potential allergens are not introduced.

Use of antidiarrheal drugs (C): antidiarrheal drugs are usually not recommended for infants without a clear diagnosis.

Antibiotics (D): not recommended unless there is clear evidence of bacterial infection.

Stop breastfeeding and use a proteolytic formula (E): This can be an option if a severe food allergy is suspected.

Considering these factors, for question 114, if the diagnosis of allergic diarrhea, the reasonable treatment may be to reduce the number of breastfeeding and use proteinytic formula (A).

114, the correct answer is: B. continue breastfeeding gradually from less to more add rice paste, rice paste and other complementary food

For children with physiological diarrhea, special treatment is generally not required, because physiological diarrhea is not a disease, but a normal physiological phenomenon. Although the children defecate more, the mental state, appetite and weight gain were not affected. If we blindly use antidiarrheal drugs or antibiotics for children, it may damage the intestinal microecology of children, but is not conducive to the healthy growth of children. Therefore, for this child, the feasible treatment is to continue breastfeeding, and gradually from less to more added complementary food, such as rice paste. This can not only meet the nutritional needs of children, but also gradually improve the diarrhea symptoms of children. Thus, option B is the correct answer. Option A "Reduce the number of breastfeeding, use proteinaceous water to degrade formula" and option E "Stop breastfeeding, use proteolytic formula" are not appropriate because breast milk is the best food for infants and the child has no intolerance or allergy to breast milk. Options C "Use antidiarrheal drugs" and options D "Use antibiotics" are also not advisable because the child does not have any conditions such as infection or inflammation.

114. Viable treatment methods:

With normal male infant weight growth and normal nutritional development, it indicates that breastfeeding can provide adequate nutrition and be beneficial to the baby. Physiological diarrhea is usually caused by the baby's digestive system is not fully mature, in this case, continuing breastfeeding and gradually add complementary food is a reasonable treatment.

Answer selection:

B. Continue to breastfeeding gradually from less to more add rice paste, rice paste and other complementary food

Female, 38 years old, contact bleeding for 1 week, regular menstruation, G3P1, gynecological examination: cervical upper lip 1.0cm×0.5cm, erosive changes, brittle quality, obvious bleeding, uterus and double attachments did not touch the abnormal uterine soft.

115. (Type A3 / A4 questions) subject

The most helpful test for making a confirmed diagnosis is

A. Gynecologic ultrasound examination

B. Cervical staining for cytology

C. Cervical tissue biopsy

D. Human papillomavirus examination

E. Examination of the vaginal secretions

Reference answer: C Your answer:

[Analysis] The patient, 38 years old, contact bleeding for 1 week, regular menstruation (no abnormal hormones), erosion changes of the upper lip 1.0cm×0.5cm, brittle quality, obvious bleeding (indicated by cervical lesions), no abnormal uterus and double appendages, and the soft (except other lesions outside the cervix). It was proved that the patient lesion mainly came from the cervix, and for further diagnosis of pathological nature, so the cervical biopsy should be taken for pathological examination, so C was selected.

[Error avoidance] uterus and double attachment are not abnormal, triple clinic is soft, gynecological ultrasound has little significance; cervical staining cytology is less authoritative in cell histology; human papillomavirus examination is mainly used to

diagnose HPV infection; vaginal discharge examination cannot further confirm the nature of the lesion.

Question 115

The most helpful test for confirming the diagnosis is a cervical biopsy.

A. Gynecological ultrasound examination: it is used to observe the structures of the uterus, accessories and accessories, but the cervical surface is not as direct as tissue examination.

B. Cervical staining cytology: useful for screening for cervical lesions, but less accurate for confirming the specific pathological state of the cervix than biopsy.

C. Cervical biopsy: the pathological changes of tissues can be directly observed, which is the gold standard for the diagnosis of cervical lesions.

D. Human papillomavirus test: mainly used for risk assessment and screening of cervical cancer, rather than direct diagnosis.

E. Vaginal discharge examination: used to detect infection and other conditions, not suitable for the diagnosis of cervical lesions.

Therefore, the best answer is a C. cervical biopsy.

115, analysis:

Young female patients, contact bleeding, gynecological examination of the cervical upper lip see erosion changes, brittle quality and easy bleeding, uterus, double appendages, side palace have no abnormalities, considered as cervical cancer. The "gold standard" for the diagnosis of cervical cancer is a cervical biopsy.

Option A Although gynecological ultrasound examination can find abnormal cervical echo mass, but can not confirm whether cervical cancer, can be ruled out.

Option B cervical staining cytology is cervical cytology or TCT and LCT, which is mainly used for CIN and early cervical cancer screening, and cannot be confirmed, so it is not selected.

Option D Human papillomavirus examination is mainly used for the examination of CIN and cervical cancer incidence factors, and it cannot be confirmed, so it is excluded.

Option E Vaginal secretion examination is mainly used for female reproductive system infection, tumor diagnosis, judgment of estrogen level and etiology examination of STD, which is not meaningful for the diagnosis of cervical cancer, so

it is not considered.

In conclusion, the correct answer is: C. Cervical biopsy.

Topic 115: The most helpful test for confirming the diagnosis is

In this case, the most helpful test to confirm the cervical lesion is the cervical biopsy. Although cervical stain cytology and HPV are also help in screening for cervical lesions, biopsies are able to provide histological diagnosis, which is the gold standard for confirmed diagnosis.

Best answer: C., cervical biopsy

116, (Type A3 / A4 questions)

If the patient is diagnosed with squamous cell carcinoma with a depth of 2mm, the stage is subject

A. IA1

B. IA2

C. IIB1

D. IIB2

E. IIIB3

Reference answer: A. Your answer:

Analysis: [Analysis] Cervical squamous cell carcinoma staging standard IA 1: measured depth of stromal invasion <3.0mm; IA2:3.0mm 5.0mm; IB1: measured depth of interstitial invasion> 5.0mm and maximum diameter <=2.0cm; IB2: maximum diameter> 2.0cm; IB3: maximum diameter> 2.0cm. So the correct answer should be A.

[Wrong avoidance] The maximum depth of IA stage infiltration is 5mm, and IB is not less than 5mm.

Question 116

If the patient was diagnosed with squamous cell carcinoma and 2mm, the stage was IA 1.

A. IA 1: Cervical squamous cell carcinoma stage IA 1 means a depth of 3mm

and a maximum transverse diameter of 7mm.

B. IA 2: infiltration depth of> 3mm and 5mm, maximum transverse diameter of 7mm.

C. B1: deeper infiltration or larger tumor, but not in this case.

D. B2: further staging of cervical cancer, inconsistent with the situation of this case.

E. B3: Higher grade cervical cancer stage, not as described in this case.

Therefore, the best answer is A. IA 1.

116, analysis:

This patient had been diagnosed as squamous cell carcinoma with subbasement invasion depth of 2mm, According to the clinical stage of cervical carcinoma, the carcinoma was localized to the cervix, namely stroma invasion depth <3mm and 7mm wide, and stage IA 1.

Option B: IA 2 is the interstitial infiltration depth of> 3mm and <5mm, with a width of 7mm, inconsistent with the problem stem description.

Option C: B1, option D: B2, both in stage B, the tumor exceeded the uterus, but did not reach the pelvic wall or 1 / 3 of the lower vagina, which did not meet the meaning of the question.

Option E: B index of tumor extension to the pelvic wall, and / or hydronephrosis, or no functional kidney, inconsistent with the title stem description.

To sum up, the correct answer is: A. IA 1.

Topic 116: If the patient is diagnosed with squamous cell carcinoma with a basement depth of 2mm, the stage is

According to the International Union of Obstetrics and Gynecology (FIGO), squamous cell carcinoma with subbasement invasion depth of less than or equal to 3mm is classified as stage IA. Further divided into IA 1 and IA 2: IA 1 is subbasement invasion depth less than or equal to 3mm and the horizontal extension did not exceed 7mm; IA 2 is subbasement invasion depth exceeding 3mm but not exceeding 5mm, and horizontal extension did not exceed 7mm.

Because the subbasement infiltration depth mentioned in the title stem was 2mm, the patient was staged as IA 1.

Best answer: A. IA 1

Female, 62 years old, G6P4, menopause for 6 years, repeated a small amount of vaginal bleeding for 4 months, and a history of diabetes for 5 years. Physical examination: BP160 / 105mmHg, height 155cm, weight 80kg, gynecological examination: smooth vaginal neck, normal uterine size, no abnormal bilateral adnexa. Transvaginal ultrasound examination: the endometrium is 1.1cm, with irregular thickening, and rich blood flow within its inner and adjacent muscle layers.

117. (Type A3 / A4 questions) subject

Factors unrelated to the disease are

A. fecundity

B. fat

C. hypertension

D. Delay of menopause

E. diabetes mellitus

Reference answer: A. Your answer:

[Analysis] The patient was 62-year-old, G6P4 (indicating postpartum), menopause for 6 years (indicating menopausal delay), a small amount of vaginal bleeding for 4 months, and a history of diabetes for 5 years (indicating a history of diabetes). BP was BP160 / 105mmHg (indicating hypertension), height 155cm, weight 80kg (excessive BMI, overweight), the main symptoms were small vaginal bleeding, smooth cervix, normal uterine size, no abnormality in bilateral adnexa. The endometrium was 1.1cm, irregular thickening, and abundant blood flow within the adjacent muscle layers. The causes of endometrial thickening can be caused by obesity, hypertension, menopause delay, diabetes and other hormone disorders lead to repeated vaginal bleeding, with a productive problem is not big. So choose A.

[Avoid mistakes] The normal menopause should be 45~55 years old, and the patient is 57 years old, which is a delayed menopause.

117 Questions:

Factors unrelated to the disease are:

A. fecundity

B. fat

C. hypertension

D. delayed menopause

E. diabetes mellitus

Factors unrelated to endometrial cancer need to be identified here. Multipartum, obesity, delayed menopause and diabetes are all considered as risk factors for endometrial cancer. The direct association between hypertension and endometrial cancer is low, so in these options, C. hypertension should be a factor unrelated to the disease.

Factors unrelated to the disease are

Answer: D. menopause delay

Analysis: The patient's elderly female, postmenopausal, postmenopausal vaginal bleeding, ultrasound showed endometrial thickening, rich blood flow, should be considered for endometrial cancer. The development of endometrial cancer is related to various factors, including endometrial hyperplasia followed by carcinogenesis under estrogen without progesterone antagonism; gene mutations such as PTEN activation; Lynch syndrome. Menopause is an inevitable process in the life process of every woman, and it has nothing to do with the occurrence of endometrial cancer.

Option A was excessive labor, option B obesity, option C hypertension, and option E diabetes, all were associated with endometrial cancer, and were excluded.

117. (A3 / A4 type problem), the factors unrelated to the disease are

A. fecundity

B. fat

C. hypertension

D. delayed menopause

E. diabetes mellitus

Answer analysis:

The condition of this patient suggests possible endometrial lesions, such as endometrial hyperplasia or endometrial cancer. Risk factors for endometrial hyperplasia and endometrial cancer include multiparity, obesity, hypertension, delayed menopause, and diabetes mellitus.

Factors not associated with endometrial hyperplasia or endometrial cancer are:

A. fecundity

118, (Type A3 / A4 questions)

The following suitable surgical methods are available subject

A. generalized hysterectomy + double apachage hysterectomy + pelvic and abdominal arterial lymphopies

B. High-dose hormone therapy

C. A hysteroscopic endometrial electrode resection

D. Exafascial total hysterectomy + double attachment hysterectomy + pelvic and abdominal arterial lymphopies

E. Endometrial ablation procedure

Refer to the answer: D. Your answer:

[Analysis] According to the medical history and signs, the patient has a history of diabetes and obesity, and endometrial thickening. The patient is 62 years old, menopause for 6 years, and has no need for fertility. Therefore for patients with total hysterectomy, extrafascial total hysterectomy is suitable for poor general condition, with other diseases can not tolerate extrafascial total hysterectomy above patients, so the patient should choose (D) extrafascial total hysterectomy + double attachment hysterectomy + pelvic cavity, abdominal artery lymphmectomy.

[Error] Extrascial total hysterectomy refers to approaching the lateral plane of the cervical separation, excluding the cervical stroma, where the uterine sacral ligament is cut at the cervical attachment, and the vaginal wall is about one centimeter. Extensive hysterectomy refers to total hysterectomy, the main uterine ligament in the pelvic wall in the anal resection, the uterosacral ligament near the lower lateral attachment to remove or retain a centimeter of the main ligament and sacral ligament, to help with the recovery of urination function, one third of the upper part of the vagina must be removed.

118 Questions:

The following suitable surgical methods are provided as follows:

A. generalized hysterectomy + double apachage hysterectomy + pelvic and abdominal arterial lymphopies

B. High-dose hormone therapy

C. A hysteroscopic endometrial electrode resection

D. Exafascial total hysterectomy + double attachment hysterectomy + pelvic and abdominal arterial lymphopies

E. Endometrial ablation procedure

Considering the patient's age, symptoms, and ultrasound findings, surgery is required if endometrial cancer is diagnosed. Among these options, generalized hysterectomy (including the uterus, adnexa, and associated lymph node resection) is the more radical treatment. However, the choice of A or D depends on the cancer stage and spread profile. Given that there is no clear information on cancer stage included in the title, but significant endometrial thickening and abundant blood flow is known, this may suggest the presence of cancer. D. Extravascial total hysterectomy + double adnexal hysterectomy + pelvic, abdominal arterial lymphopherectomy is a more thorough treatment modality that may be suitable in patients with highly suspected or confirmed endometrial cancer, especially when considering potential cancer spread.

The appropriate surgical method is the

Answer: D. Exafascial total hysterectomy + double attachment hysterectomy + pelvic and abdominal artery lymphmectomy

Analysis: The patient's elderly female, appeared postmenopausal vaginal bleeding, ultrasound showed endometrial thickening, rich blood flow, should be considered for endometrial cancer. The preferred treatment method is surgical treatment, the purpose of surgery is surgical pathological staging, determine the scope of the lesion and the factors related to the prognosis, remove the diseased uterus and other possible metastatic lesions, and determine the choice of postoperative adjuvant treatment. The patient had stage I endometrial cancer, and the suitable surgical method was total hysterectomy with bilateral adnexal excectomy, along with pelvic and para-aortic lymph node dissection.

Option A extensive hysterectomy + double attachment hysterectomy + pelvic and

abdominal artery lymphpectomy is the surgical method of cervical cancer, which is not consistent with the title.

Option B high-dose hormone therapy, option E endometrial ablation, are drug conservative therapy, mainly applicable to young patients requiring reproductive function, but the patient is menopausal and older, which is not applicable, so excluded.

Option C hysteroscopic endometrial electrode resection is mainly used for treating dysfunctional uterine bleeding, so it is not considered.

118. (A3 / A4 type questions) The following suitable surgical methods are

A. generalized hysterectomy + double apachage hysterectomy + pelvic and abdominal arterial lymphopies

B. High-dose hormone therapy

C. A hysteroscopic endometrial electrode resection

D. Exafascial total hysterectomy + double attachment hysterectomy + pelvic and abdominal arterial lymphopies

E. Endometrial ablation procedure

Answer analysis:

The 62-year-old patient had postmenopausal vaginal bleeding, 1.1cm endometrial thickness, irregular thickening, and abundant blood flow to the intima and muscle layers, which suggested possible endometrial cancer. According to best practice guidelines, surgical treatment should include resection of the potentially involved uterus, adnexa and lymph node dissection to assess the spread.

The appropriate surgical methods are:

B. Exafascial total hysterectomy + double attachment hysterectomy + pelvic and abdominal arterial lymphopies

Topic material female, 35 years old, repeated attack chest tightness, palpitation for half a year, and then send half an hour. I was under great working pressure, and I had chest tightness, palpitation and shortness of breath three times in half a year. I immediately saw emergency treatment in the hospital, checked electrocardiac CT,

myocardial zymogram and lung function examination showed no obvious abnormalities, and the symptoms were relieved after oxygen inhalation. Half an hour ago, chest tightness, shortness of breath, palpitation, palm numbness, no chest pain, no vomiting, physical examination: T37.5°C, P87 times / minute, blood pressure 120 / 70mmHg, nervous face, auscultation, cardiac rhythm, negative pathological signs.

119, (Type A3 / A4 questions) subject

Consider the diagnosis as

A. UA

B. panic disorder

C. Extensive anxiety disorder

D. somatoform disorders

E. bronchial asthma

Reference answer: B. Your answer:

[Analysis] Panic disorder is an acute anxiety disorder characterized by recurrent sudden panic experience. Typical panic attacks often occur in daily activities, and no organic lesions are seen. The patient is female, 35 years old, with recurrent attacks of chest tightness, palpitation for half a year, and another half an hour. The causes was high work pressure, sudden chest tightness, palpitation, shortness of breath (symptoms of panic disorder) three times in half a year, immediate emergency treatment, electrocardiogram examination, lung CT, myocardial enzyme spectrum, lung function examination showed no obvious abnormalities (excluding organic lesions), and the symptoms were relieved after oxygen inhalation. Half an hour ago, chest tightness, shortness of breath, palpitation, palm numbness (panic disorder symptoms), and physical examination showed no abnormality. Therefore, this patient should be diagnosed with (B) panic disorder.

[Error avoidance] Patients had no abnormal electrocardiogram and blood biochemistry, so unstable angina was excluded; generalized anxiety disorder is characterized by frequent comprehensive, nervousness and excessive without definite object or fixed content. This anxiety is not related to any specific surrounding situation; widespread anxiety disorders can manifest in abnormal skin sensation and gastrointestinal reactions without organic lesions; and bronchial asthma has many triggers and requires bronchial induction experiments for further diagnosis.

119. Consider the diagnosis of:

B. panic disorder

This patient's symptoms, including recurrent chest tightness, palpitation, shortness of breath and palm numbness, and sudden onset and relief of symptoms, were more consistent with the manifestation of panic disorder. An electrocardiogram, myocardial enzyme spectrum, and pulmonary function tests revealed no abnormalities, supporting this diagnosis rather than cardiovascular or respiratory disease.

119. Correct answer: B. Panic disorder.

Middle-aged female patients, working pressure at ordinary times, sudden chest tightness, flustered, shortness of breath, ECG, CT many times, myocardial spectrum, lung function examination did not see obvious abnormalities, symptoms relief after oxygen inhalation, half an hour ago, with palm numbness, examination no abnormal signs, according to its clinical manifestations and auxiliary examination, consider the diagnosis of panic disorder. Panic disorder, referred to as panic disorder, is an acute anxiety disorder characterized by recurrent significant autonomic symptoms such as palpitations, sweating, tremor, accompanied by a strong sense of dying or loss of control, and fear of unfortunate consequences.

Option A unstable angina Chest pain is similar to stable angina, with compression, tightness, or tightness, but the pain degree is heavier and lasts longer, and chest pain can also occur at rest, not consistent with the meaning of the question.

Option C Extensive anxiety disorder is a chronic anxiety disorder characterized by continuous significant anxiety, accompanied by autonomic excitation and excessive vigilance. Excessive vigilance and autonomic excitation are not mentioned in the problem stem, so it is not selected.

Option D Somatoform disorder is a kind of neurosis characterized by persistent worry or belief in the advantages of various physical symptoms. Patients repeatedly seek medical treatment for these symptoms, various negative medical examinations and doctor's explanation cannot dispel their doubts, which is inconsistent with the symptoms of this question.

Option E bronchial asthma typical symptoms of expiratory dyspnea with wheezing, and symptoms can occur within a few minutes, and last for several hours to several days, can be relieved after antiasthma drug treatment or self relief, night and early attack or aggravation is often an important clinical features of asthma, the patient has no related performance, so excluded.

119, and the diagnosis was considered

B. panic disorder

Explanation: The patient had recurrent attacks of chest tightness, palpitation, shortness of breath and other symptoms, and the results of each emergency examination did not see obvious abnormalities, and the symptoms were relieved after oxygen inhalation. Combined with the patient's nervous face and the attack of palpitation, palm numbness and other characteristics, more in line with the performance of panic disorder.

120, (Type A3 / A4 questions) subject

The preferred therapeutic agent is

A. A mood stabilizer

B. Amplification of blood vessels

C. antipsychotic drug

D. Spasmodic and smooth asthma drugs

E. antidepressant

Reference answer: A. Your answer:

[Analysis] Patients diagnosed with panic disorder, the preferred drug is mood stabilizer, also known as anti-manic drug. In addition to anti-manic effect, it also has a stable and preventive recurrence effect on bipolar disorder, so it is also called emotional stabilizer. The three major mood stabilizers include lithium, divalproate and lamotrigine.

[Error] Patients need anxiolytic and antimanic treatment, so mood stabilizers are needed and antidepressant drugs should be avoided.

120. The preferred therapeutic agents are:

E. antidepressant

In the treatment of panic disorder, selective serotonin reuptake inhibitors (SSRIs) are the preferred treatment option, which are antidepressants. Although these drugs were initially developed to treat depression, they have also been shown to be effective for panic disorder and other anxiety disorders.

120. Correct answer: E. Antidepressants.

Panic disorder, referred to as panic disorder, is an acute anxiety disorder characterized by recurrent significant autonomic symptoms such as palpitations, sweating, tremor, accompanied by a strong sense of dying or loss of control, and fear of unfortunate consequences. In treatment, psychotherapy is mainly used, supplemented by drug therapy. Drugs mainly choose antidepressants, benzodiazepines and β -adrenergic receptor blockers, so the preferred treatment drugs are antidepressants.

Option A mood stabilizer is mainly used for treating mania. It is the basic medication for bipolar disorder, which is inconsistent with the symptoms of the patient, so it is not selected.

Option B, option D, spasmodic drugs, are used to treat angina pectoris and bronchial asthma, which are not consistent with the patient's symptoms, and can be excluded together.

Option C Antipsychotics are mainly used to treat schizophrenia and other mental disorders with psychotic symptoms, which is inconsistent with this question.

120, the preferred therapeutic agent is

E. antidepressant

Explanation: The preferred treatment for panic disorder is usually antidepressants, especially selective serotonin reuptake inhibitors (SSRIs), such as sertraline and paroxetine, which are effective in alleviating panic attacks and preventing relapse.

121, (Type A3 / A4 questions) subject

The duration of drug treatment was

A.1 weeks

B.1 month

C.3 months

D.6 months

E.1 years

Refer to the answer: D. Your answer:

Graphic analysis: [Analysis] Mood stabilizer should be replaced with more

effective drugs after the treatment period of 6 months, and two or more therapeutic drugs are not recommended for combination.

[Error avoidance] Accurately remember the drug application time.

121. The duration of drug treatment is:

D. 6 months

For the treatment of panic disorder, medication lasting for at least 6 months is generally recommended. This is to reduce the risk of recurrence and to help patients to better manage and manage their symptoms.

121. Answer is correct: B.1 month.

Antimolytic drugs have a slow effect, and adverse reactions may occur in the initial stage of medication, so the medication should start from small doses, and then slowly increase the dosage to reduce the occurrence of adverse reactions. After 2 to 4 weeks of medication, the effect begins to start, so there is no need to change the drug without special circumstances. Generally, it can have obvious effect in about 1 month after taking the drug, so the time of drug treatment is 1 month.

Option A: One week is too short, the drug has not worked, so it is not chosen.

Option C: 3 months, option D: 6 months, option E: 1 year, both are too long for the best treatment time, so it can be excluded together.

121, the duration of drug treatment is

D. 6 months

Explanation: Drug treatment for panic disorder is usually recommended for at least 6 months to stabilize the condition and prevent recurrence of symptoms. A short duration of treatment may lead to incomplete resolution of symptoms or susceptibility to relapse.

Male, 67 years old, suffering from aplastic anemia for 5 years, corrected the anemia symptoms through blood transfusion, nearly two blood transfusions appeared fever reaction, but no low back pain, hematuria.

122, (Type A3 / A4 questions)

The transfusion fever reaction seen in this patient was

A. anaphylaxis

B. delayed hemolytic fever

C. Acute hemolytic fever

D. non-hemolytic fever

E. was treated with bacterial contamination

Refer to the answer: D. Your answer:

Graphic analysis: [analysis] non-hemolytic febrile blood transfusion reaction is a more common reaction. It refers to a kind of blood transfusion reaction in which the body temperature increases by 1°C during or after blood transfusion, and takes fever as the main clinical manifestation, and can exclude fever caused by hemolysis, bacterial contamination, serious allergy and other reasons. A 67-year-old male patient with aplastic anemia for 5 years, corrected the anemia through blood transfusion, and experienced fever in the last two blood transfusions (the patient had multiple blood transfusion history to induce non-hemolytic fever), but no low back pain, and hematuria (excluding haemolytic fever). Bacterial contamination, allergy, and hemolytic reactions were excluded, so the patient should be diagnosed as non-hemolytic fever.

[Error avoidance] The patient had a history of blood transfusion and no other skin manifestations, so anaphylaxis was excluded; the patient had no lumbago urine blood, so hemolytic reaction was excluded; the fever was not obvious, with no abnormal blood examination and no chills, so the patient could be diagnosed as non-hemolytic fever after exclusion.

Answer to question 122:

The transfusion fever reaction seen in this patient may be D. non-hemolytic fever. Non-hemolytic fever reaction is the most common side effect of blood transfusion, usually caused by white blood cells, platelets or plasma at the time of transfusion. This febrile reaction is usually not serious, and the patient does not have symptoms of low back pain and hematuria, which is usually associated with hemolytic reaction.

122. Analysis:

Elderly male patient with aplastic anemia and long-term transfusion therapy. Fever reaction occurred after the recent blood transfusion, but there were no other symptoms such as low back pain or hematuria. Based on the clinical presentation, this patient was most likely to have developed a non-hemolytic fever. Non-hemolytic fever is the most common complication of early blood transfusion, with rates of up to

2% to 10%. Most often occurred within 15 minutes to 2 hours after the start of blood transfusion. The main manifestations are chills, chills and high fever, and the body temperature can rise to 39℃ ~40℃, accompanied by headache, sweating, nausea or vomiting.

Option A allergic reaction usually occurs A few minutes after blood transfusion, but can also occur in or after blood transfusion, mainly manifested as skin limitation or systemic itching or urticaria, in severe cases, bronchospasm, breathing difficulties, chest tightness, abdominal pain, even anaphylactic shock and even coma, death, inconsistent with the question.

Option B delayed hemolytic fever, option C acute hemolytic fever, such as back pain, hemoglobinuria, etc. In severe cases, acute renal failure and DIC may occur, which are not consistent with the symptoms of the patient, so they can be excluded together.

Option E with bacterial contamination showed sepsis, and the patient did not show signs of infection and was not considered.122.D. Non-hemolytic fever;

122 The transfusion fever reaction seen in this patient was

A. anaphylaxis

B. delayed hemolytic fever

C. Acute hemolytic fever

D. non-hemolytic fever

E. was treated with bacterial contamination

Correct answer: D. Non-hemolytic fever

Non-hemolytic fever reactions are a common side effect after transfusion, usually caused by the reaction of the blood recipient during blood transfusion. Because the patient did not have low back pain and hematuria, acute and delayed hemolytic reactions were less likely, while non-hemolytic fever reactions were more likely.

123, (A3 / A4 type question) subject

The reason for the blood transfusion fever reaction in this patient was

A. The presence of a pyrogen

B. Presence of identical leukocytes and platelets in blood recipients

C. Blood group incompatibility with the common patient

D. Blood transport, preservation caused by contamination

E. Blood recipients have hemolytic disease

Reference answer: B. Your answer:

[Analysis] Non-hemololytic febrile transfusion reactions are usually related to white blood cells and / or platelet antibodies and cytokines produced in blood preservation, mostly occurring during infusion of multiple platelet concentrates (PCs) and in women with multiple transfusions or pregnancies. So this question should choose B.

[Error avoidance] There are pyrogen and blood transportation, and preservation contamination can lead to infectious fever; blood type incompatibility and hemolytic disease.

Answer to question 123:

The transfusion fever reaction may be the presence of similar white blood cells and platelets in B. recipients. This is one of the common causes of non-hemolytic fever reactions, and it may cause fever when white blood cells or platelets in a transfusion are recognized and destroyed in the blood recipient.

123. Analysis:

As for the cause of fever after blood transfusion, considering that the patient had no symptoms of hemolysis, allergy or infection, the most likely reason was the presence of pyrogen during blood transfusion, including drugs, biological products or transfusion apparatus, resulting in the patient's febrile reaction after blood transfusion.

Option B The presence of similar white blood cells and platelets in blood recipients is usually associated with immune responses after transfusion, such as fever and ineffective blood transfusion caused by HLA antibodies, but not directly leading to fever, so it is not selected.

Option C and the donor incompatibility will cause acute hemolysis, but the patient does not show symptoms of hemolysis and can be ruled out.

Option D blood transport and preservation contamination usually leads to severe infections such as sepsis, but patients do not show signs of infection and do not meet

the question.

Option E Hemytic disease usually causes hemolysis, and patients do not experience hemolysis.123.A. Presence of a pyrogen.

123 The reason for a blood transfusion fever reaction in this patient was

A. The presence of a pyrogen

B. Presence of identical leukocytes and platelets in blood recipients

C. Blood group incompatibility with the donor

D. Blood transport, preservation caused by contamination

E. Blood recipients have hemolytic disease

Correct answer: B. White blood cells and platelets exist in blood recipients

Female, 50 years old, repeated limb convulsions for 2 years, the attack of limb convulsions, accompanied by loss of consciousness, a few minutes later relief. Similar symptoms occurred 3 times within 4 months. Physical examination: T 36℃, blood pressure: 140 / 90mmH chu, cranial nerve examination was normal. Limb muscle strength grade 5, symmetrical muscle reflex, and negative pathological reflex. MRI suggests large occupancy in the parietal lobe.

124, (A3 / A4 type question) subject

What to consider

A. hematencephalon

B. brain abscess

C. ICT

D. static tremor

E. epilepsia

Reference answer: C Your answer:

[Analysis] Intracranial tumor refers to the new organism growing in the skull cavity, which can originate from the brain, meninges, nerves, blood vessels and brain accessories, or form by the transfer of other tissues or organs in the body into the skull, most of which can produce headache, intracranial hypertension and focal symptoms. The patient had repeated limb convulsions for 2 years, and the onset of limb

convulsions, with loss of consciousness, which was relieved after a few minutes (considering craniocerebral lesions, excluding static tremor). On physical examination, see T 36°C, blood pressure: 140 / 90mmHg, conscious, and normal cranial nerve examination (no abnormal discharge of brain nerve, epilepsy excluded). Limb muscle strength level 5, muscle reflex symmetry, negative pathological reflex (excluding intracerebral hemorrhage). The MRI of this patient found a large space in the parietal lobe (imaging findings of intracranial tumor, estive of intracranial tumor), which was considered as (C) intracranial tumor.

[Avoid errors] Cerebral hemorrhage and brain abscess should have corresponding imaging findings on MRI; static tremor should be manifested as muscle shaking under relaxation, loss during sleep and rhythm; patients with epilepsy will have abnormal discharge phenomenon of cranial nerve.

For question 124, considering the description of the cases and the MRI results, the most likely diagnosis is the intracranial tumor (with option C). The finding of symptomatic and space-occupying lesions on MRI suggests that this may be a tumor cause.

124. Considering the diagnosis, we should first analyze the patient's symptoms and the results of various examinations.

analyze:

Middle-aged female patients, recurrent limb convulsions and loss of consciousness, which are the typical symptoms of epilepsy. MRI suggests a large occupancy of the parietal lobe, which may be the cause of epilepsy. Taken together, the most likely diagnosis in this patient is secondary epilepsy due to an intracranial tumor. Therefore, both option C intracranial tumors and option E epilepsy are diagnoses related to the patient's condition. However, considering that MRI has clearly suggested a large occupying space of the parietal lobe, the intracranial tumor is a more direct diagnosis.

In conclusion, the correct answer is: C. intracranial tumors.

124. (Type A3 / A4 questions)

What to consider

A. hematencephalon

B. brain abscess

C. ICT

D. static tremor

E. epilepsia

Answer analysis

The patient included recurrent limb convulsions, loss of consciousness, and MRI of the parietal lobe. These features are most likely to by intracranial tumors (C) because compression or erosion of the tumor can cause seizures. Although epilepsy (E) is a symptom, the underlying cause is more likely to be an intracranial tumor.

Best answer: C. intracranial tumor

125, (A3 / A4 type question) subject

Which of the following treatments works

A. operative treatment

B. were treated with sedatives

C. Using hormonal therapy

D. Treatment with vasodilic drugs

E. Antiepileptic therapy

Reference answer: A. Your answer:

Graphic analysis: [Analysis] If the patient is diagnosed with intracranial tumor and meets the surgical indications, surgery is preferred for treatment.

[Error avoidance] BCDE can not provide A radical cure for intracranial tumors. Only A. surgical treatment and follow-up treatment according to the specific pathological results can have an appropriate effect on the disease.

For question 125, since the most likely diagnosis is intracranial tumors, the most effective treatment should be surgical treatment (option A) to remove or reduce the tumor burden and thus relieve the symptoms. Other options such as the use of sedatives, hormones, vasodilders, or antiepileptic therapy, which may be helpful for specific conditions, are not the primary treatment modality for intracranial tumors.

125, regarding the choice of treatment, we need to do it based on the above diagnosis.

analyze:

Considering that the patient's epilepsy is caused by intracranial tumors, the key to treatment is to solve this space-occupying lesion. Surgical treatment can directly remove the tumor, thus in a radical cure for epilepsy. Other treatments, such as the use of sedatives, hormones, vaso-modifying drugs or antiepileptic drugs, are only symptomatic treatment and cannot solve the fundamental problem.

In conclusion, the correct answer is: A. Surgical treatment.

125. (Type A3 / A4 questions)

Which of the following treatments works

A. operative treatment

B. were treated with sedatives

C. Using hormonal therapy

D. Treatment with vasolenic drugs

E. Antiepileptic therapy

Answer analysis

Regarding the large parietal occupying space suggested by MRI, considering intracranial tumors is the most likely diagnosis. Surgical treatment of intracranial tumors is usually preferred (A) to remove the tumor and relieve the compression on the surrounding brain tissue, thus reducing or eliminating the symptoms of seizures. Antiepileptic therapy (E) controls symptoms, but does not address the underlying problem.

Best answer: A. Surgical treatment

Pripara, 21 years old, 33 weeks gestation, dizziness, headache, blurred vision for three days, aggravated one day. Emergency blood pressure measurement 160 / 110mmHg, urinary protein (+ + +), NST unresponsive type, previously healthy.

126, (A3 / A4 type question) subject

The most likely diagnosis

A. severe preeclampsia

B. Chronic hypertension complicated with preeclampsia

C. Hypertension in pregnancy

D. Chronic hypertension combined with a complicated pregnancy

E. eclampsia

Reference answer: A. Your answer:

[Analysis] systolic or diastolic blood pressure increased with proteinuria can be diagnosed after any of the following adverse conditions: (1) blood pressure, systolic blood pressure> 160mmHg, diastolic blood pressure 110mmHg, urine protein> greater than 5g / 24h, or random urine protein> (+ + +).(2) Continuous headaches or visual disturbances, blurred viewing, dizziness, head dizziness, or blurred vision.(3) Continuous right upper abdominal pain, abnormal liver function, abnormal renal function, hypoproteinemia, abnormal blood system, heart failure, pulmonary edema, fetal intrauterine growth restriction or oligohydramnios. The patient was 33 weeks pregnant (onset of severe preeclampsia), blood pressure 160 / 110mmHg and dizziness, headache, and blurred vision (severe preeclampsia), so the patient should be diagnosed with severe preeclampsia.

[Avoid mistakes] Chronic hypertension with preeclampsia diagnosis: no proteinuria in chronic hypertension before 20 weeks of pregnancy, urinary protein quantification 0.3 g / 24 h after 20 weeks of pregnancy or random urinary protein (+), clean middle urine and rule out the confusion of low urine and increased urine proportion; or proteinuria before 20 weeks of pregnancy, urinary protein increased significantly after 20 weeks of pregnancy; or any one of the above manifestations of severe preeclampsia such as further increase in blood pressure. Gestational hypertension was: secondary or essential hypertension of various causes, different etiology, course and manifestations of various chronic hypertension, such as previous hypertension or systolic pressure 140 mmHg and (or) diastolic pressure 90 mmHg, no obvious aggravation or manifested as acute severe hypertension; or hypertension for the first time after 20 weeks of gestation but continued until 12 weeks postpartum.

Question 126: The most likely diagnosis

A. Severe preeclampsia: This diagnosis is consistent with the patient's symptoms, including hypertension (blood pressure 160 / 110mmHg), proteinuria (+ + +), headache, blurred vision, etc. Preeclampsia is a disease that occurs during pregnancy and is mainly characterized mainly by hypertension and proteinuria.

B. Chronic hypertension with preeclampsia: Given that the patient was a primiparous woman and was previously in good health without a history of chronic hypertension, this option may not be the best option.

C. Pregnancy hypertension: This usually refers to new onset hypertension during pregnancy, but in this case, the patient has other symptoms such as proteinuria, headache, etc., which point to preeclampsia.

D. Chronic hypertension combined with pregnancy: This requires a patient with a history of chronic hypertension, and no such information is mentioned in the case.

E. Eeclampsia: eclampsia is a more severe form of preeclampsia, often with convulsions or severe central nervous system problems, not mentioned in cases.

Based on the above analysis, the most likely diagnosis was that of A. severe preeclampsia.

According to the title description, the primipara, 21 years old, 33 weeks of gestation, dizziness, headache, blurred vision symptoms, blood pressure measurement results were hypertension (160 / 110mmHg), strong positive urinary protein (+ + +), fetal heart no stress test (NST) no reaction type, previously healthy. These symptoms and examination findings coincide with the typical presentation of severe preeclampsia.

Option A: Severe preeclampsia, matching the clinical findings and examination findings of the pregnant woman.

Option B: Chronic hypertension combined with preeclampsia, which usually requires a history of chronic hypertension, but the pregnant woman is previously healthy, so she is not selected.

Option C: Pregnancy hypertension, usually without significant proteinuria, dizziness and headache, was excluded.

Option D: Chronic hypertension combined with pregnancy also requires a history of chronic hypertension, which is not consistent with the description of the subject stem, so it is not selected.

Option E: eclampsia is a convulsion based on preeclampsia that cannot be explained by other reasons, and the pregnant woman did not have convulsions, so it was excluded.

In conclusion, the correct answer is: A. Severe preeclampsia.

126 The most likely diagnosis

A. severe preeclampsia

B. Chronic hypertension complicated with preeclampsia

C. Hypertension in pregnancy

D. Chronic hypertension combined with a complicated pregnancy

E. eclampsia

Answer: A. Severe pre-eclampsia

Explanation: At 33 weeks of gestation, dizziness, headache, blurred vision, blood pressure increased to 160 / 110 mmHg, urinary protein + + +, these symptoms and signs were highly suggestive of severe preeclampsia. The diagnosis of preeclampsia includes hypertension and proteinuria after 20 weeks of gestation. Severe preeclampsia is also accompanied by severe symptoms such as headache, visual impairment, and hypertension.

127, (Type A3 / A4 questions) subject

Drugs unsuitable for use

A. diazepam

B. labetalol

C. dexamethasone

D. magnesium sulfate

E. sodium nitroprussiate

Reference answer: E Your answer:

[Analysis] Deastolic blood pressure should be decreased below 90mmHg to avoid increase the risk of fetal death; sodium nitroprusside has an adverse effect on the fetus, and its use is prohibited. Therefore, (E) sodium nitroprusside should be selected.

[Eravoid avoidance] Combined with magnesium sulfate, which will produce neuromuscular blockade, myocardial inhibition and hypotension.

Question 127: Inappropriate drugs

A. Diazepam: used for the control of eclampsia or severe preeclampsia.

B. Labeolol: for reduce hypertension, for patients with preeclampsia.

C. Dexamethasone: It is used to promote fetal lung maturation and is suitable for premature infants.

D. Magnesium sulfate: used for the prevention and treatment of eclampsia episodes.

E. Sodium nitroprusside: This is a potent vasodilator that can reduce blood pressure rapidly, but should be used carefully in the treatment of hypertensive disorders of pregnancy (such as pre-eclampsia or eclampsia), as it may cause a rapid drop in blood pressure, which can affect the blood supply to the fetus.

Therefore, for this patient, the most unsuitable drug was sodium E. nitroprusside.

In pregnant women with severe preeclampsia, special attention should be paid to the choice of medication during treatment.

Option A: Diazepam, usually used for anxiolysis and sedation, may be beneficial for controlling anxiety and tension that may occur in preeclampsia and can therefore be used.

Option B: Labeelol, a α and β receptor blocker, is often used to treat hypertension during pregnancy, and therefore is also appropriate.

Option C: Dexamethasone, commonly used to promote fetal lung maturation, can be used early at a high risk of preterm birth. However, the use of dexamethasone needs severe preeclampsia in pregnant women who already have a diagnosis of severe preeclampsia, as it may aggravate hypertension and edema and is therefore not appropriate for use in this setting.

Option D: Magnesium sulfate, a medication for the prevention and treatment of eclampsia, is very important for pregnant women with severe preeclampsia.

Option E: Sodium nitroprusside, a potent vasodilator for treating severe hypertension, applies in this setting.

In conclusion, the drug drug for use is option C: dexamethasone. Because it may aggravate hypertension and oedema, it is unfavorable for the treatment of severe preeclampsia.

127. Inappropriate drugs

A. diazepam

B. labetalol

C. dexamethasone

D. magnesium sulfate

E. sodium nitroprussiate

Answer: A. Diazepam

Explanation: Diazepam is a sedative and, although in some cases can be used to control seizures, it is not the drug of choice for severe preeclampsia. Magnesium sulfate is the standard drug for the prevention and treatment of eclampsia, labeprolol and sodium nitroprusside are commonly used antihypertensive drugs, and dexamethasone is used to promote fetal lung maturation. Therefore, diazepam is not suitable for use in this setting.

Pripara, 35 years old, height 150cm, fetal weight 3500g, 38 + 5 weeks gestation, natural labor 20 hours, now contractions interval 1~2 minutes, lasting 40~60s, the patient irritability, pain, flat umbilical cord can lower uterine tenderness, fetal heart monitoring repeated early deceleration, uterine mouth near open, S= + 2,

128, (A3 / A4 type question) subject

The most likely diagnosis is the

A. Spastic uterine complex

B. protracted active phase

And C. Hypertonic uterine weakness

C. placental abruption

D. threatened rupture of uterus

Reference answer: E Your answer:

[Analysis] The threatened uterine rupture means that the fetus is hindered in the decline, and the normal uterine contraction will affect it, so that the lower part of the uterus slowly thinner, and the uterine body is slowly thicker and shorter, which is between the two will produce a contraction ring, resulting in uterine rupture. The

main performance is maternal irritability, breathing and heart rate are accelerated, the lower abdomen appears severe pain, because the uterine contraction is too strong, too frequent, the fetus can not understand, the fetal heart rate accelerated or slow or hear. Patients with primiparous, 35 years old, natural labor for 20 hours, now contractions interval 1~2 minutes, lasting 40~60s (threatened uterine rupture onset period), patients with irritability, pain, flat umbilical visible shrink complex ring (threatened uterine rupture typical manifestation), lower uterine tenderness, fetal heart monitoring repeatedly early deceleration (threatened uterine rupture fetal performance). Therefore, this patient should be diagnosed with a (E) threatened uterine rupture.

[Avoid mistakes] The difference between threatened uterine rupture and uterine rupture lies in the severity of the symptoms. If the patient is threatened with uterine rupture, the consideration is mainly will be agitated, as well as uterine contraction, abdominal falling pain and other symptoms, at the same time because the uterine contraction is more frequent, can be accompanied by acceleration and other symptoms. However, if the patient has developed uterine rupture, there may be persistent vaginal bleeding, hematuria, fetal heart disturbance, and persistent abdominal pain, and corresponding measures need to be taken in time.

Most likely diagnosis of question 128:

E. protracted active phase. This is because the patient has been delivering in the active period for a long time (20 hours) and has a full uterine opening, but the delivery fails to progress, which is consistent with the characteristics of the active period of stagnation.

The correct answer is: E. The threatened uterine rupture.

The pregnant women are advanced primary mothers, fetal weight is large, natural labor has been 20 hours, contractions are frequent and lasting for a long time, the patient has irritability, pain and other symptoms, tenderness in the lower uterine segment, fetal heart monitoring repeated early deceleration, these are the typical manifestations of threatened uterine rupture. Therefore, the most likely diagnosis is a threatened uterine rupture.

Option A uterine spastic retracted ring is the precursor of uterine rupture, but it is not equivalent to the threatened uterine rupture, and the retracted ring is not mentioned in the question stem, so it is not selected.

Item B active period stagnation refers to the cessation of the active period during childbirth, but the "near opening of the uterine mouth" was clearly mentioned in the

question, which was not consistent with the definition of active period stagnation, so it was excluded.

Option C hypertentonic uterine weakness shows the loss of symmetry, polarity and rhythm of uterine contraction, and the uterine contraction is completely uncoordinated, which belongs to abnormal uterine contractility. These manifestations are not mentioned in the topic stem, so it is not selected.

Option D Placental abruption is usually associated with abdominal pain and vaginal bleeding, which was not mentioned in the topic stem and was excluded.

Topic 128: The most likely diagnosis is

analyze:

Pyrospasm: common in labor disorders, but usually not early fetal heart deceleration.

Stagnation of active period: usually refers to the expansion of the uterine mouth does not continue, where the uterine mouth is close to full open, does not meet the diagnosis.

Hypertension uterine weakness: not consistent with clinical manifestations.

Placental abruption: usually accompanied by vaginal bleeding and abdominal pain, not mentioned.

Foresign uterine rupture: maternal irritability, pain, lower uterine tenderness, early fetal heart rate deceleration, short contractions interval, in line with the performance of threatened uterine rupture.

Best answer: E. A threatened uterine rupture

129, (Type A3 / A4 questions) subject

The most appropriate treatment is the

A. C-sect

B. Rorotate the fetal head

C. Strengthen contractions

D. Fetal labor attraction

E. Induction of labor by forceps

Reference answer: A. Your answer:

[Analysis] The patient was diagnosed as threatened uterine rupture, in line with the indications for cesarean section surgery, should be immediately performed, to avoid a threat to the patient's life.

The most appropriate treatment for the question 29 questions:

A.C-sect. Given the patient's condition, especially the failure of prolonged active delivery and fetal heart monitoring showing repeated early deceleration, the choice of cesarean section is reasonable to avoid further risks to the mother and fetus.

The correct answer is: A. cesarean section.

Given that the pregnant woman may be at risk of threatened uterine rupture and repeated early deceleration in fetal heart monitoring, this suggests possible fetal distress. In this case, the most appropriate treatment is cesarean section to ensure the safety of mother and child.

Option B freehand rotating fetal head is usually used to deal with improper fetal position. It is not applicable in this case, so it is not selected.

Option C enhanced contractions is not appropriate in this case, because the pregnant woman's contractions are already very frequent and strong, and further enhanced contractions may increase the risk of uterine rupture, so it is excluded.

Option D fetal attraction and option E forceps induction are both vaginal midwifery. Considering that the pregnant woman may face the risk of threatened uterine rupture, these midwifery methods may increase the risk of complications, so they are not the most appropriate treatment, so they are excluded together.

Topic 129: The most appropriate treatment is this

analyze:

Cesarean section: the most suitable for the case of uterine rupture, to ensure the safety of mother and baby.

Free-hand rotating fetal head: not applicable in case of threatened uterine rupture.

Strengthening contractions: This may increase the risk of uterine rupture.

Fetal attraction: not suitable for threatened uterine rupture.

Induction of forceps: not suitable for threatened uterine rupture.

Best answer: A. cesarean section

Female, 35 years old, G2P1, irregular vaginal bleeding for more than 1 month. Usually, menstruation is irregular, and the last menstruation is unknown. Physical examination: T36℃, P80 times / minute, R18 times / minute, BP100 / 70mmHg, pale examination: no abnormal vulva, vagina and cervix, uterus size at 4 months of gestation, soft, double attachment not abnormal. The ultrasound showed that the uterine size was 18cm 10cm 6cm, the uterine cavity was filled with honeycomb heterogeneous echo (+), and the chest CT showed no abnormality.

130, (Type A3 / A4 questions) subject

The next preferred treatment is the

A. fetal protection treatment

B. Drug abortion

C. chemotherapy

D. laparoscopic approach

E. Qing Palace art

Reference answer: E Your answer:

[analysis] hydatidiform mole is caused by some abnormalities of the fertilized egg during pregnancy. The early symptoms of the hydatidiform mole are usually irregular vaginal bleeding, which is similar to the symptoms of pregnancy. At the same time, imaging may show uneven honeycomb echo in the uterus and abnormal increase in hCG. Patients with irregular vaginal bleeding for more than 1 month (early symptoms of hydatidiform vaginal irregular bleeding), gynecological examination see vulva, vagina and cervix showed no abnormalities (excluding vulva, cervix), uterus such as 4 months pregnancy size, soft (hydatidiform uterus), ultrasound uterine size 18cm 10cm 6cm, uterine cavity filled with honeycomb heterogeneous echo (hydatidiform mole imaging uterine echo heterogeneous honeycomb), urinary hCG (+) (staphydiform fetal biochemical index). Considering hydatidiform mole, the first choice of hydatidiform mole is uterine surgery, so this question chooses (E) uterine surgery.

[Error avoidance] Choriocarcinoma may also appear similar clinical manifestations to hydatidiform mole, but choriocarcinoma may appear multiple cotton ball shadow in the chest, which was not seen in this case, so it was excluded.

130 Questions:

The preferred treatment should be E. Honeylet heterogenous echogenes usually suggest the possibility of hydatidiform mole, and after confirming the diagnosis, the most important management is uterine clearance to remove abnormal cells and prevent their further deterioration.

130. Analysis:

A young female patient, irregular vaginal bleeding for more than a month, irregular menstruation, physical examination showed uterine enlargement, ultrasound showed that the uterine cavity was filled with honeycomb heterogeneous echo, which was considered as hydatidiform mole. Palace surgery is the first choice of treatment of hydatidiform mole, often can not be completely clean at a time, can be cleared again after about 1 week.

Option A fetal protection treatment is mainly used for pregnant women with threatened abortion. This patient is not pregnant, and there is no need for fetal protection, so it is not selected.

Option B Drug abortion is mainly used for early pregnancy abortion, and the patient in the problem stem is not pregnant, so it is excluded.

Option C Chemotherapy is an important means for the treatment of malignant tumors. Hydatidiform mole is a benign disease without chemotherapy and can be excluded.

Option D laparoscopic exploration is invasive examination, which is not the preferred treatment for hydatidiform mole and can be ruled out.

To sum up, the correct answer is: E. Qing Palace technique.

Topic 130: The next preferred treatment is this

A. Prepreserving treatment

B. Medical abortion

C. chemotherapy

D. laparoscopic approach

E. Qing Palace art

analyze:

The 35-year-old patient, G2P1, irregular vaginal bleeding for more than 1 month, uterus such as the size of 4 months of gestation, ultrasound suggested that the uterine cavity was filled with honeycomb heterogenous echo, and these features suggested the possibility of hydatidiform mole. The first choice of treatment for hydatidiform mole is uterine clearance.

Answer: E. Qing Palace art

131, (A3 / A4 type question) subject

The next most correct step is this

A. hCG after 1 week

B. Gynecologic ultrasound was reviewed 1 week later

C. Review of the hCG after 1 month

D. Gynecologic ultrasound was reviewed 1 month later

E. HCG review after 2 months

Reference answer: A. Your answer:

[Analysis] hydatidiform follow-up content is as follows: (1) hCG quantitative measurement, after surgery once a week until hCG normal 3 consecutive times, after a month for at least 6 months, then every half a year of a year, a total of follow-up for 2 years; (2) pay attention to the presence of abnormal vaginal bleeding. Therefore, this question should choose (A) for hCG review after 1 week.

[To avoid mistakes] Note that the hydatidiform mole is changed to once a month after three hCG is normal, and then once half a year after 6 months. Do not be confused.

131 Question:

The most correct treatment was the hCG review after 1 week of A. Human chorionic gonadotropin (hCG) is an important indicator to follow the progress of hydatidiform mole treatment. After uterine clearance, hCG levels need to be monitored closely, to ensure that all abnormal tissues are cleared and to detect any potential malignant changes.

analyze:

The patient was diagnosed as hydatidiform mole and had undergone uterine

surgery. After surgery, blood hCG changes should be closely followed up to ensure early detection of trophoblastic tumors and timely treatment. Since hCG decreased to an unmeasurable level 8 weeks after the palace clearance, the hCG level should be measured every week. Therefore, the hCG review after 1 week is the most correct treatment.

Option B: reexamination of gynecological ultrasound after 1 week is of little significance for monitoring the level of hCG, so it can be ruled out.

Option C: review hCG after 1 month, option E: review hCG after 2 months, both time span is too long to closely monitor hCG level, can be excluded together.

Option D: The level of hCG cannot be monitored after the review of gynecological ultrasound after 1 month, and the uterine morphology will not change much in a short time. The review of gynecological ultrasound is of little significance, so it is not selected.

In conclusion, the correct answer is: A. Review of hCG after 1 week.

Topic 131: The most correct step is

A. hCG after 1 week

B. Gynecologic ultrasound was reviewed 1 week later

C. The hCG was reviewed 1 month later

D. Gynecologic ultrasound was reviewed 1 month later

E. HCG review after 2 months

analyze:

Patients with hydatidiform mole need regular monitoring of hCG levels after uterine clearance to ensure complete emptying and monitoring for the presence and absence of trophoblast tumors. The hCG test is usually performed at 1 week, 1 month, and 2 months after surgery.

Answer: A. Review of hCG after 1 week

Female, 48 years old, G2P1, secondary dysmenorrhea for 10 years, aggravated for 4 years, poor oral painkillers in the past two years, T36.2℃, P70 times / min, R22

times / min, no cardiopulmonary abnormalities, no cervical pain, double enlargement, posterior uterine position, uterine size such as 3 months of pregnancy, hard, tenderness, double accessories and pelvic normal.

132, (A3 / A4 type question) subject

The cause of its disease may be

A. Long-term stimulation of progesterone

B. Defects in the endometrial functional layer

C. Endometrial reflux with menstrual blood

D. The stimulation with oestrogen

E. Invasion of the endometrial basal layer into the myometrium

Reference answer: E Your answer:

Graphic resolution: [analytical] patient female, 48 (adenomyosis good age), G2P1, secondary dysmenorrhea for 10 years, aggravating 4 years (adenomyosis clinical symptoms for secondary dysmenorrhea), examination without cervical pain, bilateral accessory area without enlargement, uterine posterior, uterine size such as pregnant 3 months (adenomyosis physical examination), hard, tenderness, double attachment, pelvic cavity is normal. Adenomyosis is a benign lesion caused by the invasion of the endometrial basal layer into the myometrium. The main symptoms are secondary dysmenorrhea, menstrual disorders, frequency of urination and urgency. The patient should be diagnosed with adenomyosis, so the cause may be (E) invasion of the basal layer into the myometrium.

[Avoid avoidance] The key of this problem lies in the diagnosis of adenomyosis and its pathogenesis.

132, the cause of its onset may be

Answer: E. Invasion of the endometrial basal layer into the myometrium

This description is typical of adenomyosis, in which endometrial tissue invades into the myometrium.

B Invasion of the endometrial basal layer into the myometrium

analyze:

The patient was a middle-aged woman with secondary dysmenorrhea for many years. The uterus was enlarged, hard and tender, which was the typical clinical

manifestations and signs of adenomyosis, so the diagnosis of adenomyosis was considered. Adenomyosis is a diffuse or localized lesion of the endometrial glands and stroma, which is a common gynecological disease. Therefore, the cause of the disease is the invasion of the myometrium.

Option A progesterone long-term stimulation and option D estrogen stimulation, which are unrelated to endometriosis, so they can be excluded together.

Option B endometrial functional layer defects are common in anovulatory dysfunctional uterine bleeding and are unrelated to endometriosis, so they are not considered.

Option C endometrial flow with menstrual blood can lead to endometriosis, but adenomyosis is a special type of endometriosis, which is caused by the invasion of endometrial glands and stroma into the uterine muscle layer, and has nothing to do with the simple endometrium with menstrual blood flow, so it is not selected.

132 The cause of its onset may be

A. Long-term stimulation of progesterone: an uncommon cause.

B. Defects of endometrial functional layer: not suitable for this case.

C. Inendometrial um with menstrual blood: a main mechanism of endometriosis, but not a direct cause of adenomyosis.

D. Estrogen stimulation: a common predisposing factor, but not a direct cause.

E. Invasion of the basal layer into the myometrium: This is the main pathological mechanism of adenomyosis.

Best answer: E. Ininvasion of the myometrium

133, (A3 / A4 type question) subject

What symptoms may also be accompanied by

A. contact bleeding

B. algopareunia

C. Increased menstruation

D. frequency of urinatior

E. astriction

Reference answer: C Your answer:

[Analysis] The patient was diagnosed as adenomyosis, and one of the important

manifestations of adenomyosis is menstrual disorder, which is mainly manifested as the increase of menstrual volume and the extension of menstrual period, so the correct answer to this question should be C.

[Avoid mistakes] Frequent urination and constipation can also be one of the manifestations of adenomyosis, but it mainly occurs in the later stage of the disease, and the uterus compresses the surrounding rectum, bladder and other adjacent tissues.

133, which symptoms may also be accompanied by

Answer: B. painful sexual intercourse

Common symptoms of adenomyosis include painful sexual intercourse, dysmenorrhea, and chronic pelvic pain. In this case, painful intercourse were also possible symptoms, considering the patient's existing dysmenorrhea.

F. Increased menstruation

analyze:

Adenomyosis is mainly manifested as increased menstruation, prolonged menstruation and gradually aggravated progressive dysmenorrhea. The adenomyosis located in the posterior wall of the uterus can compress the rectum and cause constipation and other symptoms, which can lead to anemia when severe. Therefore, the patient may also have the symptoms of increased menstrual volume.

Option A Contact bleeding is more common in cervical cancer patients, which is inconsistent with the question.

Option B sexual crossing pain is mostly seen in endometriosis. Deep endometriosis can be palpable in uterine rectal notch or uterine sacral ligament nodules during gynecological examination, but the problem stem clearly indicated that the patient with double attachments and pelvic cavity were normal, so it was excluded.

Option D urinary frequency is common in urinary tract infections and is inconsistent with the patient.

Option E Although constipation can be seen in adenomyosis, when adenomyosis causes constipation, it is often combined with other compression symptoms, such as emergency weight, which is less common than the increase of menstruation, so it is not selected.

133. What other symptoms may be associated with them

A. Contact bleeding: common in cervical lesions.

B. Dyspunia: one of the common symptoms of adenomyosis.

C. Increased menstrual volume: common symptoms in patients with adenomyosis.

D. Frequency of urination: not common in adenomyosis.

E. Constipation: not common in adenomyosis.

Best answer: B. painful sexual intercourse and C. increased menstruation (this question may have multiple correct options, but need to be selected according to the actual test requirements)

What symptoms may also be accompanied by

Common concomitant symptoms of adenomyosis include increased menstrual volume and painful sexual intercourse, but rarely with contact bleeding, frequent urination, or constipation.

Correct answer: B. painful sexual intercourse

134, (A3 / A4 type question) subject

If Hb is 85 g / L, the most suitable treatment method is this

A. Place the levonorgestrel intrauterine sustained-release system

B. High-dose progesterone

C. traditional Chinese medicine

D. operative treatment

E. oral contraceptive

Reference answer: A. Your answer:

[Analysis] Levonorgestrel intrauterine sustained release system (LNG-IUS) is easy to place, can continue the sustained release of levonorgestrel for 5 years. Clinical application shows that LNG-IUS is effective in adenomyosis, dysmenorrhea and menorrhagia, and its effect is better than compound oral contraceptives. It can be used as the preferred treatment for adenomyosis patients with lunar menstruation. Therefore, we should choose (A) to place the levonorgestrel intrauterine slow-release system.

[Error] Oral contraceptives and progesterone drugs can also treat adenomyosis, but the treatment effect is slightly worse than levonorgestrel intrauterine sustained release system; Chinese medicine alone can only relieve dysmenorrhea and is prone to relapse; Hb 85g / L.

134, if Hb 85 g / L, the most suitable treatment method is

Answer: D. Surgical treatment

Analysis: Considering the patient's low hemoglobin level (85g / L), indicating that there may be severe bleeding or prolonged bleeding, which may require more aggressive intervention, such as surgical treatment, to address the underlying problem. Although medical treatment such as the intrauterine sustained-release system or progesterone may partly help to manage symptoms, surgical treatment may be more appropriate in this case, especially if considering the patient's poor response to painkillers and the severity of the long-term symptoms.

G. operative treatment

analyze:

The middle-aged female patient was diagnosed with adenomyosis with hemoglobin 85g / L, indicating anemia, so surgical hysterectomy should be used to achieve the purpose of radical cure. Therefore, the most suitable treatment method is the surgical treatment.

Option A The levonorgestrel intrauterine sustained-release system is mainly used for the treatment of menorrhagia. Although it has the effect of reducing menstrual volume, it cannot cure adenomyosis, which is inconsistent with this question.

Option B high-dose progesterone and option E oral contraceptive can be used for treating adenomyosis, but the risk of recurrence after withdrawal and side effects after long-term application, so they can be ruled out.

Option C Traditional Chinese medicine has a certain curative effect on adenomyosis, but the effect is slow, and it is not used as the preferred treatment option, so it is not considered.

134. If Hb is 85 g / L, the most suitable treatment method is

A. Placement of levonorgestrel intrauterine sustained release system: suitable for adenomyosis patients who have not plan recent surgery, but low Hb may not be suitable.

B. High-dose progesterone: short-term relief of symptoms but no radical cure.

C. Traditional Chinese medicine: to relieve symptoms but to have a slow effect.

D. Surgical treatment: the most suitable treatment for severe cases of adenomyosis and anemia.

E. Oral contraceptives: suitable for those with mild symptoms.

Best answer: D. Surgical treatment

Title material male, 22 years old, 3 years no obvious cause solitary, don't want to go out with people, curtains closed during the day, lazy life, summer can be up to half a month not take a bath, hair is very long also ignore, head C abnormal, mental check: expression, less words, deny illusion, poor thinking, emotional indifference, self-knowledge is poor.

135, (Type A3 / A4 questions) The topic treatment is

A. antidepressant drugs

B. A mood stabilizer

C. incitantia

D. antipsychotic drug

E. anxiolytic drugs

Refer to the answer: D. Your answer:

Graphic analysis: [resolution] patients male, 22 years old, 3 years have no obvious cause solitary, reluctant to go out with people, curtain closed during the day, lazy (simple schizophrenia clinical manifestations), head CT no obvious abnormalities (excluding organic lesions), mental examination see expression indifference, less words, denied hallucinations, poor thinking, emotional indifference, poor self-knowledge (simple schizophrenia physical examination visible). Simple schizophrenia is a kind of schizophrenia, patients with adolescent onset, onset, for no obvious cause, early performance as depression, inattention, insomnia, dizziness, etc., after the disease gradually progress, become isolated, passive retreat, mental decline, lazy, indifference to relatives, interest in work and study, lack of enterprise, emotional indifference, lack of corresponding response to emotional stimulation. Simple schizophrenia is mainly treated by antipsychotic drugs, and the patient should be diagnosed with simple schizophrenia, so the answer to this question should be (D) antipsychotic drugs.

[Avoid mistakes] simple schizophrenia and depression may appear low mood, sleep disorders and other symptoms, but depression patients will not have emotional and environmental disharmony performance.

For question 135, the symptoms described include social avoidance, lazy life, emotional apathy, and poor thinking, which are typical of schizophrenia. Therefore, the most suitable treatment regimen is the use of antipsychotics. Therefore, the correct answer should be that for D. antipsychotics.

135. Analysis:

Young male patients, in the past 3 years without obvious inducement of solitary, lazy life and other negative symptoms, mental examination see the patient expression is indifferent, less words, denied hallucinations, poor thinking, emotional indifference, for the simple manifestation of schizophrenia, can be diagnosed as simple schizophrenia. Antipsychotics are preferred for the treatment of the disorder.

Option A Antidepressants are the first choice for depression. Patients do not show depression, so they are not selected.

Option B mood stabilizer, or antimanic drug, was the preferred drug for mania, and patients did not show manic manifestations, so it was excluded.

Option C psychostimulant generally refers to amphetamine stimulants, has a strong central nervous system excitation effect, after taking will produce a strong mental dependence, and is determined as a drug, the patient does not need psychostimulants, can be excluded.

Option E Antianxiety drugs are mainly used to treat generalized anxiety disorder, panic disorder, etc. Patients have no anxiety symptoms and can be excluded.

In summary, the correct answer is: D. Antipsychotics.

The treatment option for question 135 was D. antipsychotics. Based on the patient's symptomatic presentation, the most consistent treatment is the use of antipsychotic drugs to improve his psychiatric symptoms.

136, (A3 / A4 type question)

After taking psychotropic drugs, the symptoms were significantly improved, but there were symptoms such as static cannot sit, hand shaking, and the next step of treatment should be subject

A. Excessive use of this psychotropic medication

B. The psychotropic drugs with other different mechanisms

C. Reduced use of this psychotropic drug

D. Discontinuation of this psychotropic medication

E. Treatment with phenenhisol

Reference answer: E Your answer:

[Analysis] Antipsychotic drugs may lead to extrapyramidal symptoms (EPS), namely hand shaking, etc., for the occurrence of EPS, we can add the anticholinergic drug benzene to relieve the symptoms.

[Error avoidance] Antipsychotic drugs cannot be stopped at will, so in order to eliminate the side effects caused by drugs, we can use benzene to reduce the side effects brought by drugs.

For question 136, the patient cannot sit and hand shaking after the use of psychotropic drugs, which may be extrapyramidal side effects, such as sitting (which is an impulsive feeling that needs to move constantly, and is a common side effect of many antipsychotic drugs). The Benzhexol (also known as Trihexyphenidyl) is a drug used to treat extrapyramidal side effects caused by drugs. Therefore, in this case, the most appropriate approach is to add benzene to control the side effects, so the correct answer is E.

136, analysis:

After taking psychotropic drugs, the patient's symptoms were significantly improved, but there were symptoms such as static, not sitting and hand shaking, which was considered as external vertebral body reaction. External vertebral reaction is the most common side effect of antipsychotic treatment, with manifestations include acute dystonia, sedentary inability, and parkinsonism. Sitting can not be manifested by the patient can not control the agitation, can not sit still, repeatedly walk or step in place, the patient is sedentary, can be treated with benzene.

Option A plus the use of this psychotropic drug may aggravate the extravertebral body reaction, so it is not selected.

Option B changes to other psychotropic drugs with different mechanisms, and option D disusing the psychotropic drug cannot guarantee the absence of the new drug, and may affect the original efficacy, so it was excluded.

Option C Reduction of this psychotropic drug will reduce the efficacy and can

not alleviate the external vertebral body reaction, which can be excluded.

In conclusion, the correct answer is: E.

The treatment option for question 136 is C. Reduction of this psychotropic drug. When patients have adverse reactions such as static sitting and hand shaking after taking psychotropic drugs, the drug should be considered to reduce the occurrence of adverse reactions, rather than immediately adding the dosage or changing other drugs.

137. (Type B1 question)

The type of abortion most susceptible to infection

A. missed abortion

B. incomplete abortion

C. threatened abortion

D. inevitable abortion

E. complete abortion

Reference answer: B. Your answer:

[Analysis] incomplete abortion: abortion is inevitable to continue to develop, some pregnancies discharged from the uterine cavity, and some residual in the uterine cavity or embedded in the cervix, or fetal discharge placenta retention uterine cavity or embedded in the cervix, affecting uterine contraction, leading to bleeding, and even shock. On gynecological examination, the cervix was dilated, with gestational blockage and persistent blood outflow, and the uterus was less than the number of menopause. Because some pregnare out of the uterine cavity, and some remain in the uterine cavity or incarcerated in the cervix,

So the most prone to infection, but also easy to cause massive bleeding (B pair).

[To avoid mistakes] (1) the most easy to cause DIC is the retention of abortion.(2) The inevitable abortion refers to the inevitable abortion. On the basis of threatened abortion, the amount of vaginal bleeding increased, paroxysmal lower abdominal pain intensified, or appear vaginal flow fluid (fetal membrane rupture). Gynecological examination of the cervix has been dilated, sometimes visible embryonic tissue or amniotic sac blocked in the cervix, the size of the uterus is basically consistent with the number of menopause or slightly small (D wrong).(3) Complete abortion means that the pregnancy has been discharged, vaginal bleeding gradually stopped, abdominal pain gradually disappeared, the gynecological examination cervix has been

closed, the uterus is close to normal size (E wrong)

For question 137, the answer to "the type of abortion most susceptible to infection" is B. incomplete abortion. In incomplete abortion, part of the embryo or placenta remains in utero and is not completely excreted, which increases the risk of infection.

Types of abortion most susceptible to infection:

Because of incomplete abortion, part of the pregnancy is discharged, and part remains in the uterine cavity, this residue can become a good culture medium for bacterial growth, so it is easy to cause infection. Therefore, B. incomplete abortion is the type of abortion prone to infection.

137. The type of abortion that is most susceptible to infection

A. missed abortion

B. incomplete abortion

C. threatened abortion

D. inevitable abortion

E. complete abortion

Answer: B. incomplete abortion

Explanation: Incomplete abortion means that part of the fetal tissue has been excreted, but still remains in the uterus. Retained tissue can become a hotbed for bacterial infection, so incomplete abortion is the type most susceptible to infection.

138. (Type B1 question)

A small amount of vaginal bleeding, B ultrasound suggests that the intrauterine fetus is alive

A. missed abortion

B. incomplete abortion

C. threatened abortion

D. inevitable abortion

E. complete abortion

Reference answer: C Your answer:

[Analysis] Seminal abortion refers to the first occurrence of a small amount of vaginal bleeding before 28 weeks of pregnancy, often dark red or bloody leucorrhea, no pregnancy discharge, followed by paroxysmal lower abdominal pain or low back pain. In gynecological examination, the cervix was not open, the membranes was not broken, and the uterine size was consistent with the number of menstrual weeks. After rest and treatment, pregnancy can continue (C pair); if vaginal bleeding increases or lower abdominal pain increases, abortion.

[To avoid mistakes] (1) inevitable abortion refers to abortion is inevitable. On the basis of threatened abortion, the amount of vaginal bleeding increased, paroxysmal lower abdominal pain intensified, or appear vaginal flow fluid (fetal membrane rupture). Gynecological examination of the cervix has been dilated, sometimes visible embryonic tissue or amniotic sac blocked in the cervix, the size of the uterus is basically consistent with the number of menopause or slightly small (D wrong).(2) Complete abortion means that the pregnancy has been discharged, the vaginal bleeding gradually stopped, the abdominal pain gradually disappeared, the gynecological examination cervix has been closed, the uterus is close to normal size (E wrong).

For question 138, "a small amount of vaginal bleeding, B ultrasound indicates that the intrauterine fetus is alive", the correct answer should be C. threatened abortion. Seminal abortion refers to the occurrence of abortion symptoms such as bleeding, but the uterine mouth is not open, the fetus is not separated, and it is possible to maintain pregnancy to continue development through appropriate treatment.

A small amount of vaginal bleeding, B ultrasound suggests that the intrauterine fetus survival is:

This condition most fits the definition of threatened abortion, where a little vaginal bleeding occurs, but the fetus is still alive. So C. threatened abortion is the correct answer.

138. A small amount of vaginal bleeding, and B ultrasound suggests that the intrauterine fetus is alive

A. missed abortion

B. incomplete abortion

C. threatened abortion

D. inevitable abortion

E. complete abortion

Answer. C. threatened abortion

Explanation: threatened abortion refers to the early pregnancy, pregnant women have a small amount of vaginal bleeding but B ultrasound examination shows that the fetus is still alive. If handled in time, threatened abortion is likely to continue the pregnancy.

139. (Type B1 question)

Infants over 3 months are susceptible to meningitis

A. meningococcus + Staphylococcus aureus

B. Streptococcus pneumoniae + Dicococcus meningitidis

C. Staphylococcus aureus

D. E.coli

E. H. influenzae + S. pneumoniae

Reference answer: E Your answer:

[Analysis] Haemophilus influenzae and Streptococcus pneumoniae are more common in infants aged 3 months to 3 years old (E pair).

[Error avoidance] Preschool and school-age children with meningitis double coccus, Streptococcus pneumoniae, Haemophilus influenzae and grape grape aureus

Cococci are more common.

For the first question (infants over 3 months of age are susceptible to meningitis pathogens), the main pathogens of meningitis in younger infants and young children include Streptococcus pneumoniae and diococcus meningitis. Therefore, option B (Streptococcus pneumoniae + Dicococcus meningitidis) is the most appropriate answer.

The pathogens that infants over 3 months who are susceptible to meningitis are:

A. Dicoccus meningitica + Staphylococcus aureus: This option does not fully meet the susceptible pathogens of infants over 3 months.

B. Streptococcus pneumoniae + meningococcus: This option is partially correct

because Streptococcus pneumoniae is one of the susceptible pathogens in infants, but diococcus meningitis is not the most susceptible pathogen at this age.

C. Staphylococcus aureus: This is not the most susceptible agent of meningitis in this age group.

D. Escherichia coli: This is also not the most susceptible agent of meningitis at this age.

E. Haemophilus influenzae + Streptococcus pneumoniae: This option is fully susceptible pathogens in infants and young children over 3 months.

Therefore, for question 139, the answer is E. influenzae + Streptococcus pneumoniae.

The pathogens that infants over 3 months who are susceptible to meningitis are:

B Streptococcus pneumoniae + double meningococcus

140. (Type B1 question)

Children over 12 are susceptible to the most common agents of meningitis

A. meningococcus + Staphylococcus aureus

B. Streptococcus pneumoniae + Dicococcus meningitidis

C. Staphylococcus aureus

D. E.coli

E. H. influenzae + S. pneumoniae

Reference answer: B. Your answer:

[Analysis] Children and adults over 12 years old are susceptible to the most common pathogen of meningitis is Streptococcus pneumoniae, meningococcus (B pair).

[Error avoidance] Preschool and school-age children with meningitis double coccus, Streptococcus pneumoniae, Haemophilus influenzae and grape grape aureus

Cococci are more common.

For the second question (children over 12 years are susceptible to the most common agent of meningitis), the most common agents in children of this age are Mesococcus meningitis and Haemophilus influenzae. Therefore, the E option (Haemophilus influenzae + S. pneumoniae) may be the closest answer, although the ideal combination should be B. meningitidis and Haemophilus influenzae. But in the

given option, E is the most appropriate choice.

The most common pathogens of meningitis in children over 12 years are:

A. Dicoccus meningitica + Staphylococcus aureus: This option does not fully meet the susceptible pathogens in children over 12 years of age.

B. Streptococcus pneumoniae + Dicococcus meningoseptica: This option is fully compatible with susceptible pathogens in children over 12 years of age.

C. Staphylococcus aureus: This is not the most susceptible agent of meningitis in this age group.

D. Escherichia coli: This is also not the most susceptible agent of meningitis at this age.

E. Haemophilus influenzae + Streptococcus pneumoniae: Haemophilus influenzae is not the most susceptible pathogen at this age group.

Therefore, for question 140, the answer was B. pneumoniae + Dicoccus meningitidis.

The most common pathogens of meningitis in children over 12 years are:

B Streptococcus pneumoniae + double meningococcus

141. (Type B1 question)

The laboratory results of neonatal hemolysis were

A. Unconjugated bilirubin increased and positive for Coombs direct test

B. unbound bilirubin and Coombs direct and indirect tests were negative

C. Abjugated bilirubin was increased with a positive antibody release test

D. Conjugated bilirubin increased with abnormal liver function

E. Unbound bilirubin was increased, and the antibody release test was negative

Reference answer: A. Your answer:

Graphic analysis: [analysis] unbound bilirubin increased, Coombs direct test positive, considered as neonatal hemolysis (A pair).

[Erstavoidance] Unbound bilirubin slightly increased, Coombs direct test and indirect test were negative, considered as hepatocellular jaundice

(B wrong). Combined with elevated bilirubin, positive antibody release test, considering hemolytic jaundice (C wrong). High direct bilirubin and positive antibody

release may be due to problems in the liver or biliary system, causing no normal discharge of bilirubin (D wrong). Antibody negative indicates that there is no relevant pathogen antibody in the body, which can basically exclude pathogen infection, and unbound bilirubin elevation is caused by noninfectious cause (E error).

For title 141, a neonatal hemolysis condition is involved. In neonatal hemolytic diseases, an increase in unconjugated (indirect) bilirubin is usually seen, because hemolysis leads to increased RBC destruction in the body, produces large amounts of hemoglobin, which is then converted to unconjugated bilirubin. If the Coombs direct test is positive, it indicates antibody-mediated RBC destruction, which fits the characteristics of immunohemolytic disease. Therefore, for topic 141, option A (unbound bilirubin increased, Coombs direct test positive) is the most appropriate answer.

For question 141, neonatal hemolysis refers to the same passive immune disease caused by the incompatibility of maternal and infant blood group, resulting in the destruction of red blood cells. It mainly shows the elevation of unconjugated bilirubin, which is because a large amount of free bilirubin is released into the blood stream after the destruction of red blood cells. The Coombs direct test is mainly used to test whether red blood cells have been coated by antibodies, and there will be agglutination reaction to the coated red blood cells, the result is positive, so option A (unbound bilirubin is increased, Coombs direct test is positive) is the correct answer.

141 The laboratory findings of neonatal hemolysis were

The most relevant answer is:

A. Unconjugated bilirubin increased and positive for Coombs direct test

explain:

Neonatal hemolysis is the destruction of red blood cells caused by maternal and infant blood group incompatibility, resulting in the increase of unconjugated bilirubin (i. e., indirect bilirubin). Coombs Direct test (direct anti-human globulin test) is used to detect whether the presence of antibodies or complement on the surface of red blood cells, and is an important detection method for the diagnosis of hemolytic diseases.

142. (Type B1 question)

The laboratory indicators of TORCH infection were

A. Unconjugated bilirubin increased and positive for Coombs direct test

B. unbound bilirubin and Coombs direct and indirect tests were negative

C. Abjugated bilirubin was increased with a positive antibody release test

D. Conjugated bilirubin increased with abnormal liver function

E. Unbound bilirubin was increased, and the antibody release test was negative

Refer to the answer: D. Your answer:

[Analysis] After TORCH infection, liver and spleen are large, jaundice, abnormal liver function, resulting in the normal discharge of bilirubin, combined with bilirubin increased (D pair).

[Fault avoidance] TORCH is composed of the initial als of a group of pathogenic microorganisms, in which T refers to Toxoplasma and O refers to it

He (others, such as Treponema pallidum, microvirus B19, etc.), R refers to rubella virus, C refers to cytomegalovirus, H mainly refers to

herpes simplex virus.

For title 142, it involved the TORCH infection. TORCH refers to toxoplasmosis (Toxoplasmosis), other (Other, such as syphilis, rubella, viruses, bacteria, etc.), rubella (Rubella), cytomegalovirus (Cytomegalovirus), and herpes simplex virus (Herpes simplex virus) infections that can be transmitted through the placenta to the fetus, leading to congenital infection. TORCH infection usually does not usually result in a significant elevation of bound or unconjugated bilirubin, and its diagnosis mainly relies mainly on specific antibody detection. Therefore, laboratory indicators of TORCH infection are not directly indicated in the title, but option C (combined with elevated bilirubin, positive antibody release test) may be linked because a positive antibody release test may point to the presence of a pathogen, which is consistent with the detection of TORCH infection.

For question 142, TORCH infection refers to a group of pathogens, including toxoplasma (TOX), rubella virus (RV), cytomegalovirus (CMV), herpes simplex virus (HSV), and other viruses (e. g., minvovirus B19). Infection with these pathogens usually does not cause significant bilirubin changes, so the bilirubin levels (whether combined or unbound) are usually not the primary indicator for diagnosing TORCH

infection. However, the presence of these pathogens may lead to the production of certain specific antibodies that can be detected by specific tests but usually do not trigger a positive response to the Coombs test. Therefore, of the options provided, option B (mild elevated bilirubin, and negative Coombs direct and indirect test) is most consistent with the laboratory indicator characteristics of TORCH infection, although this is not a diagnostic indicator of TORCH infection, but is the most appropriate for the given option.

142 The laboratory indicators of TORCH infection were

The most relevant answer is:

B. Conjugated bilirubin increased with abnormal liver function

explain:

TORCH infection (toxoplasma, other pathogens, rubella virus, cytomegalovirus, herpes simplex virus) is a common intrauterine infection in newborns. Infection may lead to abnormal liver function, manifested by elevated conjugated bilirubin (i. e. direct bilirubin) and impairment of liver function.

143. (Type B1 question)

Female patients with induced abortion surgery, sudden chest tightness, cold sweat, pale face, bradycardia, arrhythmia, the possible reason is

And A.Asherman syndrome

C. perforation of uterus

D. aeroembolism

E. induced abortion syndrome

F. Incomplete suction

Refer to the answer: D. Your answer:

[Analysis] The comprehensive reaction of induced abortion refers to the pain or local stimulation during surgery, so that the patient during or after nausea, vomiting, bradycardia, arrhythmia, pale, dizziness, chest tightness, sweating, and even severe blood pressure drop, fainting, convulsions and other vagal nerve excitement symptoms. This is related to the mood, physical condition and surgical procedure of the recipient (D pair).

[Avoid mistakes] Uterine perforation is a serious complication of induced abortion, the incidence and the operation technology of the operator and the uterus itself

Conditions (such as lactation pregnancy uterus, scar uterine pregnancy after cesarean section, etc.) are related. Suddenly feel a sense of silence during the surgery,

Or the surgical device entry depth is deeper than the original measured depth. It indicates uterine perforation, and the operation should be stopped immediately (B wrong).

Asherman Syndrome, also known as intrauterine adhesion, is caused by induced abortion or other uterine operation, damage to the endometrium

(A wrong). Air embolism is a disease caused by obstruction of cerebral vessels by gas emboli originating in the lung, usually in the periphery

When the pressure decreases (such as rising from deep water to shallow water), the swelling lung gas is common

Characterized by pain and (or) neurological symptoms. Air embolism is when the air enters the blood circulation to the lungs, blocking the pulmonary artery mainly

Access, causing severe shock (C error). Abhaia refers to the residual tissue of some pregnancy after induced abortion.and operation

Unskilled technology or abnormal uterine position, is a common complication of induced abortion, manifested as vaginal bleeding after surgery

Long time, more blood volume or repeated amount of bleeding symptoms (E wrong).

For question 143, the symptoms described include chest tightness, cold sweat, pallor, bradycardia and arrhythmia, which may be associated with air embolism. Air embolism is a serious complication that may occur during the process of induced abortion and can lead to acute cardiac and circulatory dysfunction. Therefore, the most appropriate answer is the C. air embolism.

First, we analyze question 143. The topic describes a female patient with chest tightness, cold sweat, pale face, bradycardia and arrhythmia suddenly during an induced abortion. We can analyze these symptoms:

A. Asherman syndrome: the main manifestations are menorrhagia, amenorrhea, secondary infertility, and inconsistent with the symptoms described in the topic, so it

can be excluded.

B. Uterine perforation: Although it is a complication of induced abortion, it is mainly manifested as severe pain in the lower abdomen, accompanied by nausea, vomiting, bleeding and other symptoms, which is not consistent with the cardiovascular symptoms in the topic, so it is not the best option.

C. Air embolism: Although air embolism is a serious complication, the symptoms directly related to air embolism are not mentioned in the title, and therefore can be excluded.

D. Induced abortion syndrome: the main manifestations are nausea and vomiting, bradycardia, arrhythmia, pale complexion, dizziness, chest tightness, sweating during the induced abortion or at the end of the abortion, and even severe vagal nerve excitement symptoms such as blood pressure drop, fainting and convulsions. This is highly consistent with the symptoms described by the title, and is therefore the most appropriate option.

E. Inadequate aspiration: the main manifestations are the long vaginal bleeding after the operation, the high blood volume or the reappearance of excessive bleeding after the bleeding stops, which is not consistent with the symptoms described in the topic, so it is not the best option.

In conclusion, the answer to question 143 is the D. induced abortion syndrome.

Question 143

Female patients underwent sudden chest tightness, cold sweat, pale face, bradycardia, arrhythmia during induced abortion, the possible reasons are:

These symptoms suggest an acute, life-threatening condition. Chest tightness, cold sweat, pale face, bradycardia and arrhythmia are all symptoms of acute events. Combined with induced abortion, it can be speculated as follows:

Option analysis:

A. Asherman syndrome: It usually does not cause acute symptoms and is menstrual abnormalities or infertility due to intrauterine adhesions.

B. Uterine perforation: Acute abdominal pain and bleeding may also occur, but

they are unlikely to directly cause these cardiovascular symptoms.

C. Air embolism: This is an acute and serious complication that may cause chest tightness, cold sweat, bradycardia and arrhythmia.

D. Induced abortion syndrome: also an acute complication of the vagal reflex, leading to bradycardia, arrhythmia, and other symptoms.

E. Abinsufficiency: usually postoperative bleeding, which is unlikely to cause bradycardia and arrhythmia.

Answer: D. induced abortion syndrome

144. (Type B1 question)

After induced abortion, vaginal bleeding continued for 2 weeks, and the possible diagnosis is

And A.Asherman syndrome

F. perforation of uterus

G. aeroembolism

H. induced abortion syndrome

I. Incomplete suction

Reference answer: E

[Analysis] Abinsufficiency refers to the residue of some pregnancy tissue after artificial abortion. It is related to operator incompetence or abnormal uterine position and is a common complication of induced abortion. After the operative vaginal bleeding for a long time, more blood volume or repeated bleeding after bleeding stop, should be considered as uterine insufficiency, blood or urine hCG test and ultrasound examination are helpful in diagnosis (E pair).

[Error avoidance] Uterine perforation is a serious complication of induced abortion. The incidence of surgery and the operation technique and the uterus itself

Conditions (such as lactation pregnancy uterus, scar uterine pregnancy after cesarean section, etc.) are related. Suddenly feel a sense of silence during the surgery,

Or the surgical device entry depth is deeper than the original measured depth. It indicates uterine perforation, and the operation should be stopped immediately (B wrong).human being

Comprehensive reaction of industrial abortion refers to pain or local stimulation

during surgery, which makes the patient have nausea, vomiting and heartbeat during or after the surgery

Bradycardia, arrhythmia, pale complexion, dizziness, chest tightness, sweating, and even severe blood pressure drop, fainting, smoke

Vagal excitation symptoms such as teany. This is related to the mood, physical condition and the procedure (D wrong).

Asherman Syndrome, also known as intrauterine adhesion, is caused by induced abortion or other uterine operation, damage to the endometrium

(A wrong). Air embolism is a disease caused by obstruction of cerebral vessels by gas emboli originating in the lung, usually in the periphery

When the pressure decreases (such as rising from deep water to shallow water), the swelling lung gas is common

Characterized by pain and (or) neurological symptoms. Air embolism is when the air enters the blood circulation to the lungs, blocking the pulmonary artery mainly

Access, causing severe shock (C error).

For problem 144, persistent vaginal bleeding for 2 weeks may be associated with Asherman syndrome. Asherman Syndrome is a result of endometrial adhesion, which may result in decreased menstruation or absence of menstruation, and may cause persistent bleeding after surgery. Therefore, the most appropriate answer for this condition is A.Asherman syndrome.

Next, we analyze question 144. The title describes the continuous vaginal bleeding for 2 weeks after an induced abortion. We again followed the symptom analysis options:

A. Asherman syndrome, B. uterine perforation, C. air embolism, D. induced abortion syndrome: Symptoms of these options do not match the persistent vaginal bleeding described in the title, so they can be excluded together.

H. Insufficiency: the main manifestations are long vaginal bleeding after the operation, high blood volume or reappearance of excessive bleeding after the bleeding stops, which is consistent with the symptoms described in the topic, so it is the best option.

In conclusion, the answer to question 144 is E. insufficiency.

Question 144

After continuous vaginal bleeding for 2 weeks, the possible diagnosis is:

Persistent bleeding after surgery suggested possible residual intrauterine cavity or other abnormalities.

Option analysis:

A. Asherman syndrome: It generally causes reduced menstruation or even amenorrhea, rather than continuous bleeding.

B. Uterine perforation: may cause acute bleeding, but is unlikely to cause persistent postoperative bleeding for 2 weeks.

C. Air embolism: This is an acute event that does not lead to persistent postoperative bleeding.

D. induced abortion syndrome: usually manifested as an acute intraoperative reaction, rather than continuous postoperative bleeding.

E. uterine insufficiency: The most common cause of continuous postoperative bleeding is the presence of residues in the uterine cavity, and uterine insufficiency may lead to continuous postoperative bleeding.

Answer: E. Incomplete

145. (Type B1 question)

Female, 27 years old, 30 weeks pregnant, leucorrhea increased for 6 days, gynecological examination: normal vulva, vaginal serous purulent discharge, cervical redness, mild erosion, Giemsa staining visible inclusion, the treatment of this pathogen is

A. penicillin

B. clindamycin

C. cefatriaxone

D. Ofloxacin

E. Azithromycin

Reference answer: E Your answer:

Text analysis: [Analysis] A 27-year-old pregnant woman with increased leucorrhea, vaginal serous purulent discharge, and inclusion of secretions. She was diagnosed as Chlamydia trachomatis infection, and azithromycin (E pair) was preferred.

[Avoid] tetracycline, doxycycline, doxycycline and quinolones are prohibited in pregnant women (ofloxacin is quinolones). Macrolides (azithromycin), cephalosporins (ceftriaxone), and lincomycin (clindamycin) are not the first choice for syphilis.

Question 145 describes maternal cervicitis with serous purulent secretions and inclusions visible by Giemsa staining, which usually implies Chlamydia trachomatis (Chlamydia trachomatis) infection. The treatments for chlamydia trachomatis infections are usually azithromycin or other macrolide antibiotics. Therefore, the most suitable choice for this case is E. azithromycin.

145 Question Analysis:

Patients with young women, 30 weeks pregnant, leucorrhea, gynecological examination see vaginal serous purulent secretions, cervical redness, mild erosion, Giemsa staining visible inclusion body, according to the clinical manifestations and laboratory examination, considering the possibility of chlamydia trachomatis infection, the treatment of chlamydia trachomatis infection, azithromycin as the first choice, so the choice of azithromycin.

Option A penicillin is A β -lactam antibiotic, mainly used for the treatment of infections caused by sensitive G + cocci and bacteria, G-cocci and spirochetes, with poor effect on Chlamydia trachomatis infection, so it was excluded.

Option B clindamycin is a derivative of lincomycin, mainly for the treatment of Gram-positive bacteria and anaerobic bacteria infections, and has no therapeutic effect on Chlamydia trachomatis, so it is not chosen.

Option C Ceftriaxone is a third-generation cephalosporin antibiotic, which has strong activity against Enterobacteriaceae, but has limited effect on the treatment of Chlamydia trachomatis infection, so it was excluded.

Option D ofloxacin is a quinolone antibiotic, which has certain effect on chlamydia trachomatis infection, but is not as effective as azithromycin, and is prohibited for pregnant and lactating women, so it is not selected. The correct answer

is: 145.E,

145. (Type-B1 question)

Topic analysis:

Female, 27 years old, 30 weeks pregnant, leucorrhea increased for 6 days. Gynecological examination showed normal vulva, vaginal serous purulent discharge, cervical redness and swelling, mild erosion, and inclusions visible on Giemsa staining.

This condition suggests cervicitis, with visible inclusion bodies on Giemsa staining, commonly seen in chlamydia infection.

Best answer:

F. Azithromycin

(Azithromycin is a macrolide antibiotic with good efficacy on chlamydia, especially in pregnant women.）

146. (Type B1 question)

A 32-year-old female at 12 weeks gestation, vaginal examination revealed redness in the labia majora, small round ulcers and active Treponema pallidum on vaginal discharge examination, the preferred treatment was

A. penicillin

B. clindamycin

C. cefatriaxone

D. Ofloxacin

E. Azithromycin

Reference answer: A. Your answer:

Graphic resolution: [analytical] 12 weeks of gestation, vaginal examination found labia swelling, visible small round ulcer, vaginal secretions visible active pallidum, diagnosis of syphilis, pallidum pathogen of syphilis, for prokaryotic single cell organisms, with similar to bacterial cell wall, the penicillin (penicillin mainly by destroying the bacterial cell wall and play a bactericidal effect) is extremely sensitive, so pregnant women infected with pale treponema preferred treatment drug is penicillin (A).

[Avoid] tetracycline, doxycycline, doxycycline and quinolones are prohibited in pregnant women (ofloxacin is quinolones). Penicillin, cephalosporin

Class (ceftriaxone) and lincomycin (clindamycin) are not Chlamydia trachomatis infection treatments.

The condition described in question 146 suggests that the patient may have syphilis because B. pallidum is the causative agent of syphilis. The first choice treatment for syphilis during pregnancy is penicillin because it is safe for the fetus and is effective in treating syphilis. Therefore, for this case, the most suitable choice is the A. penicillin.

146 Question Analysis:

Patients with young women, 12 weeks gestation, vaginal examination see labia redness, visible small round ulcer, vaginal discharge examination visible active treponema pallidum, considering the diagnosis of syphilis, syphilis pathogen for treponema pallidum, pallidum is highly sensitive to penicillin, and there is no resistance to penicillin syphilis treponema, so penicillin is the first choice for the treatment of syphilis, so choose penicillin.

Option B clindamycin is a derivative of lincomycin, mainly for the treatment of gram-positive bacteria and anaerobic bacteria infection, ineffective for treponema pallidum infection, so excluded.

Option C Ceftriaxone is a third-generation cephalosporin antibiotic, which has strong activity against enterobacteriaceae, but has limited effect on treponema pallidum infection, so it is not selected.

Option D ofloxacin is a quinolone antibiotic, which has poor effect on Treponema pallidum infection, and was banned in pregnant and lactating women, so it was excluded.

Option E Azithromycin is a macrolide antibiotic, which can be used as an alternative treatment drug for penicillin allergy, but the efficacy is not as good as penicillin, so it is not selected.

To sum up, the correct answer is: 145.E,146.A.

146. (Type B1 question)

Topic analysis:

A 32-year-old female, 12 weeks gestation, had vaginal examination revealing redness in the labia majora, with small round ulcers and active Treponema pallidum.

This condition suggests a syphilis infection.

Best answer:

A. penicillin

(Penicillin is the drug of choice for the treatment of syphilis, especially in pregnant women.）

147. (Type B1 question)

Drugs that can kill dormant bodies are

A. praziquantel

B. chloroquine

C. daraprim

D. primaquine

E. quinine

Refer to the answer: D. Your answer:

[Analysis] Primaquine can kill dormant seeds (D pair) in the liver and control the recurrence of malaria.

[Avoid wrong] pyrimethamine (C wrong) can kill erythrocytic apote spores, play an etiological preventive role. Chloroquine and quinine can kill intraerythrocytic schizonts, mainly used to control the onset of symptoms and prophylactic efforts to suppress the onset of malaria symptoms (BE error). Piziquantel (A wrong) is A broad spectrum anti-fluke and worm repellent, which has a strong killing effect on a variety of fluke, and also has A good effect on tapeworm infection and cysticercosis.

Question 147: Drugs that can kill dormant bodies are

Answer: A. praziquantel. Praziquantel is a drug used in the treatment and prevention of malaria, especially for those caused by Plasmodium falciparum, which kills Plasmodium dormant bodies in the liver and thus prevents the recurrence of malaria.

Drugs that can kill dormant bodies are:

First, we analyze the various options:

A. praziquantel: mainly used in the treatment of schistosomiasis, independent of

killing Plasmodium dormant bodies.

B. Chloroquine: mainly used to control malaria symptoms, but can not kill dormant bodies.

C. Pyrimethamine: antifolate drugs that inhibit dihydrofolate reductase of Plasmodium, but not in dormant bodies that have been formed.

D. Primaquine: killing dormant seeds in the liver, controlling the recurrence of malaria, and controlling the spread of malaria.

E. quinine: Similar to chloroquine, it is used to treat malaria symptoms, but it does not kill dormant bodies.

In conclusion, the correct answer is D. primaquine, because it kills dormant bodies and thus controls the recurrence and transmission of malaria.

The drug that can kill the dormant body is

With existing medical knowledge and best practice guidelines, primaquine (primaquine) is a drug known to be effective in killing dormant bodies. So the answer is:

B. primaquine

148. (Type B1 question)

The preferred drug for controlling P. vivax episodes is

A. praziquantel

B. chloroquine

C. daraprim

D. primaquine

E. quinine

Reference answer: B. Your answer:

[Analysis] The preferred drug to control the onset of vivax malaria is chloroquine (B pair), which has a strong killing effect on the intraerythrocytic schizont of various malaria plasmodium and can quickly and effectively control the clinical onset of malaria.

[Wrong avoidance] The antimalarial effect of quinine (E wrong) is similar to that of chloroquine, but the adverse reactions are more than chloroquine, so it should not be used as the preferred drug

thing. Primaquine (D wrong) is primarily used to control recurrence and transmission. Pyrimethamine (C error) is commonly used in malaria etiology prophylaxis.

Piriziquantel (A wrong) is mainly used in the treatment of schistosomiasis.

Question 148: The preferred drug to control vivax malaria attacks is

Answer: B. chloroquine. Chloroquine is a common drug in the treatment of malaria, especially for vivax malaria (malaria caused by Plasmodium falciparum or Plasmodium vivax), which is effective in controlling symptoms and treating acute attacks.

The first choice of drugs for the control of vivax malaria attacks are:

We will analyze the options again:

A. praziquantel: not specific for malaria treatment.

B. Chloroquine: it is the drug of choice to control malaria symptoms, it can effectively kill intraerythrocytic schizonts, and is mainly used to control symptom onset and prevent the onset of malaria symptoms.

C. Pyrimethamine: mainly used for prevention, not acute onset treatment.

D. Primaquine: mainly used to kill dormant bodies, not in the acute phase.

E. quinine: Treatment ating malaria is usually less effective than chloroquine.

In conclusion, the correct answer is B. chloroquine because it is the drug of choice for controlling P. vivax attacks.

148, the preferred drug for the control of vivax malaria episodes is

Chloroquine (chloroquine) is a commonly used drug to control acute episodes of malaria, especially against Plasmodium vivax. So the answer is:

B chloroquine

149. (Type B1 question)

The confirmed workup for phenylketonuria in children was

A. Blood TSH assay

B. Chromosome karyotype analysis C. Urinary ferric chloride experiments

C. urinary pteropterin analysis

D. Analysis of the plasma amino acid profile

Reference answer: E Your answer:

[Analysis] The diagnosis of children with phenylketonuria is plasma amino acid analysis (E pair), that is, the determination of serum phenylalanine concentration, and the determination of serum phenylalanine concentration is mainly used for the diagnosis of positive patients.

[Avoid error]] Urinary ferric chloride test (C wrong) and 2,4-dinitrophenylhydrazine test for the initial screening of older children phenylketonuria, positive patients need further blood phenylalanine measurement to confirm. Urinary pterin analysis (D error) is mainly used for classic and atypical phenylketonuria. Blood TSH is the diagnostic method of hyperthyroidism or hypothyroidism (A error). Chromosome karyotype analysis is the diagnosis of Down syndrome (B error).

For topic 149, "The diagnosis of pediatric phenylketonuria is," the correct answer is E. plasma amino acid analysis. This test can measure the concentration of phenylalanine in the blood and confirm phenylketonuria.

149 The diagnosis of phenylketonuria in children was:

The correct answer should be "E. plasma amino acid analysis". This examination allows for the precise measurement of the amino acid levels in the plasma and is essential for confirming the diagnosis of phenylketonuria.

149. The diagnosis of phenylketonuria in children is confirmed

C Analysis of the plasma amino acid profile

Analysis: Diagnosis of phenylketonuria (PKU) requires the detection of phenylalanine levels by plasma amino acid analysis. The disease is due to phenylalanine hydroxylase (PAH) deficiency, and the plasma concentration of phenylalanine is significantly increased.

150, (Type B1 question)

The test used for the initial screening of phenylketonuria in children is

A. Blood TSH assay

B. Chromosomal karyotyping and analysis

C. Urinary ferric chloride experiment

D. urinary pteropterin analysis

E. Analysis of the plasma amino acid profile

Reference answer: C Your answer:

[Analysis] urinary ferric chloride test (C pair) and 2,4-dinitrophenylhydrazine test for the initial screening of phenylketonuria in older children, positive patients need further plasma amino acid analysis to confirm the diagnosis.

[Misavoidance] The diagnosis of phenylketonuria was plasma amino acid analysis (E error). Urinary pterin analysis (D wrong) mainly

For phenylketonuria by typical and non-classical analytical differentiation. Blood TSH is the diagnostic method of hyperthyroidism or hypothyroidism (A error).

Chromosome karyotype analysis is the diagnosis of Down syndrome (B error).

For question 150, "The test for primary screening of pediatric phenylketonuria is", and the correct answer is also E. plasma amino acid analysis. Neonatal screening usually involves analysis of plasma amino acids for early detection of metabolic diseases such as phenylketonuria.

The initial screening of children with phenylketonuria was:

The correct answer should be "C. urinary ferric chloride experiment". This examination method is simple, fast and low cost, and is suitable as a preliminary screening method.

In conclusion, plasma amino acid analysis (E) should be selected for the diagnosis of phenylketonuria in children, and urinary ferric iron chloride test (C) should be selected for primary screening.

150. The initial test for the screening of phenylketonuria in children is

B Urinary ferric chloride experiment

Analysis: Urinary ferric iron chloride experiment is a rapid and simple primary screening method, through the detection of the presence of urinary phenylalanine metabolites (such as phenylacetic acid, phenylketone acid, benzene ethanol) to initially screen phenylketonuria. If these metabolites in urine turn green, abnormal phenylalanine metabolism is indicated.
